# Mendelian inheritance of trimodal CpG methylation sites suggests distal cis-acting genetic effects

**DOI:** 10.1186/s13148-016-0295-1

**Published:** 2016-11-22

**Authors:** Shaza B. Zaghlool, Mashael Al-Shafai, Wadha A. Al Muftah, Pankaj Kumar, Christian Gieger, Melanie Waldenberger, Mario Falchi, Karsten Suhre

**Affiliations:** 1 Department of Physiology and Biophysics, Weill Cornell Medical College in Qatar, Education City, PO Box 24144, Doha, Qatar; 2 Computer Engineering Department, Virginia Tech, Blacksburg, VA 24060 USA; 3 Department of Genomics of Common Disease, Imperial College London, London, UK; 4 Research Division, Qatar Science Leadership Program, Qatar Foundation, Doha, Qatar; 5 Department of Twin Research & Genetic Epidemiology, King’s College London, London, SE1 7EH UK; 6 Research Unit of Molecular Epidemiology, Helmholtz Zentrum München, German Research Center for Environmental Health, Neuherberg, Germany; 7 Institute of Epidemiology, Helmholtz Zentrum München, German Research Center for Environmental Health, Neuherberg, Germany; 8 Department of Biomedical Sciences, College of Health Sciences at Qatar University, Doha, Qatar

**Keywords:** Mendelian inheritance, DNA methylation, Epigenetics, CpG, Trimodal distribution, Genetic variance

## Abstract

**Background:**

Environmentally influenced phenotypes, such as obesity and insulin resistance, can be transmitted over multiple generations. Epigenetic modifications, such as methylation of DNA cytosine-guanine (CpG) pairs, may be carriers of inherited information. At the population level, the methylation state of such “heritable” CpG sites is expected to follow a trimodal distribution, and their mode of inheritance should be Mendelian.

**Methods:**

Using the Illumina Infinium 450 K DNA methylation array, we determined DNA CpG-methylation in blood cells from a family cohort 123 individuals of Arab ethnicity, including 18 elementary father-mother-child trios, we asked whether Mendelian inheritance of CpG methylation is observed, and most importantly, whether it is independent of any genetic signals. Using 40× whole genome sequencing, we therefore excluded all CpG sites with possibly confounding genetic variants (SNP) within the binding regions of the Illumina probes.

**Results:**

We identified a total of 955 CpG sites that displayed a trimodal distribution and confirmed trimodality in a study of 1805 unrelated Caucasians. Of 955 CpG sites, 99.9% observed a strict Mendelian pattern of inheritance and had no SNP within +/−110 nucleotides of the CpG site by design. However, in 97% of these cases a distal cis-acting SNP within a +/−1 Mbp window was found that explained the observed CpG distribution, excluding the hypothesis of epigenetic inheritance for these clear-cut trimodal sites. Using power analysis, we showed that in 46% of all cases, the closest CpG-associated SNP was located more than 1000 bp from the CpG site.

**Conclusions:**

Our findings suggest that CpG methylation is maintained over larger genomic distances. Furthermore, nearly half of the SNPs associated with these trimodal sites were also associated with the expression of nearby genes (*P* = 4.08 × 10^−6^), implying a regulatory effect of these trimodal CpG sites.

**Electronic supplementary material:**

The online version of this article (doi:10.1186/s13148-016-0295-1) contains supplementary material, which is available to authorized users.

## Background

Environmental factors can affect phenotype in a heritable manner without altering the DNA sequence [1], as evidenced by studies of environmental stresses such as exposure to chemicals, dietary intake, and temperature changes, among others [2–4]. Epigenetic changes, such as cytosine methylation in the context of cytosine-guanine (CpG) dinucleotides are hypothesized to occur in response to environmental challenges that indirectly affect phenotype through changes in gene expression [2, 5–8]. DNA methylation is one of the most commonly studied forms of epigenetic modification [9]. However, there is presently a gap in understanding how epigenetic marks are passed from generation to generation, and how inherited DNA methylation is re-established after meiosis.

A number of studies suggested that although methylation occurs in response to environmental changes, these epigenetic modifications can actually be inherited [10]. More recently, epigenetic heritable changes in traits such as flowering time and root length were reported in different strains of *Arabidopsis* [11, 12]. Stable inheritance of several epigenetic alterations that contribute to these complex heritable traits can propagate for more than eight generations in the absence of extensive DNA sequence polymorphisms, suggesting that the effect of loss or gain of DNA methylation can indeed be transmitted across generations [11, 12].

Trans-generational epigenetic inheritance has also been reported in animals and has been initially inspired by observations in families from the Dutch famine of 1944, where starvation in one generation led to altered body composition and poorer health in the grandchildren [13]. Moreover, diet-induced obesity in male mice led to widespread changes in DNA methylation and caused diabetes and insulin resistance in their offspring [14]. Furthermore, pregnant mice on a near-starvation diet were reported to have offspring that were significantly smaller than normal, and even though these young were well fed, their own offspring were also born unusually small and with a higher risk of diabetes [15]. In another study [16], the hereditary transmission of environmental information in the form of parental traumatic exposure was also reported to trace back to epigenetics.

Here we ask whether CpG methylation patterns that follow Mendelian inheritance can be observed in a human population. Methylation of a single CpG site in a given cell corresponds to a binary mark (either methylated or unmethylated). Because each cell has two copies of each chromosome, methods that determine CpG methylation generally distinguish between a methylated, hemi-methylated, or unmethylated state for a specific CpG site. Moreover, most methylation measurements are performed on an ensemble of cells, potentially including different cell types. Thus, the numeric value of the methylation state of a given CpG site represents the percentage of individual CpG dinucleotides that are methylated in any given sample, often referred to as the *β* value or beta [17]. In the most clear-cut situation, one would expect a trimodal distribution of the degree of methylation of a single inherited CpG site on a population scale: individuals who inherited two unmethylated CpG alleles would be expected to display low or zero overall CpG methylation (*β* value~0), individuals who inherited one unmethylated and one methylated CpG allele would show a hemi-methylated signal (*β* value~0.5), and individuals who inherited two methylated CpG alleles would show a fully methylated signal (*β* value~1).

Using the Illumina Infinium 450 K DNA methylation array to determine DNA CpG-methylation on a genome-wide scale in white blood cells from two previously published studies, a family cohort of 123 individuals of Arab ethnicity [18] [19], and from 1805 unrelated individuals of Caucasian descent, [20] we first identify all CpG sites that display a trimodal distribution in both cohorts. Using 40× whole genome sequencing performed in the Arab cohort, we then exclude all CpG sites that contain genetic variants in the vicinity of that site to eliminate cases that may correspond to a deletion of the CpG site itself or that lie in the binding regions of the Illumina probes [21]. Finally, we test the trimodal sites for Mendelian inheritance using a total of 18 nuclear father-mother-child trios in the Arab cohort.

## Methods

### Study population

A total of 123 Qatari study participants, including 72 women (mean age 39 ± 16.9 years) and 51 men (mean age 36.3 ± 17.2 years) were included. This study population has been used and described in previous work [18, 19]. Briefly, the dataset consisted of 15 families with a variety of complex pedigree structures. Seven families contained trios with one to four offspring per family, resulting in a total of 18 trios available for analysis. This study was approved by the Institutional Review Board of Weill Cornell Medical College in Qatar in concordance with the Helsinki declaration of ethical principles for medical research involving human subjects (ethical approval number 2012-003 and 2012-0025). Participants provided written informed consent.

### Methylation

A total of 7 ml of venous peripheral blood was drawn from all participants and collected in EDTA anticoagulant tubes. Genomic DNA extraction was performed in the WCMC-Q clinical laboratory with 2 ml of fresh whole blood following Qiagen protocols using the QIAamp Blood Midi Kit (Qiagen, spin protocol) catalog number (51183). Genomic DNA was quantified using the Qubit 2.0 fluorometer from Invitrogen (Qubit dsDNA BR Assay Kit; catalog numbers Q32850, Q32853). Genome-wide DNA methylation profiling was performed using the Illumina Infinium HumanMethylation450 (450 K) BeadChip array [22] for interrogating over 485,000 methylation sites per sample. The HumanMethylation450 platform uses genotyping sodium bisulphate–treated DNA at a single base resolution and incorporates two assays, Infinium I and Infinium II. Infinium I uses two 50-bp probes with a single color channel for both methylated and unmethylated CpG sites with the assumption that all methylated CpG sites match the targeted sites. Infinium II uses a single probe at each site with two different color channels (green and red) for the detection of methylated and unmethylated CpG sites. In total, we obtained methylation data for 485,577 sites.

### Whole genome sequencing

We obtained whole genome sequencing data from Illumina using the Hiseq 2500 platform for 93 subjects. The average depth of coverage that was used to obtain paired end sequence reads was 40×. We obtained variant sets by processing the sequences with the CASAVA version 1.9 pipeline (Consensus Assessment of Sequence And VAriation), which is a proprietary bioinformatics Illumina pipeline. The CASAVA pipeline includes alignment of the reads to the reference genome, sorting, indexing, realigning, and variant calling. Paired end reads were aligned to the reference human genome of NCBI build 37 using the aligner ELNAD v2 (Efficient Large-Scale Alignment of Nucleotide Database) in the CASAVA pipeline. Variant calling uses a probabilistic algorithm to call the genomic consensus sequence and compares it to the reference sequence to identify homozygous or heterozygous SNPs. For each of the variants called, CASAVA also provides quality measures. We filtered the SNPs based on the quality score provided to retain variants with an error probability less than 0.01. In total, we obtained 14,595,042 genetic variants, called in at least one individual at a quality cutoff of q20.

### Normalization and quality control

Quality control was performed on the methylation data (485,577 CpG sites) from the Qatari sample. These steps included confirmation checks to ensure sample integrity, initial filtering of the 65 SNPs from Illumina manifest and 3091 CpHs, and checking that the percentage of detected sites was over 99.5% (with a *P* value <0.01). Using the whole genome sequencing data, we also verified Mendelian inheritance in the trios. The average acceptable percentage of whole genome sequencing Mendelian violations did not exceed 10% for all trios. The overall signal intensity and the distribution of *β* values of the samples were then inspected for any abnormalities. No samples were excluded due to low signal intensity or having abnormal methylation profiles. After all of the filtering, a total of 482,421 methylation sites remained under consideration. Genetic variants or SNPs in probes or CpG sites can interfere with methylation readouts by affecting probe binding. Therefore, based on the whole genome sequencing data, all methylation data were set to missing values whenever a genetic variant existed within the probe-binding region of ±110 base pairs of the CpG. Incorporating the whole genome sequencing data ensured that no local SNPs were affecting the methylation. These accounted for about 0.5% of the methylation sites that were replaced by missing values. The data were also color bias adjusted, quantile normalized, and BMIQ normalized using the LUMI + BMIQ pipeline [23, 24].

### KORA population study

Data from 1805 subjects of the previously published KORA cohort were used. KORA is a study of a general population living in southern Germany, as described in Ref [20]. In brief, these subjects were unrelated individuals with a mean age of 60.9 ± 8.9, with 925 women and 880 men. Array-based DNA methylation data (Infinium HumanMethylation 450 BeadChip platform [22]) was obtained for these subjects. A total of 1000 ng of genomic DNA was bisulphate-converted using the EZ-96 DNA Methylation Kit (Zymo Research, Orange, CA, USA) following the manufacturer’s protocol. All samples were preprocessed using Genome Studio for background subtraction and control normalization. A total of 457,004 CpG sites were used for the analysis of this study. Various quality checks were performed on these data and have been described in studies in which they were previously used [25–27].

### Definition of trimodality and Mendelian inheritance agreement/violation

A “trimodal site” is defined here as a CpG site that had at least one individual falling into each of three predefined methylation states (methylated, hemi-methylated, and unmethylated) and in addition to at least 95% of the subjects falling inside the defined regions. The methylation states are defined as follows:

- Full methylated 0.8 < *β* ≤ 1
- Hemi-methylated 0.35 ≤ *β* ≤ 0.65
- Unmethylated 0 ≤ *β* < 0.2

We required trimodality to occur in both populations so that only CpGs that were trimodal in both studies were retained for further analysis. Mendelian agreements and violations for the methylation states were then identified. For instance, if the parents were both fully methylated (homozygous) for a given CpG, then the offspring would also have to be fully methylated to conform to Mendelian inheritance.

### Testing for SNP-methylation association in the Qatari sample

The Qatari sample DNA was sequenced by Illumina for 93 of the 123 subjects. The trimodal sites were checked for SNP-methylation associations only for those subjects for which both, methylation and sequencing data, were available. The window size was set to ±1 Mbp around each trimodal CpG locus. Contingency tables were generated for each methylation site against each SNP from the sequencing data in a specified window, considering all subjects (three rows for the three methylation states, and three columns for the three allelic states). The chi-square test of independence for count data was used to test for associations between the methylation state and genotype. *P* values were computed for every trimodal methylation site, for all SNPs in the same window. Because of the limited number of samples, the minimum obtainable *P* value was 0.0005, which made the detectability of significant associations heavily dependent on the window size (the larger the window, the greater the number of SNPs contained in that window, which is the denominator in the *P* value cutoff). The strongest associations occurred when the three methylation states segregated perfectly with the respective genotypes. A Bonferroni cutoff of significance for the *P* values was used where the significance for a given window size and a particular site was computed as follows:

$$ P\ \mathrm{value} = \frac{0.05}{\mathrm{Actual}\ \mathrm{number}\ \mathrm{of}\ \mathrm{SNPs}\ \mathrm{in}\ \mathrm{that}\ \mathrm{window}} $$

To compute the power of the chi-squared test, the “pwr.chisq.test” function in the *R* library “pwr” was used [28]. First, the effect size $w$ for two sets of *k* probabilities, *P*0 (null hypothesis), and *P*1 (alternative hypothesis) was computed. The effect size *w* was defined as follows:

$$ w=\sqrt{\sum_{i=1}^m\frac{{\left(P{0}_i-P{1}_i\right)}^2}{P{0}_i}} $$

where *P*0_*i*_ = cell frequency in the *i*th cell under *H*_0_

*P*1_*i*_ = cell frequency in the *i*th cell under *H*_1_.

The power is then a function of the total sample size *N*, the degrees of freedom, the specified significance level, and the computed effect size *w*.

We also computed the correlations between all trimodal sites and SNPs in the Qatari cohort as another measure of association using the “cor.test” routine in *R*.

### Testing for SNP-methylation association in the KORA sample

The SNP-methylation association was assessed in the KORA sample using a linear model to test for the association between DNA methylation (*β* values) and genetic variance. Three SNPs from the vicinity of the CpG site (within ±5 Mb around the CpG site) were obtained for each CpG if associations were found. The covariates used in the model included age, gender, BMI, and white blood cell coefficients, which were estimated using the method described by Houseman et al. [29]. Blood cell type coefficients included monocytes, granulocytes, NK cells, B cells, CD8+ T cells, and CD4+ T cells. The selection of the three strongest SNPs was performed iteratively and has been described previously [25]. Briefly, selection was performed iteratively by testing the association of each *β* value for additive linear dependence on every genotyped SNP with the window using the chosen covariates. Once the strongest SNP (SNP1) was determined, a second (SNP2), and third (SNP3) were selected following the same procedure but including the previously selected SNP(s) as additional covariates.

### eQTL analysis

An online tool for annotating genetic variants (SNiPA) [30] was used for the enrichment analysis of meQTLs that are associated with the trimodal sites for overlap with an expression quantitative trait locus (eQTL). SNiPA integrates eQTL data from multiple sources, including the Genotype-Tissue Expression project [31] and the seeQTL database for human eQTLs [32].

A non-biased selection of randomized SNPs is needed to test whether random SNPs have the same likelihood of being linked to an eQTL gene as meQTLs that are associated with trimodal sites. In our study, the selection of randomized SNPs was performed using the SNPsnap Web server [33], which selects SNPs with genetic properties similar to those of a provided query list of SNPs. Selection using this tool ensures appropriate matching of random SNPs and avoids biasing the enrichment. The matching is based on minor allele frequency, number of SNPs in linkage disequilibrium (LD), distance to the nearest gene, and gene density.

## Results

### Almost 1000 trimodal CpG sites are shared between the Qatar and KORA cohorts

**Fig. 1:**
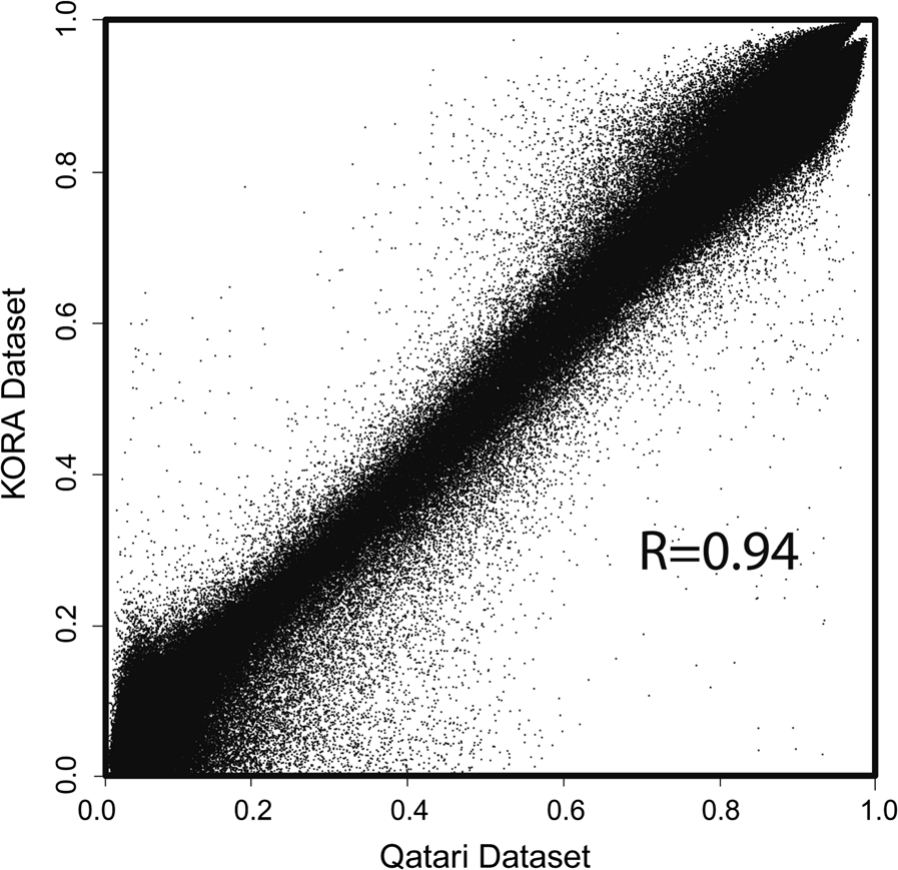
Correlation between the QMDiab study and the KORA population study. Each *point* represents the median of the methylation values for one of the 457,004 CpG sites assayed in the two cohorts

There was strong overall correlation of the median of all methylation sites between the Qatari and KORA samples (*R* = 0.94). The correlation plot of the two datasets is shown in Fig. 1. Considering both the Qatari cohort and the larger cohort KORA together, and also allowing only 5% of the subjects to have methylation values outside of the three predetermined ranges, an overlap of 955 trimodal sites was found. Additional file 1: Figure S1 shows a regional plot of the physical locations of those trimodal sites with respect to all of the methylation sites measured by the 450 K array, showing no particular location bias and that trimodal sites are dispersed genome-wide. A histogram of each of these sites can be viewed in Additional file 2: File S1.

### Mendelian inheritance is observed in 99% of the cases

**Fig. 2:**
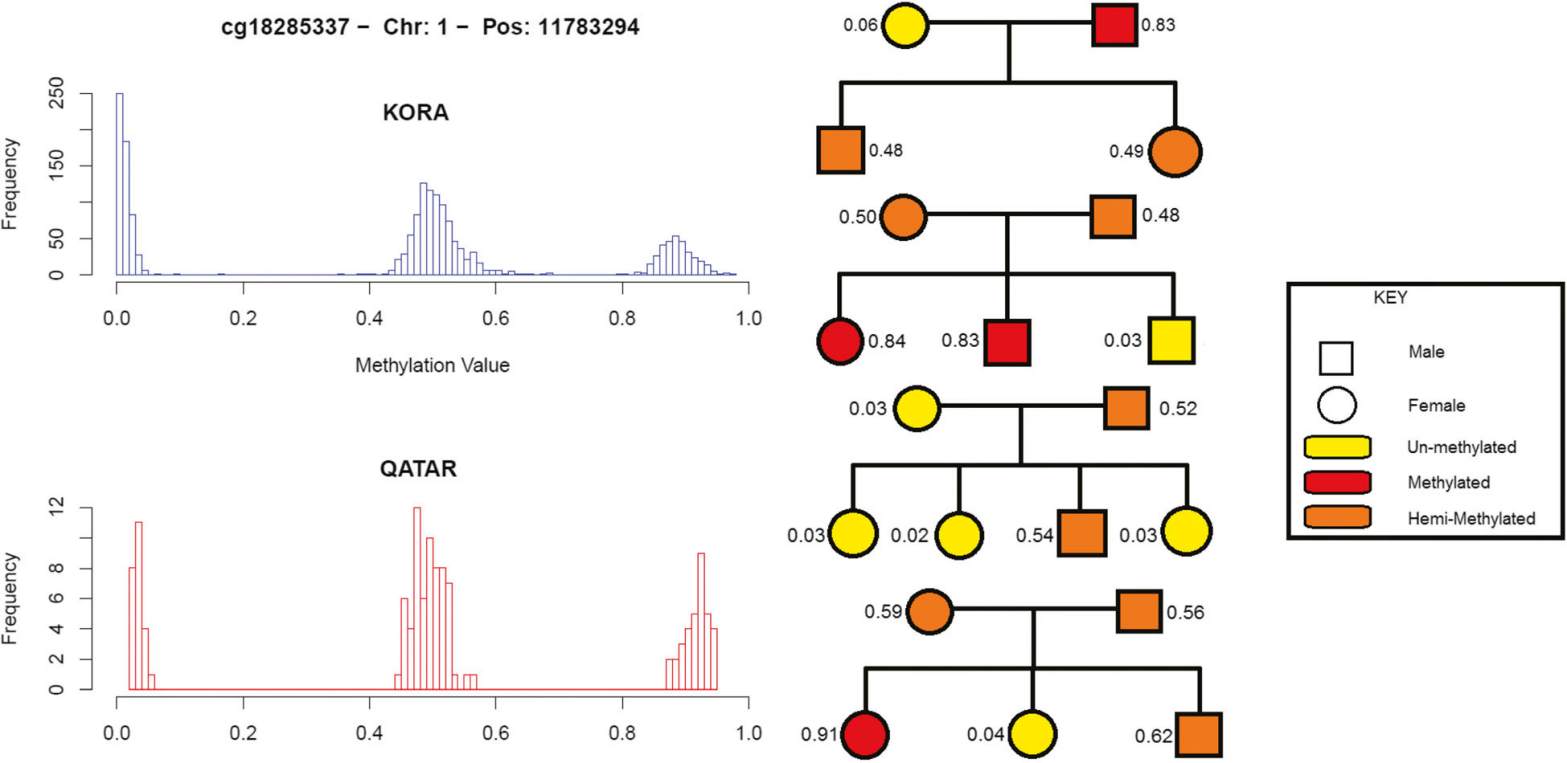
An example of Mendelian inheritance at a trimodal CpG site. The histograms on the *left* show the distribution of the methylation of CpG probe cg18285337 (*β* values) for both studies. On the *right*, examples of pedigrees from four families (constituting 12 father-mother-child trios) for this CpG are given, showing strict Mendelian inheritance of methylation *β* value

Within the 123 subjects from the Qatari cohort, 18 trios were available. The 955 trimodal sites in all 18 trios were tested for Mendelian violations. All but one of the sites showed Mendelian agreement in all trios. Using 1000 randomisations of the subjects, we found that the average number of sites violating Mendelian inheritance under the null hypothesis was 23.1% ± 2.5, making the results of study being a chance event highly unlikely (*P* = 1.19 × 10^-6^, chi-squared test). An example of a trimodal methylation site that follows Mendelian inheritance in different families having several offspring is shown in Fig. 2.

### Genetic variance that is not in the immediate vicinity of the CpG sites drives trimodality

**Fig. 3:**
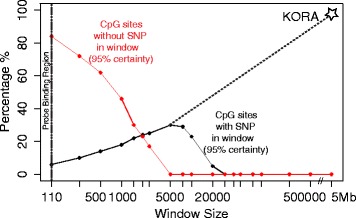
meQTL analysis. The *red line* represents cases where no SNP-CpG association was detected at 95% power within the given window. The *black line* represents cases where a SNP-CpG association was found at a 95% certainty of not falsely rejecting the null hypothesis. The actual number of SNPs tested in the respective window was used to correct for multiple testing (Bonferroni correction), effectively limiting this analysis to a maximal distance of 5000 bp from the CpG site, above which the presence of any SNP-CpG association can be neither confirmed nor rejected with 95% certainty. The region between 0–110 bp corresponds to the probe-binding region. CpGs that contain SNPs in this region were excluded from the analysis. For instance, at 95% power, only 16% of the sites have a SNP within the first 100 nucleotides from the end of the probe-binding region (210 bp from the CpG), while 54% of the sites have a SNP within the first 1000 nucleotides. In KORA, only genotyped SNPs are available; hence, a windowed approach was not possible. However, the KORA study is sufficiently powered to detect any SNP association inside a window of 5 Mb, revealing that 97% of the sites have a SNP-CpG association in that region. The *dashed line* represents a likely extrapolation of the pattern that would be expected in a similar windowed analysis in KORA

The 955 trimodal sites were tested for associations with genetic variants (SNPs) to identify methylation quantitative trait loci (meQTL) using 40× whole genome sequencing data within a +/−1 Mb window around the trimodal CpG site. Figure 3 shows the fraction of trimodal sites that do not have a meQTL that can explain the CpG trimodality as a function of window size. With increasing window size, as a consequence of testing more SNPs, the statistical power eventually drops below 95%, so that no conclusion can be drawn above this distance. Over 80% of all trimodal sites had no SNP within the direct vicinity of the CpG site (at least 100 nt from the boundary of the probe-binding region), and 46% of all trimodal sites had no meQTL within 1000 nt from the CpG site. We then checked for the presence of insertions or deletions within the probe binding regions of our 955 trimodal sites. None of the trimodal CpG sites were affected.

Since our study did not have sufficient power to evaluate window sizes larger than 5000 nt in the Qatari family cohort, we then turned to the population based KORA study. We found that within a 5-Mbp window in the KORA dataset, 97% of the trimodal sites had a meQTL association with *P* < 1 × 10^-7^ (Bonferroni 0.05/457,004 cutoff, conservatively correcting using genome-wide significance) in that region. In addition, over 90% of these meQTLs were found to be within the first 100 kbp of the trimodal CpG sites. After excluding all sites for which the trimodality could be explained by such a meQTL, only 28 CpG sites (3%) remained. Since KORA genotyping is array based and does not cover all existing variants, it is likely that a meQTL was still present for these remaining CpG sites as well (see Additional file 3: File S2 for data on those 28 sites).

### Mendelian meQTLs are enriched in eQTLs

Using SNiPA [30], we investigated enrichment of the meQTLs associated with trimodal sites for overlap with an expression quantitative trait locus (eQTL) (excluding the 28 trimodal sites where no strong meQTL association was found). We found that with a LD threshold of *r* ^2^ ≥ 0.8, 46% of the queried variants had a direct effect on regulation of gene expression through at least one cis-eQTL. These data along with the KORA SNP associations are shown in Additional file 4: File S3.

We further tested whether random SNPs have the same likelihood of being linked to an eQTL gene as meQTLs that are associated with trimodal sites or not. The method used for the selection of random SNP sets is described in the “Methods”. A total of 1000 random sets were generated, and only 15% ± 1.2 of the randomly selected SNPs were also linked to an eQTL. Therefore, there was significant enrichment in the trimodal sites being linked to an eQTL (46%) compared to a mean of 15% in the random sets (*P* = 4.08 × 10^-6^, chi-squared test). Further confirmation is shown in data from eight previous eQTL studies indicating that the number of eQTLs in all SNPs ranges between <1–19% [34], which is in agreement with our randomization.

### Trimodal CpG sites have the highest estimates of genetic heritability

For each of our triomodal CpG sites, we looked up the genetic heritability estimates of DNA methylation levels provided by McRae et al. [35]. Our set of trimodal CpG sites yielded a mean heritability of 0.80 ± 0.18. In comparison to their average genetic heritability of 0.199 for all probes together, our trimodal CpG sites all fell in the higher end of the distribution of heritability estimates.

## Discussion

The similarity in DNA methylation between relatives has been shown previously to be predominantly due to the genetic effects [35]. On a single CpG site level, most methylation *β* value profiles typically are observed to display a mono-modal (Gaussian-like) distribution near zero or one [36]. In contrast, only a small subset of CpGs is multi-modal. A population-based global analysis was designed to identify this small subset of trimodal methylation sites from the more prevalent mono-modal ones. Using 450 K methylation together with 40× whole genome sequencing data from a Qatari family study, we investigated the Mendelian inheritance of DNA methylation. We focused on DNA methylation patterns that displayed a replicated trimodal distribution at the population level. As an example, Fig. 2 shows a Mendelian inherited probe (cg18285337), whose distribution is clearly trimodal in both the Qatari familial sample and in a validation population sample from the KORA cohort. In the family presented at the top of Fig. 2, the CpG site of the mother is unmethylated while that of the father is fully methylated at cg18285337 (both alleles). Following a Mendelian mode of inheritance, the mother can only pass on an unmethylated version of that CpG site while the father can only transmit a methylated version, resulting in both children displaying a hemi-methylated site. Analogously, the other families depicted in Fig. 2 also correspond to a clear Mendelian inheritance pattern for this site.

Overall, we identified through analysis of the Qatari family sample and the KORA population study, a total of 955 trimodal loci, of which 99.9% were transmitted in a Mendelian fashion. Using whole genome sequencing data, we excluded all sites where potential interference of genetic variants in the observed CpG site itself could occur and also the possibility of spurious signals from correlation of genetic variance with the binding efficacy of the Illumina methylation probes. Then, we carried out a meQTL association study around the trimodal sites in the Qatari and KORA samples. In almost all cases (97%), methylation at these trimodal sites was strongly associated with meQTLs located within less than 1 Mb from the CpG site; in about half of the cases, these SNPs (or a strong proxy*—r* ^2^ > 0.8) were also eQTLs [30]. Although the chances of finding a single case of Mendelian inheritance by chance are relatively high (77%), the presence of underlying SNP associations for 97% of the CpG sites confirms the genetic basis of the observed Mendelian inheritance pattern. The likelihood of finding a meQTL association obviously depends on the window size, and limiting the search to cis-acting meQTLs cannot rule out the possibility of undiscovered trans-acting meQTLs anywhere in the genome that could explain the remaining 3% of trimodal CpG sites.

The role of CpG methylation in the regulation of gene expression has been well established, and several studies have shown that genetic variations are linked to quantitative changes in methylation [37–39]. Many meQTLs are also reported to be associated with changes in gene expression (eQTLs) [40], but it remains unclear whether methylation changes are a cause or consequence of altered gene expression. Genetic variants are suggested to perform a regulatory role coordinating all of the molecular changes, possibly through mechanisms leading to variations in transcription factor binding, modification of enzymes and their cofactors, or non-coding RNAs [39]. The association of changes in transcription factor binding with changes in DNA methylation contributes to the regulatory role of DNA methylation in the context of gene regulation.

Although the concept of CpG heritability has been generally addressed previously [35], to the best of our knowledge, the heritability patterns of methylation constrained to globally trimodal sites has not been explored. Previous studies identified a link between CpG methylation and genetic variation at proximal loci (cis-meQTLs) [39], but the study designs did not allow the investigation of inheritance. Trimodal sites are the most clearly heritable sites and represent the maximum level of heritability in methylation. Based on previously published data, our trimodal CpG sites have a mean heritability of 0.80 ± 0.18 [35]. Therefore, having the highest genetic heritability estimates can be attributable to the property of trimodality of these particular CpG sites. These CpG sites are more likely to also associate with meQTLs.

Why trimodality is limited to particular CpG sites while other CpG sites within the same neighbourhood or vicinity do not follow the same pattern is an intriguing question. The pattern is clearly visible in the human leukocyte antigen (HLA) region, which for its medical interest has high CpG coverage on the Illumina array. It contains 17 trimodal CpG sites that are spread out among more than 4500 other CpG sites, none of which are trimodal. The 3D structure of DNA packaging play a role through interactions between DNA loops. Because CpG sites are not physically situated in a linear space, this may allow distant CpG sites to actually become neighboring sites and vice versa.

We show that trimodality is not restricted to certain chromosomes or genes, and Additional file 1: Figure S1 further confirms the absence of any global bias in trimodal site density in the panel of sites tested by the Illumina HumanMethylation 450 K array.

In an effort to understand whether trimodality is common to specific classes of CpG sites, we tested for enrichment in CpG sites designated as regulatory elements, such as promoter or enhancers, differentially methylated regions, or cell type-specific regions, according to the Illumina manifest. In the trimodal sites, we observed significant depletion of regulatory elements (e.g., 5’UTR, 1st exon, proximity to transcription start sites TSS200 and TSS1500), promoter associated, CpG islands, and an enrichment in gene body and 3’UTR (data not shown). Further experiments are needed to explain this observed depletion; however, that is beyond the scope of this particular study.

**Fig. 4:**
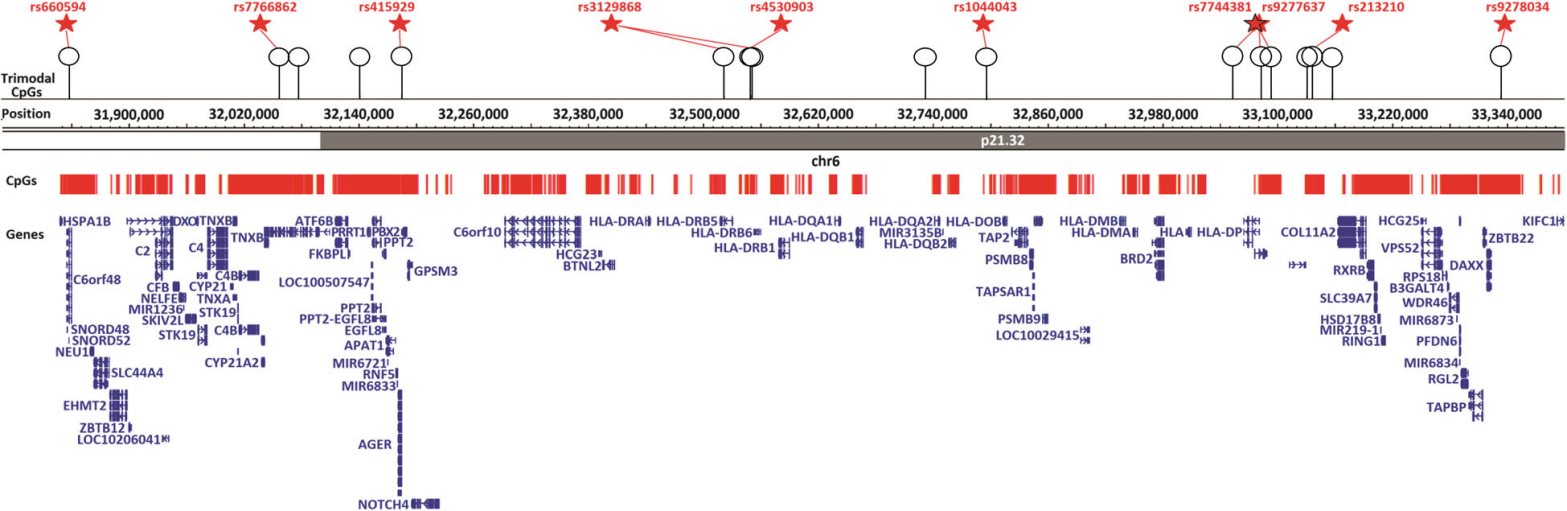
HLA Region. The 17 trimodal CpG sites found in the HLA region are shown below the eQTL SNPS (*red stars*). The *red lines* shown indicate all CpG sites in the HLA locus that are assayed by the methylation array (4754 sites). All trimodal sites in the region were strongly associated with at least one SNP (KORA). In most cases, the same SNP was also an eQTL gene. The *red stars* indicate the associated eQTL SNPs (LD > 0.8 between the SNP and the reported eQTL). The trimodal sites all had a correlation of >0.92 with the SNP while the remaining CpGs in the region had a correlation less than 0.2

Of interest, the eQTLs in the HLA region are linked with different meQTLs associated with 17 trimodal sites in chromosome 6 (Fig. 4). We focused on the major histocompatibility complex (MHC) region because it is known to have the highest estimates of genetic heritability compared to the whole genome [35]. This locus encompasses one of the most gene-dense regions of the human genome and is why it is deeply probed. MHC controls a major part of the innate and adaptive immune systems and is associated with many diseases [41]. Variants in the HLA region can have a cis effect on regulation of gene expression through cis-eQTLs and transcription, impacting both the expression level of HLA genes and methylation by giving rise to trimodal sites in these specific locations. This small subset of trimodal sites observed here is clearly due to strong genetic variations as this effect does not spill over to the entire set of CpG sites in the same region.

We can only speculate on the underlying mechanisms yielding methylation-genotype dependence. A genetic variant in a CpG site itself would lead to a trimodal distribution with a Mendelian pattern of inheritance. Unlike other studies [35] that use public data (i e., 1000 Genomes Project) where not all genetic sequence variation in the probe regions has been detected, we excluded these “trivial” cases from this study using our available 40× whole genome sequencing data that is also specific to our particular cohort under study. Changes in DNA methylation may play a role in biological feedback of regulation or in maintaining methylation between the observed trimodal CpG site and other CpG sites that are directly impacted by a genetic variant. Methylation at a particular site usually spreads out or is maintained by local methylation processes. A trimodal CpG site can be impacted by neighboring CpG sites depending on their methylation states. The first potential process leading to a genetically driven methylation site can occur when these neighboring CpG sites are affected by a SNP, resulting in the observed genotype-dependent effect being transmitted to the trimodal CpG sites as well. Hence, the observed trimodality is driven by a variant that is distal but located in cis to the CpG site. The second process that may lead to a genetically driven methylation site can be due to the mutation changing gene expression activity (eQTL) which in turn, by feedback, reflects on the methylation readout. This scenario is less likely than the third process that may lead to a genetically driven methylation site. Most likely, the genetically driven change occurs in a methylation site that resides in a regulatory region resulting in different levels of gene expression. It is also worth noting that since the majority of meQTLs (which also happen to be “coincident” with eQTLs) are actually close in distance (within 100 kbp) to the trimodal CpG sites, it is also still possible that these genetic variants and trimodal CpG sites both reside on the same haplotype only by chance. So the association may actually be due to the haplotype with the variant of the SNP being close to the CpG site and not really having a role on gene expression.

## Conclusions

**Fig. 5:**
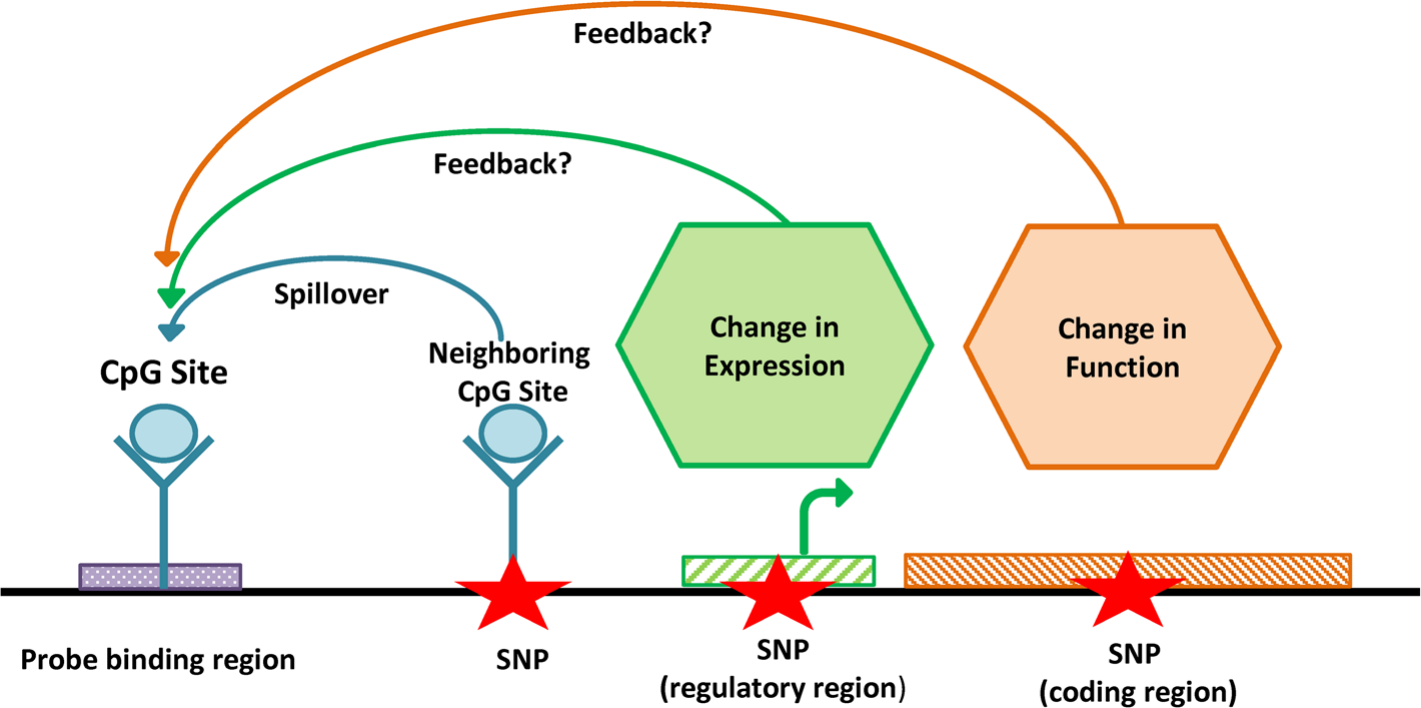
Potential processes leading to a genetically driven methylation site. Three potential processes can lead to an association of a genetic variant and a trimodal distribution of CpG methylation: (1) “Spillover from a neighboring CpG site” that is deleted by a SNP by processes of local methylation maintenance, (2) SNP-induced changes in gene expression activity may have a feedback effect on DNA methylation due to differences in the translation machinery that leaves methylation marks on the DNA, and (3) SNP-induced changes in gene function may induce different levels of gene expression that are implemented by changes in promoter gene methylation

In summary, three plausible mechanisms could lead to association between a genetic variant and a trimodal CpG site. The first possibility is a spillover of trimodal methylation from a nearby CpG that is modified by a genetic variant in the site (e.g., by a CpG-to-TpG variant) by global DNA methylation-maintaining proteins [42]. The second is a feedback mechanism in which a genetic variant modifies gene expression, and the resulting changed translation activity then leaves a modified trace in the DNA methylation level [43]. The third is a feedback mechanism in which a genetic variant modifies methylation in a promoter region inducing changes in gene expression. These potential processes are summarized in Fig. 5. In conclusion, we observed and confirmed Mendelian inheritance of CpG methylation, but almost all trimodal CpG sites are driven by genetic variance.

## Additional files

Additional file 1: Figure S1. Genome-wide location of the 955 trimodal sites. The gaps are mostly unmeasured locations on the methylation array ensuring no bias due to a technical limitation. (TIF 2639 kb)
Additional file 2: File S1. Histograms of 955 trimodal sites for both the 123 Qatari cohort and the 1805 KORA cohort. (PDF 1675 kb)
Additional file 3: File S2. Replication of 28 trimodal CpG sites that follow Mendelian inheritance in both the Qatari trios and the KORA dataset but having no Bonferroni significant underlying SNP association (based on KORA data). (PDF 200 kb)
Additional file 4: File S3. Table of eQTL SNPs for trimodal sites with known SNP associations. (TXT 53 kb)
