# Supplementary figures and images for: Mendelian inheritance of trimodal CpG methylation sites suggests distal cis-acting genetic effects
Source: Clin Epigenetics. 2016 Nov 22;8:124. doi: 10.1186/s13148-016-0295-1 (PMC5120560; doi:10.1186/s13148-016-0295-1)

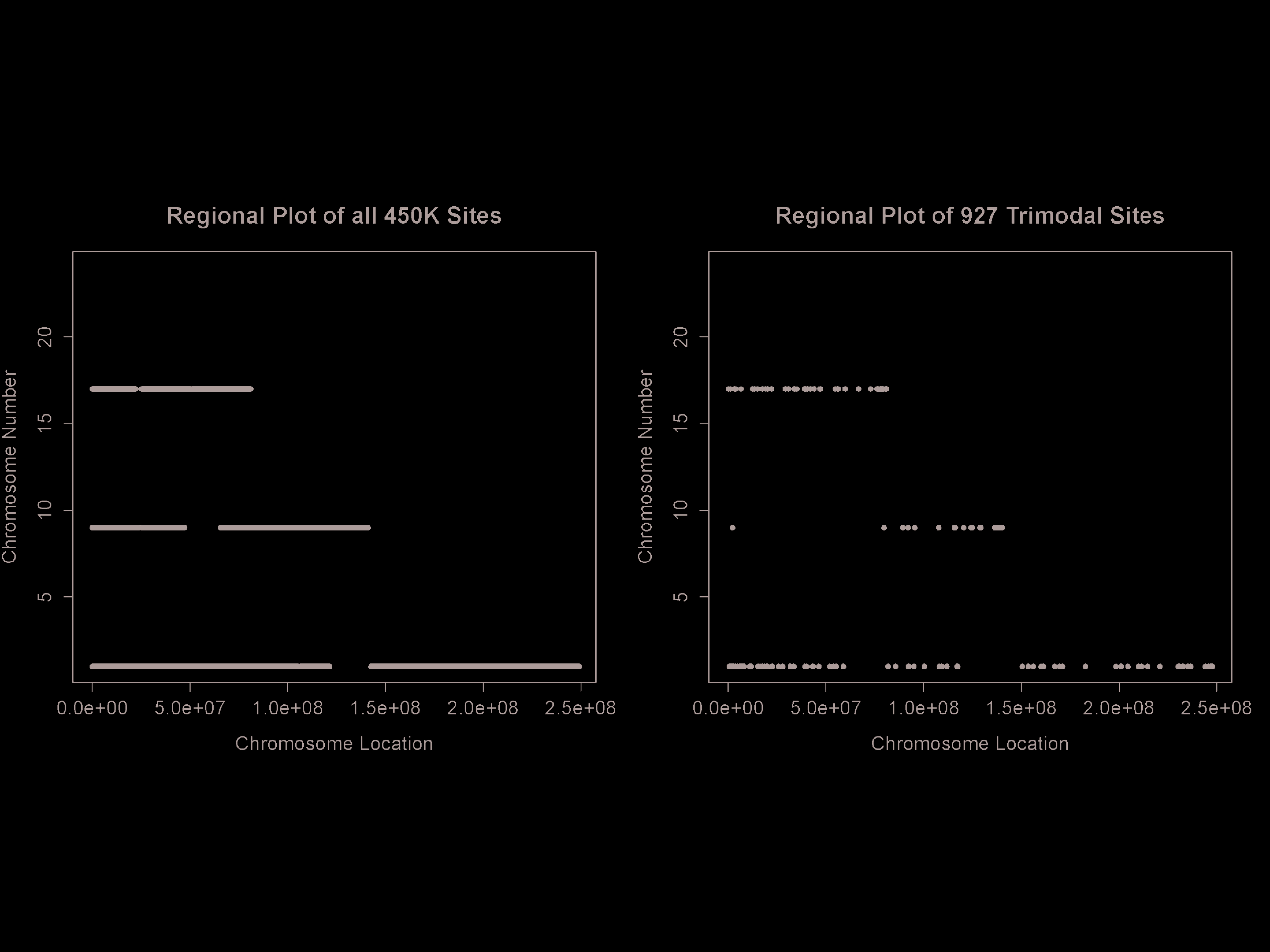

Supplement: Additional file 1: Figure S1. — Genome-wide location of the 955 trimodal sites. The gaps are mostly unmeasured locations on the methylation array ensuring no bias due to a technical limitation. (TIF 2639 kb) [file 13148_2016_295_MOESM1_ESM.tif]
