# Supplementary material for: Mendelian inheritance of trimodal CpG methylation sites suggests distal cis-acting genetic effects
Source: Clin Epigenetics. 2016 Nov 22;8:124. doi: 10.1186/s13148-016-0295-1 (PMC5120560; doi:10.1186/s13148-016-0295-1)
Supplement: Additional file 2: File S1. — Histograms of 955 trimodal sites for both the 123 Qatari cohort and the 1805 KORA cohort. (PDF 1675 kb) [file 13148_2016_295_MOESM2_ESM.pdf]

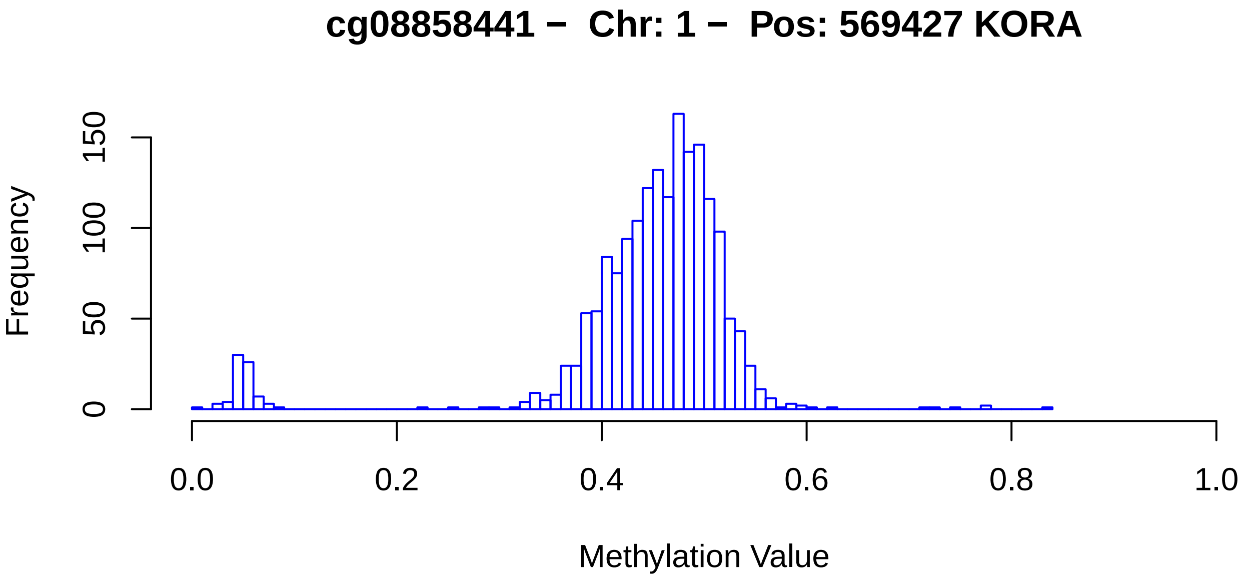
cg08858441 − Chr: 1 − Pos: 569427 KORA
Frequency
0
50
100
150
0.0
0.2
0.4
0.6
0.8
1.0
Methylation Value

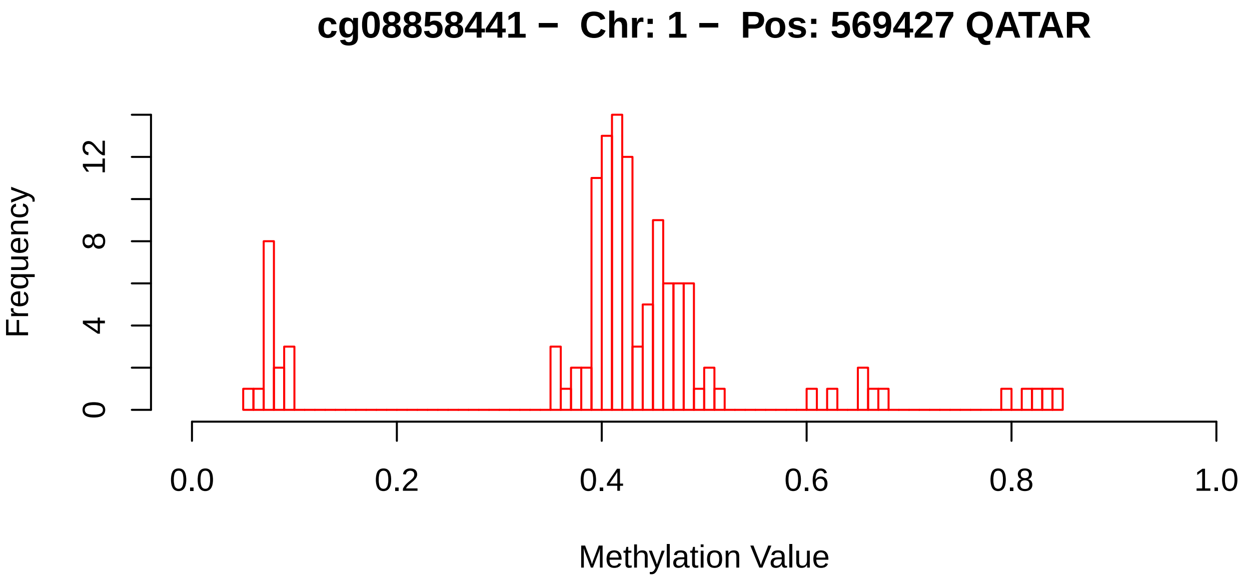
cg08858441 − Chr: 1 − Pos: 569427 QATAR
Frequency
0
4
8
12
0.0
0.2
0.4
0.6
0.8
1.0
Methylation Value

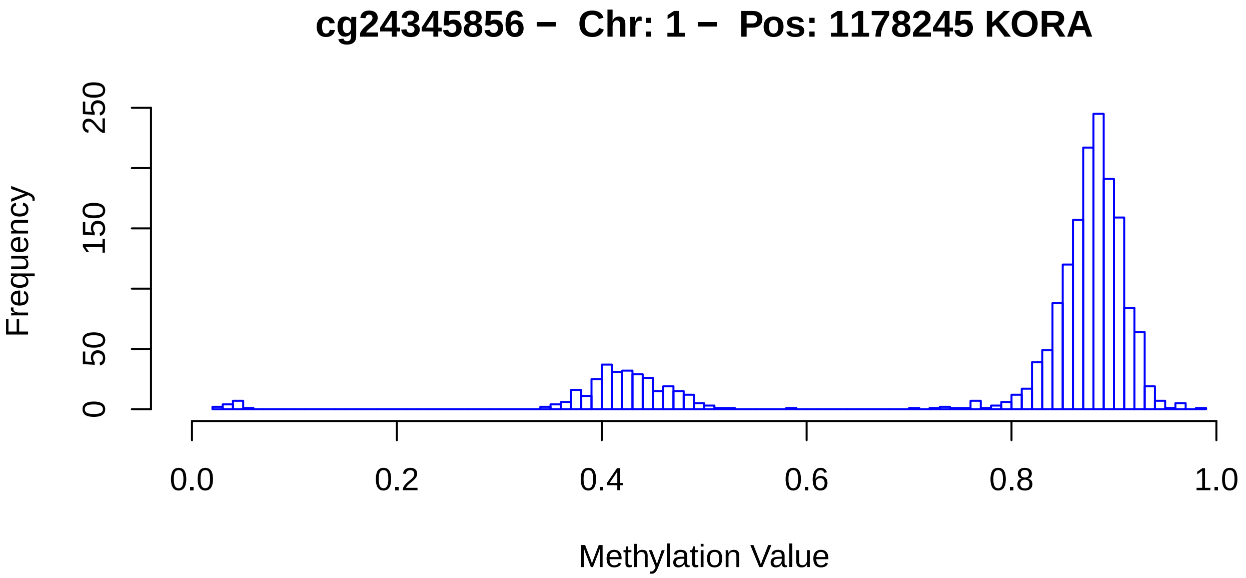
cg24345856 − Chr: 1 − Pos: 1178245 KORA
Frequency
0
50
150
250
0.0
0.2
0.4
0.6
0.8
1.0
Methylation Value

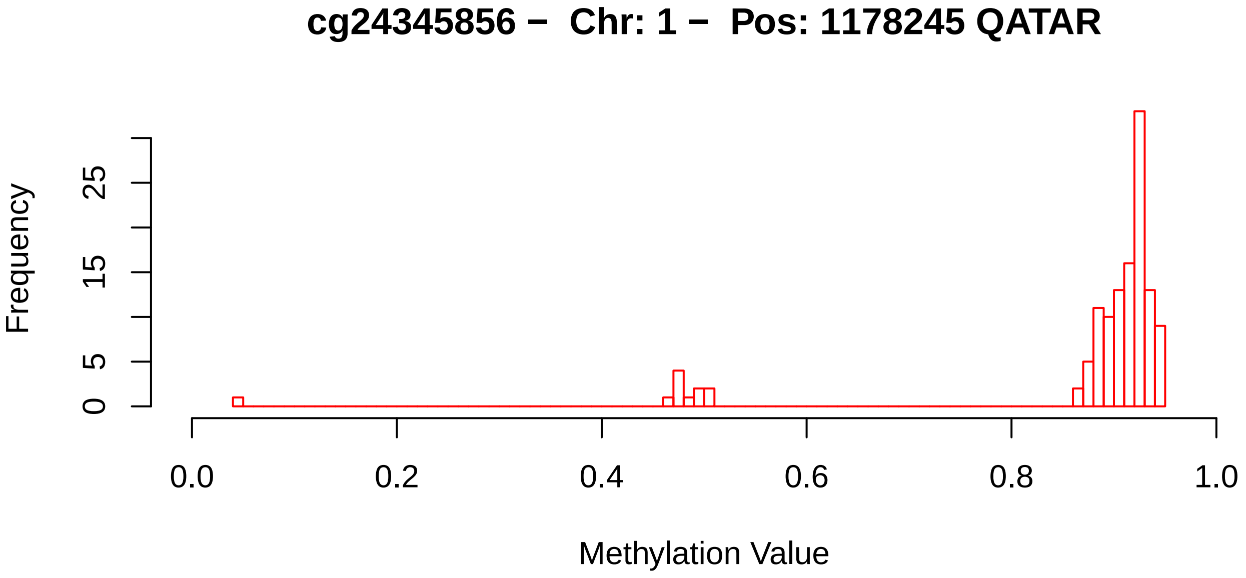
cg24345856 − Chr: 1 − Pos: 1178245 QATAR
Frequency
0
5
15
25
0.0
0.2
0.4
0.6
0.8
1.0
Methylation Value

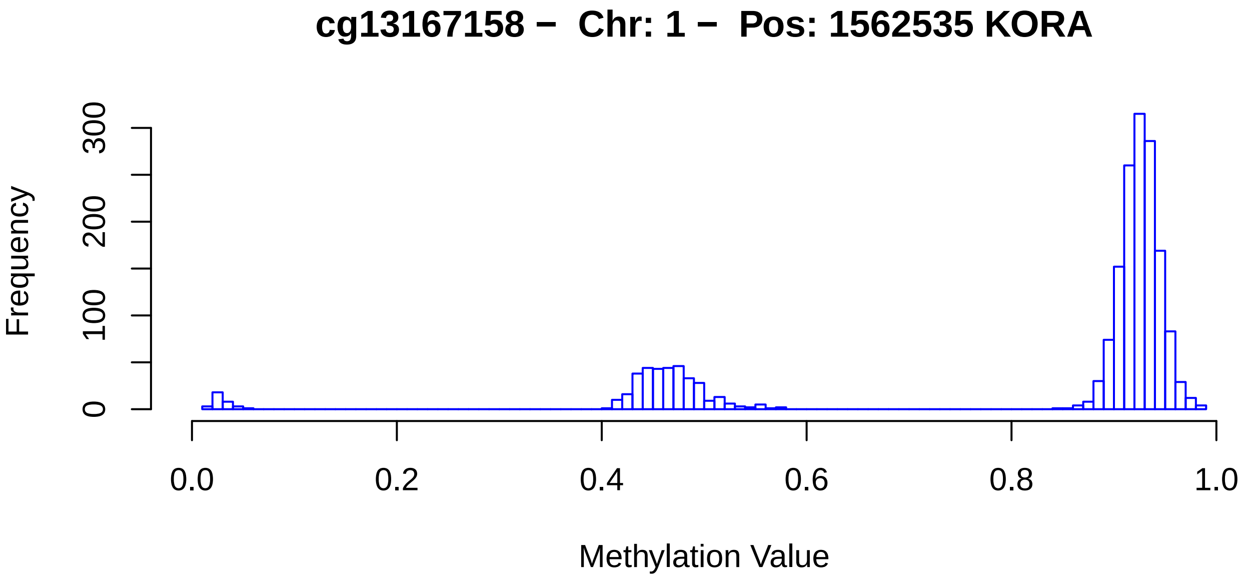
cg13167158 − Chr: 1 − Pos: 1562535 KORA
Frequency
0
100
200
300
0.0
0.2
0.4
0.6
0.8
1.0
Methylation Value

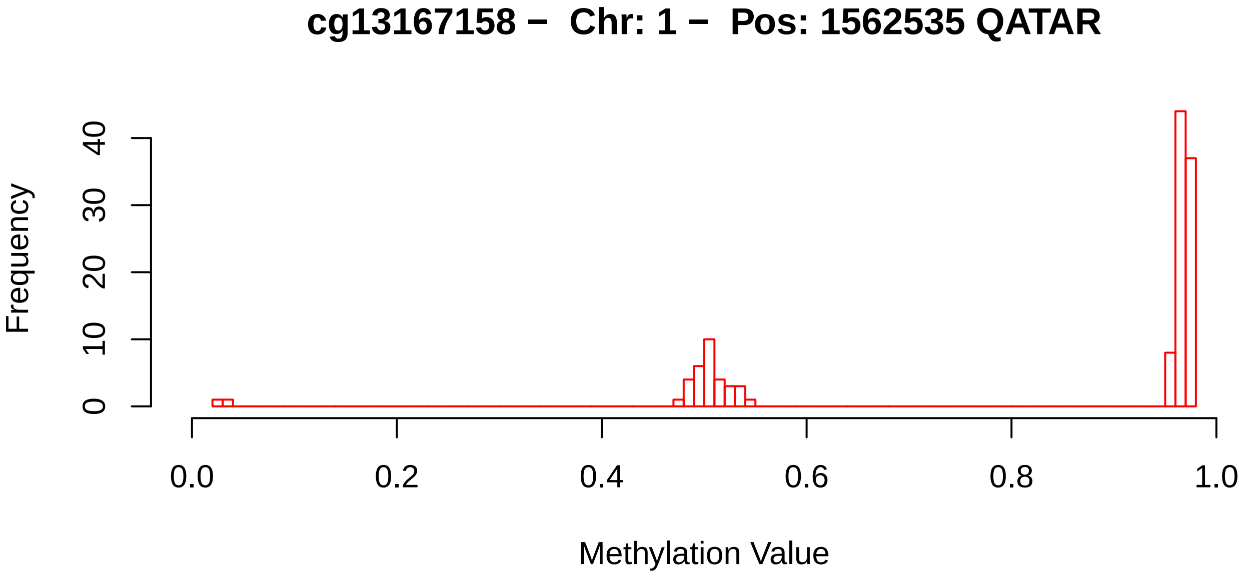
cg13167158 − Chr: 1 − Pos: 1562535 QATAR
Frequency
0
10
20
30
40
0.0
0.2
0.4
0.6
0.8
1.0
Methylation Value

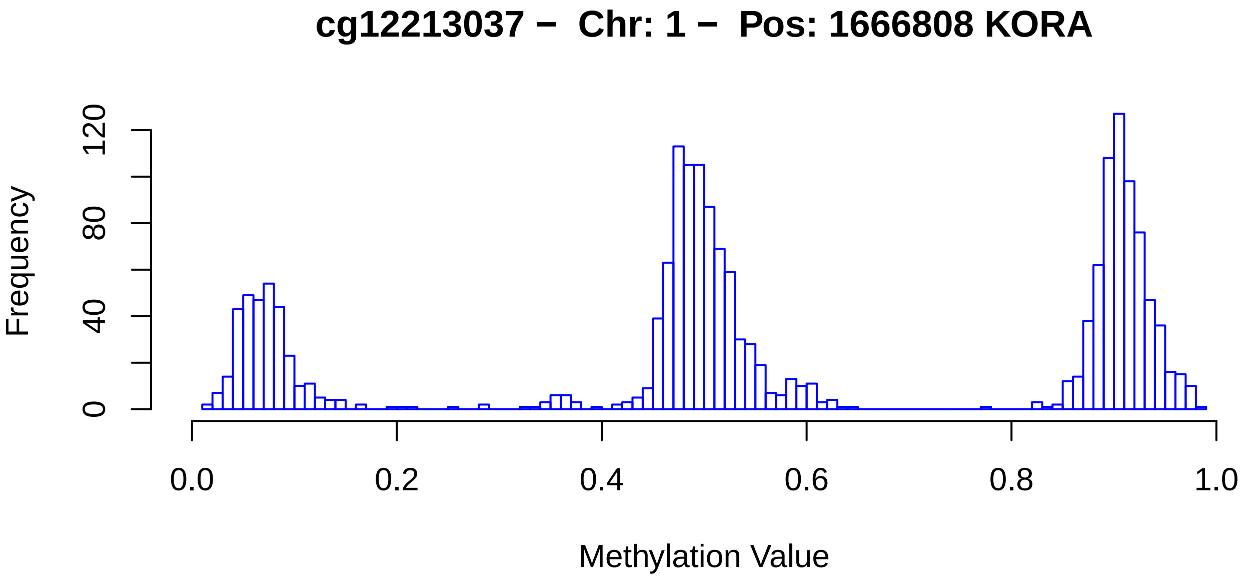
cg12213037 − Chr: 1 − Pos: 1666808 KORA
Frequency
0
40
80
120
0.0
0.2
0.4
0.6
0.8
1.0
Methylation Value

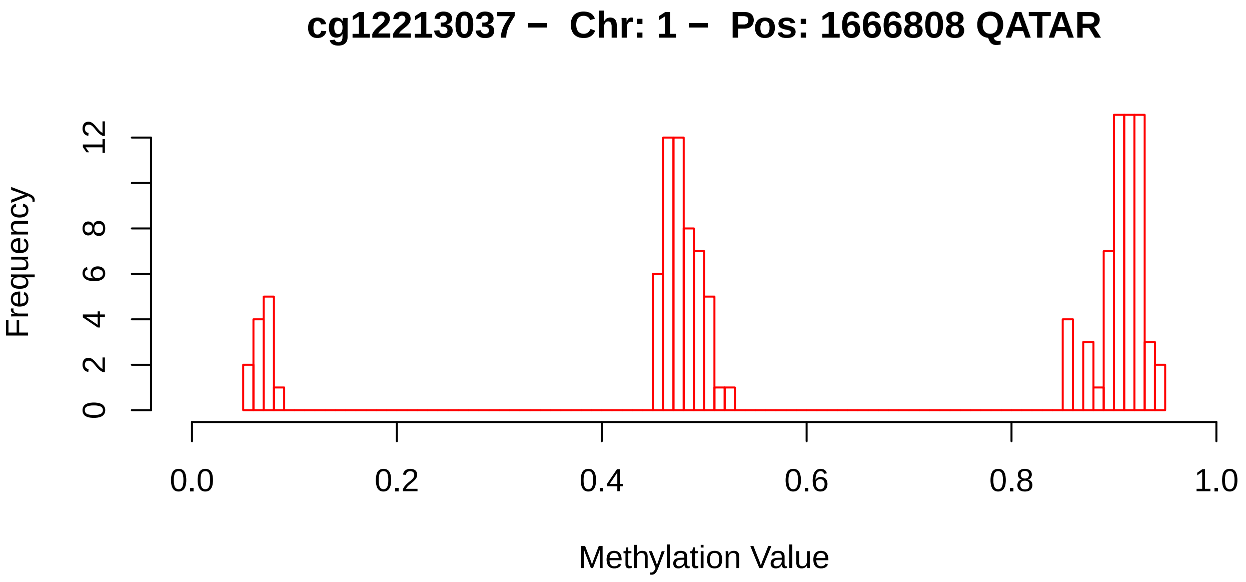
cg12213037 − Chr: 1 − Pos: 1666808 QATAR
Frequency
0
2
4
6
8
12
0.0
0.2
0.4
0.6
0.8
1.0
Methylation Value

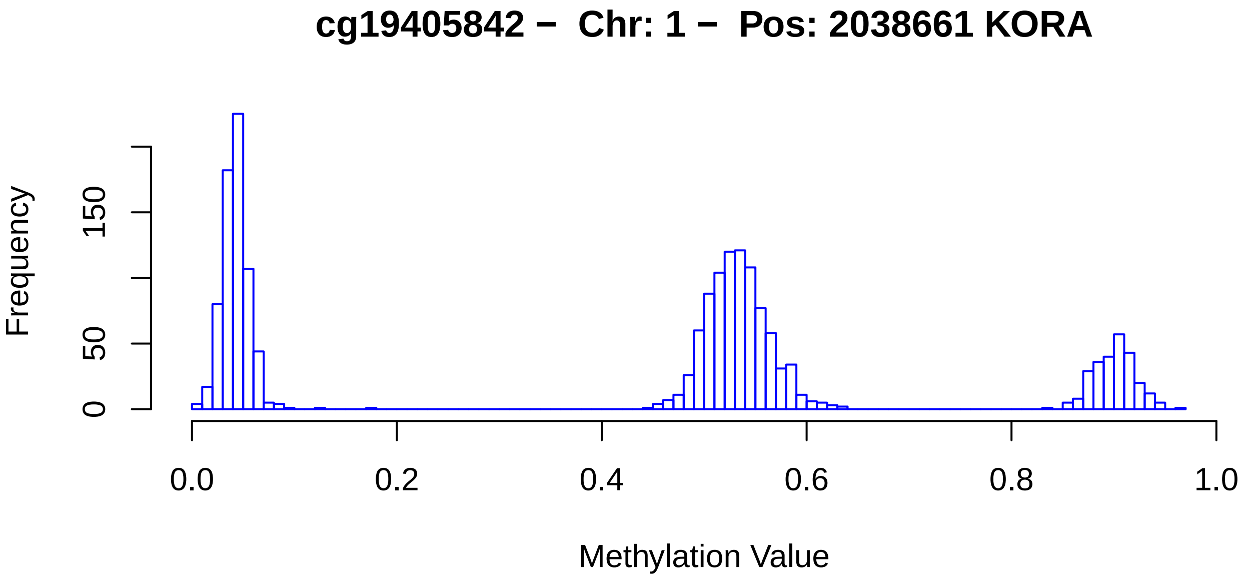
cg19405842 − Chr: 1 − Pos: 2038661 KORA
Frequency
0
50
150
0.0
0.2
0.4
0.6
0.8
1.0
Methylation Value

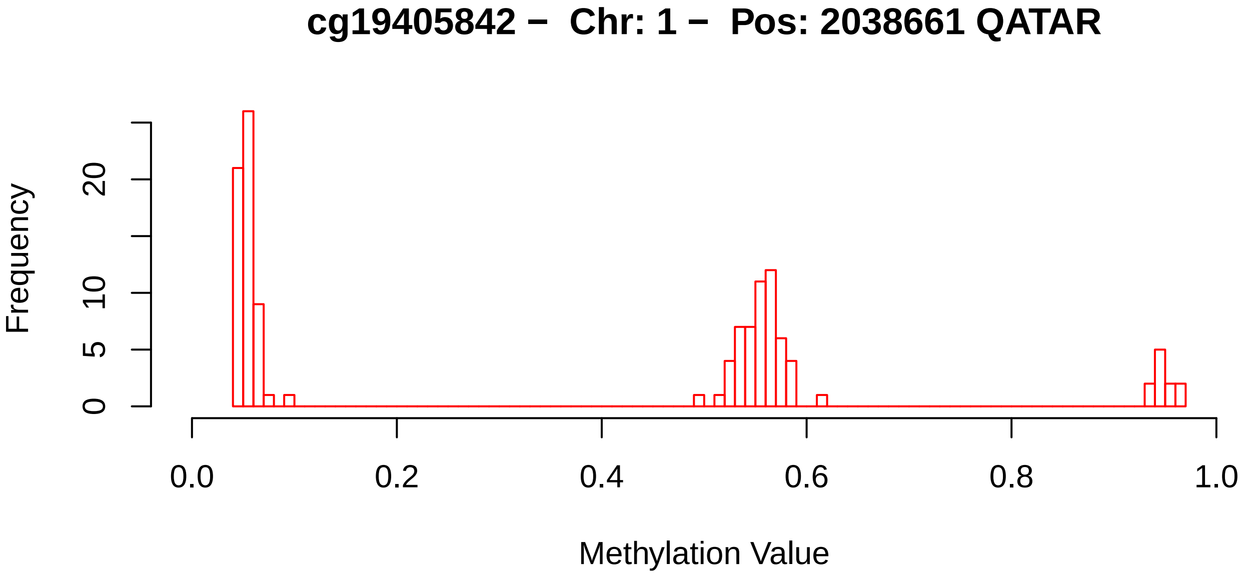
cg19405842 − Chr: 1 − Pos: 2038661 QATAR
Frequency
0
5
10
20
0.0
0.2
0.4
0.6
0.8
1.0
Methylation Value

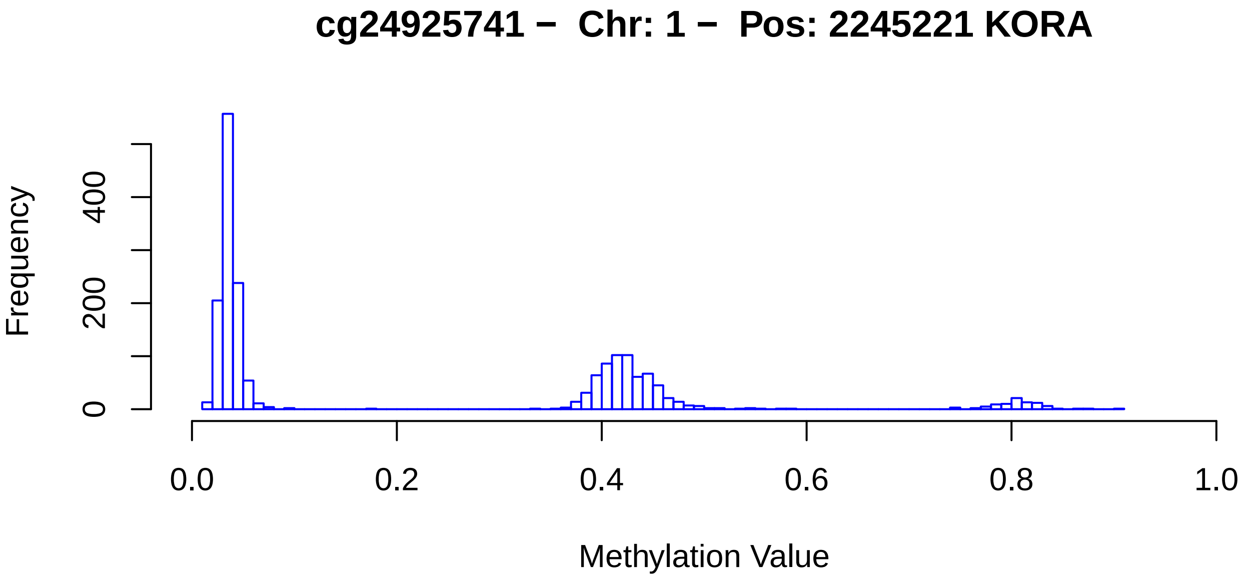
cg24925741 − Chr: 1 − Pos: 2245221 KORA
Frequency
0
200
400
0.0
0.2
0.4
0.6
0.8
1.0
Methylation Value

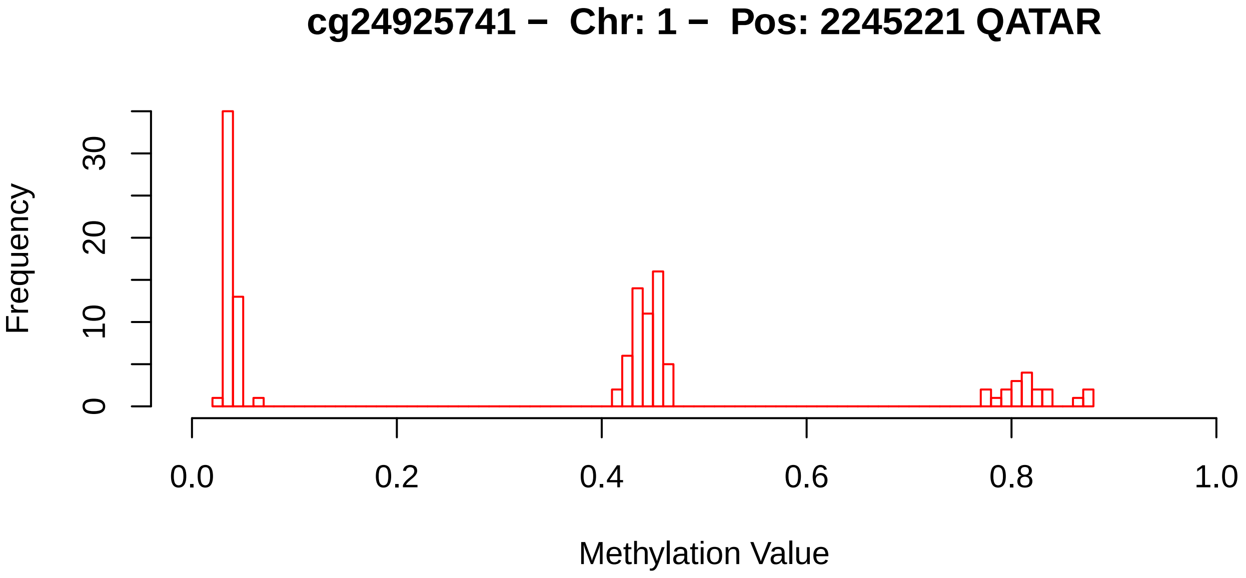
cg24925741 − Chr: 1 − Pos: 2245221 QATAR
Frequency
0
10
20
30
0.0
0.2
0.4
0.6
0.8
1.0
Methylation Value

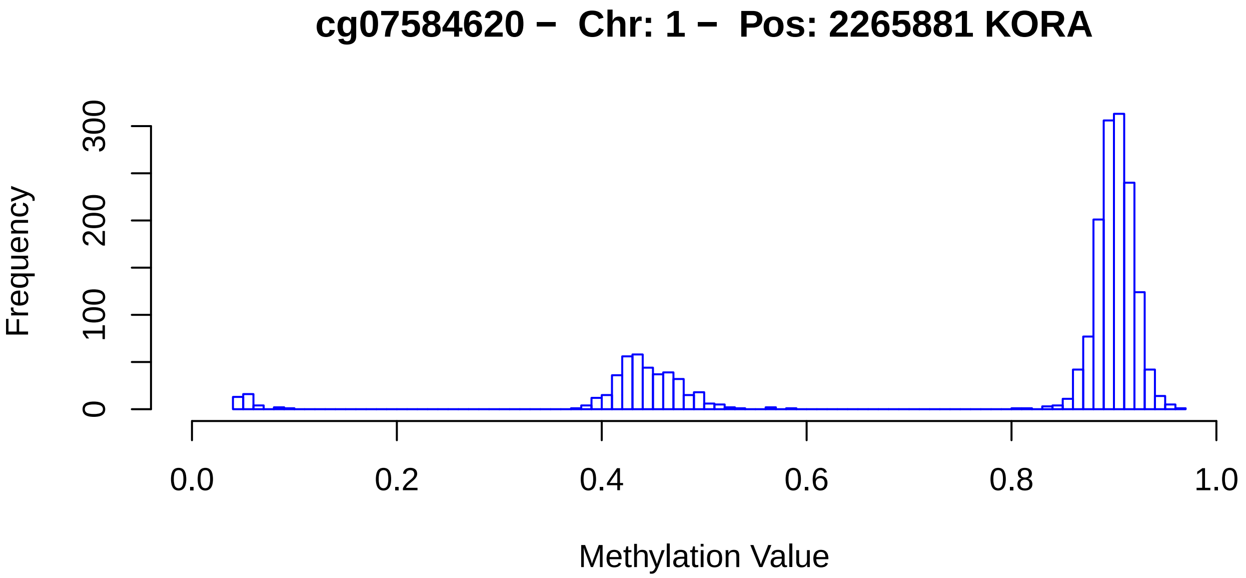
cg07584620 − Chr: 1 − Pos: 2265881 KORA
Frequency
0
100
200
300
0.0
0.2
0.4
0.6
0.8
1.0
Methylation Value

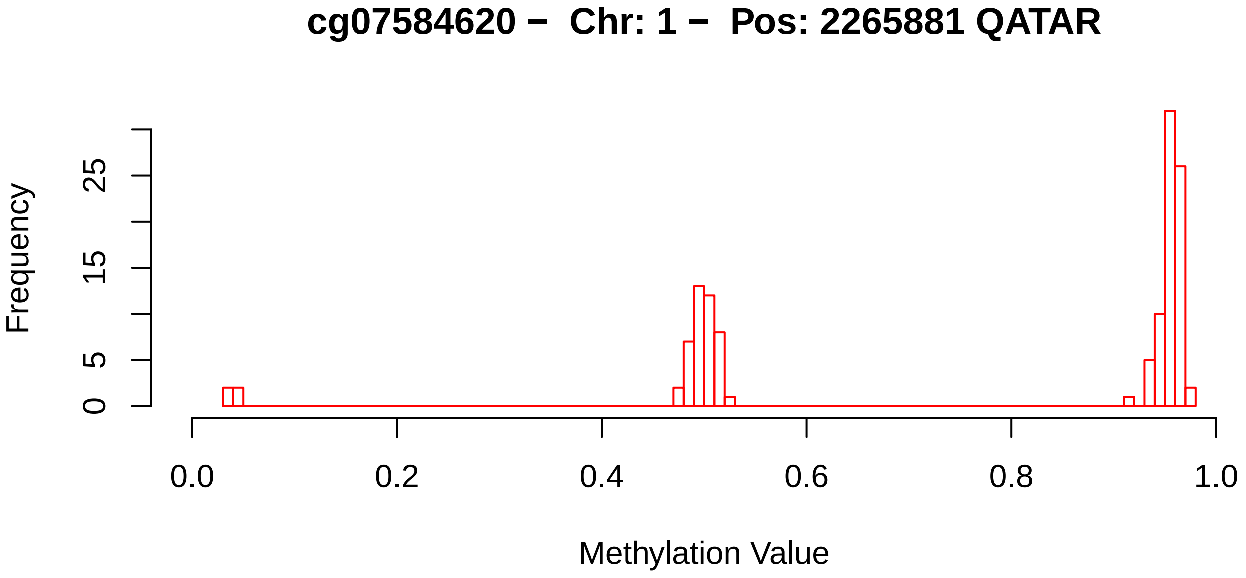
cg07584620 − Chr: 1 − Pos: 2265881 QATAR
Frequency
0
5
15
25
0.0
0.2
0.4
0.6
0.8
1.0
Methylation Value

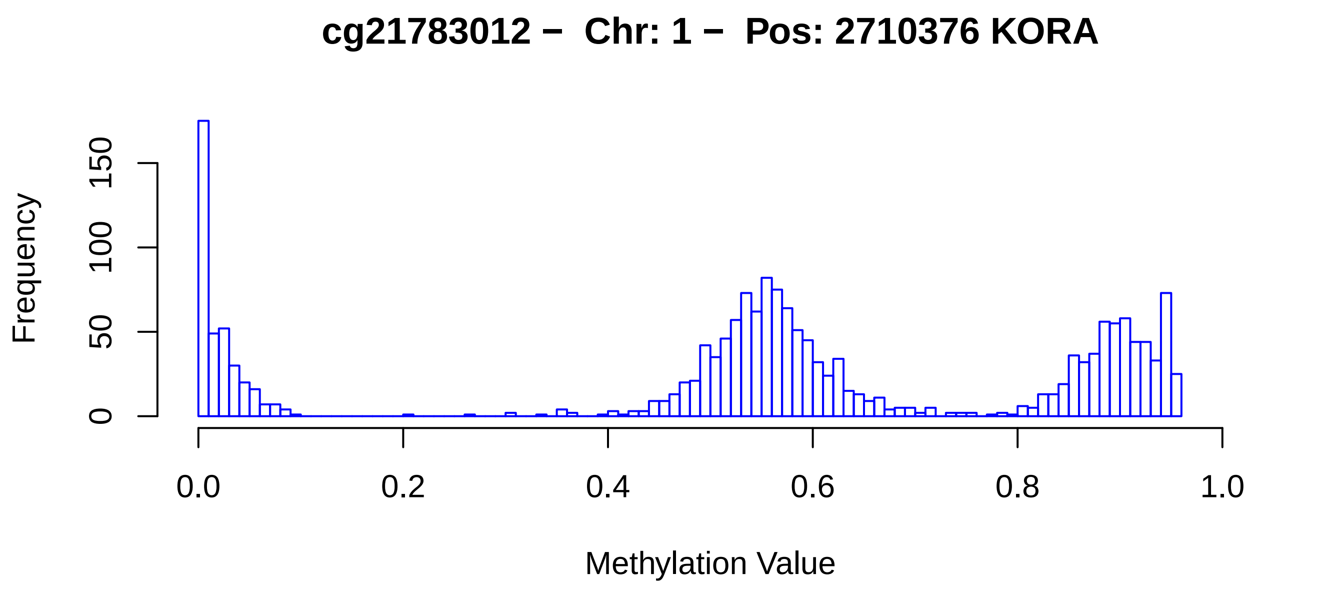
cg21783012 − Chr: 1 − Pos: 2710376 KORA
Frequency
0
50
100
150
0.0
0.2
0.4
0.6
0.8
1.0
Methylation Value

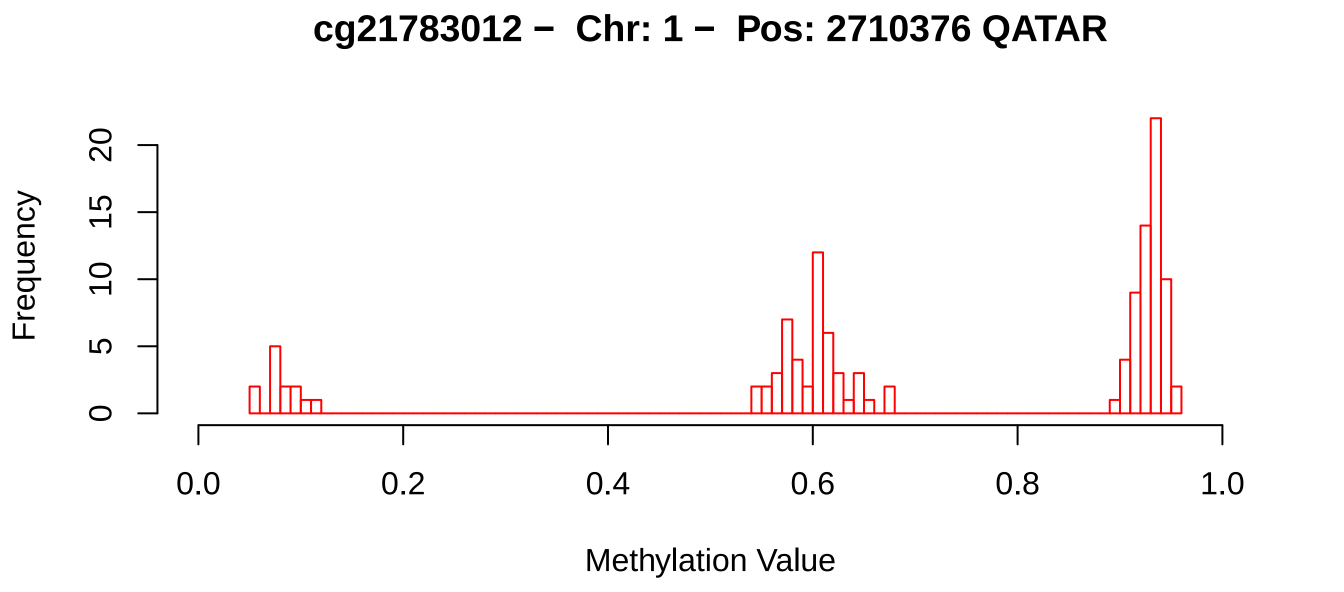
cg21783012 − Chr: 1 − Pos: 2710376 QATAR
Frequency
0
5
10
15
20
0.0
0.2
0.4
0.6
0.8
1.0
Methylation Value

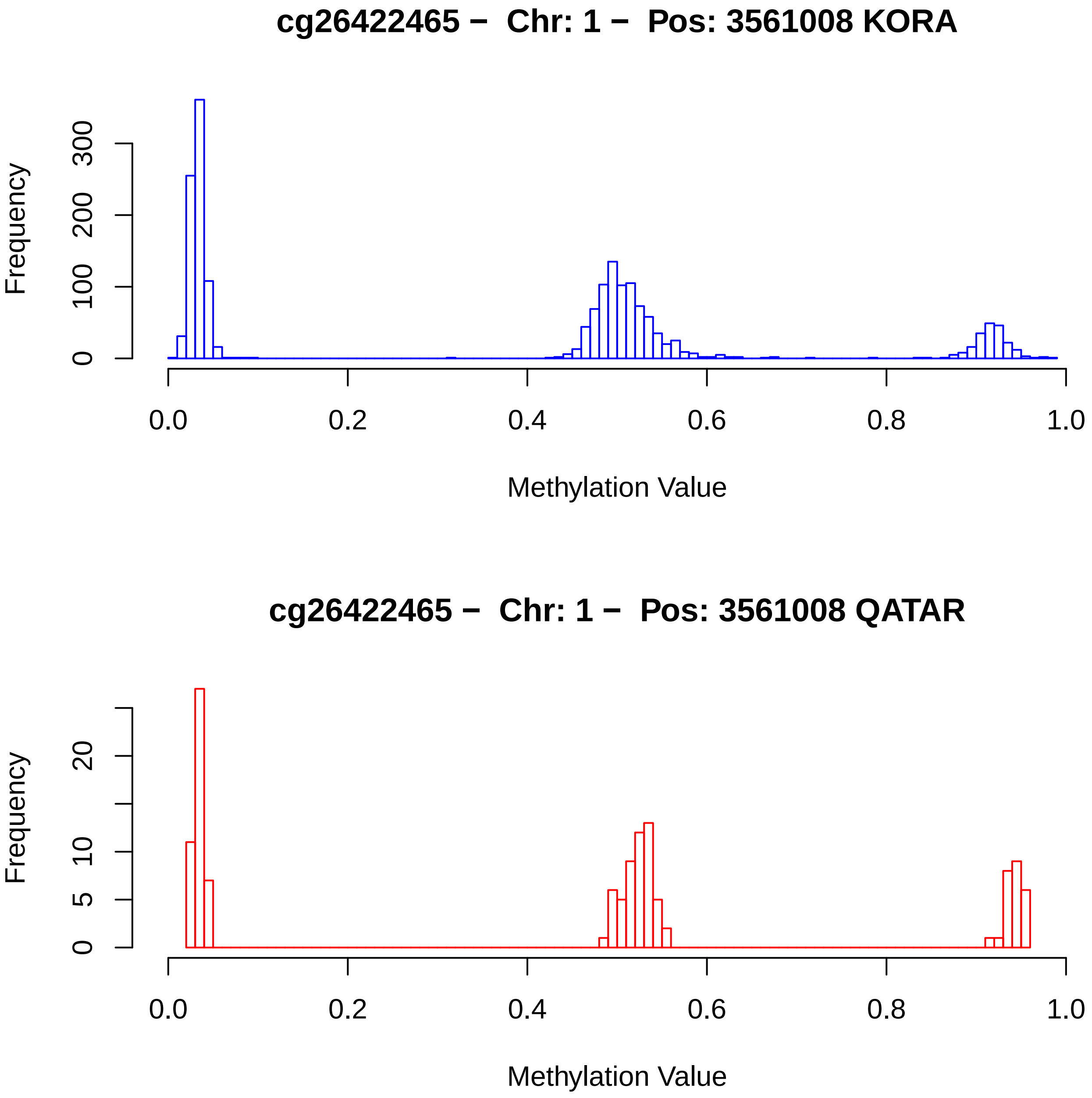
cg26422465 − Chr: 1 − Pos: 3561008 KORA
Frequency
0
100
200
300
0.0
0.2
0.4
0.6
0.8
1.0
Methylation Value
cg26422465 − Chr: 1 − Pos: 3561008 QATAR
Frequency
0
5
10
20
0.0
0.2
0.4
0.6
0.8
1.0
Methylation Value

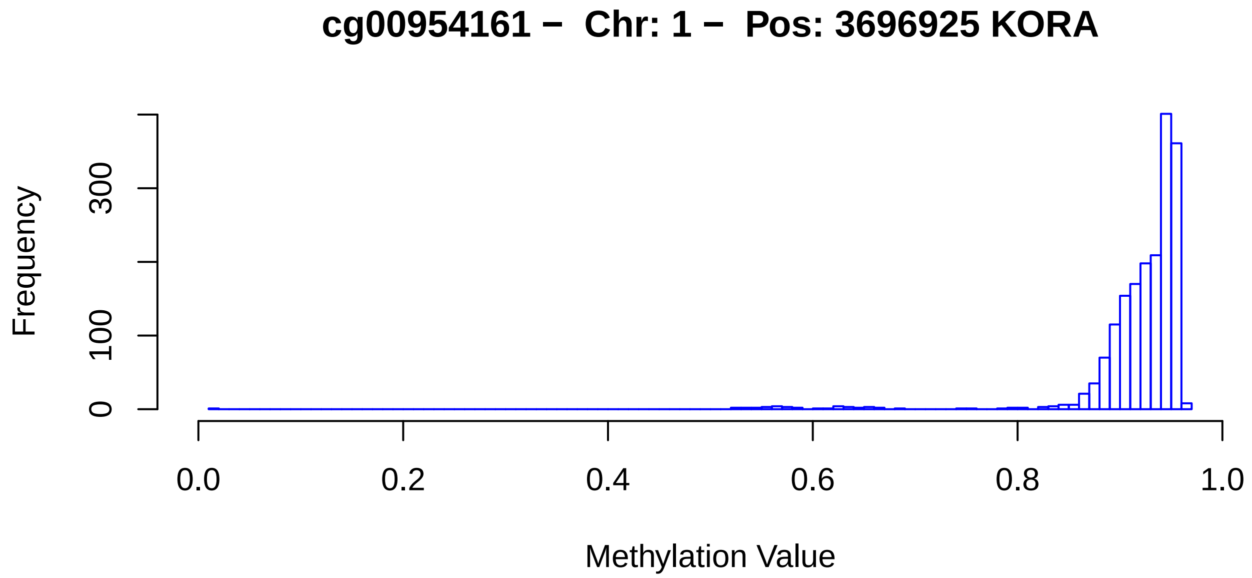
cg00954161 − Chr: 1 − Pos: 3696925 KORA
Frequency
0
100
300
0.0
0.2
0.4
0.6
0.8
1.0
Methylation Value

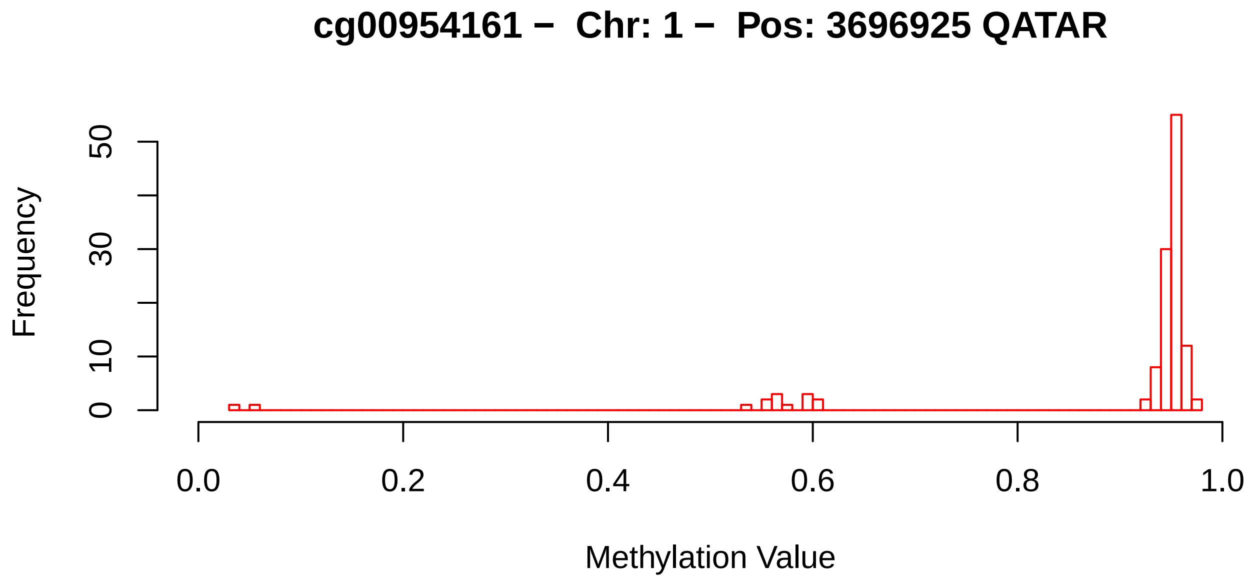
cg00954161 − Chr: 1 − Pos: 3696925 QATAR
Frequency
0
10
30
50
0.0
0.2
0.4
0.6
0.8
1.0
Methylation Value

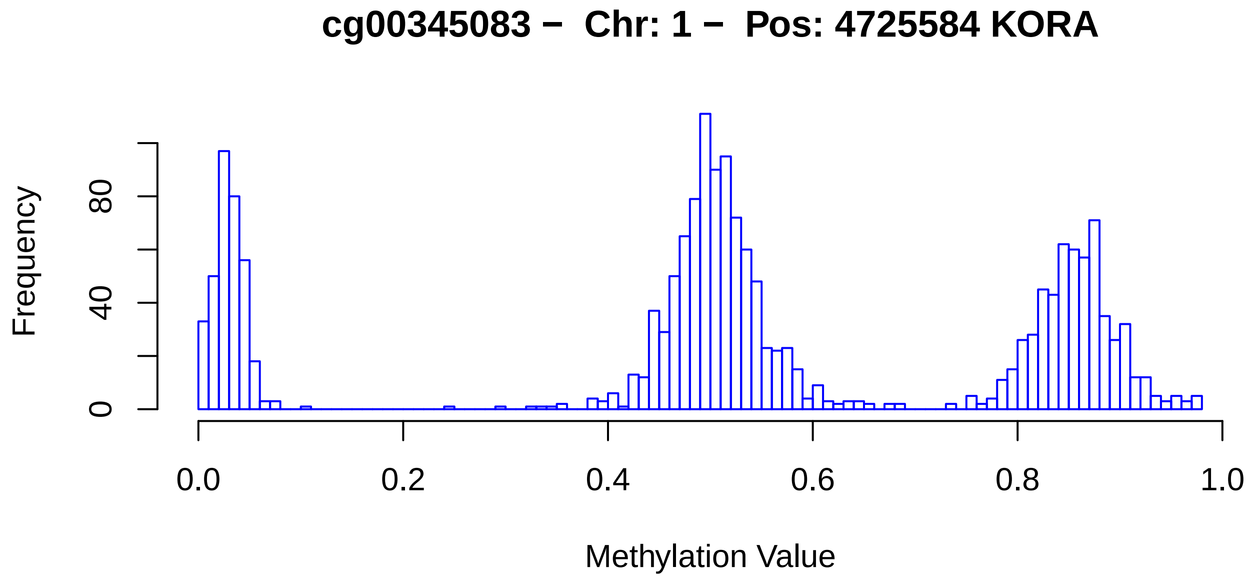
cg00345083 − Chr: 1 − Pos: 4725584 KORA
Frequency
0
40
80
0.0
0.2
0.4
0.6
0.8
1.0
Methylation Value

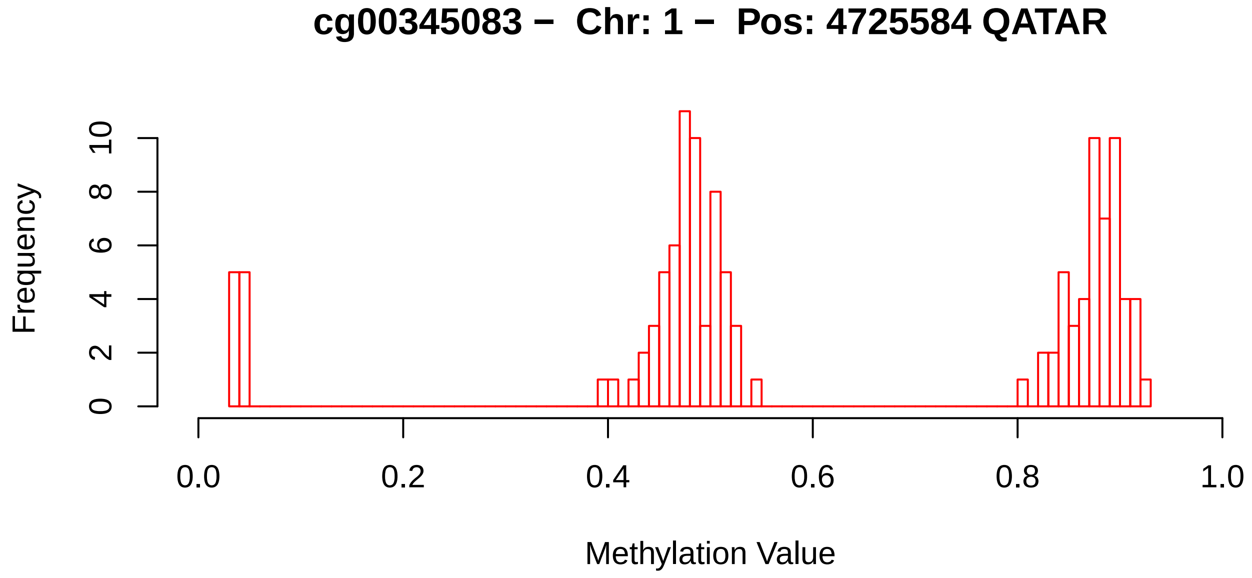
cg00345083 − Chr: 1 − Pos: 4725584 QATAR
Frequency
0
2
4
6
8
10
0.0
0.2
0.4
0.6
0.8
1.0
Methylation Value

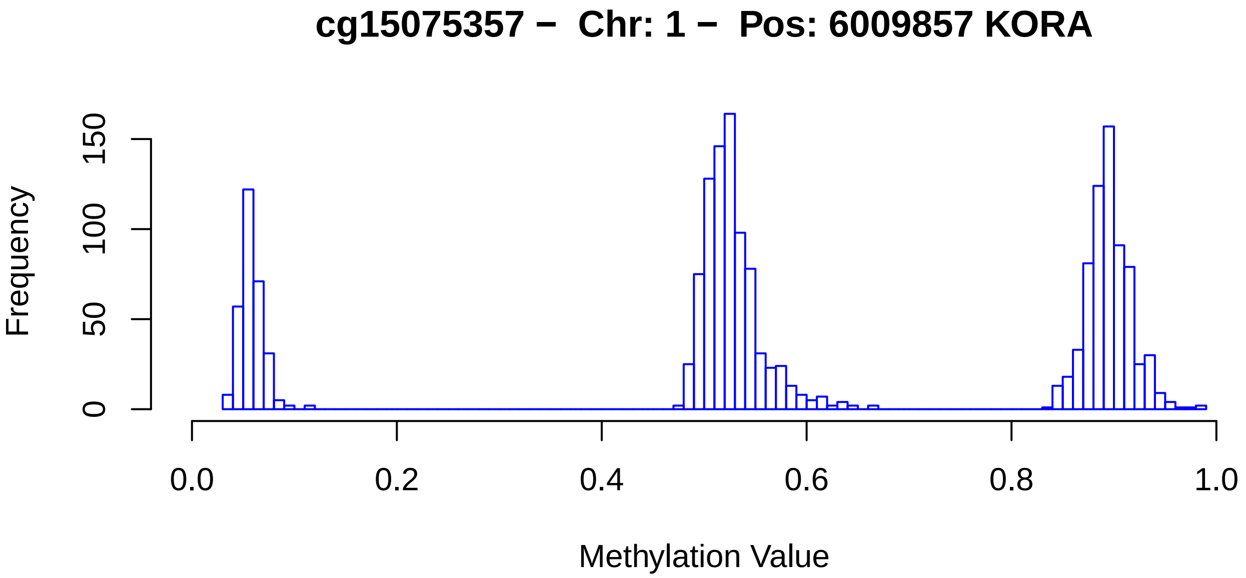
cg15075357 − Chr: 1 − Pos: 6009857 KORA
Frequency
0
50
100
150
0.0
0.2
0.4
0.6
0.8
1.0
Methylation Value

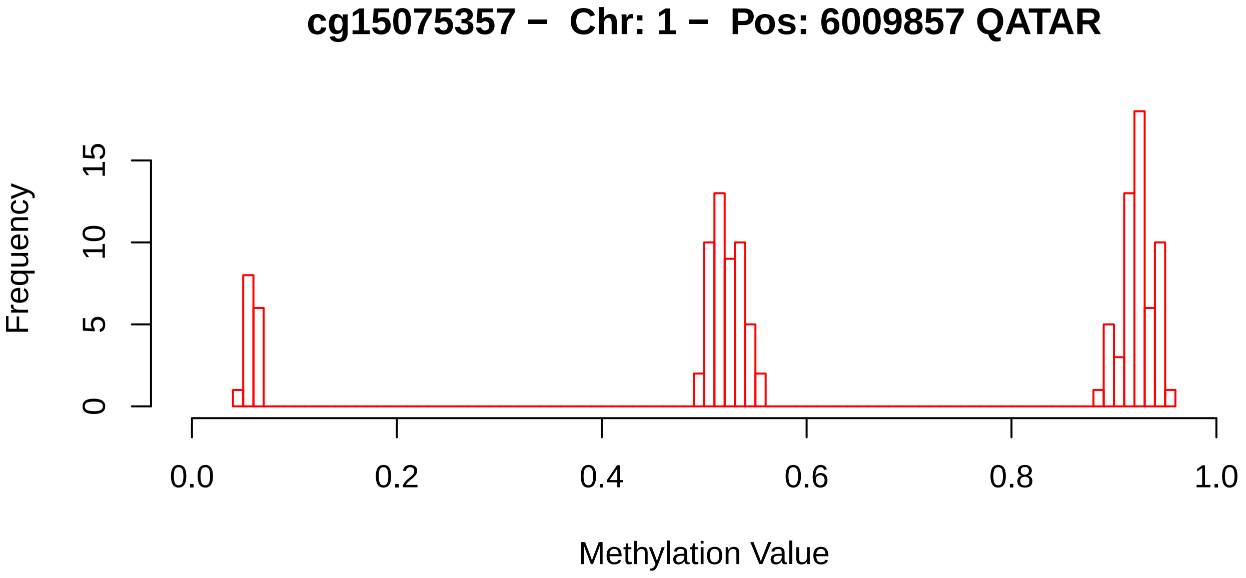
cg15075357 − Chr: 1 − Pos: 6009857 QATAR
Frequency
0
5
10
15
0.0
0.2
0.4
0.6
0.8
1.0
Methylation Value

cg12475902 – Chr: 1 – Pos: 6764205 KORA

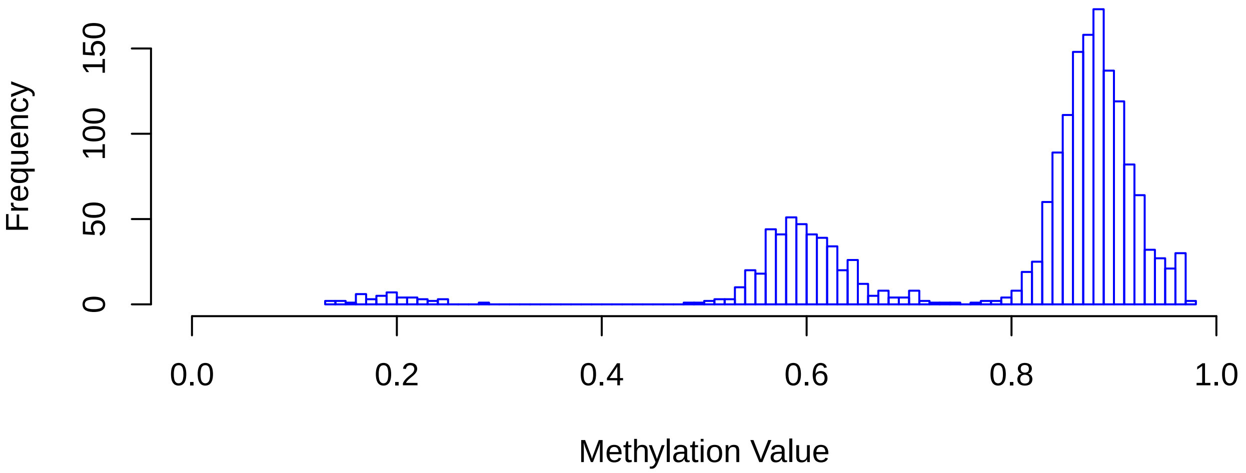

cg12475902 – Chr: 1 – Pos: 6764205 QATAR

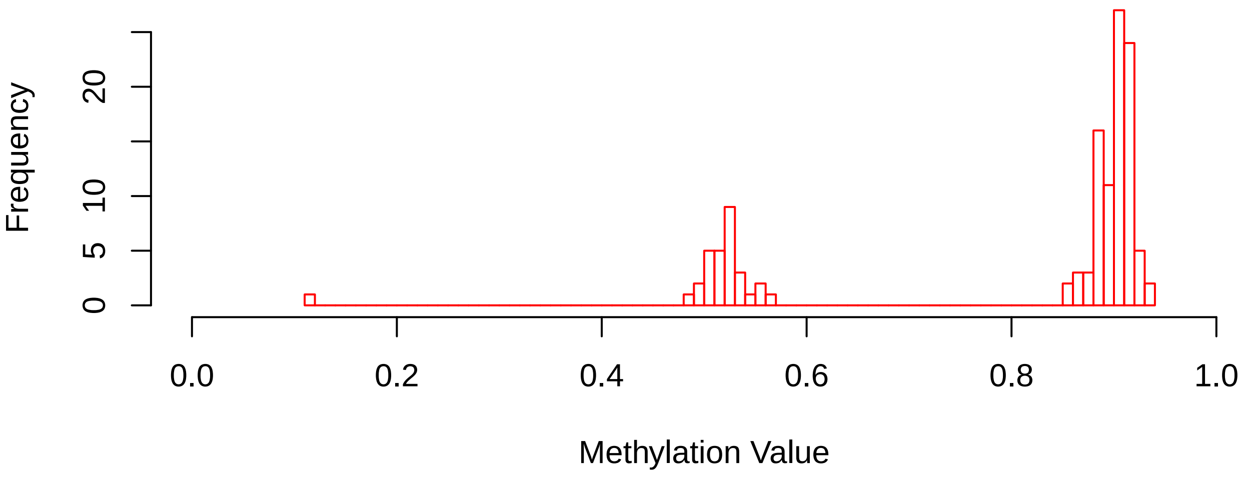

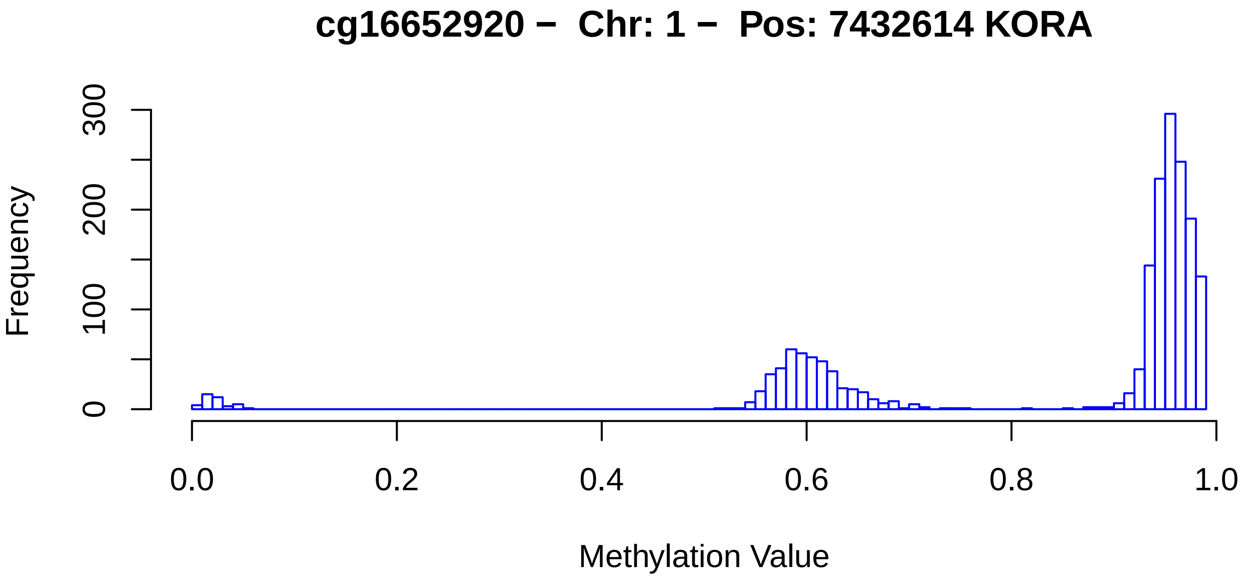
cg16652920 − Chr: 1 − Pos: 7432614 KORA
Frequency
0
100
200
300
0.0
0.2
0.4
0.6
0.8
1.0
Methylation Value

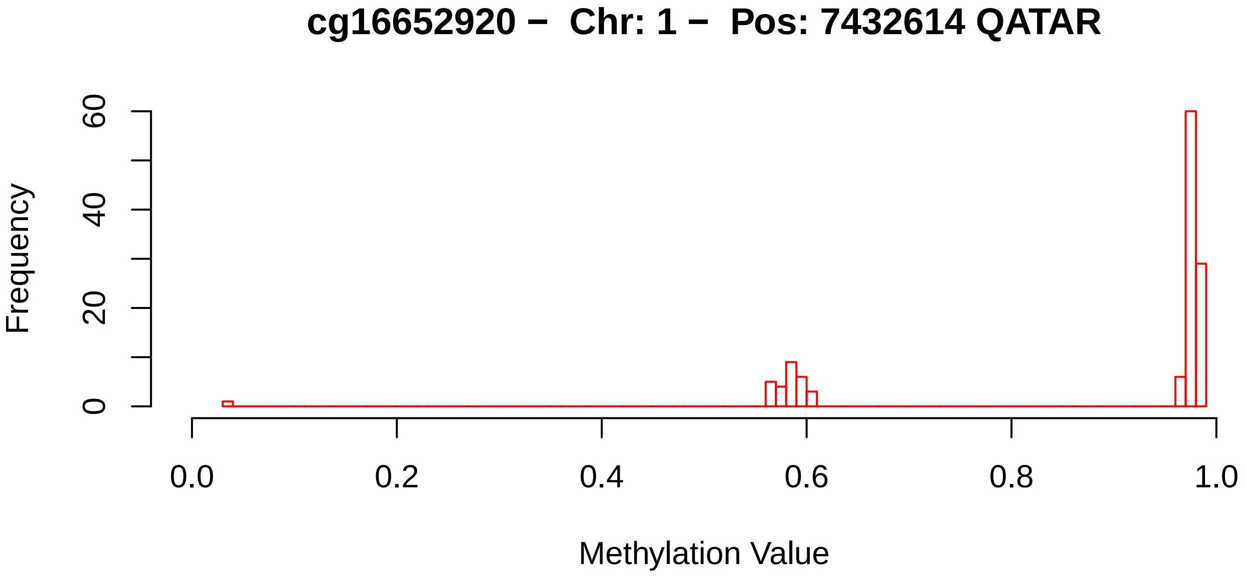
cg16652920 − Chr: 1 − Pos: 7432614 QATAR
Frequency
0
20
40
60
0.0
0.2
0.4
0.6
0.8
1.0
Methylation Value

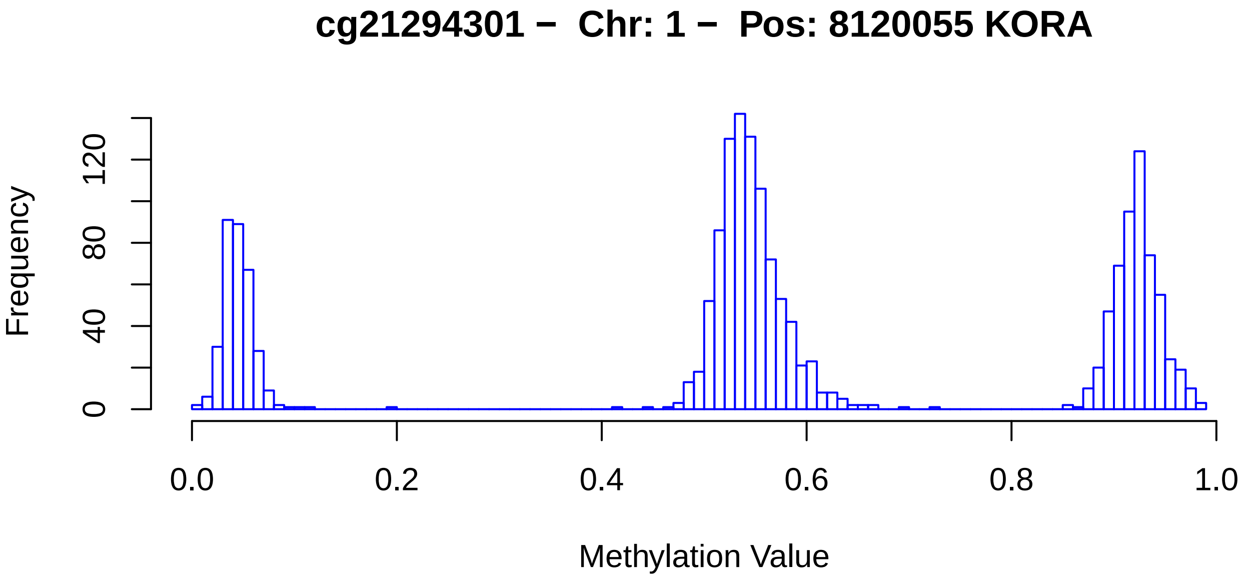
cg21294301 − Chr: 1 − Pos: 8120055 KORA
Frequency
0
40
80
120
0.0
0.2
0.4
0.6
0.8
1.0
Methylation Value

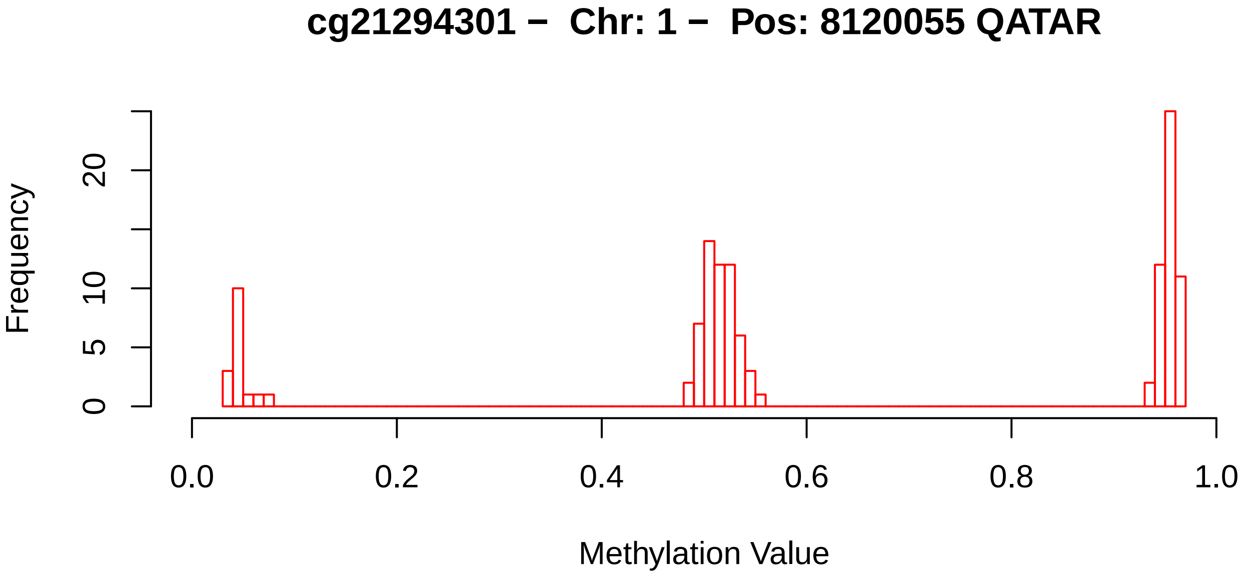
cg21294301 − Chr: 1 − Pos: 8120055 QATAR
Frequency
0
5
10
20
0.0
0.2
0.4
0.6
0.8
1.0
Methylation Value

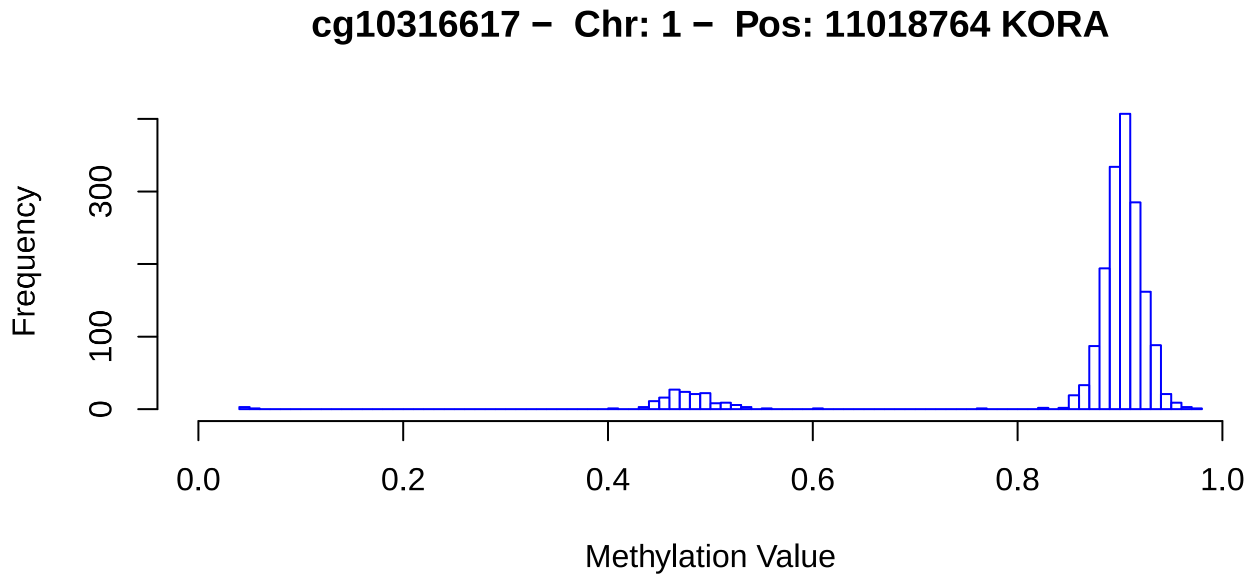
cg10316617 − Chr: 1 − Pos: 11018764 KORA
Frequency
0
100
300
0.0
0.2
0.4
0.6
0.8
1.0
Methylation Value

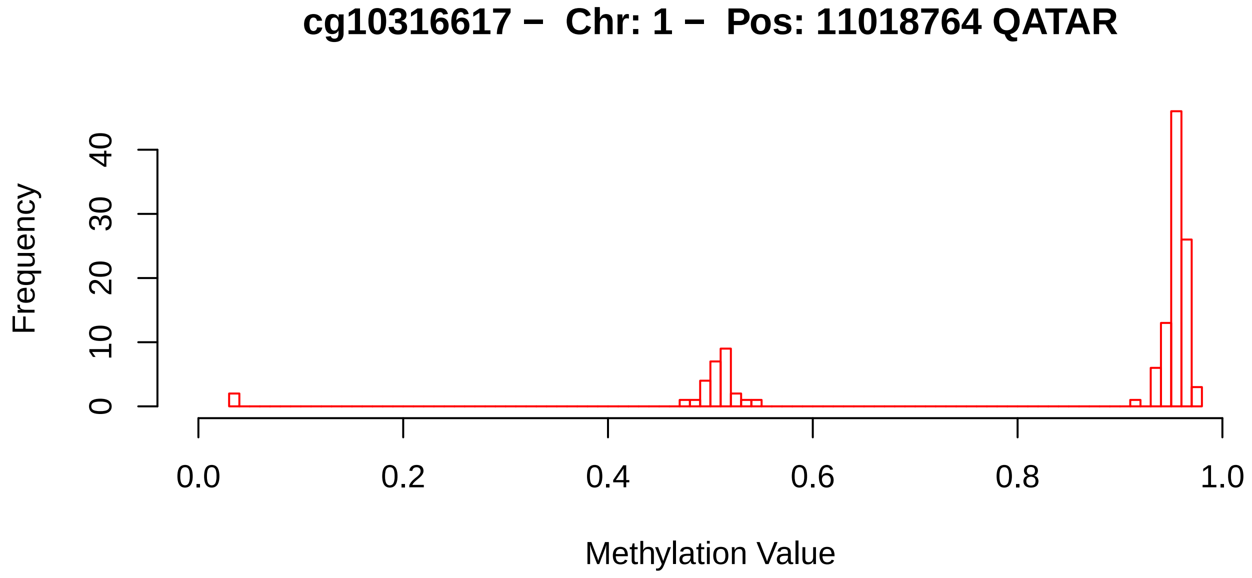
cg10316617 − Chr: 1 − Pos: 11018764 QATAR
Frequency
0
10
20
30
40
0.0
0.2
0.4
0.6
0.8
1.0
Methylation Value

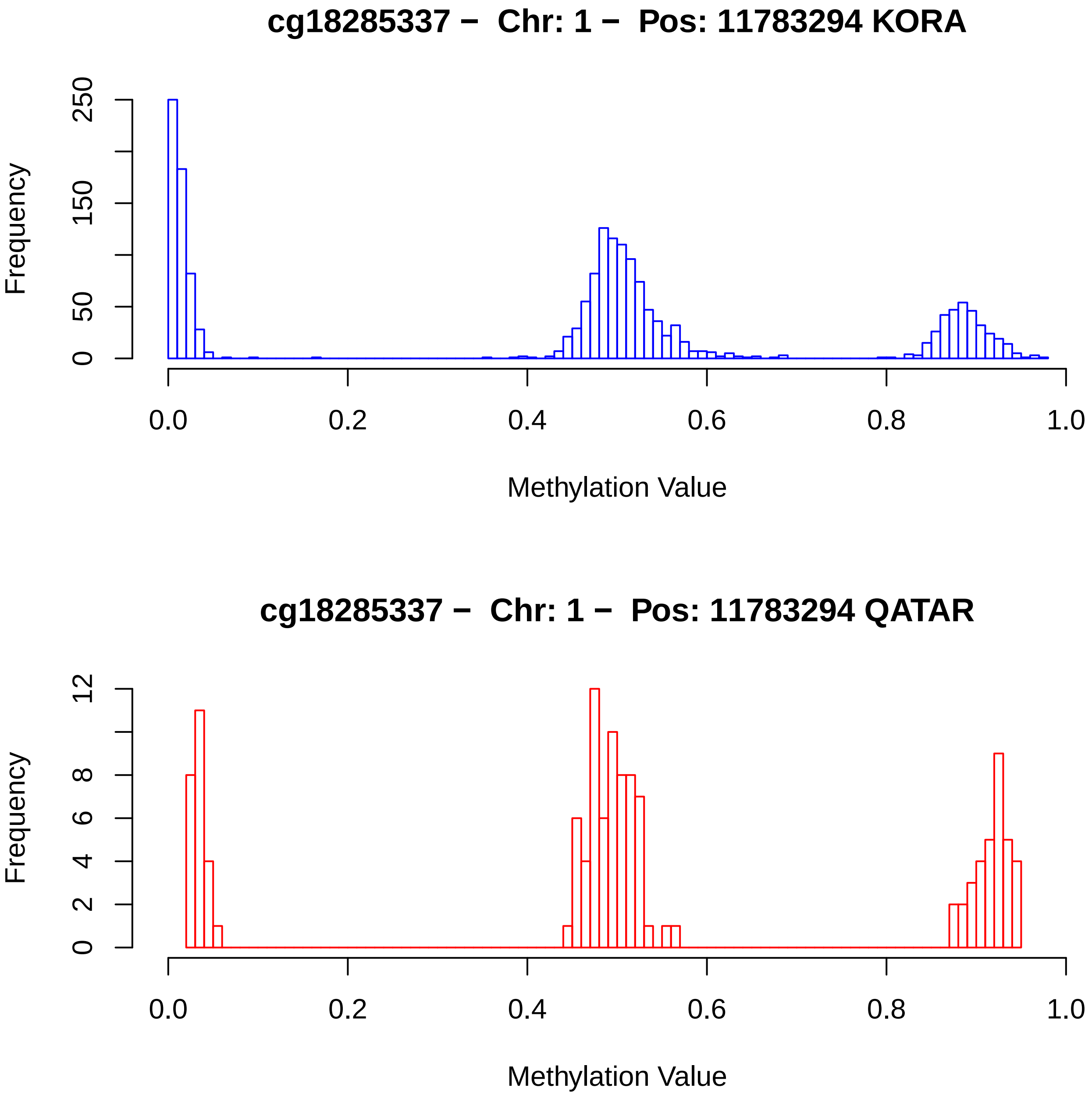
cg18285337 − Chr: 1 − Pos: 11783294 KORA
Frequency
0
50
150
250
0.0
0.2
0.4
0.6
0.8
1.0
Methylation Value
cg18285337 − Chr: 1 − Pos: 11783294 QATAR
Frequency
0
2
4
6
8
12
0.0
0.2
0.4
0.6
0.8
1.0
Methylation Value

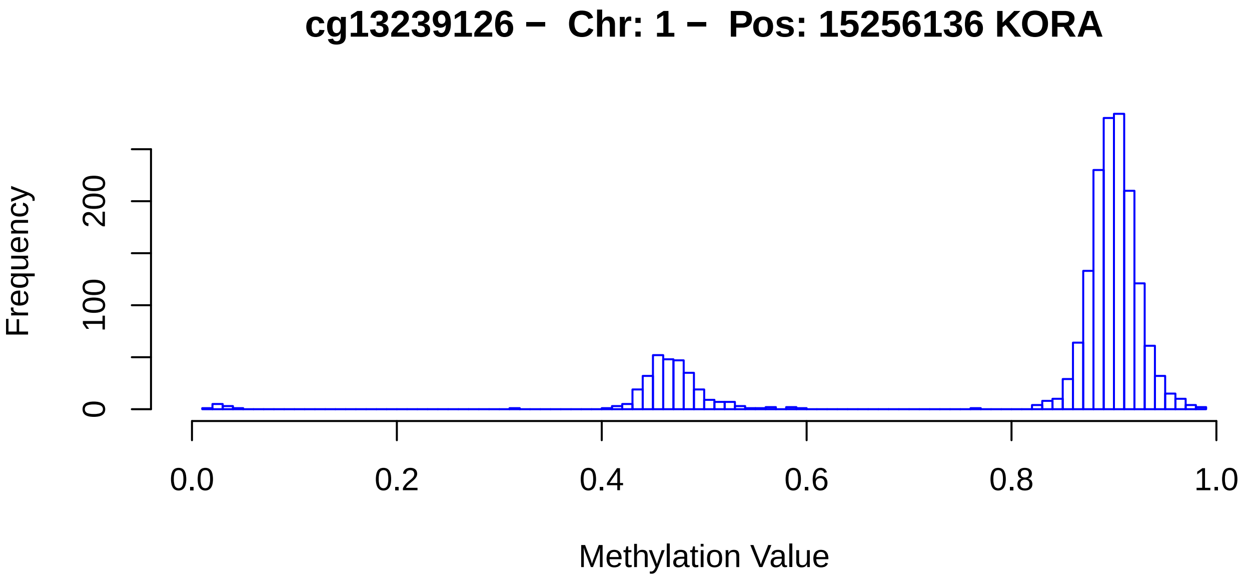
cg13239126 − Chr: 1 − Pos: 15256136 KORA
Frequency
0
100
200
0.0
0.2
0.4
0.6
0.8
1.0
Methylation Value

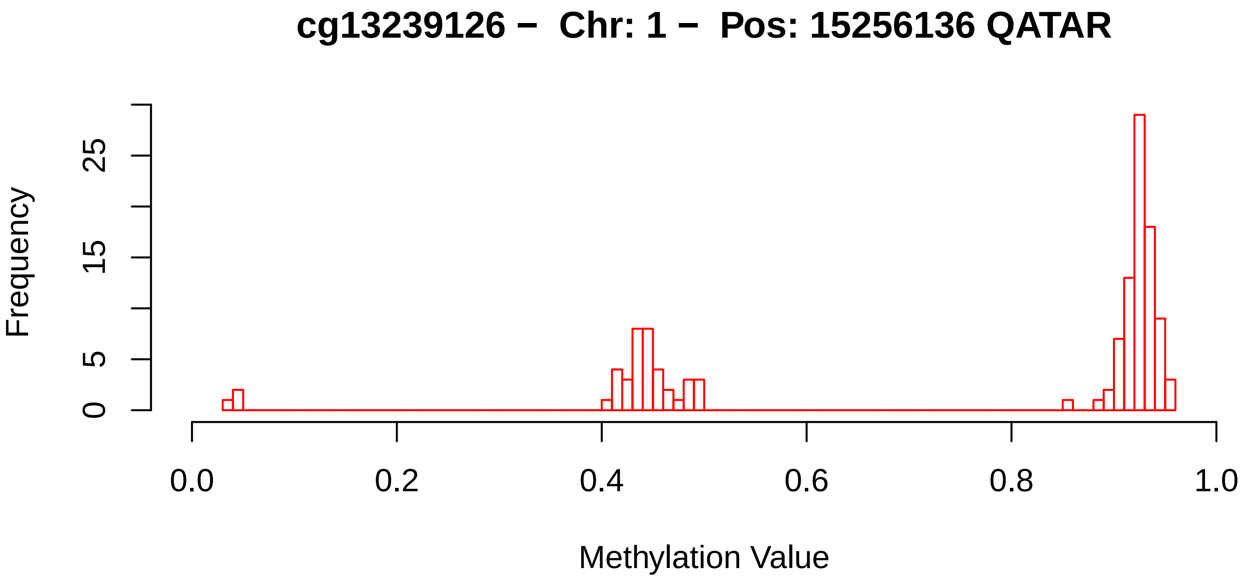
cg13239126 − Chr: 1 − Pos: 15256136 QATAR
Frequency
0
5
15
25
0.0
0.2
0.4
0.6
0.8
1.0
Methylation Value

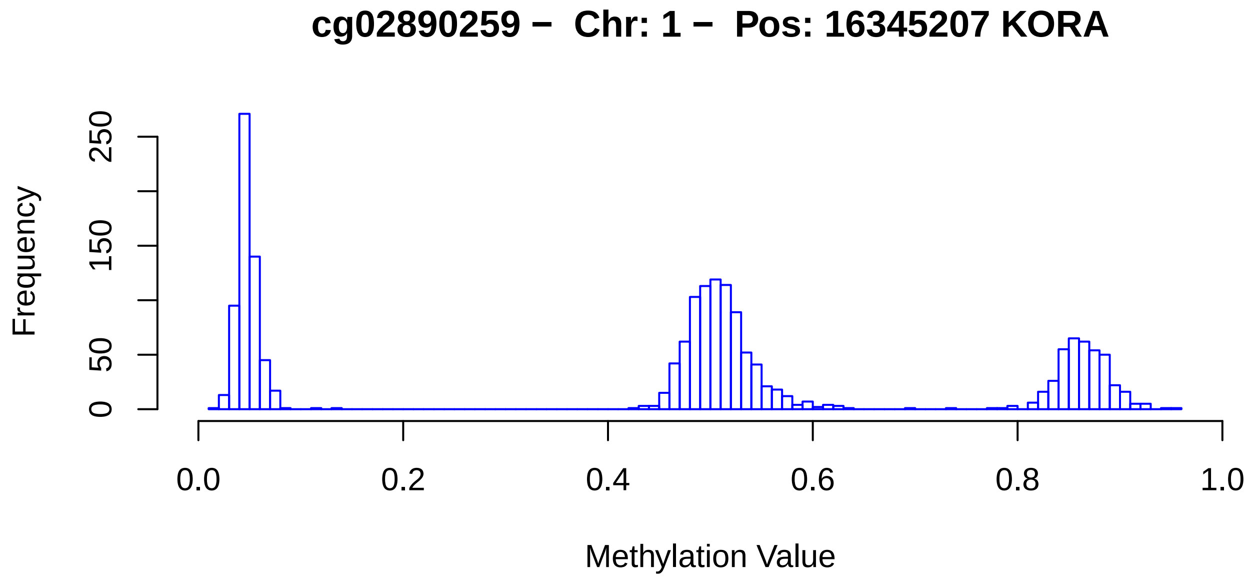
cg02890259 − Chr: 1 − Pos: 16345207 KORA
Frequency
0
50
150
250
0.0
0.2
0.4
0.6
0.8
1.0
Methylation Value

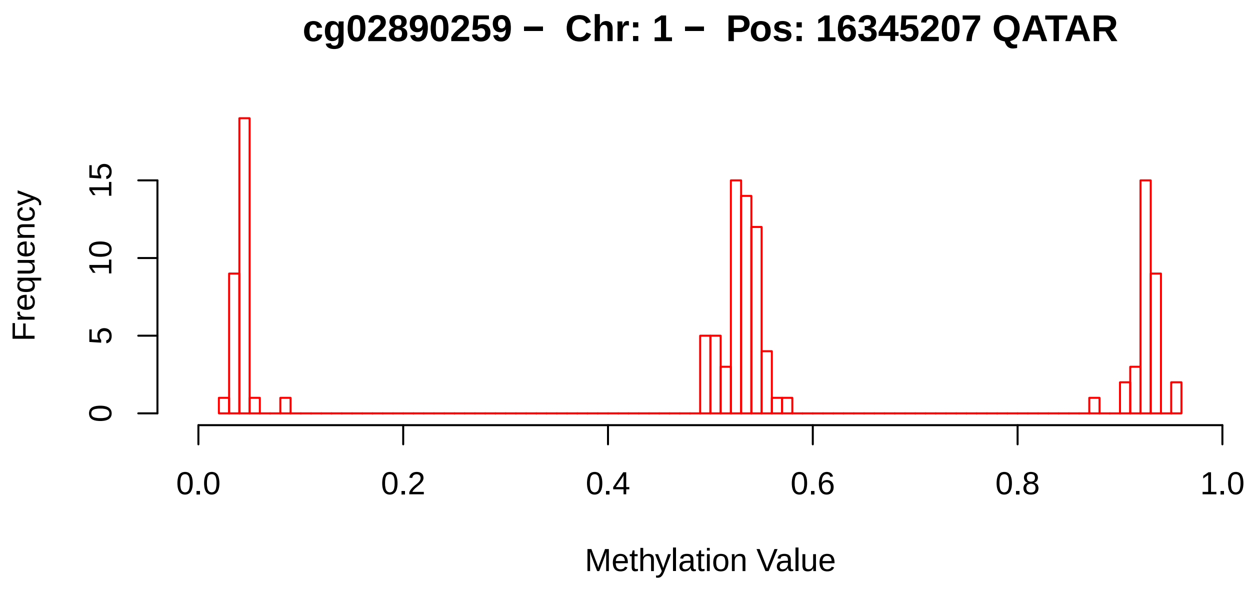
cg02890259 − Chr: 1 − Pos: 16345207 QATAR
Frequency
0
5
10
15
0.0
0.2
0.4
0.6
0.8
1.0
Methylation Value

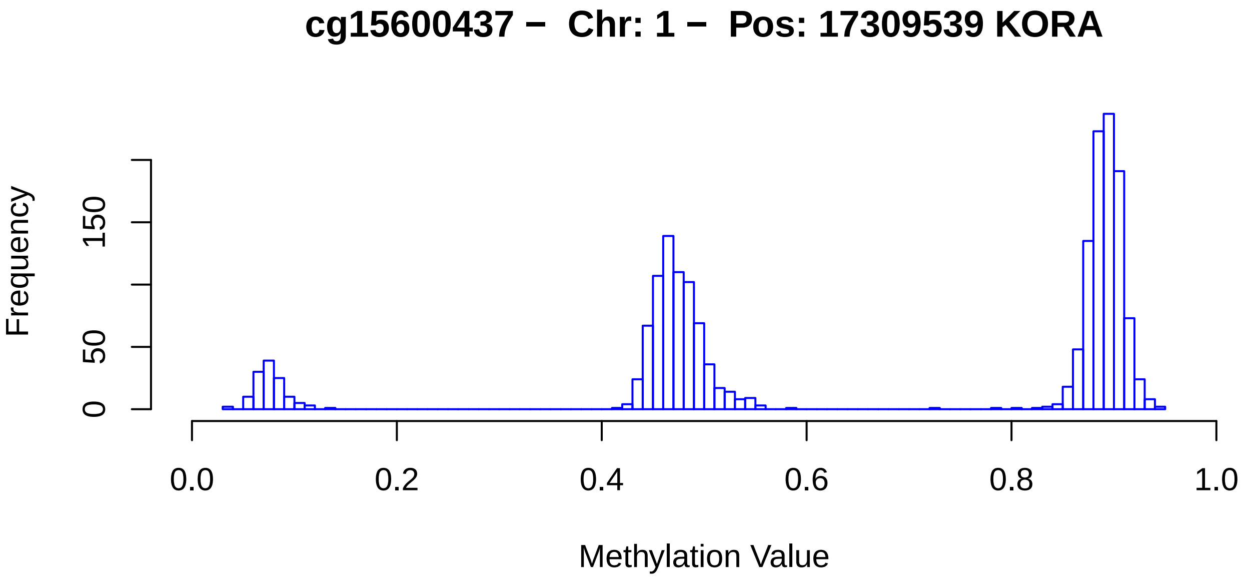
cg15600437 − Chr: 1 − Pos: 17309539 KORA
Frequency
0
50
150
0.0
0.2
0.4
0.6
0.8
1.0
Methylation Value

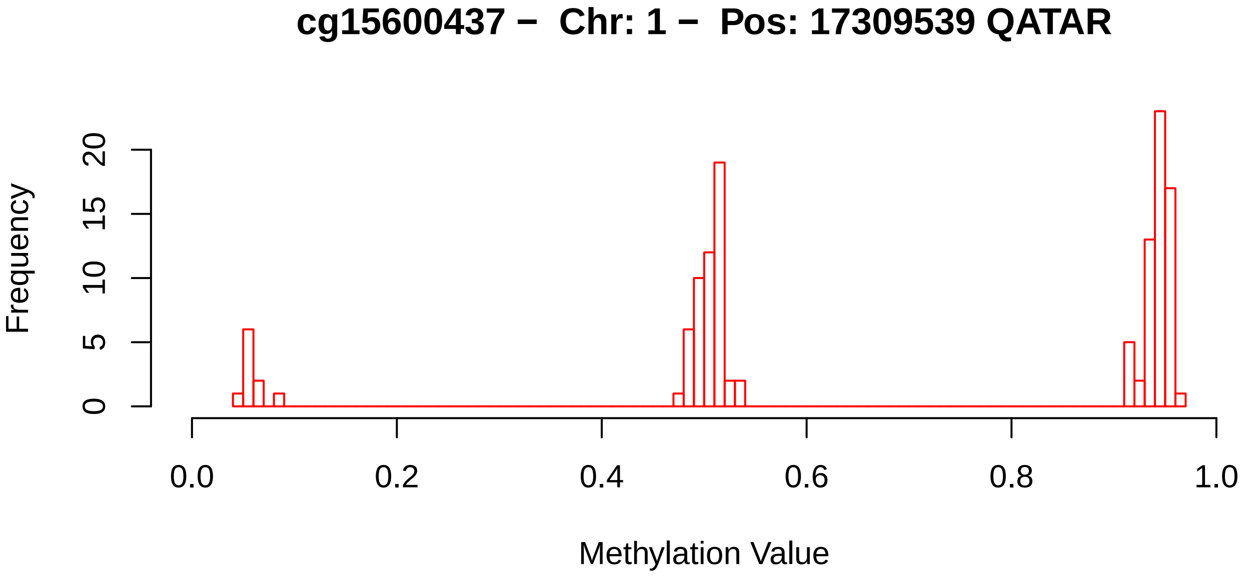
cg15600437 − Chr: 1 − Pos: 17309539 QATAR
Frequency
0
5
10
15
20
0.0
0.2
0.4
0.6
0.8
1.0
Methylation Value

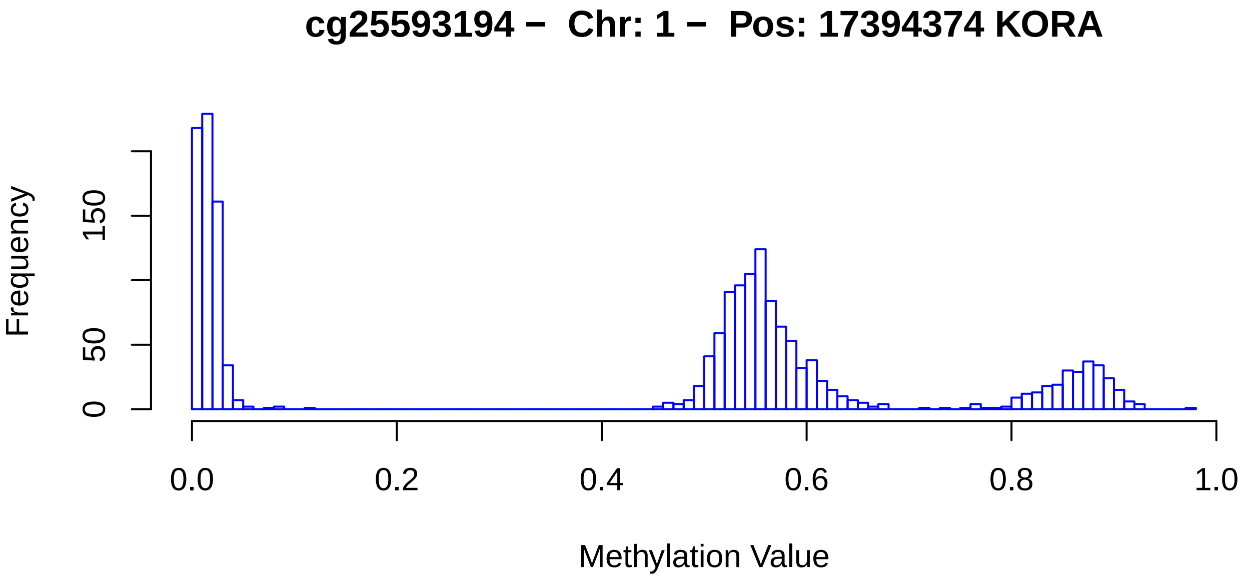
cg25593194 − Chr: 1 − Pos: 17394374 KORA
Frequency
0
50
150
0.0
0.2
0.4
0.6
0.8
1.0
Methylation Value

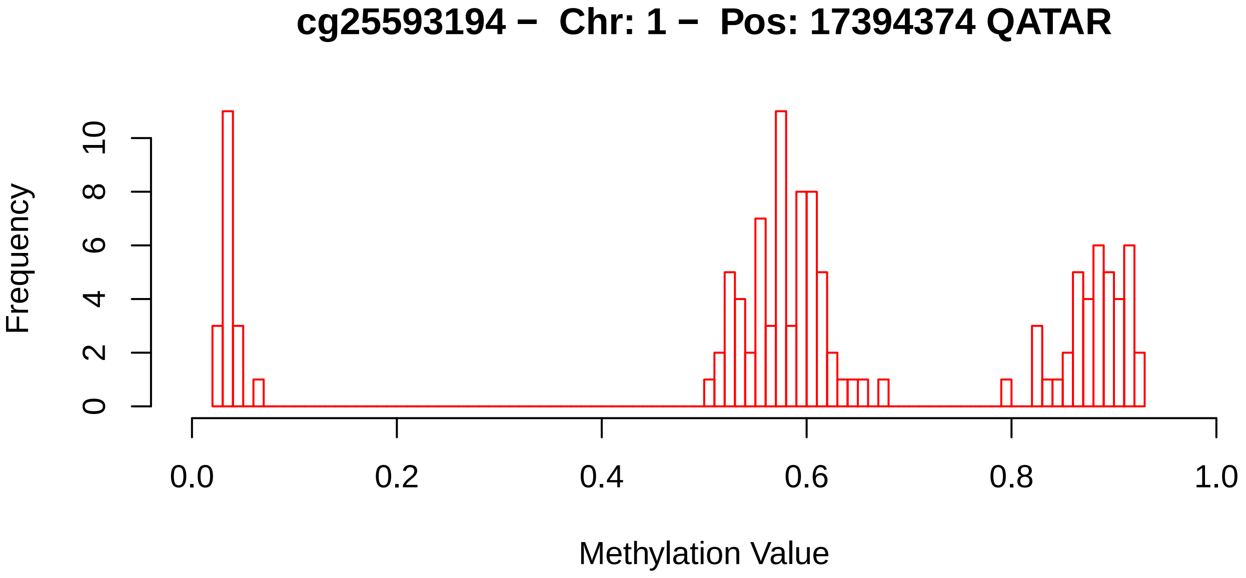
cg25593194 − Chr: 1 − Pos: 17394374 QATAR
Frequency
0
2
4
6
8
10
0.0
0.2
0.4
0.6
0.8
1.0
Methylation Value

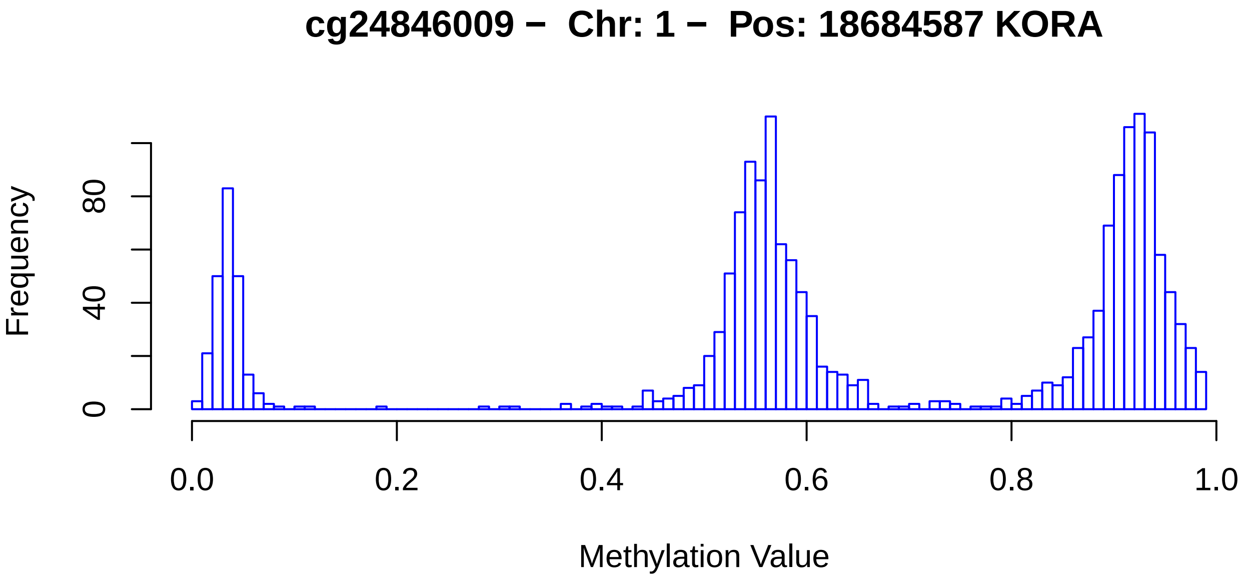
cg24846009 − Chr: 1 − Pos: 18684587 KORA
Frequency
0
40
80
0.0
0.2
0.4
0.6
0.8
1.0
Methylation Value

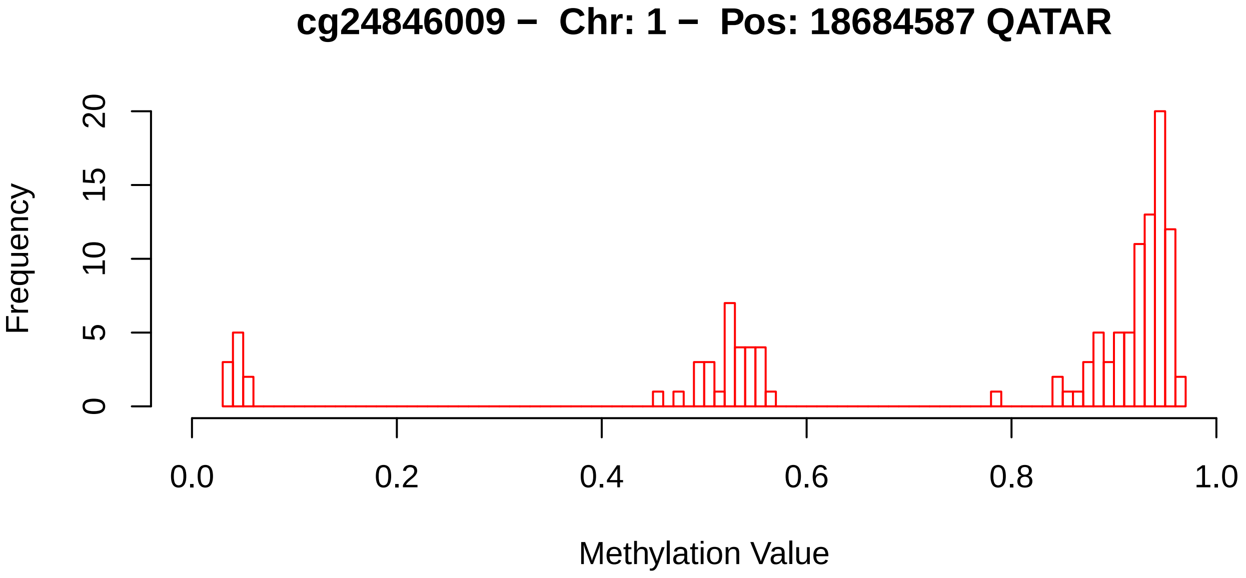
cg24846009 − Chr: 1 − Pos: 18684587 QATAR
Frequency
0
5
10
15
20
0.0
0.2
0.4
0.6
0.8
1.0
Methylation Value

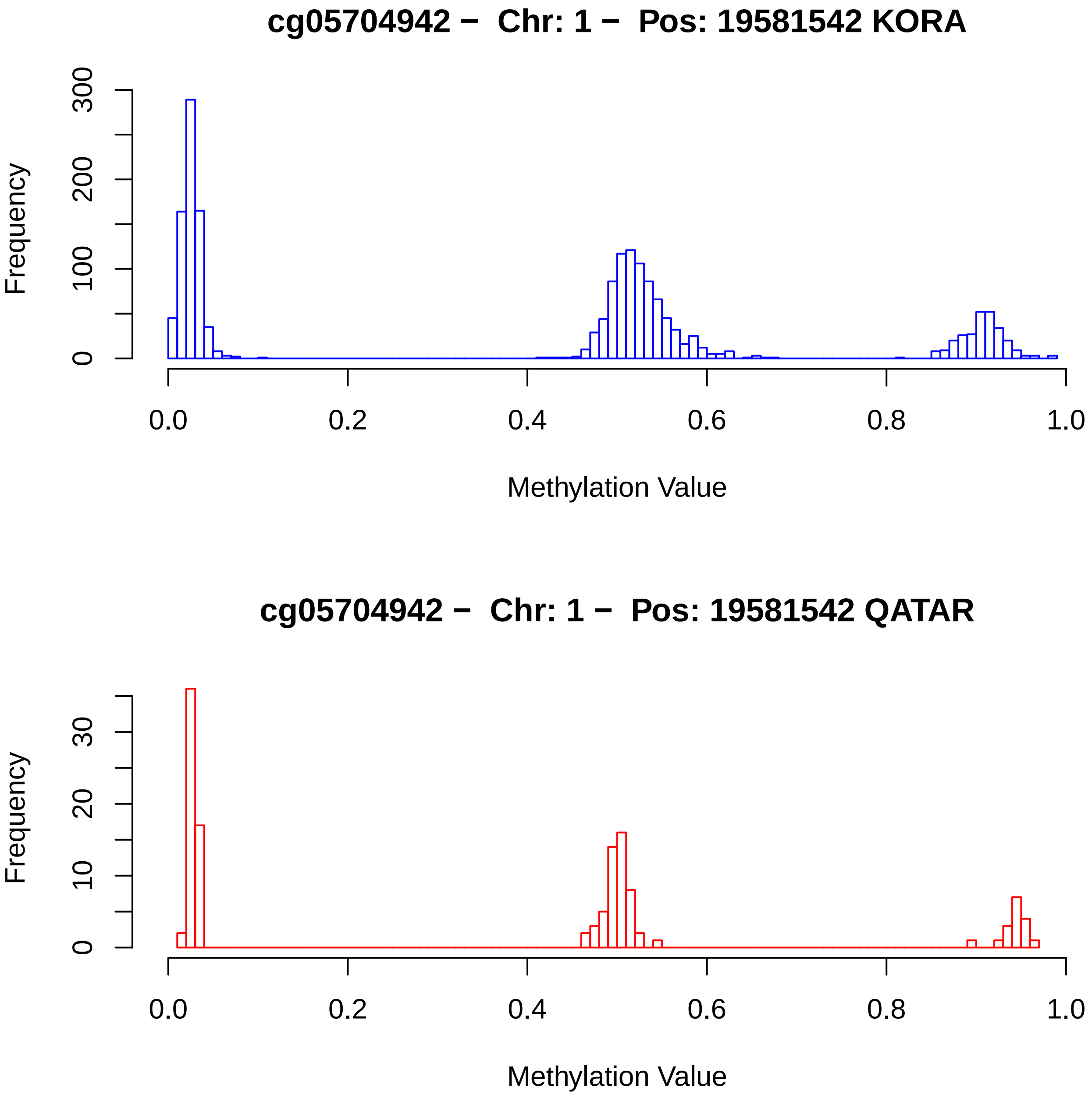
cg05704942 − Chr: 1 − Pos: 19581542 KORA
Frequency
0
100
200
300
0.0
0.2
0.4
0.6
0.8
1.0
Methylation Value
cg05704942 − Chr: 1 − Pos: 19581542 QATAR
Frequency
0
10
20
30
0.0
0.2
0.4
0.6
0.8
1.0
Methylation Value

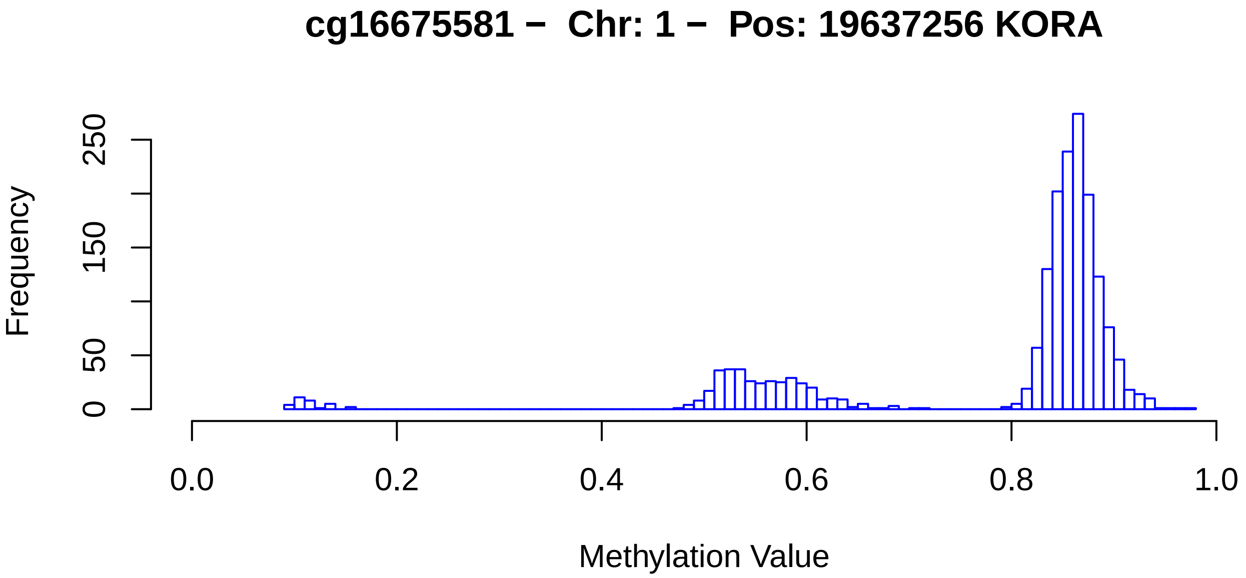
cg16675581 – Chr: 1 – Pos: 19637256 KORA
Frequency
0
50
150
250
0.0
0.2
0.4
0.6
0.8
1.0
Methylation Value
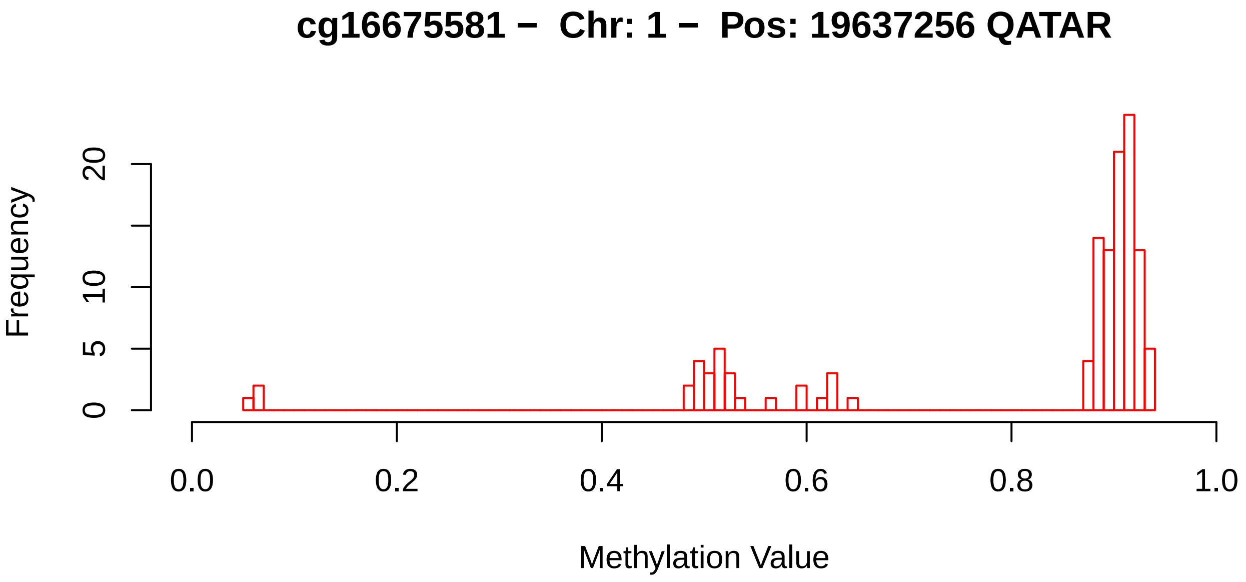
cg16675581 – Chr: 1 – Pos: 19637256 QATAR
Frequency
0
5
10
20
0.0
0.2
0.4
0.6
0.8
1.0
Methylation Value

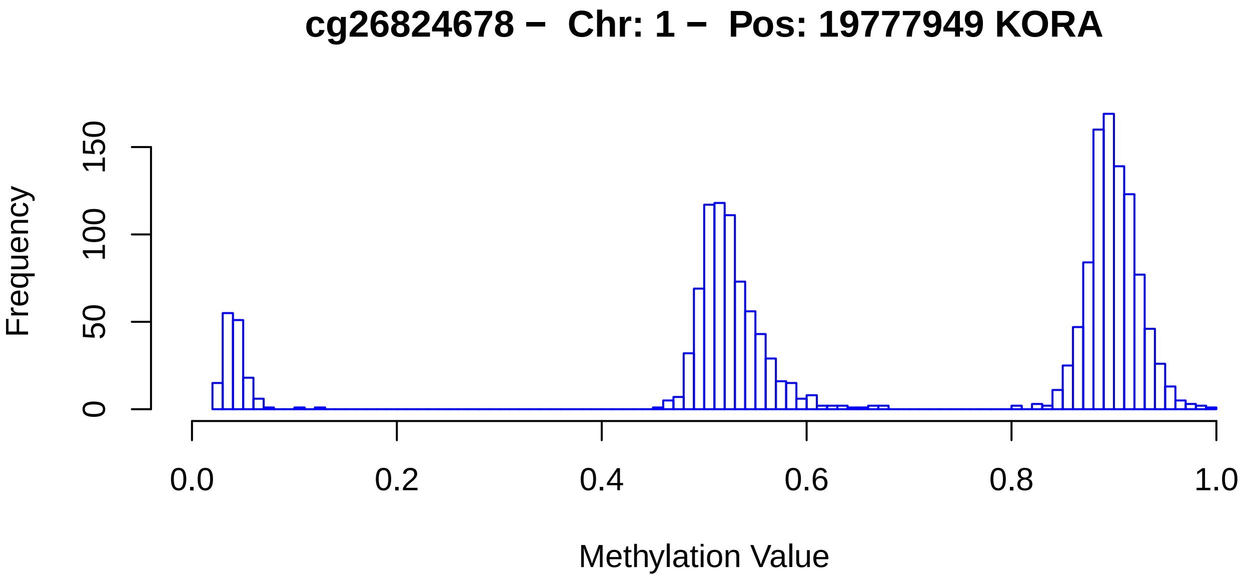
cg26824678 − Chr: 1 − Pos: 19777949 KORA
Frequency
0
50
100
150
0.0
0.2
0.4
0.6
0.8
1.0
Methylation Value

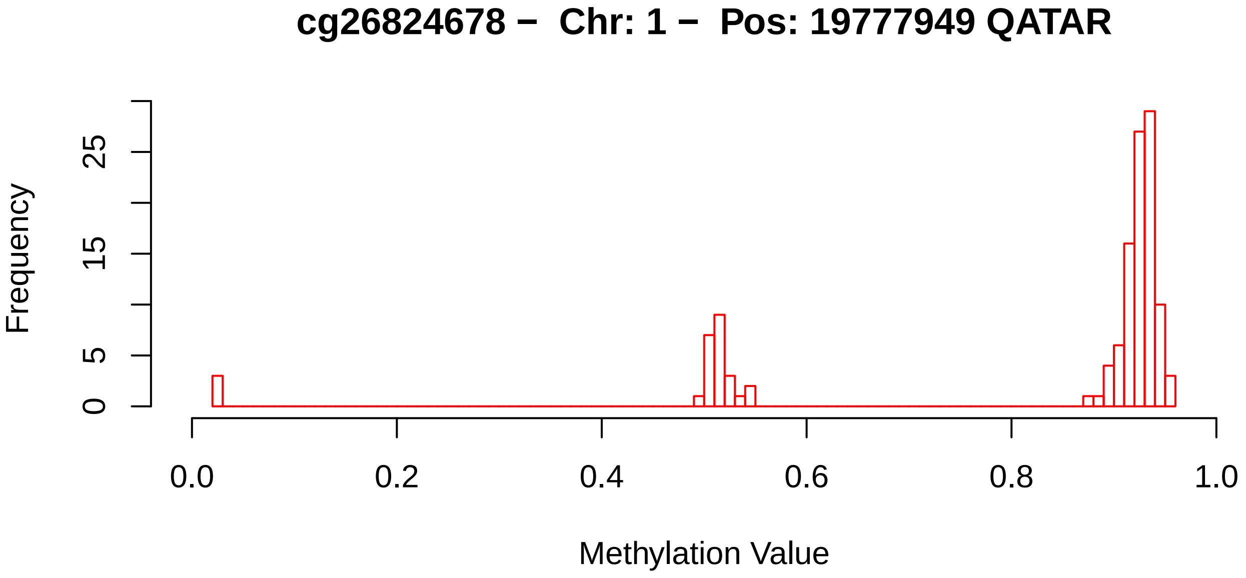
cg26824678 − Chr: 1 − Pos: 19777949 QATAR
Frequency
0
5
15
25
0.0
0.2
0.4
0.6
0.8
1.0
Methylation Value

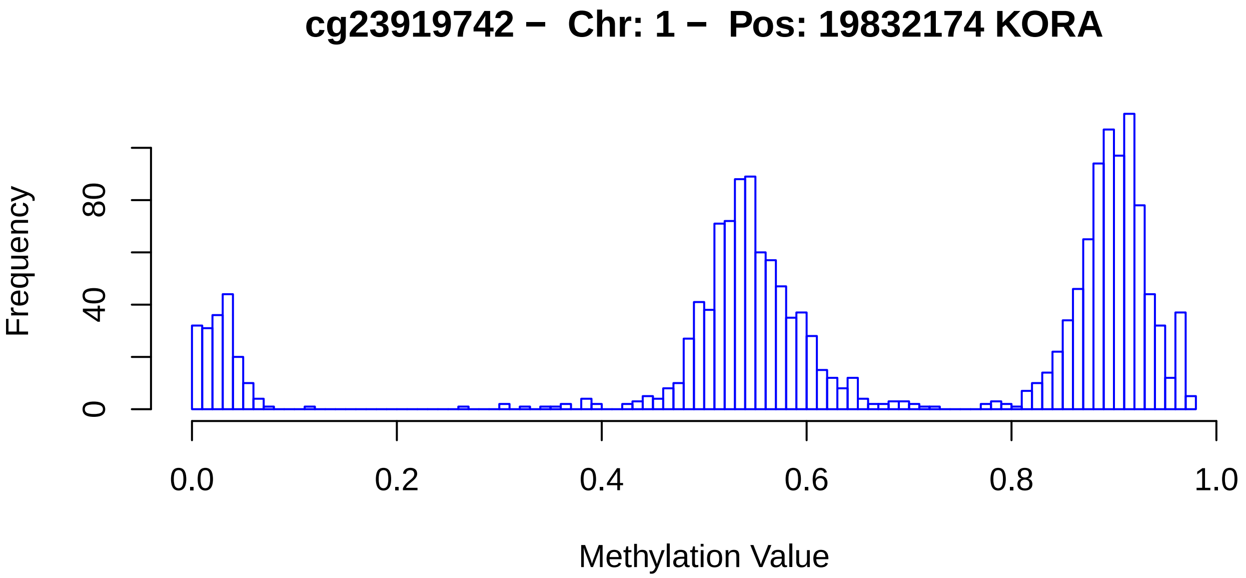
cg23919742 − Chr: 1 − Pos: 19832174 KORA
Frequency
0
40
80
0.0
0.2
0.4
0.6
0.8
1.0
Methylation Value

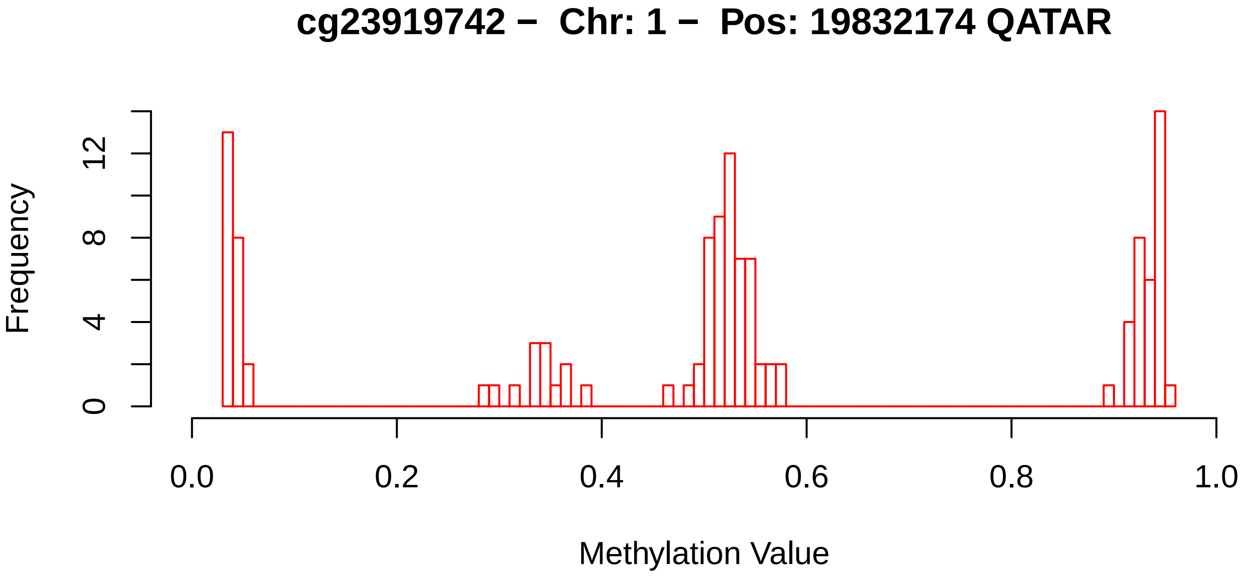
cg23919742 − Chr: 1 − Pos: 19832174 QATAR
Frequency
0
4
8
12
0.0
0.2
0.4
0.6
0.8
1.0
Methylation Value

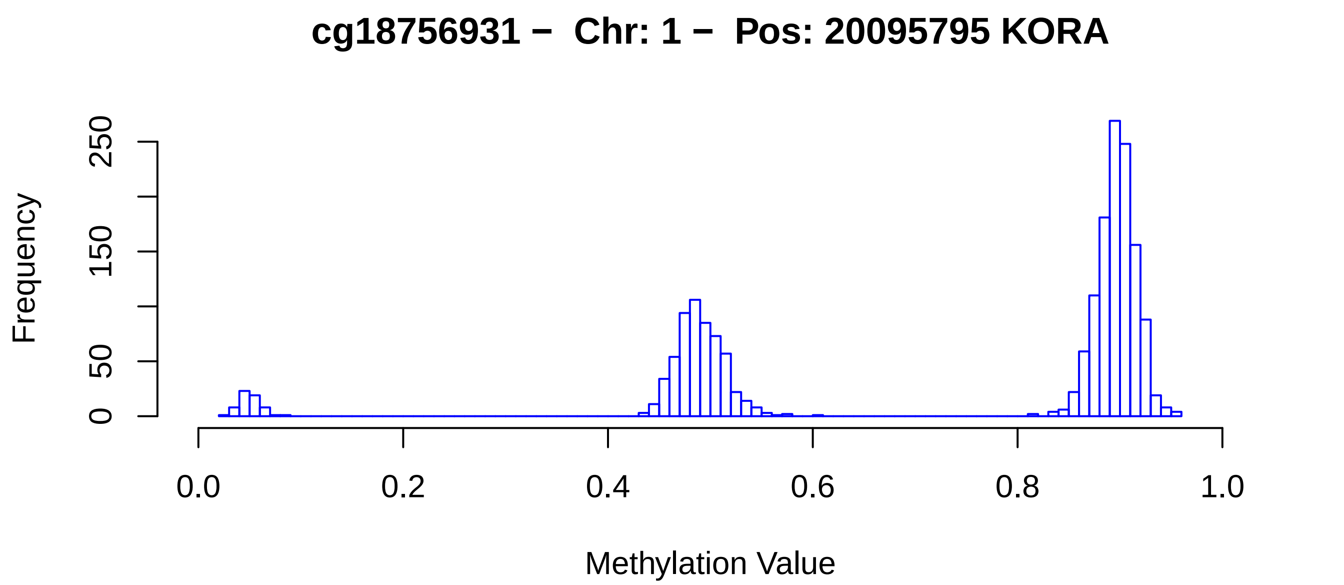
cg18756931 − Chr: 1 − Pos: 20095795 KORA
Frequency
0
50
150
250
0.0
0.2
0.4
0.6
0.8
1.0
Methylation Value

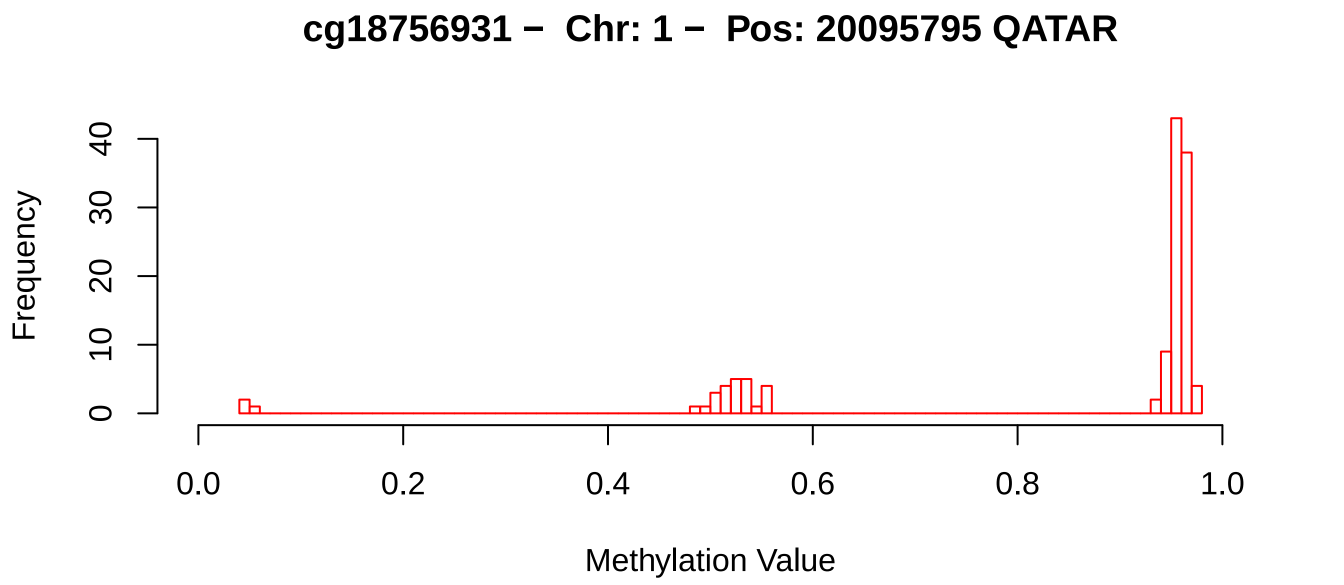
cg18756931 − Chr: 1 − Pos: 20095795 QATAR
Frequency
0
10
20
30
40
0.0
0.2
0.4
0.6
0.8
1.0
Methylation Value

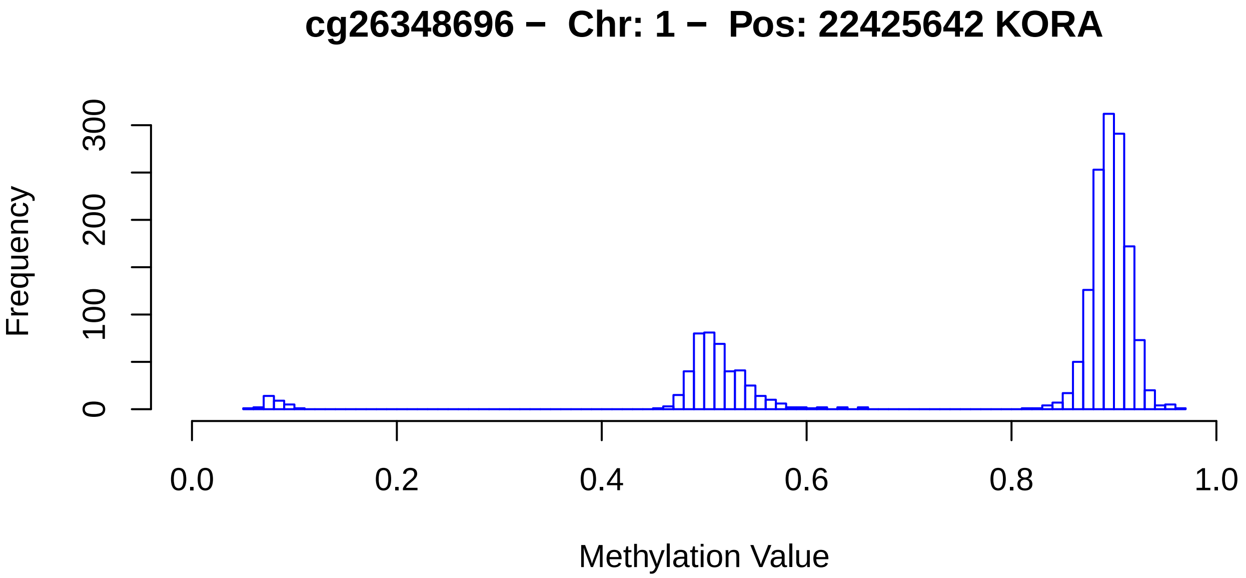
cg26348696 − Chr: 1 − Pos: 22425642 KORA
Frequency
0
100
200
300
0.0
0.2
0.4
0.6
0.8
1.0
Methylation Value

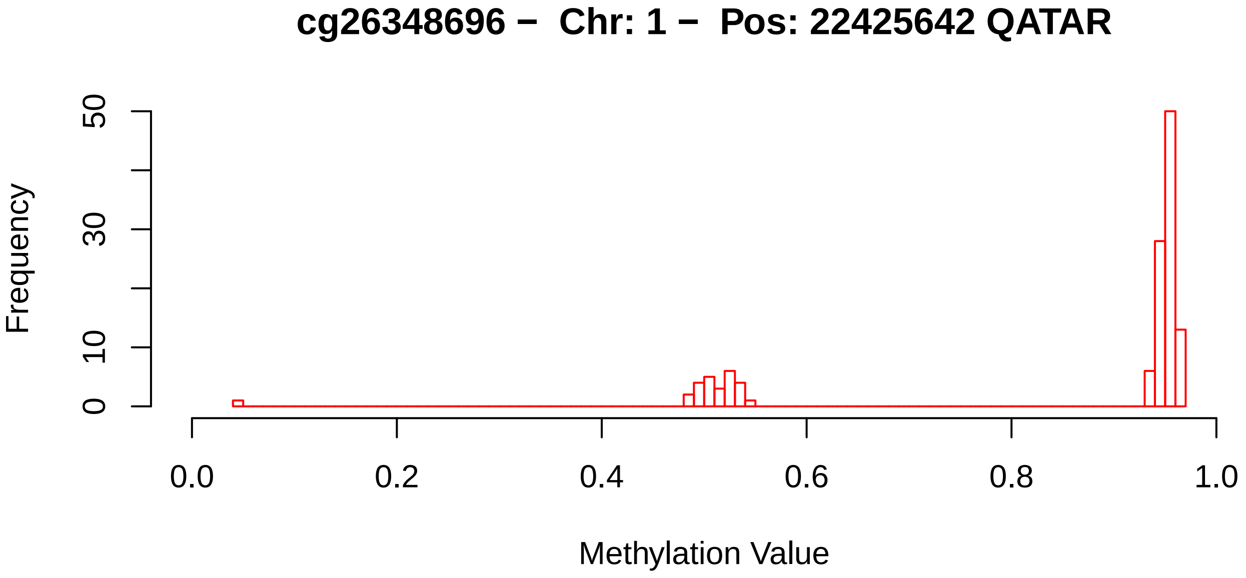
cg26348696 − Chr: 1 − Pos: 22425642 QATAR
Frequency
0
10
30
50
0.0
0.2
0.4
0.6
0.8
1.0
Methylation Value

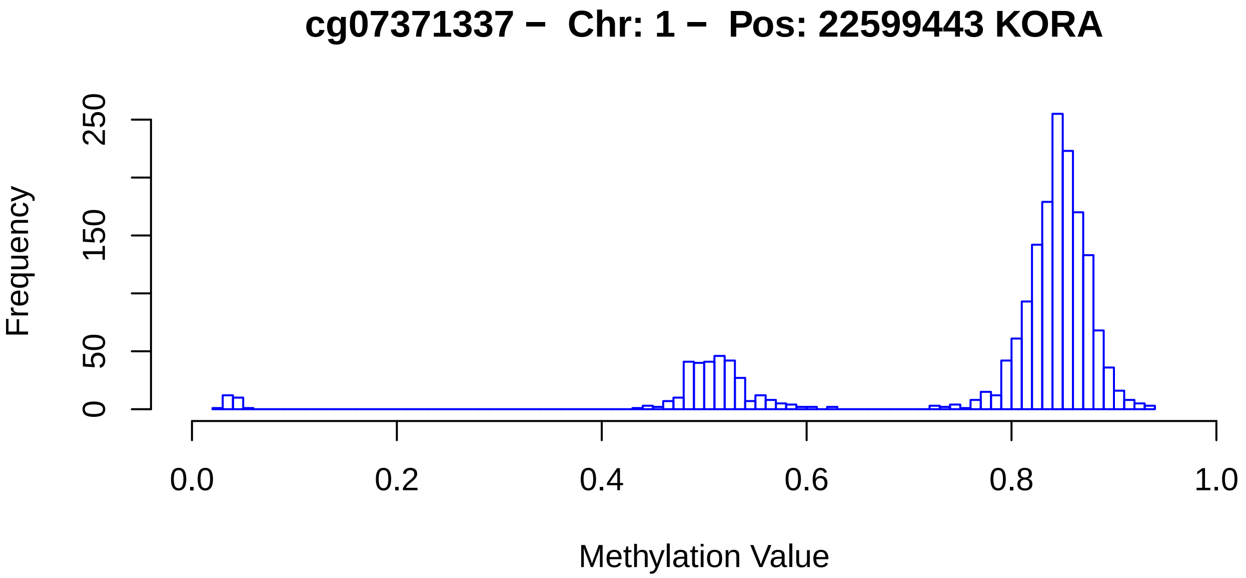
cg07371337 − Chr: 1 − Pos: 22599443 KORA
Frequency
0
50
150
250
0.0
0.2
0.4
0.6
0.8
1.0
Methylation Value

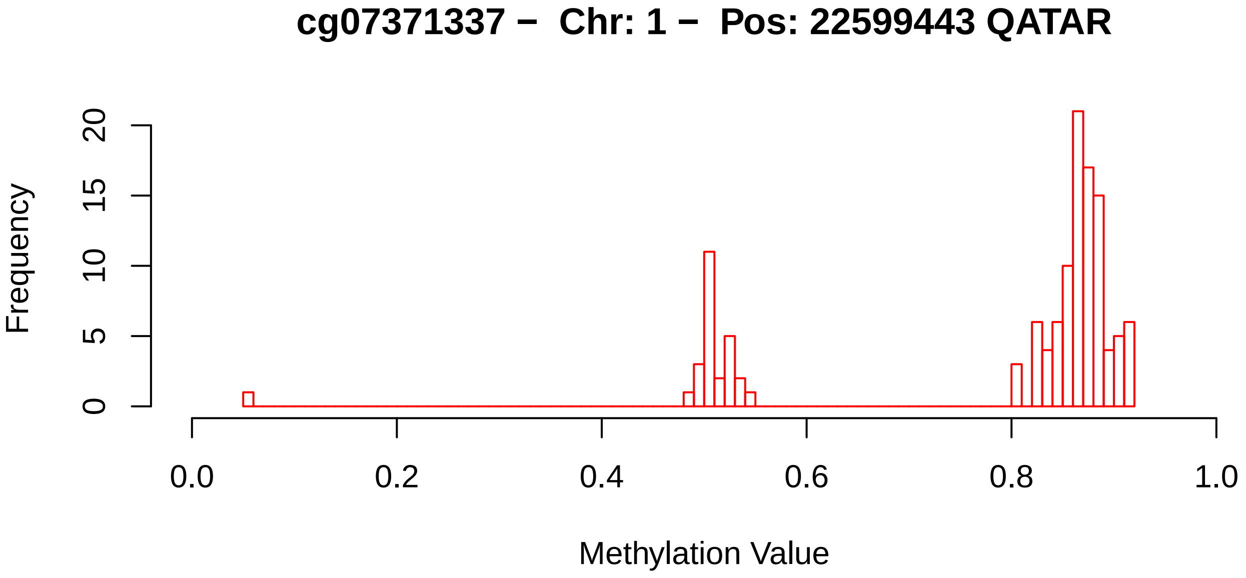
cg07371337 − Chr: 1 − Pos: 22599443 QATAR
Frequency
0
5
10
15
20
0.0
0.2
0.4
0.6
0.8
1.0
Methylation Value

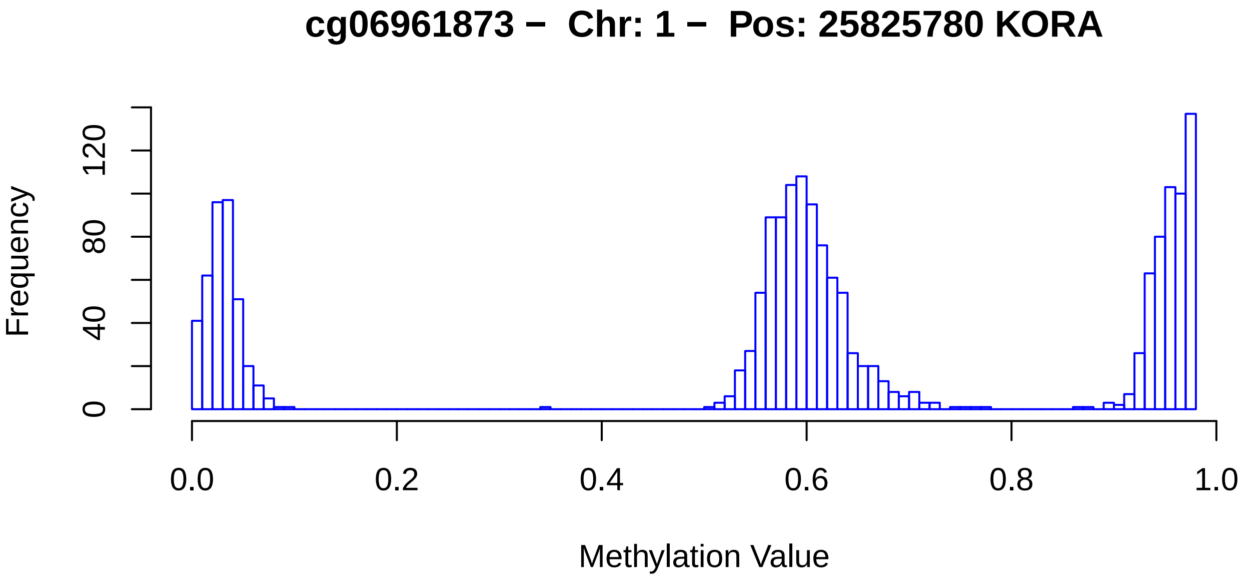

cg06961873 − Chr: 1 − Pos: 25825780 KORA
Frequency
0
40
80
120
0.0
0.2
0.4
0.6
0.8
1.0
Methylation Value

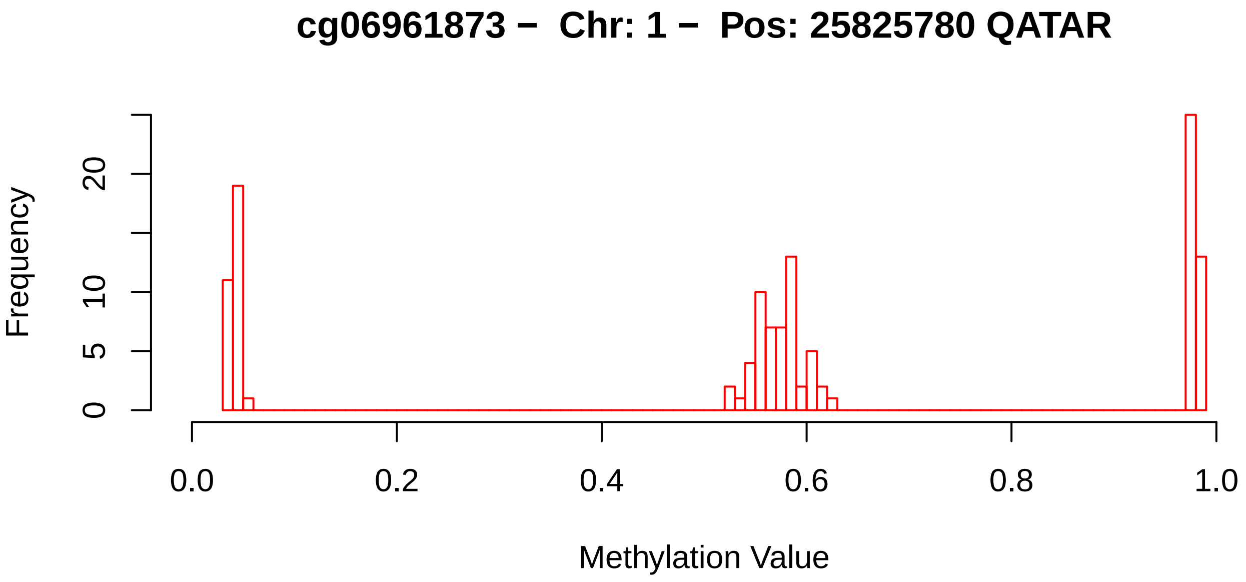

cg06961873 − Chr: 1 − Pos: 25825780 QATAR
Frequency
0
5
10
20
0.0
0.2
0.4
0.6
0.8
1.0
Methylation Value

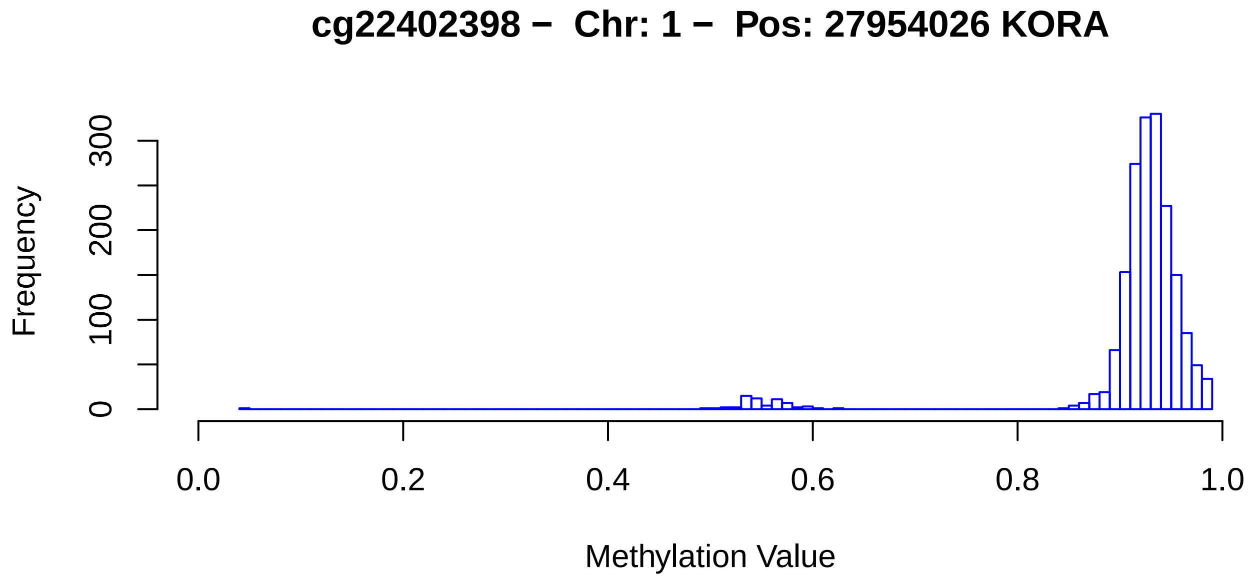
cg22402398 − Chr: 1 − Pos: 27954026 KORA
Frequency
0
100
200
300
0.0
0.2
0.4
0.6
0.8
1.0
Methylation Value

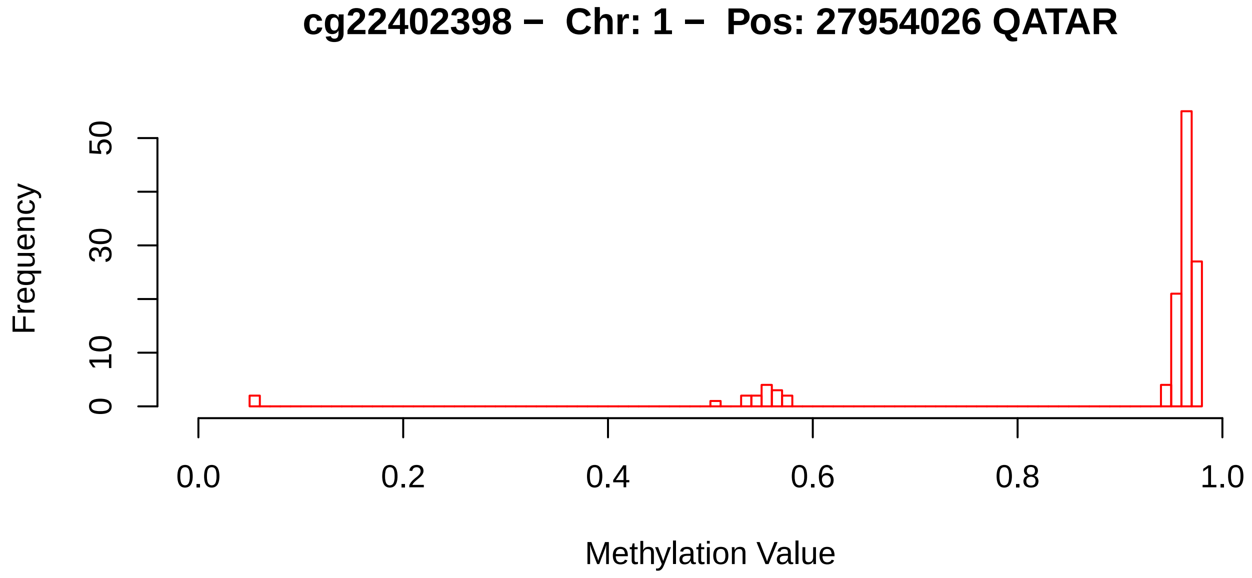
cg22402398 − Chr: 1 − Pos: 27954026 QATAR
Frequency
0
10
30
50
0.0
0.2
0.4
0.6
0.8
1.0
Methylation Value

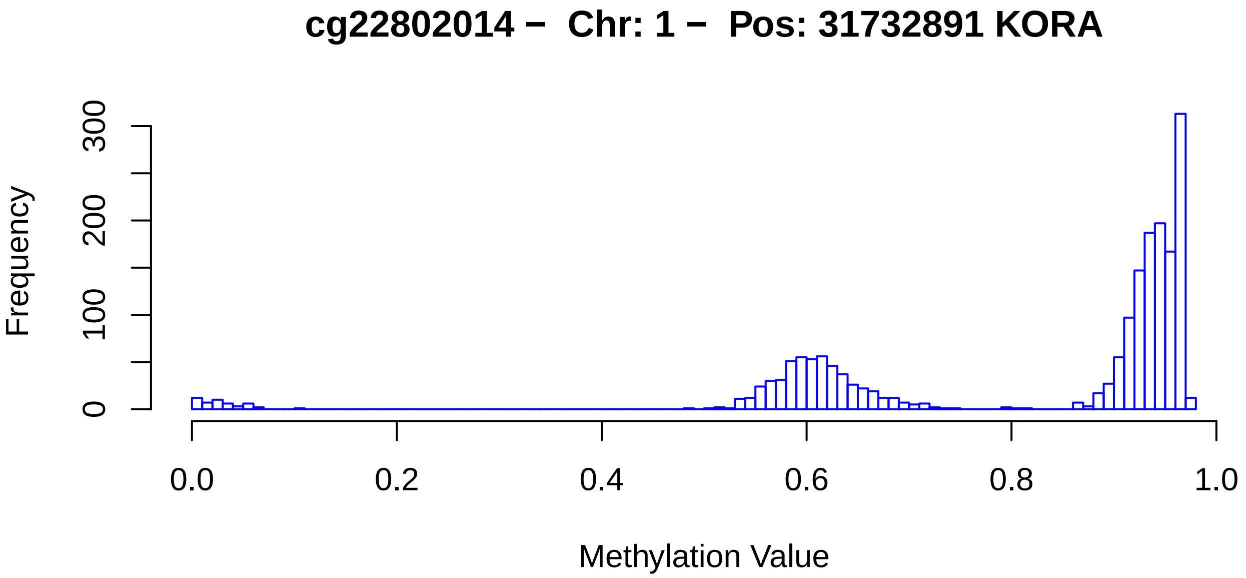
cg22802014 − Chr: 1 − Pos: 31732891 KORA
Frequency
0
100
200
300
0.0
0.2
0.4
0.6
0.8
1.0
Methylation Value

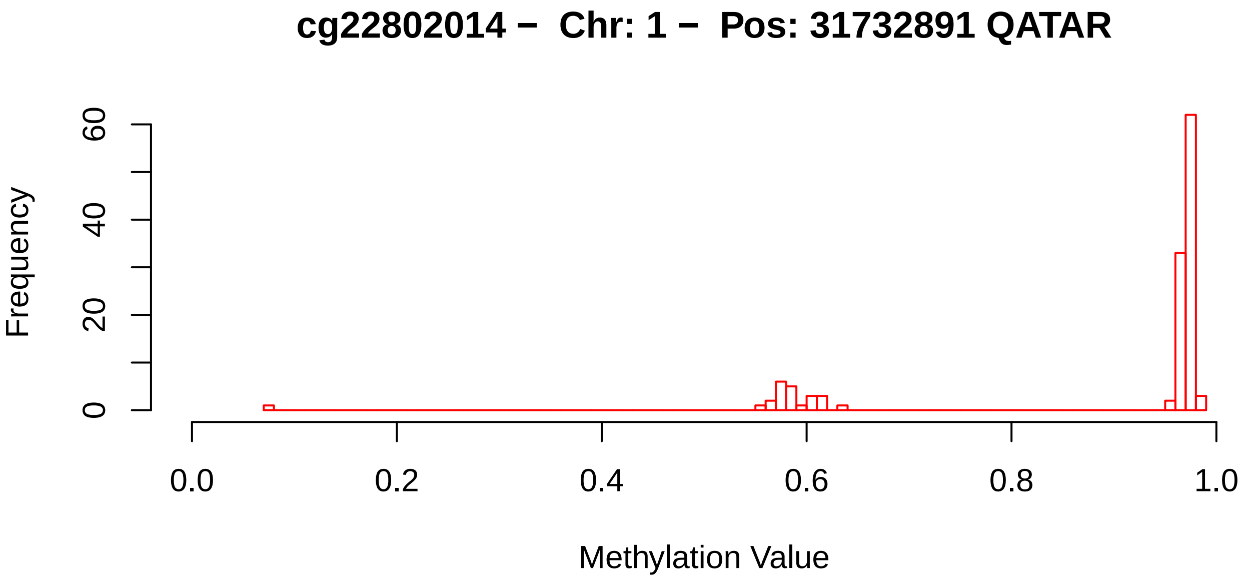
cg22802014 − Chr: 1 − Pos: 31732891 QATAR
Frequency
0
20
40
60
0.0
0.2
0.4
0.6
0.8
1.0
Methylation Value

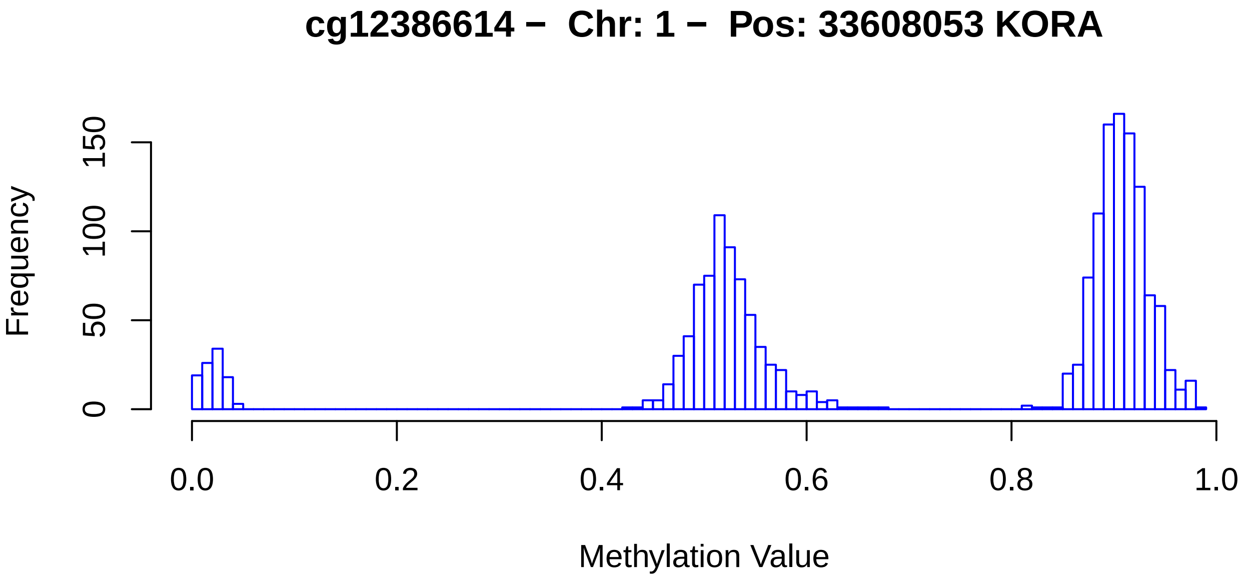
cg12386614 − Chr: 1 − Pos: 33608053 KORA
Frequency
0
50
100
150
0.0
0.2
0.4
0.6
0.8
1.0
Methylation Value

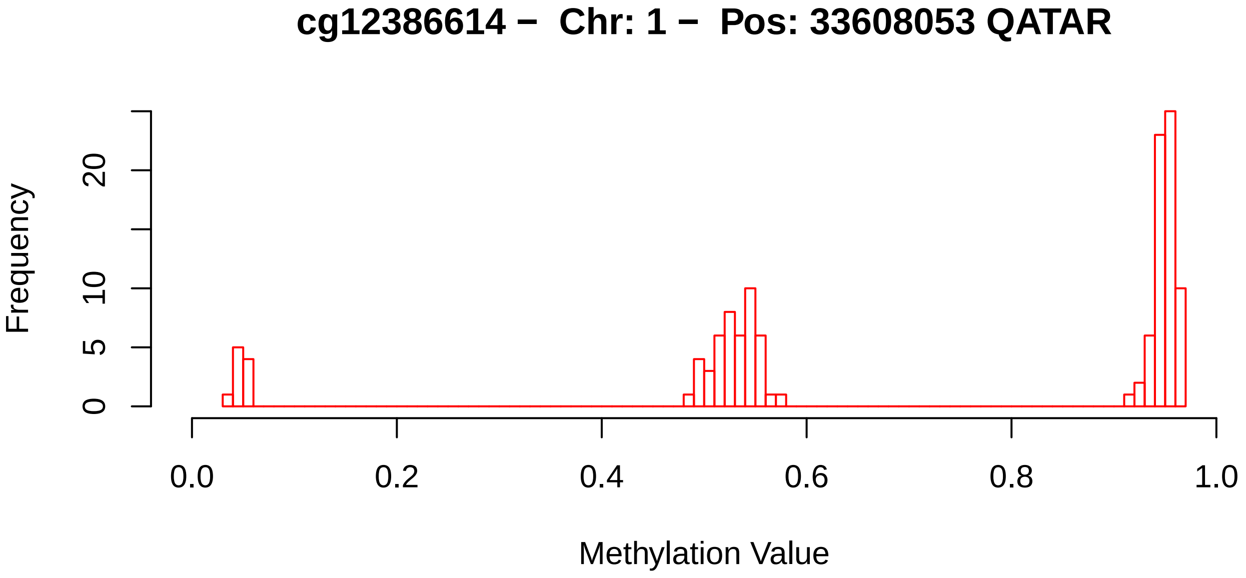
cg12386614 − Chr: 1 − Pos: 33608053 QATAR
Frequency
0
5
10
20
0.0
0.2
0.4
0.6
0.8
1.0
Methylation Value

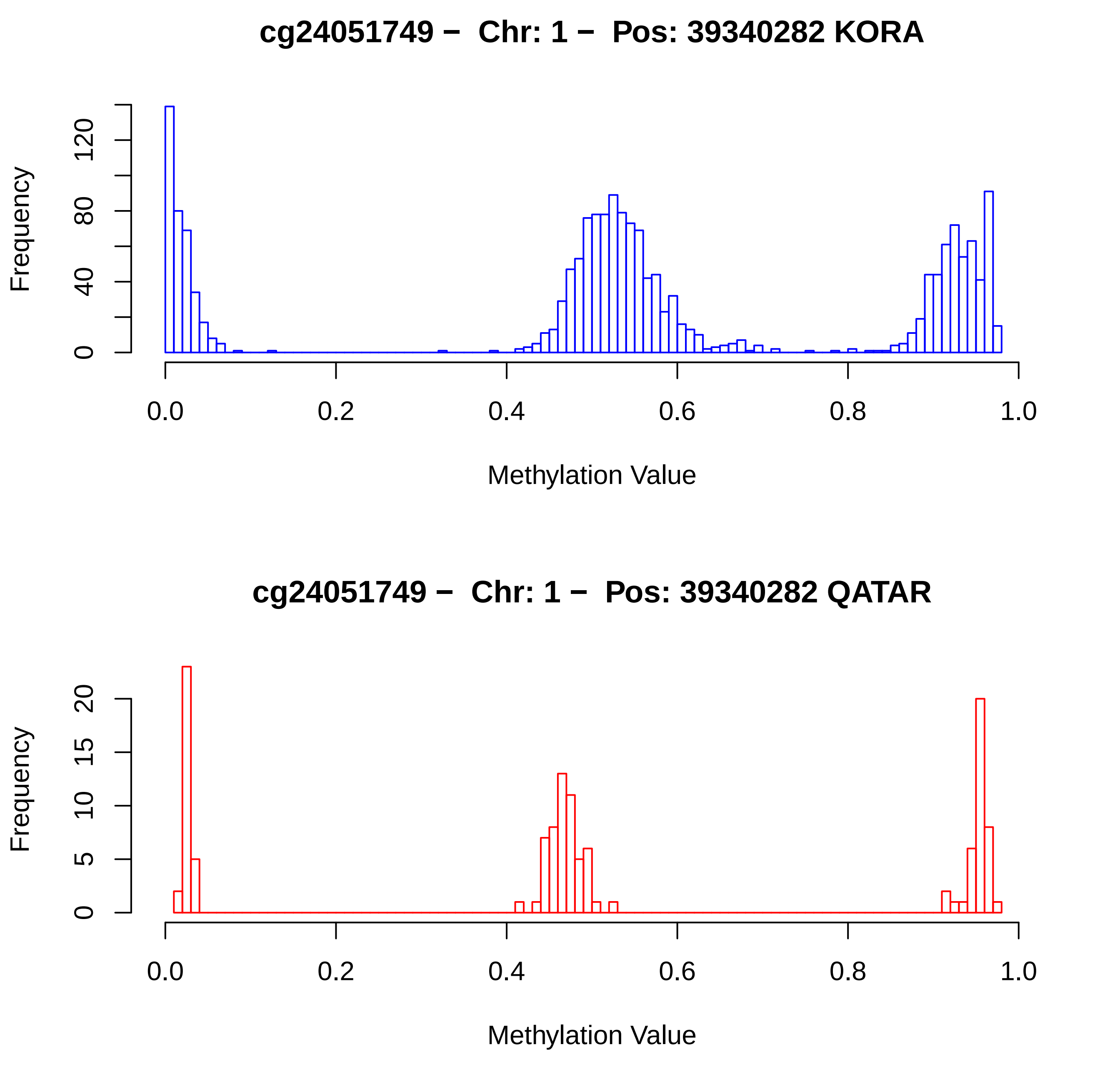
cg24051749 − Chr: 1 − Pos: 39340282 KORA
Frequency
0
40
80
120
0.0
0.2
0.4
0.6
0.8
1.0
Methylation Value
cg24051749 − Chr: 1 − Pos: 39340282 QATAR
Frequency
0
5
10
15
20
0.0
0.2
0.4
0.6
0.8
1.0
Methylation Value

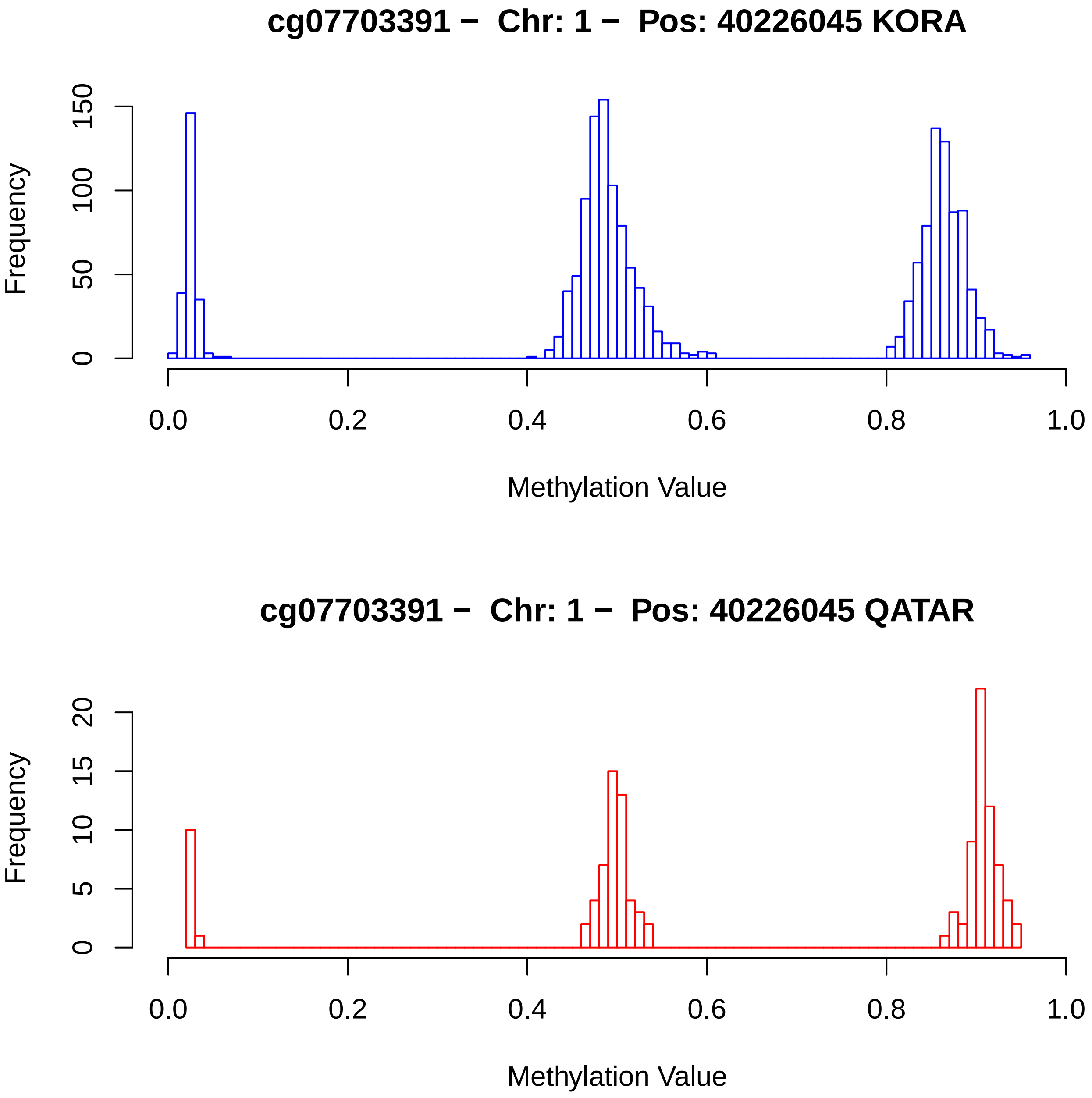
cg07703391 − Chr: 1 − Pos: 40226045 KORA
Frequency
0
50
100
150
0.0
0.2
0.4
0.6
0.8
1.0
Methylation Value
cg07703391 − Chr: 1 − Pos: 40226045 QATAR
Frequency
0
5
10
15
20
0.0
0.2
0.4
0.6
0.8
1.0
Methylation Value

cg05460975 − Chr: 1 − Pos: 43212290 KORA

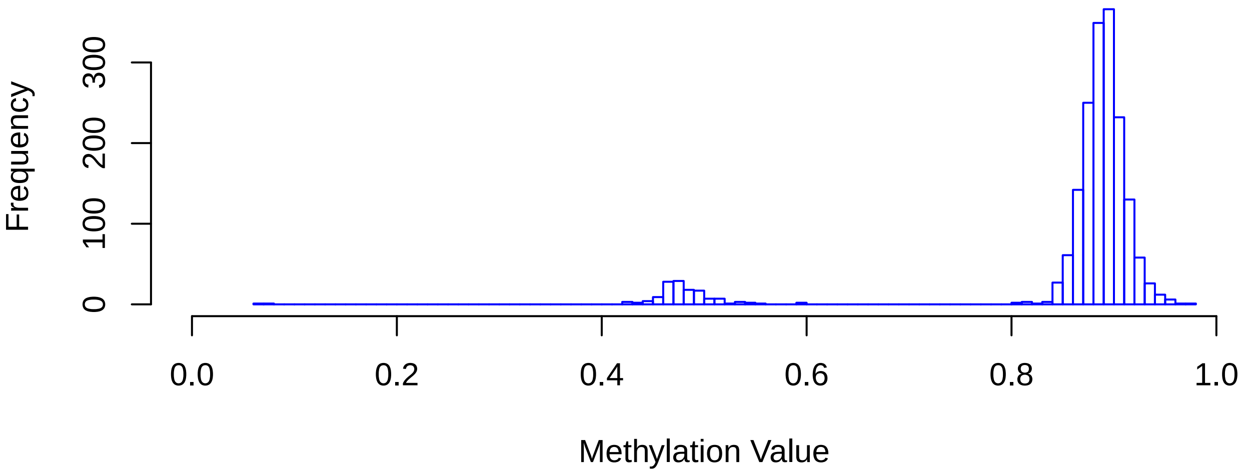

cg05460975 − Chr: 1 − Pos: 43212290 QATAR

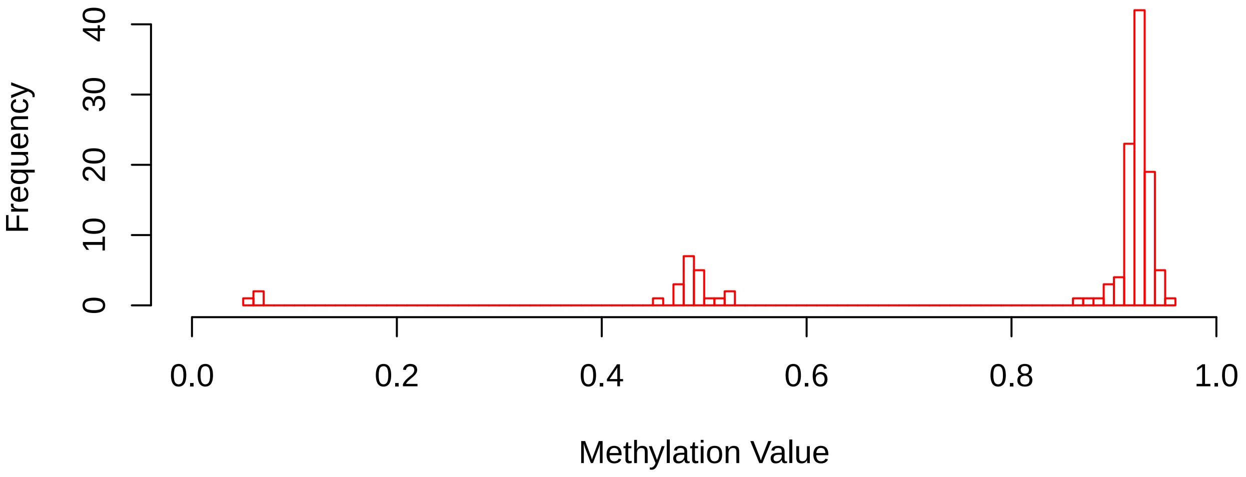

cg05712748 − Chr: 1 − Pos: 43472312 KORA

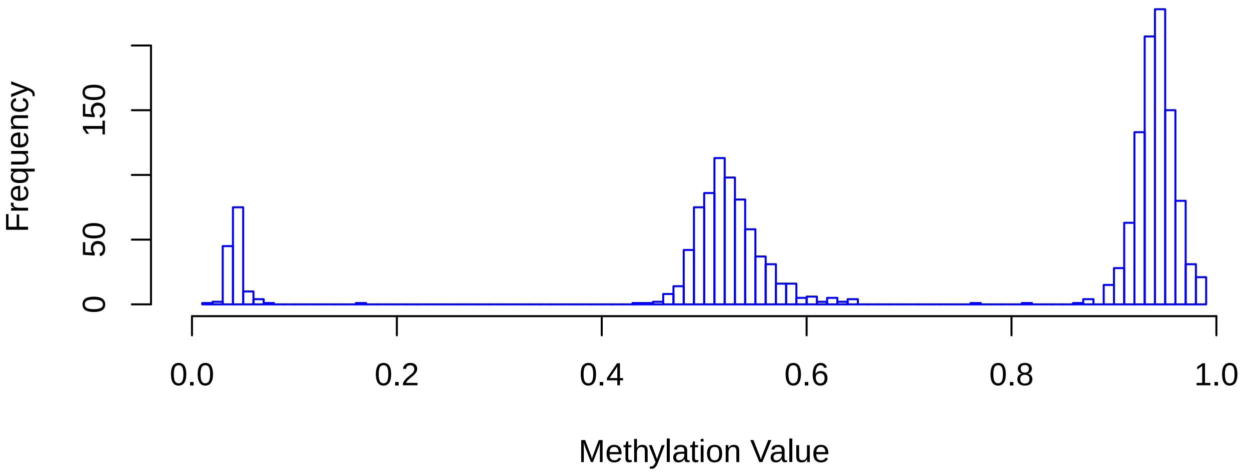

cg05712748 − Chr: 1 − Pos: 43472312 QATAR

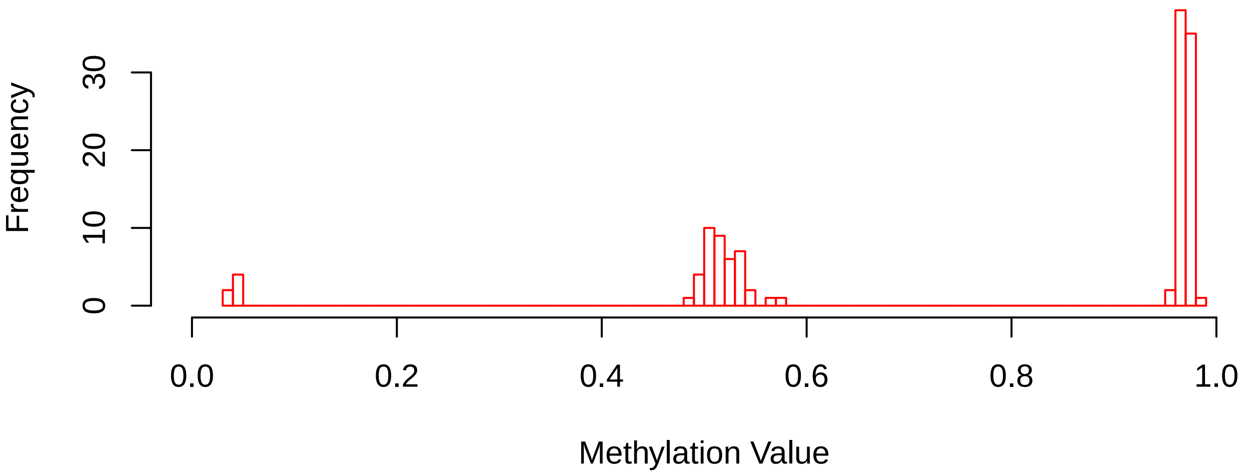

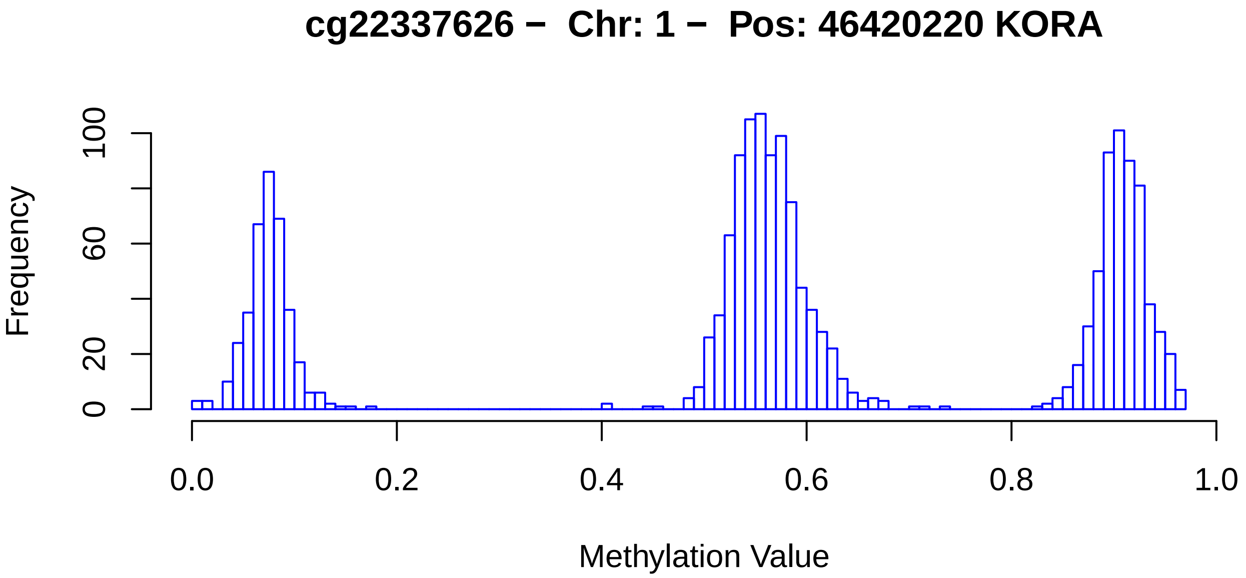
cg22337626 − Chr: 1 − Pos: 46420220 KORA
Frequency
0
20
60
100
0.0
0.2
0.4
0.6
0.8
1.0
Methylation Value

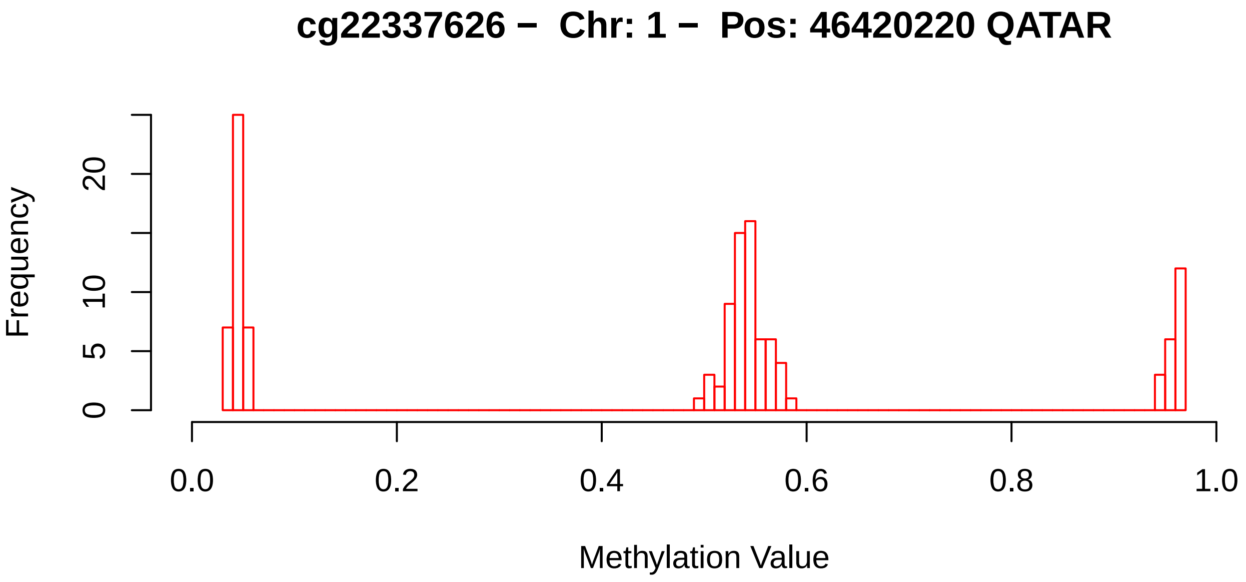
cg22337626 − Chr: 1 − Pos: 46420220 QATAR
Frequency
0
5
10
20
0.0
0.2
0.4
0.6
0.8
1.0
Methylation Value

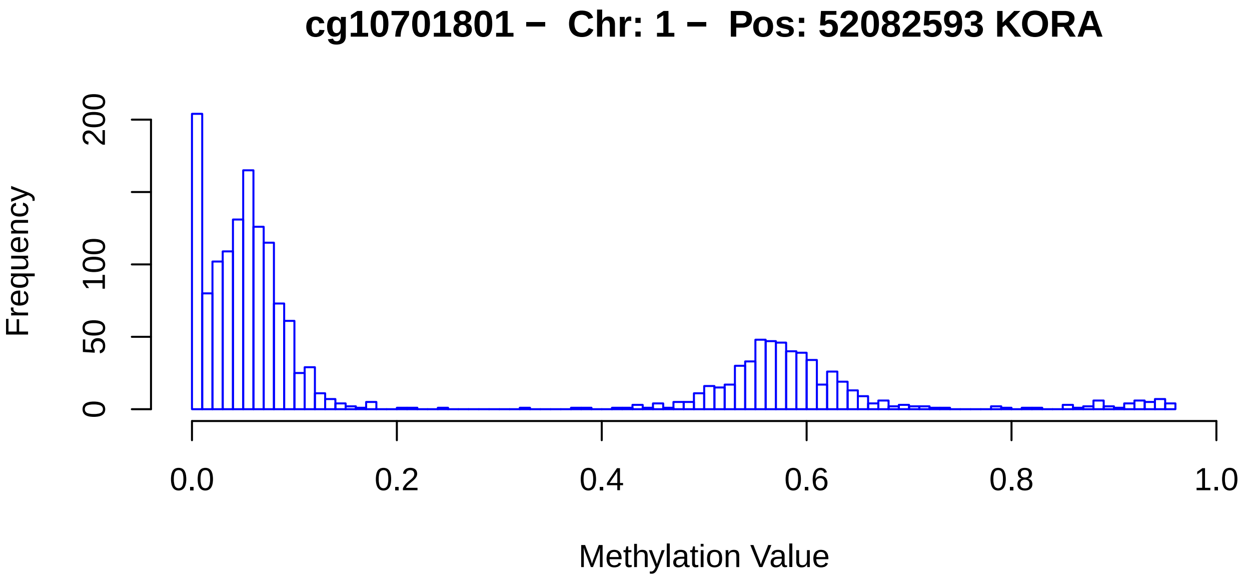
cg10701801 − Chr: 1 − Pos: 52082593 KORA
Frequency
0
50
100
200
0.0
0.2
0.4
0.6
0.8
1.0
Methylation Value

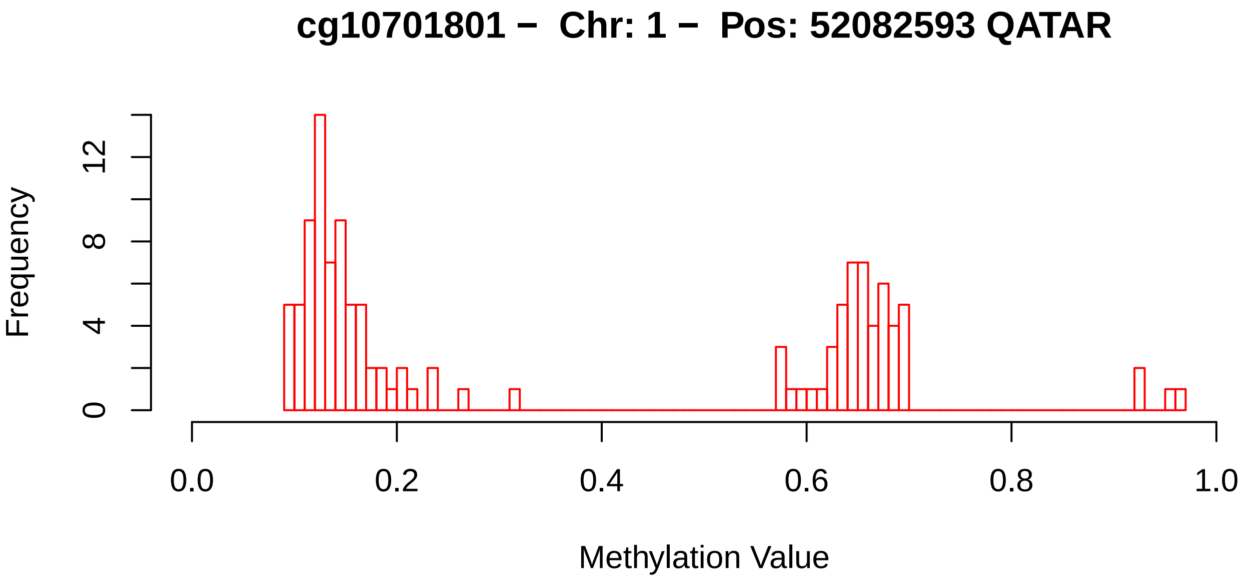
cg10701801 − Chr: 1 − Pos: 52082593 QATAR
Frequency
0
4
8
12
0.0
0.2
0.4
0.6
0.8
1.0
Methylation Value

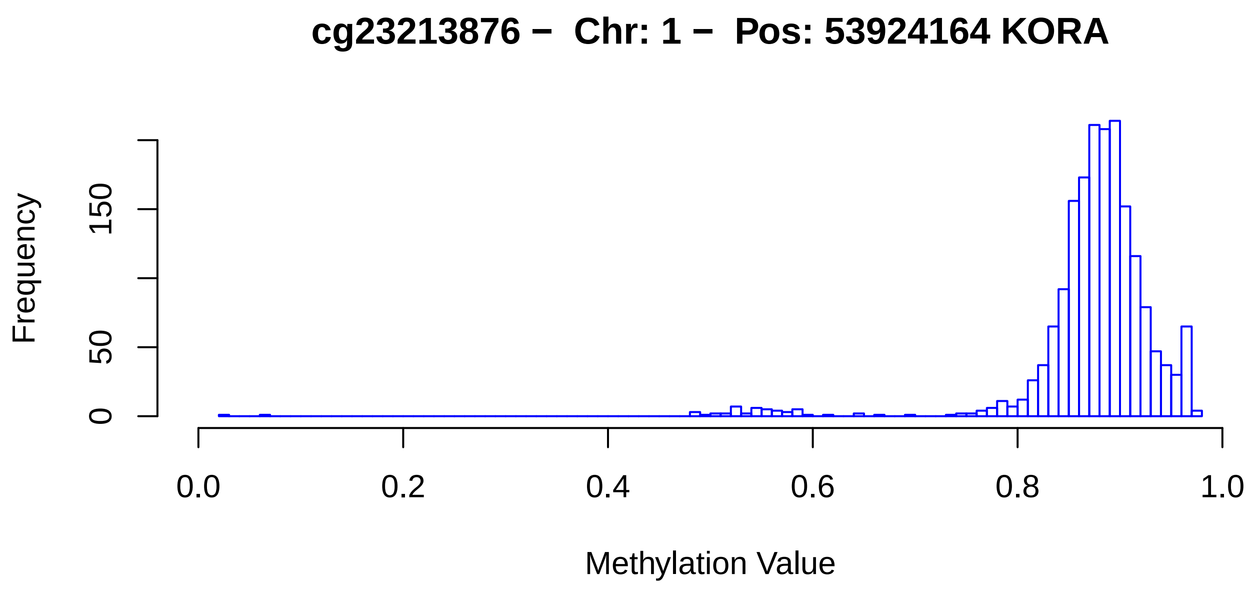
cg23213876 − Chr: 1 − Pos: 53924164 KORA
Frequency
0
50
150
0.0
0.2
0.4
0.6
0.8
1.0
Methylation Value

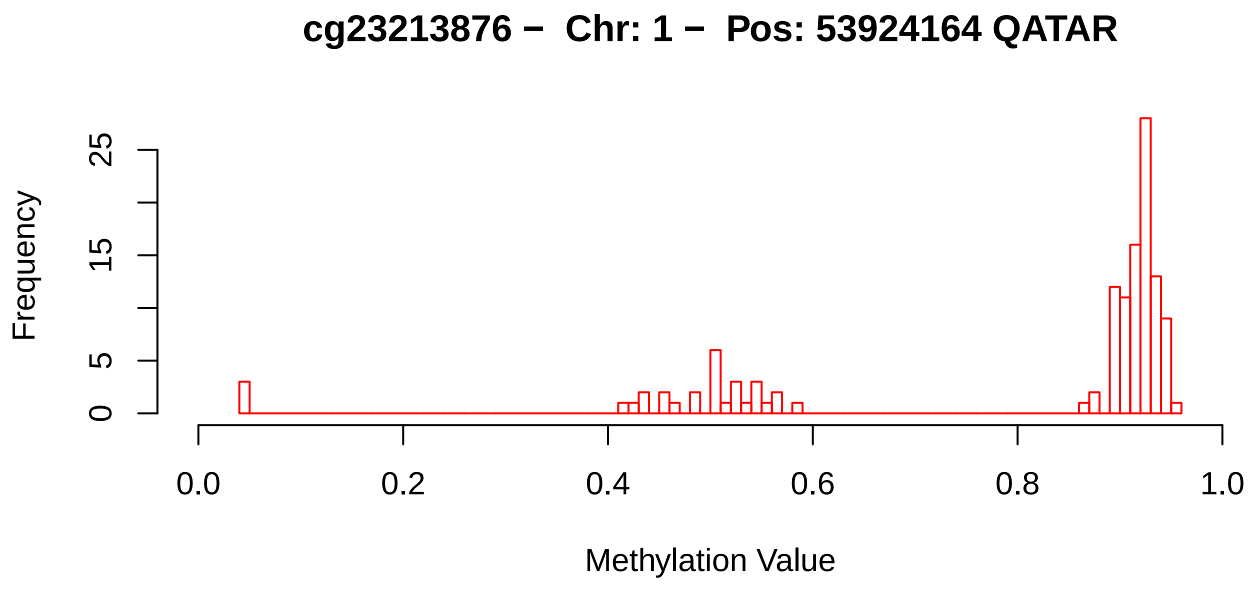
cg23213876 − Chr: 1 − Pos: 53924164 QATAR
Frequency
0
5
15
25
0.0
0.2
0.4
0.6
0.8
1.0
Methylation Value

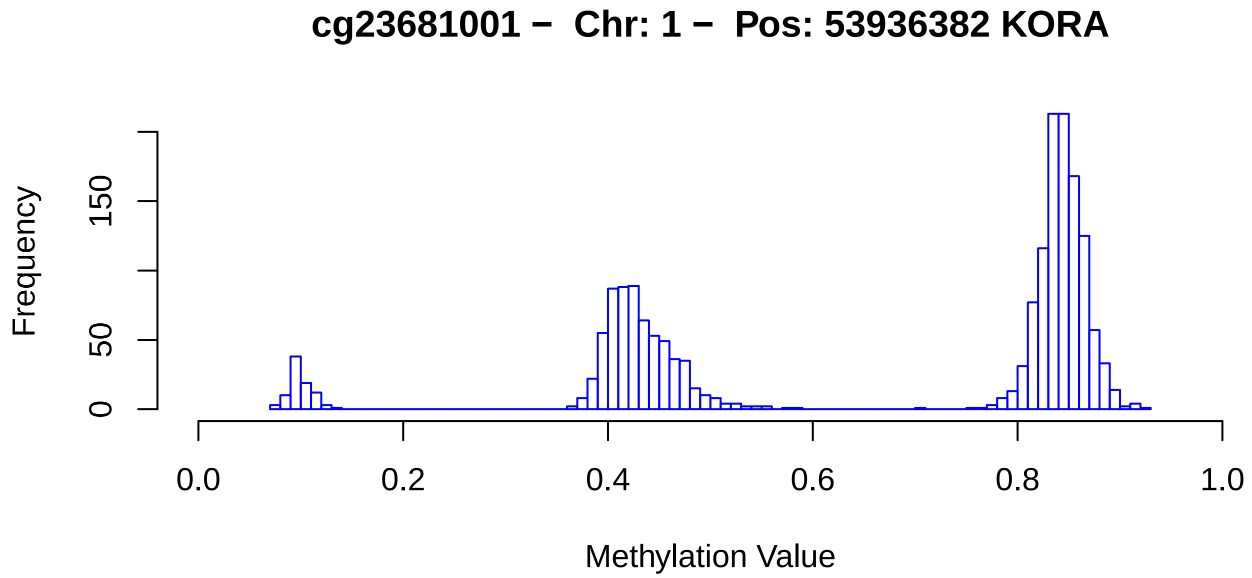
cg23681001 − Chr: 1 − Pos: 53936382 KORA
Frequency
0
50
150
0.0
0.2
0.4
0.6
0.8
1.0
Methylation Value

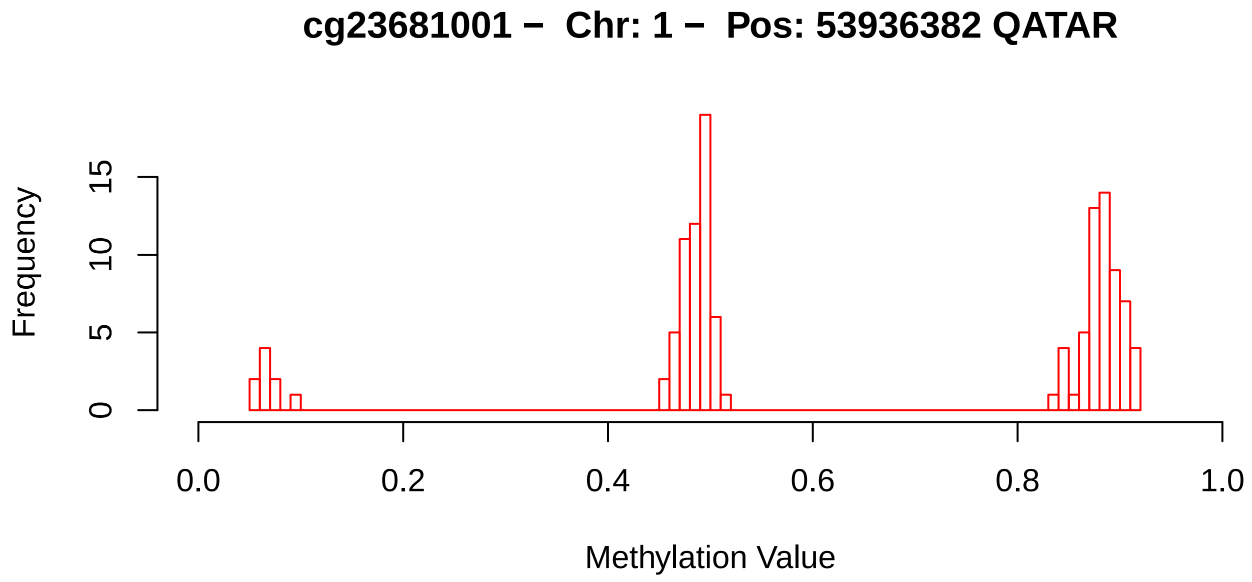
cg23681001 − Chr: 1 − Pos: 53936382 QATAR
Frequency
0
5
10
15
0.0
0.2
0.4
0.6
0.8
1.0
Methylation Value

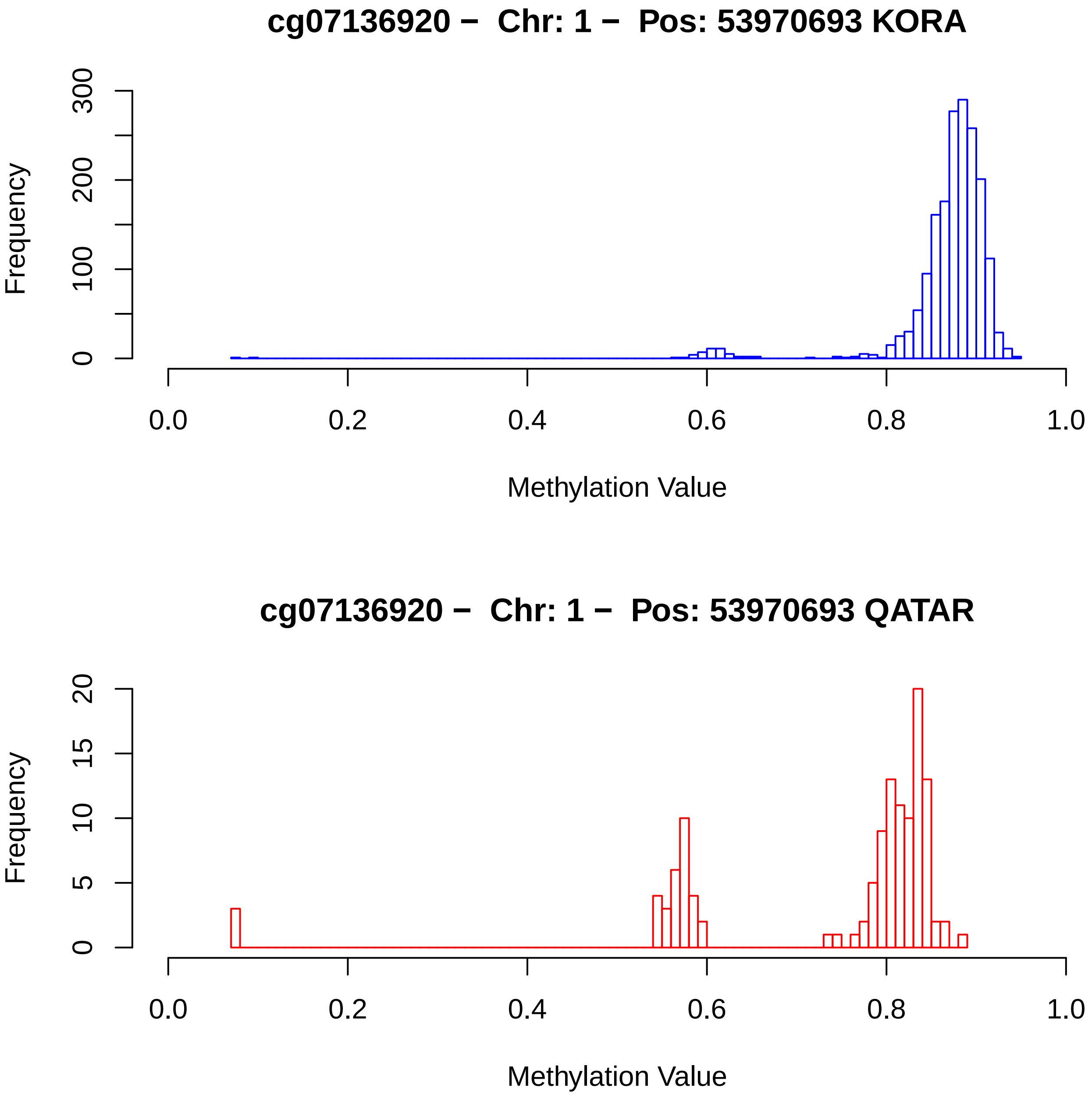
cg07136920 − Chr: 1 − Pos: 53970693 KORA
Frequency
0
100
200
300
0.0
0.2
0.4
0.6
0.8
1.0
Methylation Value
cg07136920 − Chr: 1 − Pos: 53970693 QATAR
Frequency
0
5
10
15
20
0.0
0.2
0.4
0.6
0.8
1.0
Methylation Value

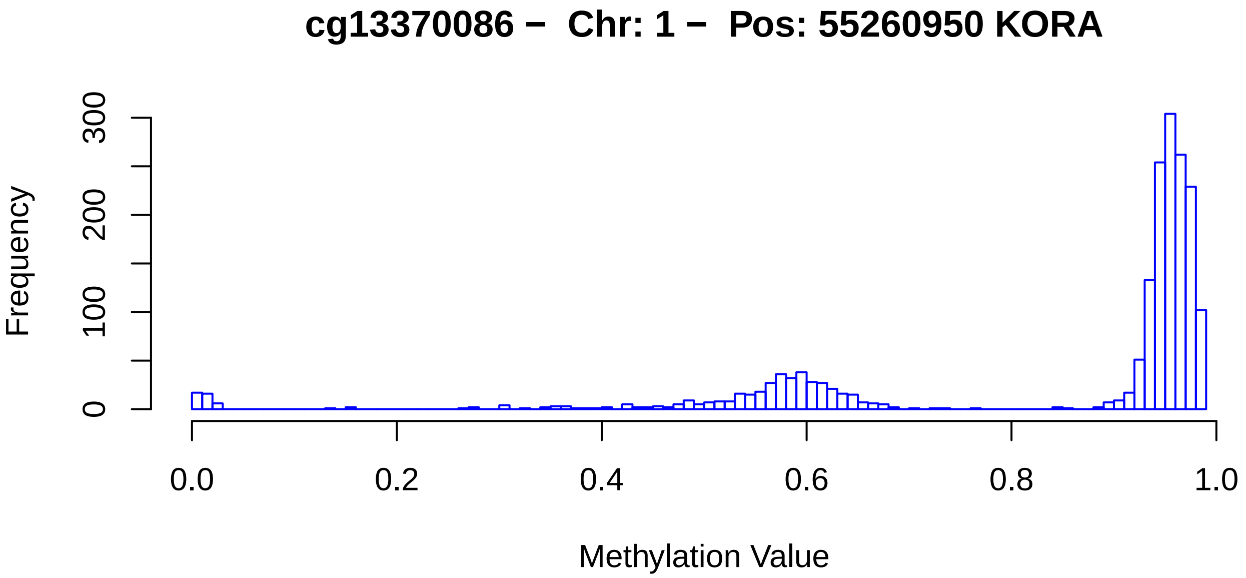
cg13370086 − Chr: 1 − Pos: 55260950 KORA
Frequency
0
100
200
300
0.0
0.2
0.4
0.6
0.8
1.0
Methylation Value

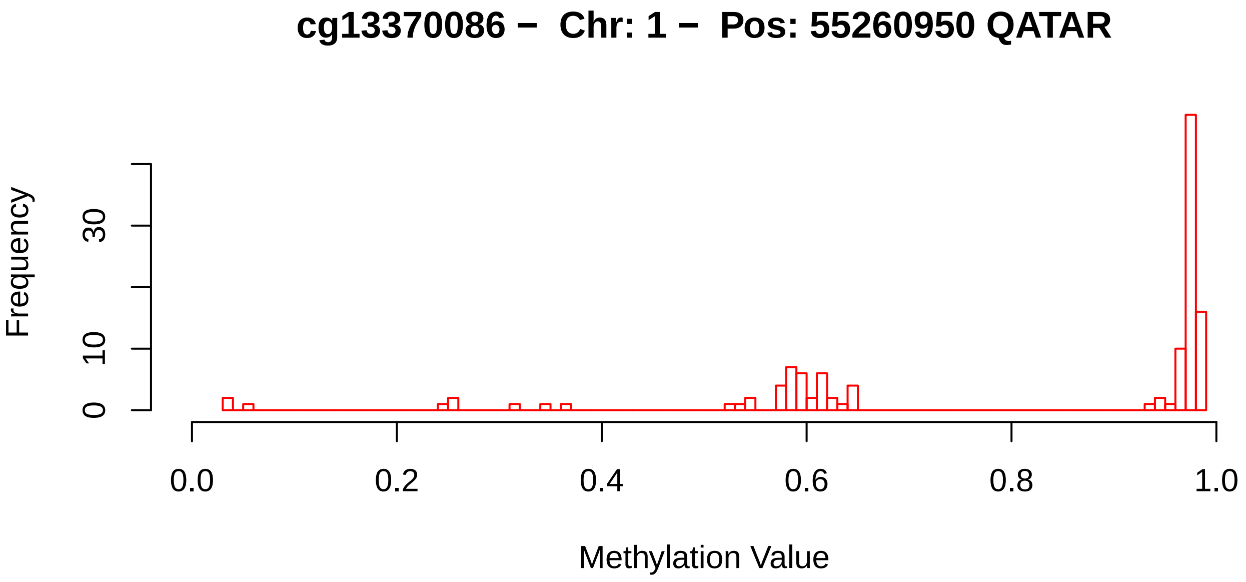
cg13370086 − Chr: 1 − Pos: 55260950 QATAR
Frequency
0
10
30
0.0
0.2
0.4
0.6
0.8
1.0
Methylation Value

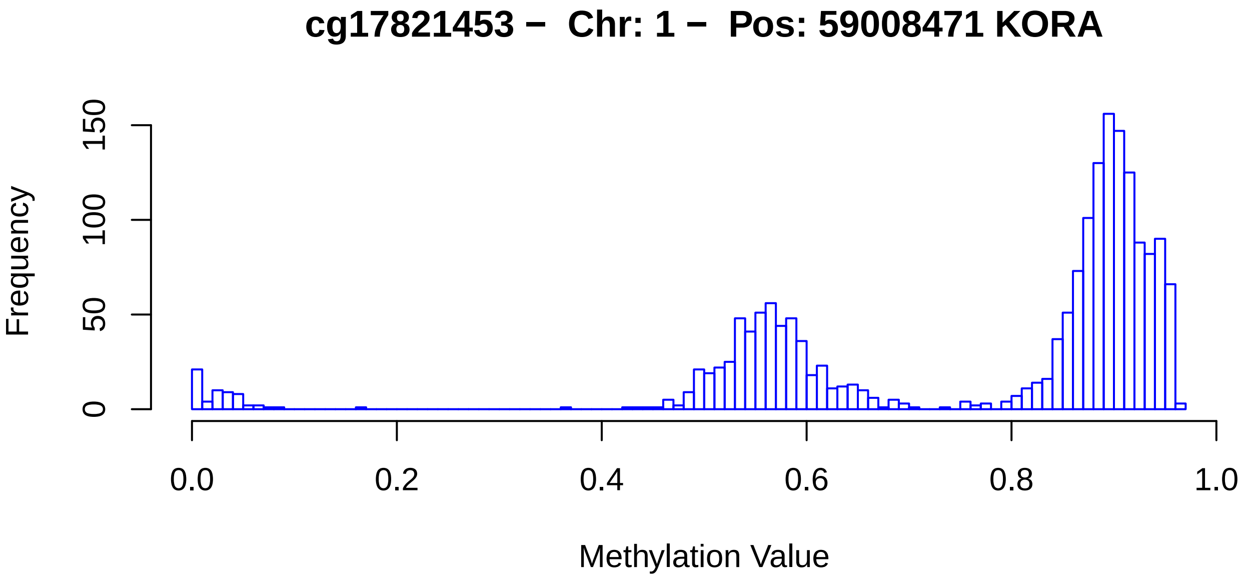
cg17821453 − Chr: 1 − Pos: 59008471 KORA
Frequency
0
50
100
150
0.0
0.2
0.4
0.6
0.8
1.0
Methylation Value

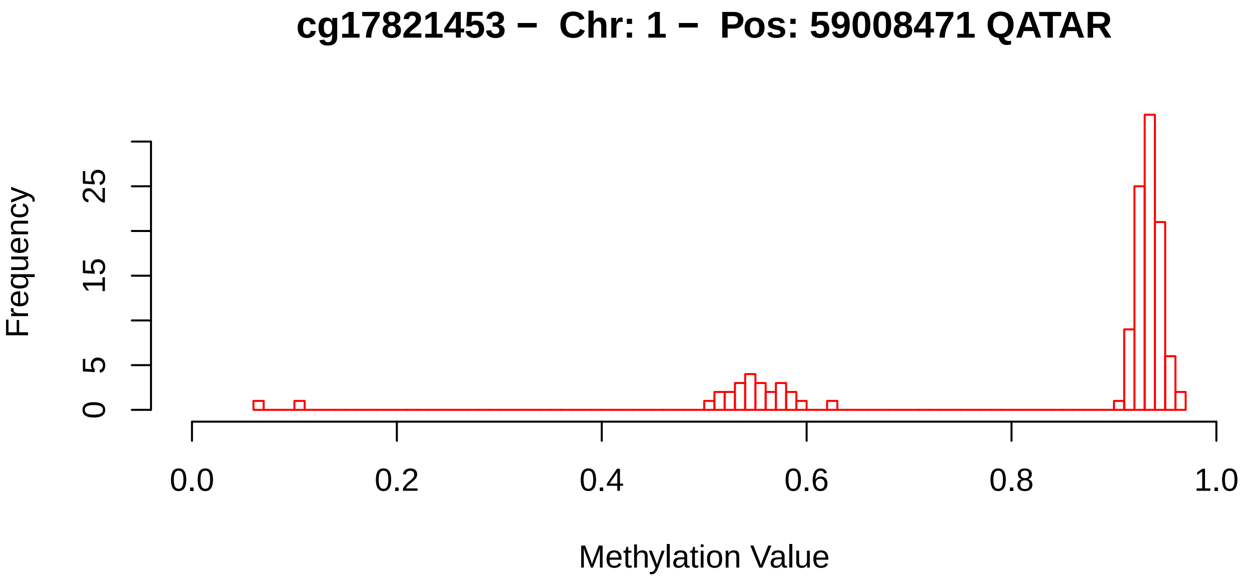
cg17821453 − Chr: 1 − Pos: 59008471 QATAR
Frequency
0
5
15
25
0.0
0.2
0.4
0.6
0.8
1.0
Methylation Value

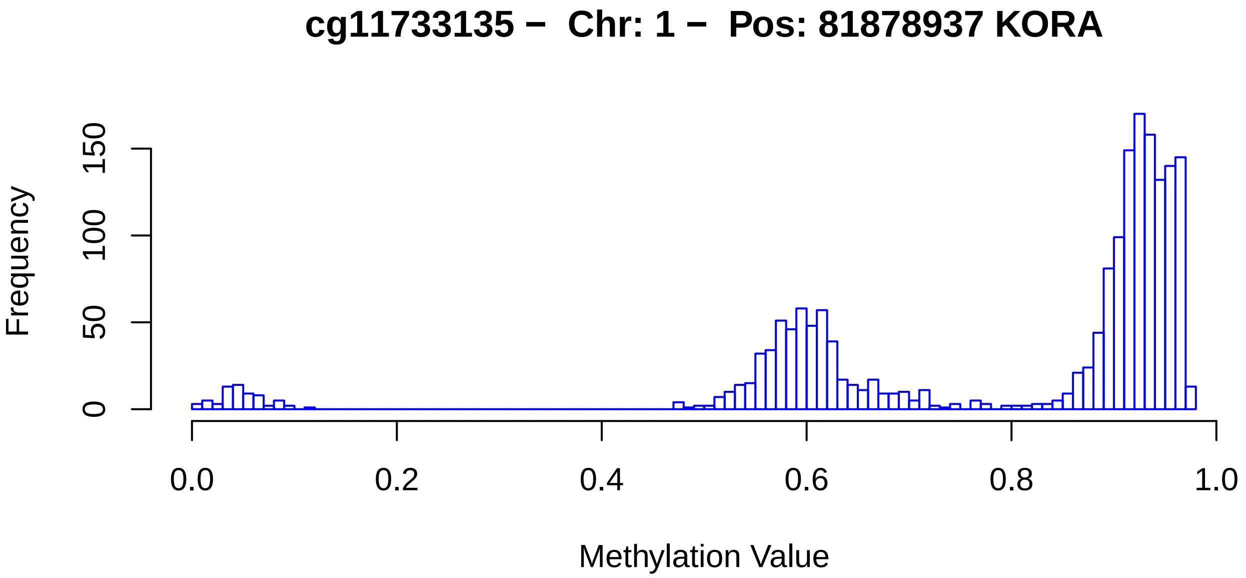
cg11733135 − Chr: 1 − Pos: 81878937 KORA
Frequency
0
50
100
150
0.0
0.2
0.4
0.6
0.8
1.0
Methylation Value

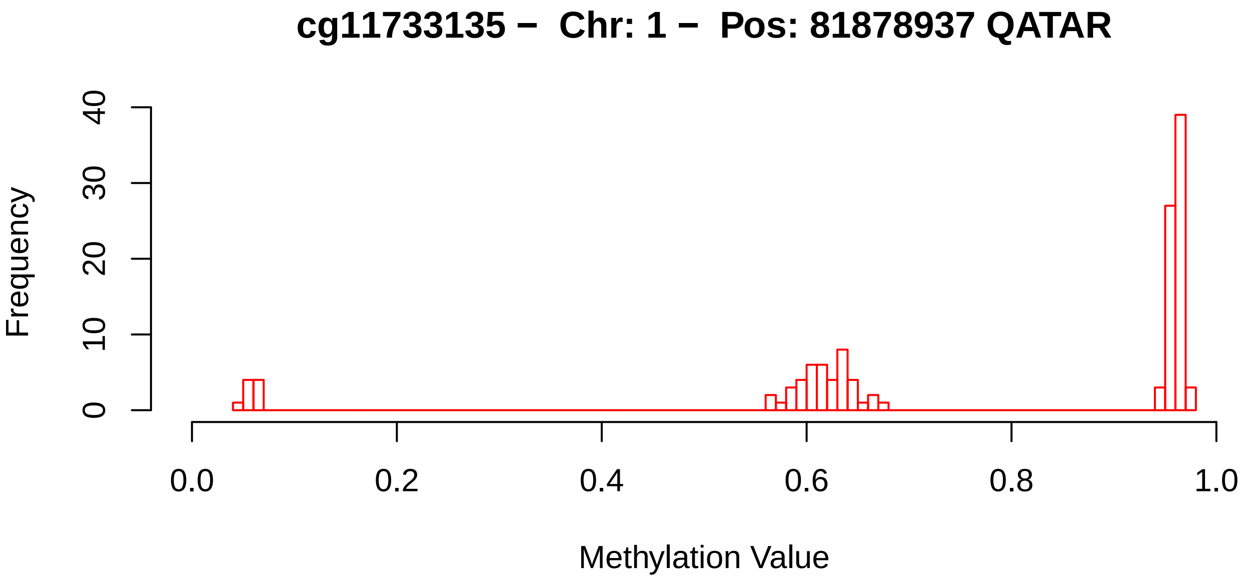
cg11733135 − Chr: 1 − Pos: 81878937 QATAR
Frequency
0
10
20
30
40
0.0
0.2
0.4
0.6
0.8
1.0
Methylation Value

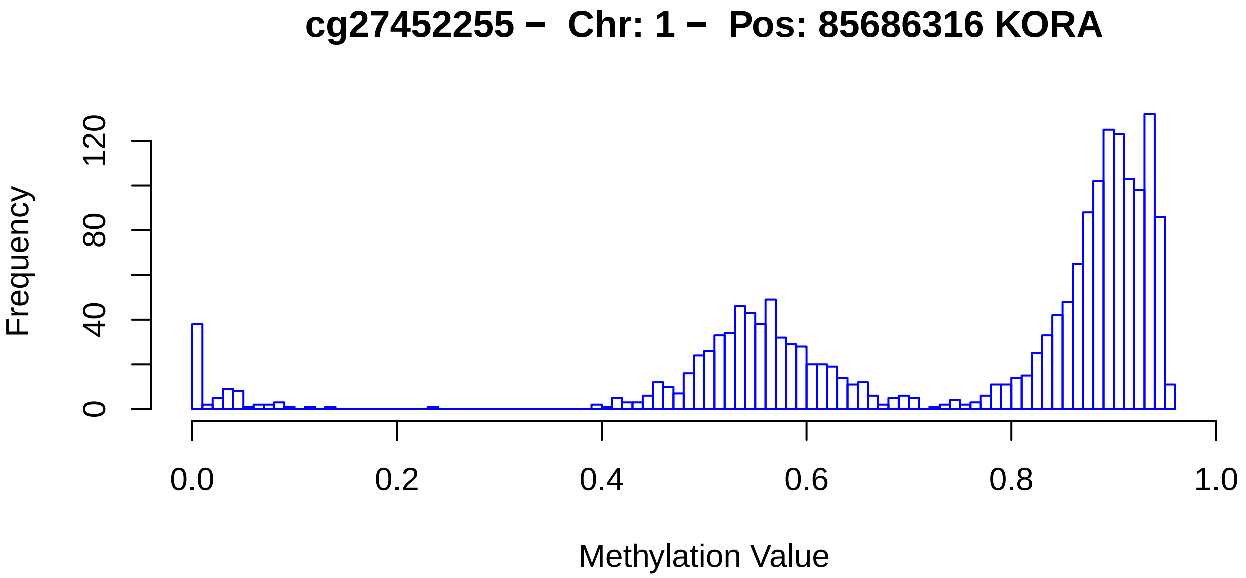
cg27452255 − Chr: 1 − Pos: 85686316 KORA
Frequency
0
40
80
120
0.0
0.2
0.4
0.6
0.8
1.0
Methylation Value

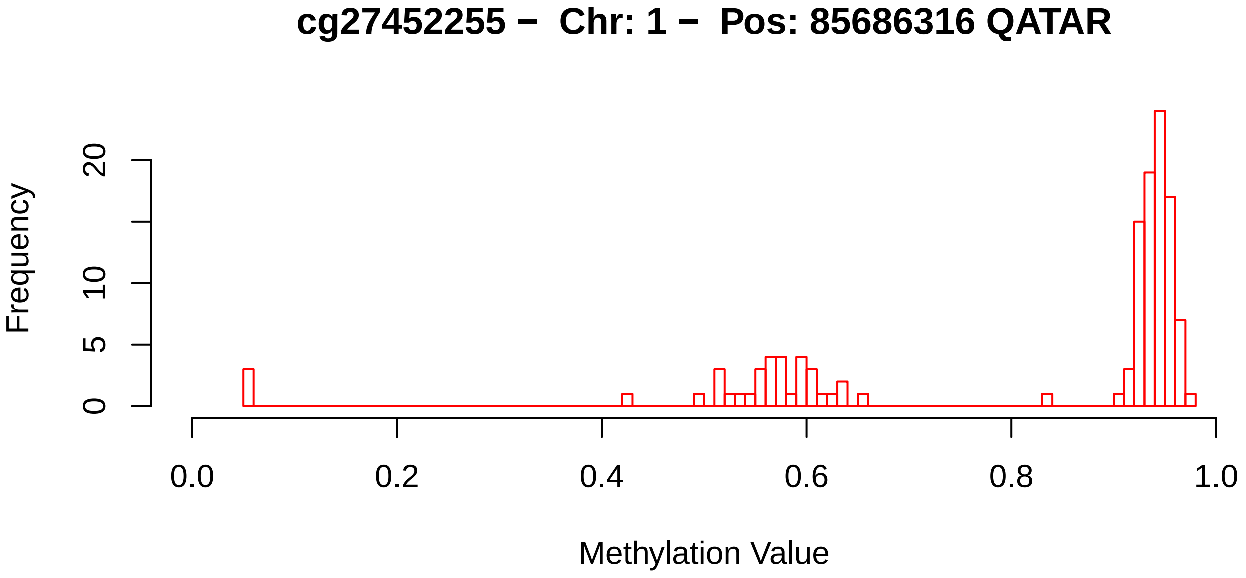
cg27452255 − Chr: 1 − Pos: 85686316 QATAR
Frequency
0
5
10
20
0.0
0.2
0.4
0.6
0.8
1.0
Methylation Value

cg13078798 − Chr: 1 − Pos: 92203667 KORA

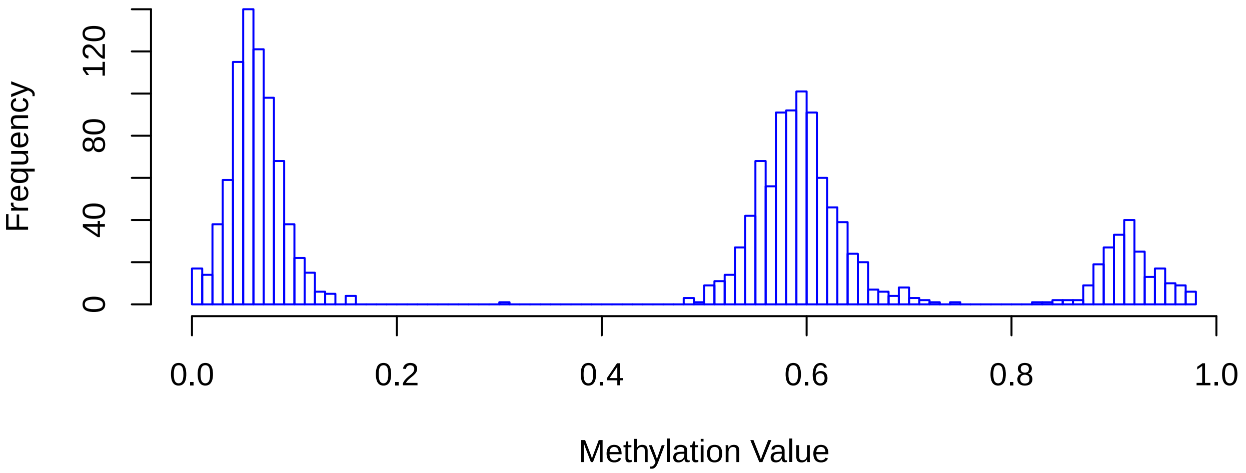

cg13078798 − Chr: 1 − Pos: 92203667 QATAR

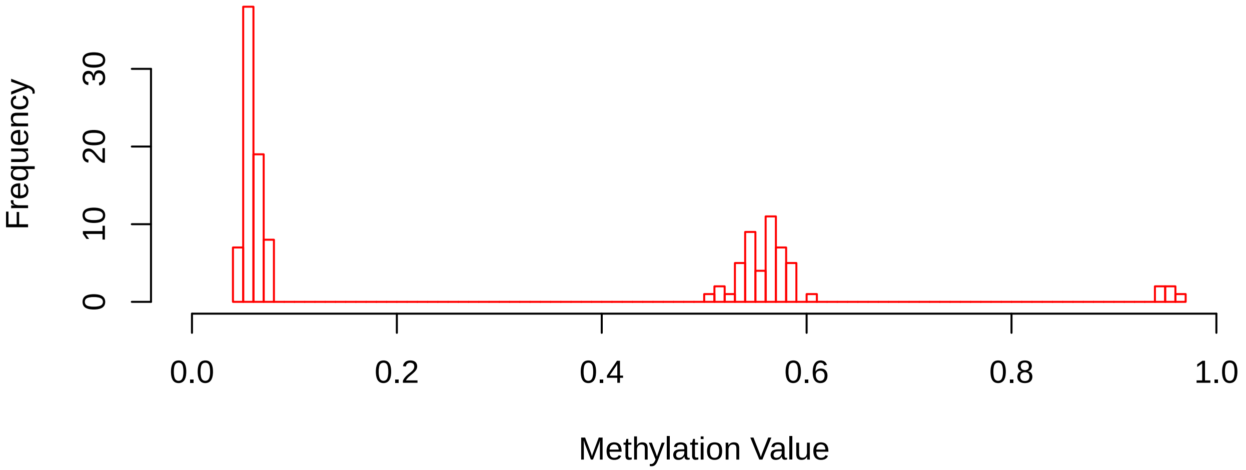

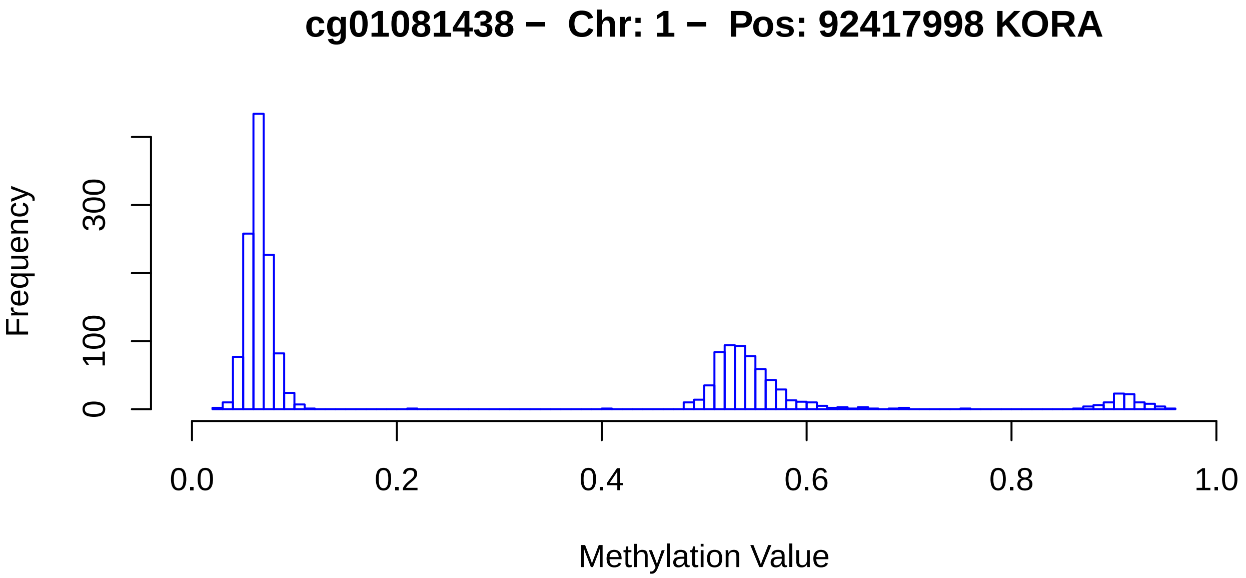

cg01081438 − Chr: 1 − Pos: 92417998 KORA
Frequency
0
100
300
0.0
0.2
0.4
0.6
0.8
1.0
Methylation Value

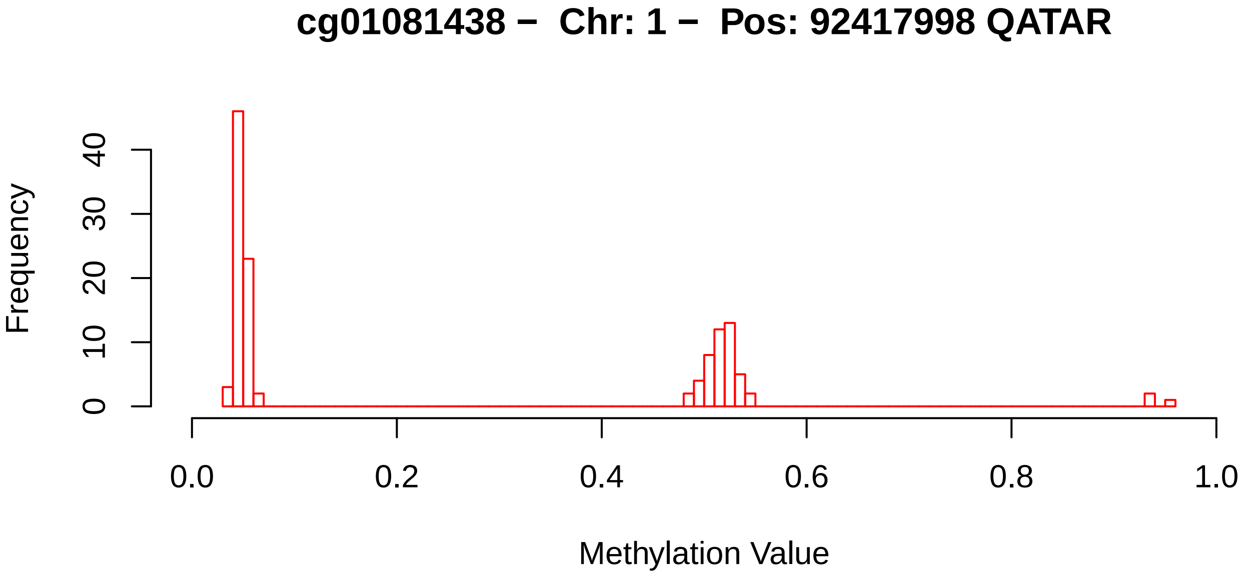

cg01081438 − Chr: 1 − Pos: 92417998 QATAR
Frequency
0
10
20
30
40
0.0
0.2
0.4
0.6
0.8
1.0
Methylation Value

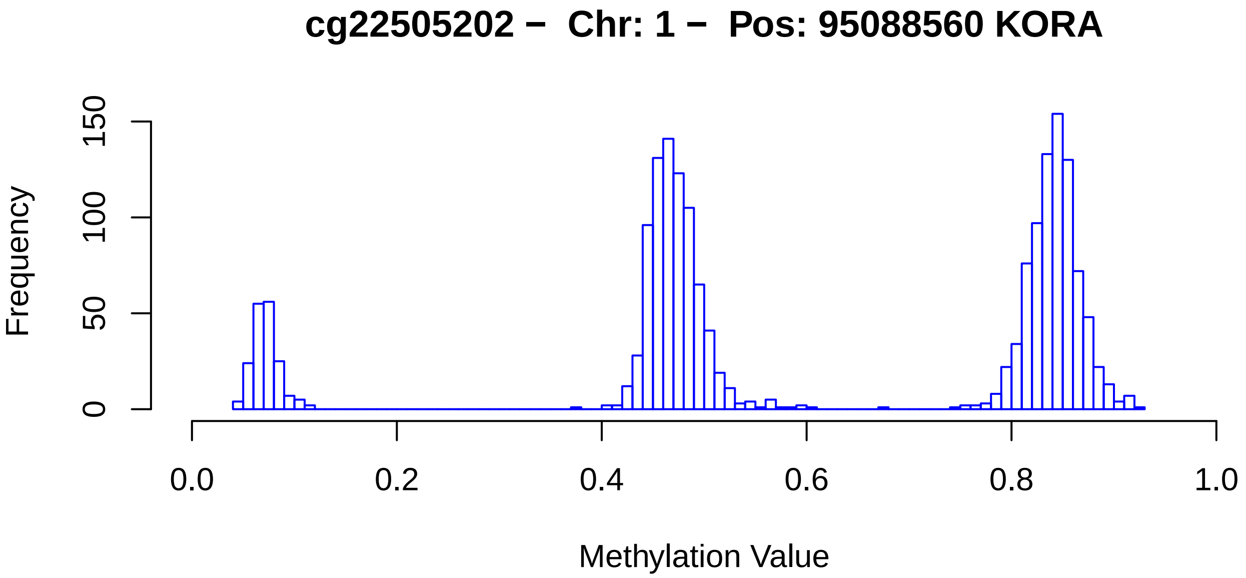
cg22505202 − Chr: 1 − Pos: 95088560 KORA
Frequency
0
50
100
150
0.0
0.2
0.4
0.6
0.8
1.0
Methylation Value

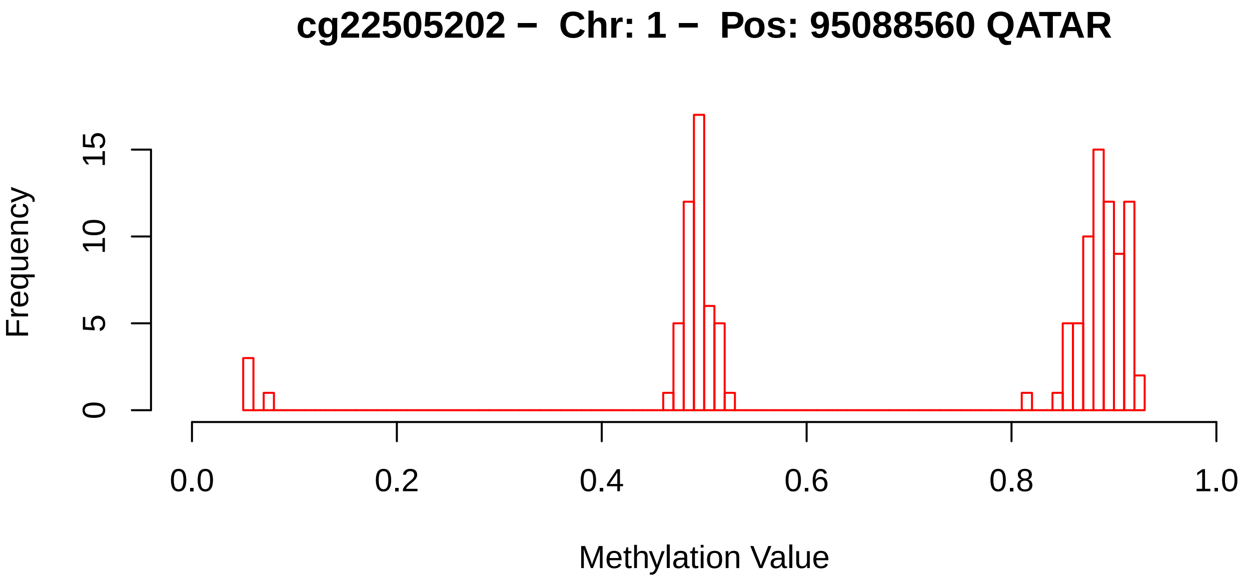
cg22505202 − Chr: 1 − Pos: 95088560 QATAR
Frequency
0
5
10
15
0.0
0.2
0.4
0.6
0.8
1.0
Methylation Value

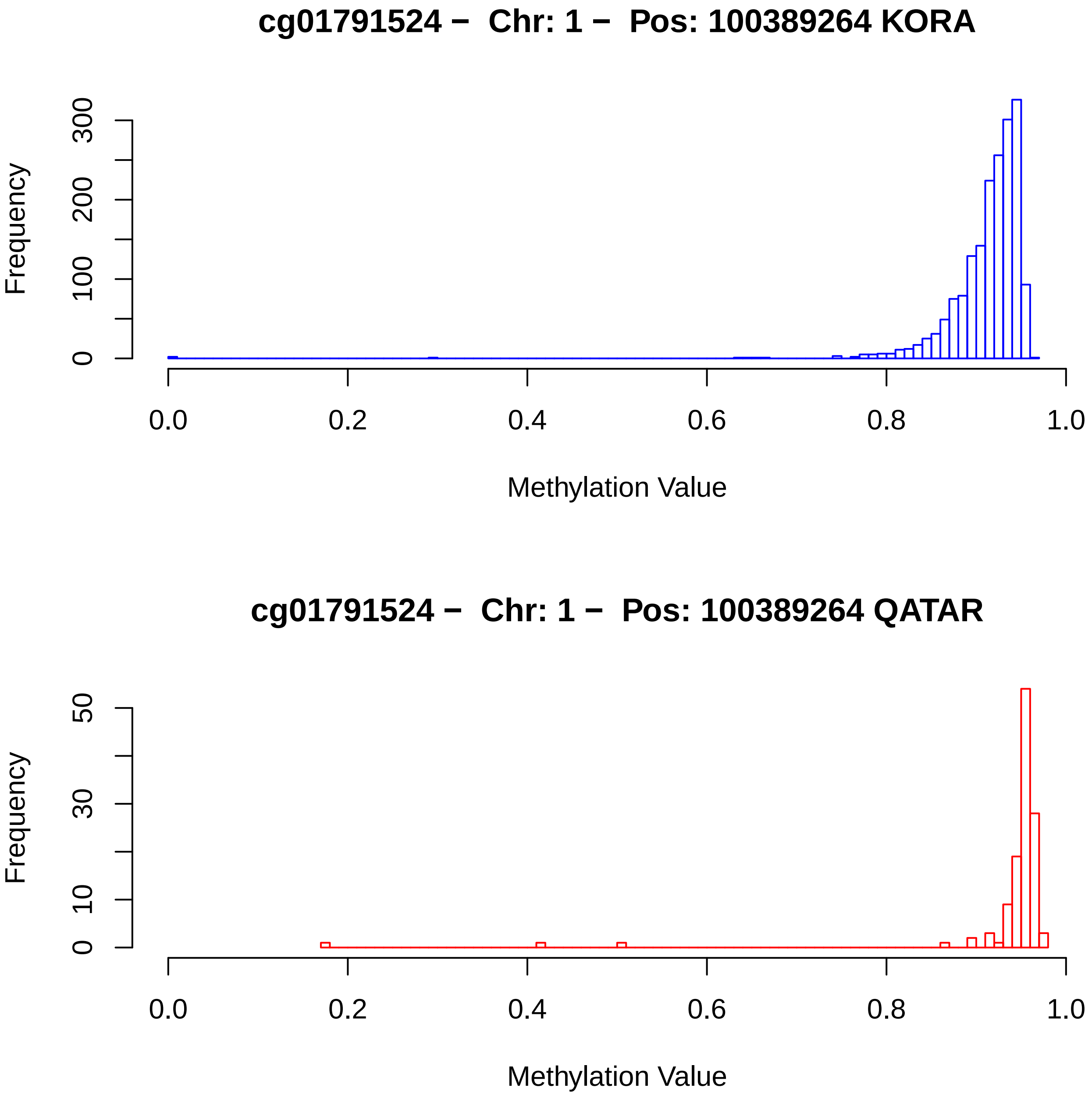
cg01791524 − Chr: 1 − Pos: 100389264 KORA
Frequency
0
100
200
300
0.0
0.2
0.4
0.6
0.8
1.0
Methylation Value
cg01791524 − Chr: 1 − Pos: 100389264 QATAR
Frequency
0
10
30
50
0.0
0.2
0.4
0.6
0.8
1.0
Methylation Value

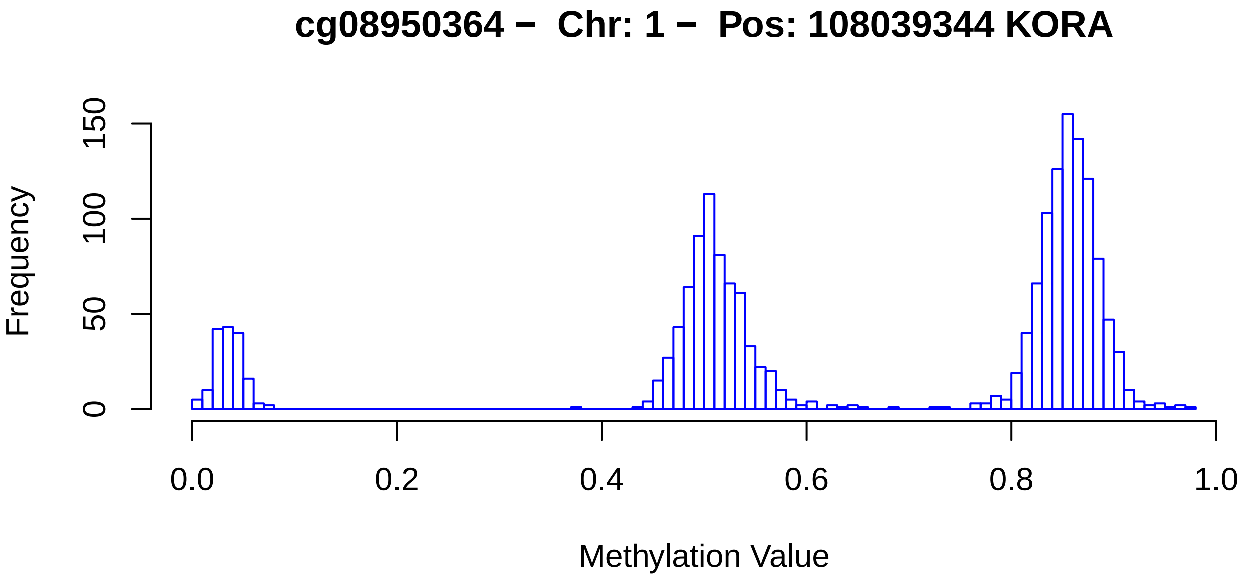
cg08950364 − Chr: 1 − Pos: 108039344 KORA
Frequency
0
50
100
150
0.0
0.2
0.4
0.6
0.8
1.0
Methylation Value

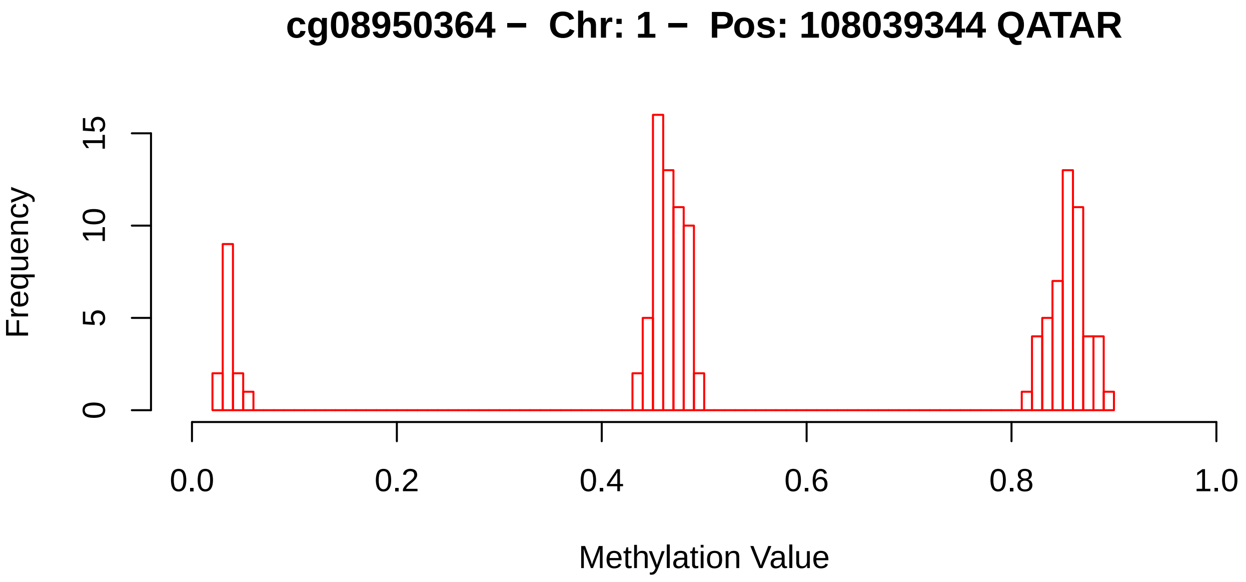
cg08950364 − Chr: 1 − Pos: 108039344 QATAR
Frequency
0
5
10
15
0.0
0.2
0.4
0.6
0.8
1.0
Methylation Value

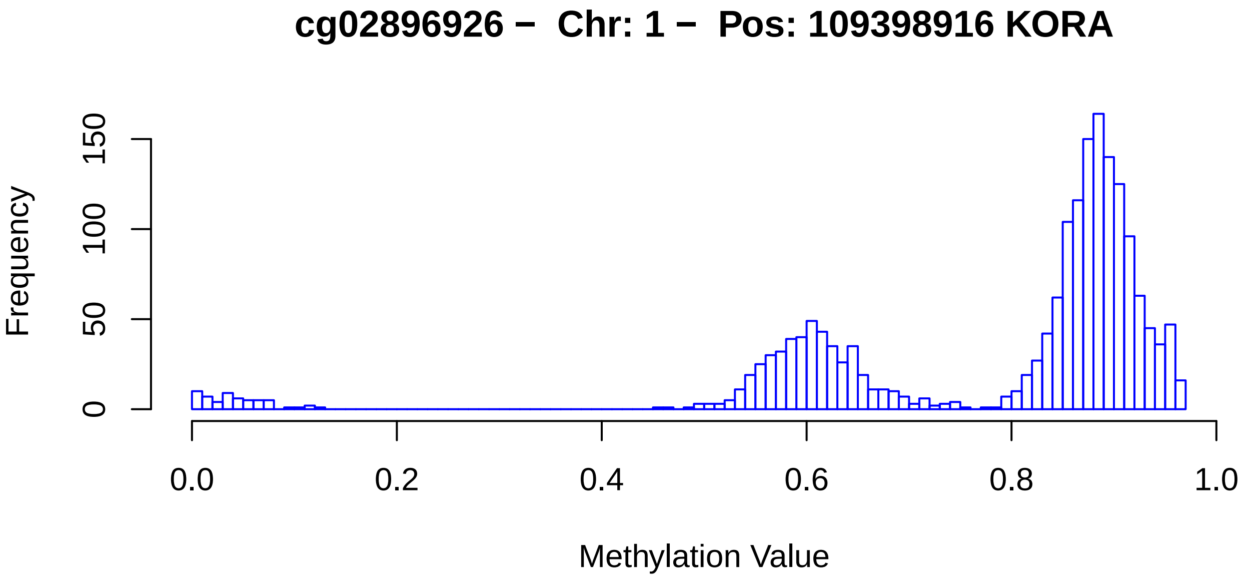
cg02896926 − Chr: 1 − Pos: 109398916 KORA
Frequency
0
50
100
150
0.0
0.2
0.4
0.6
0.8
1.0
Methylation Value

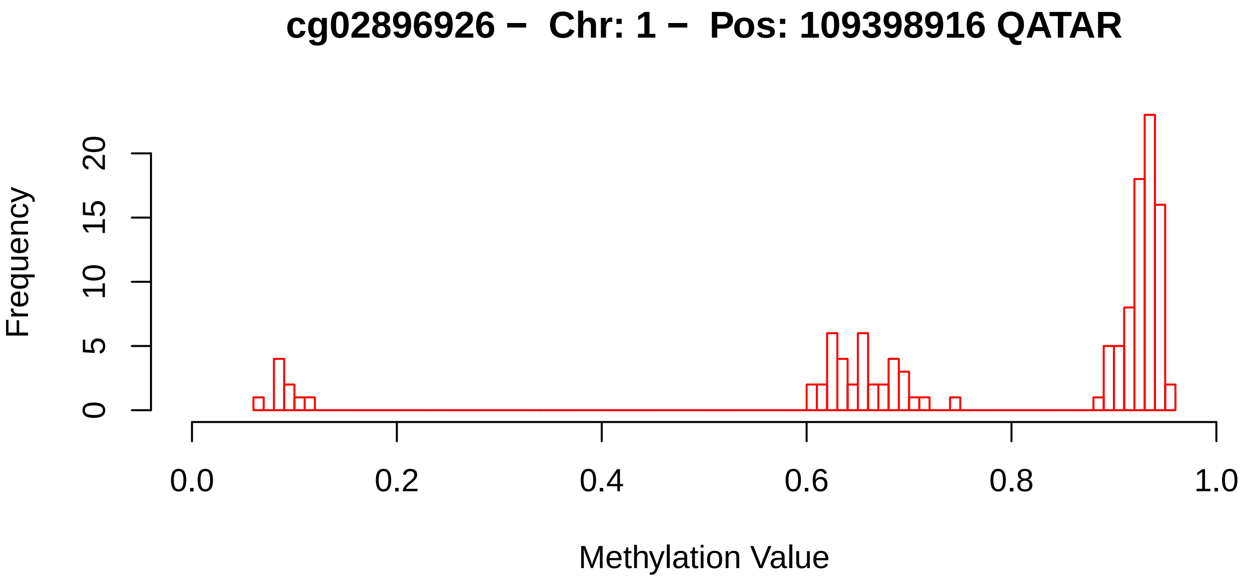
cg02896926 − Chr: 1 − Pos: 109398916 QATAR
Frequency
0
5
10
15
20
0.0
0.2
0.4
0.6
0.8
1.0
Methylation Value

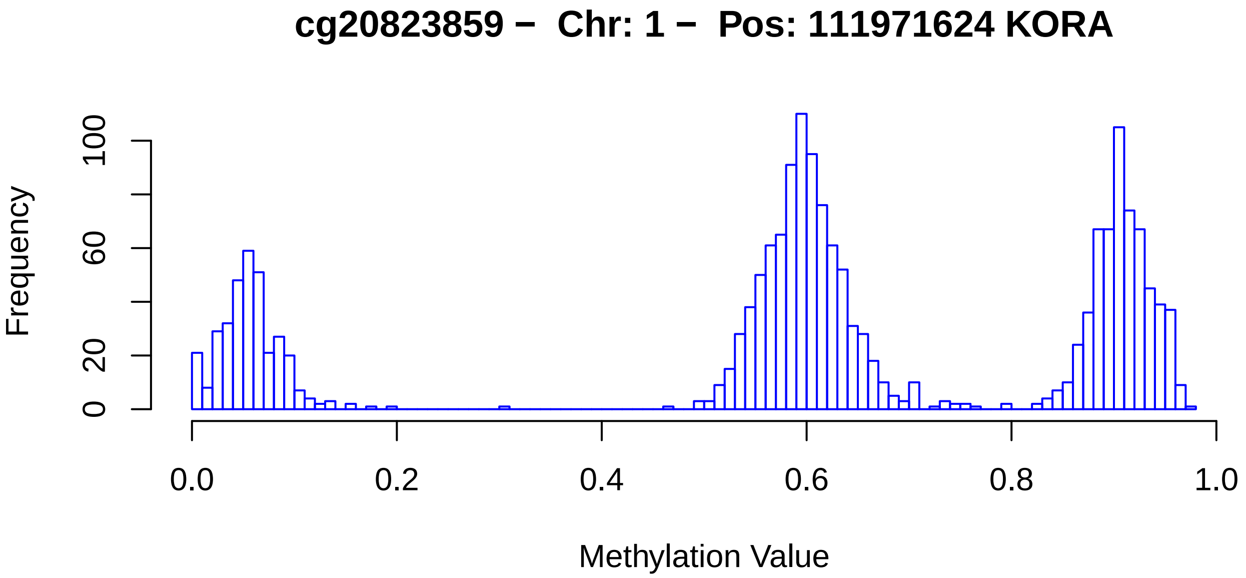
cg20823859 − Chr: 1 − Pos: 111971624 KORA
Frequency
0
20
60
100
0.0
0.2
0.4
0.6
0.8
1.0
Methylation Value

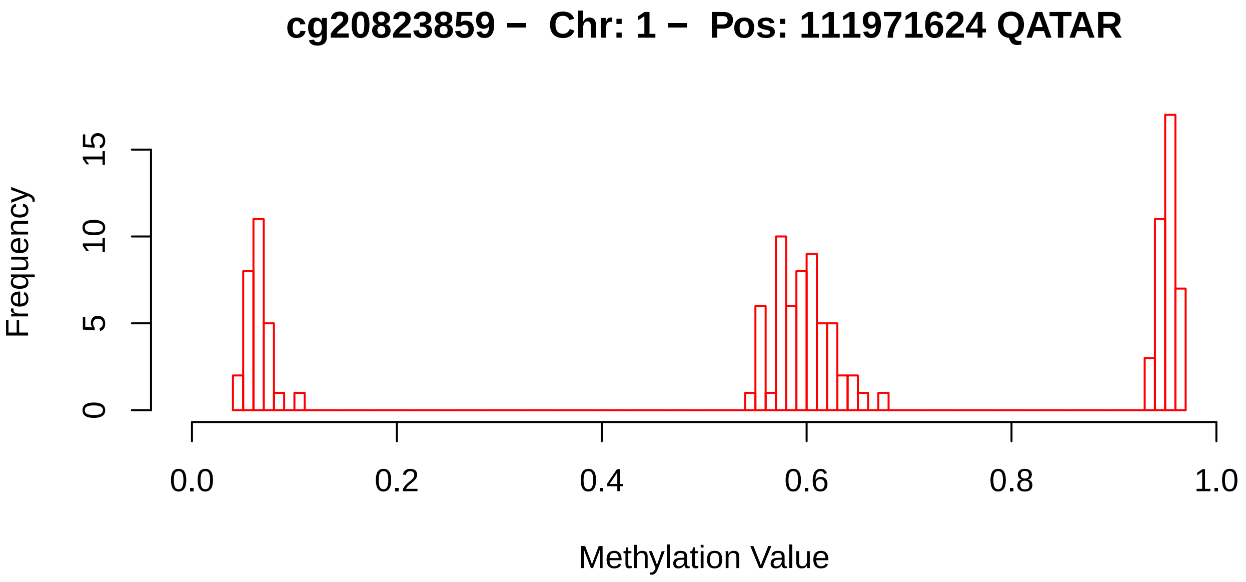
cg20823859 − Chr: 1 − Pos: 111971624 QATAR
Frequency
0
5
10
15
0.0
0.2
0.4
0.6
0.8
1.0
Methylation Value

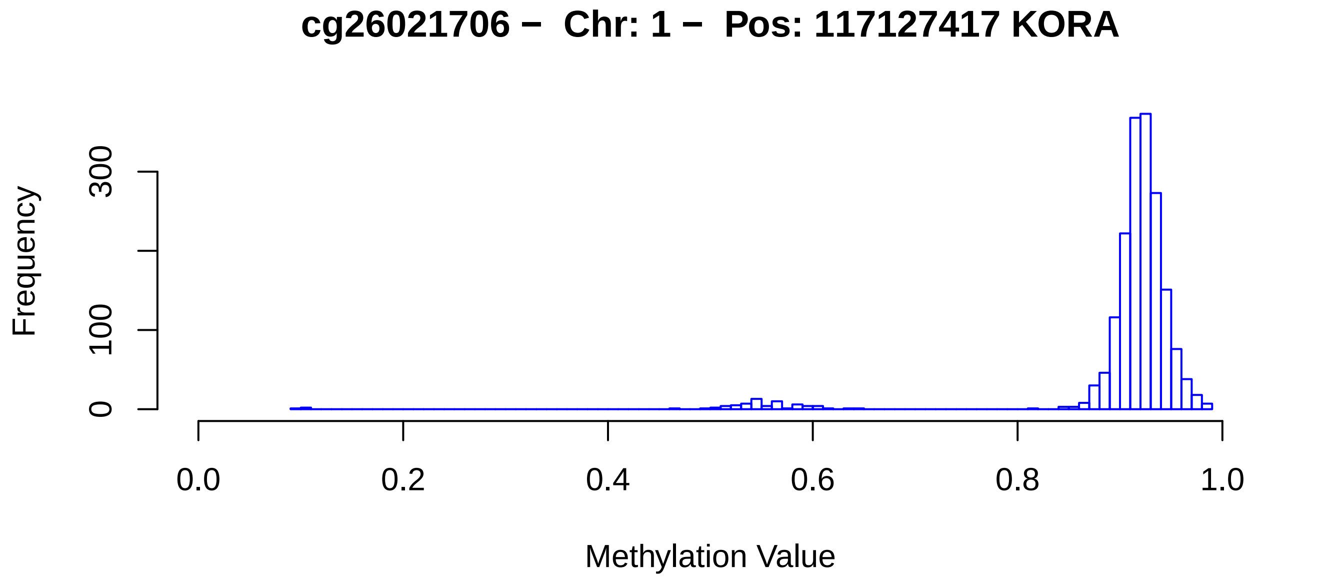
cg26021706 − Chr: 1 − Pos: 117127417 KORA
Frequency
0
100
300
0.0
0.2
0.4
0.6
0.8
1.0
Methylation Value

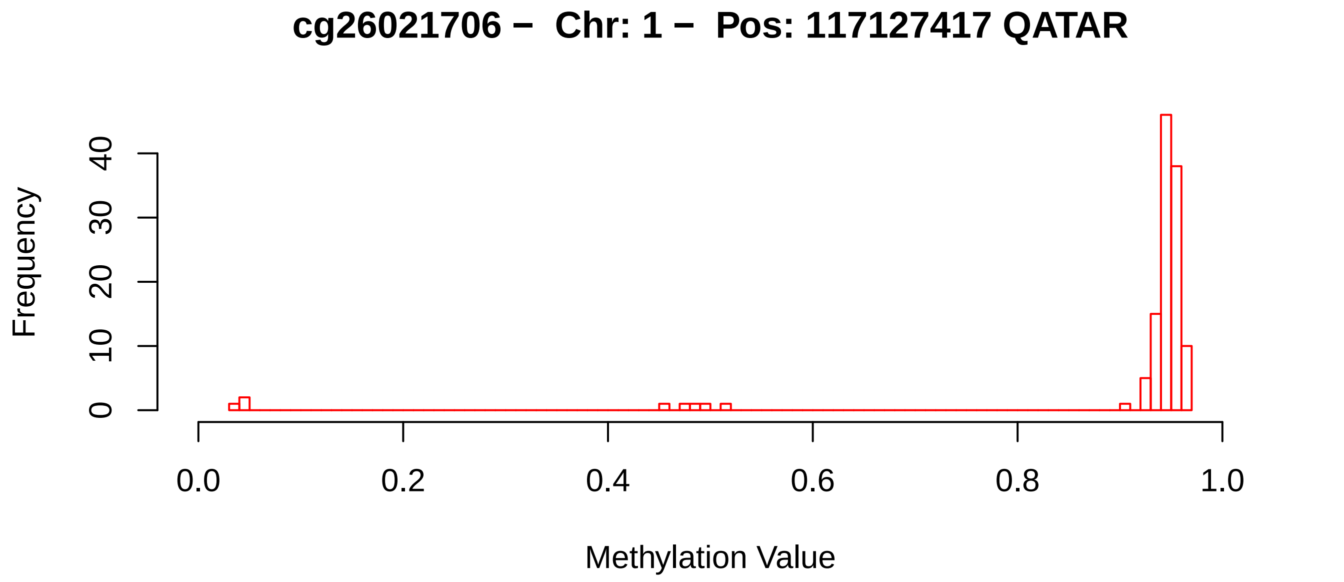
cg26021706 − Chr: 1 − Pos: 117127417 QATAR
Frequency
0
10
20
30
40
0.0
0.2
0.4
0.6
0.8
1.0
Methylation Value

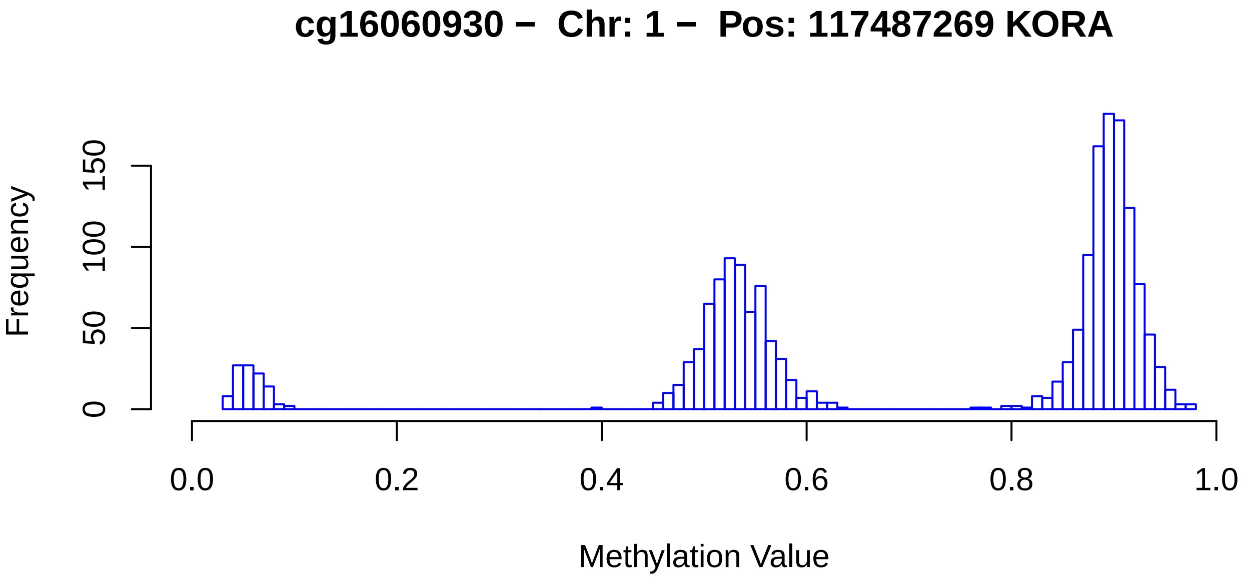
cg16060930 − Chr: 1 − Pos: 117487269 KORA
Frequency
0
50
100
150
0.0
0.2
0.4
0.6
0.8
1.0
Methylation Value

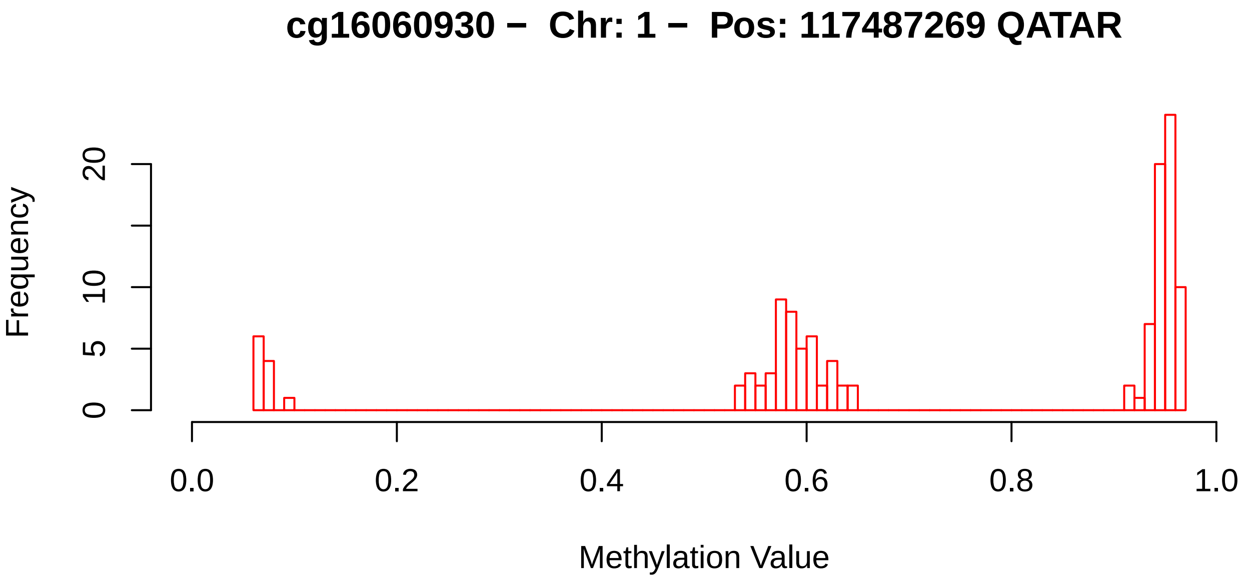
cg16060930 − Chr: 1 − Pos: 117487269 QATAR
Frequency
0
5
10
20
0.0
0.2
0.4
0.6
0.8
1.0
Methylation Value

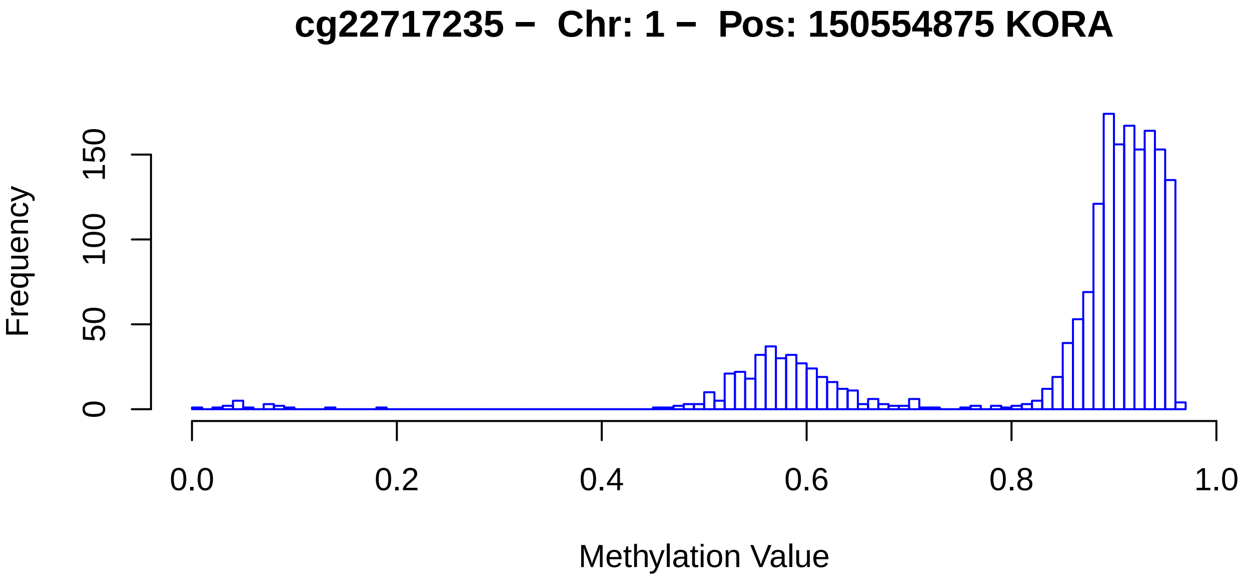
cg22717235 − Chr: 1 − Pos: 150554875 KORA
Frequency
0
50
100
150
0.0
0.2
0.4
0.6
0.8
1.0
Methylation Value

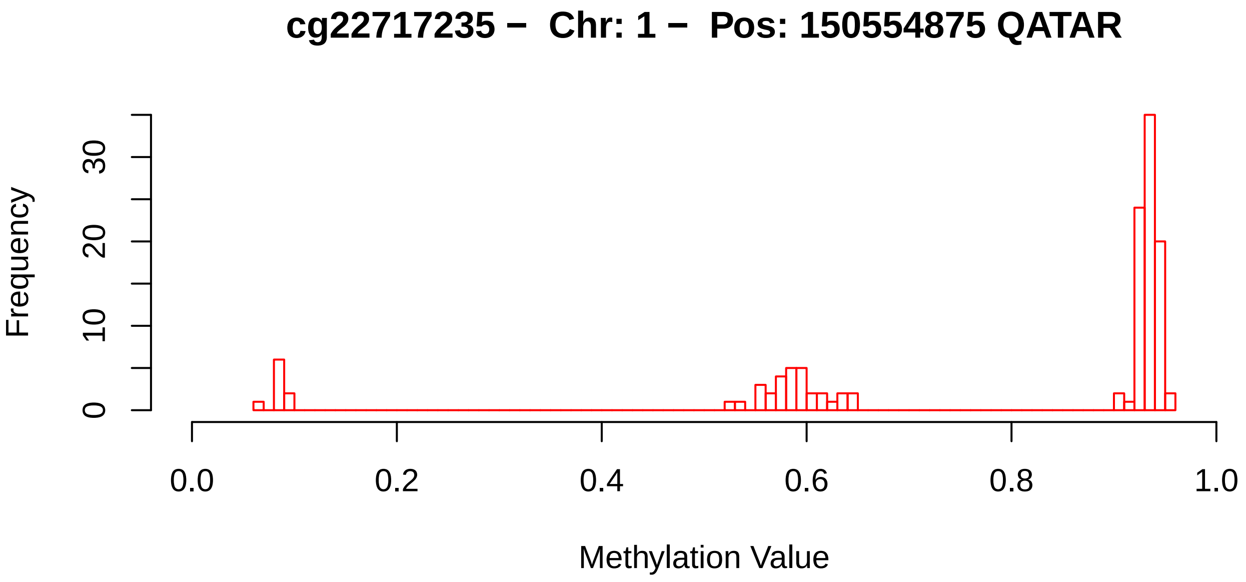
cg22717235 − Chr: 1 − Pos: 150554875 QATAR
Frequency
0
10
20
30
0.0
0.2
0.4
0.6
0.8
1.0
Methylation Value

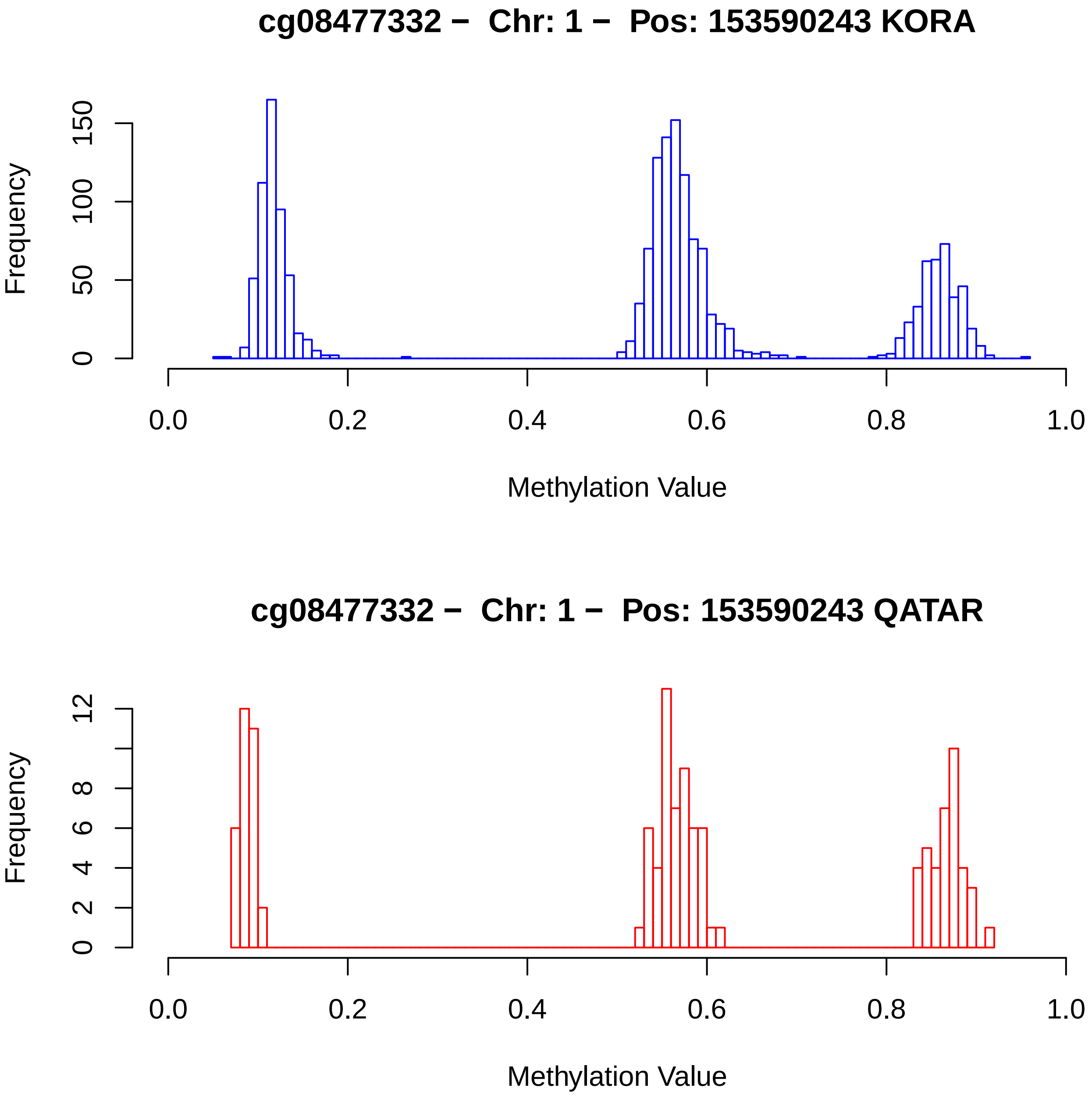
cg08477332 − Chr: 1 − Pos: 153590243 KORA
Frequency
0
50
100
150
0.0
0.2
0.4
0.6
0.8
1.0
Methylation Value
cg08477332 − Chr: 1 − Pos: 153590243 QATAR
Frequency
0
2
4
6
8
12
0.0
0.2
0.4
0.6
0.8
1.0
Methylation Value

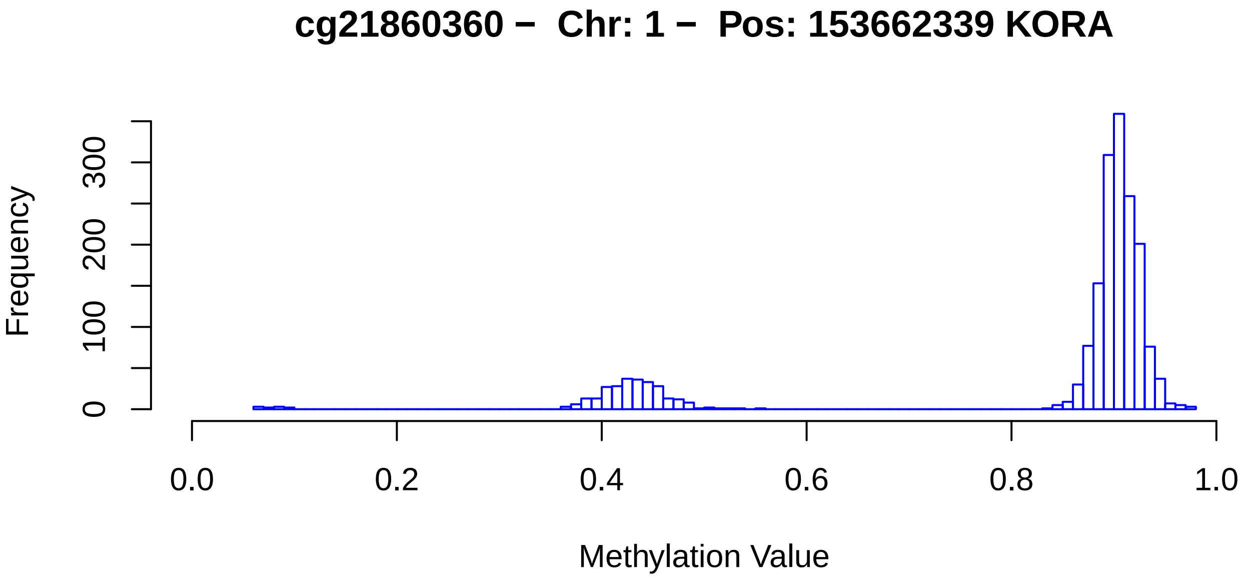

cg21860360 − Chr: 1 − Pos: 153662339 KORA
Frequency
0
100
200
300
0.0
0.2
0.4
0.6
0.8
1.0
Methylation Value

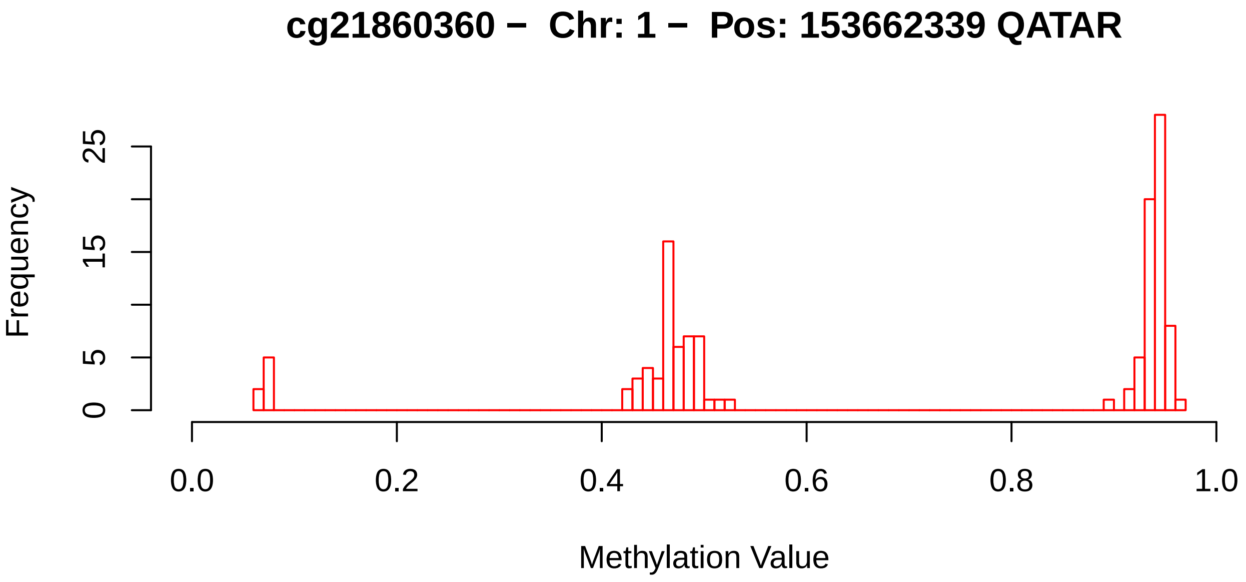

cg21860360 − Chr: 1 − Pos: 153662339 QATAR
Frequency
0
5
15
25
0.0
0.2
0.4
0.6
0.8
1.0
Methylation Value

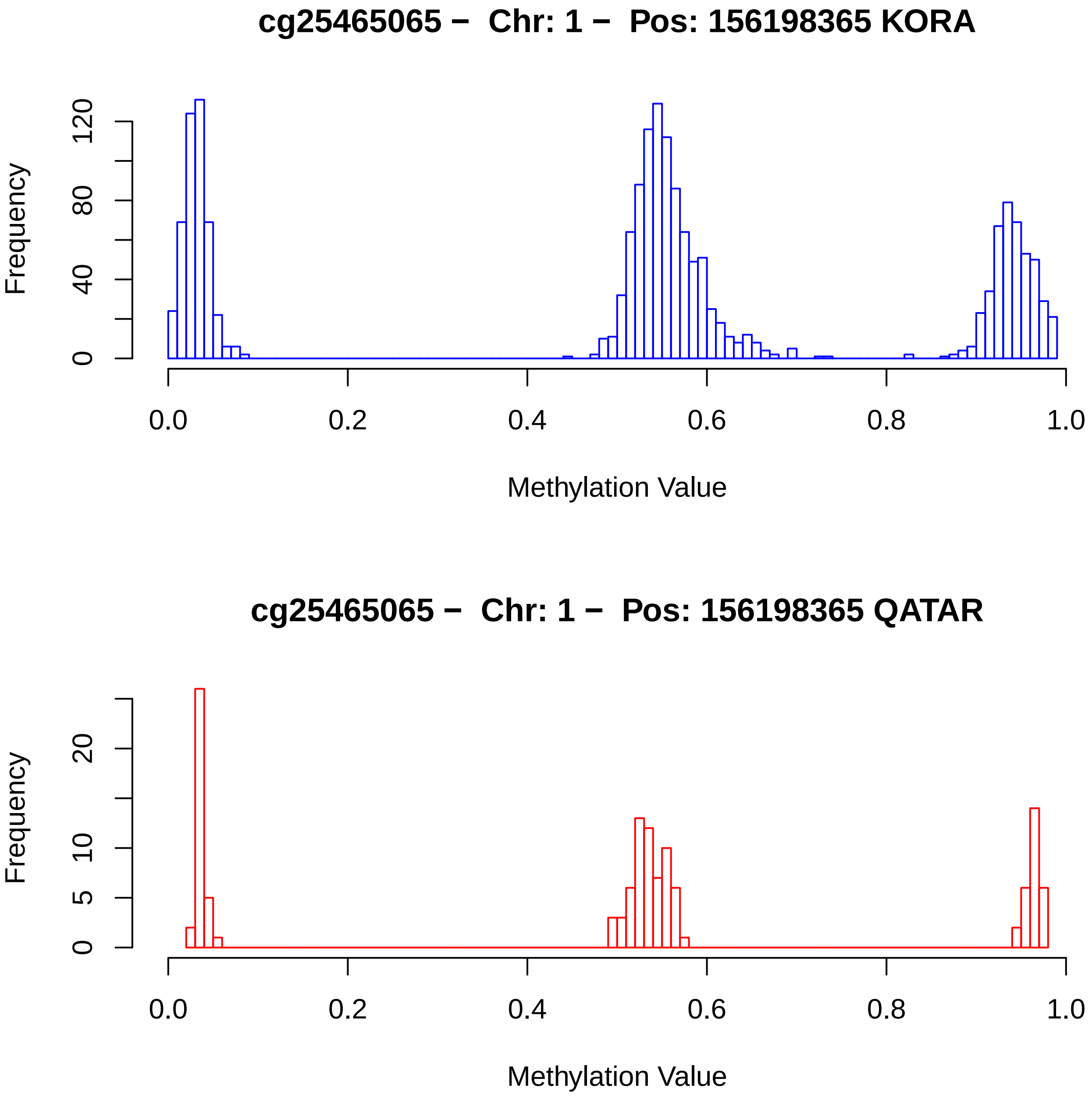
cg25465065 − Chr: 1 − Pos: 156198365 KORA
Frequency
0
40
80
120
0.0
0.2
0.4
0.6
0.8
1.0
Methylation Value
cg25465065 − Chr: 1 − Pos: 156198365 QATAR
Frequency
0
5
10
20
0.0
0.2
0.4
0.6
0.8
1.0
Methylation Value

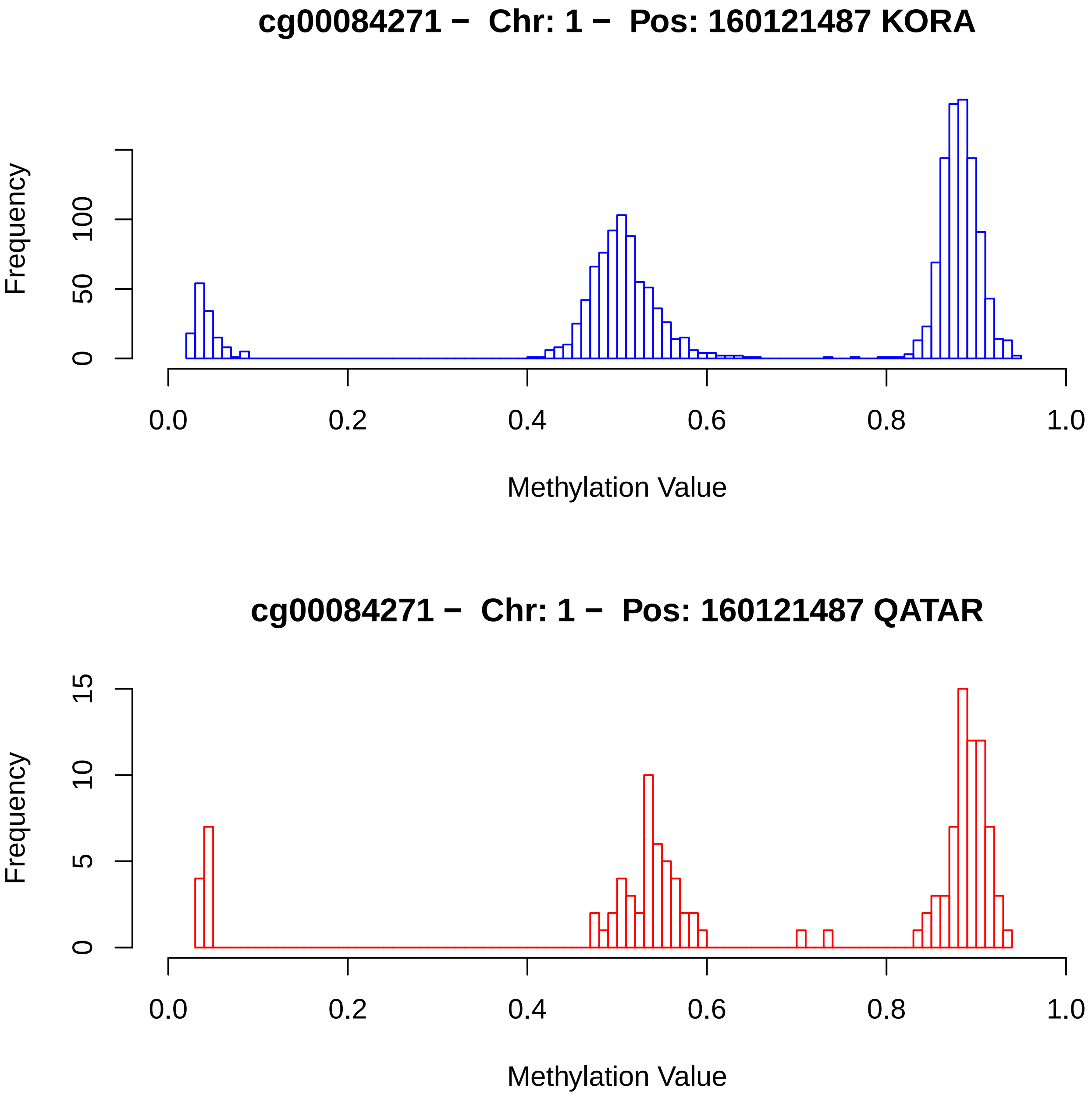

cg00084271 − Chr: 1 − Pos: 160121487 KORA
Frequency
0
50
100
0.0
0.2
0.4
0.6
0.8
1.0
Methylation Value
cg00084271 − Chr: 1 − Pos: 160121487 QATAR
Frequency
0
5
10
15
0.0
0.2
0.4
0.6
0.8
1.0
Methylation Value

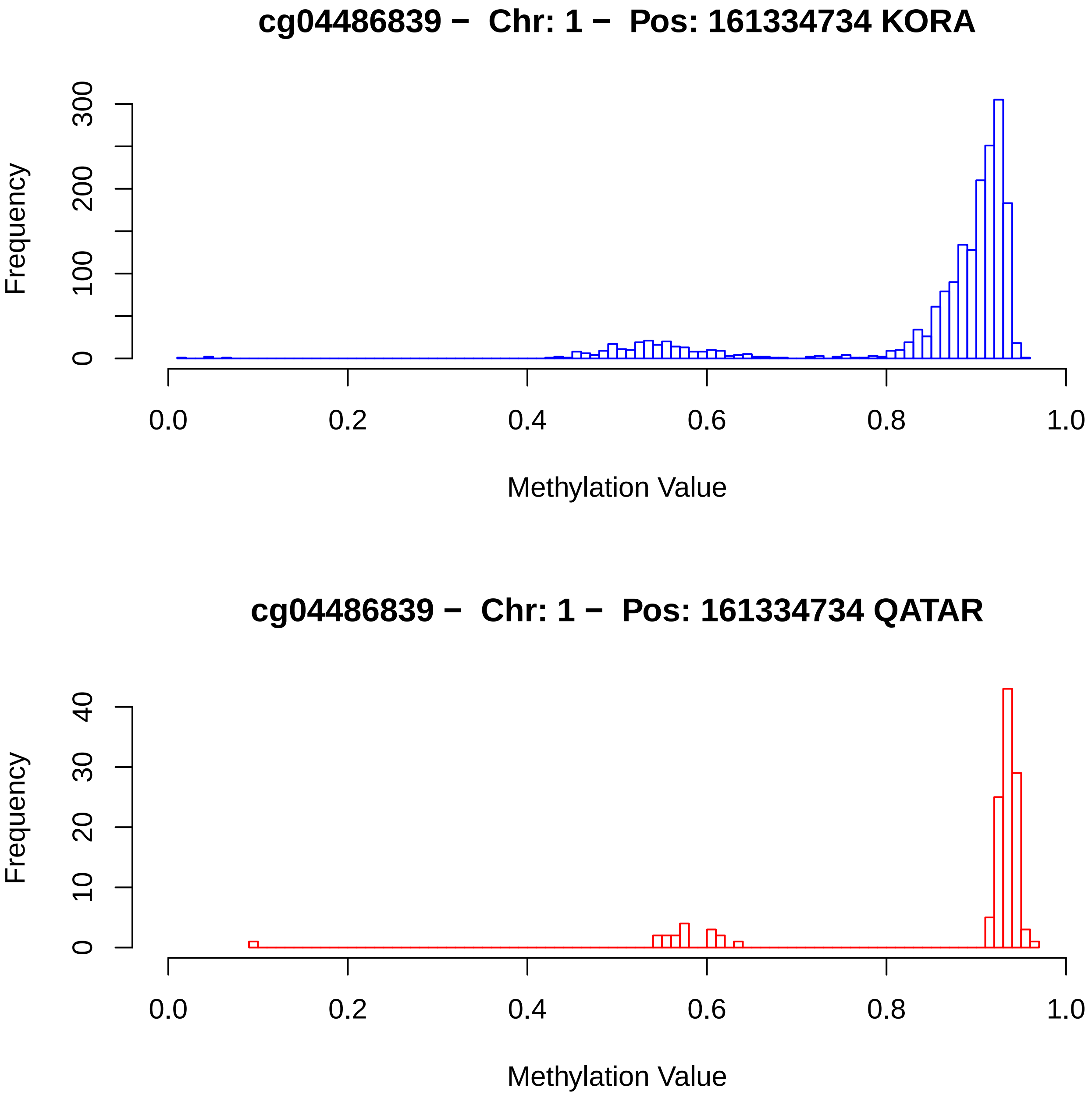
cg04486839 − Chr: 1 − Pos: 161334734 KORA
Frequency
0
100
200
300
0.0
0.2
0.4
0.6
0.8
1.0
Methylation Value
cg04486839 − Chr: 1 − Pos: 161334734 QATAR
Frequency
0
10
20
30
40
0.0
0.2
0.4
0.6
0.8
1.0
Methylation Value

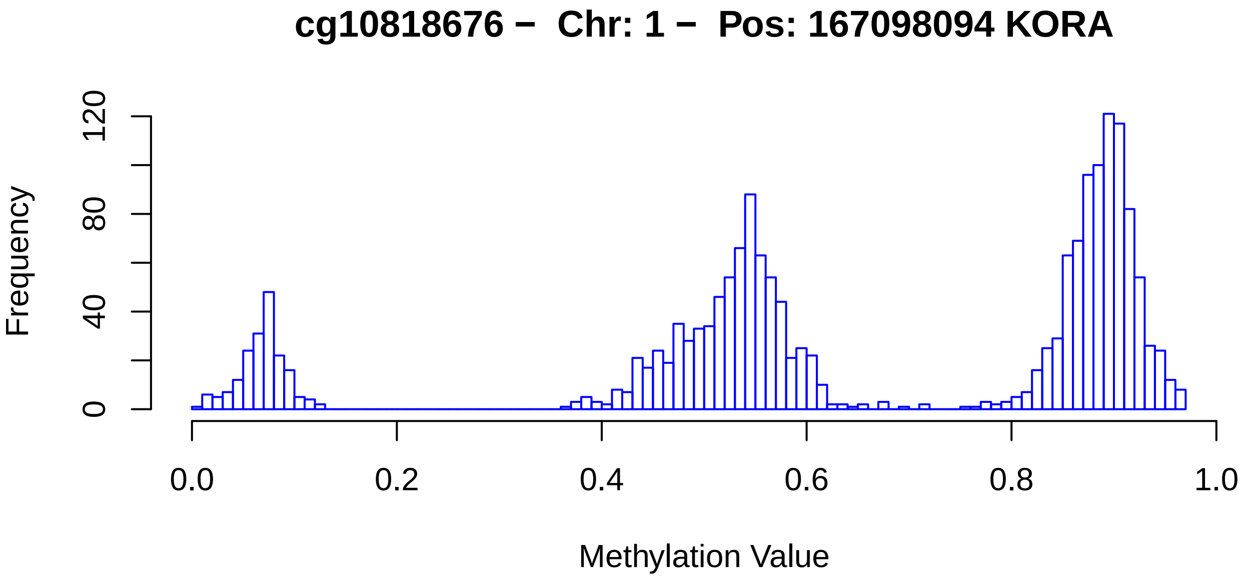
cg10818676 − Chr: 1 − Pos: 167098094 KORA
Frequency
0
40
80
120
0.0
0.2
0.4
0.6
0.8
1.0
Methylation Value

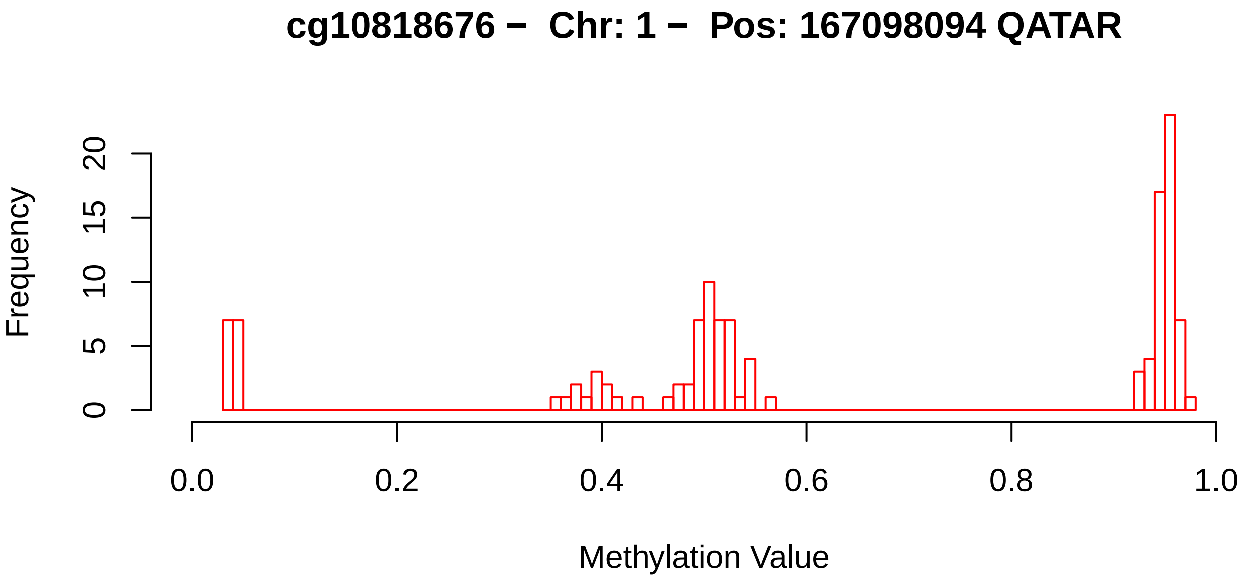
cg10818676 − Chr: 1 − Pos: 167098094 QATAR
Frequency
0
5
10
15
20
0.0
0.2
0.4
0.6
0.8
1.0
Methylation Value

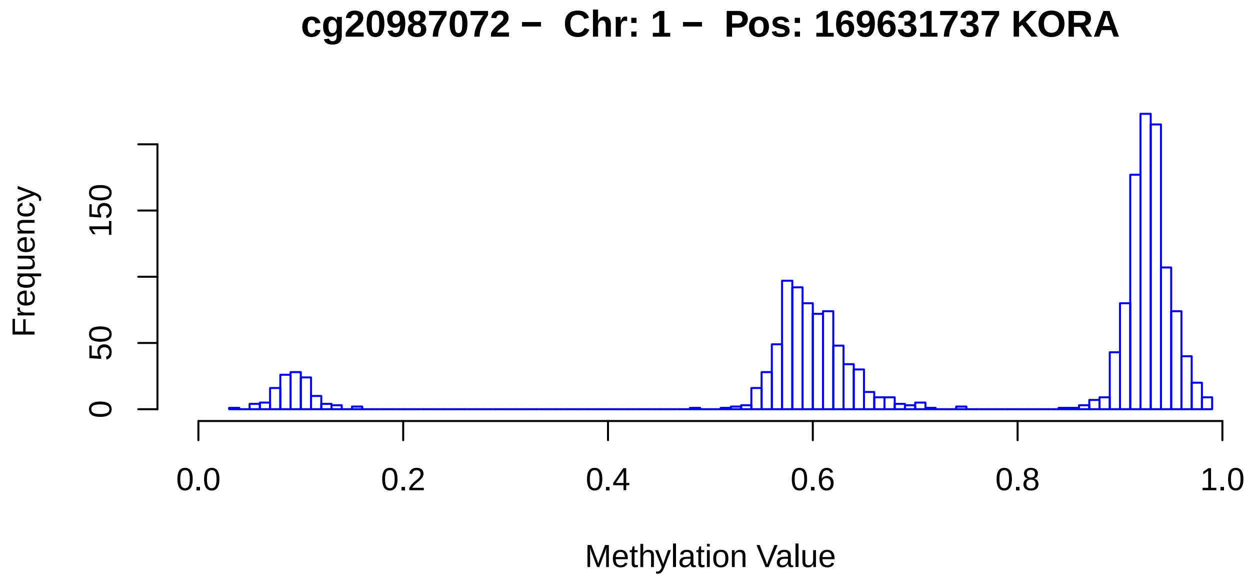
cg20987072 − Chr: 1 − Pos: 169631737 KORA
Frequency
0
50
150
0.0
0.2
0.4
0.6
0.8
1.0
Methylation Value

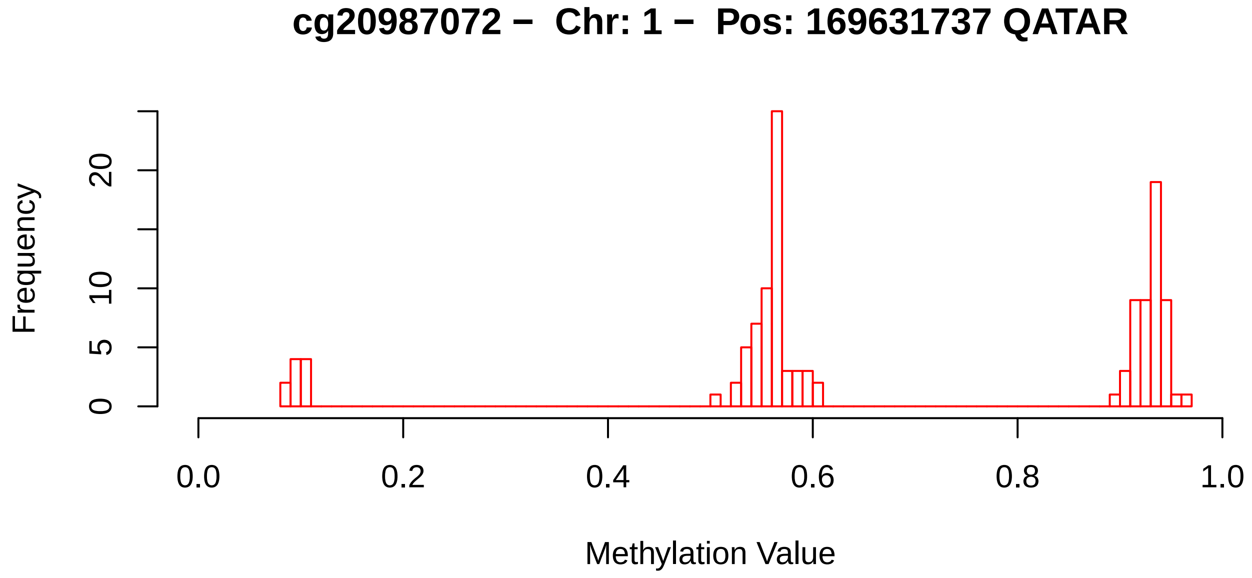
cg20987072 − Chr: 1 − Pos: 169631737 QATAR
Frequency
0
5
10
20
0.0
0.2
0.4
0.6
0.8
1.0
Methylation Value

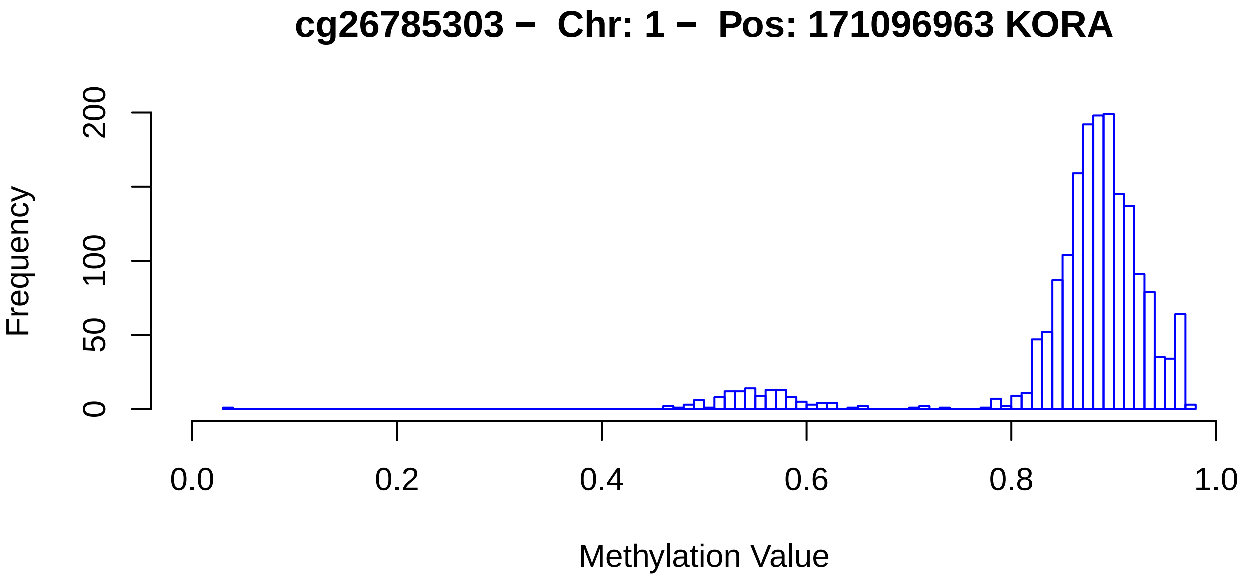

cg26785303 − Chr: 1 − Pos: 171096963 KORA
Frequency
0
50
100
200
0.0
0.2
0.4
0.6
0.8
1.0
Methylation Value

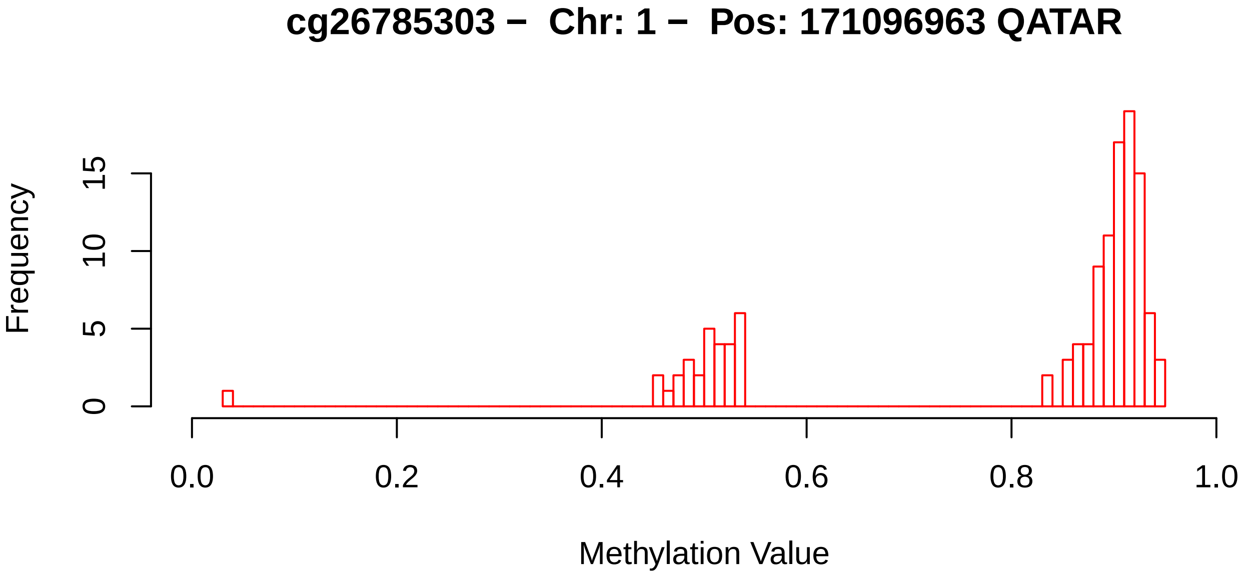

cg26785303 − Chr: 1 − Pos: 171096963 QATAR
Frequency
0
5
10
15
0.0
0.2
0.4
0.6
0.8
1.0
Methylation Value

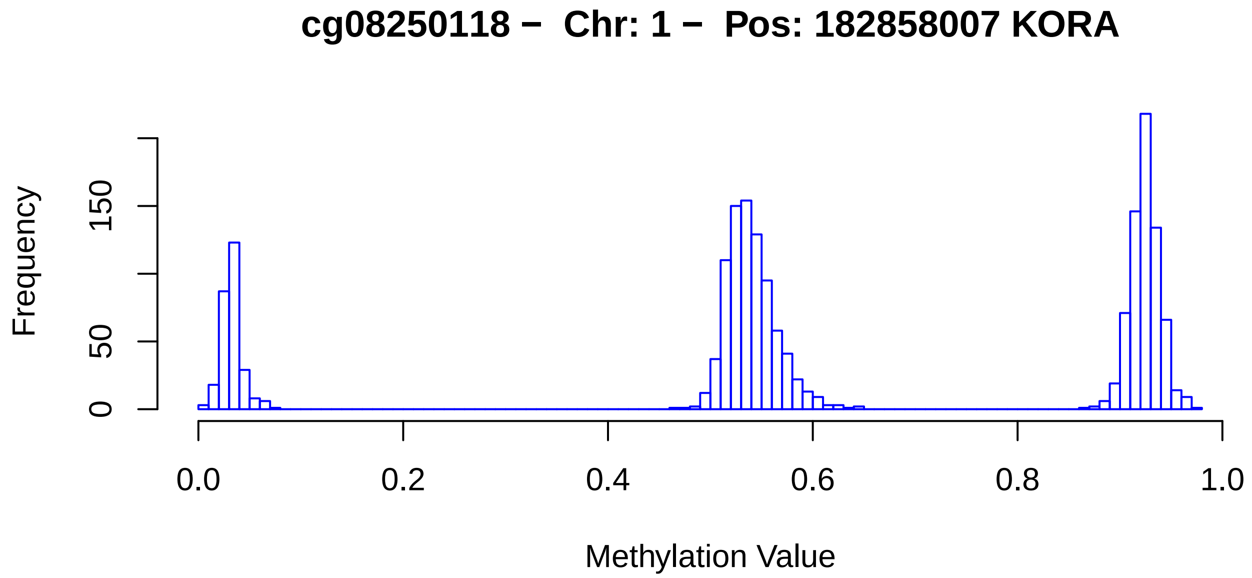
cg08250118 − Chr: 1 − Pos: 182858007 KORA
Frequency
0
50
150
0.0
0.2
0.4
0.6
0.8
1.0
Methylation Value

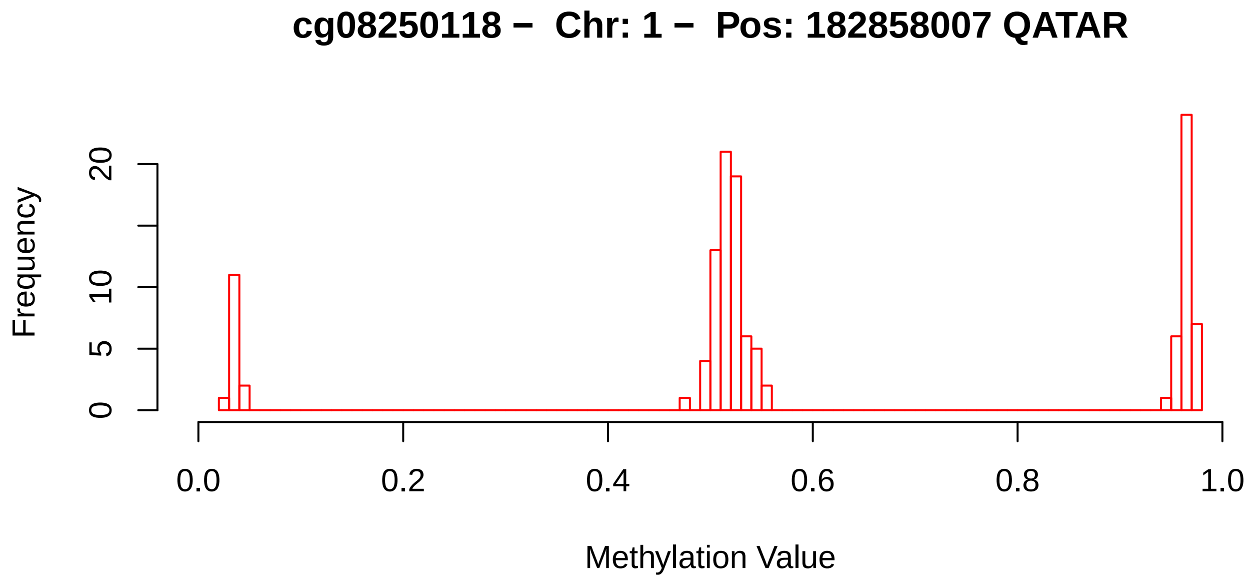
cg08250118 − Chr: 1 − Pos: 182858007 QATAR
Frequency
0
5
10
20
0.0
0.2
0.4
0.6
0.8
1.0
Methylation Value

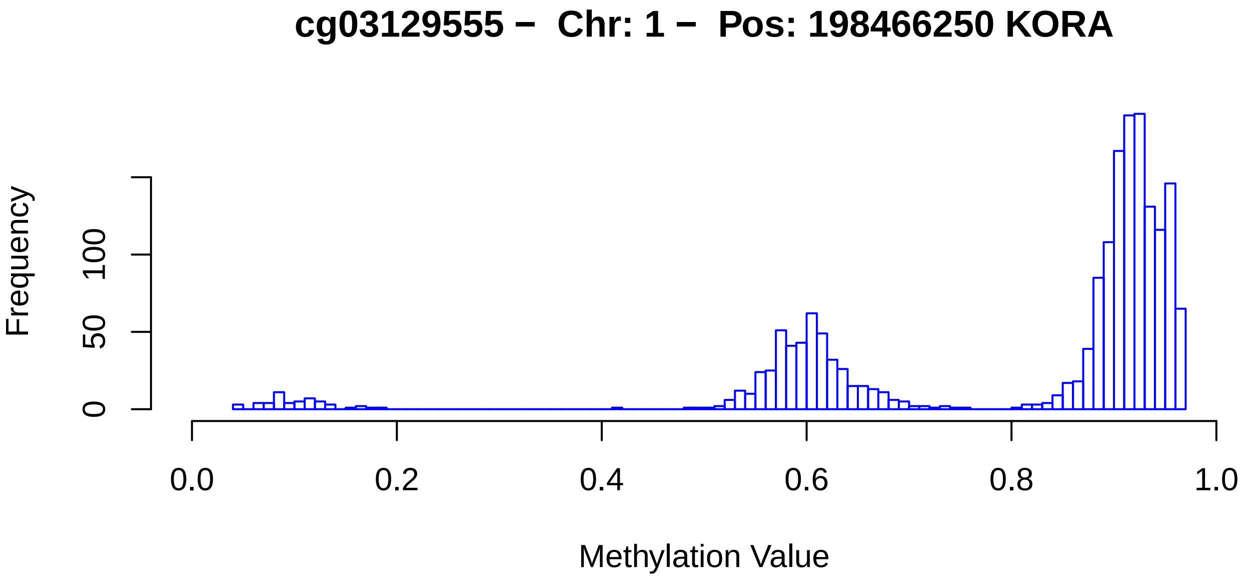
cg03129555 − Chr: 1 − Pos: 198466250 KORA
Frequency
0
50
100
0.0
0.2
0.4
0.6
0.8
1.0
Methylation Value

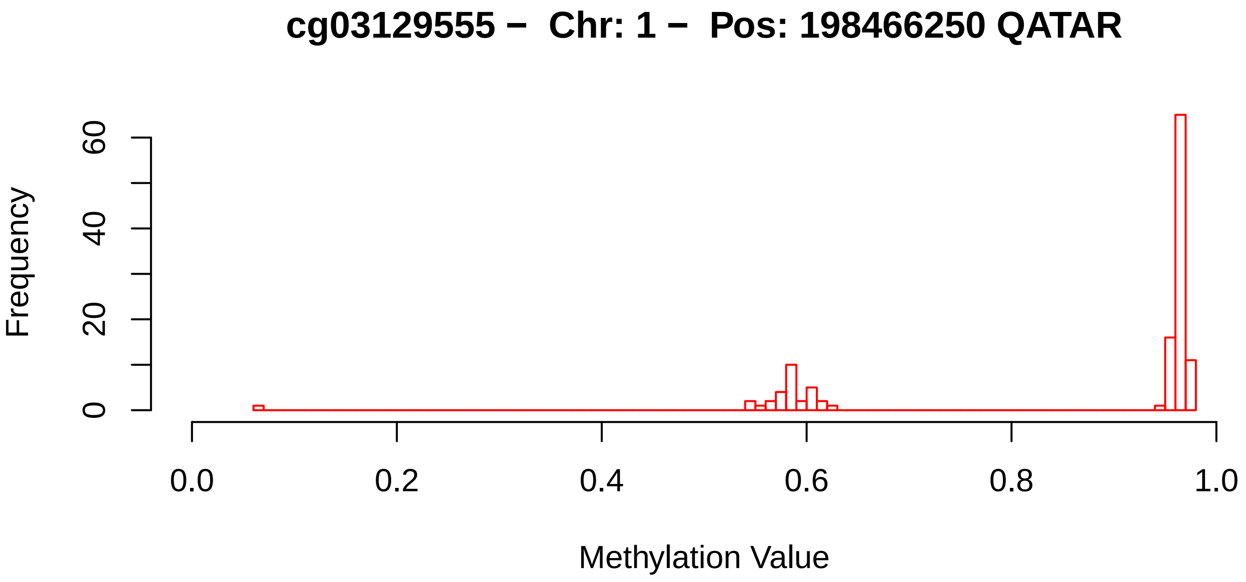
cg03129555 − Chr: 1 − Pos: 198466250 QATAR
Frequency
0
20
40
60
0.0
0.2
0.4
0.6
0.8
1.0
Methylation Value

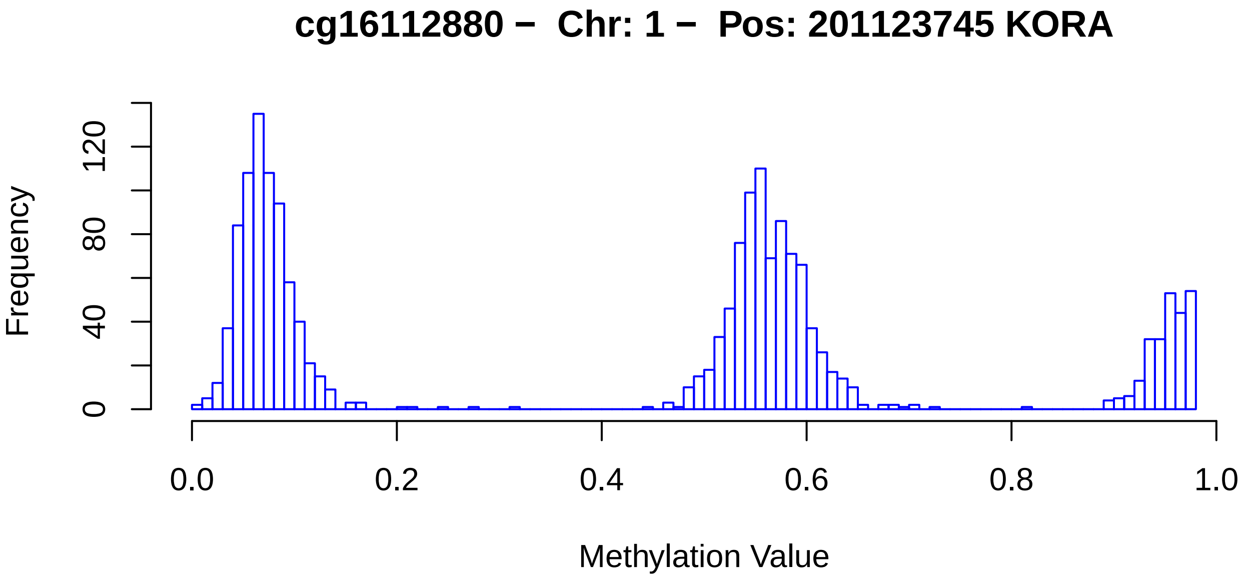
cg16112880 − Chr: 1 − Pos: 201123745 KORA
Frequency
0
40
80
120
0.0
0.2
0.4
0.6
0.8
1.0
Methylation Value

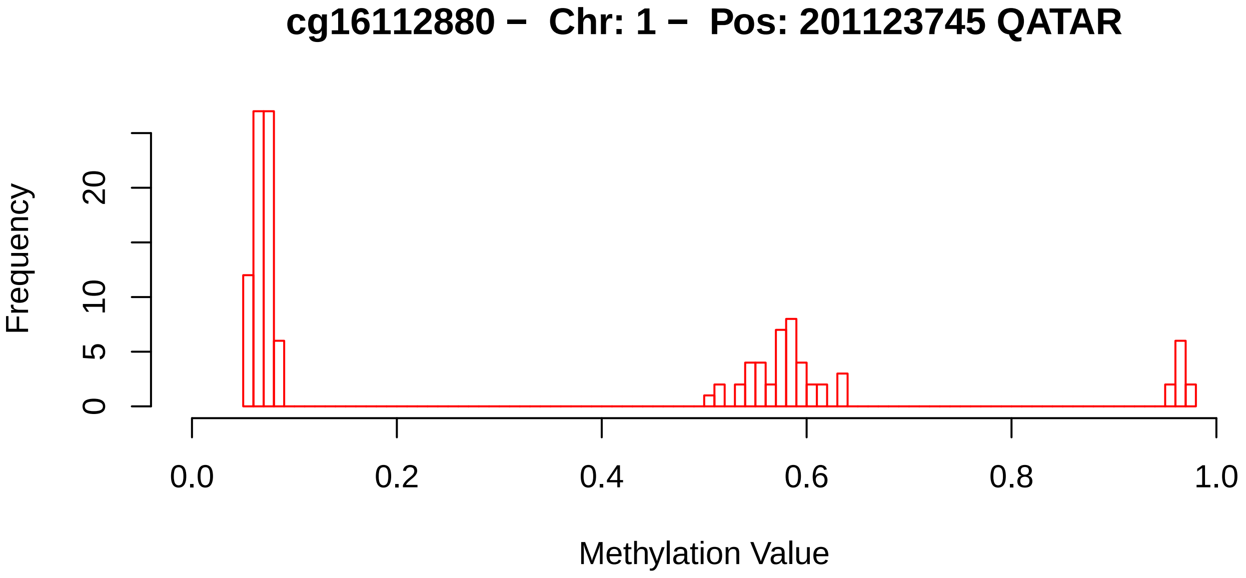
cg16112880 − Chr: 1 − Pos: 201123745 QATAR
Frequency
0
5
10
20
0.0
0.2
0.4
0.6
0.8
1.0
Methylation Value

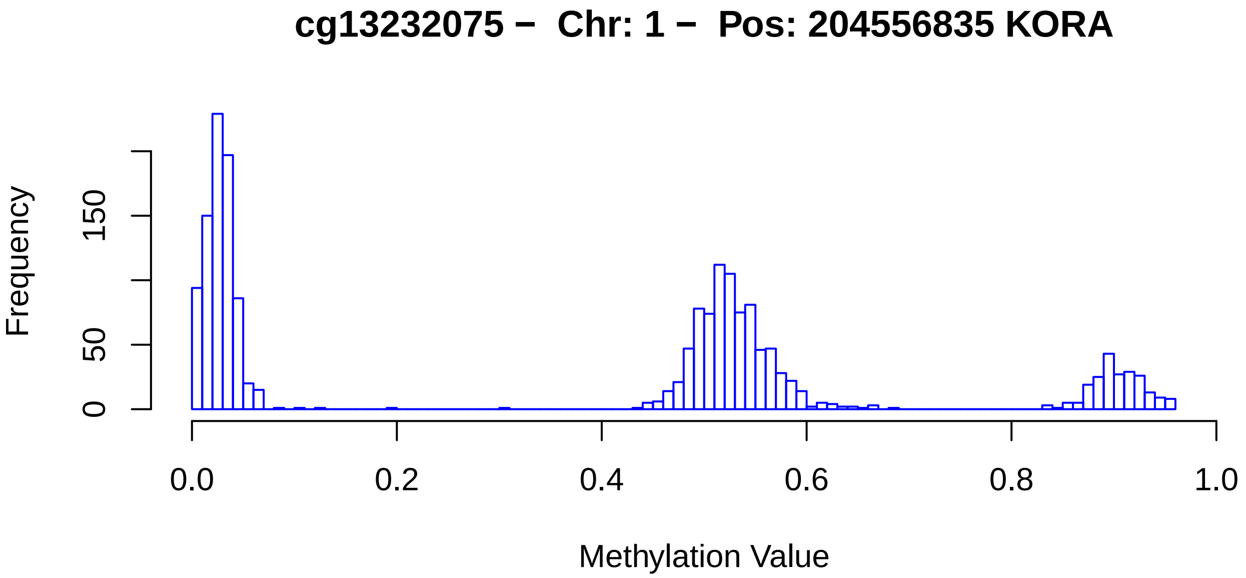
cg13232075 − Chr: 1 − Pos: 204556835 KORA
Frequency
0
50
150
0.0
0.2
0.4
0.6
0.8
1.0
Methylation Value

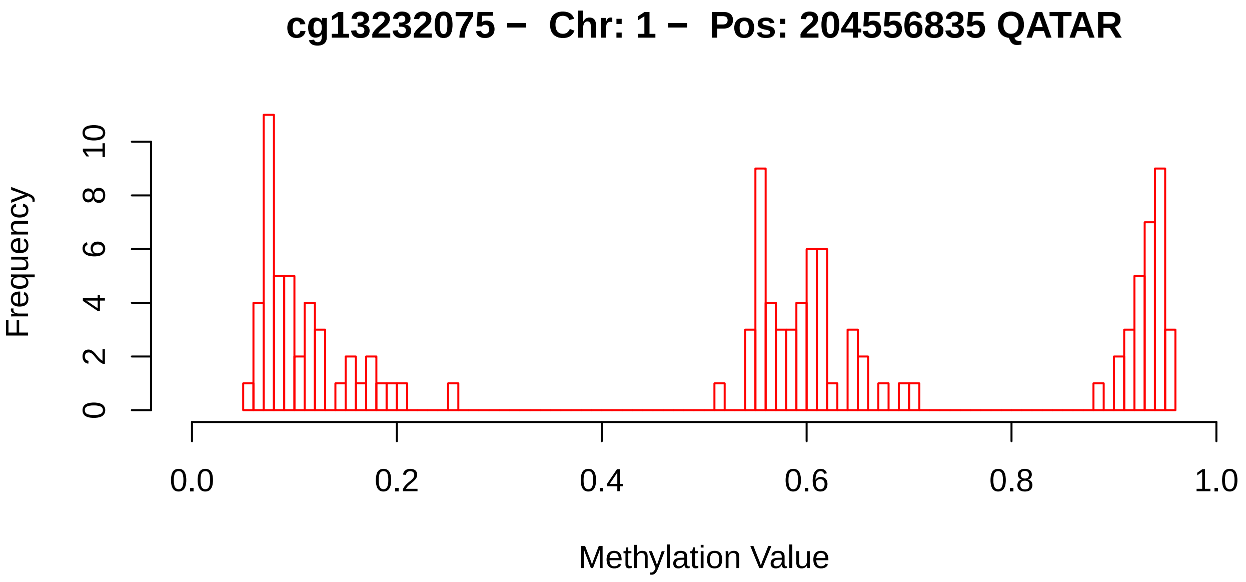
cg13232075 − Chr: 1 − Pos: 204556835 QATAR
Frequency
0
2
4
6
8
10
0.0
0.2
0.4
0.6
0.8
1.0
Methylation Value

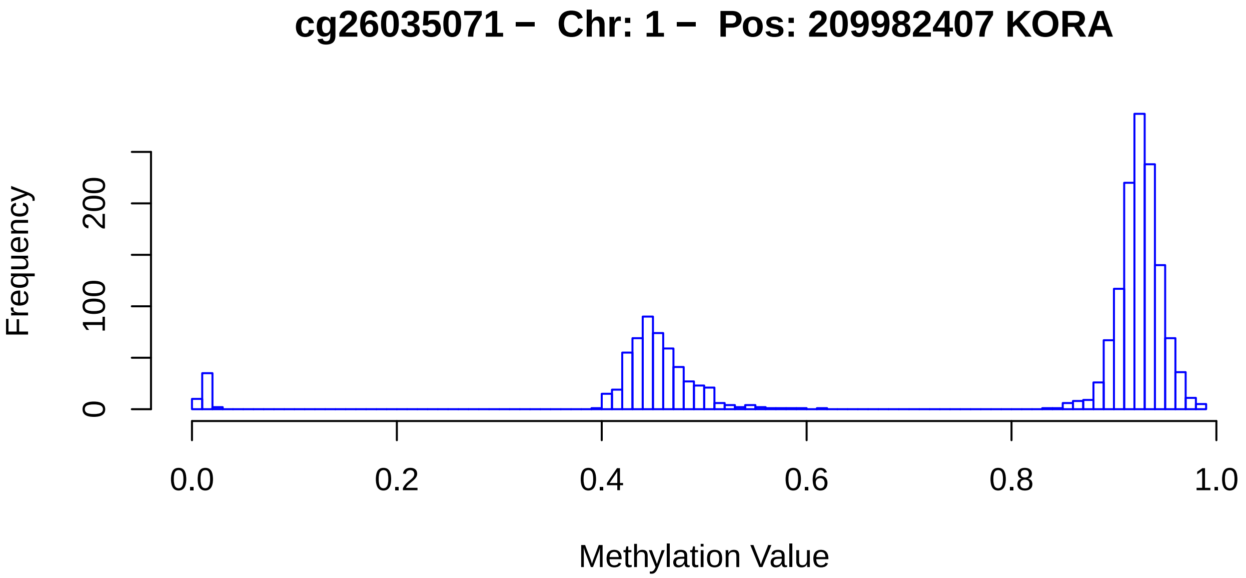
cg26035071 − Chr: 1 − Pos: 209982407 KORA
Frequency
0
100
200
0.0
0.2
0.4
0.6
0.8
1.0
Methylation Value

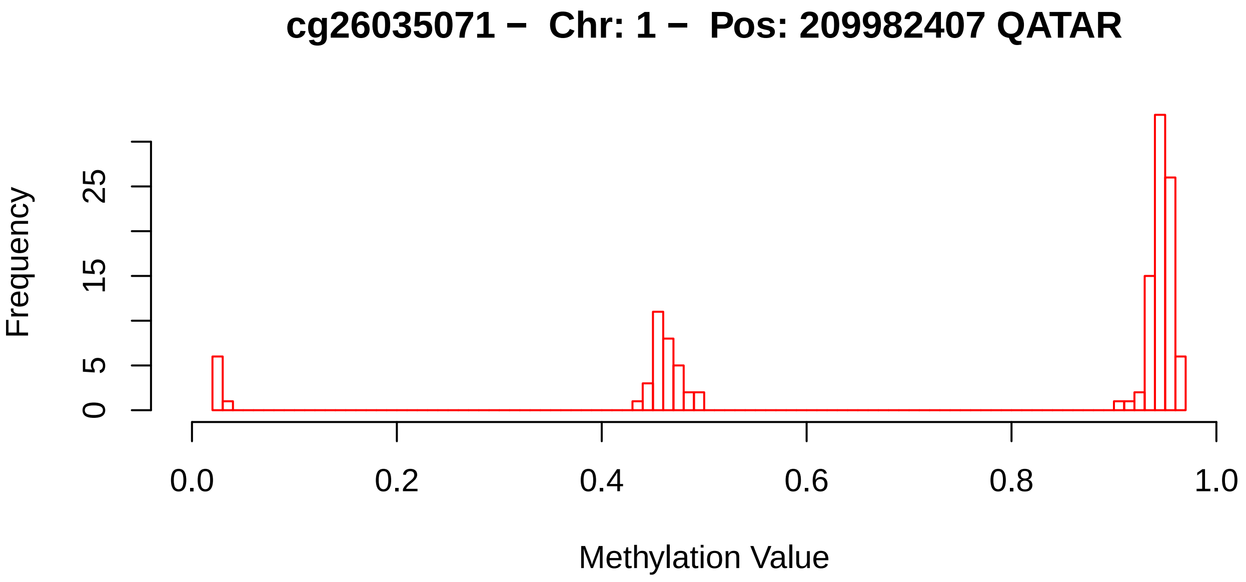
cg26035071 − Chr: 1 − Pos: 209982407 QATAR
Frequency
0
5
15
25
0.0
0.2
0.4
0.6
0.8
1.0
Methylation Value

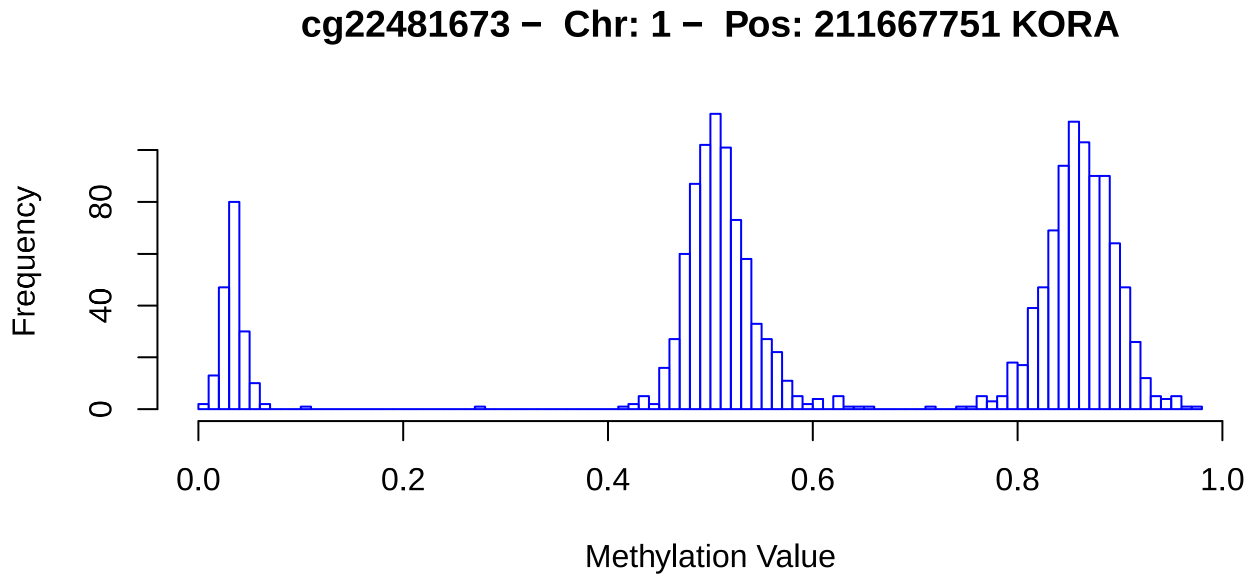
cg22481673 − Chr: 1 − Pos: 211667751 KORA
Frequency
0
40
80
0.0
0.2
0.4
0.6
0.8
1.0
Methylation Value

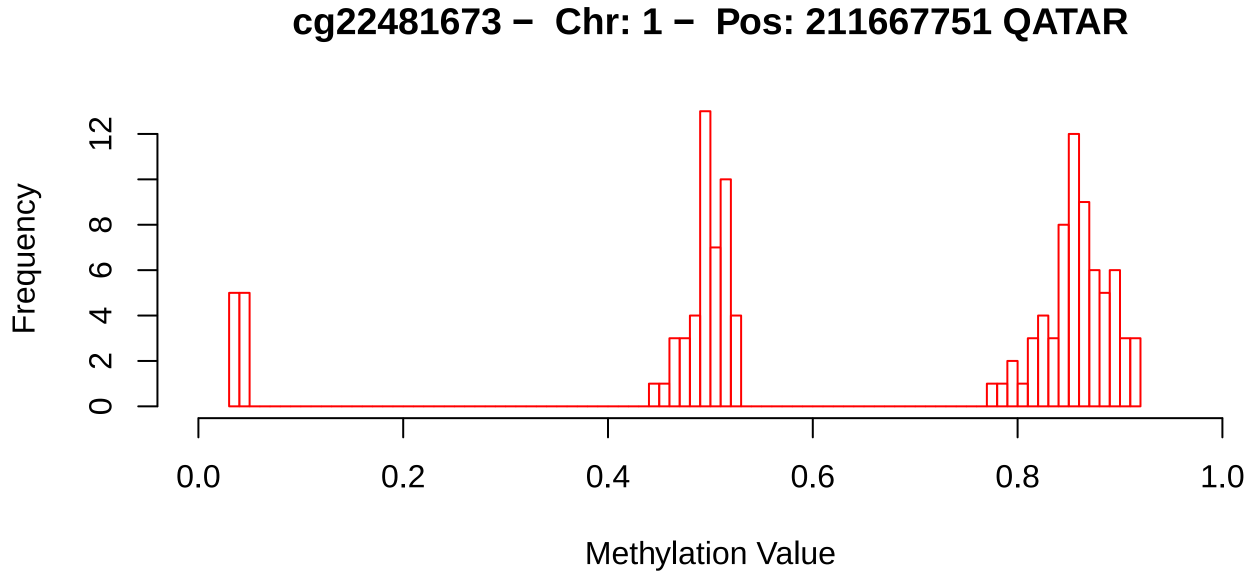
cg22481673 − Chr: 1 − Pos: 211667751 QATAR
Frequency
0
2
4
6
8
12
0.0
0.2
0.4
0.6
0.8
1.0
Methylation Value

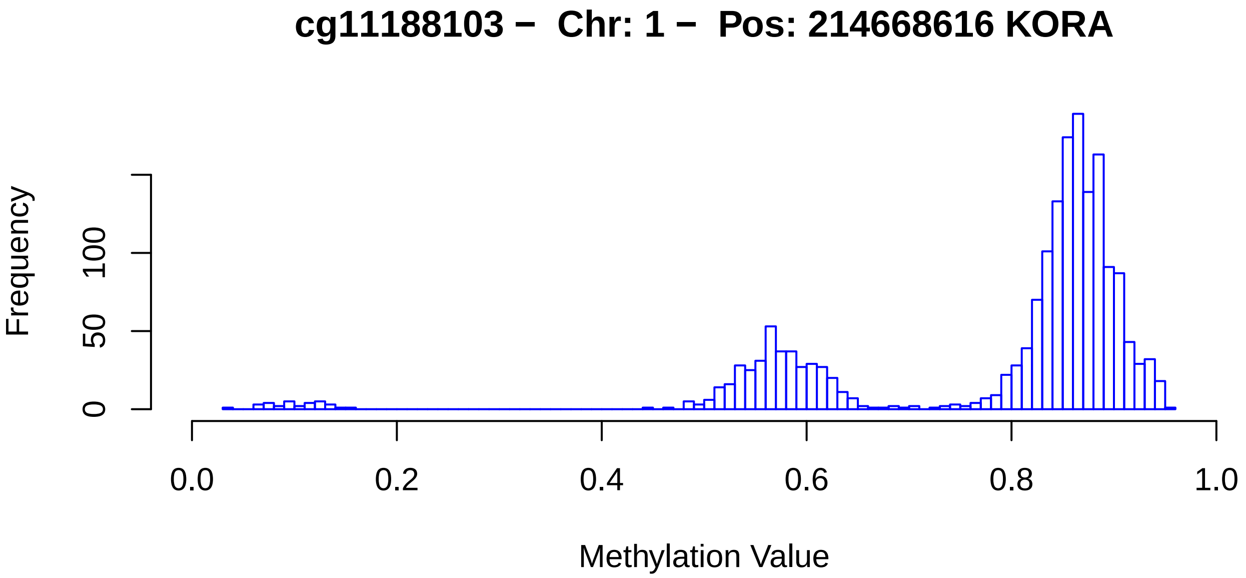
cg11188103 − Chr: 1 − Pos: 214668616 KORA
Frequency
0
50
100
0.0
0.2
0.4
0.6
0.8
1.0
Methylation Value

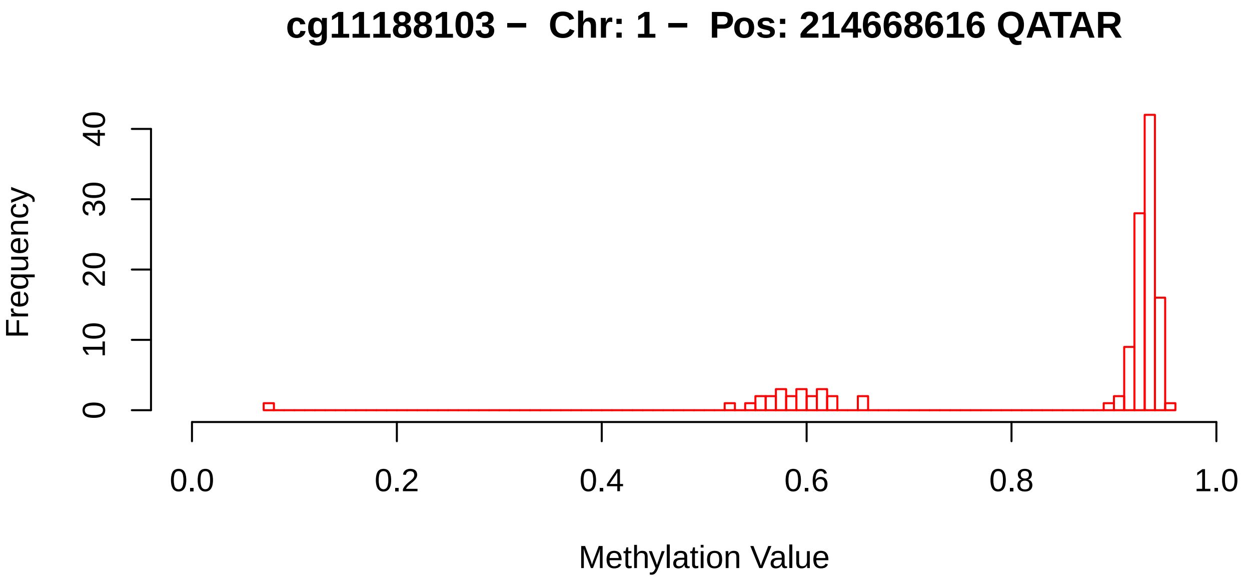
cg11188103 − Chr: 1 − Pos: 214668616 QATAR
Frequency
0
10
20
30
40
0.0
0.2
0.4
0.6
0.8
1.0
Methylation Value

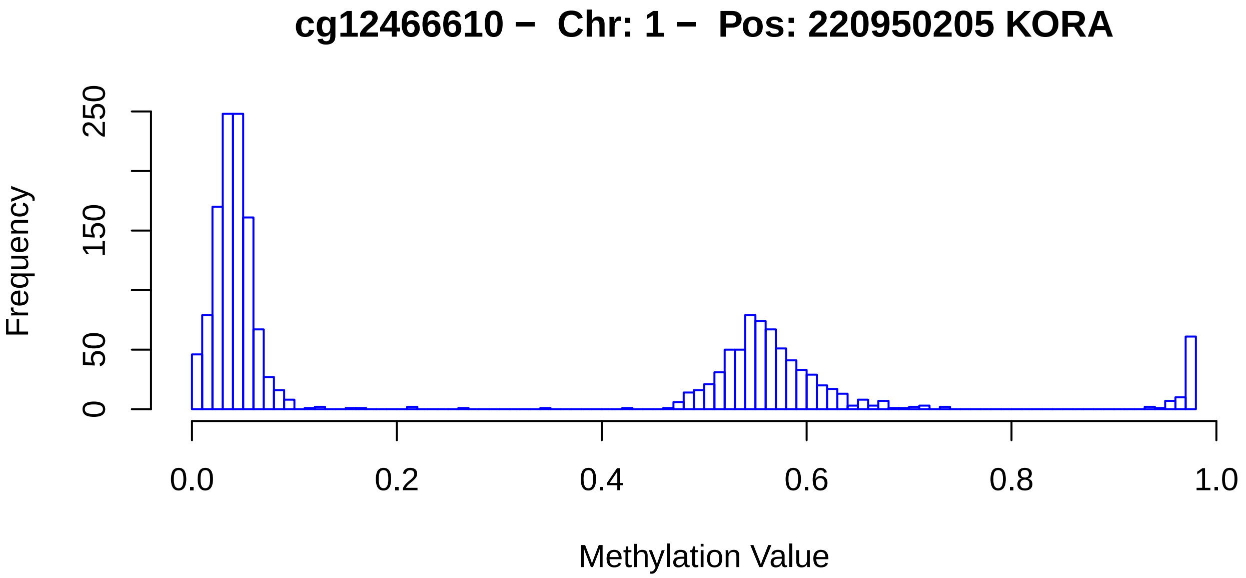
cg12466610 − Chr: 1 − Pos: 220950205 KORA
Frequency
0
50
150
250
0.0
0.2
0.4
0.6
0.8
1.0
Methylation Value

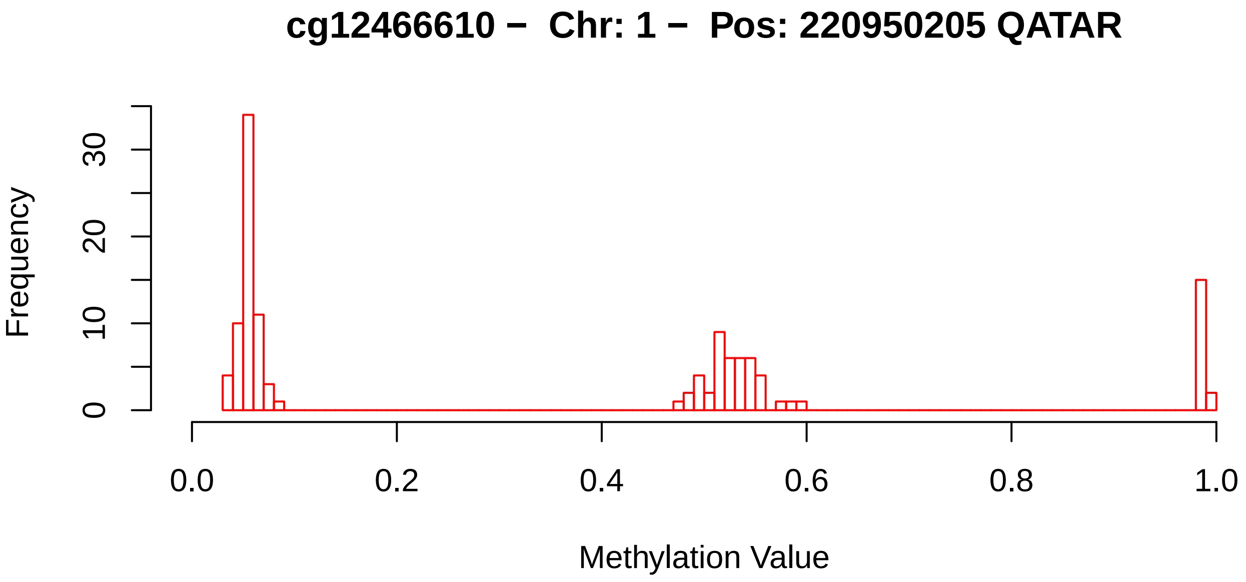
cg12466610 − Chr: 1 − Pos: 220950205 QATAR
Frequency
0
10
20
30
0.0
0.2
0.4
0.6
0.8
1.0
Methylation Value

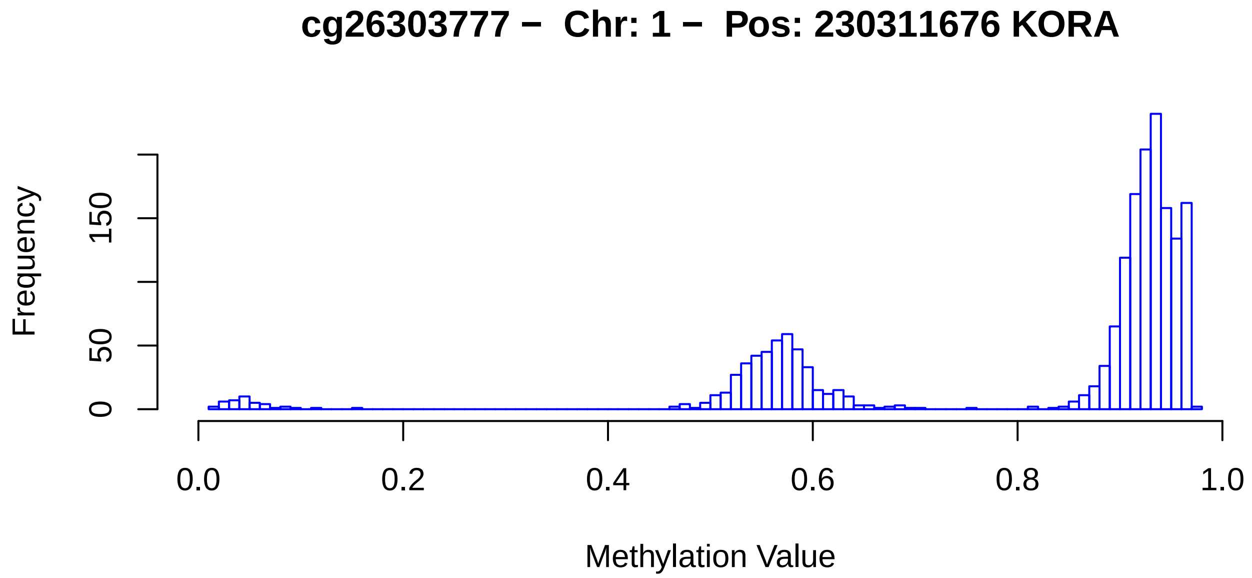
cg26303777 − Chr: 1 − Pos: 230311676 KORA
Frequency
0
50
150
0.0
0.2
0.4
0.6
0.8
1.0
Methylation Value

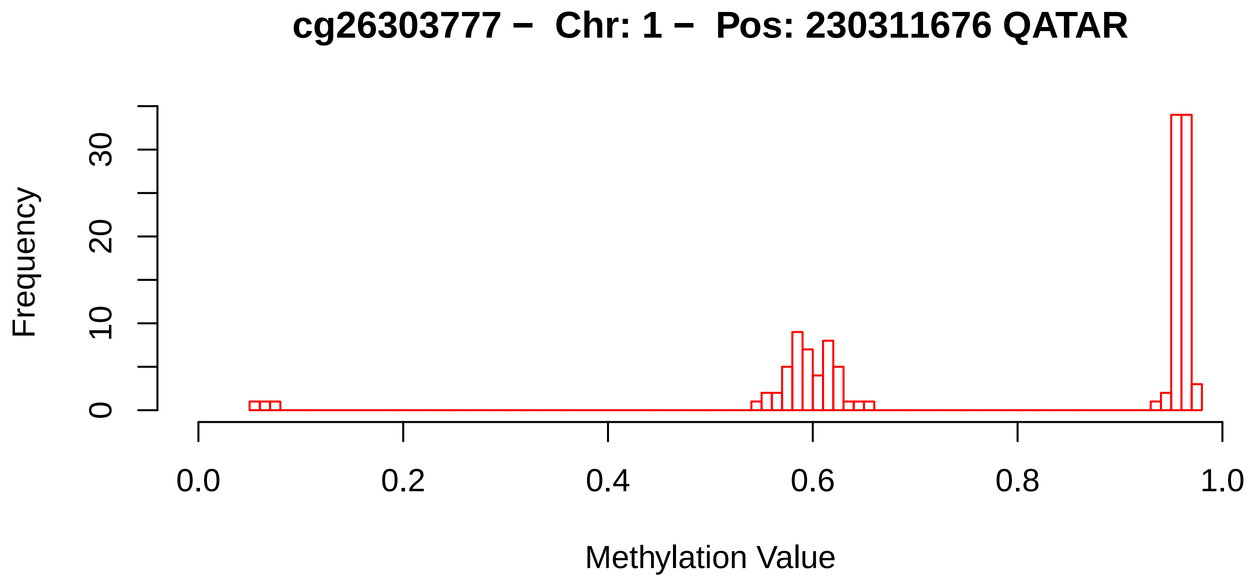
cg26303777 − Chr: 1 − Pos: 230311676 QATAR
Frequency
0
10
20
30
0.0
0.2
0.4
0.6
0.8
1.0
Methylation Value

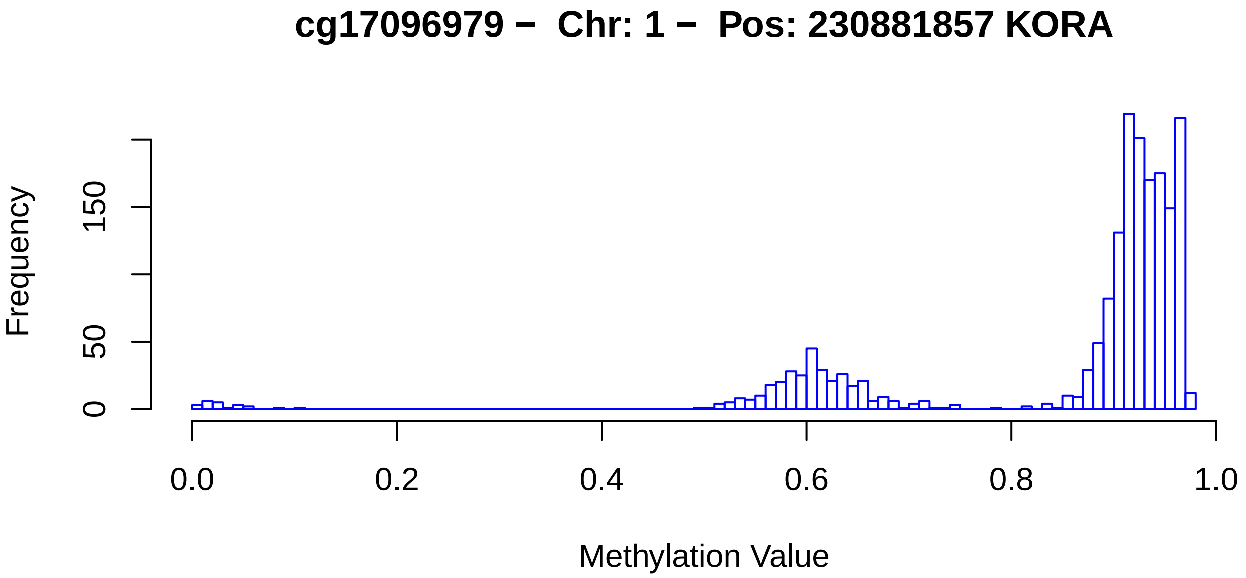

cg17096979 − Chr: 1 − Pos: 230881857 KORA
Frequency
0
50
150
0.0
0.2
0.4
0.6
0.8
1.0
Methylation Value

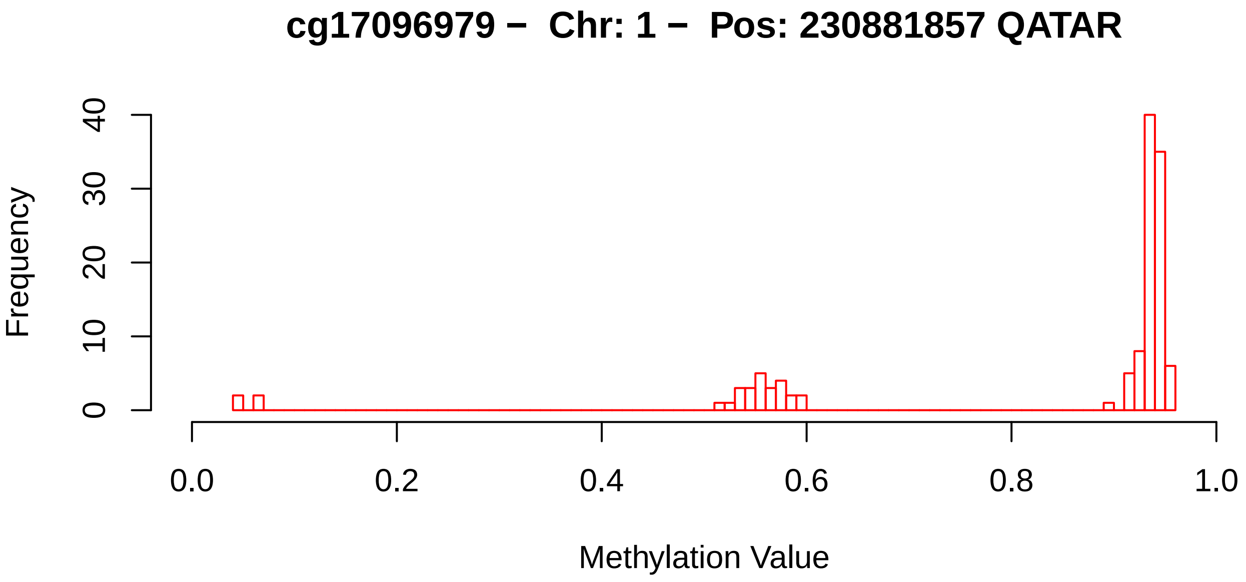

cg17096979 − Chr: 1 − Pos: 230881857 QATAR
Frequency
0
10
20
30
40
0.0
0.2
0.4
0.6
0.8
1.0
Methylation Value

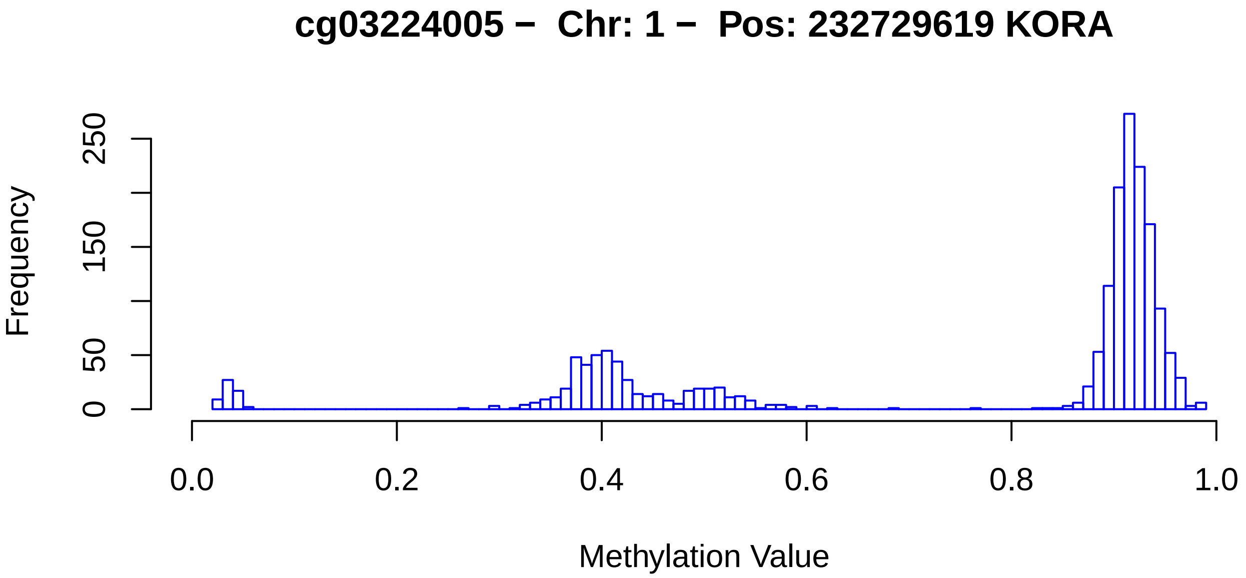
cg03224005 − Chr: 1 − Pos: 232729619 KORA
Frequency
0
50
150
250
0.0
0.2
0.4
0.6
0.8
1.0
Methylation Value

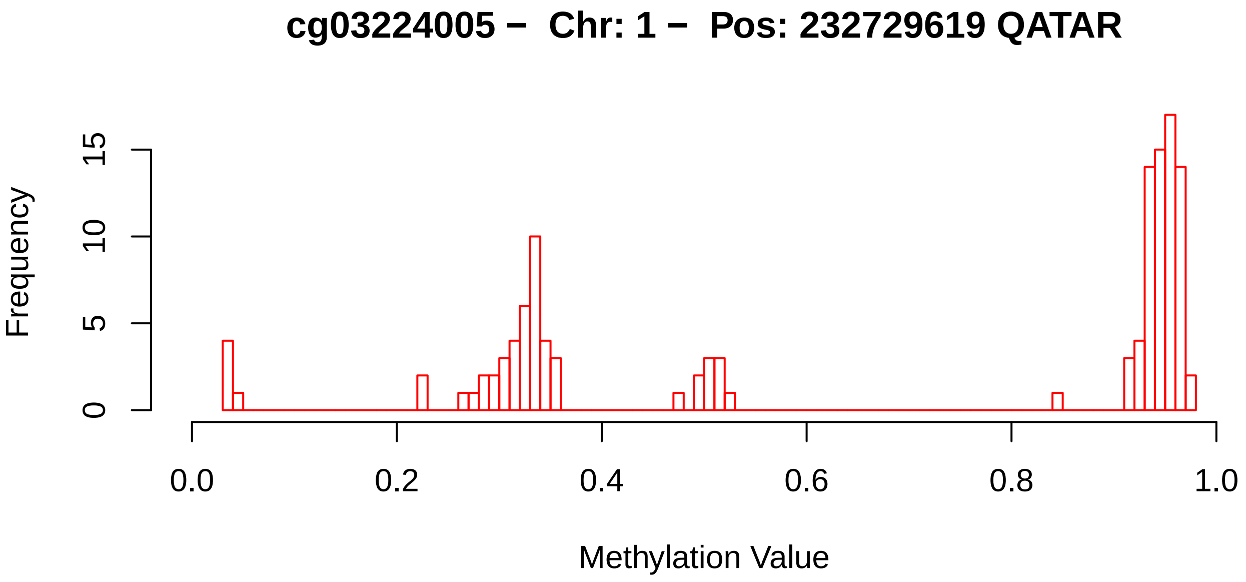
cg03224005 − Chr: 1 − Pos: 232729619 QATAR
Frequency
0
5
10
15
0.0
0.2
0.4
0.6
0.8
1.0
Methylation Value

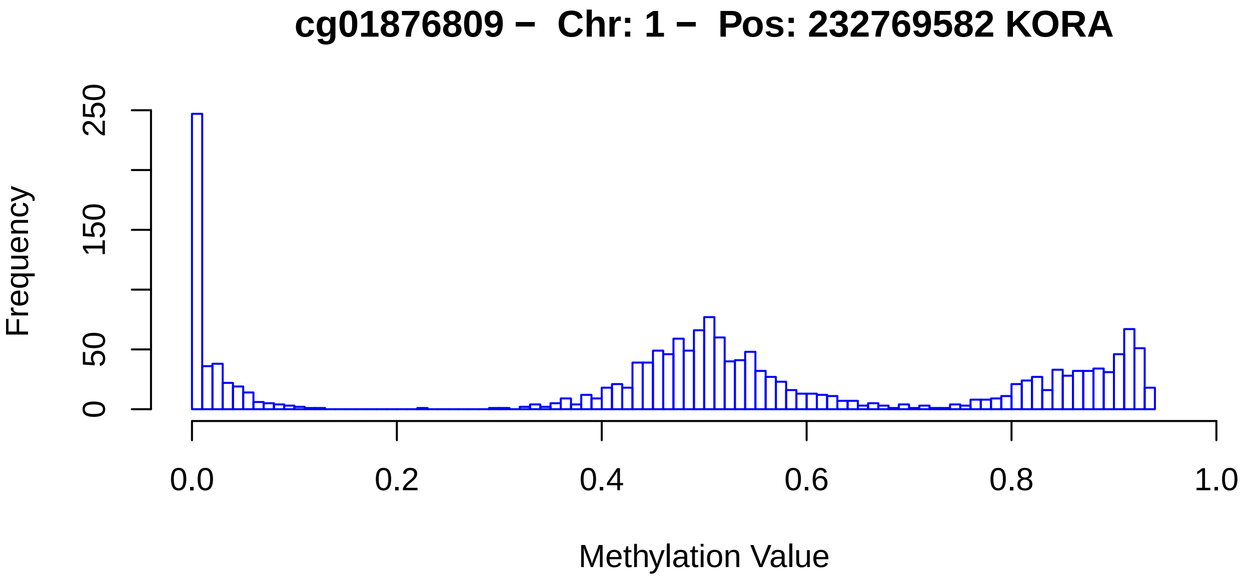
cg01876809 − Chr: 1 − Pos: 232769582 KORA
Frequency
0
50
150
250
0.0
0.2
0.4
0.6
0.8
1.0
Methylation Value

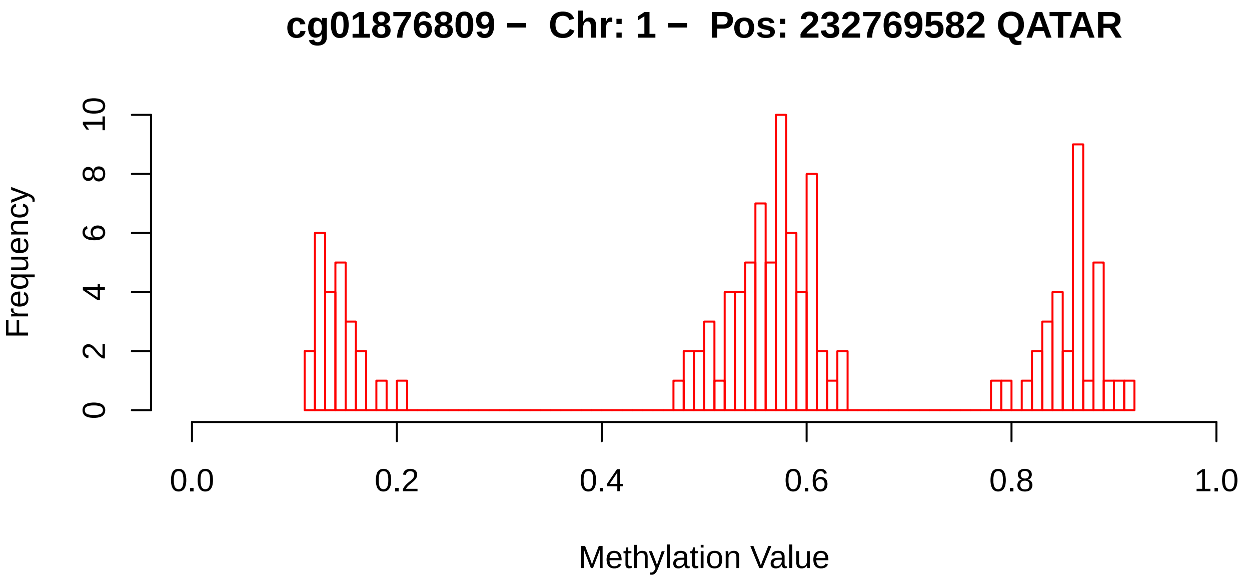
cg01876809 − Chr: 1 − Pos: 232769582 QATAR
Frequency
0
2
4
6
8
10
0.0
0.2
0.4
0.6
0.8
1.0
Methylation Value

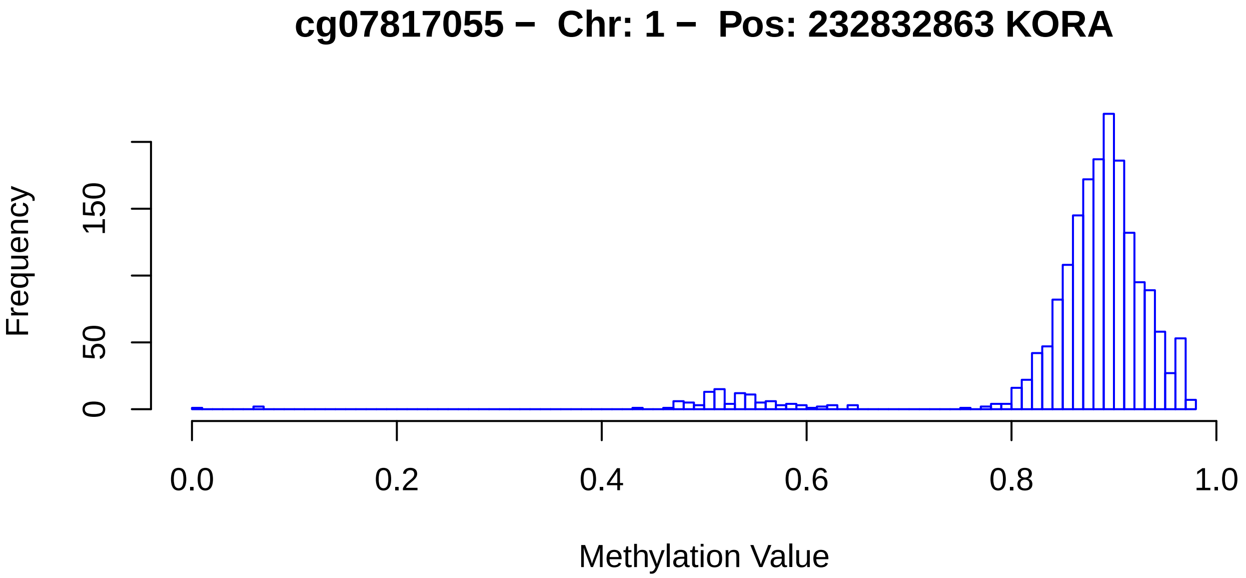
cg07817055 − Chr: 1 − Pos: 232832863 KORA
Frequency
0
50
150
0.0
0.2
0.4
0.6
0.8
1.0
Methylation Value

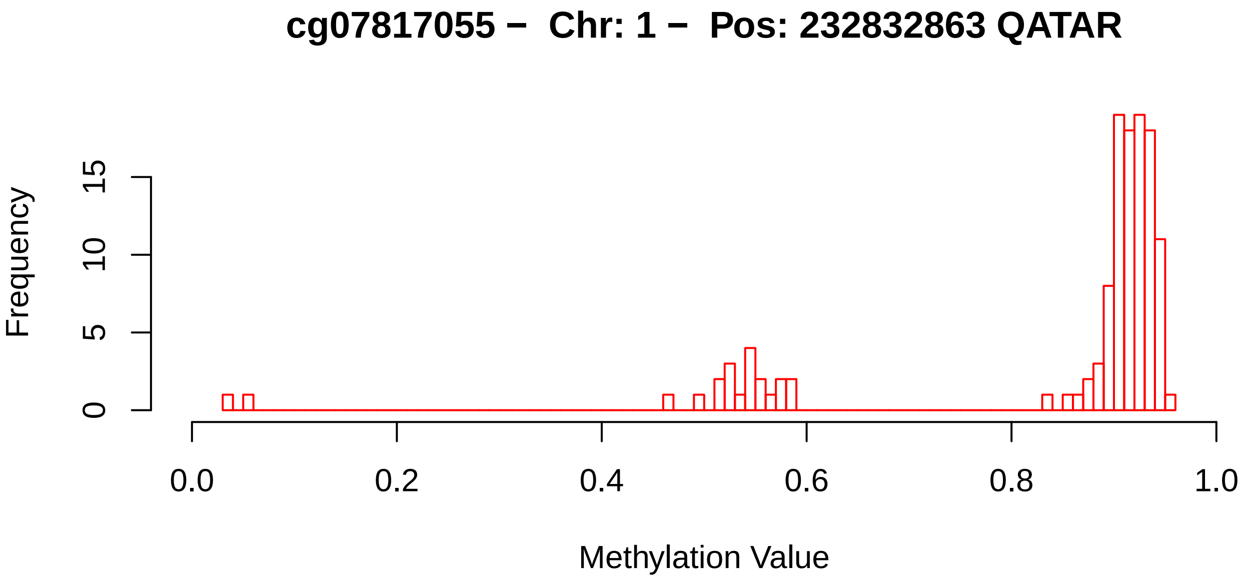
cg07817055 − Chr: 1 − Pos: 232832863 QATAR
Frequency
0
5
10
15
0.0
0.2
0.4
0.6
0.8
1.0
Methylation Value

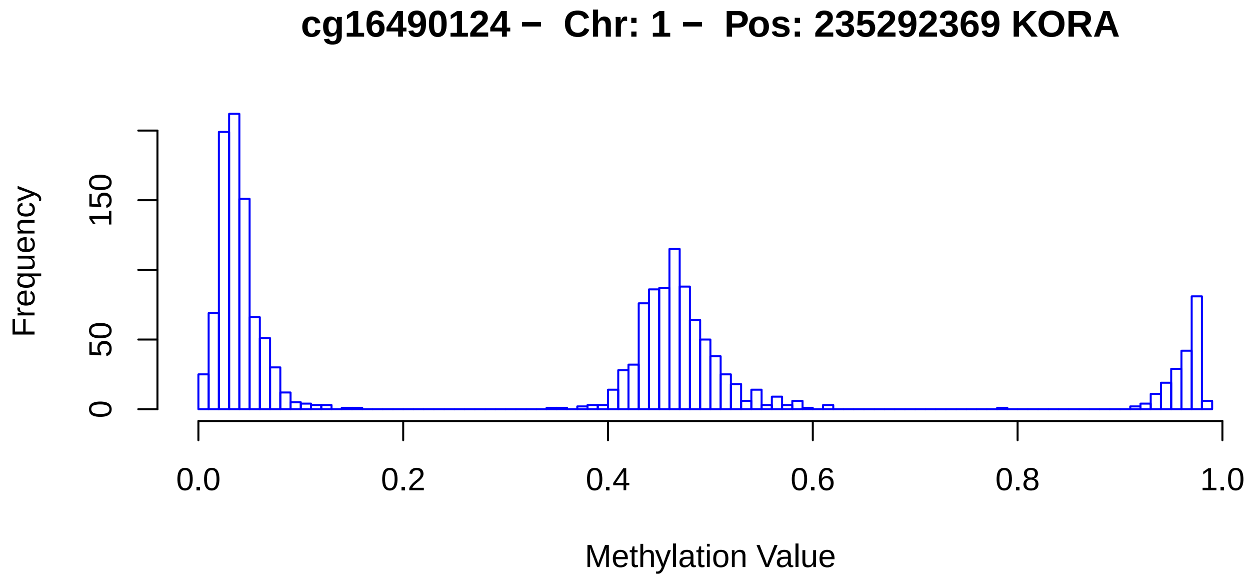
cg16490124 − Chr: 1 − Pos: 235292369 KORA
Frequency
0
50
150
0.0
0.2
0.4
0.6
0.8
1.0
Methylation Value

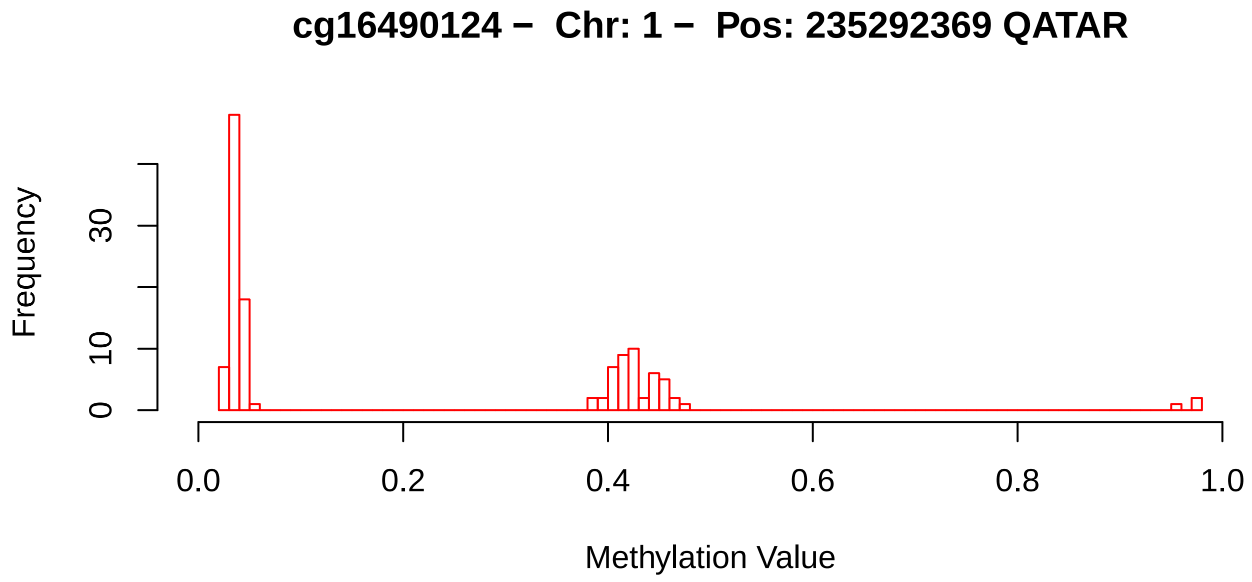
cg16490124 − Chr: 1 − Pos: 235292369 QATAR
Frequency
0
10
30
0.0
0.2
0.4
0.6
0.8
1.0
Methylation Value

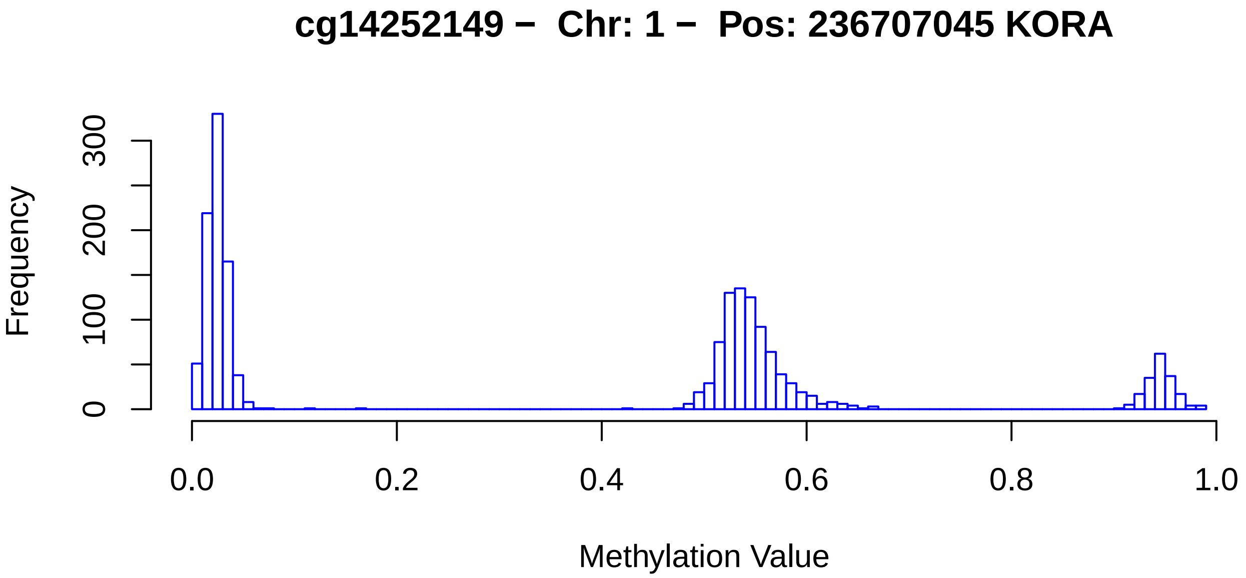
cg14252149 − Chr: 1 − Pos: 236707045 KORA
Frequency
0
100
200
300
0.0
0.2
0.4
0.6
0.8
1.0
Methylation Value

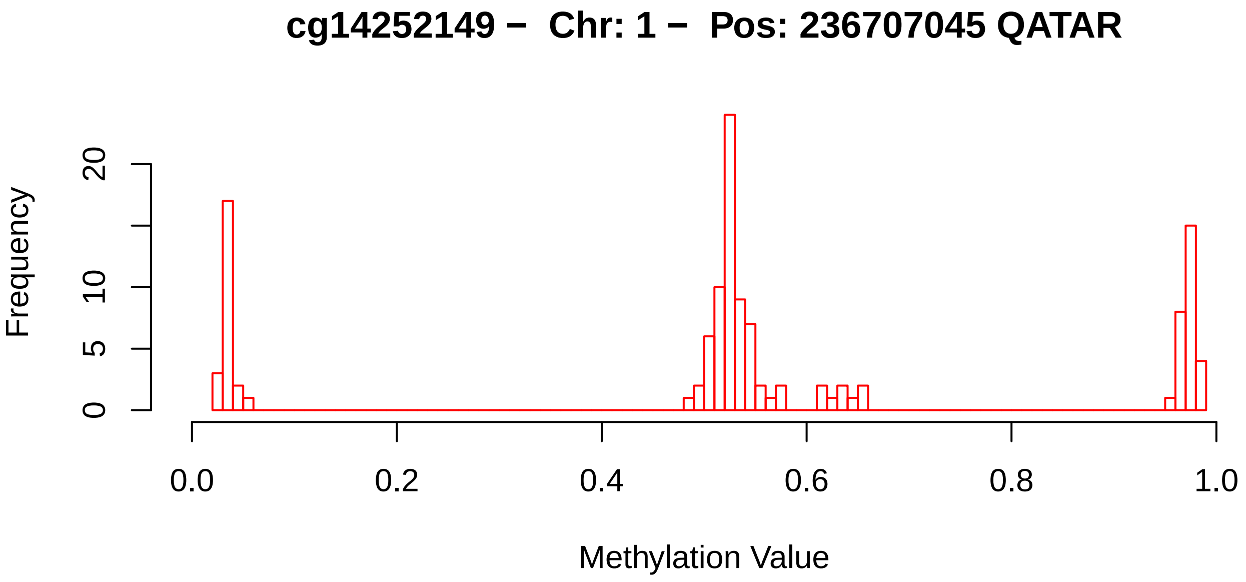
cg14252149 − Chr: 1 − Pos: 236707045 QATAR
Frequency
0
5
10
20
0.0
0.2
0.4
0.6
0.8
1.0
Methylation Value

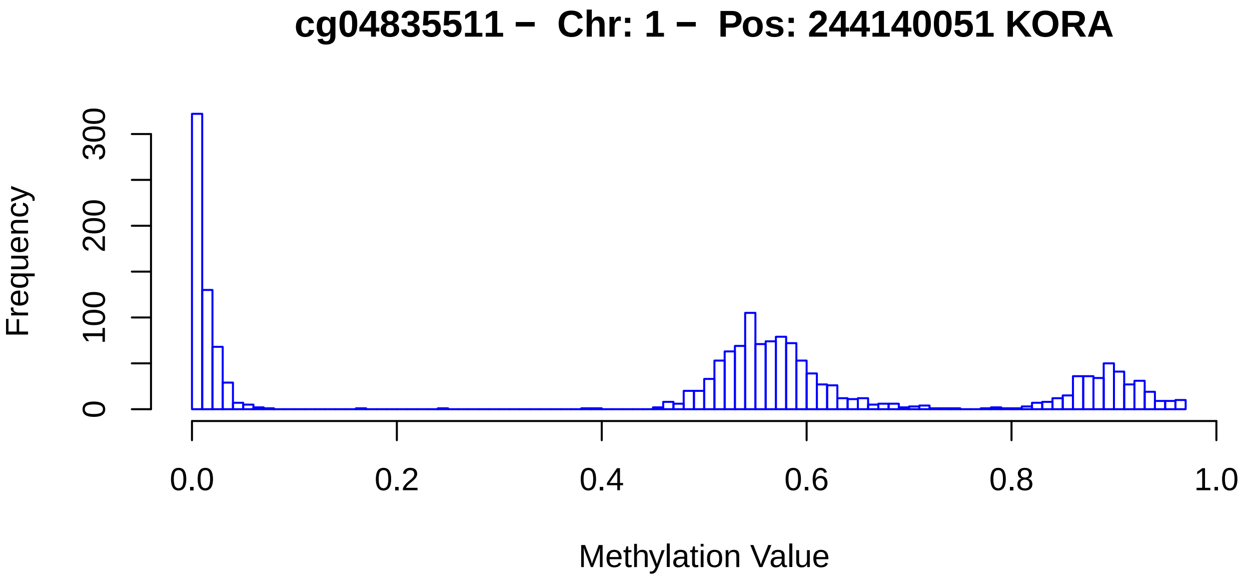
cg04835511 − Chr: 1 − Pos: 244140051 KORA
Frequency
0
100
200
300
0.0
0.2
0.4
0.6
0.8
1.0
Methylation Value

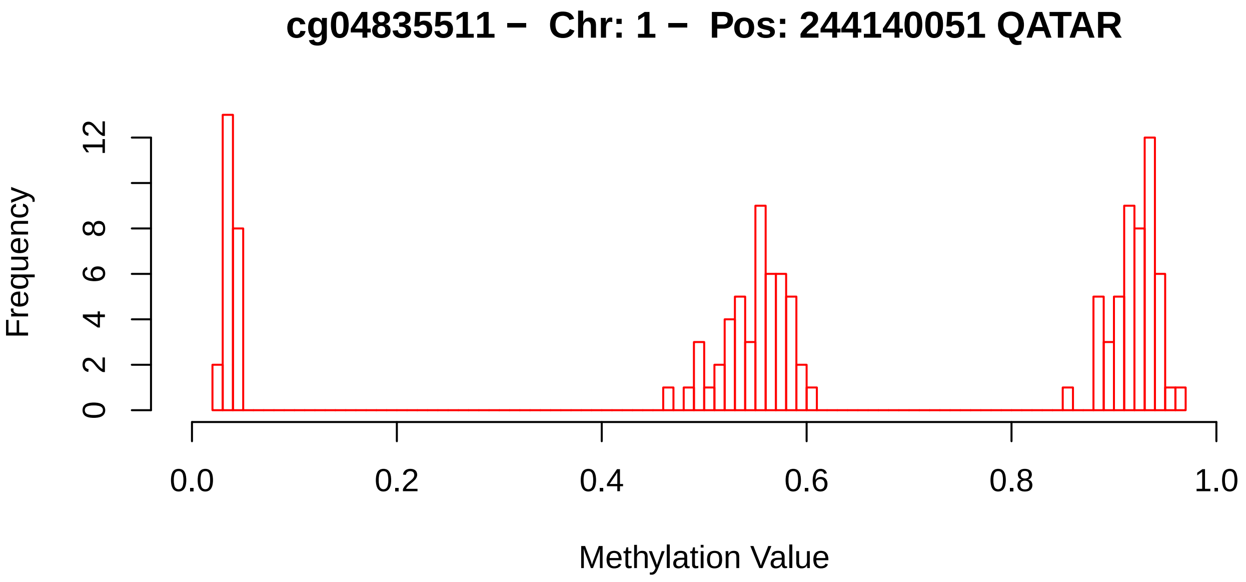
cg04835511 − Chr: 1 − Pos: 244140051 QATAR
Frequency
0
2
4
6
8
12
0.0
0.2
0.4
0.6
0.8
1.0
Methylation Value

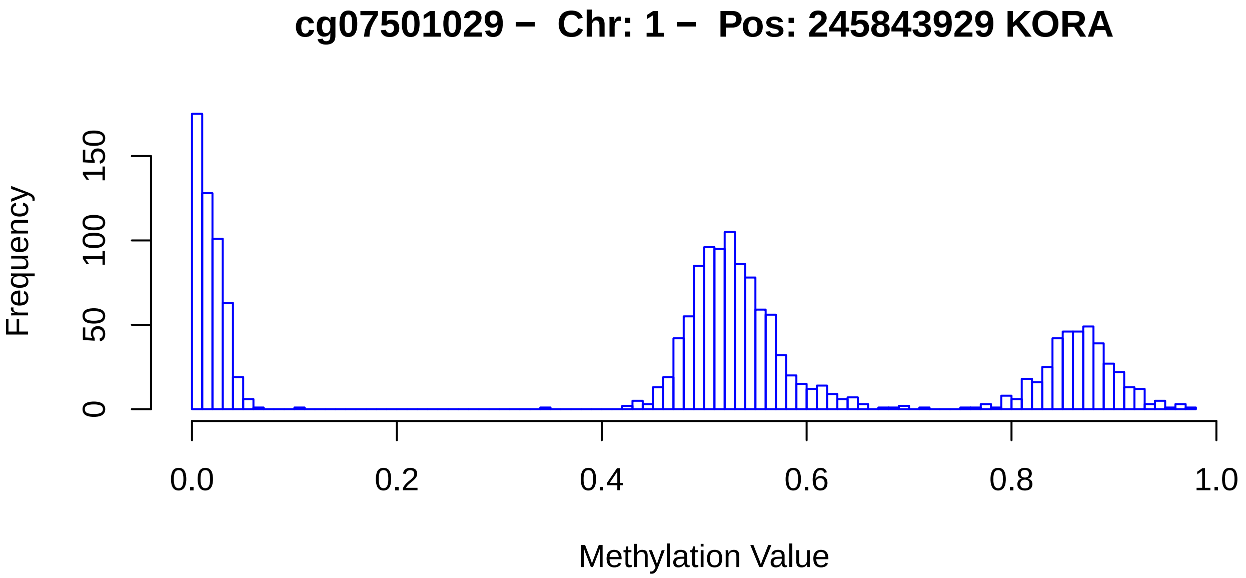
cg07501029 − Chr: 1 − Pos: 245843929 KORA
Frequency
0
50
100
150
0.0
0.2
0.4
0.6
0.8
1.0
Methylation Value

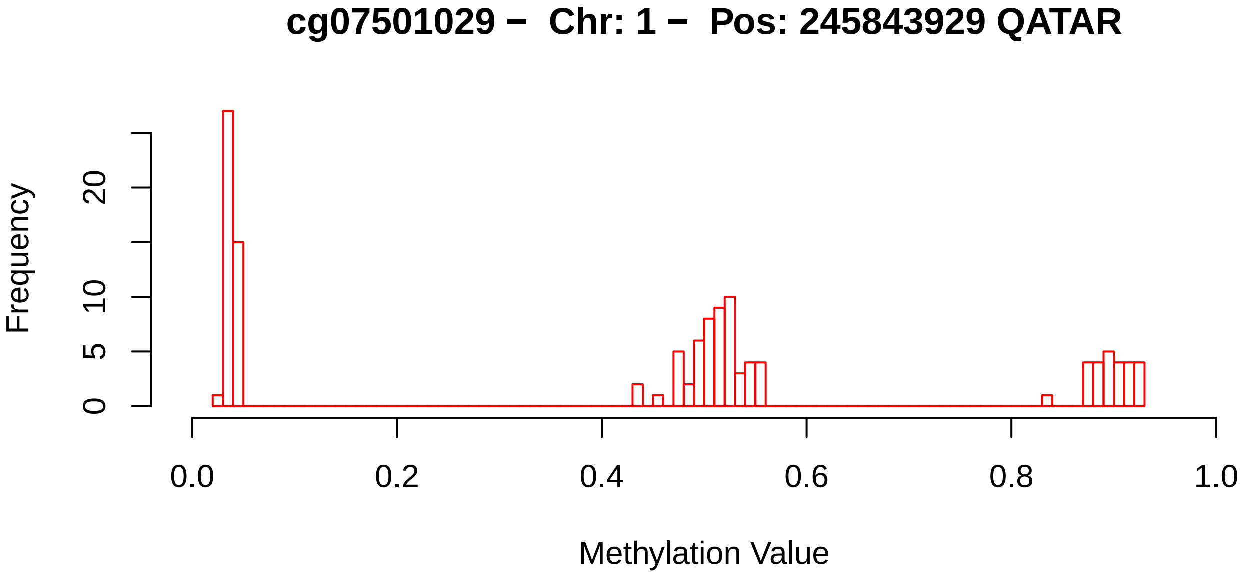
cg07501029 − Chr: 1 − Pos: 245843929 QATAR
Frequency
0
5
10
20
0.0
0.2
0.4
0.6
0.8
1.0
Methylation Value

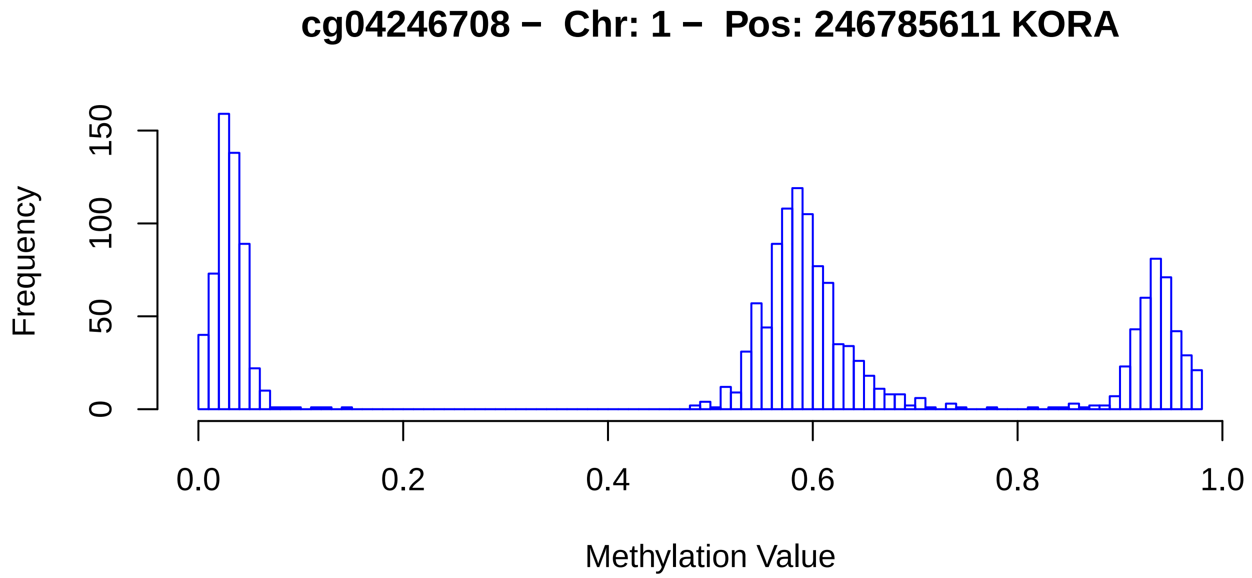
cg04246708 − Chr: 1 − Pos: 246785611 KORA
Frequency
0
50
100
150
0.0
0.2
0.4
0.6
0.8
1.0
Methylation Value

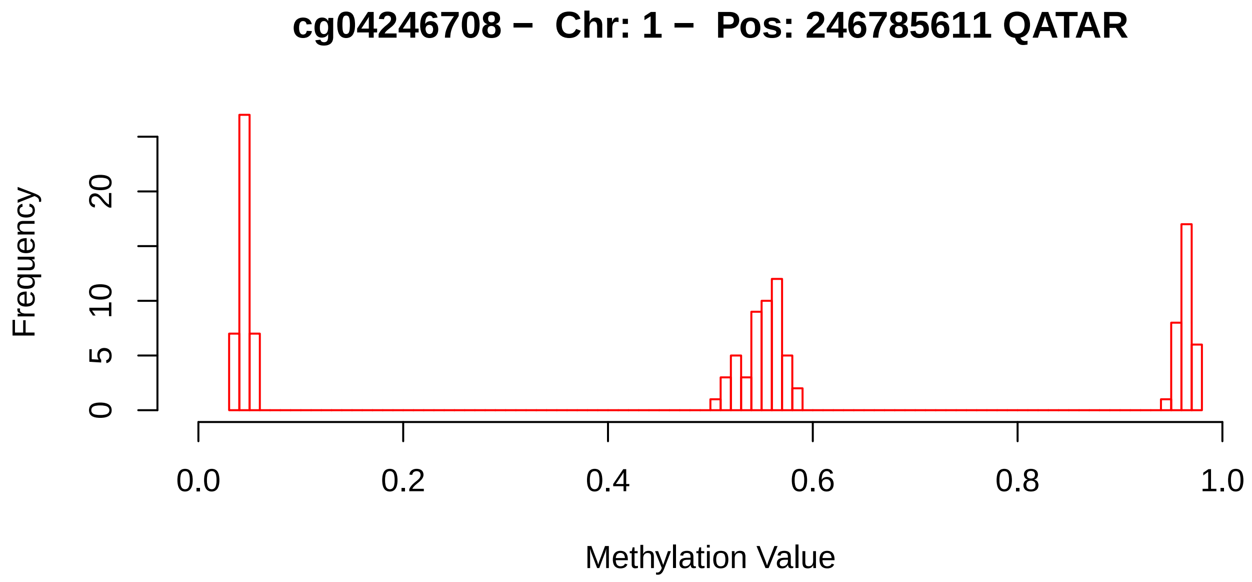
cg04246708 − Chr: 1 − Pos: 246785611 QATAR
Frequency
0
5
10
20
0.0
0.2
0.4
0.6
0.8
1.0
Methylation Value

cg25771854 − Chr: 1 − Pos: 247420421 KORA

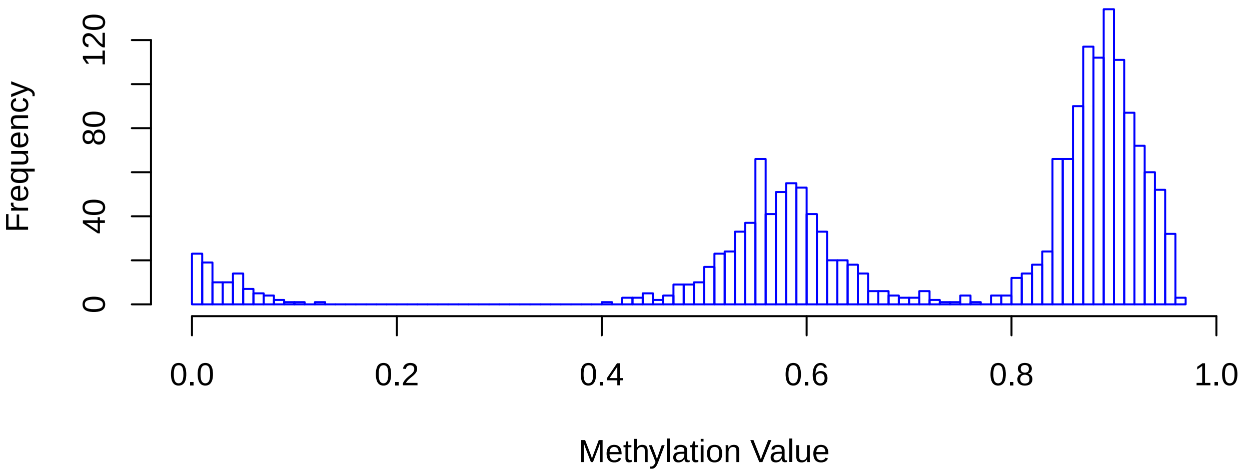

cg25771854 − Chr: 1 − Pos: 247420421 QATAR

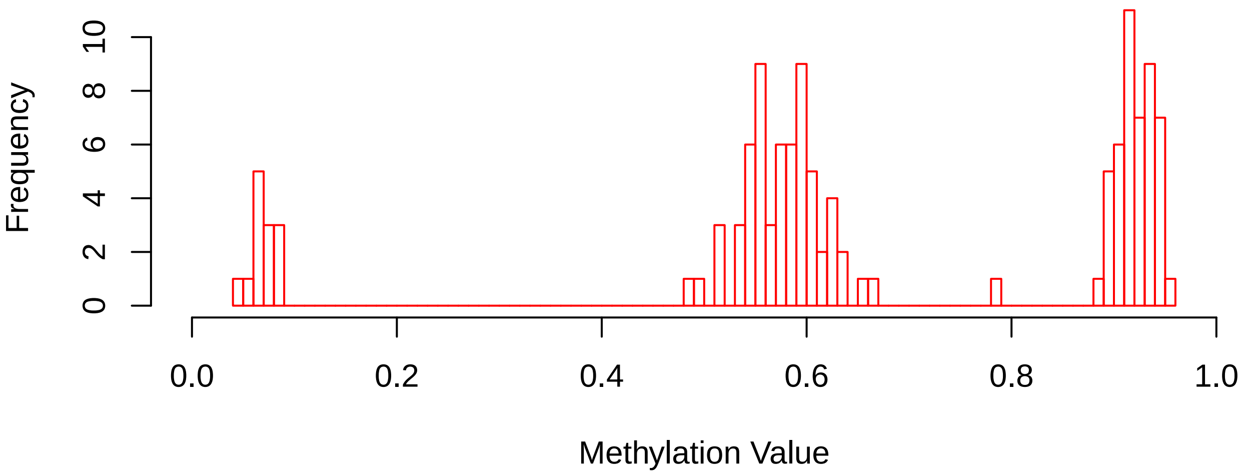

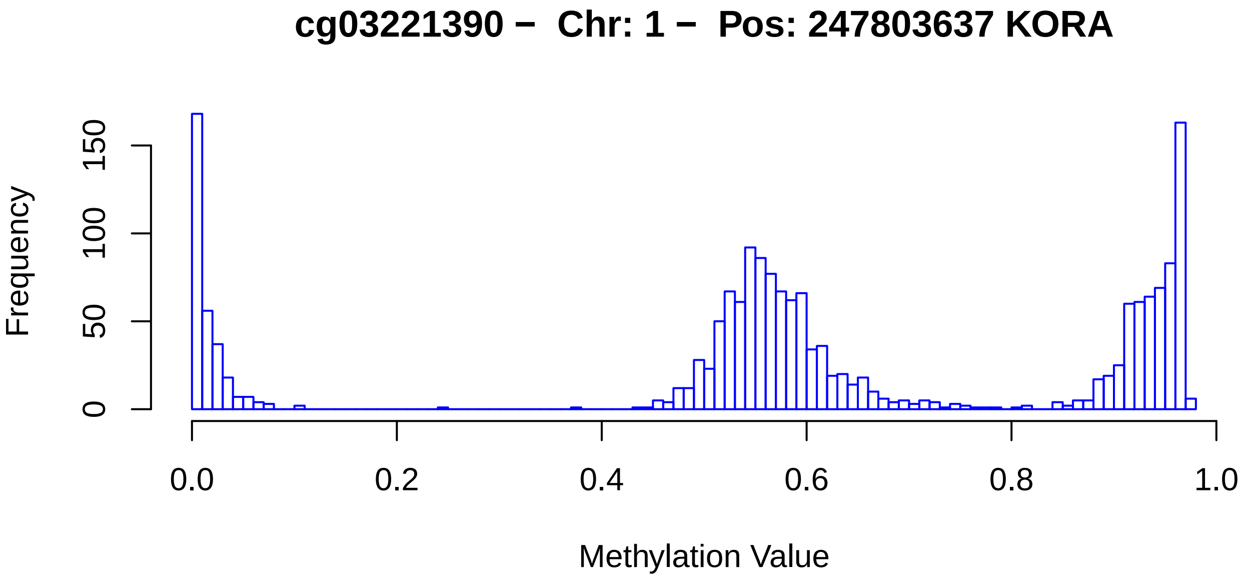
cg03221390 − Chr: 1 − Pos: 247803637 KORA
Frequency
0
50
100
150
0.0
0.2
0.4
0.6
0.8
1.0
Methylation Value

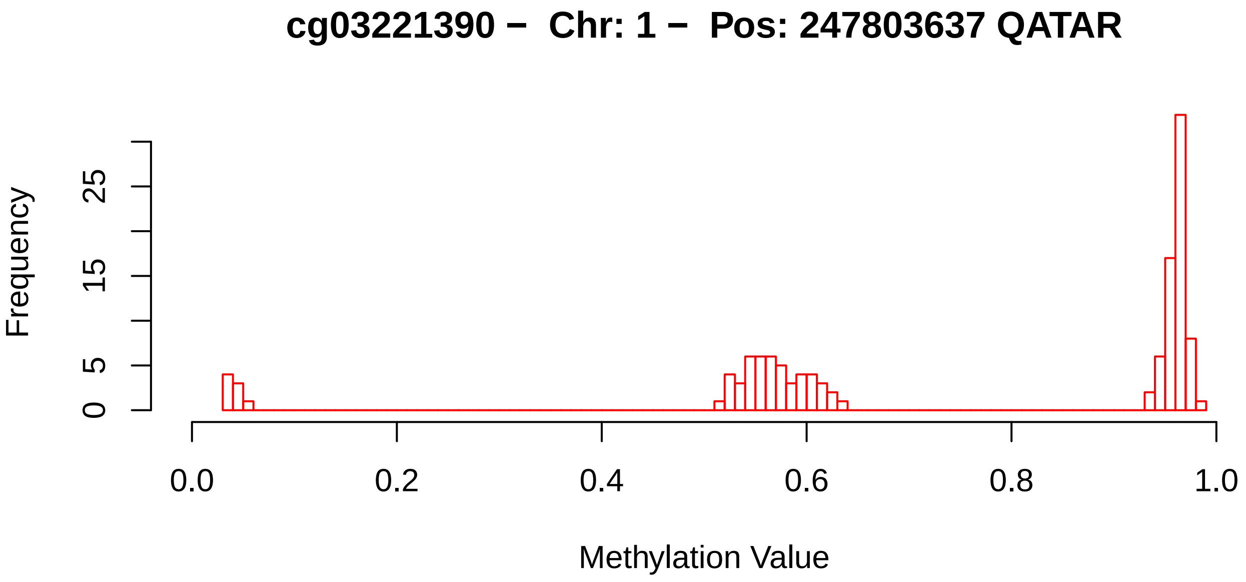
cg03221390 − Chr: 1 − Pos: 247803637 QATAR
Frequency
0
5
15
25
0.0
0.2
0.4
0.6
0.8
1.0
Methylation Value

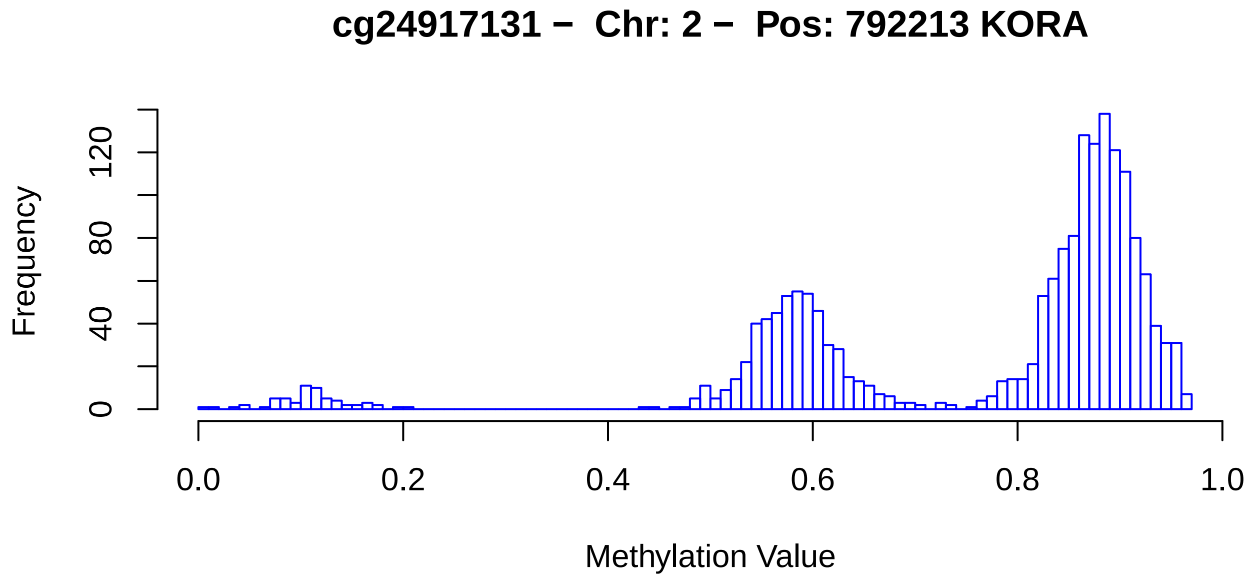
cg24917131 − Chr: 2 − Pos: 792213 KORA
Frequency
0
40
80
120
0.0
0.2
0.4
0.6
0.8
1.0
Methylation Value

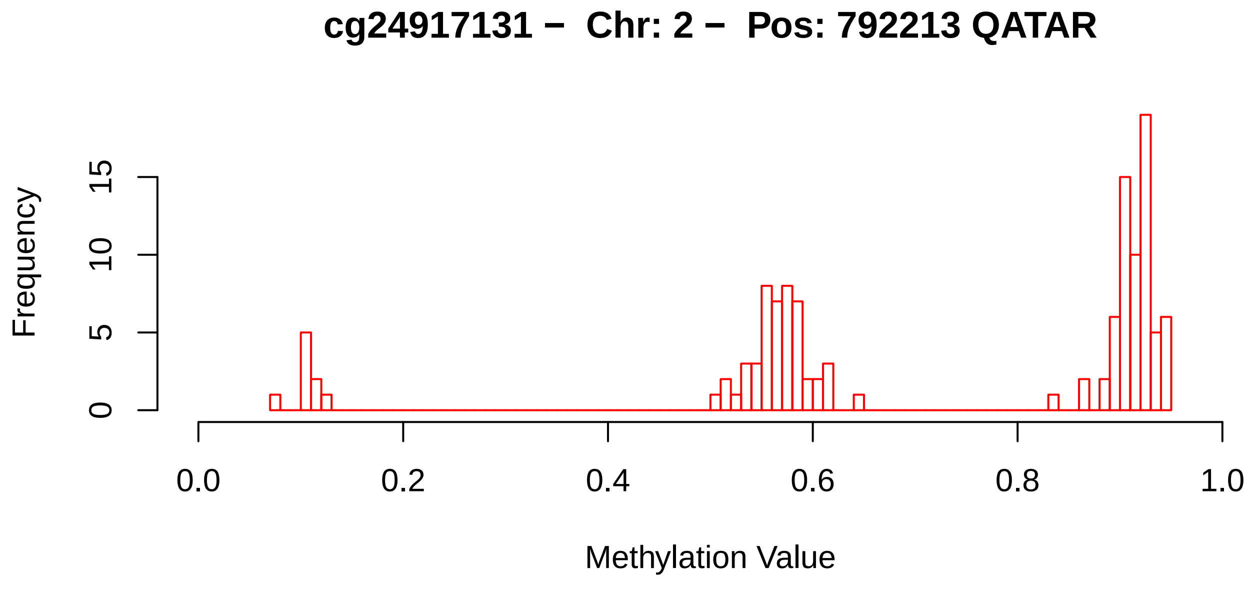
cg24917131 − Chr: 2 − Pos: 792213 QATAR
Frequency
0
5
10
15
0.0
0.2
0.4
0.6
0.8
1.0
Methylation Value

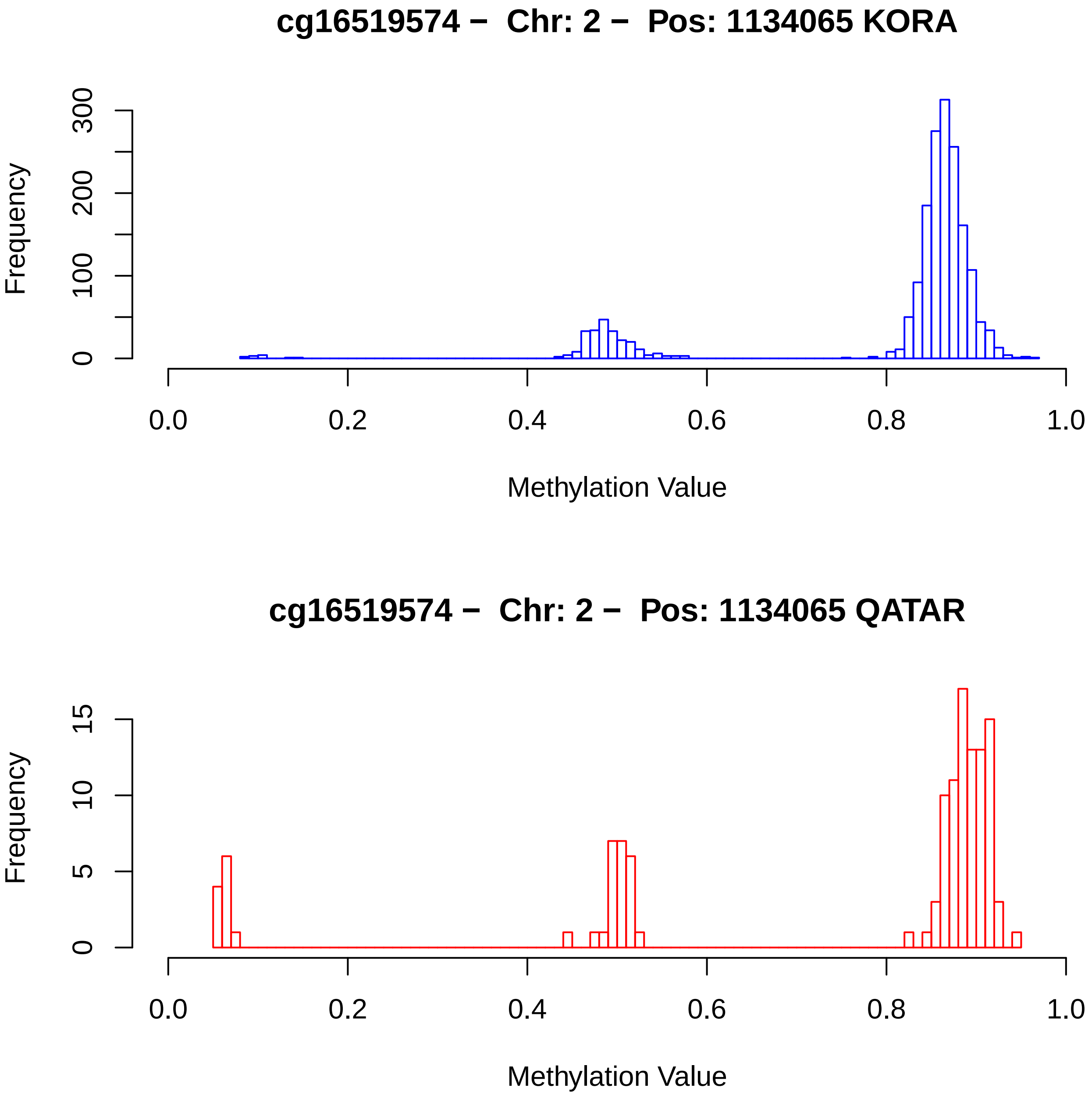

cg16519574 − Chr: 2 − Pos: 1134065 KORA
Frequency
0
100
200
300
0.0
0.2
0.4
0.6
0.8
1.0
Methylation Value
cg16519574 − Chr: 2 − Pos: 1134065 QATAR
Frequency
0
5
10
15
0.0
0.2
0.4
0.6
0.8
1.0
Methylation Value

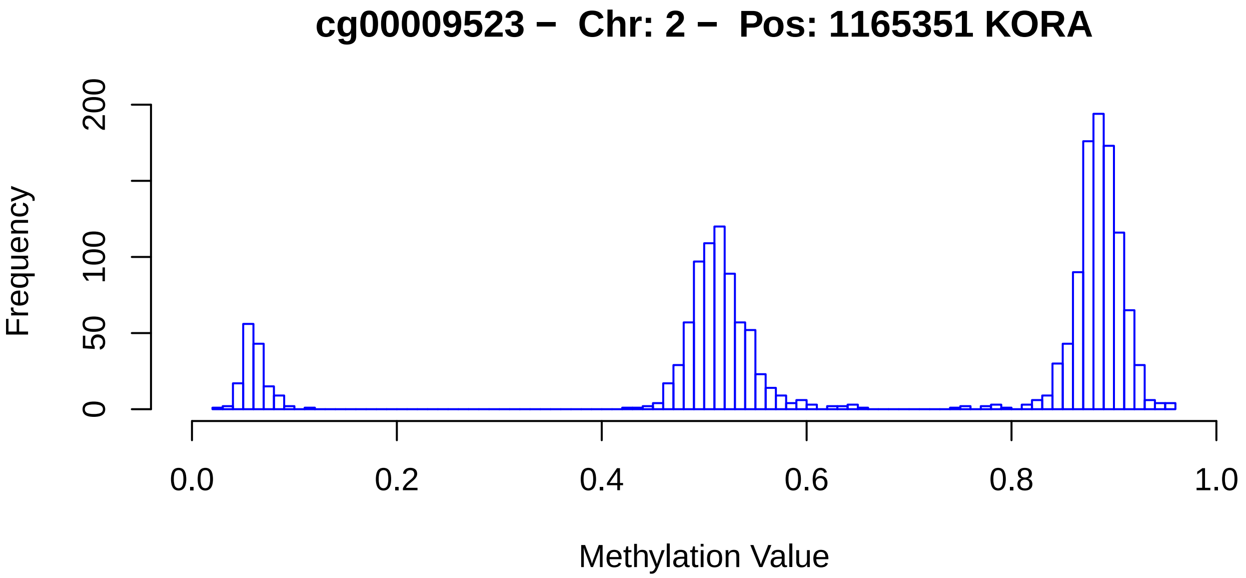
cg00009523 − Chr: 2 − Pos: 1165351 KORA
Frequency
0
50
100
200
0.0
0.2
0.4
0.6
0.8
1.0
Methylation Value

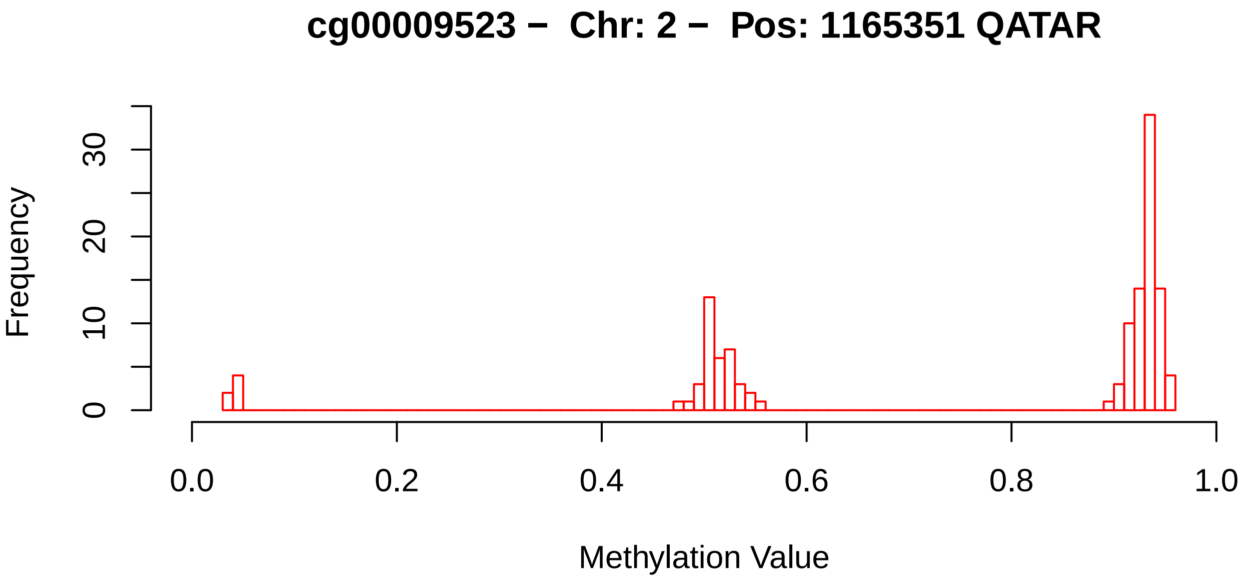
cg00009523 − Chr: 2 − Pos: 1165351 QATAR
Frequency
0
10
20
30
0.0
0.2
0.4
0.6
0.8
1.0
Methylation Value

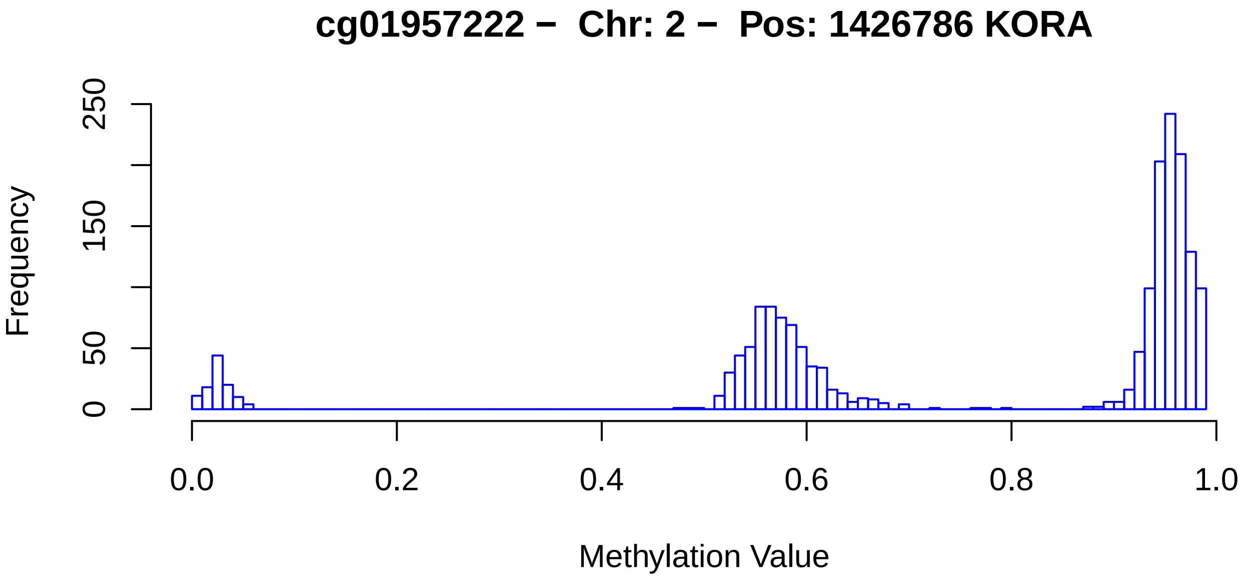
cg01957222 − Chr: 2 − Pos: 1426786 KORA
Frequency
0
50
150
250
0.0
0.2
0.4
0.6
0.8
1.0
Methylation Value

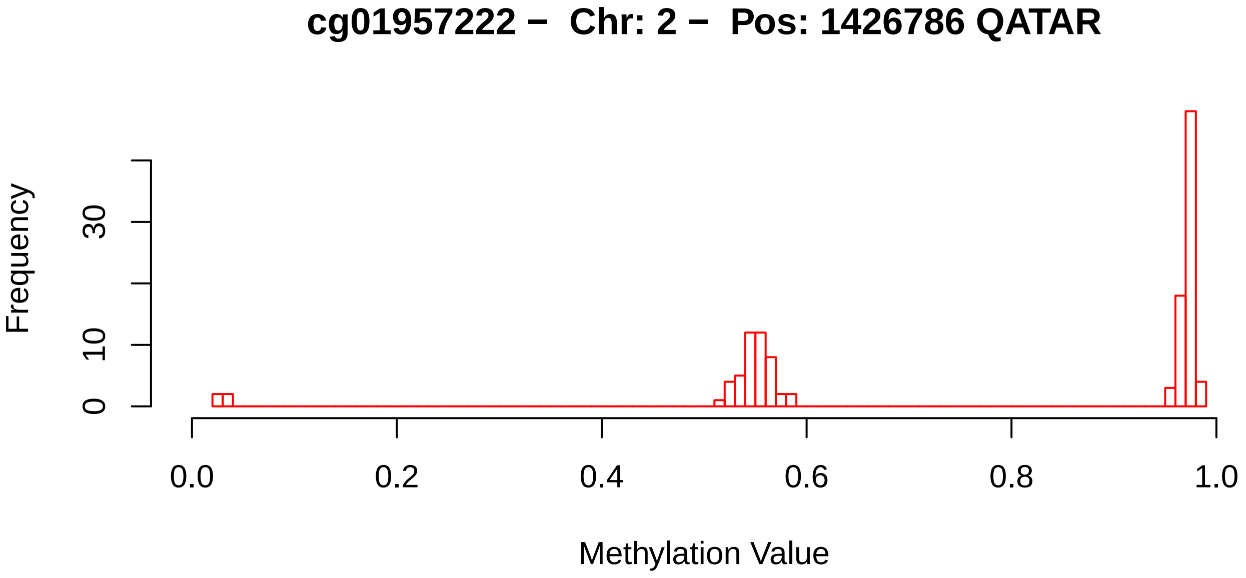
cg01957222 − Chr: 2 − Pos: 1426786 QATAR
Frequency
0
10
30
0.0
0.2
0.4
0.6
0.8
1.0
Methylation Value

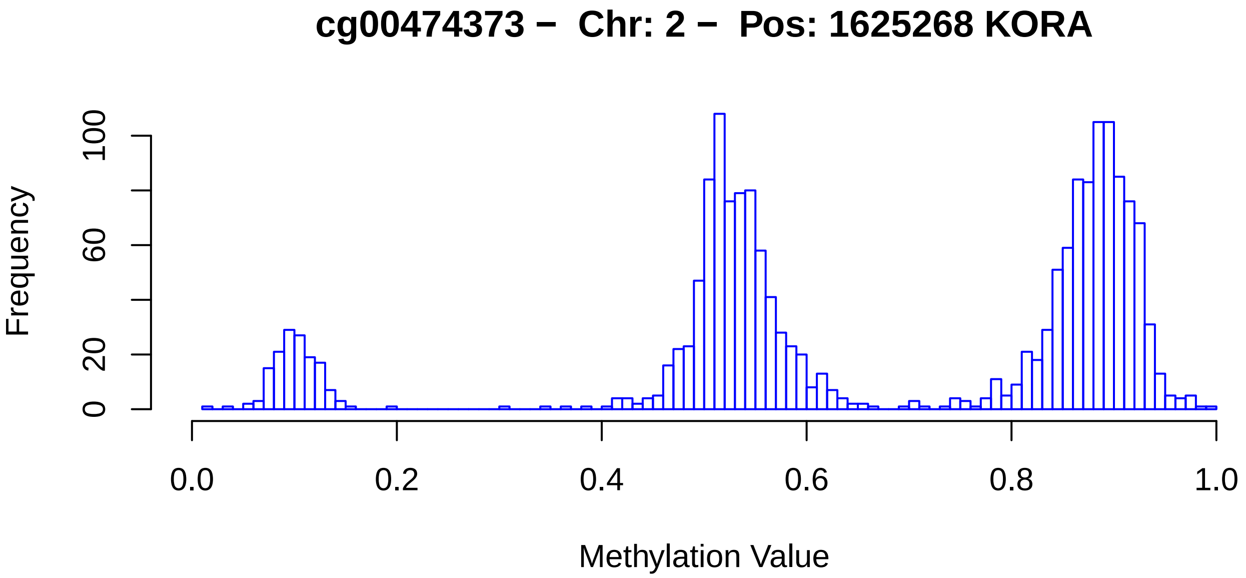
cg00474373 − Chr: 2 − Pos: 1625268 KORA
Frequency
0
20
60
100
0.0
0.2
0.4
0.6
0.8
1.0
Methylation Value

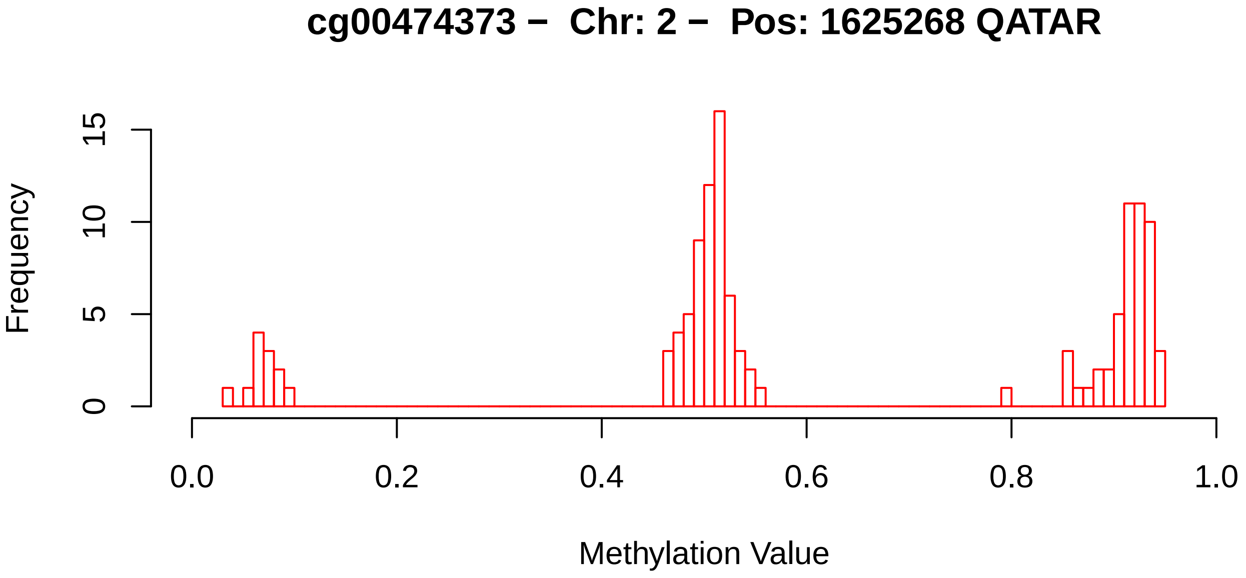
cg00474373 − Chr: 2 − Pos: 1625268 QATAR
Frequency
0
5
10
15
0.0
0.2
0.4
0.6
0.8
1.0
Methylation Value

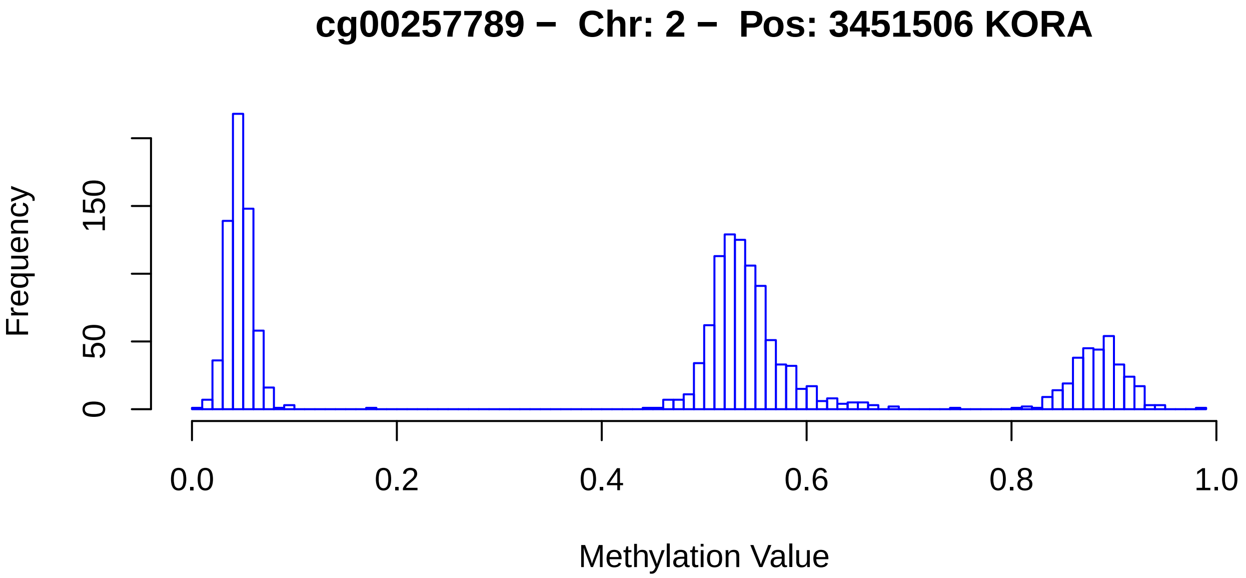
cg00257789 − Chr: 2 − Pos: 3451506 KORA
Frequency
0
50
150
0.0
0.2
0.4
0.6
0.8
1.0
Methylation Value

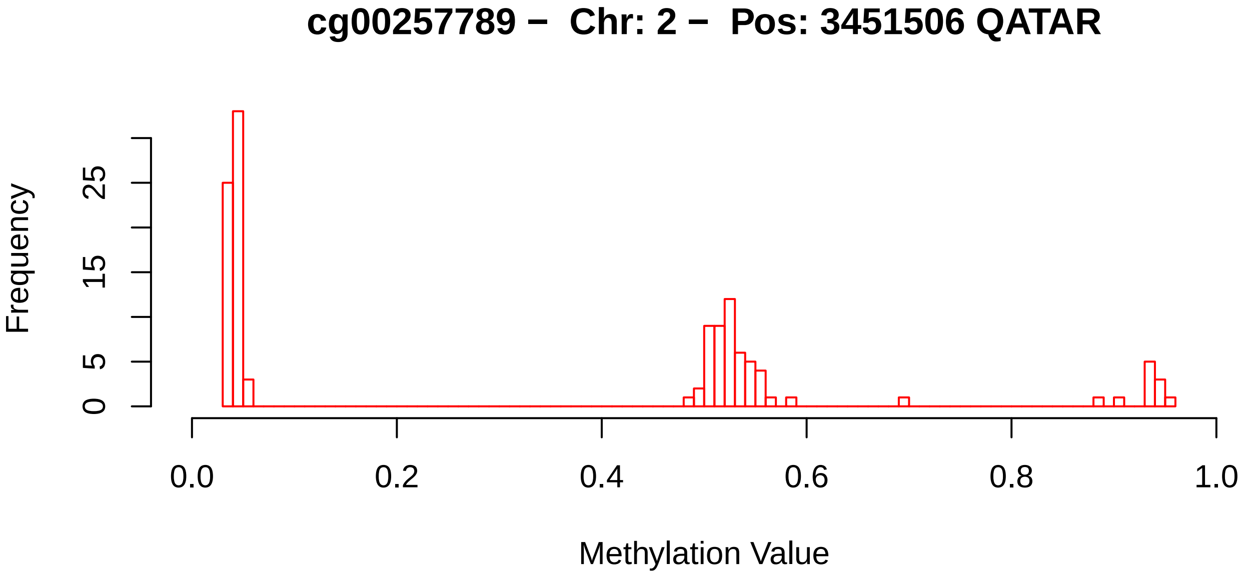
cg00257789 − Chr: 2 − Pos: 3451506 QATAR
Frequency
0
5
15
25
0.0
0.2
0.4
0.6
0.8
1.0
Methylation Value

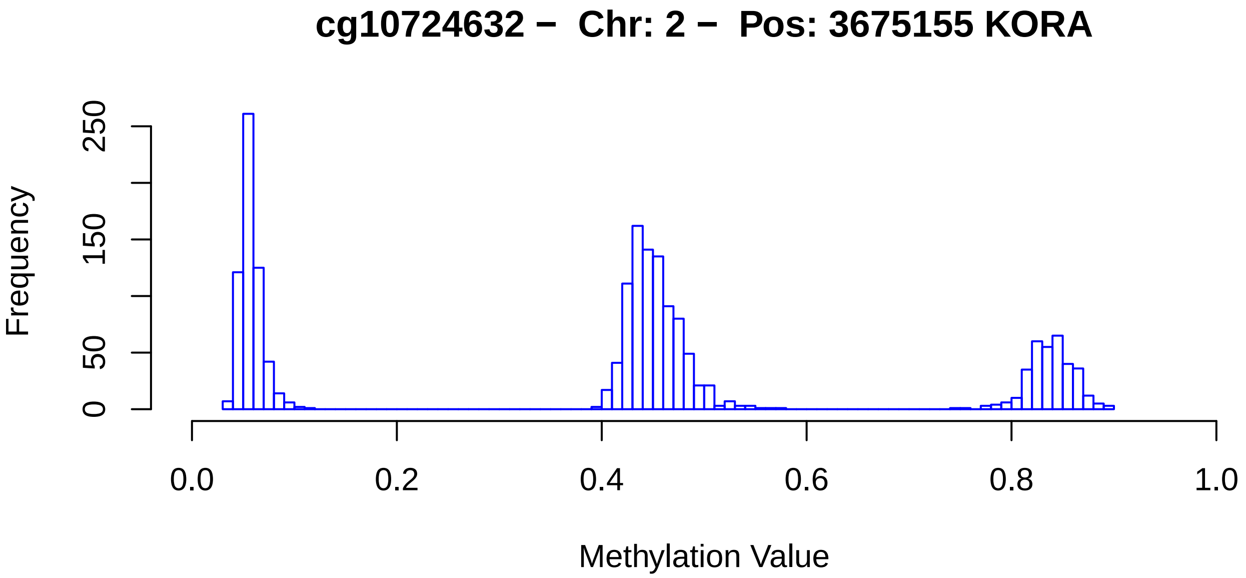
cg10724632 − Chr: 2 − Pos: 3675155 KORA
Frequency
0
50
150
250
0.0
0.2
0.4
0.6
0.8
1.0
Methylation Value

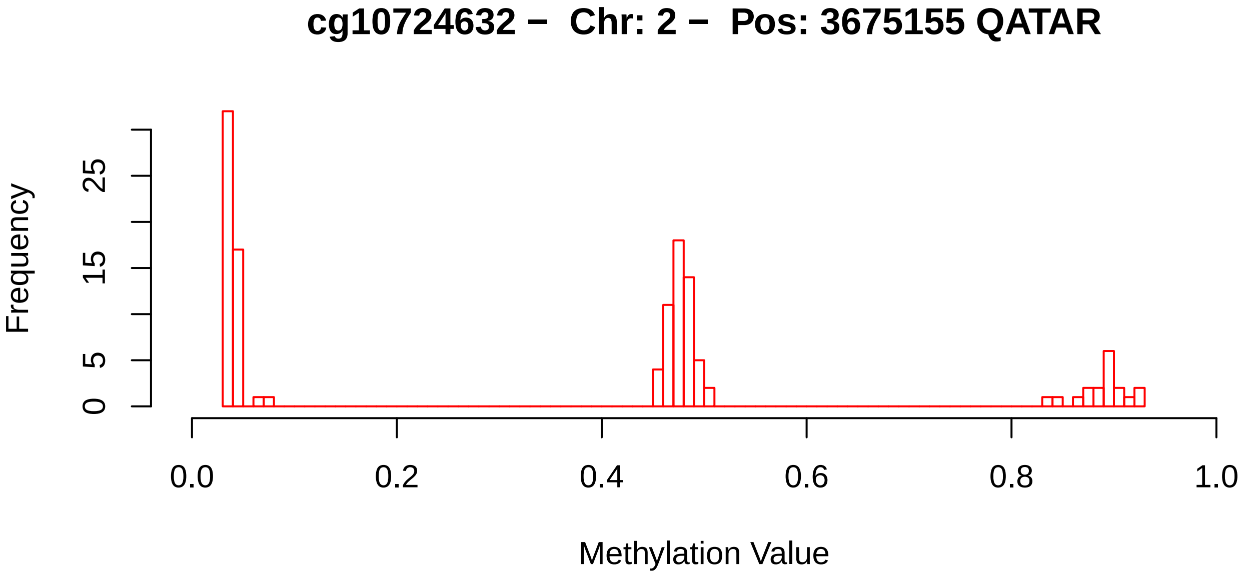
cg10724632 − Chr: 2 − Pos: 3675155 QATAR
Frequency
0
5
15
25
0.0
0.2
0.4
0.6
0.8
1.0
Methylation Value

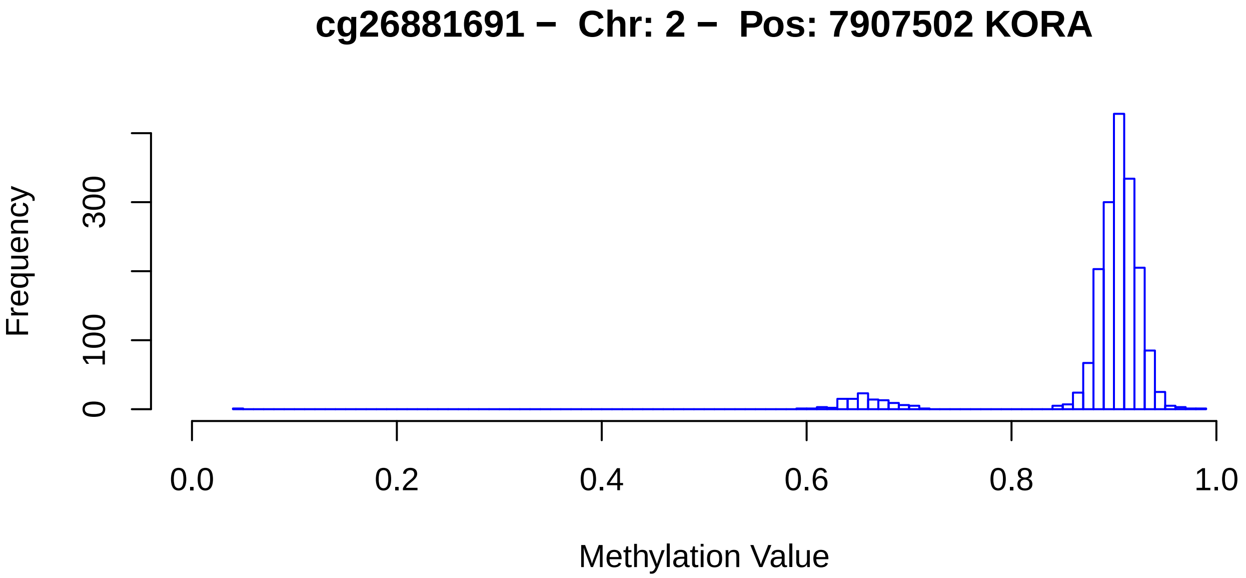
cg26881691 − Chr: 2 − Pos: 7907502 KORA
Frequency
0
100
300
0.0
0.2
0.4
0.6
0.8
1.0
Methylation Value

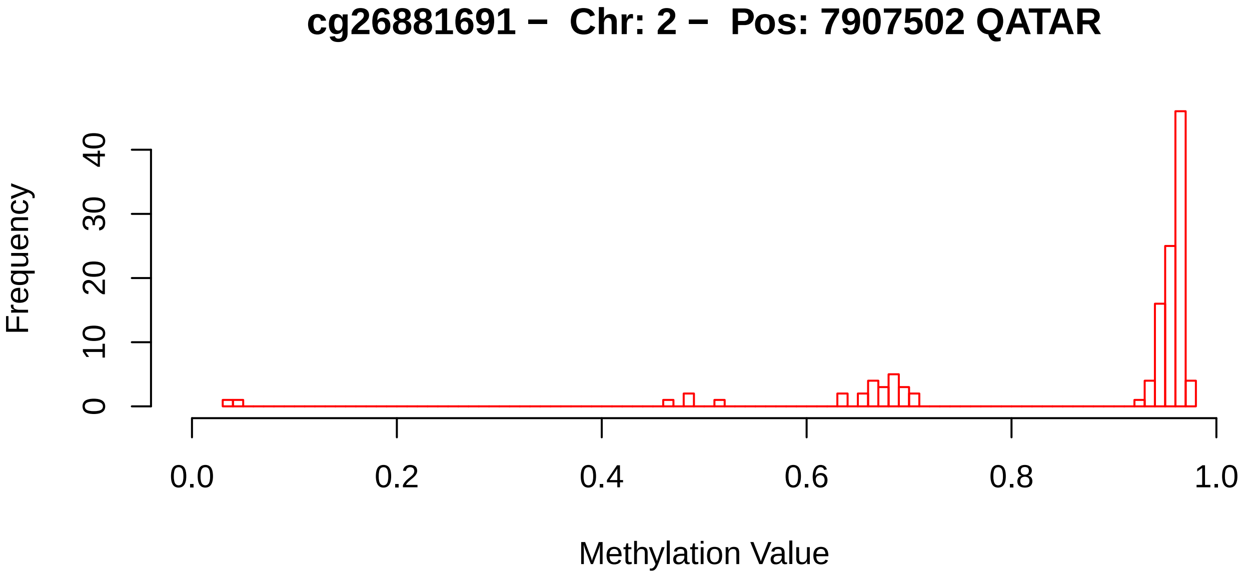
cg26881691 − Chr: 2 − Pos: 7907502 QATAR
Frequency
0
10
20
30
40
0.0
0.2
0.4
0.6
0.8
1.0
Methylation Value

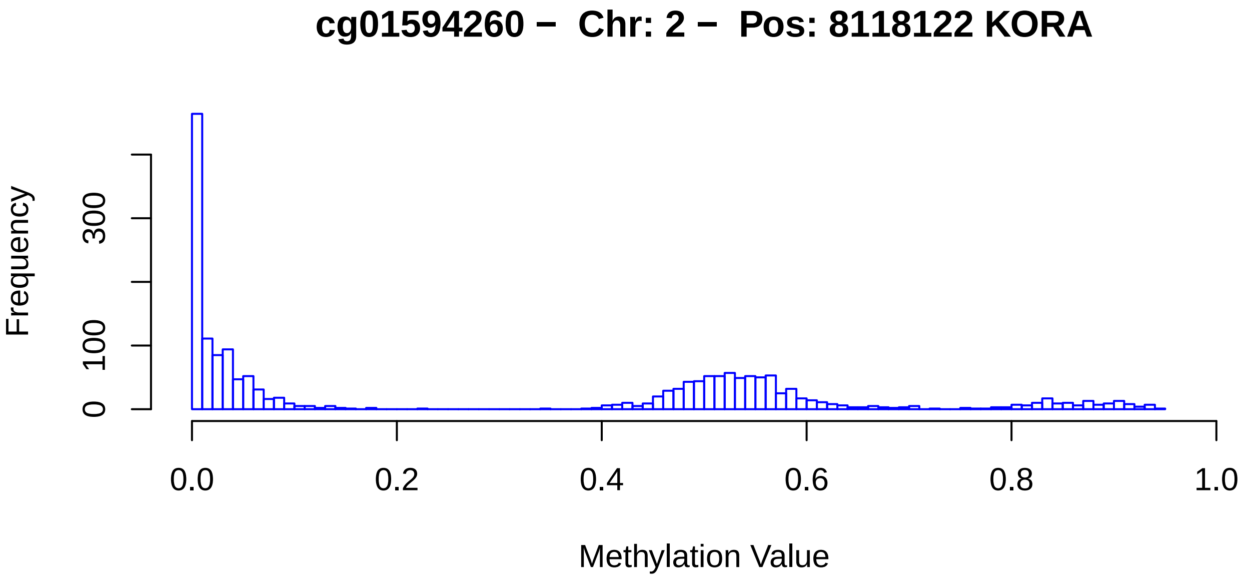
cg01594260 − Chr: 2 − Pos: 8118122 KORA
Frequency
0
100
300
0.0
0.2
0.4
0.6
0.8
1.0
Methylation Value

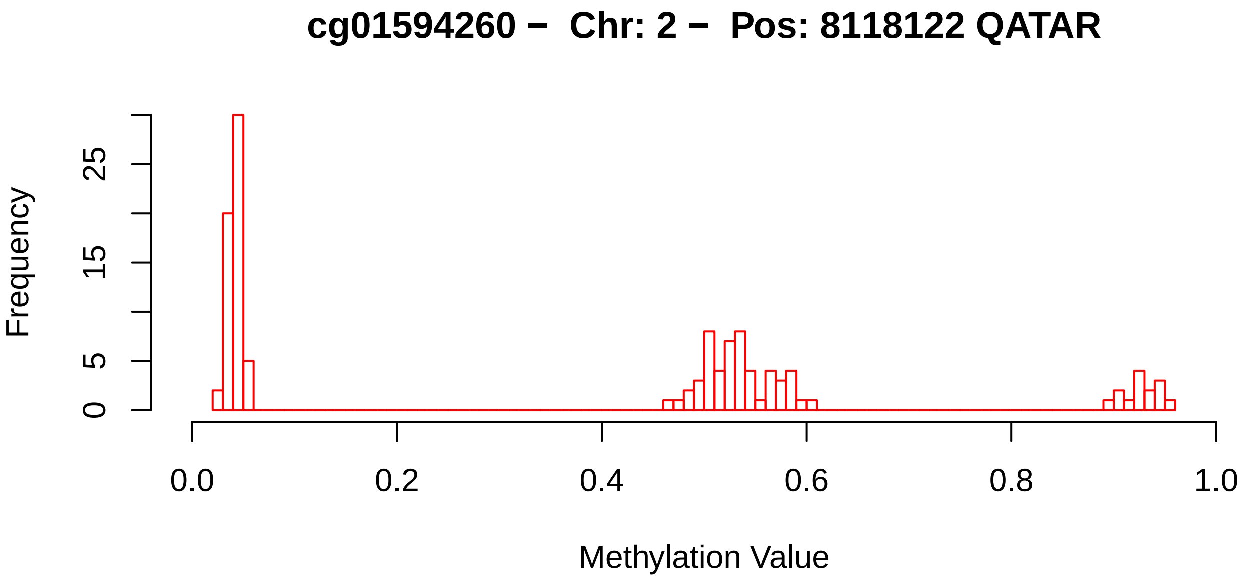
cg01594260 − Chr: 2 − Pos: 8118122 QATAR
Frequency
0
5
15
25
0.0
0.2
0.4
0.6
0.8
1.0
Methylation Value

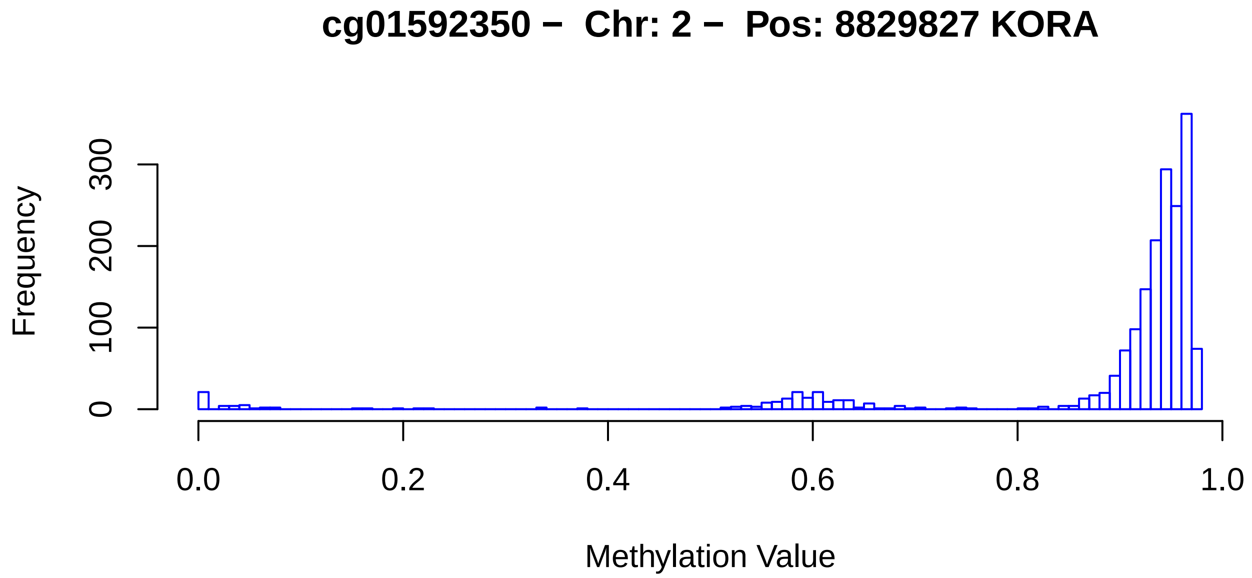
cg01592350 − Chr: 2 − Pos: 8829827 KORA
Frequency
0
100
200
300
0.0
0.2
0.4
0.6
0.8
1.0
Methylation Value

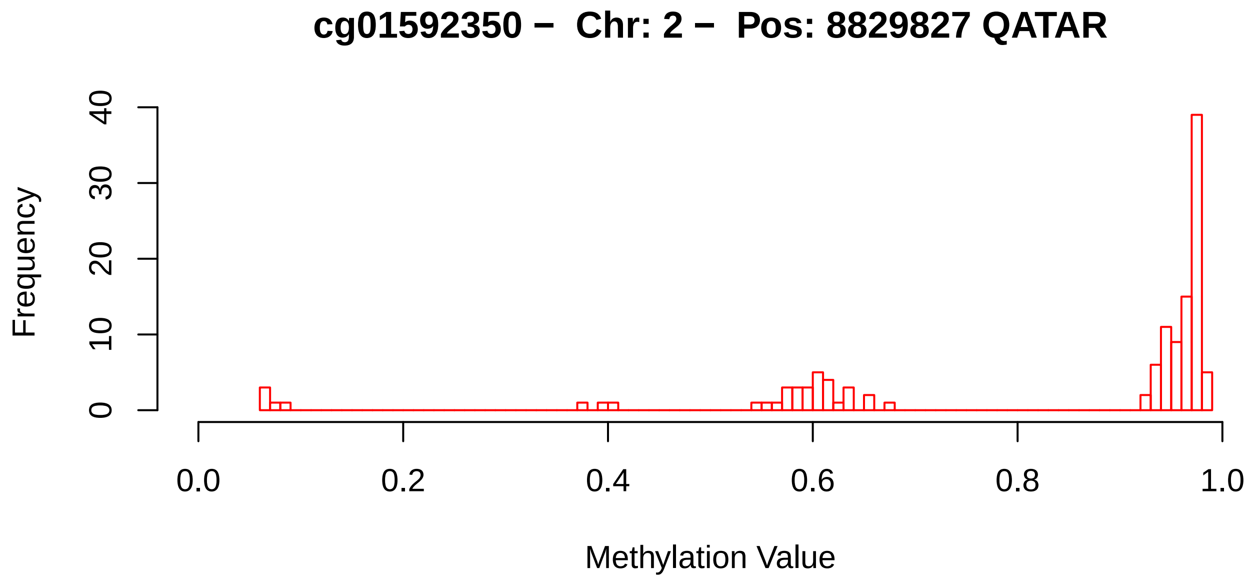
cg01592350 − Chr: 2 − Pos: 8829827 QATAR
Frequency
0
10
20
30
40
0.0
0.2
0.4
0.6
0.8
1.0
Methylation Value

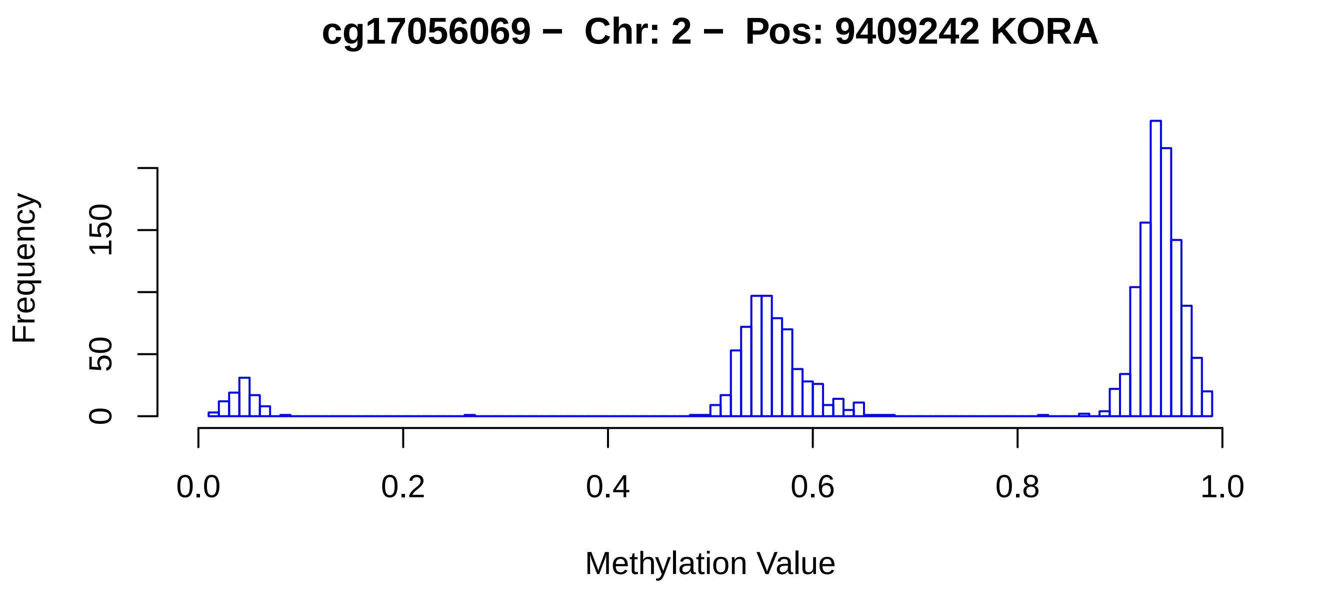
cg17056069 − Chr: 2 − Pos: 9409242 KORA
Frequency
0
50
150
0.0
0.2
0.4
0.6
0.8
1.0
Methylation Value

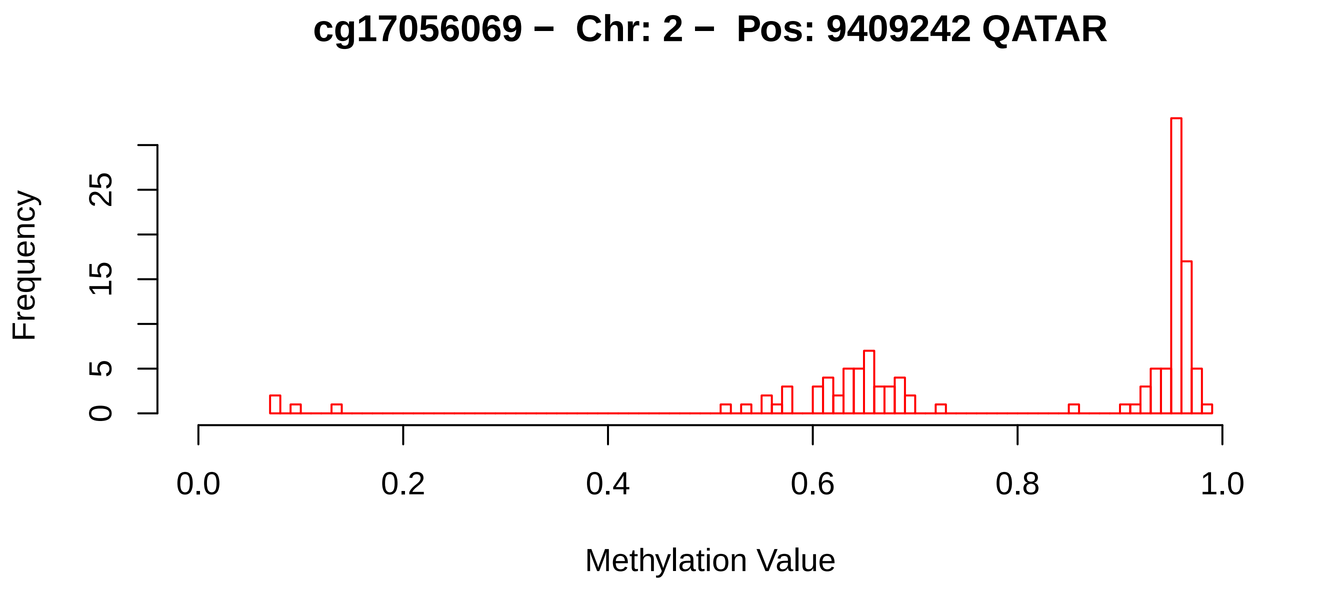
cg17056069 − Chr: 2 − Pos: 9409242 QATAR
Frequency
0
5
15
25
0.0
0.2
0.4
0.6
0.8
1.0
Methylation Value

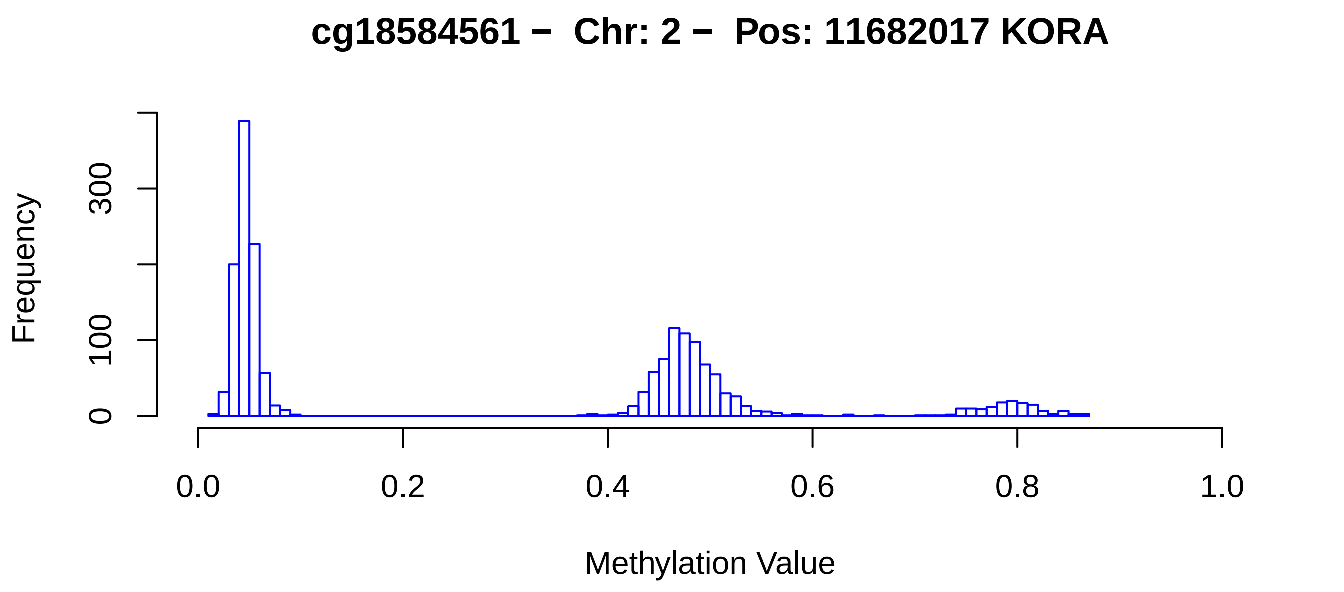

cg18584561 − Chr: 2 − Pos: 11682017 KORA
Frequency
0
100
300
0.0
0.2
0.4
0.6
0.8
1.0
Methylation Value

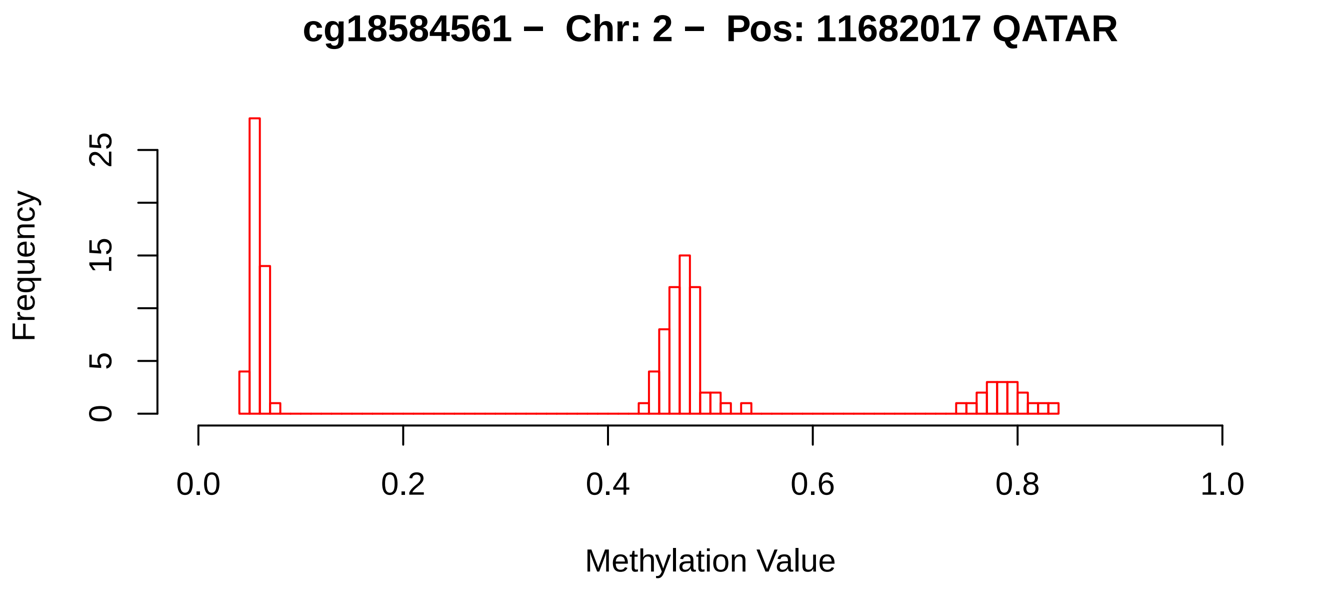

cg18584561 − Chr: 2 − Pos: 11682017 QATAR
Frequency
0
5
15
25
0.0
0.2
0.4
0.6
0.8
1.0
Methylation Value

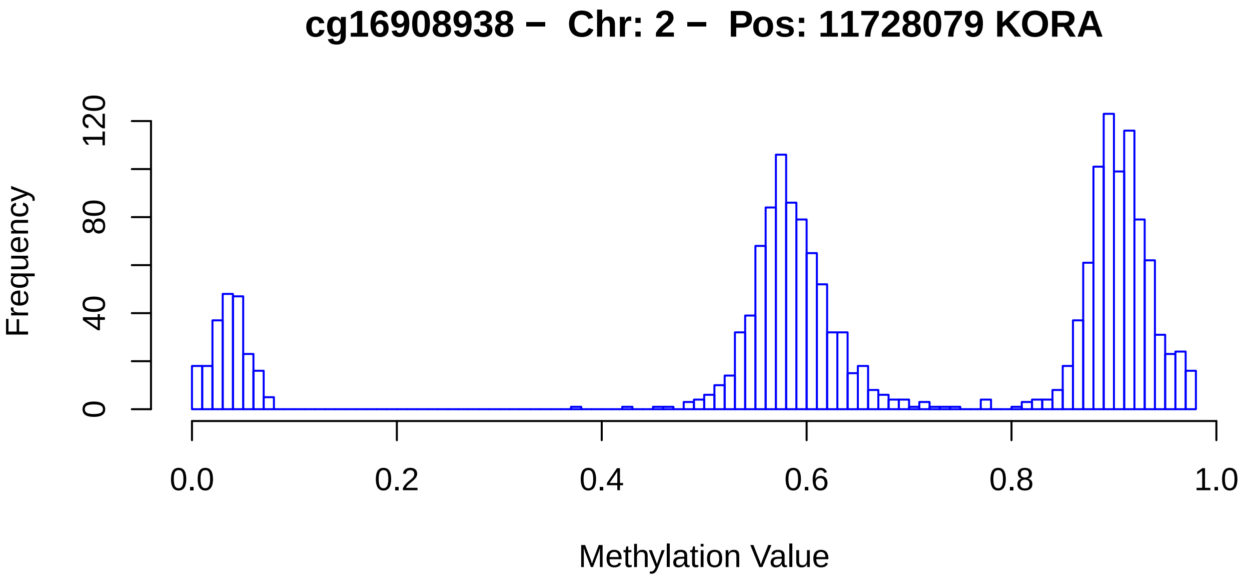
cg16908938 − Chr: 2 − Pos: 11728079 KORA
Frequency
0
40
80
120
0.0
0.2
0.4
0.6
0.8
1.0
Methylation Value

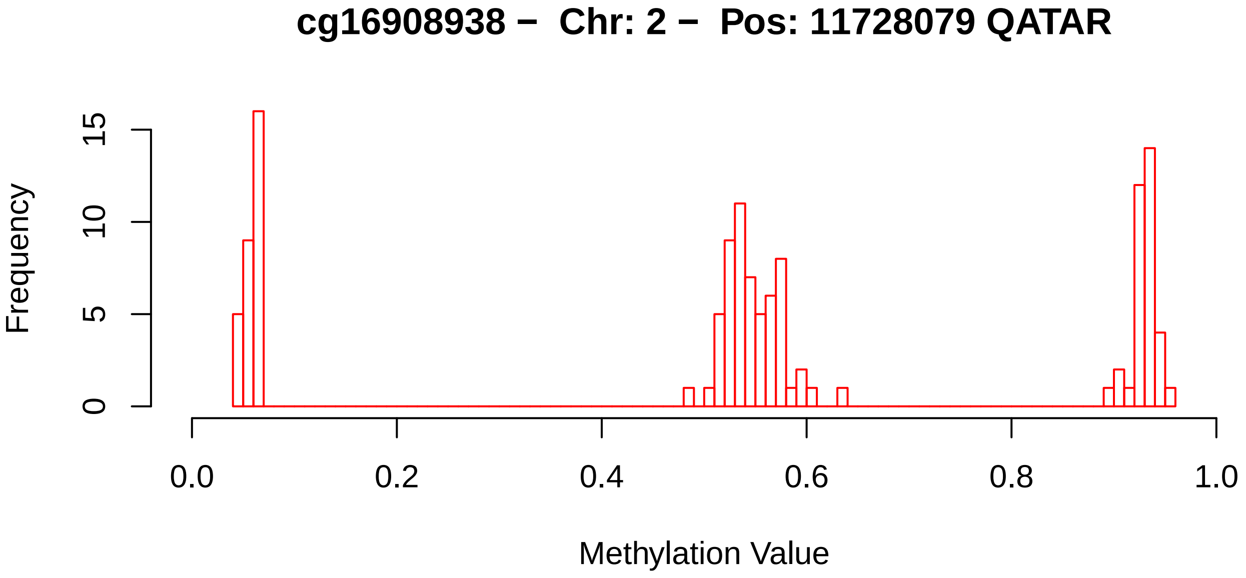
cg16908938 − Chr: 2 − Pos: 11728079 QATAR
Frequency
0
5
10
15
0.0
0.2
0.4
0.6
0.8
1.0
Methylation Value

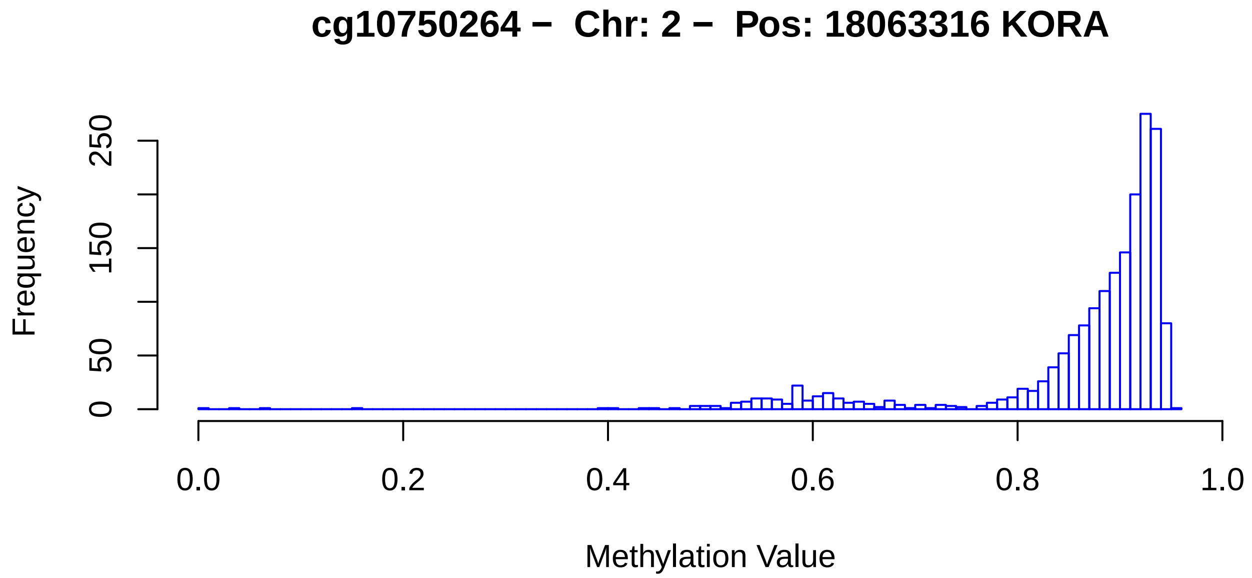
cg10750264 − Chr: 2 − Pos: 18063316 KORA
Frequency
0
50
150
250
0.0
0.2
0.4
0.6
0.8
1.0
Methylation Value

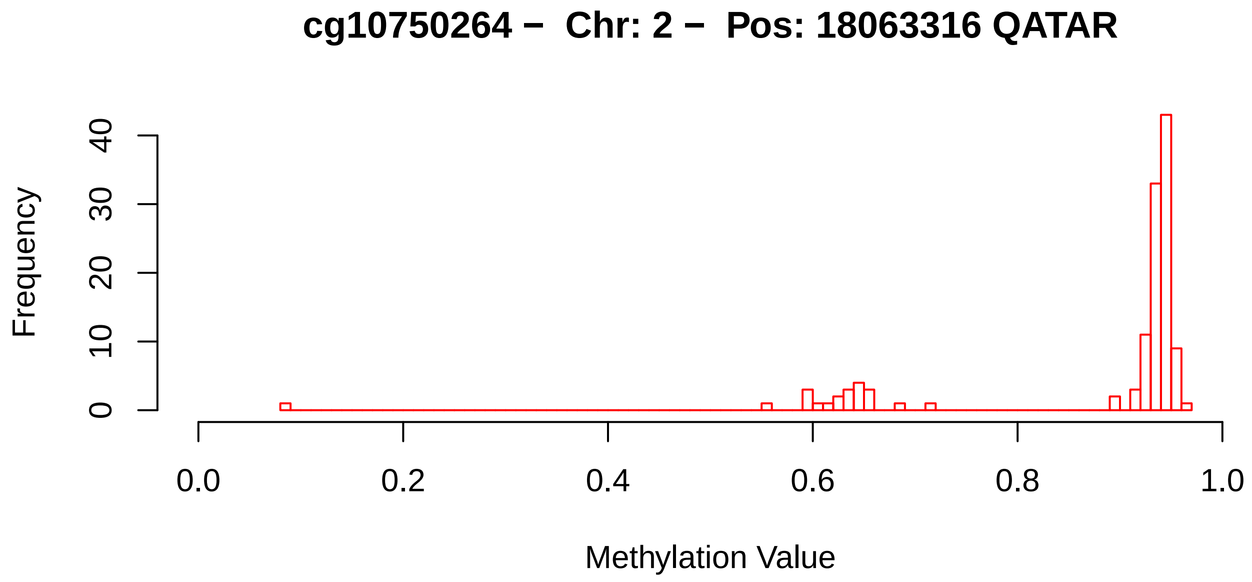
cg10750264 − Chr: 2 − Pos: 18063316 QATAR
Frequency
0
10
20
30
40
0.0
0.2
0.4
0.6
0.8
1.0
Methylation Value

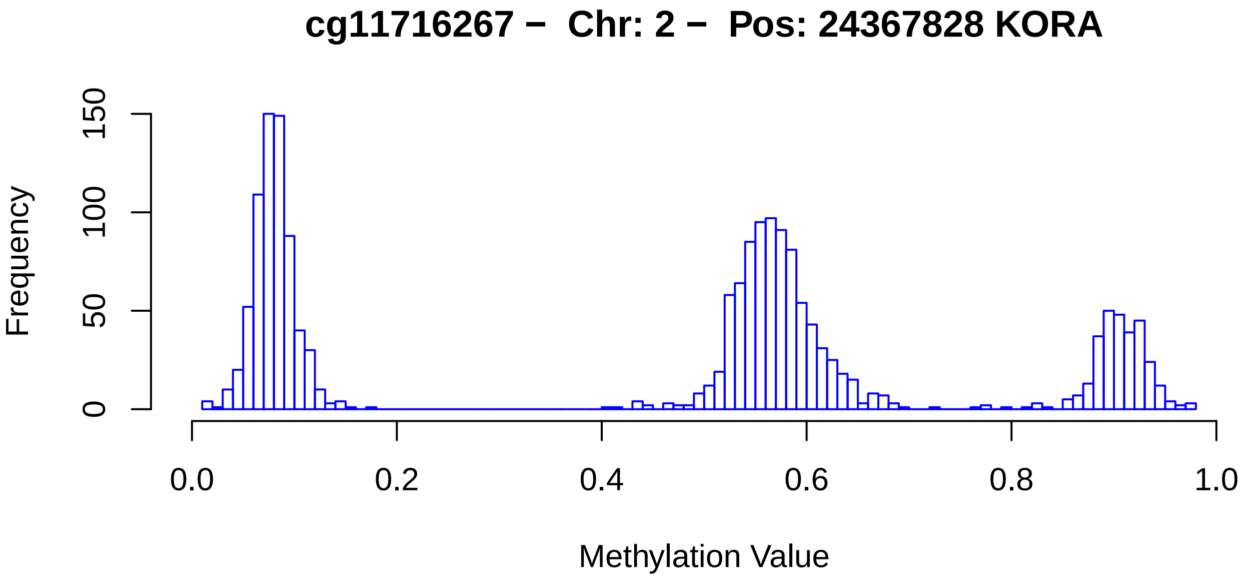
cg11716267 − Chr: 2 − Pos: 24367828 KORA
Frequency
0
50
100
150
0.0
0.2
0.4
0.6
0.8
1.0
Methylation Value

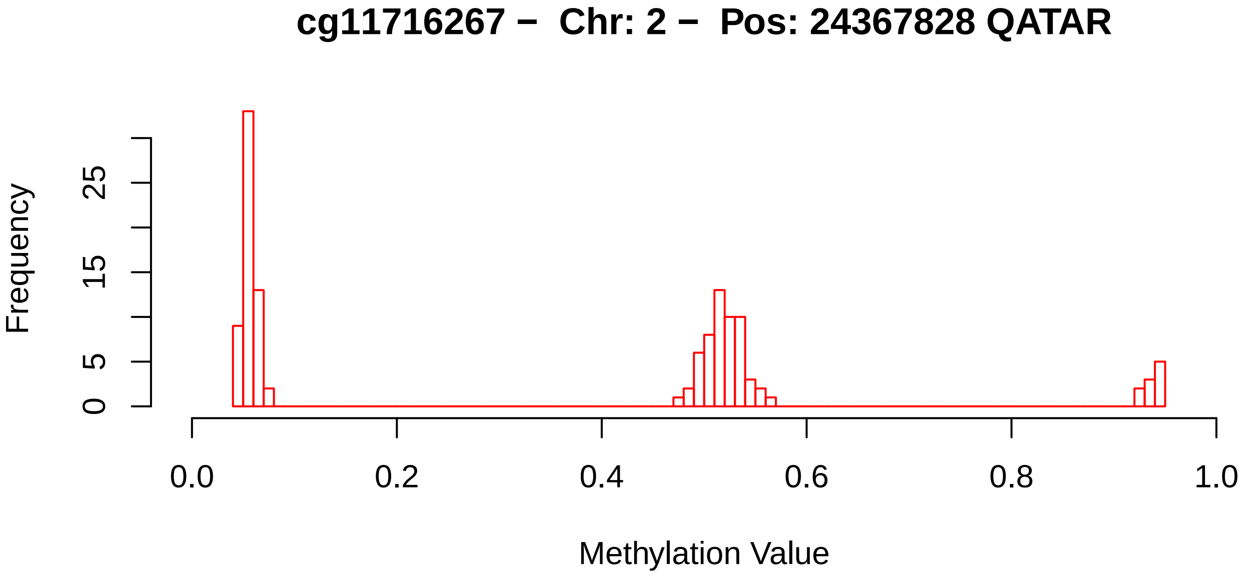
cg11716267 − Chr: 2 − Pos: 24367828 QATAR
Frequency
0
5
15
25
0.0
0.2
0.4
0.6
0.8
1.0
Methylation Value

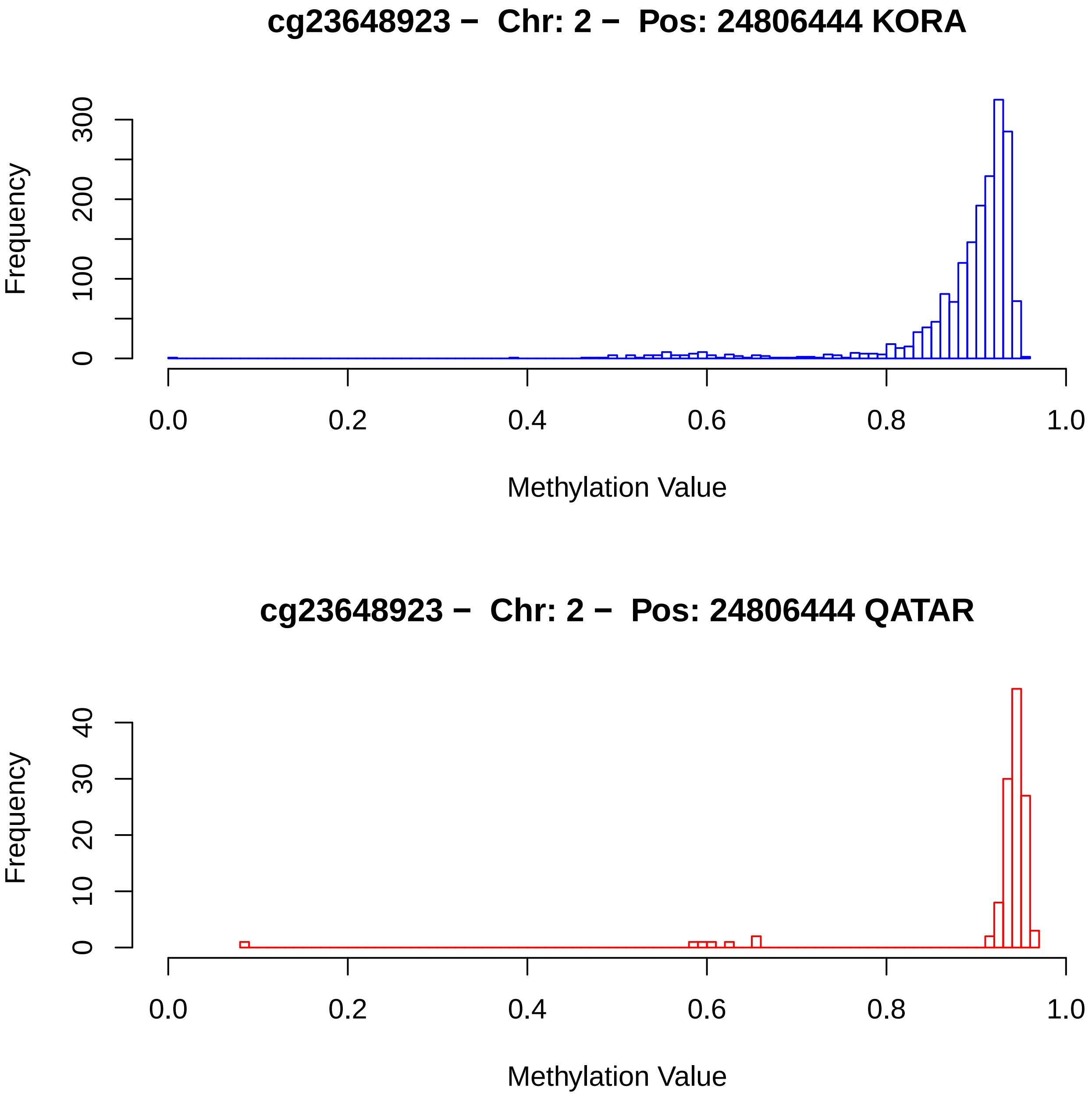
cg23648923 − Chr: 2 − Pos: 24806444 KORA
Frequency
0
100
200
300
0.0
0.2
0.4
0.6
0.8
1.0
Methylation Value
cg23648923 − Chr: 2 − Pos: 24806444 QATAR
Frequency
0
10
20
30
40
0.0
0.2
0.4
0.6
0.8
1.0
Methylation Value

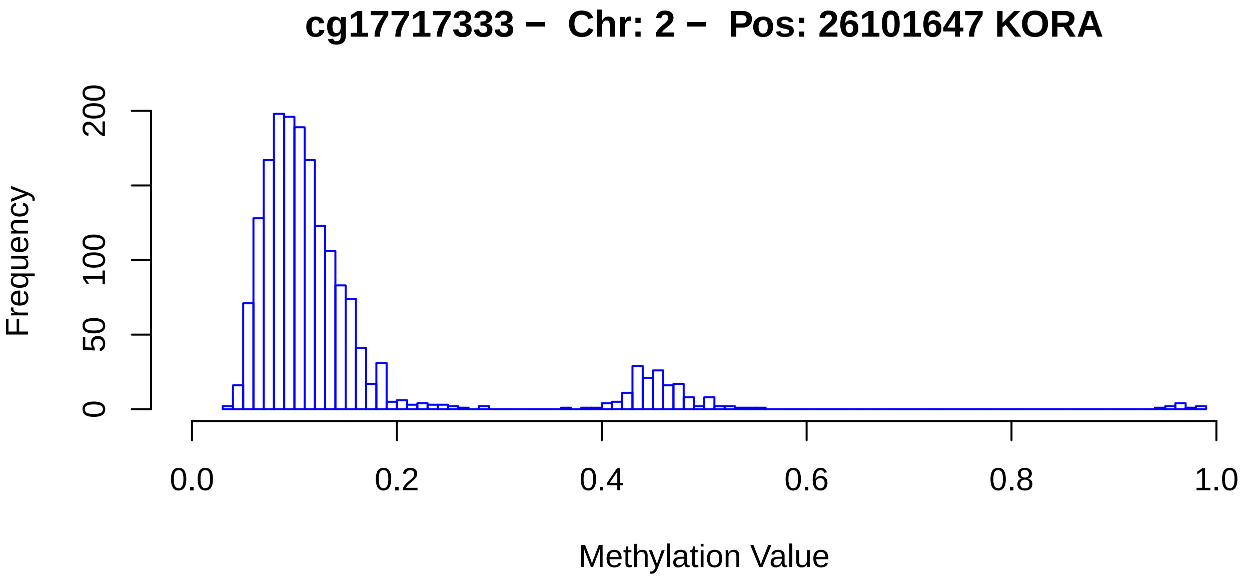

cg17717333 − Chr: 2 − Pos: 26101647 KORA
Frequency
0
50
100
200
0.0
0.2
0.4
0.6
0.8
1.0
Methylation Value

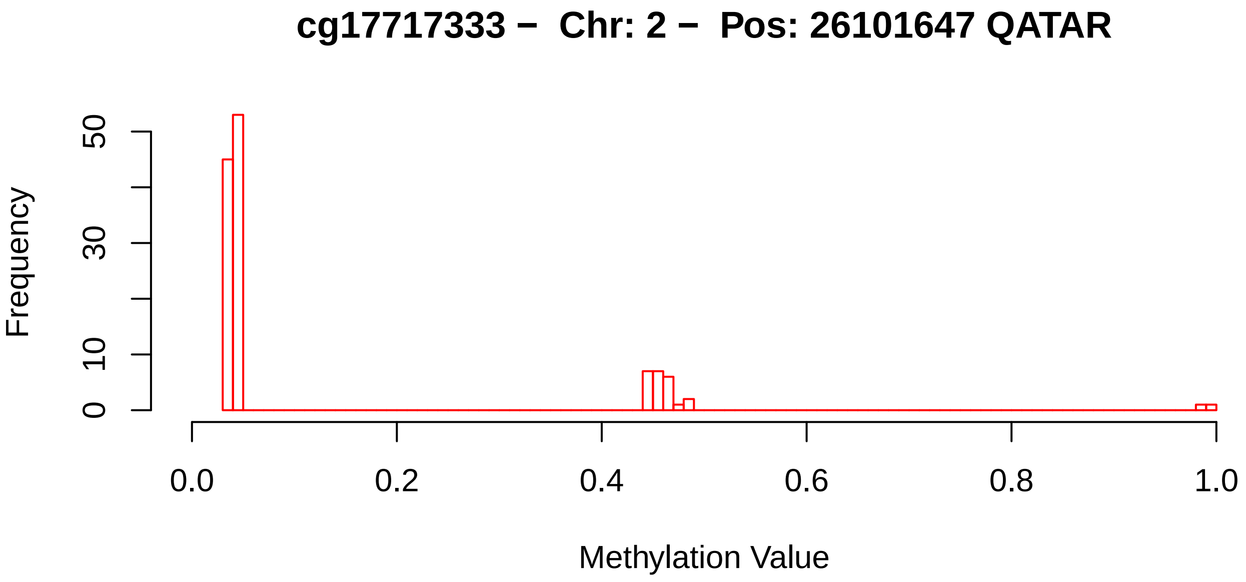

cg17717333 − Chr: 2 − Pos: 26101647 QATAR
Frequency
0
10
30
50
0.0
0.2
0.4
0.6
0.8
1.0
Methylation Value

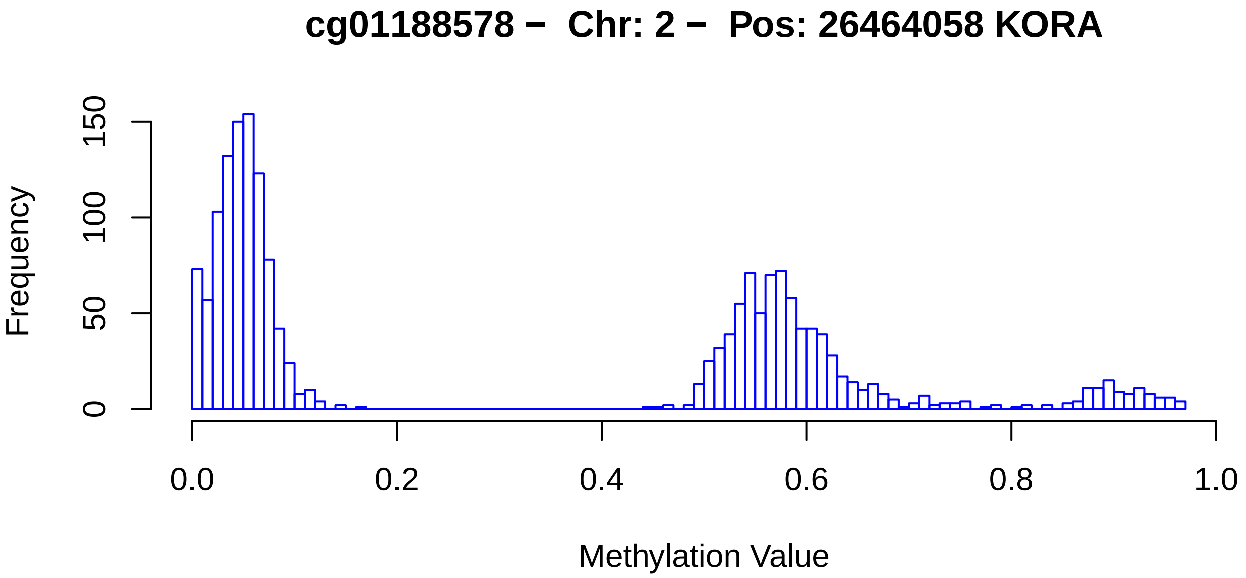
cg01188578 − Chr: 2 − Pos: 26464058 KORA
Frequency
0
50
100
150
0.0
0.2
0.4
0.6
0.8
1.0
Methylation Value

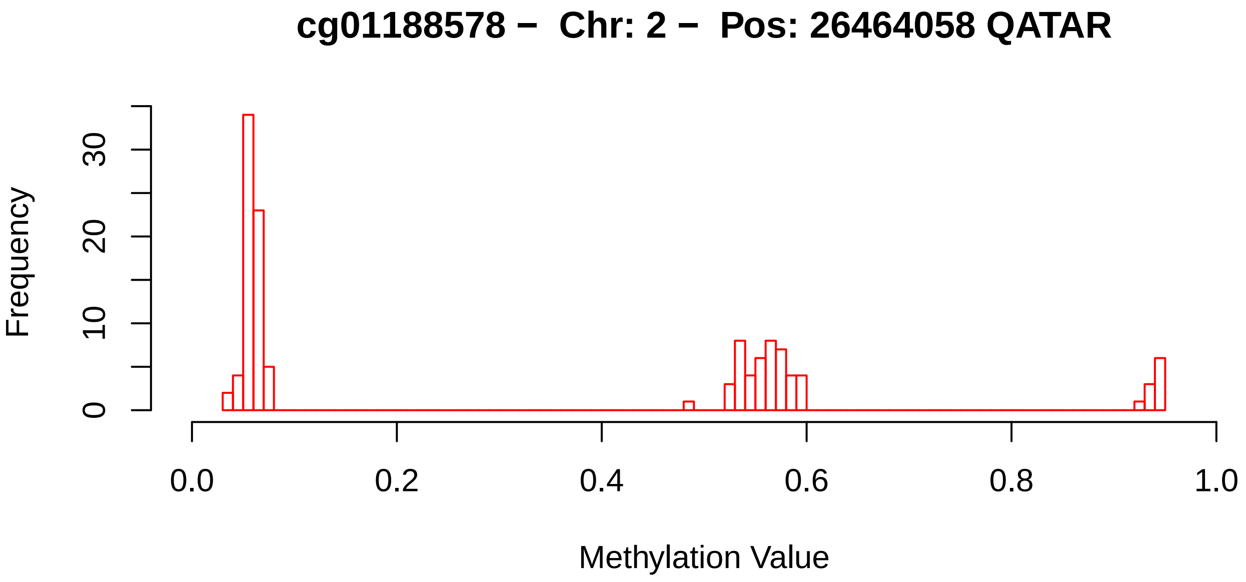
cg01188578 − Chr: 2 − Pos: 26464058 QATAR
Frequency
0
10
20
30
0.0
0.2
0.4
0.6
0.8
1.0
Methylation Value

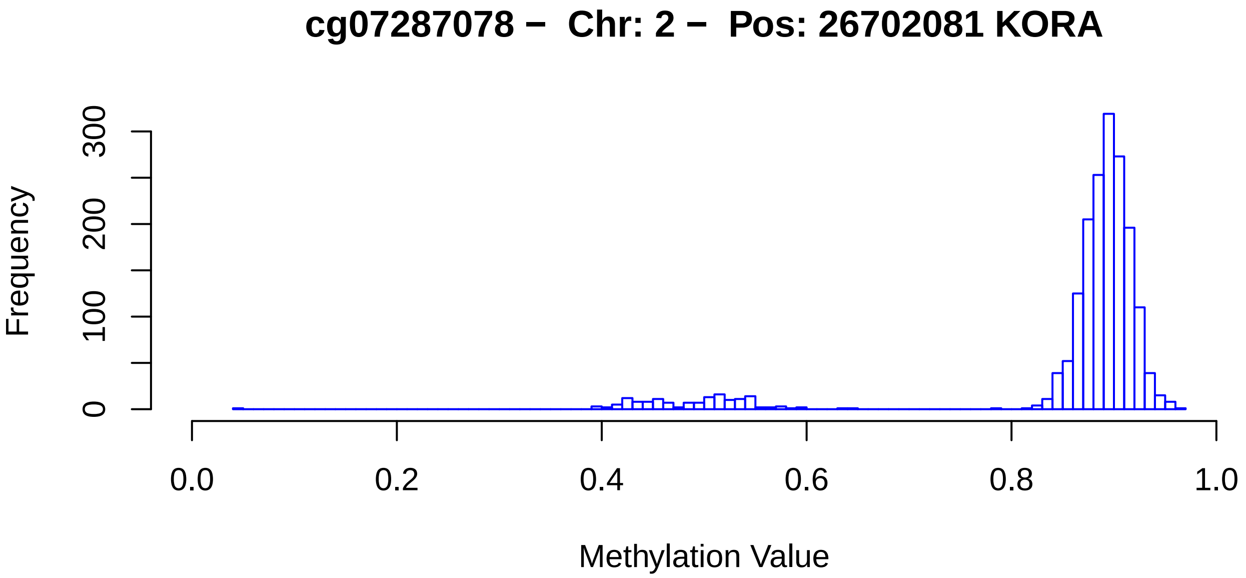
cg07287078 − Chr: 2 − Pos: 26702081 KORA
Frequency
0
100
200
300
0.0
0.2
0.4
0.6
0.8
1.0
Methylation Value

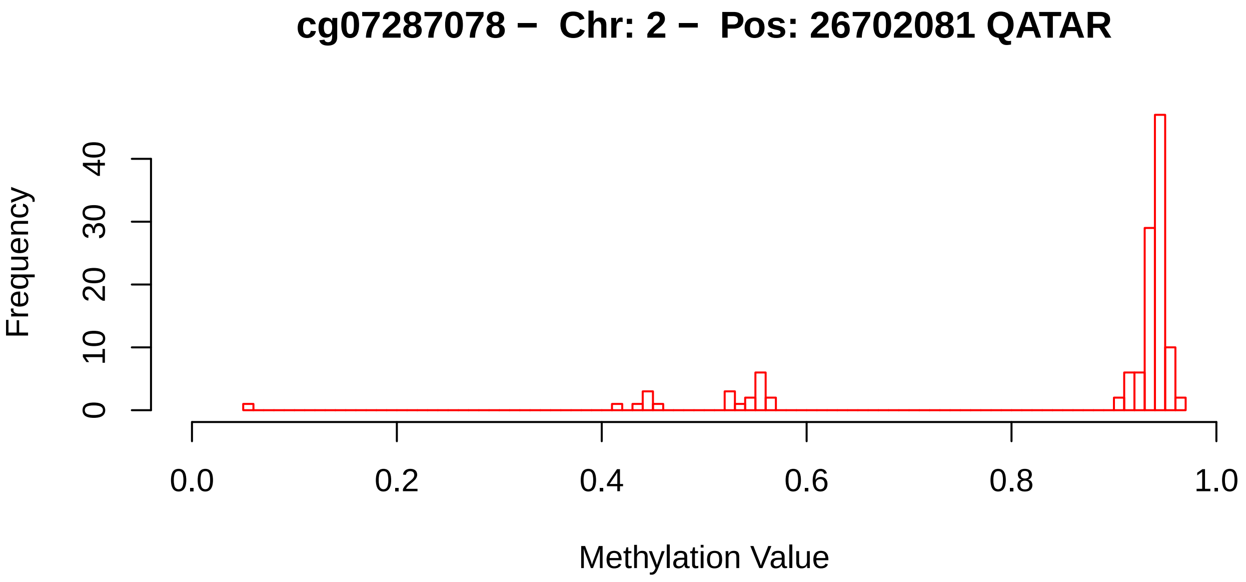
cg07287078 − Chr: 2 − Pos: 26702081 QATAR
Frequency
0
10
20
30
40
0.0
0.2
0.4
0.6
0.8
1.0
Methylation Value

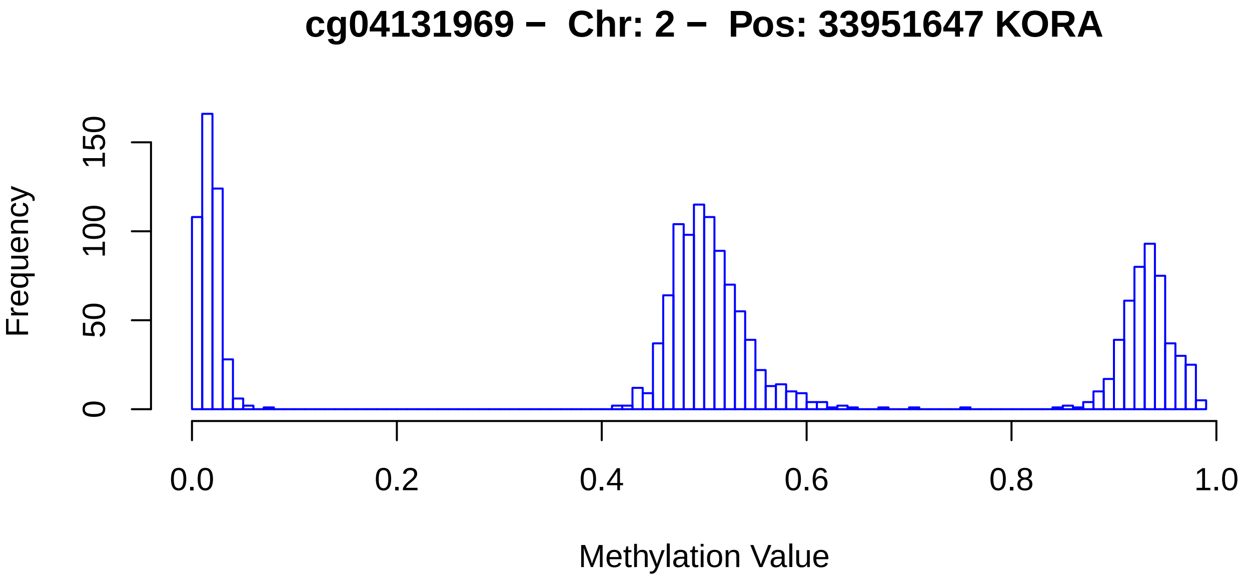
cg04131969 − Chr: 2 − Pos: 33951647 KORA
Frequency
0
50
100
150
0.0
0.2
0.4
0.6
0.8
1.0
Methylation Value

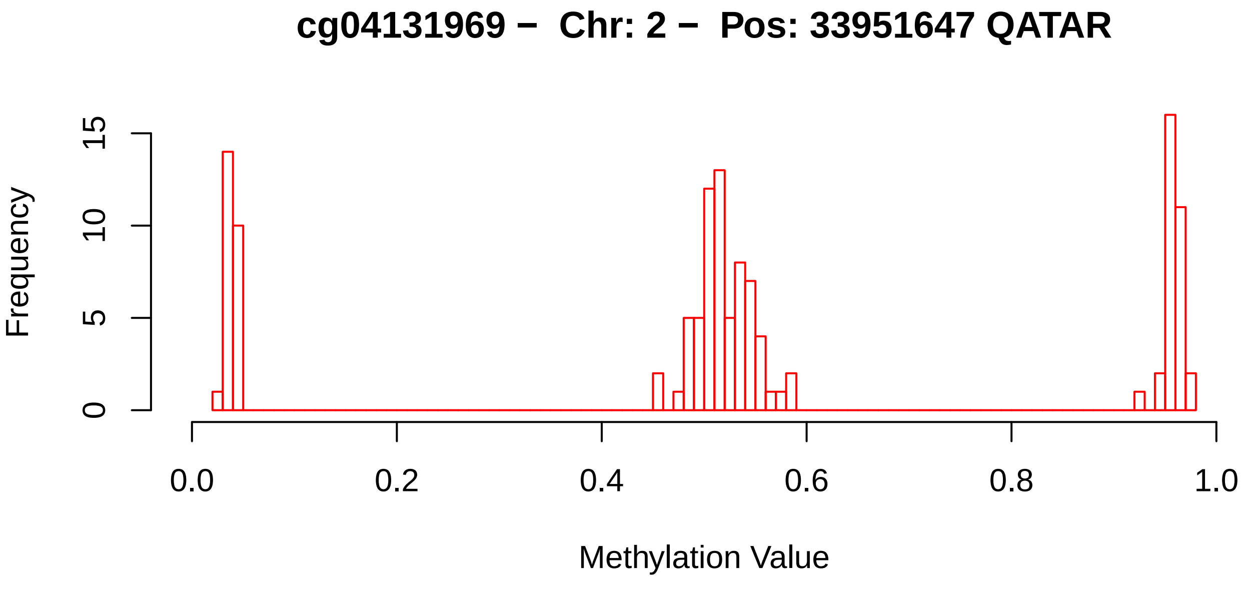
cg04131969 − Chr: 2 − Pos: 33951647 QATAR
Frequency
0
5
10
15
0.0
0.2
0.4
0.6
0.8
1.0
Methylation Value

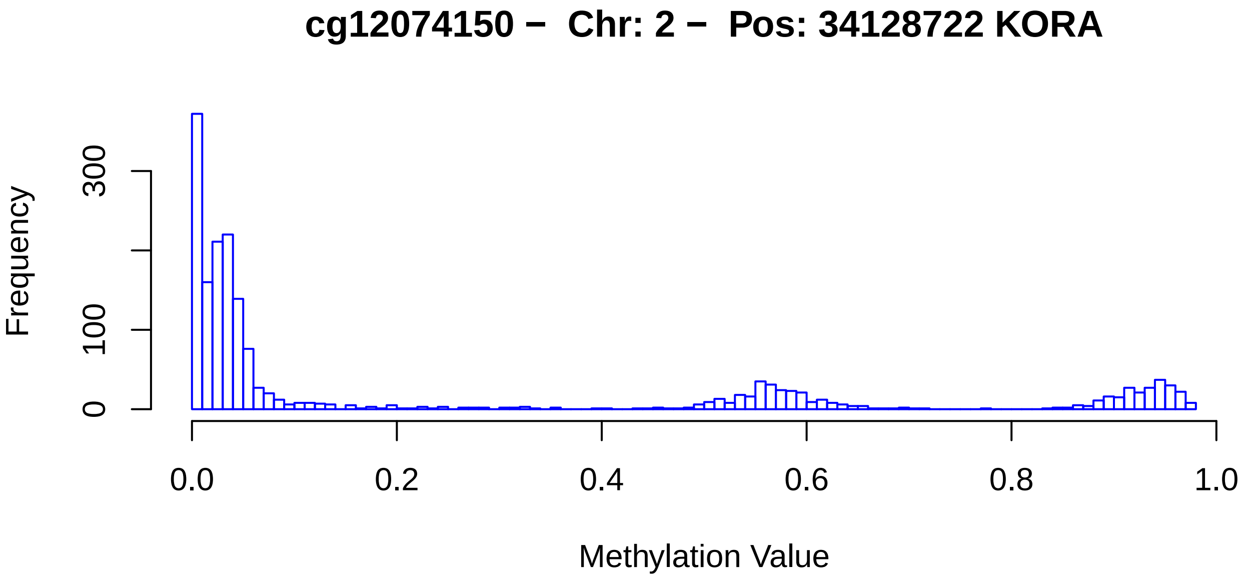
cg12074150 − Chr: 2 − Pos: 34128722 KORA
Frequency
0
100
300
0.0
0.2
0.4
0.6
0.8
1.0
Methylation Value

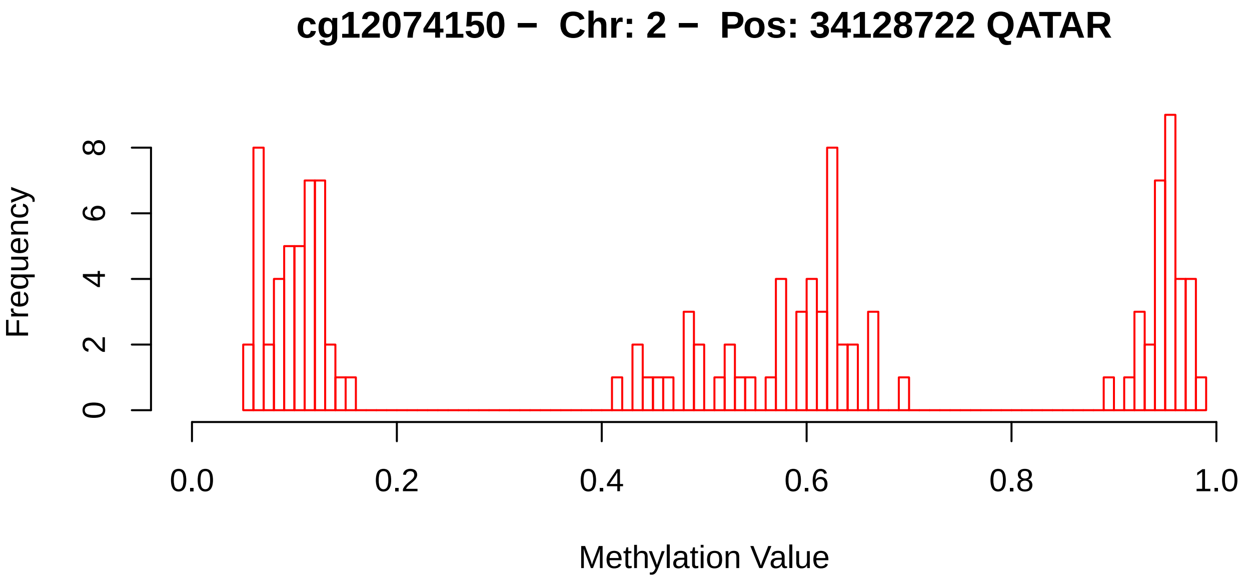
cg12074150 − Chr: 2 − Pos: 34128722 QATAR
Frequency
0
2
4
6
8
0.0
0.2
0.4
0.6
0.8
1.0
Methylation Value

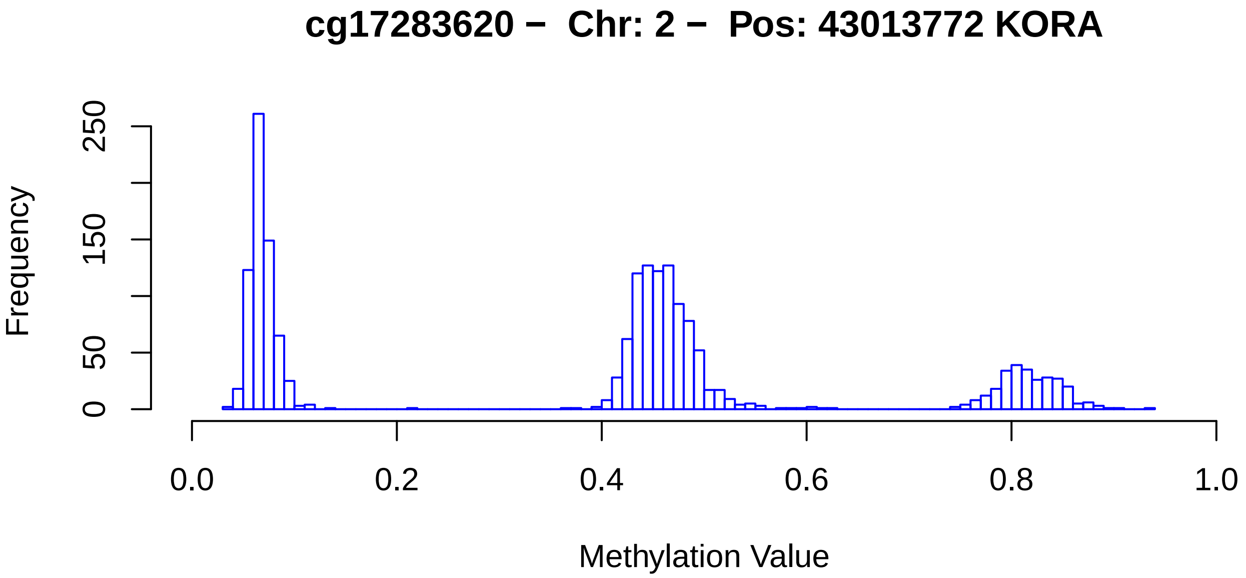
cg17283620 − Chr: 2 − Pos: 43013772 KORA
Frequency
0
50
150
250
0.0
0.2
0.4
0.6
0.8
1.0
Methylation Value

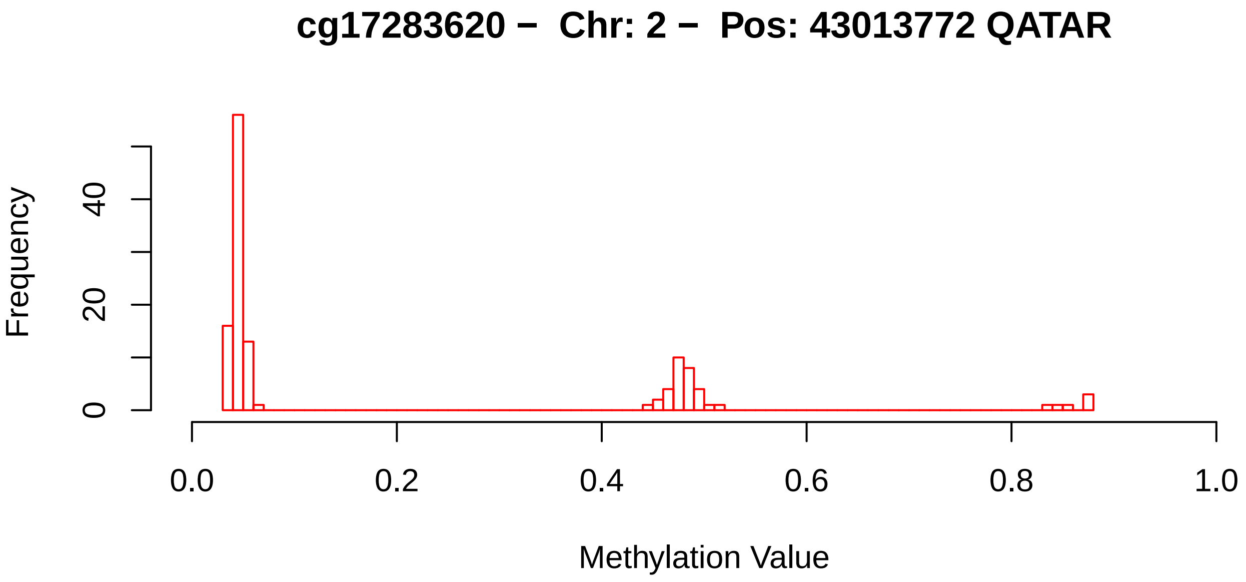
cg17283620 − Chr: 2 − Pos: 43013772 QATAR
Frequency
0
20
40
0.0
0.2
0.4
0.6
0.8
1.0
Methylation Value

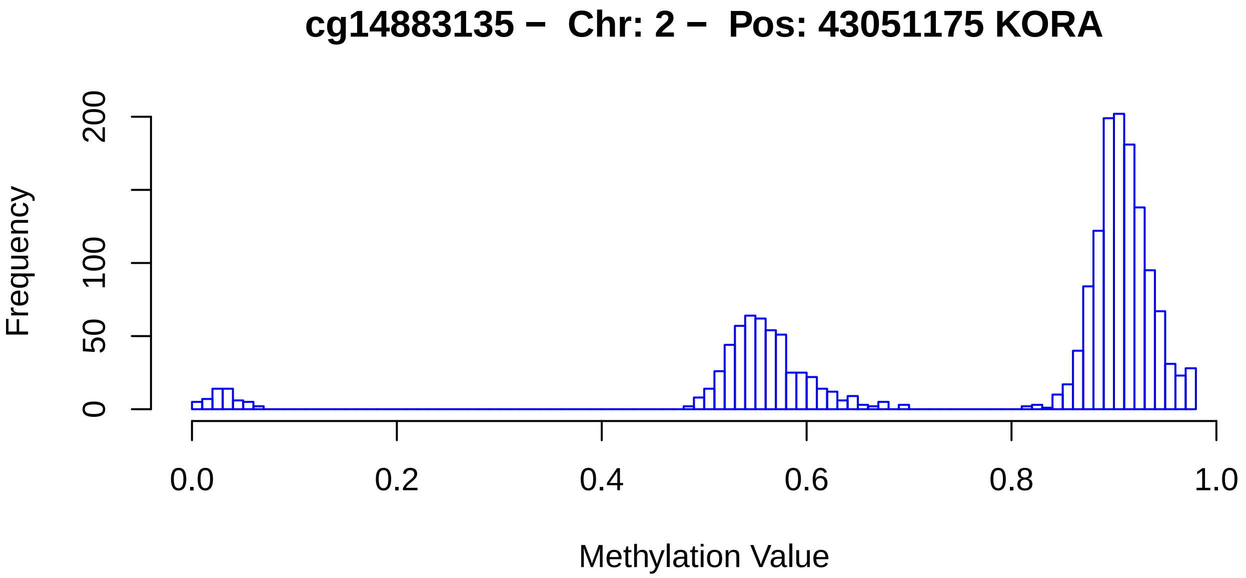

cg14883135 − Chr: 2 − Pos: 43051175 KORA
Frequency
0
50
100
200
0.0
0.2
0.4
0.6
0.8
1.0
Methylation Value

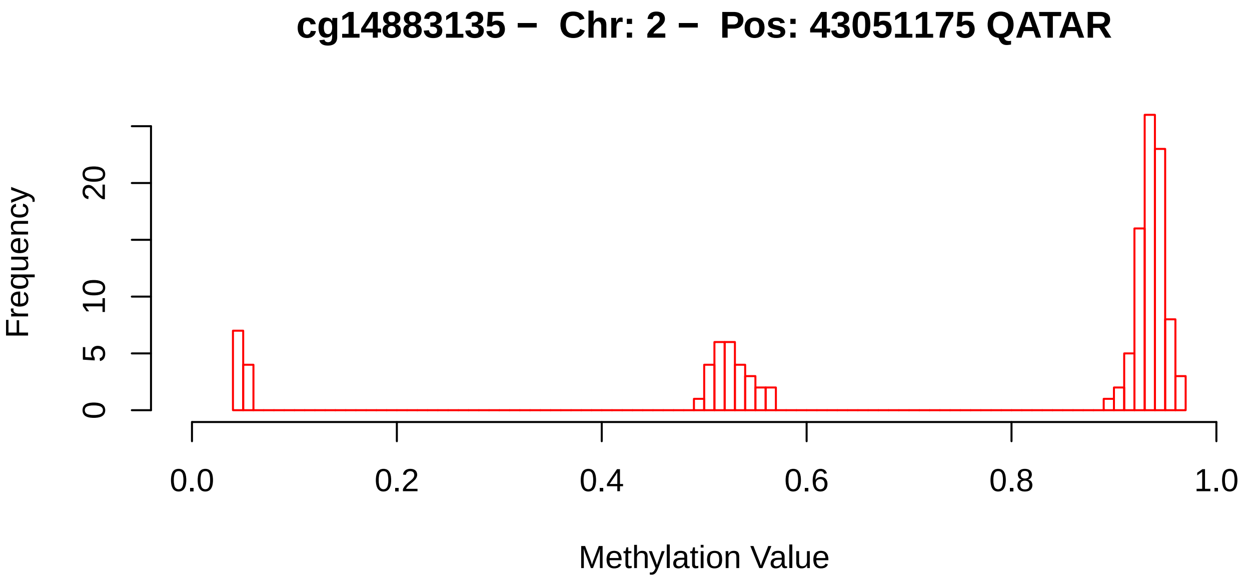

cg14883135 − Chr: 2 − Pos: 43051175 QATAR
Frequency
0
5
10
20
0.0
0.2
0.4
0.6
0.8
1.0
Methylation Value

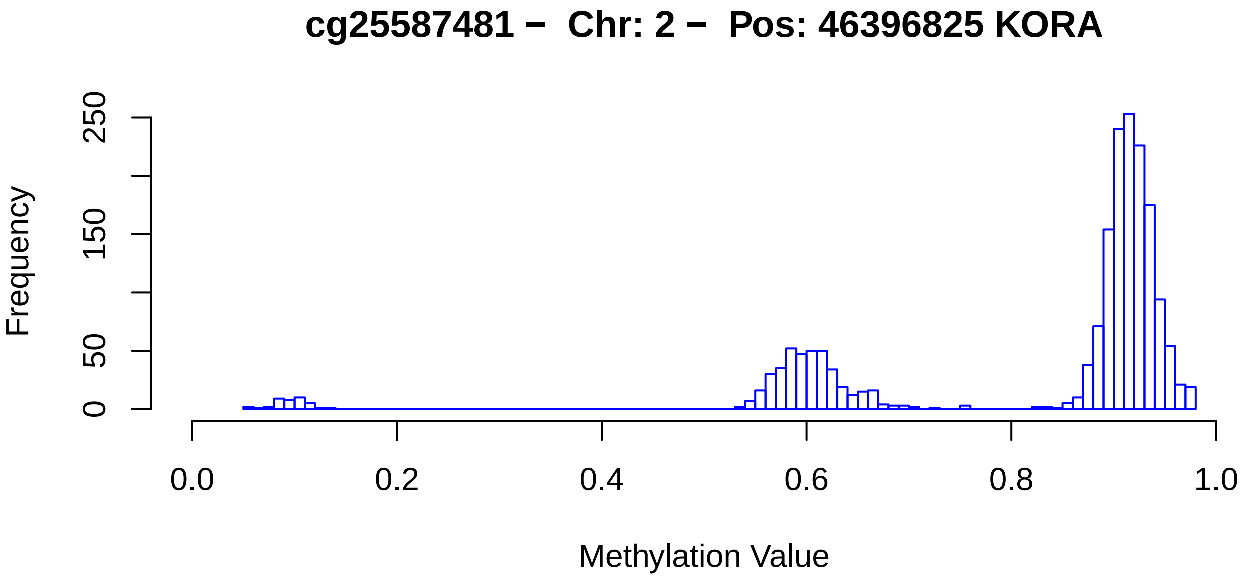
cg25587481 − Chr: 2 − Pos: 46396825 KORA
Frequency
0
50
150
250
0.0
0.2
0.4
0.6
0.8
1.0
Methylation Value

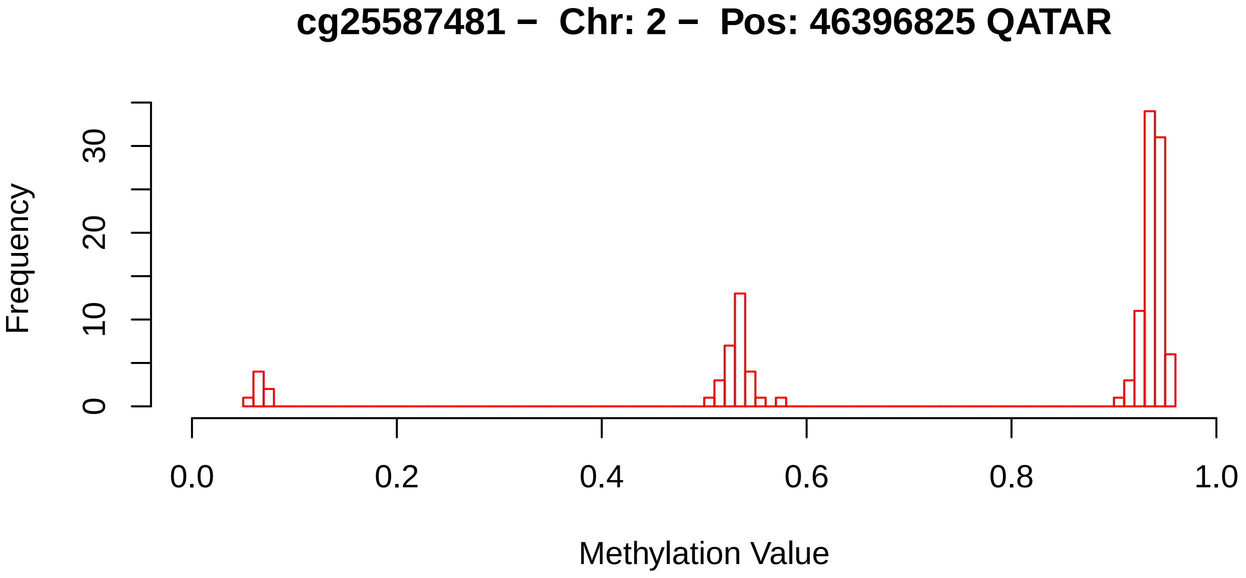
cg25587481 − Chr: 2 − Pos: 46396825 QATAR
Frequency
0
10
20
30
0.0
0.2
0.4
0.6
0.8
1.0
Methylation Value

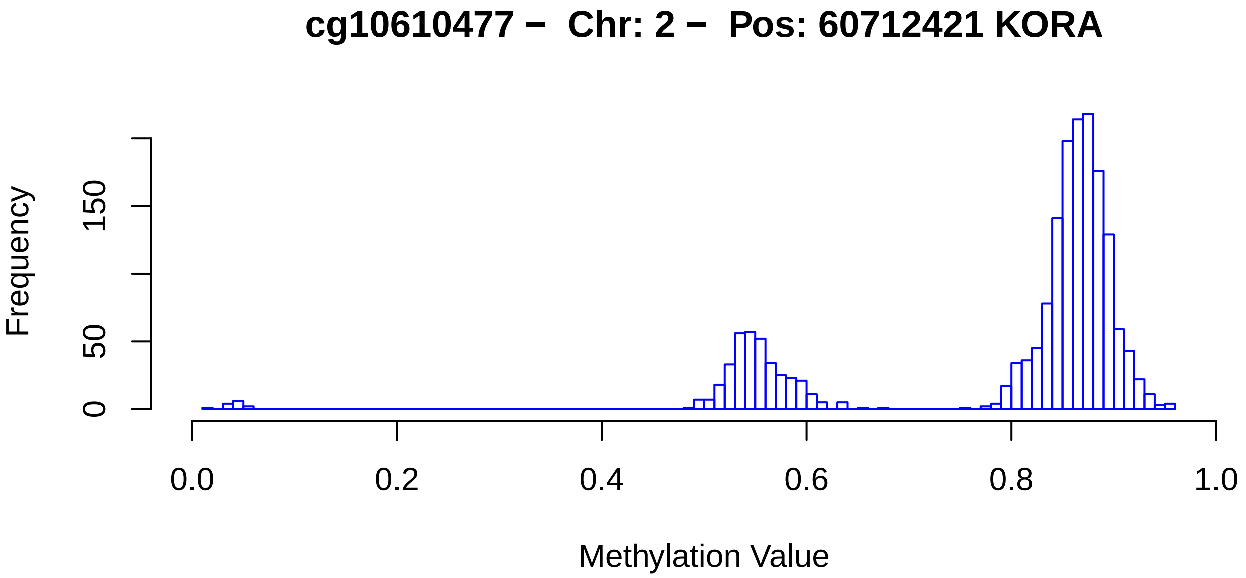
cg10610477 − Chr: 2 − Pos: 60712421 KORA
Frequency
0
50
150
0.0
0.2
0.4
0.6
0.8
1.0
Methylation Value

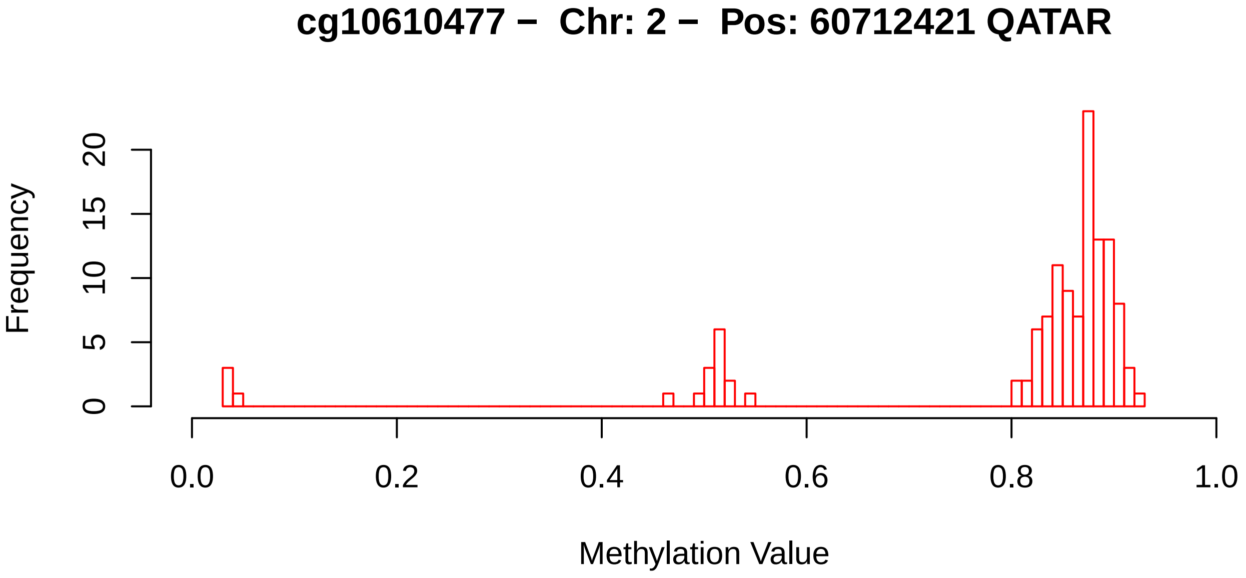
cg10610477 − Chr: 2 − Pos: 60712421 QATAR
Frequency
0
5
10
15
20
0.0
0.2
0.4
0.6
0.8
1.0
Methylation Value

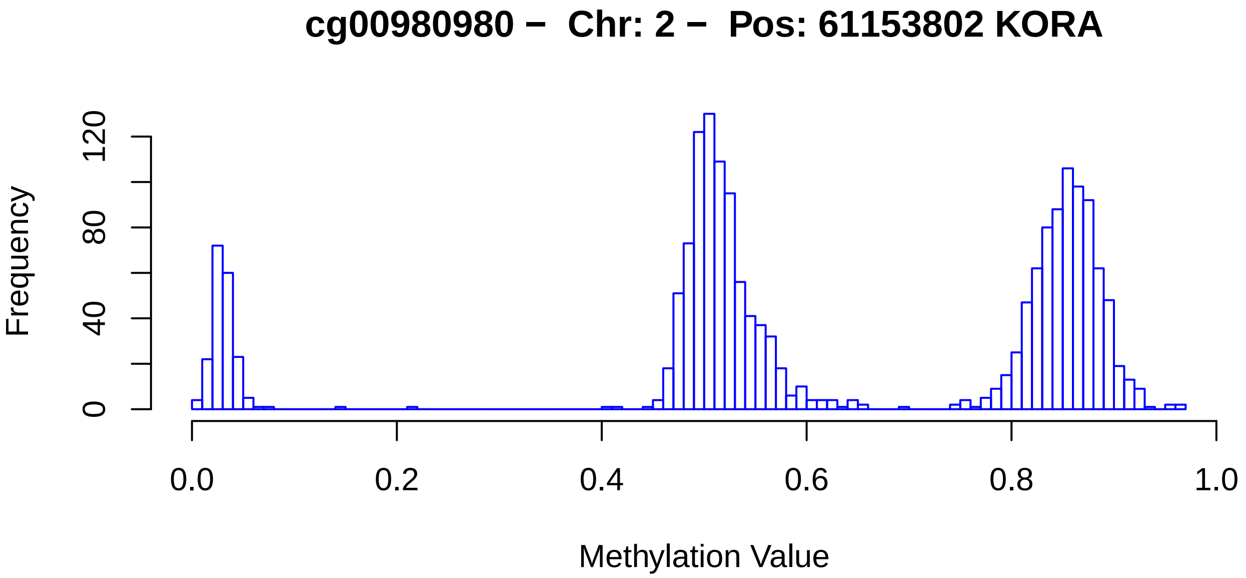
cg00980980 − Chr: 2 − Pos: 61153802 KORA
Frequency
0
40
80
120
0.0
0.2
0.4
0.6
0.8
1.0
Methylation Value

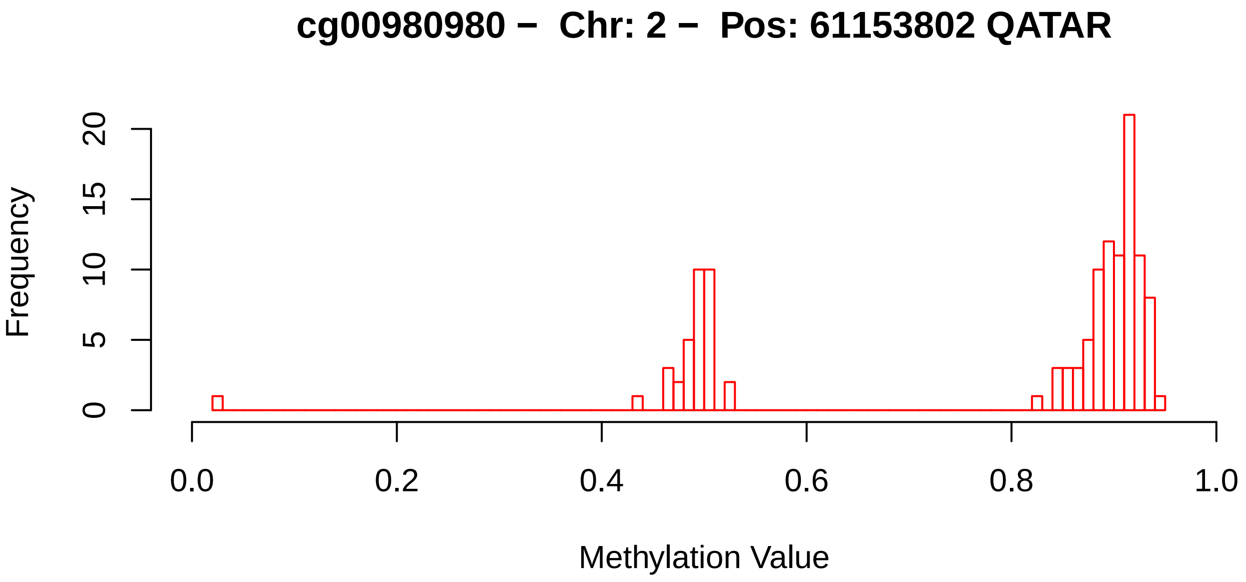
cg00980980 − Chr: 2 − Pos: 61153802 QATAR
Frequency
0
5
10
15
20
0.0
0.2
0.4
0.6
0.8
1.0
Methylation Value

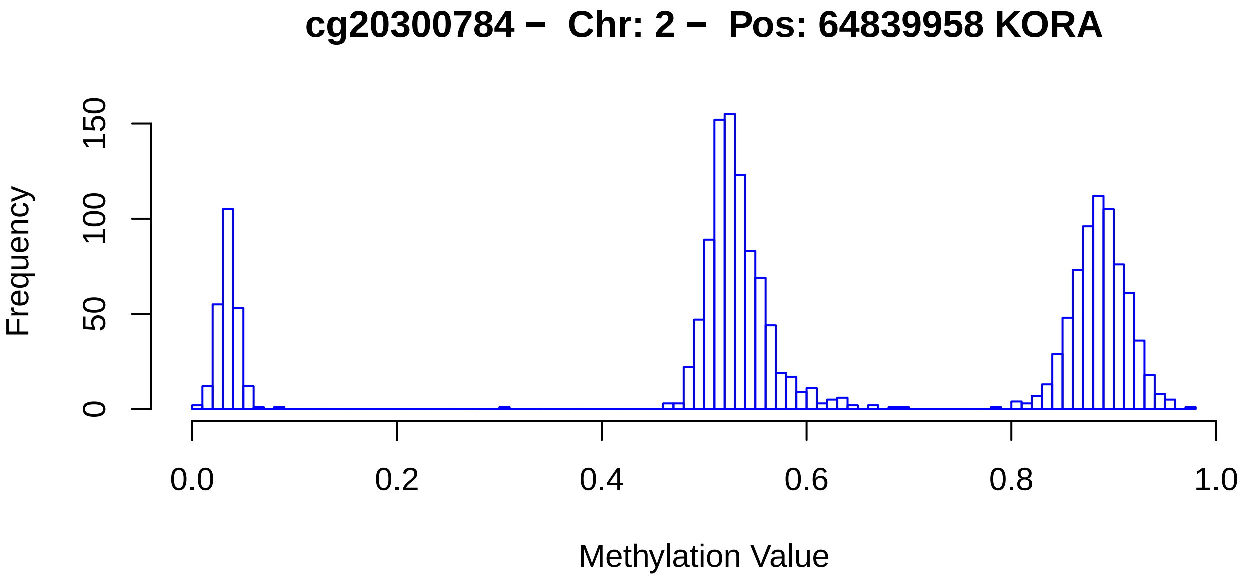
cg20300784 − Chr: 2 − Pos: 64839958 KORA
Frequency
Methylation Value
0
50
100
150
0.0
0.2
0.4
0.6
0.8
1.0

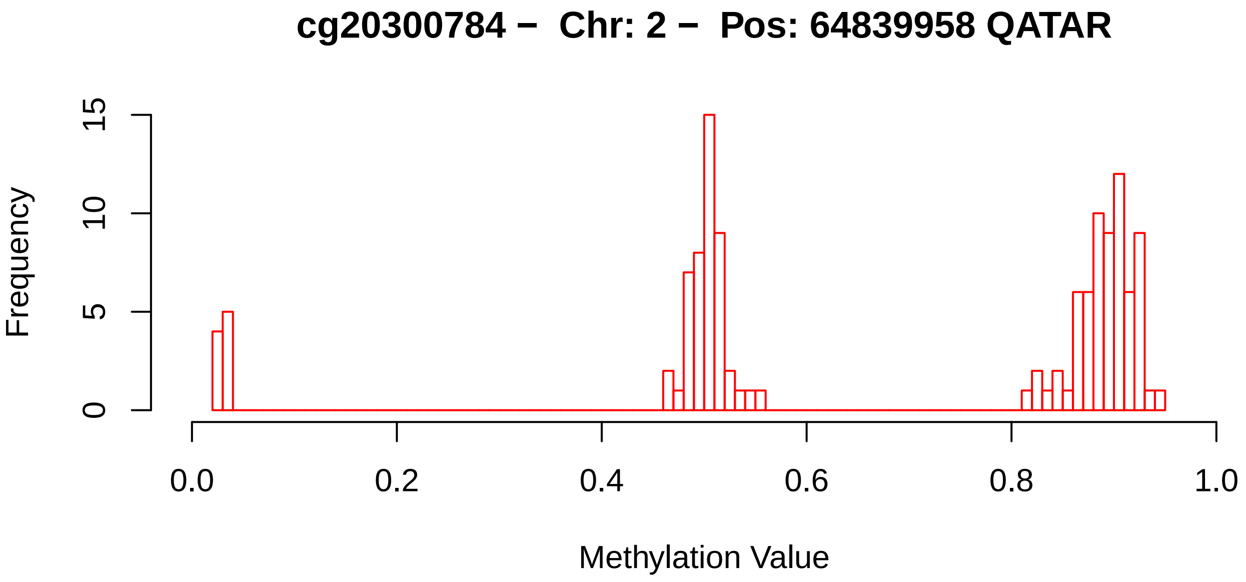
cg20300784 − Chr: 2 − Pos: 64839958 QATAR
Frequency
Methylation Value
0
5
10
15
0.0
0.2
0.4
0.6
0.8
1.0

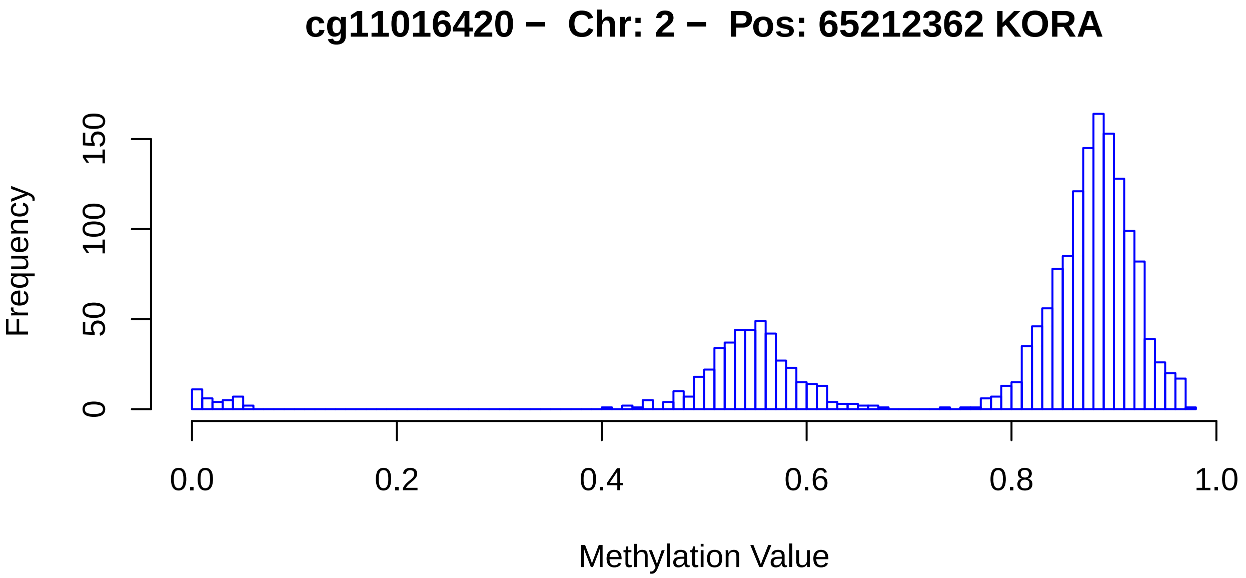
cg11016420 − Chr: 2 − Pos: 65212362 KORA
Frequency
0
50
100
150
0.0
0.2
0.4
0.6
0.8
1.0
Methylation Value

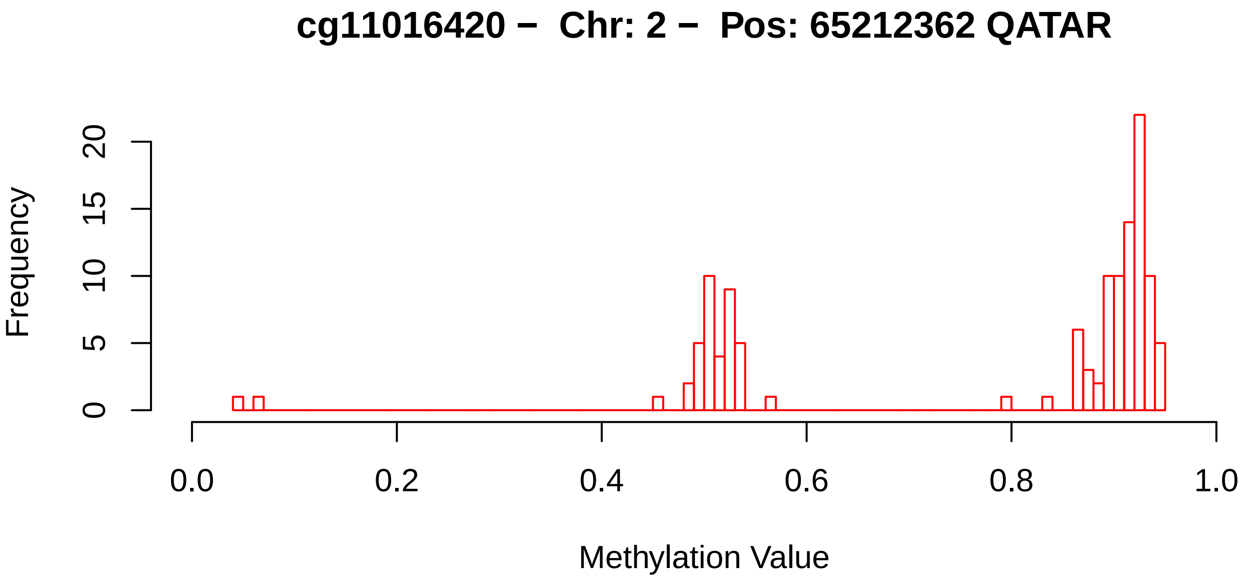
cg11016420 − Chr: 2 − Pos: 65212362 QATAR
Frequency
0
5
10
15
20
0.0
0.2
0.4
0.6
0.8
1.0
Methylation Value

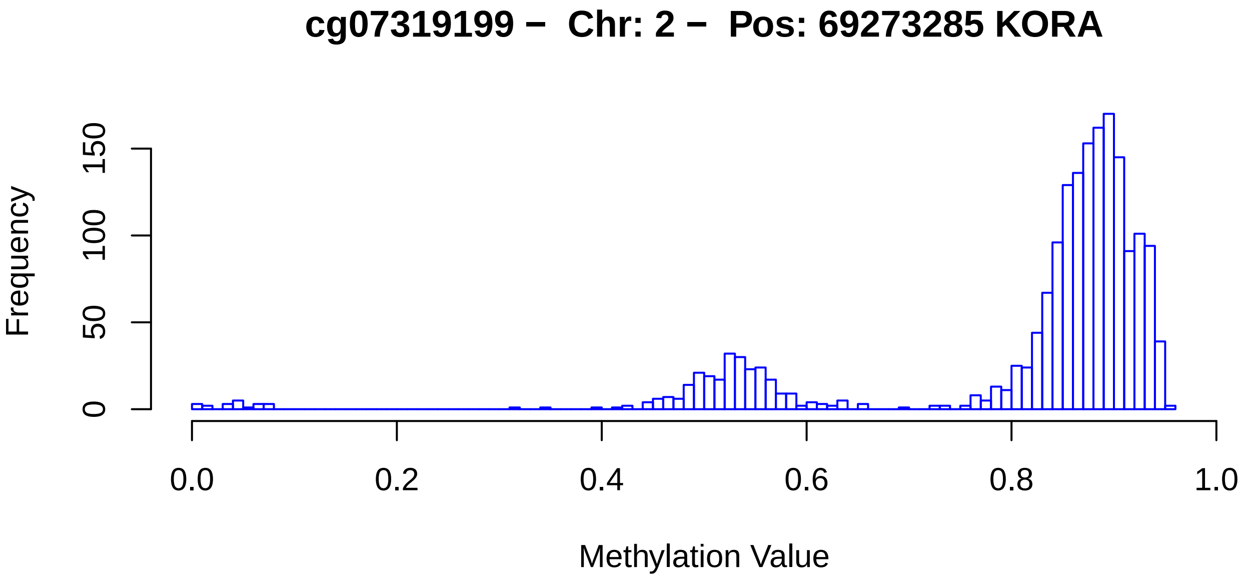
cg07319199 − Chr: 2 − Pos: 69273285 KORA
Frequency
0
50
100
150
0.0
0.2
0.4
0.6
0.8
1.0
Methylation Value

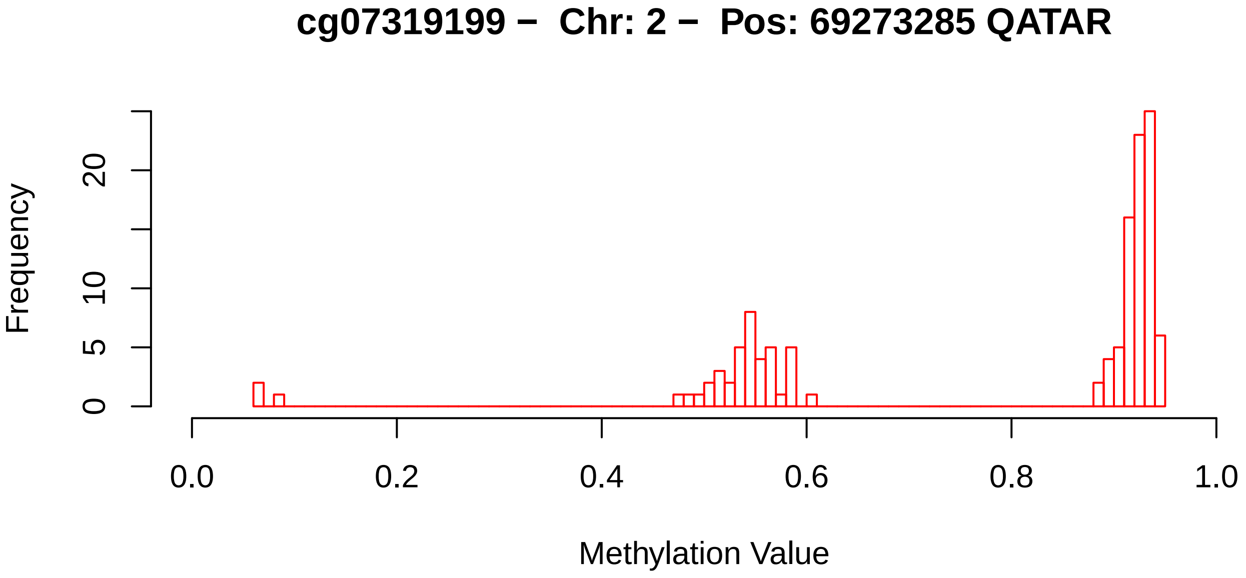
cg07319199 − Chr: 2 − Pos: 69273285 QATAR
Frequency
0
5
10
20
0.0
0.2
0.4
0.6
0.8
1.0
Methylation Value

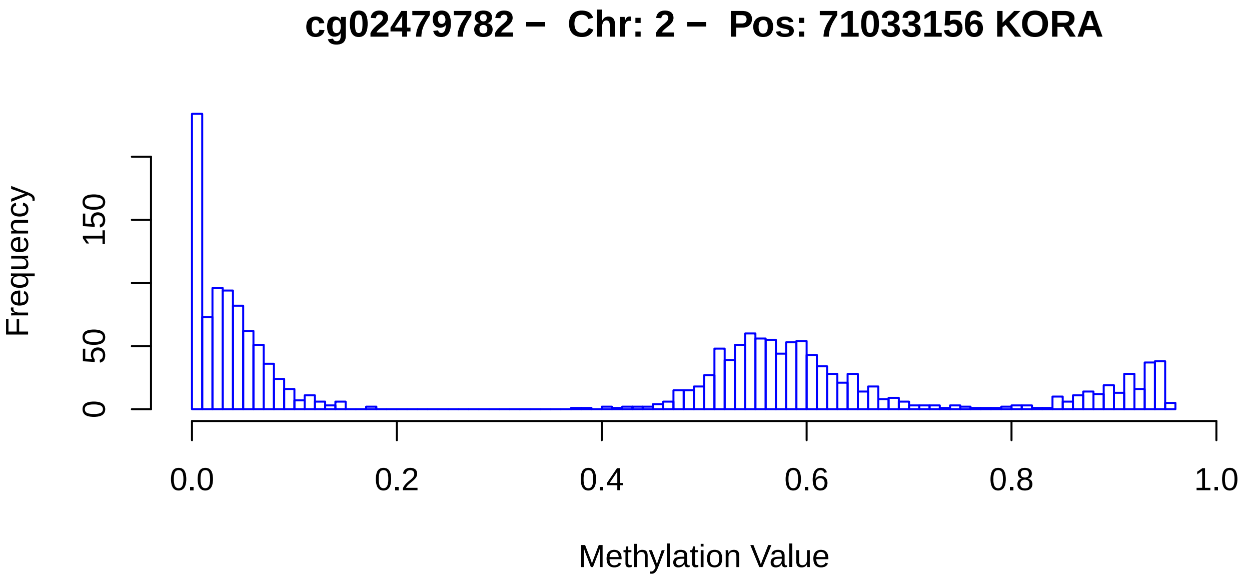
cg02479782 − Chr: 2 − Pos: 71033156 KORA
Frequency
0
50
150
0.0
0.2
0.4
0.6
0.8
1.0
Methylation Value

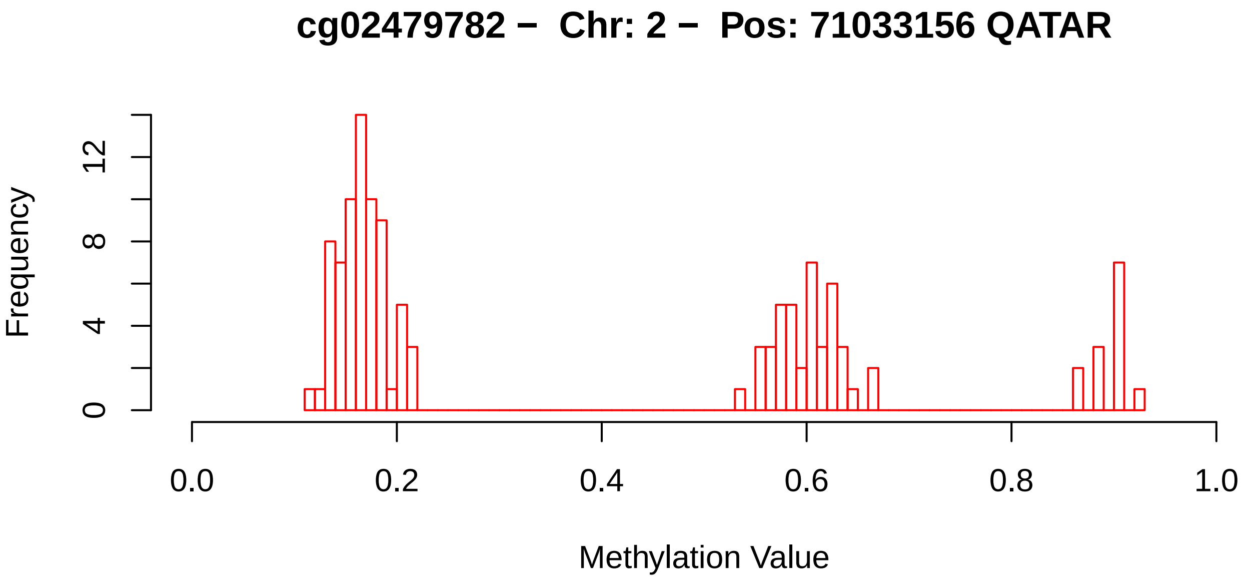
cg02479782 − Chr: 2 − Pos: 71033156 QATAR
Frequency
0
4
8
12
0.0
0.2
0.4
0.6
0.8
1.0
Methylation Value

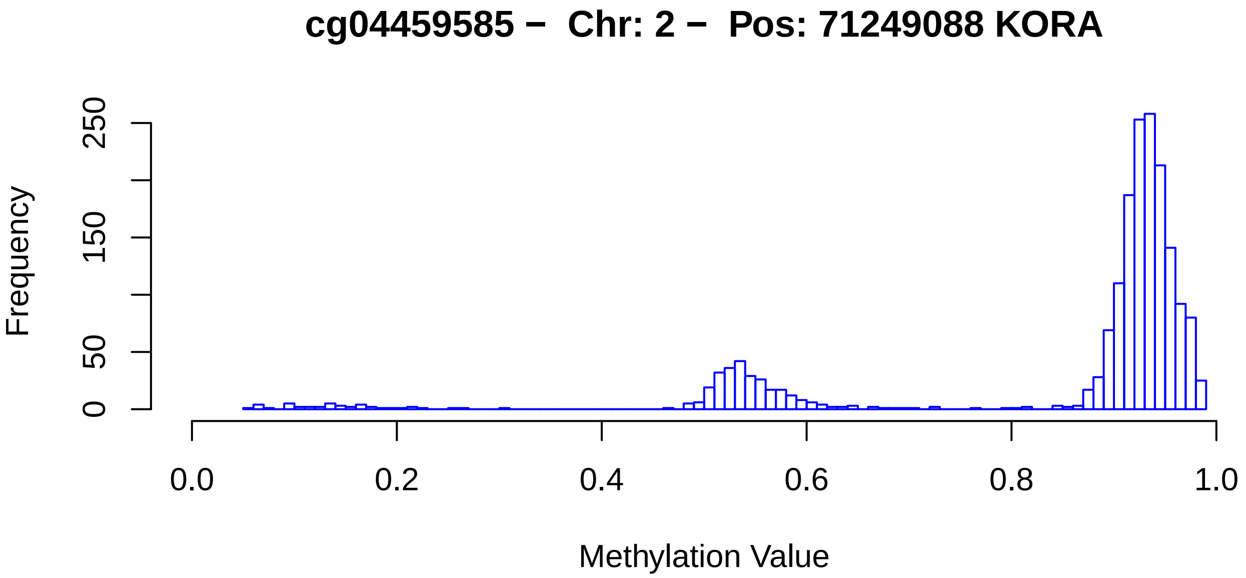
cg04459585 − Chr: 2 − Pos: 71249088 KORA
Frequency
0
50
150
250
0.0
0.2
0.4
0.6
0.8
1.0
Methylation Value

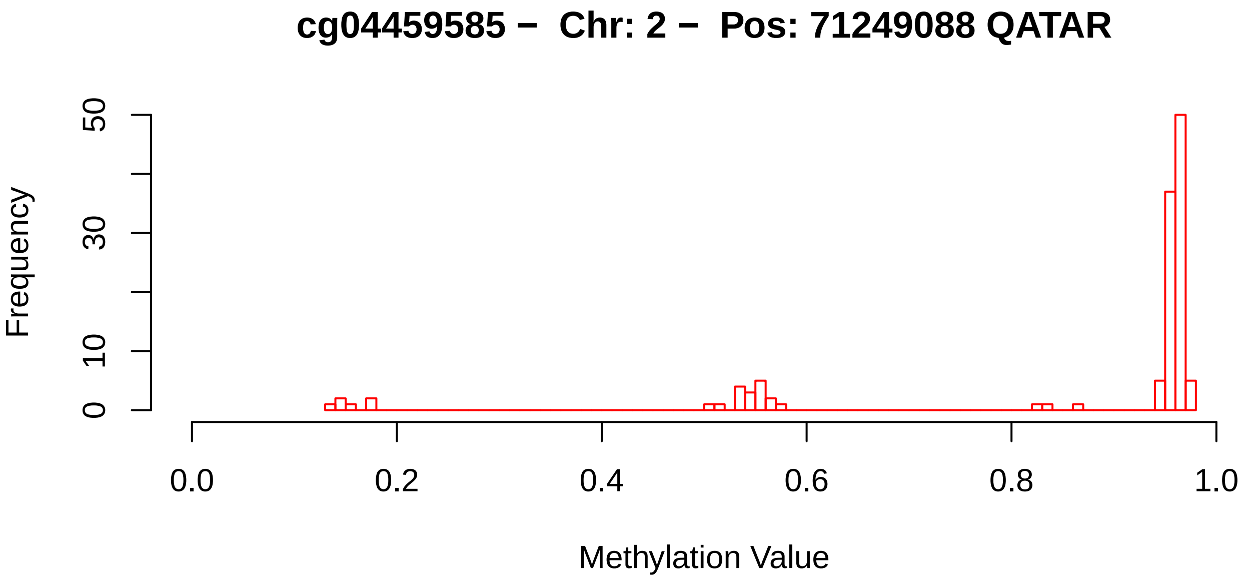
cg04459585 − Chr: 2 − Pos: 71249088 QATAR
Frequency
0
10
30
50
0.0
0.2
0.4
0.6
0.8
1.0
Methylation Value

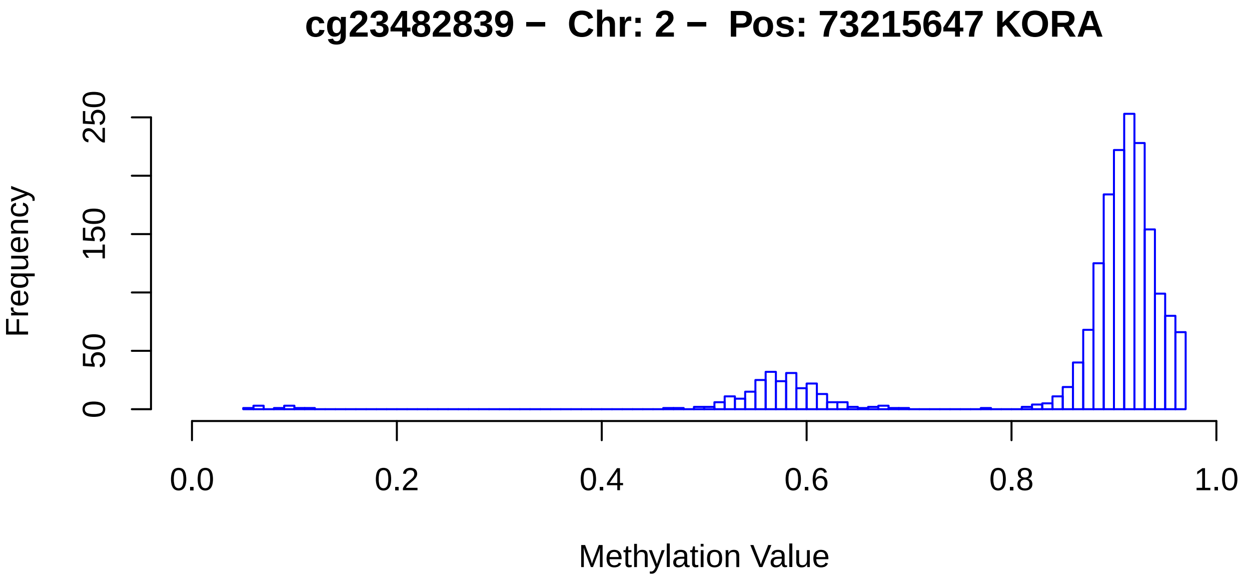
cg23482839 − Chr: 2 − Pos: 73215647 KORA
Frequency
0
50
150
250
0.0
0.2
0.4
0.6
0.8
1.0
Methylation Value

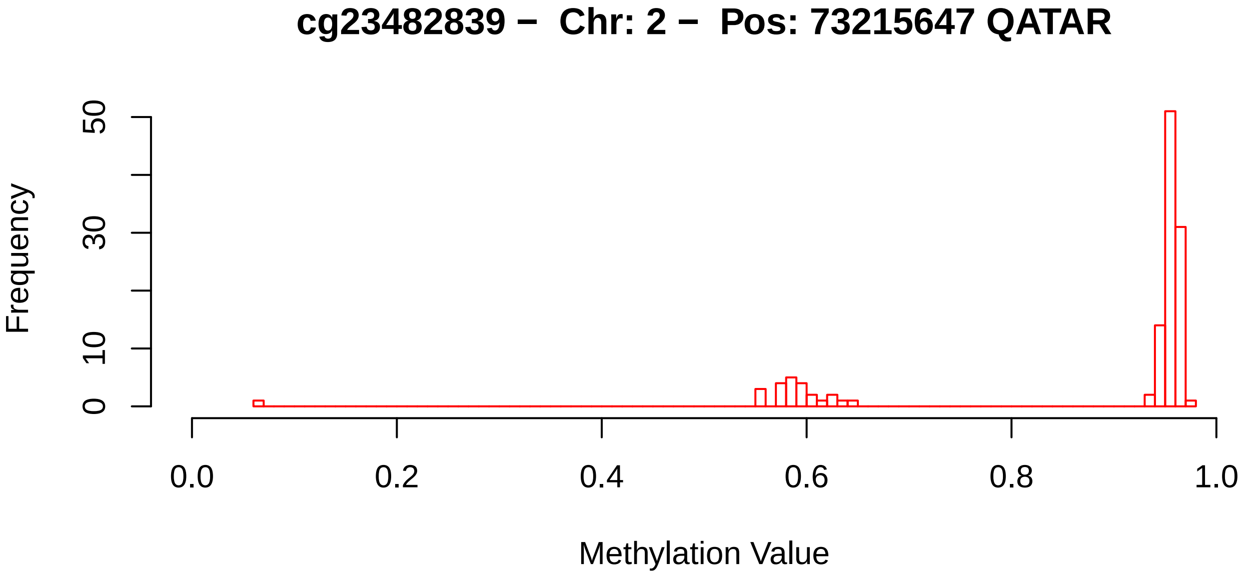
cg23482839 − Chr: 2 − Pos: 73215647 QATAR
Frequency
0
10
30
50
0.0
0.2
0.4
0.6
0.8
1.0
Methylation Value

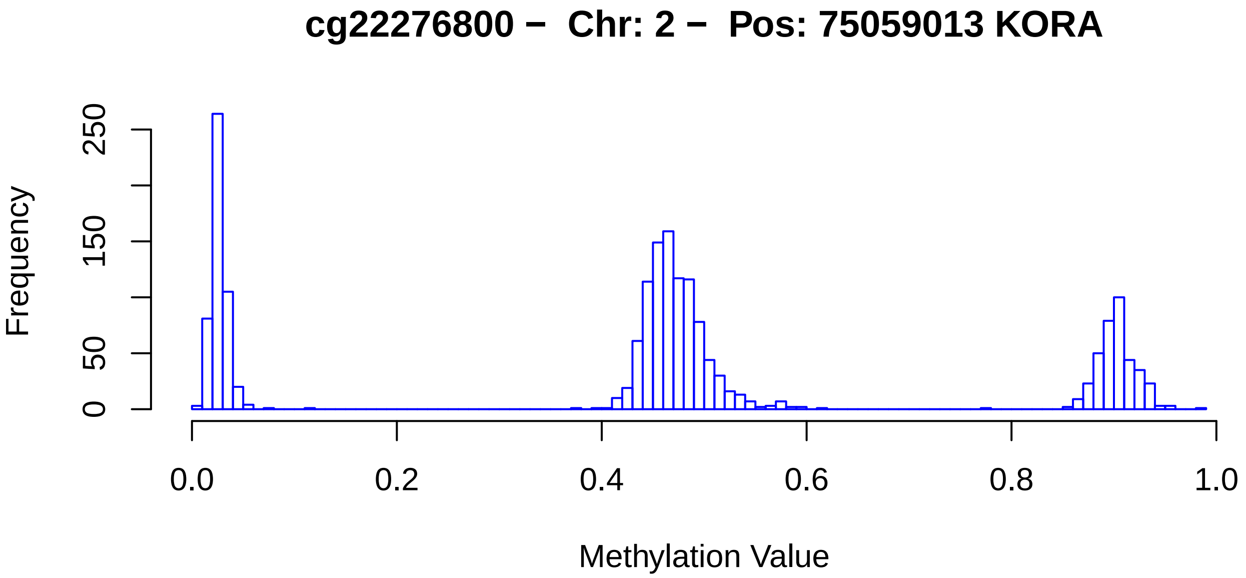
cg22276800 − Chr: 2 − Pos: 75059013 KORA
Frequency
0
50
150
250
0.0
0.2
0.4
0.6
0.8
1.0
Methylation Value

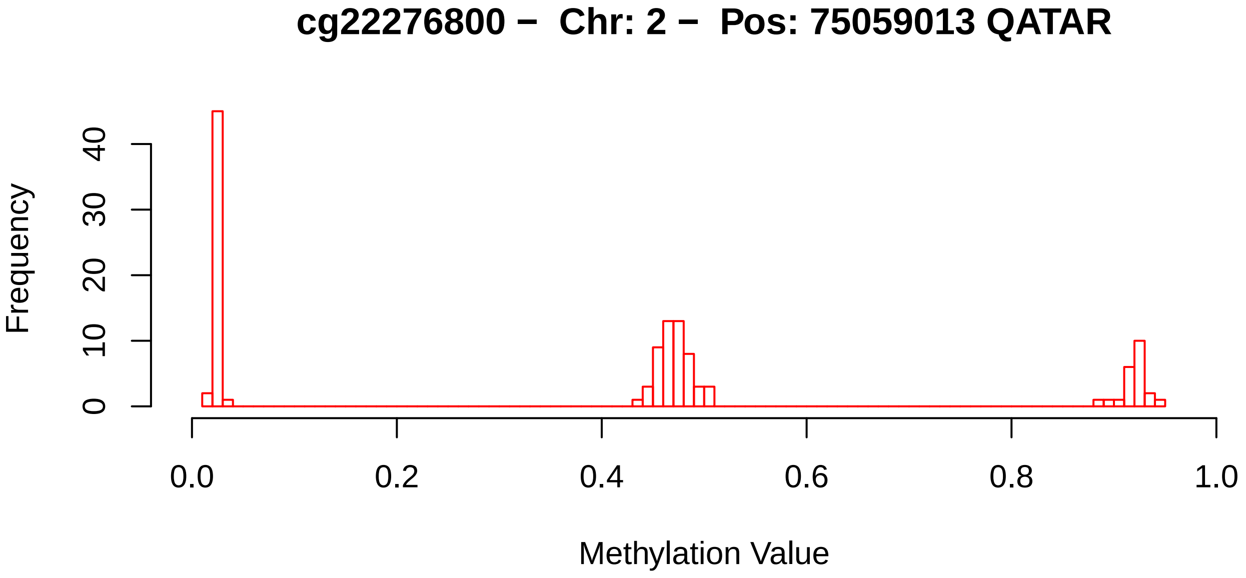
cg22276800 − Chr: 2 − Pos: 75059013 QATAR
Frequency
0
10
20
30
40
0.0
0.2
0.4
0.6
0.8
1.0
Methylation Value

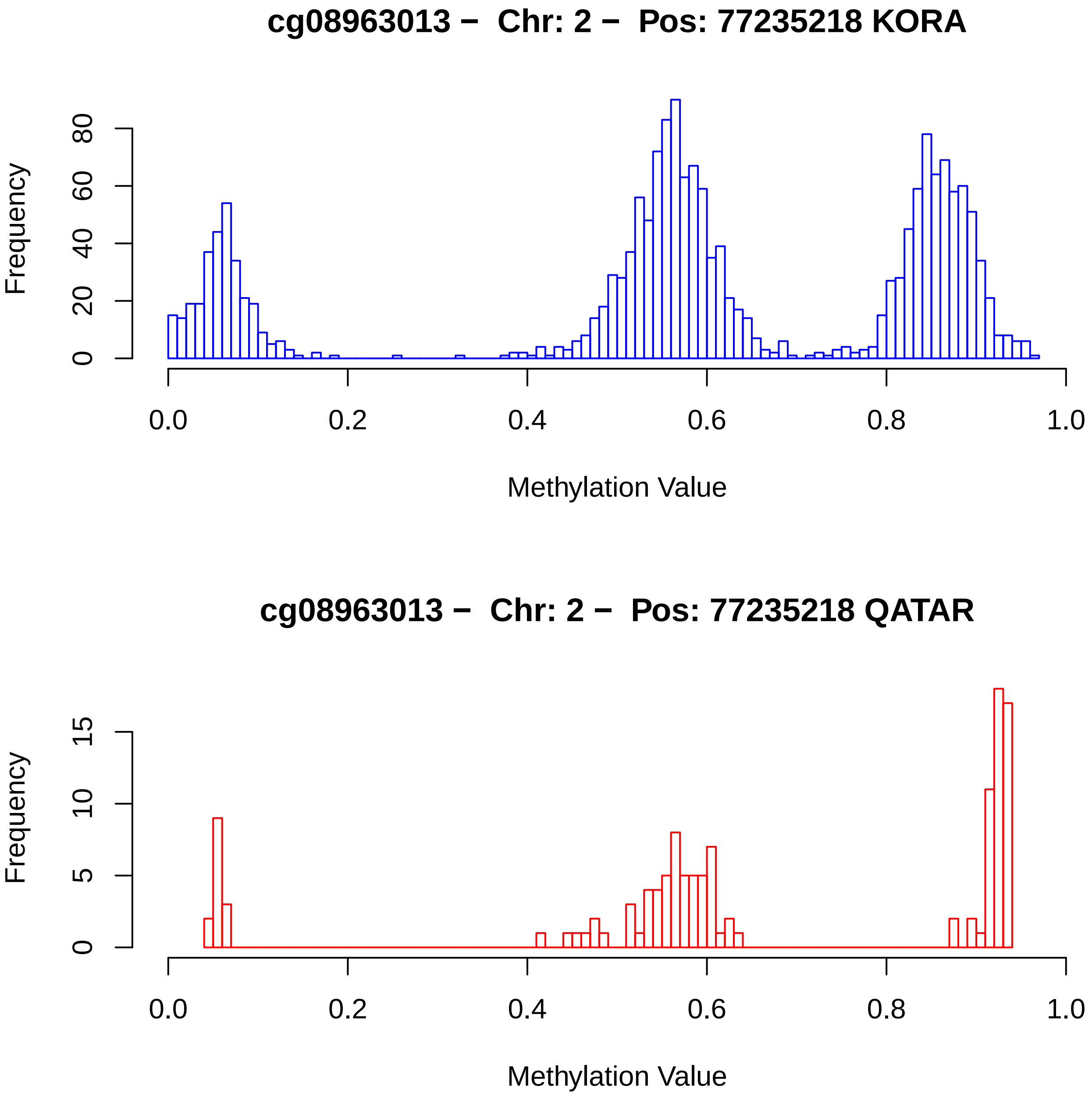
cg08963013 − Chr: 2 − Pos: 77235218 KORA
Frequency
0
20
40
60
80
0.0
0.2
0.4
0.6
0.8
1.0
Methylation Value
cg08963013 − Chr: 2 − Pos: 77235218 QATAR
Frequency
0
5
10
15
0.0
0.2
0.4
0.6
0.8
1.0
Methylation Value

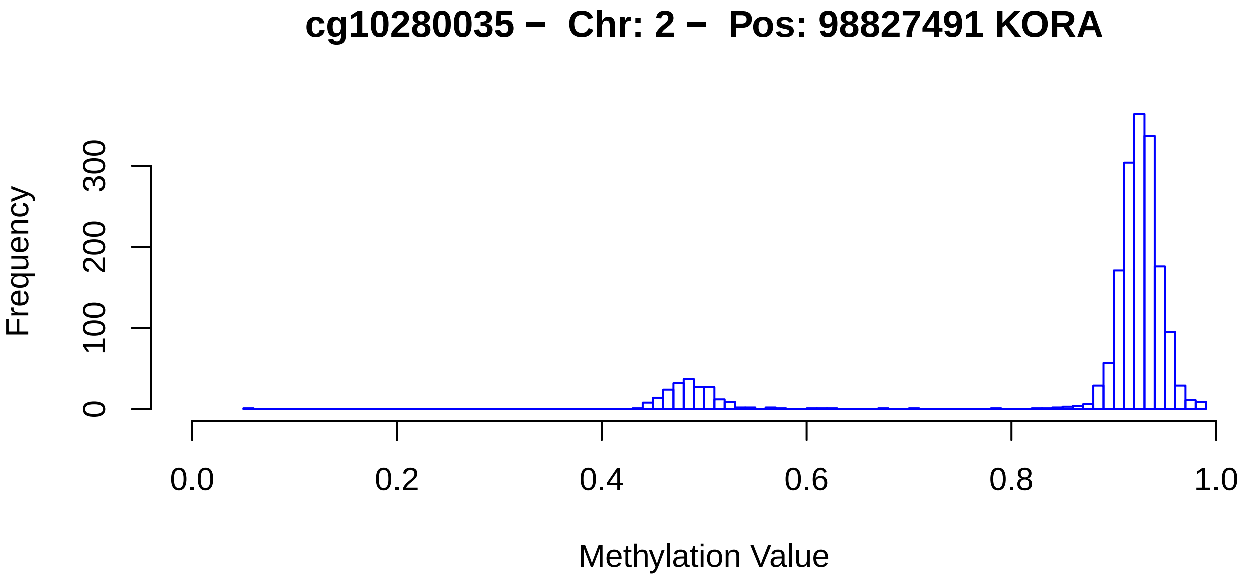
cg10280035 − Chr: 2 − Pos: 98827491 KORA
Frequency
0
100
200
300
0.0
0.2
0.4
0.6
0.8
1.0
Methylation Value

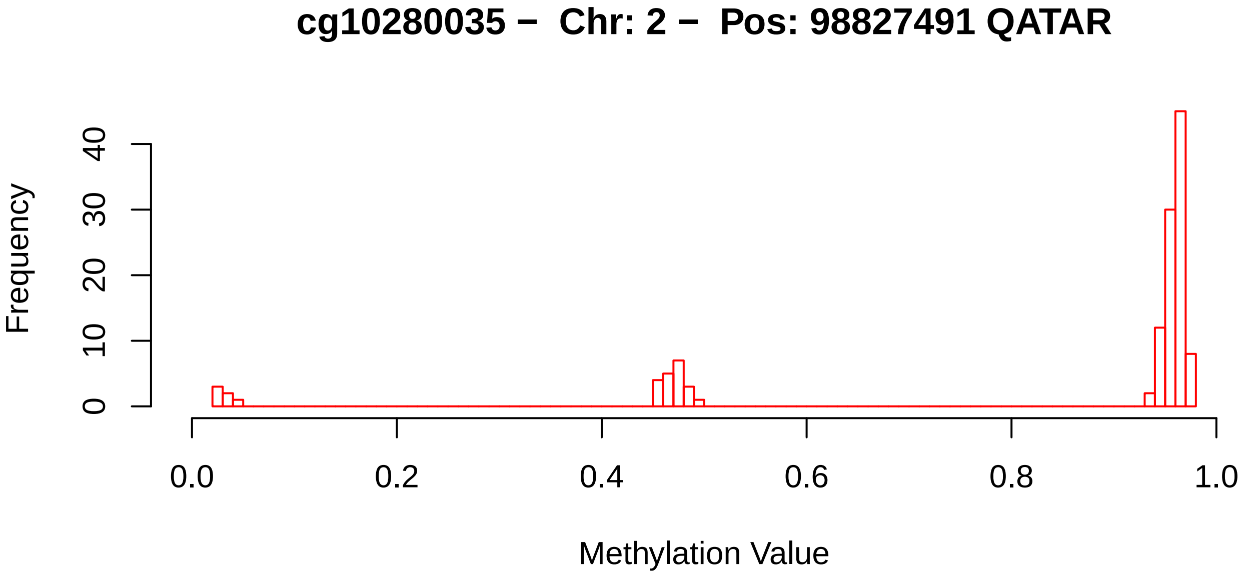
cg10280035 − Chr: 2 − Pos: 98827491 QATAR
Frequency
0
10
20
30
40
0.0
0.2
0.4
0.6
0.8
1.0
Methylation Value

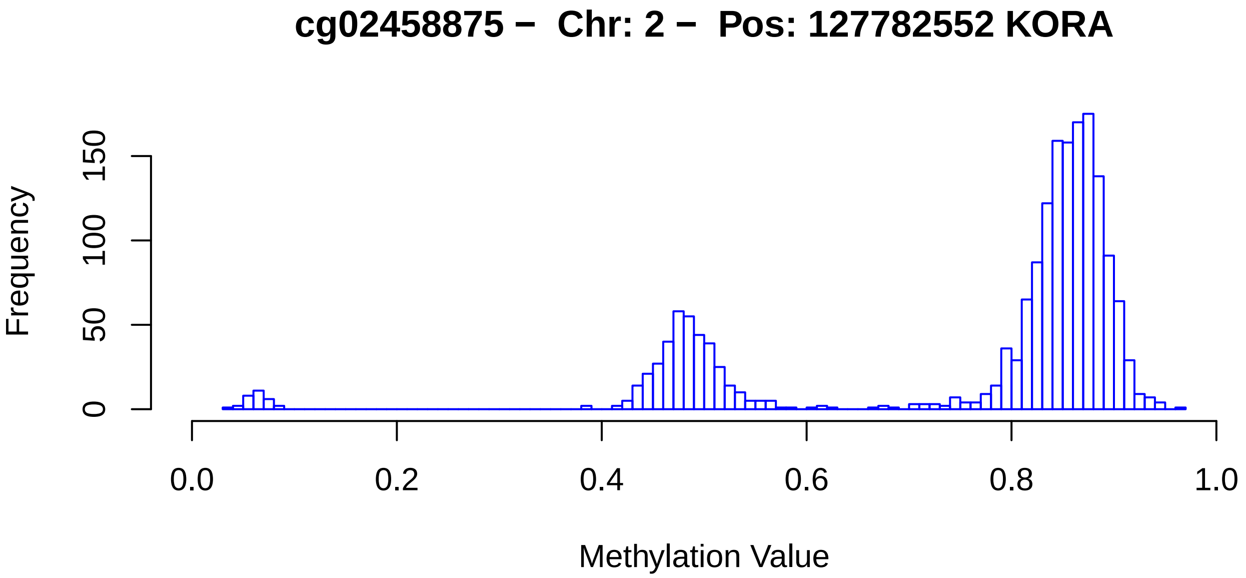
cg02458875 − Chr: 2 − Pos: 127782552 KORA
Frequency
0
50
100
150
0.0
0.2
0.4
0.6
0.8
1.0
Methylation Value

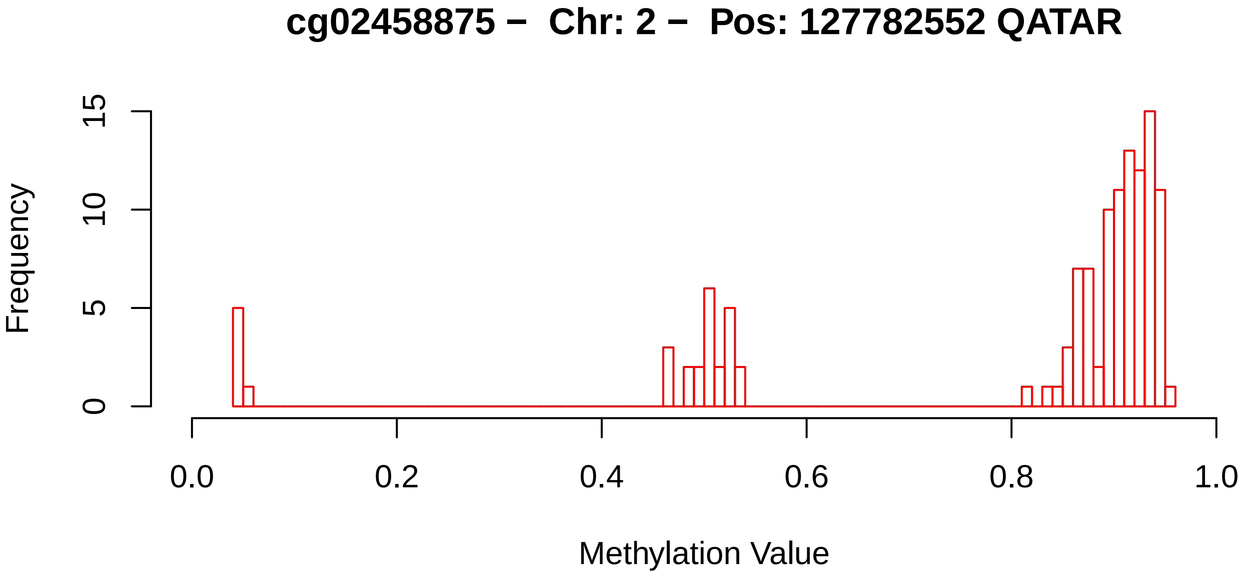
cg02458875 − Chr: 2 − Pos: 127782552 QATAR
Frequency
0
5
10
15
0.0
0.2
0.4
0.6
0.8
1.0
Methylation Value

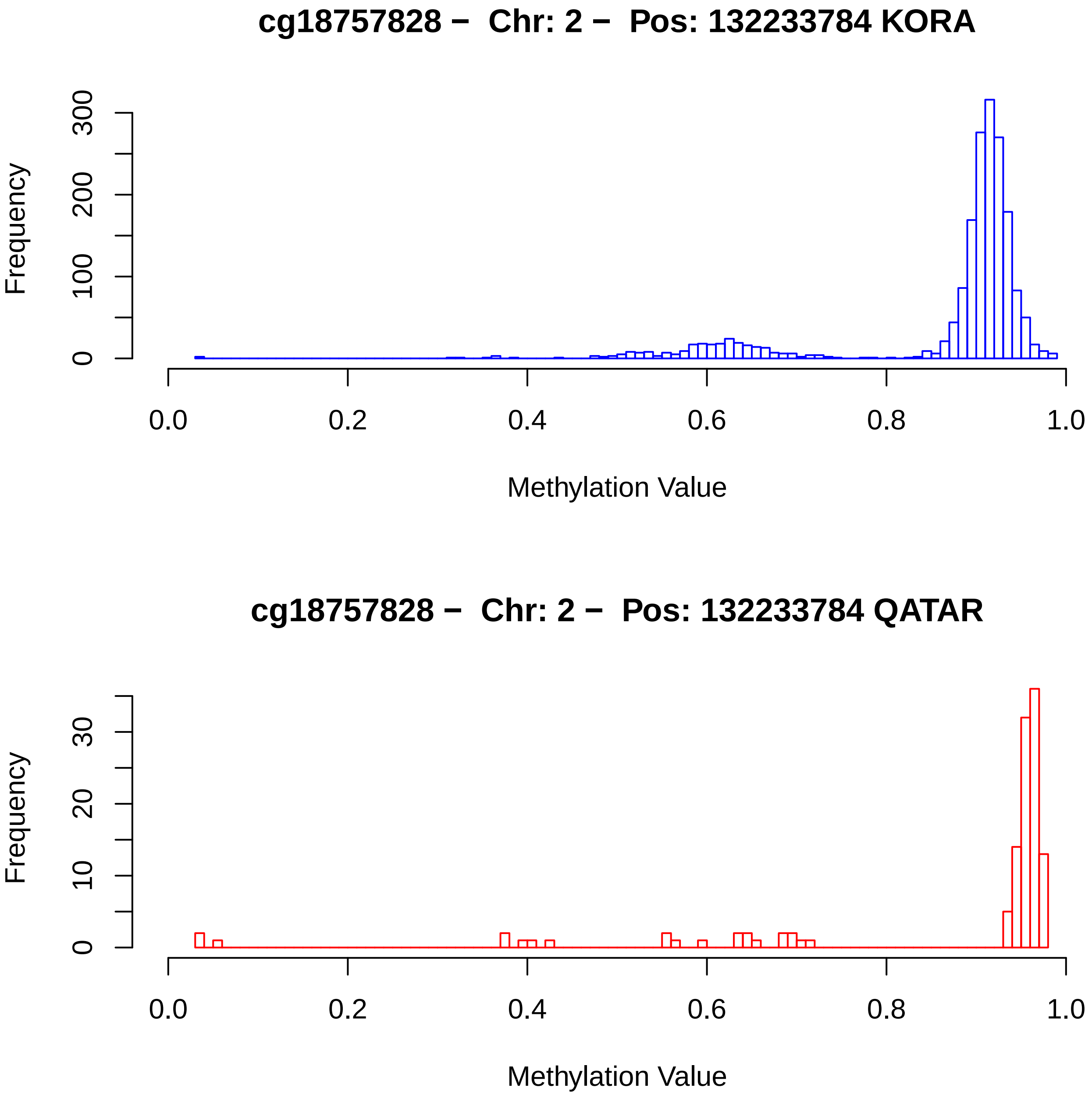
cg18757828 − Chr: 2 − Pos: 132233784 KORA
Frequency
0
100
200
300
0.0
0.2
0.4
0.6
0.8
1.0
Methylation Value
cg18757828 − Chr: 2 − Pos: 132233784 QATAR
Frequency
0
10
20
30
0.0
0.2
0.4
0.6
0.8
1.0
Methylation Value

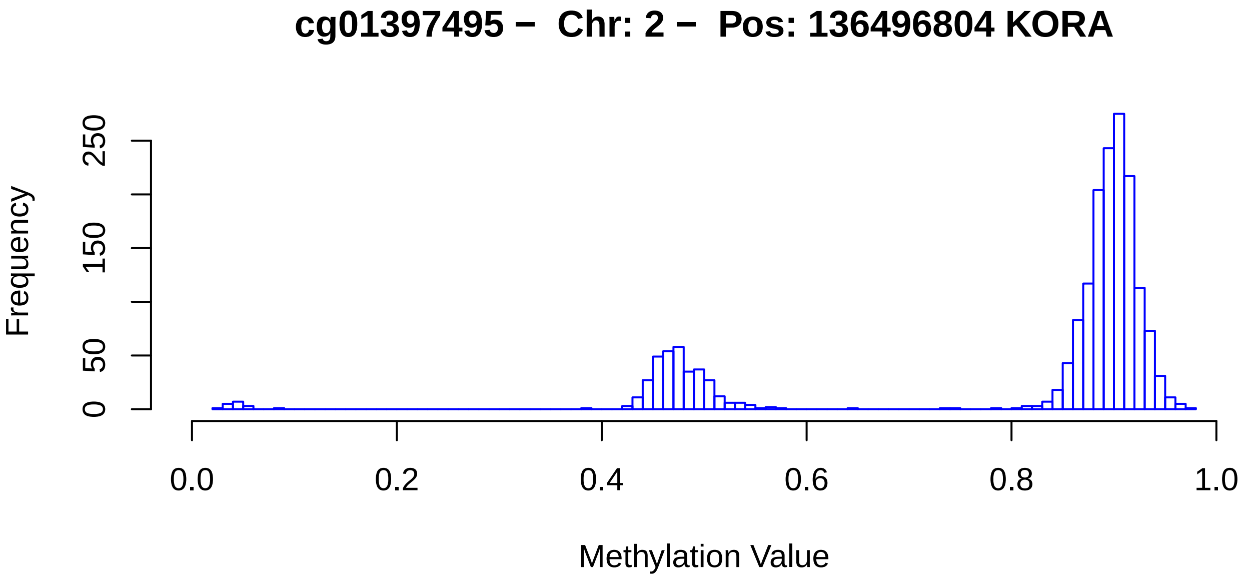
cg01397495 − Chr: 2 − Pos: 136496804 KORA
Frequency
0
50
150
250
0.0
0.2
0.4
0.6
0.8
1.0
Methylation Value

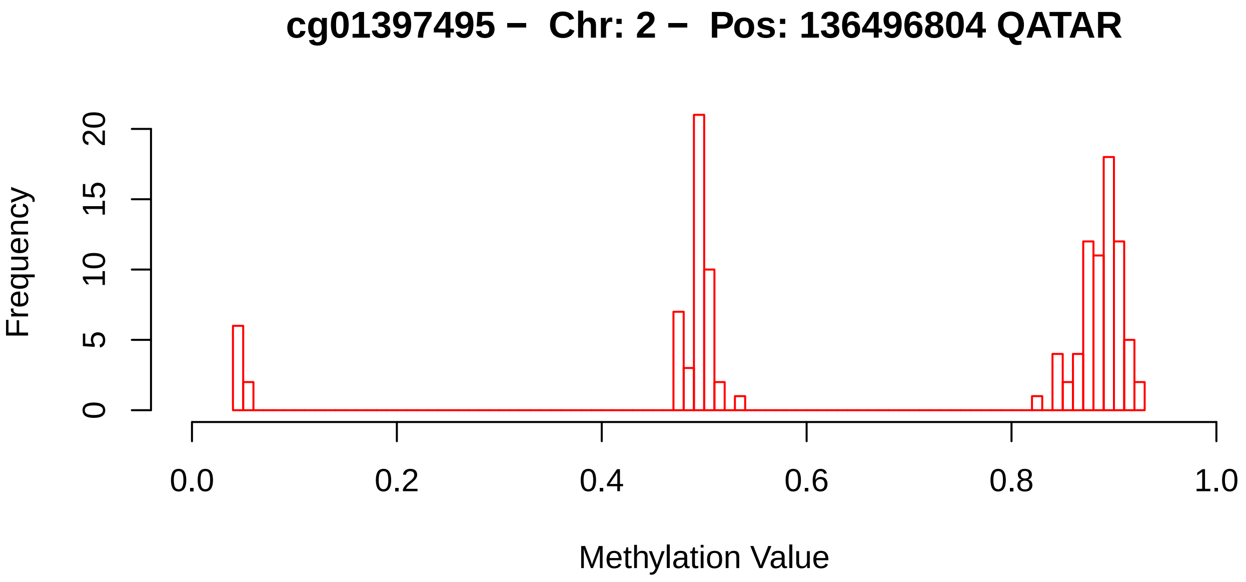
cg01397495 − Chr: 2 − Pos: 136496804 QATAR
Frequency
0
5
10
15
20
0.0
0.2
0.4
0.6
0.8
1.0
Methylation Value

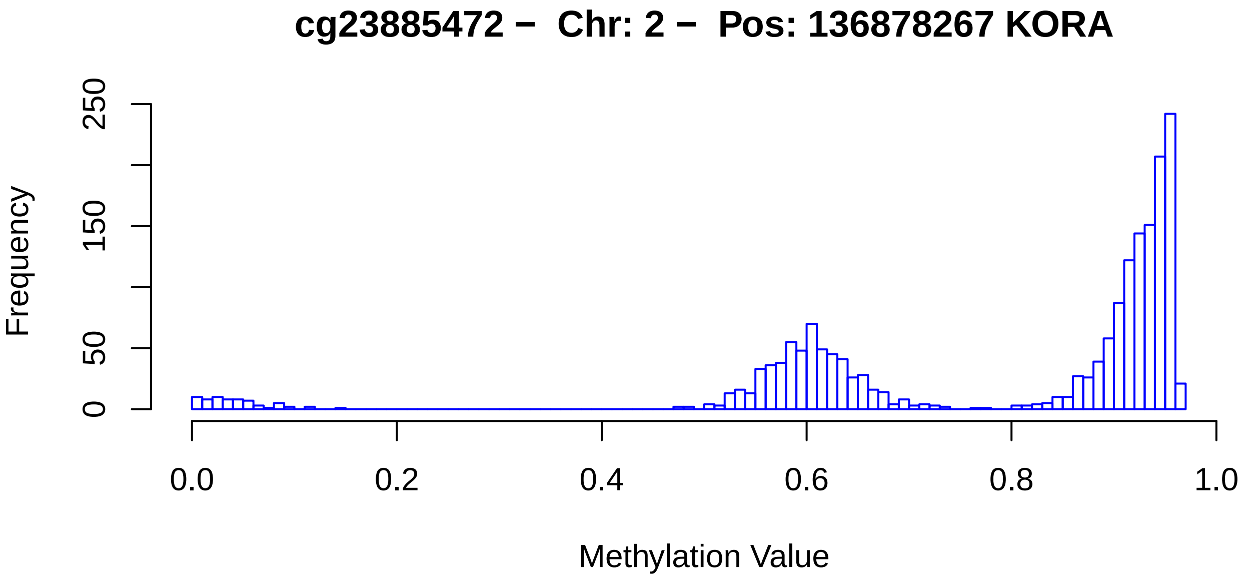
cg23885472 − Chr: 2 − Pos: 136878267 KORA
Frequency
0
50
150
250
0.0
0.2
0.4
0.6
0.8
1.0
Methylation Value

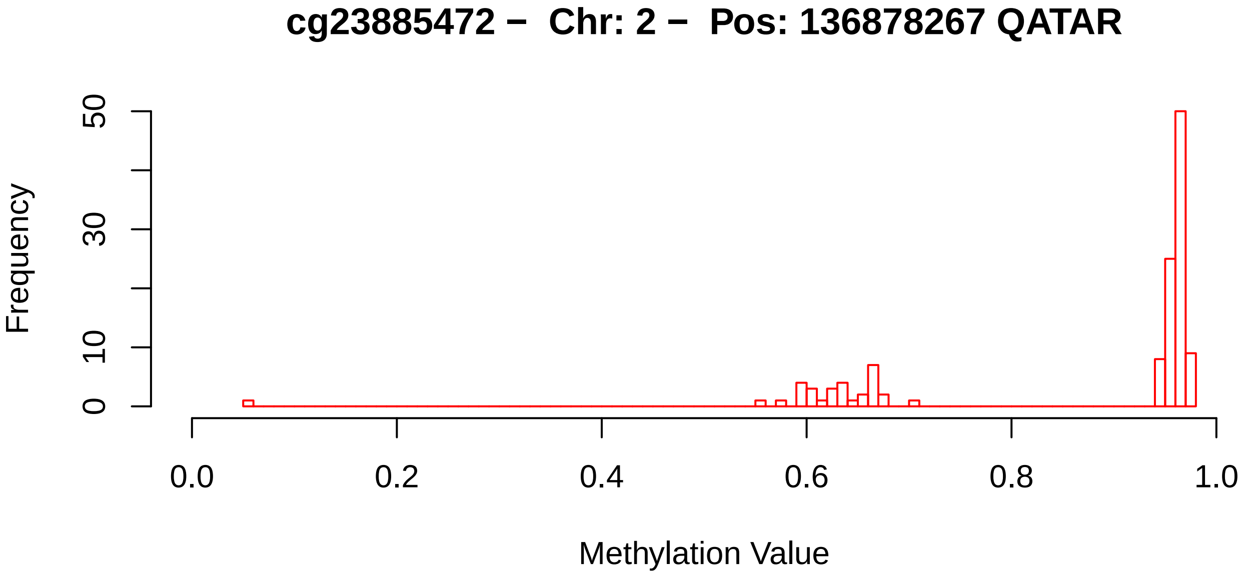
cg23885472 − Chr: 2 − Pos: 136878267 QATAR
Frequency
0
10
30
50
0.0
0.2
0.4
0.6
0.8
1.0
Methylation Value

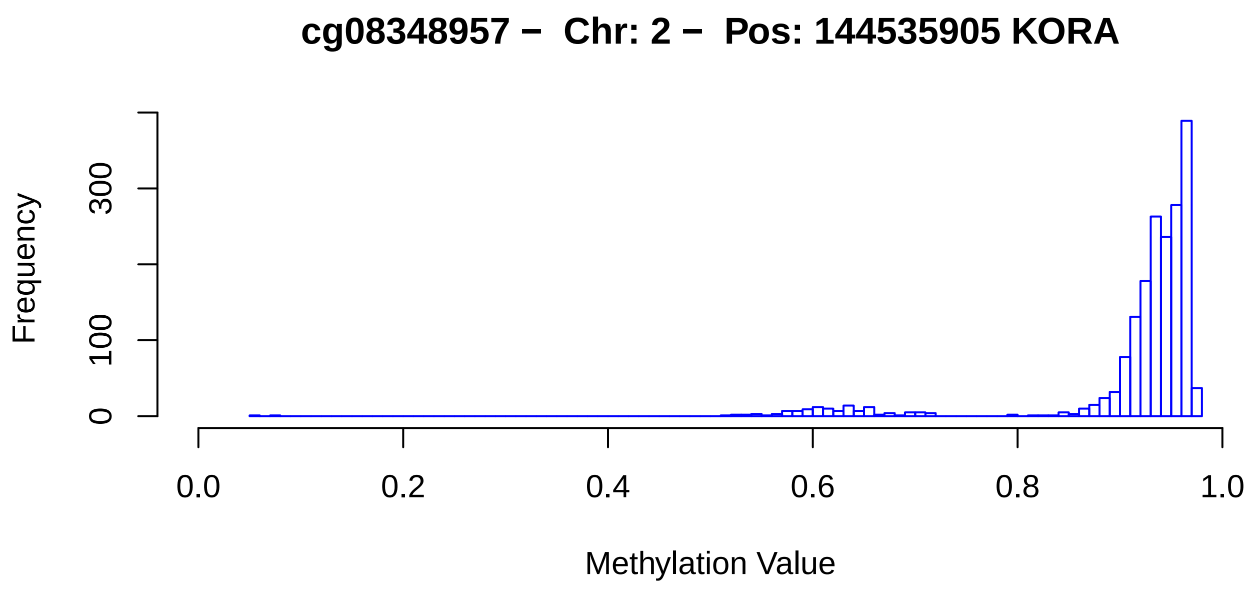
cg08348957 − Chr: 2 − Pos: 144535905 KORA
Frequency
0
100
300
0.0
0.2
0.4
0.6
0.8
1.0
Methylation Value

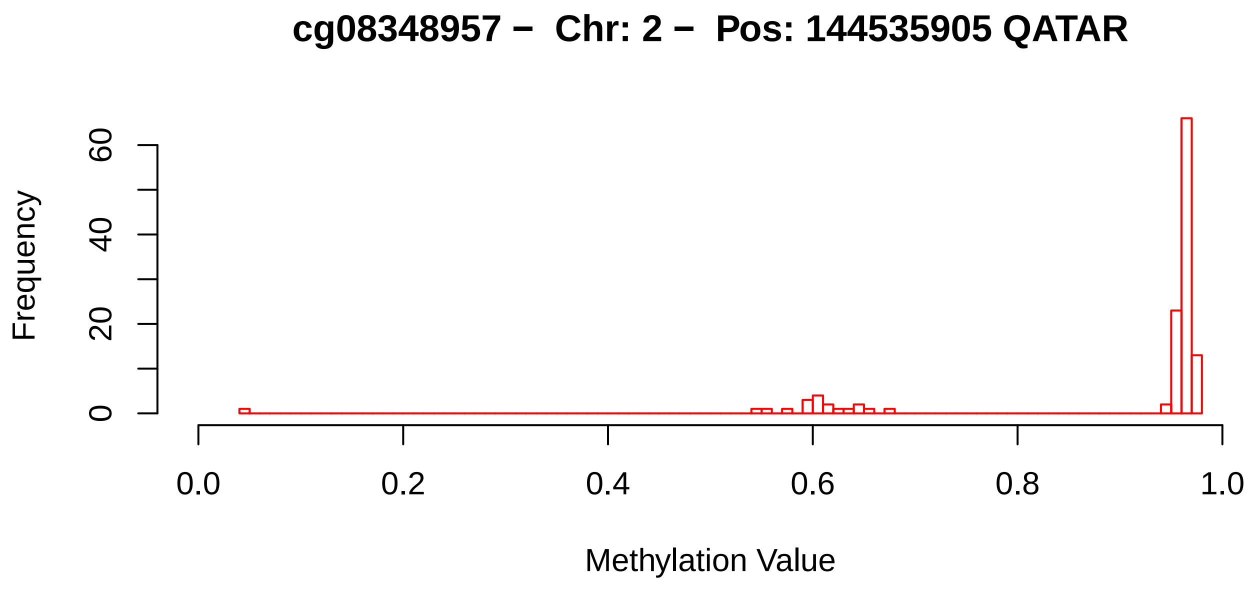
cg08348957 − Chr: 2 − Pos: 144535905 QATAR
Frequency
0
20
40
60
0.0
0.2
0.4
0.6
0.8
1.0
Methylation Value

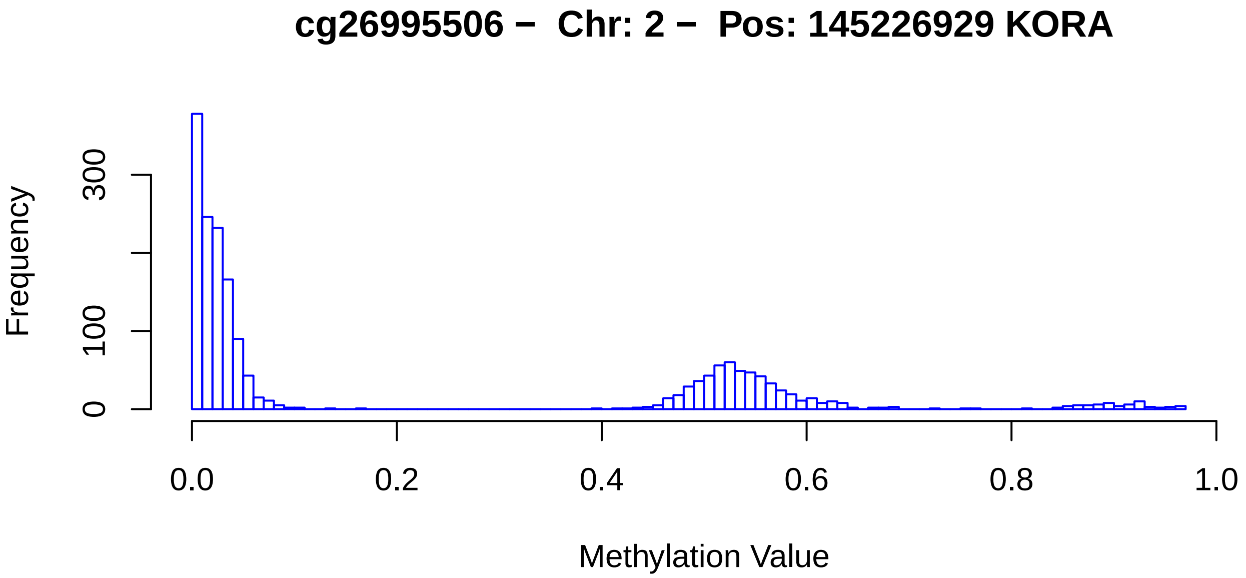
cg26995506 − Chr: 2 − Pos: 145226929 KORA
Frequency
0
100
300
0.0
0.2
0.4
0.6
0.8
1.0
Methylation Value

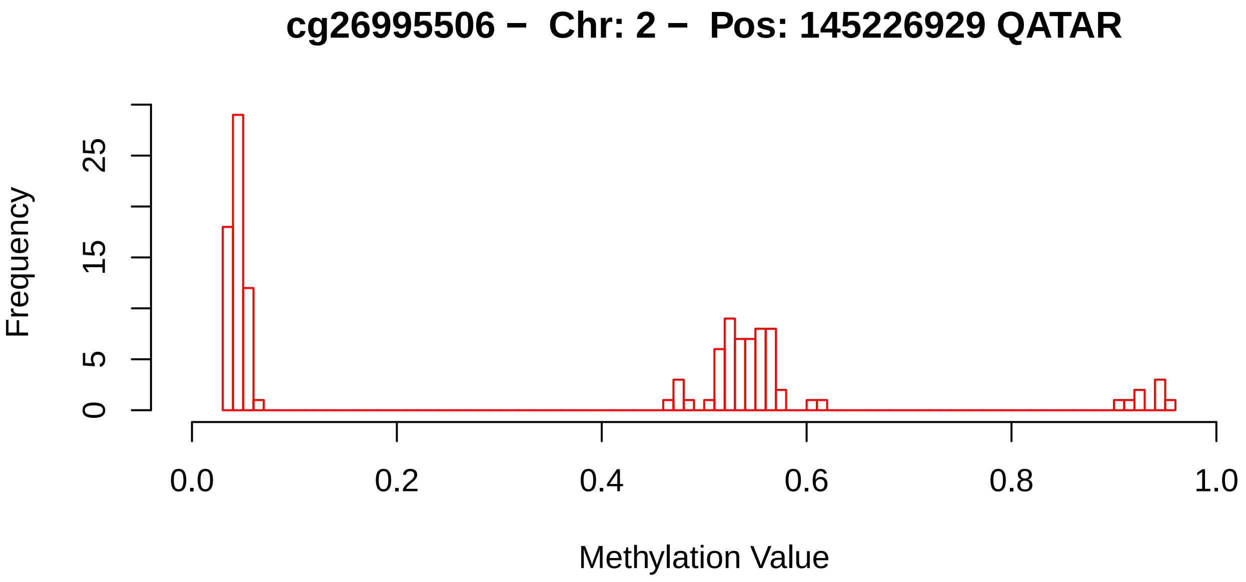
cg26995506 − Chr: 2 − Pos: 145226929 QATAR
Frequency
0
5
15
25
0.0
0.2
0.4
0.6
0.8
1.0
Methylation Value

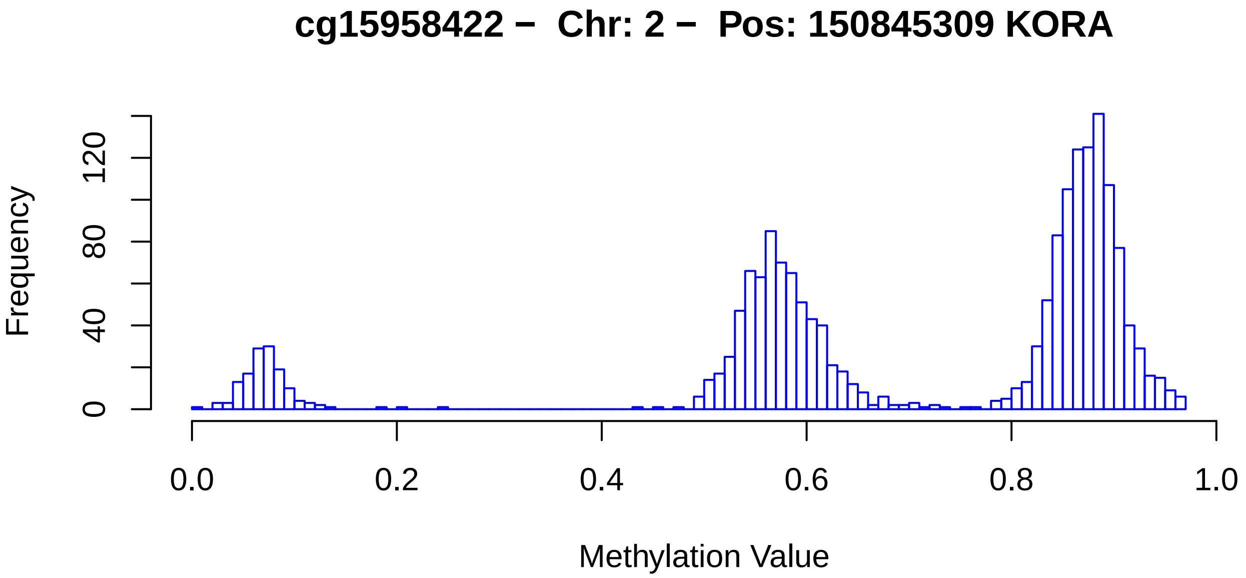
cg15958422 − Chr: 2 − Pos: 150845309 KORA
Frequency
0
40
80
120
0.0
0.2
0.4
0.6
0.8
1.0
Methylation Value

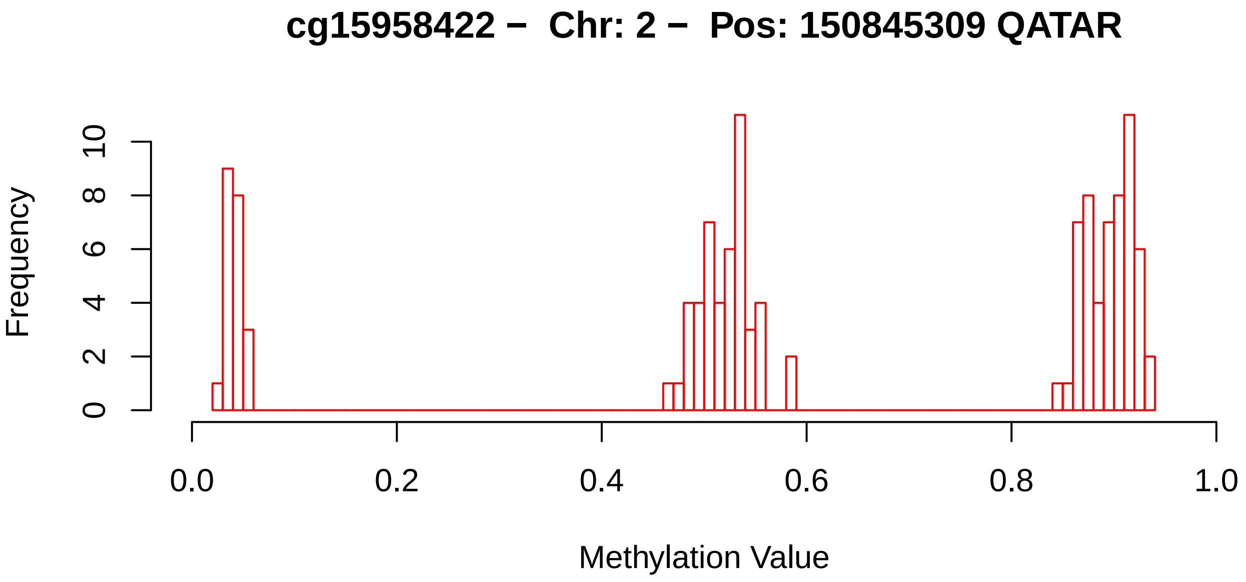
cg15958422 − Chr: 2 − Pos: 150845309 QATAR
Frequency
0
2
4
6
8
10
0.0
0.2
0.4
0.6
0.8
1.0
Methylation Value

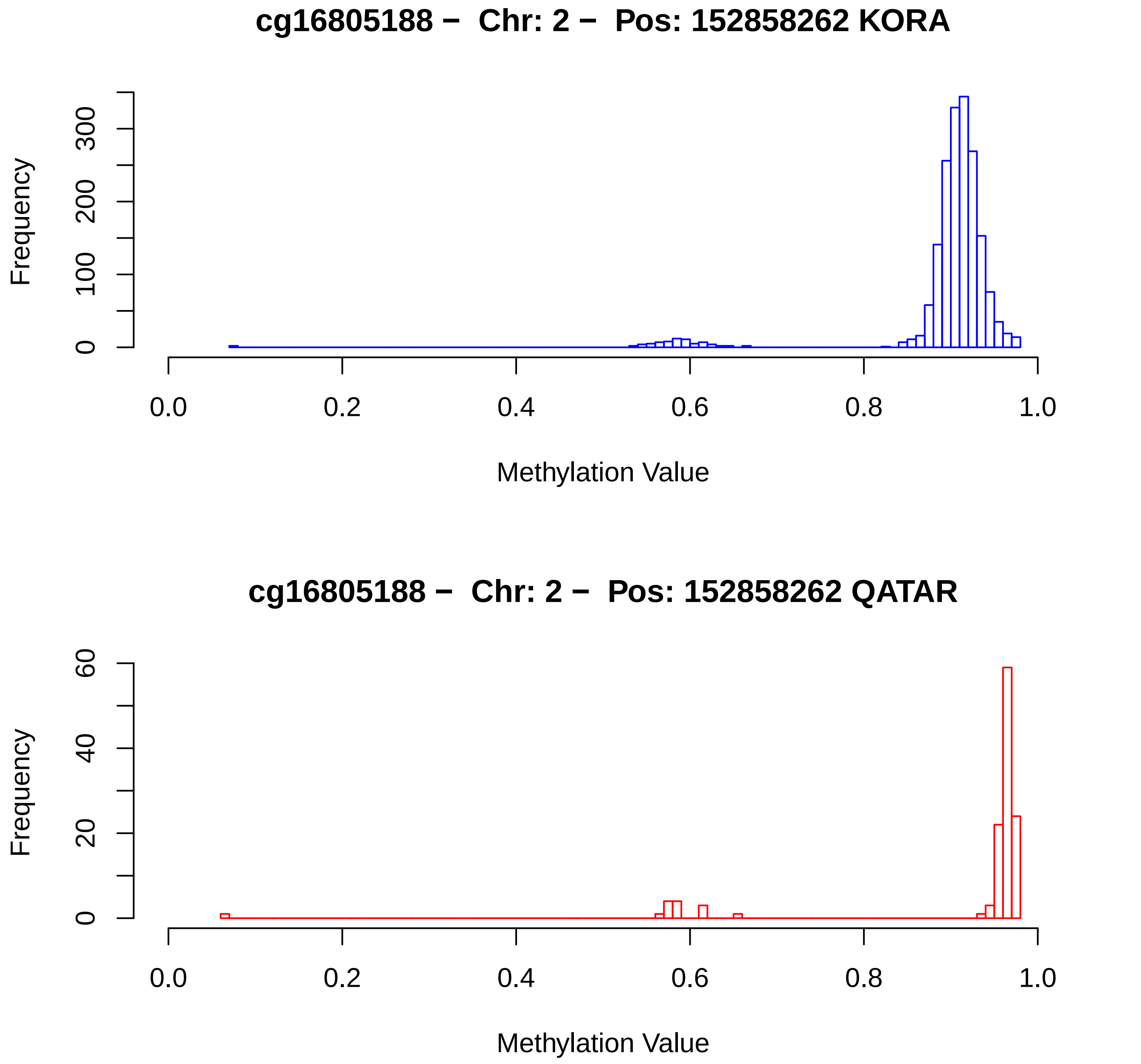
cg16805188 − Chr: 2 − Pos: 152858262 KORA
Frequency
0
100
200
300
0.0
0.2
0.4
0.6
0.8
1.0
Methylation Value
cg16805188 − Chr: 2 − Pos: 152858262 QATAR
Frequency
0
20
40
60
0.0
0.2
0.4
0.6
0.8
1.0
Methylation Value

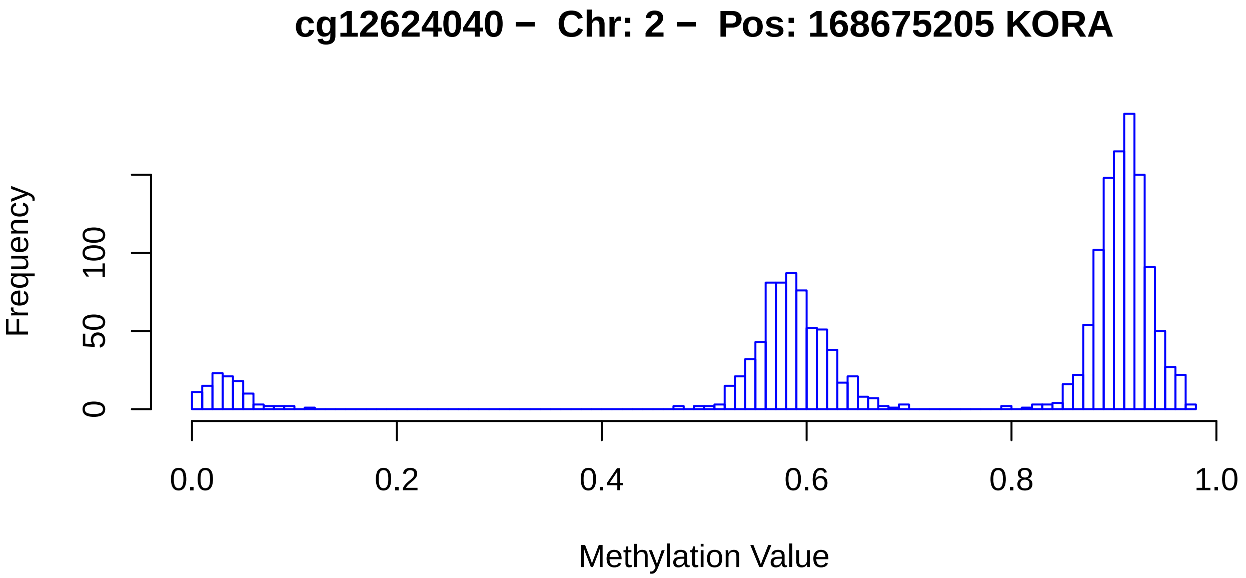

cg12624040 − Chr: 2 − Pos: 168675205 KORA
Frequency
0
50
100
0.0
0.2
0.4
0.6
0.8
1.0
Methylation Value

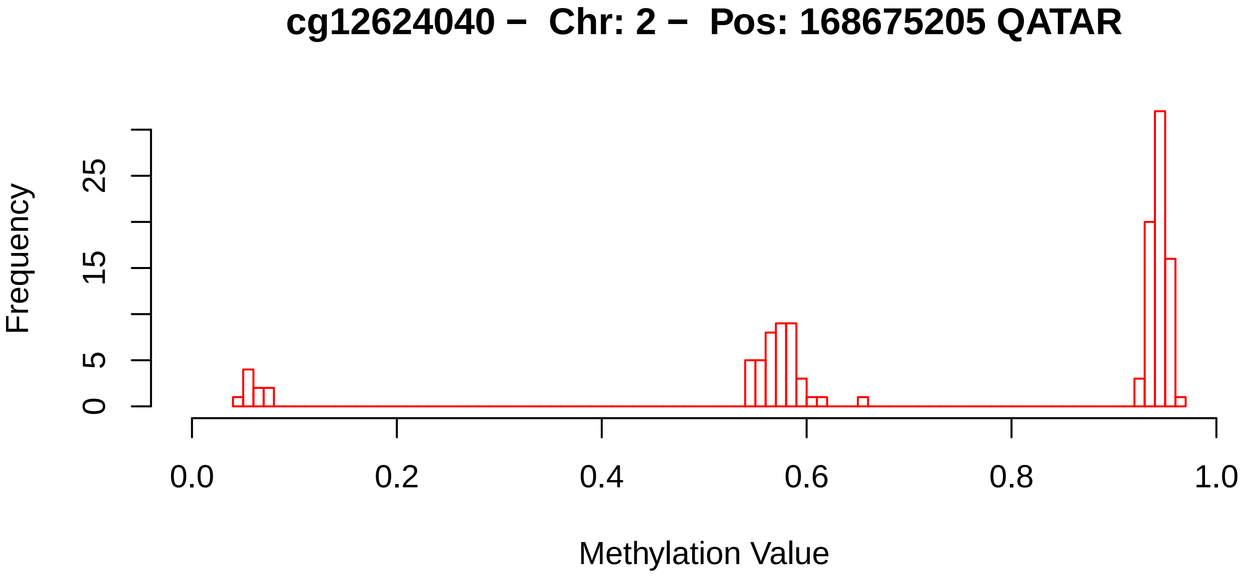

cg12624040 − Chr: 2 − Pos: 168675205 QATAR
Frequency
0
5
15
25
0.0
0.2
0.4
0.6
0.8
1.0
Methylation Value

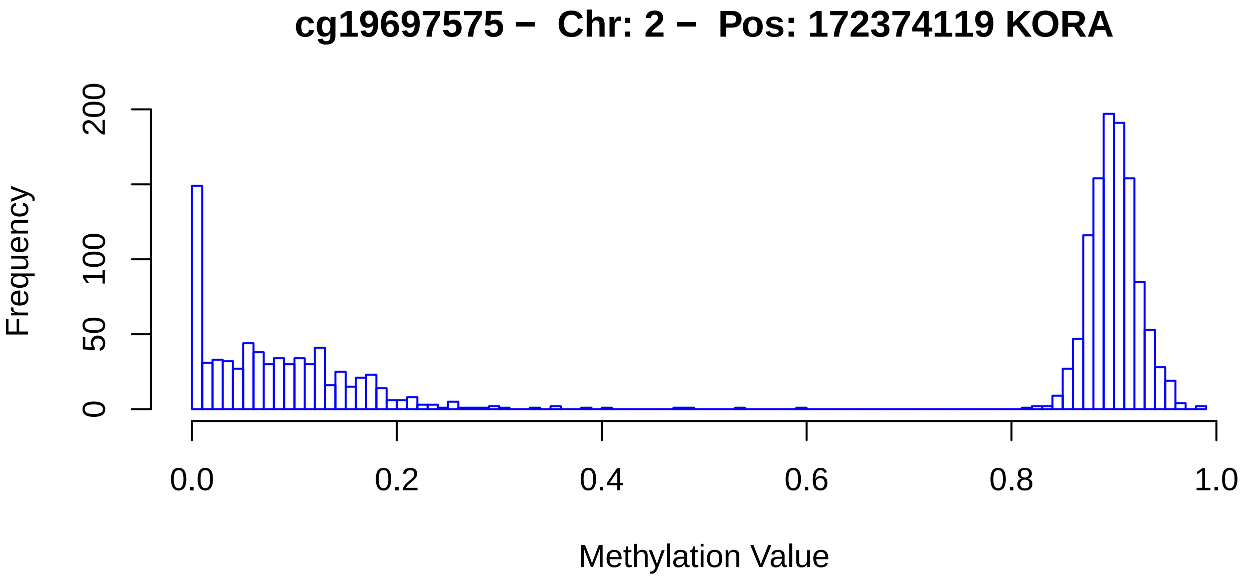
cg19697575 − Chr: 2 − Pos: 172374119 KORA
Frequency
0
50
100
200
0.0
0.2
0.4
0.6
0.8
1.0
Methylation Value

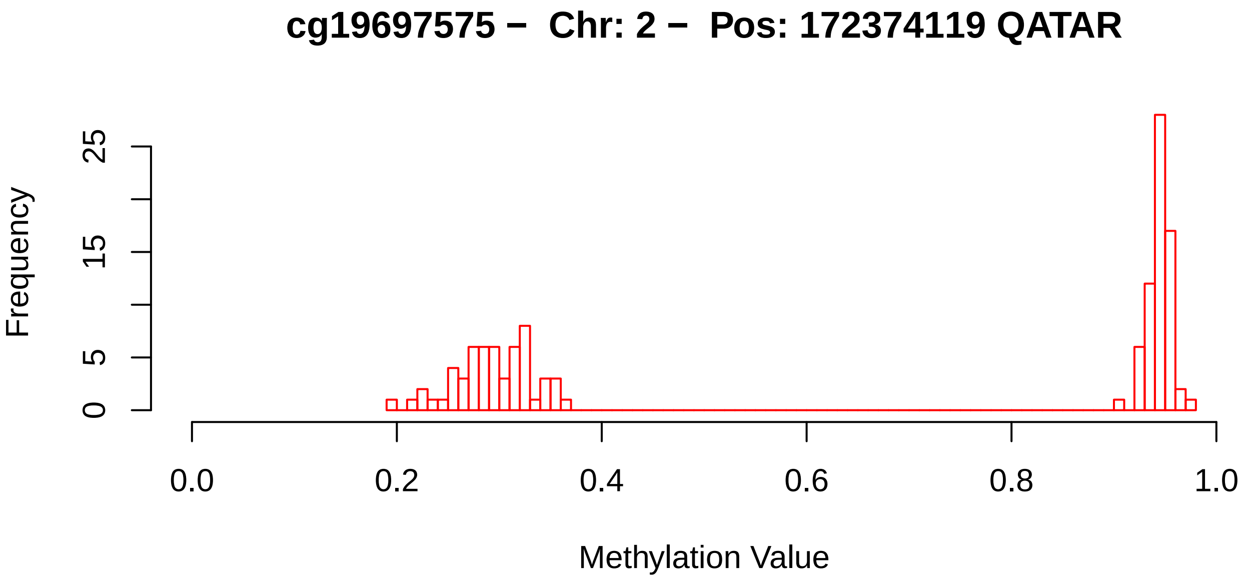
cg19697575 − Chr: 2 − Pos: 172374119 QATAR
Frequency
0
5
15
25
0.0
0.2
0.4
0.6
0.8
1.0
Methylation Value

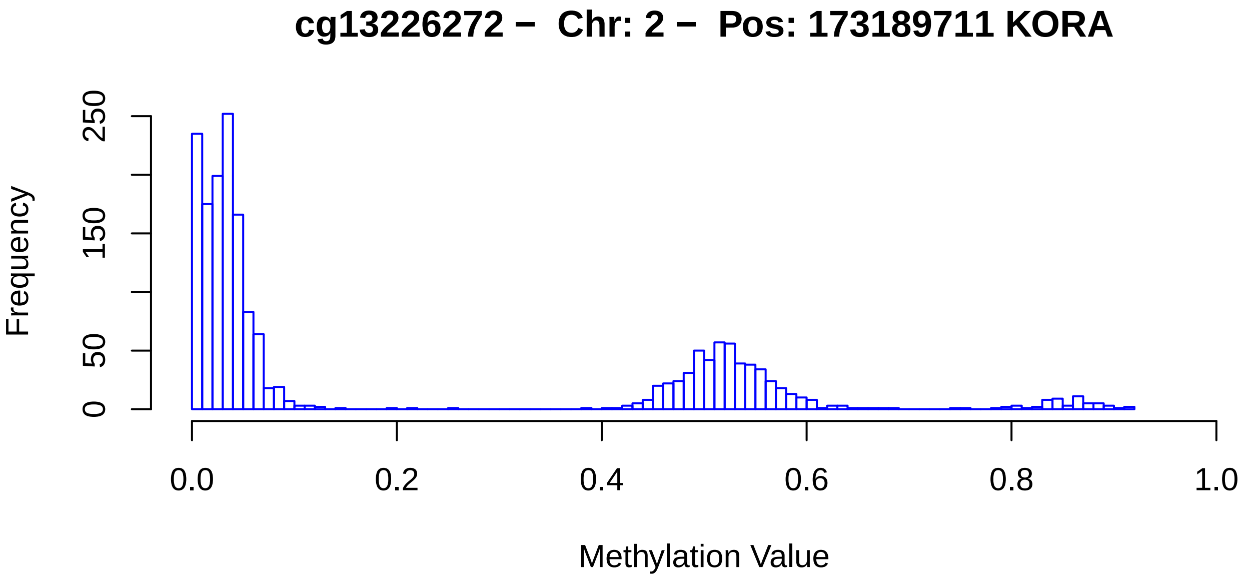

cg13226272 − Chr: 2 − Pos: 173189711 KORA
Frequency
0
50
150
250
0.0
0.2
0.4
0.6
0.8
1.0
Methylation Value

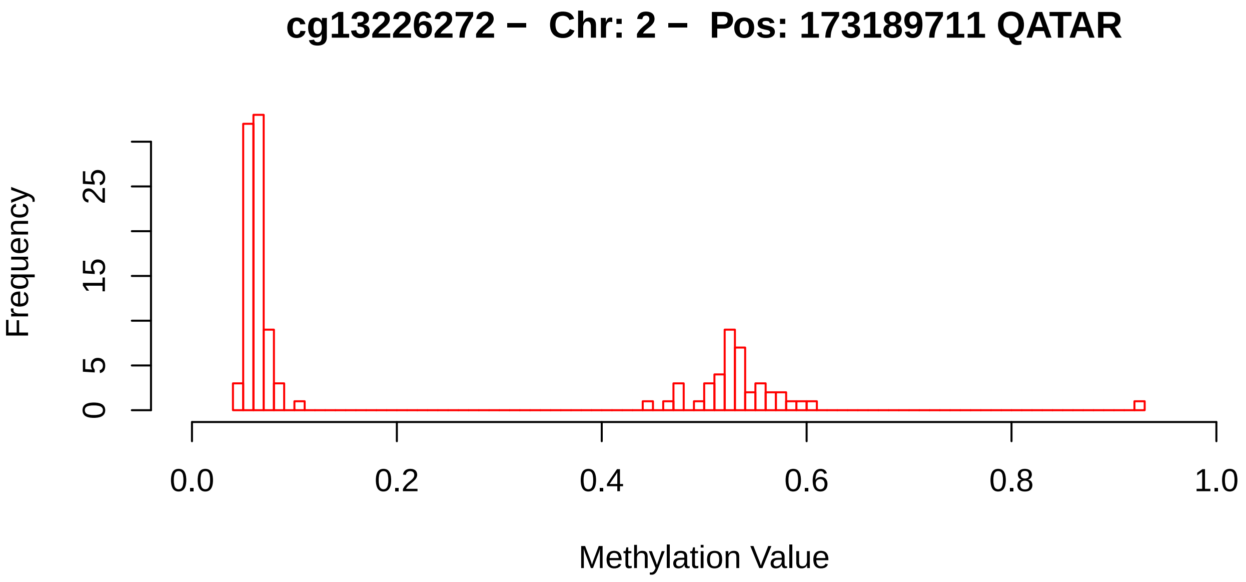

cg13226272 − Chr: 2 − Pos: 173189711 QATAR
Frequency
0
5
15
25
0.0
0.2
0.4
0.6
0.8
1.0
Methylation Value

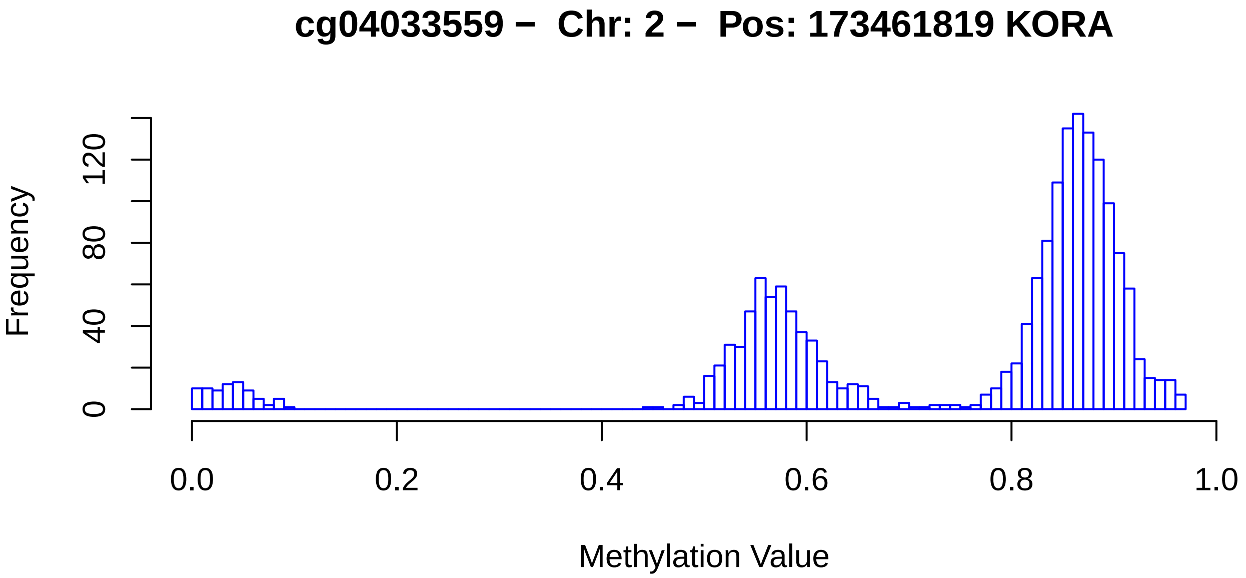
cg04033559 − Chr: 2 − Pos: 173461819 KORA
Frequency
0
40
80
120
0.0
0.2
0.4
0.6
0.8
1.0
Methylation Value

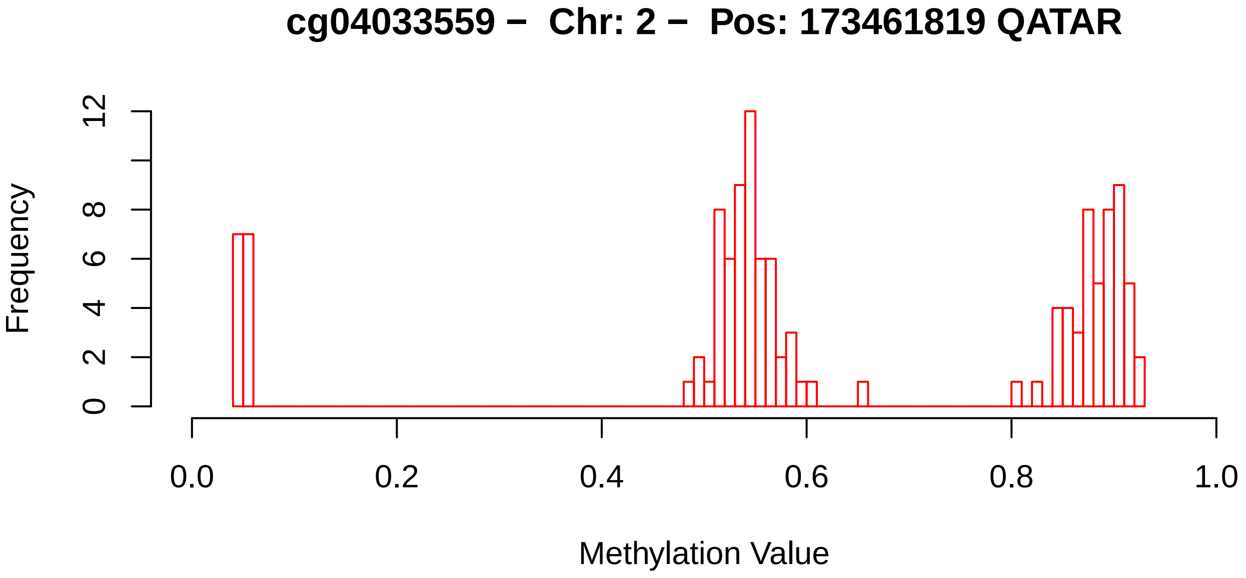
cg04033559 − Chr: 2 − Pos: 173461819 QATAR
Frequency
0
2
4
6
8
12
0.0
0.2
0.4
0.6
0.8
1.0
Methylation Value

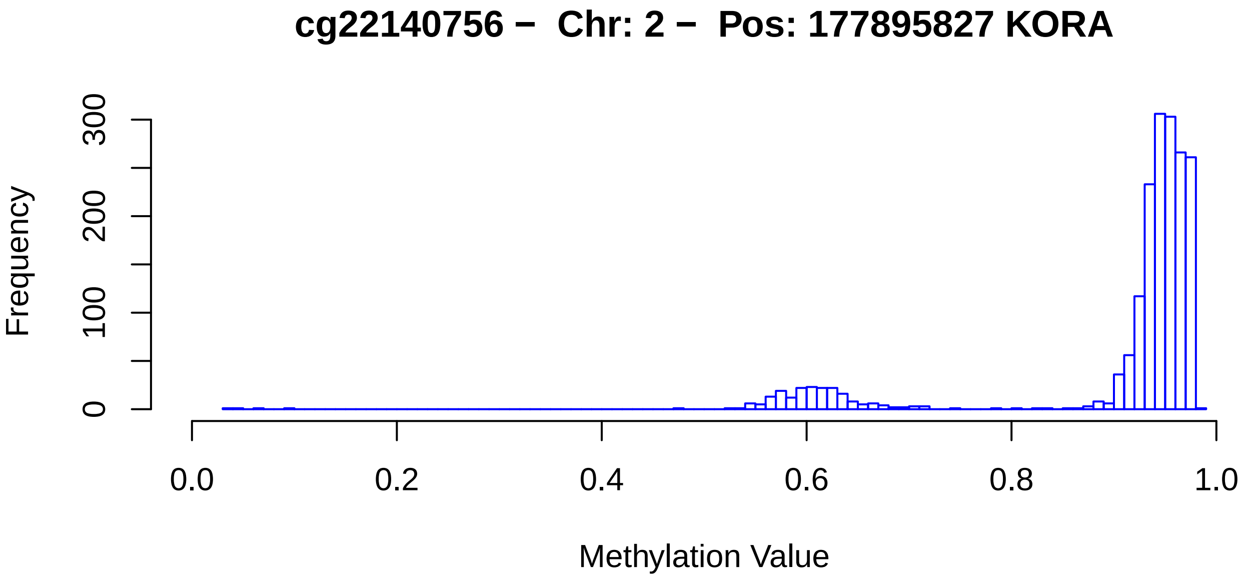
cg22140756 − Chr: 2 − Pos: 177895827 KORA
Frequency
0
100
200
300
0.0
0.2
0.4
0.6
0.8
1.0
Methylation Value

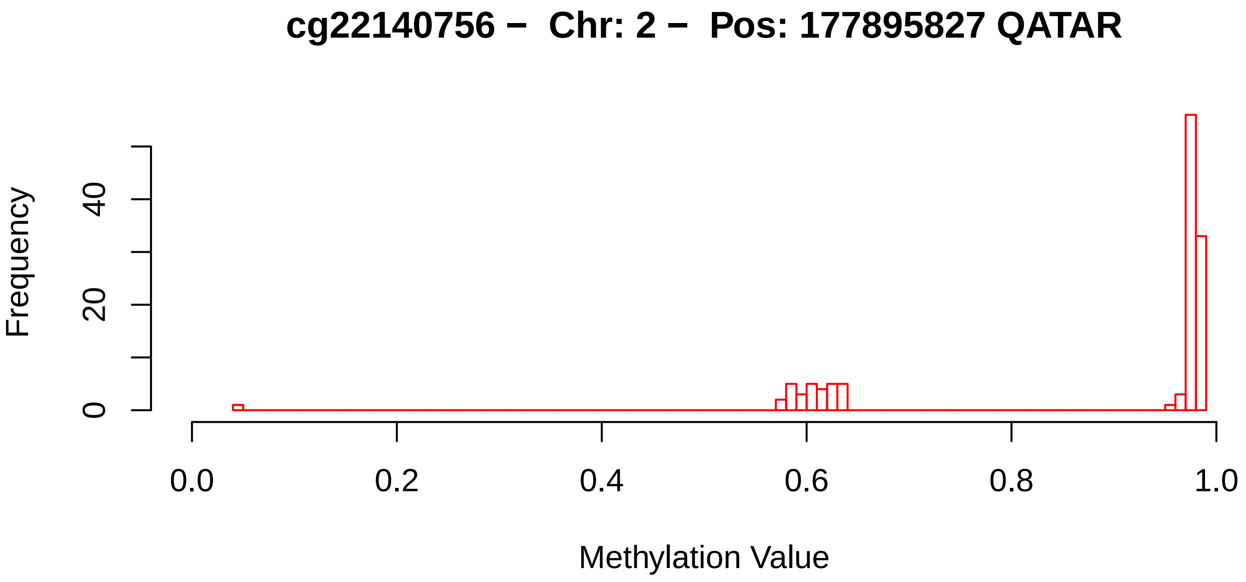
cg22140756 − Chr: 2 − Pos: 177895827 QATAR
Frequency
0
20
40
0.0
0.2
0.4
0.6
0.8
1.0
Methylation Value

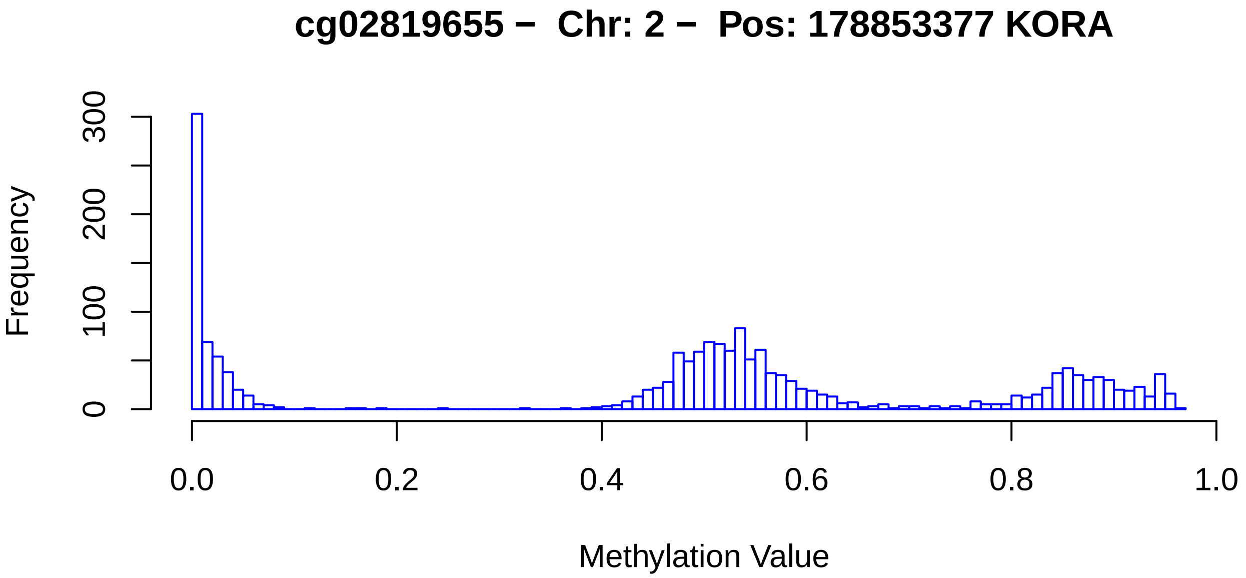
cg02819655 − Chr: 2 − Pos: 178853377 KORA
Frequency
0
100
200
300
0.0
0.2
0.4
0.6
0.8
1.0
Methylation Value

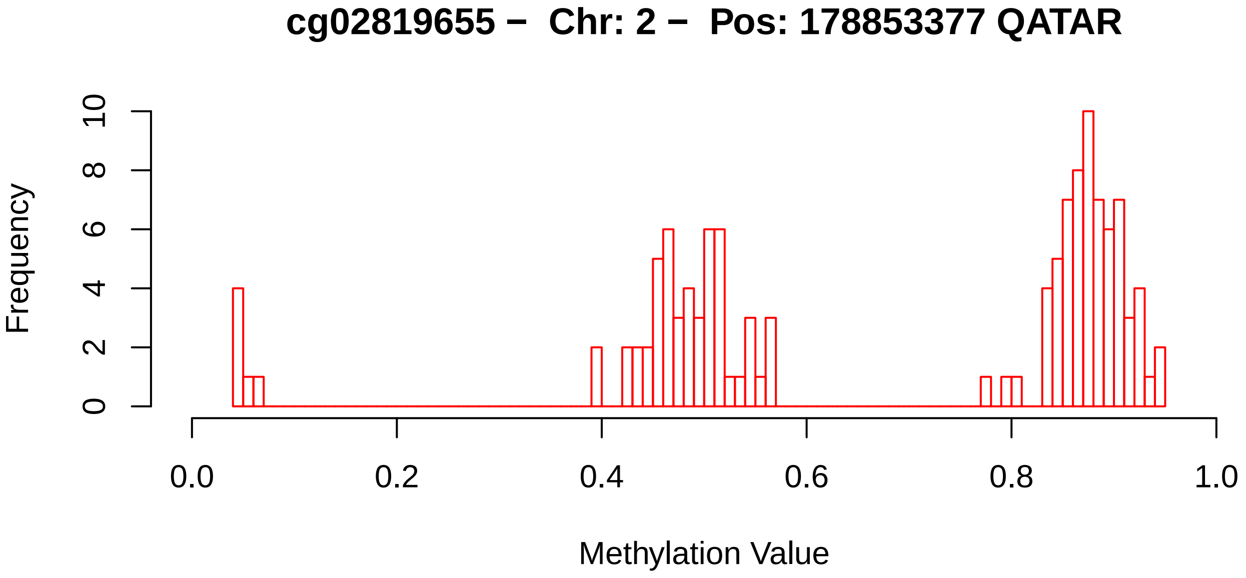
cg02819655 − Chr: 2 − Pos: 178853377 QATAR
Frequency
0
2
4
6
8
10
0.0
0.2
0.4
0.6
0.8
1.0
Methylation Value

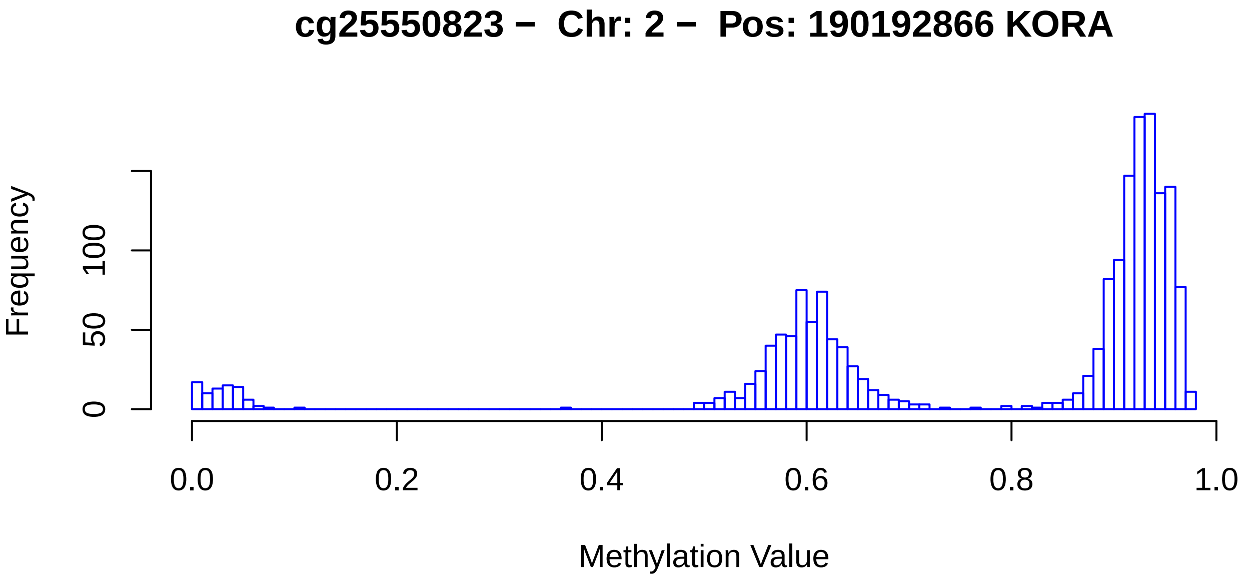
cg25550823 − Chr: 2 − Pos: 190192866 KORA
Frequency
0
50
100
0.0
0.2
0.4
0.6
0.8
1.0
Methylation Value

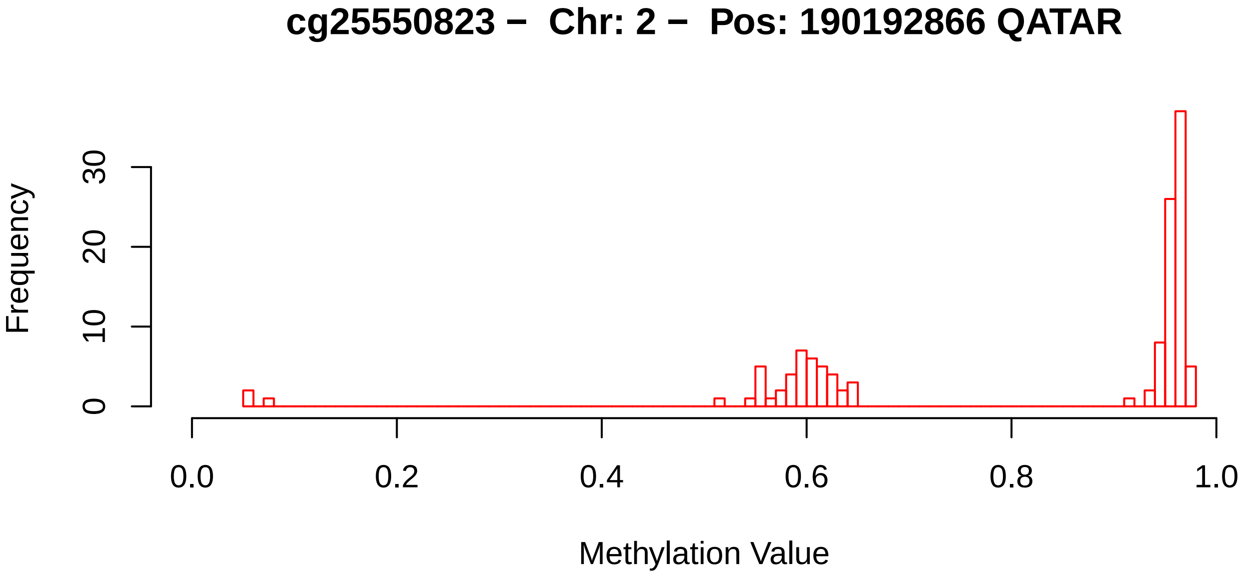
cg25550823 − Chr: 2 − Pos: 190192866 QATAR
Frequency
0
10
20
30
0.0
0.2
0.4
0.6
0.8
1.0
Methylation Value

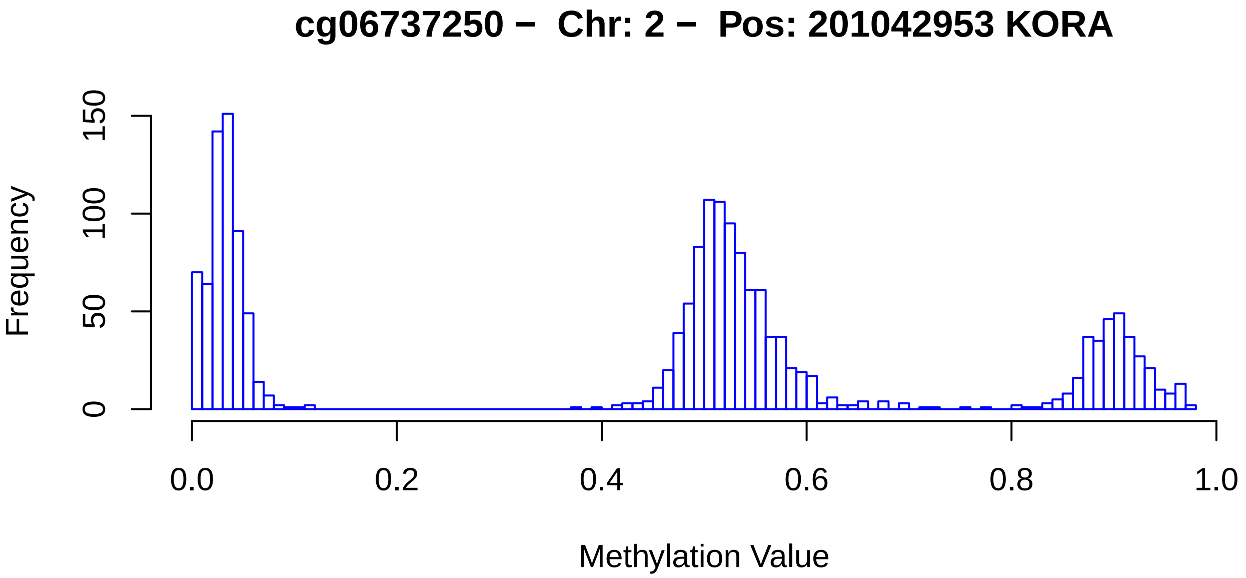
cg06737250 − Chr: 2 − Pos: 201042953 KORA
Frequency
0
50
100
150
0.0
0.2
0.4
0.6
0.8
1.0
Methylation Value

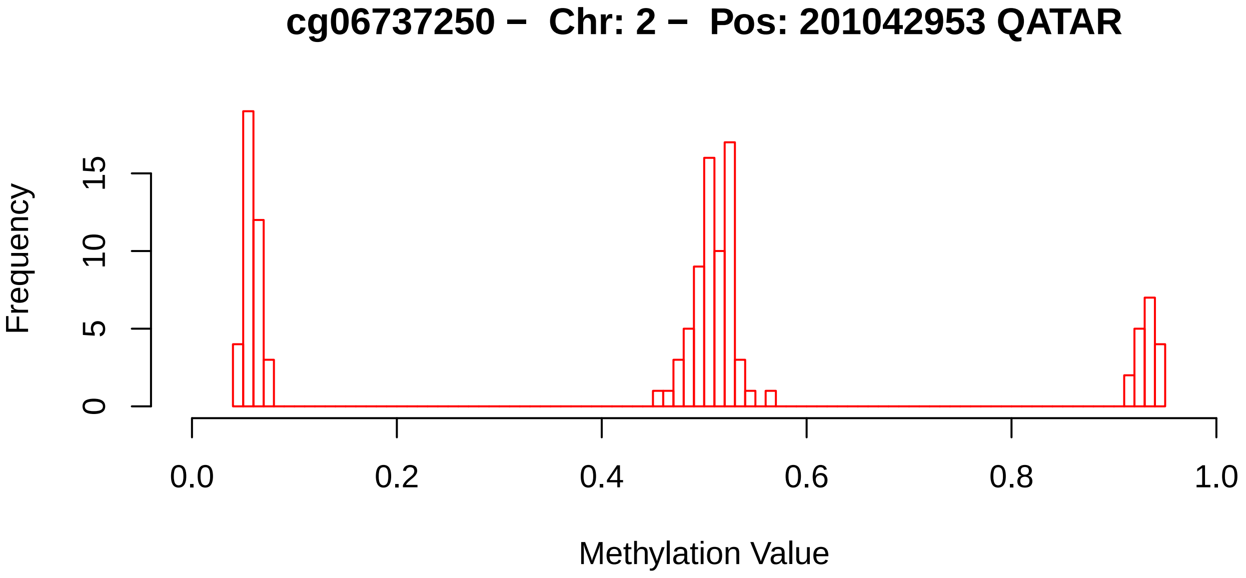
cg06737250 − Chr: 2 − Pos: 201042953 QATAR
Frequency
0
5
10
15
0.0
0.2
0.4
0.6
0.8
1.0
Methylation Value

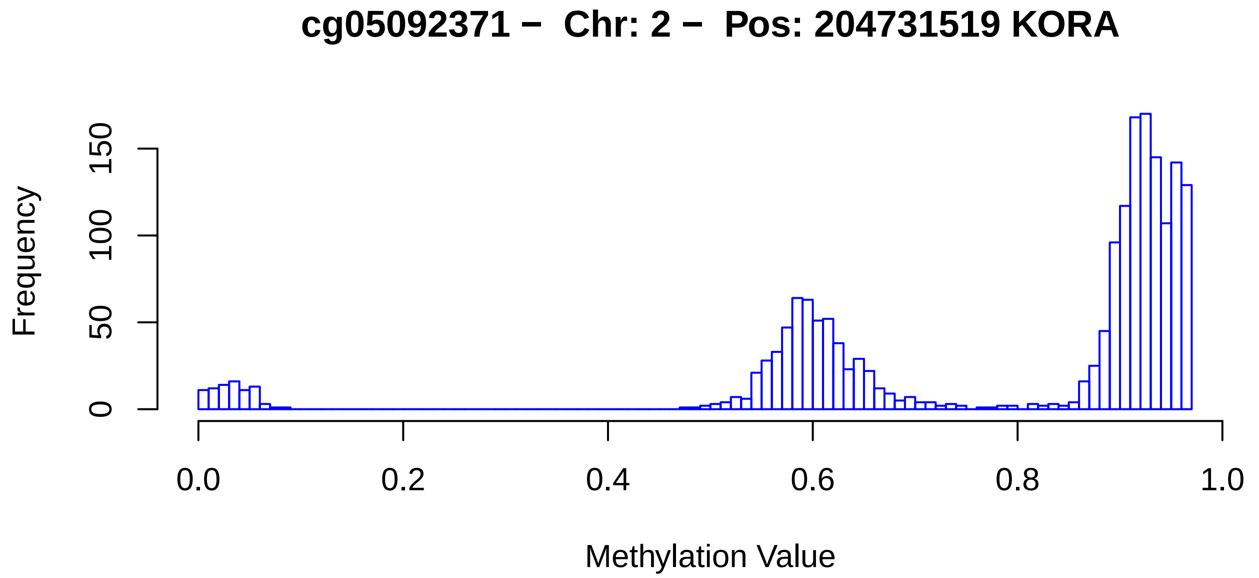
cg05092371 − Chr: 2 − Pos: 204731519 KORA
Frequency
0
50
100
150
0.0
0.2
0.4
0.6
0.8
1.0
Methylation Value

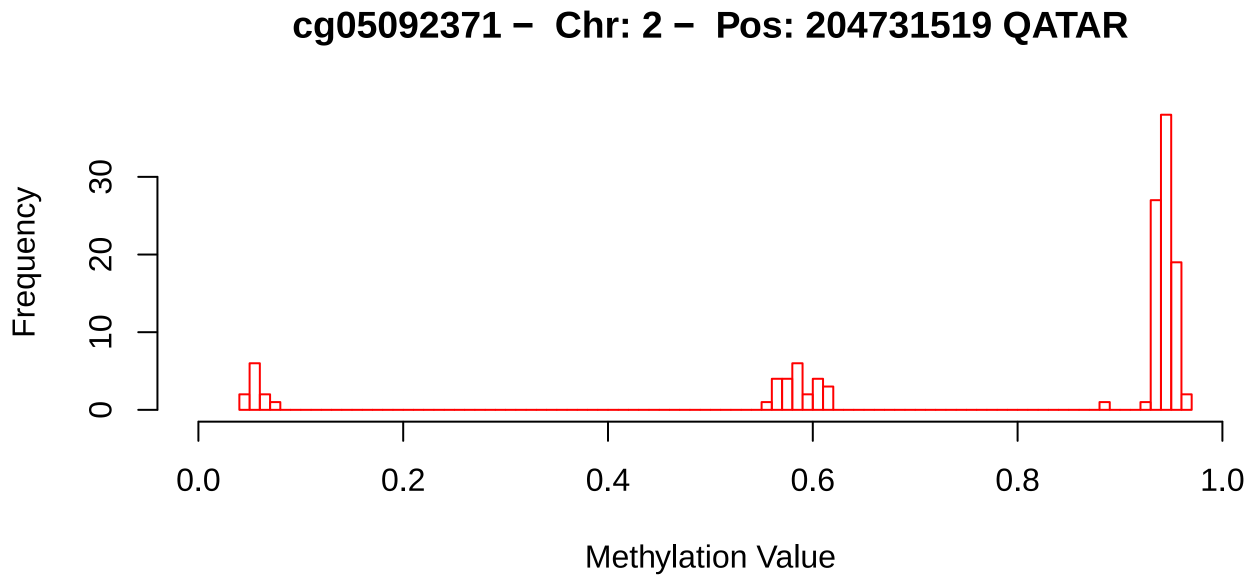
cg05092371 − Chr: 2 − Pos: 204731519 QATAR
Frequency
0
10
20
30
0.0
0.2
0.4
0.6
0.8
1.0
Methylation Value

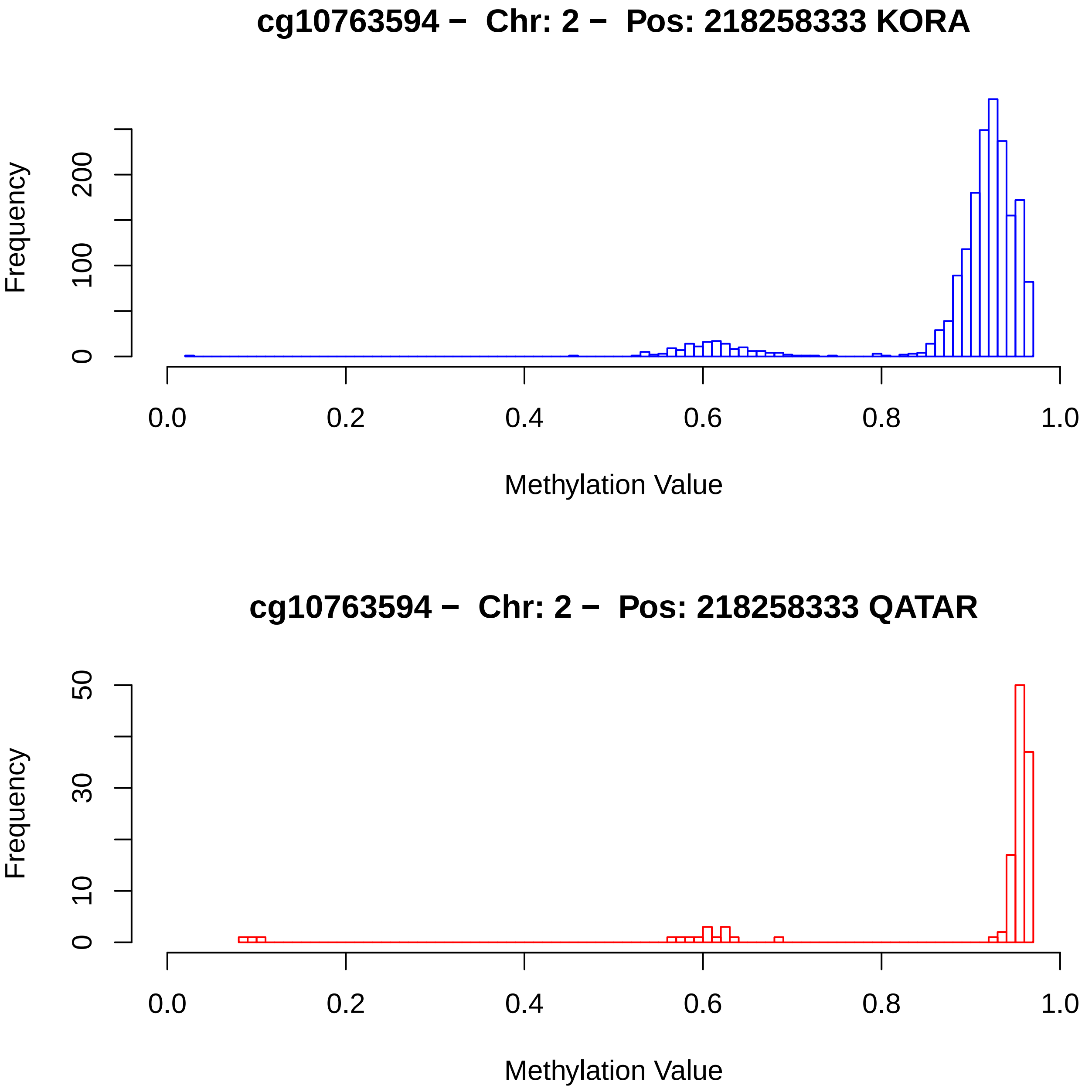
cg10763594 − Chr: 2 − Pos: 218258333 KORA
Frequency
0
100
200
0.0
0.2
0.4
0.6
0.8
1.0
Methylation Value
cg10763594 − Chr: 2 − Pos: 218258333 QATAR
Frequency
0
10
30
50
0.0
0.2
0.4
0.6
0.8
1.0
Methylation Value

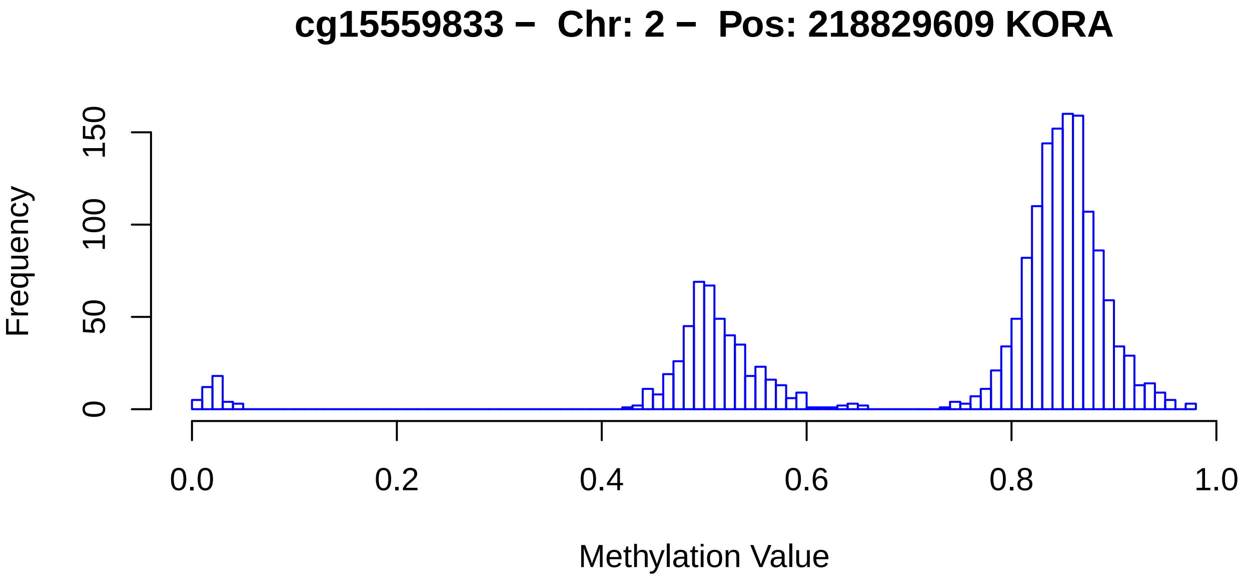

cg15559833 − Chr: 2 − Pos: 218829609 KORA
Frequency
0
50
100
150
0.0
0.2
0.4
0.6
0.8
1.0
Methylation Value

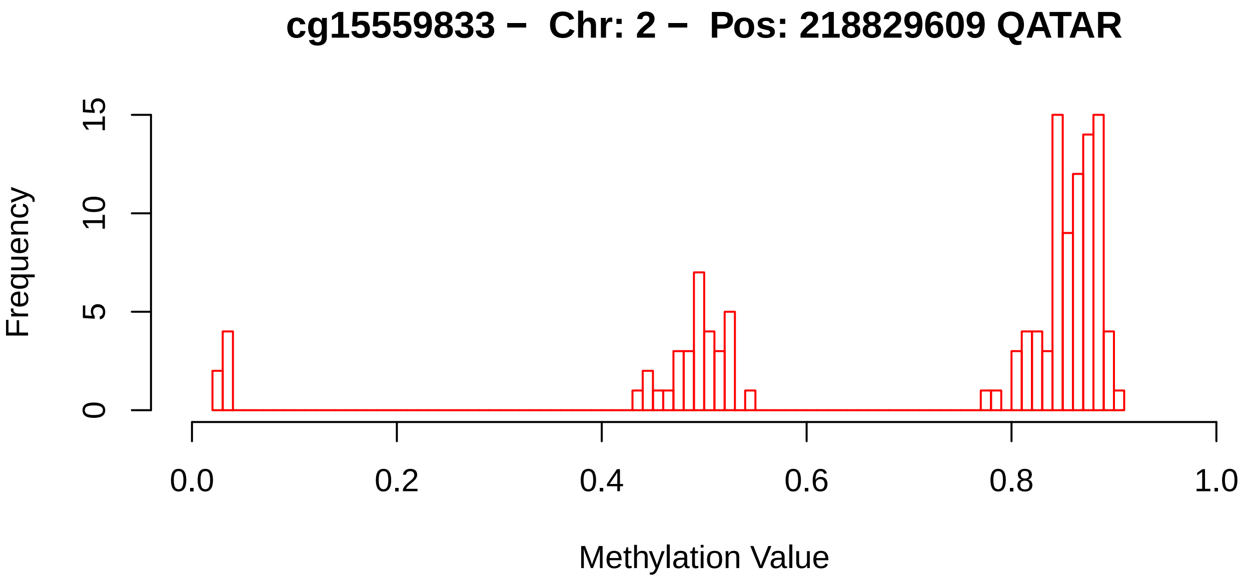

cg15559833 − Chr: 2 − Pos: 218829609 QATAR
Frequency
0
5
10
15
0.0
0.2
0.4
0.6
0.8
1.0
Methylation Value

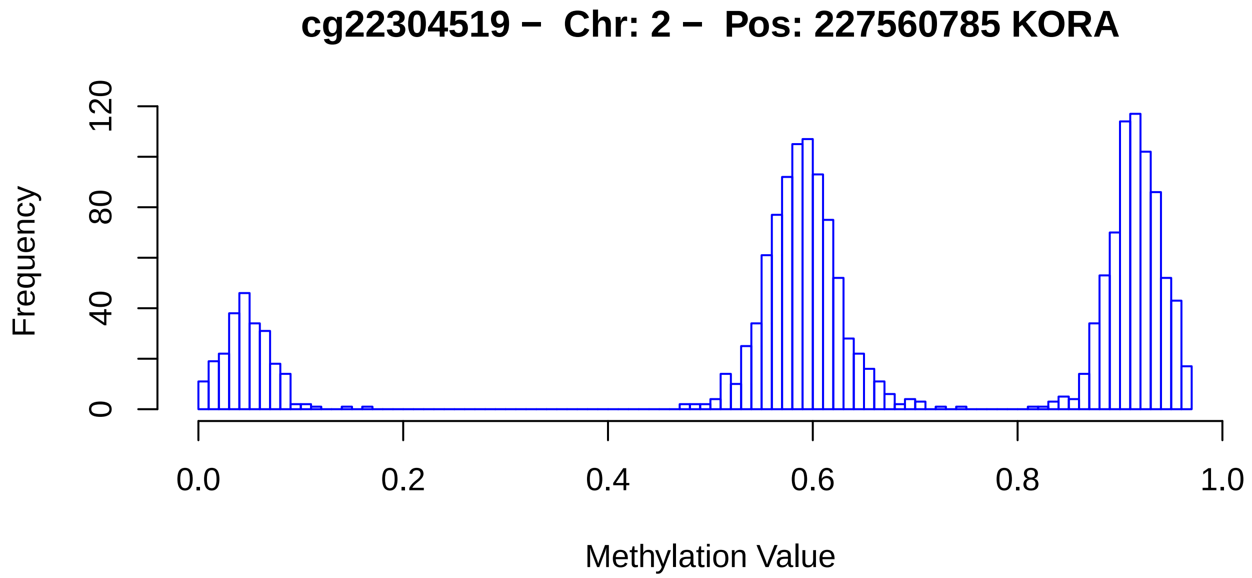
cg22304519 − Chr: 2 − Pos: 227560785 KORA
Frequency
0
40
80
120
0.0
0.2
0.4
0.6
0.8
1.0
Methylation Value

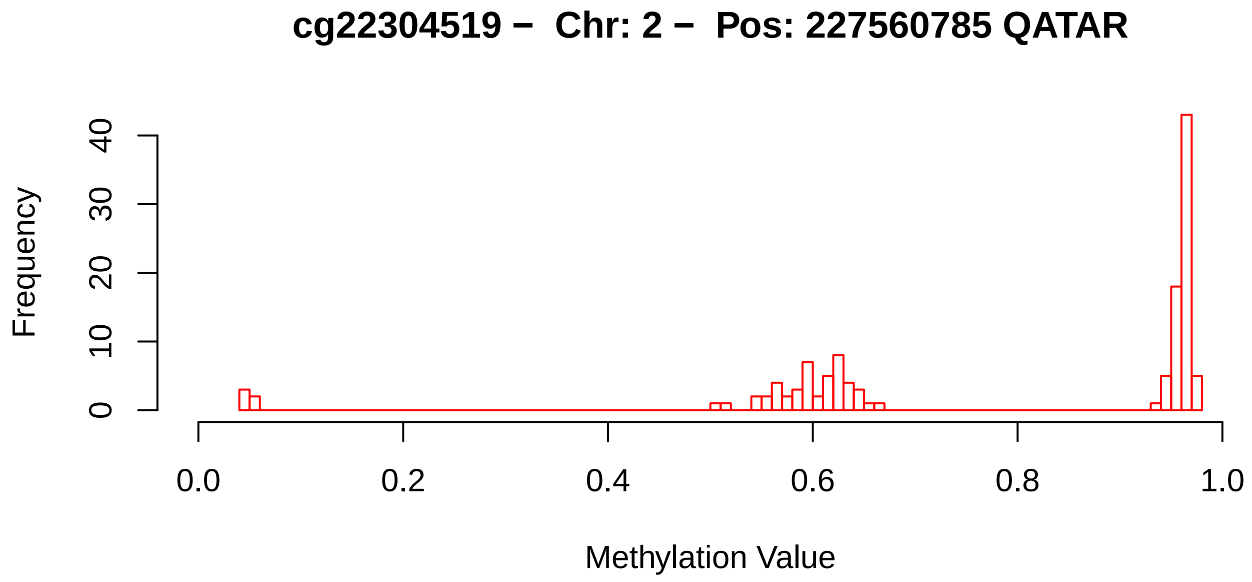
cg22304519 − Chr: 2 − Pos: 227560785 QATAR
Frequency
0
10
20
30
40
0.0
0.2
0.4
0.6
0.8
1.0
Methylation Value

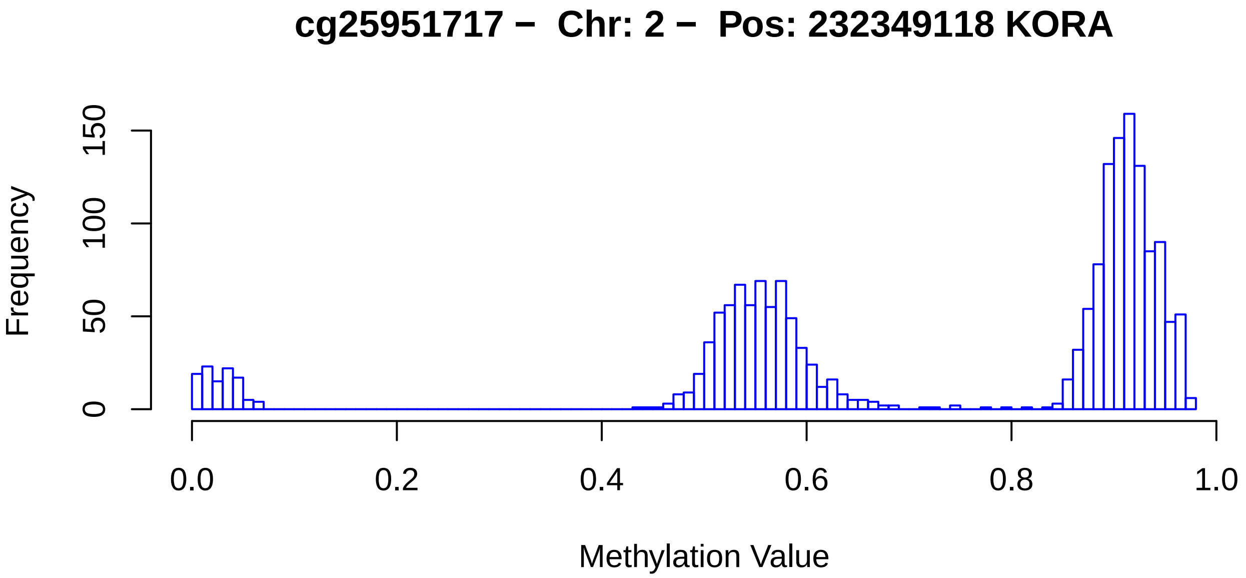

cg25951717 − Chr: 2 − Pos: 232349118 KORA
Frequency
0
50
100
150
0.0
0.2
0.4
0.6
0.8
1.0
Methylation Value

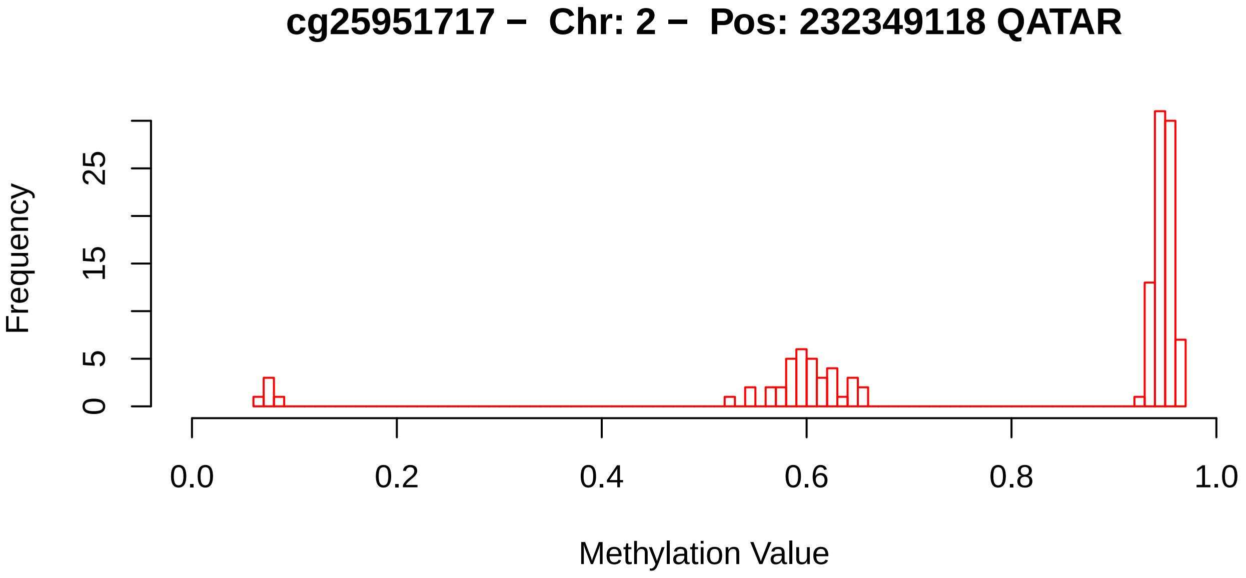

cg25951717 − Chr: 2 − Pos: 232349118 QATAR
Frequency
0
5
15
25
0.0
0.2
0.4
0.6
0.8
1.0
Methylation Value

cg20421295 − Chr: 2 − Pos: 233749913 KORA

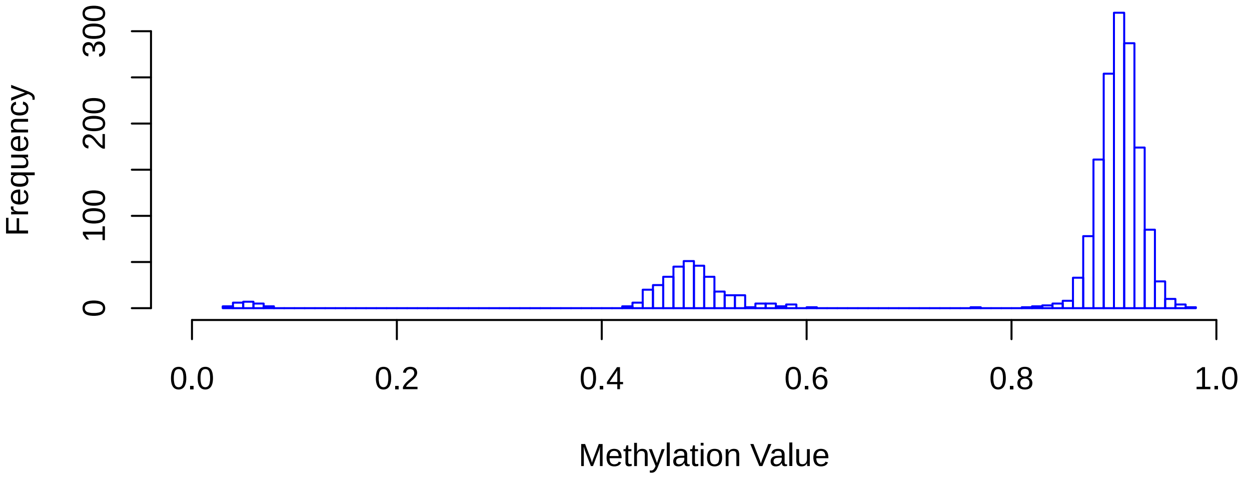

cg20421295 − Chr: 2 − Pos: 233749913 QATAR

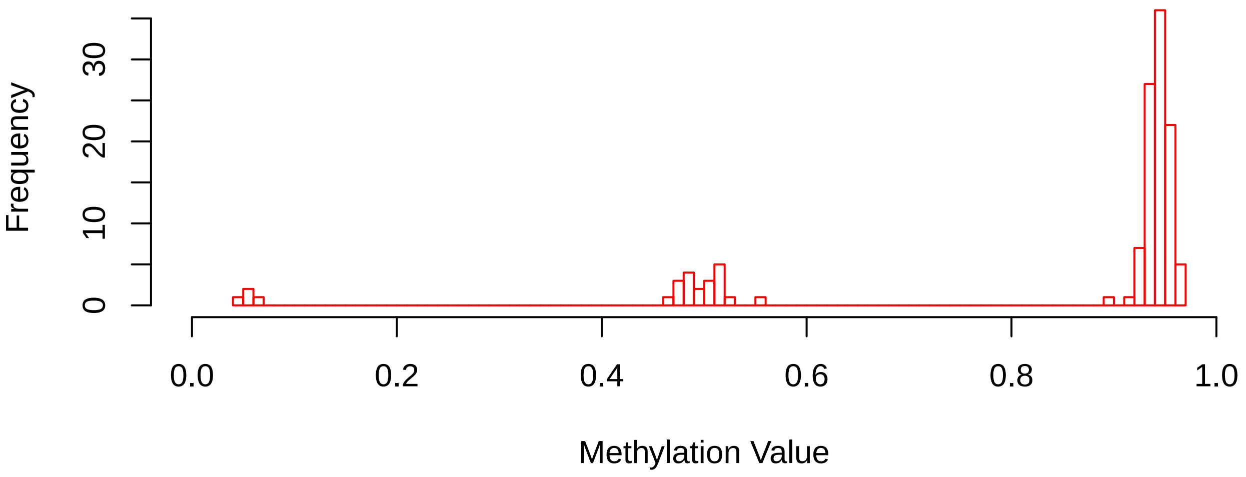

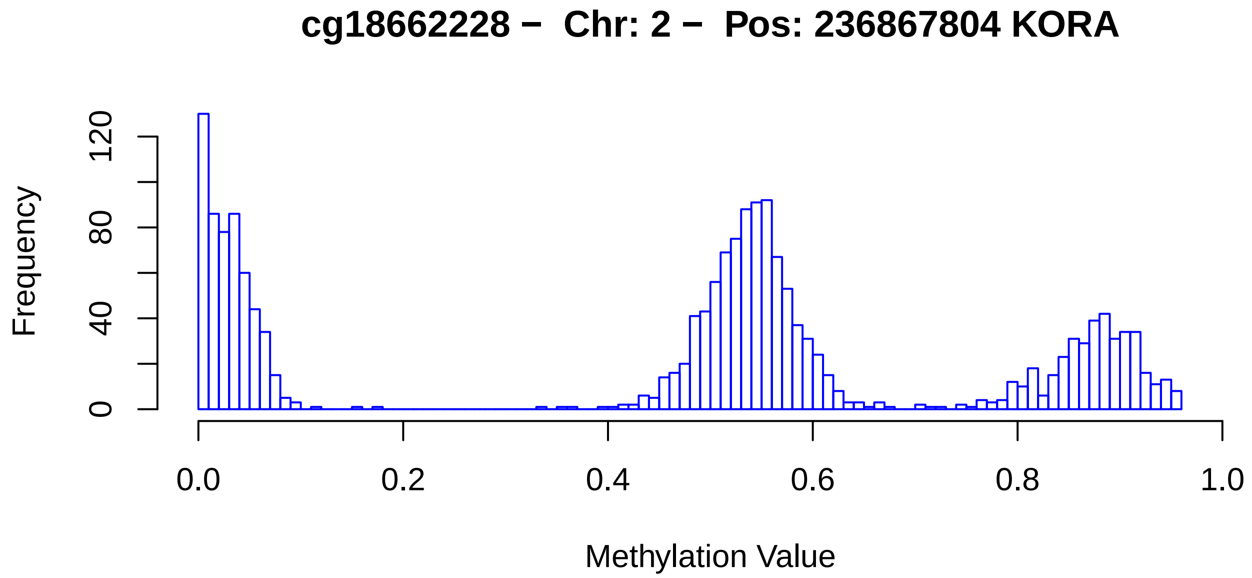
cg18662228 − Chr: 2 − Pos: 236867804 KORA
Frequency
0
40
80
120
0.0
0.2
0.4
0.6
0.8
1.0
Methylation Value

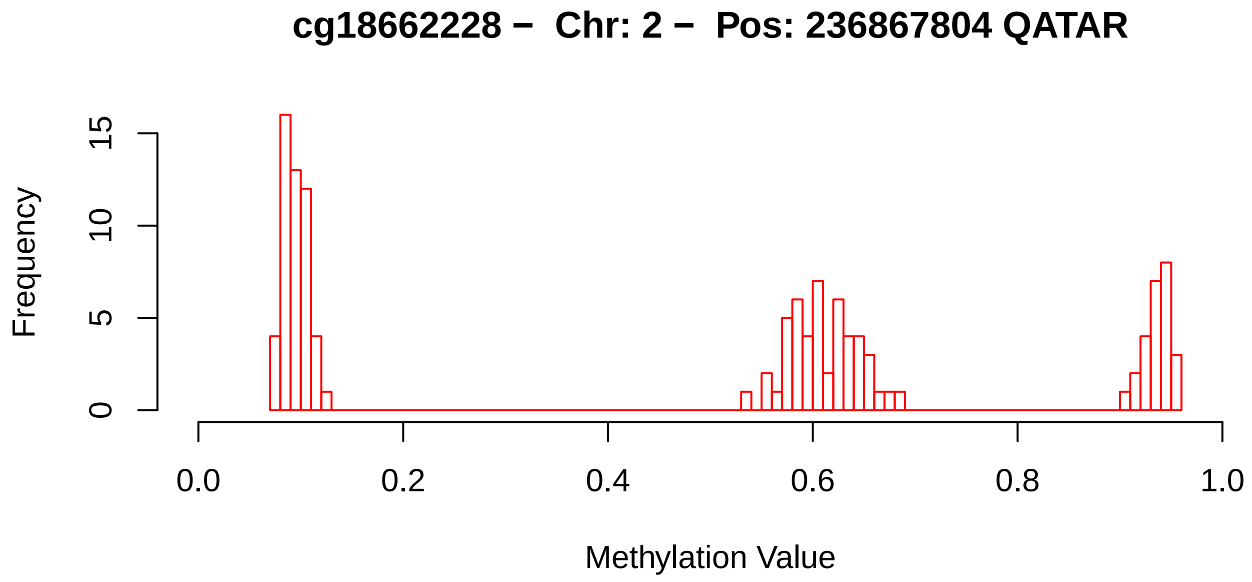
cg18662228 − Chr: 2 − Pos: 236867804 QATAR
Frequency
0
5
10
15
0.0
0.2
0.4
0.6
0.8
1.0
Methylation Value

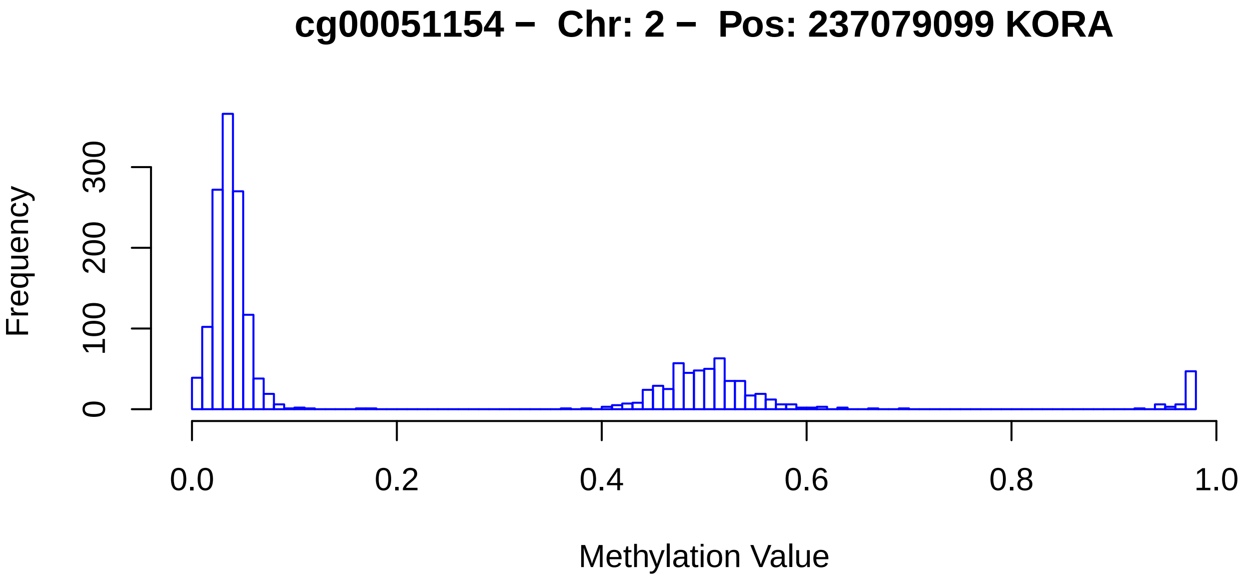
cg00051154 − Chr: 2 − Pos: 237079099 KORA
Frequency
0
100
200
300
0.0
0.2
0.4
0.6
0.8
1.0
Methylation Value

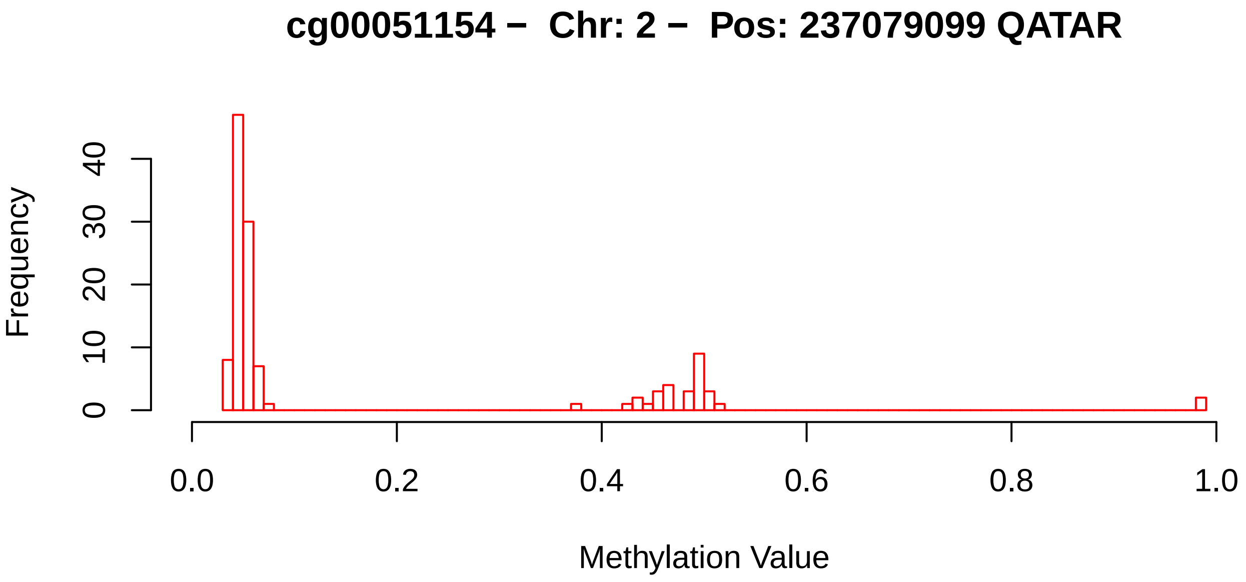
cg00051154 − Chr: 2 − Pos: 237079099 QATAR
Frequency
0
10
20
30
40
0.0
0.2
0.4
0.6
0.8
1.0
Methylation Value

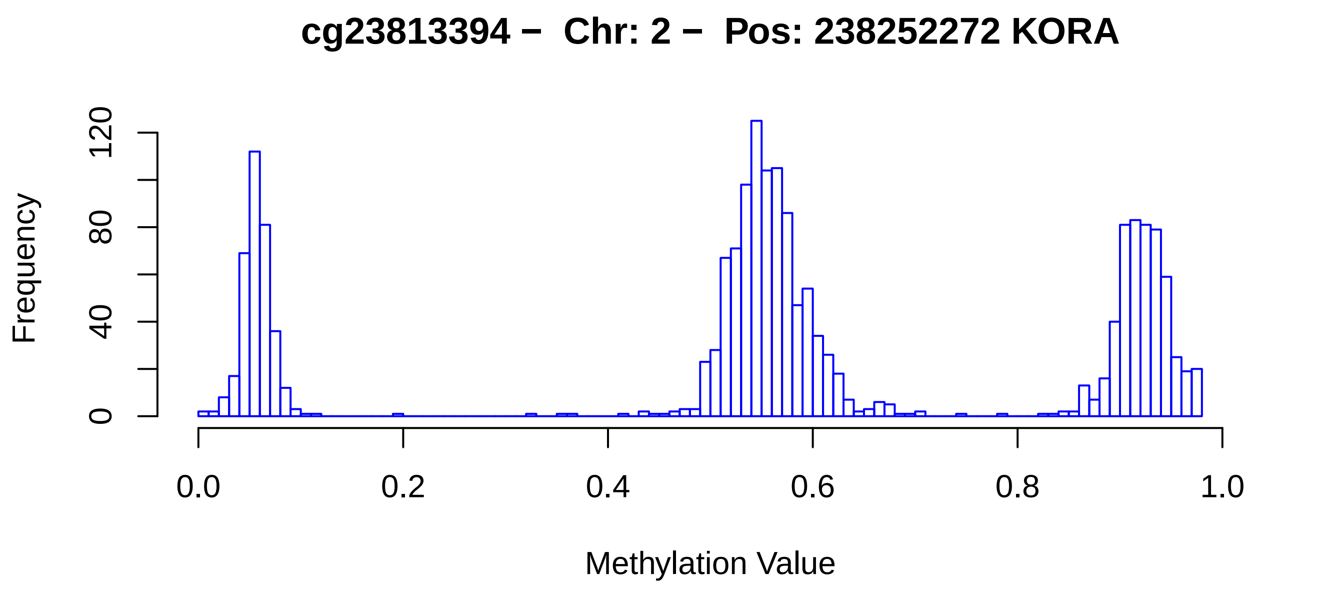
cg23813394 − Chr: 2 − Pos: 238252272 KORA
Frequency
0
40
80
120
0.0
0.2
0.4
0.6
0.8
1.0
Methylation Value

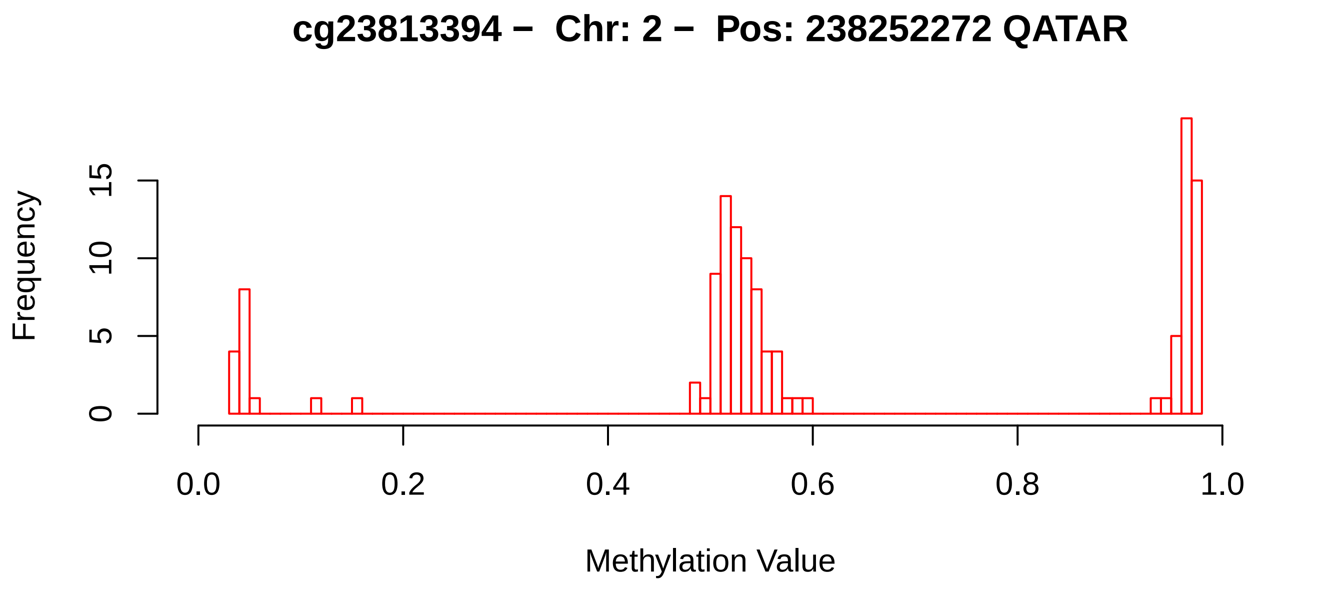
cg23813394 − Chr: 2 − Pos: 238252272 QATAR
Frequency
0
5
10
15
0.0
0.2
0.4
0.6
0.8
1.0
Methylation Value

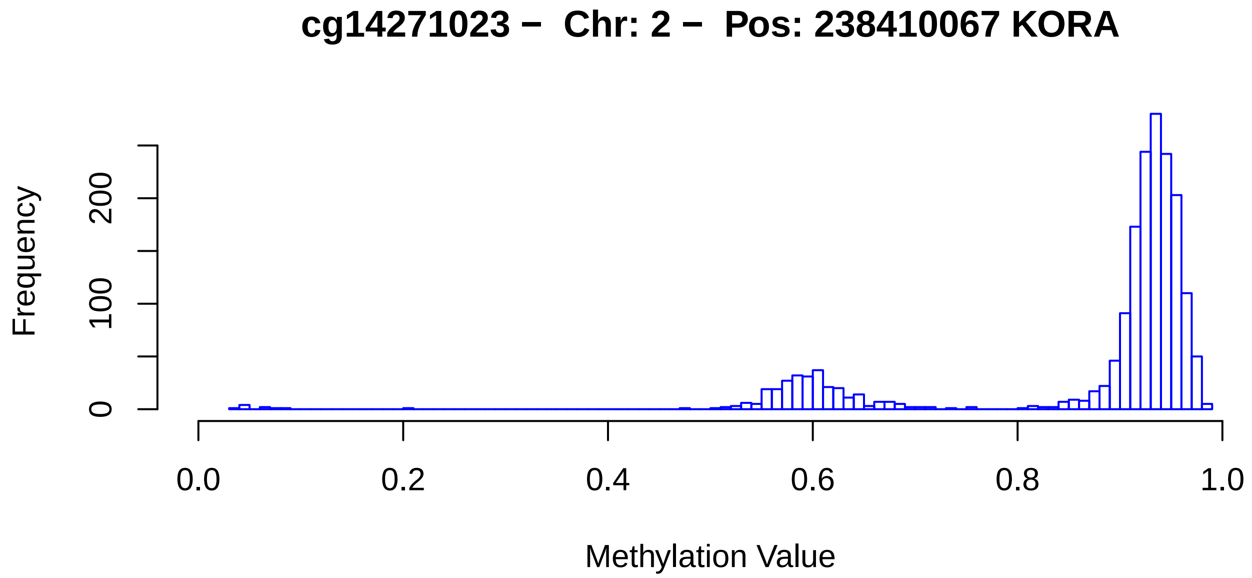
cg14271023 − Chr: 2 − Pos: 238410067 KORA
Frequency
0
100
200
0.0
0.2
0.4
0.6
0.8
1.0
Methylation Value

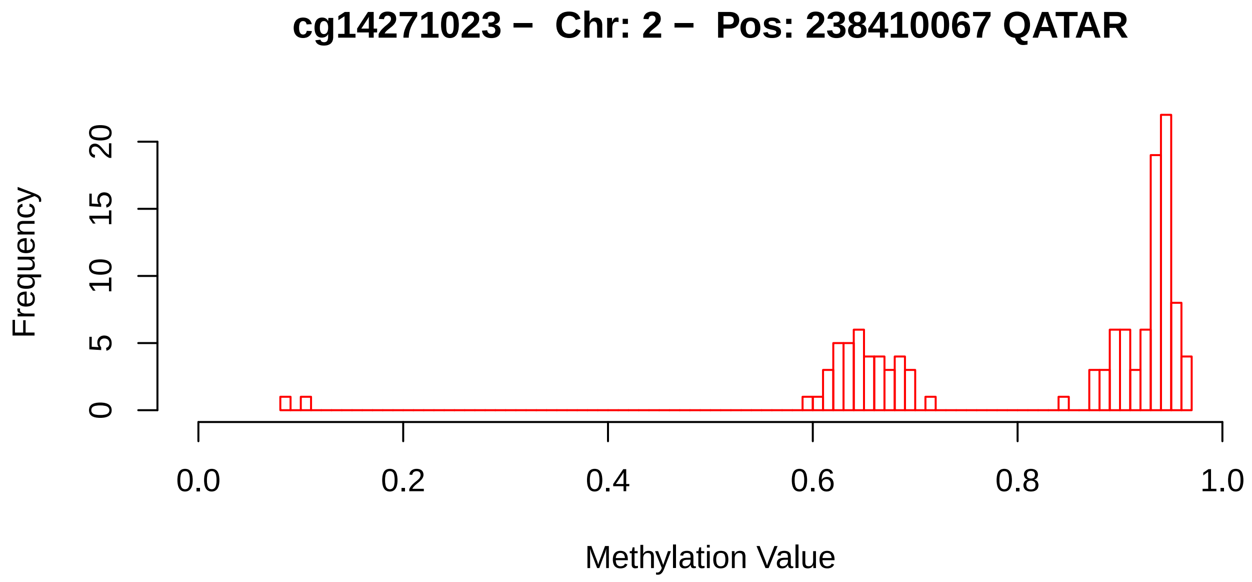
cg14271023 − Chr: 2 − Pos: 238410067 QATAR
Frequency
0
5
10
15
20
0.0
0.2
0.4
0.6
0.8
1.0
Methylation Value

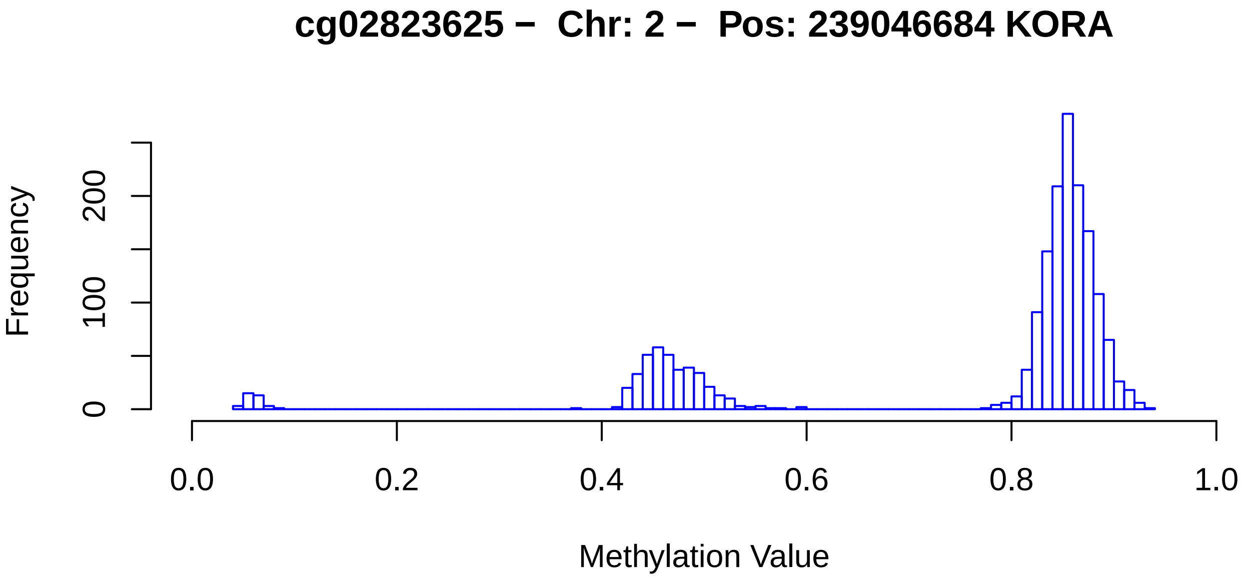
cg02823625 − Chr: 2 − Pos: 239046684 KORA
Frequency
0
100
200
0.0
0.2
0.4
0.6
0.8
1.0
Methylation Value

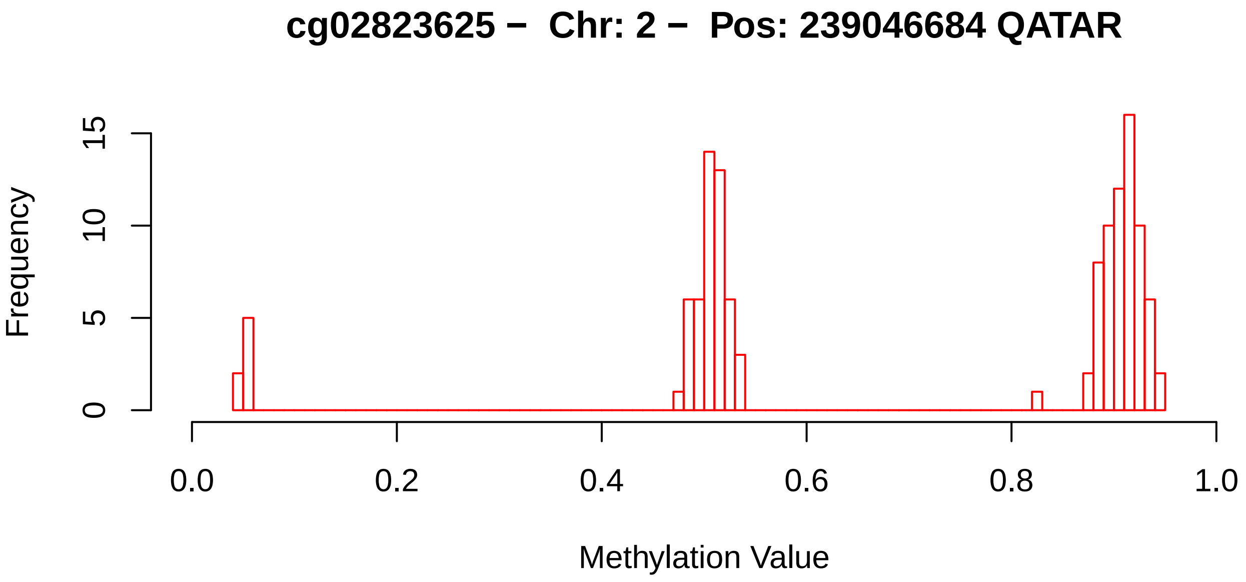
cg02823625 − Chr: 2 − Pos: 239046684 QATAR
Frequency
0
5
10
15
0.0
0.2
0.4
0.6
0.8
1.0
Methylation Value

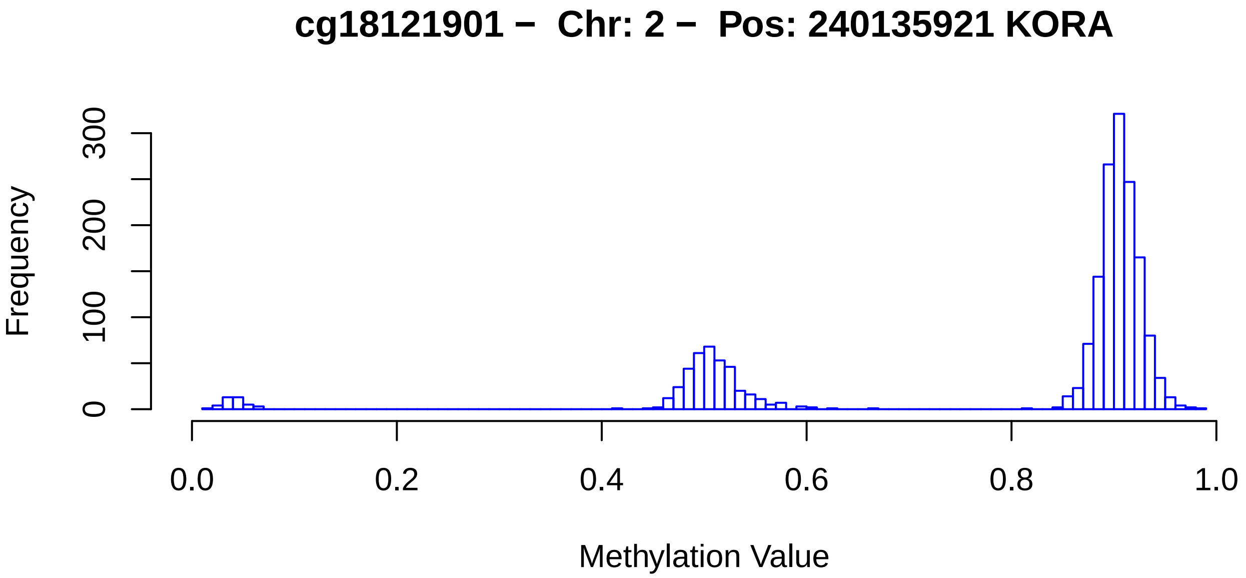

cg18121901 − Chr: 2 − Pos: 240135921 KORA
Frequency
0
100
200
300
0.0
0.2
0.4
0.6
0.8
1.0
Methylation Value

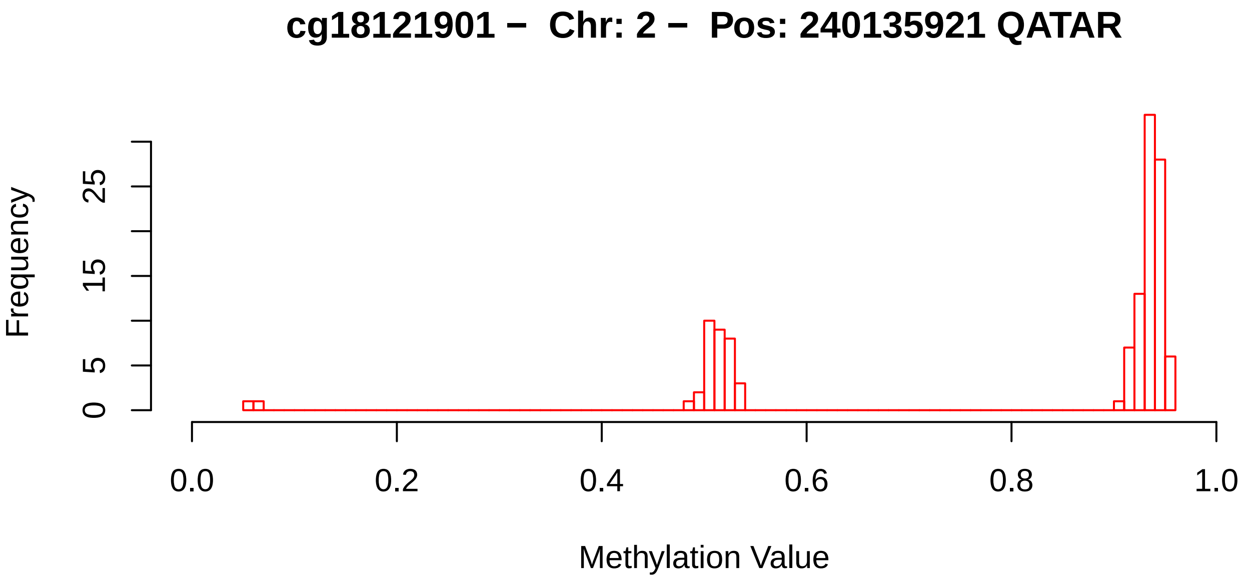

cg18121901 − Chr: 2 − Pos: 240135921 QATAR
Frequency
0
5
15
25
0.0
0.2
0.4
0.6
0.8
1.0
Methylation Value

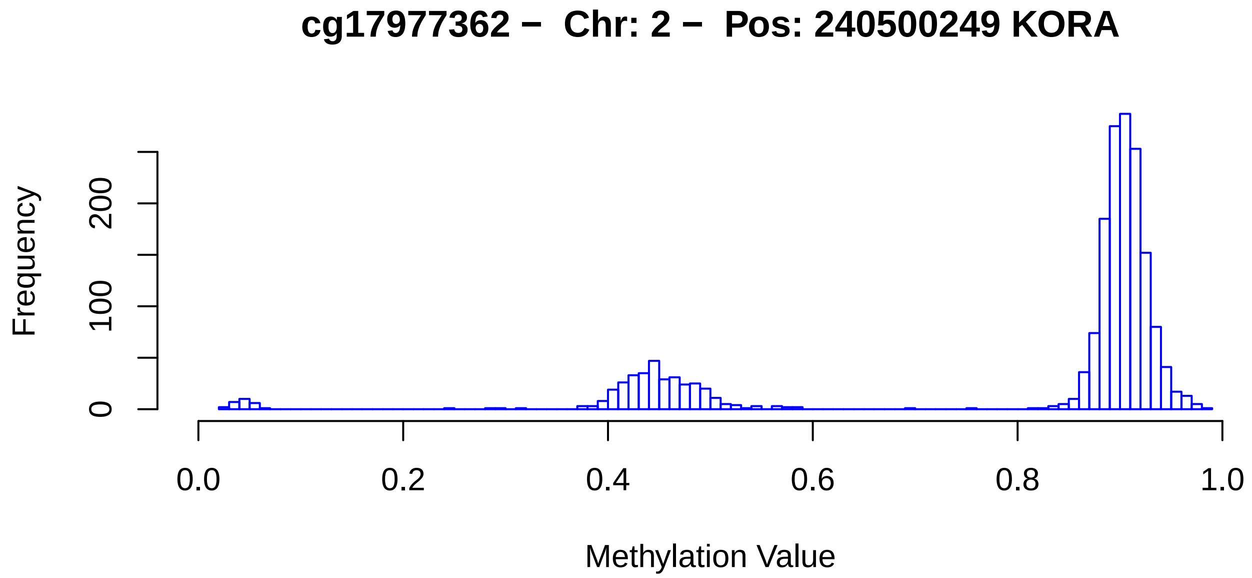
cg17977362 − Chr: 2 − Pos: 240500249 KORA
Frequency
0
100
200
0.0
0.2
0.4
0.6
0.8
1.0
Methylation Value

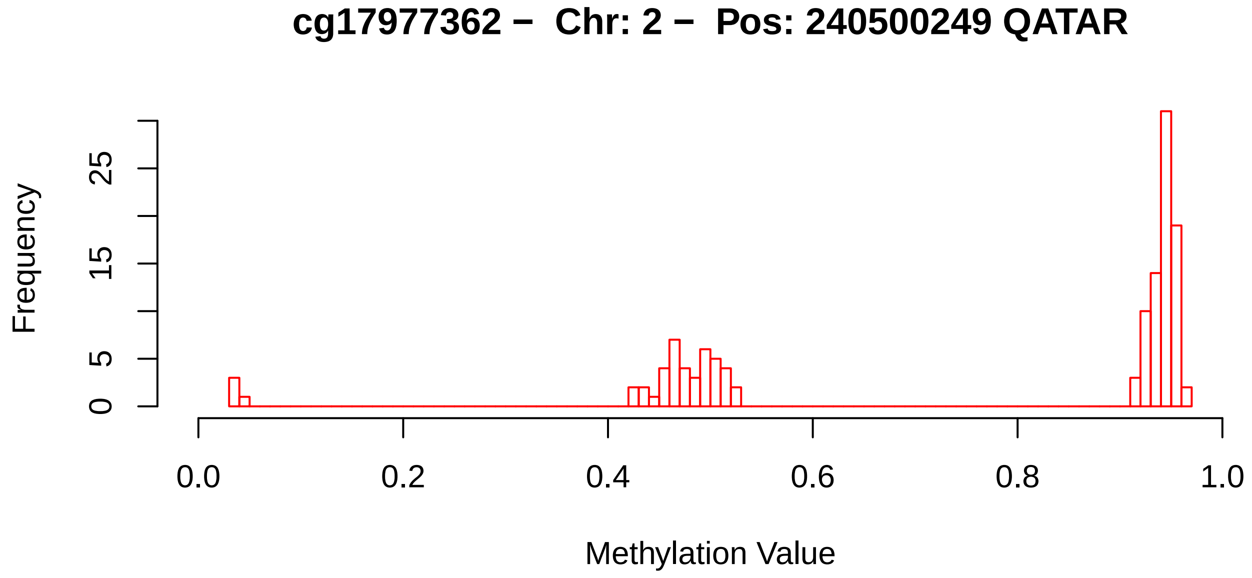
cg17977362 − Chr: 2 − Pos: 240500249 QATAR
Frequency
0
5
15
25
0.0
0.2
0.4
0.6
0.8
1.0
Methylation Value

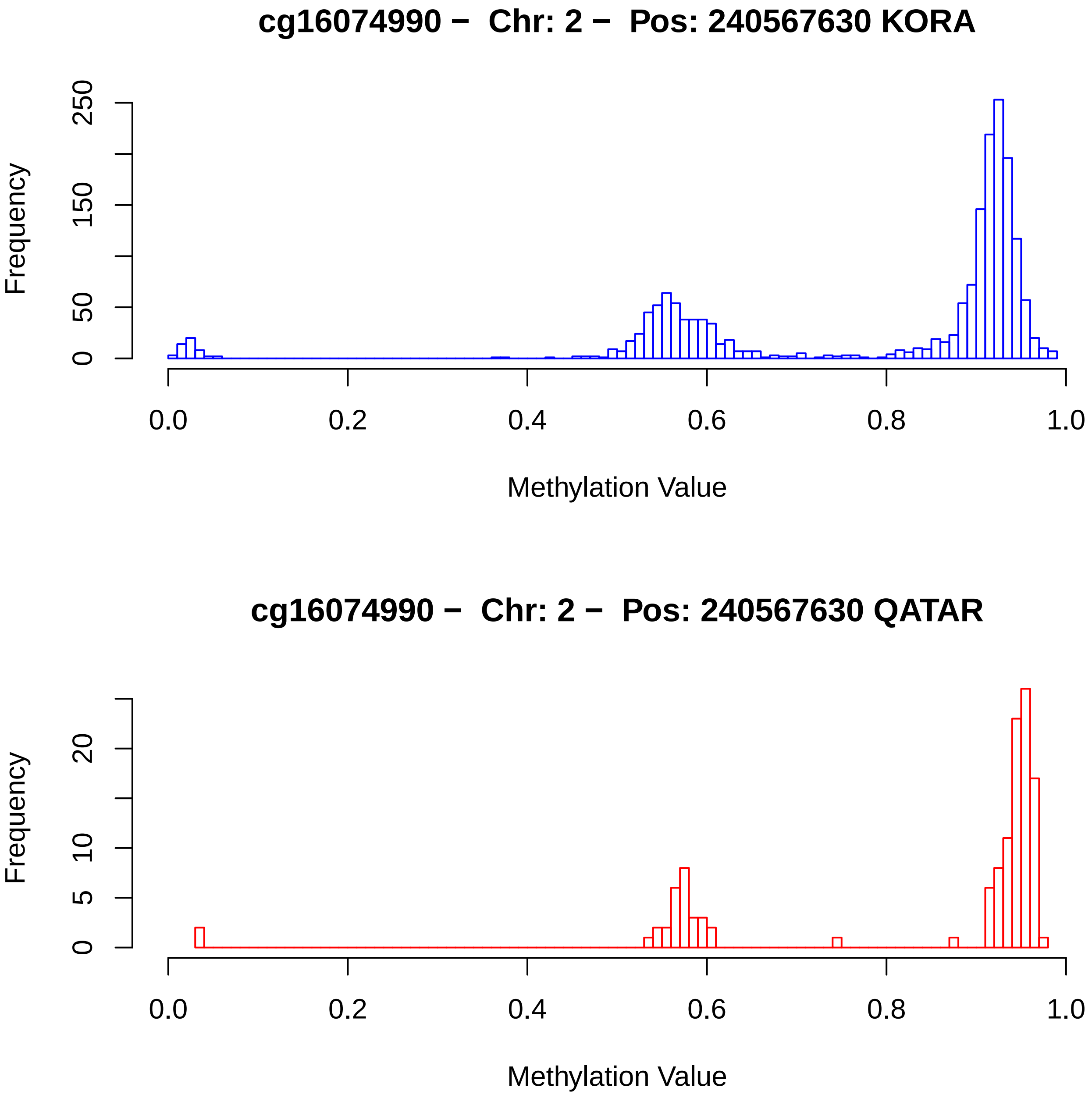
cg16074990 − Chr: 2 − Pos: 240567630 KORA
Frequency
0
50
150
250
0.0
0.2
0.4
0.6
0.8
1.0
Methylation Value
cg16074990 − Chr: 2 − Pos: 240567630 QATAR
Frequency
0
5
10
20
0.0
0.2
0.4
0.6
0.8
1.0
Methylation Value

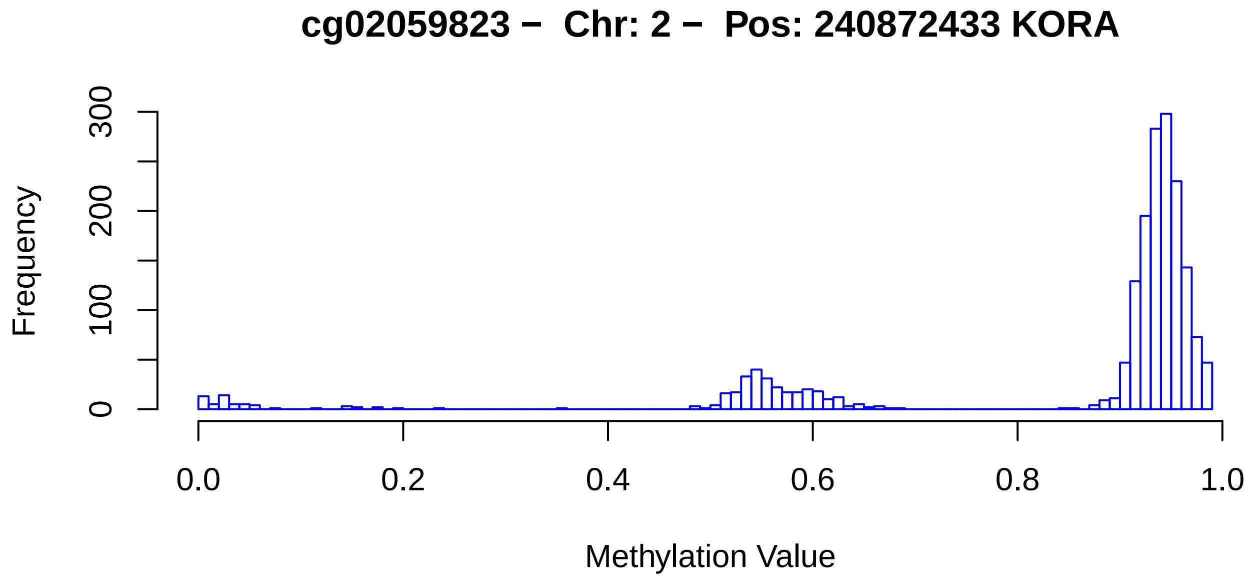
cg02059823 − Chr: 2 − Pos: 240872433 KORA
Frequency
0
100
200
300
0.0
0.2
0.4
0.6
0.8
1.0
Methylation Value

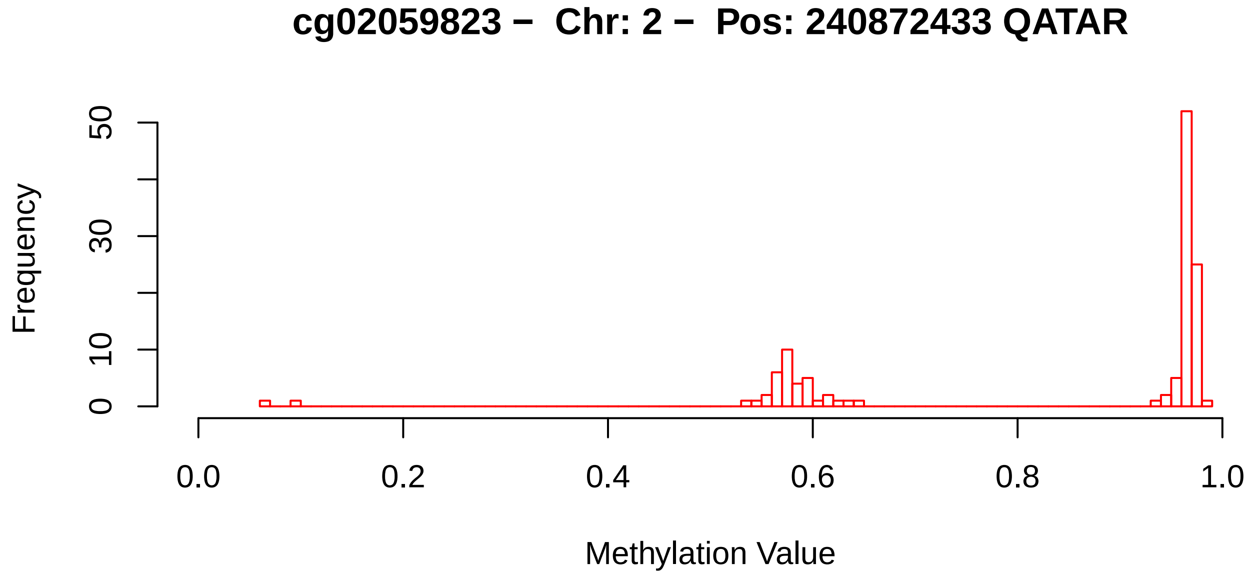
cg02059823 − Chr: 2 − Pos: 240872433 QATAR
Frequency
0
10
30
50
0.0
0.2
0.4
0.6
0.8
1.0
Methylation Value

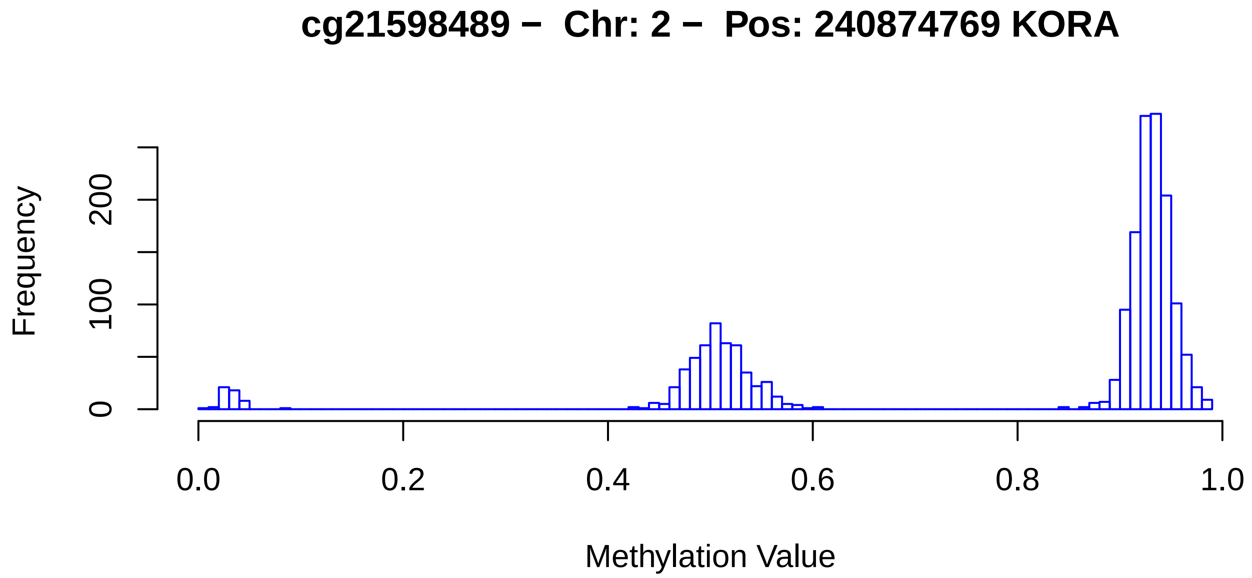
cg21598489 − Chr: 2 − Pos: 240874769 KORA
Frequency
0
100
200
0.0
0.2
0.4
0.6
0.8
1.0
Methylation Value

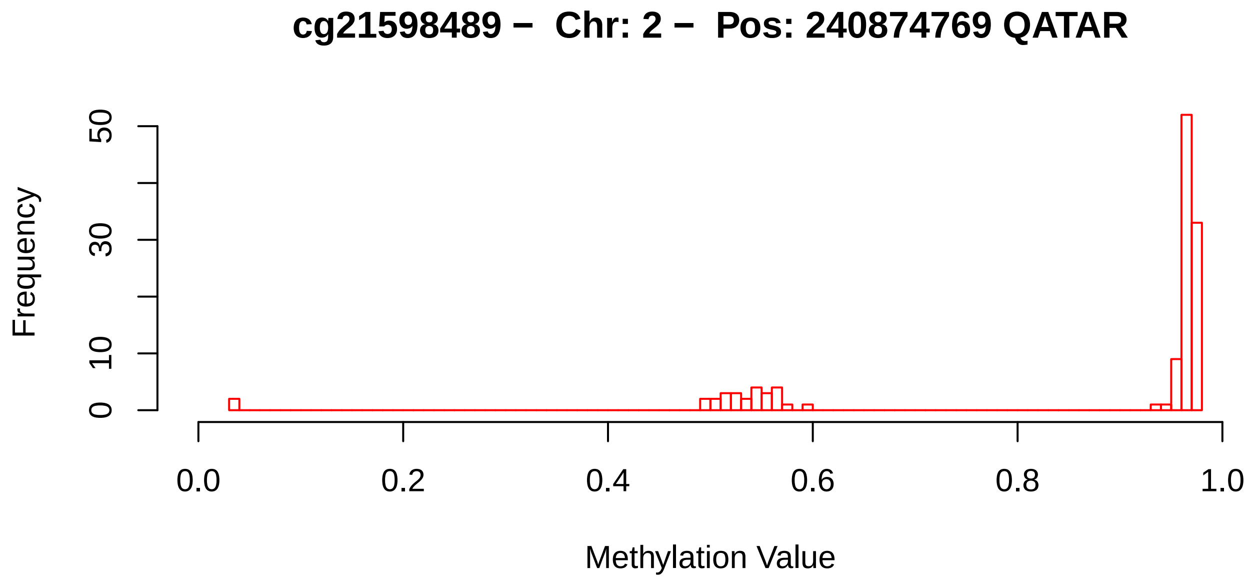
cg21598489 − Chr: 2 − Pos: 240874769 QATAR
Frequency
0
10
30
50
0.0
0.2
0.4
0.6
0.8
1.0
Methylation Value

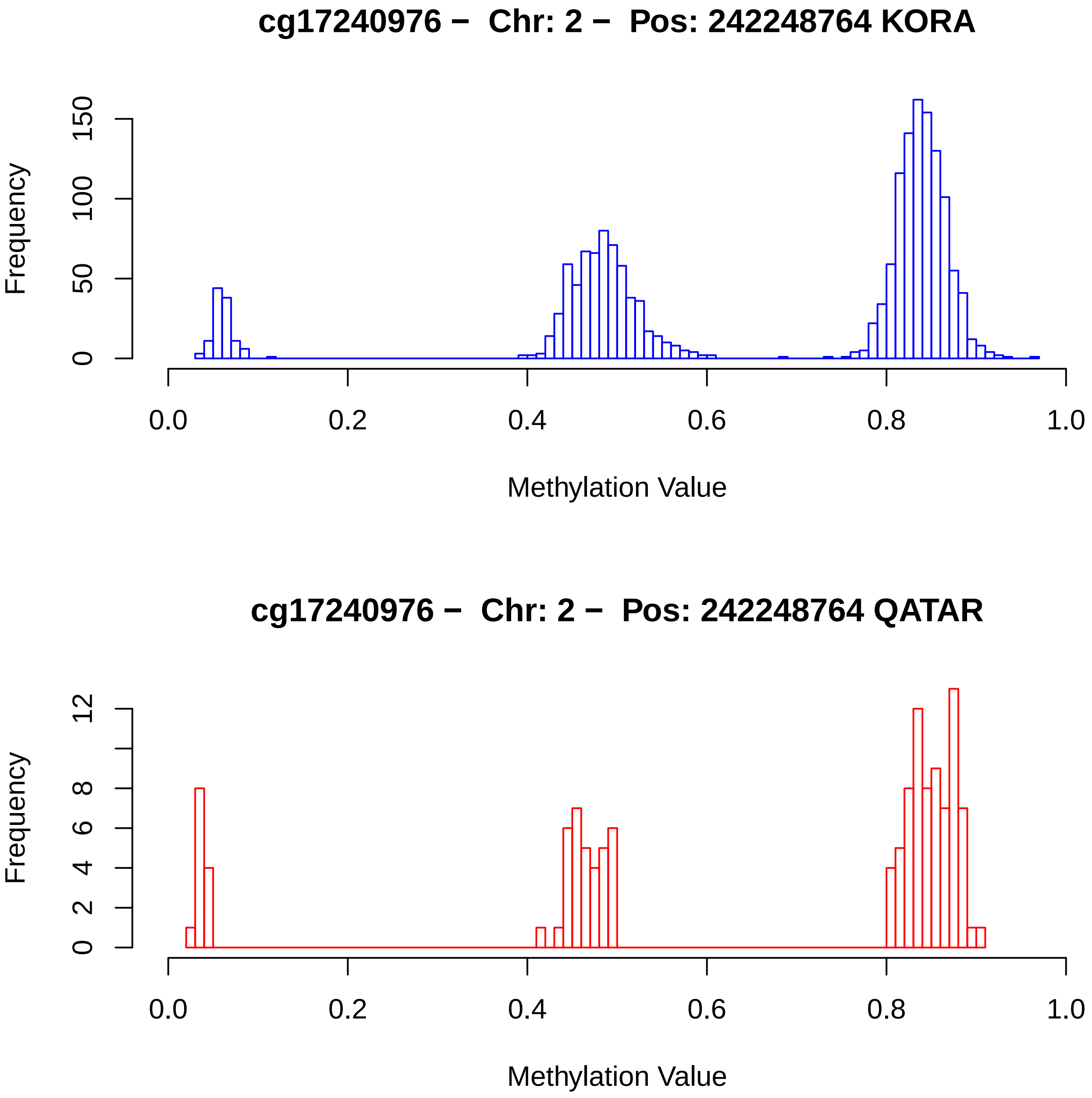

cg17240976 − Chr: 2 − Pos: 242248764 KORA
Frequency
0
50
100
150
0.0
0.2
0.4
0.6
0.8
1.0
Methylation Value
cg17240976 − Chr: 2 − Pos: 242248764 QATAR
Frequency
0
2
4
6
8
12
0.0
0.2
0.4
0.6
0.8
1.0
Methylation Value

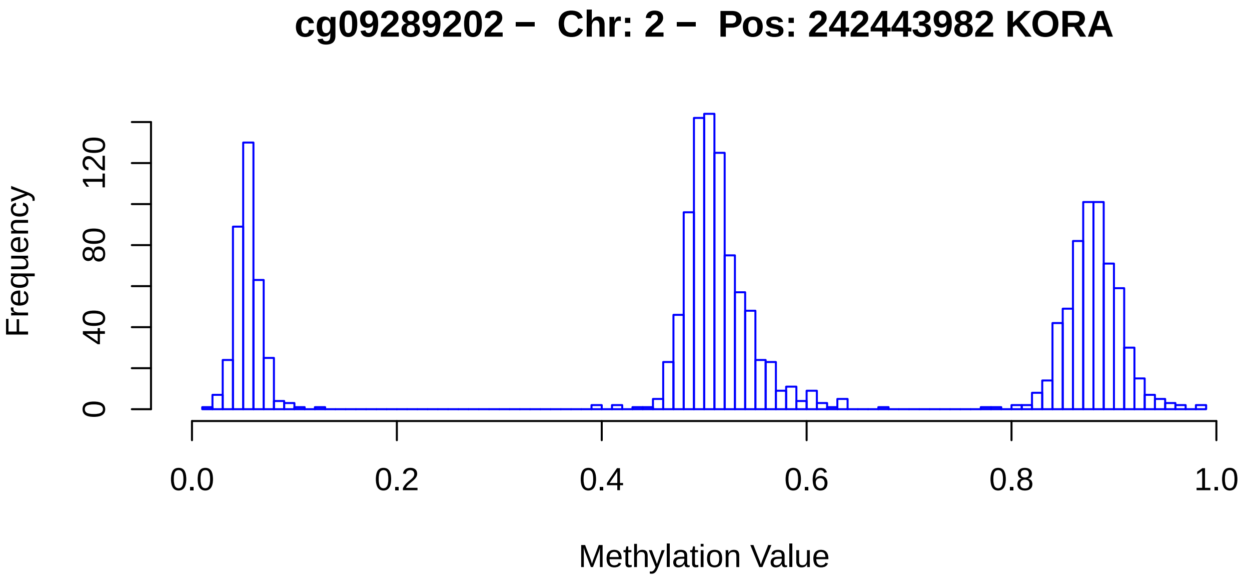
cg09289202 − Chr: 2 − Pos: 242443982 KORA
Frequency
0
40
80
120
0.0
0.2
0.4
0.6
0.8
1.0
Methylation Value

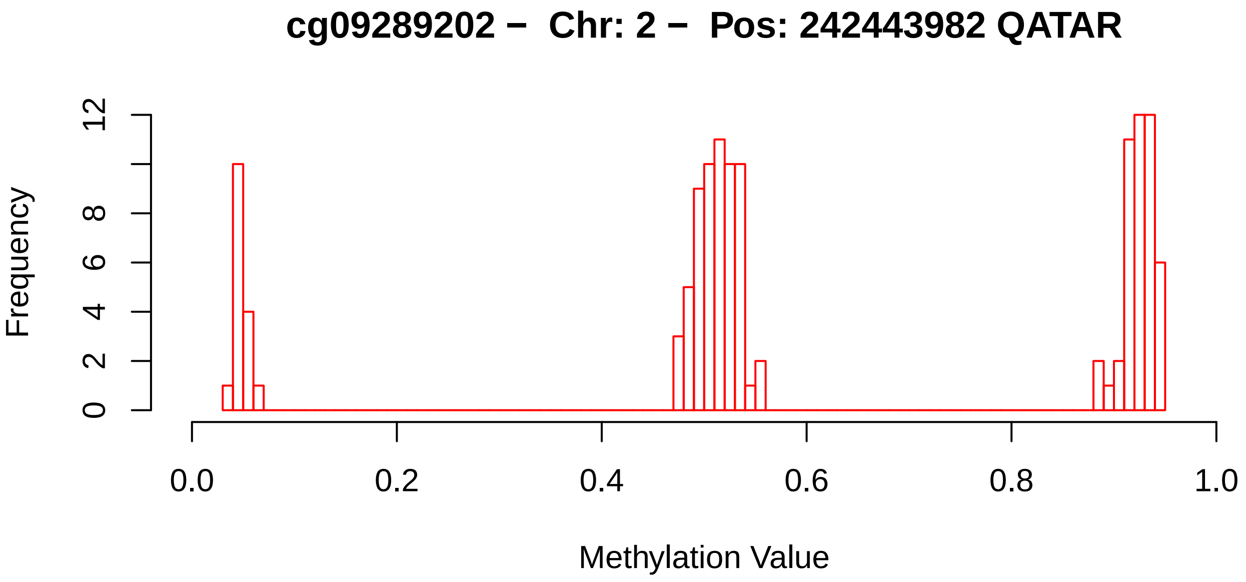
cg09289202 − Chr: 2 − Pos: 242443982 QATAR
Frequency
0
2
4
6
8
12
0.0
0.2
0.4
0.6
0.8
1.0
Methylation Value

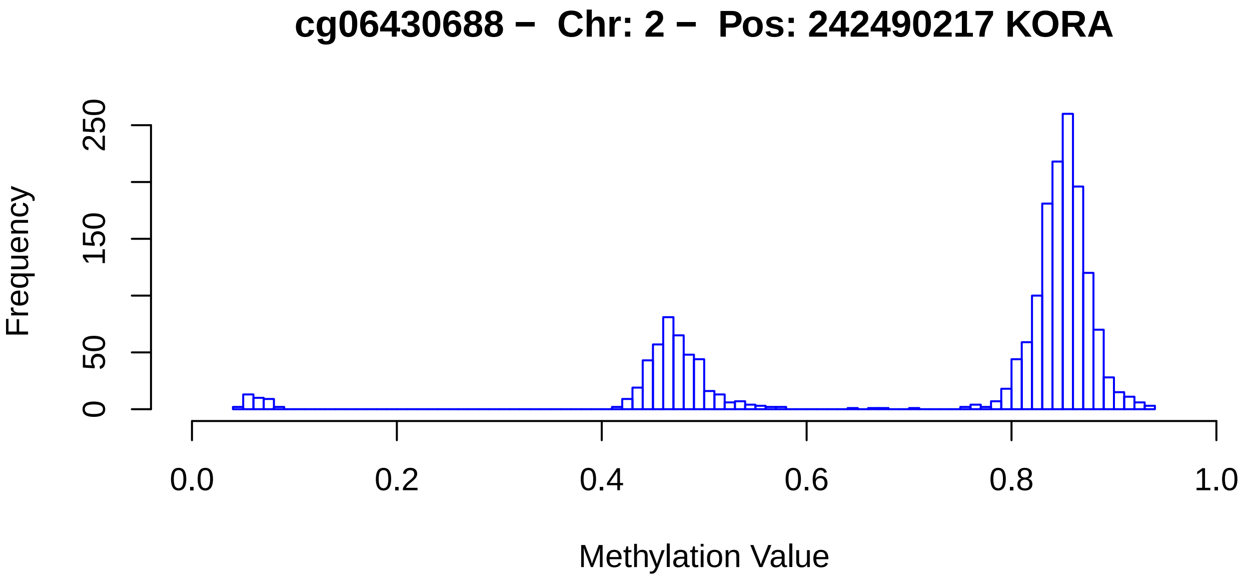
cg06430688 − Chr: 2 − Pos: 242490217 KORA
Frequency
0
50
150
250
0.0
0.2
0.4
0.6
0.8
1.0
Methylation Value

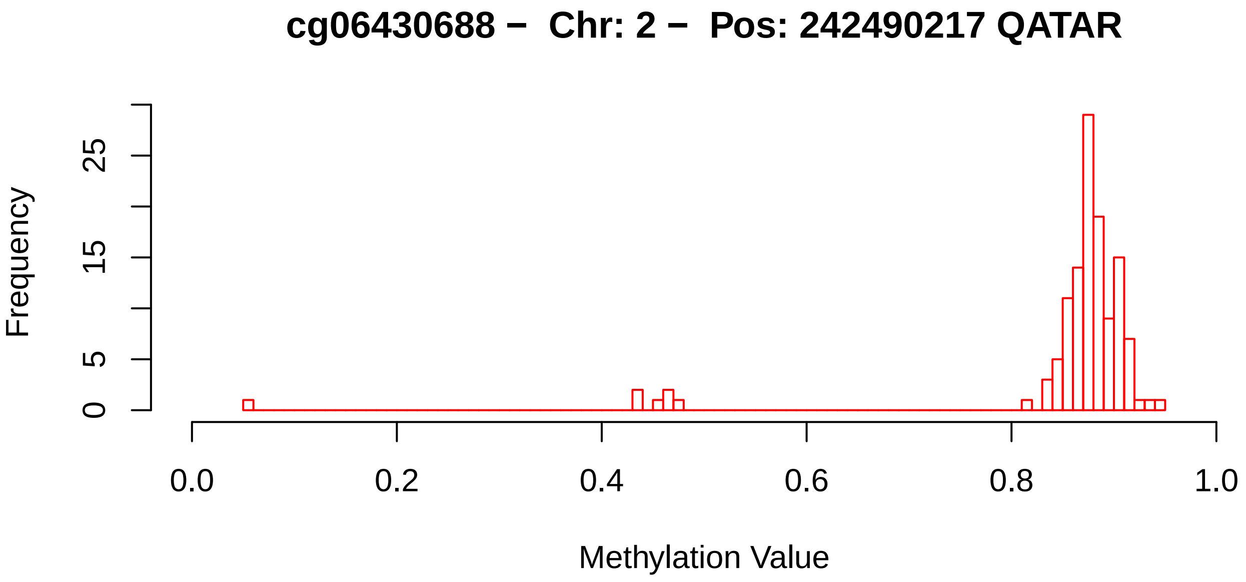
cg06430688 − Chr: 2 − Pos: 242490217 QATAR
Frequency
0
5
15
25
0.0
0.2
0.4
0.6
0.8
1.0
Methylation Value

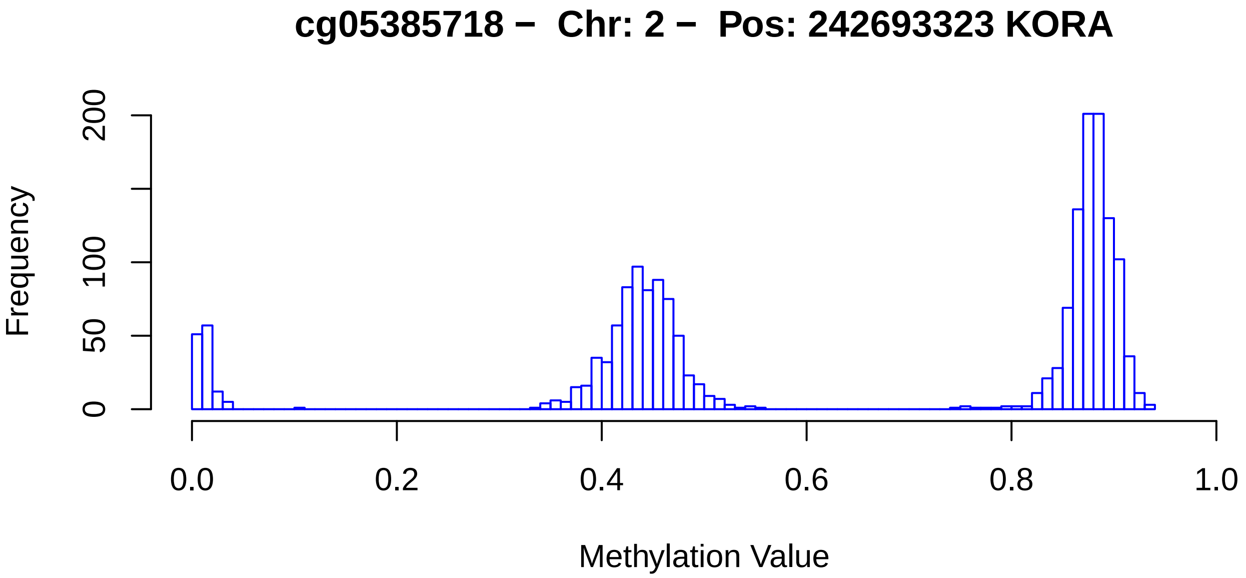
cg05385718 − Chr: 2 − Pos: 242693323 KORA
Frequency
0
50
100
200
0.0
0.2
0.4
0.6
0.8
1.0
Methylation Value

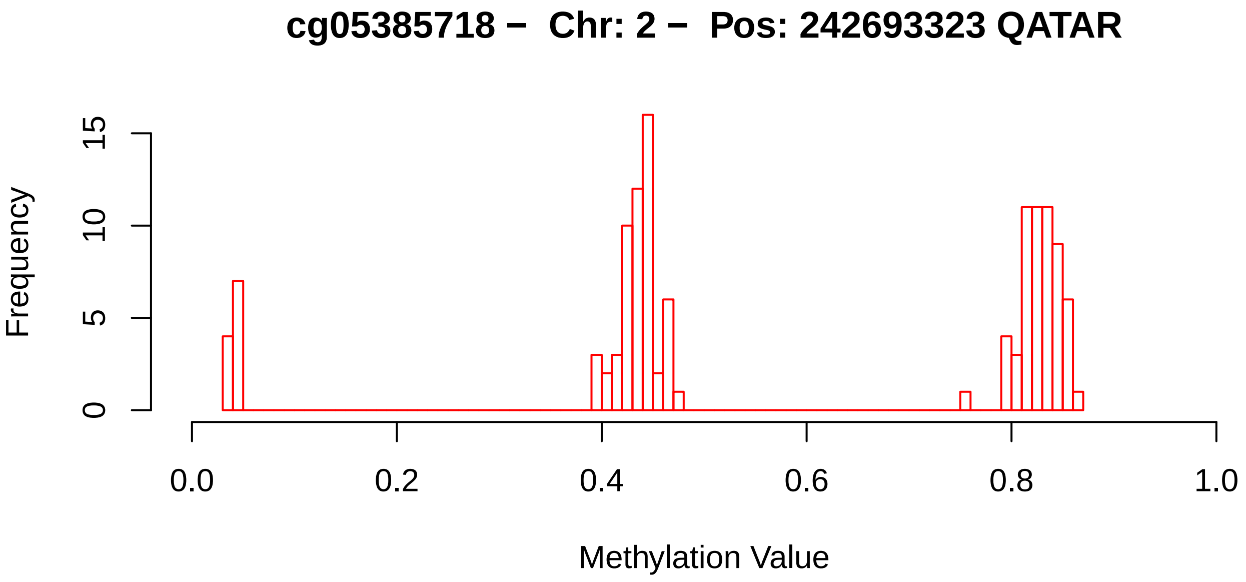
cg05385718 − Chr: 2 − Pos: 242693323 QATAR
Frequency
0
5
10
15
0.0
0.2
0.4
0.6
0.8
1.0
Methylation Value

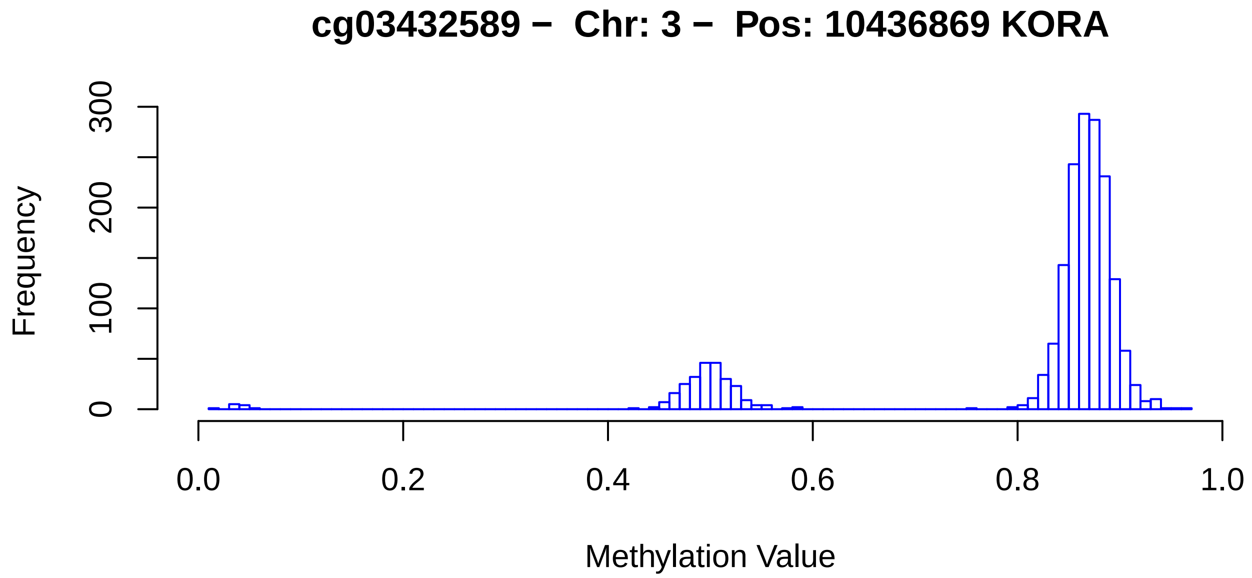
cg03432589 − Chr: 3 − Pos: 10436869 KORA
Frequency
0
100
200
300
0.0
0.2
0.4
0.6
0.8
1.0
Methylation Value

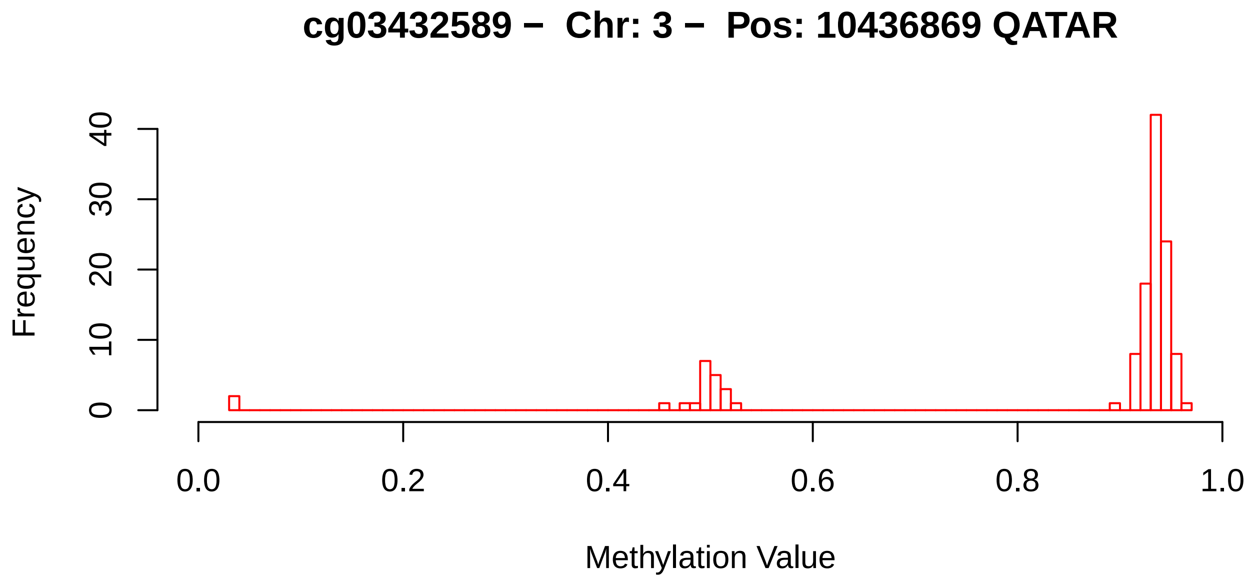
cg03432589 − Chr: 3 − Pos: 10436869 QATAR
Frequency
0
10
20
30
40
0.0
0.2
0.4
0.6
0.8
1.0
Methylation Value

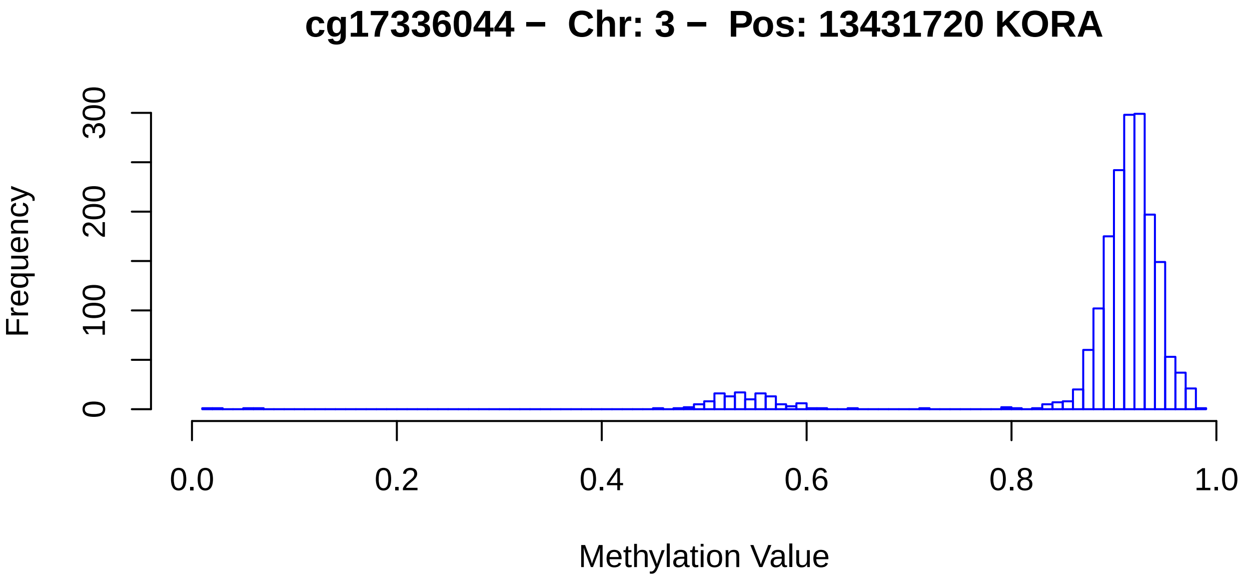
cg17336044 − Chr: 3 − Pos: 13431720 KORA
Frequency
0
100
200
300
0.0
0.2
0.4
0.6
0.8
1.0
Methylation Value

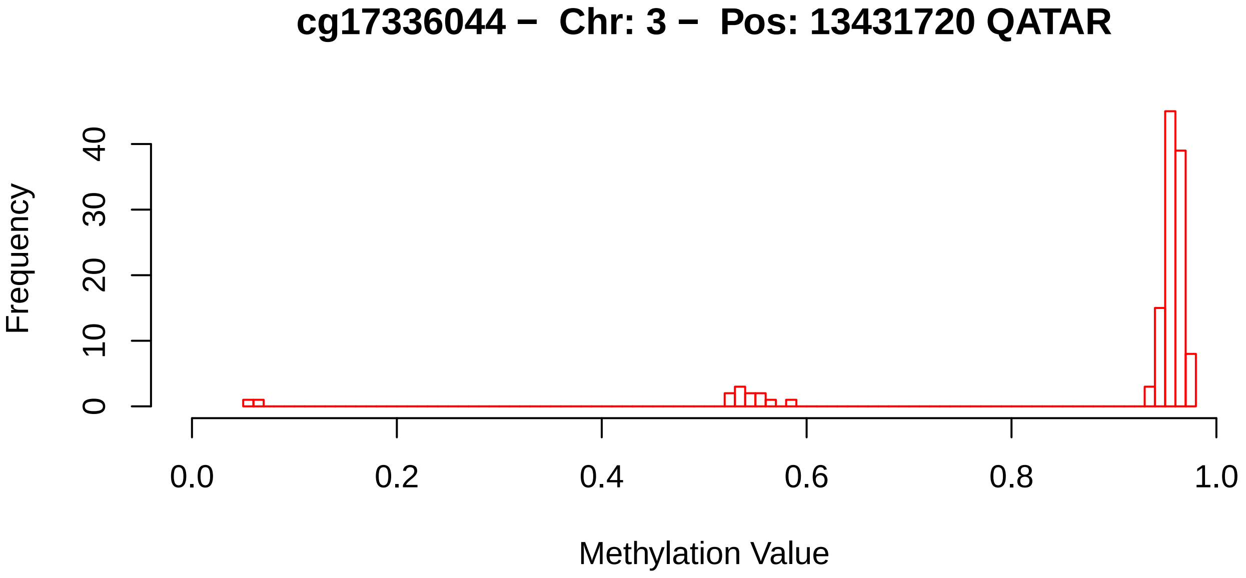
cg17336044 − Chr: 3 − Pos: 13431720 QATAR
Frequency
0
10
20
30
40
0.0
0.2
0.4
0.6
0.8
1.0
Methylation Value

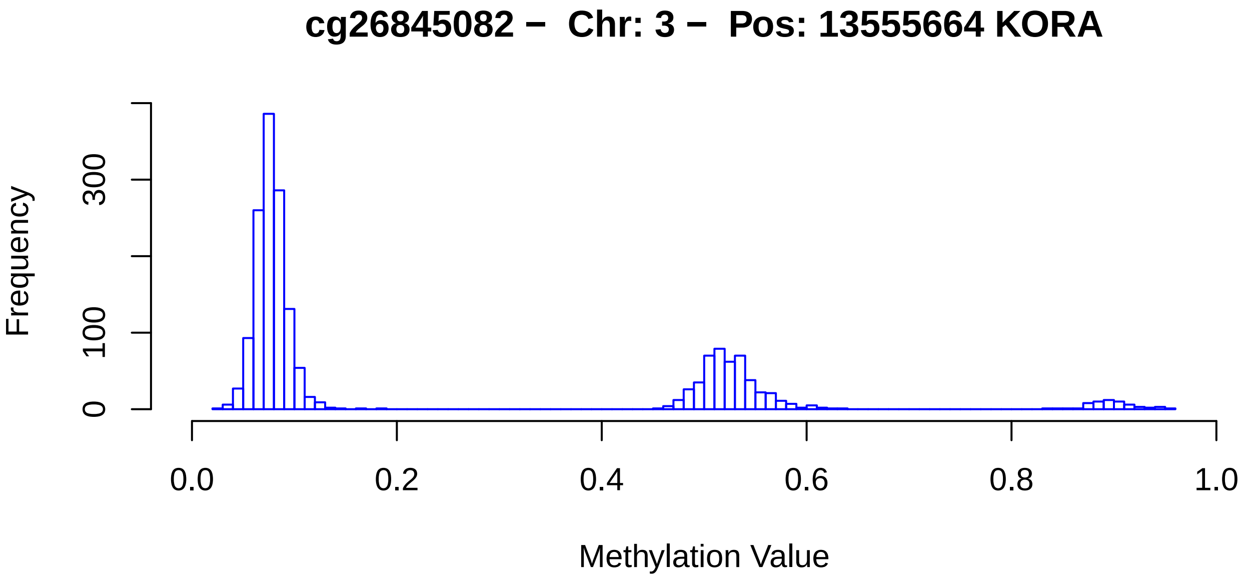
cg26845082 − Chr: 3 − Pos: 13555664 KORA
Frequency
0
100
300
0.0
0.2
0.4
0.6
0.8
1.0
Methylation Value

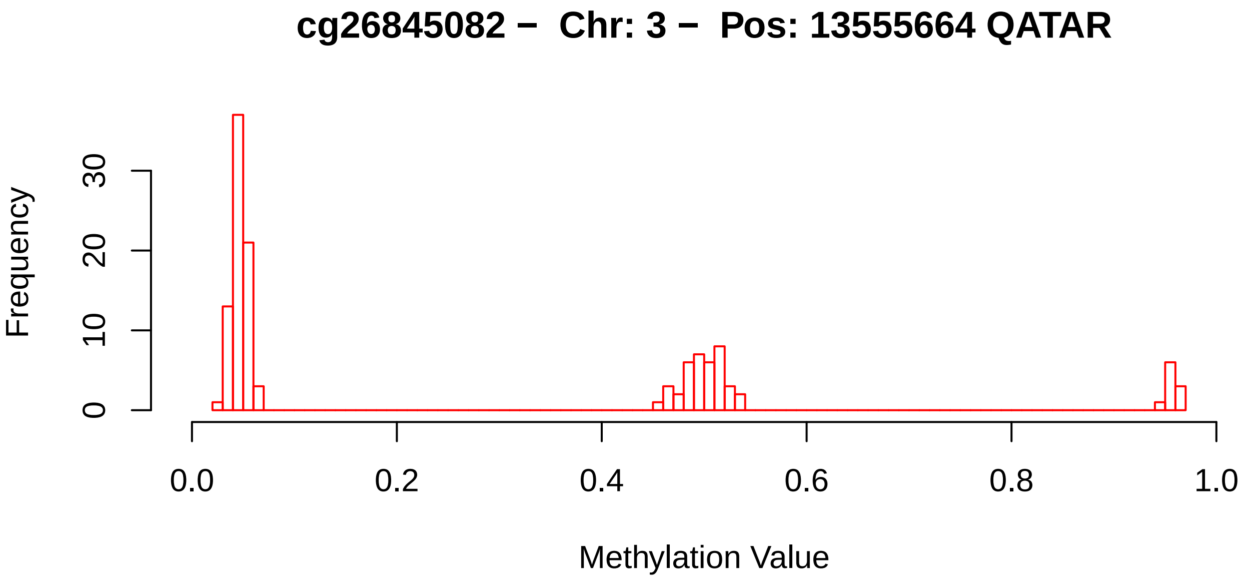
cg26845082 − Chr: 3 − Pos: 13555664 QATAR
Frequency
0
10
20
30
0.0
0.2
0.4
0.6
0.8
1.0
Methylation Value

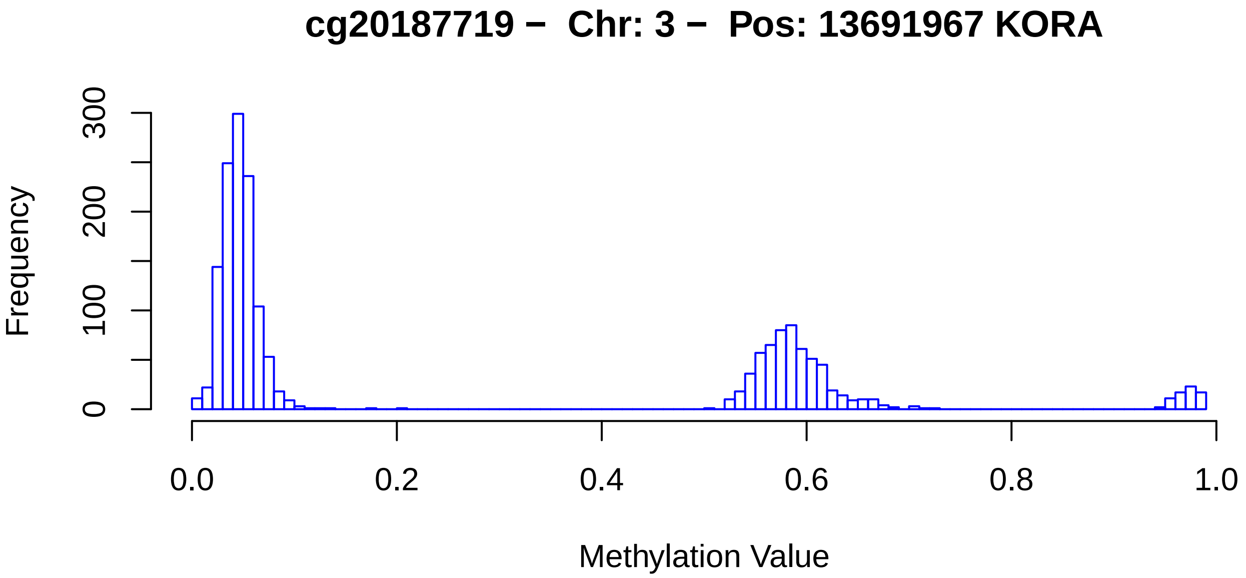
cg20187719 − Chr: 3 − Pos: 13691967 KORA
Frequency
0
100
200
300
0.0
0.2
0.4
0.6
0.8
1.0
Methylation Value

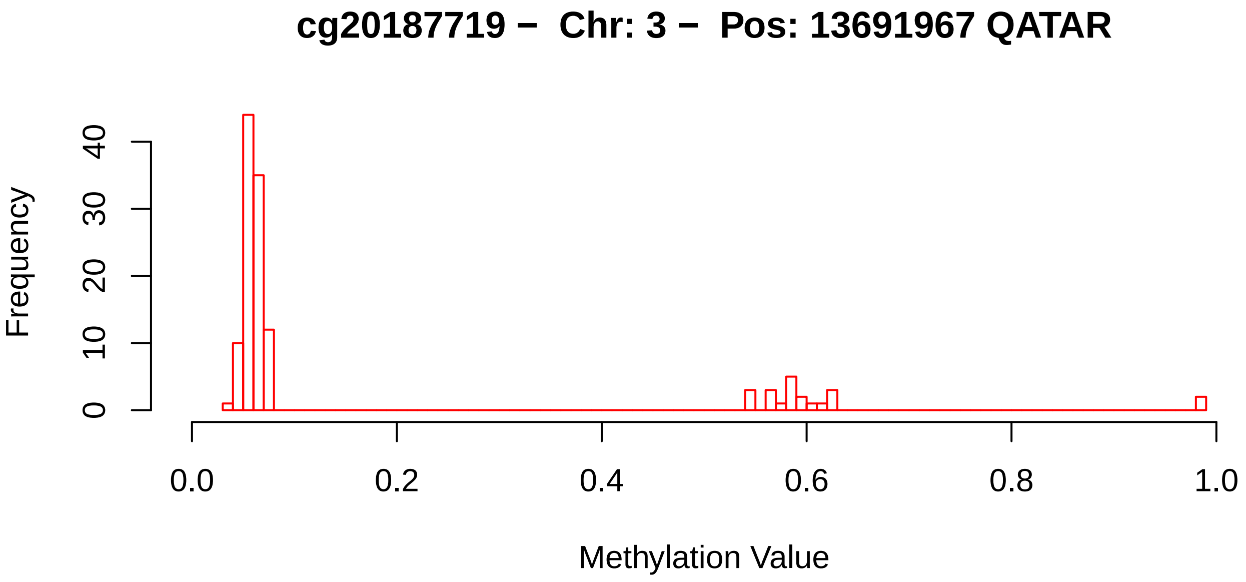
cg20187719 − Chr: 3 − Pos: 13691967 QATAR
Frequency
0
10
20
30
40
0.0
0.2
0.4
0.6
0.8
1.0
Methylation Value

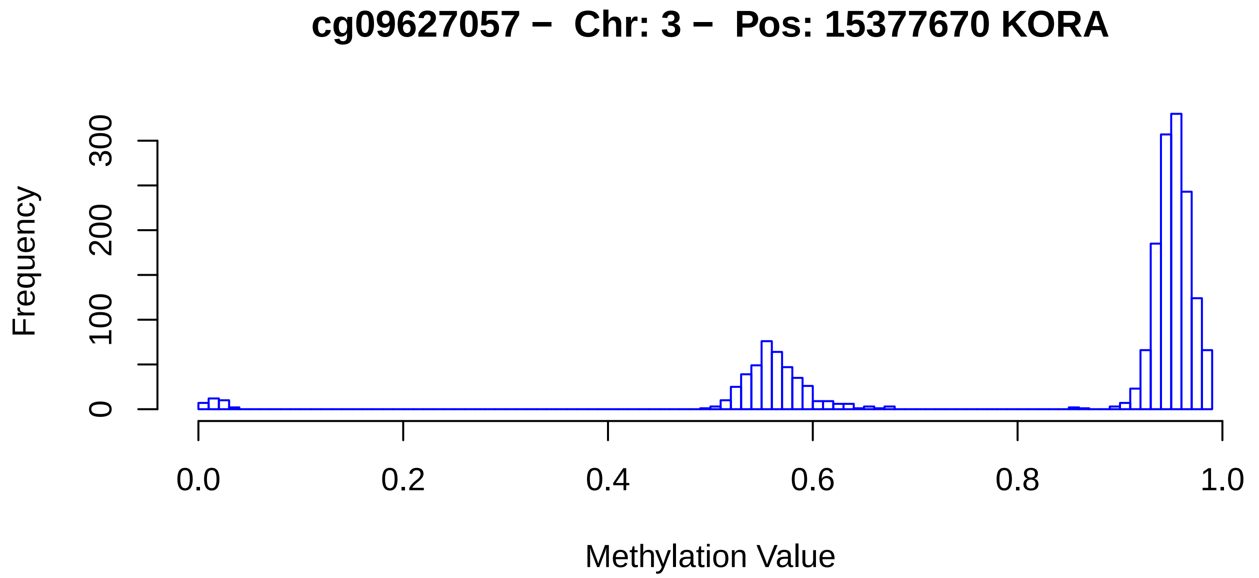
cg09627057 − Chr: 3 − Pos: 15377670 KORA
Frequency
0
100
200
300
0.0
0.2
0.4
0.6
0.8
1.0
Methylation Value

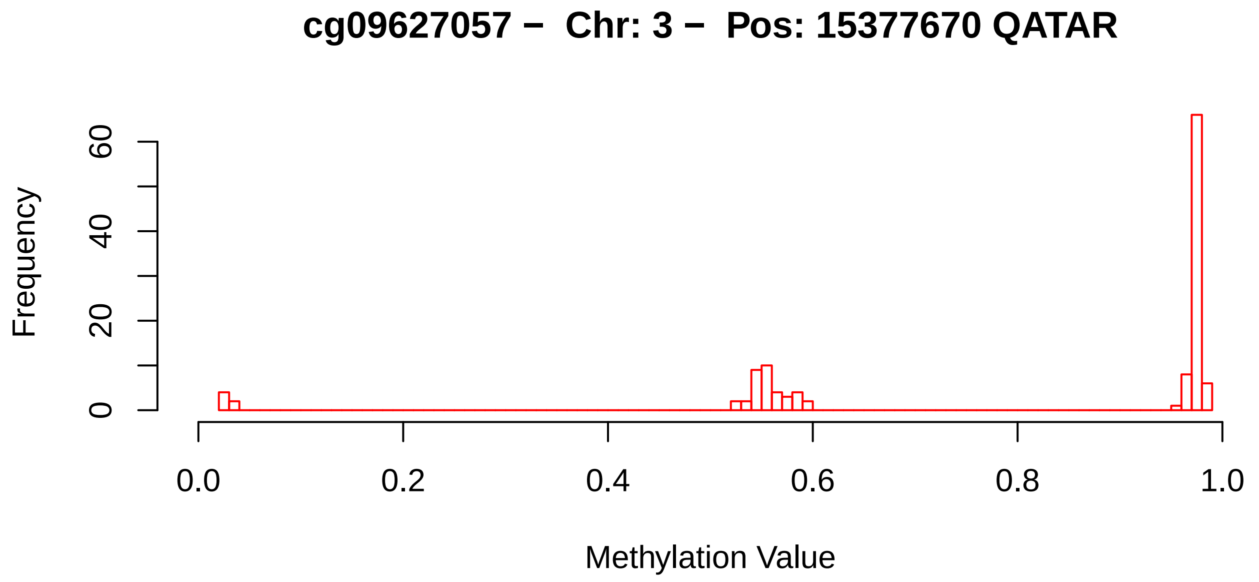
cg09627057 − Chr: 3 − Pos: 15377670 QATAR
Frequency
0
20
40
60
0.0
0.2
0.4
0.6
0.8
1.0
Methylation Value

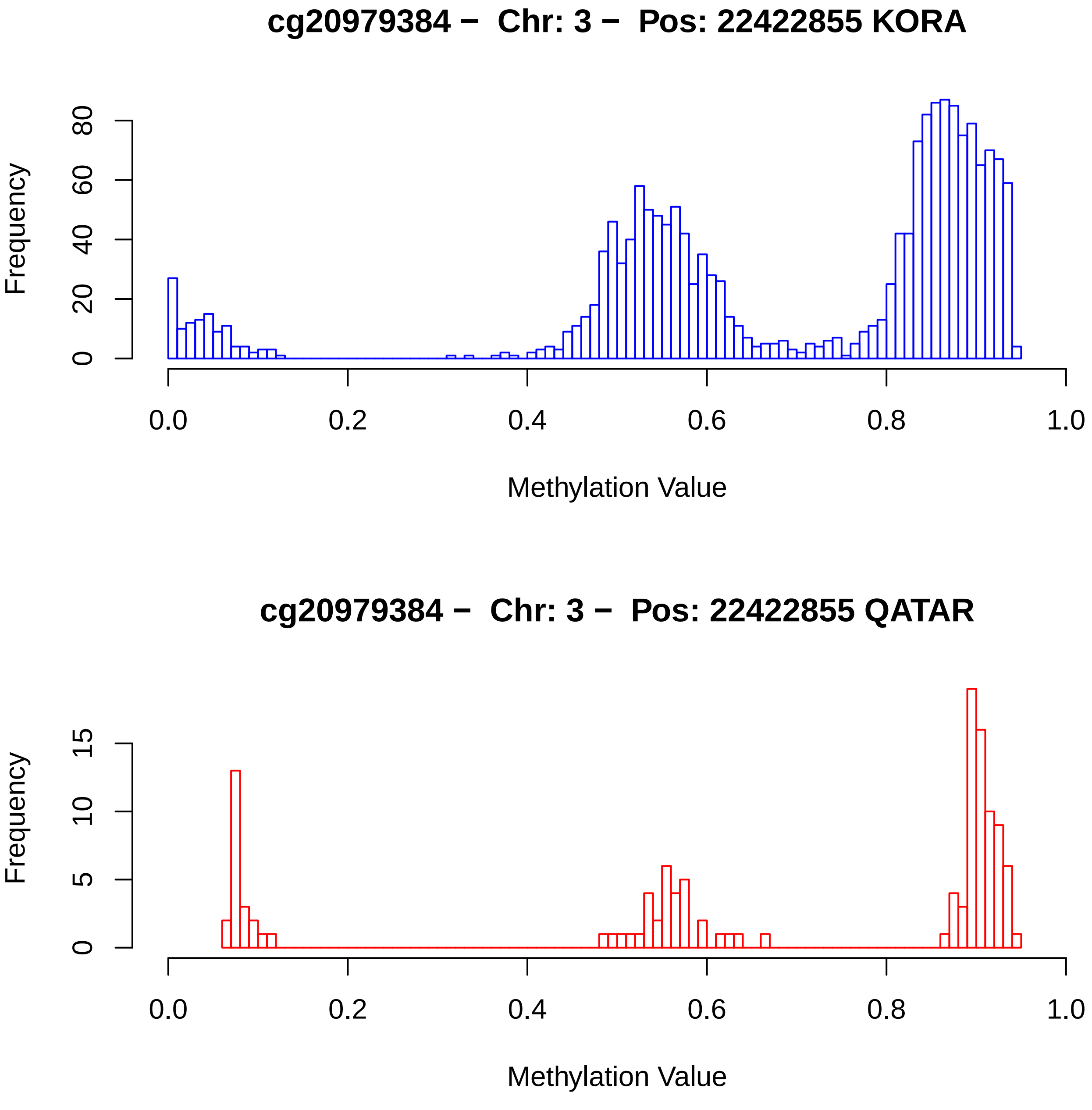
cg20979384 − Chr: 3 − Pos: 22422855 KORA
Frequency
0
20
40
60
80
0.0
0.2
0.4
0.6
0.8
1.0
Methylation Value
cg20979384 − Chr: 3 − Pos: 22422855 QATAR
Frequency
0
5
10
15
0.0
0.2
0.4
0.6
0.8
1.0
Methylation Value

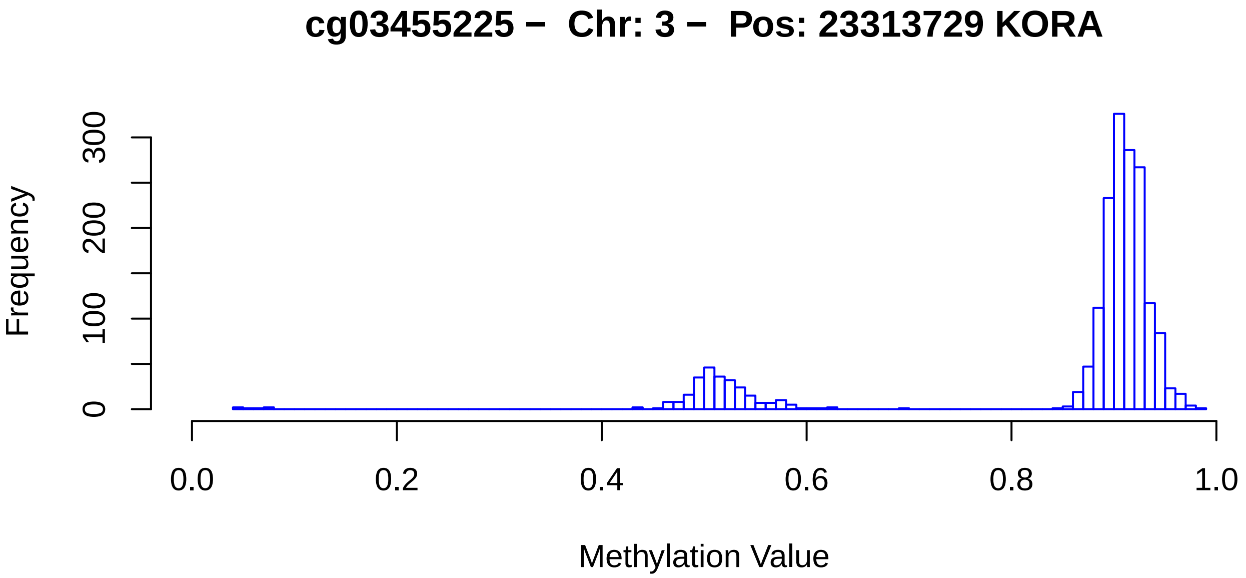
cg03455225 − Chr: 3 − Pos: 23313729 KORA
Frequency
0
100
200
300
0.0
0.2
0.4
0.6
0.8
1.0
Methylation Value

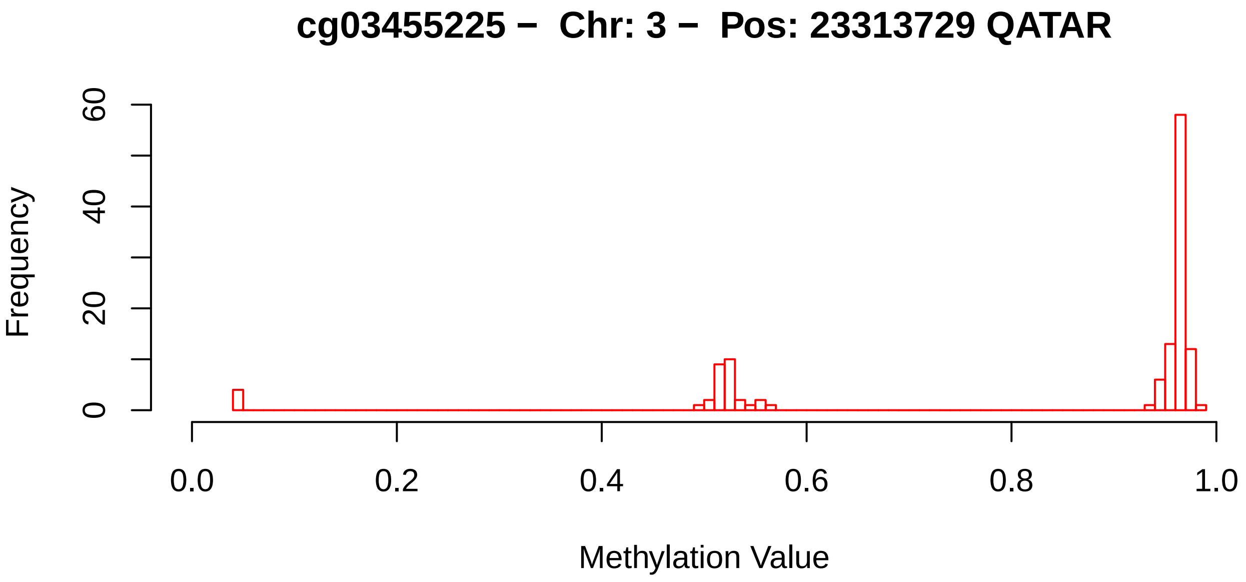
cg03455225 − Chr: 3 − Pos: 23313729 QATAR
Frequency
0
20
40
60
0.0
0.2
0.4
0.6
0.8
1.0
Methylation Value

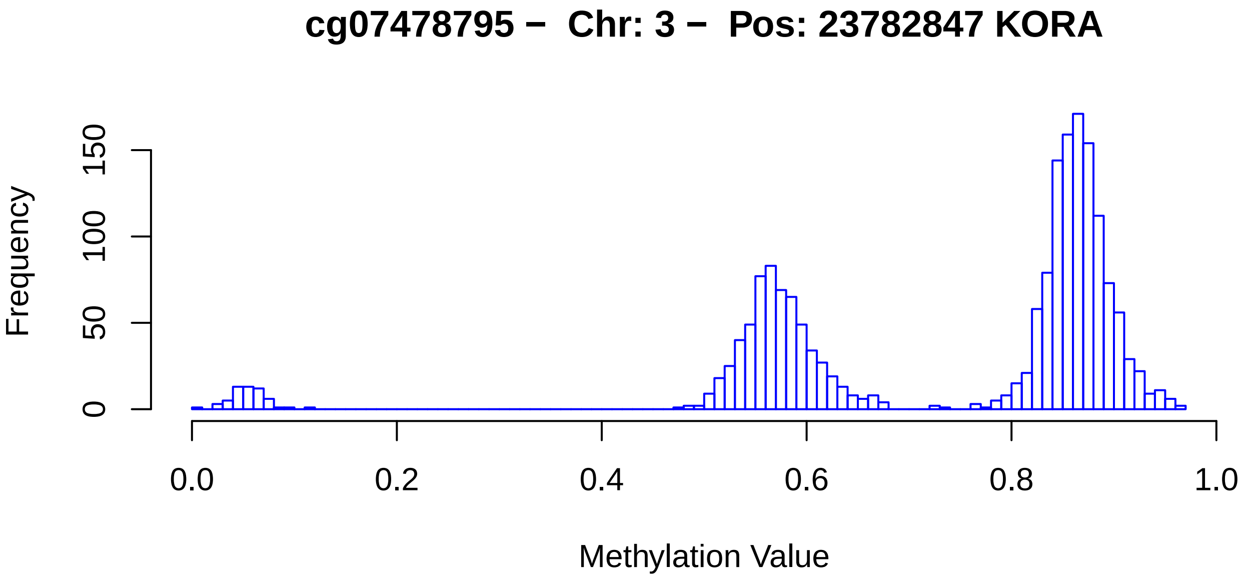
cg07478795 − Chr: 3 − Pos: 23782847 KORA
Frequency
0
50
100
150
0.0
0.2
0.4
0.6
0.8
1.0
Methylation Value

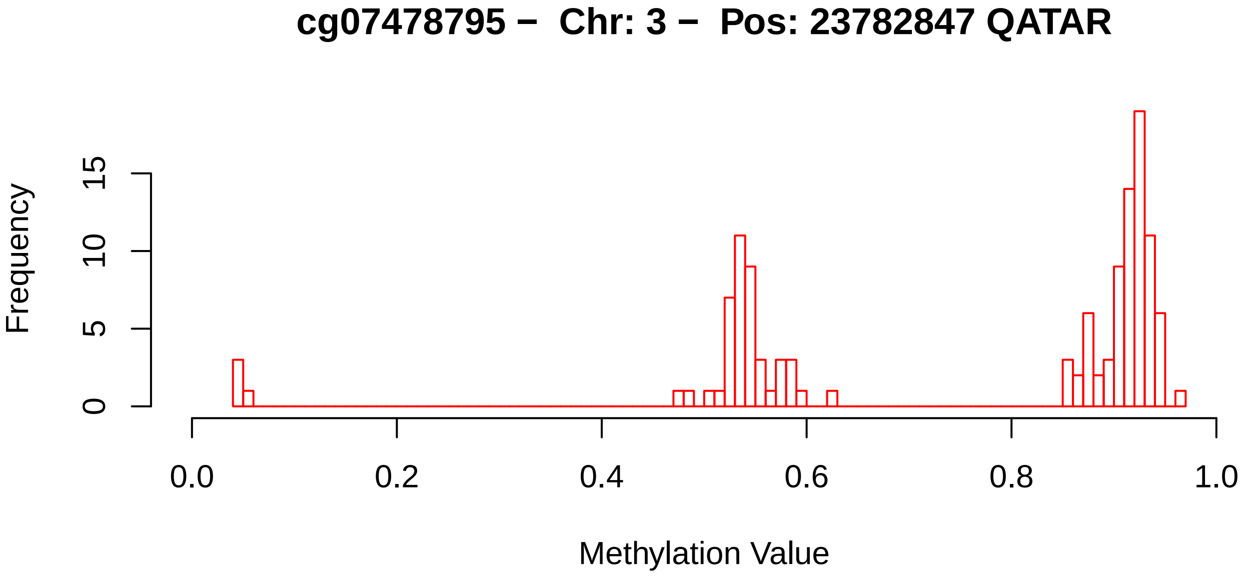
cg07478795 − Chr: 3 − Pos: 23782847 QATAR
Frequency
0
5
10
15
0.0
0.2
0.4
0.6
0.8
1.0
Methylation Value

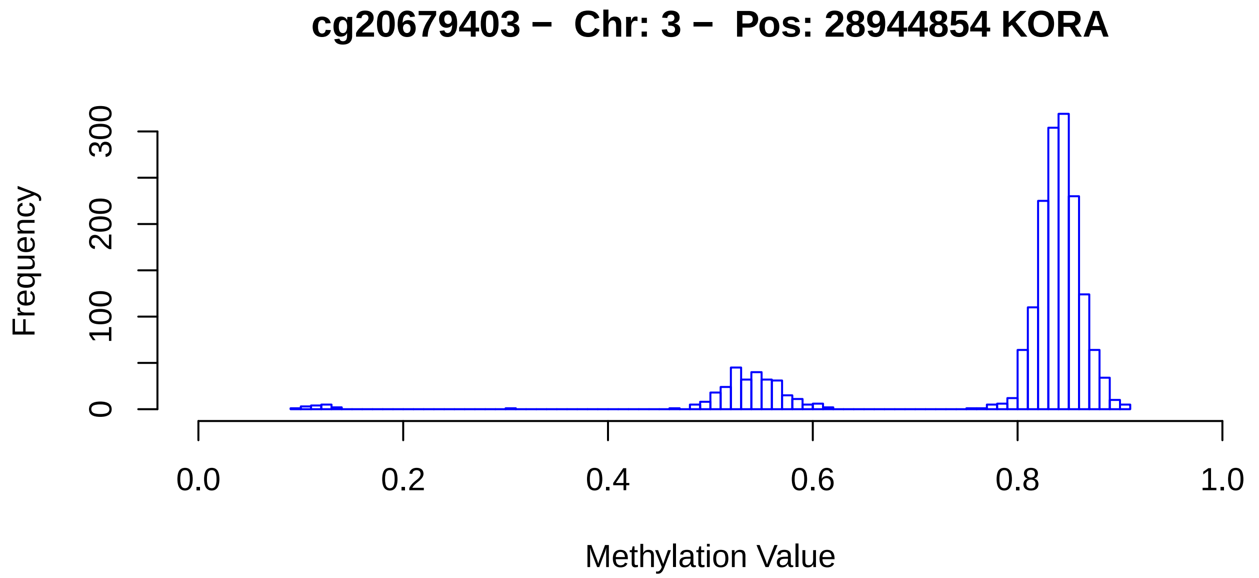

cg20679403 − Chr: 3 − Pos: 28944854 KORA
Frequency
0
100
200
300
0.0
0.2
0.4
0.6
0.8
1.0
Methylation Value

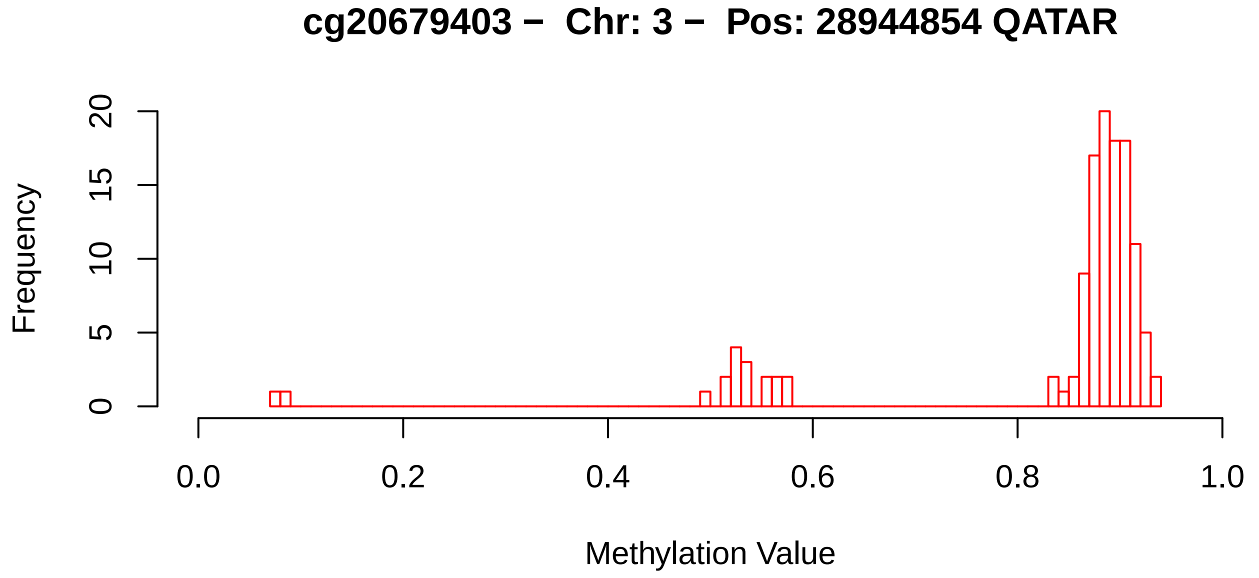

cg20679403 − Chr: 3 − Pos: 28944854 QATAR
Frequency
0
5
10
15
20
0.0
0.2
0.4
0.6
0.8
1.0
Methylation Value

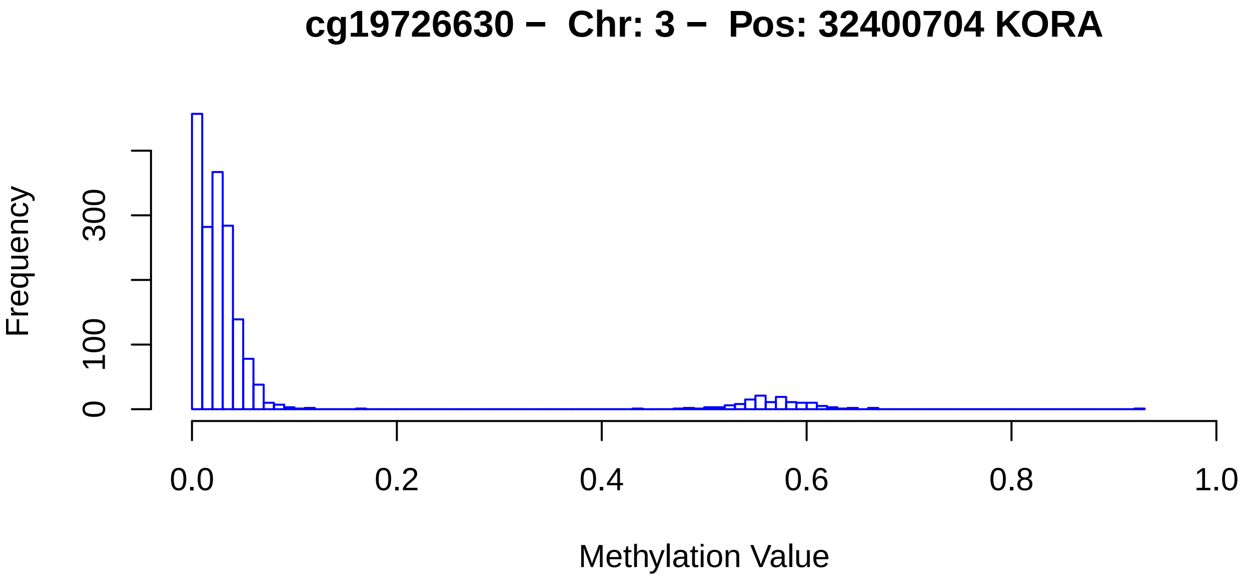
cg19726630 − Chr: 3 − Pos: 32400704 KORA
Frequency
0
100
300
0.0
0.2
0.4
0.6
0.8
1.0
Methylation Value

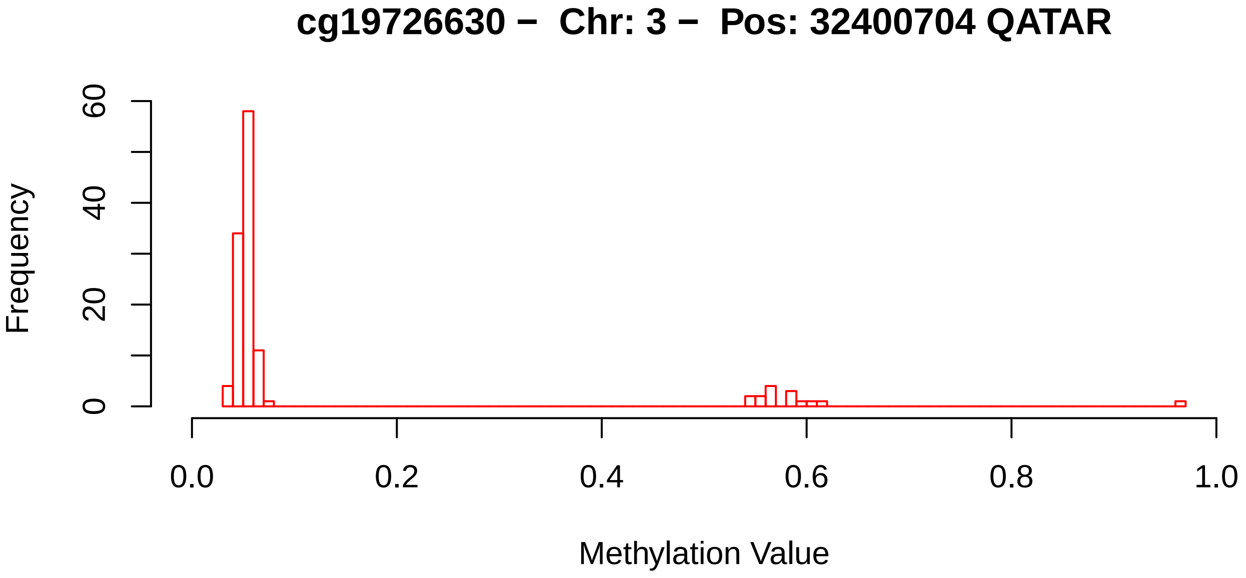
cg19726630 − Chr: 3 − Pos: 32400704 QATAR
Frequency
0
20
40
60
0.0
0.2
0.4
0.6
0.8
1.0
Methylation Value

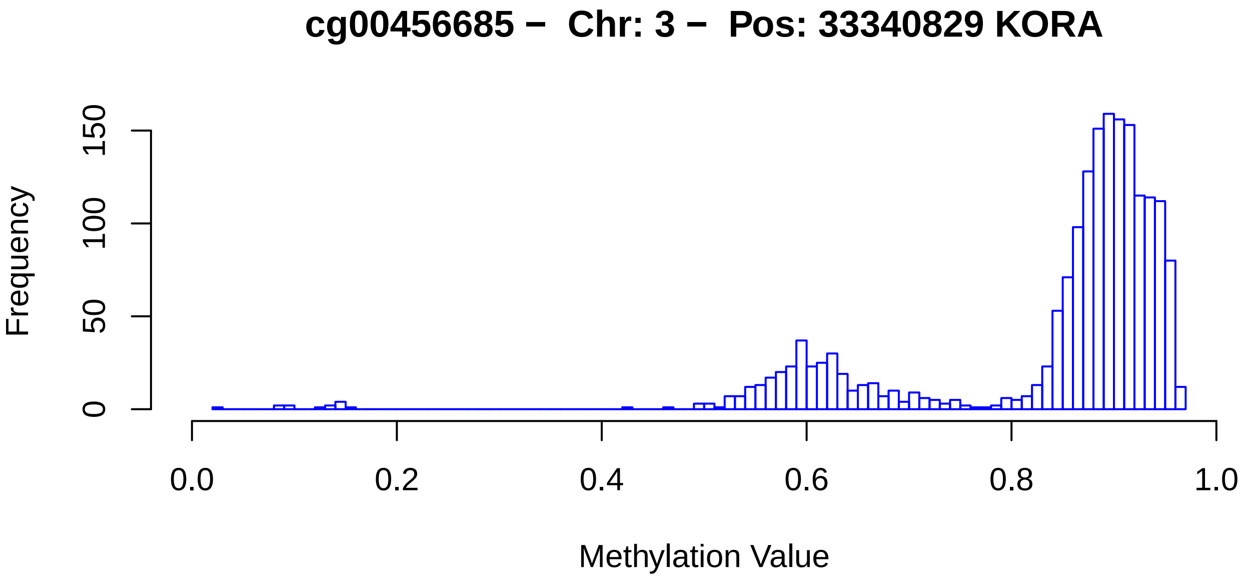
cg00456685 − Chr: 3 − Pos: 33340829 KORA
Frequency
0
50
100
150
0.0
0.2
0.4
0.6
0.8
1.0
Methylation Value

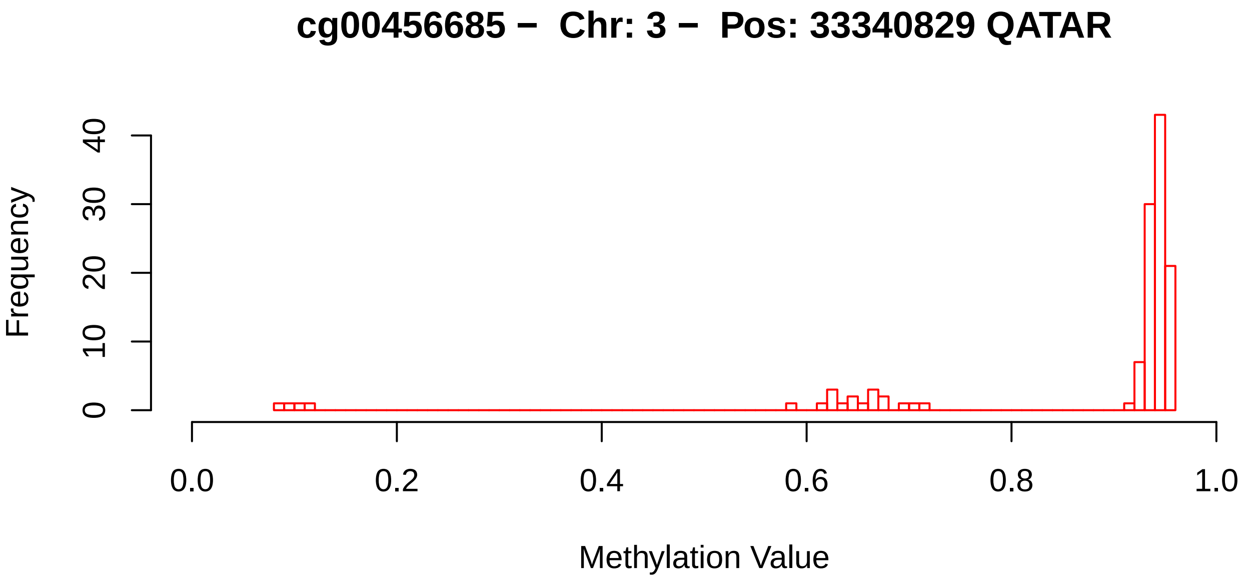
cg00456685 − Chr: 3 − Pos: 33340829 QATAR
Frequency
0
10
20
30
40
0.0
0.2
0.4
0.6
0.8
1.0
Methylation Value

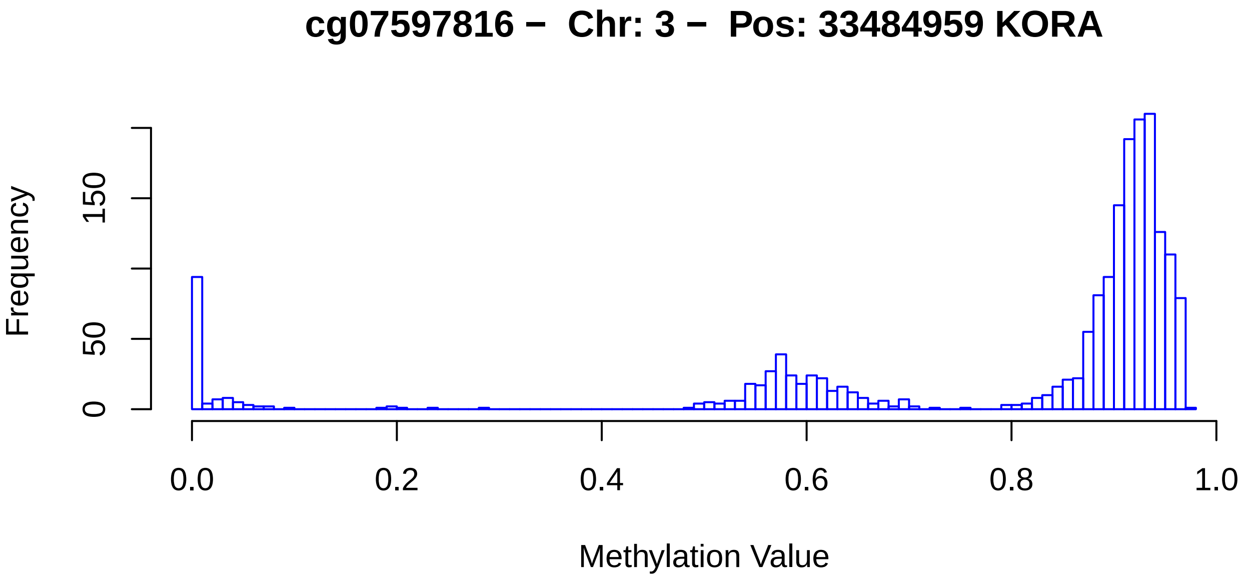
cg07597816 − Chr: 3 − Pos: 33484959 KORA
Frequency
0
50
150
0.0
0.2
0.4
0.6
0.8
1.0
Methylation Value

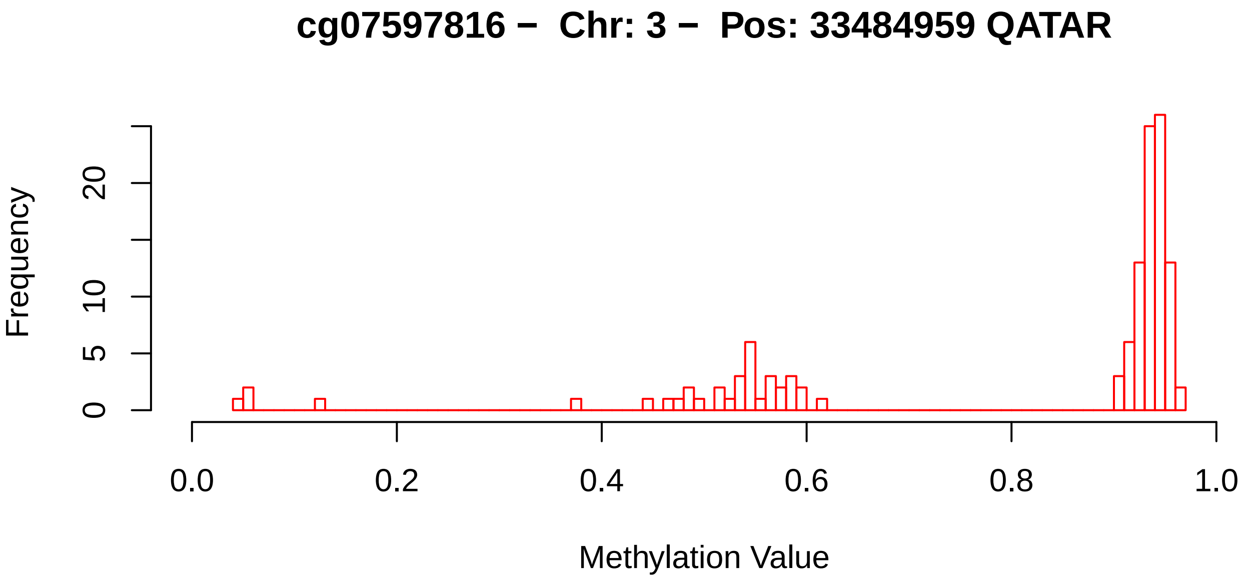
cg07597816 − Chr: 3 − Pos: 33484959 QATAR
Frequency
0
5
10
20
0.0
0.2
0.4
0.6
0.8
1.0
Methylation Value

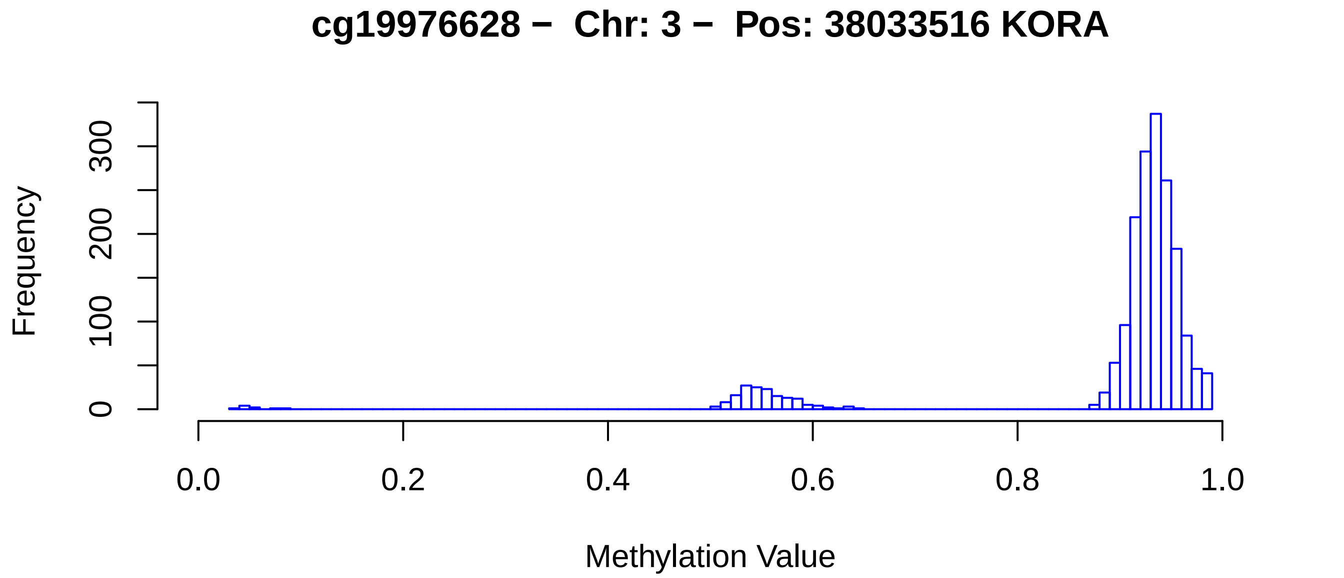
cg19976628 − Chr: 3 − Pos: 38033516 KORA
Frequency
0
100
200
300
0.0
0.2
0.4
0.6
0.8
1.0
Methylation Value

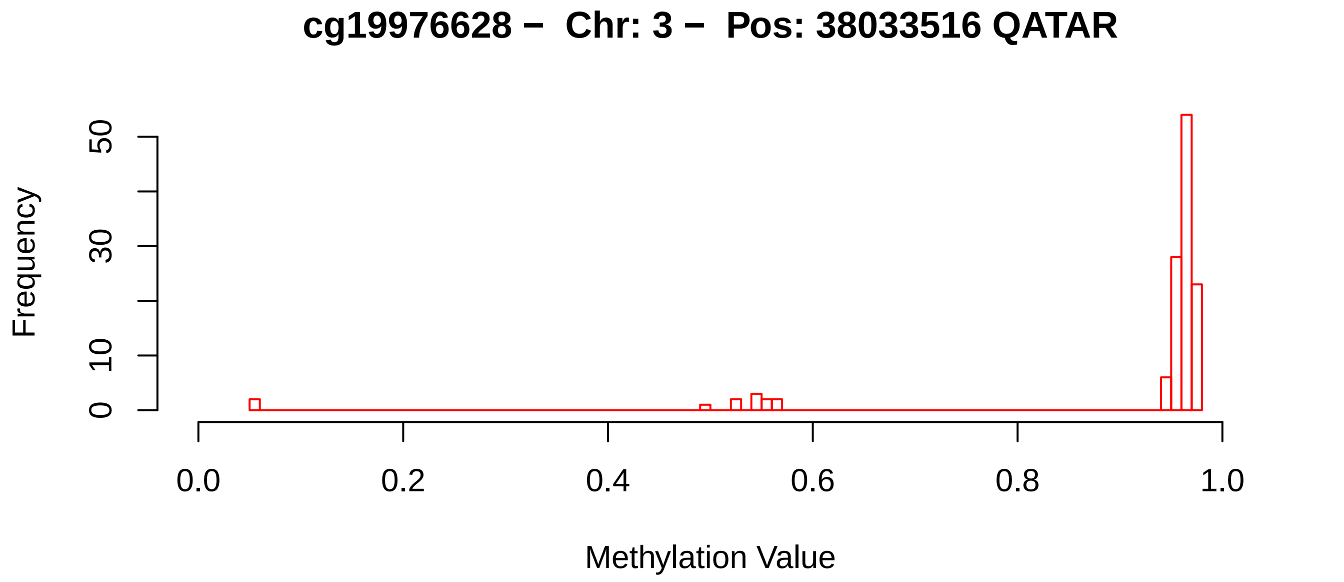
cg19976628 − Chr: 3 − Pos: 38033516 QATAR
Frequency
0
10
30
50
0.0
0.2
0.4
0.6
0.8
1.0
Methylation Value

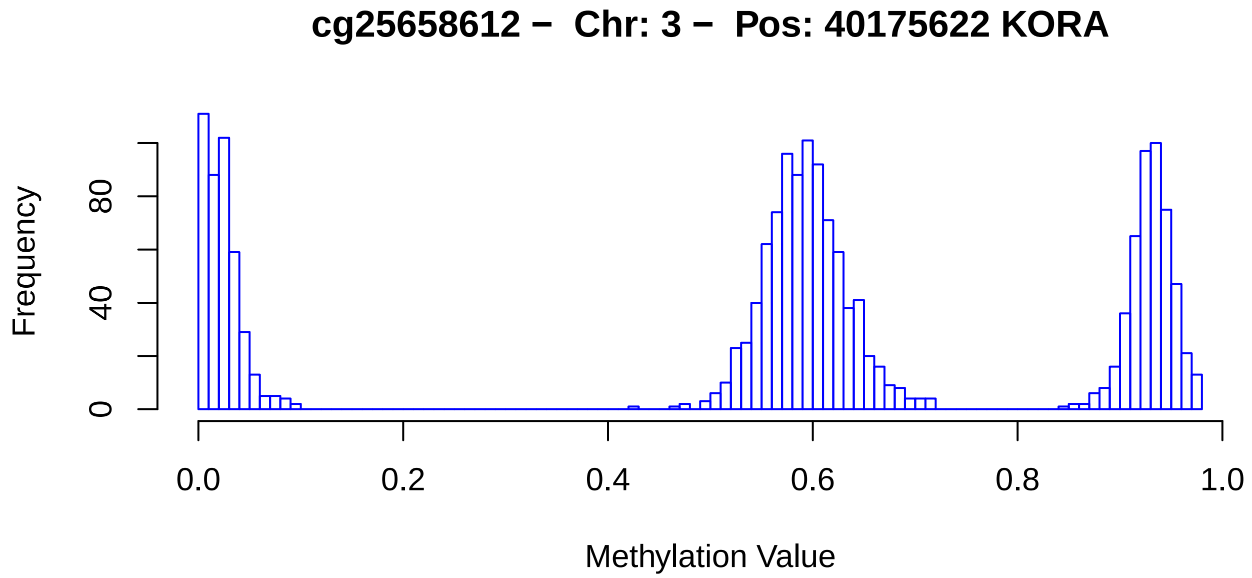
cg25658612 − Chr: 3 − Pos: 40175622 KORA
Frequency
0
40
80
0.0
0.2
0.4
0.6
0.8
1.0
Methylation Value

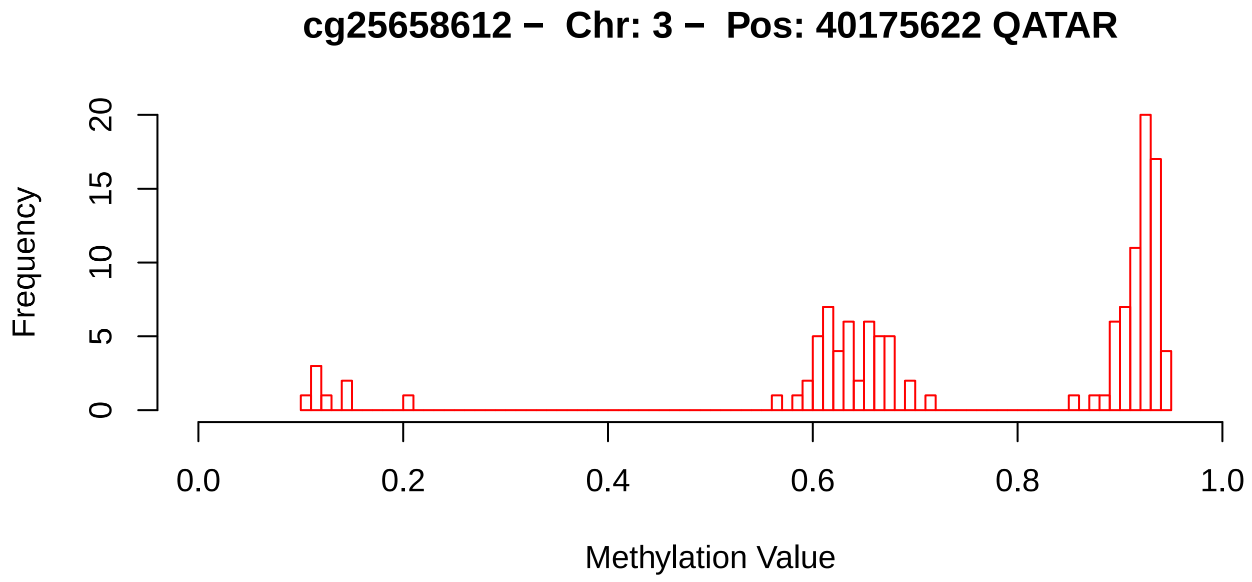
cg25658612 − Chr: 3 − Pos: 40175622 QATAR
Frequency
0
5
10
15
20
0.0
0.2
0.4
0.6
0.8
1.0
Methylation Value

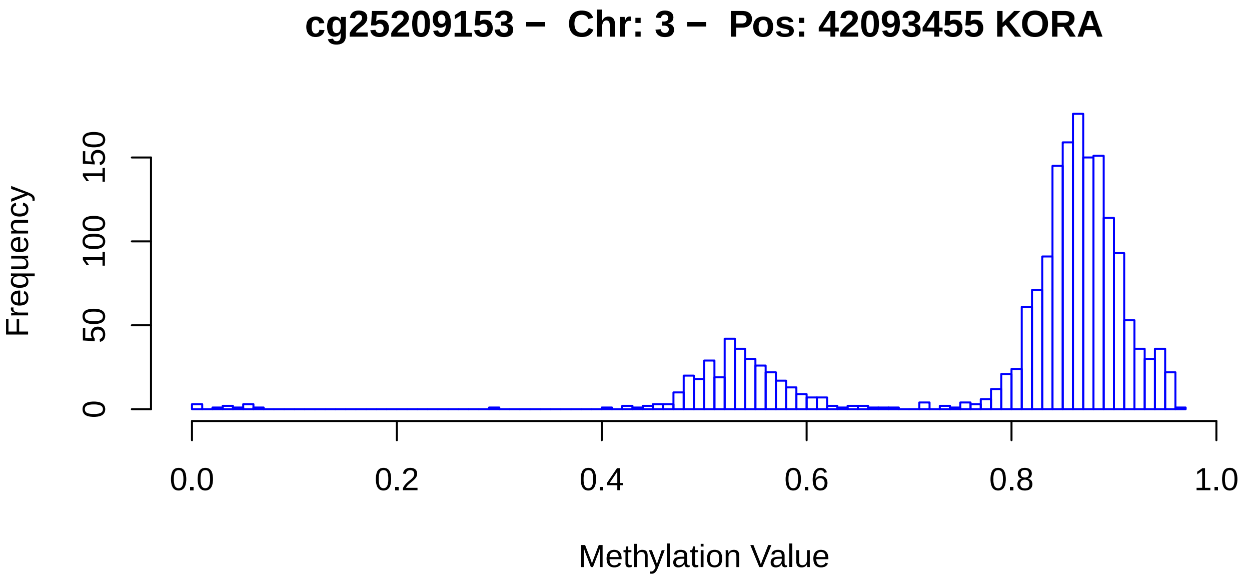
cg25209153 − Chr: 3 − Pos: 42093455 KORA
Frequency
0
50
100
150
0.0
0.2
0.4
0.6
0.8
1.0
Methylation Value

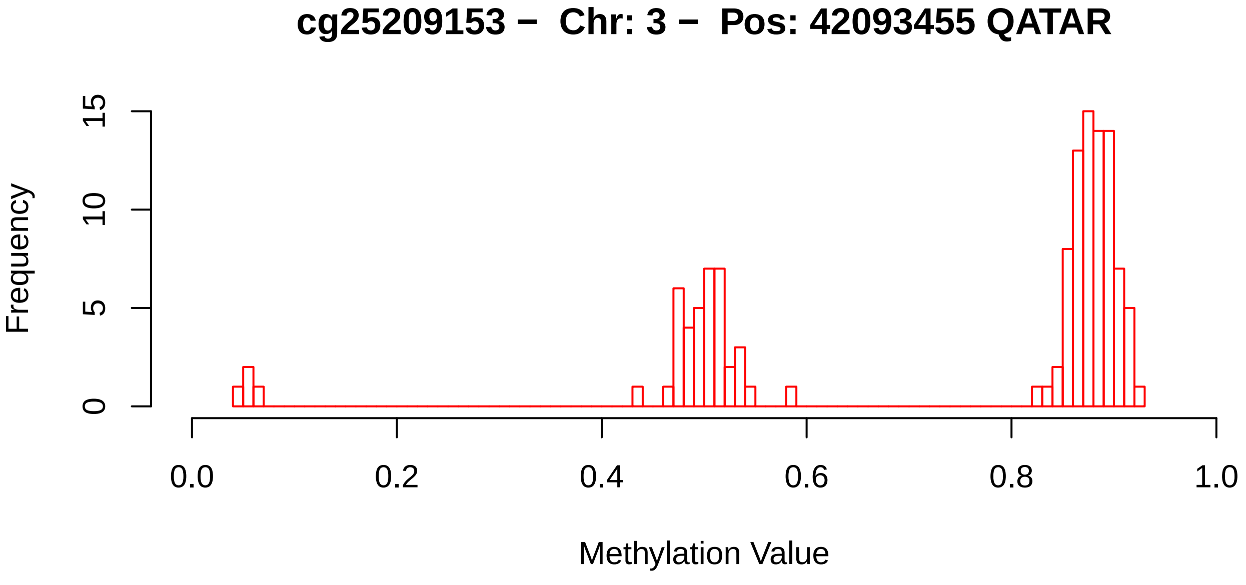
cg25209153 − Chr: 3 − Pos: 42093455 QATAR
Frequency
0
5
10
15
0.0
0.2
0.4
0.6
0.8
1.0
Methylation Value

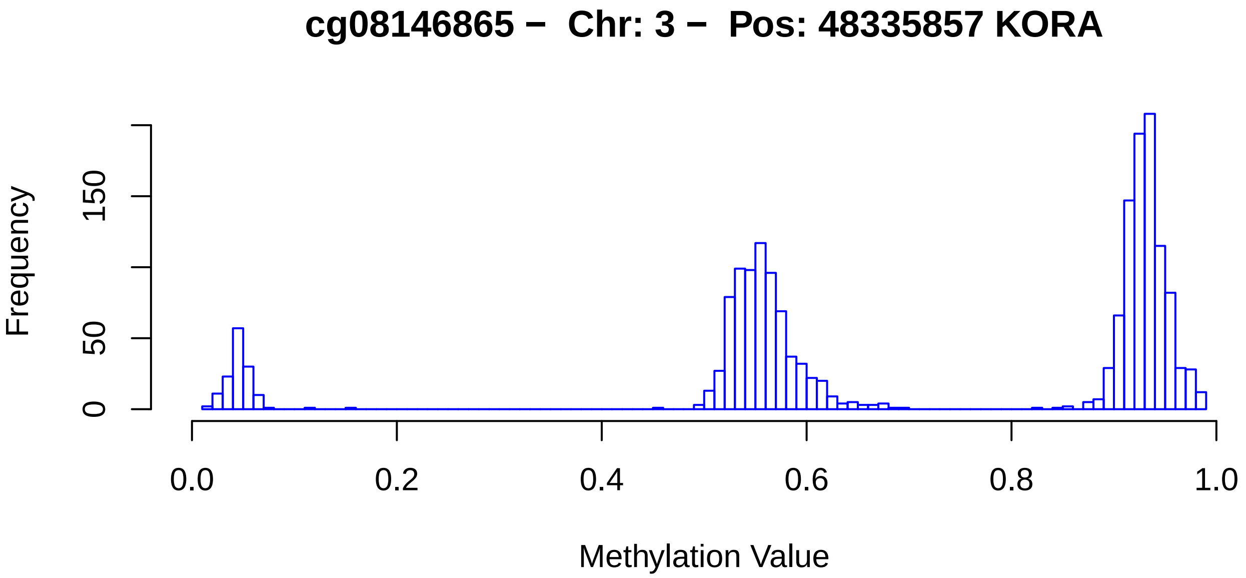
cg08146865 − Chr: 3 − Pos: 48335857 KORA
Frequency
0
50
150
0.0
0.2
0.4
0.6
0.8
1.0
Methylation Value

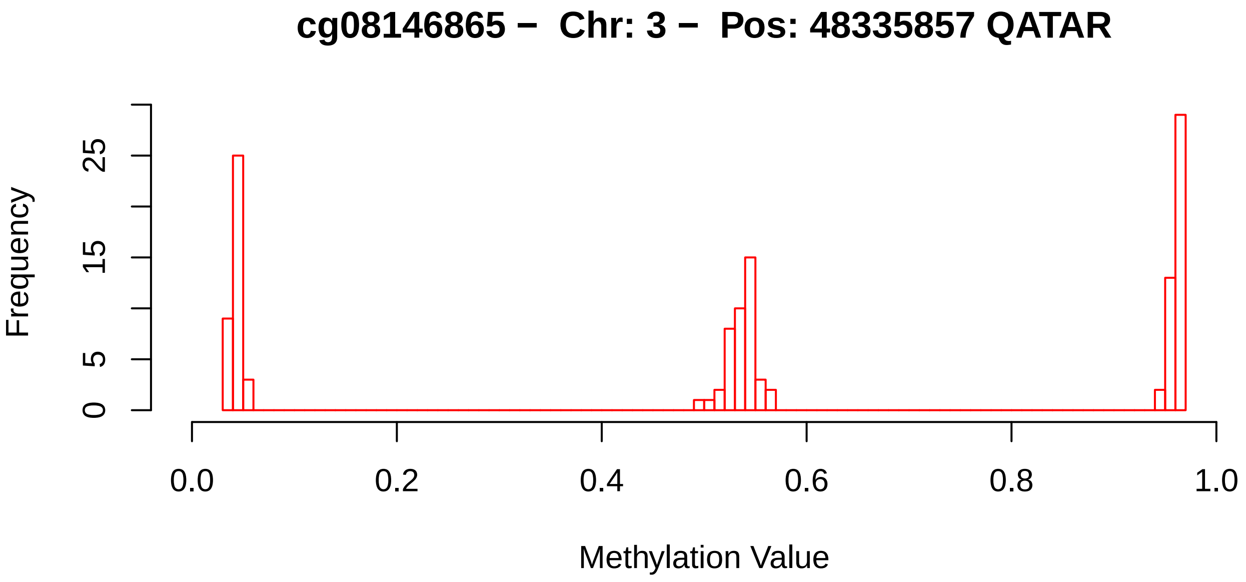
cg08146865 − Chr: 3 − Pos: 48335857 QATAR
Frequency
0
5
15
25
0.0
0.2
0.4
0.6
0.8
1.0
Methylation Value

cg16364629 − Chr: 3 − Pos: 52303212 KORA

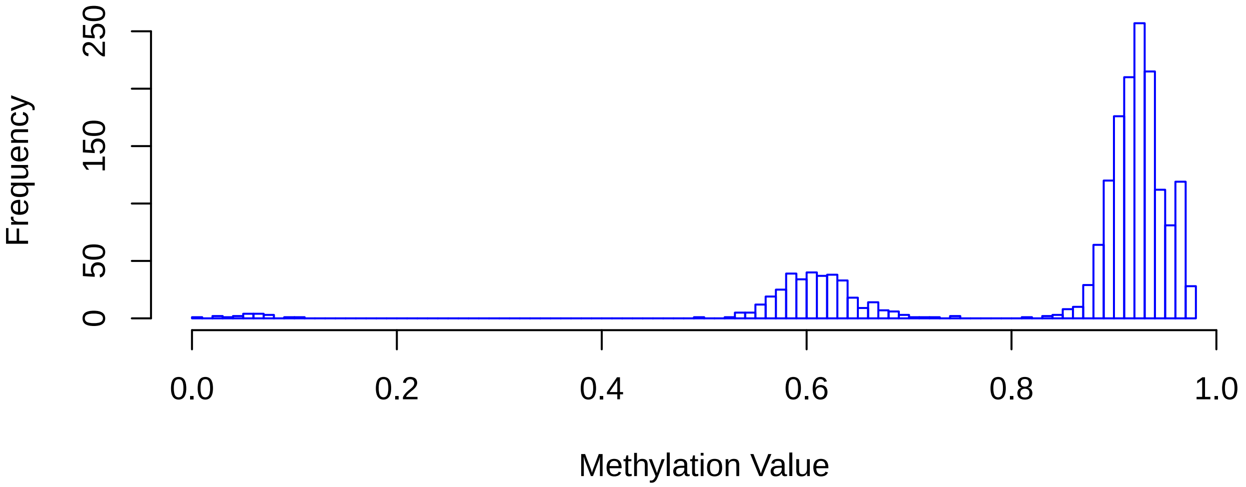

cg16364629 − Chr: 3 − Pos: 52303212 QATAR

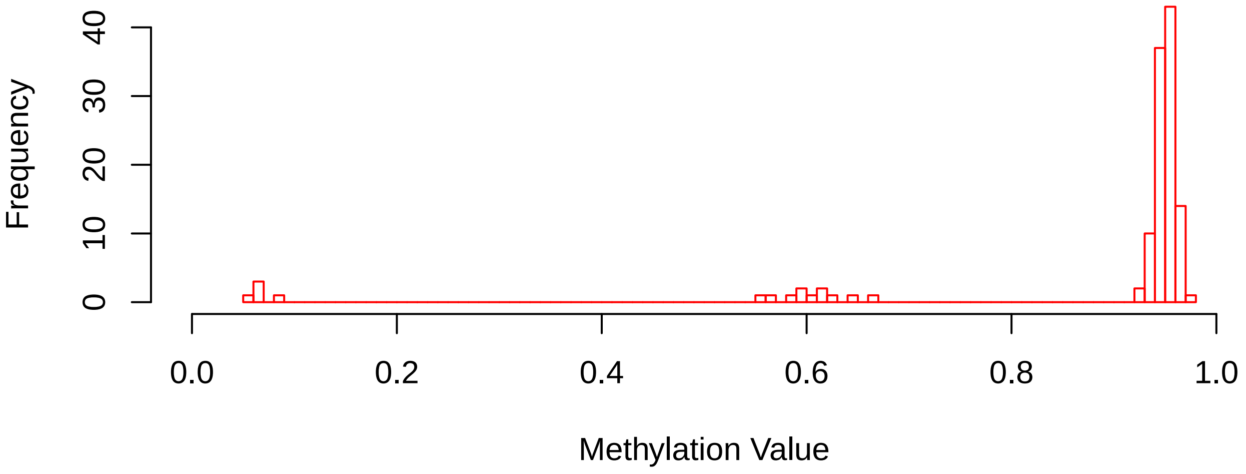

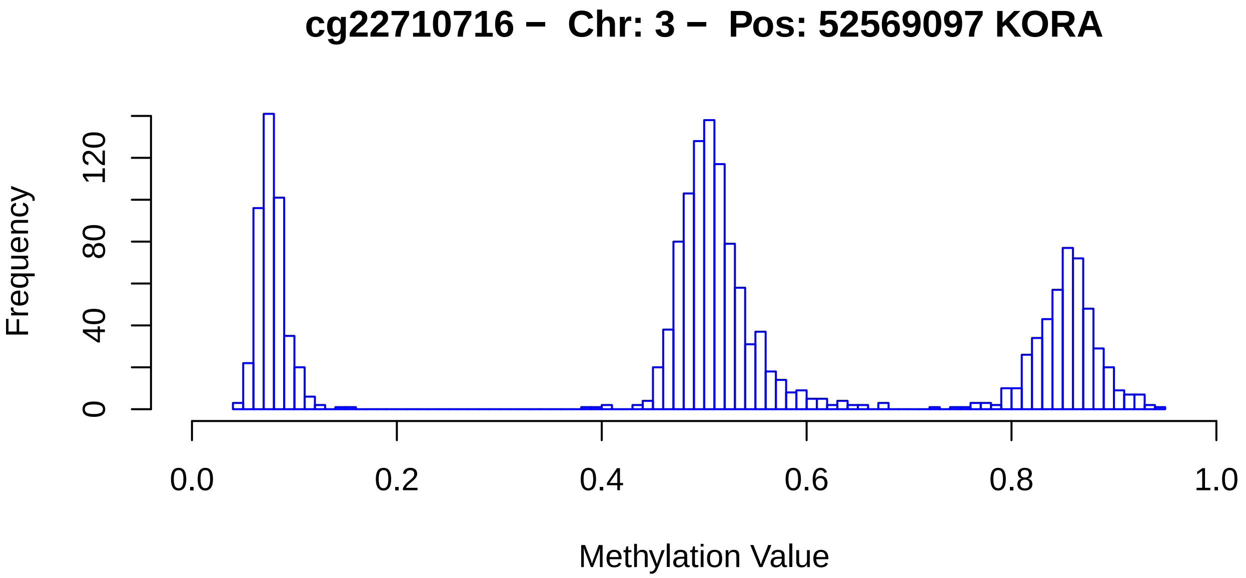

cg22710716 − Chr: 3 − Pos: 52569097 KORA
Frequency
0
40
80
120
0.0
0.2
0.4
0.6
0.8
1.0
Methylation Value

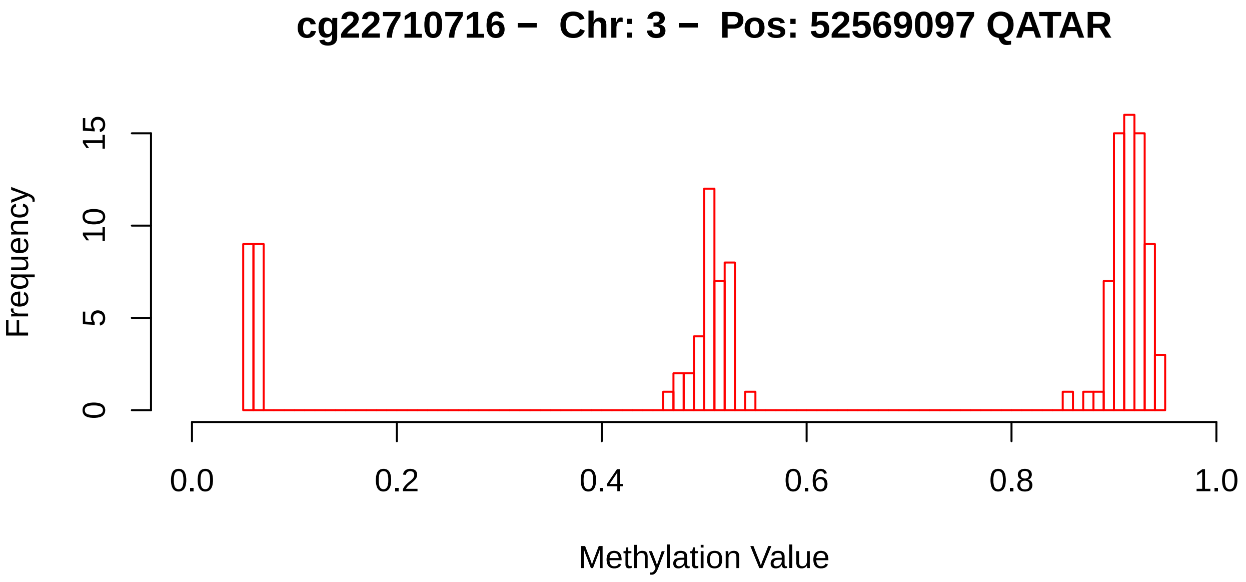

cg22710716 − Chr: 3 − Pos: 52569097 QATAR
Frequency
0
5
10
15
0.0
0.2
0.4
0.6
0.8
1.0
Methylation Value

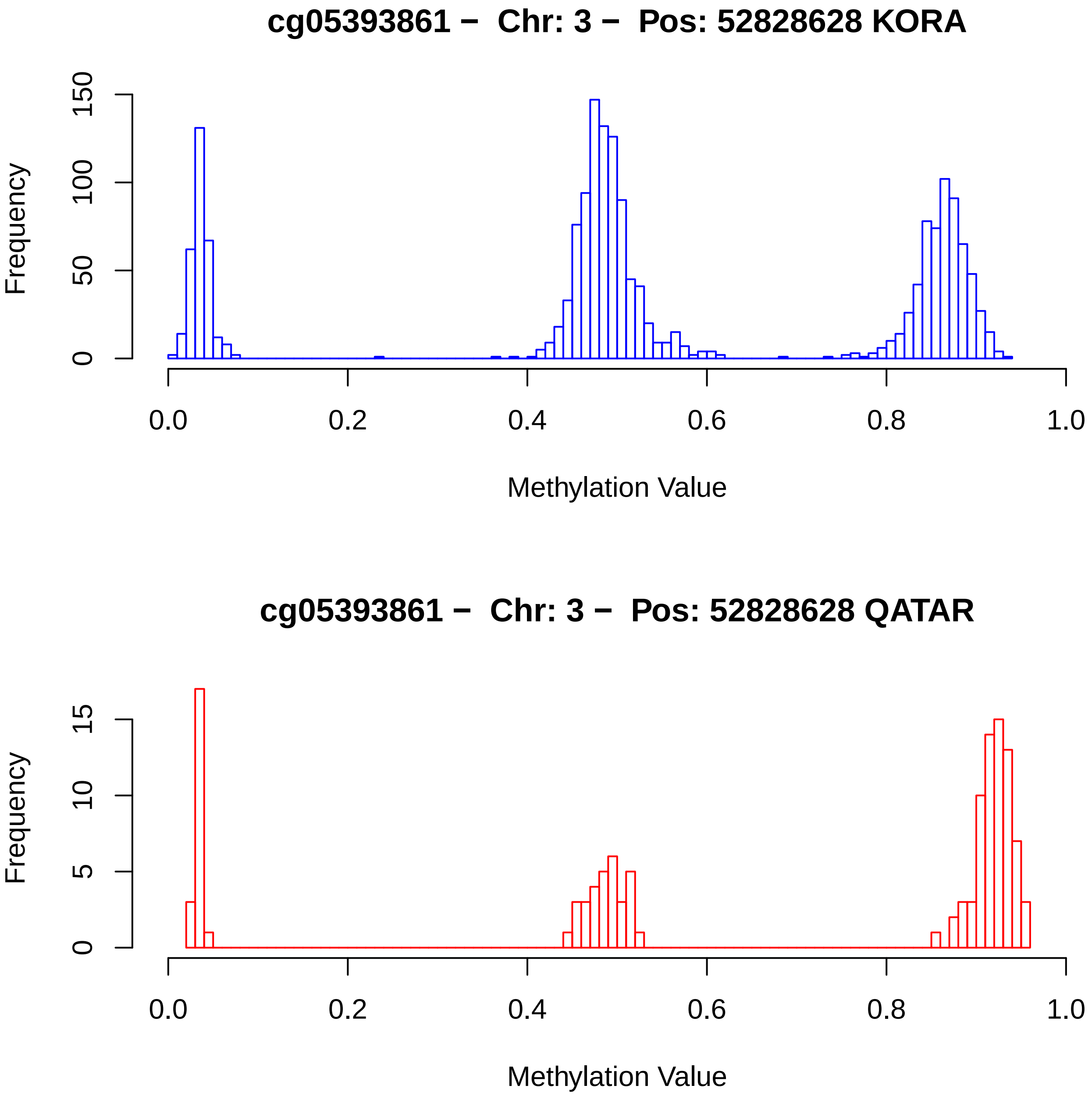
cg05393861 − Chr: 3 − Pos: 52828628 KORA
Frequency
0
50
100
150
0.0
0.2
0.4
0.6
0.8
1.0
Methylation Value
cg05393861 − Chr: 3 − Pos: 52828628 QATAR
Frequency
0
5
10
15
0.0
0.2
0.4
0.6
0.8
1.0
Methylation Value

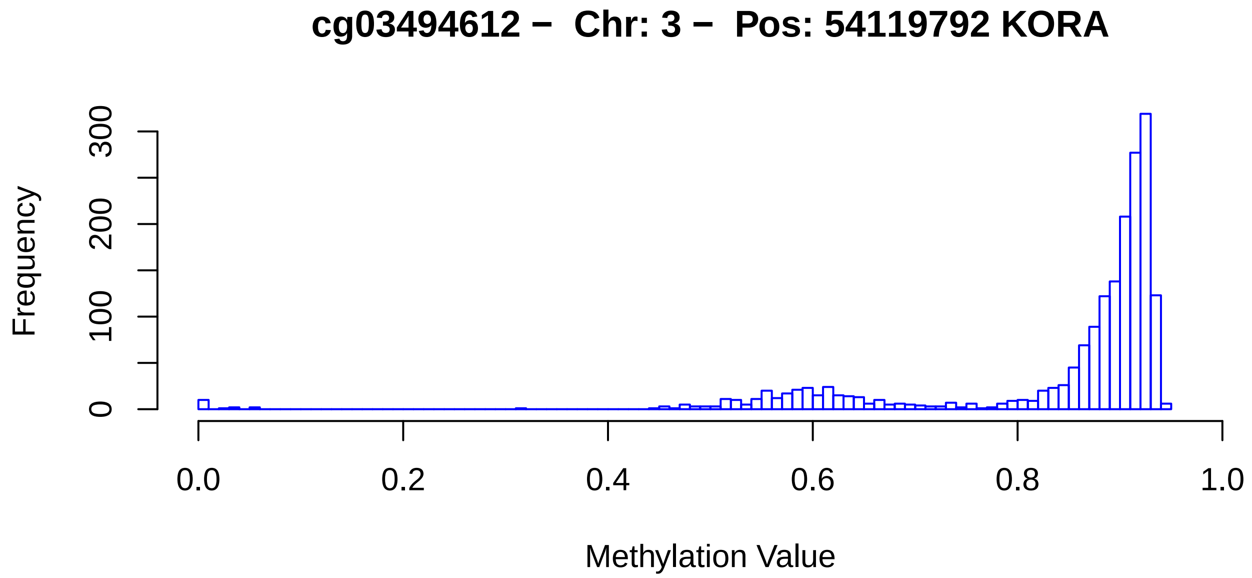
cg03494612 − Chr: 3 − Pos: 54119792 KORA
Frequency
0
100
200
300
0.0
0.2
0.4
0.6
0.8
1.0
Methylation Value

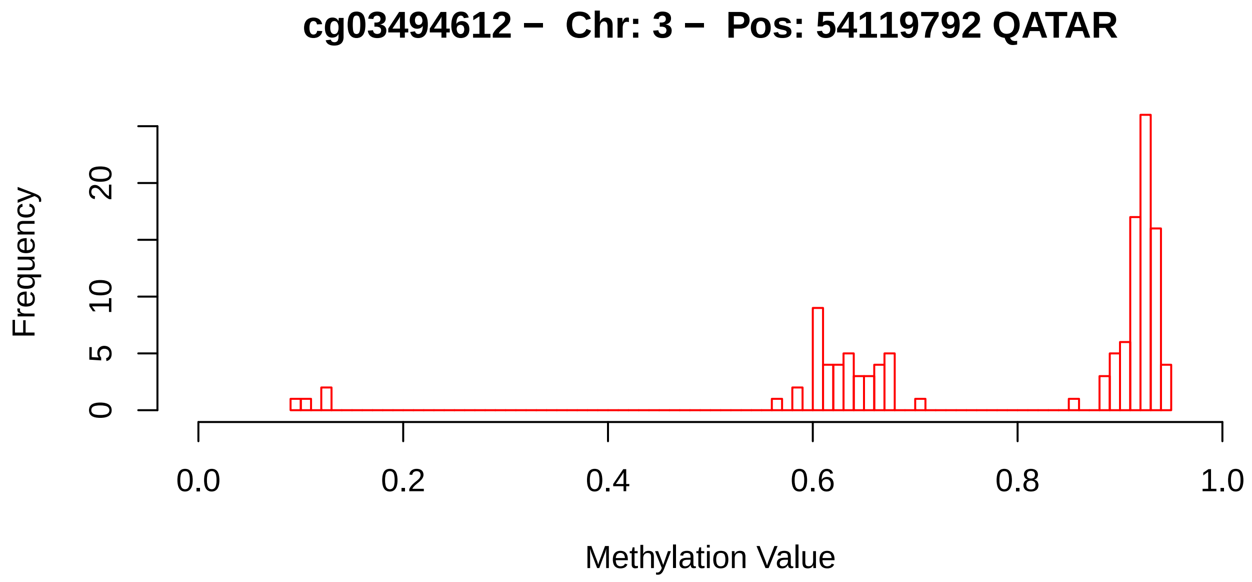
cg03494612 − Chr: 3 − Pos: 54119792 QATAR
Frequency
0
5
10
20
0.0
0.2
0.4
0.6
0.8
1.0
Methylation Value

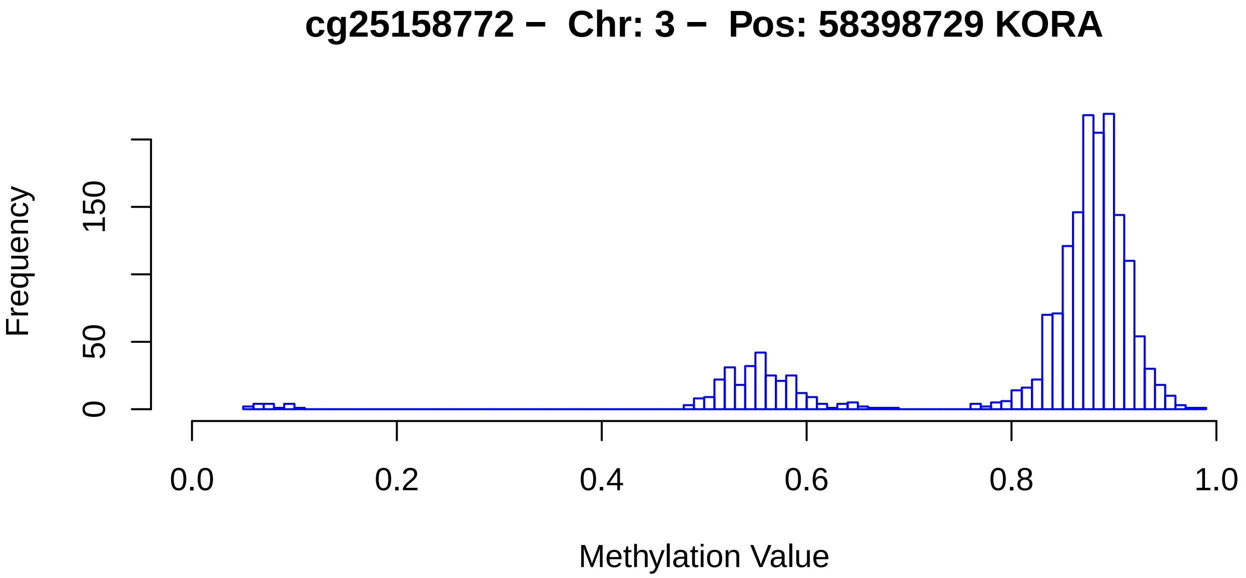
cg25158772 − Chr: 3 − Pos: 58398729 KORA
Frequency
0
50
150
0.0
0.2
0.4
0.6
0.8
1.0
Methylation Value

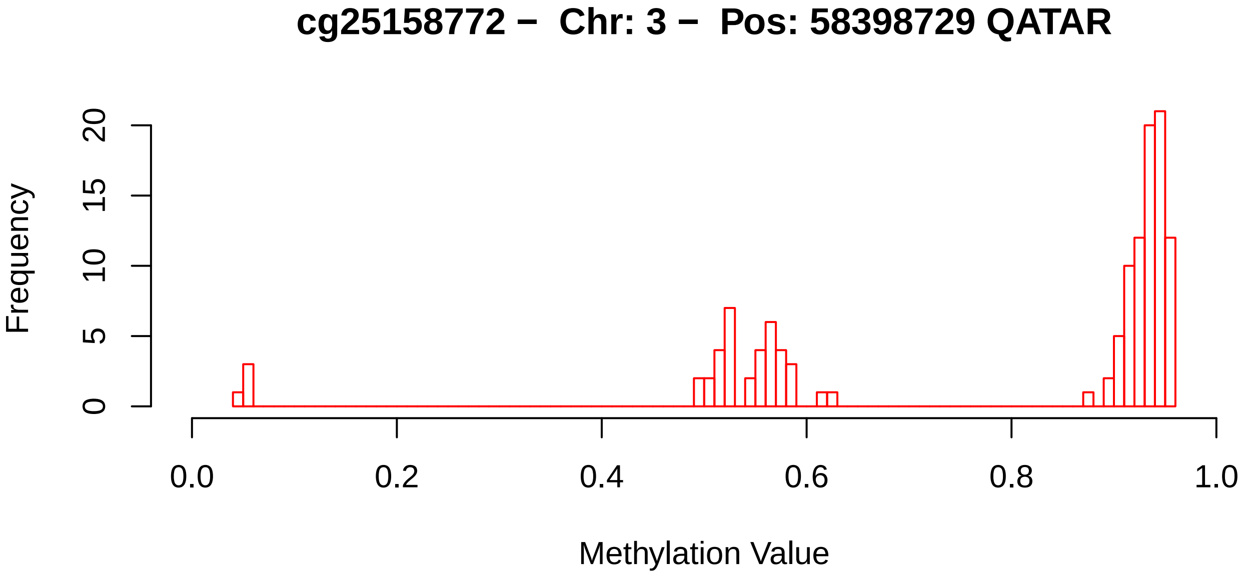
cg25158772 − Chr: 3 − Pos: 58398729 QATAR
Frequency
0
5
10
15
20
0.0
0.2
0.4
0.6
0.8
1.0
Methylation Value

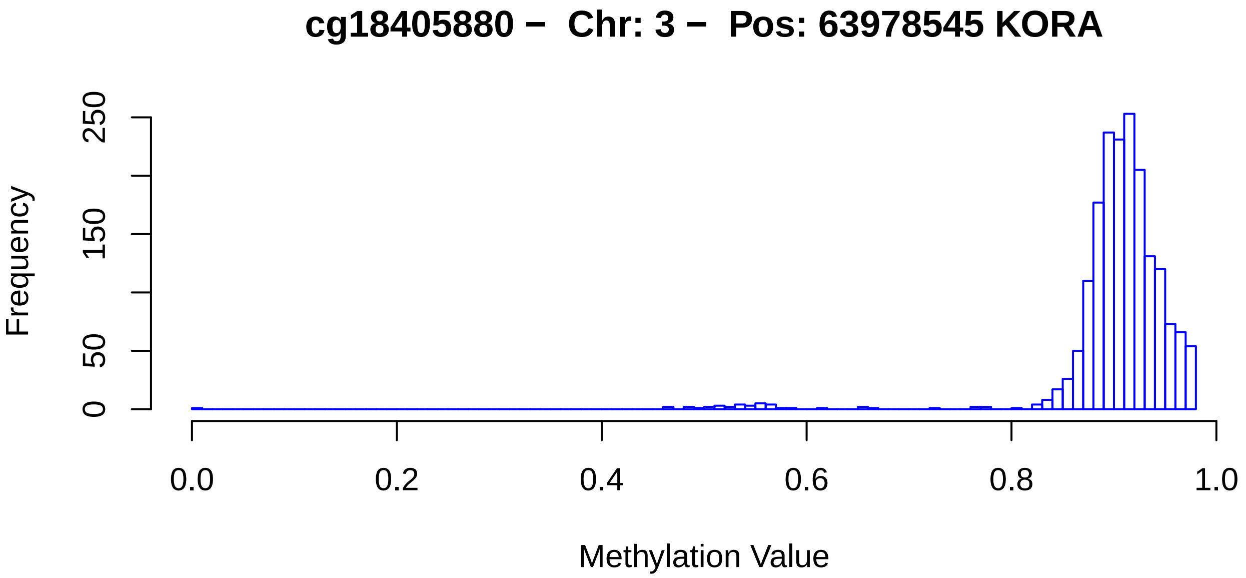
cg18405880 − Chr: 3 − Pos: 63978545 KORA
Frequency
0
50
150
250
0.0
0.2
0.4
0.6
0.8
1.0
Methylation Value

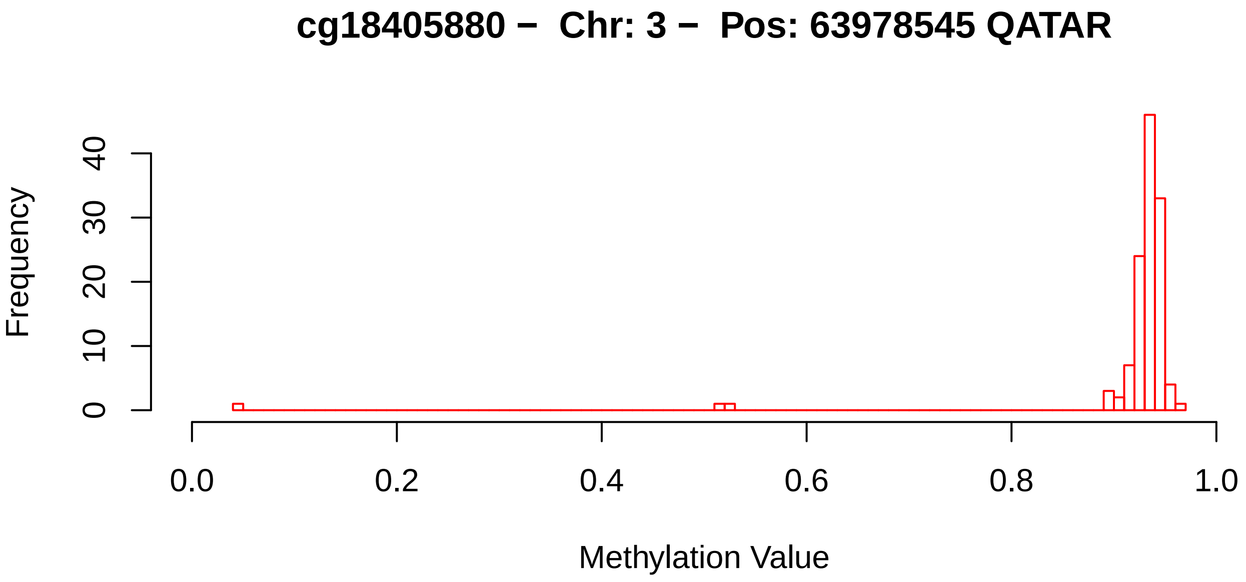
cg18405880 − Chr: 3 − Pos: 63978545 QATAR
Frequency
0
10
20
30
40
0.0
0.2
0.4
0.6
0.8
1.0
Methylation Value

cg12403190 − Chr: 3 − Pos: 65345027 KORA

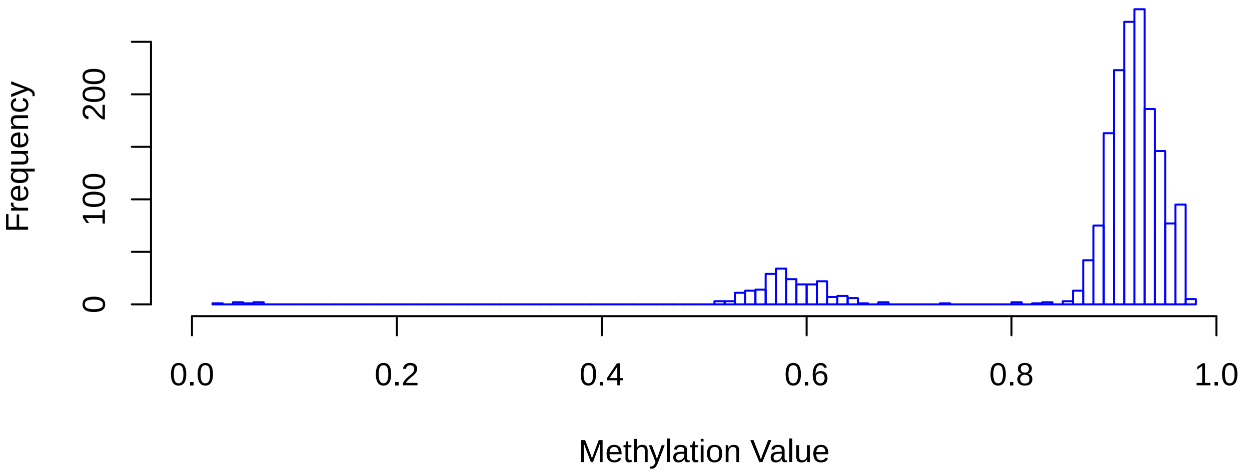

cg12403190 − Chr: 3 − Pos: 65345027 QATAR

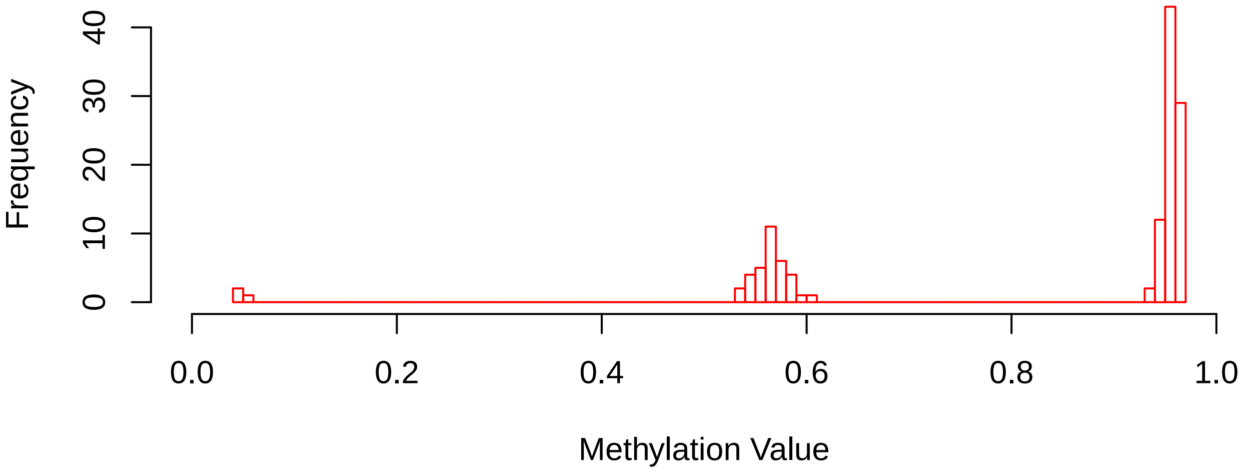

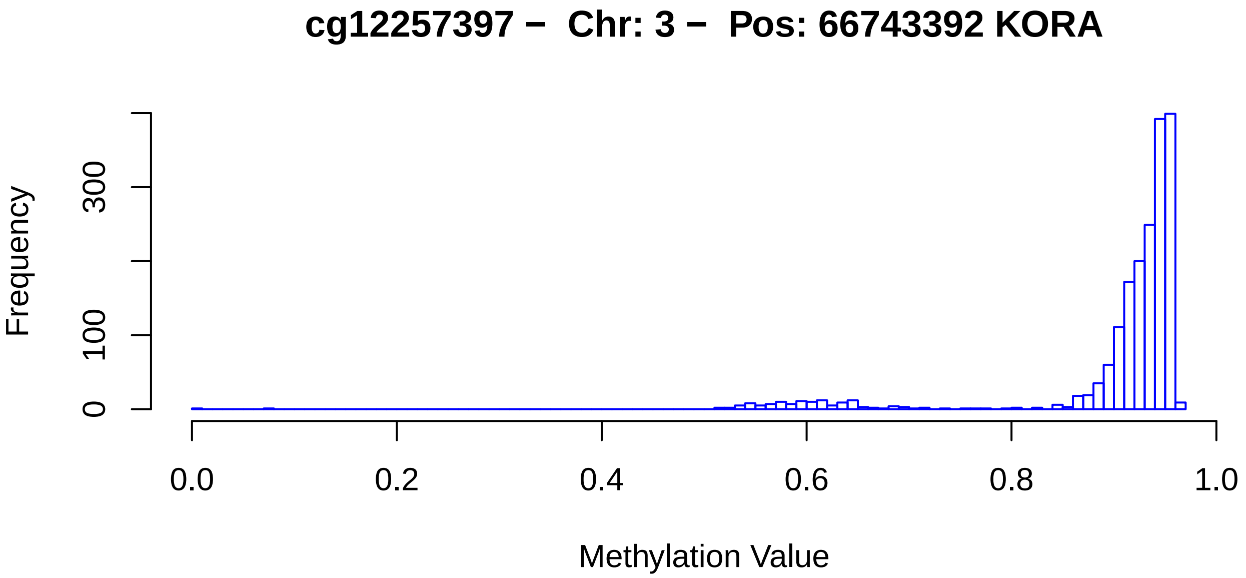
cg12257397 − Chr: 3 − Pos: 66743392 KORA
Frequency
0
100
300
0.0
0.2
0.4
0.6
0.8
1.0
Methylation Value

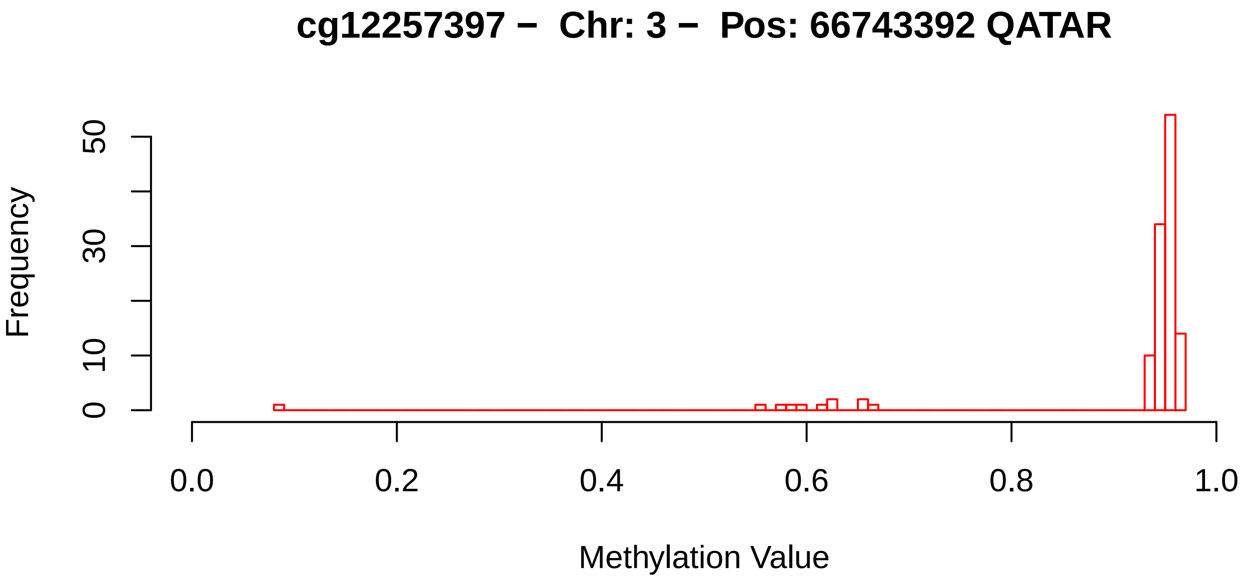
cg12257397 − Chr: 3 − Pos: 66743392 QATAR
Frequency
0
10
30
50
0.0
0.2
0.4
0.6
0.8
1.0
Methylation Value

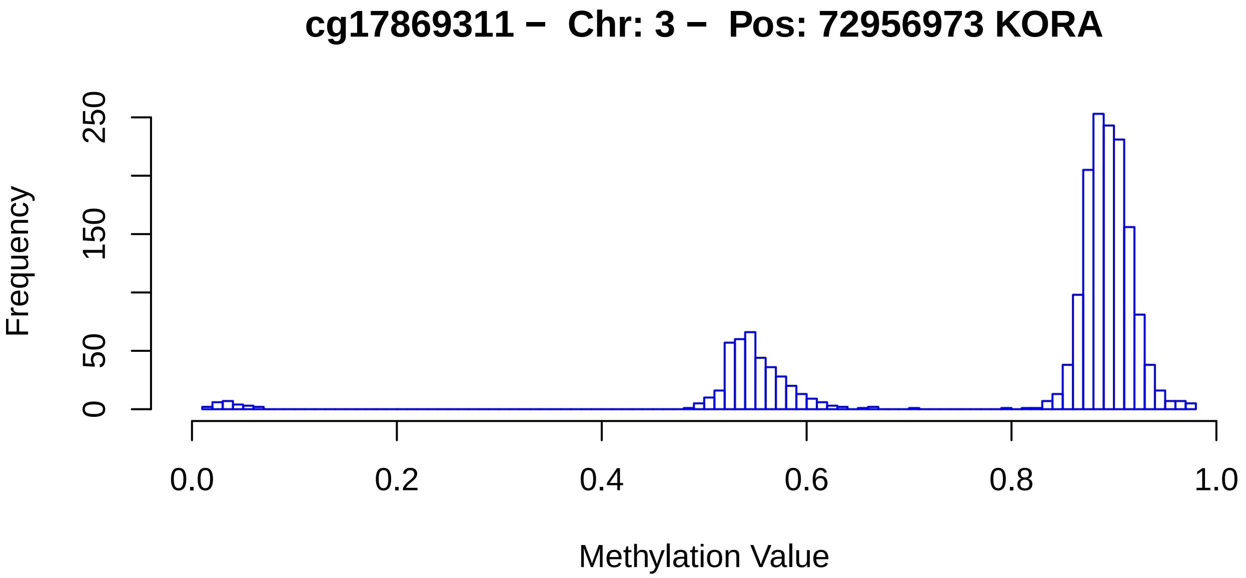

cg17869311 − Chr: 3 − Pos: 72956973 KORA
Frequency
0
50
150
250
0.0
0.2
0.4
0.6
0.8
1.0
Methylation Value

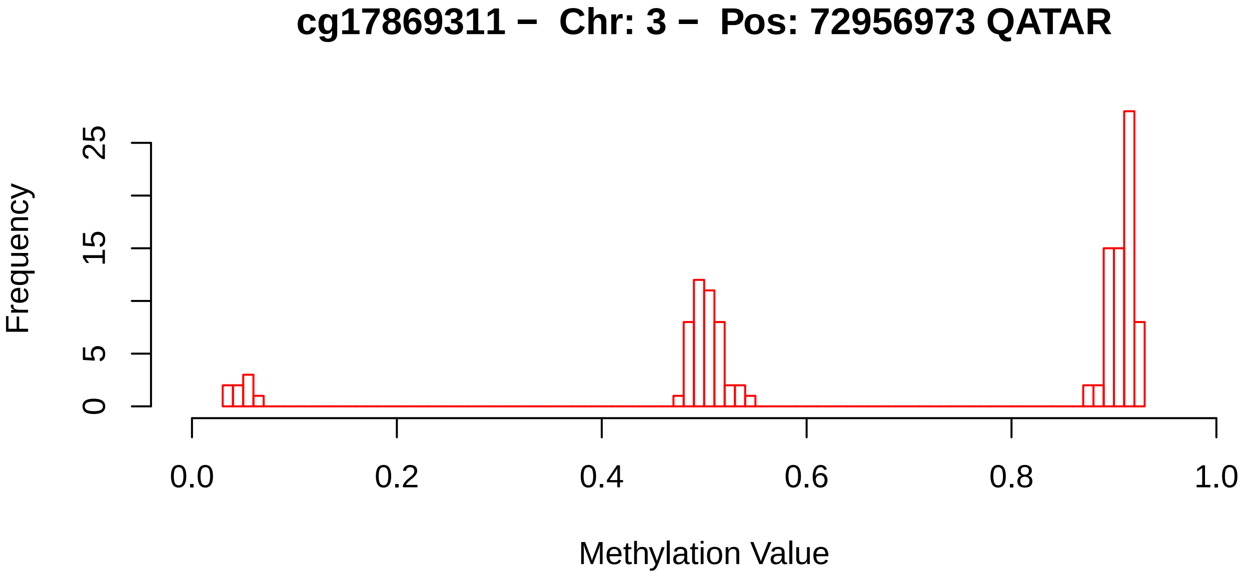

cg17869311 − Chr: 3 − Pos: 72956973 QATAR
Frequency
0
5
15
25
0.0
0.2
0.4
0.6
0.8
1.0
Methylation Value

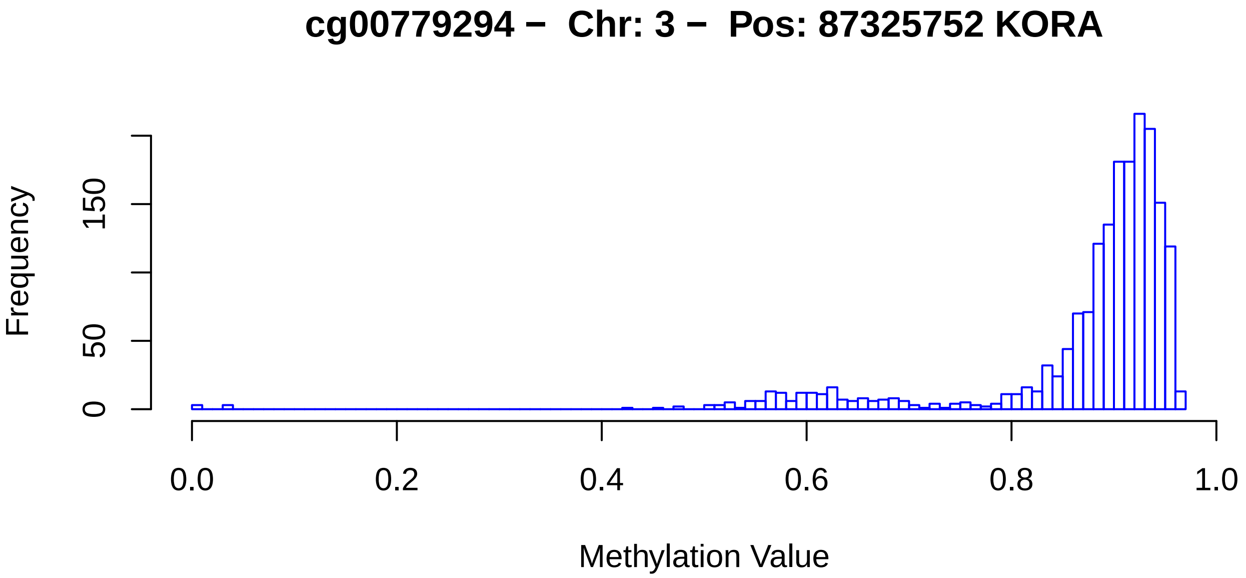
cg00779294 − Chr: 3 − Pos: 87325752 KORA
Frequency
0
50
150
0.0
0.2
0.4
0.6
0.8
1.0
Methylation Value

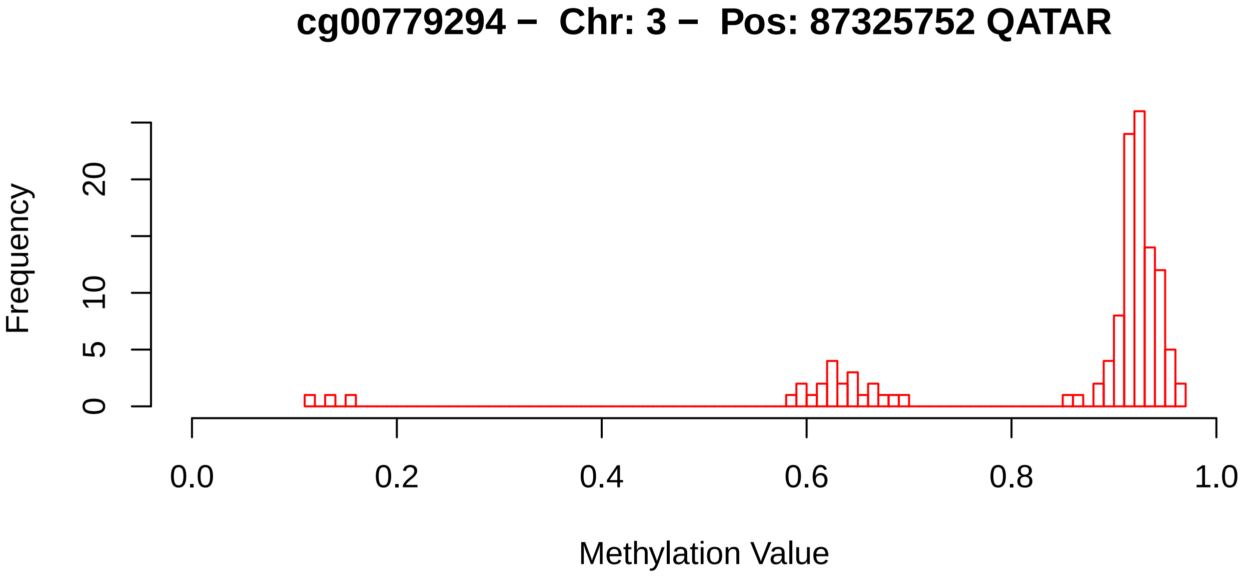
cg00779294 − Chr: 3 − Pos: 87325752 QATAR
Frequency
0
5
10
20
0.0
0.2
0.4
0.6
0.8
1.0
Methylation Value

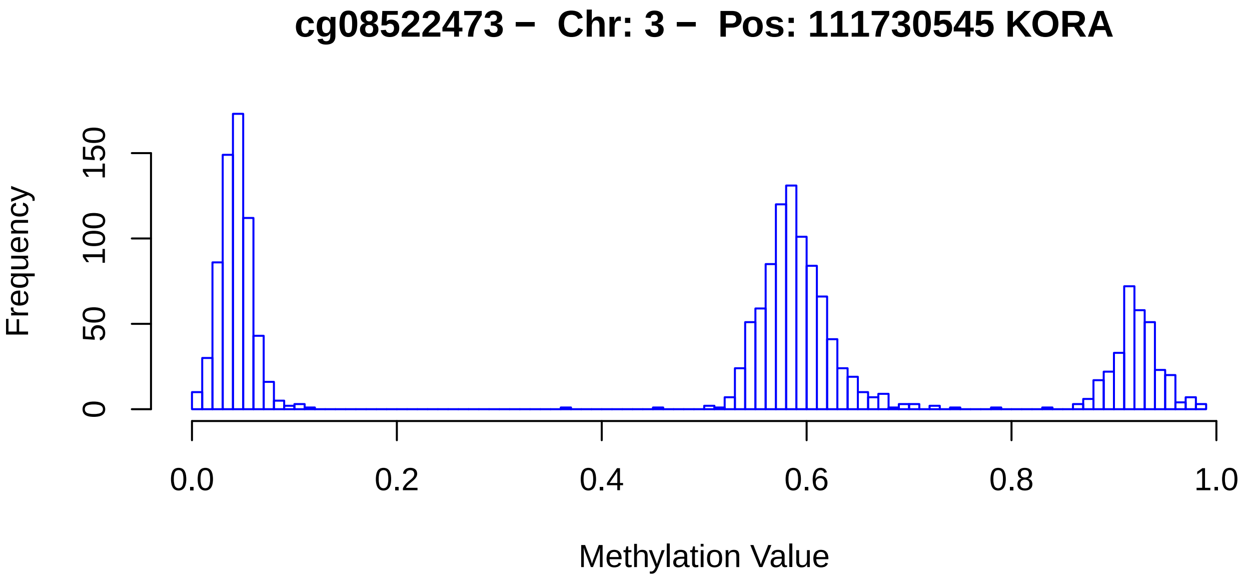
cg08522473 − Chr: 3 − Pos: 111730545 KORA
Frequency
0
50
100
150
0.0
0.2
0.4
0.6
0.8
1.0
Methylation Value

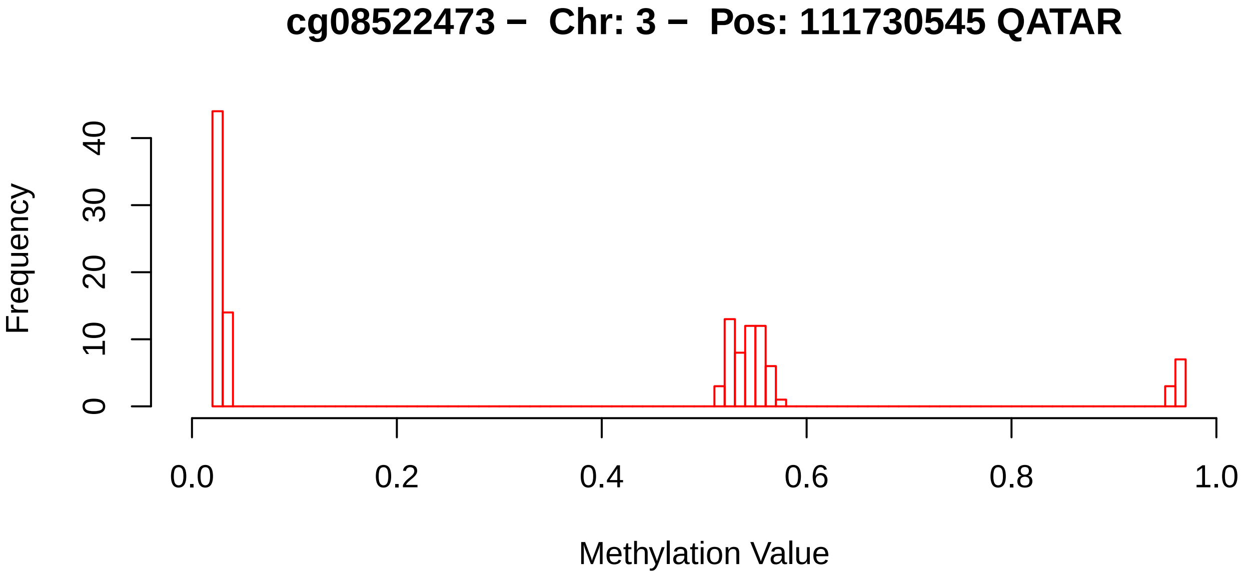
cg08522473 − Chr: 3 − Pos: 111730545 QATAR
Frequency
0
10
20
30
40
0.0
0.2
0.4
0.6
0.8
1.0
Methylation Value

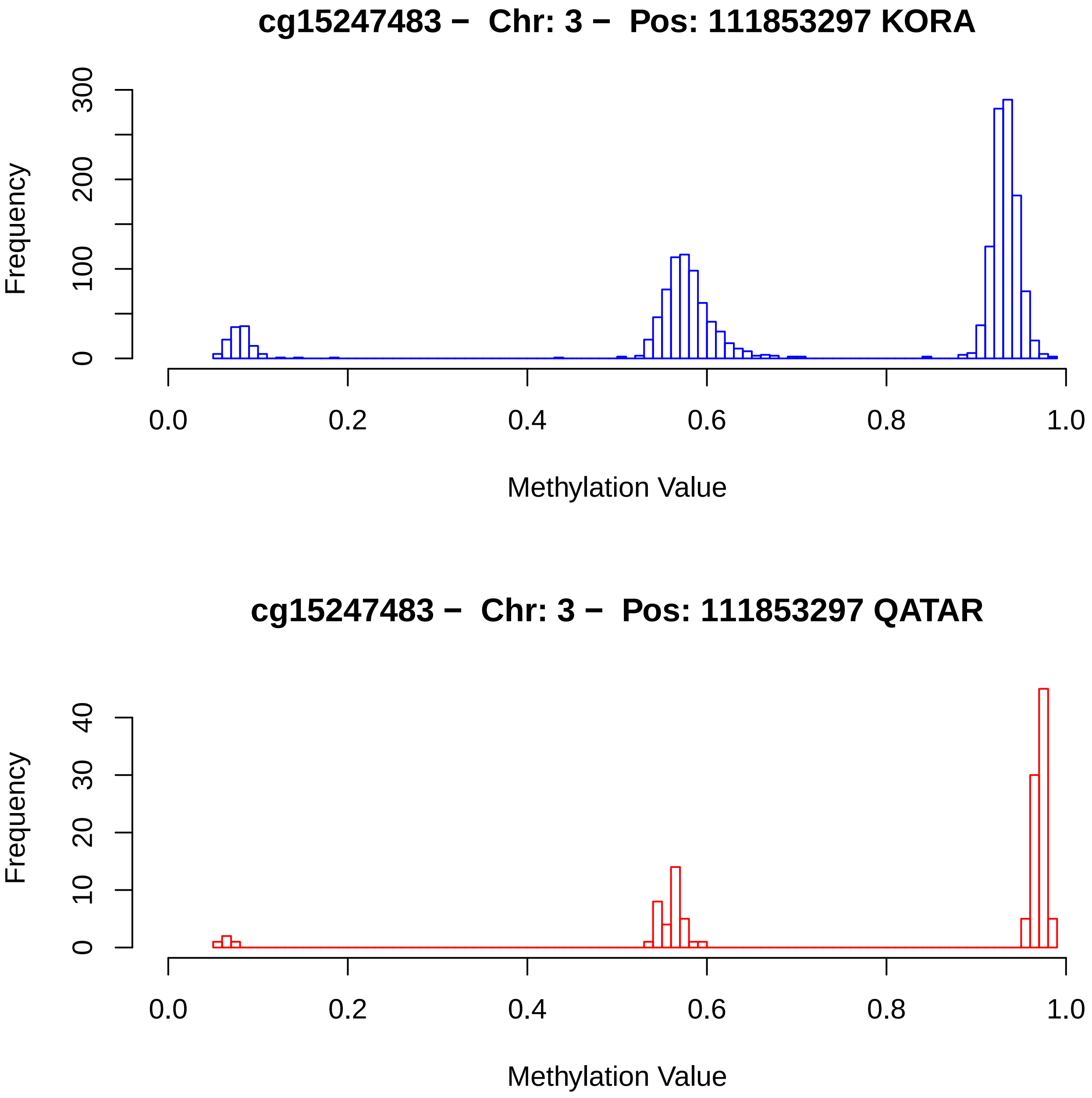

cg15247483 − Chr: 3 − Pos: 111853297 KORA
Frequency
0
100
200
300
0.0
0.2
0.4
0.6
0.8
1.0
Methylation Value
cg15247483 − Chr: 3 − Pos: 111853297 QATAR
Frequency
0
10
20
30
40
0.0
0.2
0.4
0.6
0.8
1.0
Methylation Value

cg06300880 − Chr: 3 − Pos: 119279147 KORA

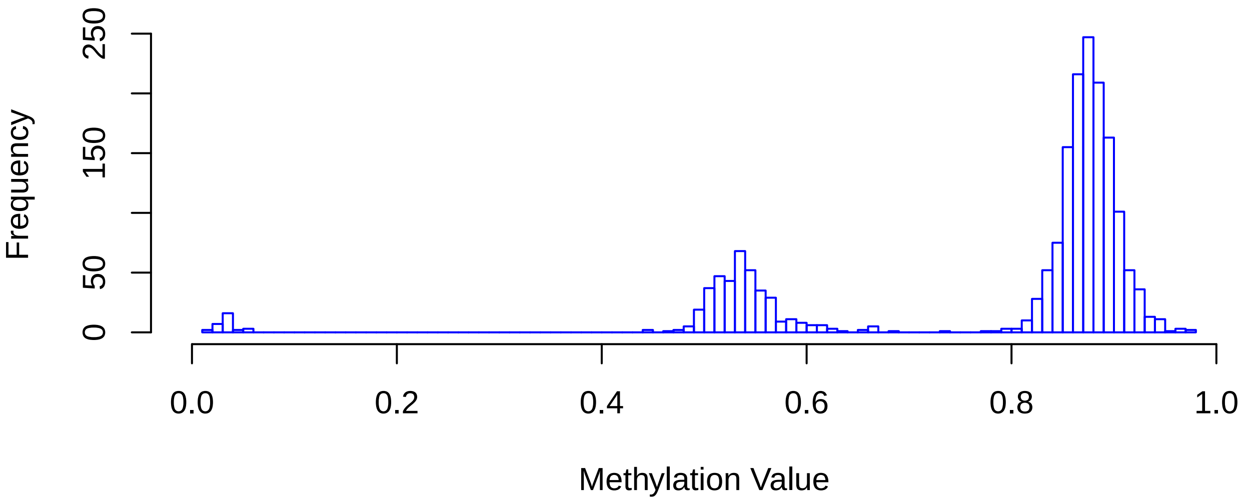

cg06300880 − Chr: 3 − Pos: 119279147 QATAR

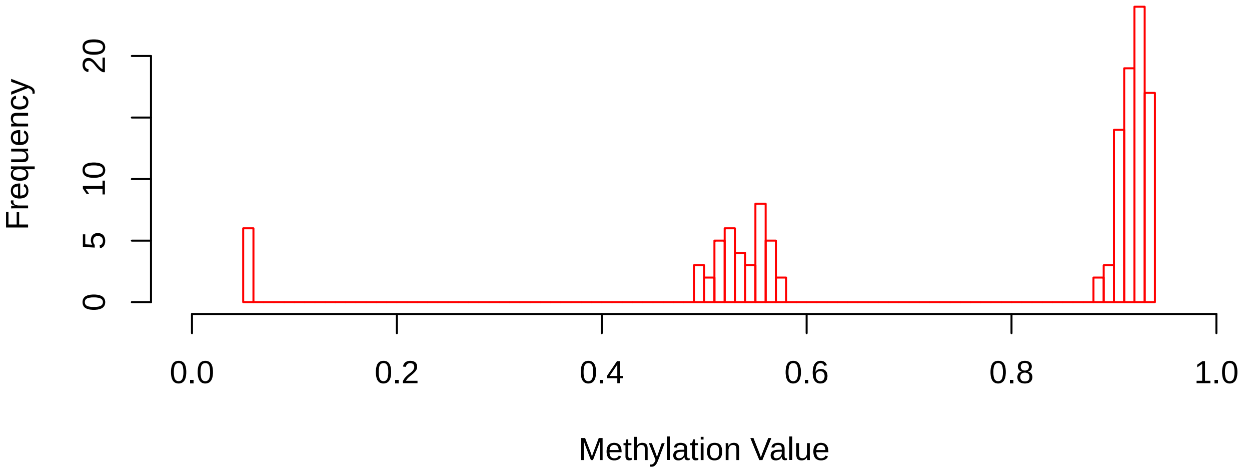

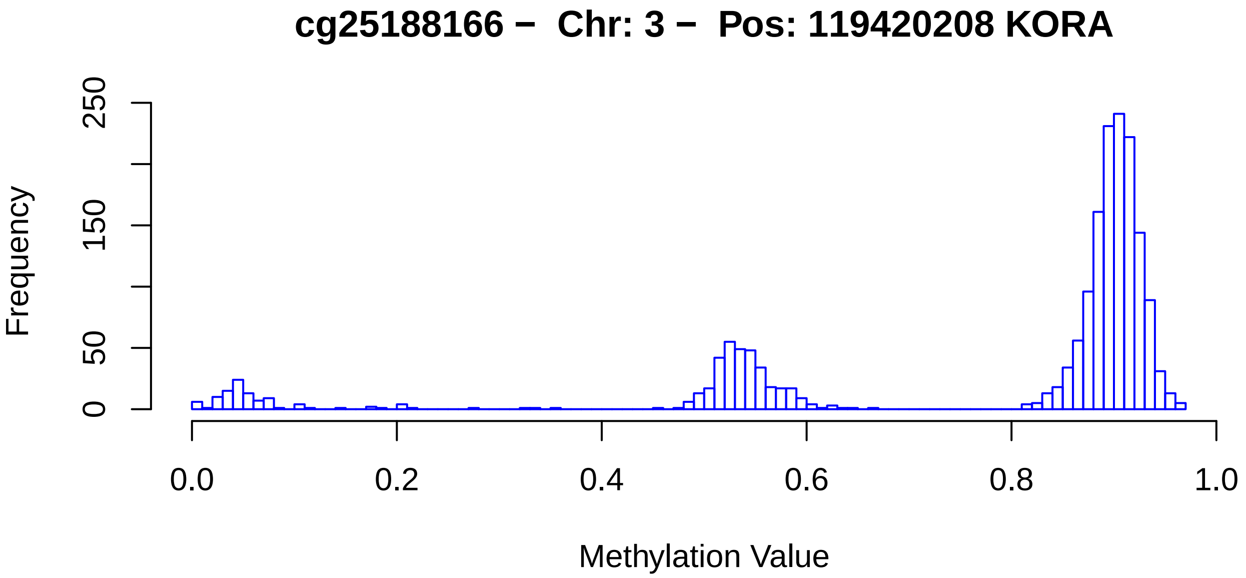
cg25188166 − Chr: 3 − Pos: 119420208 KORA
Frequency
0
50
150
250
0.0
0.2
0.4
0.6
0.8
1.0
Methylation Value

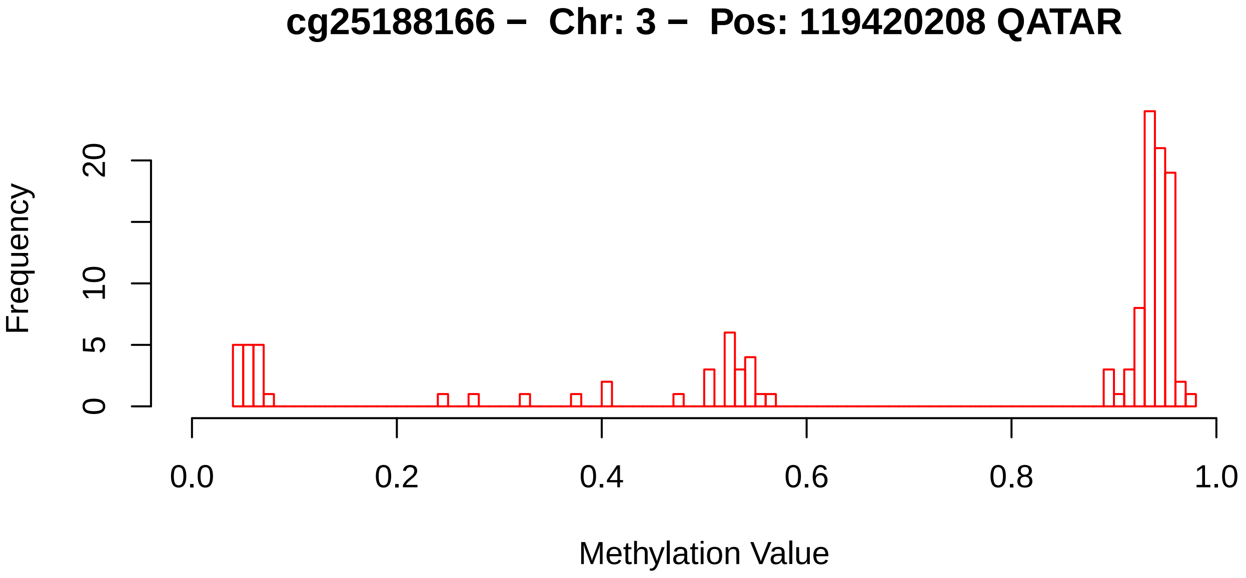
cg25188166 − Chr: 3 − Pos: 119420208 QATAR
Frequency
0
5
10
20
0.0
0.2
0.4
0.6
0.8
1.0
Methylation Value

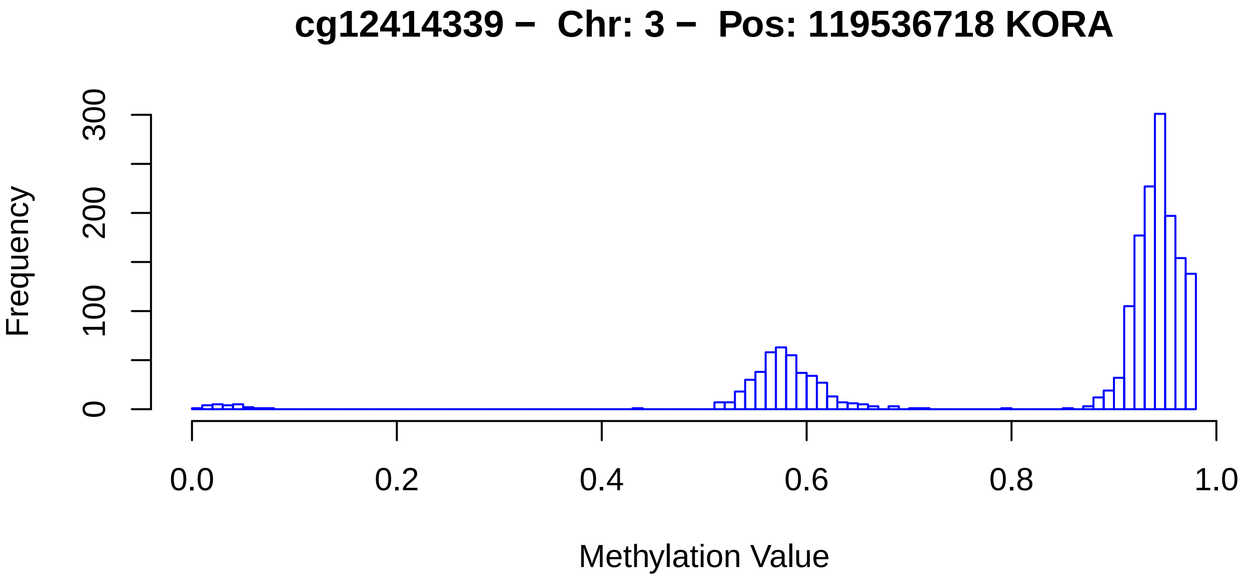
cg12414339 − Chr: 3 − Pos: 119536718 KORA
Frequency
0
100
200
300
0.0
0.2
0.4
0.6
0.8
1.0
Methylation Value

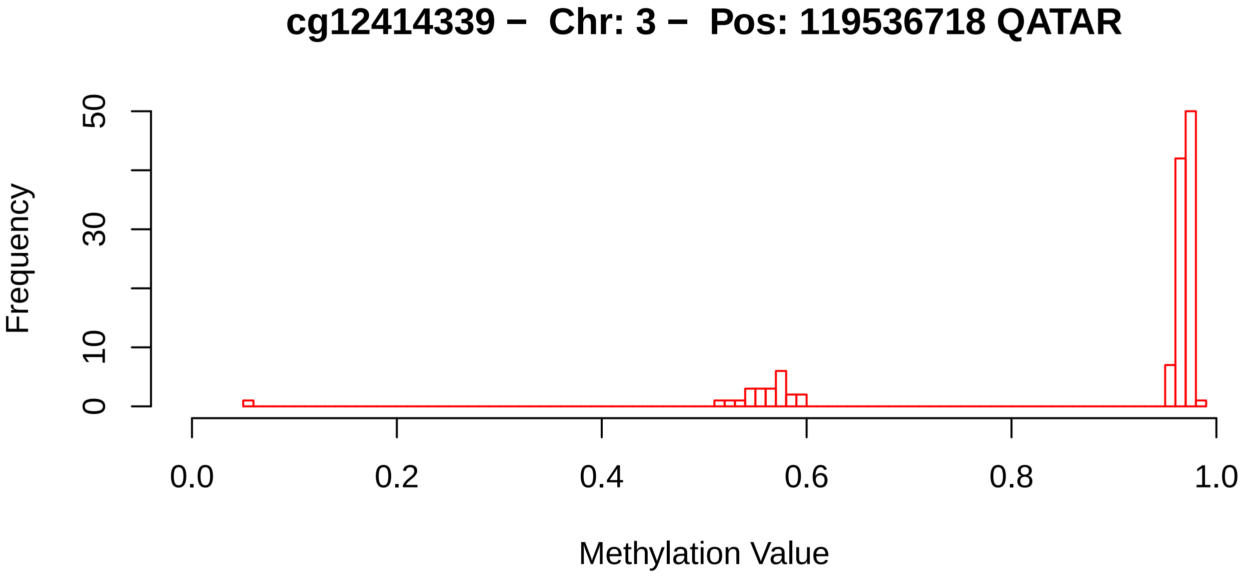
cg12414339 − Chr: 3 − Pos: 119536718 QATAR
Frequency
0
10
30
50
0.0
0.2
0.4
0.6
0.8
1.0
Methylation Value

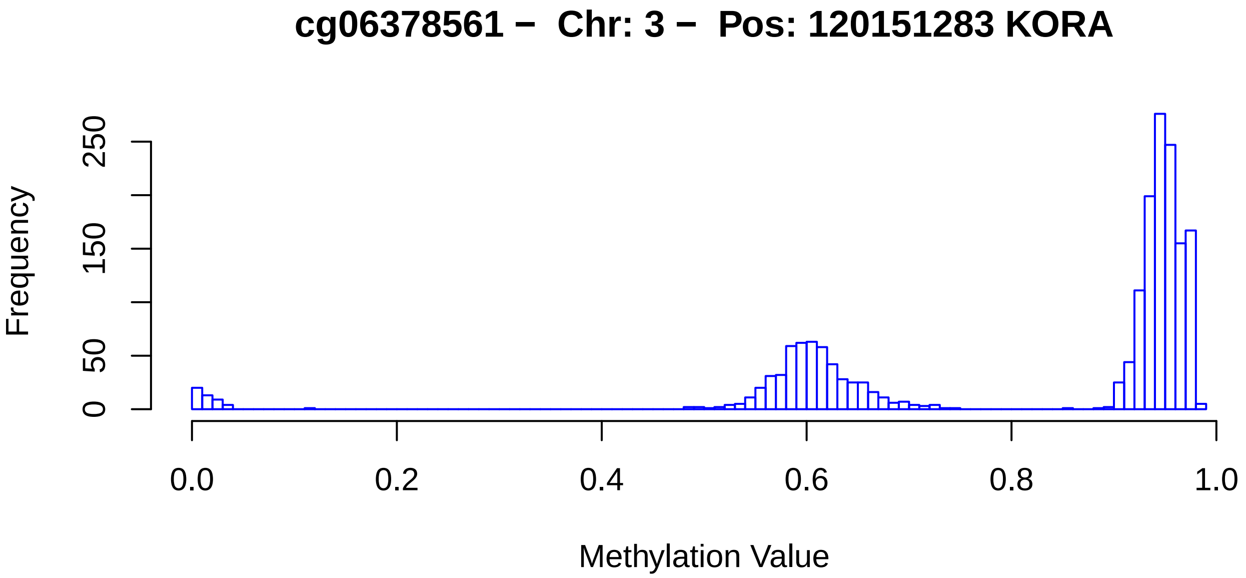

cg06378561 − Chr: 3 − Pos: 120151283 KORA
Frequency
0
50
150
250
0.0
0.2
0.4
0.6
0.8
1.0
Methylation Value

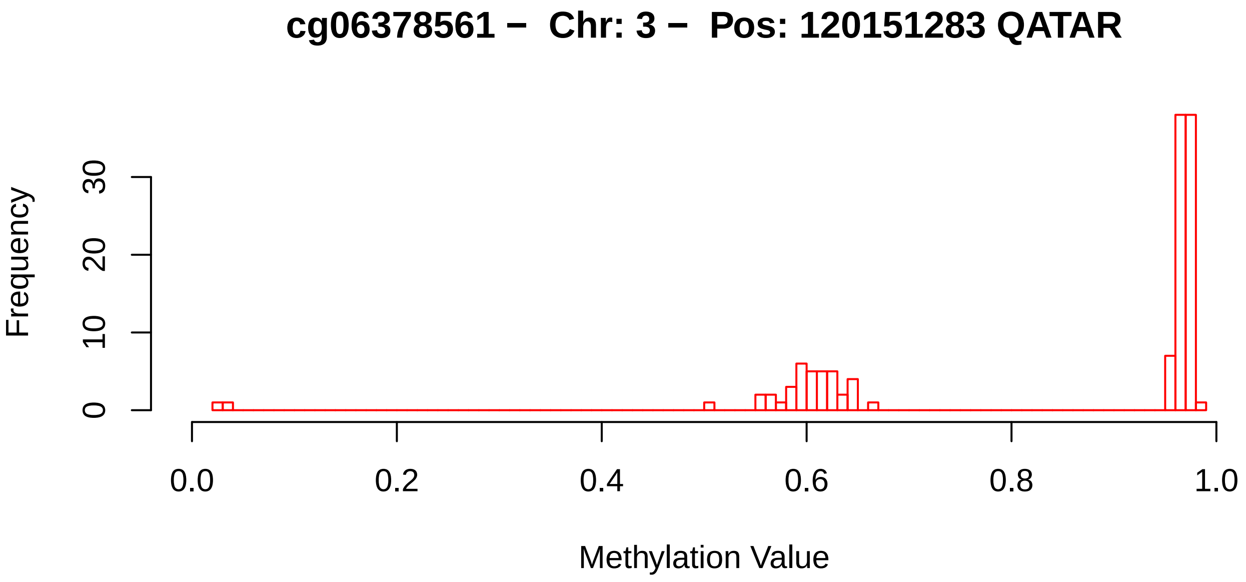

cg06378561 − Chr: 3 − Pos: 120151283 QATAR
Frequency
0
10
20
30
0.0
0.2
0.4
0.6
0.8
1.0
Methylation Value

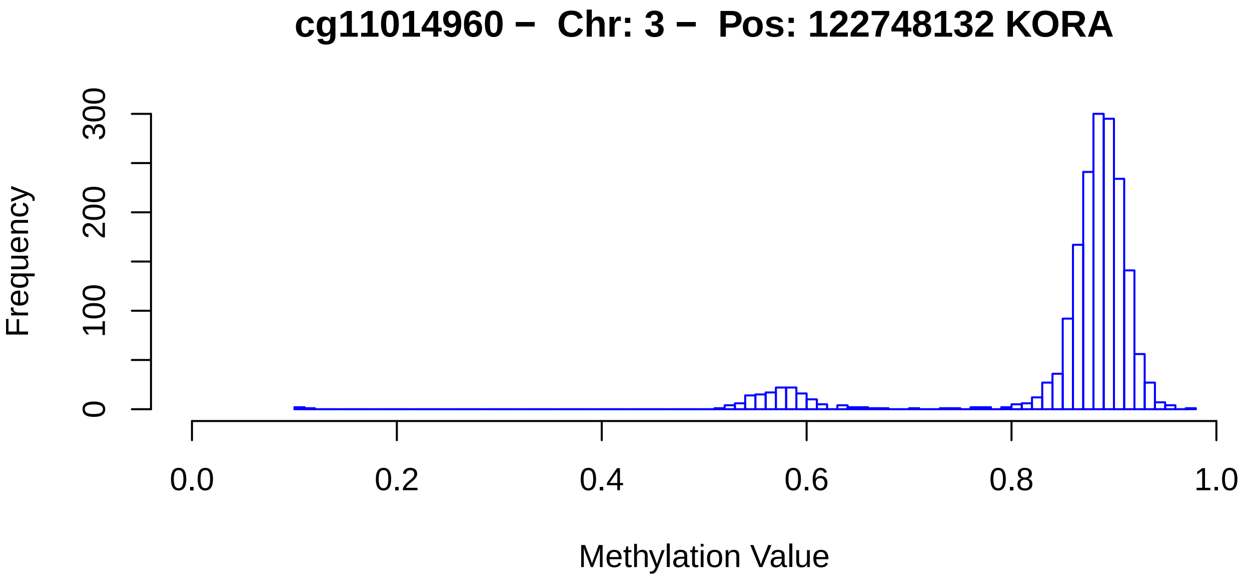
cg11014960 − Chr: 3 − Pos: 122748132 KORA
Frequency
0
100
200
300
0.0
0.2
0.4
0.6
0.8
1.0
Methylation Value

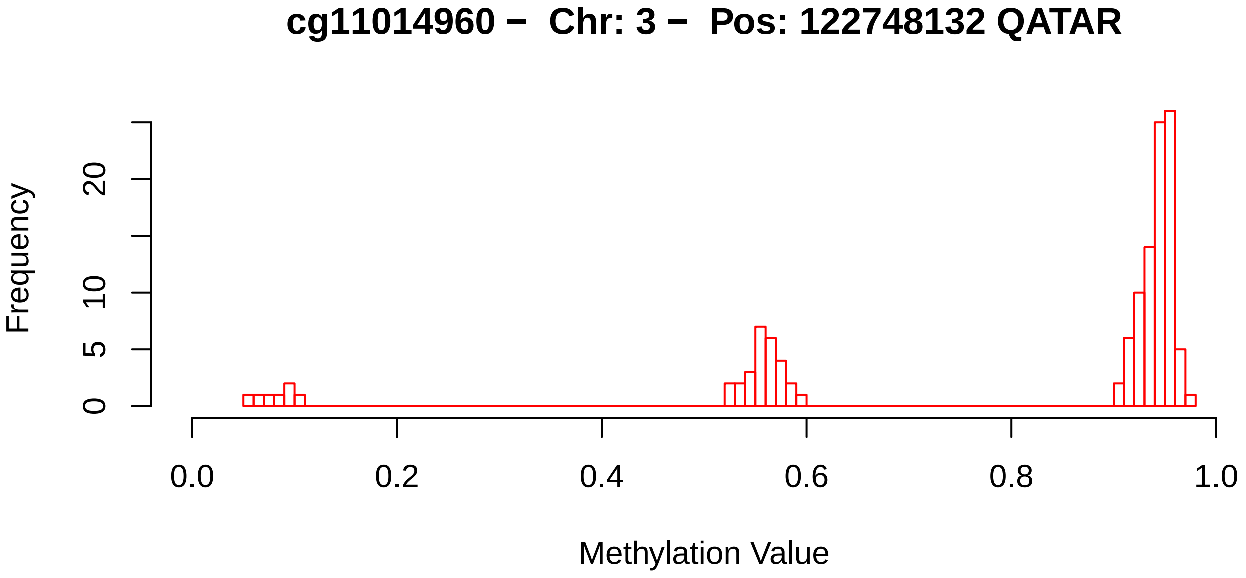
cg11014960 − Chr: 3 − Pos: 122748132 QATAR
Frequency
0
5
10
20
0.0
0.2
0.4
0.6
0.8
1.0
Methylation Value

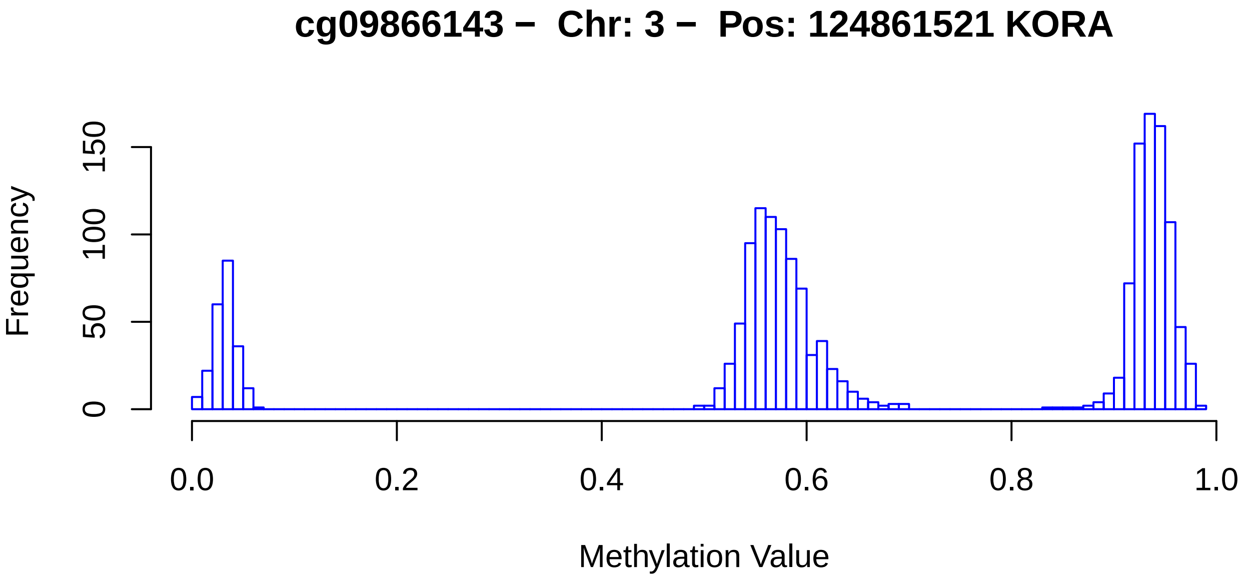

cg09866143 − Chr: 3 − Pos: 124861521 KORA
Frequency
0
50
100
150
0.0
0.2
0.4
0.6
0.8
1.0
Methylation Value

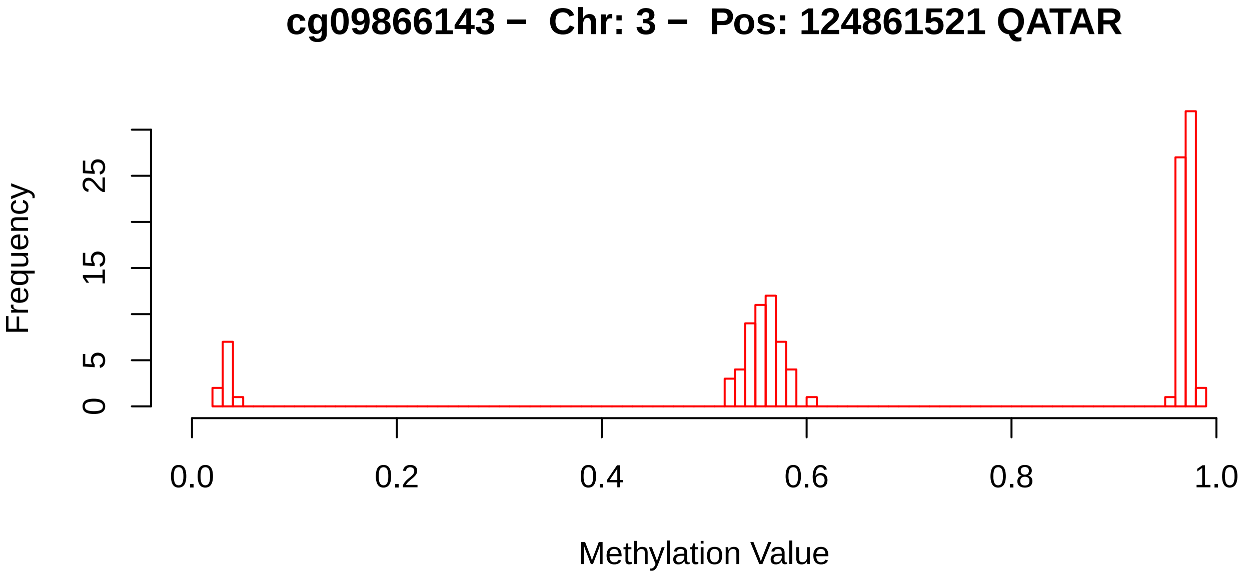

cg09866143 − Chr: 3 − Pos: 124861521 QATAR
Frequency
0
5
15
25
0.0
0.2
0.4
0.6
0.8
1.0
Methylation Value

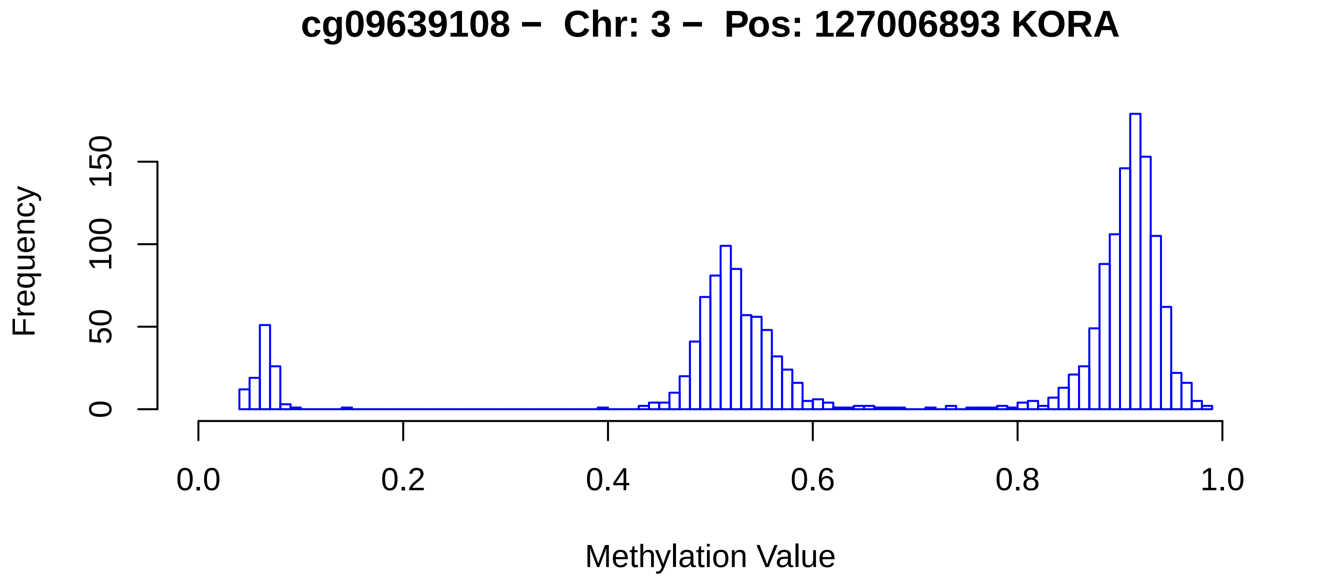
cg09639108 − Chr: 3 − Pos: 127006893 KORA
Frequency
0
50
100
150
0.0
0.2
0.4
0.6
0.8
1.0
Methylation Value

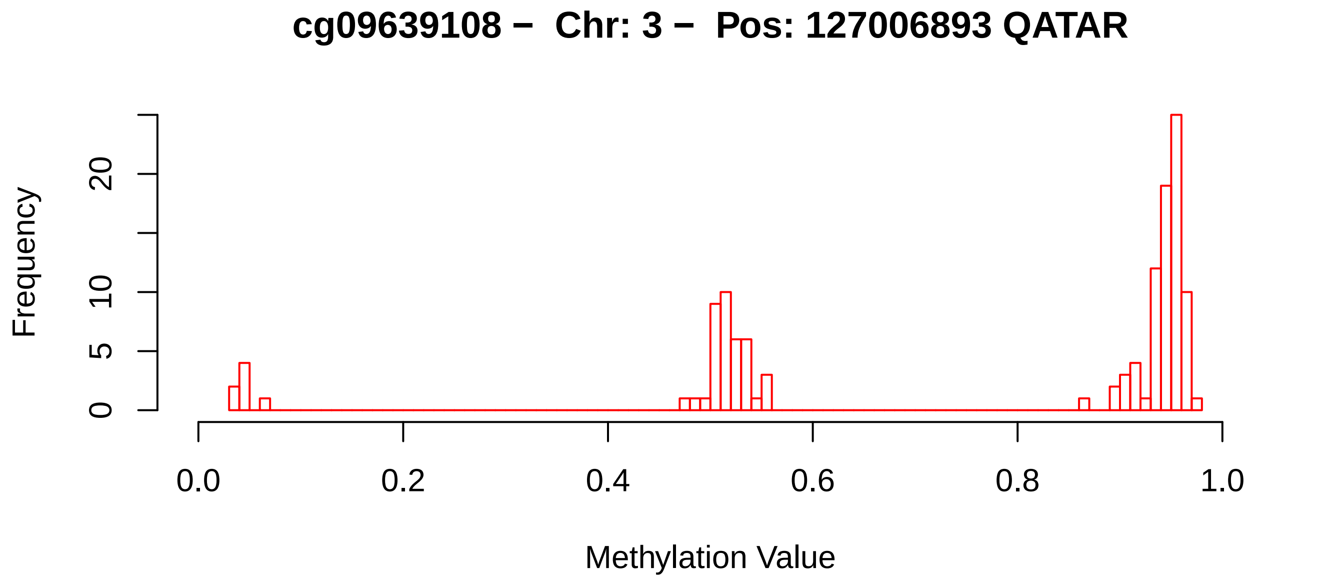
cg09639108 − Chr: 3 − Pos: 127006893 QATAR
Frequency
0
5
10
20
0.0
0.2
0.4
0.6
0.8
1.0
Methylation Value

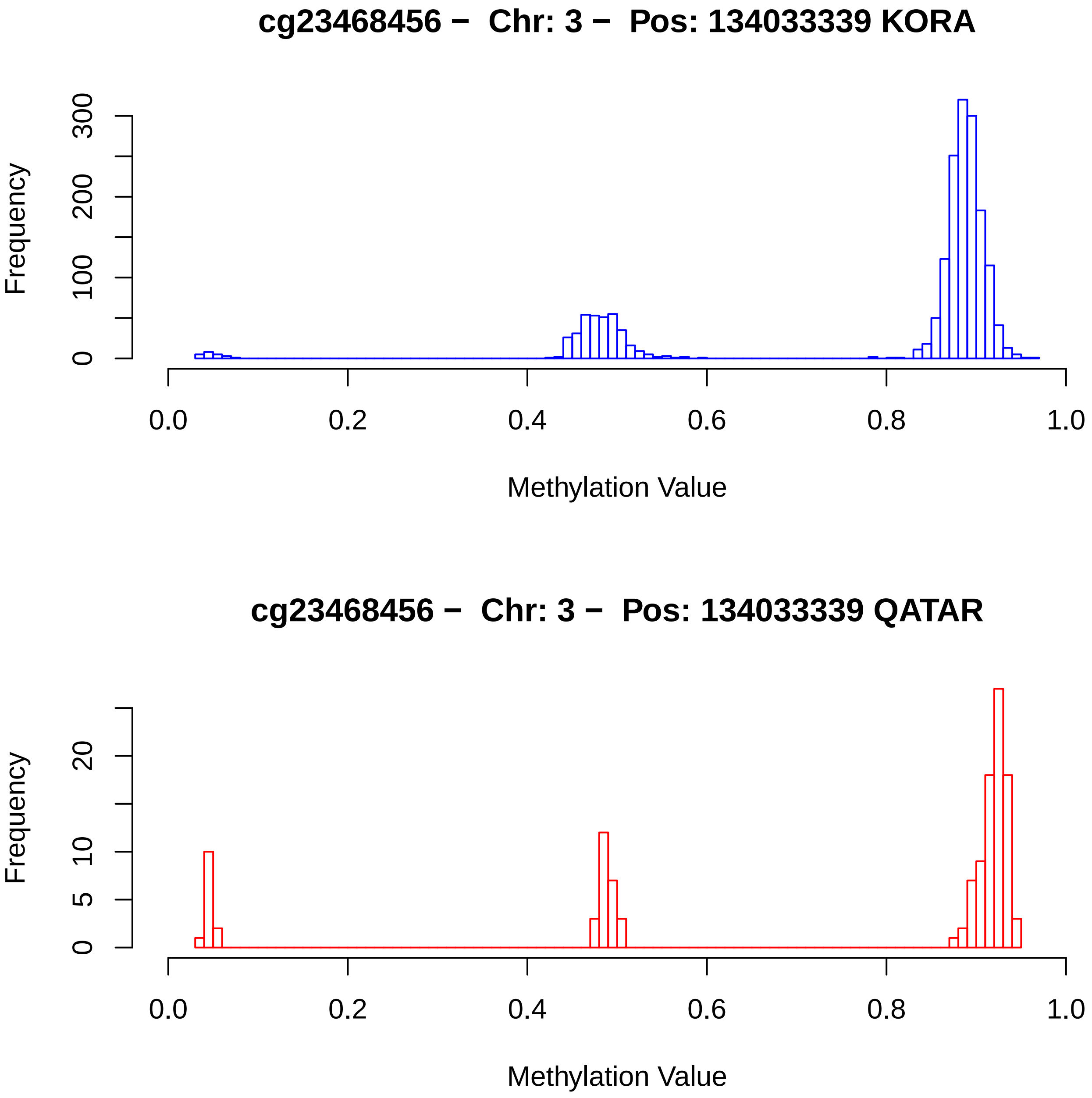
cg23468456 − Chr: 3 − Pos: 134033339 KORA
Frequency
0
100
200
300
0.0
0.2
0.4
0.6
0.8
1.0
Methylation Value
cg23468456 − Chr: 3 − Pos: 134033339 QATAR
Frequency
0
5
10
20
0.0
0.2
0.4
0.6
0.8
1.0
Methylation Value

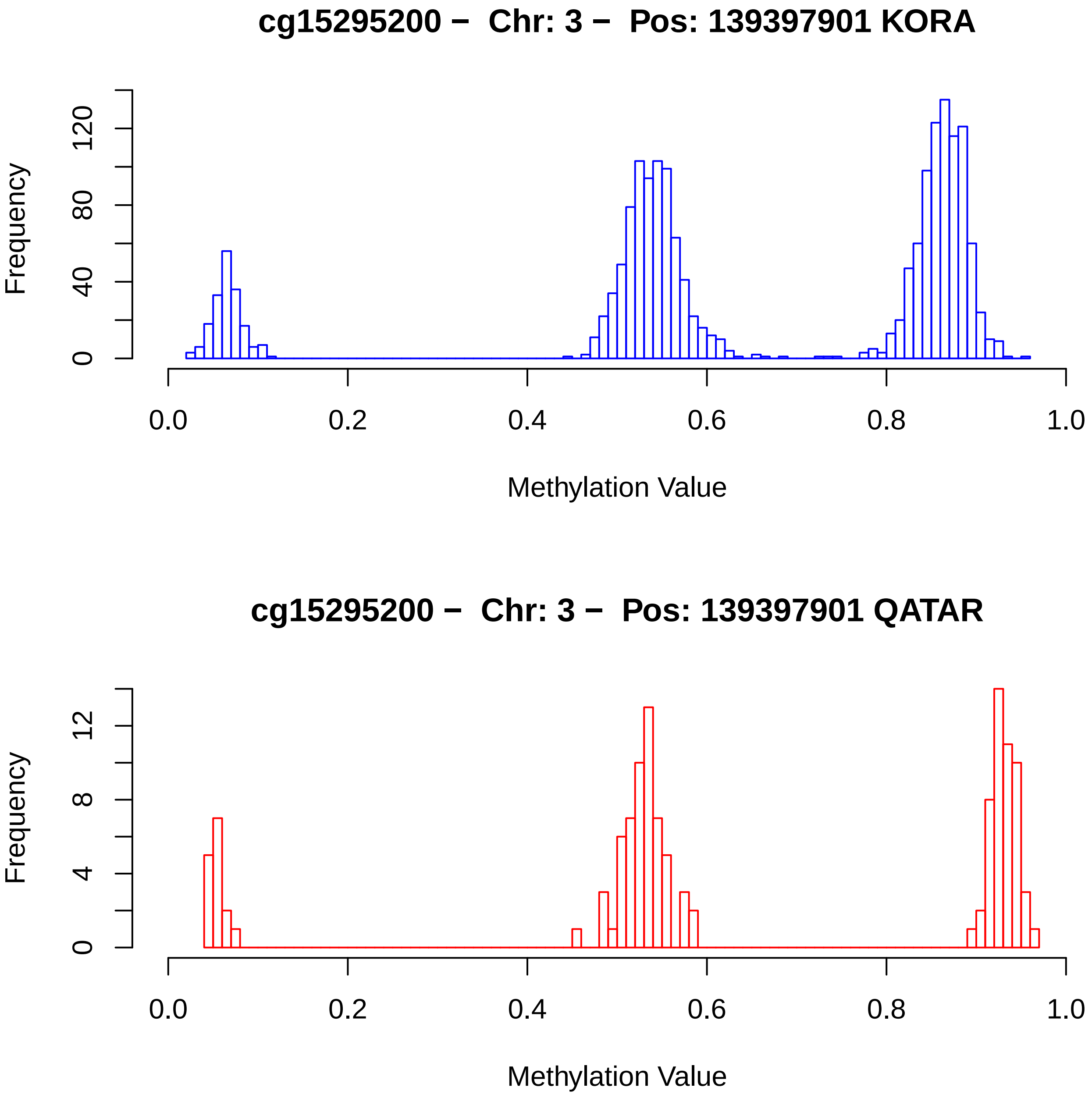
cg15295200 − Chr: 3 − Pos: 139397901 KORA
Frequency
0
40
80
120
0.0
0.2
0.4
0.6
0.8
1.0
Methylation Value
cg15295200 − Chr: 3 − Pos: 139397901 QATAR
Frequency
0
4
8
12
0.0
0.2
0.4
0.6
0.8
1.0
Methylation Value

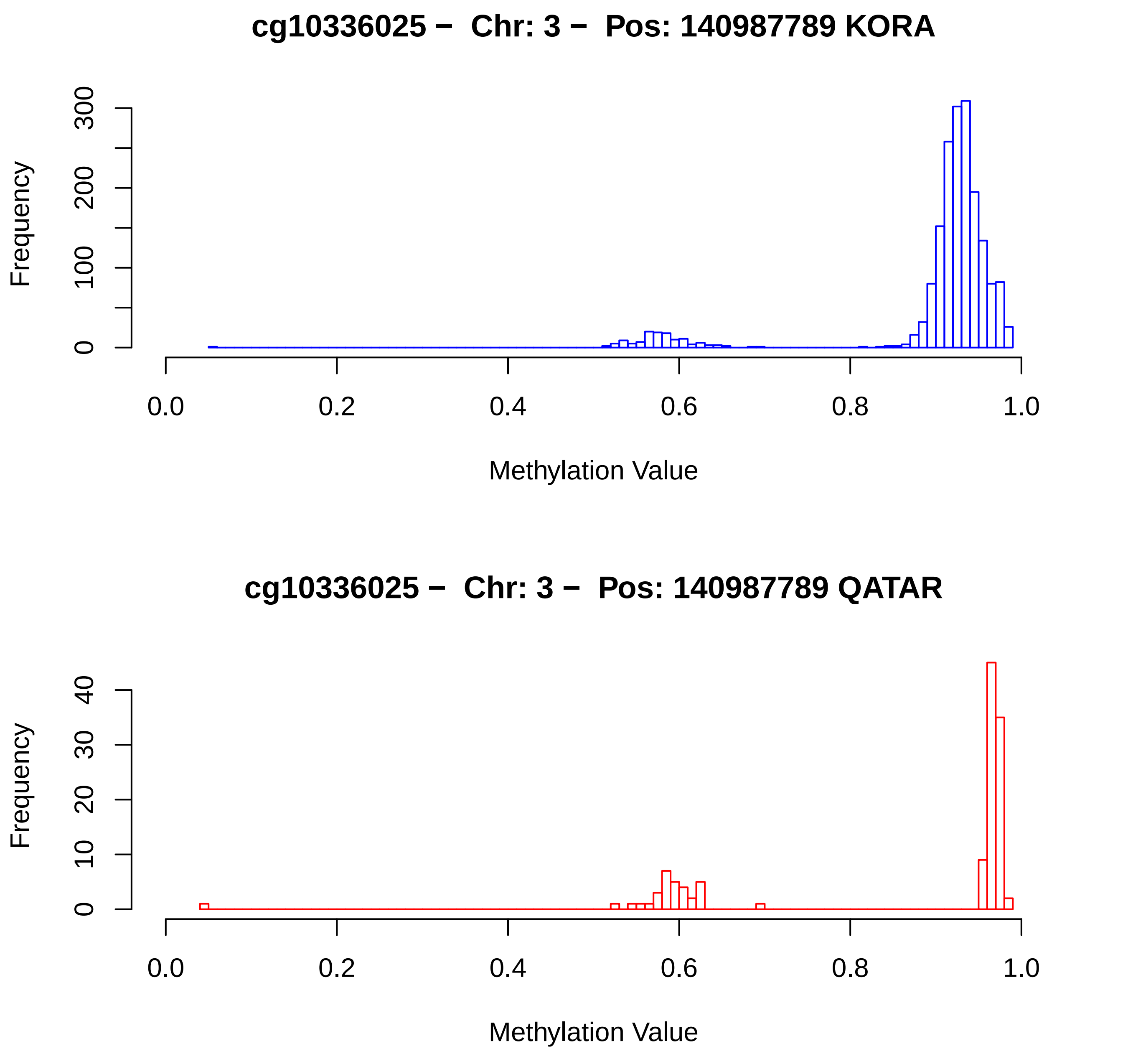

cg10336025 − Chr: 3 − Pos: 140987789 KORA
Frequency
0
100
200
300
0.0
0.2
0.4
0.6
0.8
1.0
Methylation Value
cg10336025 − Chr: 3 − Pos: 140987789 QATAR
Frequency
0
10
20
30
40
0.0
0.2
0.4
0.6
0.8
1.0
Methylation Value

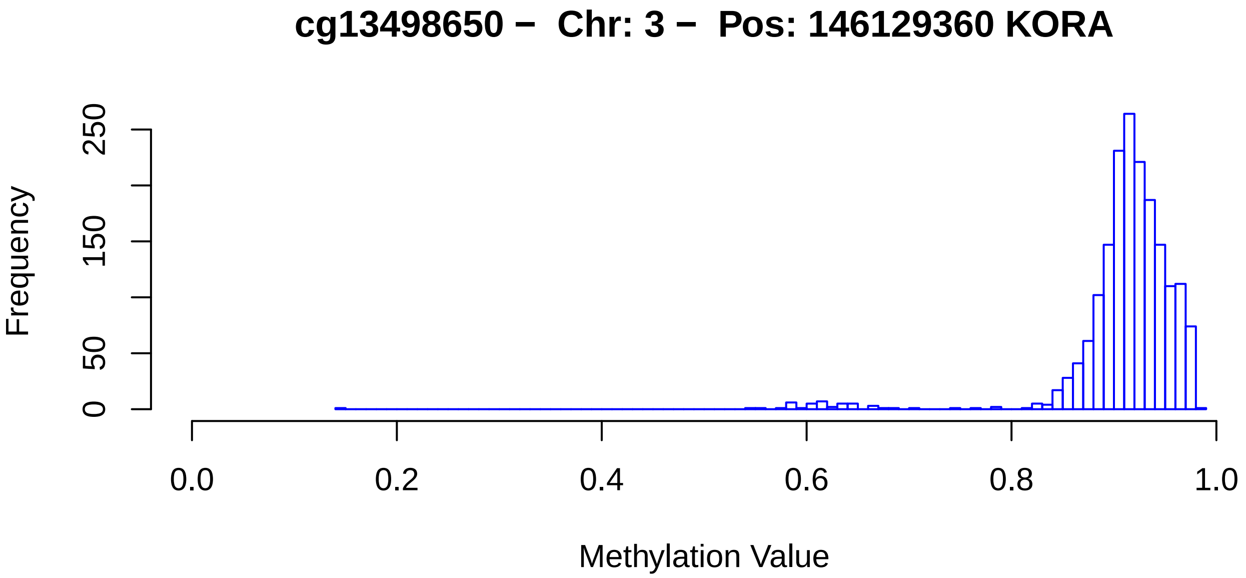
cg13498650 − Chr: 3 − Pos: 146129360 KORA
Frequency
0
50
150
250
0.0
0.2
0.4
0.6
0.8
1.0
Methylation Value

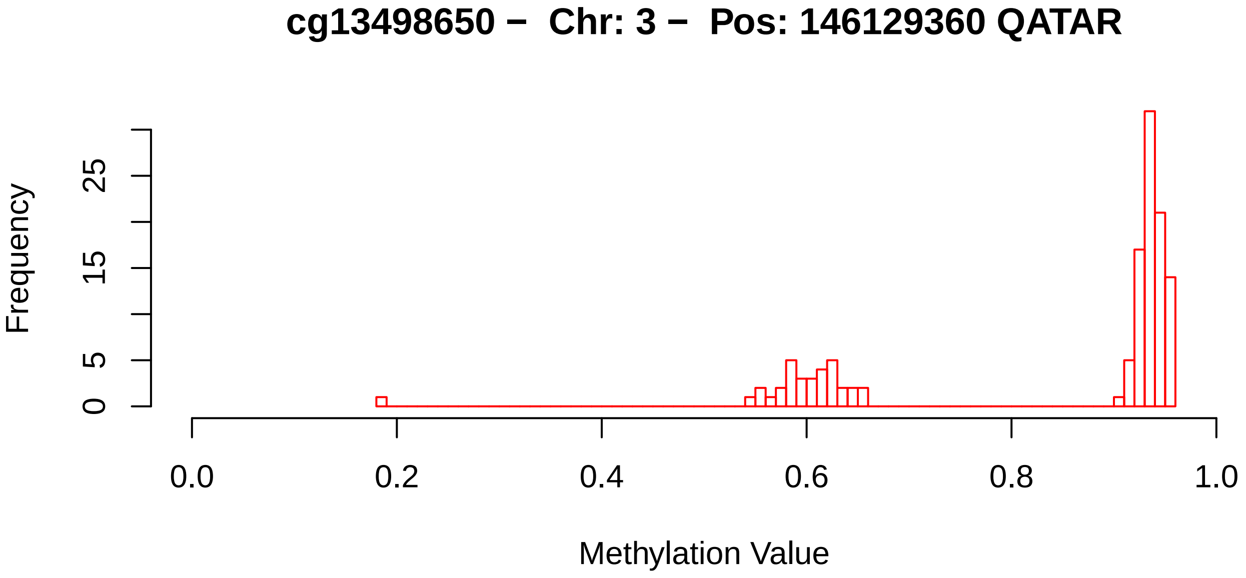
cg13498650 − Chr: 3 − Pos: 146129360 QATAR
Frequency
0
5
15
25
0.0
0.2
0.4
0.6
0.8
1.0
Methylation Value

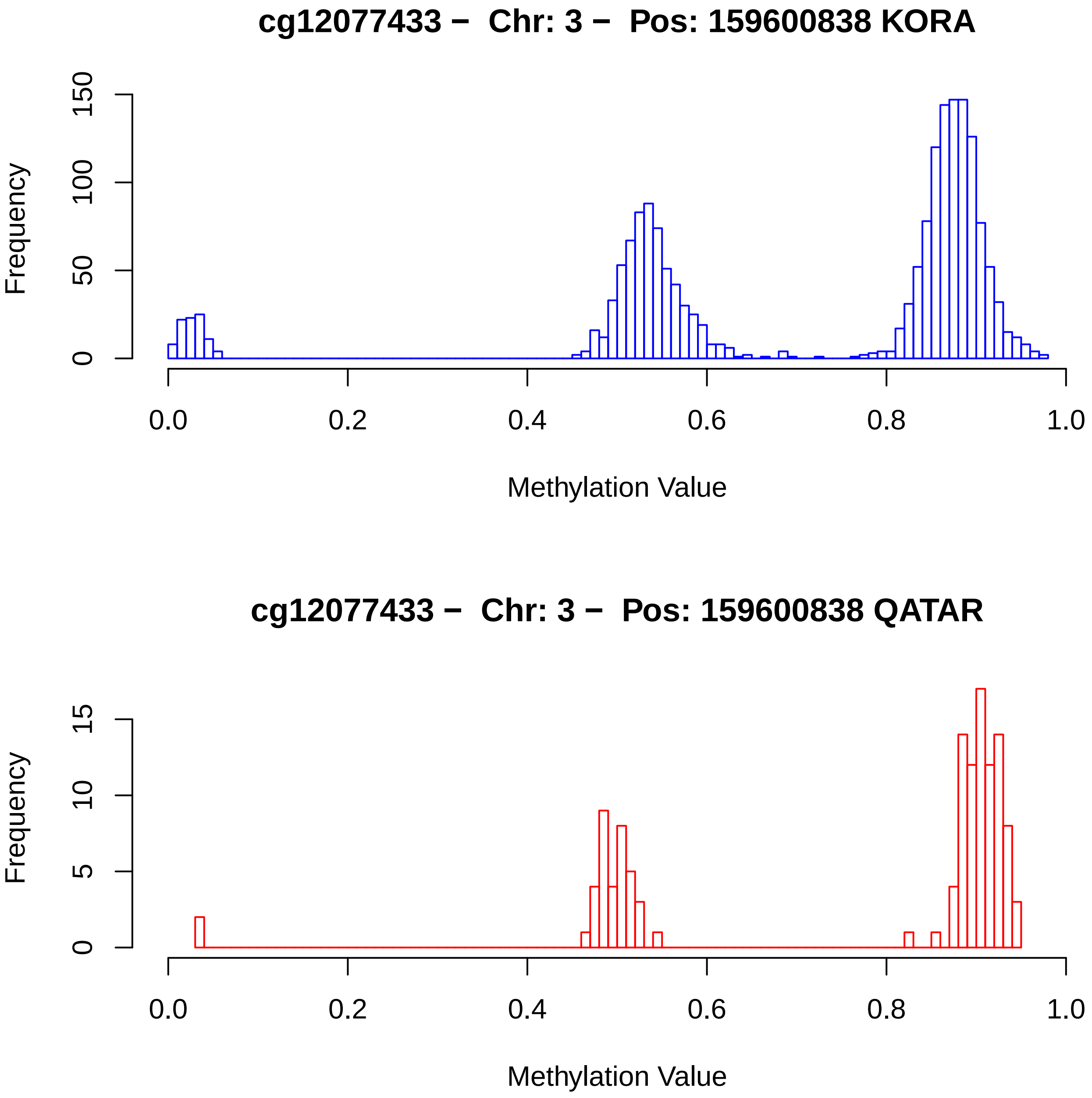
cg12077433 − Chr: 3 − Pos: 159600838 KORA
Frequency
0
50
100
150
0.0
0.2
0.4
0.6
0.8
1.0
Methylation Value
cg12077433 − Chr: 3 − Pos: 159600838 QATAR
Frequency
0
5
10
15
0.0
0.2
0.4
0.6
0.8
1.0
Methylation Value

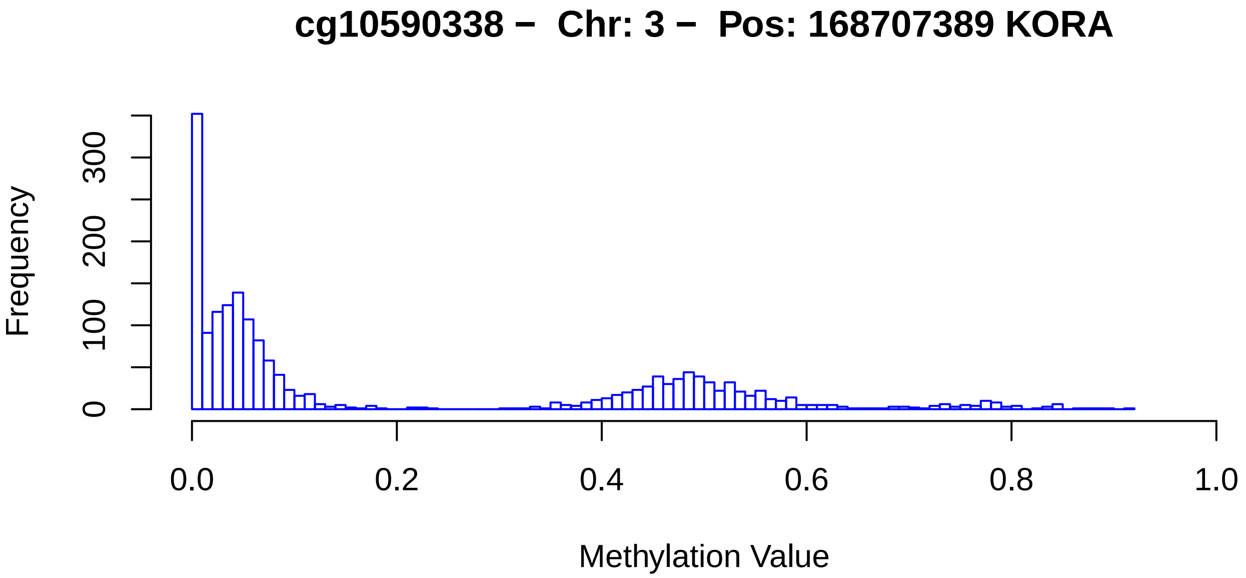
cg10590338 − Chr: 3 − Pos: 168707389 KORA
Frequency
0
100
200
300
0.0
0.2
0.4
0.6
0.8
1.0
Methylation Value

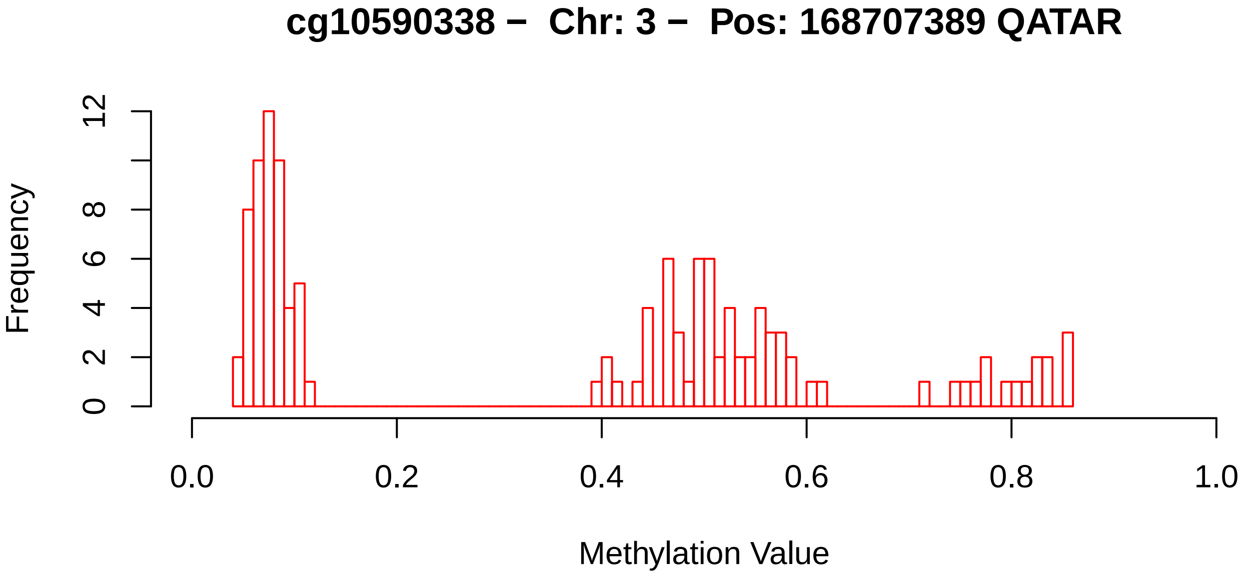
cg10590338 − Chr: 3 − Pos: 168707389 QATAR
Frequency
0
2
4
6
8
12
0.0
0.2
0.4
0.6
0.8
1.0
Methylation Value

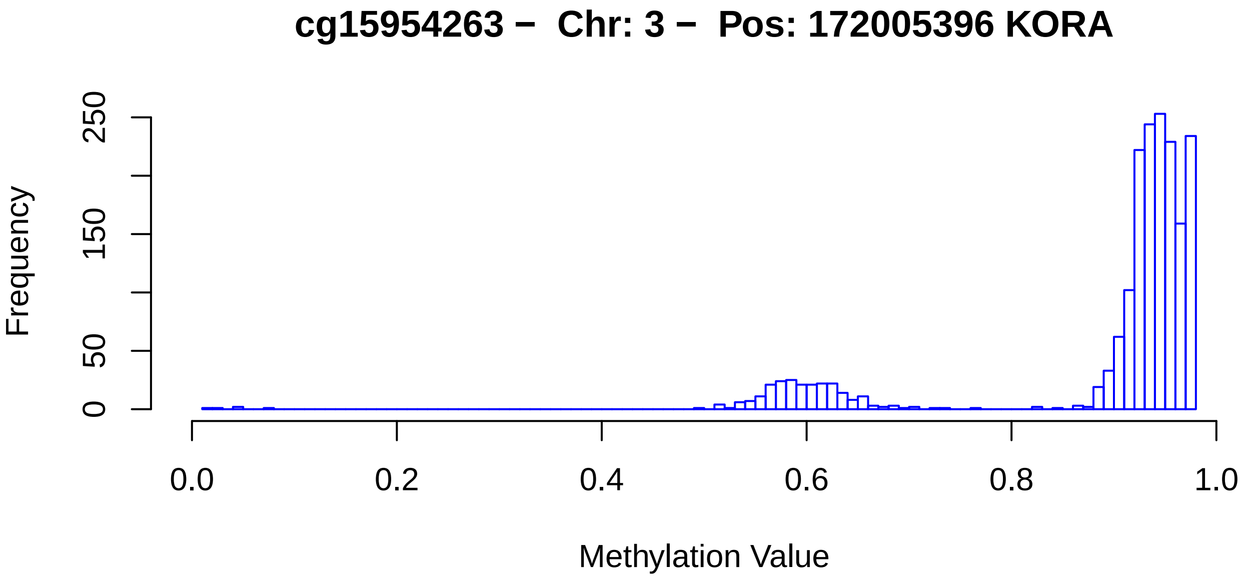
cg15954263 − Chr: 3 − Pos: 172005396 KORA
Frequency
0
50
150
250
0.0
0.2
0.4
0.6
0.8
1.0
Methylation Value

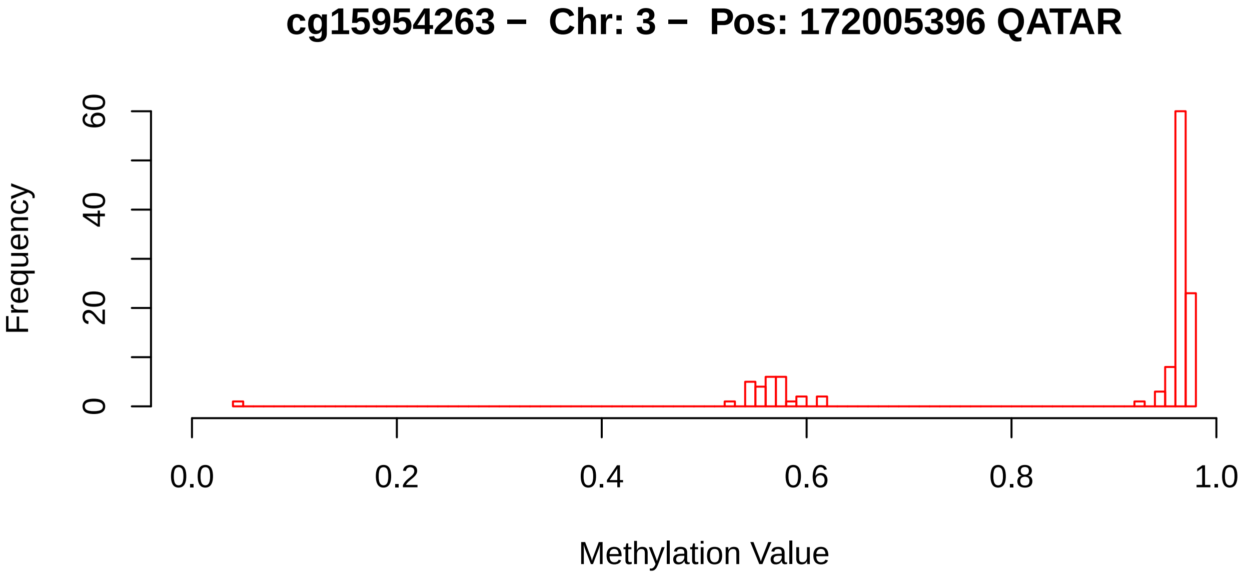
cg15954263 − Chr: 3 − Pos: 172005396 QATAR
Frequency
0
20
40
60
0.0
0.2
0.4
0.6
0.8
1.0
Methylation Value

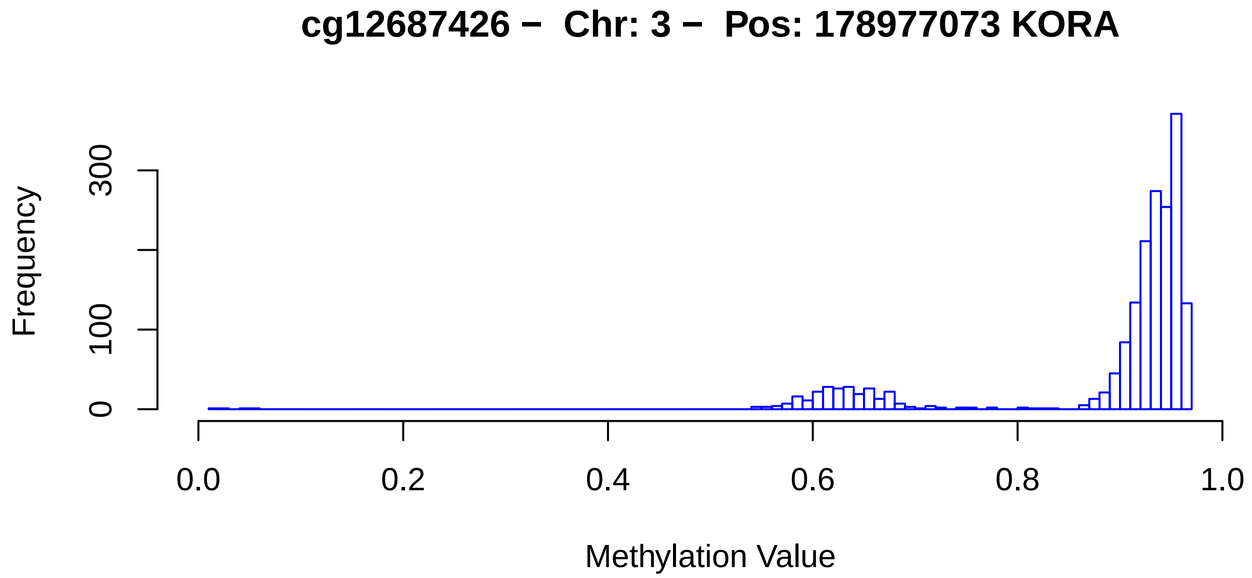
cg12687426 − Chr: 3 − Pos: 178977073 KORA
Frequency
0
100
300
0.0
0.2
0.4
0.6
0.8
1.0
Methylation Value

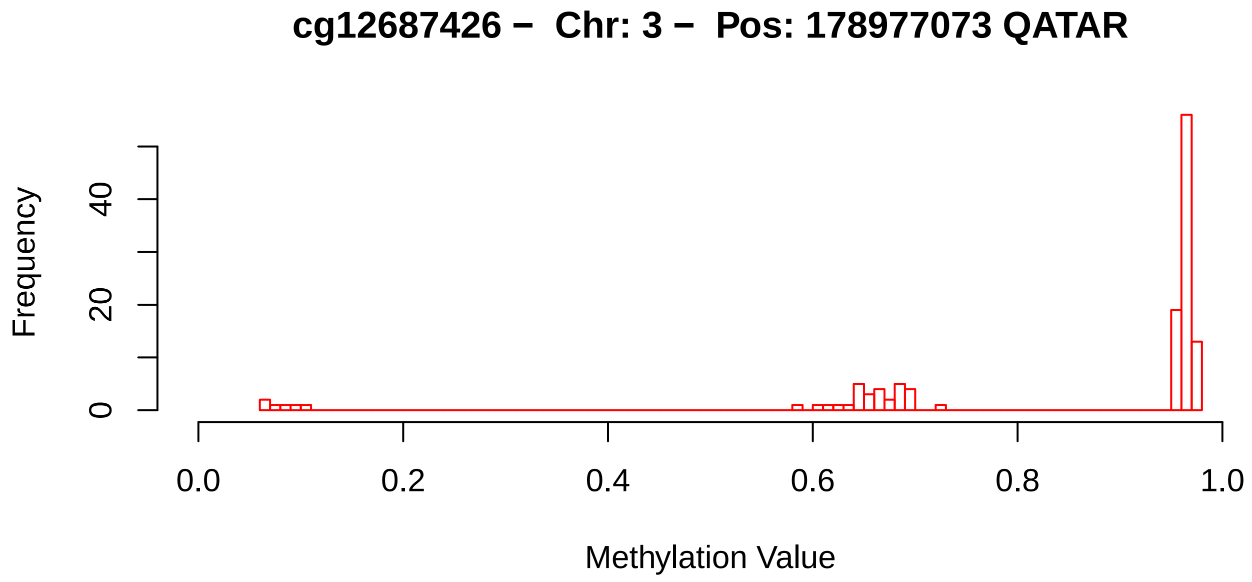
cg12687426 − Chr: 3 − Pos: 178977073 QATAR
Frequency
0
20
40
0.0
0.2
0.4
0.6
0.8
1.0
Methylation Value

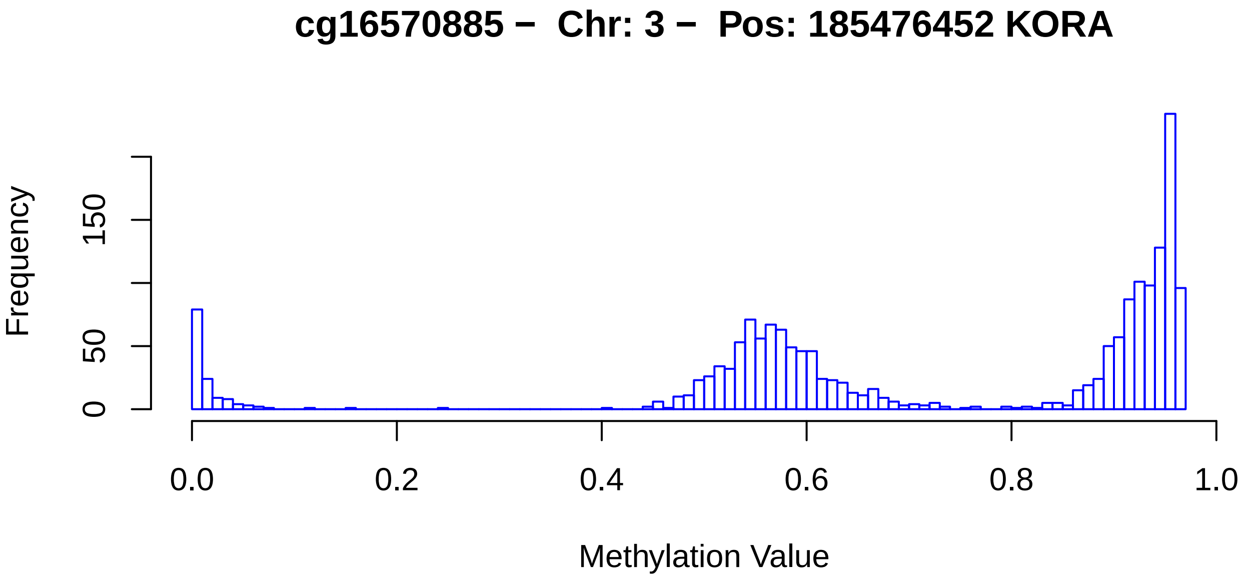

cg16570885 − Chr: 3 − Pos: 185476452 KORA
Frequency
0
50
150
0.0
0.2
0.4
0.6
0.8
1.0
Methylation Value

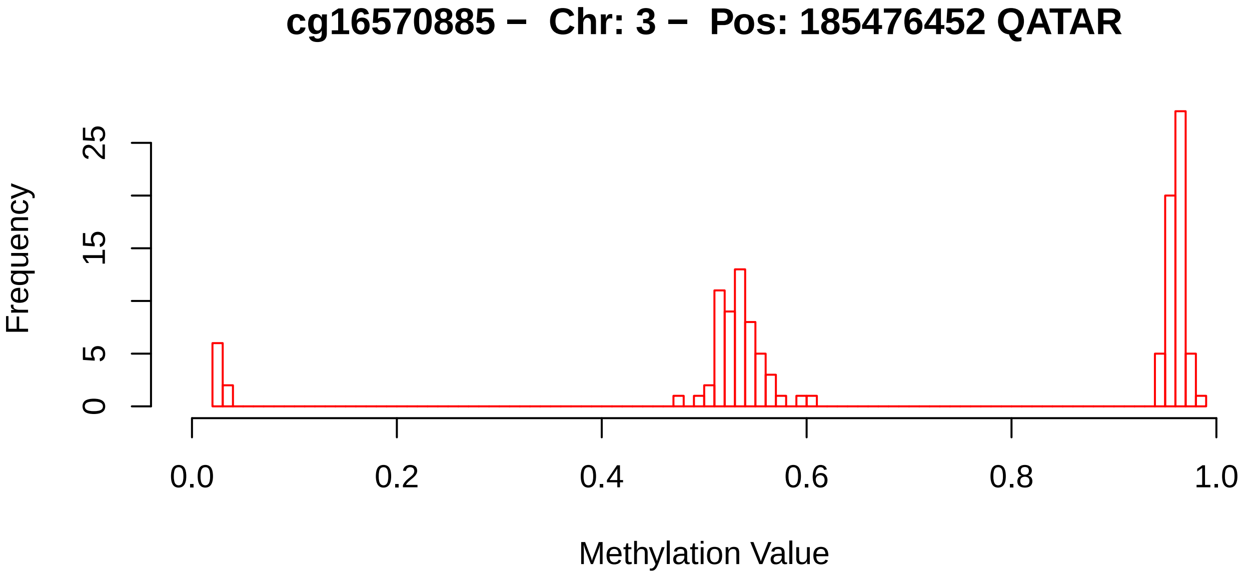

cg16570885 − Chr: 3 − Pos: 185476452 QATAR
Frequency
0
5
15
25
0.0
0.2
0.4
0.6
0.8
1.0
Methylation Value

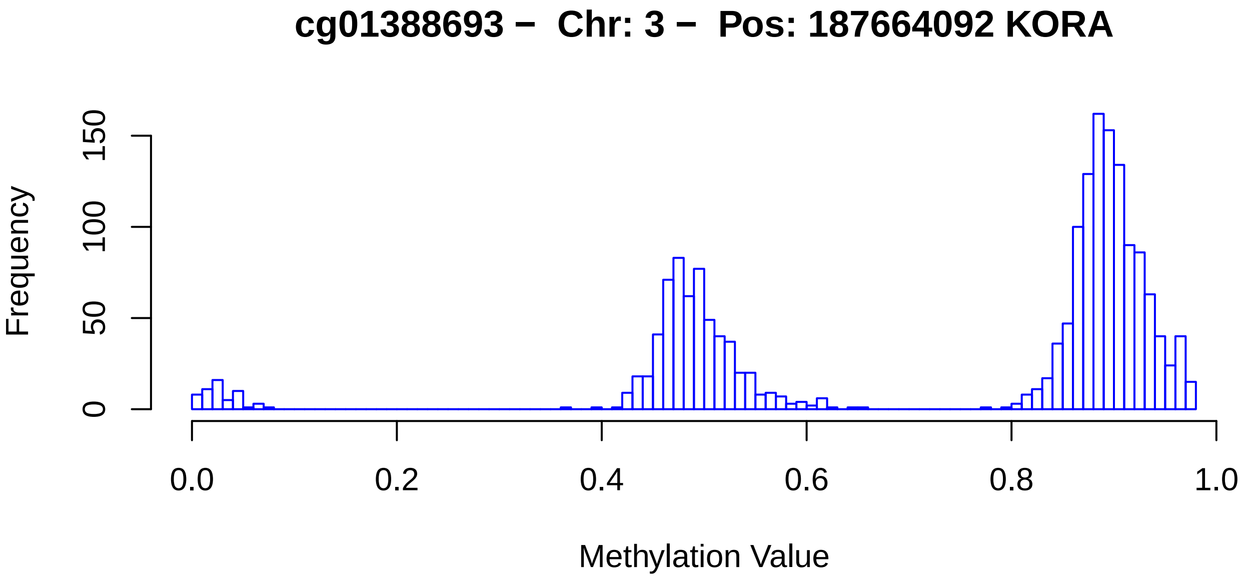
cg01388693 − Chr: 3 − Pos: 187664092 KORA
Frequency
0
50
100
150
0.0
0.2
0.4
0.6
0.8
1.0
Methylation Value

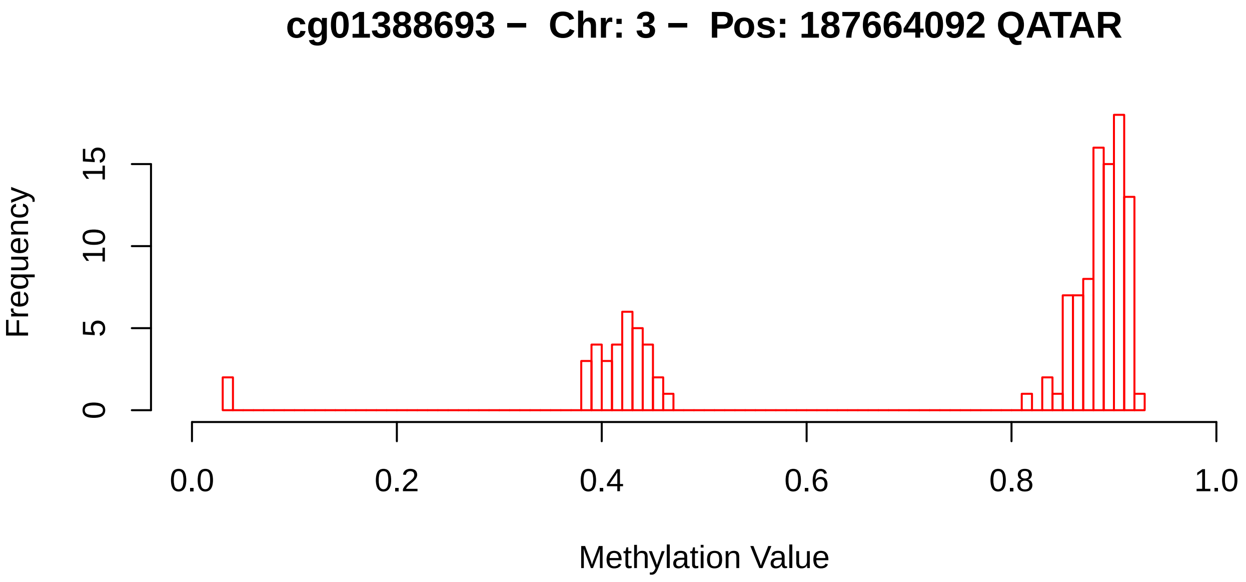
cg01388693 − Chr: 3 − Pos: 187664092 QATAR
Frequency
0
5
10
15
0.0
0.2
0.4
0.6
0.8
1.0
Methylation Value

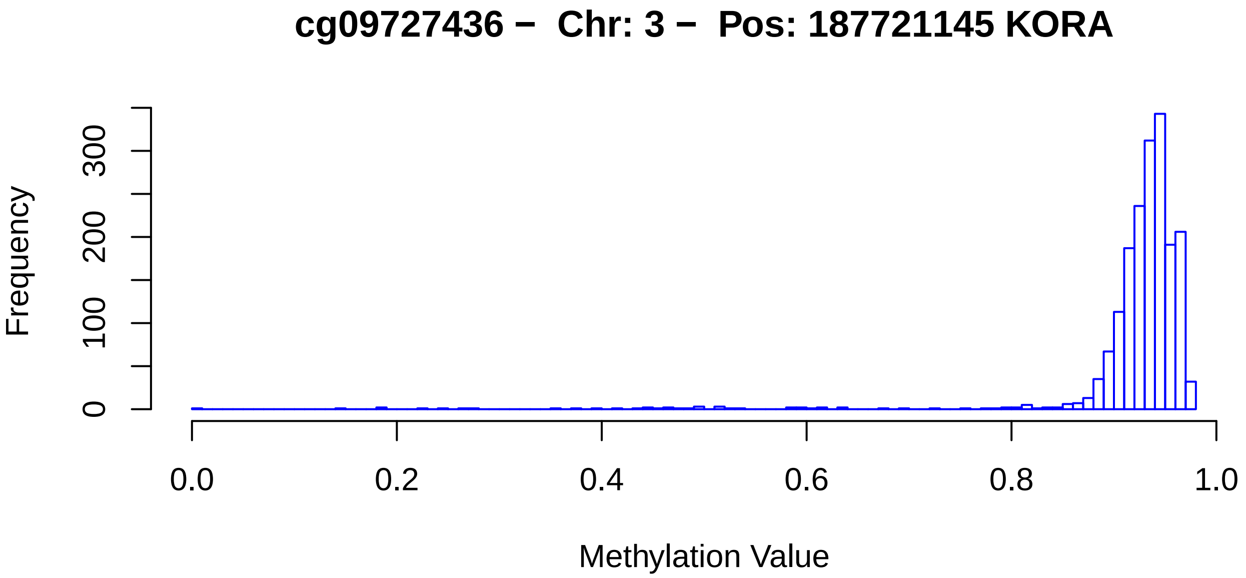

cg09727436 − Chr: 3 − Pos: 187721145 KORA
Frequency
0
100
200
300
0.0
0.2
0.4
0.6
0.8
1.0
Methylation Value

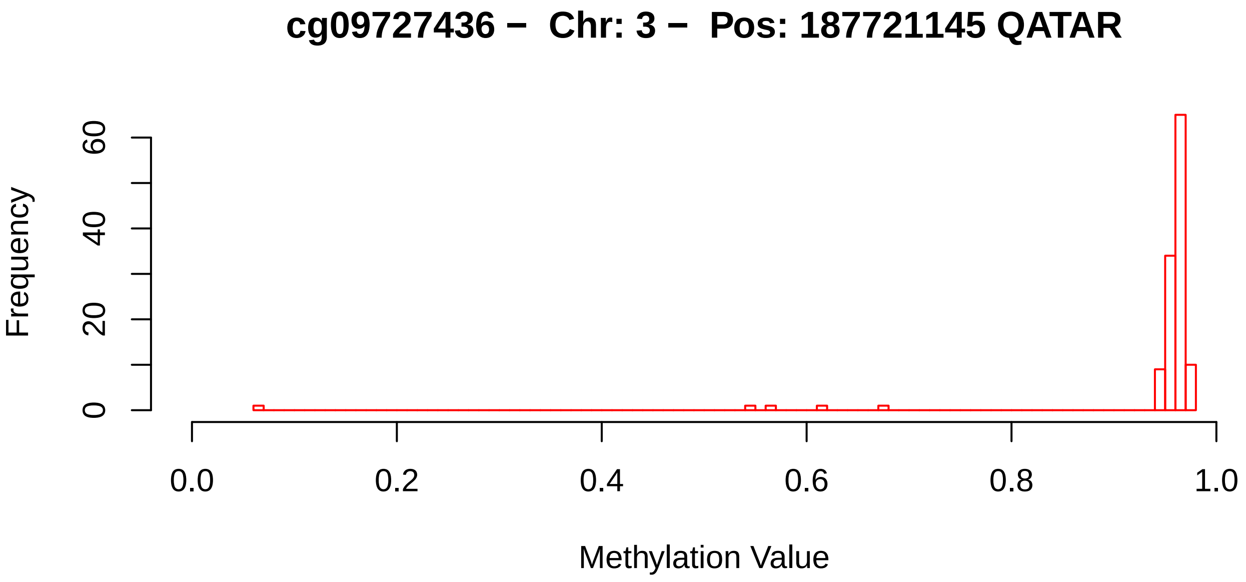

cg09727436 − Chr: 3 − Pos: 187721145 QATAR
Frequency
0
20
40
60
0.0
0.2
0.4
0.6
0.8
1.0
Methylation Value

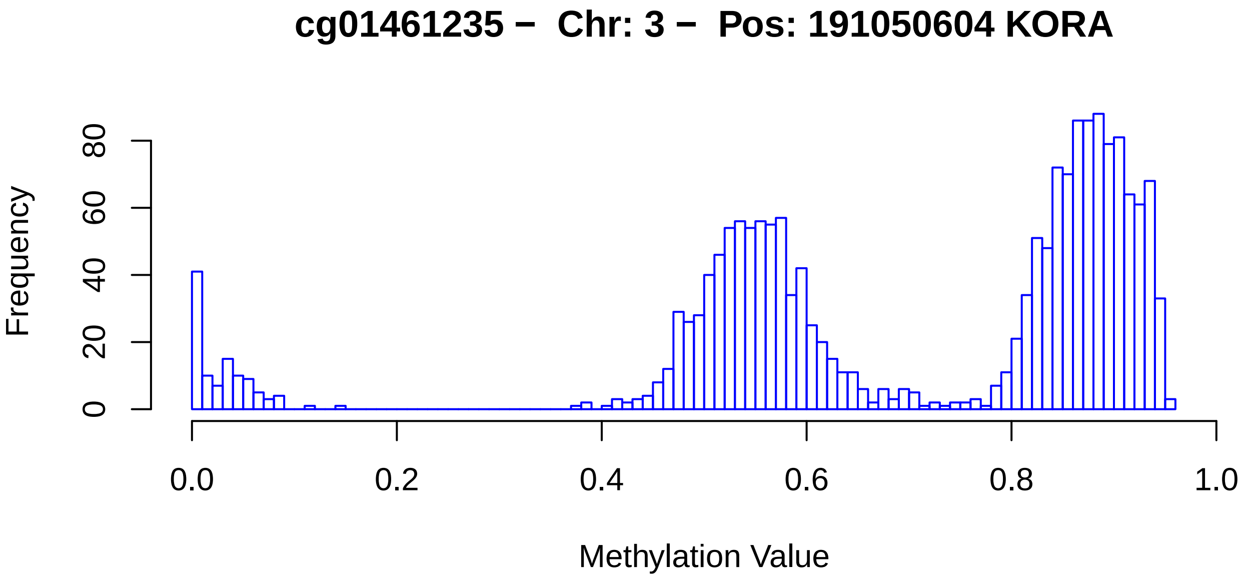

cg01461235 − Chr: 3 − Pos: 191050604 KORA
Frequency
Methylation Value
0
20
40
60
80
0.0
0.2
0.4
0.6
0.8
1.0

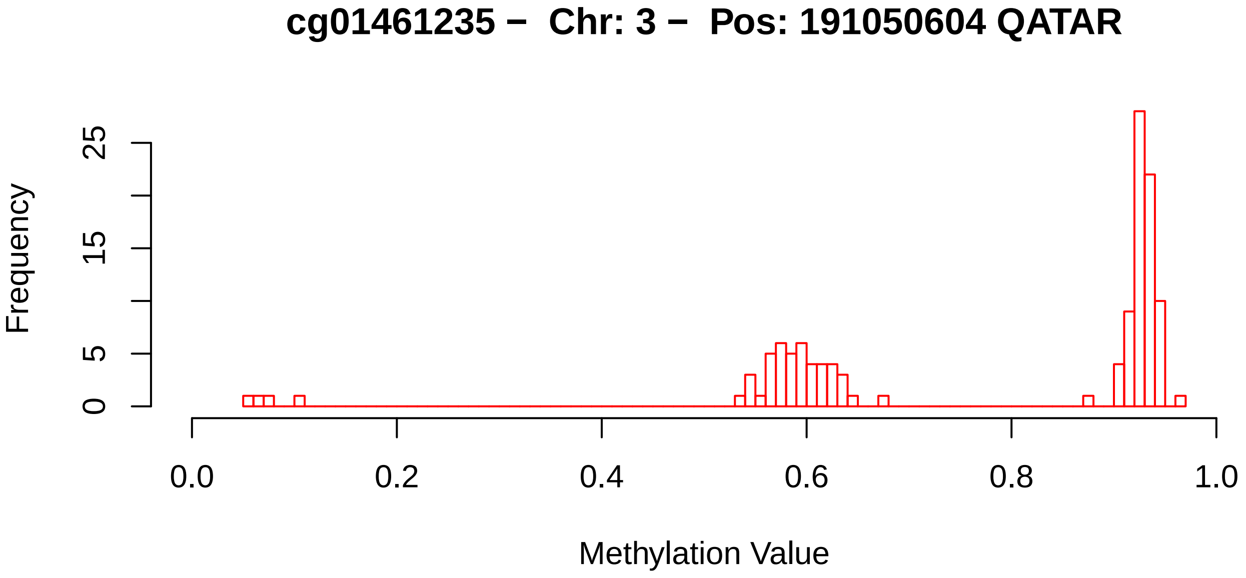

cg01461235 − Chr: 3 − Pos: 191050604 QATAR
Frequency
Methylation Value
0
5
15
25
0.0
0.2
0.4
0.6
0.8
1.0

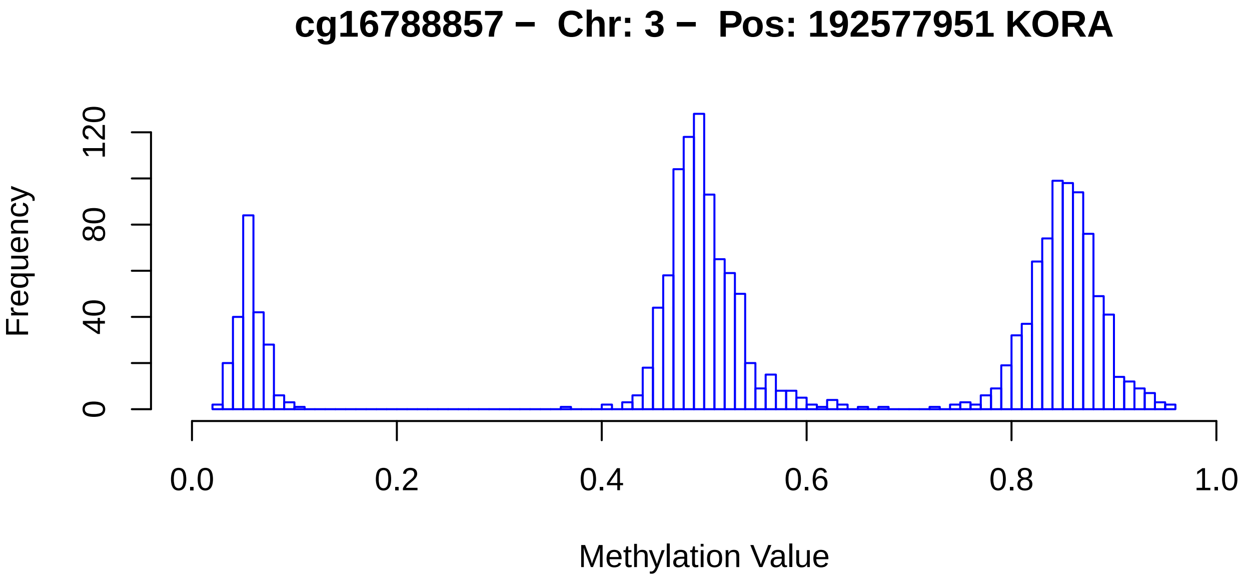
cg16788857 − Chr: 3 − Pos: 192577951 KORA
Frequency
0
40
80
120
0.0
0.2
0.4
0.6
0.8
1.0
Methylation Value

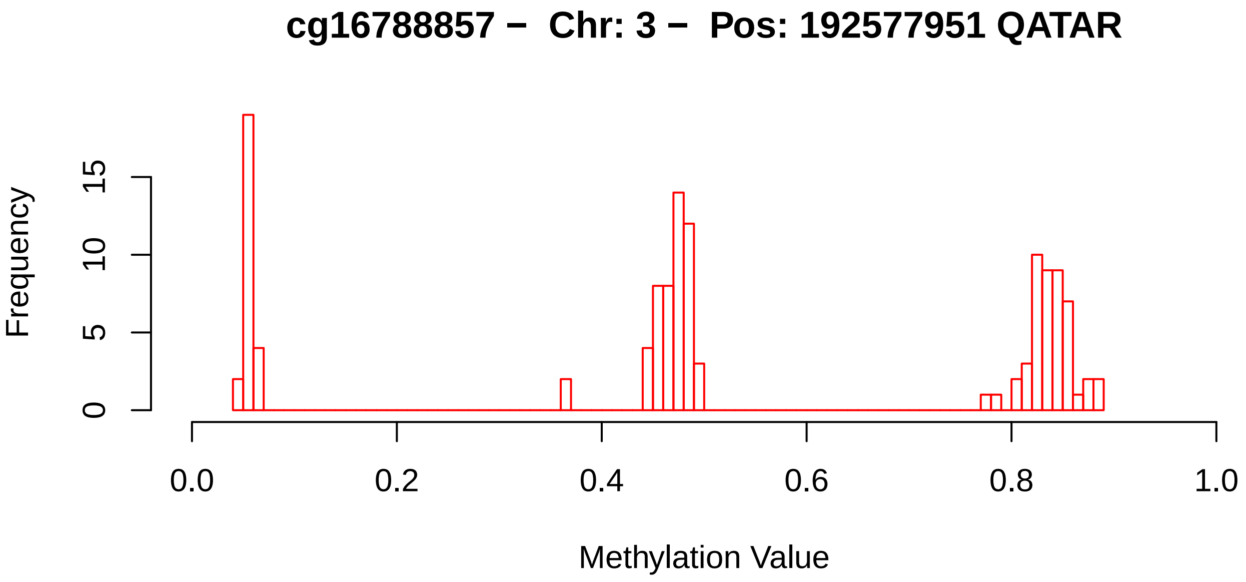
cg16788857 − Chr: 3 − Pos: 192577951 QATAR
Frequency
0
5
10
15
0.0
0.2
0.4
0.6
0.8
1.0
Methylation Value

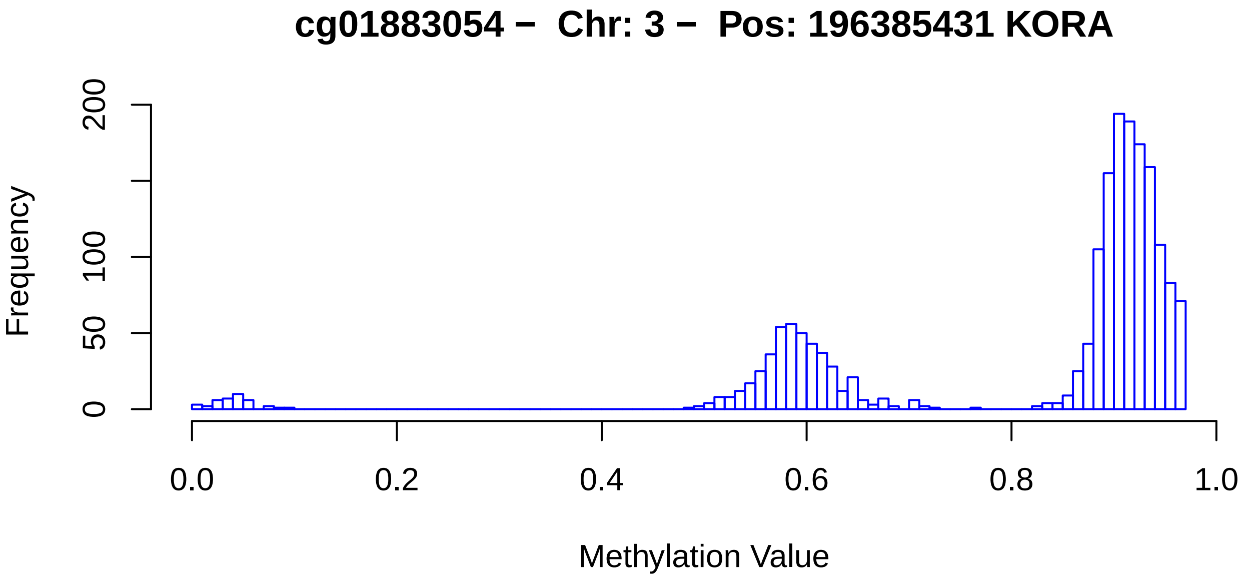
cg01883054 − Chr: 3 − Pos: 196385431 KORA
Frequency
0
50
100
200
0.0
0.2
0.4
0.6
0.8
1.0
Methylation Value

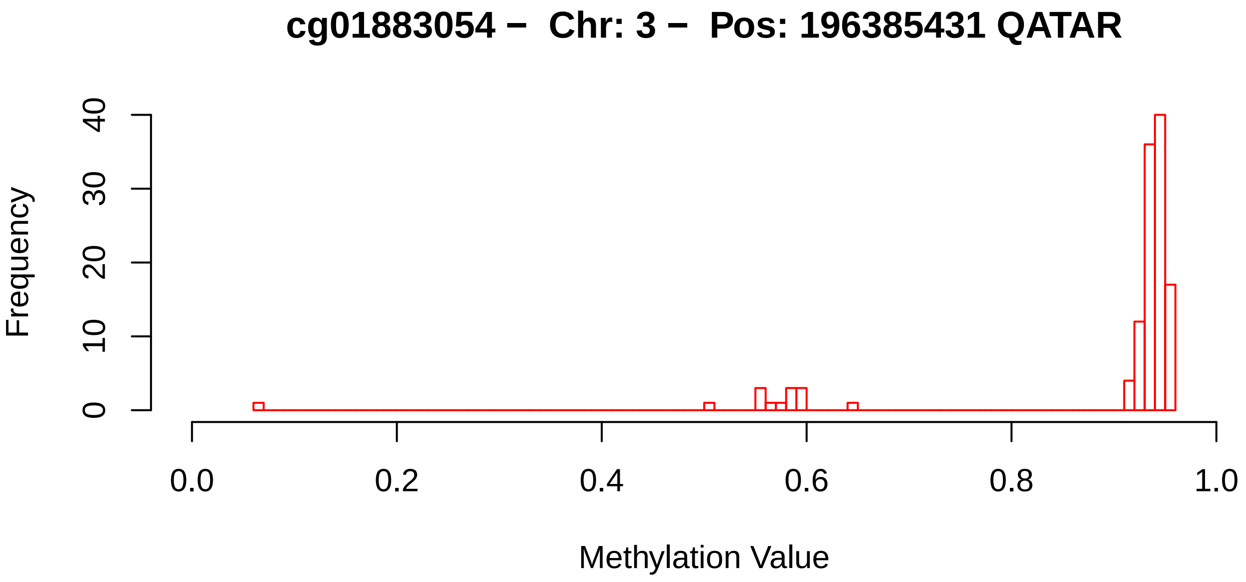
cg01883054 − Chr: 3 − Pos: 196385431 QATAR
Frequency
0
10
20
30
40
0.0
0.2
0.4
0.6
0.8
1.0
Methylation Value

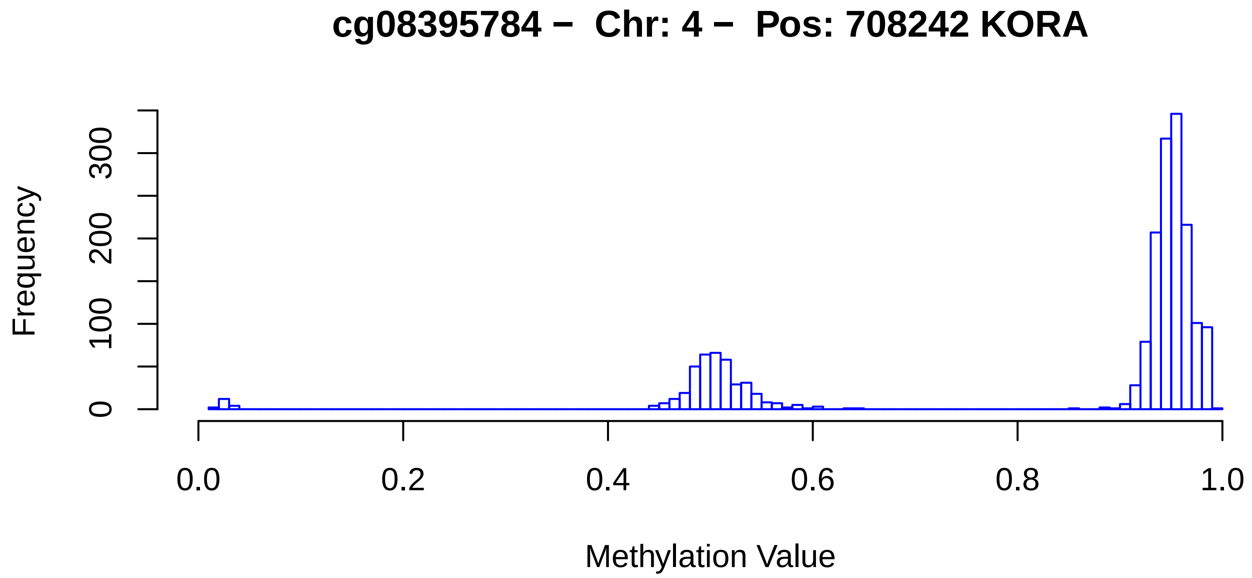
cg08395784 − Chr: 4 − Pos: 708242 KORA
Frequency
0
100
200
300
0.0
0.2
0.4
0.6
0.8
1.0
Methylation Value

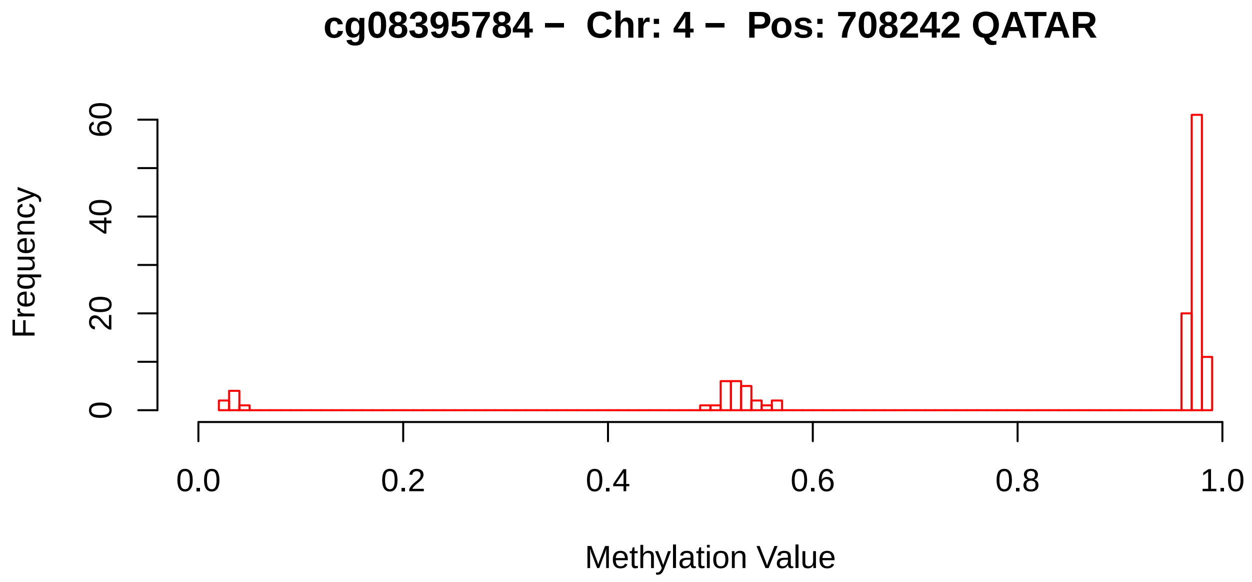
cg08395784 − Chr: 4 − Pos: 708242 QATAR
Frequency
0
20
40
60
0.0
0.2
0.4
0.6
0.8
1.0
Methylation Value

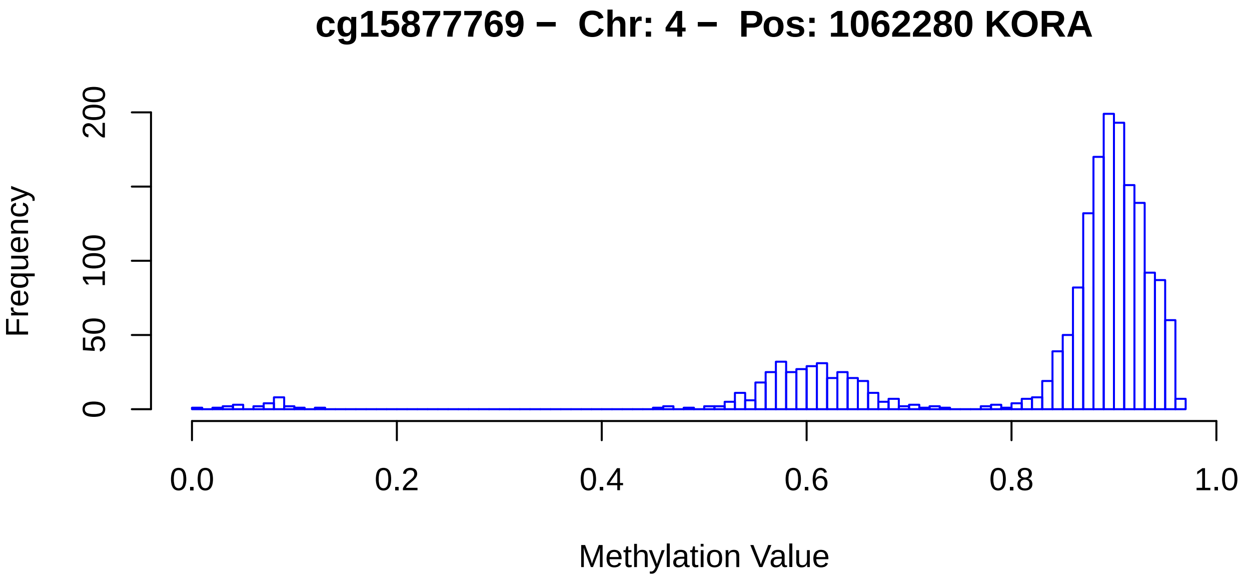
cg15877769 − Chr: 4 − Pos: 1062280 KORA
Frequency
0
50
100
200
0.0
0.2
0.4
0.6
0.8
1.0
Methylation Value

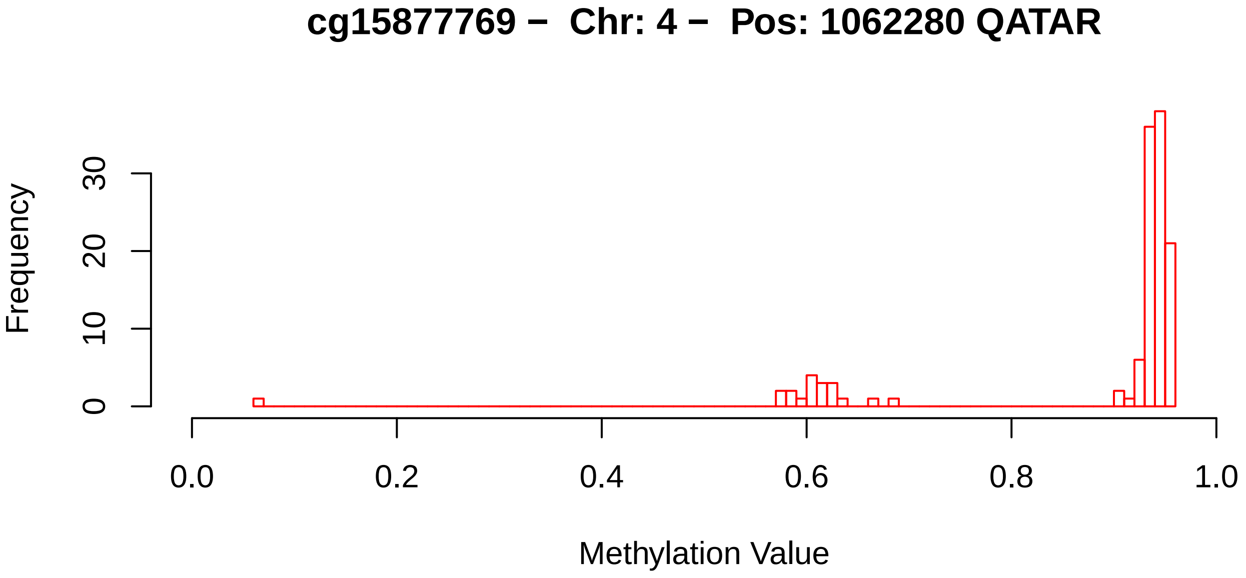
cg15877769 − Chr: 4 − Pos: 1062280 QATAR
Frequency
0
10
20
30
0.0
0.2
0.4
0.6
0.8
1.0
Methylation Value

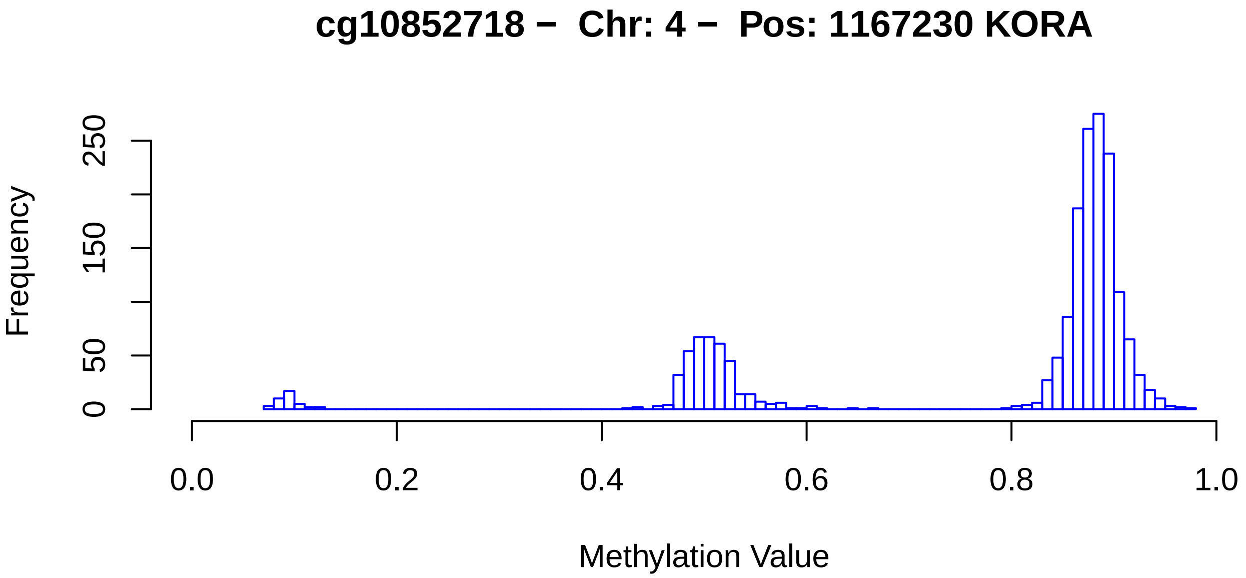
cg10852718 − Chr: 4 − Pos: 1167230 KORA
Frequency
0
50
150
250
0.0
0.2
0.4
0.6
0.8
1.0
Methylation Value

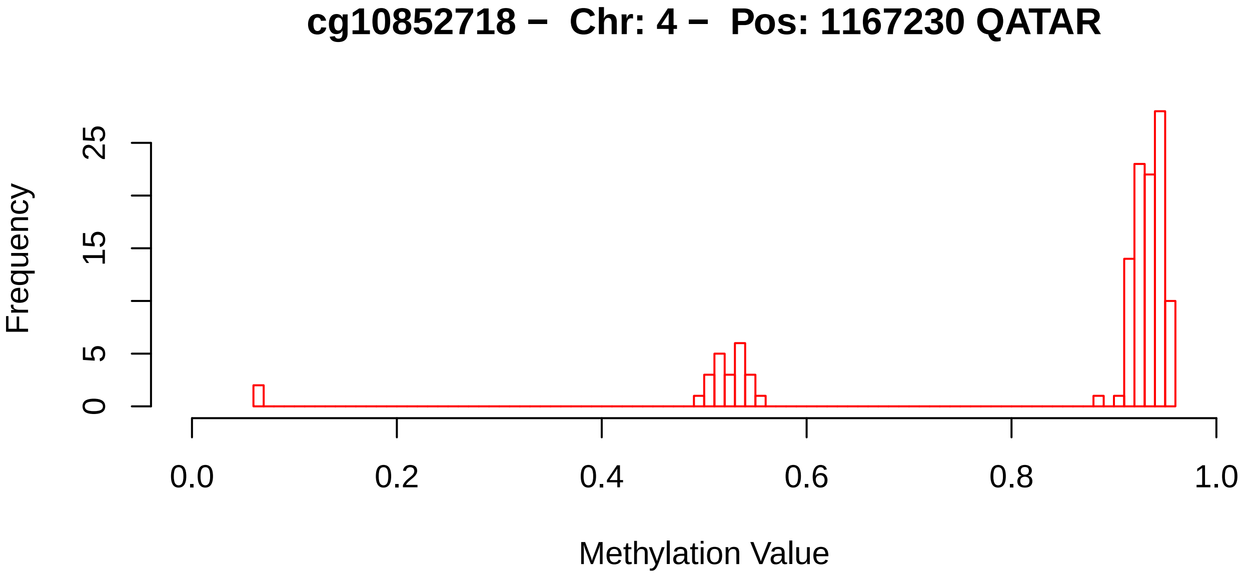
cg10852718 − Chr: 4 − Pos: 1167230 QATAR
Frequency
0
5
15
25
0.0
0.2
0.4
0.6
0.8
1.0
Methylation Value

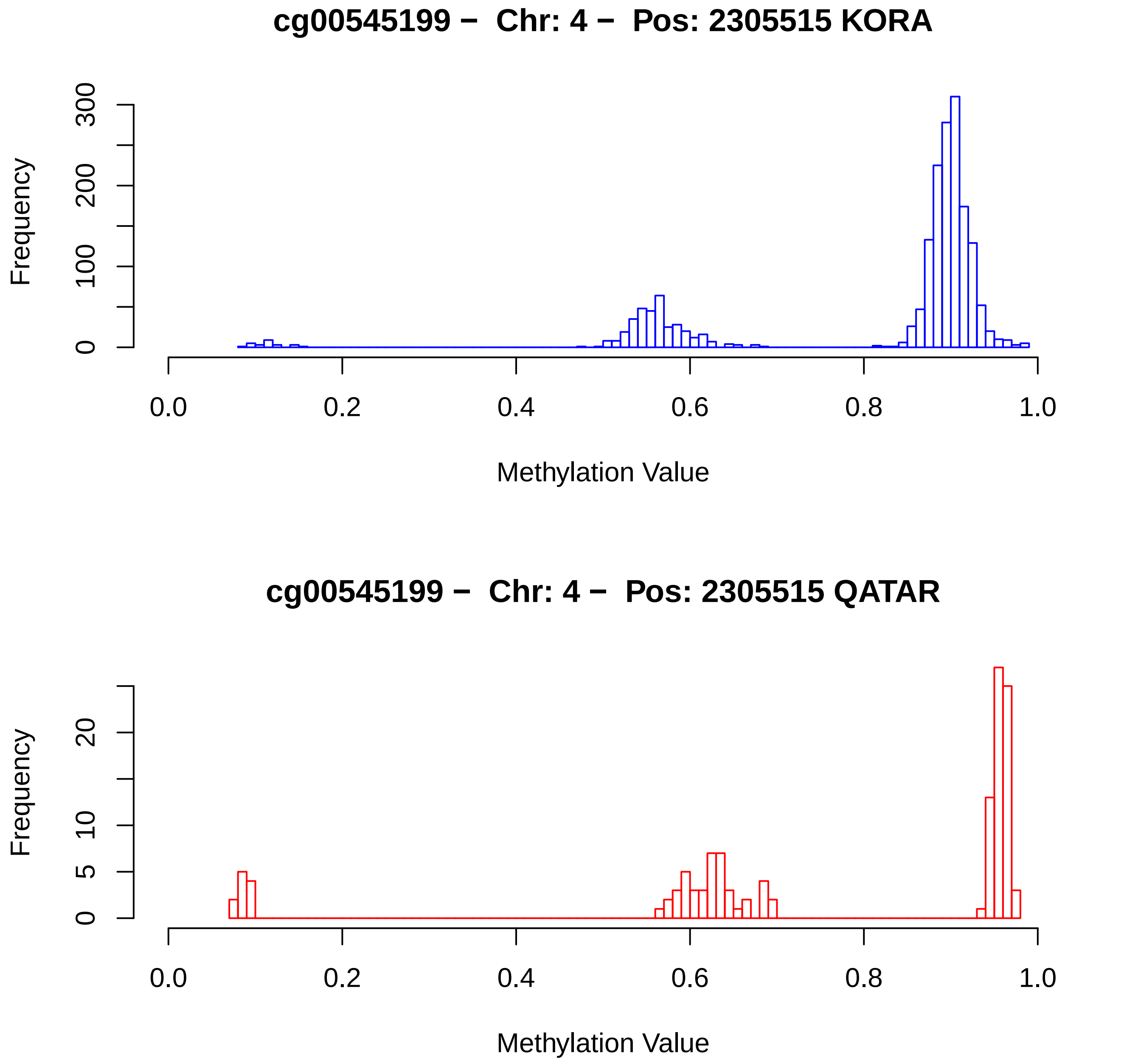
cg00545199 − Chr: 4 − Pos: 2305515 KORA
Frequency
0
100
200
300
0.0
0.2
0.4
0.6
0.8
1.0
Methylation Value
cg00545199 − Chr: 4 − Pos: 2305515 QATAR
Frequency
0
5
10
20
0.0
0.2
0.4
0.6
0.8
1.0
Methylation Value

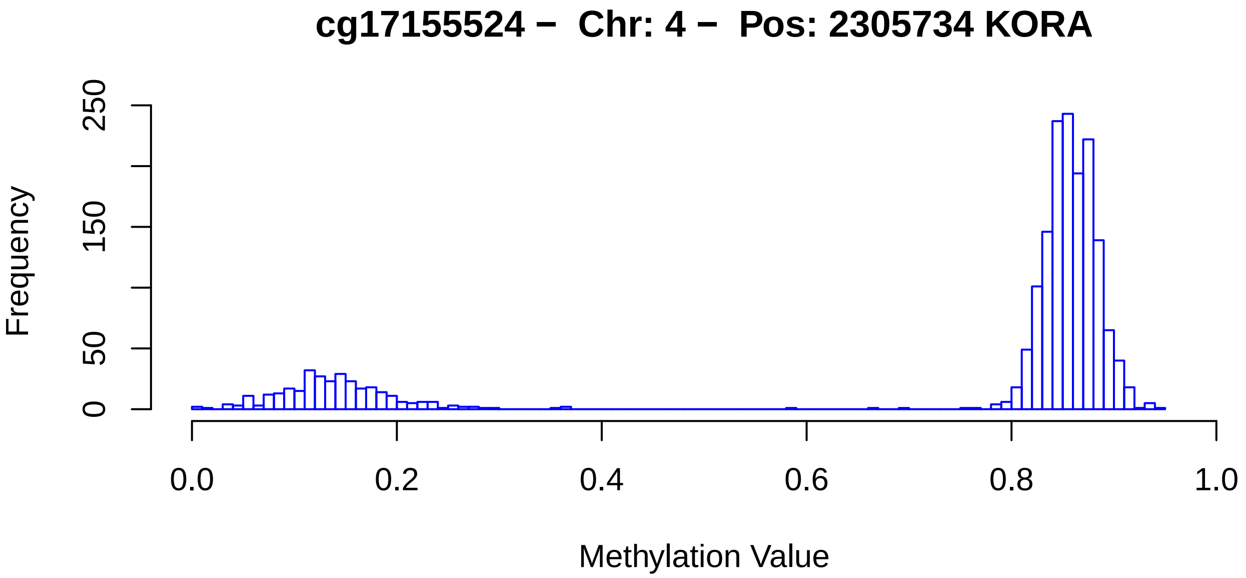
cg17155524 − Chr: 4 − Pos: 2305734 KORA
Frequency
0
50
150
250
0.0
0.2
0.4
0.6
0.8
1.0
Methylation Value

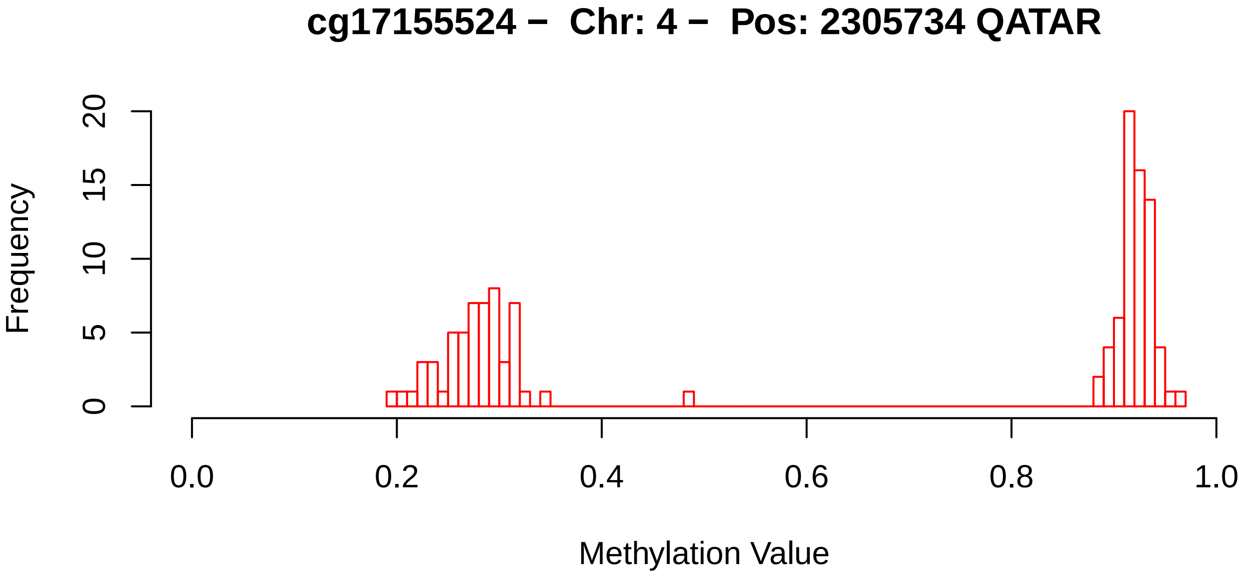
cg17155524 − Chr: 4 − Pos: 2305734 QATAR
Frequency
0
5
10
15
20
0.0
0.2
0.4
0.6
0.8
1.0
Methylation Value

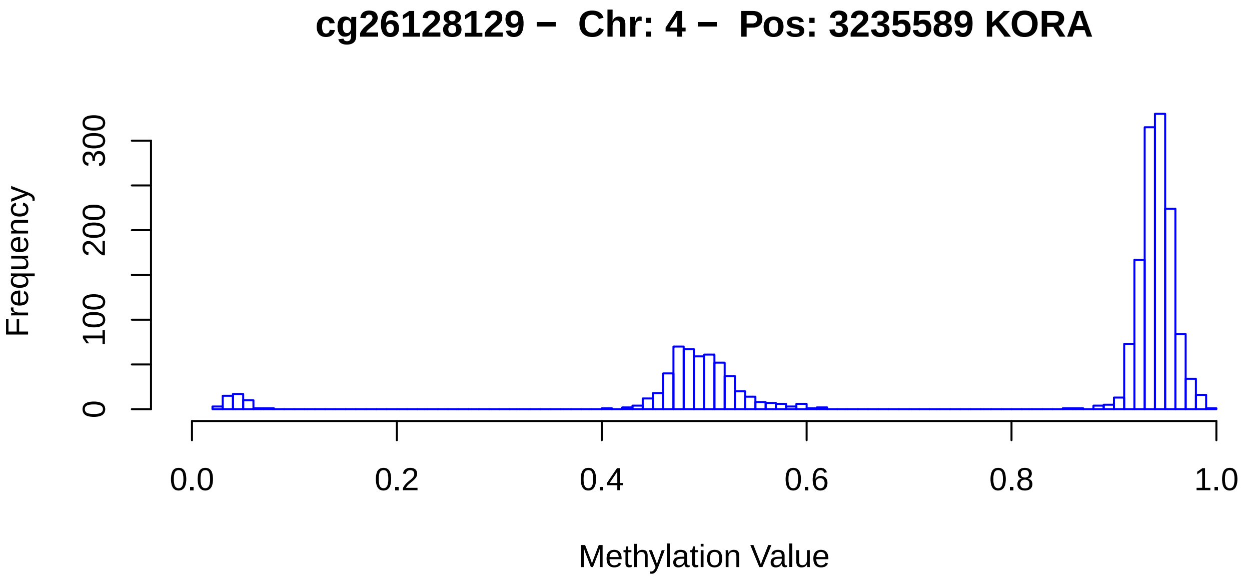
cg26128129 − Chr: 4 − Pos: 3235589 KORA
Frequency
0
100
200
300
0.0
0.2
0.4
0.6
0.8
1.0
Methylation Value

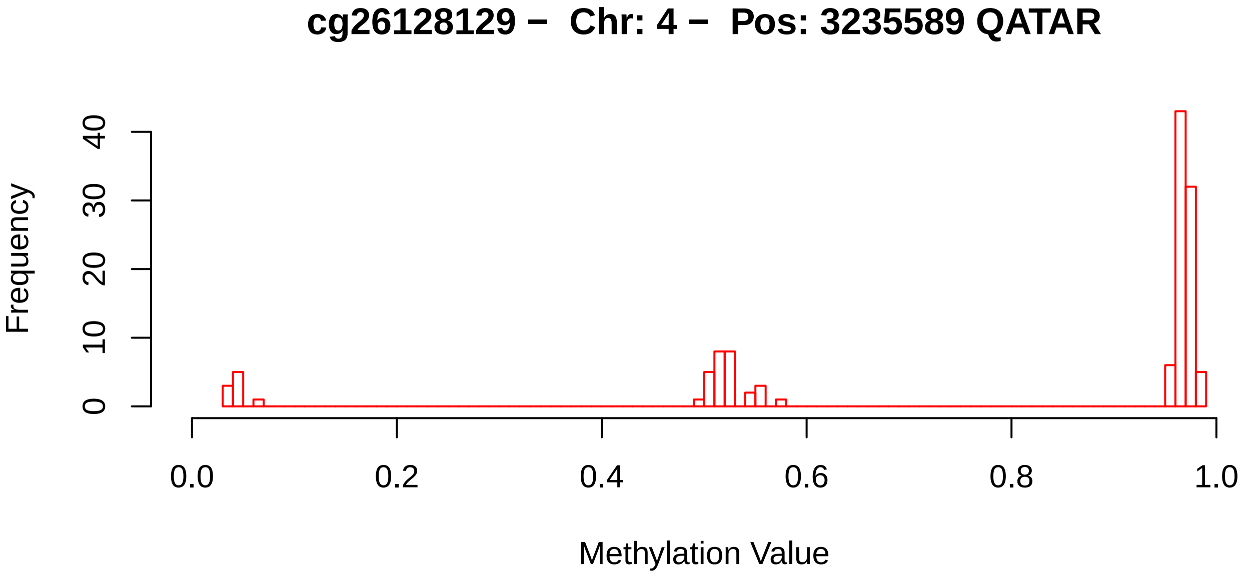
cg26128129 − Chr: 4 − Pos: 3235589 QATAR
Frequency
0
10
20
30
40
0.0
0.2
0.4
0.6
0.8
1.0
Methylation Value

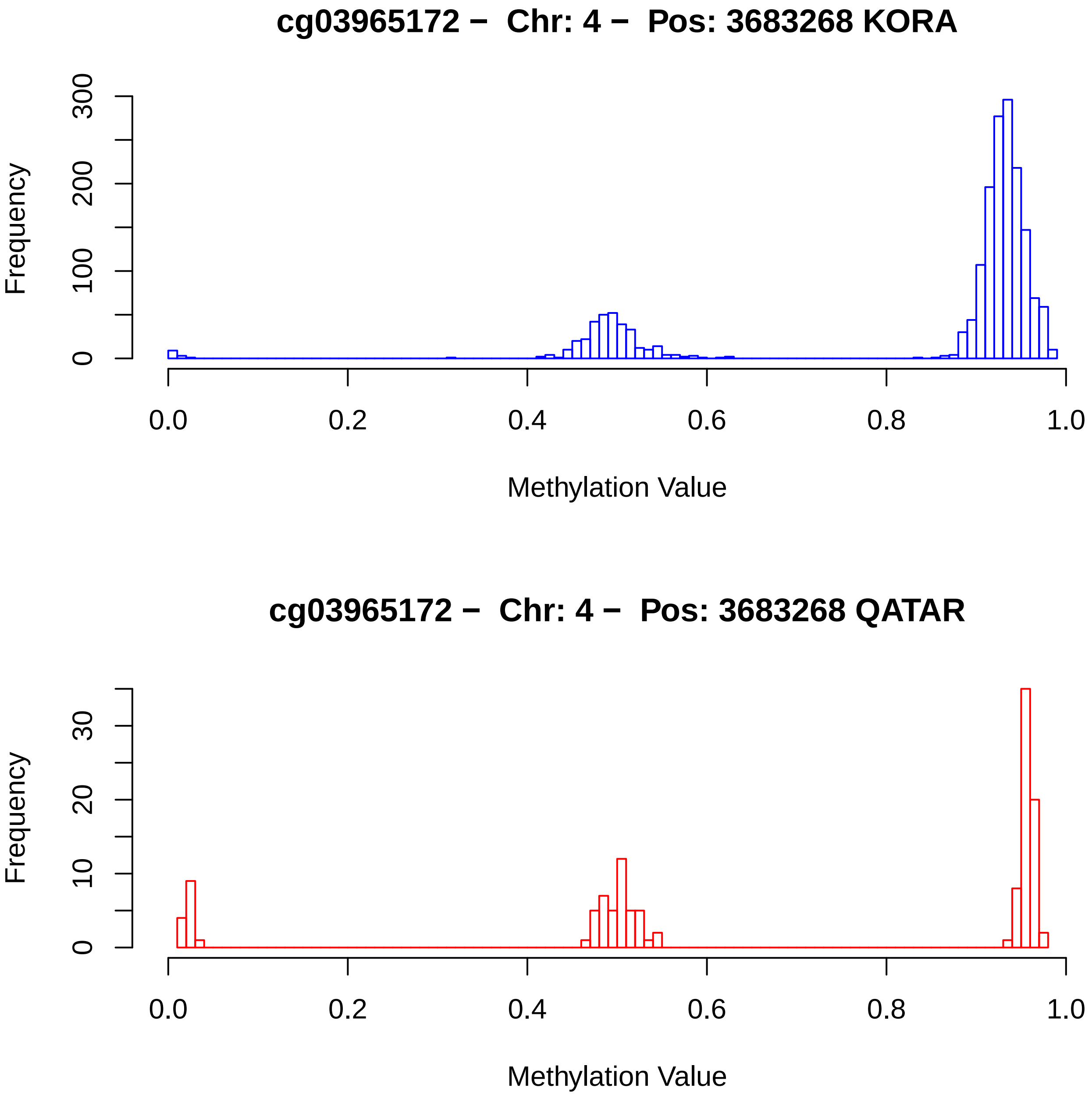
cg03965172 − Chr: 4 − Pos: 3683268 KORA
Frequency
0
100
200
300
0.0
0.2
0.4
0.6
0.8
1.0
Methylation Value
cg03965172 − Chr: 4 − Pos: 3683268 QATAR
Frequency
0
10
20
30
0.0
0.2
0.4
0.6
0.8
1.0
Methylation Value

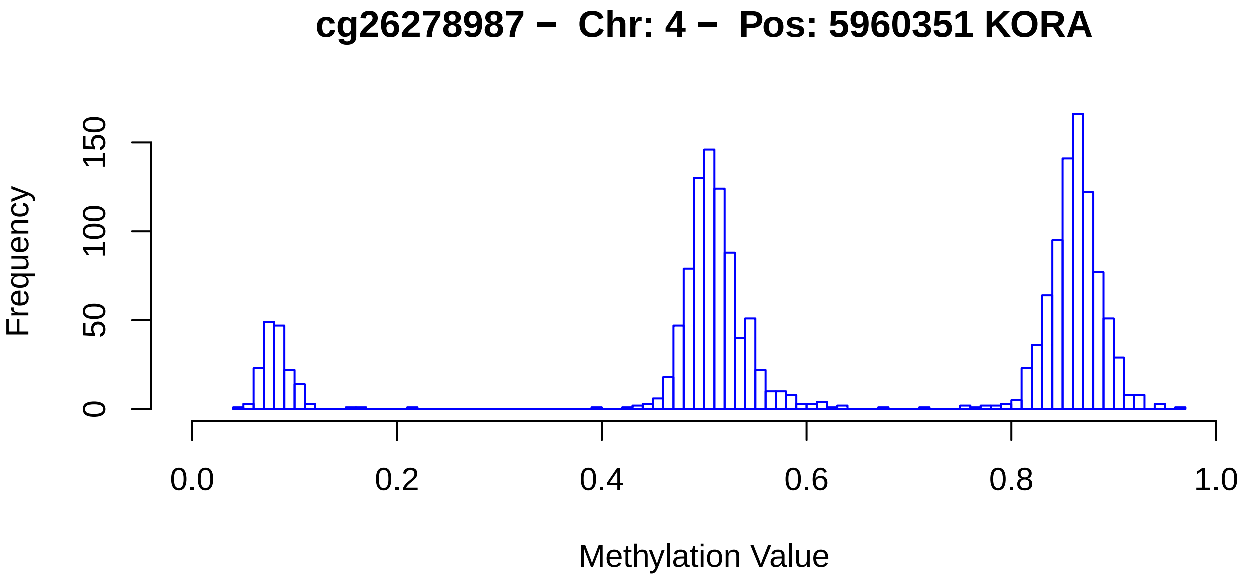
cg26278987 − Chr: 4 − Pos: 5960351 KORA
Frequency
0
50
100
150
0.0
0.2
0.4
0.6
0.8
1.0
Methylation Value

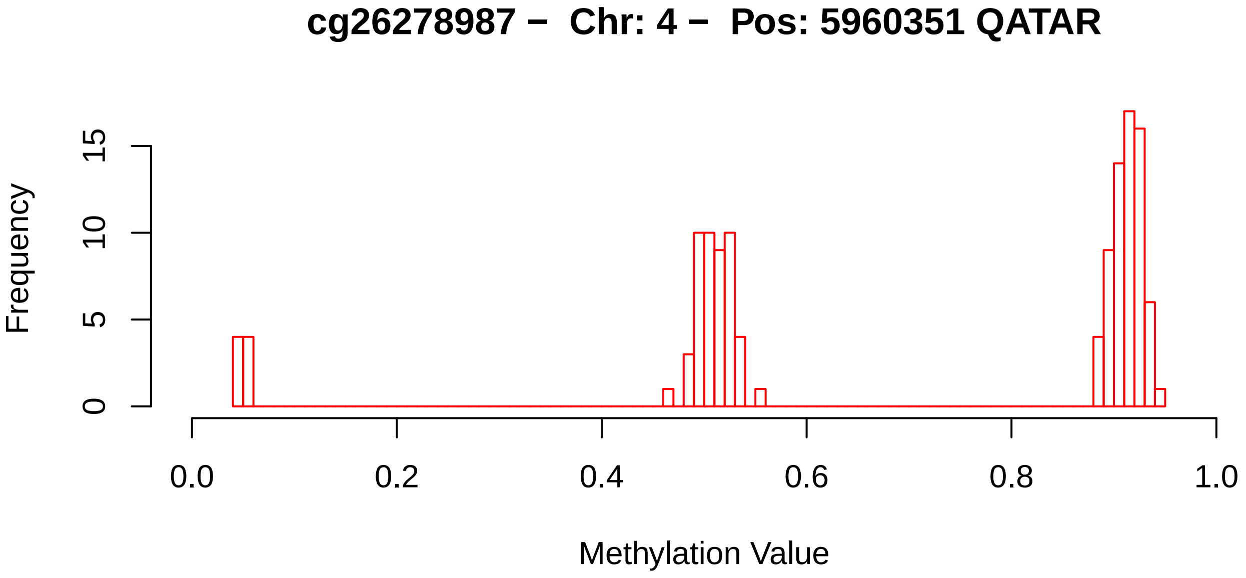
cg26278987 − Chr: 4 − Pos: 5960351 QATAR
Frequency
0
5
10
15
0.0
0.2
0.4
0.6
0.8
1.0
Methylation Value

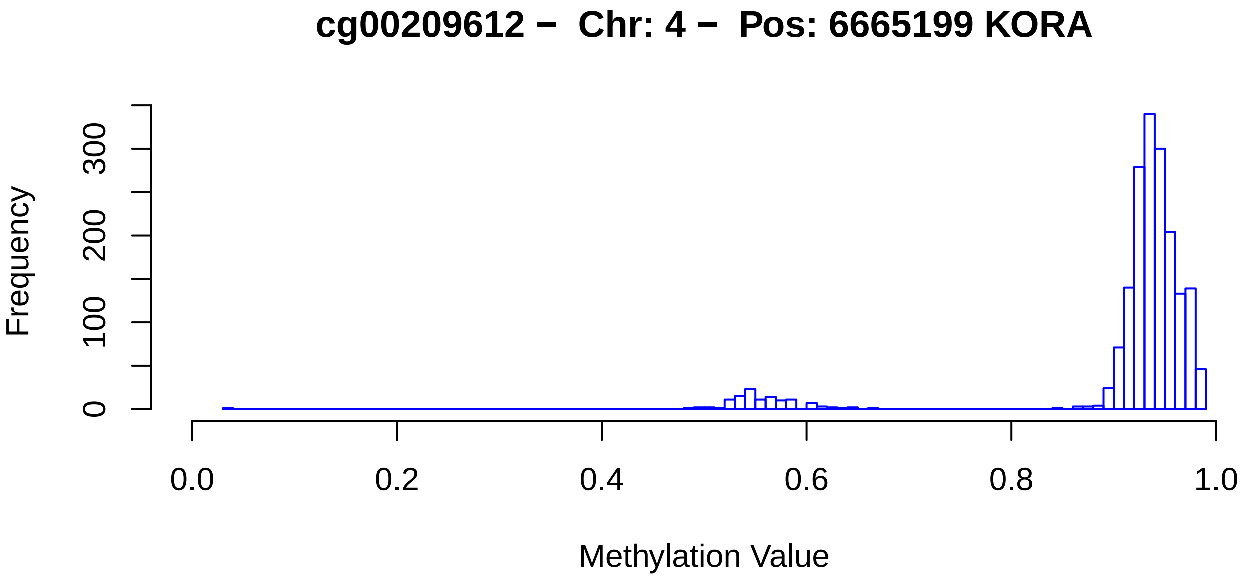
cg00209612 − Chr: 4 − Pos: 6665199 KORA
Frequency
0
100
200
300
0.0
0.2
0.4
0.6
0.8
1.0
Methylation Value

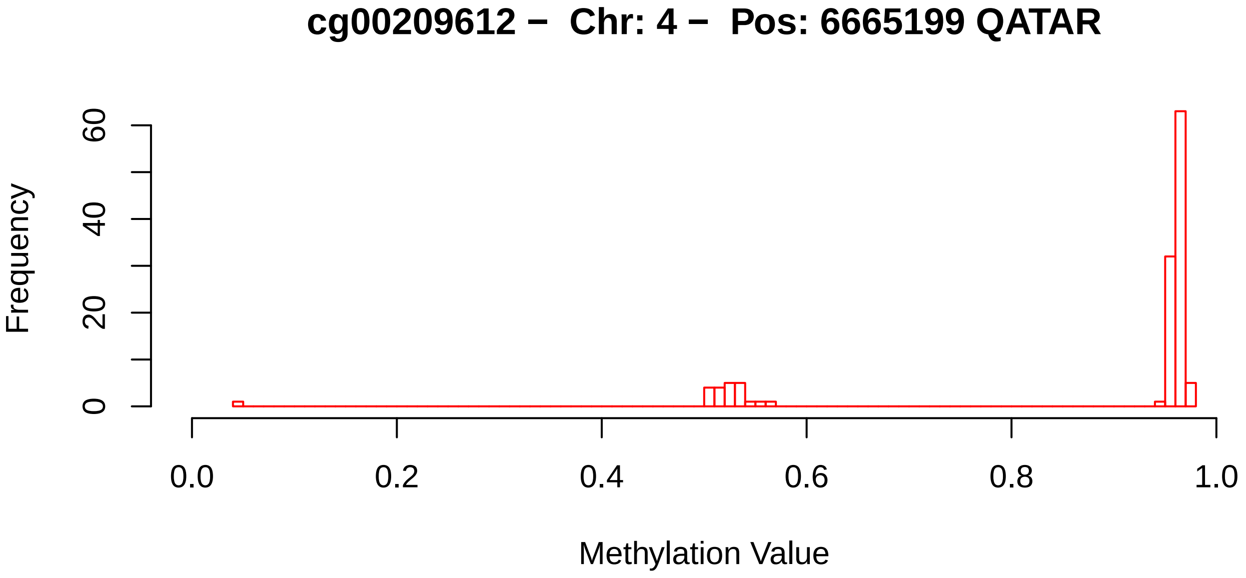
cg00209612 − Chr: 4 − Pos: 6665199 QATAR
Frequency
0
20
40
60
0.0
0.2
0.4
0.6
0.8
1.0
Methylation Value

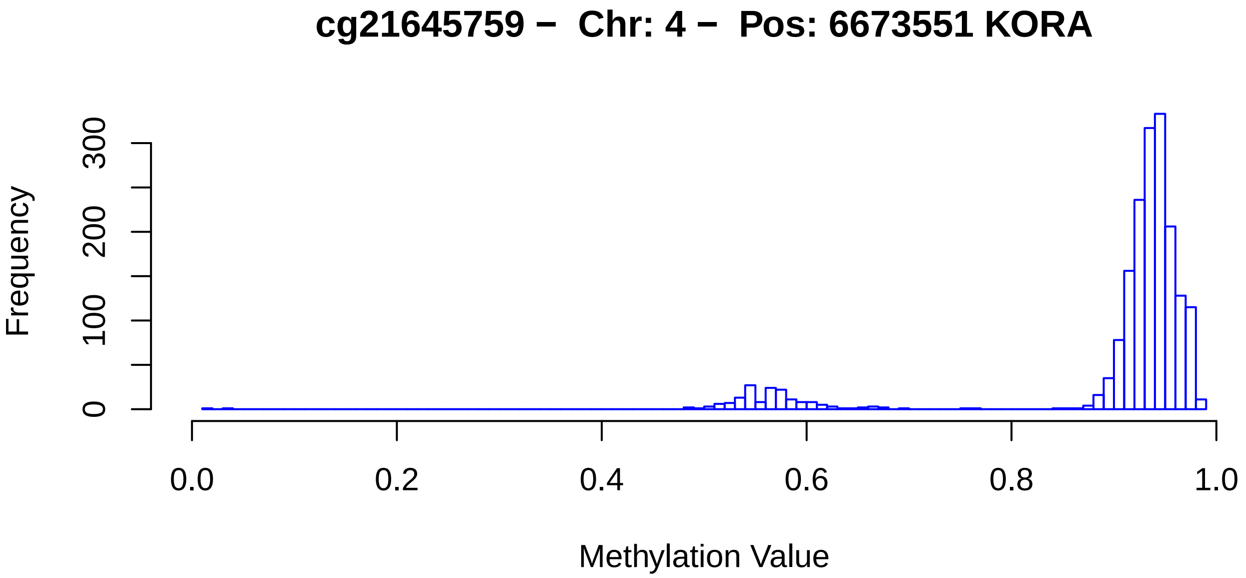
cg21645759 − Chr: 4 − Pos: 6673551 KORA
Frequency
0
100
200
300
0.0
0.2
0.4
0.6
0.8
1.0
Methylation Value

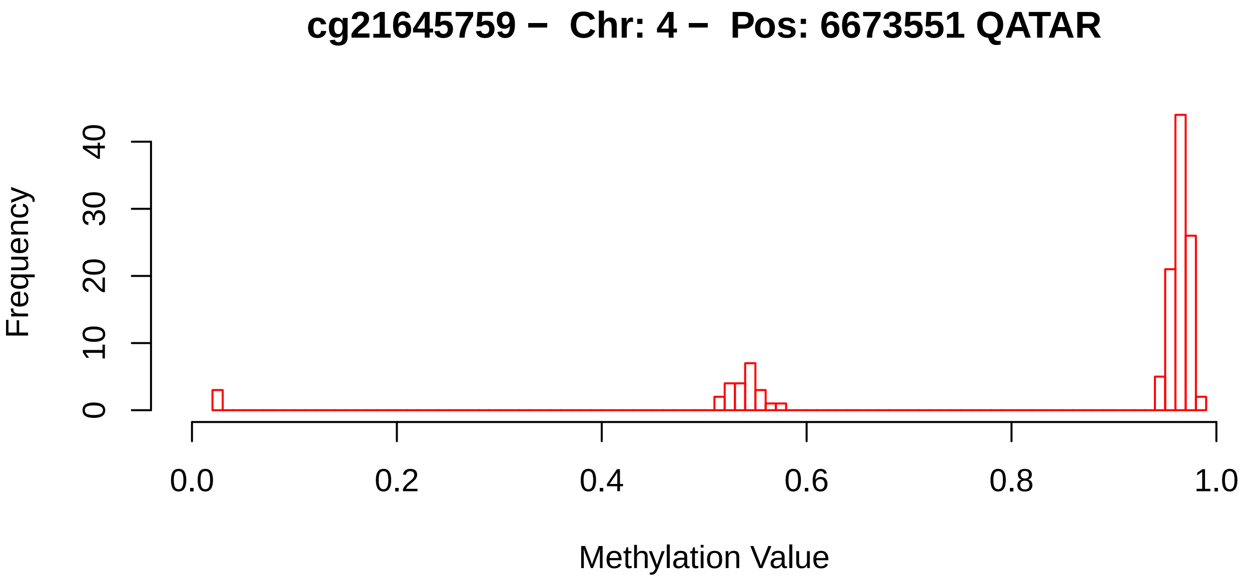
cg21645759 − Chr: 4 − Pos: 6673551 QATAR
Frequency
0
10
20
30
40
0.0
0.2
0.4
0.6
0.8
1.0
Methylation Value

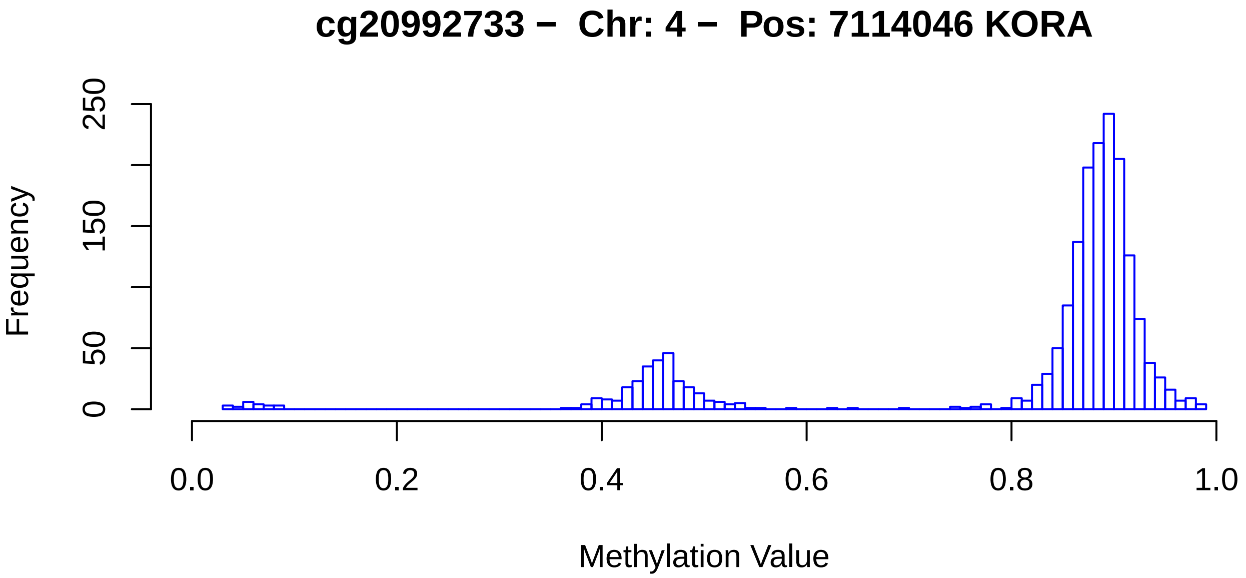
cg20992733 − Chr: 4 − Pos: 7114046 KORA
Frequency
0
50
150
250
0.0
0.2
0.4
0.6
0.8
1.0
Methylation Value

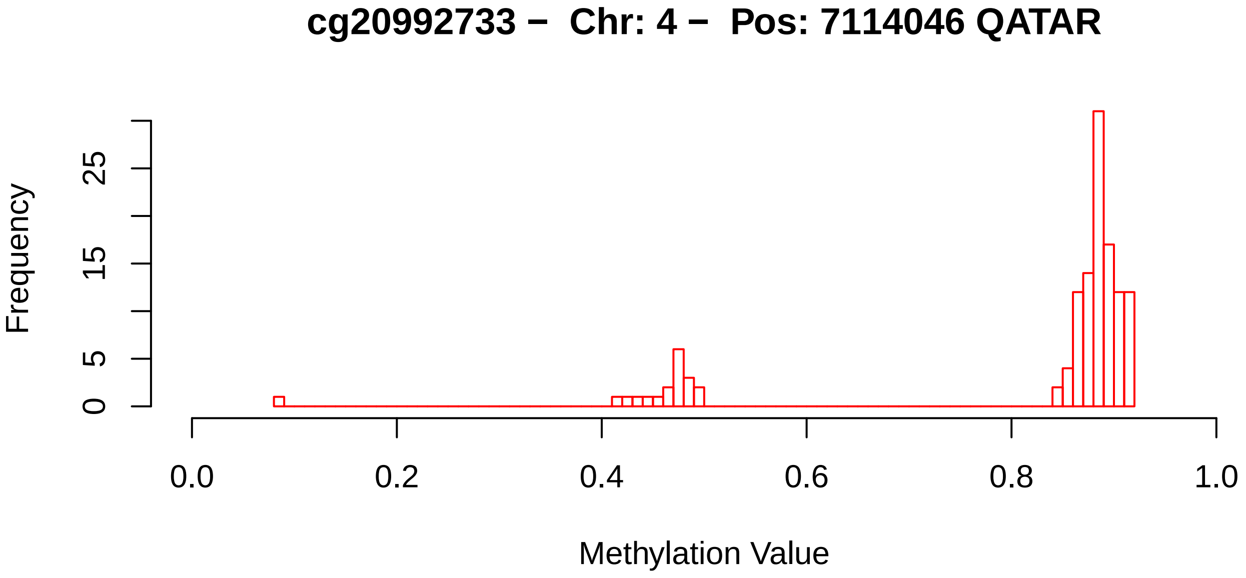
cg20992733 − Chr: 4 − Pos: 7114046 QATAR
Frequency
0
5
15
25
0.0
0.2
0.4
0.6
0.8
1.0
Methylation Value

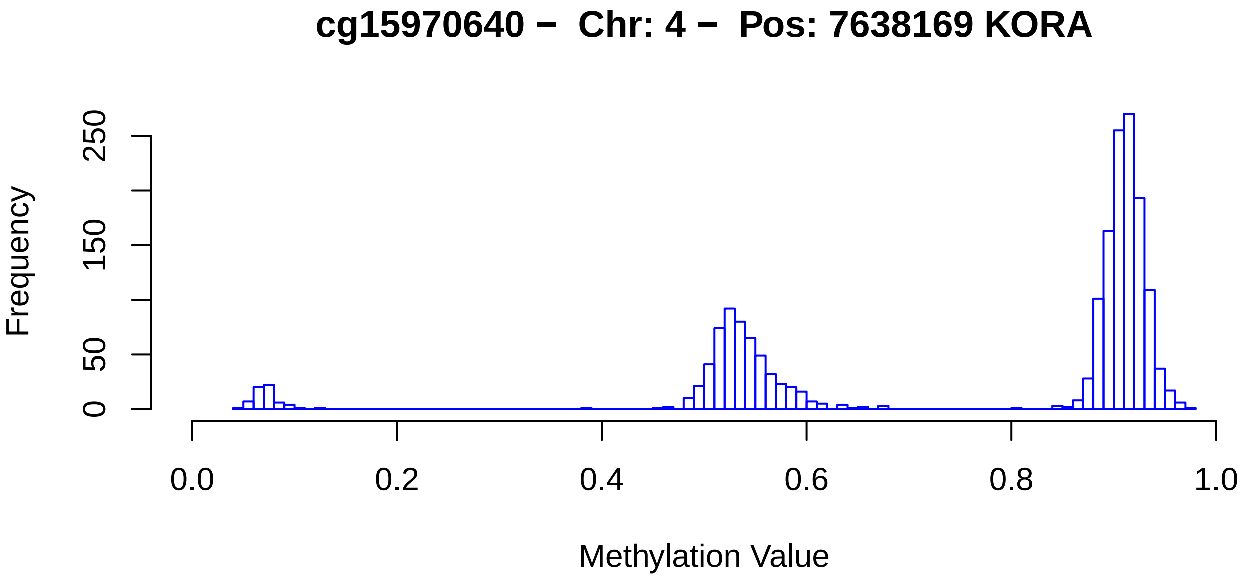
cg15970640 − Chr: 4 − Pos: 7638169 KORA
Frequency
0
50
150
250
0.0
0.2
0.4
0.6
0.8
1.0
Methylation Value

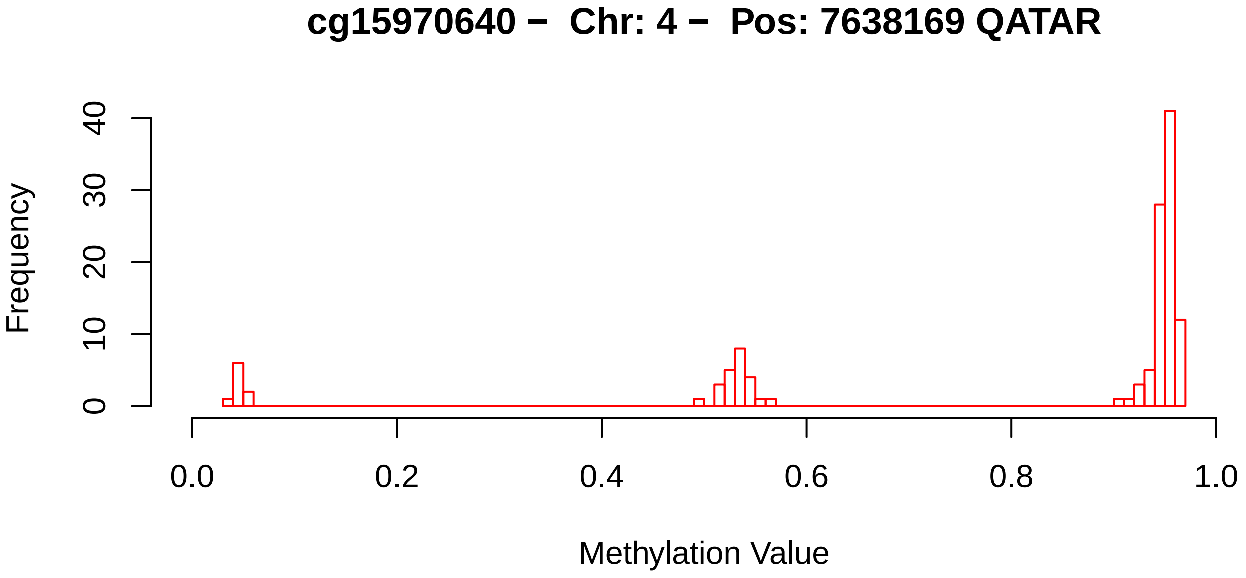
cg15970640 − Chr: 4 − Pos: 7638169 QATAR
Frequency
0
10
20
30
40
0.0
0.2
0.4
0.6
0.8
1.0
Methylation Value

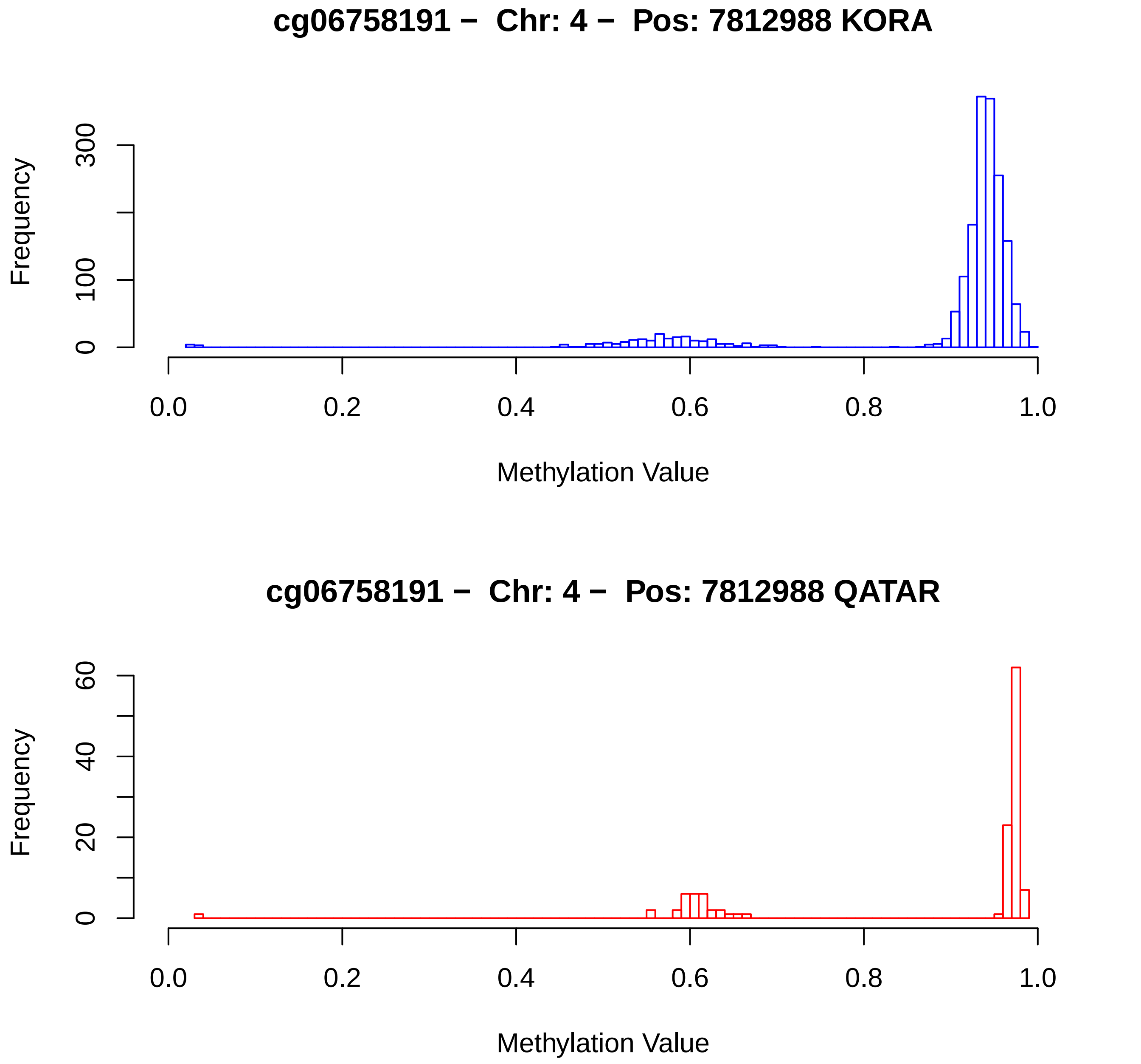
cg06758191 − Chr: 4 − Pos: 7812988 KORA
Frequency
0
100
300
0.0
0.2
0.4
0.6
0.8
1.0
Methylation Value
cg06758191 − Chr: 4 − Pos: 7812988 QATAR
Frequency
0
20
40
60
0.0
0.2
0.4
0.6
0.8
1.0
Methylation Value

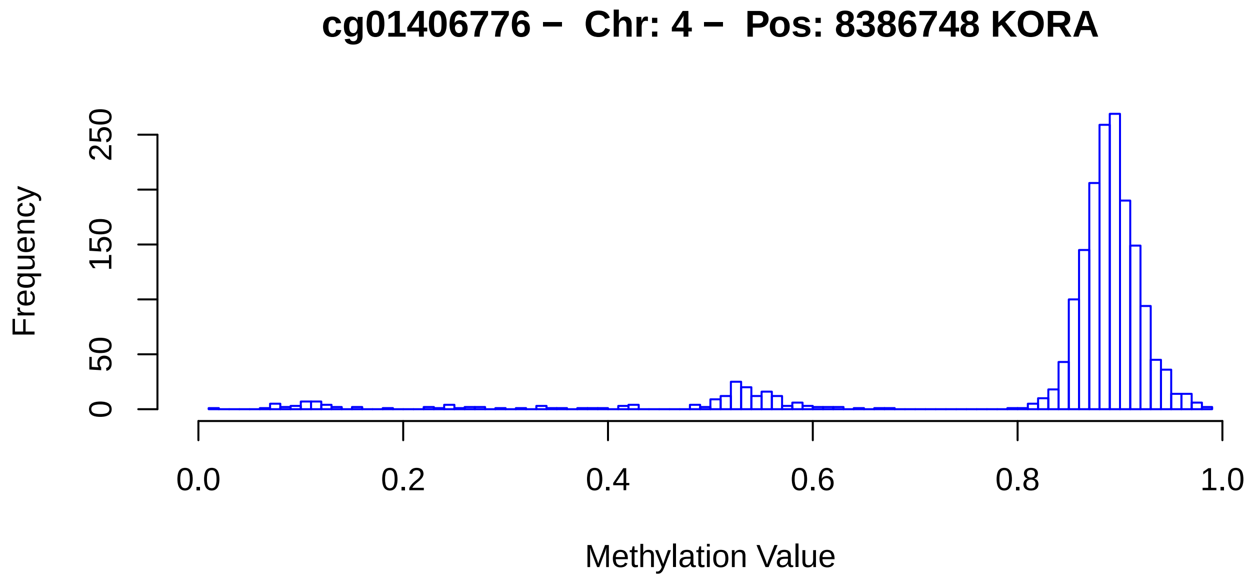
cg01406776 − Chr: 4 − Pos: 8386748 KORA
Frequency
0
50
150
250
0.0
0.2
0.4
0.6
0.8
1.0
Methylation Value

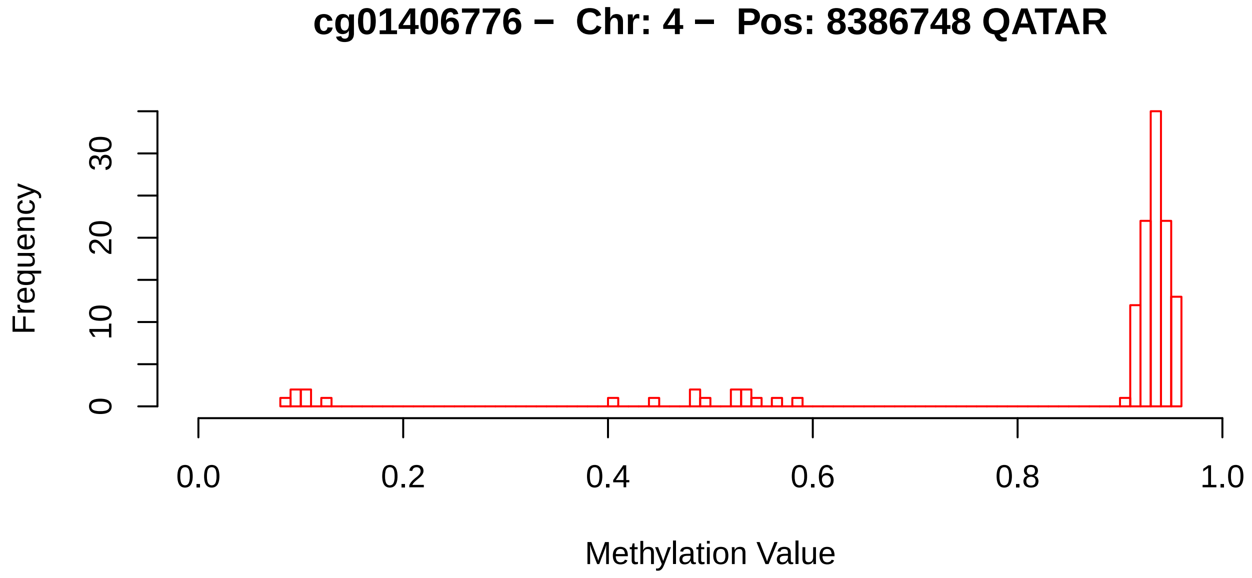
cg01406776 − Chr: 4 − Pos: 8386748 QATAR
Frequency
0
10
20
30
0.0
0.2
0.4
0.6
0.8
1.0
Methylation Value

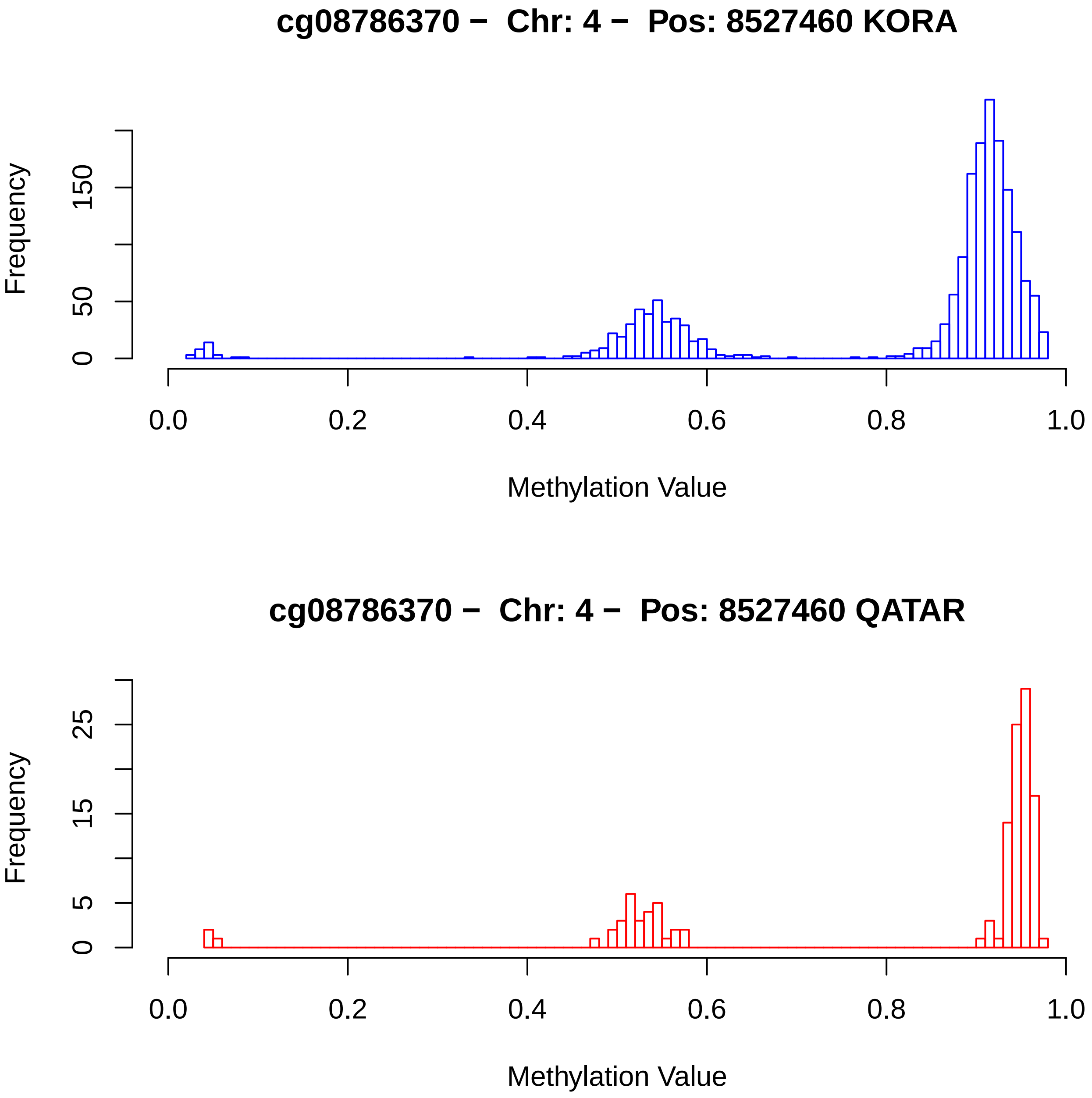
cg08786370 − Chr: 4 − Pos: 8527460 KORA
Frequency
0
50
150
0.0
0.2
0.4
0.6
0.8
1.0
Methylation Value
cg08786370 − Chr: 4 − Pos: 8527460 QATAR
Frequency
0
5
15
25
0.0
0.2
0.4
0.6
0.8
1.0
Methylation Value

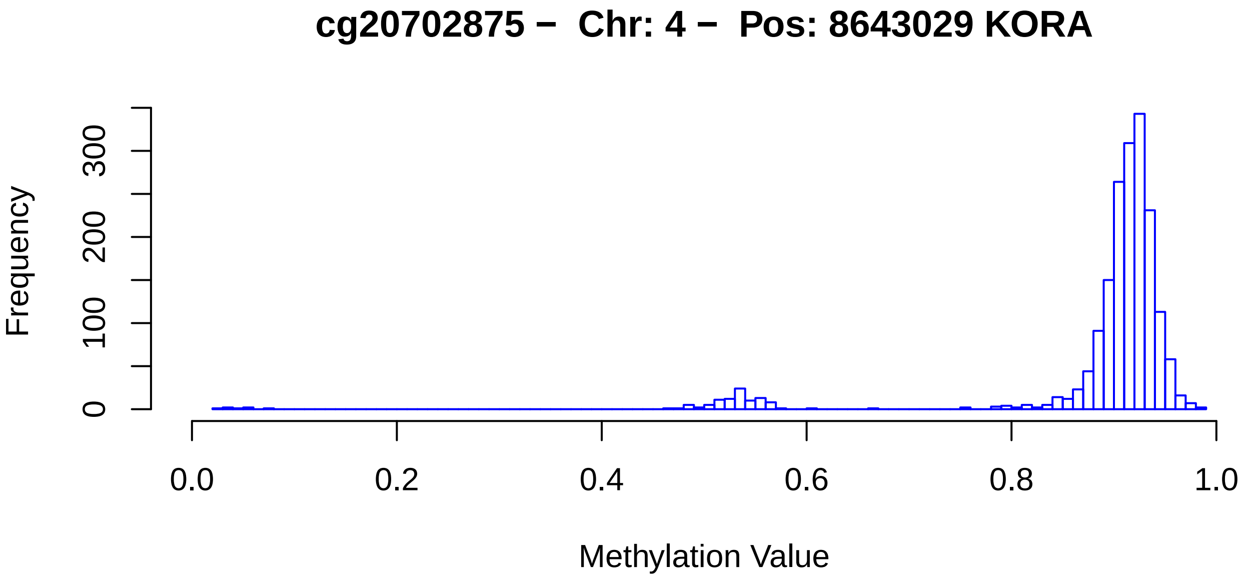

cg20702875 − Chr: 4 − Pos: 8643029 KORA
Frequency
0
100
200
300
0.0
0.2
0.4
0.6
0.8
1.0
Methylation Value

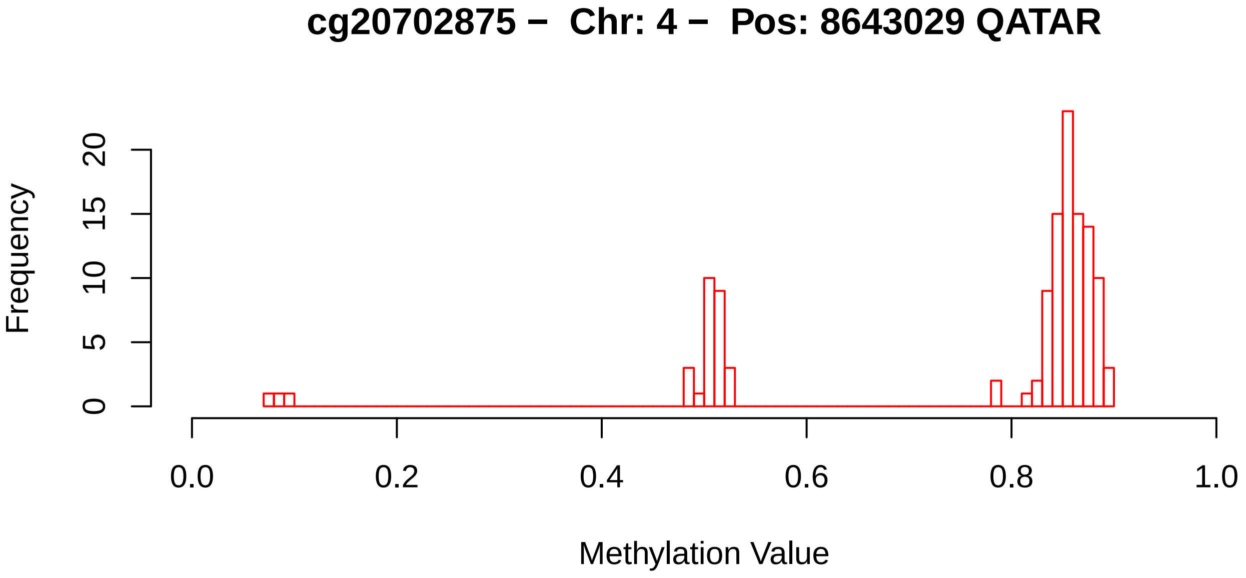

cg20702875 − Chr: 4 − Pos: 8643029 QATAR
Frequency
0
5
10
15
20
0.0
0.2
0.4
0.6
0.8
1.0
Methylation Value

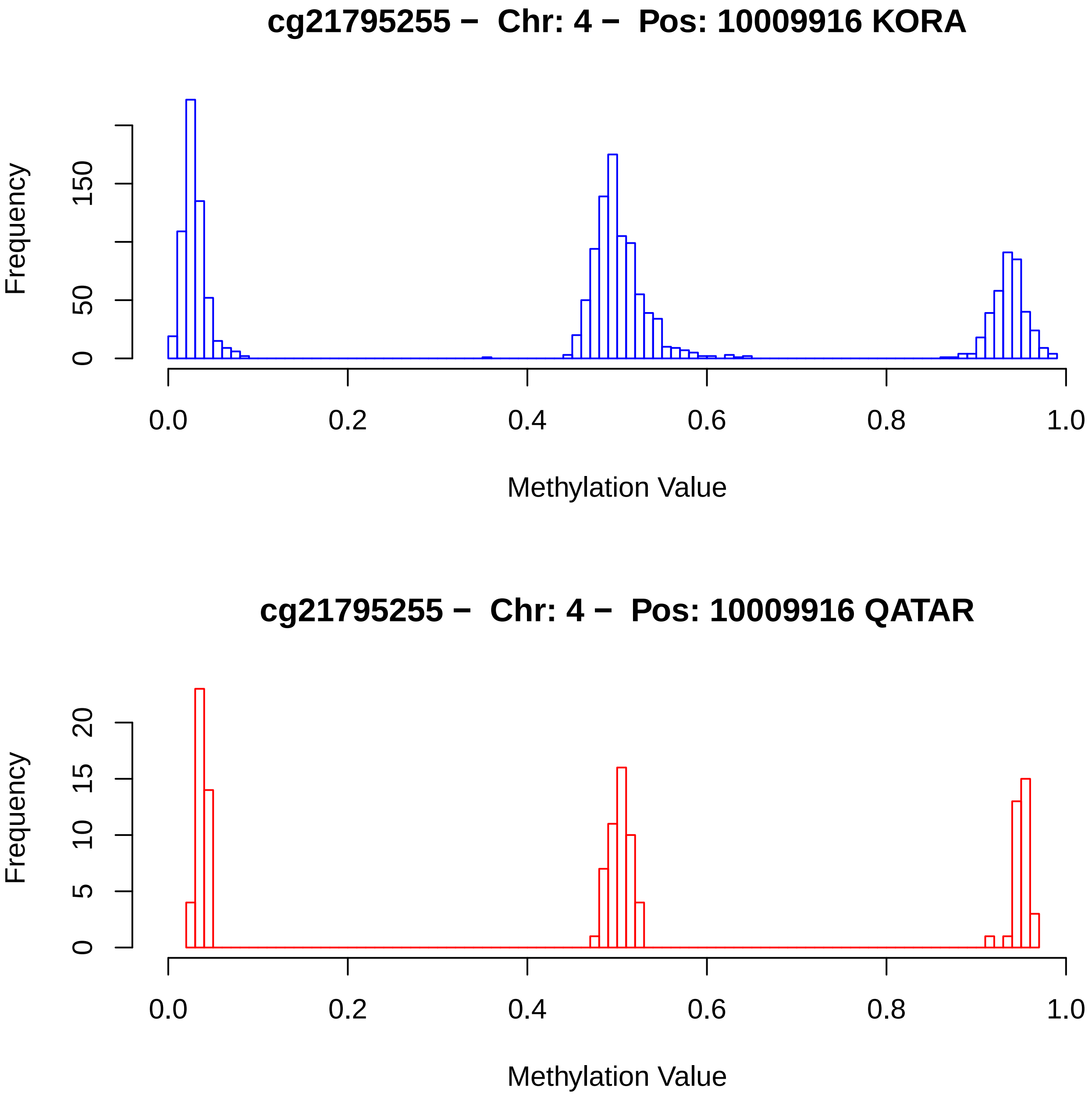
cg21795255 − Chr: 4 − Pos: 10009916 KORA
Frequency
0
50
150
0.0
0.2
0.4
0.6
0.8
1.0
Methylation Value
cg21795255 − Chr: 4 − Pos: 10009916 QATAR
Frequency
0
5
10
15
20
0.0
0.2
0.4
0.6
0.8
1.0
Methylation Value

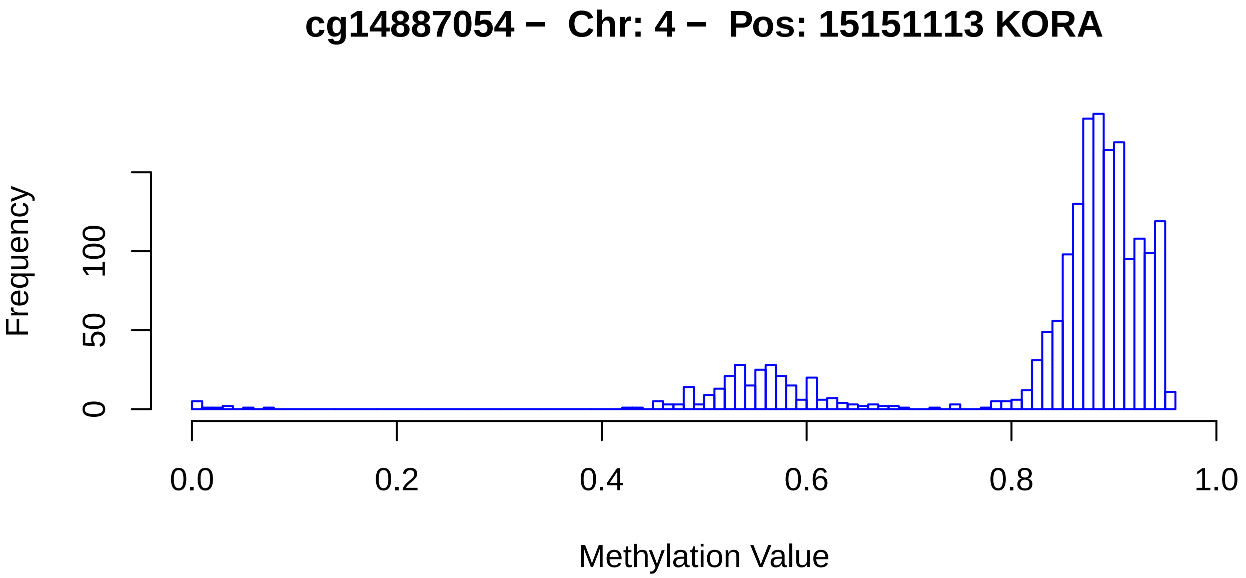
cg14887054 − Chr: 4 − Pos: 15151113 KORA
Frequency
0
50
100
0.0
0.2
0.4
0.6
0.8
1.0
Methylation Value

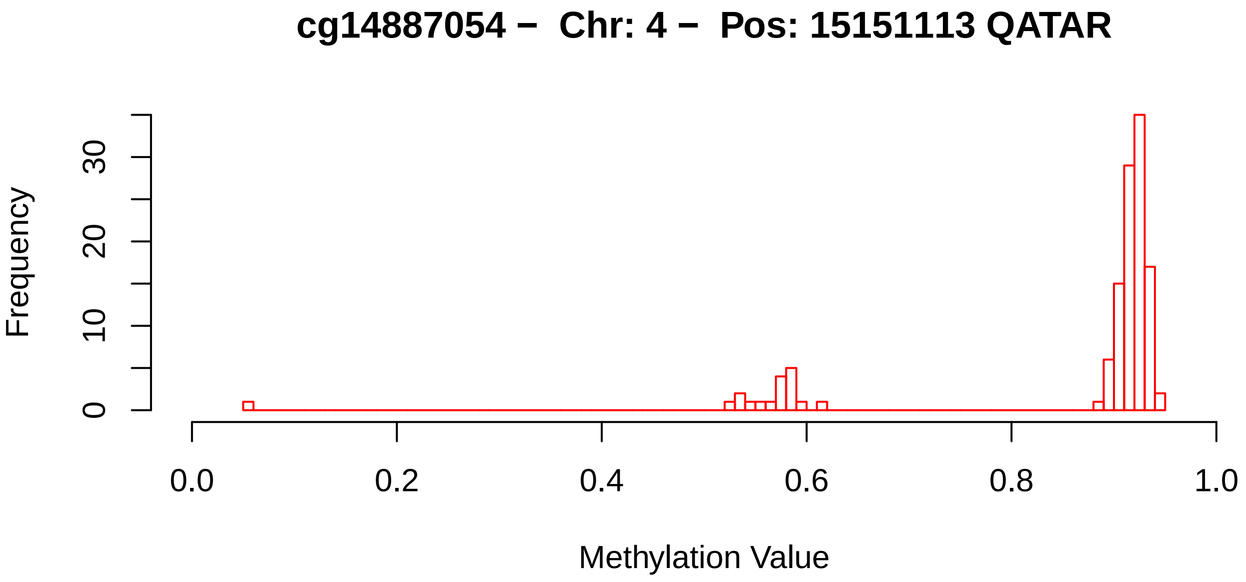
cg14887054 − Chr: 4 − Pos: 15151113 QATAR
Frequency
0
10
20
30
0.0
0.2
0.4
0.6
0.8
1.0
Methylation Value

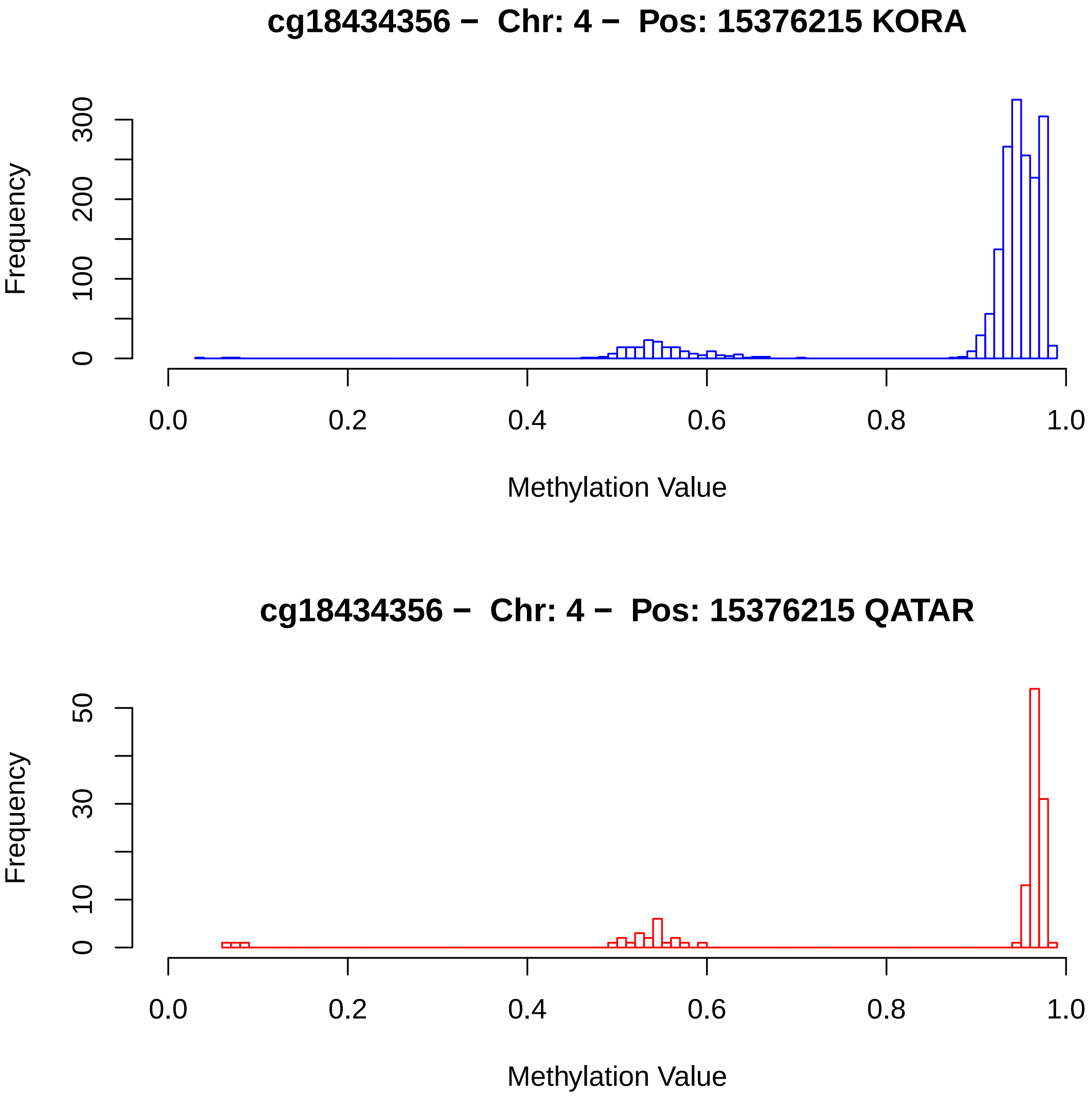
cg18434356 − Chr: 4 − Pos: 15376215 KORA
Frequency
0
100
200
300
0.0
0.2
0.4
0.6
0.8
1.0
Methylation Value
cg18434356 − Chr: 4 − Pos: 15376215 QATAR
Frequency
0
10
30
50
0.0
0.2
0.4
0.6
0.8
1.0
Methylation Value

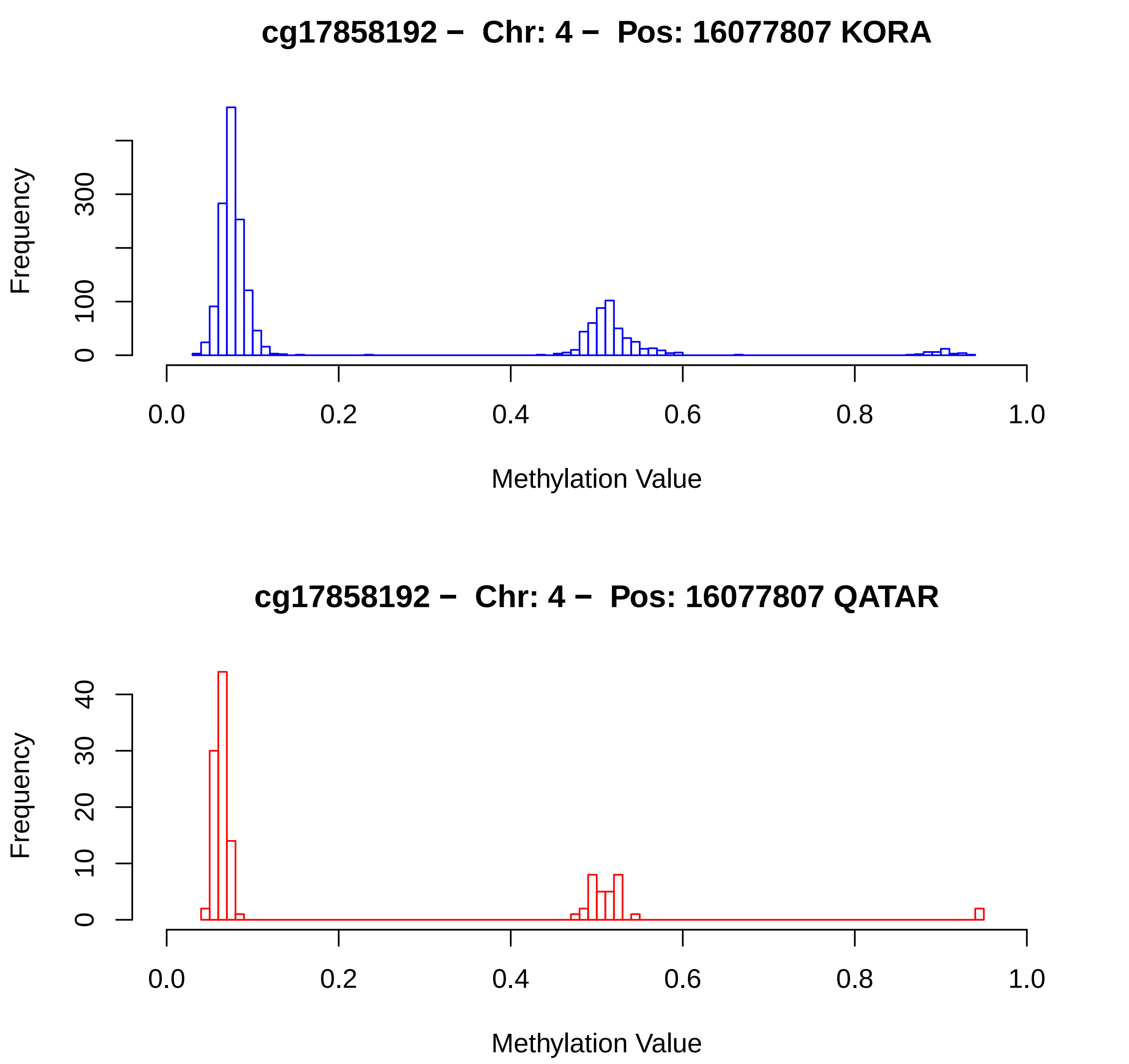
cg17858192 − Chr: 4 − Pos: 16077807 KORA
Frequency
0
100
300
0.0
0.2
0.4
0.6
0.8
1.0
Methylation Value
cg17858192 − Chr: 4 − Pos: 16077807 QATAR
Frequency
0
10
20
30
40
0.0
0.2
0.4
0.6
0.8
1.0
Methylation Value

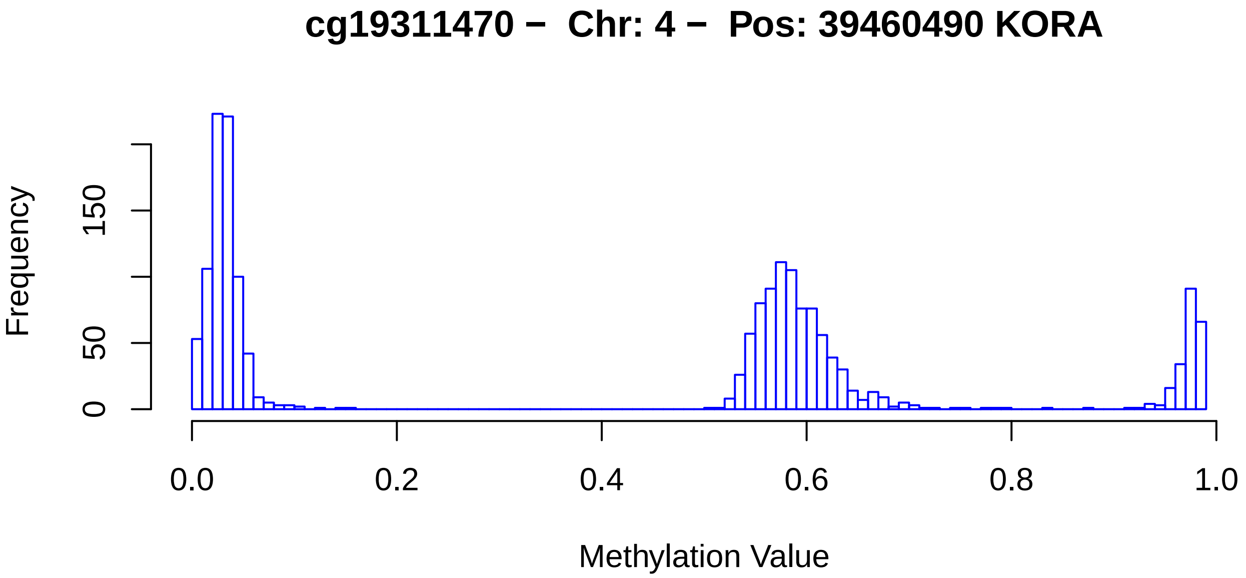
cg19311470 − Chr: 4 − Pos: 39460490 KORA
Frequency
0
50
150
0.0
0.2
0.4
0.6
0.8
1.0
Methylation Value

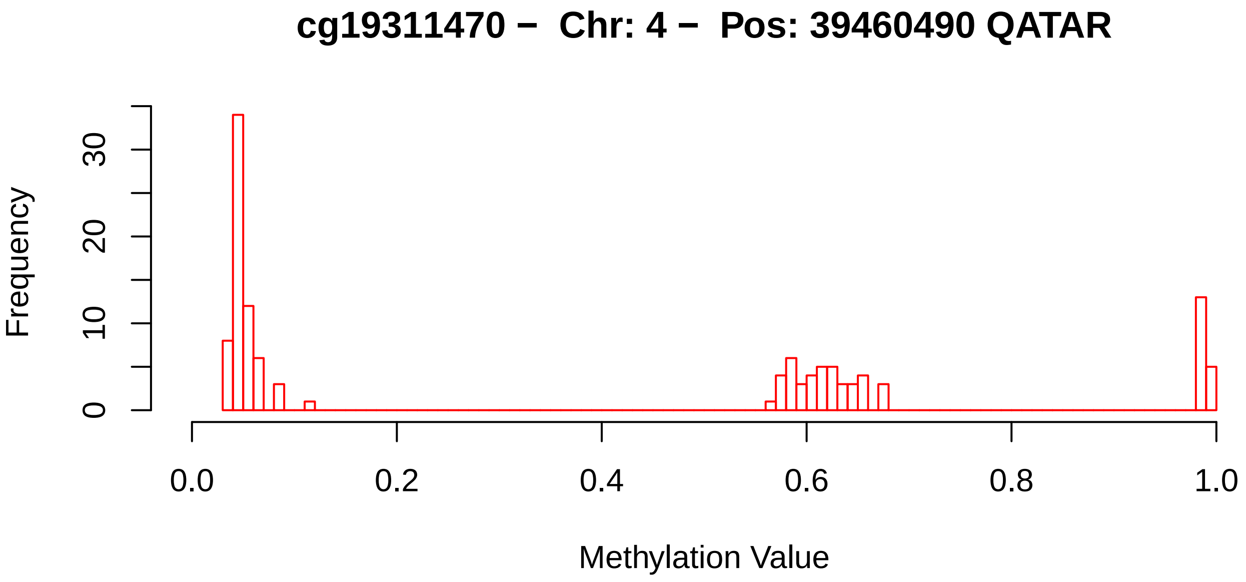
cg19311470 − Chr: 4 − Pos: 39460490 QATAR
Frequency
0
10
20
30
0.0
0.2
0.4
0.6
0.8
1.0
Methylation Value

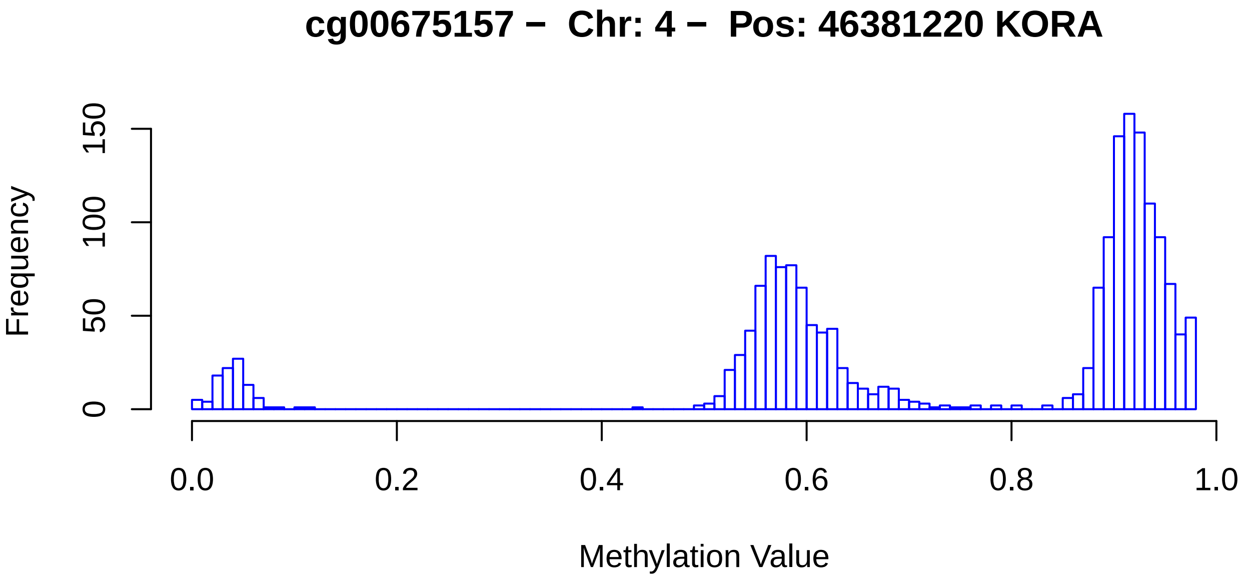
cg00675157 − Chr: 4 − Pos: 46381220 KORA
Frequency
0
50
100
150
0.0
0.2
0.4
0.6
0.8
1.0
Methylation Value

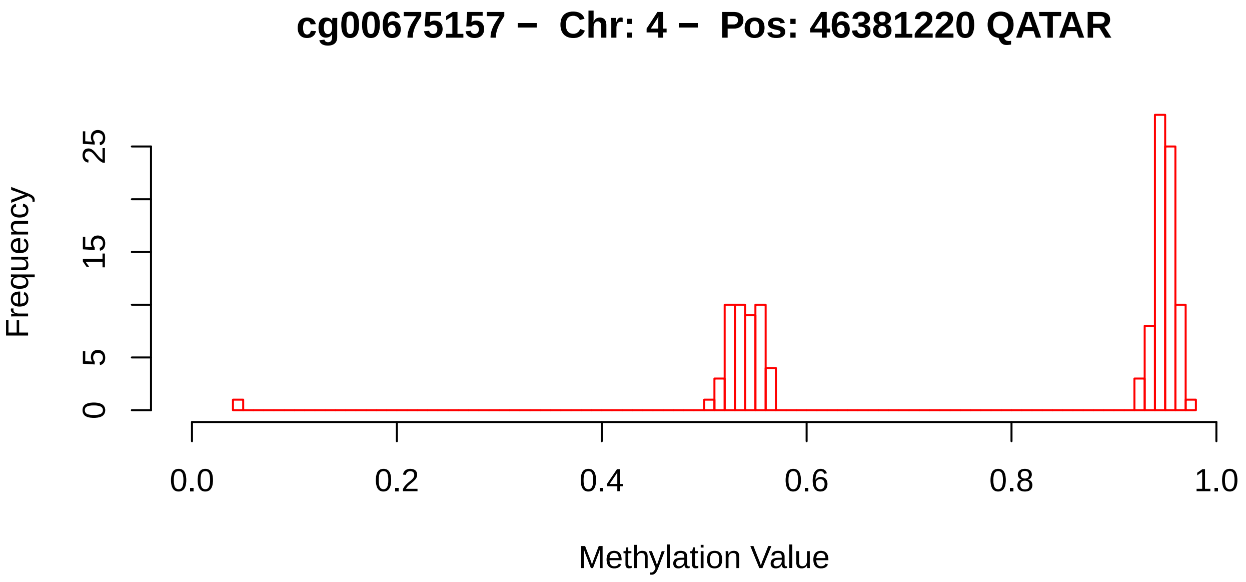
cg00675157 − Chr: 4 − Pos: 46381220 QATAR
Frequency
0
5
15
25
0.0
0.2
0.4
0.6
0.8
1.0
Methylation Value

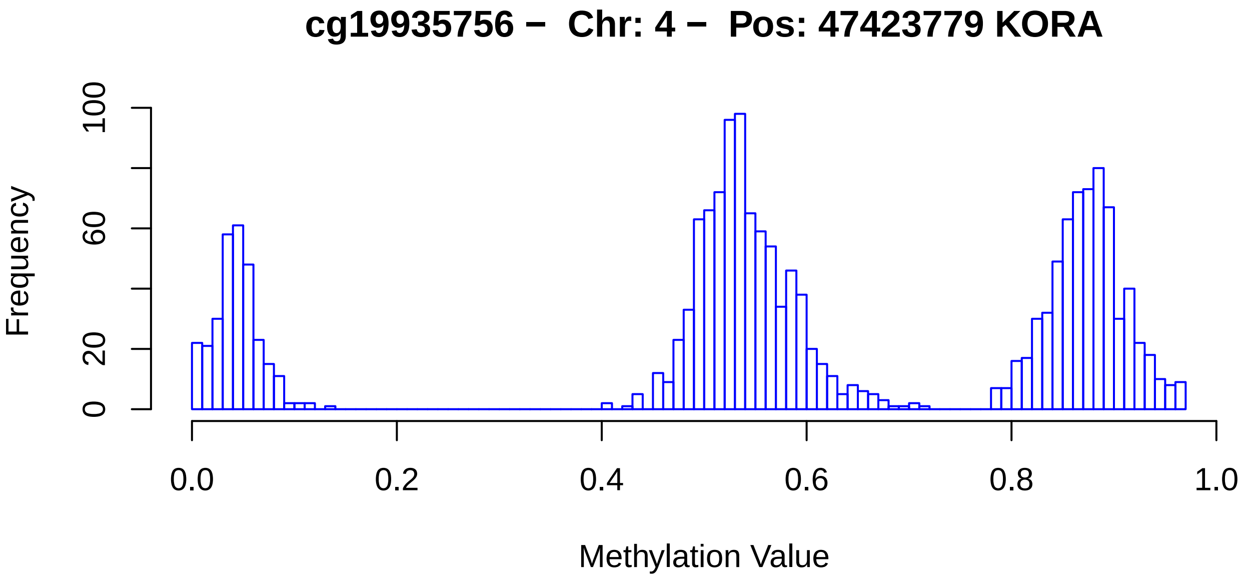
cg19935756 − Chr: 4 − Pos: 47423779 KORA
Frequency
0
20
60
100
0.0
0.2
0.4
0.6
0.8
1.0
Methylation Value

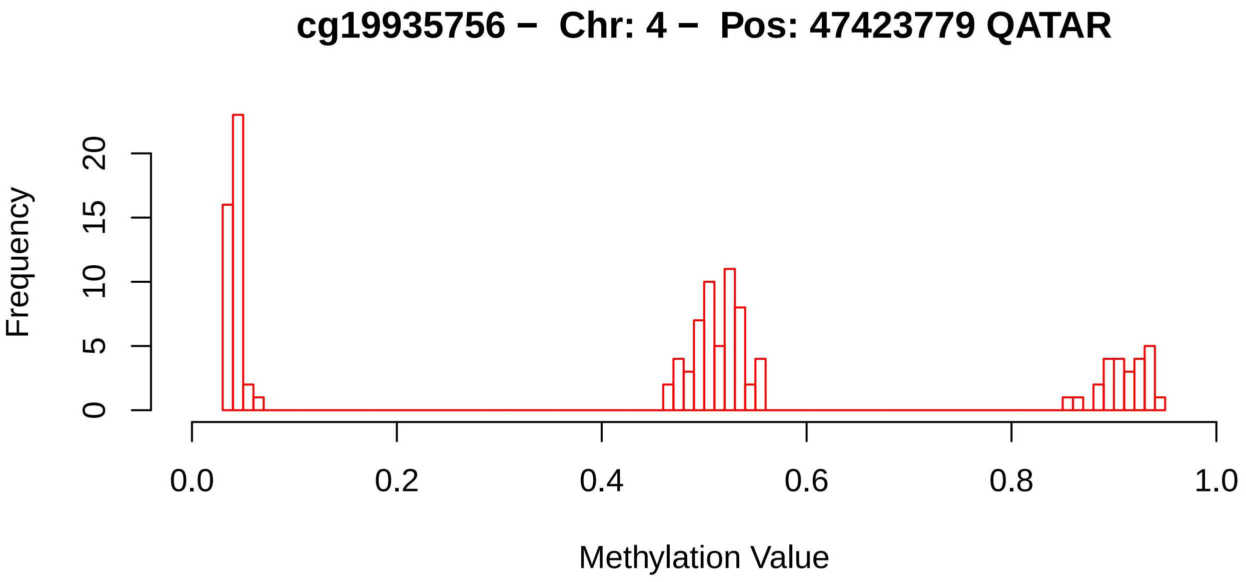
cg19935756 − Chr: 4 − Pos: 47423779 QATAR
Frequency
0
5
10
15
20
0.0
0.2
0.4
0.6
0.8
1.0
Methylation Value

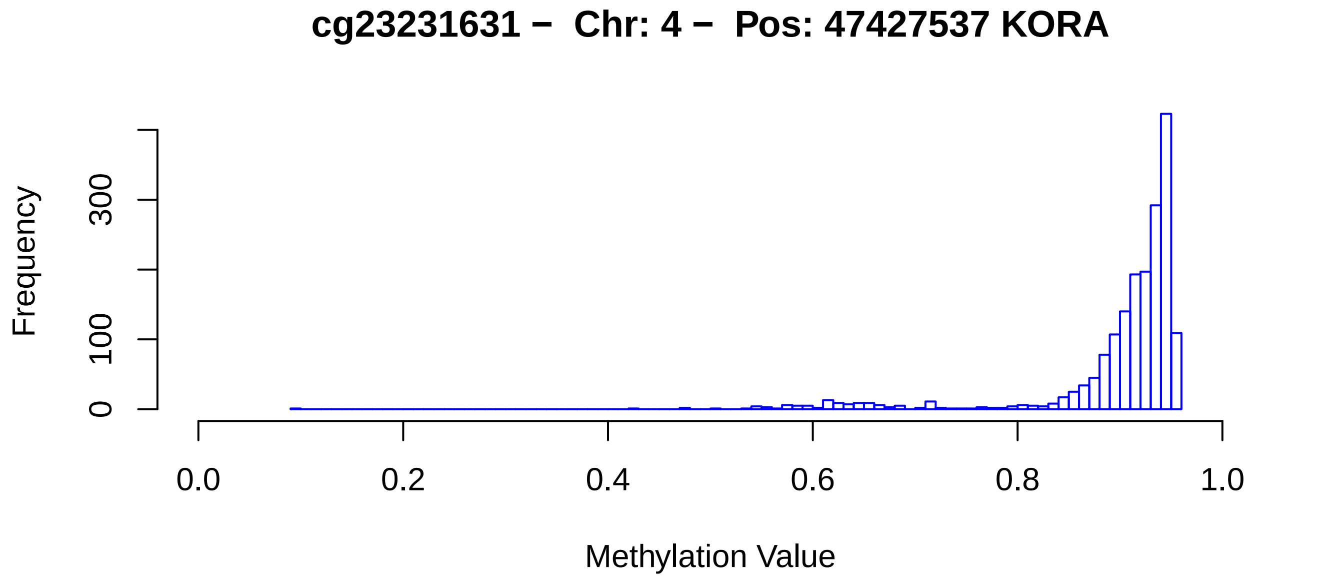
cg23231631 − Chr: 4 − Pos: 47427537 KORA
Frequency
0
100
300
0.0
0.2
0.4
0.6
0.8
1.0
Methylation Value

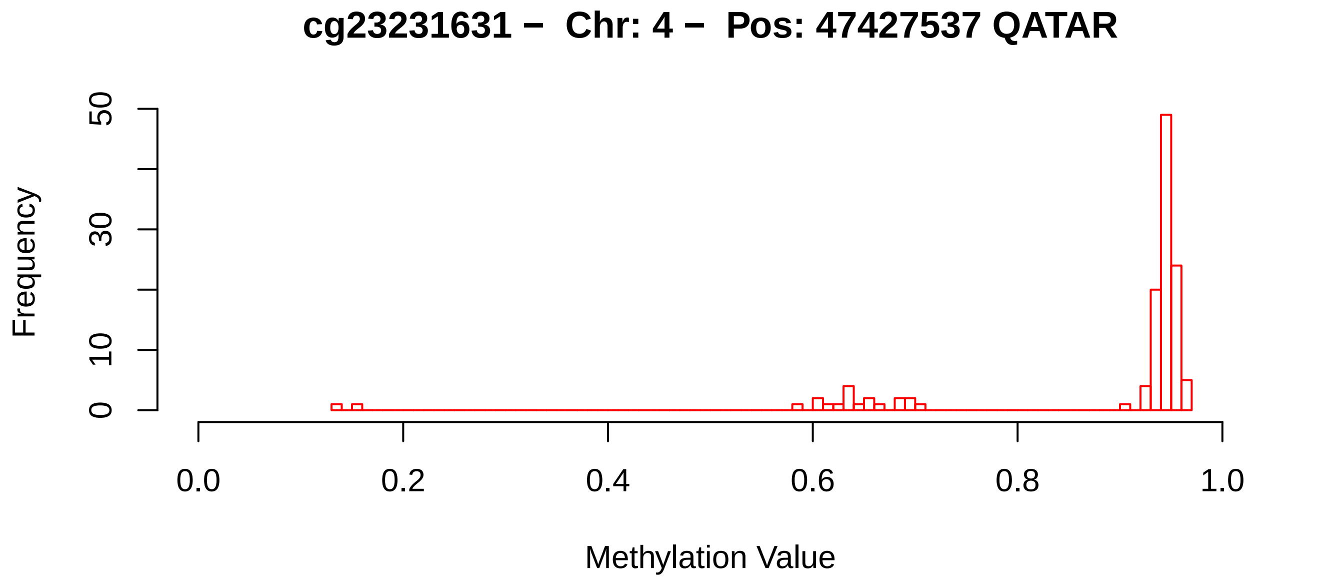
cg23231631 − Chr: 4 − Pos: 47427537 QATAR
Frequency
0
10
30
50
0.0
0.2
0.4
0.6
0.8
1.0
Methylation Value

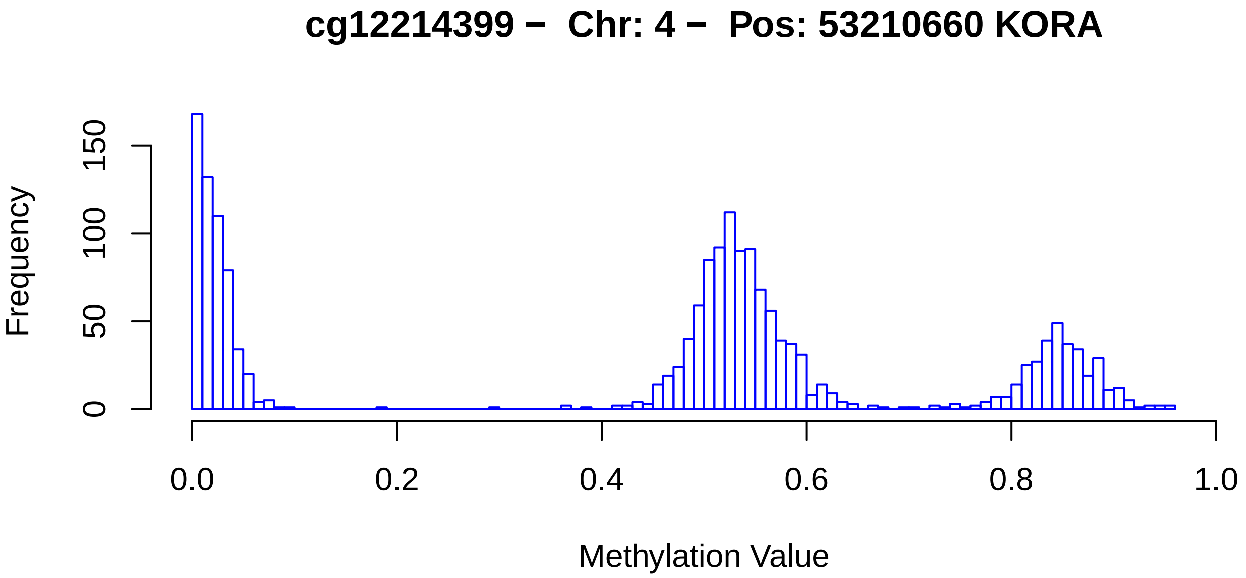
cg12214399 − Chr: 4 − Pos: 53210660 KORA
Frequency
0
50
100
150
0.0
0.2
0.4
0.6
0.8
1.0
Methylation Value

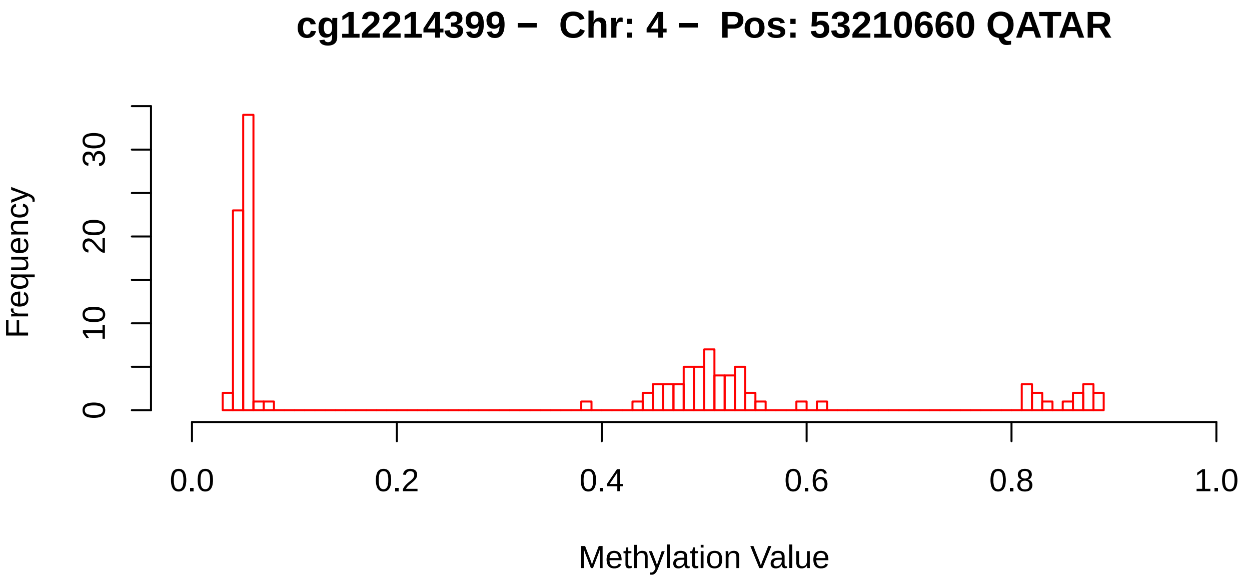
cg12214399 − Chr: 4 − Pos: 53210660 QATAR
Frequency
0
10
20
30
0.0
0.2
0.4
0.6
0.8
1.0
Methylation Value

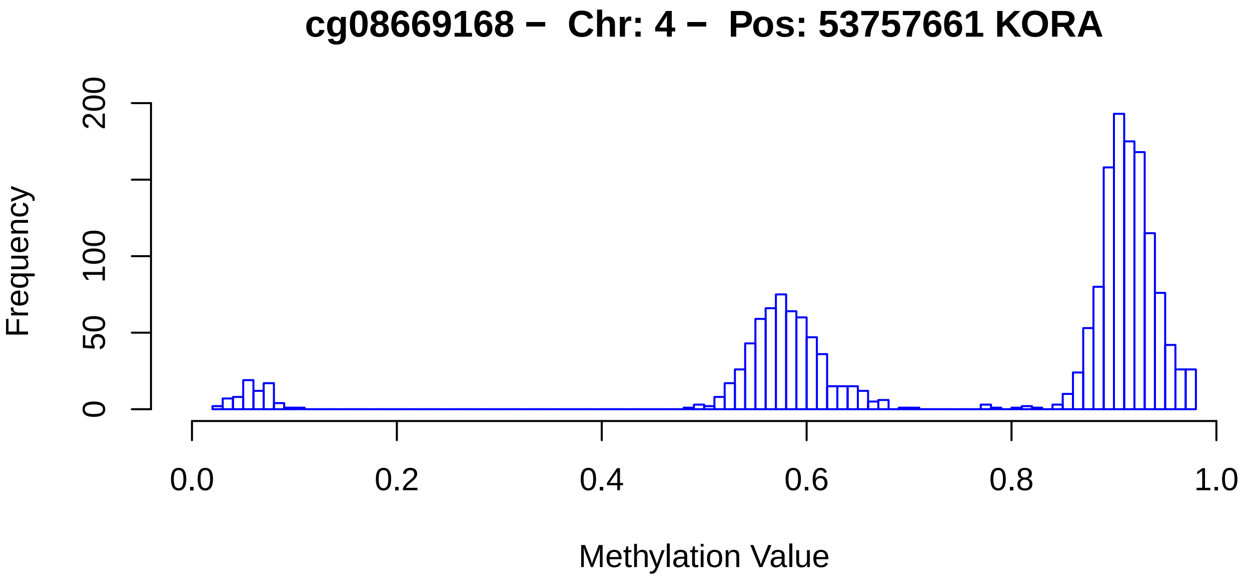
cg08669168 − Chr: 4 − Pos: 53757661 KORA
Frequency
0
50
100
200
0.0
0.2
0.4
0.6
0.8
1.0
Methylation Value

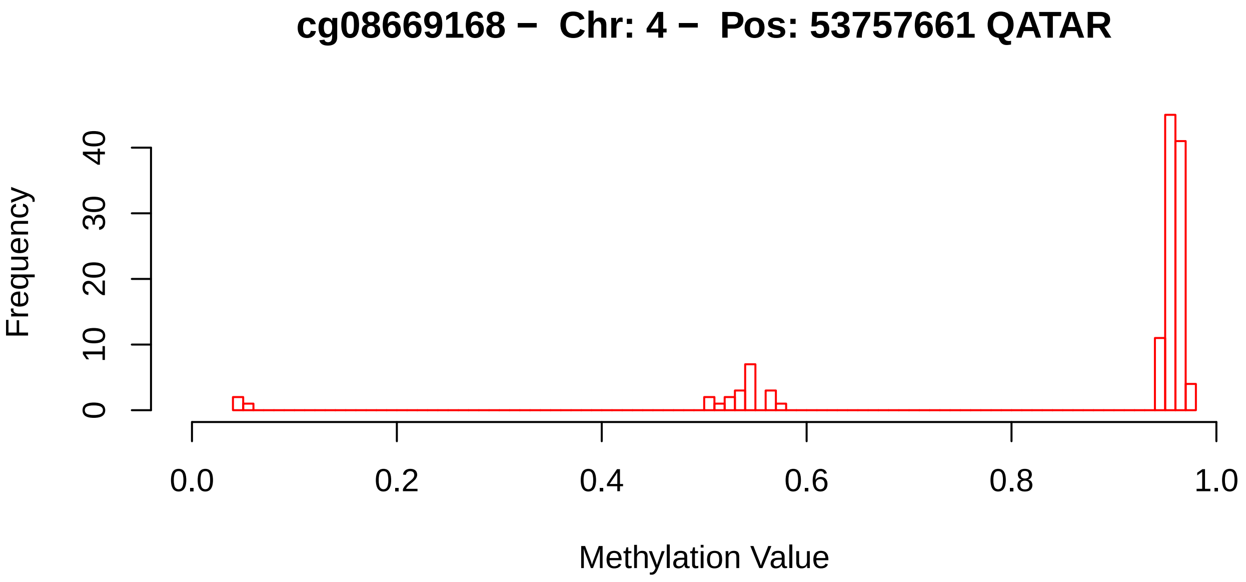
cg08669168 − Chr: 4 − Pos: 53757661 QATAR
Frequency
0
10
20
30
40
0.0
0.2
0.4
0.6
0.8
1.0
Methylation Value

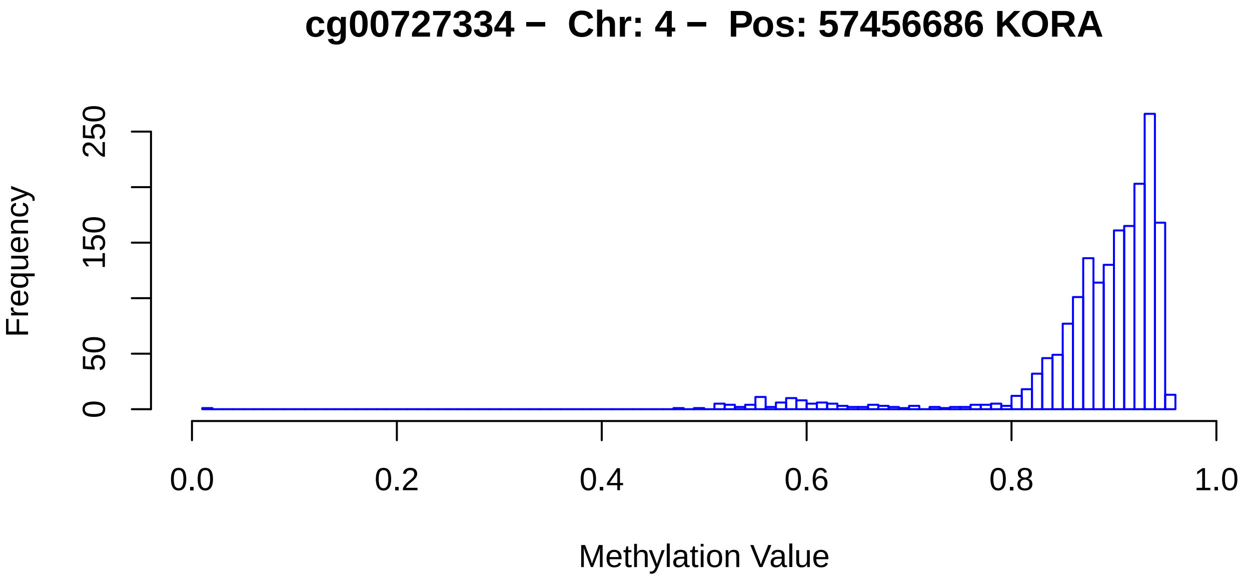
cg00727334 − Chr: 4 − Pos: 57456686 KORA
Frequency
0
50
150
250
0.0
0.2
0.4
0.6
0.8
1.0
Methylation Value

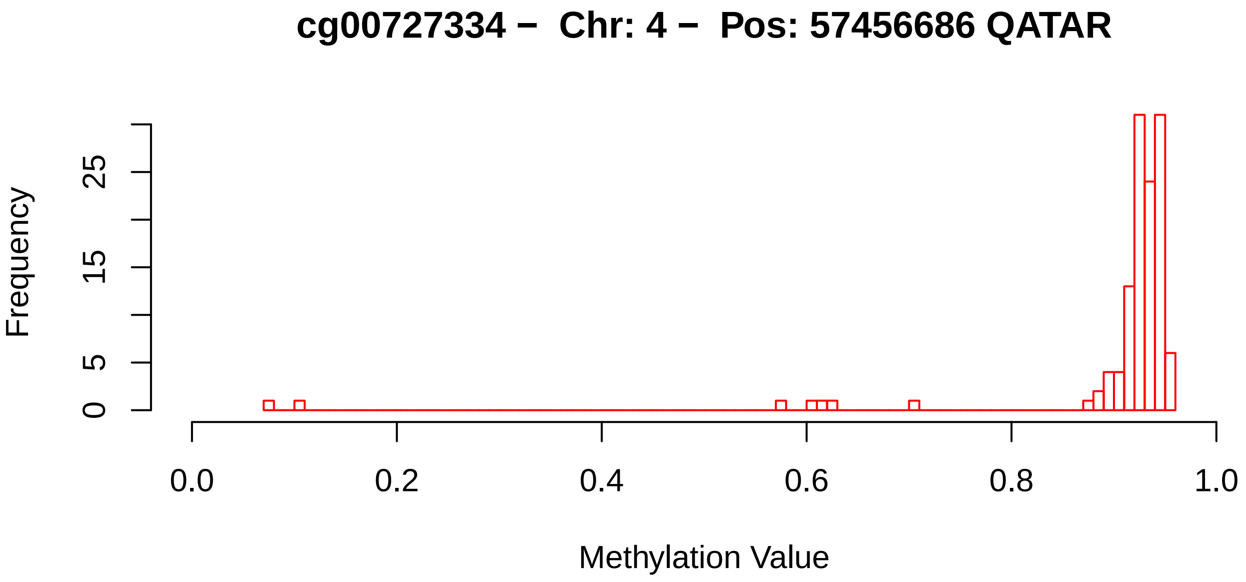
cg00727334 − Chr: 4 − Pos: 57456686 QATAR
Frequency
0
5
15
25
0.0
0.2
0.4
0.6
0.8
1.0
Methylation Value

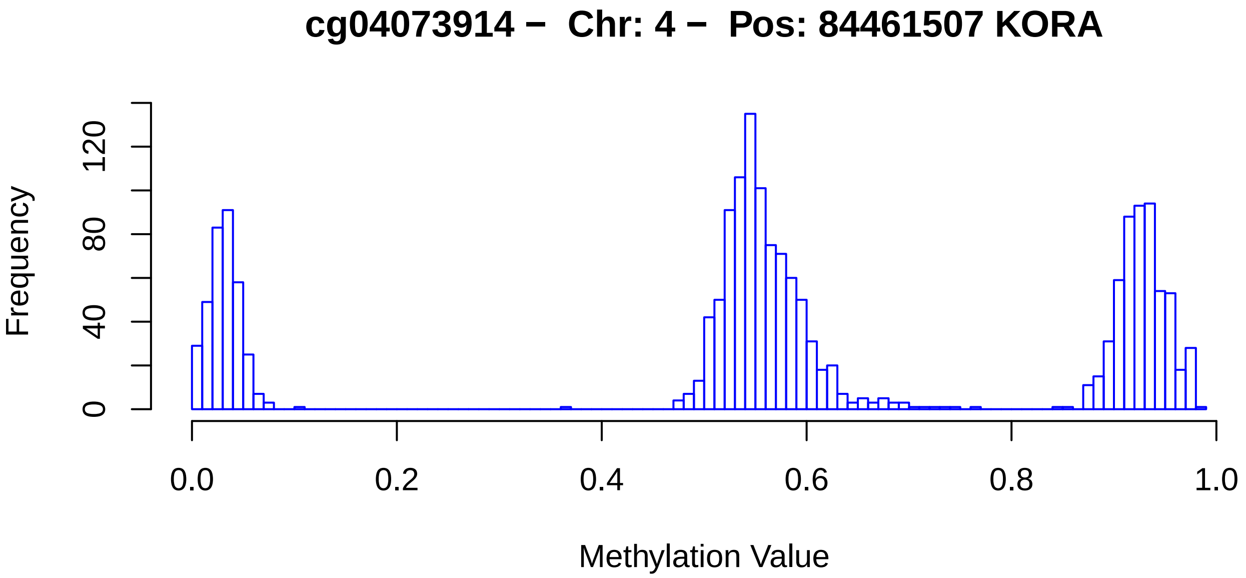
cg04073914 − Chr: 4 − Pos: 84461507 KORA
Frequency
0
40
80
120
0.0
0.2
0.4
0.6
0.8
1.0
Methylation Value

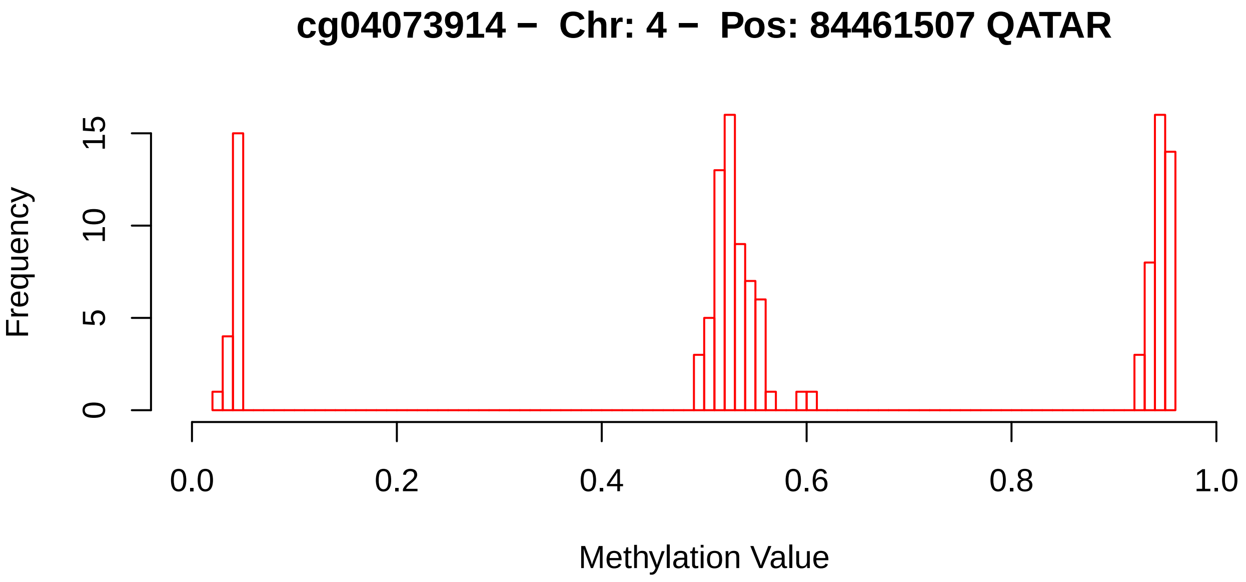
cg04073914 − Chr: 4 − Pos: 84461507 QATAR
Frequency
0
5
10
15
0.0
0.2
0.4
0.6
0.8
1.0
Methylation Value

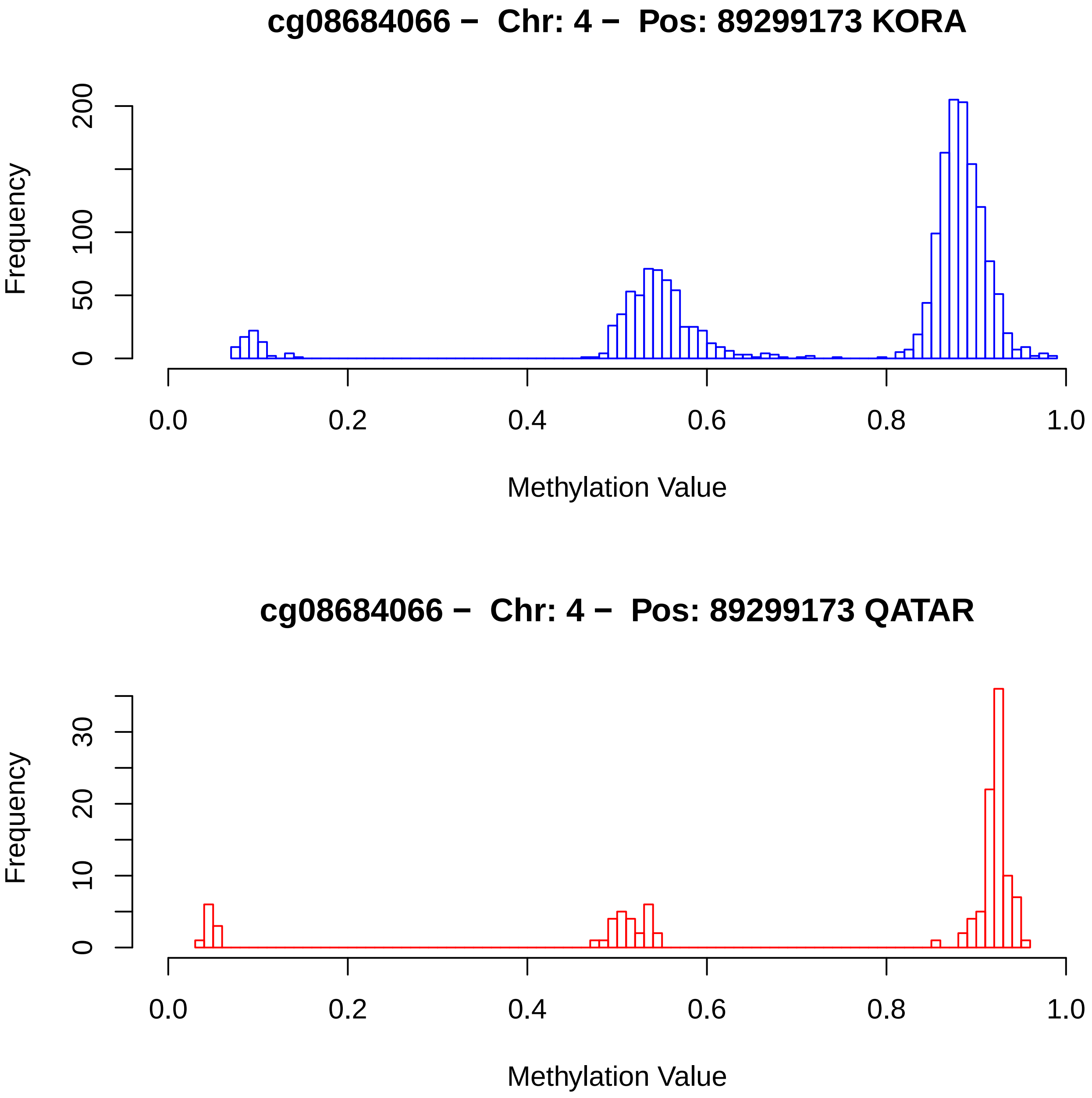
cg08684066 − Chr: 4 − Pos: 89299173 KORA
Frequency
0
50
100
200
0.0
0.2
0.4
0.6
0.8
1.0
Methylation Value
cg08684066 − Chr: 4 − Pos: 89299173 QATAR
Frequency
0
10
20
30
0.0
0.2
0.4
0.6
0.8
1.0
Methylation Value

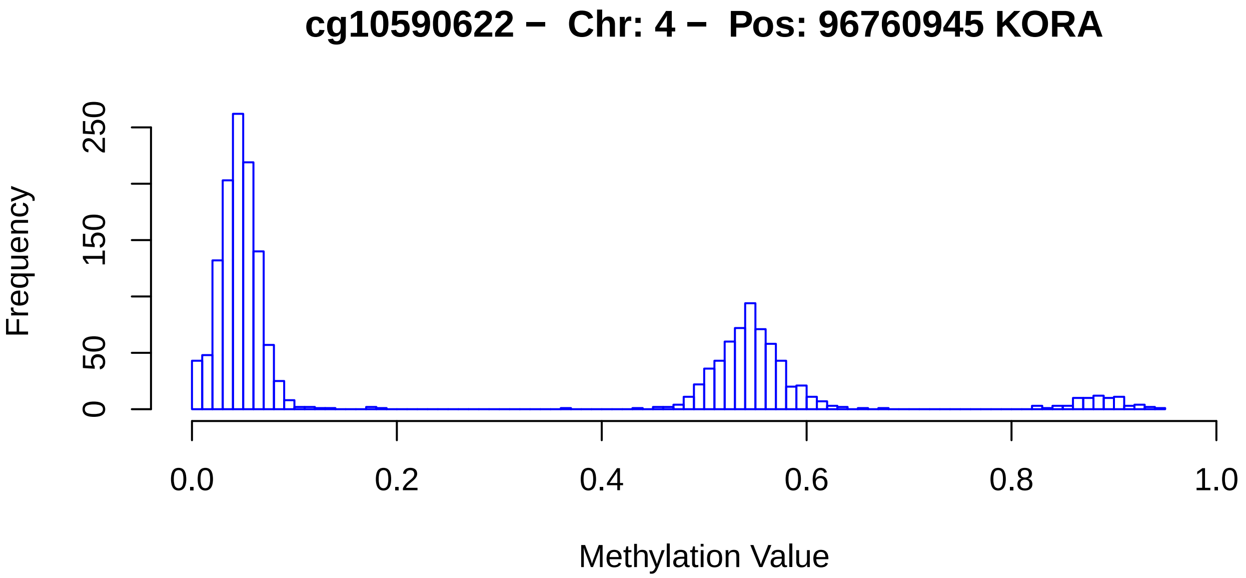
cg10590622 − Chr: 4 − Pos: 96760945 KORA
Frequency
0
50
150
250
0.0
0.2
0.4
0.6
0.8
1.0
Methylation Value

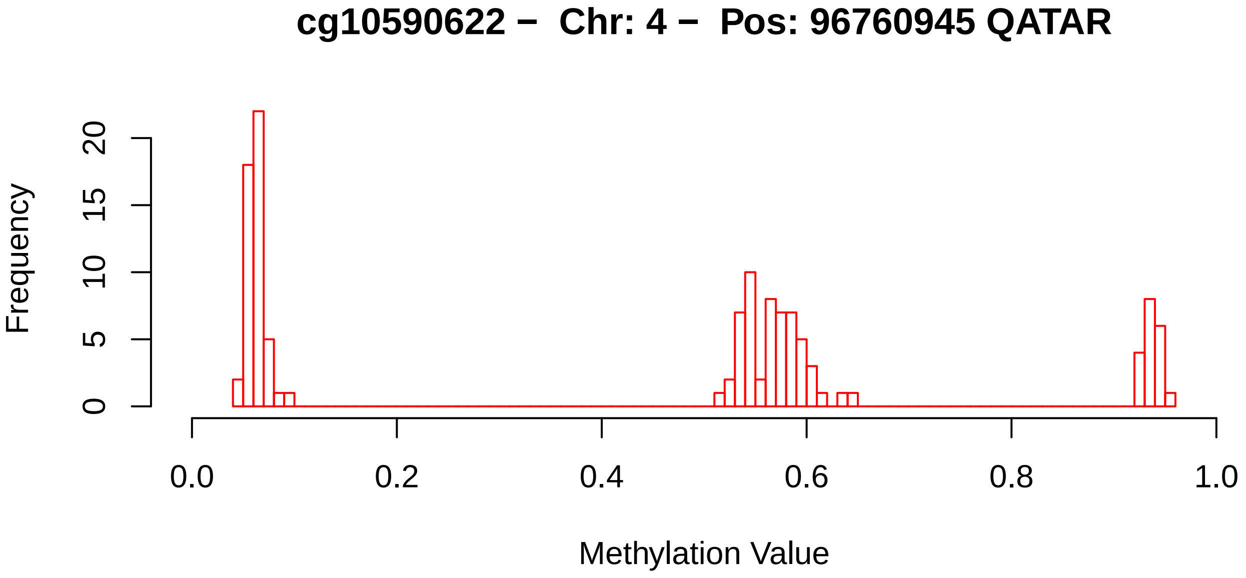
cg10590622 − Chr: 4 − Pos: 96760945 QATAR
Frequency
0
5
10
15
20
0.0
0.2
0.4
0.6
0.8
1.0
Methylation Value

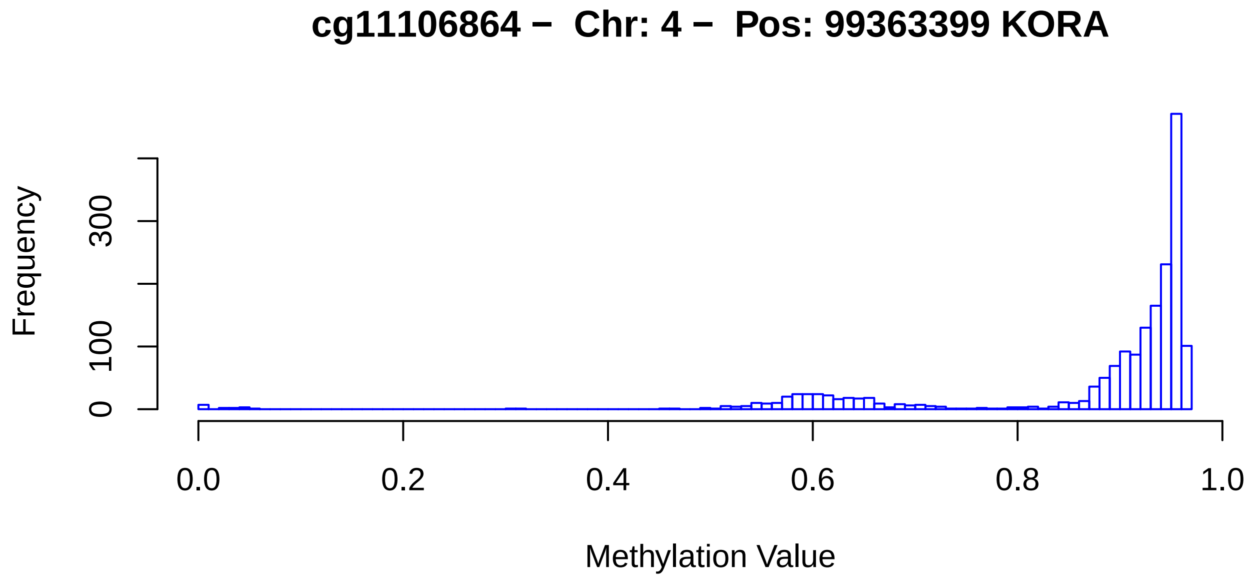
cg11106864 − Chr: 4 − Pos: 99363399 KORA
Frequency
0
100
300
0.0
0.2
0.4
0.6
0.8
1.0
Methylation Value

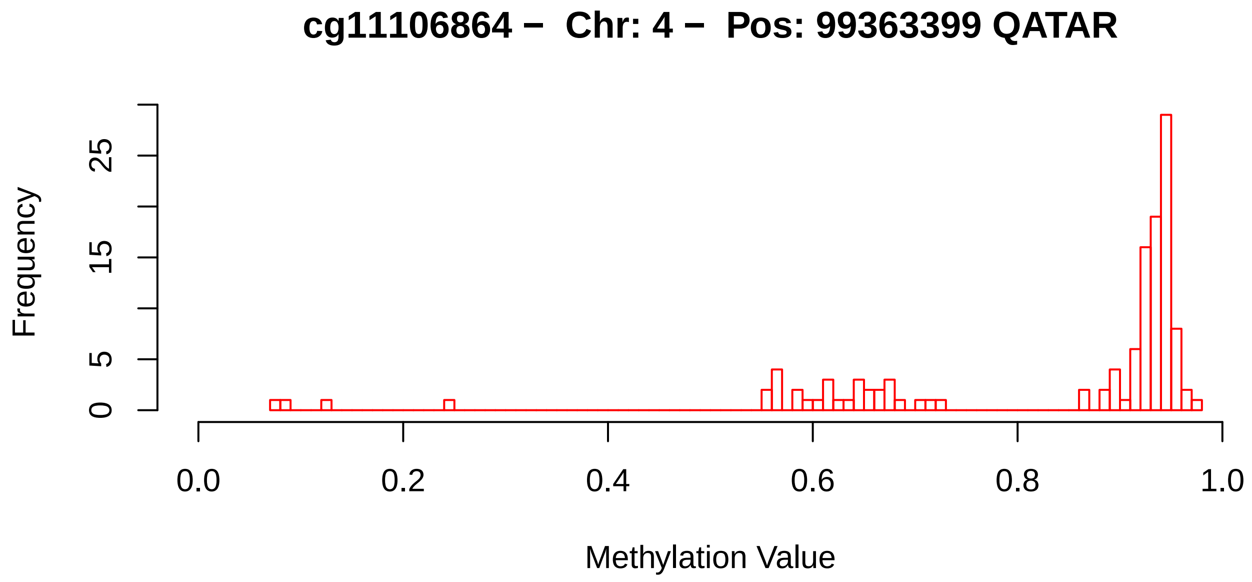
cg11106864 − Chr: 4 − Pos: 99363399 QATAR
Frequency
0
5
15
25
0.0
0.2
0.4
0.6
0.8
1.0
Methylation Value

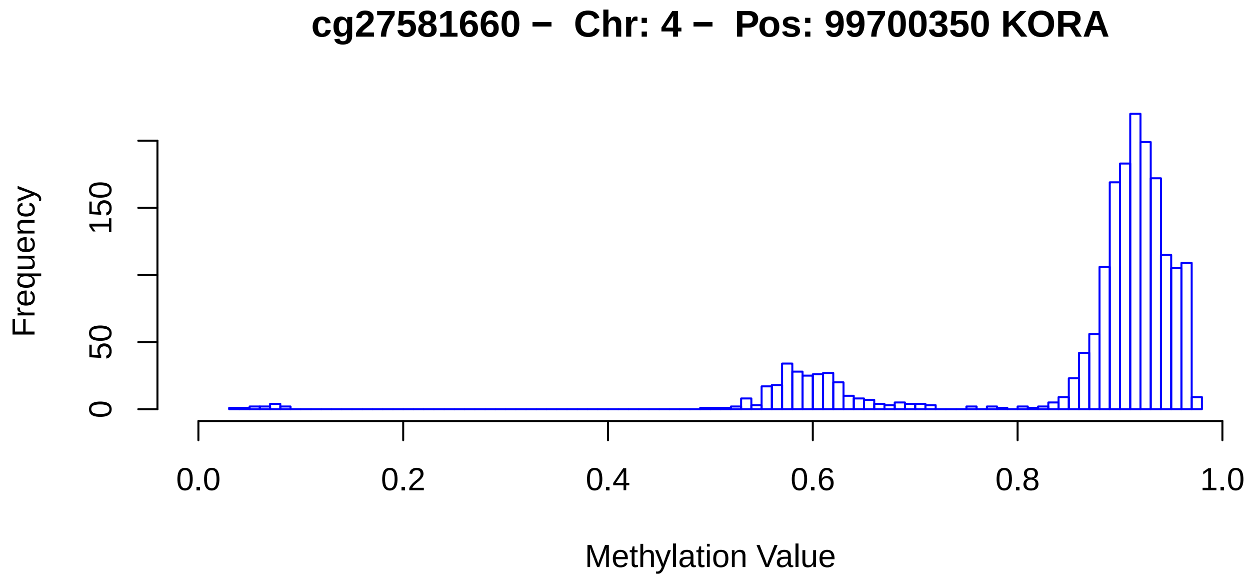
cg27581660 − Chr: 4 − Pos: 99700350 KORA
Frequency
0
50
150
0.0
0.2
0.4
0.6
0.8
1.0
Methylation Value

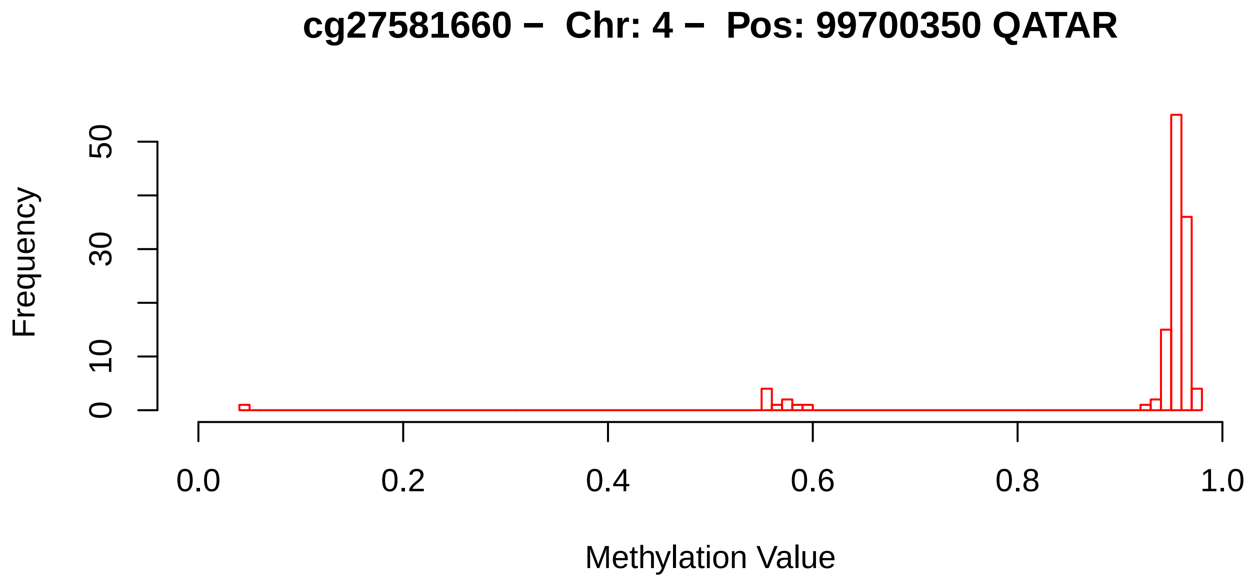
cg27581660 − Chr: 4 − Pos: 99700350 QATAR
Frequency
0
10
30
50
0.0
0.2
0.4
0.6
0.8
1.0
Methylation Value

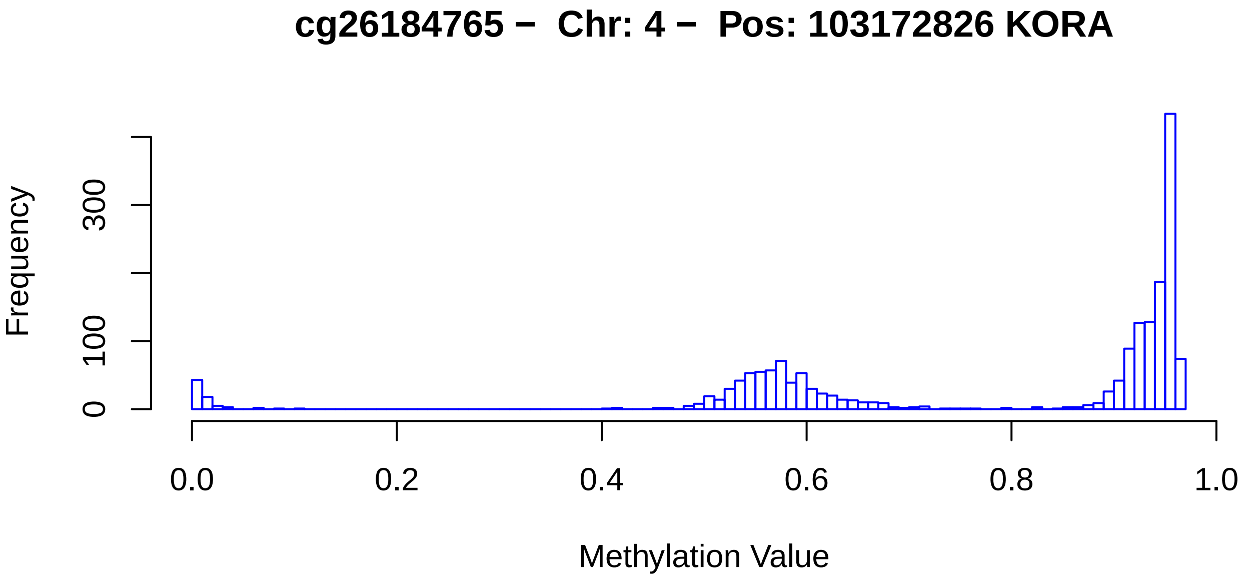
cg26184765 − Chr: 4 − Pos: 103172826 KORA
Frequency
0
100
300
0.0
0.2
0.4
0.6
0.8
1.0
Methylation Value

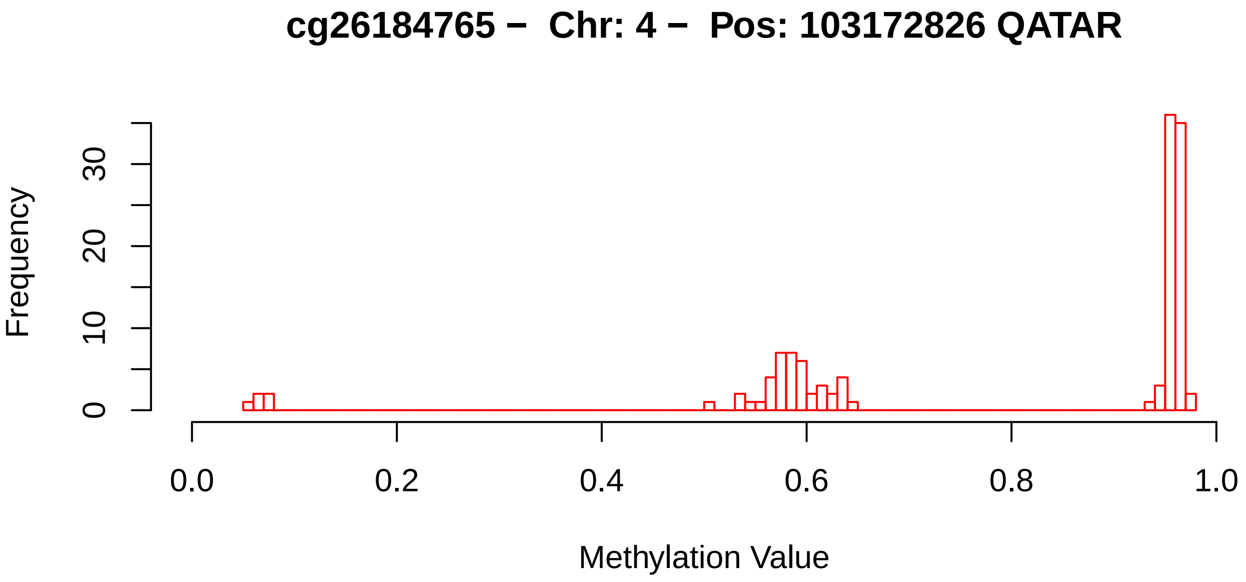
cg26184765 − Chr: 4 − Pos: 103172826 QATAR
Frequency
0
10
20
30
0.0
0.2
0.4
0.6
0.8
1.0
Methylation Value

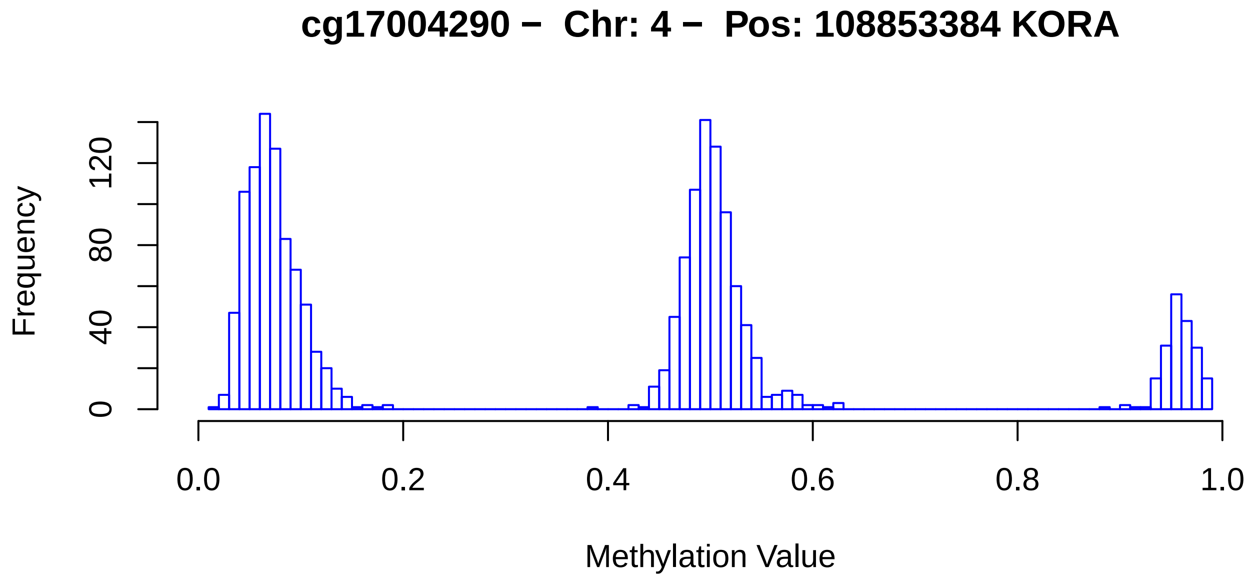
cg17004290 − Chr: 4 − Pos: 108853384 KORA
Frequency
0
40
80
120
0.0
0.2
0.4
0.6
0.8
1.0
Methylation Value

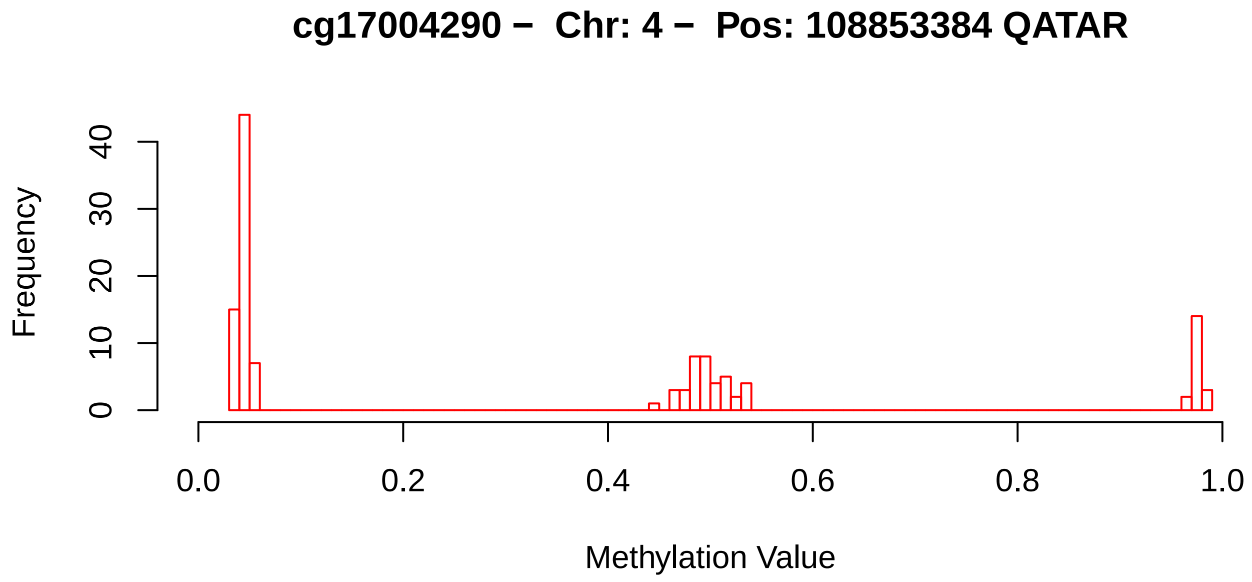
cg17004290 − Chr: 4 − Pos: 108853384 QATAR
Frequency
0
10
20
30
40
0.0
0.2
0.4
0.6
0.8
1.0
Methylation Value

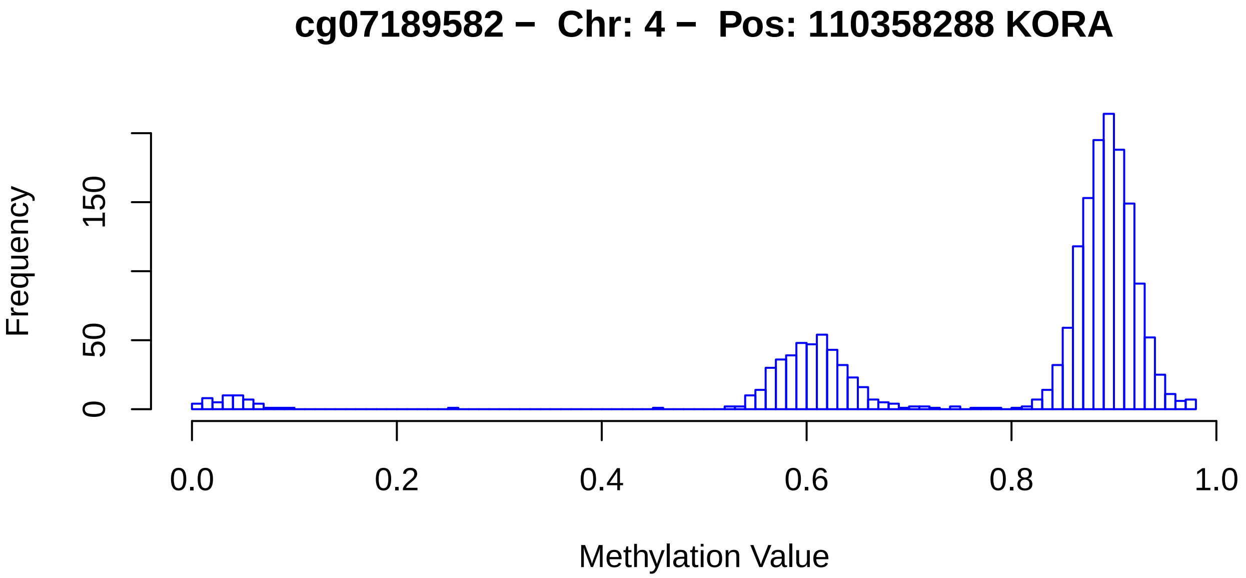

cg07189582 − Chr: 4 − Pos: 110358288 KORA
Frequency
0
50
150
0.0
0.2
0.4
0.6
0.8
1.0
Methylation Value

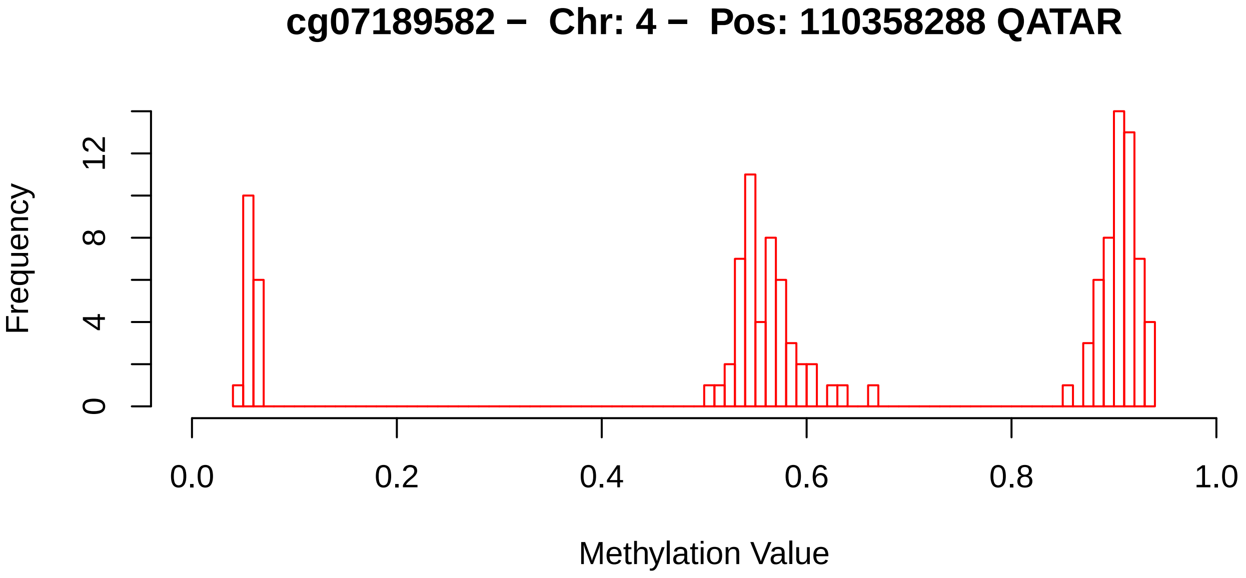

cg07189582 − Chr: 4 − Pos: 110358288 QATAR
Frequency
0
4
8
12
0.0
0.2
0.4
0.6
0.8
1.0
Methylation Value

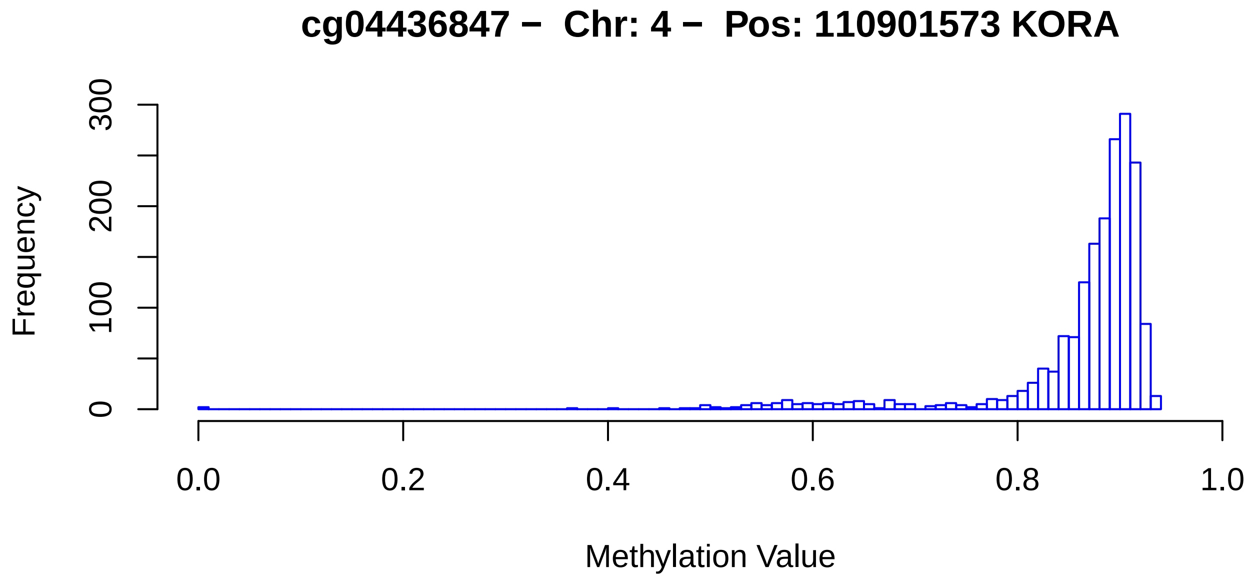
cg04436847 − Chr: 4 − Pos: 110901573 KORA
Frequency
0
100
200
300
0.0
0.2
0.4
0.6
0.8
1.0
Methylation Value

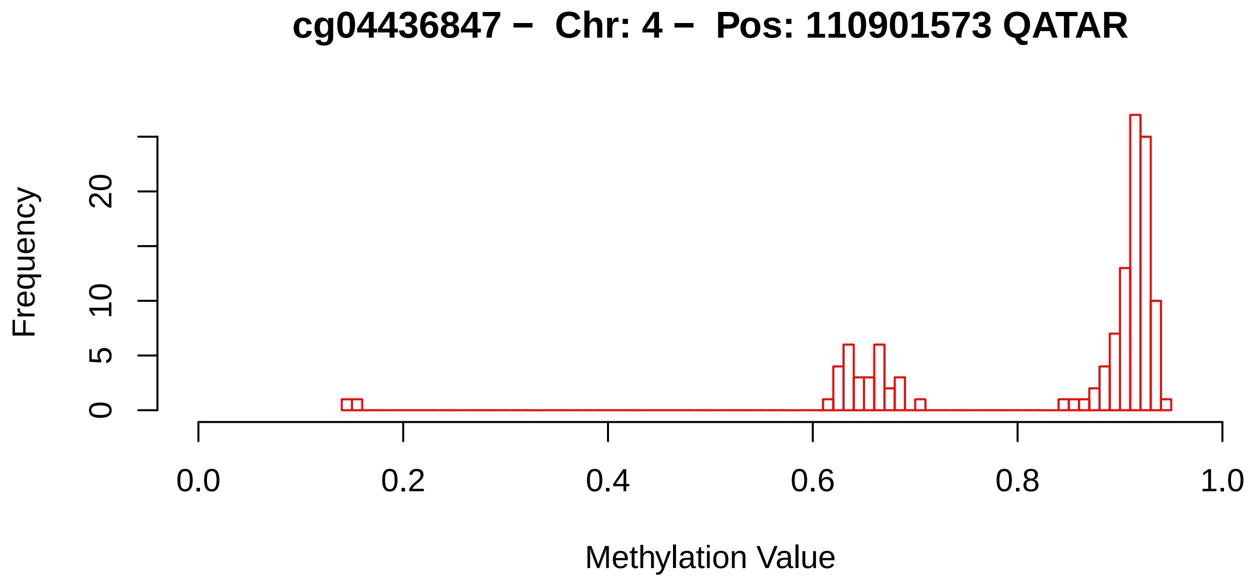
cg04436847 − Chr: 4 − Pos: 110901573 QATAR
Frequency
0
5
10
20
0.0
0.2
0.4
0.6
0.8
1.0
Methylation Value

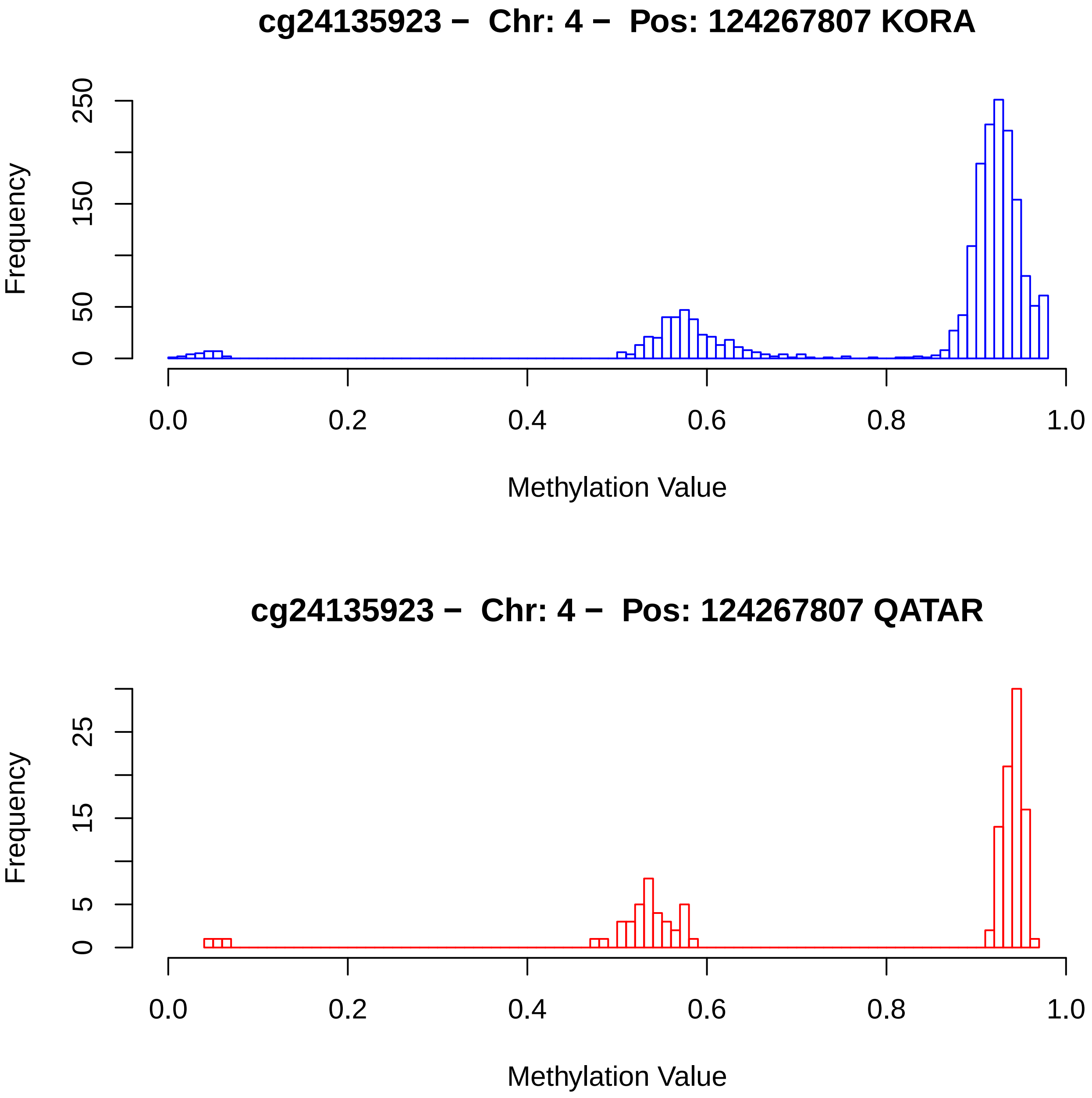
cg24135923 − Chr: 4 − Pos: 124267807 KORA
Frequency
0
50
150
250
0.0
0.2
0.4
0.6
0.8
1.0
Methylation Value
cg24135923 − Chr: 4 − Pos: 124267807 QATAR
Frequency
0
5
15
25
0.0
0.2
0.4
0.6
0.8
1.0
Methylation Value

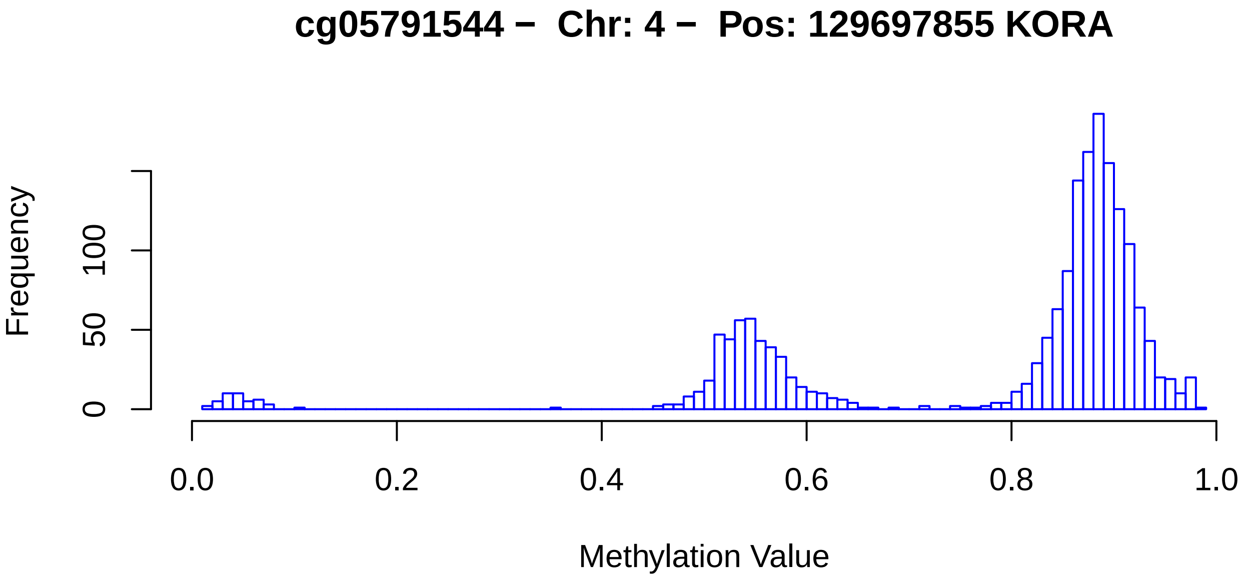
cg05791544 − Chr: 4 − Pos: 129697855 KORA
Frequency
0
50
100
0.0
0.2
0.4
0.6
0.8
1.0
Methylation Value

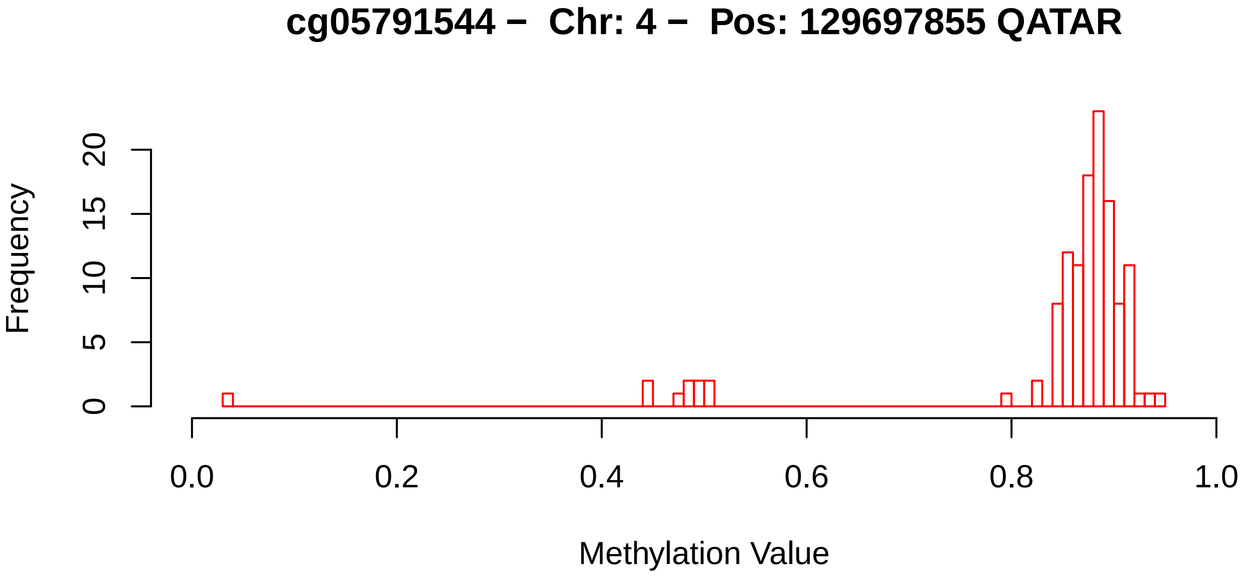
cg05791544 − Chr: 4 − Pos: 129697855 QATAR
Frequency
0
5
10
15
20
0.0
0.2
0.4
0.6
0.8
1.0
Methylation Value

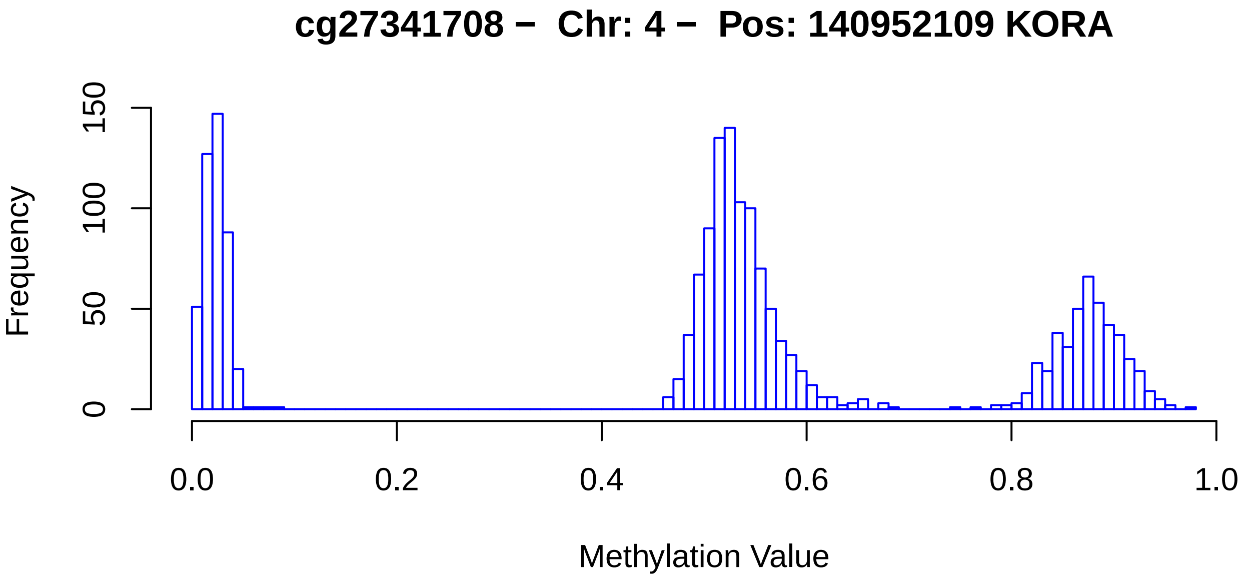
cg27341708 − Chr: 4 − Pos: 140952109 KORA
Frequency
0
50
100
150
0.0
0.2
0.4
0.6
0.8
1.0
Methylation Value

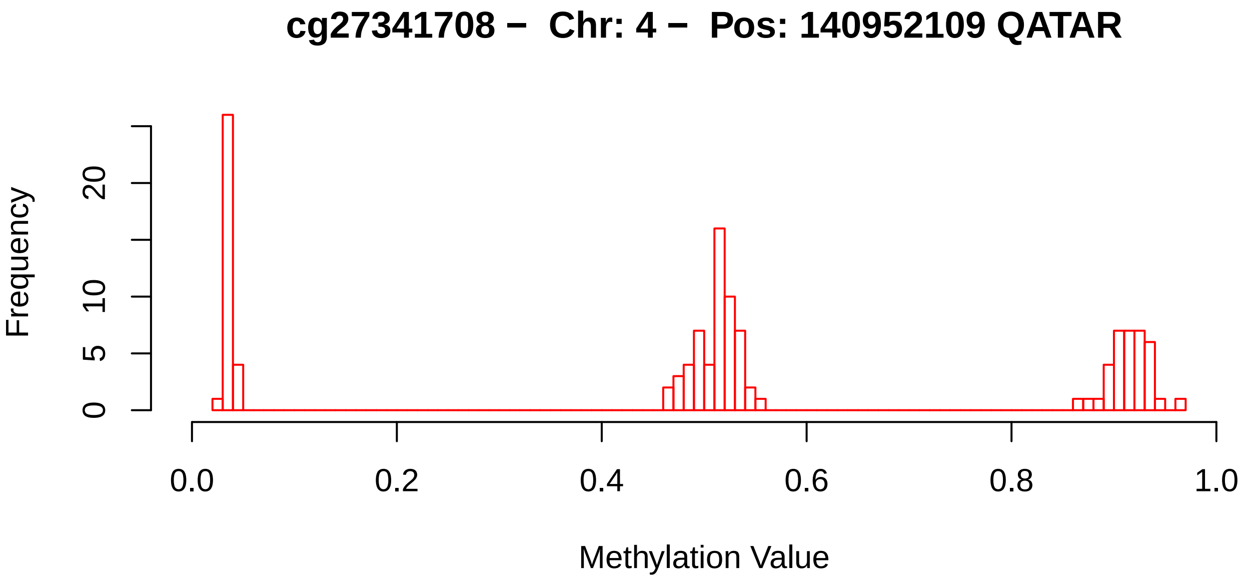
cg27341708 − Chr: 4 − Pos: 140952109 QATAR
Frequency
0
5
10
20
0.0
0.2
0.4
0.6
0.8
1.0
Methylation Value

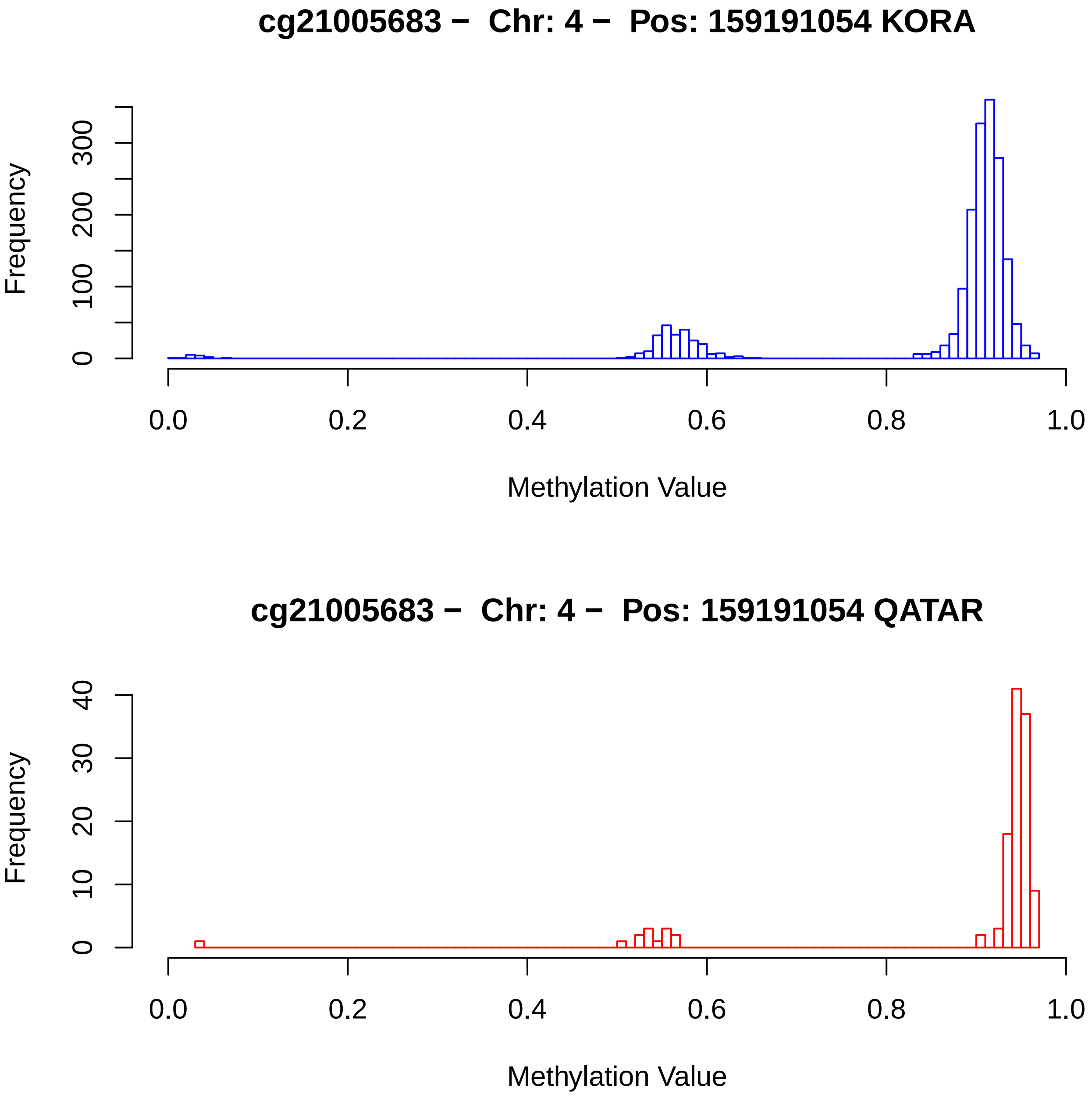
cg21005683 − Chr: 4 − Pos: 159191054 KORA
Frequency
0
100
200
300
0.0
0.2
0.4
0.6
0.8
1.0
Methylation Value
cg21005683 − Chr: 4 − Pos: 159191054 QATAR
Frequency
0
10
20
30
40
0.0
0.2
0.4
0.6
0.8
1.0
Methylation Value

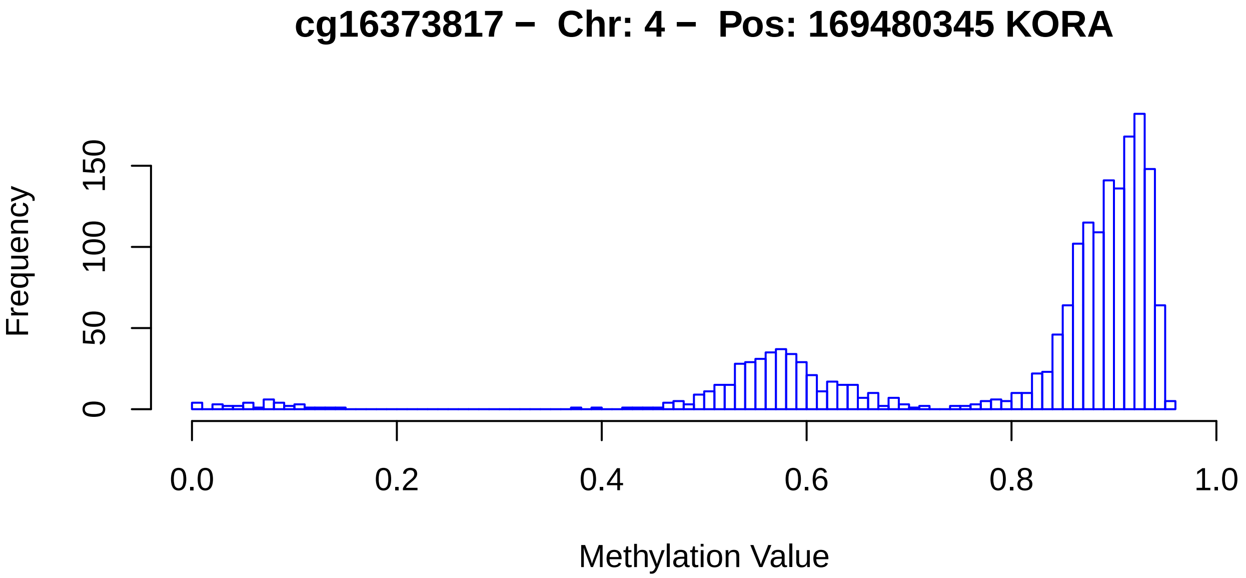

cg16373817 − Chr: 4 − Pos: 169480345 KORA
Frequency
0
50
100
150
0.0
0.2
0.4
0.6
0.8
1.0
Methylation Value

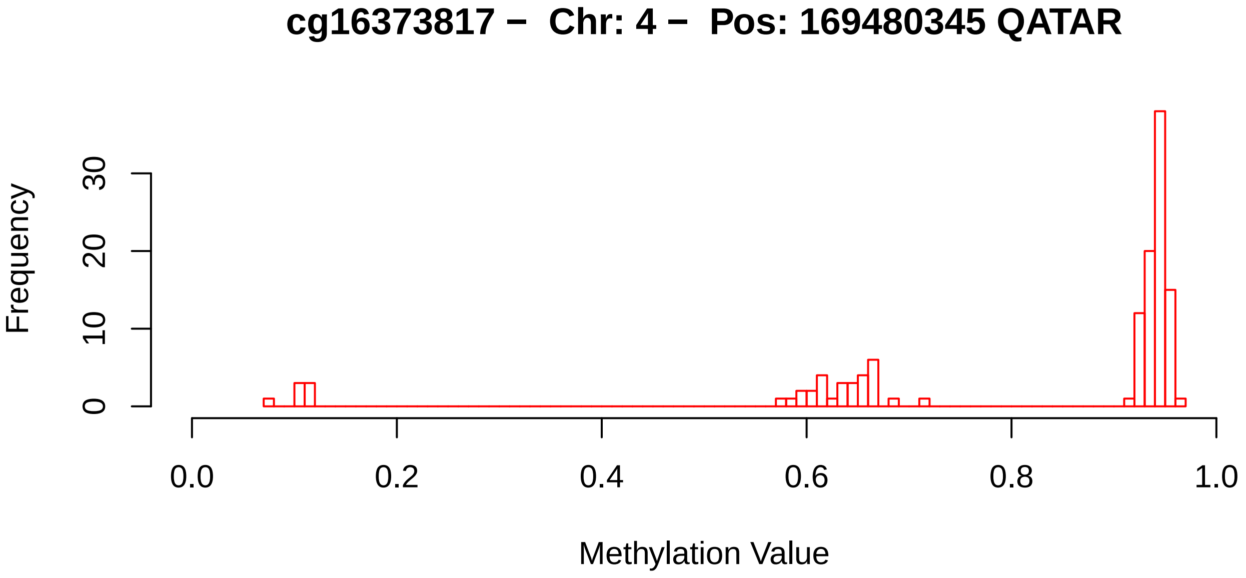

cg16373817 − Chr: 4 − Pos: 169480345 QATAR
Frequency
0
10
20
30
0.0
0.2
0.4
0.6
0.8
1.0
Methylation Value

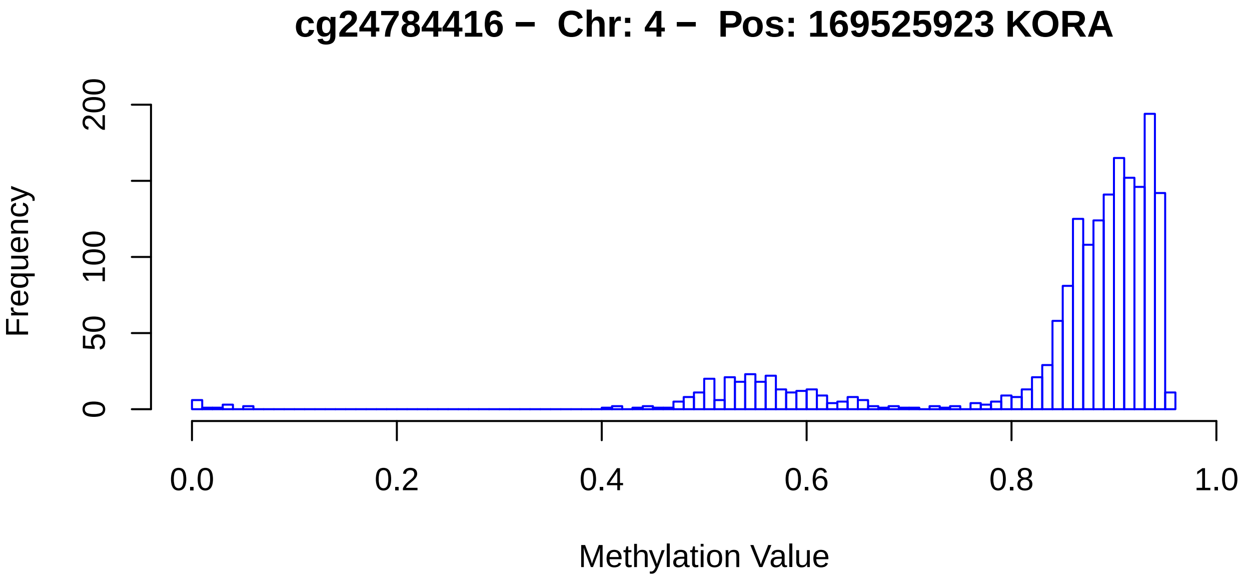
cg24784416 − Chr: 4 − Pos: 169525923 KORA
Frequency
0
50
100
200
0.0
0.2
0.4
0.6
0.8
1.0
Methylation Value

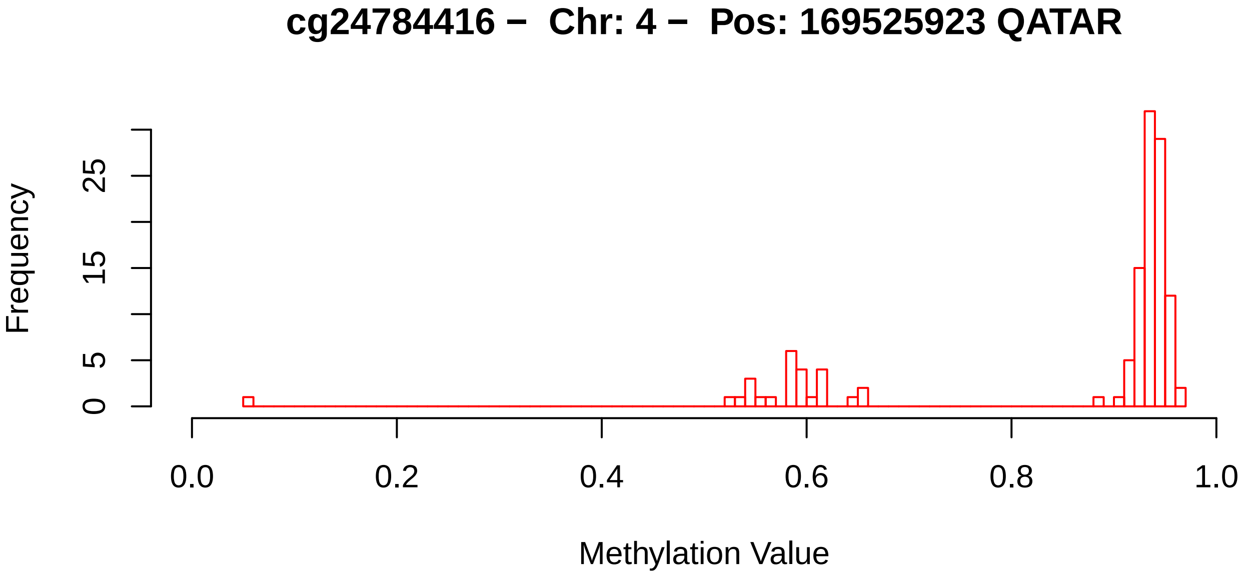
cg24784416 − Chr: 4 − Pos: 169525923 QATAR
Frequency
0
5
15
25
0.0
0.2
0.4
0.6
0.8
1.0
Methylation Value

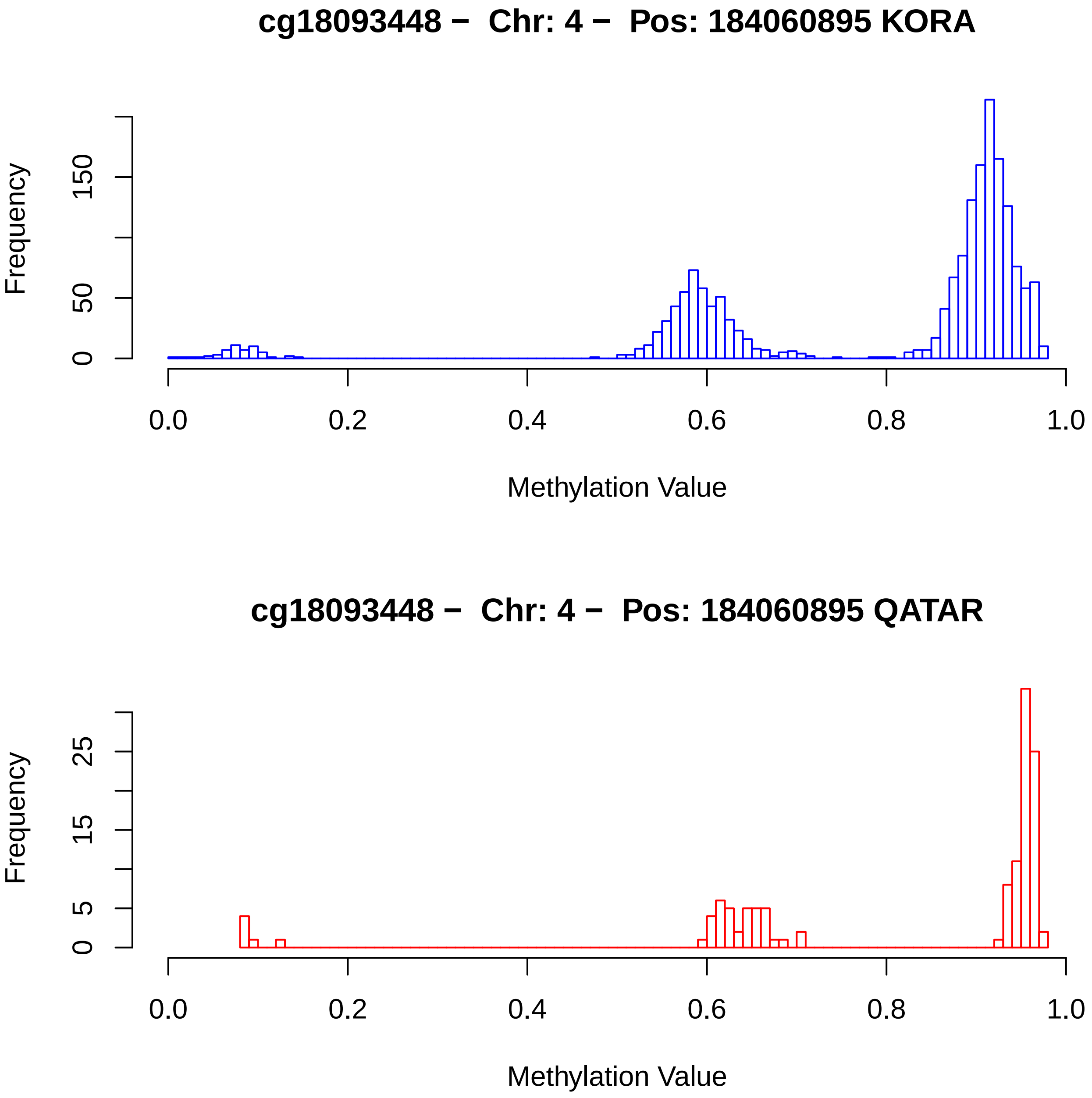

cg18093448 − Chr: 4 − Pos: 184060895 KORA
Frequency
0
50
150
0.0
0.2
0.4
0.6
0.8
1.0
Methylation Value
cg18093448 − Chr: 4 − Pos: 184060895 QATAR
Frequency
0
5
15
25
0.0
0.2
0.4
0.6
0.8
1.0
Methylation Value

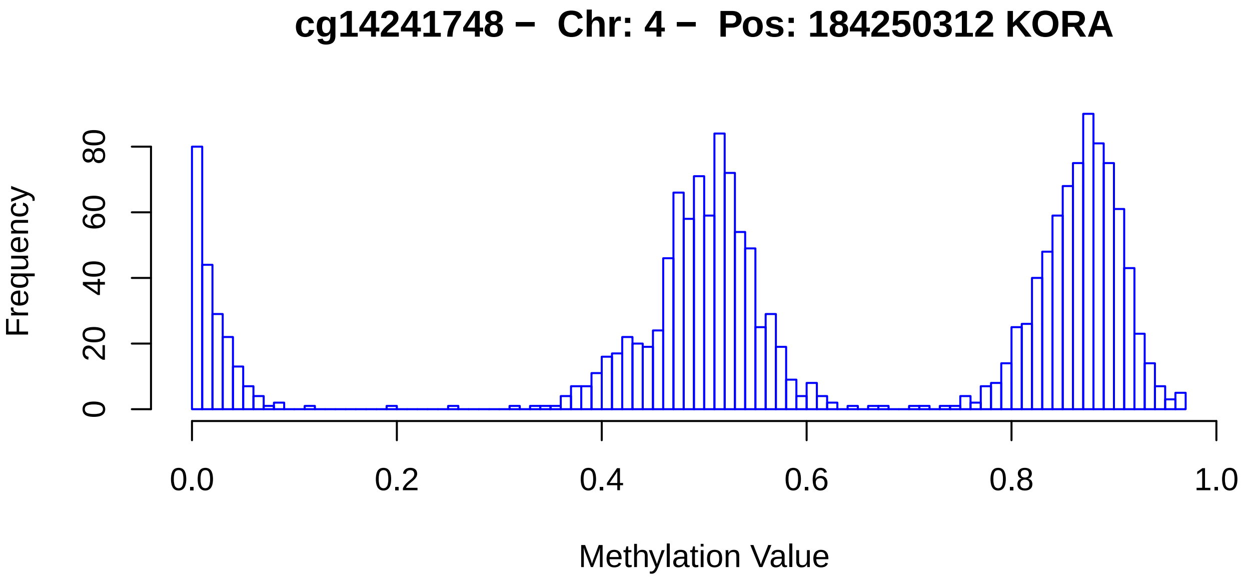
cg14241748 − Chr: 4 − Pos: 184250312 KORA
Frequency
0
20
40
60
80
0.0
0.2
0.4
0.6
0.8
1.0
Methylation Value

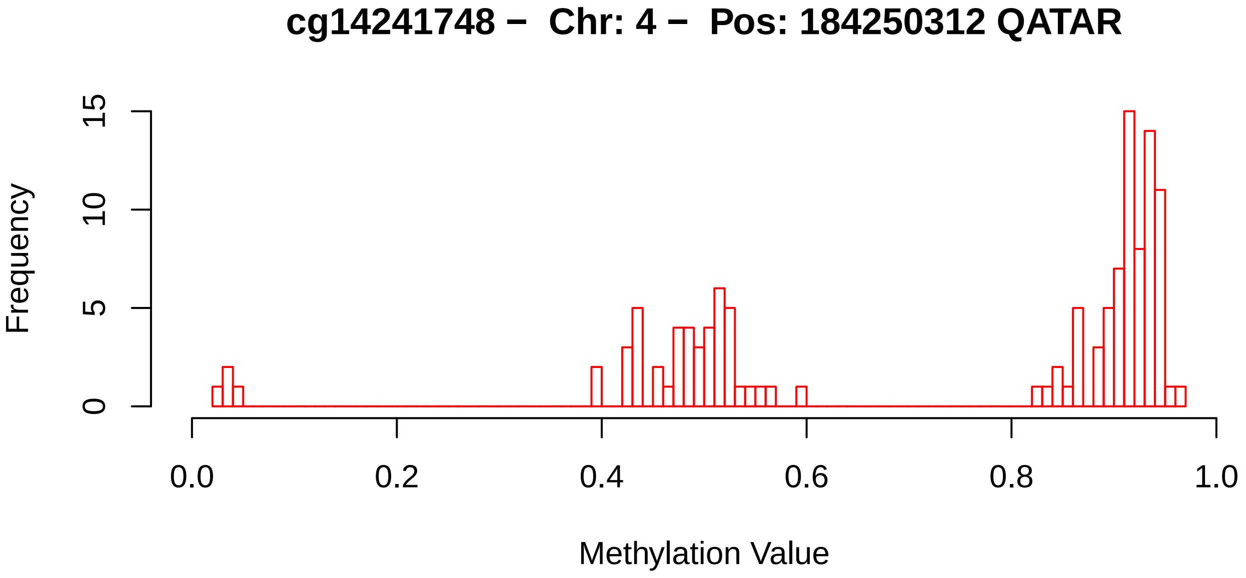
cg14241748 − Chr: 4 − Pos: 184250312 QATAR
Frequency
0
5
10
15
0.0
0.2
0.4
0.6
0.8
1.0
Methylation Value

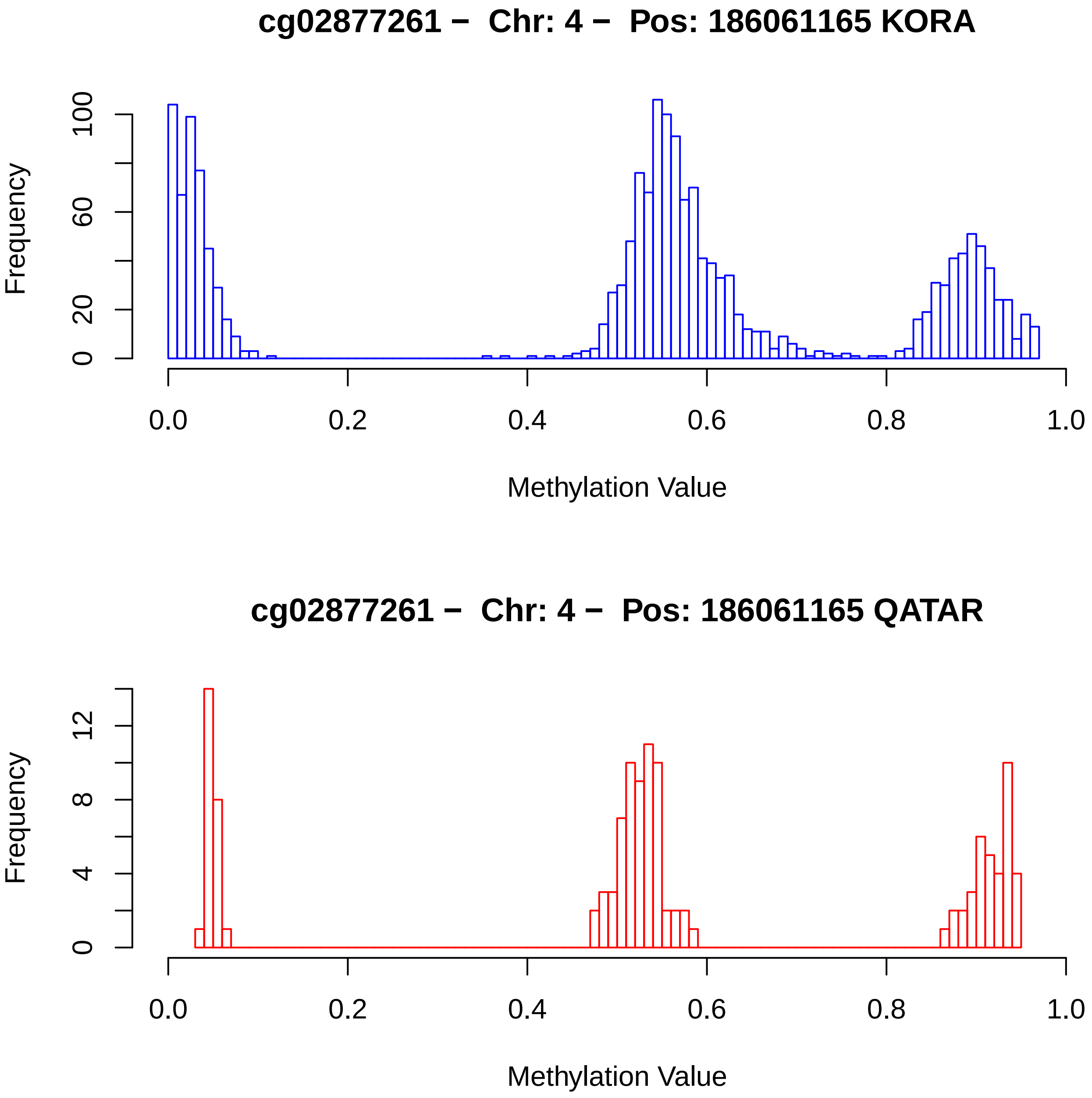
cg02877261 − Chr: 4 − Pos: 186061165 KORA
Frequency
0
20
60
100
0.0
0.2
0.4
0.6
0.8
1.0
Methylation Value
cg02877261 − Chr: 4 − Pos: 186061165 QATAR
Frequency
0
4
8
12
0.0
0.2
0.4
0.6
0.8
1.0
Methylation Value

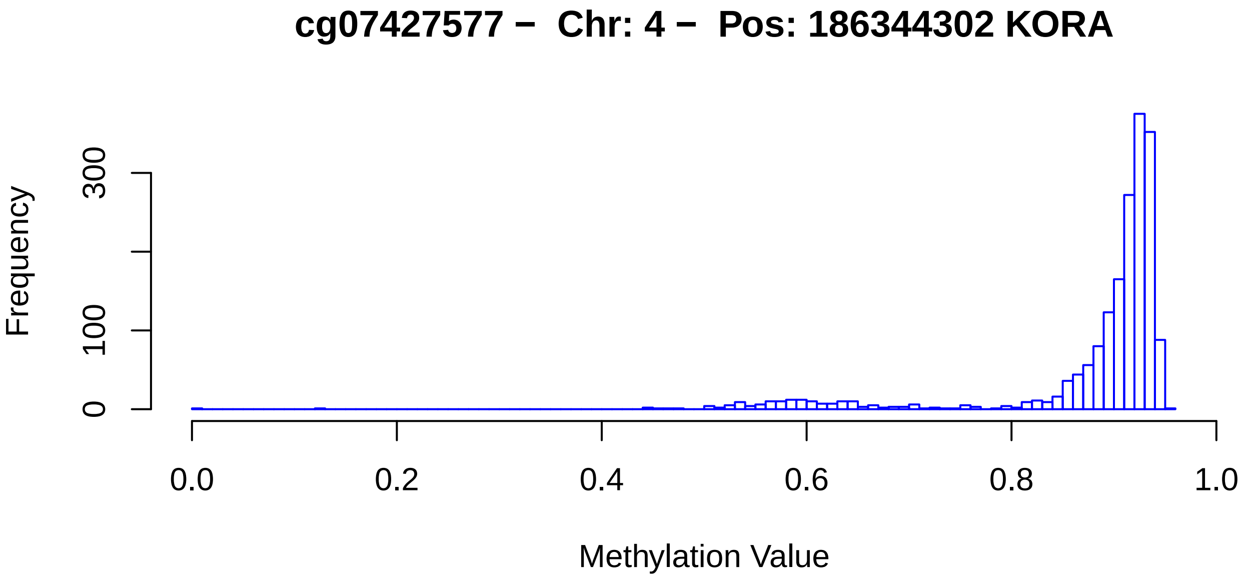

cg07427577 − Chr: 4 − Pos: 186344302 KORA
Frequency
0
100
300
0.0
0.2
0.4
0.6
0.8
1.0
Methylation Value

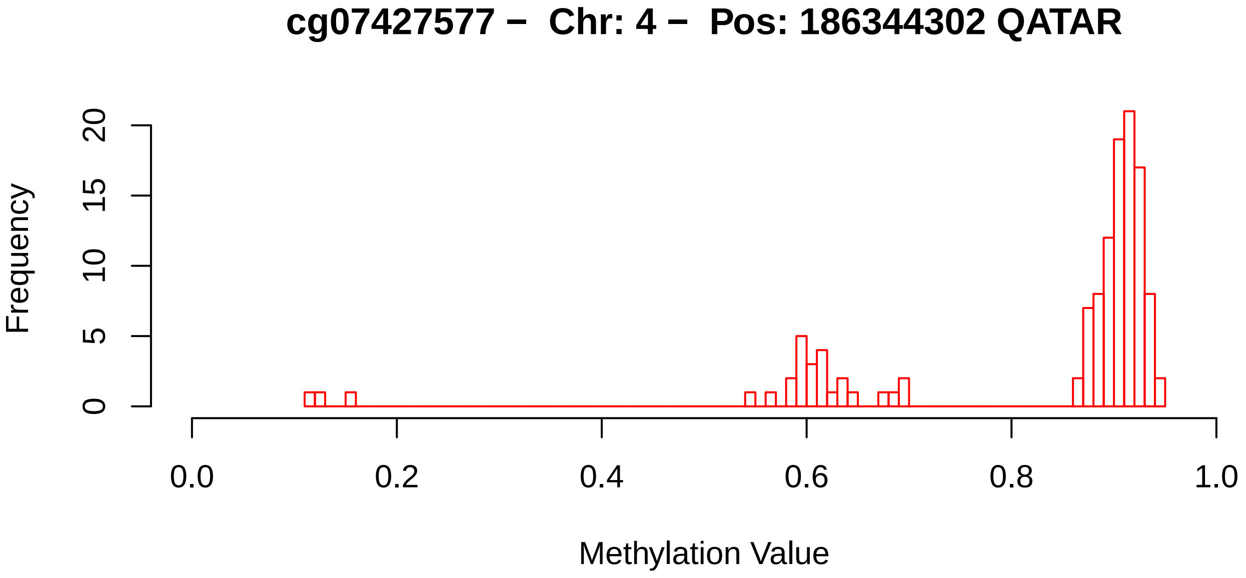

cg07427577 − Chr: 4 − Pos: 186344302 QATAR
Frequency
0
5
10
15
20
0.0
0.2
0.4
0.6
0.8
1.0
Methylation Value

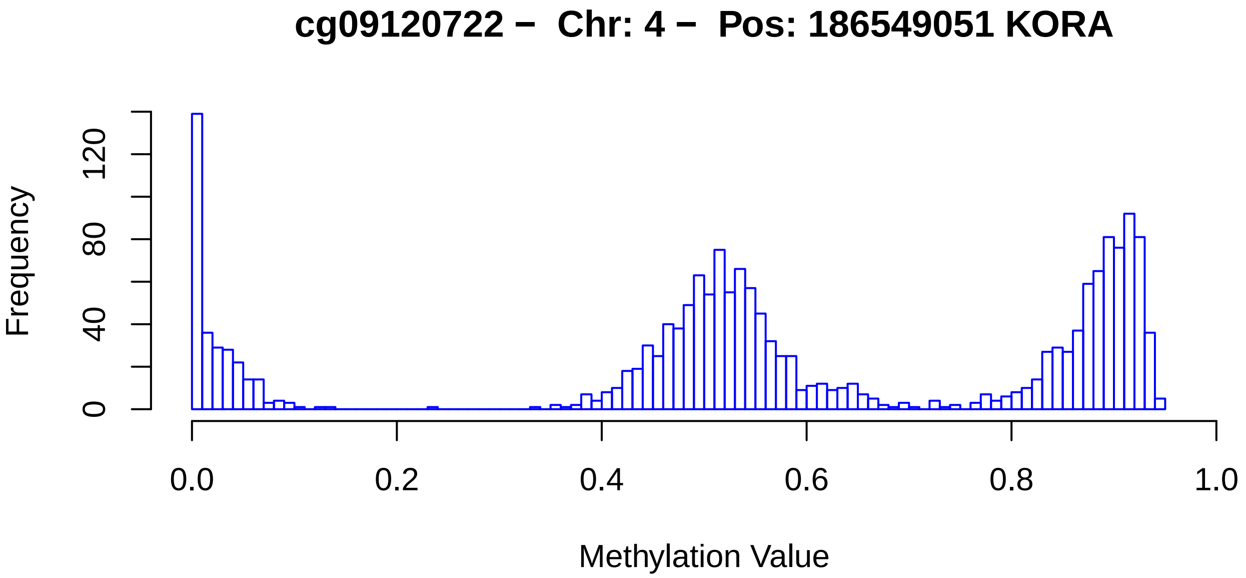
cg09120722 − Chr: 4 − Pos: 186549051 KORA
Frequency
0
40
80
120
0.0
0.2
0.4
0.6
0.8
1.0
Methylation Value

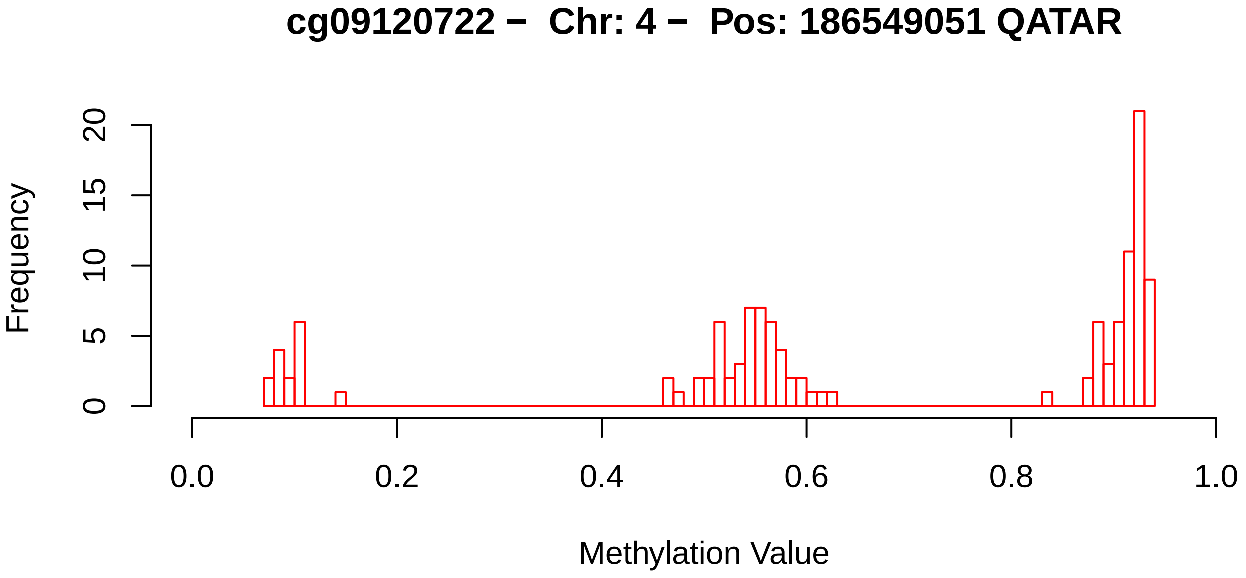
cg09120722 − Chr: 4 − Pos: 186549051 QATAR
Frequency
0
5
10
15
20
0.0
0.2
0.4
0.6
0.8
1.0
Methylation Value

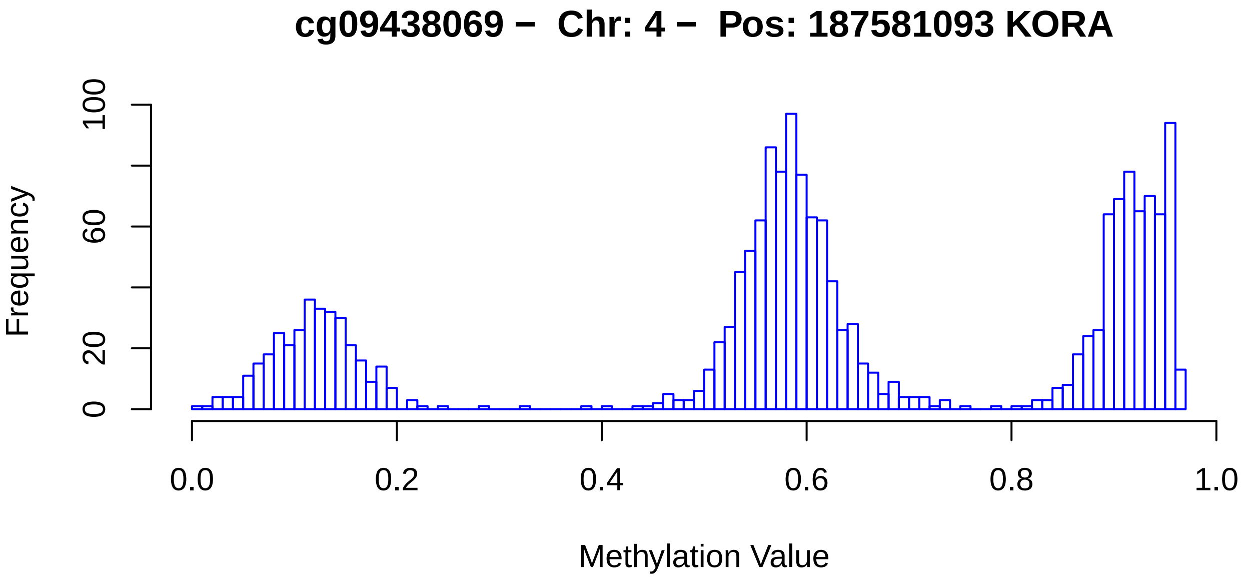
cg09438069 − Chr: 4 − Pos: 187581093 KORA
Frequency
0
20
60
100
0.0
0.2
0.4
0.6
0.8
1.0
Methylation Value

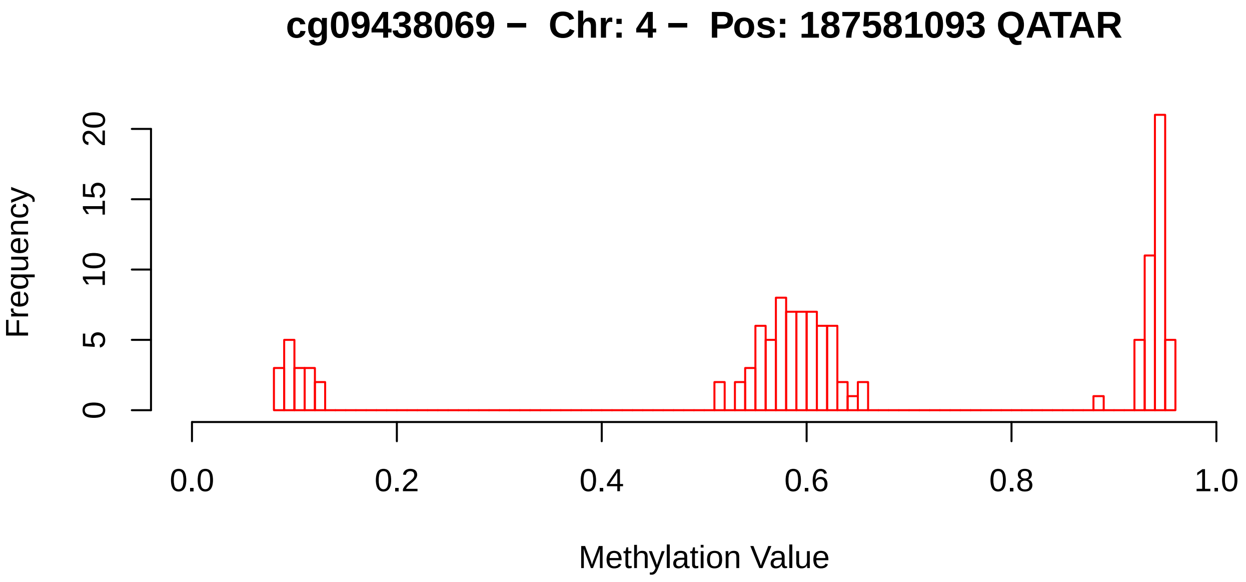
cg09438069 − Chr: 4 − Pos: 187581093 QATAR
Frequency
0
5
10
15
20
0.0
0.2
0.4
0.6
0.8
1.0
Methylation Value

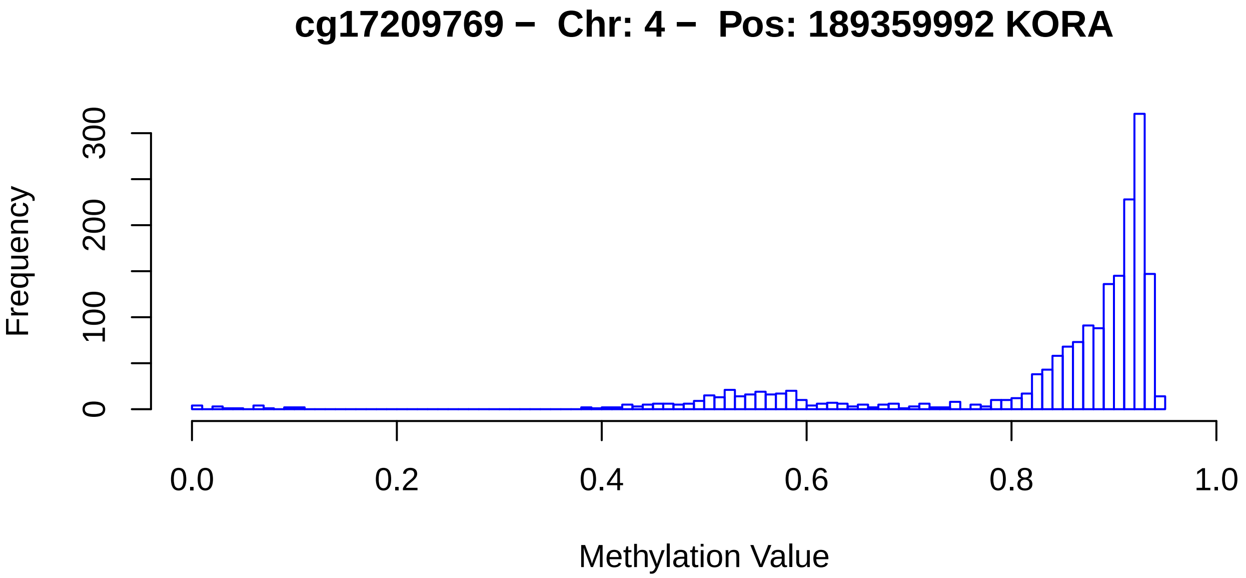
cg17209769 − Chr: 4 − Pos: 189359992 KORA
Frequency
0
100
200
300
0.0
0.2
0.4
0.6
0.8
1.0
Methylation Value

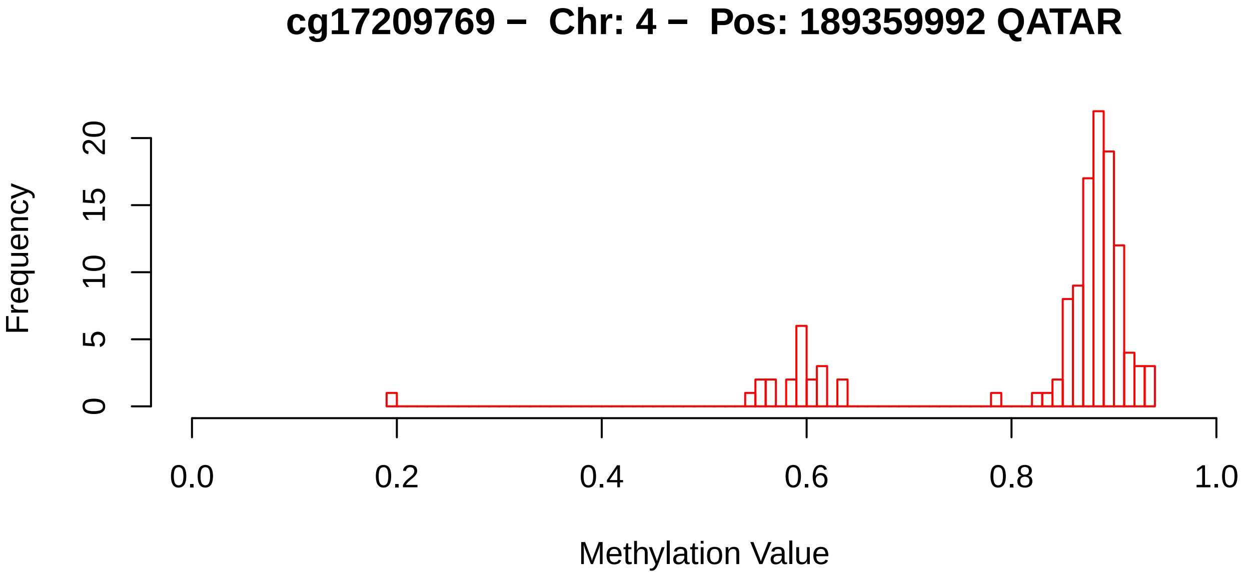
cg17209769 − Chr: 4 − Pos: 189359992 QATAR
Frequency
0
5
10
15
20
0.0
0.2
0.4
0.6
0.8
1.0
Methylation Value

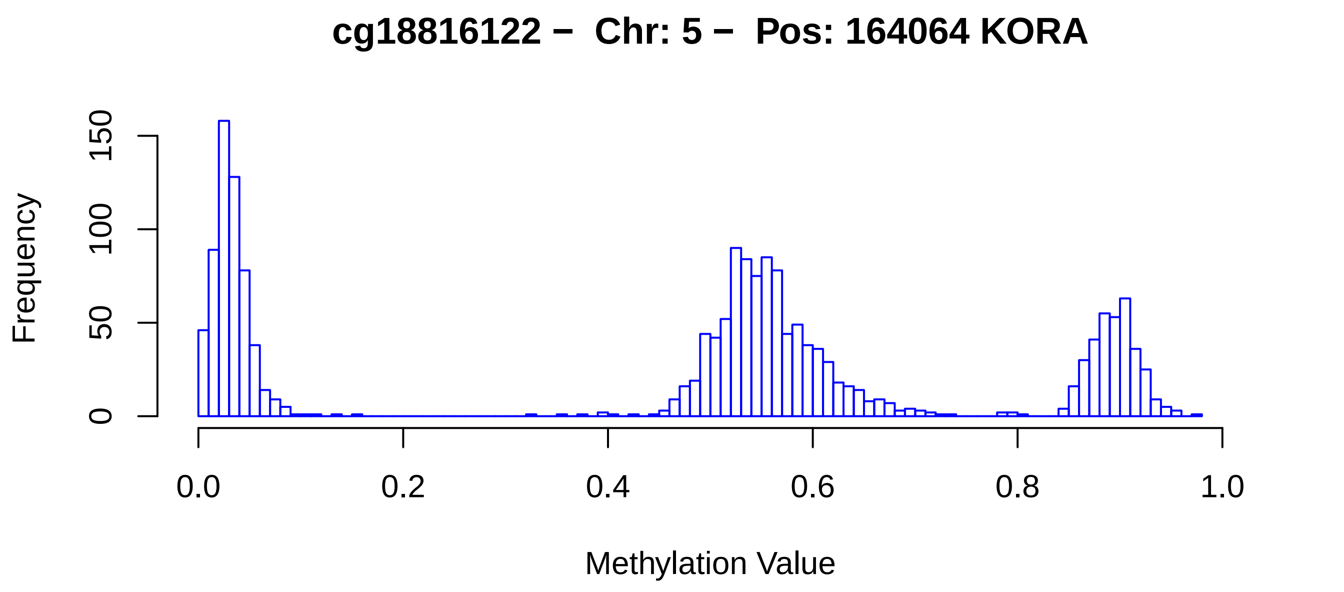
cg18816122 − Chr: 5 − Pos: 164064 KORA
Frequency
0
50
100
150
0.0
0.2
0.4
0.6
0.8
1.0
Methylation Value

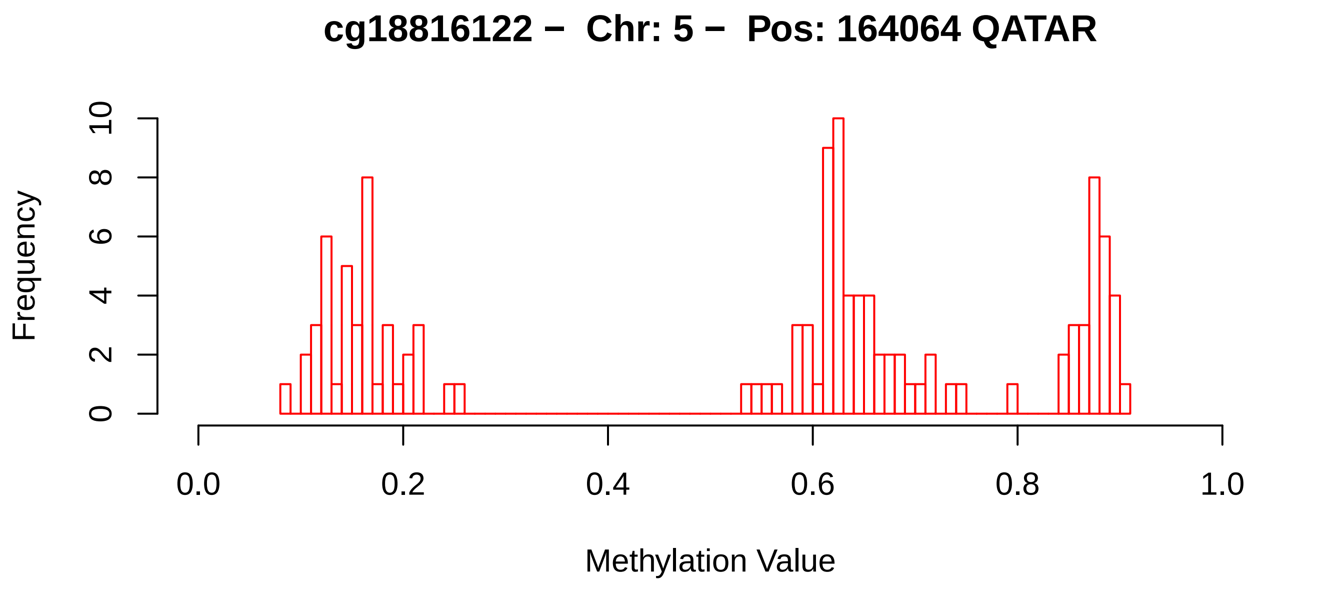
cg18816122 − Chr: 5 − Pos: 164064 QATAR
Frequency
0
2
4
6
8
10
0.0
0.2
0.4
0.6
0.8
1.0
Methylation Value

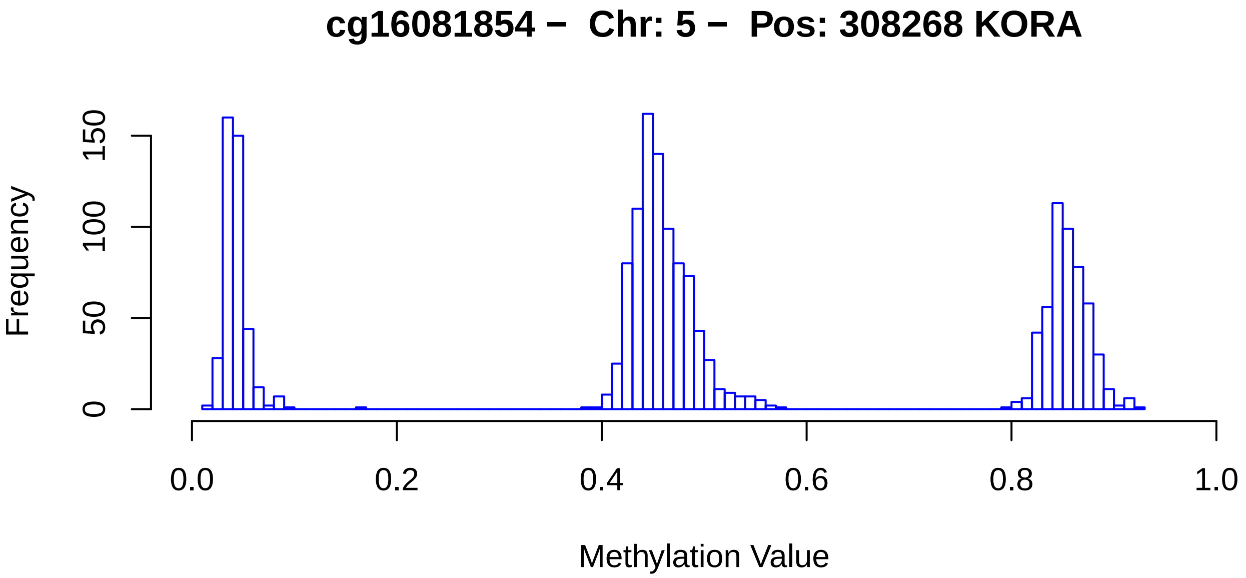
cg16081854 − Chr: 5 − Pos: 308268 KORA
Frequency
0
50
100
150
0.0
0.2
0.4
0.6
0.8
1.0
Methylation Value

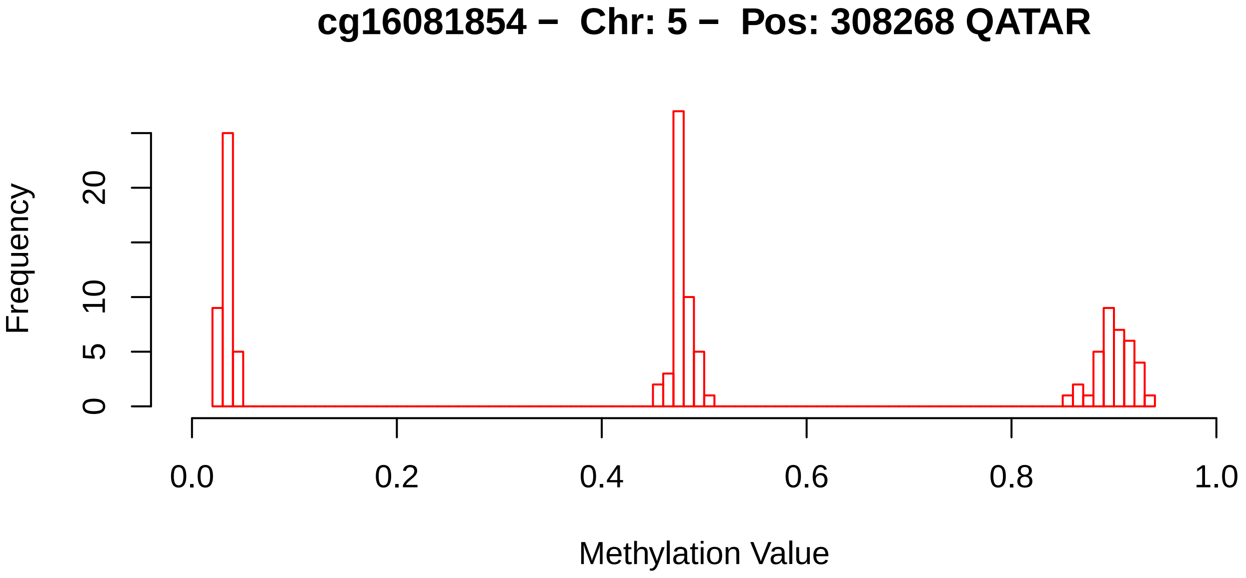
cg16081854 − Chr: 5 − Pos: 308268 QATAR
Frequency
0
5
10
20
0.0
0.2
0.4
0.6
0.8
1.0
Methylation Value

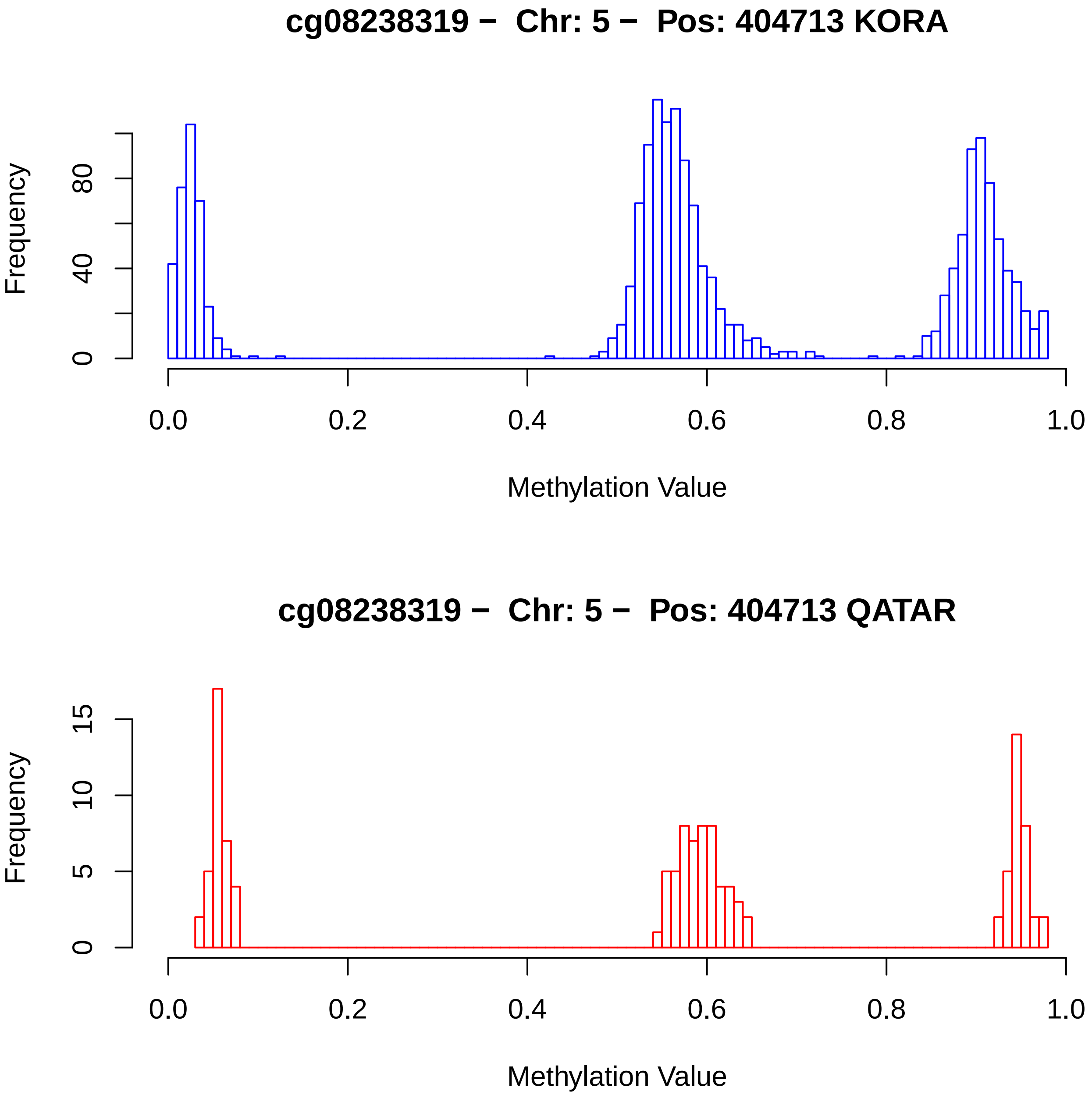
cg08238319 − Chr: 5 − Pos: 404713 KORA
Frequency
0
40
80
0.0
0.2
0.4
0.6
0.8
1.0
Methylation Value
cg08238319 − Chr: 5 − Pos: 404713 QATAR
Frequency
0
5
10
15
0.0
0.2
0.4
0.6
0.8
1.0
Methylation Value

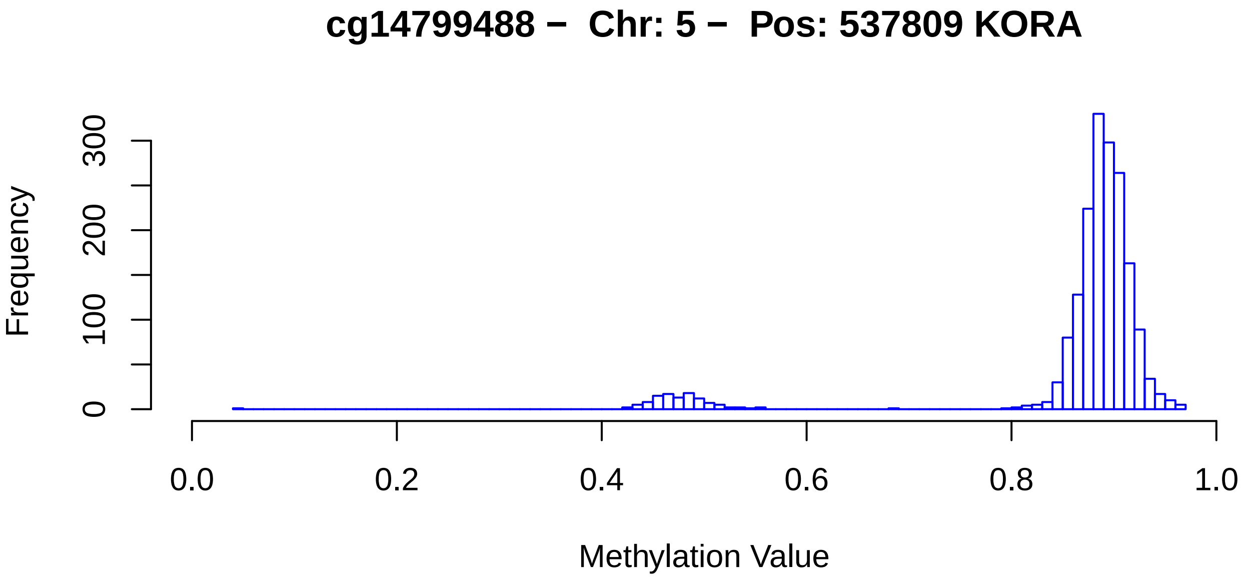
cg14799488 − Chr: 5 − Pos: 537809 KORA
Frequency
0
100
200
300
0.0
0.2
0.4
0.6
0.8
1.0
Methylation Value

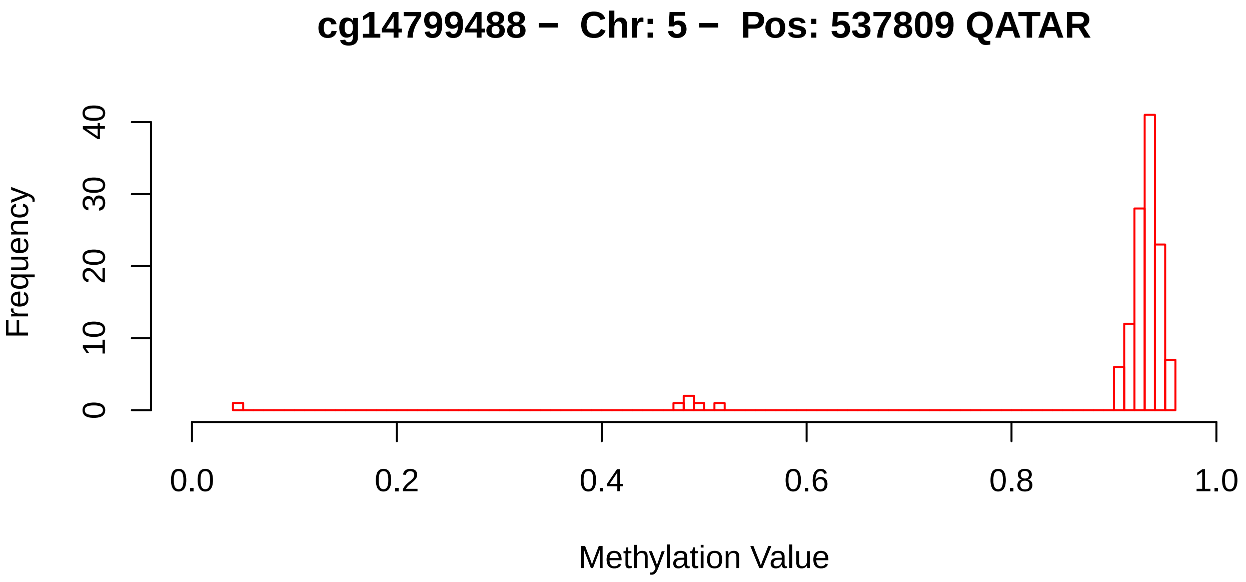
cg14799488 − Chr: 5 − Pos: 537809 QATAR
Frequency
0
10
20
30
40
0.0
0.2
0.4
0.6
0.8
1.0
Methylation Value

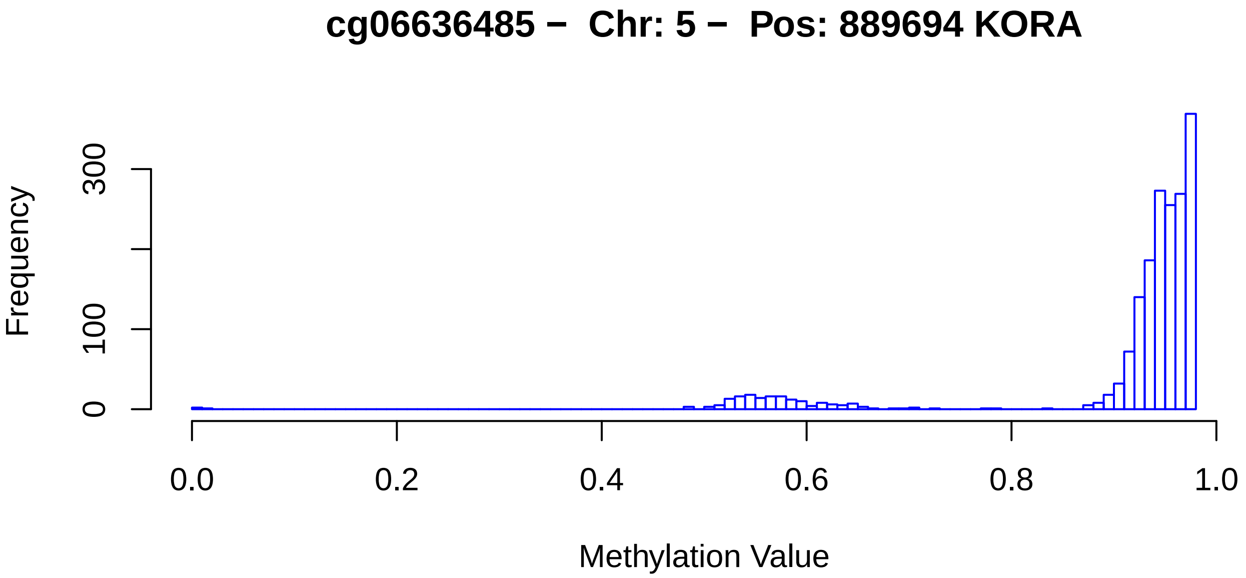
cg06636485 − Chr: 5 − Pos: 889694 KORA
Frequency
0
100
300
0.0
0.2
0.4
0.6
0.8
1.0
Methylation Value

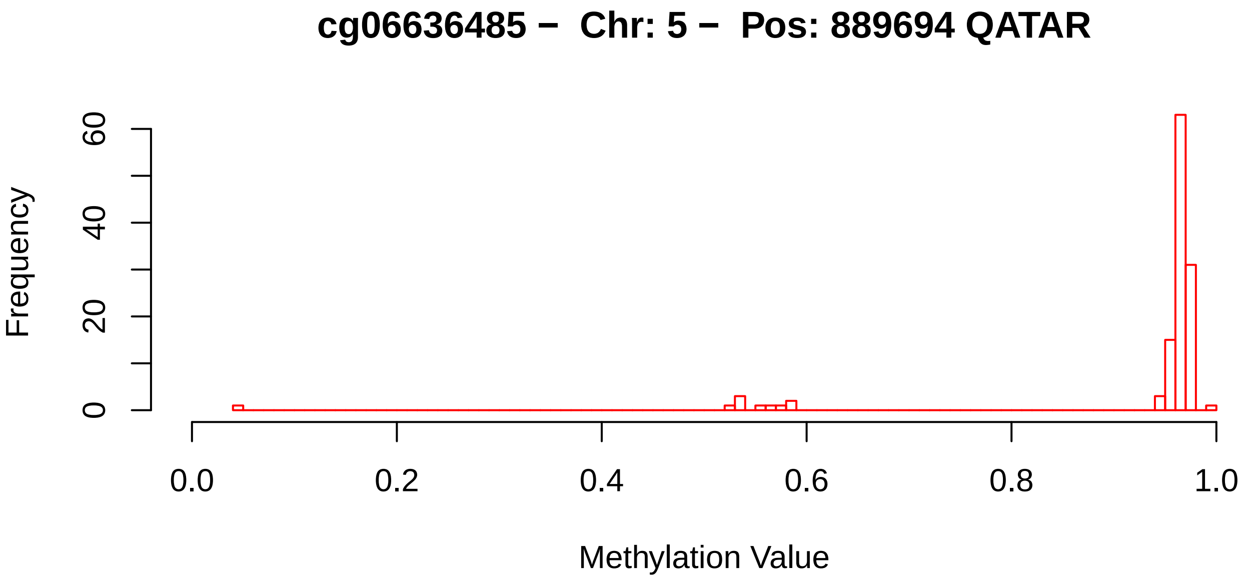
cg06636485 − Chr: 5 − Pos: 889694 QATAR
Frequency
0
20
40
60
0.0
0.2
0.4
0.6
0.8
1.0
Methylation Value

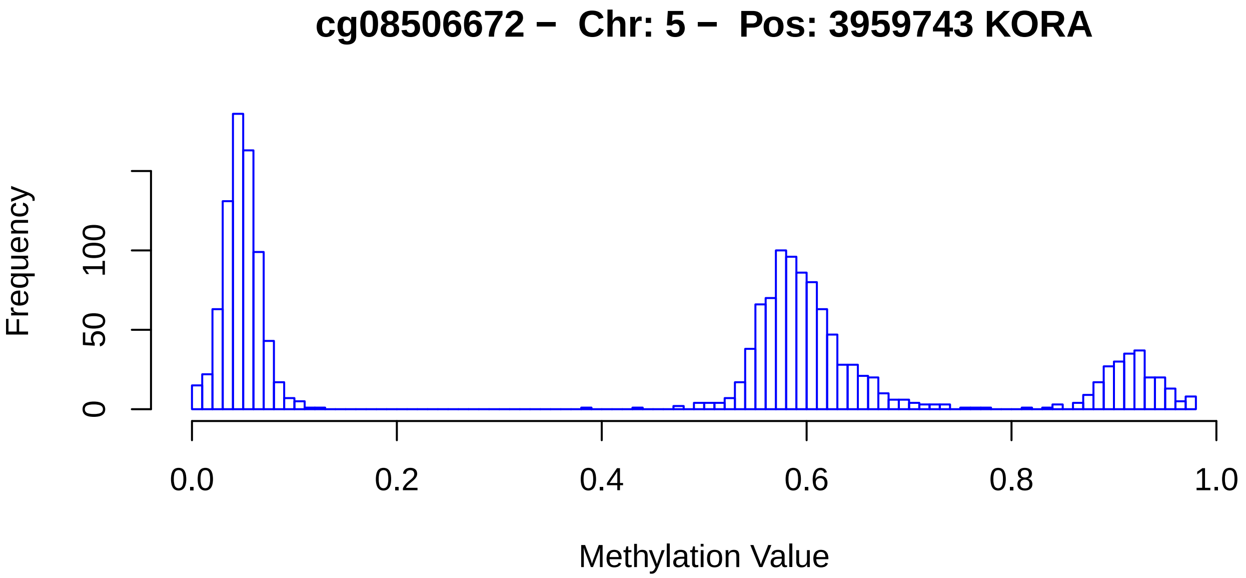
cg08506672 − Chr: 5 − Pos: 3959743 KORA
Frequency
0
50
100
0.0
0.2
0.4
0.6
0.8
1.0
Methylation Value

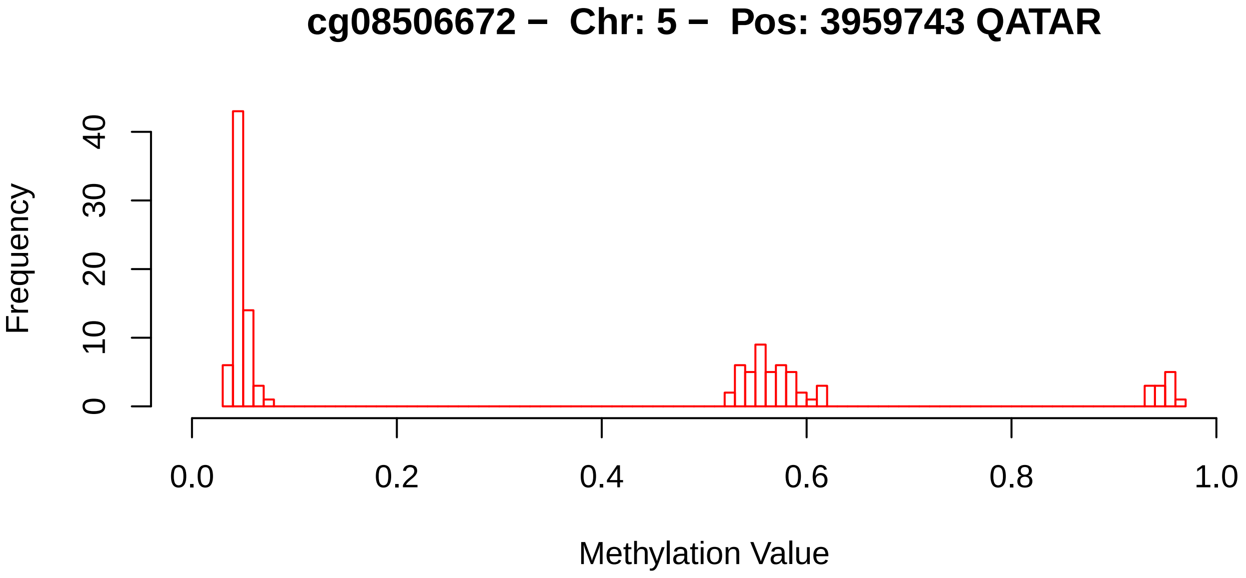
cg08506672 − Chr: 5 − Pos: 3959743 QATAR
Frequency
0
10
20
30
40
0.0
0.2
0.4
0.6
0.8
1.0
Methylation Value

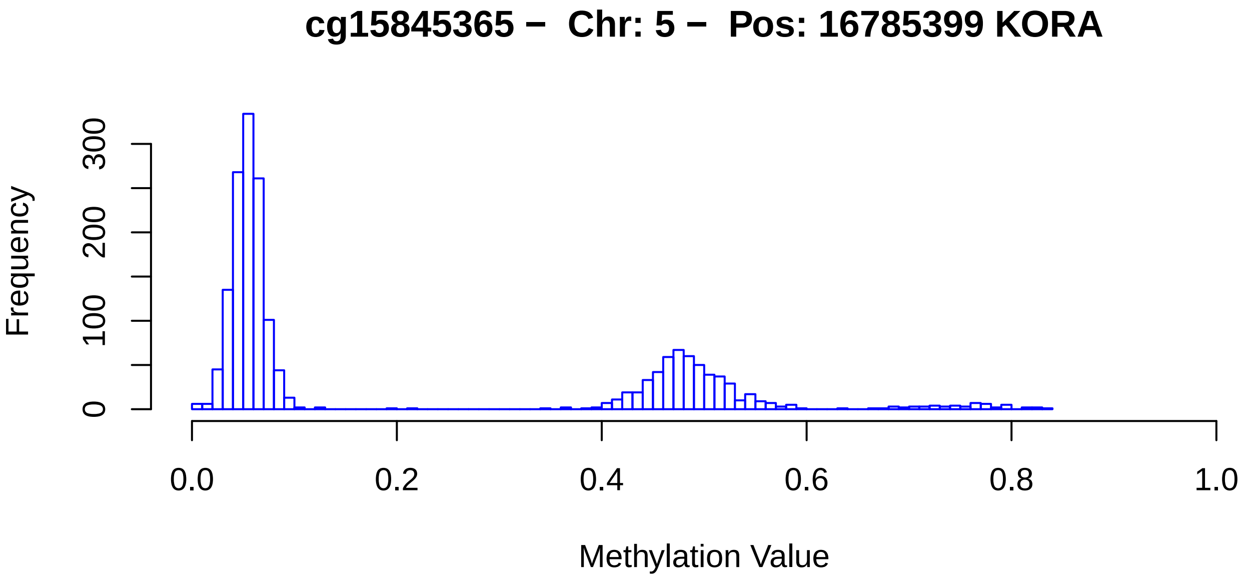
cg15845365 − Chr: 5 − Pos: 16785399 KORA
Frequency
0
100
200
300
0.0
0.2
0.4
0.6
0.8
1.0
Methylation Value

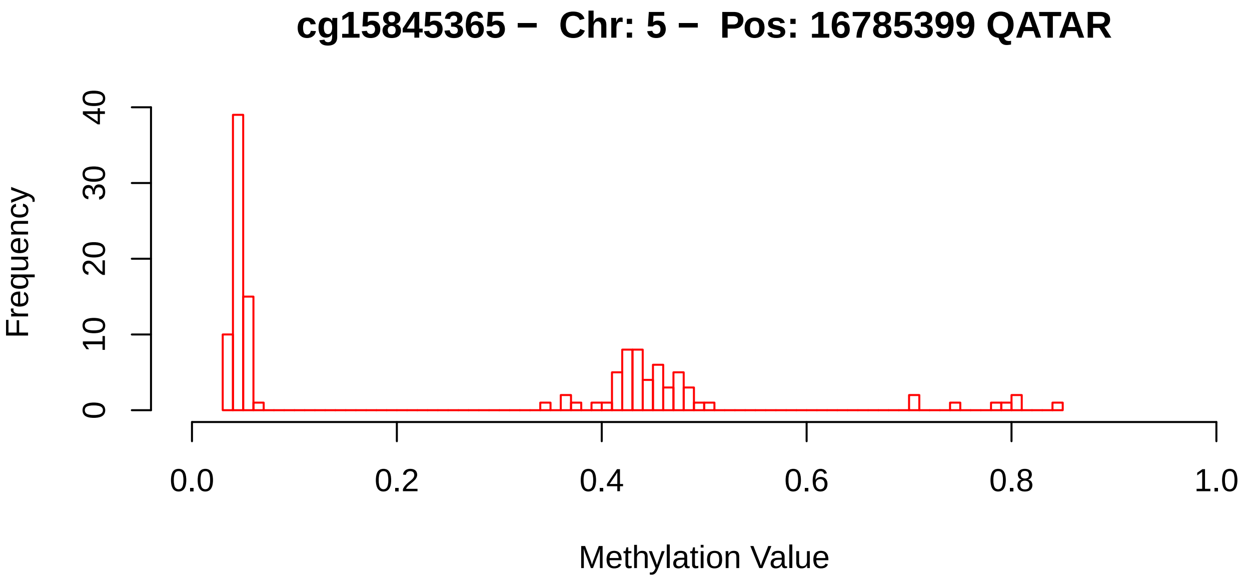
cg15845365 − Chr: 5 − Pos: 16785399 QATAR
Frequency
0
10
20
30
40
0.0
0.2
0.4
0.6
0.8
1.0
Methylation Value

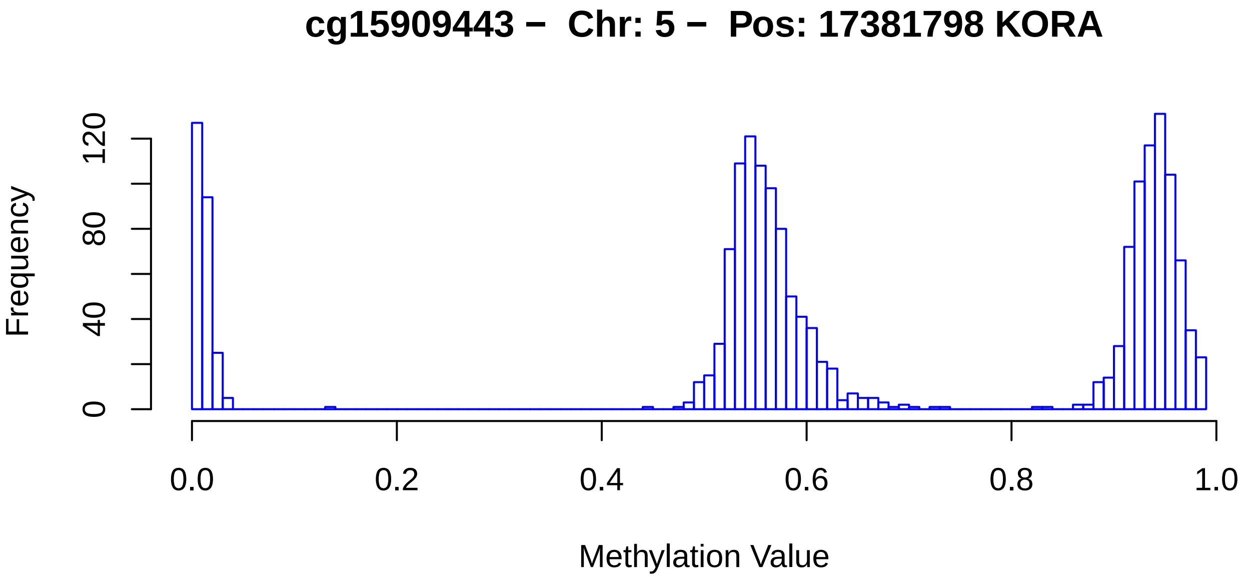
cg15909443 − Chr: 5 − Pos: 17381798 KORA
Frequency
0
40
80
120
0.0
0.2
0.4
0.6
0.8
1.0
Methylation Value

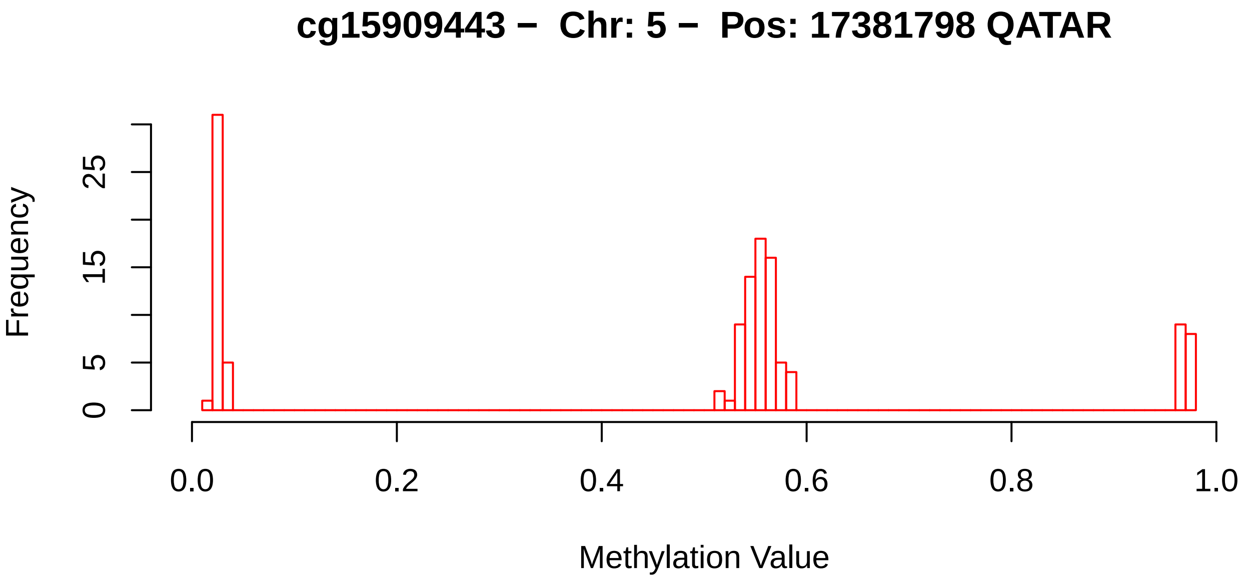
cg15909443 − Chr: 5 − Pos: 17381798 QATAR
Frequency
0
5
15
25
0.0
0.2
0.4
0.6
0.8
1.0
Methylation Value

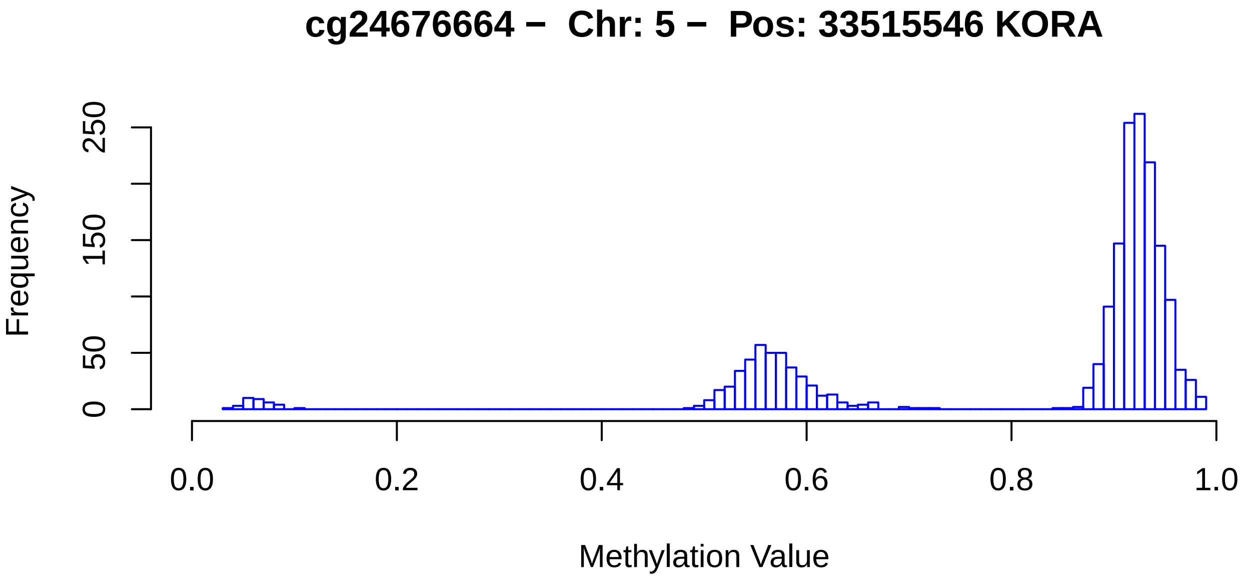
cg24676664 − Chr: 5 − Pos: 33515546 KORA
Frequency
0
50
150
250
0.0
0.2
0.4
0.6
0.8
1.0
Methylation Value

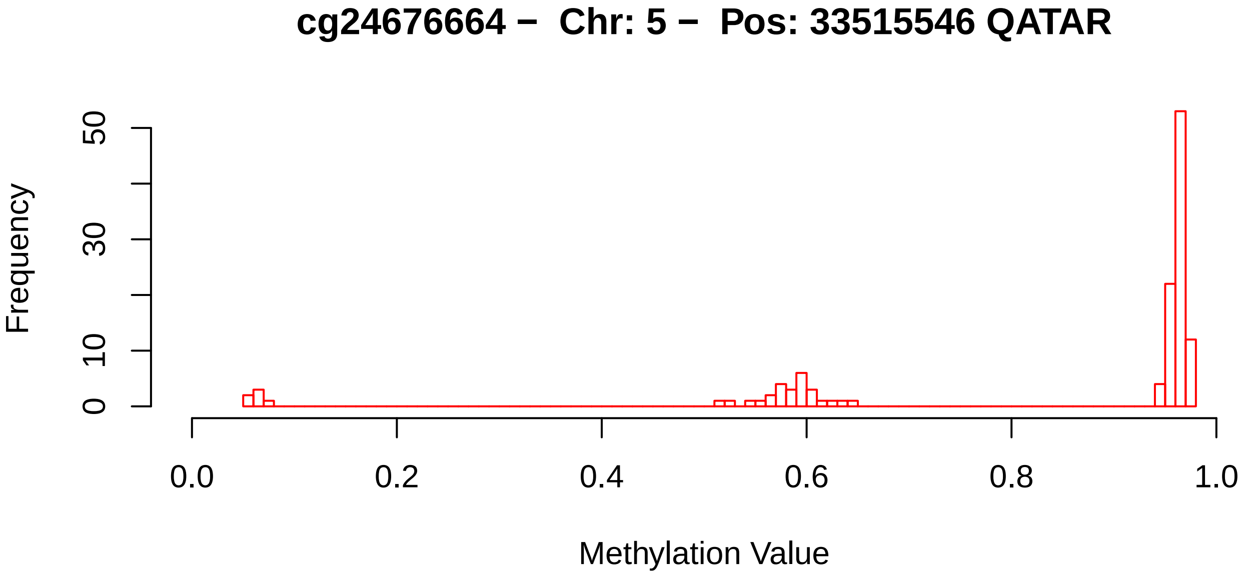
cg24676664 − Chr: 5 − Pos: 33515546 QATAR
Frequency
0
10
30
50
0.0
0.2
0.4
0.6
0.8
1.0
Methylation Value

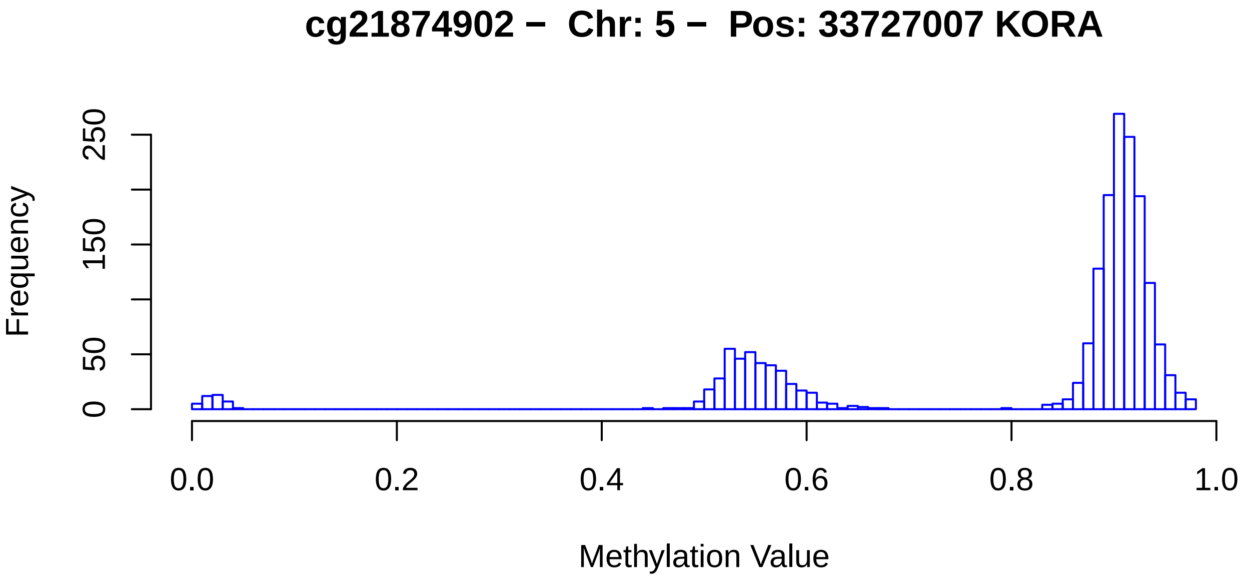
cg21874902 − Chr: 5 − Pos: 33727007 KORA
Frequency
0
50
150
250
0.0
0.2
0.4
0.6
0.8
1.0
Methylation Value

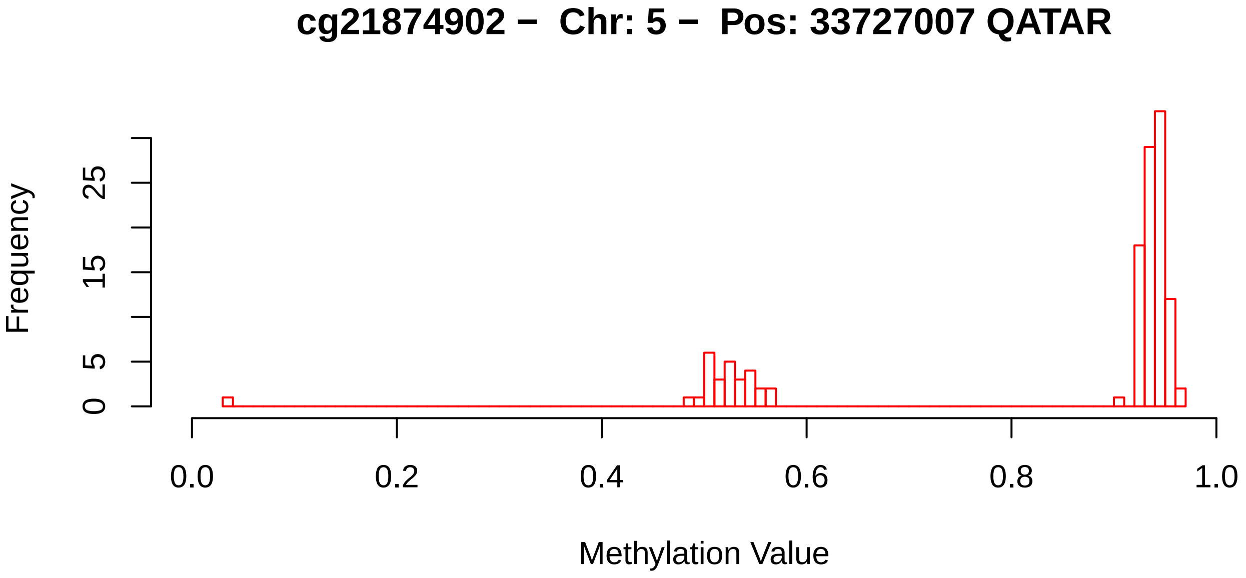
cg21874902 − Chr: 5 − Pos: 33727007 QATAR
Frequency
0
5
15
25
0.0
0.2
0.4
0.6
0.8
1.0
Methylation Value

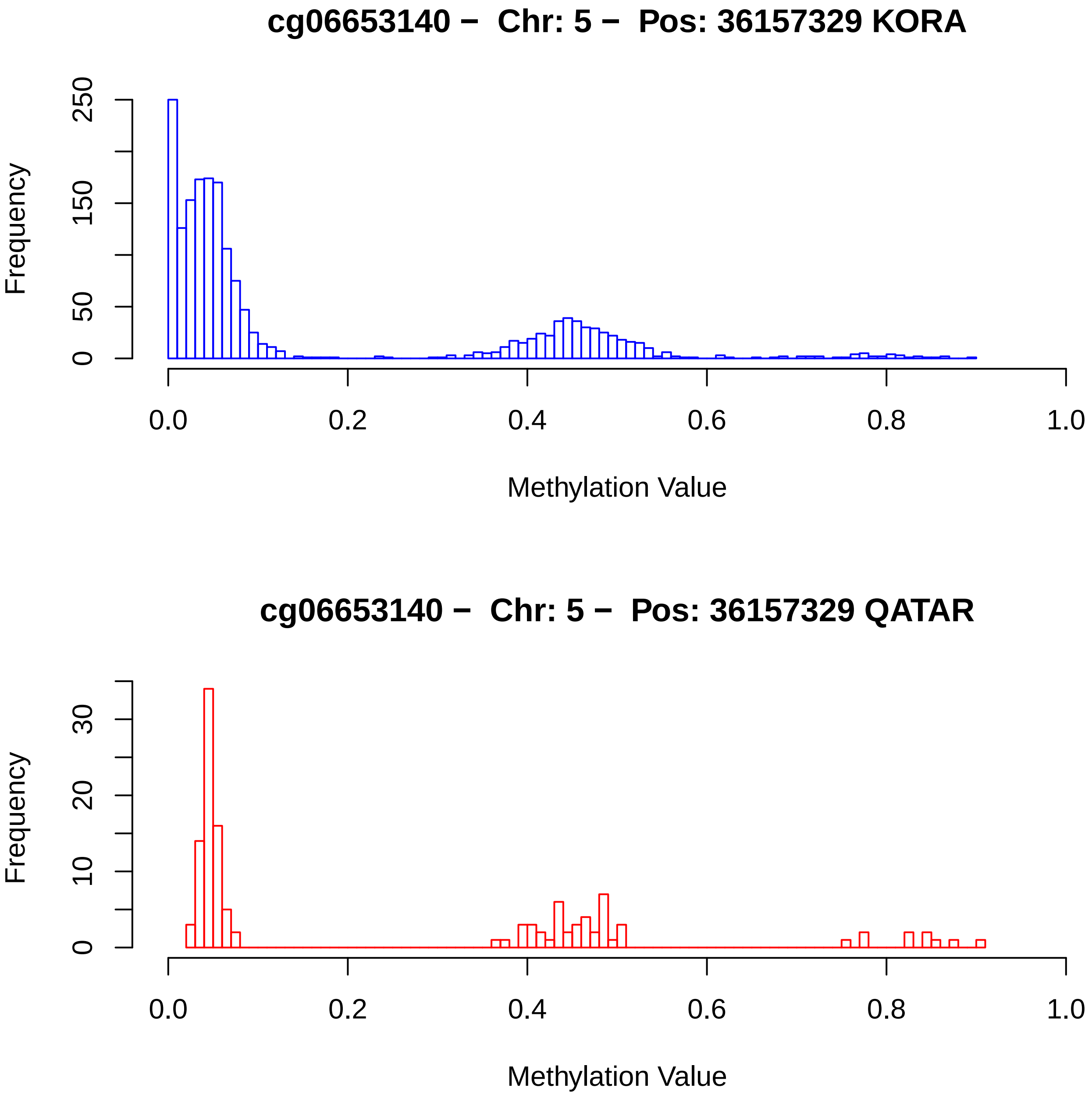
cg06653140 − Chr: 5 − Pos: 36157329 KORA
Frequency
0
50
150
250
0.0
0.2
0.4
0.6
0.8
1.0
Methylation Value
cg06653140 − Chr: 5 − Pos: 36157329 QATAR
Frequency
0
10
20
30
0.0
0.2
0.4
0.6
0.8
1.0
Methylation Value

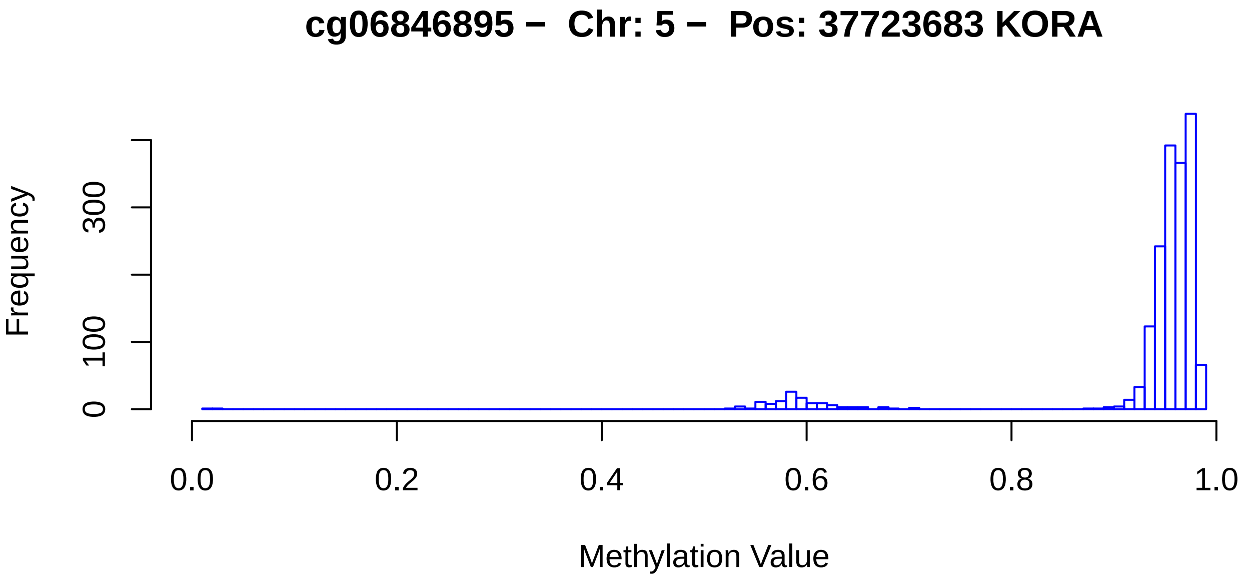
cg06846895 − Chr: 5 − Pos: 37723683 KORA
Frequency
0
100
300
0.0
0.2
0.4
0.6
0.8
1.0
Methylation Value

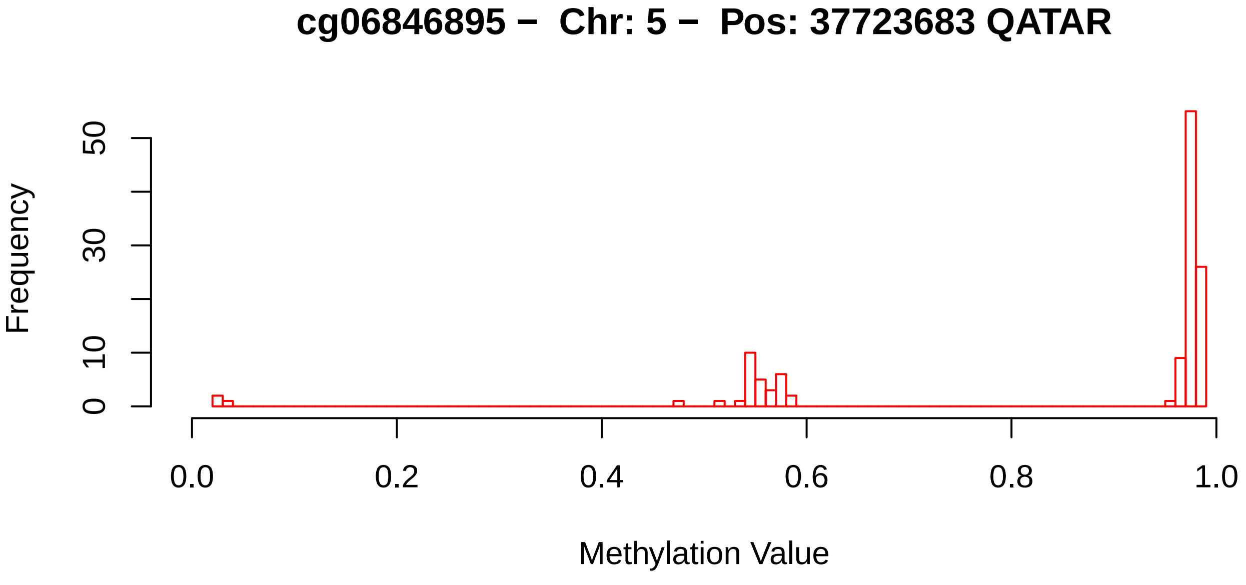
cg06846895 − Chr: 5 − Pos: 37723683 QATAR
Frequency
0
10
30
50
0.0
0.2
0.4
0.6
0.8
1.0
Methylation Value

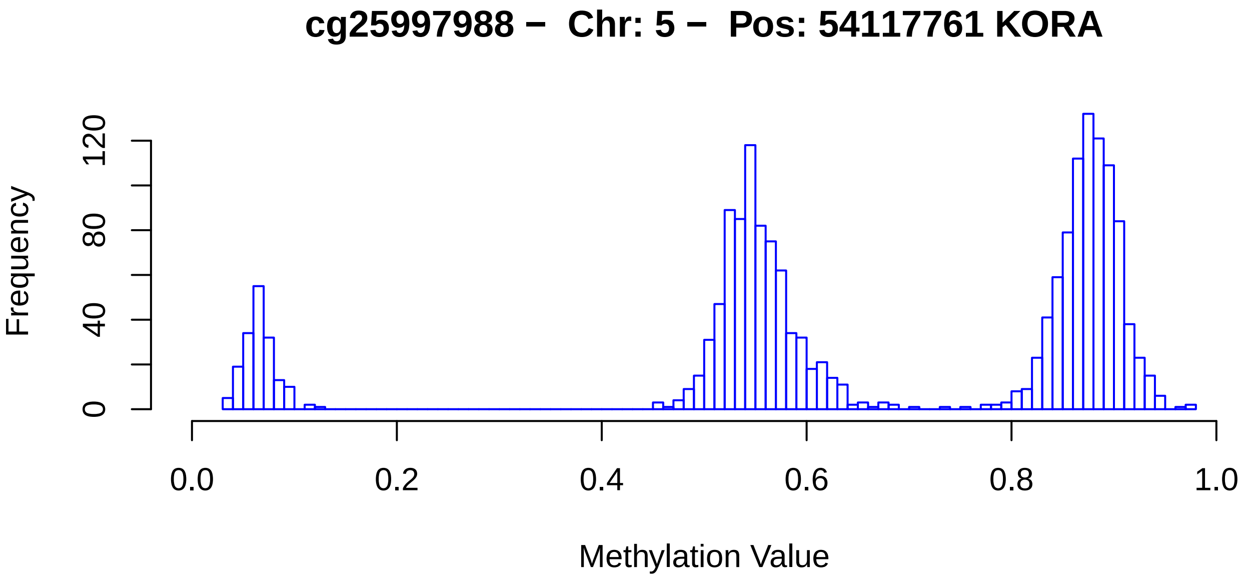
cg25997988 − Chr: 5 − Pos: 54117761 KORA
Frequency
0
40
80
120
0.0
0.2
0.4
0.6
0.8
1.0
Methylation Value

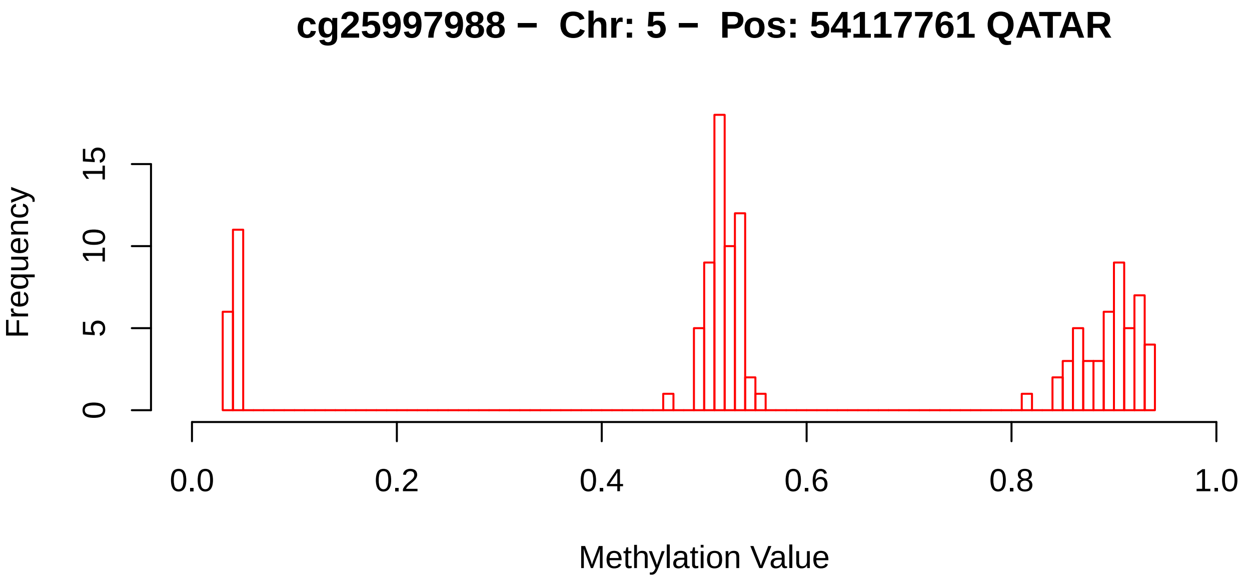
cg25997988 − Chr: 5 − Pos: 54117761 QATAR
Frequency
0
5
10
15
0.0
0.2
0.4
0.6
0.8
1.0
Methylation Value

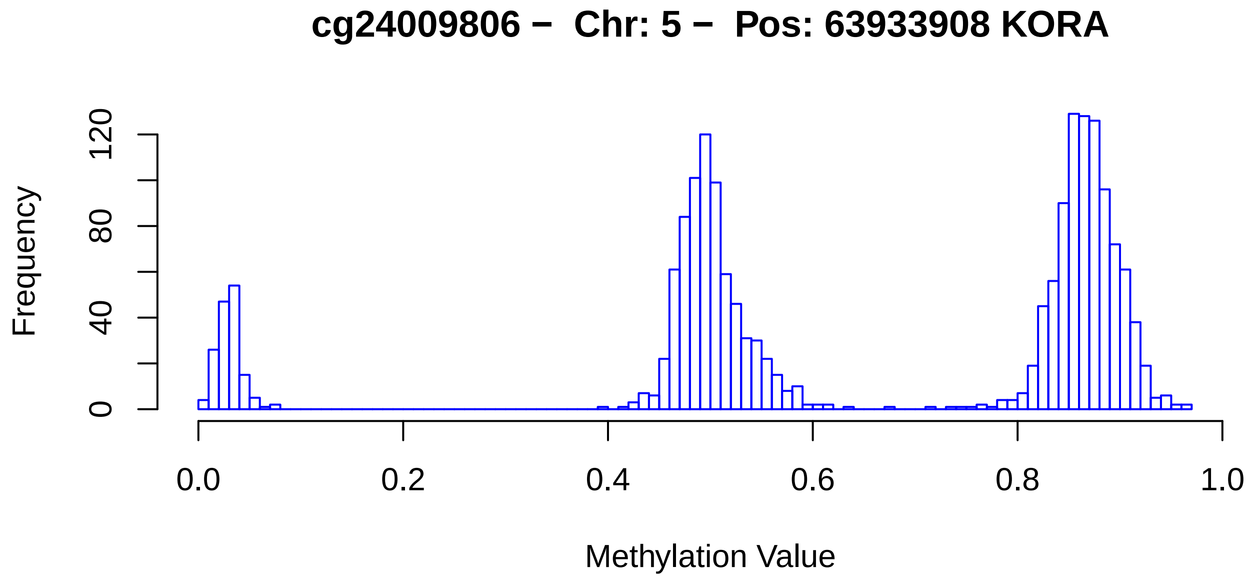
cg24009806 − Chr: 5 − Pos: 63933908 KORA
Frequency
0
40
80
120
0.0
0.2
0.4
0.6
0.8
1.0
Methylation Value

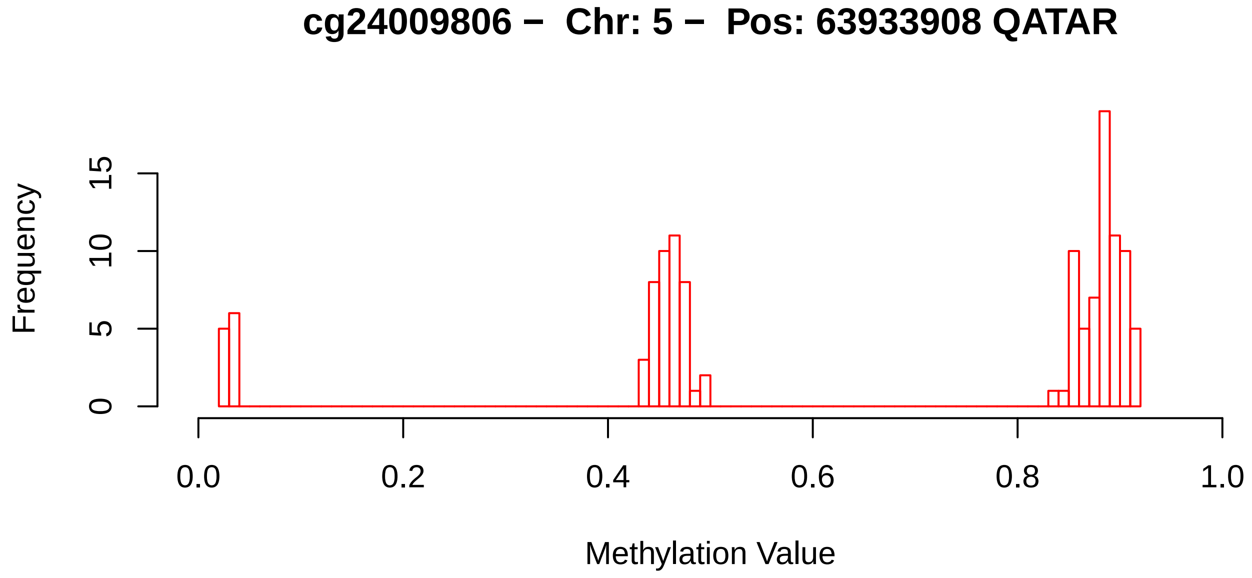
cg24009806 − Chr: 5 − Pos: 63933908 QATAR
Frequency
0
5
10
15
0.0
0.2
0.4
0.6
0.8
1.0
Methylation Value

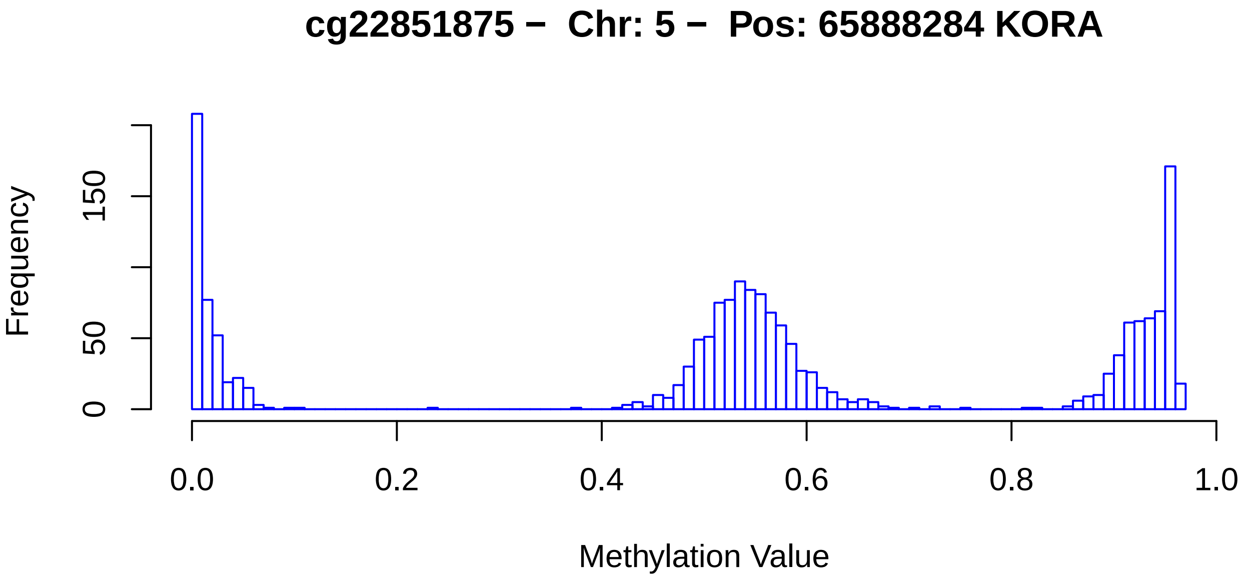
cg22851875 − Chr: 5 − Pos: 65888284 KORA
Frequency
0
50
150
0.0
0.2
0.4
0.6
0.8
1.0
Methylation Value

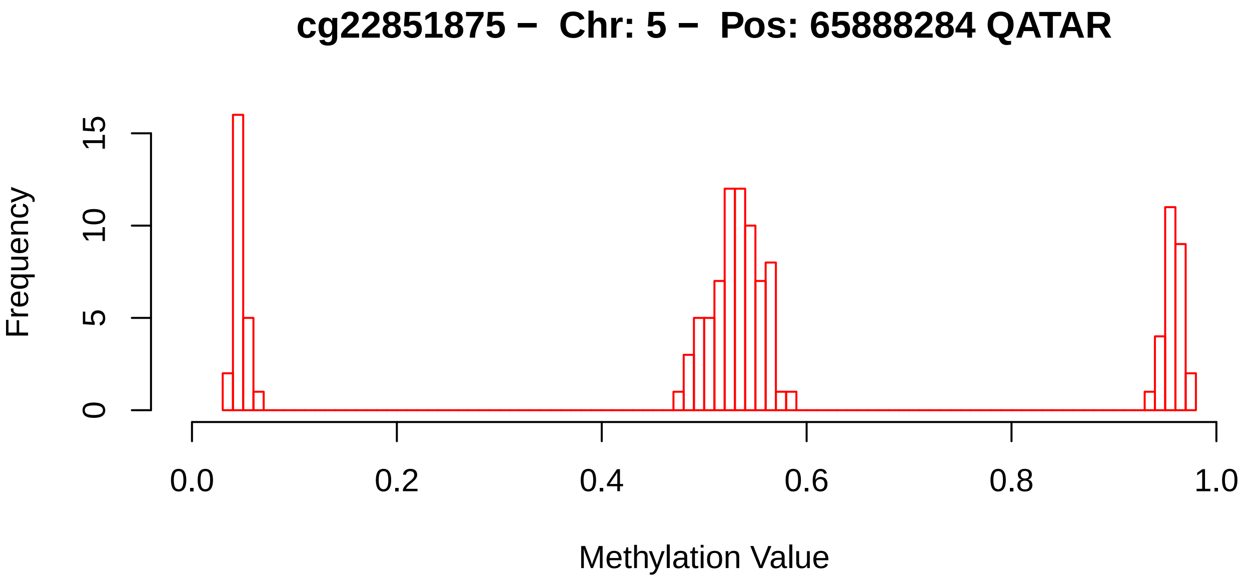
cg22851875 − Chr: 5 − Pos: 65888284 QATAR
Frequency
0
5
10
15
0.0
0.2
0.4
0.6
0.8
1.0
Methylation Value

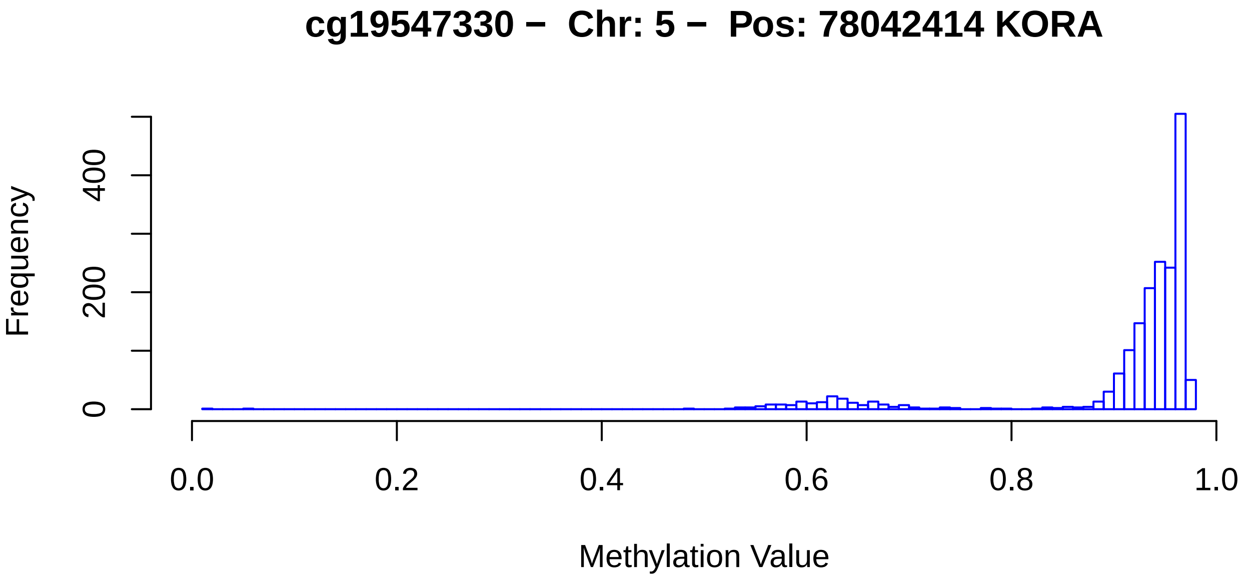
cg19547330 − Chr: 5 − Pos: 78042414 KORA
Frequency
0
200
400
0.0
0.2
0.4
0.6
0.8
1.0
Methylation Value

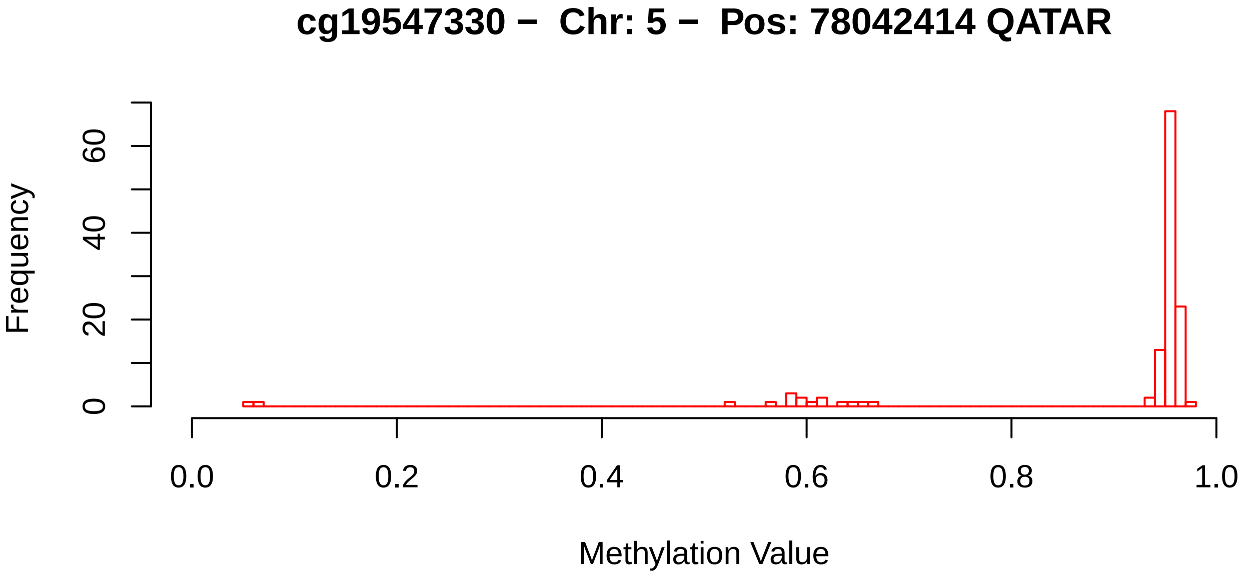
cg19547330 − Chr: 5 − Pos: 78042414 QATAR
Frequency
0
20
40
60
0.0
0.2
0.4
0.6
0.8
1.0
Methylation Value

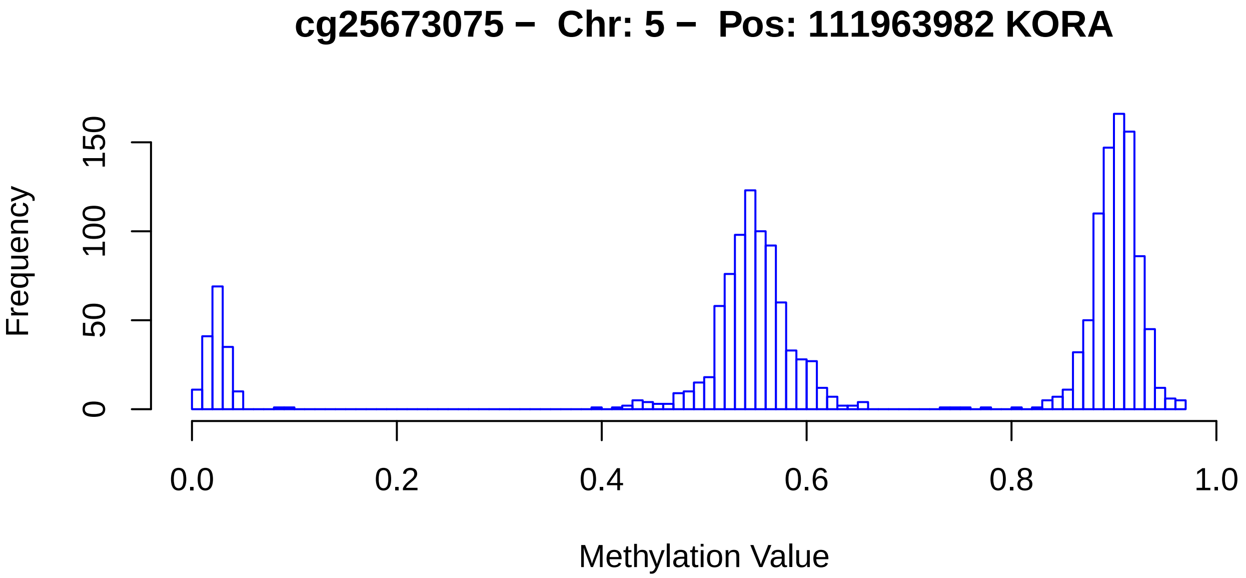
cg25673075 − Chr: 5 − Pos: 111963982 KORA
Frequency
0
50
100
150
0.0
0.2
0.4
0.6
0.8
1.0
Methylation Value

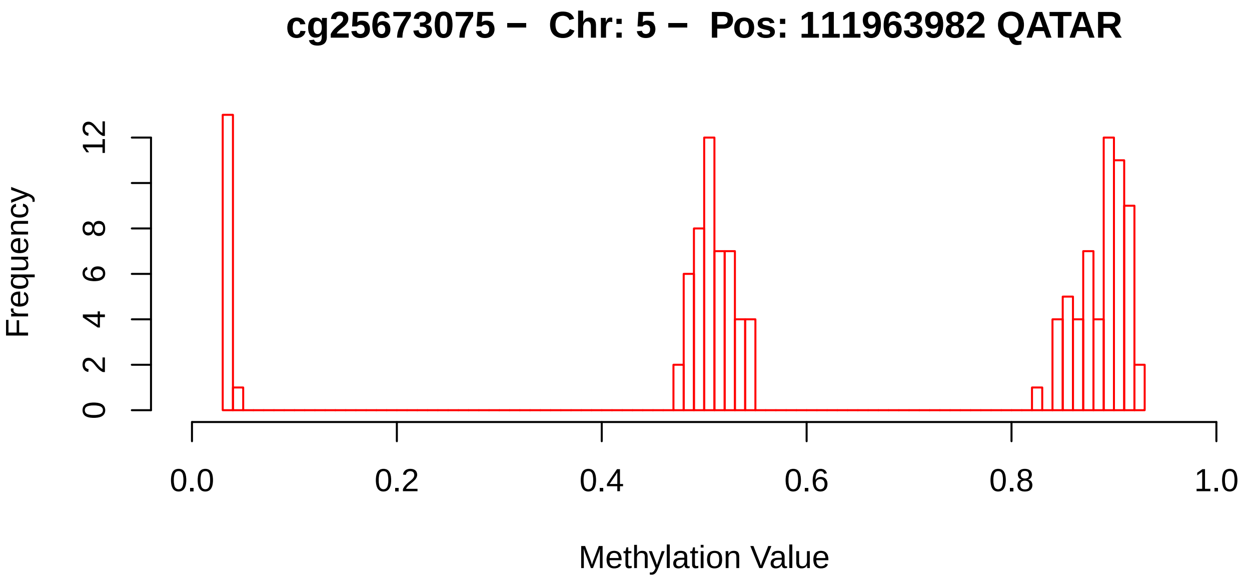
cg25673075 − Chr: 5 − Pos: 111963982 QATAR
Frequency
0
2
4
6
8
12
0.0
0.2
0.4
0.6
0.8
1.0
Methylation Value

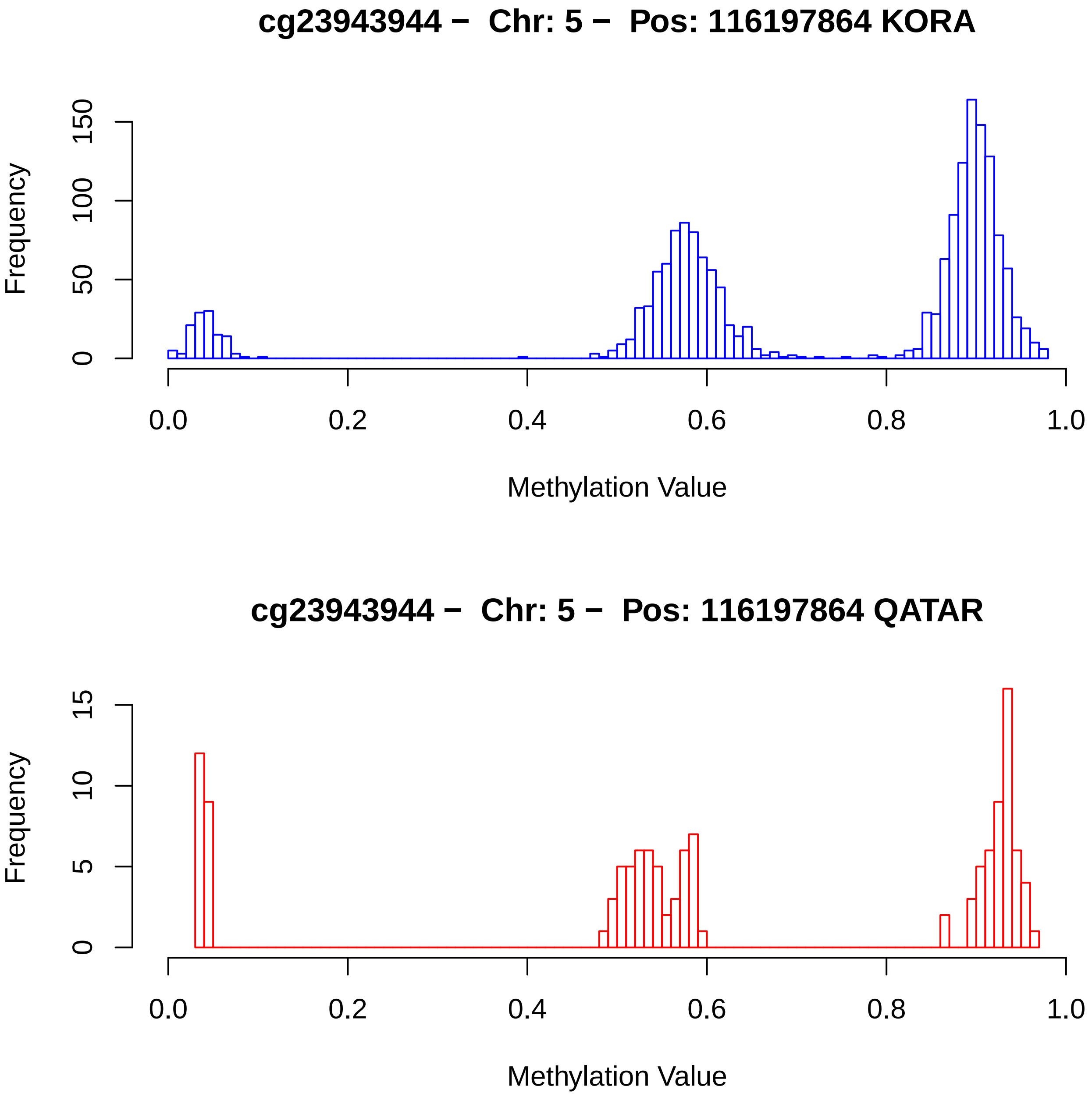

cg23943944 − Chr: 5 − Pos: 116197864 KORA
Frequency
0
50
100
150
0.0
0.2
0.4
0.6
0.8
1.0
Methylation Value
cg23943944 − Chr: 5 − Pos: 116197864 QATAR
Frequency
0
5
10
15
0.0
0.2
0.4
0.6
0.8
1.0
Methylation Value

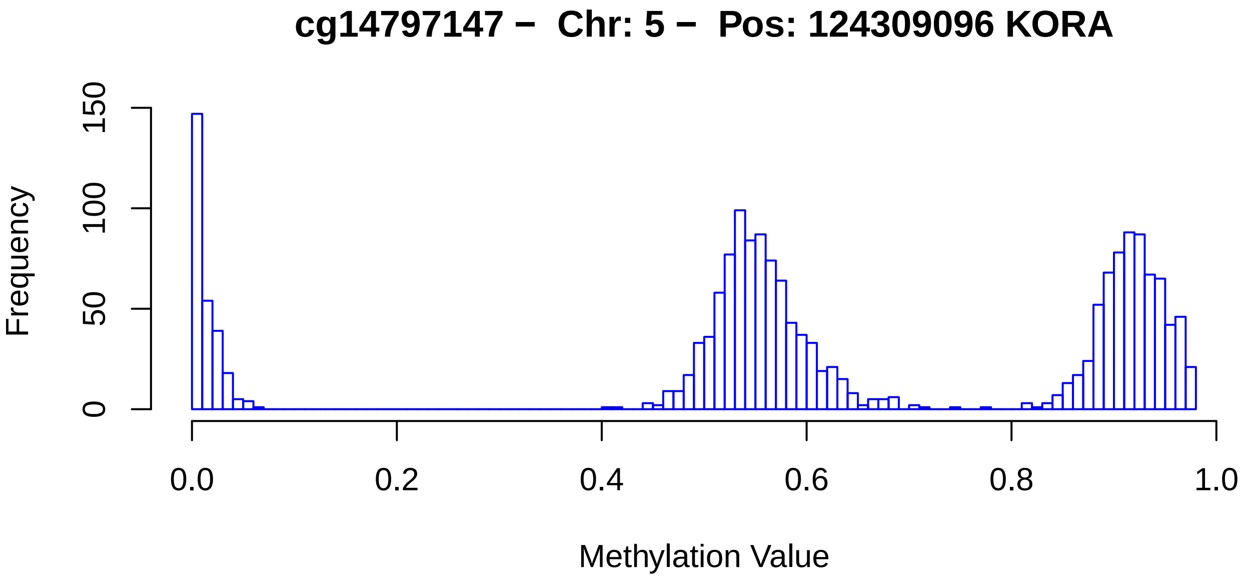
cg14797147 − Chr: 5 − Pos: 124309096 KORA
Frequency
0
50
100
150
0.0
0.2
0.4
0.6
0.8
1.0
Methylation Value

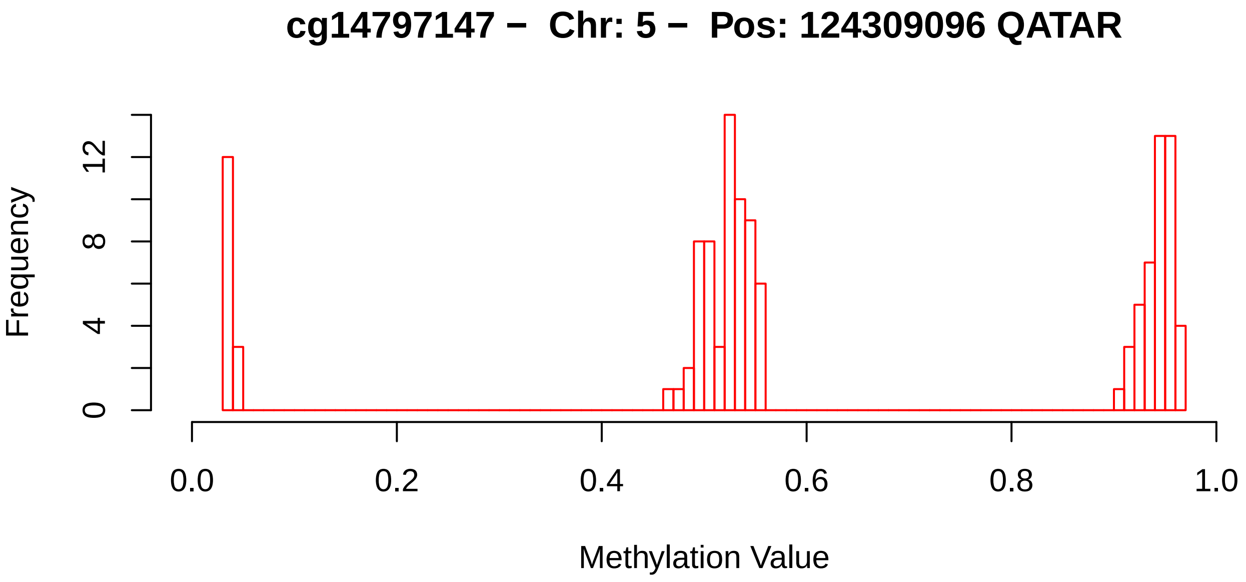
cg14797147 − Chr: 5 − Pos: 124309096 QATAR
Frequency
0
4
8
12
0.0
0.2
0.4
0.6
0.8
1.0
Methylation Value

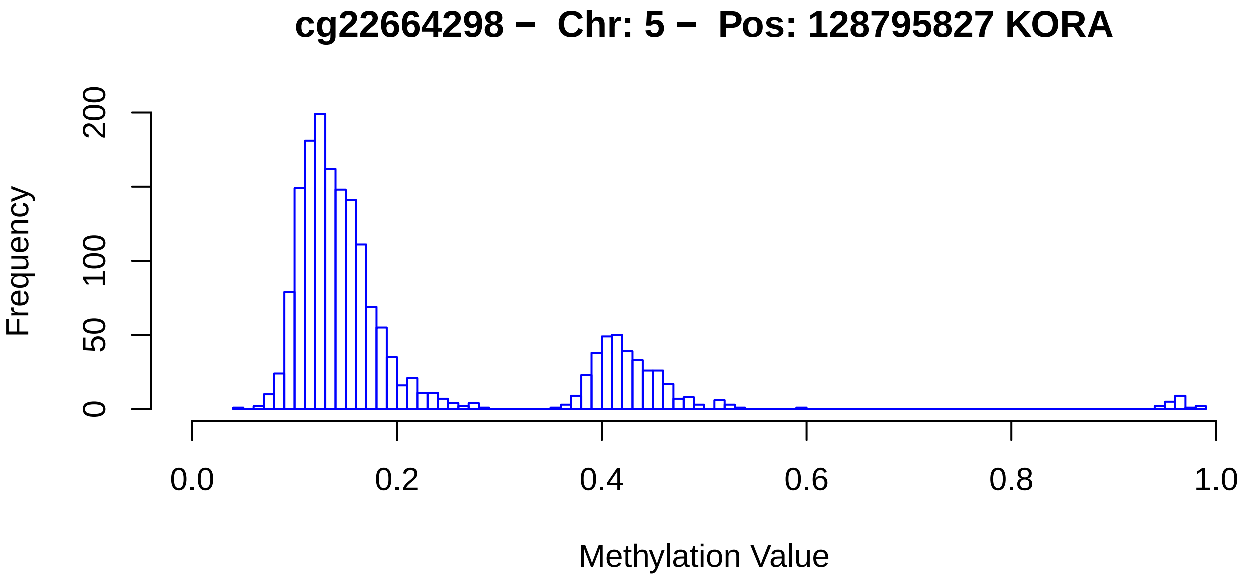
cg22664298 − Chr: 5 − Pos: 128795827 KORA
Frequency
0
50
100
200
0.0
0.2
0.4
0.6
0.8
1.0
Methylation Value

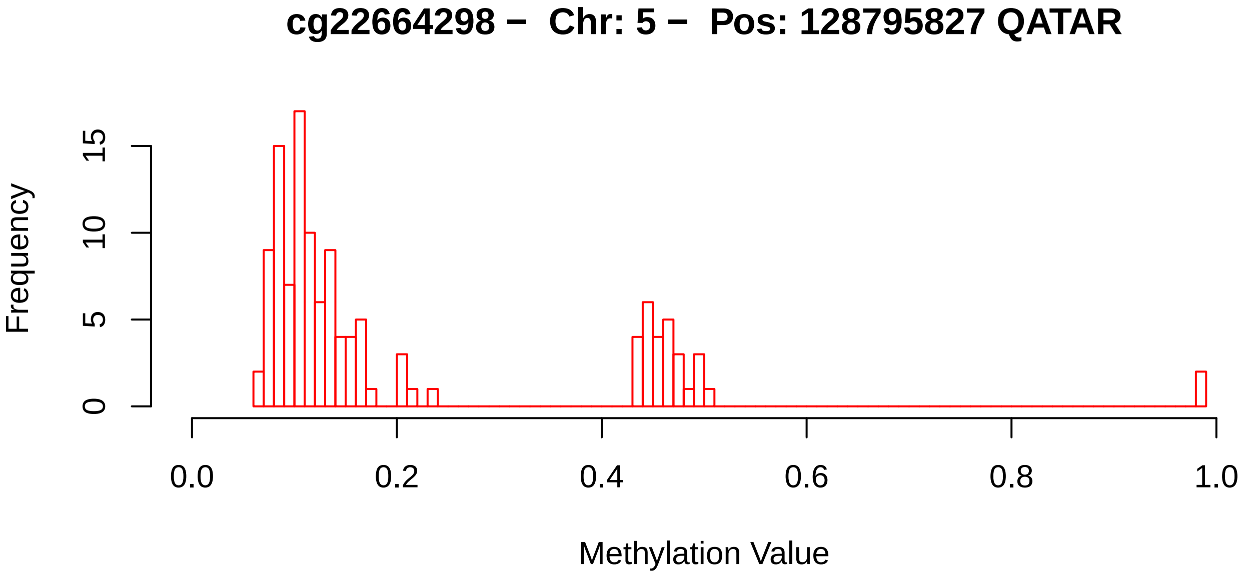
cg22664298 − Chr: 5 − Pos: 128795827 QATAR
Frequency
0
5
10
15
0.0
0.2
0.4
0.6
0.8
1.0
Methylation Value

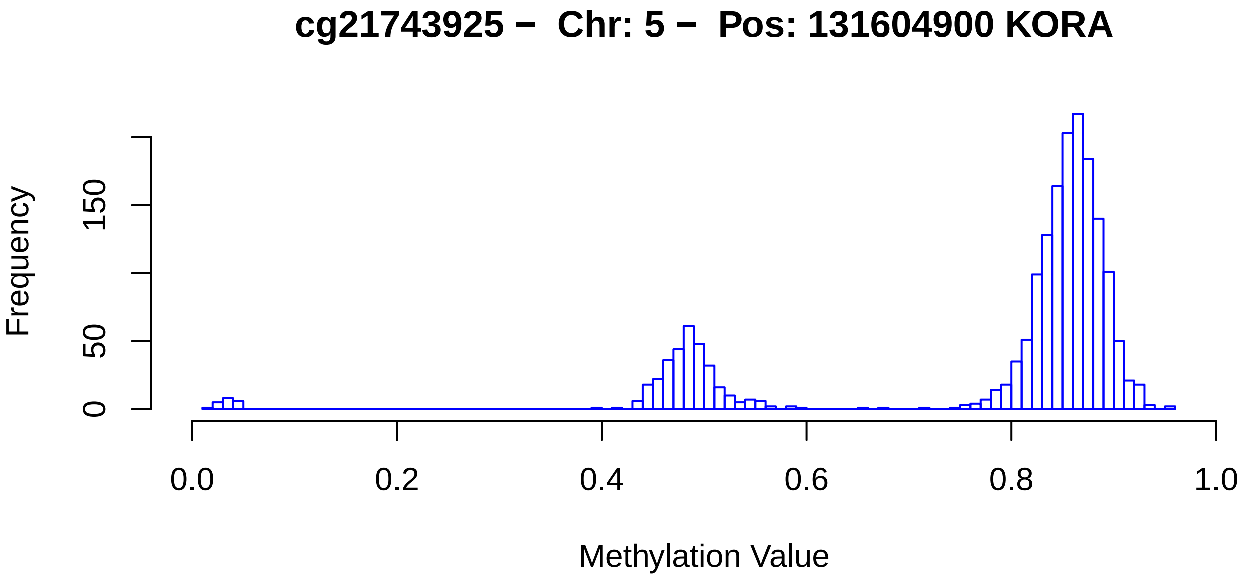
cg21743925 − Chr: 5 − Pos: 131604900 KORA
Frequency
0
50
150
0.0
0.2
0.4
0.6
0.8
1.0
Methylation Value

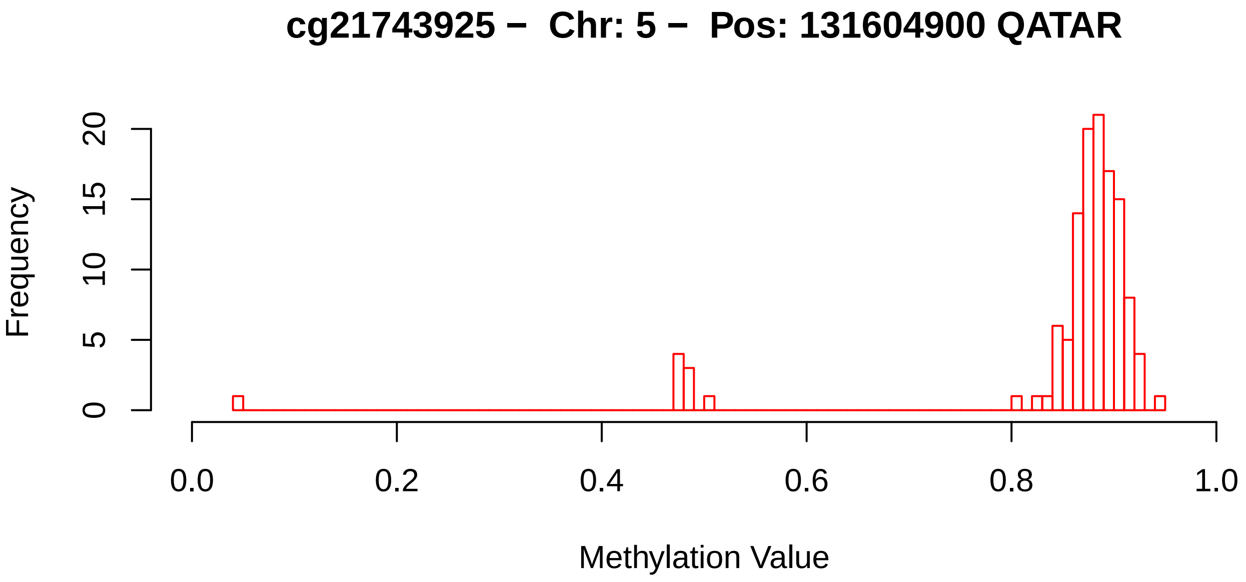
cg21743925 − Chr: 5 − Pos: 131604900 QATAR
Frequency
0
5
10
15
20
0.0
0.2
0.4
0.6
0.8
1.0
Methylation Value

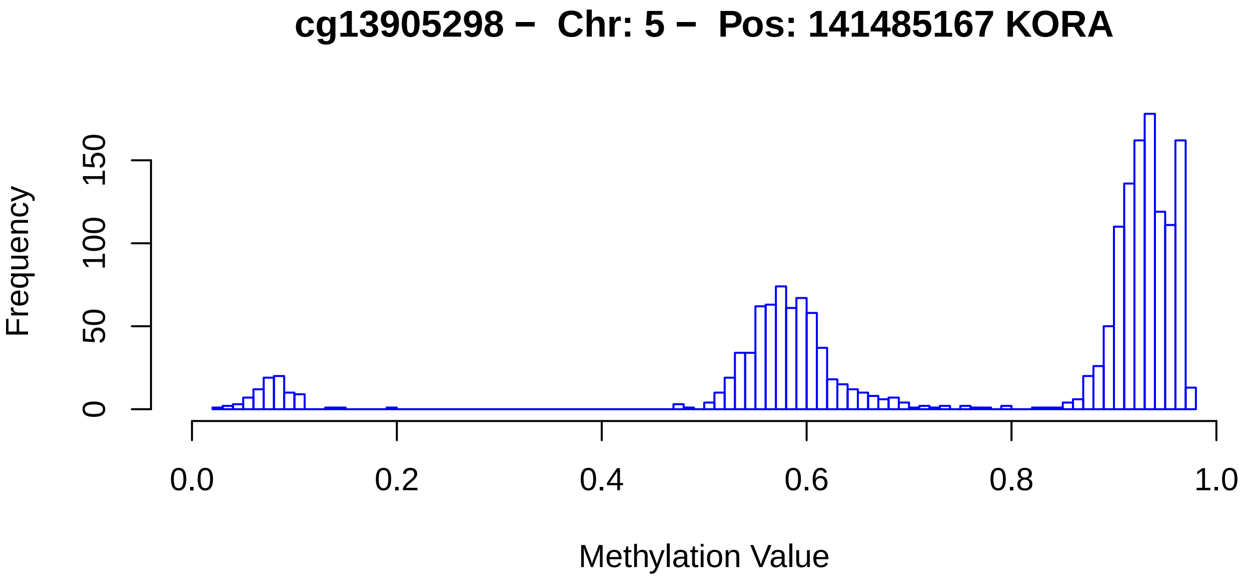
cg13905298 − Chr: 5 − Pos: 141485167 KORA
Frequency
0
50
100
150
0.0
0.2
0.4
0.6
0.8
1.0
Methylation Value

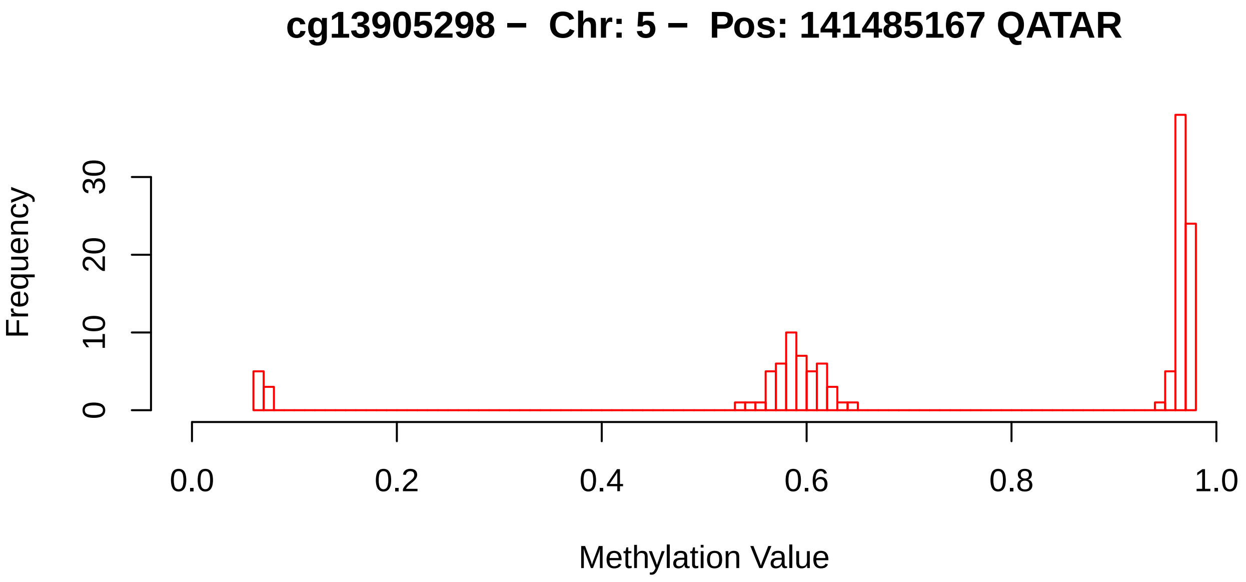
cg13905298 − Chr: 5 − Pos: 141485167 QATAR
Frequency
0
10
20
30
0.0
0.2
0.4
0.6
0.8
1.0
Methylation Value

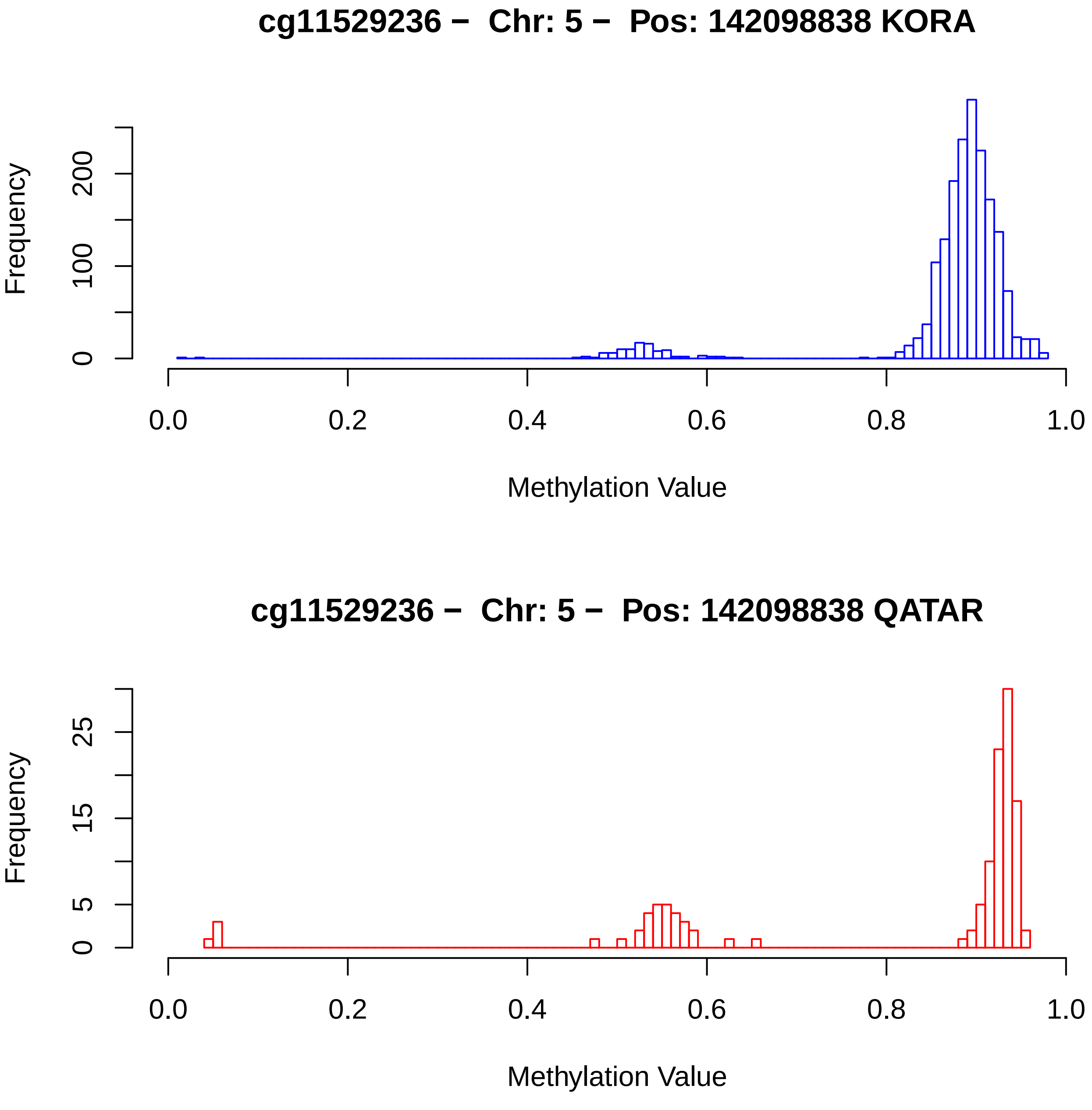
cg11529236 − Chr: 5 − Pos: 142098838 KORA
Frequency
0
100
200
0.0
0.2
0.4
0.6
0.8
1.0
Methylation Value
cg11529236 − Chr: 5 − Pos: 142098838 QATAR
Frequency
0
5
15
25
0.0
0.2
0.4
0.6
0.8
1.0
Methylation Value

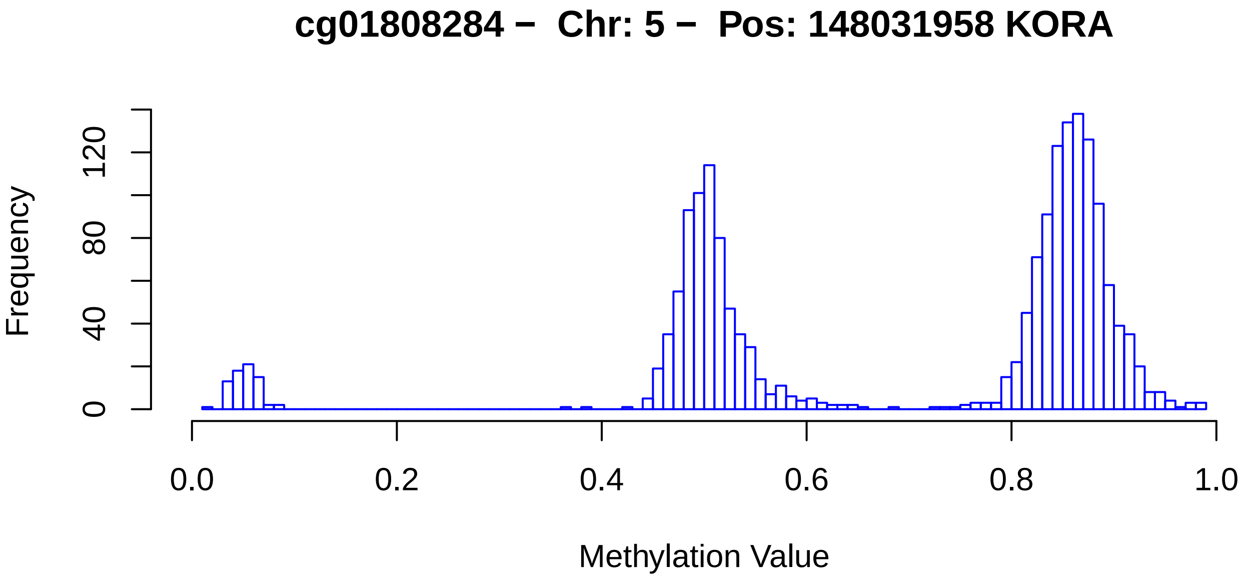
cg01808284 − Chr: 5 − Pos: 148031958 KORA
Frequency
0
40
80
120
0.0
0.2
0.4
0.6
0.8
1.0
Methylation Value

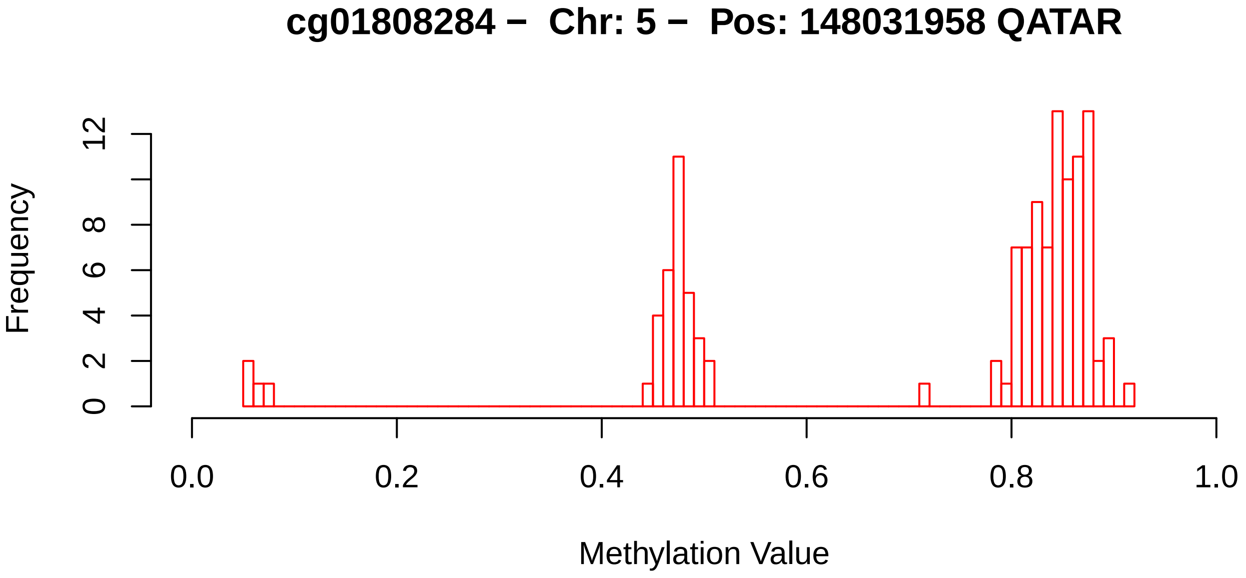
cg01808284 − Chr: 5 − Pos: 148031958 QATAR
Frequency
0
2
4
6
8
12
0.0
0.2
0.4
0.6
0.8
1.0
Methylation Value

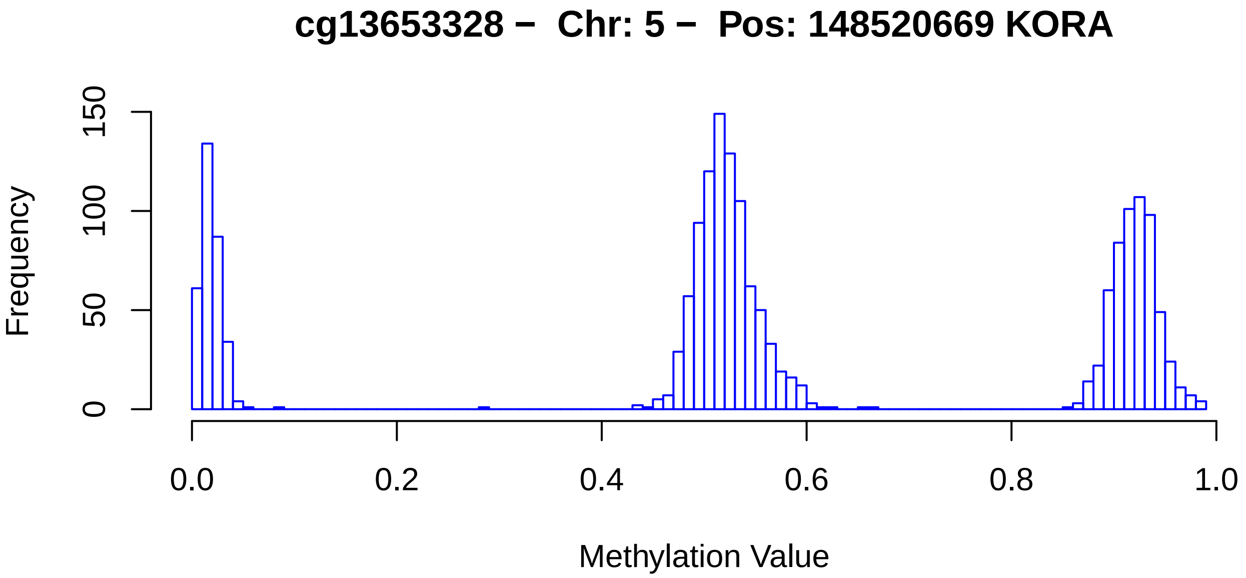
cg13653328 − Chr: 5 − Pos: 148520669 KORA
Frequency
0
50
100
150
0.0
0.2
0.4
0.6
0.8
1.0
Methylation Value

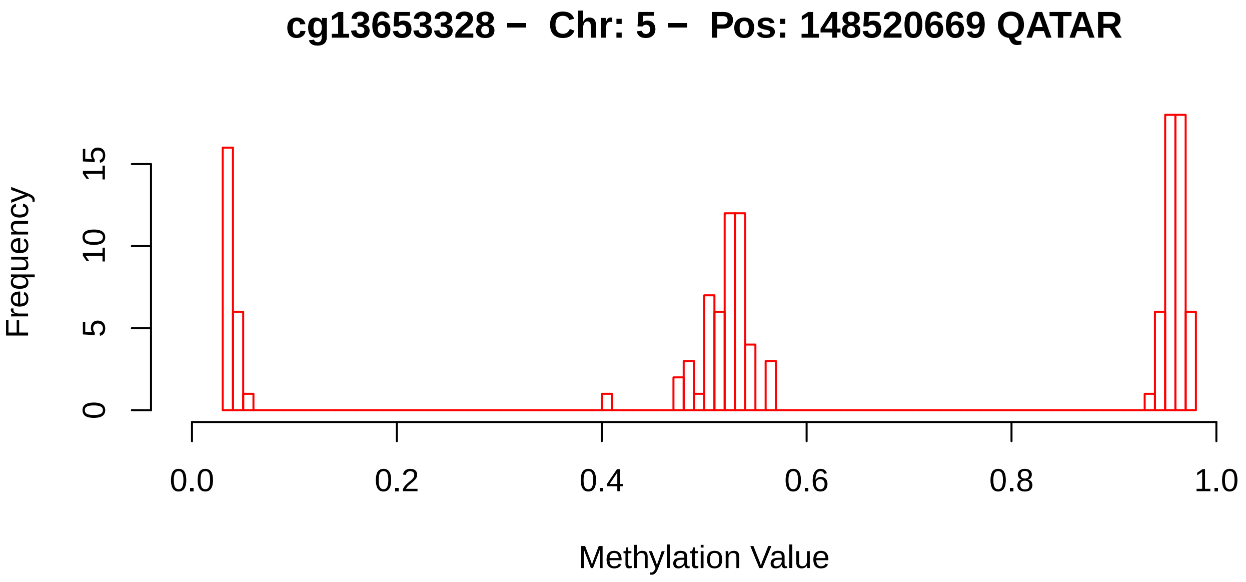
cg13653328 − Chr: 5 − Pos: 148520669 QATAR
Frequency
0
5
10
15
0.0
0.2
0.4
0.6
0.8
1.0
Methylation Value

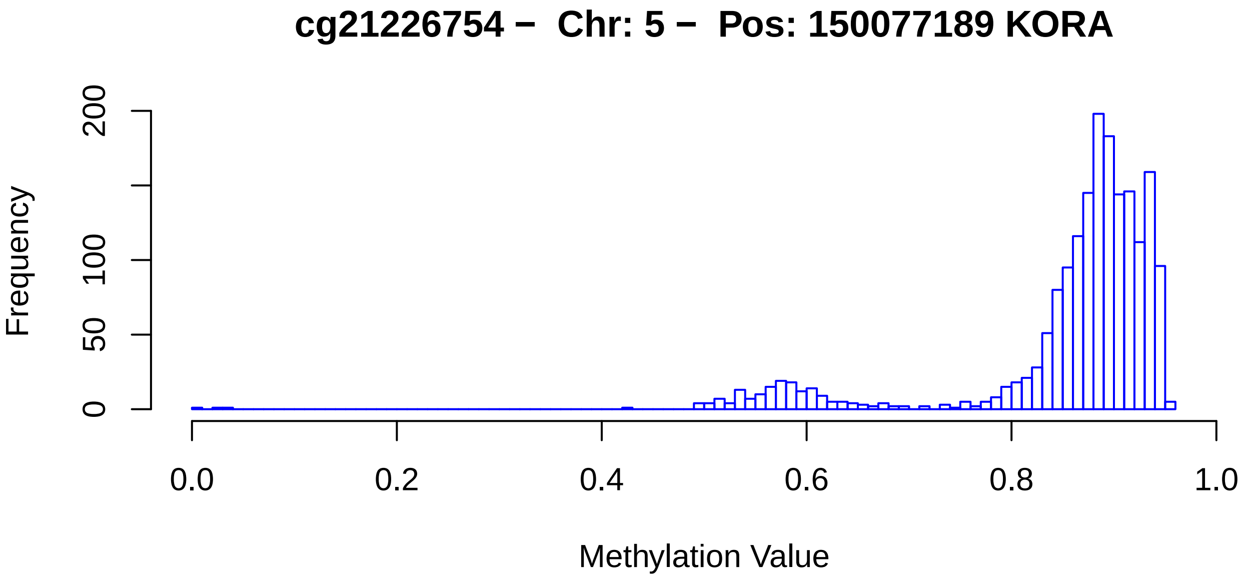
cg21226754 − Chr: 5 − Pos: 150077189 KORA
Frequency
0
50
100
200
0.0
0.2
0.4
0.6
0.8
1.0
Methylation Value

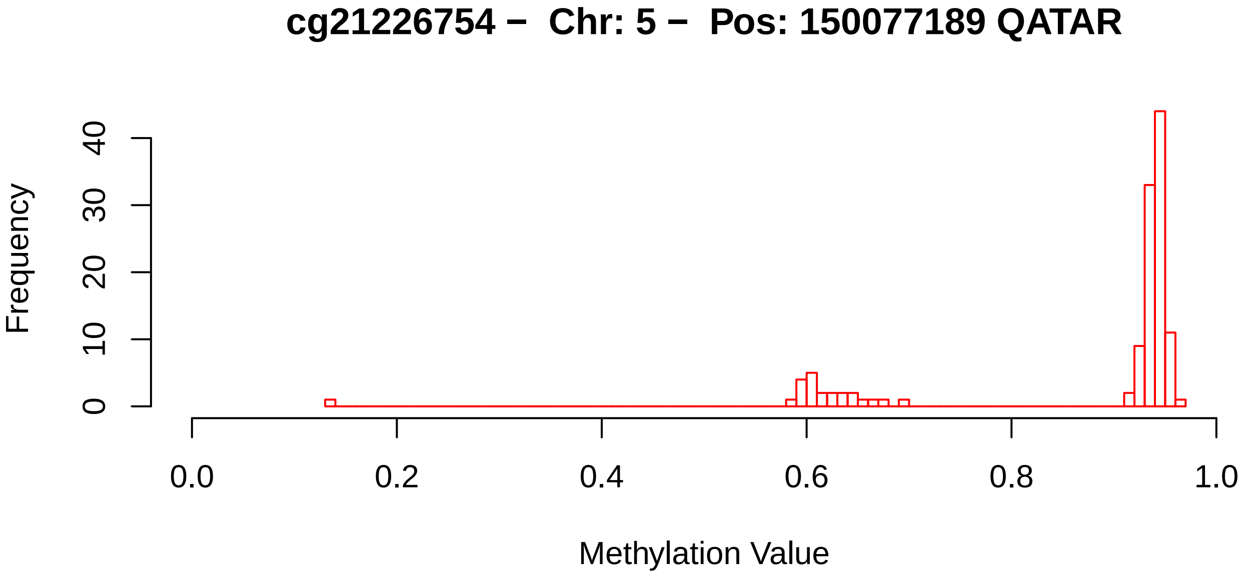
cg21226754 − Chr: 5 − Pos: 150077189 QATAR
Frequency
0
10
20
30
40
0.0
0.2
0.4
0.6
0.8
1.0
Methylation Value

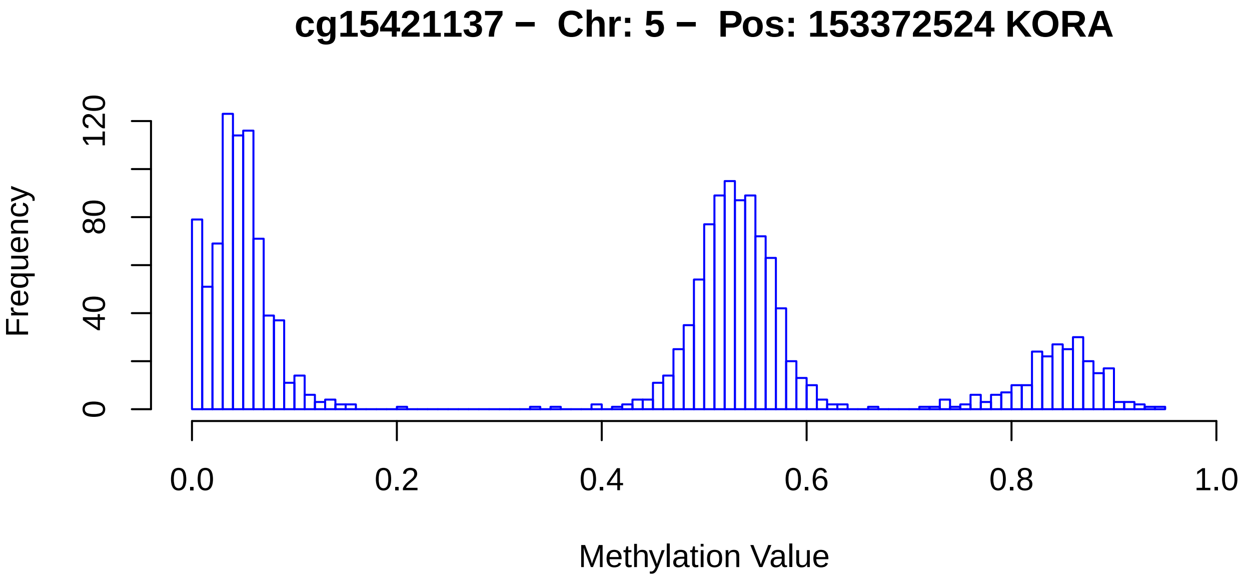

cg15421137 − Chr: 5 − Pos: 153372524 KORA
Frequency
0
40
80
120
0.0
0.2
0.4
0.6
0.8
1.0
Methylation Value

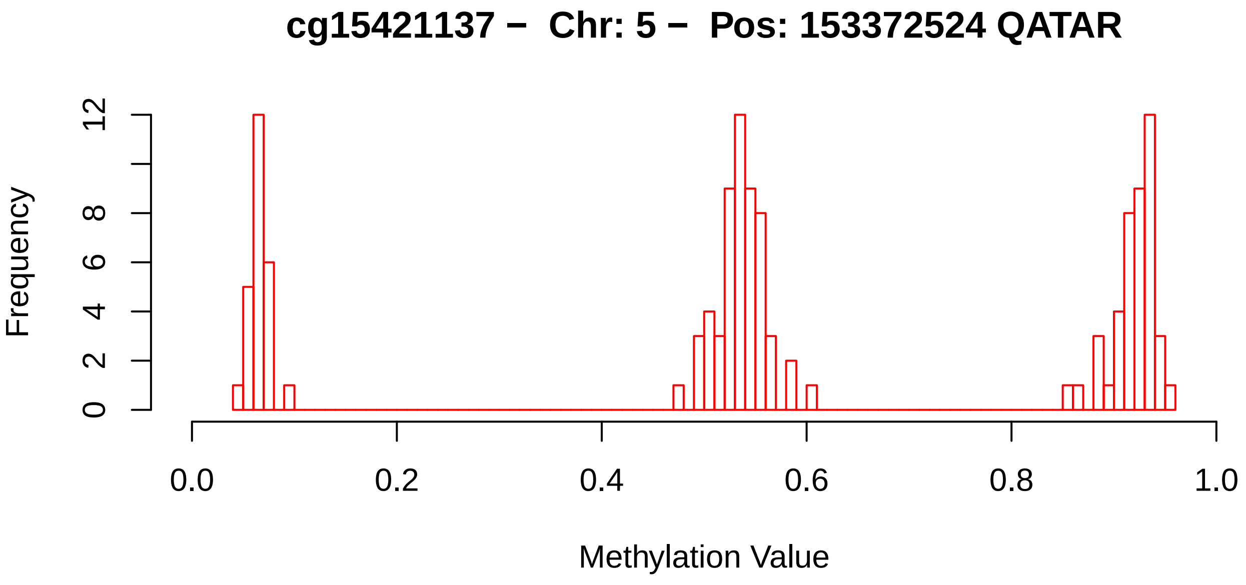

cg15421137 − Chr: 5 − Pos: 153372524 QATAR
Frequency
0
2
4
6
8
12
0.0
0.2
0.4
0.6
0.8
1.0
Methylation Value

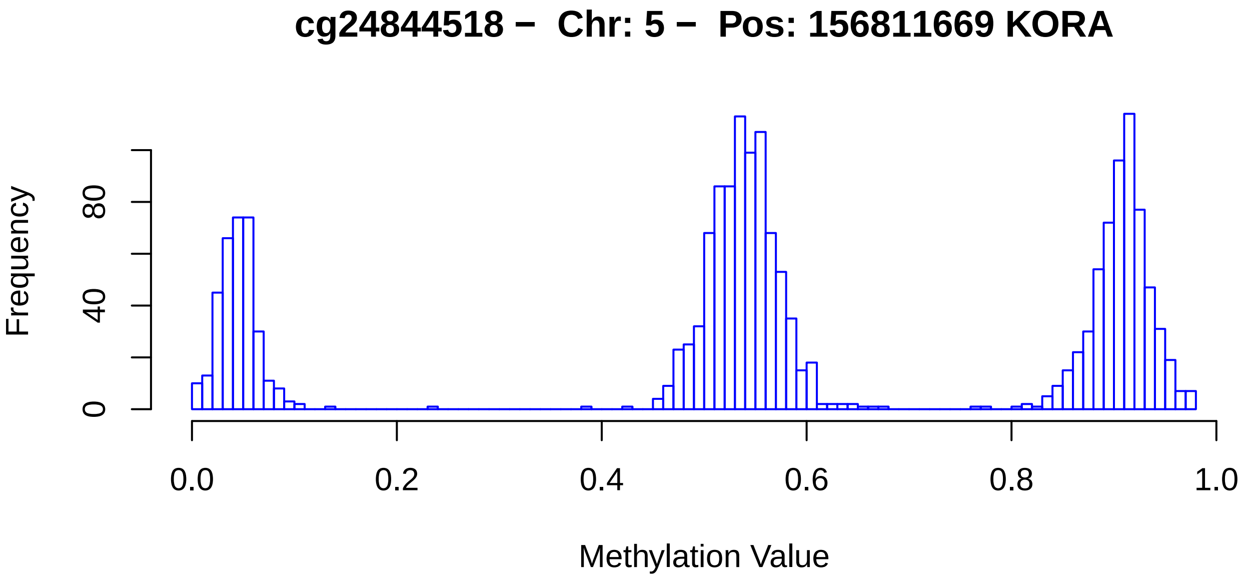
cg24844518 − Chr: 5 − Pos: 156811669 KORA
Frequency
0
40
80
0.0
0.2
0.4
0.6
0.8
1.0
Methylation Value

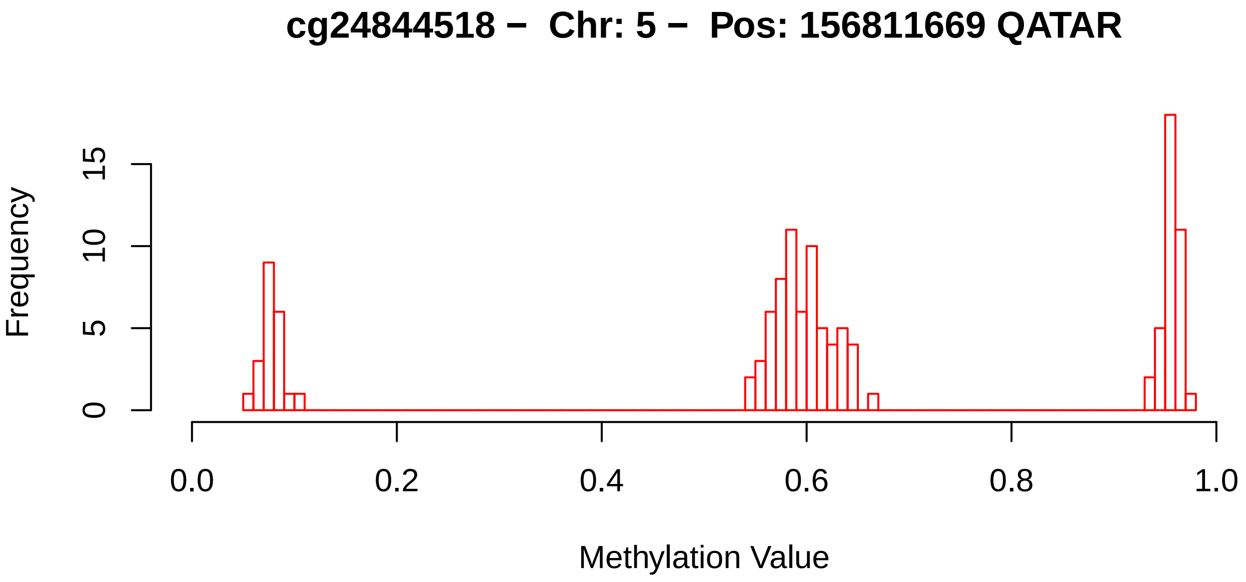
cg24844518 − Chr: 5 − Pos: 156811669 QATAR
Frequency
0
5
10
15
0.0
0.2
0.4
0.6
0.8
1.0
Methylation Value

cg13386926 − Chr: 5 − Pos: 156890399 KORA

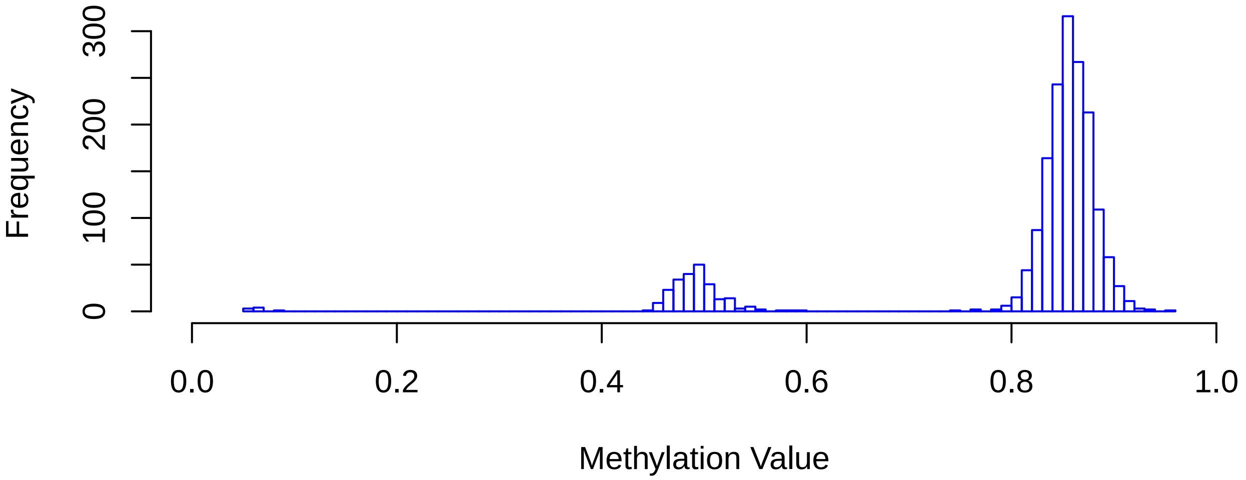

cg13386926 − Chr: 5 − Pos: 156890399 QATAR

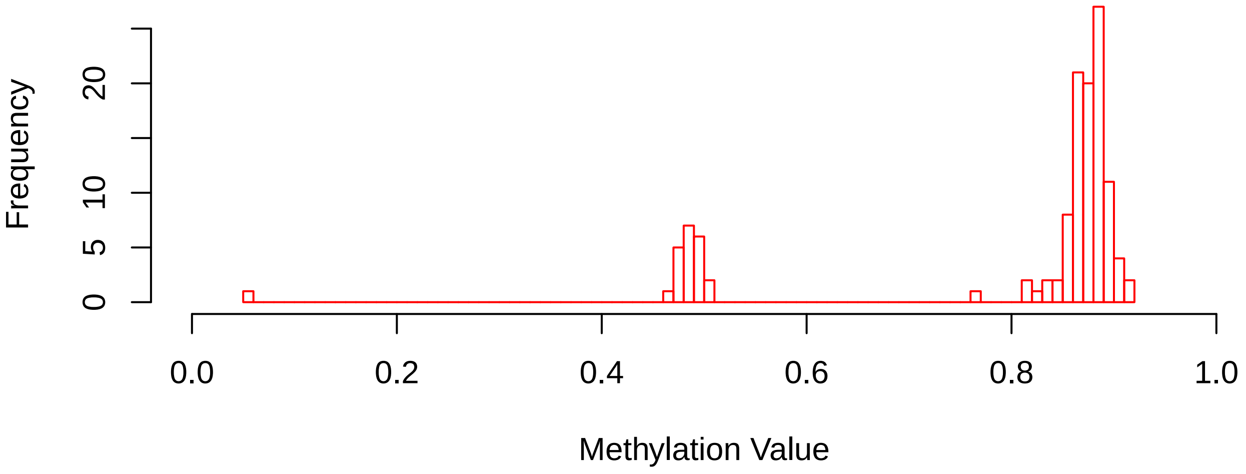

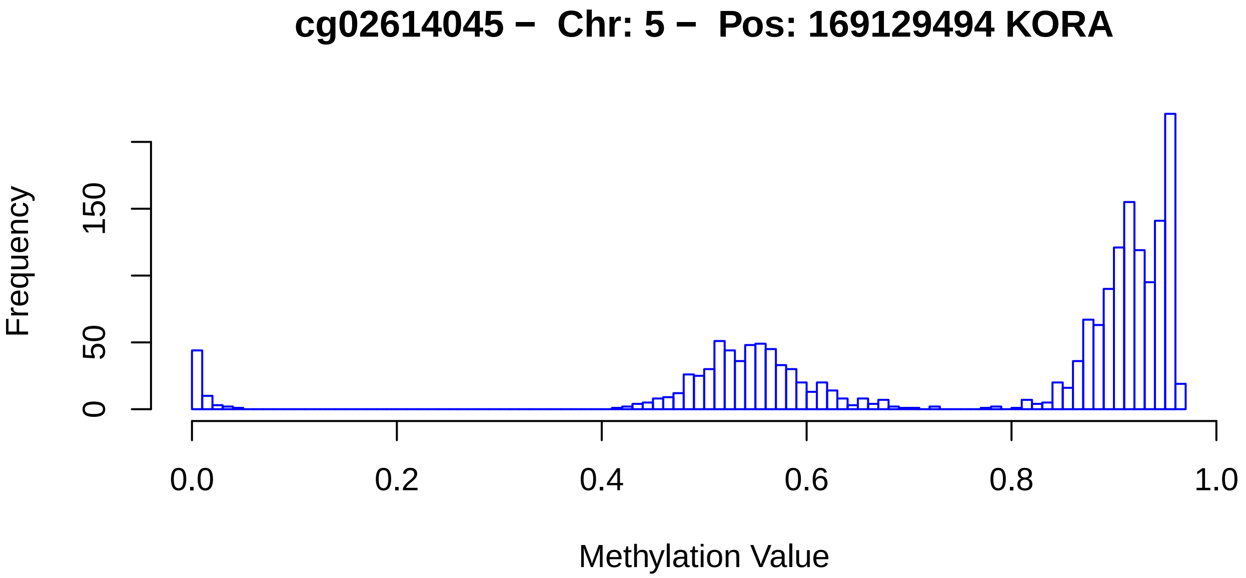
cg02614045 − Chr: 5 − Pos: 169129494 KORA
Frequency
0
50
150
0.0
0.2
0.4
0.6
0.8
1.0
Methylation Value

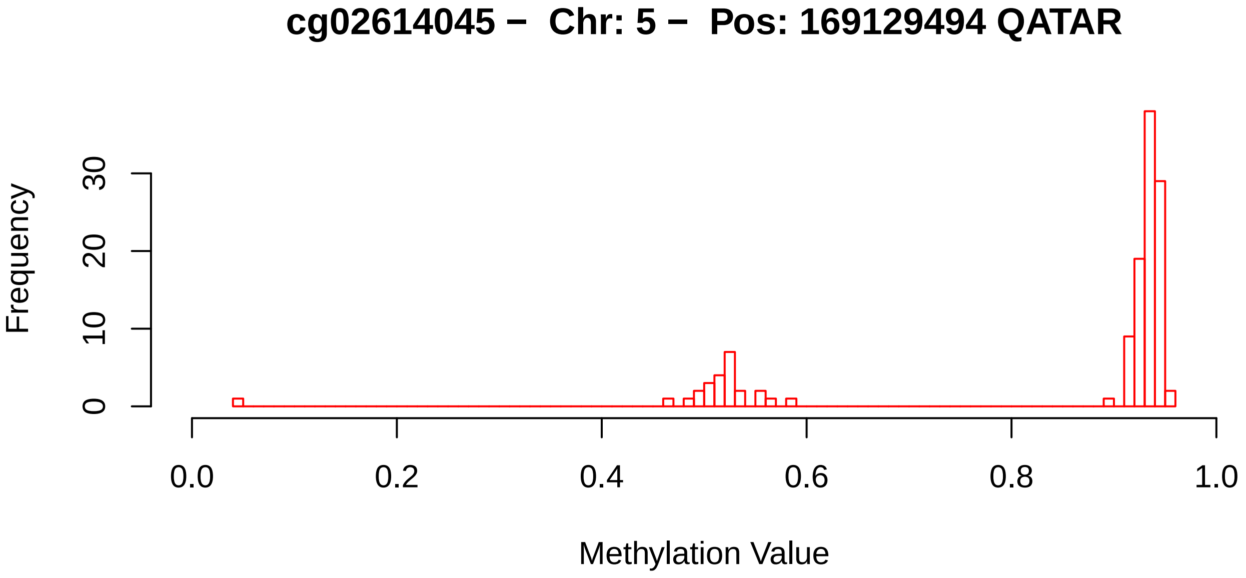
cg02614045 − Chr: 5 − Pos: 169129494 QATAR
Frequency
0
10
20
30
0.0
0.2
0.4
0.6
0.8
1.0
Methylation Value

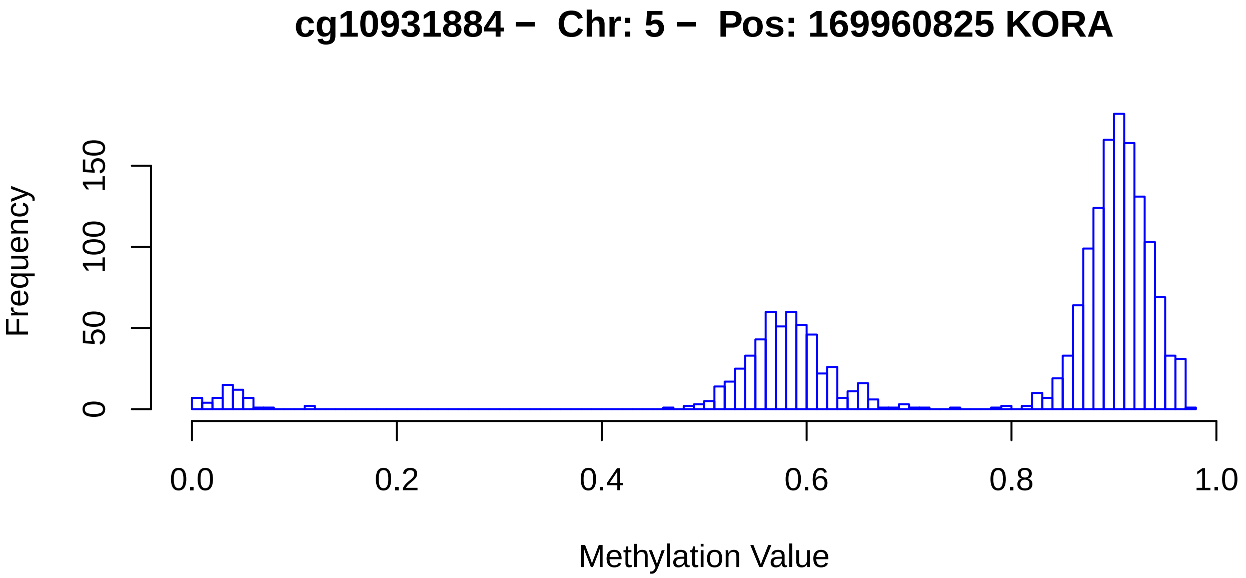
cg10931884 − Chr: 5 − Pos: 169960825 KORA
Frequency
0
50
100
150
0.0
0.2
0.4
0.6
0.8
1.0
Methylation Value

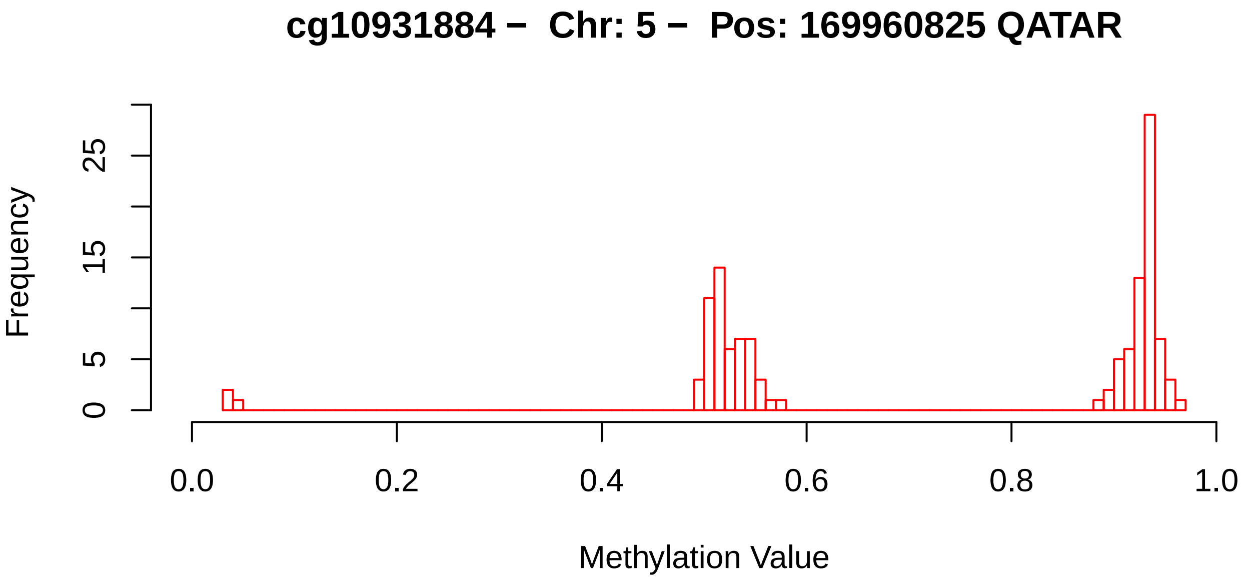
cg10931884 − Chr: 5 − Pos: 169960825 QATAR
Frequency
0
5
15
25
0.0
0.2
0.4
0.6
0.8
1.0
Methylation Value

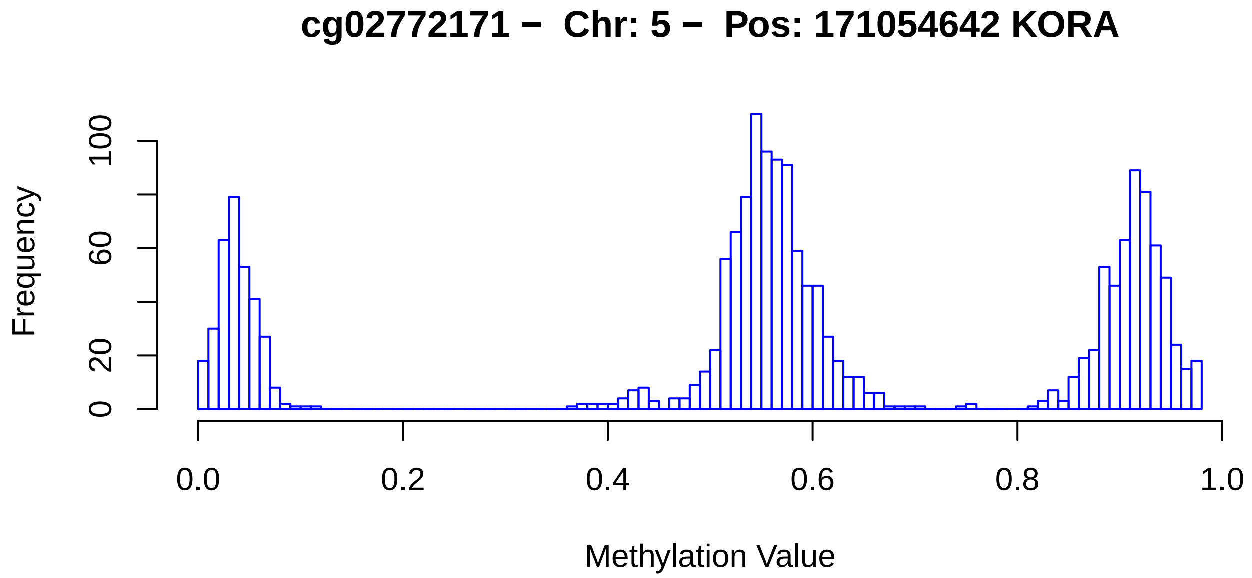
cg02772171 − Chr: 5 − Pos: 171054642 KORA
Frequency
0
20
60
100
0.0
0.2
0.4
0.6
0.8
1.0
Methylation Value

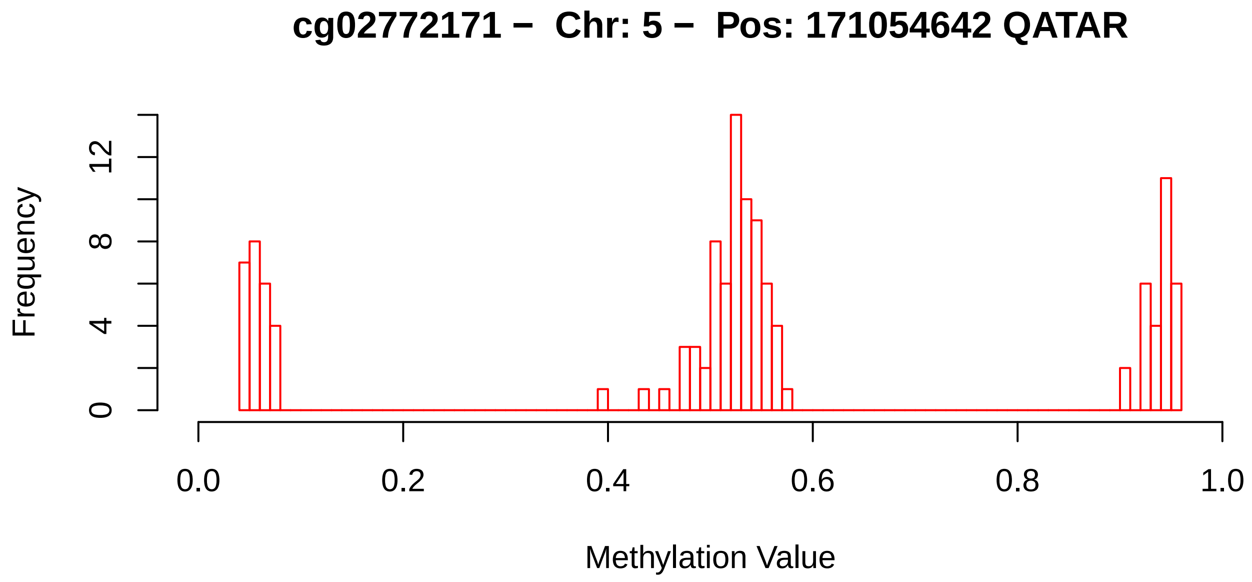
cg02772171 − Chr: 5 − Pos: 171054642 QATAR
Frequency
0
4
8
12
0.0
0.2
0.4
0.6
0.8
1.0
Methylation Value

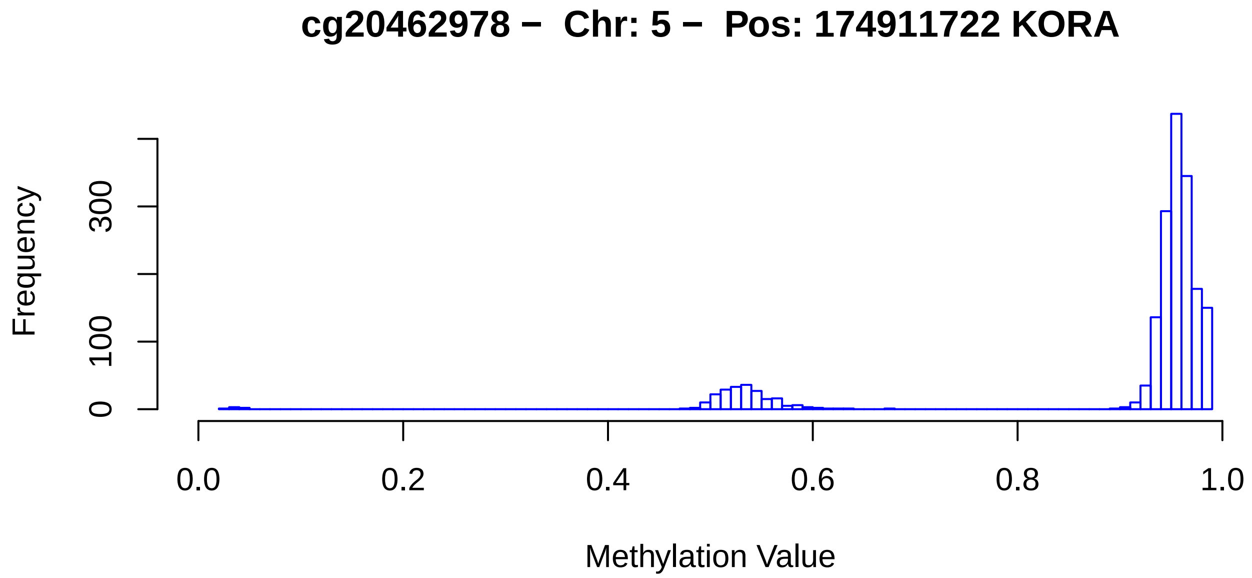
cg20462978 − Chr: 5 − Pos: 174911722 KORA
Frequency
0
100
300
0.0
0.2
0.4
0.6
0.8
1.0
Methylation Value

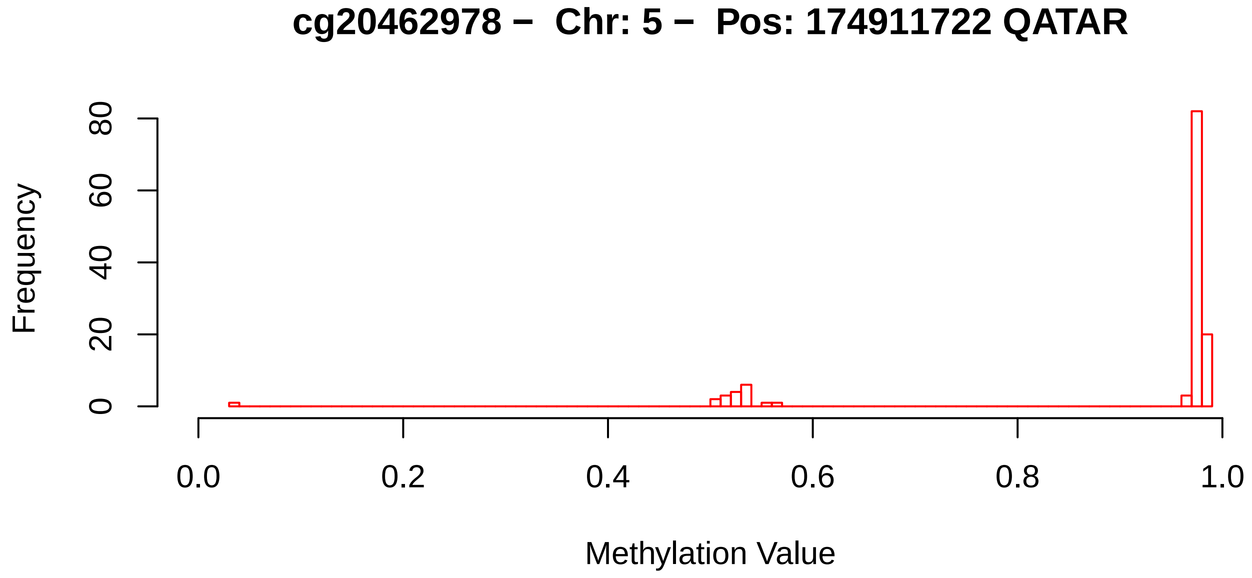
cg20462978 − Chr: 5 − Pos: 174911722 QATAR
Frequency
0
20
40
60
80
0.0
0.2
0.4
0.6
0.8
1.0
Methylation Value

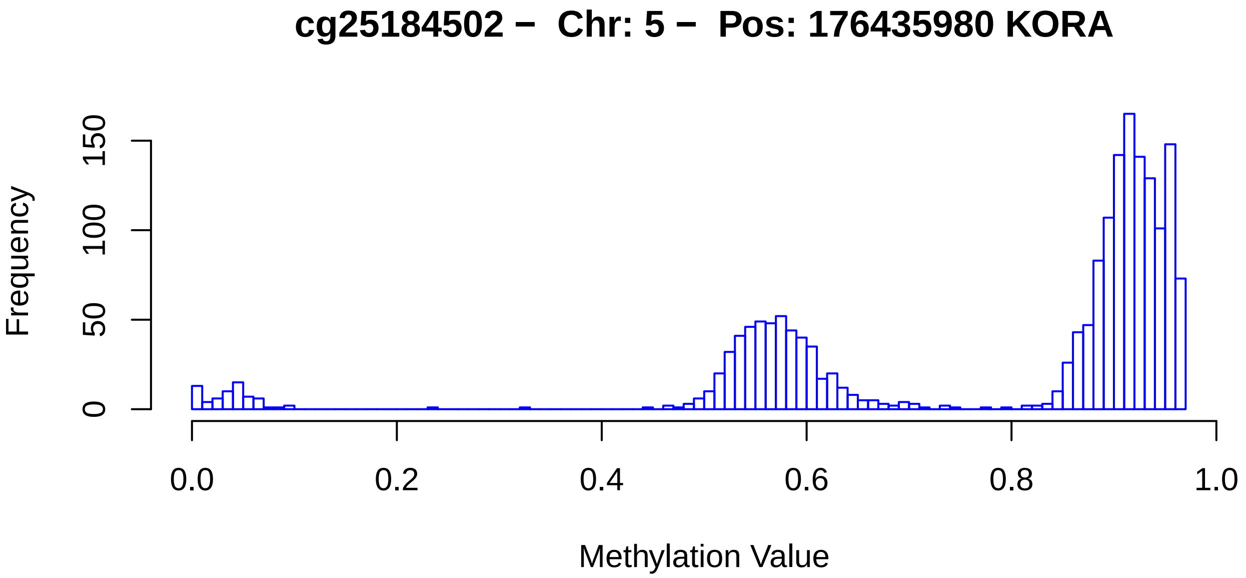
cg25184502 − Chr: 5 − Pos: 176435980 KORA
Frequency
0
50
100
150
0.0
0.2
0.4
0.6
0.8
1.0
Methylation Value

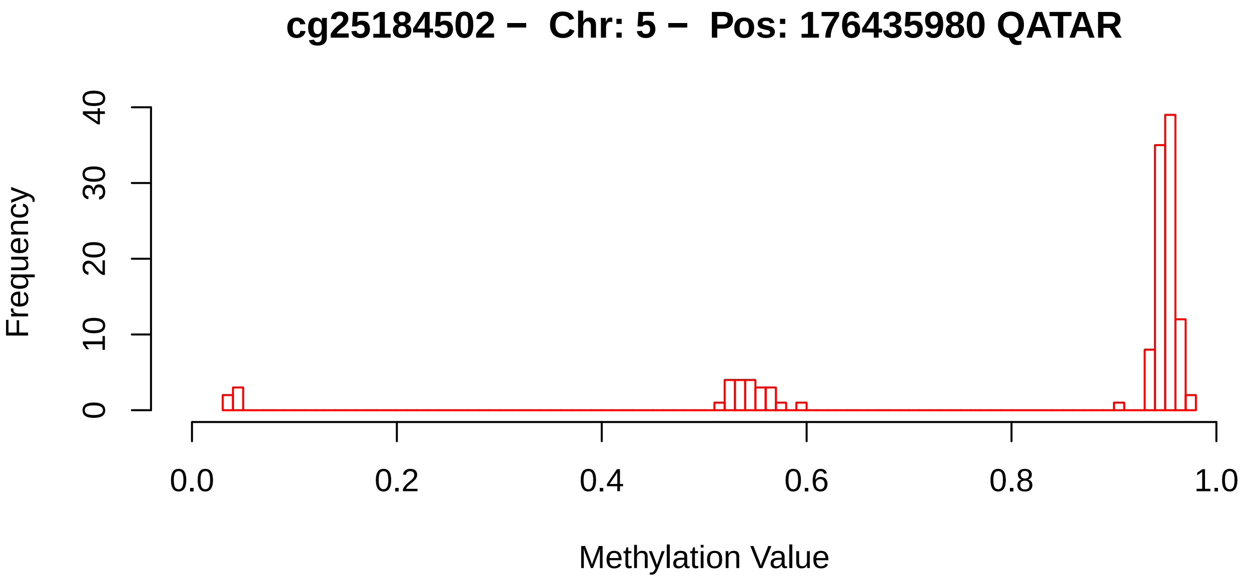
cg25184502 − Chr: 5 − Pos: 176435980 QATAR
Frequency
0
10
20
30
40
0.0
0.2
0.4
0.6
0.8
1.0
Methylation Value

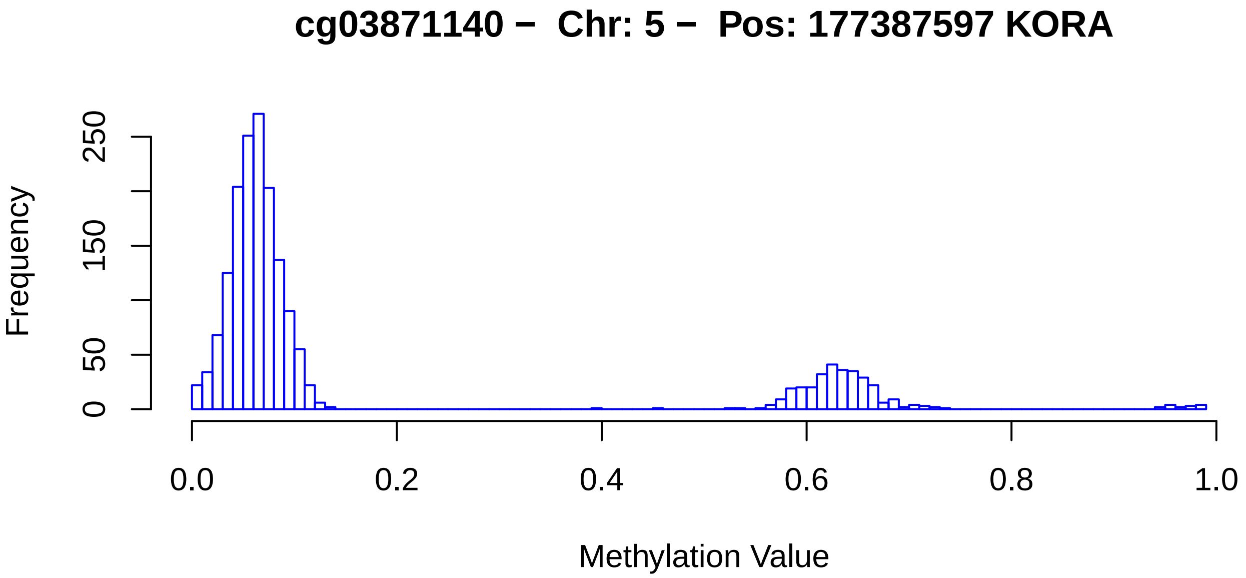
cg03871140 − Chr: 5 − Pos: 177387597 KORA
Frequency
0
50
150
250
0.0
0.2
0.4
0.6
0.8
1.0
Methylation Value

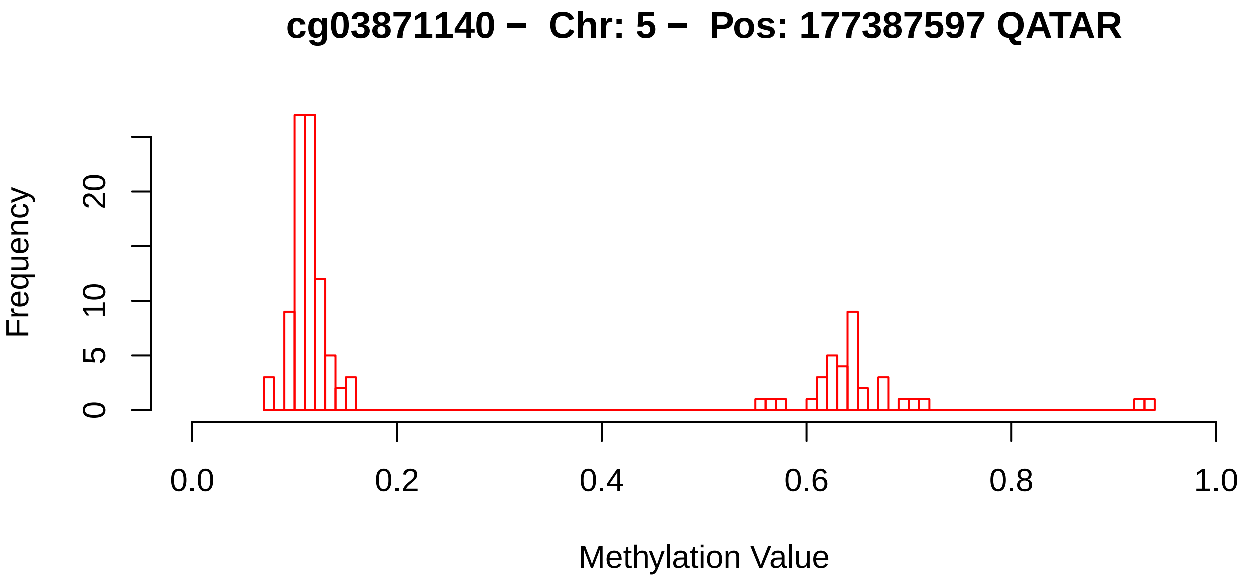
cg03871140 − Chr: 5 − Pos: 177387597 QATAR
Frequency
0
5
10
20
0.0
0.2
0.4
0.6
0.8
1.0
Methylation Value

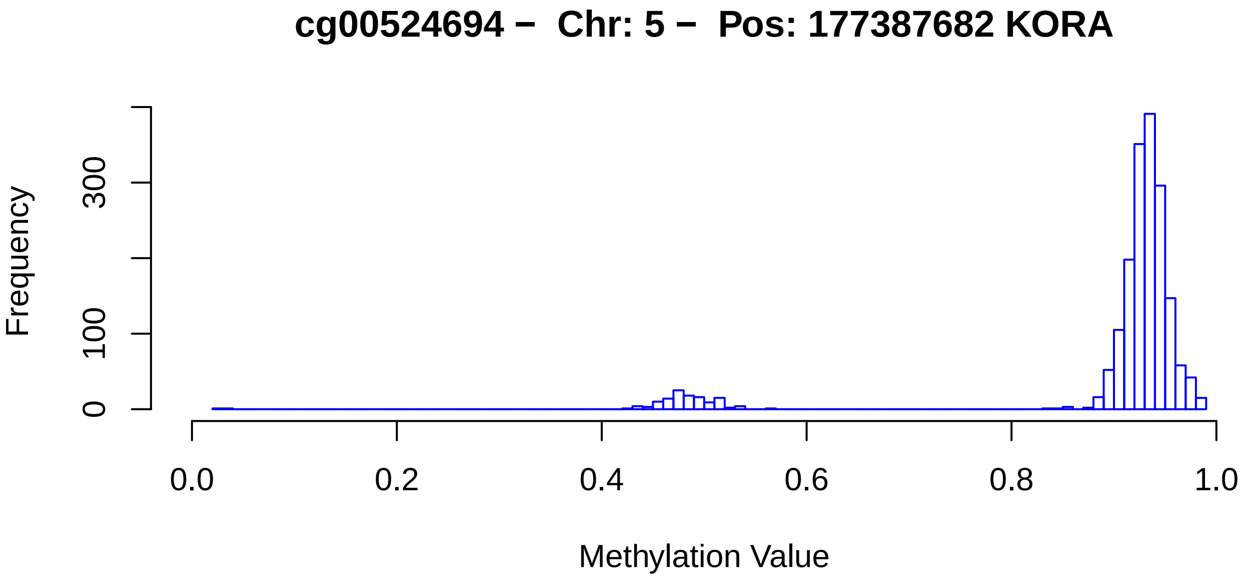
cg00524694 − Chr: 5 − Pos: 177387682 KORA
Frequency
0
100
300
0.0
0.2
0.4
0.6
0.8
1.0
Methylation Value

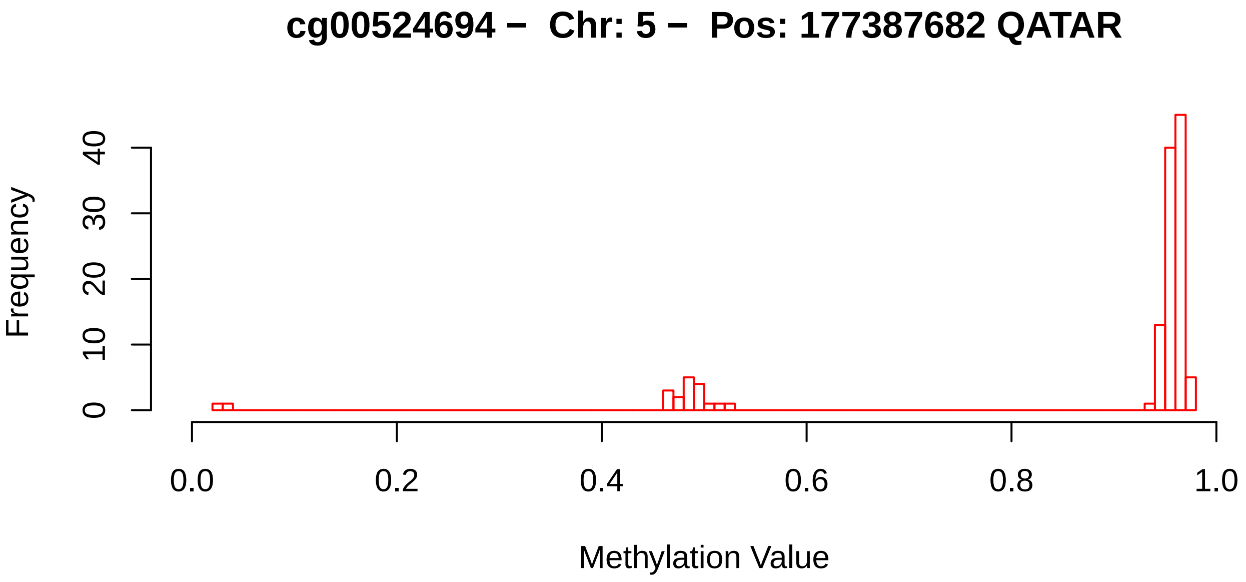
cg00524694 − Chr: 5 − Pos: 177387682 QATAR
Frequency
0
10
20
30
40
0.0
0.2
0.4
0.6
0.8
1.0
Methylation Value

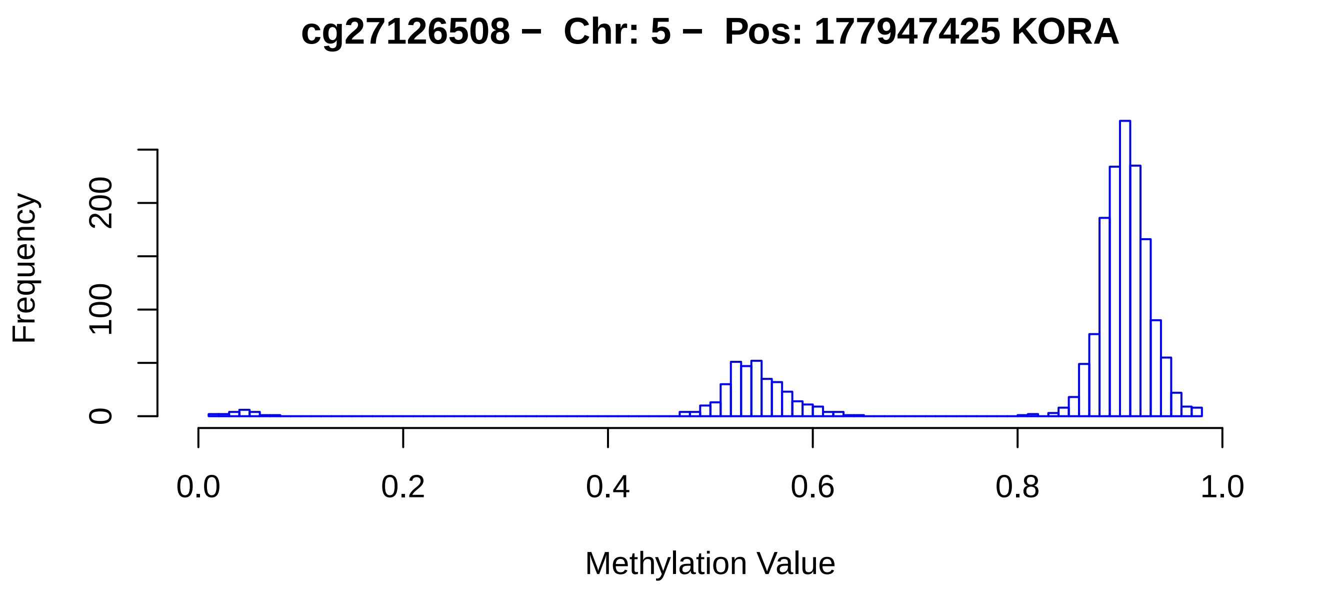
cg27126508 − Chr: 5 − Pos: 177947425 KORA
Frequency
0
100
200
0.0
0.2
0.4
0.6
0.8
1.0
Methylation Value

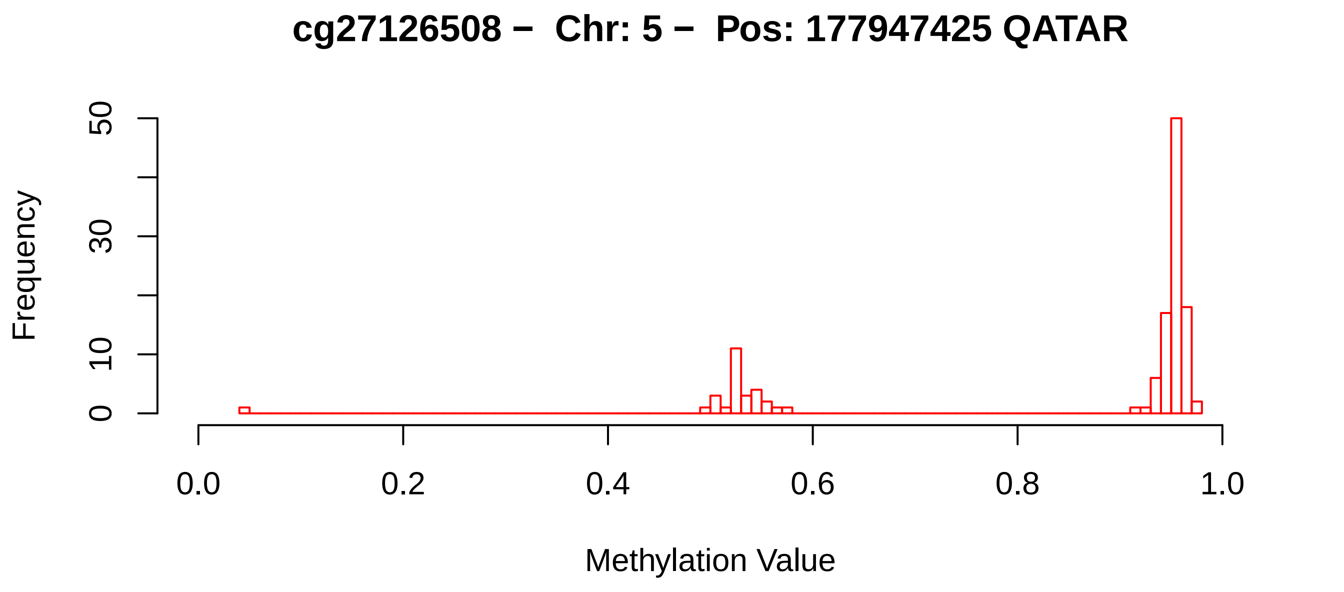
cg27126508 − Chr: 5 − Pos: 177947425 QATAR
Frequency
0
10
30
50
0.0
0.2
0.4
0.6
0.8
1.0
Methylation Value

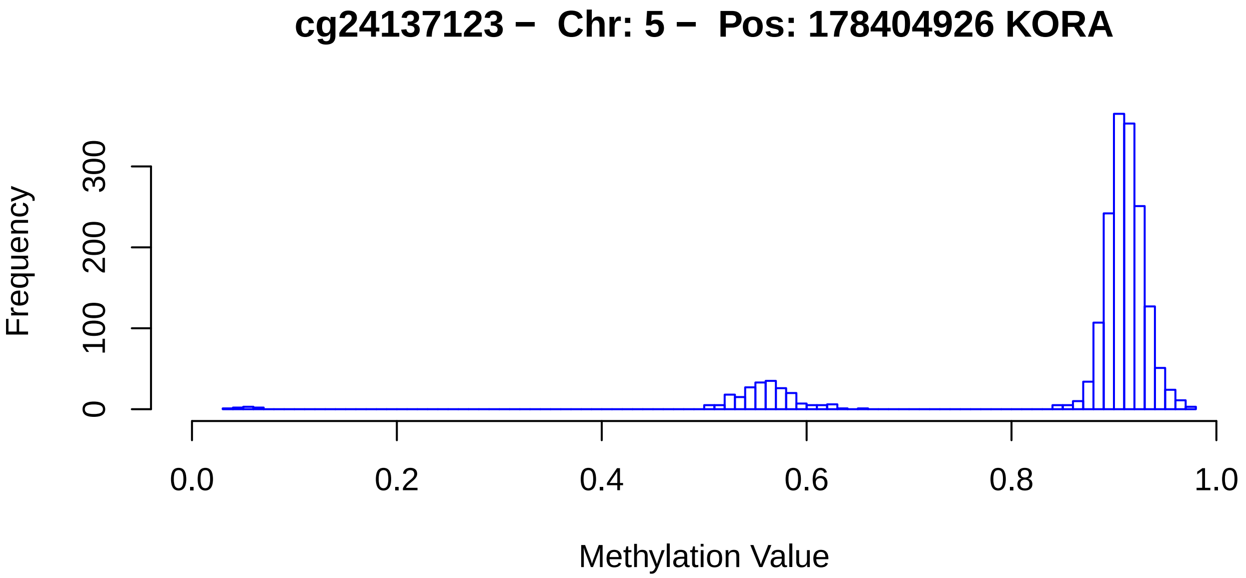
cg24137123 − Chr: 5 − Pos: 178404926 KORA
Frequency
0
100
200
300
0.0
0.2
0.4
0.6
0.8
1.0
Methylation Value

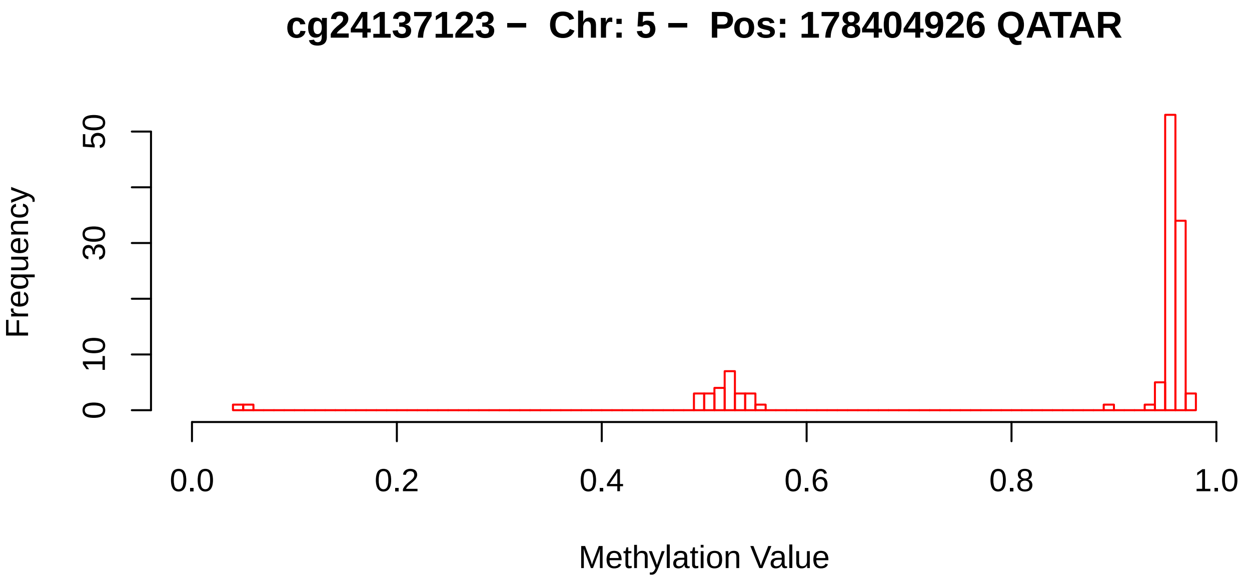
cg24137123 − Chr: 5 − Pos: 178404926 QATAR
Frequency
0
10
30
50
0.0
0.2
0.4
0.6
0.8
1.0
Methylation Value

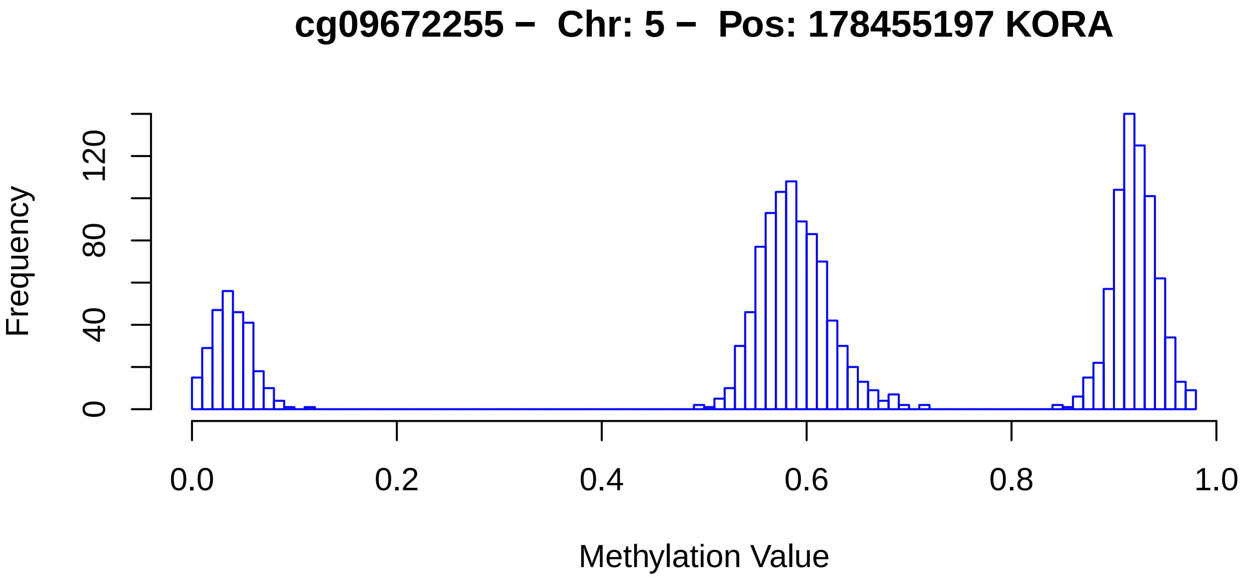

cg09672255 − Chr: 5 − Pos: 178455197 KORA
Frequency
0
40
80
120
0.0
0.2
0.4
0.6
0.8
1.0
Methylation Value

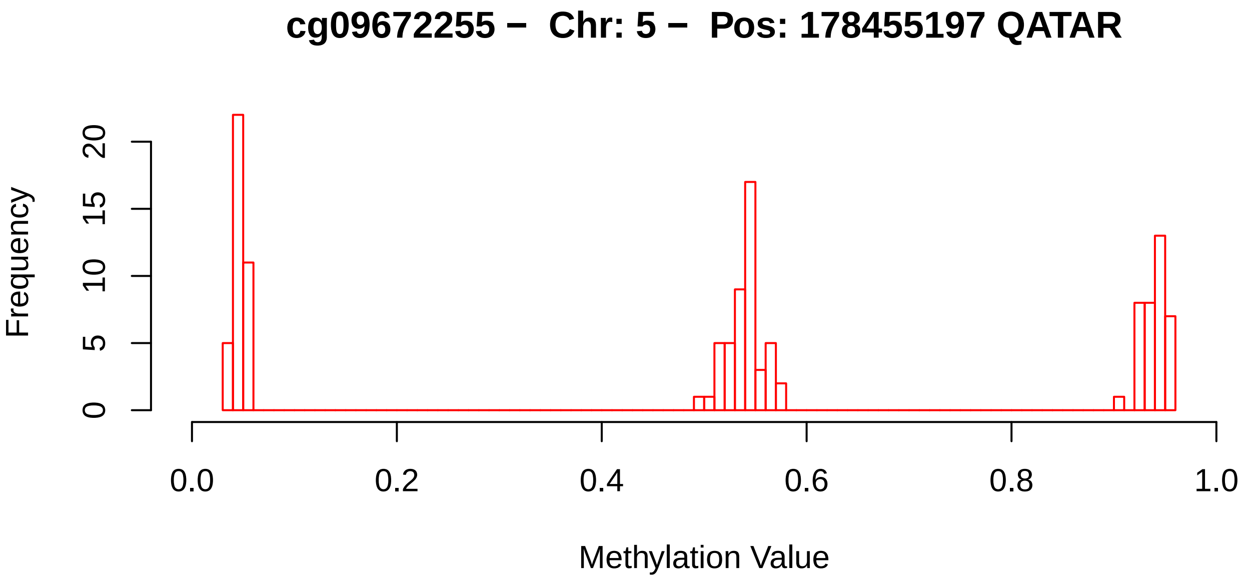

cg09672255 − Chr: 5 − Pos: 178455197 QATAR
Frequency
0
5
10
15
20
0.0
0.2
0.4
0.6
0.8
1.0
Methylation Value

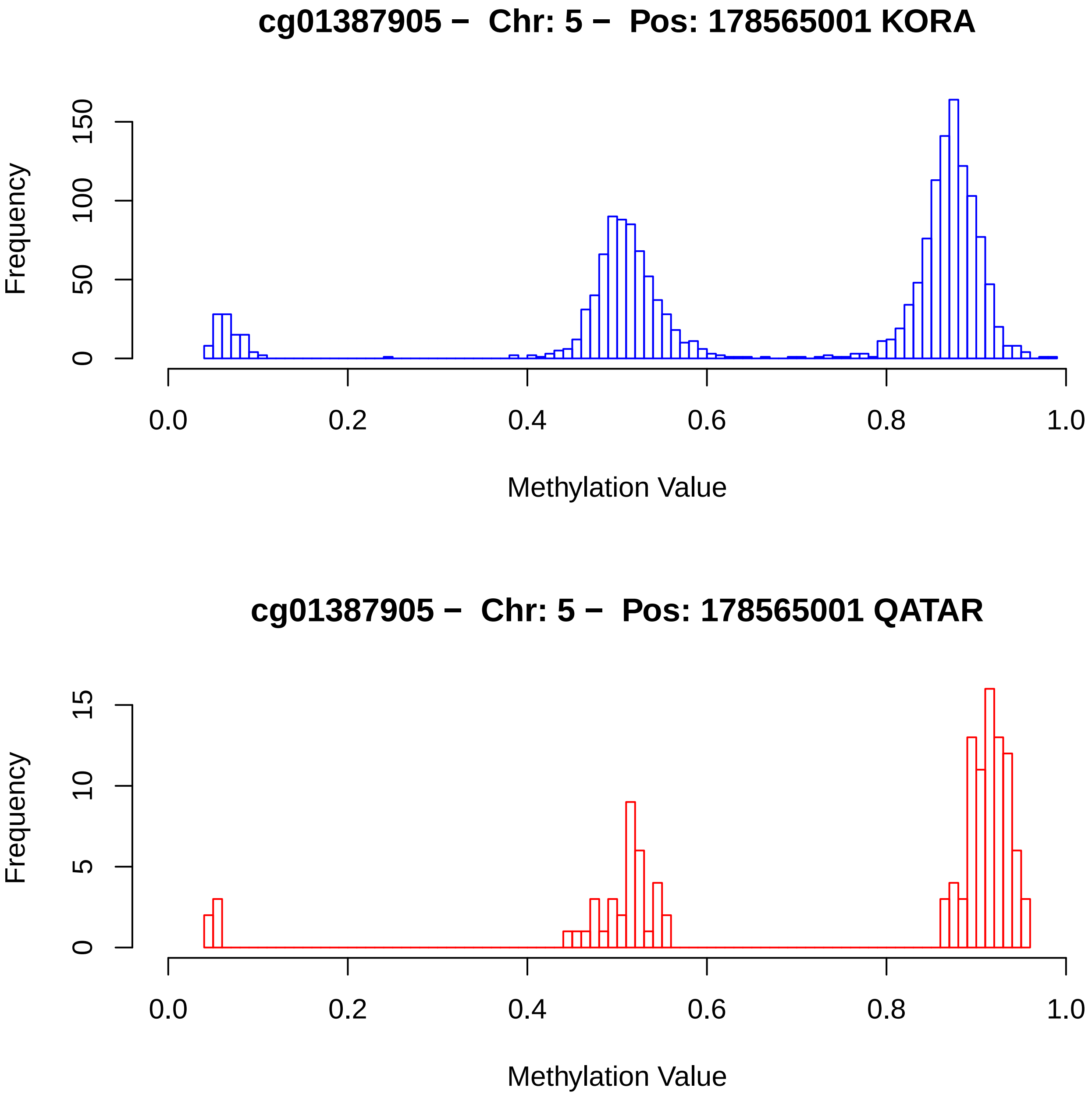
cg01387905 − Chr: 5 − Pos: 178565001 KORA
Frequency
0
50
100
150
0.0
0.2
0.4
0.6
0.8
1.0
Methylation Value
cg01387905 − Chr: 5 − Pos: 178565001 QATAR
Frequency
0
5
10
15
0.0
0.2
0.4
0.6
0.8
1.0
Methylation Value

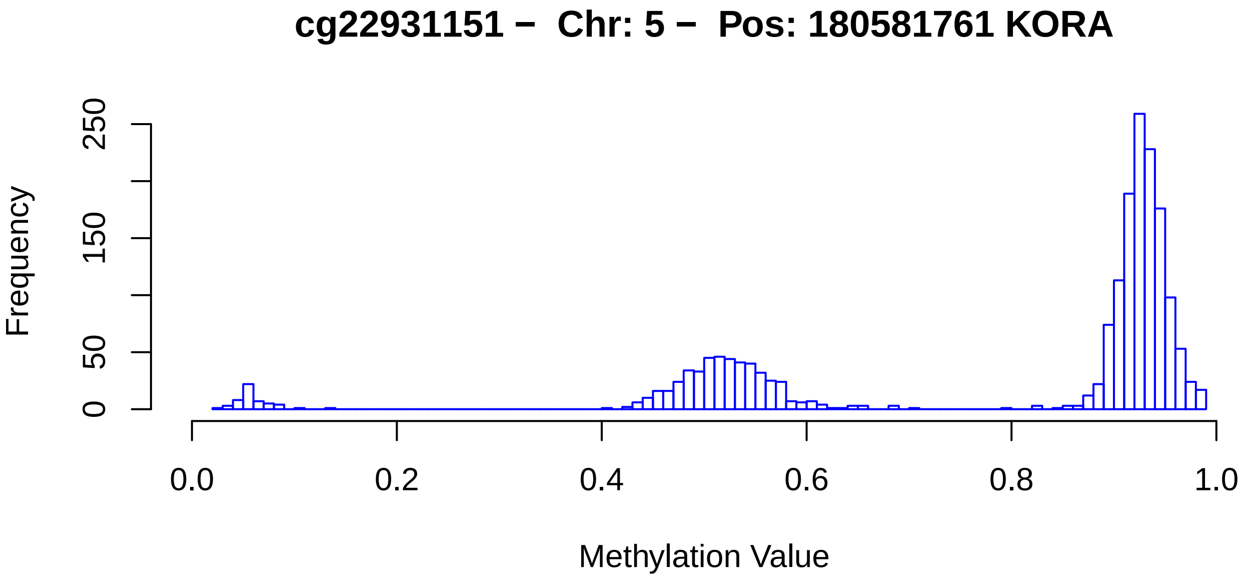
cg22931151 − Chr: 5 − Pos: 180581761 KORA
Frequency
0
50
150
250
0.0
0.2
0.4
0.6
0.8
1.0
Methylation Value

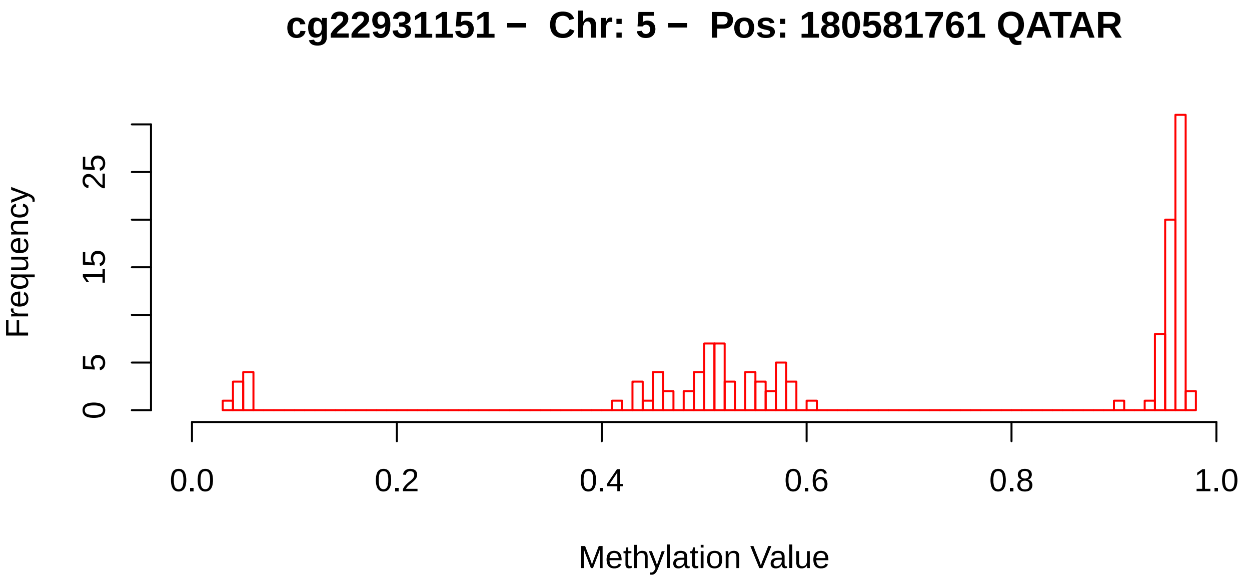
cg22931151 − Chr: 5 − Pos: 180581761 QATAR
Frequency
0
5
15
25
0.0
0.2
0.4
0.6
0.8
1.0
Methylation Value

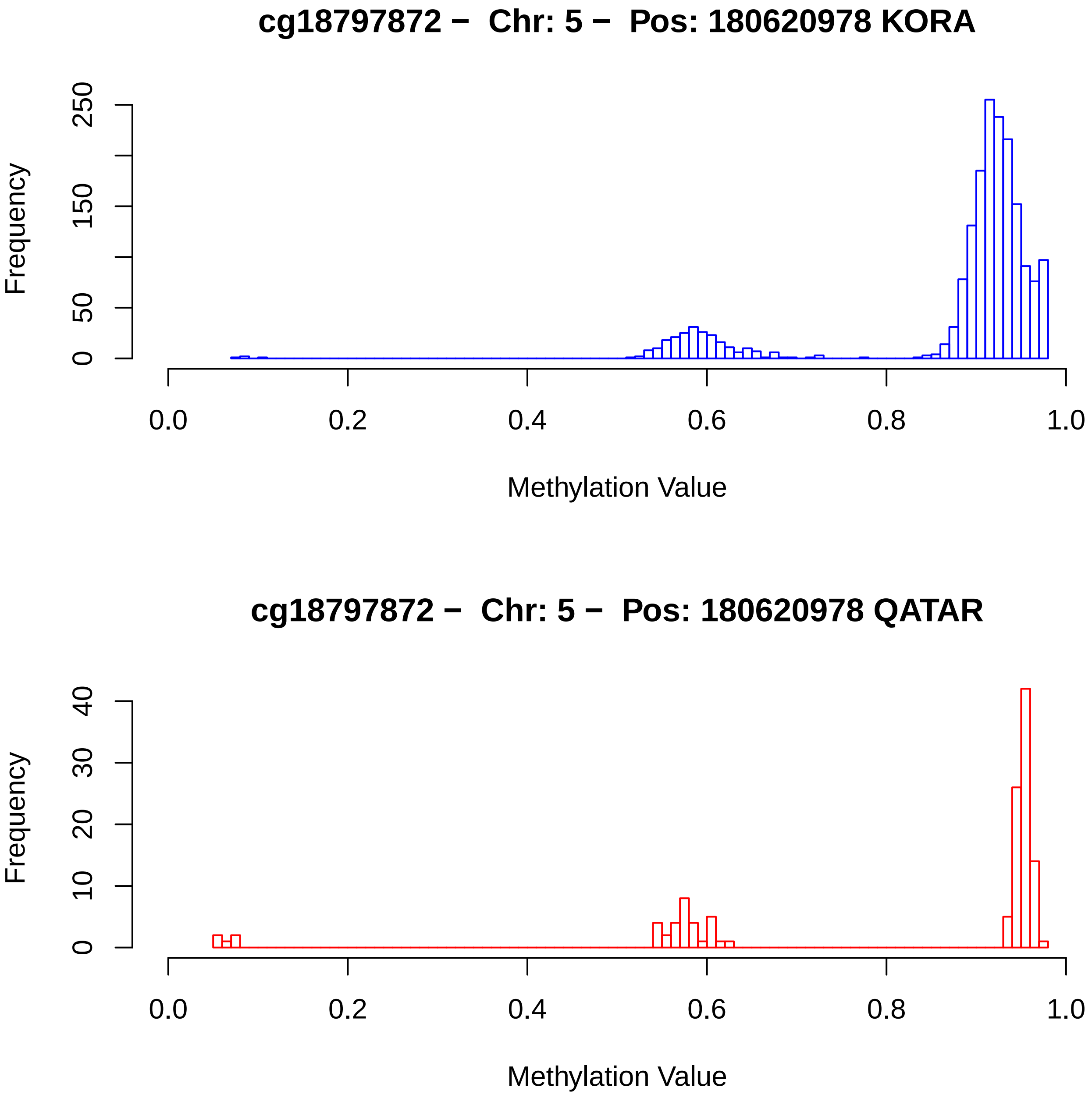
cg18797872 − Chr: 5 − Pos: 180620978 KORA
Frequency
0
50
150
250
0.0
0.2
0.4
0.6
0.8
1.0
Methylation Value
cg18797872 − Chr: 5 − Pos: 180620978 QATAR
Frequency
0
10
20
30
40
0.0
0.2
0.4
0.6
0.8
1.0
Methylation Value

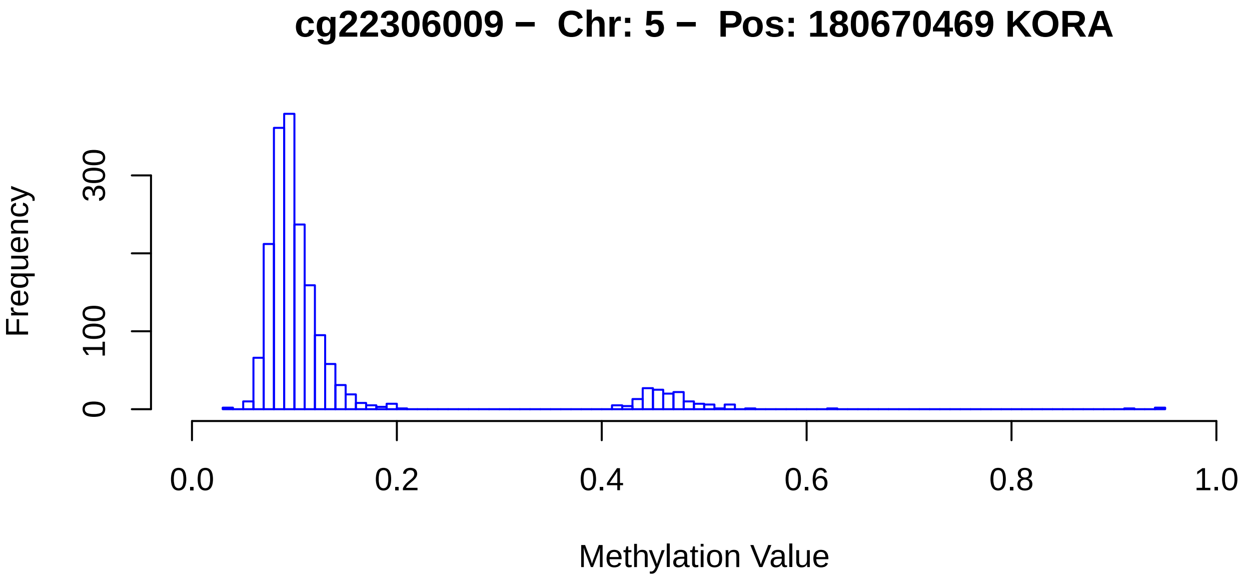
cg22306009 − Chr: 5 − Pos: 180670469 KORA
Frequency
0
100
300
0.0
0.2
0.4
0.6
0.8
1.0
Methylation Value

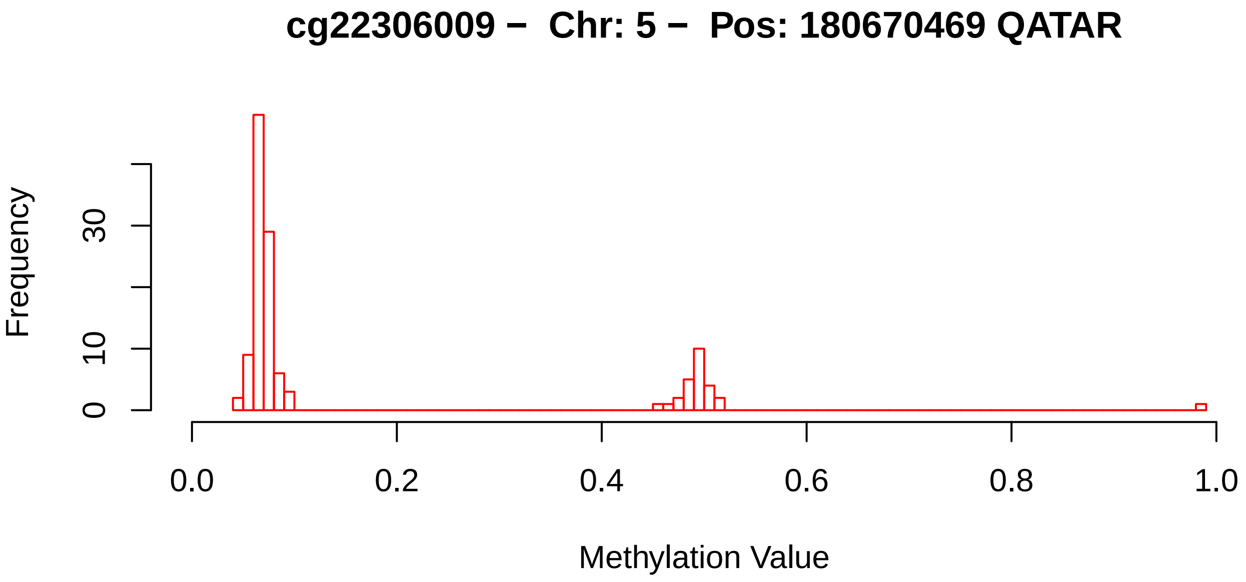
cg22306009 − Chr: 5 − Pos: 180670469 QATAR
Frequency
0
10
30
0.0
0.2
0.4
0.6
0.8
1.0
Methylation Value

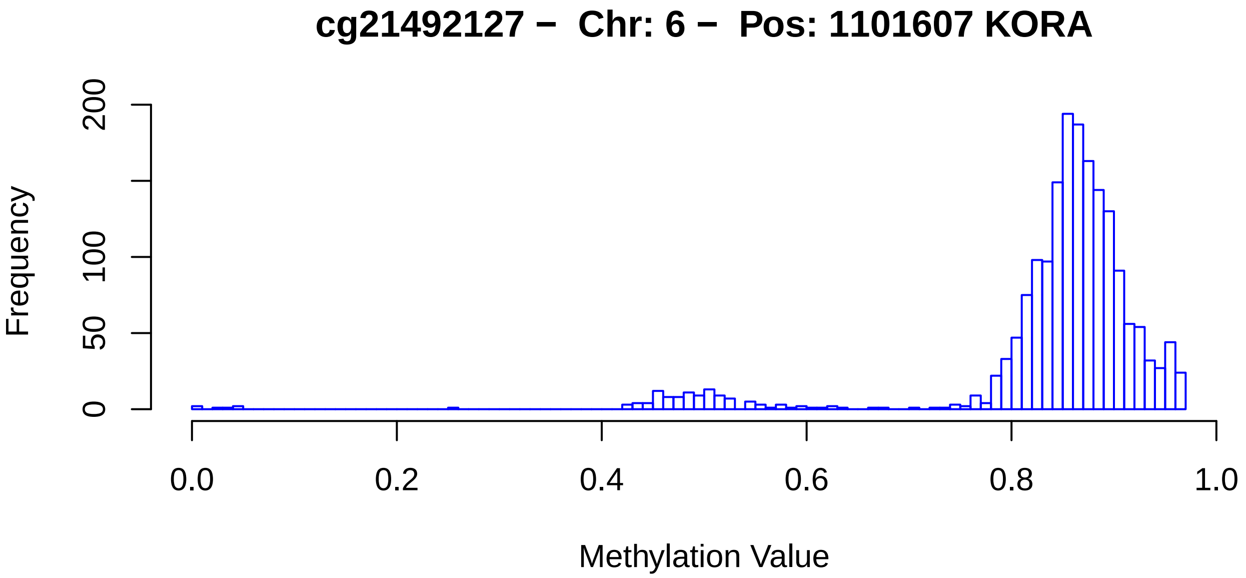
cg21492127 − Chr: 6 − Pos: 1101607 KORA
Frequency
0
50
100
200
0.0
0.2
0.4
0.6
0.8
1.0
Methylation Value

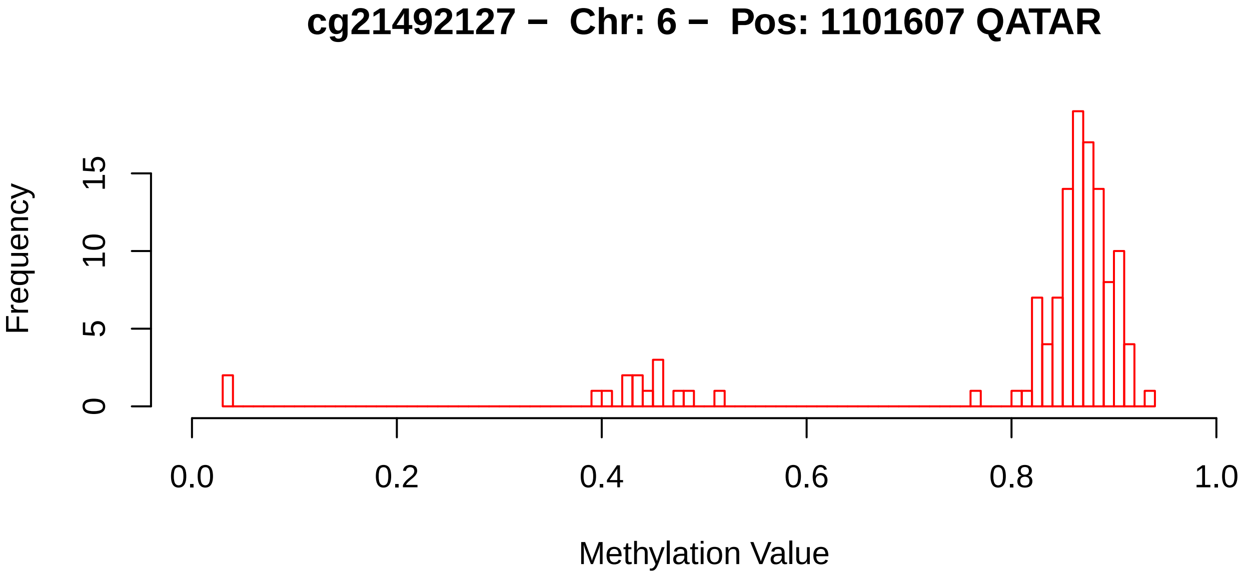
cg21492127 − Chr: 6 − Pos: 1101607 QATAR
Frequency
0
5
10
15
0.0
0.2
0.4
0.6
0.8
1.0
Methylation Value

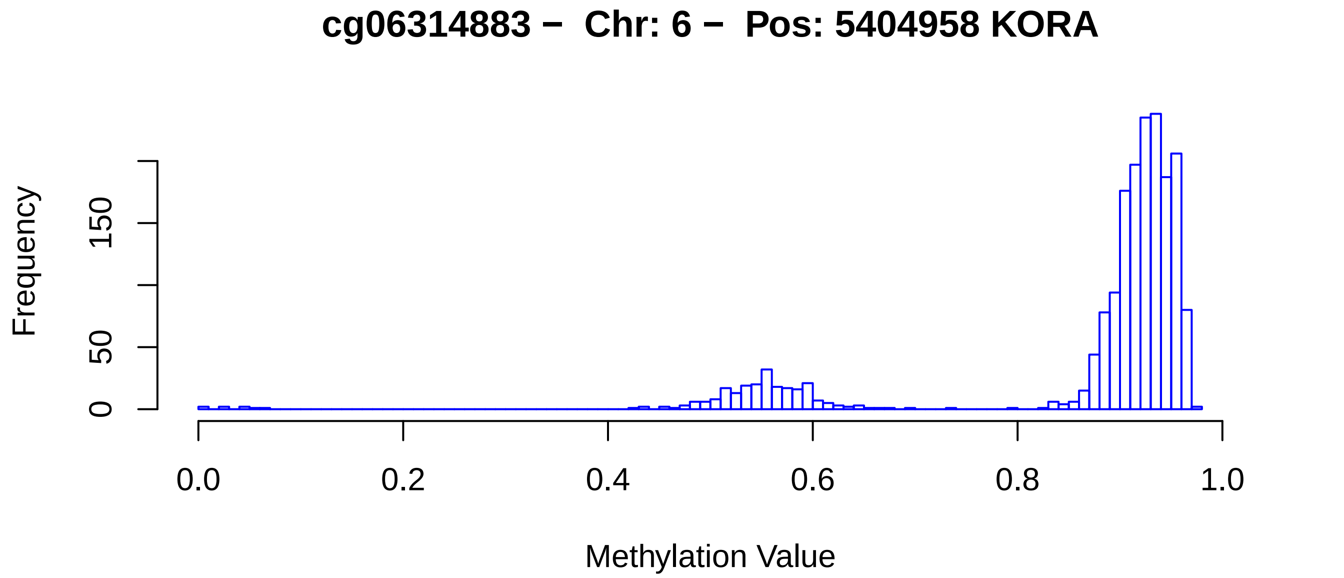
cg06314883 − Chr: 6 − Pos: 5404958 KORA
Frequency
0
50
150
0.0
0.2
0.4
0.6
0.8
1.0
Methylation Value

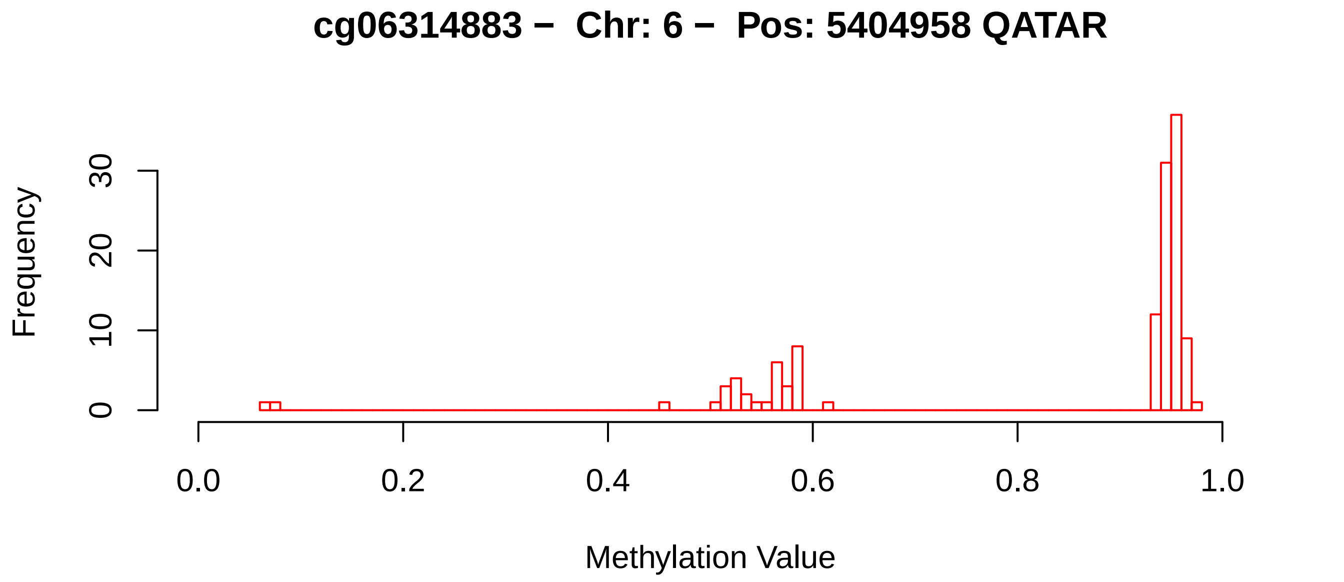
cg06314883 − Chr: 6 − Pos: 5404958 QATAR
Frequency
0
10
20
30
0.0
0.2
0.4
0.6
0.8
1.0
Methylation Value

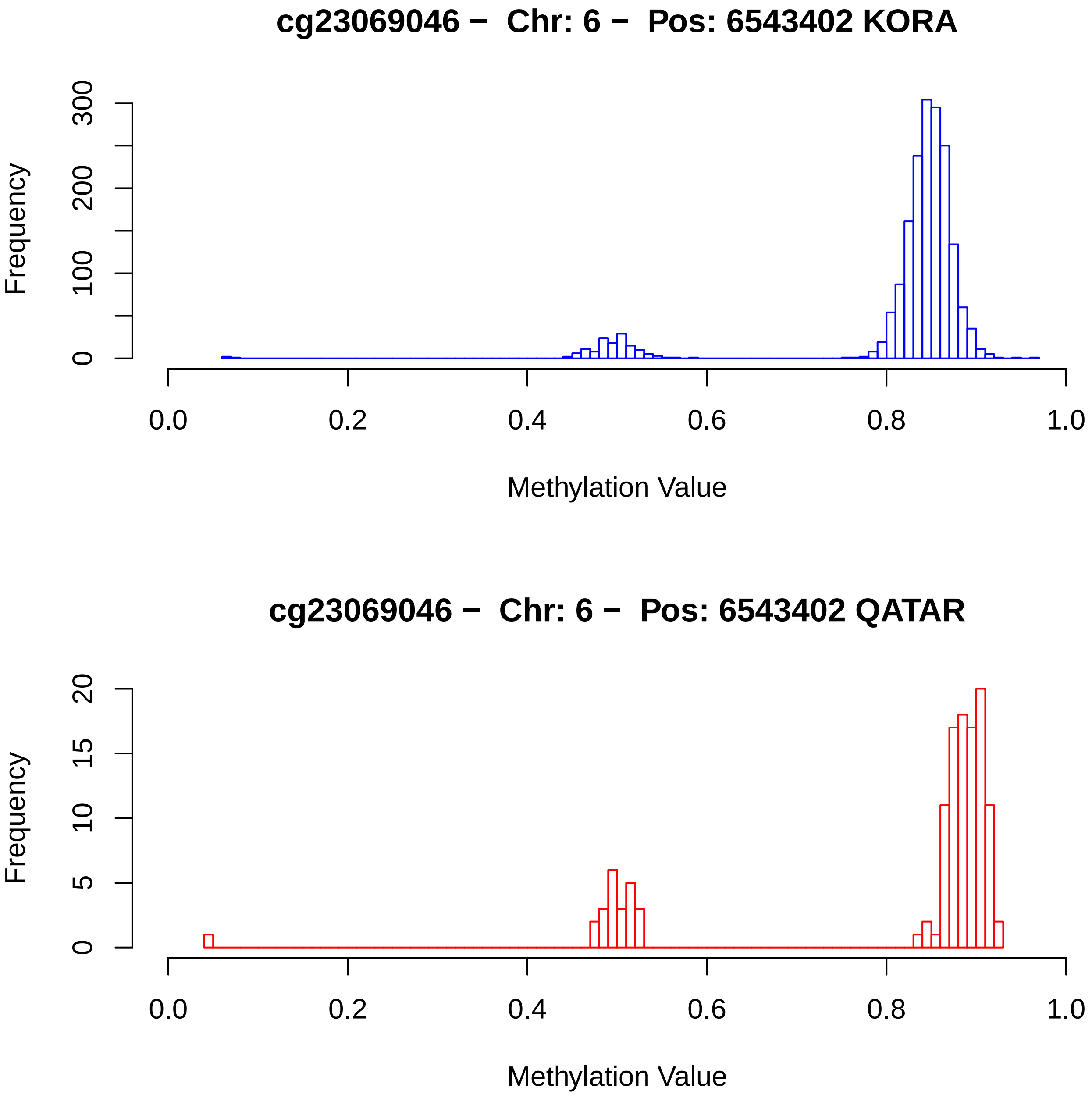
cg23069046 − Chr: 6 − Pos: 6543402 KORA
Frequency
0
100
200
300
0.0
0.2
0.4
0.6
0.8
1.0
Methylation Value
cg23069046 − Chr: 6 − Pos: 6543402 QATAR
Frequency
0
5
10
15
20
0.0
0.2
0.4
0.6
0.8
1.0
Methylation Value

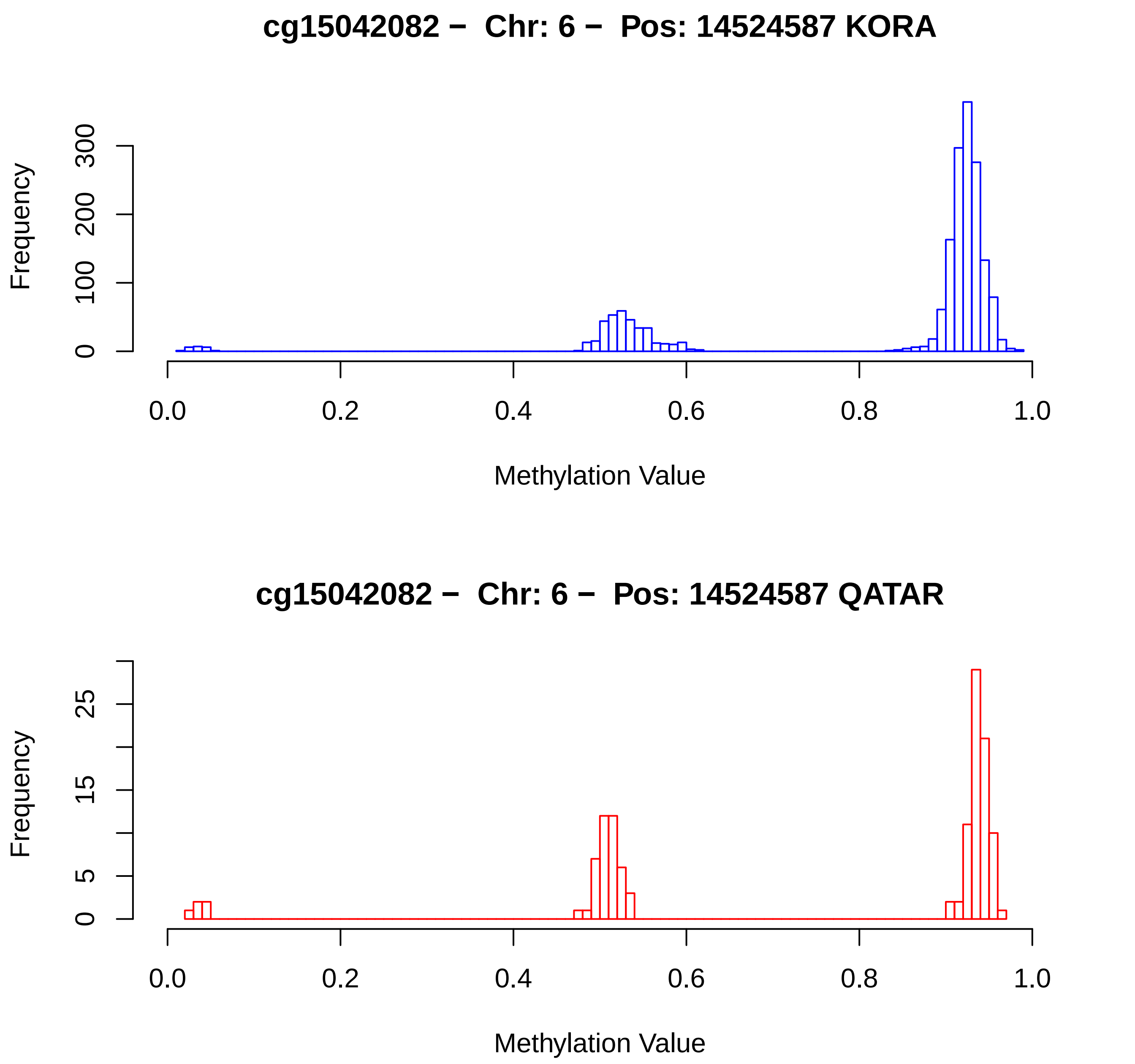
cg15042082 − Chr: 6 − Pos: 14524587 KORA
Frequency
0
100
200
300
0.0
0.2
0.4
0.6
0.8
1.0
Methylation Value
cg15042082 − Chr: 6 − Pos: 14524587 QATAR
Frequency
0
5
15
25
0.0
0.2
0.4
0.6
0.8
1.0
Methylation Value

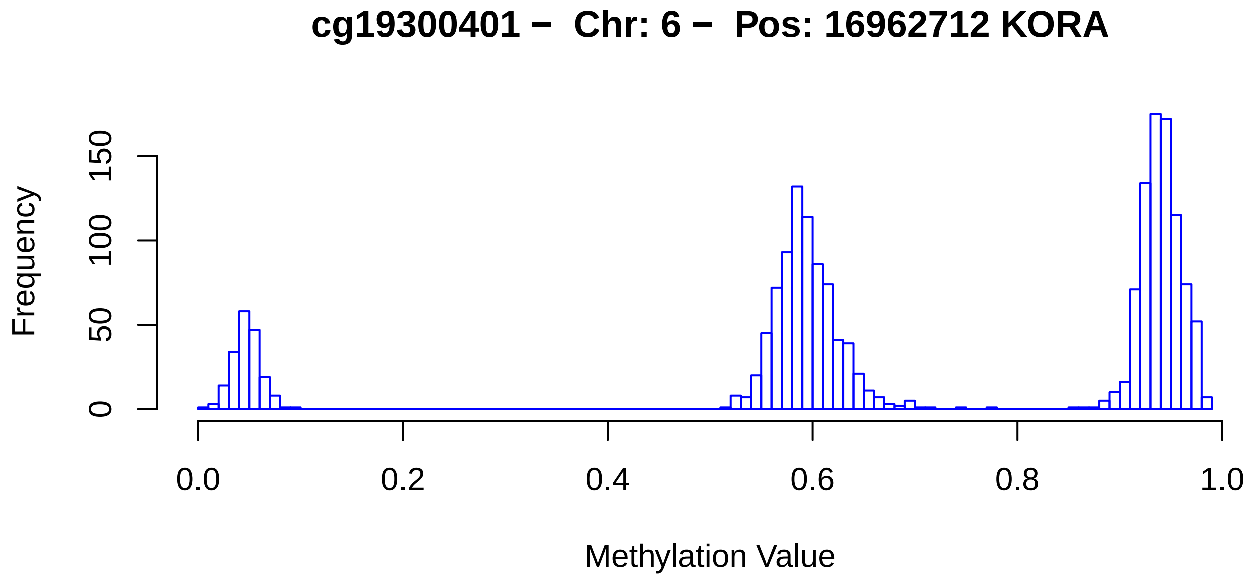
cg19300401 − Chr: 6 − Pos: 16962712 KORA
Frequency
0
50
100
150
0.0
0.2
0.4
0.6
0.8
1.0
Methylation Value

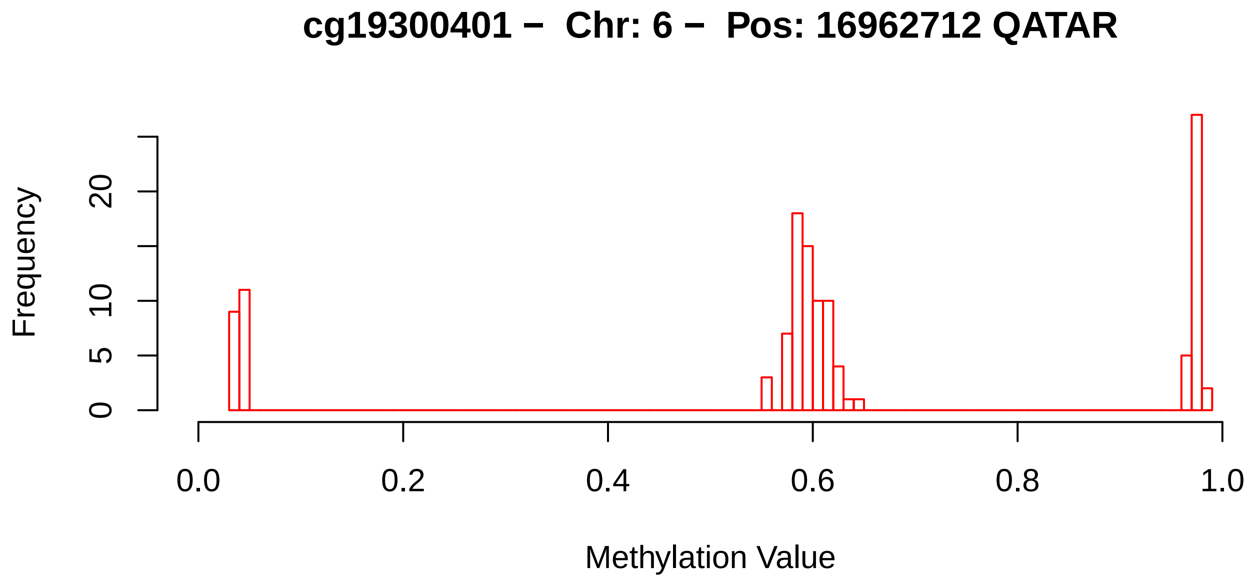
cg19300401 − Chr: 6 − Pos: 16962712 QATAR
Frequency
0
5
10
20
0.0
0.2
0.4
0.6
0.8
1.0
Methylation Value

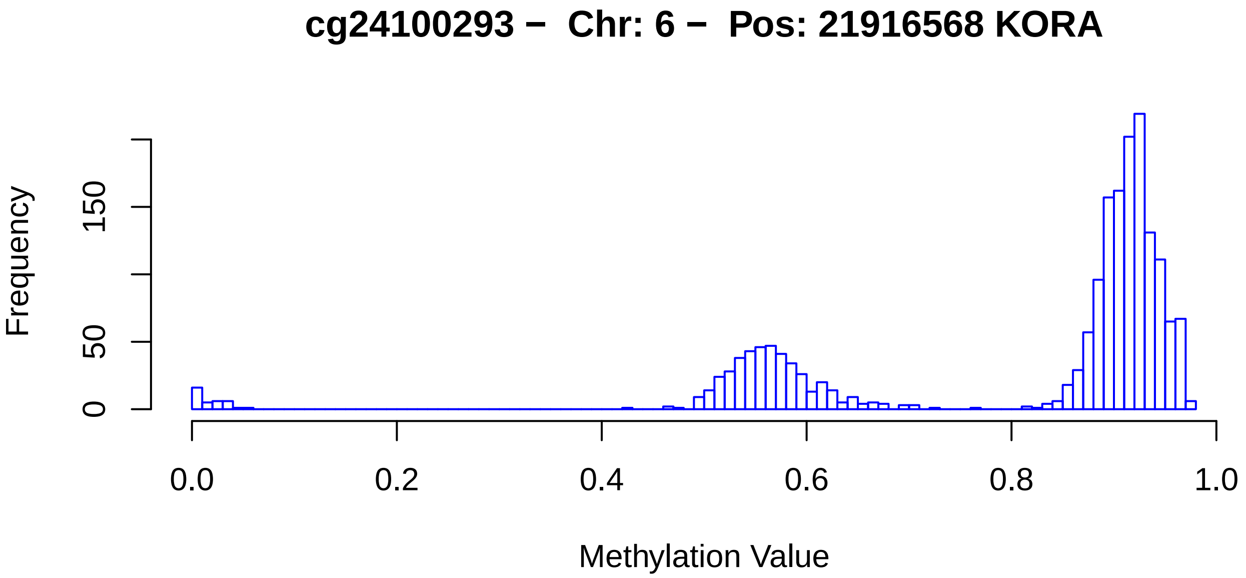
cg24100293 − Chr: 6 − Pos: 21916568 KORA
Frequency
0
50
150
0.0
0.2
0.4
0.6
0.8
1.0
Methylation Value

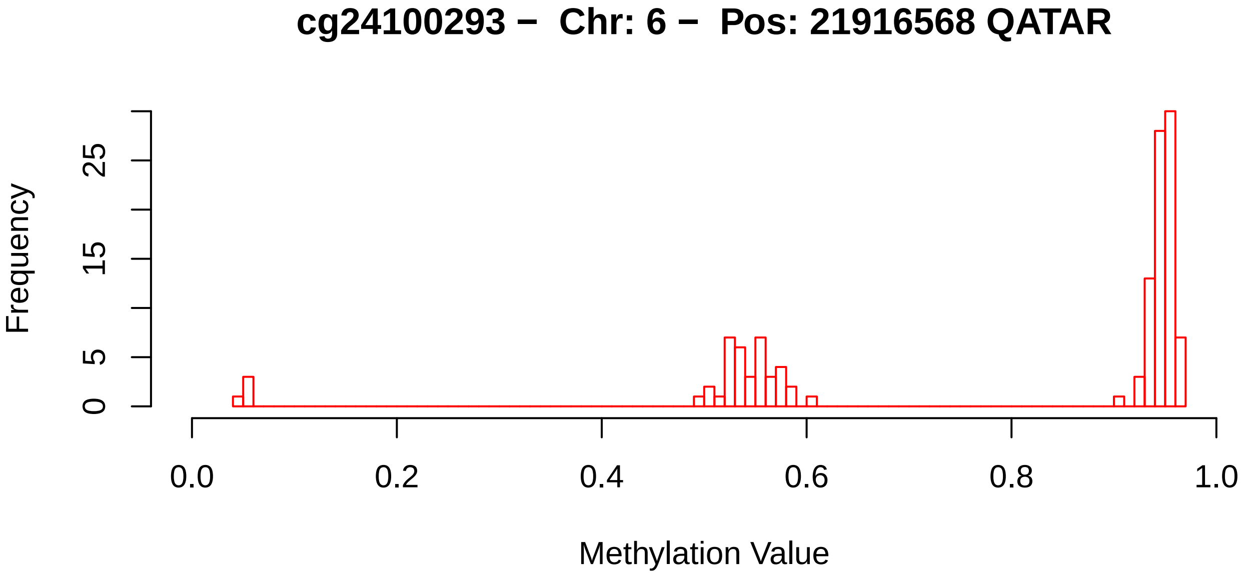
cg24100293 − Chr: 6 − Pos: 21916568 QATAR
Frequency
0
5
15
25
0.0
0.2
0.4
0.6
0.8
1.0
Methylation Value

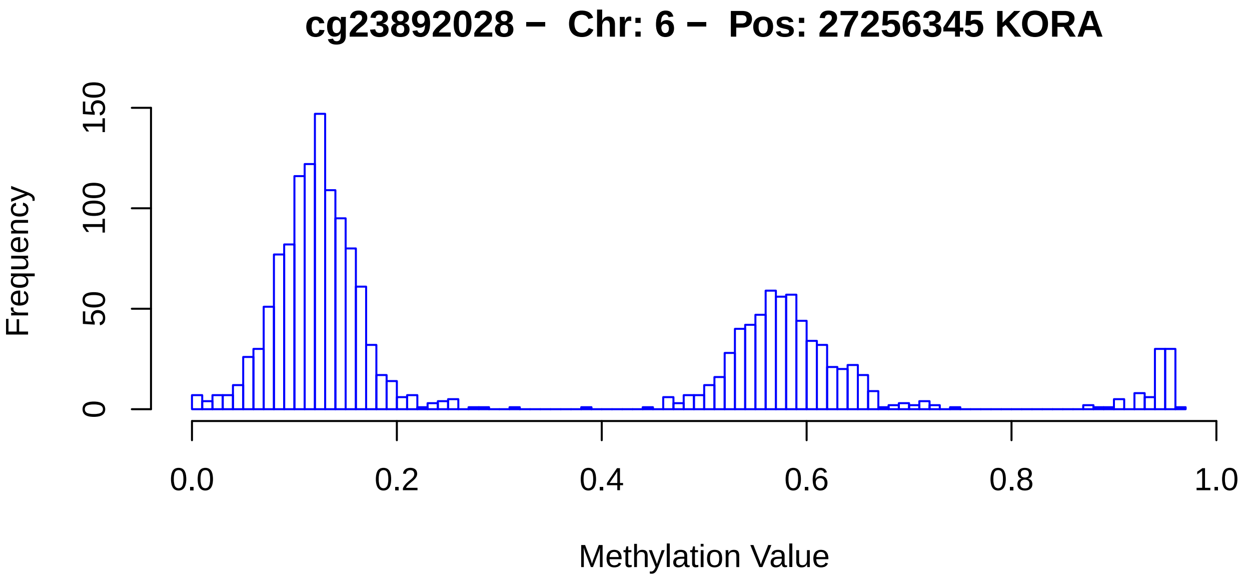
cg23892028 − Chr: 6 − Pos: 27256345 KORA
Frequency
0
50
100
150
0.0
0.2
0.4
0.6
0.8
1.0
Methylation Value

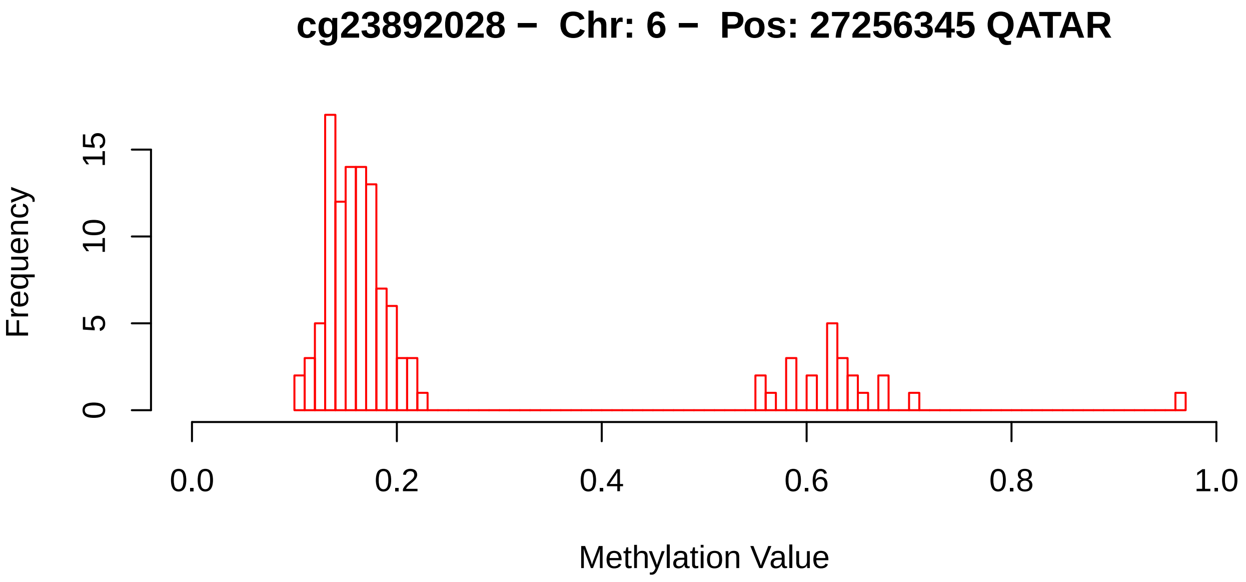
cg23892028 − Chr: 6 − Pos: 27256345 QATAR
Frequency
0
5
10
15
0.0
0.2
0.4
0.6
0.8
1.0
Methylation Value

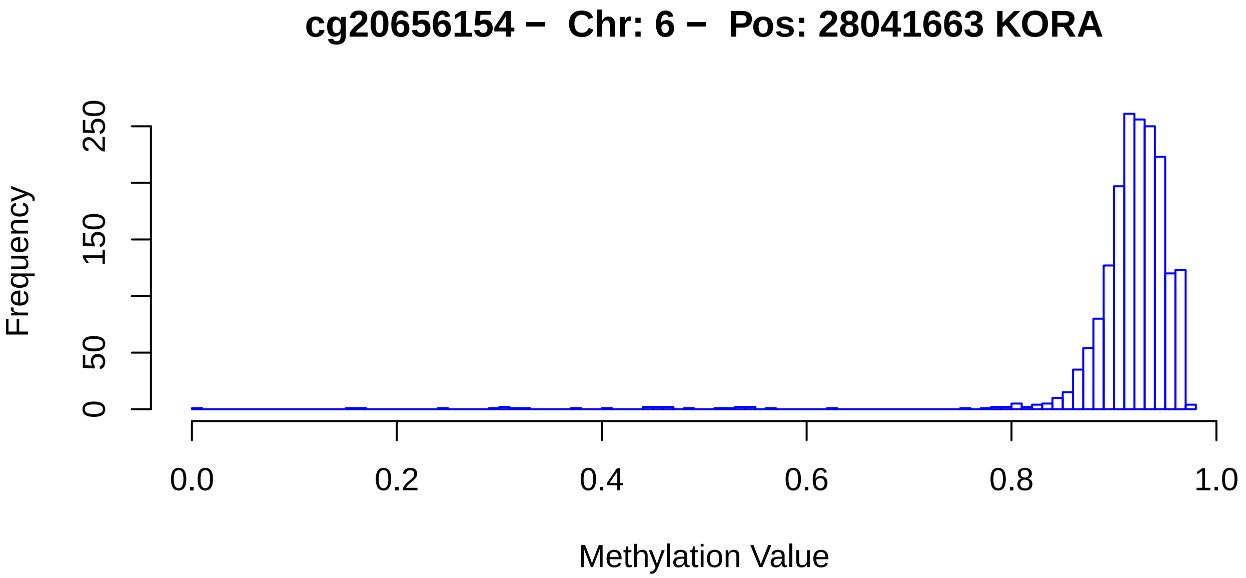
cg20656154 − Chr: 6 − Pos: 28041663 KORA
Frequency
0
50
150
250
0.0
0.2
0.4
0.6
0.8
1.0
Methylation Value

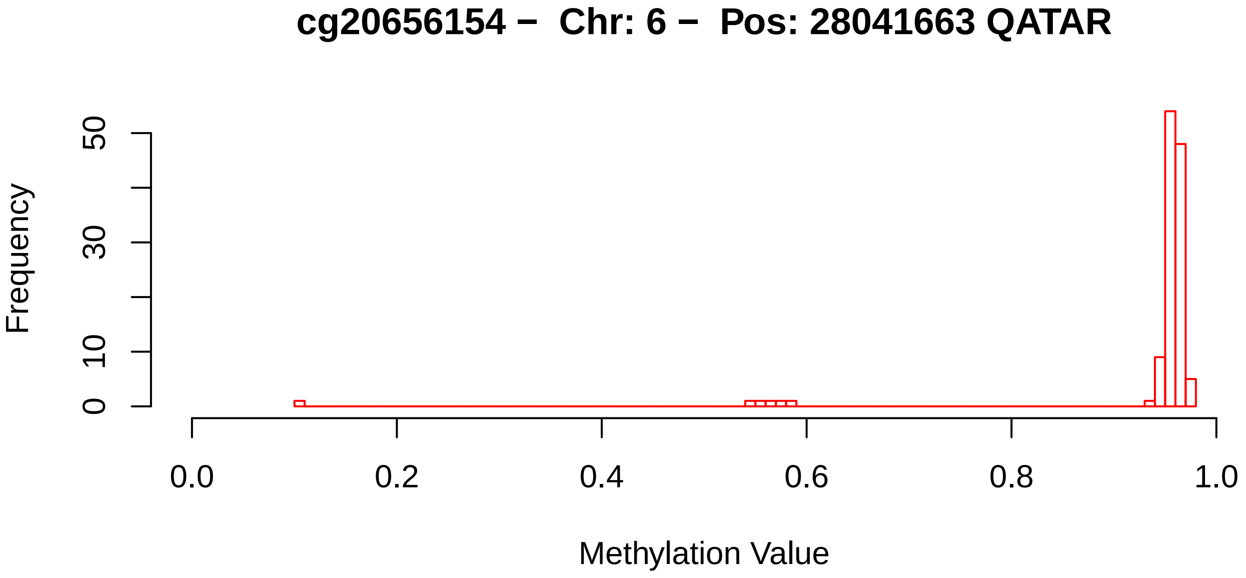
cg20656154 − Chr: 6 − Pos: 28041663 QATAR
Frequency
0
10
30
50
0.0
0.2
0.4
0.6
0.8
1.0
Methylation Value

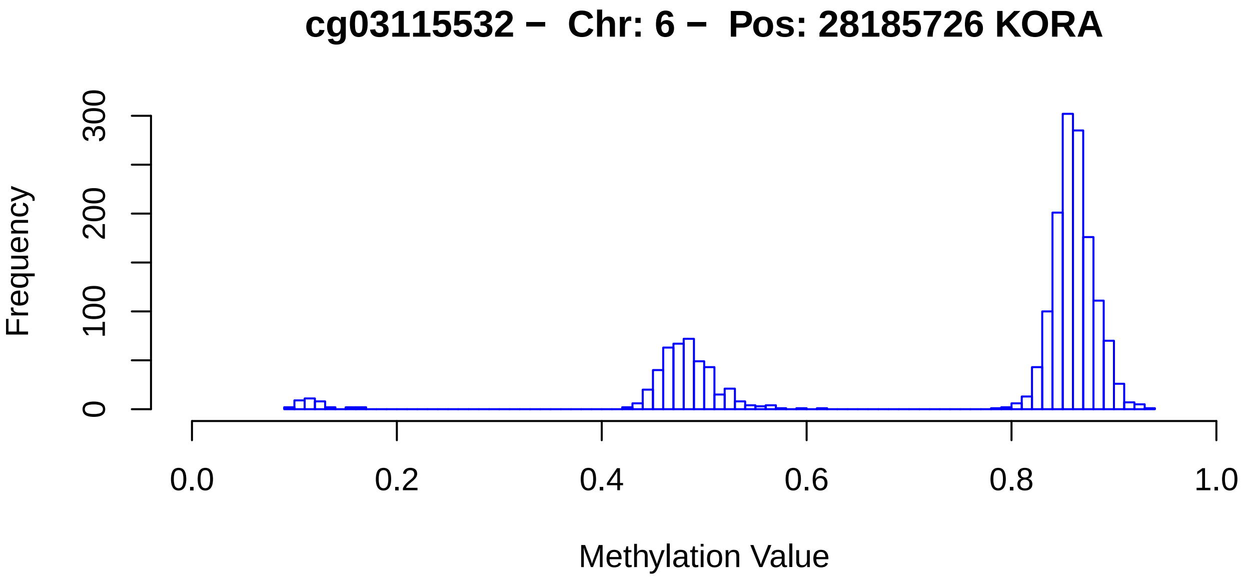

cg03115532 − Chr: 6 − Pos: 28185726 KORA
Frequency
0
100
200
300
0.0
0.2
0.4
0.6
0.8
1.0
Methylation Value

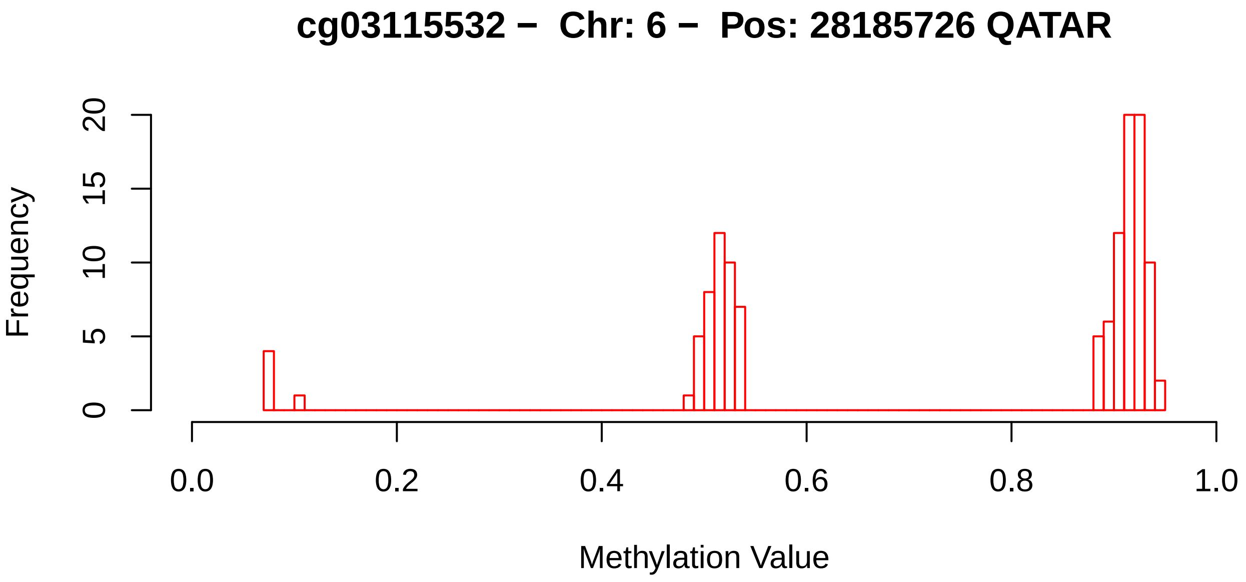

cg03115532 − Chr: 6 − Pos: 28185726 QATAR
Frequency
0
5
10
15
20
0.0
0.2
0.4
0.6
0.8
1.0
Methylation Value

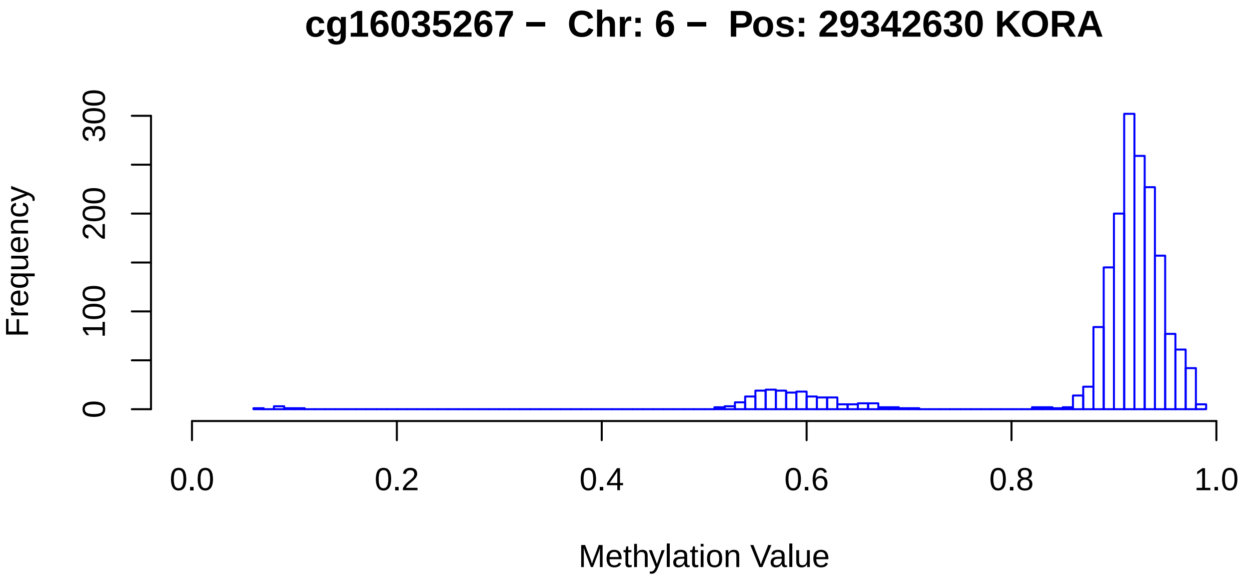
cg16035267 − Chr: 6 − Pos: 29342630 KORA
Frequency
0
100
200
300
0.0
0.2
0.4
0.6
0.8
1.0
Methylation Value

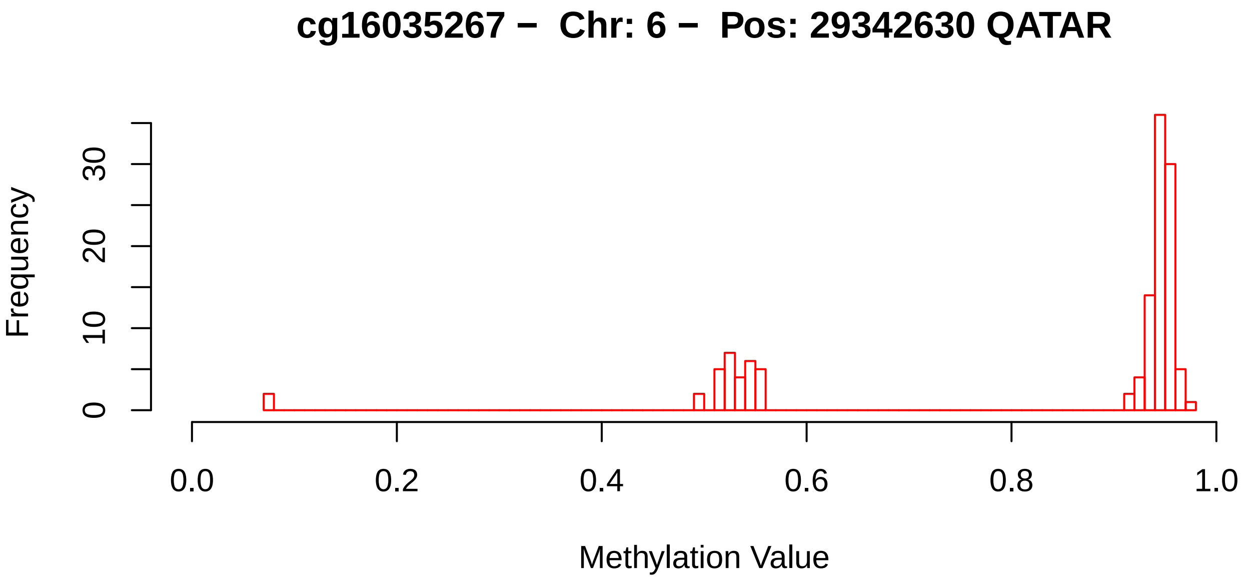
cg16035267 − Chr: 6 − Pos: 29342630 QATAR
Frequency
0
10
20
30
0.0
0.2
0.4
0.6
0.8
1.0
Methylation Value

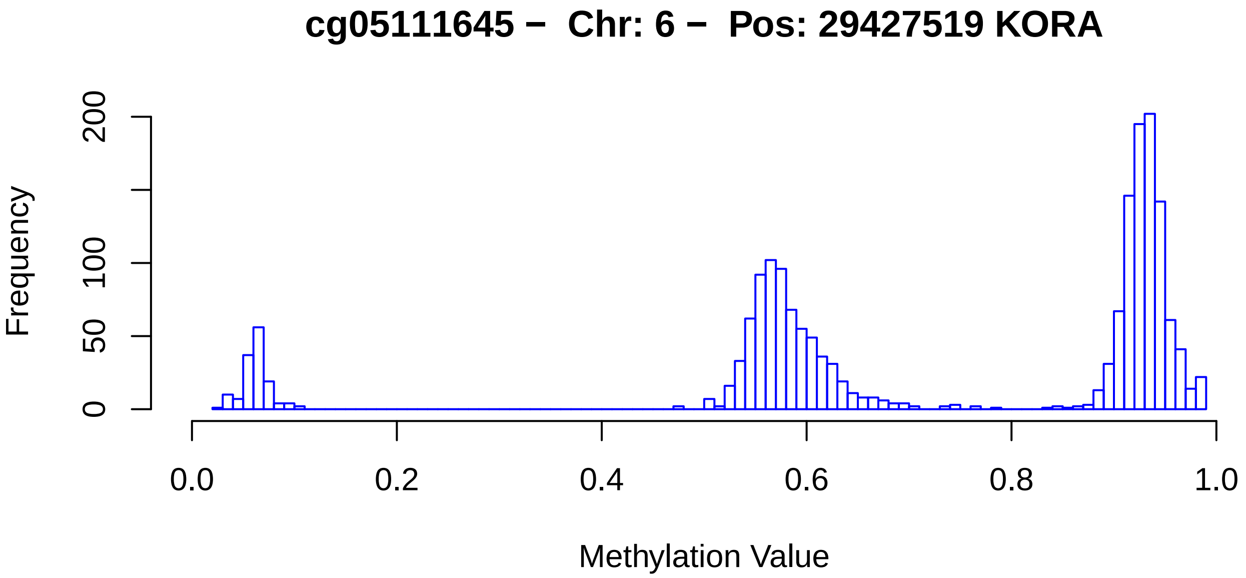
cg05111645 − Chr: 6 − Pos: 29427519 KORA
Frequency
0
50
100
200
0.0
0.2
0.4
0.6
0.8
1.0
Methylation Value

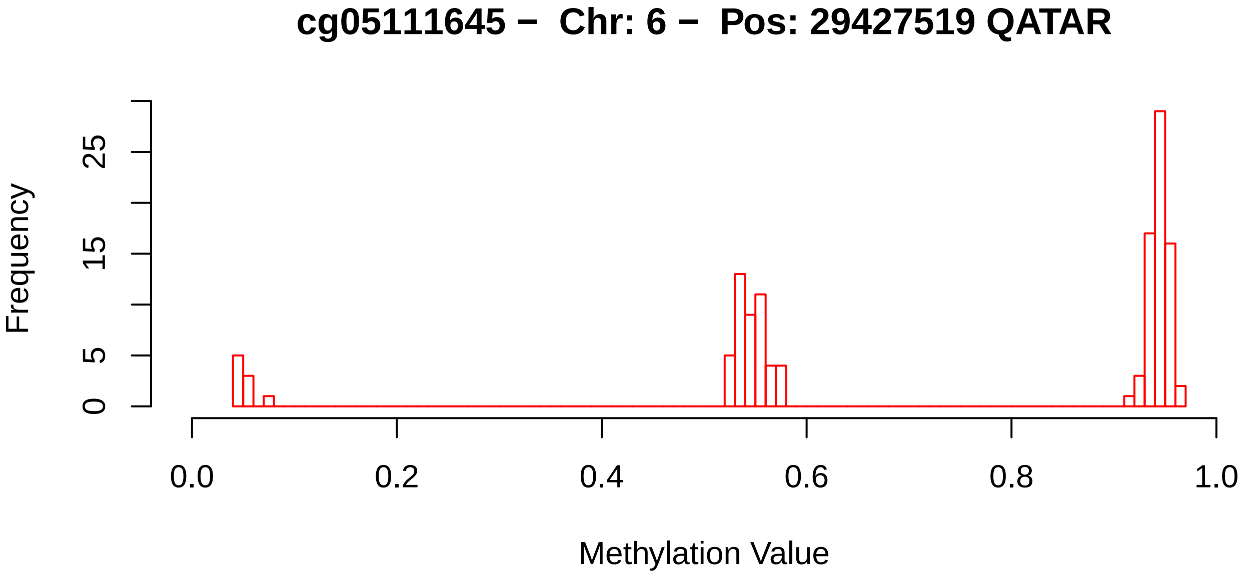
cg05111645 − Chr: 6 − Pos: 29427519 QATAR
Frequency
0
5
15
25
0.0
0.2
0.4
0.6
0.8
1.0
Methylation Value

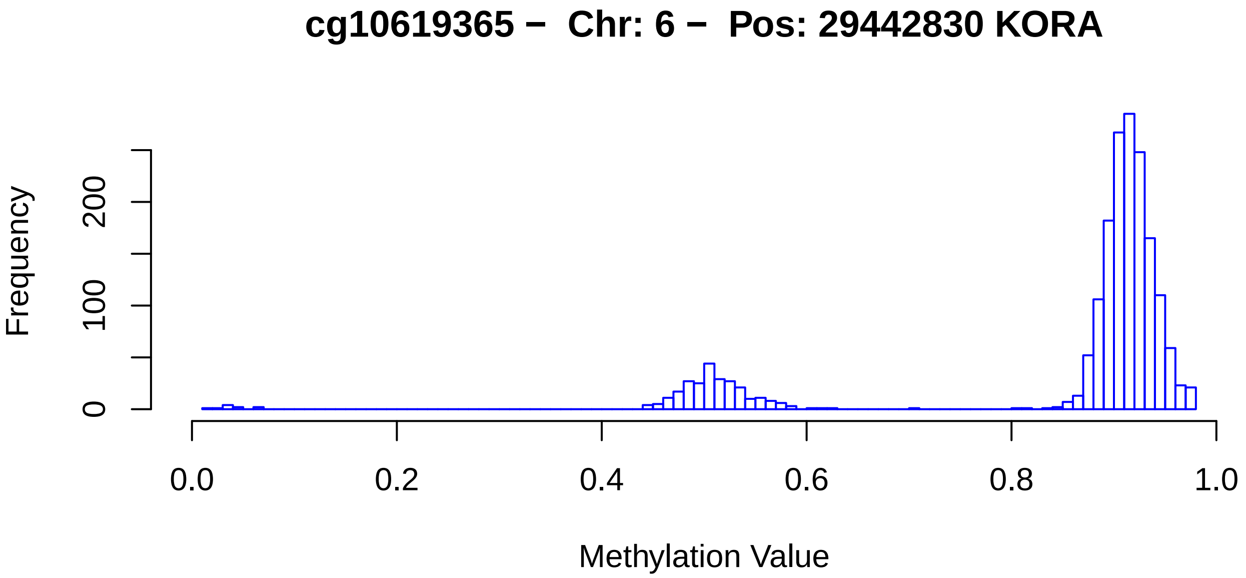
cg10619365 − Chr: 6 − Pos: 29442830 KORA
Frequency
0
100
200
0.0
0.2
0.4
0.6
0.8
1.0
Methylation Value

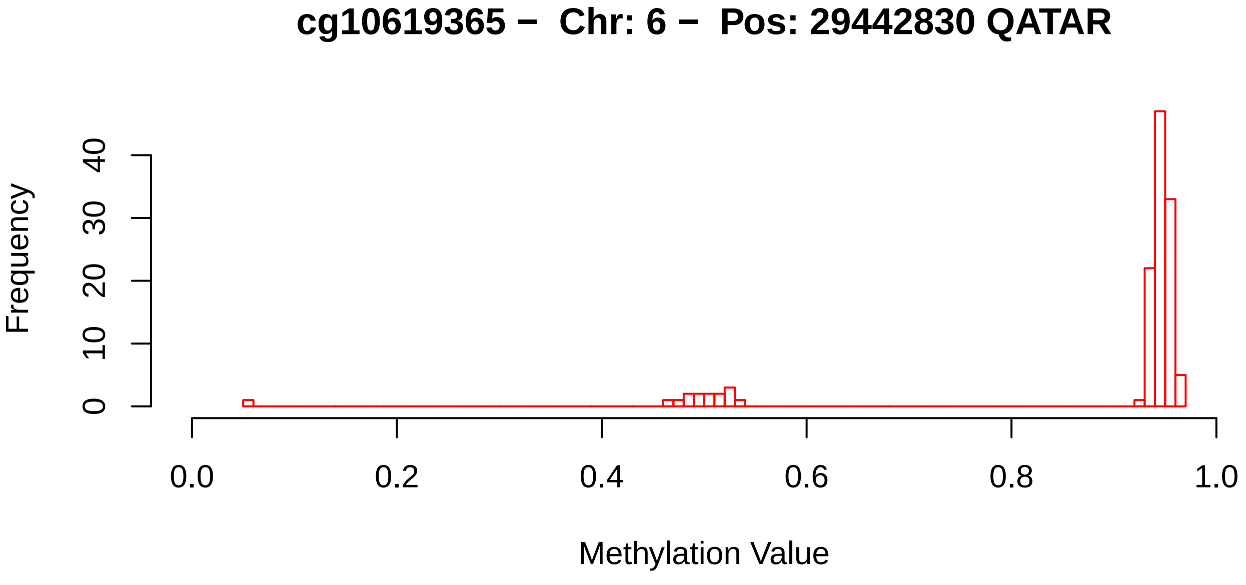
cg10619365 − Chr: 6 − Pos: 29442830 QATAR
Frequency
0
10
20
30
40
0.0
0.2
0.4
0.6
0.8
1.0
Methylation Value

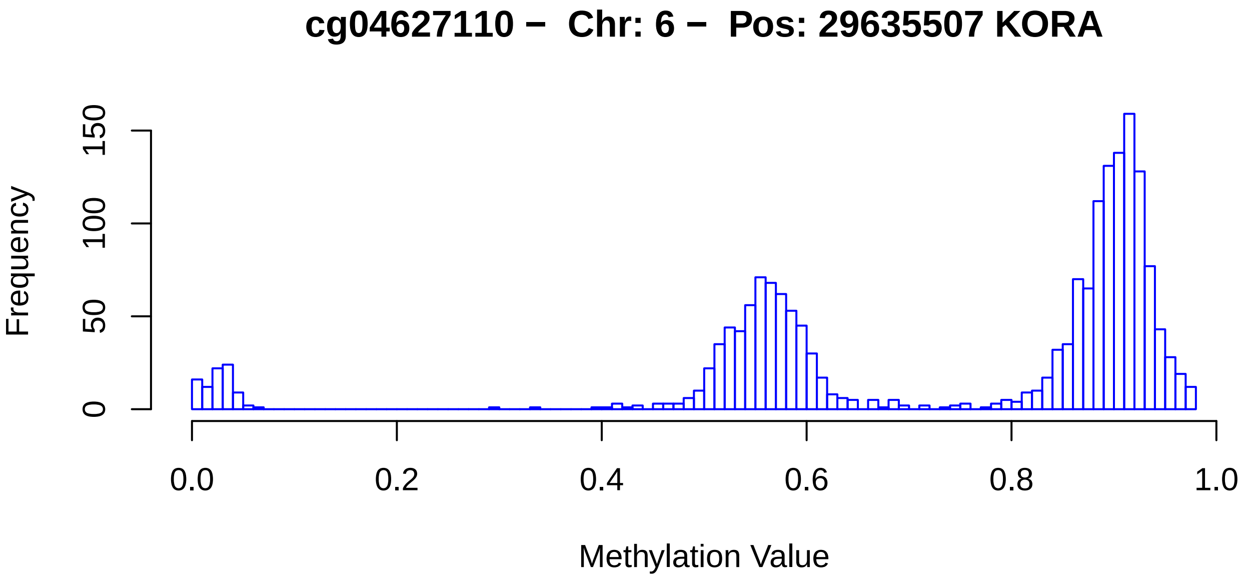

cg04627110 − Chr: 6 − Pos: 29635507 KORA
Frequency
0
50
100
150
0.0
0.2
0.4
0.6
0.8
1.0
Methylation Value

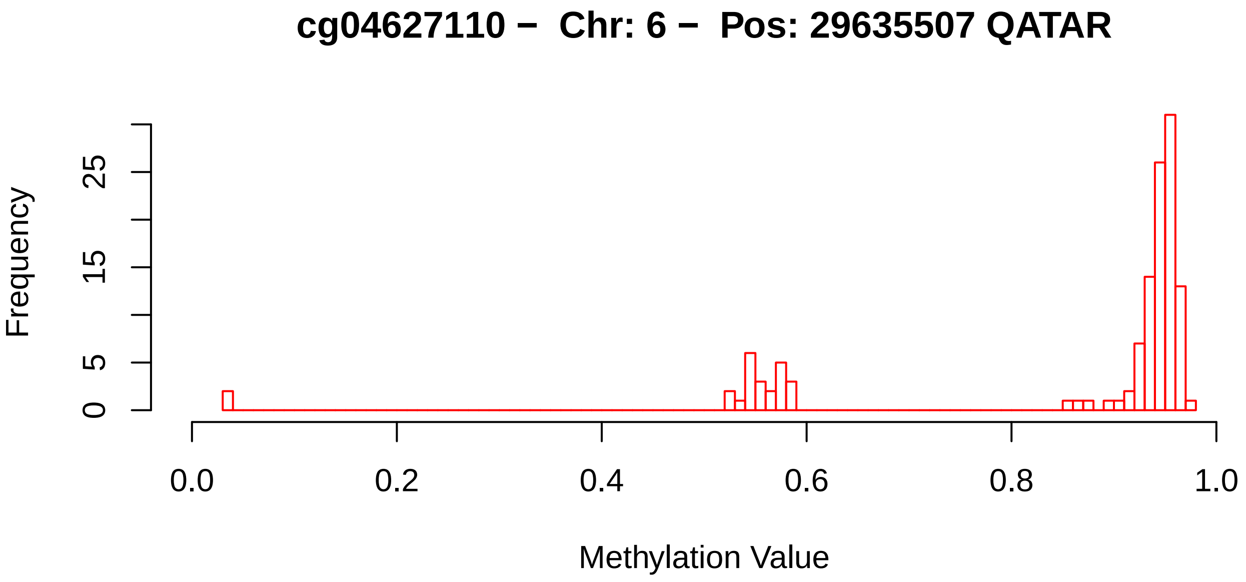

cg04627110 − Chr: 6 − Pos: 29635507 QATAR
Frequency
0
5
15
25
0.0
0.2
0.4
0.6
0.8
1.0
Methylation Value

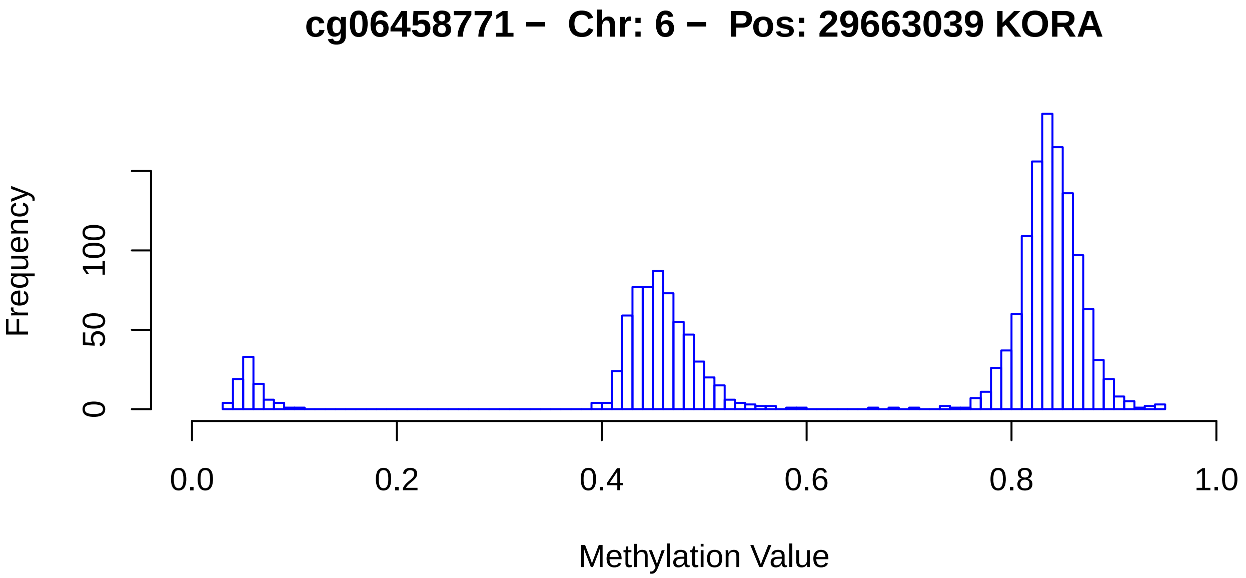

cg06458771 − Chr: 6 − Pos: 29663039 KORA
Frequency
0
50
100
0.0
0.2
0.4
0.6
0.8
1.0
Methylation Value

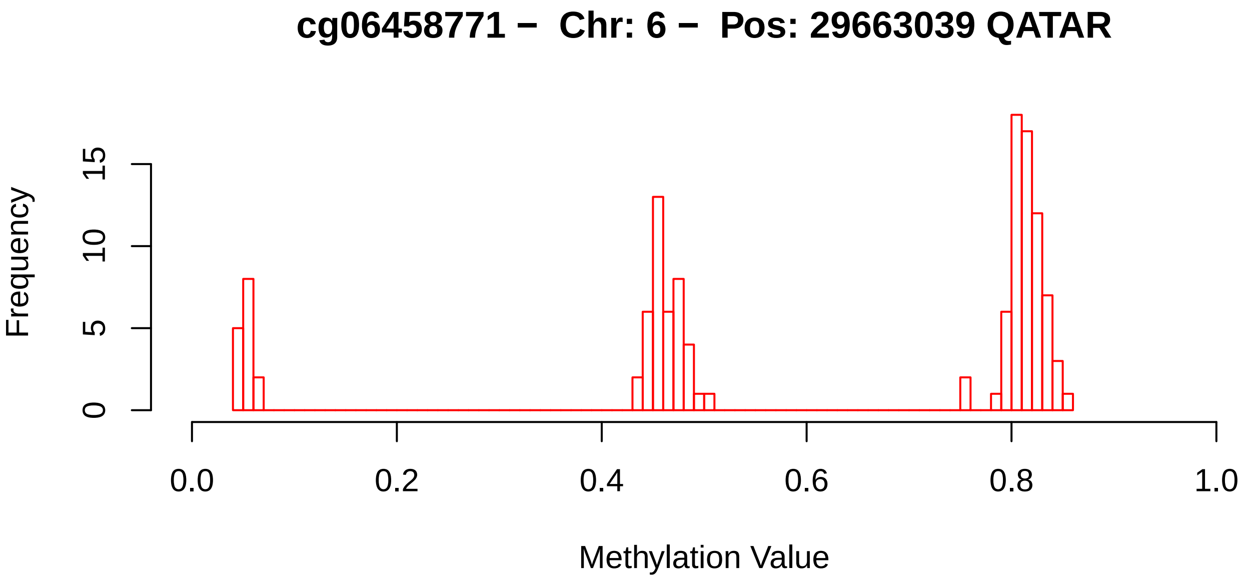

cg06458771 − Chr: 6 − Pos: 29663039 QATAR
Frequency
0
5
10
15
0.0
0.2
0.4
0.6
0.8
1.0
Methylation Value

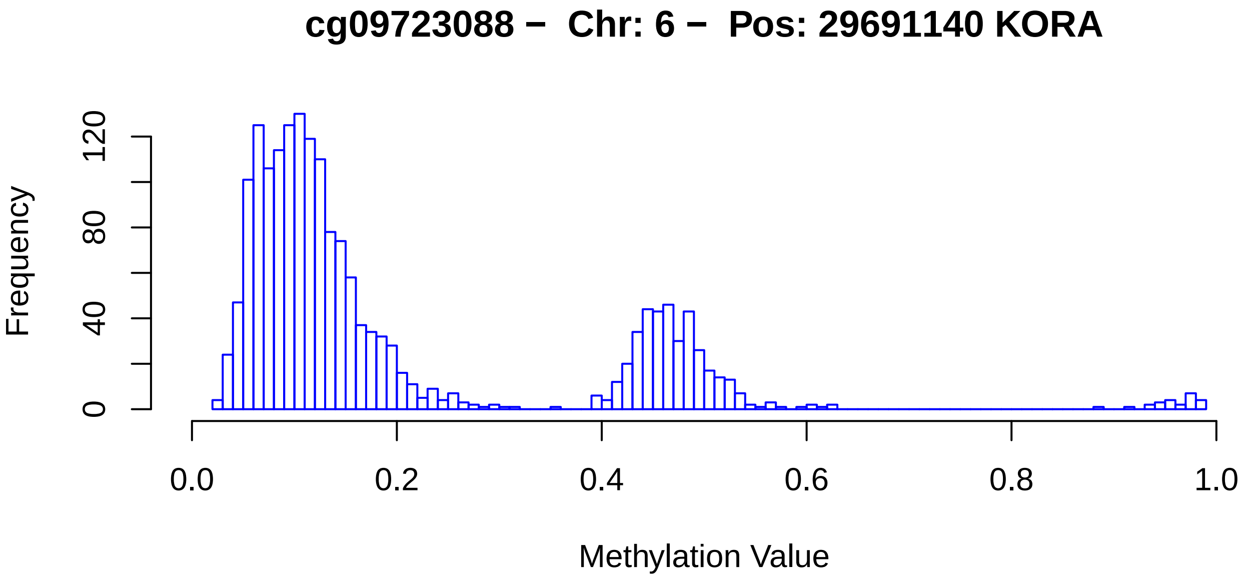
cg09723088 − Chr: 6 − Pos: 29691140 KORA
Frequency
0
40
80
120
0.0
0.2
0.4
0.6
0.8
1.0
Methylation Value

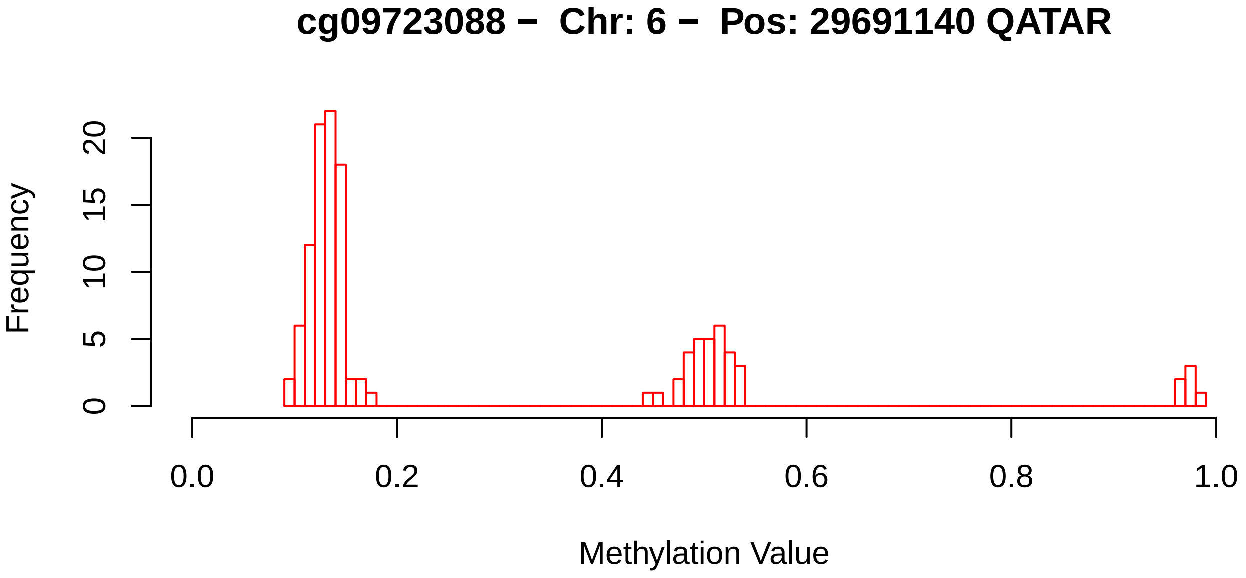
cg09723088 − Chr: 6 − Pos: 29691140 QATAR
Frequency
0
5
10
15
20
0.0
0.2
0.4
0.6
0.8
1.0
Methylation Value

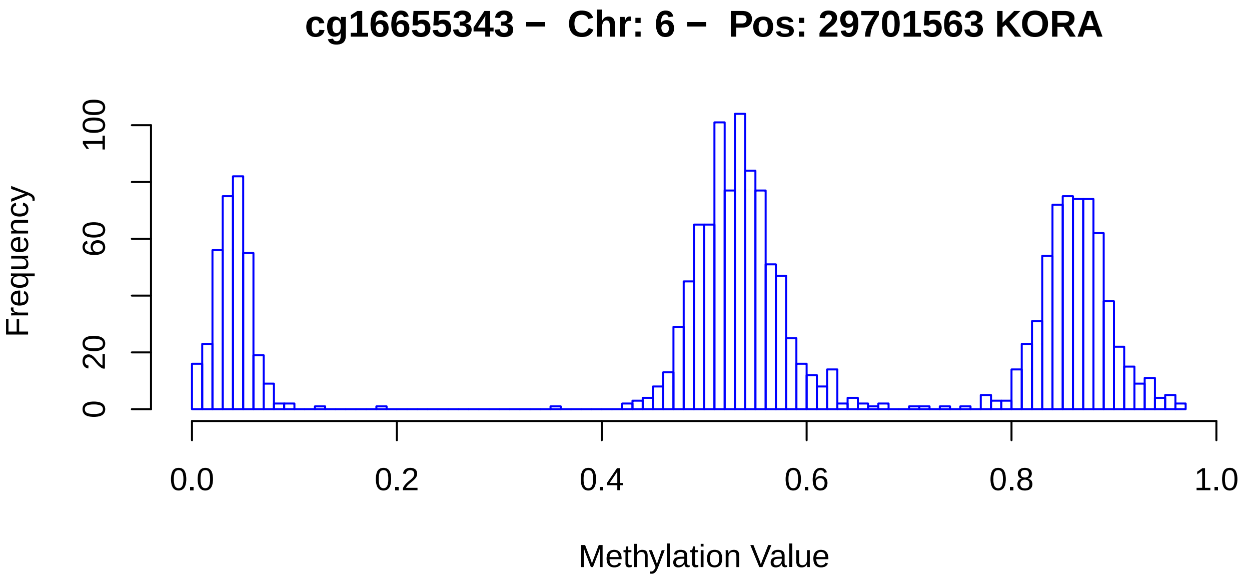
cg16655343 − Chr: 6 − Pos: 29701563 KORA
Frequency
0
20
60
100
0.0
0.2
0.4
0.6
0.8
1.0
Methylation Value

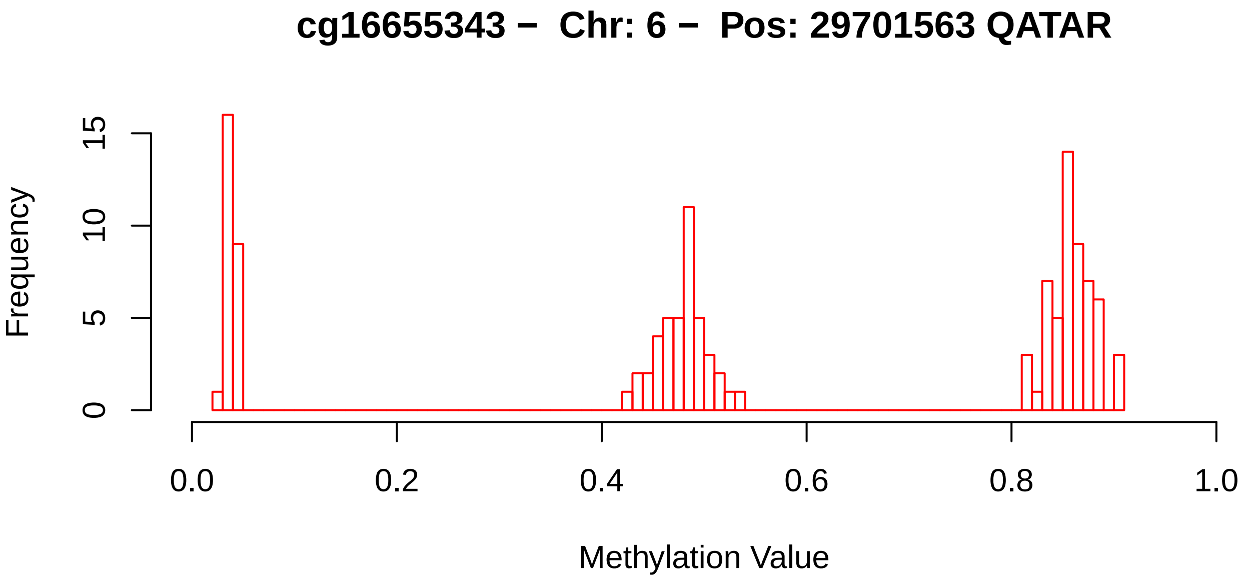
cg16655343 − Chr: 6 − Pos: 29701563 QATAR
Frequency
0
5
10
15
0.0
0.2
0.4
0.6
0.8
1.0
Methylation Value

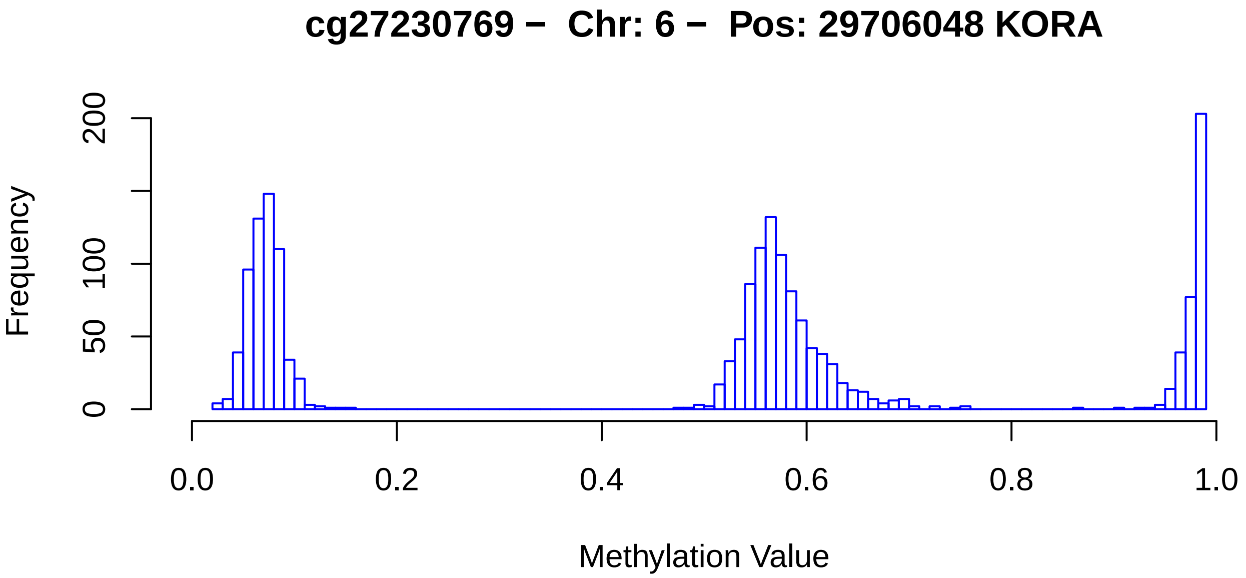
cg27230769 − Chr: 6 − Pos: 29706048 KORA
Frequency
0
50
100
200
0.0
0.2
0.4
0.6
0.8
1.0
Methylation Value

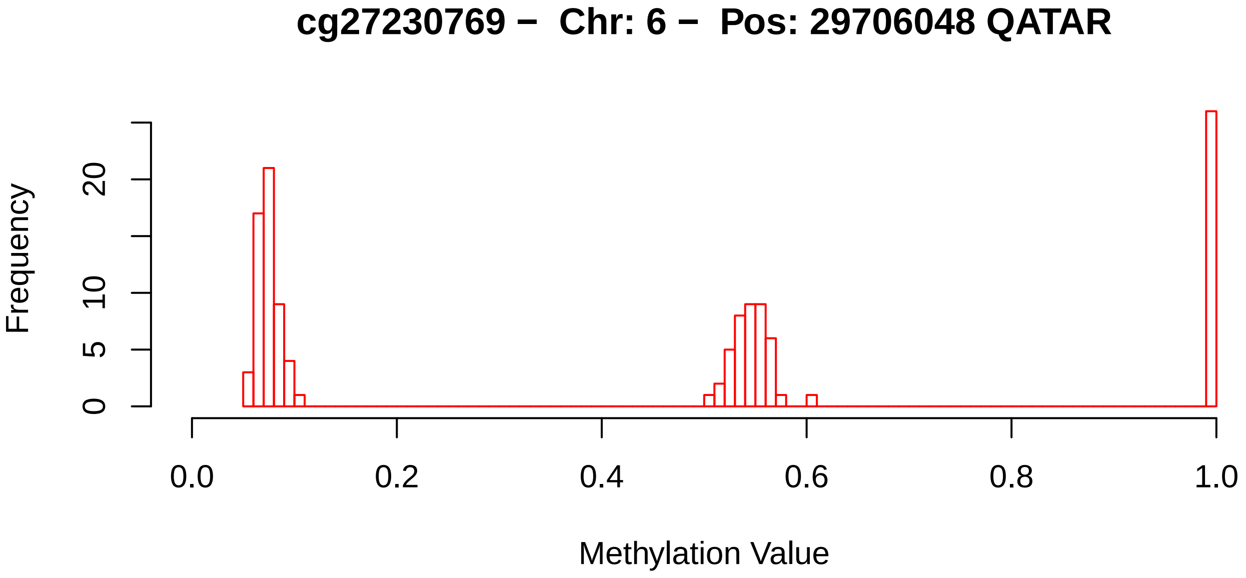
cg27230769 − Chr: 6 − Pos: 29706048 QATAR
Frequency
0
5
10
20
0.0
0.2
0.4
0.6
0.8
1.0
Methylation Value

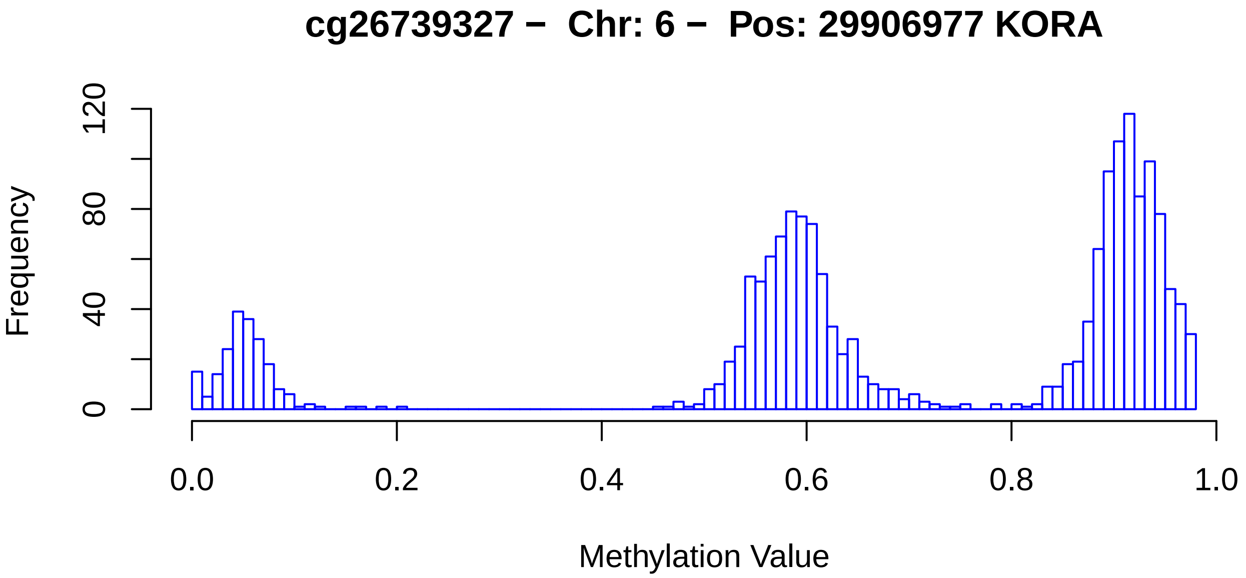
cg26739327 − Chr: 6 − Pos: 29906977 KORA
Frequency
0
40
80
120
0.0
0.2
0.4
0.6
0.8
1.0
Methylation Value

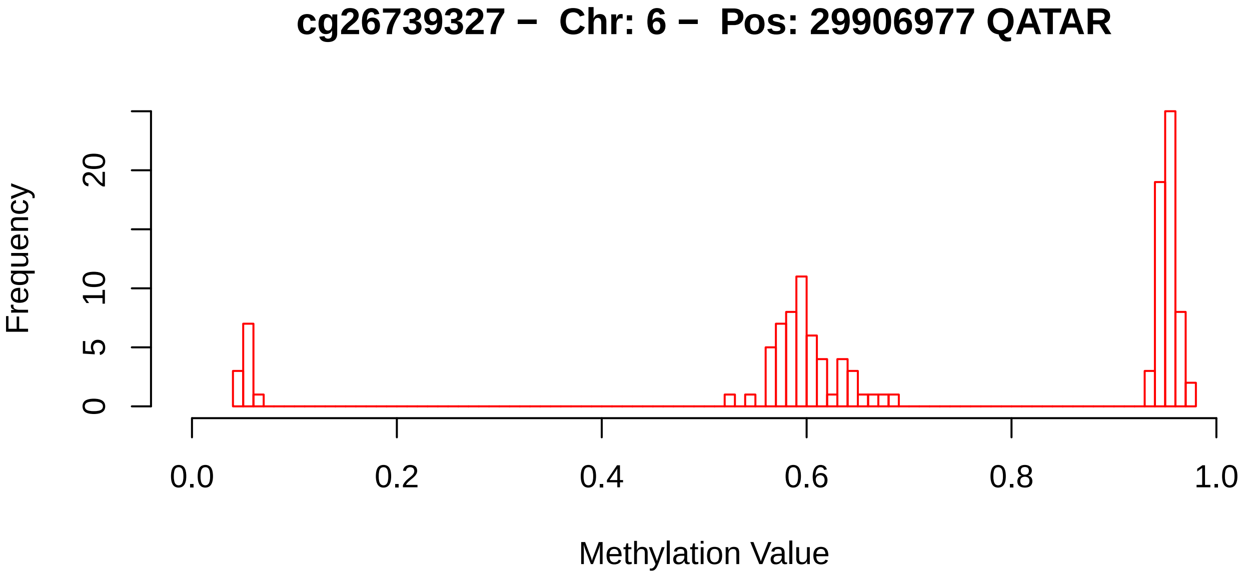
cg26739327 − Chr: 6 − Pos: 29906977 QATAR
Frequency
0
5
10
20
0.0
0.2
0.4
0.6
0.8
1.0
Methylation Value

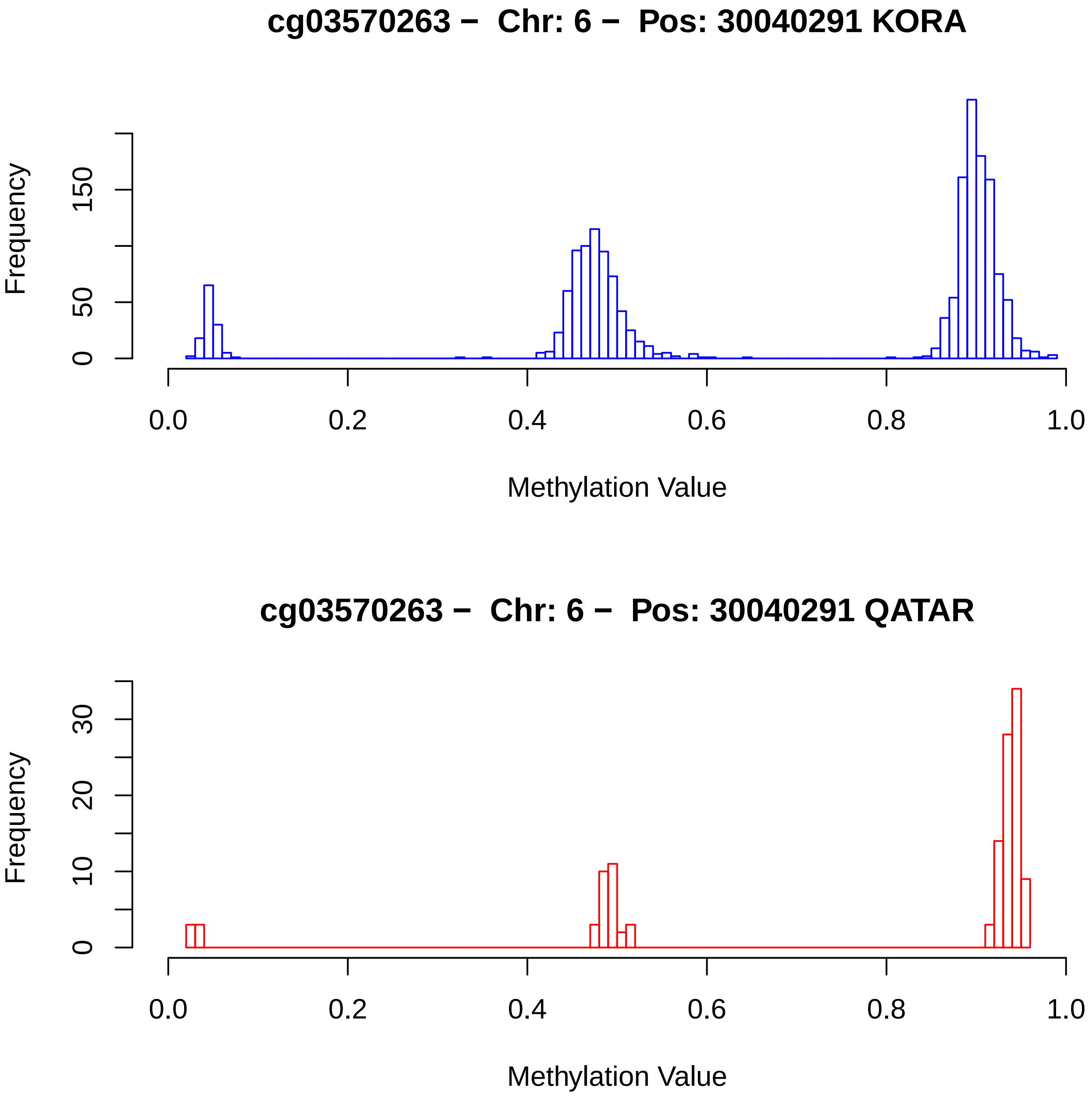
cg03570263 − Chr: 6 − Pos: 30040291 KORA
Frequency
0
50
150
0.0
0.2
0.4
0.6
0.8
1.0
Methylation Value
cg03570263 − Chr: 6 − Pos: 30040291 QATAR
Frequency
0
10
20
30
0.0
0.2
0.4
0.6
0.8
1.0
Methylation Value

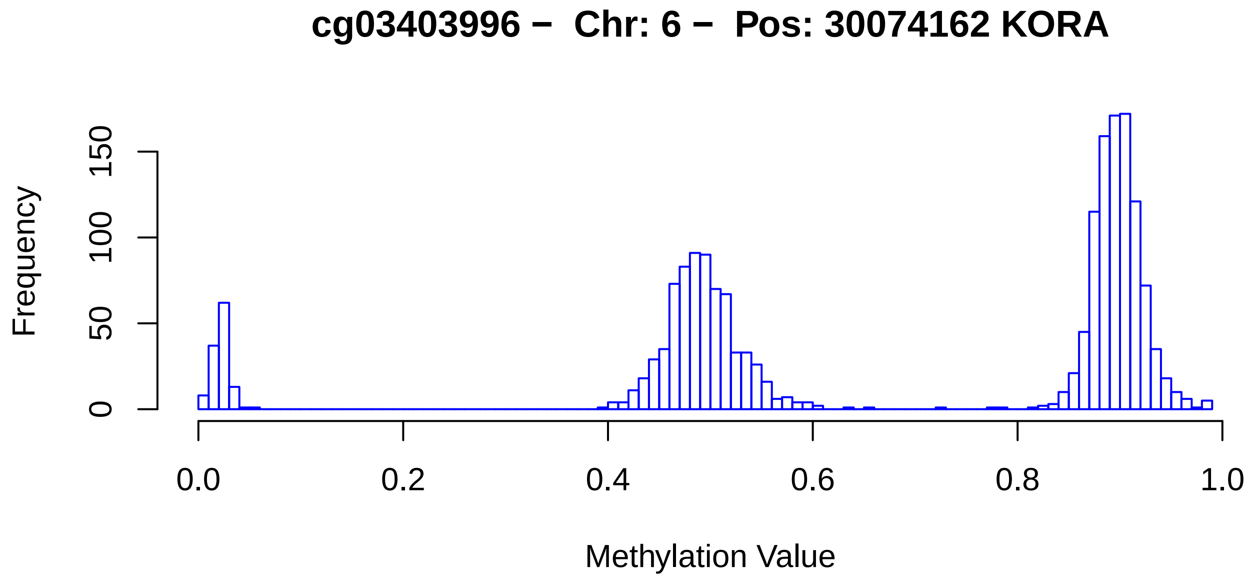
cg03403996 − Chr: 6 − Pos: 30074162 KORA
Frequency
0
50
100
150
0.0
0.2
0.4
0.6
0.8
1.0
Methylation Value

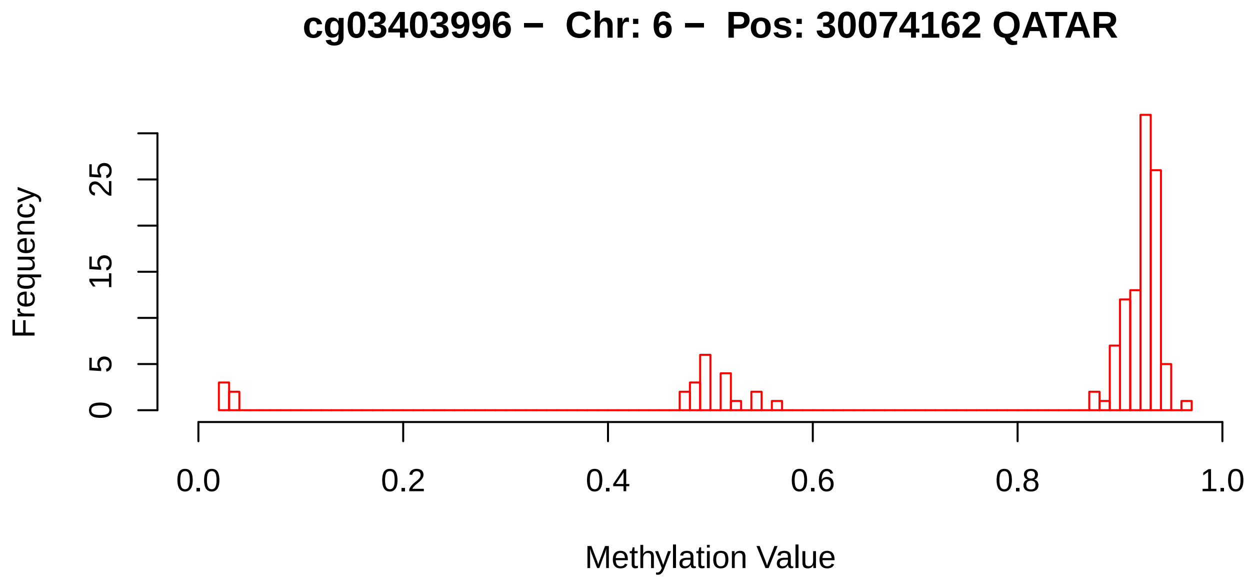
cg03403996 − Chr: 6 − Pos: 30074162 QATAR
Frequency
0
5
15
25
0.0
0.2
0.4
0.6
0.8
1.0
Methylation Value

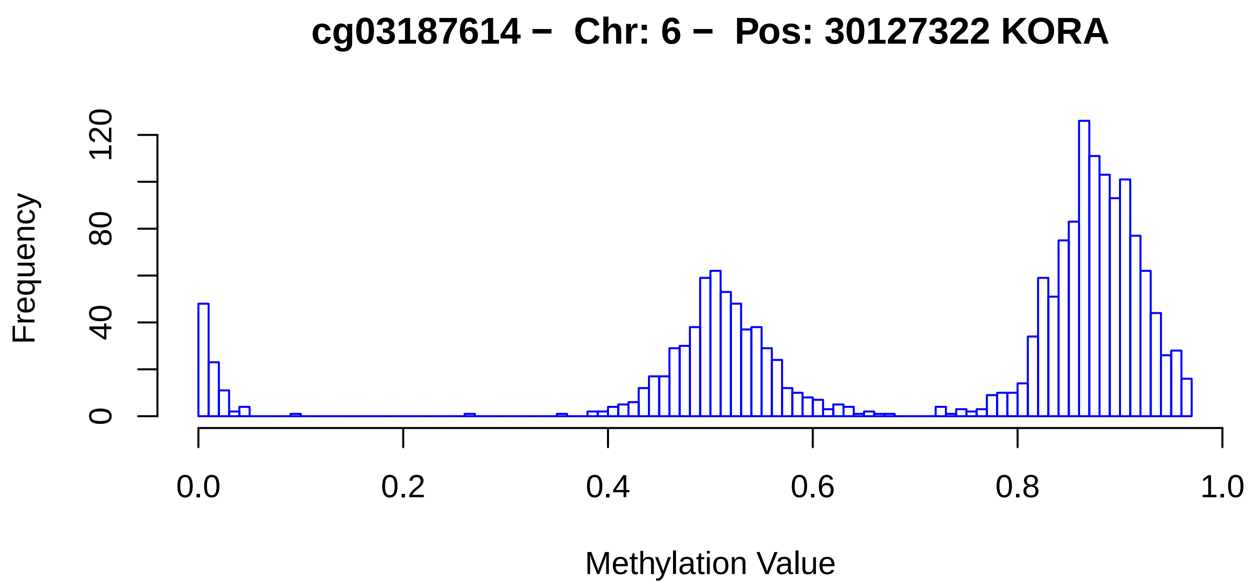
cg03187614 − Chr: 6 − Pos: 30127322 KORA
Frequency
0
40
80
120
0.0
0.2
0.4
0.6
0.8
1.0
Methylation Value

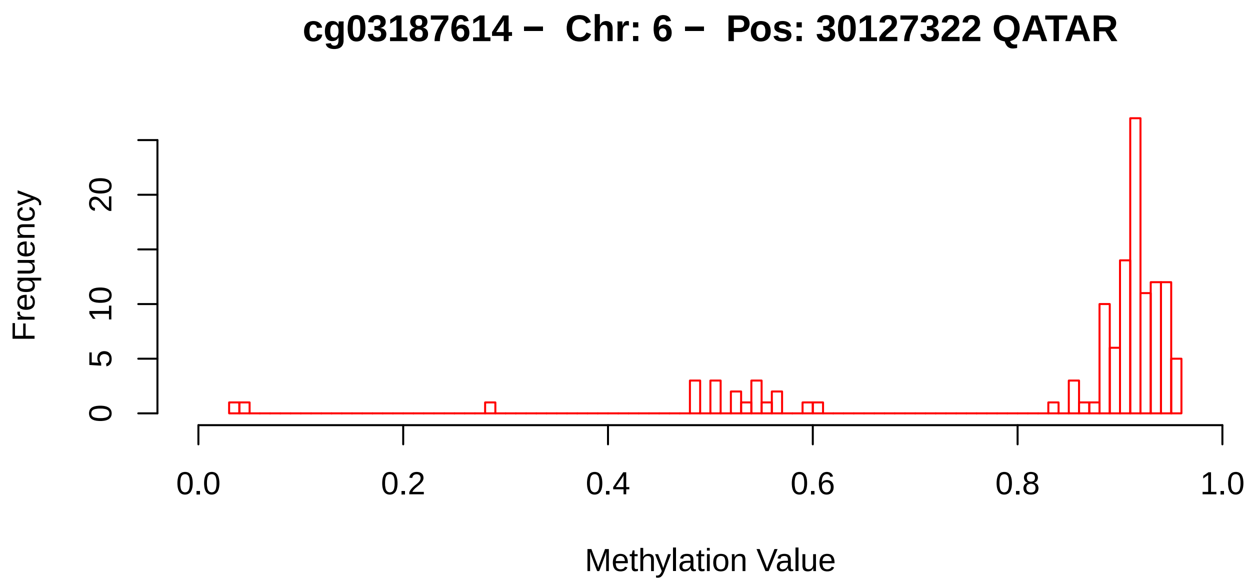
cg03187614 − Chr: 6 − Pos: 30127322 QATAR
Frequency
0
5
10
20
0.0
0.2
0.4
0.6
0.8
1.0
Methylation Value

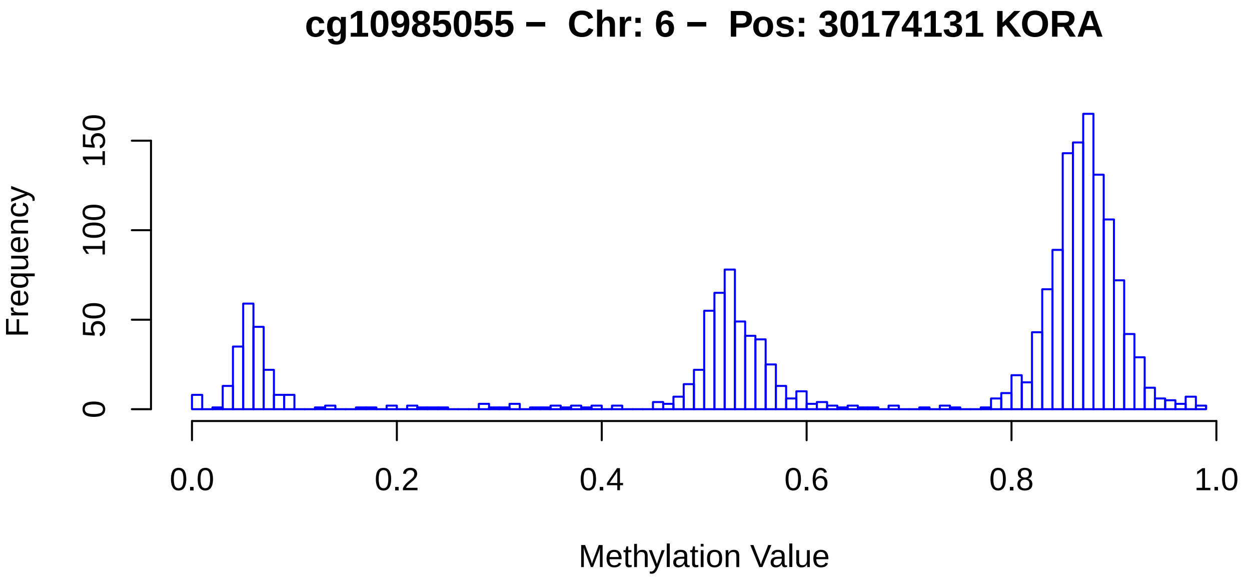
cg10985055 − Chr: 6 − Pos: 30174131 KORA
Frequency
0
50
100
150
0.0
0.2
0.4
0.6
0.8
1.0
Methylation Value

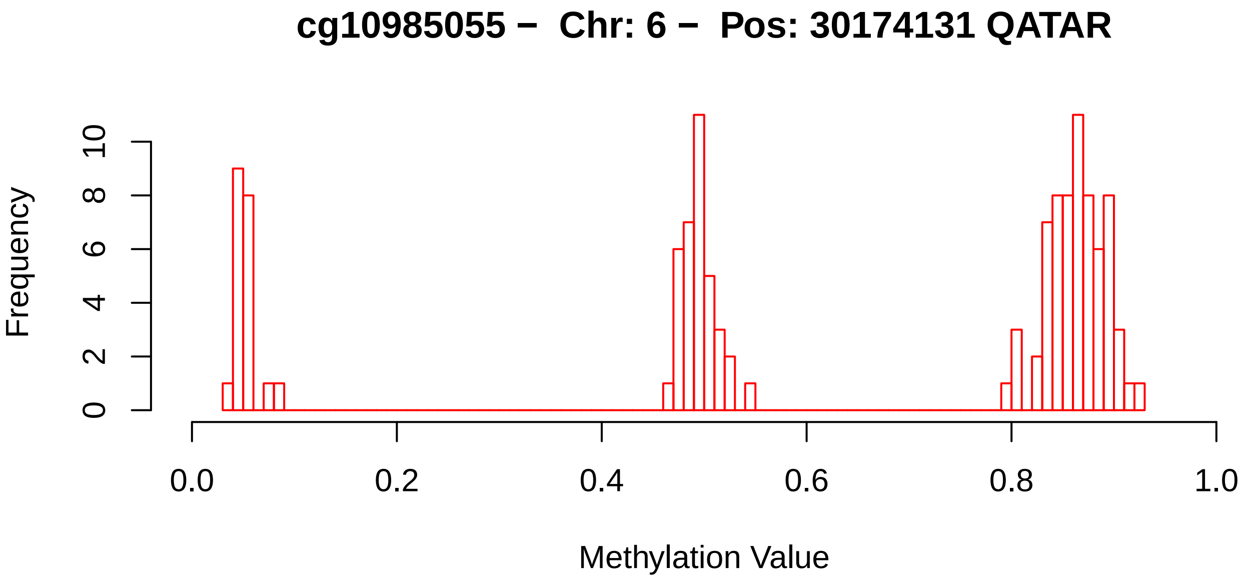
cg10985055 − Chr: 6 − Pos: 30174131 QATAR
Frequency
0
2
4
6
8
10
0.0
0.2
0.4
0.6
0.8
1.0
Methylation Value

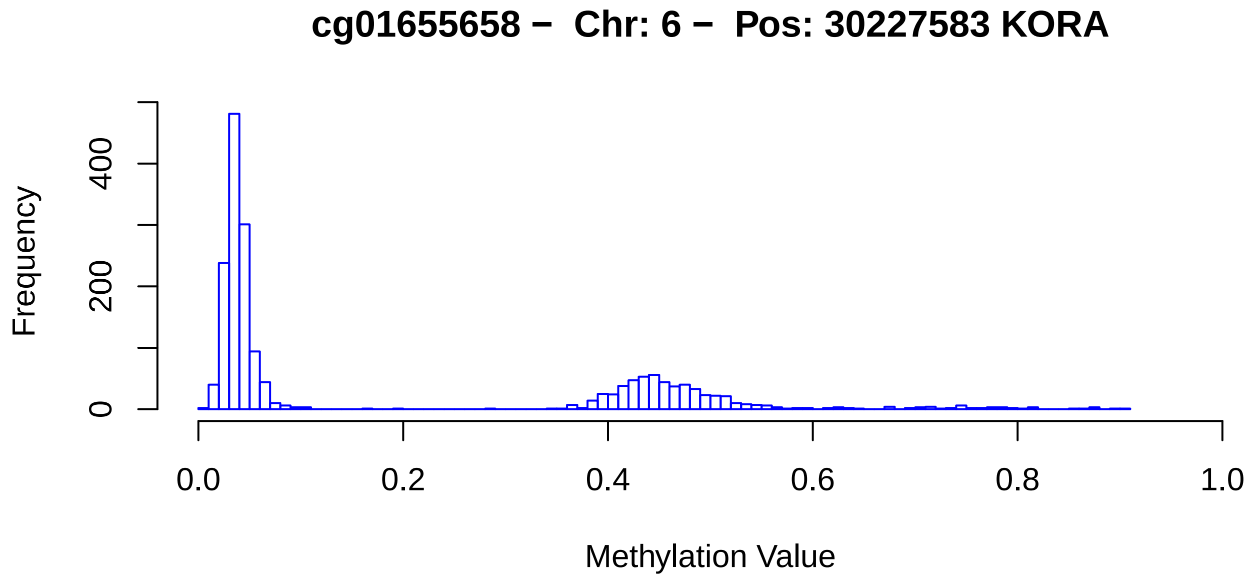
cg01655658 − Chr: 6 − Pos: 30227583 KORA
Frequency
0
200
400
0.0
0.2
0.4
0.6
0.8
1.0
Methylation Value

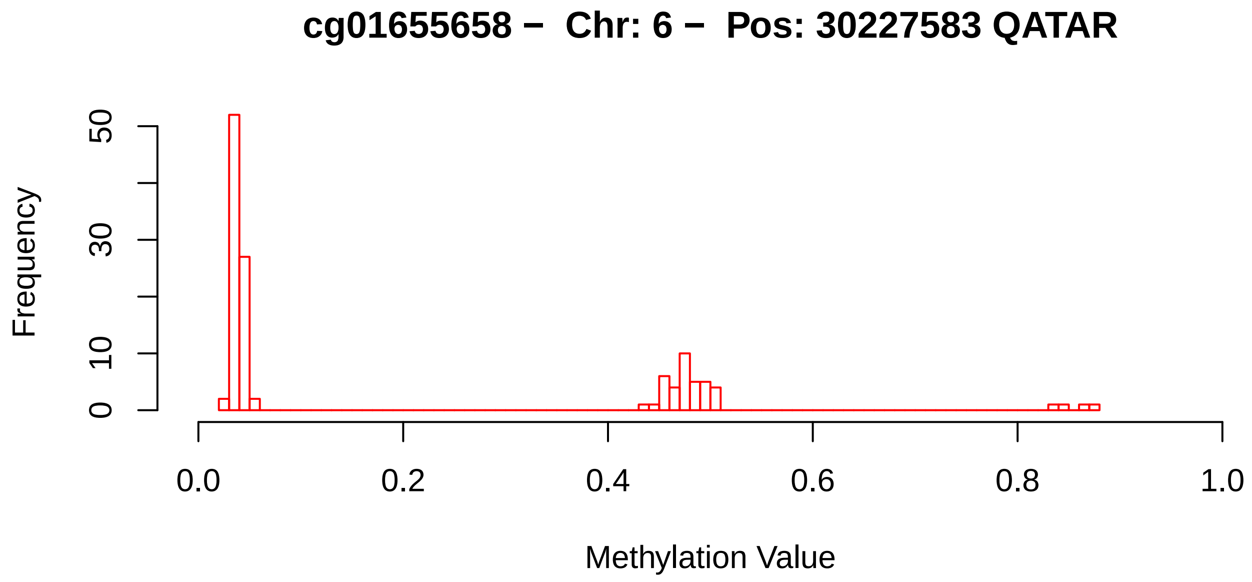
cg01655658 − Chr: 6 − Pos: 30227583 QATAR
Frequency
0
10
30
50
0.0
0.2
0.4
0.6
0.8
1.0
Methylation Value

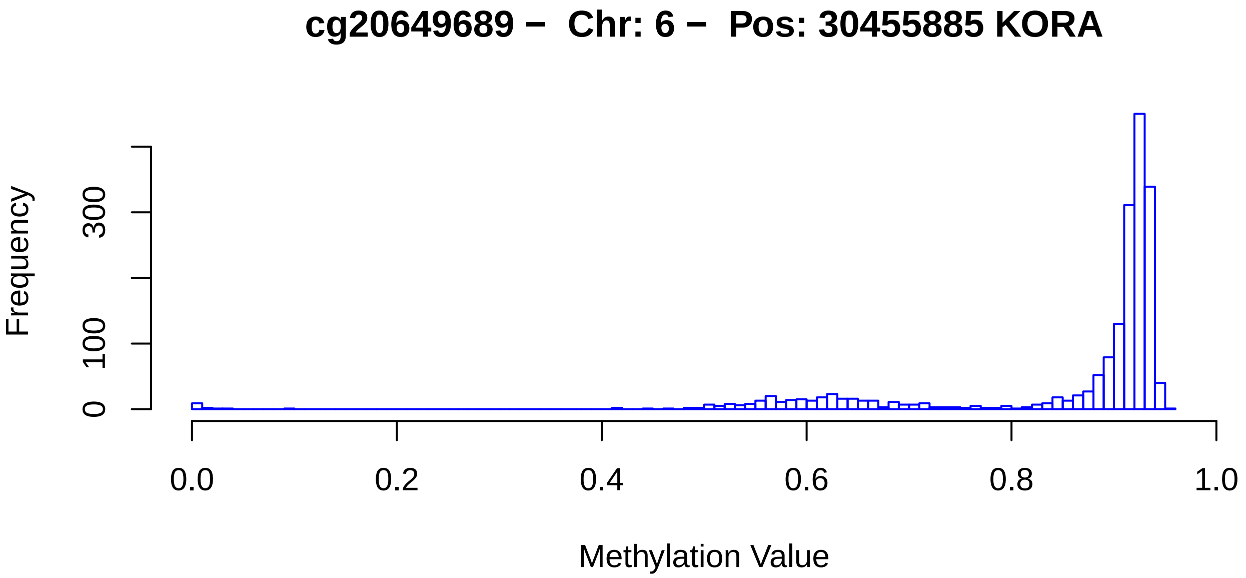
cg20649689 − Chr: 6 − Pos: 30455885 KORA
Frequency
0
100
300
0.0
0.2
0.4
0.6
0.8
1.0
Methylation Value

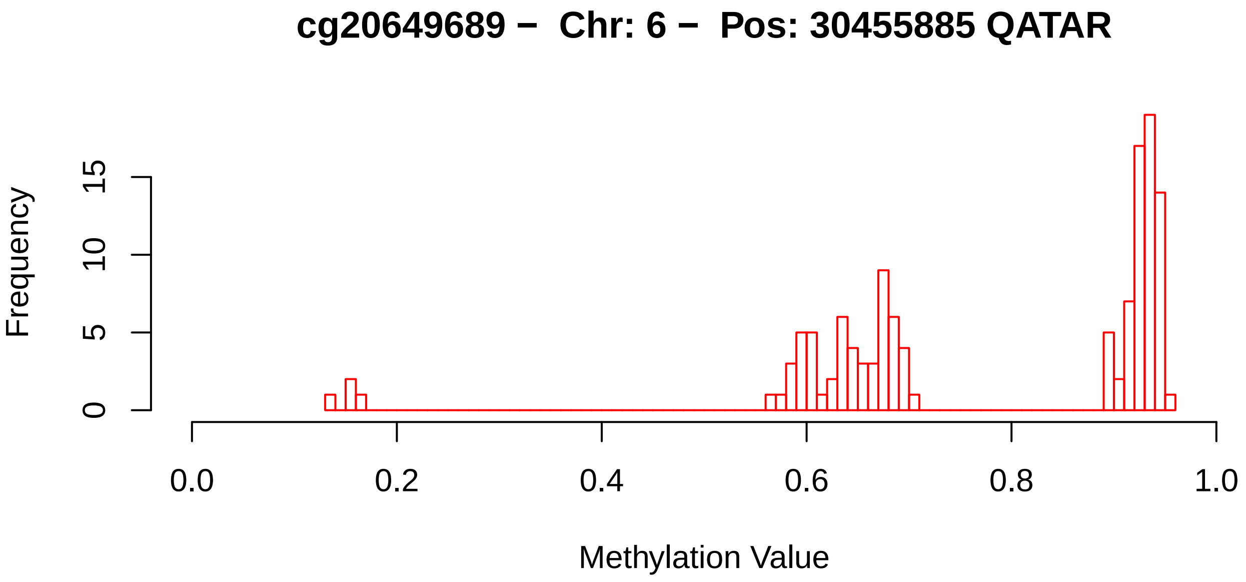
cg20649689 − Chr: 6 − Pos: 30455885 QATAR
Frequency
0
5
10
15
0.0
0.2
0.4
0.6
0.8
1.0
Methylation Value

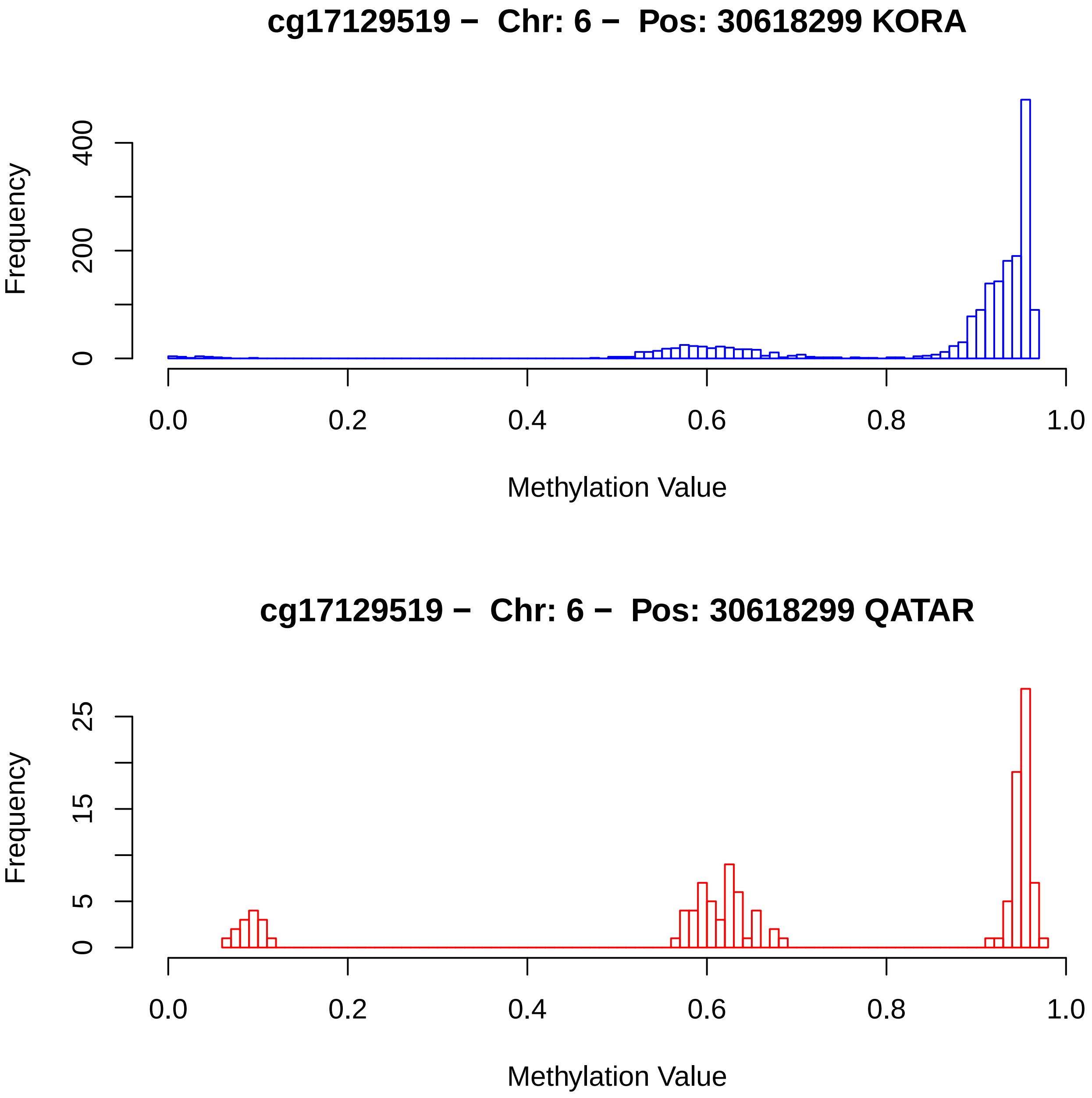
cg17129519 − Chr: 6 − Pos: 30618299 KORA
Frequency
0
200
400
0.0
0.2
0.4
0.6
0.8
1.0
Methylation Value
cg17129519 − Chr: 6 − Pos: 30618299 QATAR
Frequency
0
5
15
25
0.0
0.2
0.4
0.6
0.8
1.0
Methylation Value

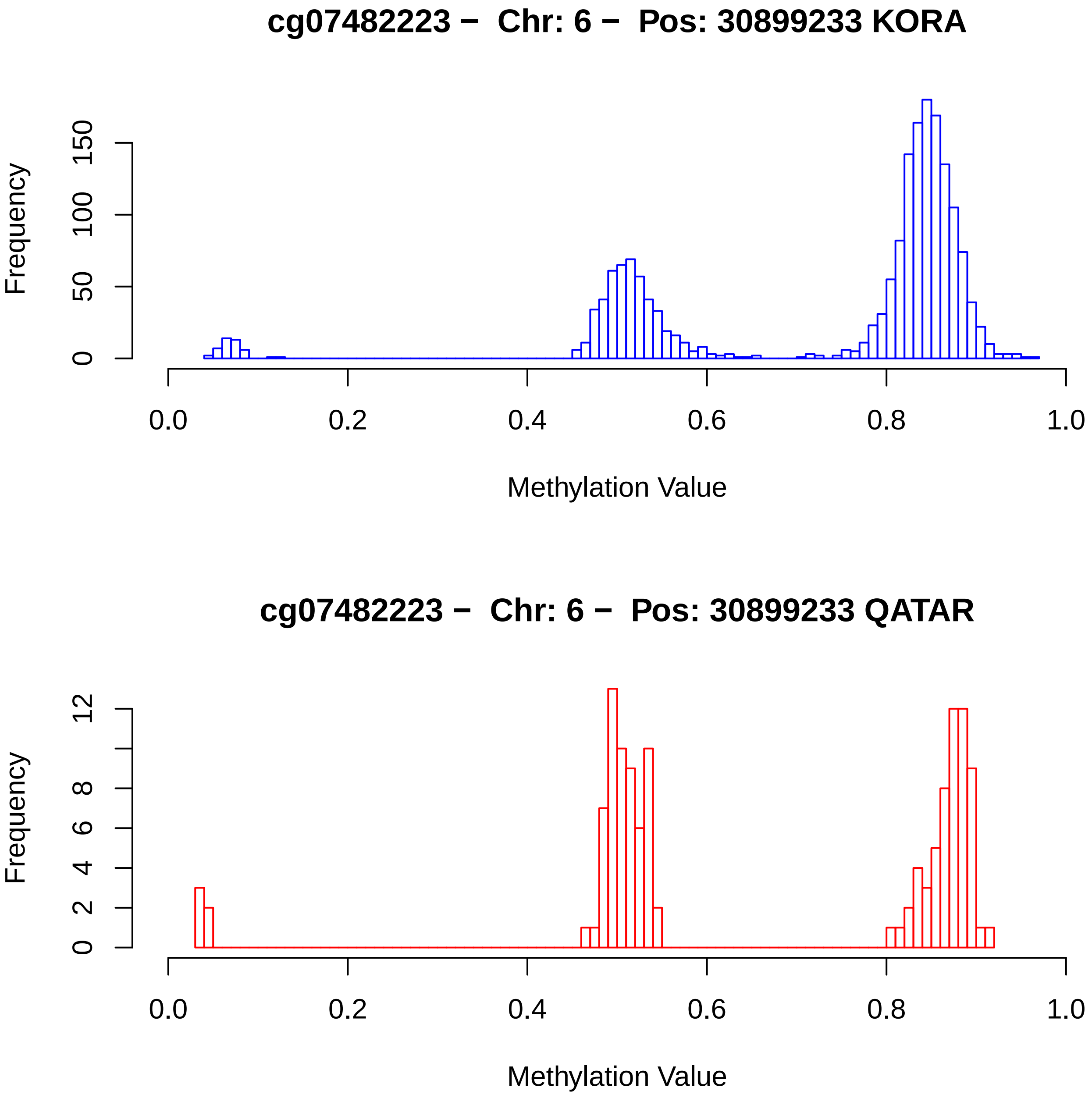

cg07482223 − Chr: 6 − Pos: 30899233 KORA
Frequency
Methylation Value
0
50
100
150
0.0
0.2
0.4
0.6
0.8
1.0
cg07482223 − Chr: 6 − Pos: 30899233 QATAR
Frequency
Methylation Value
0
2
4
6
8
12
0.0
0.2
0.4
0.6
0.8
1.0

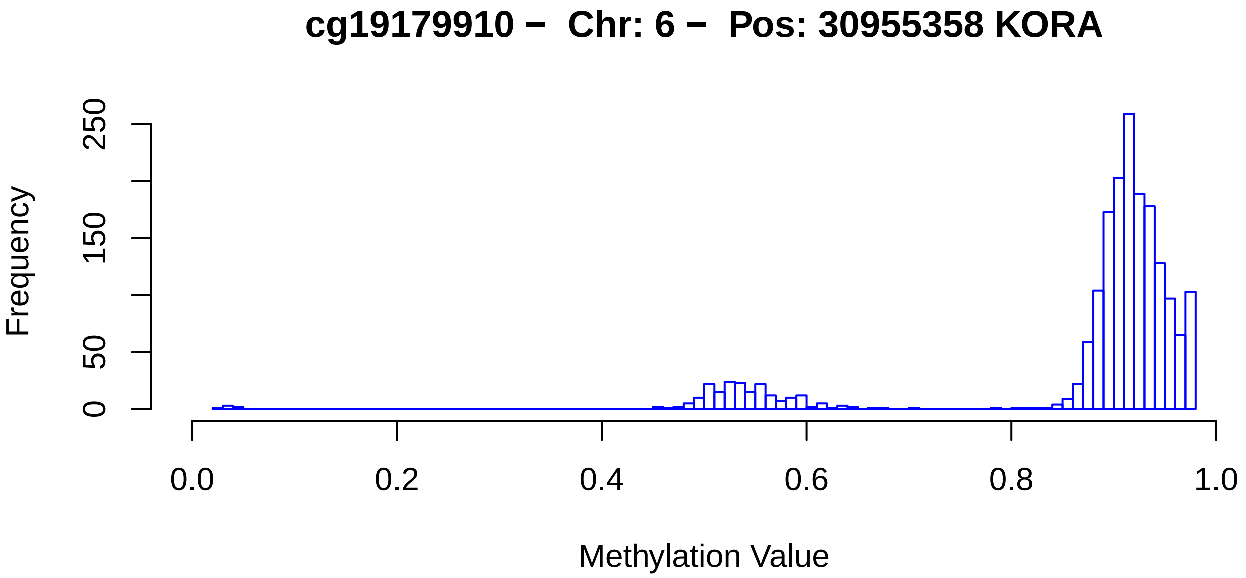
cg19179910 − Chr: 6 − Pos: 30955358 KORA
Frequency
0
50
150
250
0.0
0.2
0.4
0.6
0.8
1.0
Methylation Value

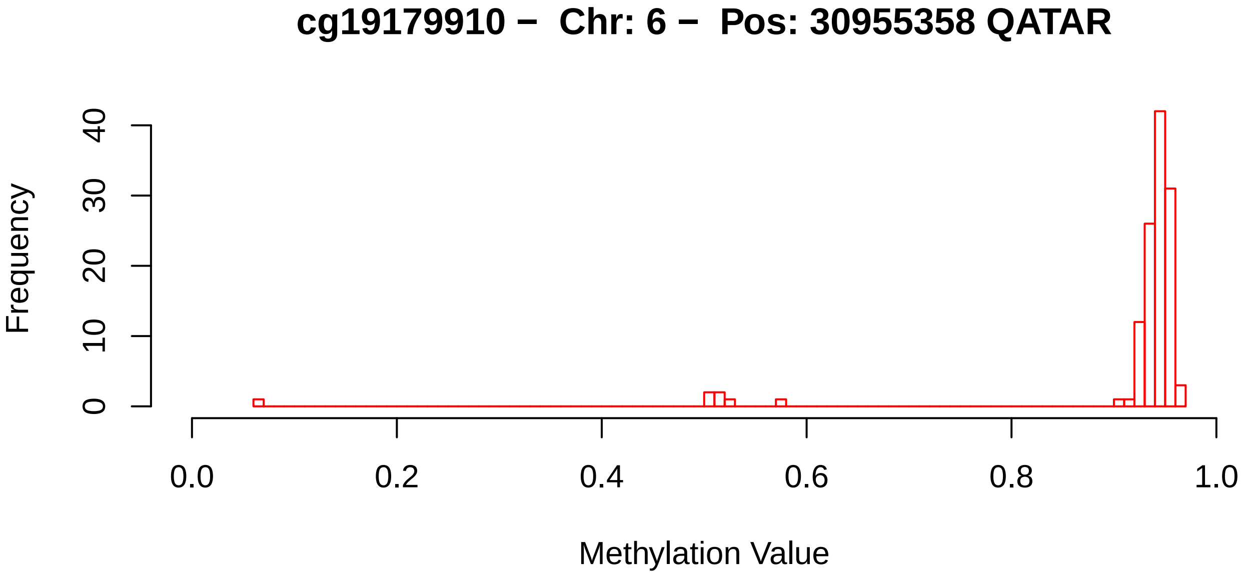
cg19179910 − Chr: 6 − Pos: 30955358 QATAR
Frequency
0
10
20
30
40
0.0
0.2
0.4
0.6
0.8
1.0
Methylation Value

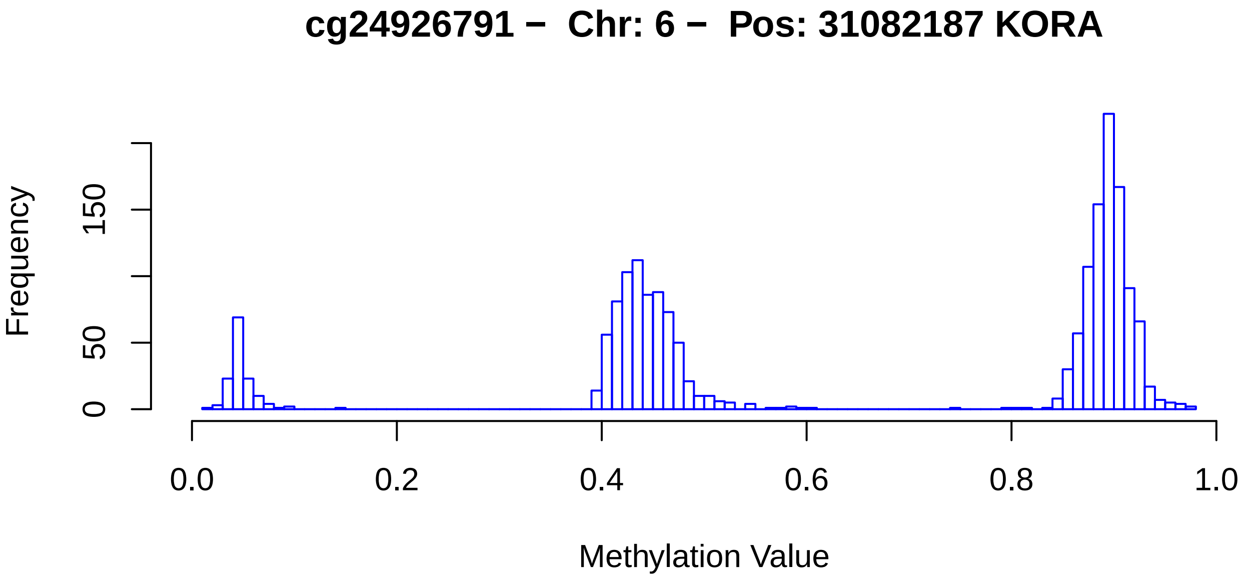
cg24926791 − Chr: 6 − Pos: 31082187 KORA
Frequency
0
50
150
0.0
0.2
0.4
0.6
0.8
1.0
Methylation Value

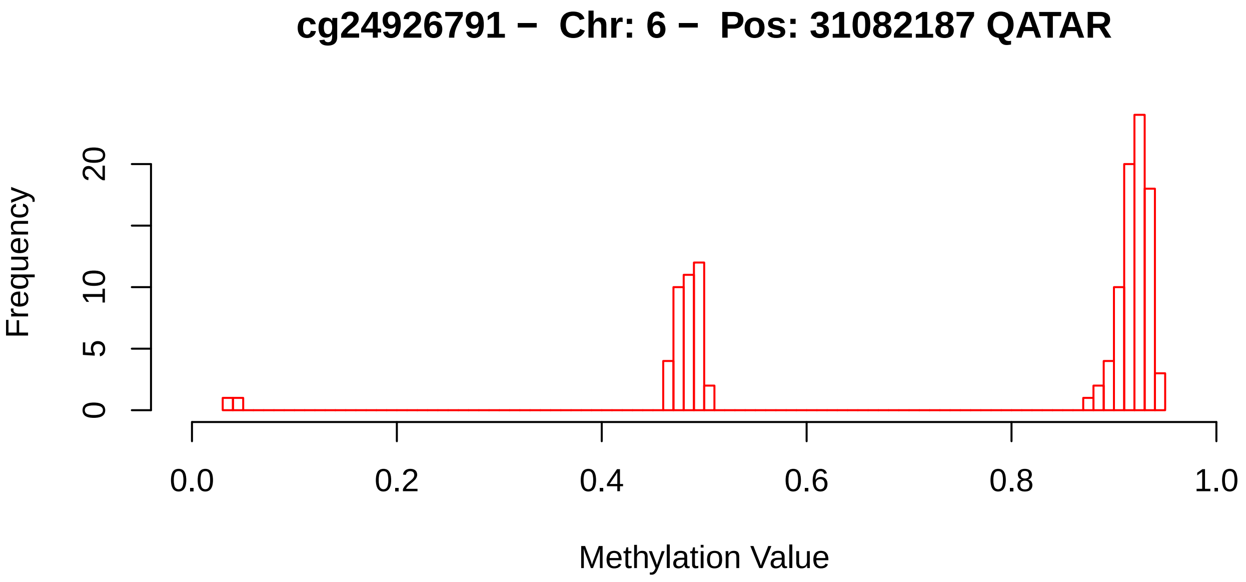
cg24926791 − Chr: 6 − Pos: 31082187 QATAR
Frequency
0
5
10
20
0.0
0.2
0.4
0.6
0.8
1.0
Methylation Value

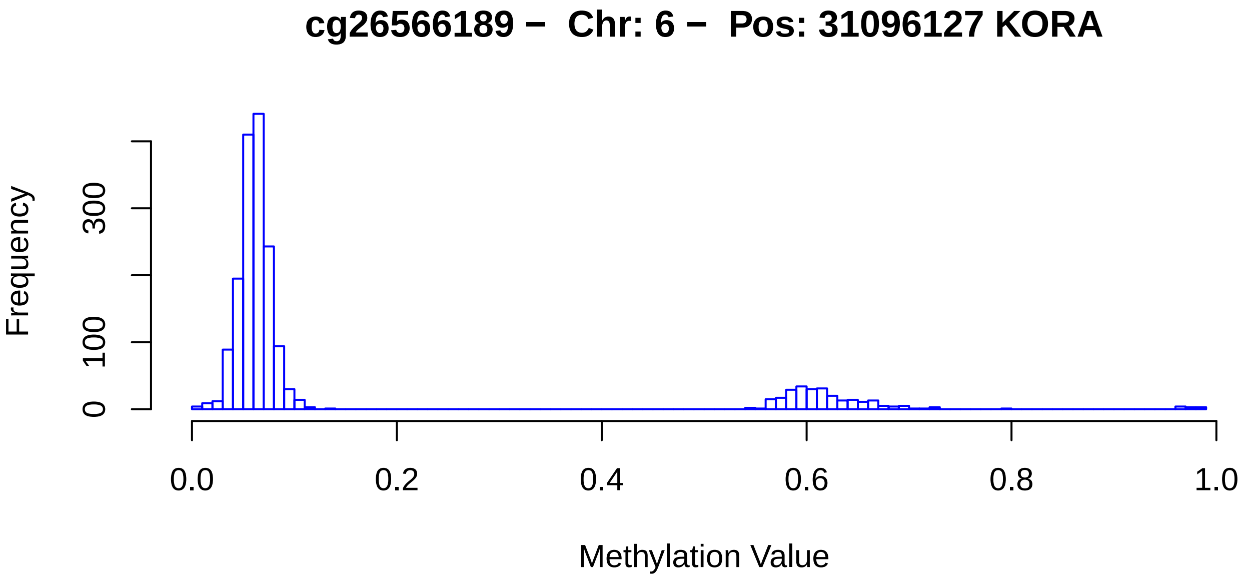
cg26566189 − Chr: 6 − Pos: 31096127 KORA
Frequency
0
100
300
0.0
0.2
0.4
0.6
0.8
1.0
Methylation Value

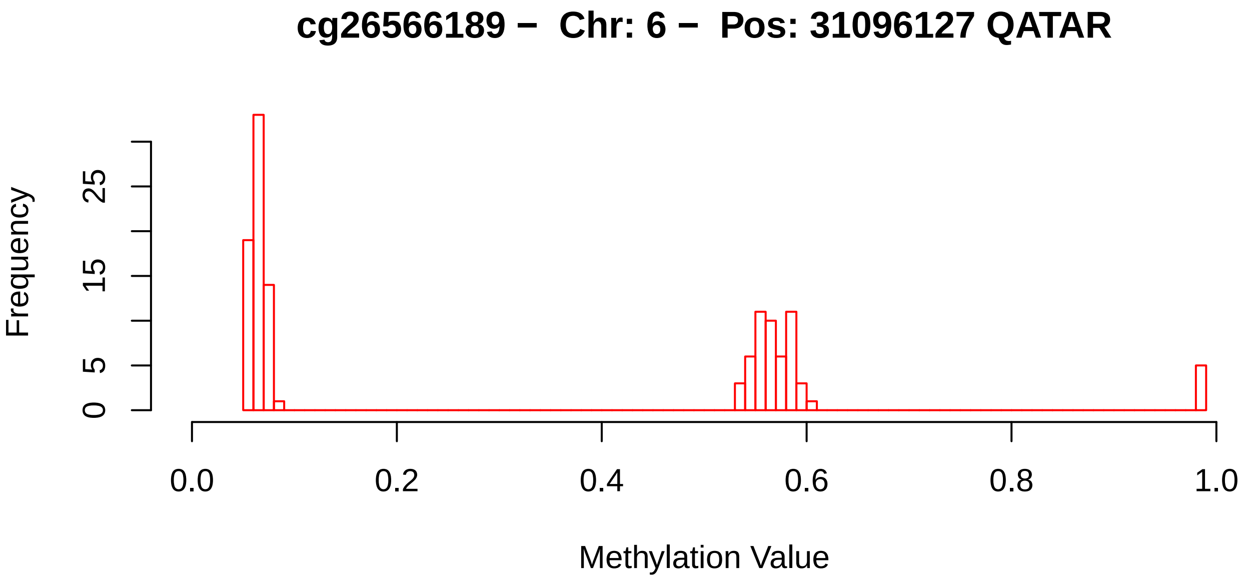
cg26566189 − Chr: 6 − Pos: 31096127 QATAR
Frequency
0
5
15
25
0.0
0.2
0.4
0.6
0.8
1.0
Methylation Value

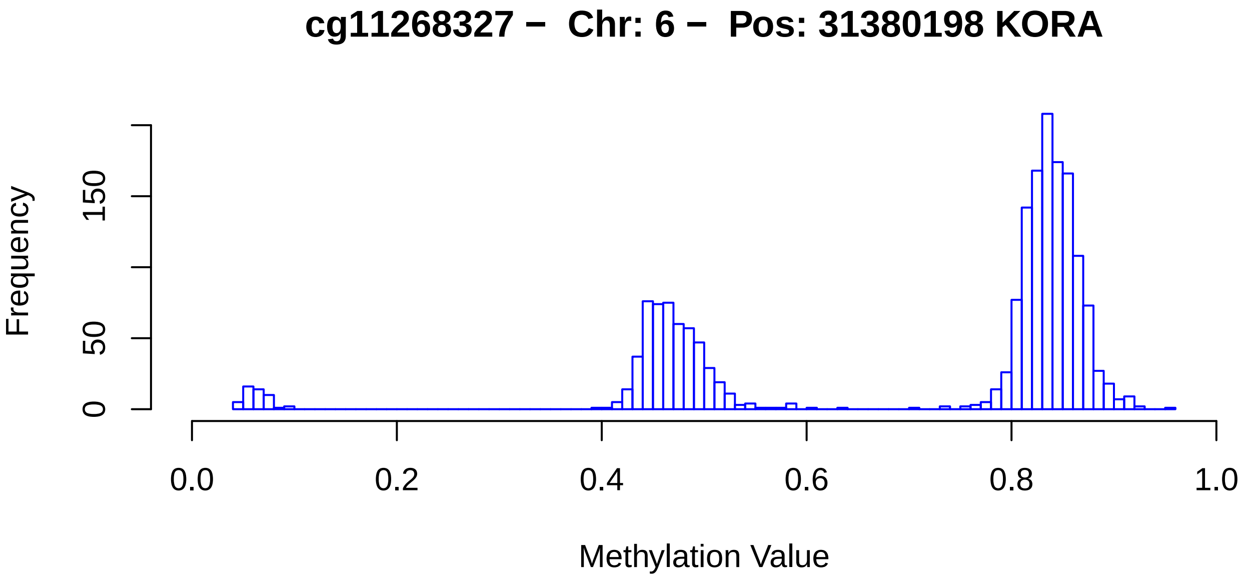
cg11268327 − Chr: 6 − Pos: 31380198 KORA
Frequency
0
50
150
0.0
0.2
0.4
0.6
0.8
1.0
Methylation Value

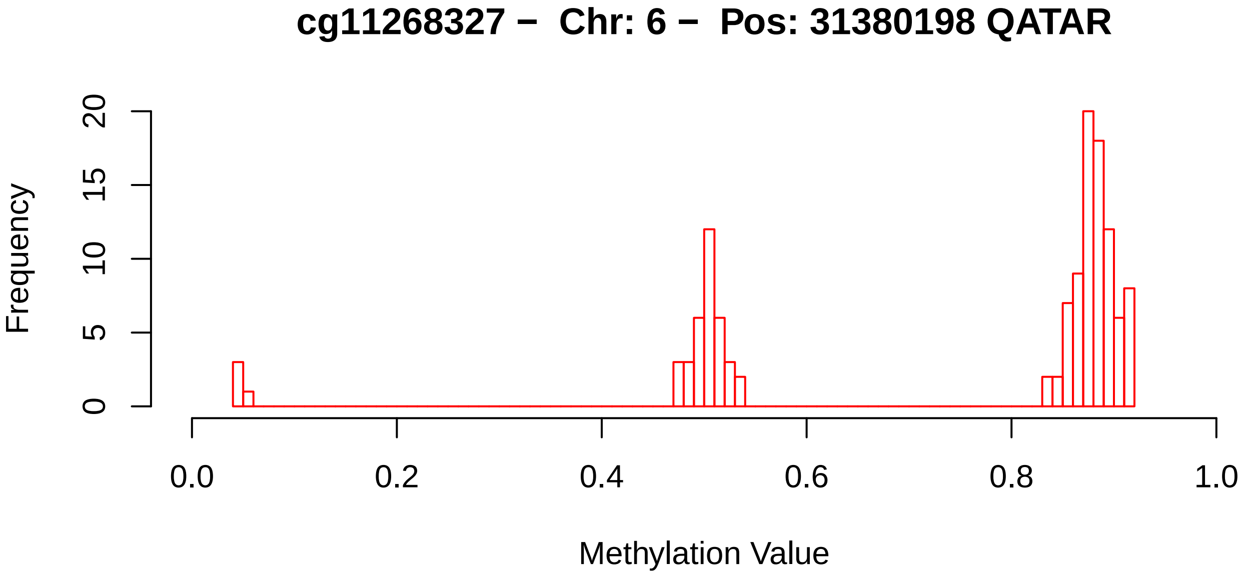
cg11268327 − Chr: 6 − Pos: 31380198 QATAR
Frequency
0
5
10
15
20
0.0
0.2
0.4
0.6
0.8
1.0
Methylation Value

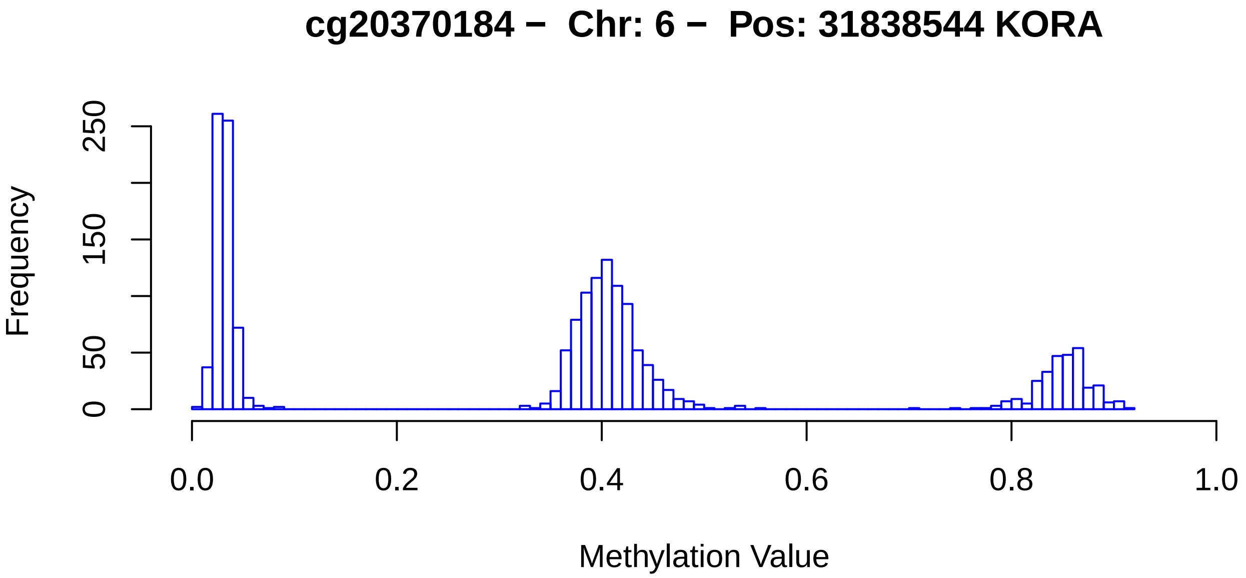
cg20370184 − Chr: 6 − Pos: 31838544 KORA
Frequency
0
50
150
250
0.0
0.2
0.4
0.6
0.8
1.0
Methylation Value

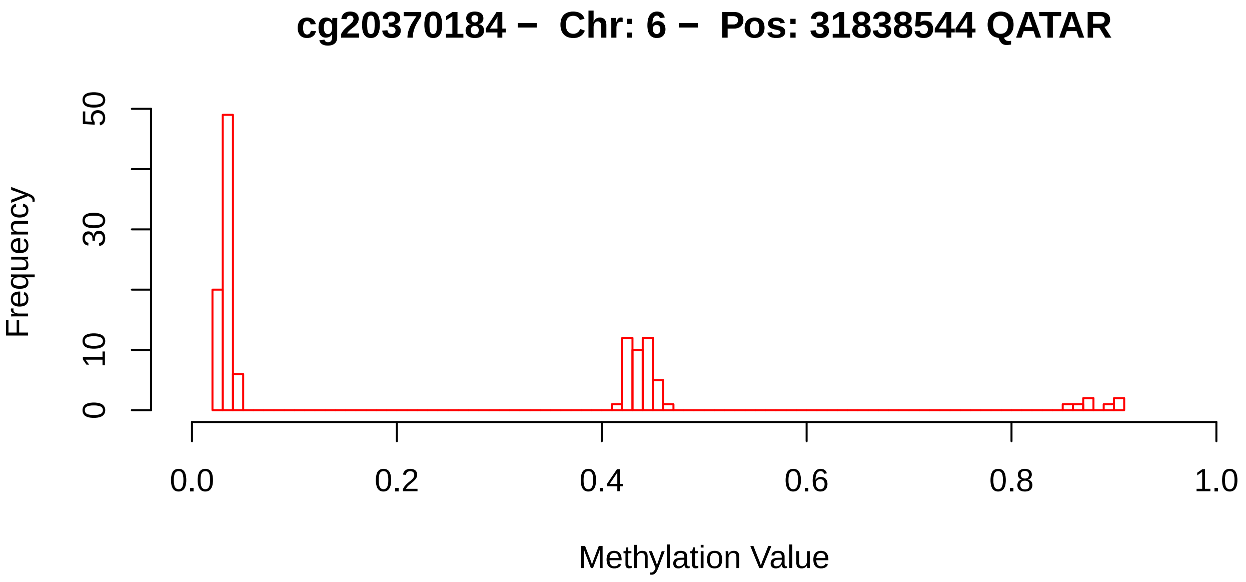
cg20370184 − Chr: 6 − Pos: 31838544 QATAR
Frequency
0
10
30
50
0.0
0.2
0.4
0.6
0.8
1.0
Methylation Value

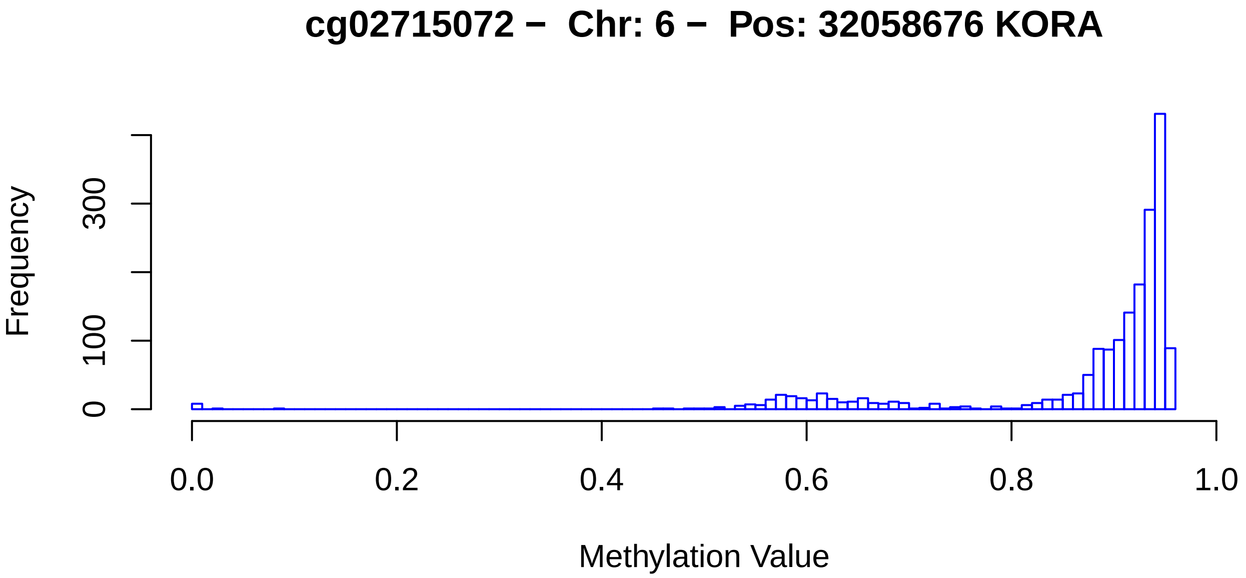
cg02715072 − Chr: 6 − Pos: 32058676 KORA
Frequency
0
100
300
0.0
0.2
0.4
0.6
0.8
1.0
Methylation Value

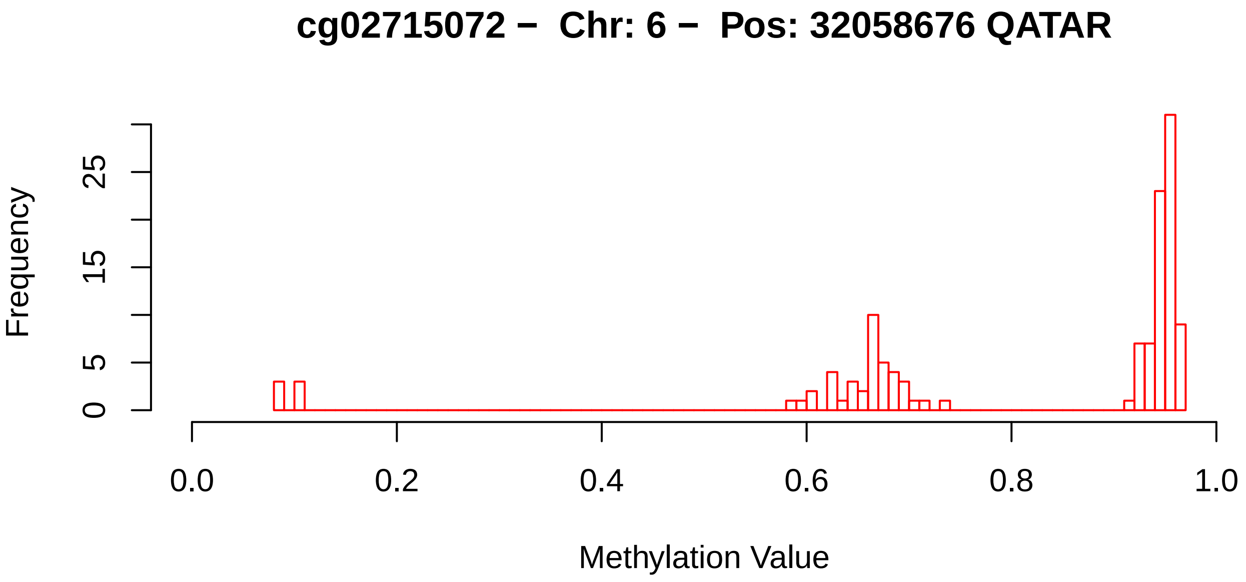
cg02715072 − Chr: 6 − Pos: 32058676 QATAR
Frequency
0
5
15
25
0.0
0.2
0.4
0.6
0.8
1.0
Methylation Value

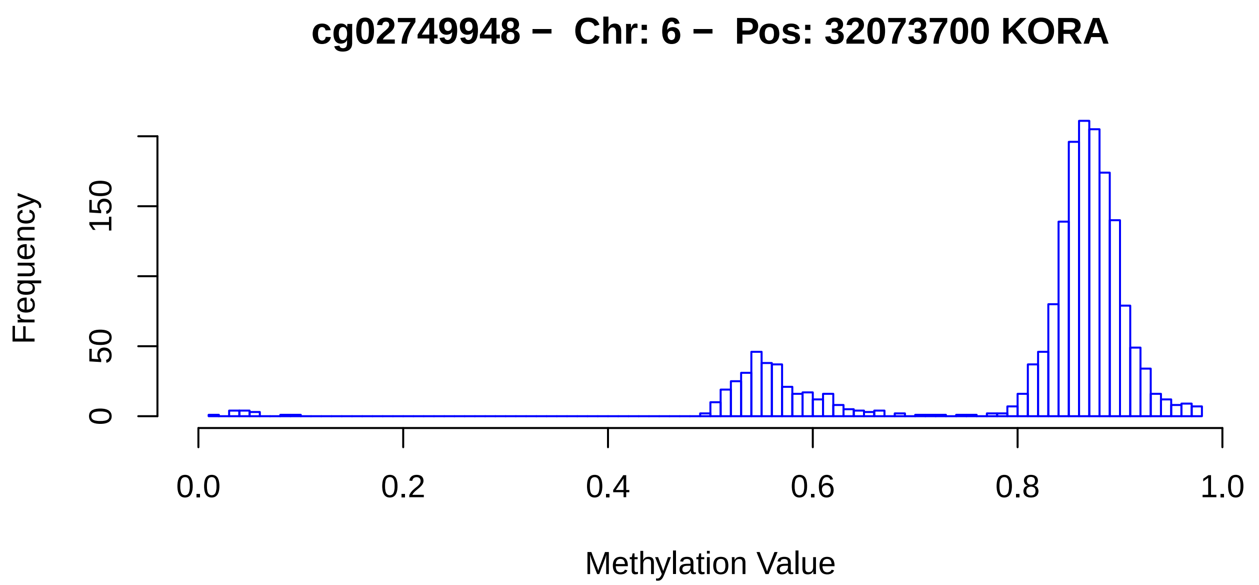
cg02749948 − Chr: 6 − Pos: 32073700 KORA
Frequency
0
50
150
0.0
0.2
0.4
0.6
0.8
1.0
Methylation Value

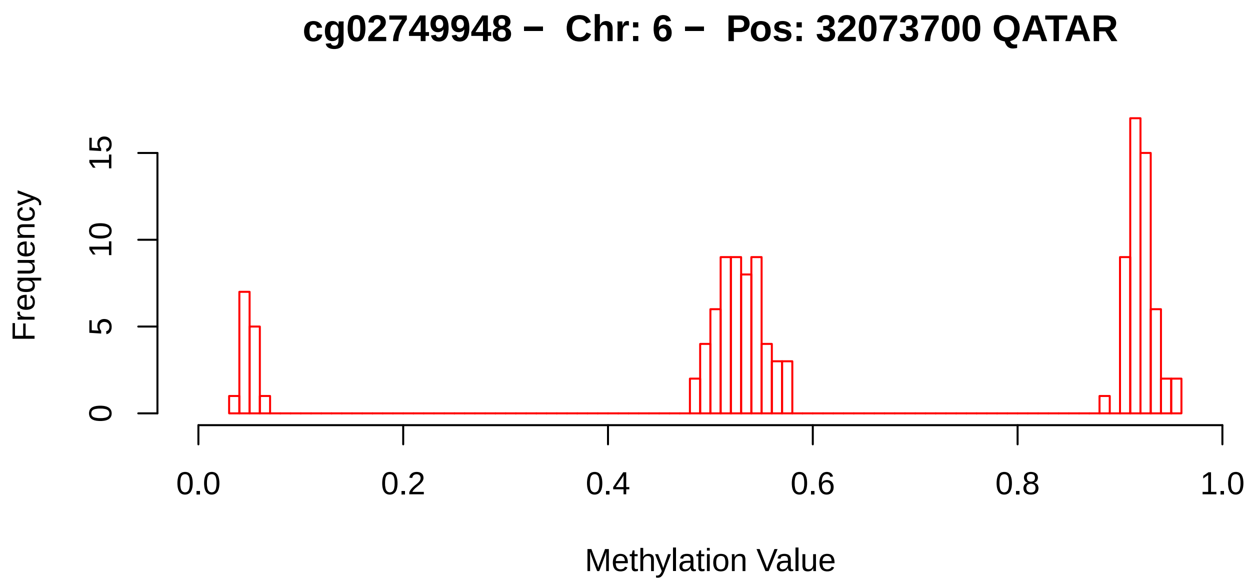
cg02749948 − Chr: 6 − Pos: 32073700 QATAR
Frequency
0
5
10
15
0.0
0.2
0.4
0.6
0.8
1.0
Methylation Value

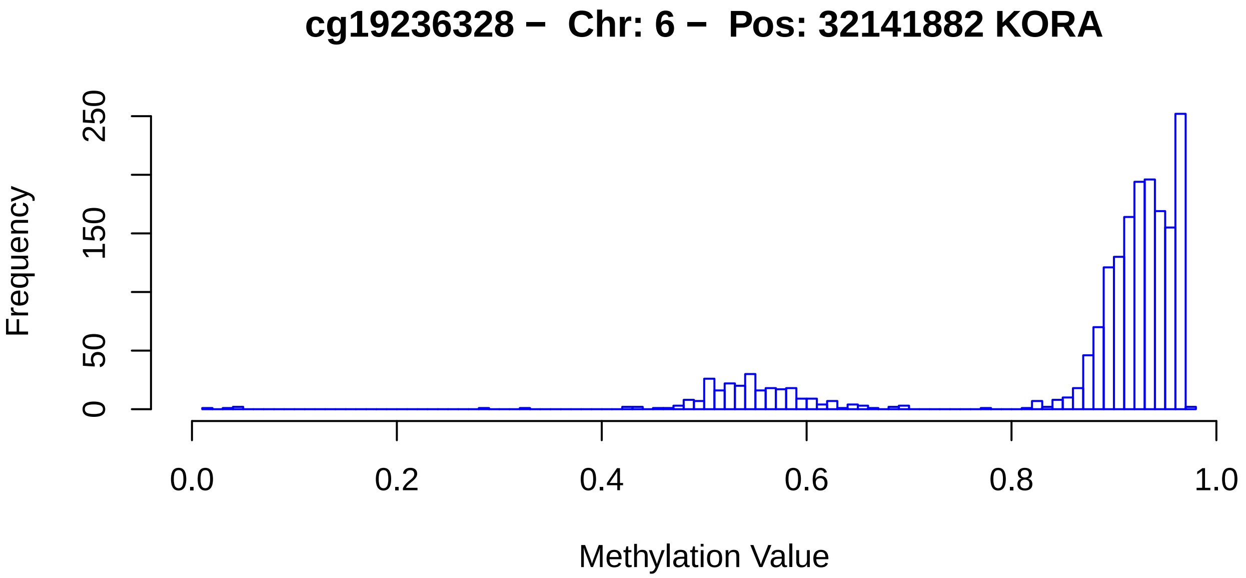
cg19236328 − Chr: 6 − Pos: 32141882 KORA
Frequency
0
50
150
250
0.0
0.2
0.4
0.6
0.8
1.0
Methylation Value

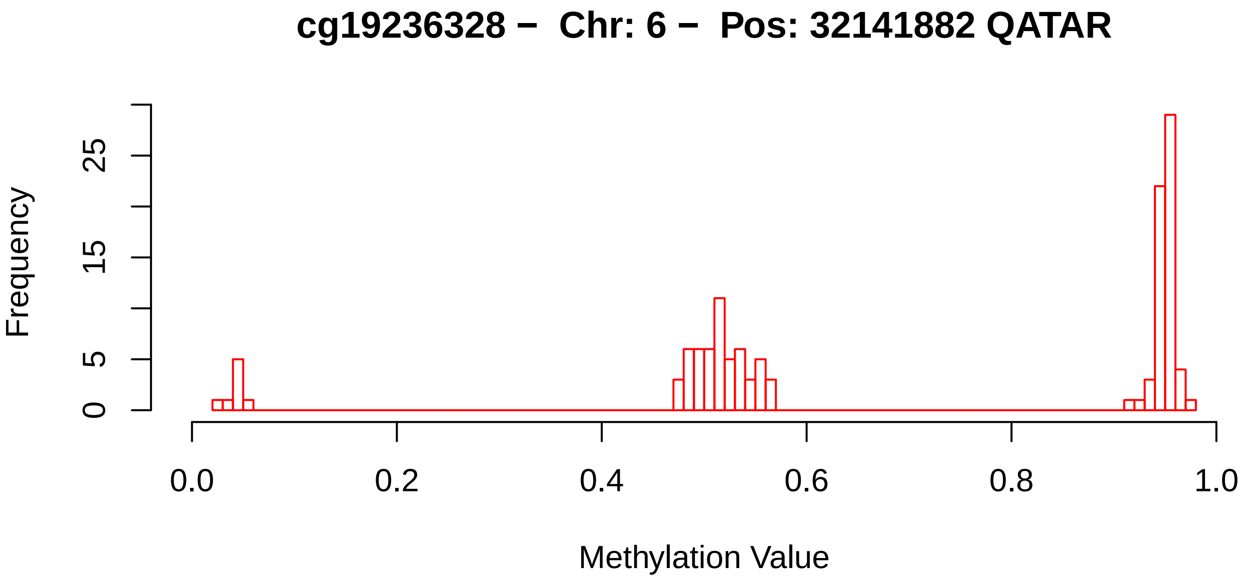
cg19236328 − Chr: 6 − Pos: 32141882 QATAR
Frequency
0
5
15
25
0.0
0.2
0.4
0.6
0.8
1.0
Methylation Value

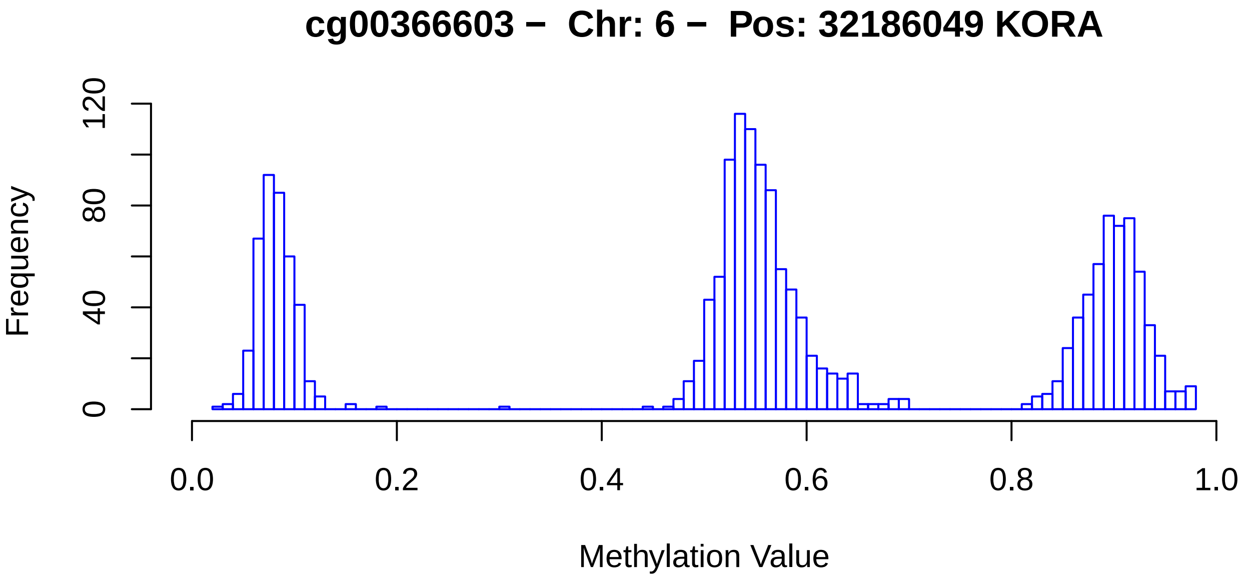
cg00366603 − Chr: 6 − Pos: 32186049 KORA
Frequency
0
40
80
120
0.0
0.2
0.4
0.6
0.8
1.0
Methylation Value

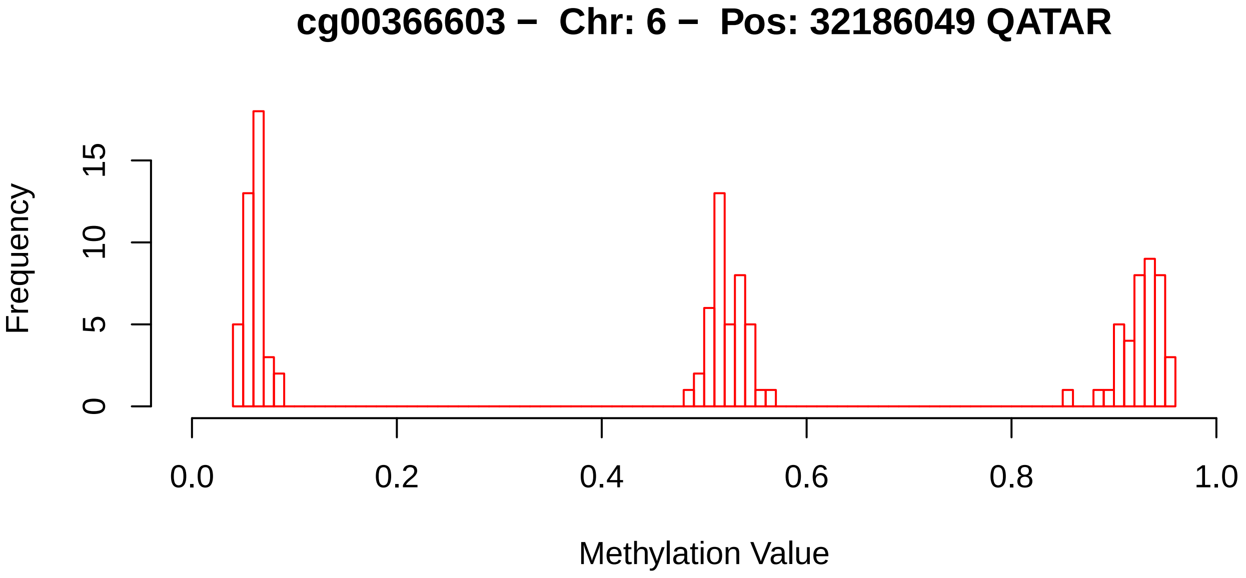
cg00366603 − Chr: 6 − Pos: 32186049 QATAR
Frequency
0
5
10
15
0.0
0.2
0.4
0.6
0.8
1.0
Methylation Value

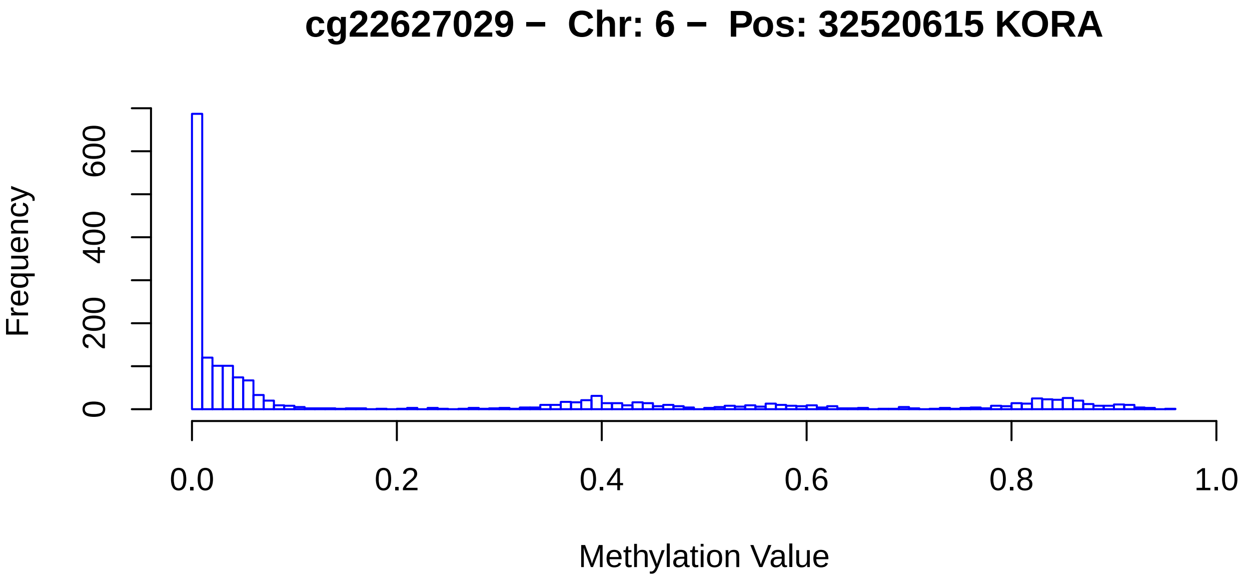
cg22627029 − Chr: 6 − Pos: 32520615 KORA
Frequency
0
200
400
600
0.0
0.2
0.4
0.6
0.8
1.0
Methylation Value

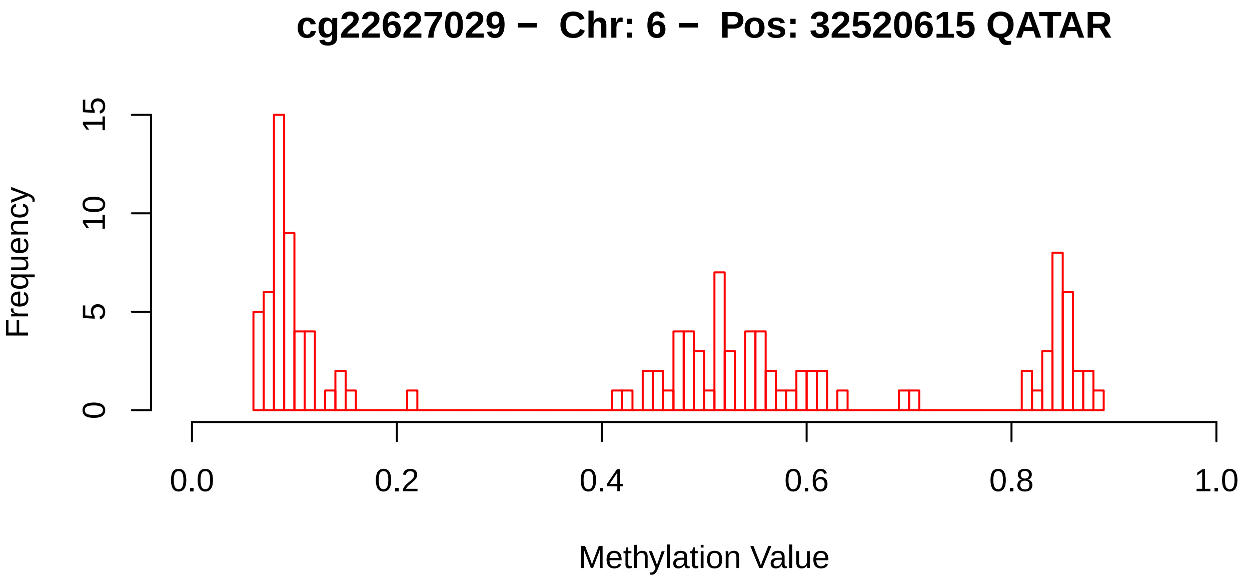
cg22627029 − Chr: 6 − Pos: 32520615 QATAR
Frequency
0
5
10
15
0.0
0.2
0.4
0.6
0.8
1.0
Methylation Value

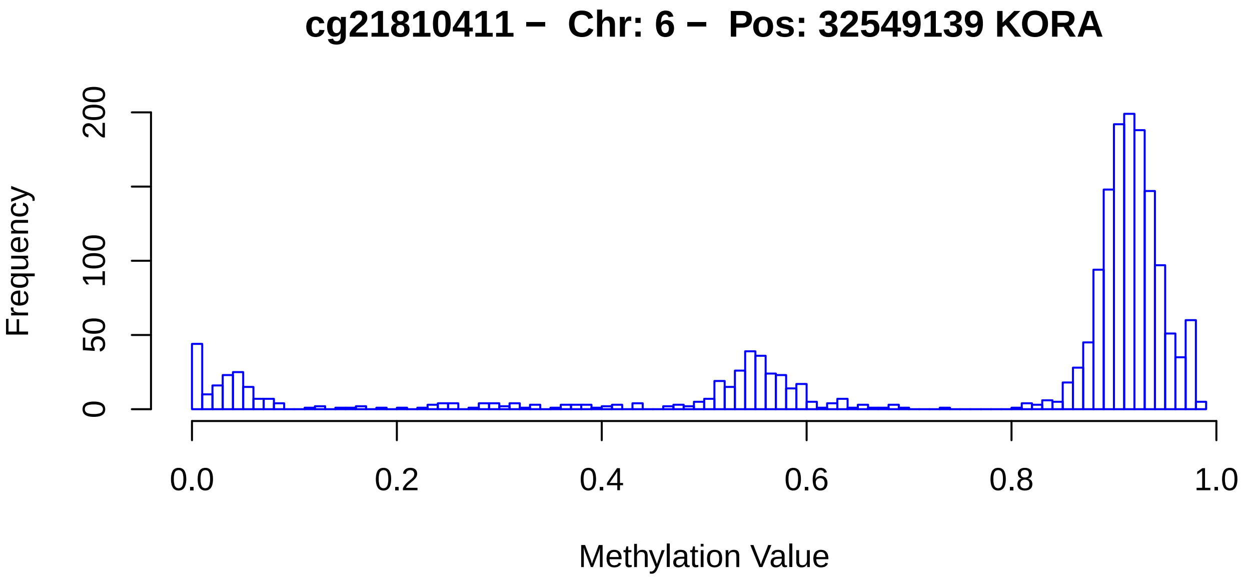
cg21810411 − Chr: 6 − Pos: 32549139 KORA
Frequency
0
50
100
200
0.0
0.2
0.4
0.6
0.8
1.0
Methylation Value

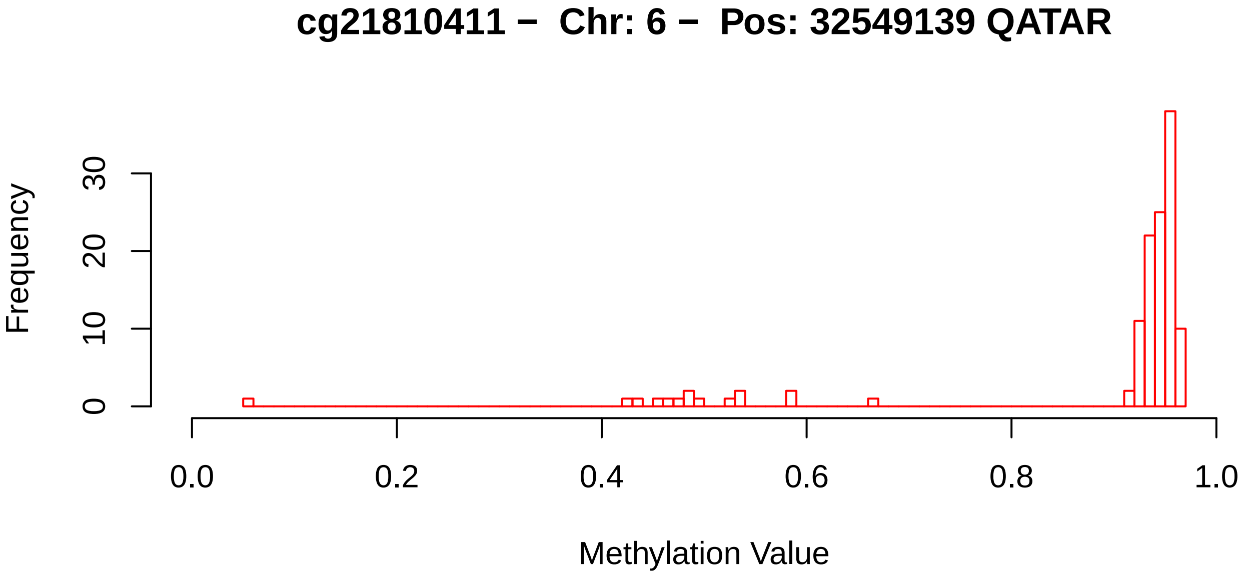
cg21810411 − Chr: 6 − Pos: 32549139 QATAR
Frequency
0
10
20
30
0.0
0.2
0.4
0.6
0.8
1.0
Methylation Value

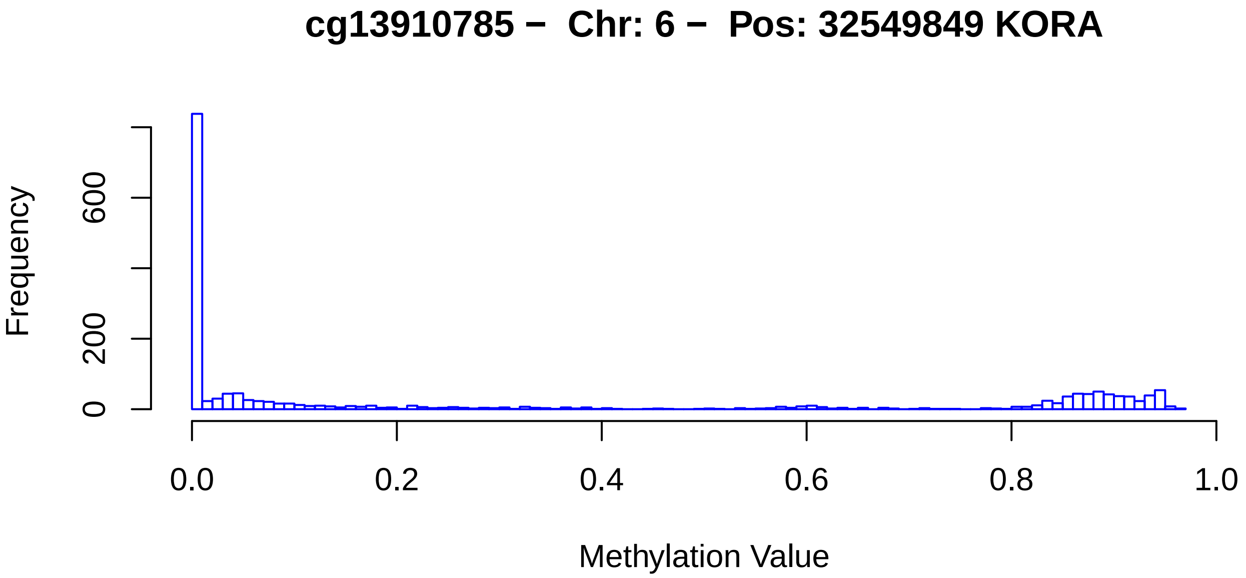
cg13910785 − Chr: 6 − Pos: 32549849 KORA
Frequency
0
200
600
0.0
0.2
0.4
0.6
0.8
1.0
Methylation Value

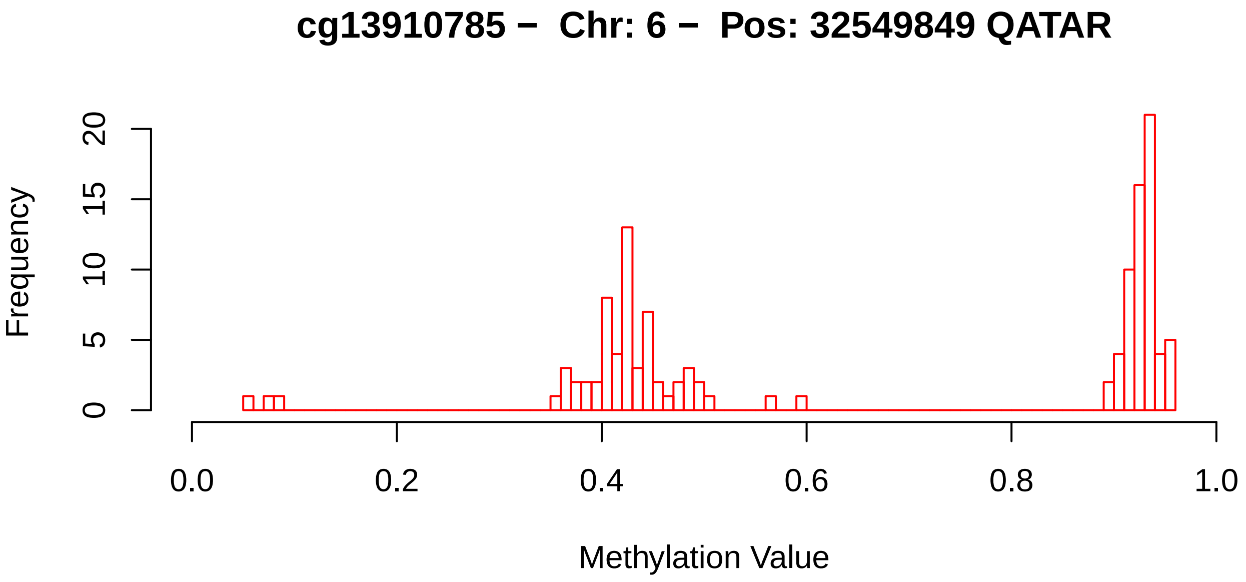
cg13910785 − Chr: 6 − Pos: 32549849 QATAR
Frequency
0
5
10
15
20
0.0
0.2
0.4
0.6
0.8
1.0
Methylation Value

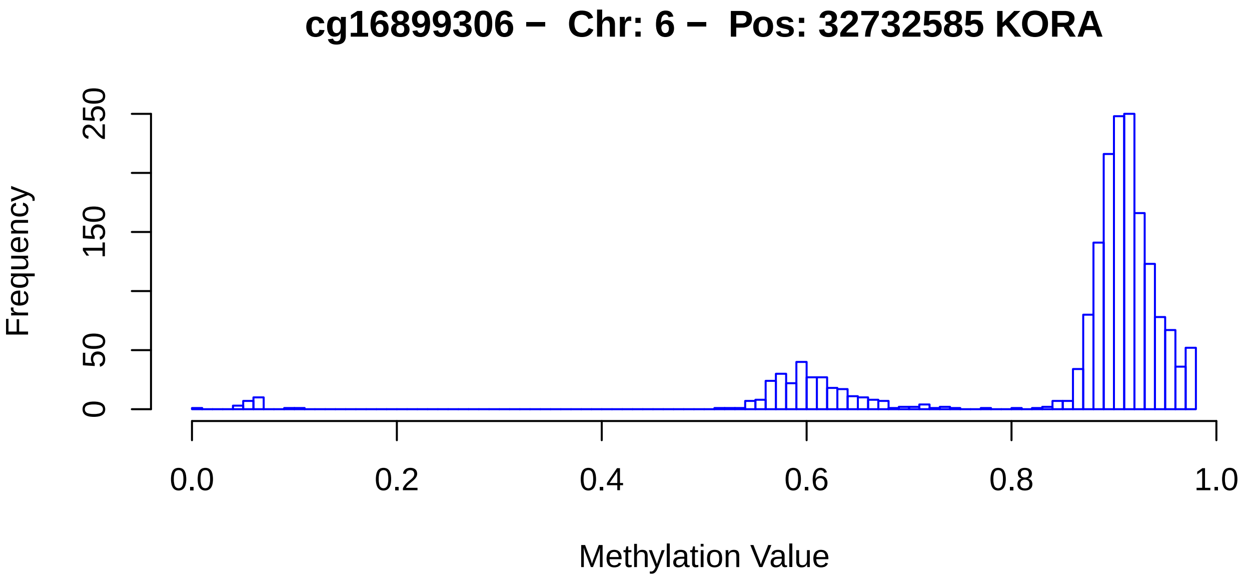
cg16899306 − Chr: 6 − Pos: 32732585 KORA
Frequency
0
50
150
250
0.0
0.2
0.4
0.6
0.8
1.0
Methylation Value

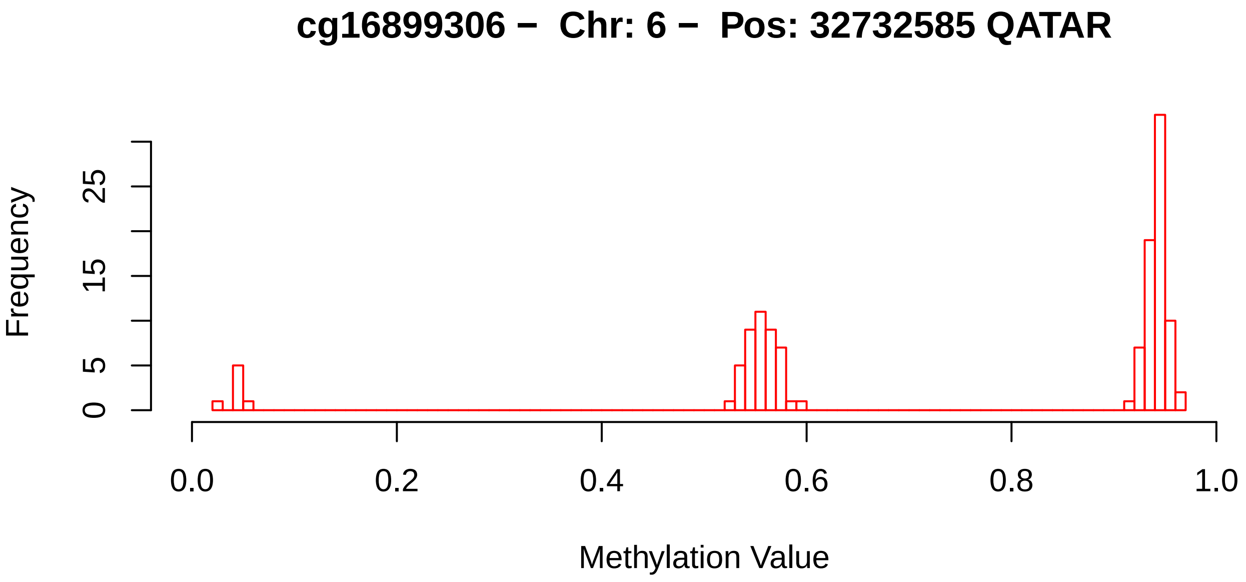
cg16899306 − Chr: 6 − Pos: 32732585 QATAR
Frequency
0
5
15
25
0.0
0.2
0.4
0.6
0.8
1.0
Methylation Value

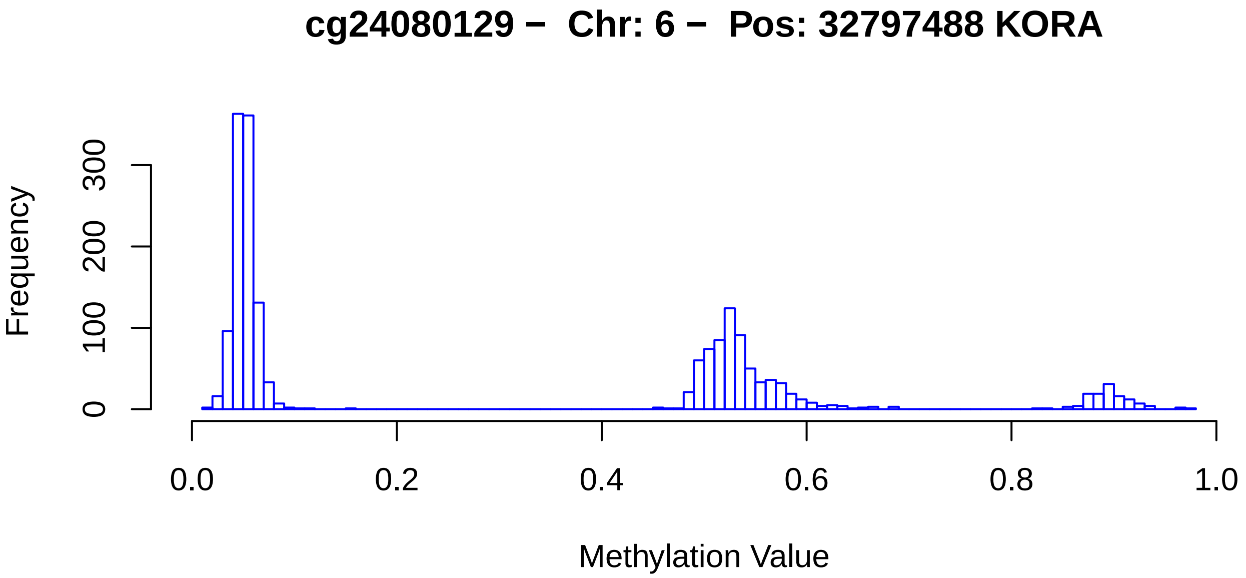
cg24080129 − Chr: 6 − Pos: 32797488 KORA
Frequency
0
100
200
300
0.0
0.2
0.4
0.6
0.8
1.0
Methylation Value

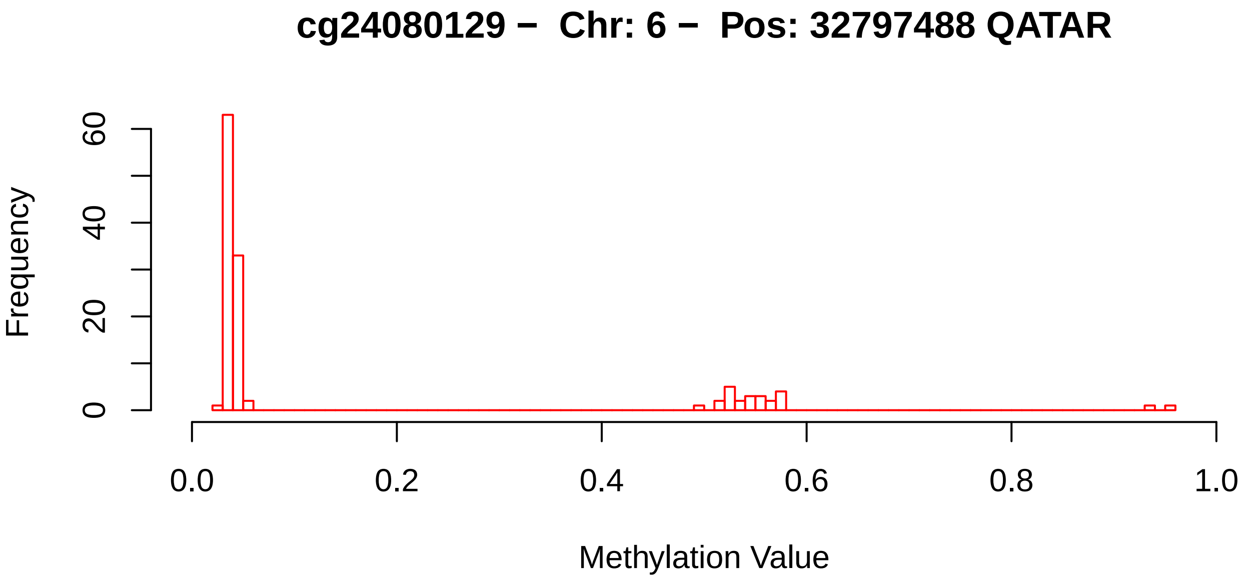
cg24080129 − Chr: 6 − Pos: 32797488 QATAR
Frequency
0
20
40
60
0.0
0.2
0.4
0.6
0.8
1.0
Methylation Value

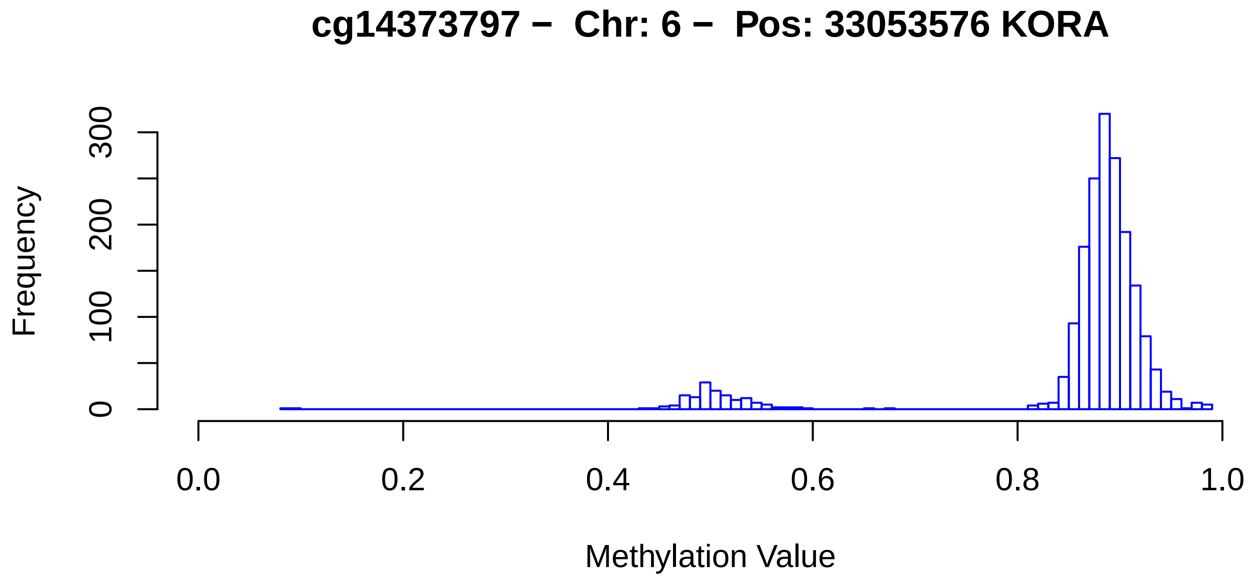
cg14373797 − Chr: 6 − Pos: 33053576 KORA
Frequency
0
100
200
300
0.0
0.2
0.4
0.6
0.8
1.0
Methylation Value

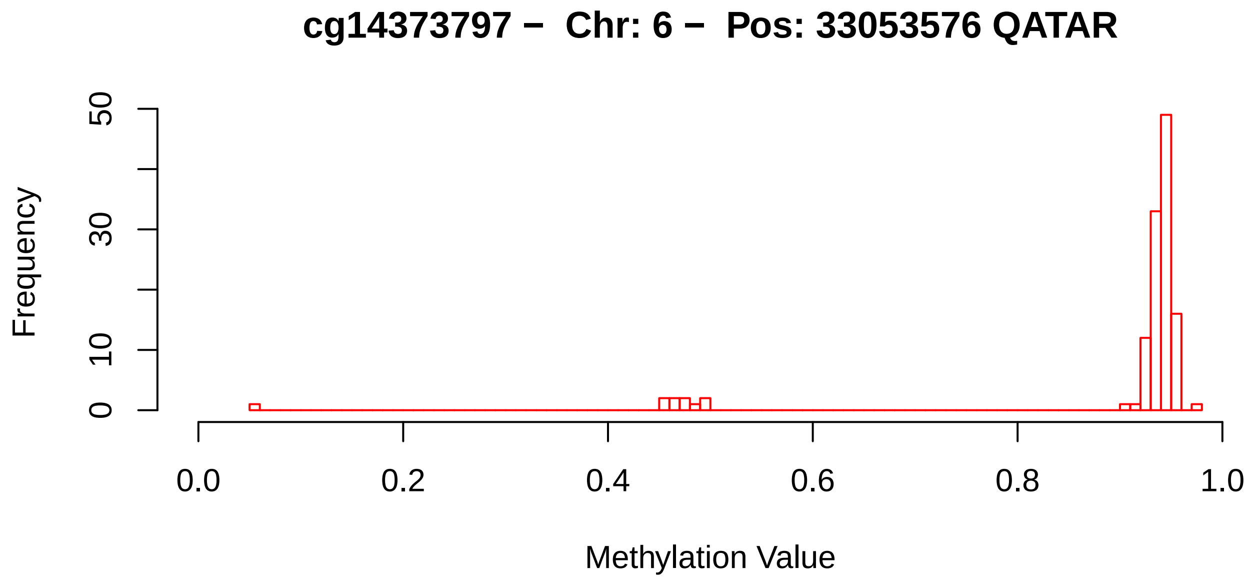
cg14373797 − Chr: 6 − Pos: 33053576 QATAR
Frequency
0
10
30
50
0.0
0.2
0.4
0.6
0.8
1.0
Methylation Value

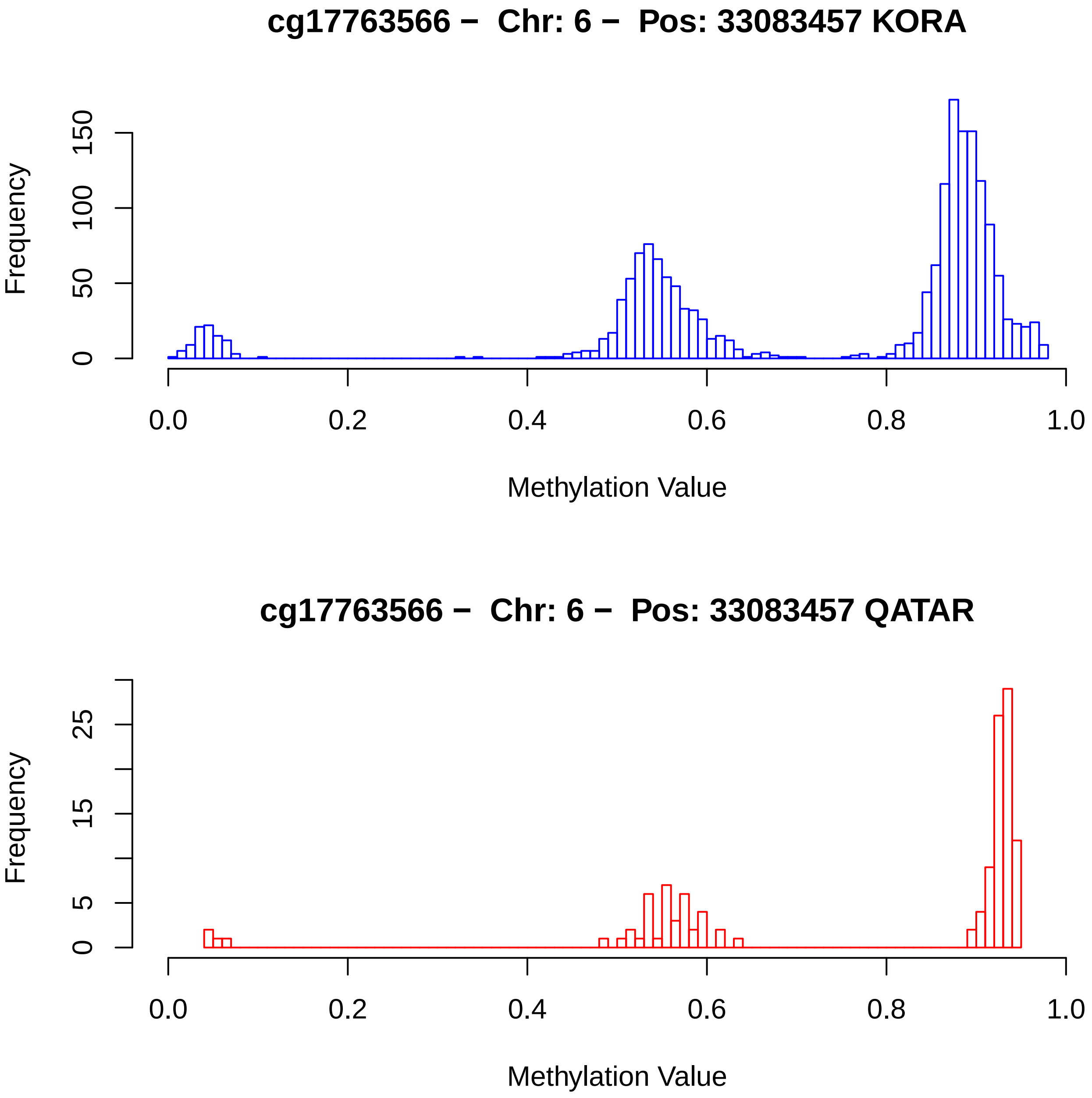
cg17763566 − Chr: 6 − Pos: 33083457 KORA
Frequency
0
50
100
150
0.0
0.2
0.4
0.6
0.8
1.0
Methylation Value
cg17763566 − Chr: 6 − Pos: 33083457 QATAR
Frequency
0
5
15
25
0.0
0.2
0.4
0.6
0.8
1.0
Methylation Value

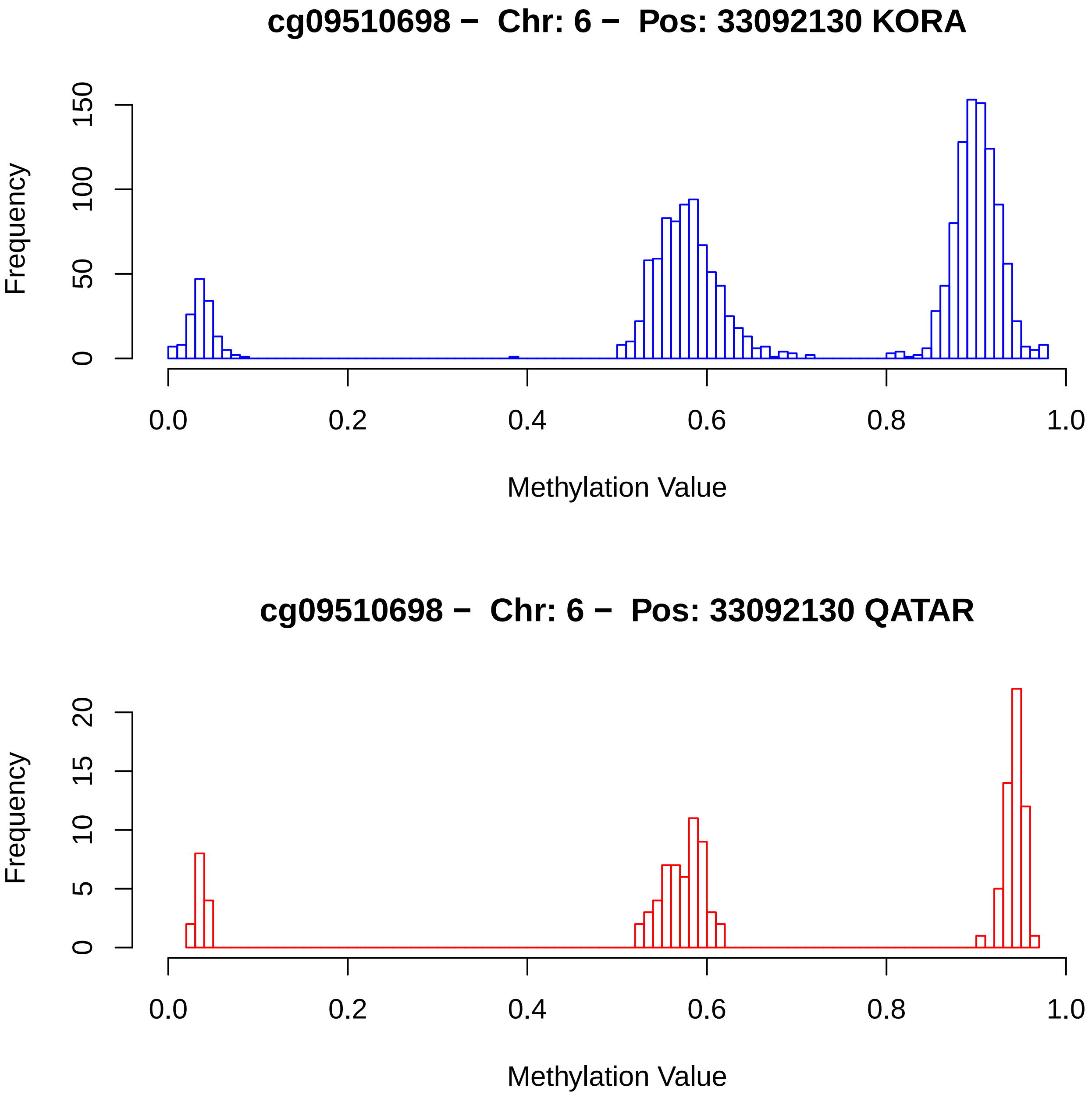
cg09510698 − Chr: 6 − Pos: 33092130 KORA
Frequency
0
50
100
150
0.0
0.2
0.4
0.6
0.8
1.0
Methylation Value
cg09510698 − Chr: 6 − Pos: 33092130 QATAR
Frequency
0
5
10
15
20
0.0
0.2
0.4
0.6
0.8
1.0
Methylation Value

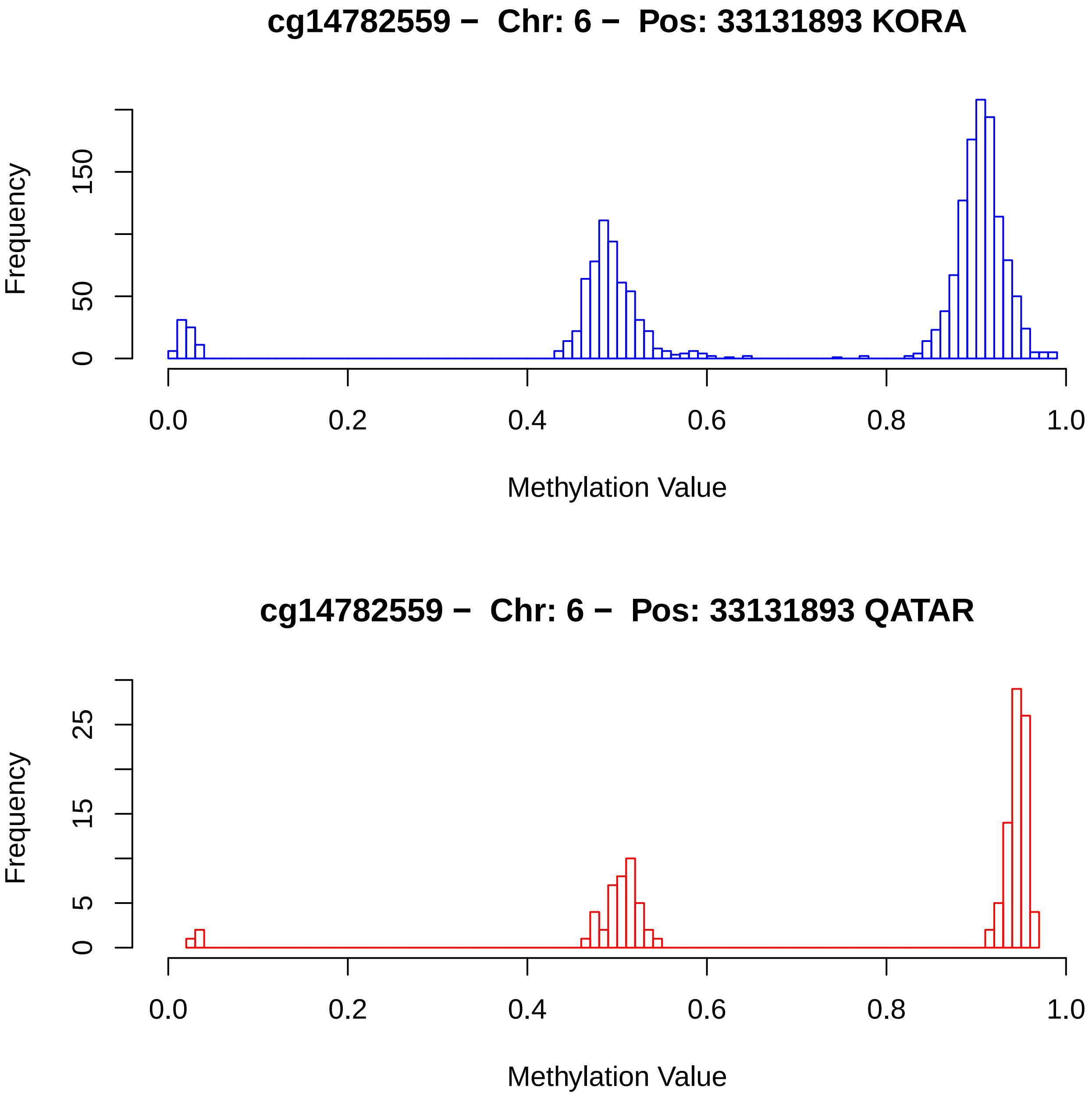

cg14782559 − Chr: 6 − Pos: 33131893 KORA
Frequency
0
50
150
0.0
0.2
0.4
0.6
0.8
1.0
Methylation Value
cg14782559 − Chr: 6 − Pos: 33131893 QATAR
Frequency
0
5
15
25
0.0
0.2
0.4
0.6
0.8
1.0
Methylation Value

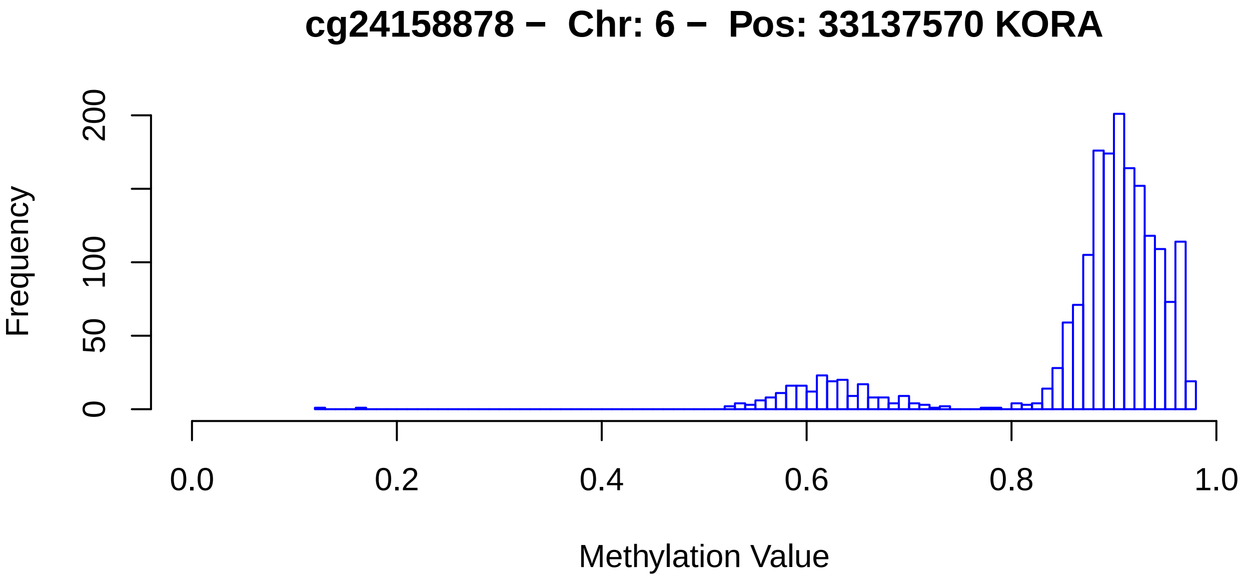
cg24158878 − Chr: 6 − Pos: 33137570 KORA
Frequency
0
50
100
200
0.0
0.2
0.4
0.6
0.8
1.0
Methylation Value

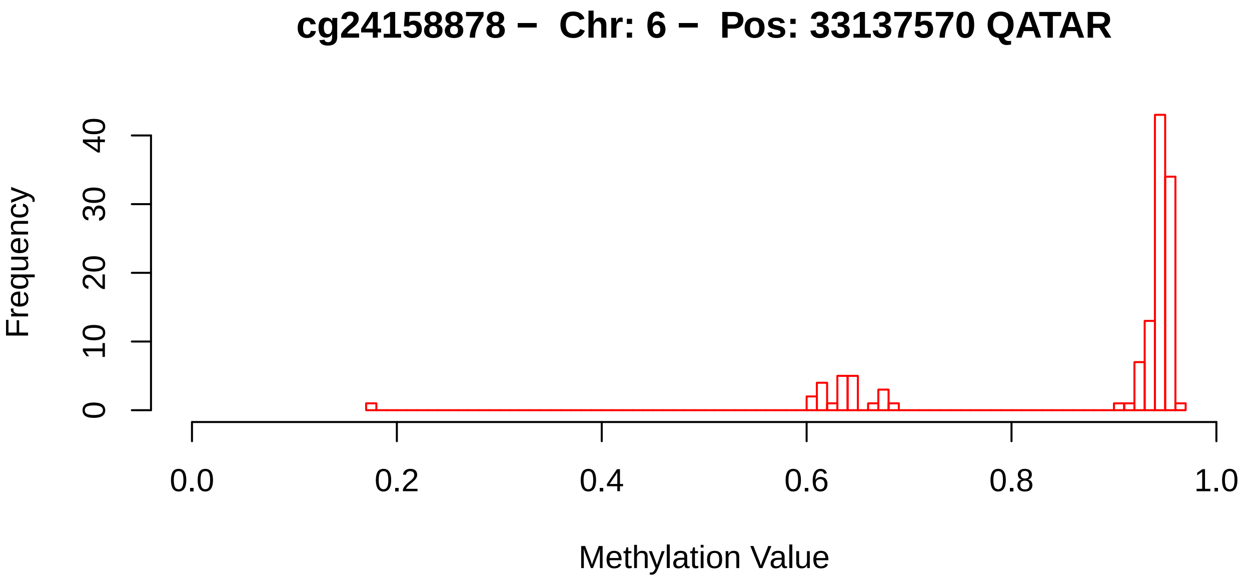
cg24158878 − Chr: 6 − Pos: 33137570 QATAR
Frequency
0
10
20
30
40
0.0
0.2
0.4
0.6
0.8
1.0
Methylation Value

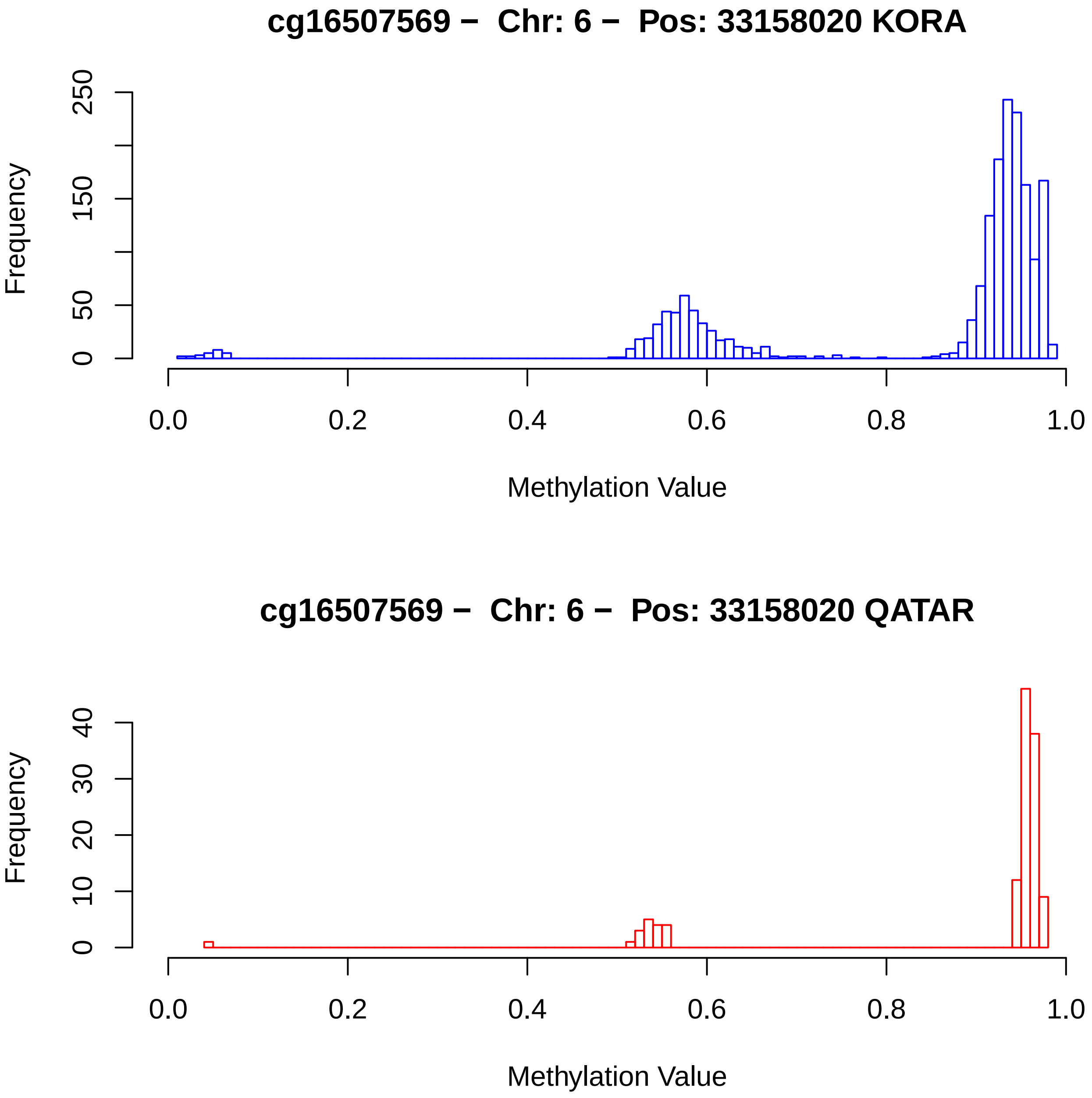

cg16507569 − Chr: 6 − Pos: 33158020 KORA
Frequency
0
50
150
250
0.0
0.2
0.4
0.6
0.8
1.0
Methylation Value
cg16507569 − Chr: 6 − Pos: 33158020 QATAR
Frequency
0
10
20
30
40
0.0
0.2
0.4
0.6
0.8
1.0
Methylation Value

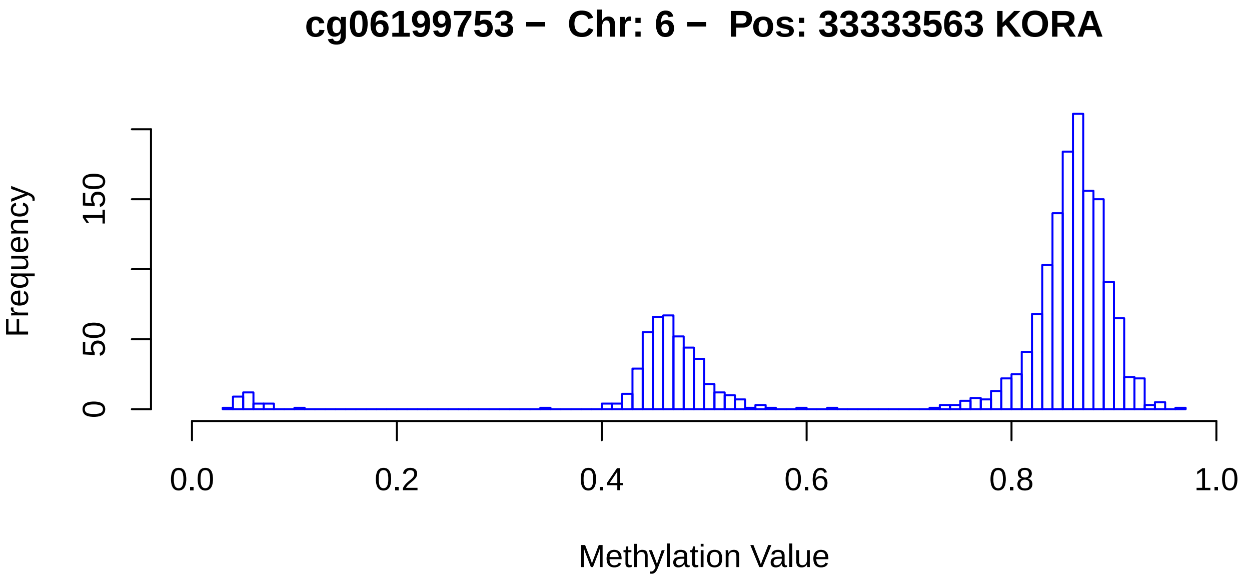
cg06199753 − Chr: 6 − Pos: 33333563 KORA
Frequency
0
50
150
0.0
0.2
0.4
0.6
0.8
1.0
Methylation Value

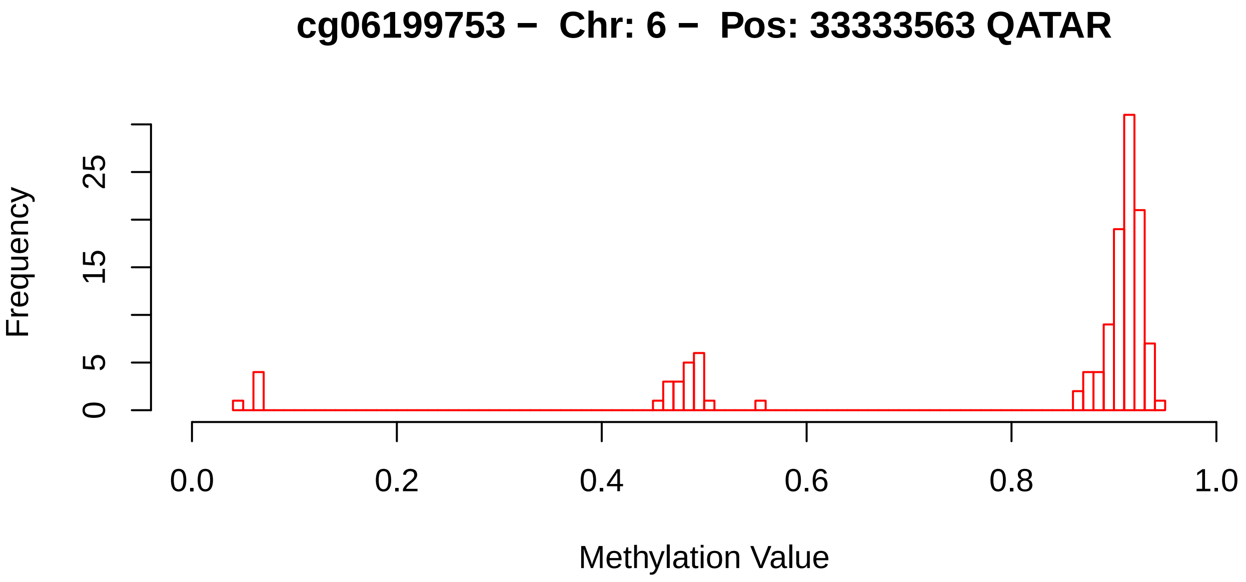
cg06199753 − Chr: 6 − Pos: 33333563 QATAR
Frequency
0
5
15
25
0.0
0.2
0.4
0.6
0.8
1.0
Methylation Value

cg16527629 − Chr: 6 − Pos: 34524698 KORA

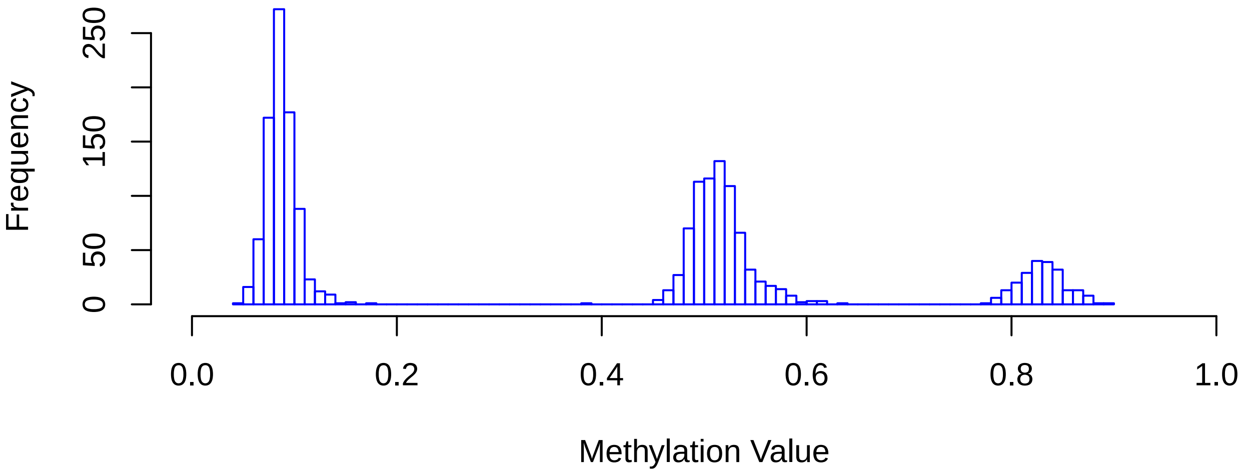

cg16527629 − Chr: 6 − Pos: 34524698 QATAR

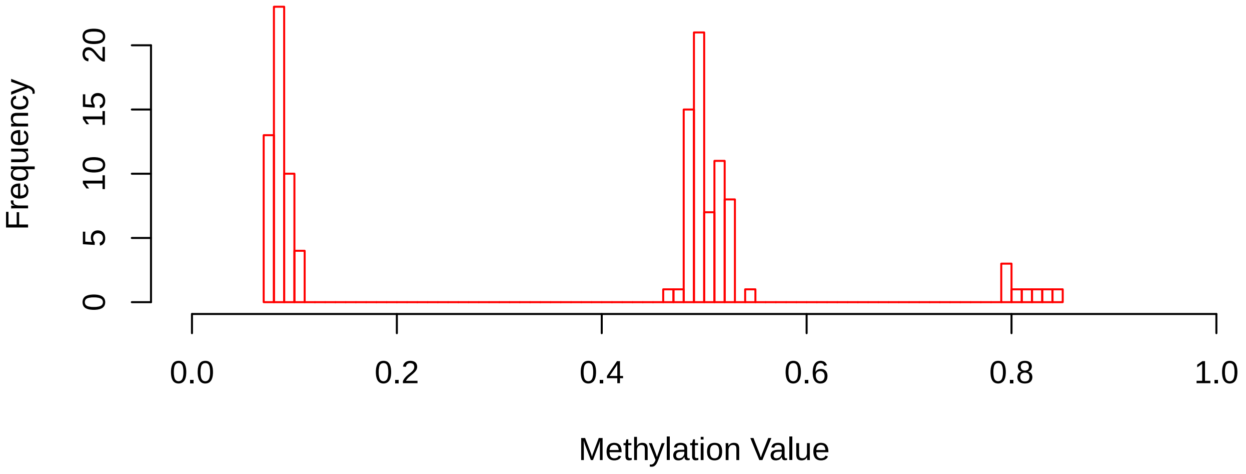

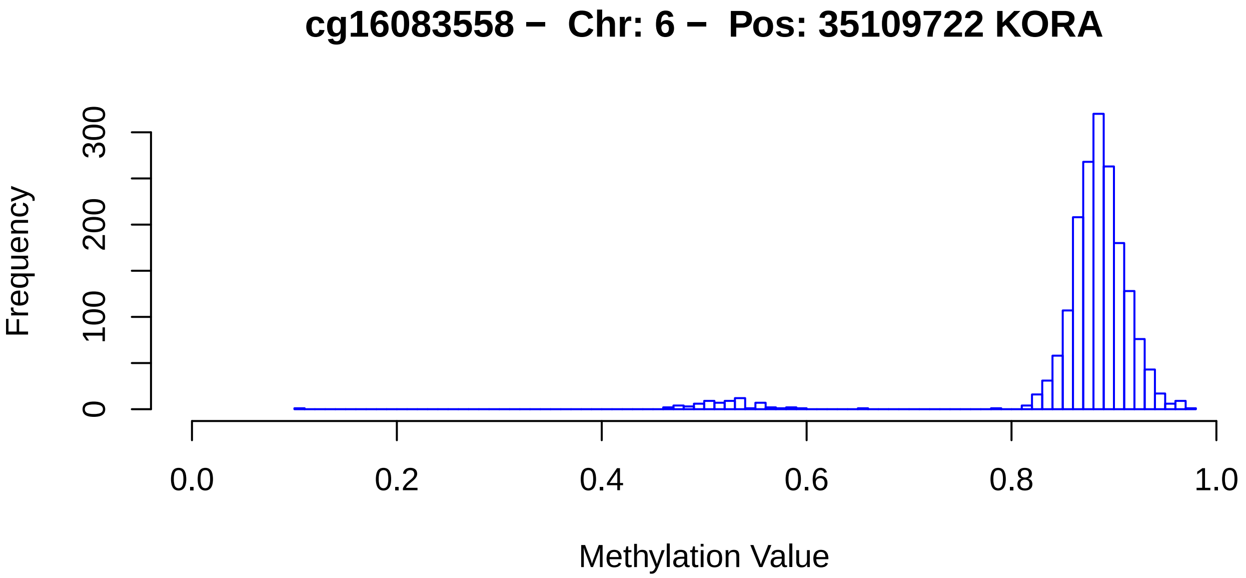
cg16083558 − Chr: 6 − Pos: 35109722 KORA
Frequency
0
100
200
300
0.0
0.2
0.4
0.6
0.8
1.0
Methylation Value

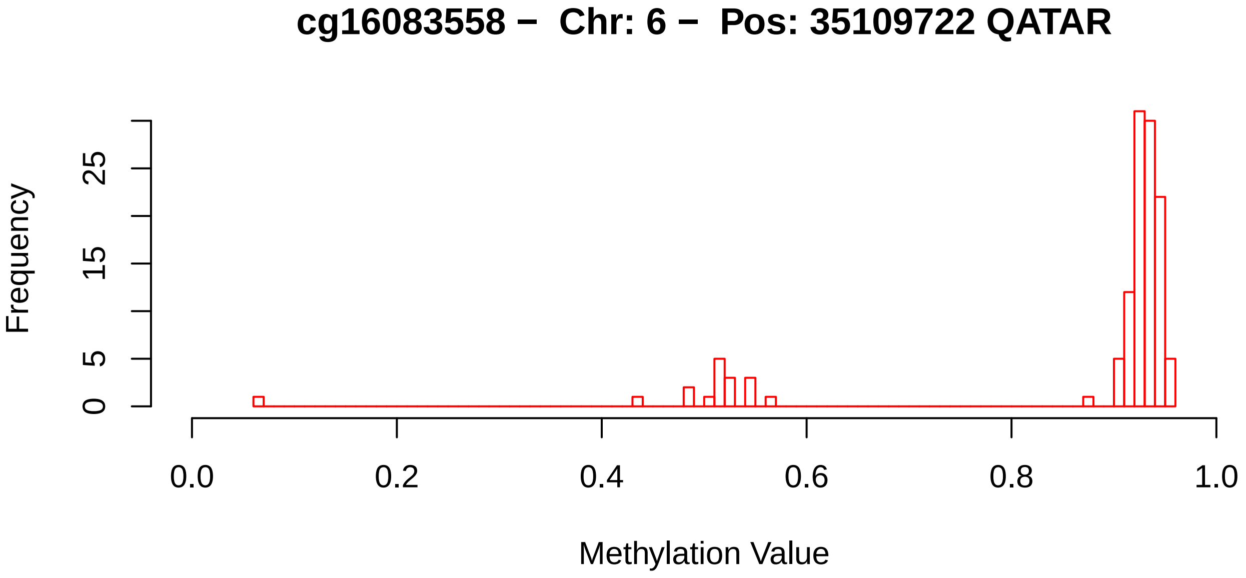
cg16083558 − Chr: 6 − Pos: 35109722 QATAR
Frequency
0
5
15
25
0.0
0.2
0.4
0.6
0.8
1.0
Methylation Value

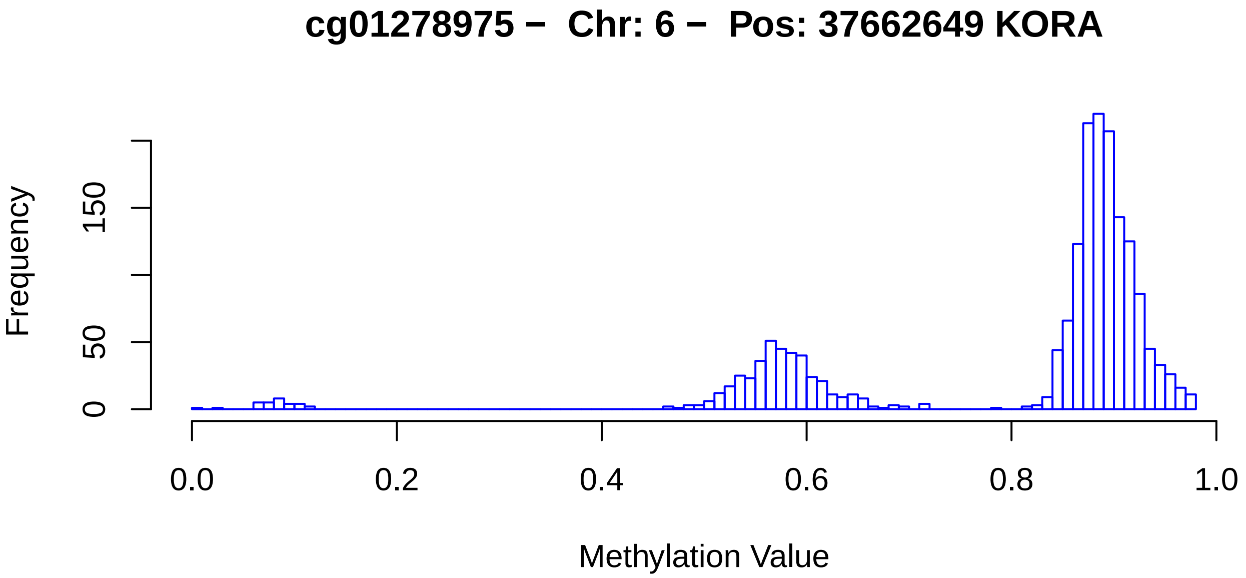
cg01278975 − Chr: 6 − Pos: 37662649 KORA
Frequency
0
50
150
0.0
0.2
0.4
0.6
0.8
1.0
Methylation Value

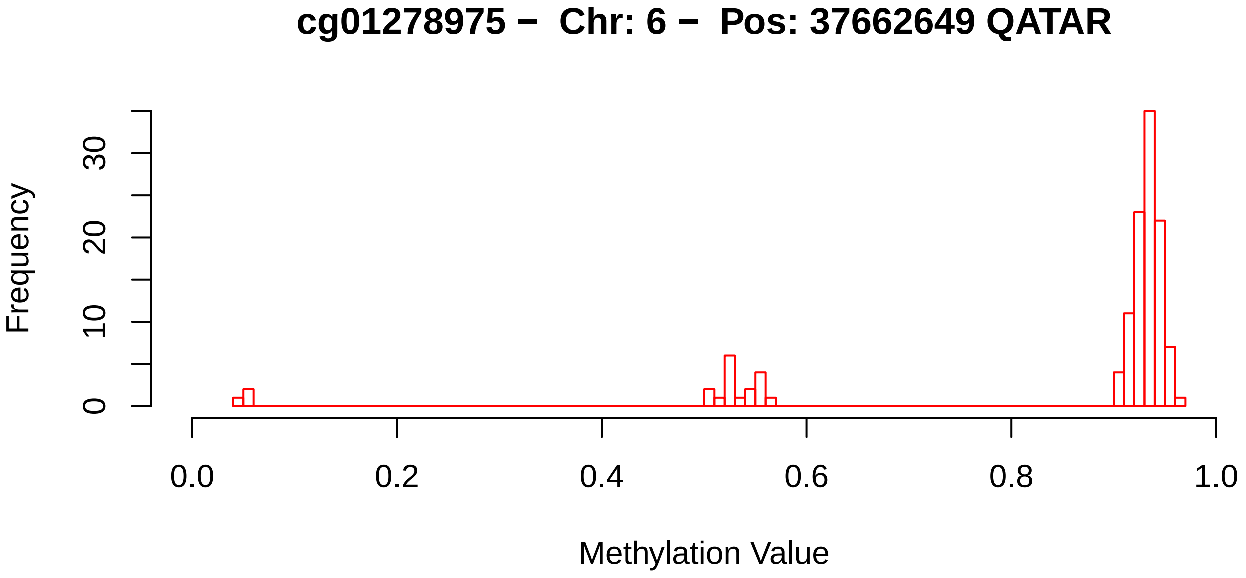
cg01278975 − Chr: 6 − Pos: 37662649 QATAR
Frequency
0
10
20
30
0.0
0.2
0.4
0.6
0.8
1.0
Methylation Value

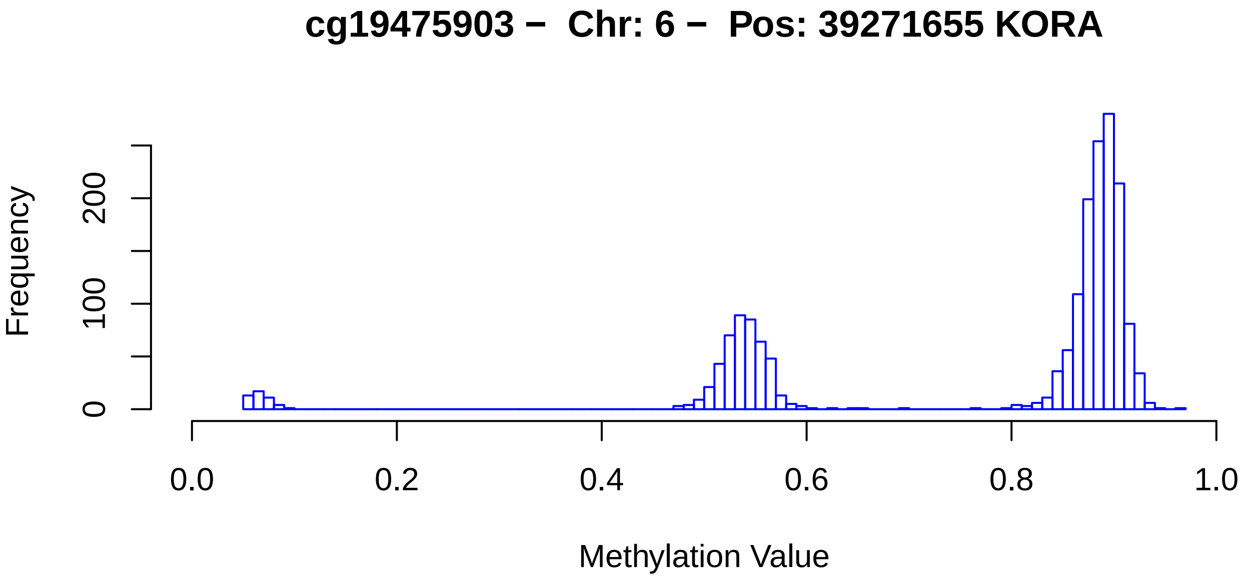
cg19475903 − Chr: 6 − Pos: 39271655 KORA
Frequency
0
100
200
0.0
0.2
0.4
0.6
0.8
1.0
Methylation Value

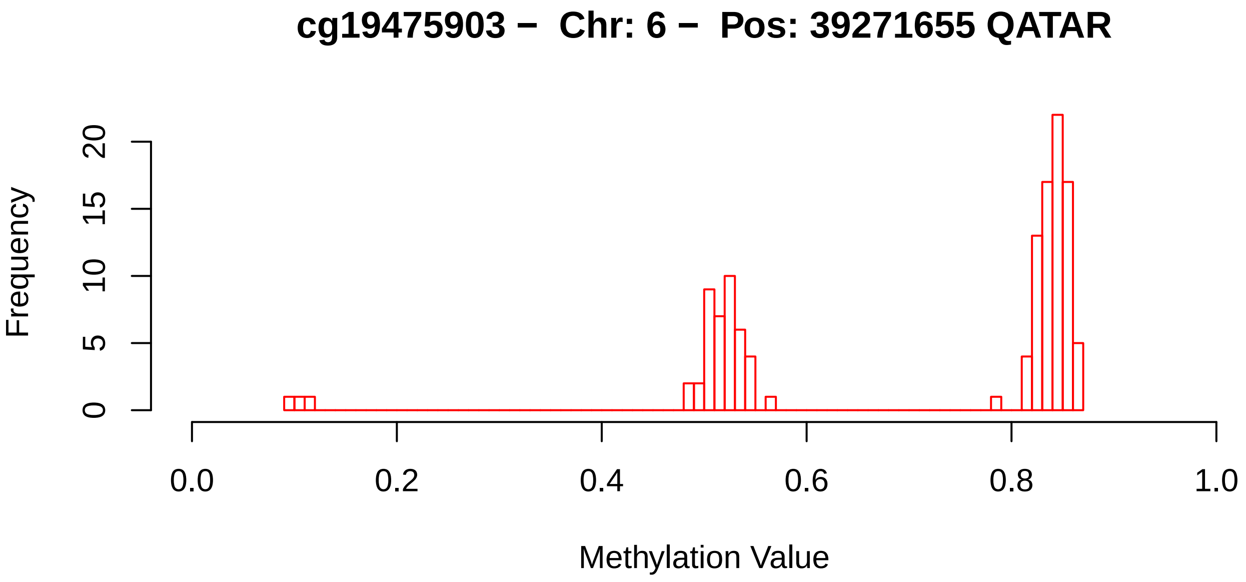
cg19475903 − Chr: 6 − Pos: 39271655 QATAR
Frequency
0
5
10
15
20
0.0
0.2
0.4
0.6
0.8
1.0
Methylation Value

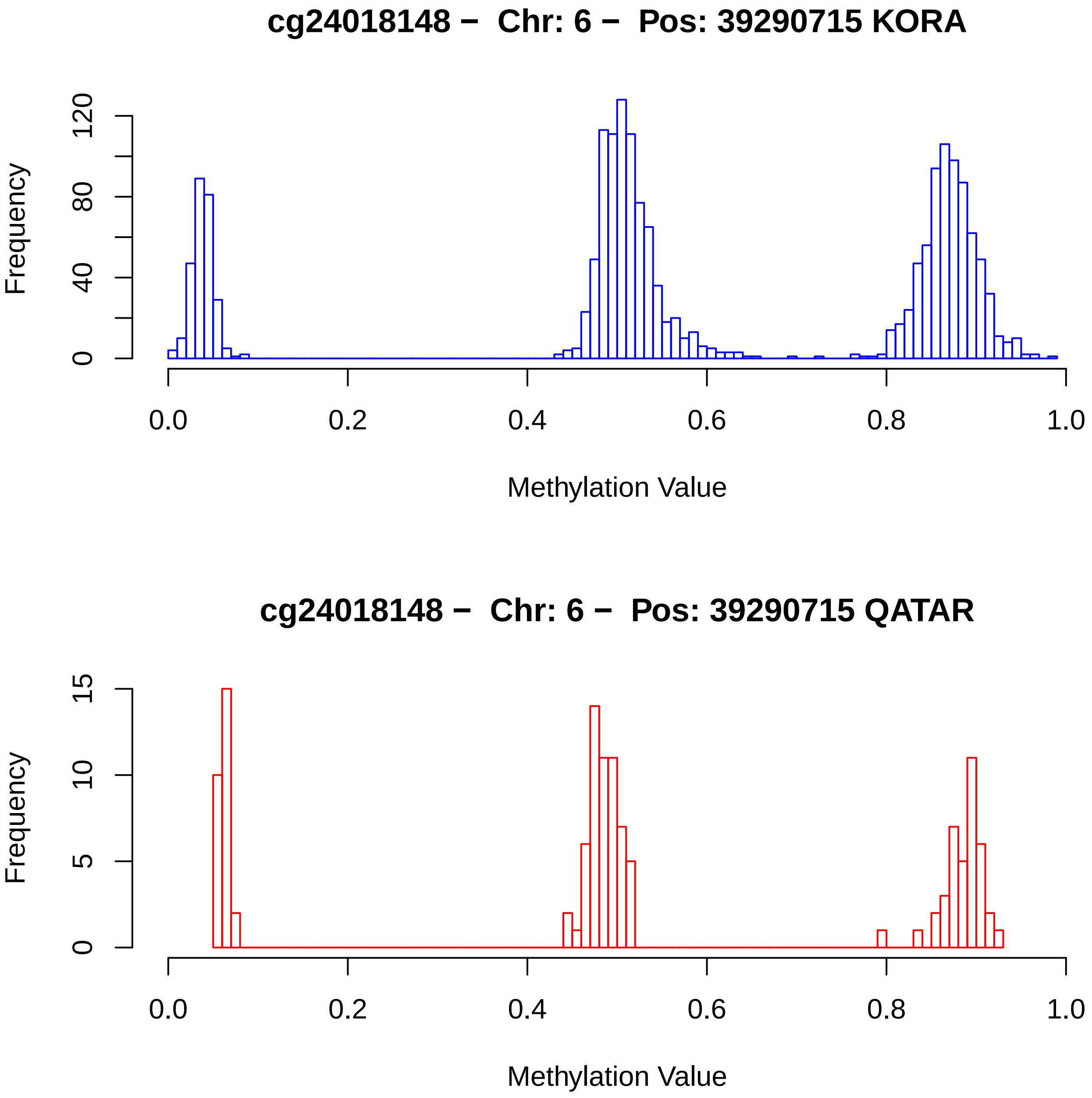

cg24018148 − Chr: 6 − Pos: 39290715 KORA
Frequency
0
40
80
120
0.0
0.2
0.4
0.6
0.8
1.0
Methylation Value
cg24018148 − Chr: 6 − Pos: 39290715 QATAR
Frequency
0
5
10
15
0.0
0.2
0.4
0.6
0.8
1.0
Methylation Value

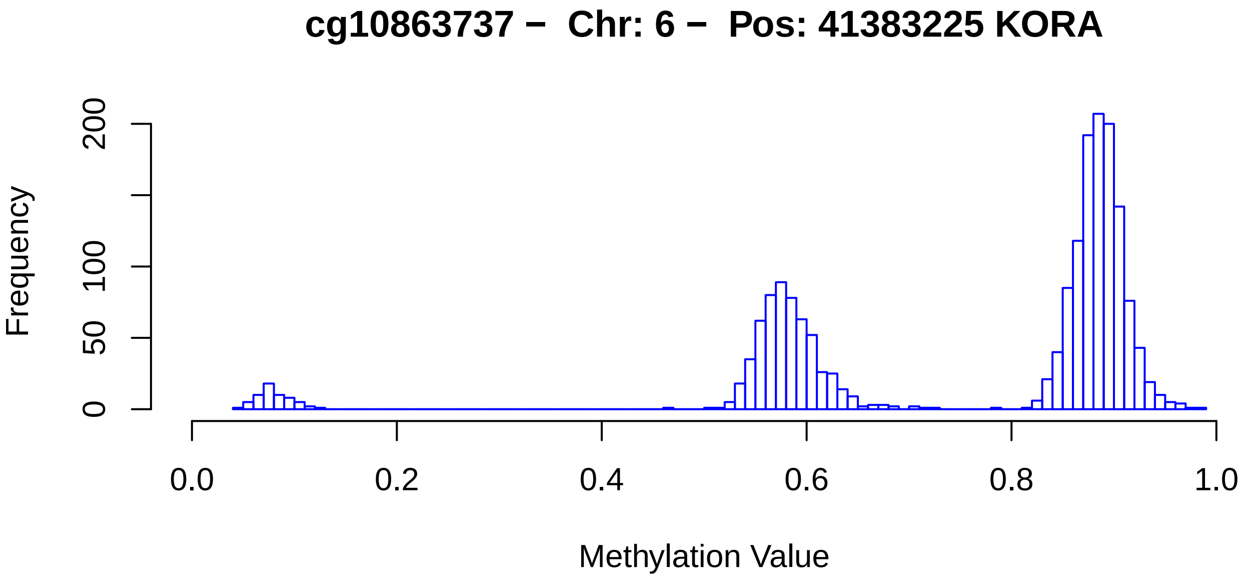
cg10863737 − Chr: 6 − Pos: 41383225 KORA
Frequency
0
50
100
200
0.0
0.2
0.4
0.6
0.8
1.0
Methylation Value

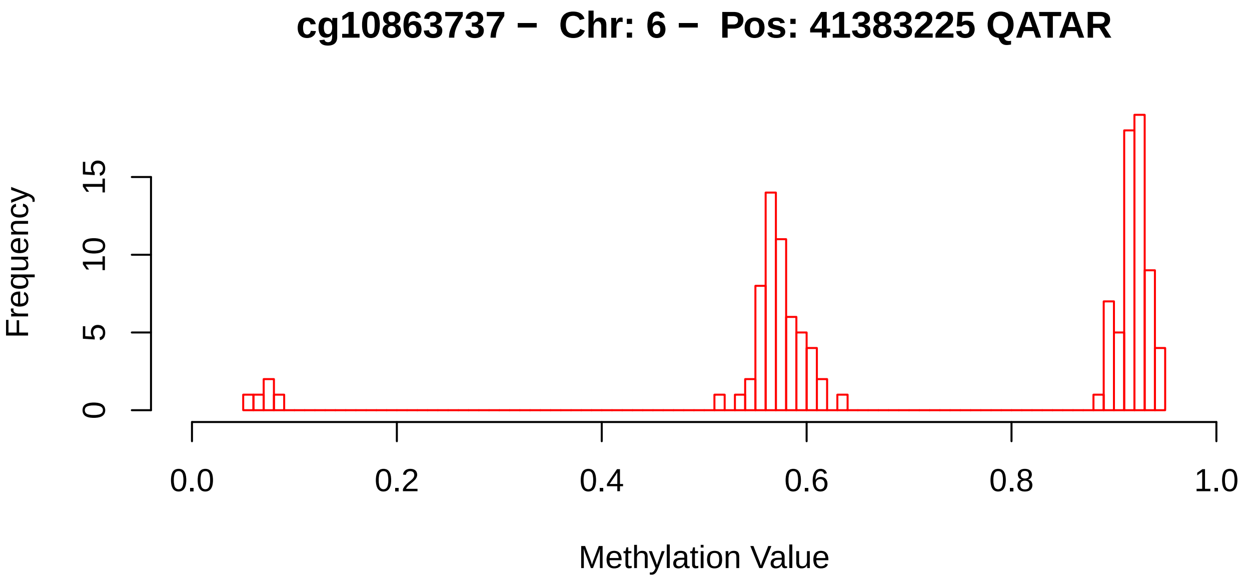
cg10863737 − Chr: 6 − Pos: 41383225 QATAR
Frequency
0
5
10
15
0.0
0.2
0.4
0.6
0.8
1.0
Methylation Value

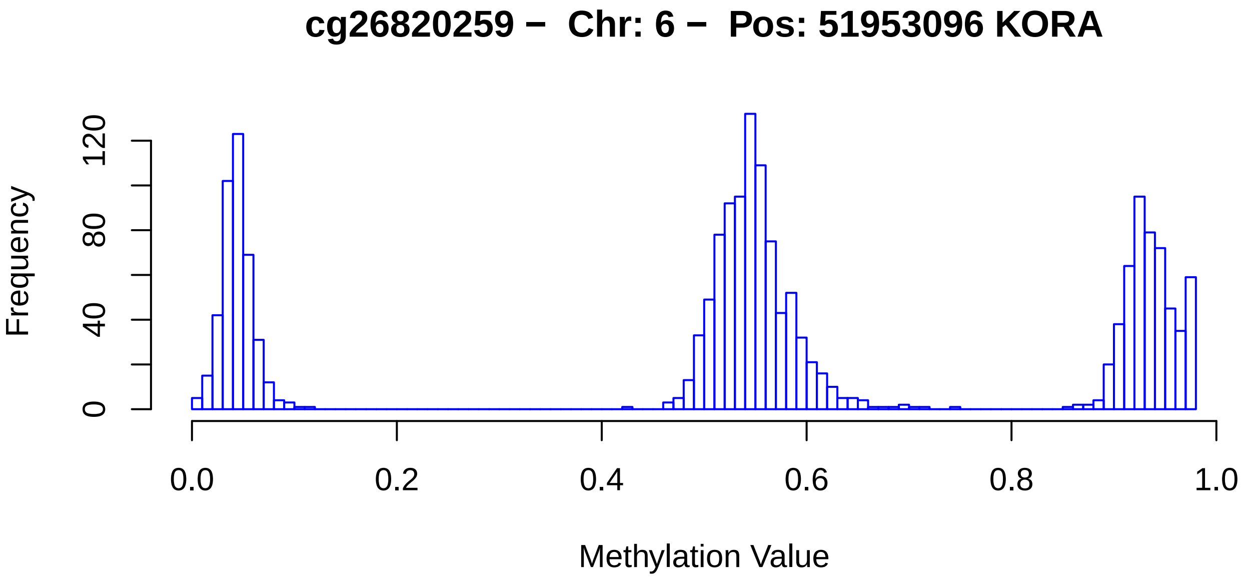
cg26820259 − Chr: 6 − Pos: 51953096 KORA
Frequency
0
40
80
120
0.0
0.2
0.4
0.6
0.8
1.0
Methylation Value

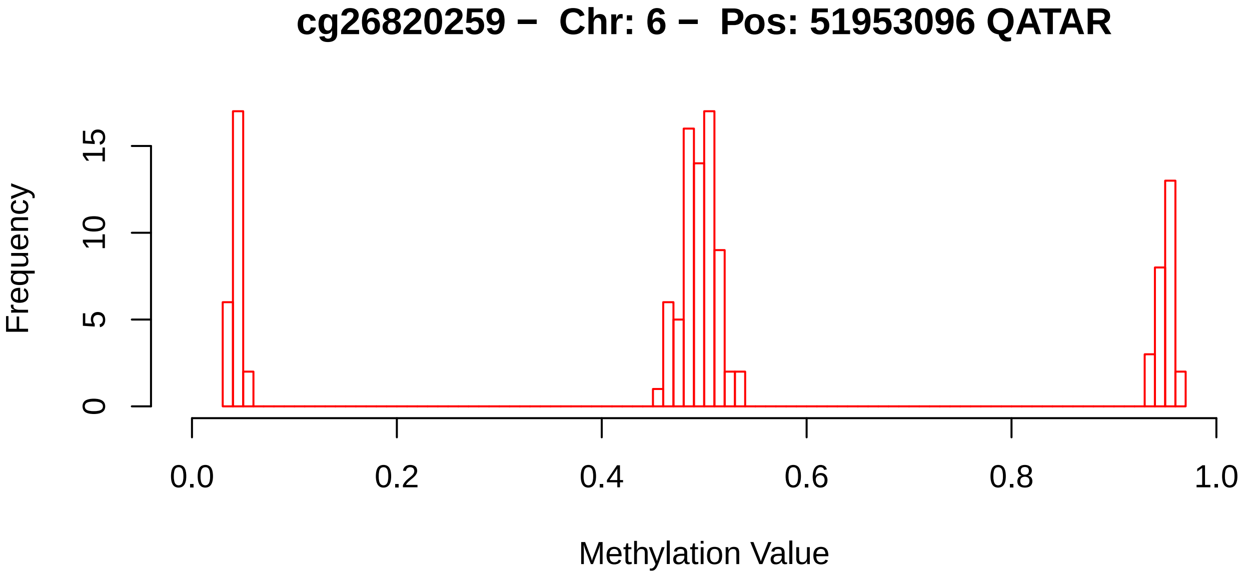
cg26820259 − Chr: 6 − Pos: 51953096 QATAR
Frequency
0
5
10
15
0.0
0.2
0.4
0.6
0.8
1.0
Methylation Value

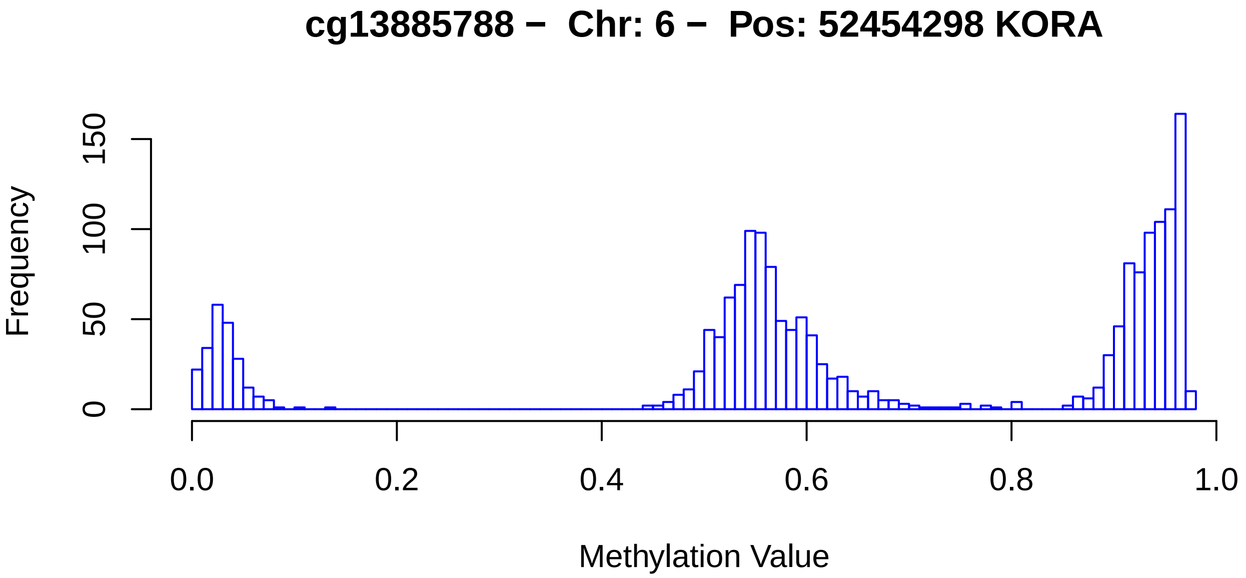
cg13885788 − Chr: 6 − Pos: 52454298 KORA
Frequency
0
50
100
150
0.0
0.2
0.4
0.6
0.8
1.0
Methylation Value
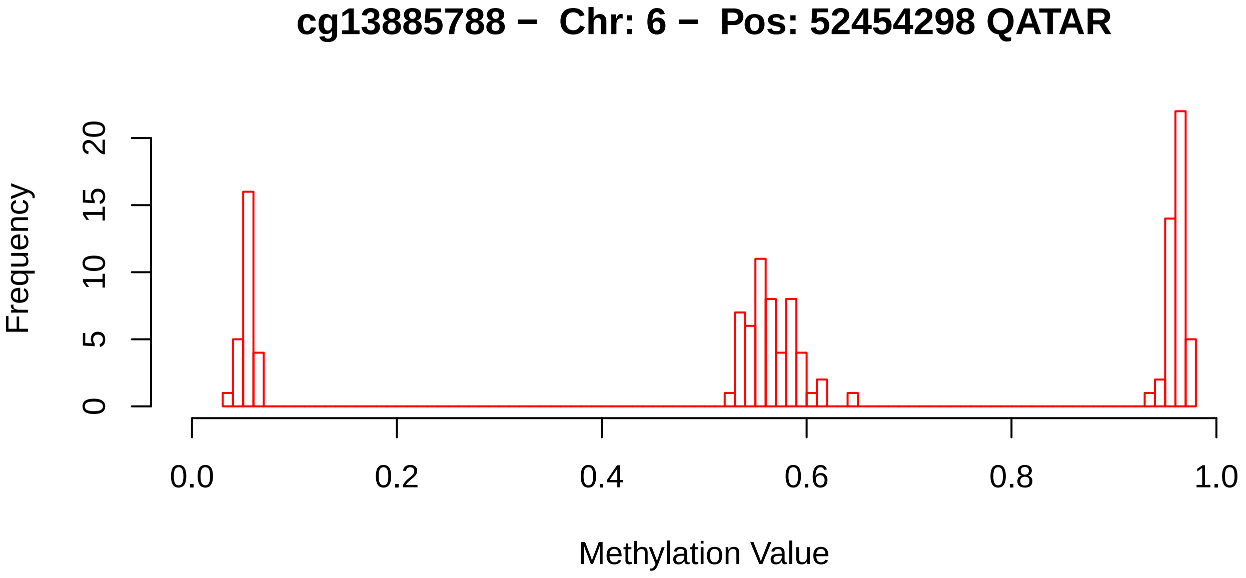
cg13885788 − Chr: 6 − Pos: 52454298 QATAR
Frequency
0
5
10
15
20
0.0
0.2
0.4
0.6
0.8
1.0
Methylation Value

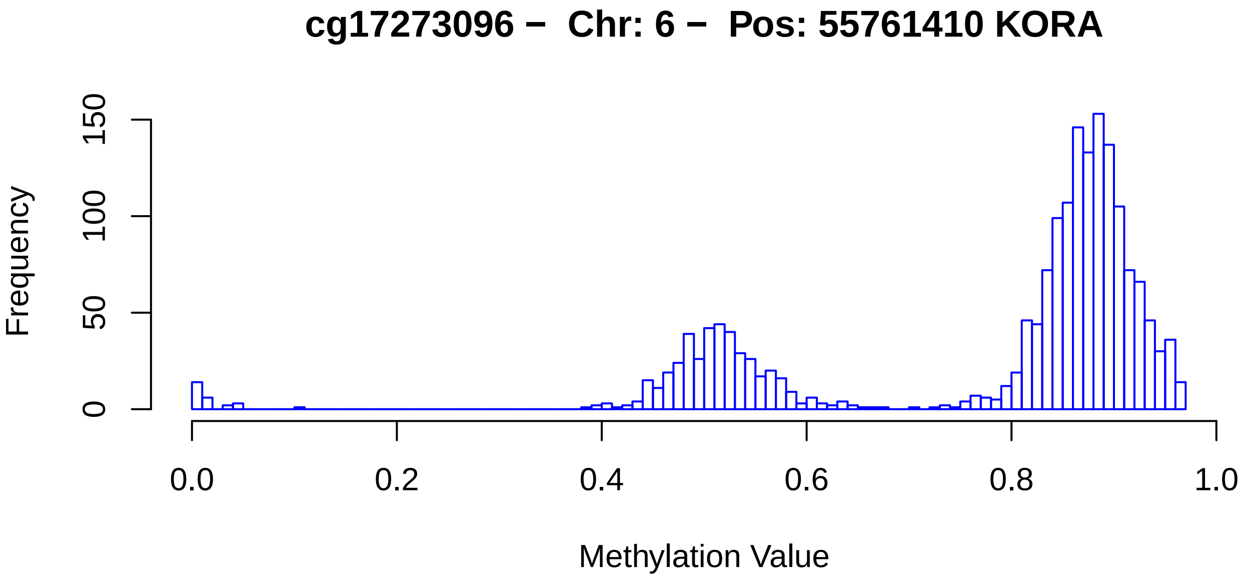
cg17273096 – Chr: 6 – Pos: 55761410 KORA
Frequency
0
50
100
150
0.0
0.2
0.4
0.6
0.8
1.0
Methylation Value

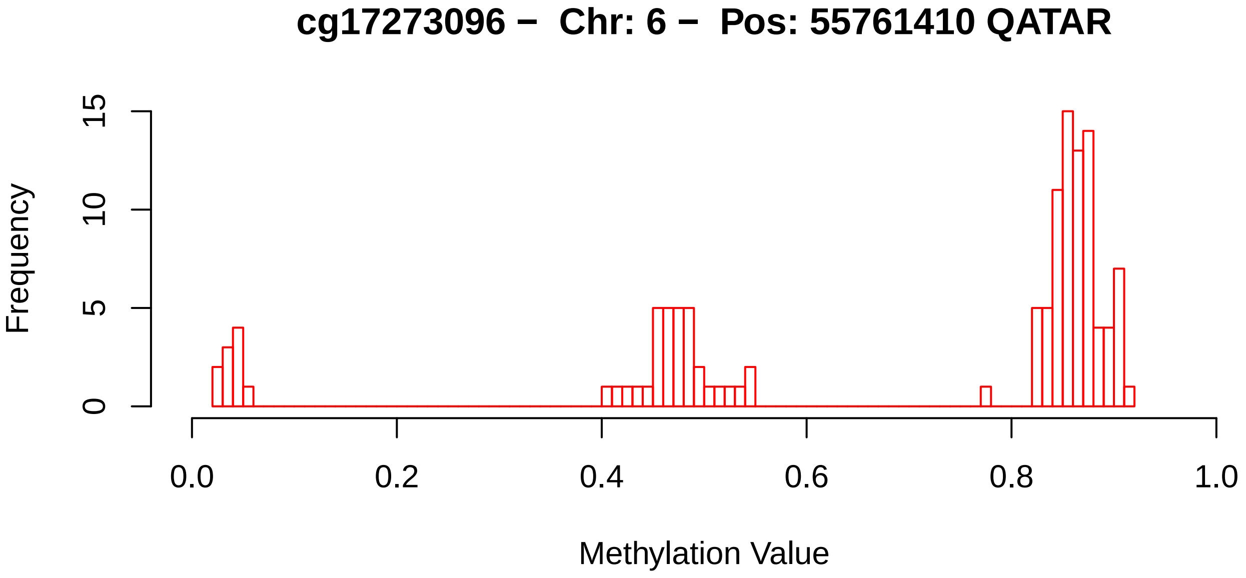
cg17273096 – Chr: 6 – Pos: 55761410 QATAR
Frequency
0
5
10
15
0.0
0.2
0.4
0.6
0.8
1.0
Methylation Value

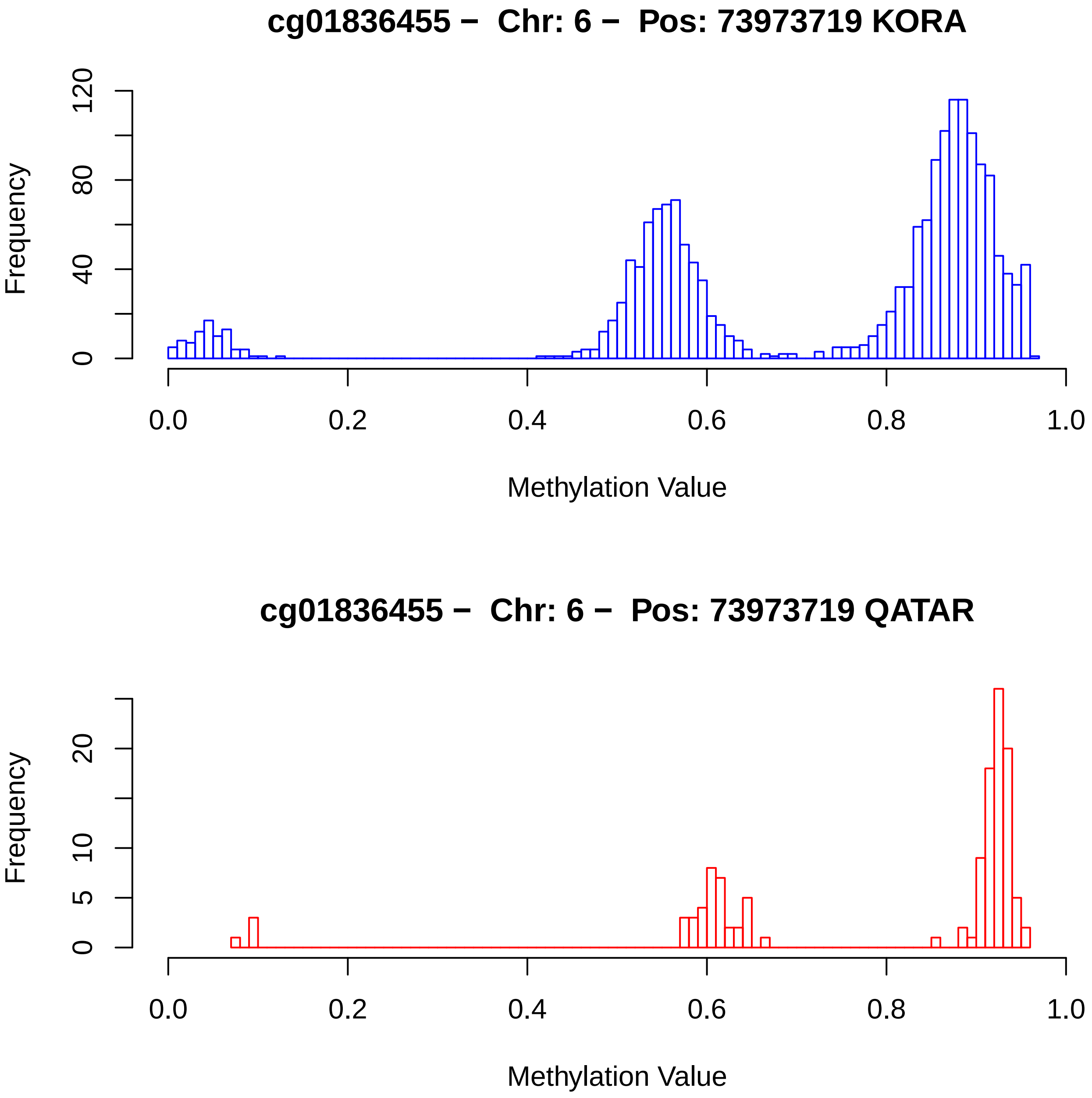
cg01836455 − Chr: 6 − Pos: 73973719 KORA
Frequency
0
40
80
120
0.0
0.2
0.4
0.6
0.8
1.0
Methylation Value
cg01836455 − Chr: 6 − Pos: 73973719 QATAR
Frequency
0
5
10
20
0.0
0.2
0.4
0.6
0.8
1.0
Methylation Value

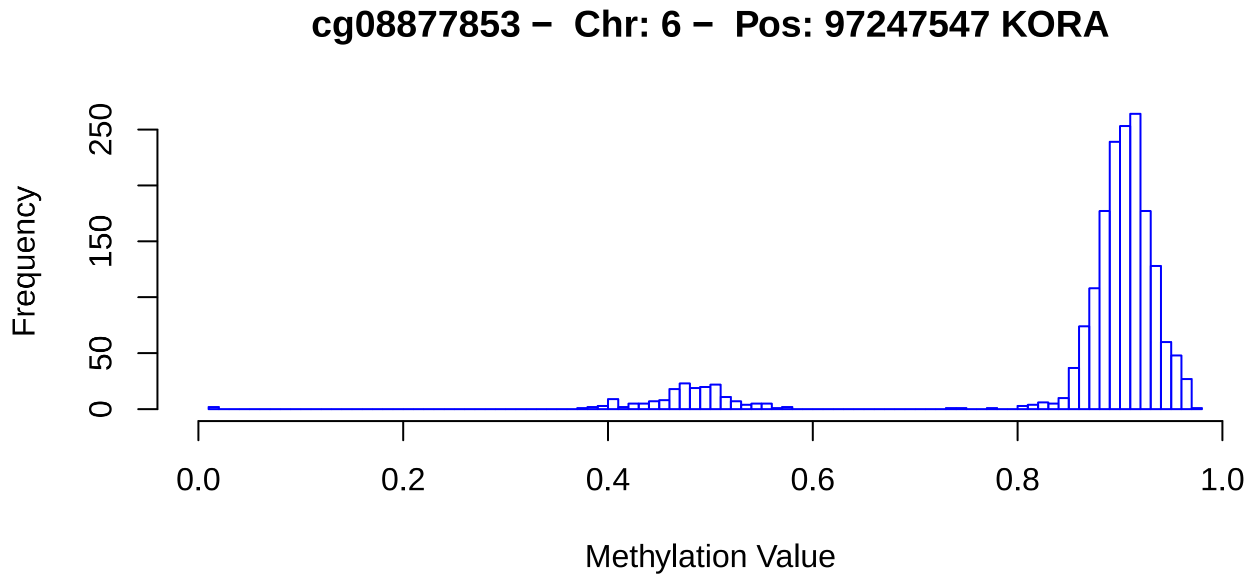
cg08877853 − Chr: 6 − Pos: 97247547 KORA
Frequency
0
50
150
250
0.0
0.2
0.4
0.6
0.8
1.0
Methylation Value

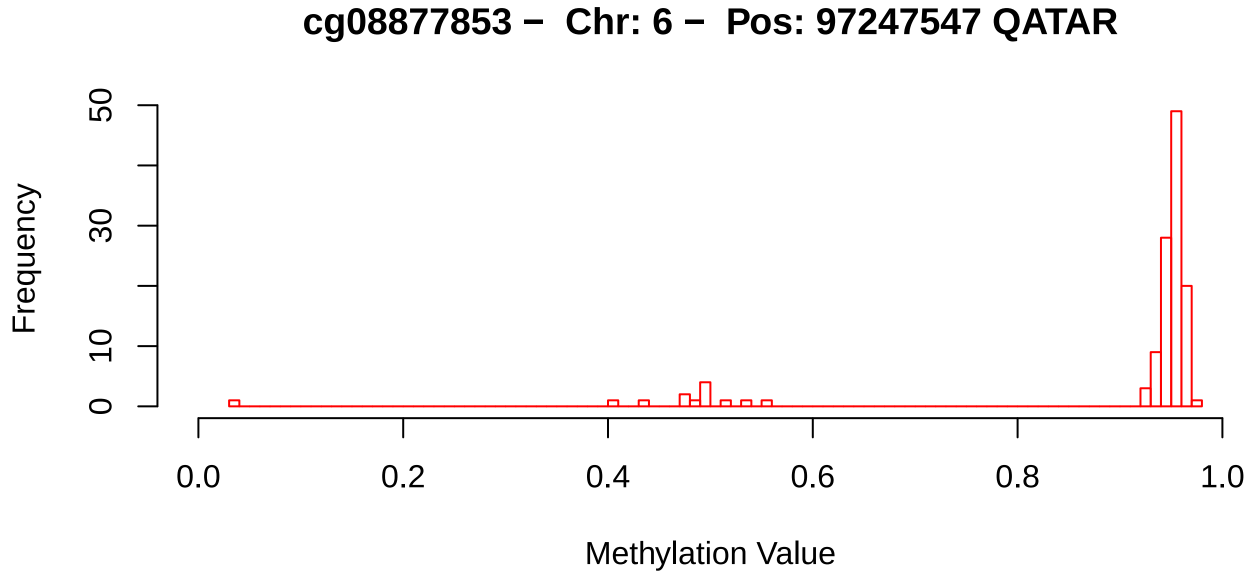
cg08877853 − Chr: 6 − Pos: 97247547 QATAR
Frequency
0
10
30
50
0.0
0.2
0.4
0.6
0.8
1.0
Methylation Value

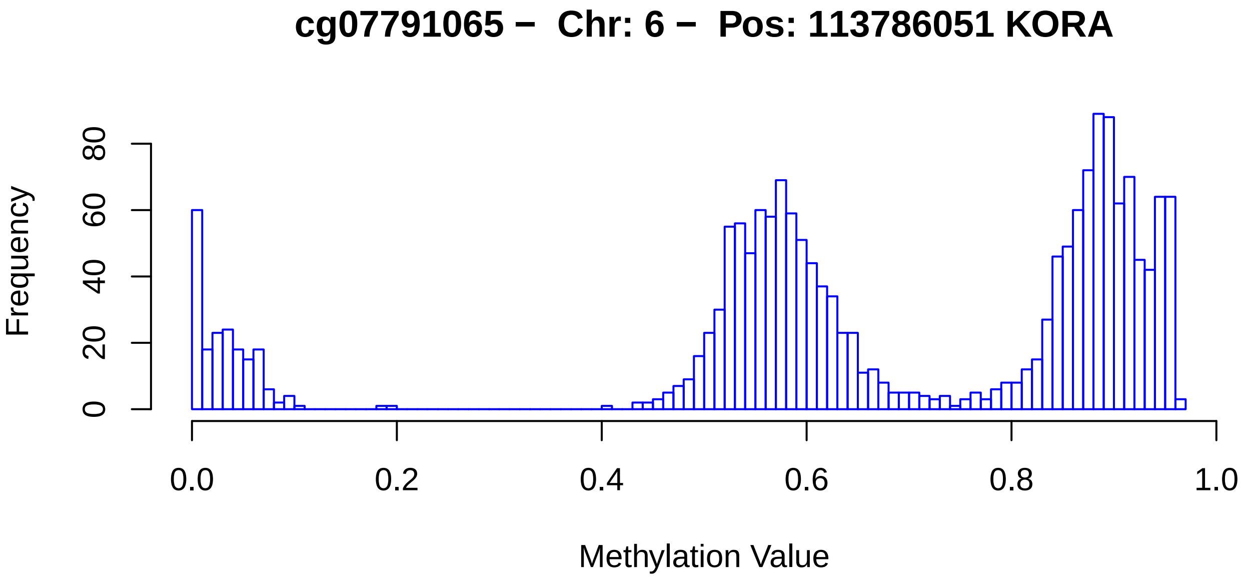
cg07791065 − Chr: 6 − Pos: 113786051 KORA
Frequency
0
20
40
60
80
0.0
0.2
0.4
0.6
0.8
1.0
Methylation Value

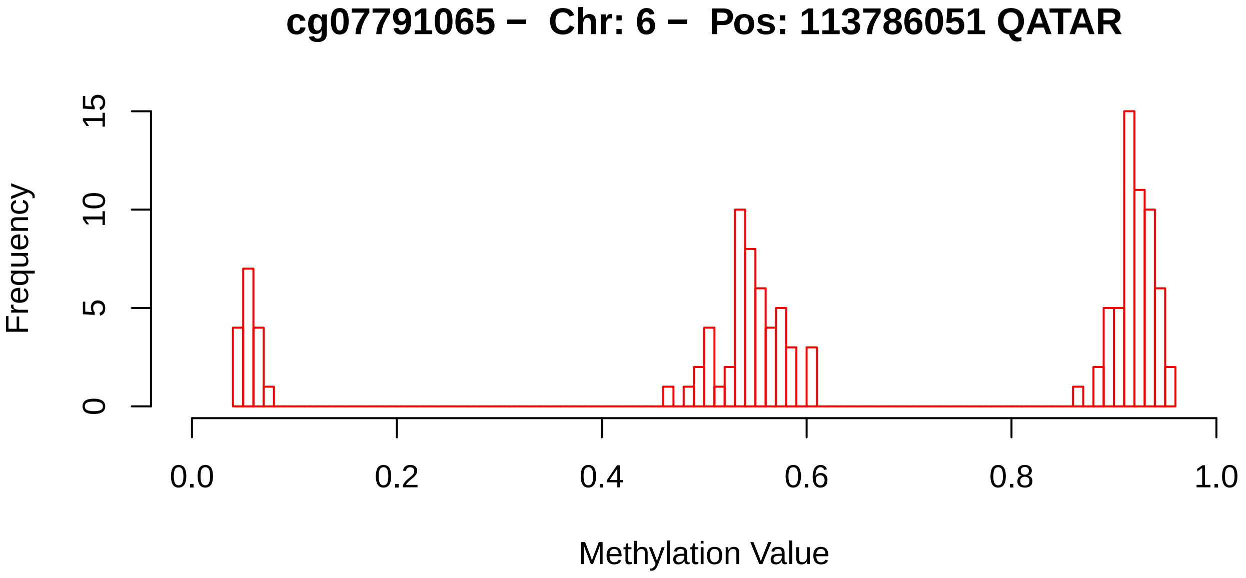
cg07791065 − Chr: 6 − Pos: 113786051 QATAR
Frequency
0
5
10
15
0.0
0.2
0.4
0.6
0.8
1.0
Methylation Value

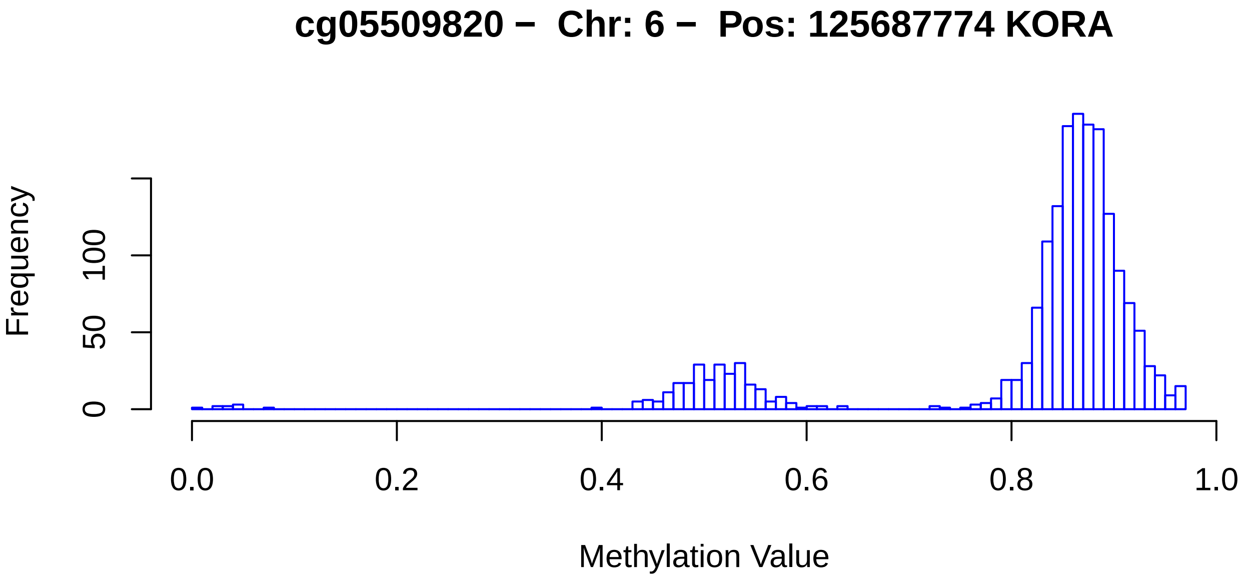
cg05509820 − Chr: 6 − Pos: 125687774 KORA
Frequency
0
50
100
0.0
0.2
0.4
0.6
0.8
1.0
Methylation Value

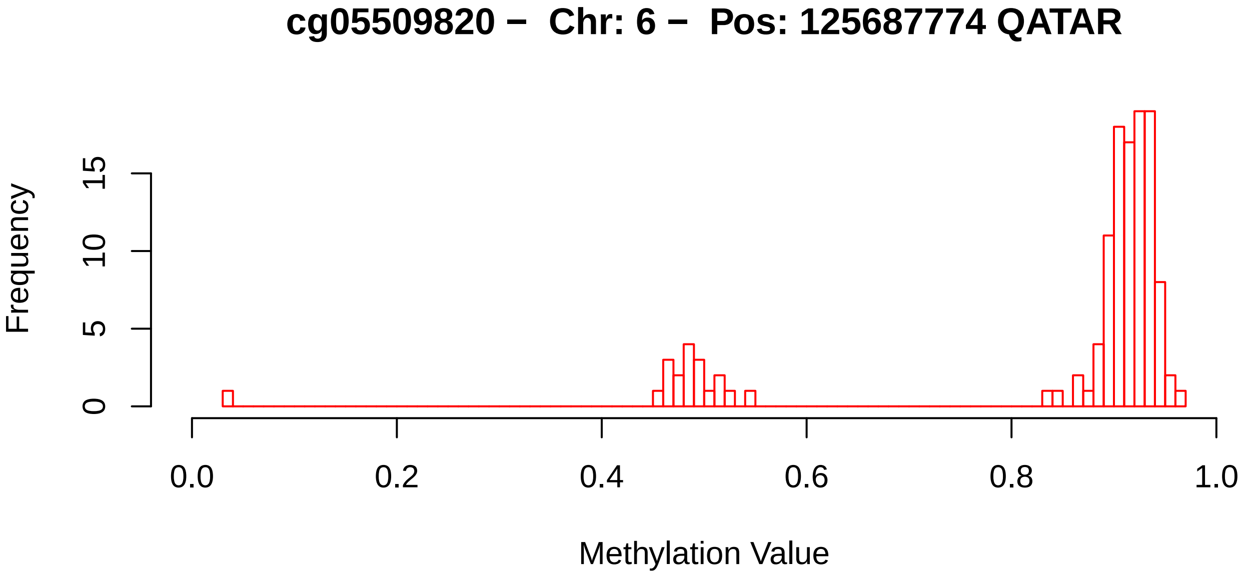
cg05509820 − Chr: 6 − Pos: 125687774 QATAR
Frequency
0
5
10
15
0.0
0.2
0.4
0.6
0.8
1.0
Methylation Value

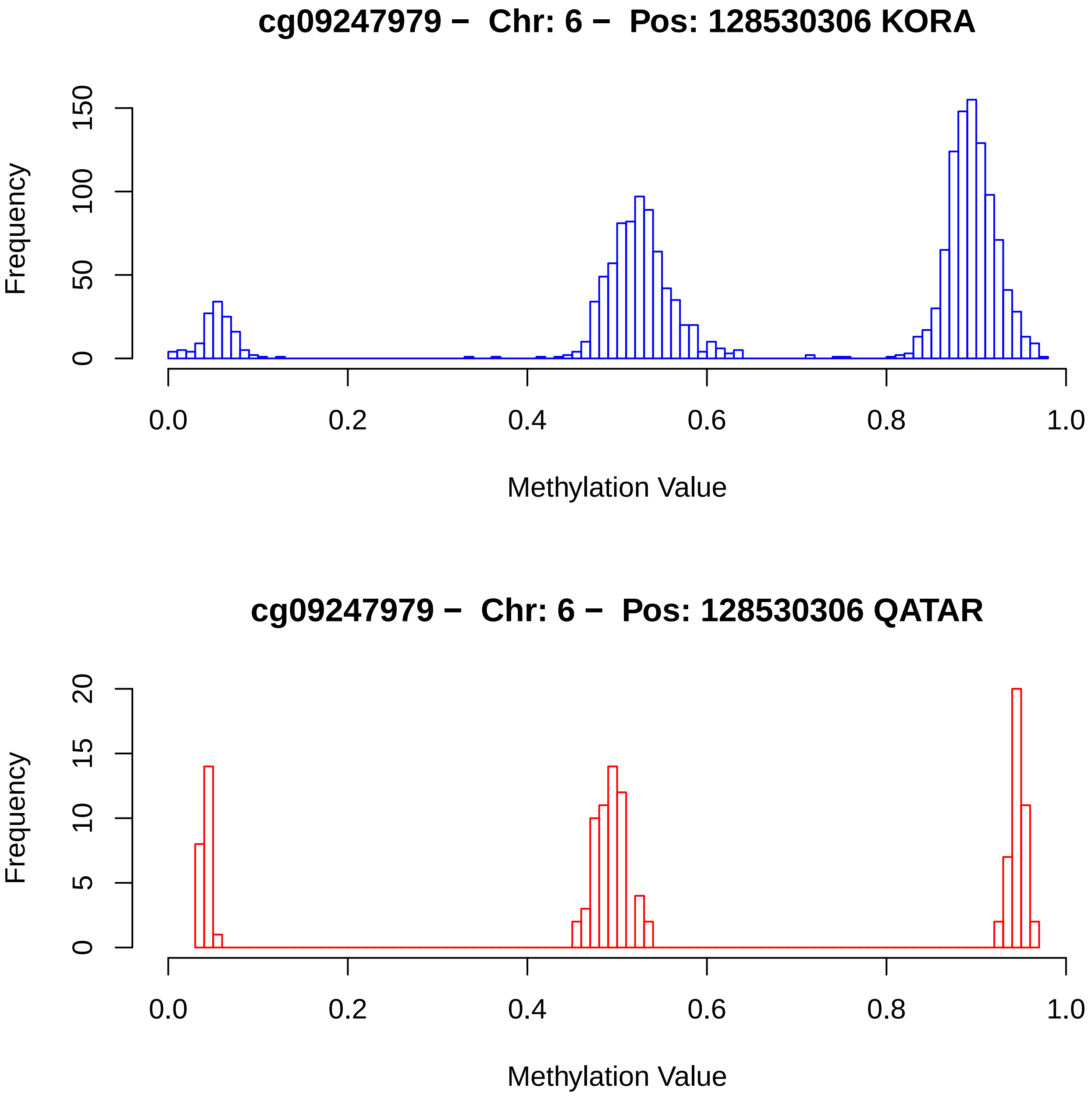

cg09247979 − Chr: 6 − Pos: 128530306 KORA
Frequency
0
50
100
150
0.0
0.2
0.4
0.6
0.8
1.0
Methylation Value
cg09247979 − Chr: 6 − Pos: 128530306 QATAR
Frequency
0
5
10
15
20
0.0
0.2
0.4
0.6
0.8
1.0
Methylation Value

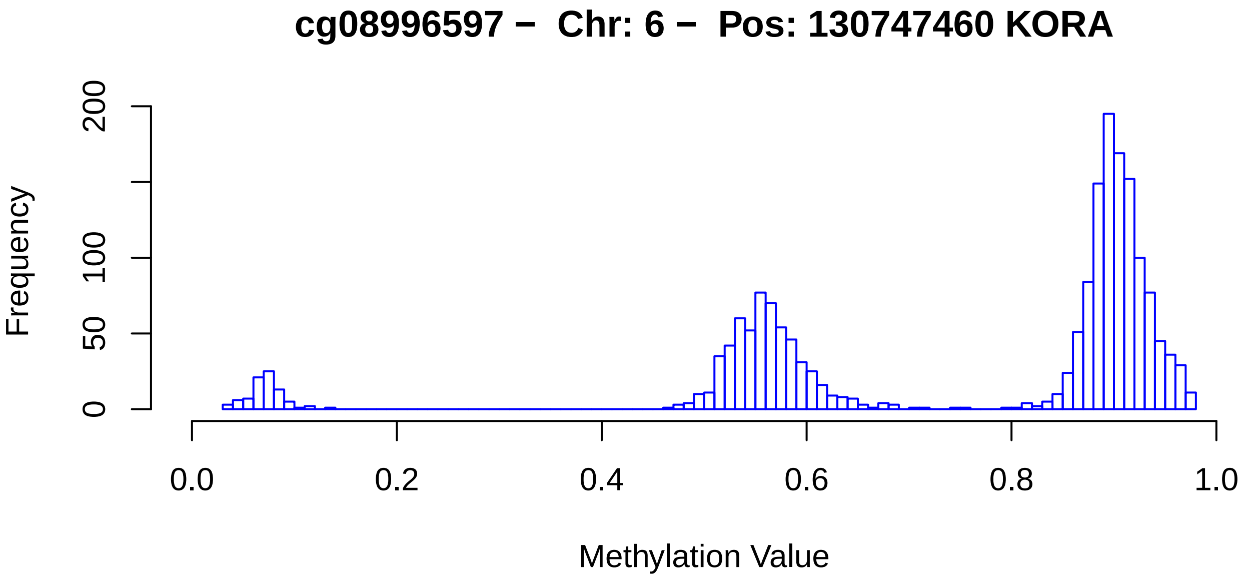
cg08996597 − Chr: 6 − Pos: 130747460 KORA
Frequency
0
50
100
200
0.0
0.2
0.4
0.6
0.8
1.0
Methylation Value

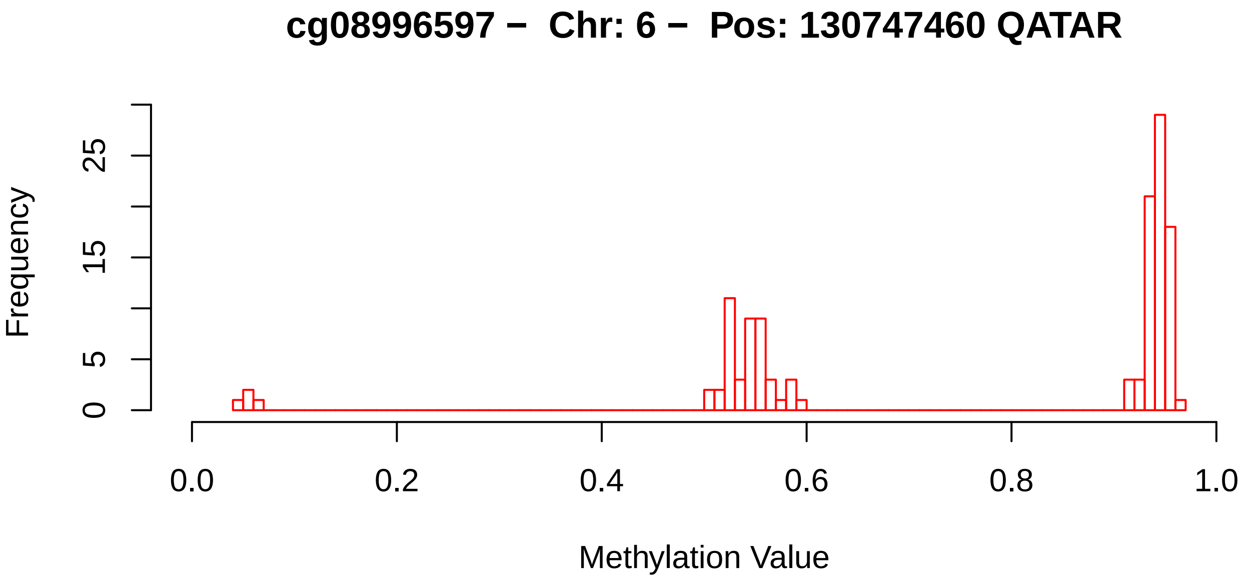
cg08996597 − Chr: 6 − Pos: 130747460 QATAR
Frequency
0
5
15
25
0.0
0.2
0.4
0.6
0.8
1.0
Methylation Value

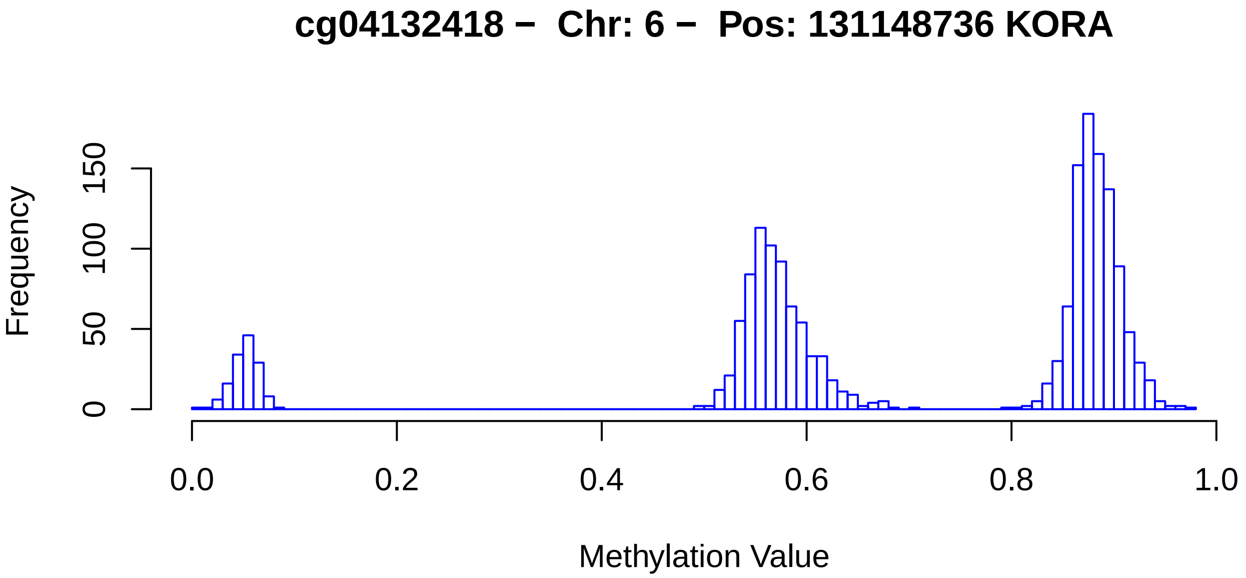
cg04132418 − Chr: 6 − Pos: 131148736 KORA
Frequency
0
50
100
150
0.0
0.2
0.4
0.6
0.8
1.0
Methylation Value

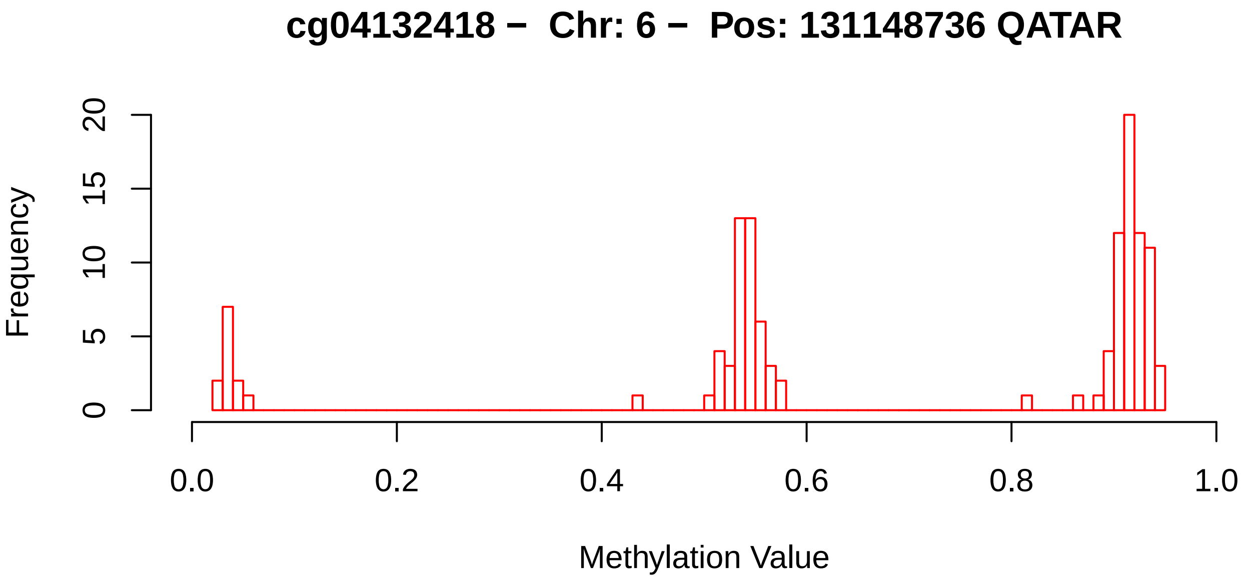
cg04132418 − Chr: 6 − Pos: 131148736 QATAR
Frequency
0
5
10
15
20
0.0
0.2
0.4
0.6
0.8
1.0
Methylation Value

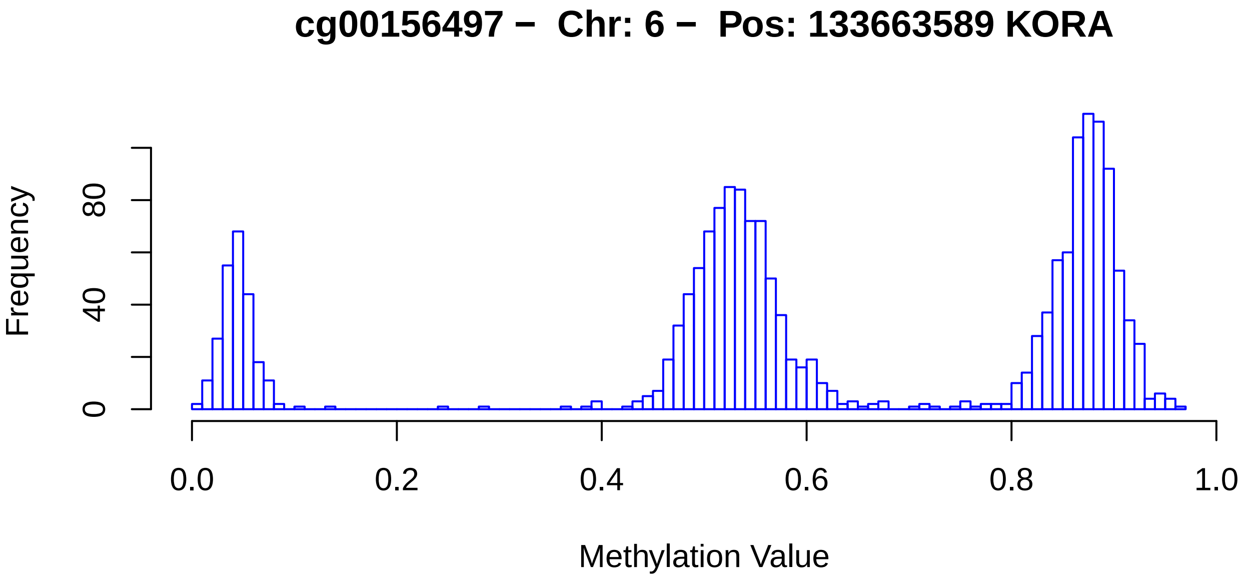
cg00156497 − Chr: 6 − Pos: 133663589 KORA
Frequency
0
40
80
0.0
0.2
0.4
0.6
0.8
1.0
Methylation Value

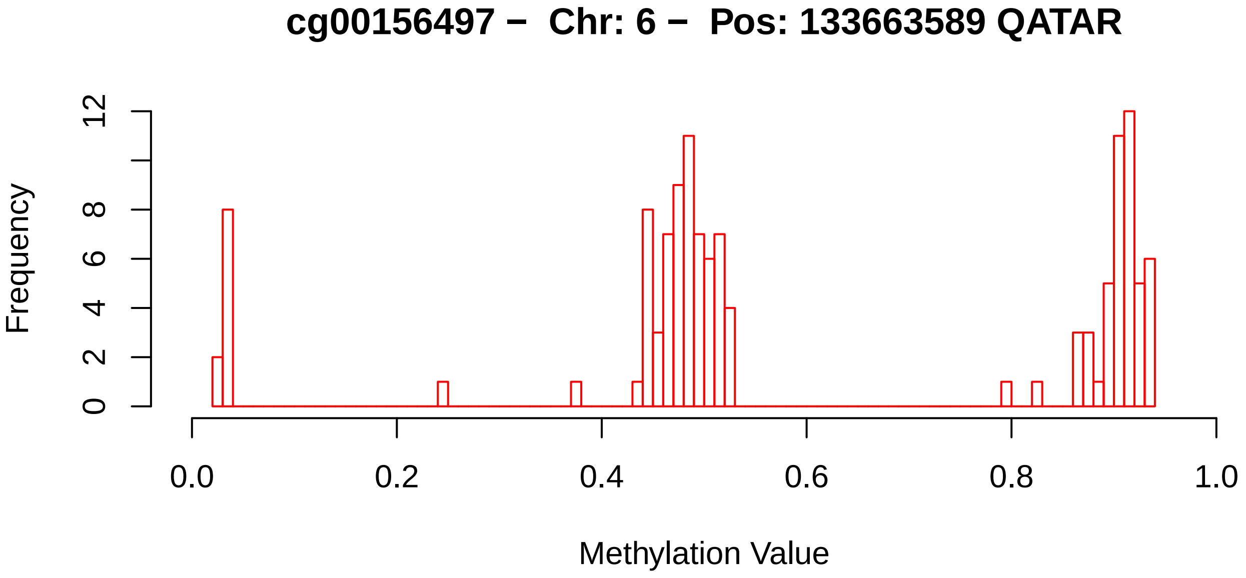
cg00156497 − Chr: 6 − Pos: 133663589 QATAR
Frequency
0
2
4
6
8
12
0.0
0.2
0.4
0.6
0.8
1.0
Methylation Value

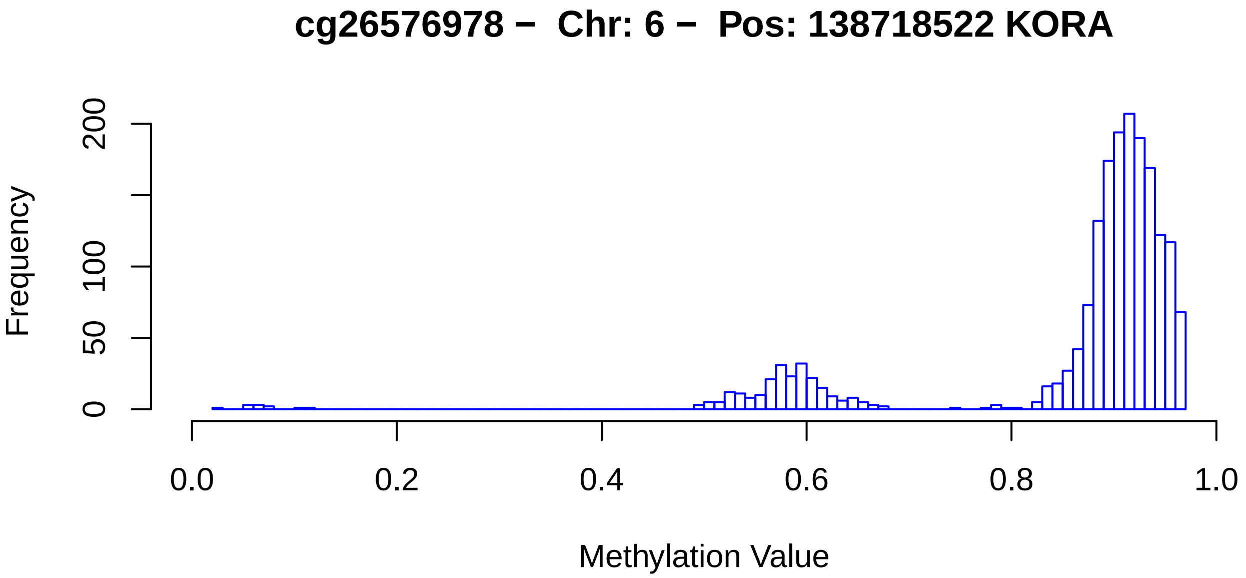

cg26576978 − Chr: 6 − Pos: 138718522 KORA
Frequency
0
50
100
200
0.0
0.2
0.4
0.6
0.8
1.0
Methylation Value

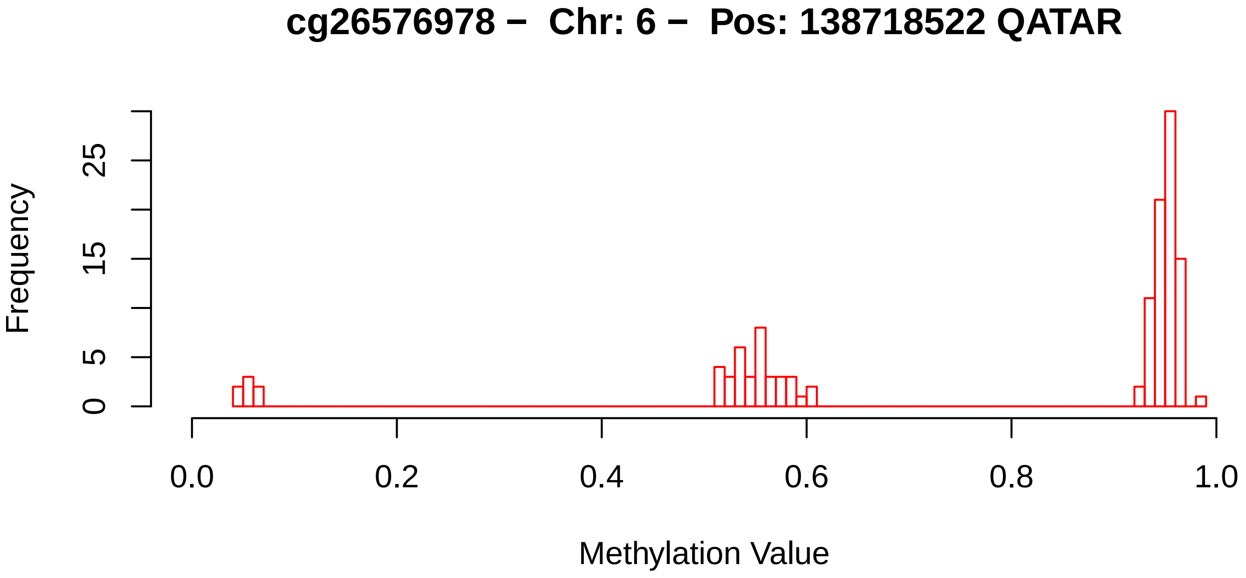

cg26576978 − Chr: 6 − Pos: 138718522 QATAR
Frequency
0
5
15
25
0.0
0.2
0.4
0.6
0.8
1.0
Methylation Value

cg15029183 − Chr: 6 − Pos: 143586006 KORA

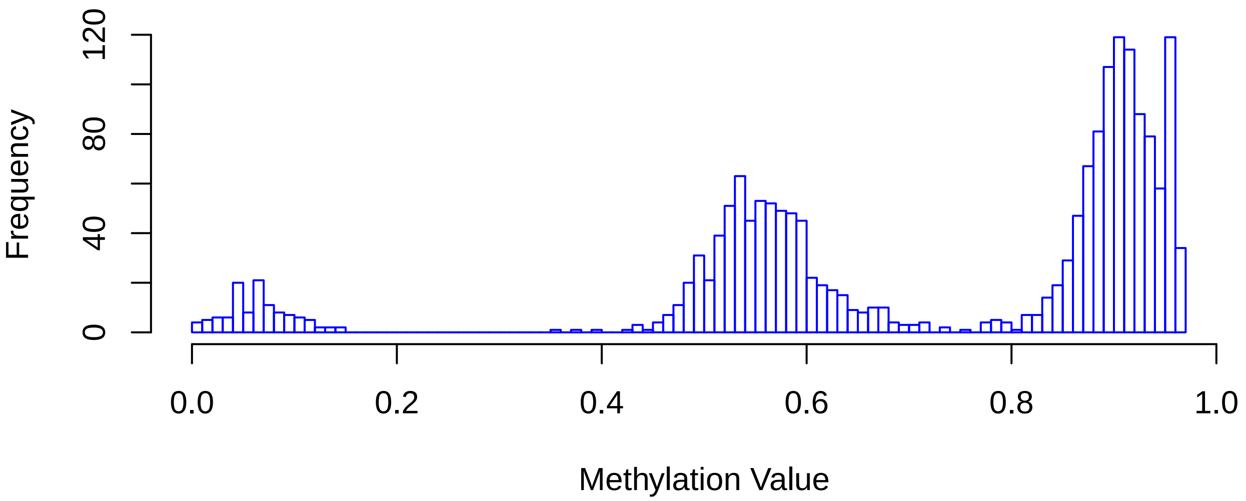

cg15029183 − Chr: 6 − Pos: 143586006 QATAR

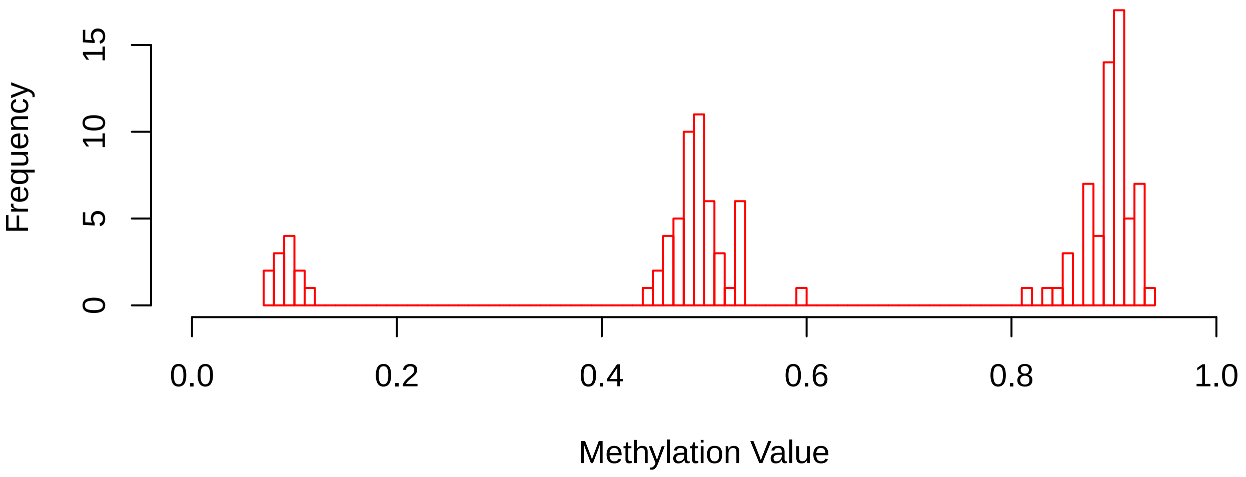

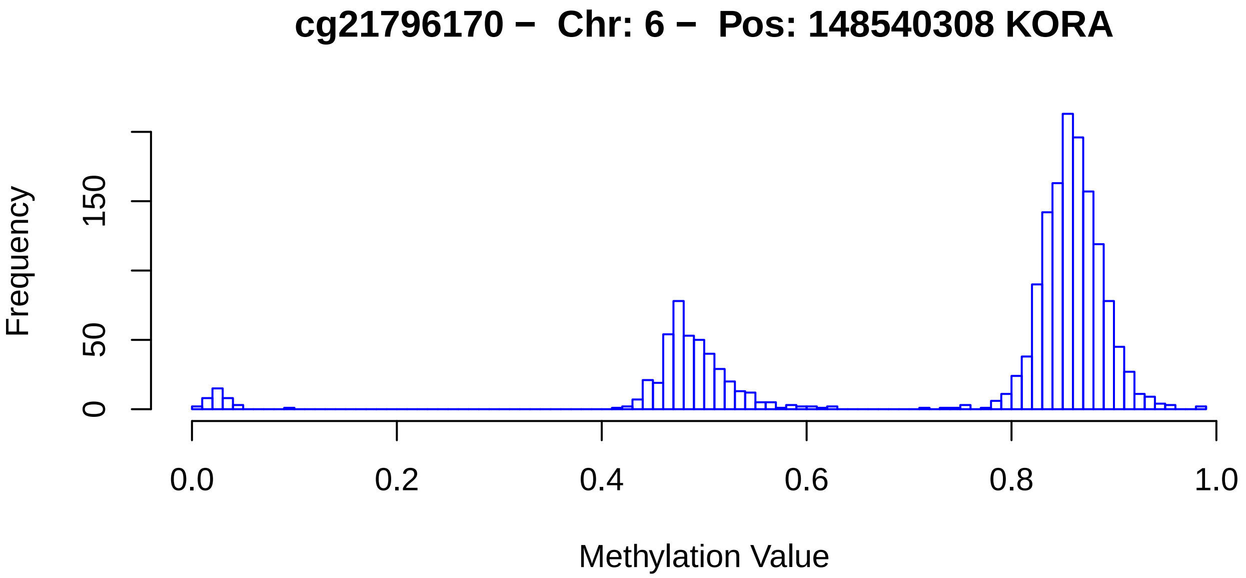
cg21796170 − Chr: 6 − Pos: 148540308 KORA
Frequency
0
50
150
0.0
0.2
0.4
0.6
0.8
1.0
Methylation Value

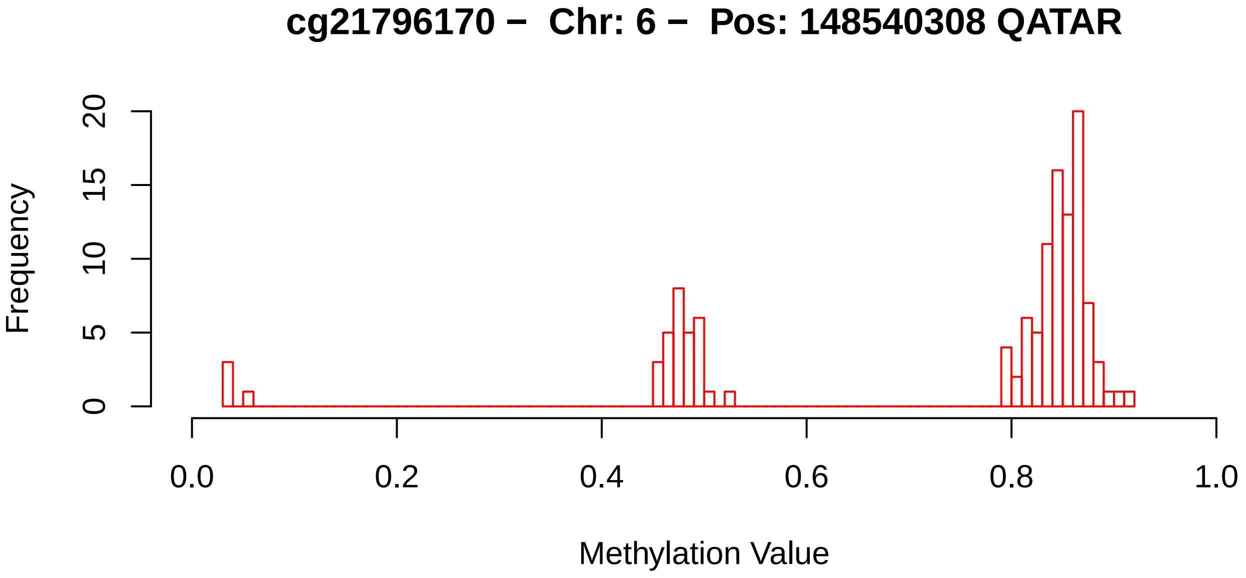
cg21796170 − Chr: 6 − Pos: 148540308 QATAR
Frequency
0
5
10
15
20
0.0
0.2
0.4
0.6
0.8
1.0
Methylation Value

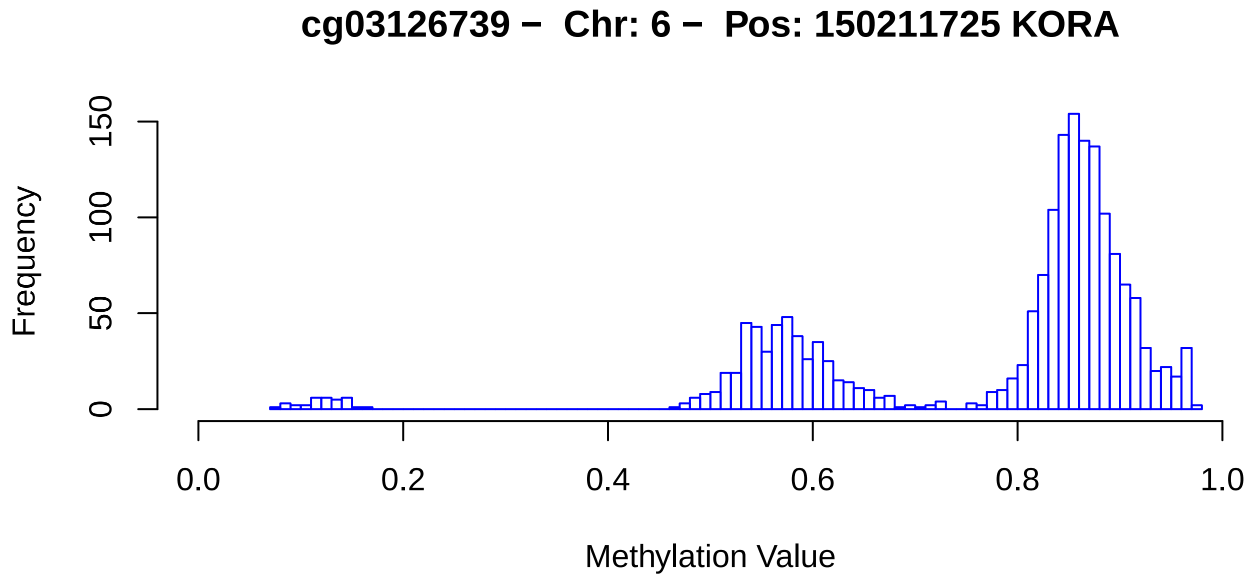
cg03126739 − Chr: 6 − Pos: 150211725 KORA
Frequency
0
50
100
150
0.0
0.2
0.4
0.6
0.8
1.0
Methylation Value

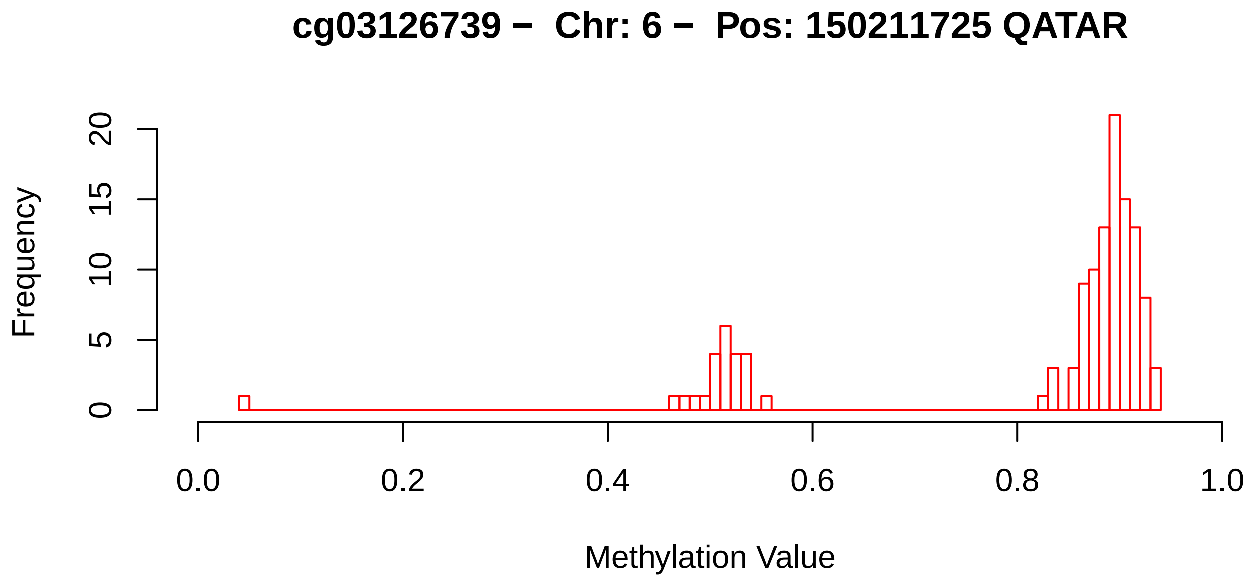
cg03126739 − Chr: 6 − Pos: 150211725 QATAR
Frequency
0
5
10
15
20
0.0
0.2
0.4
0.6
0.8
1.0
Methylation Value

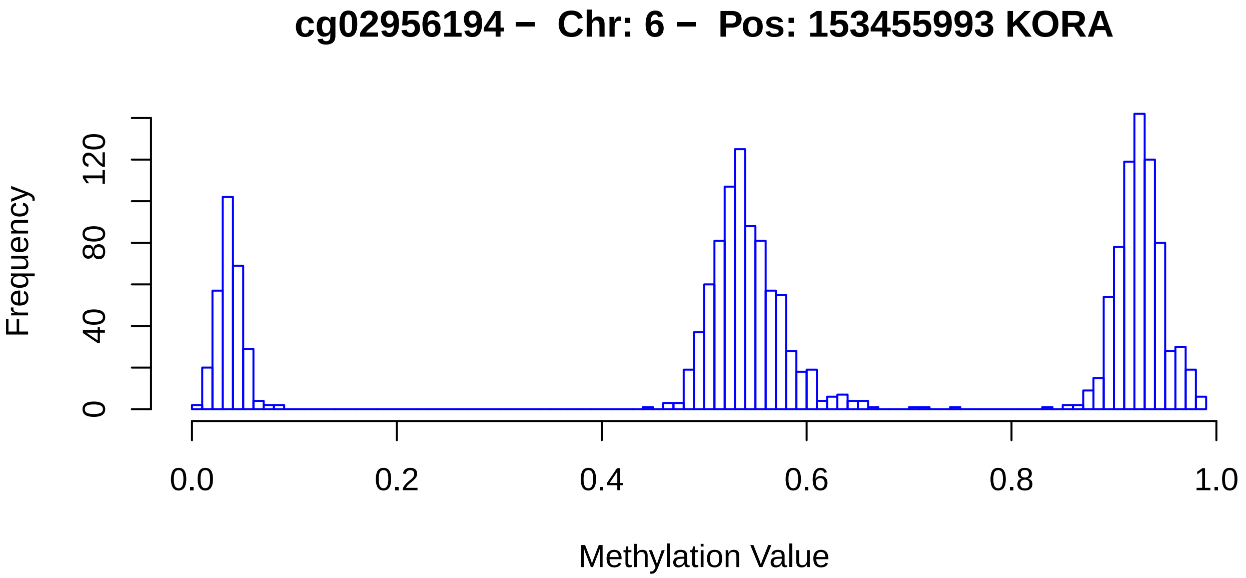
cg02956194 − Chr: 6 − Pos: 153455993 KORA
Frequency
0
40
80
120
0.0
0.2
0.4
0.6
0.8
1.0
Methylation Value

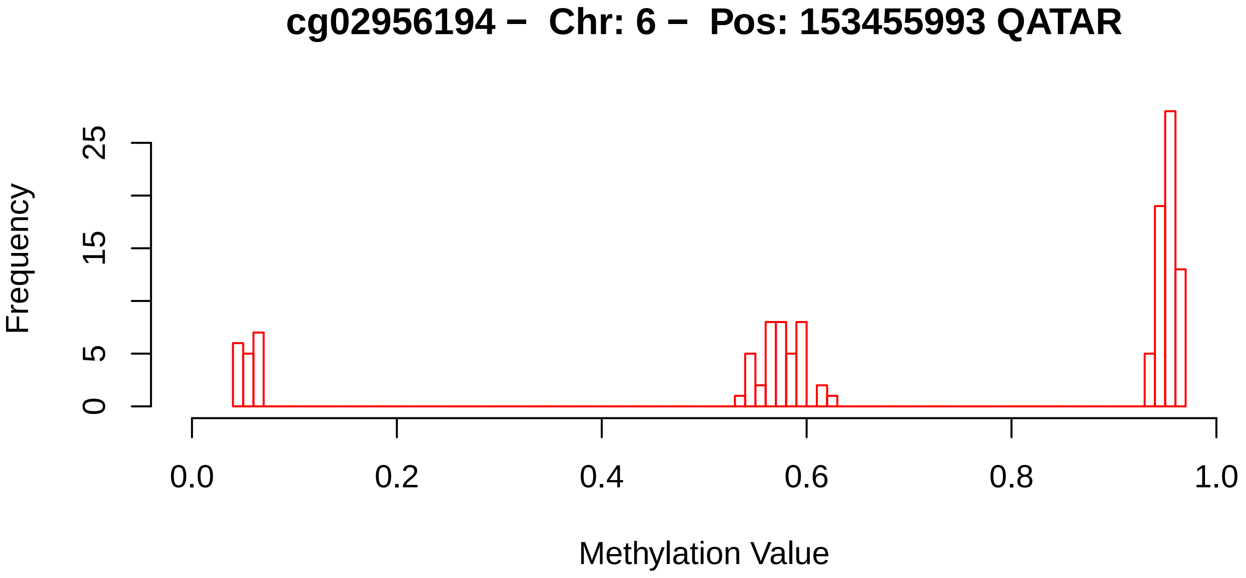
cg02956194 − Chr: 6 − Pos: 153455993 QATAR
Frequency
0
5
15
25
0.0
0.2
0.4
0.6
0.8
1.0
Methylation Value

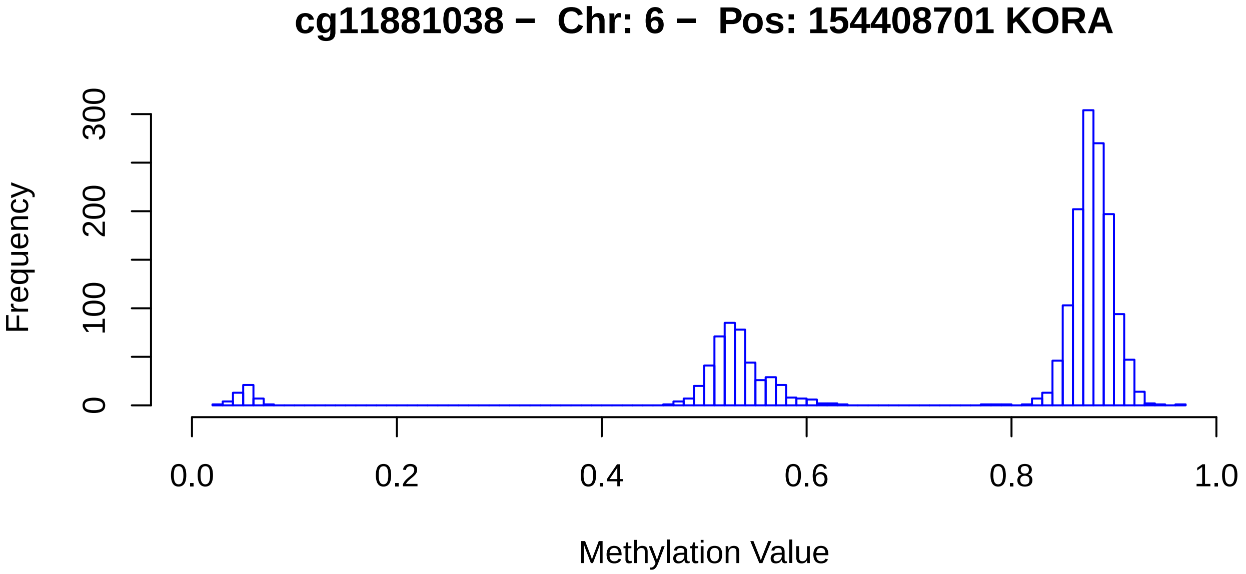

cg11881038 − Chr: 6 − Pos: 154408701 KORA
Frequency
0
100
200
300
0.0
0.2
0.4
0.6
0.8
1.0
Methylation Value

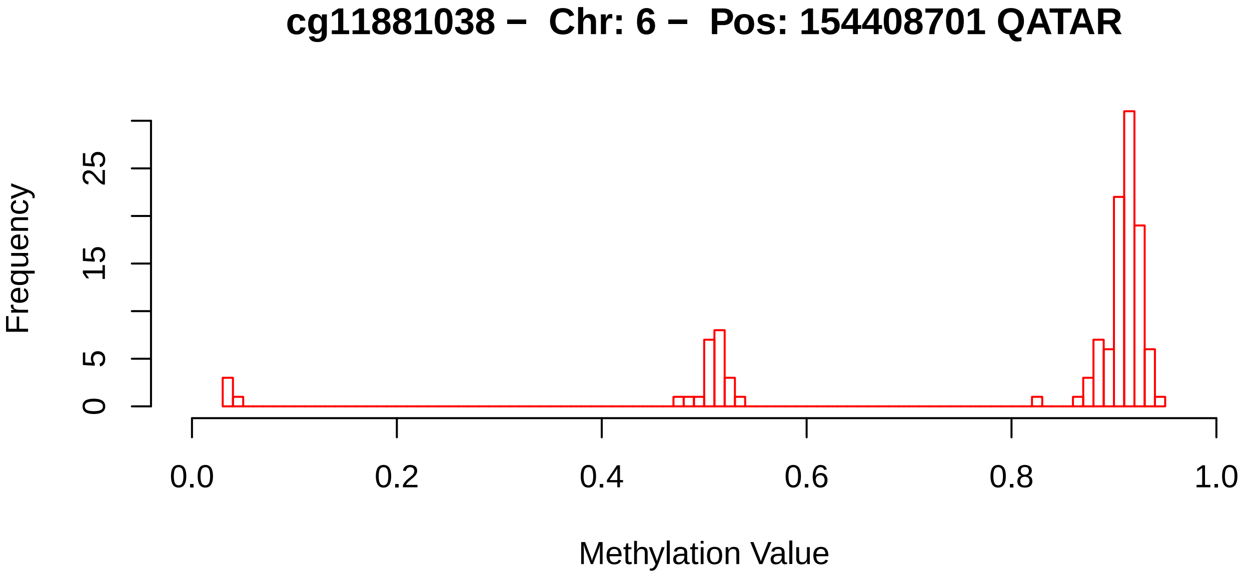

cg11881038 − Chr: 6 − Pos: 154408701 QATAR
Frequency
0
5
15
25
0.0
0.2
0.4
0.6
0.8
1.0
Methylation Value

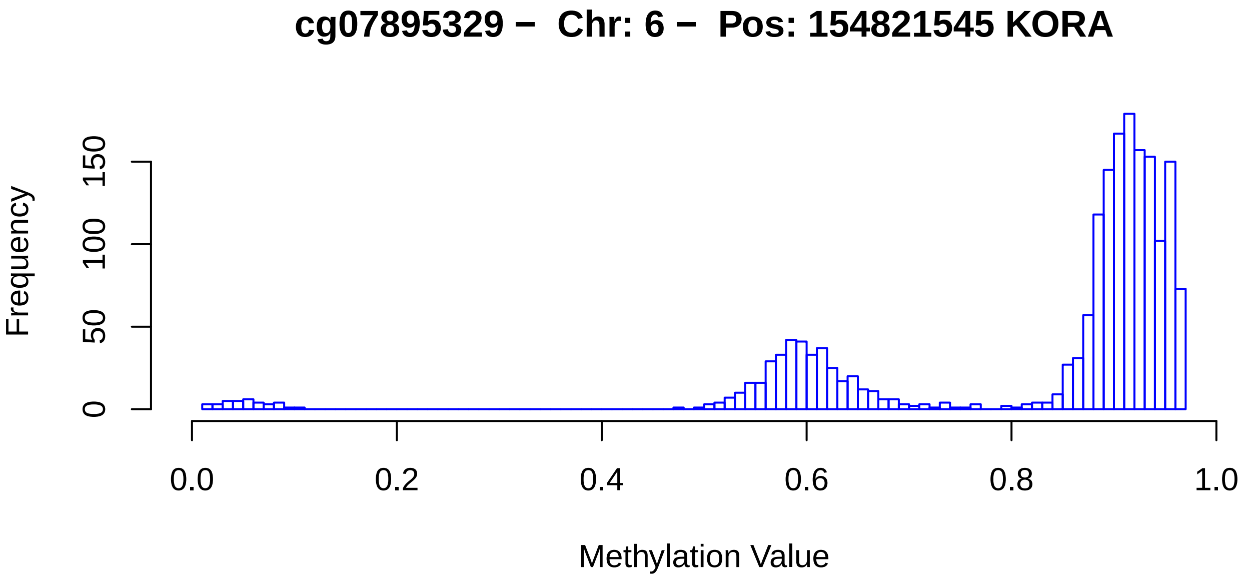

cg07895329 − Chr: 6 − Pos: 154821545 KORA
Frequency
0
50
100
150
0.0
0.2
0.4
0.6
0.8
1.0
Methylation Value

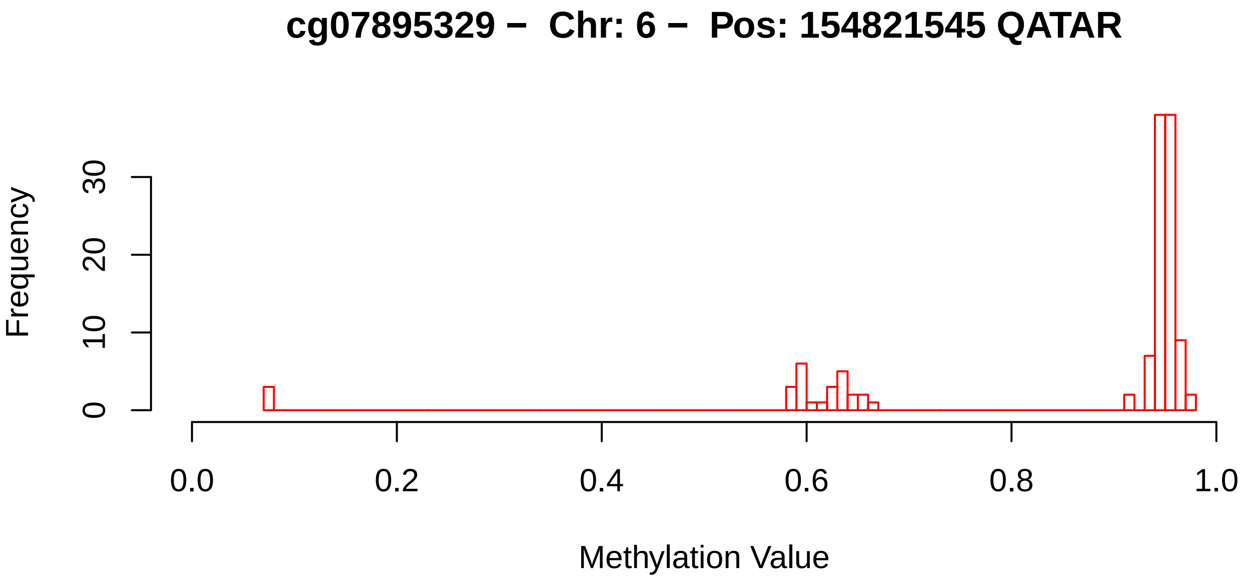

cg07895329 − Chr: 6 − Pos: 154821545 QATAR
Frequency
0
10
20
30
0.0
0.2
0.4
0.6
0.8
1.0
Methylation Value

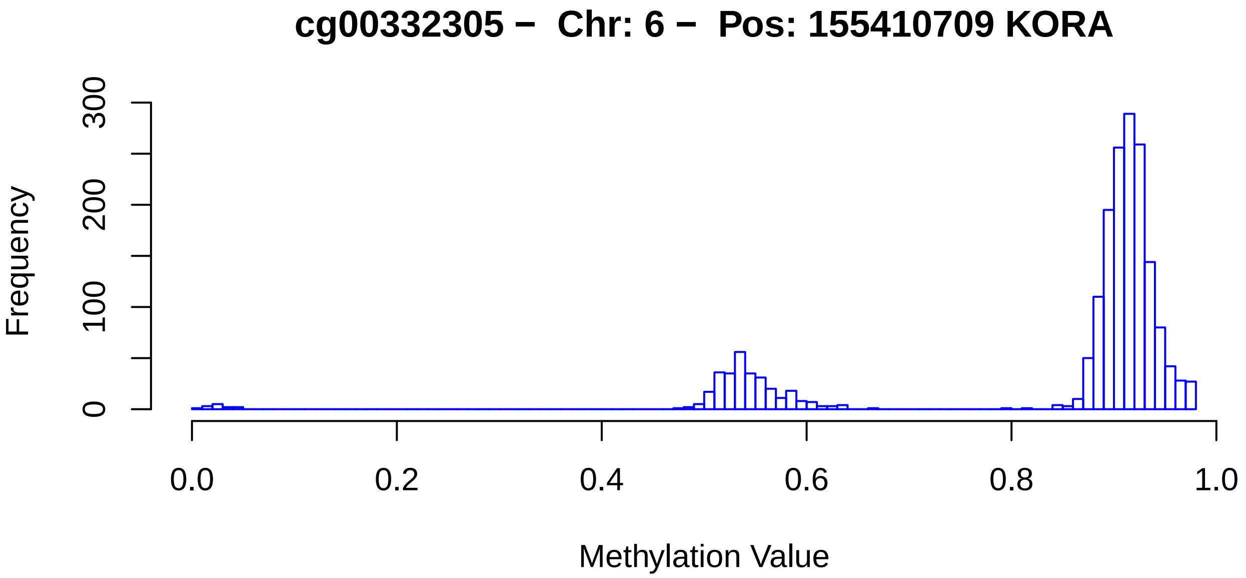
cg00332305 − Chr: 6 − Pos: 155410709 KORA
Frequency
0
100
200
300
0.0
0.2
0.4
0.6
0.8
1.0
Methylation Value

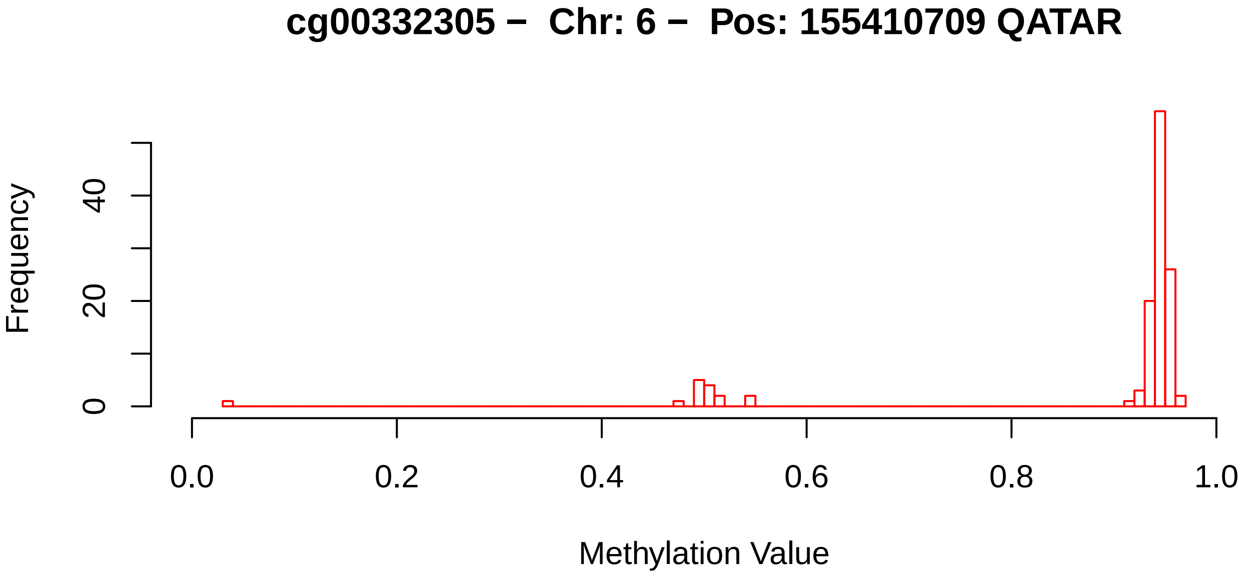
cg00332305 − Chr: 6 − Pos: 155410709 QATAR
Frequency
0
20
40
0.0
0.2
0.4
0.6
0.8
1.0
Methylation Value

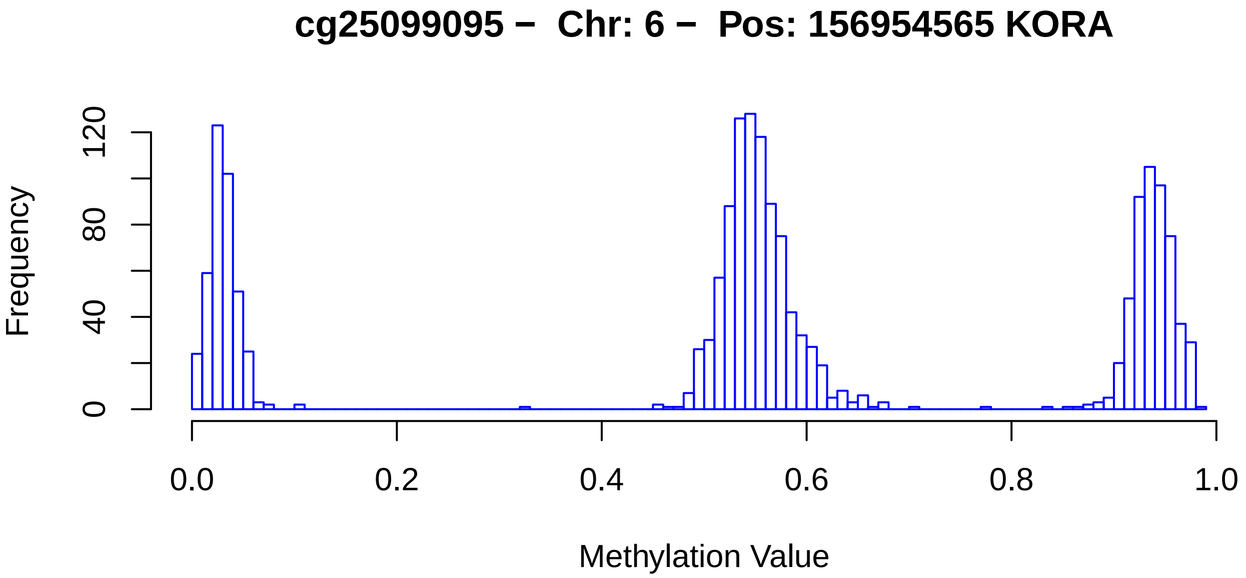
cg25099095 − Chr: 6 − Pos: 156954565 KORA
Frequency
0
40
80
120
0.0
0.2
0.4
0.6
0.8
1.0
Methylation Value

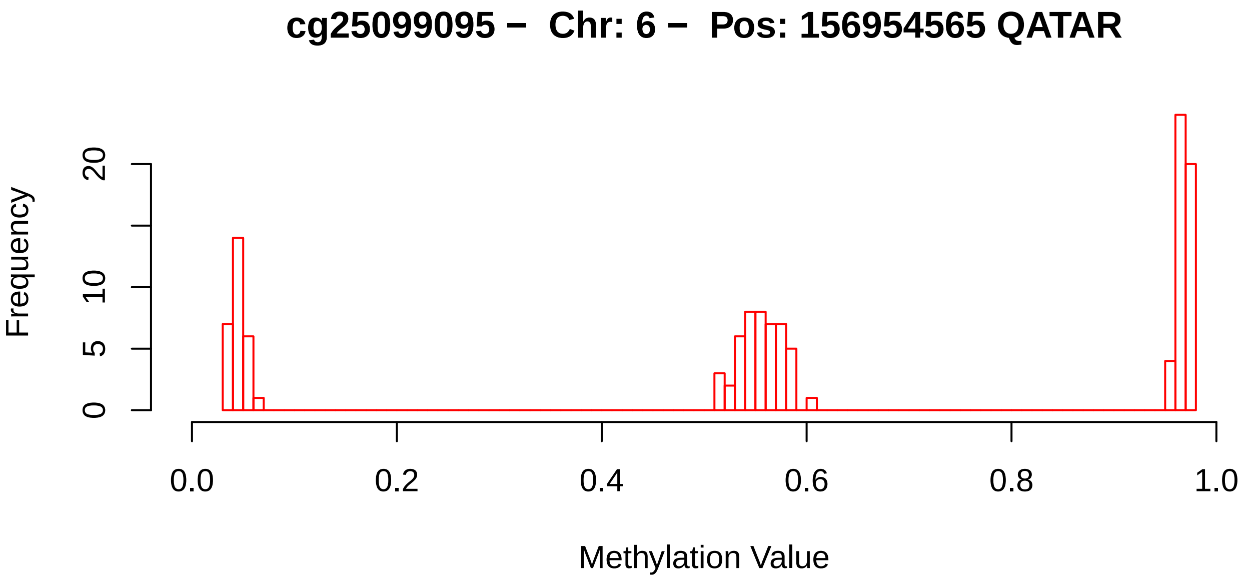
cg25099095 − Chr: 6 − Pos: 156954565 QATAR
Frequency
0
5
10
20
0.0
0.2
0.4
0.6
0.8
1.0
Methylation Value

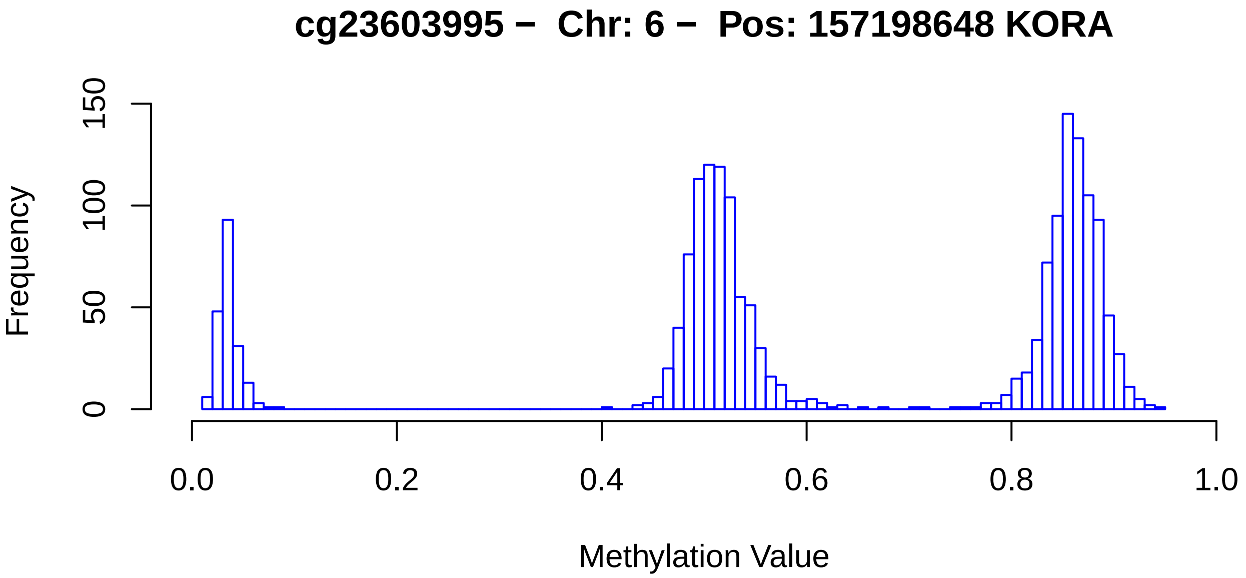
cg23603995 − Chr: 6 − Pos: 157198648 KORA
Frequency
0
50
100
150
0.0
0.2
0.4
0.6
0.8
1.0
Methylation Value

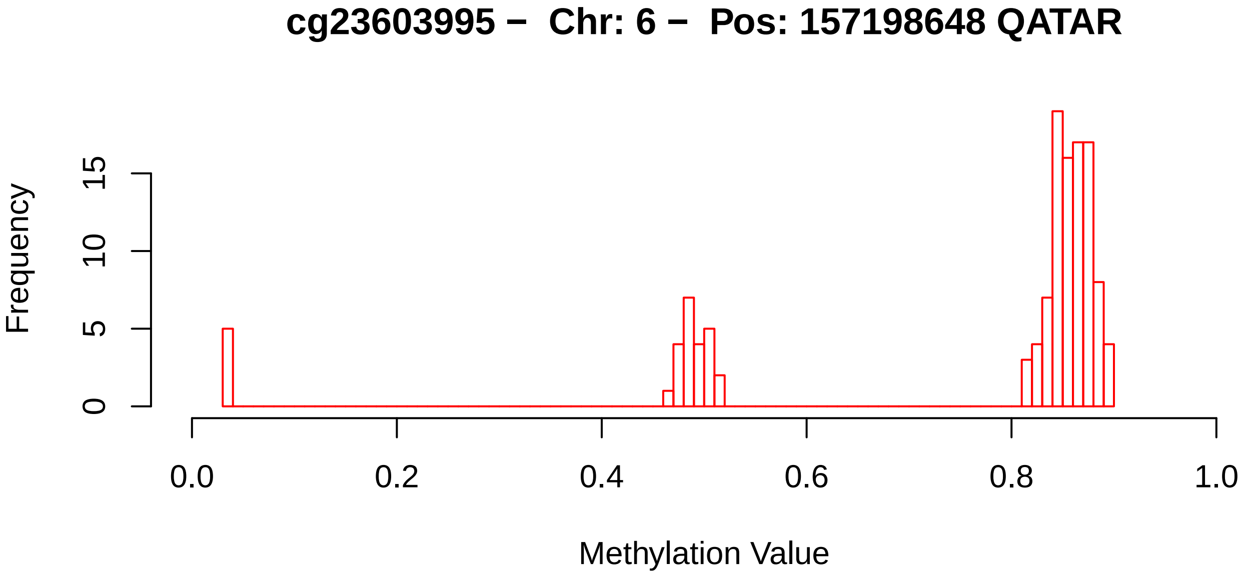
cg23603995 − Chr: 6 − Pos: 157198648 QATAR
Frequency
0
5
10
15
0.0
0.2
0.4
0.6
0.8
1.0
Methylation Value

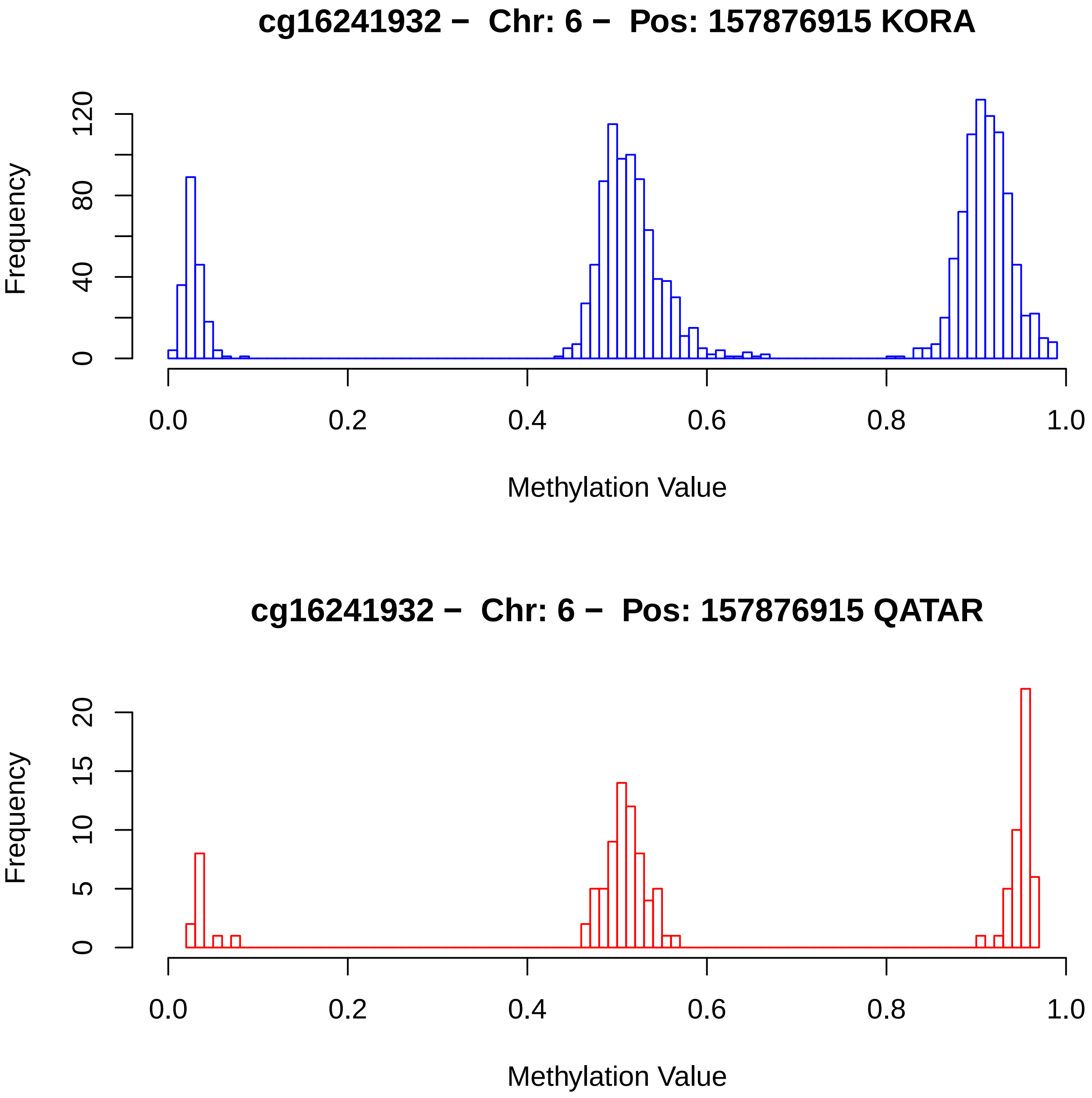
cg16241932 − Chr: 6 − Pos: 157876915 KORA
Frequency
0
40
80
120
0.0
0.2
0.4
0.6
0.8
1.0
Methylation Value
cg16241932 − Chr: 6 − Pos: 157876915 QATAR
Frequency
0
5
10
15
20
0.0
0.2
0.4
0.6
0.8
1.0
Methylation Value

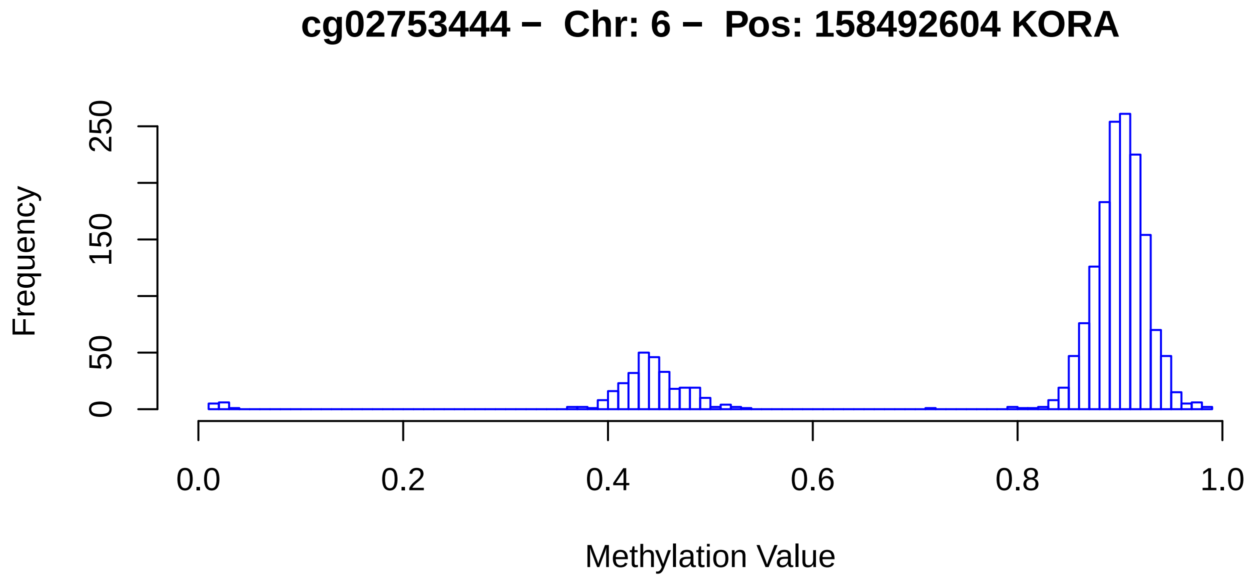
cg02753444 − Chr: 6 − Pos: 158492604 KORA
Frequency
0
50
150
250
0.0
0.2
0.4
0.6
0.8
1.0
Methylation Value

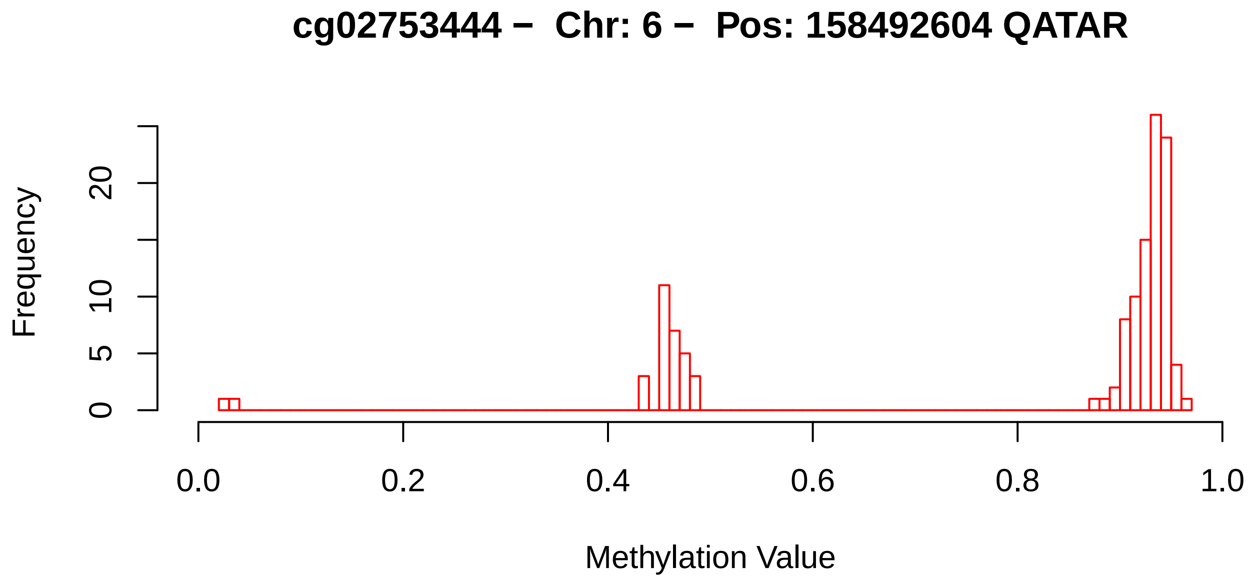
cg02753444 − Chr: 6 − Pos: 158492604 QATAR
Frequency
0
5
10
20
0.0
0.2
0.4
0.6
0.8
1.0
Methylation Value

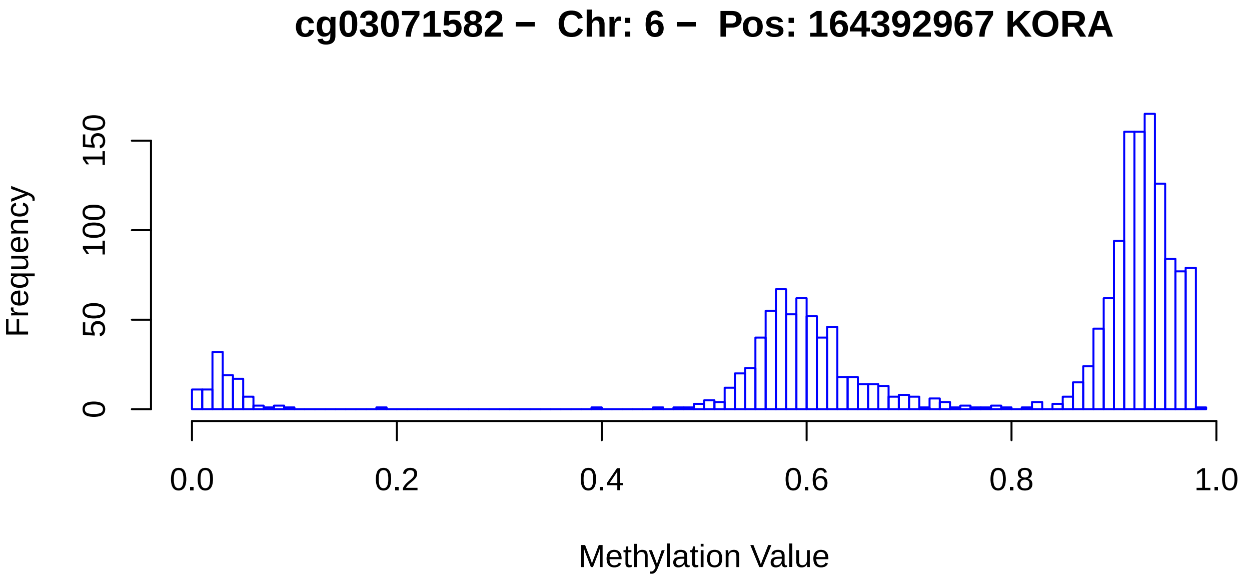
cg03071582 − Chr: 6 − Pos: 164392967 KORA
Frequency
0
50
100
150
0.0
0.2
0.4
0.6
0.8
1.0
Methylation Value

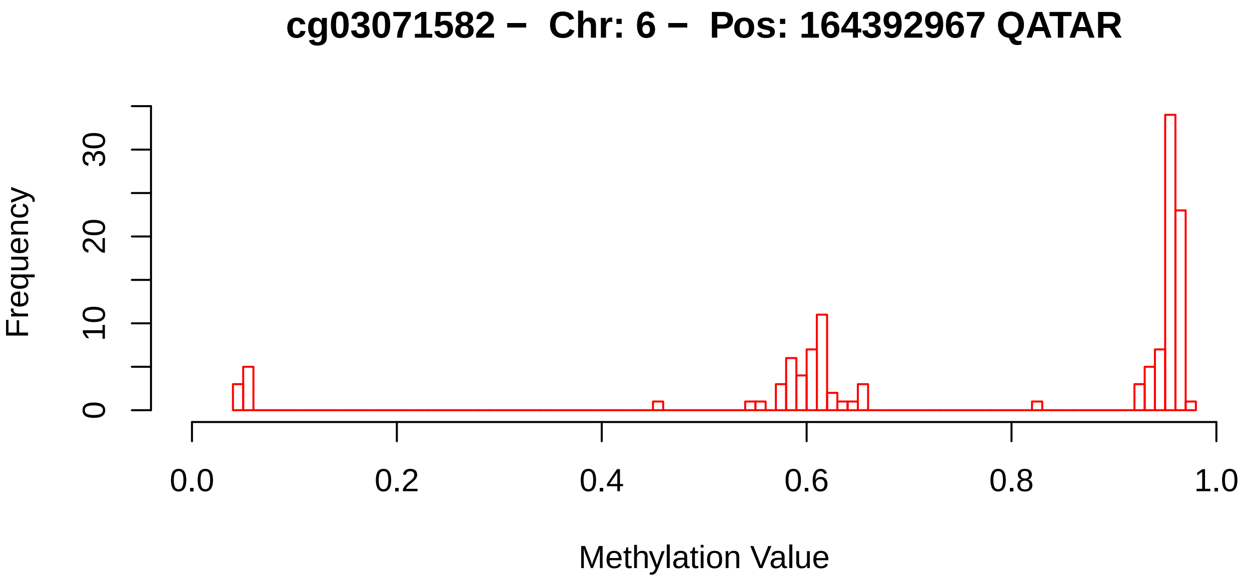
cg03071582 − Chr: 6 − Pos: 164392967 QATAR
Frequency
0
10
20
30
0.0
0.2
0.4
0.6
0.8
1.0
Methylation Value

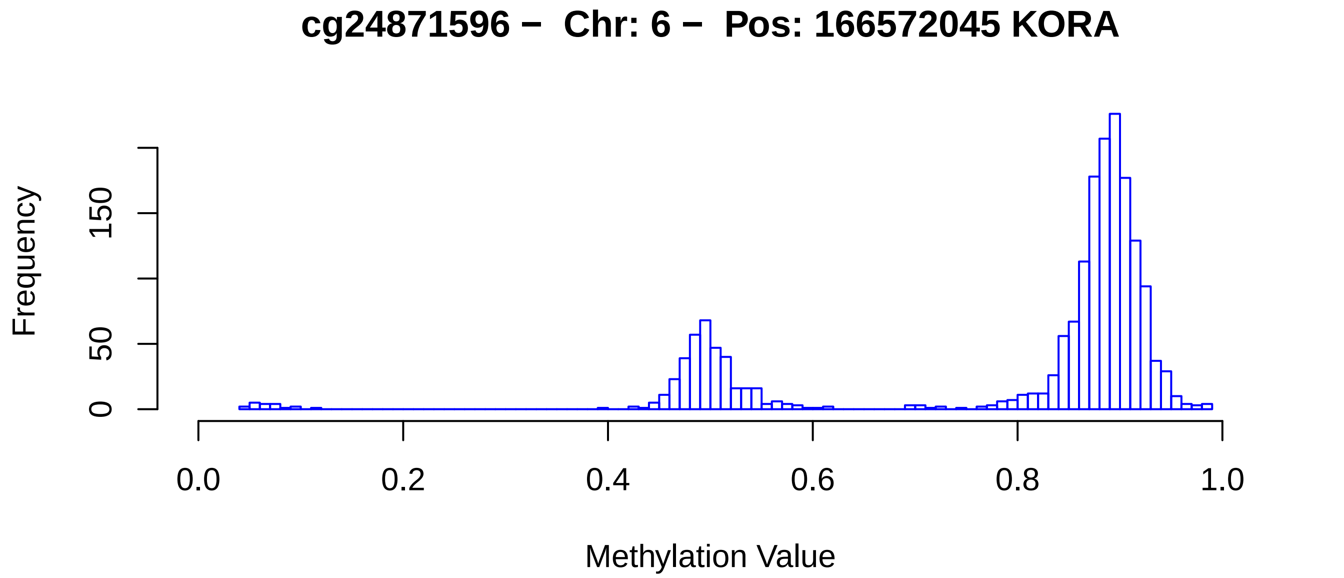
cg24871596 − Chr: 6 − Pos: 166572045 KORA
Frequency
0
50
150
0.0
0.2
0.4
0.6
0.8
1.0
Methylation Value

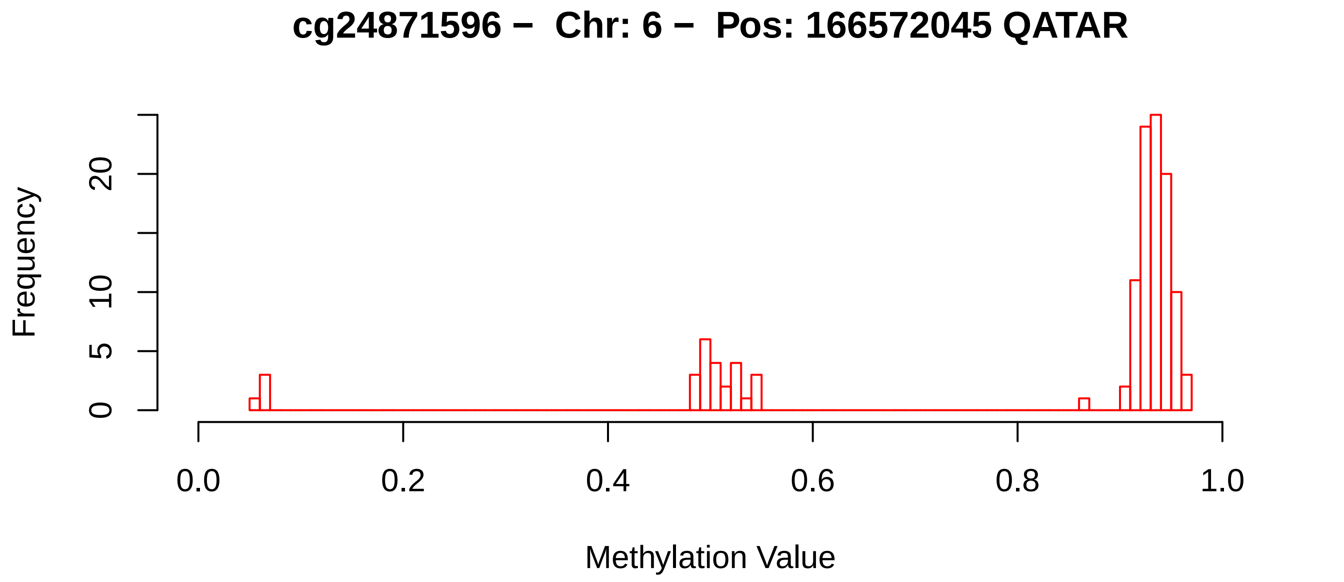
cg24871596 − Chr: 6 − Pos: 166572045 QATAR
Frequency
0
5
10
20
0.0
0.2
0.4
0.6
0.8
1.0
Methylation Value

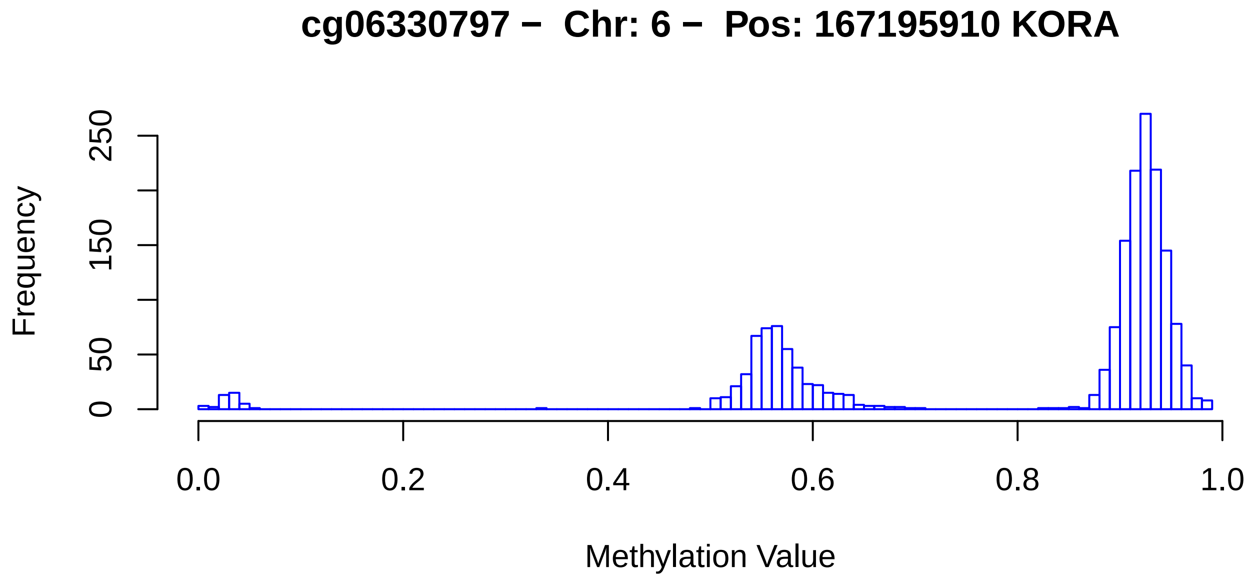
cg06330797 − Chr: 6 − Pos: 167195910 KORA
Frequency
0
50
150
250
0.0
0.2
0.4
0.6
0.8
1.0
Methylation Value

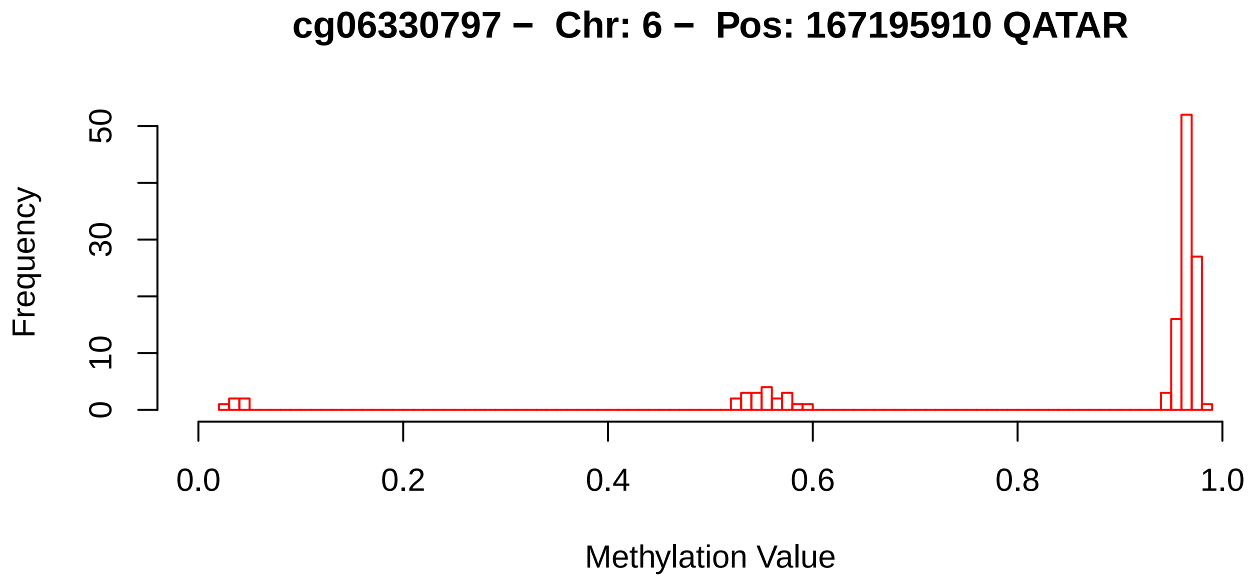
cg06330797 − Chr: 6 − Pos: 167195910 QATAR
Frequency
0
10
30
50
0.0
0.2
0.4
0.6
0.8
1.0
Methylation Value

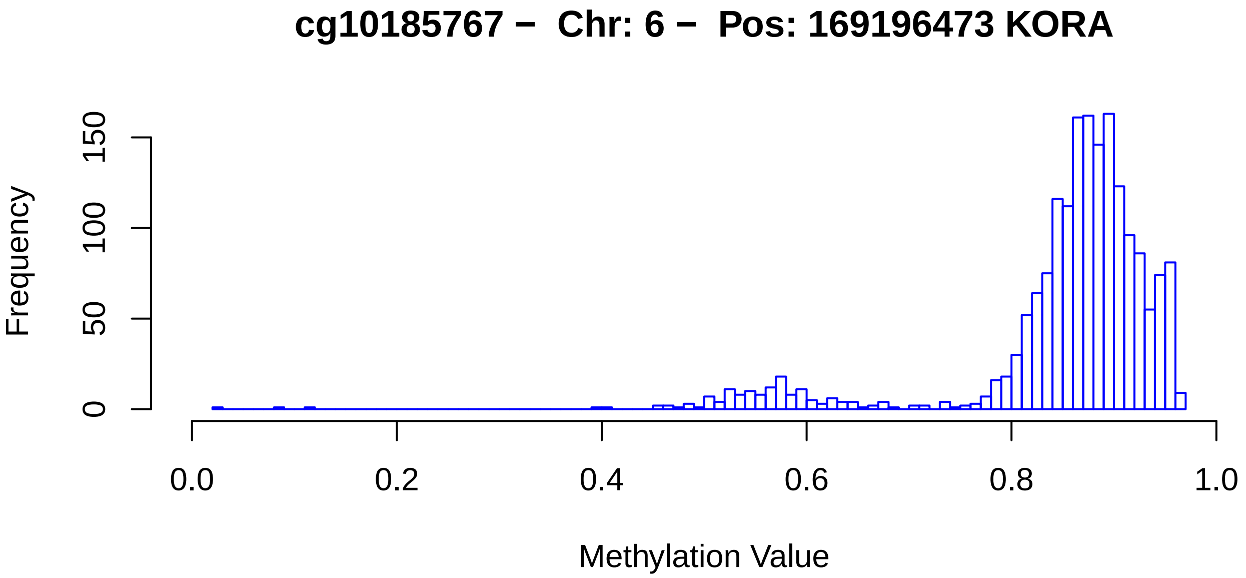

cg10185767 − Chr: 6 − Pos: 169196473 KORA
Frequency
0
50
100
150
0.0
0.2
0.4
0.6
0.8
1.0
Methylation Value

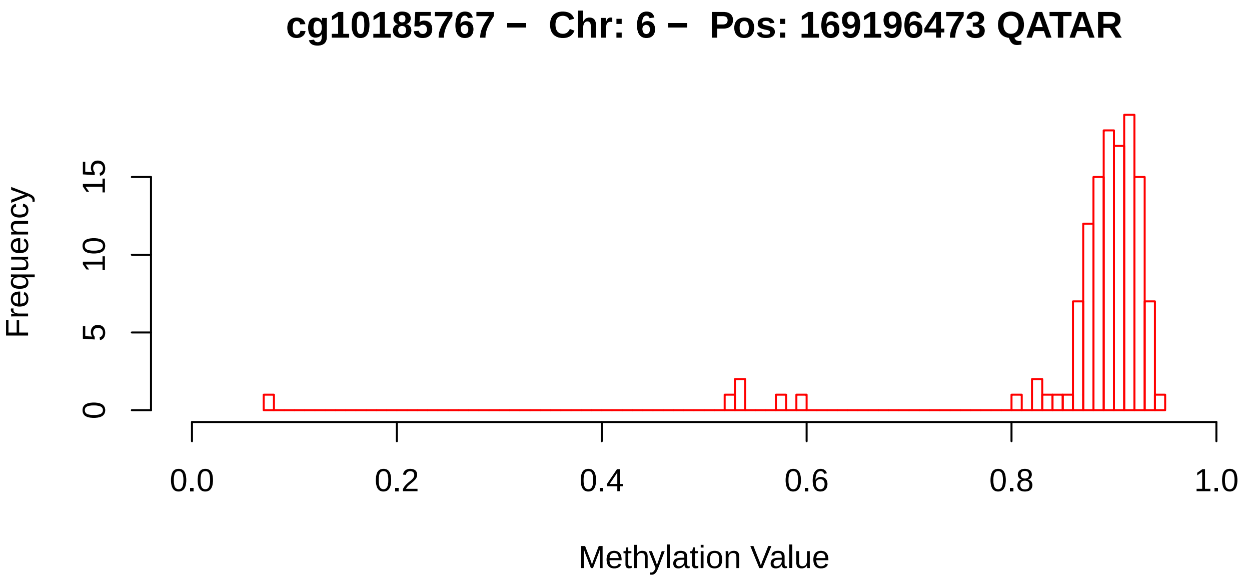

cg10185767 − Chr: 6 − Pos: 169196473 QATAR
Frequency
0
5
10
15
0.0
0.2
0.4
0.6
0.8
1.0
Methylation Value

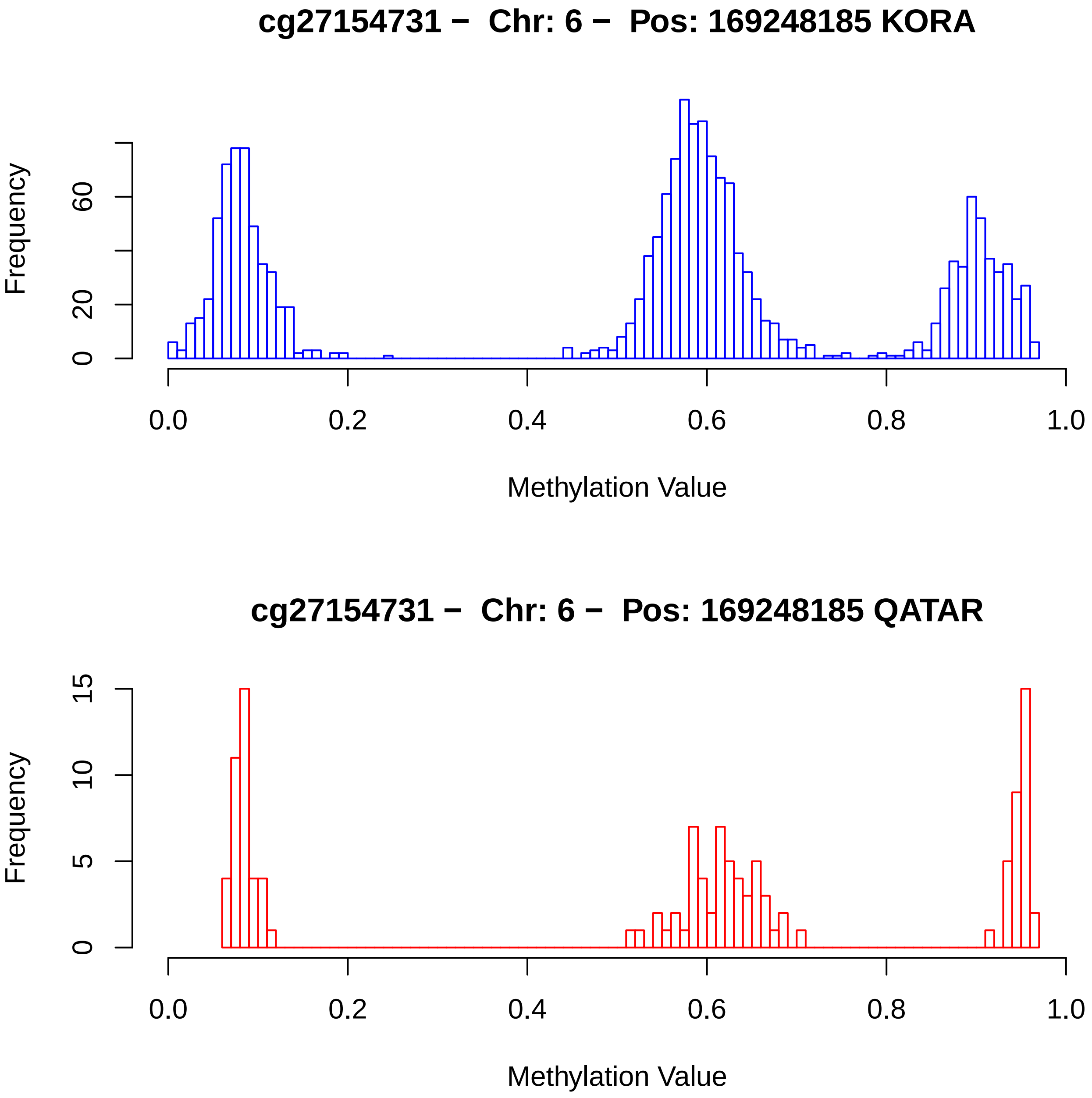
cg27154731 – Chr: 6 – Pos: 169248185 KORA
Frequency
0
20
60
0.0
0.2
0.4
0.6
0.8
1.0
Methylation Value
cg27154731 – Chr: 6 – Pos: 169248185 QATAR
Frequency
0
5
10
15
0.0
0.2
0.4
0.6
0.8
1.0
Methylation Value

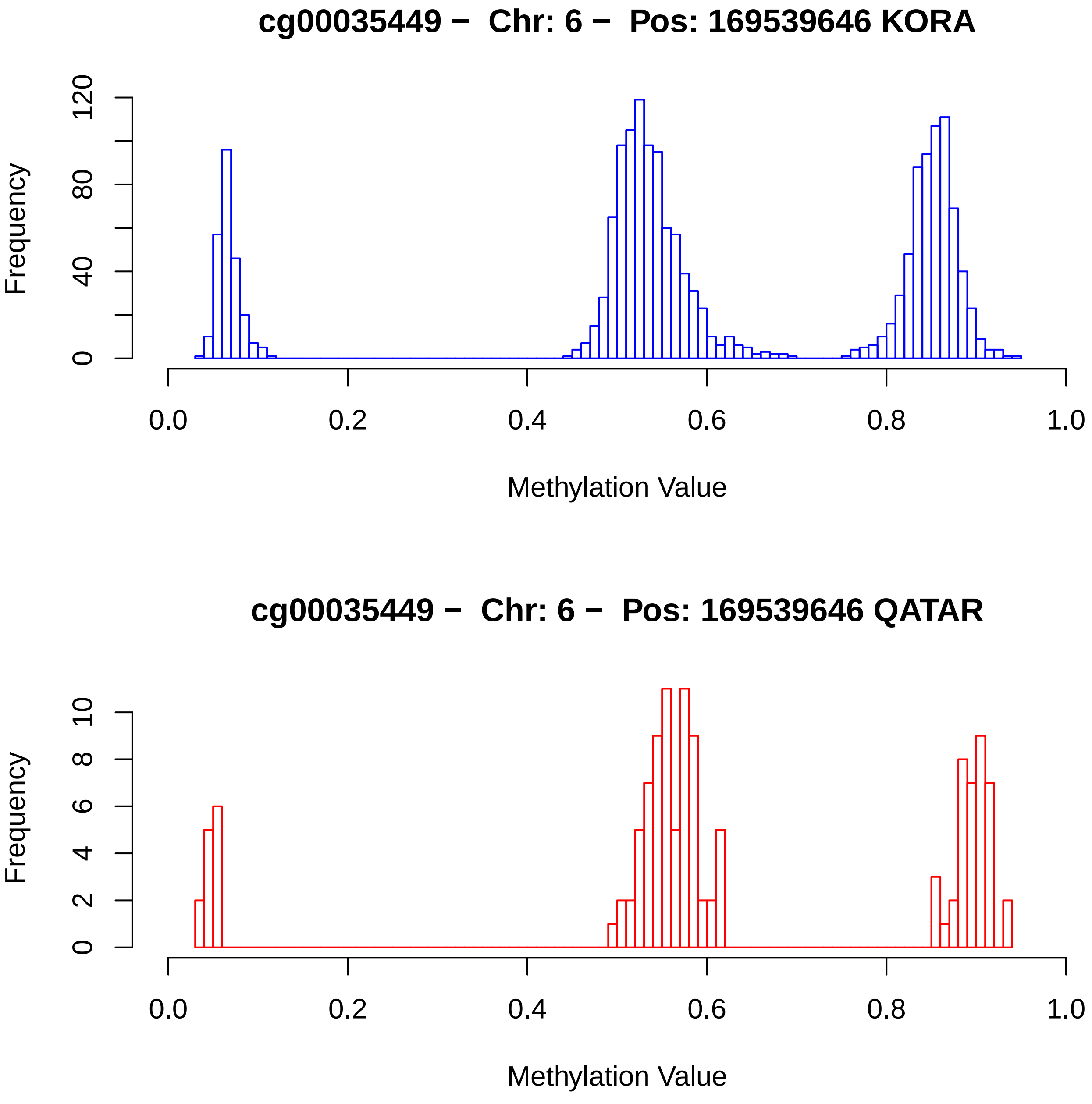
cg00035449 − Chr: 6 − Pos: 169539646 KORA
Frequency
0
40
80
120
0.0
0.2
0.4
0.6
0.8
1.0
Methylation Value
cg00035449 − Chr: 6 − Pos: 169539646 QATAR
Frequency
0
2
4
6
8
10
0.0
0.2
0.4
0.6
0.8
1.0
Methylation Value

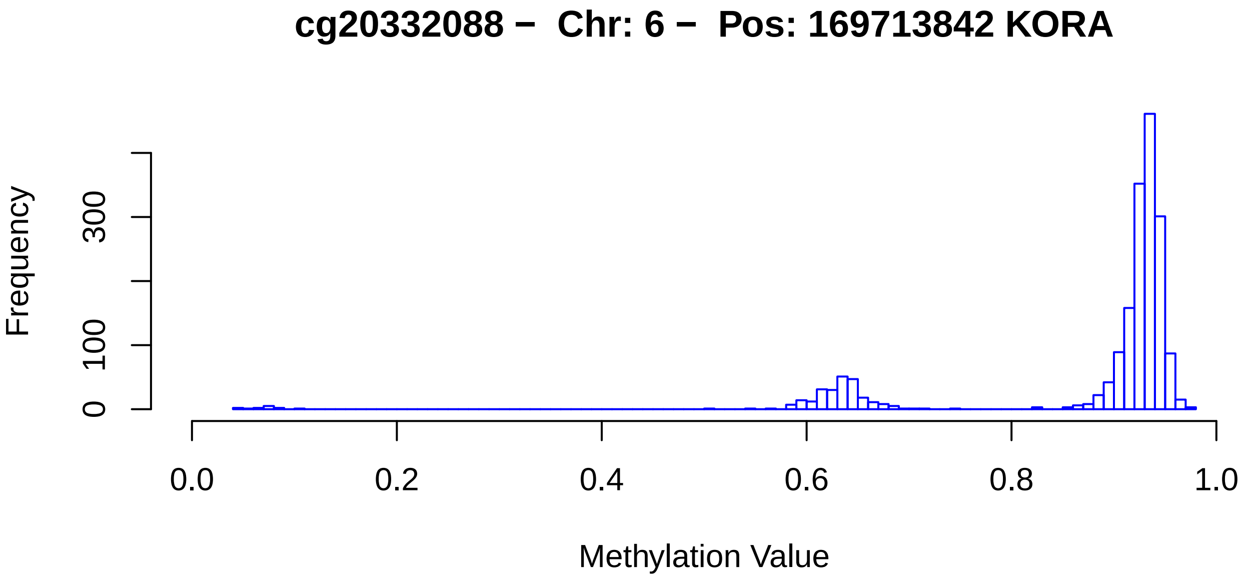
cg20332088 − Chr: 6 − Pos: 169713842 KORA
Frequency
0
100
300
0.0
0.2
0.4
0.6
0.8
1.0
Methylation Value

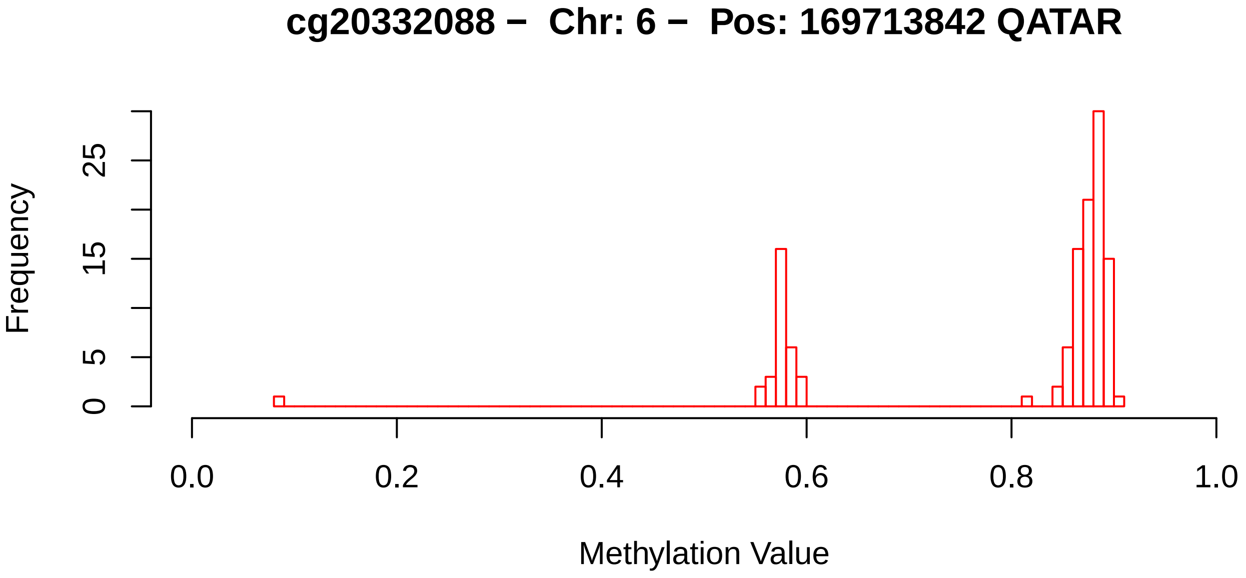
cg20332088 − Chr: 6 − Pos: 169713842 QATAR
Frequency
0
5
15
25
0.0
0.2
0.4
0.6
0.8
1.0
Methylation Value

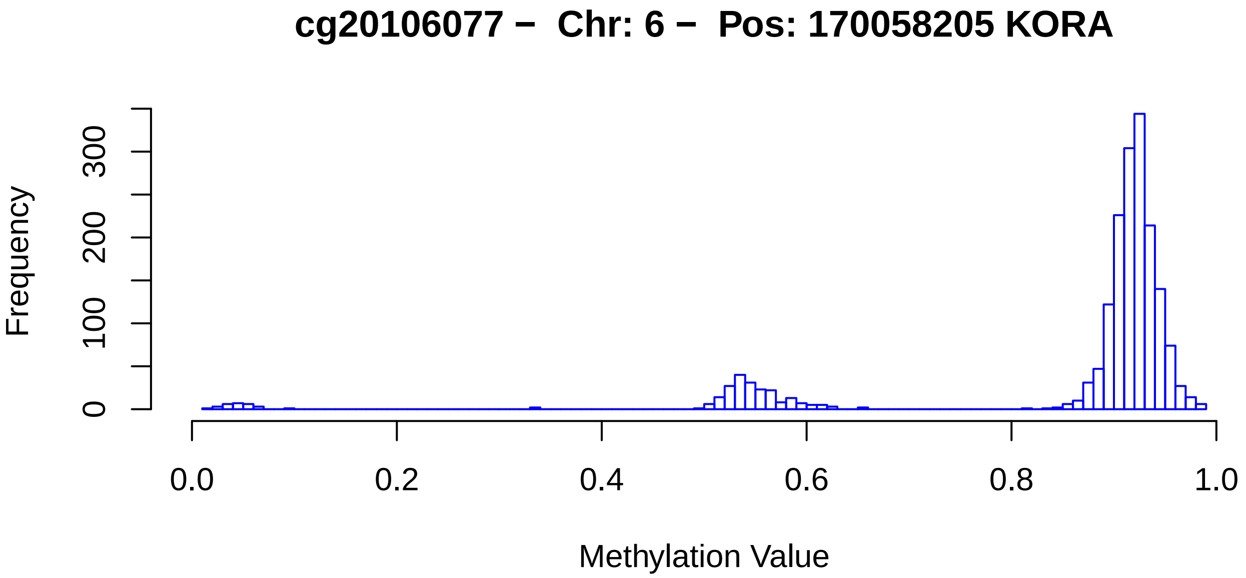

cg20106077 − Chr: 6 − Pos: 170058205 KORA
Frequency
0
100
200
300
0.0
0.2
0.4
0.6
0.8
1.0
Methylation Value

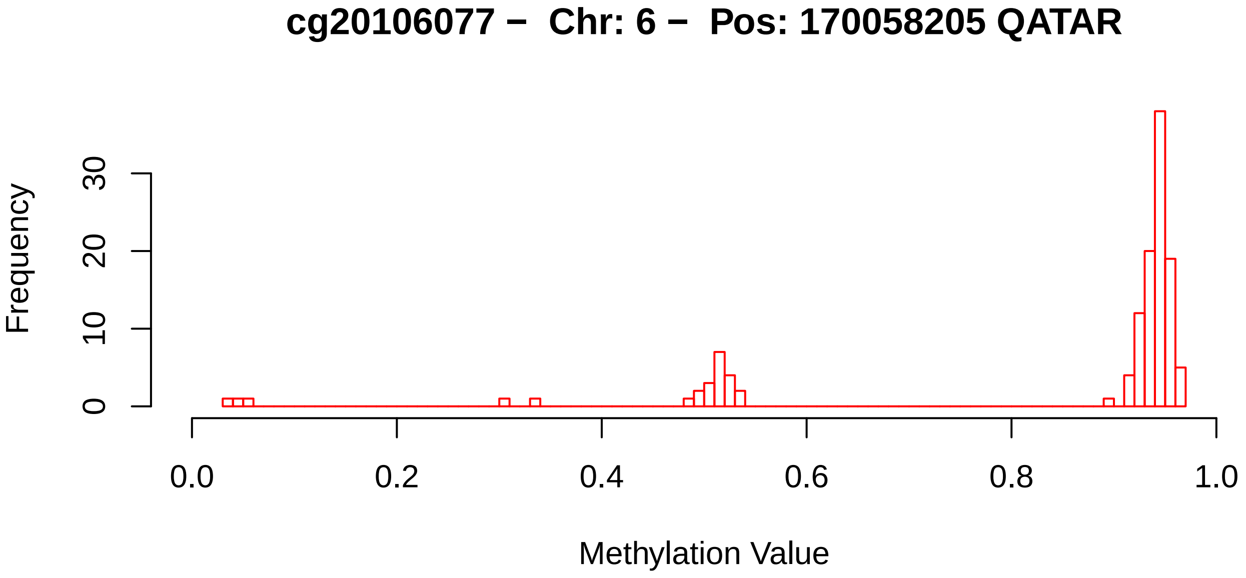

cg20106077 − Chr: 6 − Pos: 170058205 QATAR
Frequency
0
10
20
30
0.0
0.2
0.4
0.6
0.8
1.0
Methylation Value

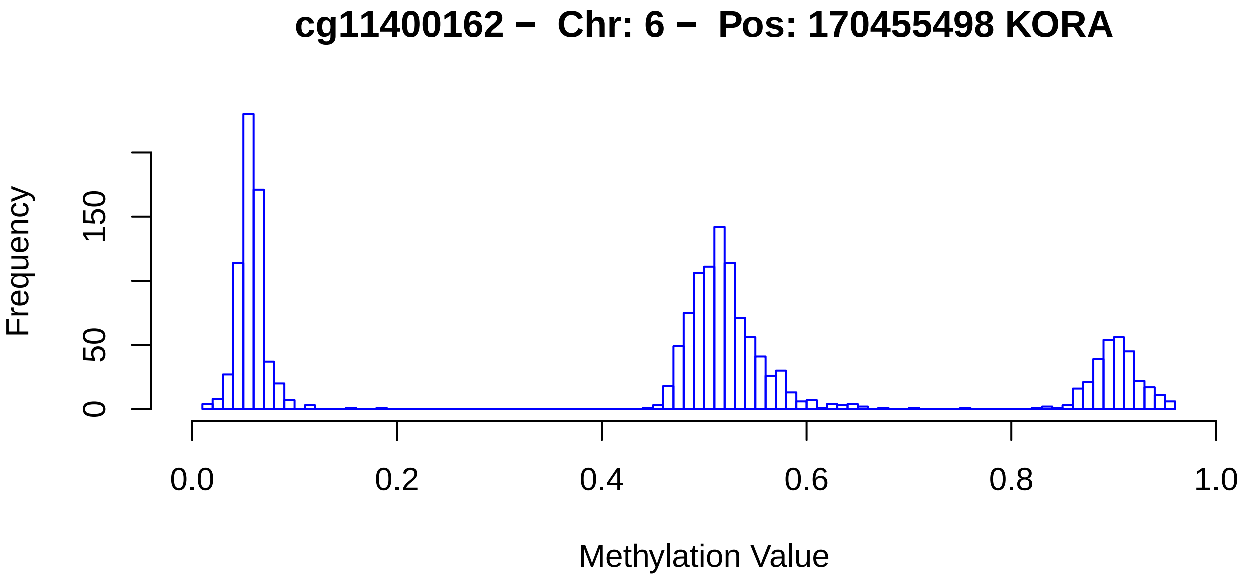
cg11400162 − Chr: 6 − Pos: 170455498 KORA
Frequency
0
50
150
0.0
0.2
0.4
0.6
0.8
1.0
Methylation Value

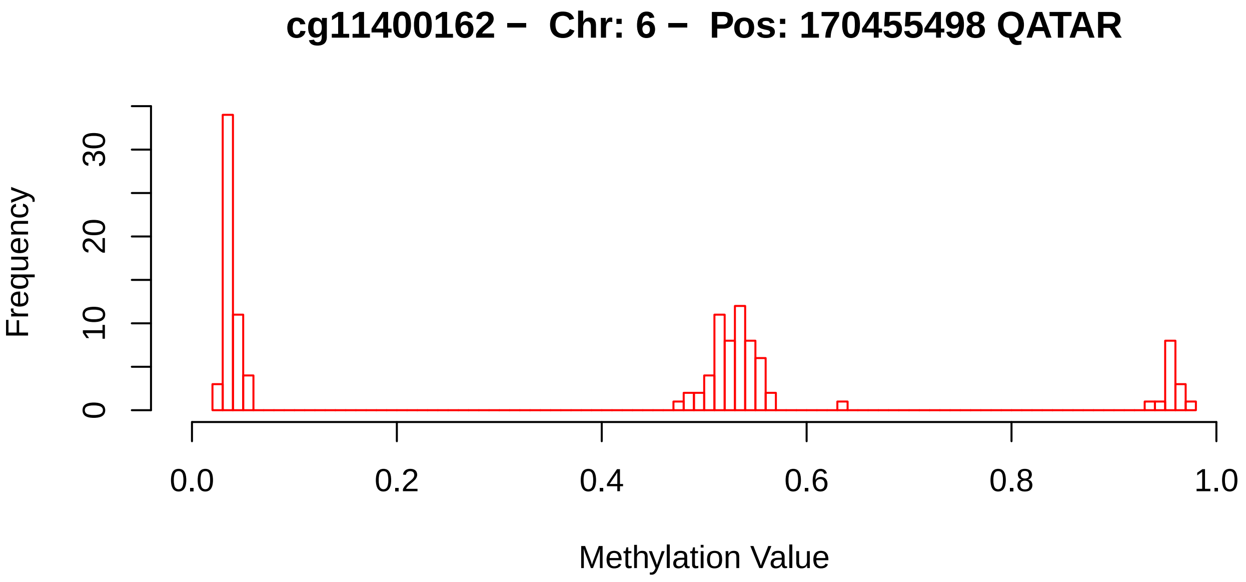
cg11400162 − Chr: 6 − Pos: 170455498 QATAR
Frequency
0
10
20
30
0.0
0.2
0.4
0.6
0.8
1.0
Methylation Value

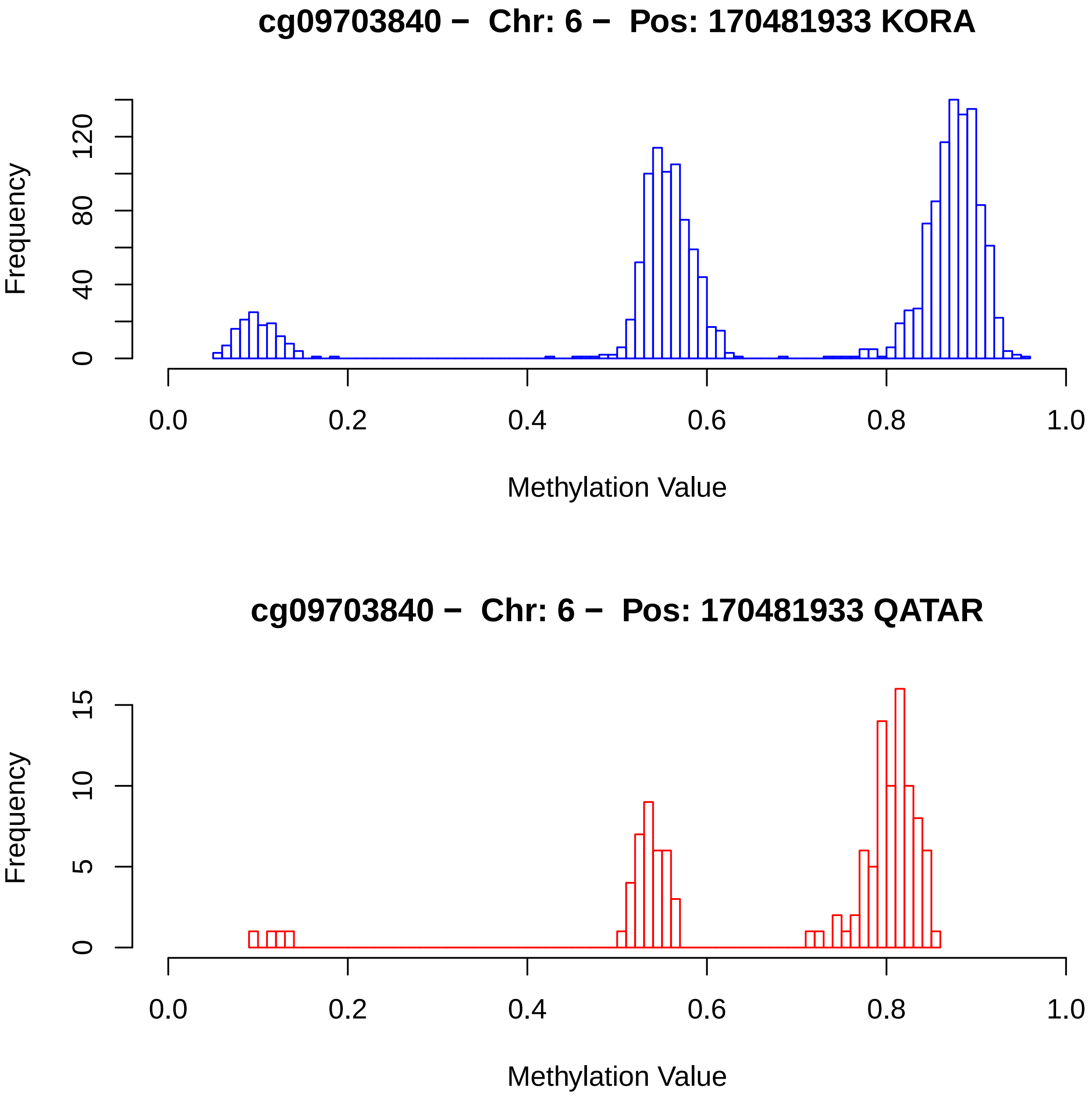
cg09703840 − Chr: 6 − Pos: 170481933 KORA
Frequency
0
40
80
120
0.0
0.2
0.4
0.6
0.8
1.0
Methylation Value
cg09703840 − Chr: 6 − Pos: 170481933 QATAR
Frequency
0
5
10
15
0.0
0.2
0.4
0.6
0.8
1.0
Methylation Value

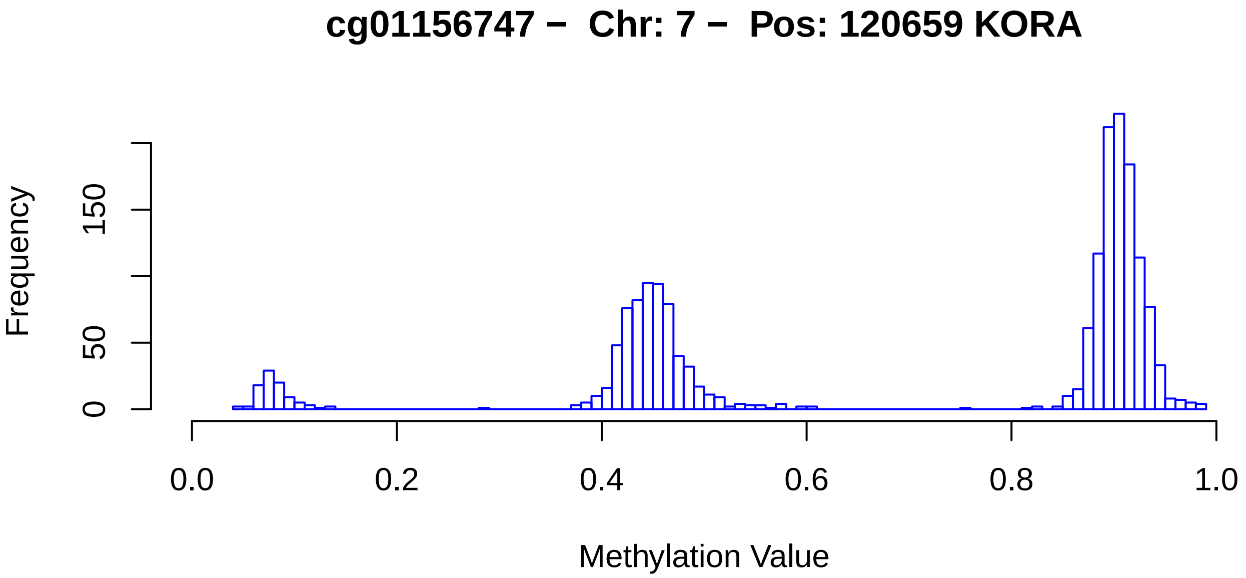
cg01156747 − Chr: 7 − Pos: 120659 KORA
Frequency
0
50
150
0.0
0.2
0.4
0.6
0.8
1.0
Methylation Value

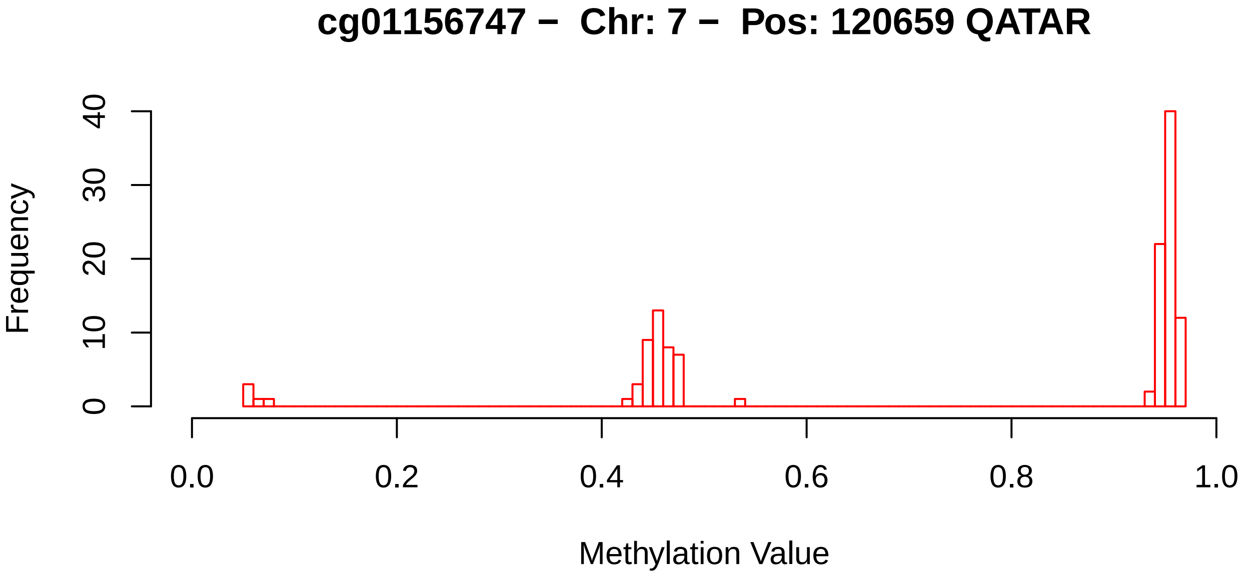
cg01156747 − Chr: 7 − Pos: 120659 QATAR
Frequency
0
10
20
30
40
0.0
0.2
0.4
0.6
0.8
1.0
Methylation Value

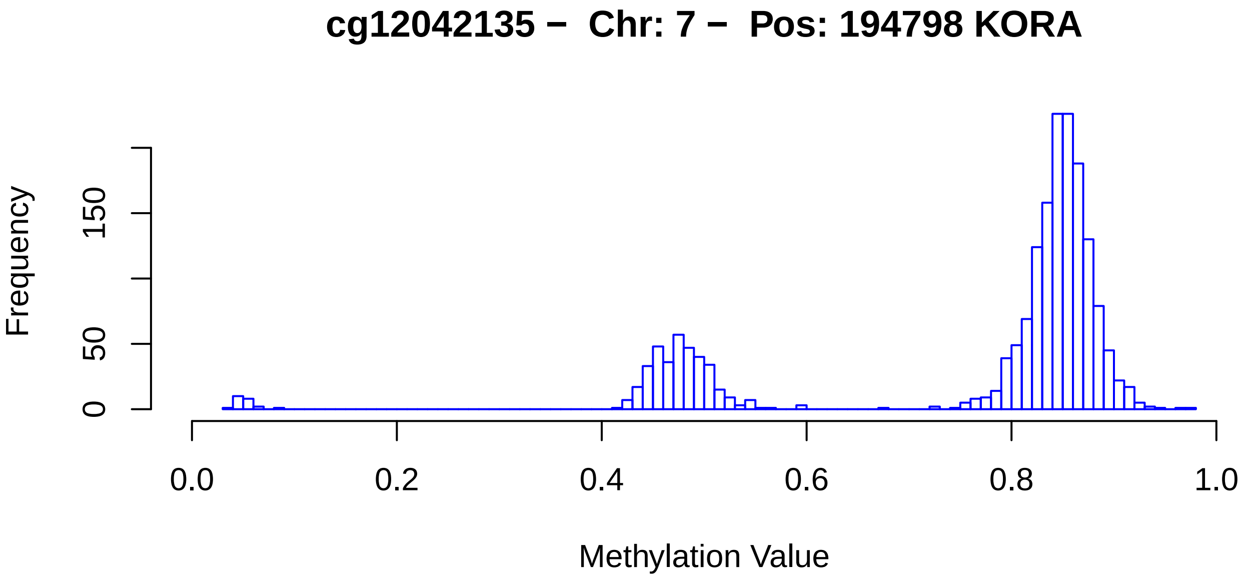
cg12042135 − Chr: 7 − Pos: 194798 KORA
Frequency
0
50
150
0.0
0.2
0.4
0.6
0.8
1.0
Methylation Value

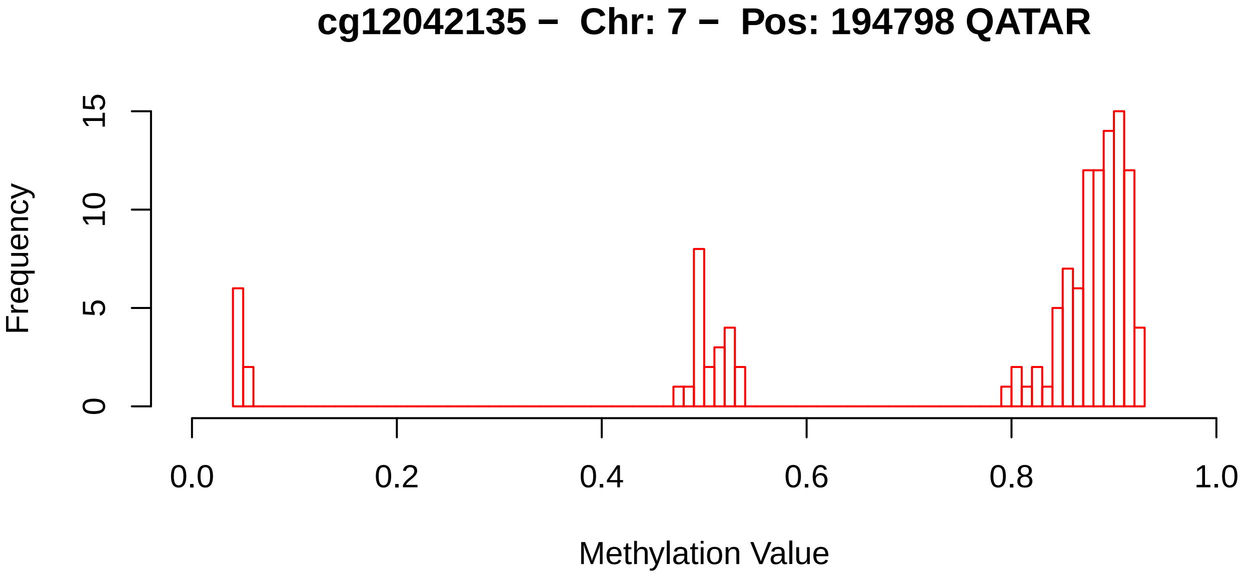
cg12042135 − Chr: 7 − Pos: 194798 QATAR
Frequency
0
5
10
15
0.0
0.2
0.4
0.6
0.8
1.0
Methylation Value

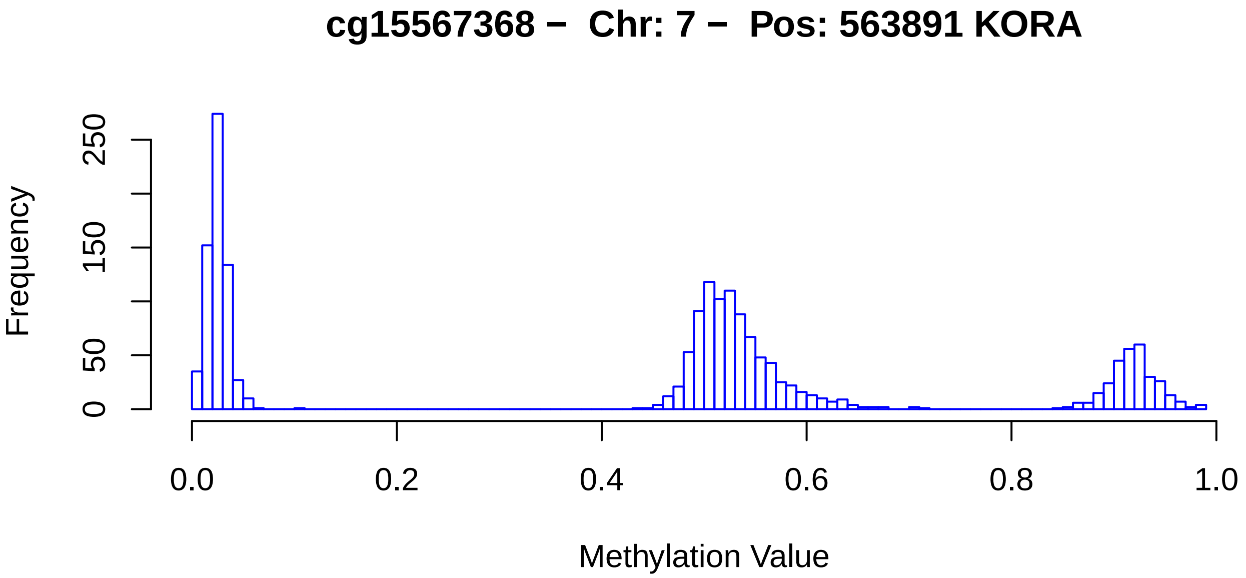
cg15567368 − Chr: 7 − Pos: 563891 KORA
Frequency
0
50
150
250
0.0
0.2
0.4
0.6
0.8
1.0
Methylation Value

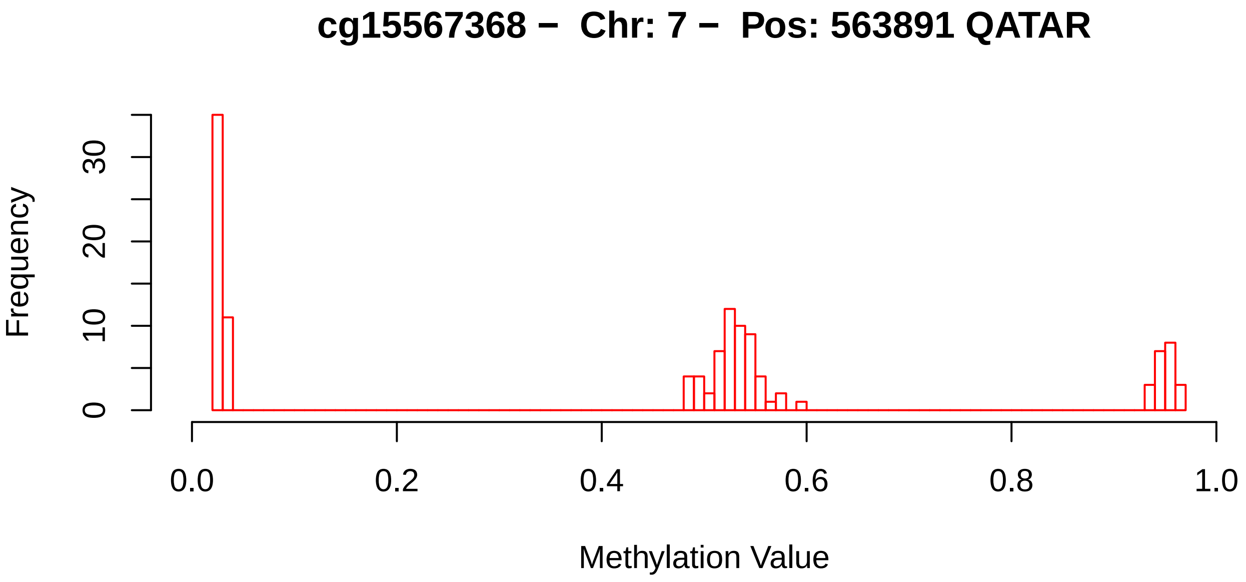
cg15567368 − Chr: 7 − Pos: 563891 QATAR
Frequency
0
10
20
30
0.0
0.2
0.4
0.6
0.8
1.0
Methylation Value

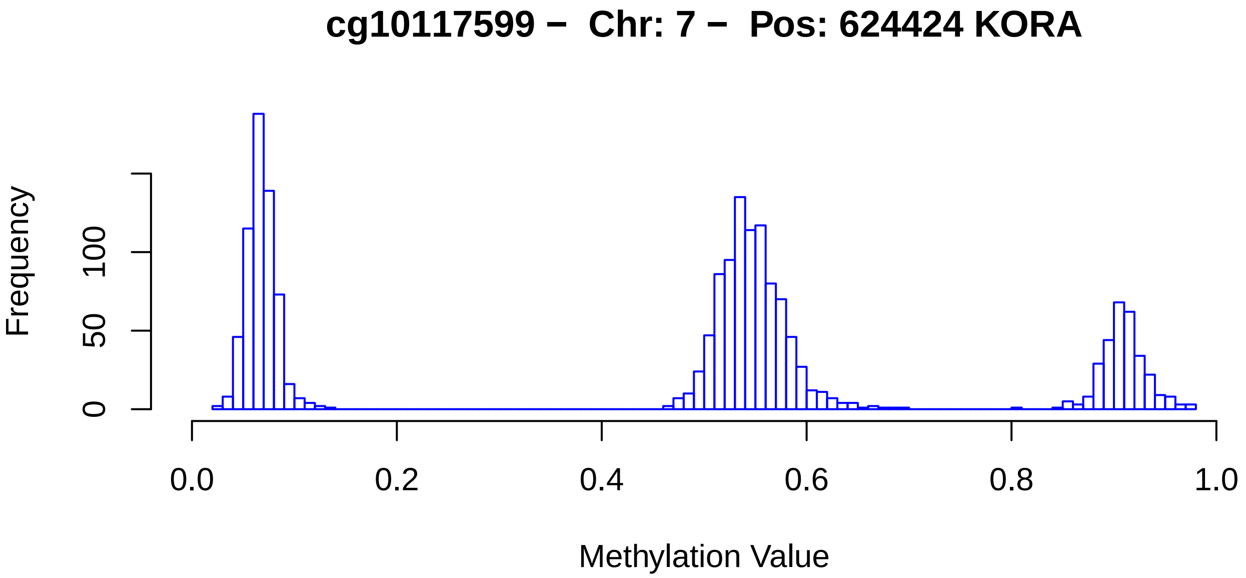
cg10117599 − Chr: 7 − Pos: 624424 KORA
Frequency
0
50
100
0.0
0.2
0.4
0.6
0.8
1.0
Methylation Value

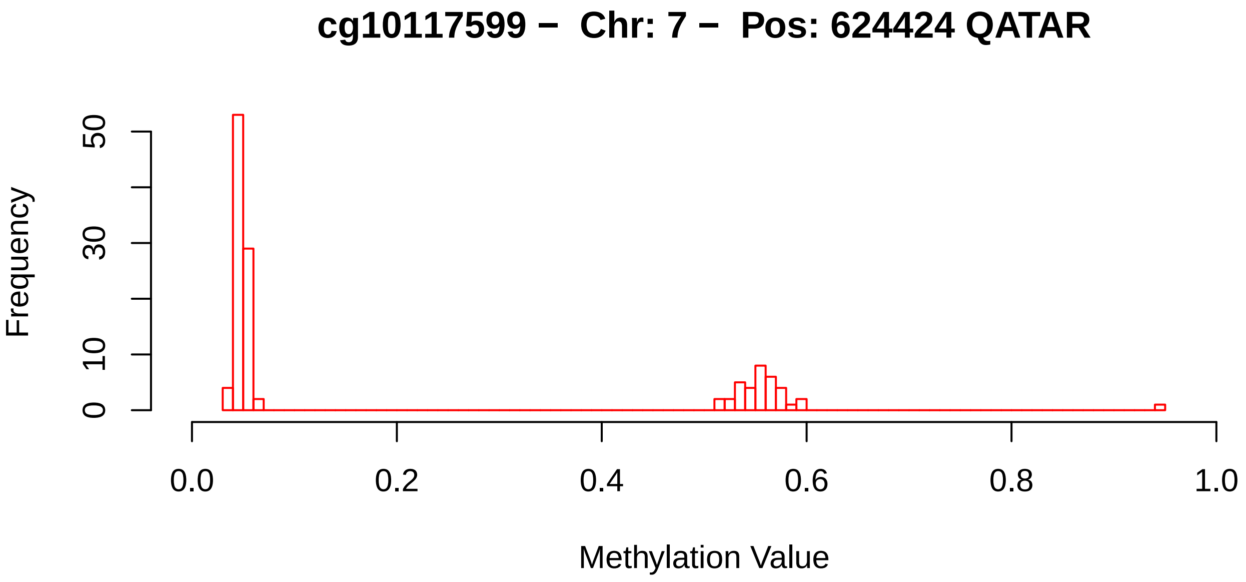
cg10117599 − Chr: 7 − Pos: 624424 QATAR
Frequency
0
10
30
50
0.0
0.2
0.4
0.6
0.8
1.0
Methylation Value

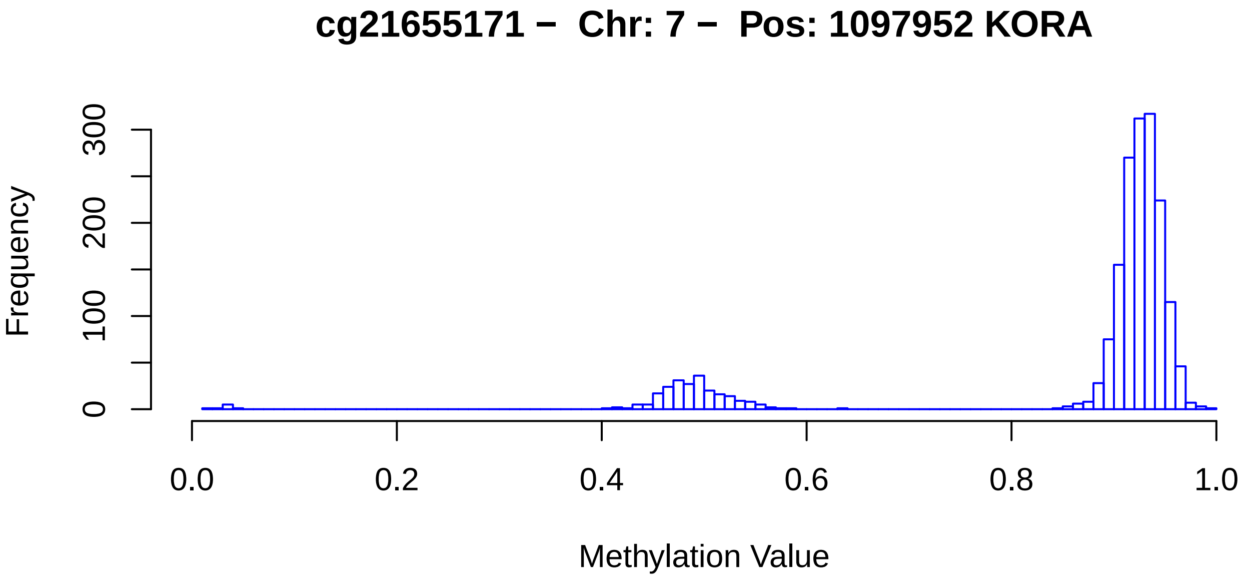
cg21655171 − Chr: 7 − Pos: 1097952 KORA
Frequency
0
100
200
300
0.0
0.2
0.4
0.6
0.8
1.0
Methylation Value

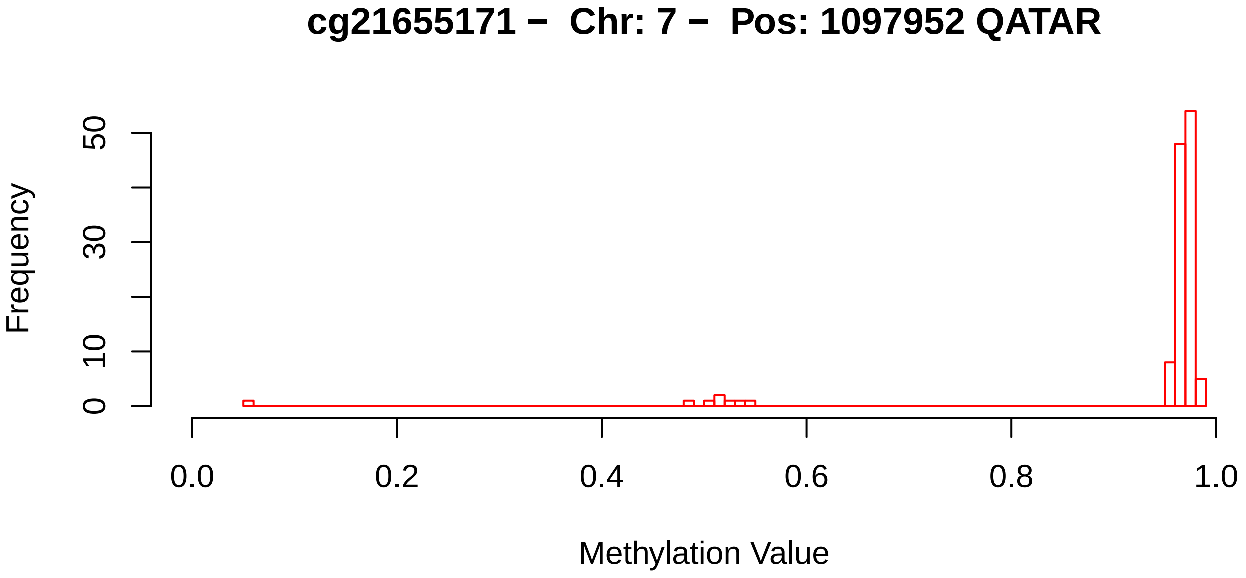
cg21655171 − Chr: 7 − Pos: 1097952 QATAR
Frequency
0
10
30
50
0.0
0.2
0.4
0.6
0.8
1.0
Methylation Value

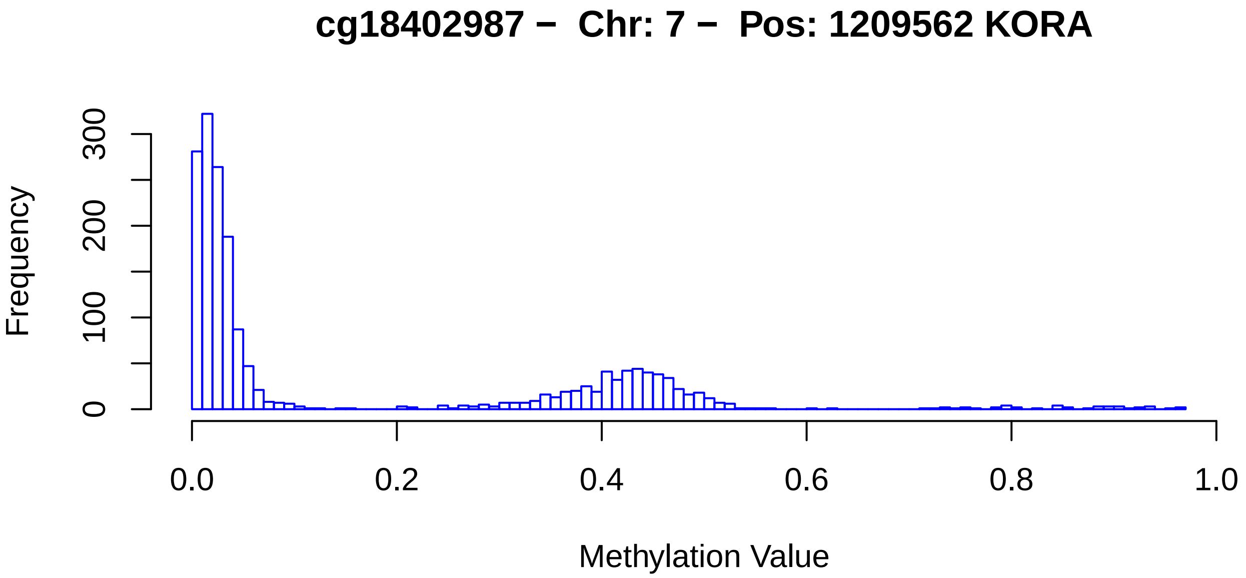

cg18402987 − Chr: 7 − Pos: 1209562 KORA
Frequency
0
100
200
300
0.0
0.2
0.4
0.6
0.8
1.0
Methylation Value

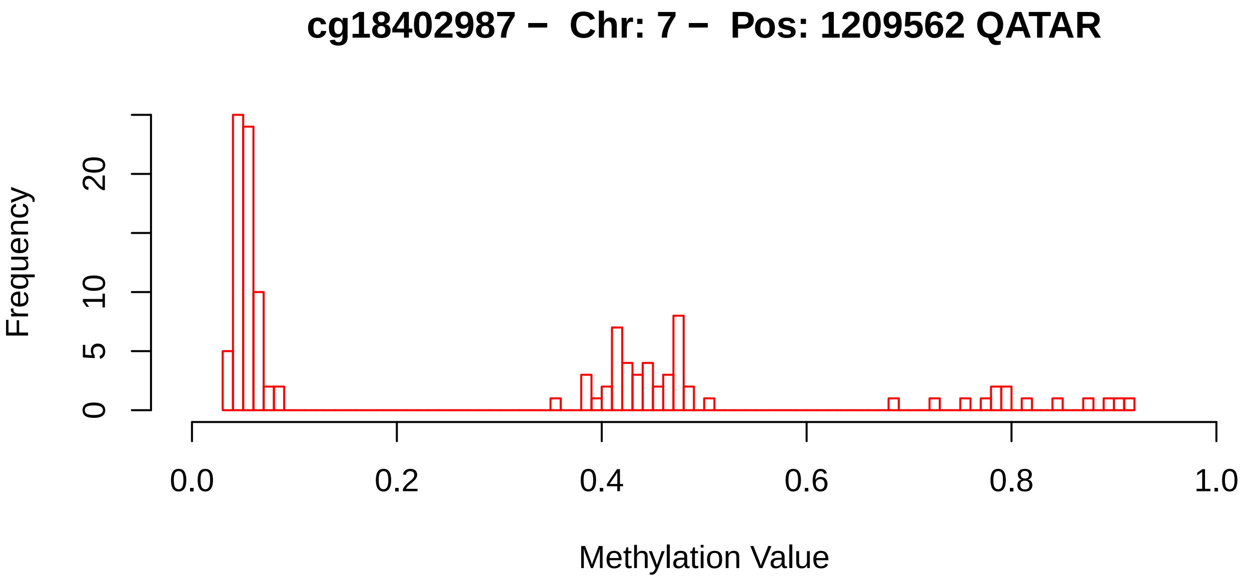

cg18402987 − Chr: 7 − Pos: 1209562 QATAR
Frequency
0
5
10
20
0.0
0.2
0.4
0.6
0.8
1.0
Methylation Value

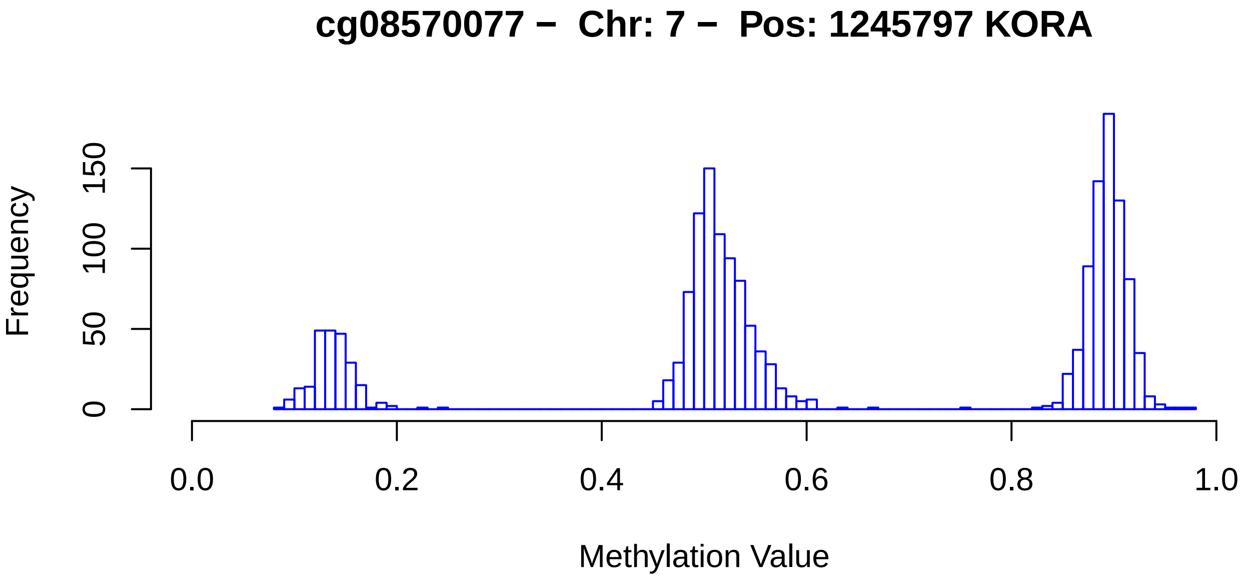
cg08570077 − Chr: 7 − Pos: 1245797 KORA
Frequency
0
50
100
150
0.0
0.2
0.4
0.6
0.8
1.0
Methylation Value

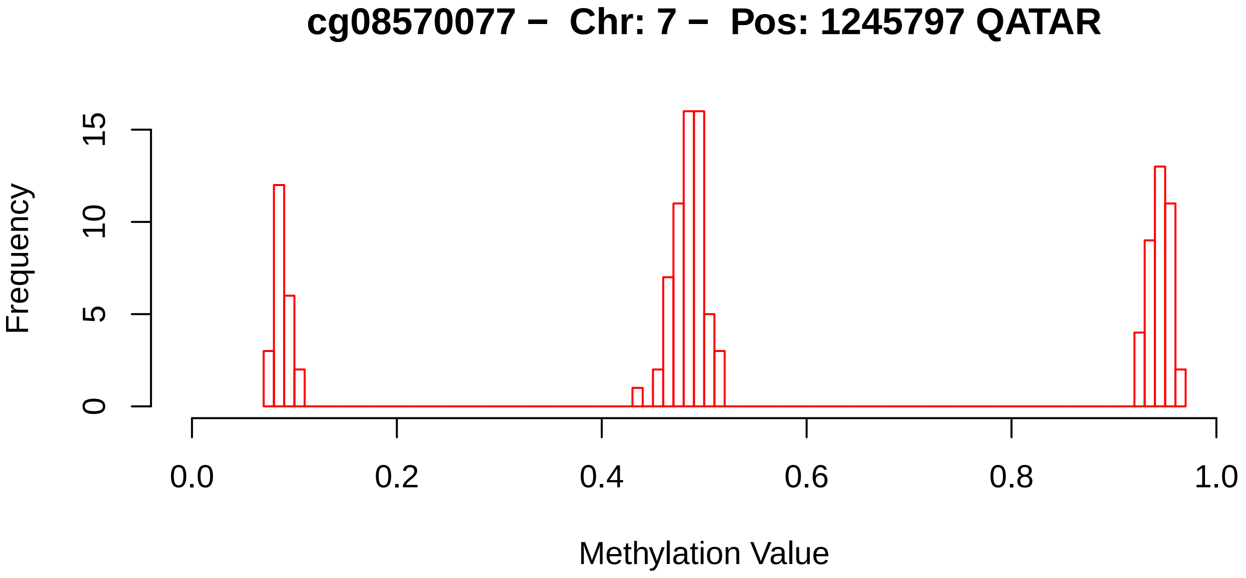
cg08570077 − Chr: 7 − Pos: 1245797 QATAR
Frequency
0
5
10
15
0.0
0.2
0.4
0.6
0.8
1.0
Methylation Value

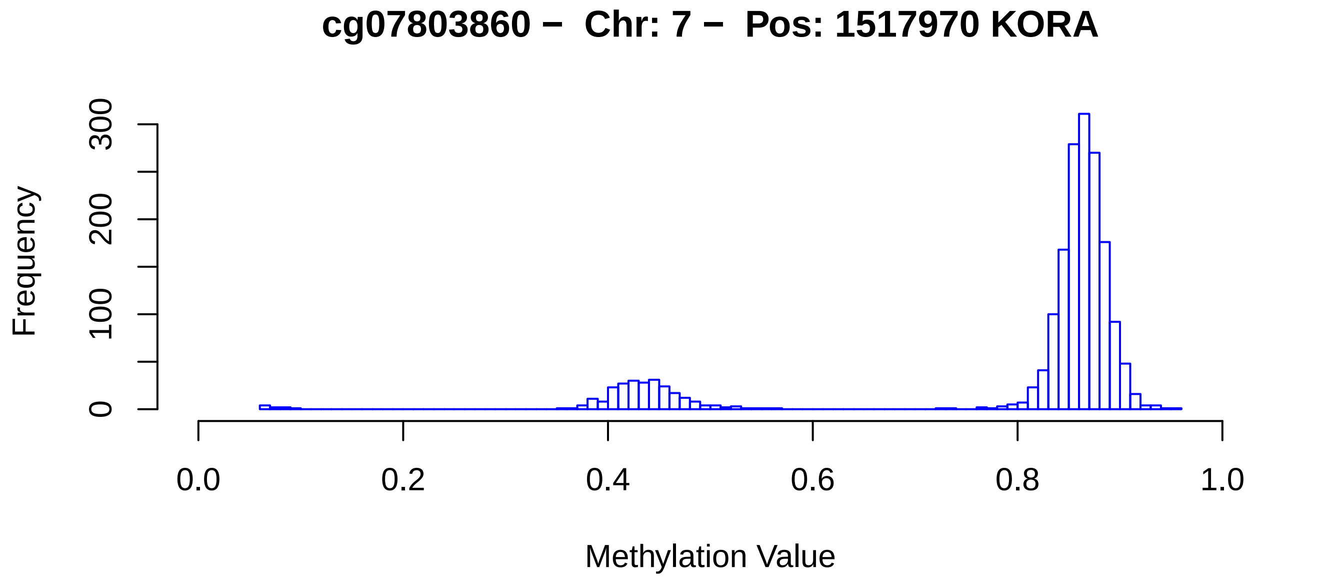

cg07803860 − Chr: 7 − Pos: 1517970 KORA
Frequency
0
100
200
300
0.0
0.2
0.4
0.6
0.8
1.0
Methylation Value

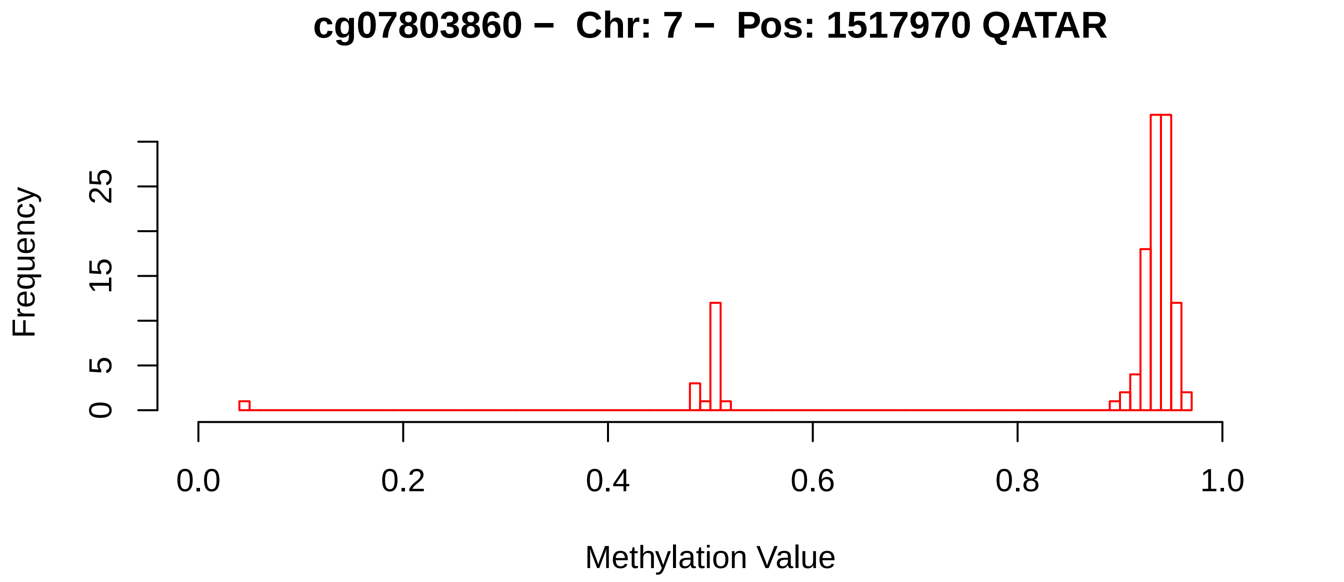

cg07803860 − Chr: 7 − Pos: 1517970 QATAR
Frequency
0
5
15
25
0.0
0.2
0.4
0.6
0.8
1.0
Methylation Value

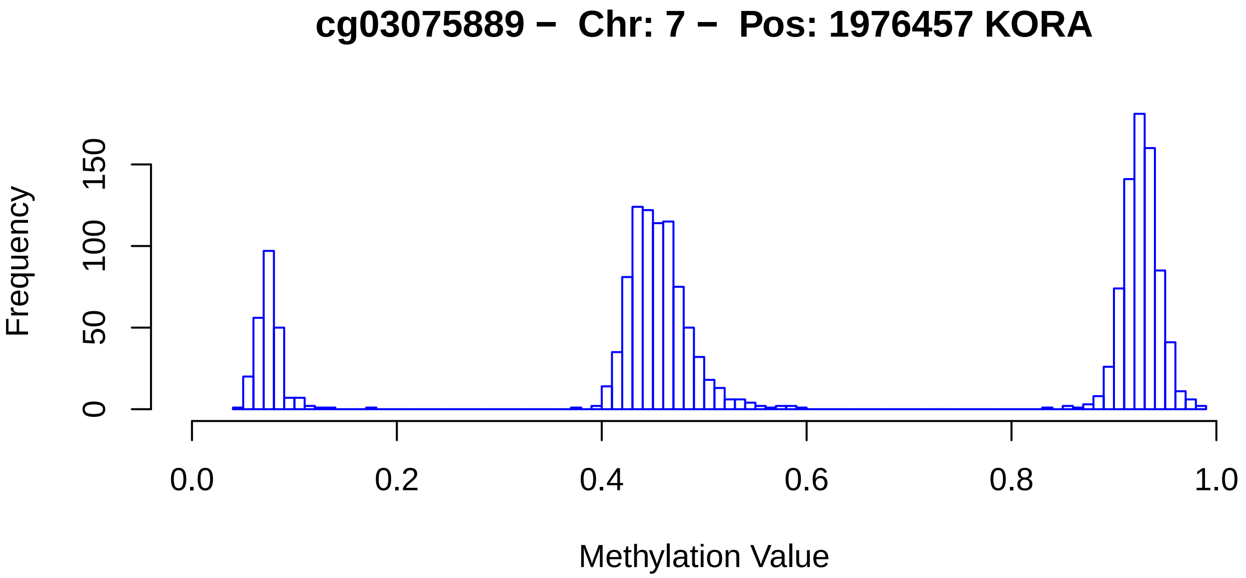
cg03075889 − Chr: 7 − Pos: 1976457 KORA
Frequency
0
50
100
150
0.0
0.2
0.4
0.6
0.8
1.0
Methylation Value

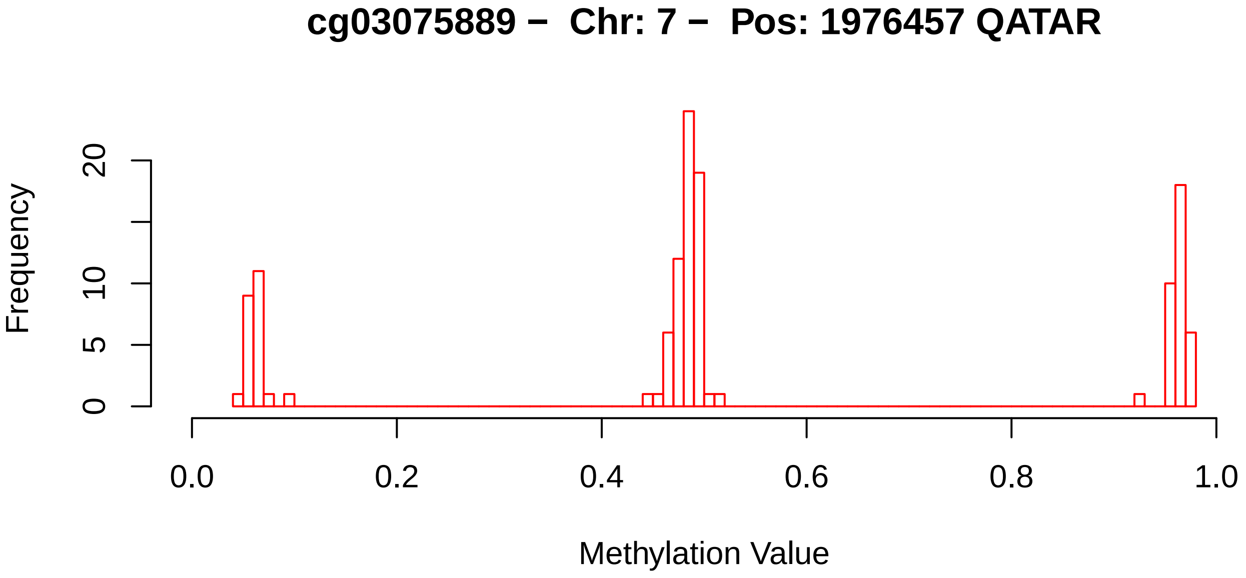
cg03075889 − Chr: 7 − Pos: 1976457 QATAR
Frequency
0
5
10
20
0.0
0.2
0.4
0.6
0.8
1.0
Methylation Value

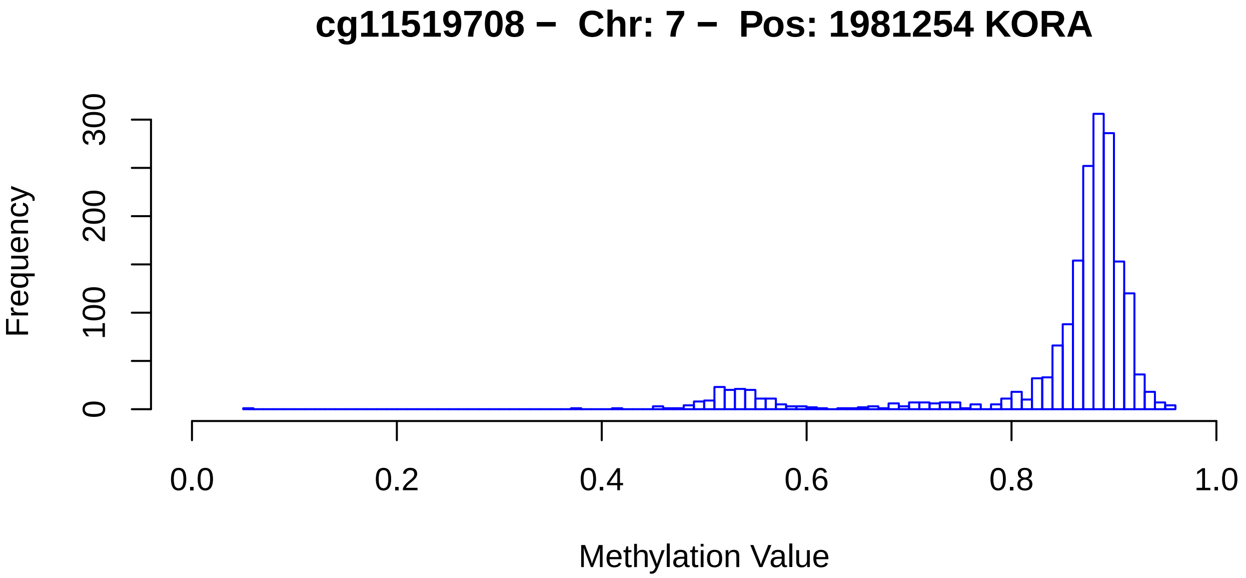
cg11519708 − Chr: 7 − Pos: 1981254 KORA
Frequency
0
100
200
300
0.0
0.2
0.4
0.6
0.8
1.0
Methylation Value

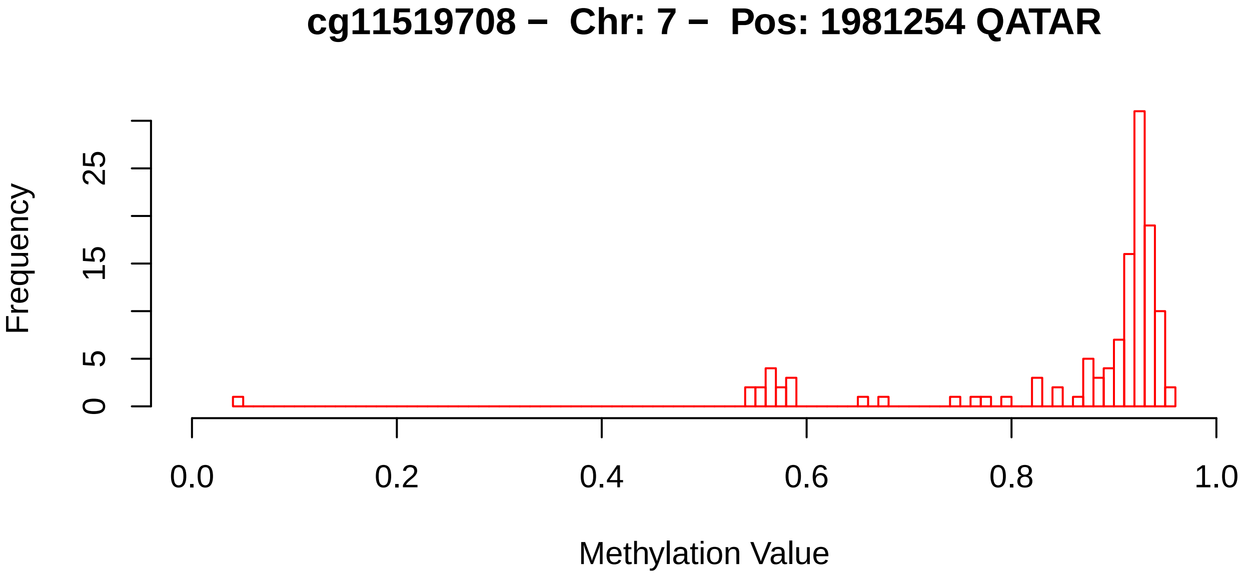
cg11519708 − Chr: 7 − Pos: 1981254 QATAR
Frequency
0
5
15
25
0.0
0.2
0.4
0.6
0.8
1.0
Methylation Value

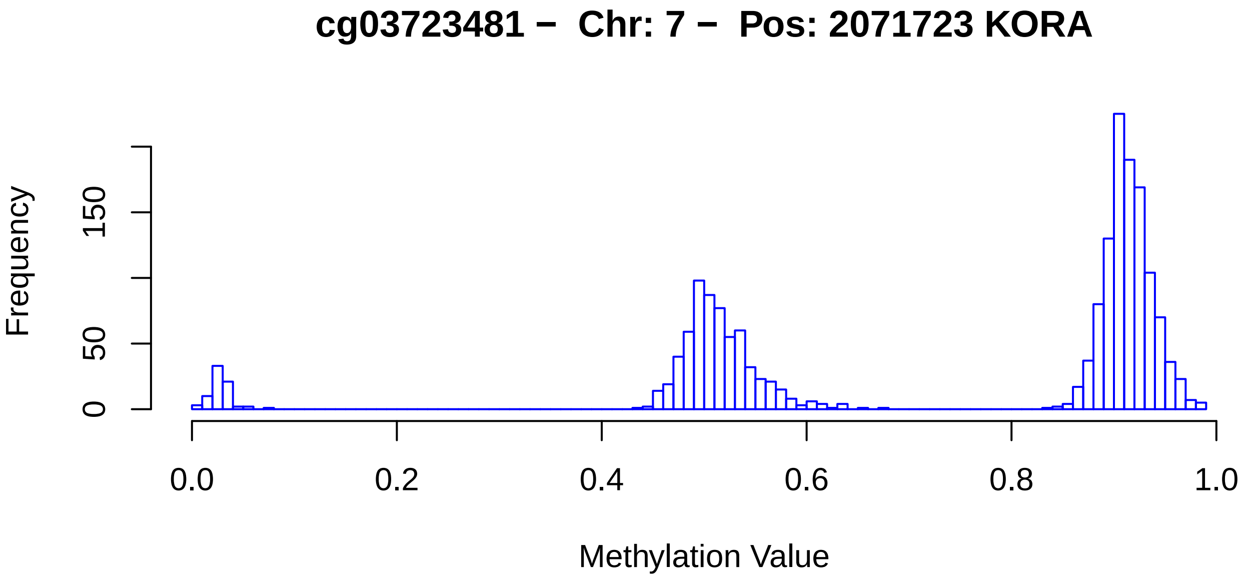
cg03723481 − Chr: 7 − Pos: 2071723 KORA
Frequency
0
50
150
0.0
0.2
0.4
0.6
0.8
1.0
Methylation Value

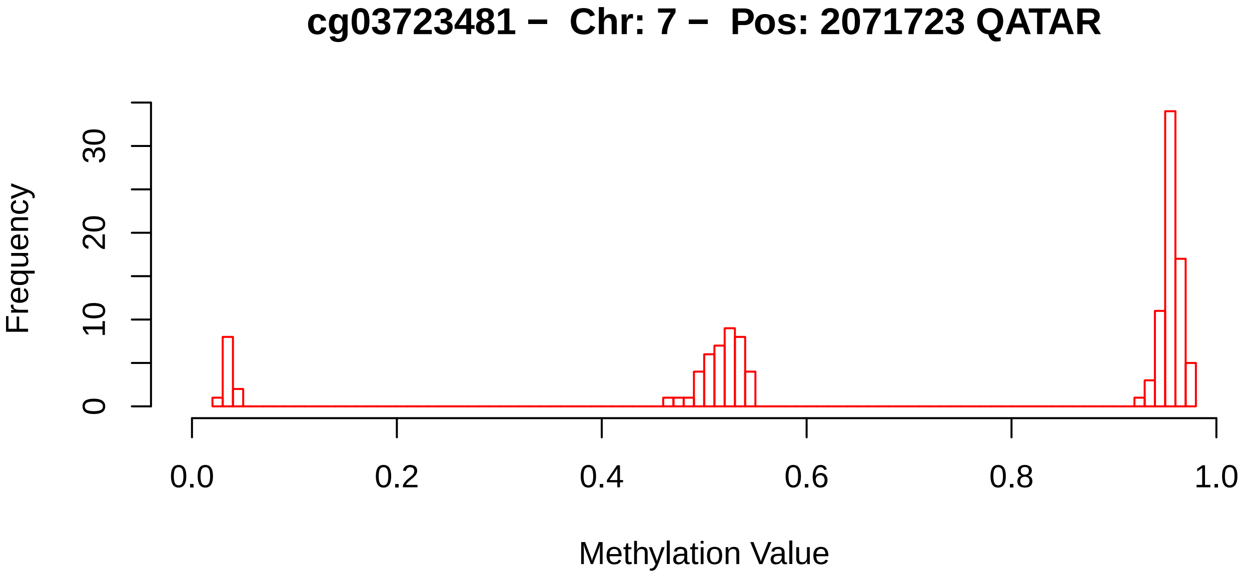
cg03723481 − Chr: 7 − Pos: 2071723 QATAR
Frequency
0
10
20
30
0.0
0.2
0.4
0.6
0.8
1.0
Methylation Value

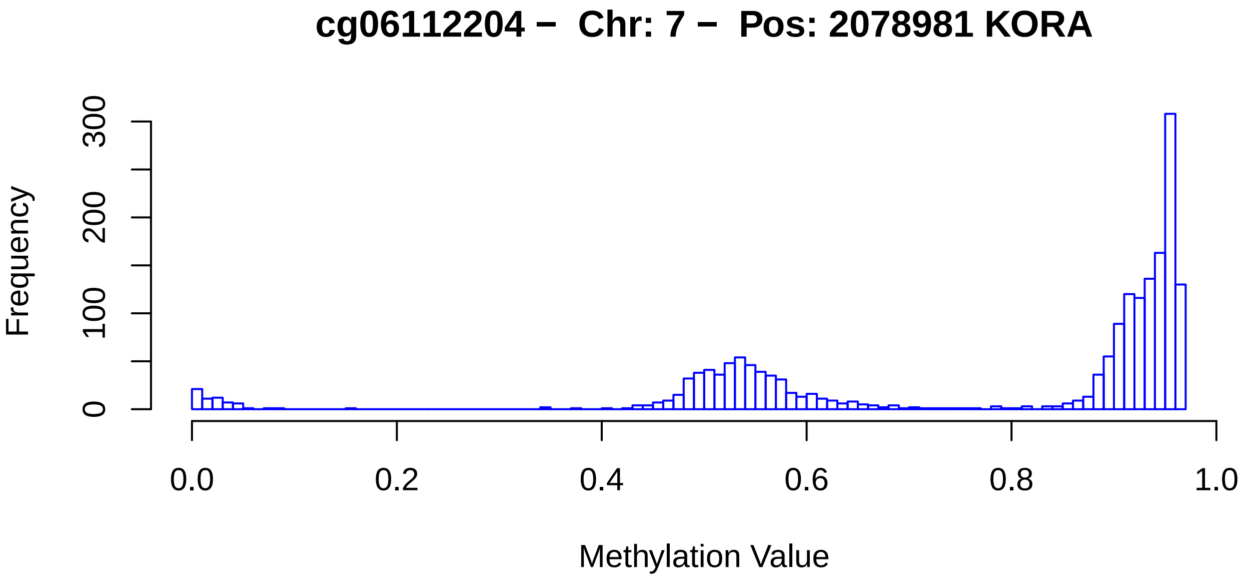
cg06112204 − Chr: 7 − Pos: 2078981 KORA
Frequency
0
100
200
300
0.0
0.2
0.4
0.6
0.8
1.0
Methylation Value

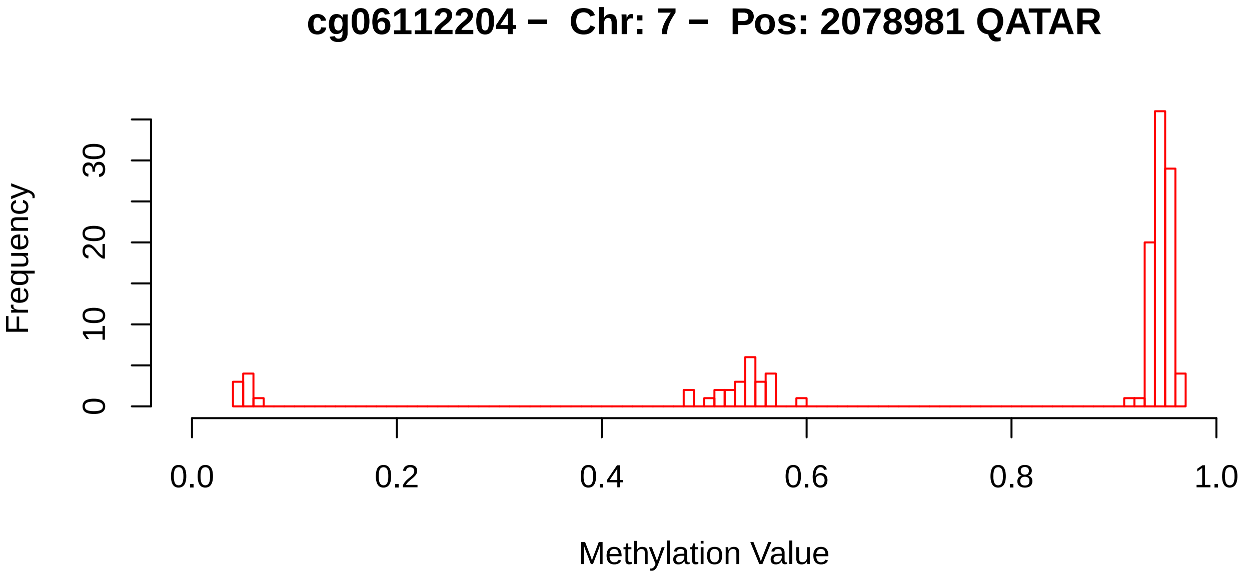
cg06112204 − Chr: 7 − Pos: 2078981 QATAR
Frequency
0
10
20
30
0.0
0.2
0.4
0.6
0.8
1.0
Methylation Value

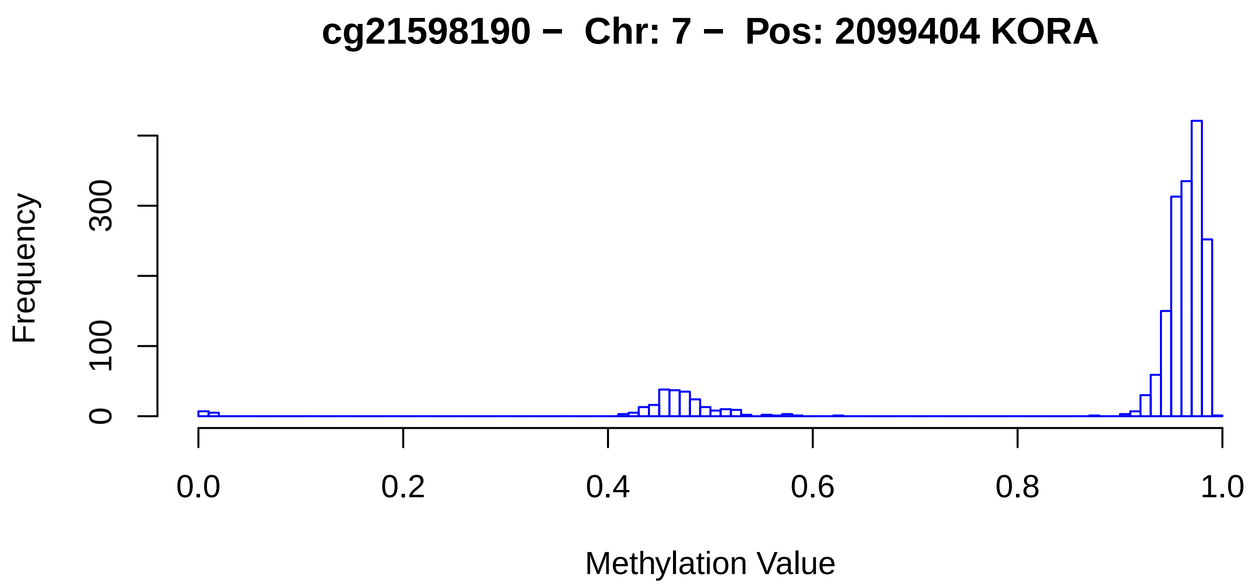
cg21598190 − Chr: 7 − Pos: 2099404 KORA
Frequency
0
100
300
0.0
0.2
0.4
0.6
0.8
1.0
Methylation Value

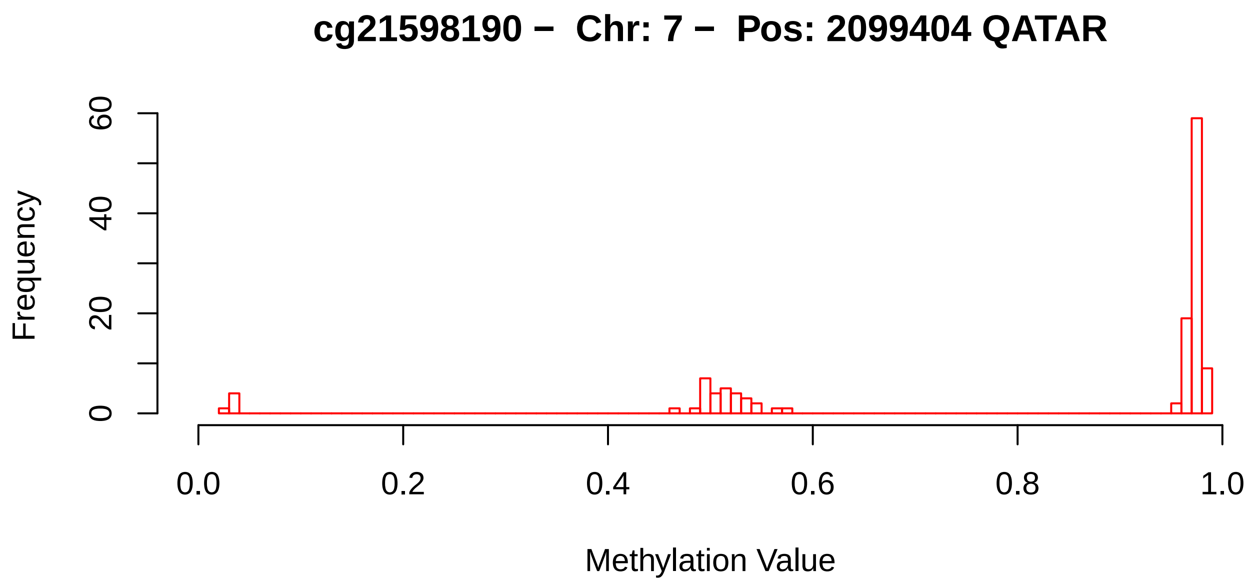
cg21598190 − Chr: 7 − Pos: 2099404 QATAR
Frequency
0
20
40
60
0.0
0.2
0.4
0.6
0.8
1.0
Methylation Value

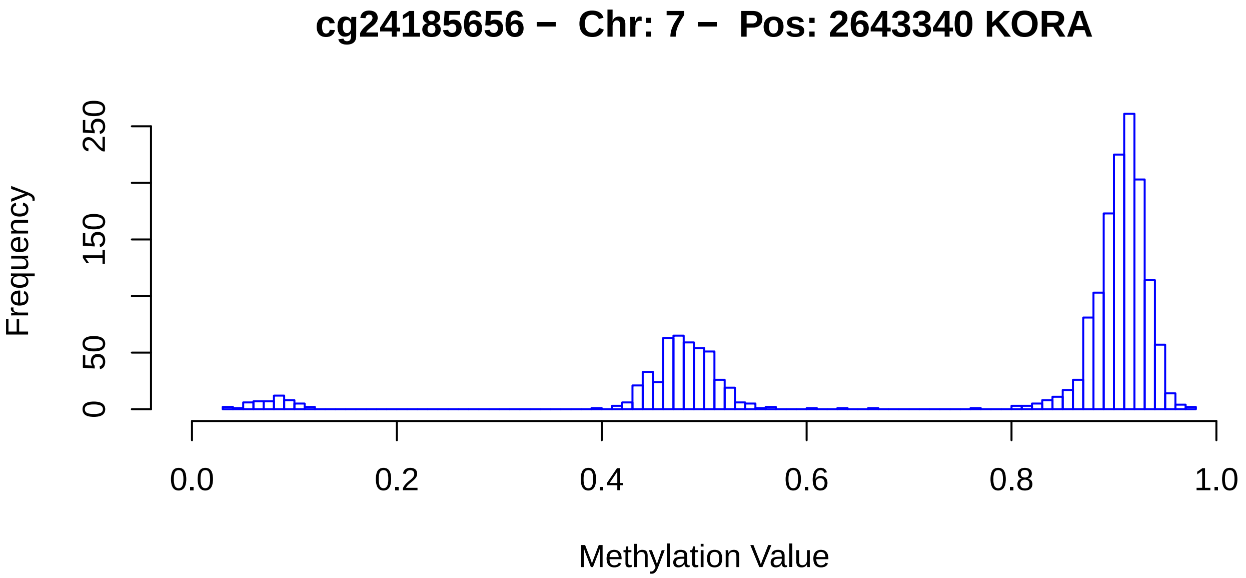
cg24185656 − Chr: 7 − Pos: 2643340 KORA
Frequency
0
50
150
250
0.0
0.2
0.4
0.6
0.8
1.0
Methylation Value

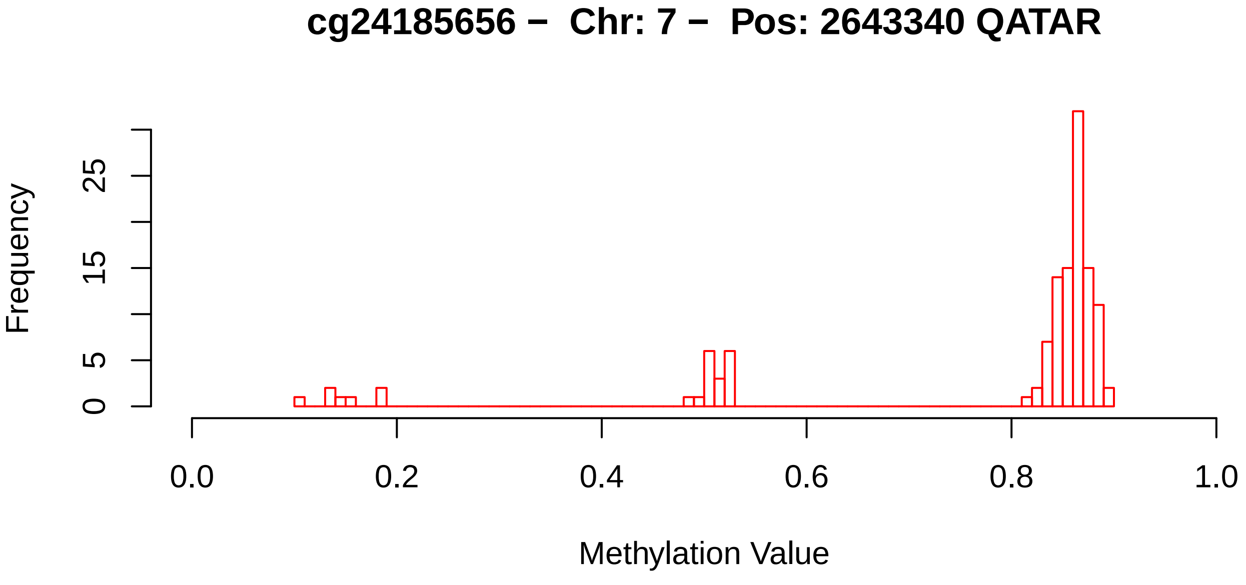
cg24185656 − Chr: 7 − Pos: 2643340 QATAR
Frequency
0
5
15
25
0.0
0.2
0.4
0.6
0.8
1.0
Methylation Value

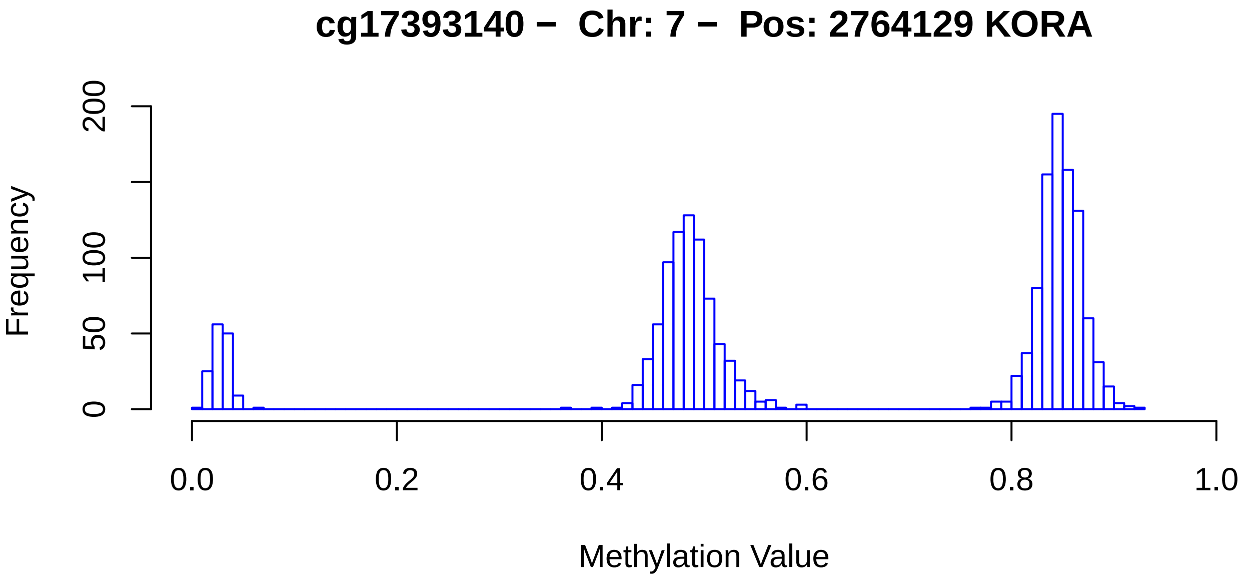
cg17393140 − Chr: 7 − Pos: 2764129 KORA
Frequency
0
50
100
200
0.0
0.2
0.4
0.6
0.8
1.0
Methylation Value

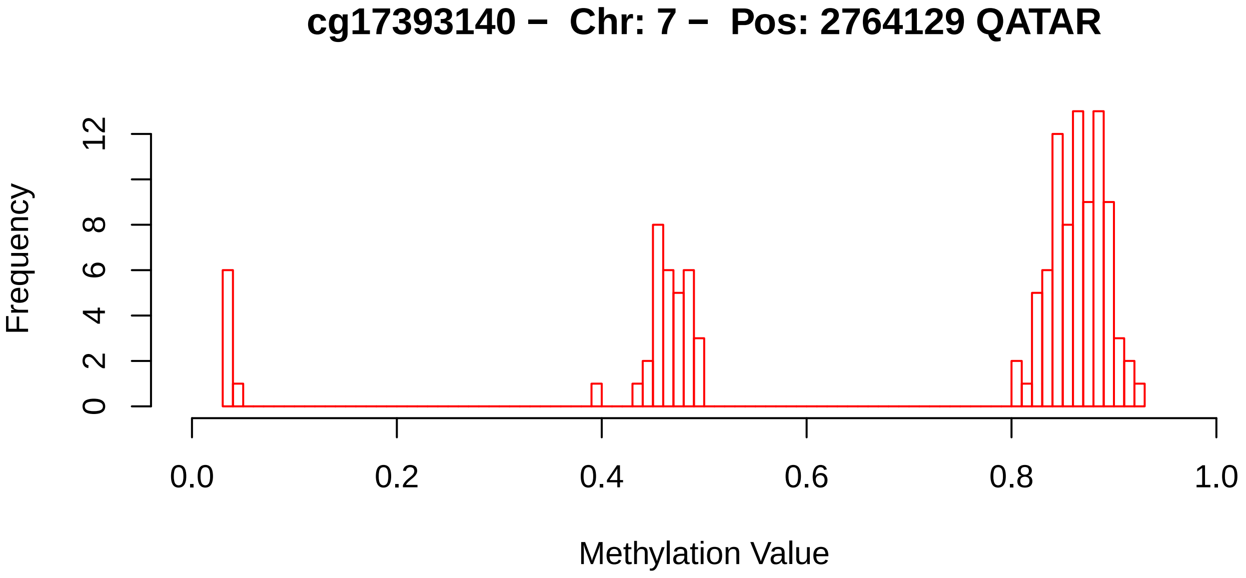
cg17393140 − Chr: 7 − Pos: 2764129 QATAR
Frequency
0
2
4
6
8
12
0.0
0.2
0.4
0.6
0.8
1.0
Methylation Value

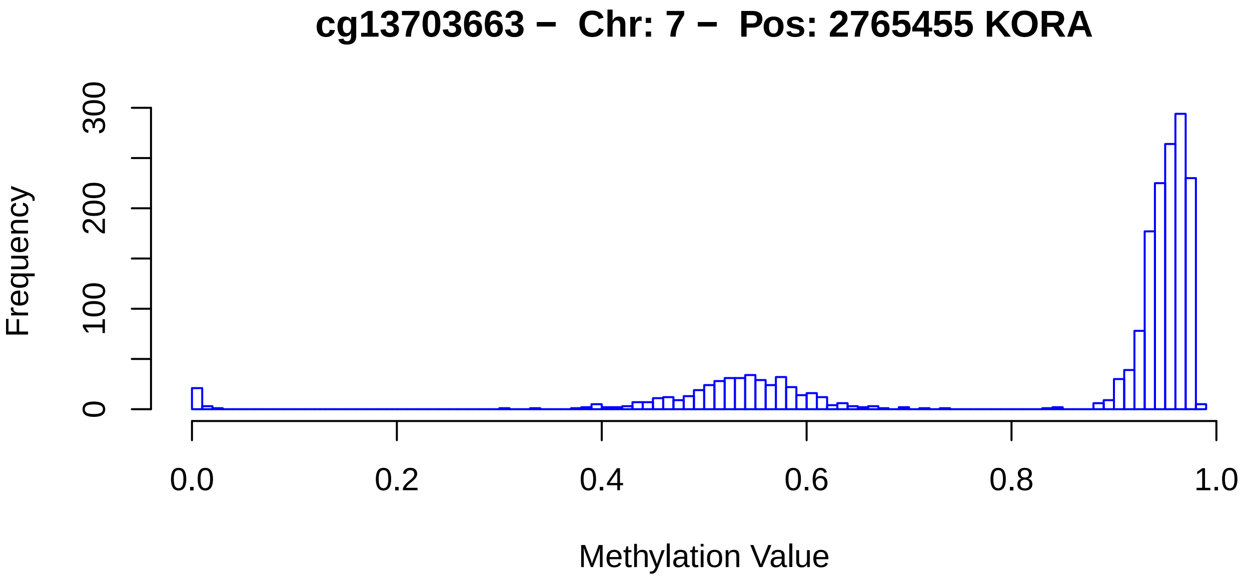
cg13703663 − Chr: 7 − Pos: 2765455 KORA
Frequency
0
100
200
300
0.0
0.2
0.4
0.6
0.8
1.0
Methylation Value

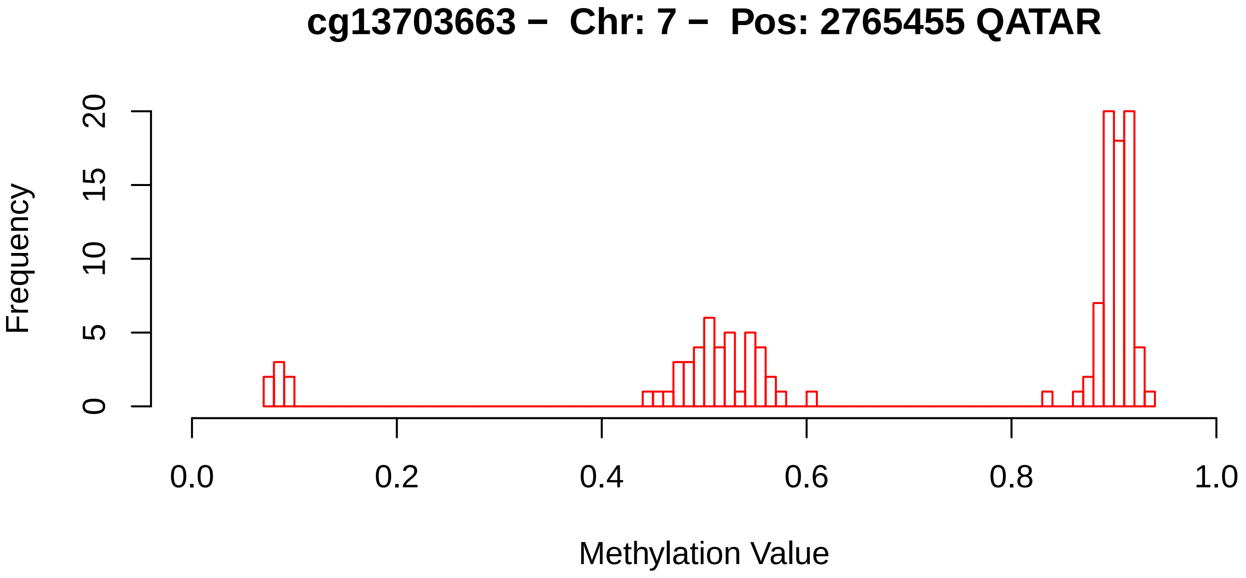
cg13703663 − Chr: 7 − Pos: 2765455 QATAR
Frequency
0
5
10
15
20
0.0
0.2
0.4
0.6
0.8
1.0
Methylation Value

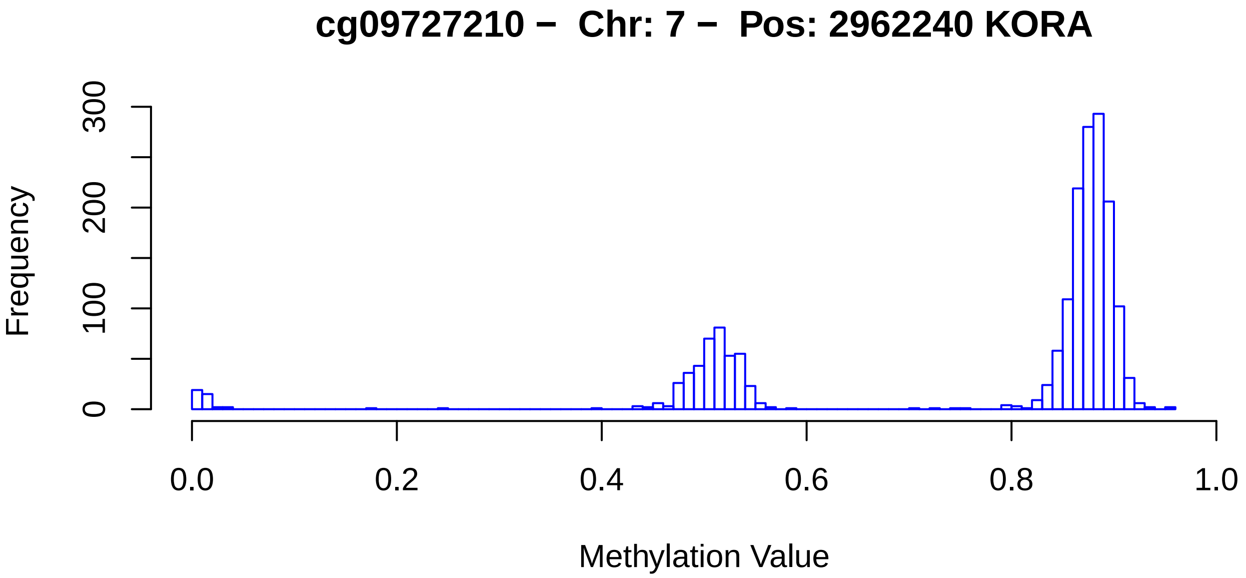
cg09727210 − Chr: 7 − Pos: 2962240 KORA
Frequency
0
100
200
300
0.0
0.2
0.4
0.6
0.8
1.0
Methylation Value

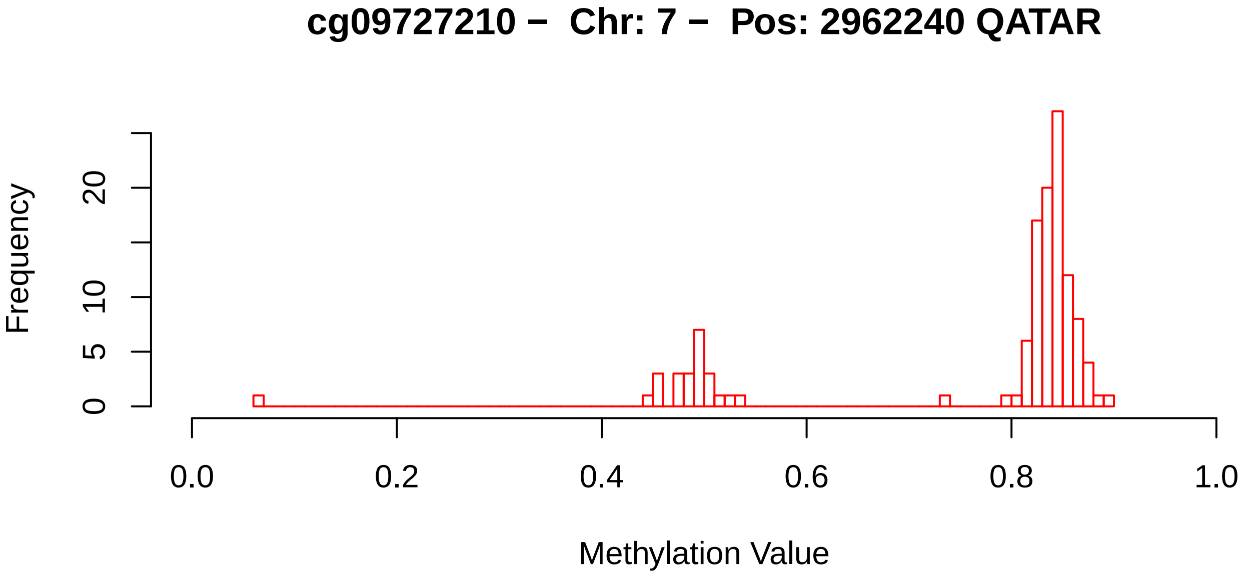
cg09727210 − Chr: 7 − Pos: 2962240 QATAR
Frequency
0
5
10
20
0.0
0.2
0.4
0.6
0.8
1.0
Methylation Value

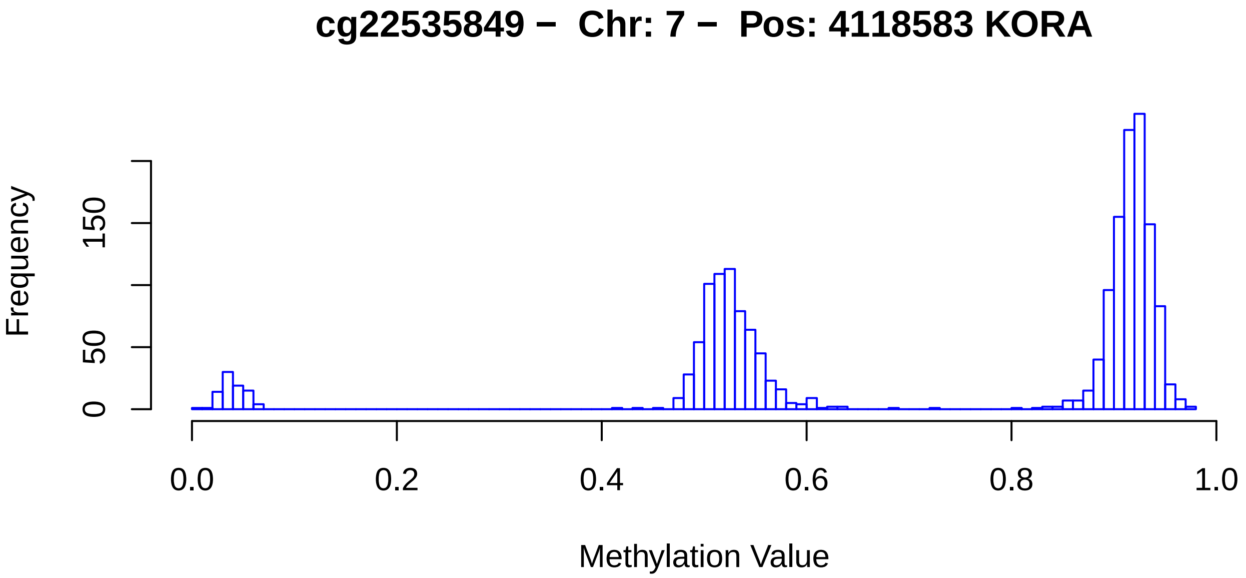
cg22535849 − Chr: 7 − Pos: 4118583 KORA
Frequency
0
50
150
0.0
0.2
0.4
0.6
0.8
1.0
Methylation Value

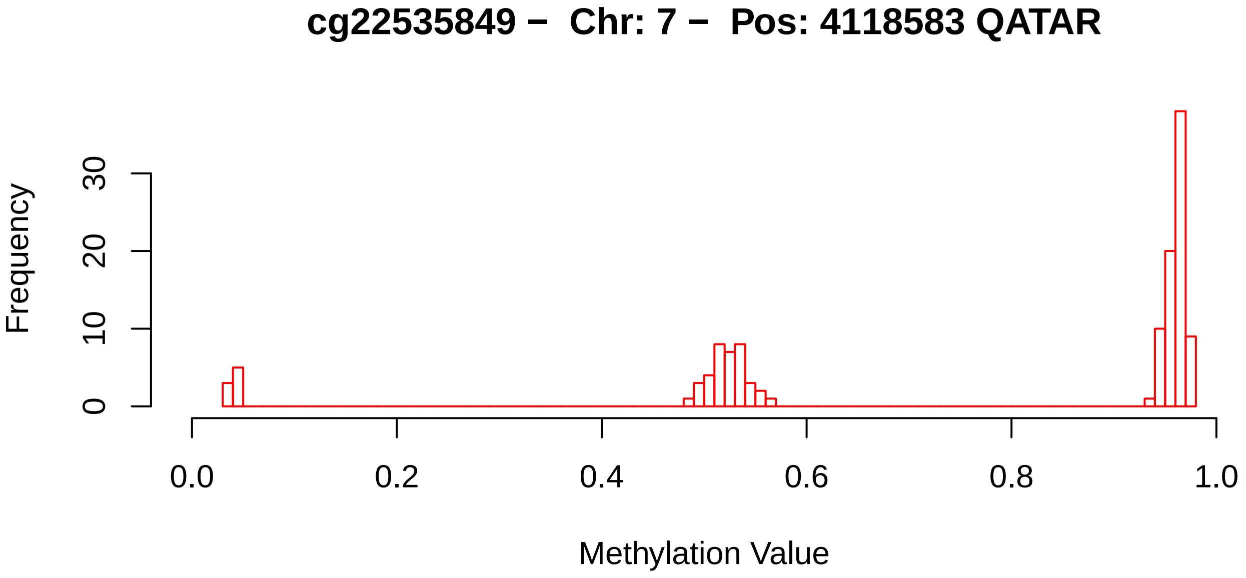
cg22535849 − Chr: 7 − Pos: 4118583 QATAR
Frequency
0
10
20
30
0.0
0.2
0.4
0.6
0.8
1.0
Methylation Value

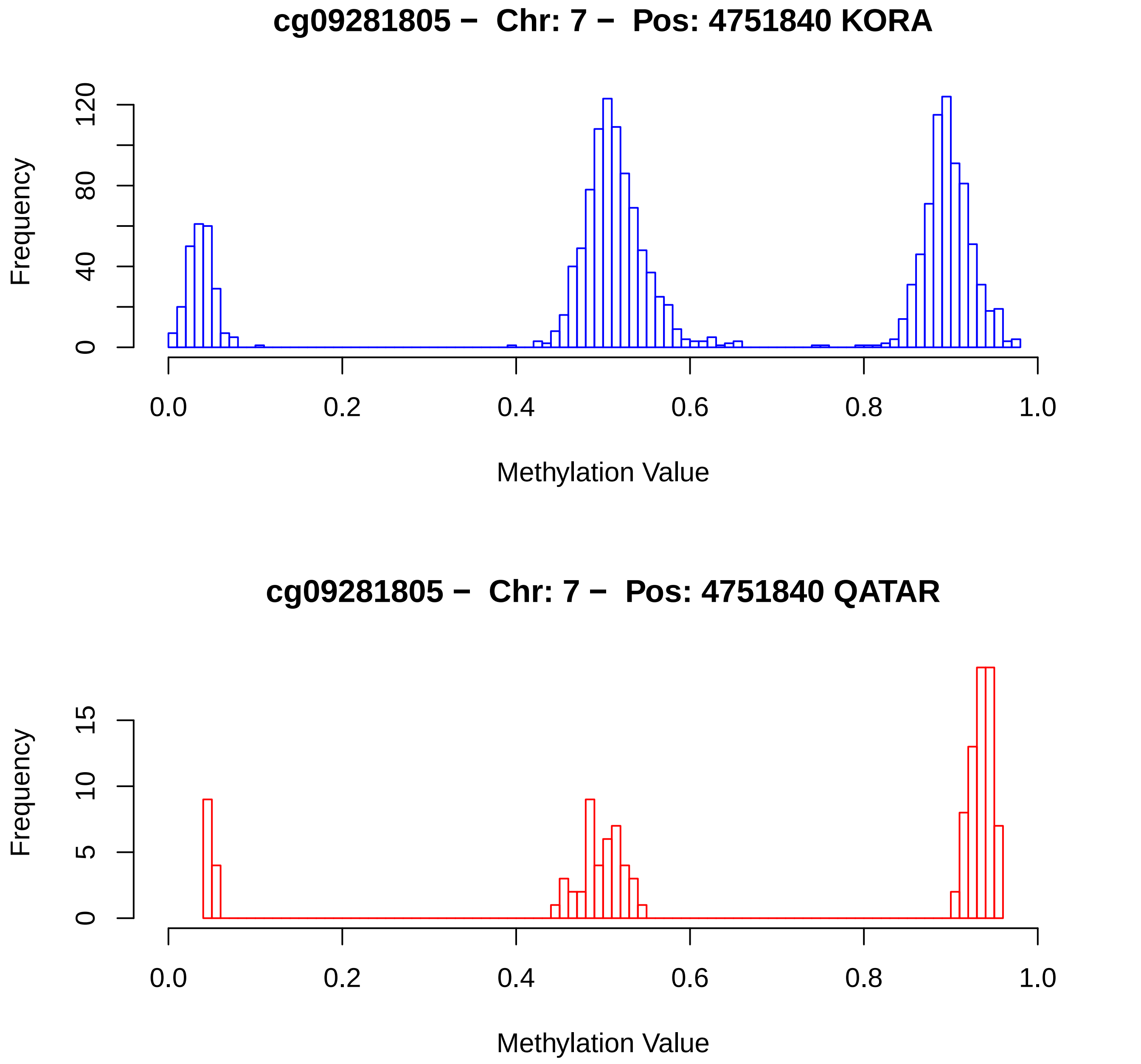

cg09281805 − Chr: 7 − Pos: 4751840 KORA
Frequency
0
40
80
120
0.0
0.2
0.4
0.6
0.8
1.0
Methylation Value
cg09281805 − Chr: 7 − Pos: 4751840 QATAR
Frequency
0
5
10
15
0.0
0.2
0.4
0.6
0.8
1.0
Methylation Value

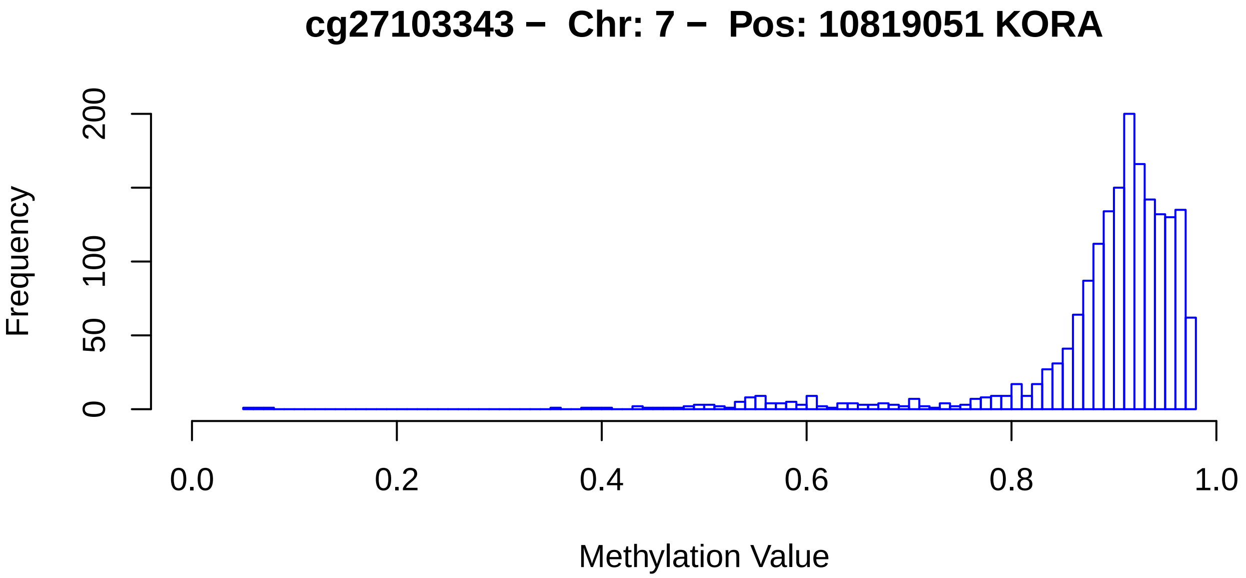
cg27103343 − Chr: 7 − Pos: 10819051 KORA
Frequency
0
50
100
200
0.0
0.2
0.4
0.6
0.8
1.0
Methylation Value

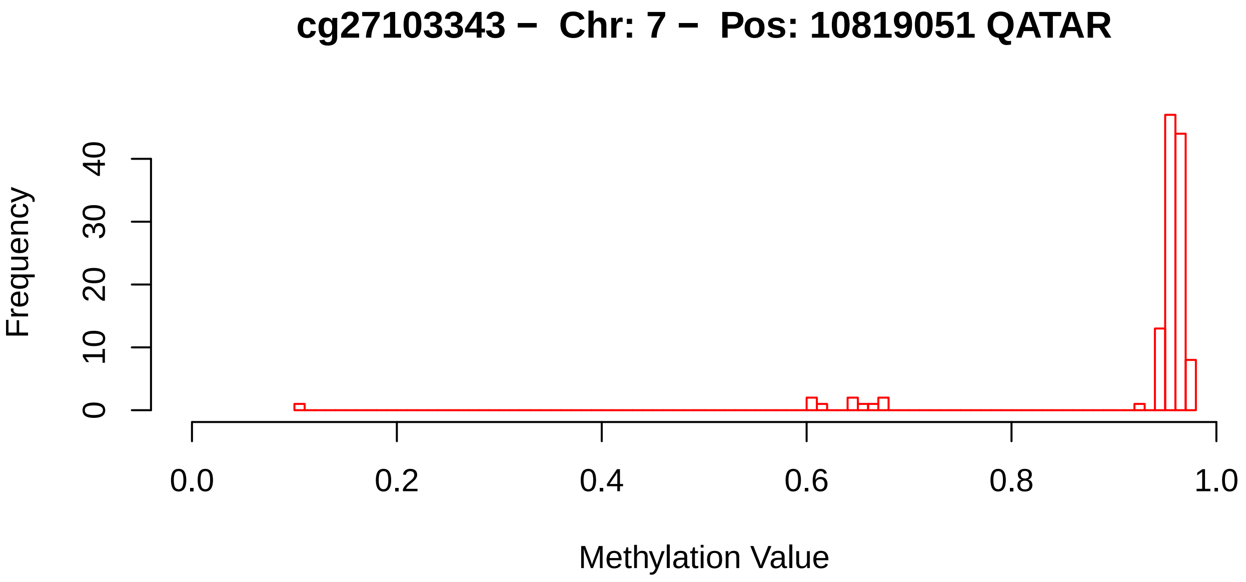
cg27103343 − Chr: 7 − Pos: 10819051 QATAR
Frequency
0
10
20
30
40
0.0
0.2
0.4
0.6
0.8
1.0
Methylation Value

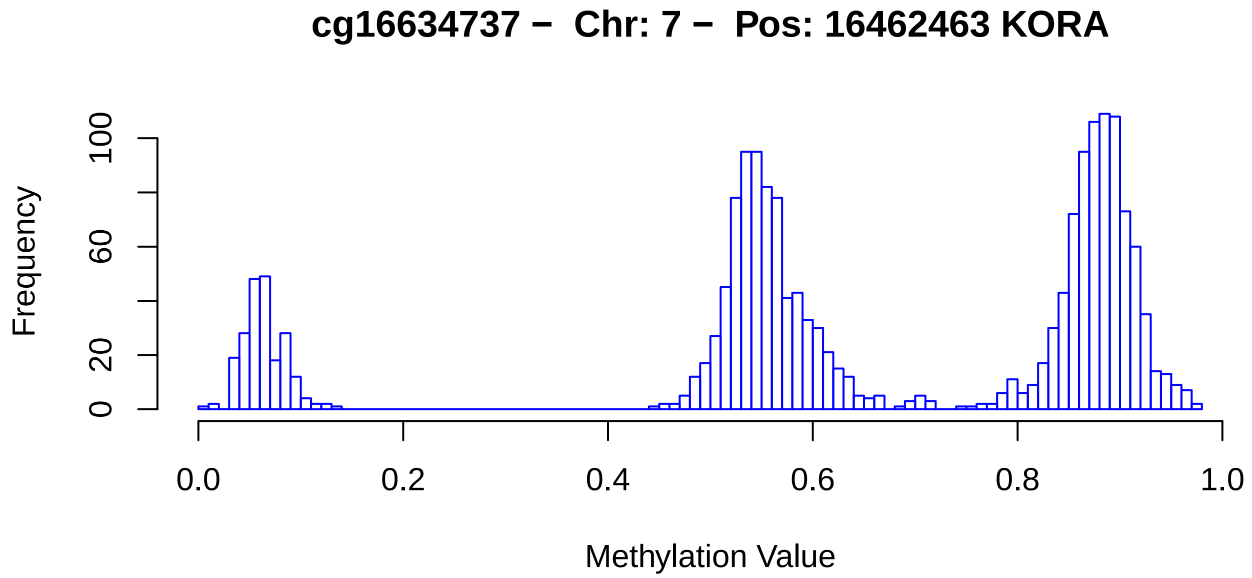
cg16634737 − Chr: 7 − Pos: 16462463 KORA
Frequency
0
20
60
100
0.0
0.2
0.4
0.6
0.8
1.0
Methylation Value

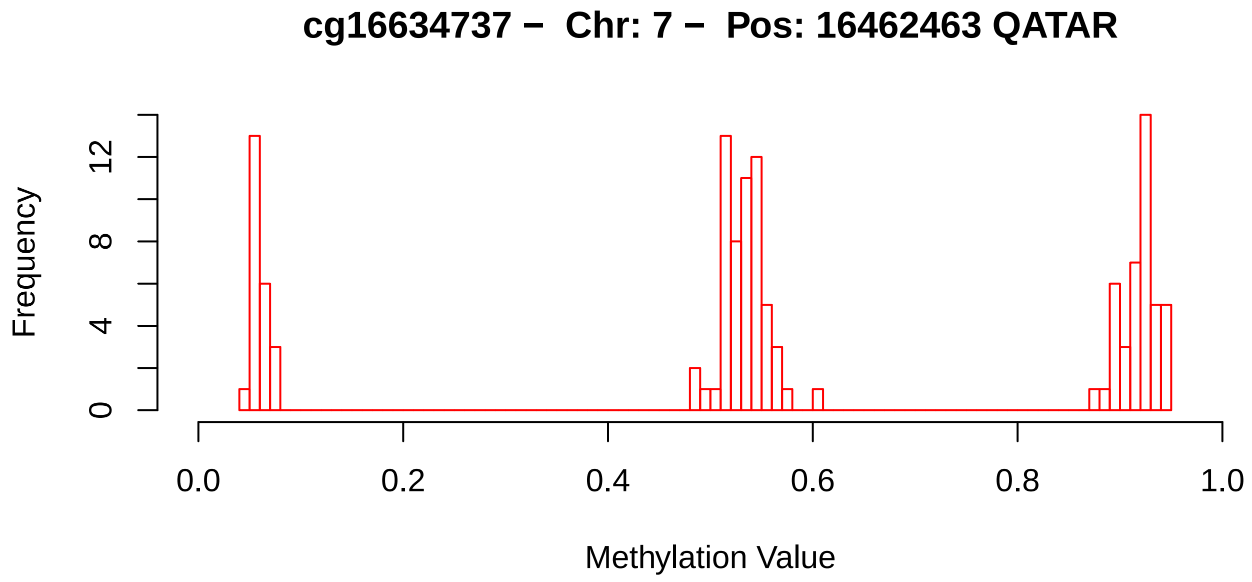
cg16634737 − Chr: 7 − Pos: 16462463 QATAR
Frequency
0
4
8
12
0.0
0.2
0.4
0.6
0.8
1.0
Methylation Value

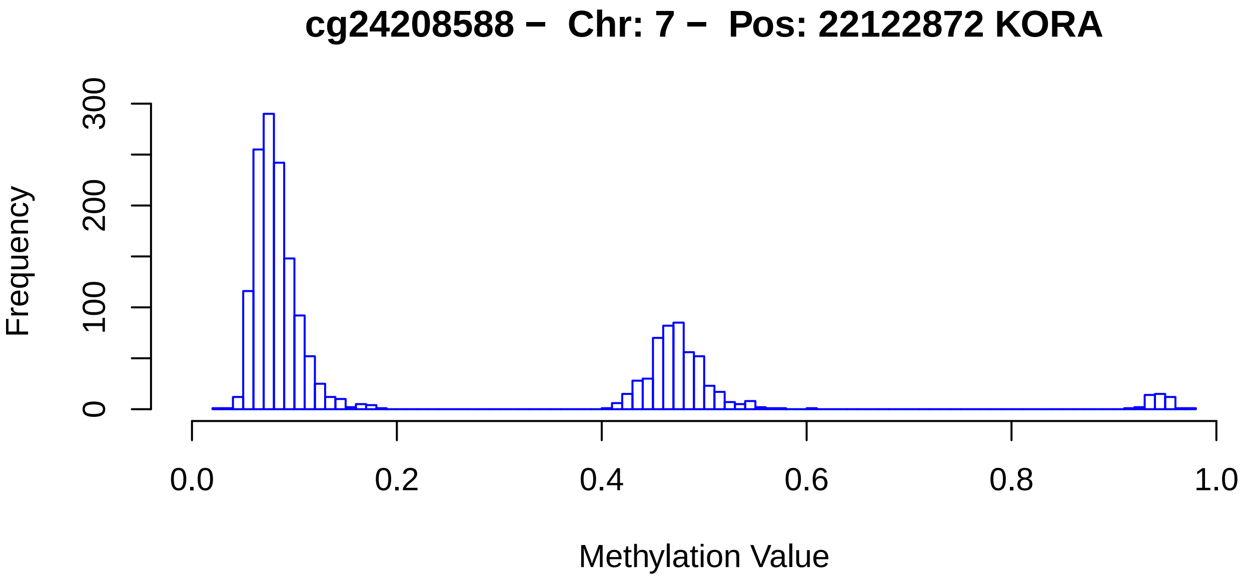
cg24208588 − Chr: 7 − Pos: 22122872 KORA
Frequency
0
100
200
300
0.0
0.2
0.4
0.6
0.8
1.0
Methylation Value

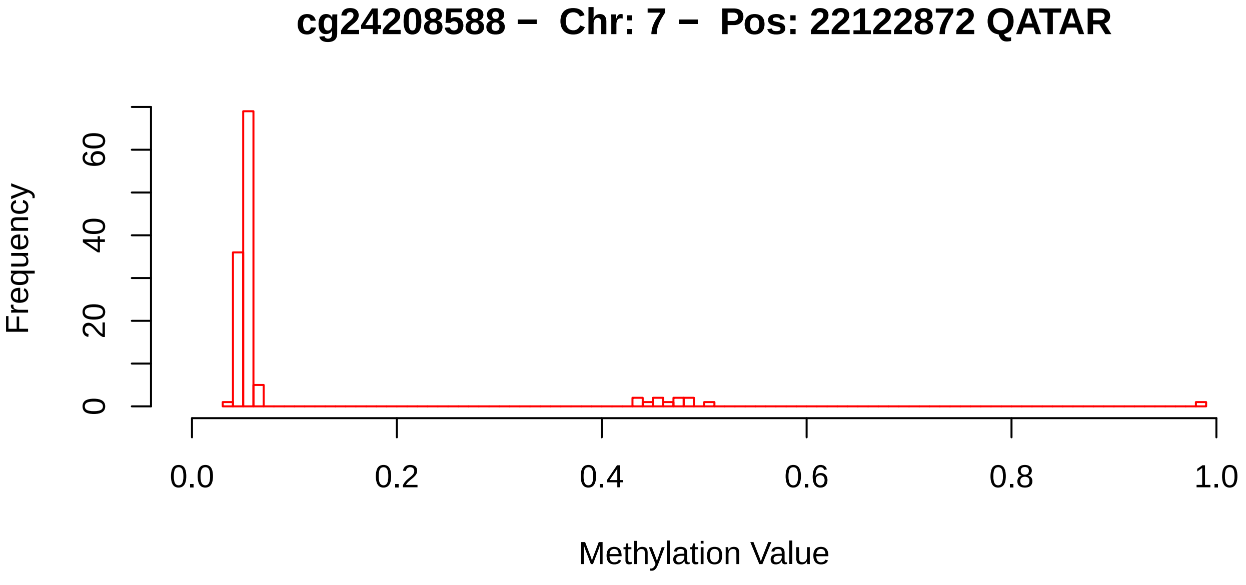
cg24208588 − Chr: 7 − Pos: 22122872 QATAR
Frequency
0
20
40
60
0.0
0.2
0.4
0.6
0.8
1.0
Methylation Value

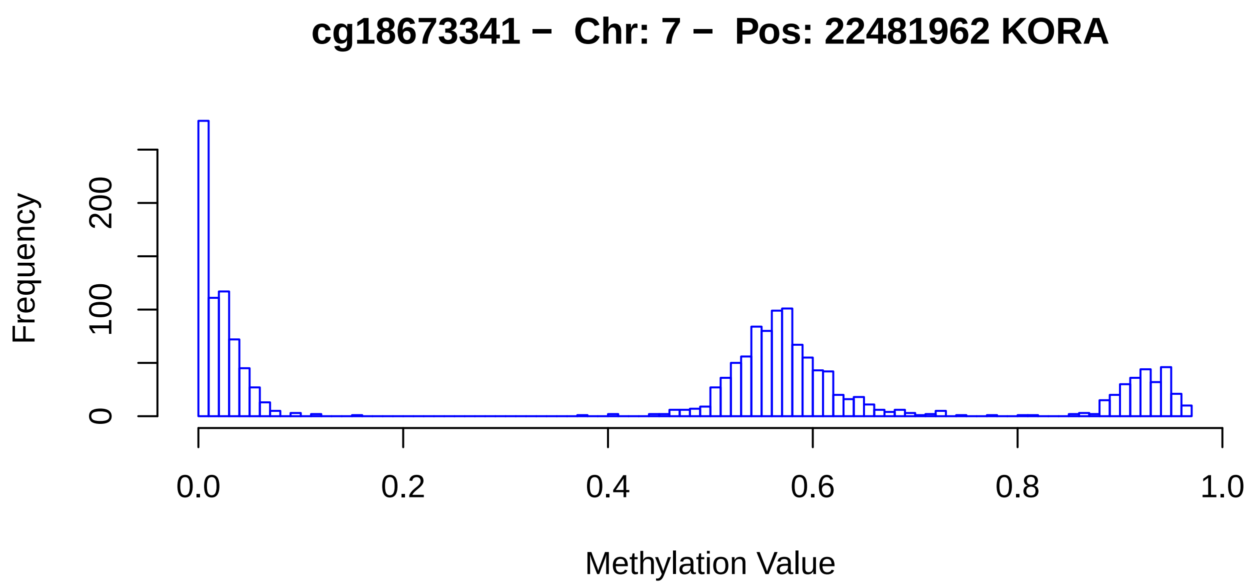
cg18673341 − Chr: 7 − Pos: 22481962 KORA
Frequency
0
100
200
0.0
0.2
0.4
0.6
0.8
1.0
Methylation Value

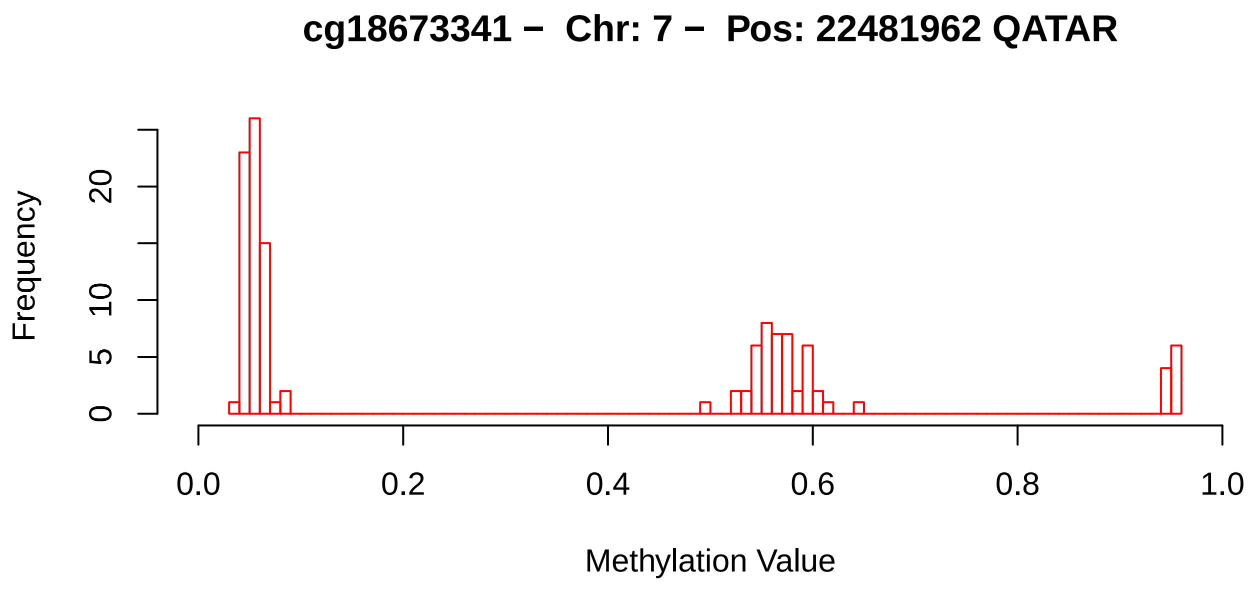
cg18673341 − Chr: 7 − Pos: 22481962 QATAR
Frequency
0
5
10
20
0.0
0.2
0.4
0.6
0.8
1.0
Methylation Value

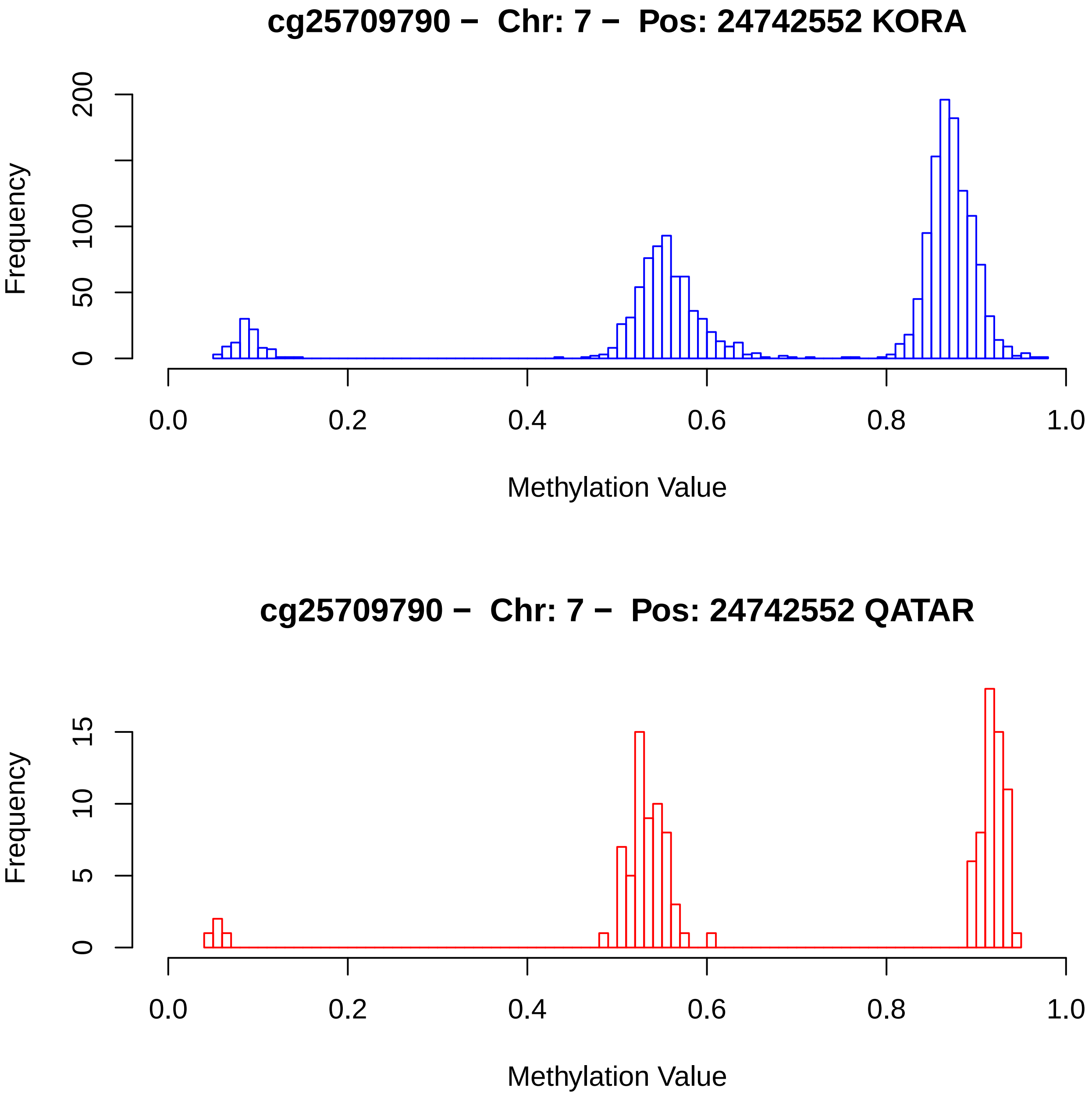

cg25709790 − Chr: 7 − Pos: 24742552 KORA
Frequency
0
50
100
200
0.0
0.2
0.4
0.6
0.8
1.0
Methylation Value
cg25709790 − Chr: 7 − Pos: 24742552 QATAR
Frequency
0
5
10
15
0.0
0.2
0.4
0.6
0.8
1.0
Methylation Value

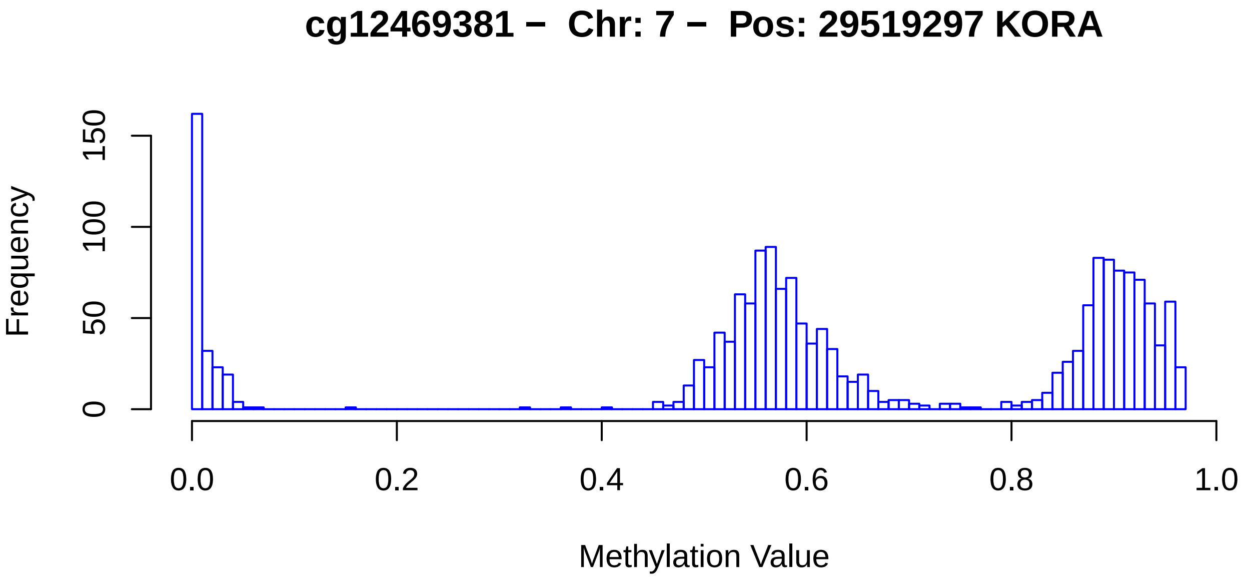
cg12469381 − Chr: 7 − Pos: 29519297 KORA
Frequency
0
50
100
150
0.0
0.2
0.4
0.6
0.8
1.0
Methylation Value

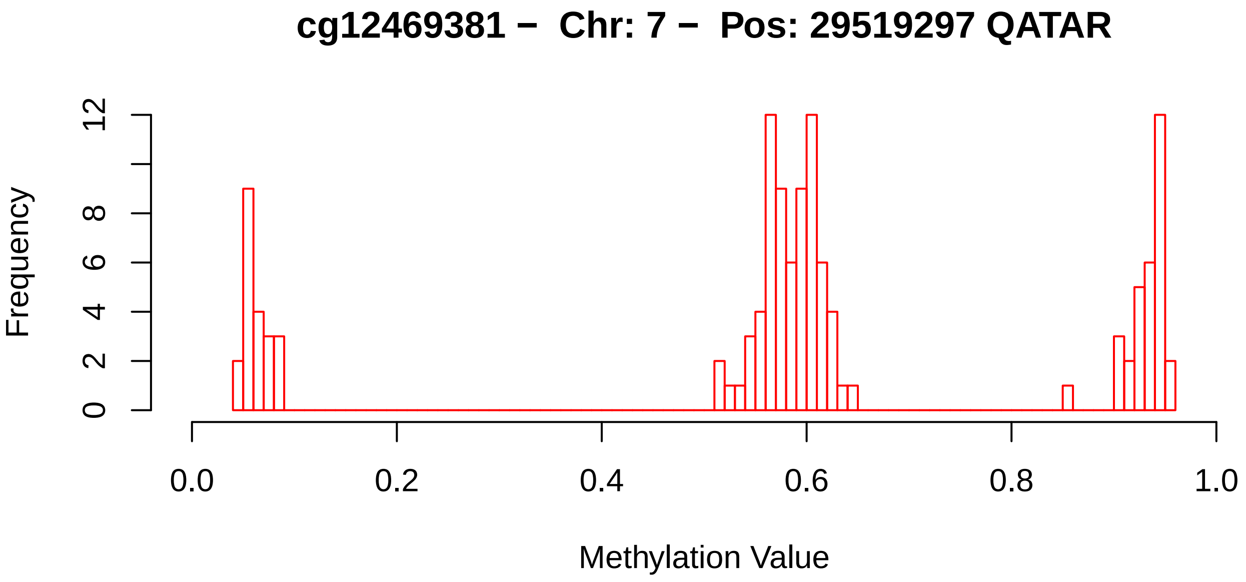
cg12469381 − Chr: 7 − Pos: 29519297 QATAR
Frequency
0
2
4
6
8
12
0.0
0.2
0.4
0.6
0.8
1.0
Methylation Value

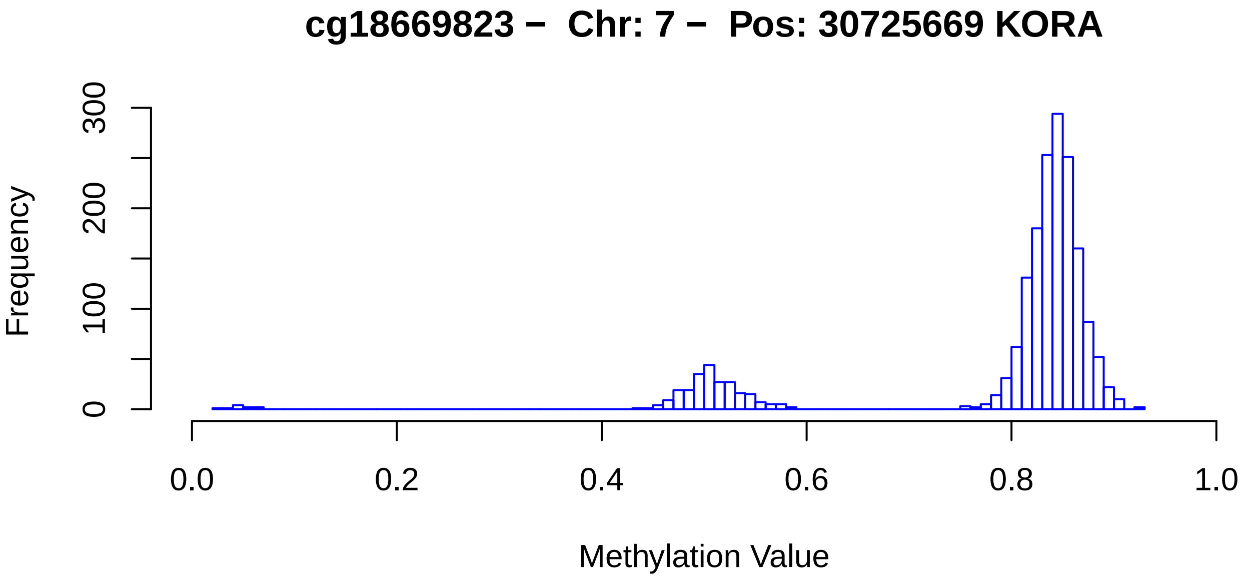
cg18669823 − Chr: 7 − Pos: 30725669 KORA
Frequency
0
100
200
300
0.0
0.2
0.4
0.6
0.8
1.0
Methylation Value

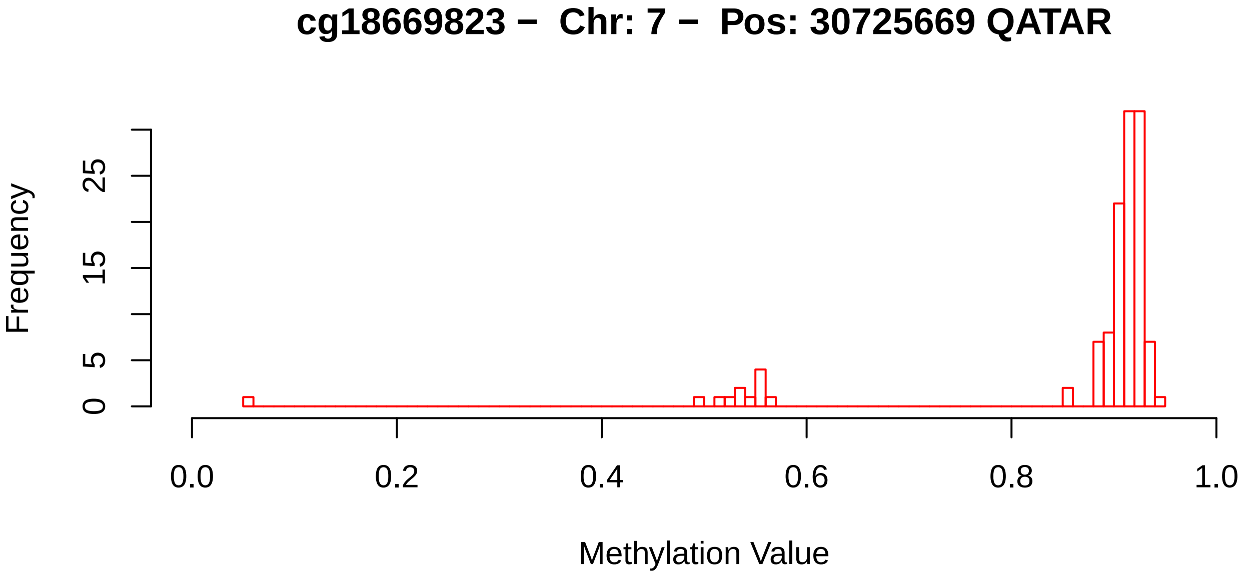
cg18669823 − Chr: 7 − Pos: 30725669 QATAR
Frequency
0
5
15
25
0.0
0.2
0.4
0.6
0.8
1.0
Methylation Value

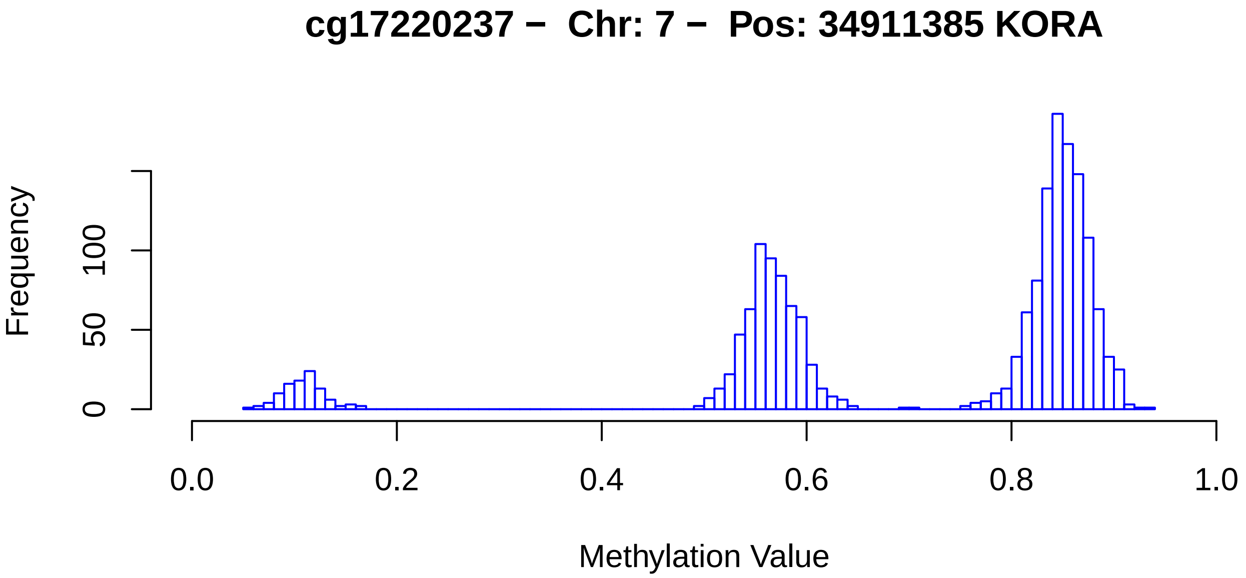
cg17220237 – Chr: 7 – Pos: 34911385 KORA
Frequency
0
50
100
0.0
0.2
0.4
0.6
0.8
1.0
Methylation Value

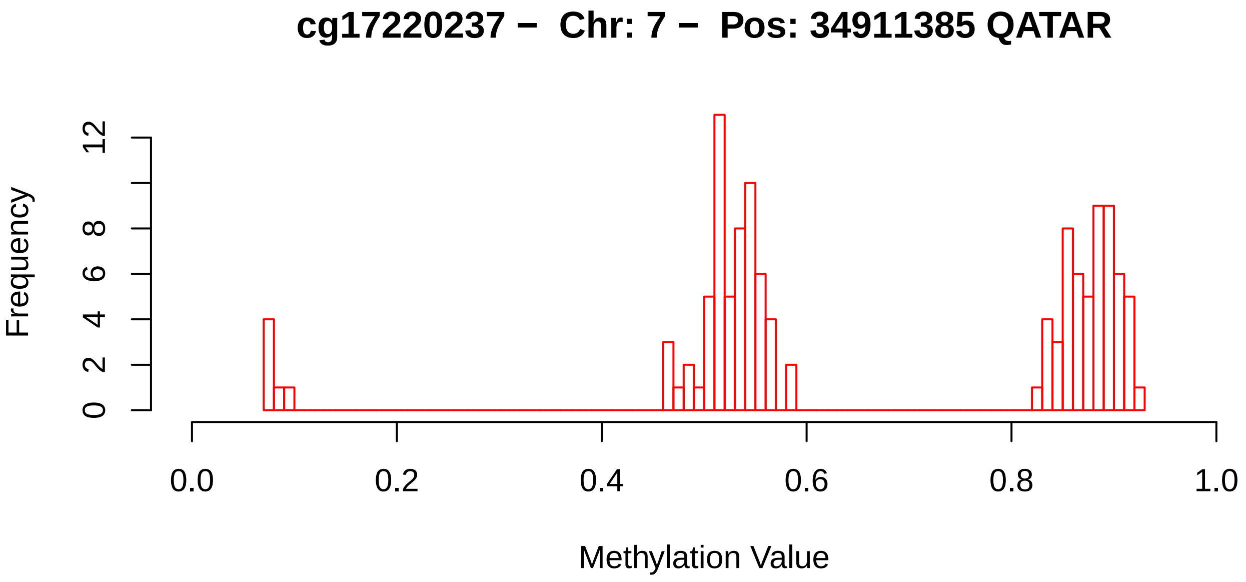
cg17220237 – Chr: 7 – Pos: 34911385 QATAR
Frequency
0
2
4
6
8
12
0.0
0.2
0.4
0.6
0.8
1.0
Methylation Value

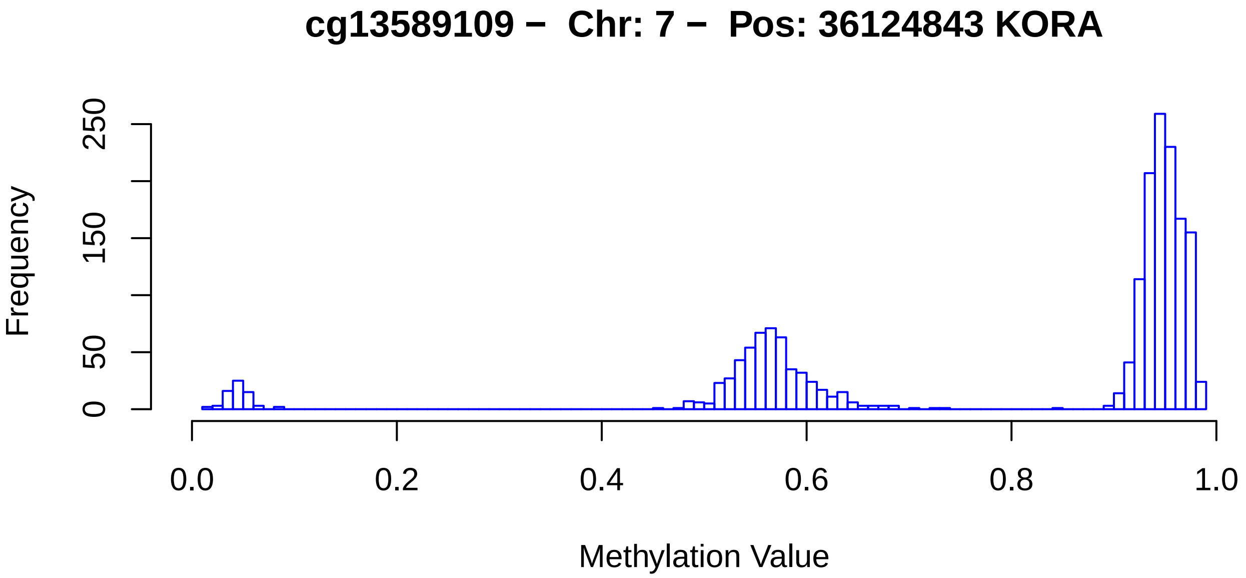

cg13589109 − Chr: 7 − Pos: 36124843 KORA
Frequency
0
50
150
250
0.0
0.2
0.4
0.6
0.8
1.0
Methylation Value

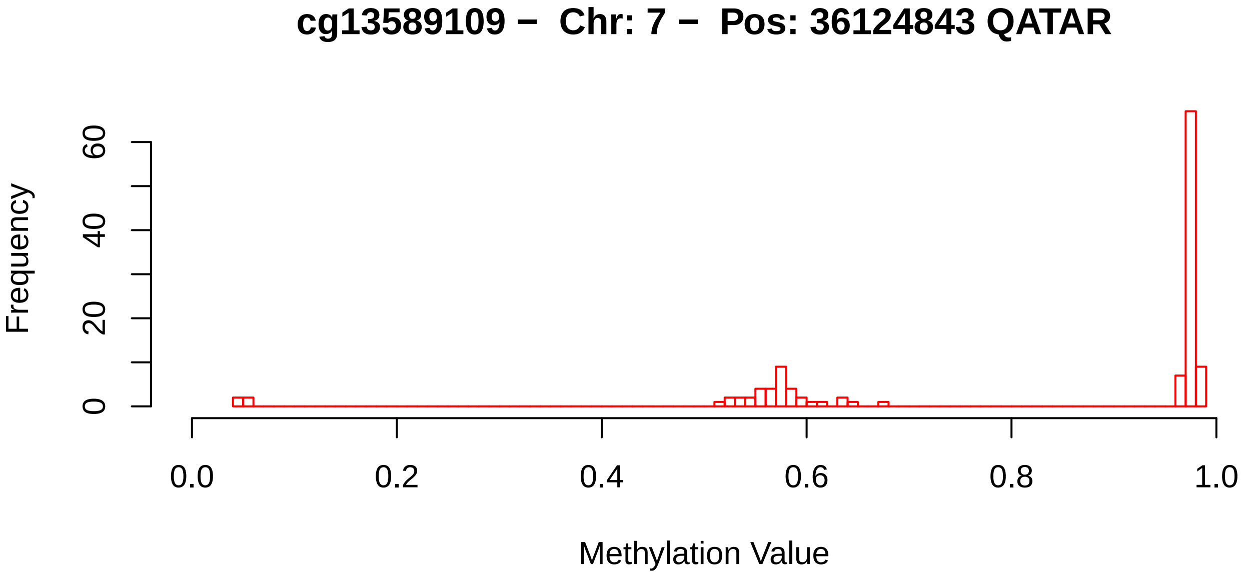

cg13589109 − Chr: 7 − Pos: 36124843 QATAR
Frequency
0
20
40
60
0.0
0.2
0.4
0.6
0.8
1.0
Methylation Value

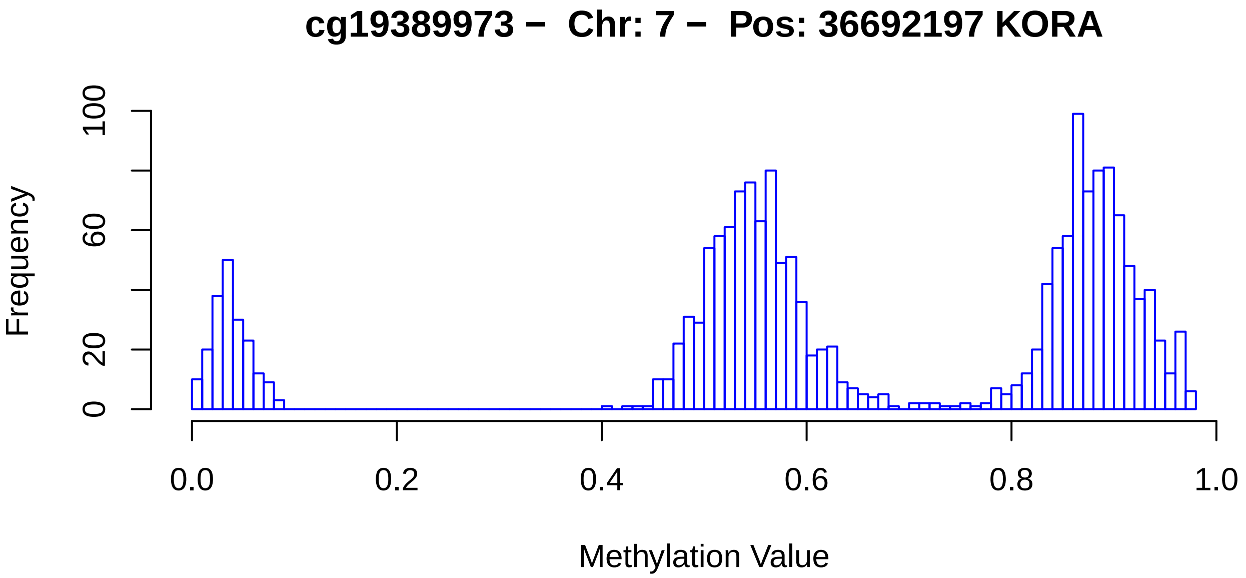

cg19389973 − Chr: 7 − Pos: 36692197 KORA
Frequency
0
20
60
100
0.0
0.2
0.4
0.6
0.8
1.0
Methylation Value

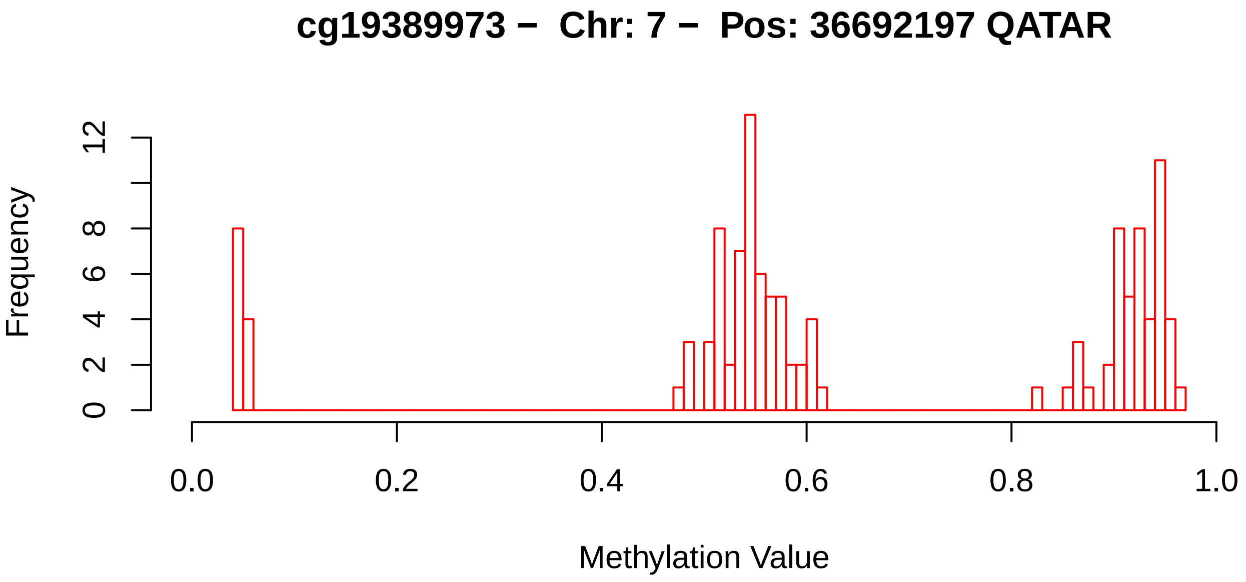

cg19389973 − Chr: 7 − Pos: 36692197 QATAR
Frequency
0
2
4
6
8
12
0.0
0.2
0.4
0.6
0.8
1.0
Methylation Value

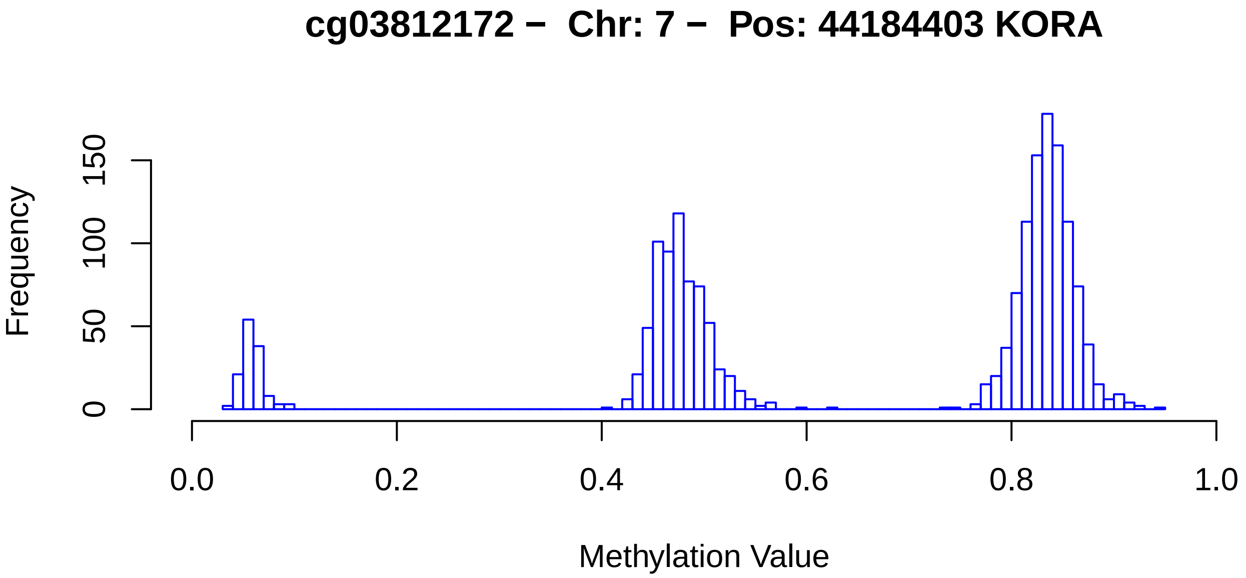
cg03812172 − Chr: 7 − Pos: 44184403 KORA
Frequency
0
50
100
150
0.0
0.2
0.4
0.6
0.8
1.0
Methylation Value

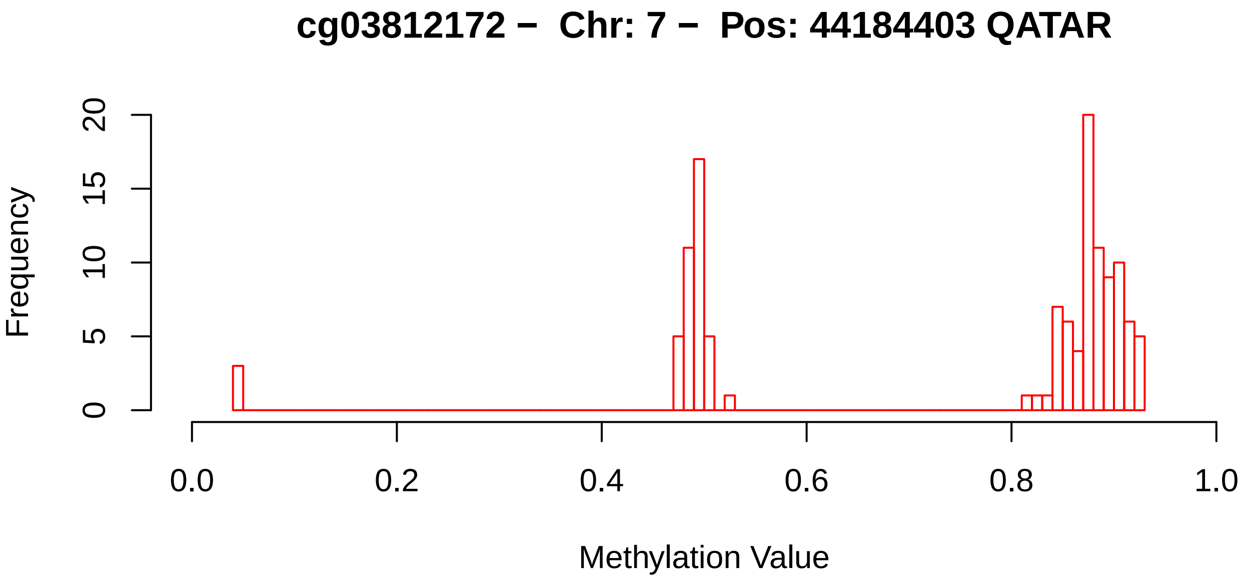
cg03812172 − Chr: 7 − Pos: 44184403 QATAR
Frequency
0
5
10
15
20
0.0
0.2
0.4
0.6
0.8
1.0
Methylation Value

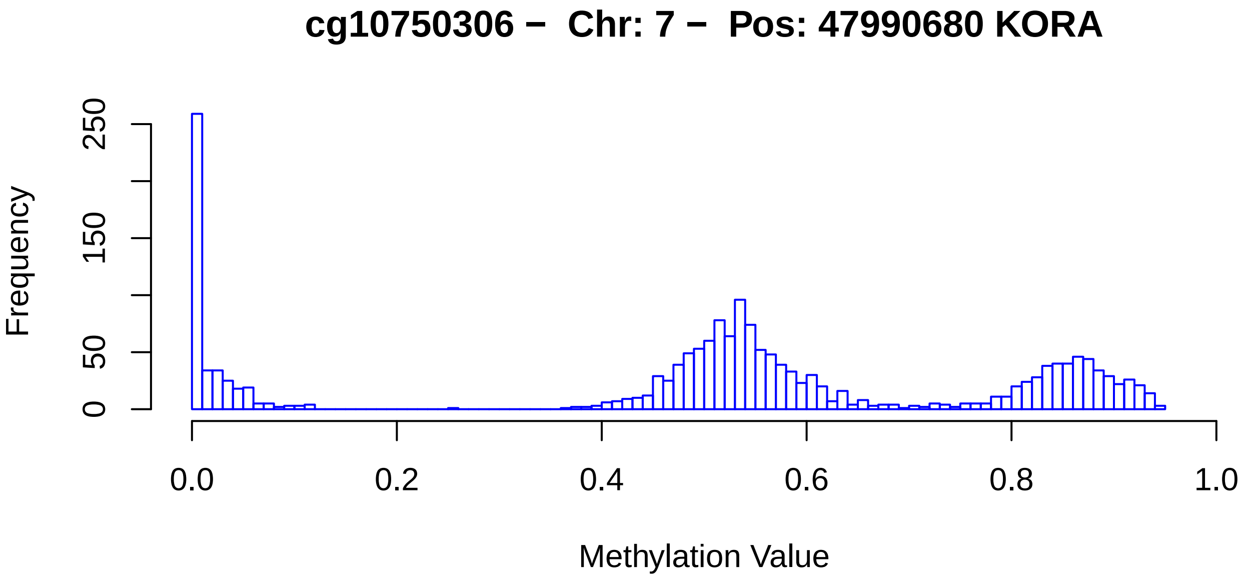
cg10750306 − Chr: 7 − Pos: 47990680 KORA
Frequency
0
50
150
250
0.0
0.2
0.4
0.6
0.8
1.0
Methylation Value

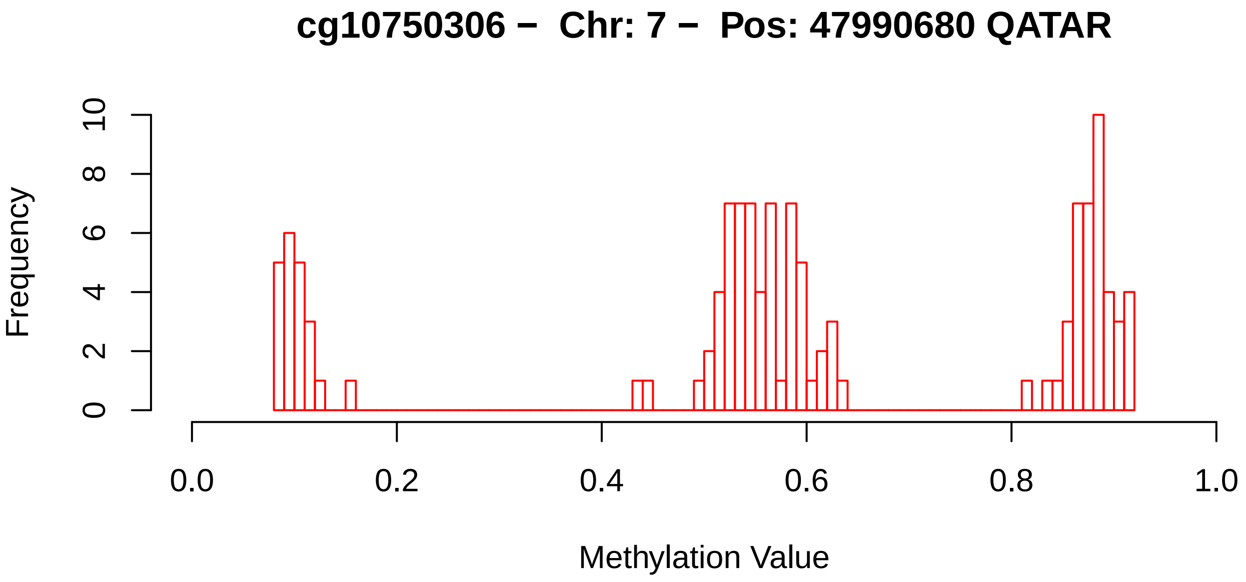
cg10750306 − Chr: 7 − Pos: 47990680 QATAR
Frequency
0
2
4
6
8
10
0.0
0.2
0.4
0.6
0.8
1.0
Methylation Value

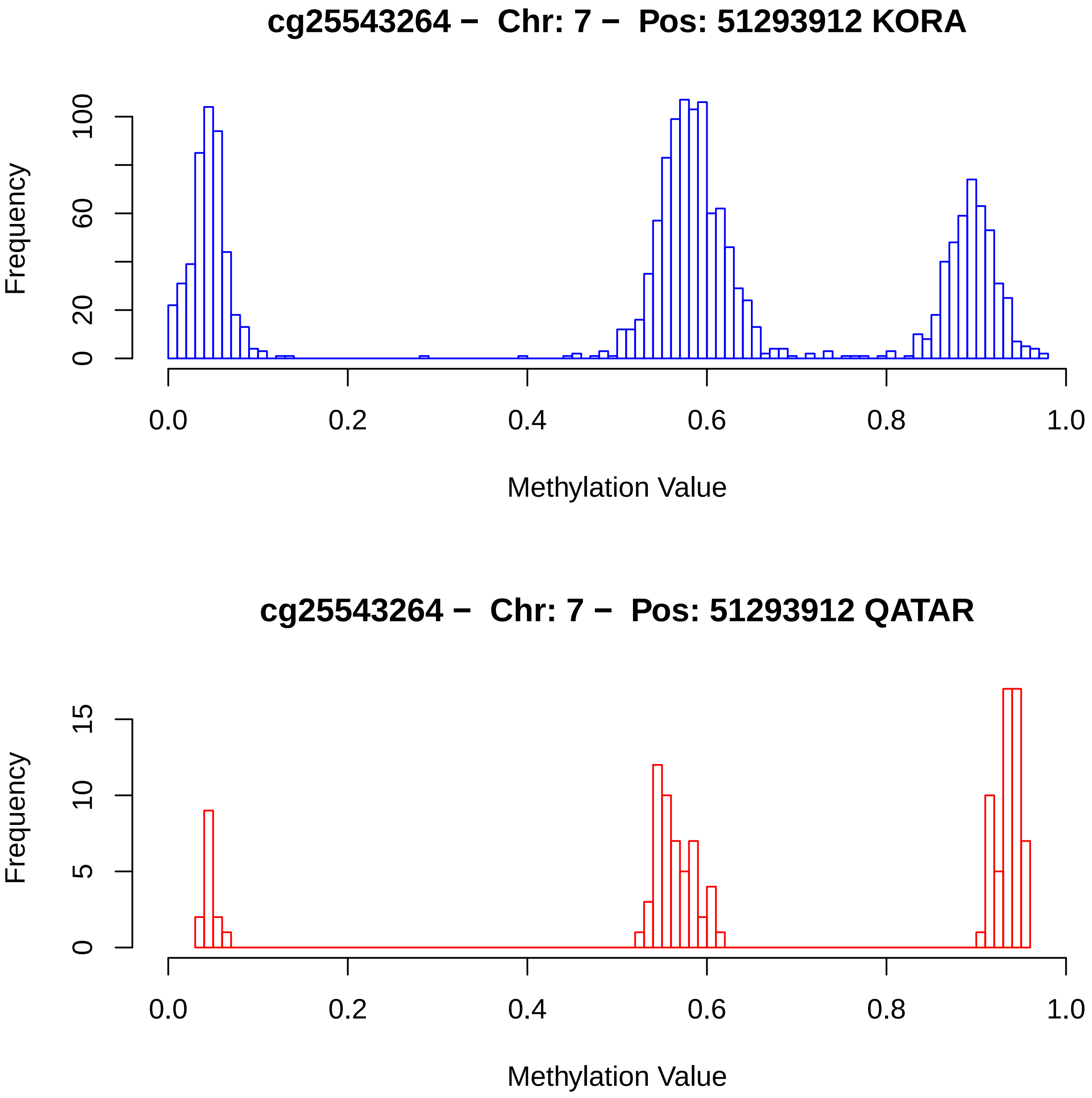
cg25543264 – Chr: 7 – Pos: 51293912 KORA
Frequency
0
20
60
100
0.0
0.2
0.4
0.6
0.8
1.0
Methylation Value
cg25543264 – Chr: 7 – Pos: 51293912 QATAR
Frequency
0
5
10
15
0.0
0.2
0.4
0.6
0.8
1.0
Methylation Value

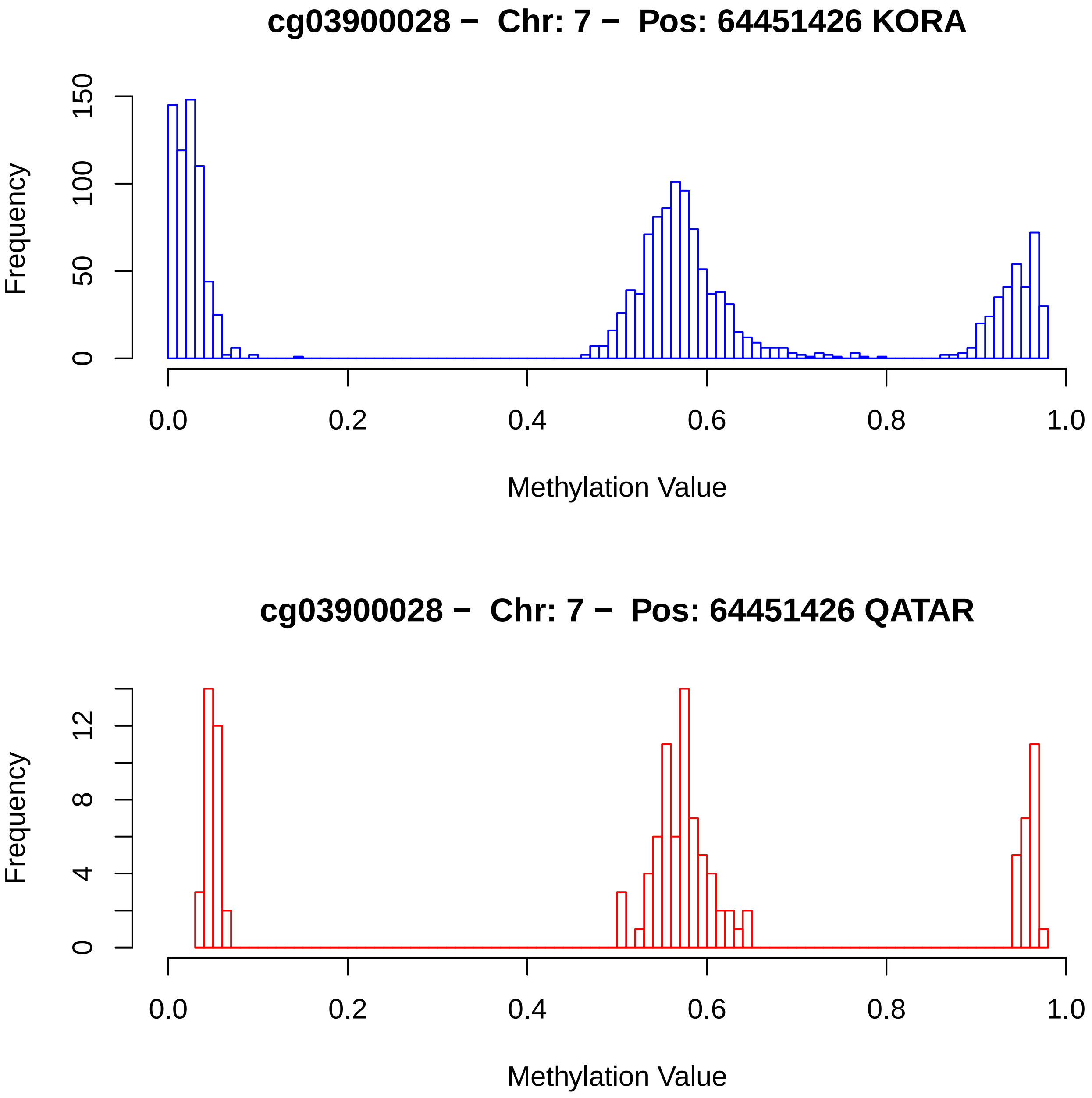
cg03900028 − Chr: 7 − Pos: 64451426 KORA
Frequency
0
50
100
150
0.0
0.2
0.4
0.6
0.8
1.0
Methylation Value
cg03900028 − Chr: 7 − Pos: 64451426 QATAR
Frequency
0
4
8
12
0.0
0.2
0.4
0.6
0.8
1.0
Methylation Value

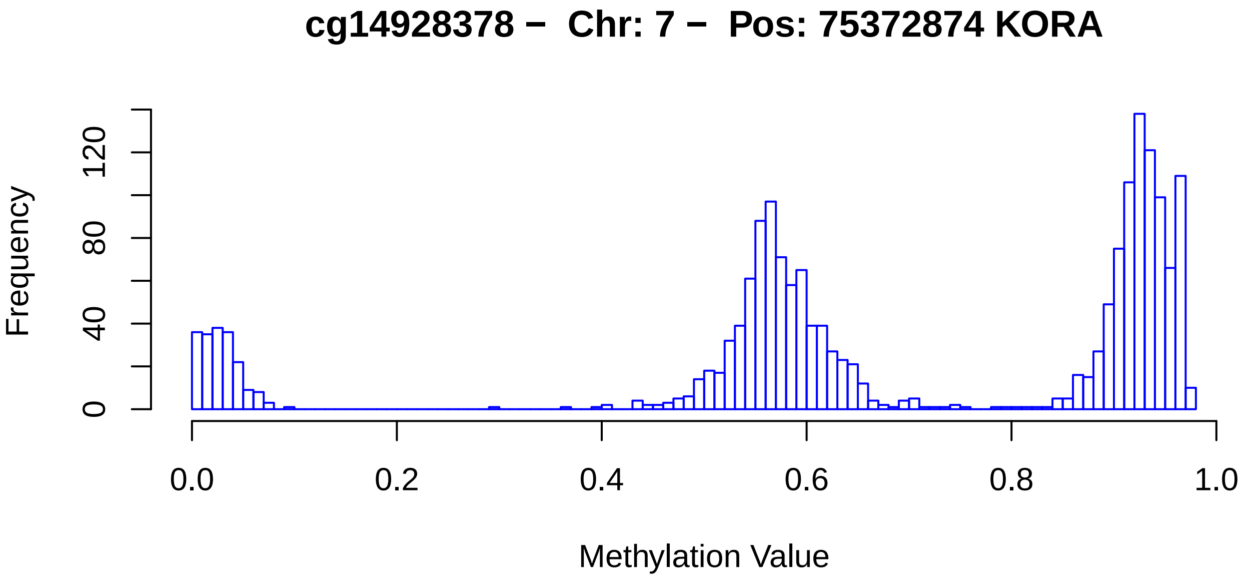

cg14928378 − Chr: 7 − Pos: 75372874 KORA
Frequency
0
40
80
120
0.0
0.2
0.4
0.6
0.8
1.0
Methylation Value

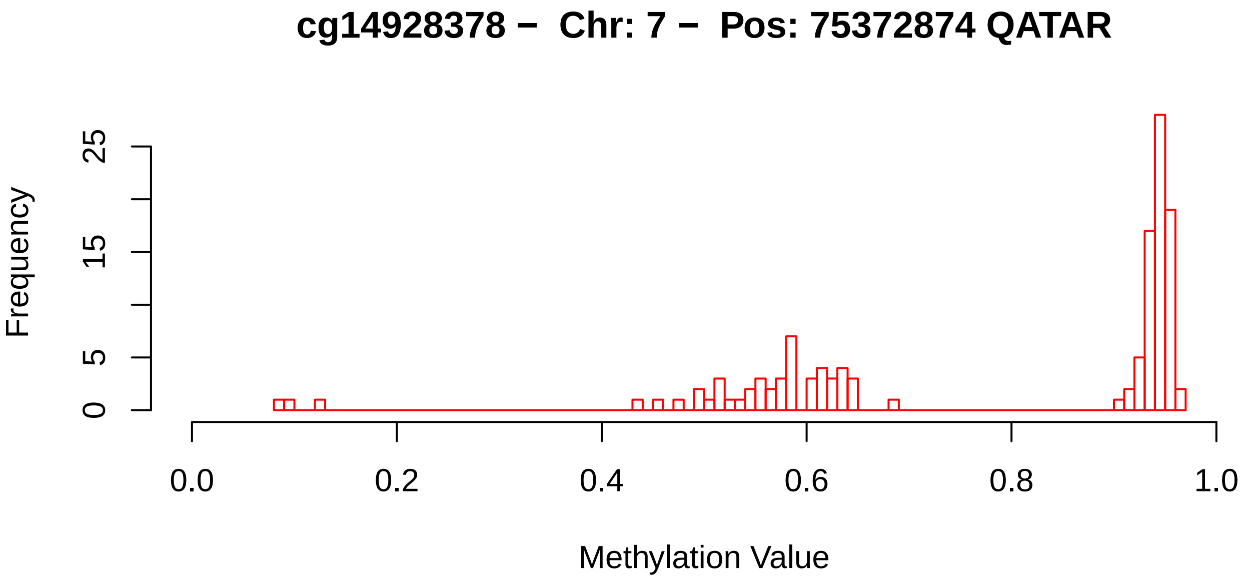

cg14928378 − Chr: 7 − Pos: 75372874 QATAR
Frequency
0
5
15
25
0.0
0.2
0.4
0.6
0.8
1.0
Methylation Value

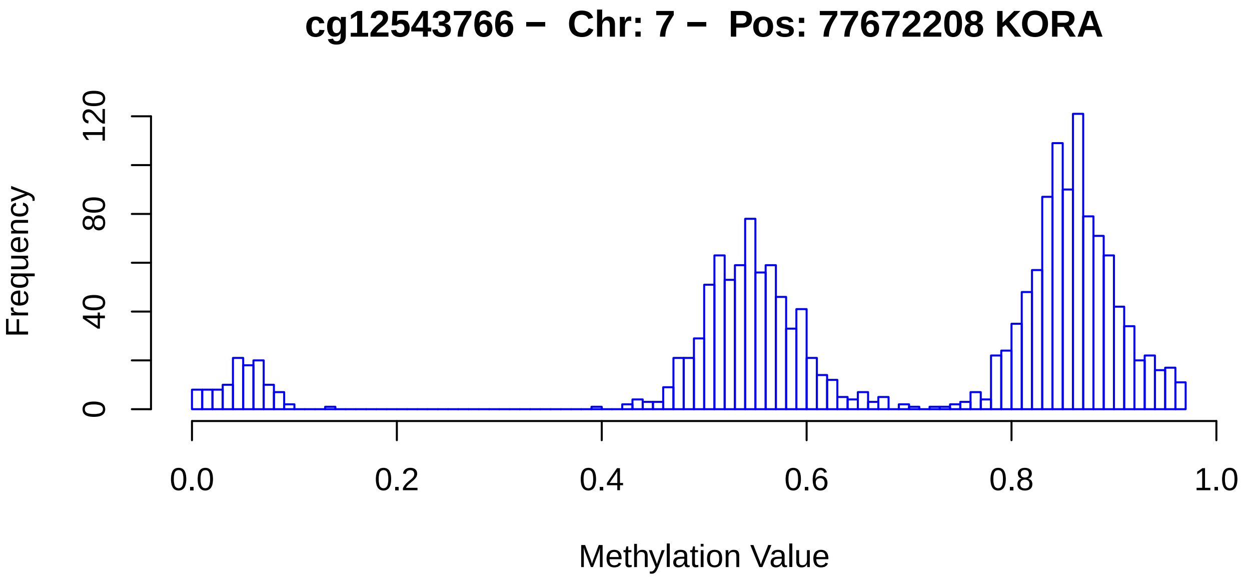
cg12543766 − Chr: 7 − Pos: 77672208 KORA
Frequency
0
40
80
120
0.0
0.2
0.4
0.6
0.8
1.0
Methylation Value

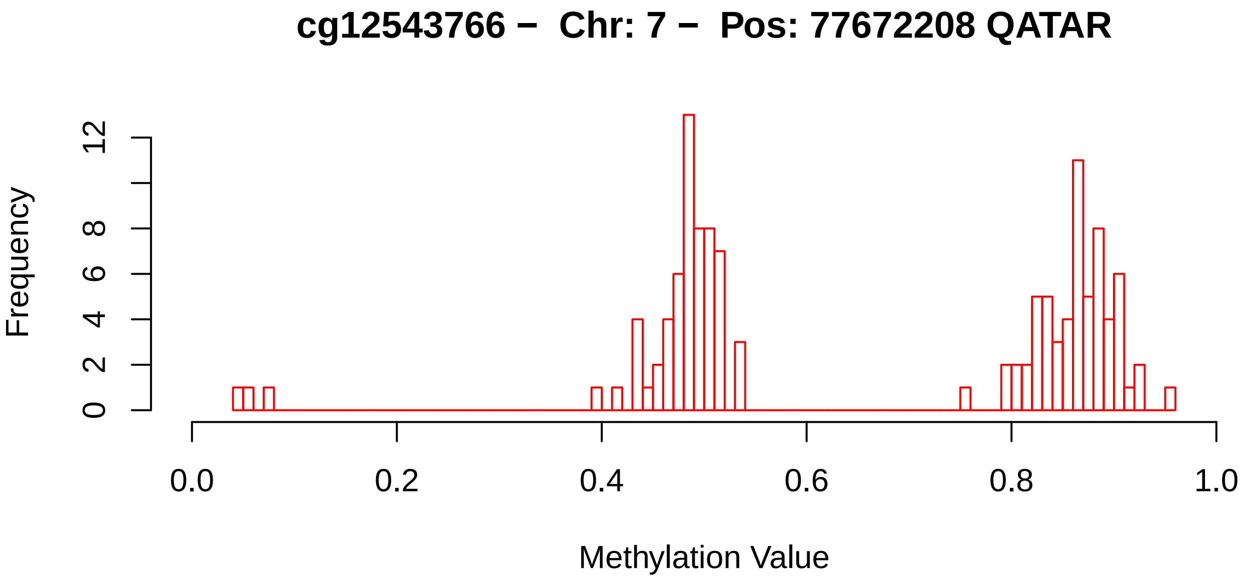
cg12543766 − Chr: 7 − Pos: 77672208 QATAR
Frequency
0
2
4
6
8
12
0.0
0.2
0.4
0.6
0.8
1.0
Methylation Value

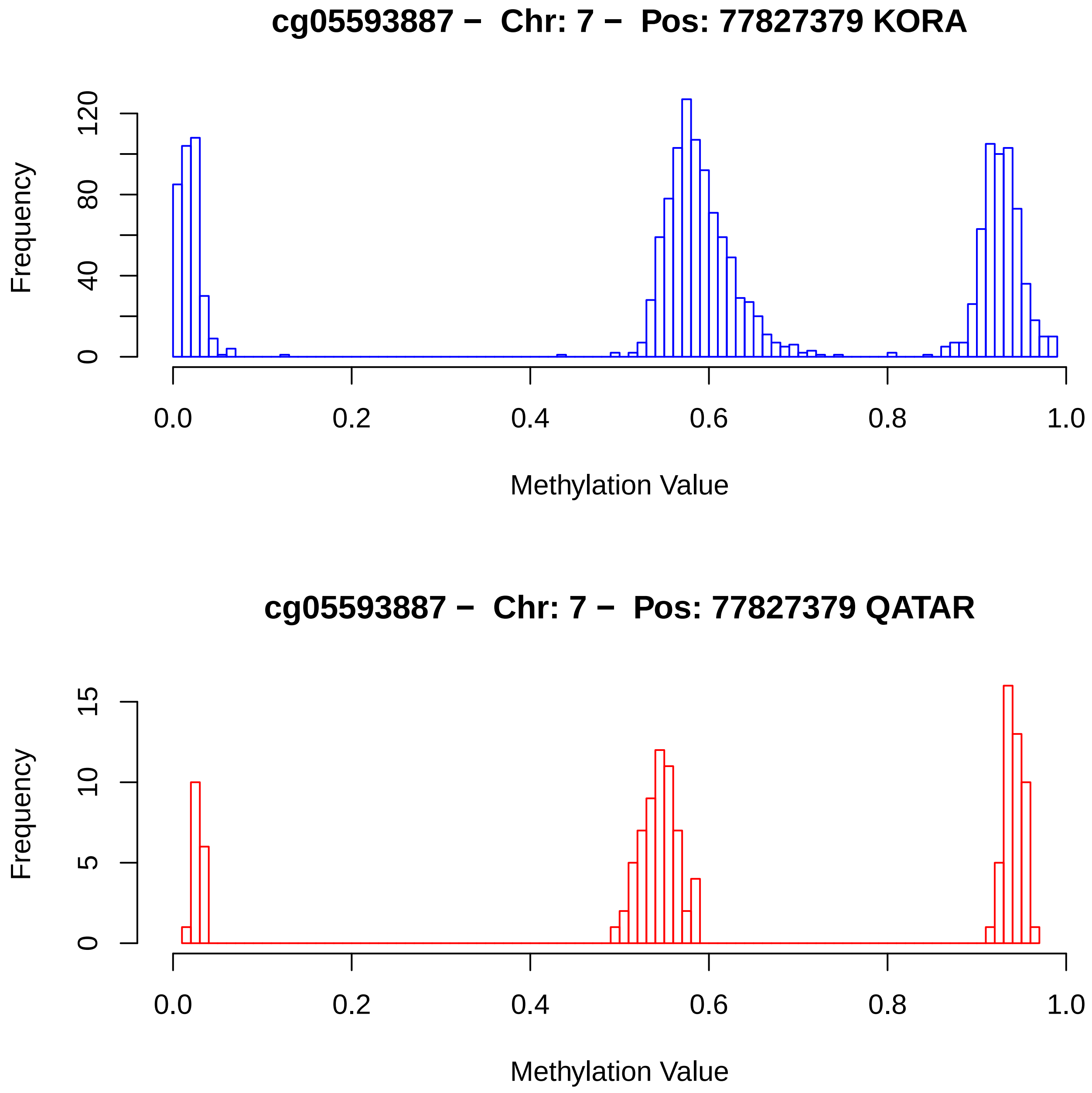
cg05593887 − Chr: 7 − Pos: 77827379 KORA
Frequency
0
40
80
120
0.0
0.2
0.4
0.6
0.8
1.0
Methylation Value
cg05593887 − Chr: 7 − Pos: 77827379 QATAR
Frequency
0
5
10
15
0.0
0.2
0.4
0.6
0.8
1.0
Methylation Value

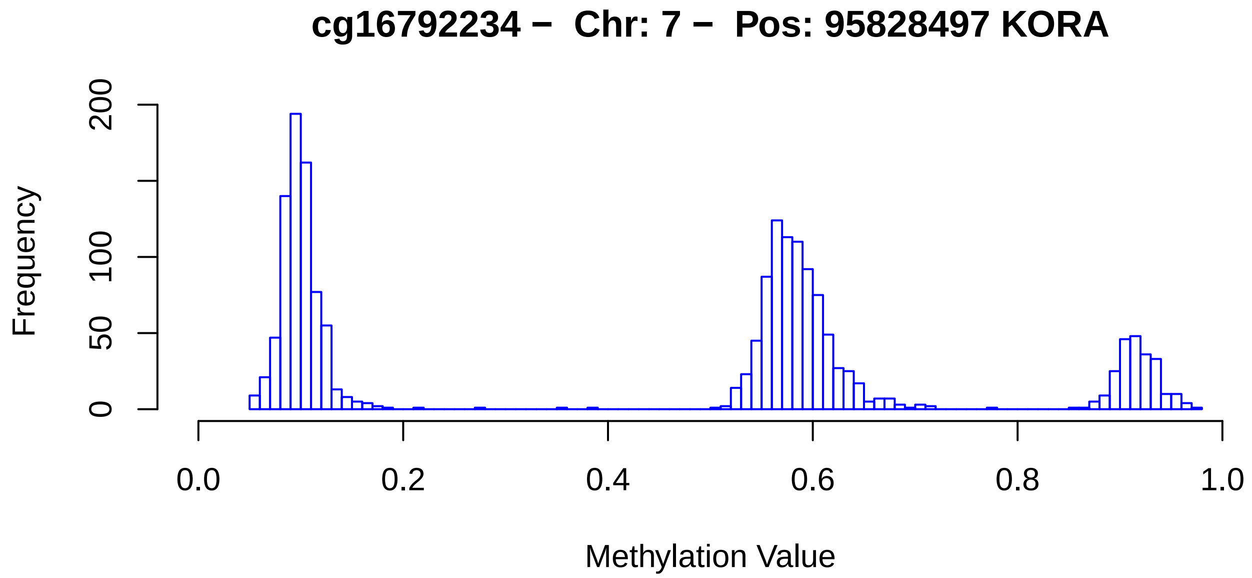
cg16792234 − Chr: 7 − Pos: 95828497 KORA
Frequency
0
50
100
200
0.0
0.2
0.4
0.6
0.8
1.0
Methylation Value

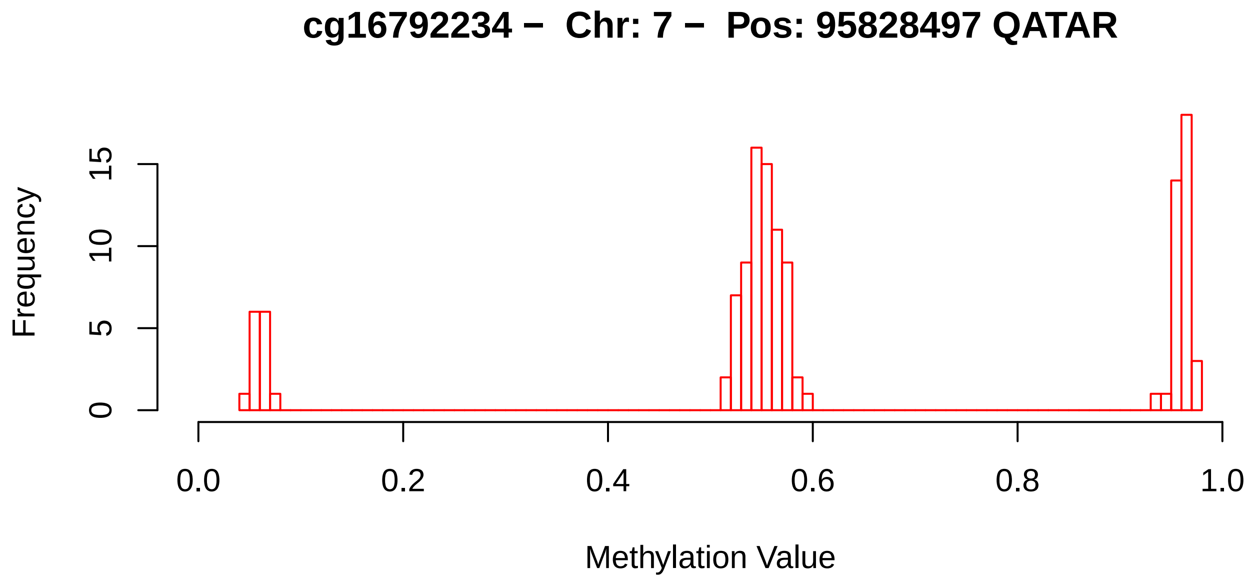
cg16792234 − Chr: 7 − Pos: 95828497 QATAR
Frequency
0
5
10
15
0.0
0.2
0.4
0.6
0.8
1.0
Methylation Value

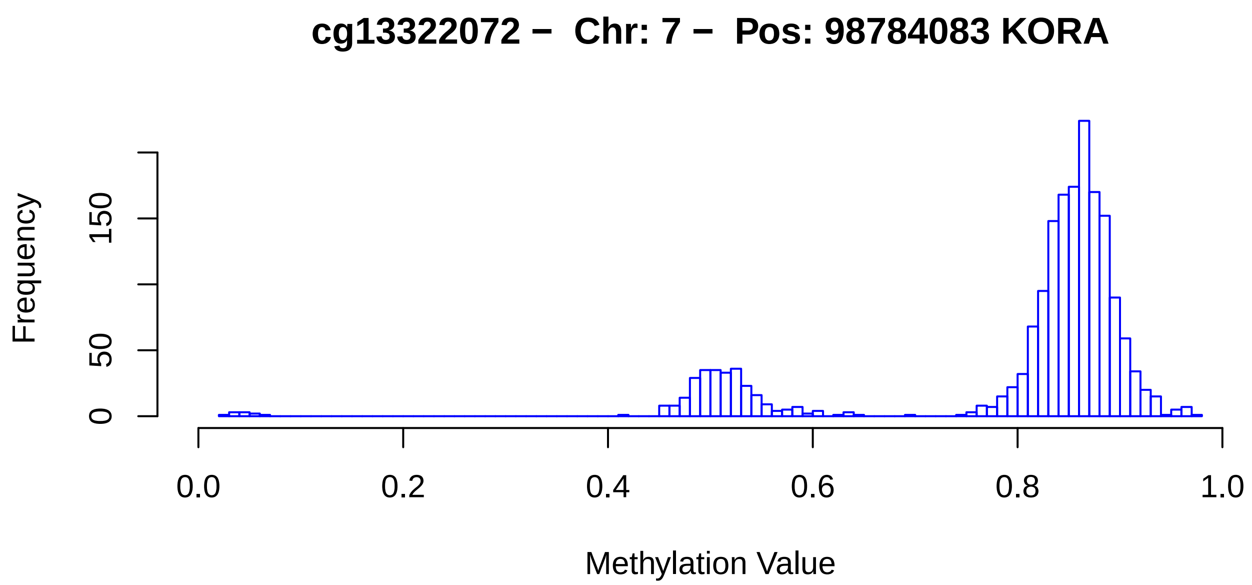
cg13322072 − Chr: 7 − Pos: 98784083 KORA
Frequency
0
50
150
0.0
0.2
0.4
0.6
0.8
1.0
Methylation Value

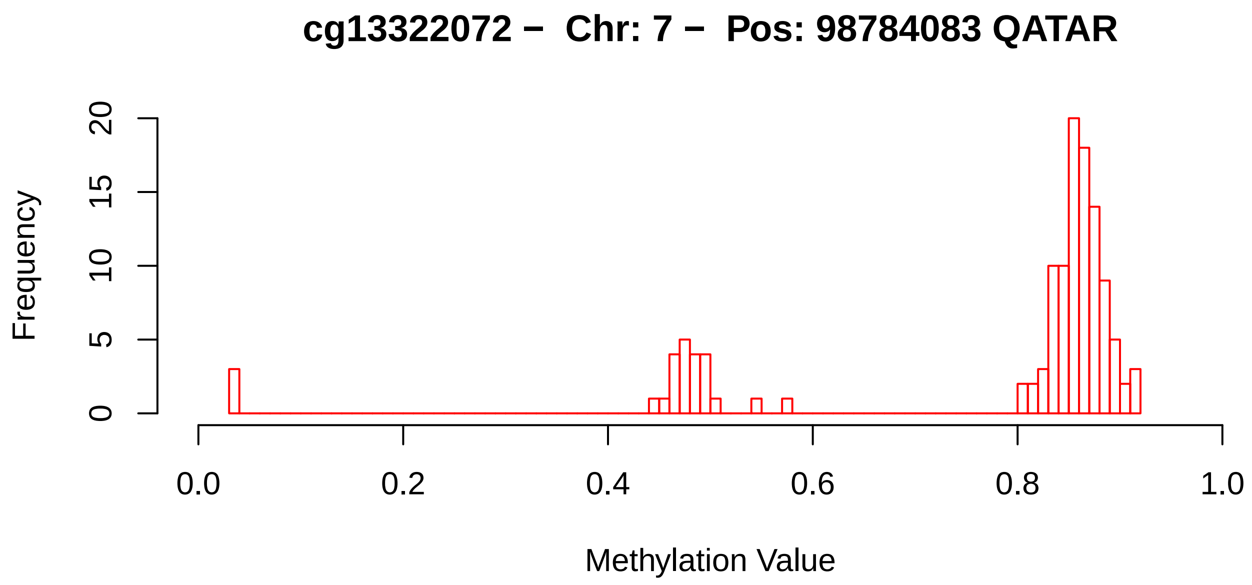
cg13322072 − Chr: 7 − Pos: 98784083 QATAR
Frequency
0
5
10
15
20
0.0
0.2
0.4
0.6
0.8
1.0
Methylation Value

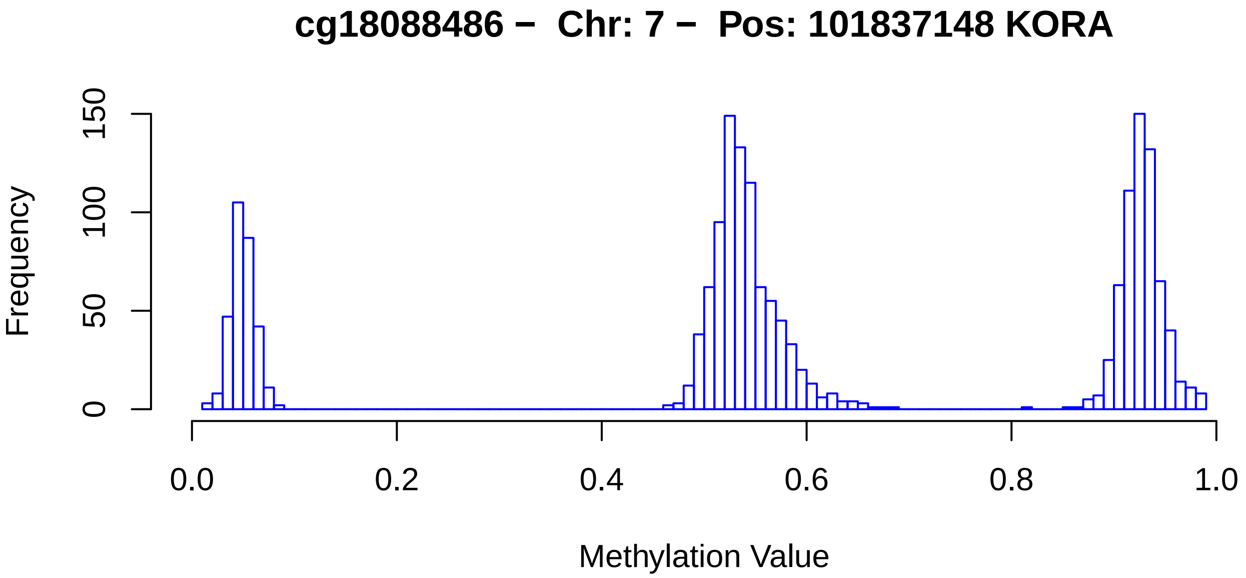
cg18088486 − Chr: 7 − Pos: 101837148 KORA
Frequency
0
50
100
150
0.0
0.2
0.4
0.6
0.8
1.0
Methylation Value

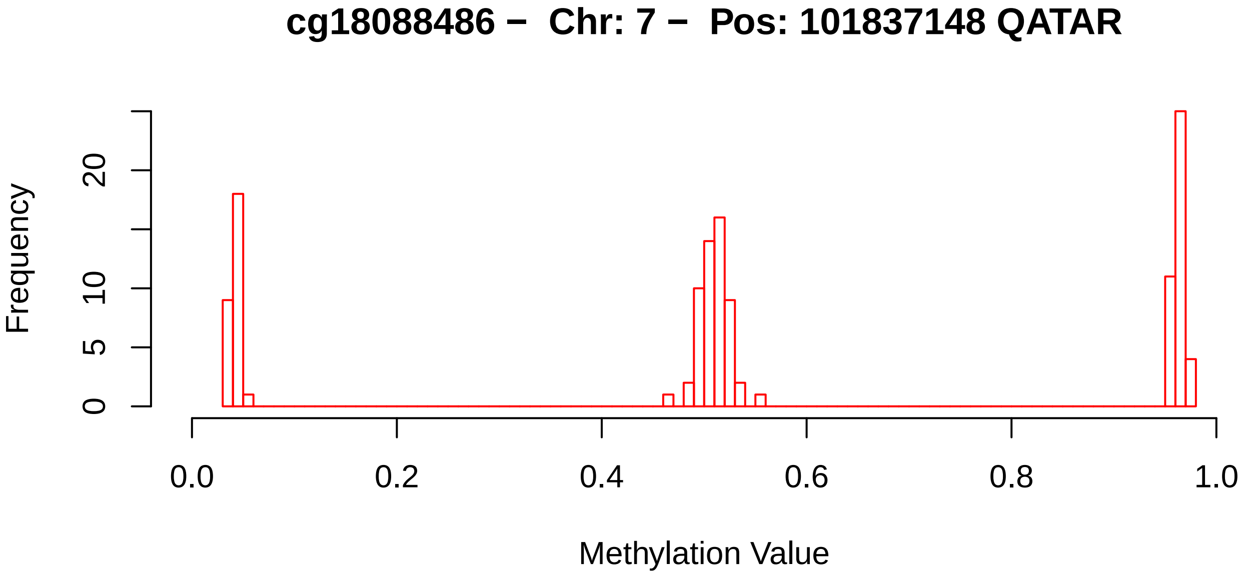
cg18088486 − Chr: 7 − Pos: 101837148 QATAR
Frequency
0
5
10
20
0.0
0.2
0.4
0.6
0.8
1.0
Methylation Value

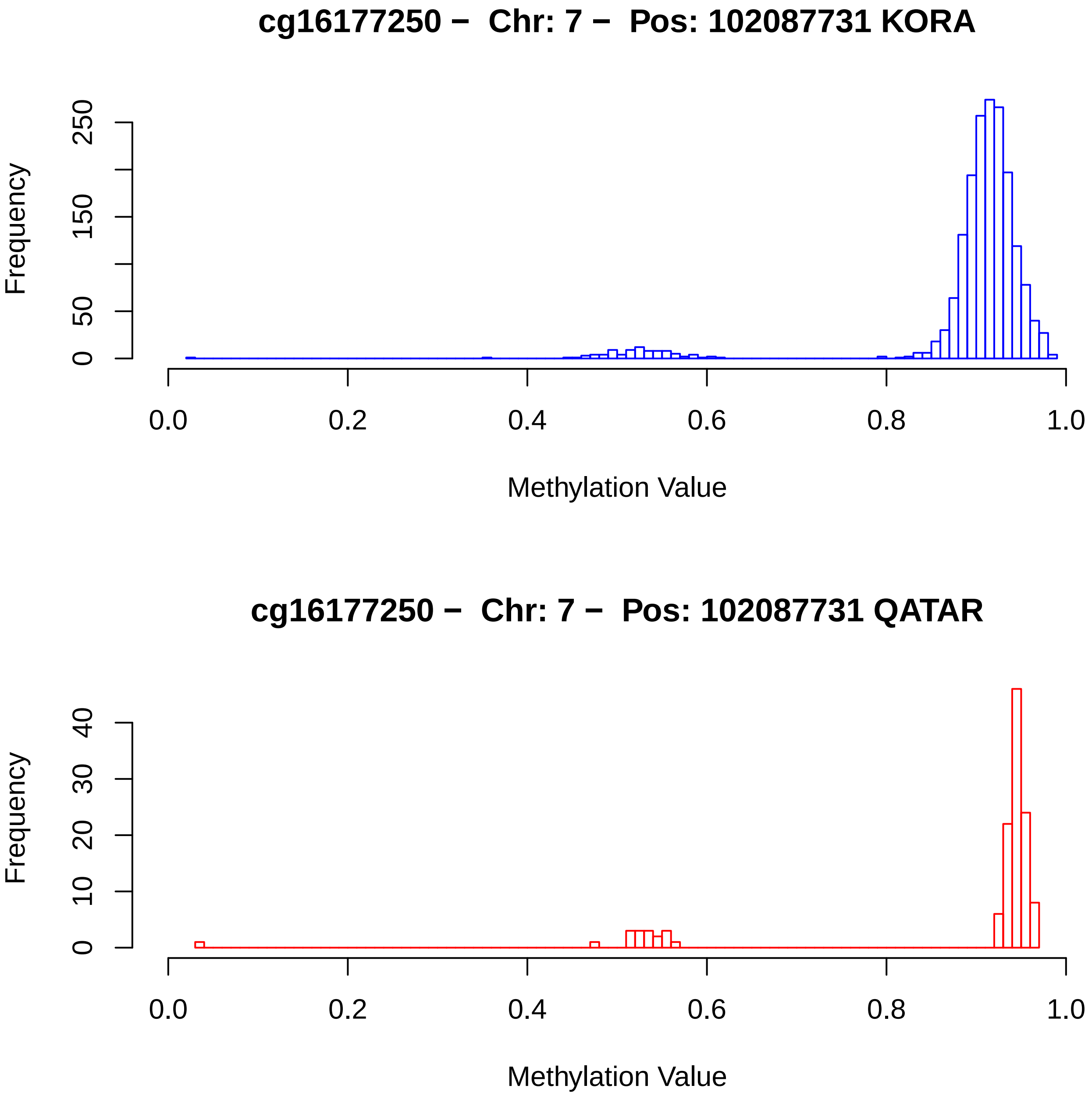
cg16177250 − Chr: 7 − Pos: 102087731 KORA
Frequency
0
50
150
250
0.0
0.2
0.4
0.6
0.8
1.0
Methylation Value
cg16177250 − Chr: 7 − Pos: 102087731 QATAR
Frequency
0
10
20
30
40
0.0
0.2
0.4
0.6
0.8
1.0
Methylation Value

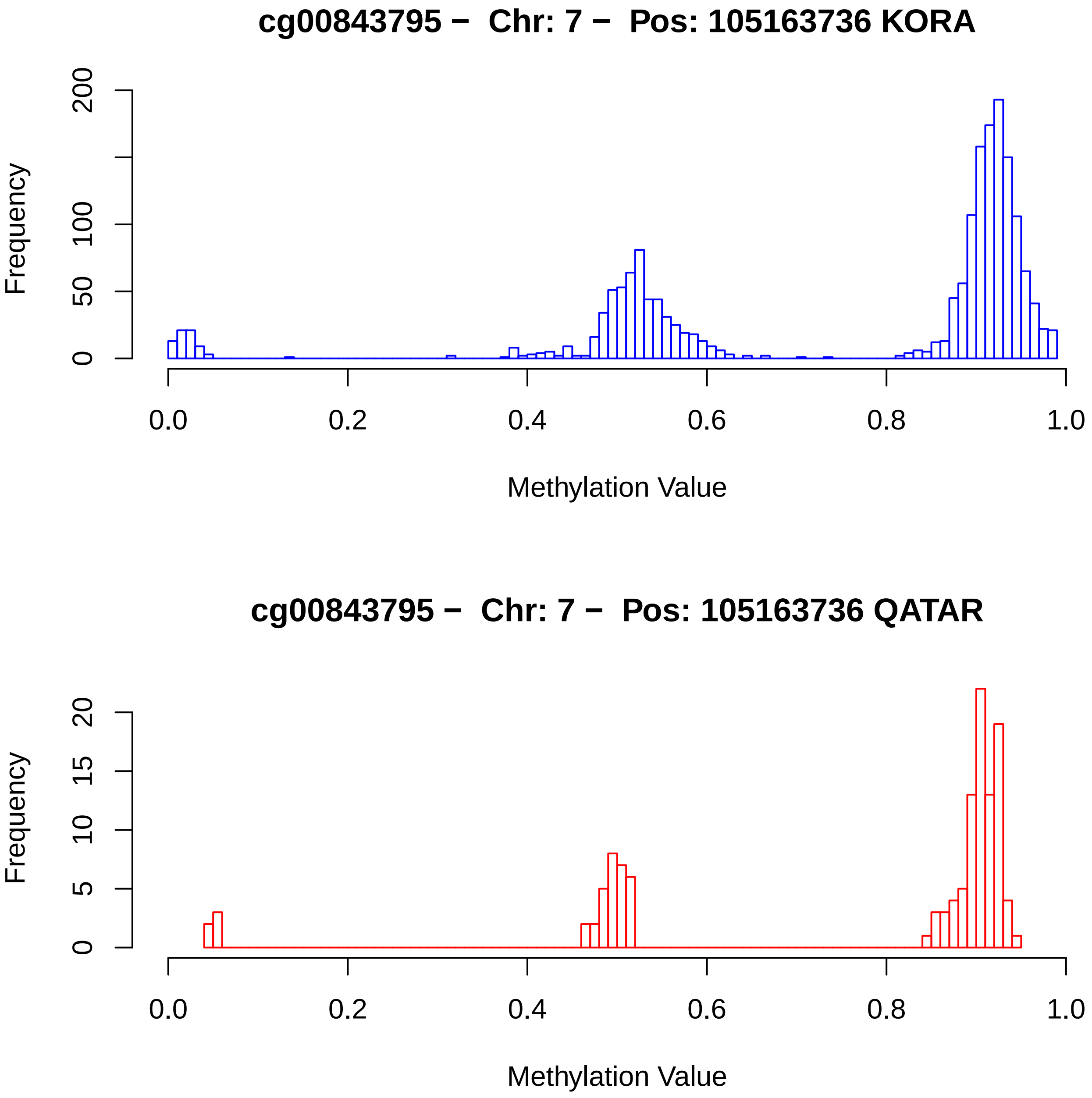
cg00843795 − Chr: 7 − Pos: 105163736 KORA
Frequency
0
50
100
200
0.0
0.2
0.4
0.6
0.8
1.0
Methylation Value
cg00843795 − Chr: 7 − Pos: 105163736 QATAR
Frequency
0
5
10
15
20
0.0
0.2
0.4
0.6
0.8
1.0
Methylation Value

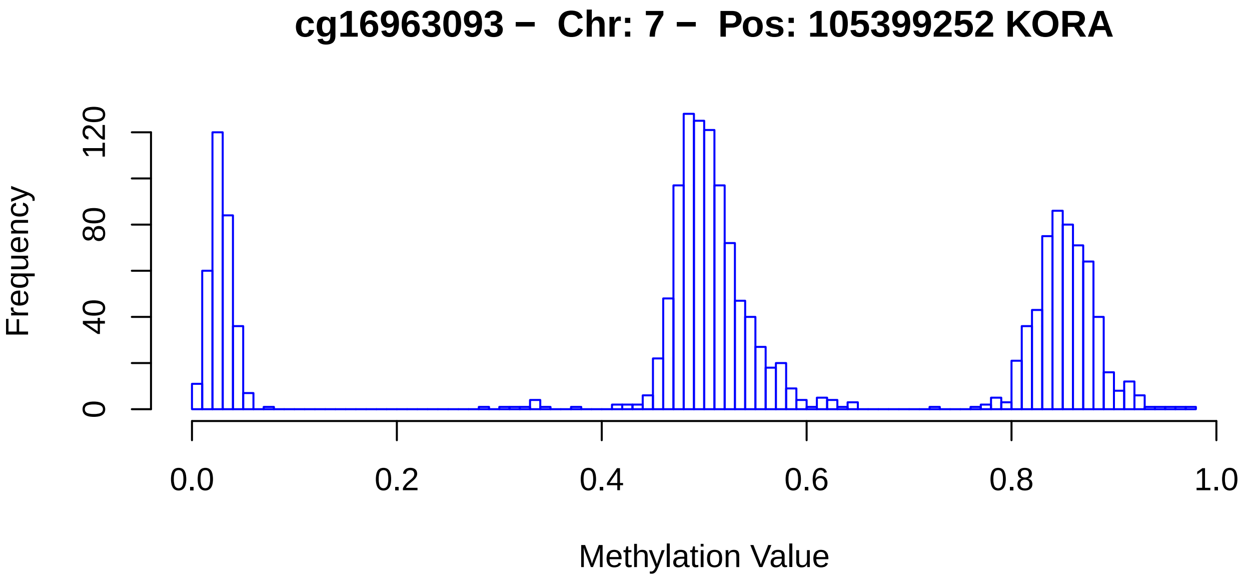
cg16963093 − Chr: 7 − Pos: 105399252 KORA
Frequency
0
40
80
120
0.0
0.2
0.4
0.6
0.8
1.0
Methylation Value

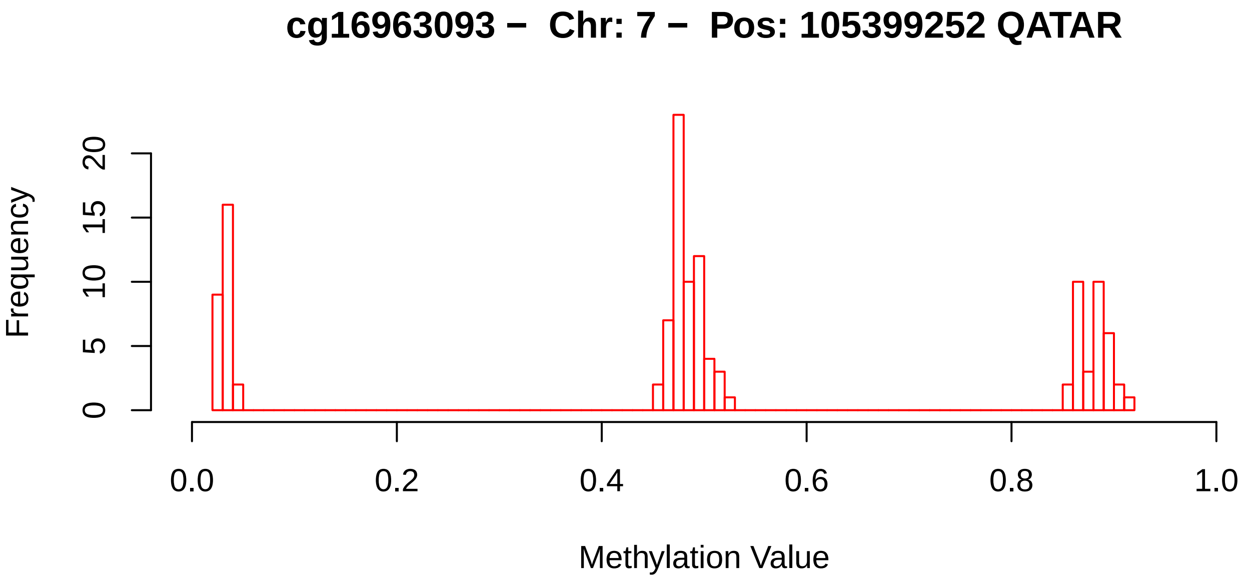
cg16963093 − Chr: 7 − Pos: 105399252 QATAR
Frequency
0
5
10
15
20
0.0
0.2
0.4
0.6
0.8
1.0
Methylation Value

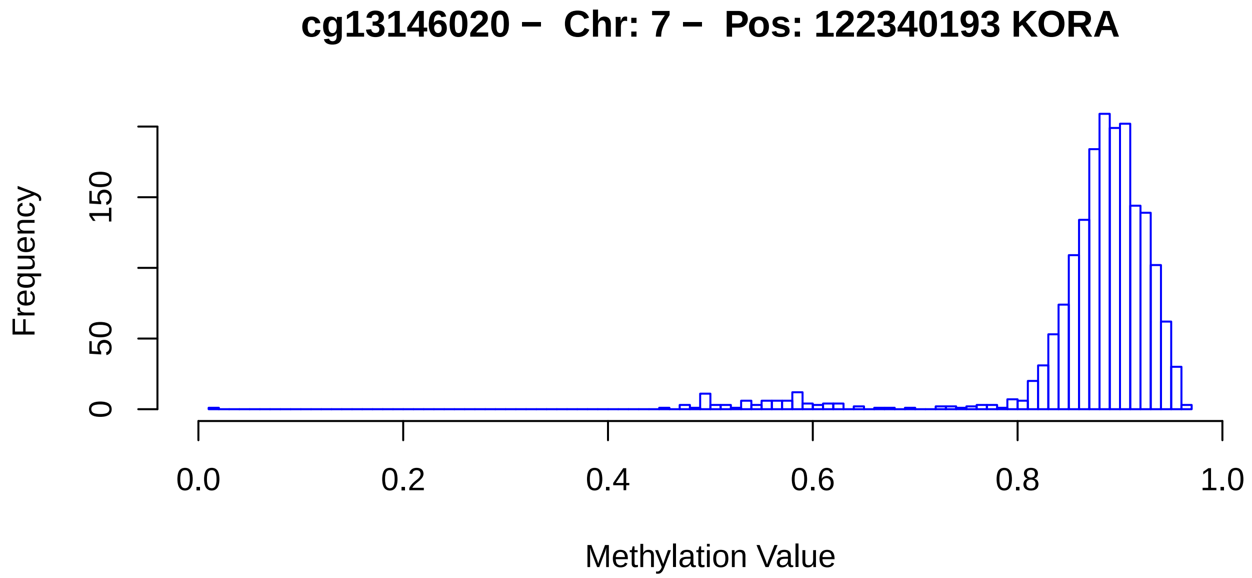
cg13146020 – Chr: 7 – Pos: 122340193 KORA
Frequency
0
50
150
0.0
0.2
0.4
0.6
0.8
1.0
Methylation Value

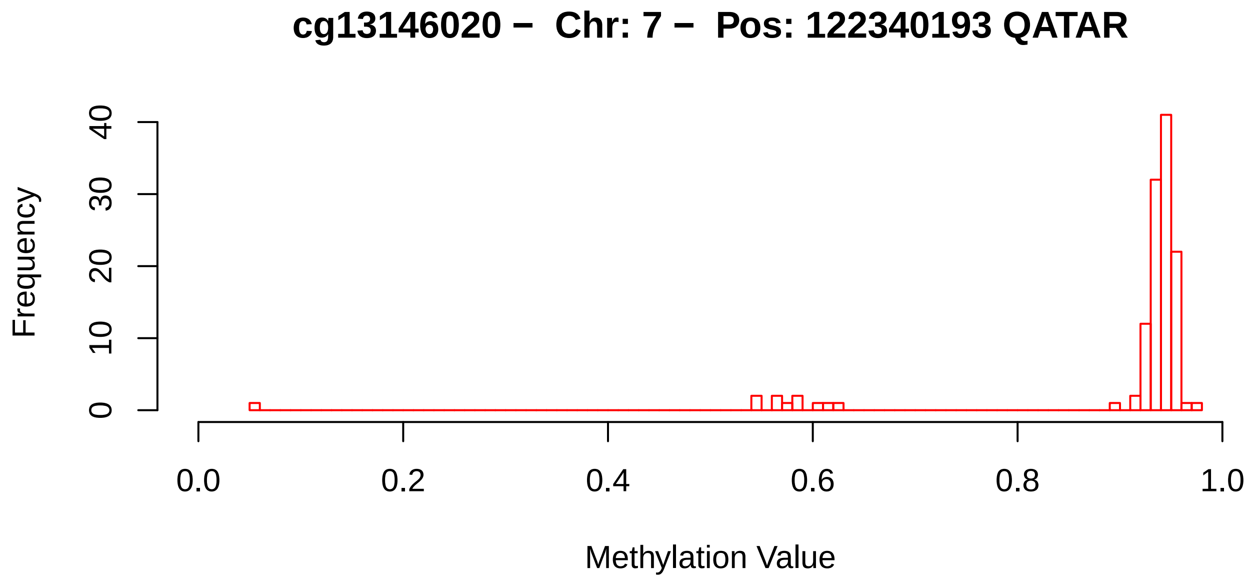
cg13146020 – Chr: 7 – Pos: 122340193 QATAR
Frequency
0
10
20
30
40
0.0
0.2
0.4
0.6
0.8
1.0
Methylation Value

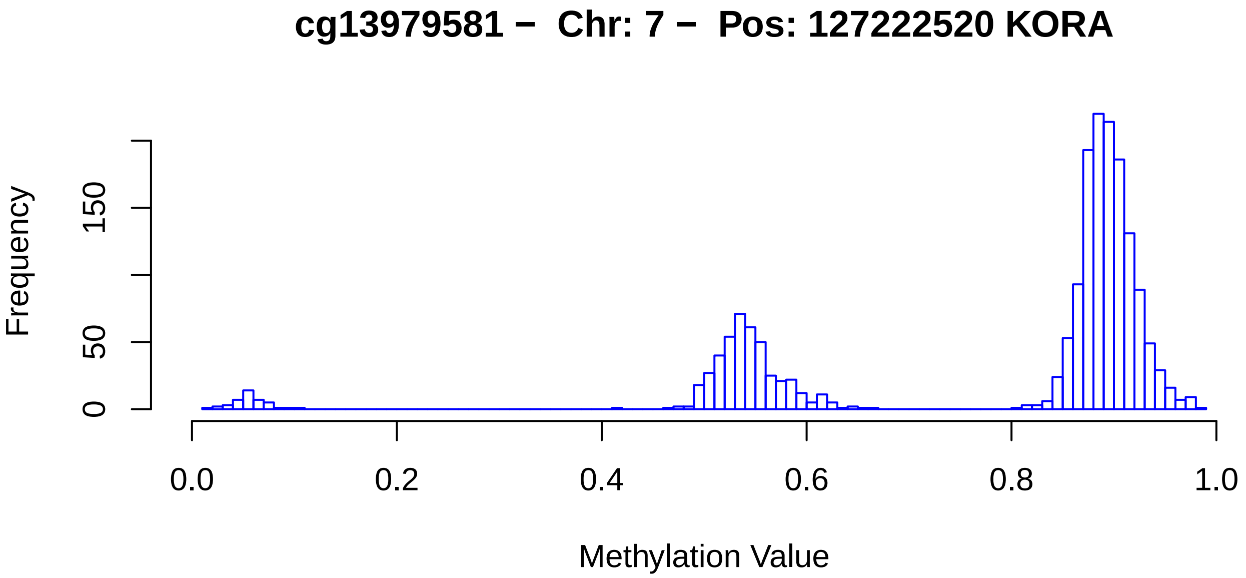
cg13979581 − Chr: 7 − Pos: 127222520 KORA
Frequency
0
50
150
0.0
0.2
0.4
0.6
0.8
1.0
Methylation Value

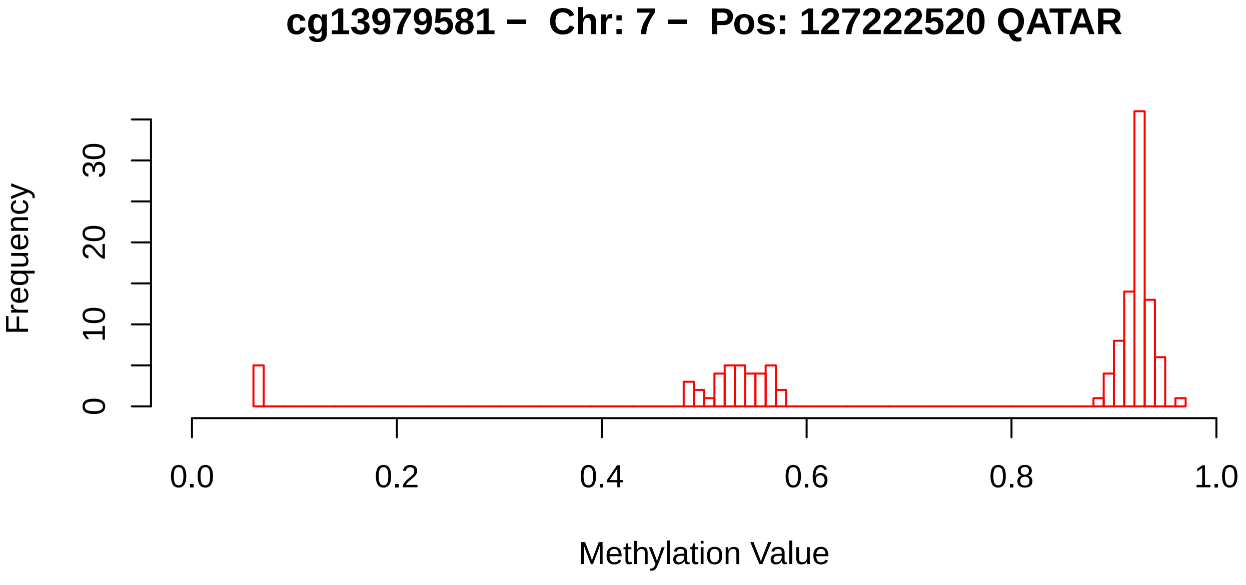
cg13979581 − Chr: 7 − Pos: 127222520 QATAR
Frequency
0
10
20
30
0.0
0.2
0.4
0.6
0.8
1.0
Methylation Value

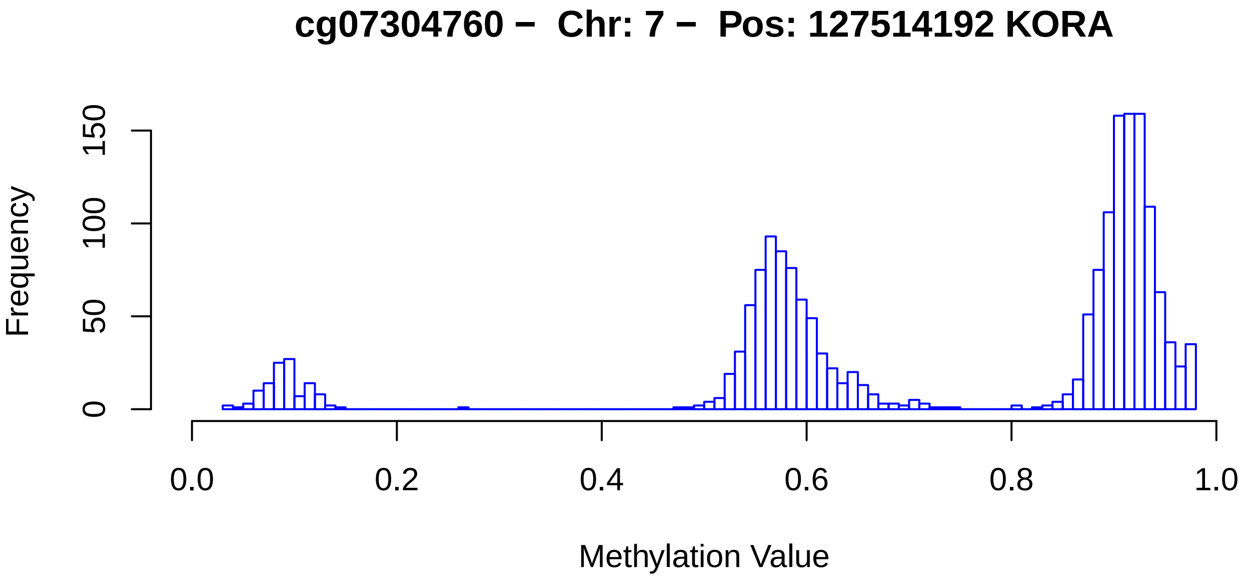
cg07304760 − Chr: 7 − Pos: 127514192 KORA
Frequency
0
50
100
150
0.0
0.2
0.4
0.6
0.8
1.0
Methylation Value

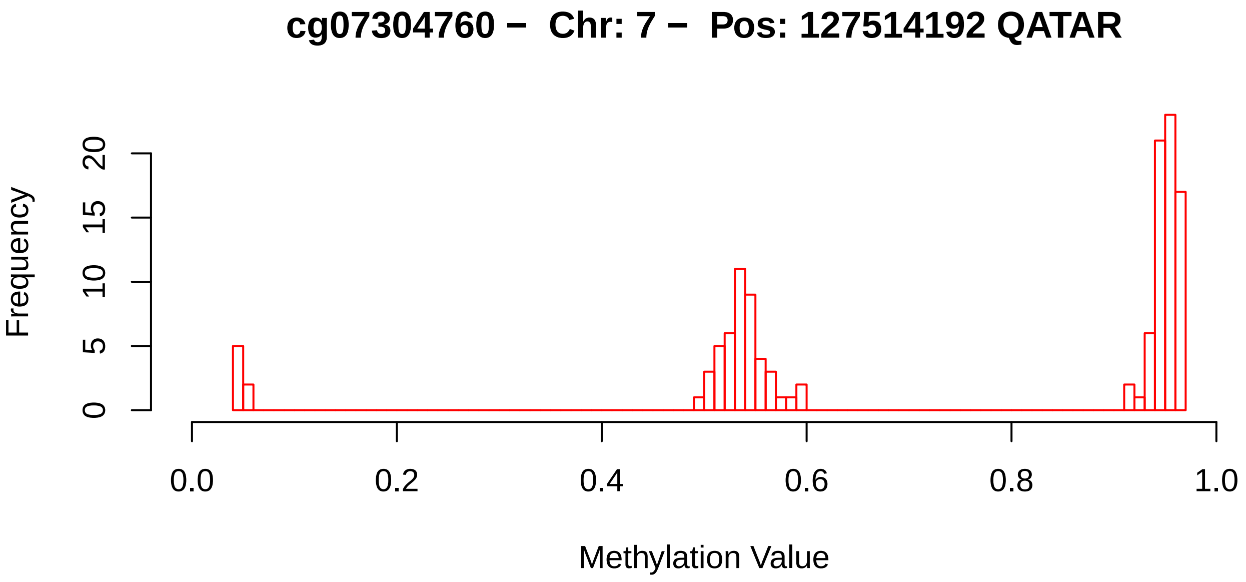
cg07304760 − Chr: 7 − Pos: 127514192 QATAR
Frequency
0
5
10
15
20
0.0
0.2
0.4
0.6
0.8
1.0
Methylation Value

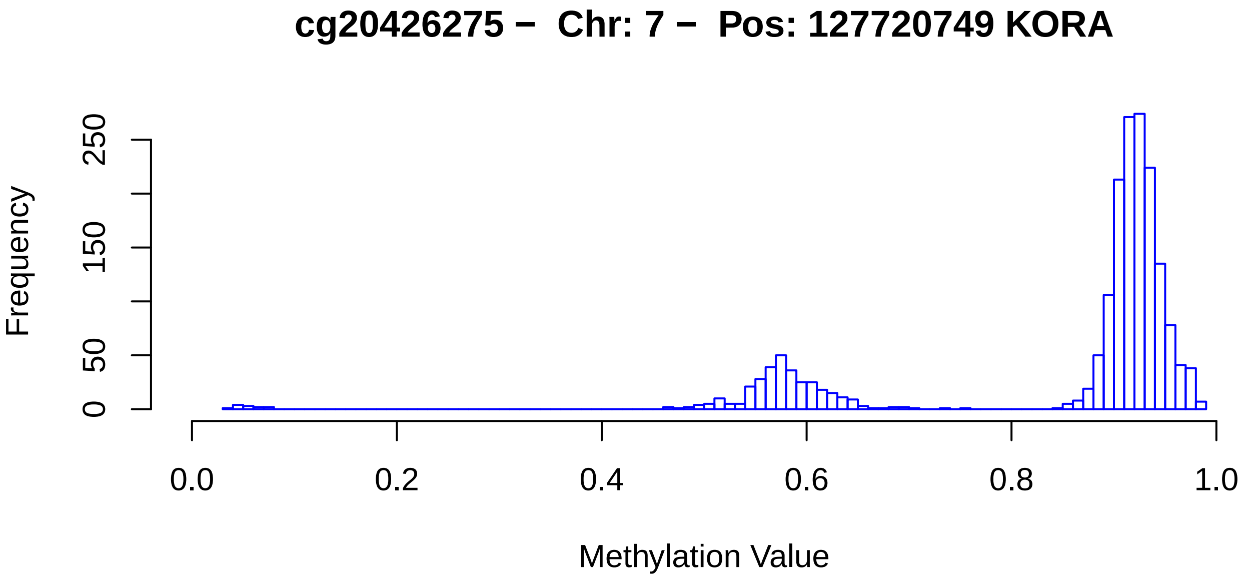
cg20426275 − Chr: 7 − Pos: 127720749 KORA
Frequency
0
50
150
250
0.0
0.2
0.4
0.6
0.8
1.0
Methylation Value

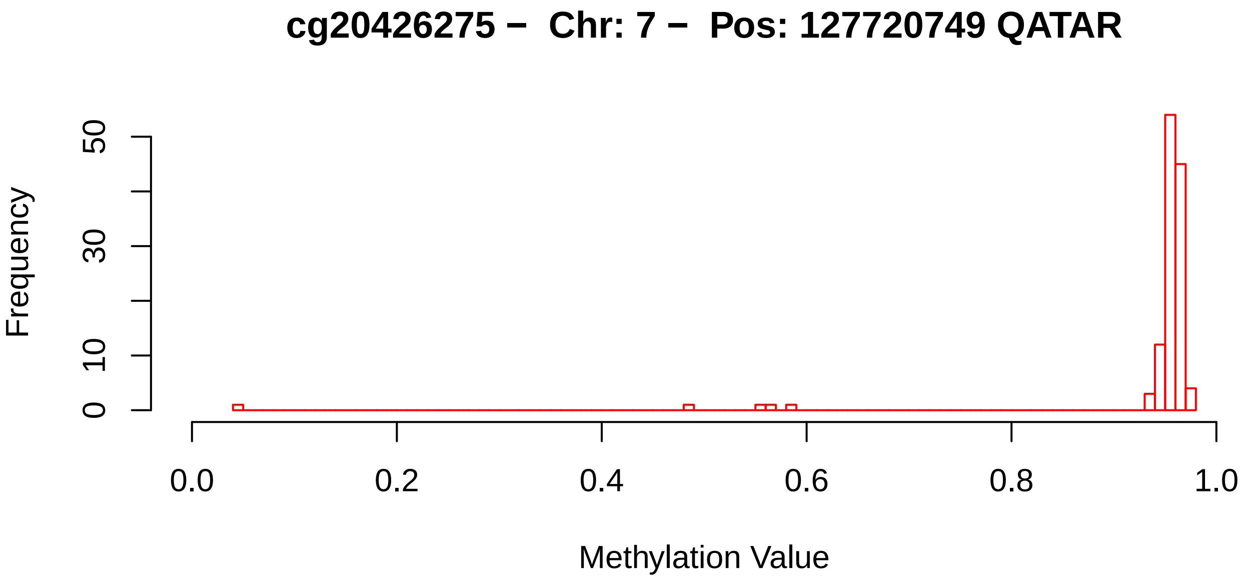
cg20426275 − Chr: 7 − Pos: 127720749 QATAR
Frequency
0
10
30
50
0.0
0.2
0.4
0.6
0.8
1.0
Methylation Value

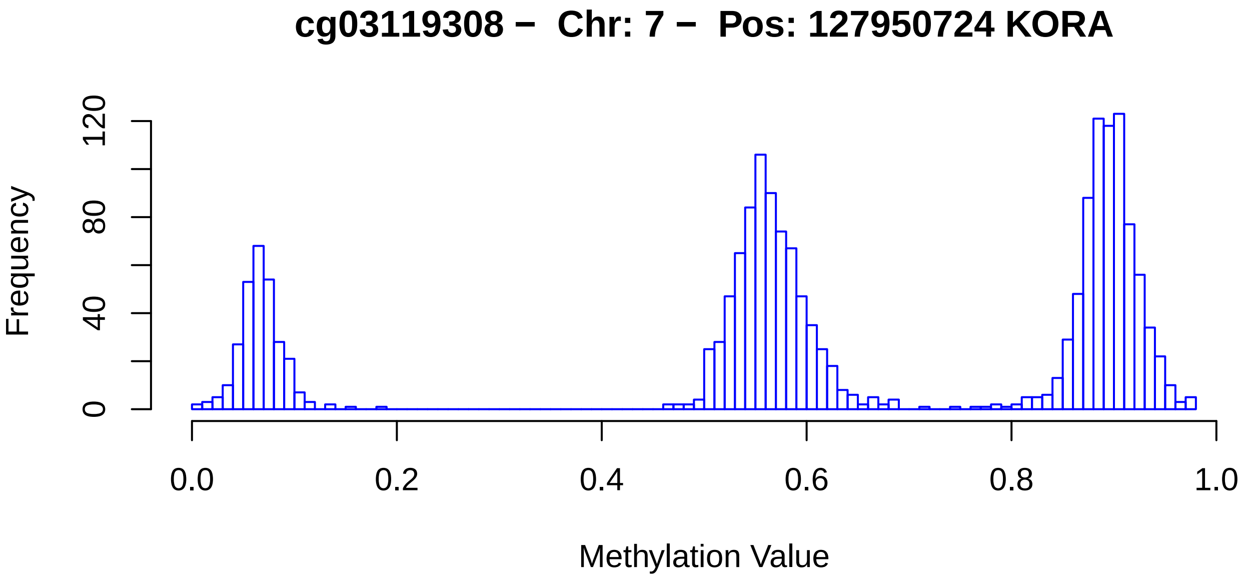
cg03119308 − Chr: 7 − Pos: 127950724 KORA
Frequency
0
40
80
120
0.0
0.2
0.4
0.6
0.8
1.0
Methylation Value

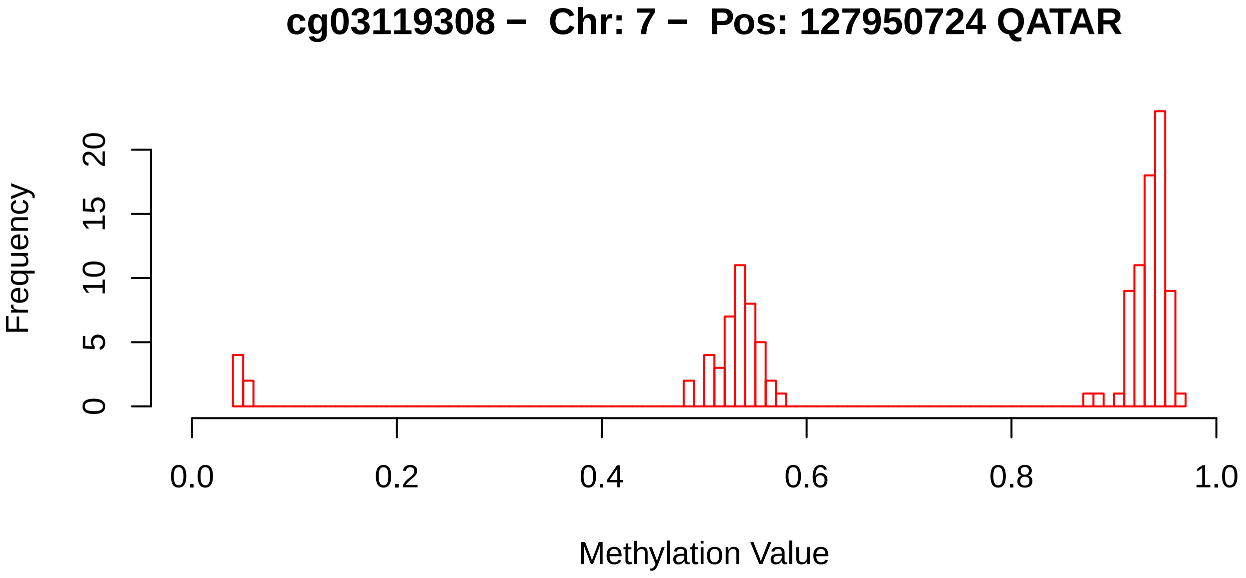
cg03119308 − Chr: 7 − Pos: 127950724 QATAR
Frequency
0
5
10
15
20
0.0
0.2
0.4
0.6
0.8
1.0
Methylation Value

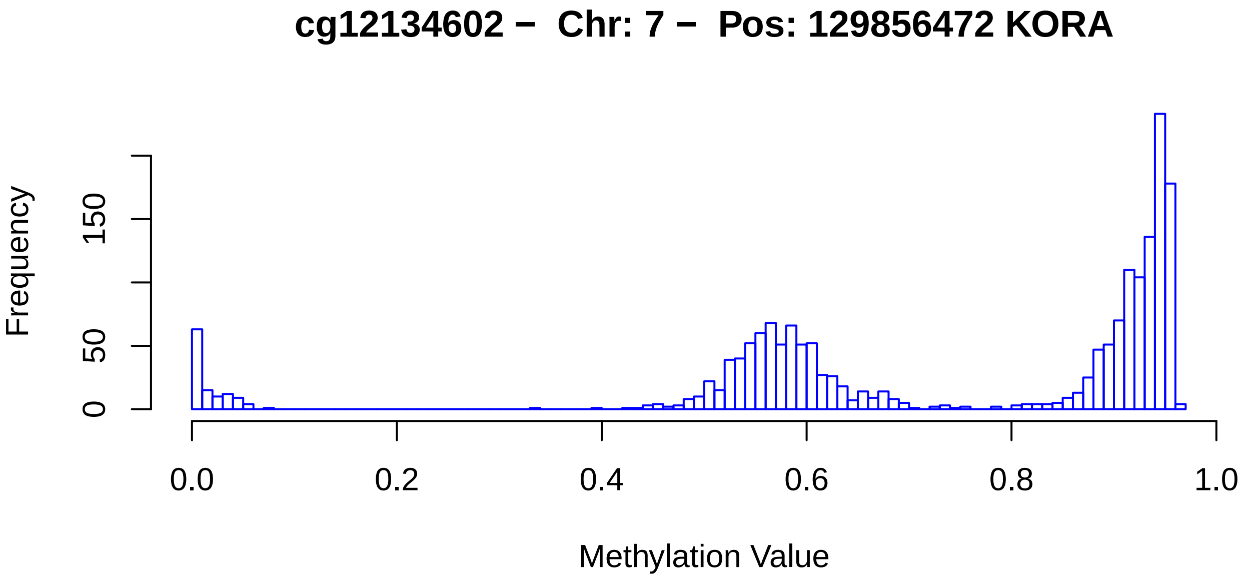
cg12134602 − Chr: 7 − Pos: 129856472 KORA
Frequency
0
50
150
0.0
0.2
0.4
0.6
0.8
1.0
Methylation Value

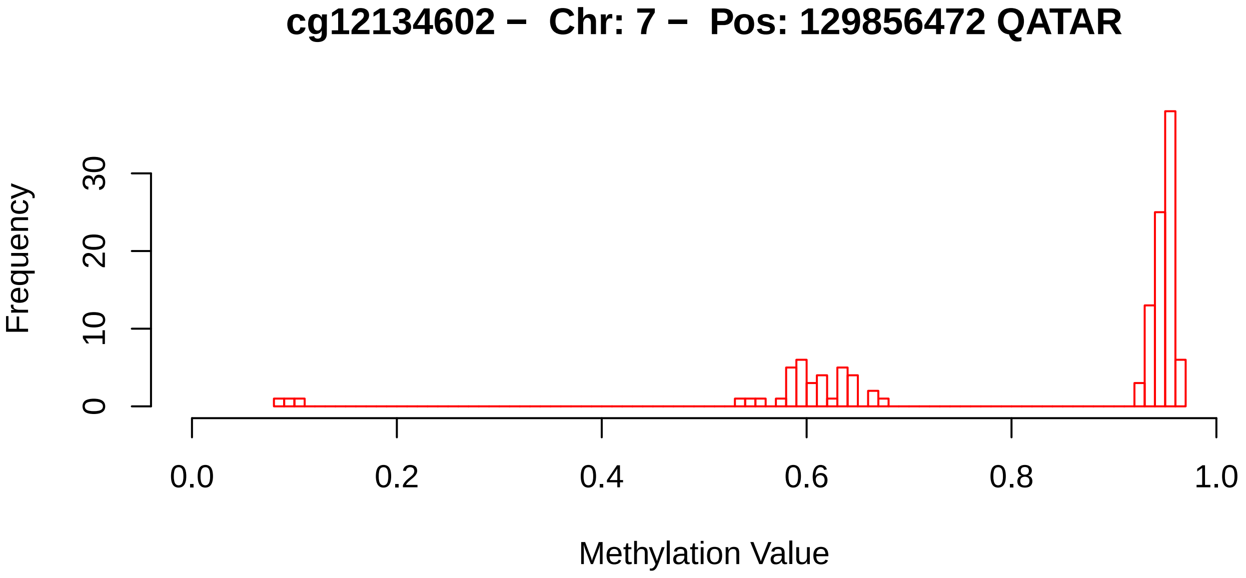
cg12134602 − Chr: 7 − Pos: 129856472 QATAR
Frequency
0
10
20
30
0.0
0.2
0.4
0.6
0.8
1.0
Methylation Value

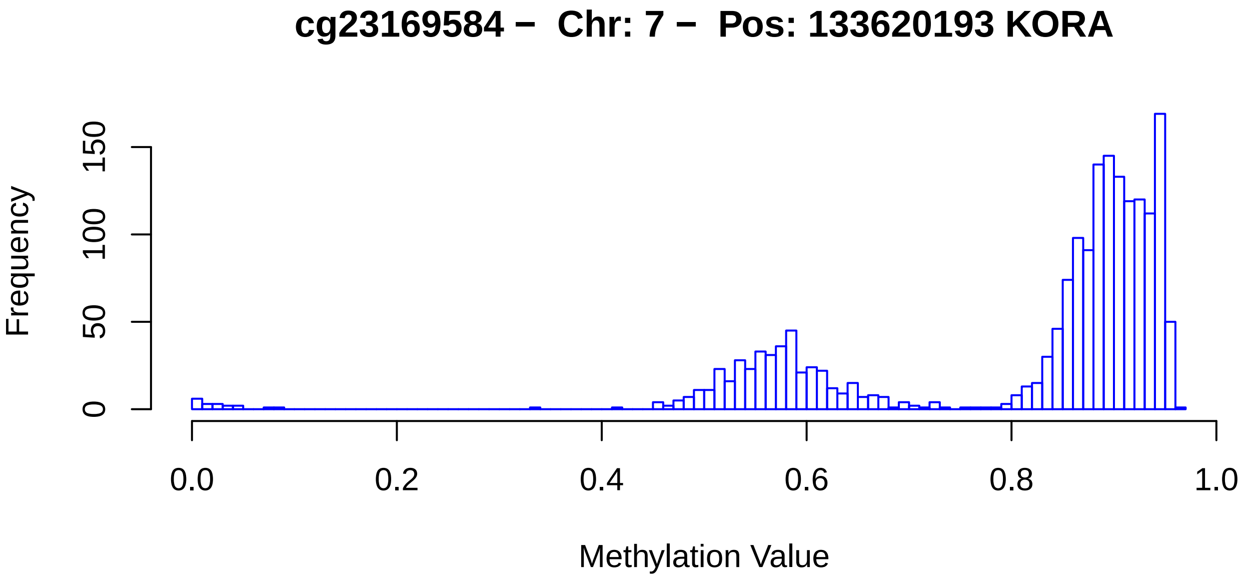
cg23169584 − Chr: 7 − Pos: 133620193 KORA
Frequency
0
50
100
150
0.0
0.2
0.4
0.6
0.8
1.0
Methylation Value

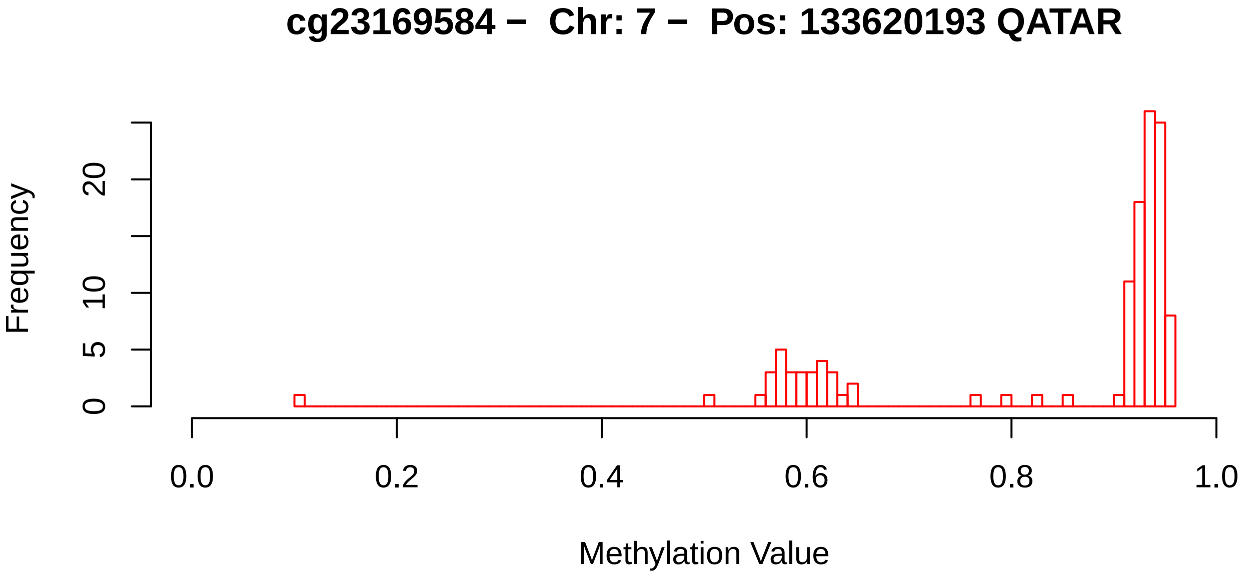
cg23169584 − Chr: 7 − Pos: 133620193 QATAR
Frequency
0
5
10
20
0.0
0.2
0.4
0.6
0.8
1.0
Methylation Value

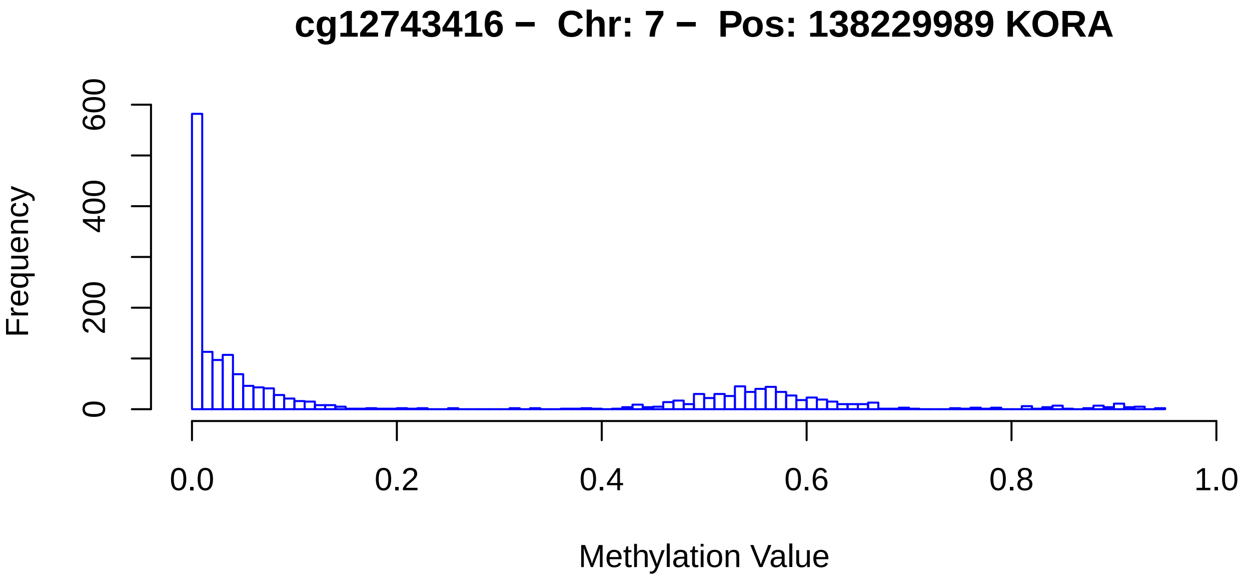

cg12743416 − Chr: 7 − Pos: 138229989 KORA
Frequency
0
200
400
600
0.0
0.2
0.4
0.6
0.8
1.0
Methylation Value

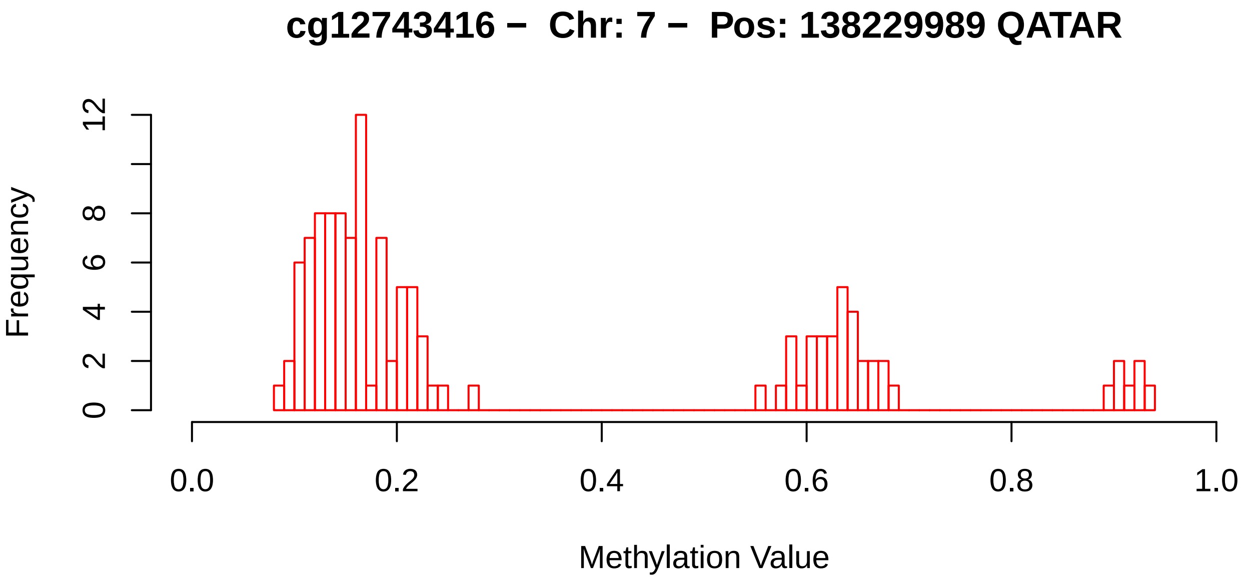

cg12743416 − Chr: 7 − Pos: 138229989 QATAR
Frequency
0
2
4
6
8
12
0.0
0.2
0.4
0.6
0.8
1.0
Methylation Value

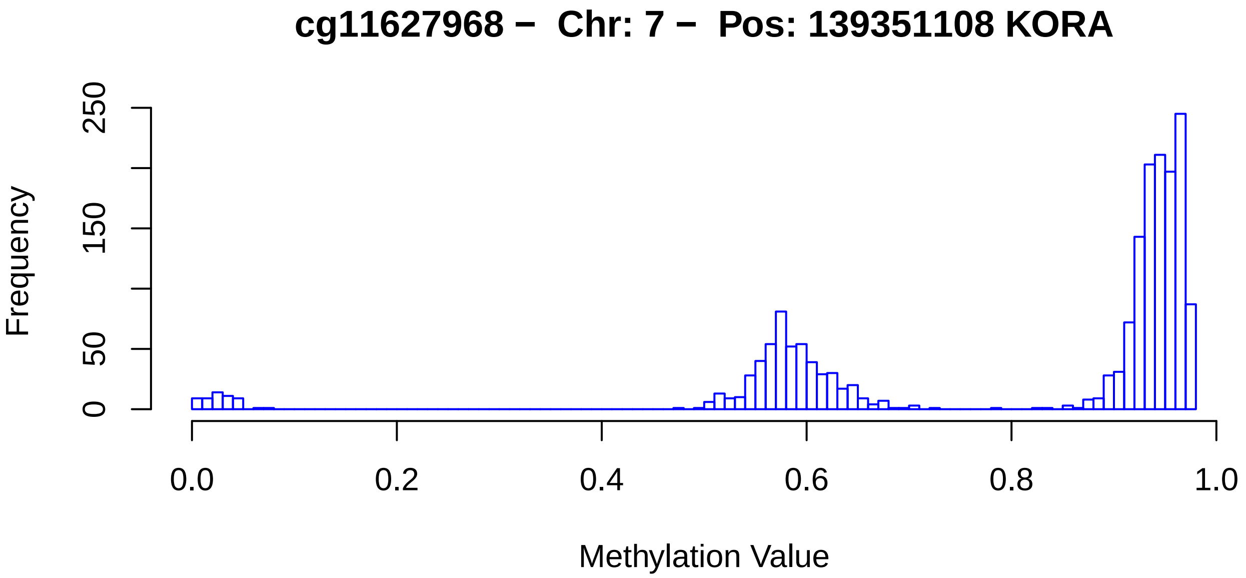
cg11627968 − Chr: 7 − Pos: 139351108 KORA
Frequency
0
50
150
250
0.0
0.2
0.4
0.6
0.8
1.0
Methylation Value

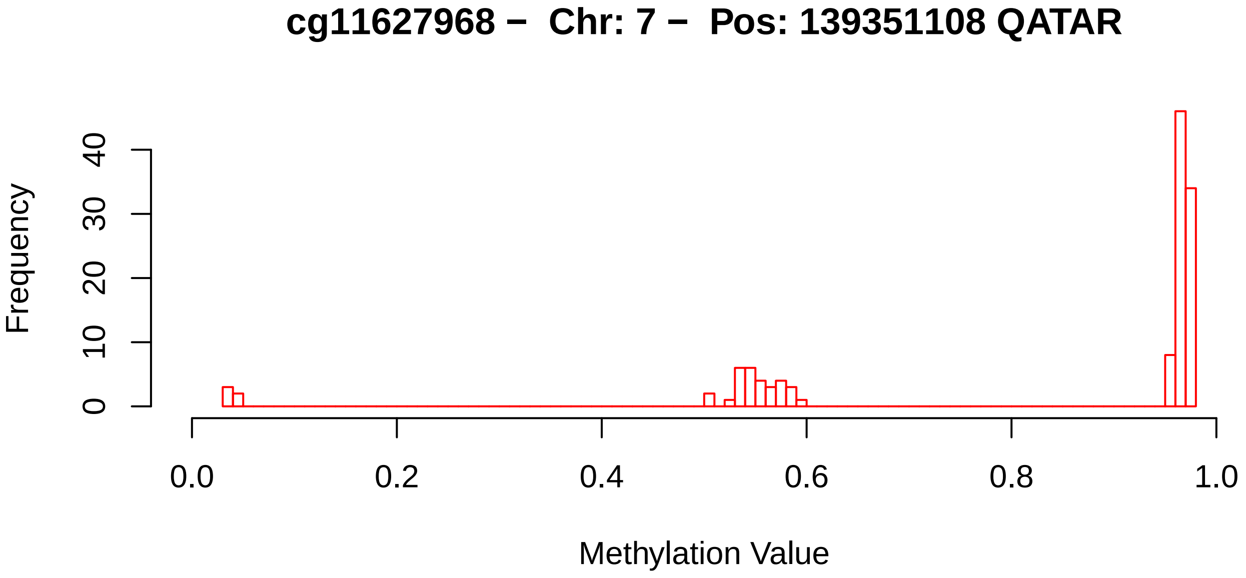
cg11627968 − Chr: 7 − Pos: 139351108 QATAR
Frequency
0
10
20
30
40
0.0
0.2
0.4
0.6
0.8
1.0
Methylation Value

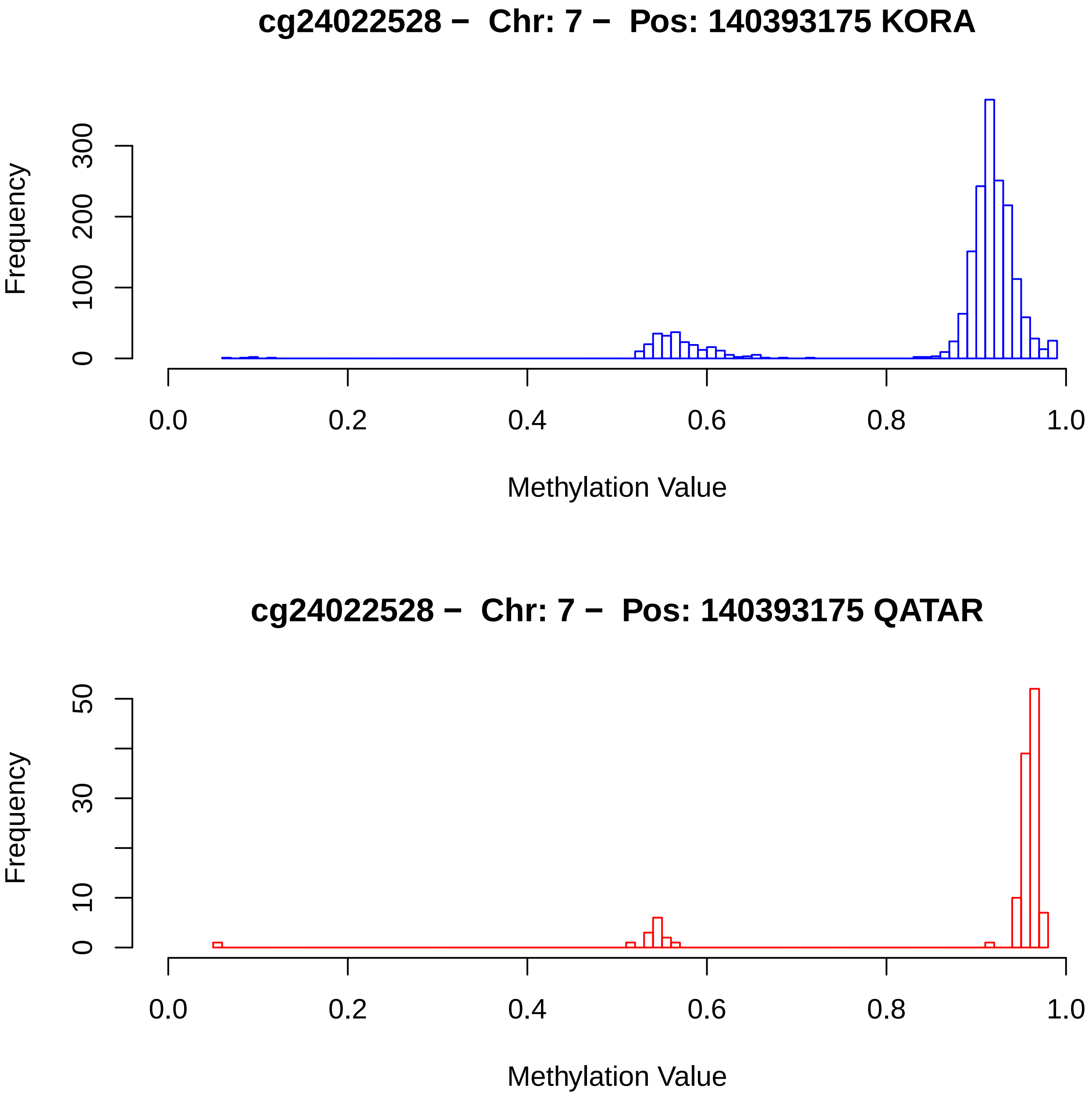

cg24022528 − Chr: 7 − Pos: 140393175 KORA
Frequency
0
100
200
300
0.0
0.2
0.4
0.6
0.8
1.0
Methylation Value
cg24022528 − Chr: 7 − Pos: 140393175 QATAR
Frequency
0
10
30
50
0.0
0.2
0.4
0.6
0.8
1.0
Methylation Value

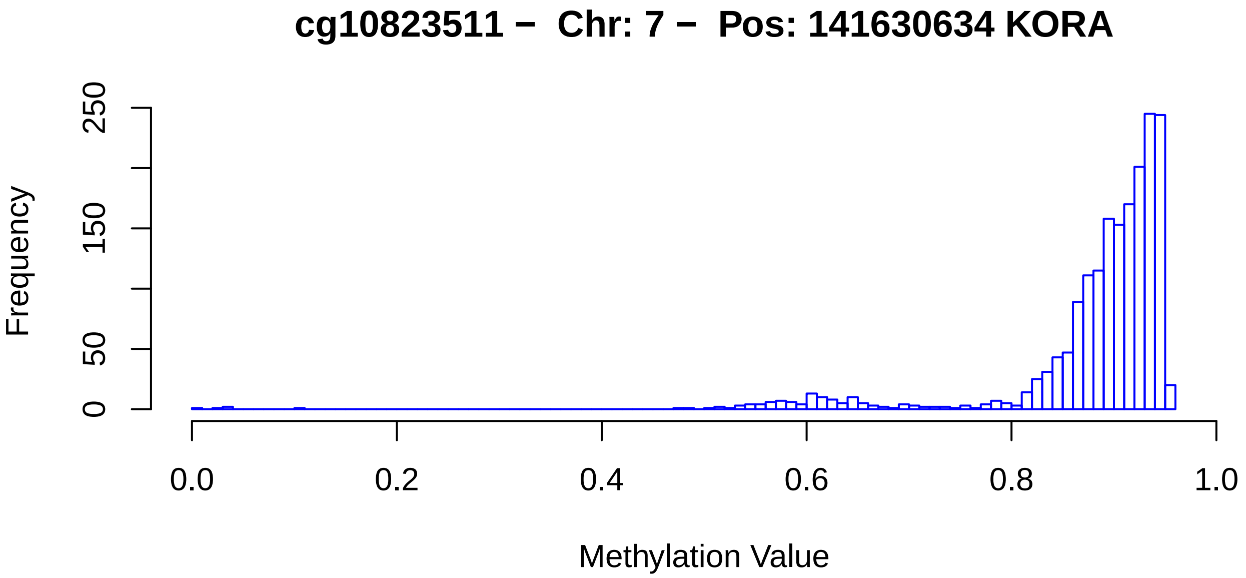
cg10823511 − Chr: 7 − Pos: 141630634 KORA
Frequency
0
50
150
250
0.0
0.2
0.4
0.6
0.8
1.0
Methylation Value

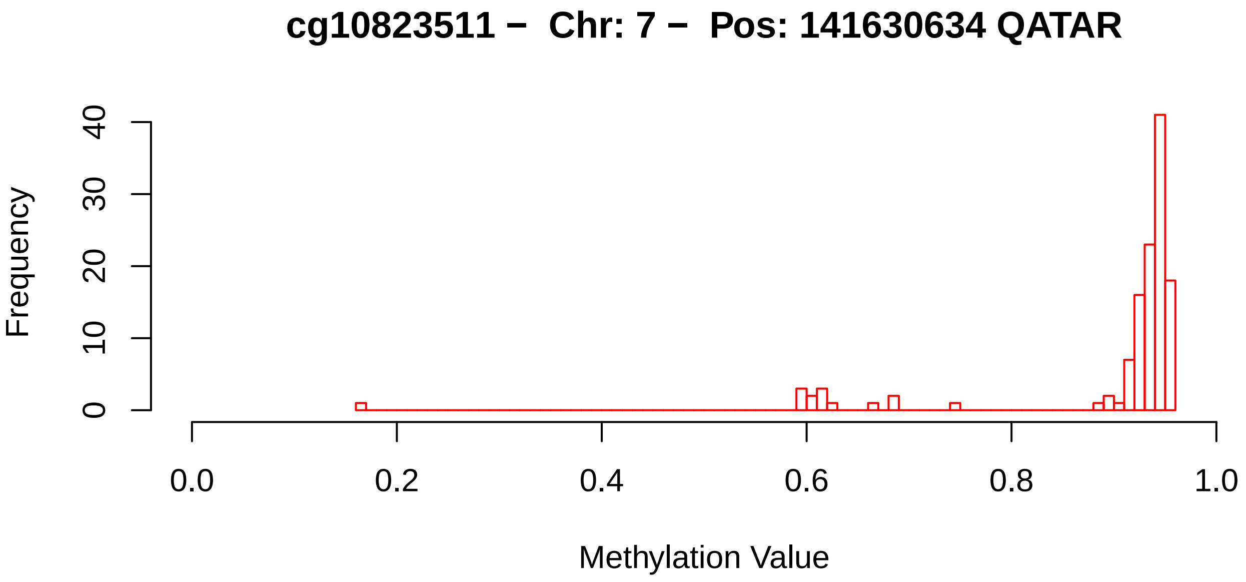
cg10823511 − Chr: 7 − Pos: 141630634 QATAR
Frequency
0
10
20
30
40
0.0
0.2
0.4
0.6
0.8
1.0
Methylation Value

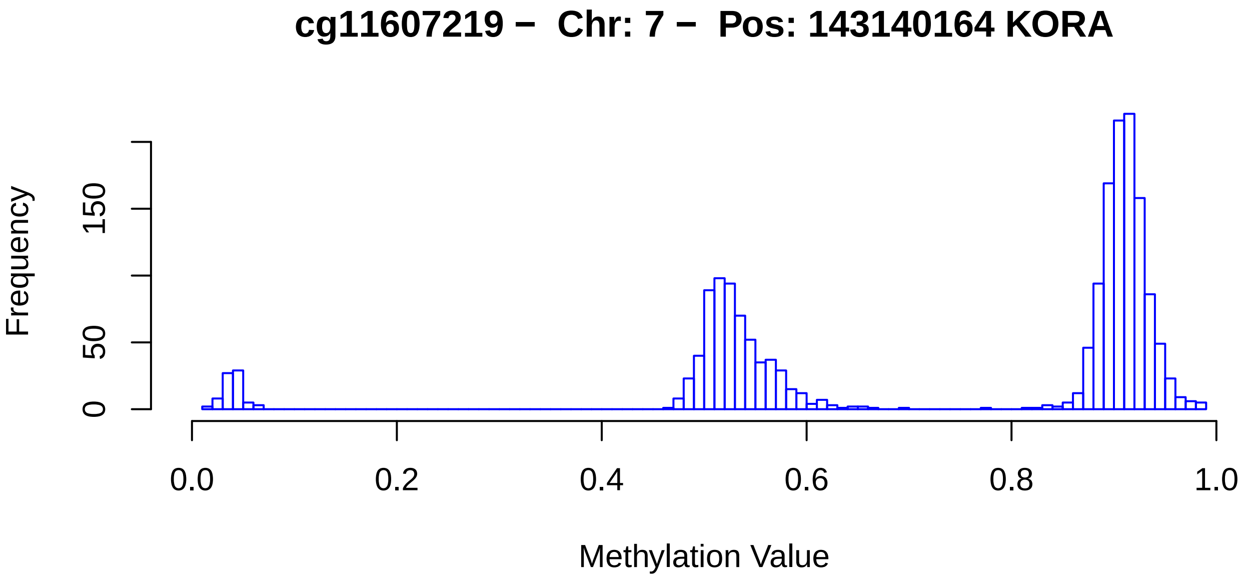
cg11607219 − Chr: 7 − Pos: 143140164 KORA
Frequency
0
50
150
0.0
0.2
0.4
0.6
0.8
1.0
Methylation Value

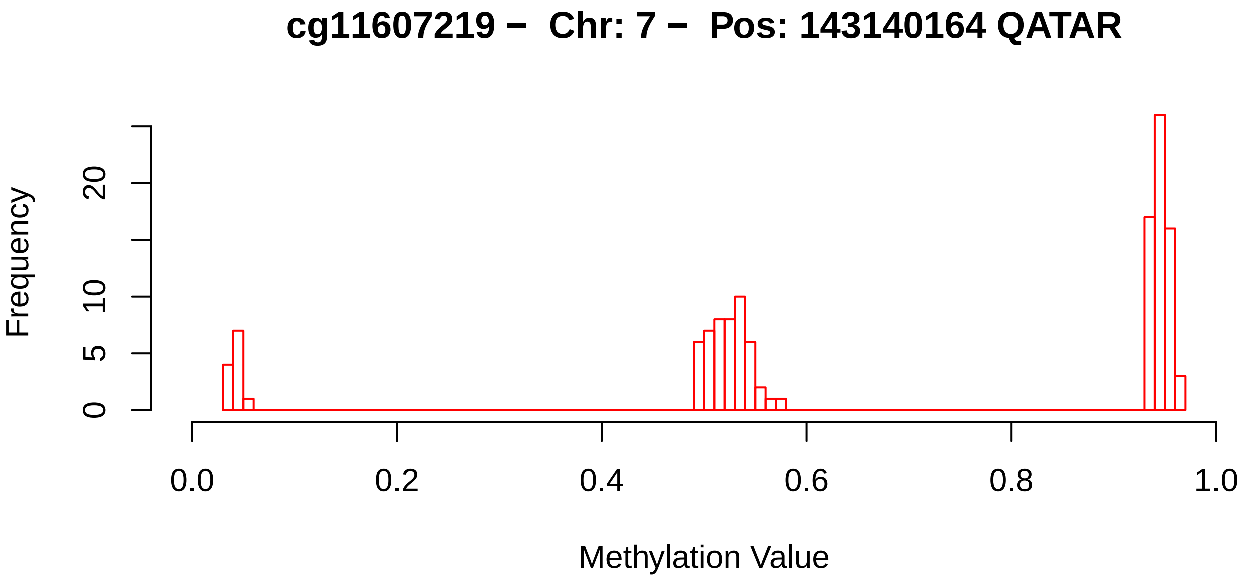
cg11607219 − Chr: 7 − Pos: 143140164 QATAR
Frequency
0
5
10
20
0.0
0.2
0.4
0.6
0.8
1.0
Methylation Value

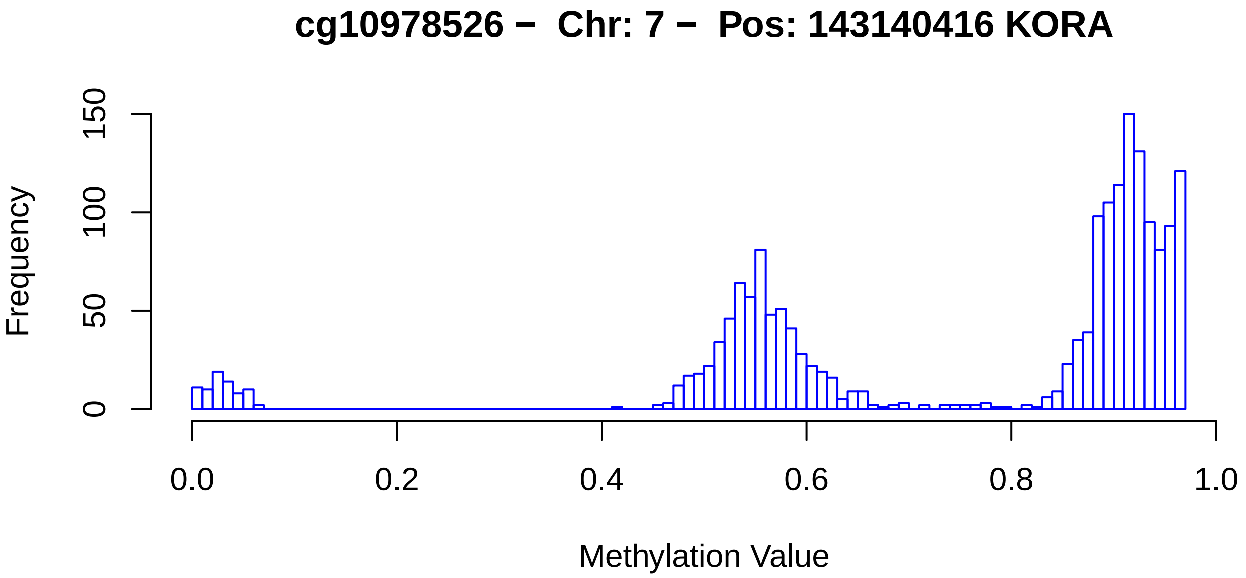
cg10978526 − Chr: 7 − Pos: 143140416 KORA
Frequency
0
50
100
150
0.0
0.2
0.4
0.6
0.8
1.0
Methylation Value

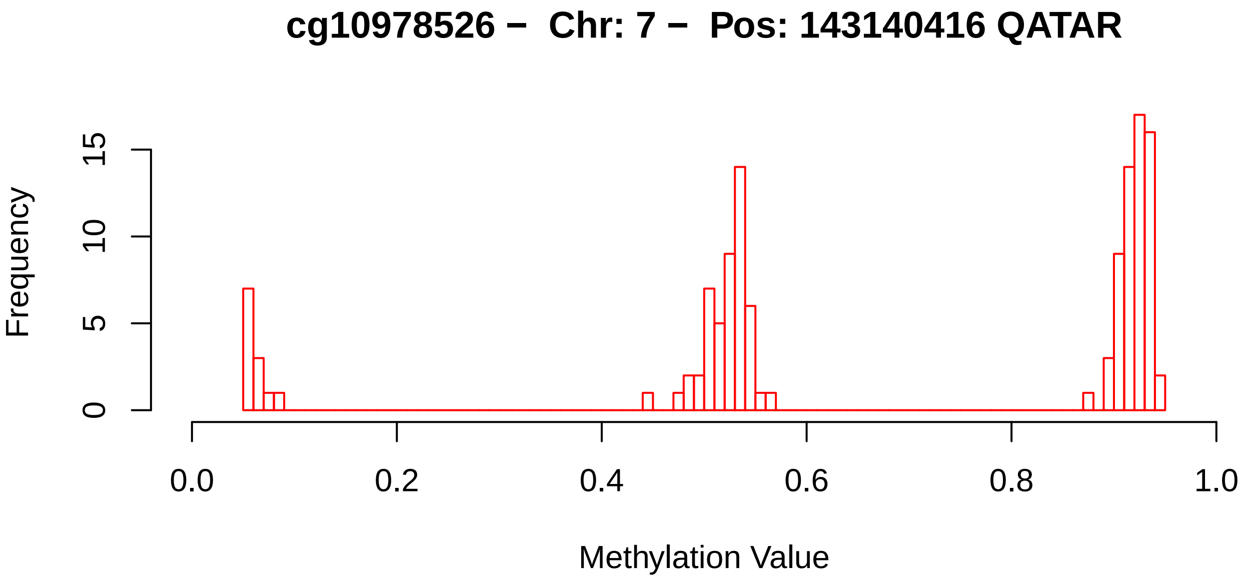
cg10978526 − Chr: 7 − Pos: 143140416 QATAR
Frequency
0
5
10
15
0.0
0.2
0.4
0.6
0.8
1.0
Methylation Value

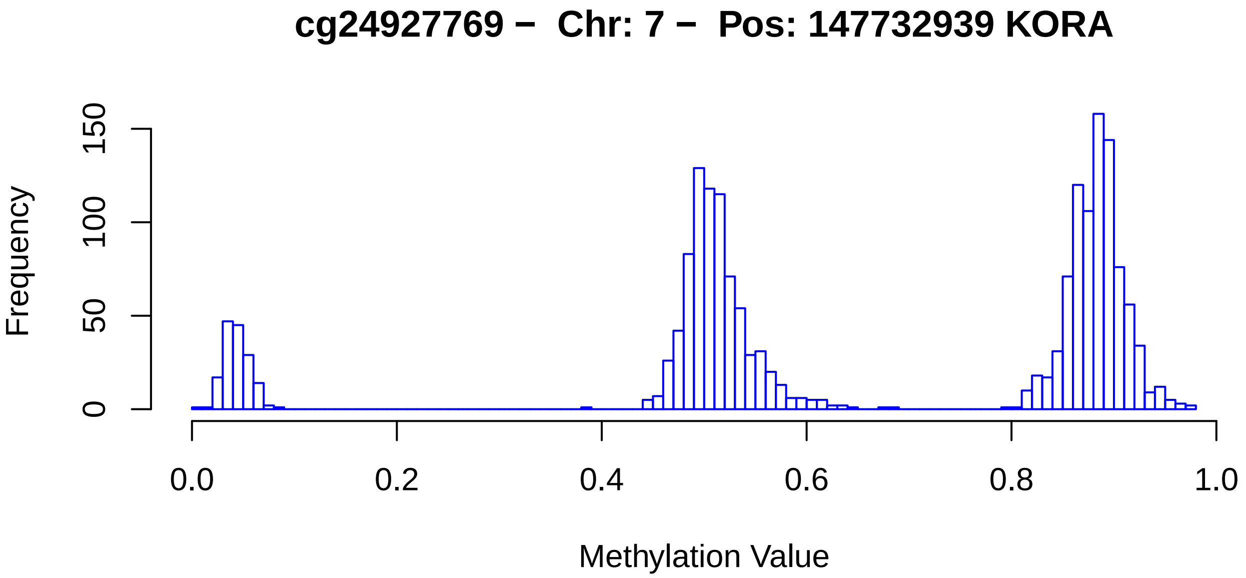

cg24927769 − Chr: 7 − Pos: 147732939 KORA
Frequency
0
50
100
150
0.0
0.2
0.4
0.6
0.8
1.0
Methylation Value

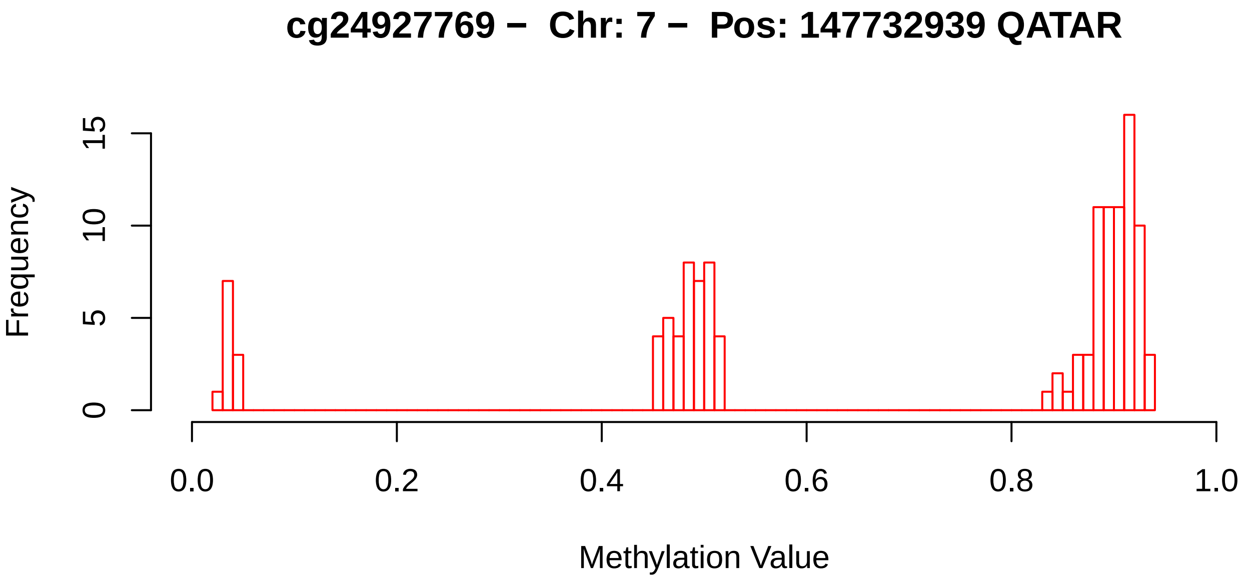

cg24927769 − Chr: 7 − Pos: 147732939 QATAR
Frequency
0
5
10
15
0.0
0.2
0.4
0.6
0.8
1.0
Methylation Value

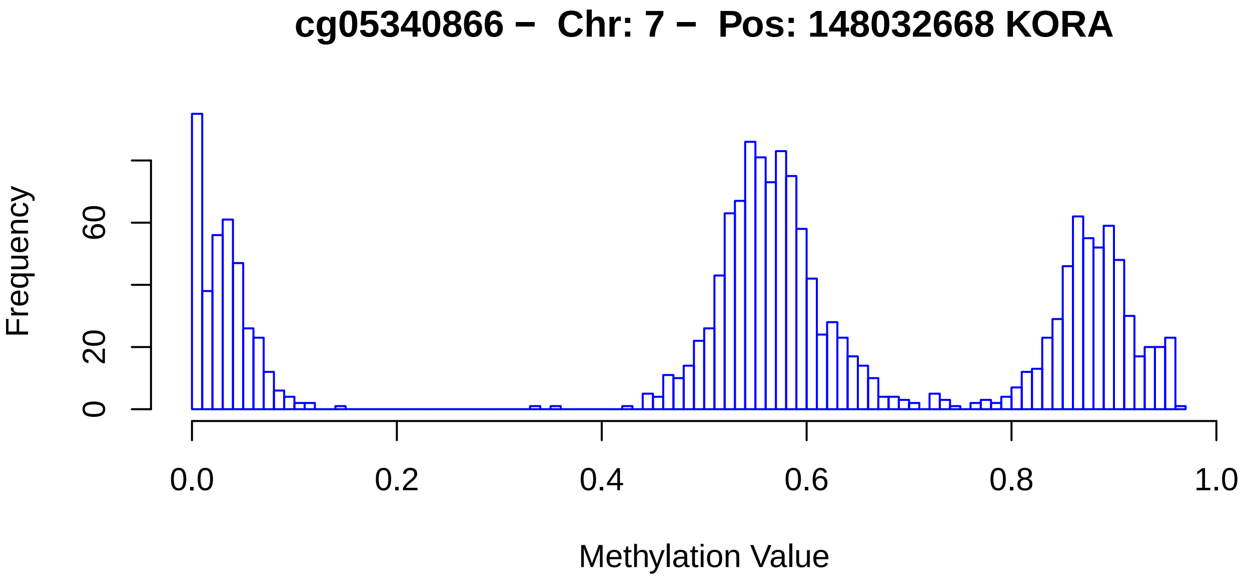
cg05340866 − Chr: 7 − Pos: 148032668 KORA
Frequency
0
20
60
0.0
0.2
0.4
0.6
0.8
1.0
Methylation Value

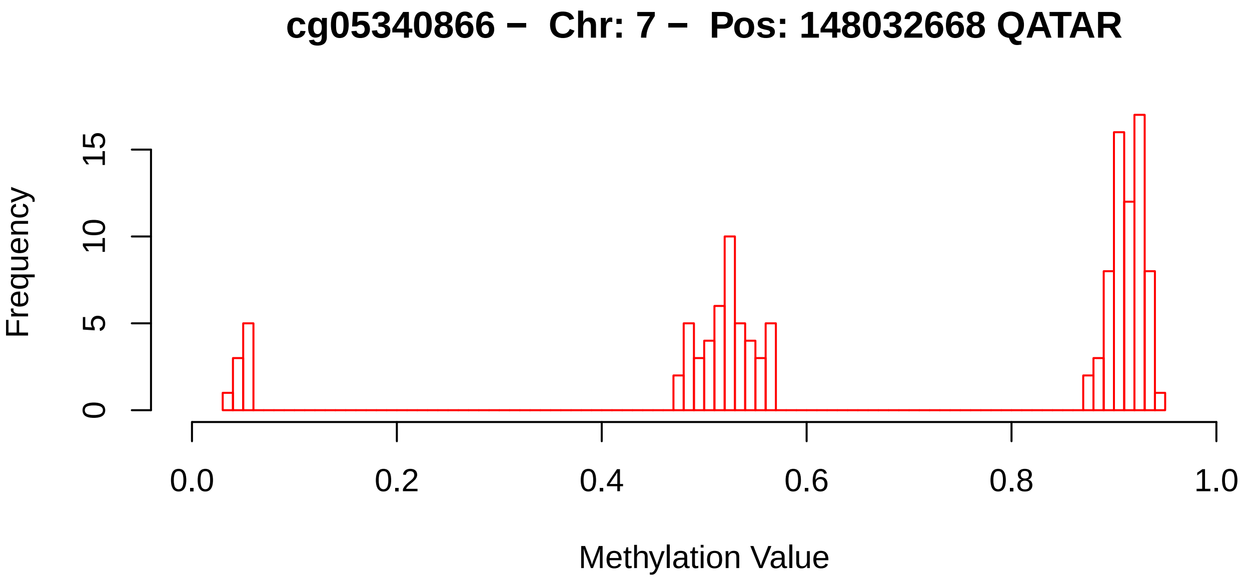
cg05340866 − Chr: 7 − Pos: 148032668 QATAR
Frequency
0
5
10
15
0.0
0.2
0.4
0.6
0.8
1.0
Methylation Value

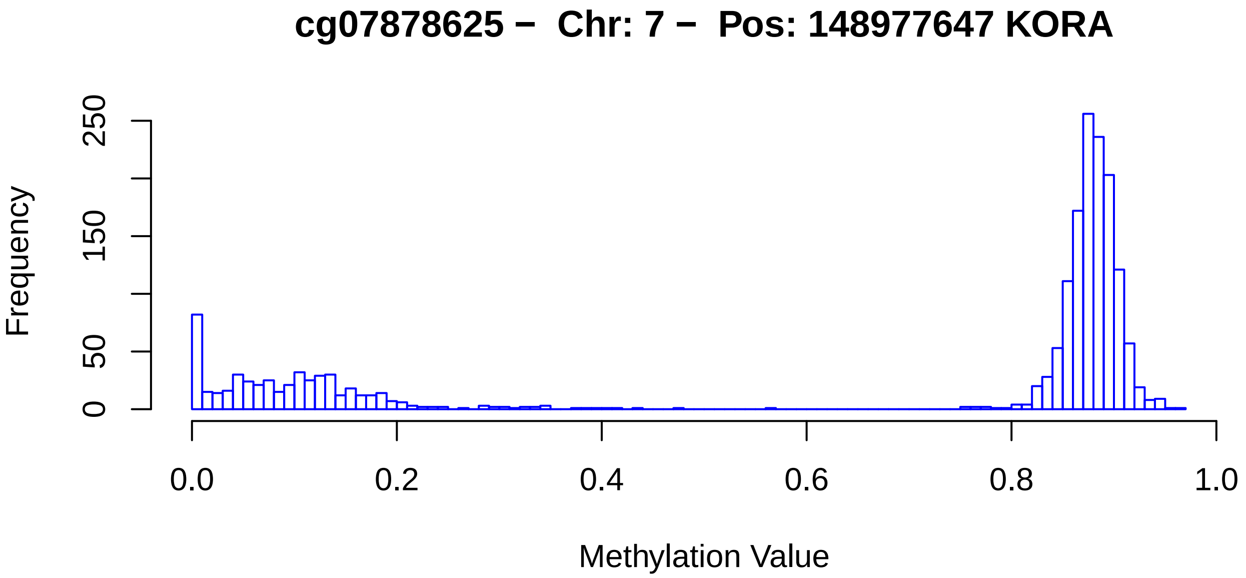
cg07878625 − Chr: 7 − Pos: 148977647 KORA
Frequency
0
50
150
250
0.0
0.2
0.4
0.6
0.8
1.0
Methylation Value

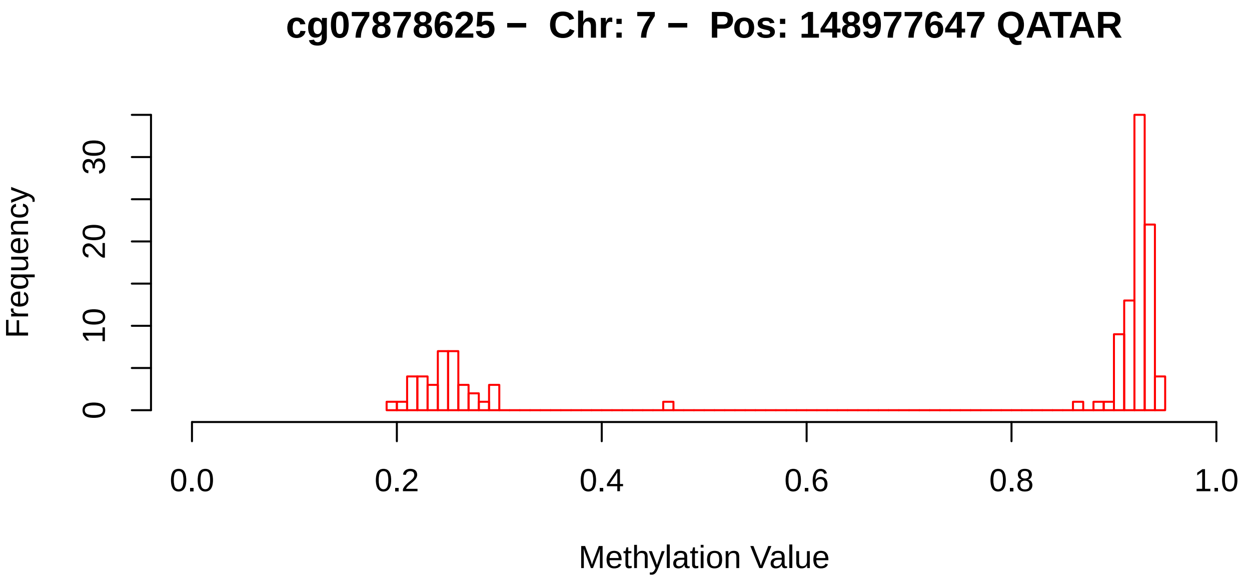
cg07878625 − Chr: 7 − Pos: 148977647 QATAR
Frequency
0
10
20
30
0.0
0.2
0.4
0.6
0.8
1.0
Methylation Value

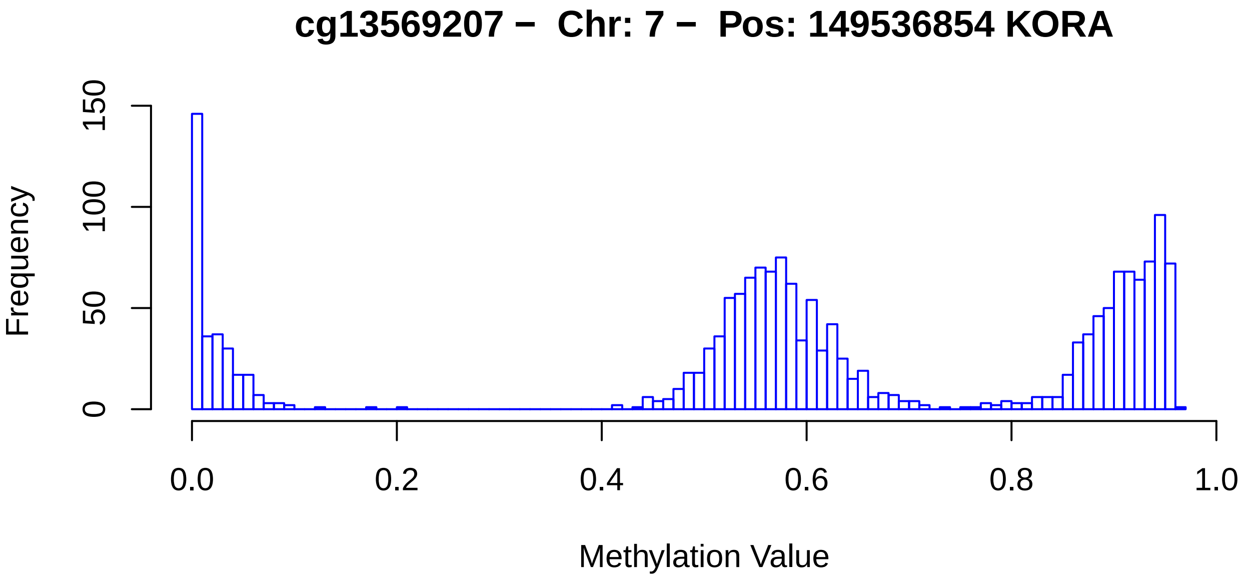
cg13569207 − Chr: 7 − Pos: 149536854 KORA
Frequency
0
50
100
150
0.0
0.2
0.4
0.6
0.8
1.0
Methylation Value

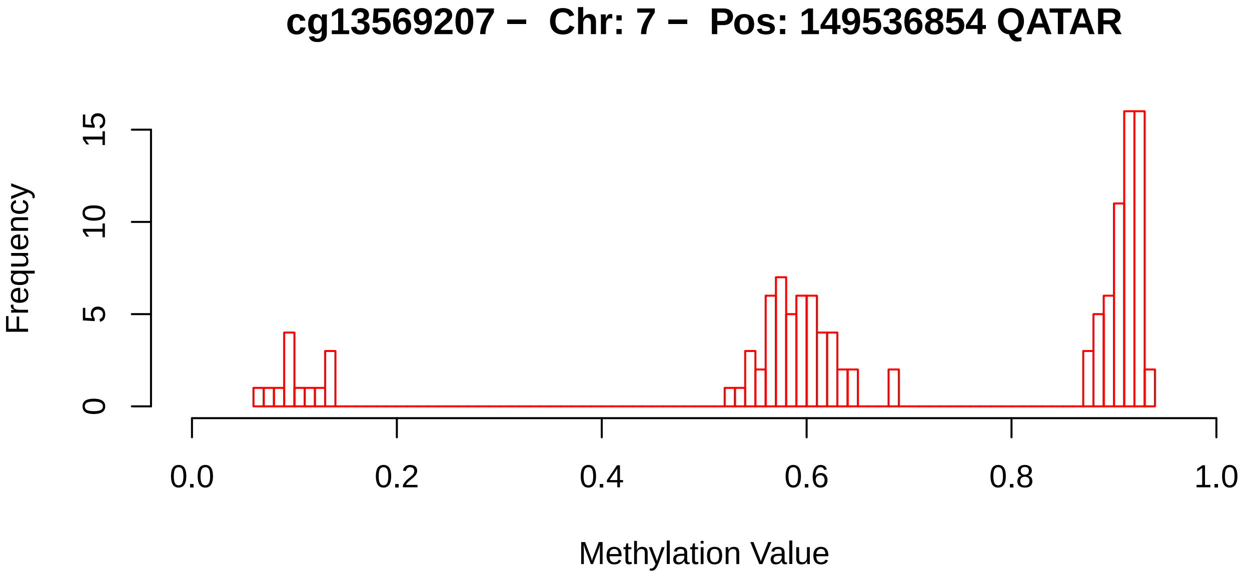
cg13569207 − Chr: 7 − Pos: 149536854 QATAR
Frequency
0
5
10
15
0.0
0.2
0.4
0.6
0.8
1.0
Methylation Value

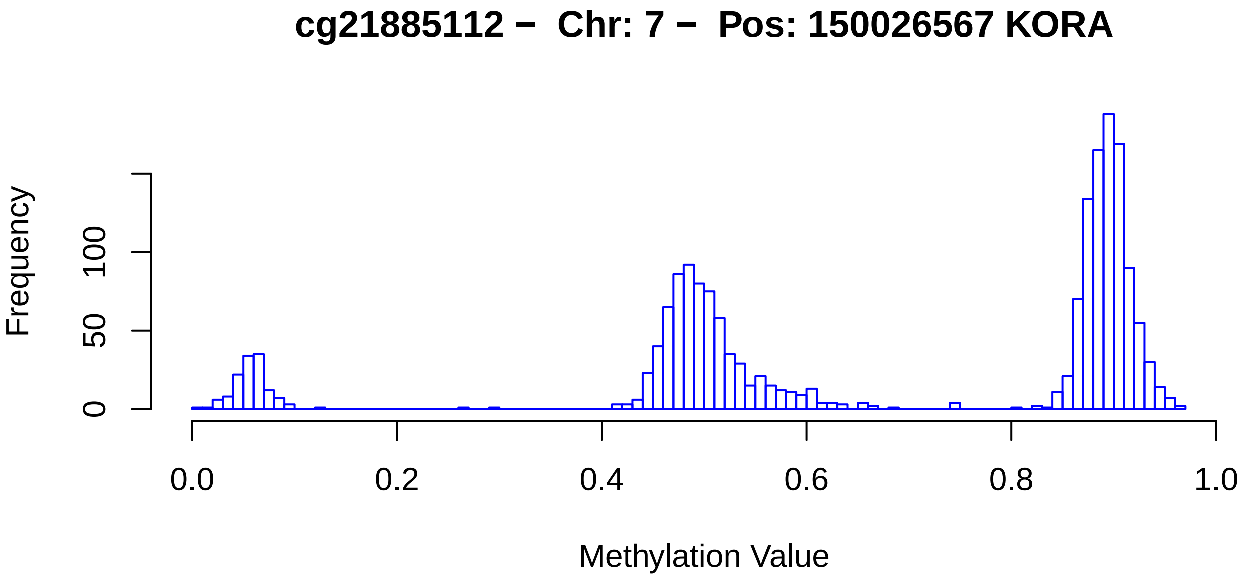
cg21885112 − Chr: 7 − Pos: 150026567 KORA
Frequency
0
50
100
0.0
0.2
0.4
0.6
0.8
1.0
Methylation Value

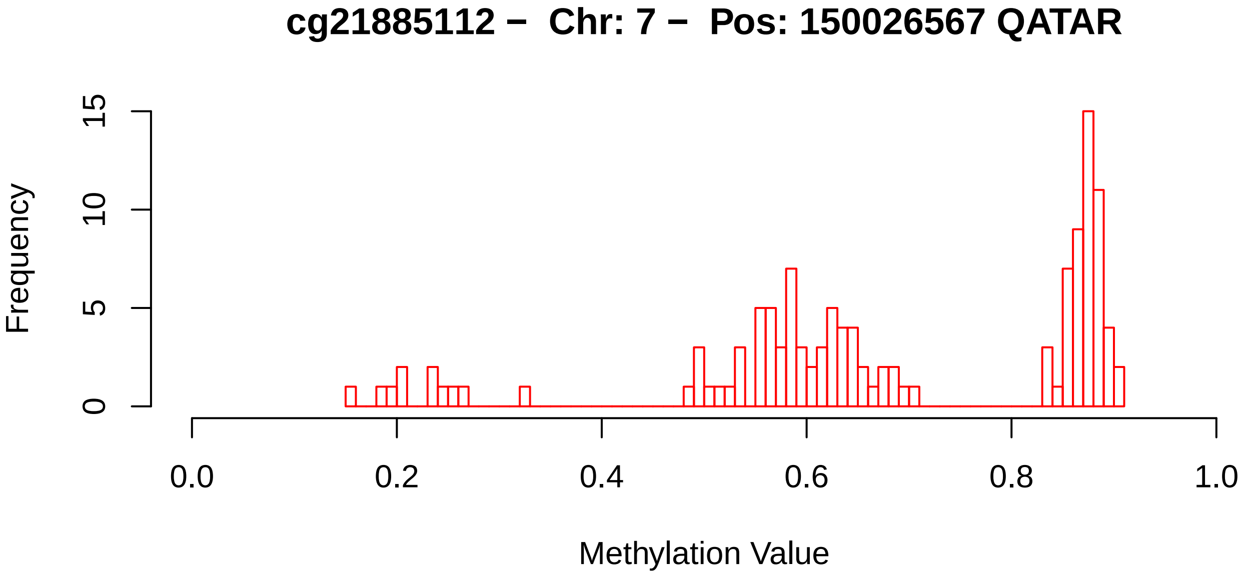
cg21885112 − Chr: 7 − Pos: 150026567 QATAR
Frequency
0
5
10
15
0.0
0.2
0.4
0.6
0.8
1.0
Methylation Value

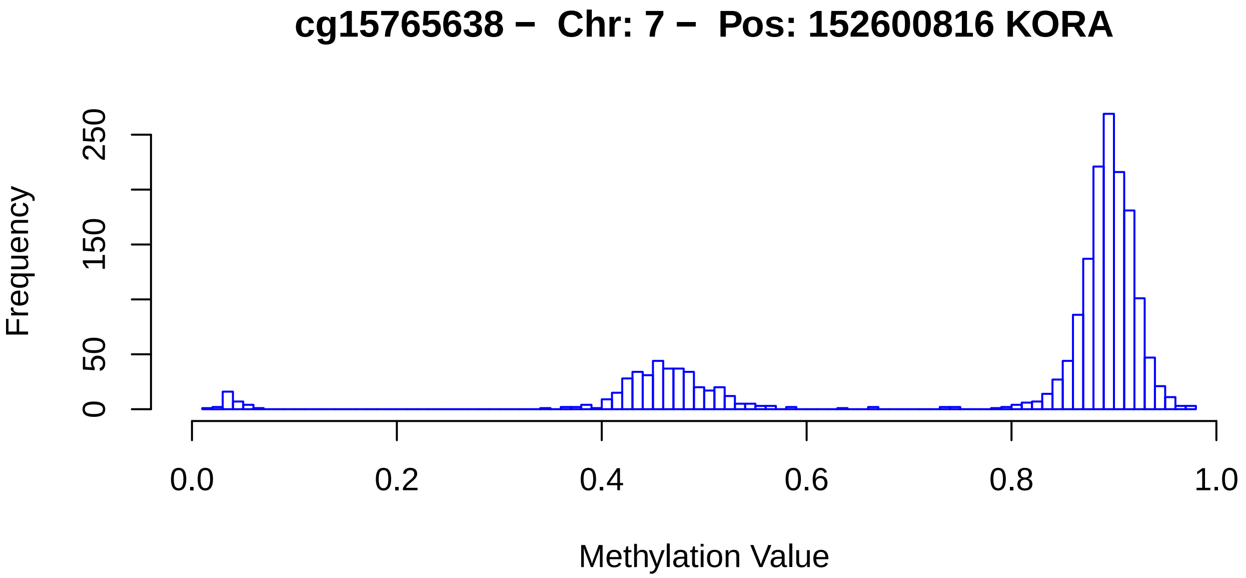
cg15765638 − Chr: 7 − Pos: 152600816 KORA
Frequency
0
50
150
250
0.0
0.2
0.4
0.6
0.8
1.0
Methylation Value

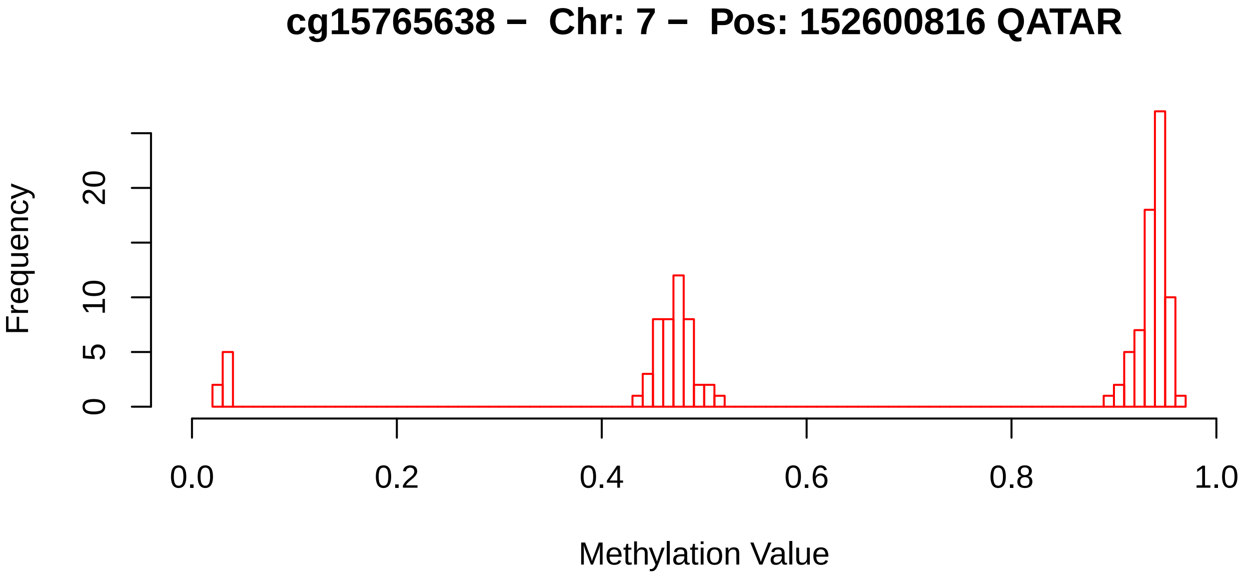
cg15765638 − Chr: 7 − Pos: 152600816 QATAR
Frequency
0
5
10
20
0.0
0.2
0.4
0.6
0.8
1.0
Methylation Value

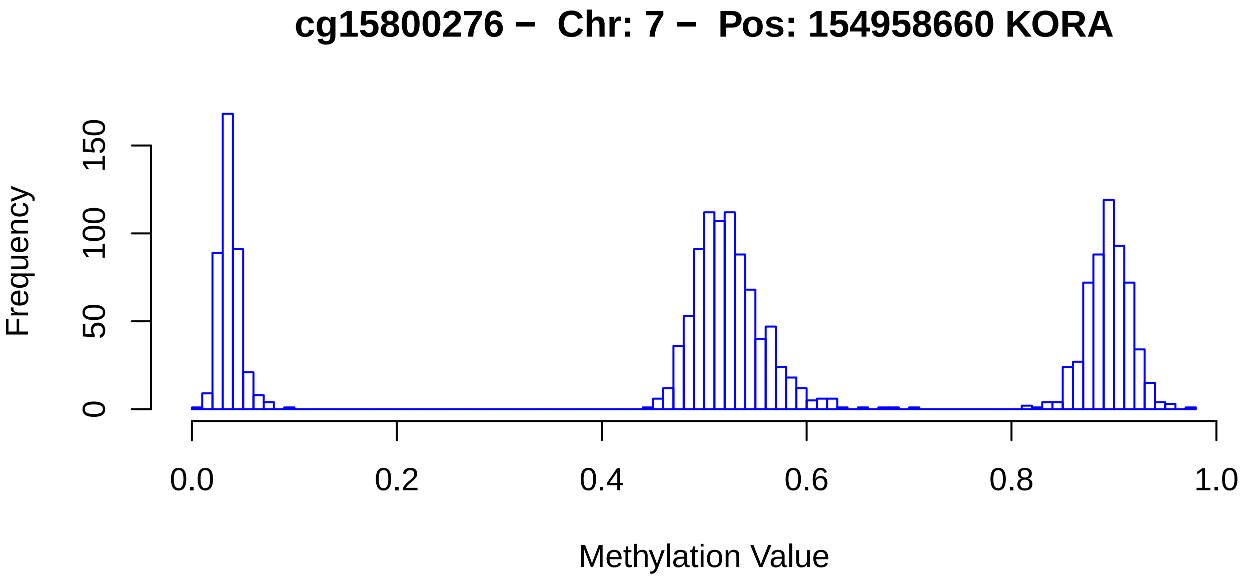
cg15800276 − Chr: 7 − Pos: 154958660 KORA
Frequency
0
50
100
150
0.0
0.2
0.4
0.6
0.8
1.0
Methylation Value

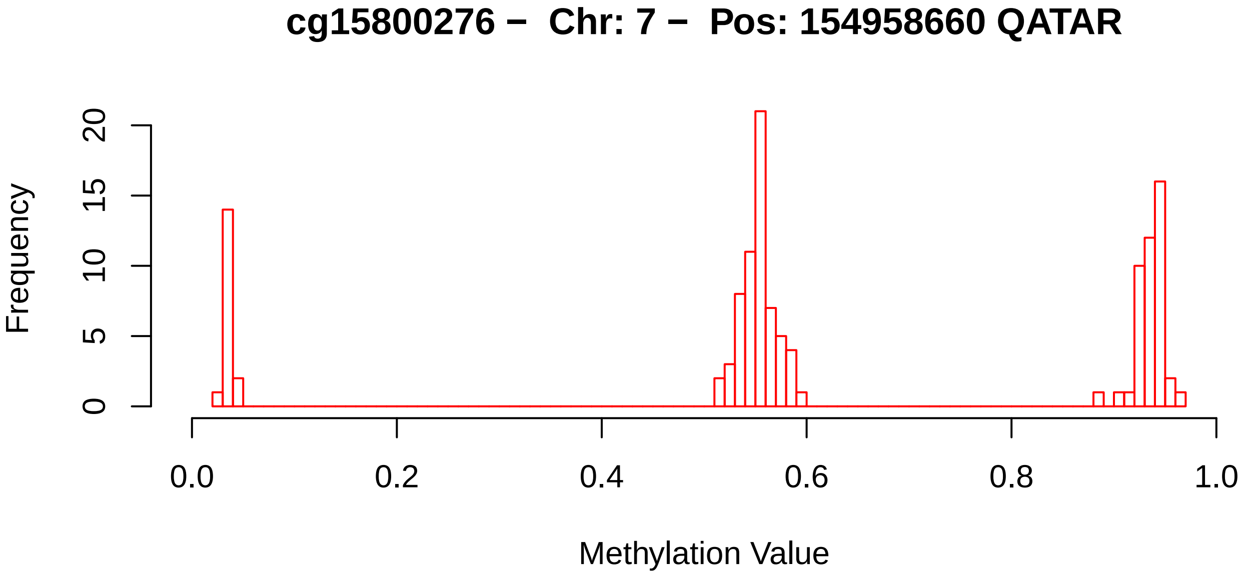
cg15800276 − Chr: 7 − Pos: 154958660 QATAR
Frequency
0
5
10
15
20
0.0
0.2
0.4
0.6
0.8
1.0
Methylation Value

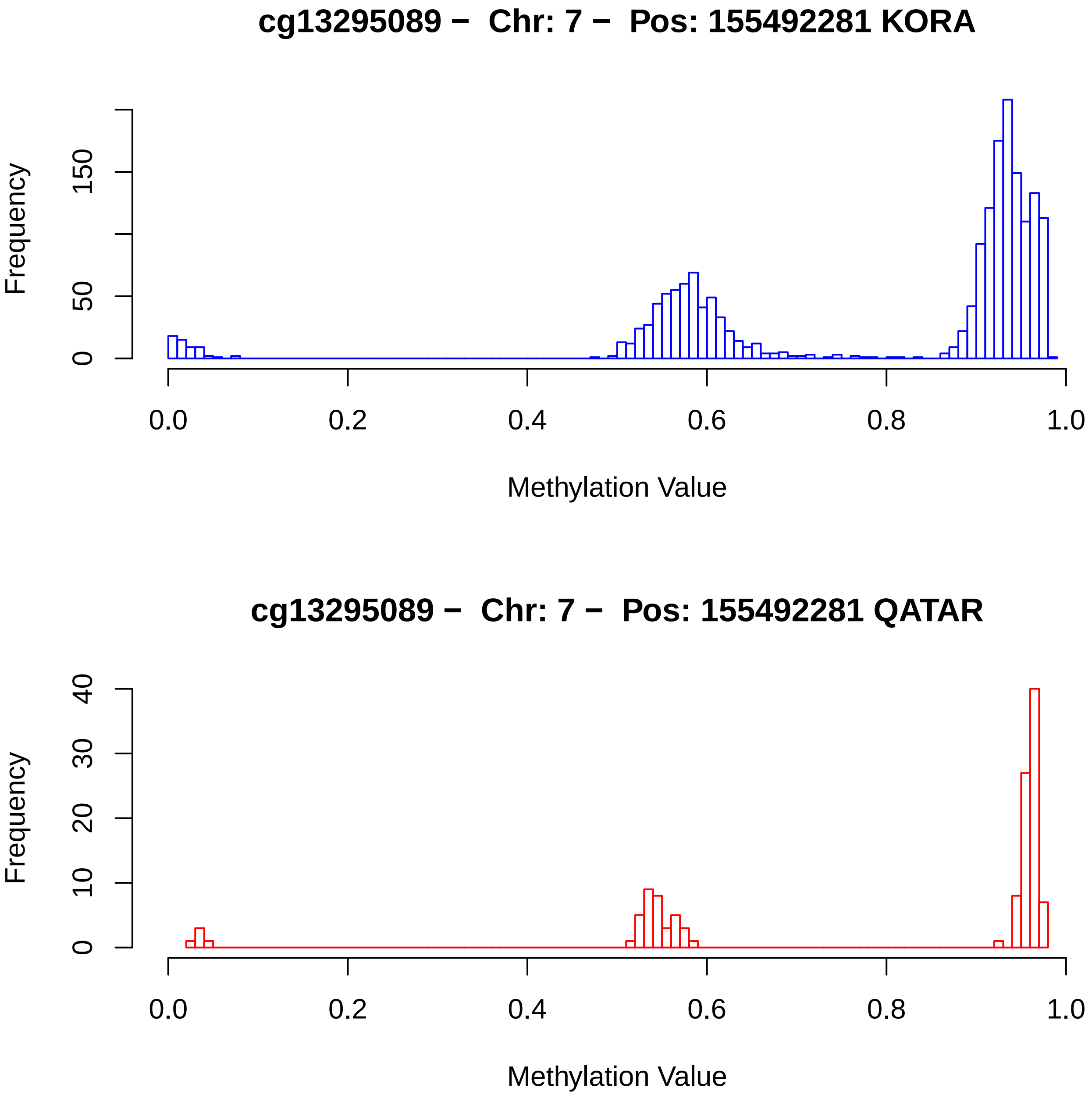

cg13295089 − Chr: 7 − Pos: 155492281 KORA
Frequency
0
50
150
0.0
0.2
0.4
0.6
0.8
1.0
Methylation Value
cg13295089 − Chr: 7 − Pos: 155492281 QATAR
Frequency
0
10
20
30
40
0.0
0.2
0.4
0.6
0.8
1.0
Methylation Value

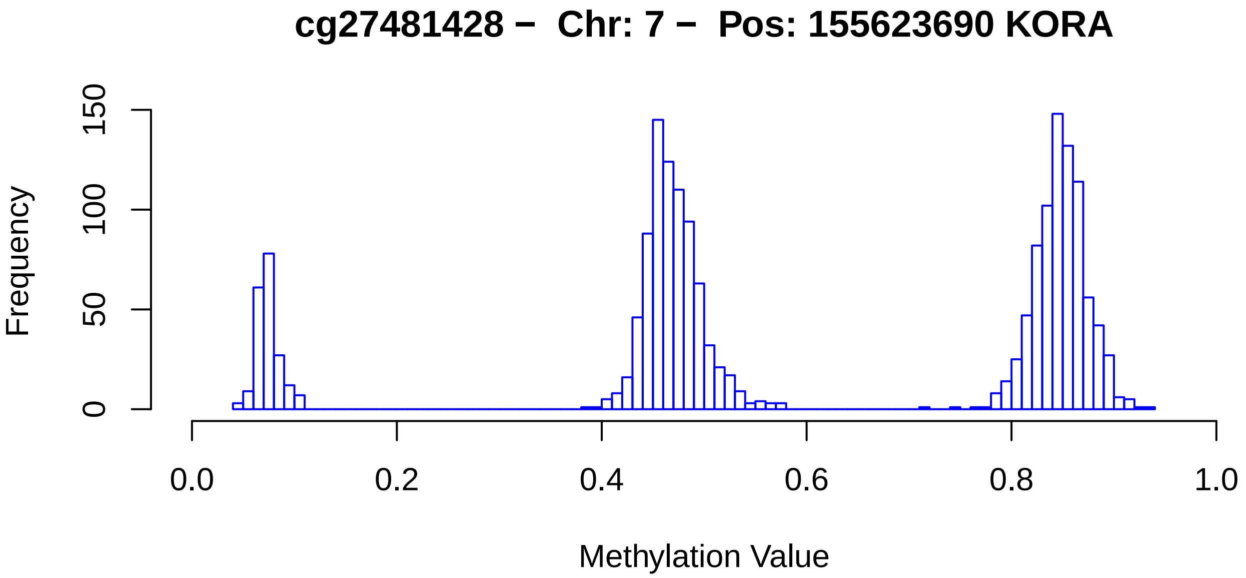
cg27481428 − Chr: 7 − Pos: 155623690 KORA
Frequency
0
50
100
150
0.0
0.2
0.4
0.6
0.8
1.0
Methylation Value

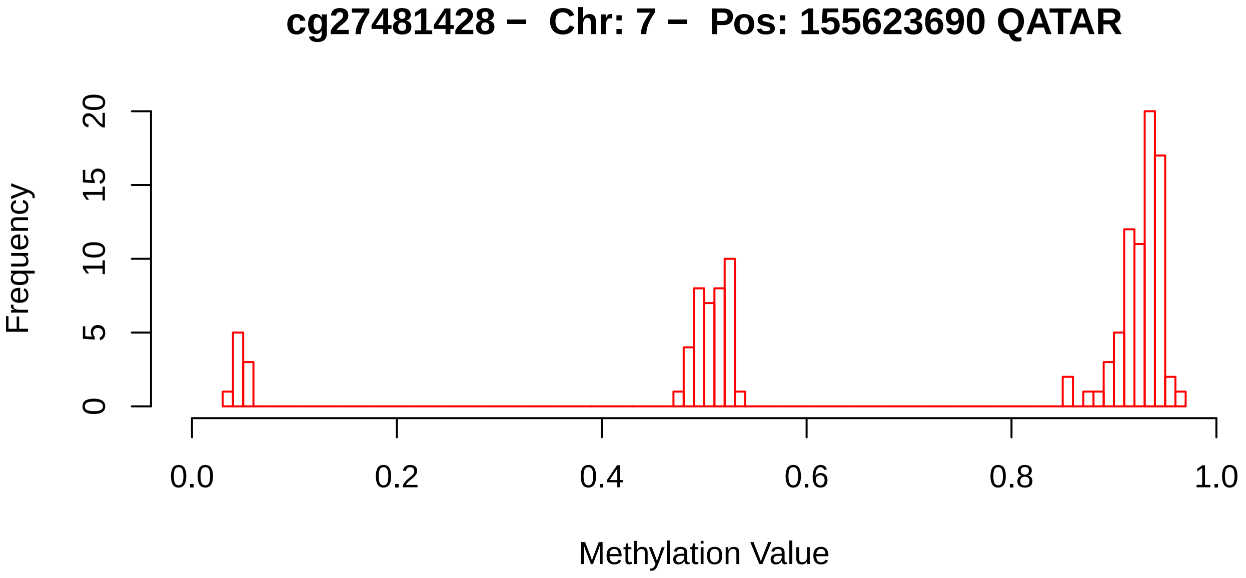
cg27481428 − Chr: 7 − Pos: 155623690 QATAR
Frequency
0
5
10
15
20
0.0
0.2
0.4
0.6
0.8
1.0
Methylation Value

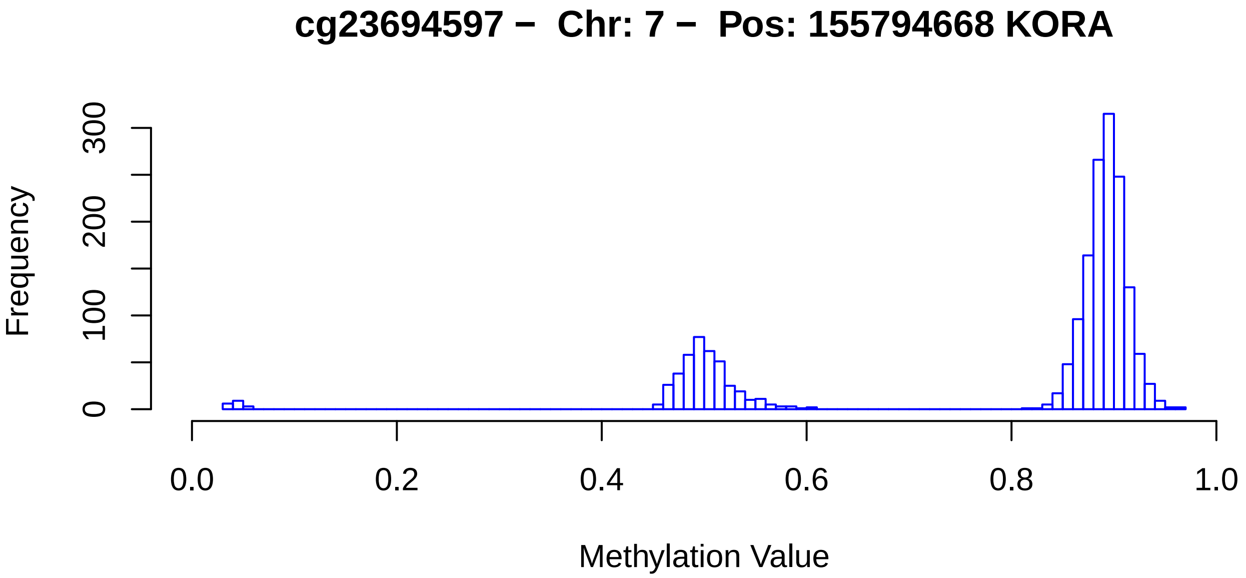
cg23694597 − Chr: 7 − Pos: 155794668 KORA
Frequency
0
100
200
300
0.0
0.2
0.4
0.6
0.8
1.0
Methylation Value

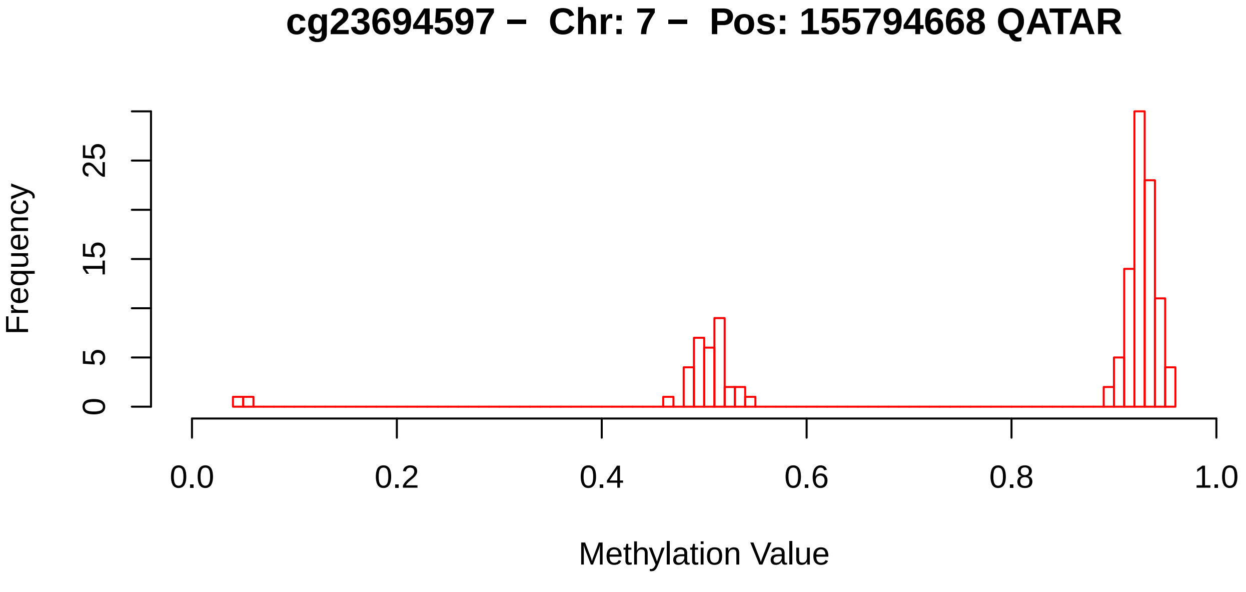
cg23694597 − Chr: 7 − Pos: 155794668 QATAR
Frequency
0
5
15
25
0.0
0.2
0.4
0.6
0.8
1.0
Methylation Value

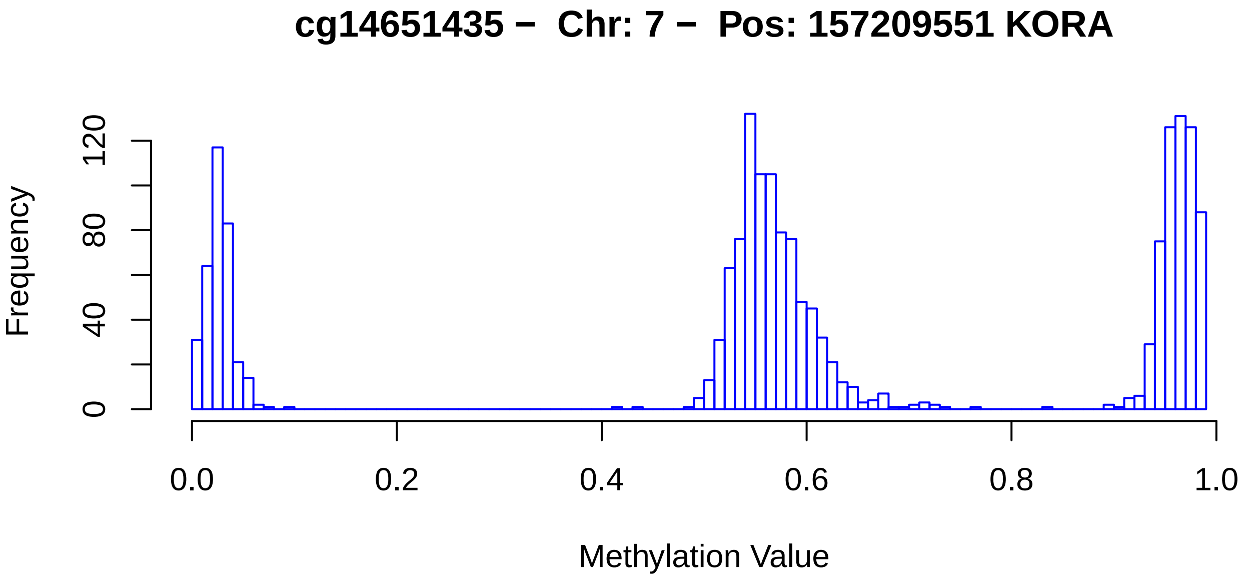
cg14651435 − Chr: 7 − Pos: 157209551 KORA
Frequency
0
40
80
120
0.0
0.2
0.4
0.6
0.8
1.0
Methylation Value

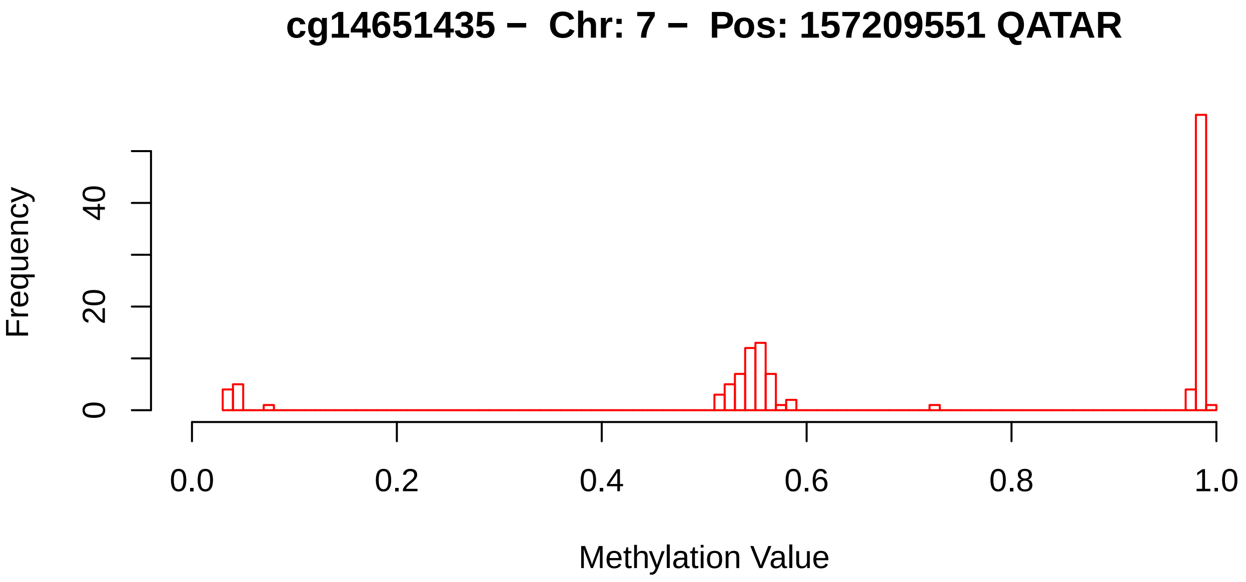
cg14651435 − Chr: 7 − Pos: 157209551 QATAR
Frequency
0
20
40
0.0
0.2
0.4
0.6
0.8
1.0
Methylation Value

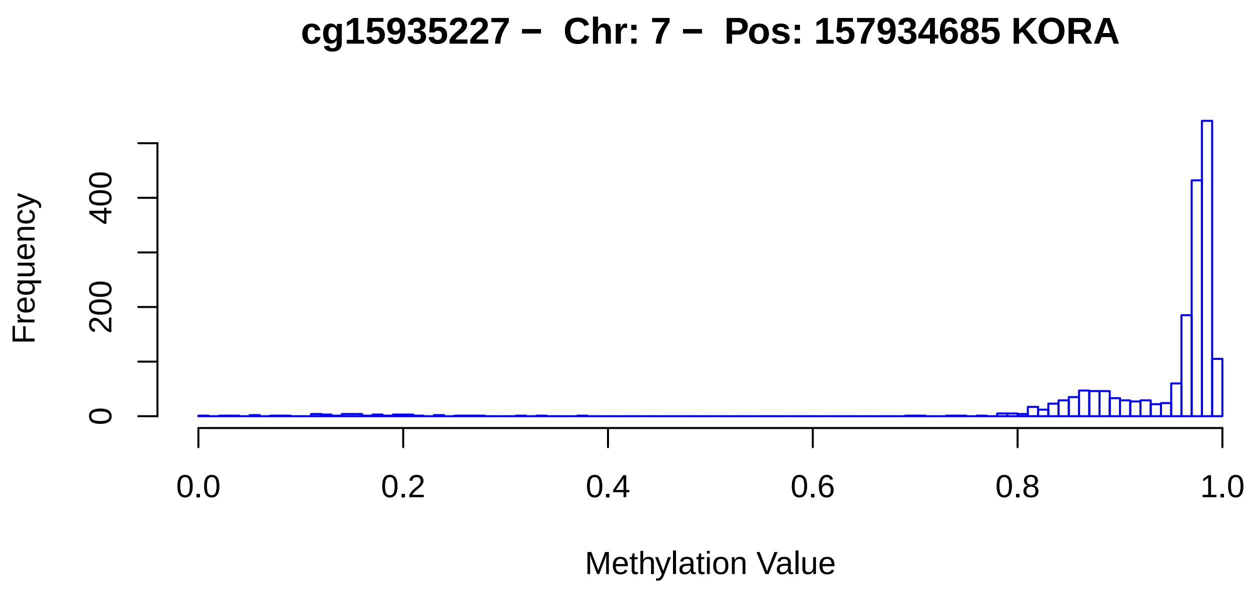
cg15935227 − Chr: 7 − Pos: 157934685 KORA
Frequency
0
200
400
0.0
0.2
0.4
0.6
0.8
1.0
Methylation Value

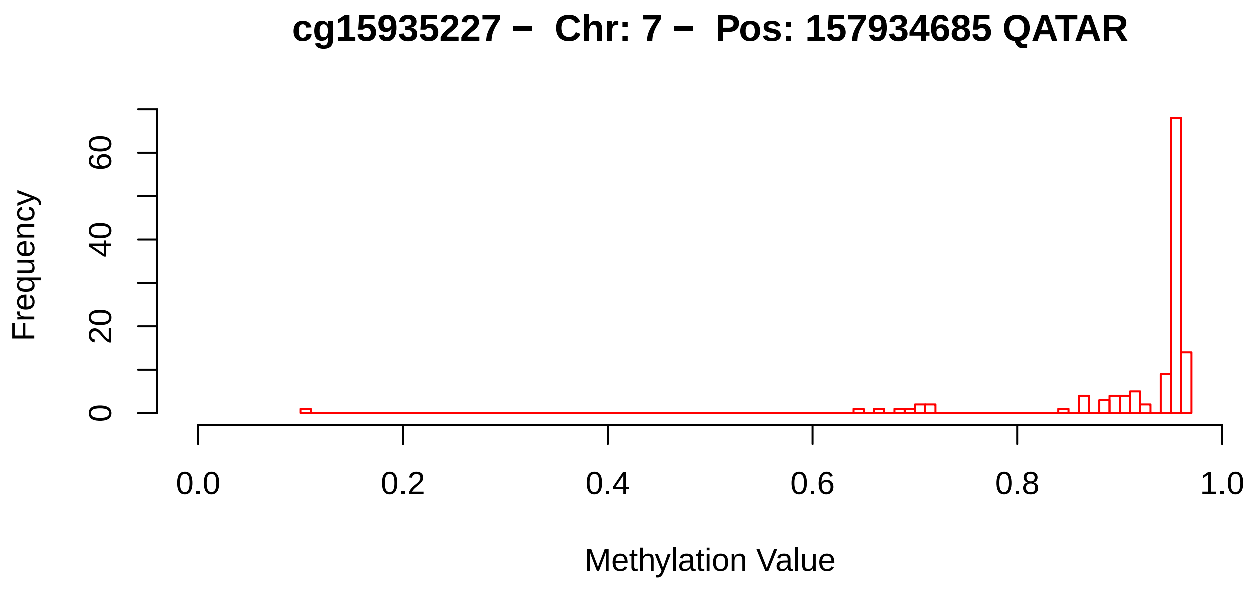
cg15935227 − Chr: 7 − Pos: 157934685 QATAR
Frequency
0
20
40
60
0.0
0.2
0.4
0.6
0.8
1.0
Methylation Value

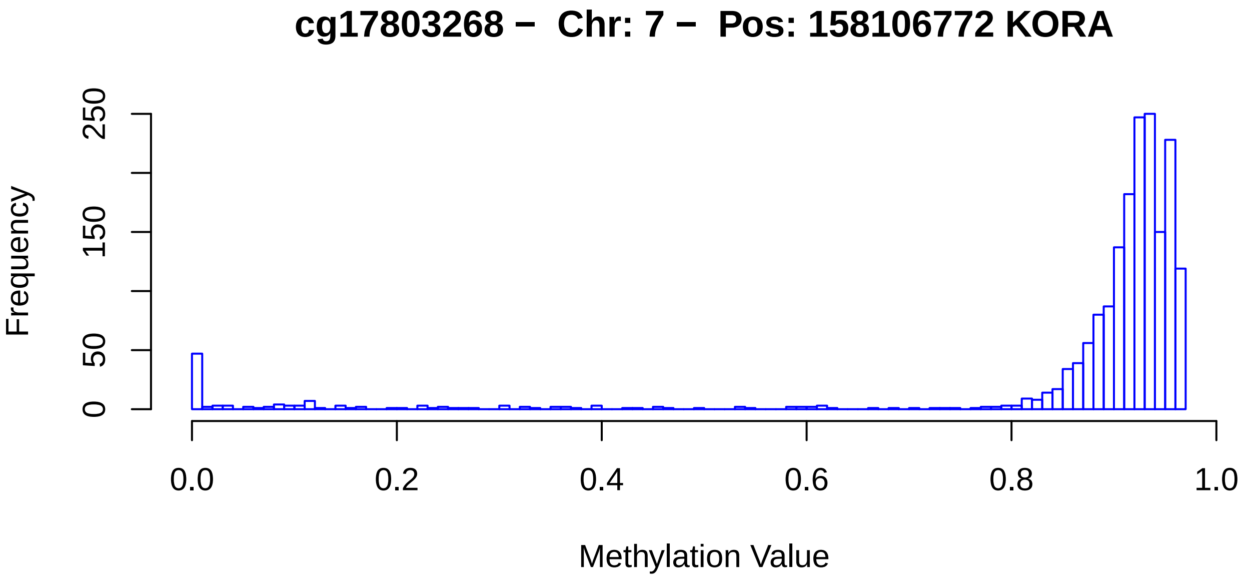
cg17803268 − Chr: 7 − Pos: 158106772 KORA
Frequency
0
50
150
250
0.0
0.2
0.4
0.6
0.8
1.0
Methylation Value

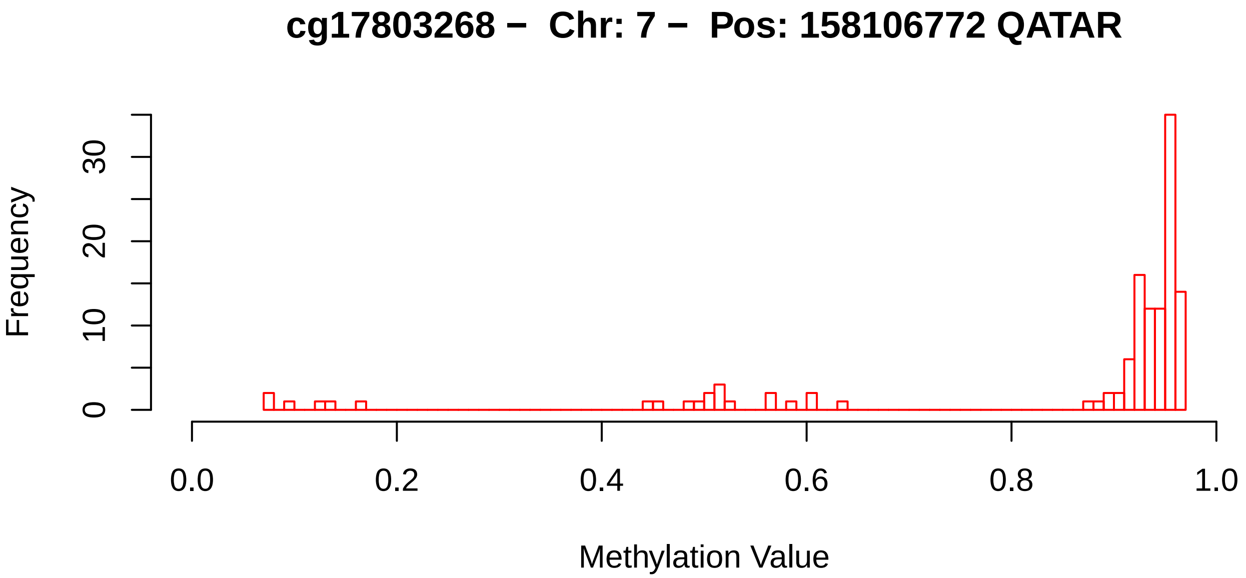
cg17803268 − Chr: 7 − Pos: 158106772 QATAR
Frequency
0
10
20
30
0.0
0.2
0.4
0.6
0.8
1.0
Methylation Value

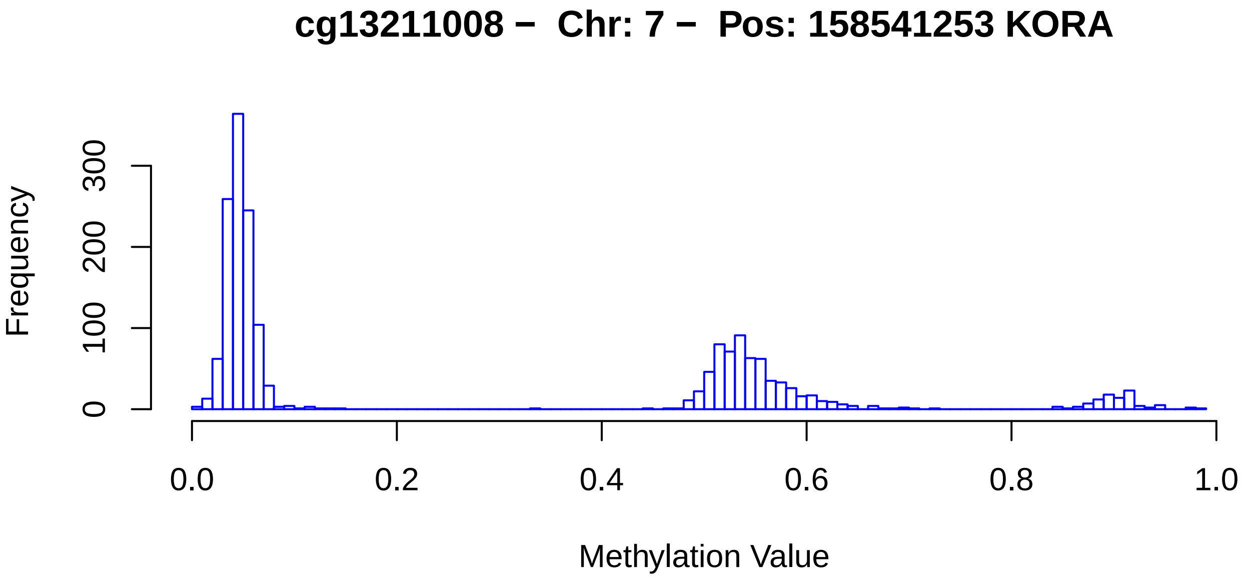
cg13211008 − Chr: 7 − Pos: 158541253 KORA
Frequency
0
100
200
300
0.0
0.2
0.4
0.6
0.8
1.0
Methylation Value

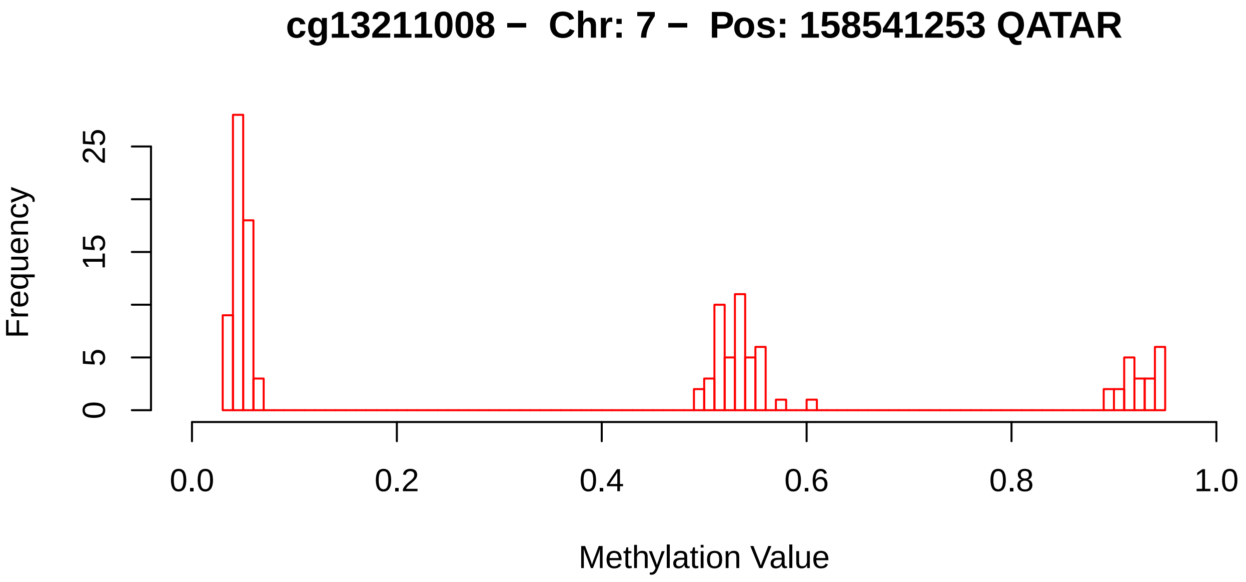
cg13211008 − Chr: 7 − Pos: 158541253 QATAR
Frequency
0
5
15
25
0.0
0.2
0.4
0.6
0.8
1.0
Methylation Value

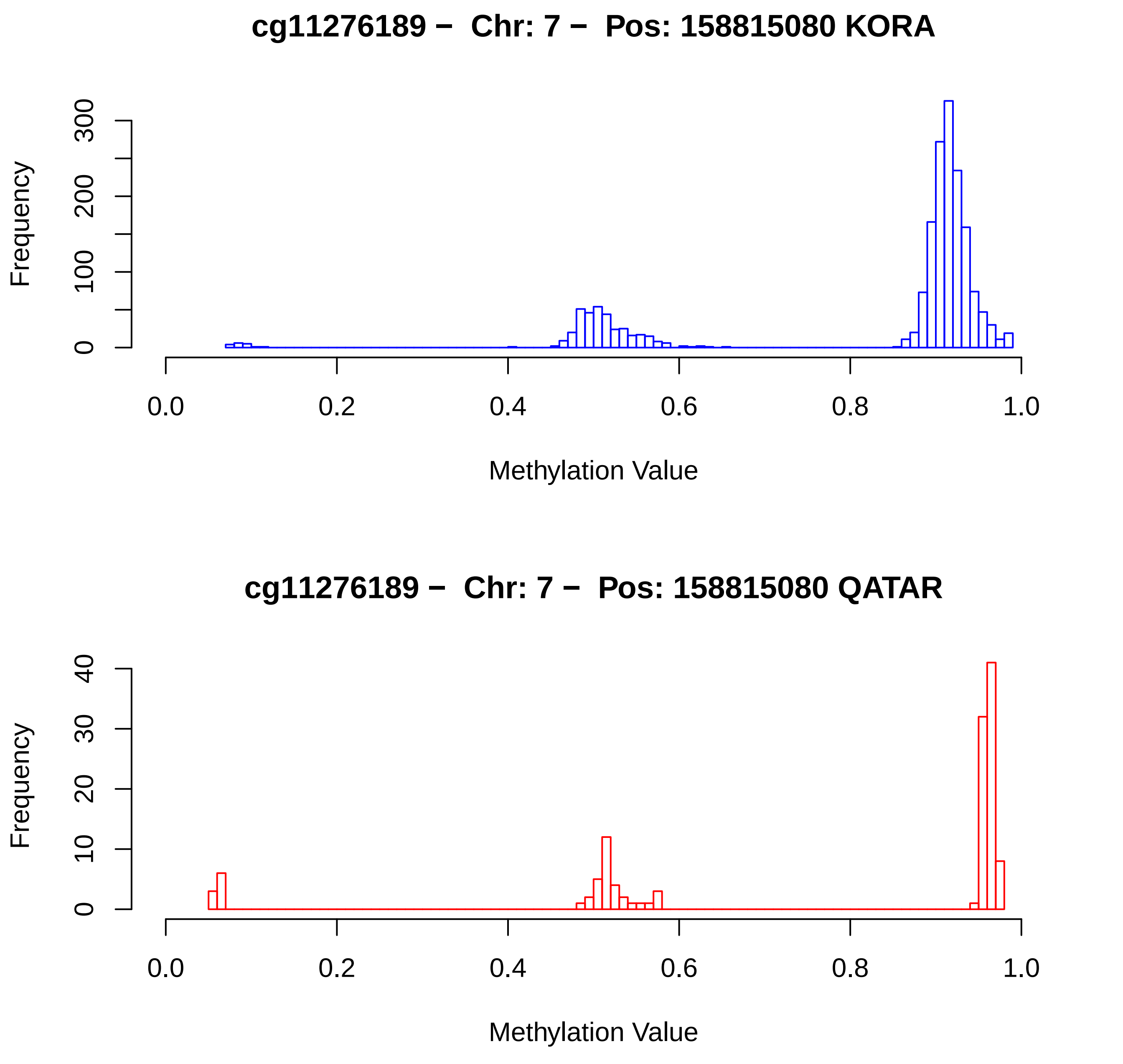
cg11276189 − Chr: 7 − Pos: 158815080 KORA
Frequency
0
100
200
300
0.0
0.2
0.4
0.6
0.8
1.0
Methylation Value
cg11276189 − Chr: 7 − Pos: 158815080 QATAR
Frequency
0
10
20
30
40
0.0
0.2
0.4
0.6
0.8
1.0
Methylation Value

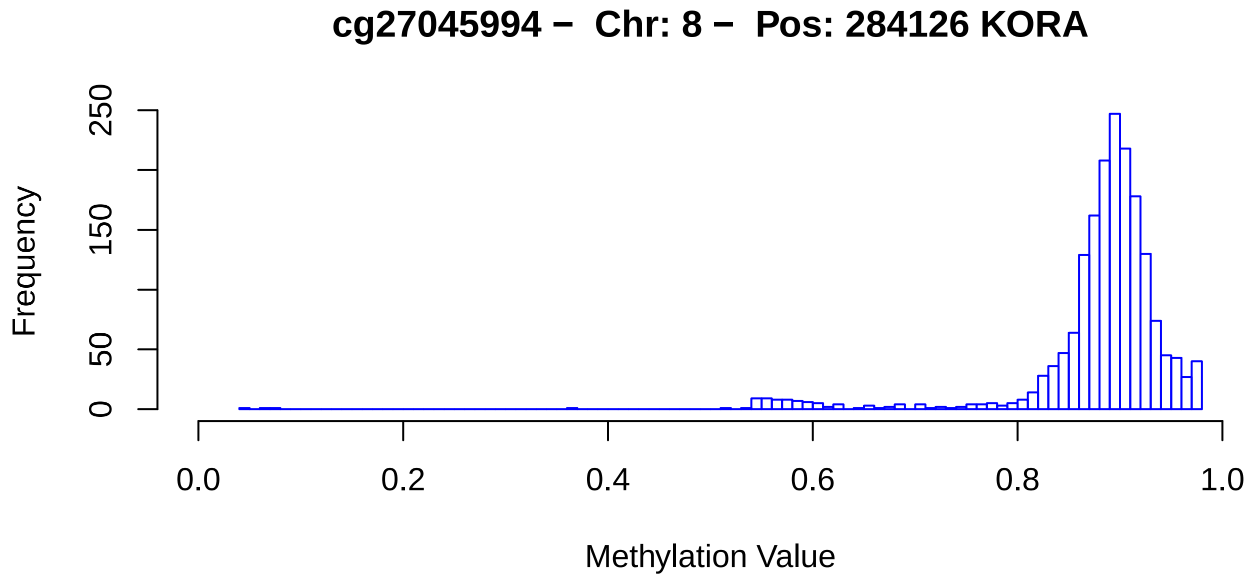
cg27045994 − Chr: 8 − Pos: 284126 KORA
Frequency
0
50
150
250
0.0
0.2
0.4
0.6
0.8
1.0
Methylation Value

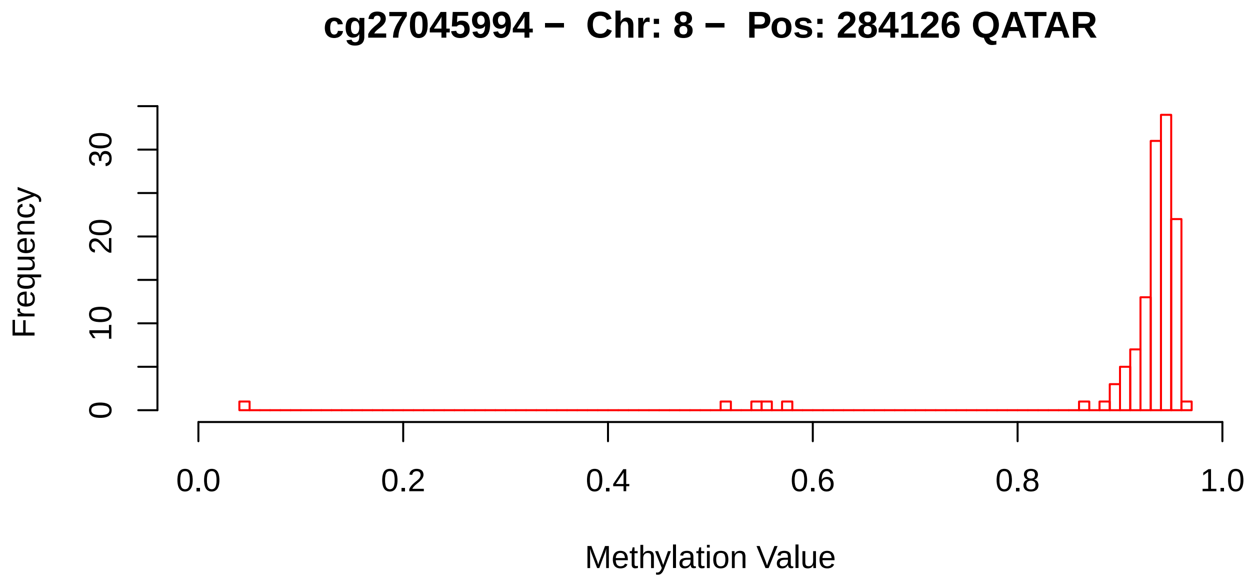
cg27045994 − Chr: 8 − Pos: 284126 QATAR
Frequency
0
10
20
30
0.0
0.2
0.4
0.6
0.8
1.0
Methylation Value

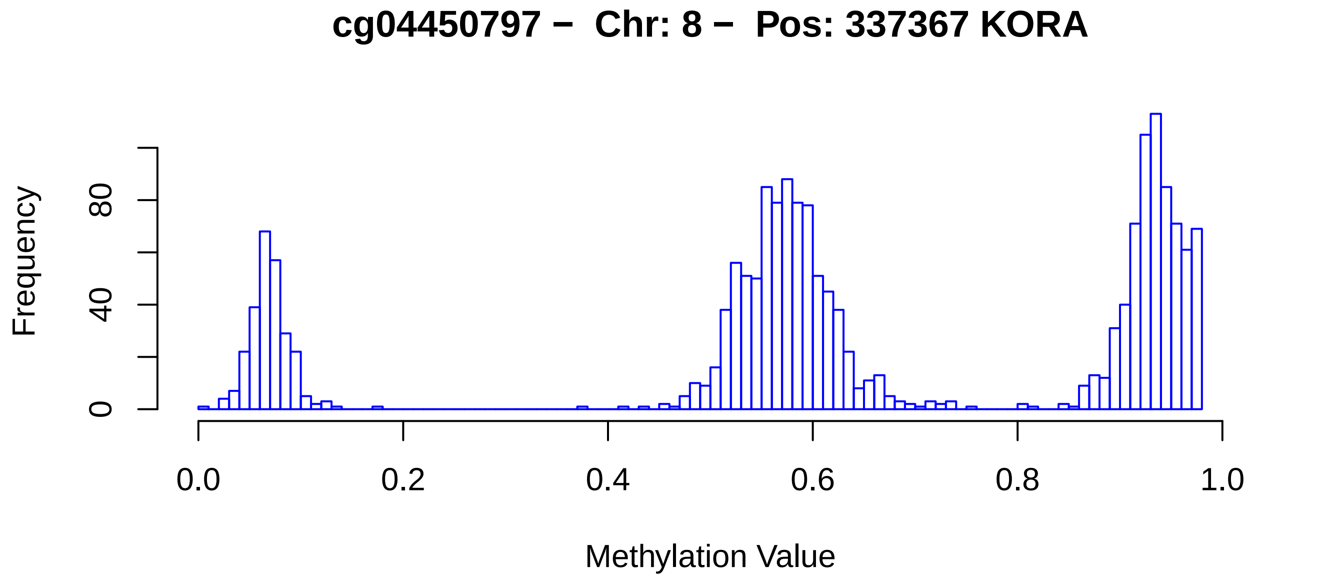

cg04450797 − Chr: 8 − Pos: 337367 KORA
Frequency
0
40
80
0.0
0.2
0.4
0.6
0.8
1.0
Methylation Value

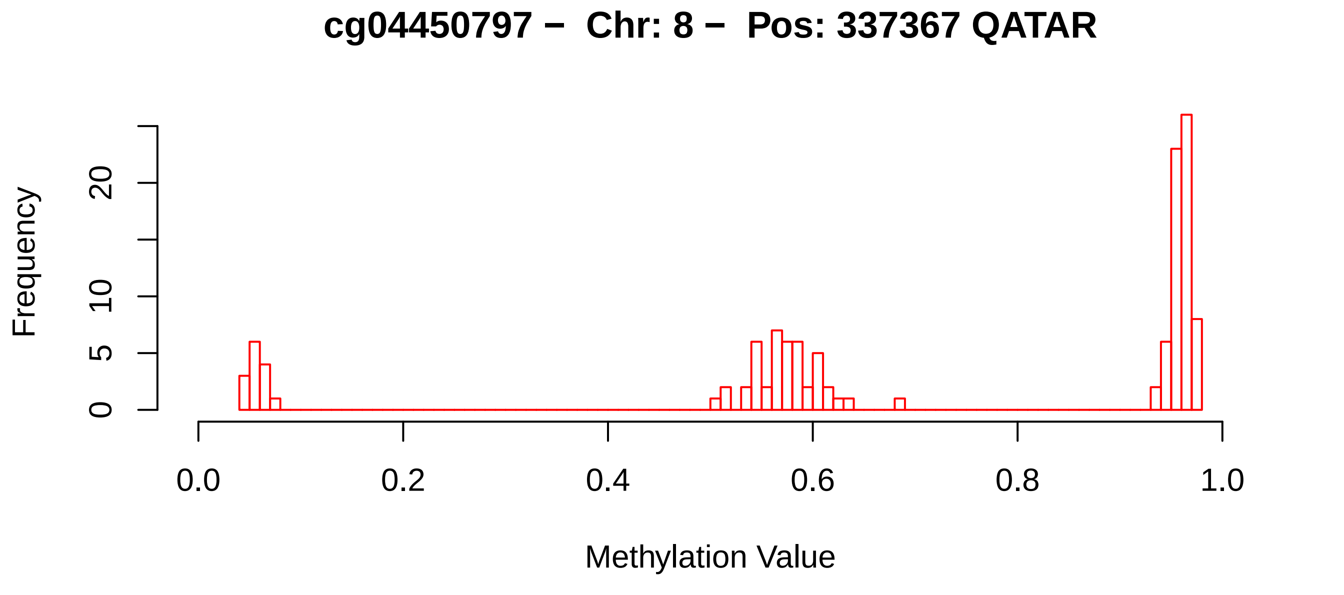

cg04450797 − Chr: 8 − Pos: 337367 QATAR
Frequency
0
5
10
20
0.0
0.2
0.4
0.6
0.8
1.0
Methylation Value

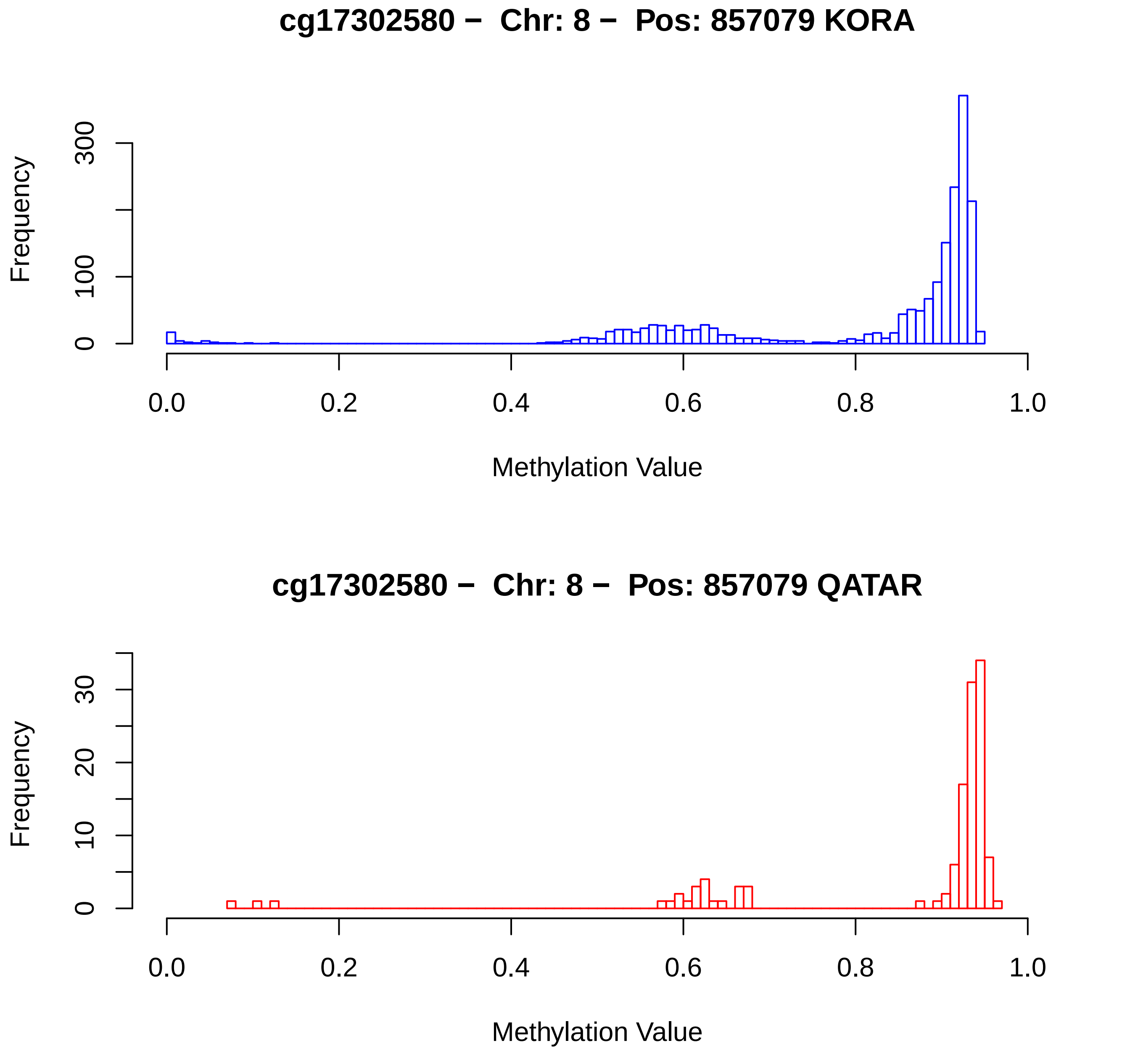
cg17302580 − Chr: 8 − Pos: 857079 KORA
Frequency
0
100
300
0.0
0.2
0.4
0.6
0.8
1.0
Methylation Value
cg17302580 − Chr: 8 − Pos: 857079 QATAR
Frequency
0
10
20
30
0.0
0.2
0.4
0.6
0.8
1.0
Methylation Value

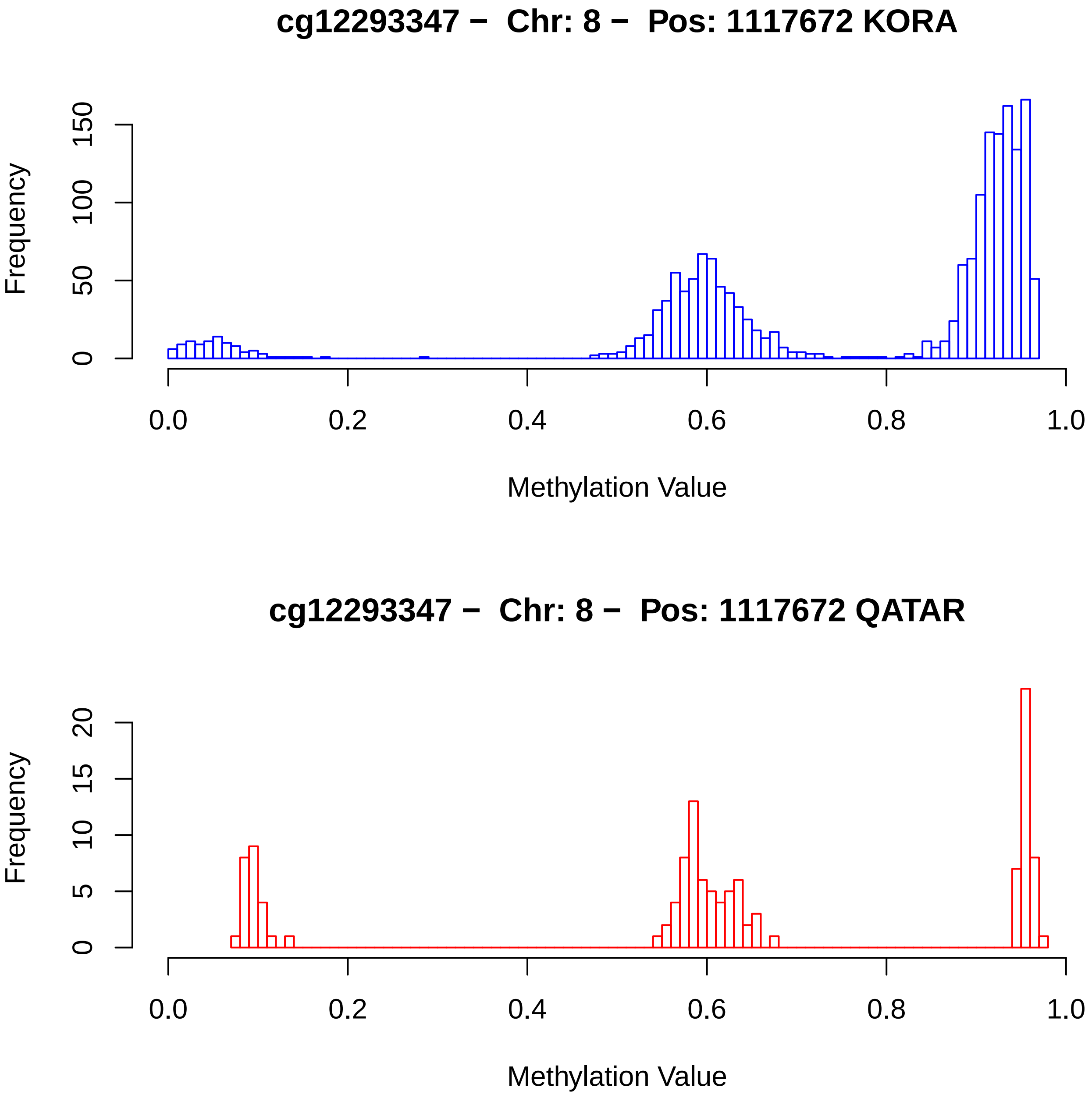
cg12293347 − Chr: 8 − Pos: 1117672 KORA
Frequency
0
50
100
150
0.0
0.2
0.4
0.6
0.8
1.0
Methylation Value
cg12293347 − Chr: 8 − Pos: 1117672 QATAR
Frequency
0
5
10
15
20
0.0
0.2
0.4
0.6
0.8
1.0
Methylation Value

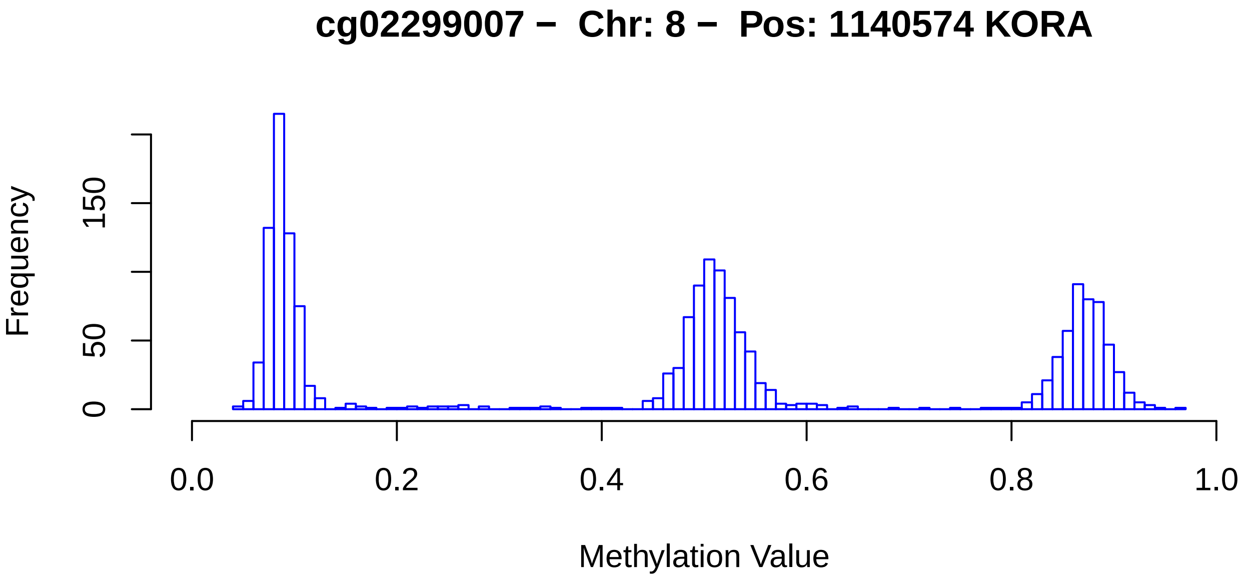
cg02299007 − Chr: 8 − Pos: 1140574 KORA
Frequency
0
50
150
0.0
0.2
0.4
0.6
0.8
1.0
Methylation Value

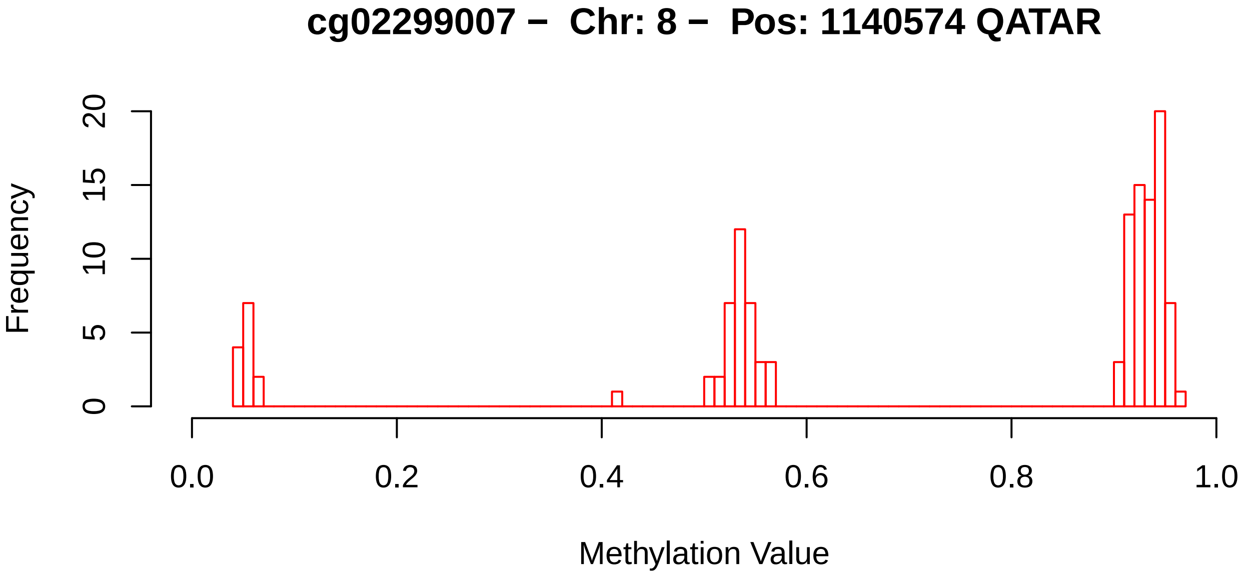
cg02299007 − Chr: 8 − Pos: 1140574 QATAR
Frequency
0
5
10
15
20
0.0
0.2
0.4
0.6
0.8
1.0
Methylation Value

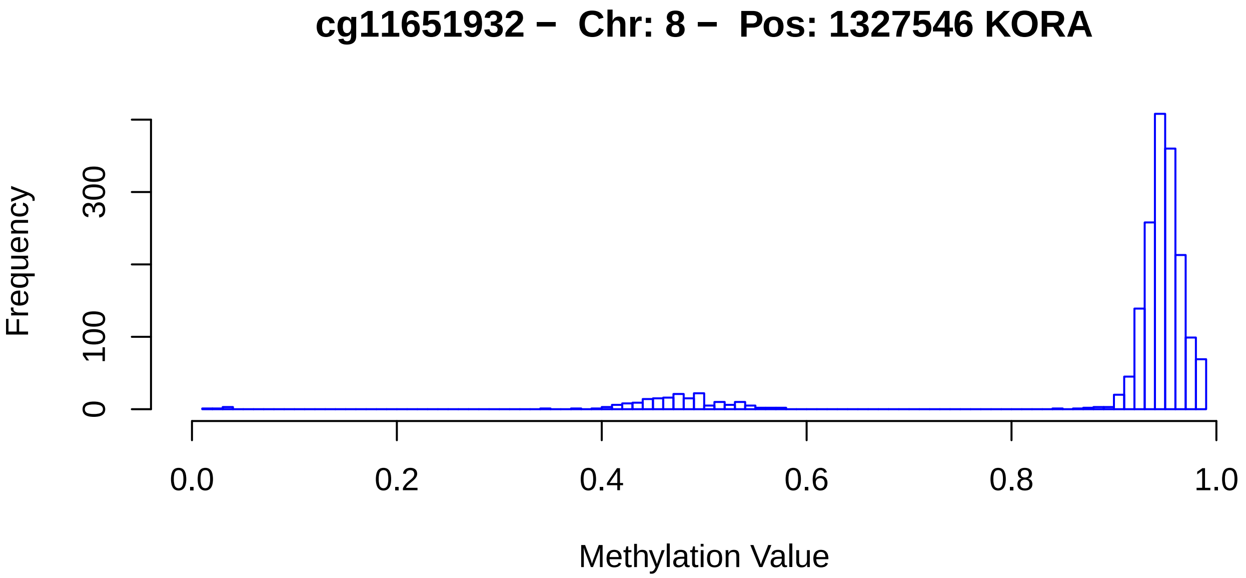
cg11651932 − Chr: 8 − Pos: 1327546 KORA
Frequency
0
100
300
0.0
0.2
0.4
0.6
0.8
1.0
Methylation Value

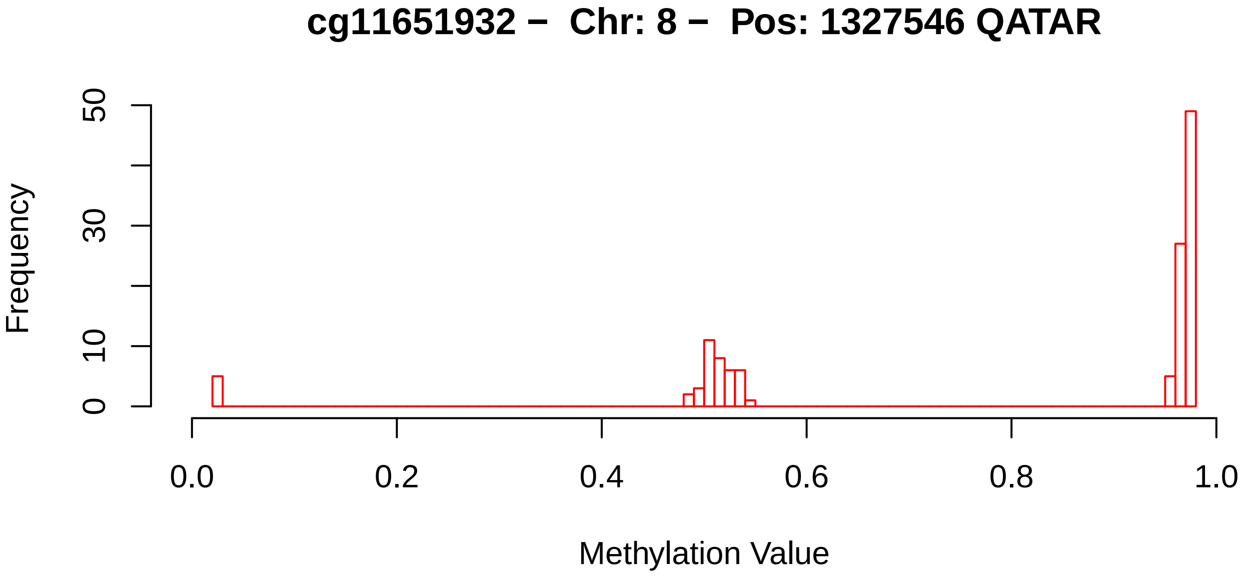
cg11651932 − Chr: 8 − Pos: 1327546 QATAR
Frequency
0
10
30
50
0.0
0.2
0.4
0.6
0.8
1.0
Methylation Value

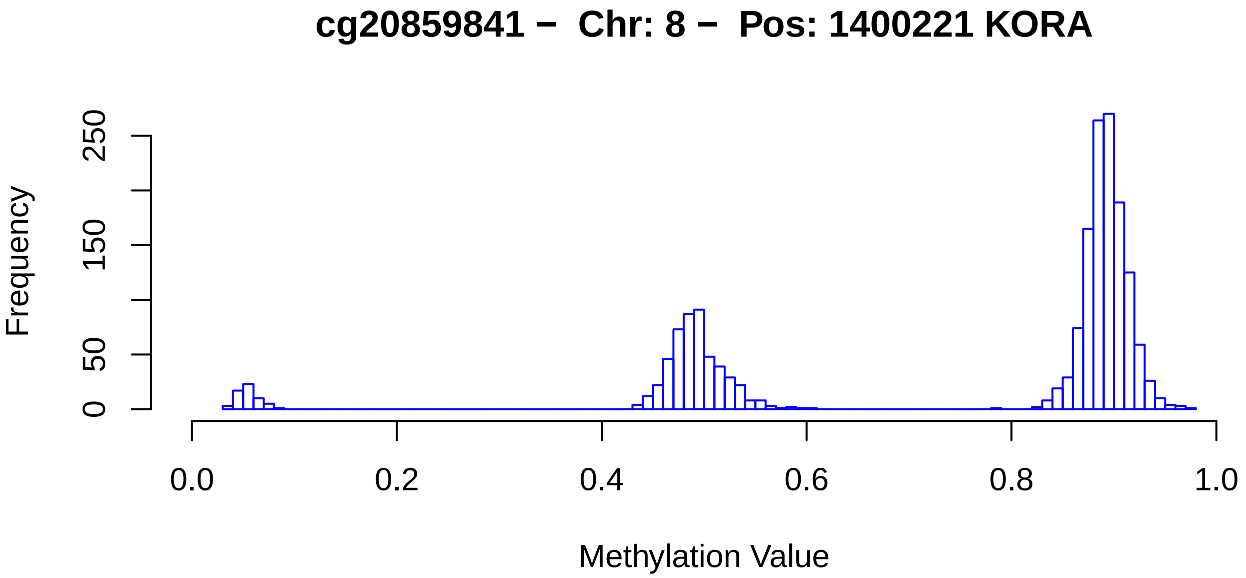
cg20859841 − Chr: 8 − Pos: 1400221 KORA
Frequency
0
50
150
250
0.0
0.2
0.4
0.6
0.8
1.0
Methylation Value

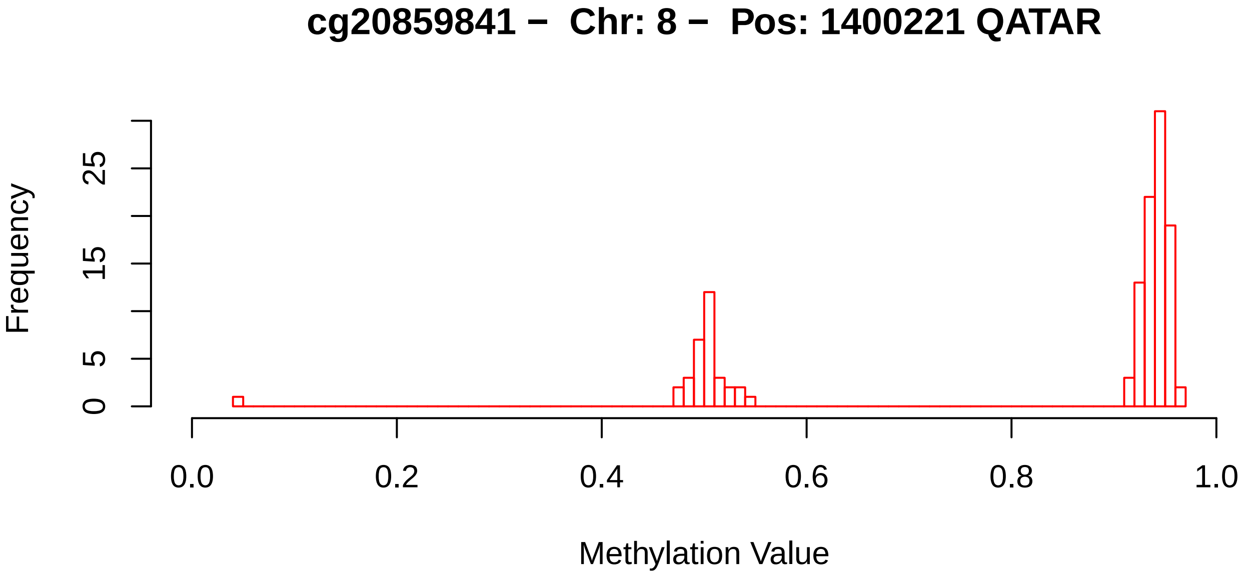
cg20859841 − Chr: 8 − Pos: 1400221 QATAR
Frequency
0
5
15
25
0.0
0.2
0.4
0.6
0.8
1.0
Methylation Value

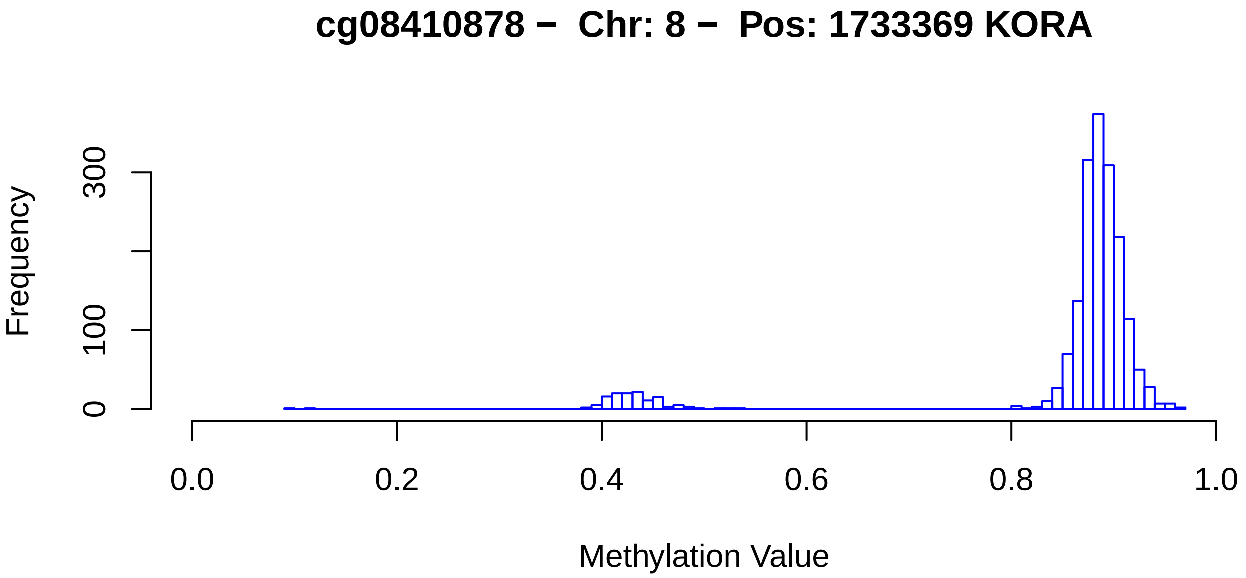
cg08410878 − Chr: 8 − Pos: 1733369 KORA
Frequency
0
100
300
0.0
0.2
0.4
0.6
0.8
1.0
Methylation Value

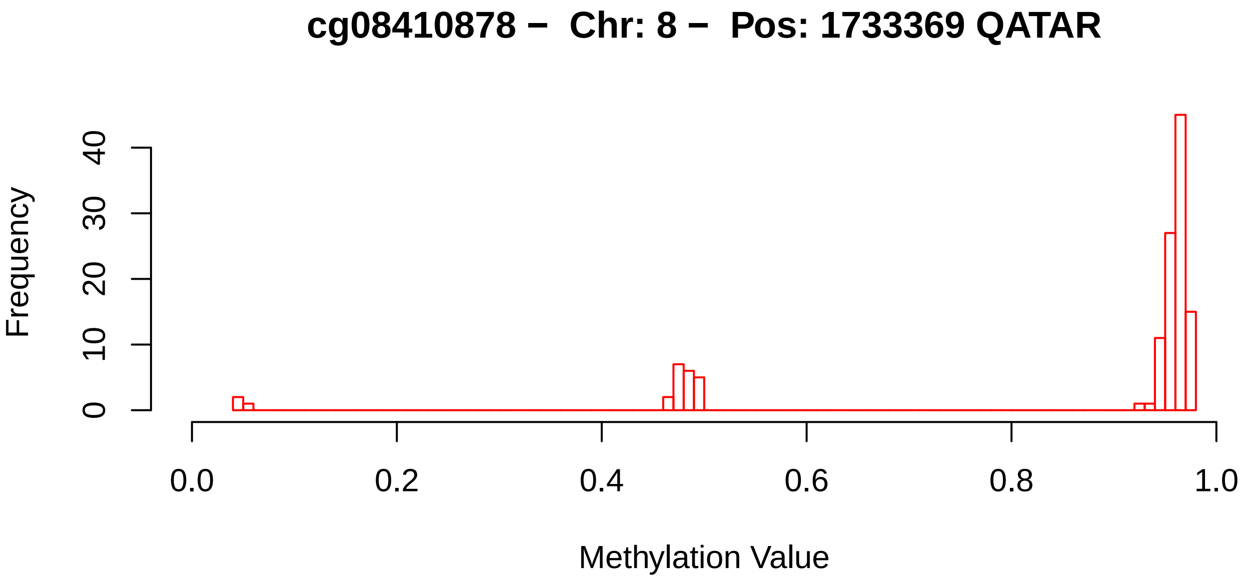
cg08410878 − Chr: 8 − Pos: 1733369 QATAR
Frequency
0
10
20
30
40
0.0
0.2
0.4
0.6
0.8
1.0
Methylation Value

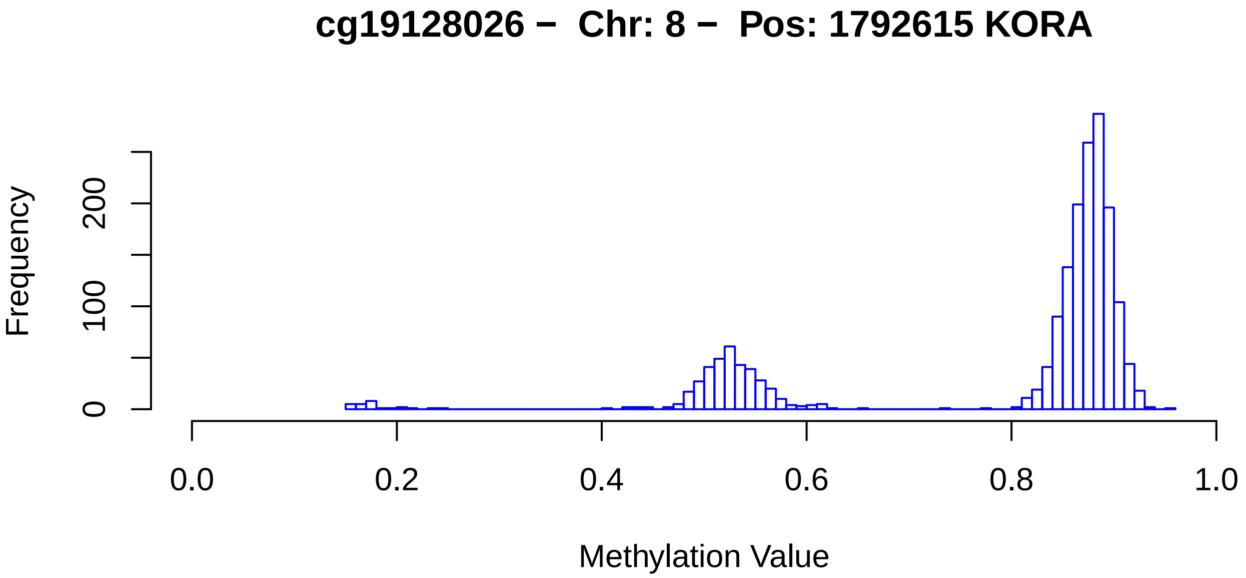
cg19128026 − Chr: 8 − Pos: 1792615 KORA
Frequency
0
100
200
0.0
0.2
0.4
0.6
0.8
1.0
Methylation Value

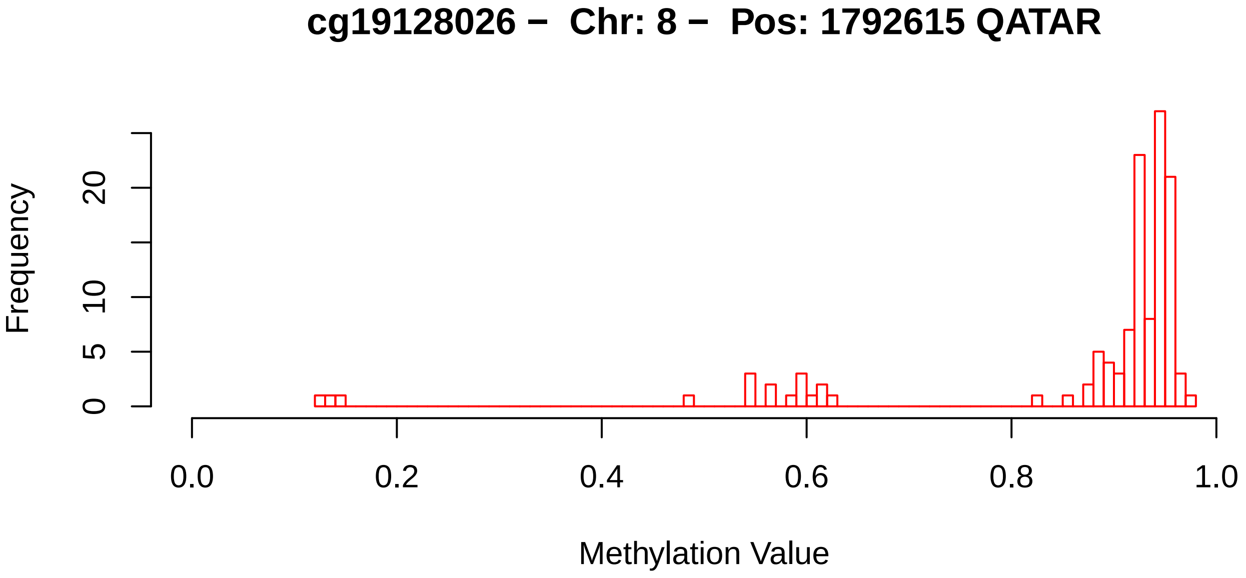
cg19128026 − Chr: 8 − Pos: 1792615 QATAR
Frequency
0
5
10
20
0.0
0.2
0.4
0.6
0.8
1.0
Methylation Value

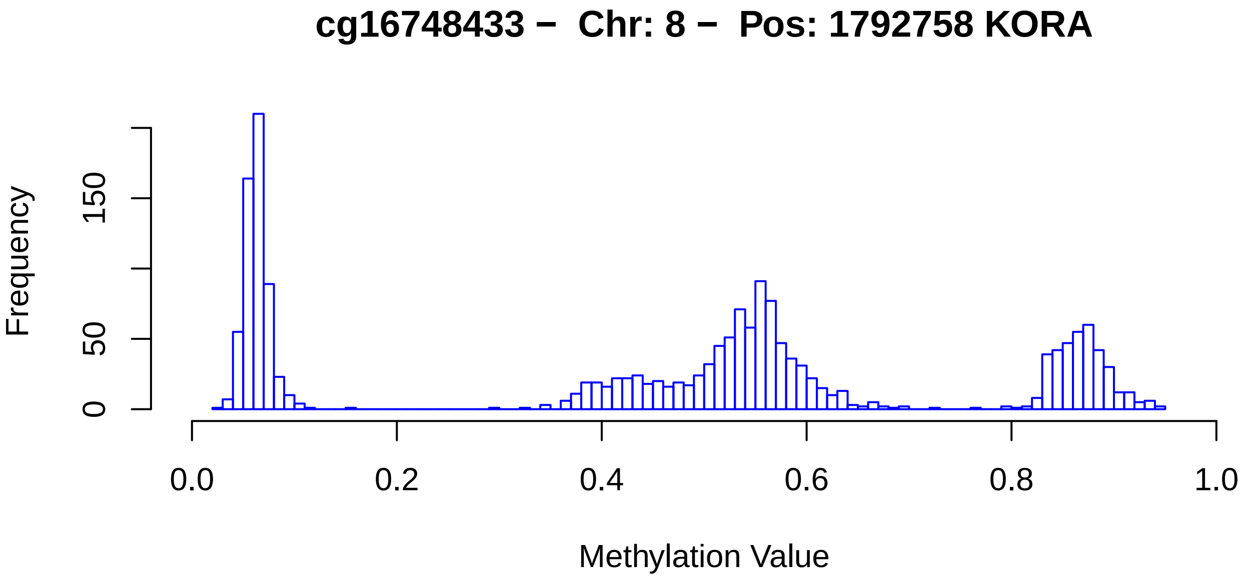
cg16748433 − Chr: 8 − Pos: 1792758 KORA
Frequency
0
50
150
0.0
0.2
0.4
0.6
0.8
1.0
Methylation Value

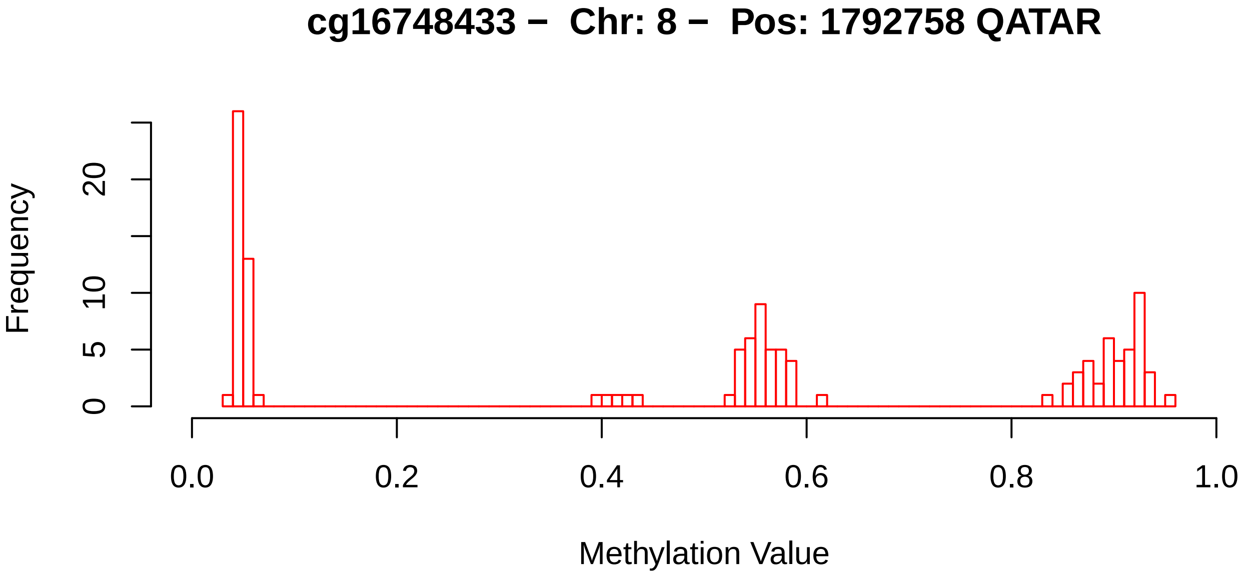
cg16748433 − Chr: 8 − Pos: 1792758 QATAR
Frequency
0
5
10
20
0.0
0.2
0.4
0.6
0.8
1.0
Methylation Value

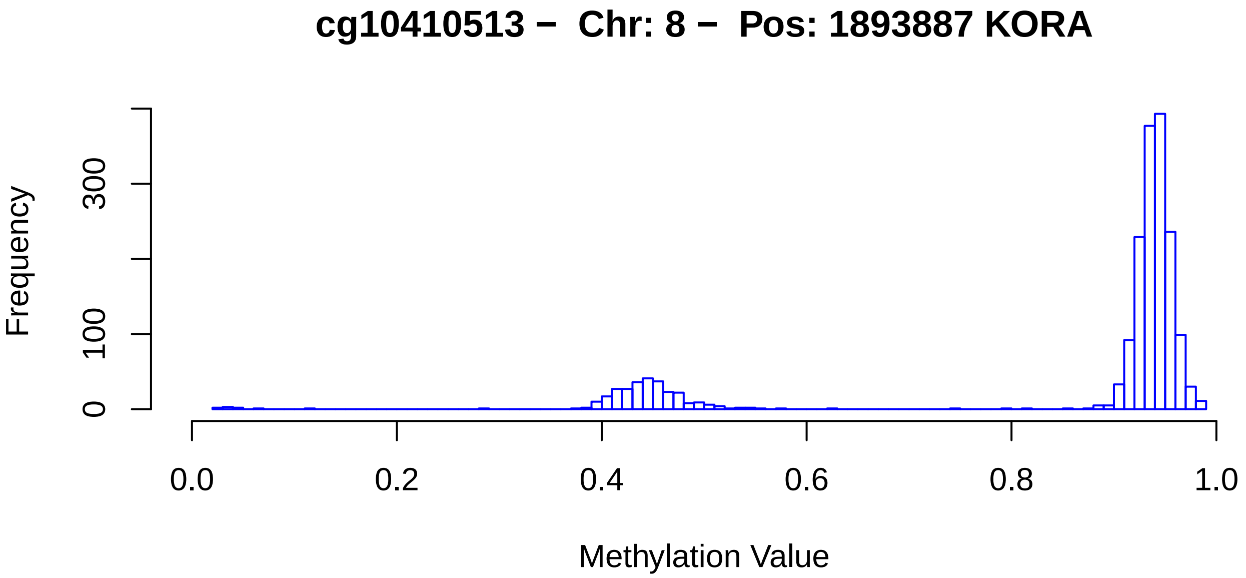

cg10410513 − Chr: 8 − Pos: 1893887 KORA
Frequency
0
100
300
0.0
0.2
0.4
0.6
0.8
1.0
Methylation Value

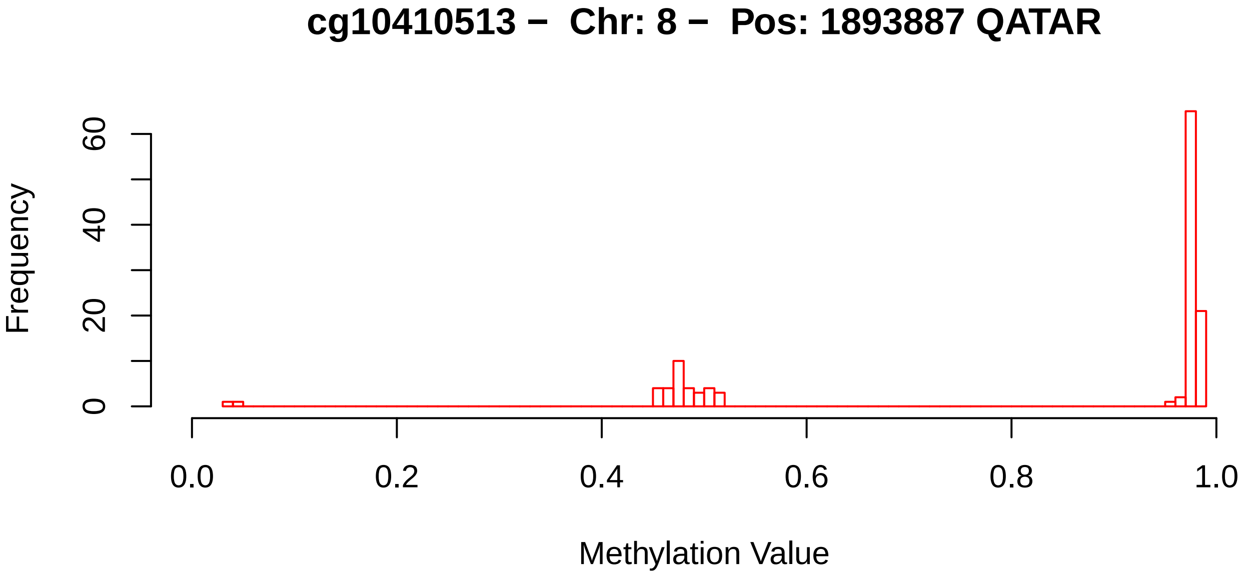

cg10410513 − Chr: 8 − Pos: 1893887 QATAR
Frequency
0
20
40
60
0.0
0.2
0.4
0.6
0.8
1.0
Methylation Value

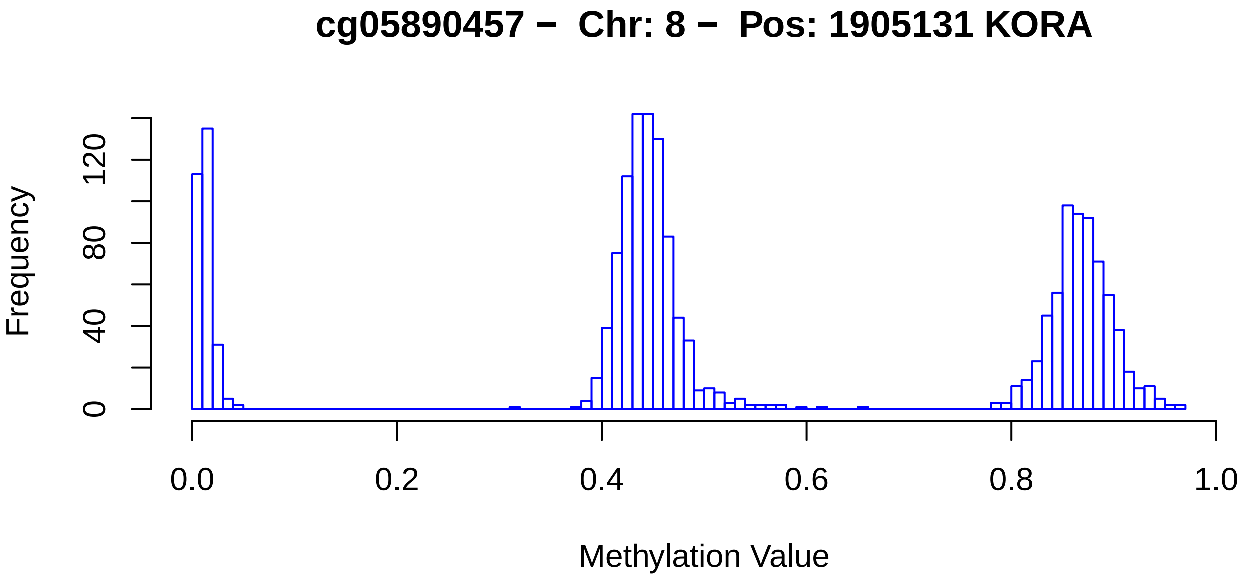
cg05890457 − Chr: 8 − Pos: 1905131 KORA
Frequency
0
40
80
120
0.0
0.2
0.4
0.6
0.8
1.0
Methylation Value

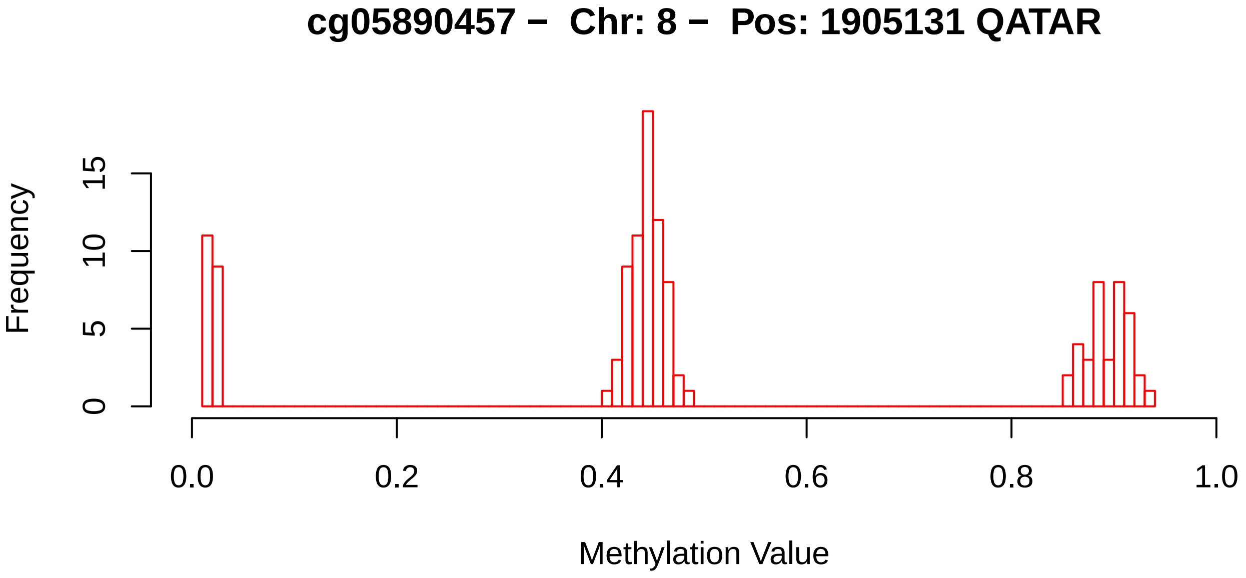
cg05890457 − Chr: 8 − Pos: 1905131 QATAR
Frequency
0
5
10
15
0.0
0.2
0.4
0.6
0.8
1.0
Methylation Value

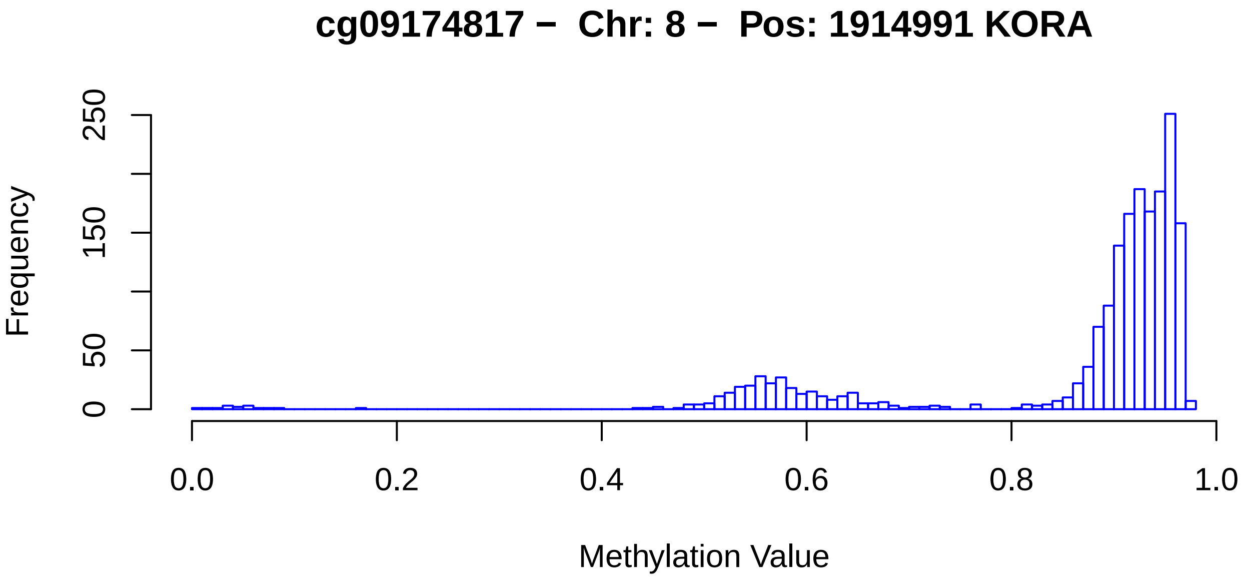
cg09174817 − Chr: 8 − Pos: 1914991 KORA
Frequency
0
50
150
250
0.0
0.2
0.4
0.6
0.8
1.0
Methylation Value

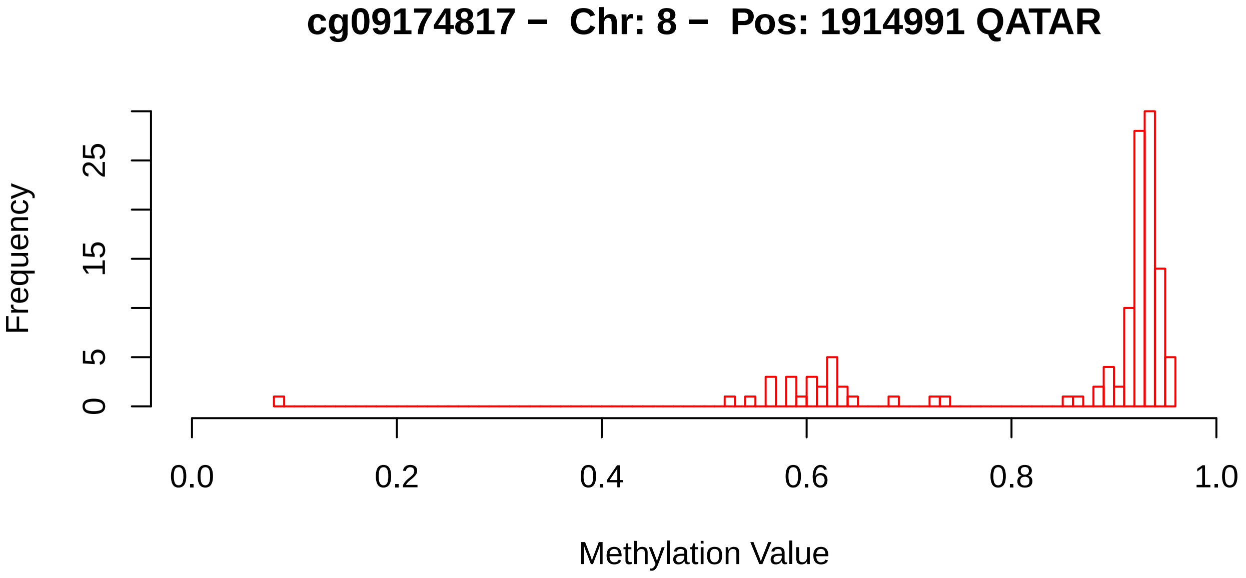
cg09174817 − Chr: 8 − Pos: 1914991 QATAR
Frequency
0
5
15
25
0.0
0.2
0.4
0.6
0.8
1.0
Methylation Value

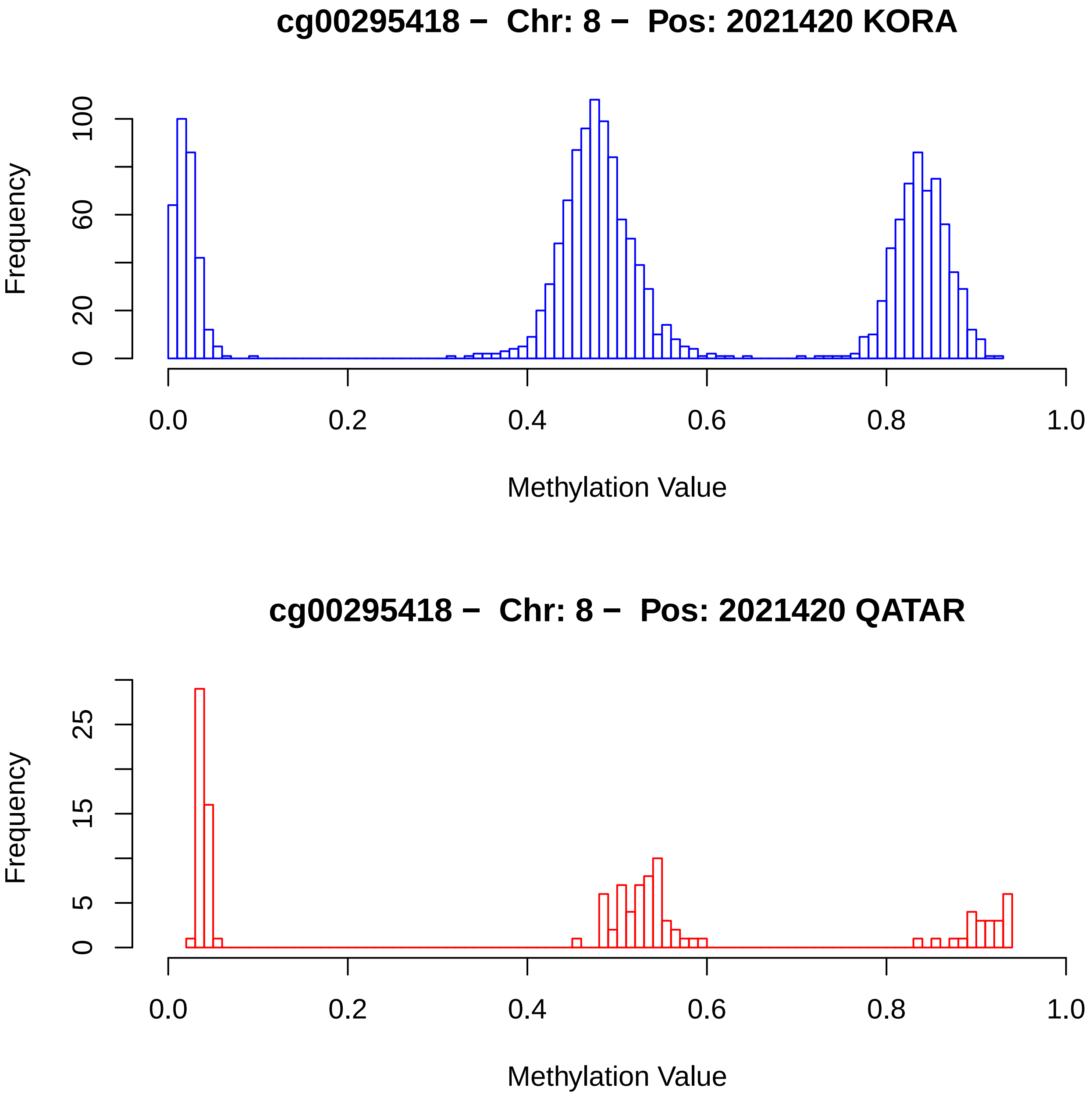
cg00295418 − Chr: 8 − Pos: 2021420 KORA
Frequency
0
20
60
100
0.0
0.2
0.4
0.6
0.8
1.0
Methylation Value
cg00295418 − Chr: 8 − Pos: 2021420 QATAR
Frequency
0
5
15
25
0.0
0.2
0.4
0.6
0.8
1.0
Methylation Value

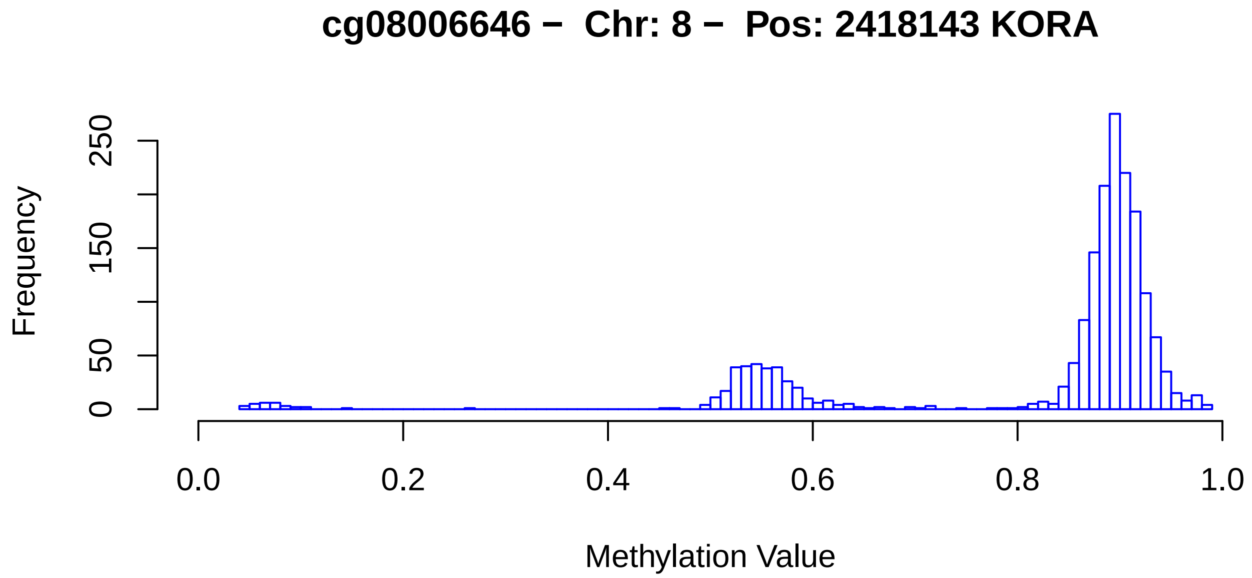

cg08006646 − Chr: 8 − Pos: 2418143 KORA
Frequency
0
50
150
250
0.0
0.2
0.4
0.6
0.8
1.0
Methylation Value

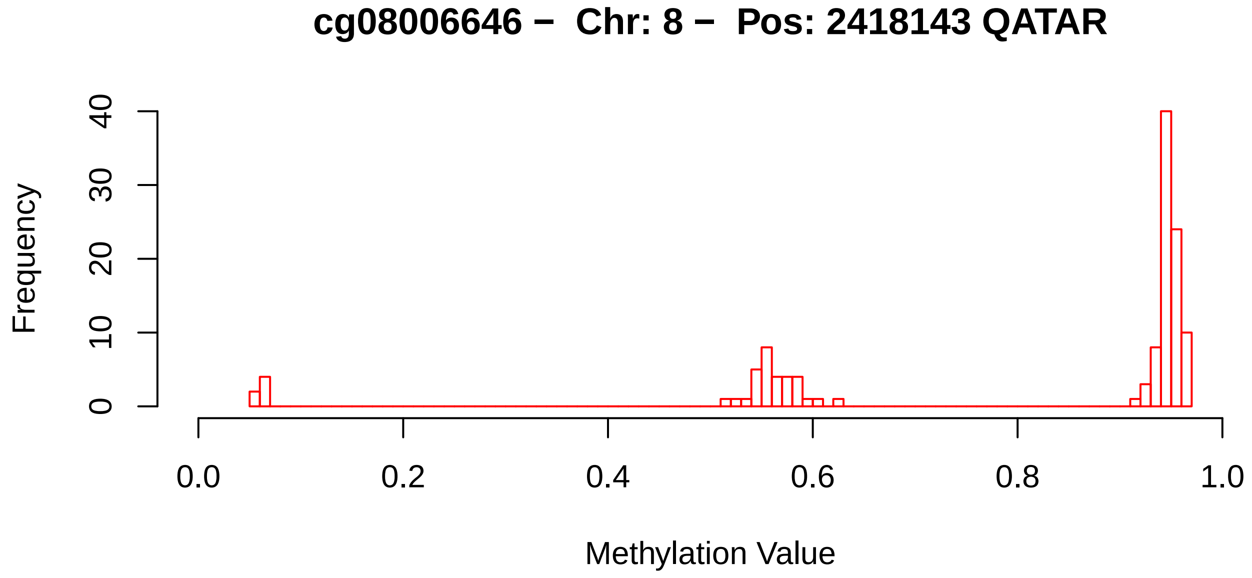

cg08006646 − Chr: 8 − Pos: 2418143 QATAR
Frequency
0
10
20
30
40
0.0
0.2
0.4
0.6
0.8
1.0
Methylation Value

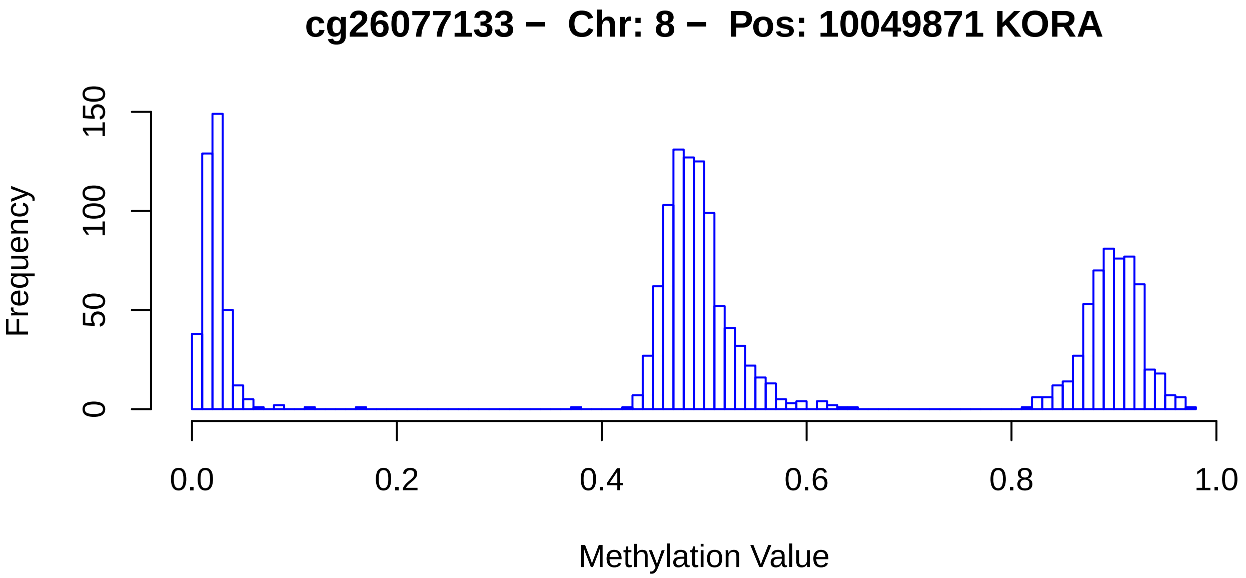
cg26077133 − Chr: 8 − Pos: 10049871 KORA
Frequency
0
50
100
150
0.0
0.2
0.4
0.6
0.8
1.0
Methylation Value

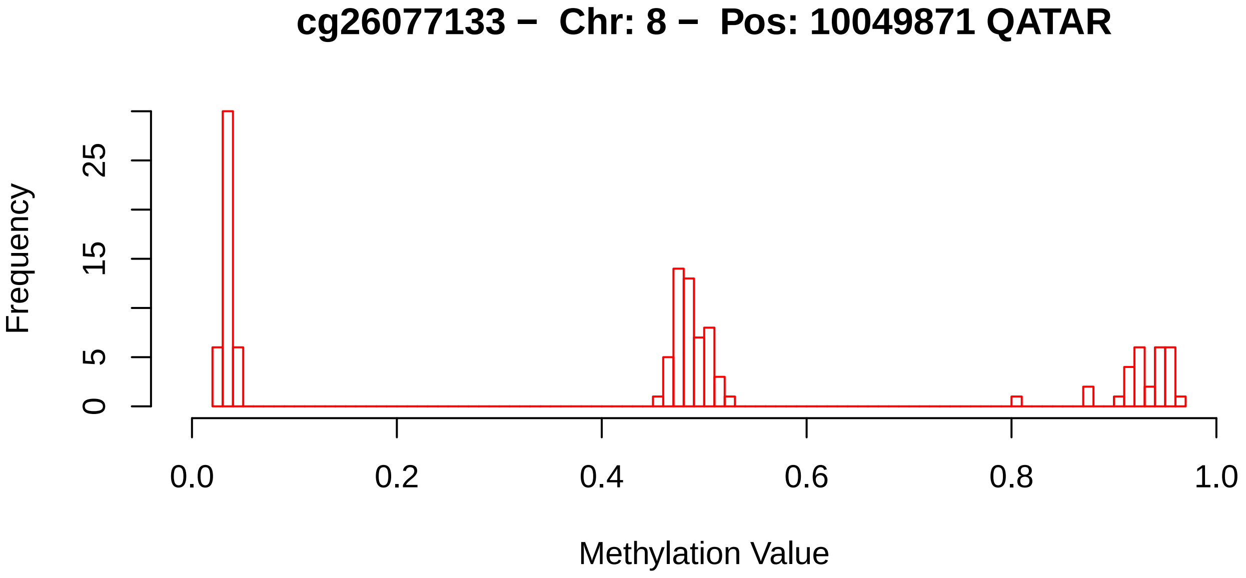
cg26077133 − Chr: 8 − Pos: 10049871 QATAR
Frequency
0
5
15
25
0.0
0.2
0.4
0.6
0.8
1.0
Methylation Value

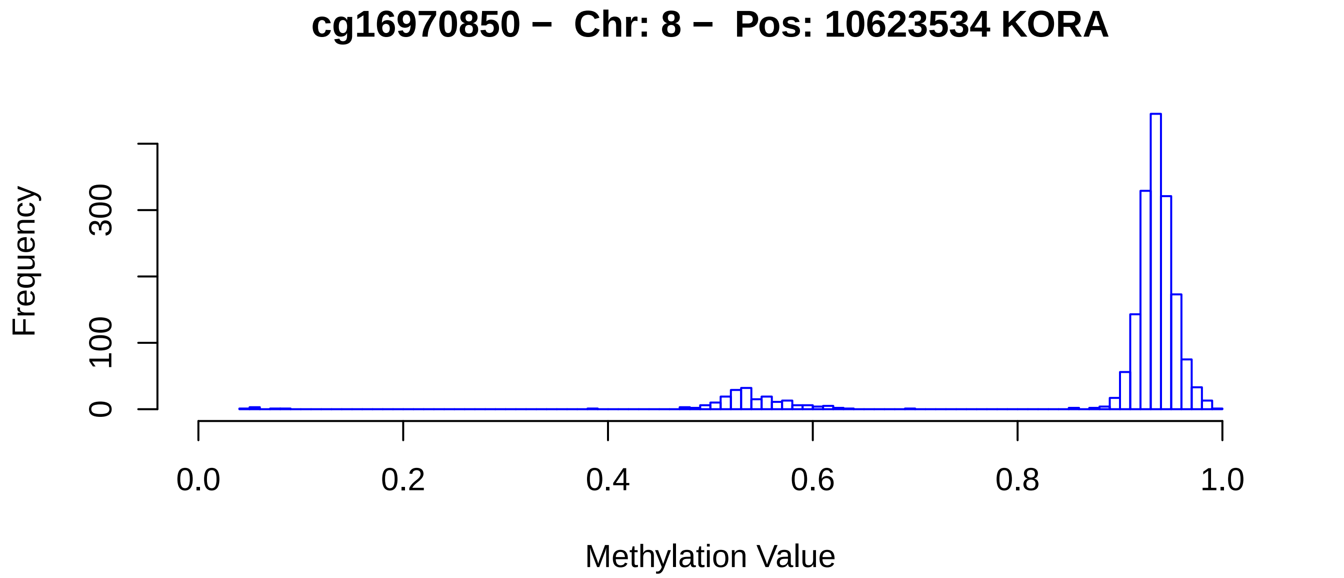
cg16970850 − Chr: 8 − Pos: 10623534 KORA
Frequency
0
100
300
0.0
0.2
0.4
0.6
0.8
1.0
Methylation Value

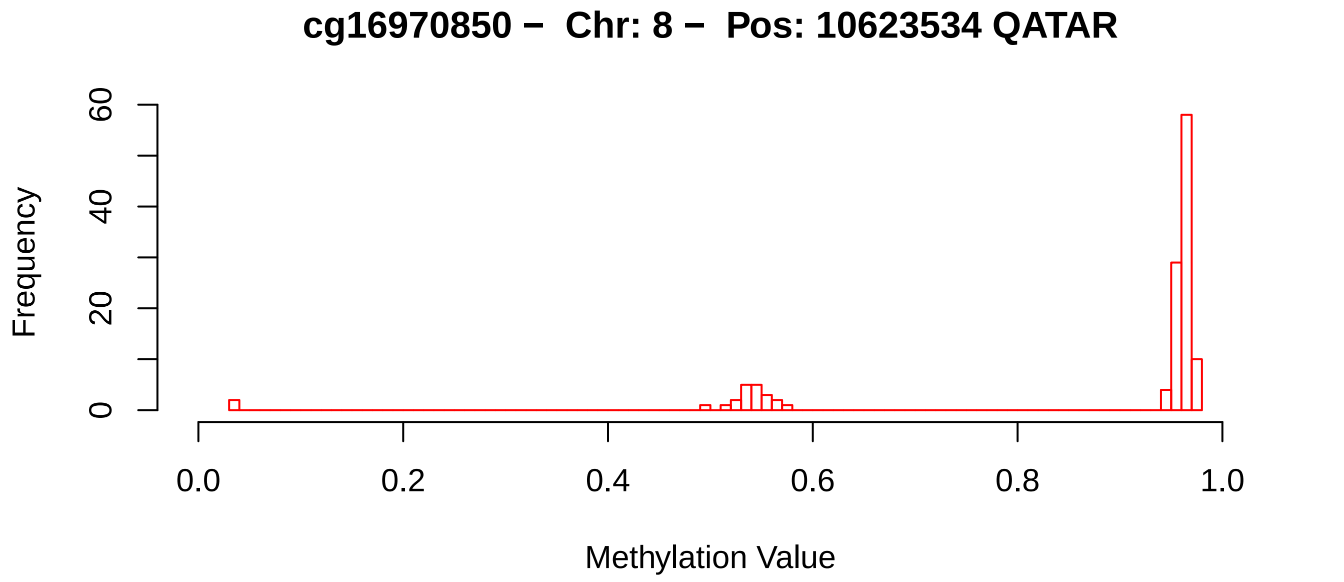
cg16970850 − Chr: 8 − Pos: 10623534 QATAR
Frequency
0
20
40
60
0.0
0.2
0.4
0.6
0.8
1.0
Methylation Value

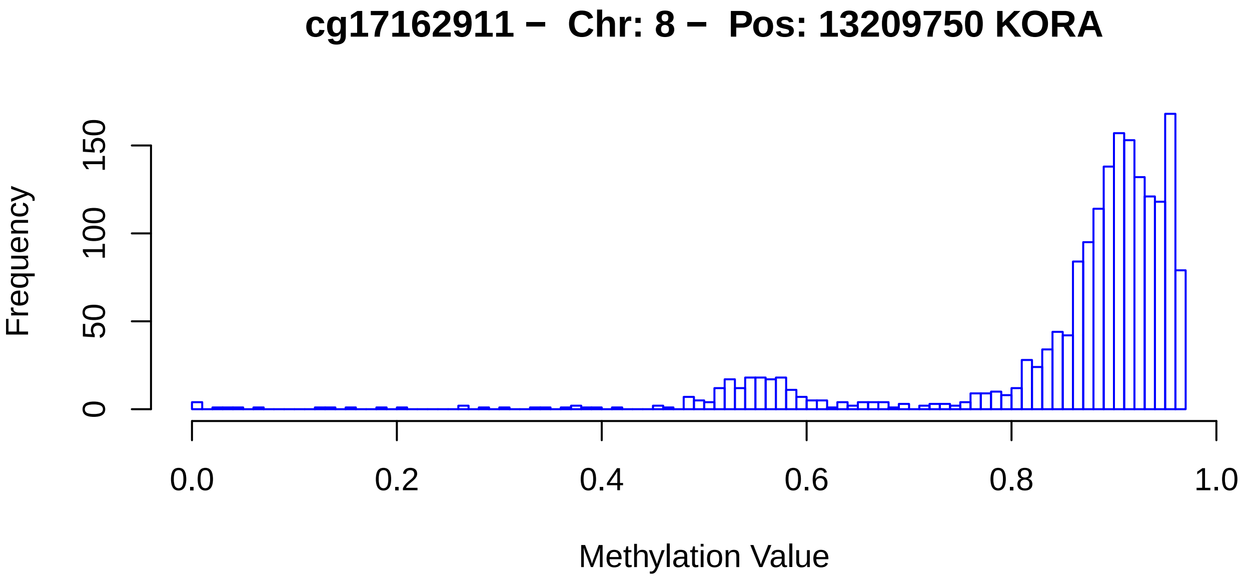
cg17162911 − Chr: 8 − Pos: 13209750 KORA
Frequency
0
50
100
150
0.0
0.2
0.4
0.6
0.8
1.0
Methylation Value

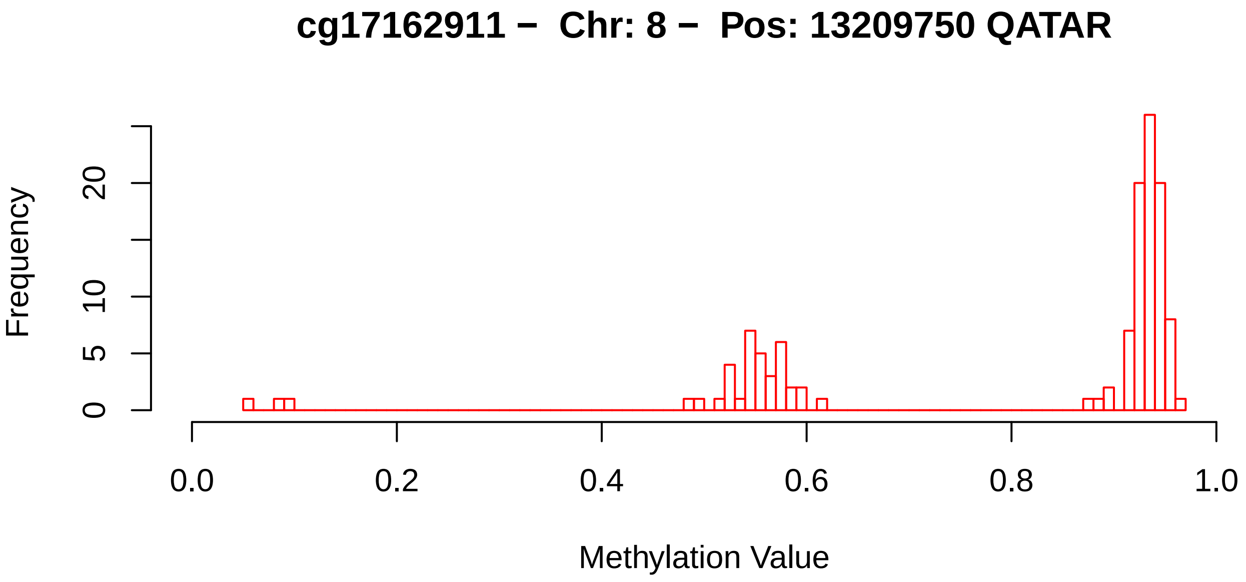
cg17162911 − Chr: 8 − Pos: 13209750 QATAR
Frequency
0
5
10
20
0.0
0.2
0.4
0.6
0.8
1.0
Methylation Value

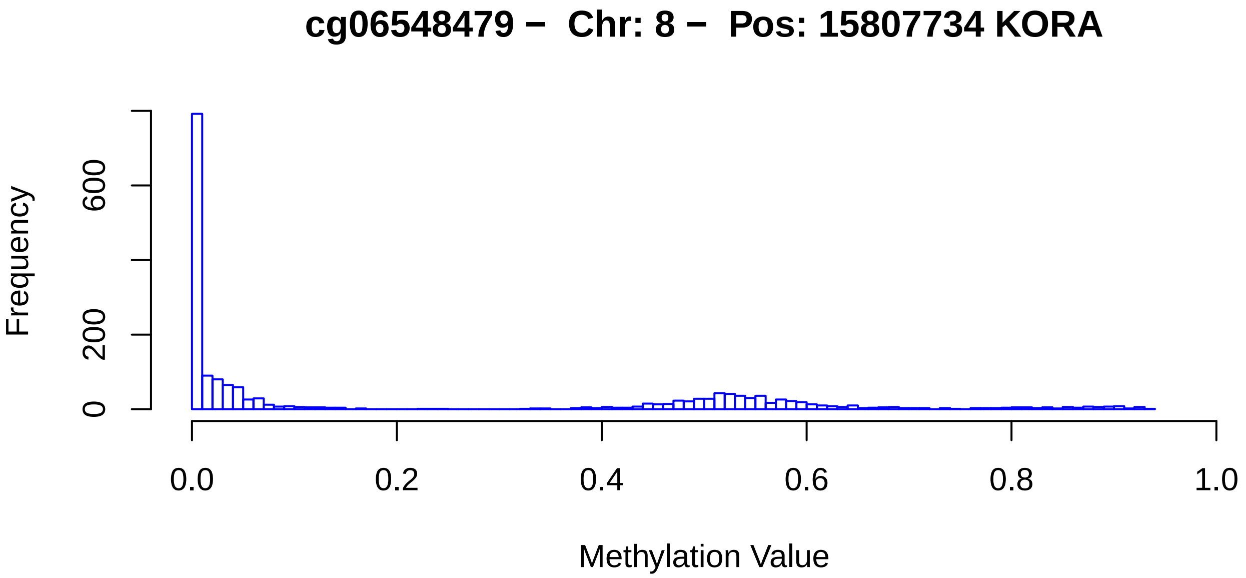
cg06548479 − Chr: 8 − Pos: 15807734 KORA
Frequency
0
200
600
0.0
0.2
0.4
0.6
0.8
1.0
Methylation Value

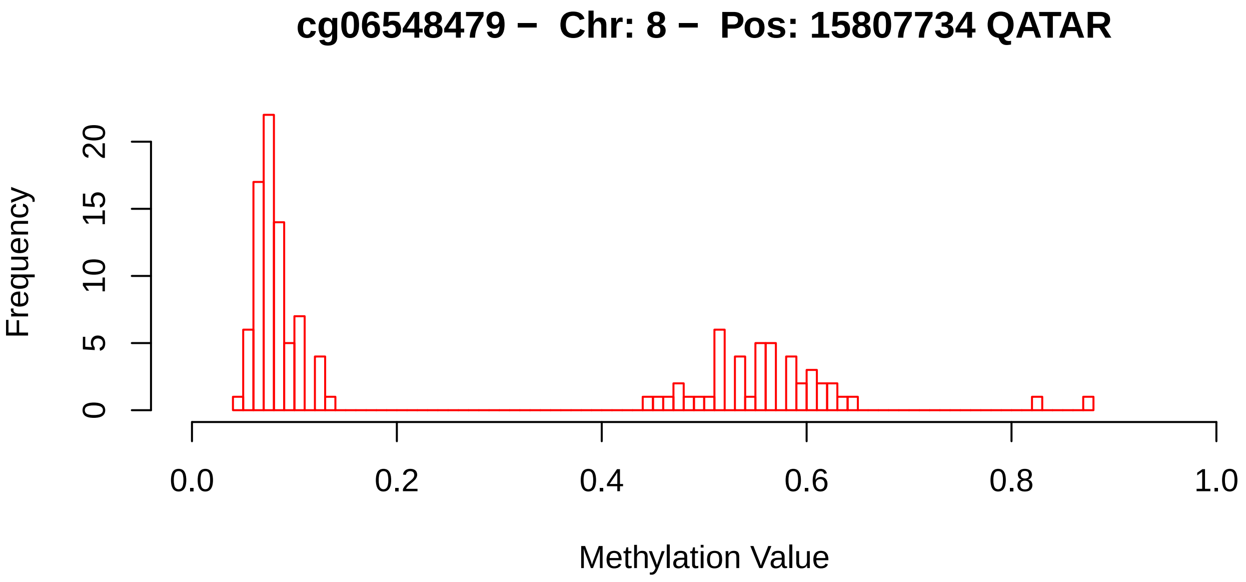
cg06548479 − Chr: 8 − Pos: 15807734 QATAR
Frequency
0
5
10
15
20
0.0
0.2
0.4
0.6
0.8
1.0
Methylation Value

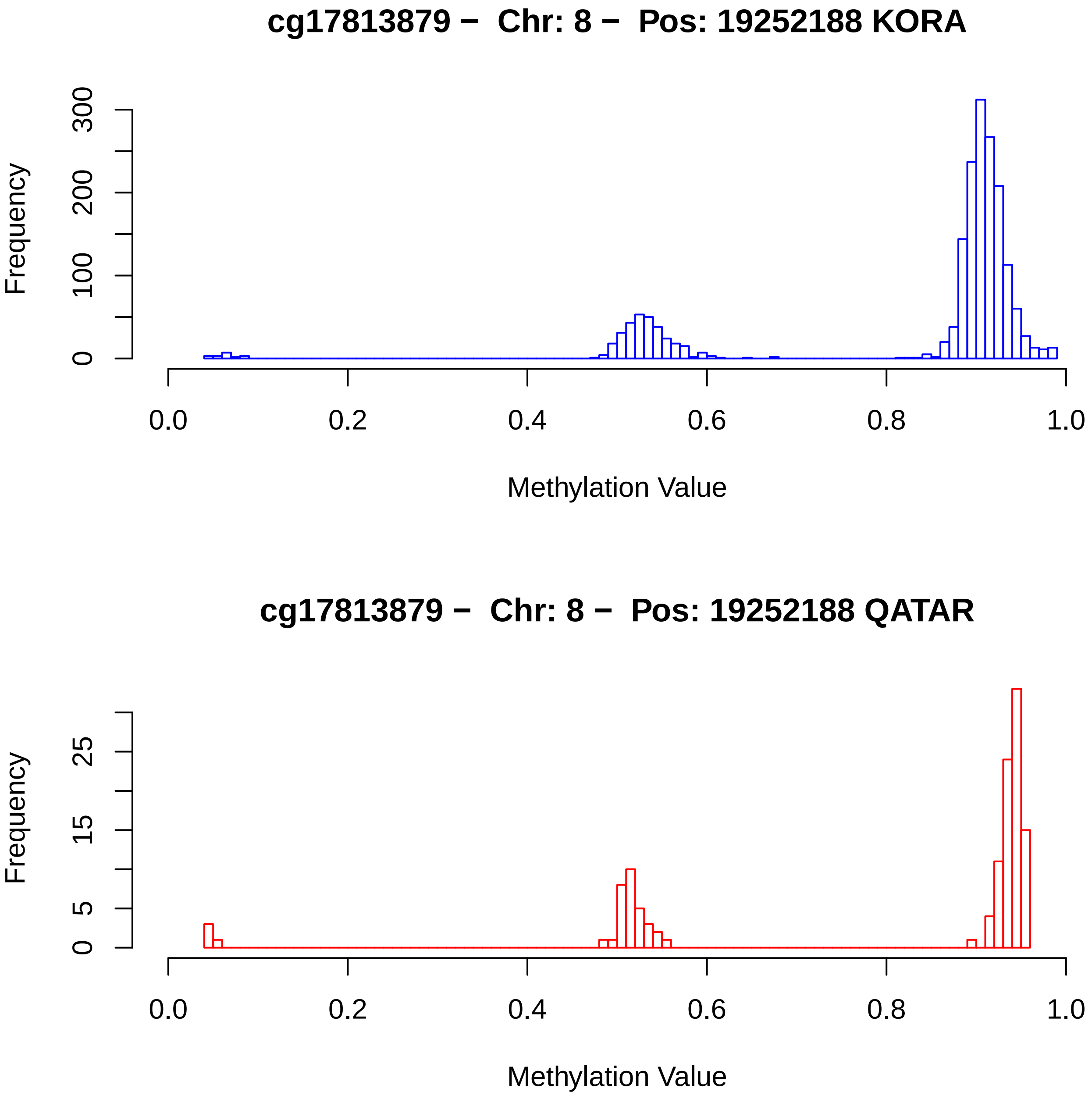

cg17813879 − Chr: 8 − Pos: 19252188 KORA
Frequency
0
100
200
300
0.0
0.2
0.4
0.6
0.8
1.0
Methylation Value
cg17813879 − Chr: 8 − Pos: 19252188 QATAR
Frequency
0
5
15
25
0.0
0.2
0.4
0.6
0.8
1.0
Methylation Value

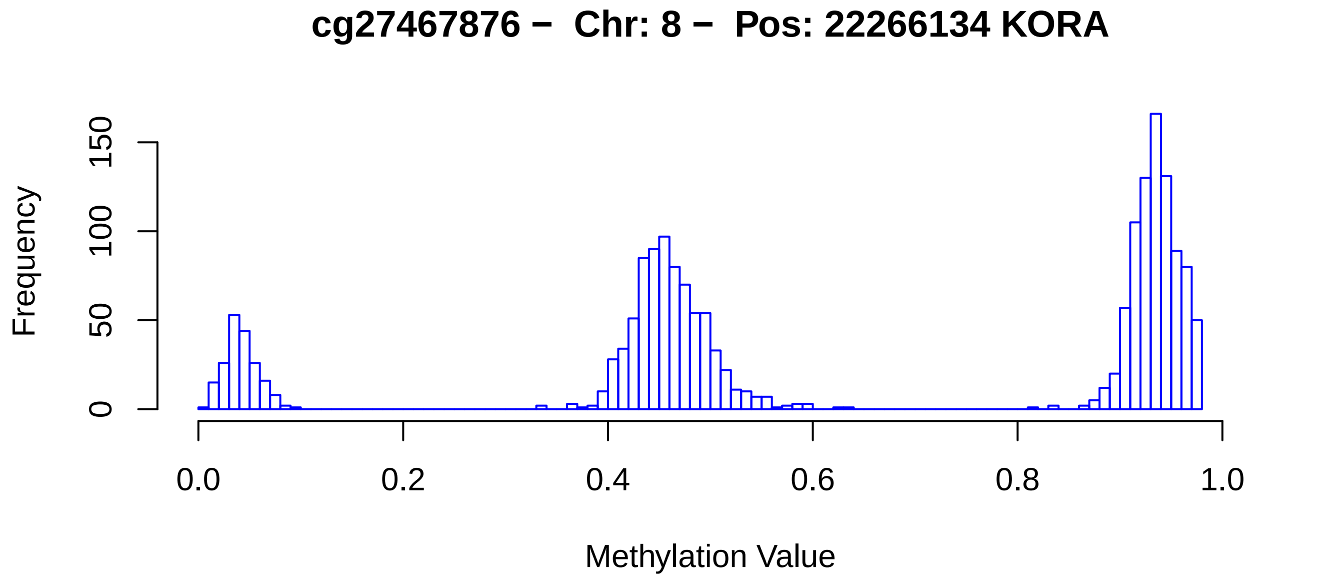
cg27467876 − Chr: 8 − Pos: 22266134 KORA
Frequency
0
50
100
150
0.0
0.2
0.4
0.6
0.8
1.0
Methylation Value

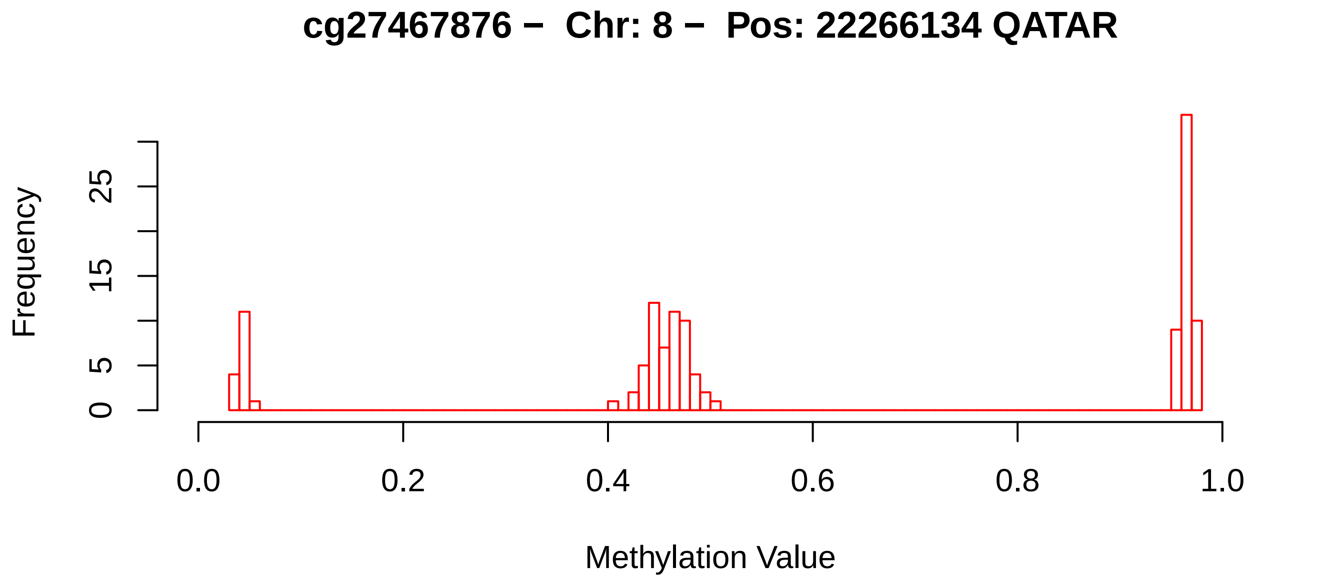
cg27467876 − Chr: 8 − Pos: 22266134 QATAR
Frequency
0
5
15
25
0.0
0.2
0.4
0.6
0.8
1.0
Methylation Value

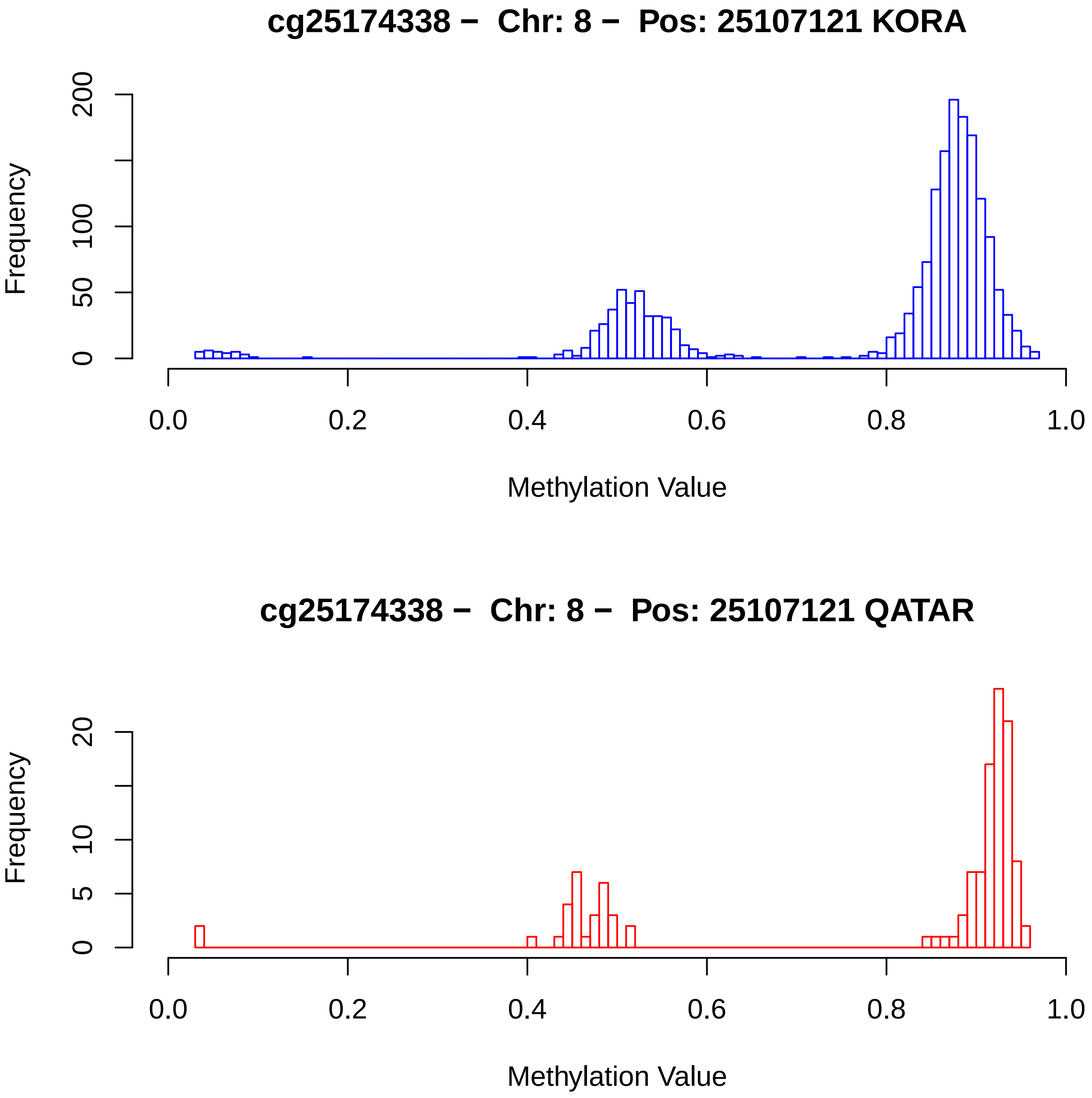

cg25174338 − Chr: 8 − Pos: 25107121 KORA
Frequency
0
50
100
200
0.0
0.2
0.4
0.6
0.8
1.0
Methylation Value
cg25174338 − Chr: 8 − Pos: 25107121 QATAR
Frequency
0
5
10
20
0.0
0.2
0.4
0.6
0.8
1.0
Methylation Value

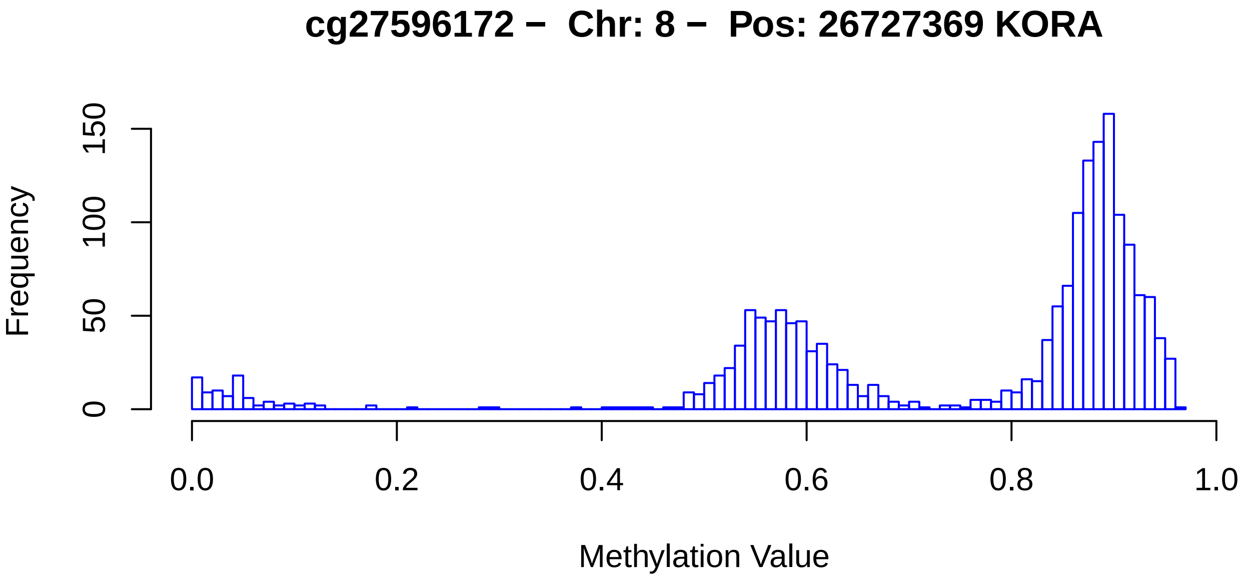
cg27596172 − Chr: 8 − Pos: 26727369 KORA
Frequency
0
50
100
150
0.0
0.2
0.4
0.6
0.8
1.0
Methylation Value

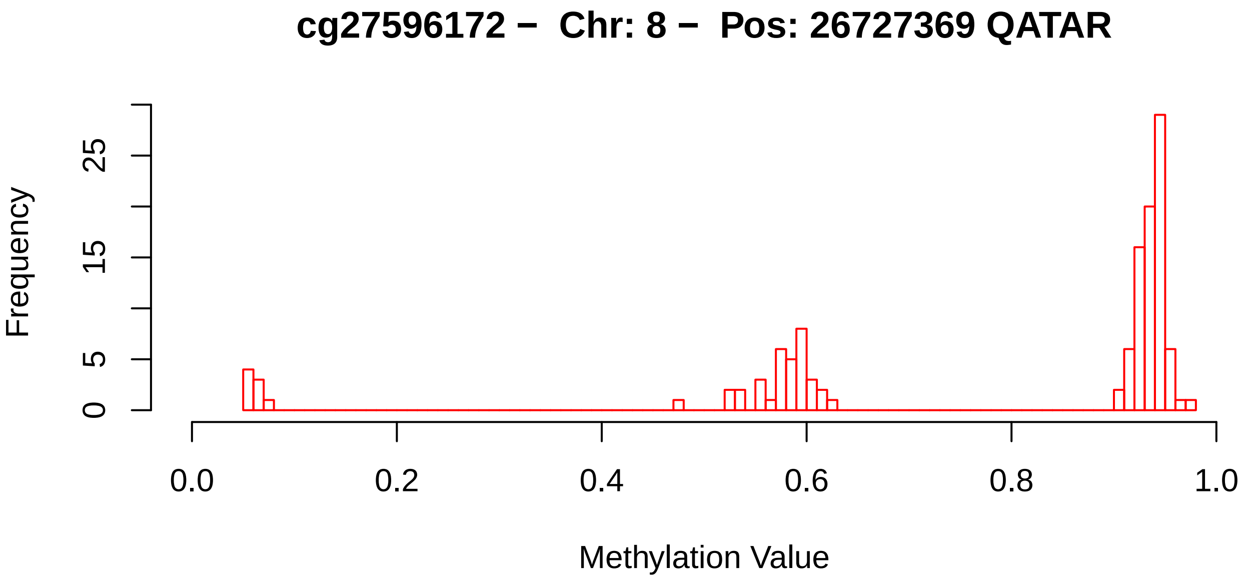
cg27596172 − Chr: 8 − Pos: 26727369 QATAR
Frequency
0
5
15
25
0.0
0.2
0.4
0.6
0.8
1.0
Methylation Value

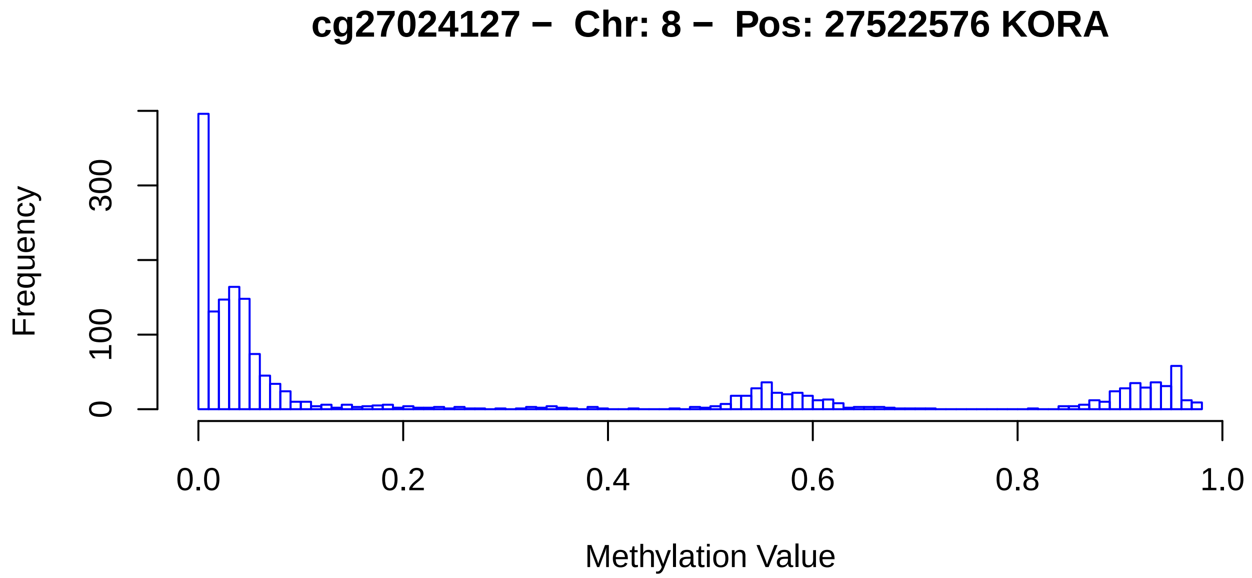
cg27024127 − Chr: 8 − Pos: 27522576 KORA
Frequency
0
100
300
0.0
0.2
0.4
0.6
0.8
1.0
Methylation Value

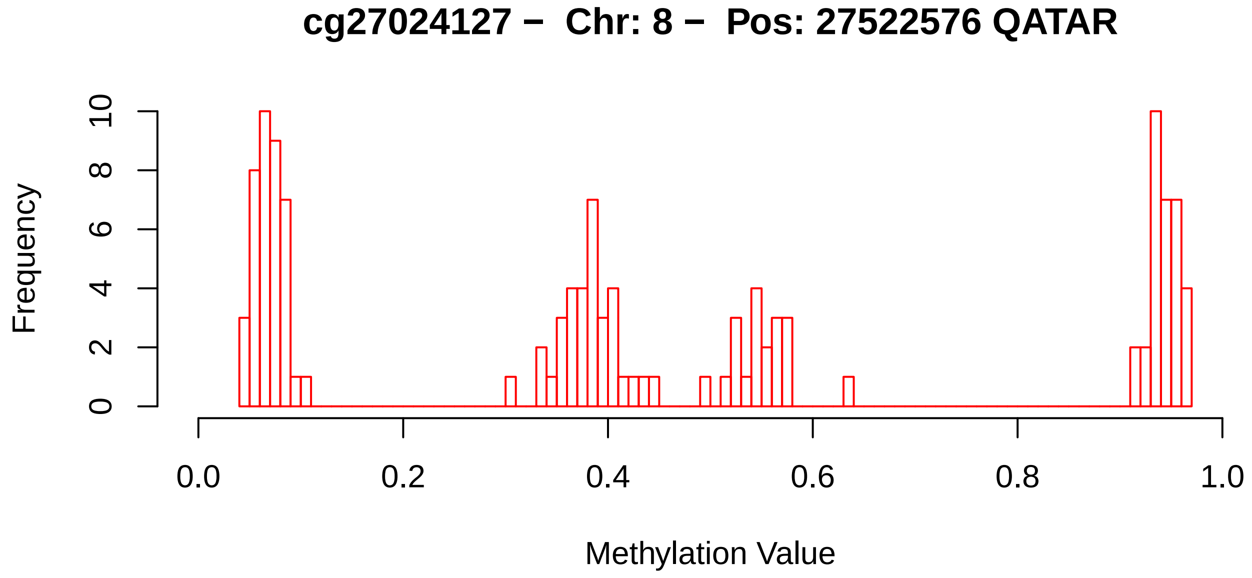
cg27024127 − Chr: 8 − Pos: 27522576 QATAR
Frequency
0
2
4
6
8
10
0.0
0.2
0.4
0.6
0.8
1.0
Methylation Value

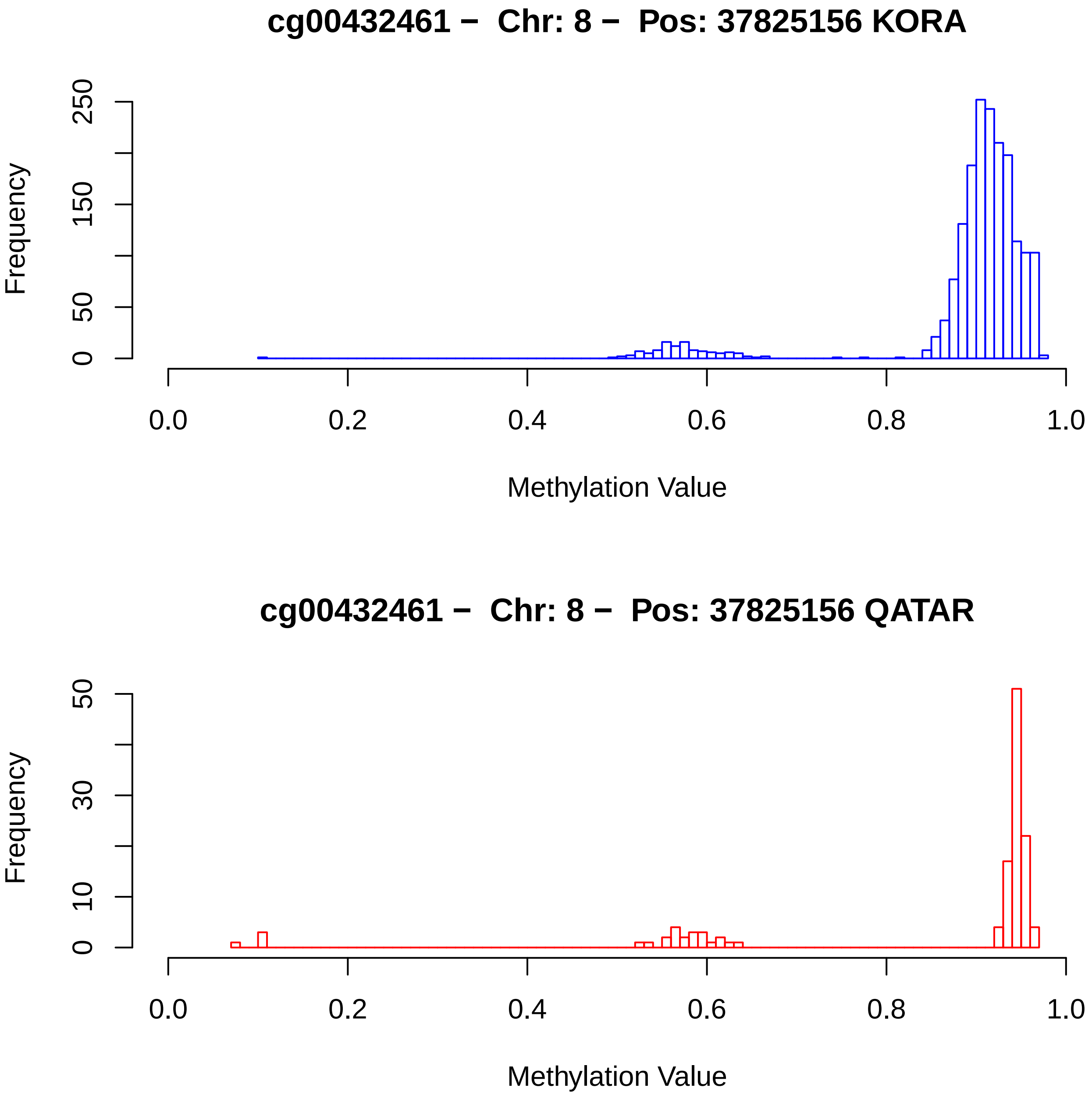

cg00432461 − Chr: 8 − Pos: 37825156 KORA
Frequency
0
50
150
250
0.0
0.2
0.4
0.6
0.8
1.0
Methylation Value
cg00432461 − Chr: 8 − Pos: 37825156 QATAR
Frequency
0
10
30
50
0.0
0.2
0.4
0.6
0.8
1.0
Methylation Value

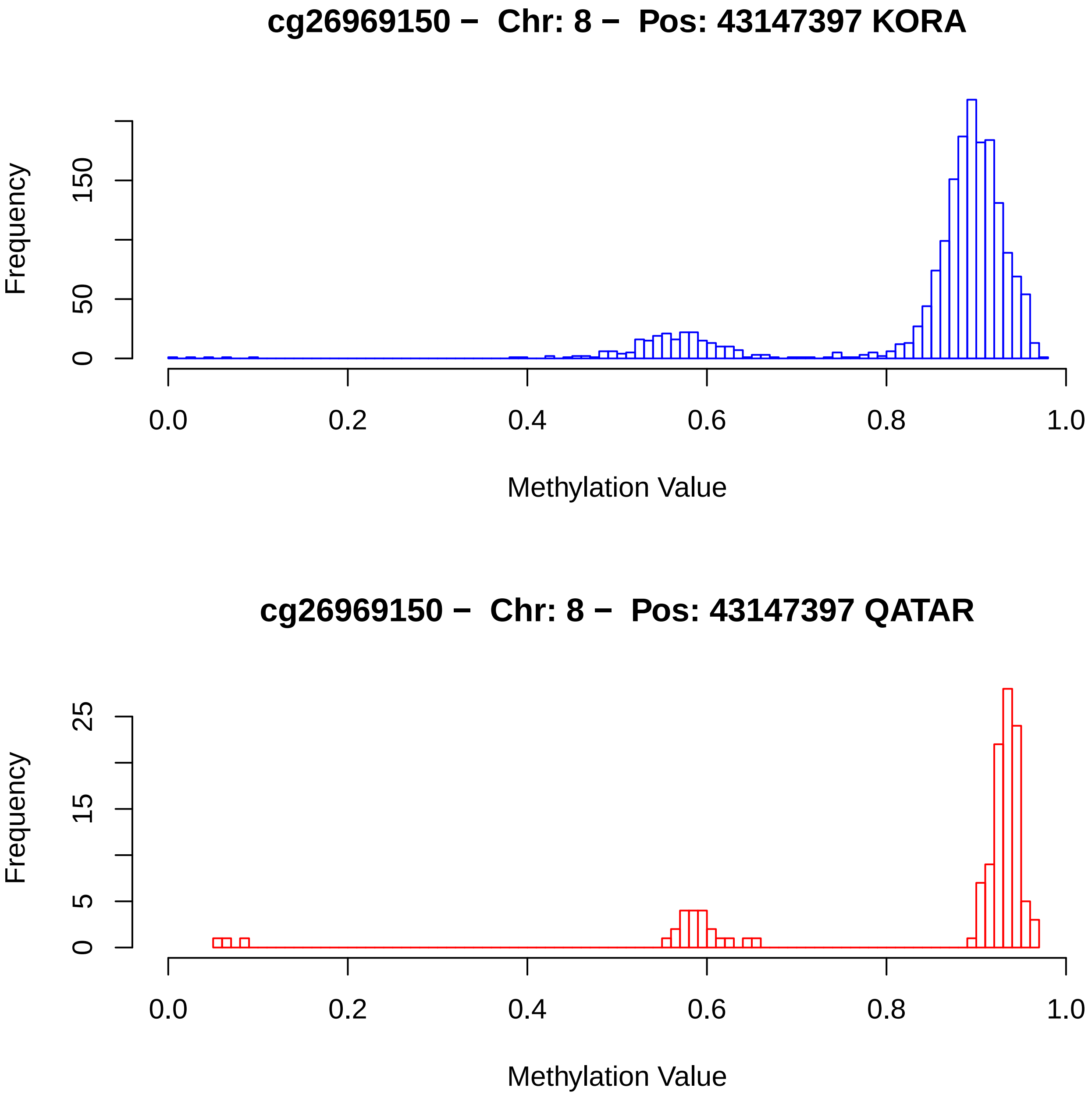
cg26969150 − Chr: 8 − Pos: 43147397 KORA
Frequency
0
50
150
0.0
0.2
0.4
0.6
0.8
1.0
Methylation Value
cg26969150 − Chr: 8 − Pos: 43147397 QATAR
Frequency
0
5
15
25
0.0
0.2
0.4
0.6
0.8
1.0
Methylation Value

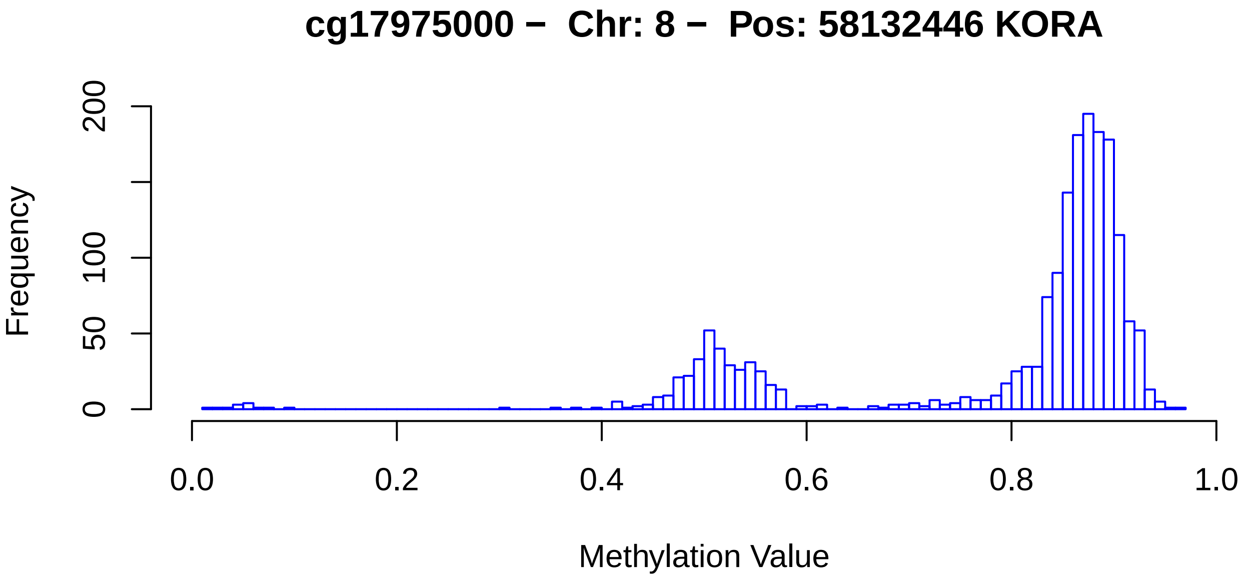
cg17975000 − Chr: 8 − Pos: 58132446 KORA
Frequency
0
50
100
200
0.0
0.2
0.4
0.6
0.8
1.0
Methylation Value

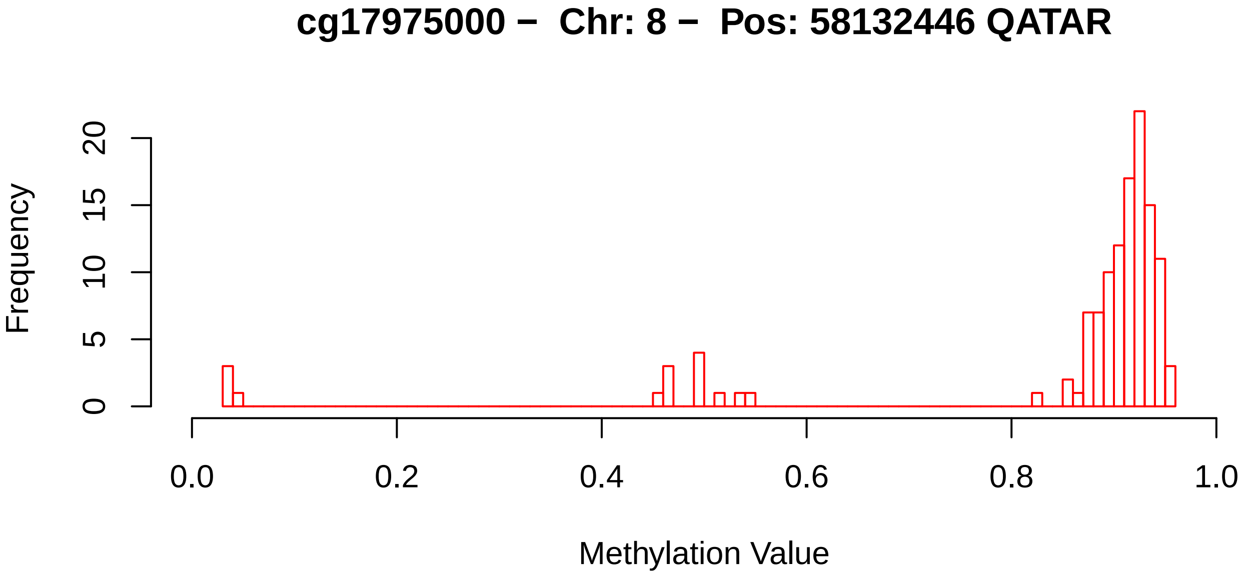
cg17975000 − Chr: 8 − Pos: 58132446 QATAR
Frequency
0
5
10
15
20
0.0
0.2
0.4
0.6
0.8
1.0
Methylation Value

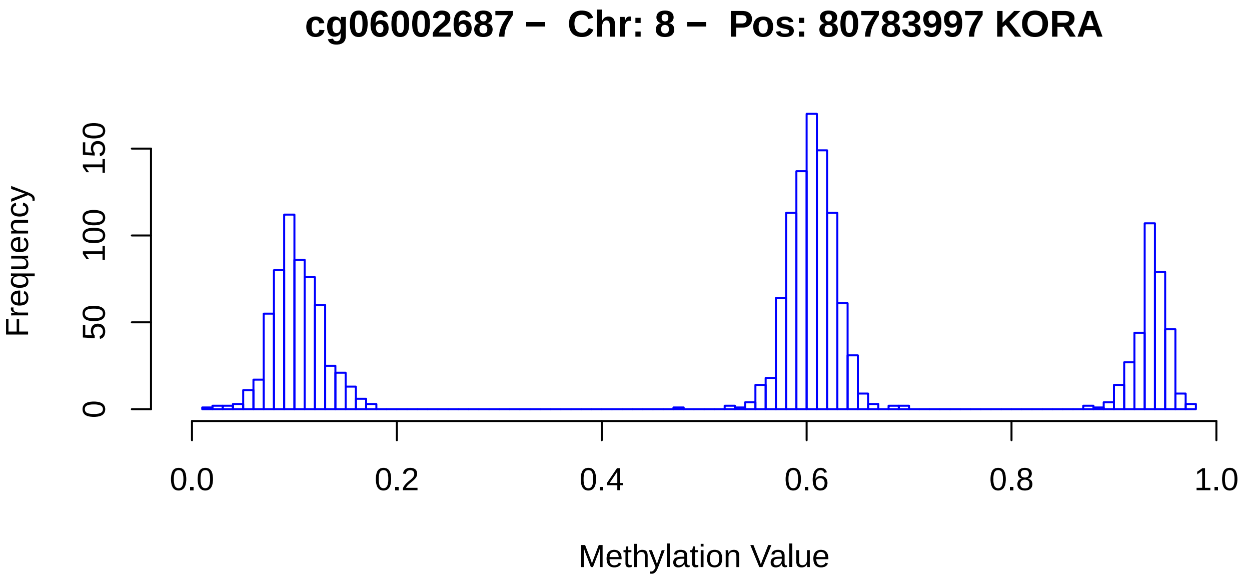
cg06002687 − Chr: 8 − Pos: 80783997 KORA
Frequency
0
50
100
150
0.0
0.2
0.4
0.6
0.8
1.0
Methylation Value

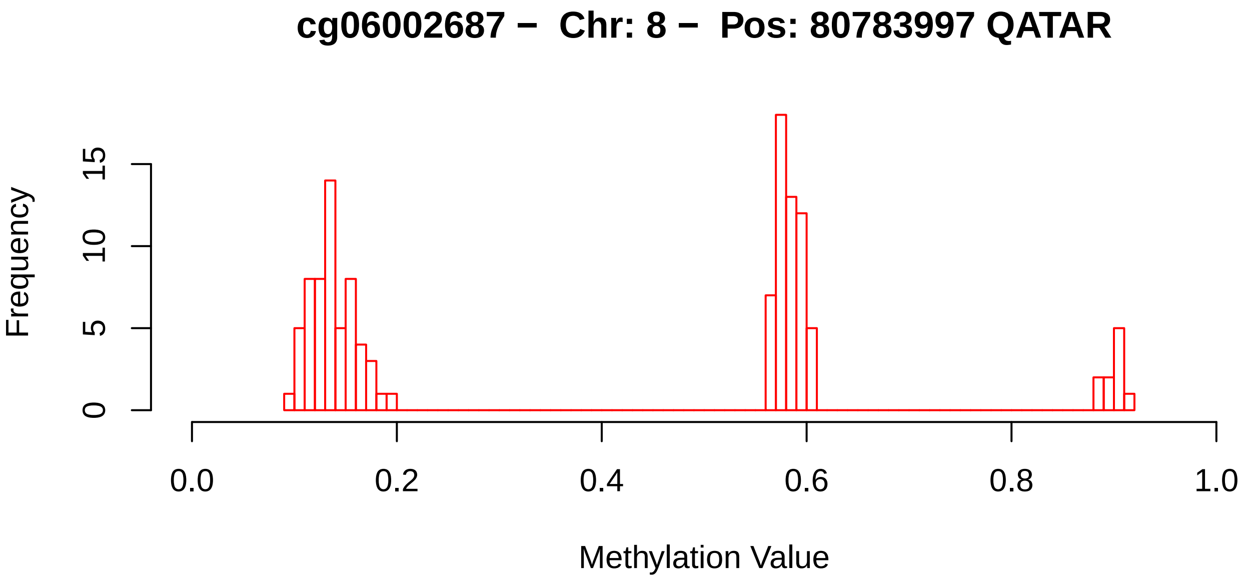
cg06002687 − Chr: 8 − Pos: 80783997 QATAR
Frequency
0
5
10
15
0.0
0.2
0.4
0.6
0.8
1.0
Methylation Value

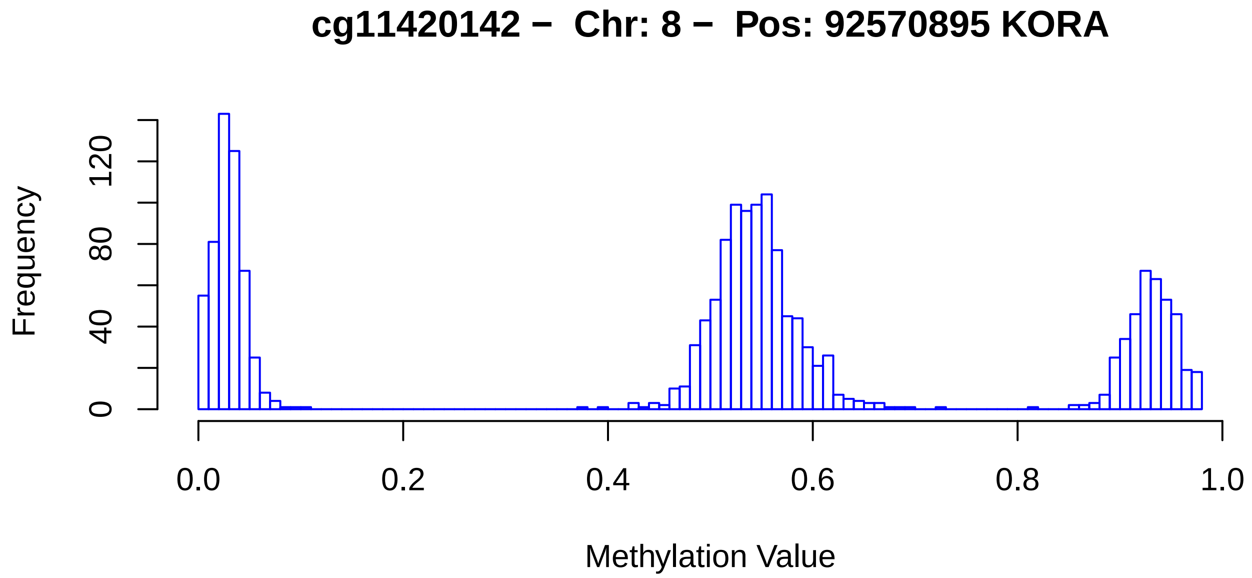
cg11420142 − Chr: 8 − Pos: 92570895 KORA
Frequency
0
40
80
120
0.0
0.2
0.4
0.6
0.8
1.0
Methylation Value

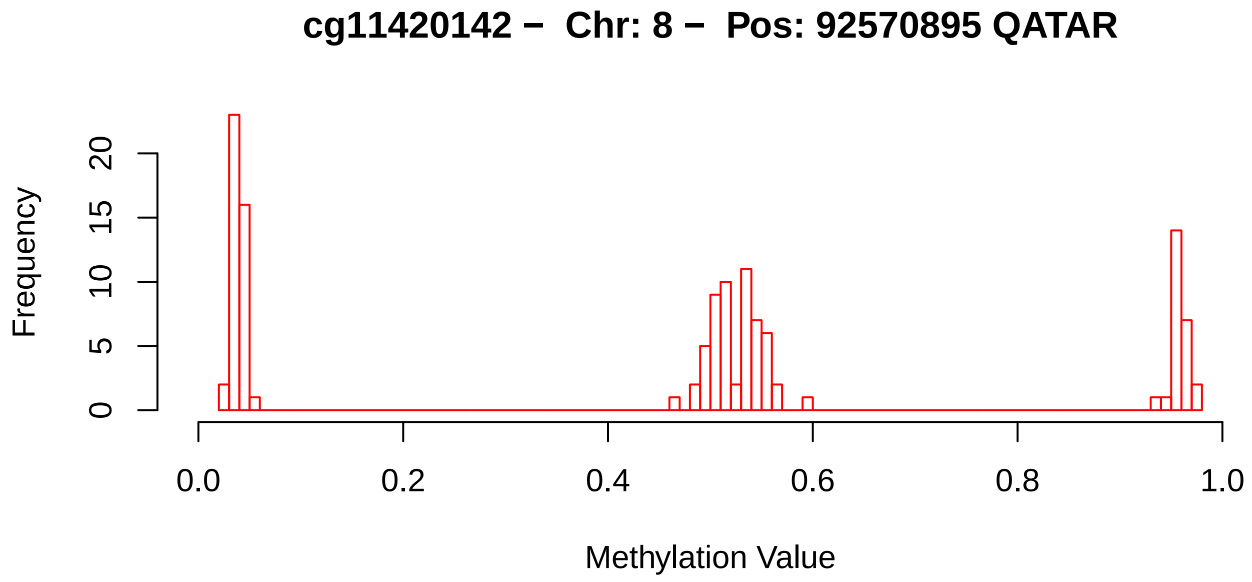
cg11420142 − Chr: 8 − Pos: 92570895 QATAR
Frequency
0
5
10
15
20
0.0
0.2
0.4
0.6
0.8
1.0
Methylation Value

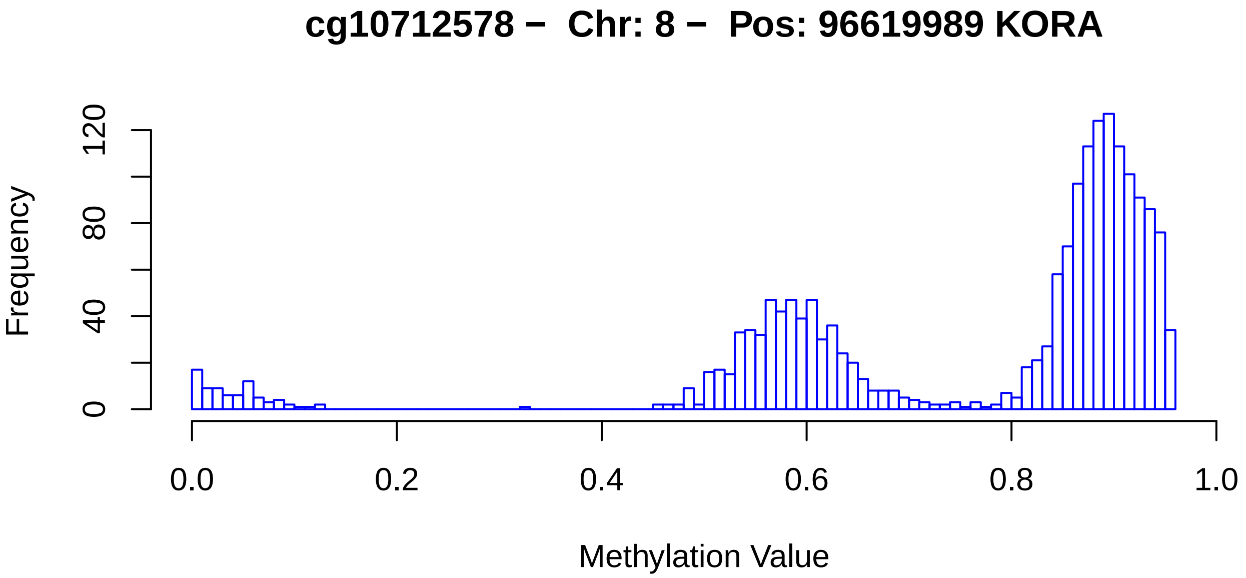
cg10712578 − Chr: 8 − Pos: 96619989 KORA
Frequency
0
40
80
120
0.0
0.2
0.4
0.6
0.8
1.0
Methylation Value

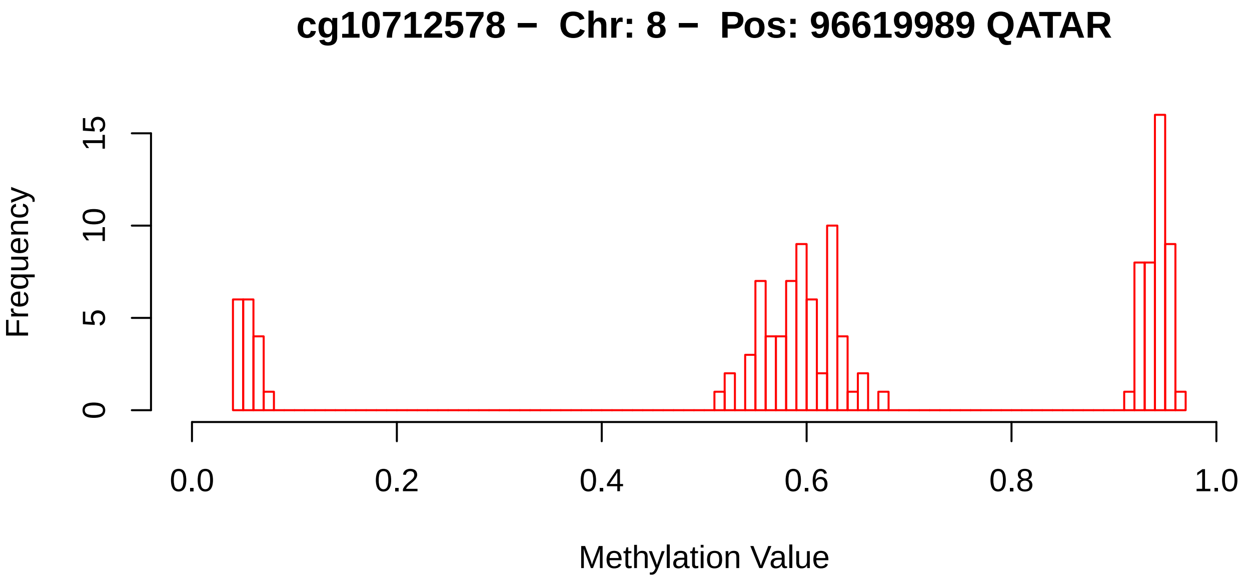
cg10712578 − Chr: 8 − Pos: 96619989 QATAR
Frequency
0
5
10
15
0.0
0.2
0.4
0.6
0.8
1.0
Methylation Value

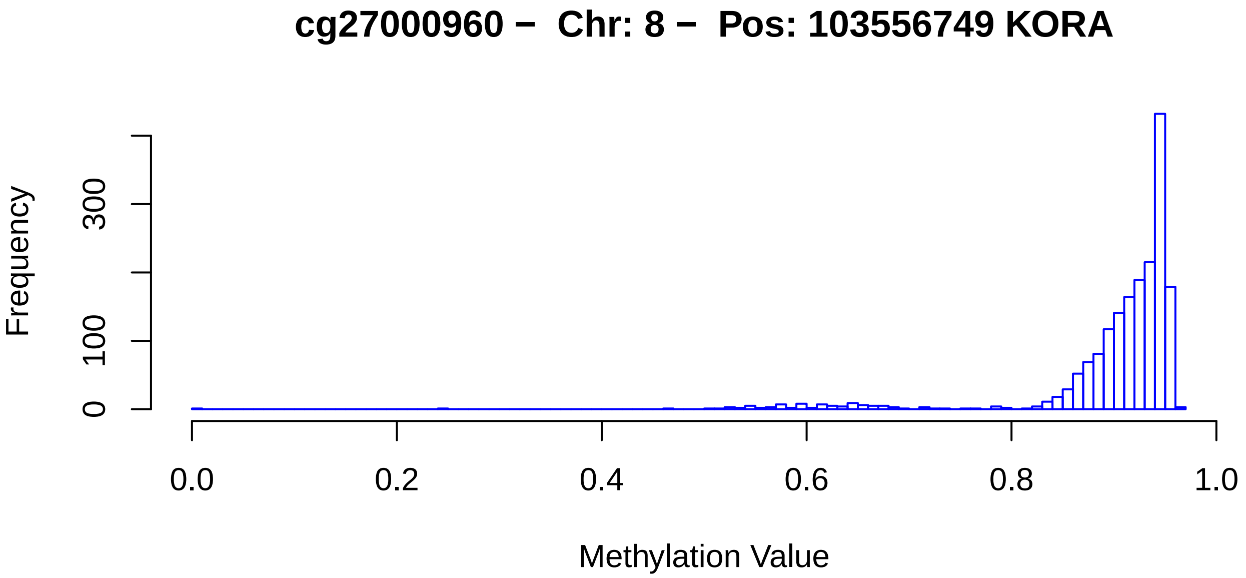
cg27000960 − Chr: 8 − Pos: 103556749 KORA
Frequency
0
100
300
0.0
0.2
0.4
0.6
0.8
1.0
Methylation Value

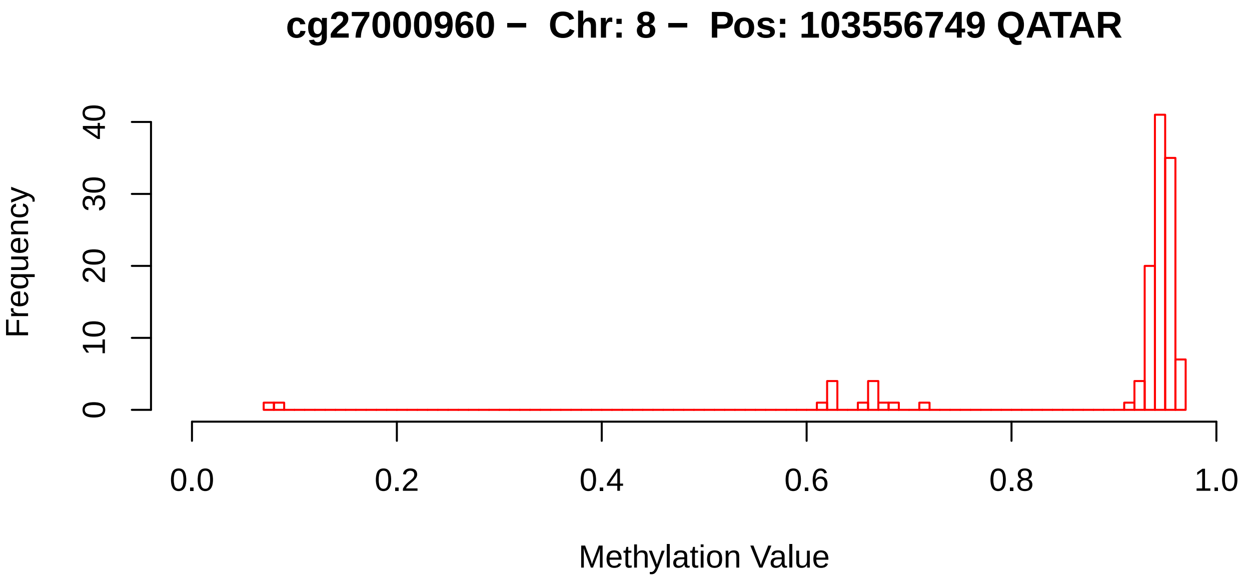
cg27000960 − Chr: 8 − Pos: 103556749 QATAR
Frequency
0
10
20
30
40
0.0
0.2
0.4
0.6
0.8
1.0
Methylation Value

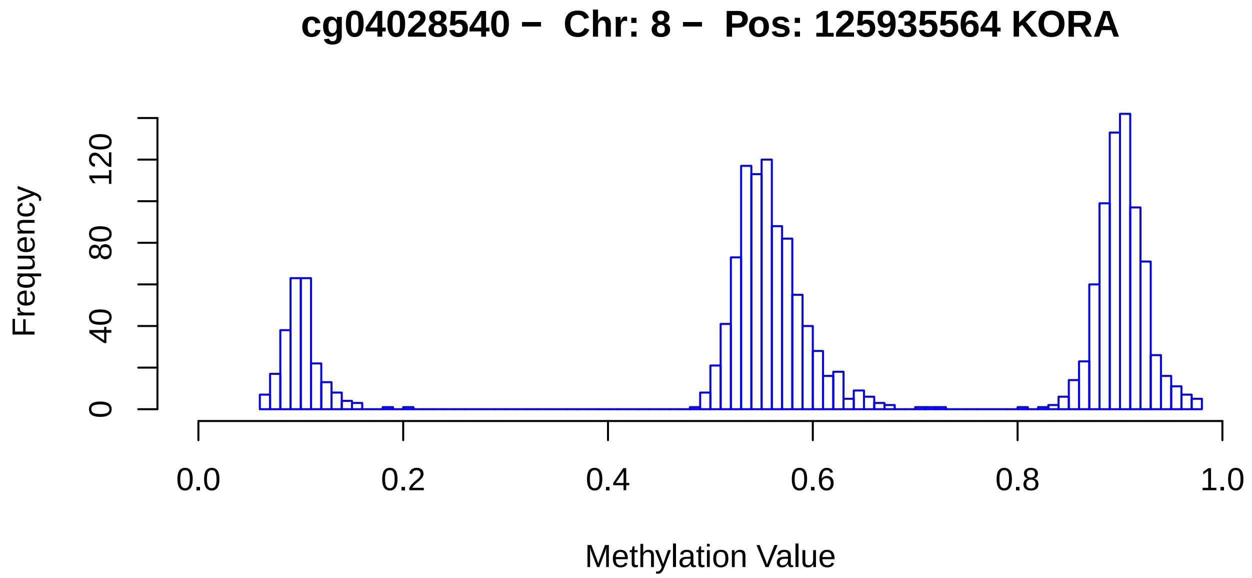
cg04028540 − Chr: 8 − Pos: 125935564 KORA
Frequency
0
40
80
120
0.0
0.2
0.4
0.6
0.8
1.0
Methylation Value

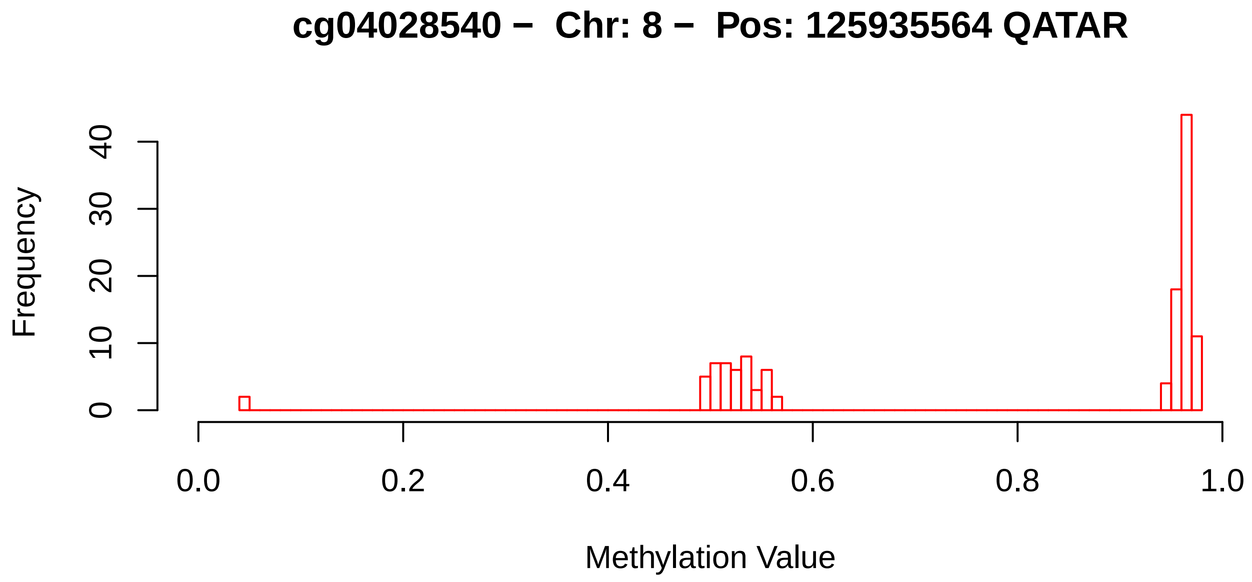
cg04028540 − Chr: 8 − Pos: 125935564 QATAR
Frequency
0
10
20
30
40
0.0
0.2
0.4
0.6
0.8
1.0
Methylation Value

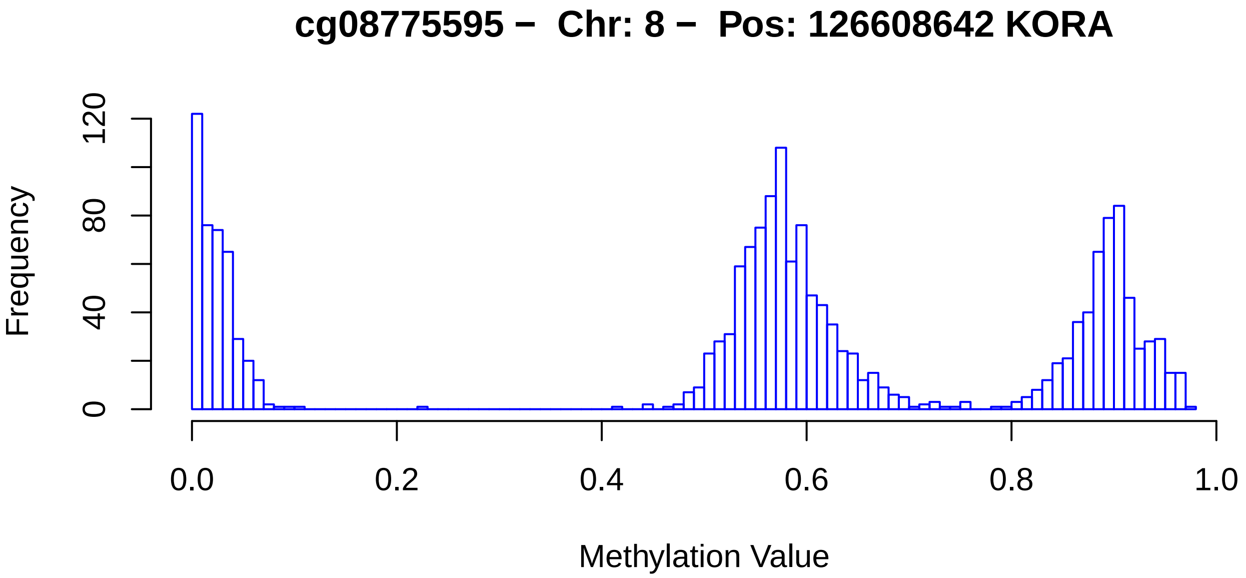
cg08775595 − Chr: 8 − Pos: 126608642 KORA
Frequency
0
40
80
120
0.0
0.2
0.4
0.6
0.8
1.0
Methylation Value

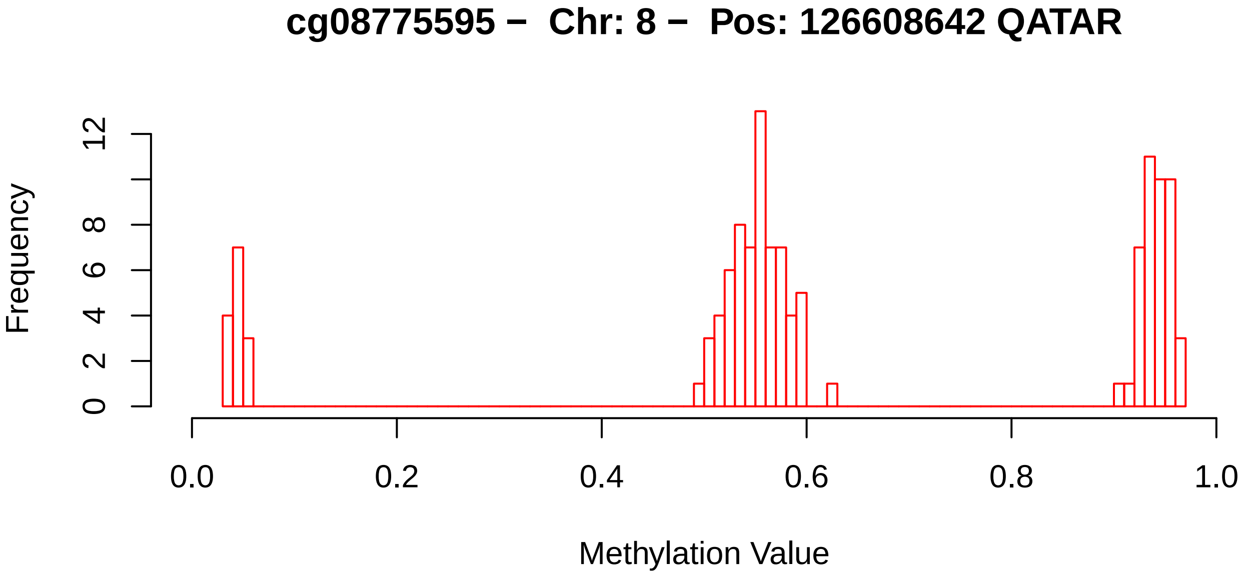
cg08775595 − Chr: 8 − Pos: 126608642 QATAR
Frequency
0
2
4
6
8
12
0.0
0.2
0.4
0.6
0.8
1.0
Methylation Value

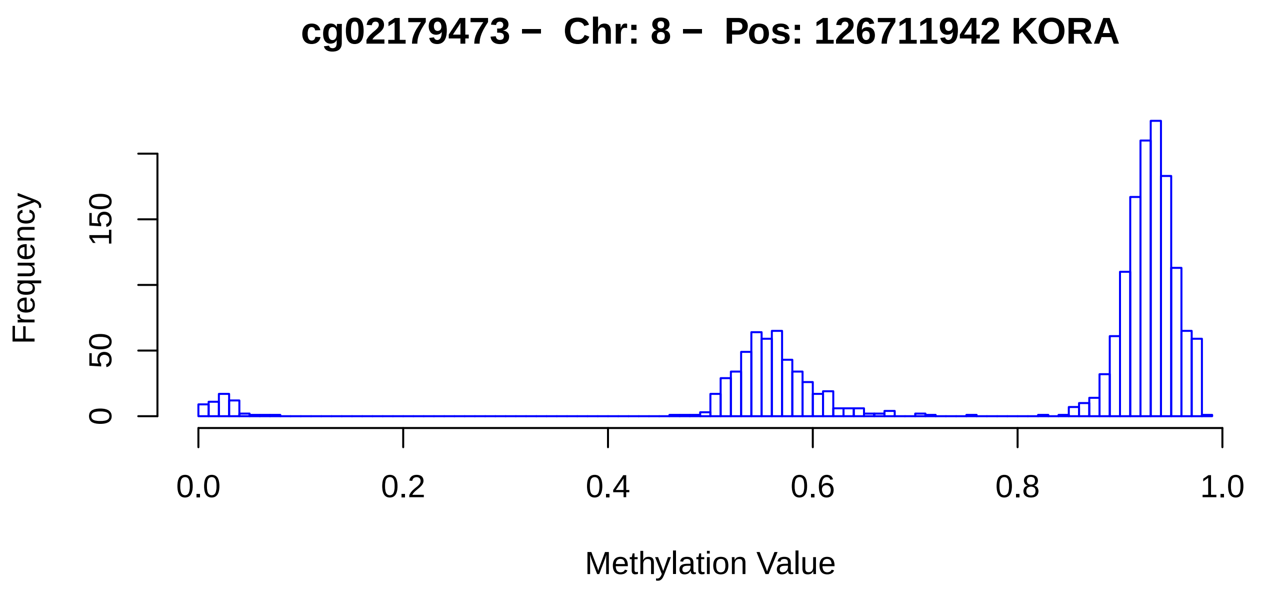
cg02179473 − Chr: 8 − Pos: 126711942 KORA
Frequency
0
50
150
0.0
0.2
0.4
0.6
0.8
1.0
Methylation Value

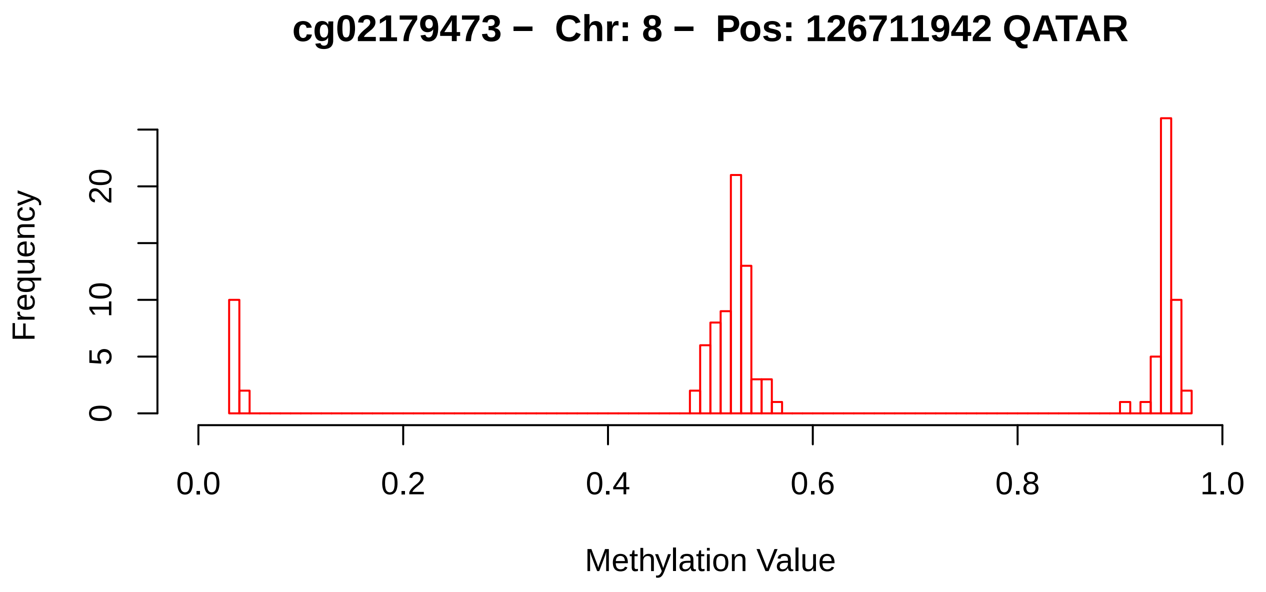
cg02179473 − Chr: 8 − Pos: 126711942 QATAR
Frequency
0
5
10
20
0.0
0.2
0.4
0.6
0.8
1.0
Methylation Value

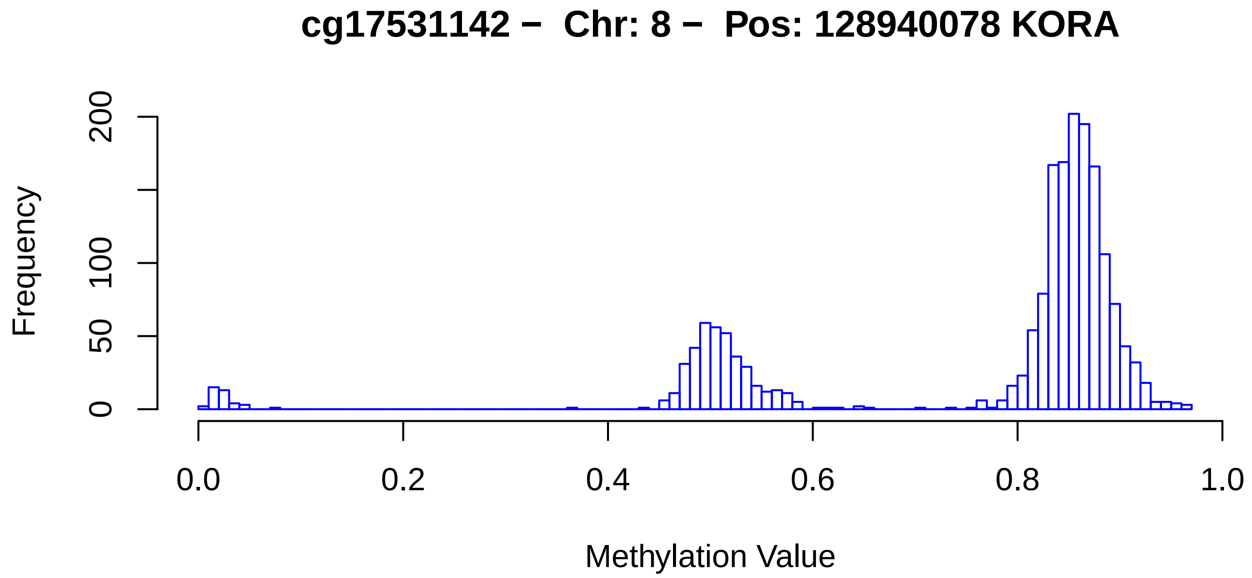

cg17531142 − Chr: 8 − Pos: 128940078 KORA
Frequency
0
50
100
200
0.0
0.2
0.4
0.6
0.8
1.0
Methylation Value

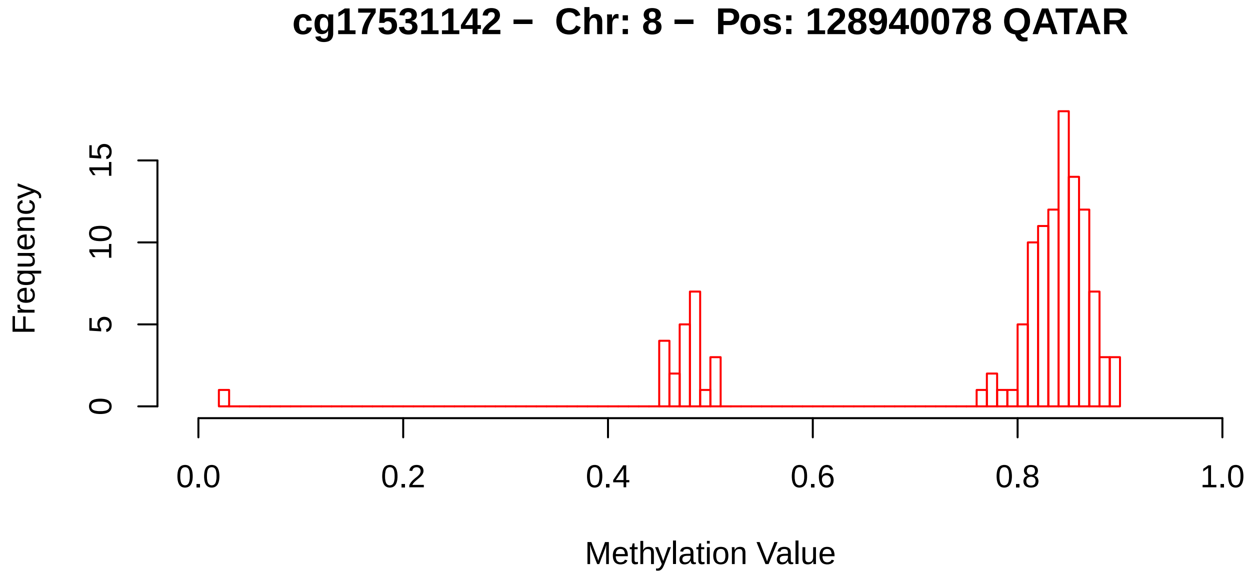

cg17531142 − Chr: 8 − Pos: 128940078 QATAR
Frequency
0
5
10
15
0.0
0.2
0.4
0.6
0.8
1.0
Methylation Value

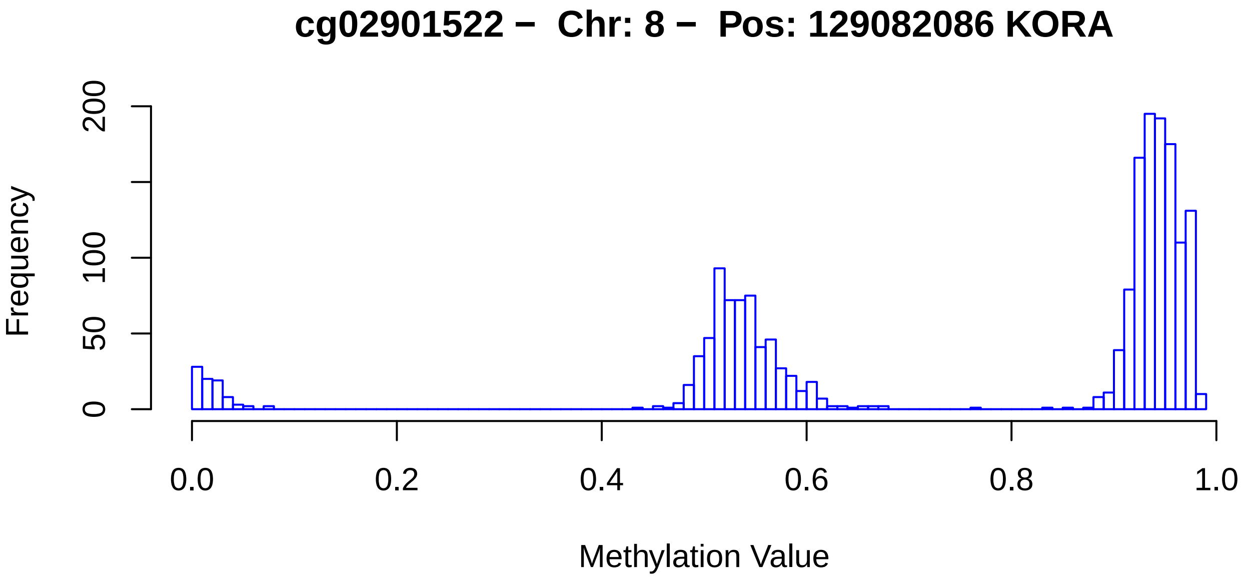
cg02901522 − Chr: 8 − Pos: 129082086 KORA
Frequency
0
50
100
200
0.0
0.2
0.4
0.6
0.8
1.0
Methylation Value

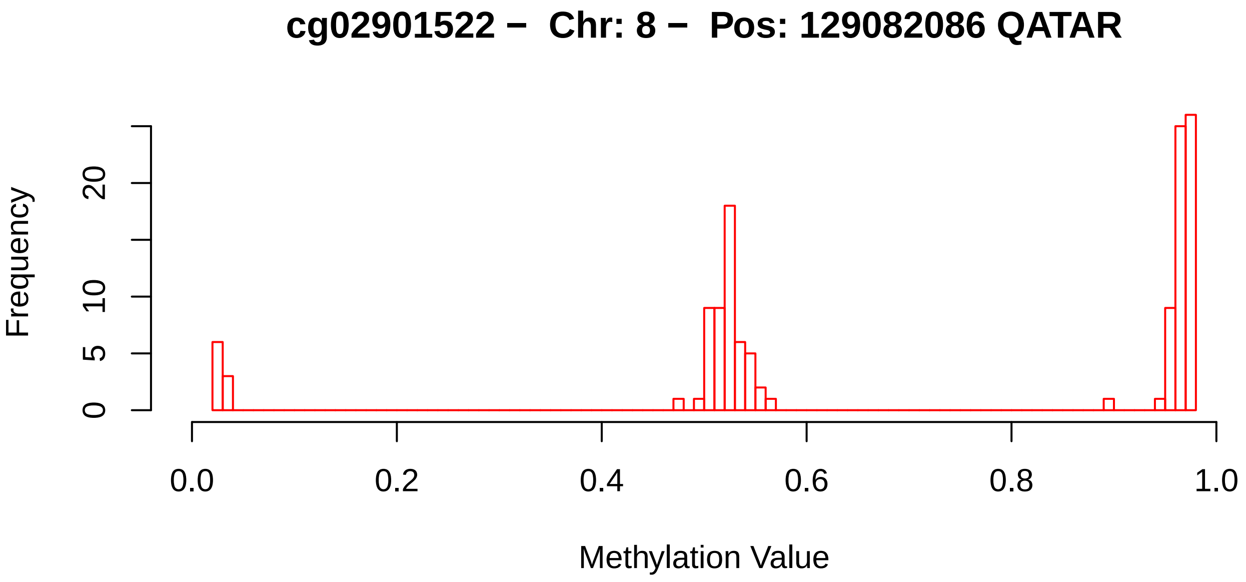
cg02901522 − Chr: 8 − Pos: 129082086 QATAR
Frequency
0
5
10
20
0.0
0.2
0.4
0.6
0.8
1.0
Methylation Value

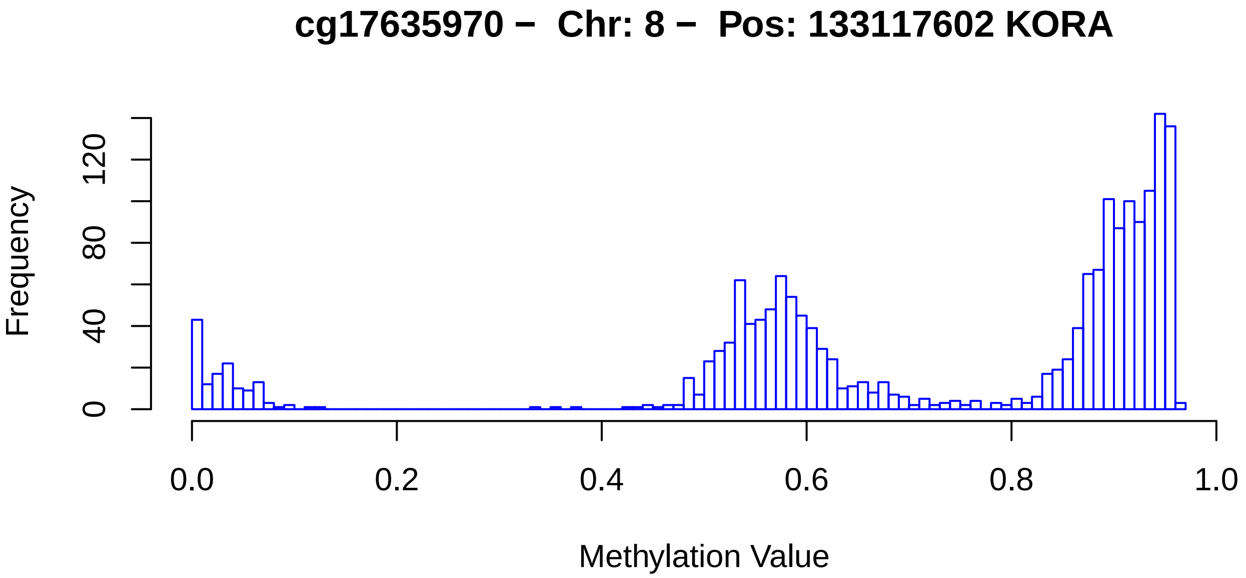
cg17635970 − Chr: 8 − Pos: 133117602 KORA
Frequency
0
40
80
120
0.0
0.2
0.4
0.6
0.8
1.0
Methylation Value

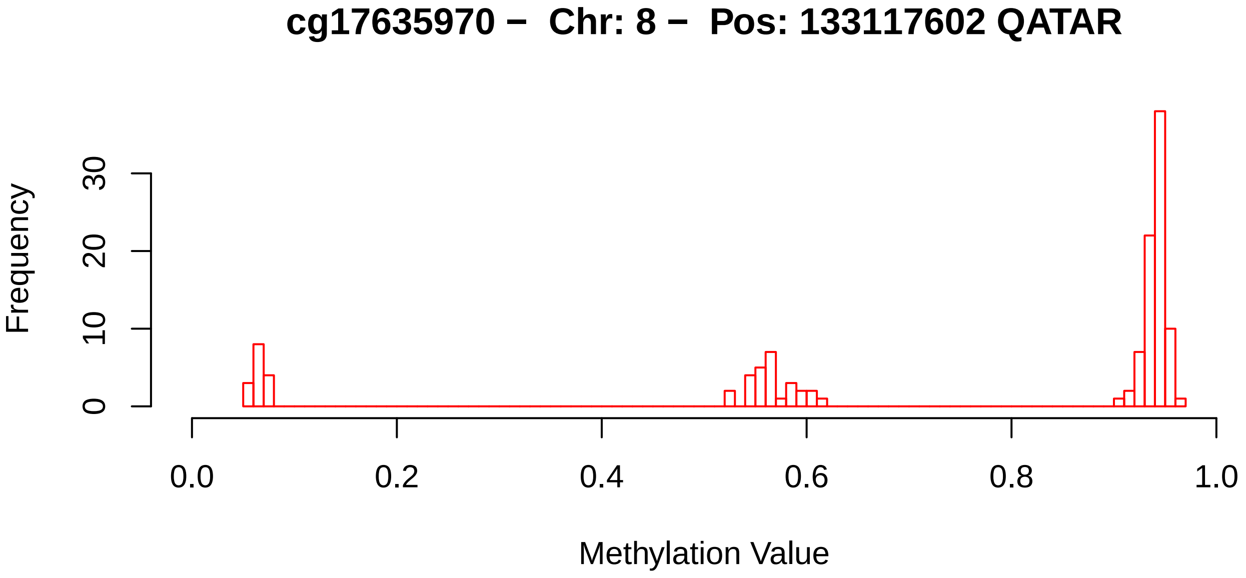
cg17635970 − Chr: 8 − Pos: 133117602 QATAR
Frequency
0
10
20
30
0.0
0.2
0.4
0.6
0.8
1.0
Methylation Value

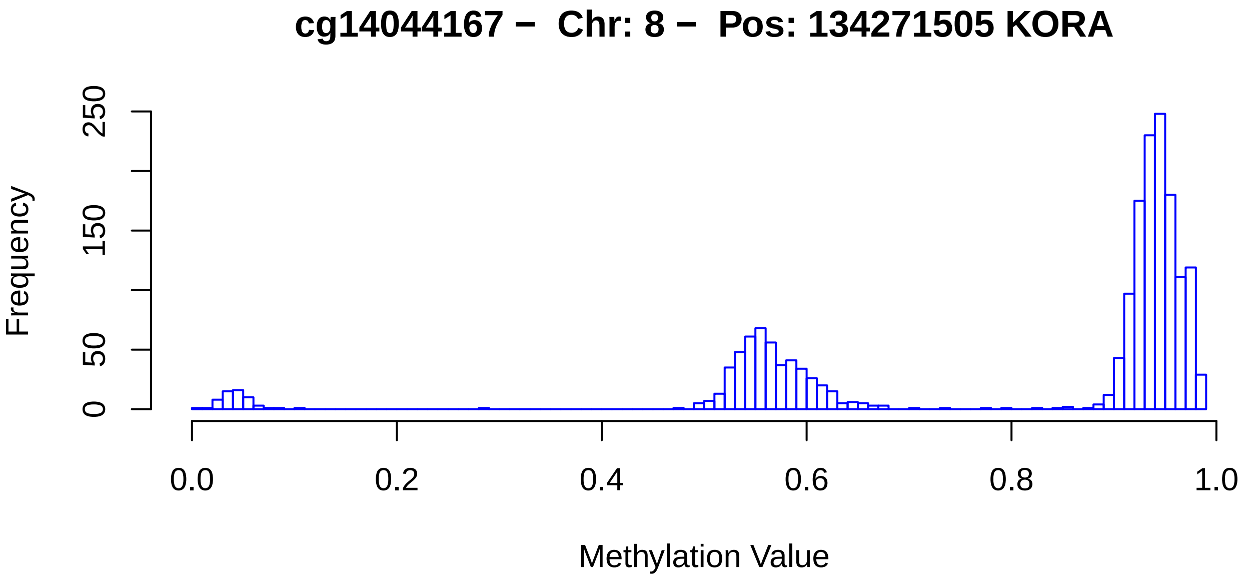
cg14044167 − Chr: 8 − Pos: 134271505 KORA
Frequency
0
50
150
250
0.0
0.2
0.4
0.6
0.8
1.0
Methylation Value

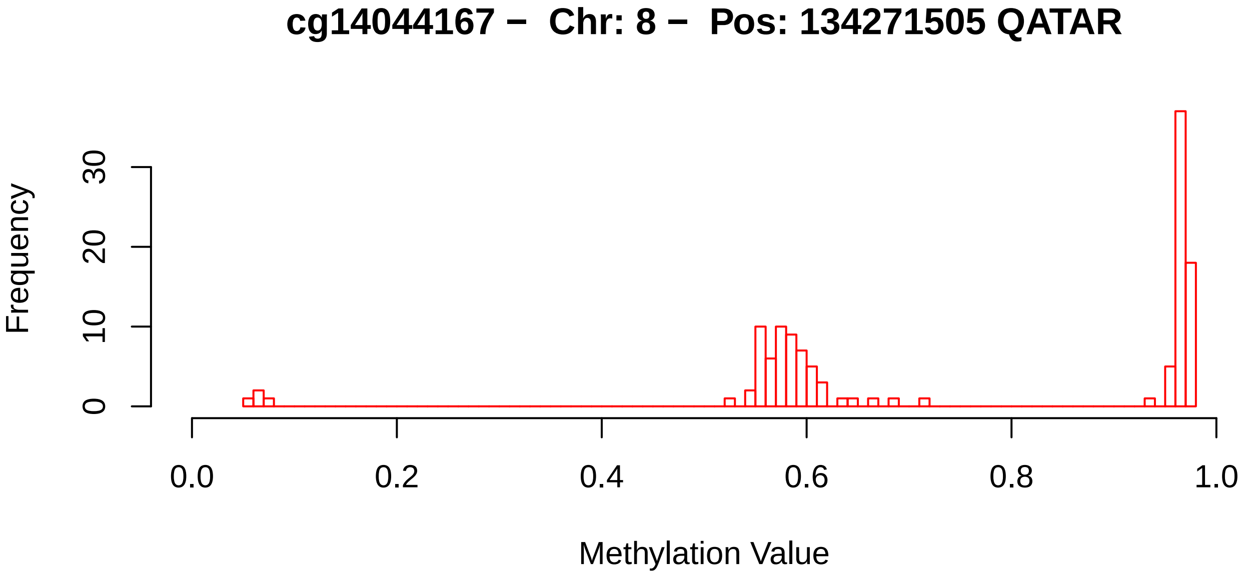
cg14044167 − Chr: 8 − Pos: 134271505 QATAR
Frequency
0
10
20
30
0.0
0.2
0.4
0.6
0.8
1.0
Methylation Value

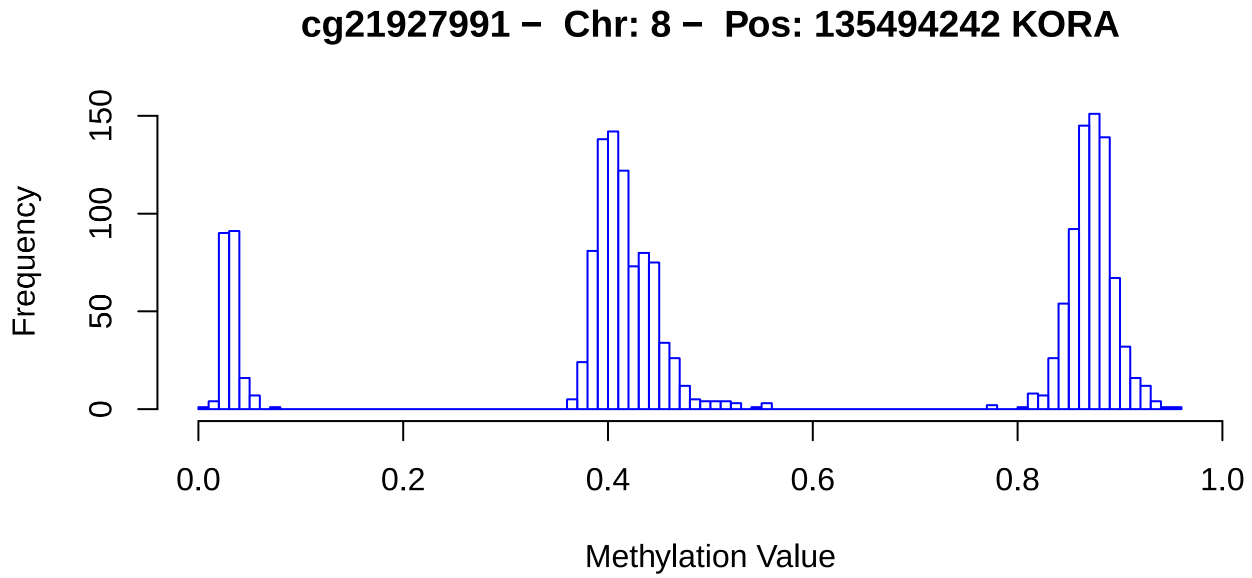
cg21927991 − Chr: 8 − Pos: 135494242 KORA
Frequency
0
50
100
150
0.0
0.2
0.4
0.6
0.8
1.0
Methylation Value

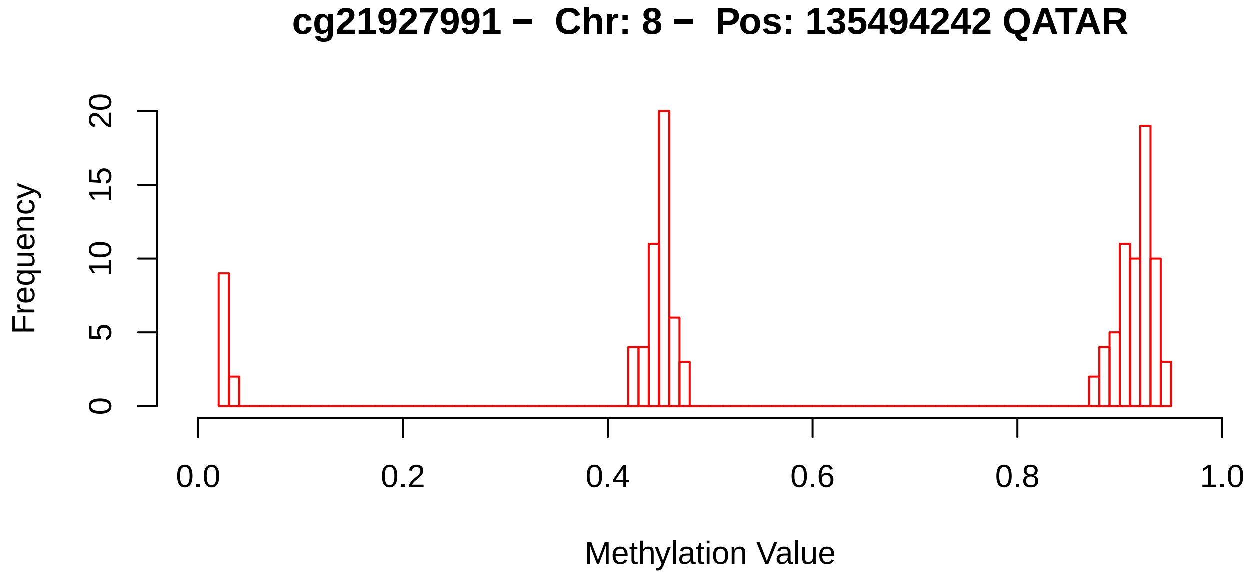
cg21927991 − Chr: 8 − Pos: 135494242 QATAR
Frequency
0
5
10
15
20
0.0
0.2
0.4
0.6
0.8
1.0
Methylation Value

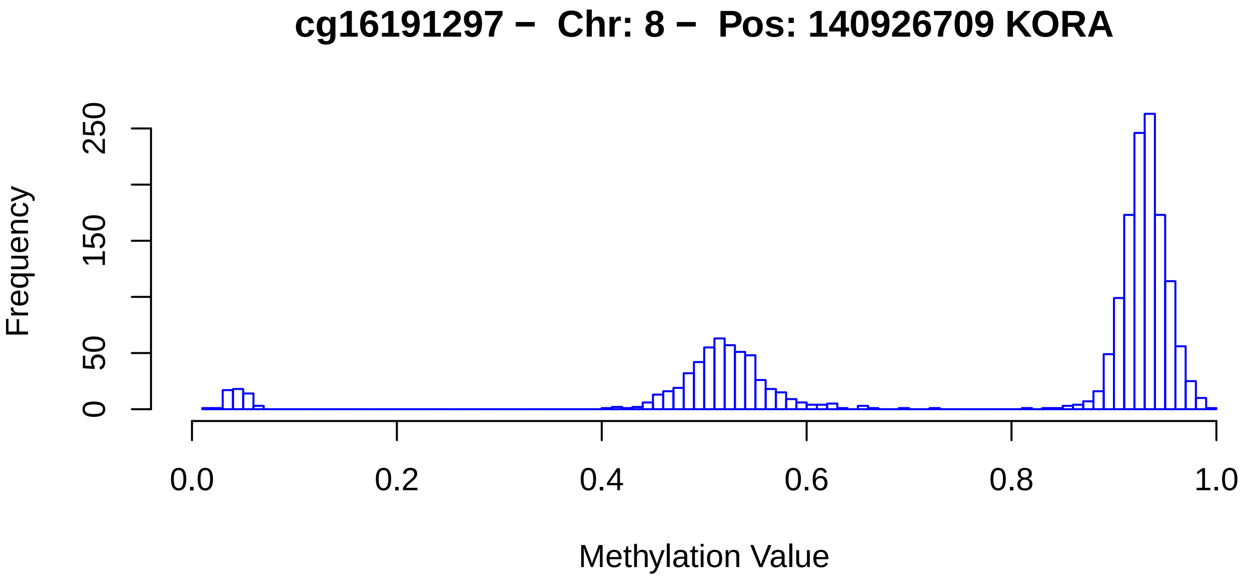
cg16191297 − Chr: 8 − Pos: 140926709 KORA
Frequency
0
50
150
250
0.0
0.2
0.4
0.6
0.8
1.0
Methylation Value

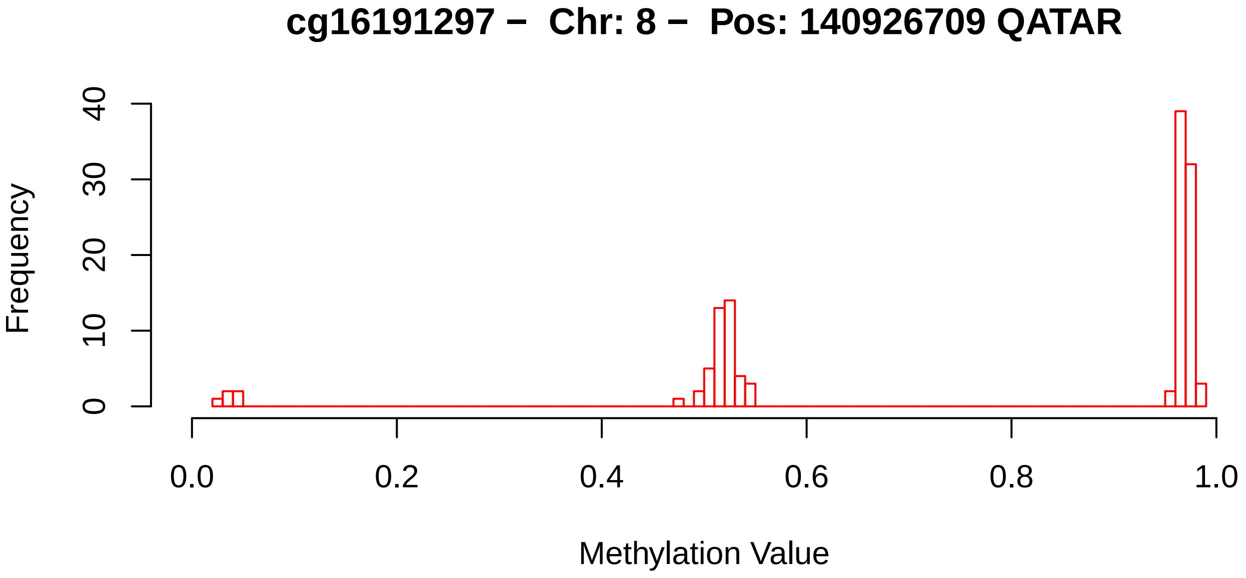
cg16191297 − Chr: 8 − Pos: 140926709 QATAR
Frequency
0
10
20
30
40
0.0
0.2
0.4
0.6
0.8
1.0
Methylation Value

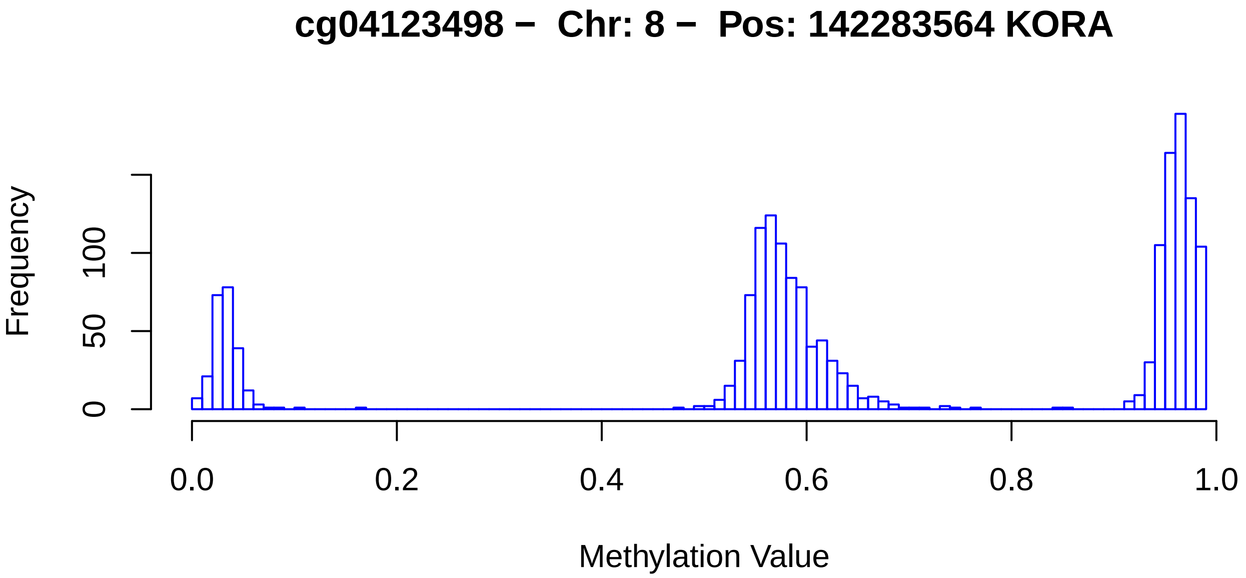
cg04123498 − Chr: 8 − Pos: 142283564 KORA
Frequency
0
50
100
0.0
0.2
0.4
0.6
0.8
1.0
Methylation Value

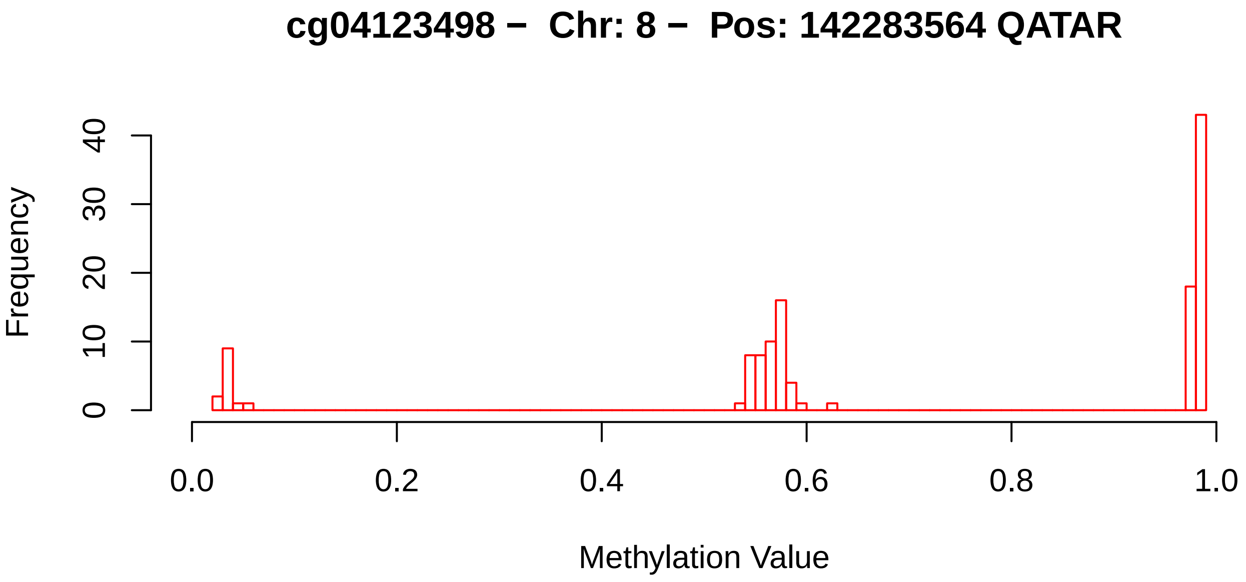
cg04123498 − Chr: 8 − Pos: 142283564 QATAR
Frequency
0
10
20
30
40
0.0
0.2
0.4
0.6
0.8
1.0
Methylation Value

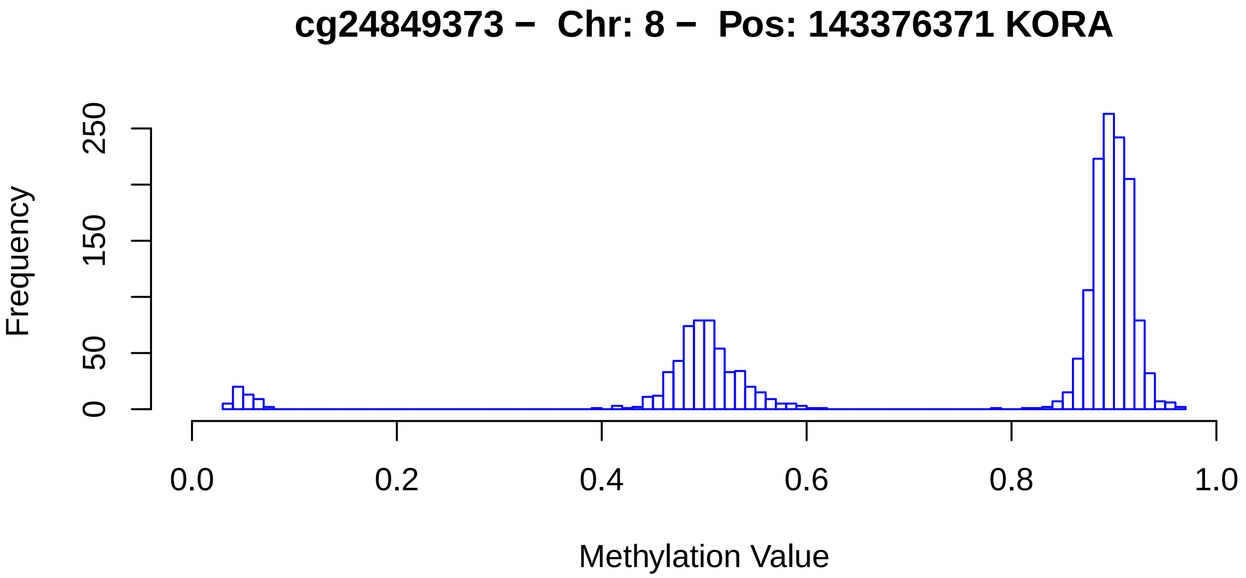
cg24849373 − Chr: 8 − Pos: 143376371 KORA
Frequency
0
50
150
250
0.0
0.2
0.4
0.6
0.8
1.0
Methylation Value

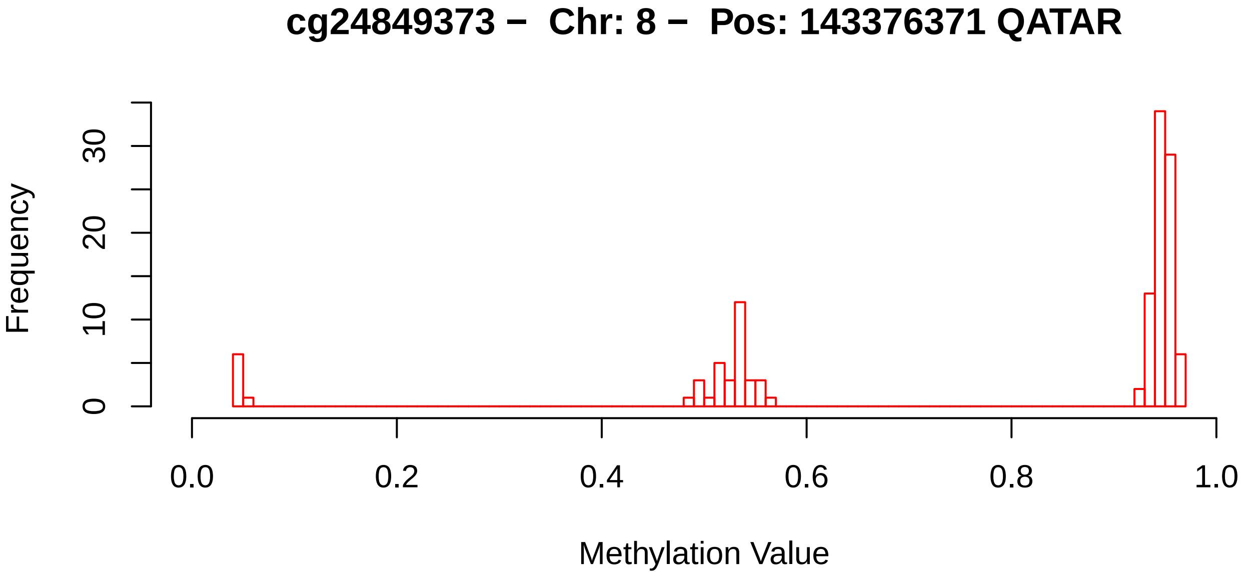
cg24849373 − Chr: 8 − Pos: 143376371 QATAR
Frequency
0
10
20
30
0.0
0.2
0.4
0.6
0.8
1.0
Methylation Value

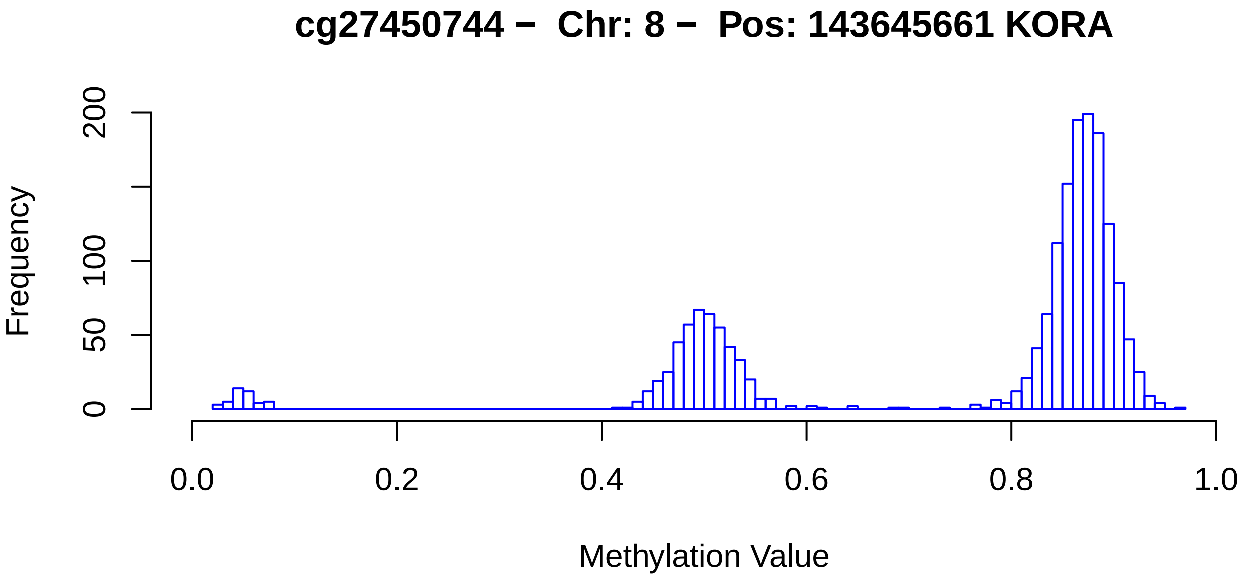

cg27450744 − Chr: 8 − Pos: 143645661 KORA
Frequency
0
50
100
200
0.0
0.2
0.4
0.6
0.8
1.0
Methylation Value

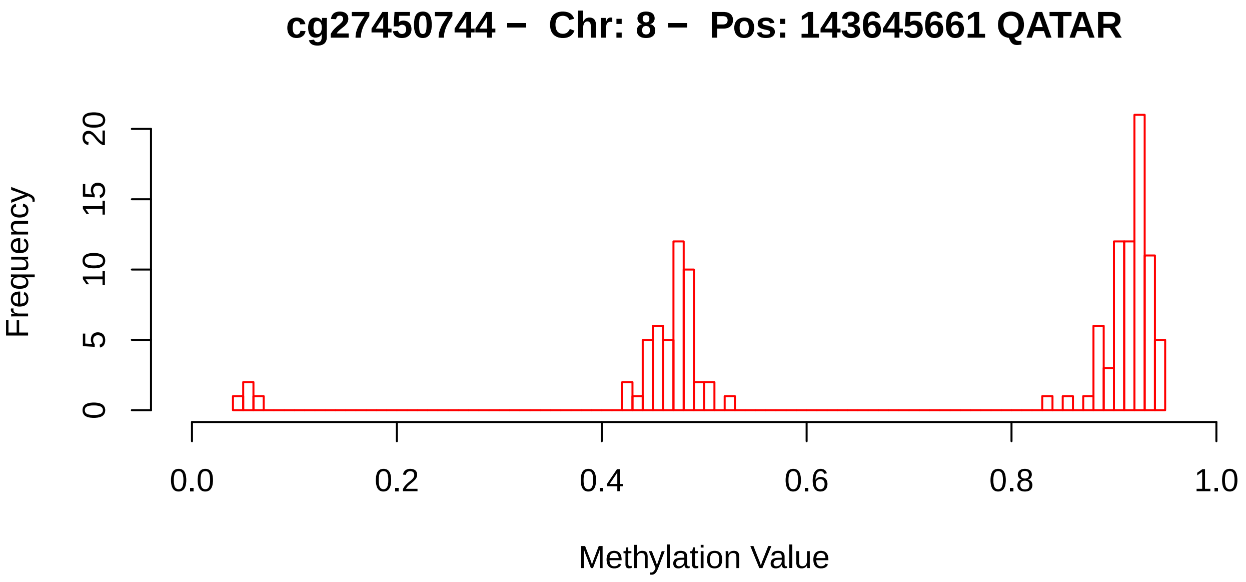

cg27450744 − Chr: 8 − Pos: 143645661 QATAR
Frequency
0
5
10
15
20
0.0
0.2
0.4
0.6
0.8
1.0
Methylation Value

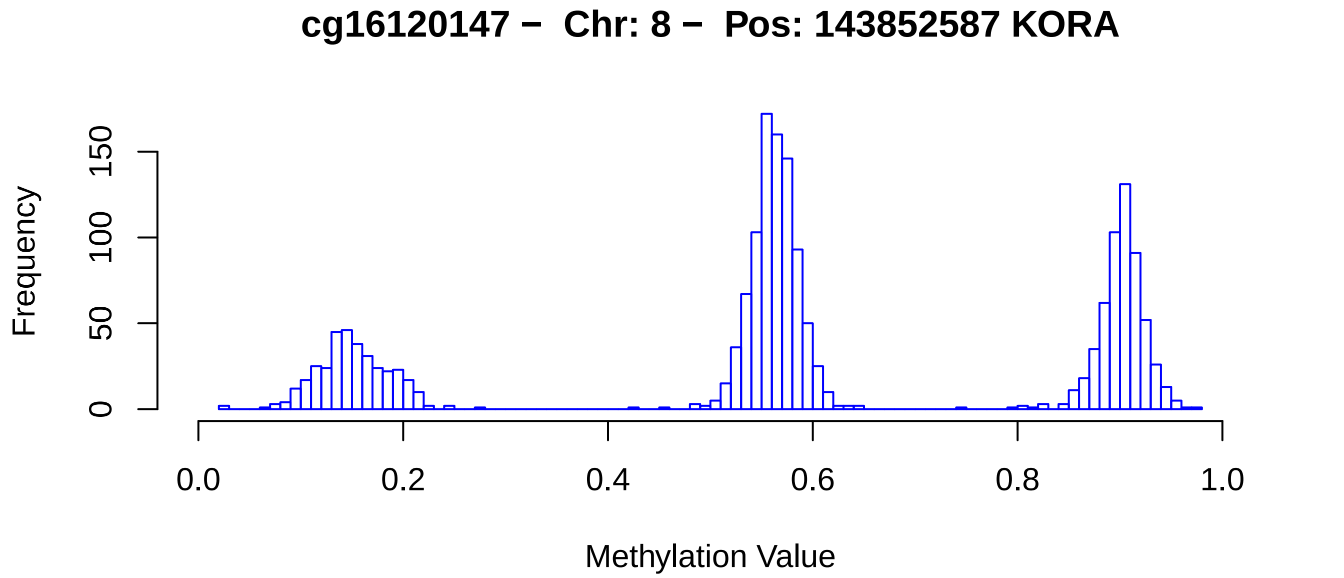
cg16120147 − Chr: 8 − Pos: 143852587 KORA
Frequency
0
50
100
150
0.0
0.2
0.4
0.6
0.8
1.0
Methylation Value

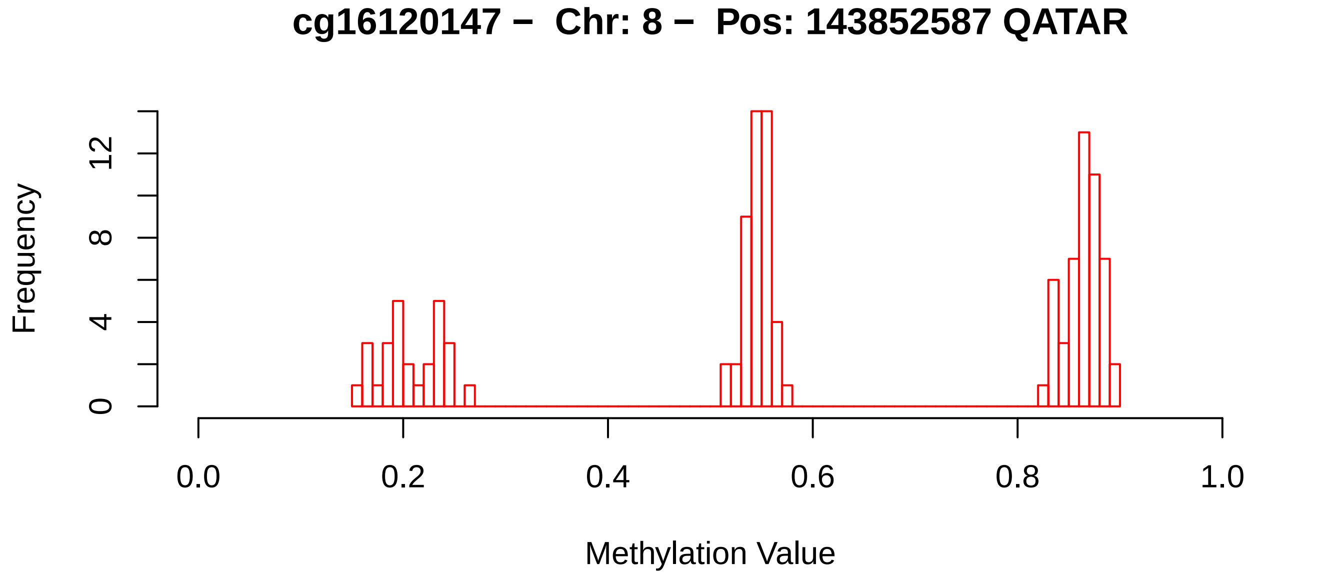
cg16120147 − Chr: 8 − Pos: 143852587 QATAR
Frequency
0
4
8
12
0.0
0.2
0.4
0.6
0.8
1.0
Methylation Value

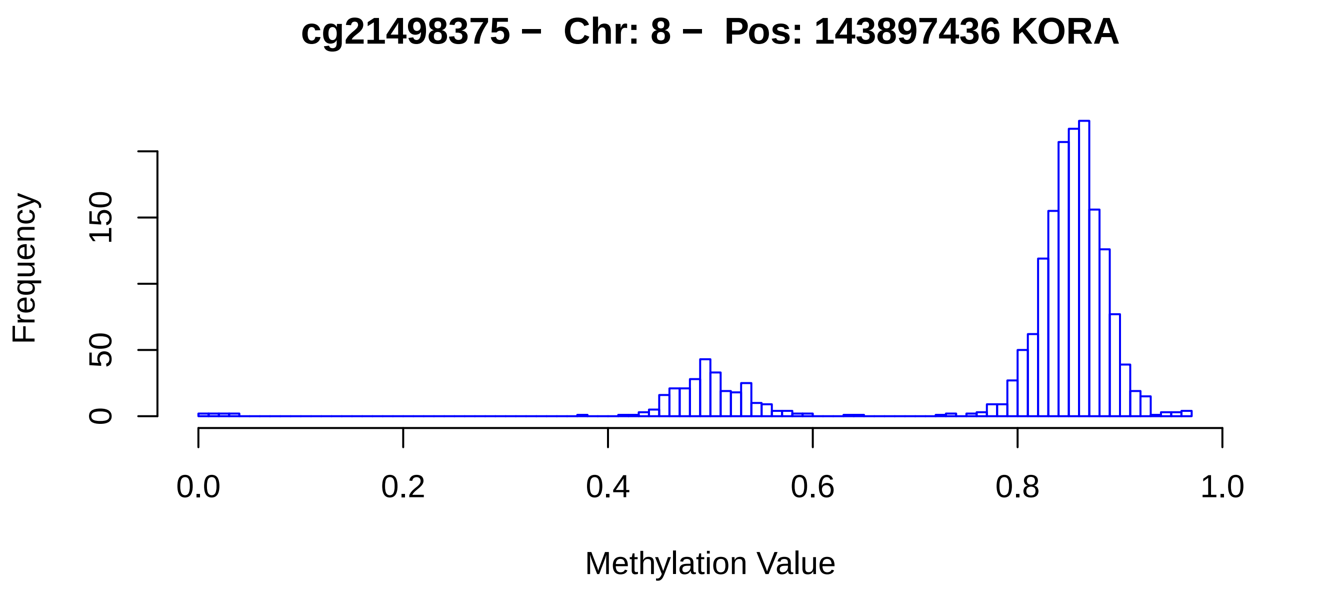
cg21498375 − Chr: 8 − Pos: 143897436 KORA
Frequency
0
50
150
0.0
0.2
0.4
0.6
0.8
1.0
Methylation Value

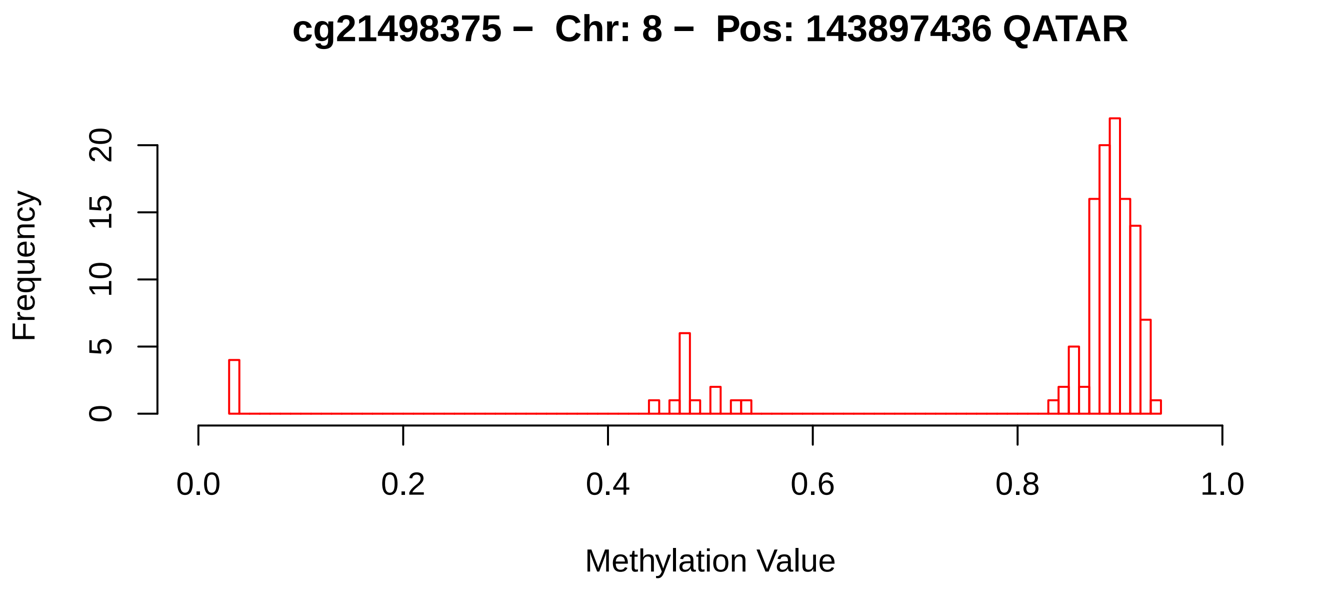
cg21498375 − Chr: 8 − Pos: 143897436 QATAR
Frequency
0
5
10
15
20
0.0
0.2
0.4
0.6
0.8
1.0
Methylation Value

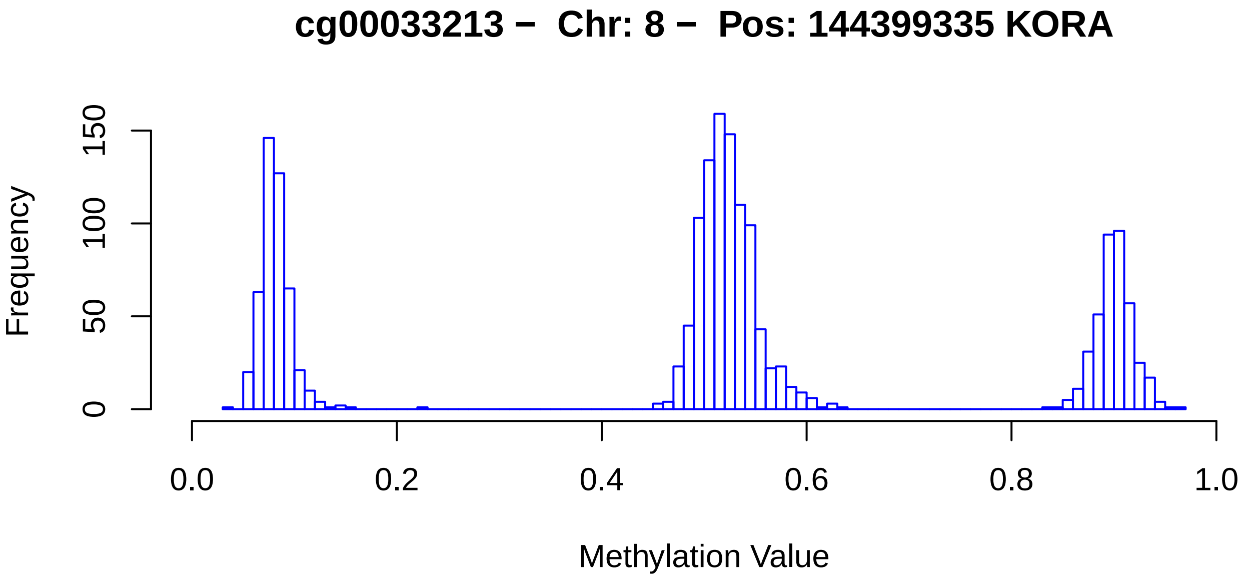
cg00033213 − Chr: 8 − Pos: 144399335 KORA
Frequency
0
50
100
150
0.0
0.2
0.4
0.6
0.8
1.0
Methylation Value

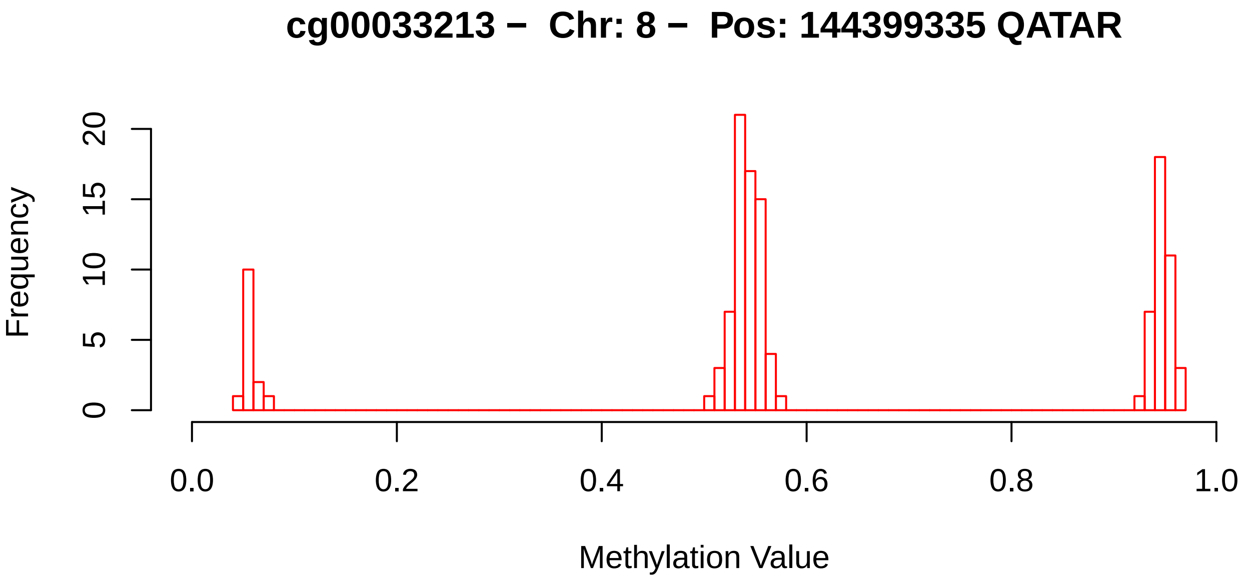
cg00033213 − Chr: 8 − Pos: 144399335 QATAR
Frequency
0
5
10
15
20
0.0
0.2
0.4
0.6
0.8
1.0
Methylation Value

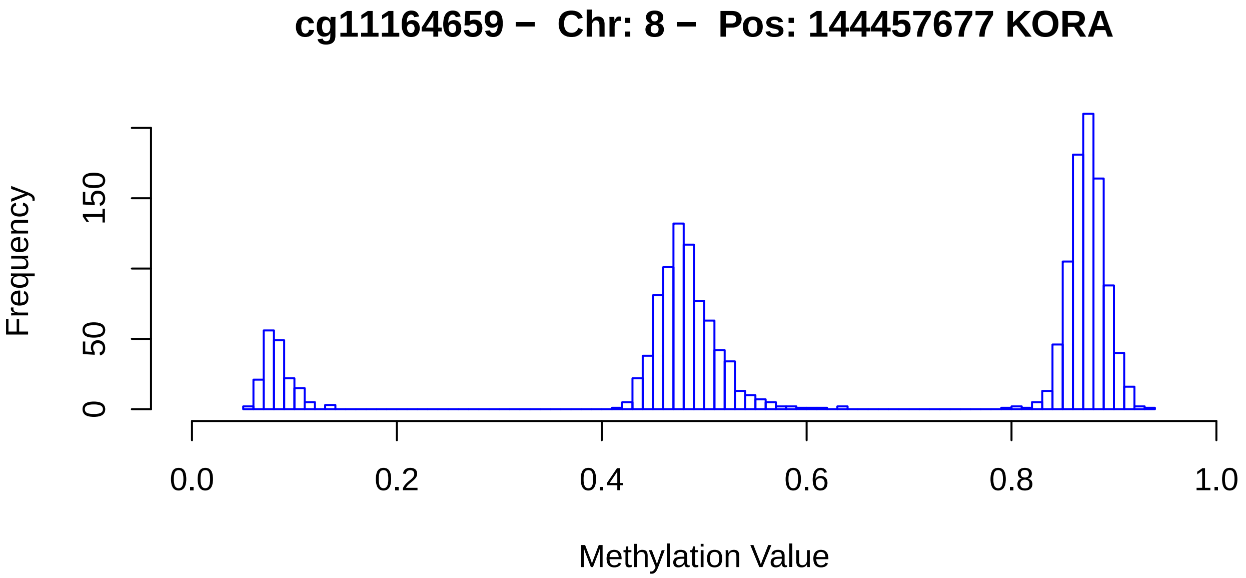
cg11164659 − Chr: 8 − Pos: 144457677 KORA
Frequency
0
50
150
0.0
0.2
0.4
0.6
0.8
1.0
Methylation Value

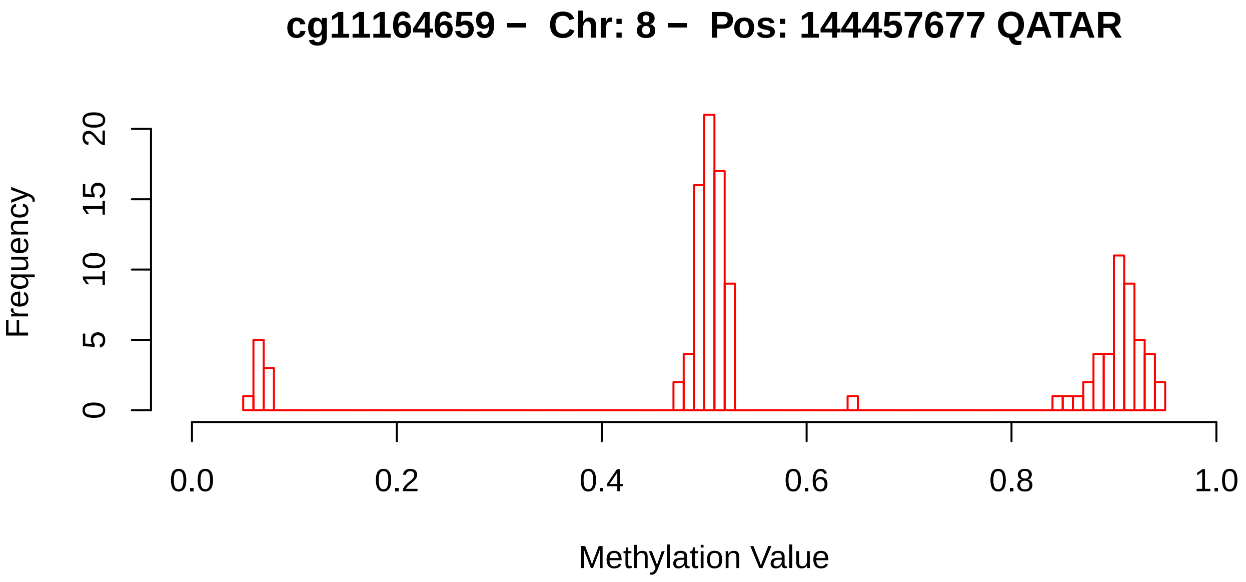
cg11164659 − Chr: 8 − Pos: 144457677 QATAR
Frequency
0
5
10
15
20
0.0
0.2
0.4
0.6
0.8
1.0
Methylation Value

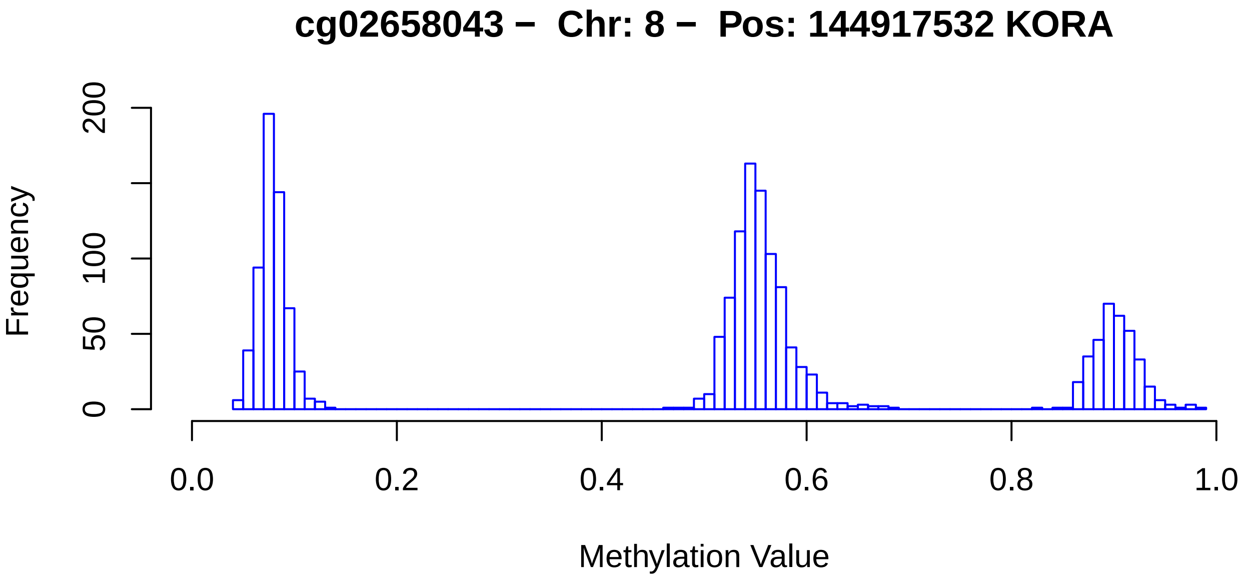
cg02658043 − Chr: 8 − Pos: 144917532 KORA
Frequency
0
50
100
200
0.0
0.2
0.4
0.6
0.8
1.0
Methylation Value

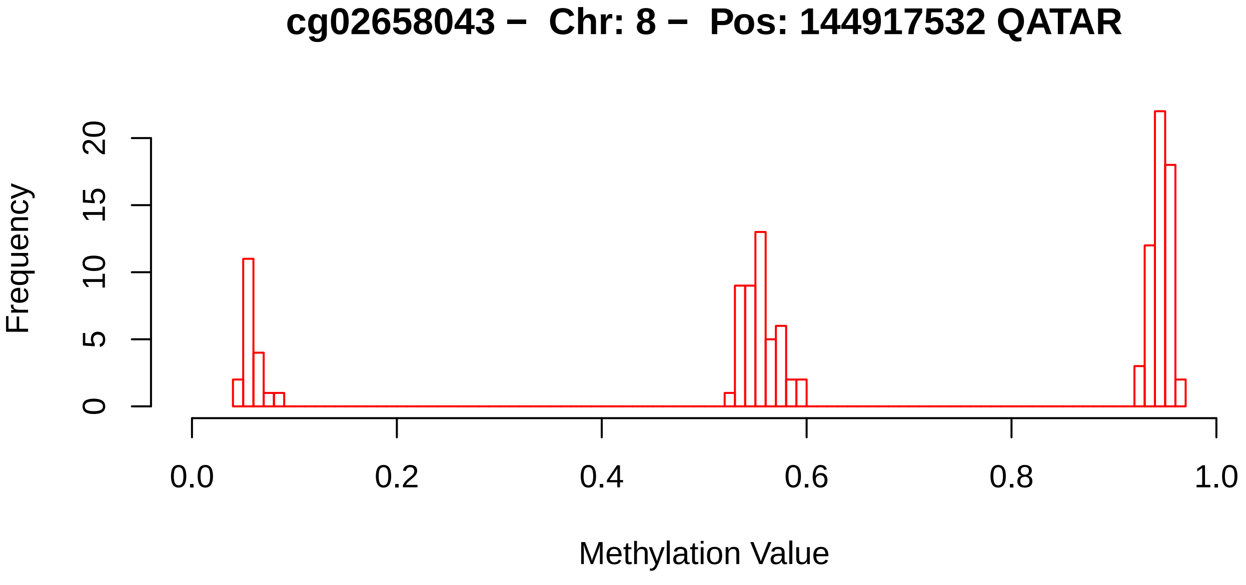
cg02658043 − Chr: 8 − Pos: 144917532 QATAR
Frequency
0
5
10
15
20
0.0
0.2
0.4
0.6
0.8
1.0
Methylation Value

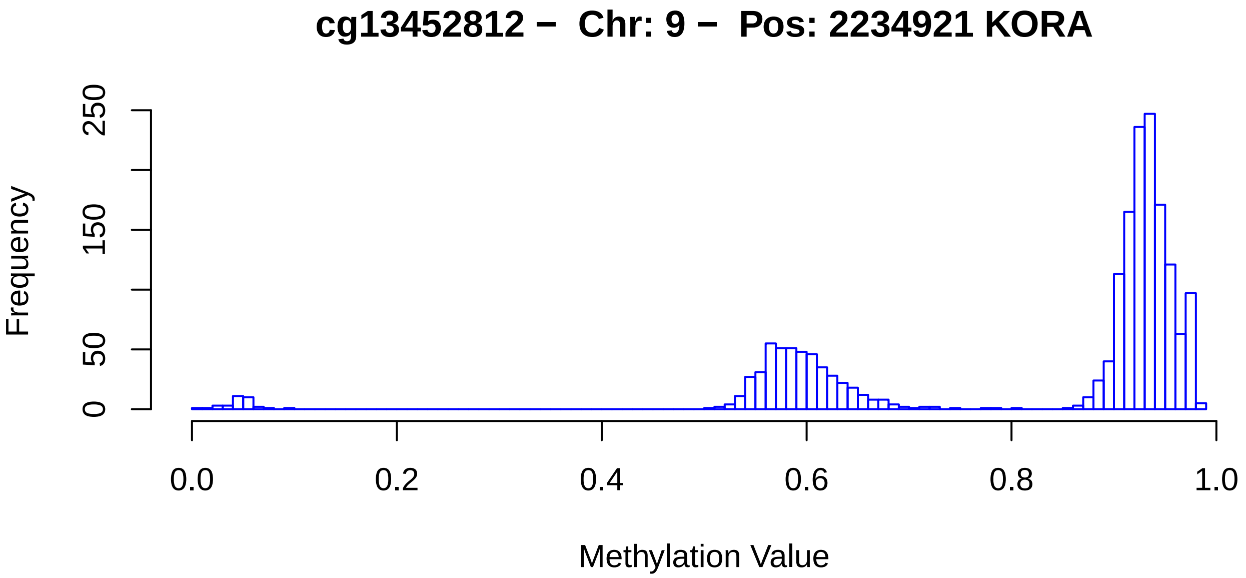
cg13452812 − Chr: 9 − Pos: 2234921 KORA
Frequency
0
50
150
250
0.0
0.2
0.4
0.6
0.8
1.0
Methylation Value

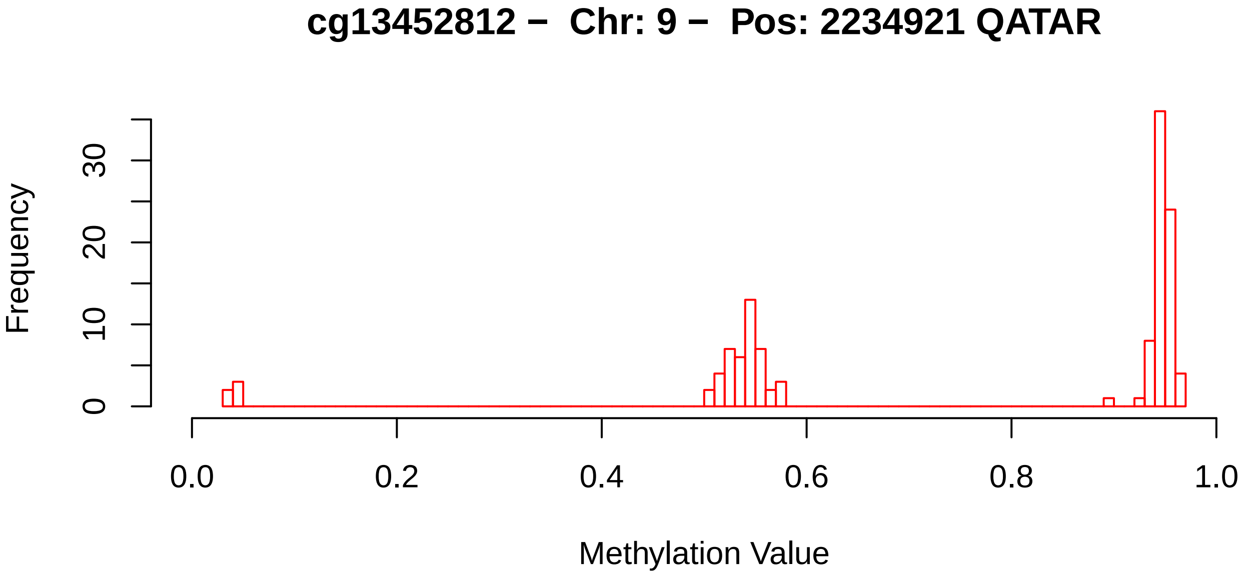
cg13452812 − Chr: 9 − Pos: 2234921 QATAR
Frequency
0
10
20
30
0.0
0.2
0.4
0.6
0.8
1.0
Methylation Value

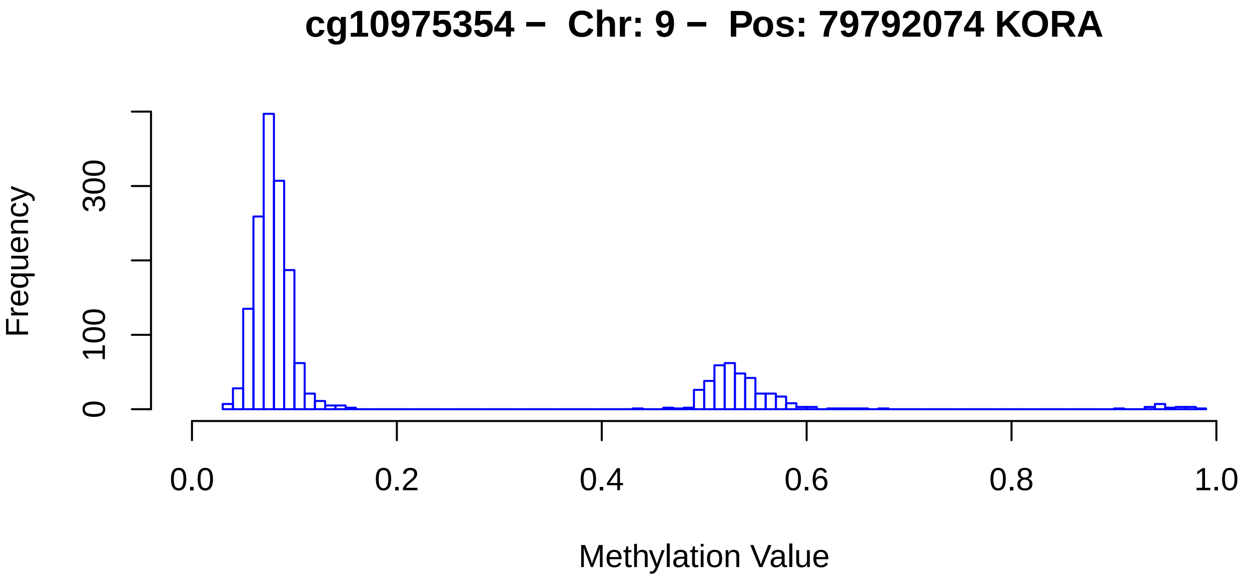
cg10975354 − Chr: 9 − Pos: 79792074 KORA
Frequency
0
100
300
0.0
0.2
0.4
0.6
0.8
1.0
Methylation Value

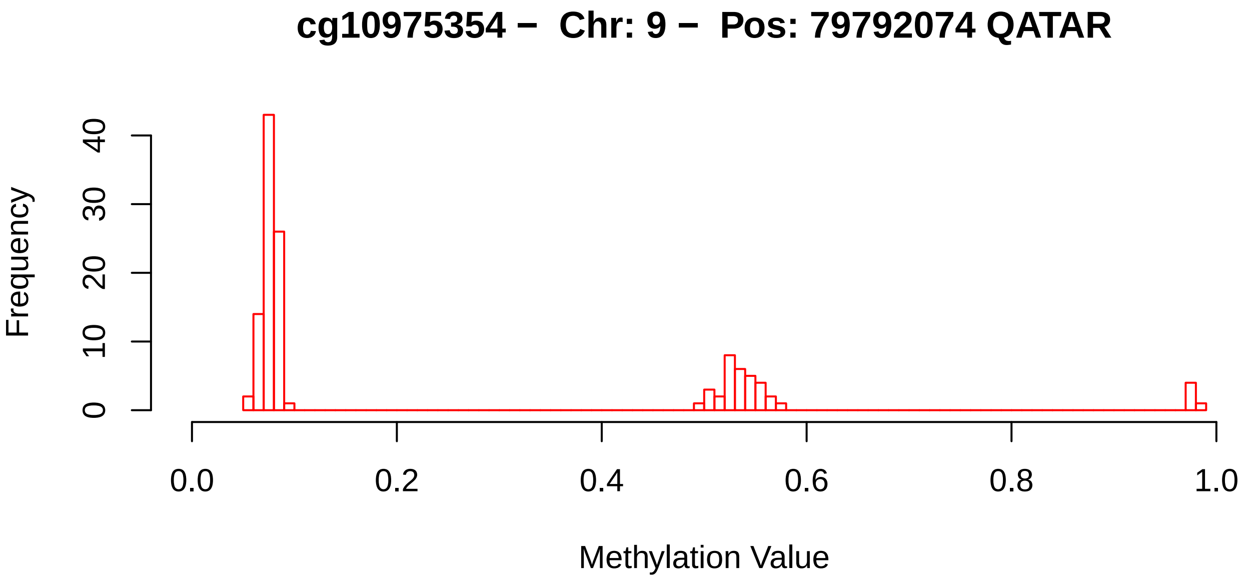
cg10975354 − Chr: 9 − Pos: 79792074 QATAR
Frequency
0
10
20
30
40
0.0
0.2
0.4
0.6
0.8
1.0
Methylation Value

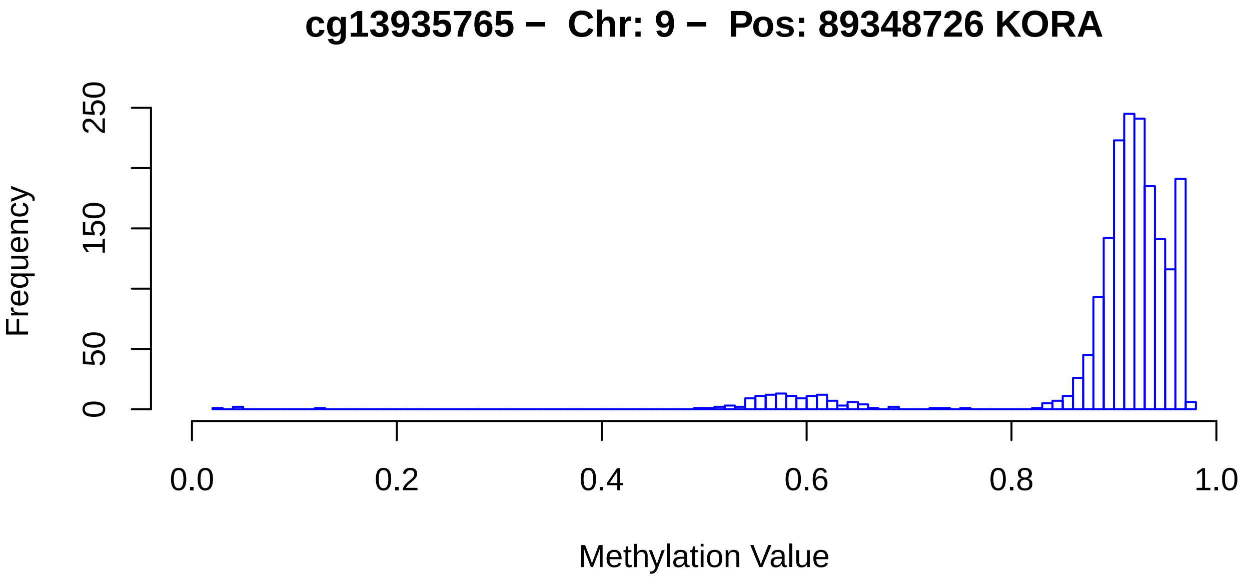
cg13935765 − Chr: 9 − Pos: 89348726 KORA
Frequency
0
50
150
250
0.0
0.2
0.4
0.6
0.8
1.0
Methylation Value

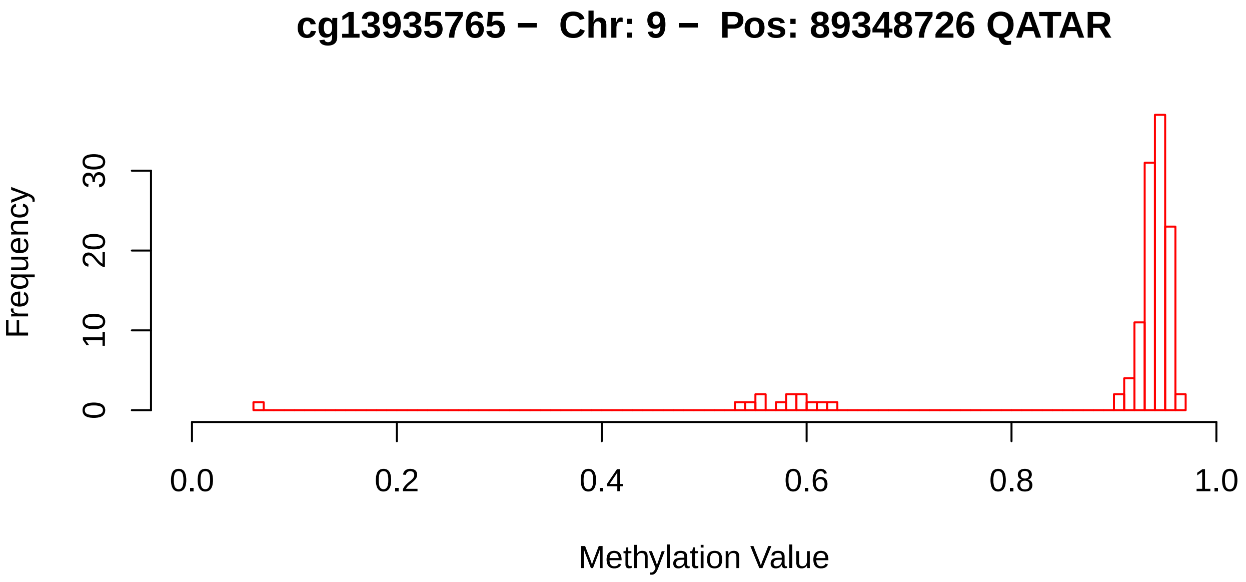
cg13935765 − Chr: 9 − Pos: 89348726 QATAR
Frequency
0
10
20
30
0.0
0.2
0.4
0.6
0.8
1.0
Methylation Value

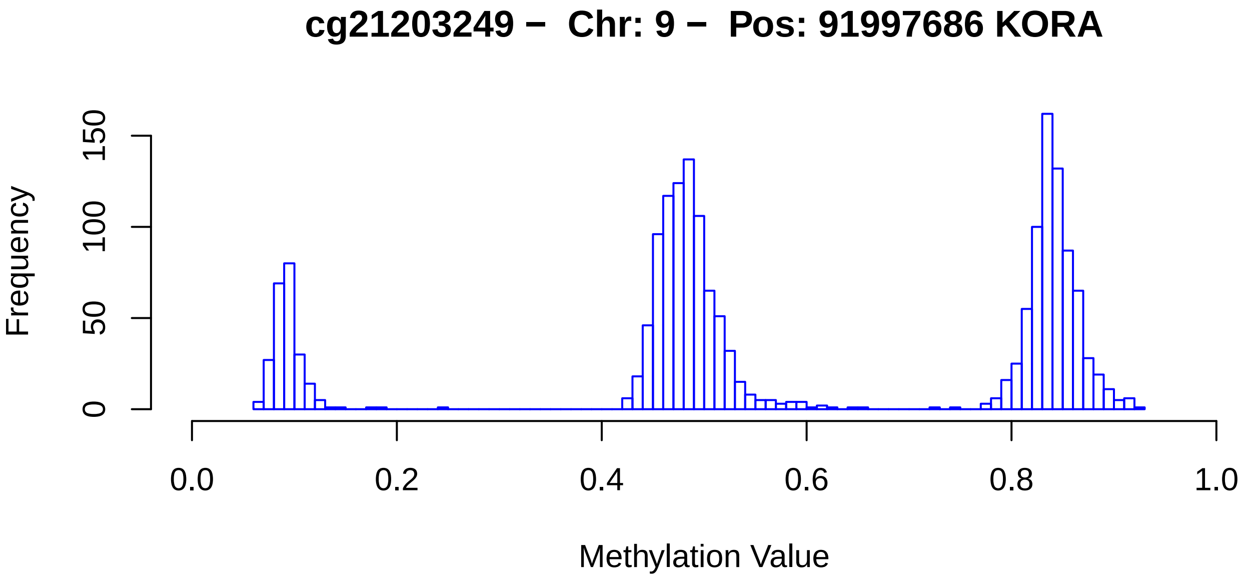
cg21203249 − Chr: 9 − Pos: 91997686 KORA
Frequency
0
50
100
150
0.0
0.2
0.4
0.6
0.8
1.0
Methylation Value

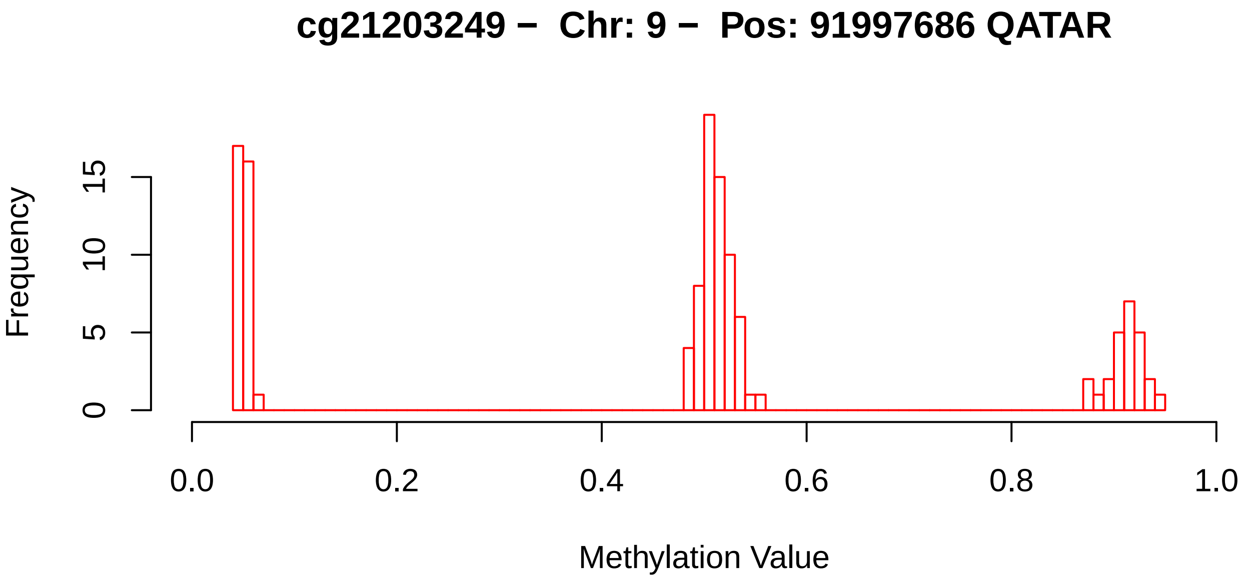
cg21203249 − Chr: 9 − Pos: 91997686 QATAR
Frequency
0
5
10
15
0.0
0.2
0.4
0.6
0.8
1.0
Methylation Value

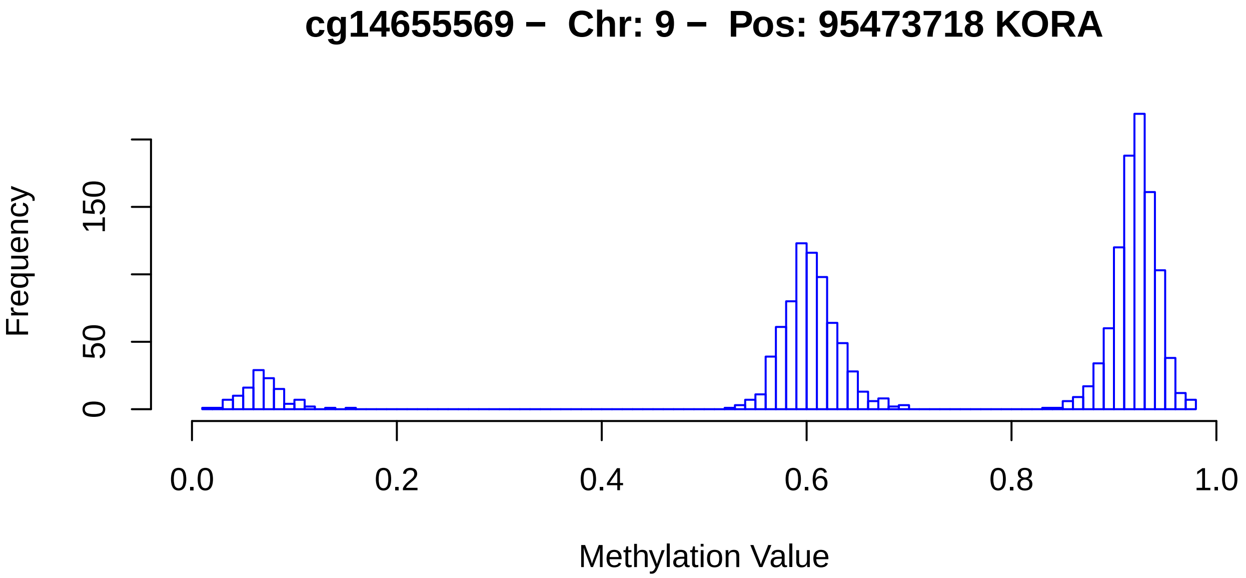
cg14655569 − Chr: 9 − Pos: 95473718 KORA
Frequency
0
50
150
0.0
0.2
0.4
0.6
0.8
1.0
Methylation Value

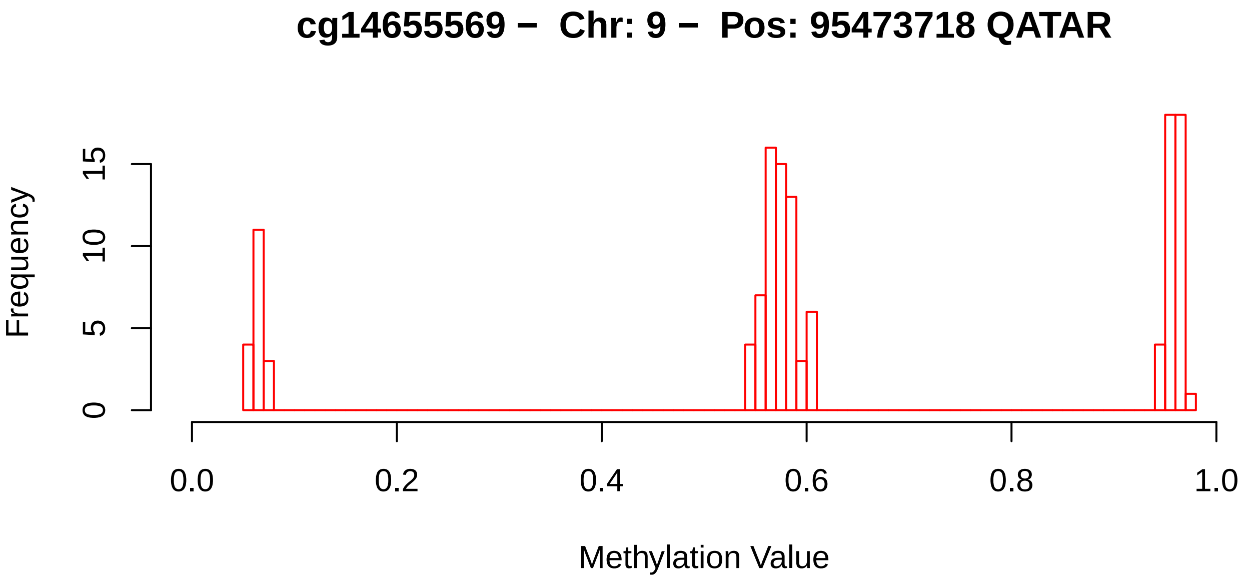
cg14655569 − Chr: 9 − Pos: 95473718 QATAR
Frequency
0
5
10
15
0.0
0.2
0.4
0.6
0.8
1.0
Methylation Value

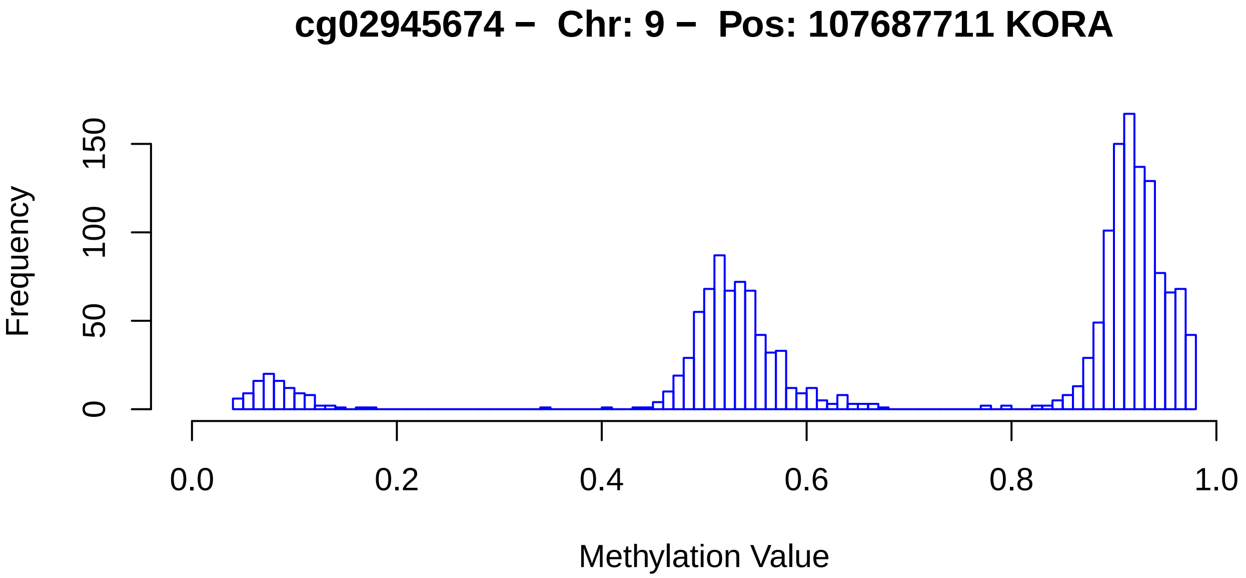
cg02945674 − Chr: 9 − Pos: 107687711 KORA
Frequency
0
50
100
150
0.0
0.2
0.4
0.6
0.8
1.0
Methylation Value

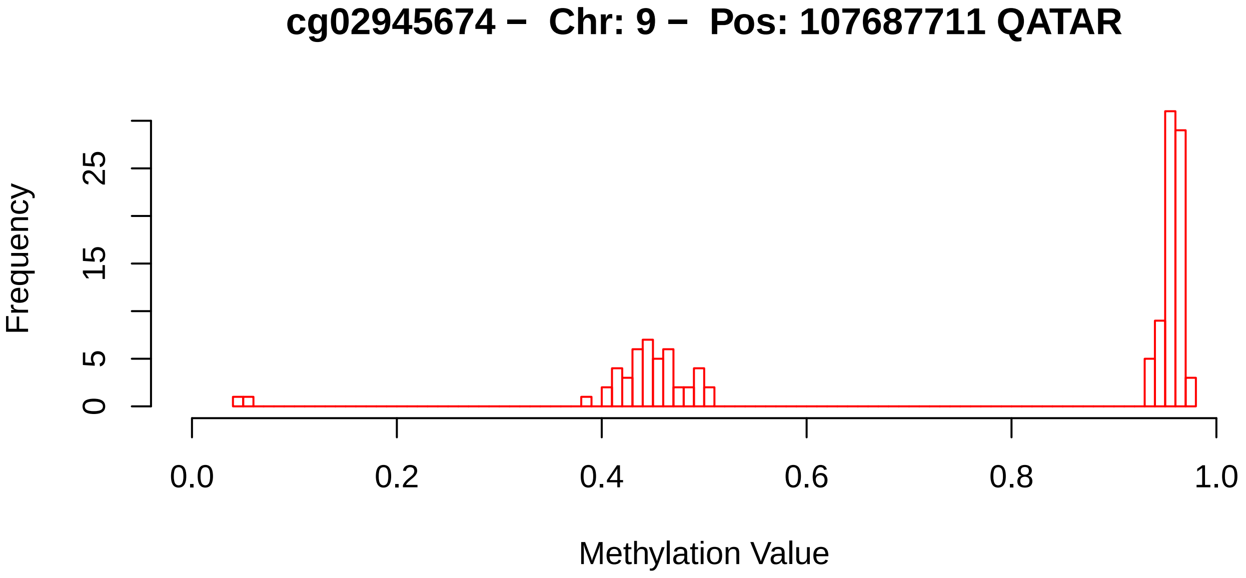
cg02945674 − Chr: 9 − Pos: 107687711 QATAR
Frequency
0
5
15
25
0.0
0.2
0.4
0.6
0.8
1.0
Methylation Value

cg14056849 − Chr: 9 − Pos: 115771599 KORA

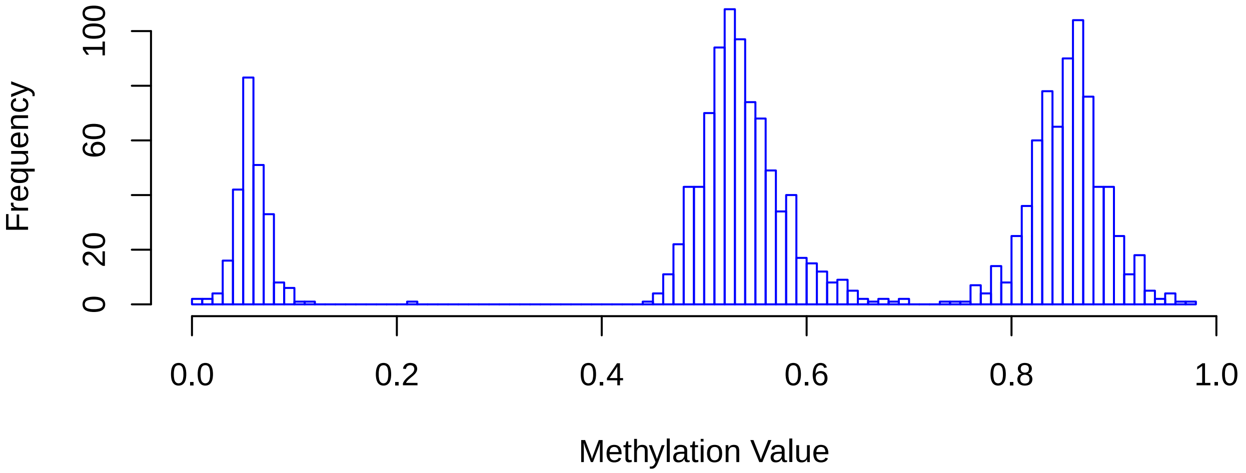

cg14056849 − Chr: 9 − Pos: 115771599 QATAR

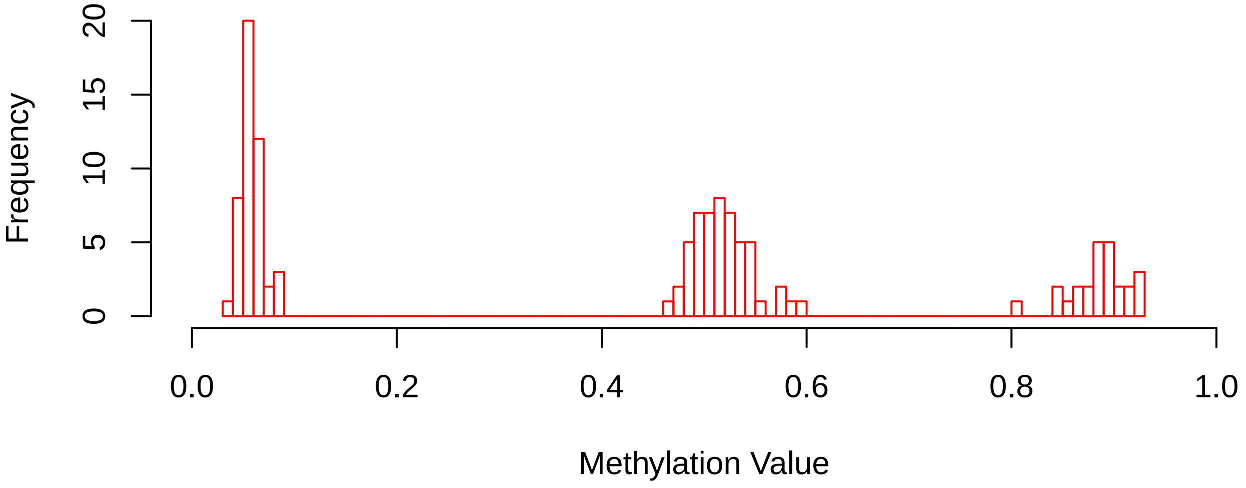

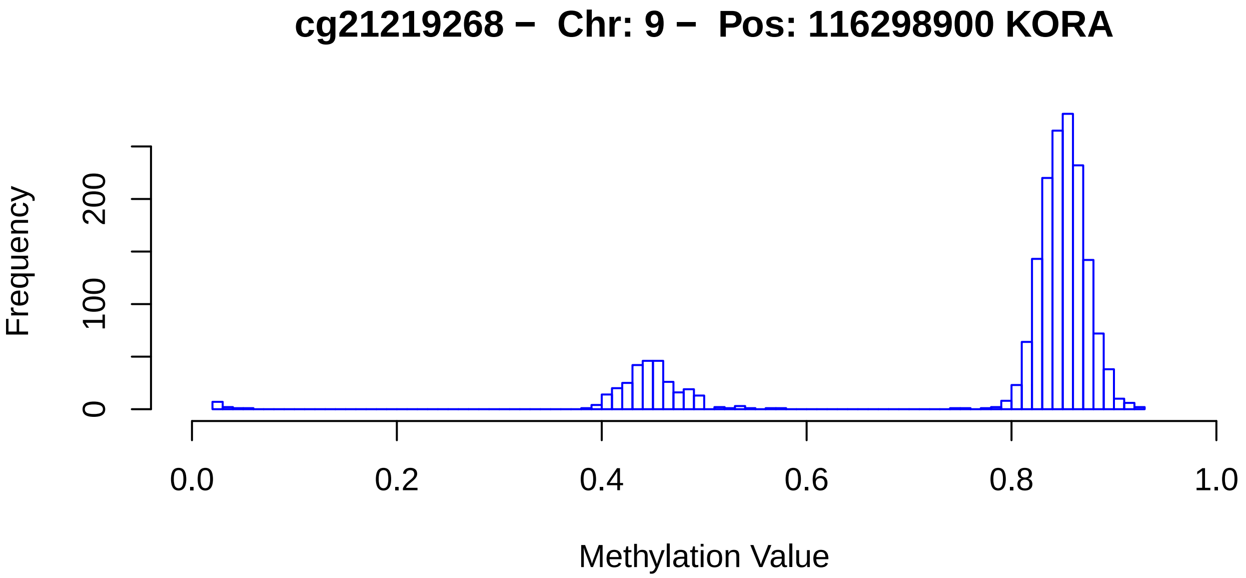
cg21219268 − Chr: 9 − Pos: 116298900 KORA
Frequency
0
100
200
0.0
0.2
0.4
0.6
0.8
1.0
Methylation Value

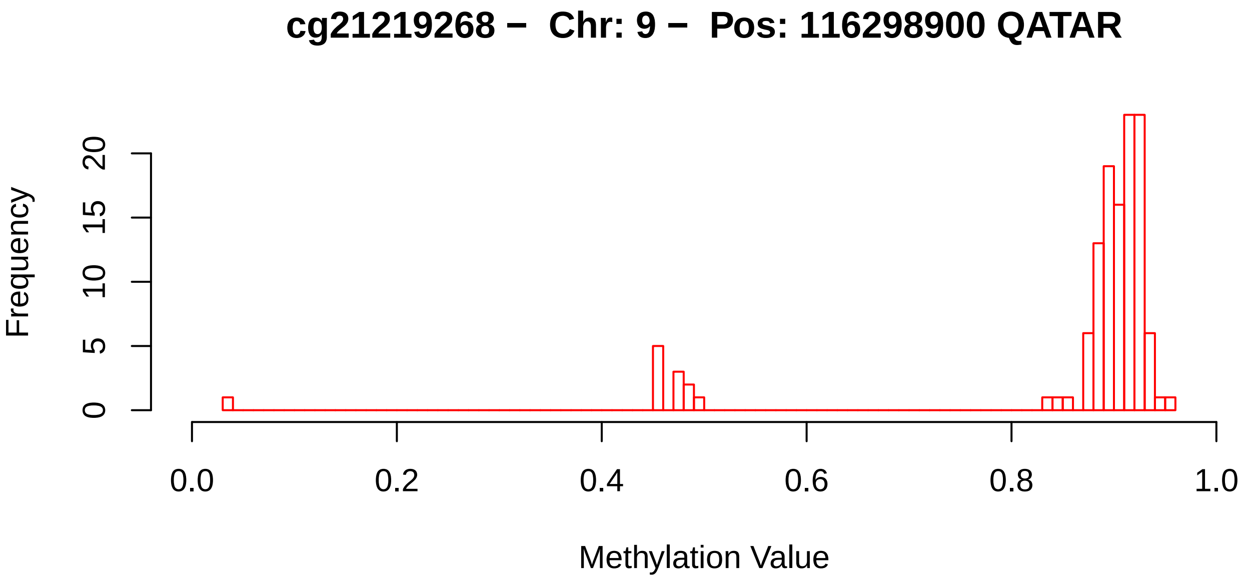
cg21219268 − Chr: 9 − Pos: 116298900 QATAR
Frequency
0
5
10
15
20
0.0
0.2
0.4
0.6
0.8
1.0
Methylation Value

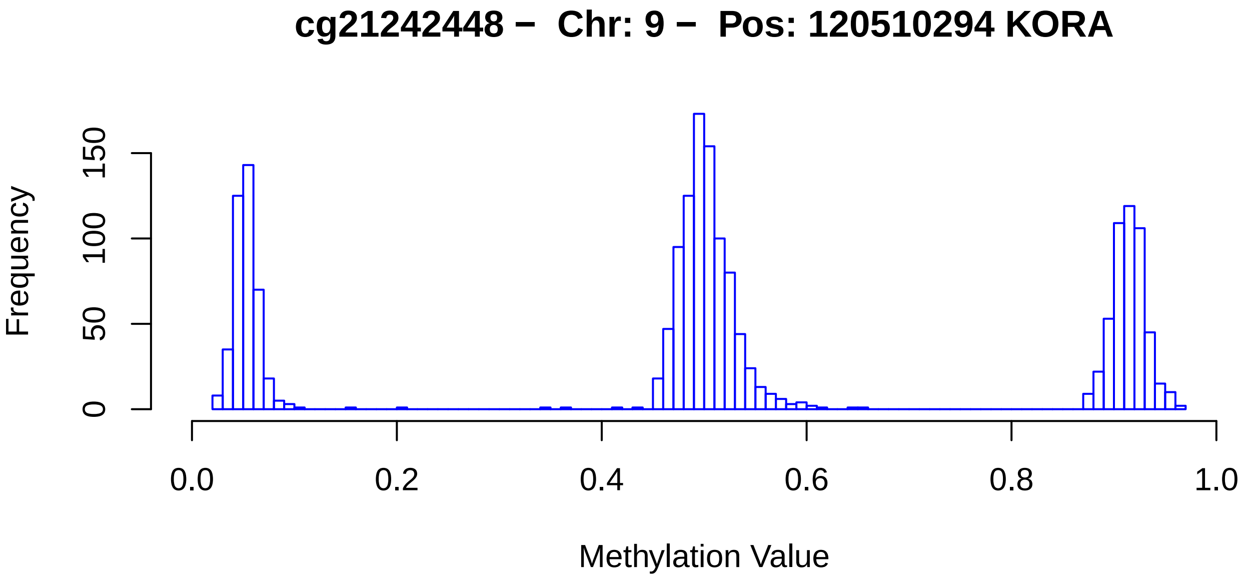
cg21242448 − Chr: 9 − Pos: 120510294 KORA
Frequency
0
50
100
150
0.0
0.2
0.4
0.6
0.8
1.0
Methylation Value

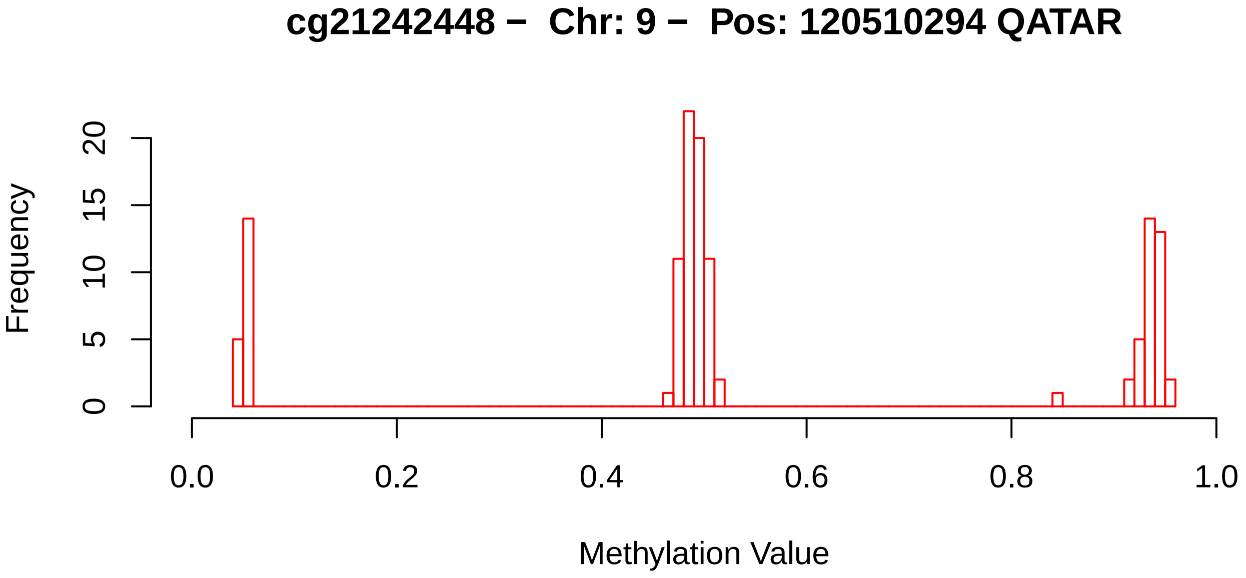
cg21242448 − Chr: 9 − Pos: 120510294 QATAR
Frequency
0
5
10
15
20
0.0
0.2
0.4
0.6
0.8
1.0
Methylation Value

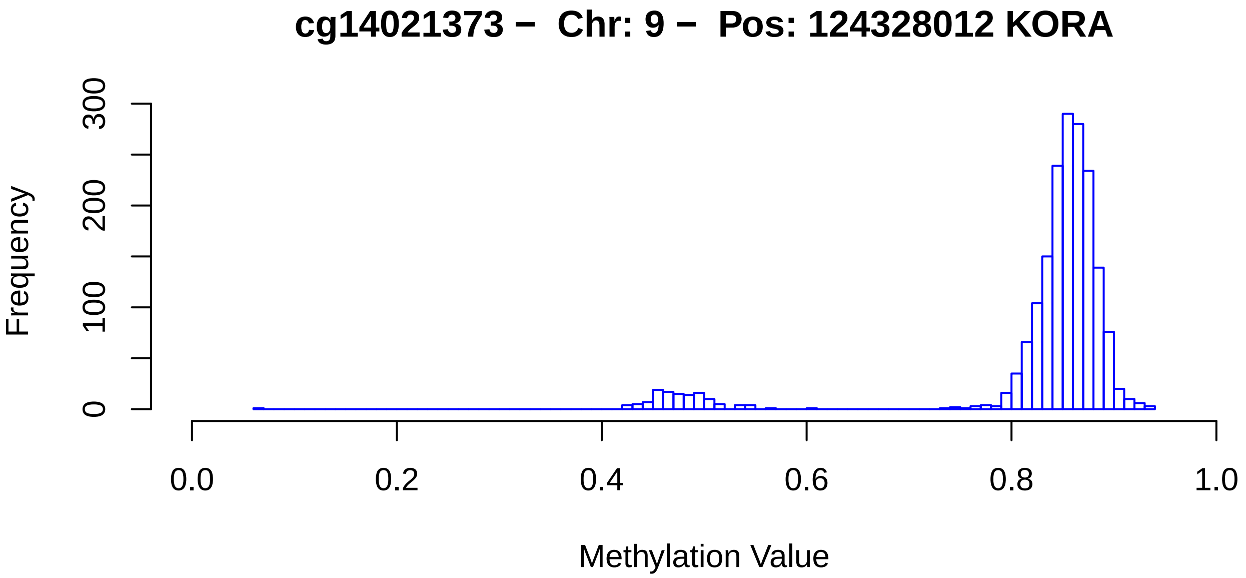
cg14021373 − Chr: 9 − Pos: 124328012 KORA
Frequency
0
100
200
300
0.0
0.2
0.4
0.6
0.8
1.0
Methylation Value

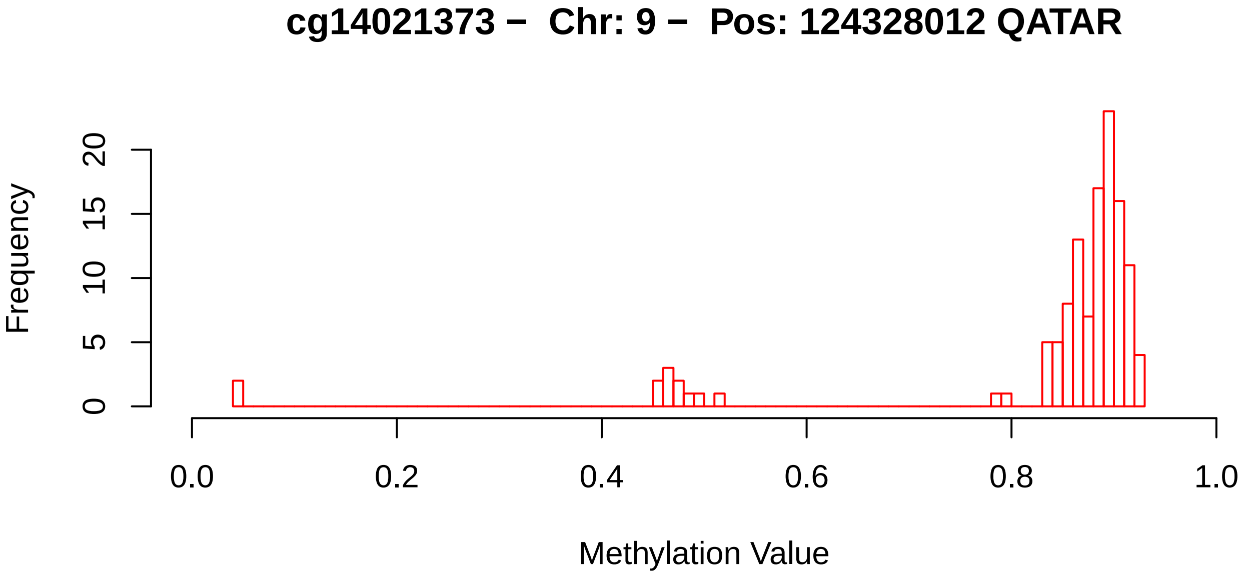
cg14021373 − Chr: 9 − Pos: 124328012 QATAR
Frequency
0
5
10
15
20
0.0
0.2
0.4
0.6
0.8
1.0
Methylation Value

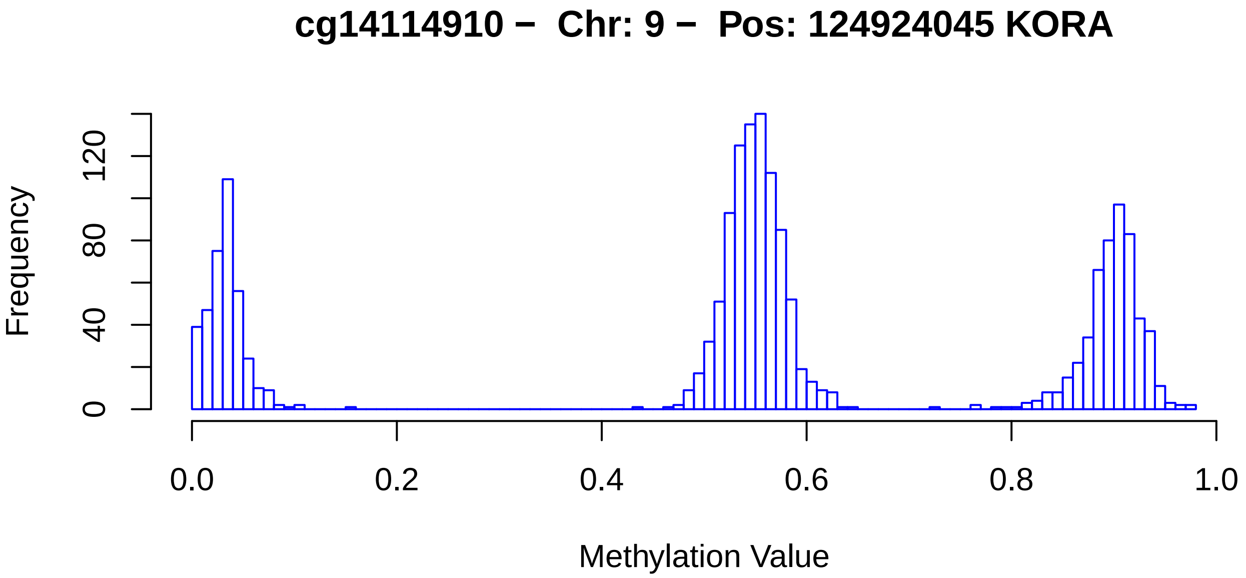
cg14114910 − Chr: 9 − Pos: 124924045 KORA
Frequency
0
40
80
120
0.0
0.2
0.4
0.6
0.8
1.0
Methylation Value

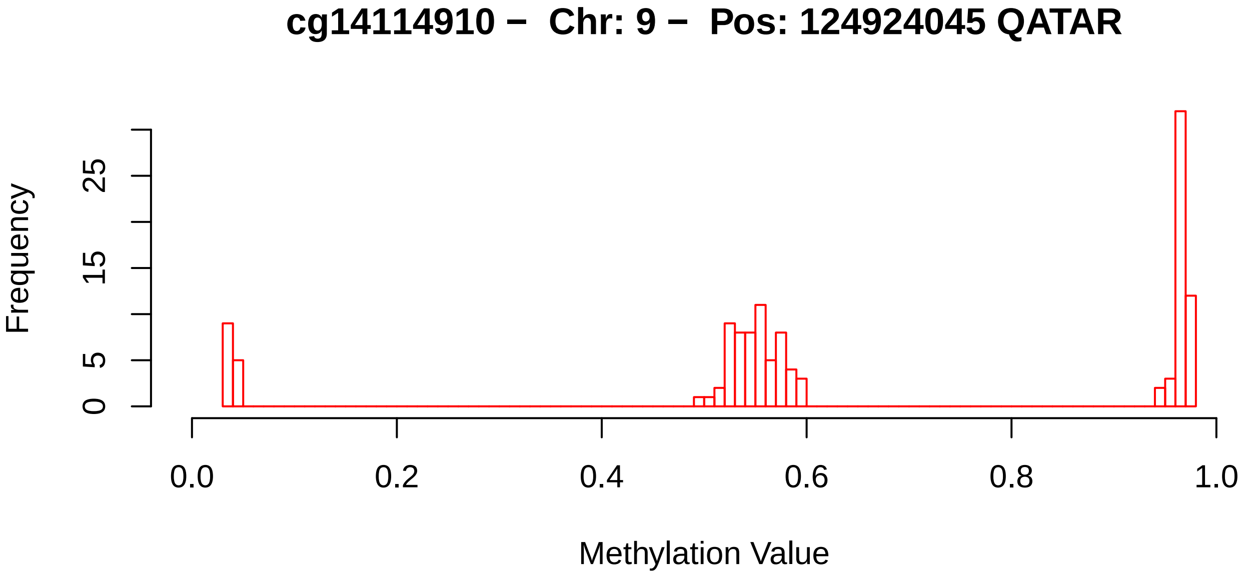
cg14114910 − Chr: 9 − Pos: 124924045 QATAR
Frequency
0
5
15
25
0.0
0.2
0.4
0.6
0.8
1.0
Methylation Value

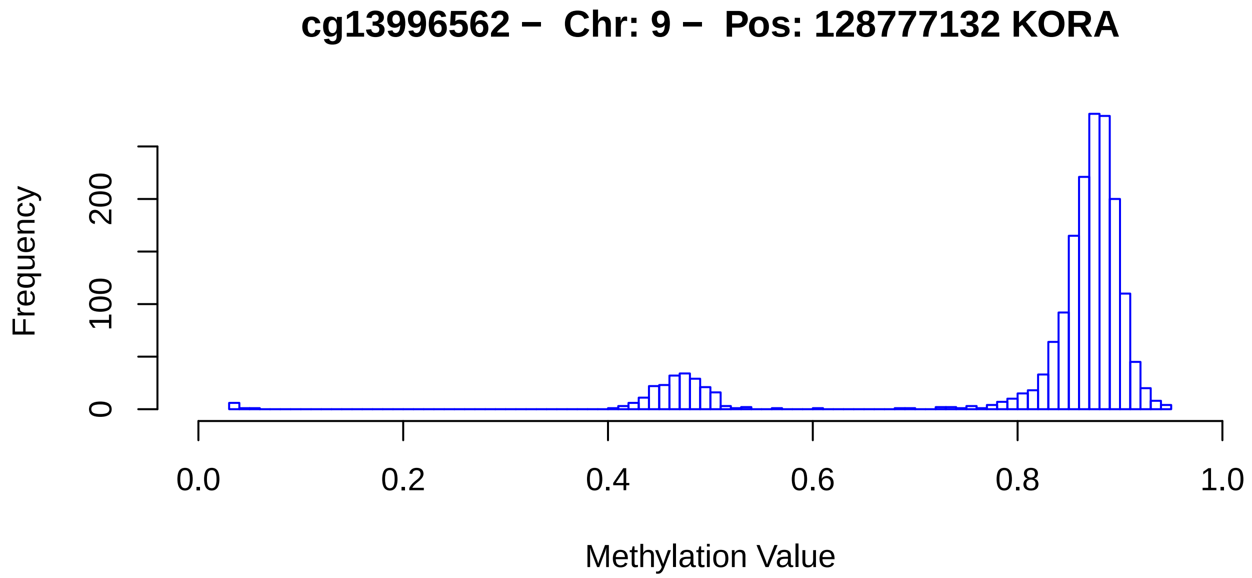
cg13996562 − Chr: 9 − Pos: 128777132 KORA
Frequency
0
100
200
0.0
0.2
0.4
0.6
0.8
1.0
Methylation Value

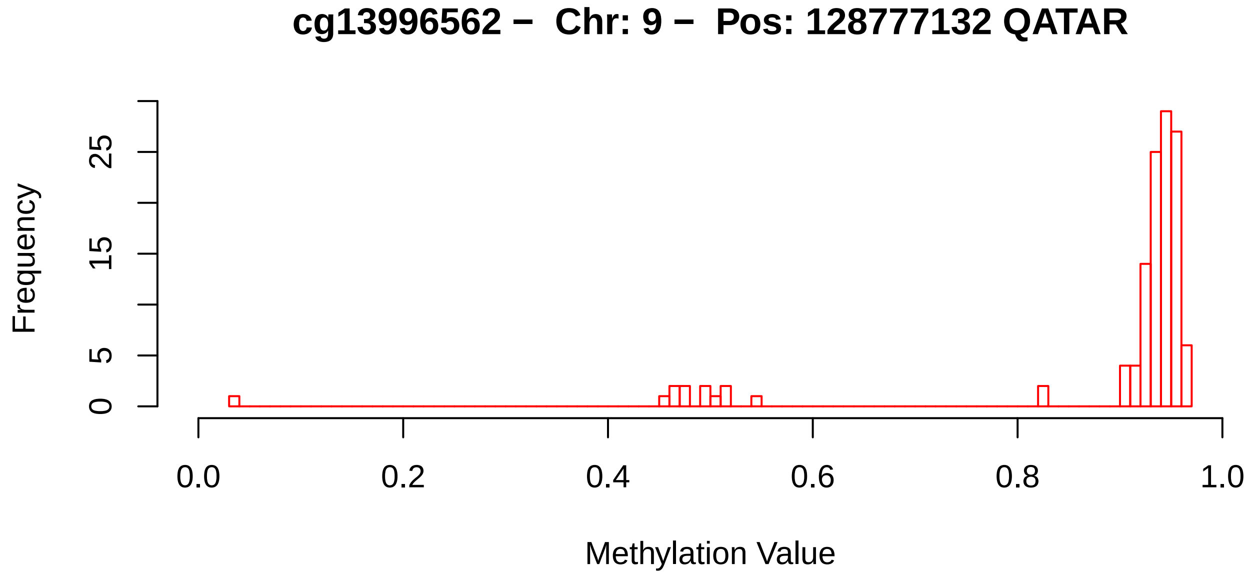
cg13996562 − Chr: 9 − Pos: 128777132 QATAR
Frequency
0
5
15
25
0.0
0.2
0.4
0.6
0.8
1.0
Methylation Value

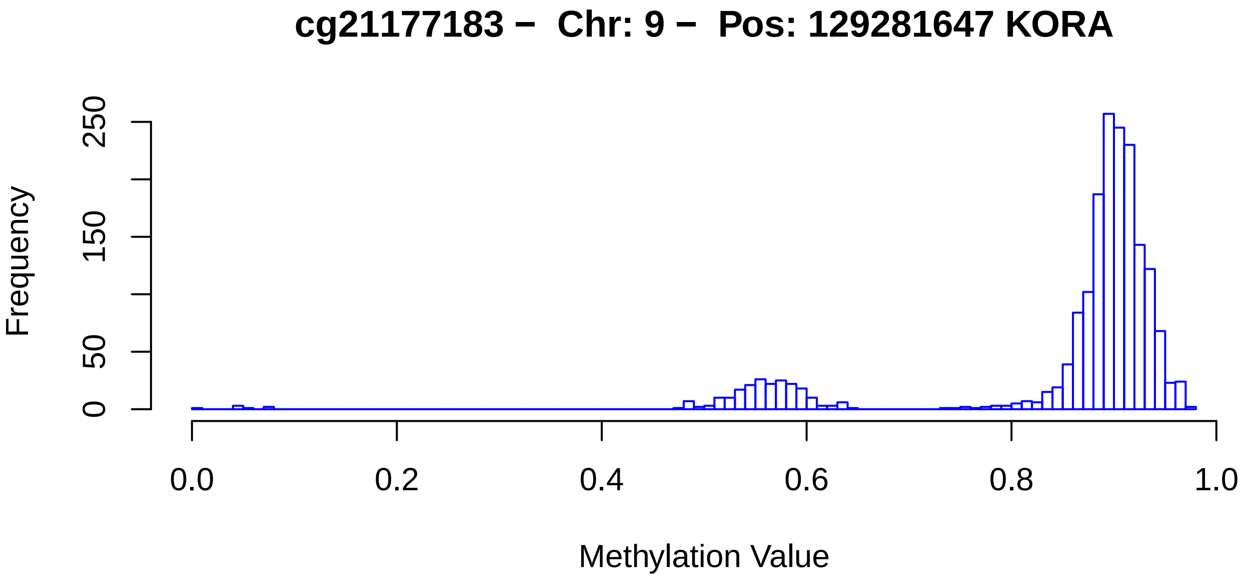
cg21177183 − Chr: 9 − Pos: 129281647 KORA
Frequency
0
50
150
250
0.0
0.2
0.4
0.6
0.8
1.0
Methylation Value

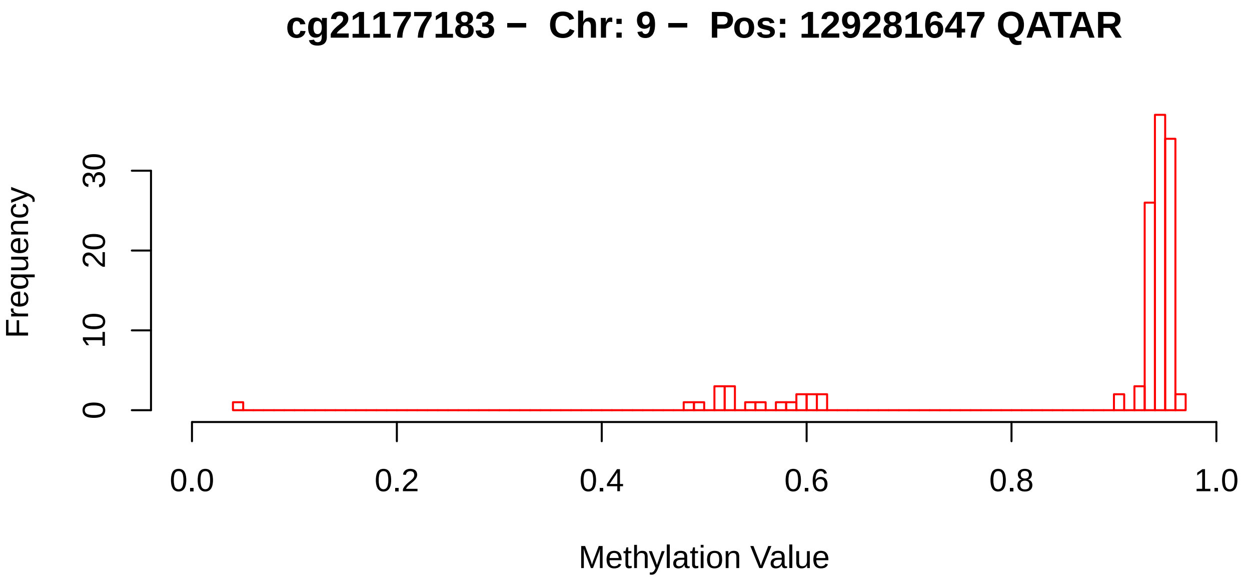
cg21177183 − Chr: 9 − Pos: 129281647 QATAR
Frequency
0
10
20
30
0.0
0.2
0.4
0.6
0.8
1.0
Methylation Value

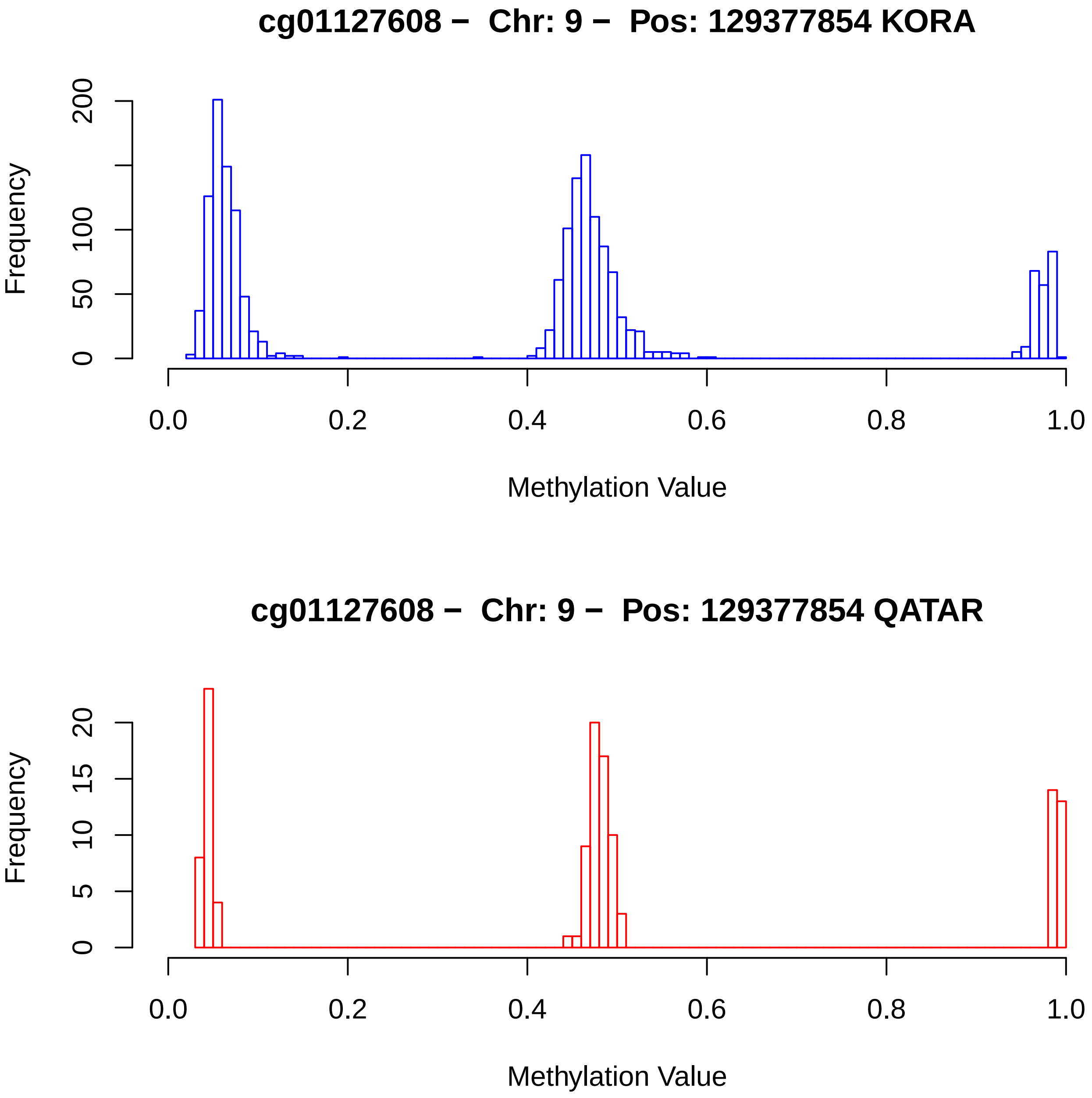
cg01127608 − Chr: 9 − Pos: 129377854 KORA
Frequency
0
50
100
200
0.0
0.2
0.4
0.6
0.8
1.0
Methylation Value
cg01127608 − Chr: 9 − Pos: 129377854 QATAR
Frequency
0
5
10
15
20
0.0
0.2
0.4
0.6
0.8
1.0
Methylation Value

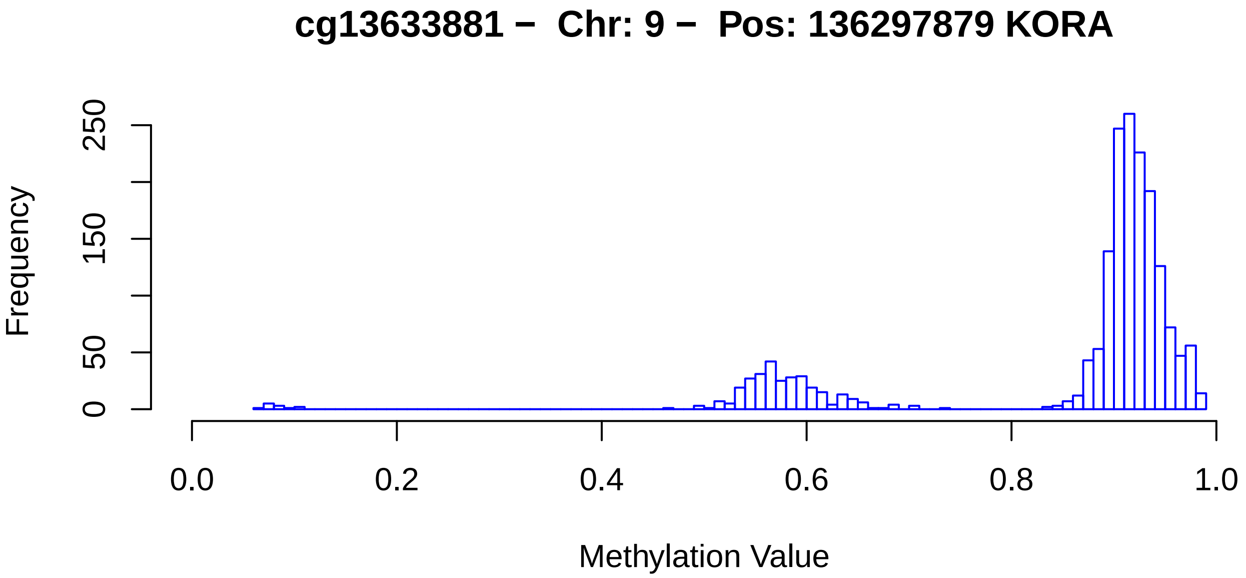
cg13633881 − Chr: 9 − Pos: 136297879 KORA
Frequency
0
50
150
250
0.0
0.2
0.4
0.6
0.8
1.0
Methylation Value

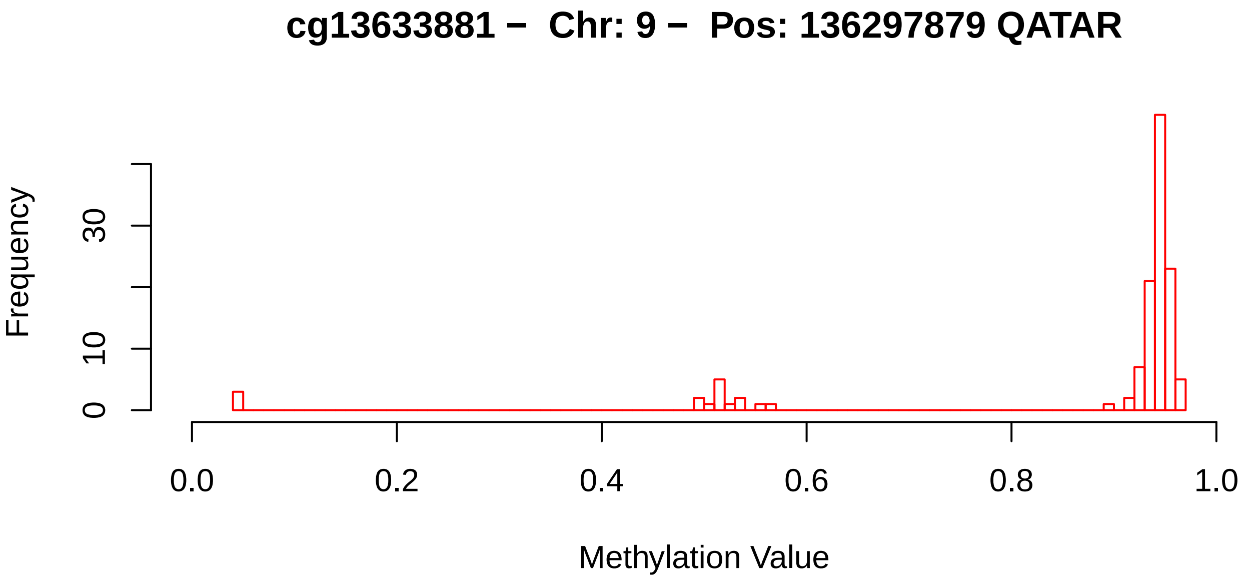
cg13633881 − Chr: 9 − Pos: 136297879 QATAR
Frequency
0
10
30
0.0
0.2
0.4
0.6
0.8
1.0
Methylation Value

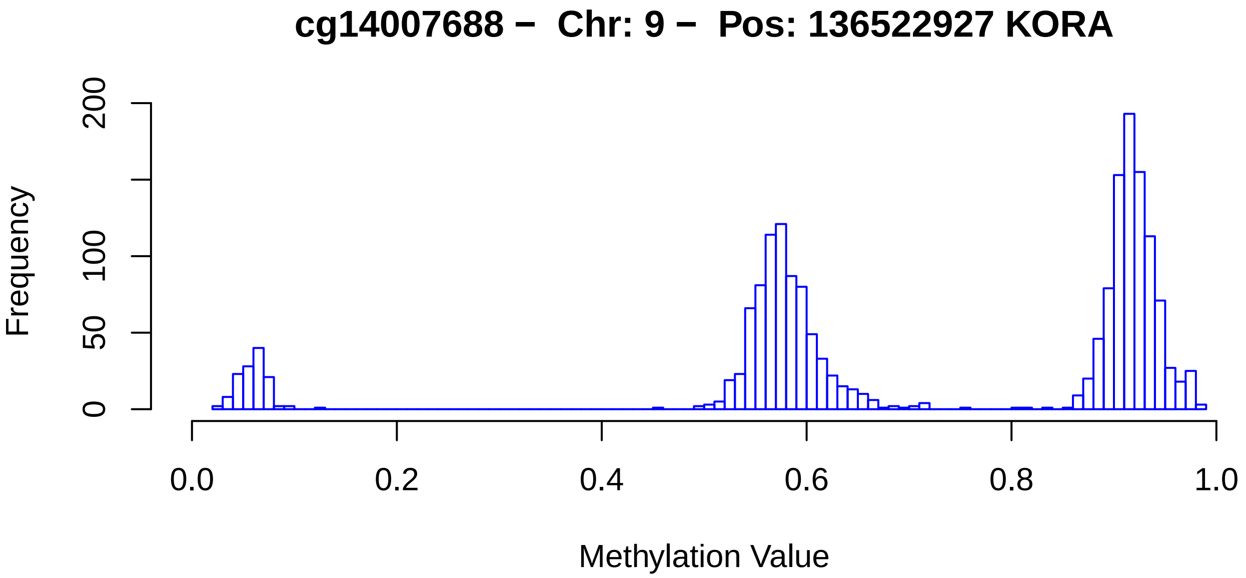
cg14007688 − Chr: 9 − Pos: 136522927 KORA
Frequency
0
50
100
200
0.0
0.2
0.4
0.6
0.8
1.0
Methylation Value

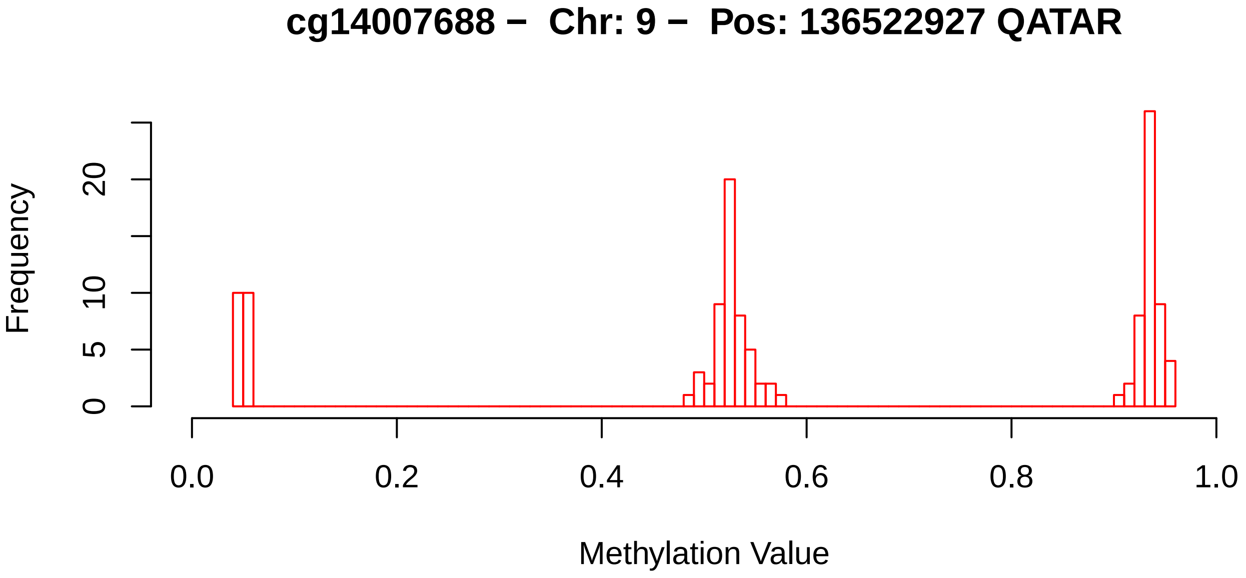
cg14007688 − Chr: 9 − Pos: 136522927 QATAR
Frequency
0
5
10
20
0.0
0.2
0.4
0.6
0.8
1.0
Methylation Value

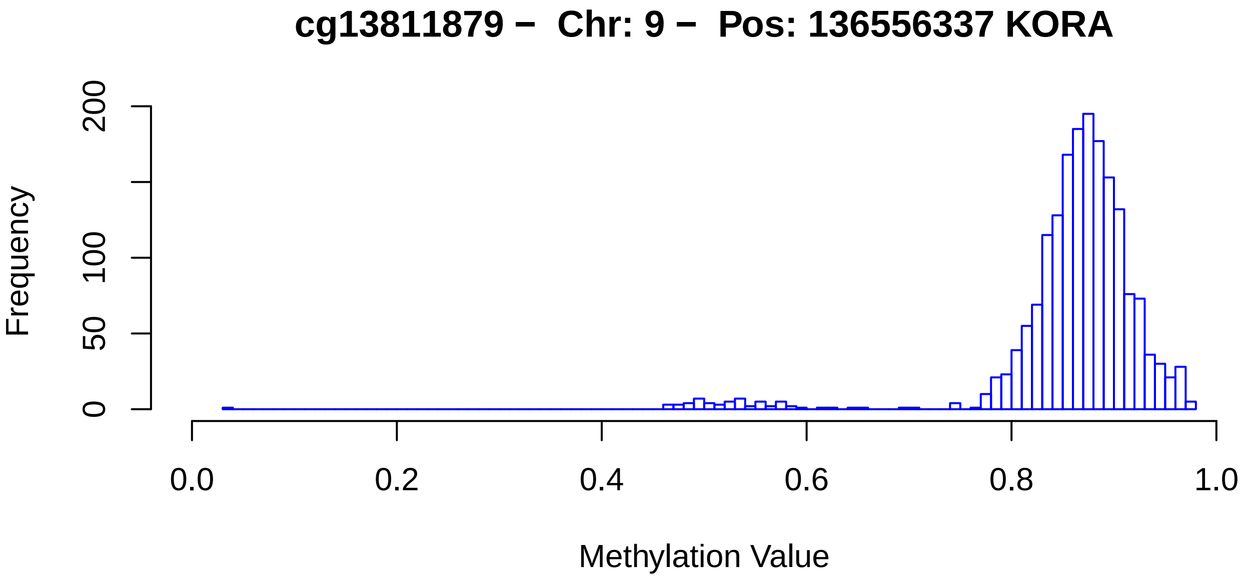
cg13811879 − Chr: 9 − Pos: 136556337 KORA
Frequency
0
50
100
200
0.0
0.2
0.4
0.6
0.8
1.0
Methylation Value

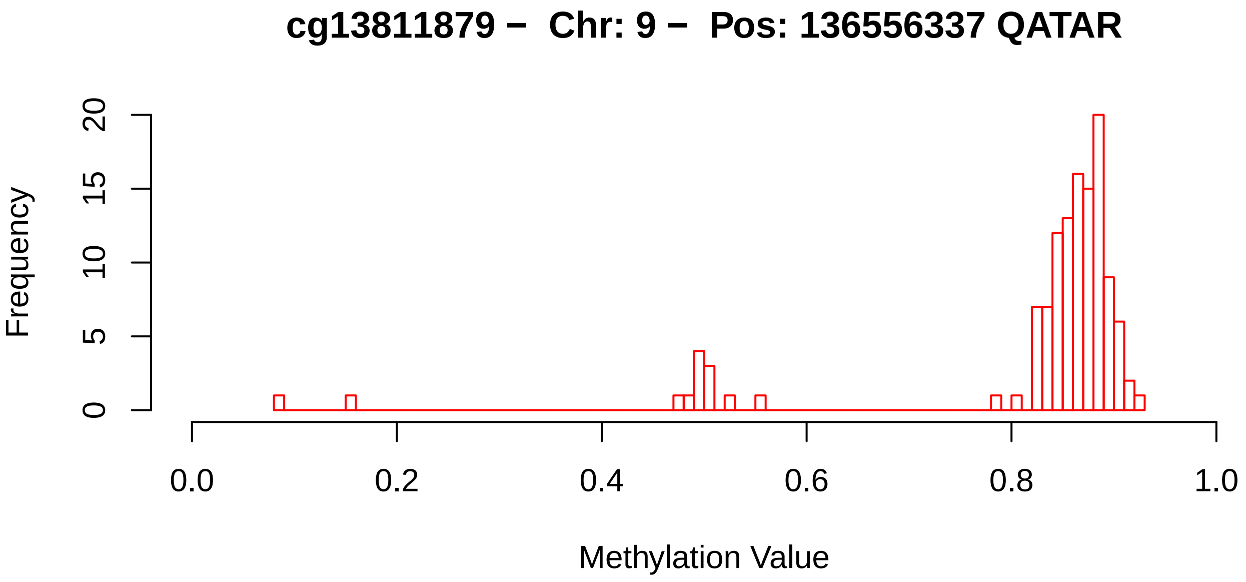
cg13811879 − Chr: 9 − Pos: 136556337 QATAR
Frequency
0
5
10
15
20
0.0
0.2
0.4
0.6
0.8
1.0
Methylation Value

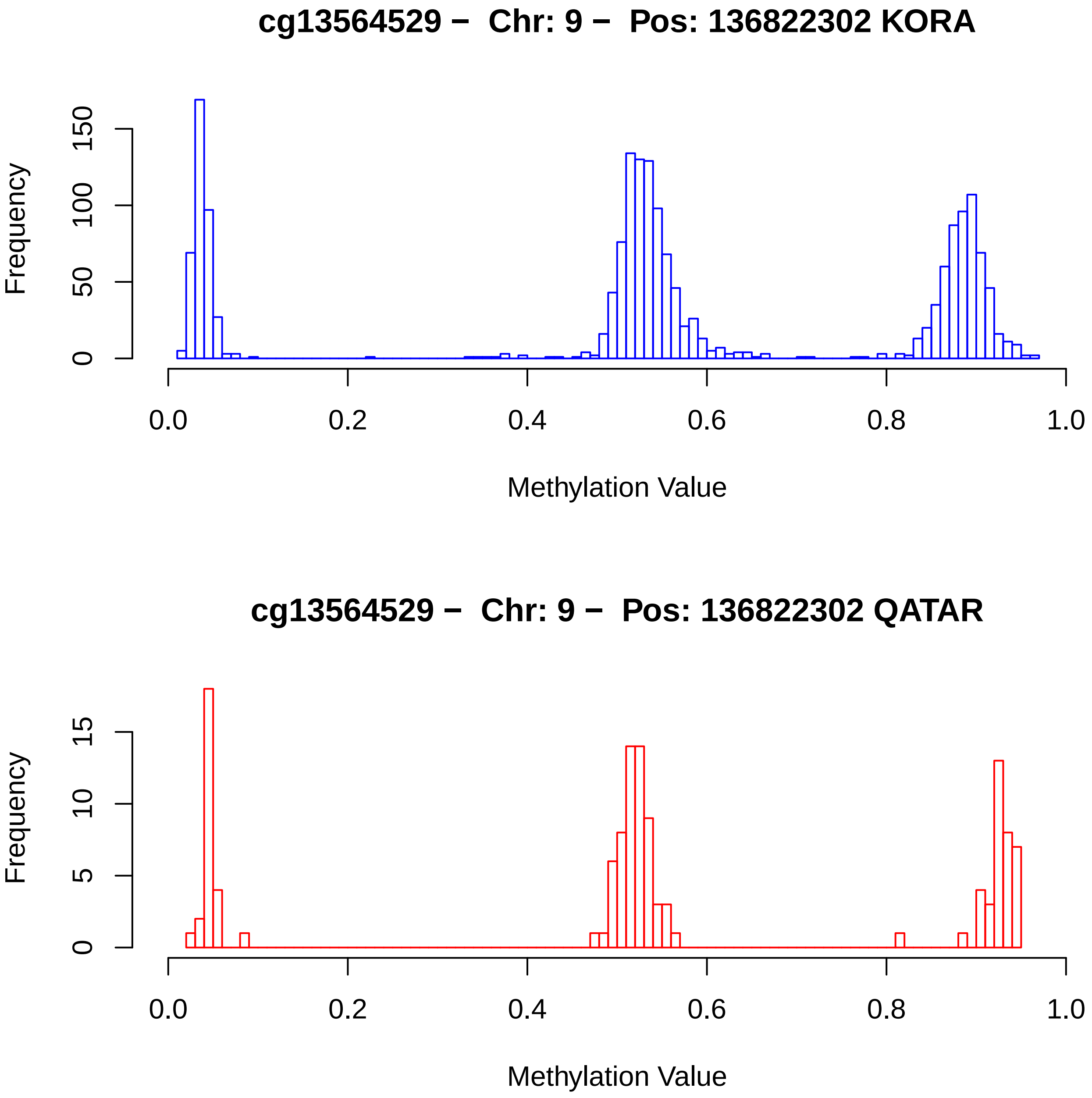
cg13564529 − Chr: 9 − Pos: 136822302 KORA
Frequency
0
50
100
150
0.0
0.2
0.4
0.6
0.8
1.0
Methylation Value
cg13564529 − Chr: 9 − Pos: 136822302 QATAR
Frequency
0
5
10
15
0.0
0.2
0.4
0.6
0.8
1.0
Methylation Value

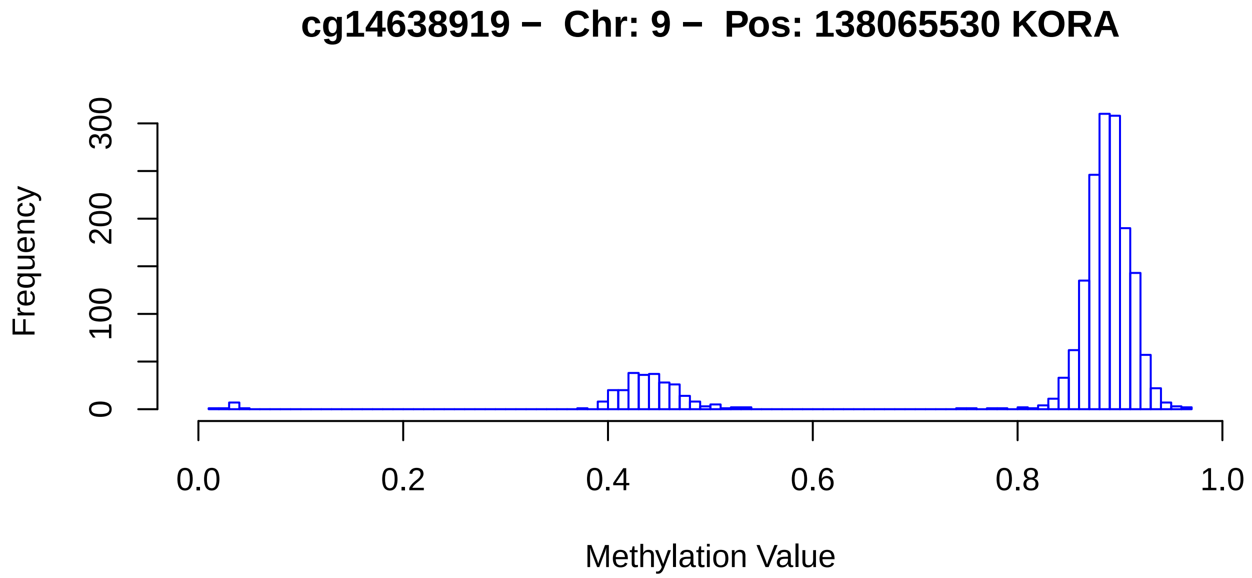
cg14638919 − Chr: 9 − Pos: 138065530 KORA
Frequency
0
100
200
300
0.0
0.2
0.4
0.6
0.8
1.0
Methylation Value

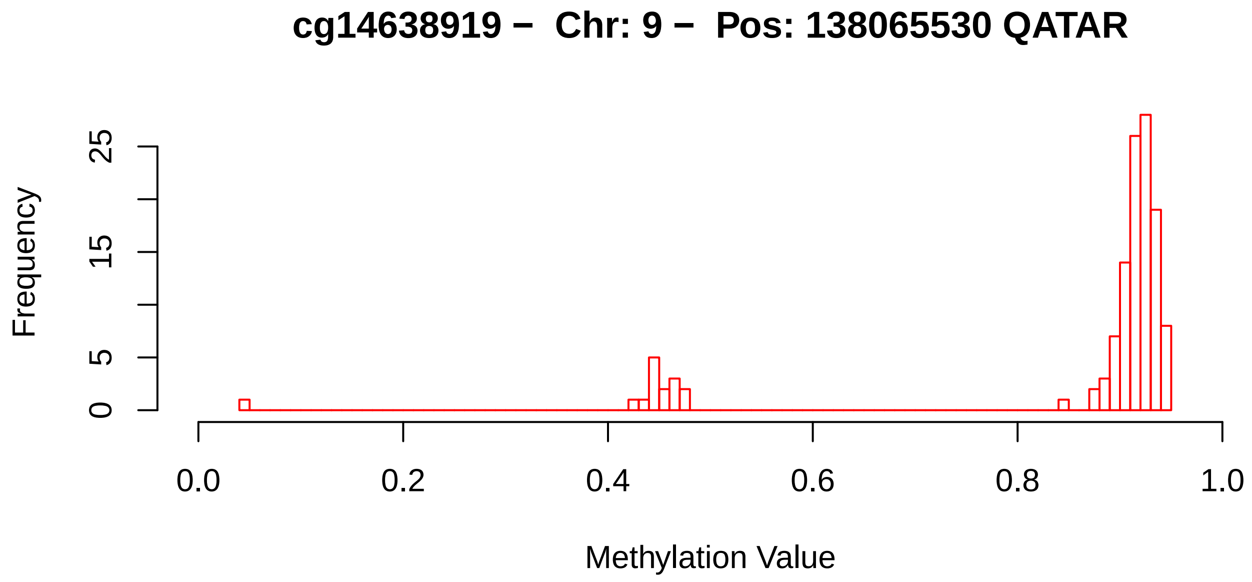
cg14638919 − Chr: 9 − Pos: 138065530 QATAR
Frequency
0
5
15
25
0.0
0.2
0.4
0.6
0.8
1.0
Methylation Value

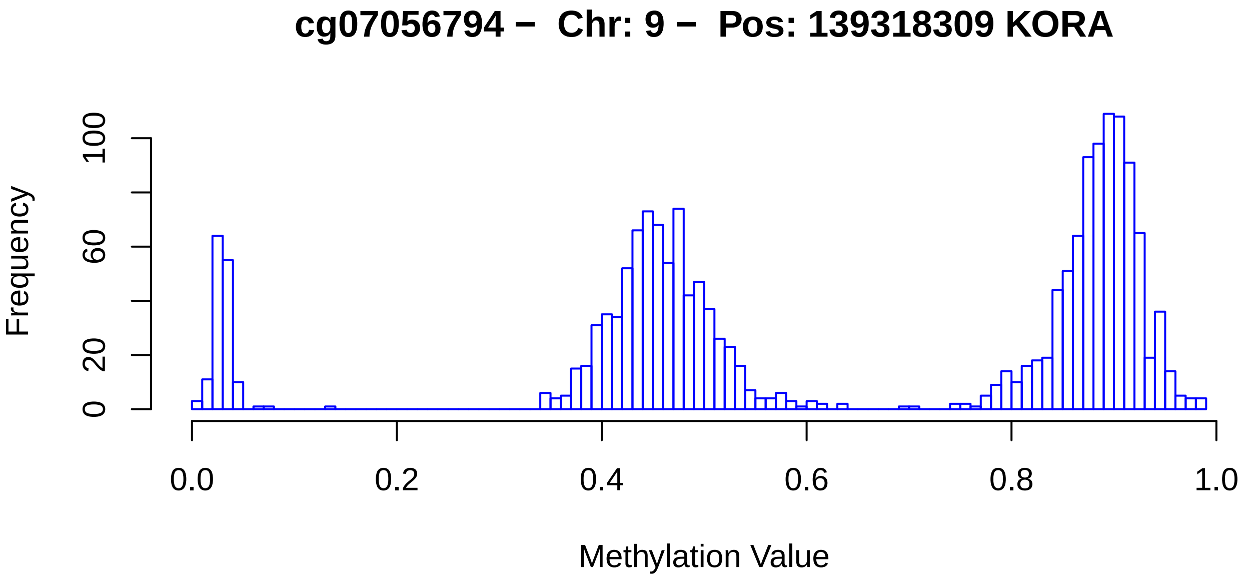
cg07056794 − Chr: 9 − Pos: 139318309 KORA
Frequency
0
20
60
100
0.0
0.2
0.4
0.6
0.8
1.0
Methylation Value

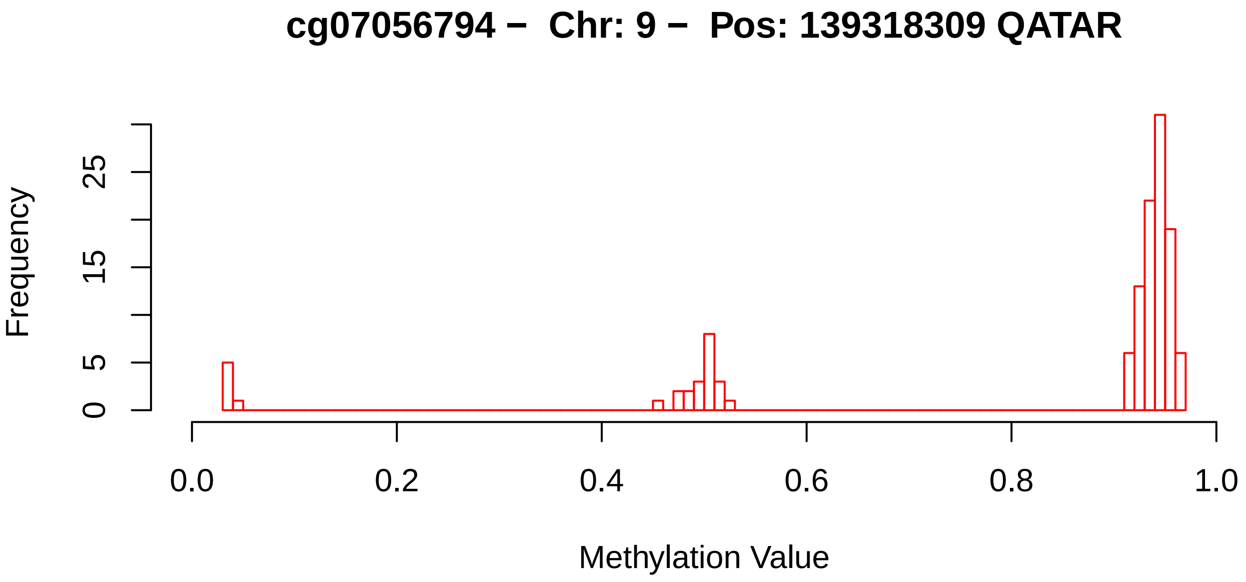
cg07056794 − Chr: 9 − Pos: 139318309 QATAR
Frequency
0
5
15
25
0.0
0.2
0.4
0.6
0.8
1.0
Methylation Value

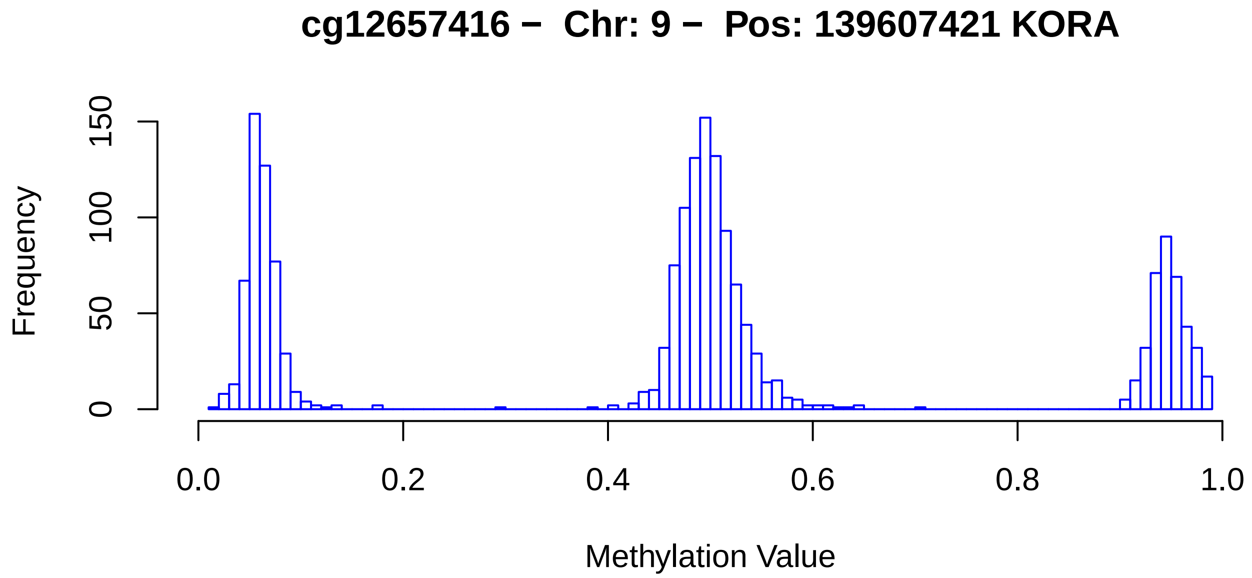
cg12657416 − Chr: 9 − Pos: 139607421 KORA
Frequency
0
50
100
150
0.0
0.2
0.4
0.6
0.8
1.0
Methylation Value

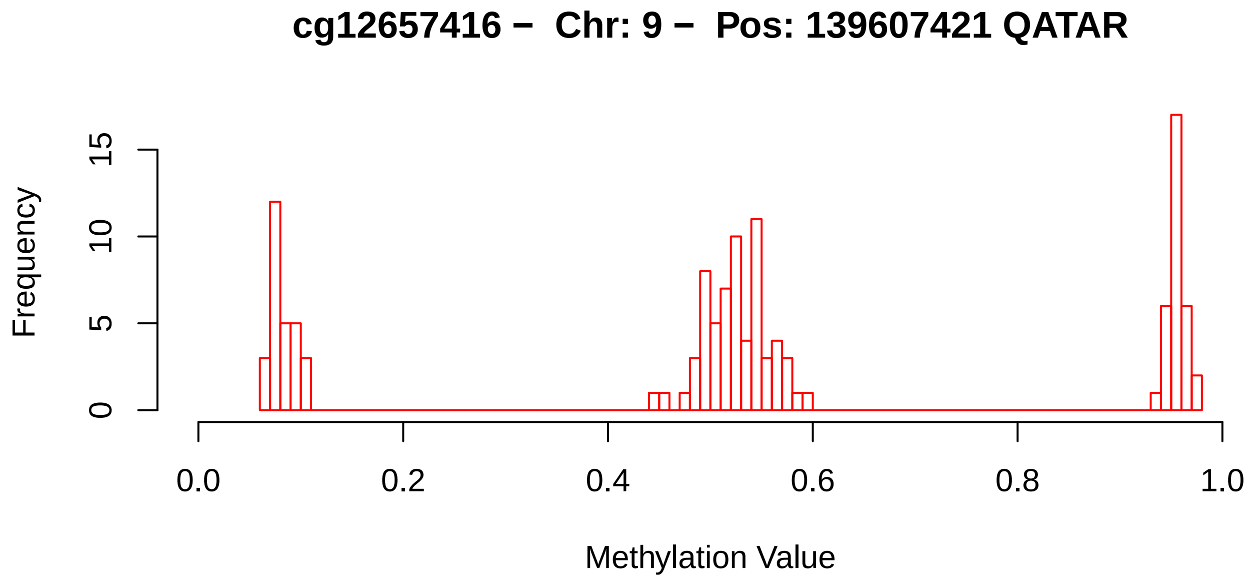
cg12657416 − Chr: 9 − Pos: 139607421 QATAR
Frequency
0
5
10
15
0.0
0.2
0.4
0.6
0.8
1.0
Methylation Value

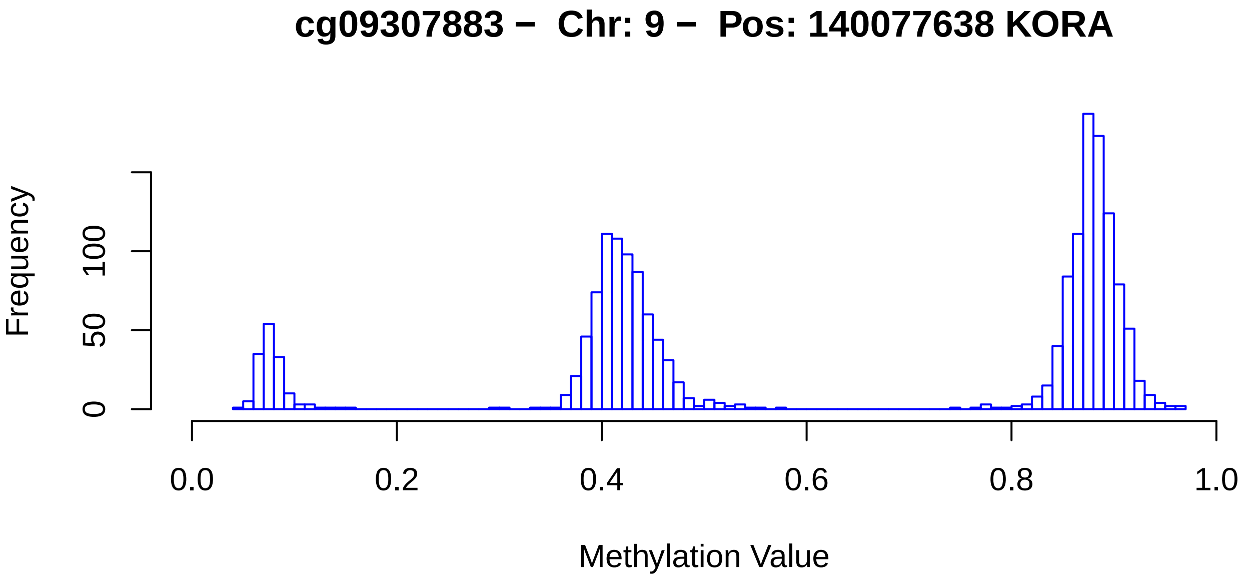
cg09307883 − Chr: 9 − Pos: 140077638 KORA
Frequency
0
50
100
0.0
0.2
0.4
0.6
0.8
1.0
Methylation Value

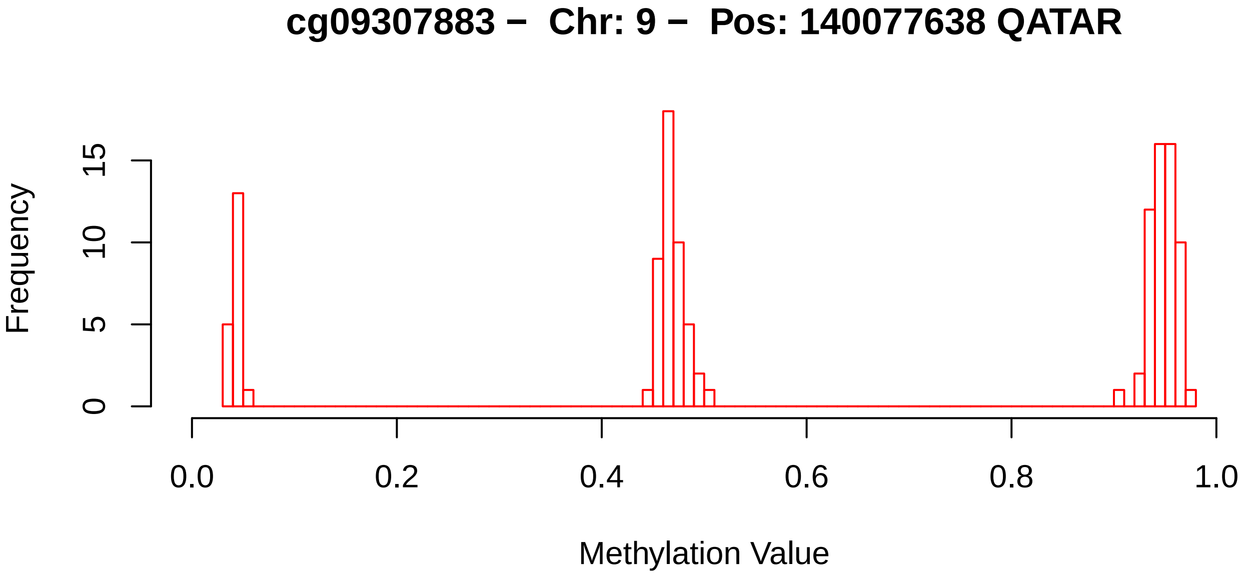
cg09307883 − Chr: 9 − Pos: 140077638 QATAR
Frequency
0
5
10
15
0.0
0.2
0.4
0.6
0.8
1.0
Methylation Value

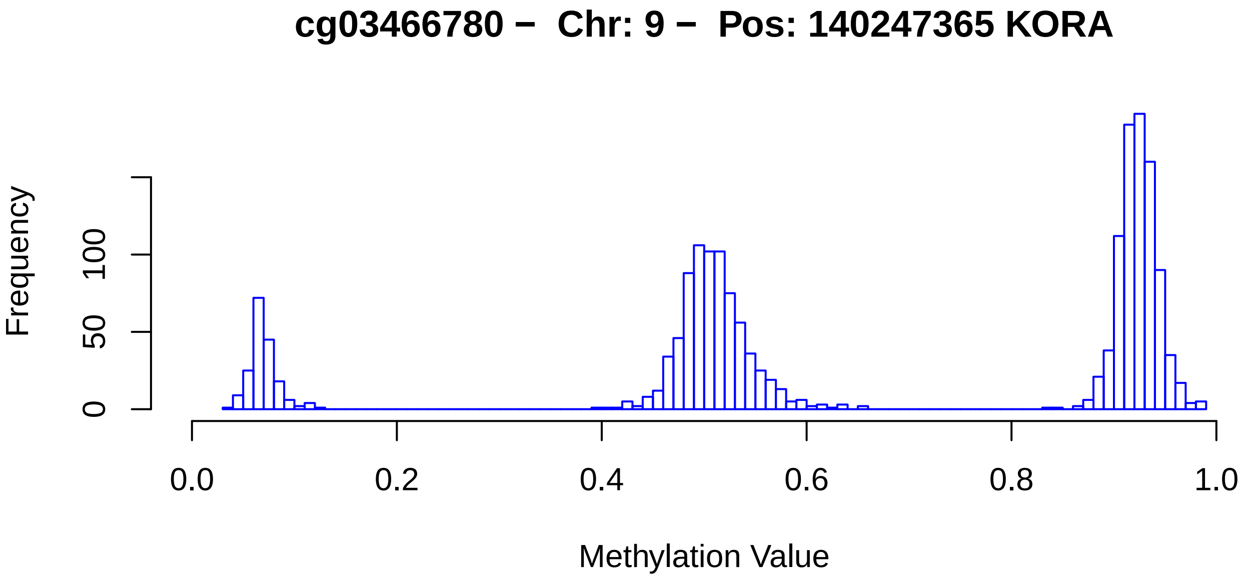
cg03466780 − Chr: 9 − Pos: 140247365 KORA
Frequency
0
50
100
0.0
0.2
0.4
0.6
0.8
1.0
Methylation Value

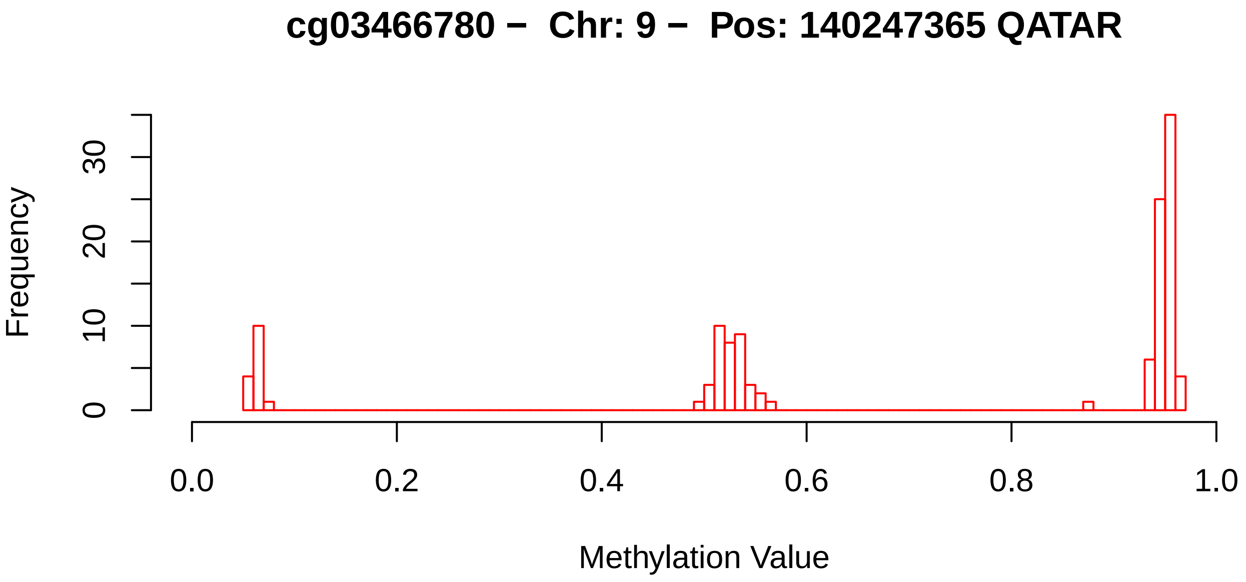
cg03466780 − Chr: 9 − Pos: 140247365 QATAR
Frequency
0
10
20
30
0.0
0.2
0.4
0.6
0.8
1.0
Methylation Value

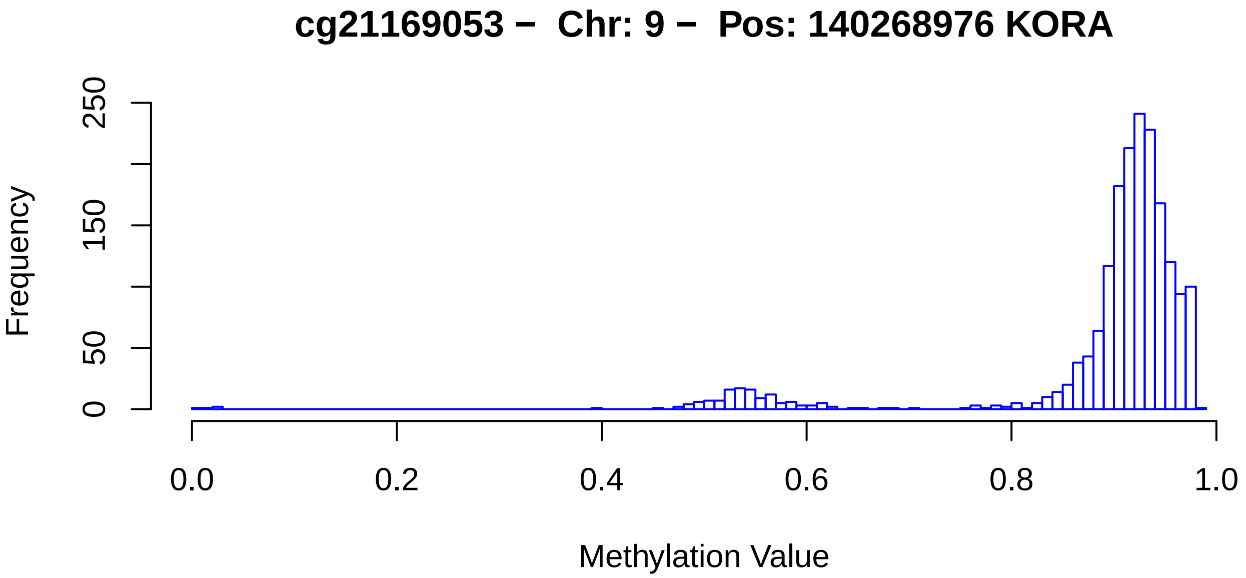

cg21169053 − Chr: 9 − Pos: 140268976 KORA
Frequency
0
50
150
250
0.0
0.2
0.4
0.6
0.8
1.0
Methylation Value

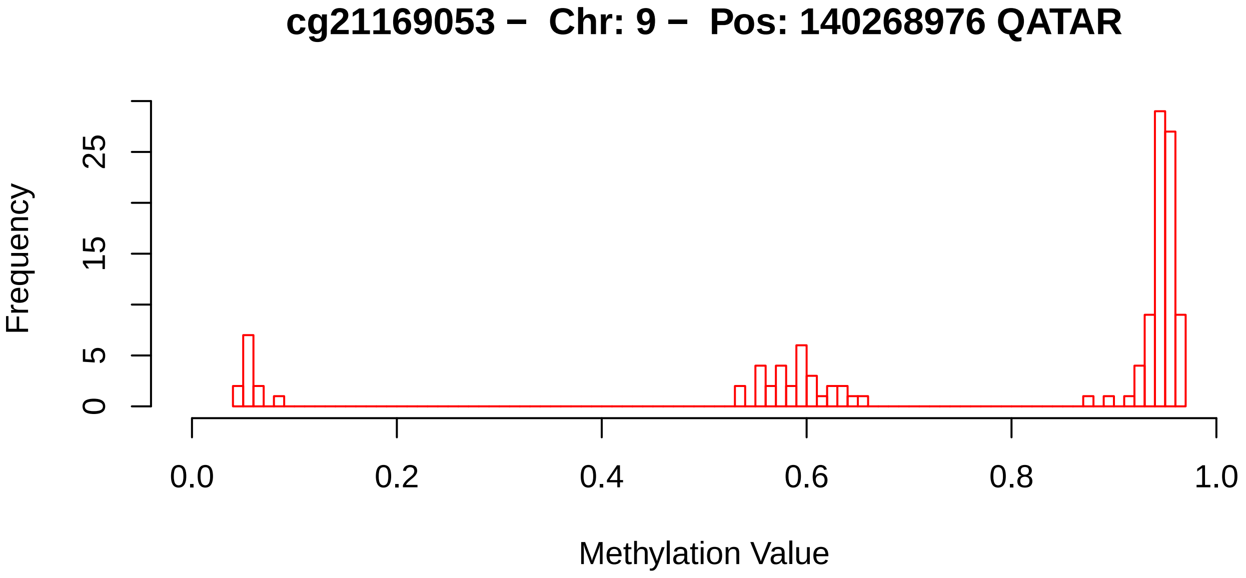

cg21169053 − Chr: 9 − Pos: 140268976 QATAR
Frequency
0
5
15
25
0.0
0.2
0.4
0.6
0.8
1.0
Methylation Value

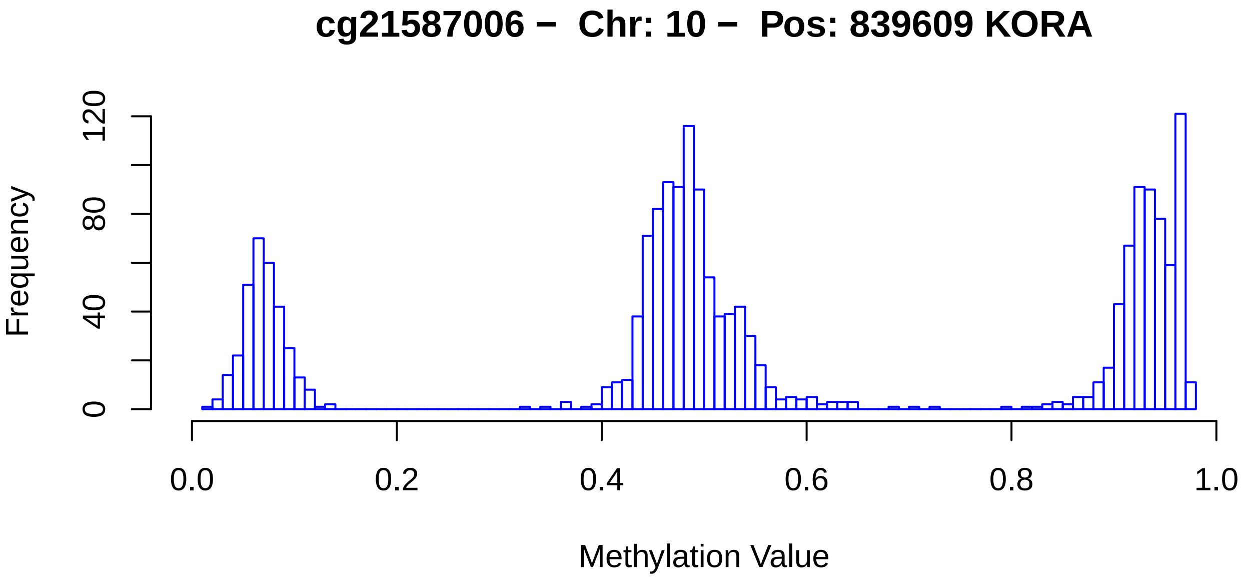
cg21587006 − Chr: 10 − Pos: 839609 KORA
Frequency
0
40
80
120
0.0
0.2
0.4
0.6
0.8
1.0
Methylation Value

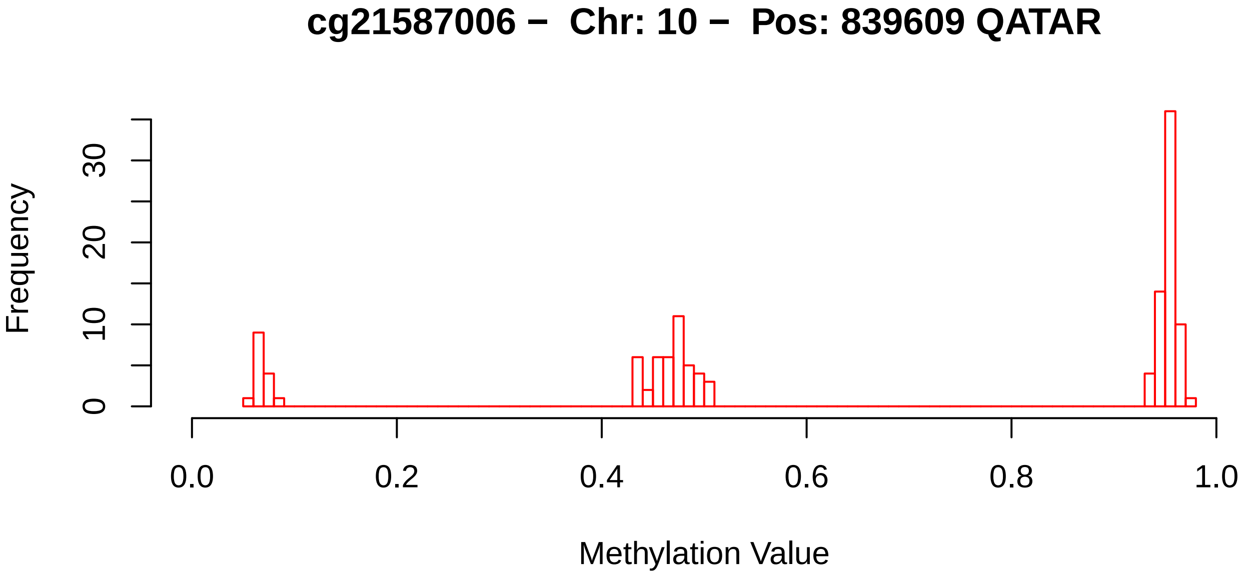
cg21587006 − Chr: 10 − Pos: 839609 QATAR
Frequency
0
10
20
30
0.0
0.2
0.4
0.6
0.8
1.0
Methylation Value

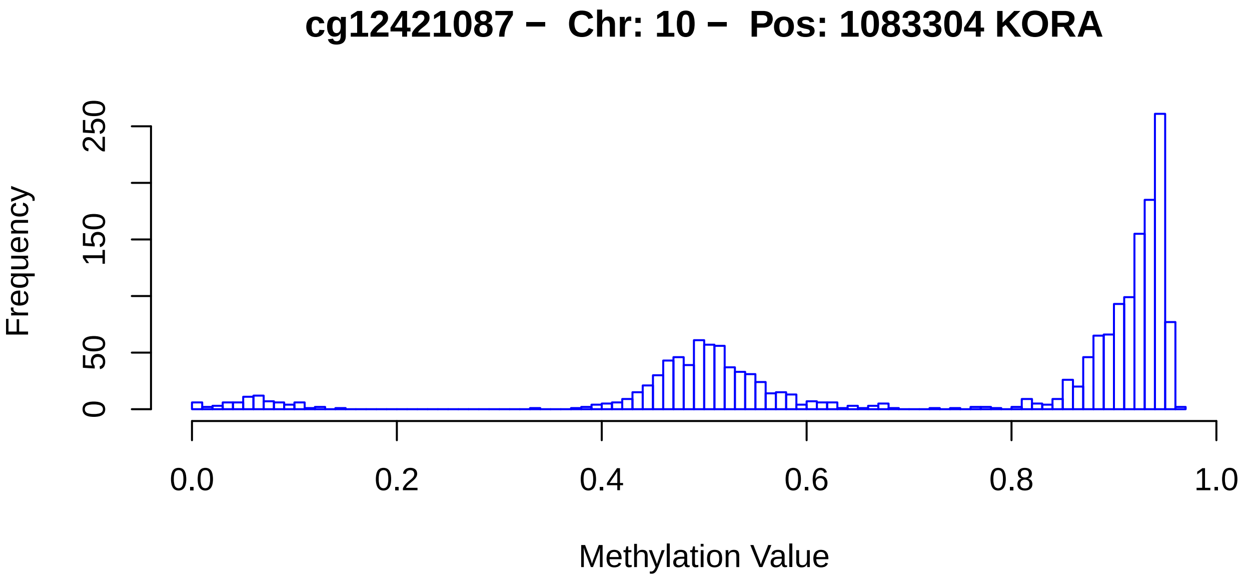
cg12421087 − Chr: 10 − Pos: 1083304 KORA
Frequency
0
50
150
250
0.0
0.2
0.4
0.6
0.8
1.0
Methylation Value

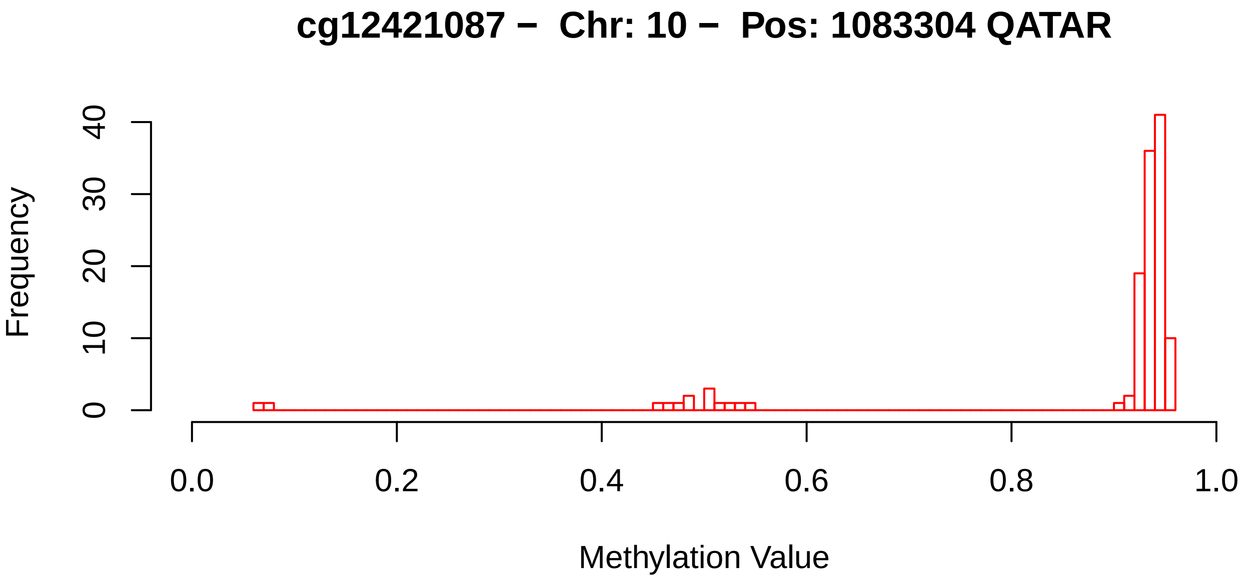
cg12421087 − Chr: 10 − Pos: 1083304 QATAR
Frequency
0
10
20
30
40
0.0
0.2
0.4
0.6
0.8
1.0
Methylation Value

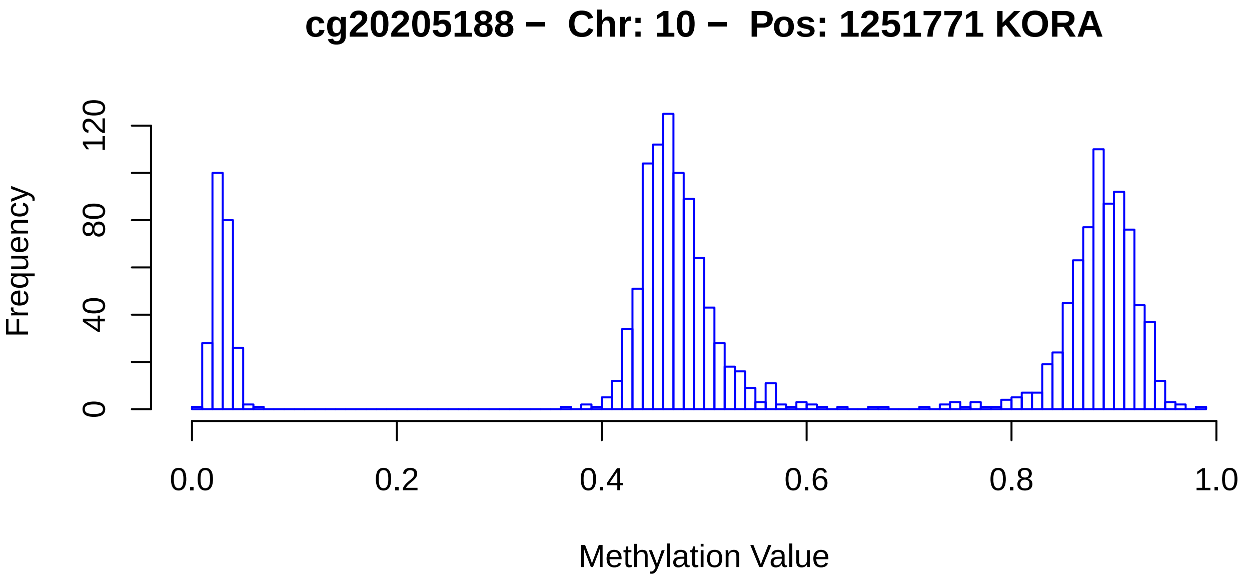
cg20205188 − Chr: 10 − Pos: 1251771 KORA
Frequency
0
40
80
120
0.0
0.2
0.4
0.6
0.8
1.0
Methylation Value

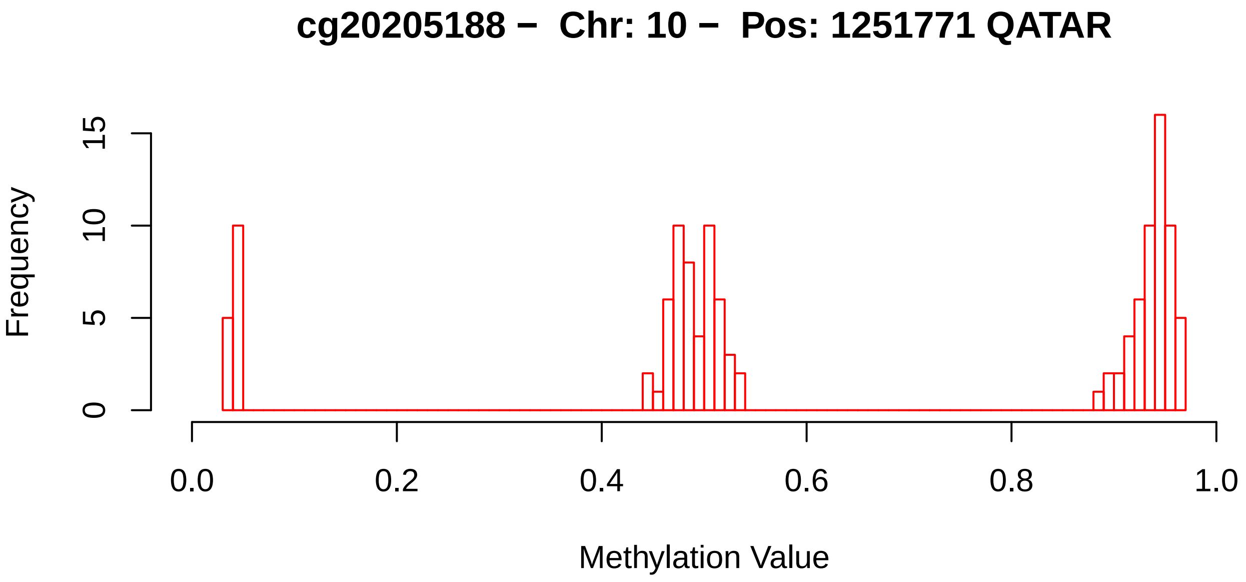
cg20205188 − Chr: 10 − Pos: 1251771 QATAR
Frequency
0
5
10
15
0.0
0.2
0.4
0.6
0.8
1.0
Methylation Value

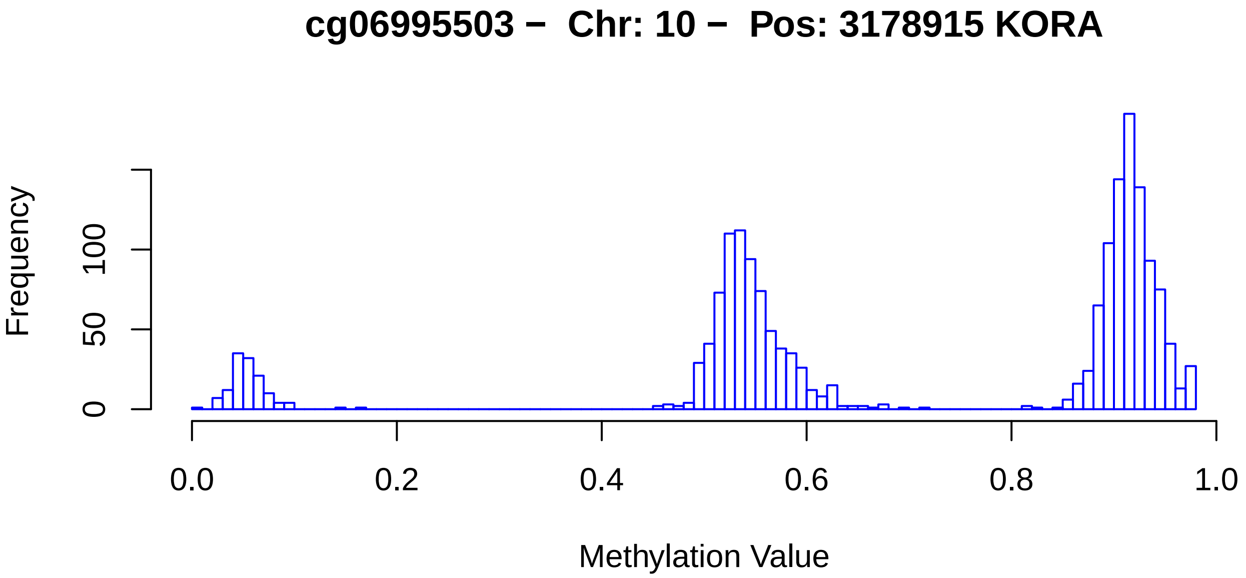
cg06995503 − Chr: 10 − Pos: 3178915 KORA
Frequency
0
50
100
0.0
0.2
0.4
0.6
0.8
1.0
Methylation Value

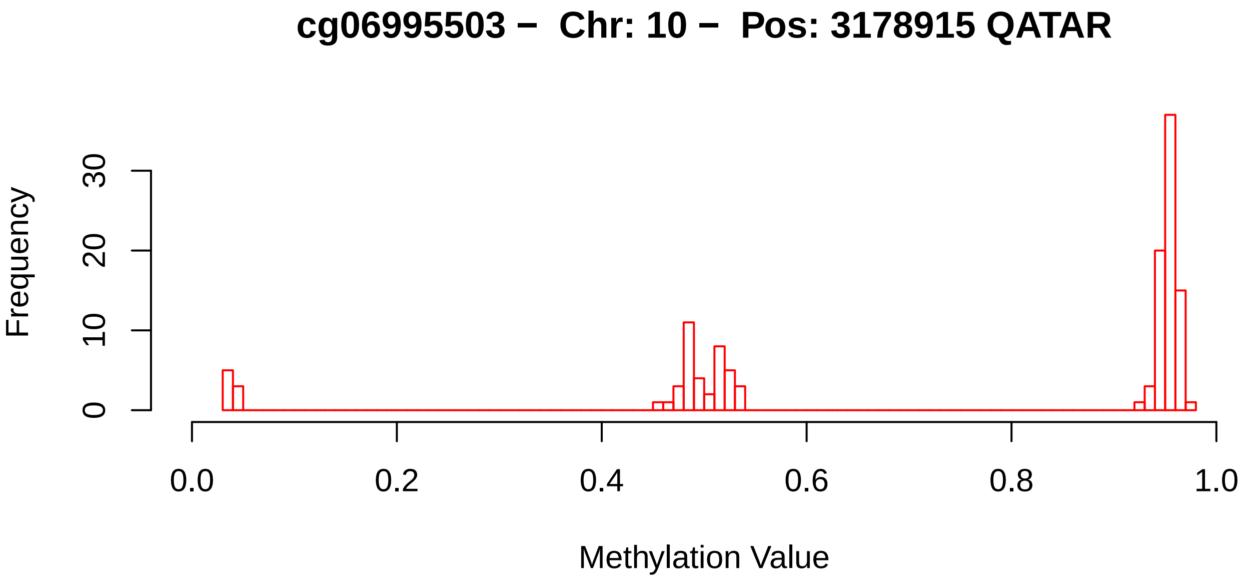
cg06995503 − Chr: 10 − Pos: 3178915 QATAR
Frequency
0
10
20
30
0.0
0.2
0.4
0.6
0.8
1.0
Methylation Value

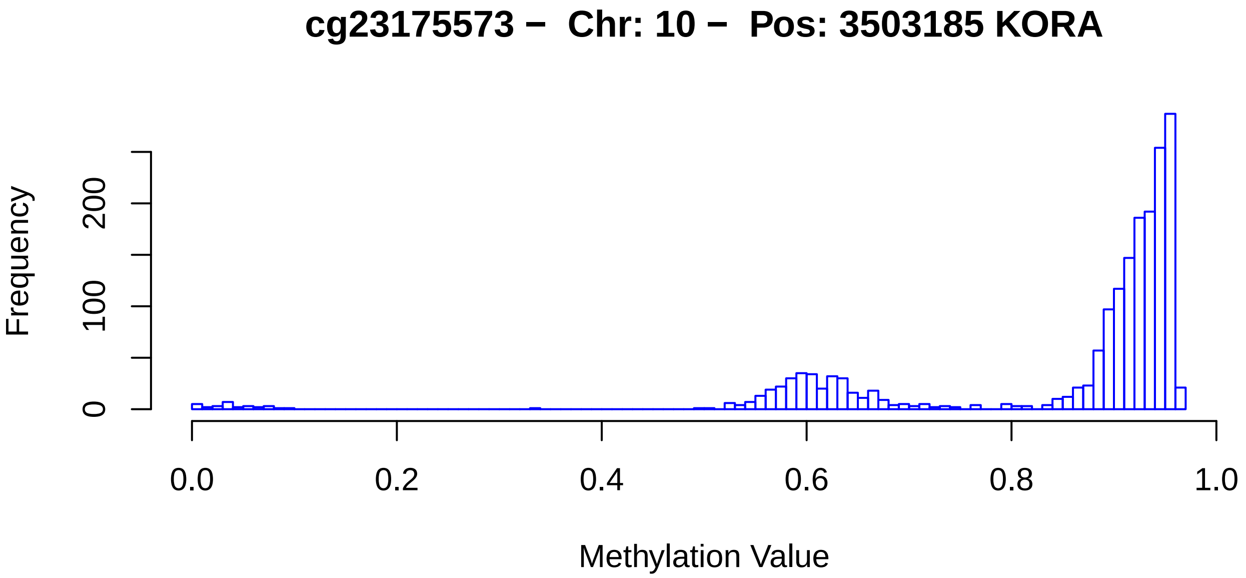

cg23175573 − Chr: 10 − Pos: 3503185 KORA
Frequency
0
100
200
0.0
0.2
0.4
0.6
0.8
1.0
Methylation Value

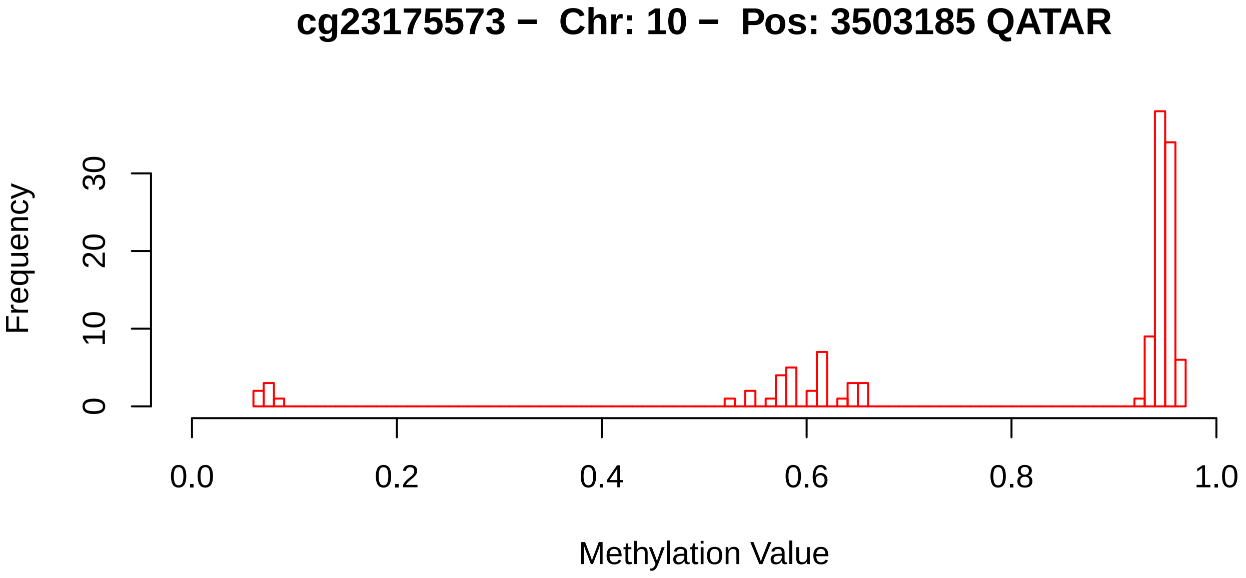

cg23175573 − Chr: 10 − Pos: 3503185 QATAR
Frequency
0
10
20
30
0.0
0.2
0.4
0.6
0.8
1.0
Methylation Value

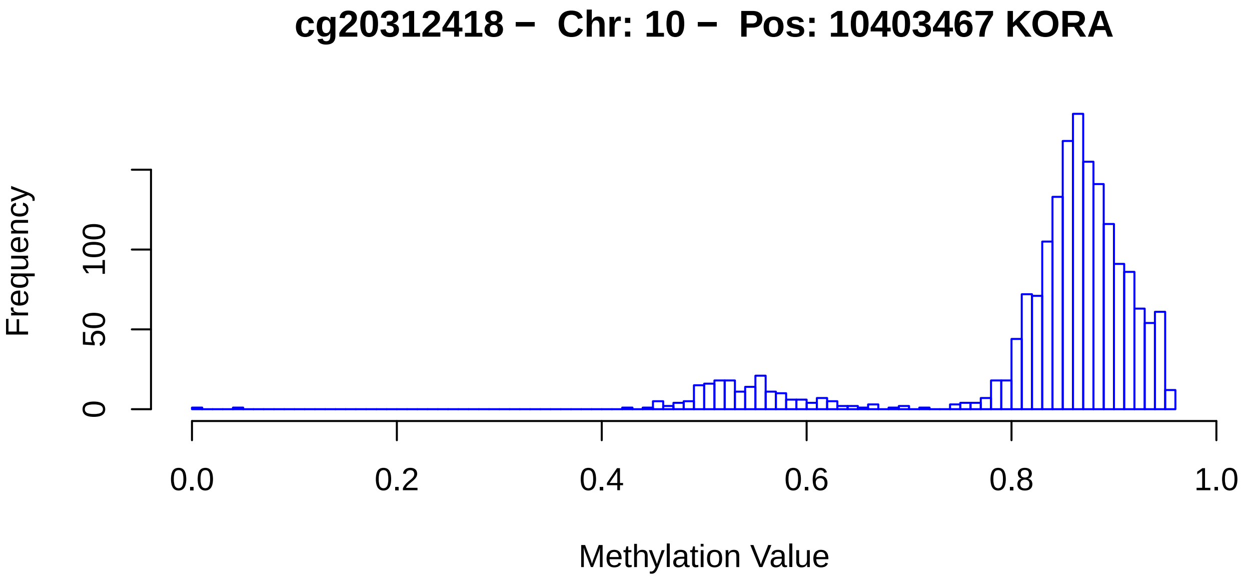

cg20312418 − Chr: 10 − Pos: 10403467 KORA
Frequency
0
50
100
0.0
0.2
0.4
0.6
0.8
1.0
Methylation Value

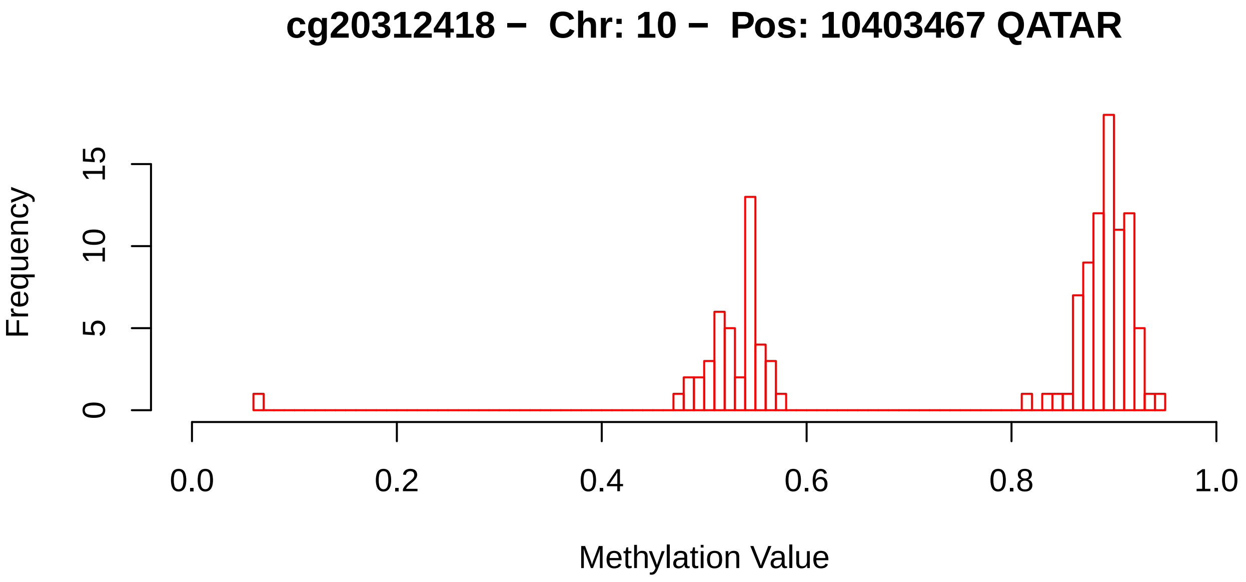

cg20312418 − Chr: 10 − Pos: 10403467 QATAR
Frequency
0
5
10
15
0.0
0.2
0.4
0.6
0.8
1.0
Methylation Value

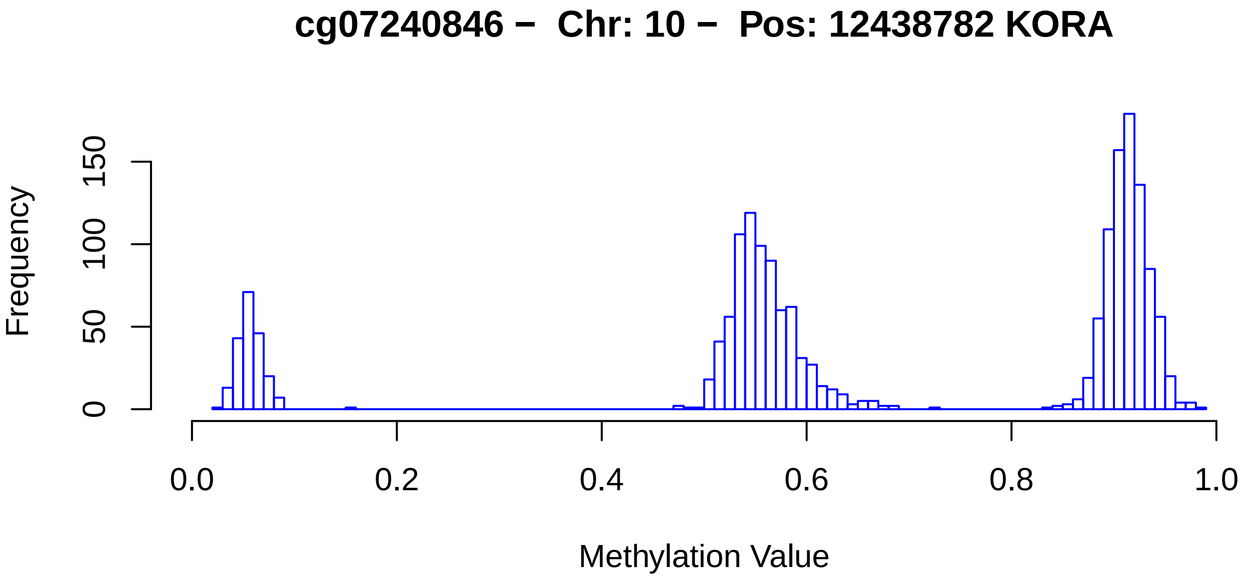
cg07240846 − Chr: 10 − Pos: 12438782 KORA
Frequency
0
50
100
150
0.0
0.2
0.4
0.6
0.8
1.0
Methylation Value

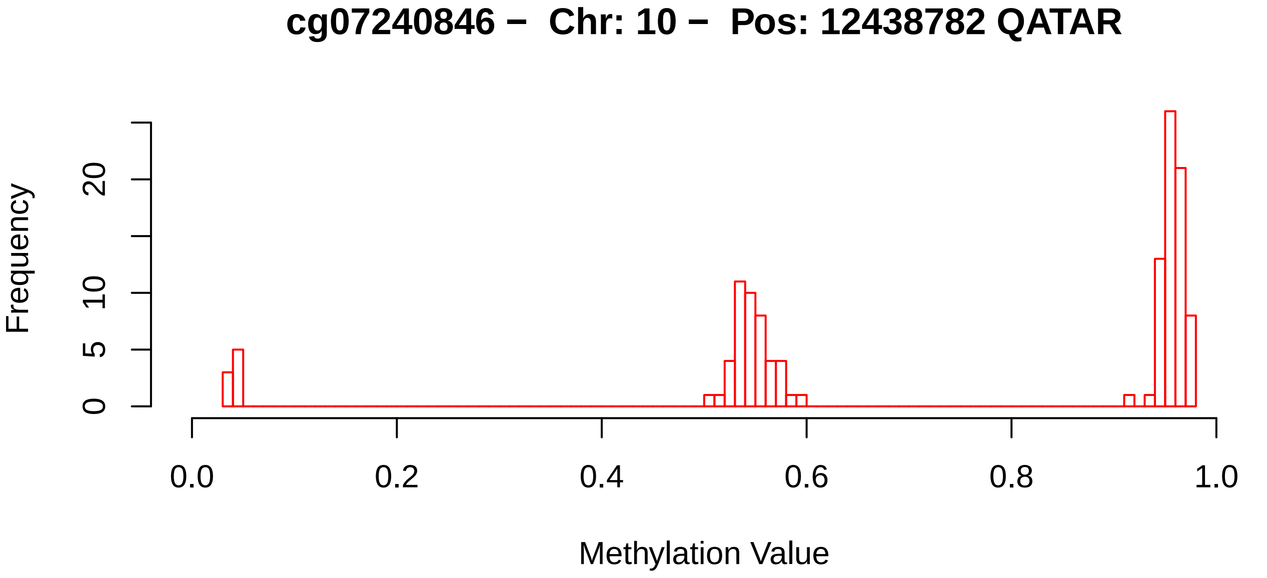
cg07240846 − Chr: 10 − Pos: 12438782 QATAR
Frequency
0
5
10
20
0.0
0.2
0.4
0.6
0.8
1.0
Methylation Value

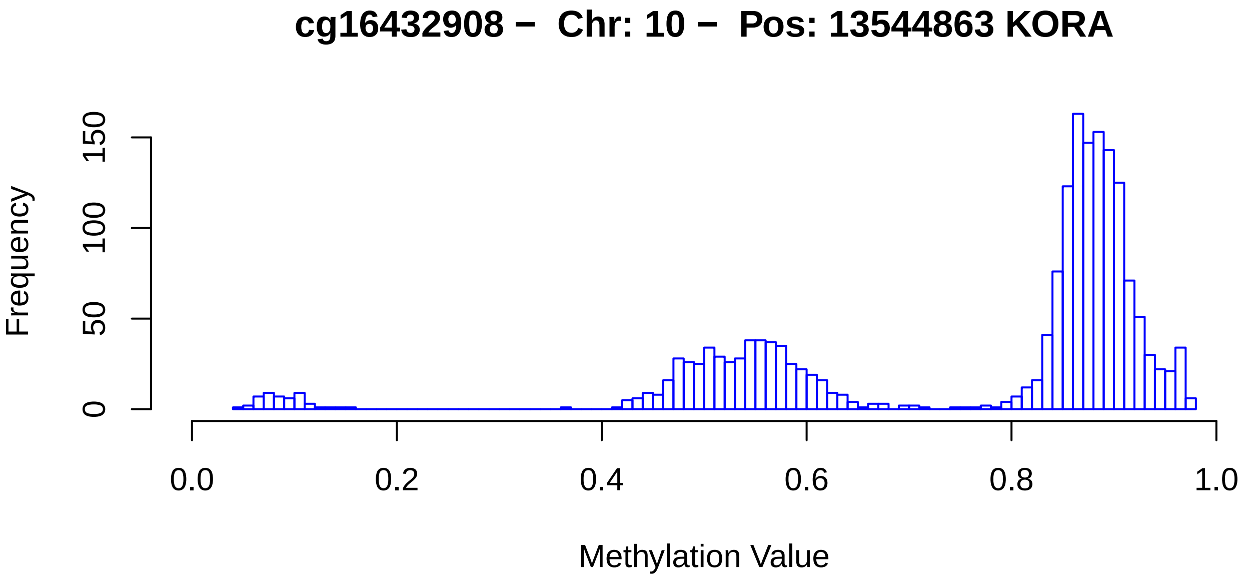
cg16432908 − Chr: 10 − Pos: 13544863 KORA
Frequency
0
50
100
150
0.0
0.2
0.4
0.6
0.8
1.0
Methylation Value

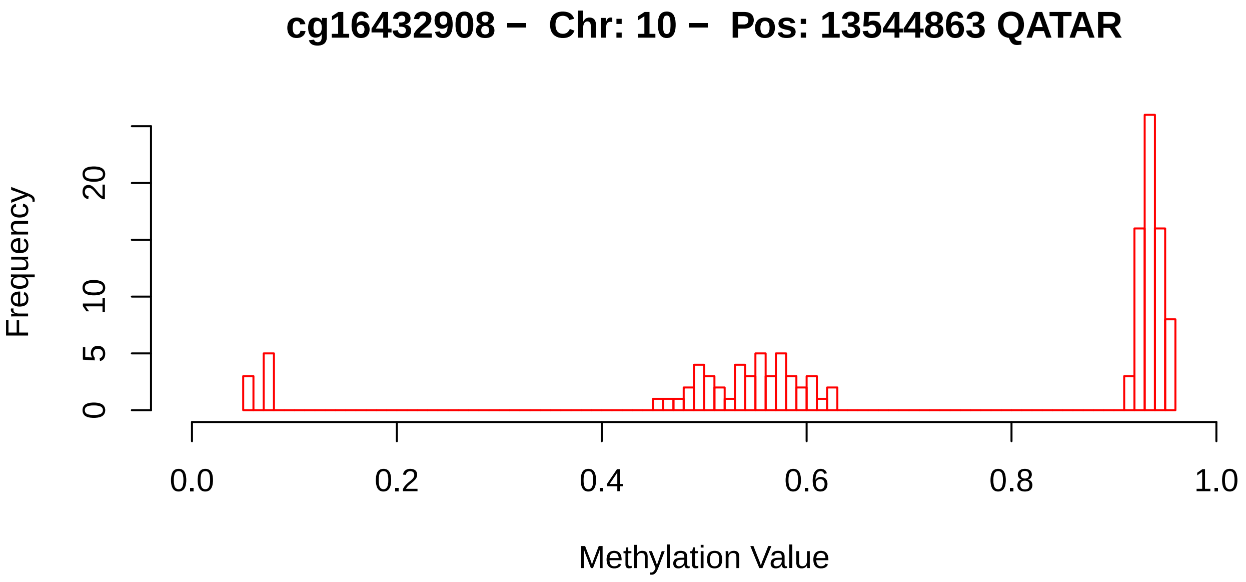
cg16432908 − Chr: 10 − Pos: 13544863 QATAR
Frequency
0
5
10
20
0.0
0.2
0.4
0.6
0.8
1.0
Methylation Value

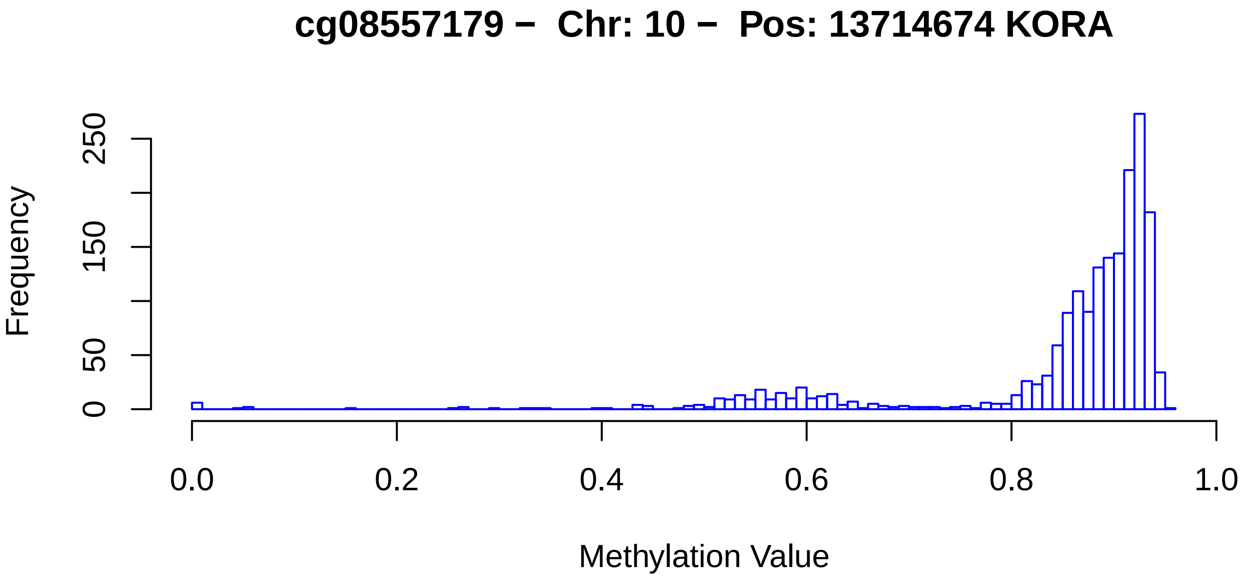

cg08557179 − Chr: 10 − Pos: 13714674 KORA
Frequency
0
50
150
250
0.0
0.2
0.4
0.6
0.8
1.0
Methylation Value

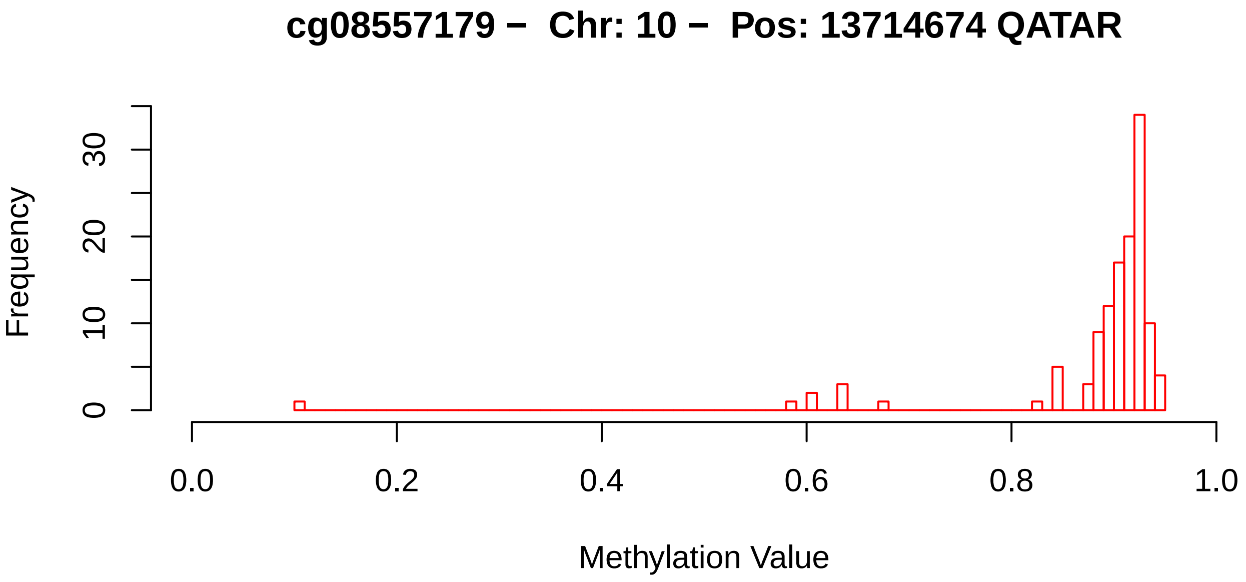

cg08557179 − Chr: 10 − Pos: 13714674 QATAR
Frequency
0
10
20
30
0.0
0.2
0.4
0.6
0.8
1.0
Methylation Value

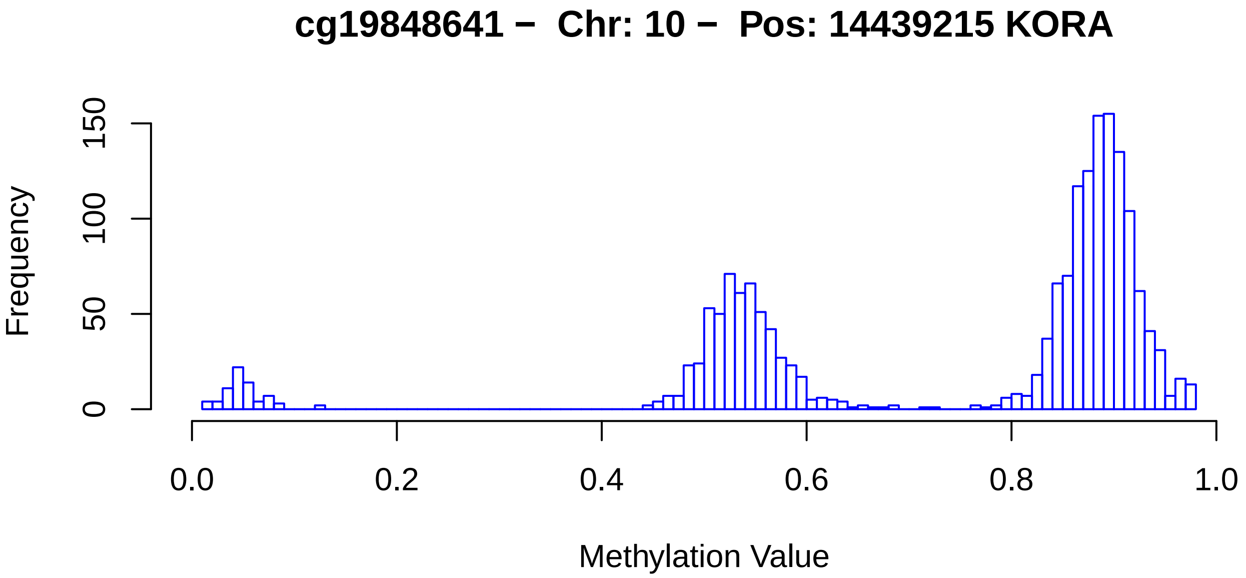
cg19848641 − Chr: 10 − Pos: 14439215 KORA
Frequency
0
50
100
150
0.0
0.2
0.4
0.6
0.8
1.0
Methylation Value

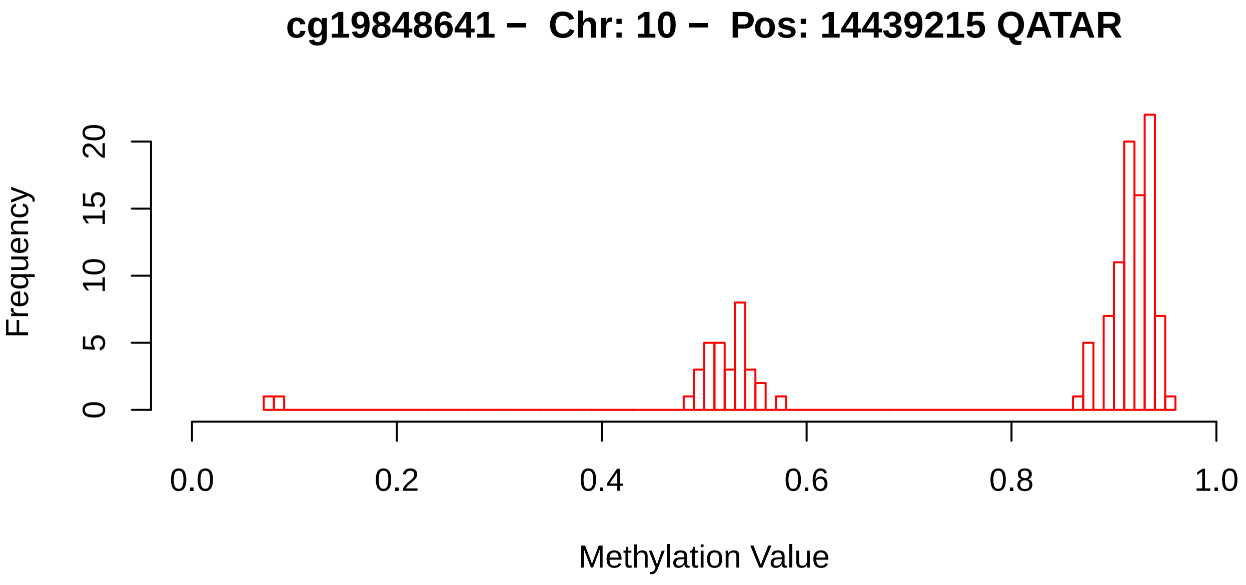
cg19848641 − Chr: 10 − Pos: 14439215 QATAR
Frequency
0
5
10
15
20
0.0
0.2
0.4
0.6
0.8
1.0
Methylation Value

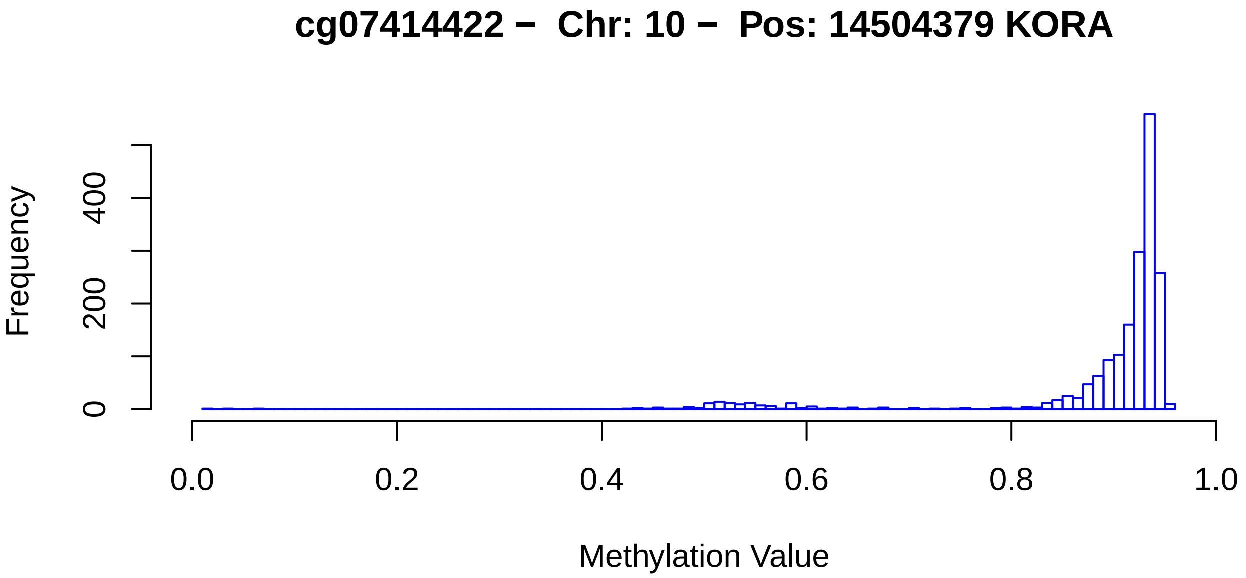

cg07414422 − Chr: 10 − Pos: 14504379 KORA
Frequency
0
200
400
0.0
0.2
0.4
0.6
0.8
1.0
Methylation Value

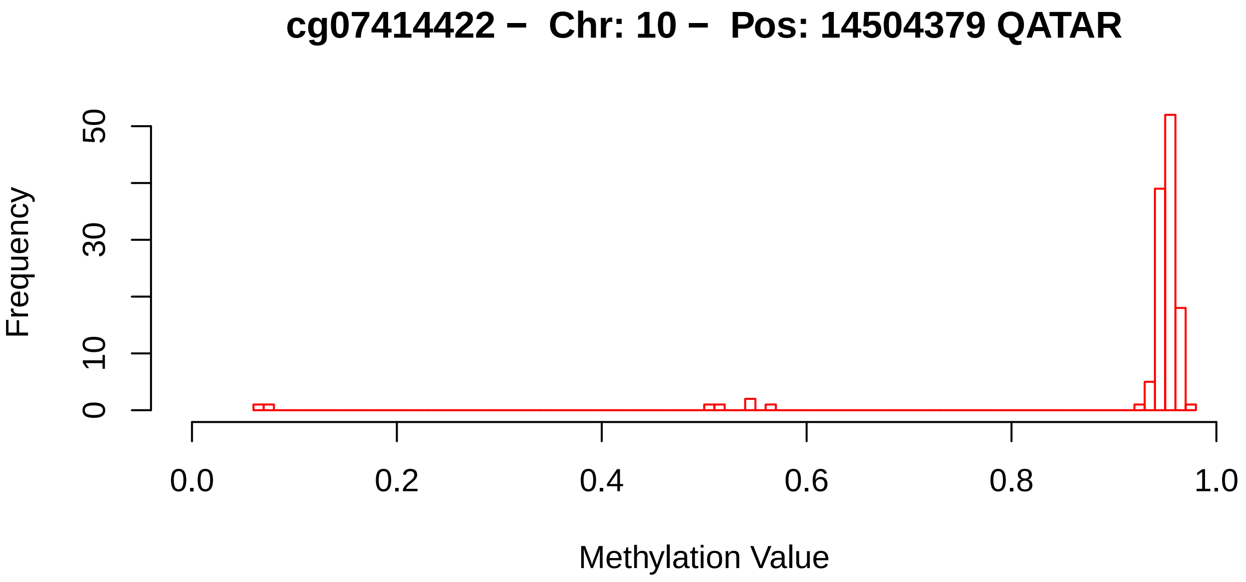

cg07414422 − Chr: 10 − Pos: 14504379 QATAR
Frequency
0
10
30
50
0.0
0.2
0.4
0.6
0.8
1.0
Methylation Value

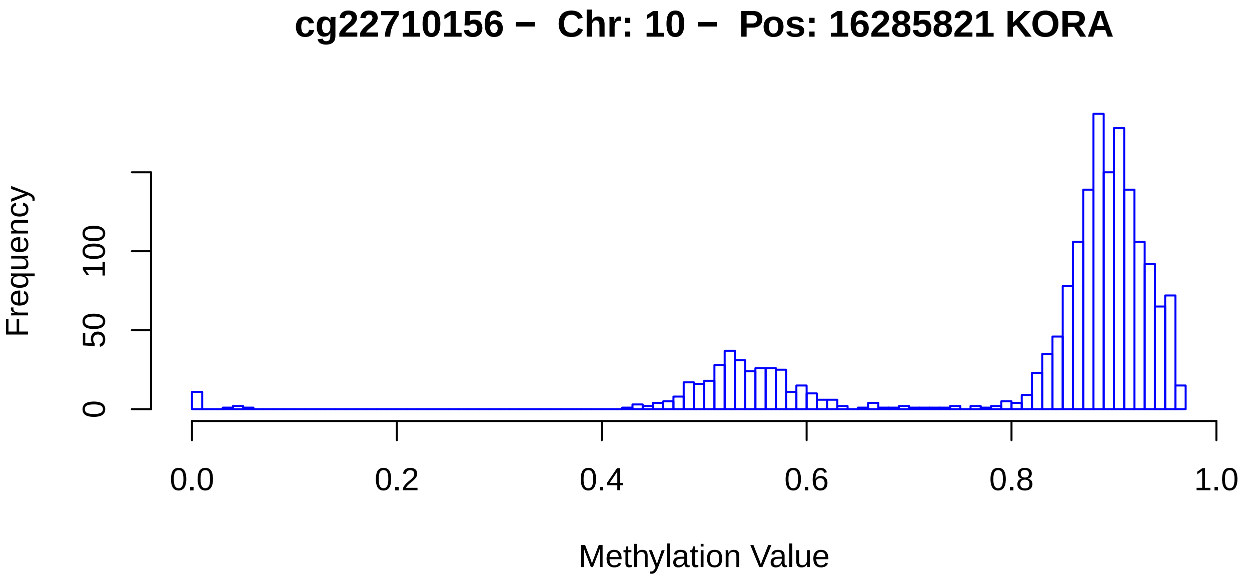
cg22710156 − Chr: 10 − Pos: 16285821 KORA
Frequency
0
50
100
0.0
0.2
0.4
0.6
0.8
1.0
Methylation Value

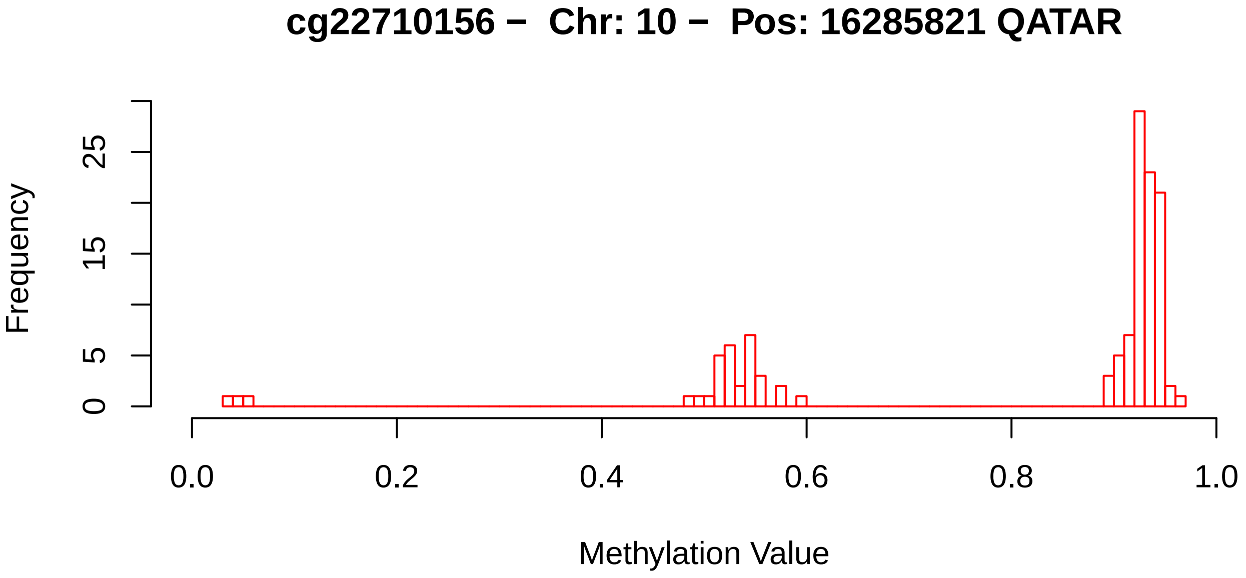
cg22710156 − Chr: 10 − Pos: 16285821 QATAR
Frequency
0
5
15
25
0.0
0.2
0.4
0.6
0.8
1.0
Methylation Value

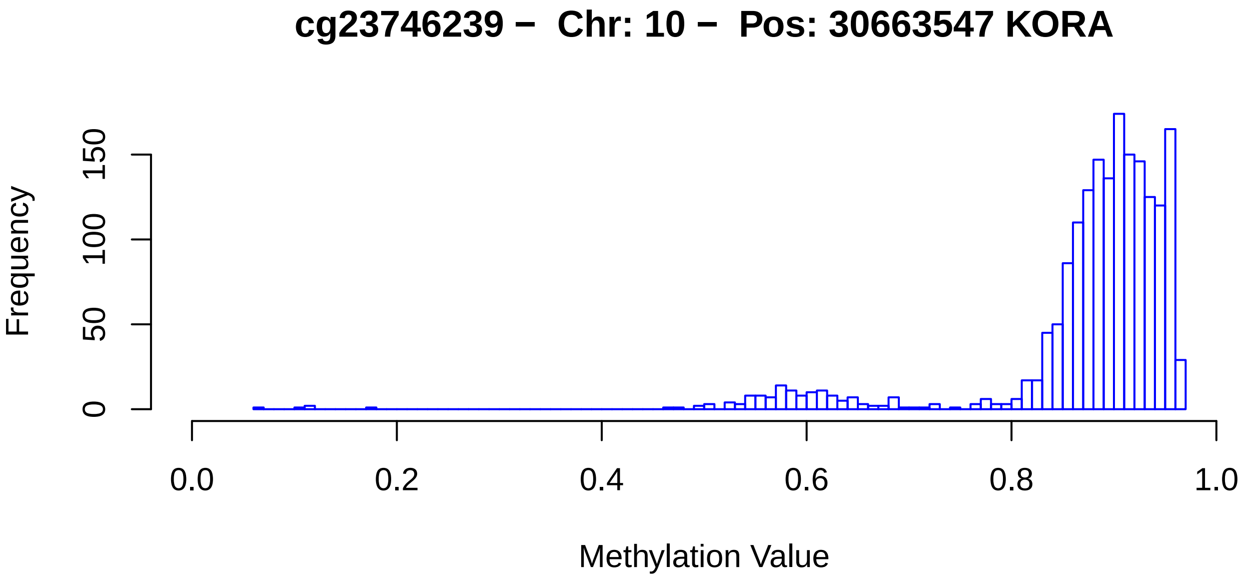
cg23746239 − Chr: 10 − Pos: 30663547 KORA
Frequency
0
50
100
150
0.0
0.2
0.4
0.6
0.8
1.0
Methylation Value

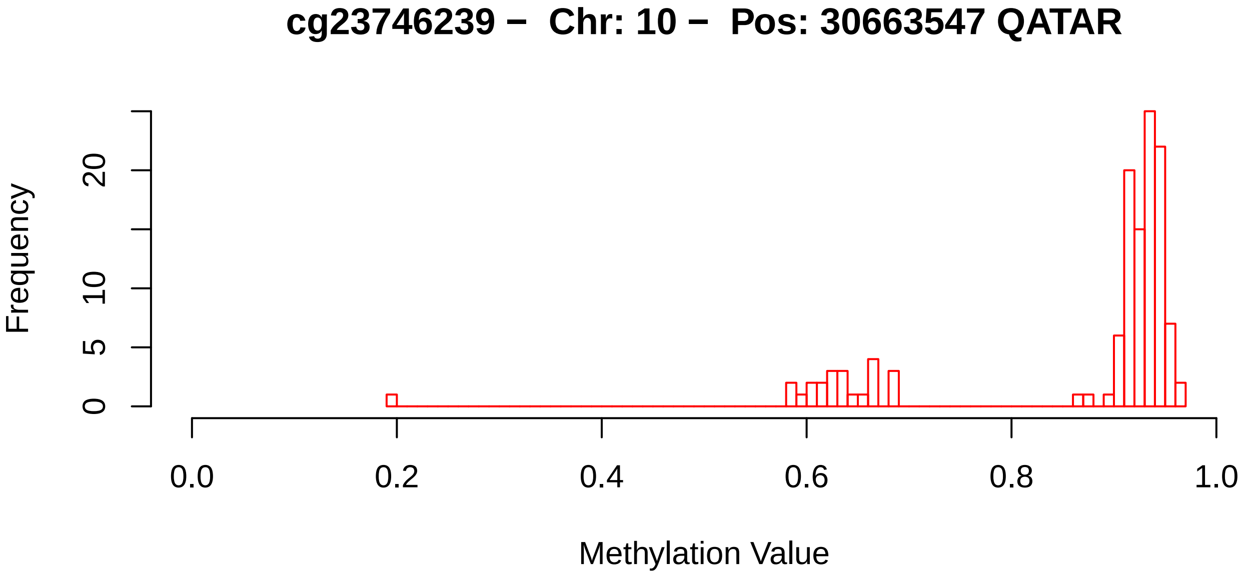
cg23746239 − Chr: 10 − Pos: 30663547 QATAR
Frequency
0
5
10
20
0.0
0.2
0.4
0.6
0.8
1.0
Methylation Value

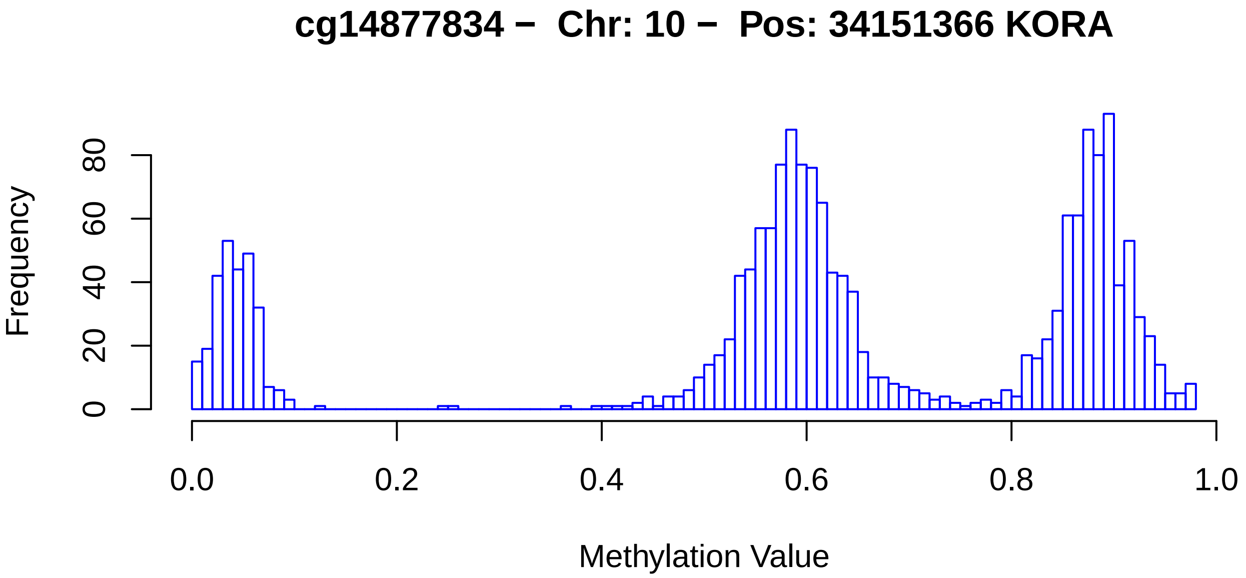
cg14877834 − Chr: 10 − Pos: 34151366 KORA
Frequency
0
20
40
60
80
0.0
0.2
0.4
0.6
0.8
1.0
Methylation Value

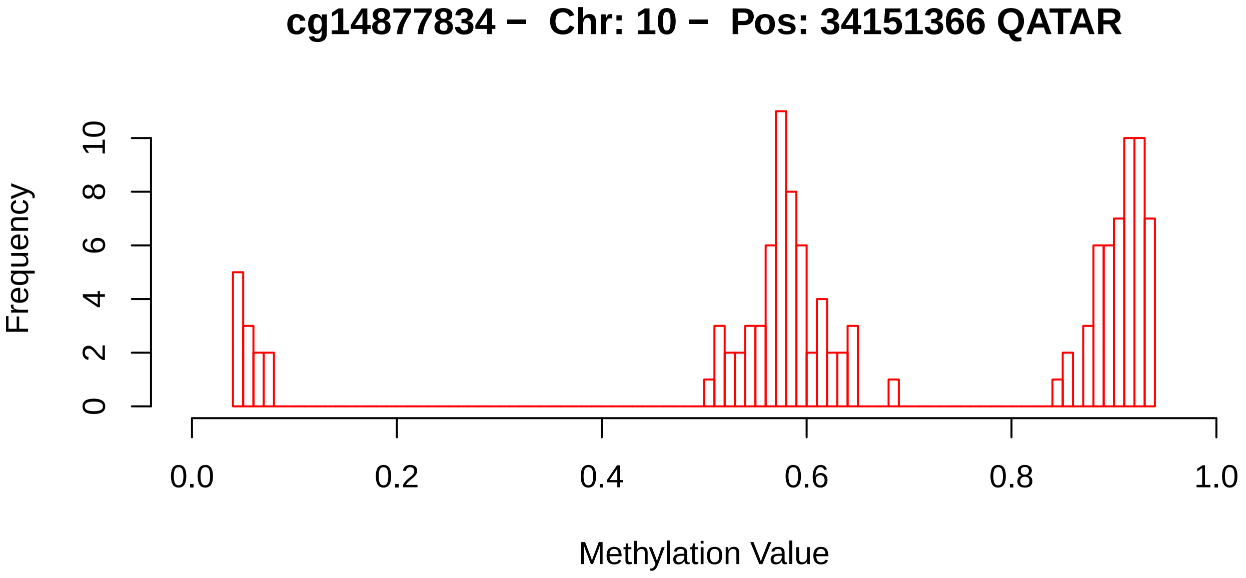
cg14877834 − Chr: 10 − Pos: 34151366 QATAR
Frequency
0
2
4
6
8
10
0.0
0.2
0.4
0.6
0.8
1.0
Methylation Value

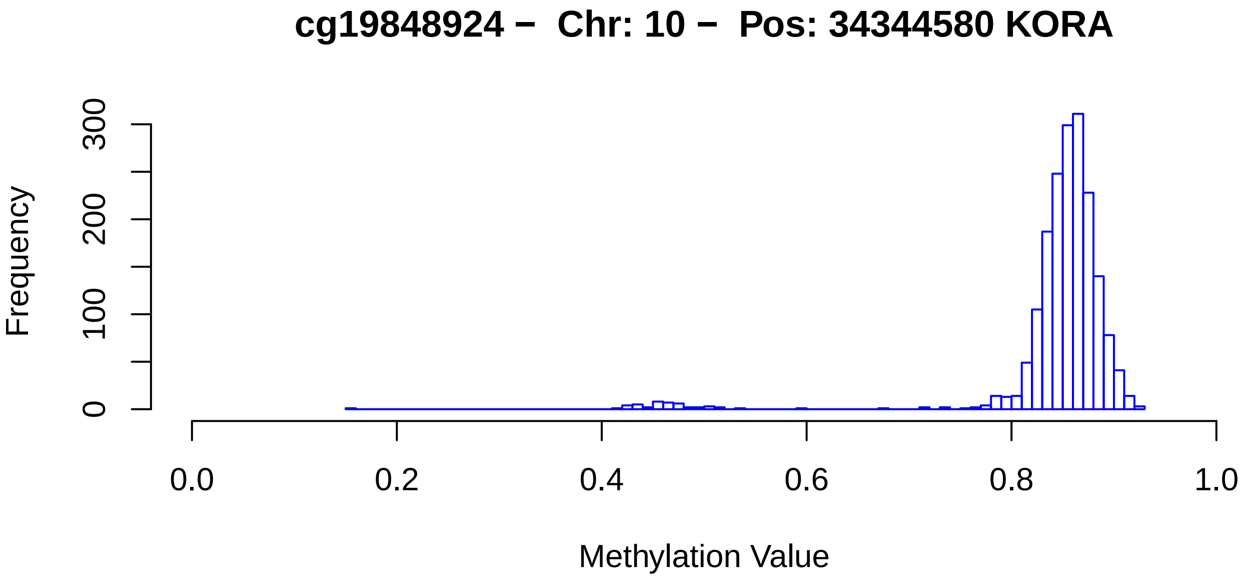

cg19848924 − Chr: 10 − Pos: 34344580 KORA
Frequency
0
100
200
300
0.0
0.2
0.4
0.6
0.8
1.0
Methylation Value

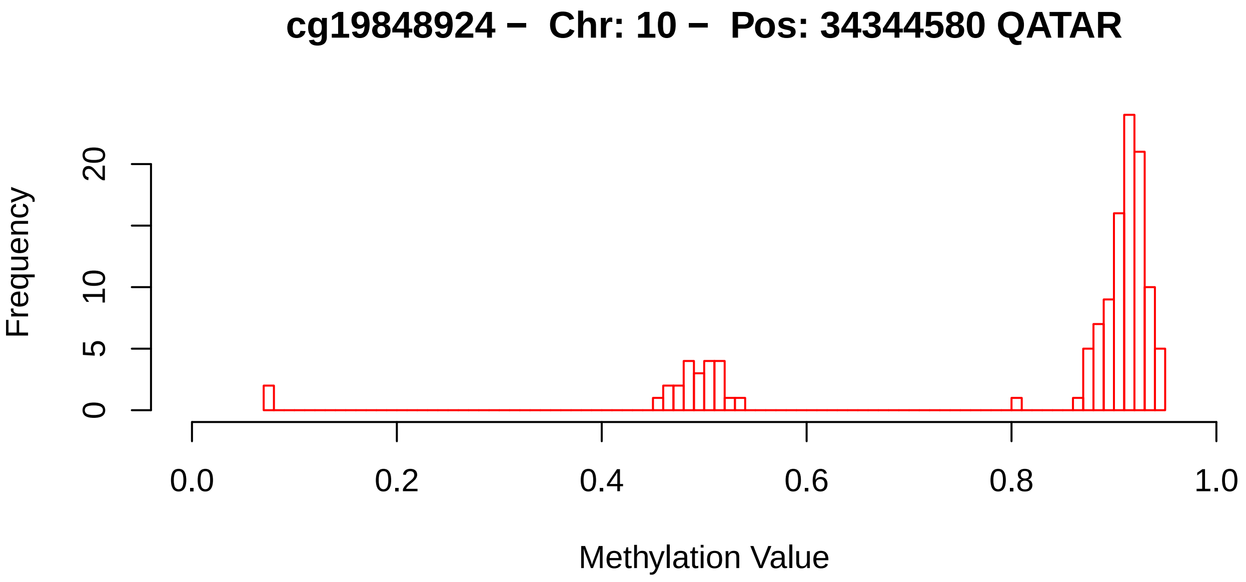

cg19848924 − Chr: 10 − Pos: 34344580 QATAR
Frequency
0
5
10
20
0.0
0.2
0.4
0.6
0.8
1.0
Methylation Value

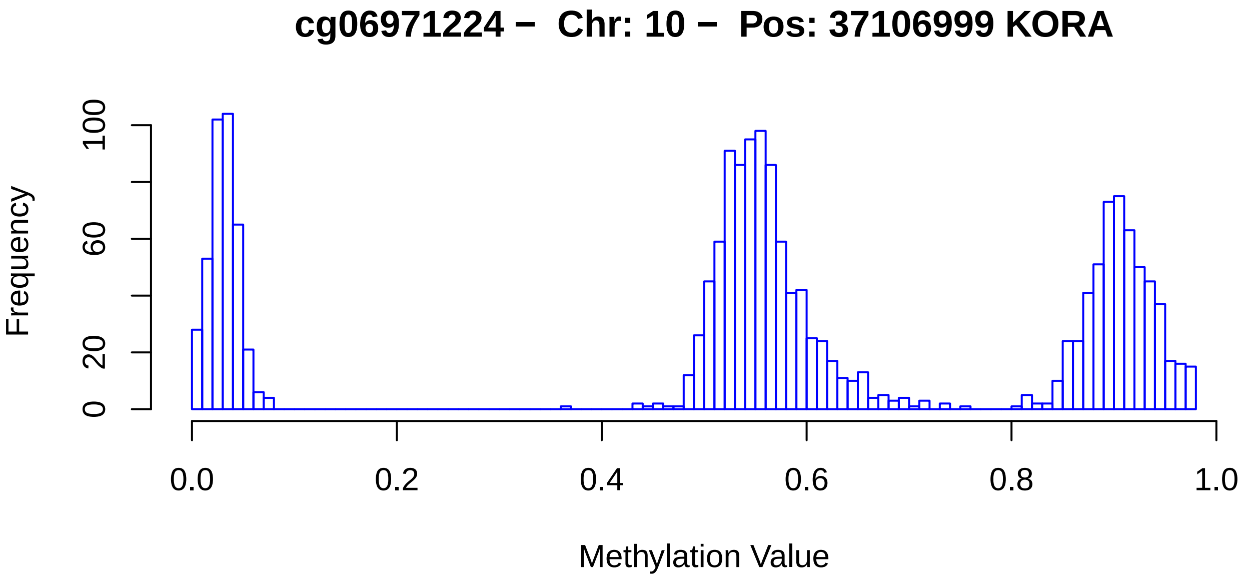
cg06971224 − Chr: 10 − Pos: 37106999 KORA
Frequency
0
20
60
100
0.0
0.2
0.4
0.6
0.8
1.0
Methylation Value

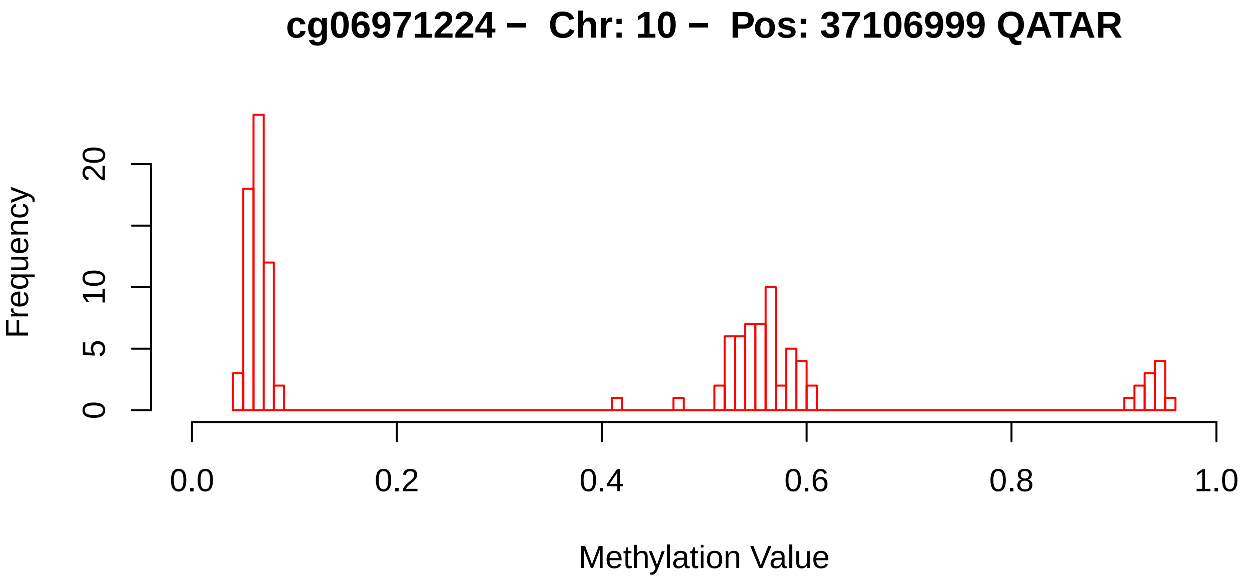
cg06971224 − Chr: 10 − Pos: 37106999 QATAR
Frequency
0
5
10
20
0.0
0.2
0.4
0.6
0.8
1.0
Methylation Value

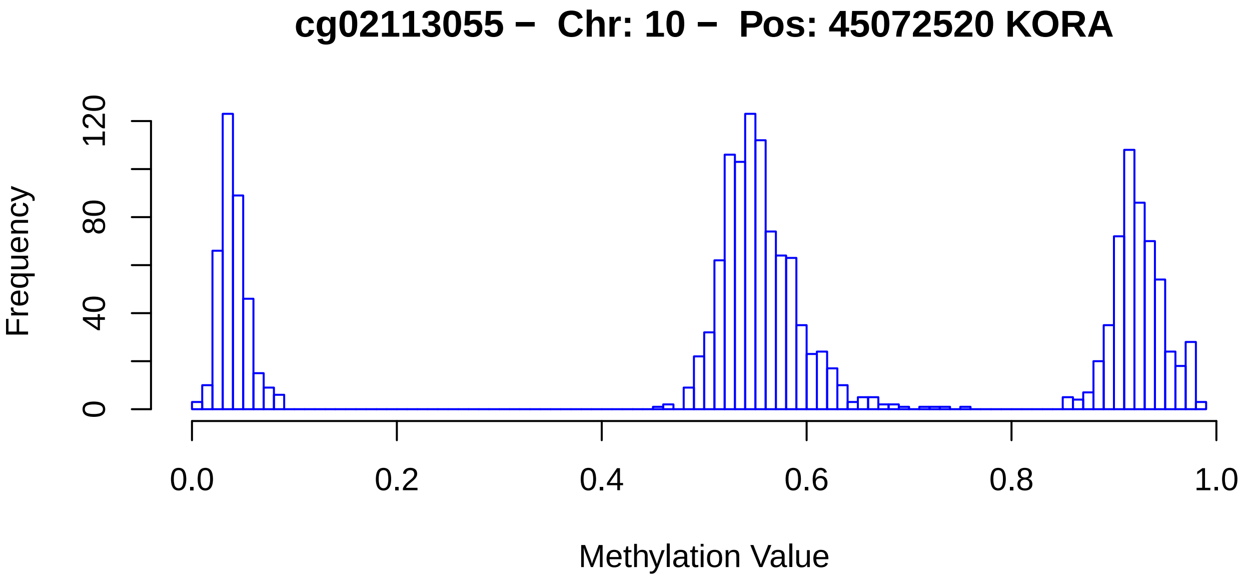
cg02113055 − Chr: 10 − Pos: 45072520 KORA
Frequency
0
40
80
120
0.0
0.2
0.4
0.6
0.8
1.0
Methylation Value

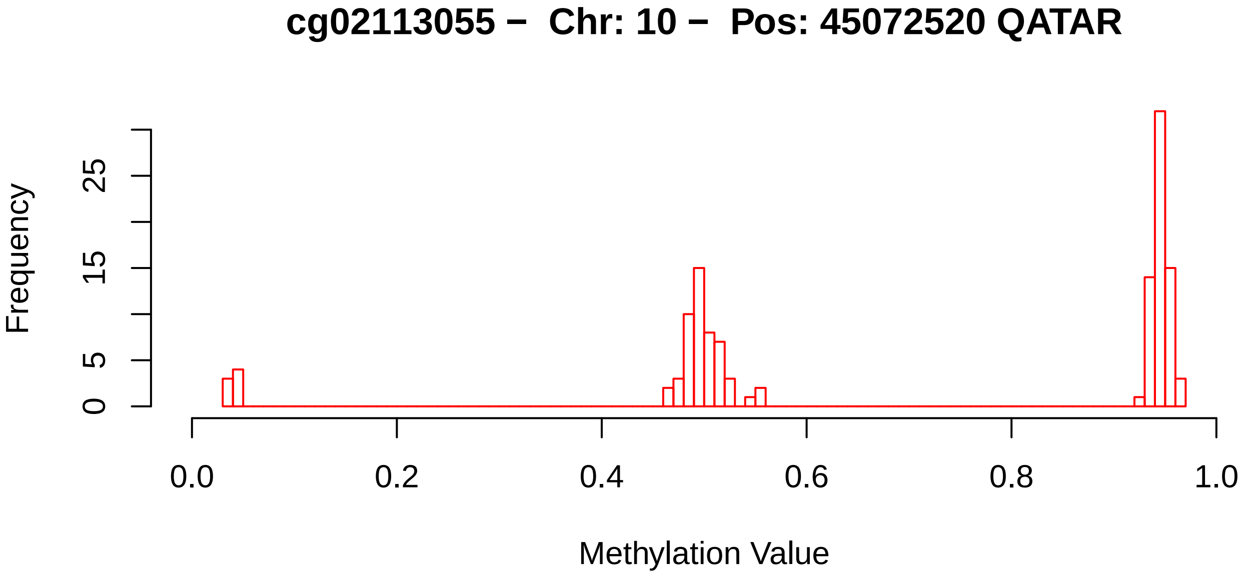
cg02113055 − Chr: 10 − Pos: 45072520 QATAR
Frequency
0
5
15
25
0.0
0.2
0.4
0.6
0.8
1.0
Methylation Value

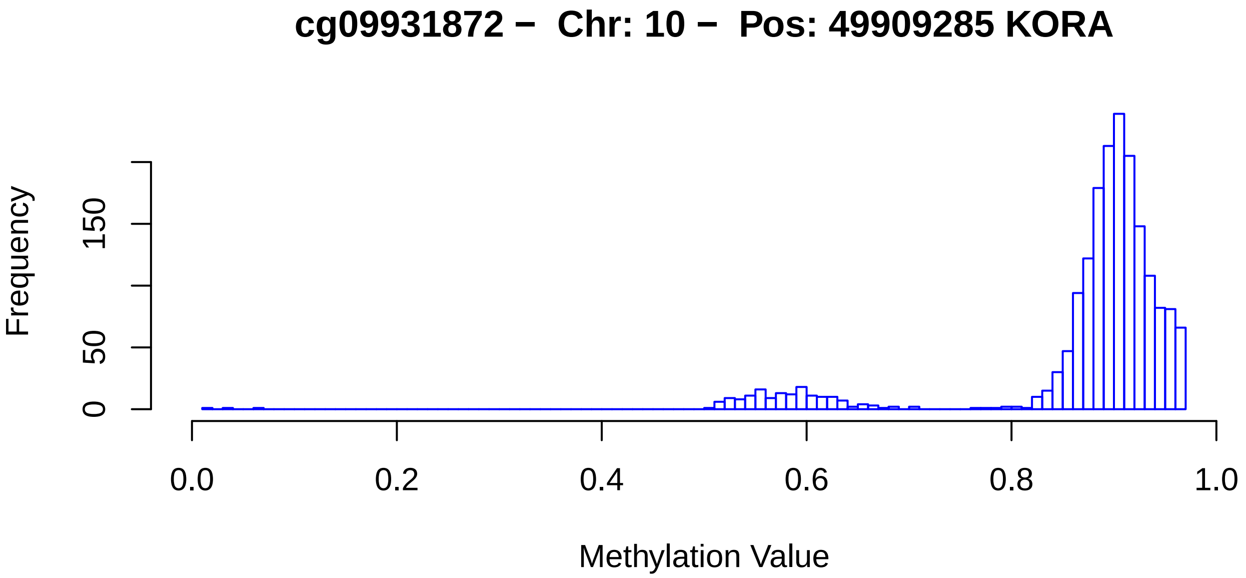
cg09931872 − Chr: 10 − Pos: 49909285 KORA
Frequency
0
50
150
0.0
0.2
0.4
0.6
0.8
1.0
Methylation Value

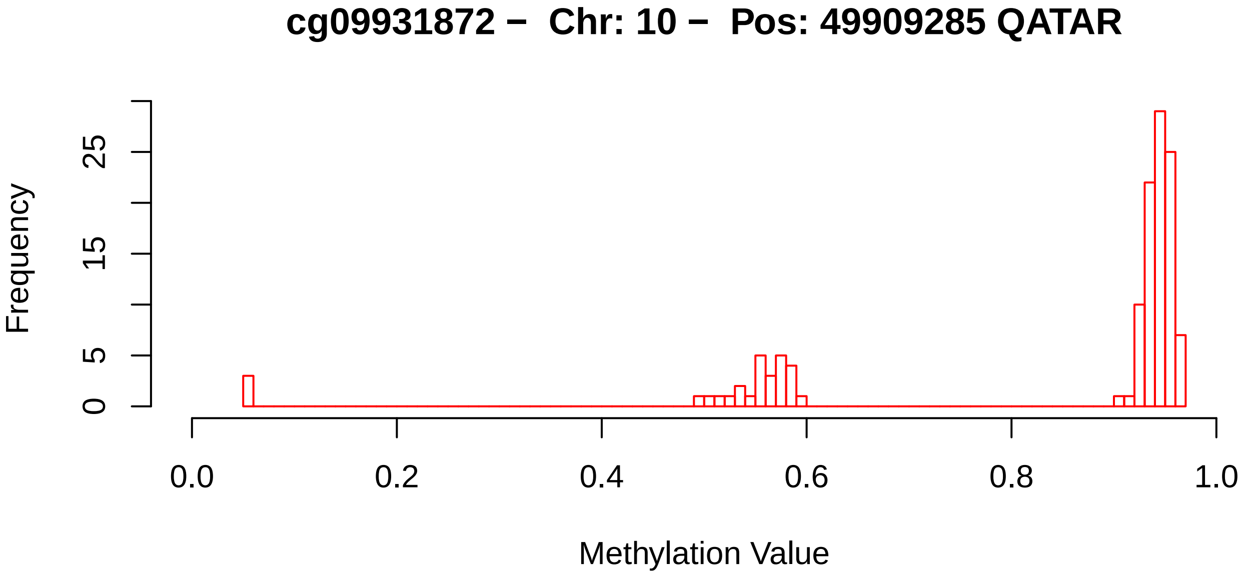
cg09931872 − Chr: 10 − Pos: 49909285 QATAR
Frequency
0
5
15
25
0.0
0.2
0.4
0.6
0.8
1.0
Methylation Value

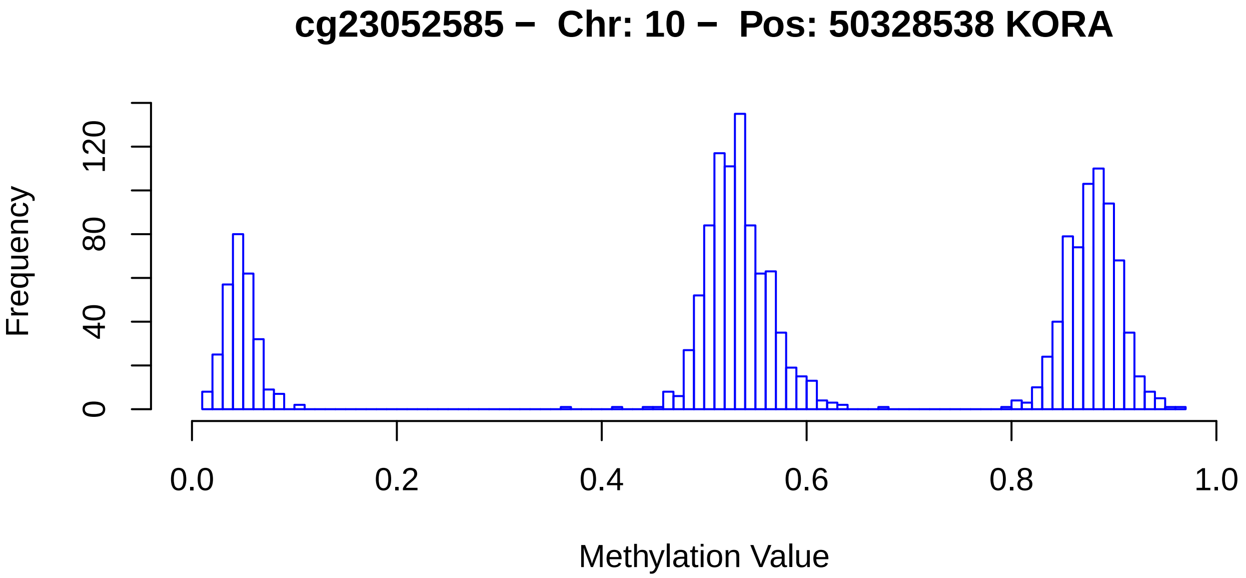
cg23052585 – Chr: 10 – Pos: 50328538 KORA
Frequency
0
40
80
120
0.0
0.2
0.4
0.6
0.8
1.0
Methylation Value

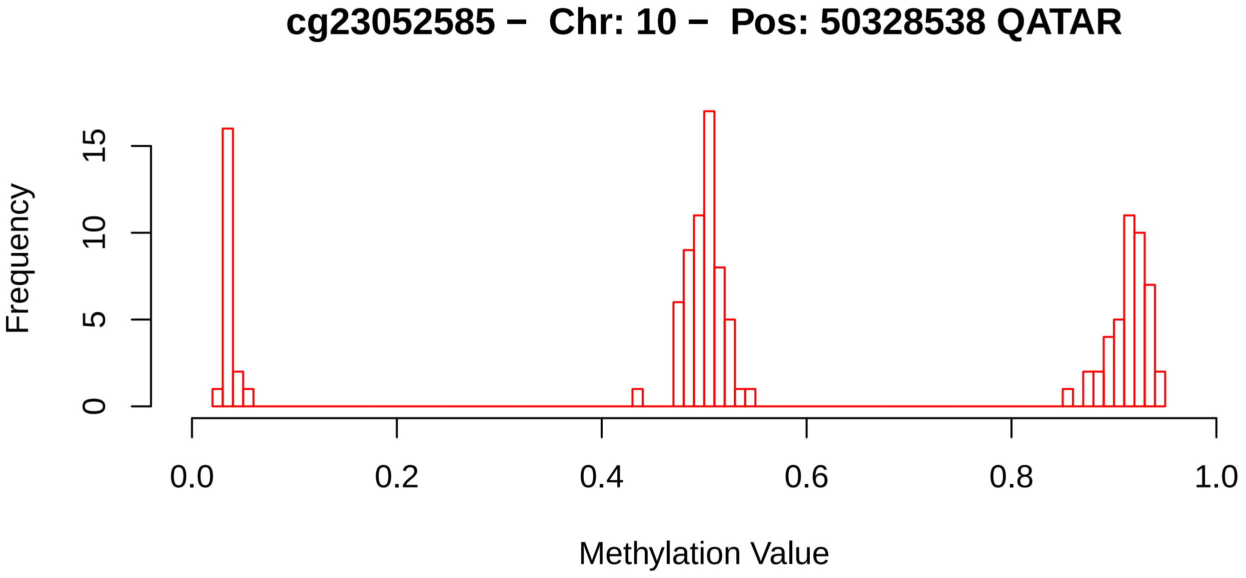
cg23052585 – Chr: 10 – Pos: 50328538 QATAR
Frequency
0
5
10
15
0.0
0.2
0.4
0.6
0.8
1.0
Methylation Value

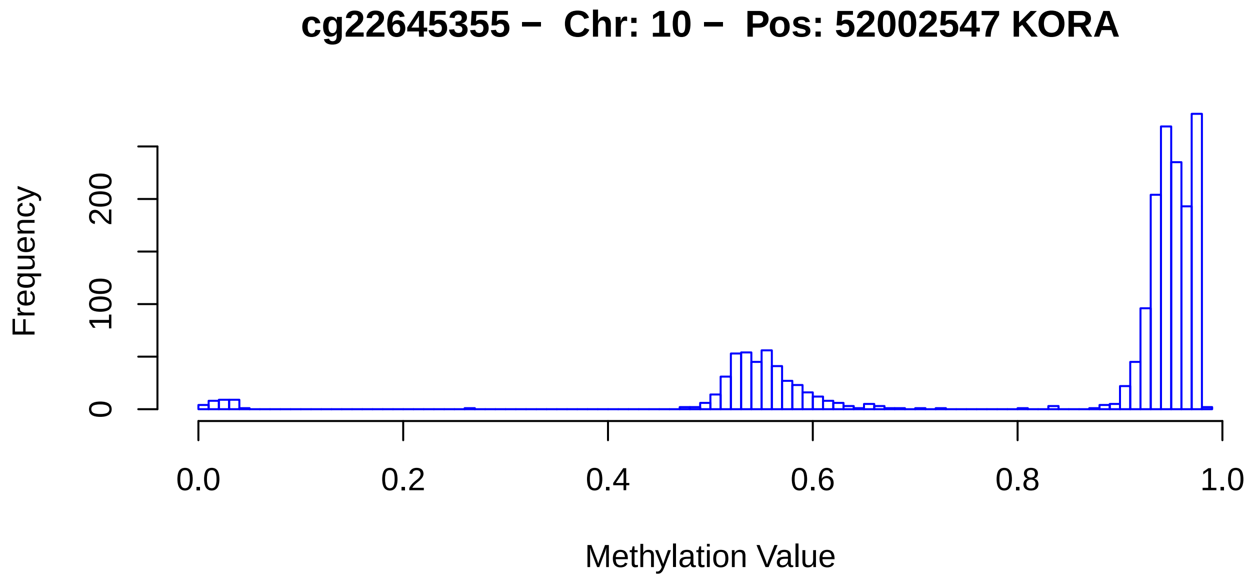

cg22645355 − Chr: 10 − Pos: 52002547 KORA
Frequency
0
100
200
0.0
0.2
0.4
0.6
0.8
1.0
Methylation Value

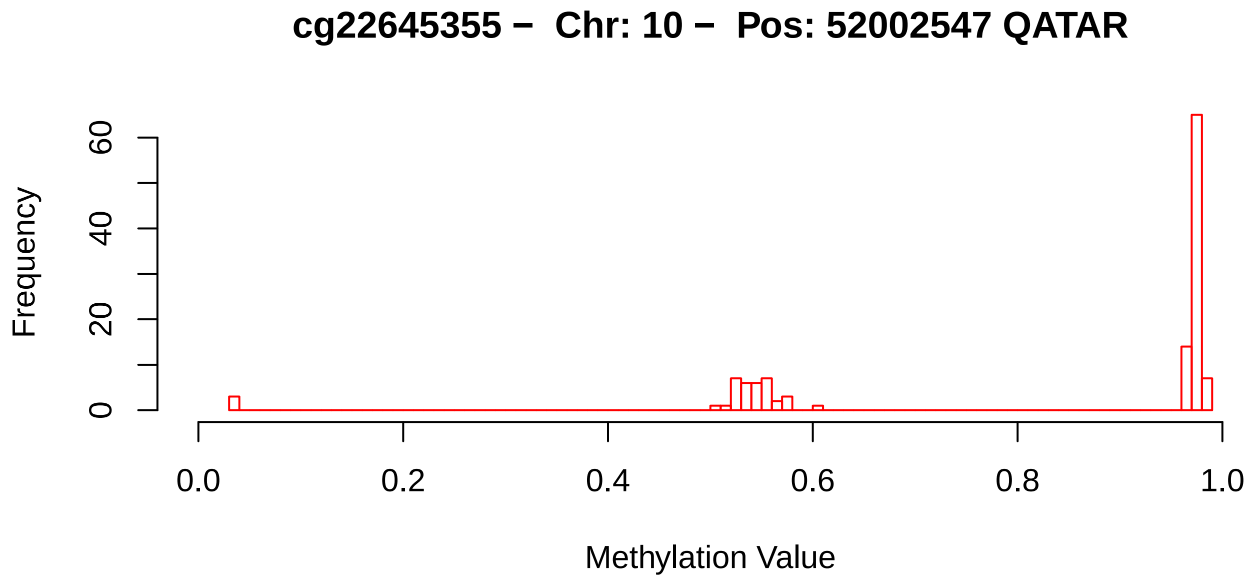

cg22645355 − Chr: 10 − Pos: 52002547 QATAR
Frequency
0
20
40
60
0.0
0.2
0.4
0.6
0.8
1.0
Methylation Value

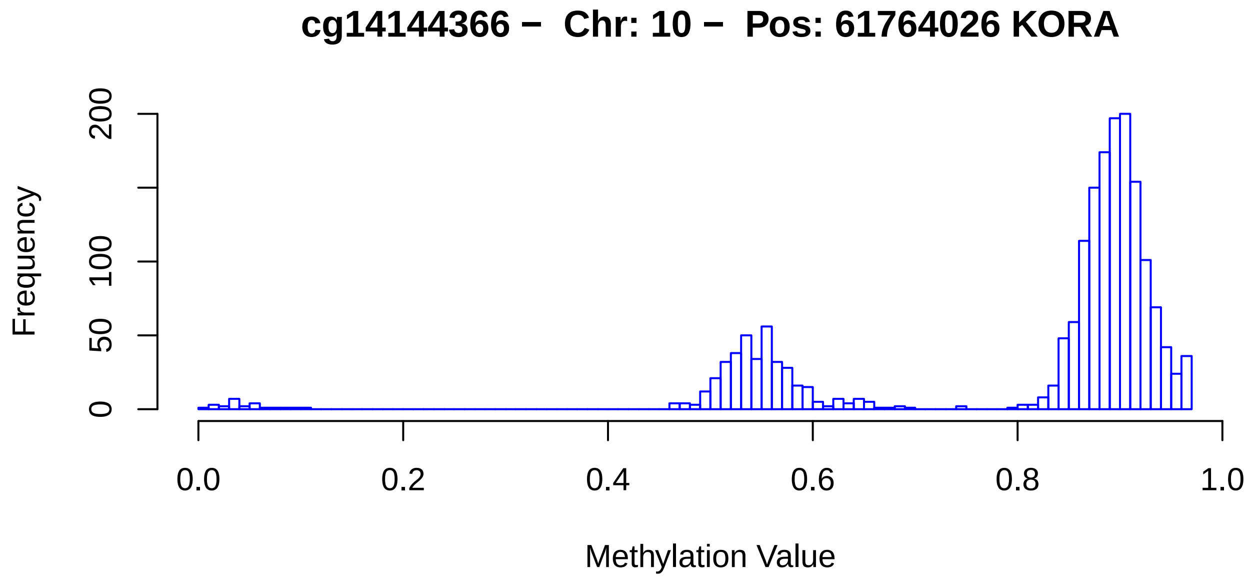
cg14144366 − Chr: 10 − Pos: 61764026 KORA
Frequency
0
50
100
200
0.0
0.2
0.4
0.6
0.8
1.0
Methylation Value

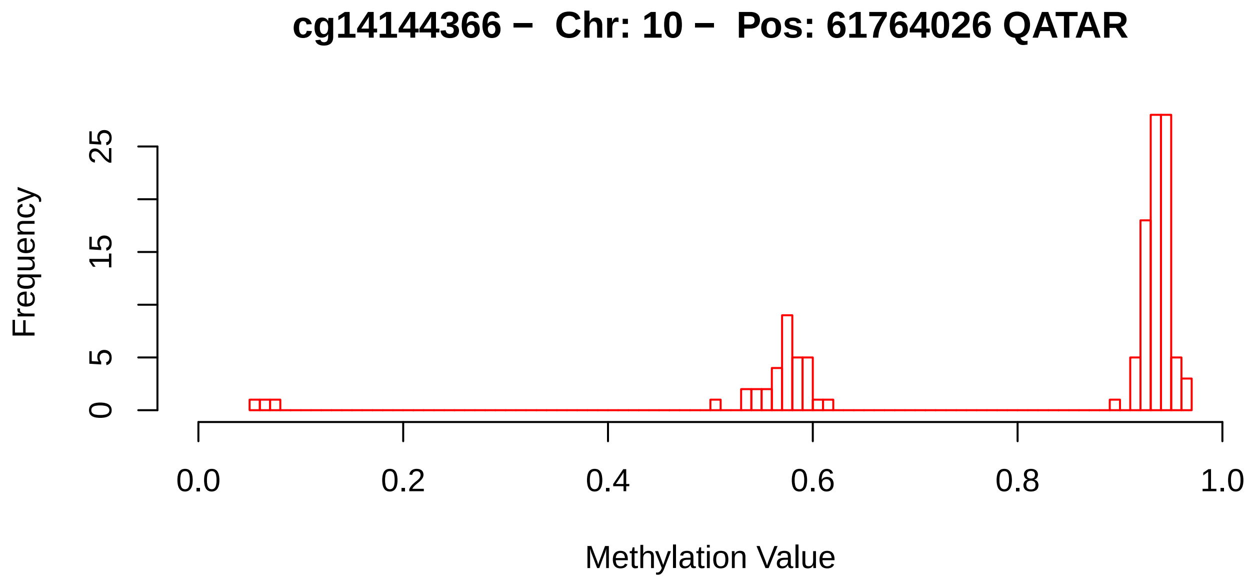
cg14144366 − Chr: 10 − Pos: 61764026 QATAR
Frequency
0
5
15
25
0.0
0.2
0.4
0.6
0.8
1.0
Methylation Value

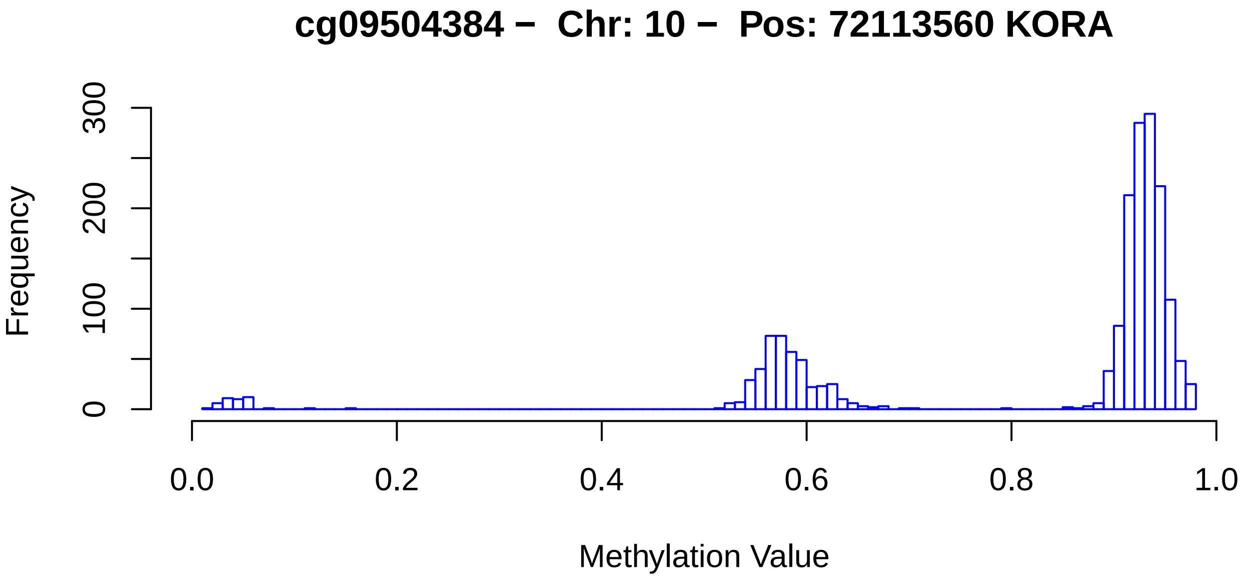
cg09504384 – Chr: 10 – Pos: 72113560 KORA
Frequency
0
100
200
300
0.0
0.2
0.4
0.6
0.8
1.0
Methylation Value

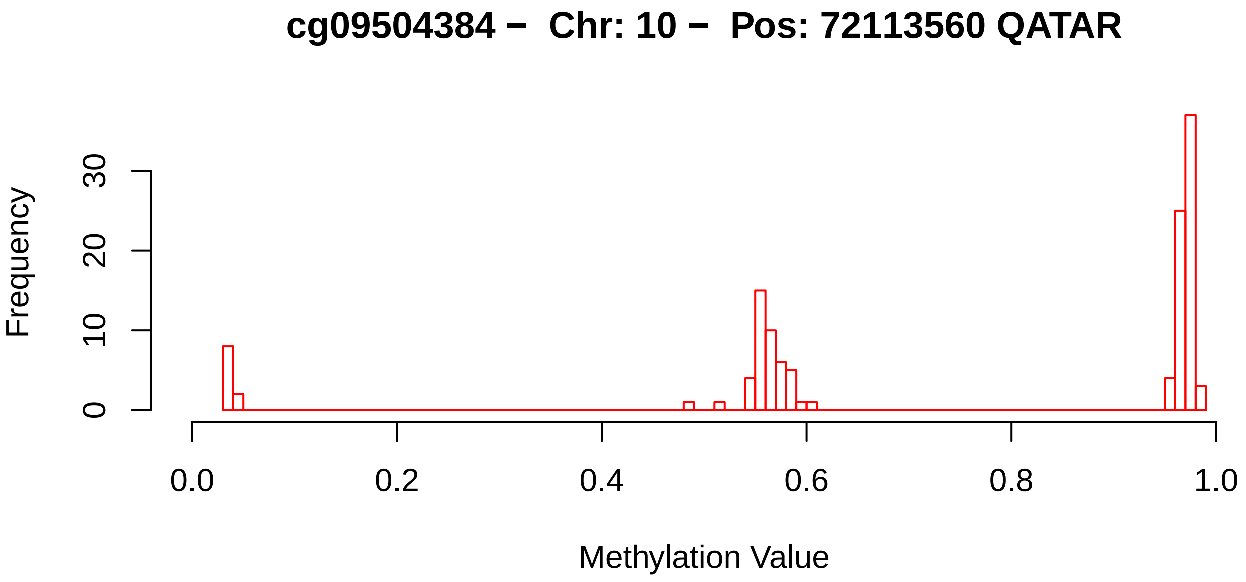
cg09504384 – Chr: 10 – Pos: 72113560 QATAR
Frequency
0
10
20
30
0.0
0.2
0.4
0.6
0.8
1.0
Methylation Value

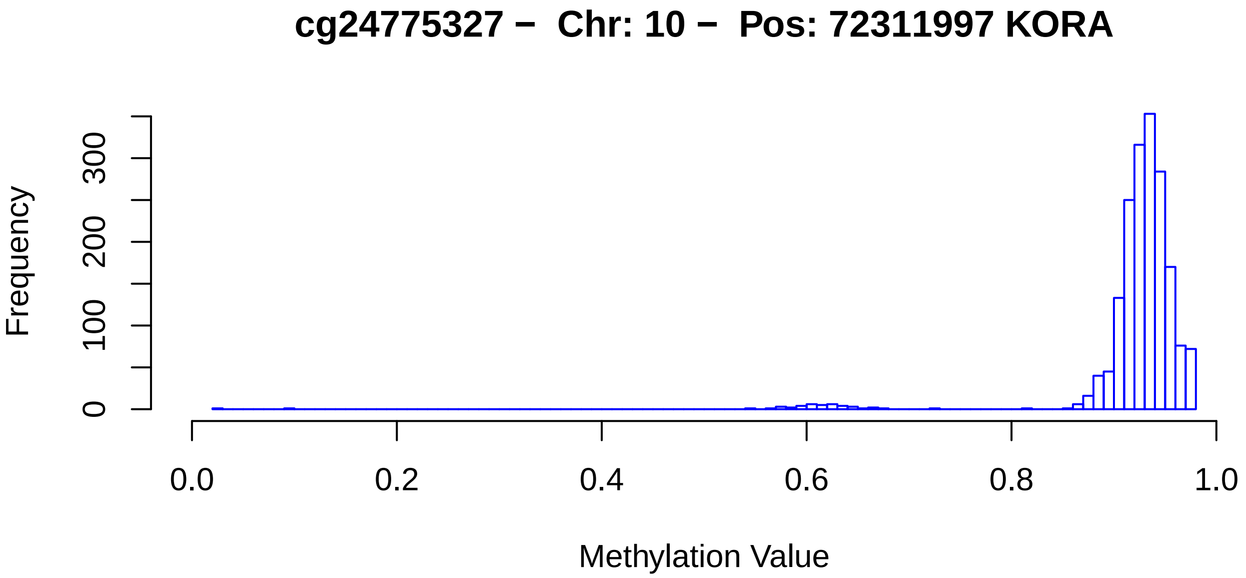
cg24775327 − Chr: 10 − Pos: 72311997 KORA
Frequency
0
100
200
300
0.0
0.2
0.4
0.6
0.8
1.0
Methylation Value

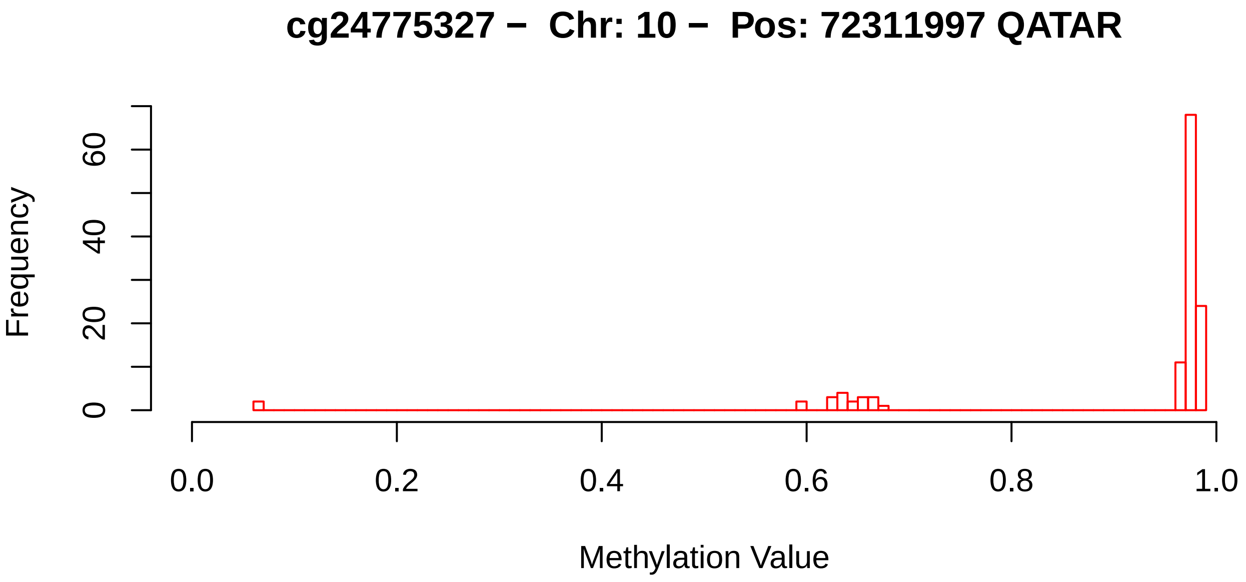
cg24775327 − Chr: 10 − Pos: 72311997 QATAR
Frequency
0
20
40
60
0.0
0.2
0.4
0.6
0.8
1.0
Methylation Value

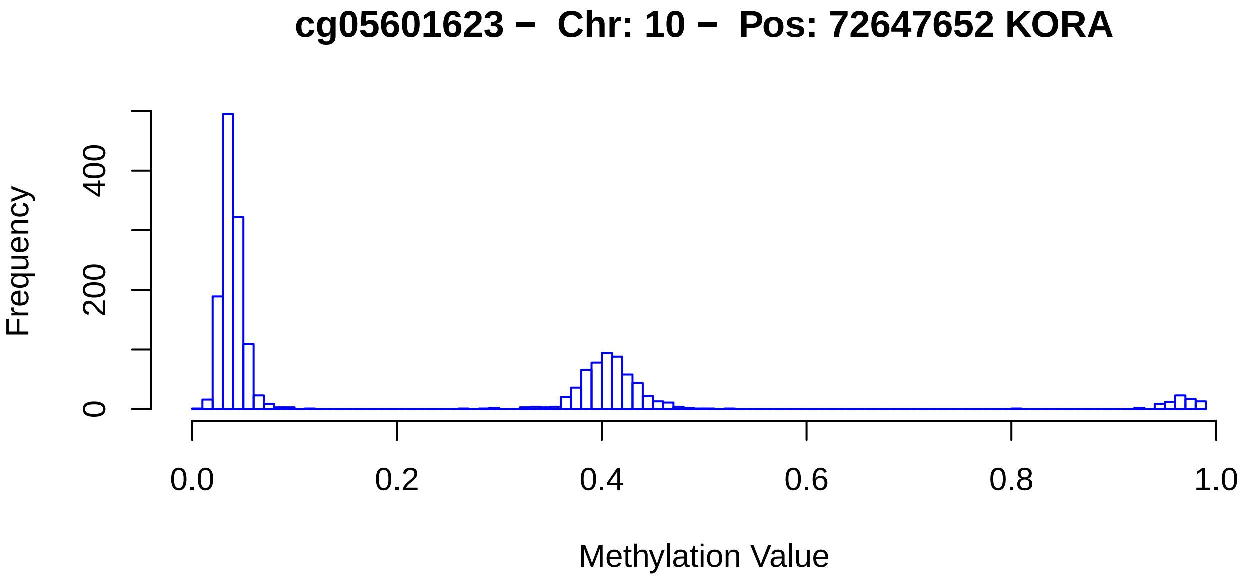
cg05601623 − Chr: 10 − Pos: 72647652 KORA
Frequency
0
200
400
0.0
0.2
0.4
0.6
0.8
1.0
Methylation Value

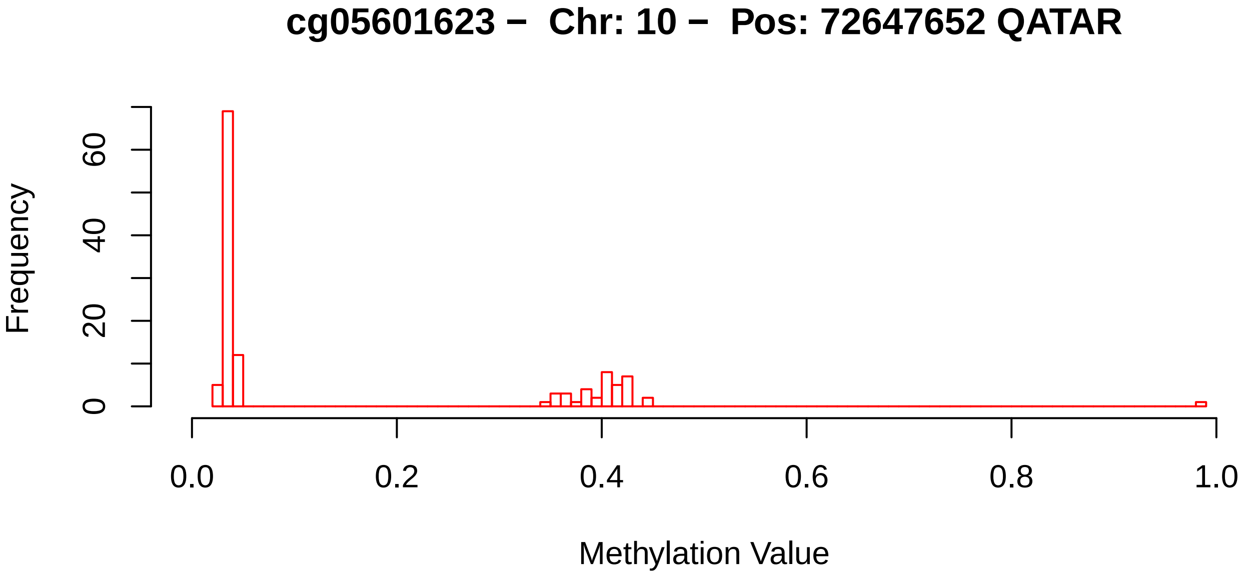
cg05601623 − Chr: 10 − Pos: 72647652 QATAR
Frequency
0
20
40
60
0.0
0.2
0.4
0.6
0.8
1.0
Methylation Value

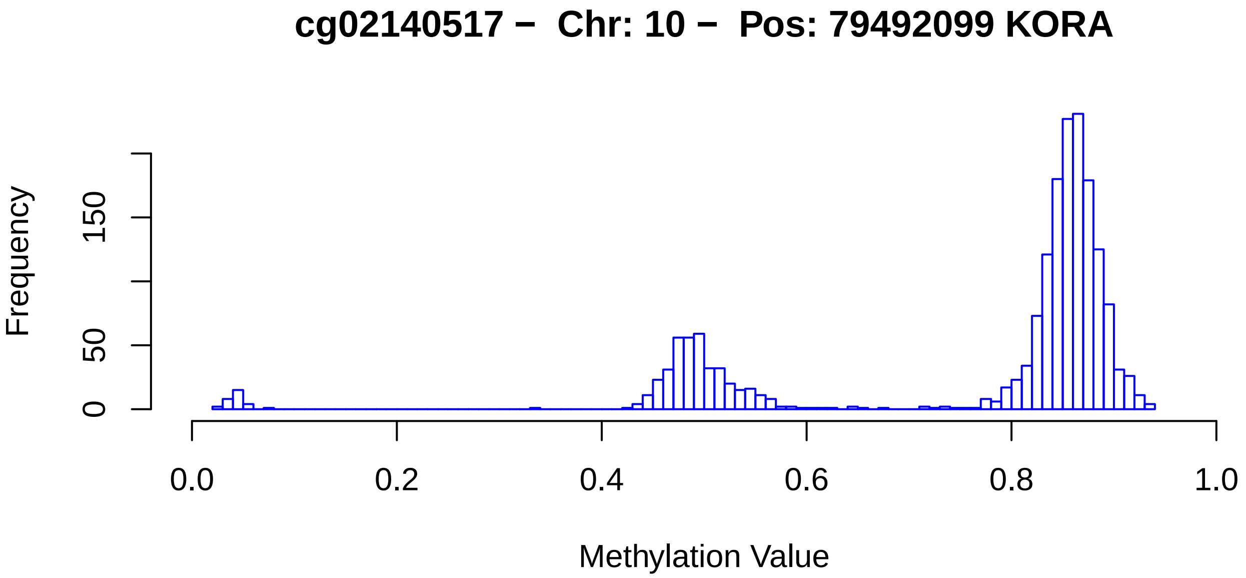
cg02140517 − Chr: 10 − Pos: 79492099 KORA
Frequency
0
50
150
0.0
0.2
0.4
0.6
0.8
1.0
Methylation Value

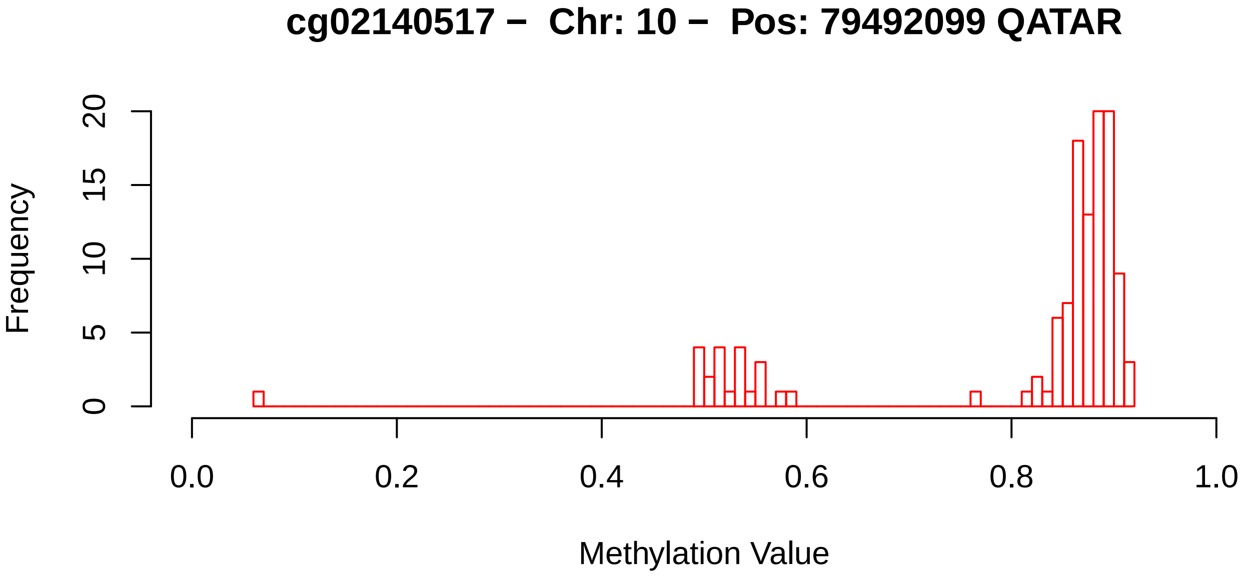
cg02140517 − Chr: 10 − Pos: 79492099 QATAR
Frequency
0
5
10
15
20
0.0
0.2
0.4
0.6
0.8
1.0
Methylation Value

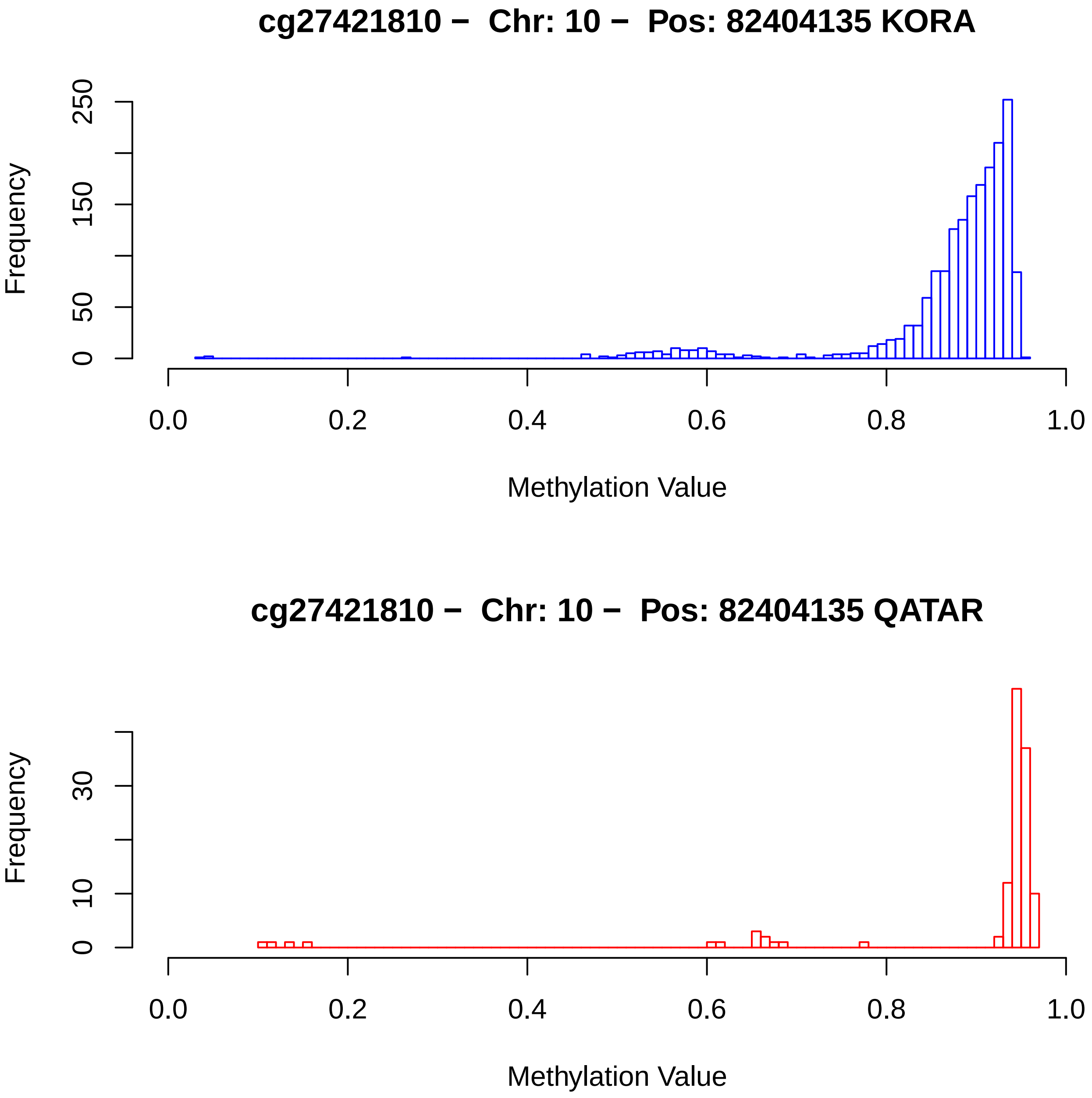
cg27421810 − Chr: 10 − Pos: 82404135 KORA
Frequency
0
50
150
250
0.0
0.2
0.4
0.6
0.8
1.0
Methylation Value
cg27421810 − Chr: 10 − Pos: 82404135 QATAR
Frequency
0
10
30
0.0
0.2
0.4
0.6
0.8
1.0
Methylation Value

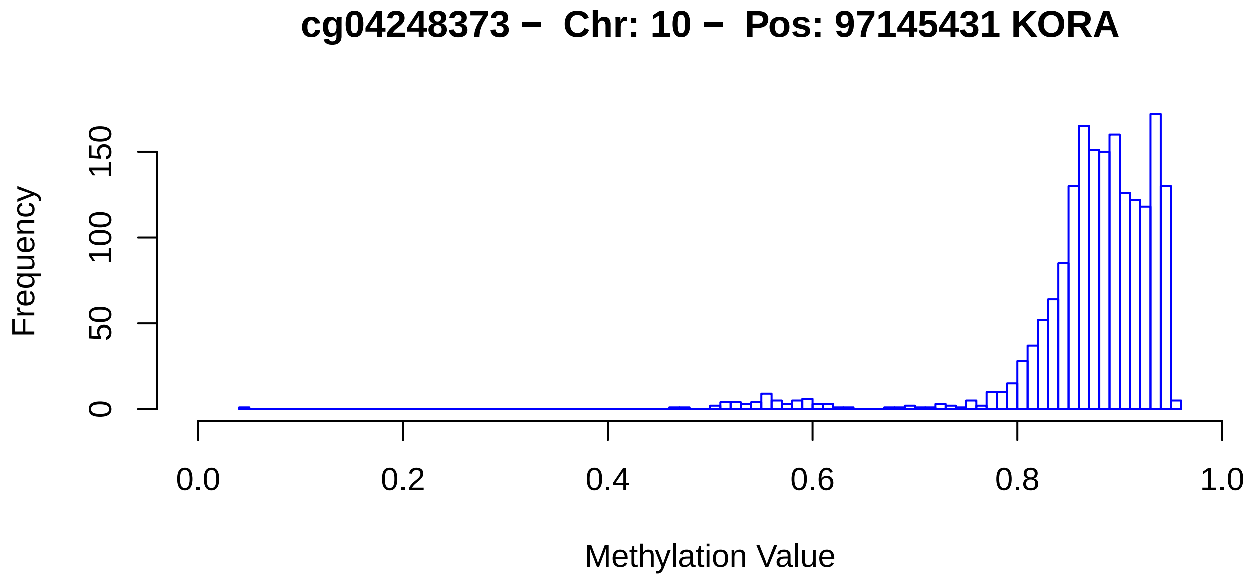
cg04248373 − Chr: 10 − Pos: 97145431 KORA
Frequency
0
50
100
150
0.0
0.2
0.4
0.6
0.8
1.0
Methylation Value

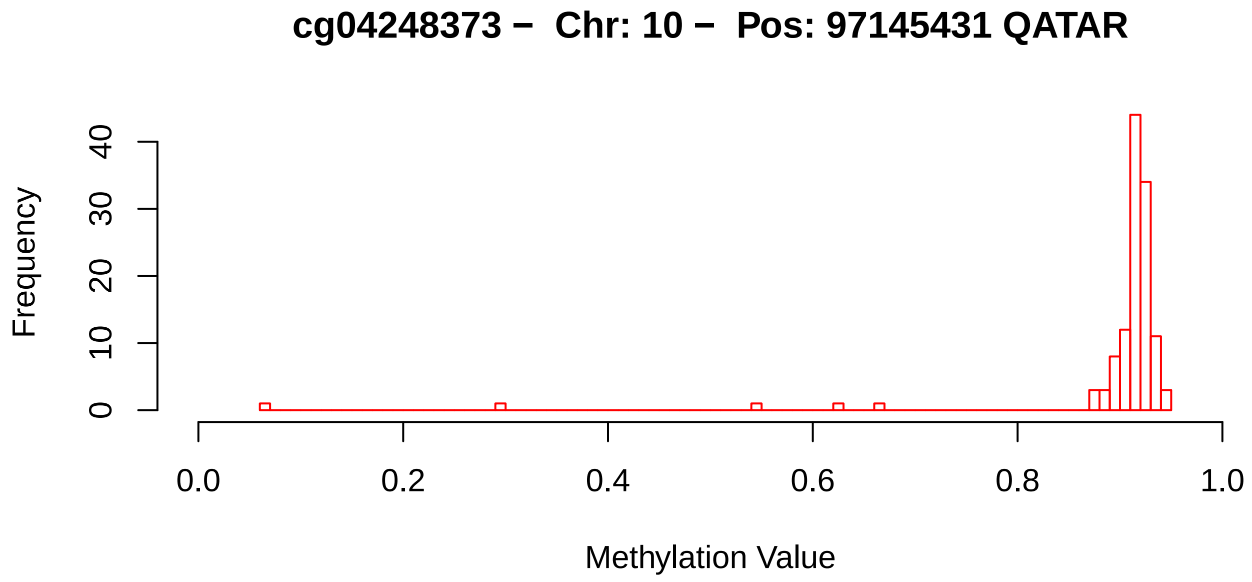
cg04248373 − Chr: 10 − Pos: 97145431 QATAR
Frequency
0
10
20
30
40
0.0
0.2
0.4
0.6
0.8
1.0
Methylation Value

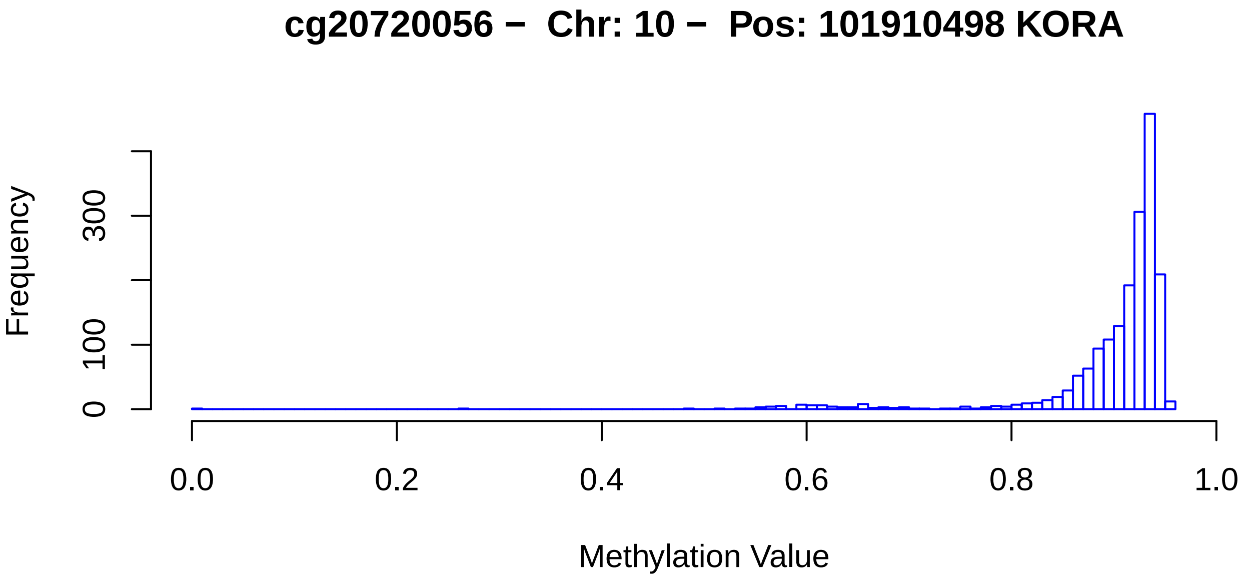
cg20720056 − Chr: 10 − Pos: 101910498 KORA
Frequency
0
100
300
0.0
0.2
0.4
0.6
0.8
1.0
Methylation Value

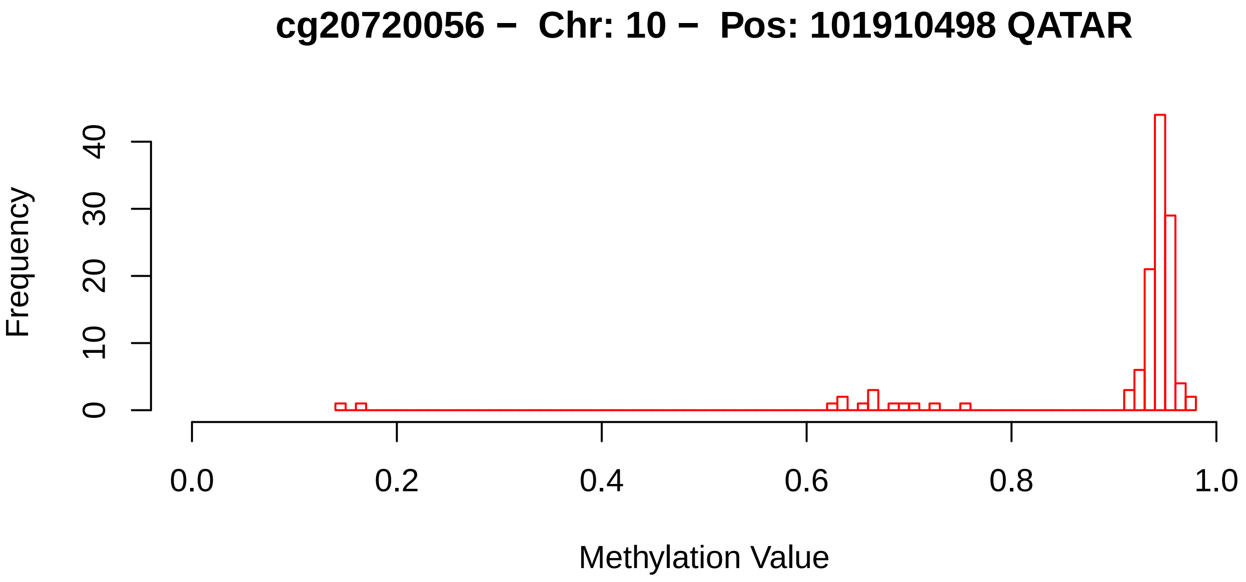
cg20720056 − Chr: 10 − Pos: 101910498 QATAR
Frequency
0
10
20
30
40
0.0
0.2
0.4
0.6
0.8
1.0
Methylation Value

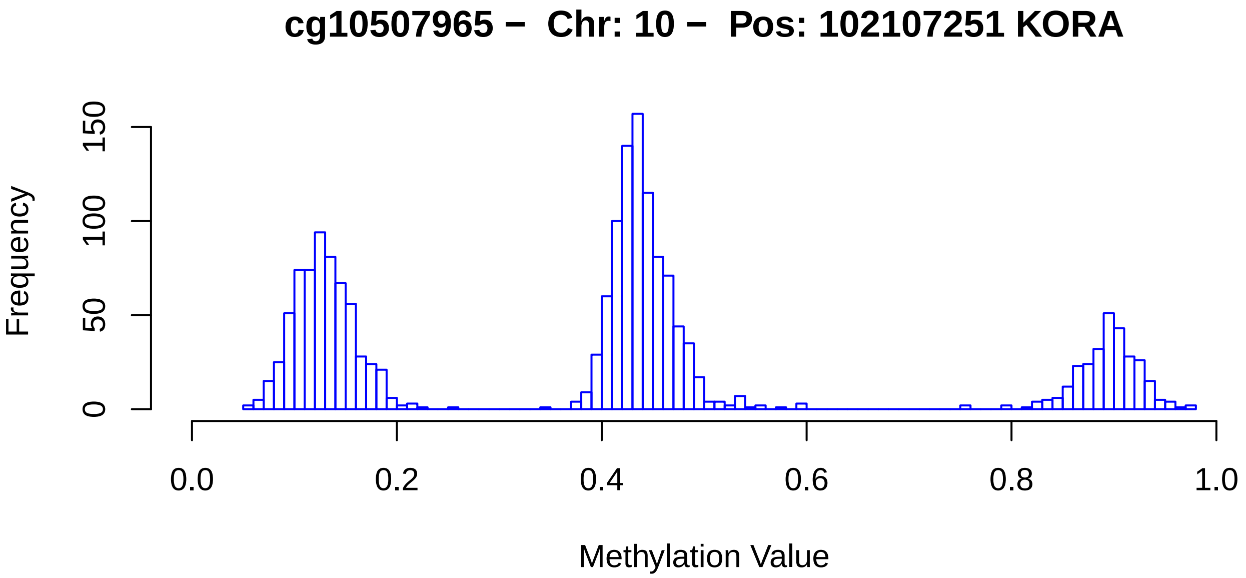
cg10507965 − Chr: 10 − Pos: 102107251 KORA
Frequency
Methylation Value
0
50
100
150
0.0
0.2
0.4
0.6
0.8
1.0

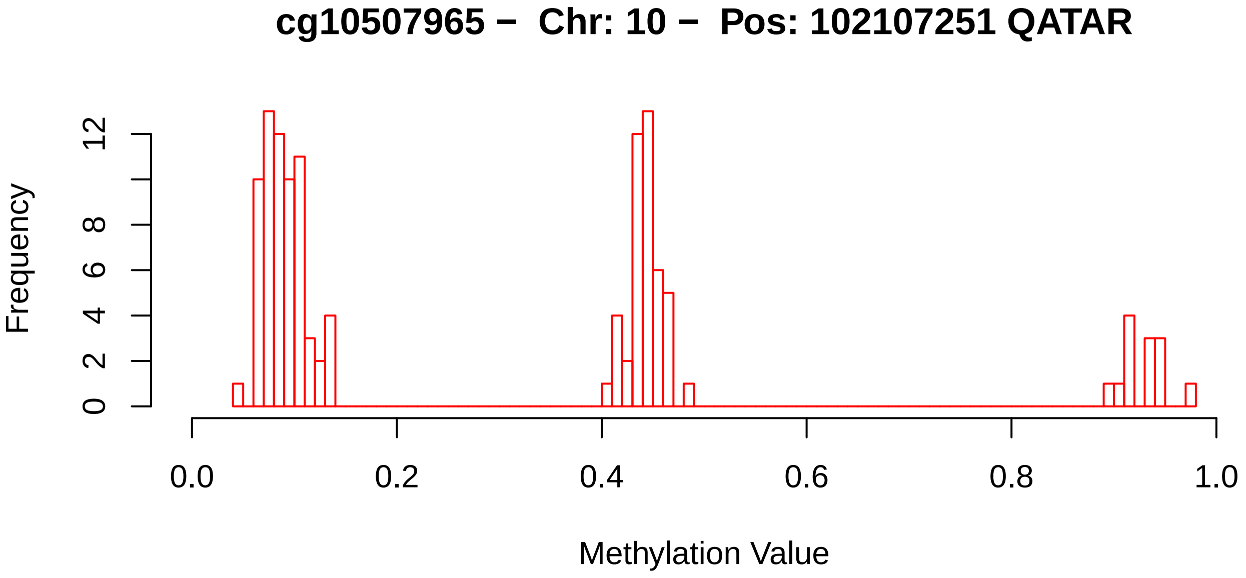
cg10507965 − Chr: 10 − Pos: 102107251 QATAR
Frequency
Methylation Value
0
2
4
6
8
12
0.0
0.2
0.4
0.6
0.8
1.0

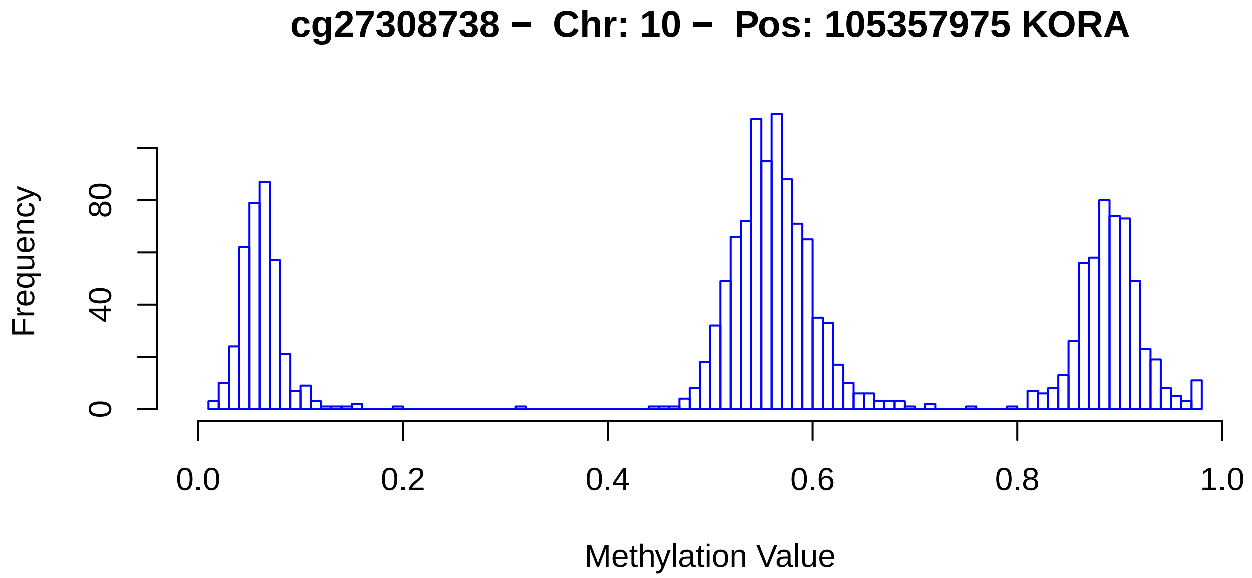
cg27308738 − Chr: 10 − Pos: 105357975 KORA
Frequency
0
40
80
0.0
0.2
0.4
0.6
0.8
1.0
Methylation Value

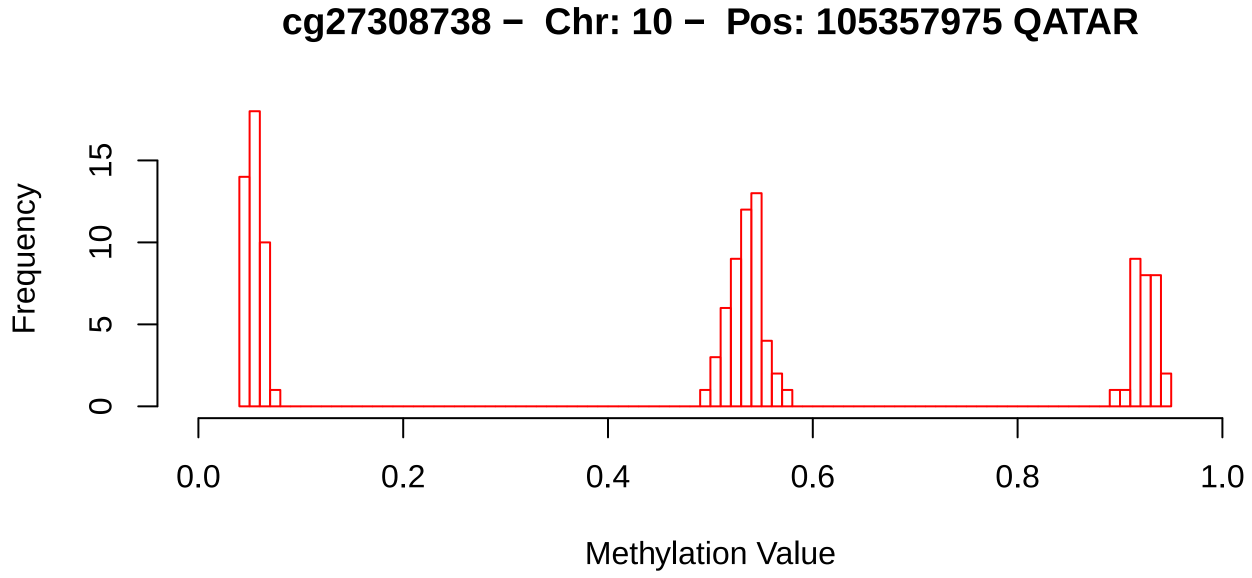
cg27308738 − Chr: 10 − Pos: 105357975 QATAR
Frequency
0
5
10
15
0.0
0.2
0.4
0.6
0.8
1.0
Methylation Value

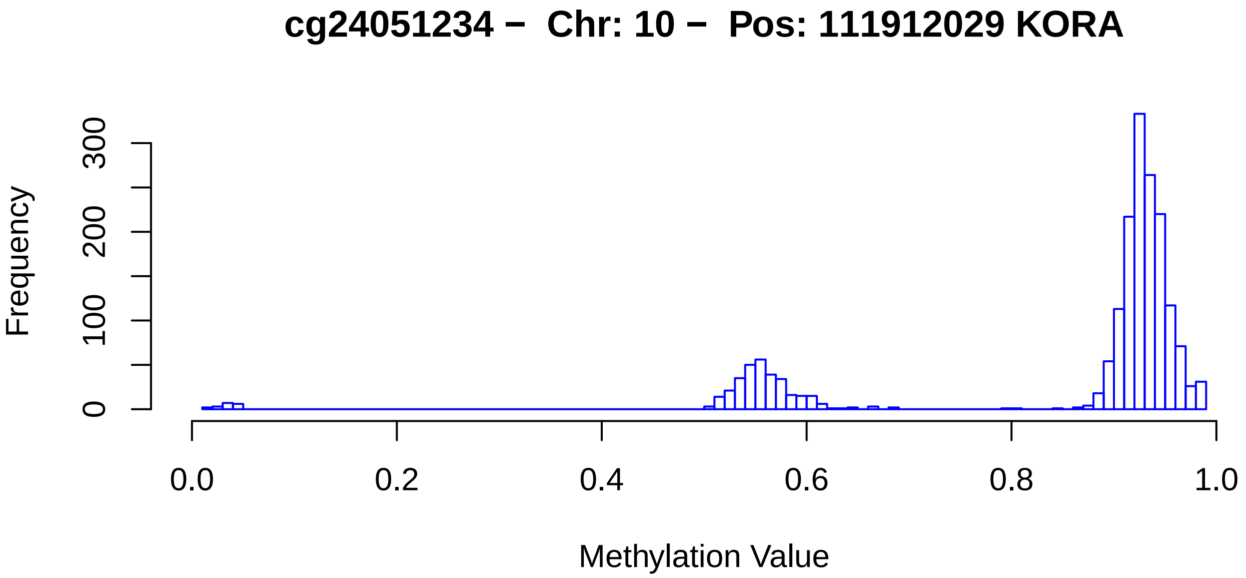

cg24051234 − Chr: 10 − Pos: 111912029 KORA
Frequency
0
100
200
300
0.0
0.2
0.4
0.6
0.8
1.0
Methylation Value

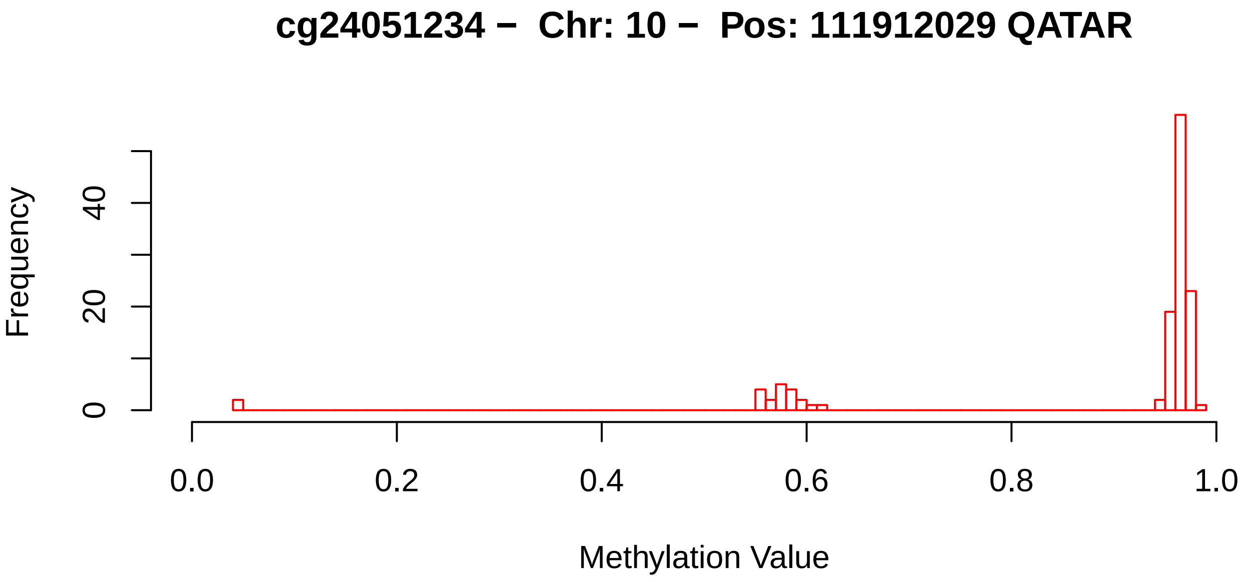

cg24051234 − Chr: 10 − Pos: 111912029 QATAR
Frequency
0
20
40
0.0
0.2
0.4
0.6
0.8
1.0
Methylation Value

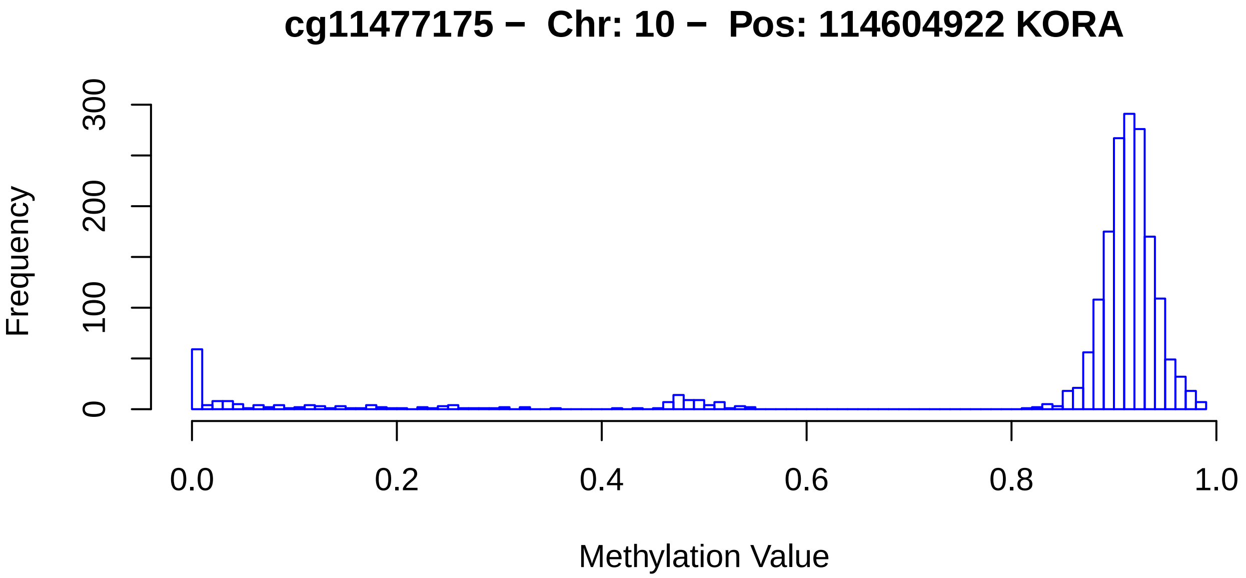

cg11477175 − Chr: 10 − Pos: 114604922 KORA
Frequency
0
100
200
300
0.0
0.2
0.4
0.6
0.8
1.0
Methylation Value

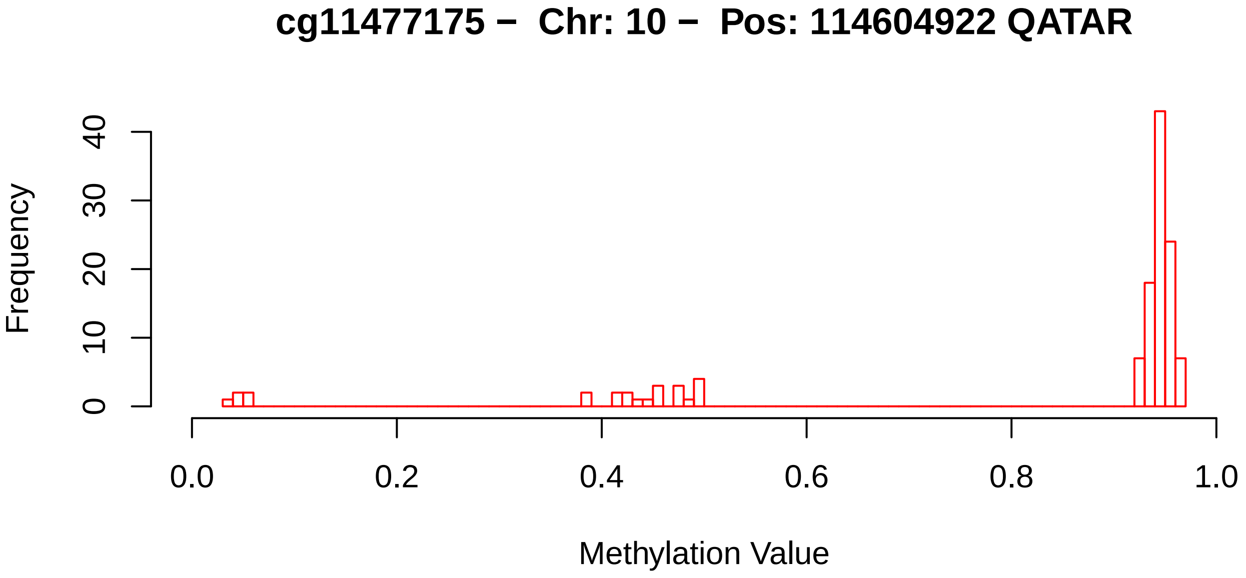

cg11477175 − Chr: 10 − Pos: 114604922 QATAR
Frequency
0
10
20
30
40
0.0
0.2
0.4
0.6
0.8
1.0
Methylation Value

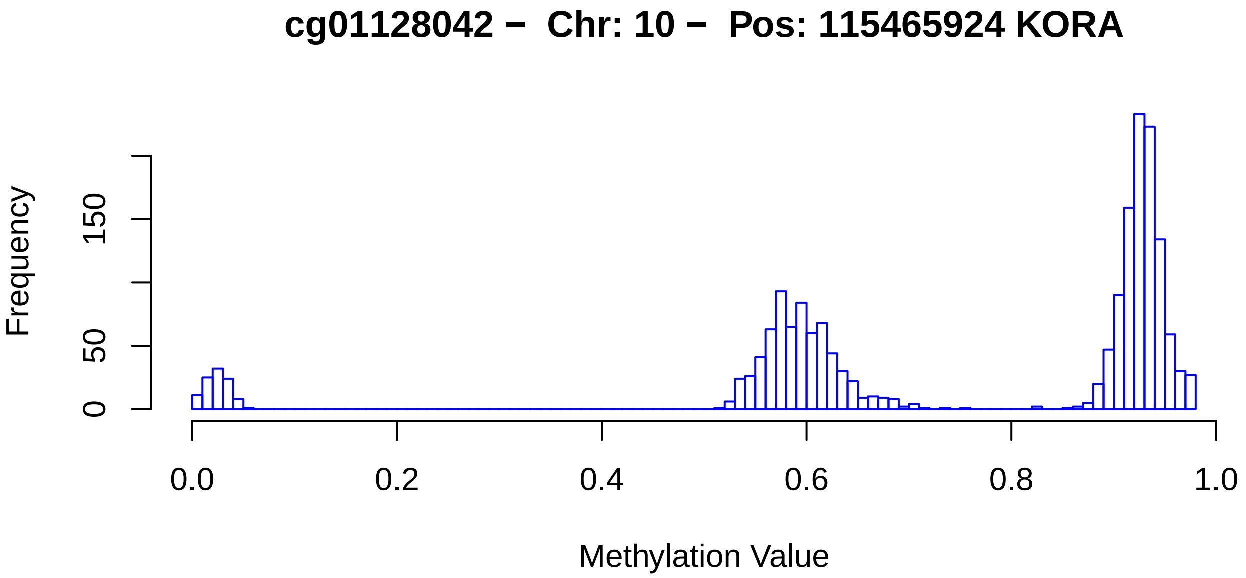
cg01128042 − Chr: 10 − Pos: 115465924 KORA
Frequency
0
50
150
0.0
0.2
0.4
0.6
0.8
1.0
Methylation Value

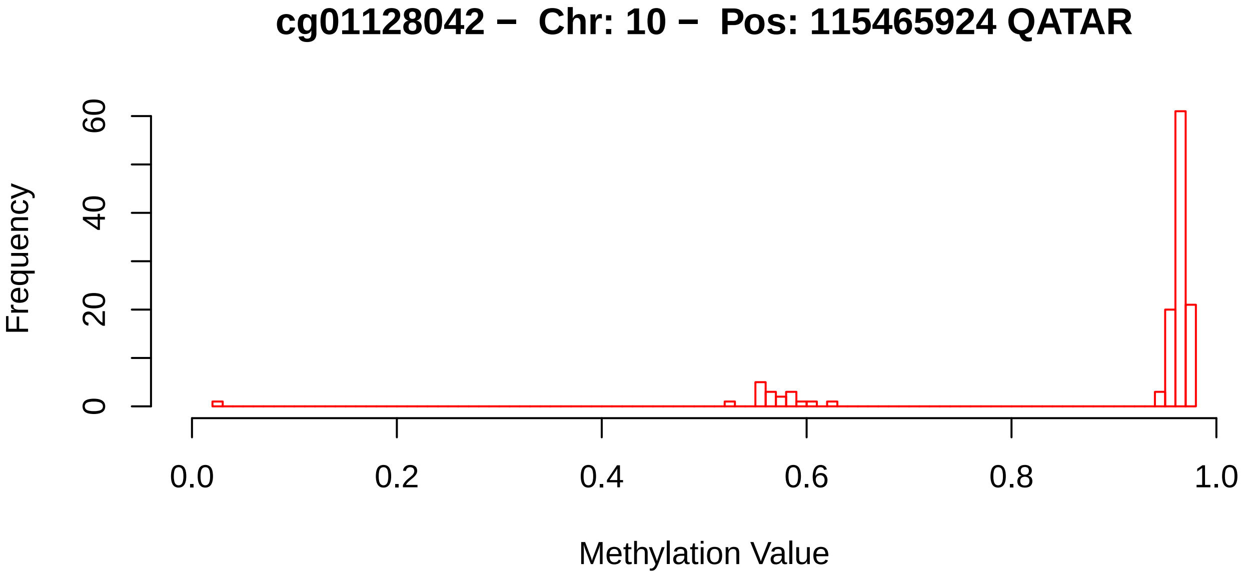
cg01128042 − Chr: 10 − Pos: 115465924 QATAR
Frequency
0
20
40
60
0.0
0.2
0.4
0.6
0.8
1.0
Methylation Value

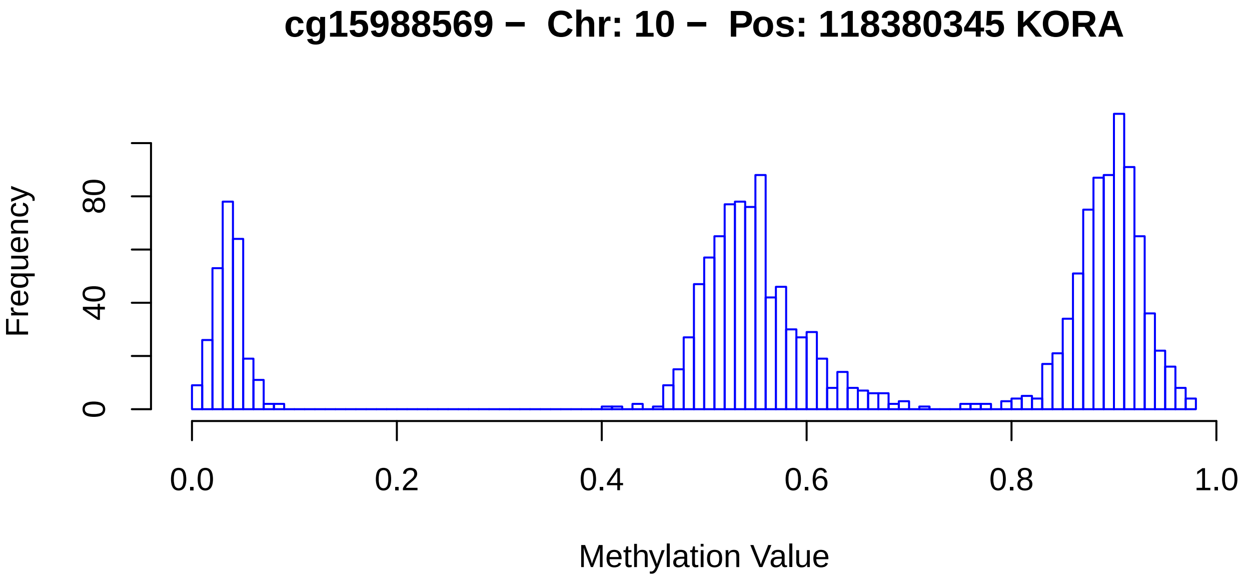
cg15988569 − Chr: 10 − Pos: 118380345 KORA
Frequency
0
40
80
0.0
0.2
0.4
0.6
0.8
1.0
Methylation Value

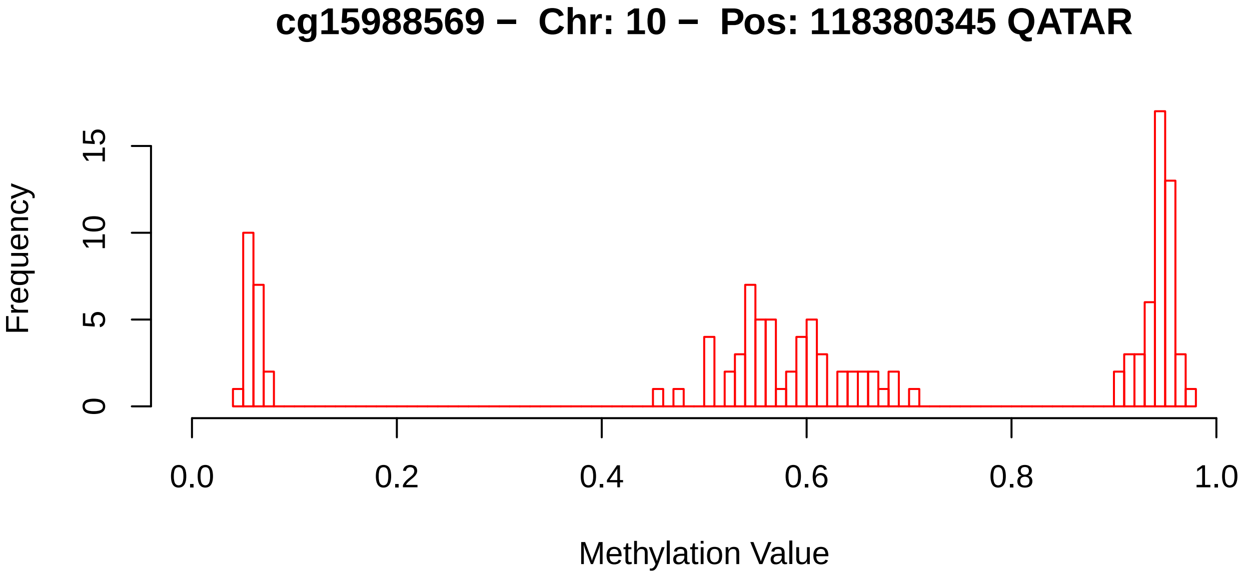
cg15988569 − Chr: 10 − Pos: 118380345 QATAR
Frequency
0
5
10
15
0.0
0.2
0.4
0.6
0.8
1.0
Methylation Value

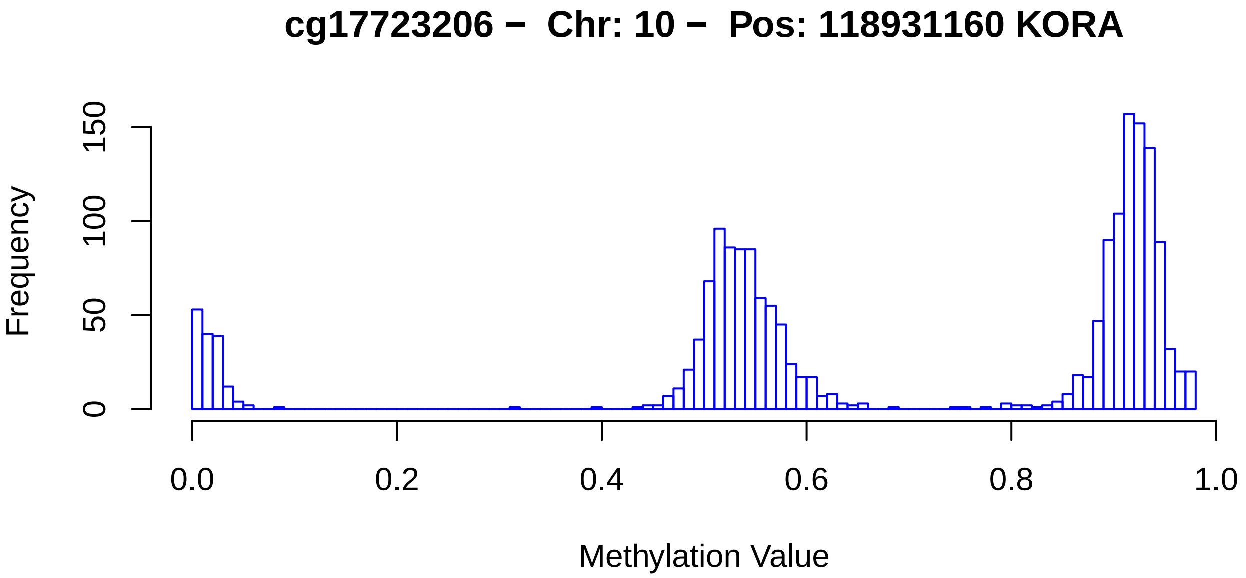
cg17723206 − Chr: 10 − Pos: 118931160 KORA
Frequency
0
50
100
150
0.0
0.2
0.4
0.6
0.8
1.0
Methylation Value

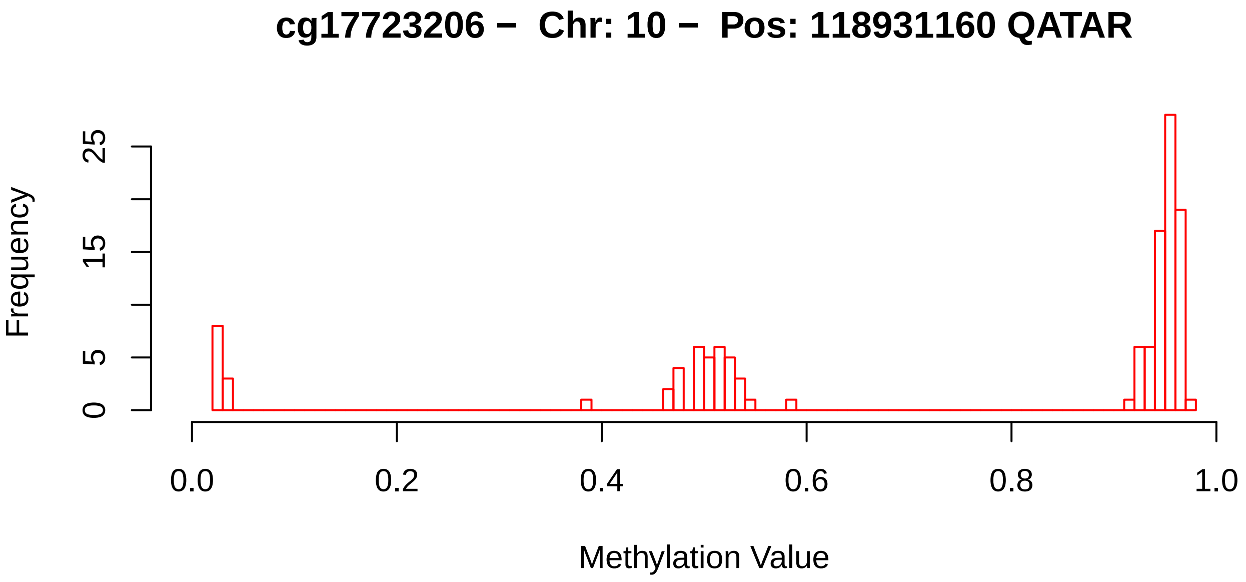
cg17723206 − Chr: 10 − Pos: 118931160 QATAR
Frequency
0
5
15
25
0.0
0.2
0.4
0.6
0.8
1.0
Methylation Value

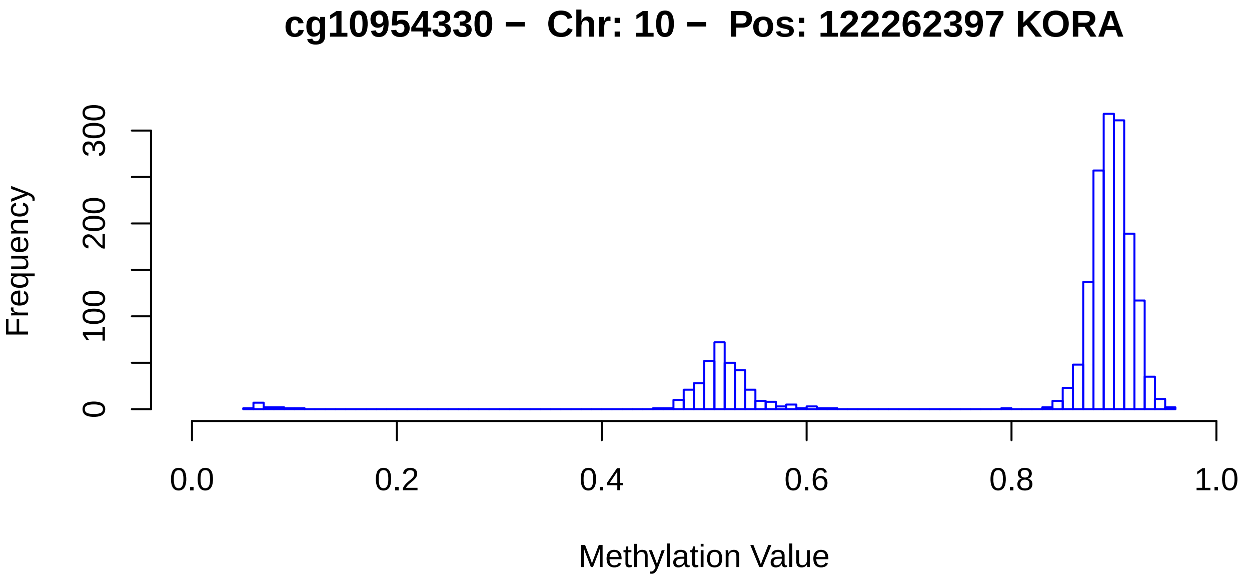
cg10954330 − Chr: 10 − Pos: 122262397 KORA
Frequency
0
100
200
300
0.0
0.2
0.4
0.6
0.8
1.0
Methylation Value

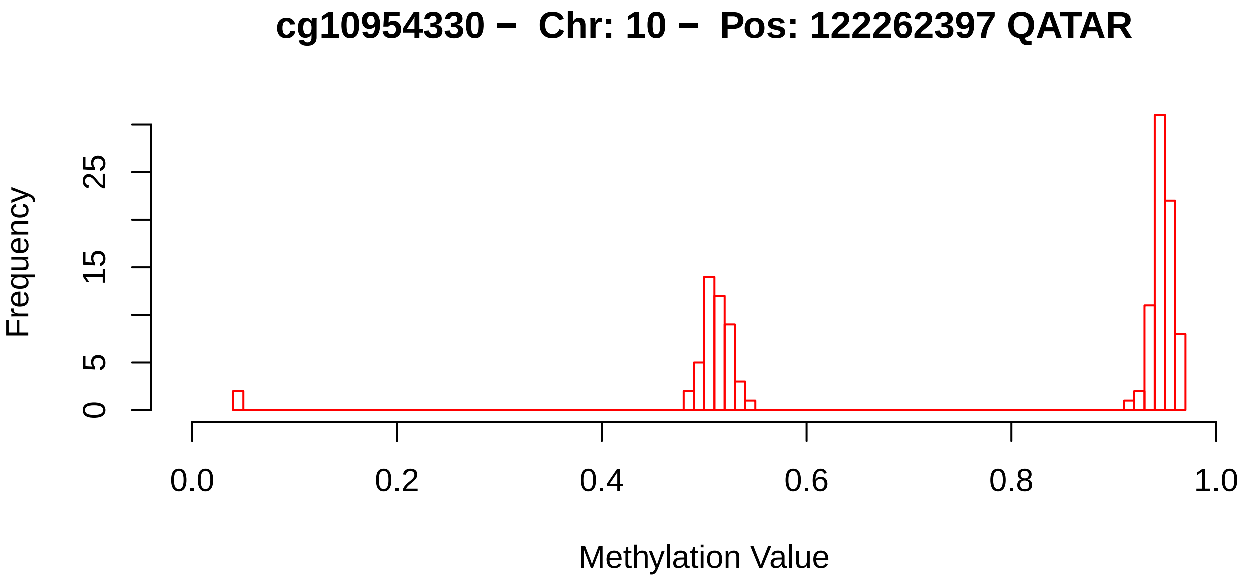
cg10954330 − Chr: 10 − Pos: 122262397 QATAR
Frequency
0
5
15
25
0.0
0.2
0.4
0.6
0.8
1.0
Methylation Value

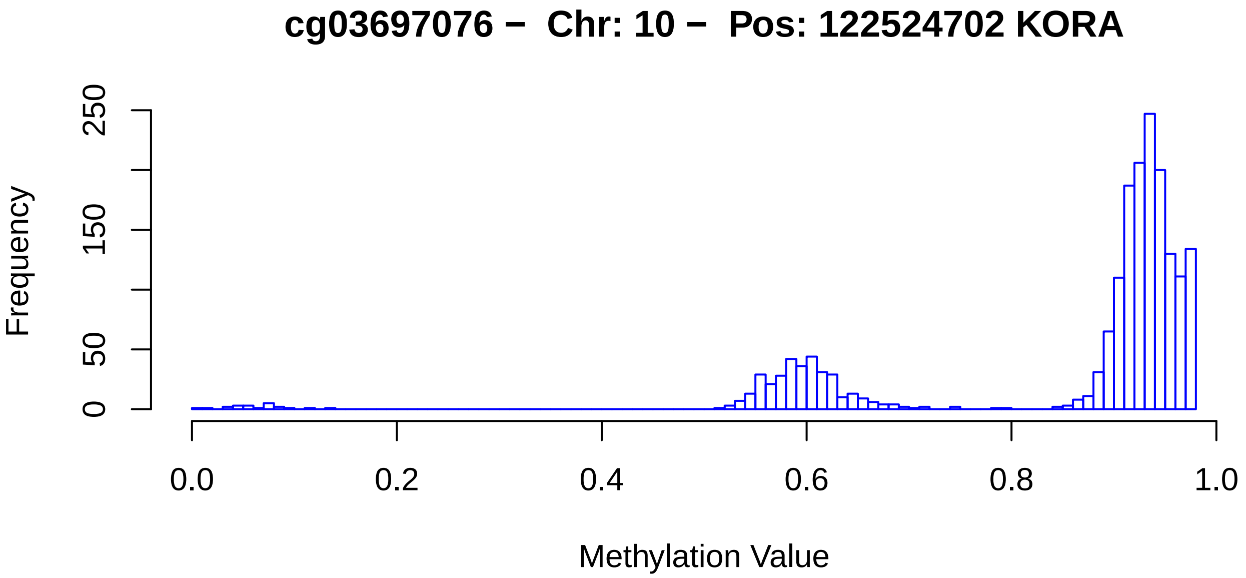

cg03697076 − Chr: 10 − Pos: 122524702 KORA
Frequency
0
50
150
250
0.0
0.2
0.4
0.6
0.8
1.0
Methylation Value

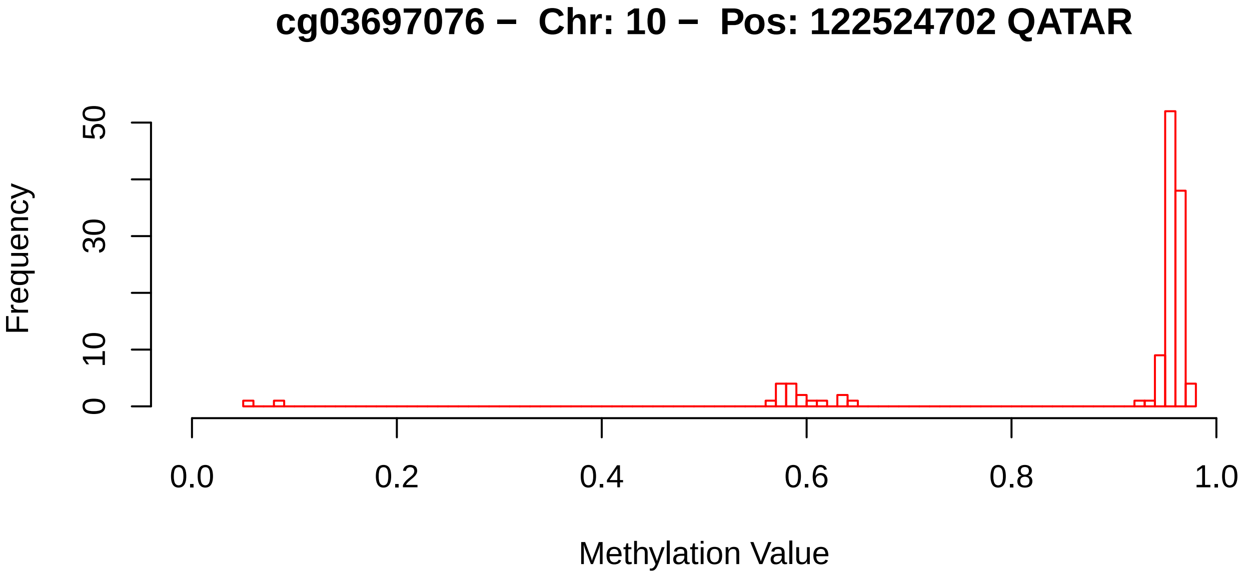

cg03697076 − Chr: 10 − Pos: 122524702 QATAR
Frequency
0
10
30
50
0.0
0.2
0.4
0.6
0.8
1.0
Methylation Value

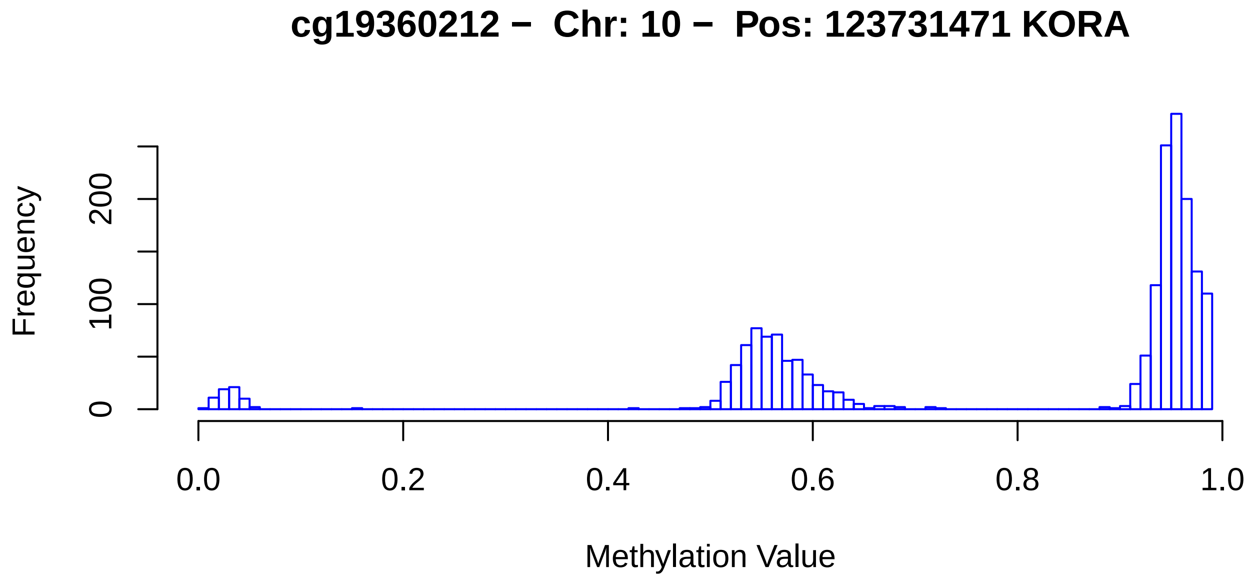
cg19360212 − Chr: 10 − Pos: 123731471 KORA
Frequency
0
100
200
0.0
0.2
0.4
0.6
0.8
1.0
Methylation Value

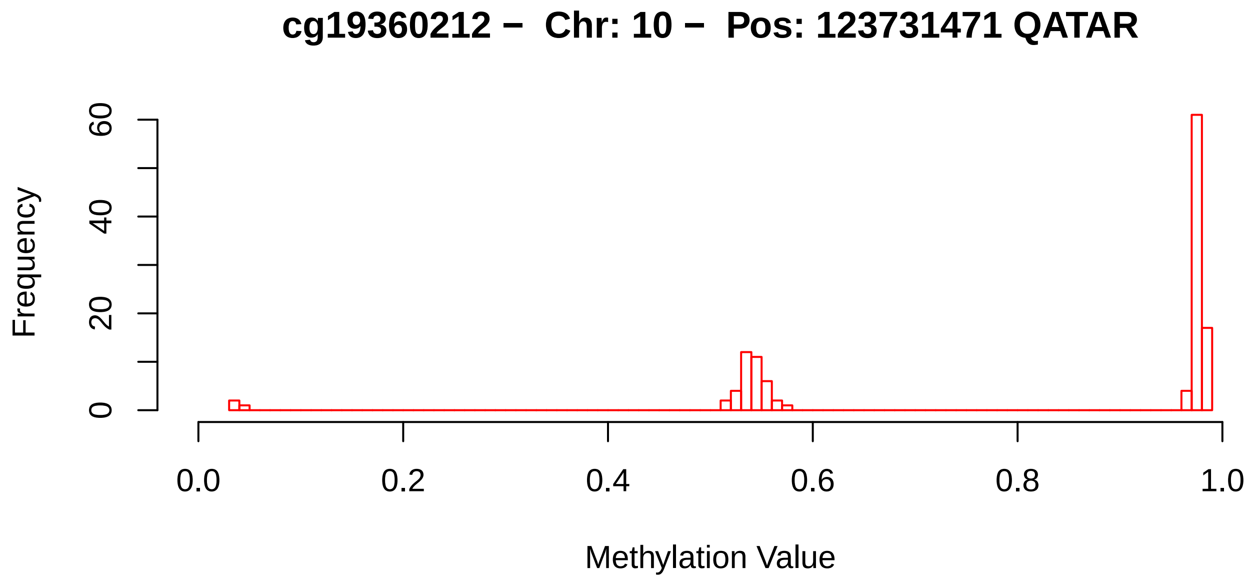
cg19360212 − Chr: 10 − Pos: 123731471 QATAR
Frequency
0
20
40
60
0.0
0.2
0.4
0.6
0.8
1.0
Methylation Value

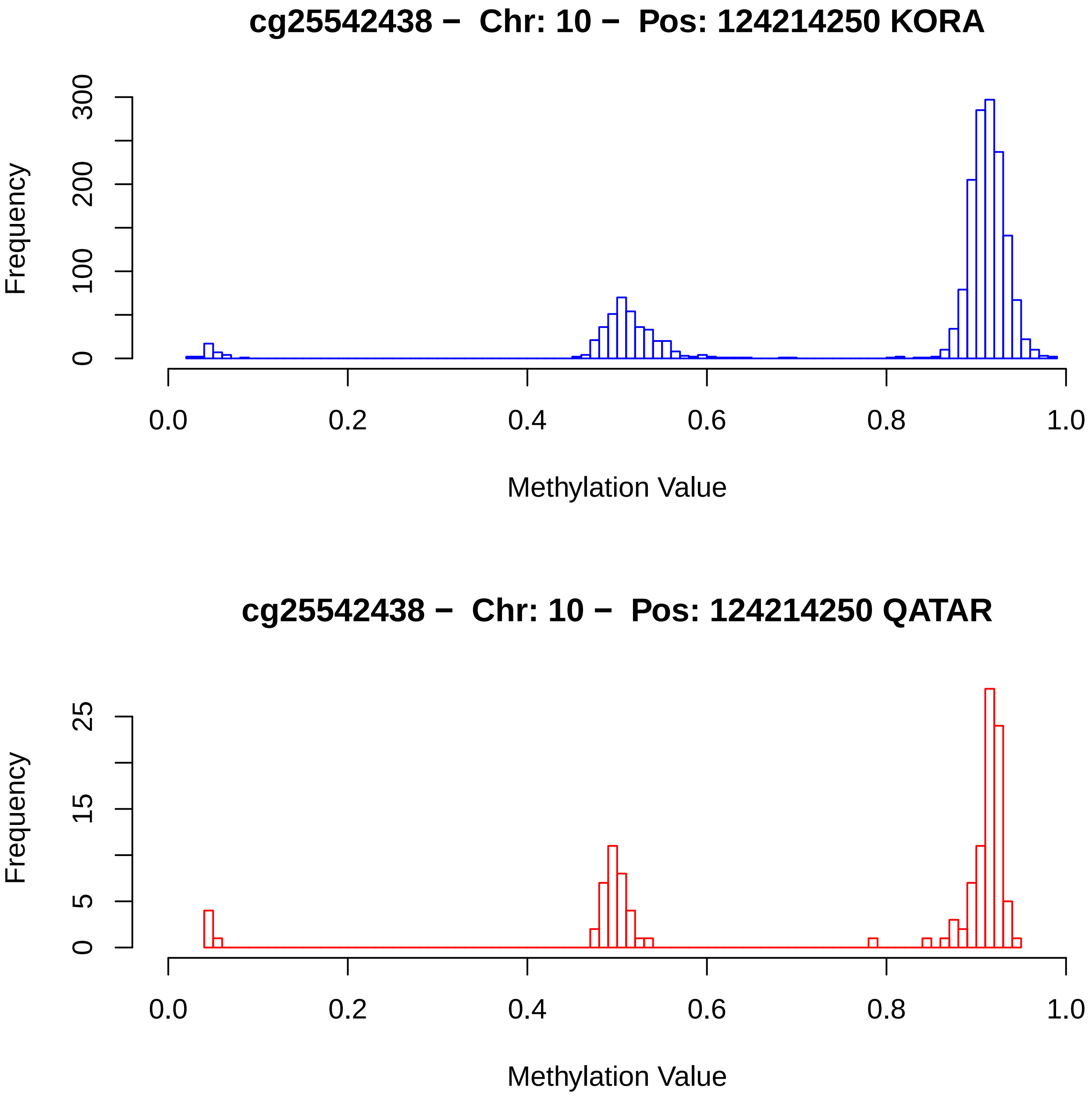

cg25542438 − Chr: 10 − Pos: 124214250 KORA
Frequency
0
100
200
300
0.0
0.2
0.4
0.6
0.8
1.0
Methylation Value
cg25542438 − Chr: 10 − Pos: 124214250 QATAR
Frequency
0
5
15
25
0.0
0.2
0.4
0.6
0.8
1.0
Methylation Value

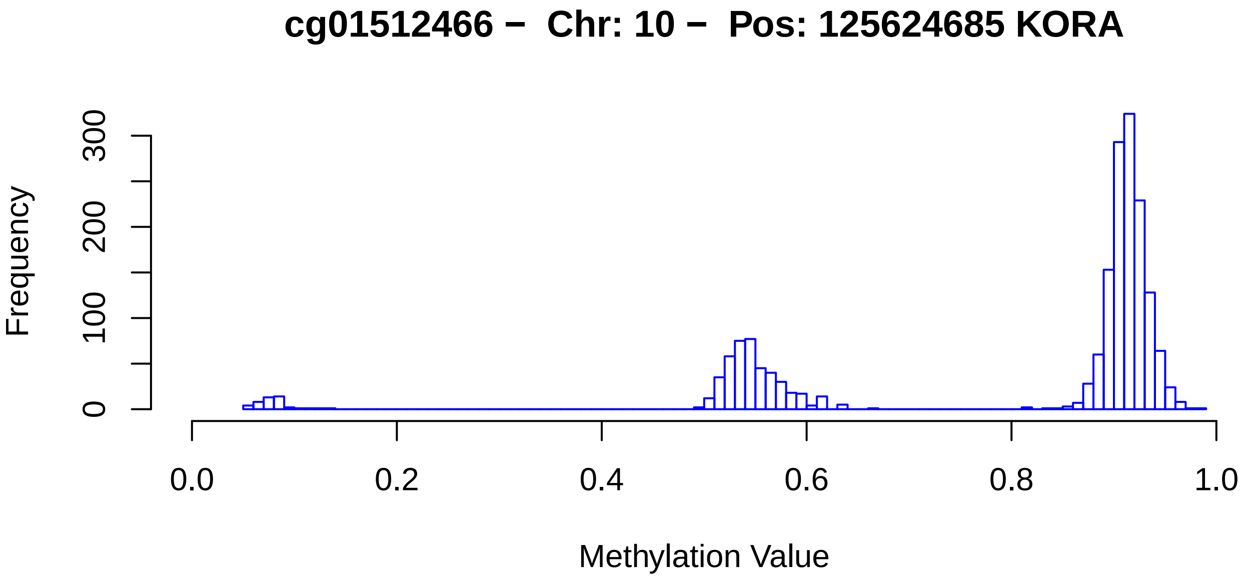
cg01512466 − Chr: 10 − Pos: 125624685 KORA
Frequency
0
100
200
300
0.0
0.2
0.4
0.6
0.8
1.0
Methylation Value

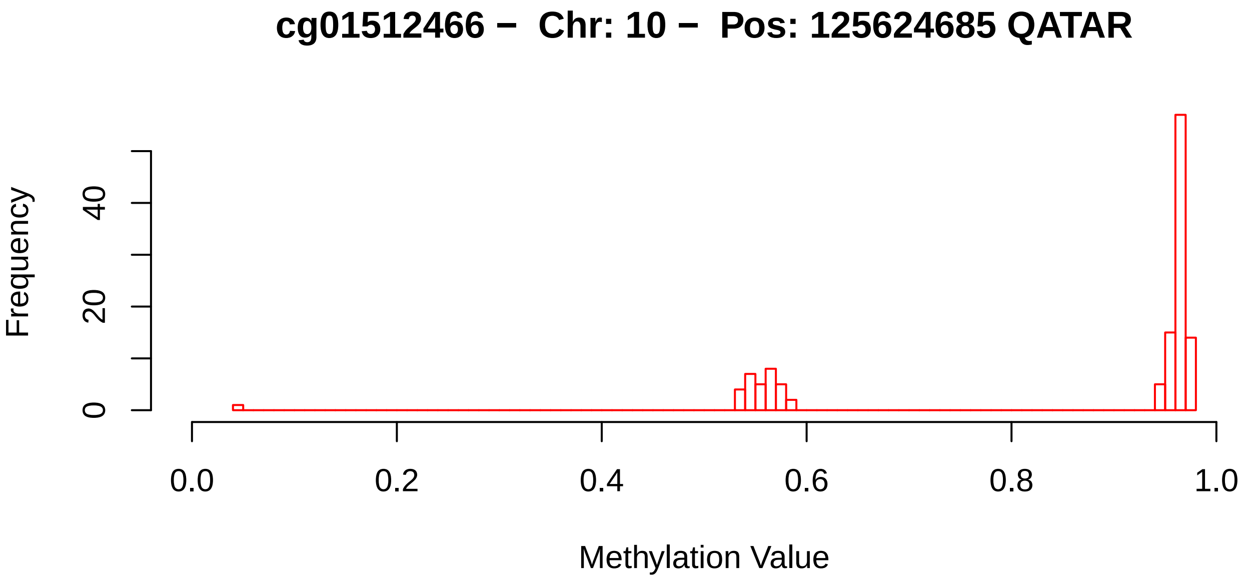
cg01512466 − Chr: 10 − Pos: 125624685 QATAR
Frequency
0
20
40
0.0
0.2
0.4
0.6
0.8
1.0
Methylation Value

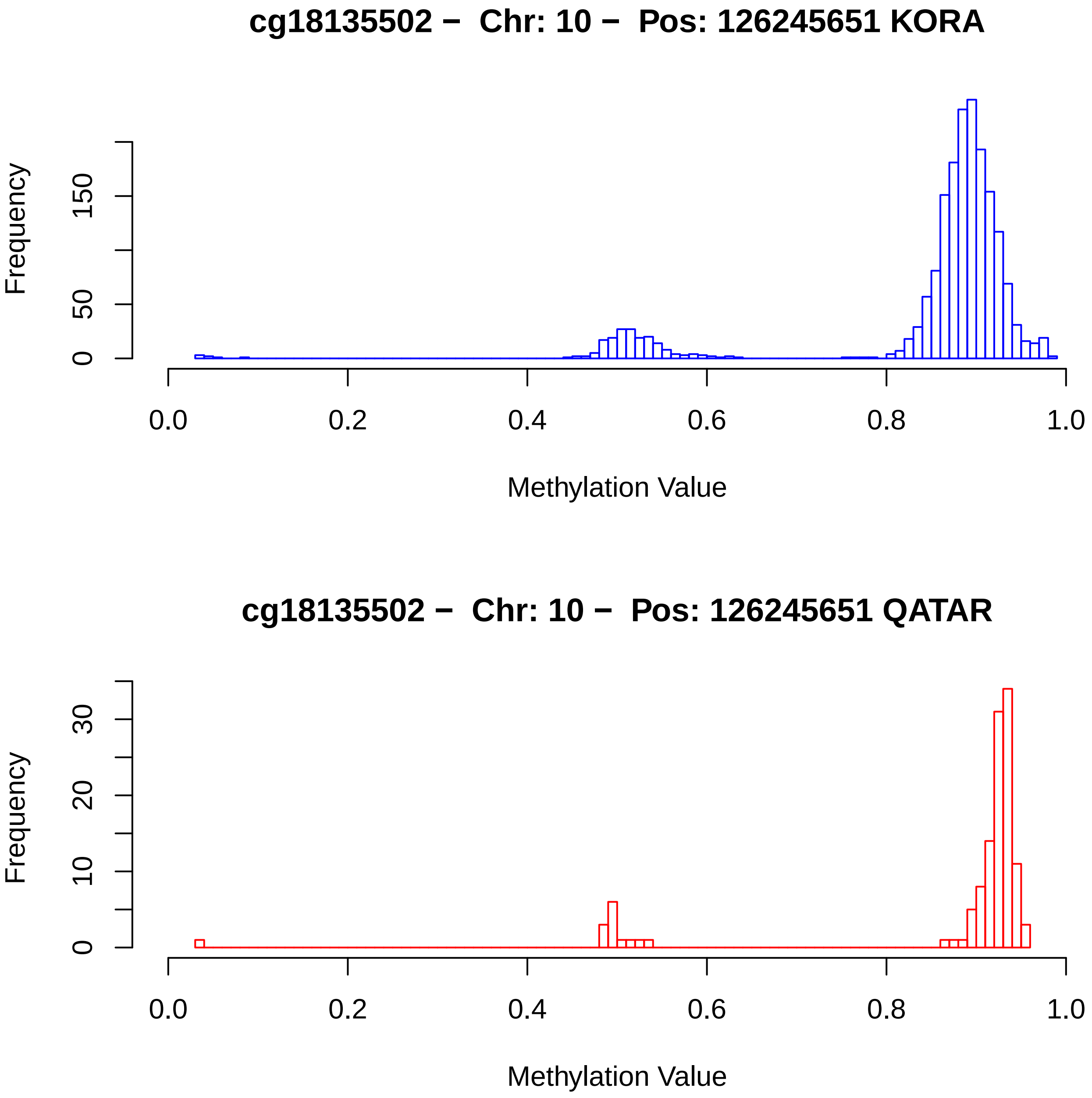
cg18135502 − Chr: 10 − Pos: 126245651 KORA
Frequency
0
50
150
0.0
0.2
0.4
0.6
0.8
1.0
Methylation Value
cg18135502 − Chr: 10 − Pos: 126245651 QATAR
Frequency
0
10
20
30
0.0
0.2
0.4
0.6
0.8
1.0
Methylation Value

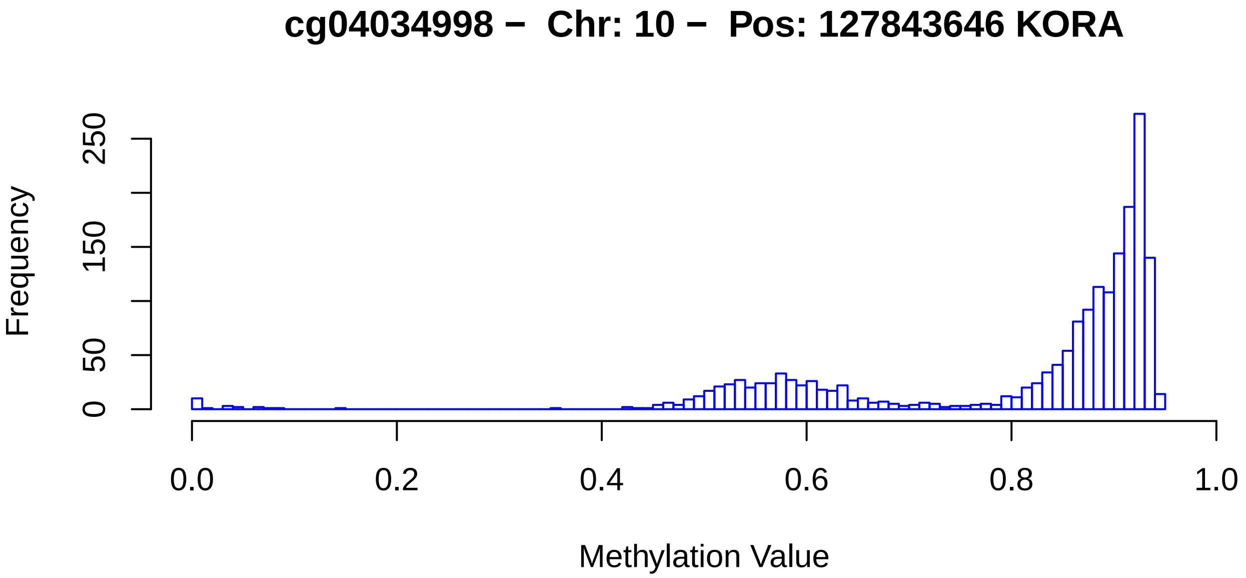
cg04034998 − Chr: 10 − Pos: 127843646 KORA
Frequency
0
50
150
250
0.0
0.2
0.4
0.6
0.8
1.0
Methylation Value

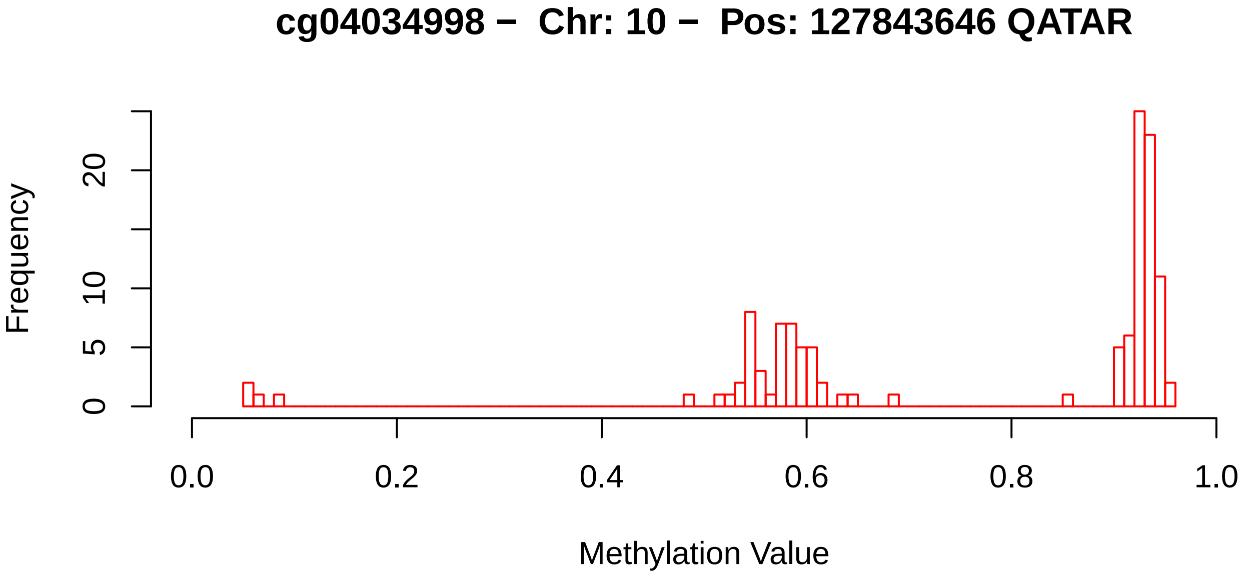
cg04034998 − Chr: 10 − Pos: 127843646 QATAR
Frequency
0
5
10
20
0.0
0.2
0.4
0.6
0.8
1.0
Methylation Value

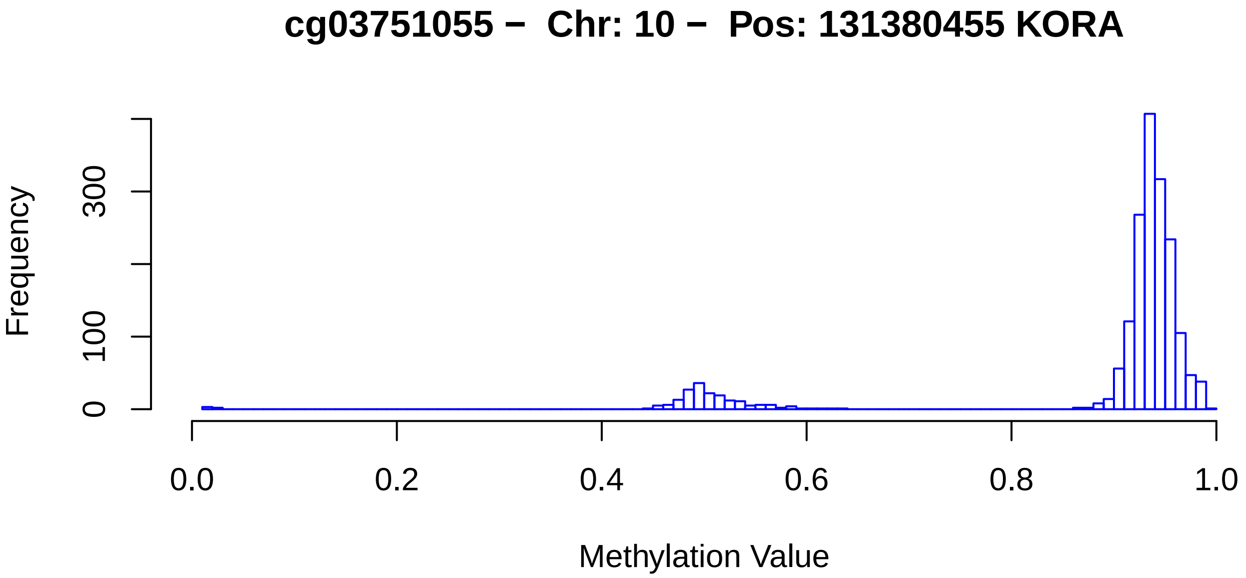
cg03751055 − Chr: 10 − Pos: 131380455 KORA
Frequency
0
100
300
0.0
0.2
0.4
0.6
0.8
1.0
Methylation Value

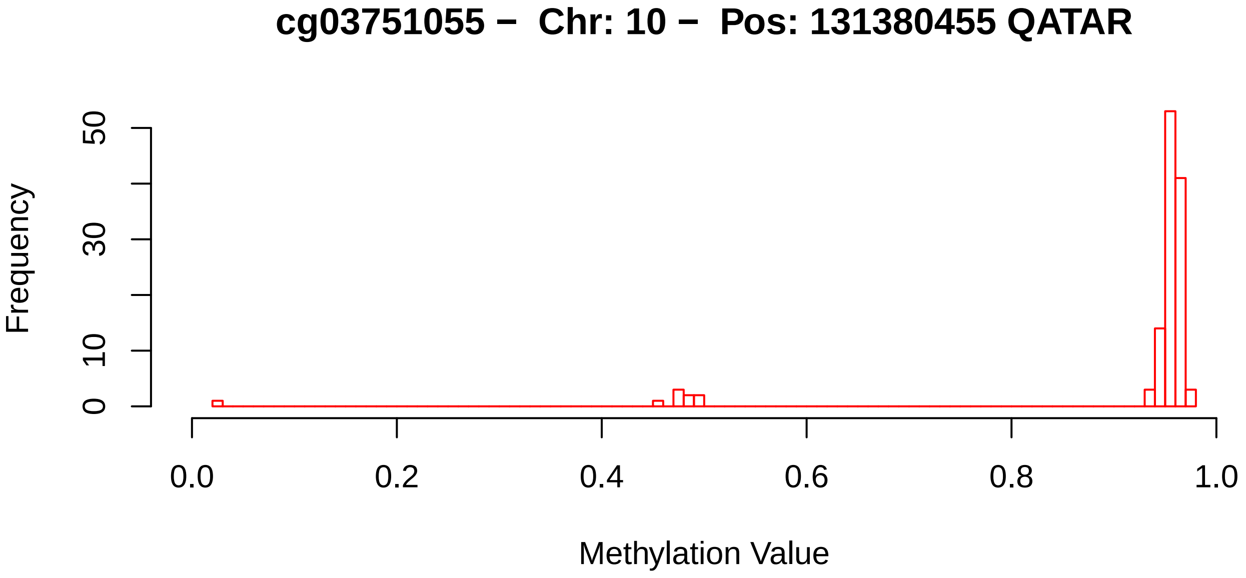
cg03751055 − Chr: 10 − Pos: 131380455 QATAR
Frequency
0
10
30
50
0.0
0.2
0.4
0.6
0.8
1.0
Methylation Value

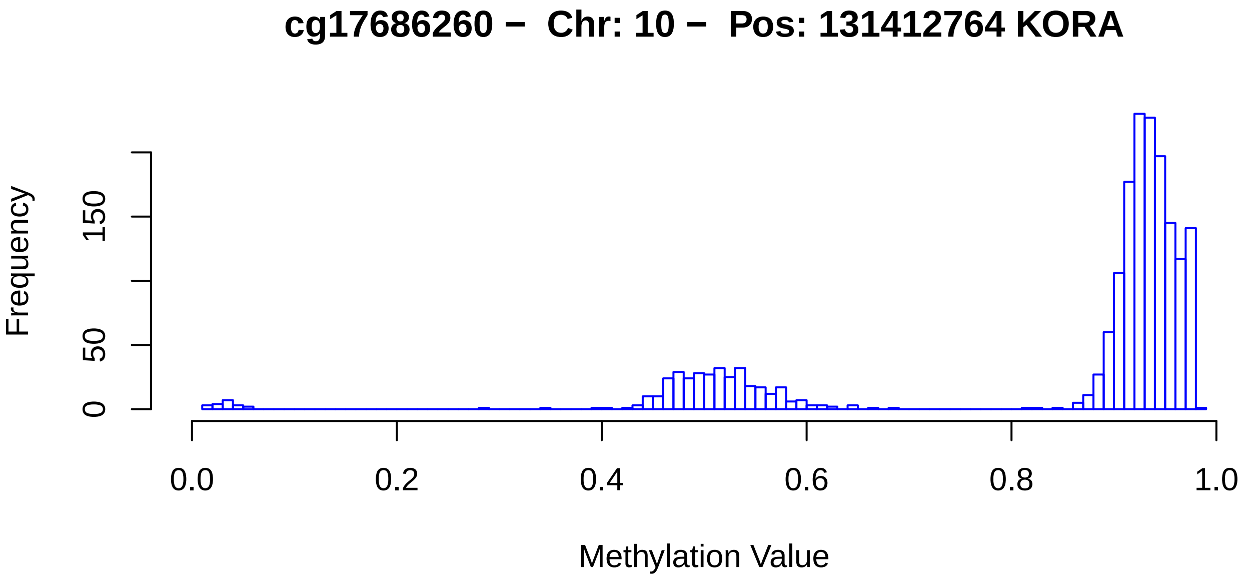
cg17686260 − Chr: 10 − Pos: 131412764 KORA
Frequency
0
50
150
0.0
0.2
0.4
0.6
0.8
1.0
Methylation Value

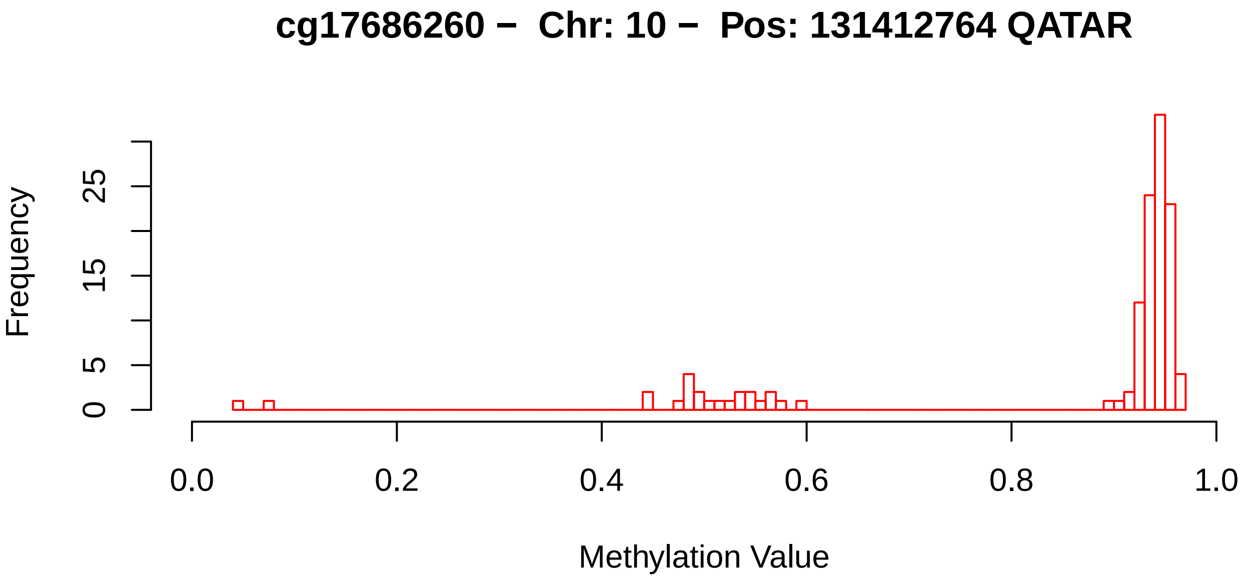
cg17686260 − Chr: 10 − Pos: 131412764 QATAR
Frequency
0
5
15
25
0.0
0.2
0.4
0.6
0.8
1.0
Methylation Value

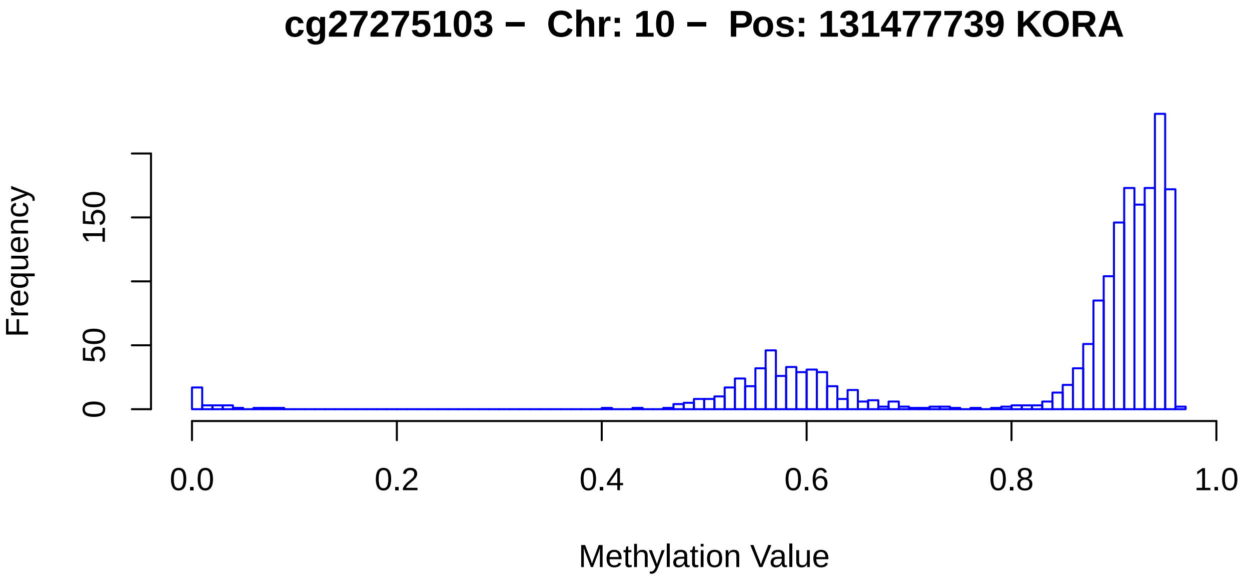
cg27275103 − Chr: 10 − Pos: 131477739 KORA
Frequency
0
50
150
0.0
0.2
0.4
0.6
0.8
1.0
Methylation Value

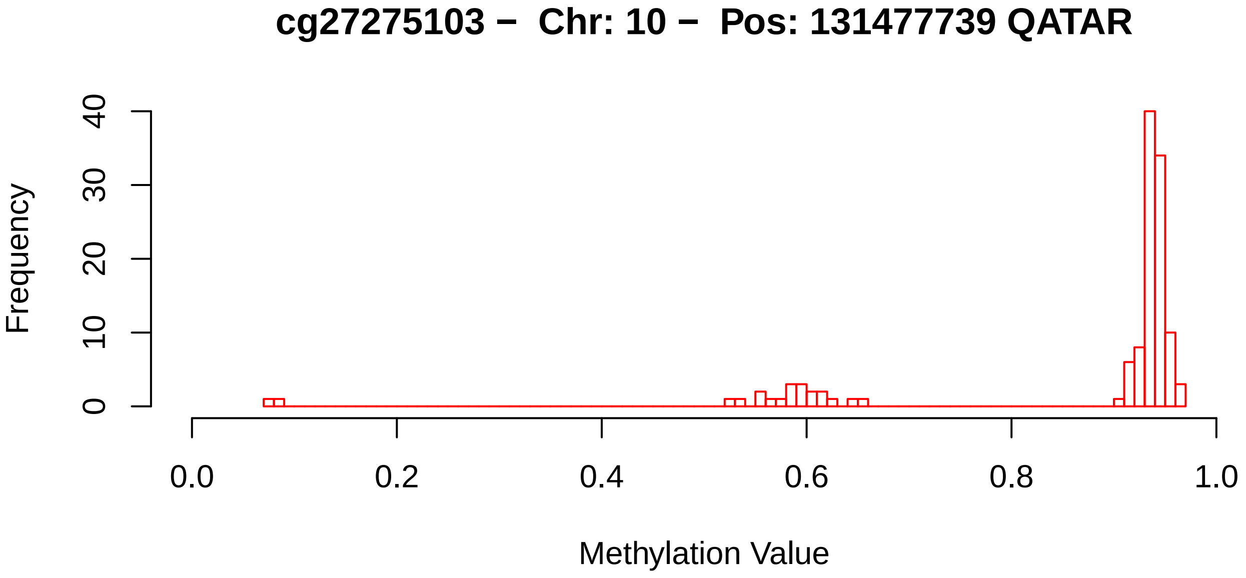
cg27275103 − Chr: 10 − Pos: 131477739 QATAR
Frequency
0
10
20
30
40
0.0
0.2
0.4
0.6
0.8
1.0
Methylation Value

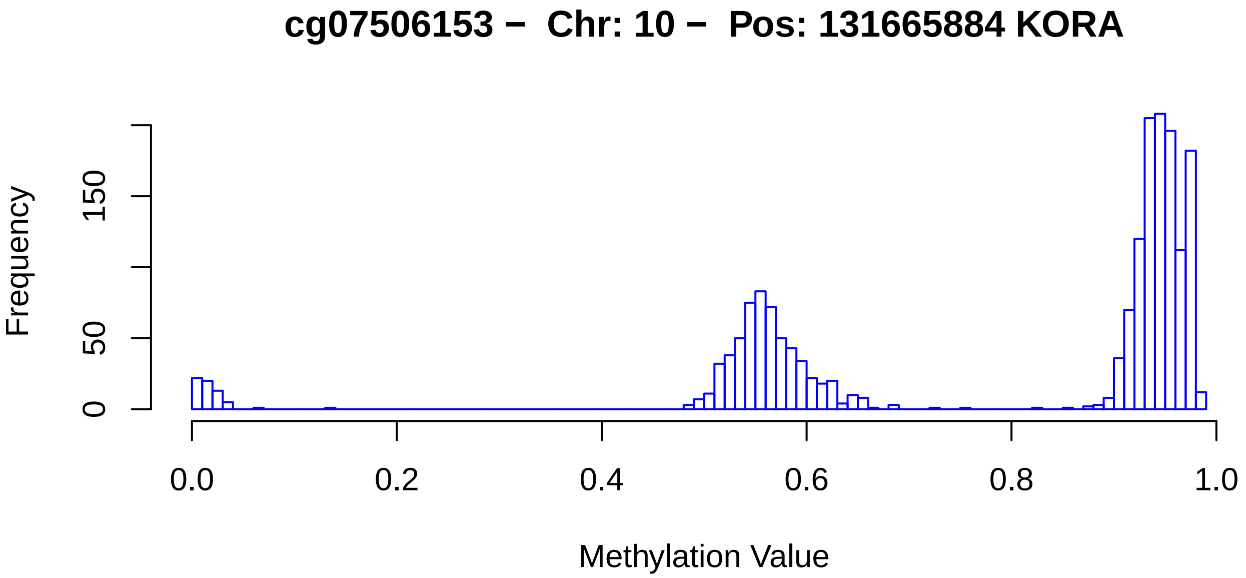
cg07506153 − Chr: 10 − Pos: 131665884 KORA
Frequency
0
50
150
0.0
0.2
0.4
0.6
0.8
1.0
Methylation Value

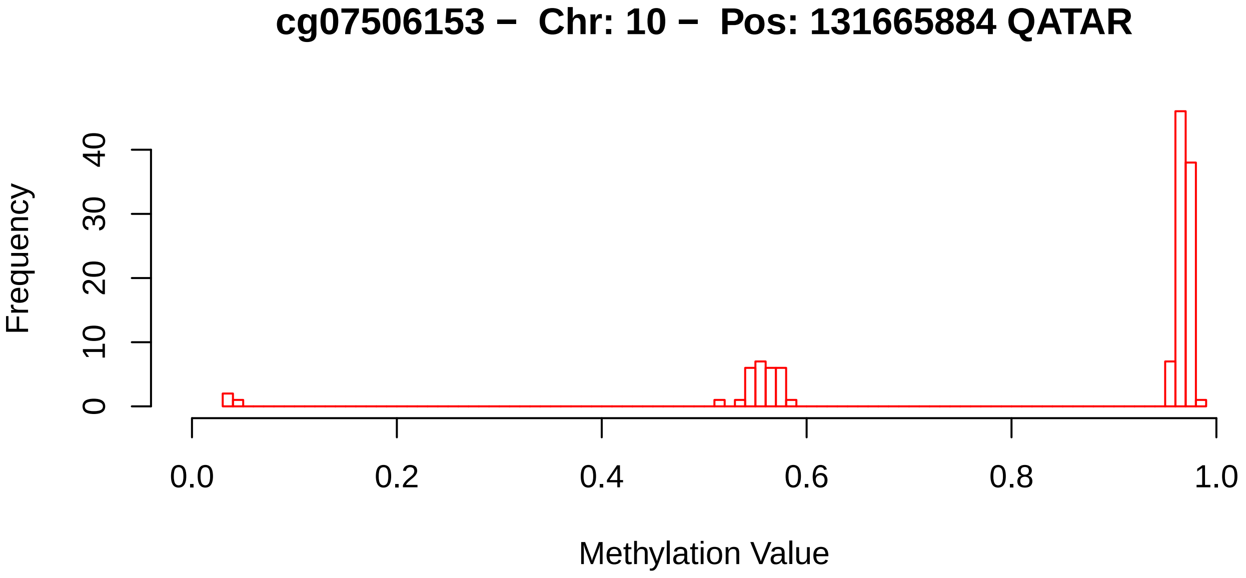
cg07506153 − Chr: 10 − Pos: 131665884 QATAR
Frequency
0
10
20
30
40
0.0
0.2
0.4
0.6
0.8
1.0
Methylation Value

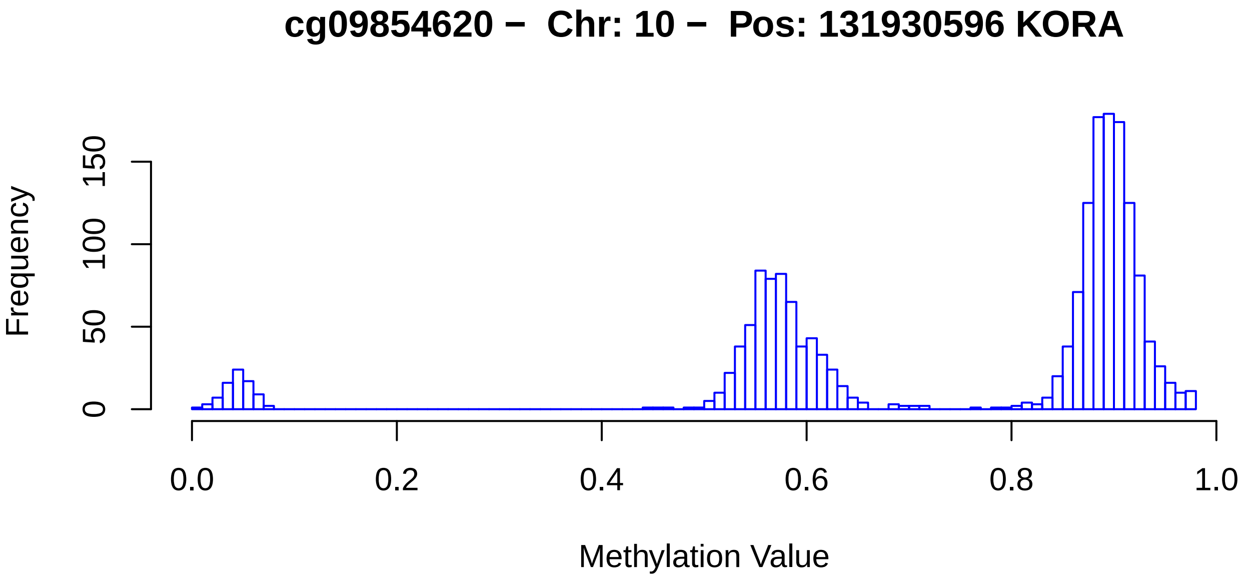
cg09854620 − Chr: 10 − Pos: 131930596 KORA
Frequency
0
50
100
150
0.0
0.2
0.4
0.6
0.8
1.0
Methylation Value

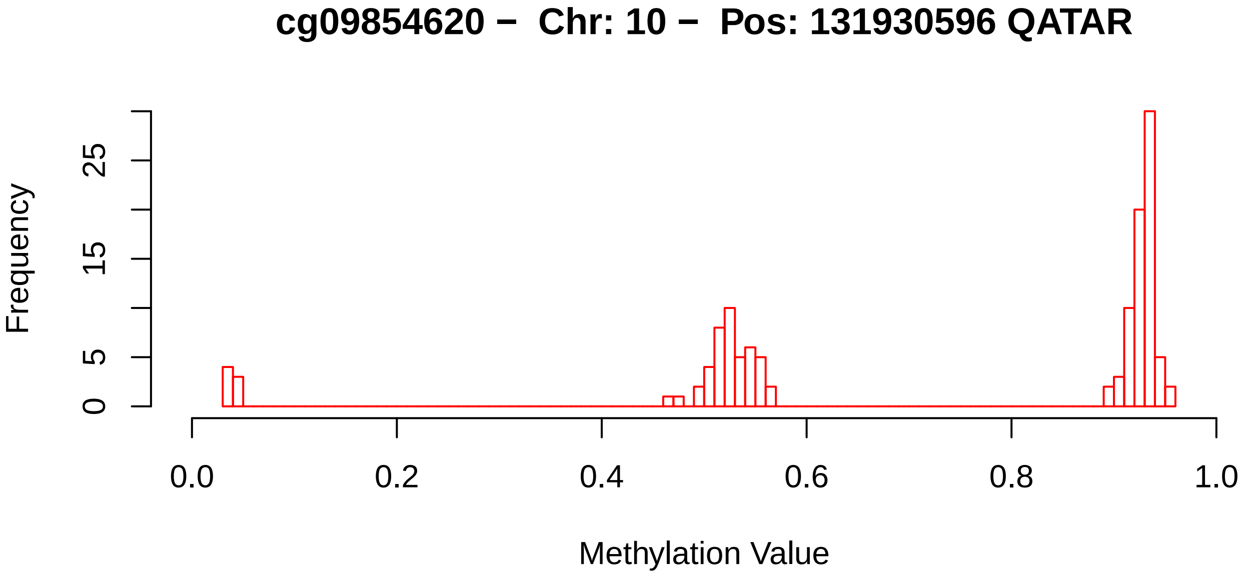
cg09854620 − Chr: 10 − Pos: 131930596 QATAR
Frequency
0
5
15
25
0.0
0.2
0.4
0.6
0.8
1.0
Methylation Value

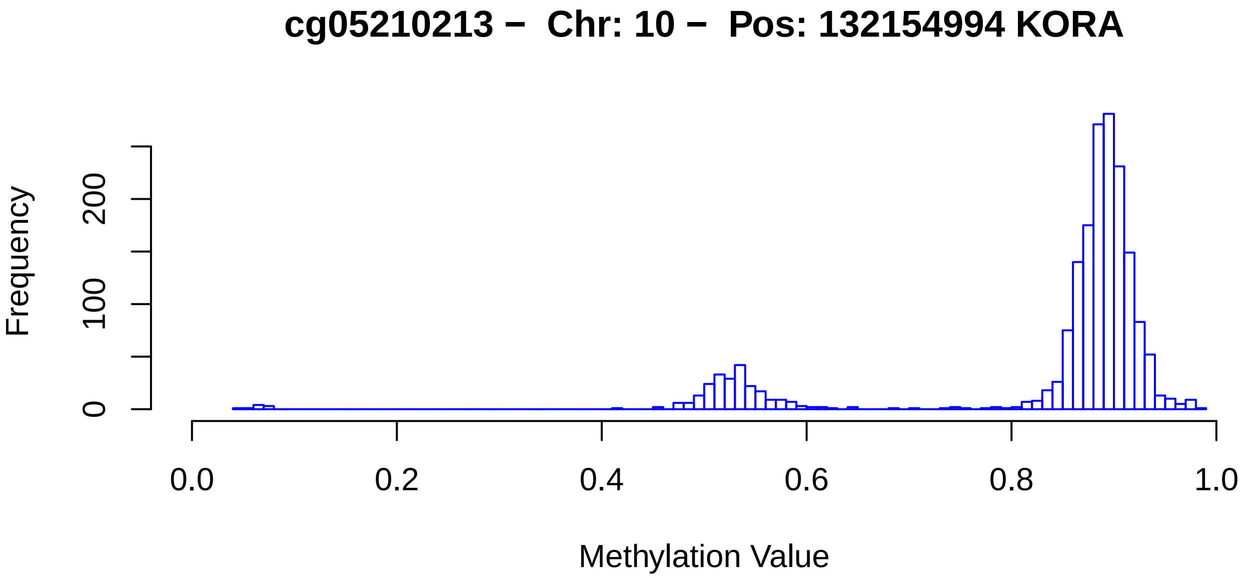
cg05210213 − Chr: 10 − Pos: 132154994 KORA
Frequency
0
100
200
0.0
0.2
0.4
0.6
0.8
1.0
Methylation Value

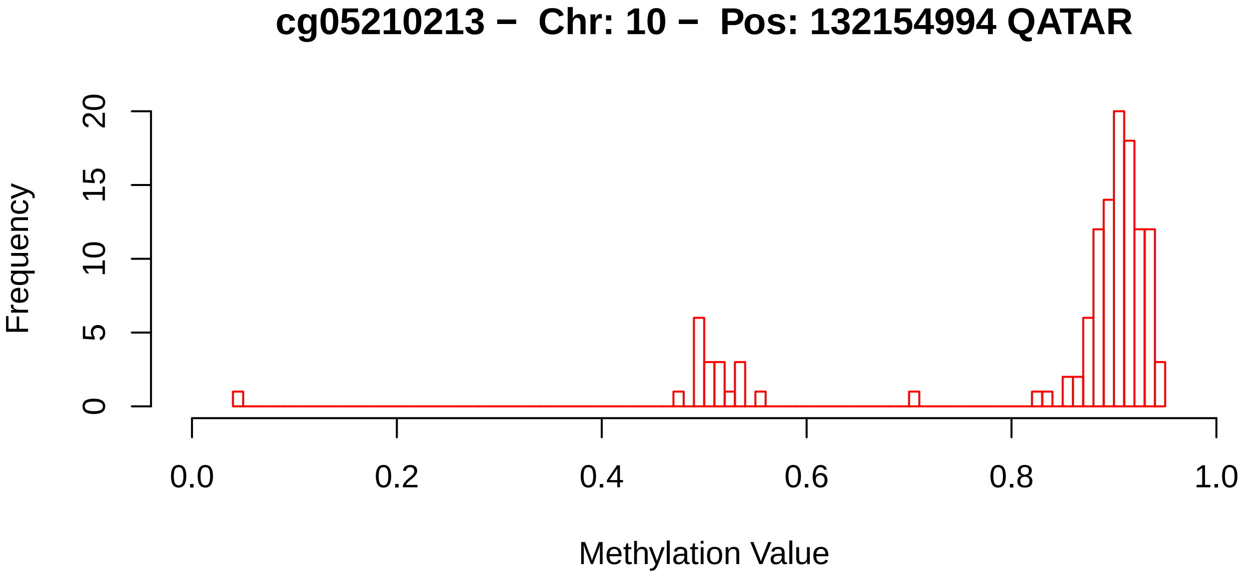
cg05210213 − Chr: 10 − Pos: 132154994 QATAR
Frequency
0
5
10
15
20
0.0
0.2
0.4
0.6
0.8
1.0
Methylation Value

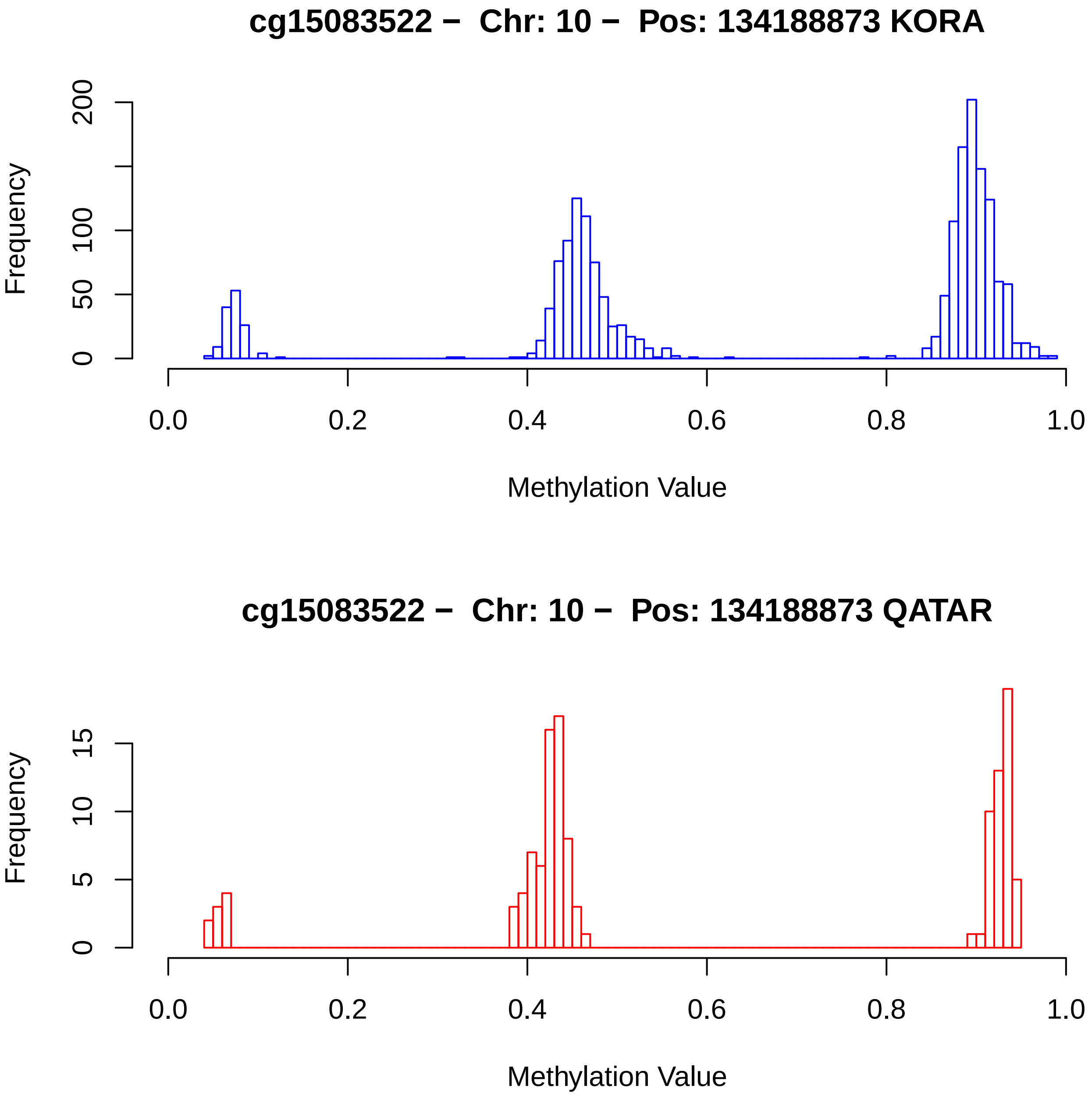
cg15083522 − Chr: 10 − Pos: 134188873 KORA
Frequency
0
50
100
200
0.0
0.2
0.4
0.6
0.8
1.0
Methylation Value
cg15083522 − Chr: 10 − Pos: 134188873 QATAR
Frequency
0
5
10
15
0.0
0.2
0.4
0.6
0.8
1.0
Methylation Value

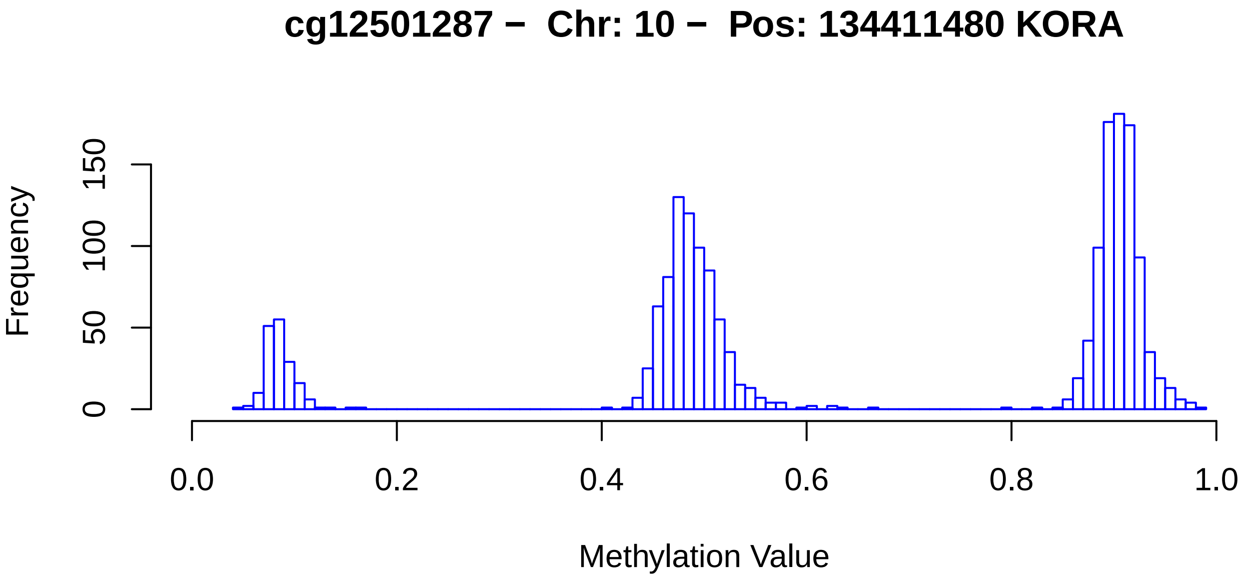

cg12501287 − Chr: 10 − Pos: 134411480 KORA
Frequency
0
50
100
150
0.0
0.2
0.4
0.6
0.8
1.0
Methylation Value

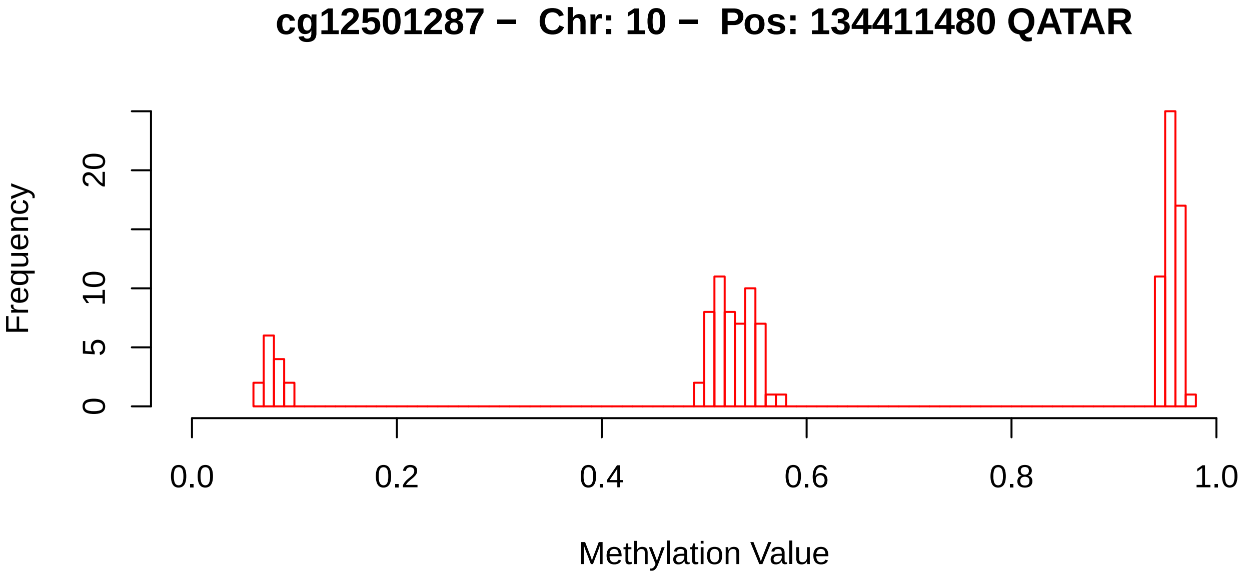

cg12501287 − Chr: 10 − Pos: 134411480 QATAR
Frequency
0
5
10
20
0.0
0.2
0.4
0.6
0.8
1.0
Methylation Value

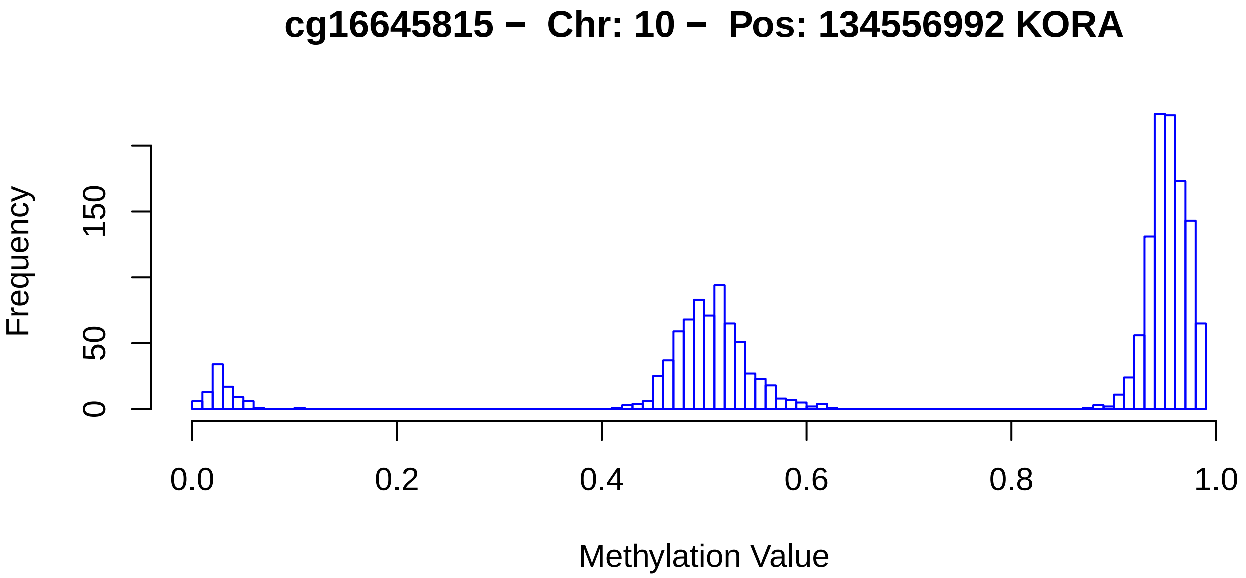
cg16645815 − Chr: 10 − Pos: 134556992 KORA
Frequency
0
50
150
0.0
0.2
0.4
0.6
0.8
1.0
Methylation Value

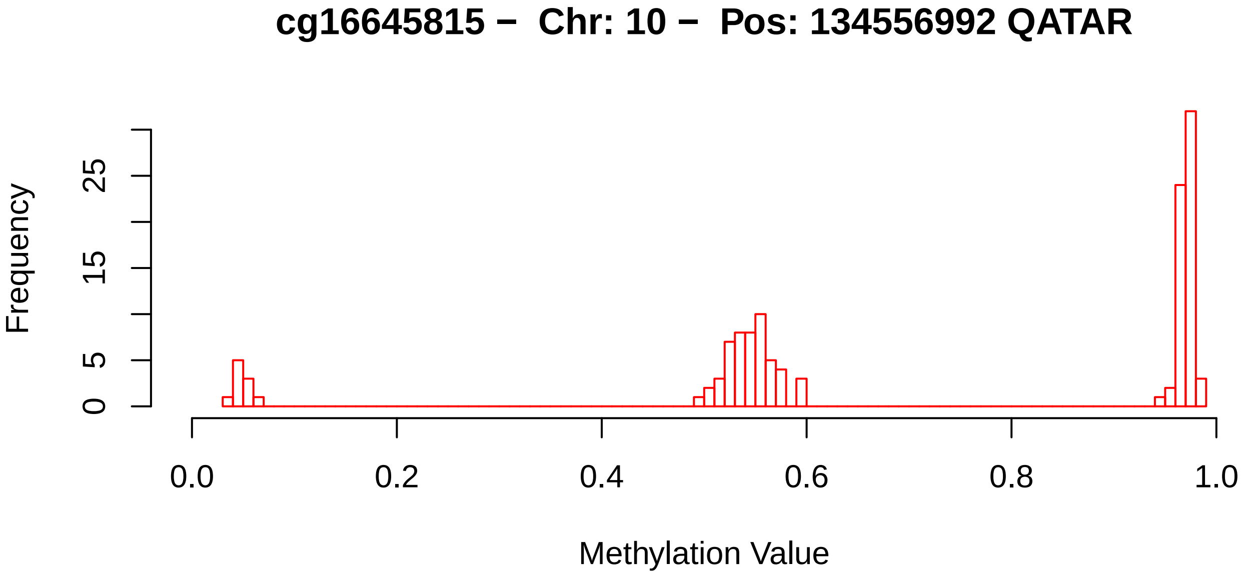
cg16645815 − Chr: 10 − Pos: 134556992 QATAR
Frequency
0
5
15
25
0.0
0.2
0.4
0.6
0.8
1.0
Methylation Value

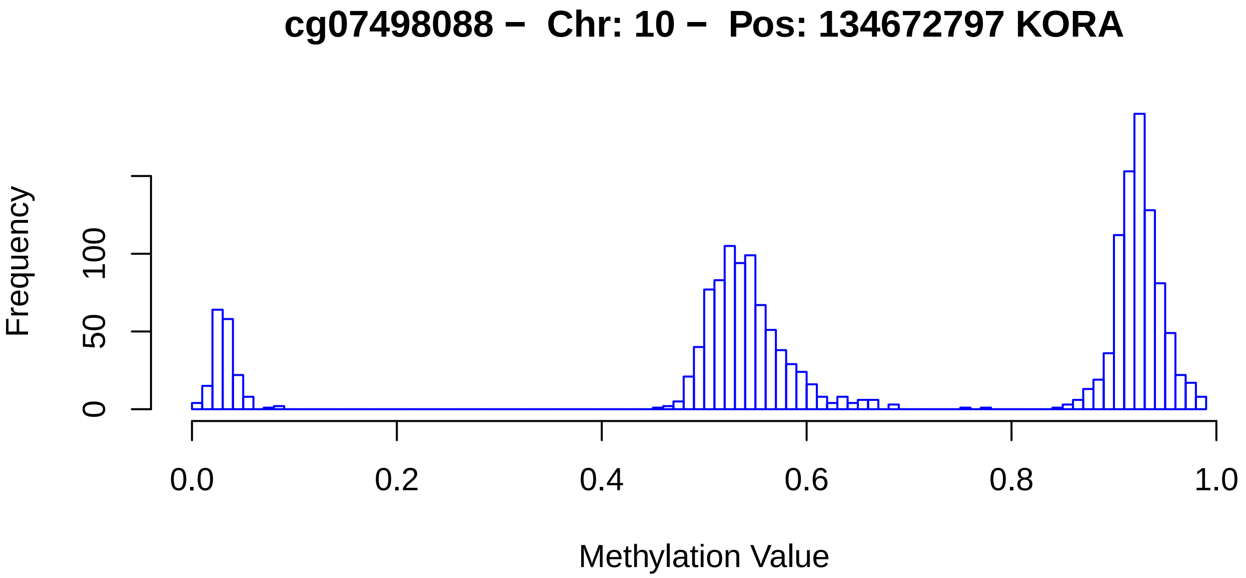
cg07498088 − Chr: 10 − Pos: 134672797 KORA
Frequency
0
50
100
0.0
0.2
0.4
0.6
0.8
1.0
Methylation Value

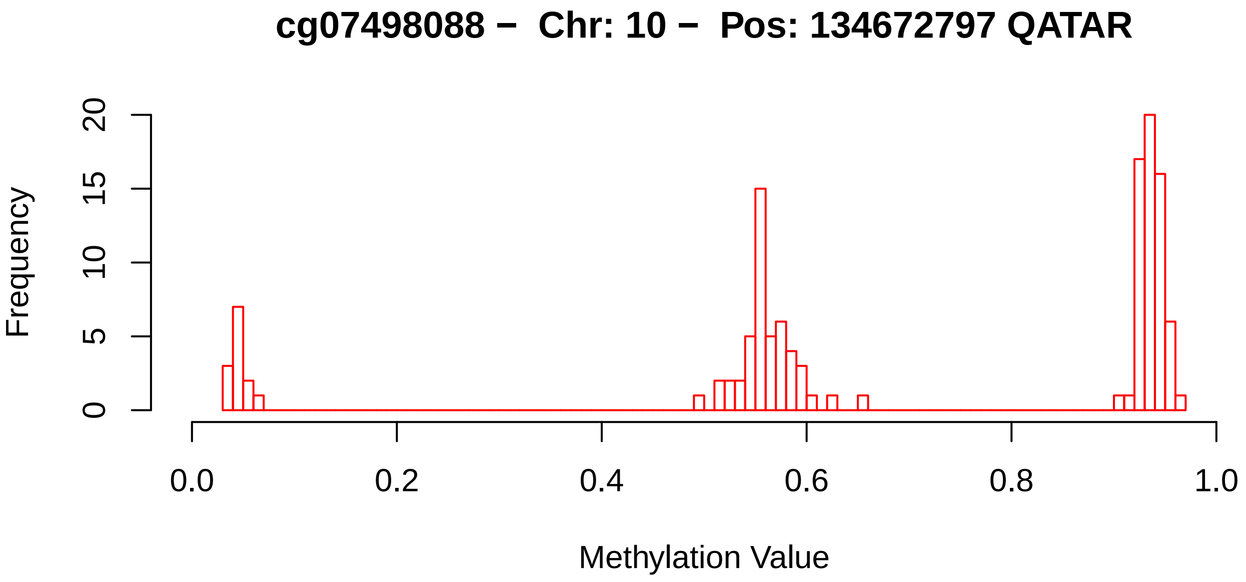
cg07498088 − Chr: 10 − Pos: 134672797 QATAR
Frequency
0
5
10
15
20
0.0
0.2
0.4
0.6
0.8
1.0
Methylation Value

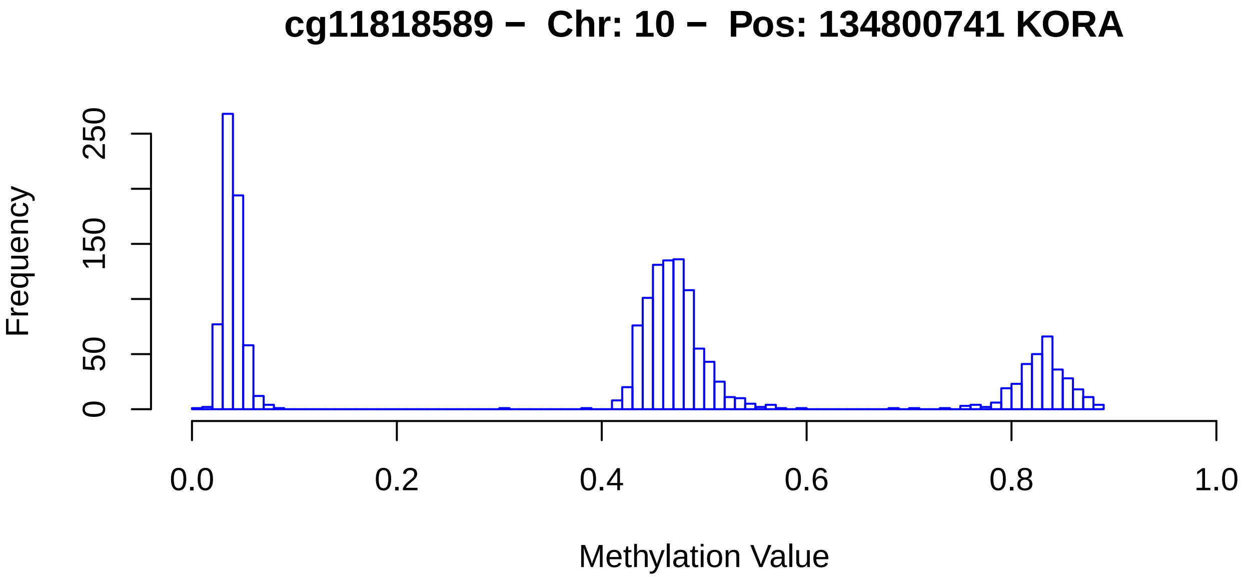
cg11818589 − Chr: 10 − Pos: 134800741 KORA
Frequency
0
50
150
250
0.0
0.2
0.4
0.6
0.8
1.0
Methylation Value

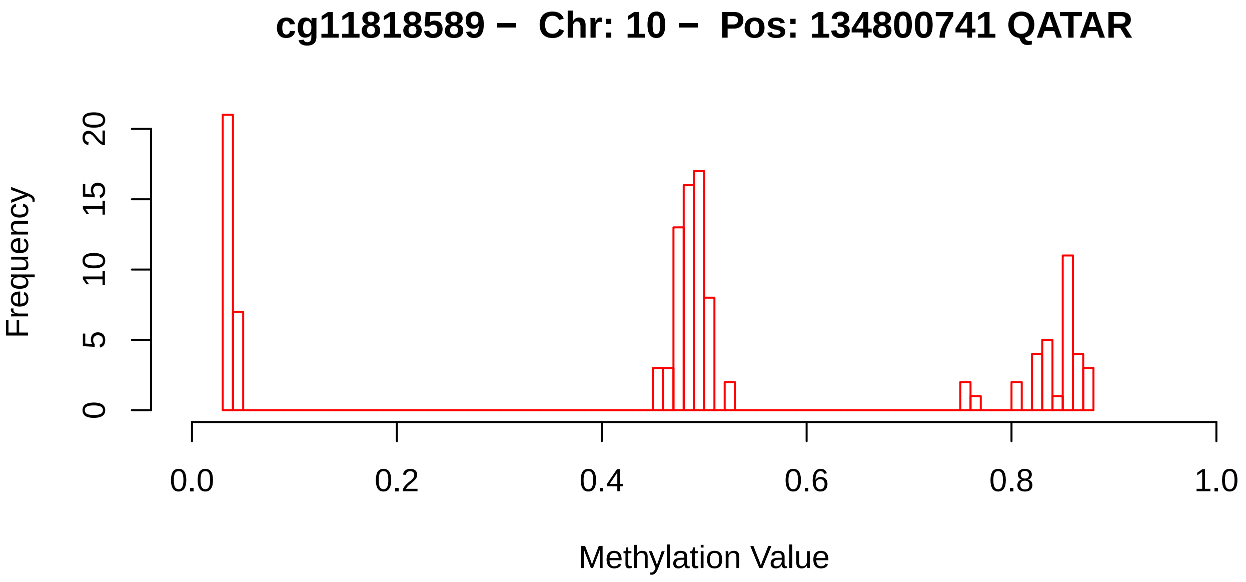
cg11818589 − Chr: 10 − Pos: 134800741 QATAR
Frequency
0
5
10
15
20
0.0
0.2
0.4
0.6
0.8
1.0
Methylation Value

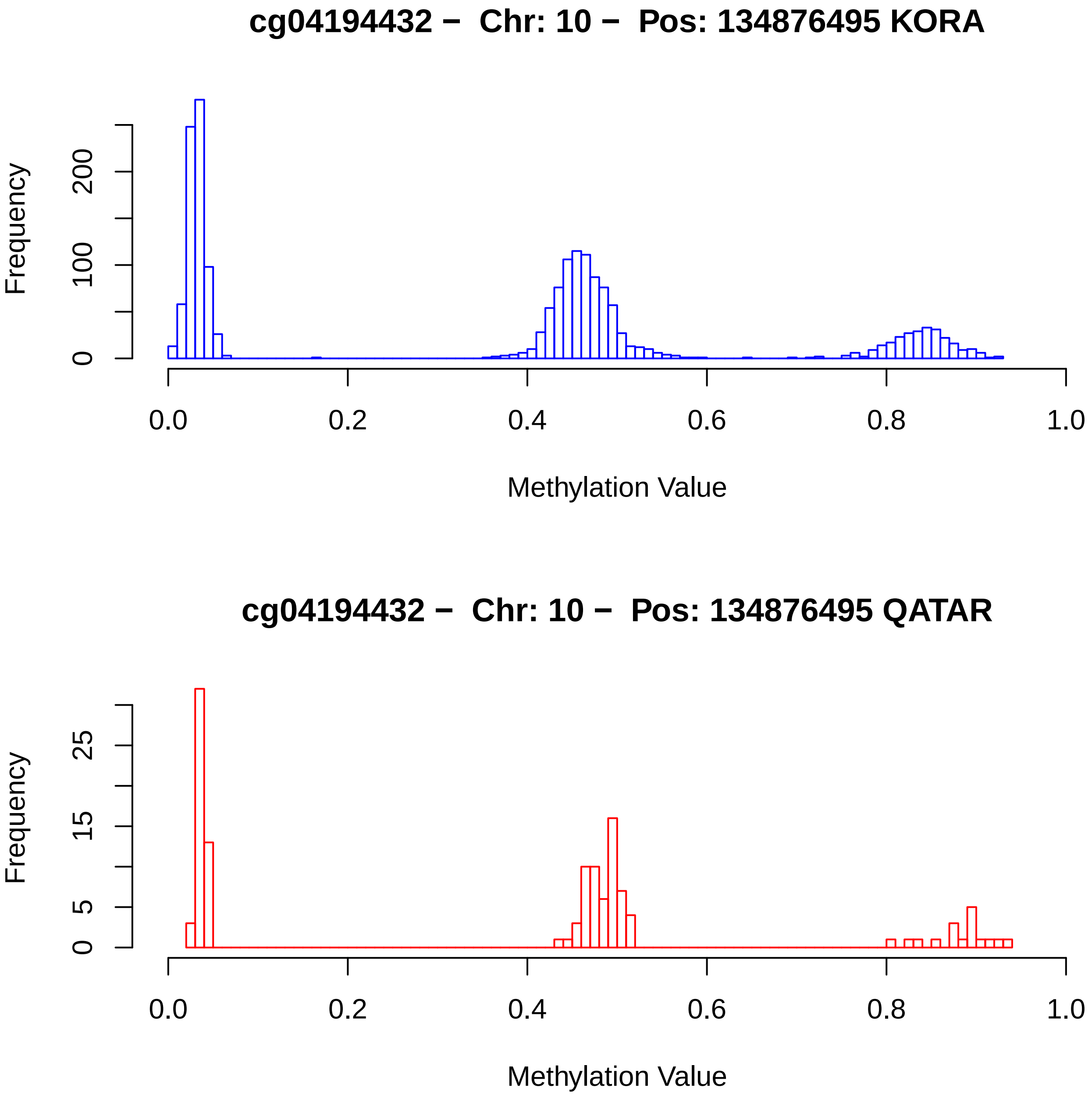
cg04194432 − Chr: 10 − Pos: 134876495 KORA
Frequency
0
100
200
0.0
0.2
0.4
0.6
0.8
1.0
Methylation Value
cg04194432 − Chr: 10 − Pos: 134876495 QATAR
Frequency
0
5
15
25
0.0
0.2
0.4
0.6
0.8
1.0
Methylation Value

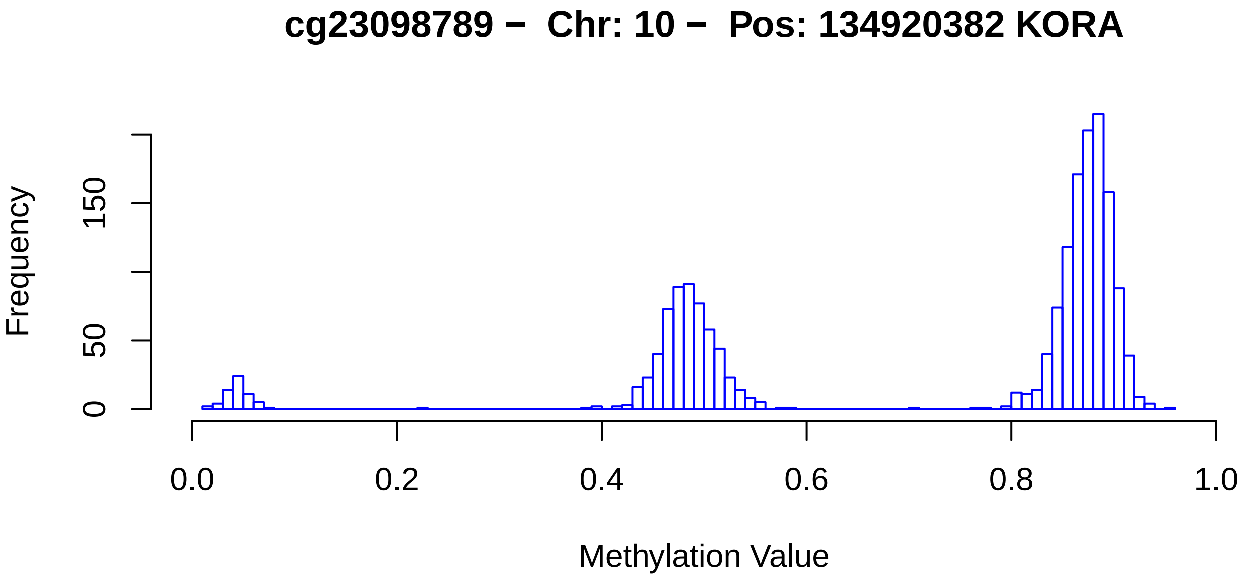
cg23098789 − Chr: 10 − Pos: 134920382 KORA
Frequency
0
50
150
0.0
0.2
0.4
0.6
0.8
1.0
Methylation Value

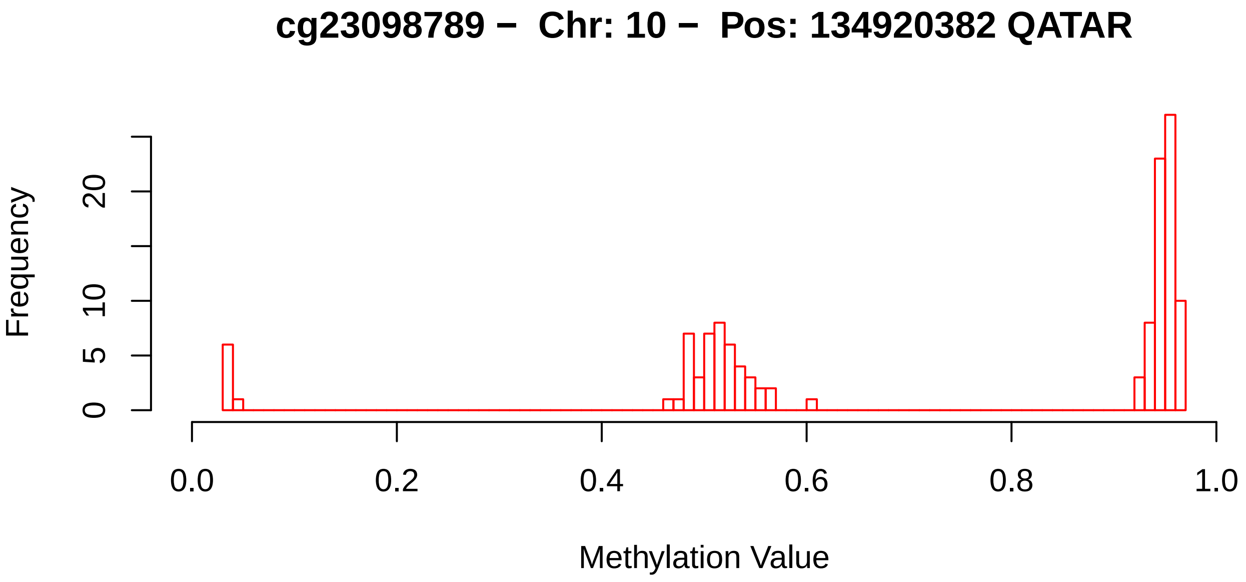
cg23098789 − Chr: 10 − Pos: 134920382 QATAR
Frequency
0
5
10
20
0.0
0.2
0.4
0.6
0.8
1.0
Methylation Value

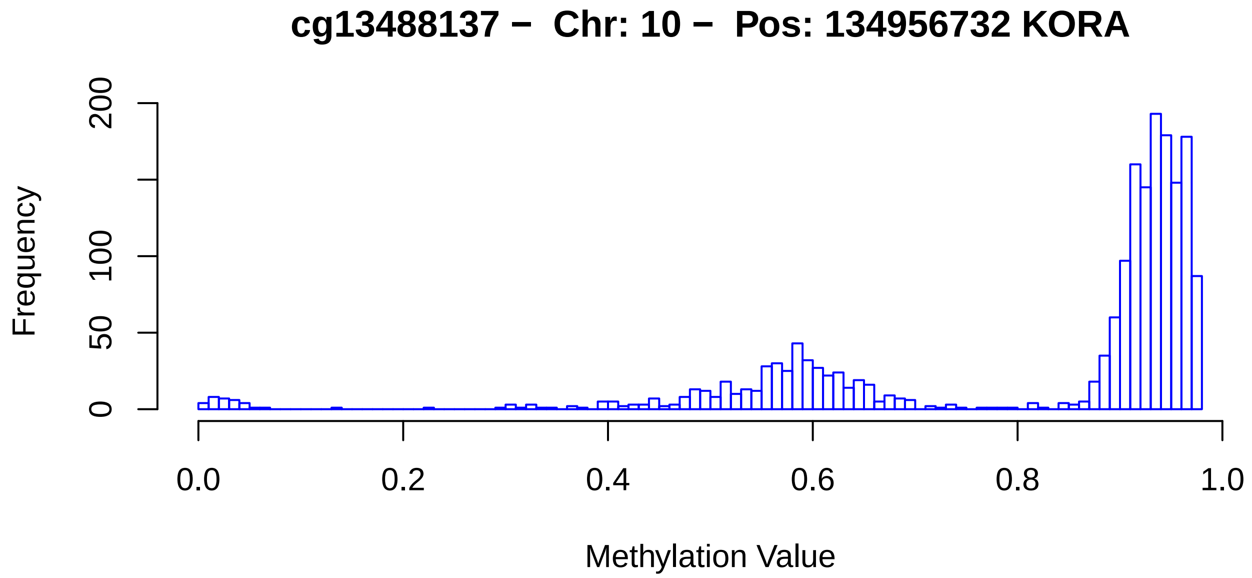
cg13488137 − Chr: 10 − Pos: 134956732 KORA
Frequency
0
50
100
200
0.0
0.2
0.4
0.6
0.8
1.0
Methylation Value

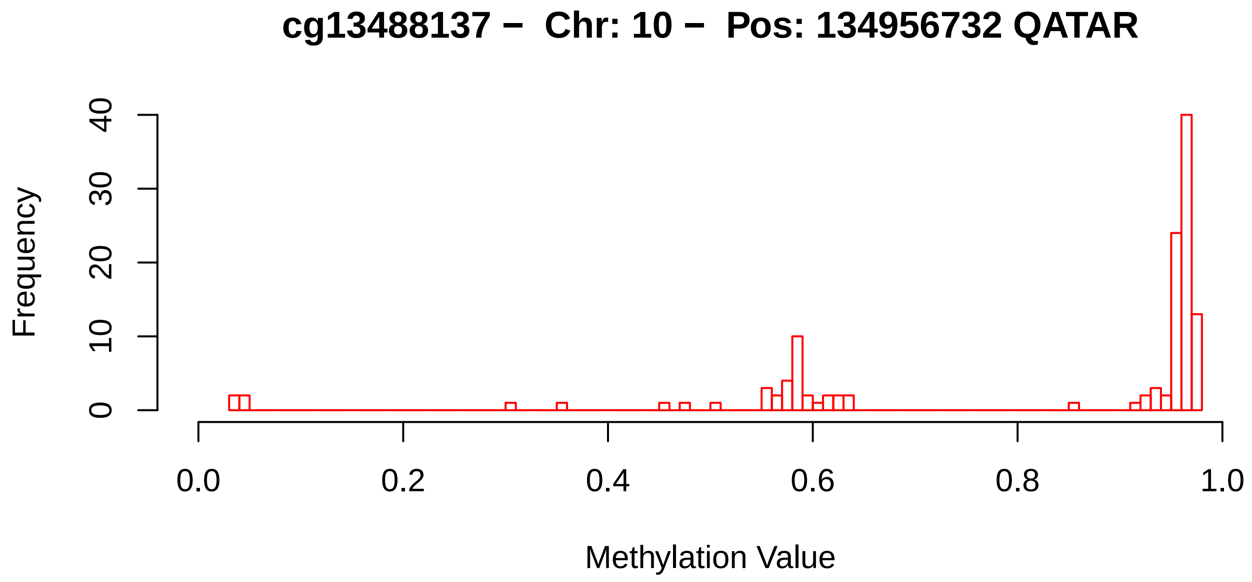
cg13488137 − Chr: 10 − Pos: 134956732 QATAR
Frequency
0
10
20
30
40
0.0
0.2
0.4
0.6
0.8
1.0
Methylation Value

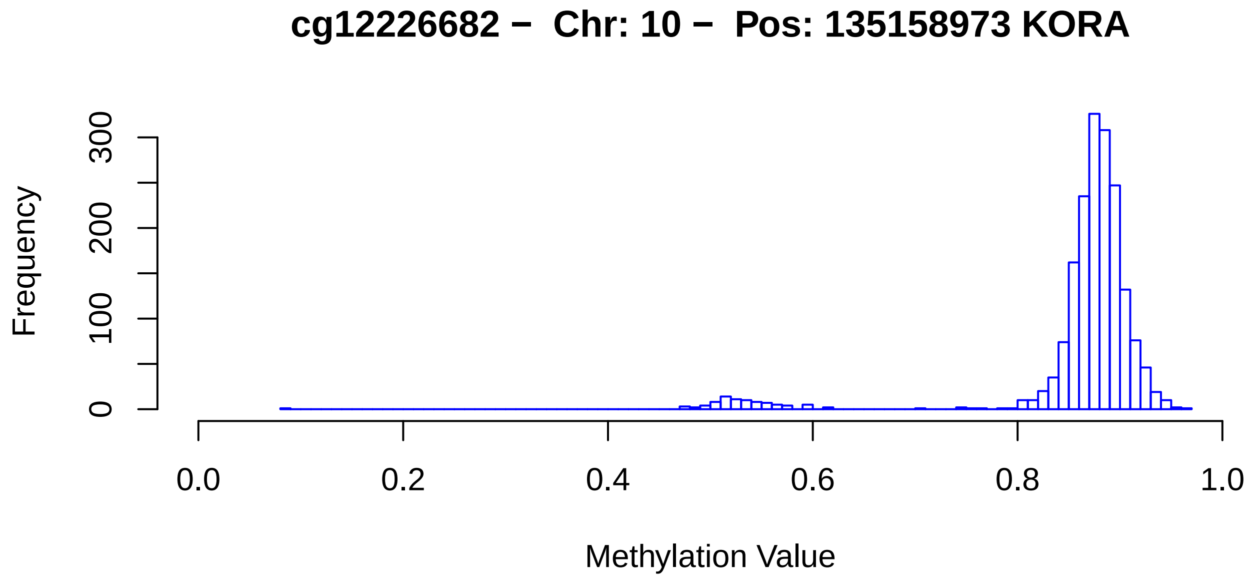
cg12226682 − Chr: 10 − Pos: 135158973 KORA
Frequency
0
100
200
300
0.0
0.2
0.4
0.6
0.8
1.0
Methylation Value

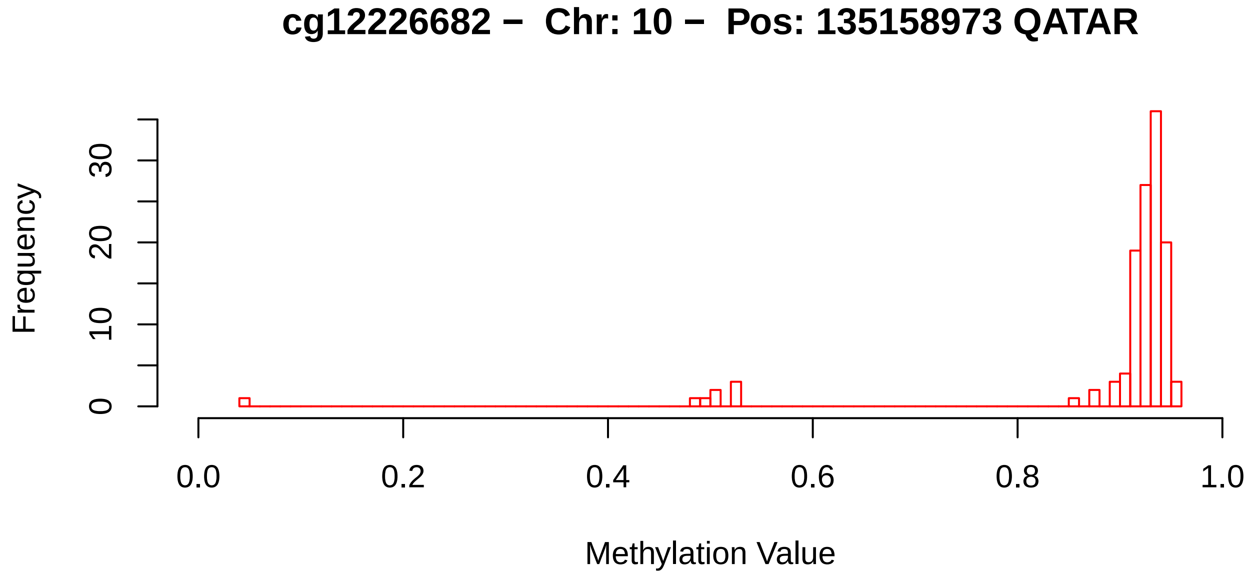
cg12226682 − Chr: 10 − Pos: 135158973 QATAR
Frequency
0
10
20
30
0.0
0.2
0.4
0.6
0.8
1.0
Methylation Value

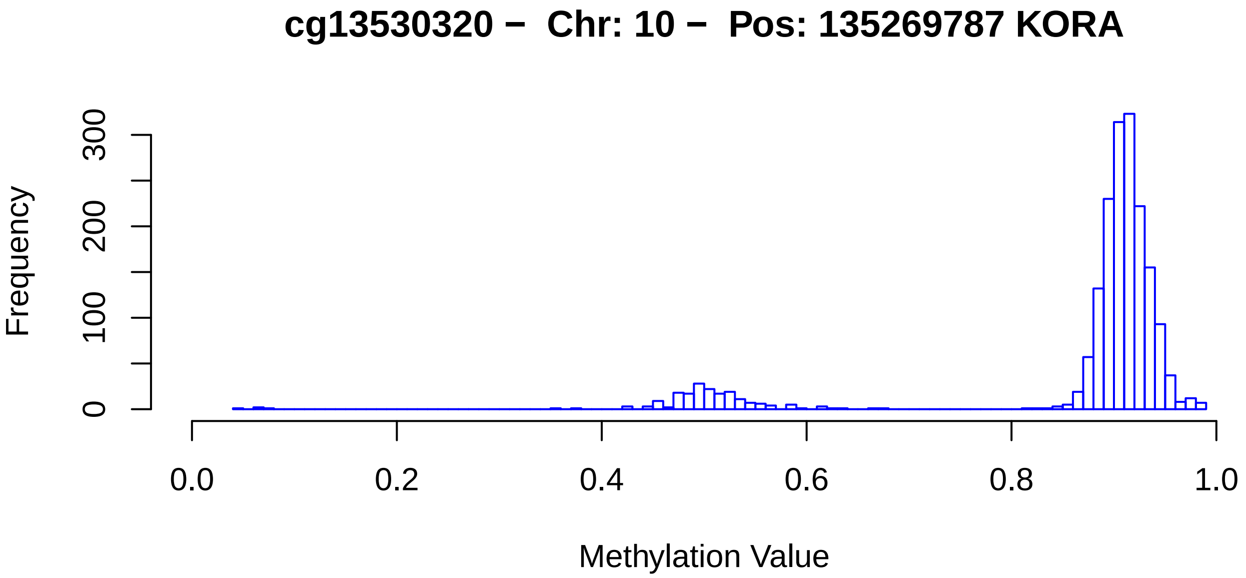
cg13530320 − Chr: 10 − Pos: 135269787 KORA
Frequency
0
100
200
300
0.0
0.2
0.4
0.6
0.8
1.0
Methylation Value

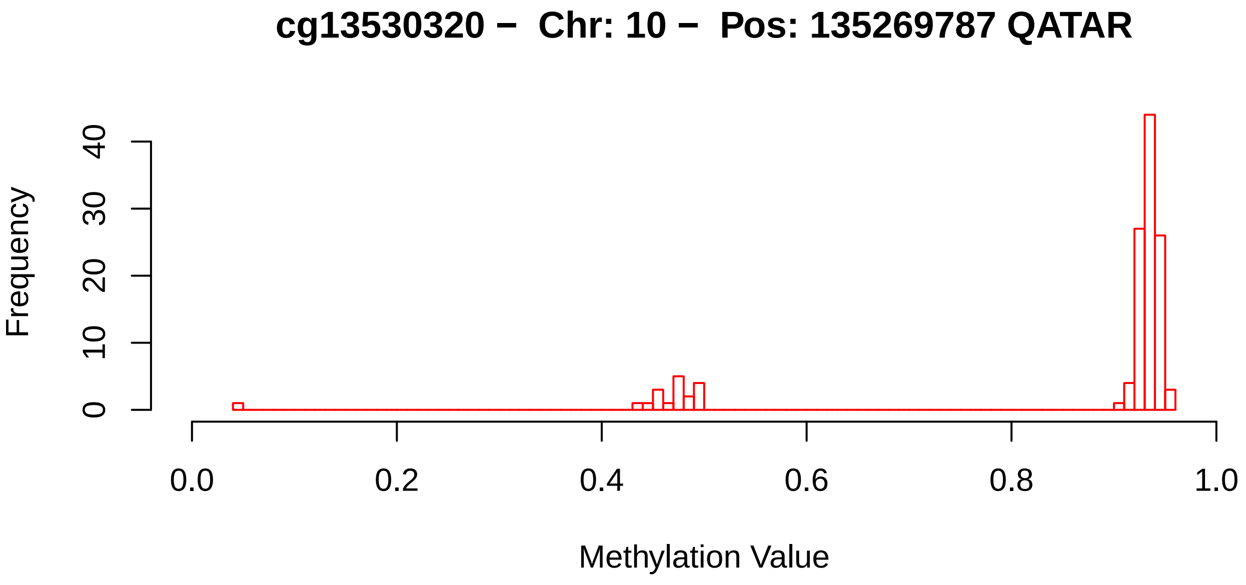
cg13530320 − Chr: 10 − Pos: 135269787 QATAR
Frequency
0
10
20
30
40
0.0
0.2
0.4
0.6
0.8
1.0
Methylation Value

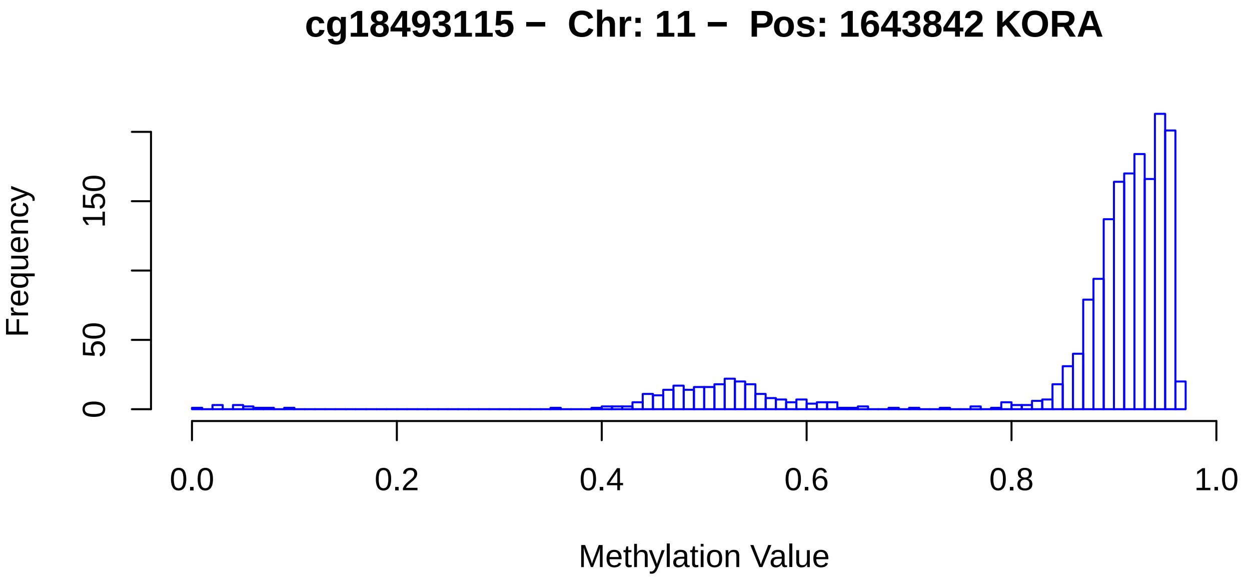
cg18493115 − Chr: 11 − Pos: 1643842 KORA
Frequency
0
50
150
0.0
0.2
0.4
0.6
0.8
1.0
Methylation Value

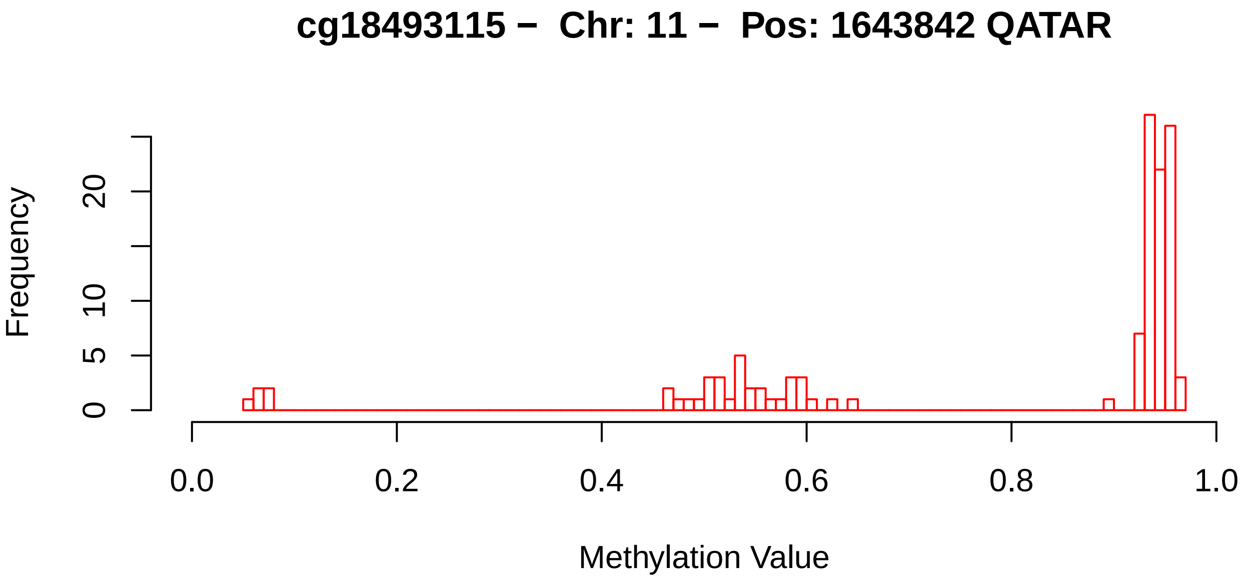
cg18493115 − Chr: 11 − Pos: 1643842 QATAR
Frequency
0
5
10
20
0.0
0.2
0.4
0.6
0.8
1.0
Methylation Value

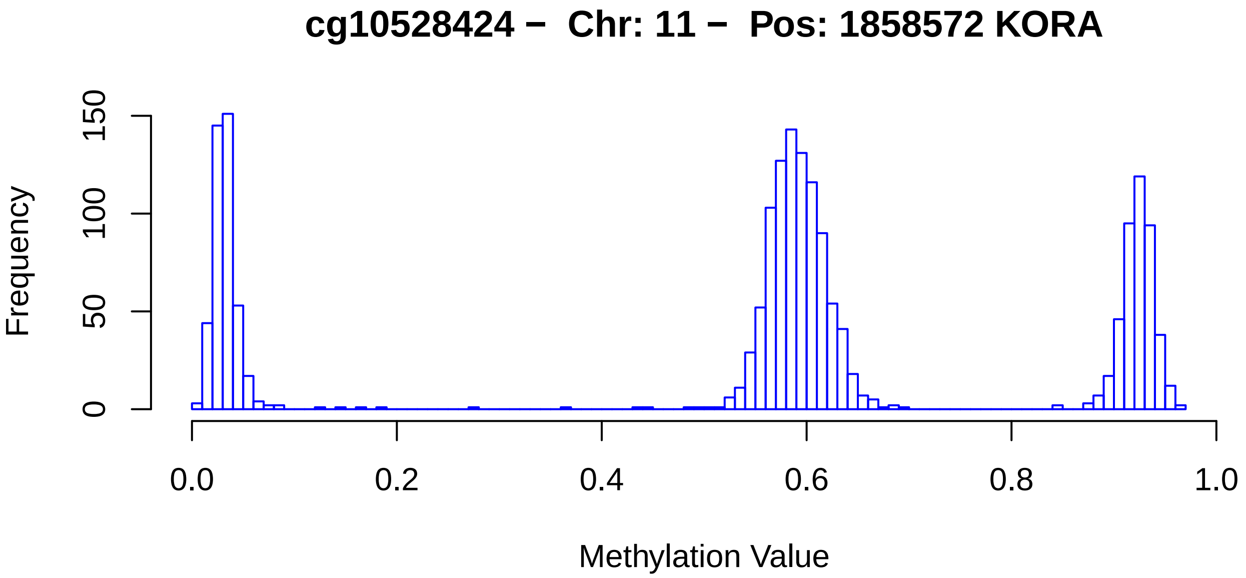
cg10528424 − Chr: 11 − Pos: 1858572 KORA
Frequency
0
50
100
150
0.0
0.2
0.4
0.6
0.8
1.0
Methylation Value

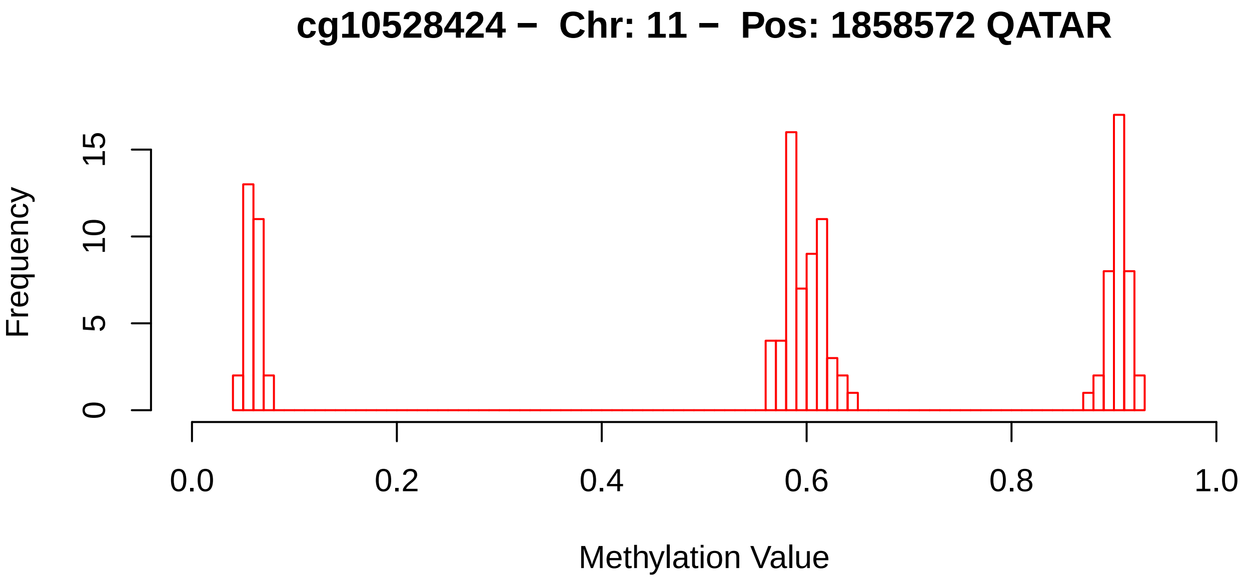
cg10528424 − Chr: 11 − Pos: 1858572 QATAR
Frequency
0
5
10
15
0.0
0.2
0.4
0.6
0.8
1.0
Methylation Value

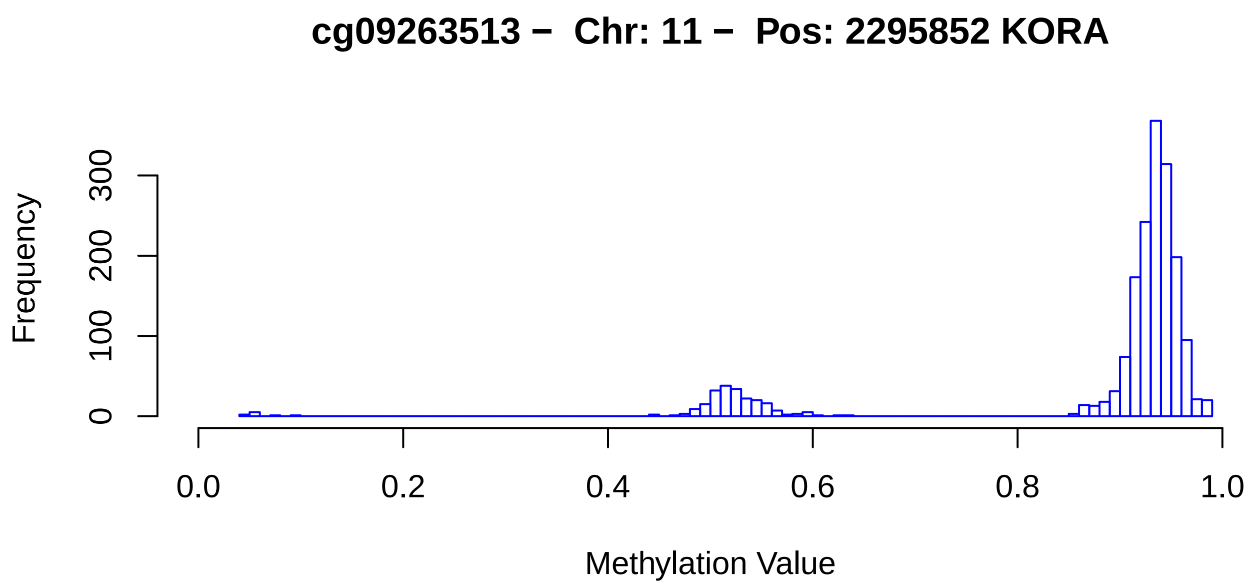
cg09263513 − Chr: 11 − Pos: 2295852 KORA
Frequency
0
100
200
300
0.0
0.2
0.4
0.6
0.8
1.0
Methylation Value

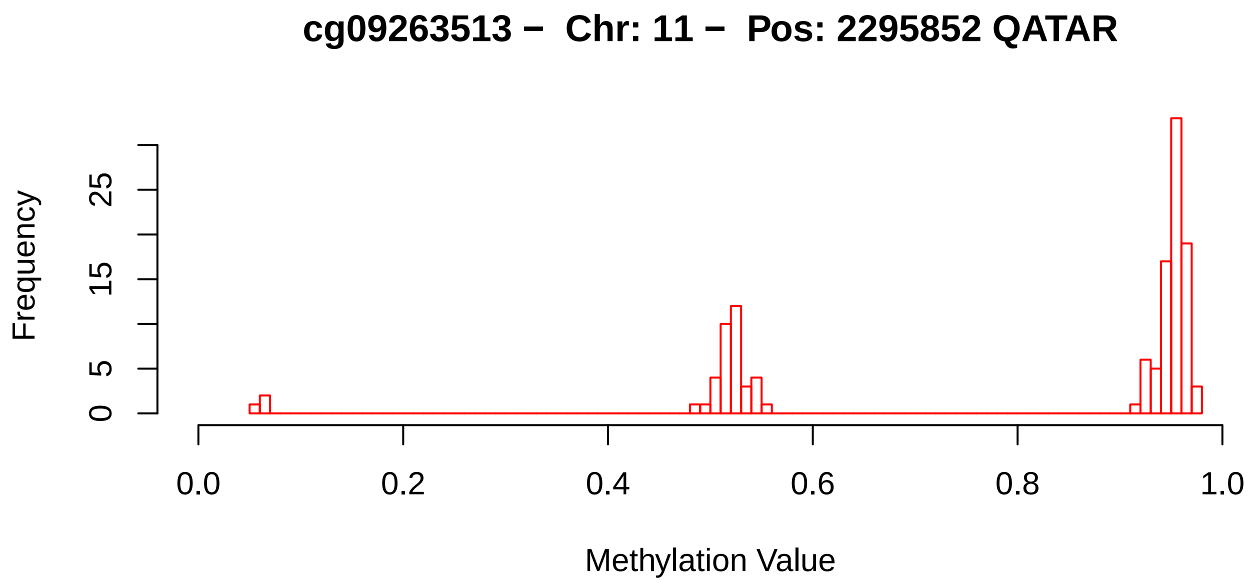
cg09263513 − Chr: 11 − Pos: 2295852 QATAR
Frequency
0
5
15
25
0.0
0.2
0.4
0.6
0.8
1.0
Methylation Value

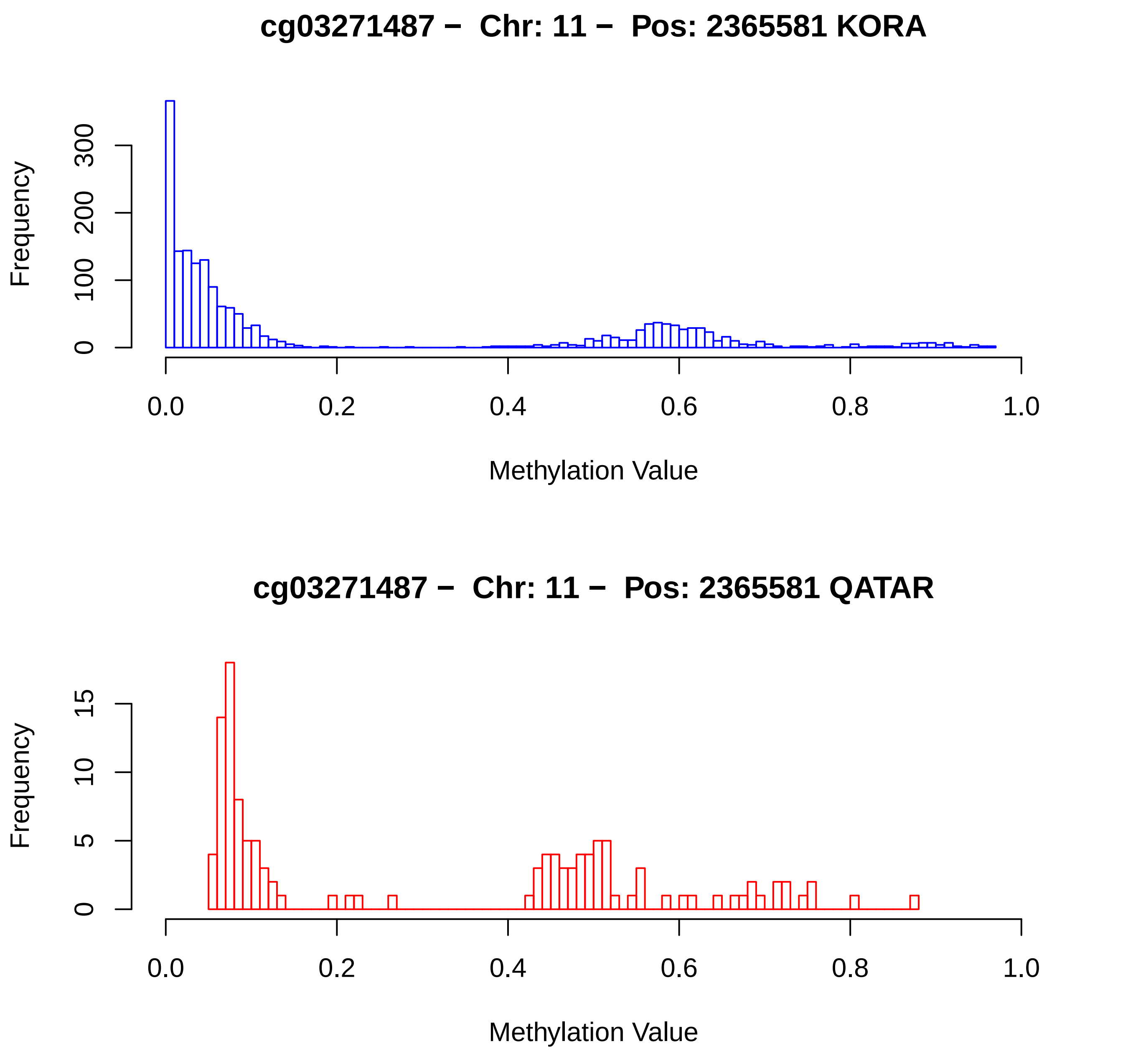
cg03271487 − Chr: 11 − Pos: 2365581 KORA
Frequency
0
100
200
300
0.0
0.2
0.4
0.6
0.8
1.0
Methylation Value
cg03271487 − Chr: 11 − Pos: 2365581 QATAR
Frequency
0
5
10
15
0.0
0.2
0.4
0.6
0.8
1.0
Methylation Value

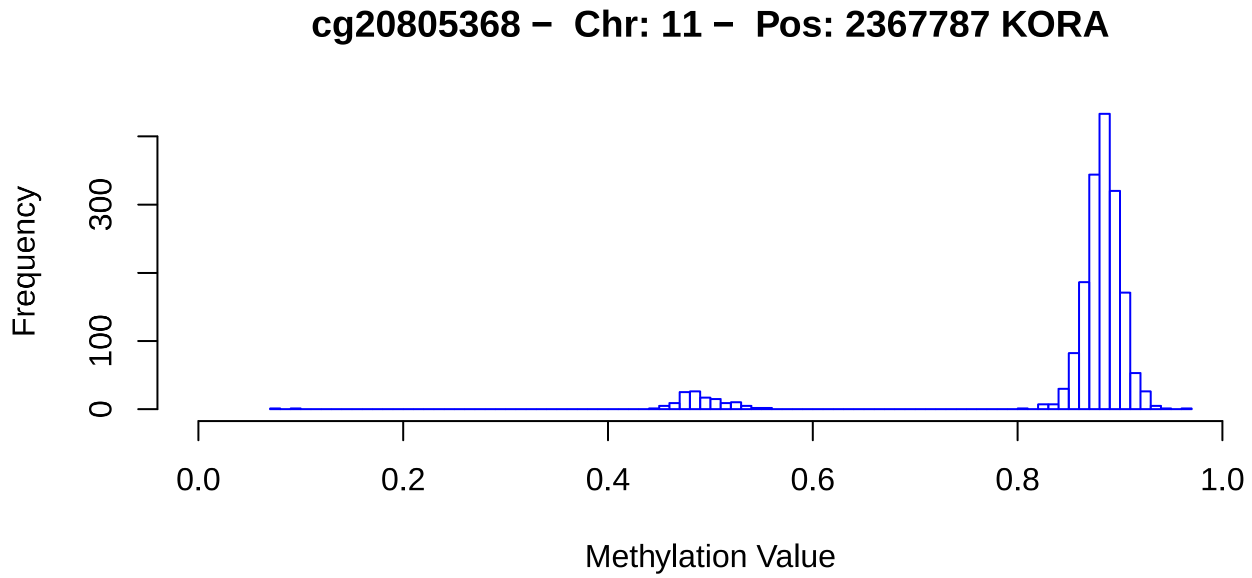
cg20805368 − Chr: 11 − Pos: 2367787 KORA
Frequency
0
100
300
0.0
0.2
0.4
0.6
0.8
1.0
Methylation Value

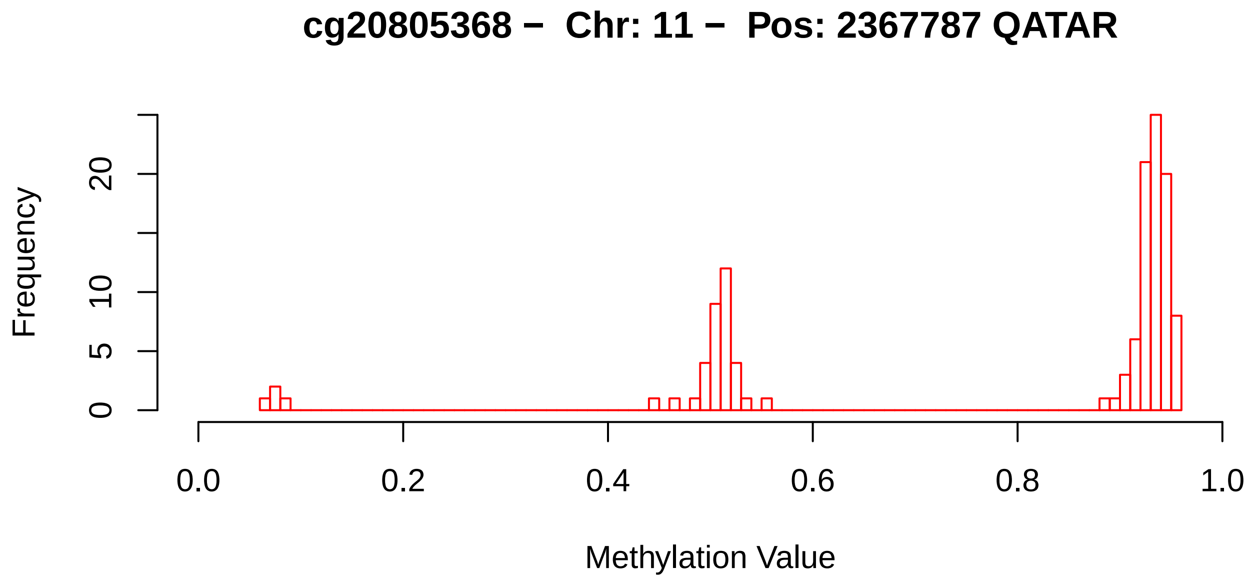
cg20805368 − Chr: 11 − Pos: 2367787 QATAR
Frequency
0
5
10
20
0.0
0.2
0.4
0.6
0.8
1.0
Methylation Value

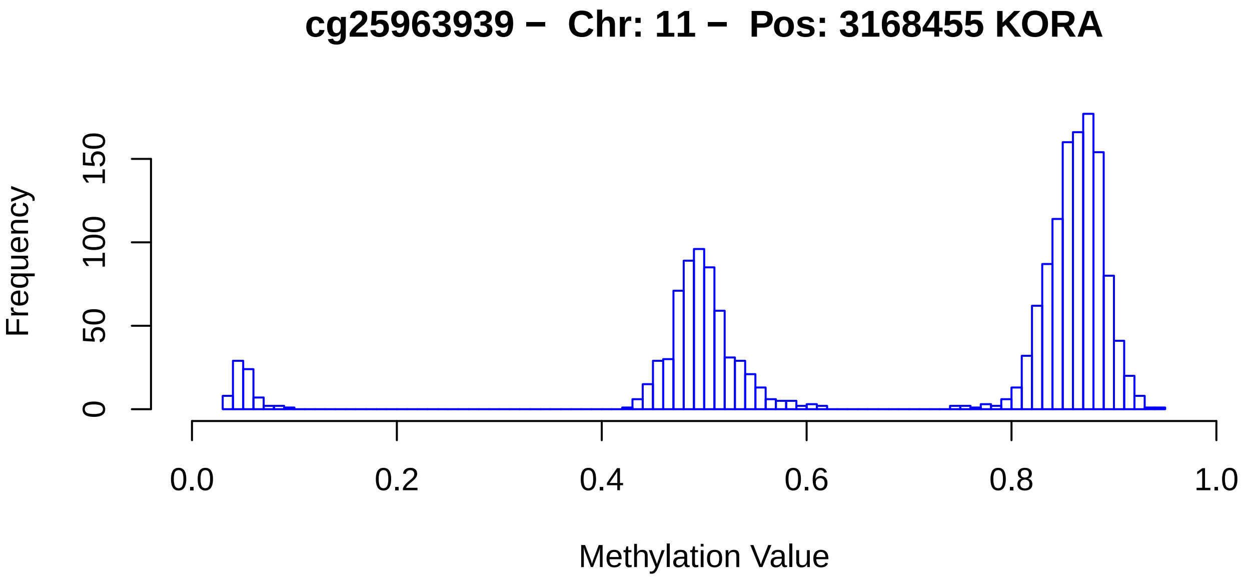
cg25963939 − Chr: 11 − Pos: 3168455 KORA
Frequency
0
50
100
150
0.0
0.2
0.4
0.6
0.8
1.0
Methylation Value

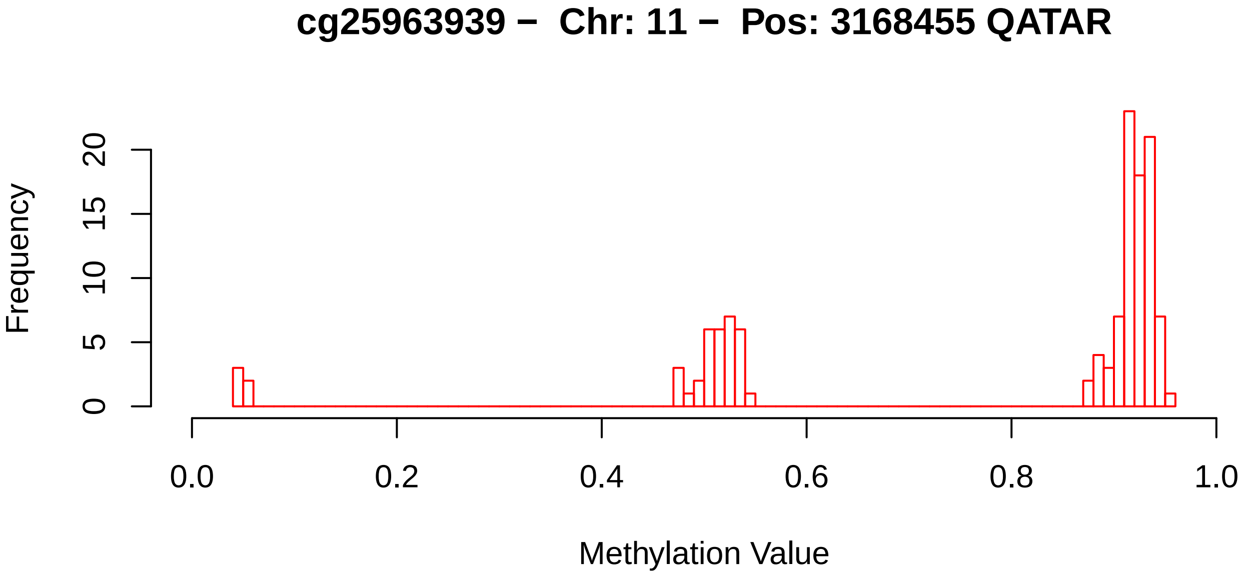
cg25963939 − Chr: 11 − Pos: 3168455 QATAR
Frequency
0
5
10
15
20
0.0
0.2
0.4
0.6
0.8
1.0
Methylation Value

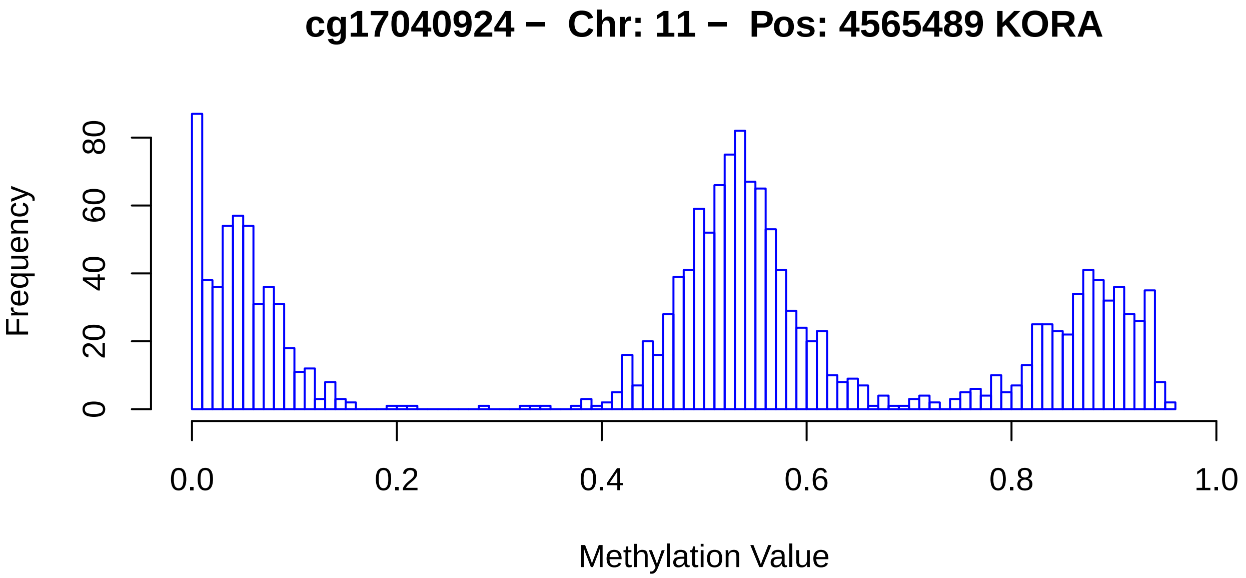

cg17040924 − Chr: 11 − Pos: 4565489 KORA
Frequency
0
20
40
60
80
0.0
0.2
0.4
0.6
0.8
1.0
Methylation Value

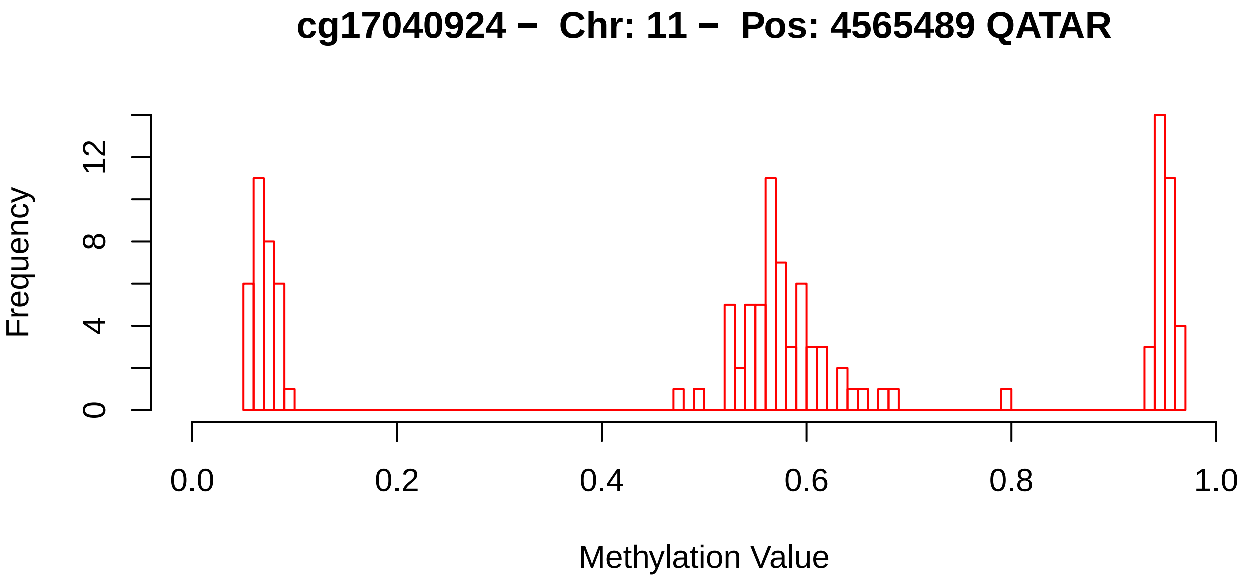

cg17040924 − Chr: 11 − Pos: 4565489 QATAR
Frequency
0
4
8
12
0.0
0.2
0.4
0.6
0.8
1.0
Methylation Value

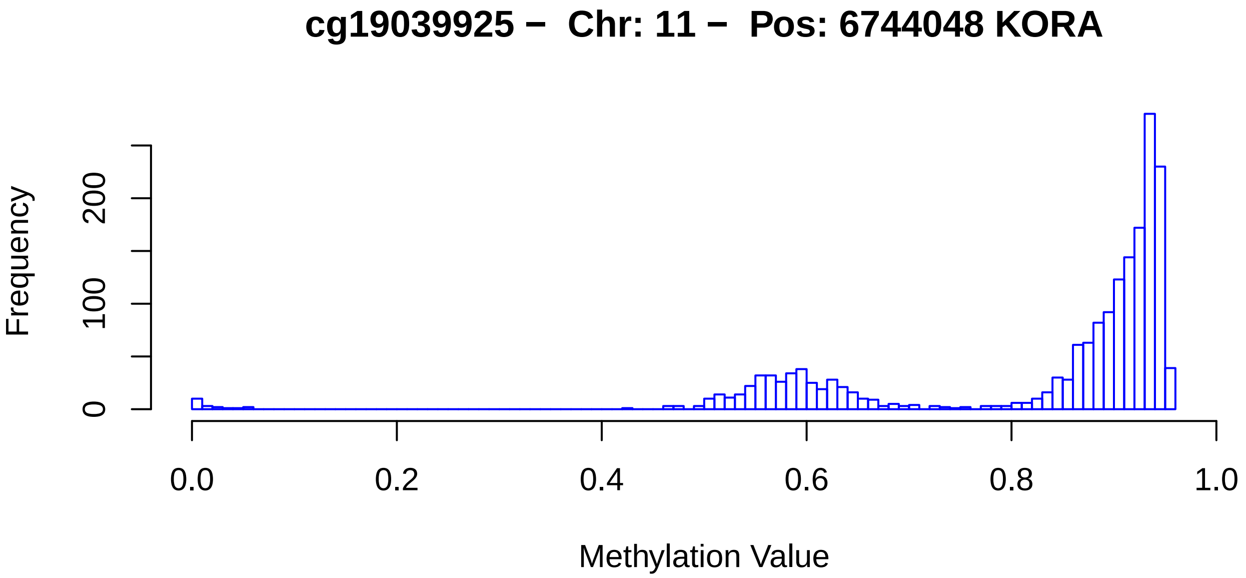
cg19039925 − Chr: 11 − Pos: 6744048 KORA
Frequency
0
100
200
0.0
0.2
0.4
0.6
0.8
1.0
Methylation Value

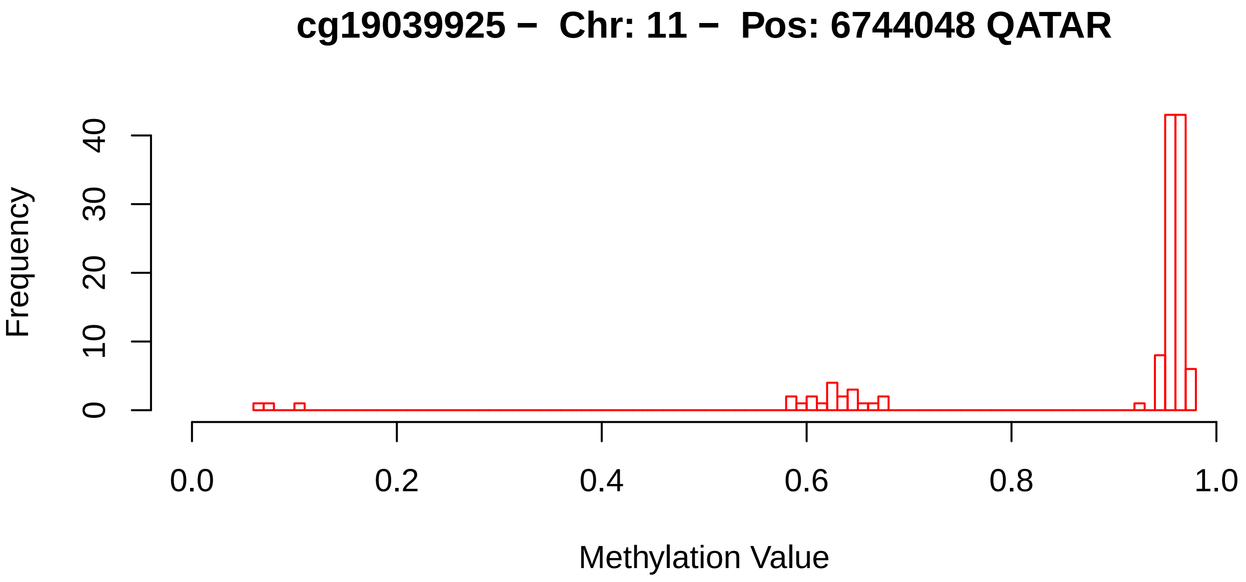
cg19039925 − Chr: 11 − Pos: 6744048 QATAR
Frequency
0
10
20
30
40
0.0
0.2
0.4
0.6
0.8
1.0
Methylation Value

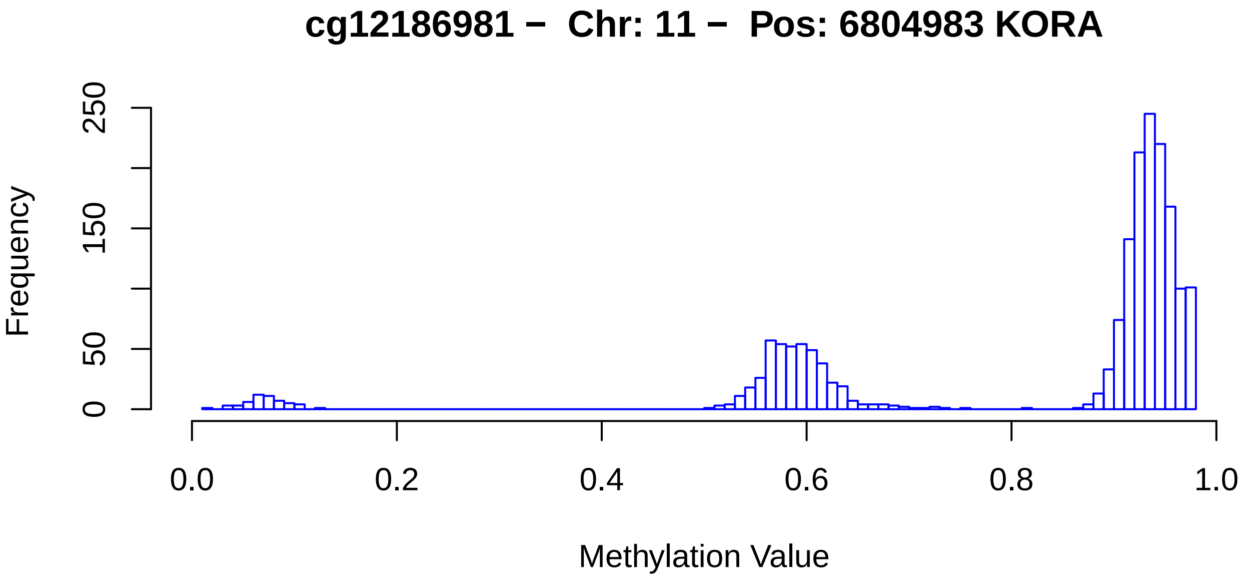
cg12186981 − Chr: 11 − Pos: 6804983 KORA
Frequency
0
50
150
250
0.0
0.2
0.4
0.6
0.8
1.0
Methylation Value

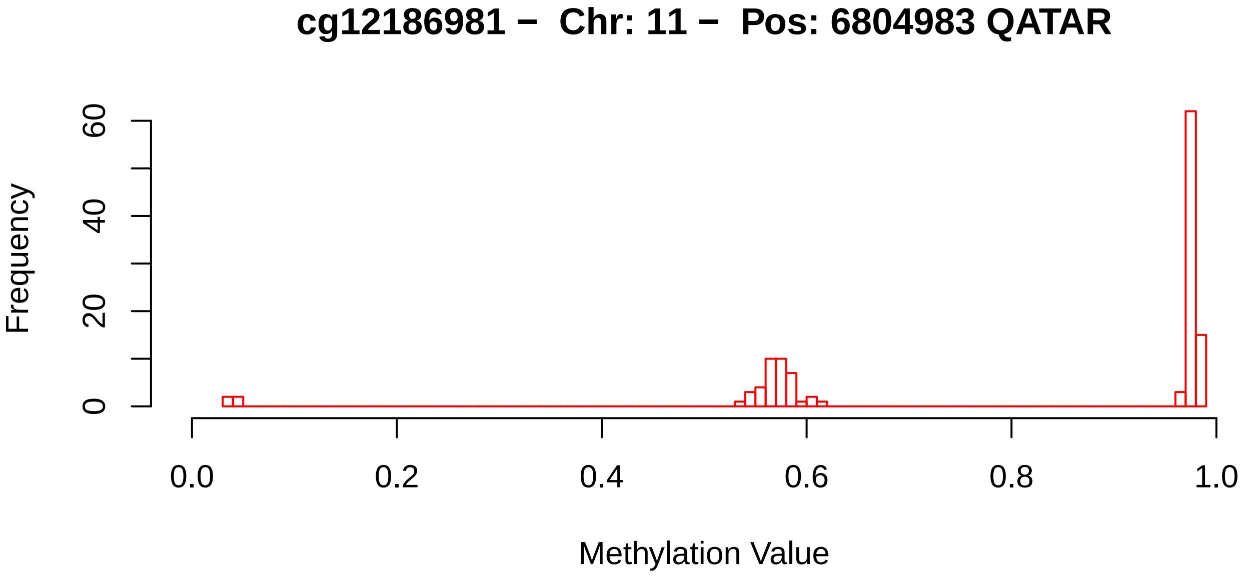
cg12186981 − Chr: 11 − Pos: 6804983 QATAR
Frequency
0
20
40
60
0.0
0.2
0.4
0.6
0.8
1.0
Methylation Value

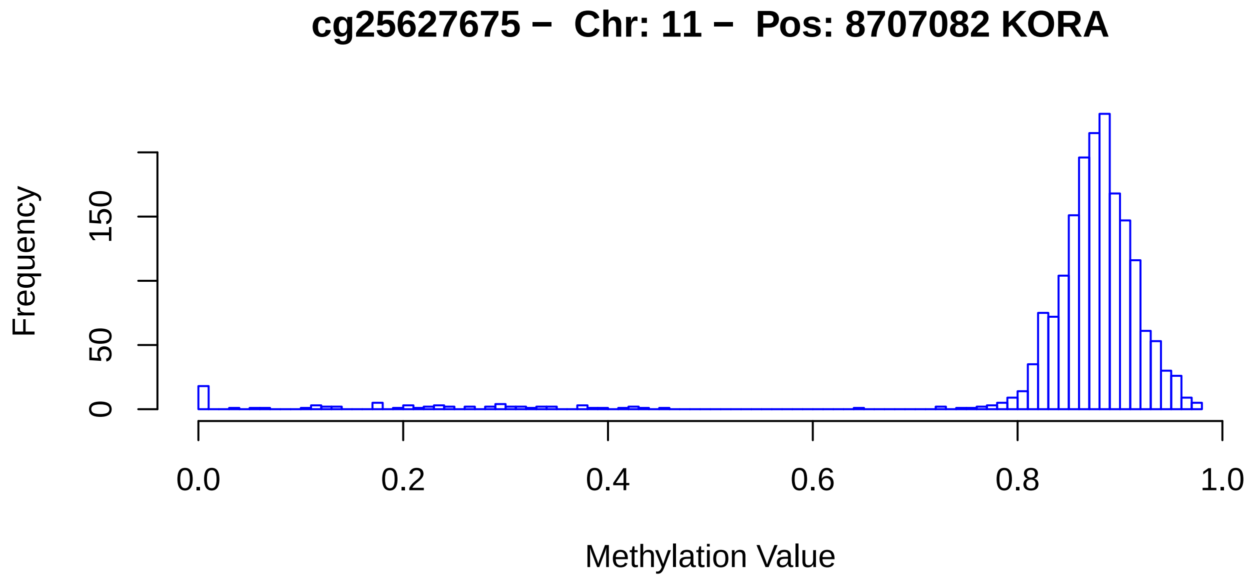
cg25627675 − Chr: 11 − Pos: 8707082 KORA
Frequency
0
50
150
0.0
0.2
0.4
0.6
0.8
1.0
Methylation Value

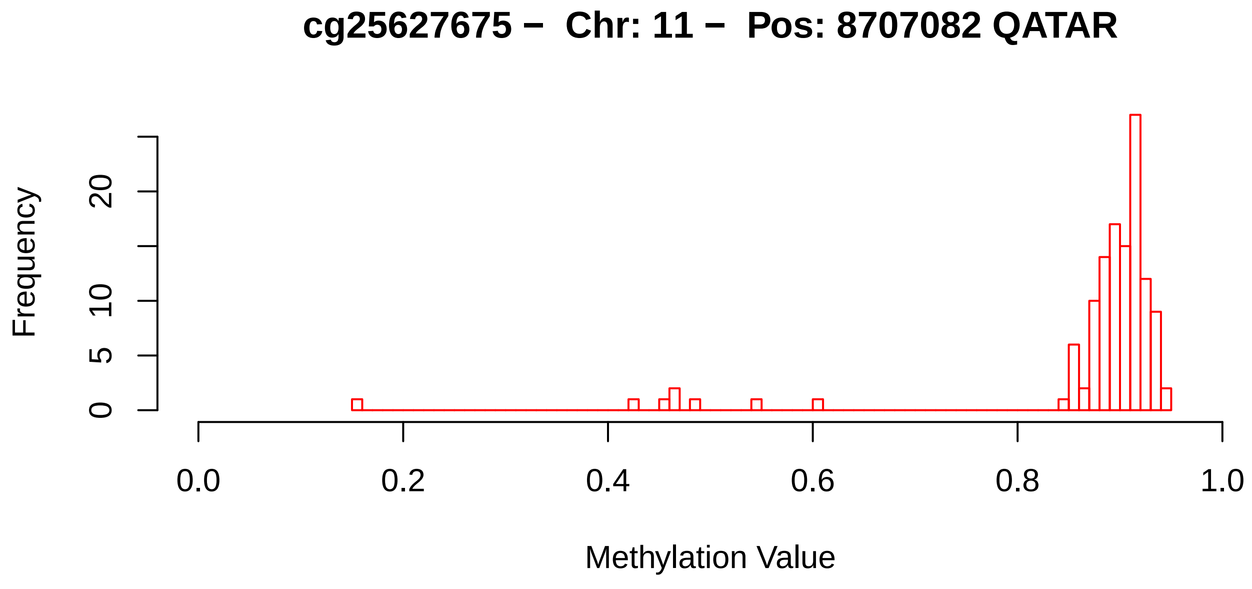
cg25627675 − Chr: 11 − Pos: 8707082 QATAR
Frequency
0
5
10
20
0.0
0.2
0.4
0.6
0.8
1.0
Methylation Value

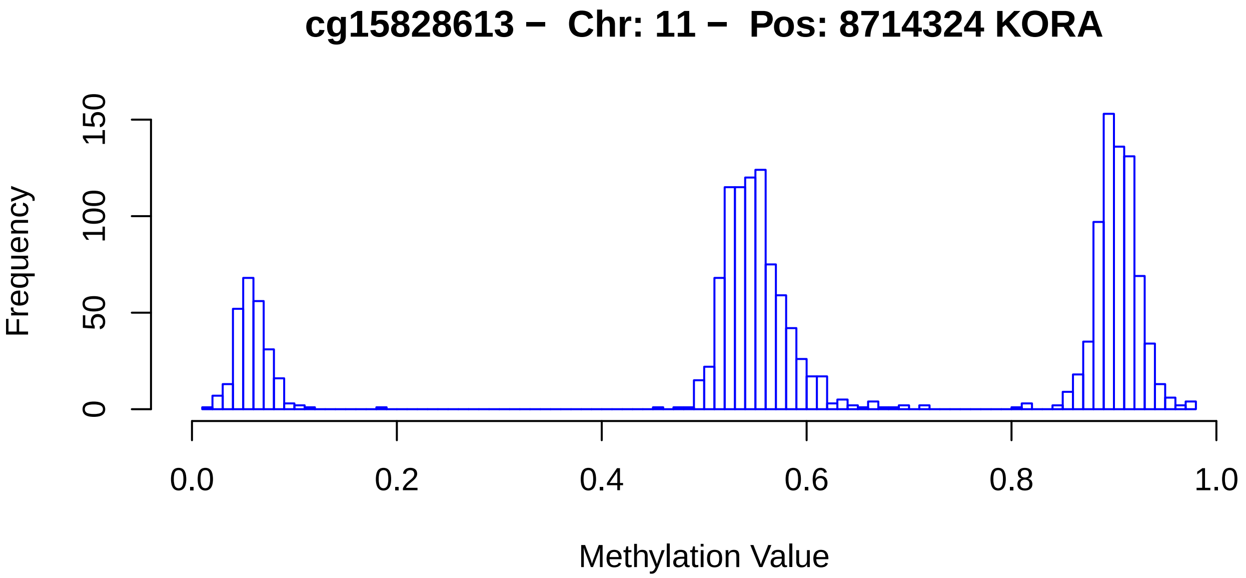
cg15828613 − Chr: 11 − Pos: 8714324 KORA
Frequency
0
50
100
150
0.0
0.2
0.4
0.6
0.8
1.0
Methylation Value

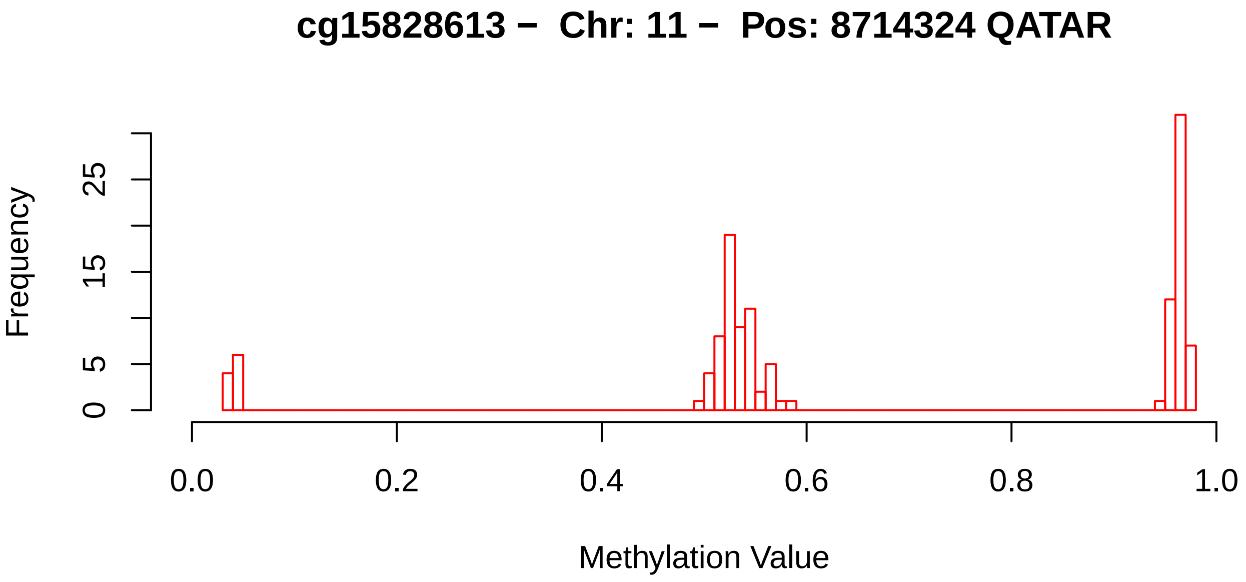
cg15828613 − Chr: 11 − Pos: 8714324 QATAR
Frequency
0
5
15
25
0.0
0.2
0.4
0.6
0.8
1.0
Methylation Value

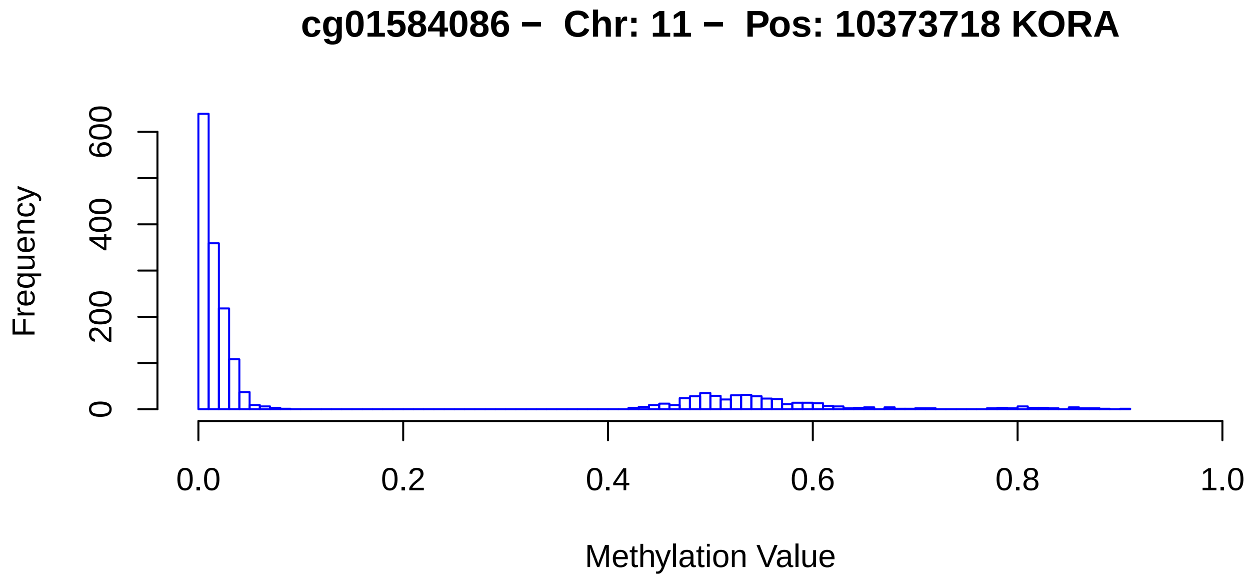

cg01584086 − Chr: 11 − Pos: 10373718 KORA
Frequency
0
200
400
600
0.0
0.2
0.4
0.6
0.8
1.0
Methylation Value

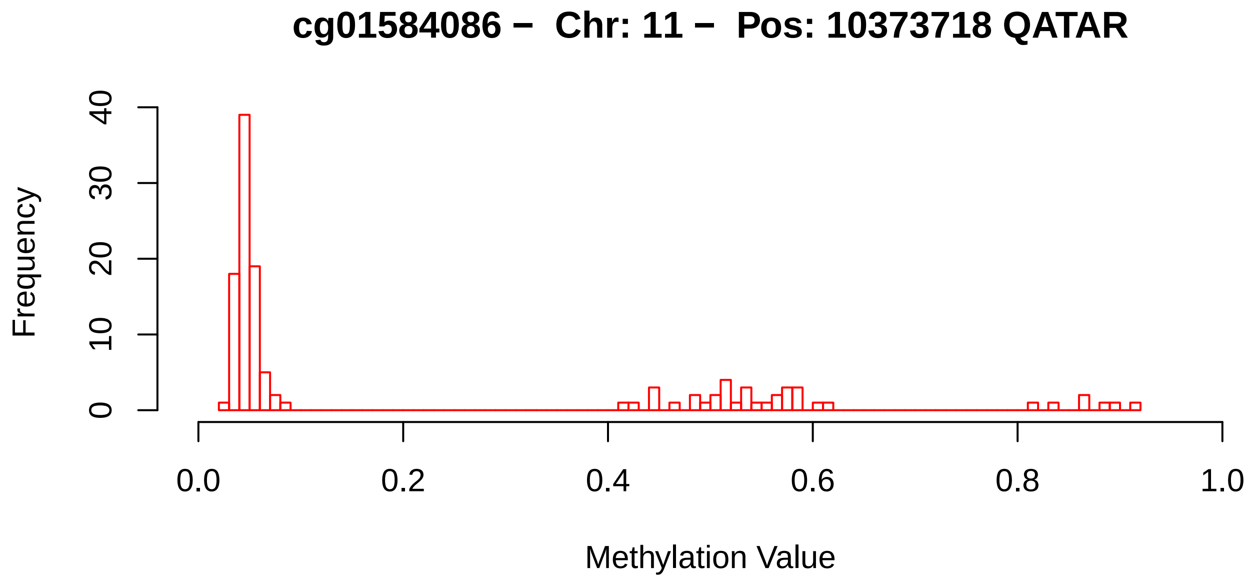

cg01584086 − Chr: 11 − Pos: 10373718 QATAR
Frequency
0
10
20
30
40
0.0
0.2
0.4
0.6
0.8
1.0
Methylation Value

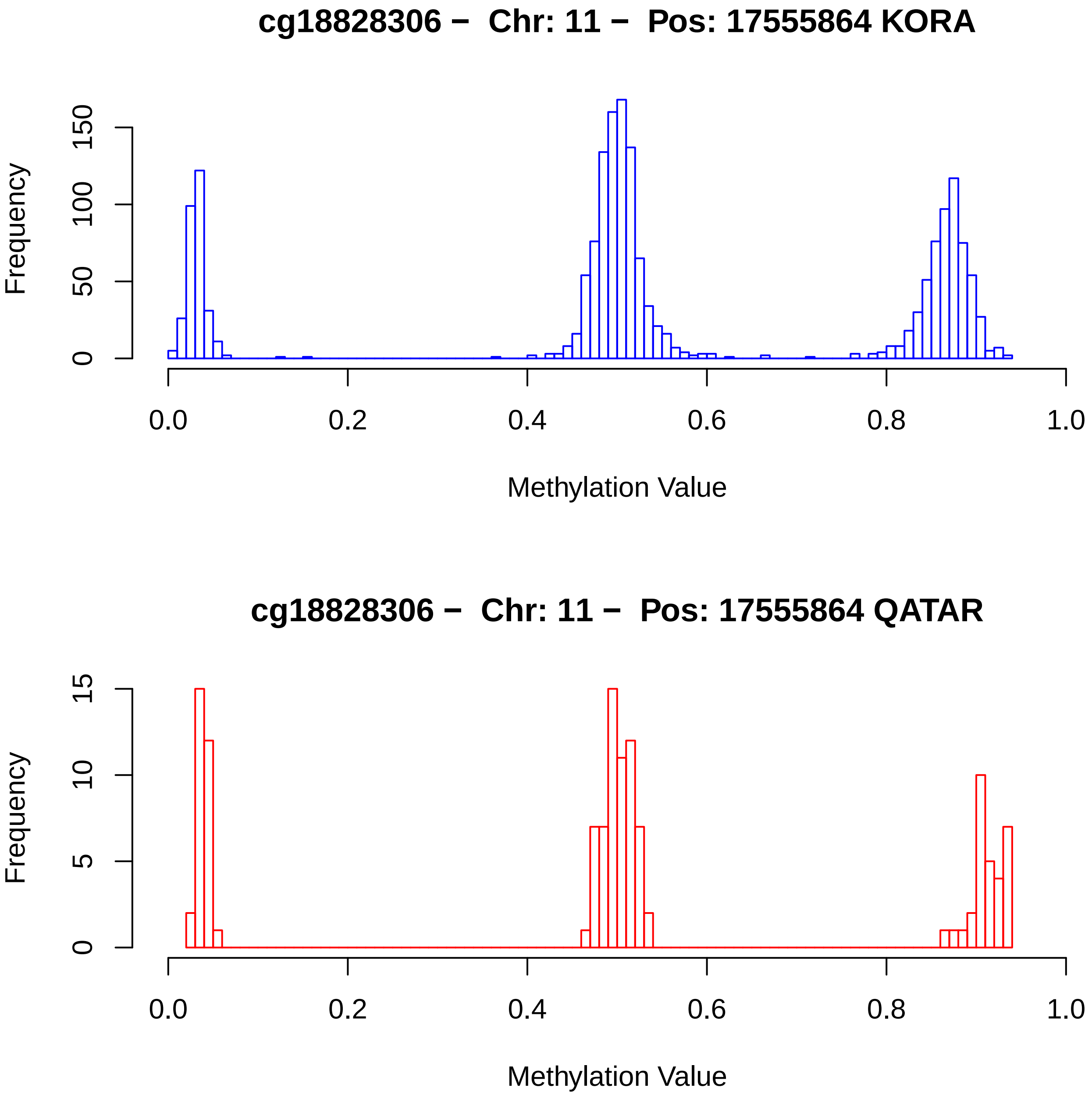
cg18828306 − Chr: 11 − Pos: 17555864 KORA
Frequency
0
50
100
150
0.0
0.2
0.4
0.6
0.8
1.0
Methylation Value
cg18828306 − Chr: 11 − Pos: 17555864 QATAR
Frequency
0
5
10
15
0.0
0.2
0.4
0.6
0.8
1.0
Methylation Value

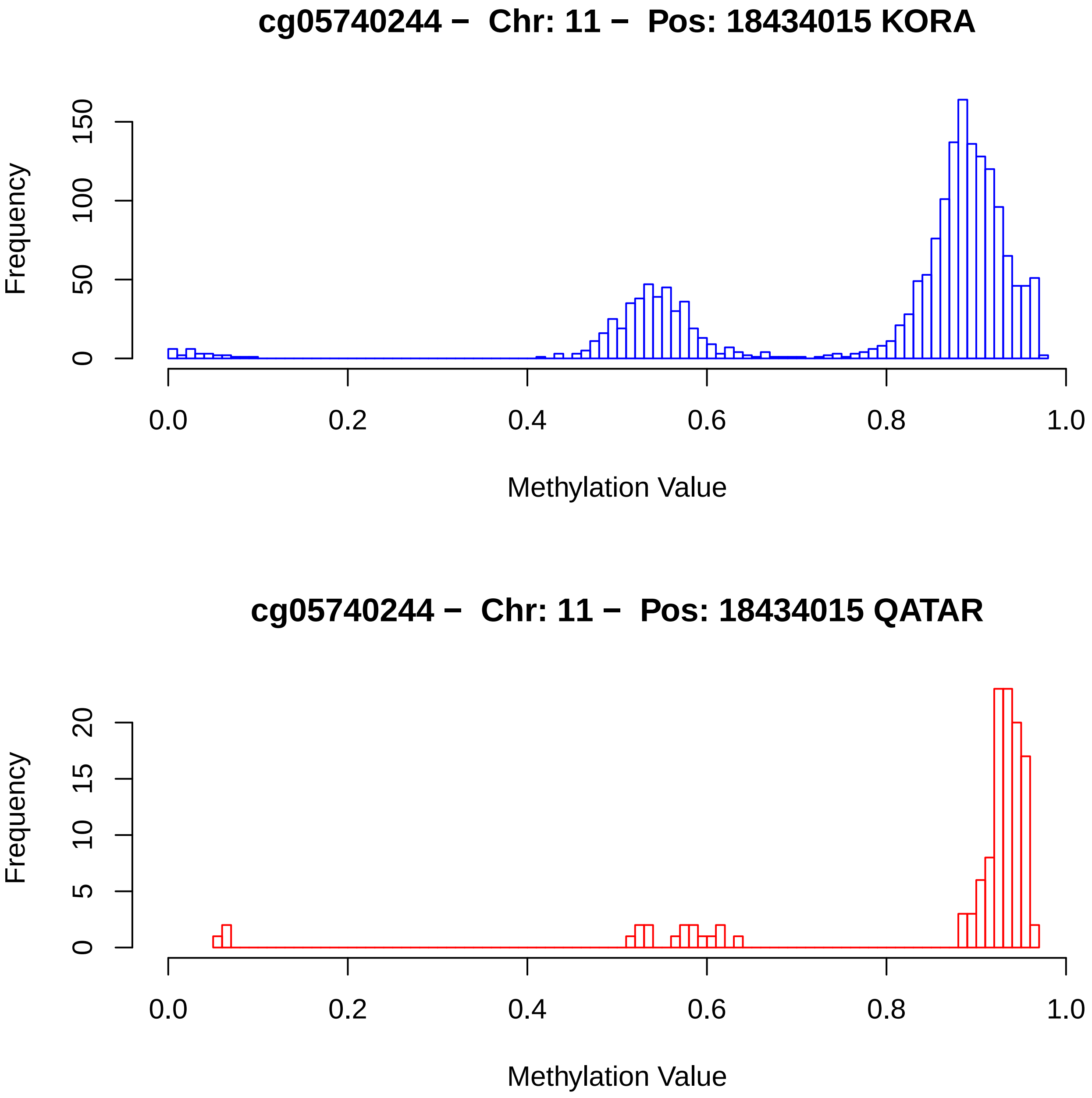
cg05740244 − Chr: 11 − Pos: 18434015 KORA
Frequency
0
50
100
150
0.0
0.2
0.4
0.6
0.8
1.0
Methylation Value
cg05740244 − Chr: 11 − Pos: 18434015 QATAR
Frequency
0
5
10
15
20
0.0
0.2
0.4
0.6
0.8
1.0
Methylation Value

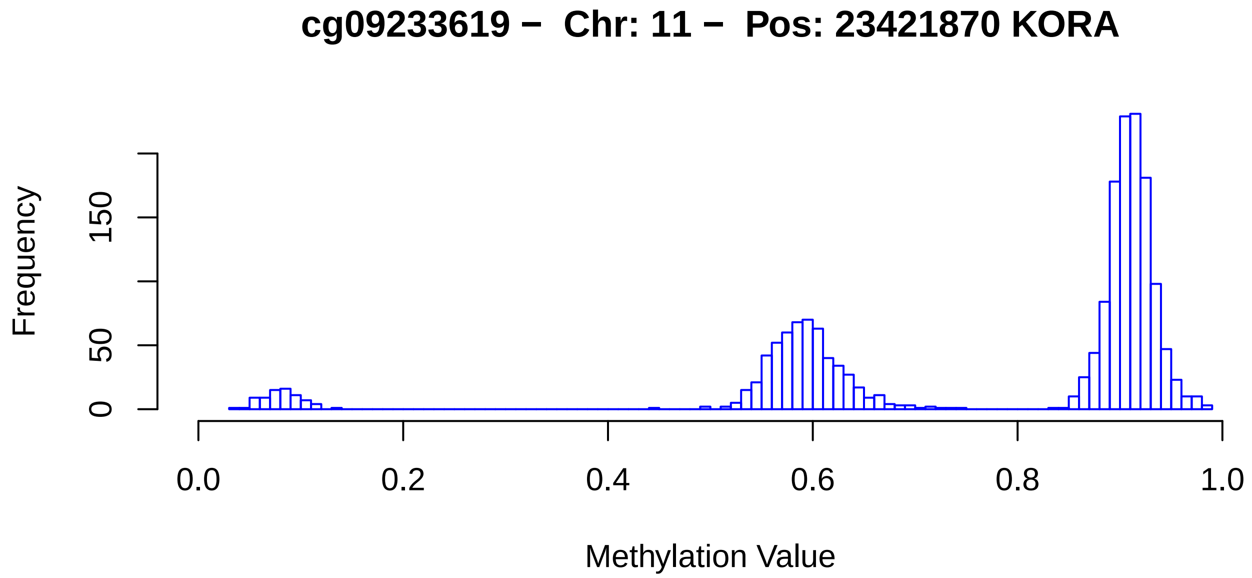
cg09233619 − Chr: 11 − Pos: 23421870 KORA
Frequency
0
50
150
0.0
0.2
0.4
0.6
0.8
1.0
Methylation Value

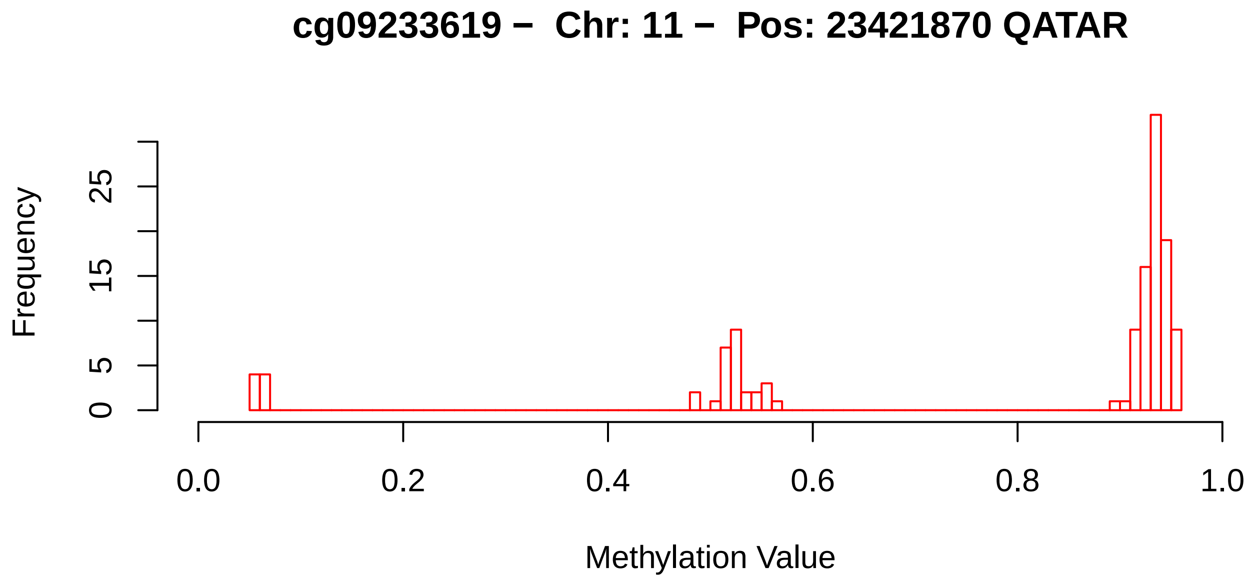
cg09233619 − Chr: 11 − Pos: 23421870 QATAR
Frequency
0
5
15
25
0.0
0.2
0.4
0.6
0.8
1.0
Methylation Value

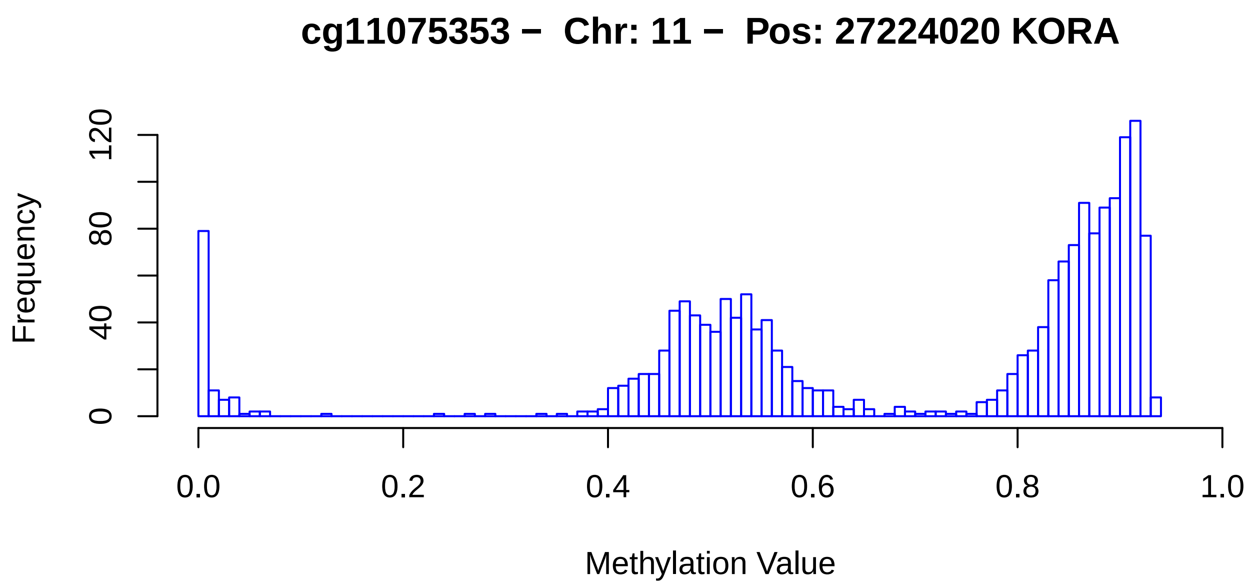
cg11075353 − Chr: 11 − Pos: 27224020 KORA
Frequency
0
40
80
120
0.0
0.2
0.4
0.6
0.8
1.0
Methylation Value

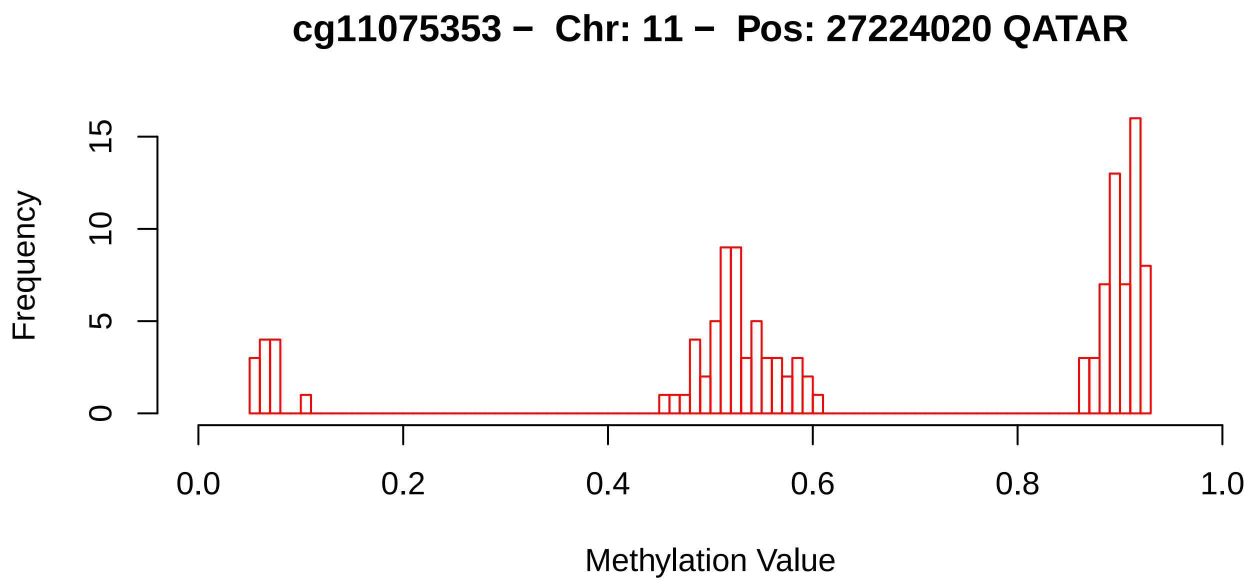
cg11075353 − Chr: 11 − Pos: 27224020 QATAR
Frequency
0
5
10
15
0.0
0.2
0.4
0.6
0.8
1.0
Methylation Value

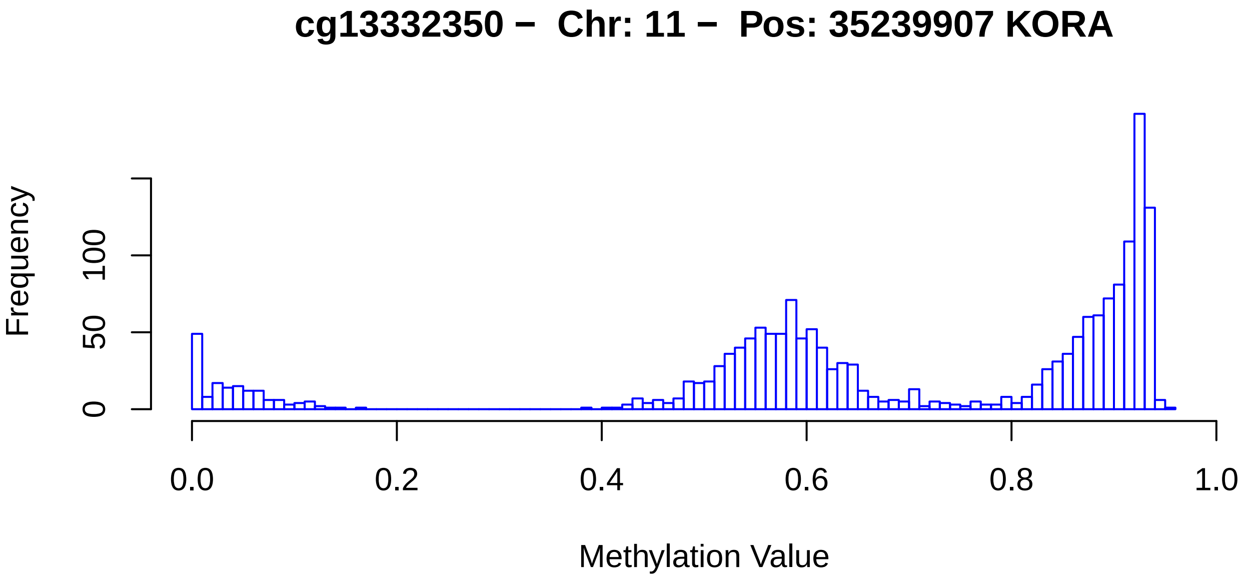
cg13332350 − Chr: 11 − Pos: 35239907 KORA
Frequency
0
50
100
0.0
0.2
0.4
0.6
0.8
1.0
Methylation Value

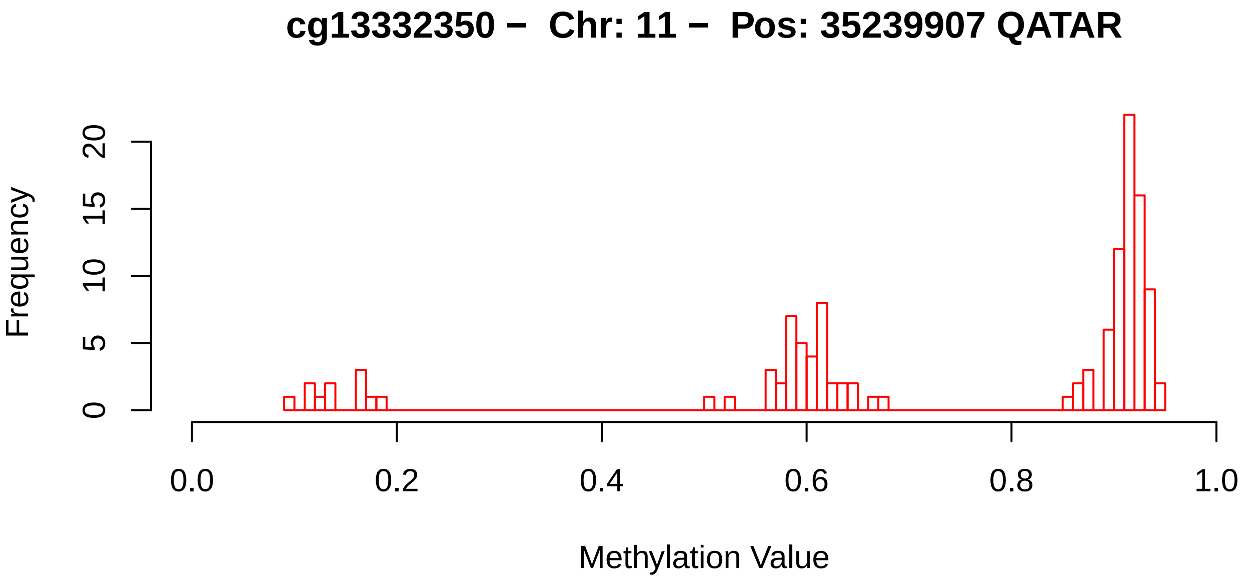
cg13332350 − Chr: 11 − Pos: 35239907 QATAR
Frequency
0
5
10
15
20
0.0
0.2
0.4
0.6
0.8
1.0
Methylation Value

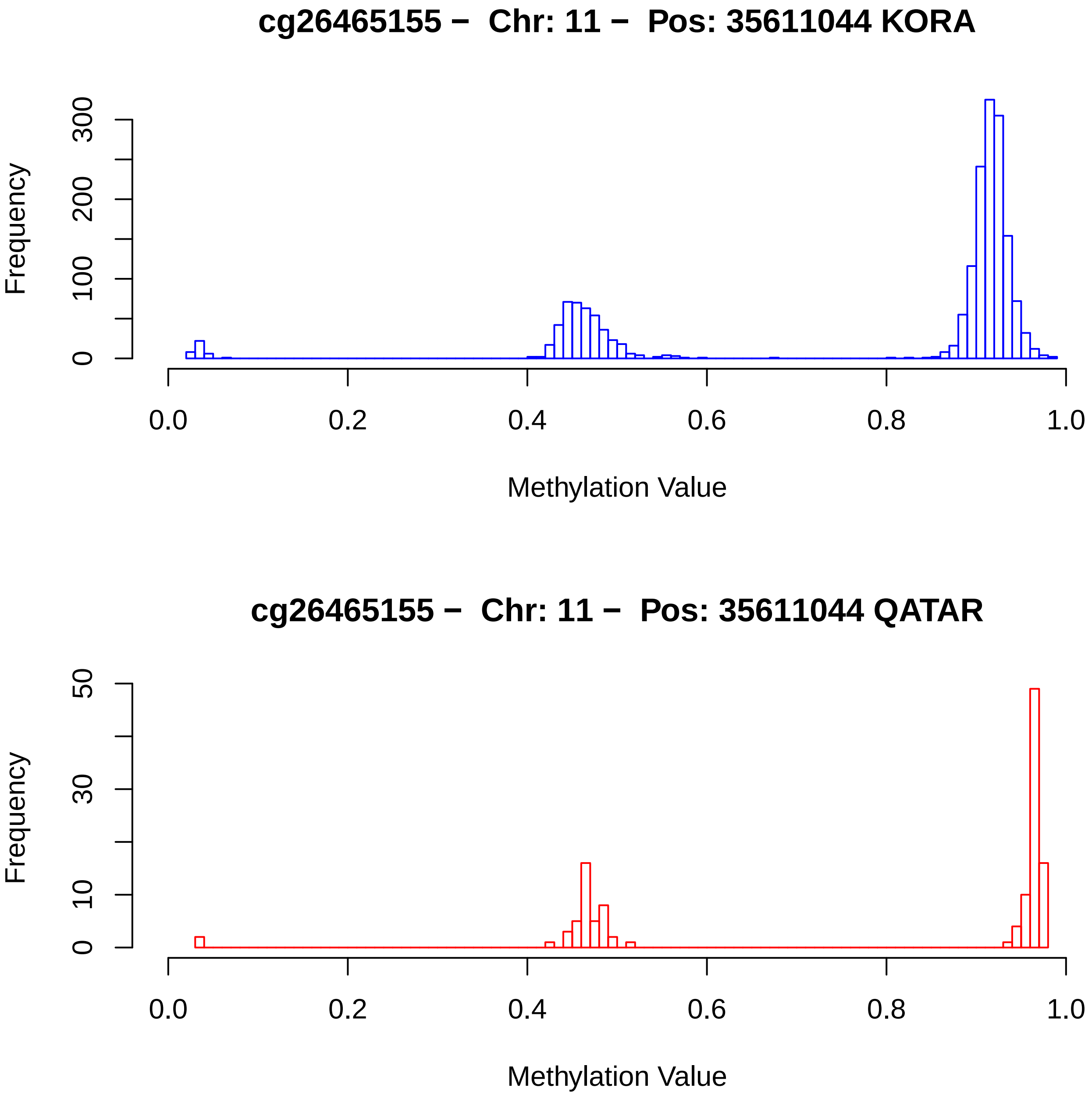

cg26465155 − Chr: 11 − Pos: 35611044 KORA
Frequency
0
100
200
300
0.0
0.2
0.4
0.6
0.8
1.0
Methylation Value
cg26465155 − Chr: 11 − Pos: 35611044 QATAR
Frequency
0
10
30
50
0.0
0.2
0.4
0.6
0.8
1.0
Methylation Value

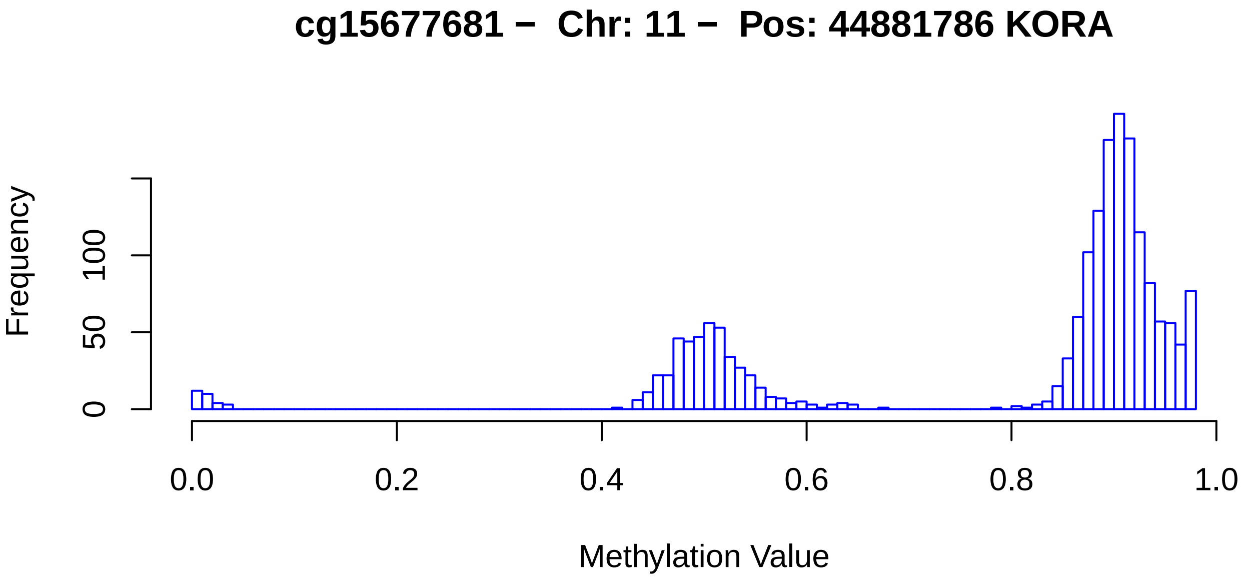
cg15677681 − Chr: 11 − Pos: 44881786 KORA
Frequency
0
50
100
0.0
0.2
0.4
0.6
0.8
1.0
Methylation Value

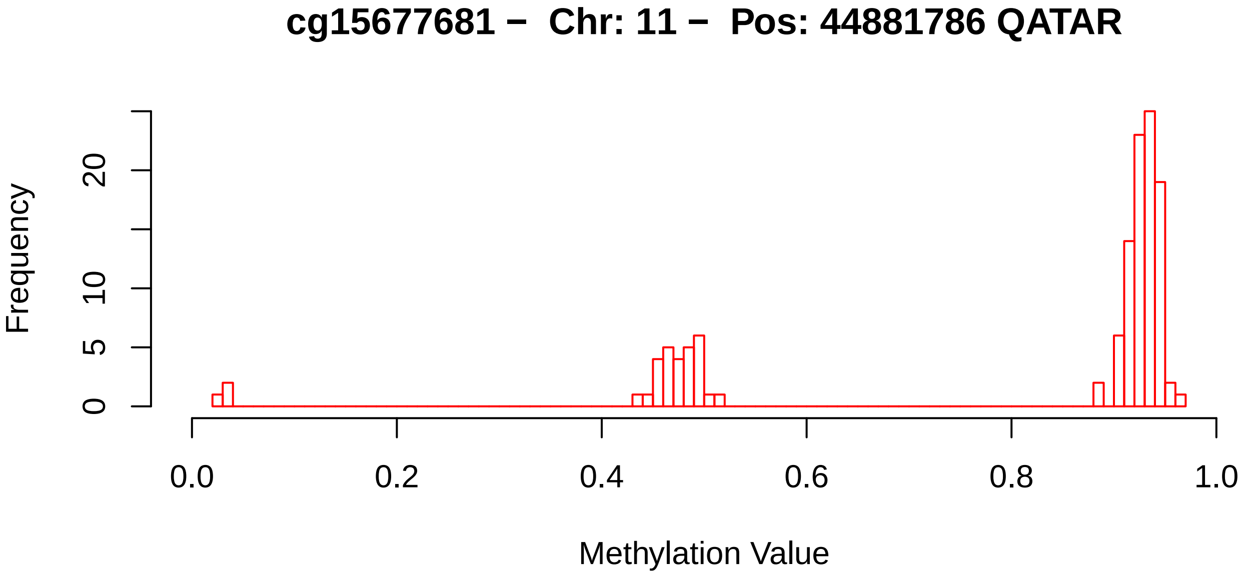
cg15677681 − Chr: 11 − Pos: 44881786 QATAR
Frequency
0
5
10
20
0.0
0.2
0.4
0.6
0.8
1.0
Methylation Value

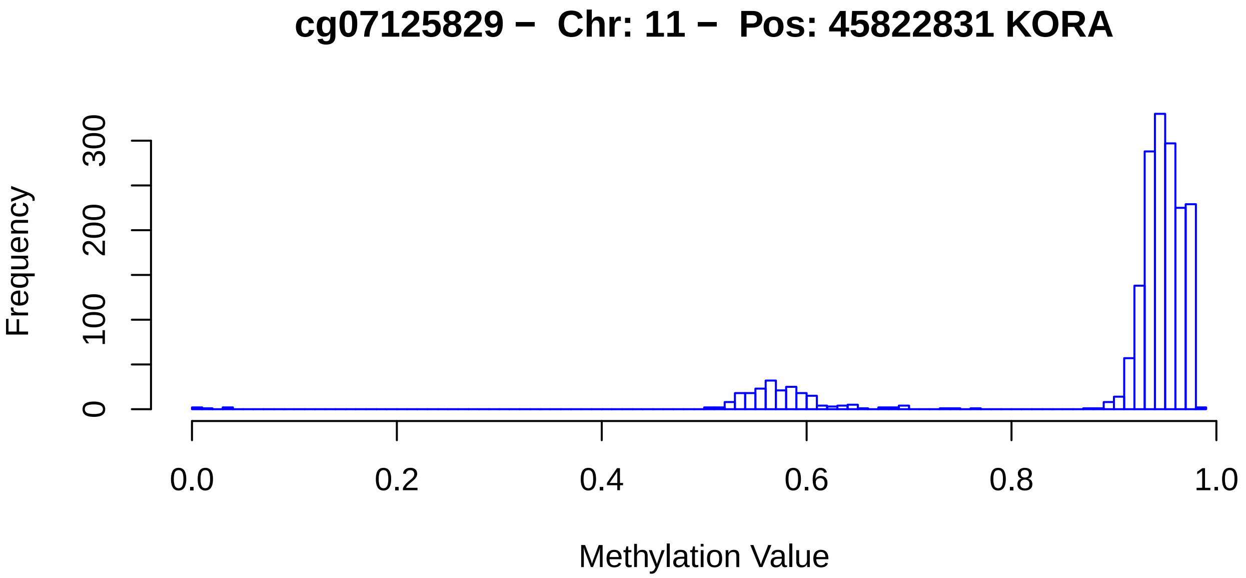
cg07125829 − Chr: 11 − Pos: 45822831 KORA
Frequency
0
100
200
300
0.0
0.2
0.4
0.6
0.8
1.0
Methylation Value

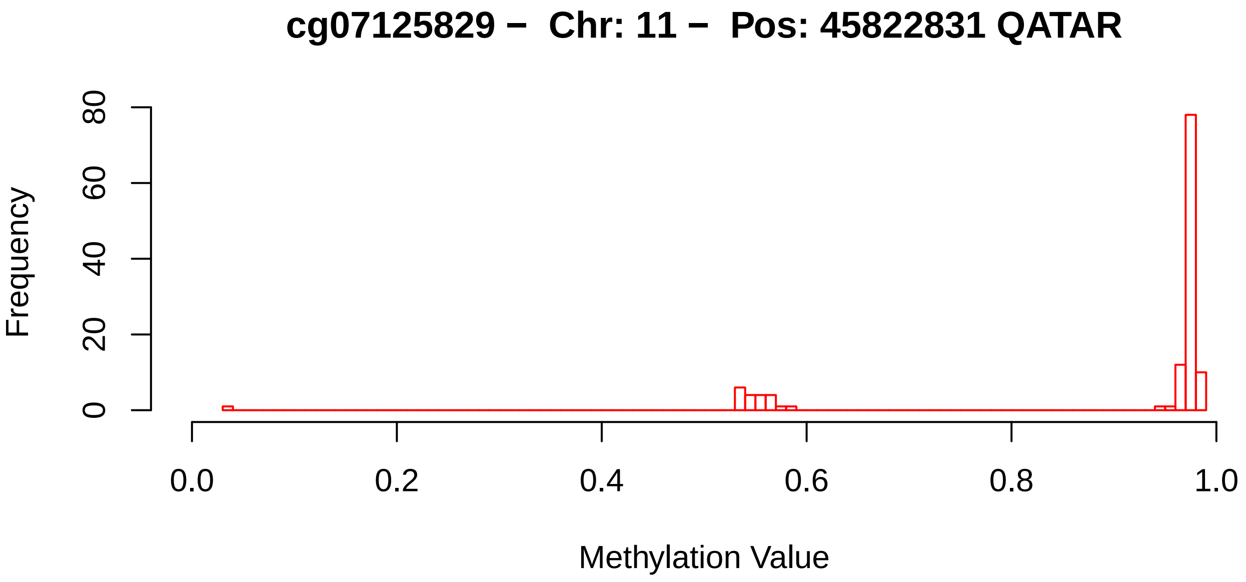
cg07125829 − Chr: 11 − Pos: 45822831 QATAR
Frequency
0
20
40
60
80
0.0
0.2
0.4
0.6
0.8
1.0
Methylation Value

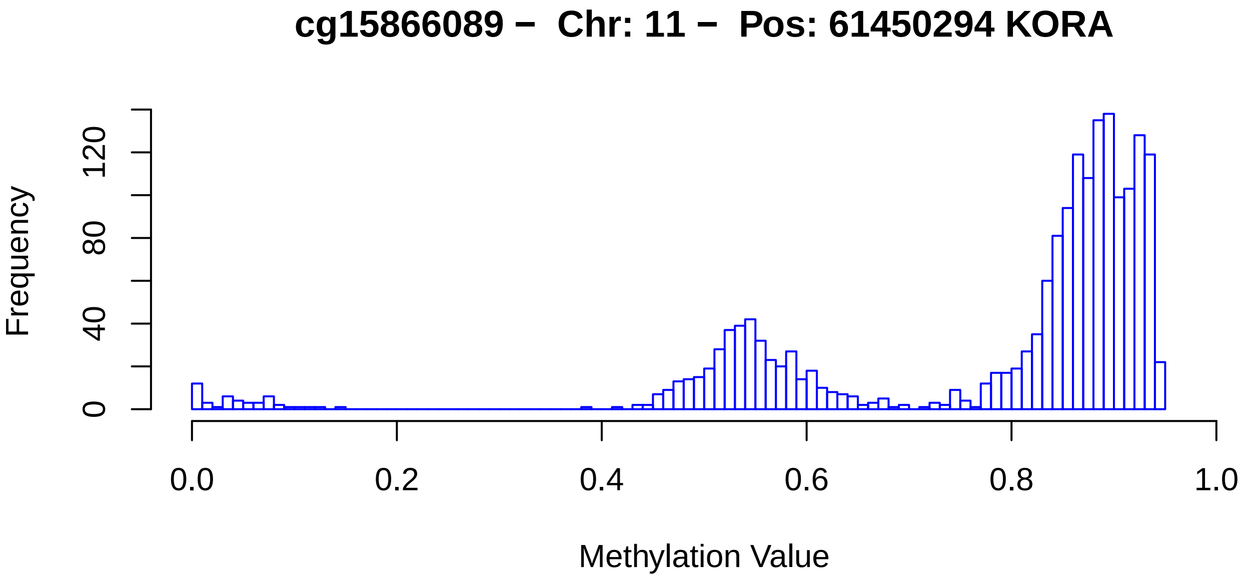
cg15866089 − Chr: 11 − Pos: 61450294 KORA
Frequency
0
40
80
120
0.0
0.2
0.4
0.6
0.8
1.0
Methylation Value

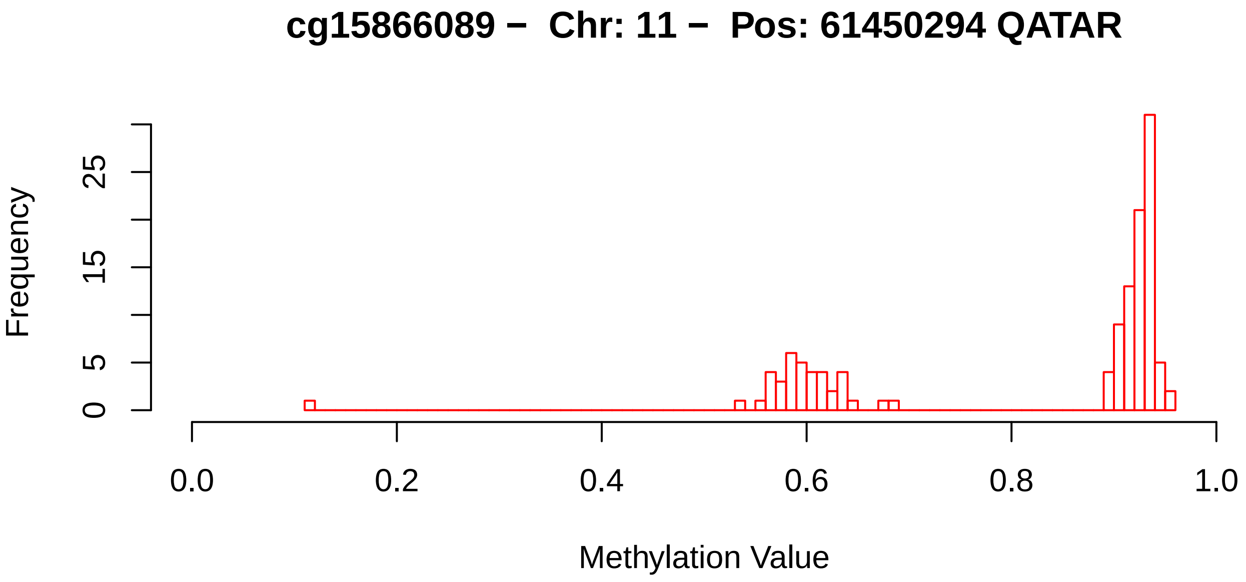
cg15866089 − Chr: 11 − Pos: 61450294 QATAR
Frequency
0
5
15
25
0.0
0.2
0.4
0.6
0.8
1.0
Methylation Value

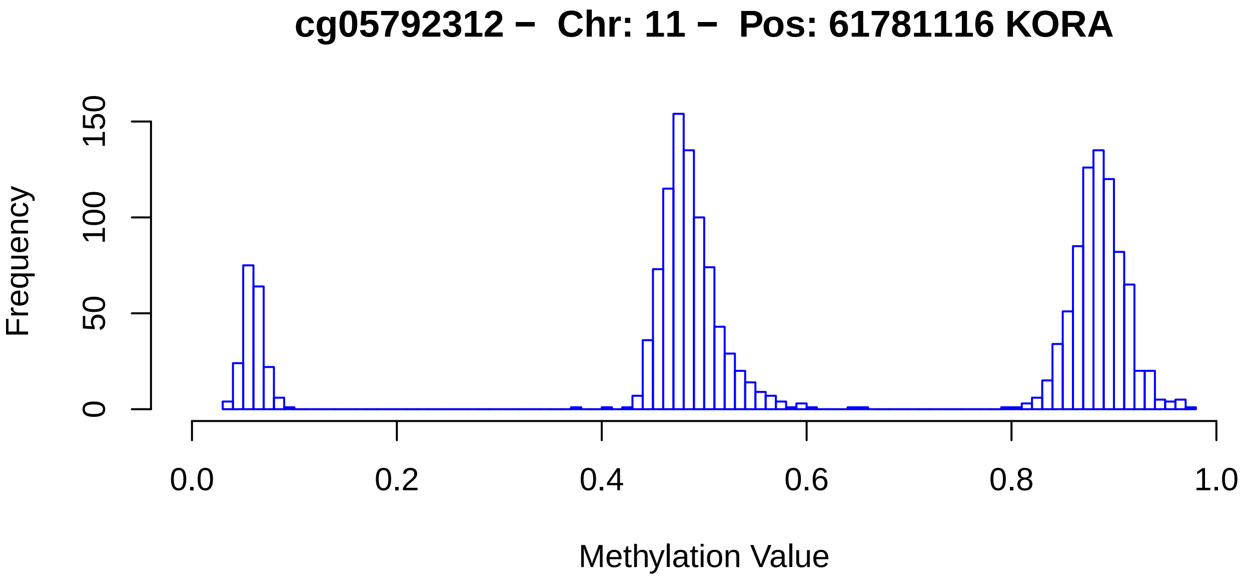

cg05792312 − Chr: 11 − Pos: 61781116 KORA
Frequency
0
50
100
150
0.0
0.2
0.4
0.6
0.8
1.0
Methylation Value

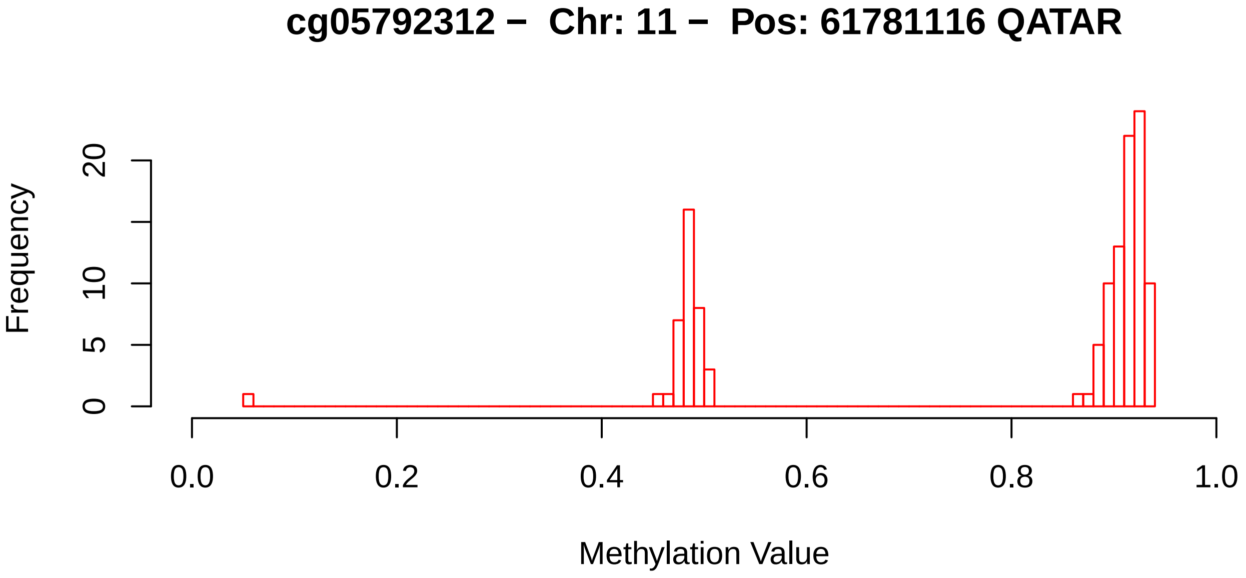

cg05792312 − Chr: 11 − Pos: 61781116 QATAR
Frequency
0
5
10
20
0.0
0.2
0.4
0.6
0.8
1.0
Methylation Value

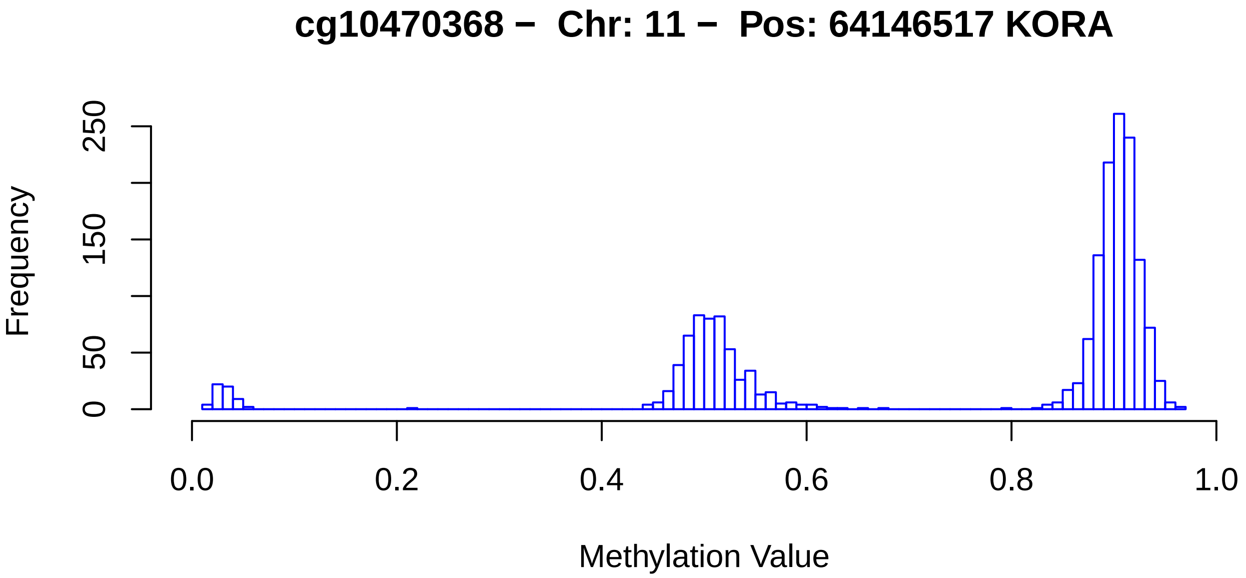
cg10470368 − Chr: 11 − Pos: 64146517 KORA
Frequency
0
50
150
250
0.0
0.2
0.4
0.6
0.8
1.0
Methylation Value

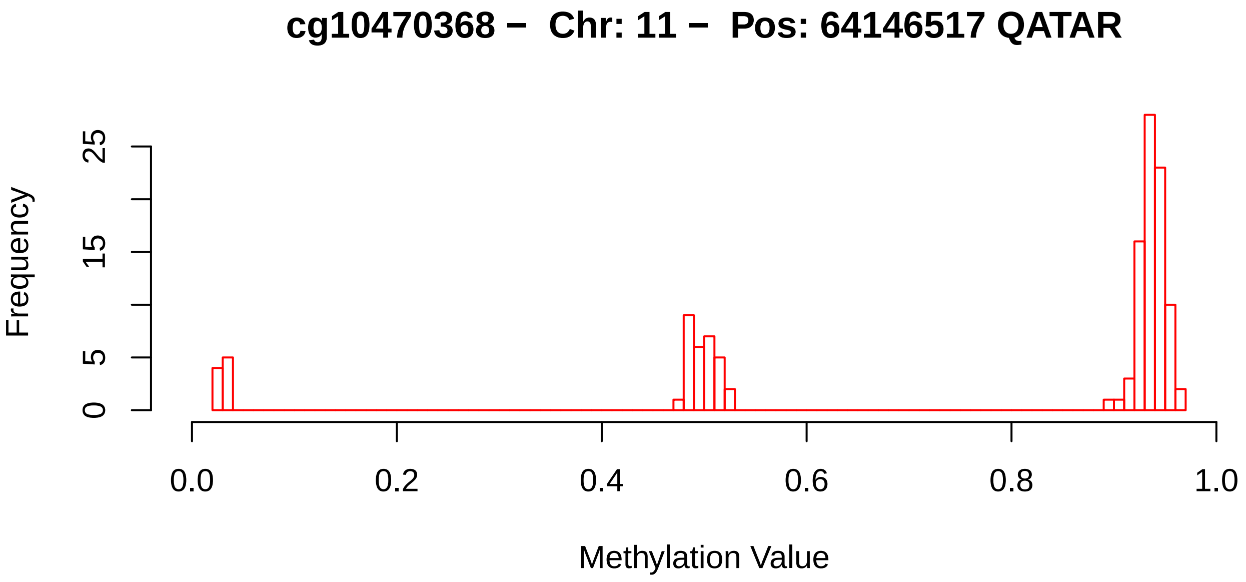
cg10470368 − Chr: 11 − Pos: 64146517 QATAR
Frequency
0
5
15
25
0.0
0.2
0.4
0.6
0.8
1.0
Methylation Value

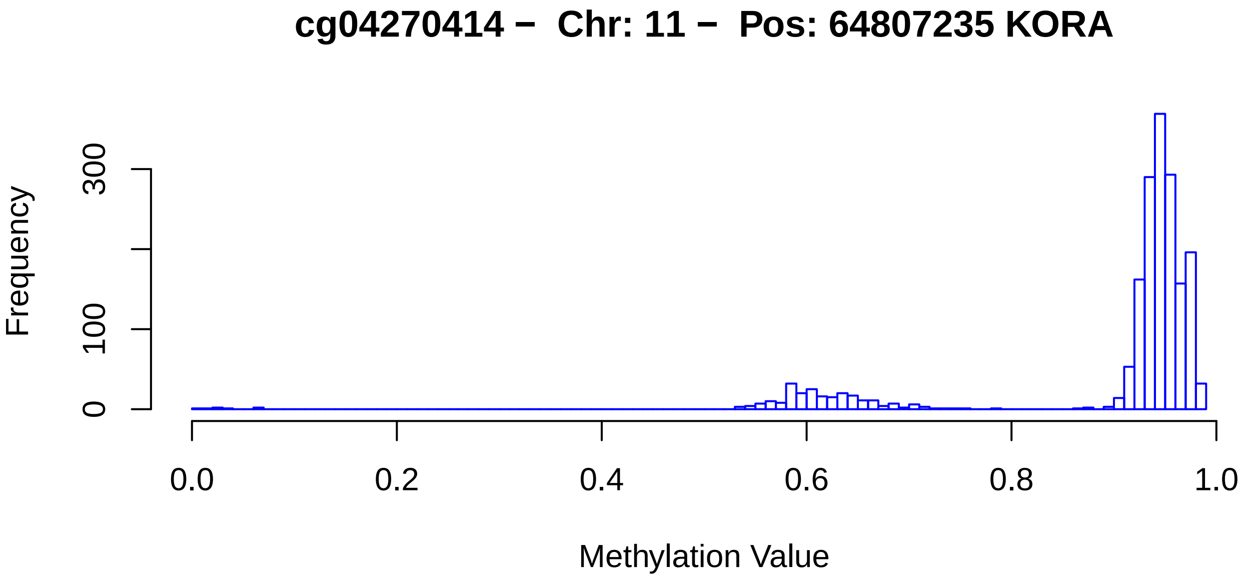
cg04270414 − Chr: 11 − Pos: 64807235 KORA
Frequency
0
100
300
0.0
0.2
0.4
0.6
0.8
1.0
Methylation Value

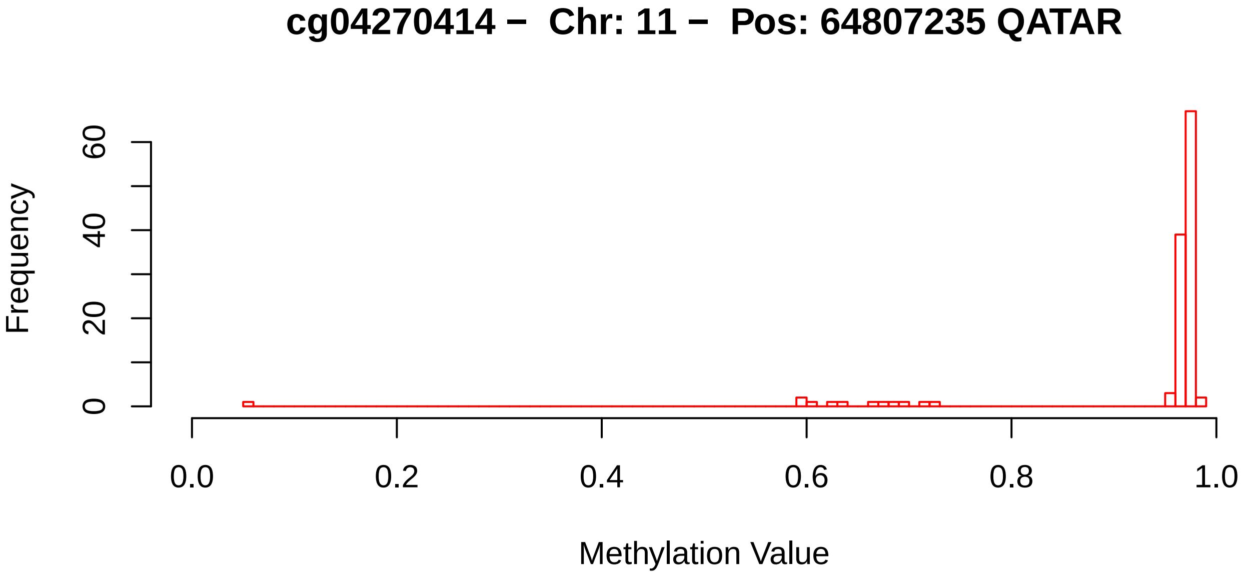
cg04270414 − Chr: 11 − Pos: 64807235 QATAR
Frequency
0
20
40
60
0.0
0.2
0.4
0.6
0.8
1.0
Methylation Value

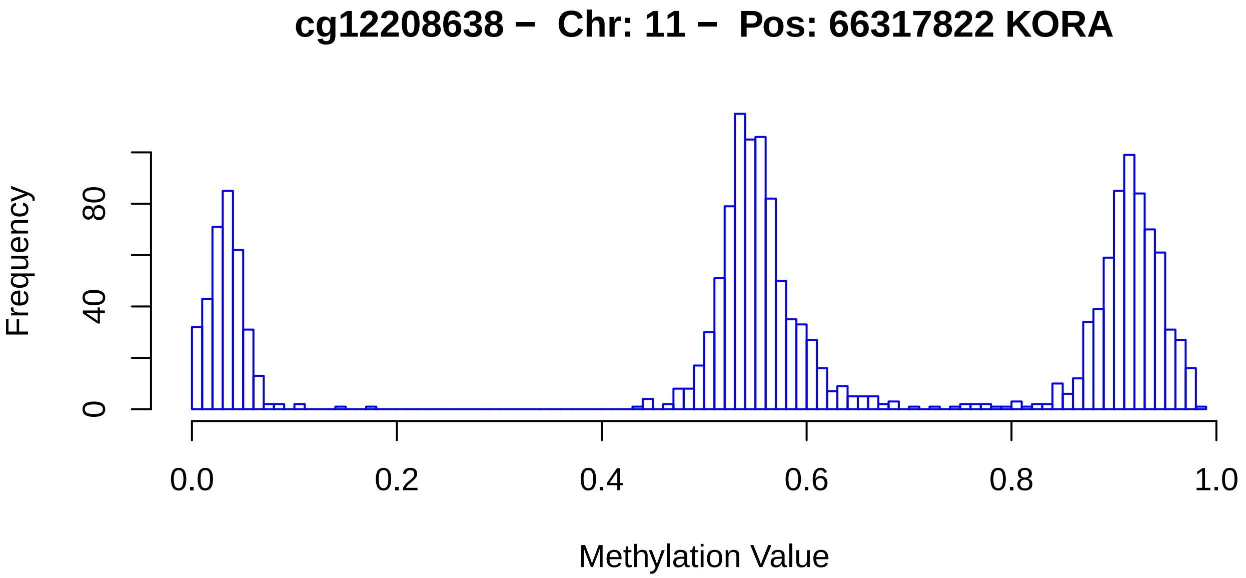
cg12208638 − Chr: 11 − Pos: 66317822 KORA
Frequency
0
40
80
0.0
0.2
0.4
0.6
0.8
1.0
Methylation Value

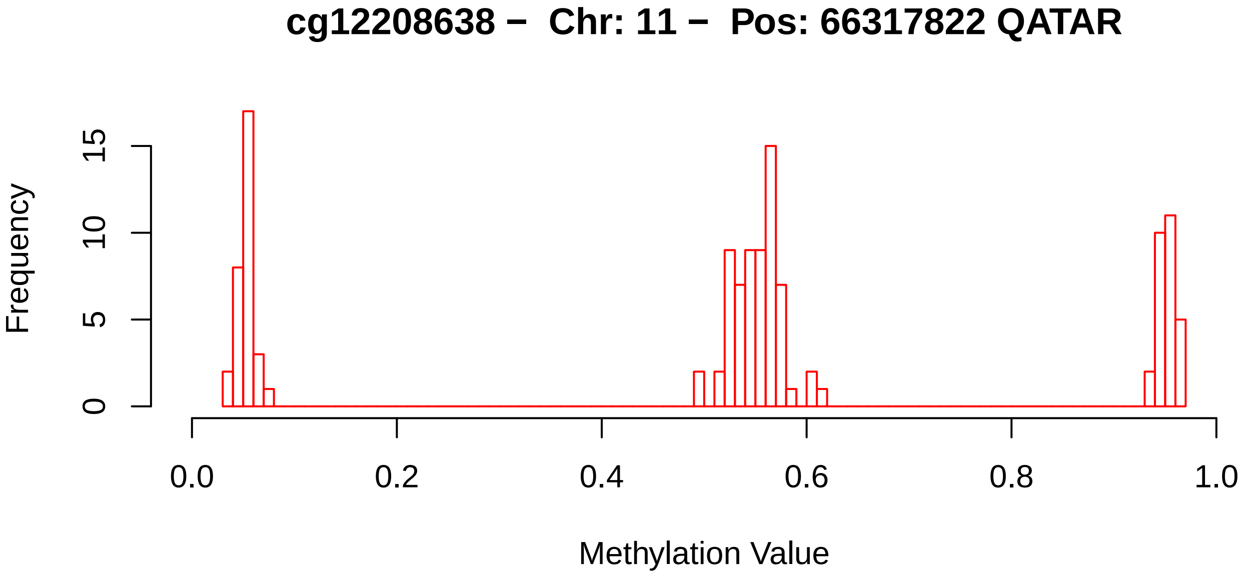
cg12208638 − Chr: 11 − Pos: 66317822 QATAR
Frequency
0
5
10
15
0.0
0.2
0.4
0.6
0.8
1.0
Methylation Value

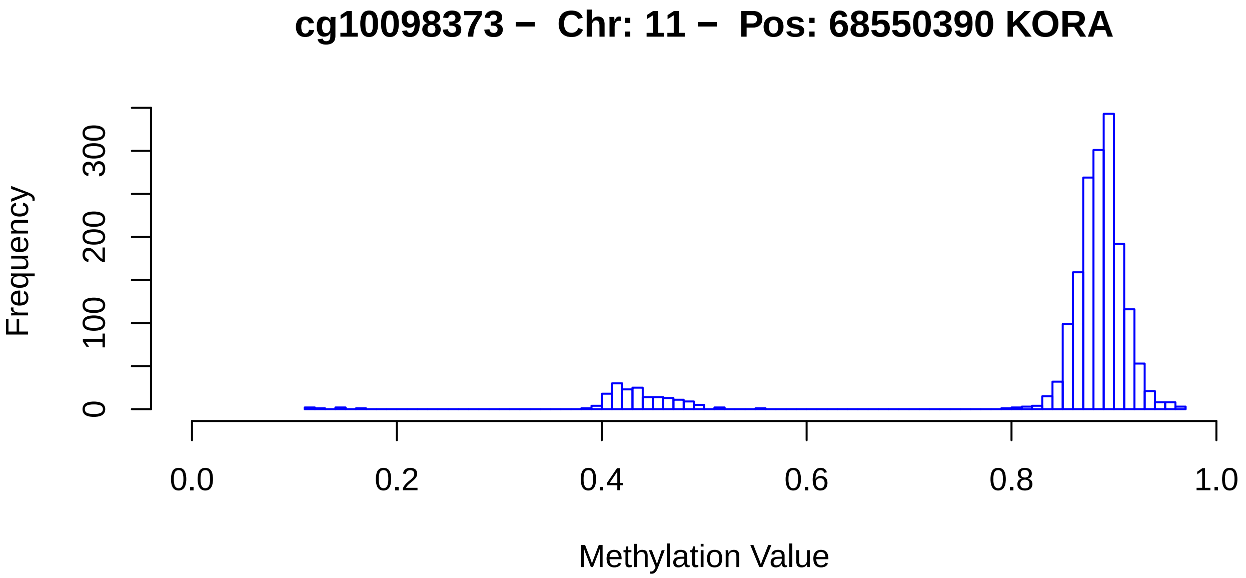
cg10098373 − Chr: 11 − Pos: 68550390 KORA
Frequency
0
100
200
300
0.0
0.2
0.4
0.6
0.8
1.0
Methylation Value

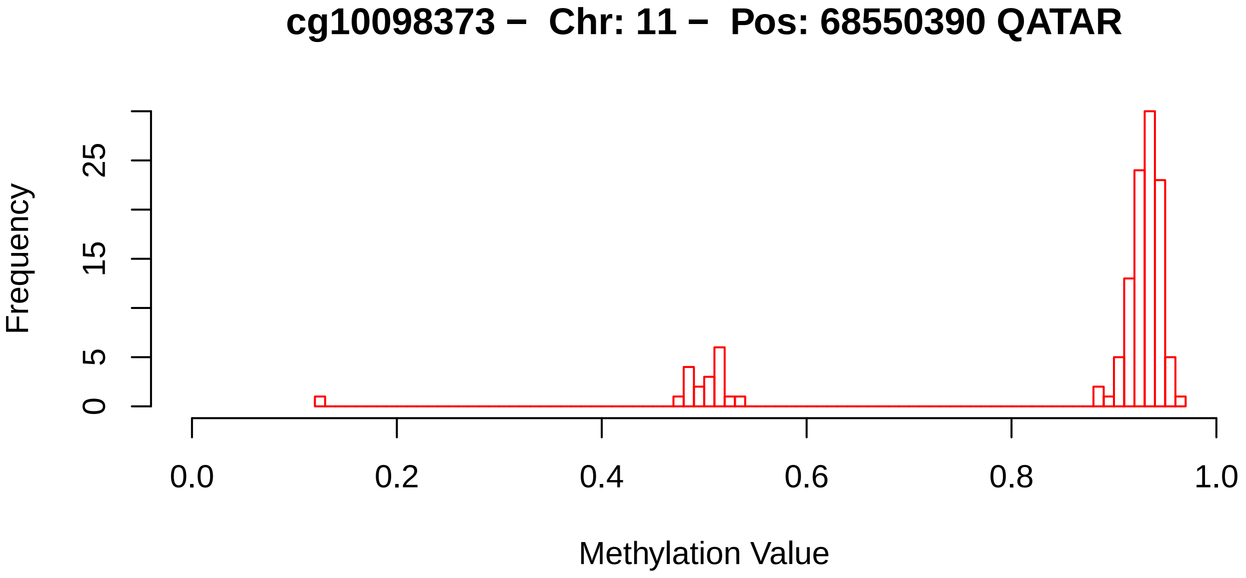
cg10098373 − Chr: 11 − Pos: 68550390 QATAR
Frequency
0
5
15
25
0.0
0.2
0.4
0.6
0.8
1.0
Methylation Value

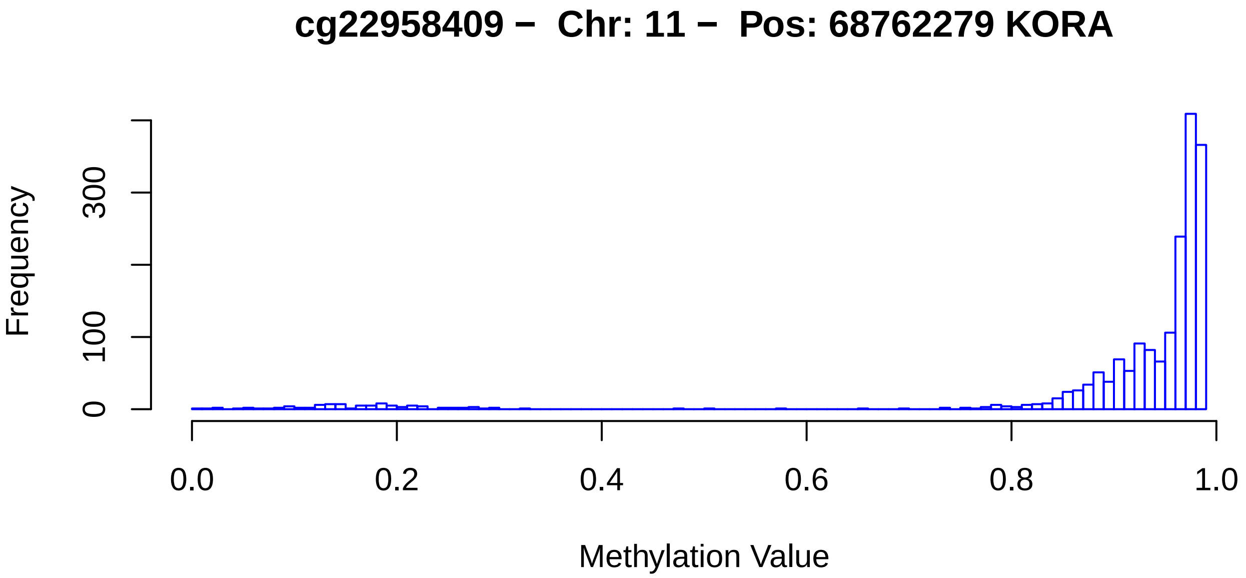
cg22958409 − Chr: 11 − Pos: 68762279 KORA
Frequency
0
100
300
0.0
0.2
0.4
0.6
0.8
1.0
Methylation Value

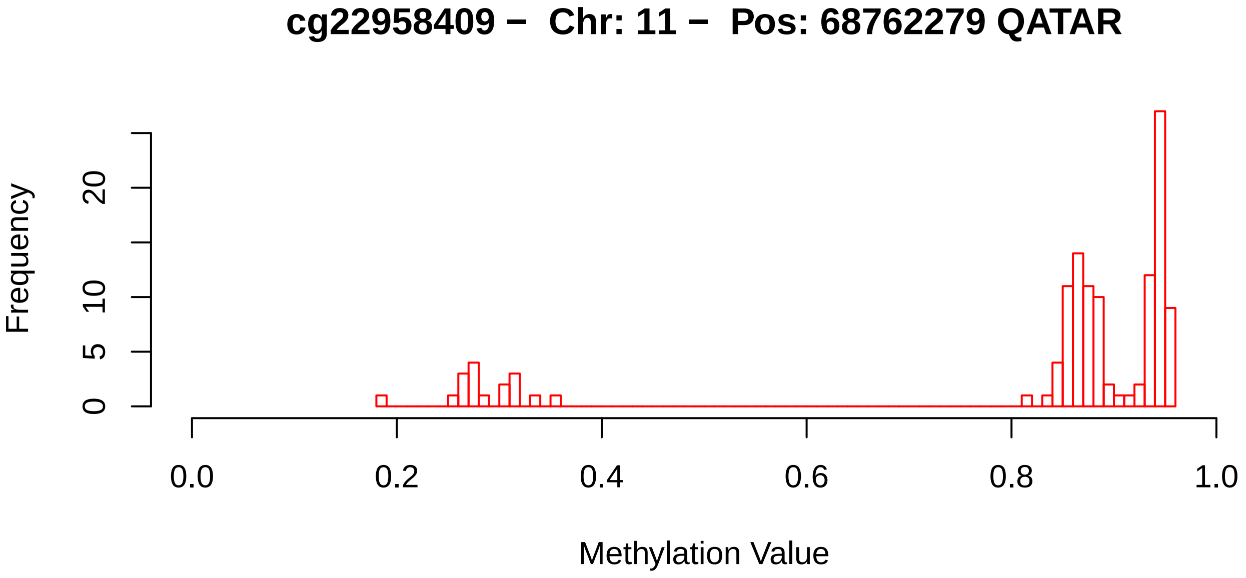
cg22958409 − Chr: 11 − Pos: 68762279 QATAR
Frequency
0
5
10
20
0.0
0.2
0.4
0.6
0.8
1.0
Methylation Value

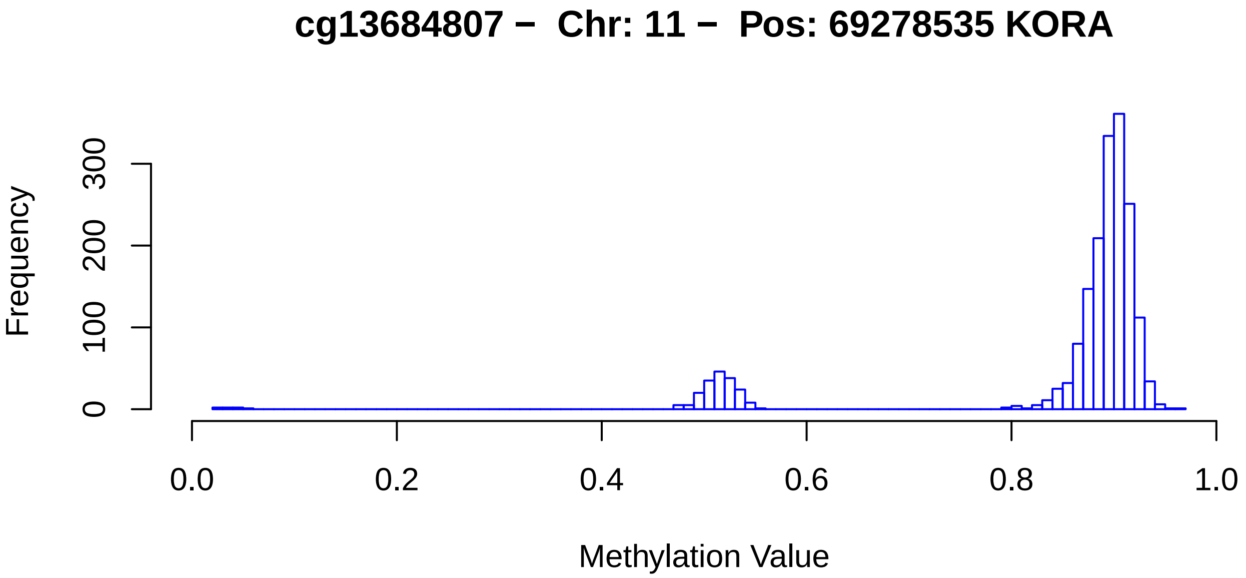
cg13684807 − Chr: 11 − Pos: 69278535 KORA
Frequency
0
100
200
300
0.0
0.2
0.4
0.6
0.8
1.0
Methylation Value

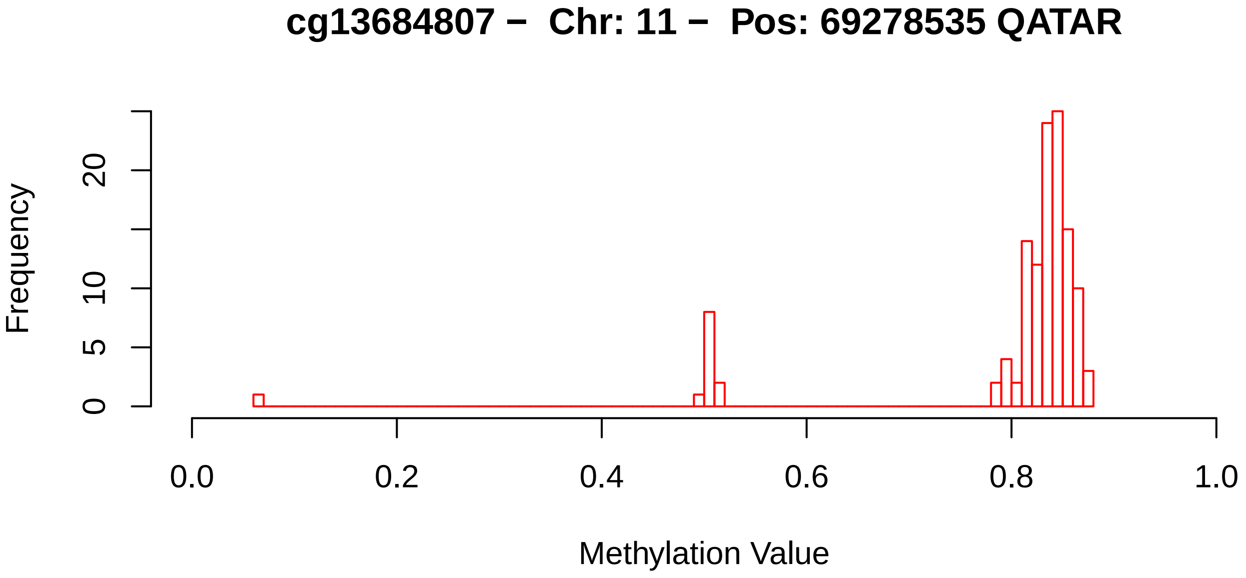
cg13684807 − Chr: 11 − Pos: 69278535 QATAR
Frequency
0
5
10
20
0.0
0.2
0.4
0.6
0.8
1.0
Methylation Value

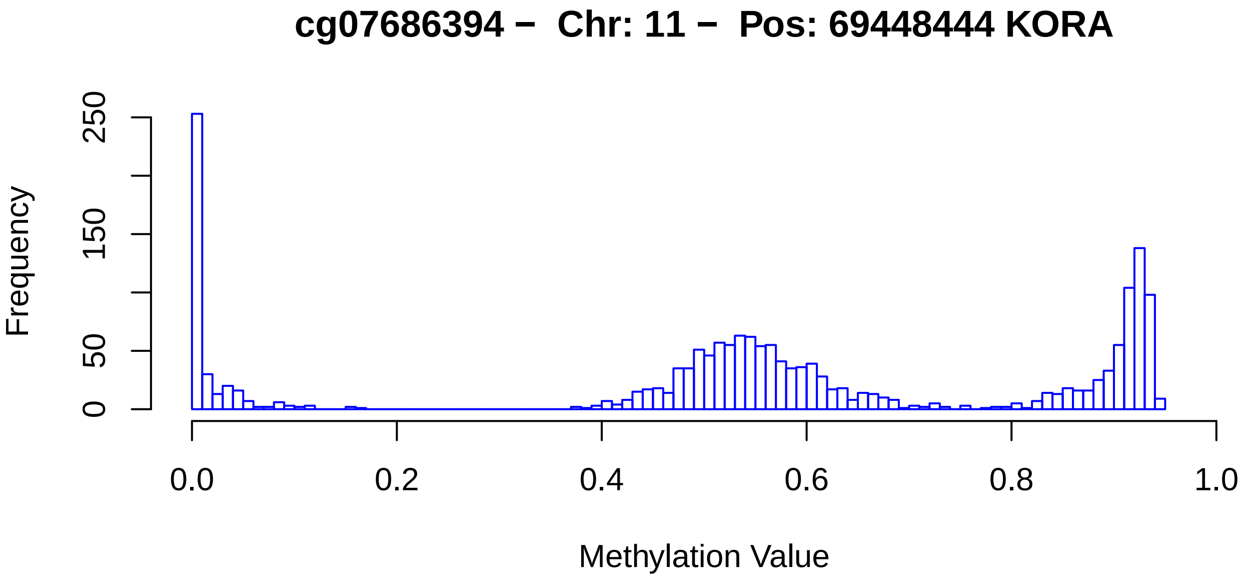
cg07686394 − Chr: 11 − Pos: 69448444 KORA
Frequency
0
50
150
250
0.0
0.2
0.4
0.6
0.8
1.0
Methylation Value

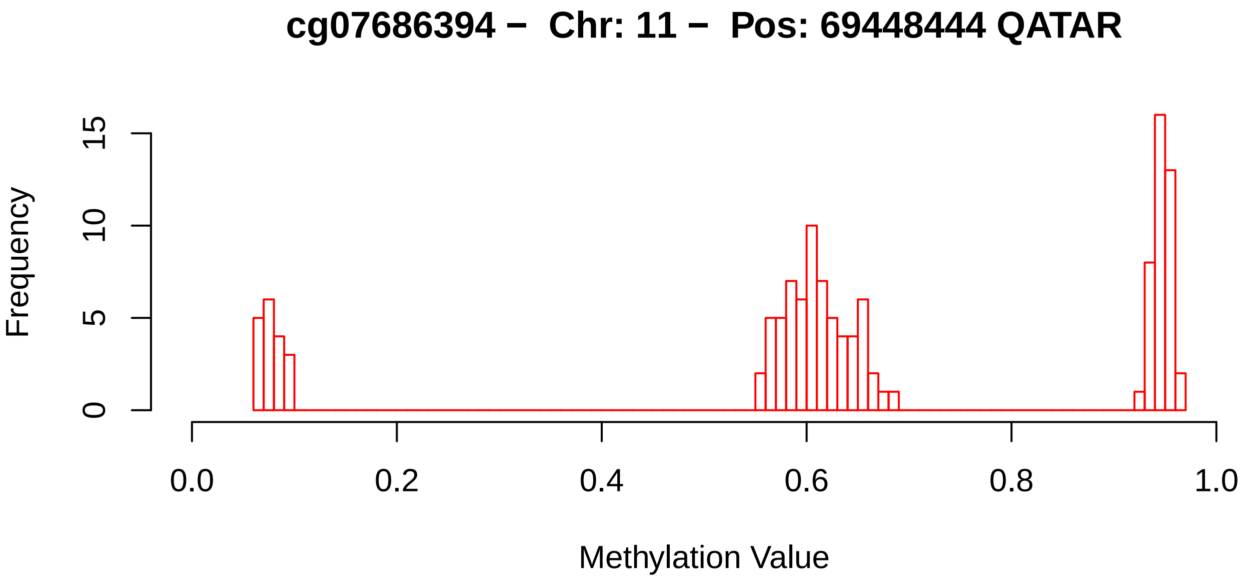
cg07686394 − Chr: 11 − Pos: 69448444 QATAR
Frequency
0
5
10
15
0.0
0.2
0.4
0.6
0.8
1.0
Methylation Value

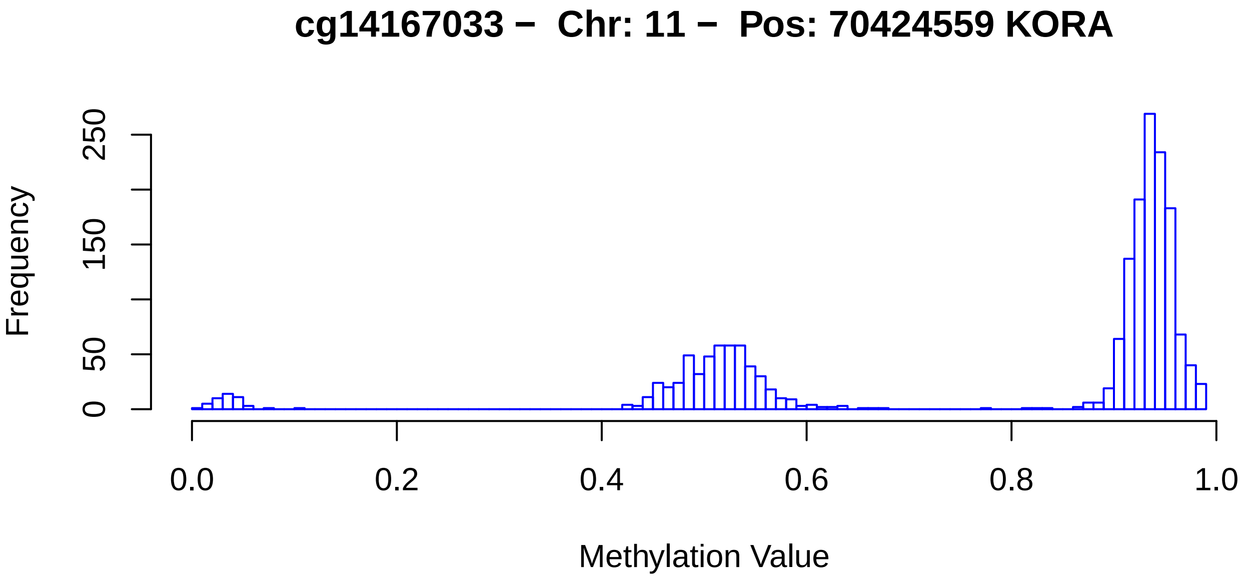

cg14167033 − Chr: 11 − Pos: 70424559 KORA
Frequency
0
50
150
250
0.0
0.2
0.4
0.6
0.8
1.0
Methylation Value

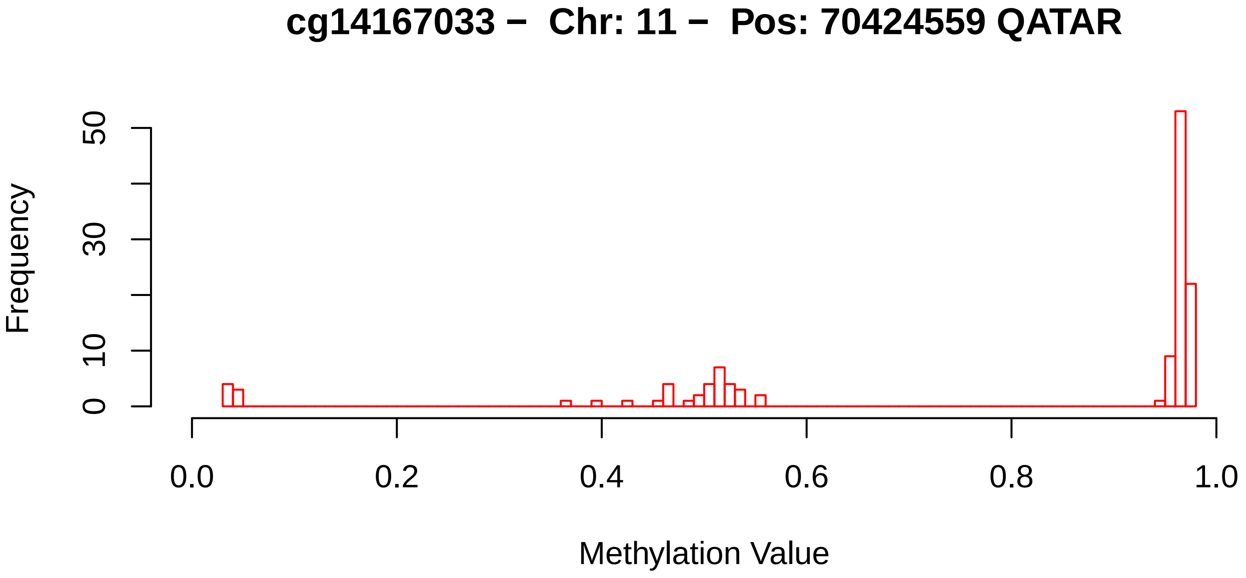

cg14167033 − Chr: 11 − Pos: 70424559 QATAR
Frequency
0
10
30
50
0.0
0.2
0.4
0.6
0.8
1.0
Methylation Value

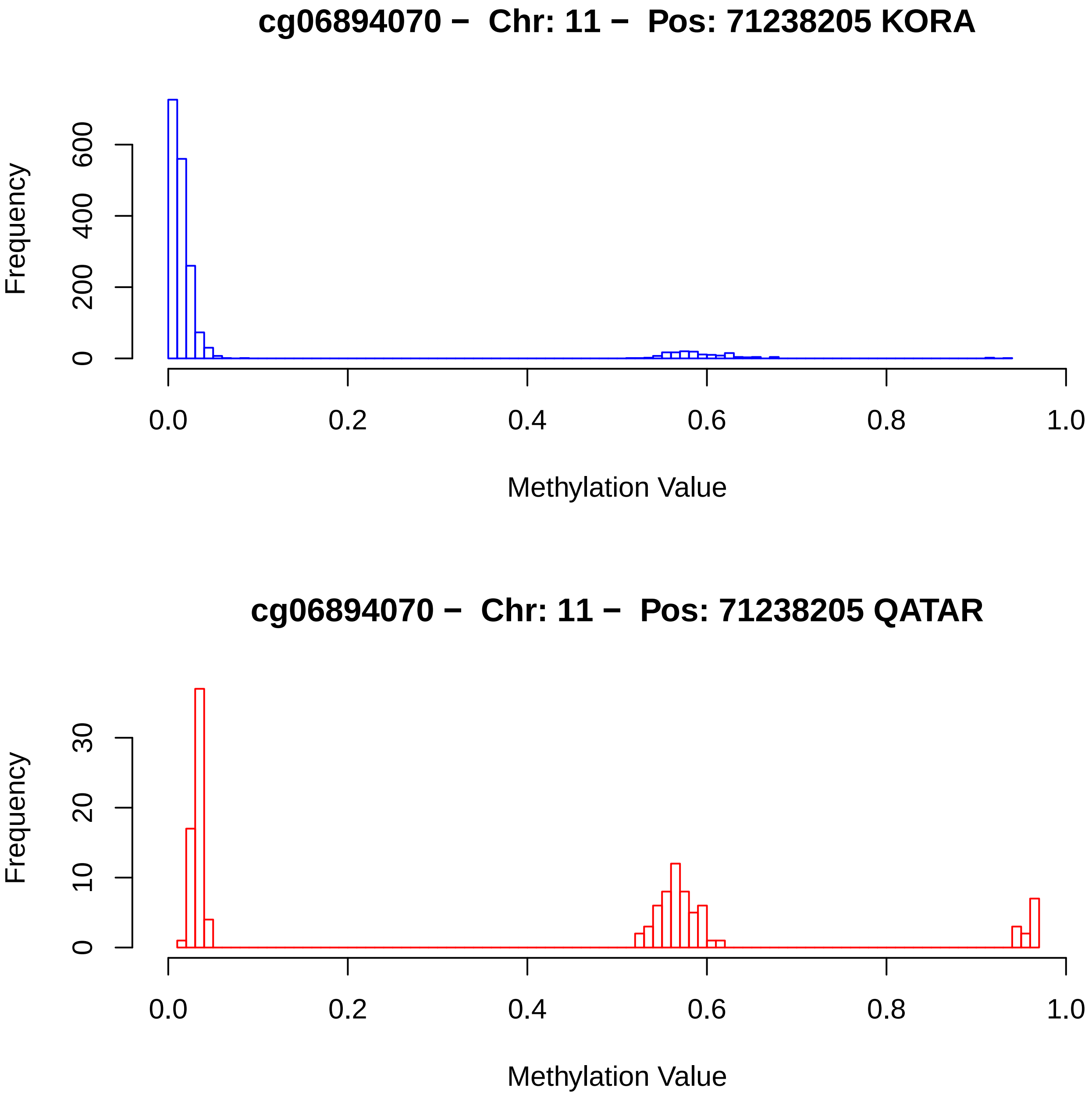
cg06894070 − Chr: 11 − Pos: 71238205 KORA
Frequency
0
200
400
600
0.0
0.2
0.4
0.6
0.8
1.0
Methylation Value
cg06894070 − Chr: 11 − Pos: 71238205 QATAR
Frequency
0
10
20
30
0.0
0.2
0.4
0.6
0.8
1.0
Methylation Value

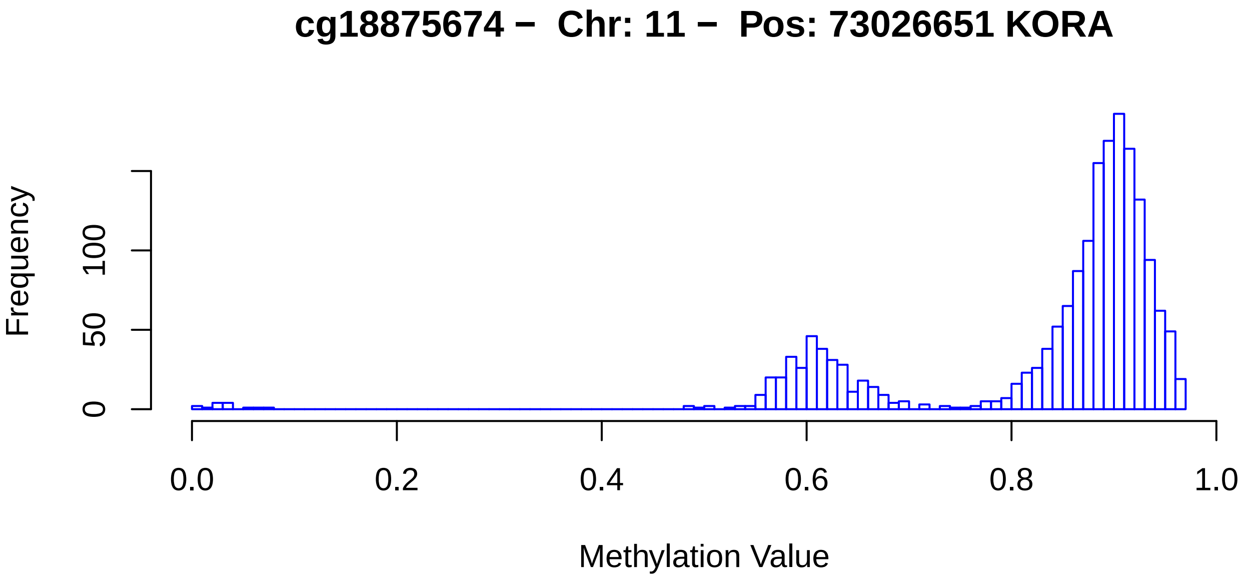
cg18875674 − Chr: 11 − Pos: 73026651 KORA
Frequency
0
50
100
0.0
0.2
0.4
0.6
0.8
1.0
Methylation Value

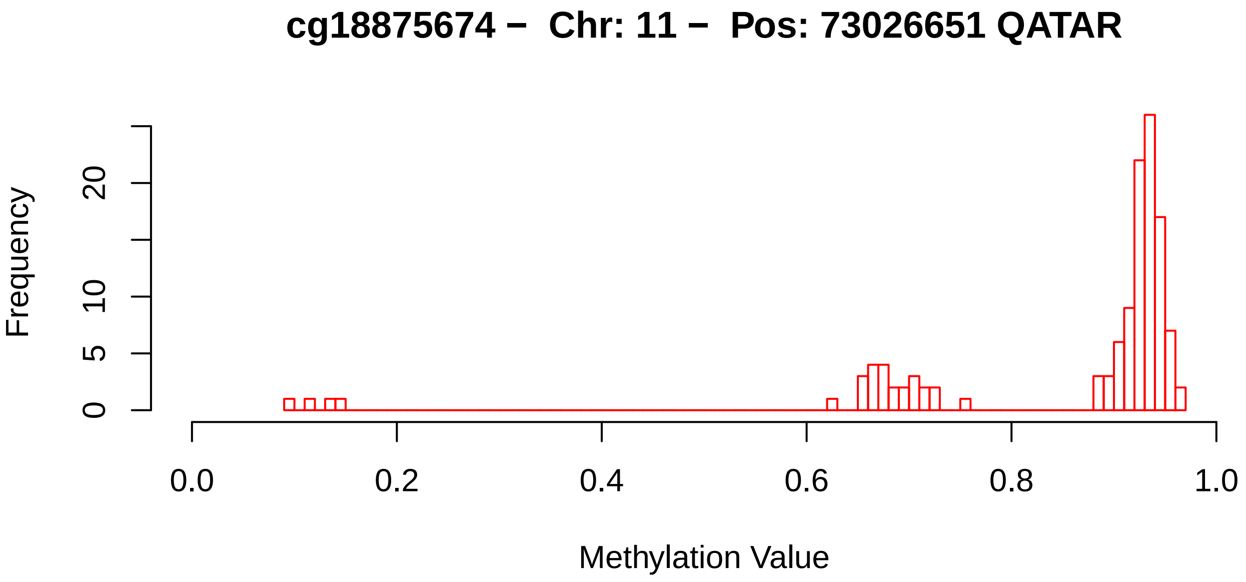
cg18875674 − Chr: 11 − Pos: 73026651 QATAR
Frequency
0
5
10
20
0.0
0.2
0.4
0.6
0.8
1.0
Methylation Value

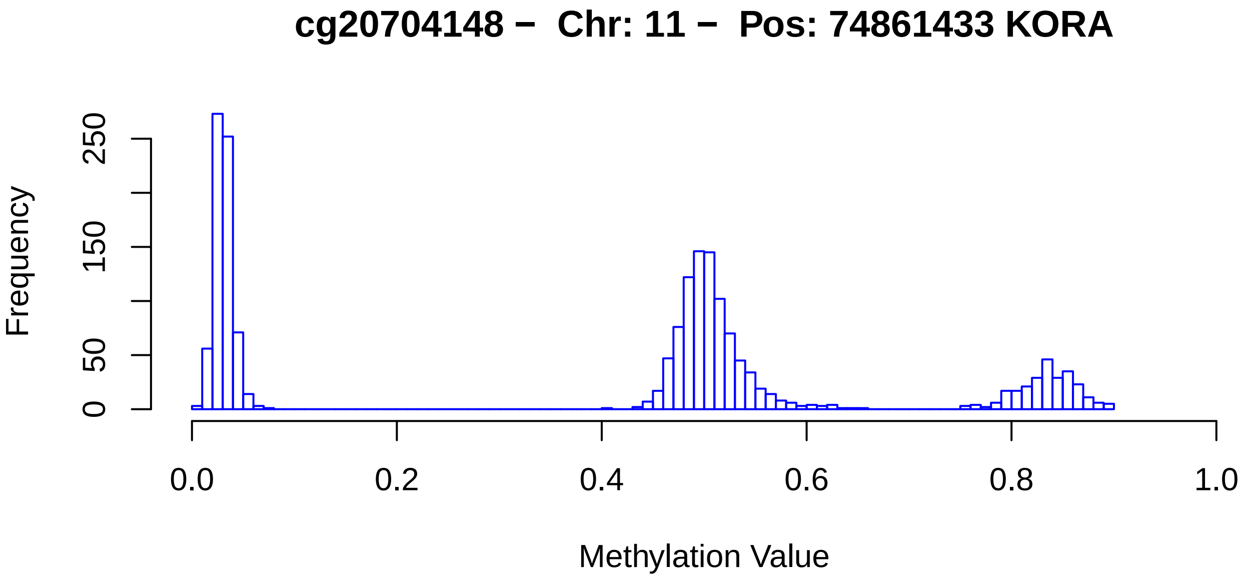
cg20704148 − Chr: 11 − Pos: 74861433 KORA
Frequency
0
50
150
250
0.0
0.2
0.4
0.6
0.8
1.0
Methylation Value

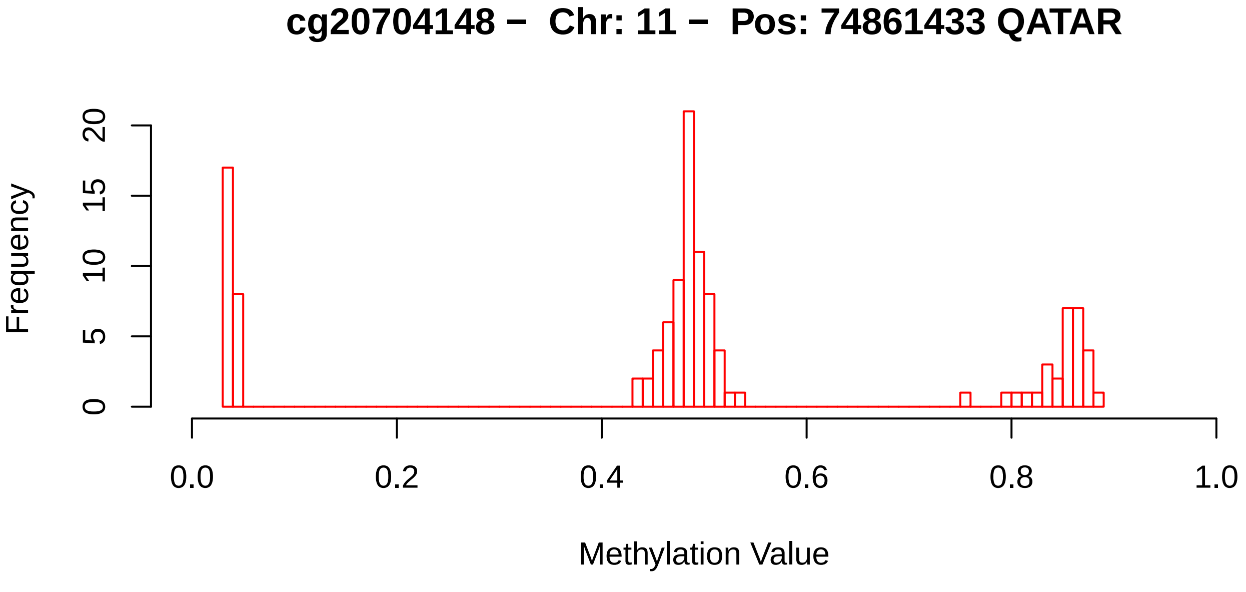
cg20704148 − Chr: 11 − Pos: 74861433 QATAR
Frequency
0
5
10
15
20
0.0
0.2
0.4
0.6
0.8
1.0
Methylation Value

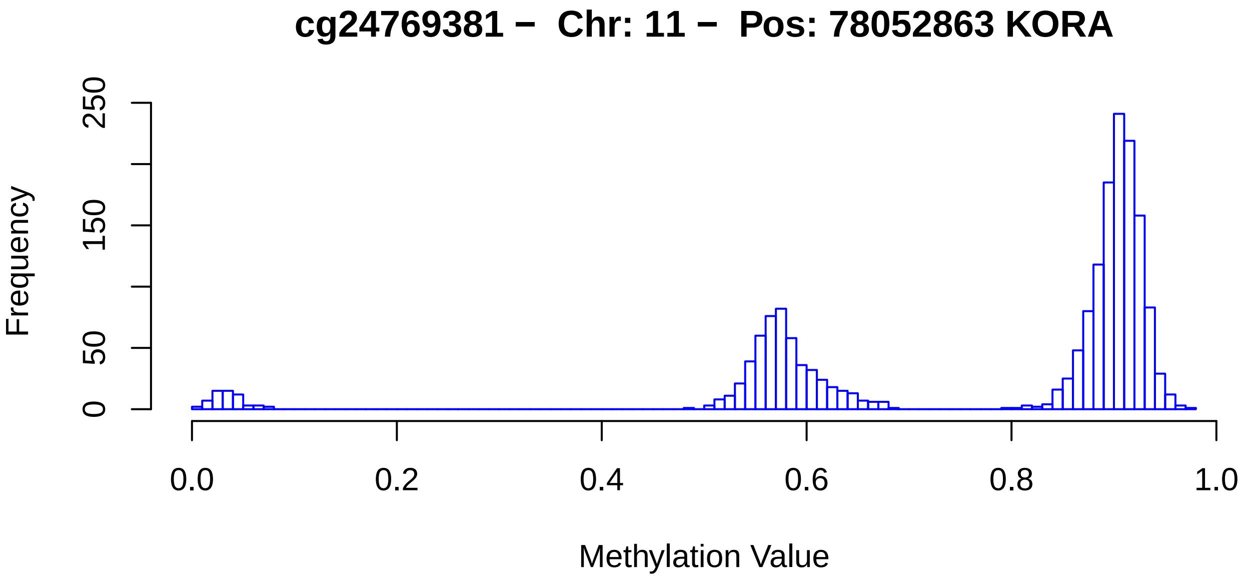
cg24769381 − Chr: 11 − Pos: 78052863 KORA
Frequency
0
50
150
250
0.0
0.2
0.4
0.6
0.8
1.0
Methylation Value

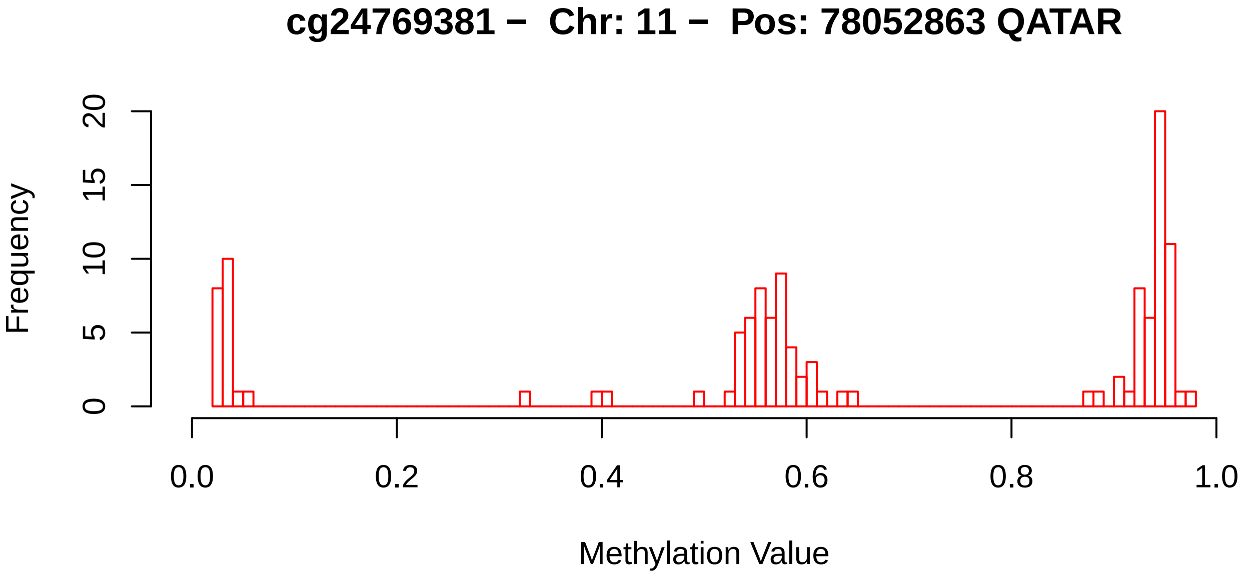
cg24769381 − Chr: 11 − Pos: 78052863 QATAR
Frequency
0
5
10
15
20
0.0
0.2
0.4
0.6
0.8
1.0
Methylation Value

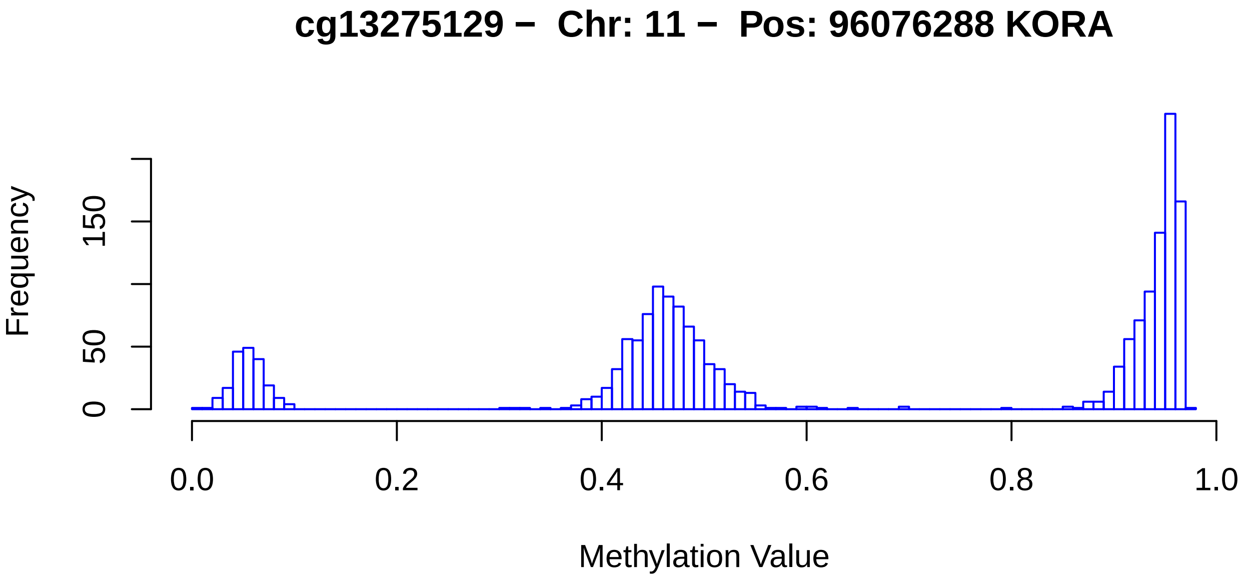
cg13275129 − Chr: 11 − Pos: 96076288 KORA
Frequency
0
50
150
0.0
0.2
0.4
0.6
0.8
1.0
Methylation Value

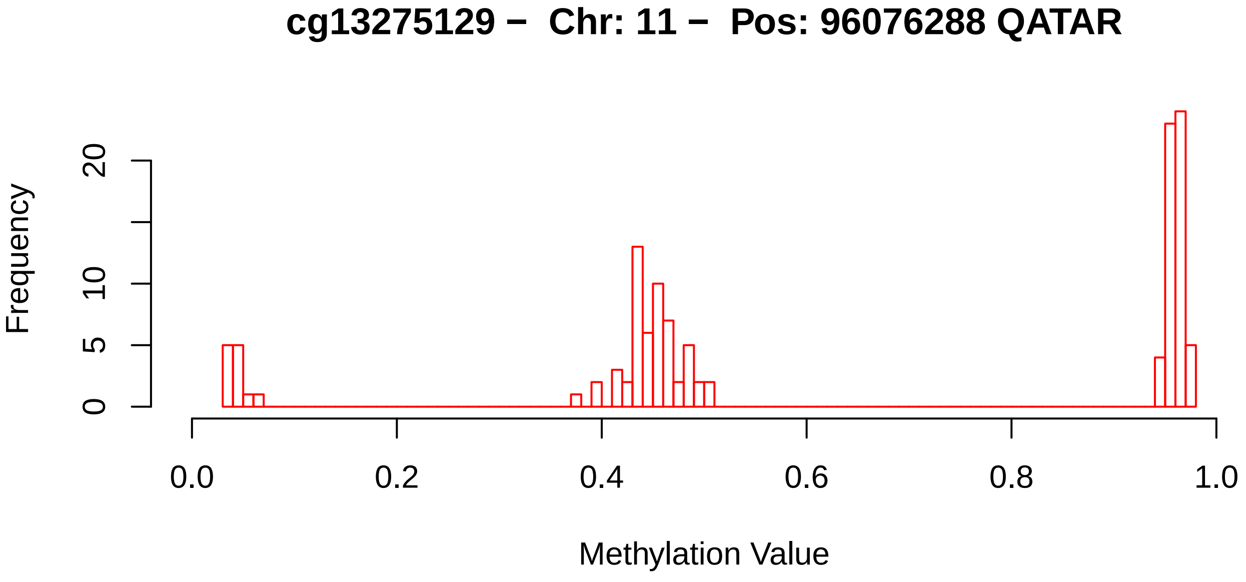
cg13275129 − Chr: 11 − Pos: 96076288 QATAR
Frequency
0
5
10
20
0.0
0.2
0.4
0.6
0.8
1.0
Methylation Value

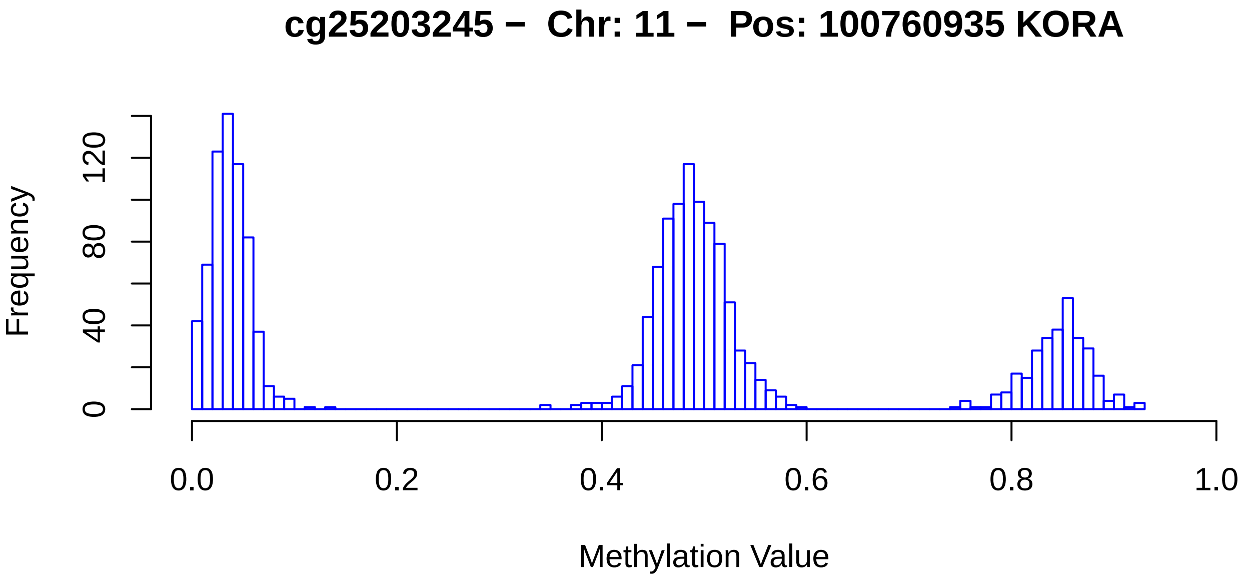
cg25203245 − Chr: 11 − Pos: 100760935 KORA
Frequency
0
40
80
120
0.0
0.2
0.4
0.6
0.8
1.0
Methylation Value

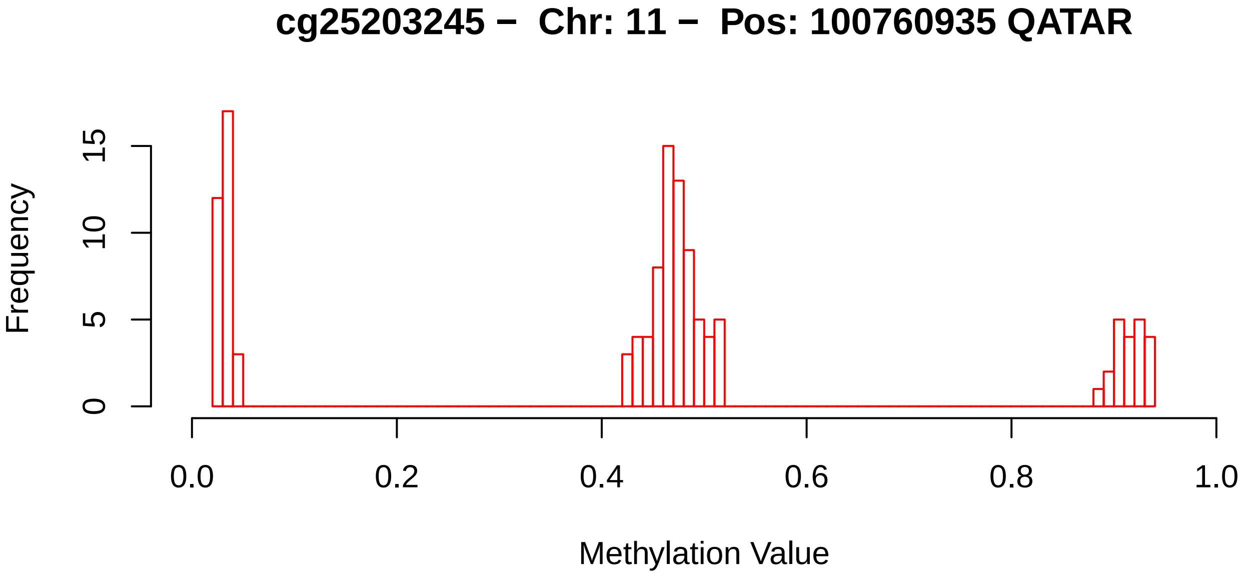
cg25203245 − Chr: 11 − Pos: 100760935 QATAR
Frequency
0
5
10
15
0.0
0.2
0.4
0.6
0.8
1.0
Methylation Value

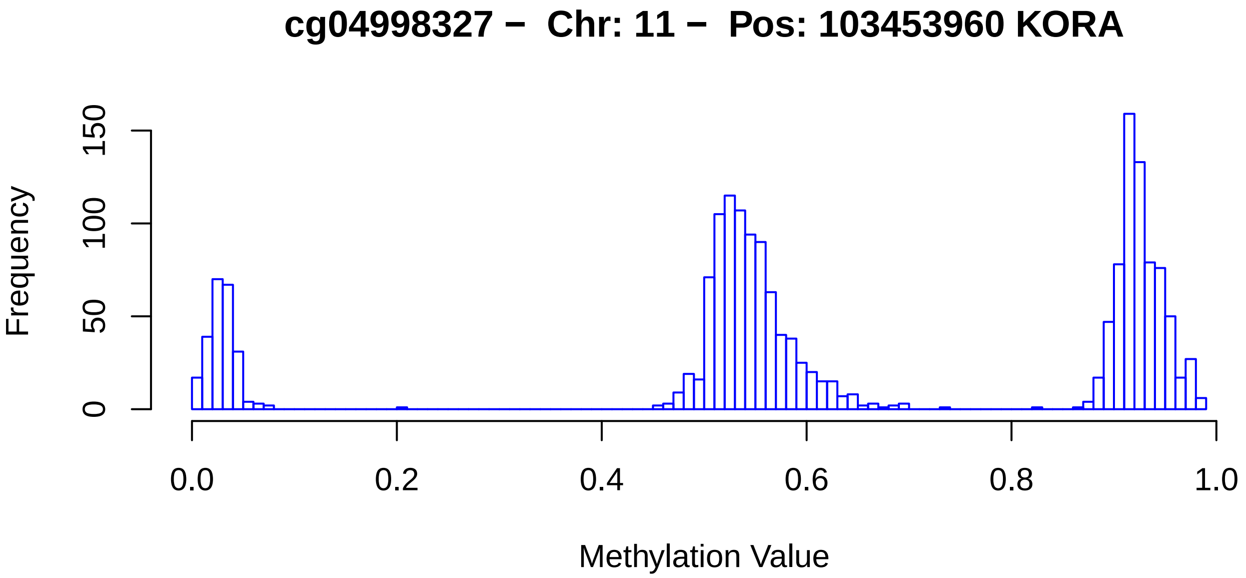
cg04998327 − Chr: 11 − Pos: 103453960 KORA
Frequency
0
50
100
150
0.0
0.2
0.4
0.6
0.8
1.0
Methylation Value

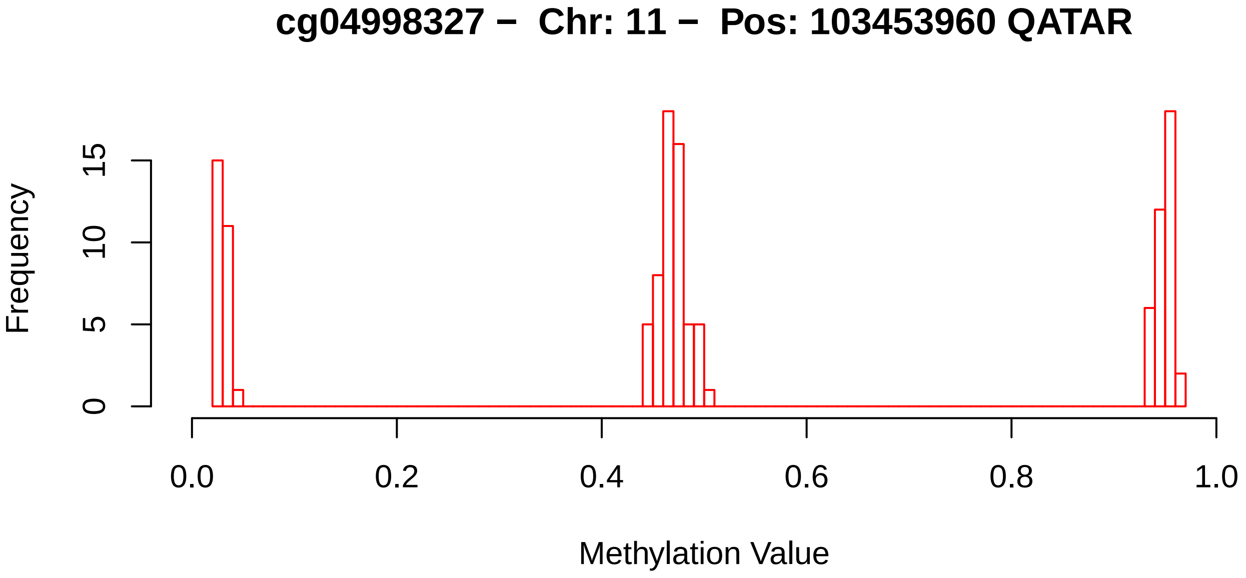
cg04998327 − Chr: 11 − Pos: 103453960 QATAR
Frequency
0
5
10
15
0.0
0.2
0.4
0.6
0.8
1.0
Methylation Value

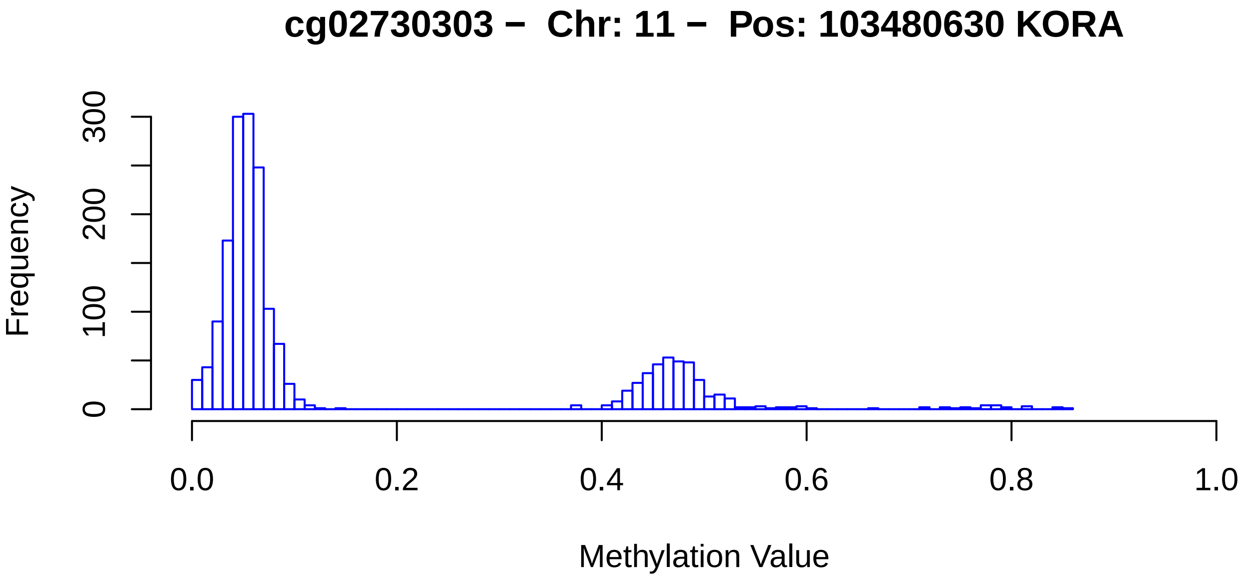
cg02730303 − Chr: 11 − Pos: 103480630 KORA
Frequency
0
100
200
300
0.0
0.2
0.4
0.6
0.8
1.0
Methylation Value

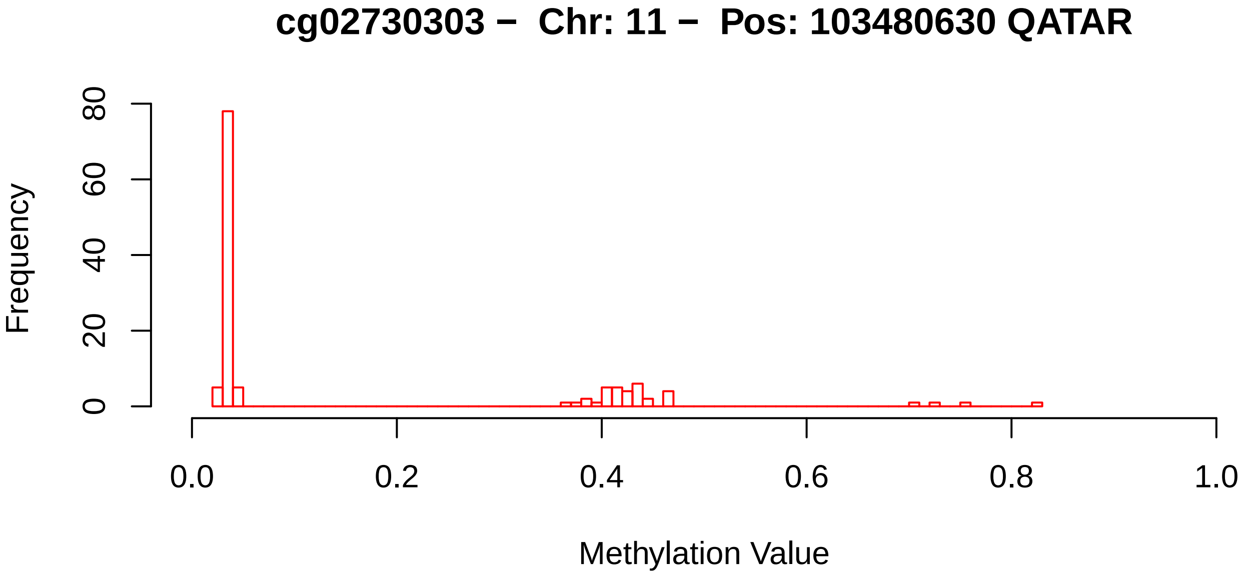
cg02730303 − Chr: 11 − Pos: 103480630 QATAR
Frequency
0
20
40
60
80
0.0
0.2
0.4
0.6
0.8
1.0
Methylation Value

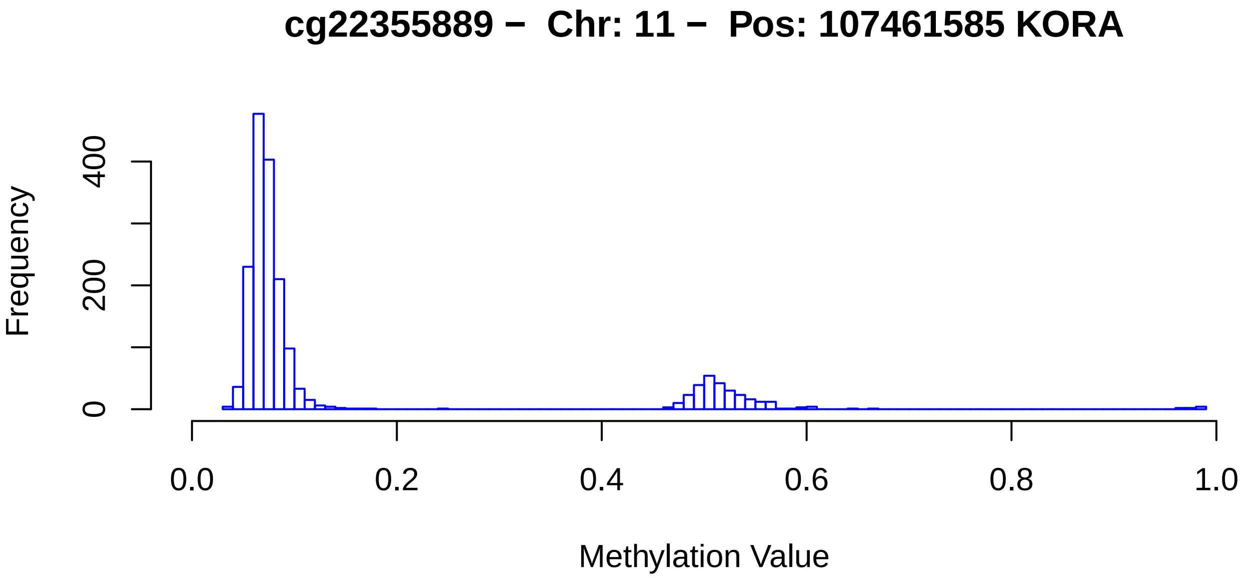
cg22355889 − Chr: 11 − Pos: 107461585 KORA
Frequency
0
200
400
0.0
0.2
0.4
0.6
0.8
1.0
Methylation Value

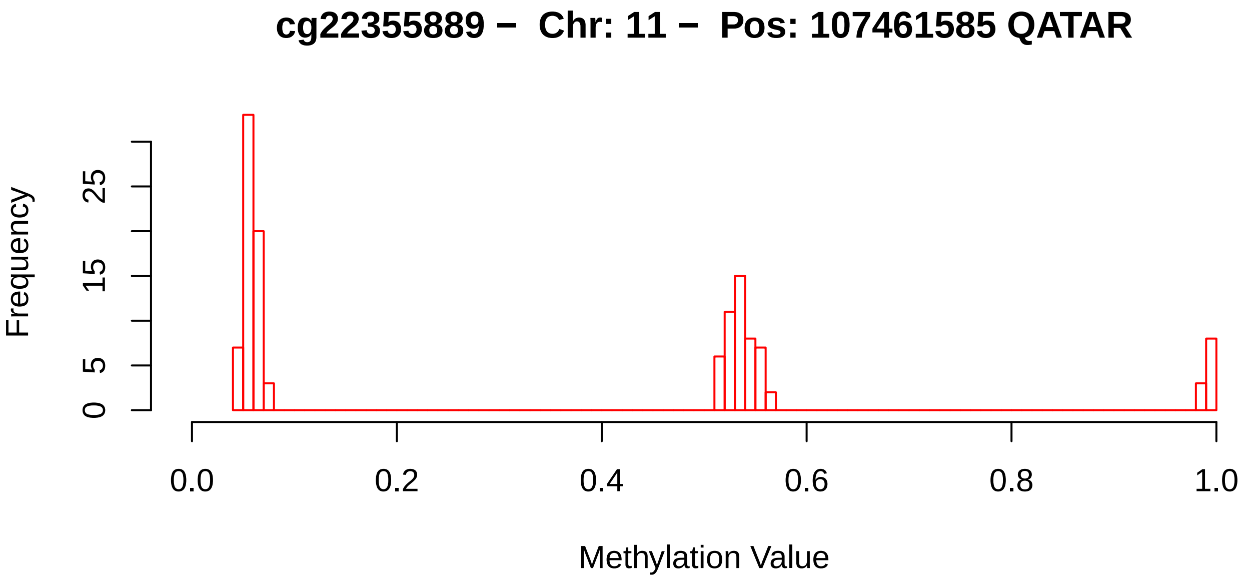
cg22355889 − Chr: 11 − Pos: 107461585 QATAR
Frequency
0
5
15
25
0.0
0.2
0.4
0.6
0.8
1.0
Methylation Value

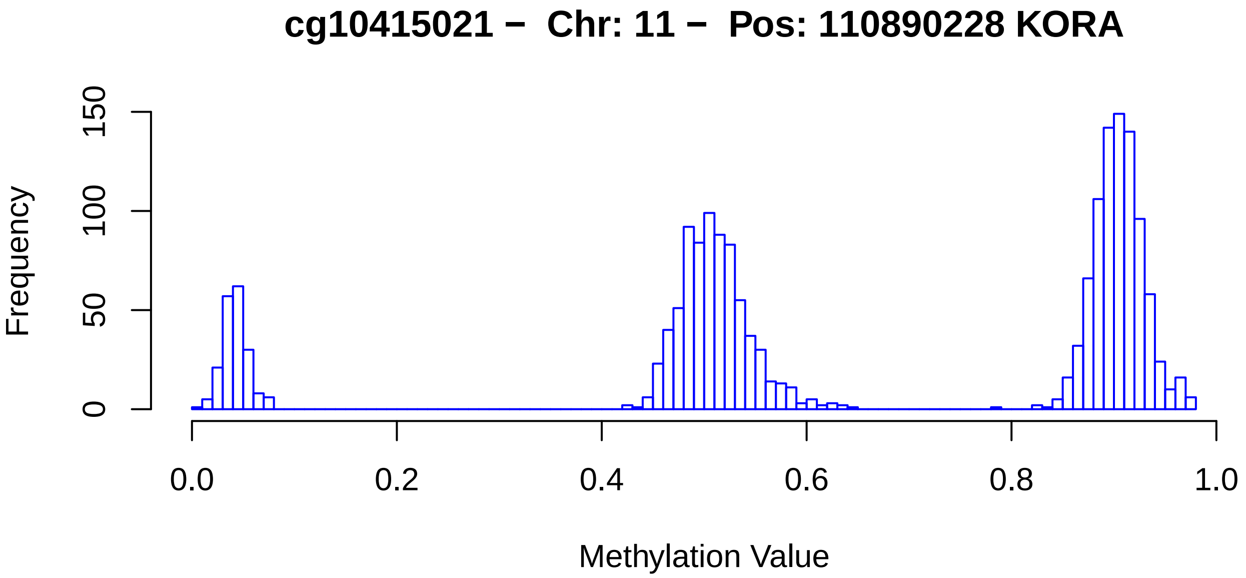
cg10415021 − Chr: 11 − Pos: 110890228 KORA
Frequency
0
50
100
150
0.0
0.2
0.4
0.6
0.8
1.0
Methylation Value

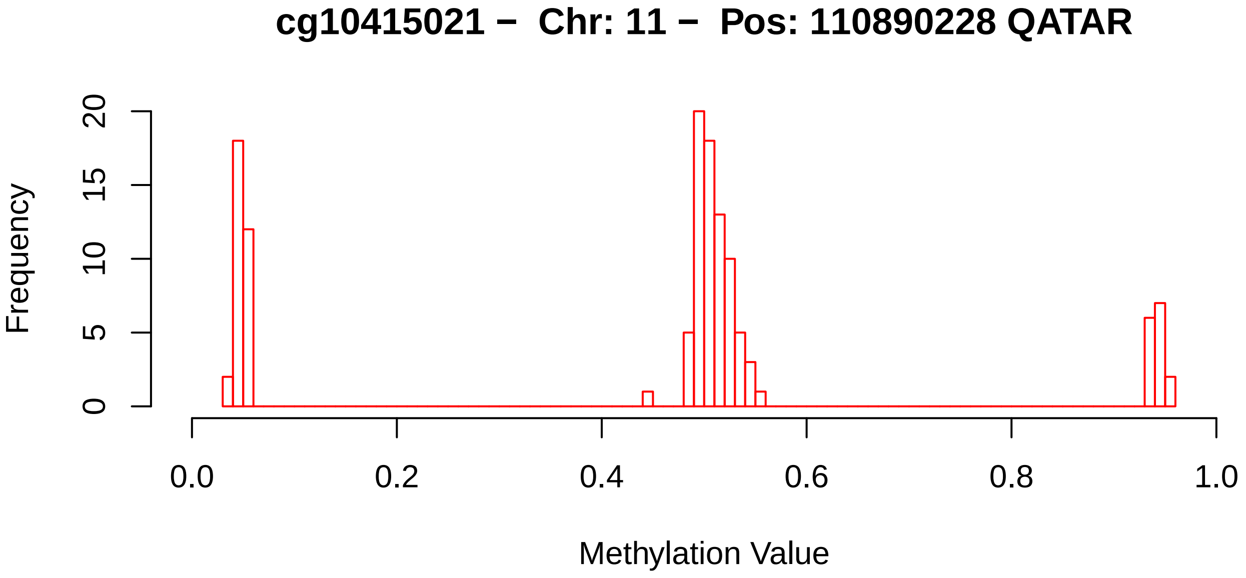
cg10415021 − Chr: 11 − Pos: 110890228 QATAR
Frequency
0
5
10
15
20
0.0
0.2
0.4
0.6
0.8
1.0
Methylation Value

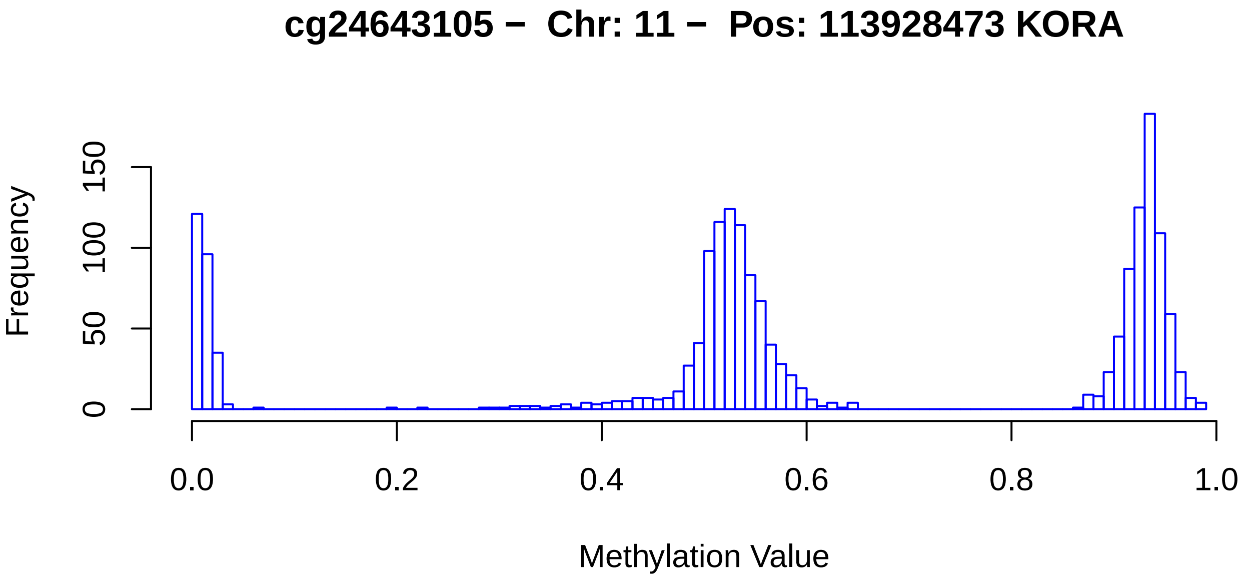
cg24643105 − Chr: 11 − Pos: 113928473 KORA
Frequency
0
50
100
150
0.0
0.2
0.4
0.6
0.8
1.0
Methylation Value

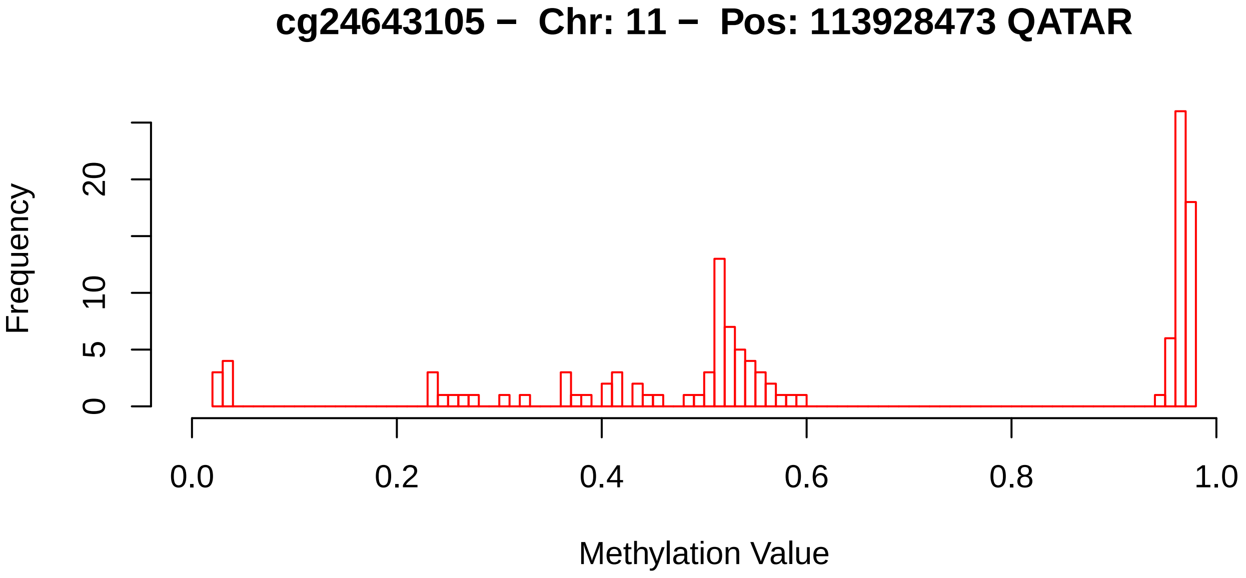
cg24643105 − Chr: 11 − Pos: 113928473 QATAR
Frequency
0
5
10
20
0.0
0.2
0.4
0.6
0.8
1.0
Methylation Value

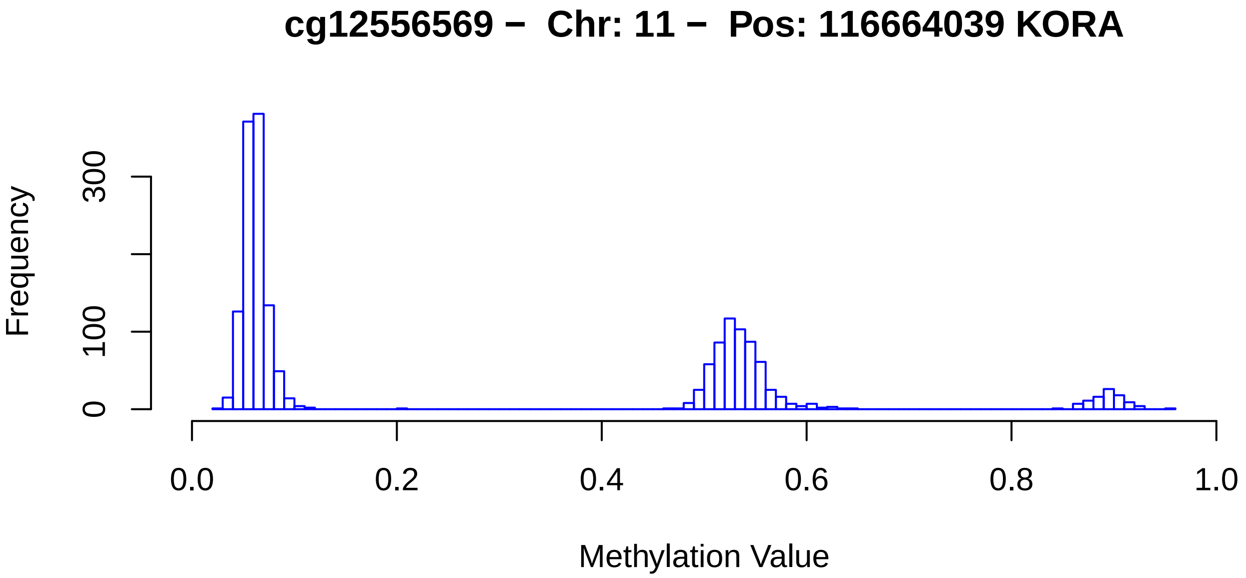
cg12556569 − Chr: 11 − Pos: 116664039 KORA
Frequency
0
100
300
0.0
0.2
0.4
0.6
0.8
1.0
Methylation Value

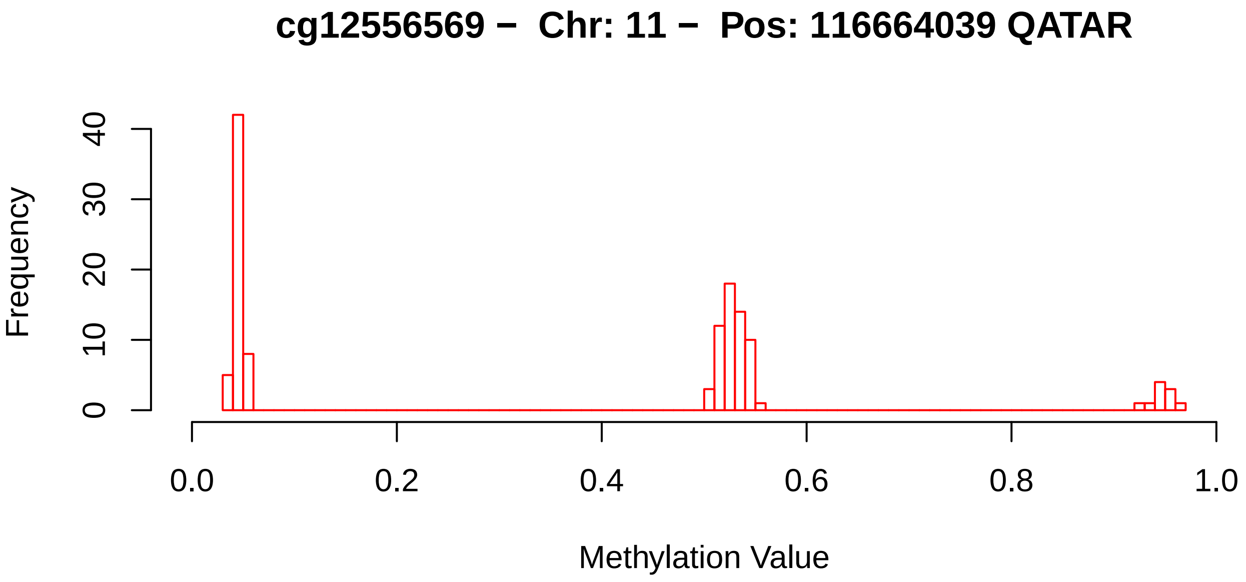
cg12556569 − Chr: 11 − Pos: 116664039 QATAR
Frequency
0
10
20
30
40
0.0
0.2
0.4
0.6
0.8
1.0
Methylation Value

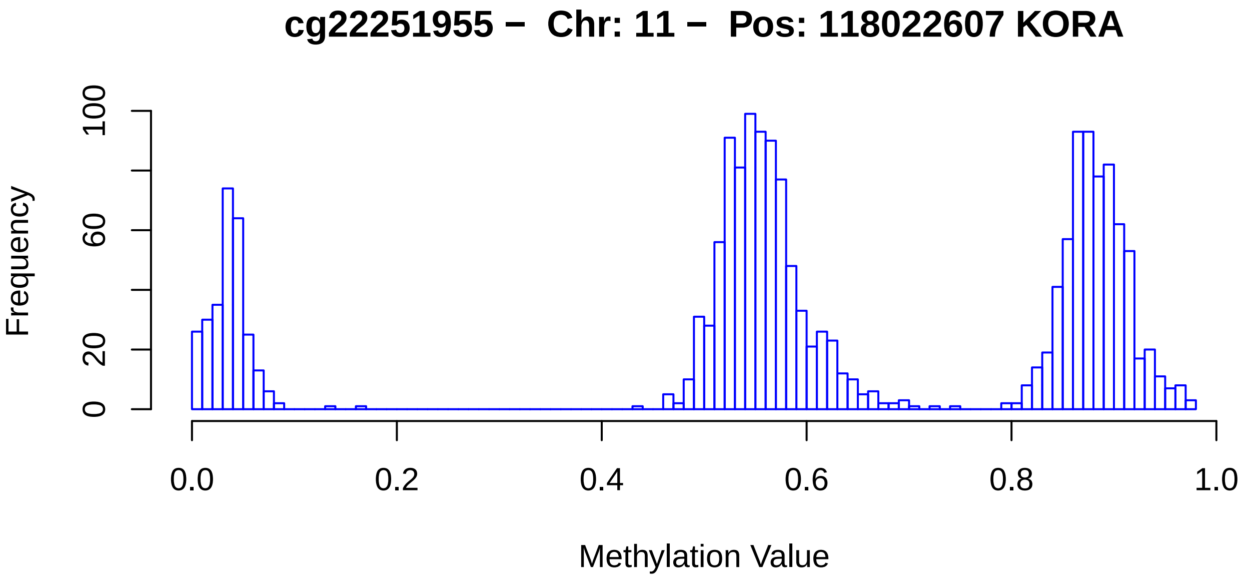
cg22251955 − Chr: 11 − Pos: 118022607 KORA
Frequency
0
20
60
100
0.0
0.2
0.4
0.6
0.8
1.0
Methylation Value

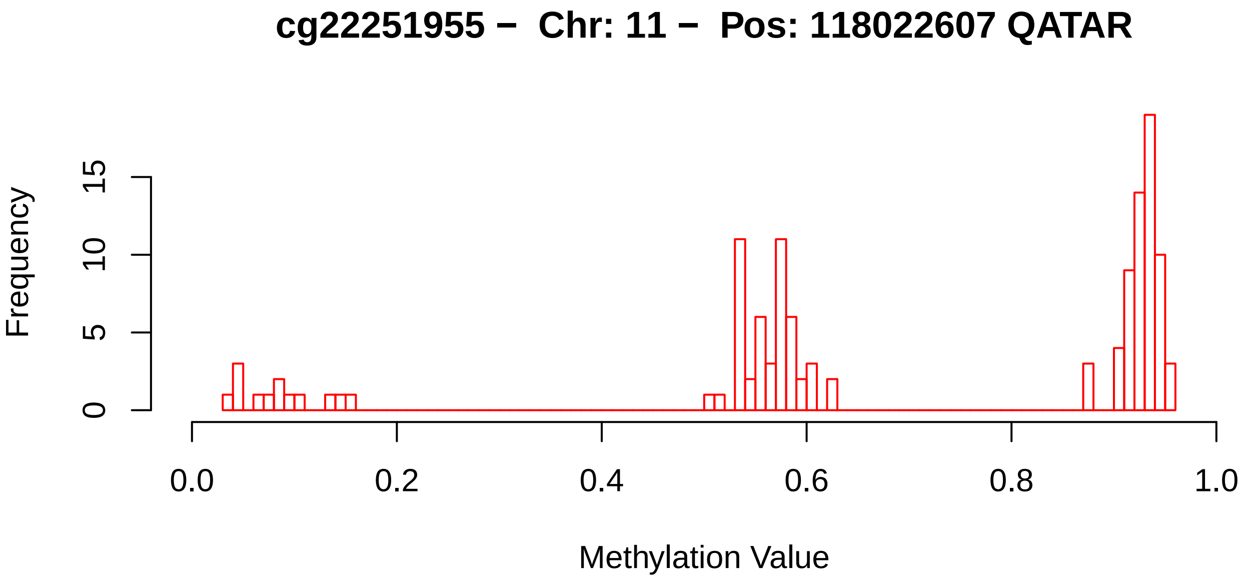
cg22251955 − Chr: 11 − Pos: 118022607 QATAR
Frequency
0
5
10
15
0.0
0.2
0.4
0.6
0.8
1.0
Methylation Value

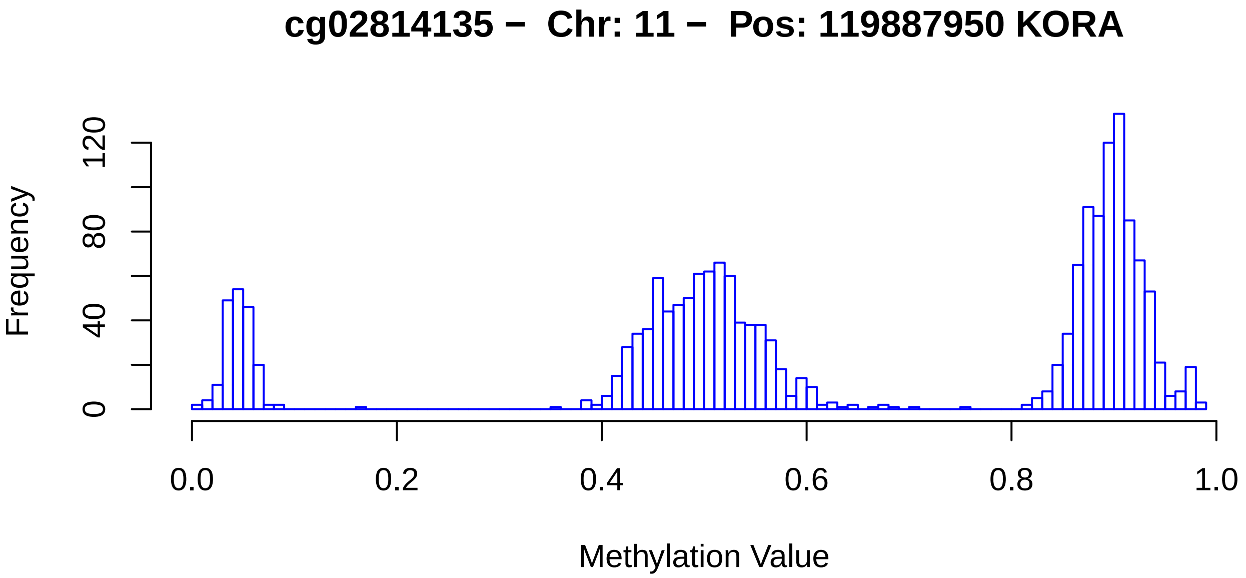

cg02814135 − Chr: 11 − Pos: 119887950 KORA
Frequency
0
40
80
120
0.0
0.2
0.4
0.6
0.8
1.0
Methylation Value

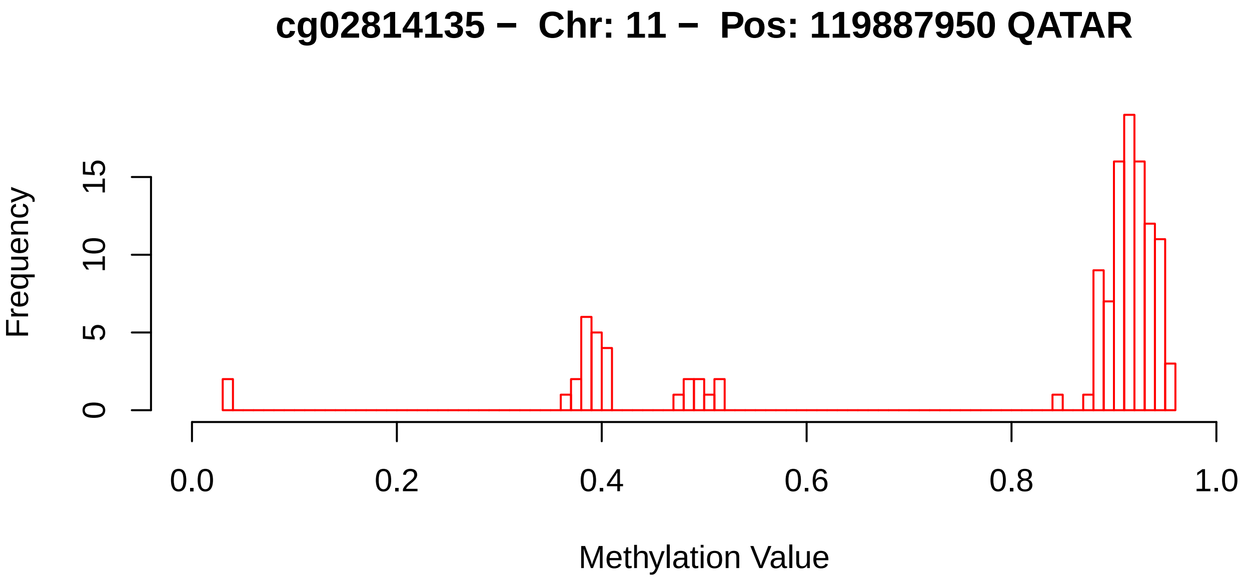

cg02814135 − Chr: 11 − Pos: 119887950 QATAR
Frequency
0
5
10
15
0.0
0.2
0.4
0.6
0.8
1.0
Methylation Value

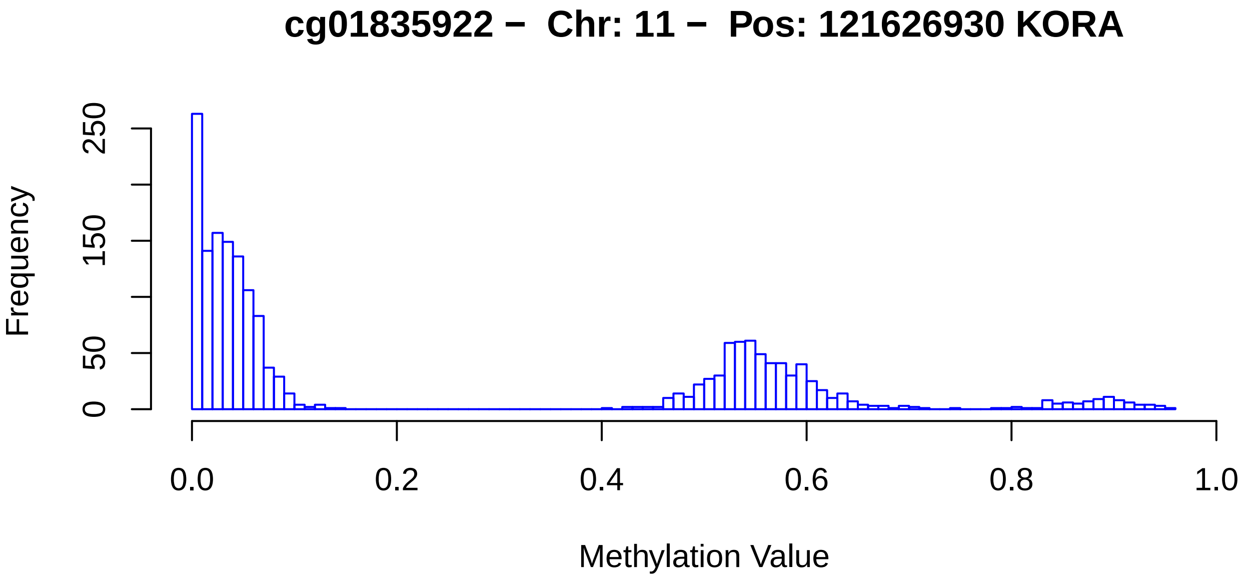
cg01835922 − Chr: 11 − Pos: 121626930 KORA
Frequency
0
50
150
250
0.0
0.2
0.4
0.6
0.8
1.0
Methylation Value

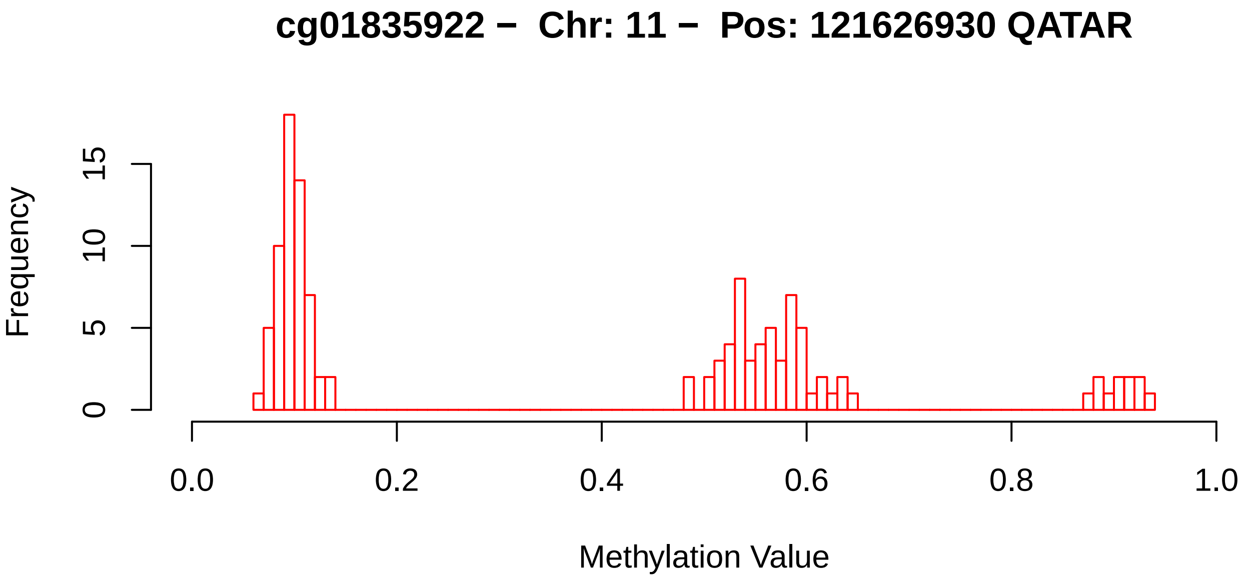
cg01835922 − Chr: 11 − Pos: 121626930 QATAR
Frequency
0
5
10
15
0.0
0.2
0.4
0.6
0.8
1.0
Methylation Value

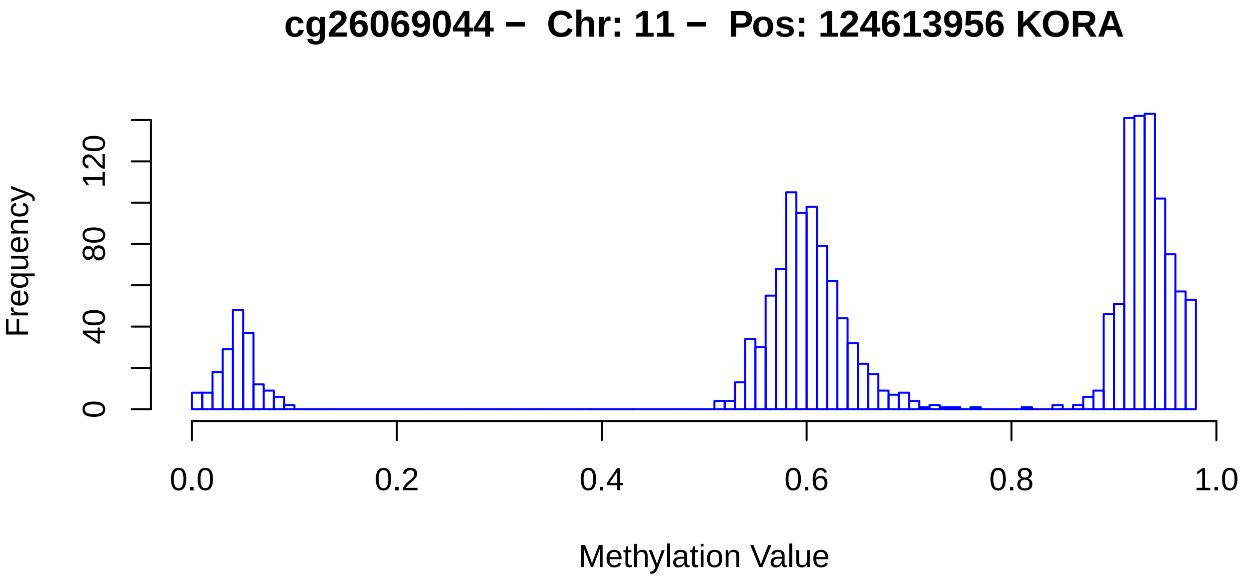
cg26069044 − Chr: 11 − Pos: 124613956 KORA
Frequency
0
40
80
120
0.0
0.2
0.4
0.6
0.8
1.0
Methylation Value

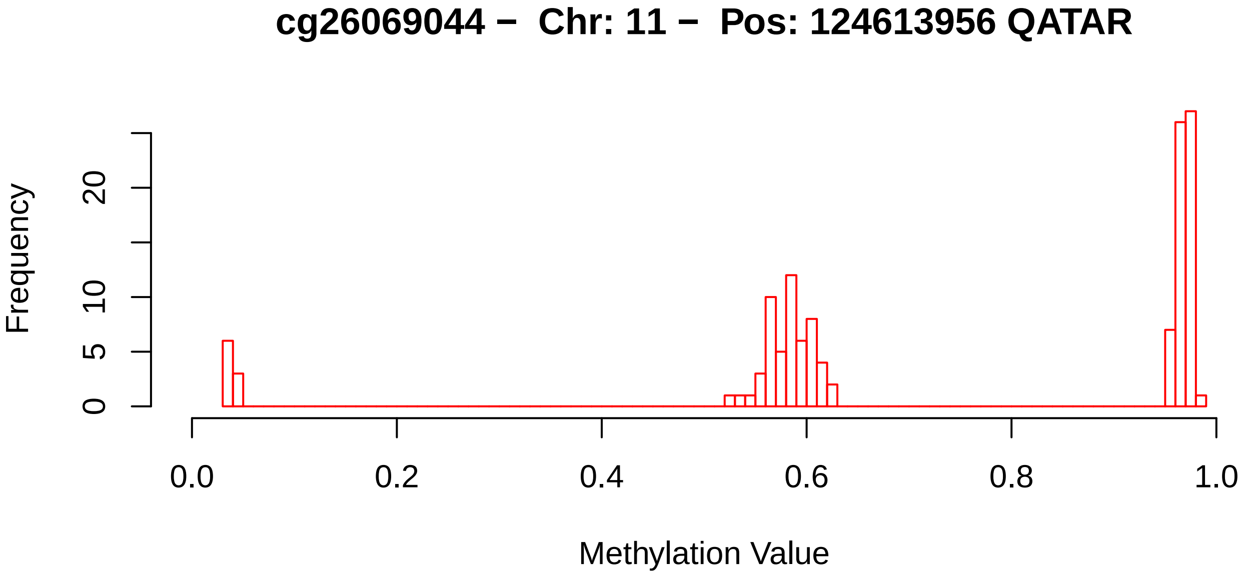
cg26069044 − Chr: 11 − Pos: 124613956 QATAR
Frequency
0
5
10
20
0.0
0.2
0.4
0.6
0.8
1.0
Methylation Value

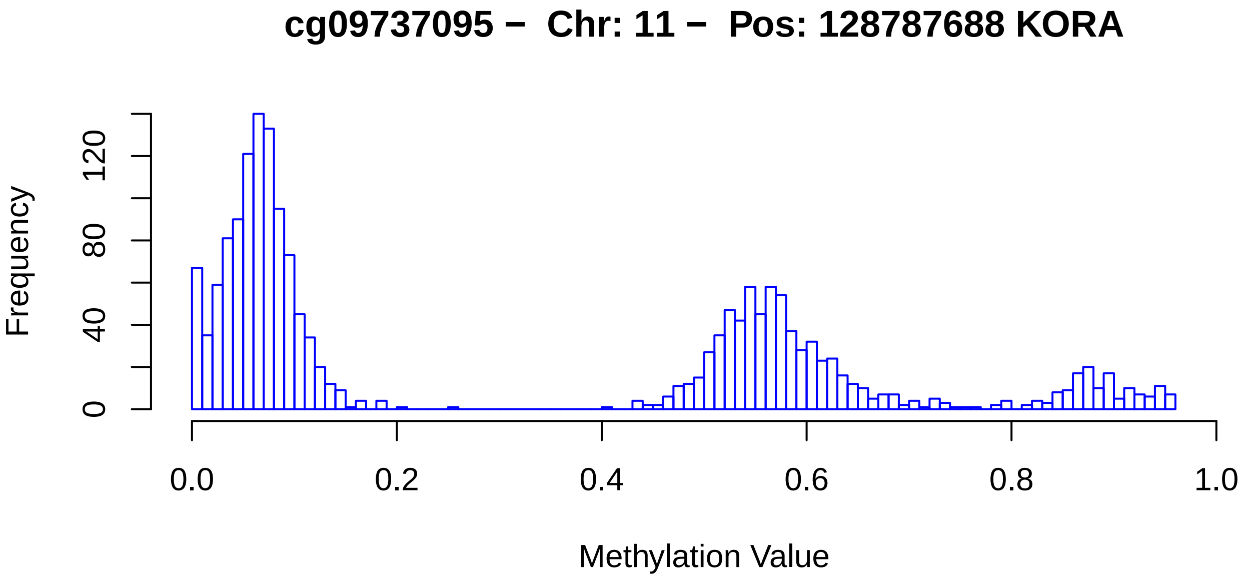
cg09737095 − Chr: 11 − Pos: 128787688 KORA
Frequency
0
40
80
120
0.0
0.2
0.4
0.6
0.8
1.0
Methylation Value

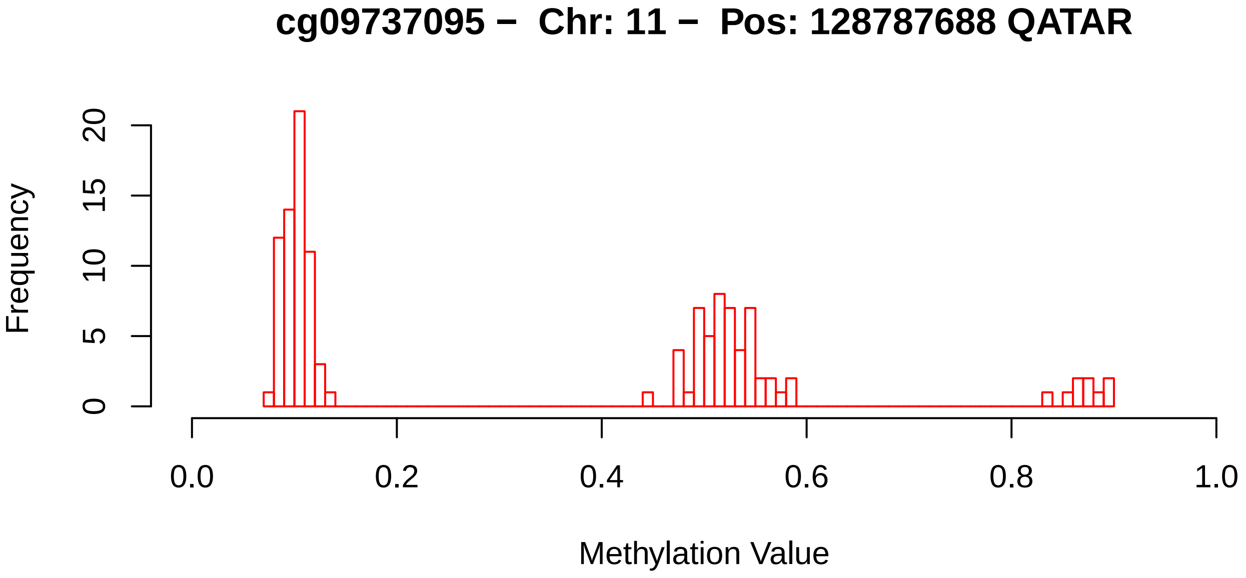
cg09737095 − Chr: 11 − Pos: 128787688 QATAR
Frequency
0
5
10
15
20
0.0
0.2
0.4
0.6
0.8
1.0
Methylation Value

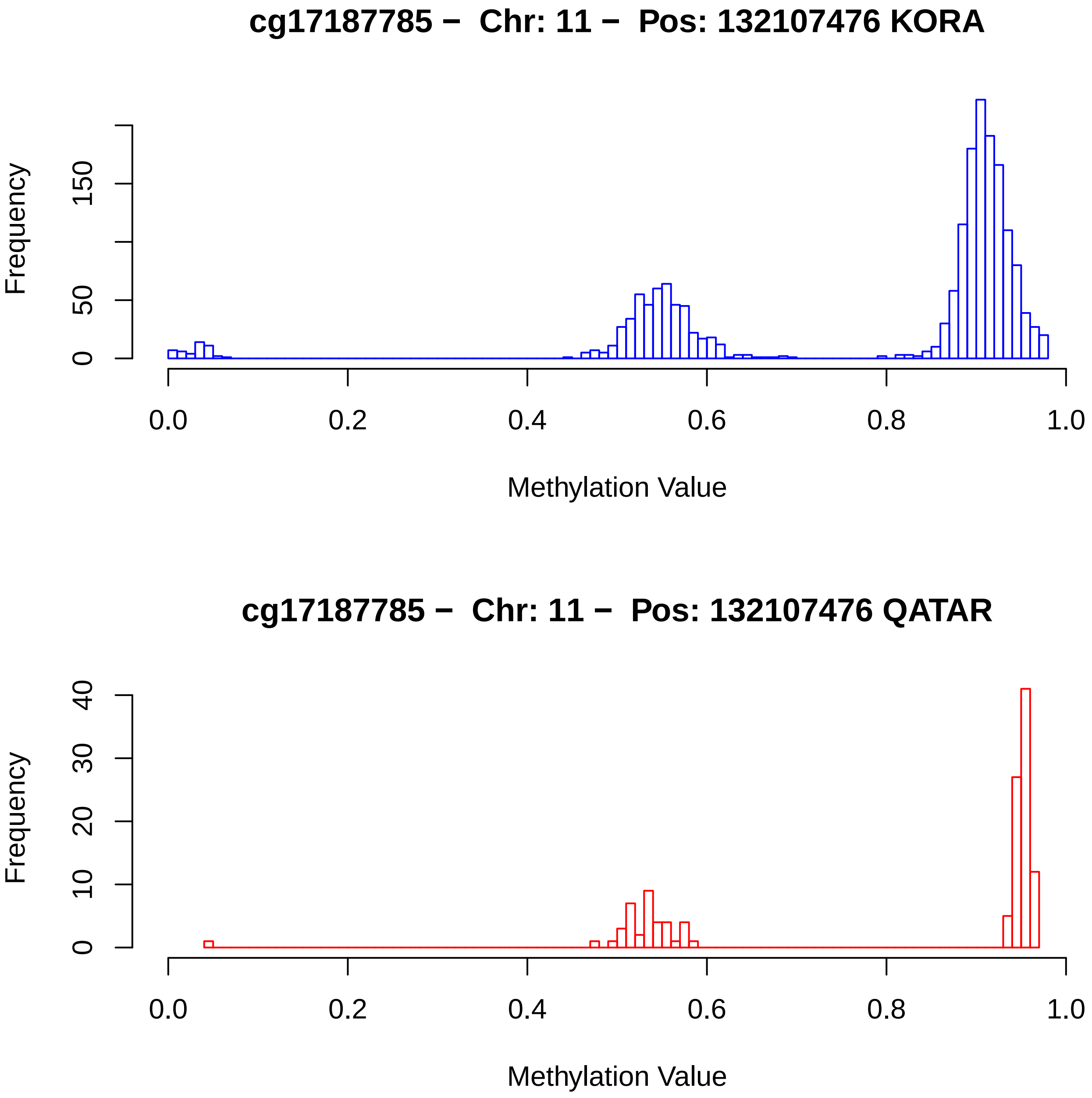
cg17187785 − Chr: 11 − Pos: 132107476 KORA
Frequency
0
50
150
0.0
0.2
0.4
0.6
0.8
1.0
Methylation Value
cg17187785 − Chr: 11 − Pos: 132107476 QATAR
Frequency
0
10
20
30
40
0.0
0.2
0.4
0.6
0.8
1.0
Methylation Value

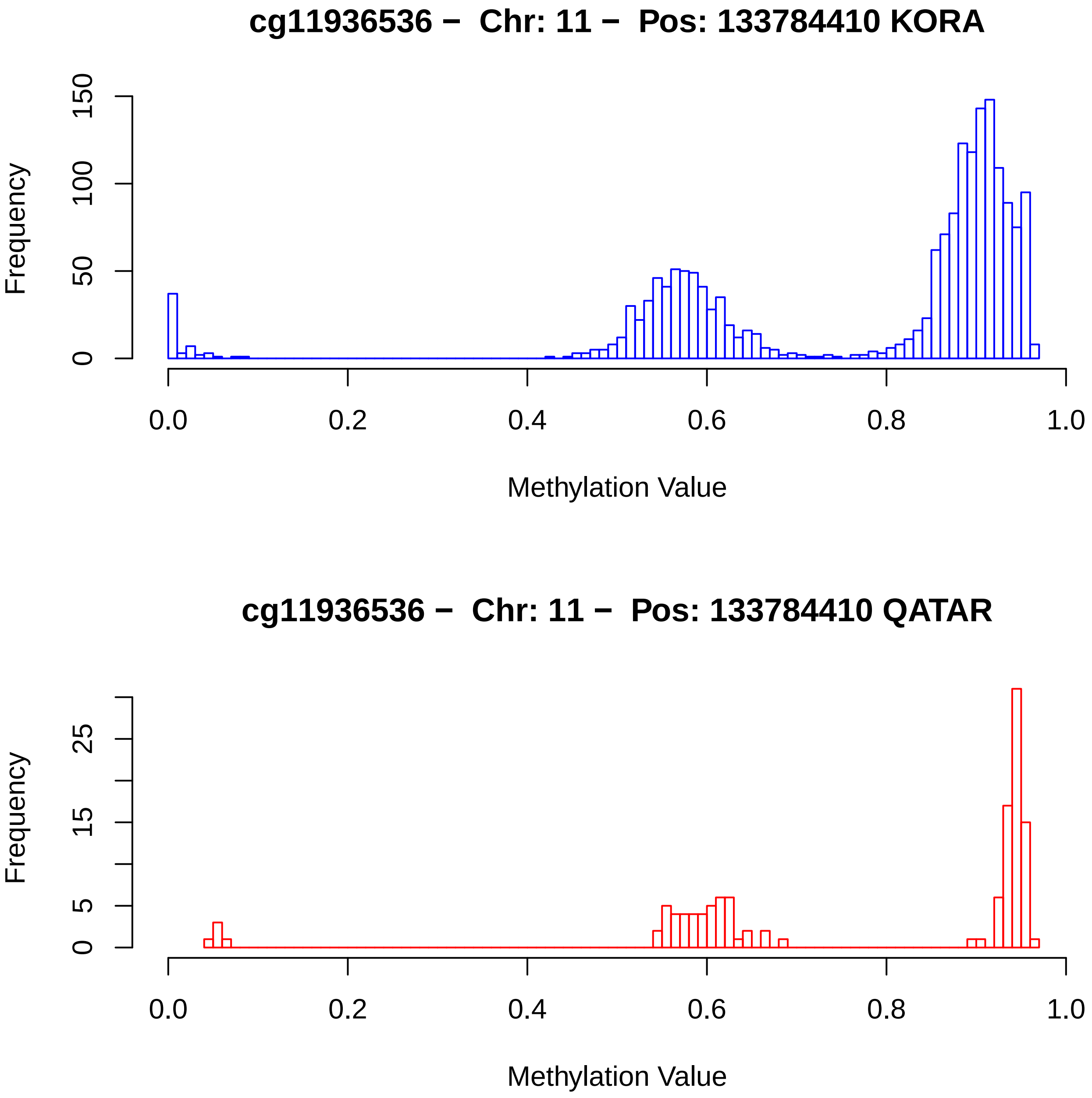
cg11936536 − Chr: 11 − Pos: 133784410 KORA
Frequency
0
50
100
150
0.0
0.2
0.4
0.6
0.8
1.0
Methylation Value
cg11936536 − Chr: 11 − Pos: 133784410 QATAR
Frequency
0
5
15
25
0.0
0.2
0.4
0.6
0.8
1.0
Methylation Value

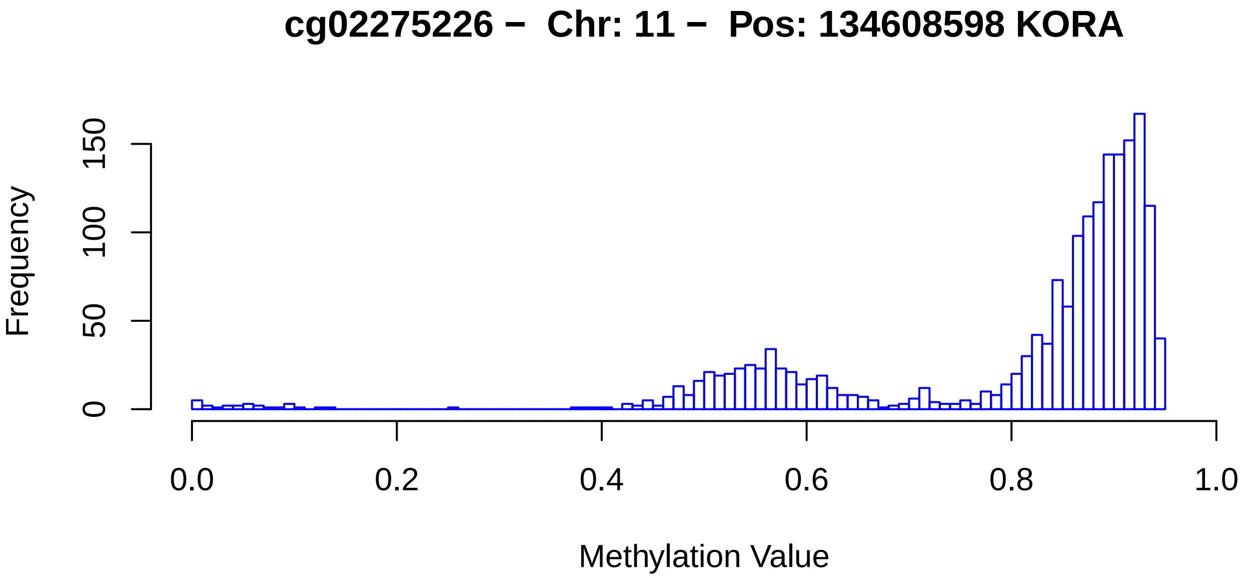
cg02275226 − Chr: 11 − Pos: 134608598 KORA
Frequency
0
50
100
150
0.0
0.2
0.4
0.6
0.8
1.0
Methylation Value

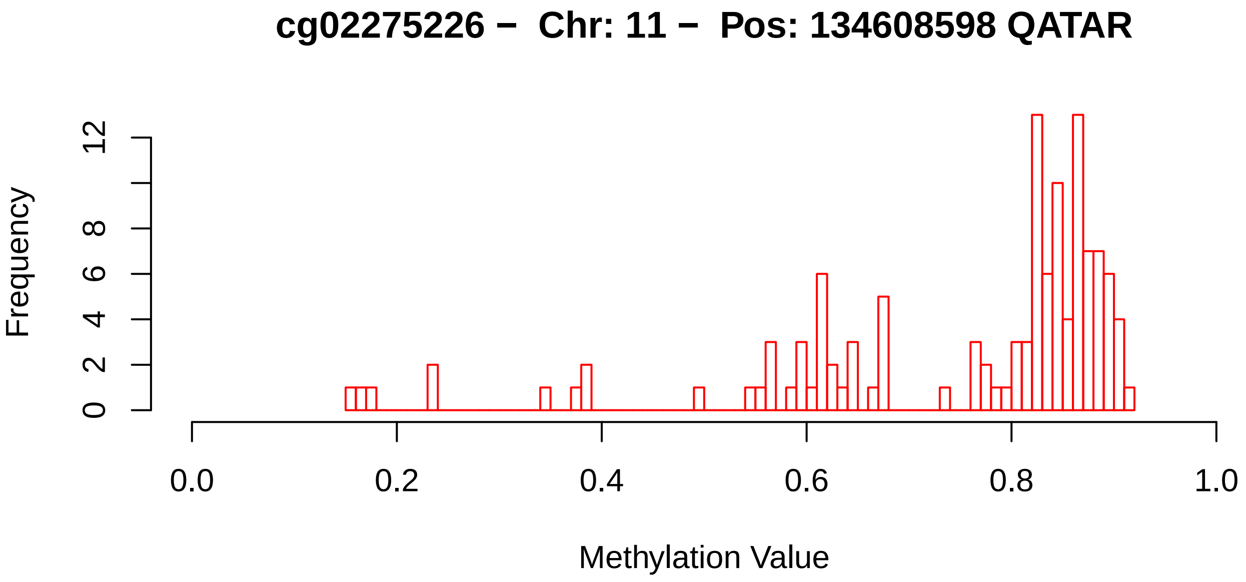
cg02275226 − Chr: 11 − Pos: 134608598 QATAR
Frequency
0
2
4
6
8
12
0.0
0.2
0.4
0.6
0.8
1.0
Methylation Value

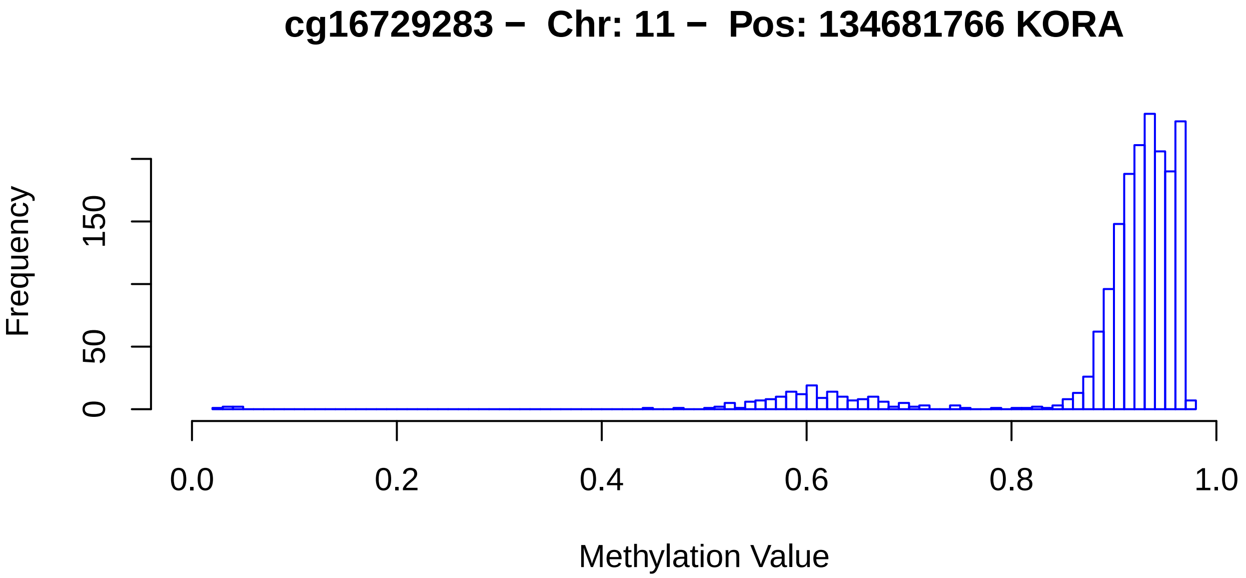
cg16729283 − Chr: 11 − Pos: 134681766 KORA
Frequency
0
50
150
0.0
0.2
0.4
0.6
0.8
1.0
Methylation Value

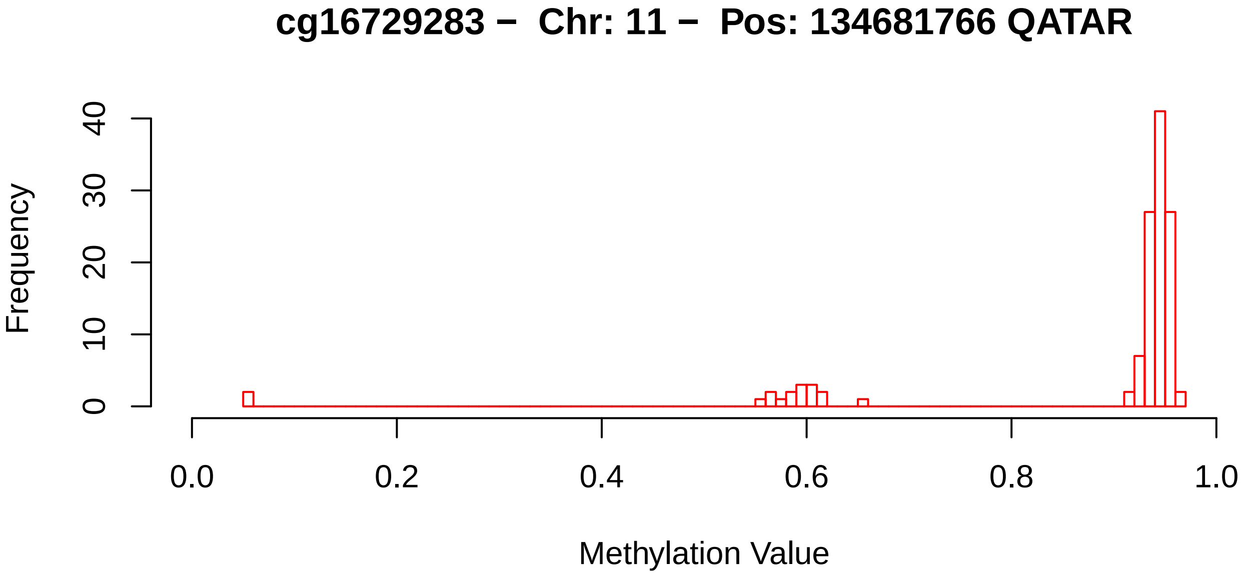
cg16729283 − Chr: 11 − Pos: 134681766 QATAR
Frequency
0
10
20
30
40
0.0
0.2
0.4
0.6
0.8
1.0
Methylation Value

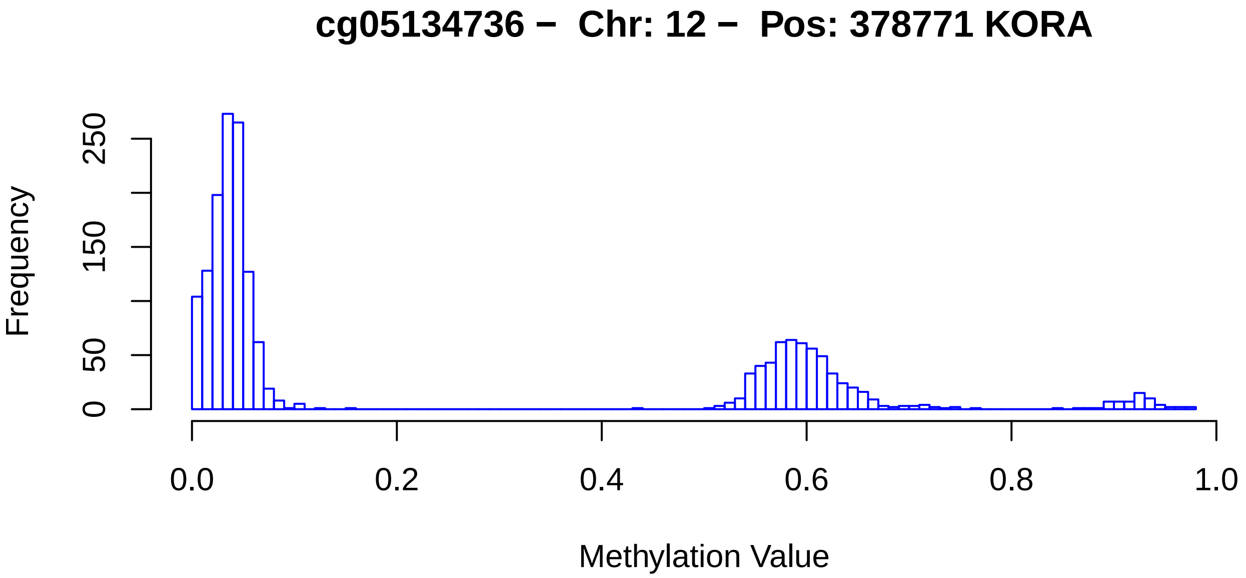
cg05134736 − Chr: 12 − Pos: 378771 KORA
Frequency
0
50
150
250
0.0
0.2
0.4
0.6
0.8
1.0
Methylation Value

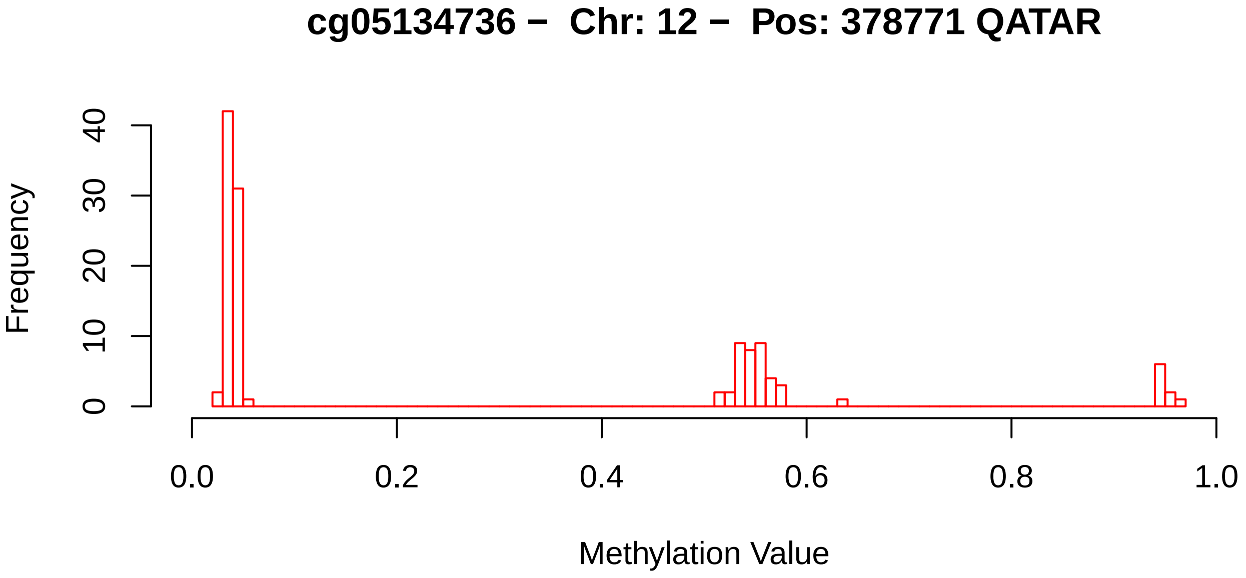
cg05134736 − Chr: 12 − Pos: 378771 QATAR
Frequency
0
10
20
30
40
0.0
0.2
0.4
0.6
0.8
1.0
Methylation Value

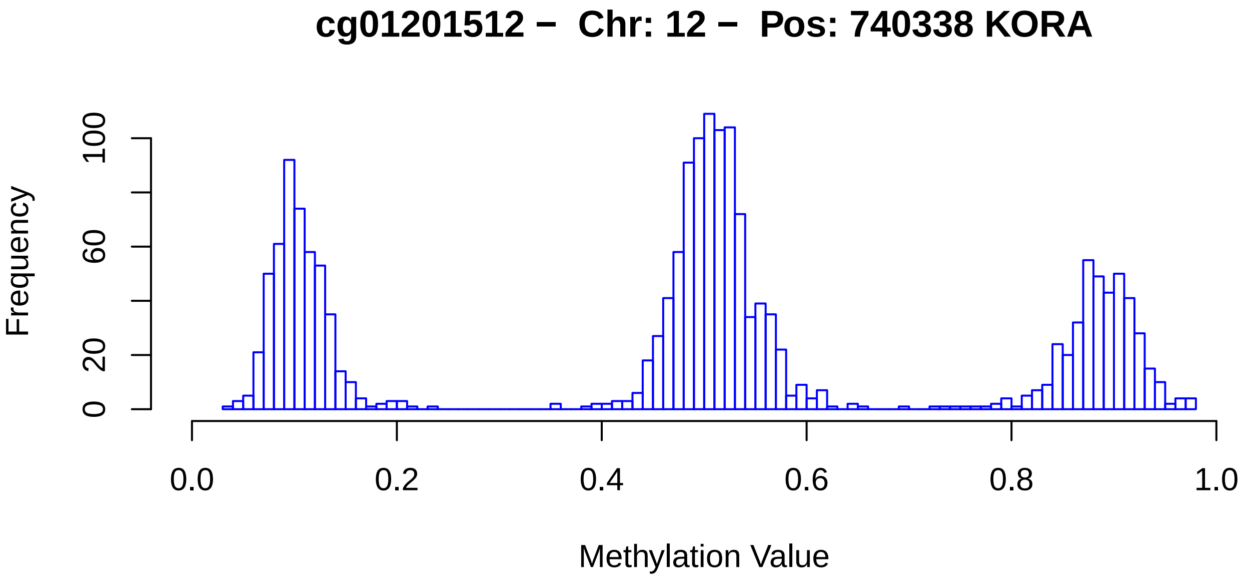
cg01201512 − Chr: 12 − Pos: 740338 KORA
Frequency
0
20
60
100
0.0
0.2
0.4
0.6
0.8
1.0
Methylation Value

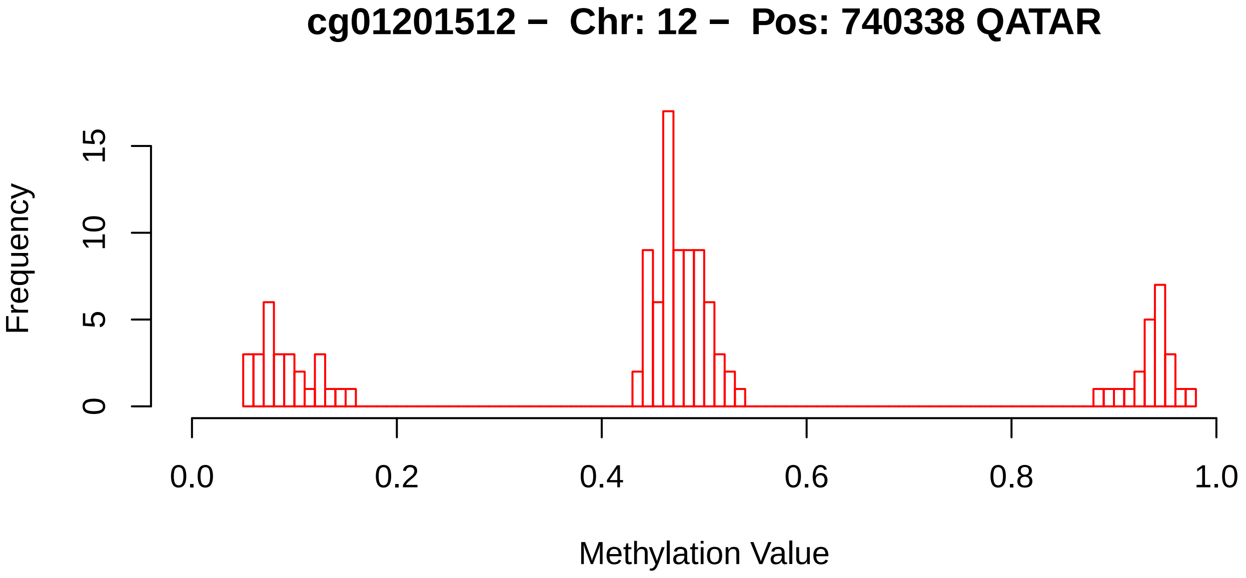
cg01201512 − Chr: 12 − Pos: 740338 QATAR
Frequency
0
5
10
15
0.0
0.2
0.4
0.6
0.8
1.0
Methylation Value

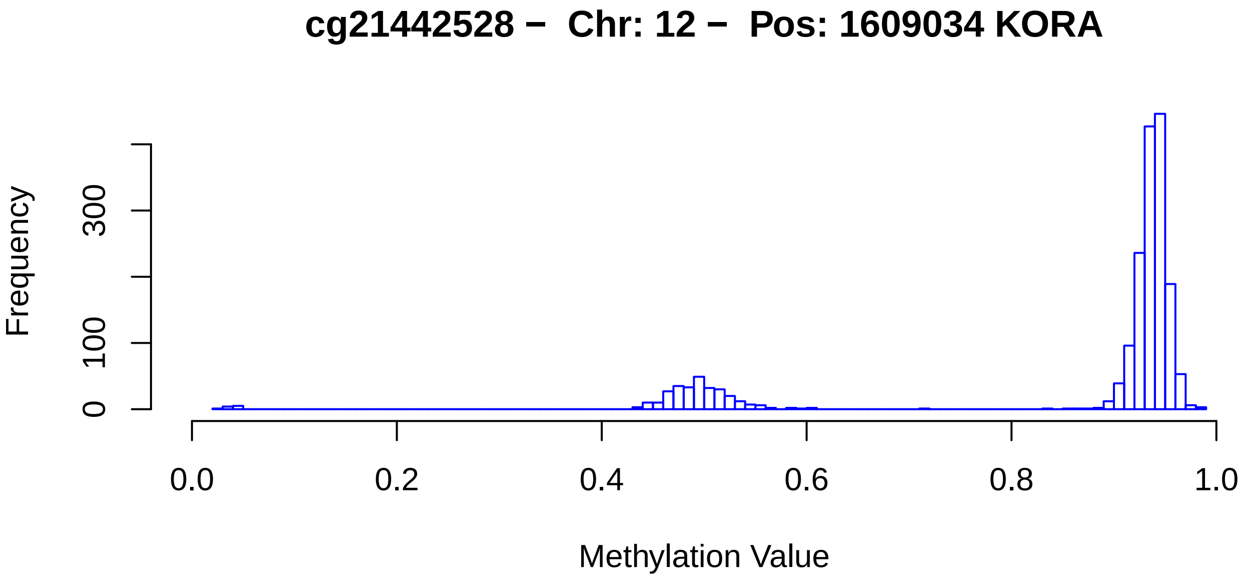
cg21442528 − Chr: 12 − Pos: 1609034 KORA
Frequency
0
100
300
0.0
0.2
0.4
0.6
0.8
1.0
Methylation Value

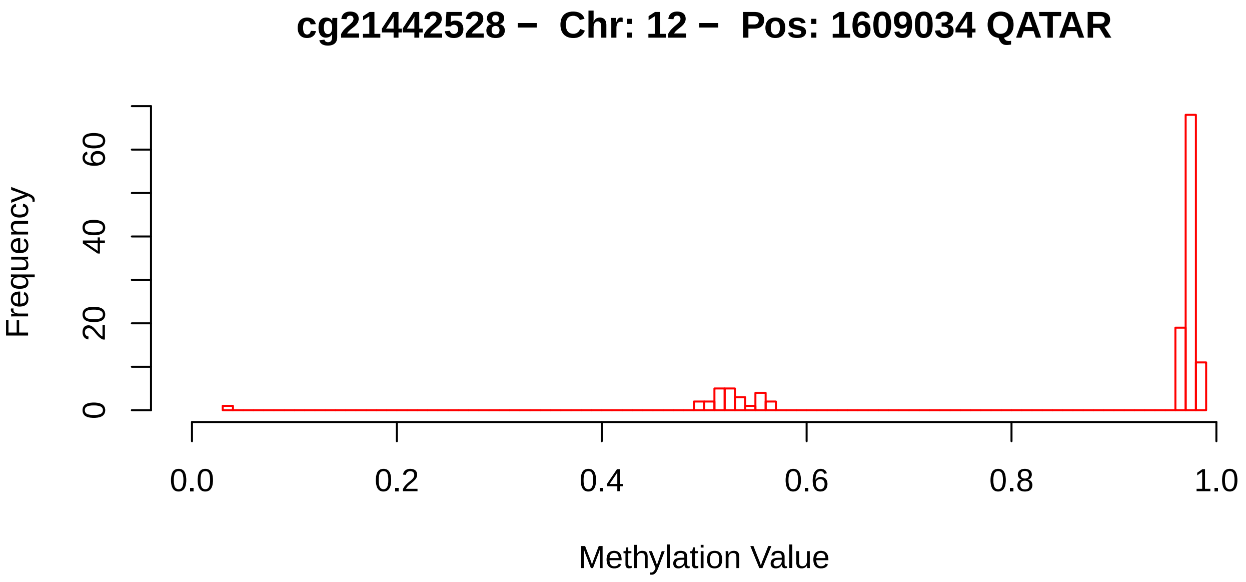
cg21442528 − Chr: 12 − Pos: 1609034 QATAR
Frequency
0
20
40
60
0.0
0.2
0.4
0.6
0.8
1.0
Methylation Value

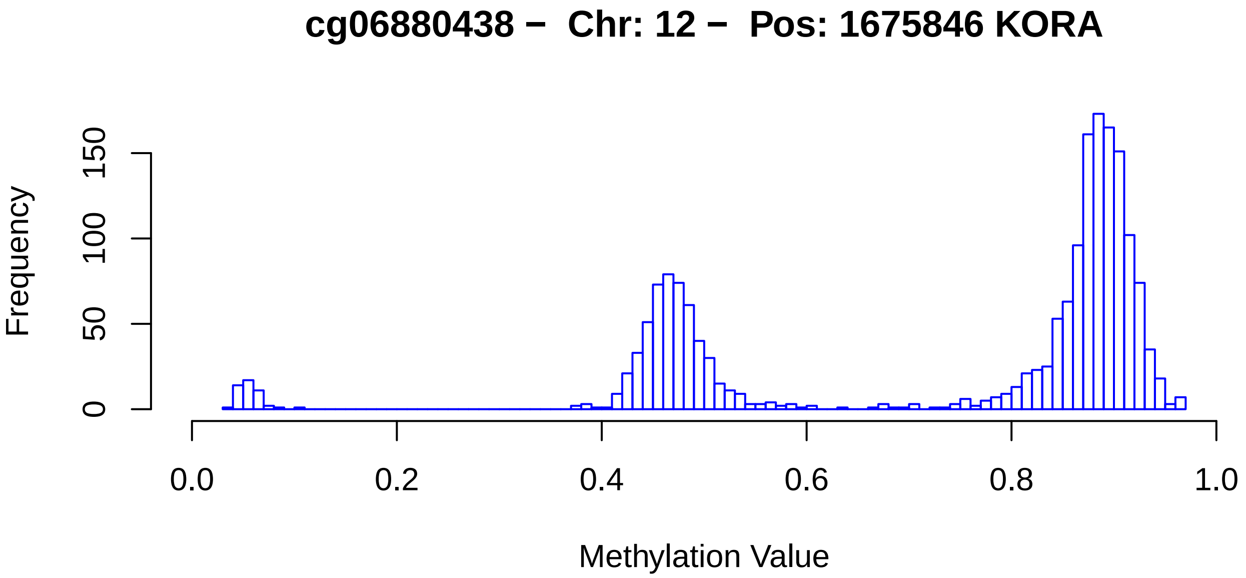
cg06880438 − Chr: 12 − Pos: 1675846 KORA
Frequency
0
50
100
150
0.0
0.2
0.4
0.6
0.8
1.0
Methylation Value

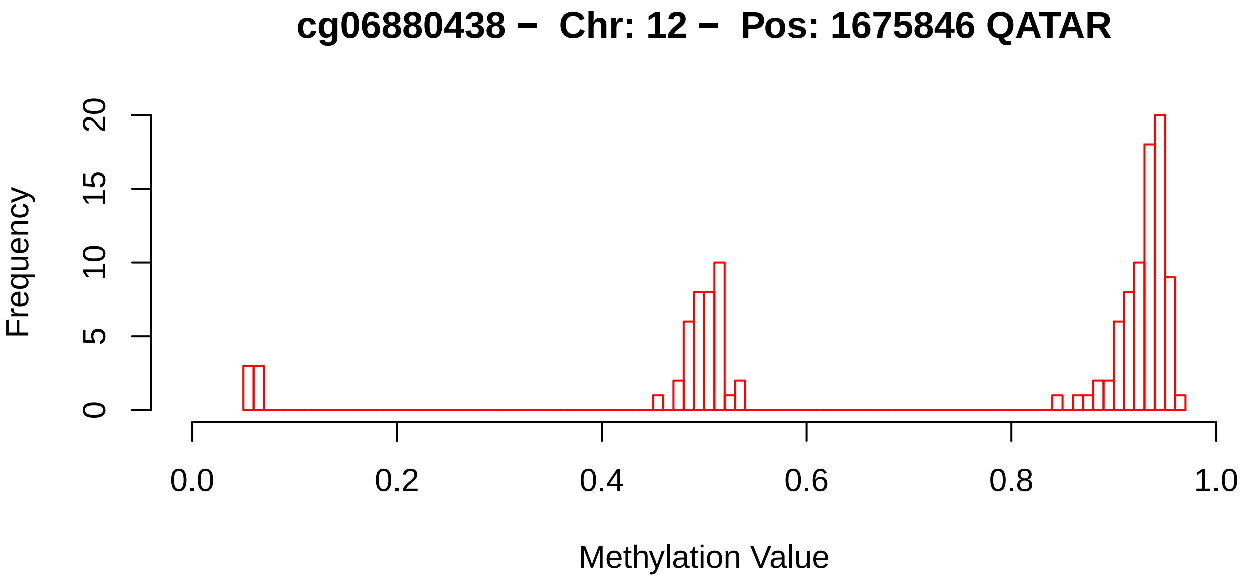
cg06880438 − Chr: 12 − Pos: 1675846 QATAR
Frequency
0
5
10
15
20
0.0
0.2
0.4
0.6
0.8
1.0
Methylation Value

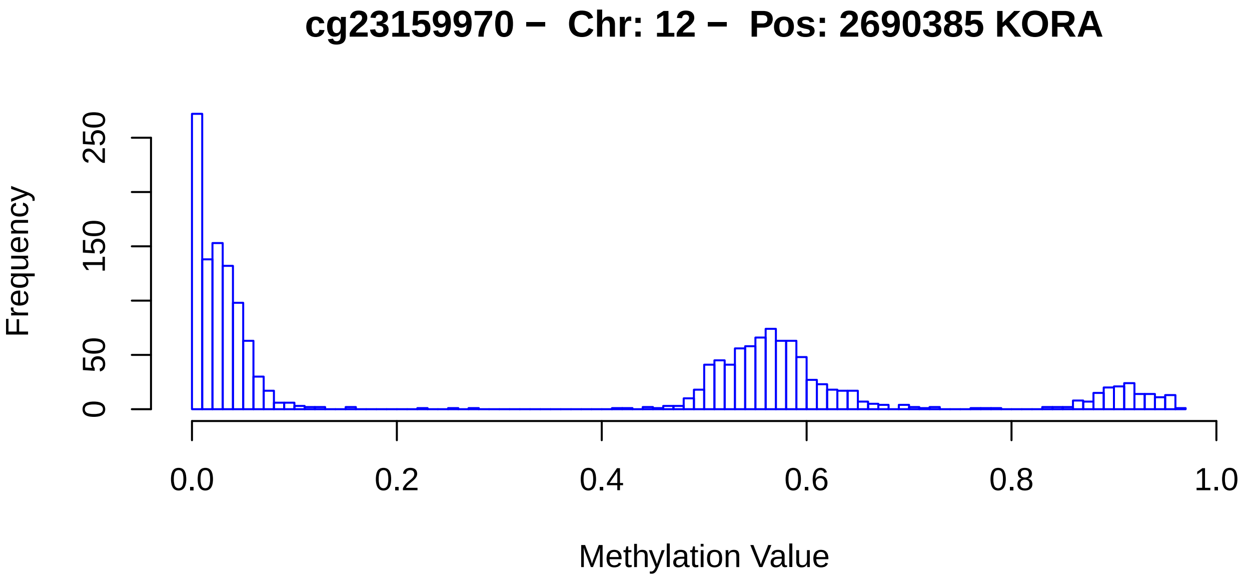
cg23159970 − Chr: 12 − Pos: 2690385 KORA
Frequency
0
50
150
250
0.0
0.2
0.4
0.6
0.8
1.0
Methylation Value

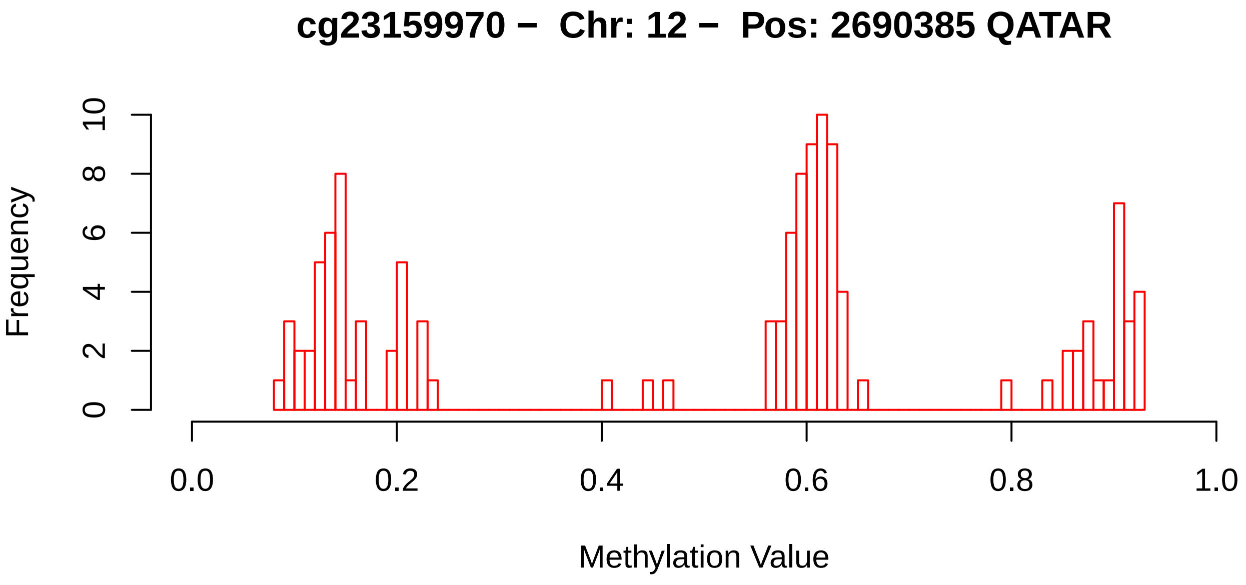
cg23159970 − Chr: 12 − Pos: 2690385 QATAR
Frequency
0
2
4
6
8
10
0.0
0.2
0.4
0.6
0.8
1.0
Methylation Value

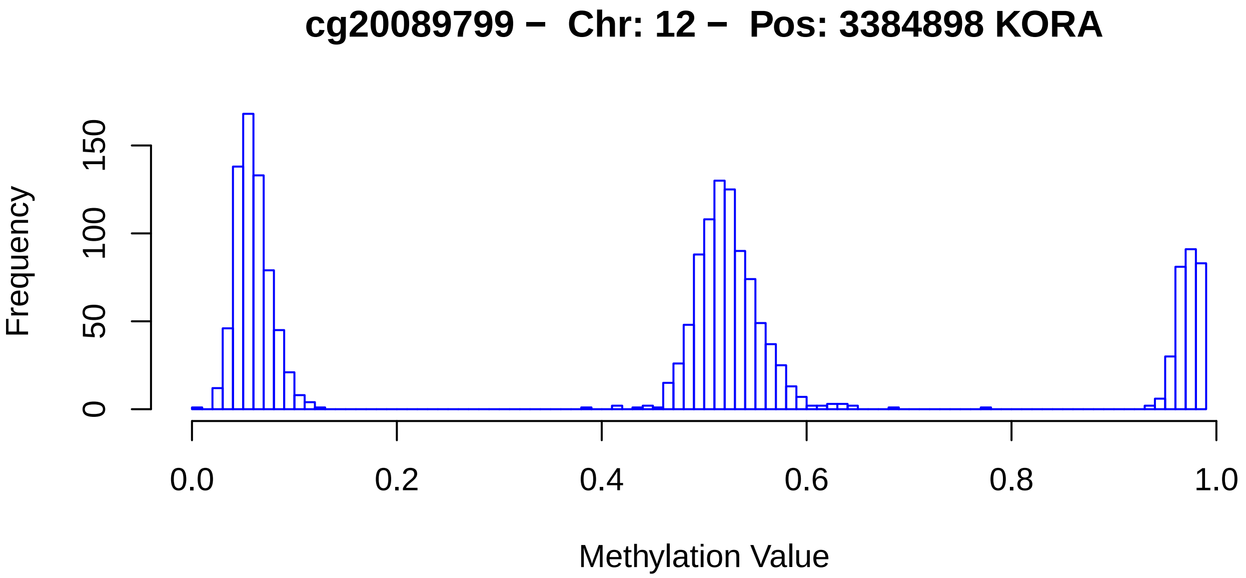
cg20089799 − Chr: 12 − Pos: 3384898 KORA
Frequency
0
50
100
150
0.0
0.2
0.4
0.6
0.8
1.0
Methylation Value

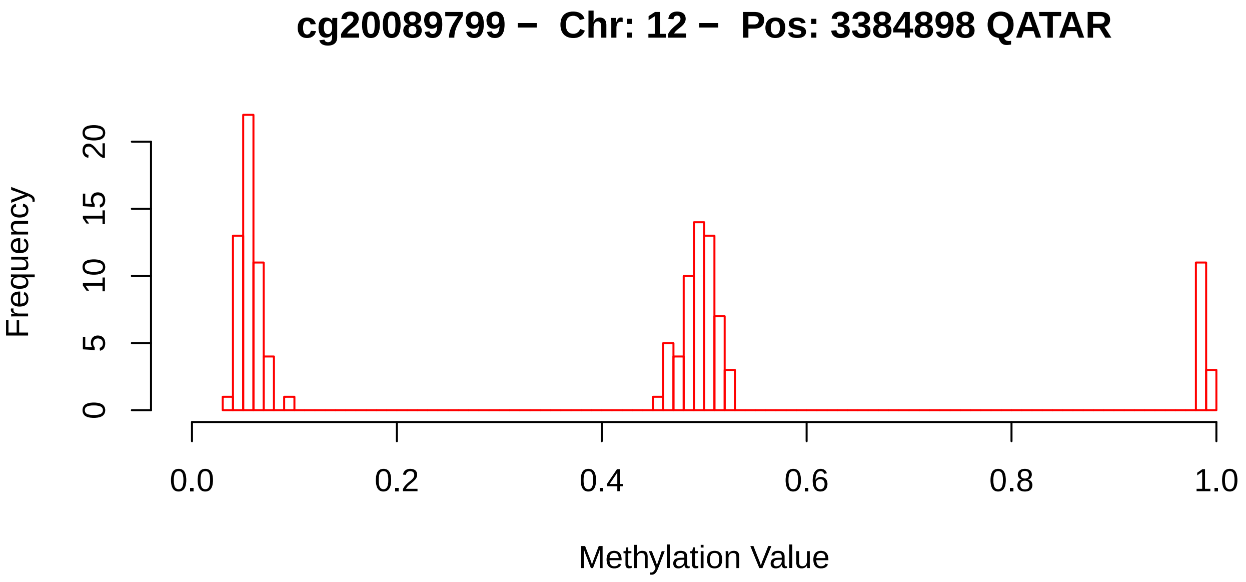
cg20089799 − Chr: 12 − Pos: 3384898 QATAR
Frequency
0
5
10
15
20
0.0
0.2
0.4
0.6
0.8
1.0
Methylation Value

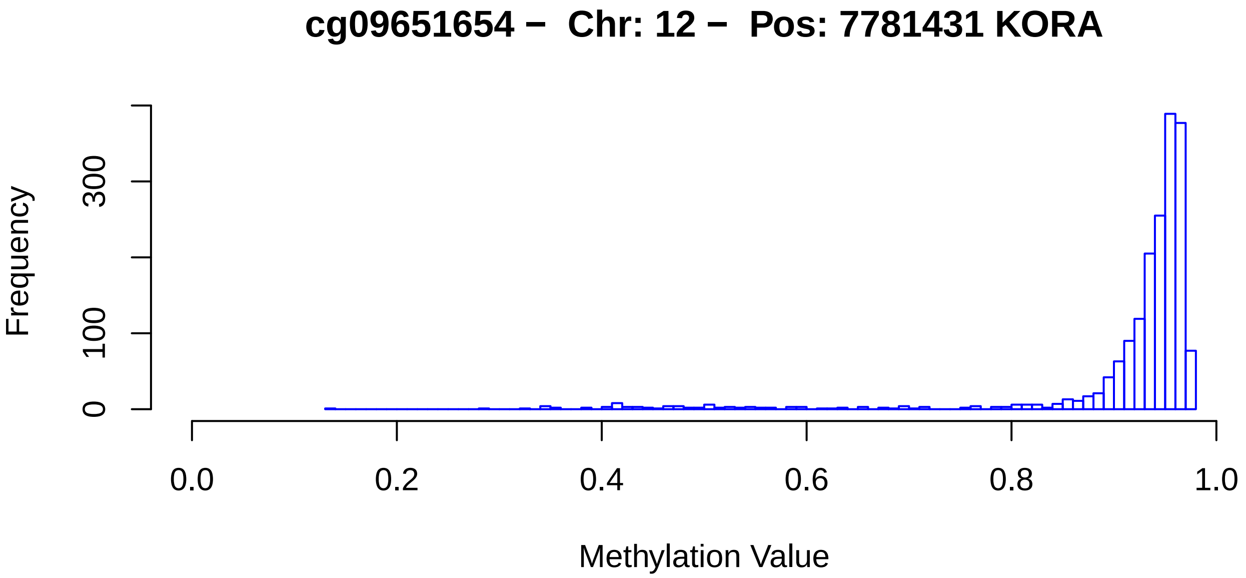

cg09651654 − Chr: 12 − Pos: 7781431 KORA
Frequency
0
100
300
0.0
0.2
0.4
0.6
0.8
1.0
Methylation Value

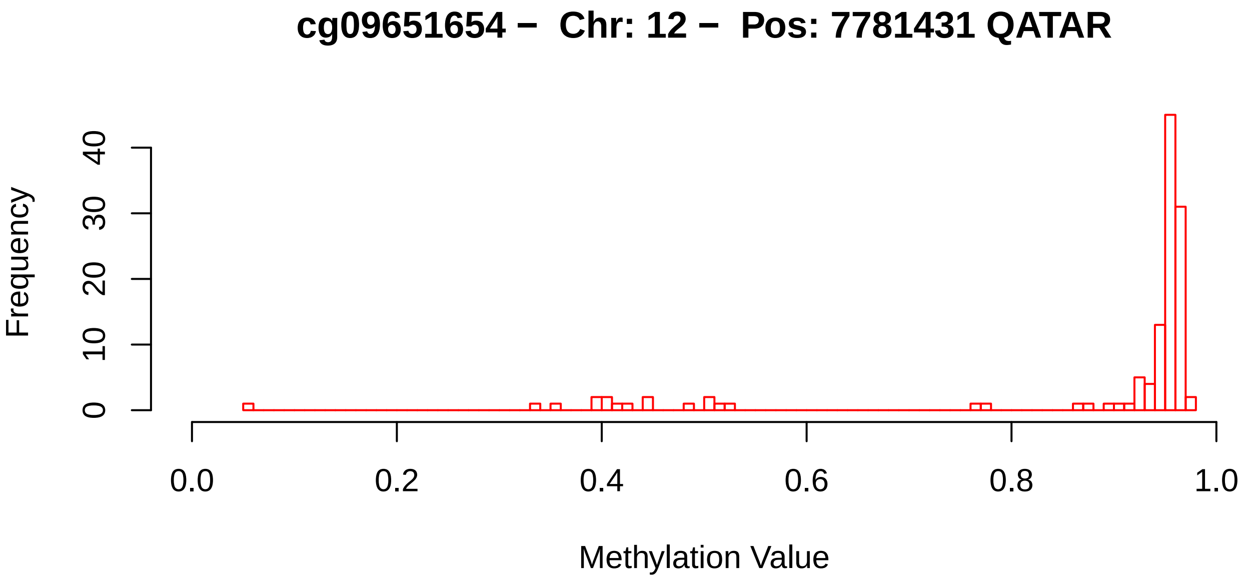

cg09651654 − Chr: 12 − Pos: 7781431 QATAR
Frequency
0
10
20
30
40
0.0
0.2
0.4
0.6
0.8
1.0
Methylation Value

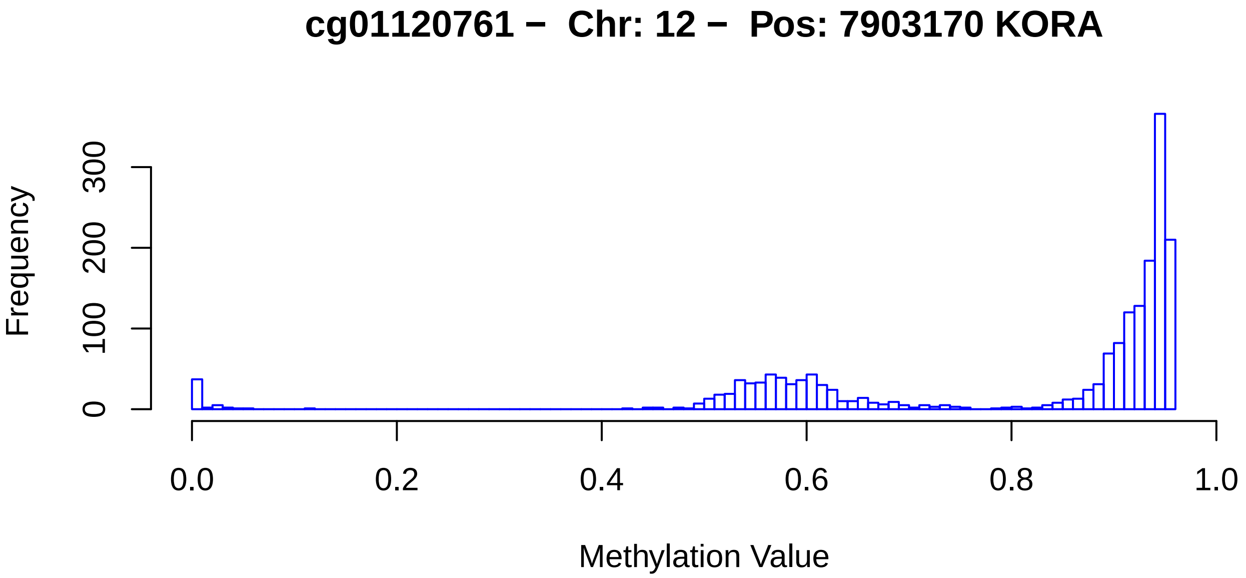

cg01120761 − Chr: 12 − Pos: 7903170 KORA
Frequency
0
100
200
300
0.0
0.2
0.4
0.6
0.8
1.0
Methylation Value

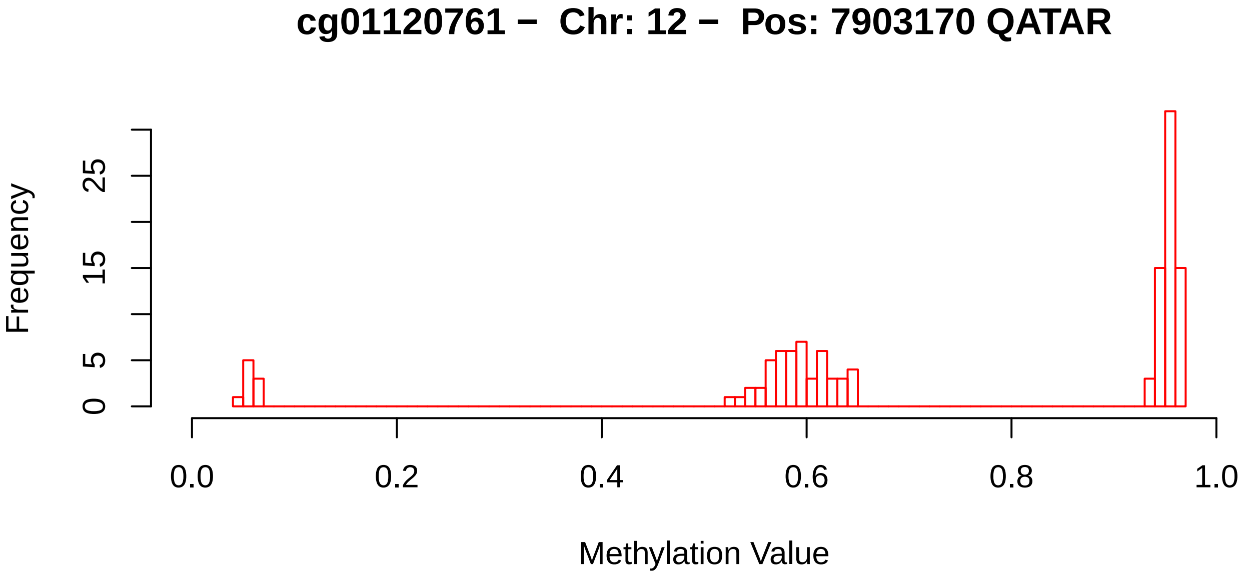

cg01120761 − Chr: 12 − Pos: 7903170 QATAR
Frequency
0
5
15
25
0.0
0.2
0.4
0.6
0.8
1.0
Methylation Value

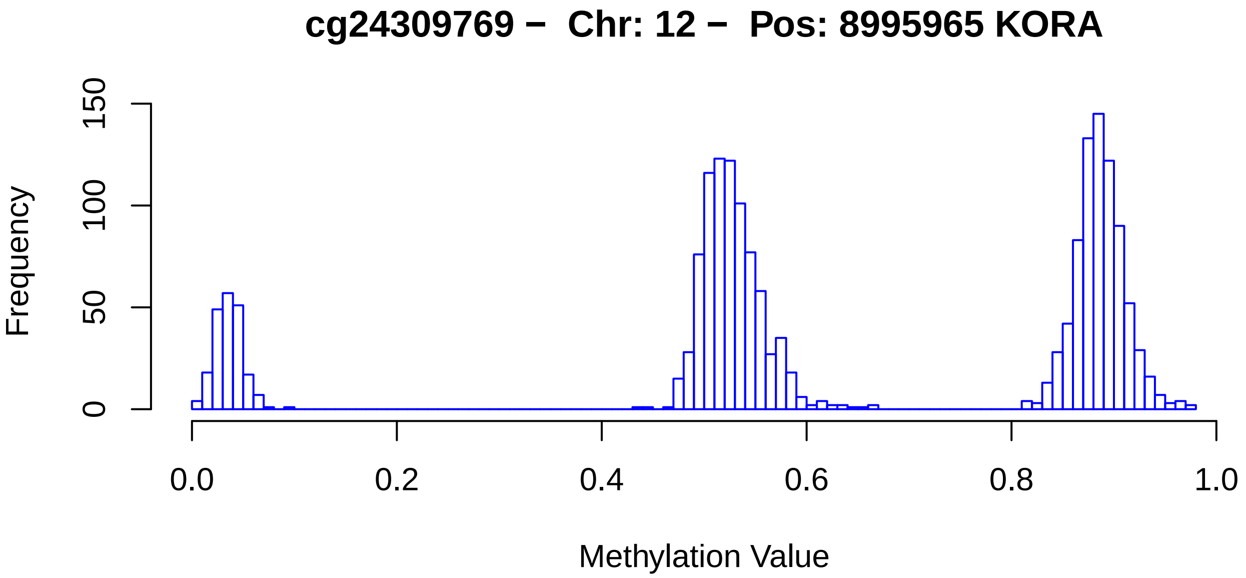
cg24309769 − Chr: 12 − Pos: 8995965 KORA
Frequency
0
50
100
150
0.0
0.2
0.4
0.6
0.8
1.0
Methylation Value

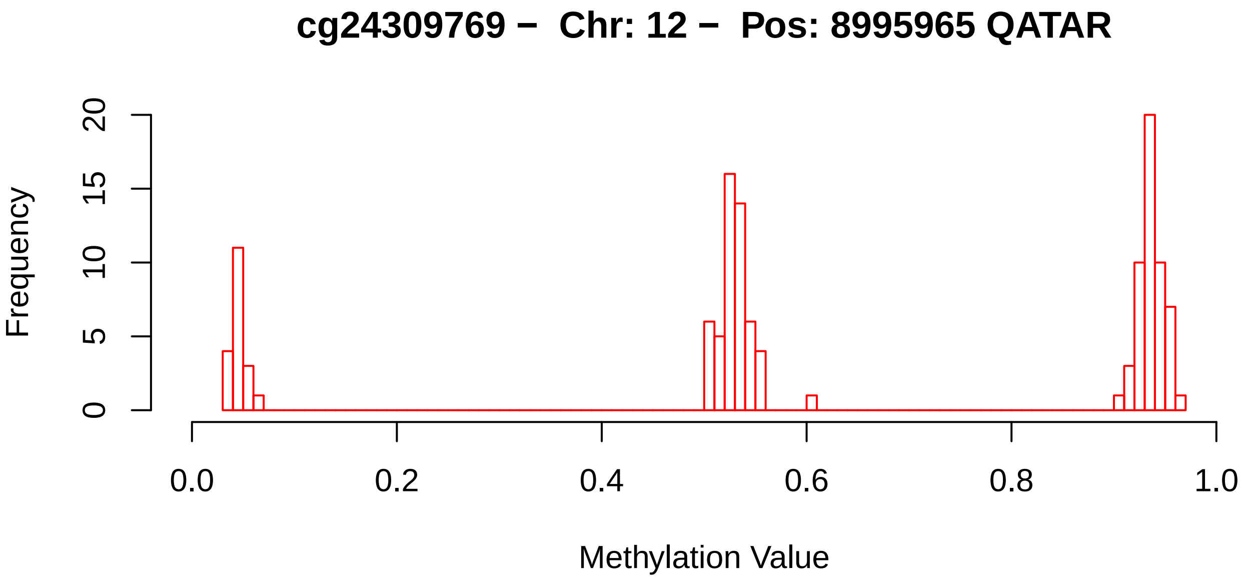
cg24309769 − Chr: 12 − Pos: 8995965 QATAR
Frequency
0
5
10
15
20
0.0
0.2
0.4
0.6
0.8
1.0
Methylation Value

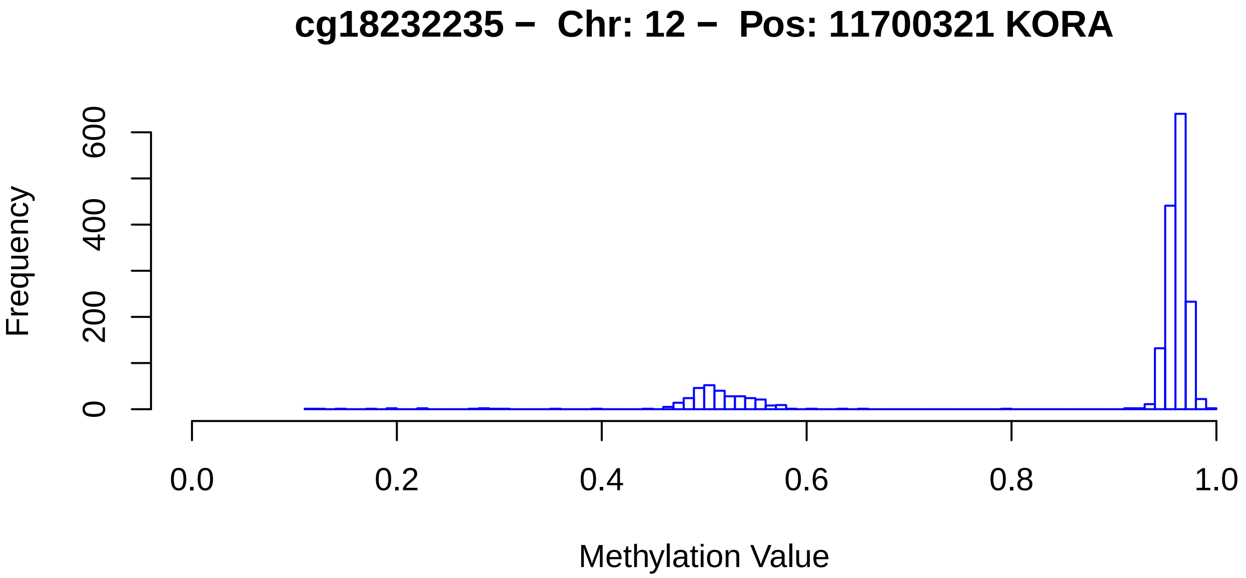
cg18232235 − Chr: 12 − Pos: 11700321 KORA
Frequency
0
200
400
600
0.0
0.2
0.4
0.6
0.8
1.0
Methylation Value

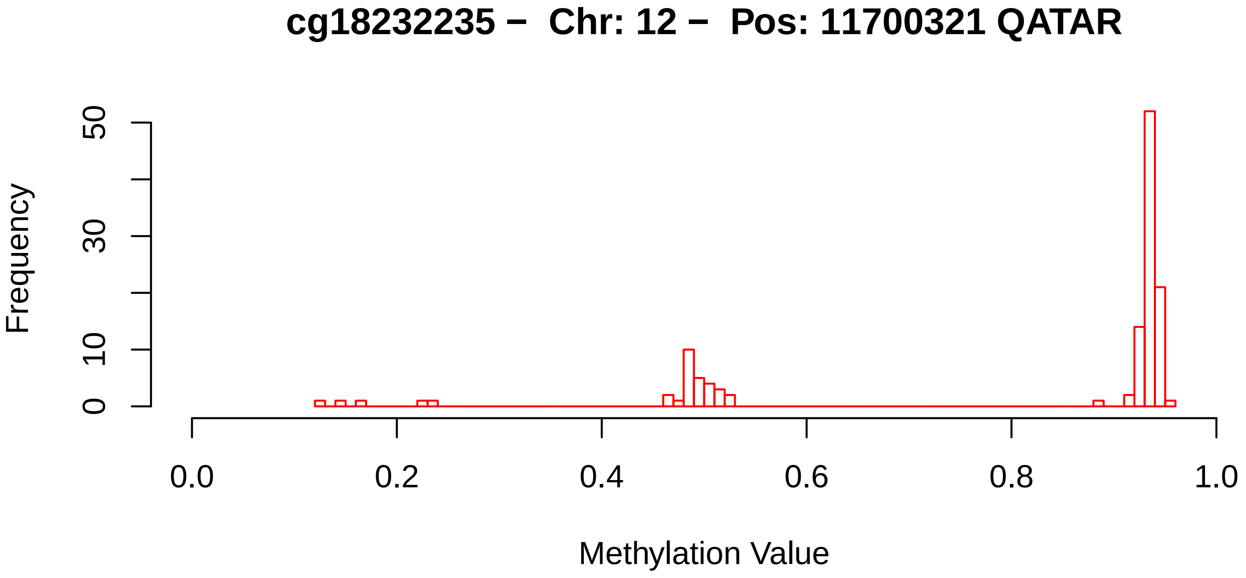
cg18232235 − Chr: 12 − Pos: 11700321 QATAR
Frequency
0
10
30
50
0.0
0.2
0.4
0.6
0.8
1.0
Methylation Value

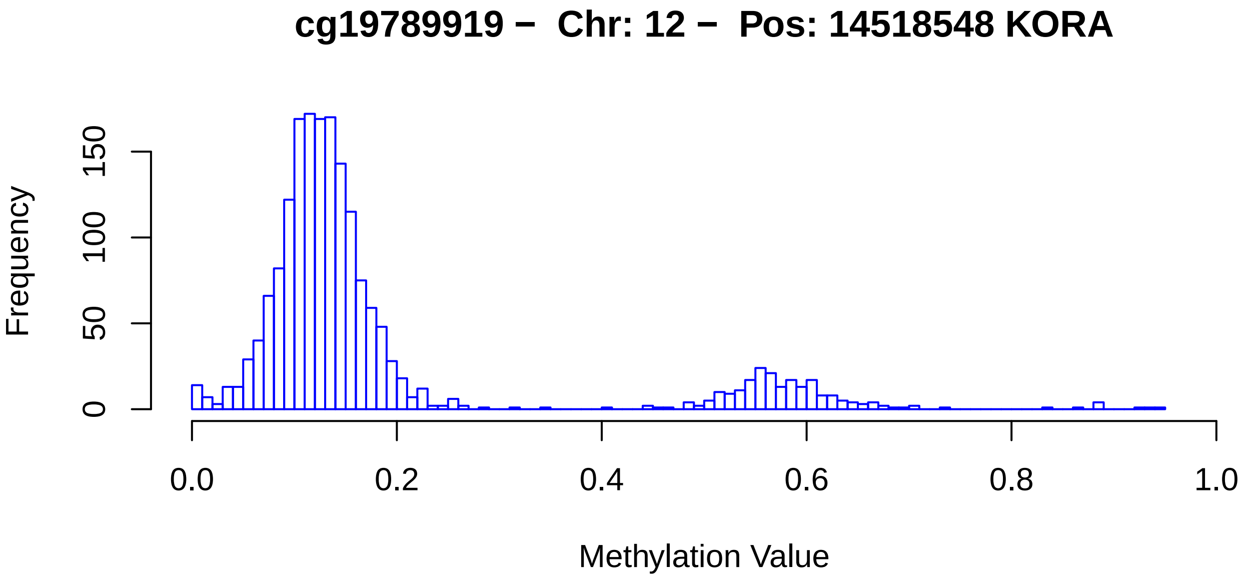
cg19789919 − Chr: 12 − Pos: 14518548 KORA
Frequency
0
50
100
150
0.0
0.2
0.4
0.6
0.8
1.0
Methylation Value

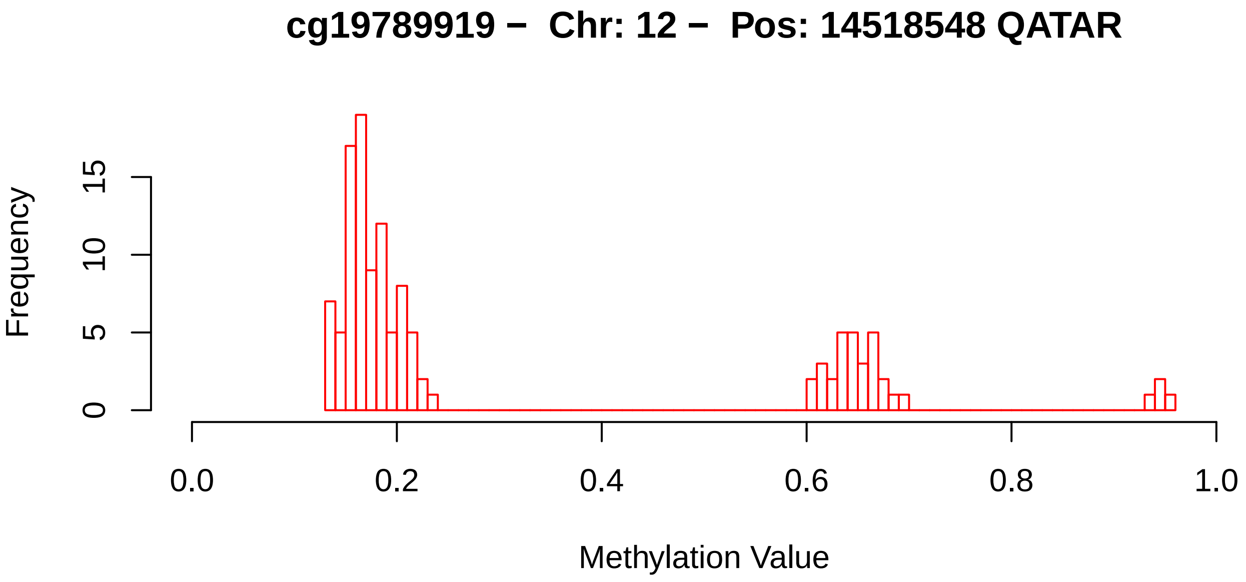
cg19789919 − Chr: 12 − Pos: 14518548 QATAR
Frequency
0
5
10
15
0.0
0.2
0.4
0.6
0.8
1.0
Methylation Value

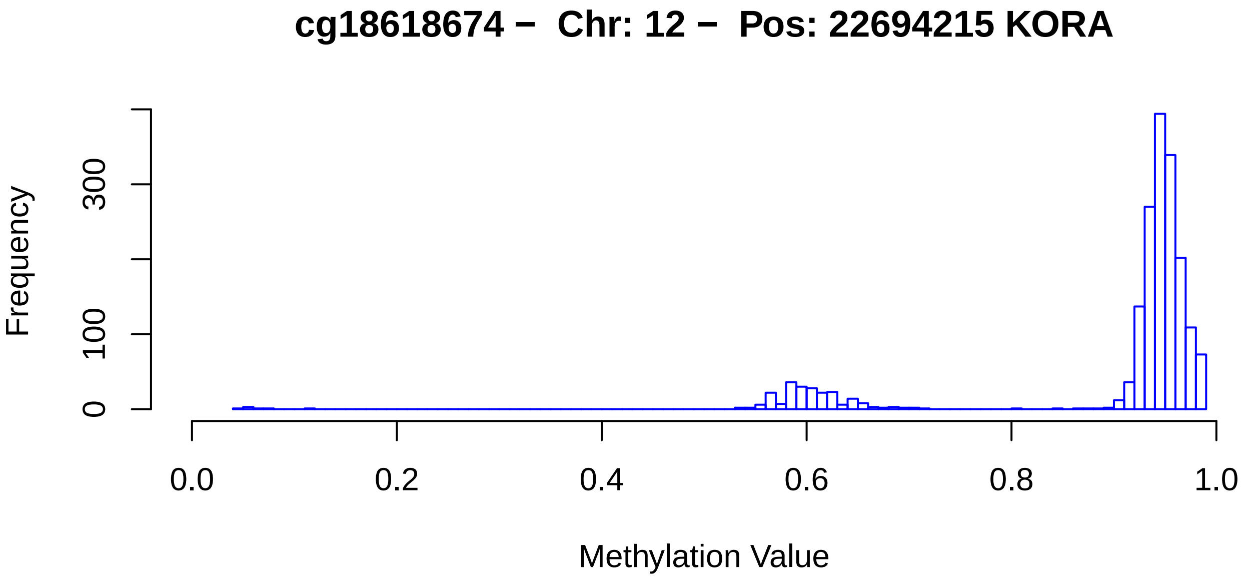
cg18618674 − Chr: 12 − Pos: 22694215 KORA
Frequency
0
100
300
0.0
0.2
0.4
0.6
0.8
1.0
Methylation Value

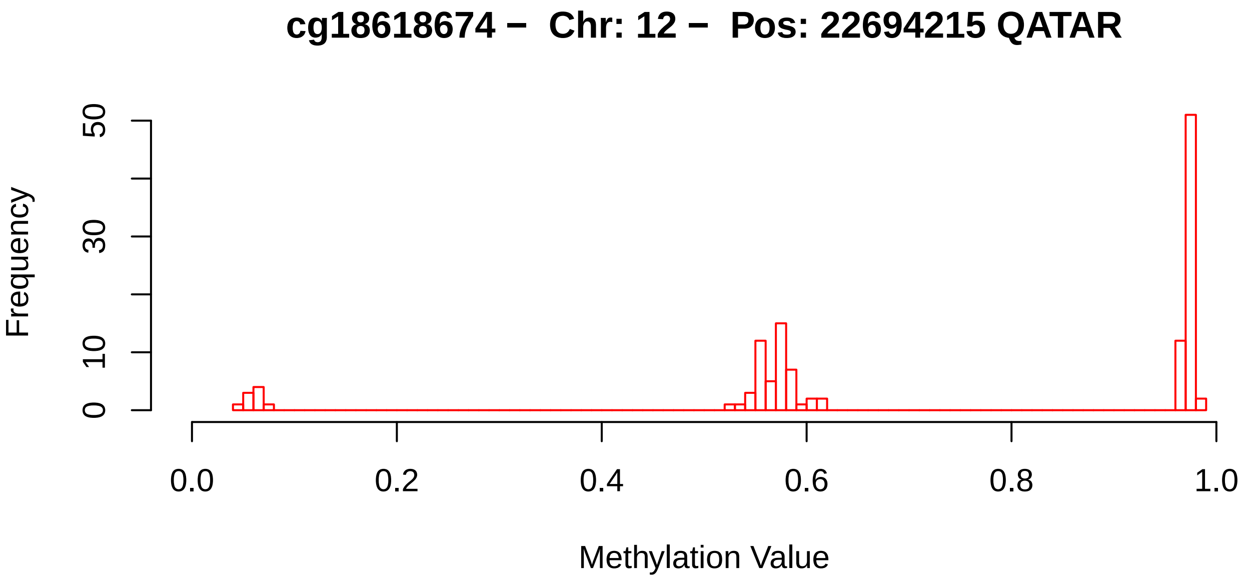
cg18618674 − Chr: 12 − Pos: 22694215 QATAR
Frequency
0
10
30
50
0.0
0.2
0.4
0.6
0.8
1.0
Methylation Value

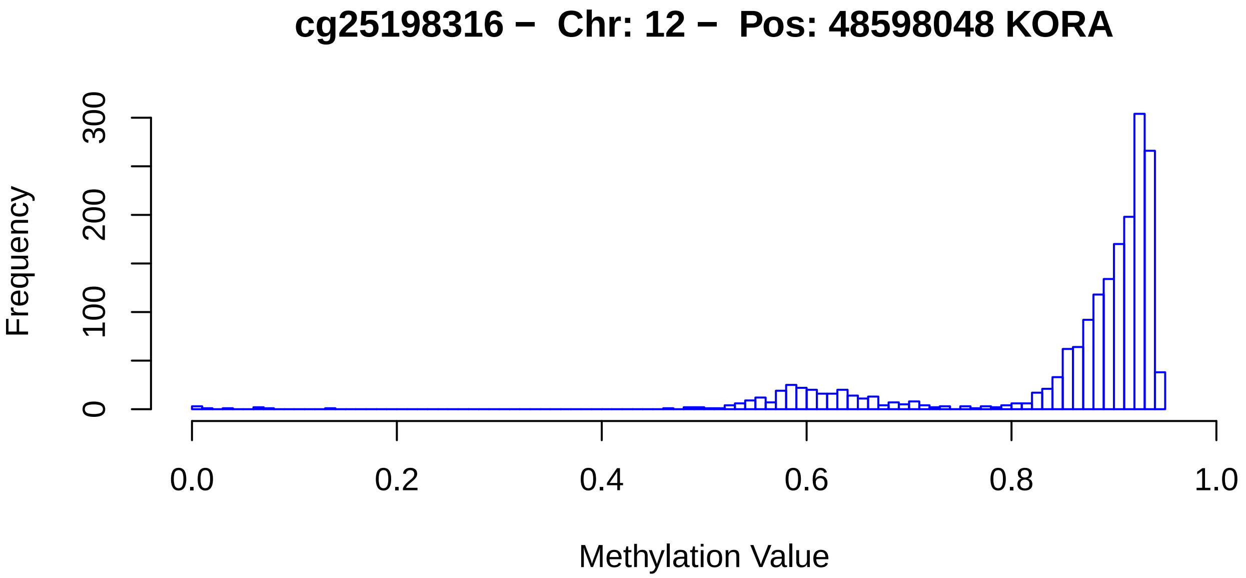
cg25198316 − Chr: 12 − Pos: 48598048 KORA
Frequency
0
100
200
300
0.0
0.2
0.4
0.6
0.8
1.0
Methylation Value

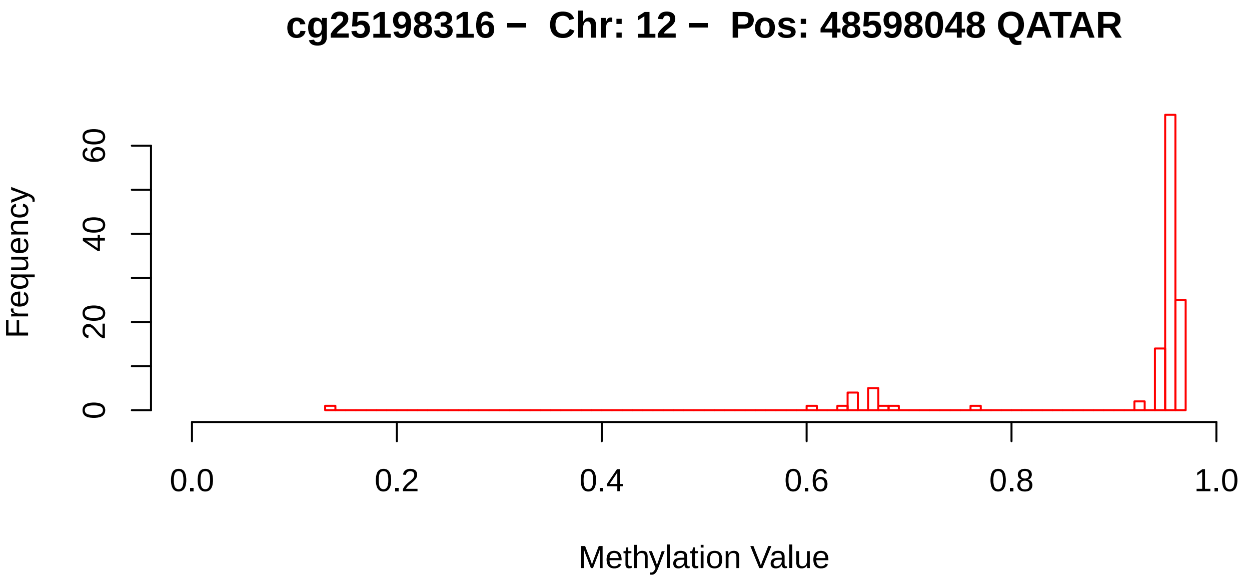
cg25198316 − Chr: 12 − Pos: 48598048 QATAR
Frequency
0
20
40
60
0.0
0.2
0.4
0.6
0.8
1.0
Methylation Value

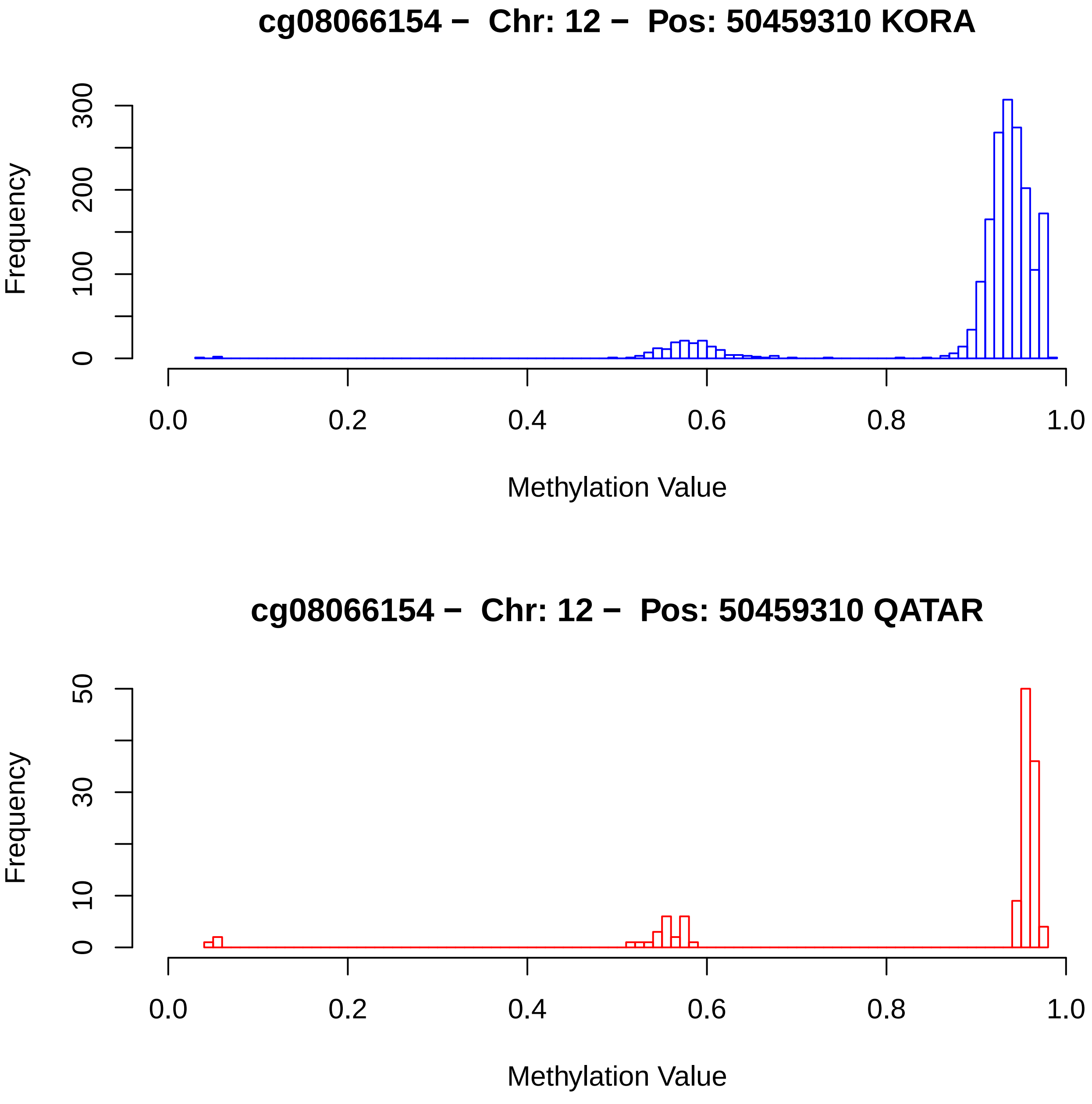
cg08066154 − Chr: 12 − Pos: 50459310 KORA
Frequency
0
100
200
300
0.0
0.2
0.4
0.6
0.8
1.0
Methylation Value
cg08066154 − Chr: 12 − Pos: 50459310 QATAR
Frequency
0
10
30
50
0.0
0.2
0.4
0.6
0.8
1.0
Methylation Value

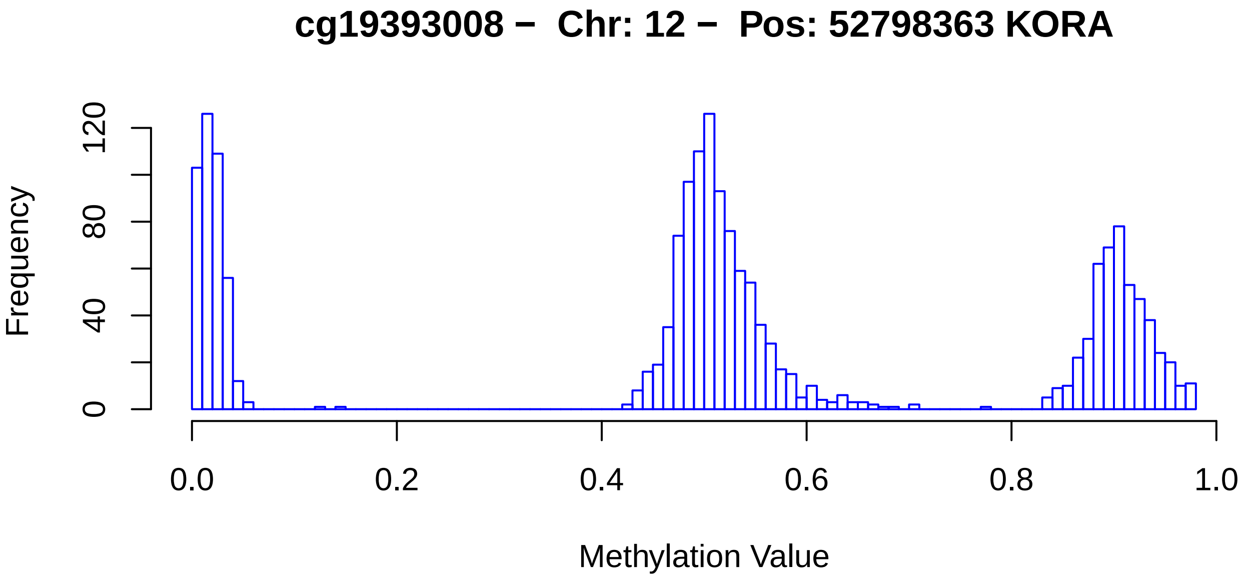
cg19393008 − Chr: 12 − Pos: 52798363 KORA
Frequency
0
40
80
120
0.0
0.2
0.4
0.6
0.8
1.0
Methylation Value

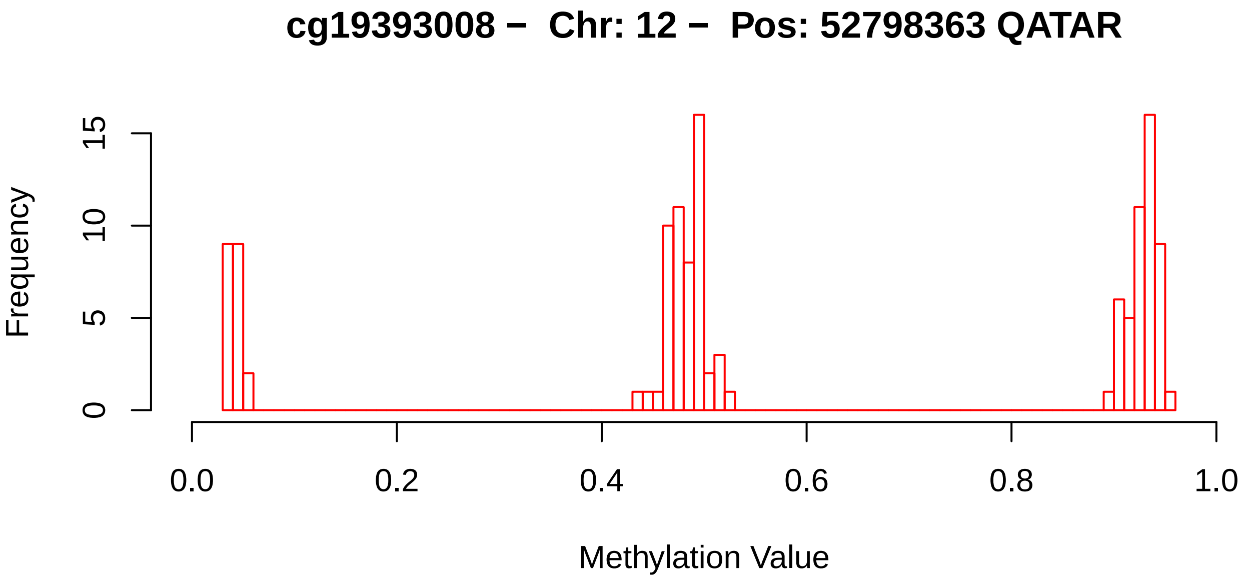
cg19393008 − Chr: 12 − Pos: 52798363 QATAR
Frequency
0
5
10
15
0.0
0.2
0.4
0.6
0.8
1.0
Methylation Value

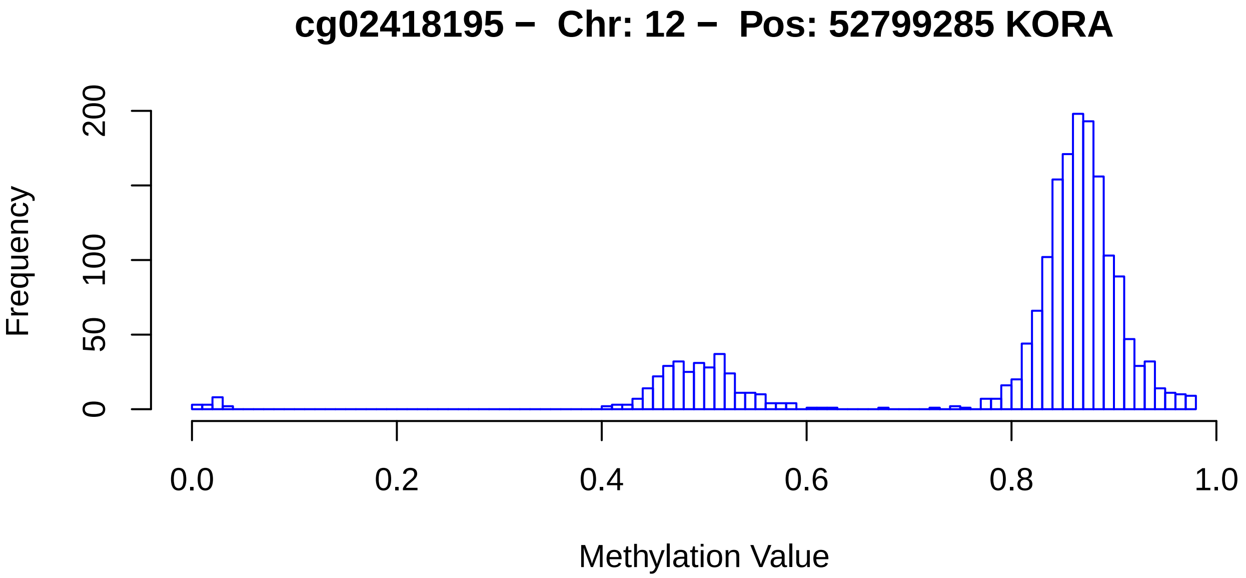

cg02418195 − Chr: 12 − Pos: 52799285 KORA
Frequency
0
50
100
200
0.0
0.2
0.4
0.6
0.8
1.0
Methylation Value

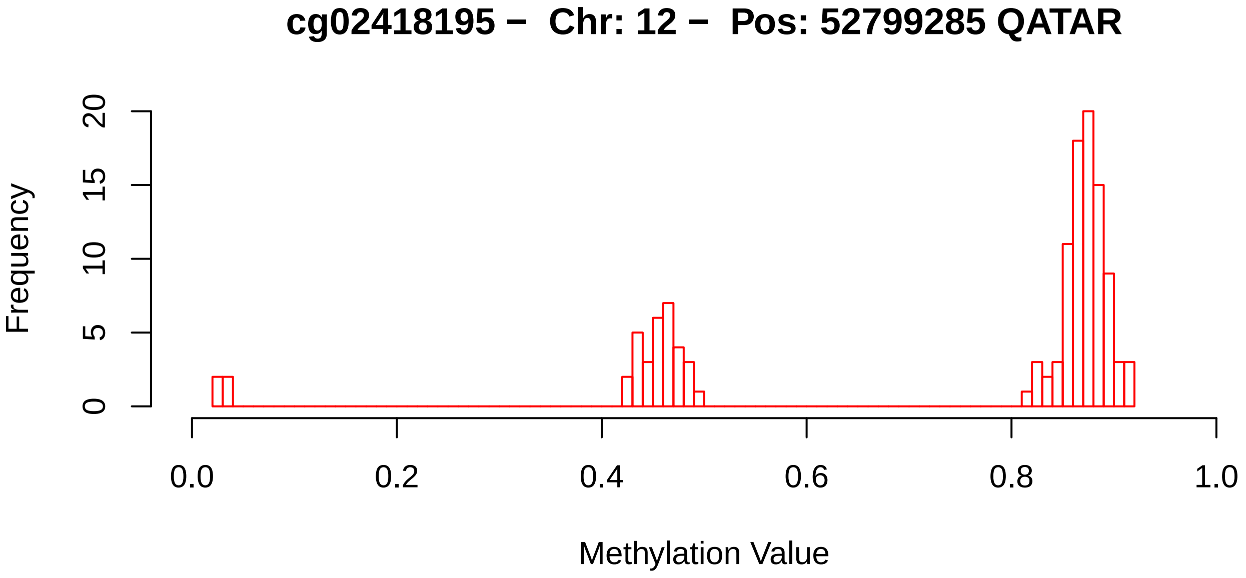

cg02418195 − Chr: 12 − Pos: 52799285 QATAR
Frequency
0
5
10
15
20
0.0
0.2
0.4
0.6
0.8
1.0
Methylation Value

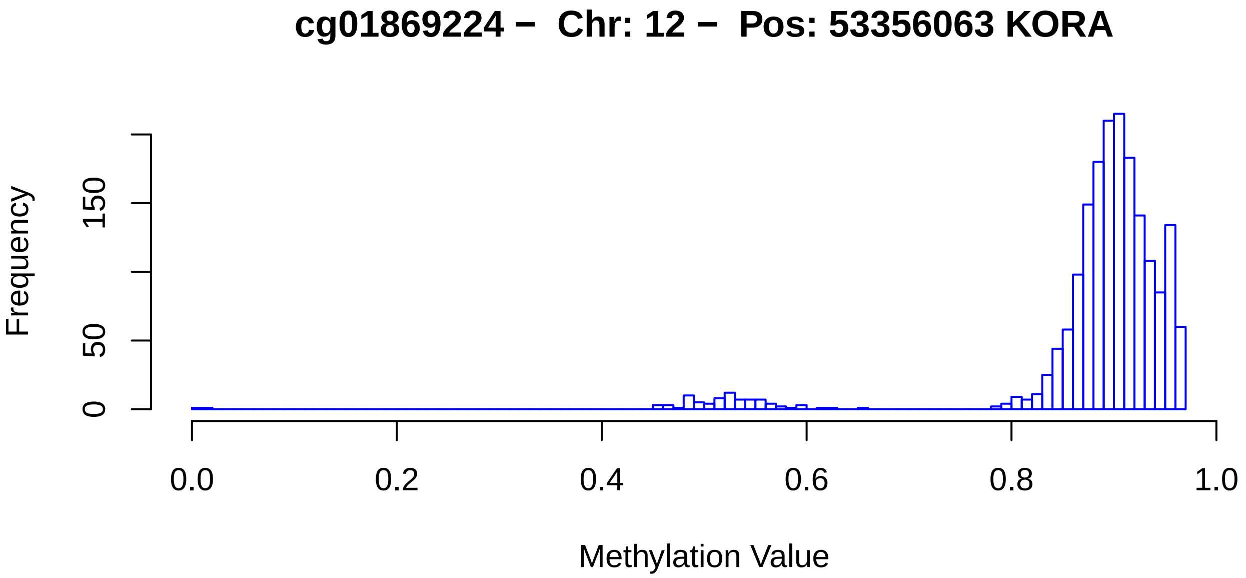
cg01869224 − Chr: 12 − Pos: 53356063 KORA
Frequency
0
50
150
0.0
0.2
0.4
0.6
0.8
1.0
Methylation Value

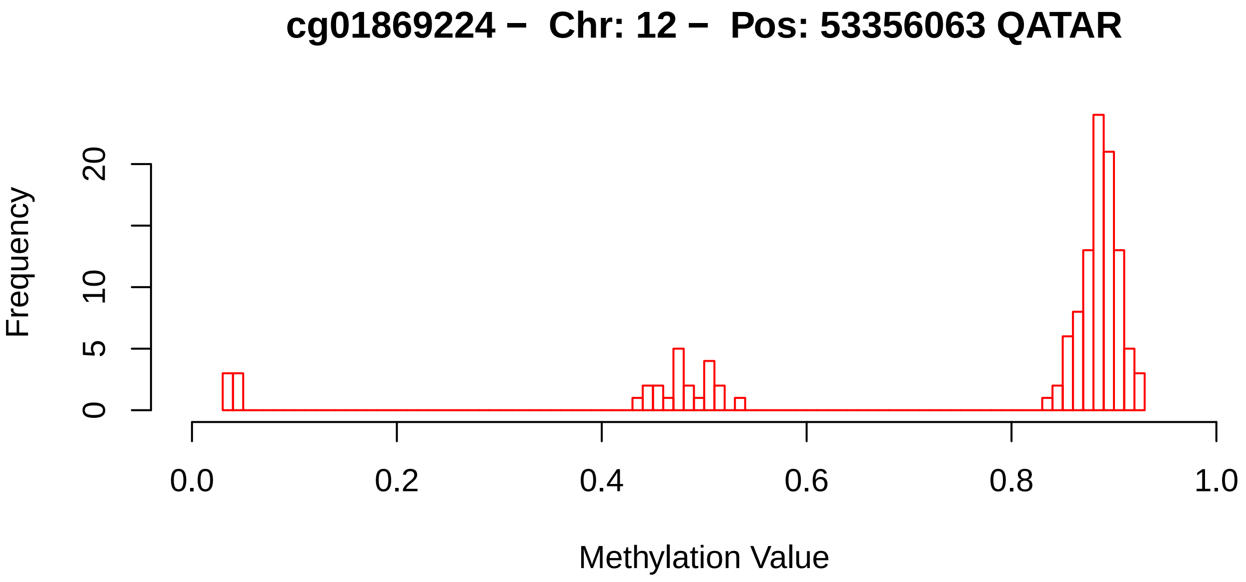
cg01869224 − Chr: 12 − Pos: 53356063 QATAR
Frequency
0
5
10
20
0.0
0.2
0.4
0.6
0.8
1.0
Methylation Value

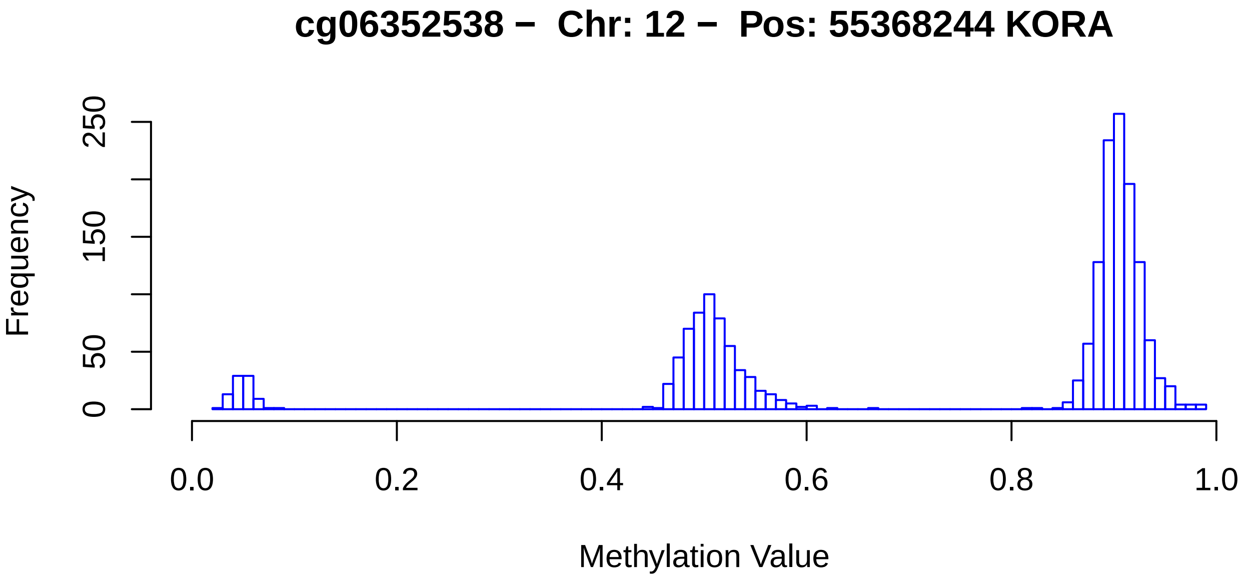
cg06352538 − Chr: 12 − Pos: 55368244 KORA
Frequency
0
50
150
250
0.0
0.2
0.4
0.6
0.8
1.0
Methylation Value

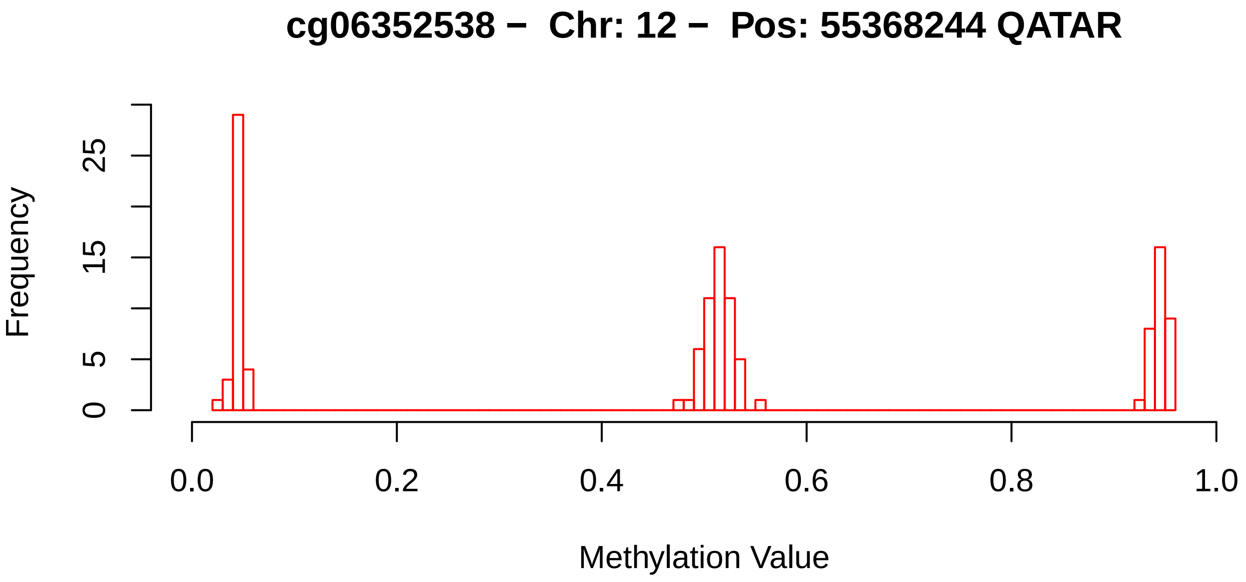
cg06352538 − Chr: 12 − Pos: 55368244 QATAR
Frequency
0
5
15
25
0.0
0.2
0.4
0.6
0.8
1.0
Methylation Value

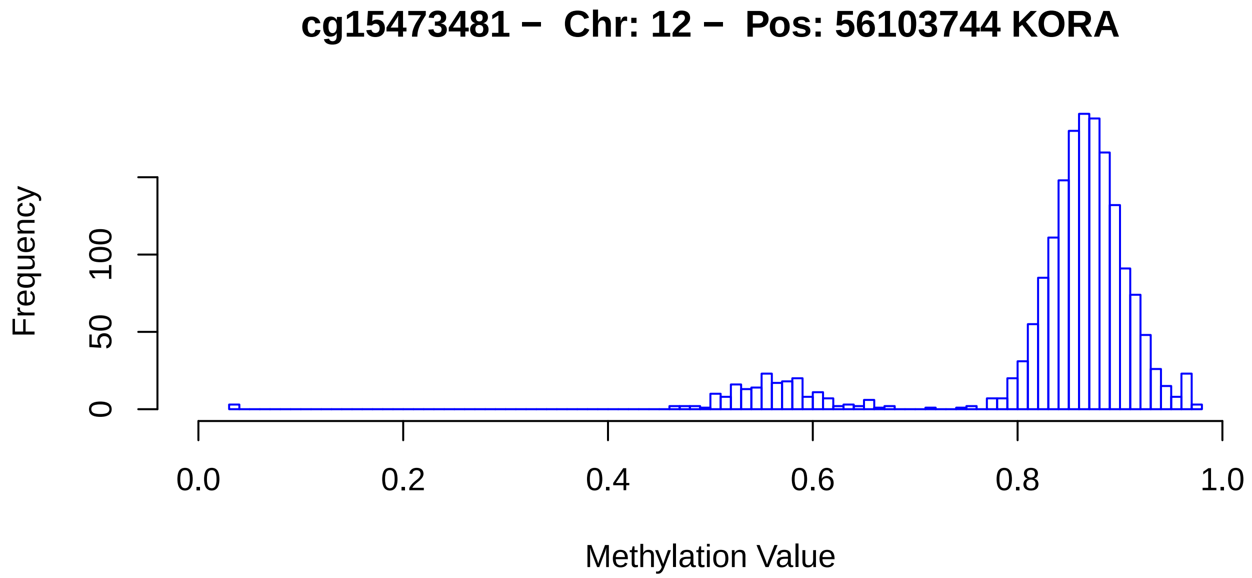
cg15473481 − Chr: 12 − Pos: 56103744 KORA
Frequency
0
50
100
0.0
0.2
0.4
0.6
0.8
1.0
Methylation Value

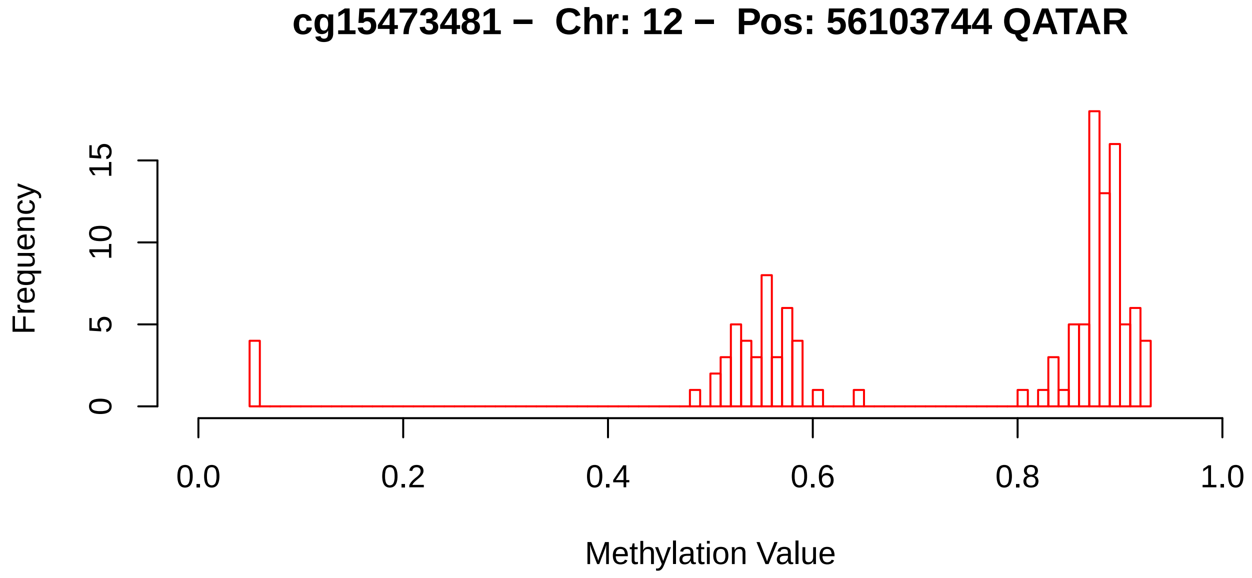
cg15473481 − Chr: 12 − Pos: 56103744 QATAR
Frequency
0
5
10
15
0.0
0.2
0.4
0.6
0.8
1.0
Methylation Value

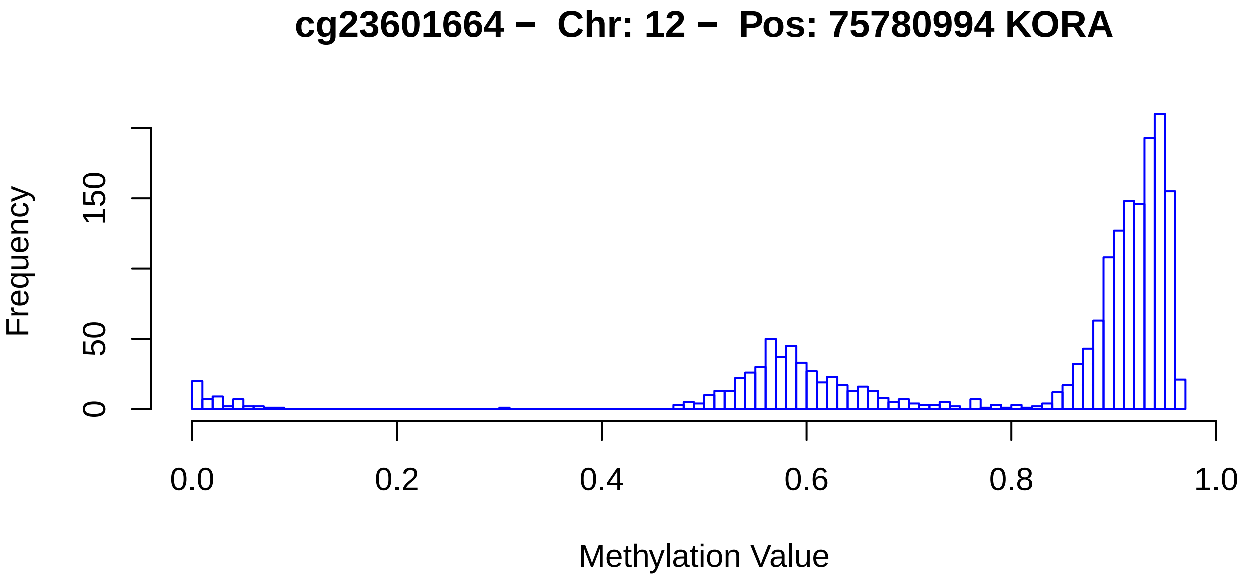
cg23601664 − Chr: 12 − Pos: 75780994 KORA
Frequency
0
50
150
0.0
0.2
0.4
0.6
0.8
1.0
Methylation Value

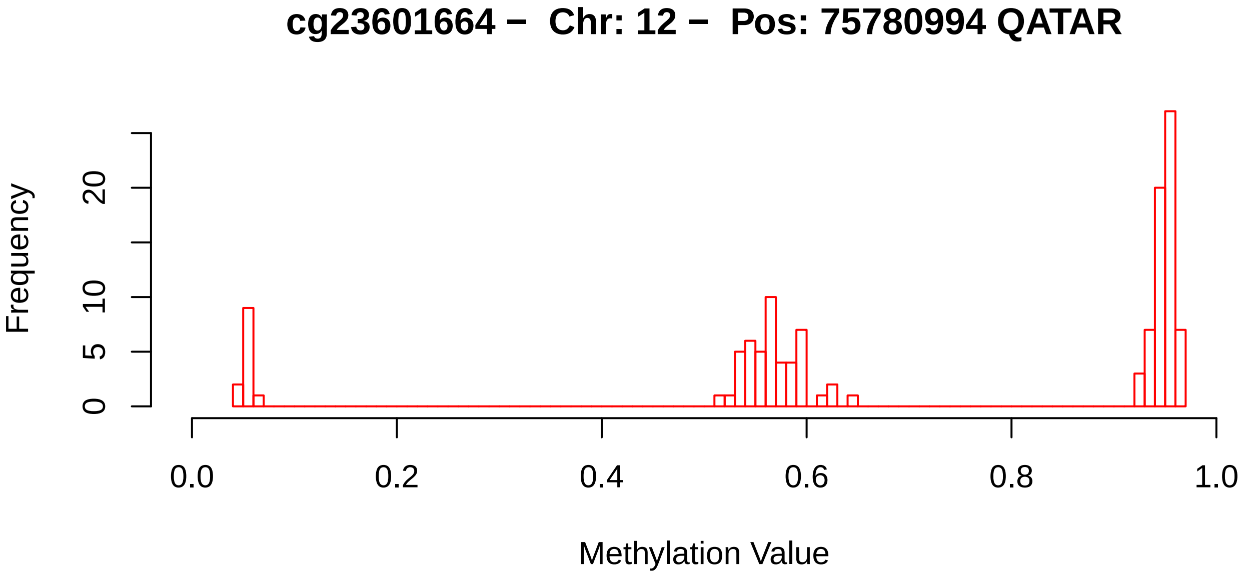
cg23601664 − Chr: 12 − Pos: 75780994 QATAR
Frequency
0
5
10
20
0.0
0.2
0.4
0.6
0.8
1.0
Methylation Value

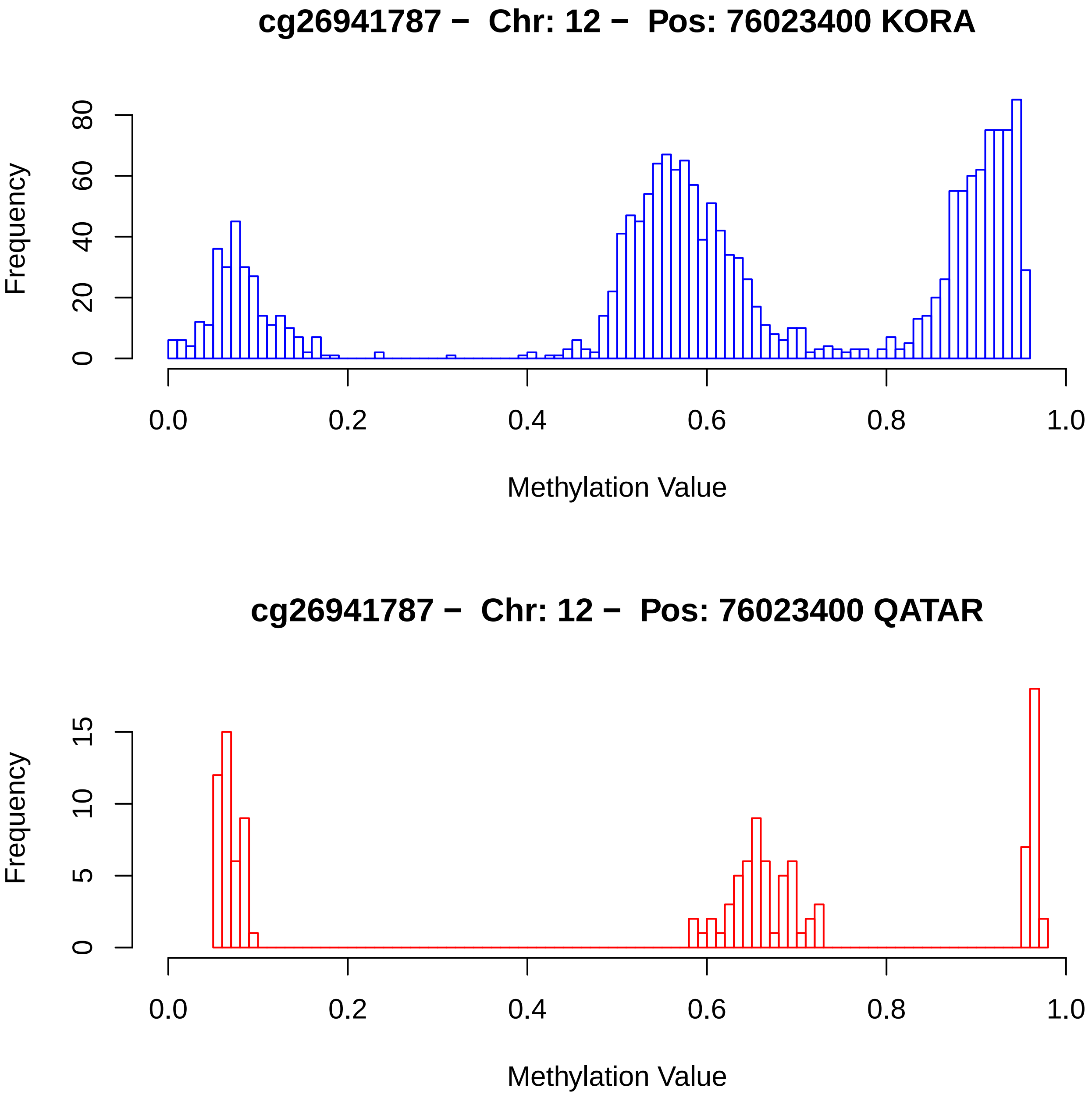
cg26941787 − Chr: 12 − Pos: 76023400 KORA
Frequency
0
20
40
60
80
0.0
0.2
0.4
0.6
0.8
1.0
Methylation Value
cg26941787 − Chr: 12 − Pos: 76023400 QATAR
Frequency
0
5
10
15
0.0
0.2
0.4
0.6
0.8
1.0
Methylation Value

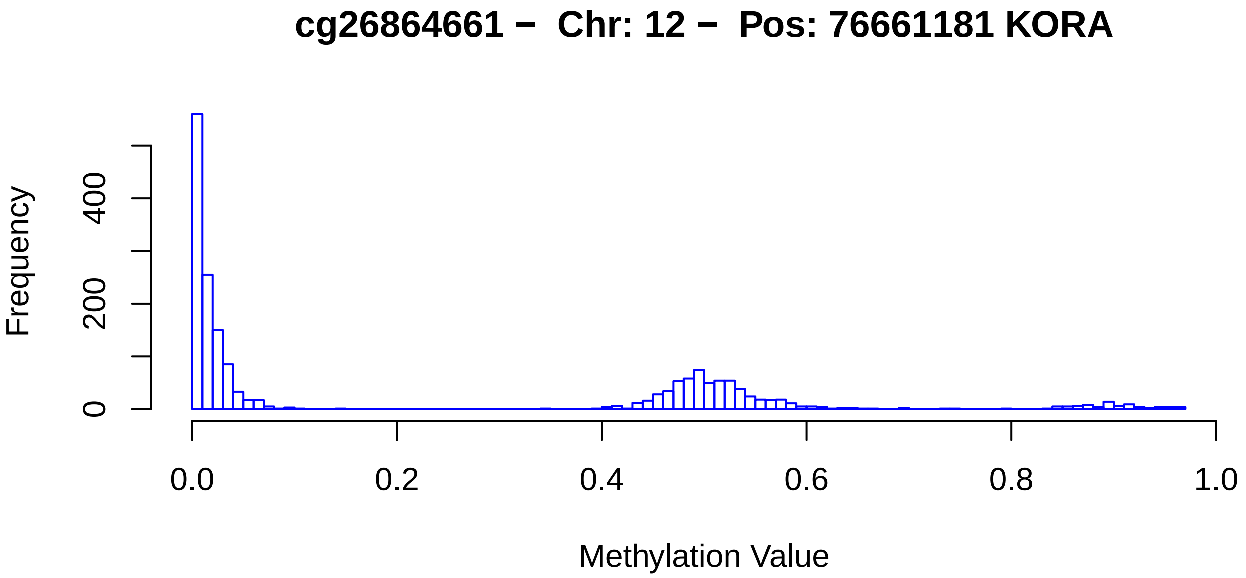
cg26864661 − Chr: 12 − Pos: 76661181 KORA
Frequency
0
200
400
0.0
0.2
0.4
0.6
0.8
1.0
Methylation Value

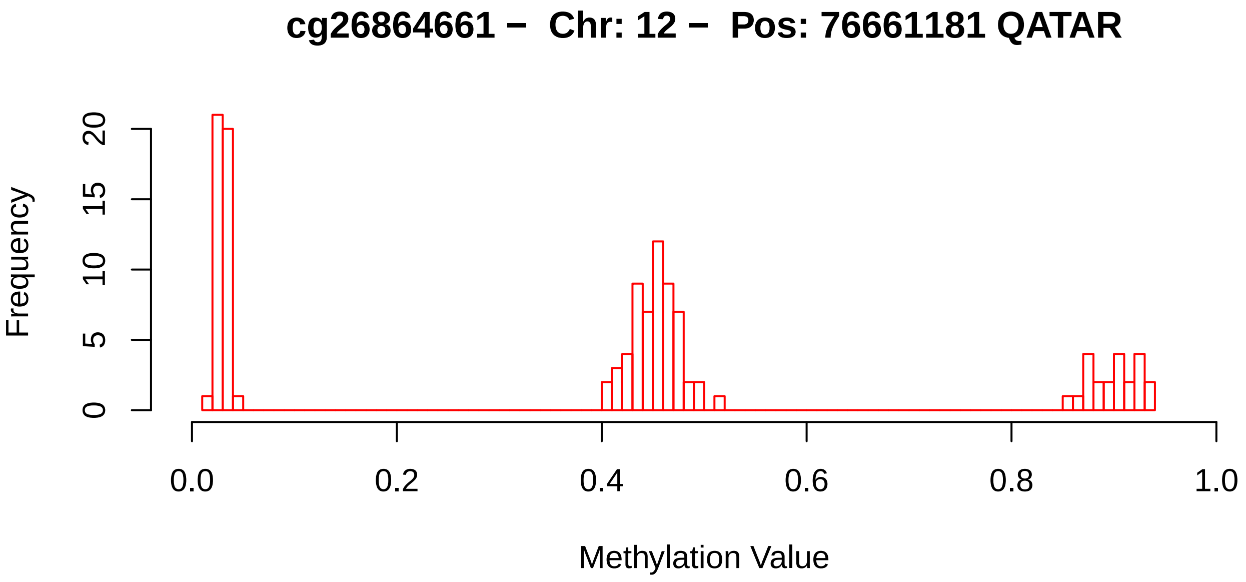
cg26864661 − Chr: 12 − Pos: 76661181 QATAR
Frequency
0
5
10
15
20
0.0
0.2
0.4
0.6
0.8
1.0
Methylation Value

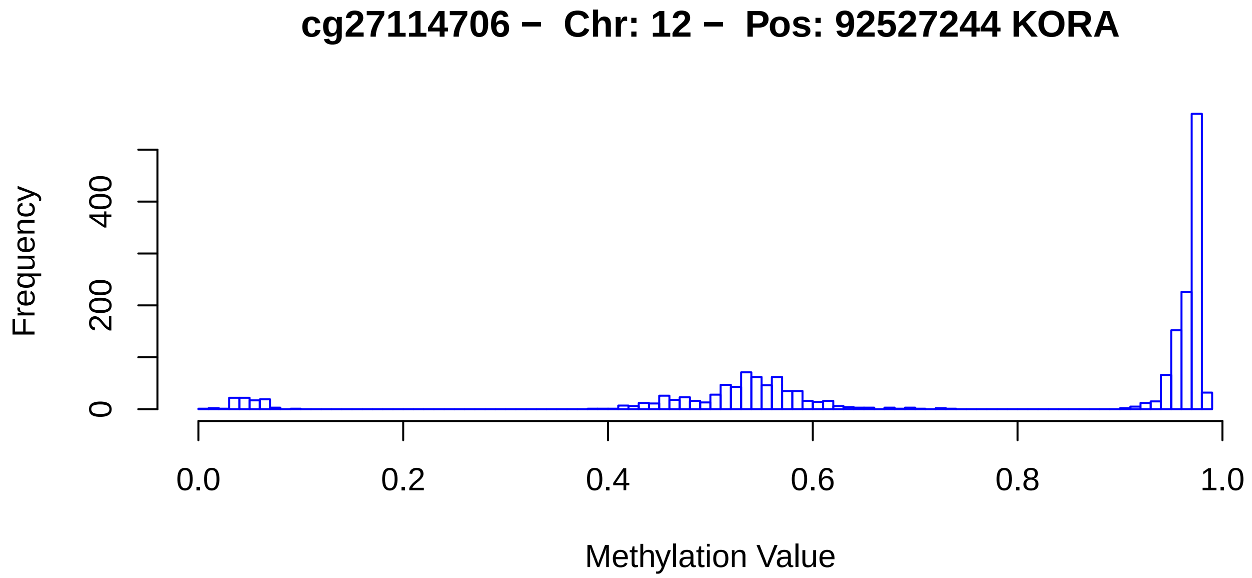

cg27114706 − Chr: 12 − Pos: 92527244 KORA
Frequency
0
200
400
0.0
0.2
0.4
0.6
0.8
1.0
Methylation Value

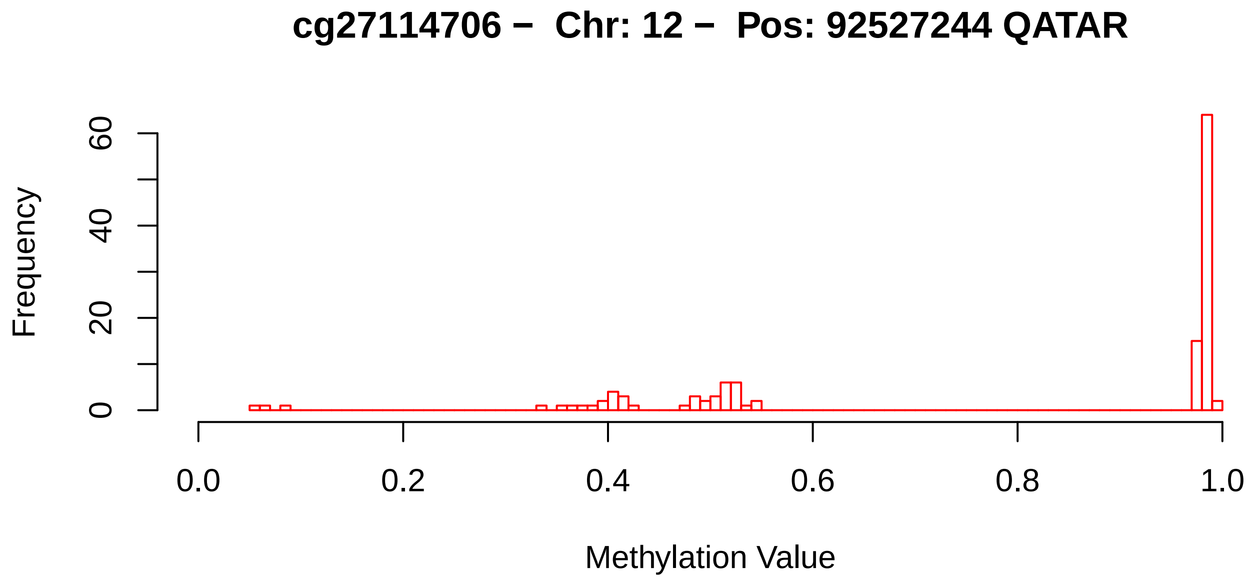

cg27114706 − Chr: 12 − Pos: 92527244 QATAR
Frequency
0
20
40
60
0.0
0.2
0.4
0.6
0.8
1.0
Methylation Value

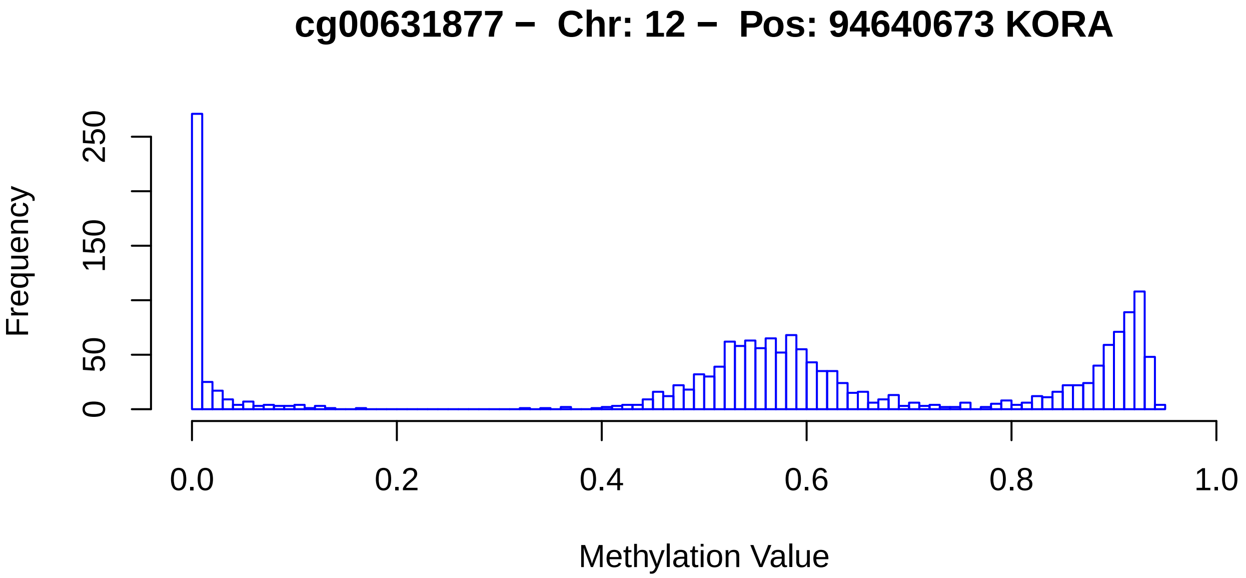
cg00631877 − Chr: 12 − Pos: 94640673 KORA
Frequency
0
50
150
250
0.0
0.2
0.4
0.6
0.8
1.0
Methylation Value

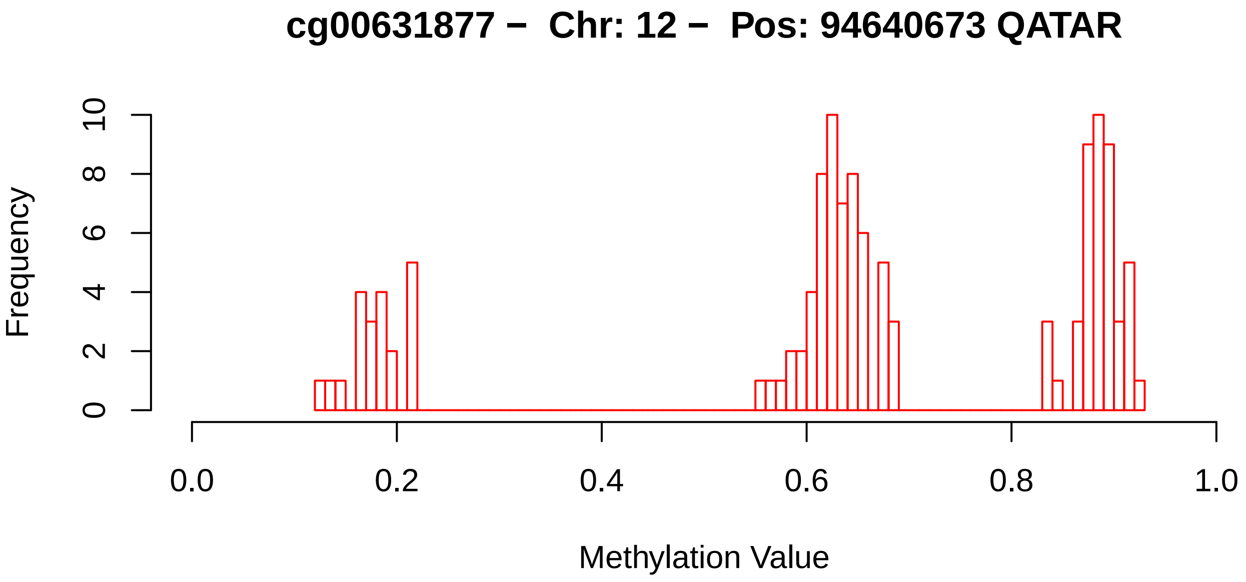
cg00631877 − Chr: 12 − Pos: 94640673 QATAR
Frequency
0
2
4
6
8
10
0.0
0.2
0.4
0.6
0.8
1.0
Methylation Value

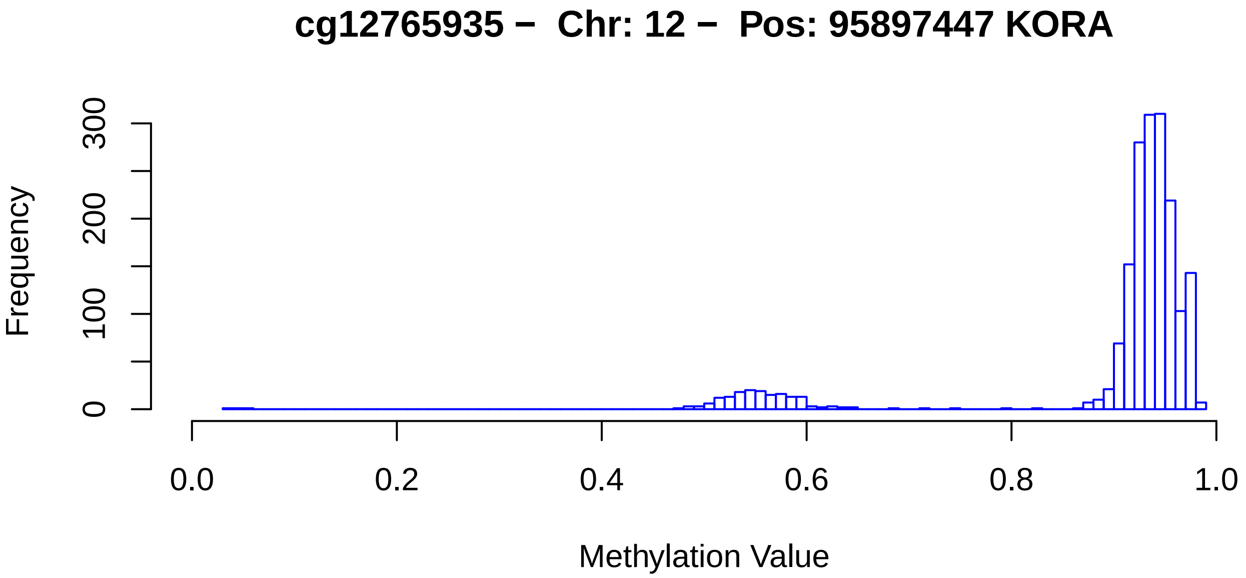
cg12765935 − Chr: 12 − Pos: 95897447 KORA
Frequency
0
100
200
300
0.0
0.2
0.4
0.6
0.8
1.0
Methylation Value

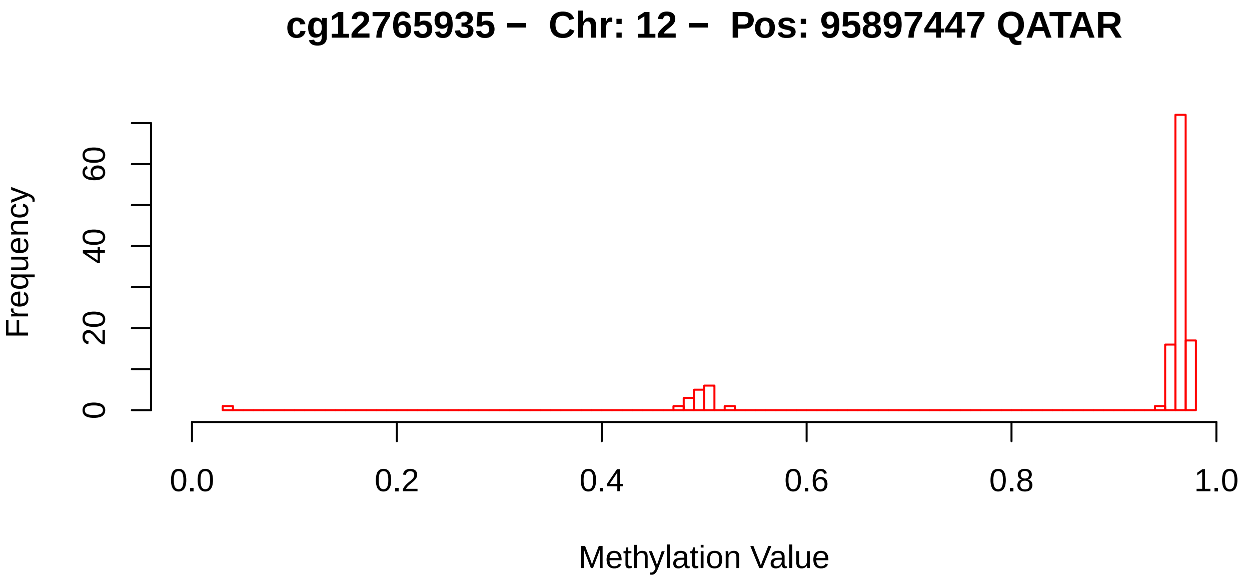
cg12765935 − Chr: 12 − Pos: 95897447 QATAR
Frequency
0
20
40
60
0.0
0.2
0.4
0.6
0.8
1.0
Methylation Value

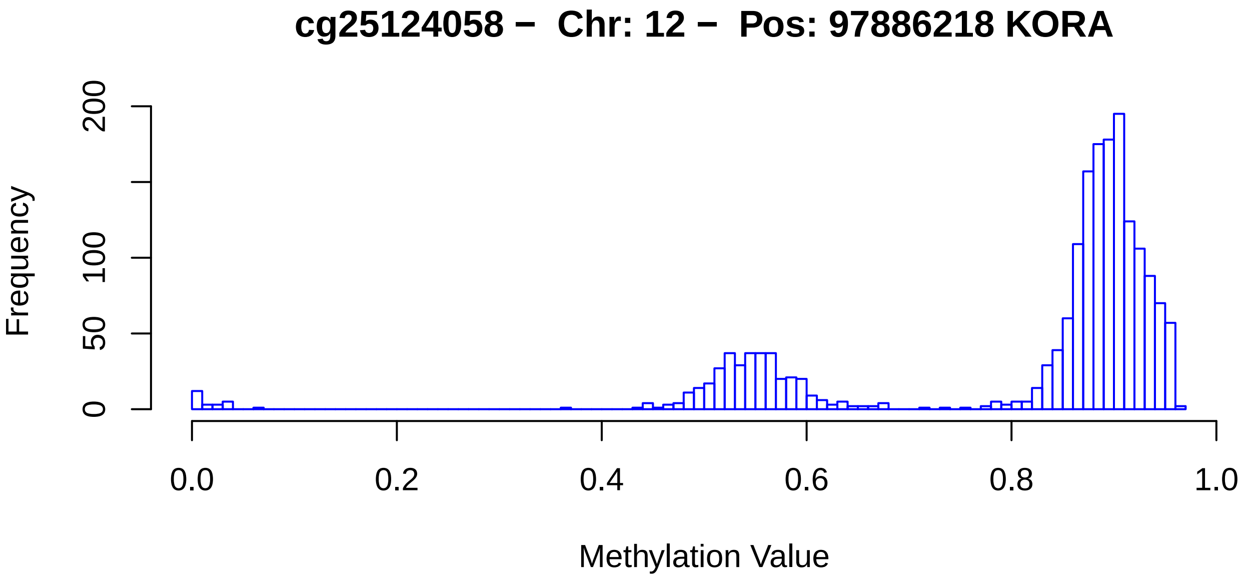
cg25124058 − Chr: 12 − Pos: 97886218 KORA
Frequency
0
50
100
200
0.0
0.2
0.4
0.6
0.8
1.0
Methylation Value

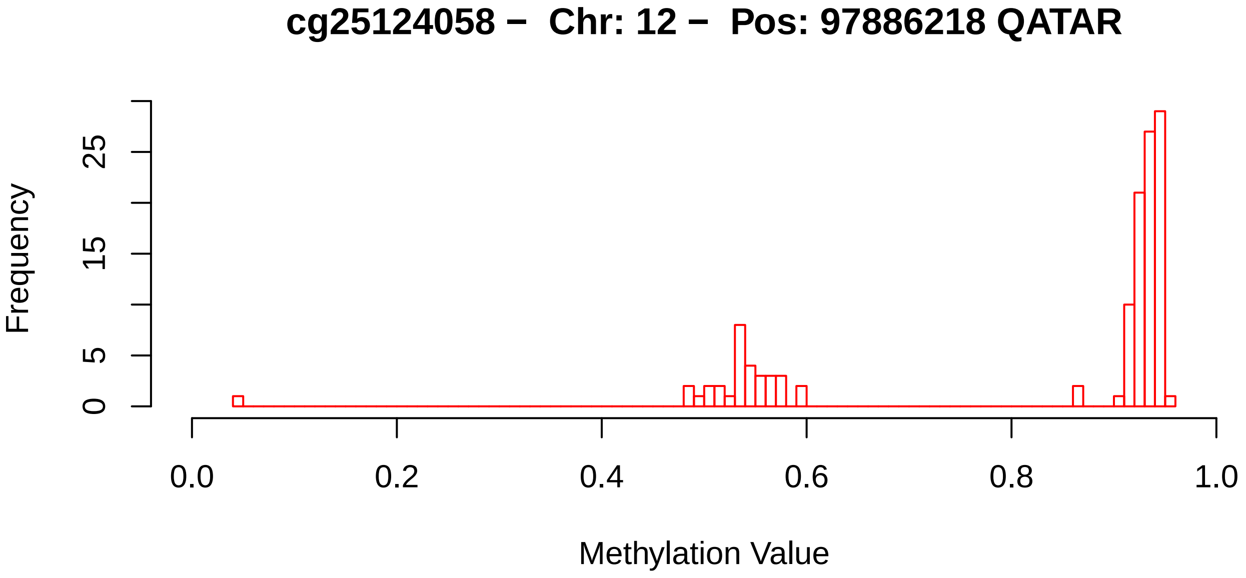
cg25124058 − Chr: 12 − Pos: 97886218 QATAR
Frequency
0
5
15
25
0.0
0.2
0.4
0.6
0.8
1.0
Methylation Value

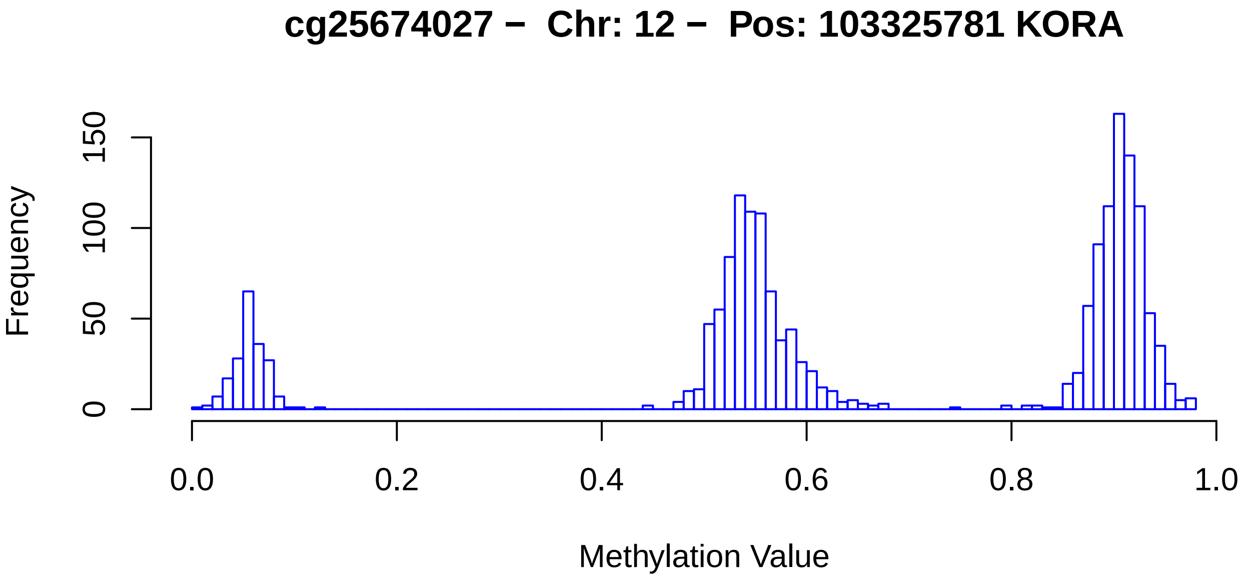
cg25674027 − Chr: 12 − Pos: 103325781 KORA
Frequency
0
50
100
150
0.0
0.2
0.4
0.6
0.8
1.0
Methylation Value

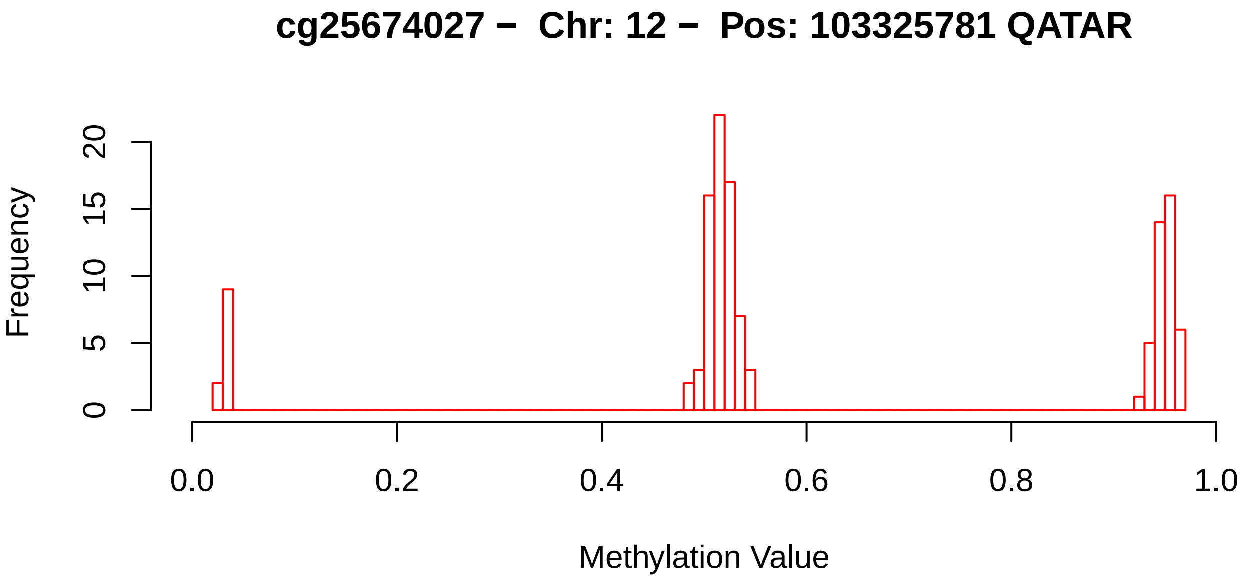
cg25674027 − Chr: 12 − Pos: 103325781 QATAR
Frequency
0
5
10
15
20
0.0
0.2
0.4
0.6
0.8
1.0
Methylation Value

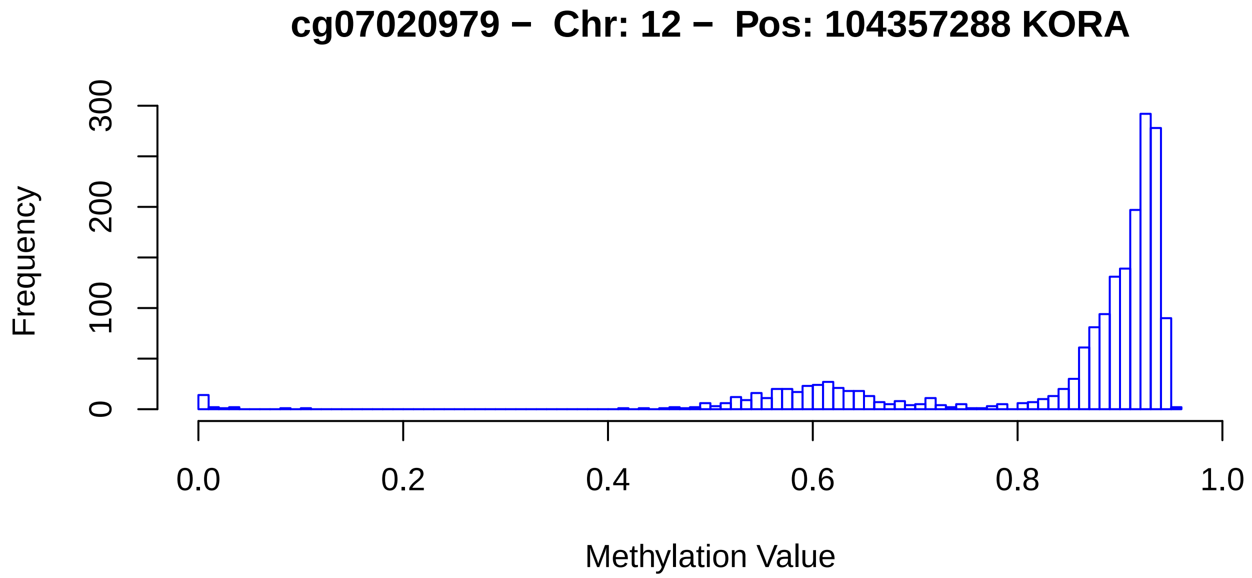
cg07020979 − Chr: 12 − Pos: 104357288 KORA
Frequency
0
100
200
300
0.0
0.2
0.4
0.6
0.8
1.0
Methylation Value

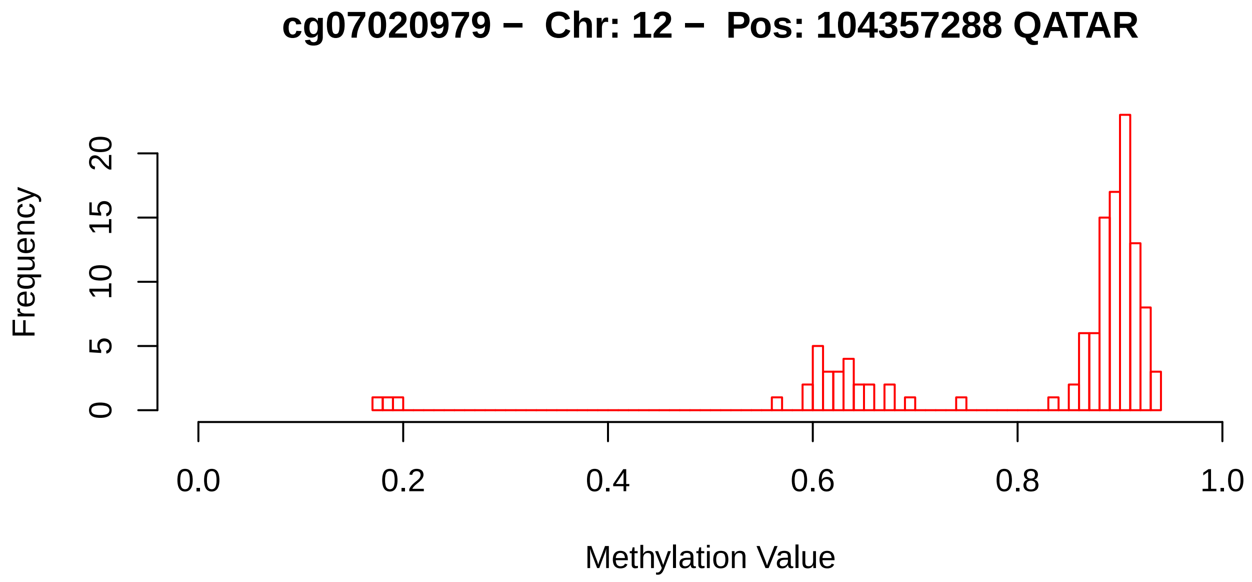
cg07020979 − Chr: 12 − Pos: 104357288 QATAR
Frequency
0
5
10
15
20
0.0
0.2
0.4
0.6
0.8
1.0
Methylation Value

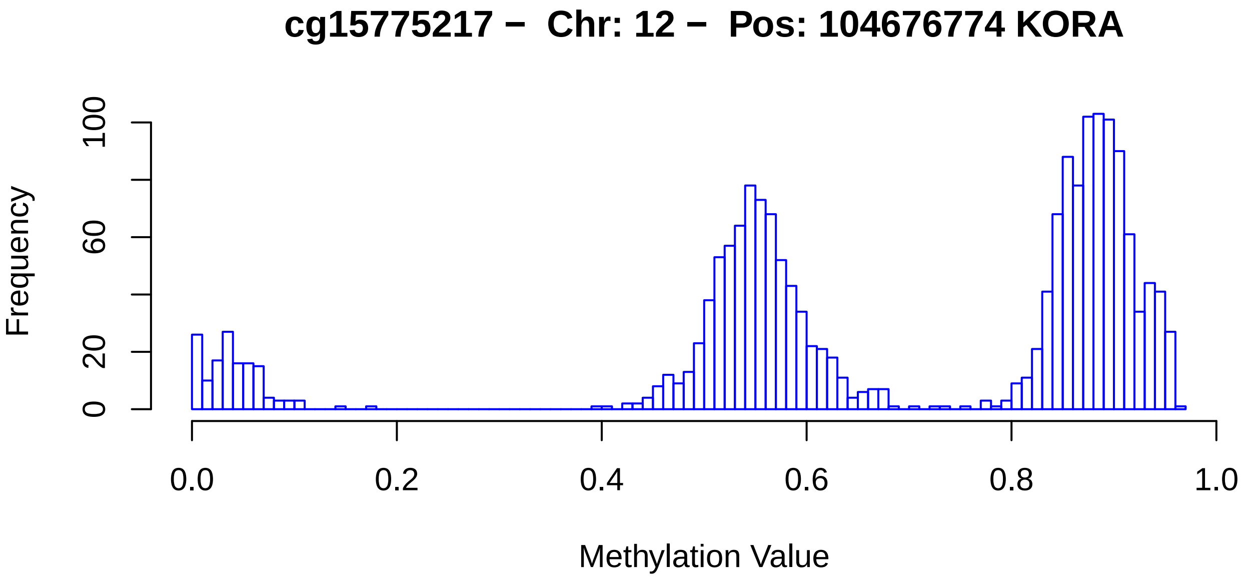
cg15775217 − Chr: 12 − Pos: 104676774 KORA
Frequency
0
20
60
100
0.0
0.2
0.4
0.6
0.8
1.0
Methylation Value

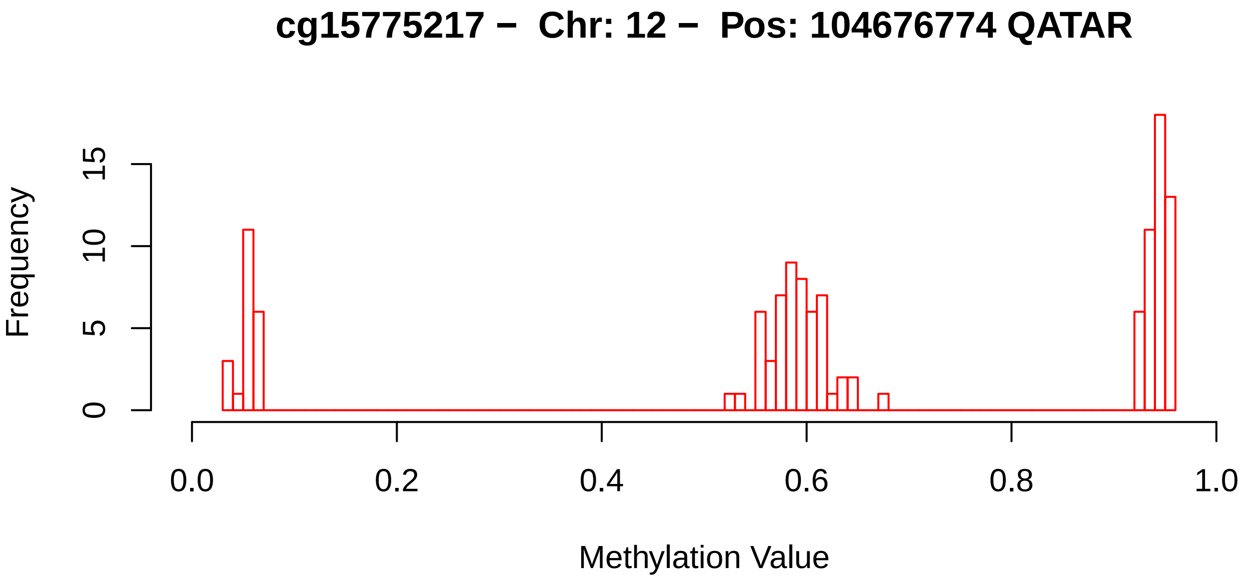
cg15775217 − Chr: 12 − Pos: 104676774 QATAR
Frequency
0
5
10
15
0.0
0.2
0.4
0.6
0.8
1.0
Methylation Value

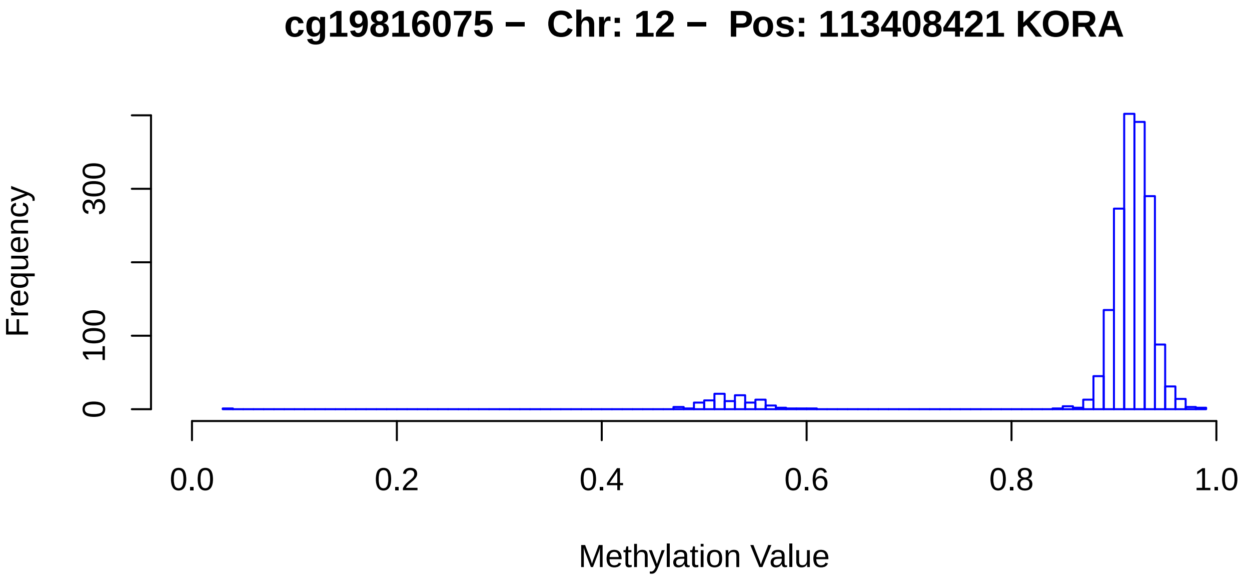
cg19816075 − Chr: 12 − Pos: 113408421 KORA
Frequency
0
100
300
0.0
0.2
0.4
0.6
0.8
1.0
Methylation Value

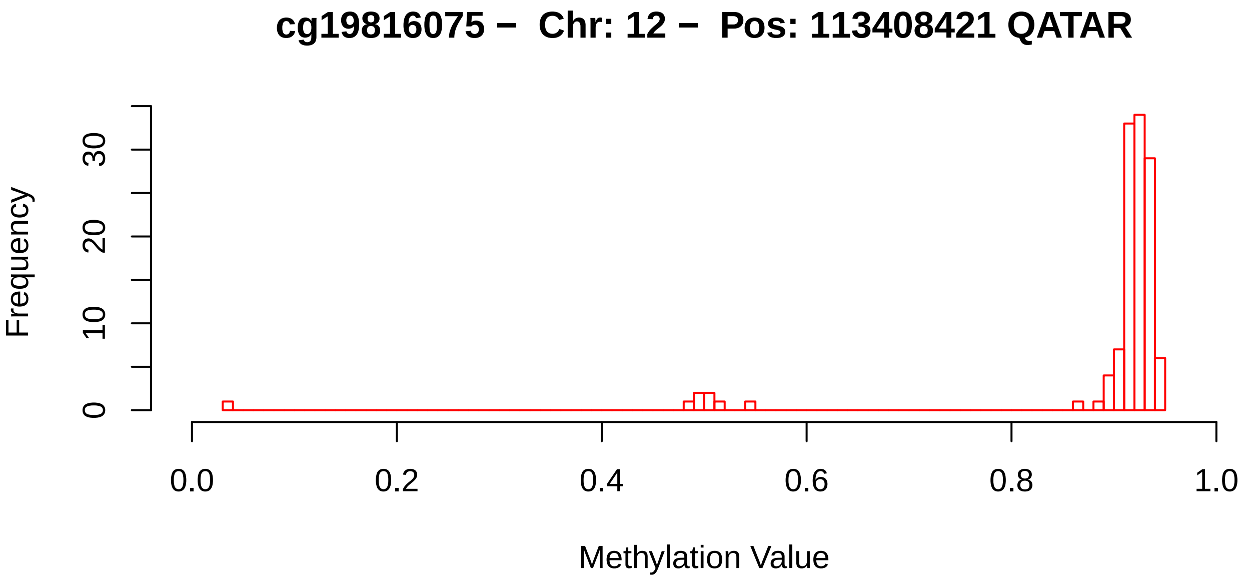
cg19816075 − Chr: 12 − Pos: 113408421 QATAR
Frequency
0
10
20
30
0.0
0.2
0.4
0.6
0.8
1.0
Methylation Value

cg19353052 − Chr: 12 − Pos: 113516445 KORA

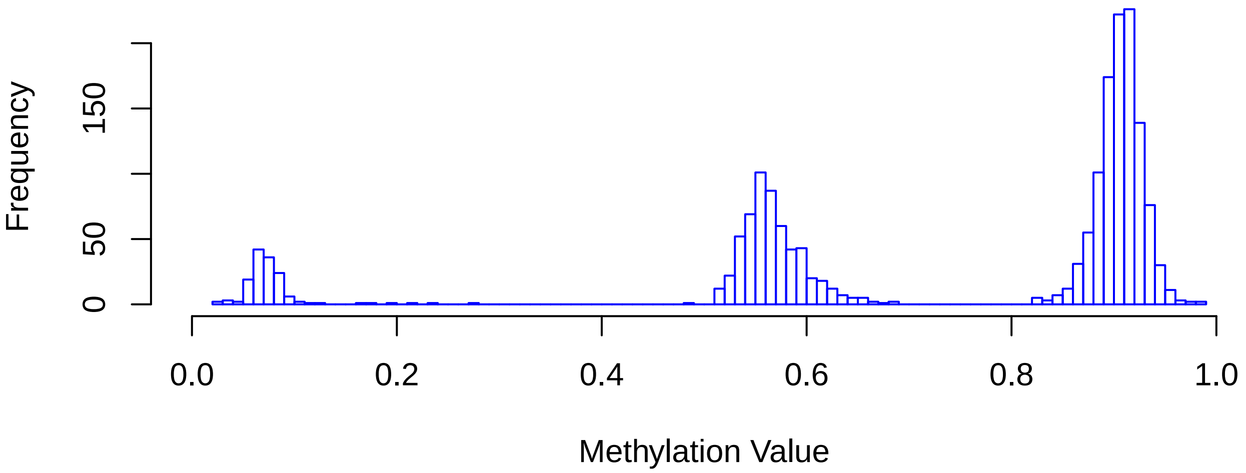

cg19353052 − Chr: 12 − Pos: 113516445 QATAR

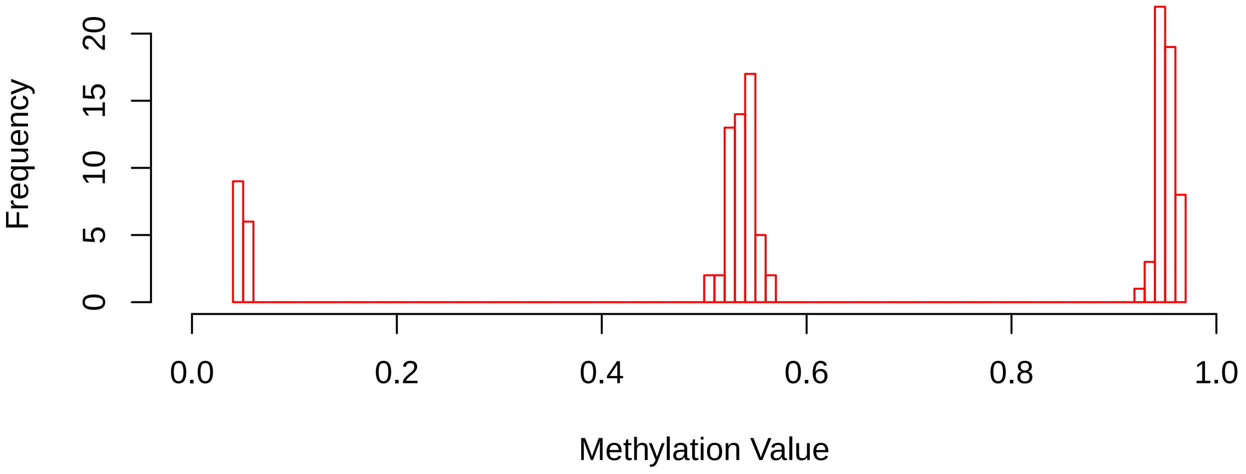

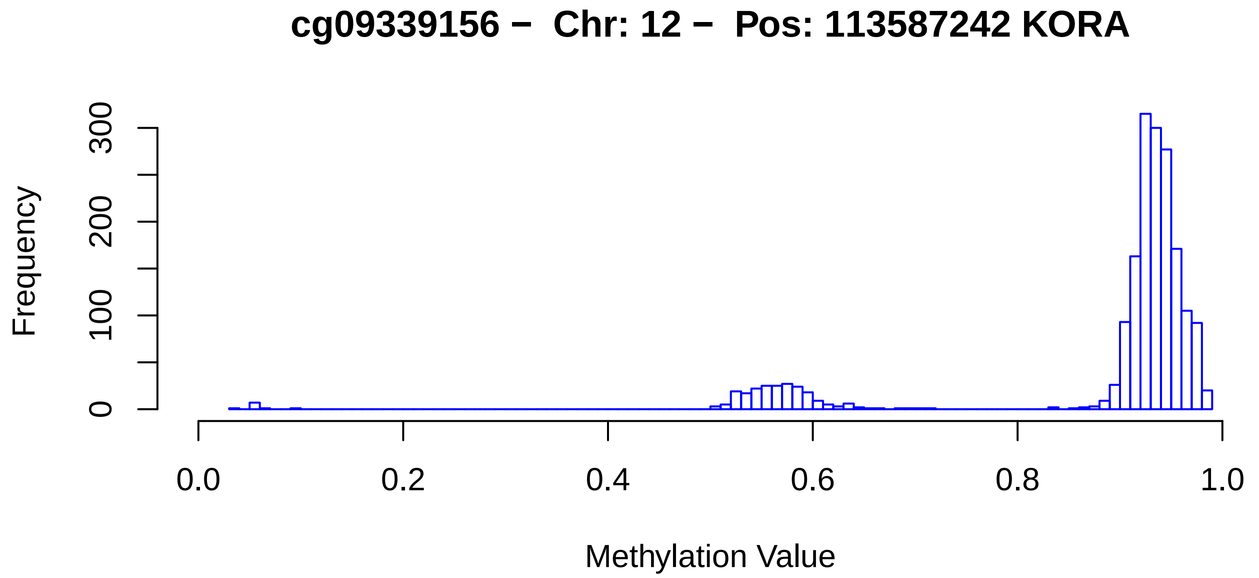
cg09339156 − Chr: 12 − Pos: 113587242 KORA
Frequency
0
100
200
300
0.0
0.2
0.4
0.6
0.8
1.0
Methylation Value

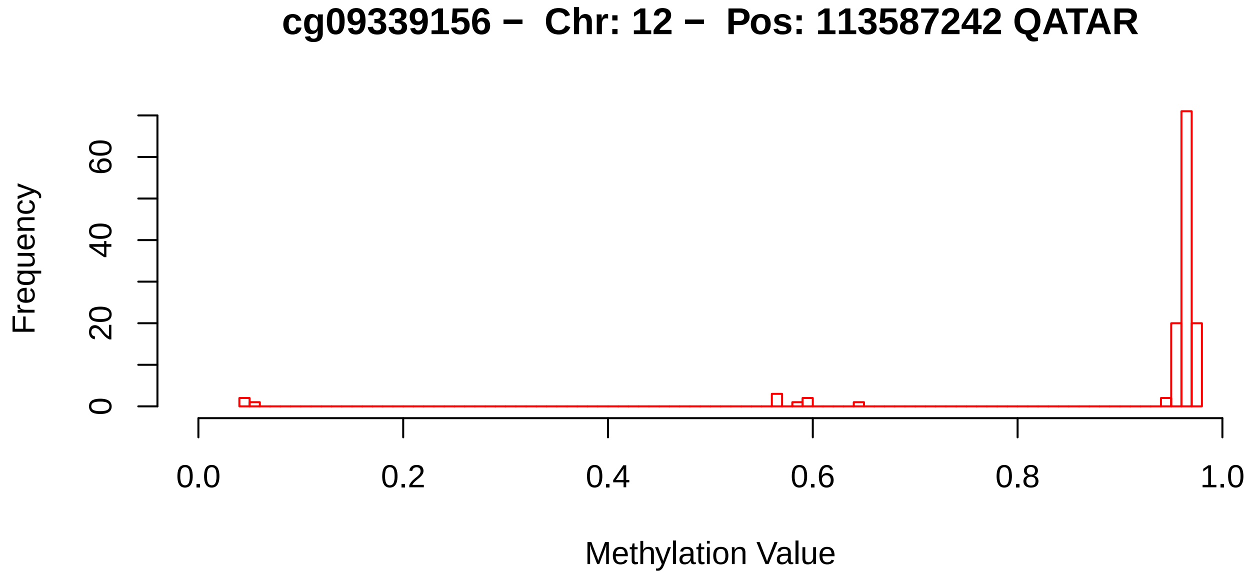
cg09339156 − Chr: 12 − Pos: 113587242 QATAR
Frequency
0
20
40
60
0.0
0.2
0.4
0.6
0.8
1.0
Methylation Value

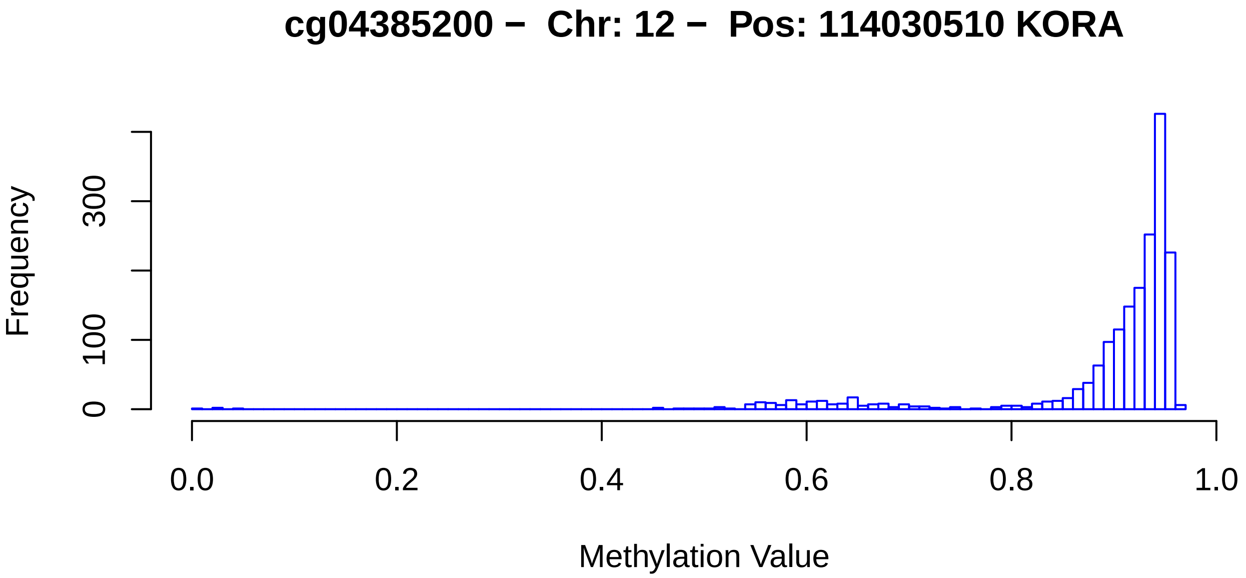
cg04385200 − Chr: 12 − Pos: 114030510 KORA
Frequency
0
100
300
0.0
0.2
0.4
0.6
0.8
1.0
Methylation Value

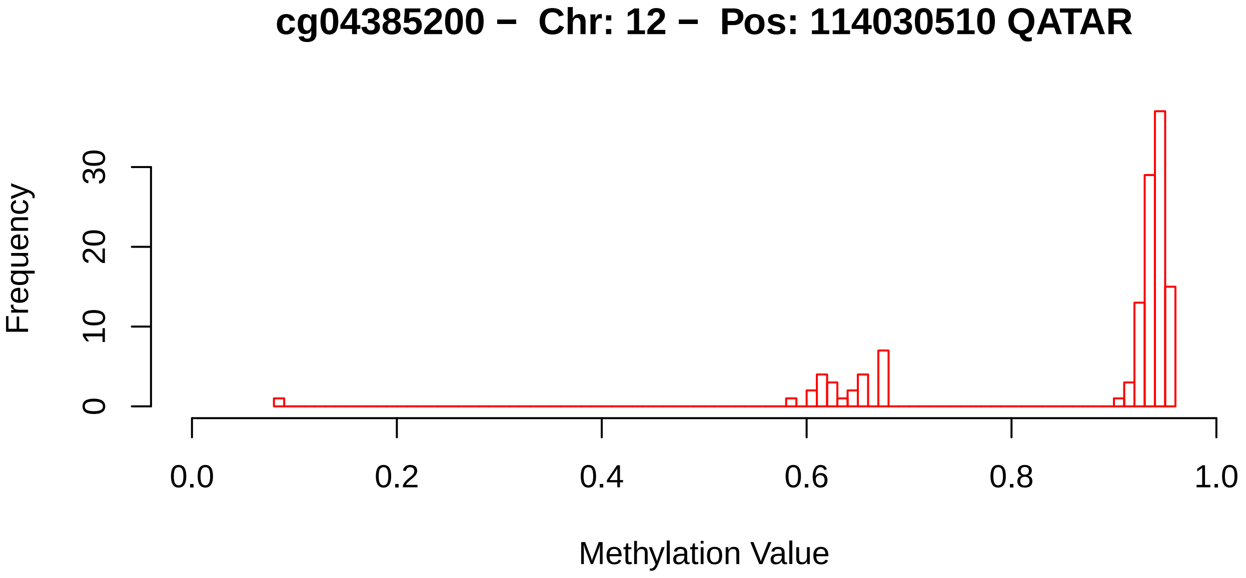
cg04385200 − Chr: 12 − Pos: 114030510 QATAR
Frequency
0
10
20
30
0.0
0.2
0.4
0.6
0.8
1.0
Methylation Value

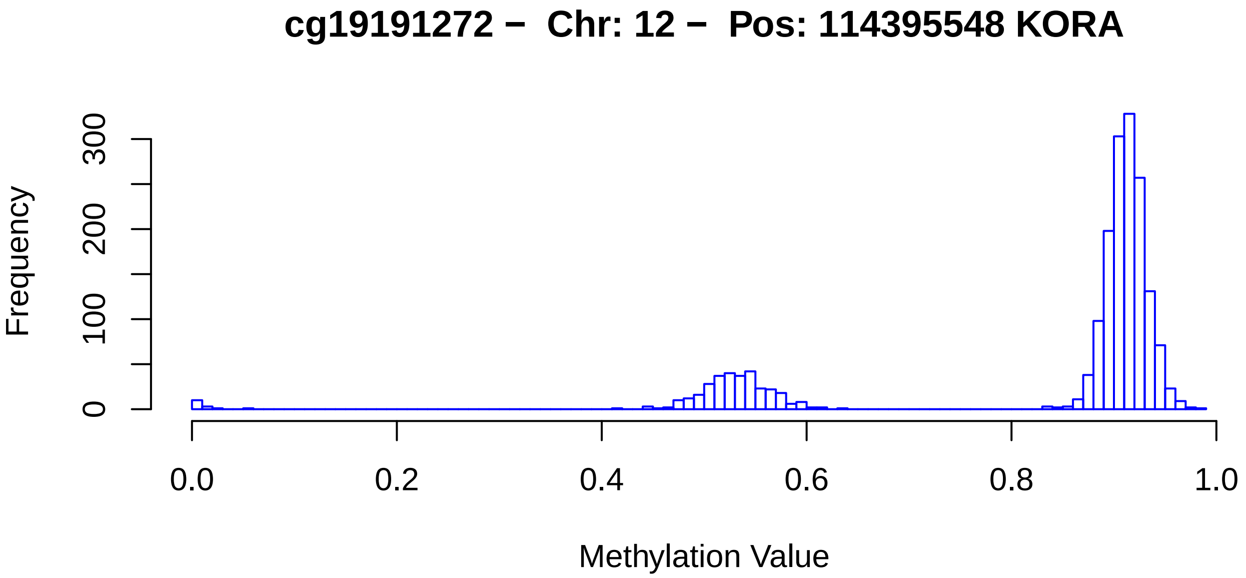
cg19191272 − Chr: 12 − Pos: 114395548 KORA
Frequency
0
100
200
300
0.0
0.2
0.4
0.6
0.8
1.0
Methylation Value

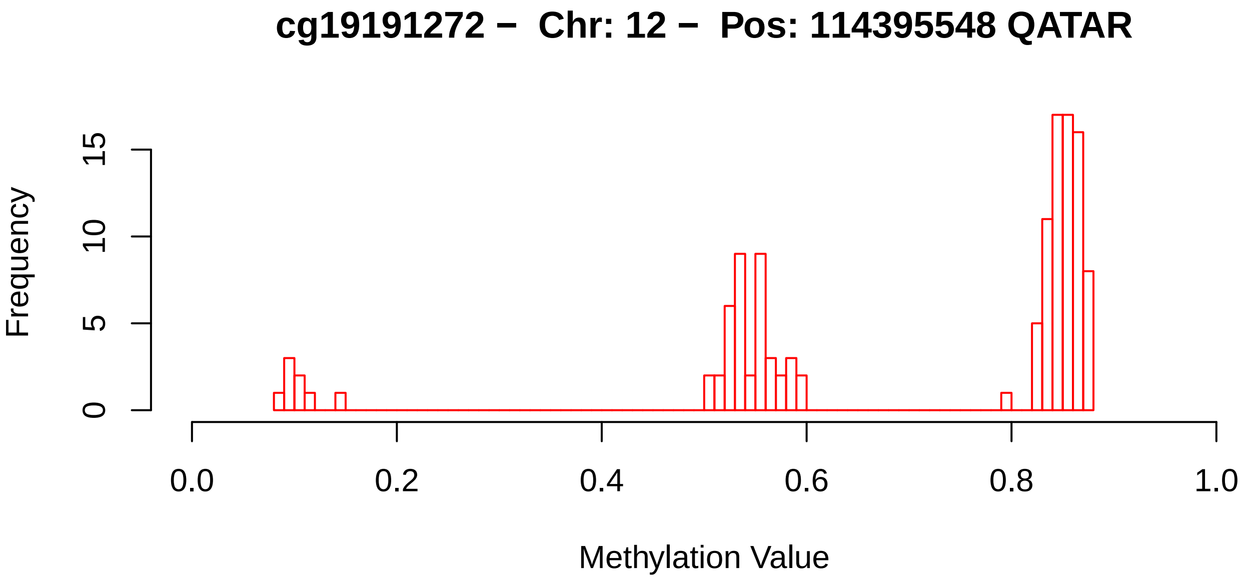
cg19191272 − Chr: 12 − Pos: 114395548 QATAR
Frequency
0
5
10
15
0.0
0.2
0.4
0.6
0.8
1.0
Methylation Value

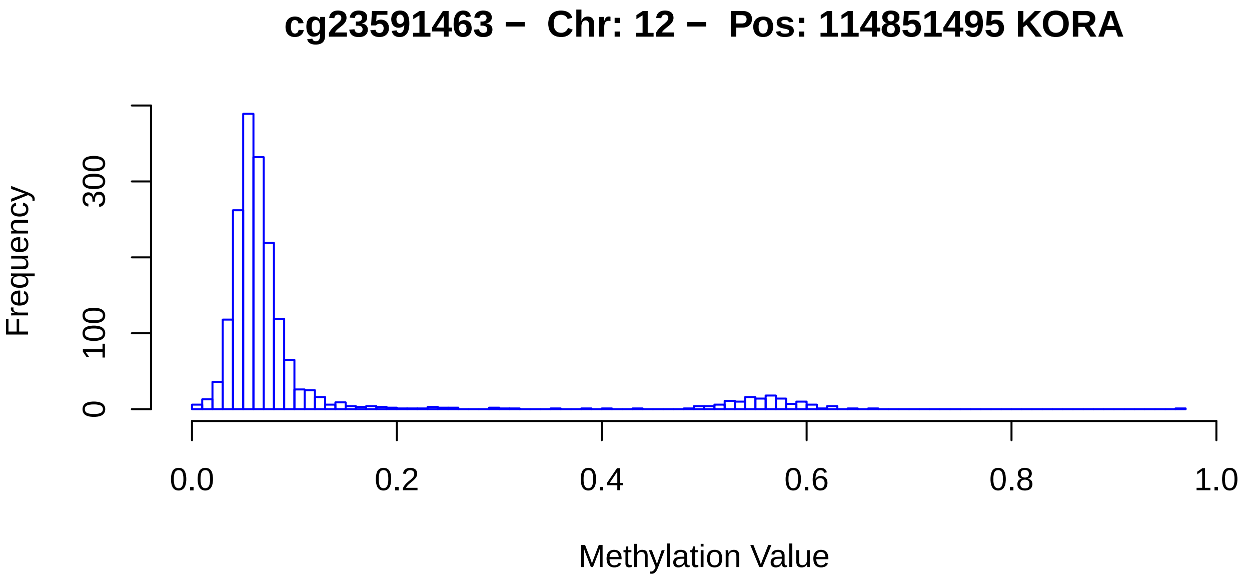
cg23591463 − Chr: 12 − Pos: 114851495 KORA
Frequency
0
100
300
0.0
0.2
0.4
0.6
0.8
1.0
Methylation Value

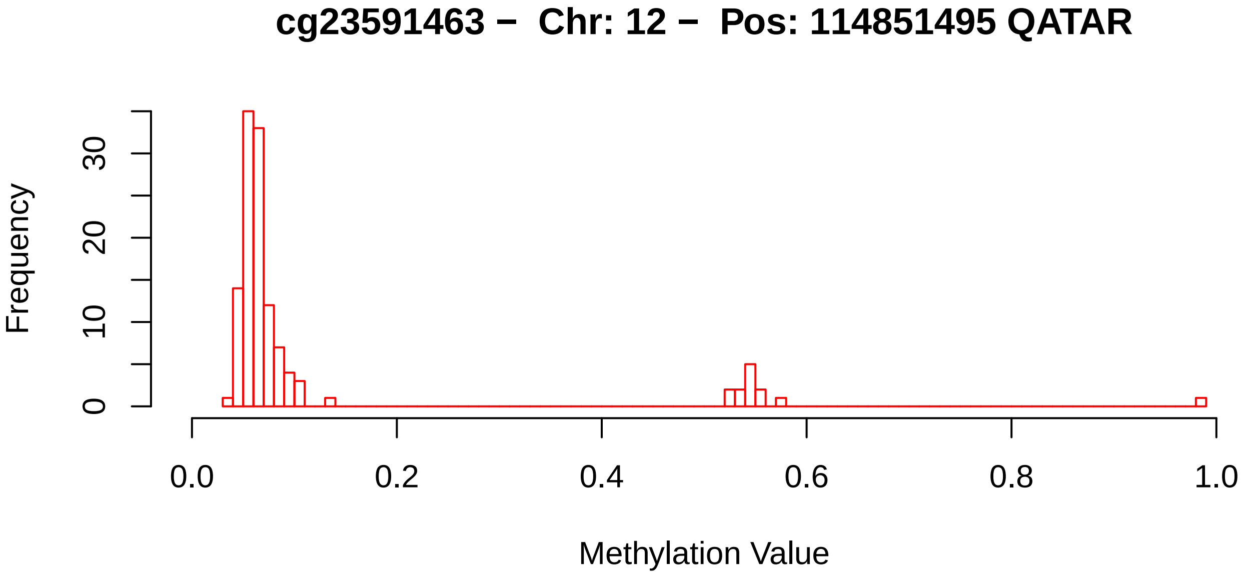
cg23591463 − Chr: 12 − Pos: 114851495 QATAR
Frequency
0
10
20
30
0.0
0.2
0.4
0.6
0.8
1.0
Methylation Value

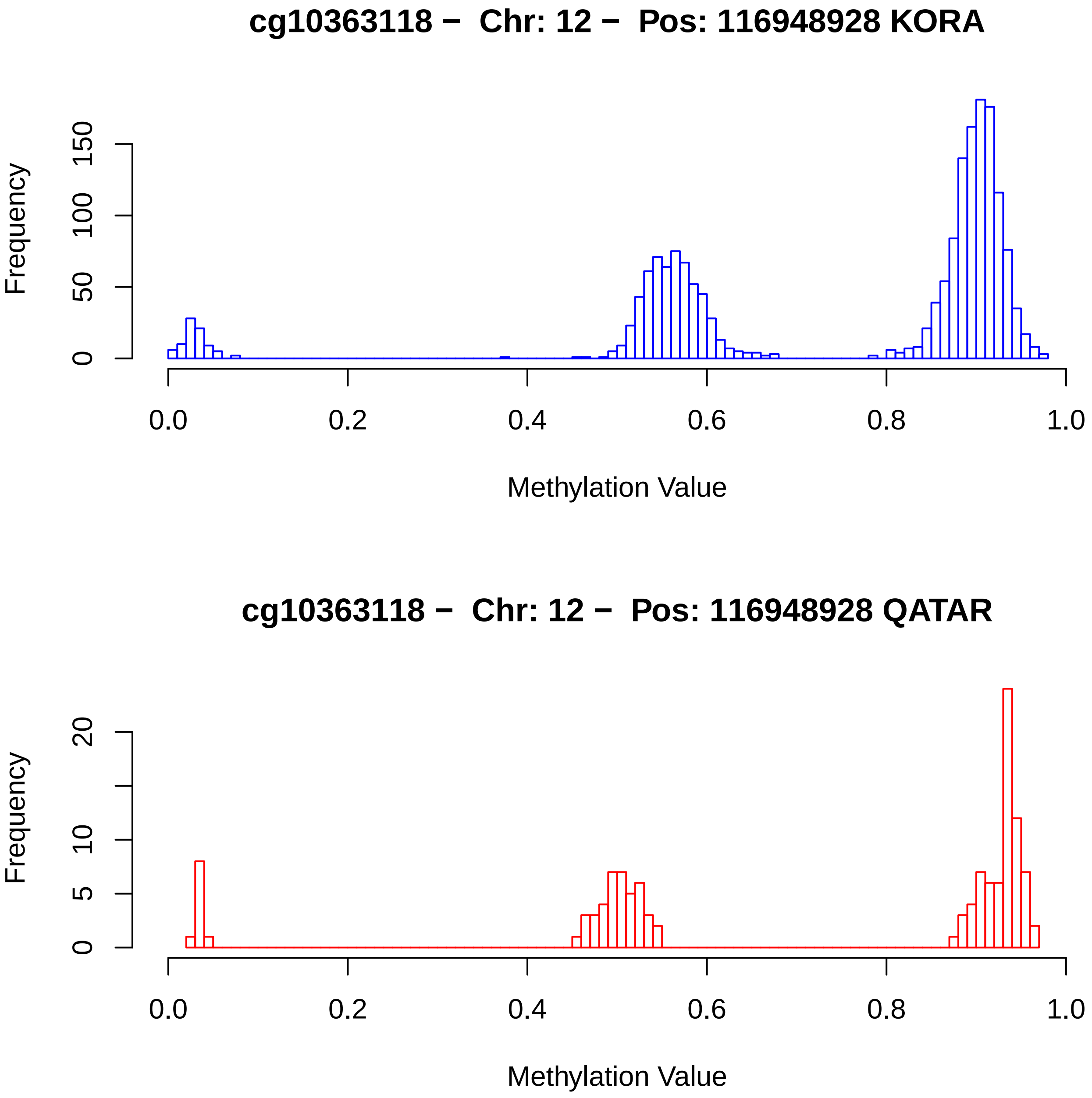
cg10363118 − Chr: 12 − Pos: 116948928 KORA
Frequency
0
50
100
150
0.0
0.2
0.4
0.6
0.8
1.0
Methylation Value
cg10363118 − Chr: 12 − Pos: 116948928 QATAR
Frequency
0
5
10
20
0.0
0.2
0.4
0.6
0.8
1.0
Methylation Value

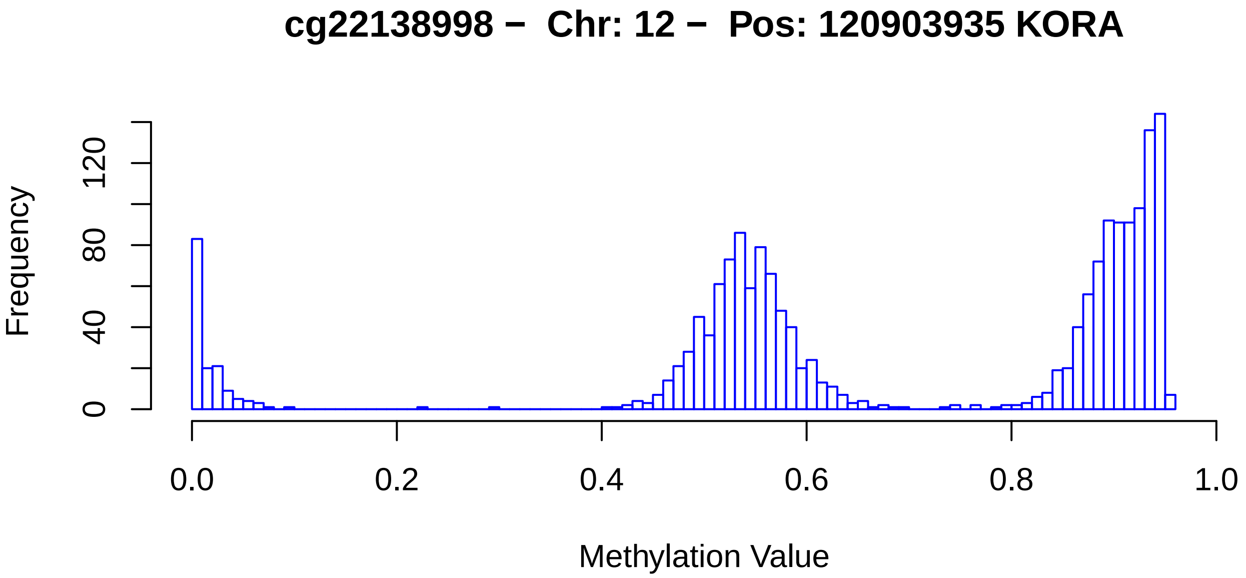

cg22138998 − Chr: 12 − Pos: 120903935 KORA
Frequency
0
40
80
120
0.0
0.2
0.4
0.6
0.8
1.0
Methylation Value

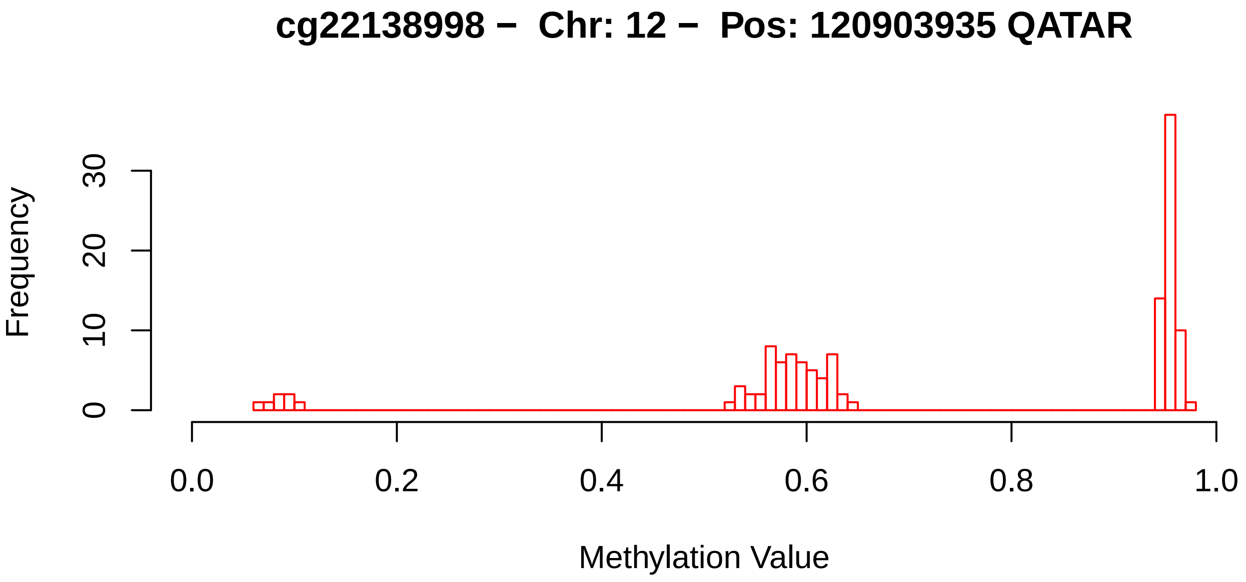

cg22138998 − Chr: 12 − Pos: 120903935 QATAR
Frequency
0
10
20
30
0.0
0.2
0.4
0.6
0.8
1.0
Methylation Value

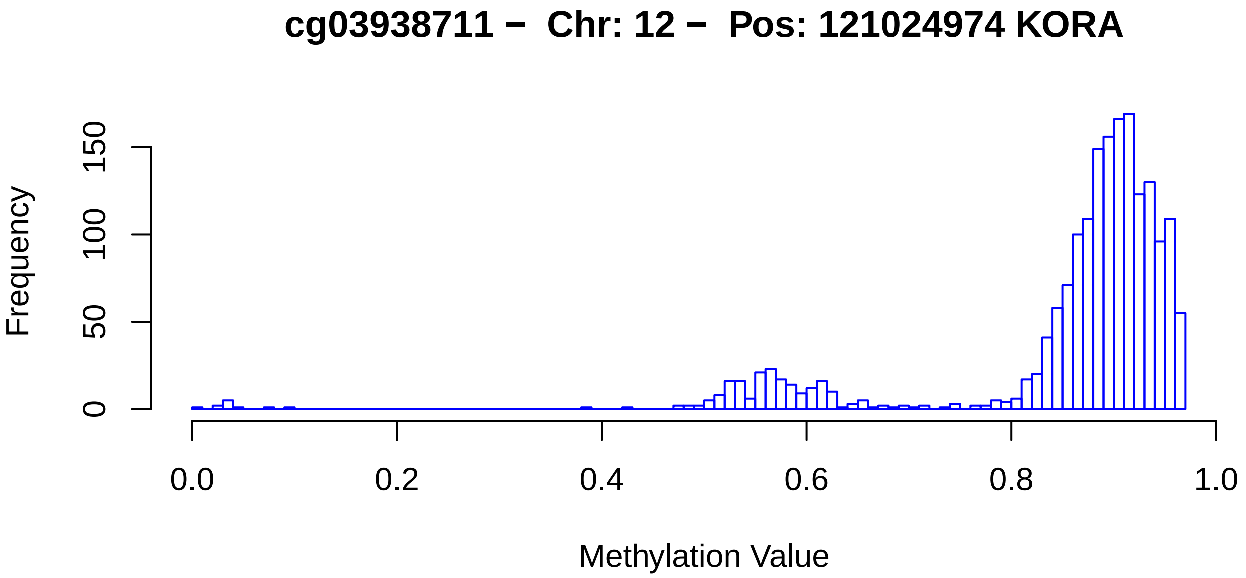
cg03938711 − Chr: 12 − Pos: 121024974 KORA
Frequency
0
50
100
150
0.0
0.2
0.4
0.6
0.8
1.0
Methylation Value

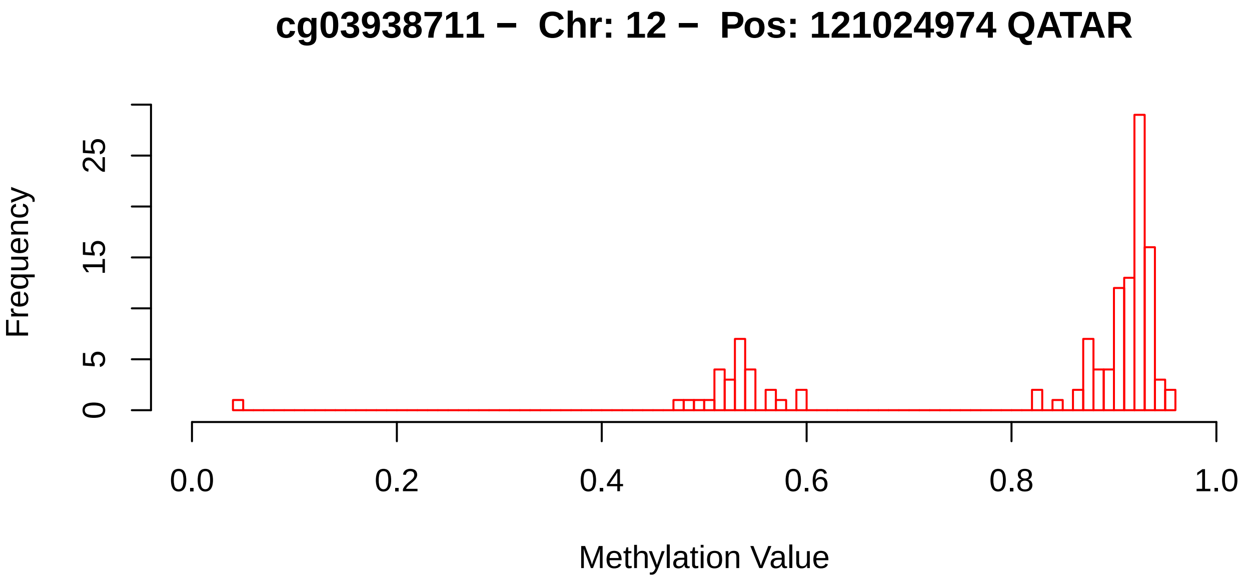
cg03938711 − Chr: 12 − Pos: 121024974 QATAR
Frequency
0
5
15
25
0.0
0.2
0.4
0.6
0.8
1.0
Methylation Value

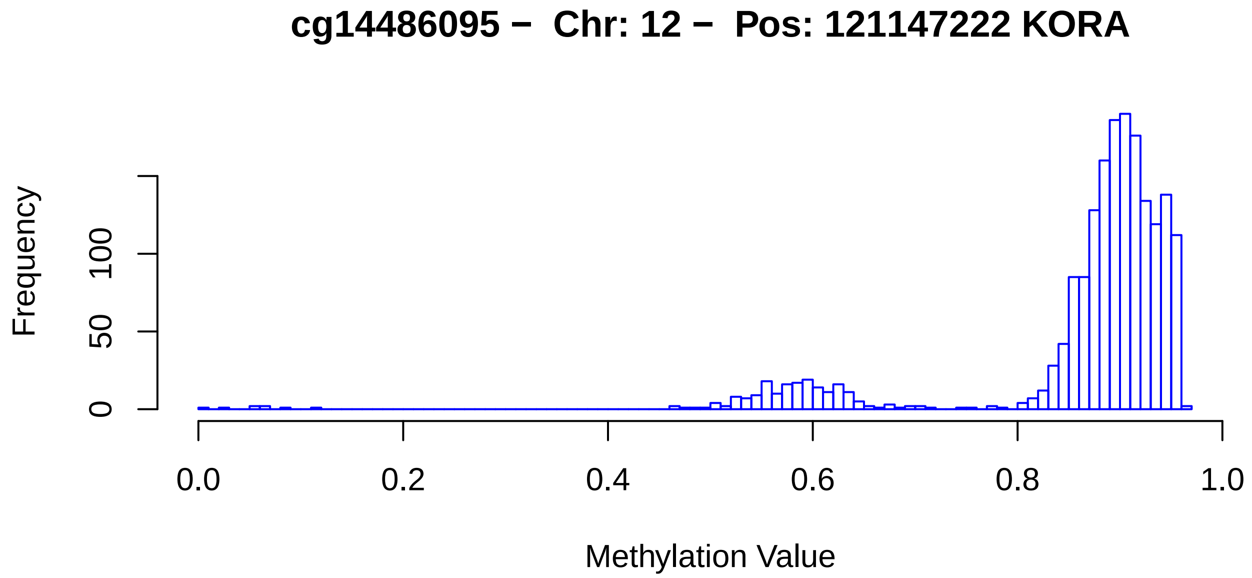
cg14486095 − Chr: 12 − Pos: 121147222 KORA
Frequency
0
50
100
0.0
0.2
0.4
0.6
0.8
1.0
Methylation Value

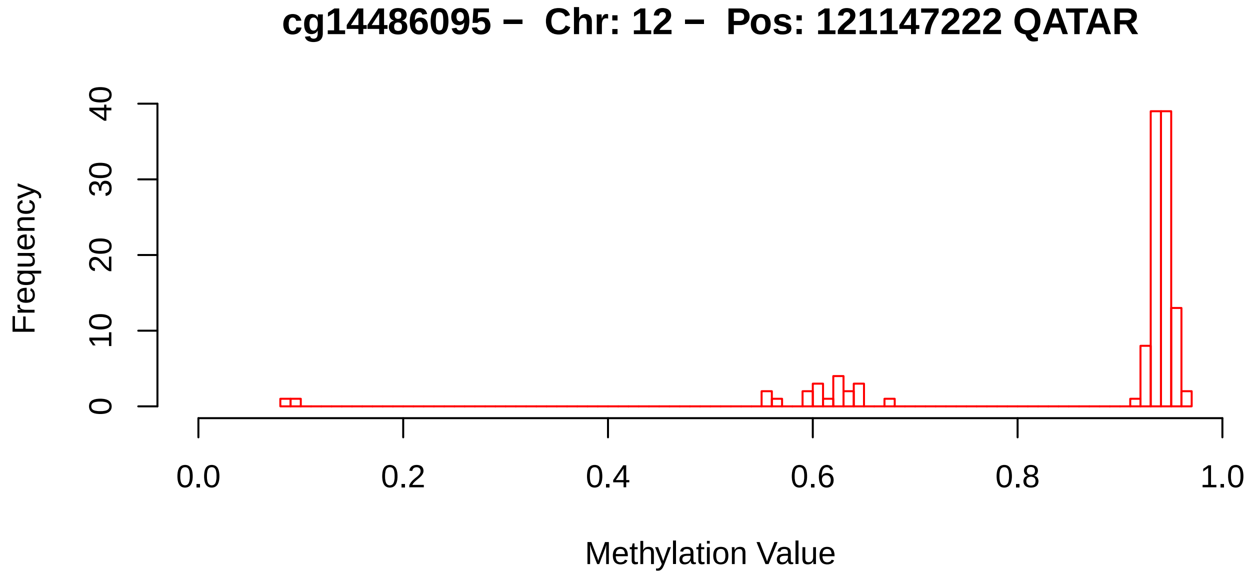
cg14486095 − Chr: 12 − Pos: 121147222 QATAR
Frequency
0
10
20
30
40
0.0
0.2
0.4
0.6
0.8
1.0
Methylation Value

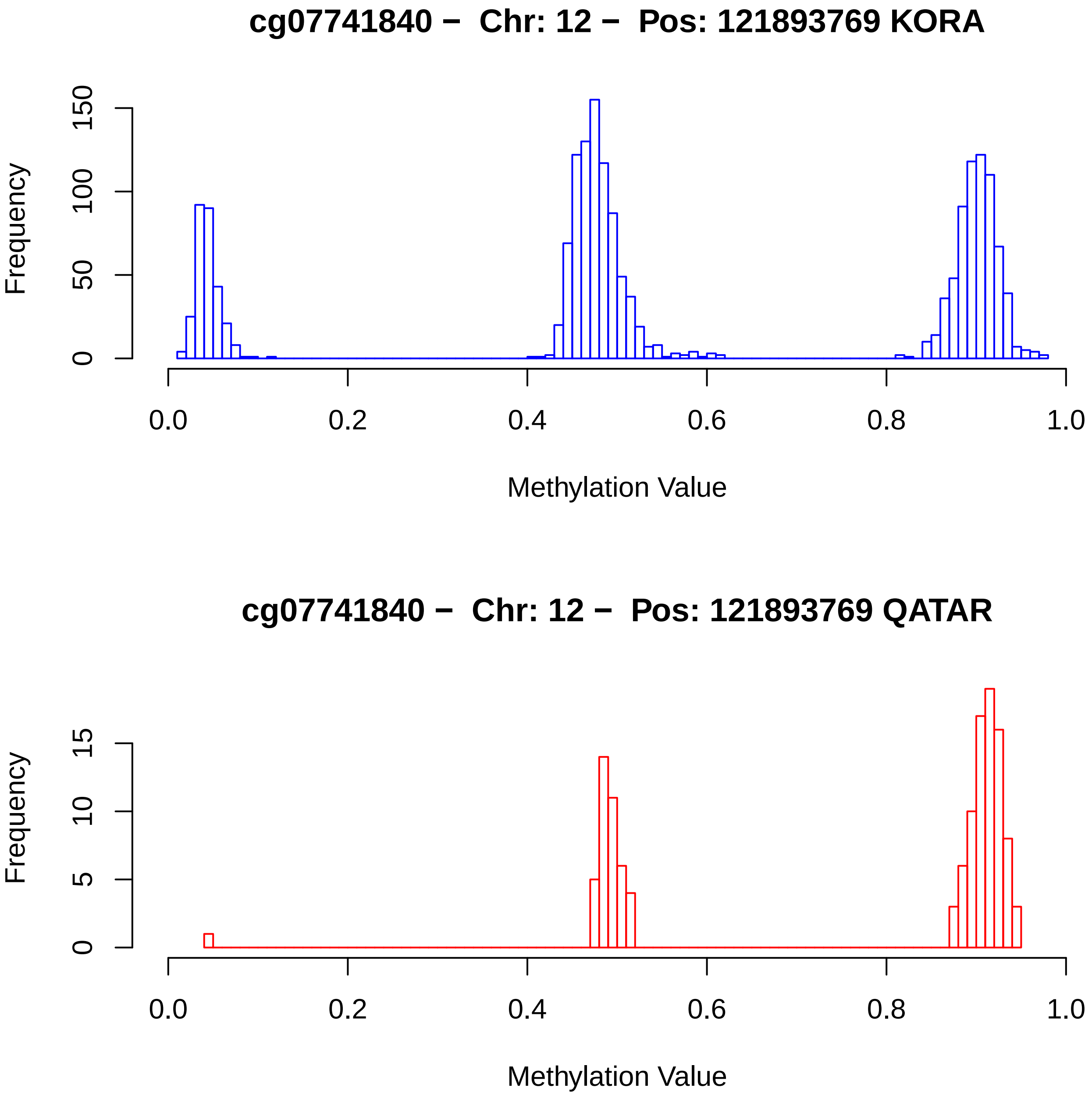
cg07741840 − Chr: 12 − Pos: 121893769 KORA
Frequency
0
50
100
150
0.0
0.2
0.4
0.6
0.8
1.0
Methylation Value
cg07741840 − Chr: 12 − Pos: 121893769 QATAR
Frequency
0
5
10
15
0.0
0.2
0.4
0.6
0.8
1.0
Methylation Value

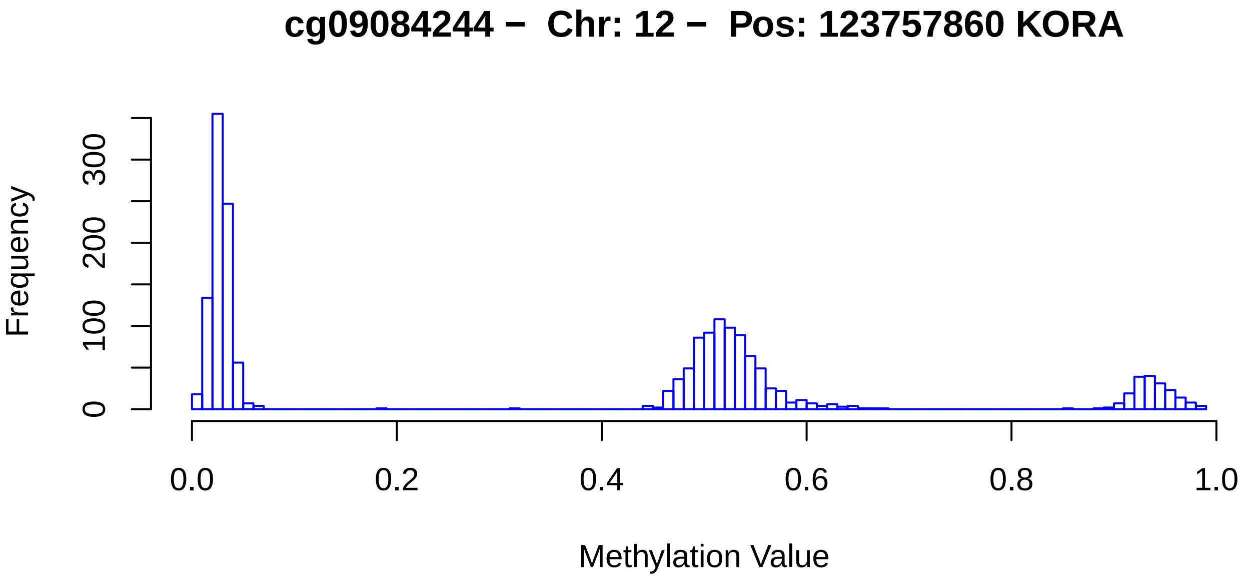
cg09084244 − Chr: 12 − Pos: 123757860 KORA
Frequency
0
100
200
300
0.0
0.2
0.4
0.6
0.8
1.0
Methylation Value

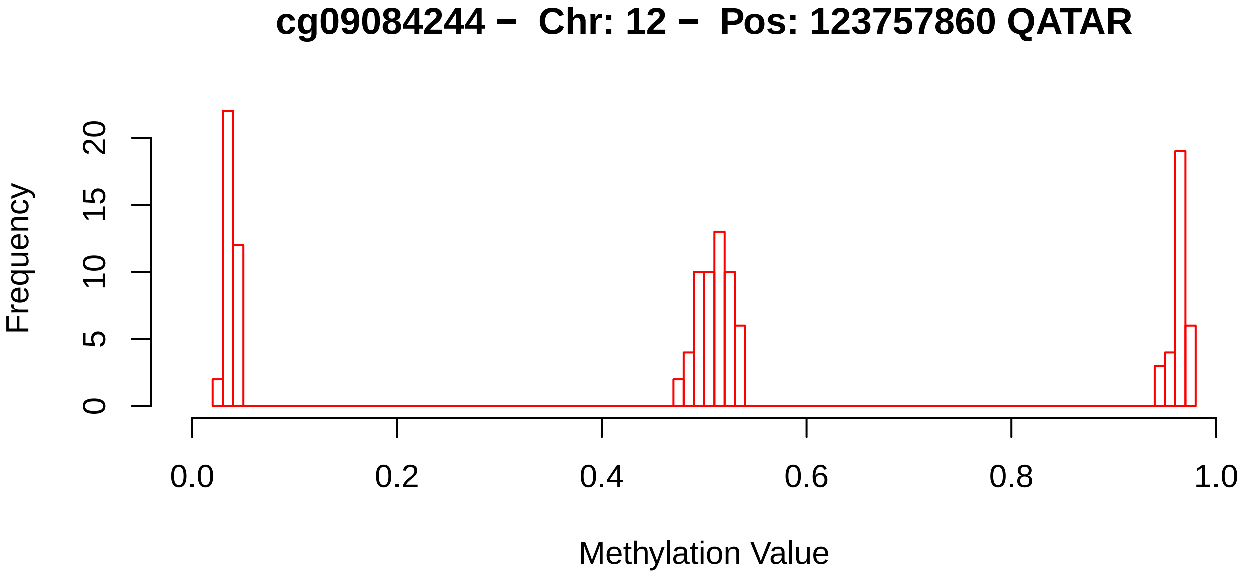
cg09084244 − Chr: 12 − Pos: 123757860 QATAR
Frequency
0
5
10
15
20
0.0
0.2
0.4
0.6
0.8
1.0
Methylation Value

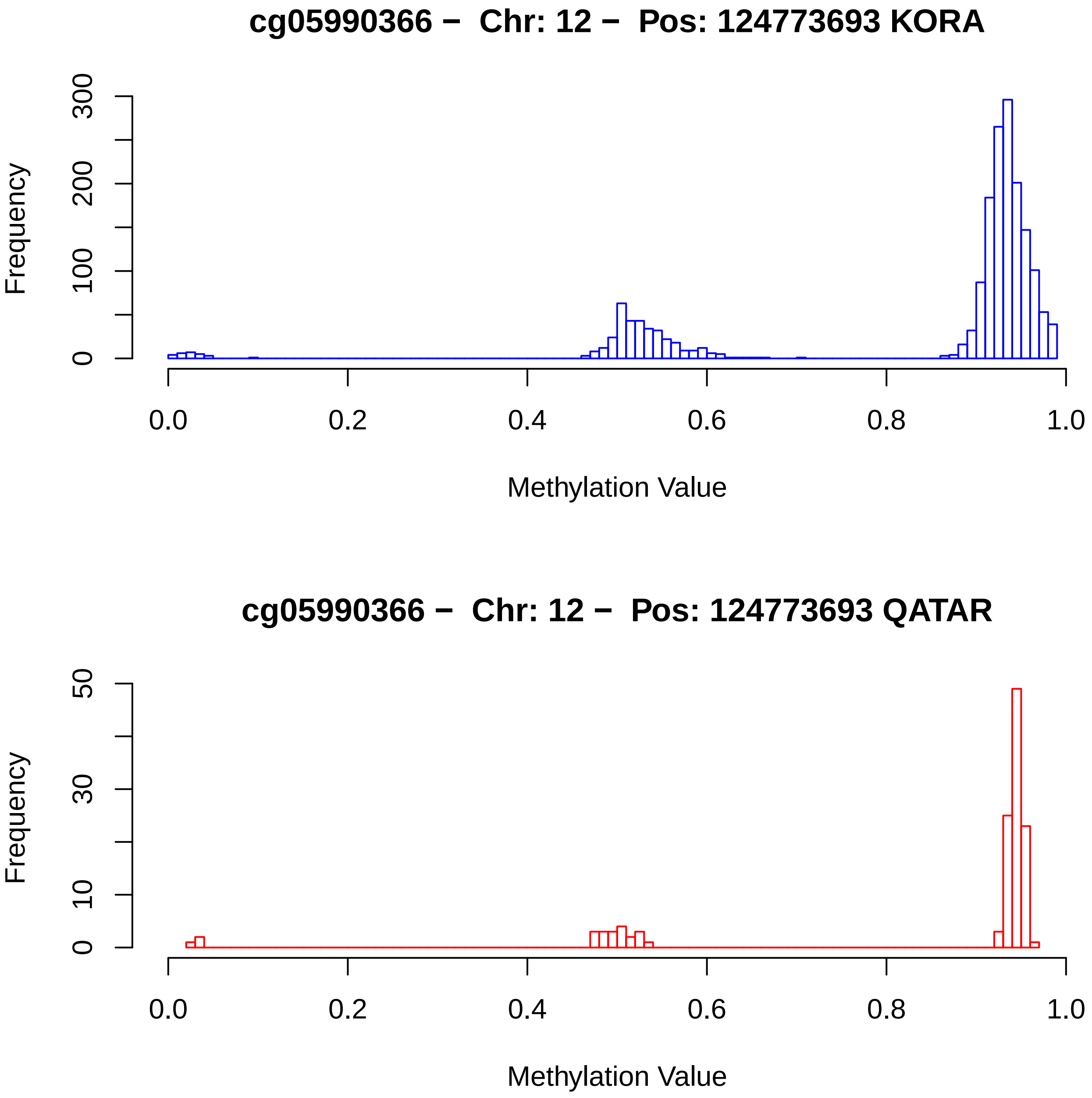
cg05990366 − Chr: 12 − Pos: 124773693 KORA
Frequency
0
100
200
300
0.0
0.2
0.4
0.6
0.8
1.0
Methylation Value
cg05990366 − Chr: 12 − Pos: 124773693 QATAR
Frequency
0
10
30
50
0.0
0.2
0.4
0.6
0.8
1.0
Methylation Value

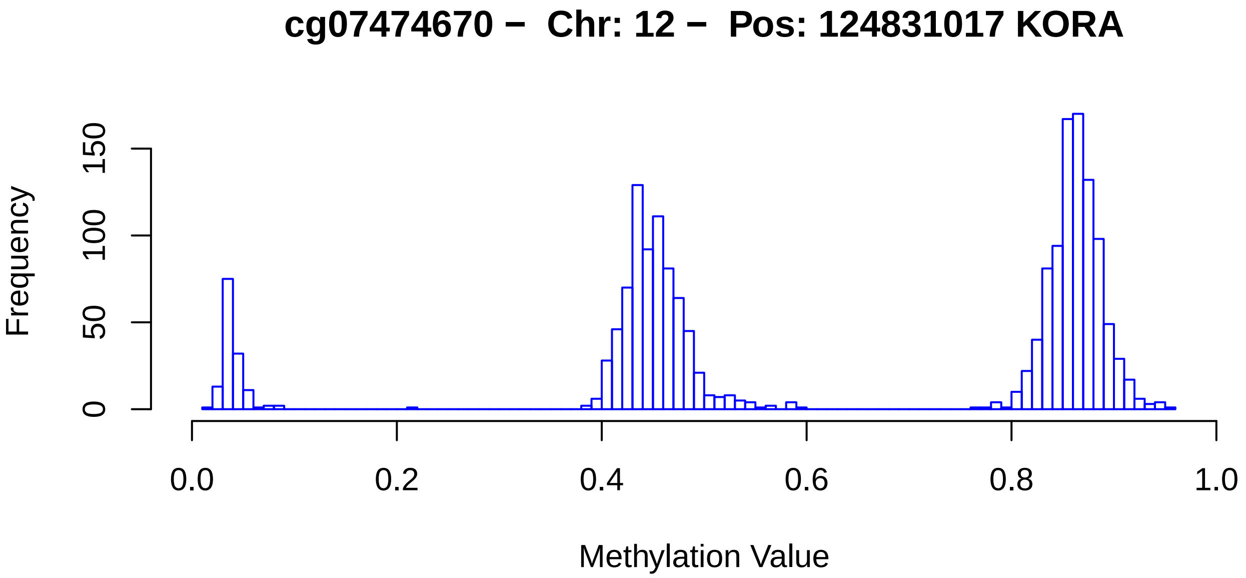
cg07474670 − Chr: 12 − Pos: 124831017 KORA
Frequency
0
50
100
150
0.0
0.2
0.4
0.6
0.8
1.0
Methylation Value

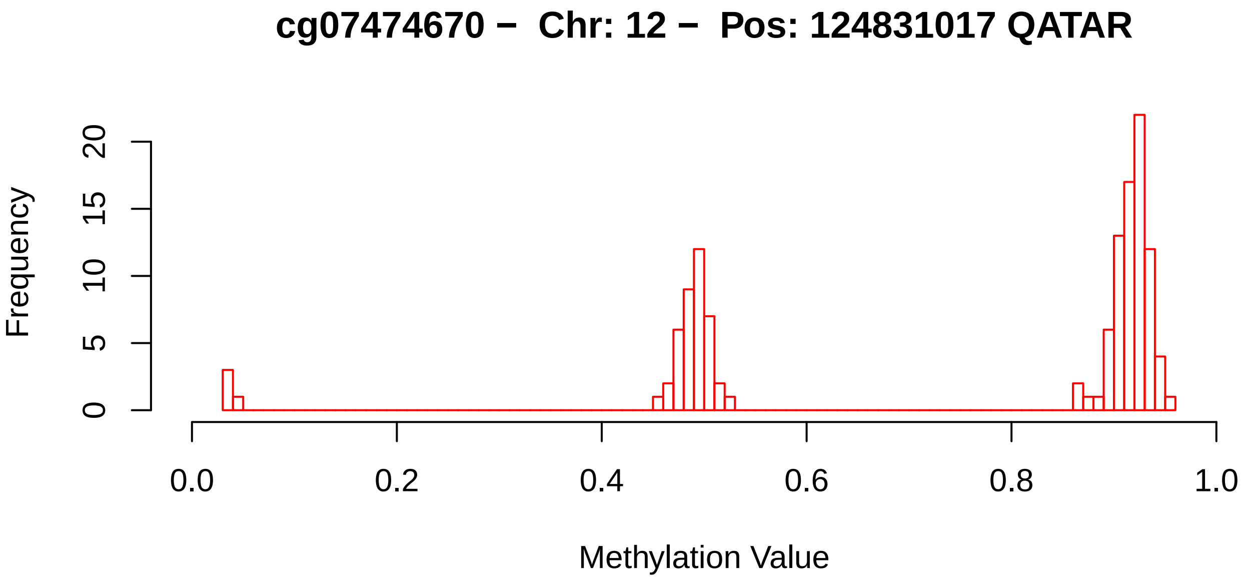
cg07474670 − Chr: 12 − Pos: 124831017 QATAR
Frequency
0
5
10
15
20
0.0
0.2
0.4
0.6
0.8
1.0
Methylation Value

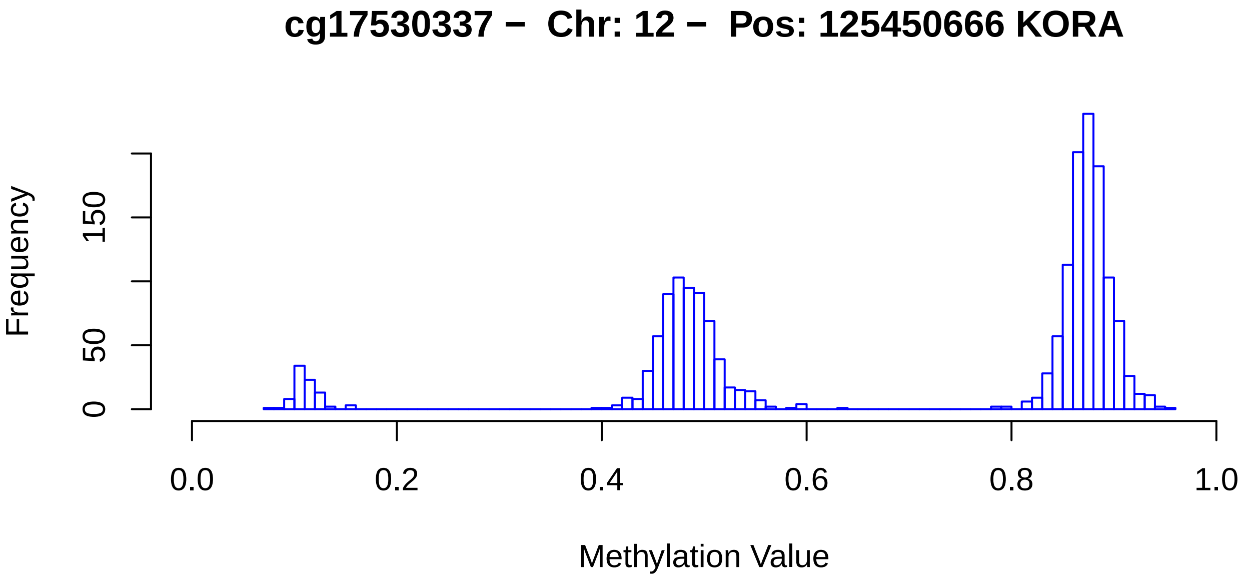
cg17530337 − Chr: 12 − Pos: 125450666 KORA
Frequency
0
50
150
0.0
0.2
0.4
0.6
0.8
1.0
Methylation Value

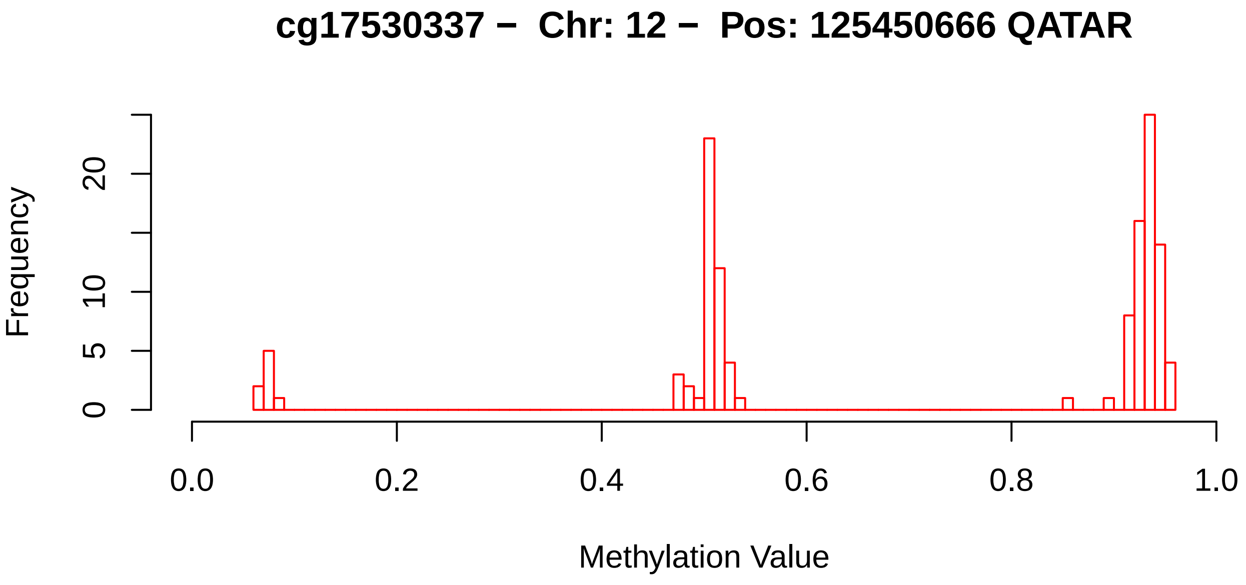
cg17530337 − Chr: 12 − Pos: 125450666 QATAR
Frequency
0
5
10
20
0.0
0.2
0.4
0.6
0.8
1.0
Methylation Value

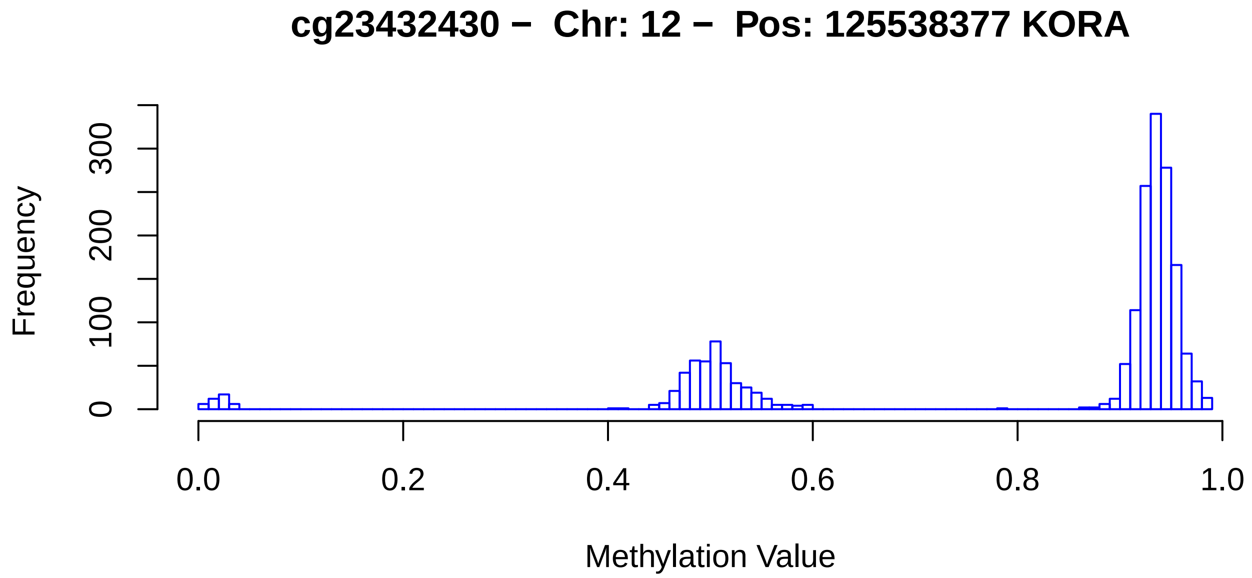
cg23432430 − Chr: 12 − Pos: 125538377 KORA
Frequency
0
100
200
300
0.0
0.2
0.4
0.6
0.8
1.0
Methylation Value

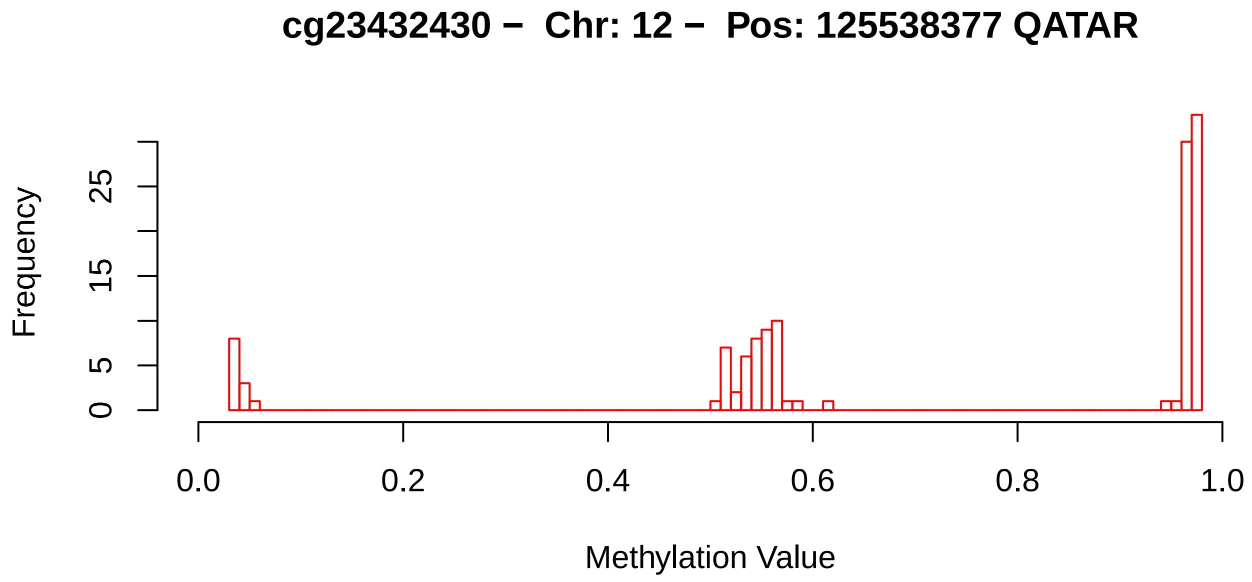
cg23432430 − Chr: 12 − Pos: 125538377 QATAR
Frequency
0
5
15
25
0.0
0.2
0.4
0.6
0.8
1.0
Methylation Value

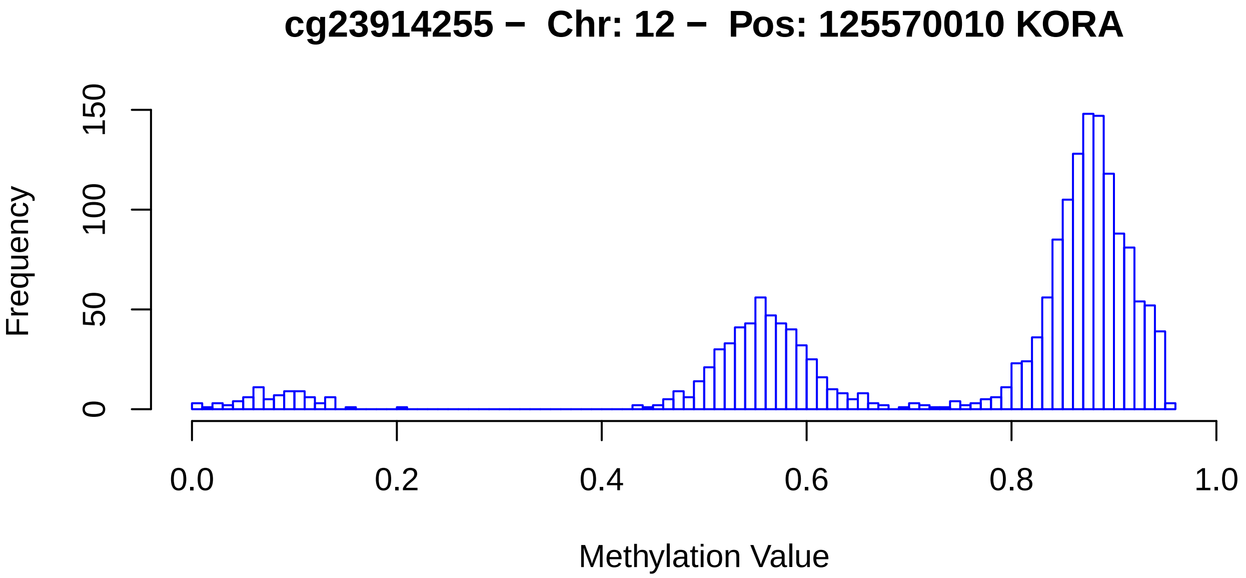
cg23914255 − Chr: 12 − Pos: 125570010 KORA
Frequency
0
50
100
150
0.0
0.2
0.4
0.6
0.8
1.0
Methylation Value

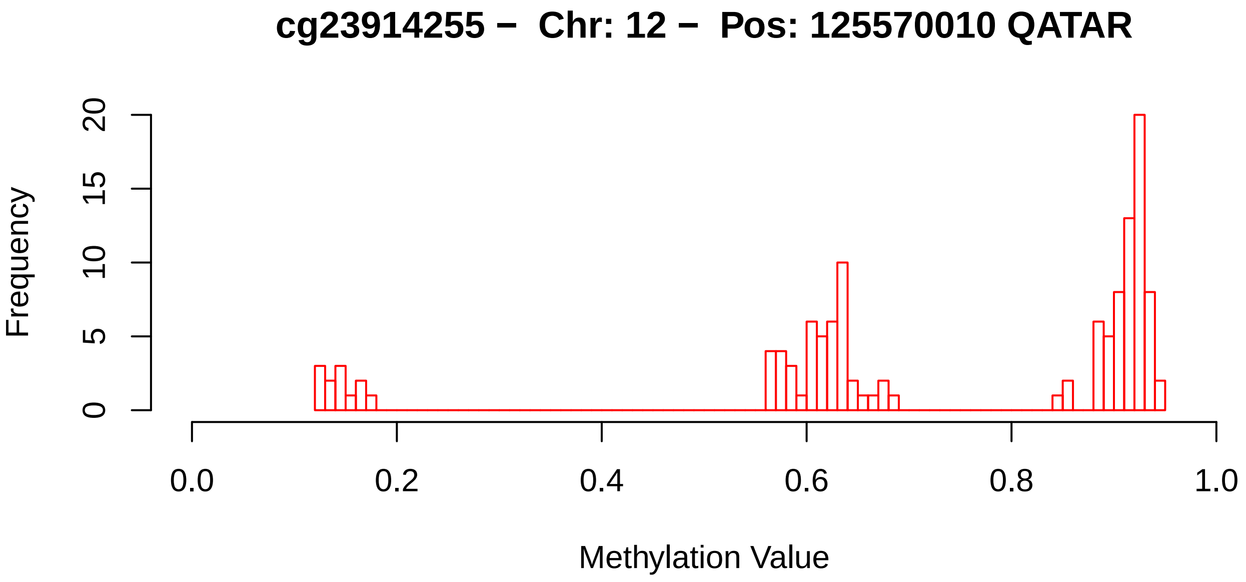
cg23914255 − Chr: 12 − Pos: 125570010 QATAR
Frequency
0
5
10
15
20
0.0
0.2
0.4
0.6
0.8
1.0
Methylation Value

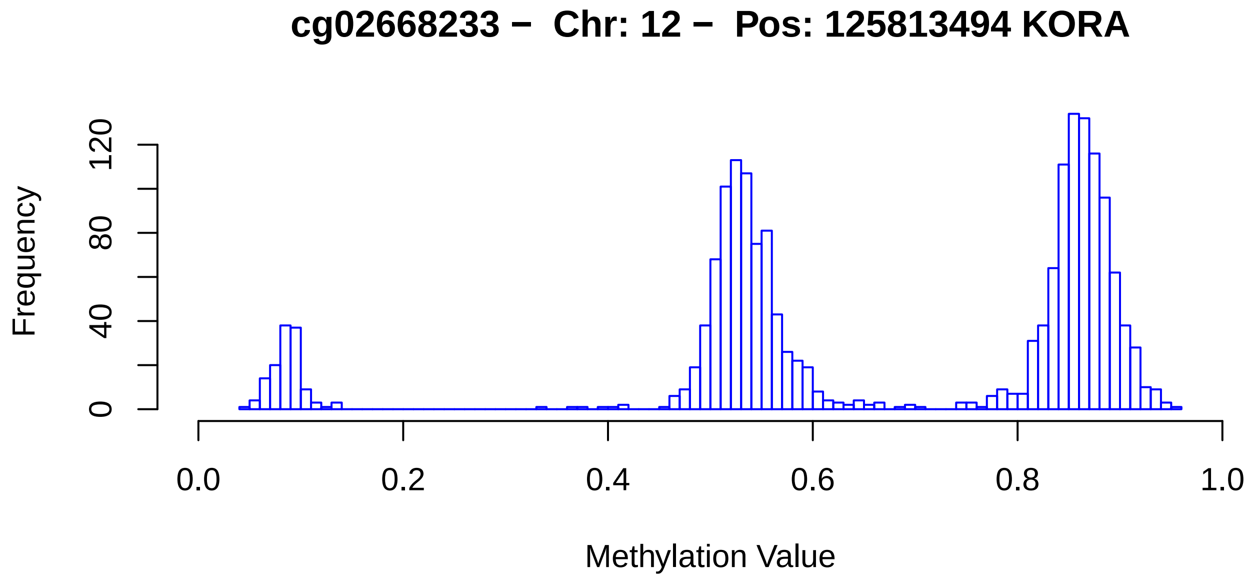
cg02668233 − Chr: 12 − Pos: 125813494 KORA
Frequency
0
40
80
120
0.0
0.2
0.4
0.6
0.8
1.0
Methylation Value

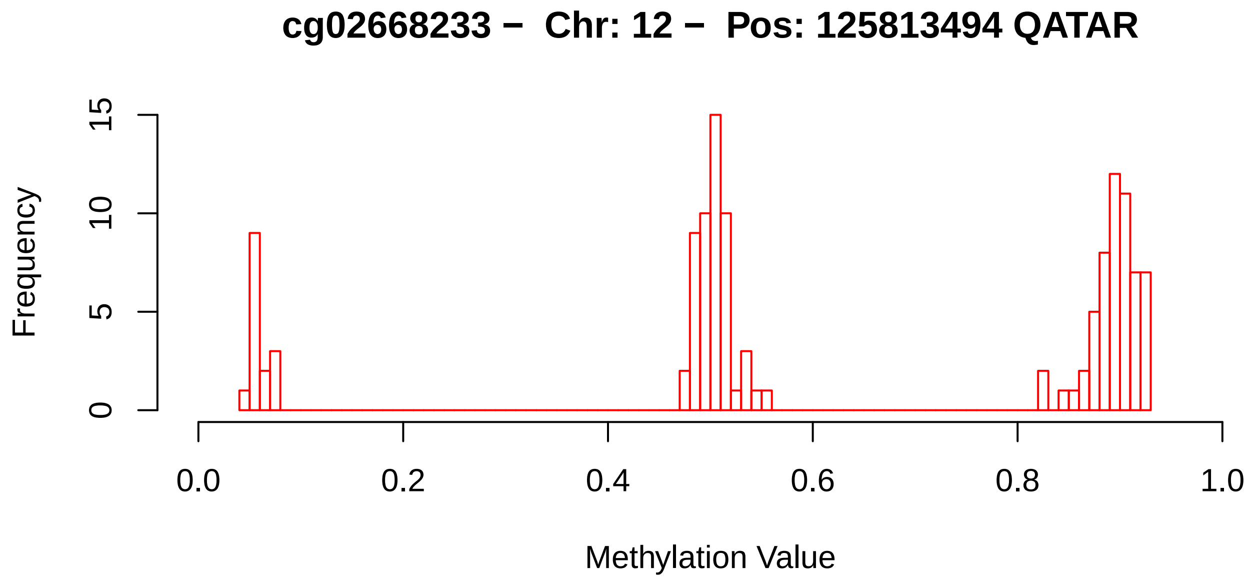
cg02668233 − Chr: 12 − Pos: 125813494 QATAR
Frequency
0
5
10
15
0.0
0.2
0.4
0.6
0.8
1.0
Methylation Value

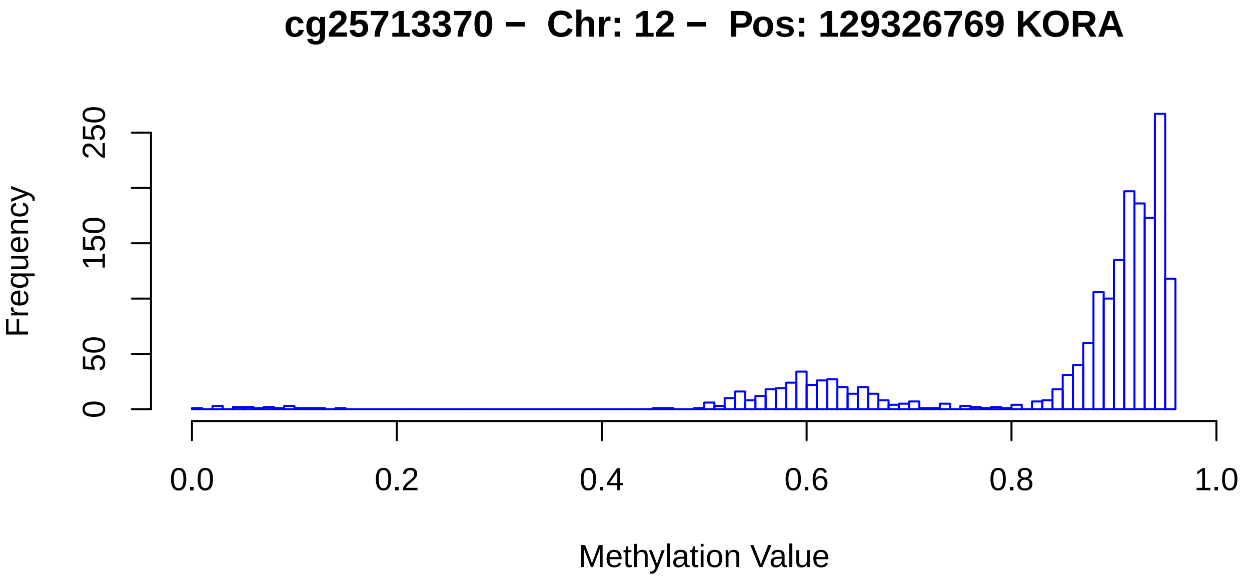

cg25713370 − Chr: 12 − Pos: 129326769 KORA
Frequency
0
50
150
250
0.0
0.2
0.4
0.6
0.8
1.0
Methylation Value

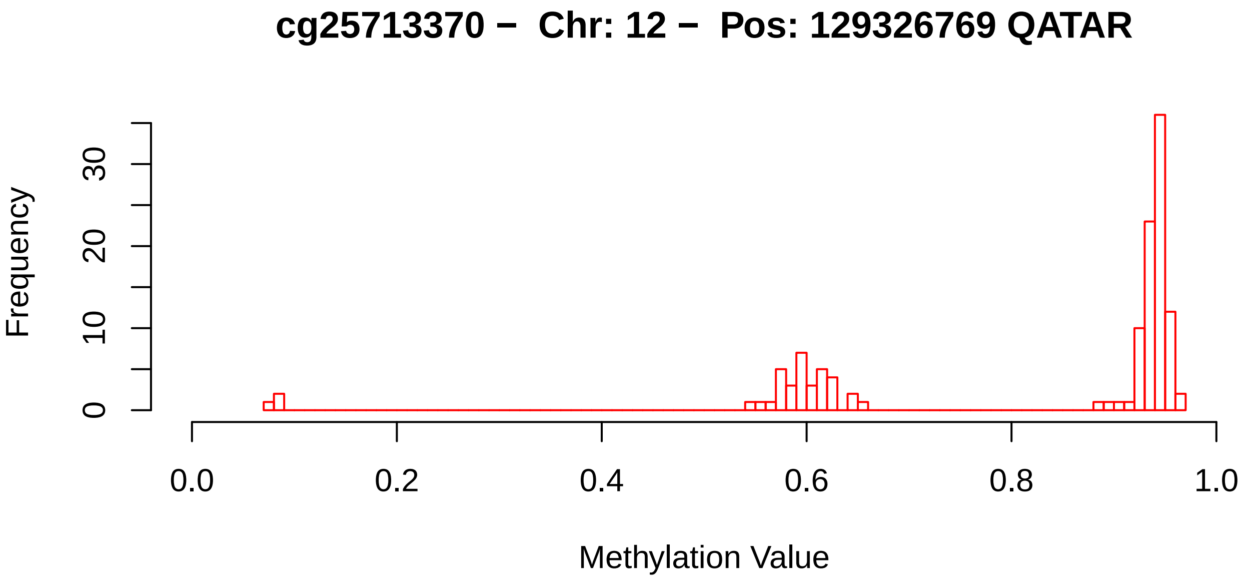

cg25713370 − Chr: 12 − Pos: 129326769 QATAR
Frequency
0
10
20
30
0.0
0.2
0.4
0.6
0.8
1.0
Methylation Value

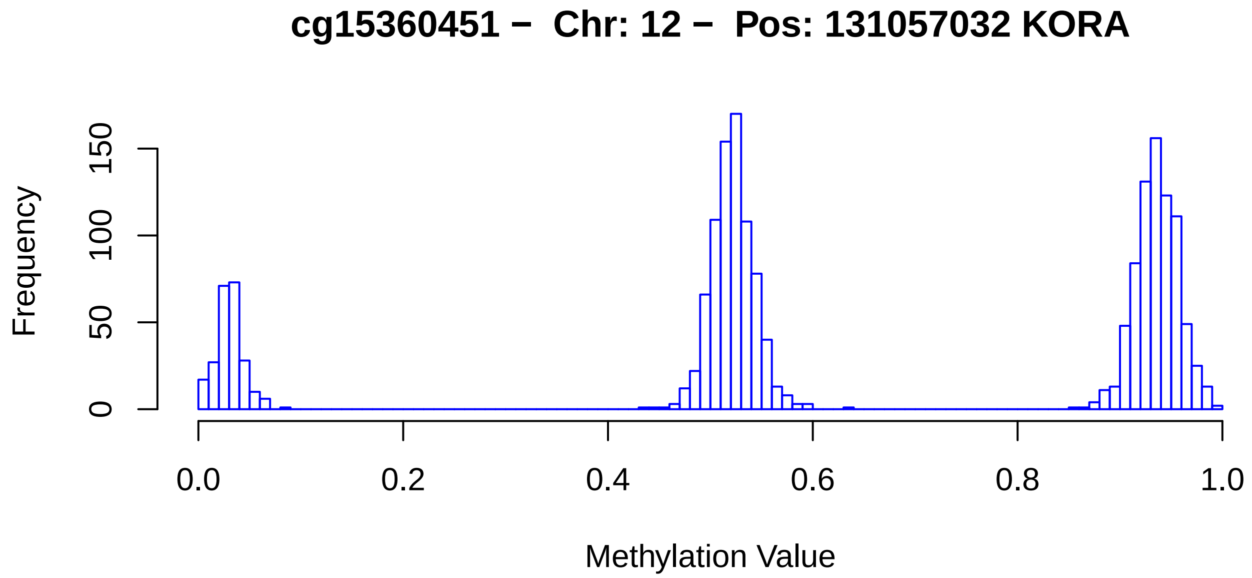
cg15360451 − Chr: 12 − Pos: 131057032 KORA
Frequency
0
50
100
150
0.0
0.2
0.4
0.6
0.8
1.0
Methylation Value

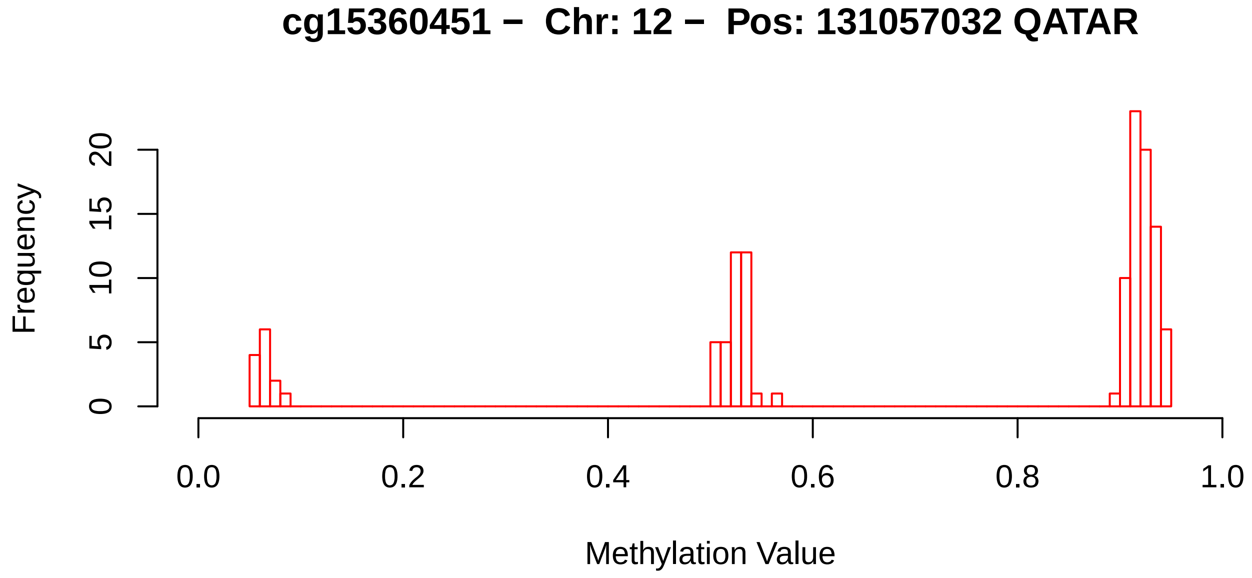
cg15360451 − Chr: 12 − Pos: 131057032 QATAR
Frequency
0
5
10
15
20
0.0
0.2
0.4
0.6
0.8
1.0
Methylation Value

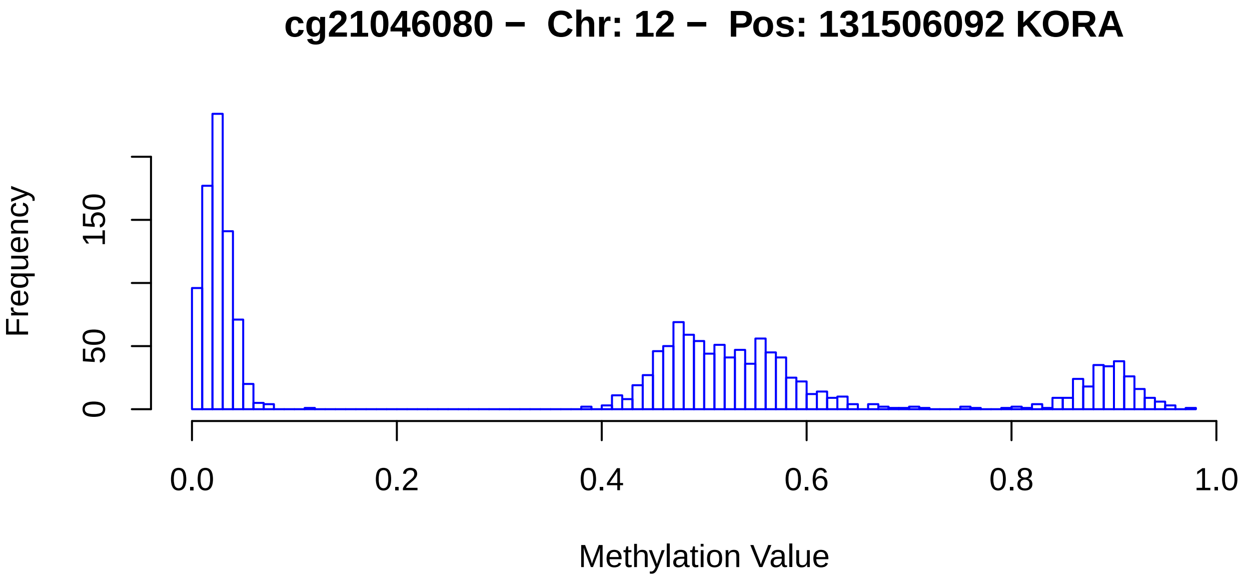

cg21046080 − Chr: 12 − Pos: 131506092 KORA
Frequency
0
50
150
0.0
0.2
0.4
0.6
0.8
1.0
Methylation Value

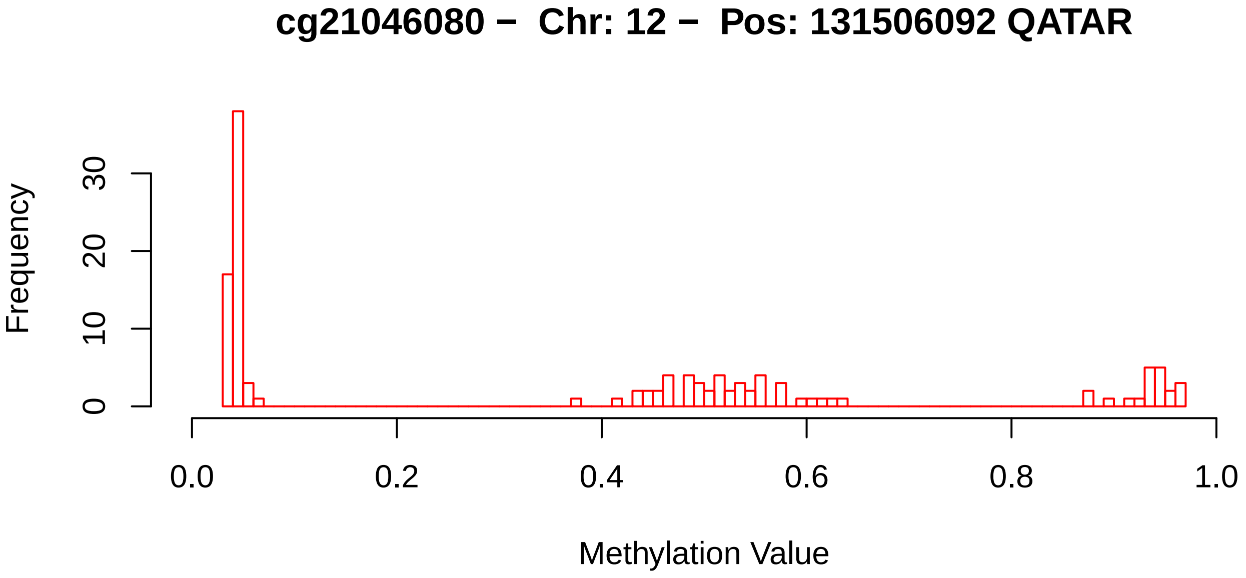

cg21046080 − Chr: 12 − Pos: 131506092 QATAR
Frequency
0
10
20
30
0.0
0.2
0.4
0.6
0.8
1.0
Methylation Value

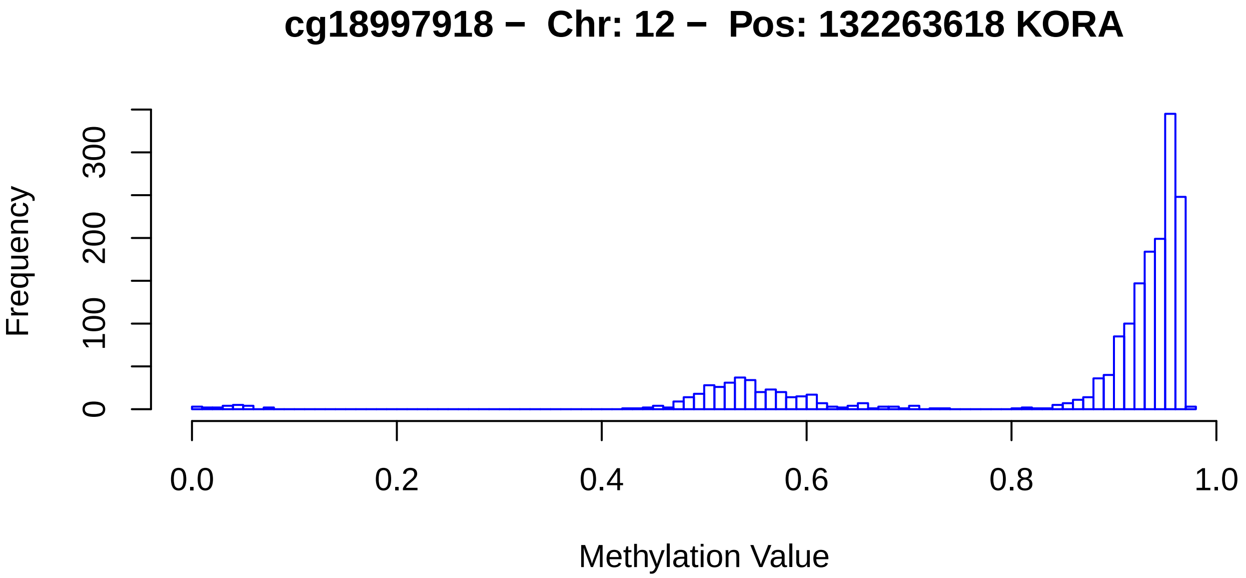
cg18997918 − Chr: 12 − Pos: 132263618 KORA
Frequency
0
100
200
300
0.0
0.2
0.4
0.6
0.8
1.0
Methylation Value

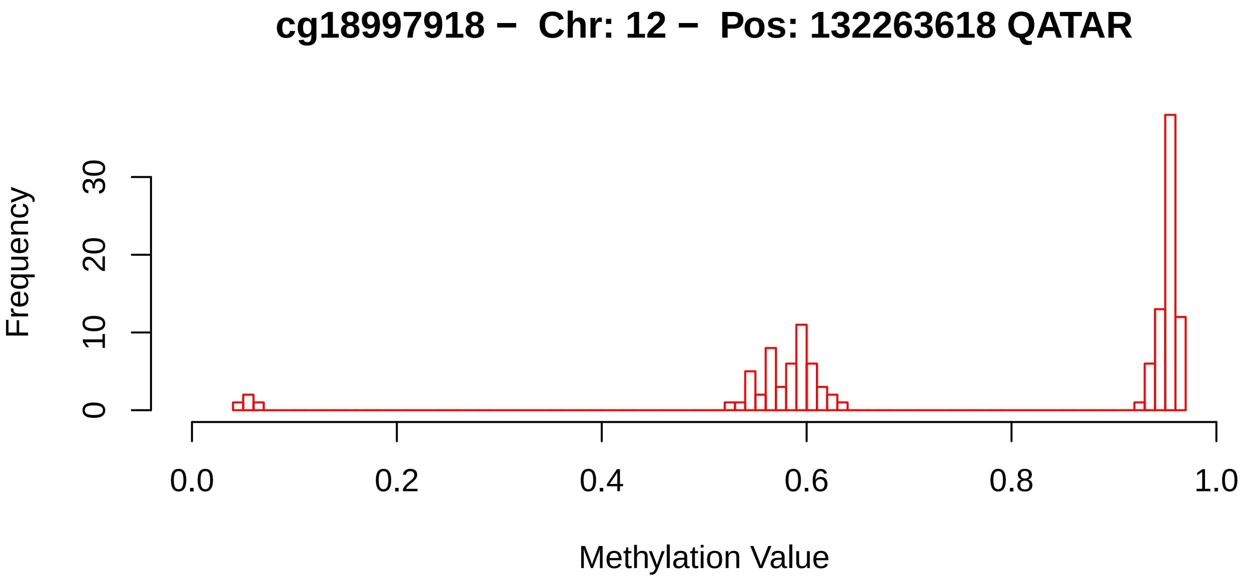
cg18997918 − Chr: 12 − Pos: 132263618 QATAR
Frequency
0
10
20
30
0.0
0.2
0.4
0.6
0.8
1.0
Methylation Value

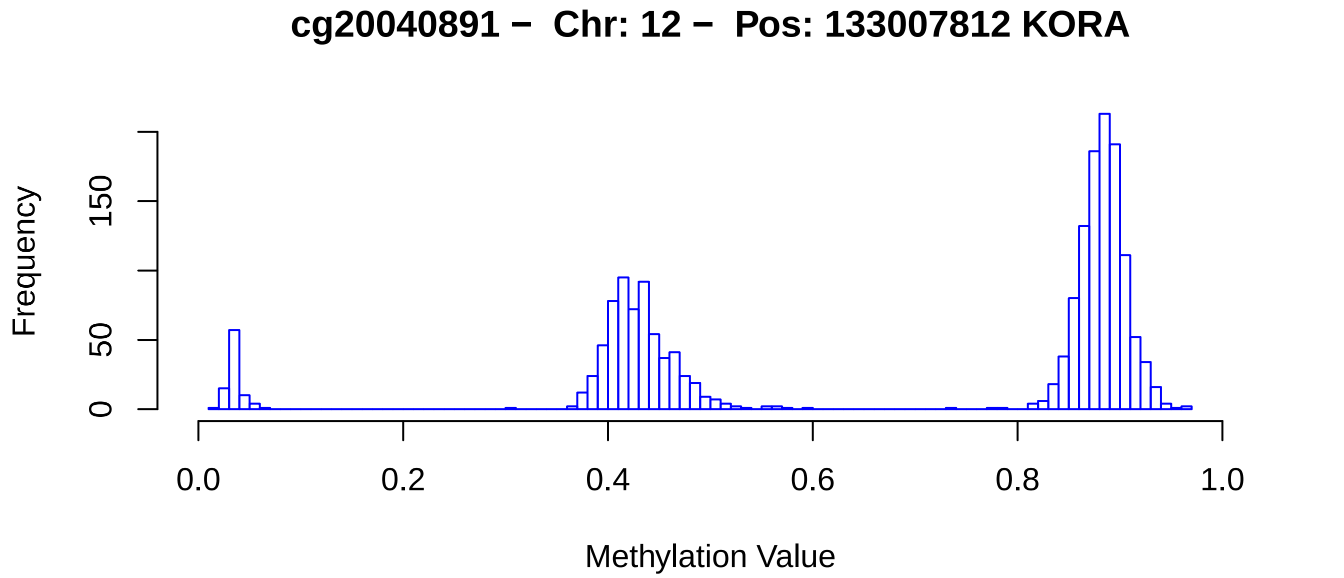
cg20040891 − Chr: 12 − Pos: 133007812 KORA
Frequency
0
50
150
0.0
0.2
0.4
0.6
0.8
1.0
Methylation Value

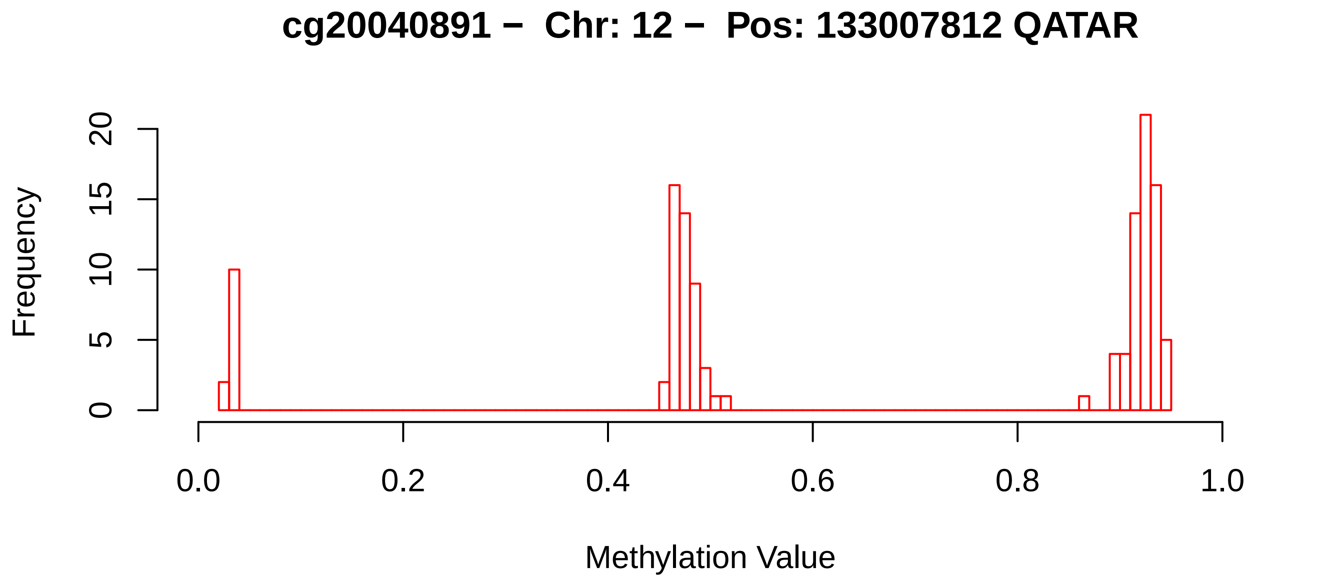
cg20040891 − Chr: 12 − Pos: 133007812 QATAR
Frequency
0
5
10
15
20
0.0
0.2
0.4
0.6
0.8
1.0
Methylation Value

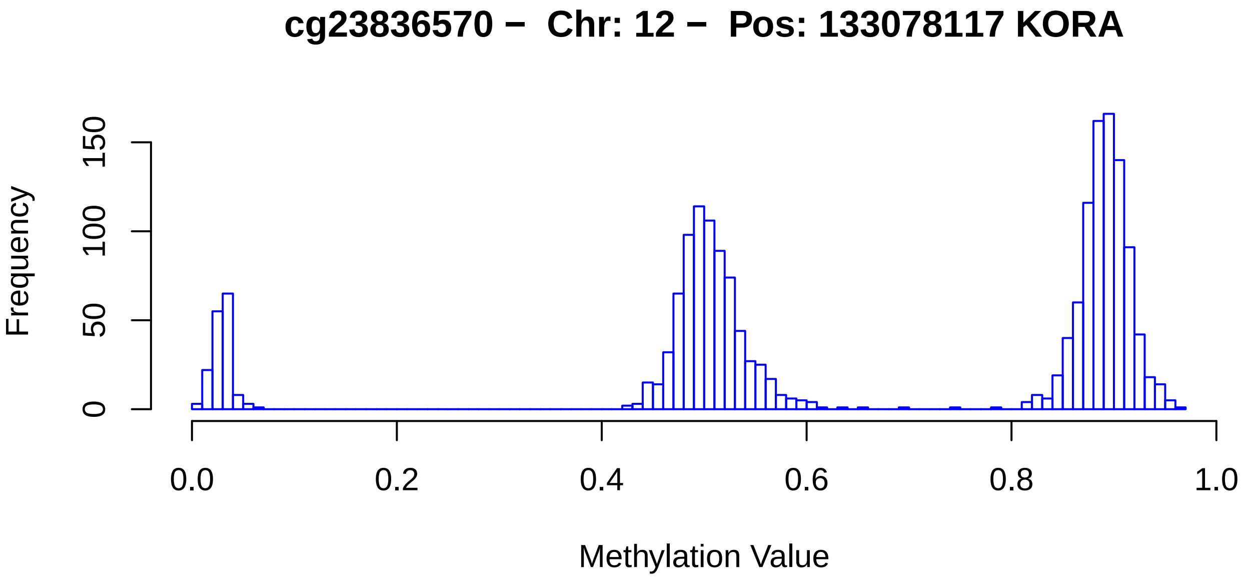
cg23836570 − Chr: 12 − Pos: 133078117 KORA
Frequency
0
50
100
150
0.0
0.2
0.4
0.6
0.8
1.0
Methylation Value

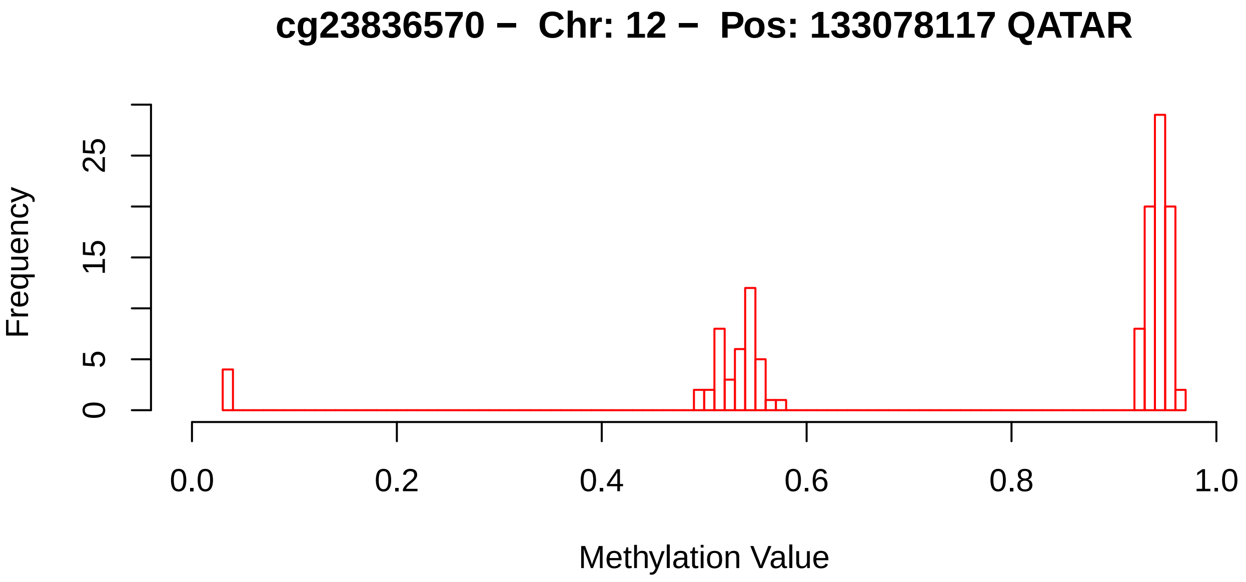
cg23836570 − Chr: 12 − Pos: 133078117 QATAR
Frequency
0
5
15
25
0.0
0.2
0.4
0.6
0.8
1.0
Methylation Value

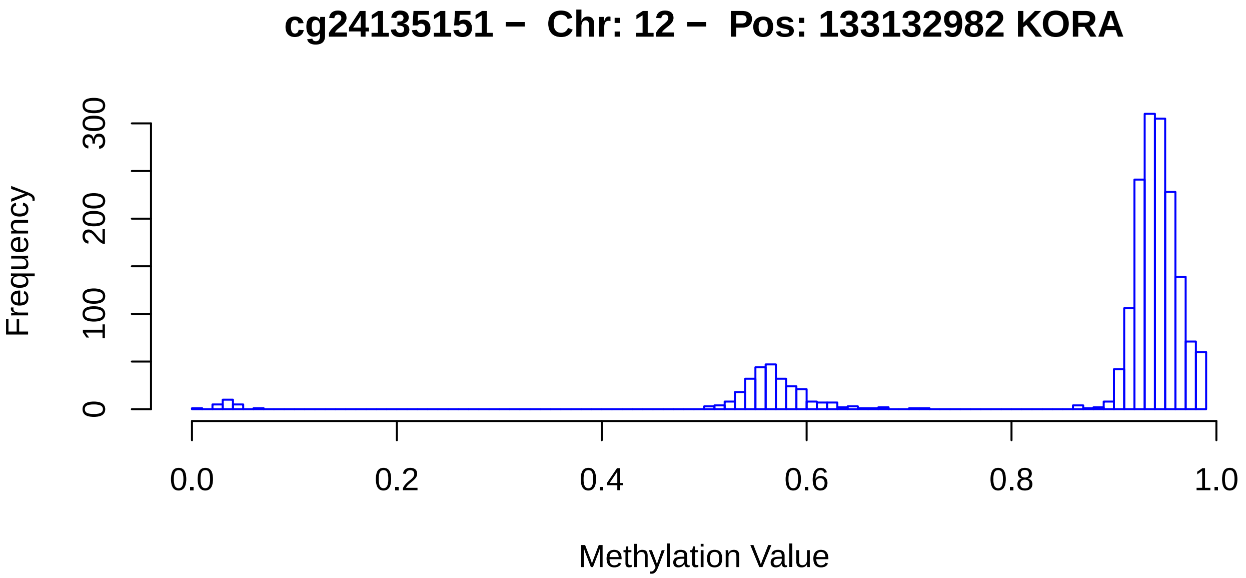
cg24135151 − Chr: 12 − Pos: 133132982 KORA
Frequency
0
100
200
300
0.0
0.2
0.4
0.6
0.8
1.0
Methylation Value

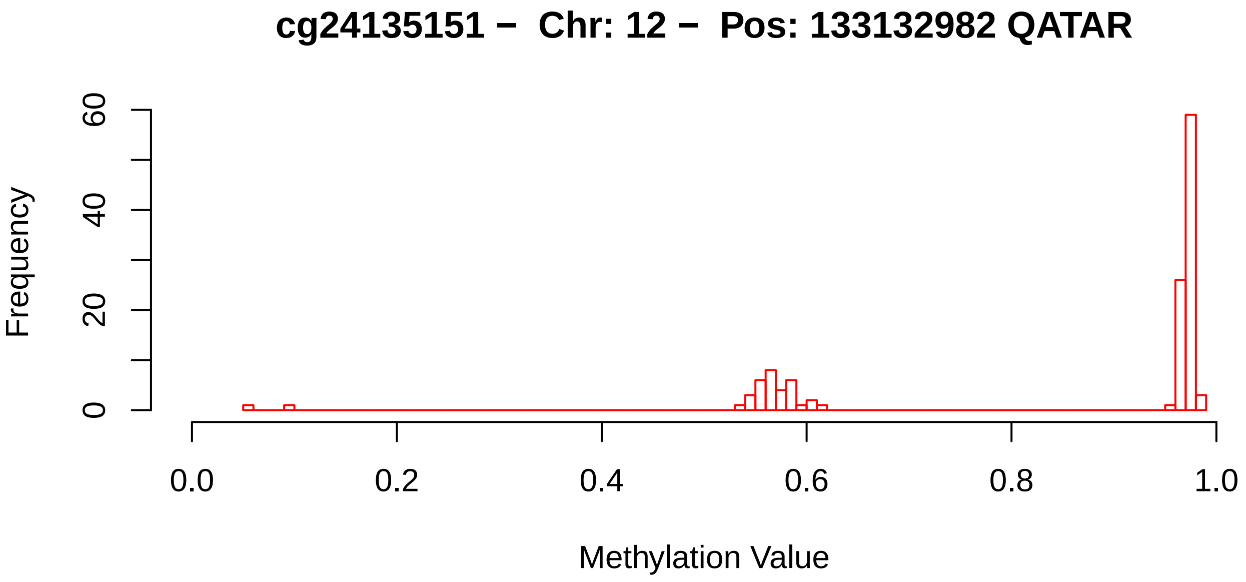
cg24135151 − Chr: 12 − Pos: 133132982 QATAR
Frequency
0
20
40
60
0.0
0.2
0.4
0.6
0.8
1.0
Methylation Value

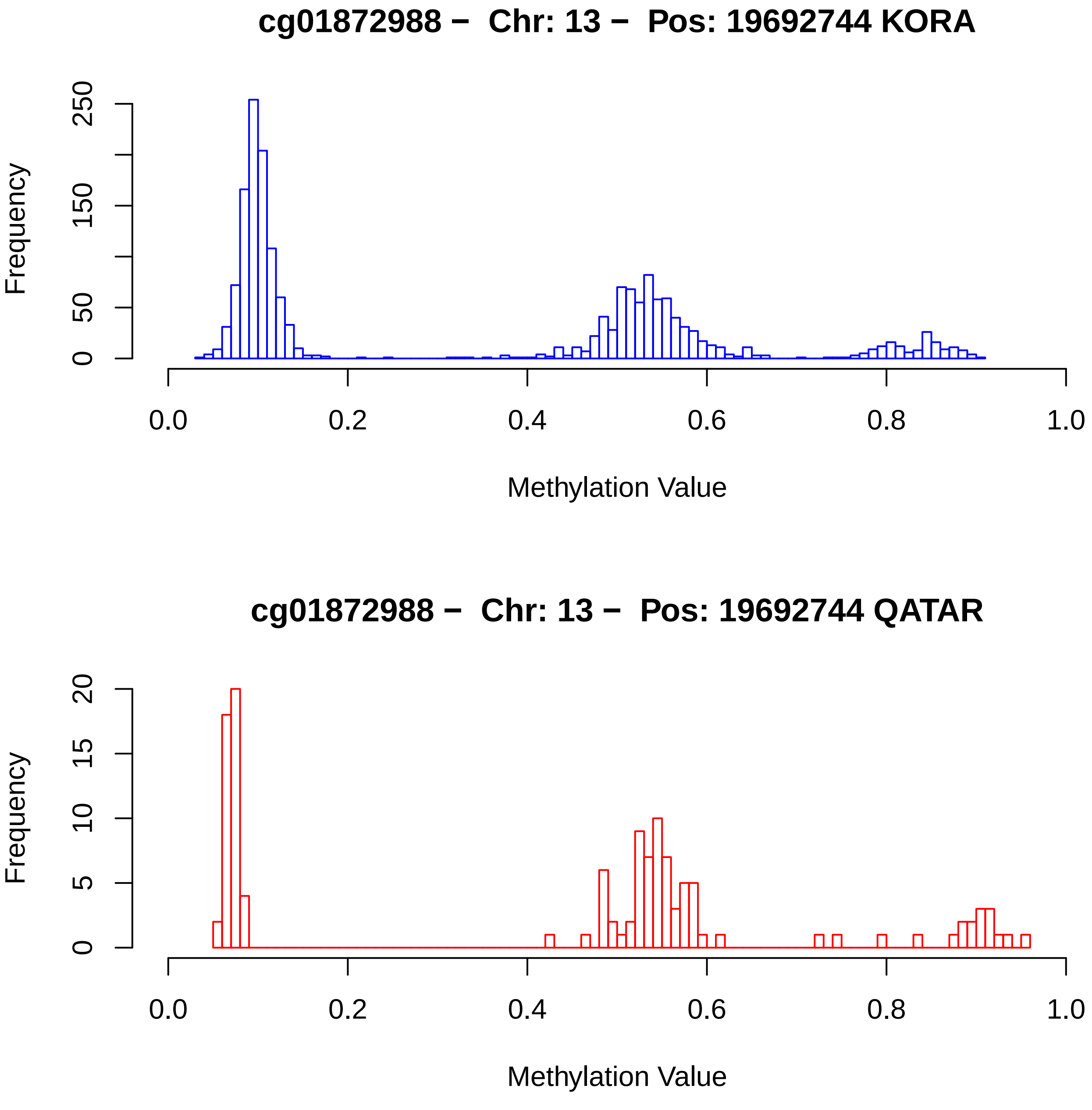
cg01872988 − Chr: 13 − Pos: 19692744 KORA
Frequency
0
50
150
250
0.0
0.2
0.4
0.6
0.8
1.0
Methylation Value
cg01872988 − Chr: 13 − Pos: 19692744 QATAR
Frequency
0
5
10
15
20
0.0
0.2
0.4
0.6
0.8
1.0
Methylation Value

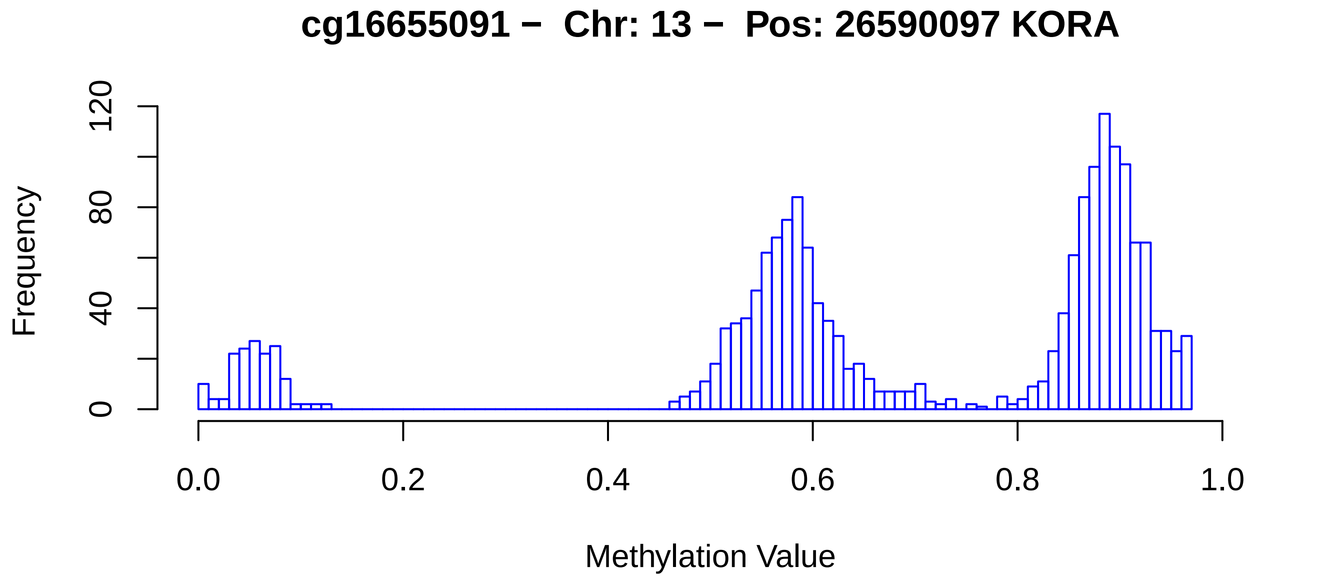
cg16655091 − Chr: 13 − Pos: 26590097 KORA
Frequency
0
40
80
120
0.0
0.2
0.4
0.6
0.8
1.0
Methylation Value

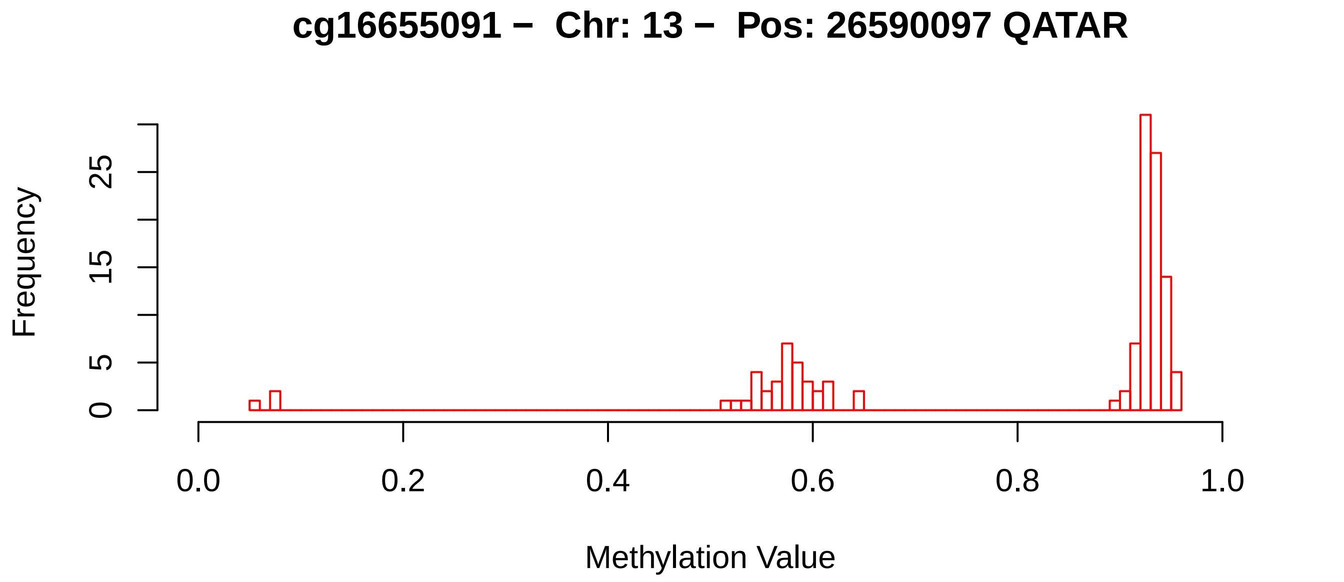
cg16655091 − Chr: 13 − Pos: 26590097 QATAR
Frequency
0
5
15
25
0.0
0.2
0.4
0.6
0.8
1.0
Methylation Value

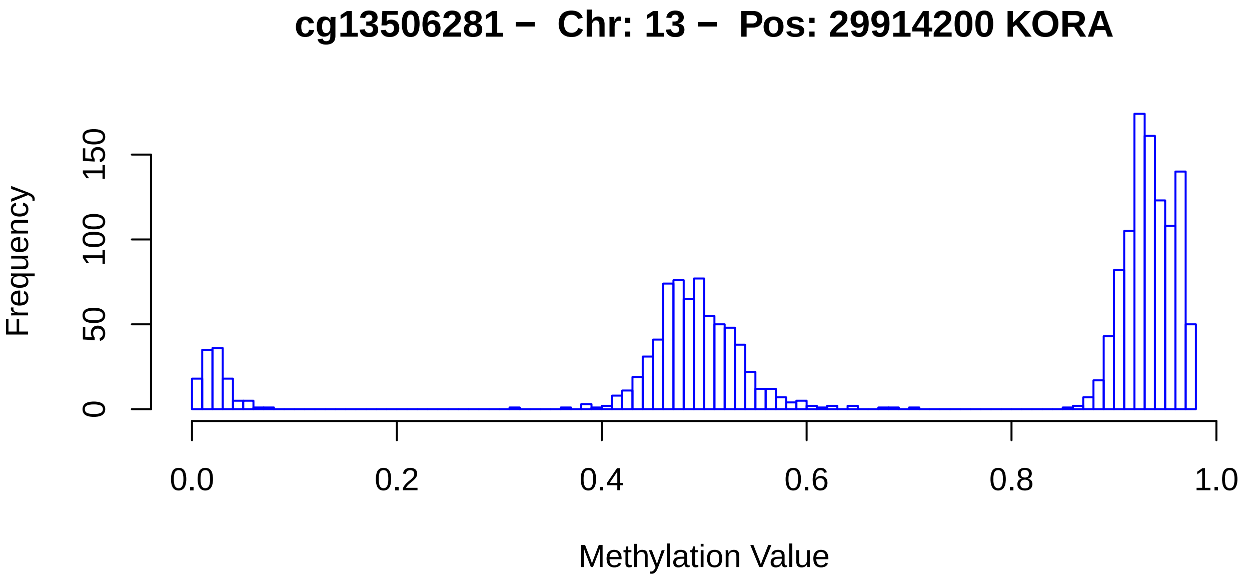

cg13506281 − Chr: 13 − Pos: 29914200 KORA
Frequency
0
50
100
150
0.0
0.2
0.4
0.6
0.8
1.0
Methylation Value

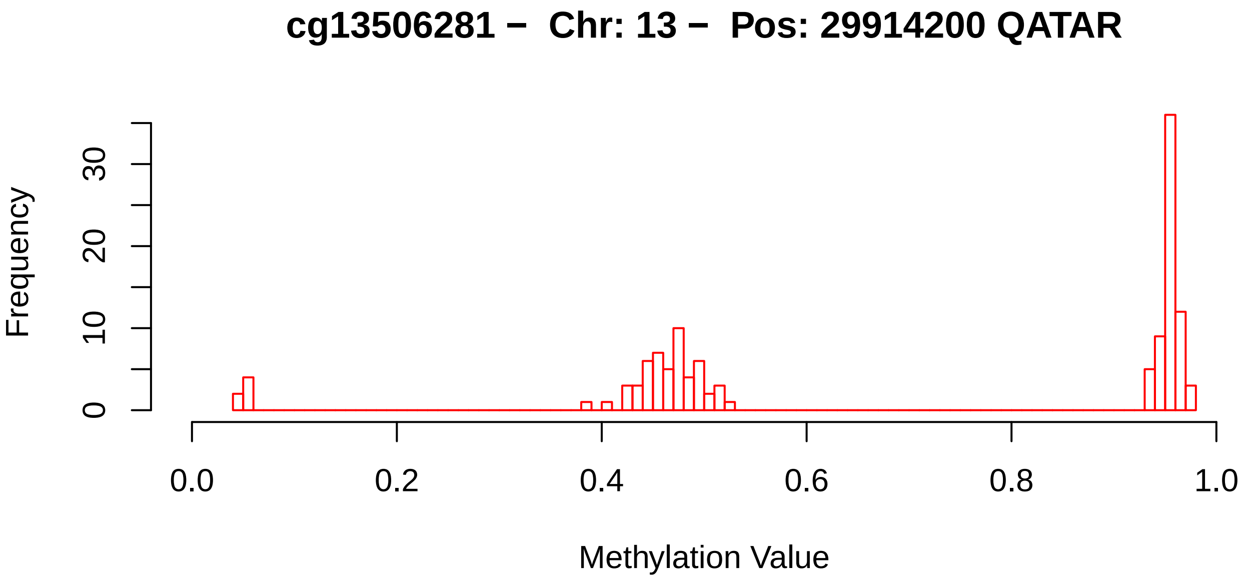

cg13506281 − Chr: 13 − Pos: 29914200 QATAR
Frequency
0
10
20
30
0.0
0.2
0.4
0.6
0.8
1.0
Methylation Value

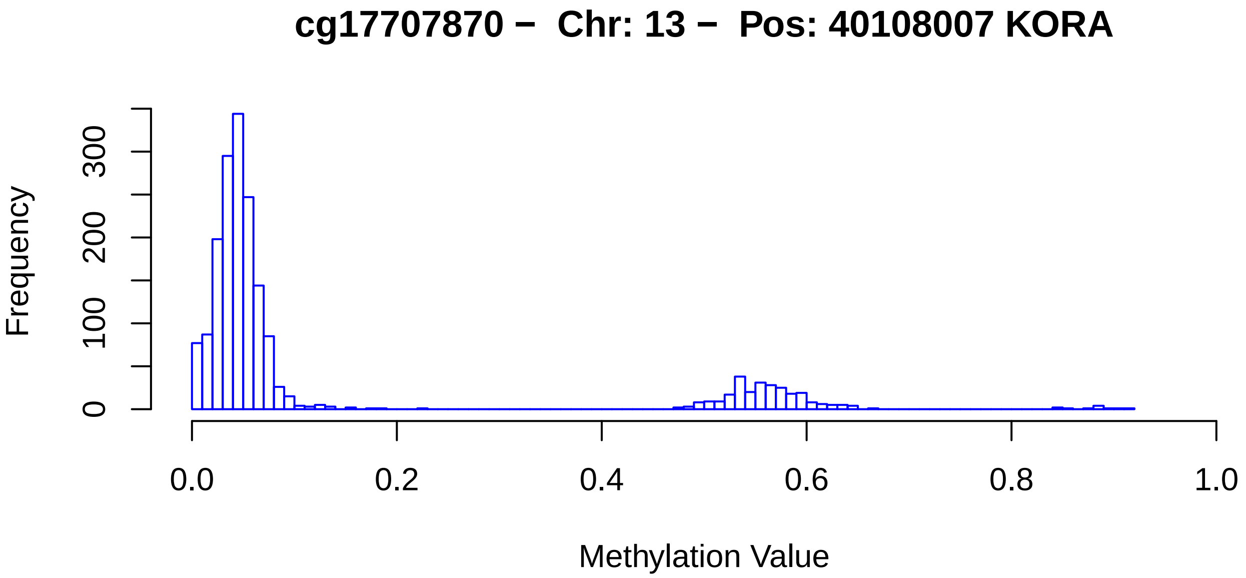
cg17707870 − Chr: 13 − Pos: 40108007 KORA
Frequency
0
100
200
300
0.0
0.2
0.4
0.6
0.8
1.0
Methylation Value

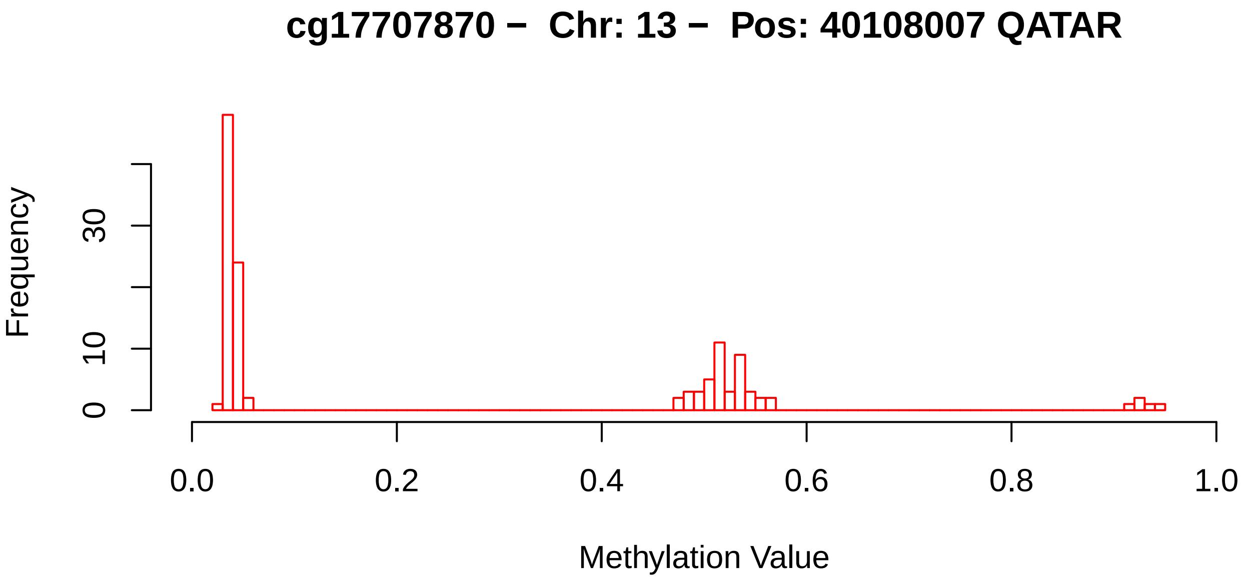
cg17707870 − Chr: 13 − Pos: 40108007 QATAR
Frequency
0
10
30
0.0
0.2
0.4
0.6
0.8
1.0
Methylation Value

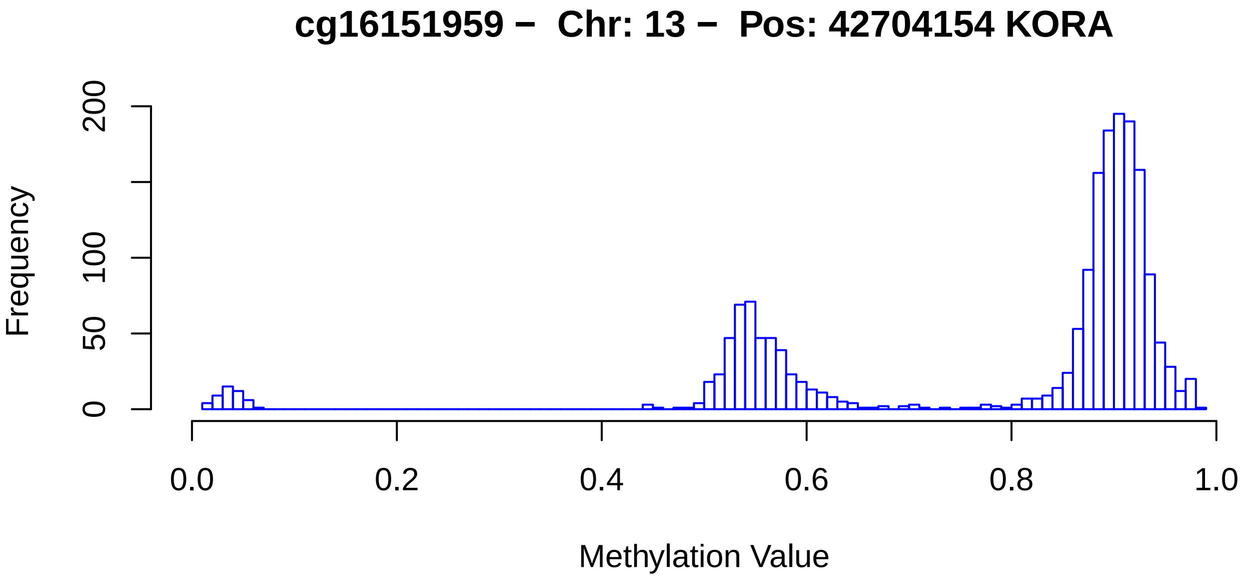
cg16151959 − Chr: 13 − Pos: 42704154 KORA
Frequency
0
50
100
200
0.0
0.2
0.4
0.6
0.8
1.0
Methylation Value

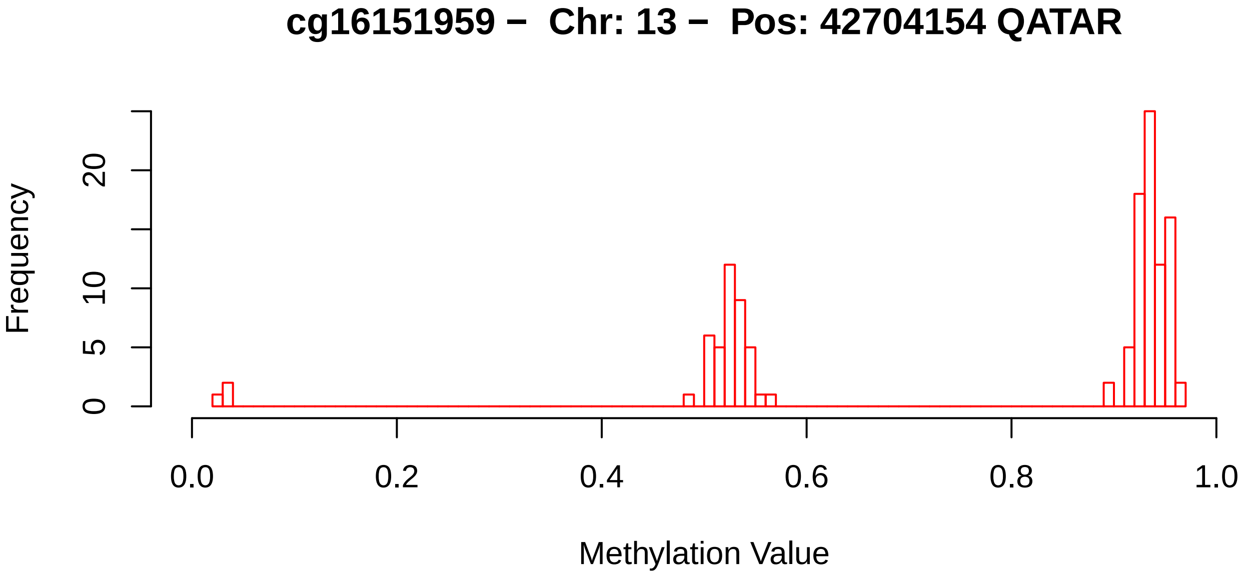
cg16151959 − Chr: 13 − Pos: 42704154 QATAR
Frequency
0
5
10
20
0.0
0.2
0.4
0.6
0.8
1.0
Methylation Value

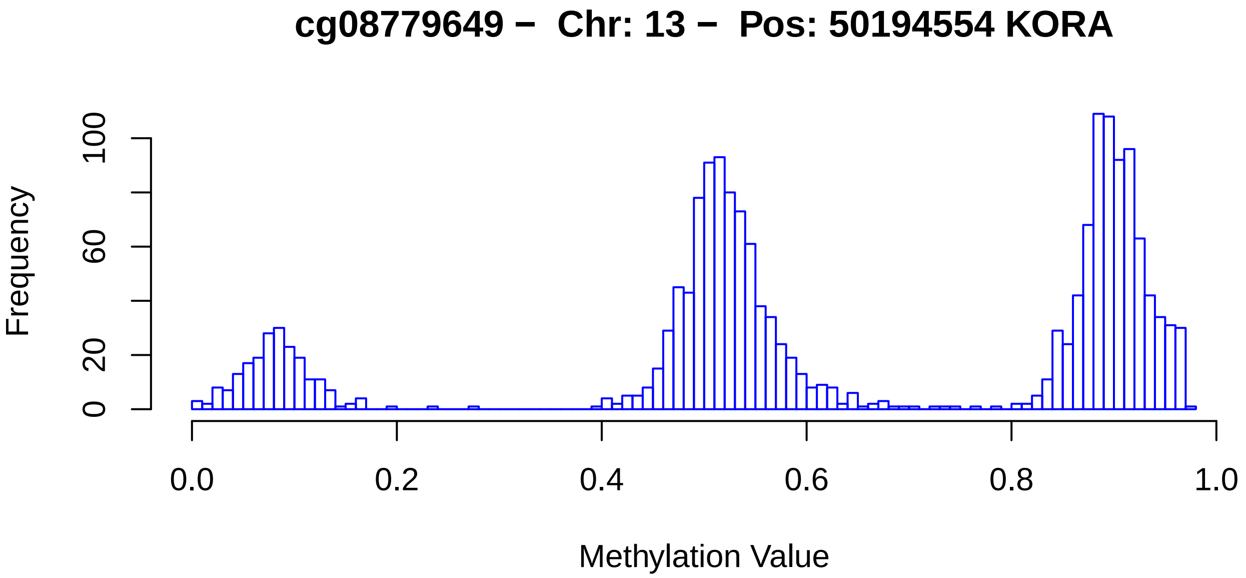

cg08779649 − Chr: 13 − Pos: 50194554 KORA
Frequency
0
20
60
100
0.0
0.2
0.4
0.6
0.8
1.0
Methylation Value

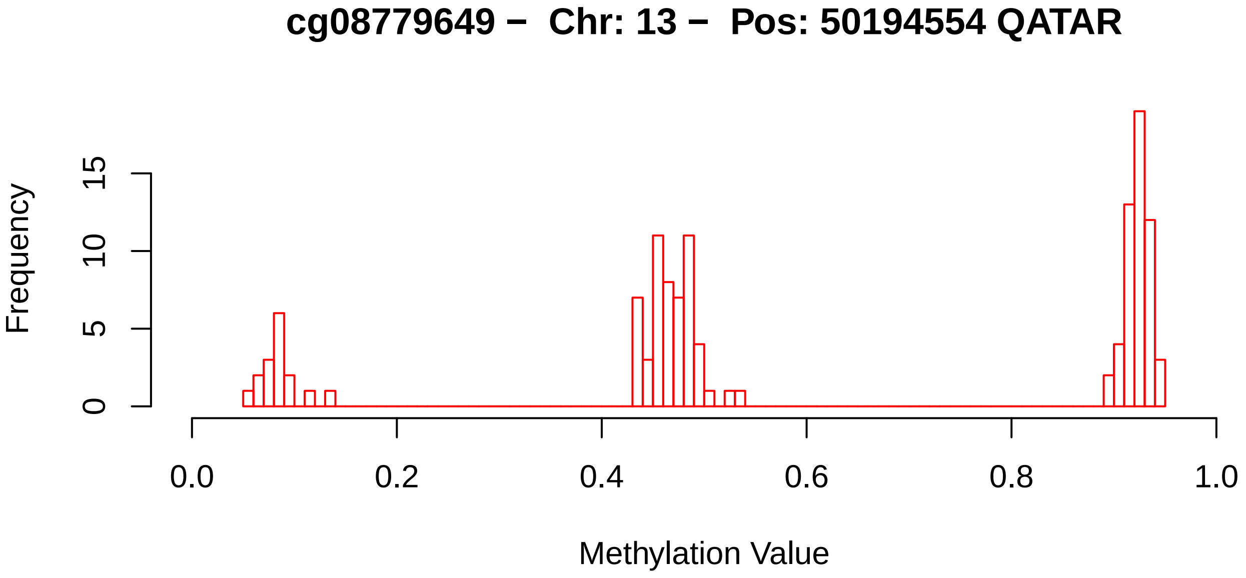

cg08779649 − Chr: 13 − Pos: 50194554 QATAR
Frequency
0
5
10
15
0.0
0.2
0.4
0.6
0.8
1.0
Methylation Value

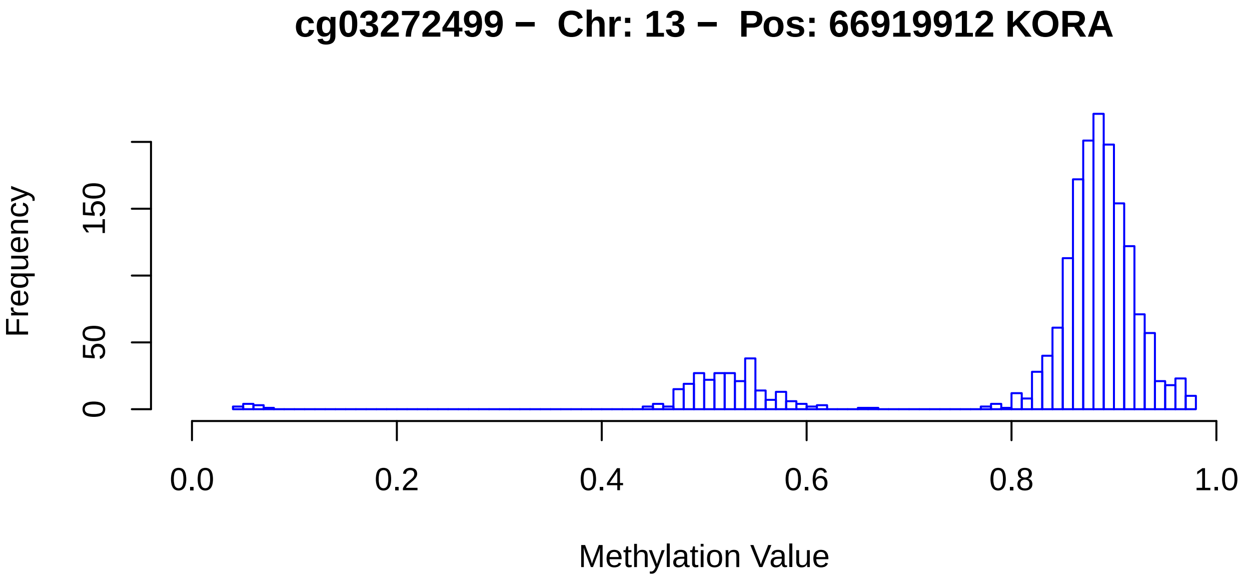
cg03272499 − Chr: 13 − Pos: 66919912 KORA
Frequency
0
50
150
0.0
0.2
0.4
0.6
0.8
1.0
Methylation Value

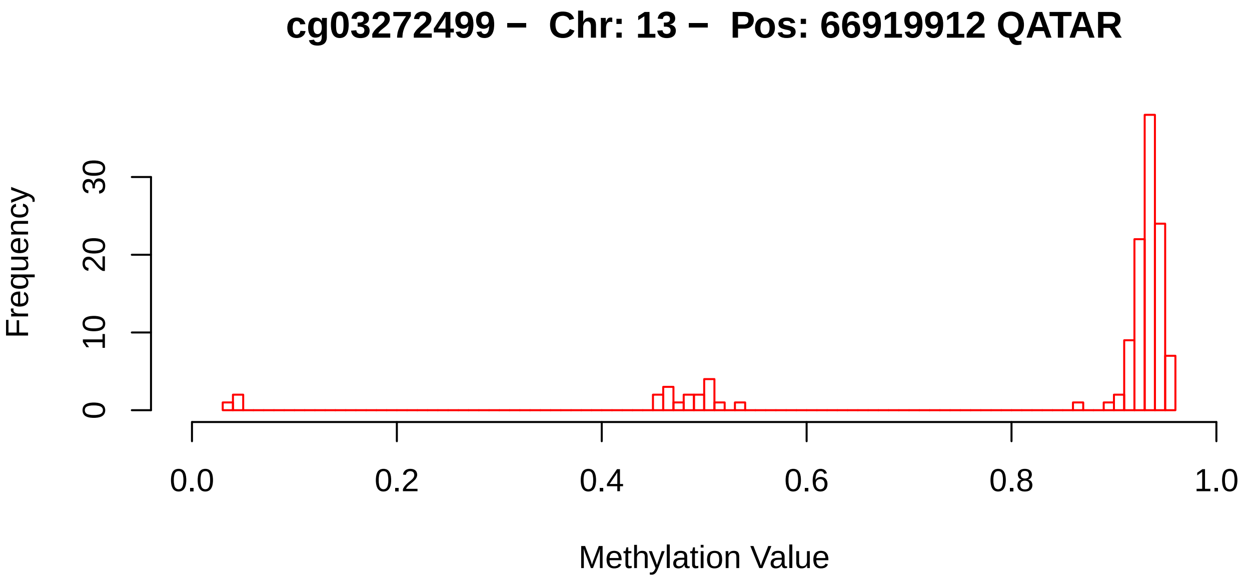
cg03272499 − Chr: 13 − Pos: 66919912 QATAR
Frequency
0
10
20
30
0.0
0.2
0.4
0.6
0.8
1.0
Methylation Value

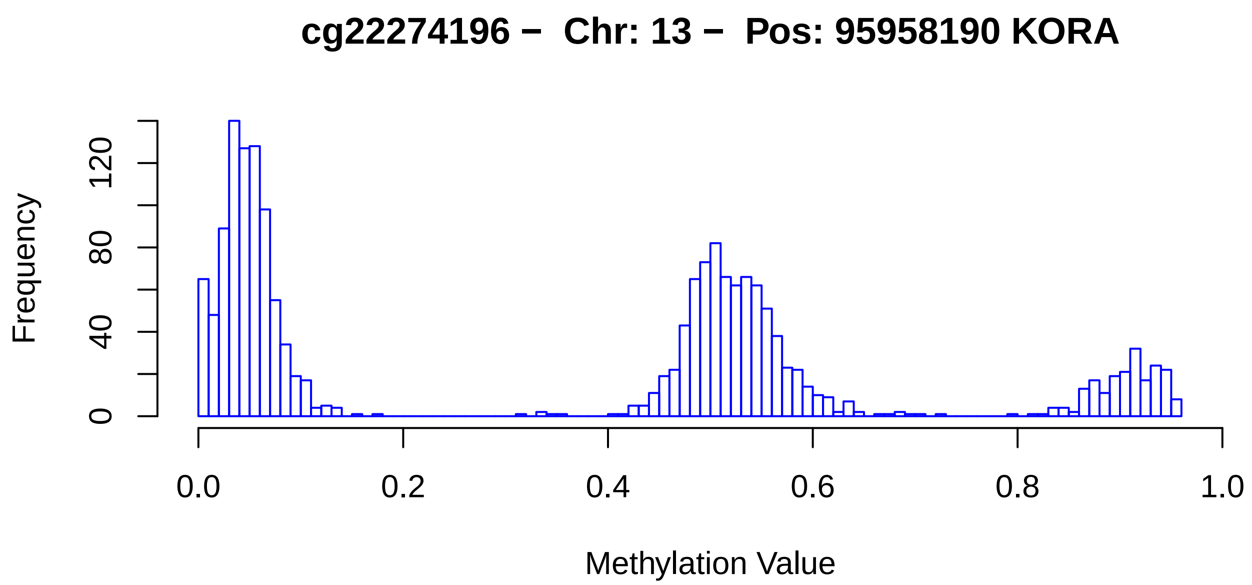
cg22274196 − Chr: 13 − Pos: 95958190 KORA
Frequency
0
40
80
120
0.0
0.2
0.4
0.6
0.8
1.0
Methylation Value

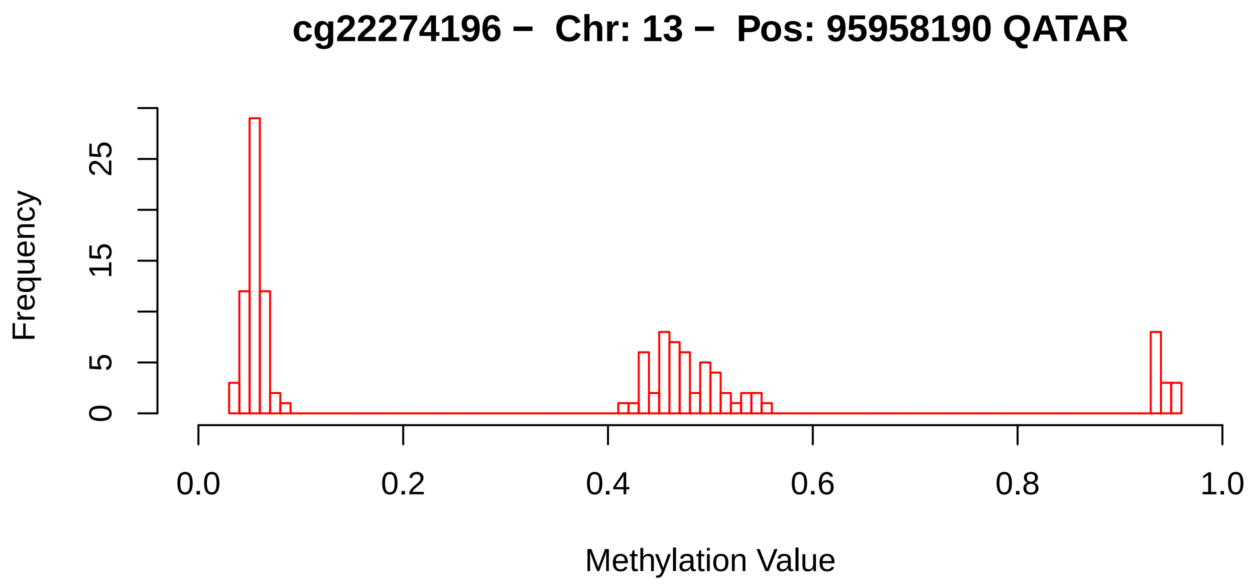
cg22274196 − Chr: 13 − Pos: 95958190 QATAR
Frequency
0
5
15
25
0.0
0.2
0.4
0.6
0.8
1.0
Methylation Value

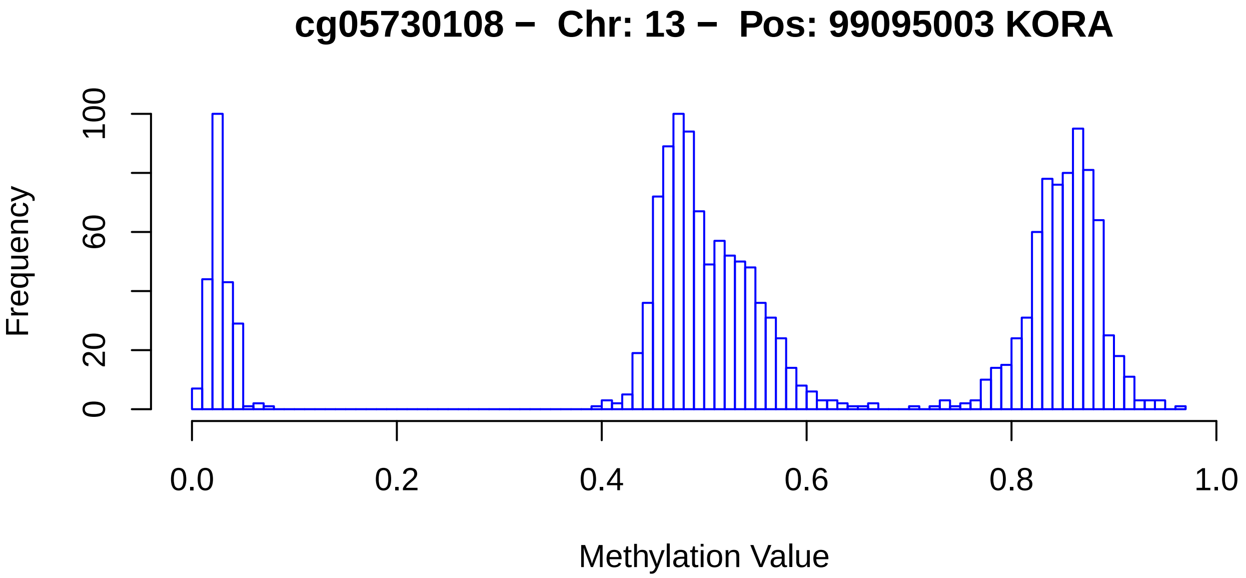
cg05730108 − Chr: 13 − Pos: 99095003 KORA
Frequency
0
20
60
100
0.0
0.2
0.4
0.6
0.8
1.0
Methylation Value

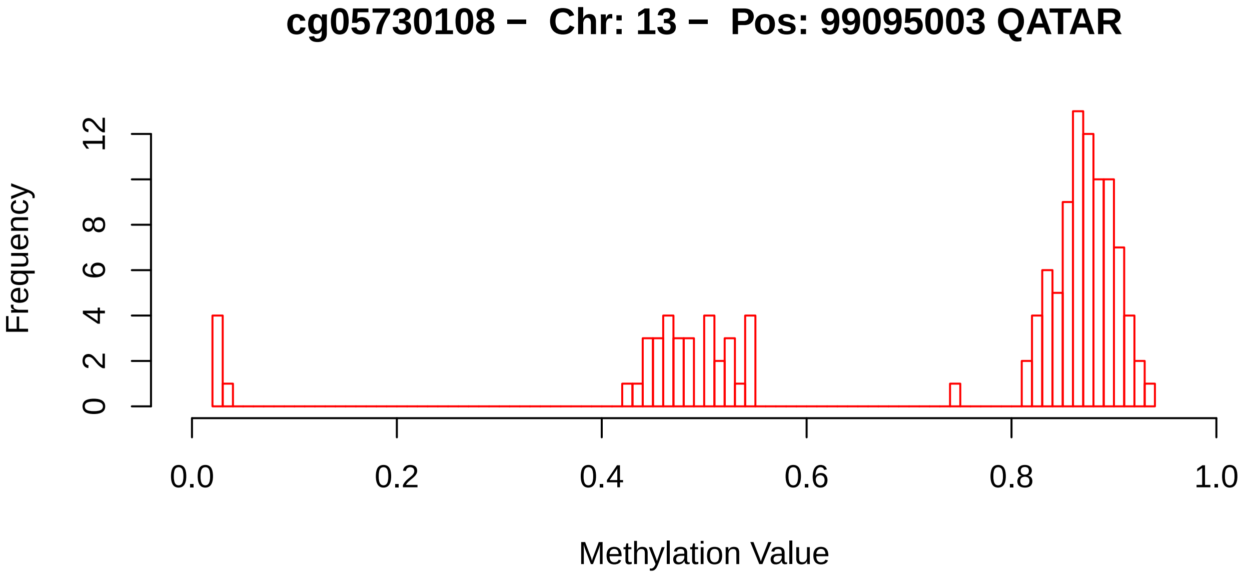
cg05730108 − Chr: 13 − Pos: 99095003 QATAR
Frequency
0
2
4
6
8
12
0.0
0.2
0.4
0.6
0.8
1.0
Methylation Value

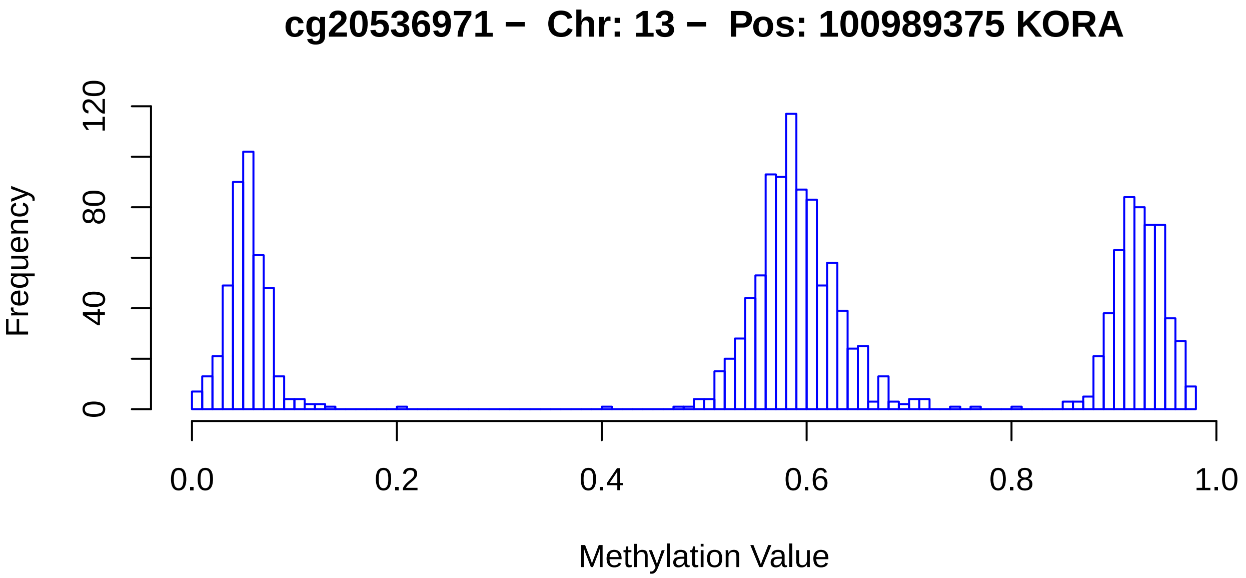
cg20536971 − Chr: 13 − Pos: 100989375 KORA
Frequency
0
40
80
120
0.0
0.2
0.4
0.6
0.8
1.0
Methylation Value

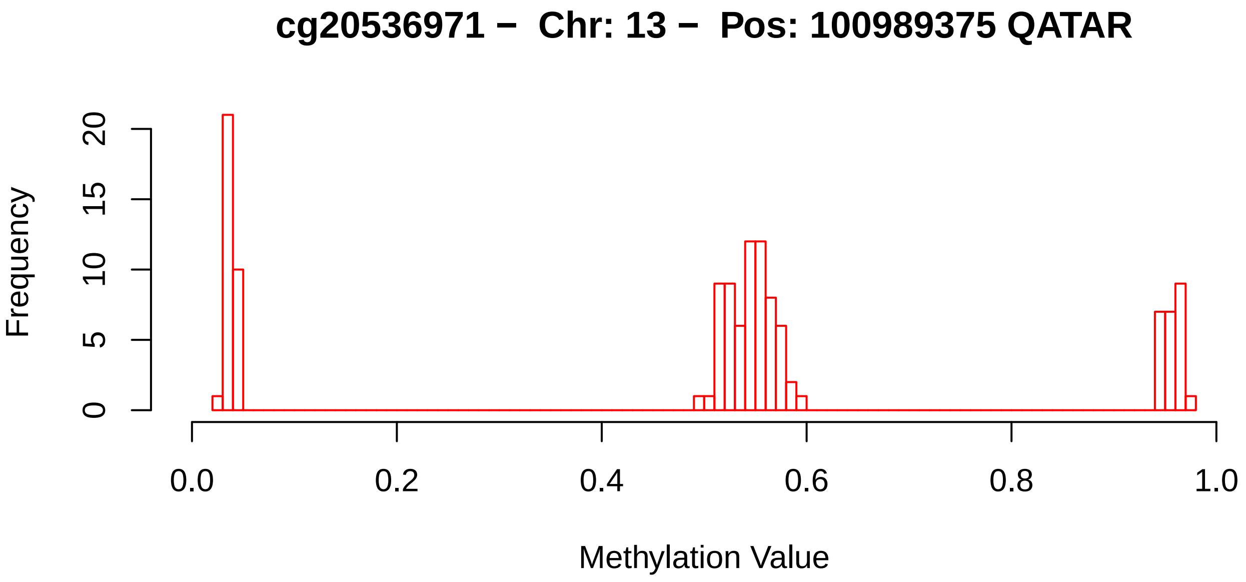
cg20536971 − Chr: 13 − Pos: 100989375 QATAR
Frequency
0
5
10
15
20
0.0
0.2
0.4
0.6
0.8
1.0
Methylation Value

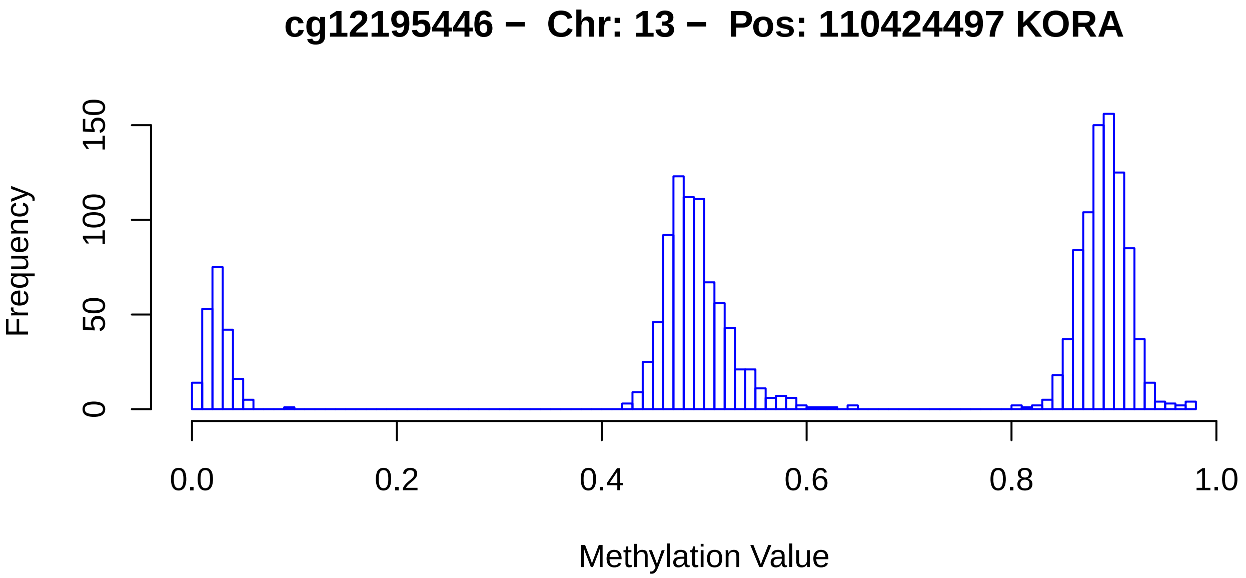
cg12195446 − Chr: 13 − Pos: 110424497 KORA
Frequency
0
50
100
150
0.0
0.2
0.4
0.6
0.8
1.0
Methylation Value

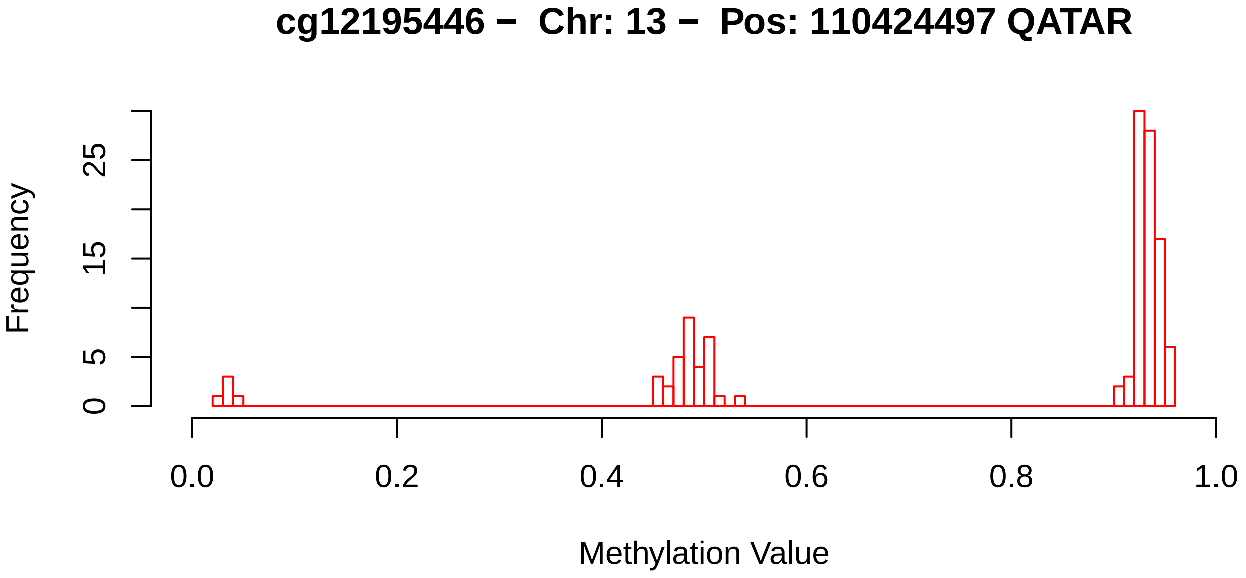
cg12195446 − Chr: 13 − Pos: 110424497 QATAR
Frequency
0
5
15
25
0.0
0.2
0.4
0.6
0.8
1.0
Methylation Value

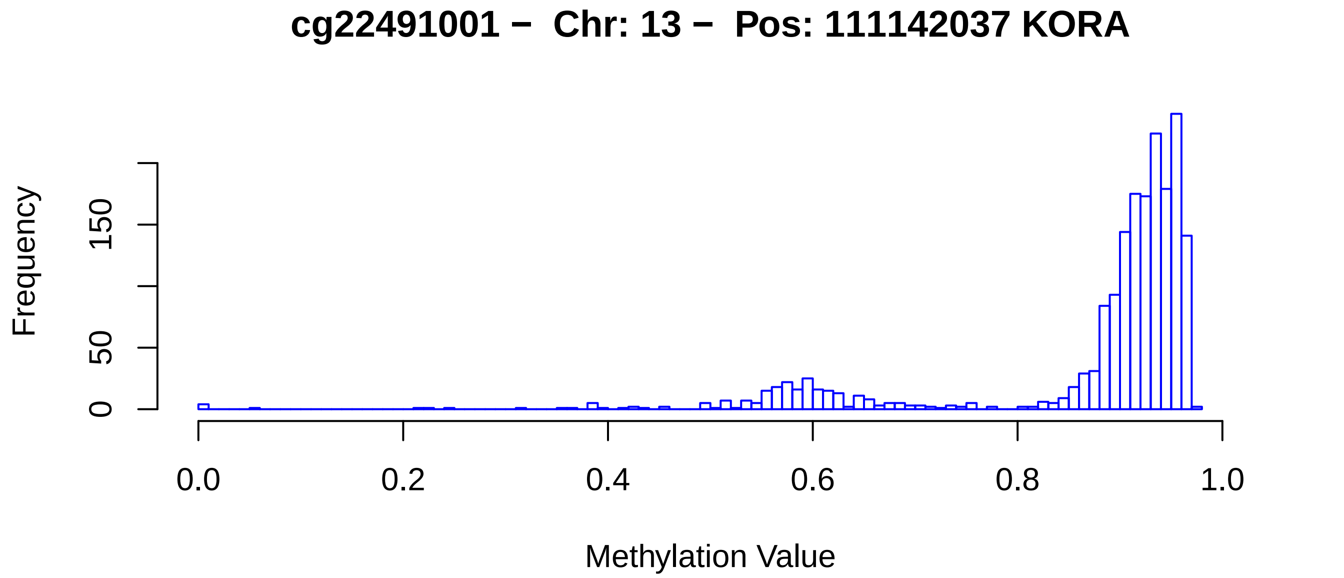
cg22491001 − Chr: 13 − Pos: 111142037 KORA
Frequency
0
50
150
0.0
0.2
0.4
0.6
0.8
1.0
Methylation Value

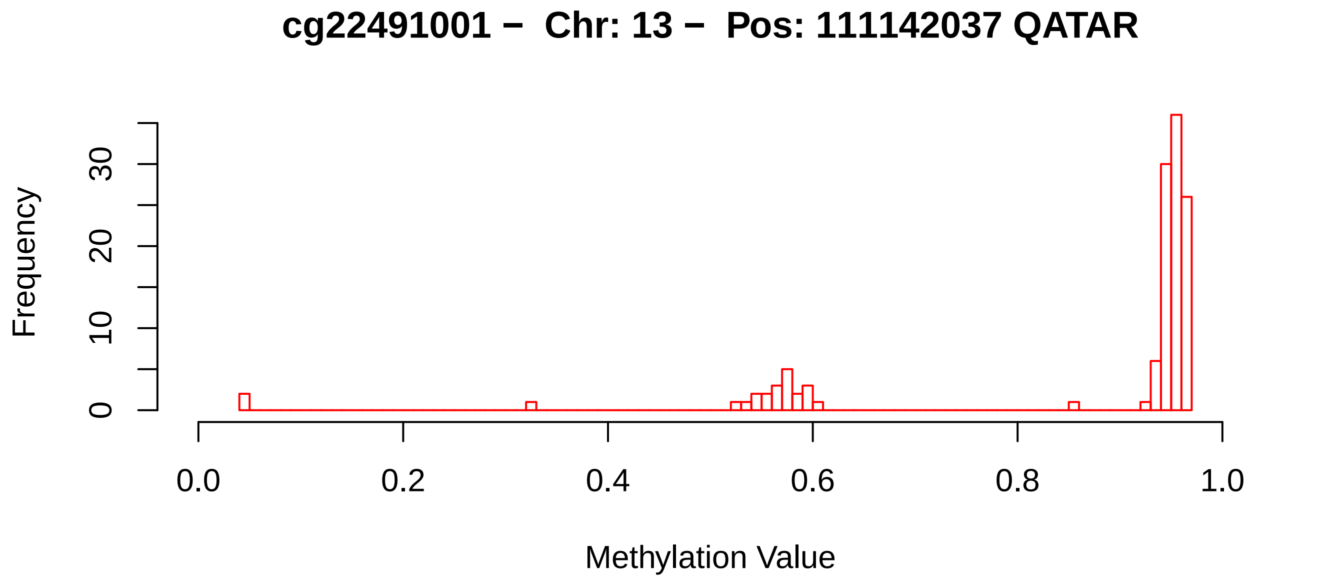
cg22491001 − Chr: 13 − Pos: 111142037 QATAR
Frequency
0
10
20
30
0.0
0.2
0.4
0.6
0.8
1.0
Methylation Value

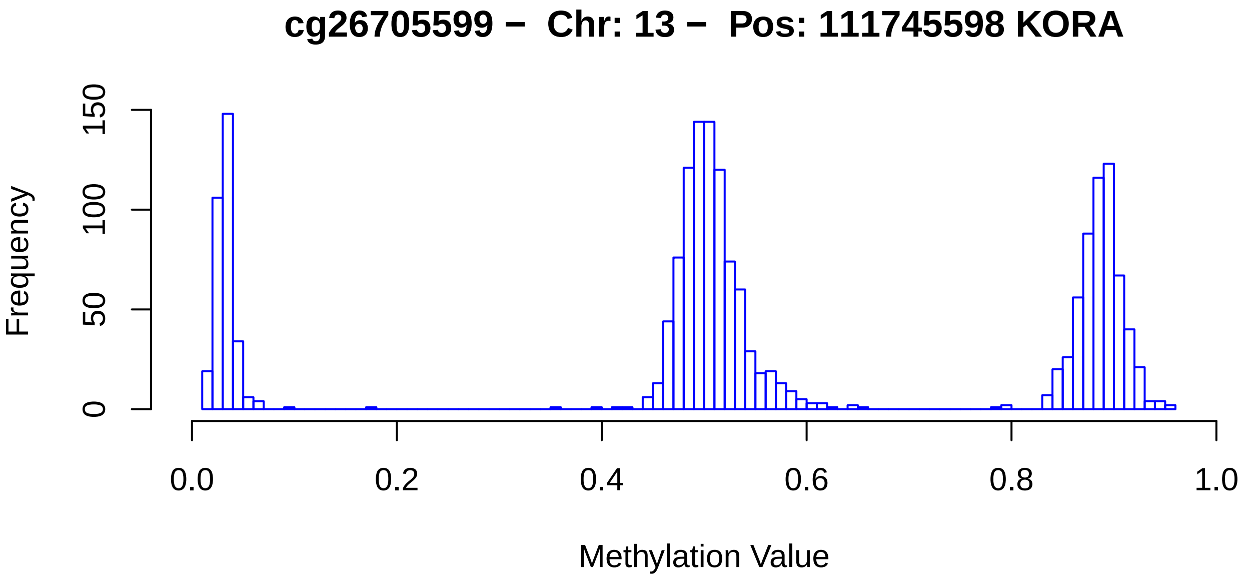

cg26705599 − Chr: 13 − Pos: 111745598 KORA
Frequency
0
50
100
150
0.0
0.2
0.4
0.6
0.8
1.0
Methylation Value

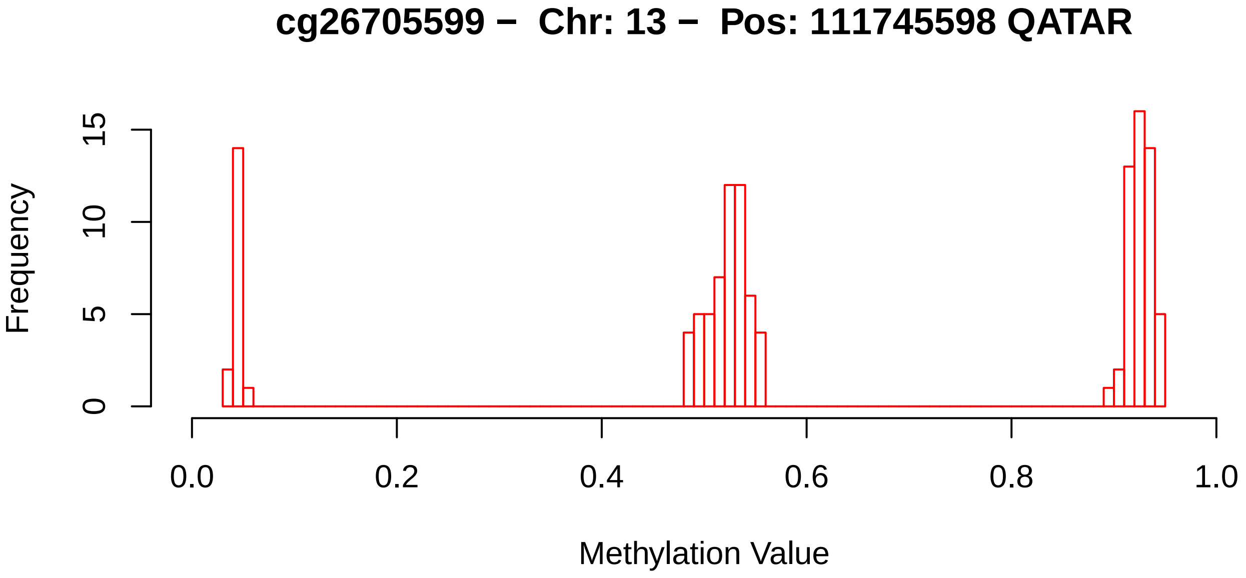

cg26705599 − Chr: 13 − Pos: 111745598 QATAR
Frequency
0
5
10
15
0.0
0.2
0.4
0.6
0.8
1.0
Methylation Value

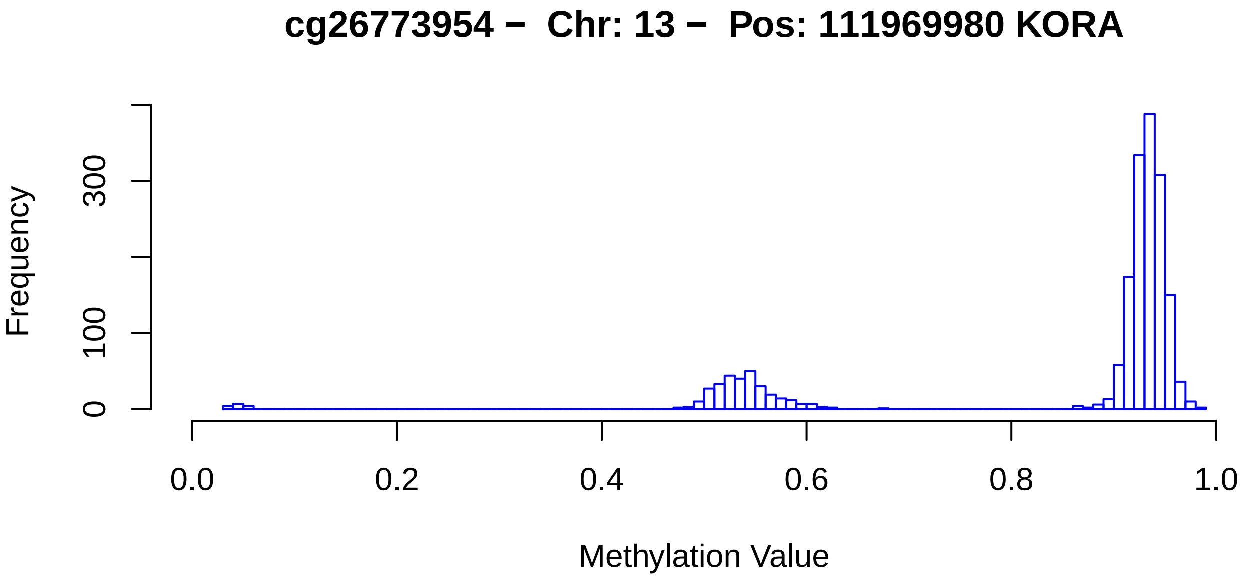
cg26773954 − Chr: 13 − Pos: 111969980 KORA
Frequency
0
100
300
0.0
0.2
0.4
0.6
0.8
1.0
Methylation Value

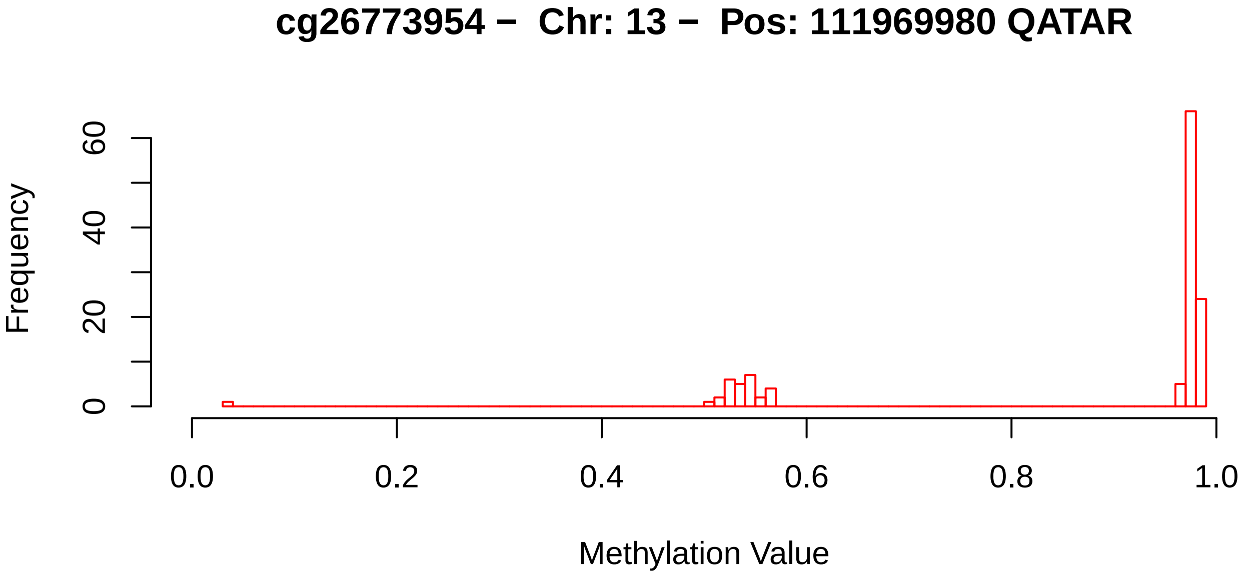
cg26773954 − Chr: 13 − Pos: 111969980 QATAR
Frequency
0
20
40
60
0.0
0.2
0.4
0.6
0.8
1.0
Methylation Value

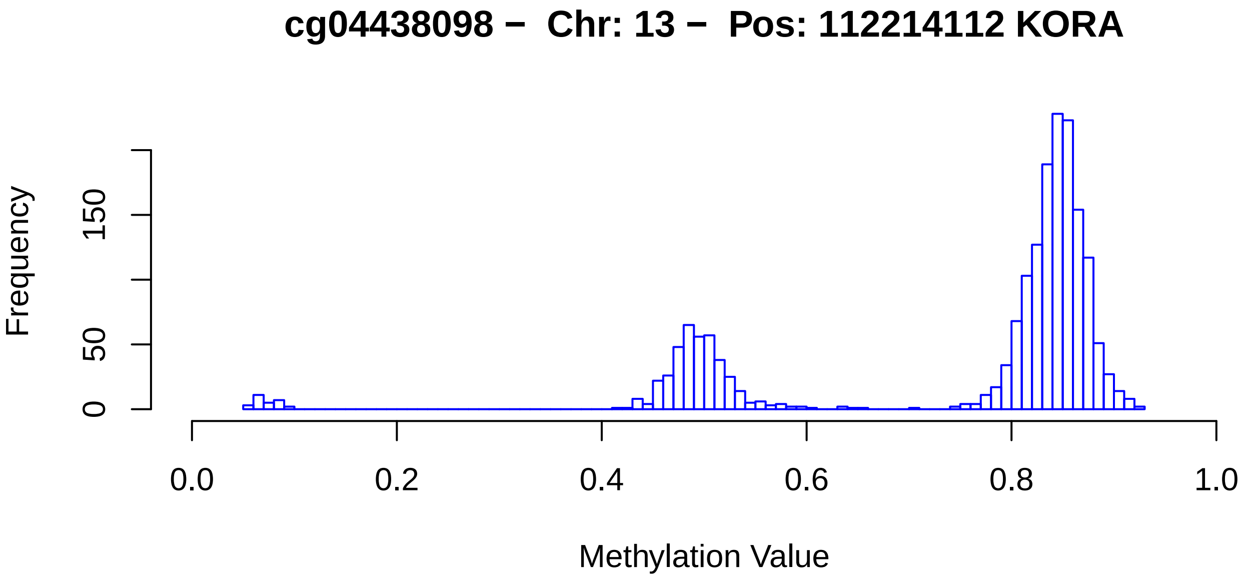
cg04438098 − Chr: 13 − Pos: 112214112 KORA
Frequency
0
50
150
0.0
0.2
0.4
0.6
0.8
1.0
Methylation Value

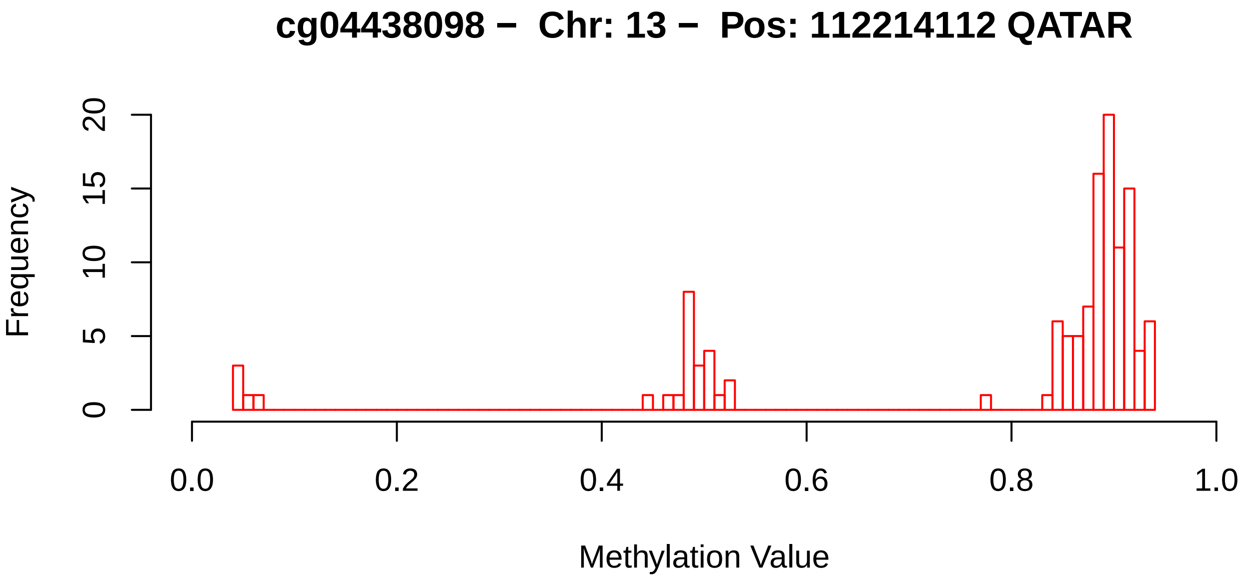
cg04438098 − Chr: 13 − Pos: 112214112 QATAR
Frequency
0
5
10
15
20
0.0
0.2
0.4
0.6
0.8
1.0
Methylation Value

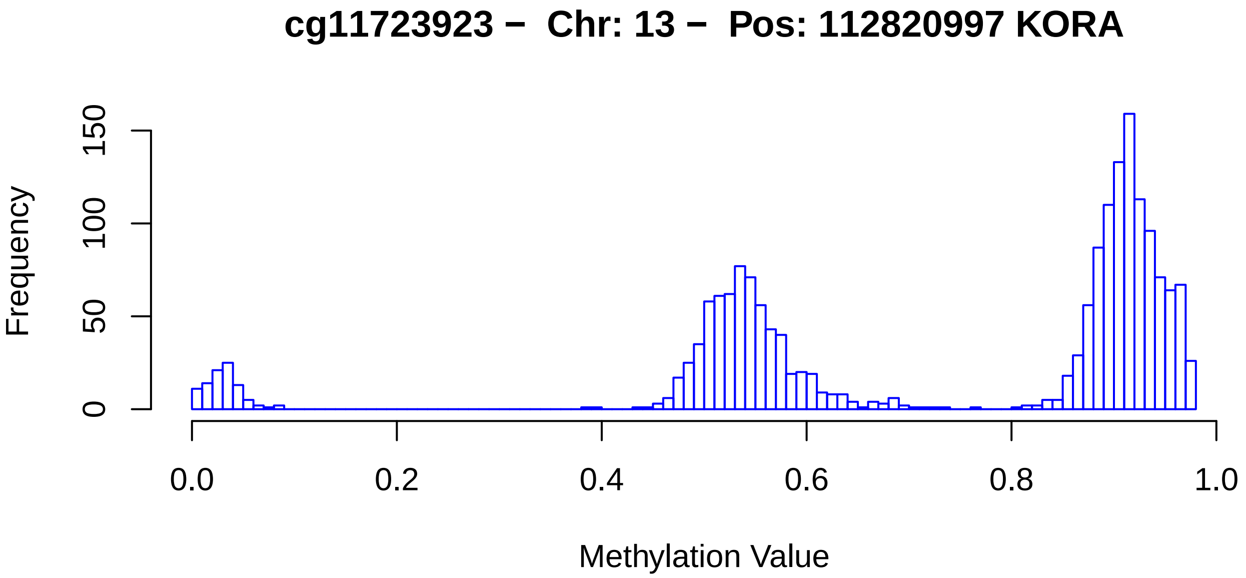
cg11723923 − Chr: 13 − Pos: 112820997 KORA
Frequency
0
50
100
150
0.0
0.2
0.4
0.6
0.8
1.0
Methylation Value

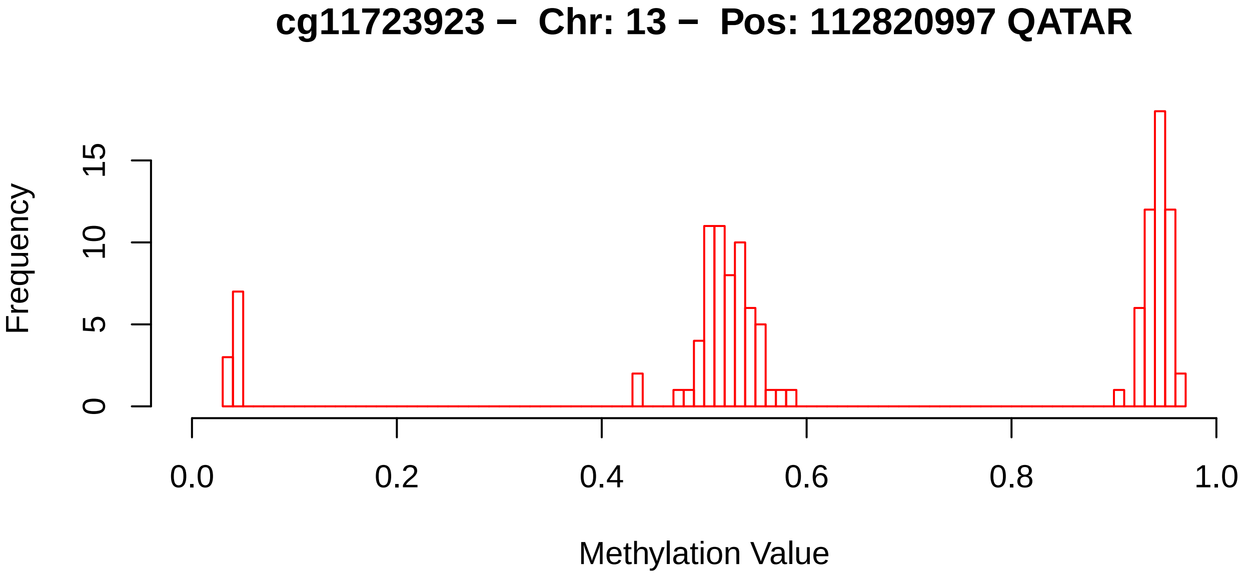
cg11723923 − Chr: 13 − Pos: 112820997 QATAR
Frequency
0
5
10
15
0.0
0.2
0.4
0.6
0.8
1.0
Methylation Value

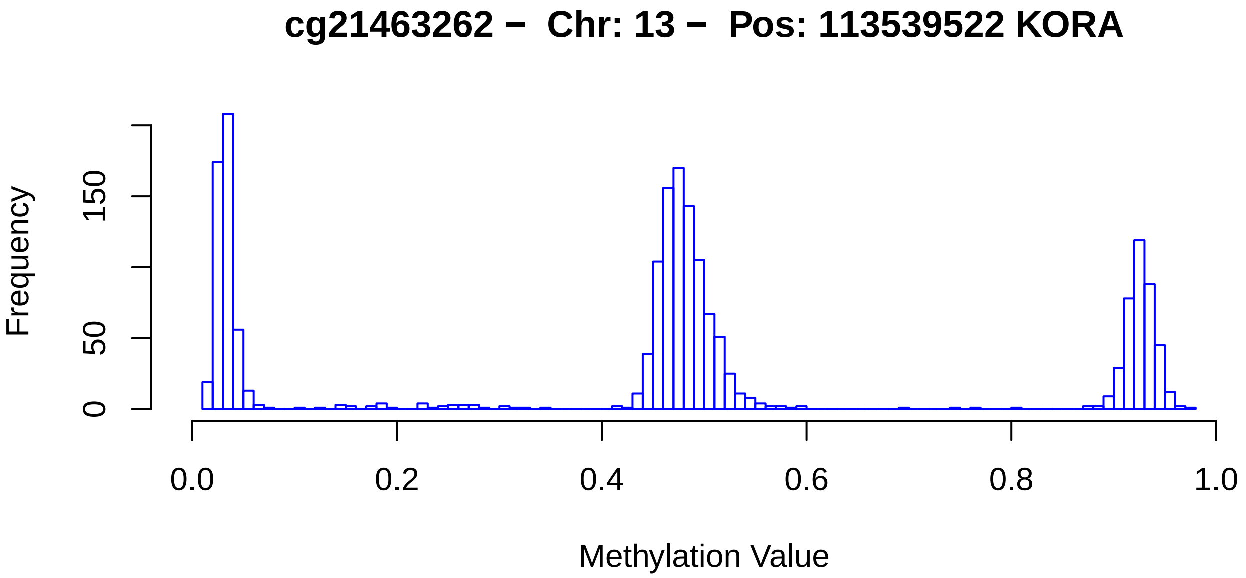
cg21463262 − Chr: 13 − Pos: 113539522 KORA
Frequency
0
50
150
0.0
0.2
0.4
0.6
0.8
1.0
Methylation Value

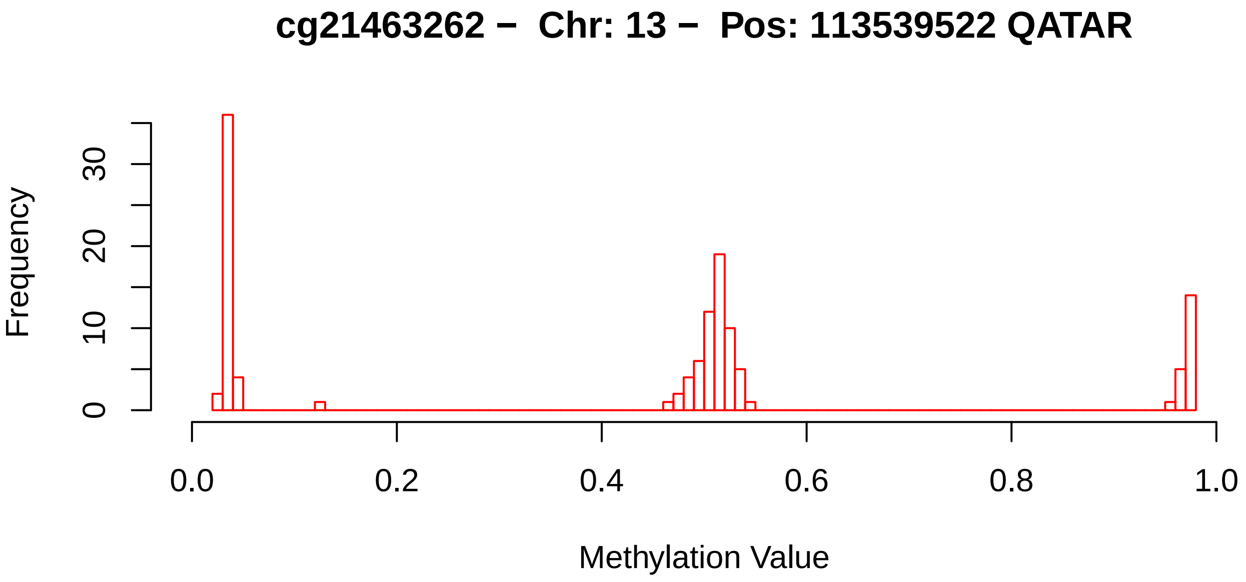
cg21463262 − Chr: 13 − Pos: 113539522 QATAR
Frequency
0
10
20
30
0.0
0.2
0.4
0.6
0.8
1.0
Methylation Value

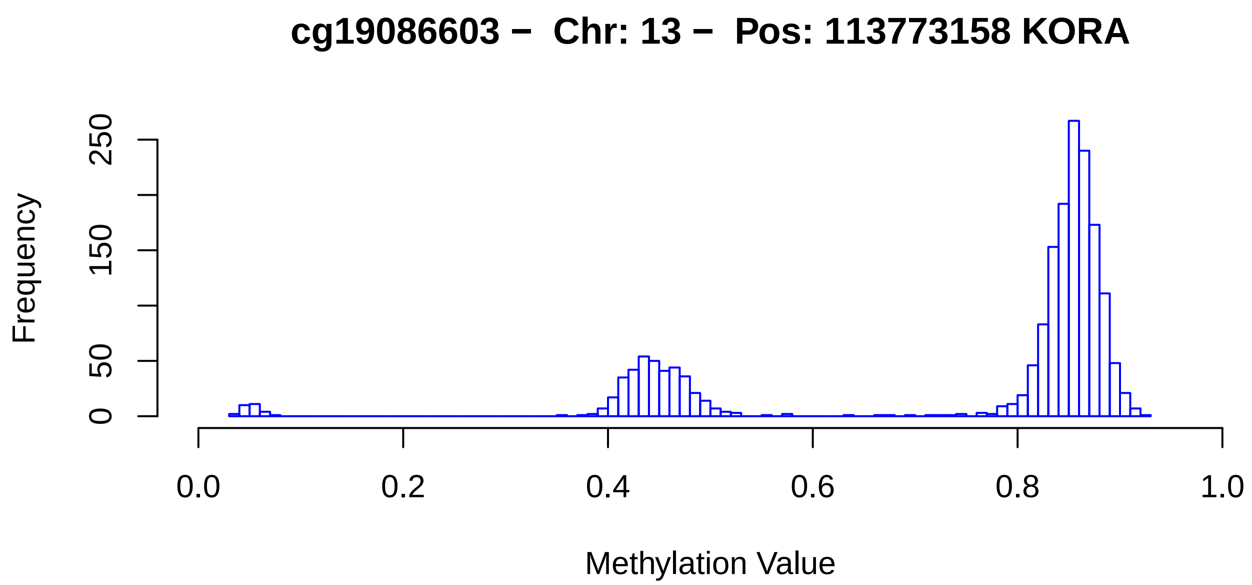
cg19086603 − Chr: 13 − Pos: 113773158 KORA
Frequency
0
50
150
250
0.0
0.2
0.4
0.6
0.8
1.0
Methylation Value

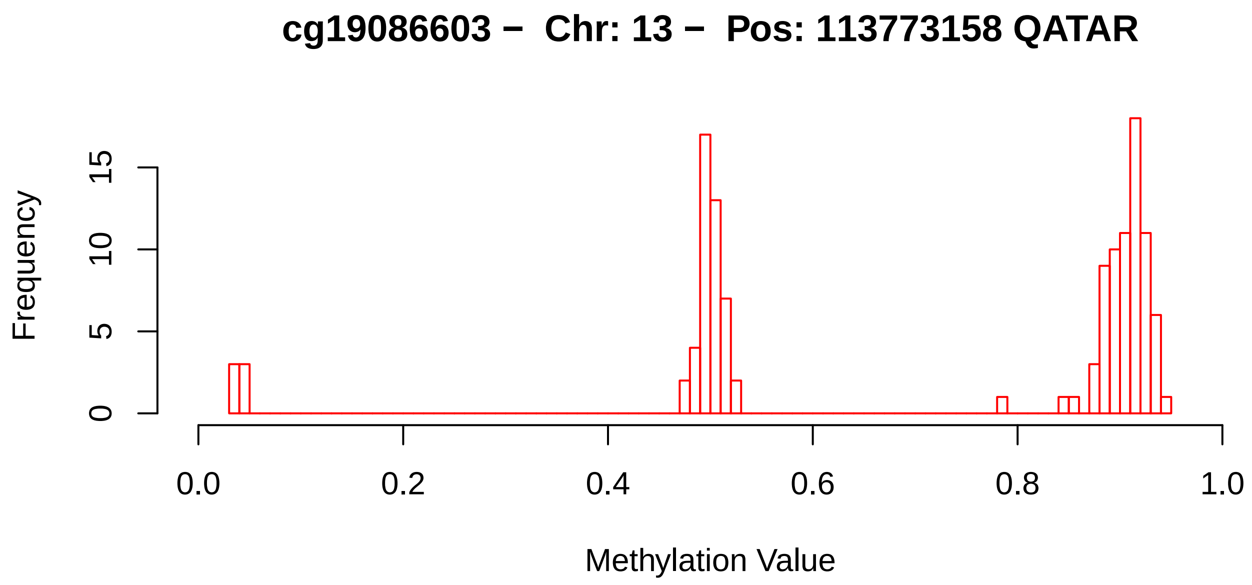
cg19086603 − Chr: 13 − Pos: 113773158 QATAR
Frequency
0
5
10
15
0.0
0.2
0.4
0.6
0.8
1.0
Methylation Value

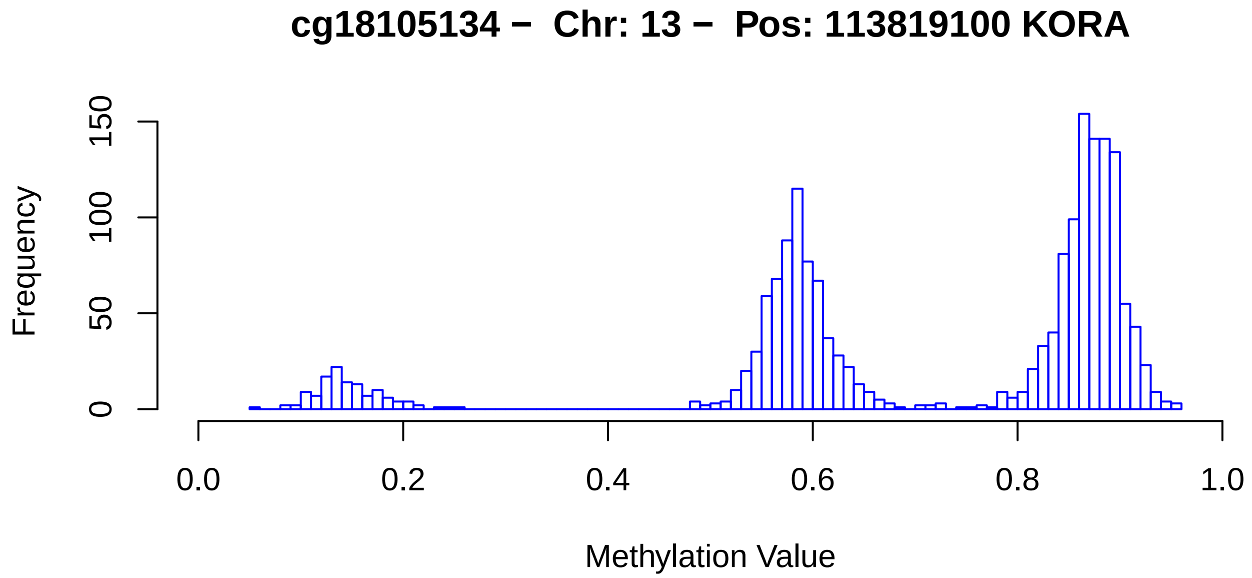
cg18105134 − Chr: 13 − Pos: 113819100 KORA
Frequency
0
50
100
150
0.0
0.2
0.4
0.6
0.8
1.0
Methylation Value

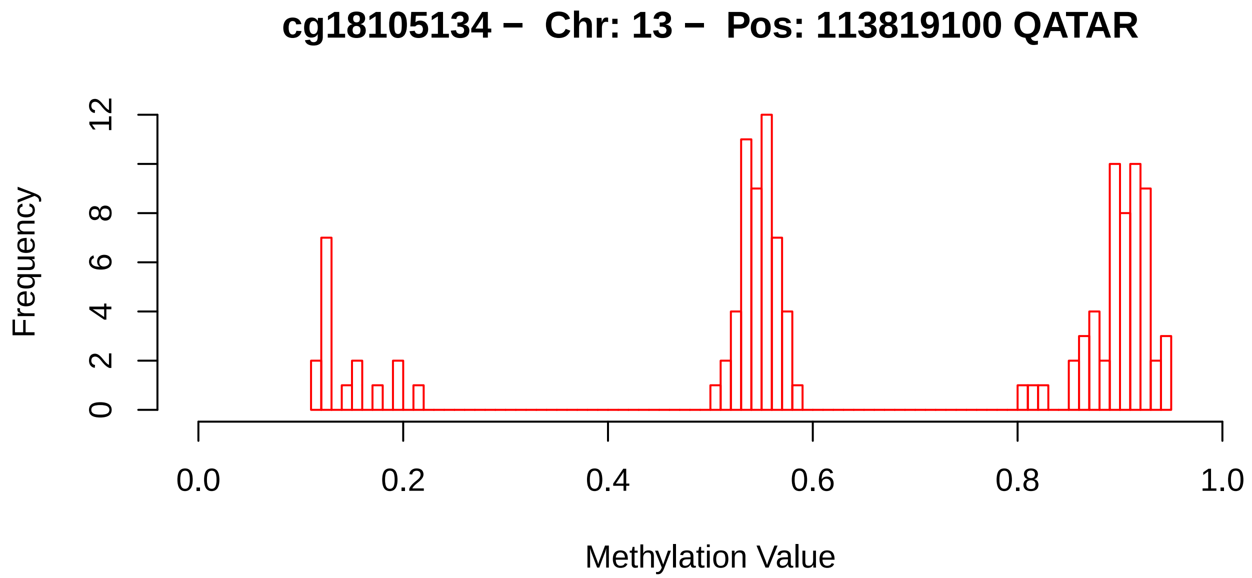
cg18105134 − Chr: 13 − Pos: 113819100 QATAR
Frequency
0
2
4
6
8
12
0.0
0.2
0.4
0.6
0.8
1.0
Methylation Value

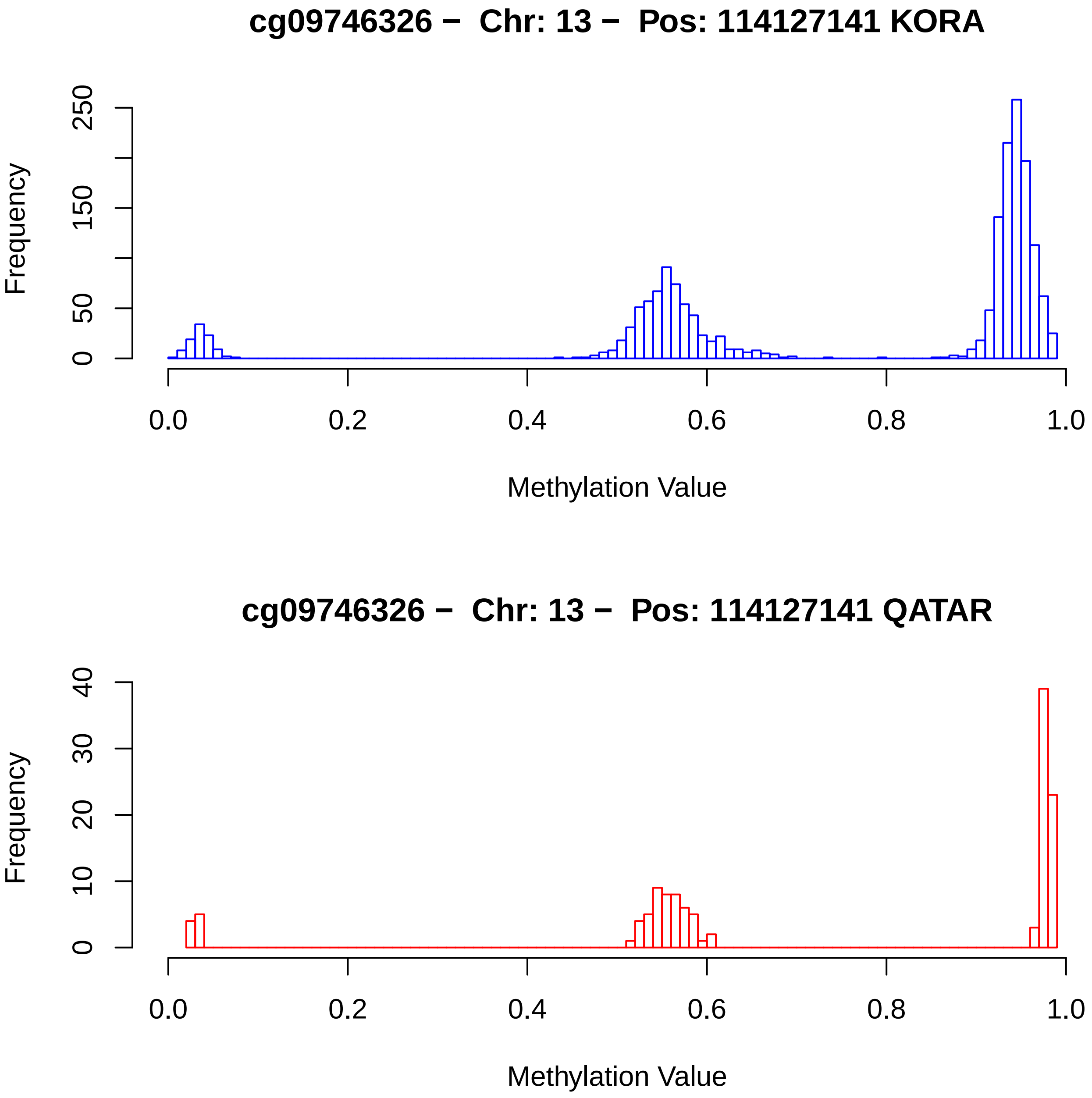
cg09746326 − Chr: 13 − Pos: 114127141 KORA
Frequency
0
50
150
250
0.0
0.2
0.4
0.6
0.8
1.0
Methylation Value
cg09746326 − Chr: 13 − Pos: 114127141 QATAR
Frequency
0
10
20
30
40
0.0
0.2
0.4
0.6
0.8
1.0
Methylation Value

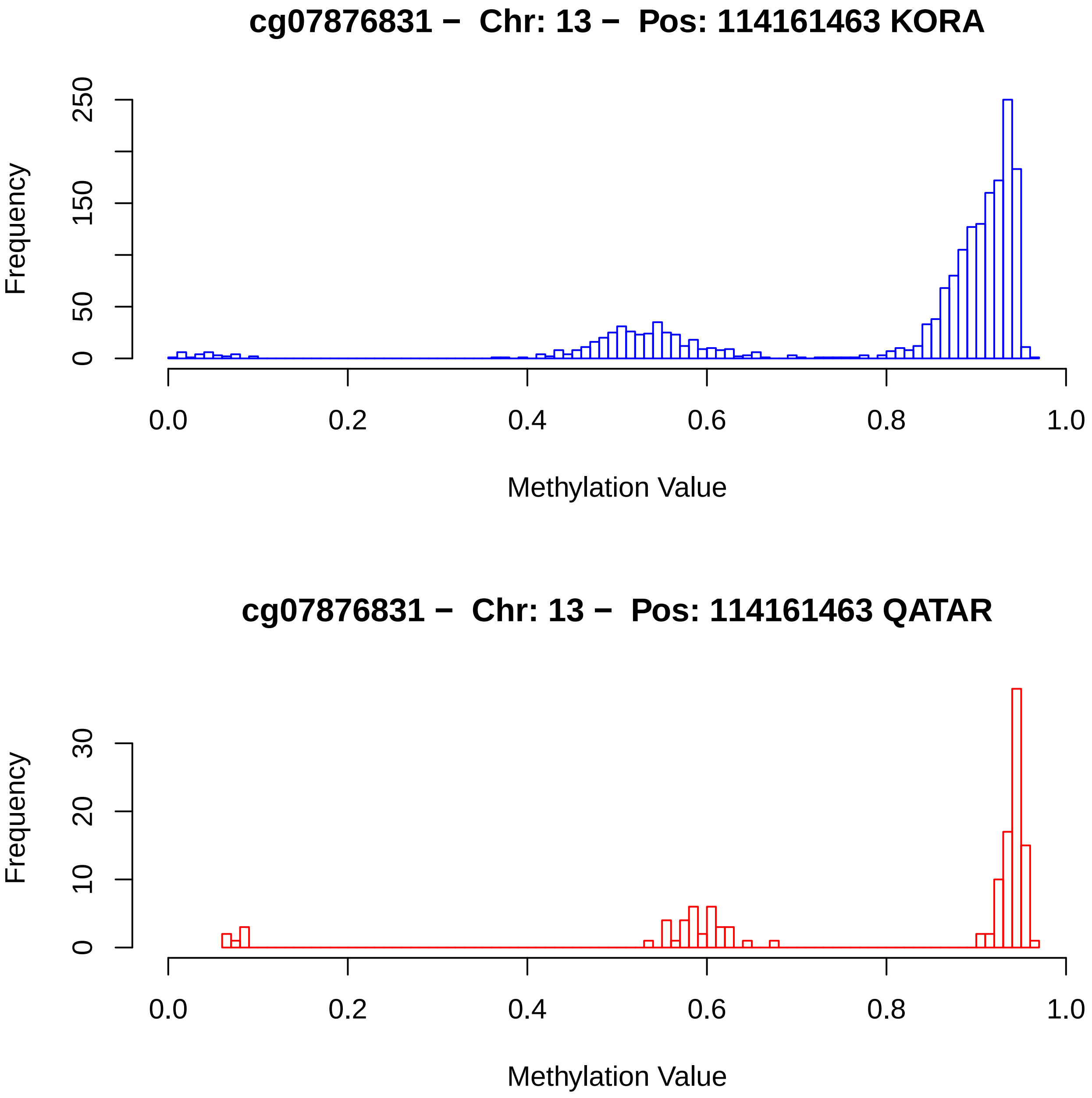
cg07876831 − Chr: 13 − Pos: 114161463 KORA
Frequency
0
50
150
250
0.0
0.2
0.4
0.6
0.8
1.0
Methylation Value
cg07876831 − Chr: 13 − Pos: 114161463 QATAR
Frequency
0
10
20
30
0.0
0.2
0.4
0.6
0.8
1.0
Methylation Value

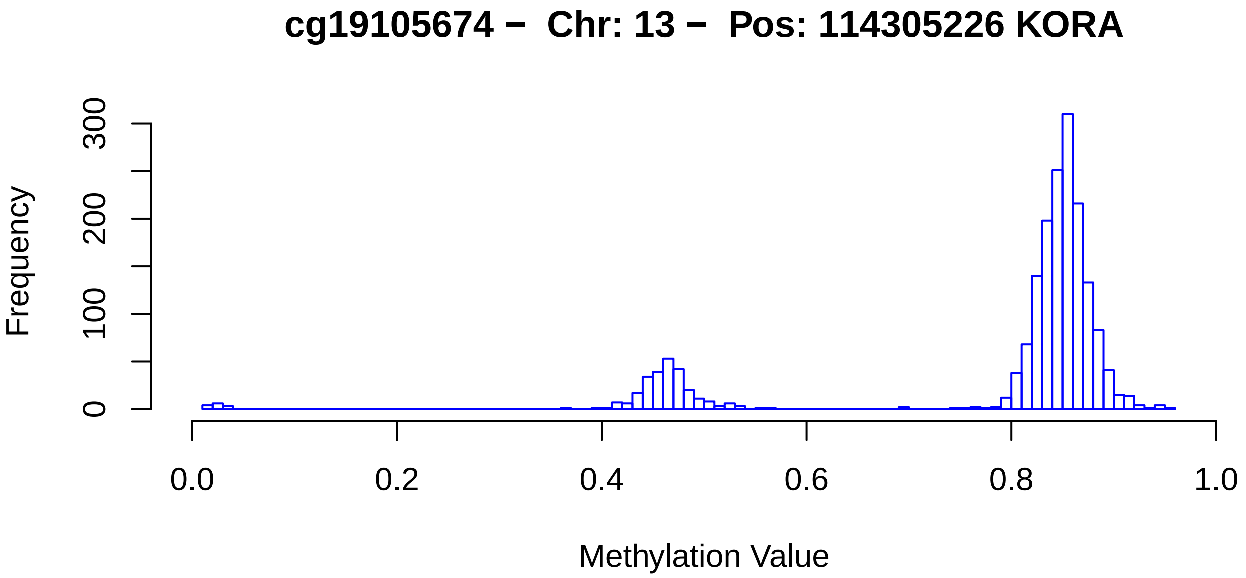
cg19105674 − Chr: 13 − Pos: 114305226 KORA
Frequency
0
100
200
300
0.0
0.2
0.4
0.6
0.8
1.0
Methylation Value

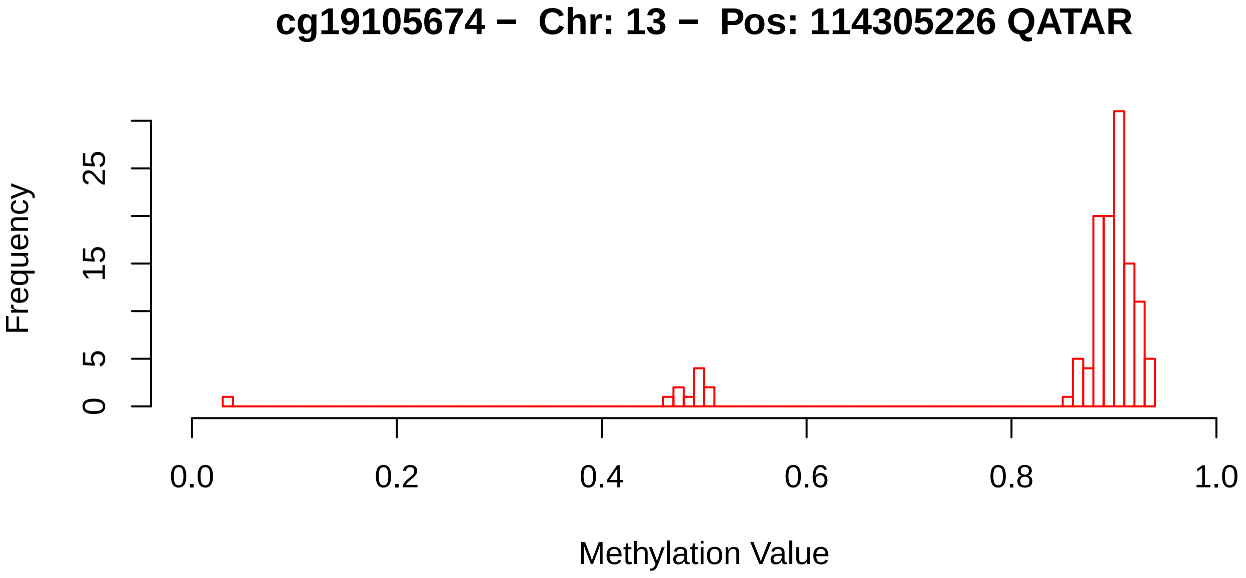
cg19105674 − Chr: 13 − Pos: 114305226 QATAR
Frequency
0
5
15
25
0.0
0.2
0.4
0.6
0.8
1.0
Methylation Value

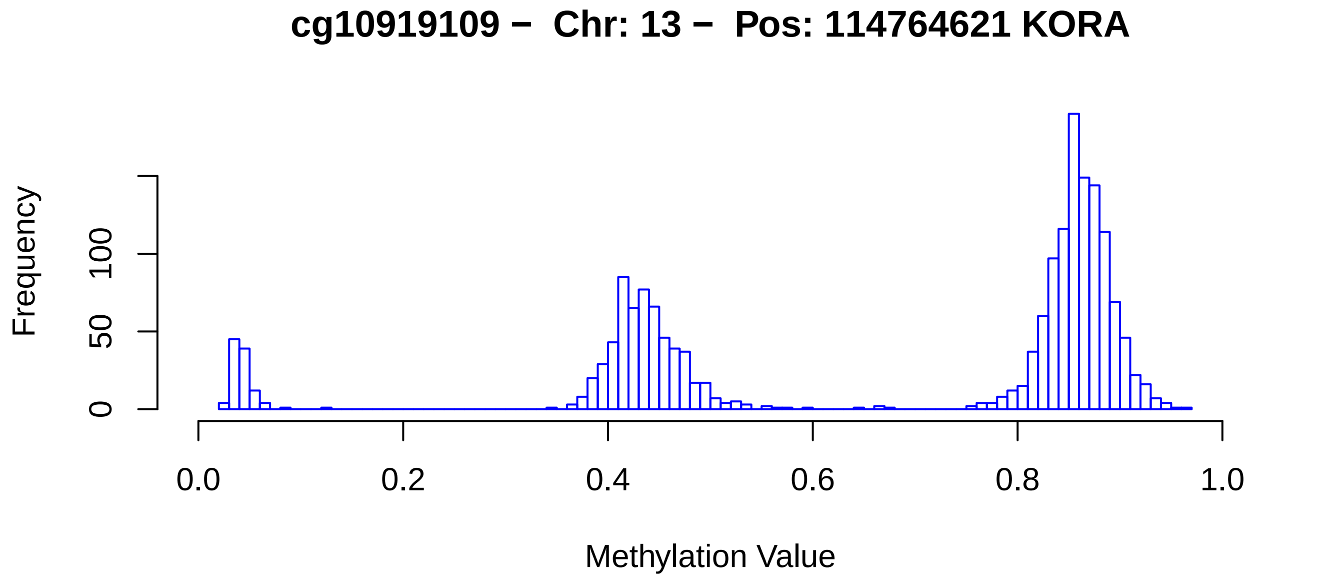
cg10919109 − Chr: 13 − Pos: 114764621 KORA
Frequency
0
50
100
0.0
0.2
0.4
0.6
0.8
1.0
Methylation Value

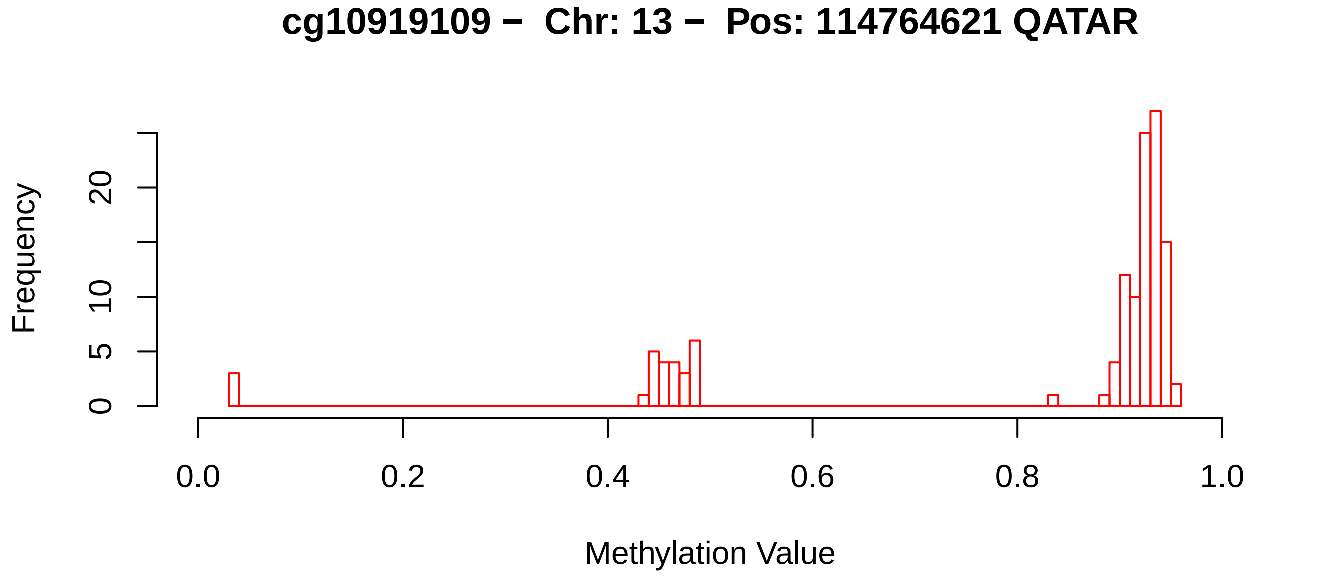
cg10919109 − Chr: 13 − Pos: 114764621 QATAR
Frequency
0
5
10
20
0.0
0.2
0.4
0.6
0.8
1.0
Methylation Value

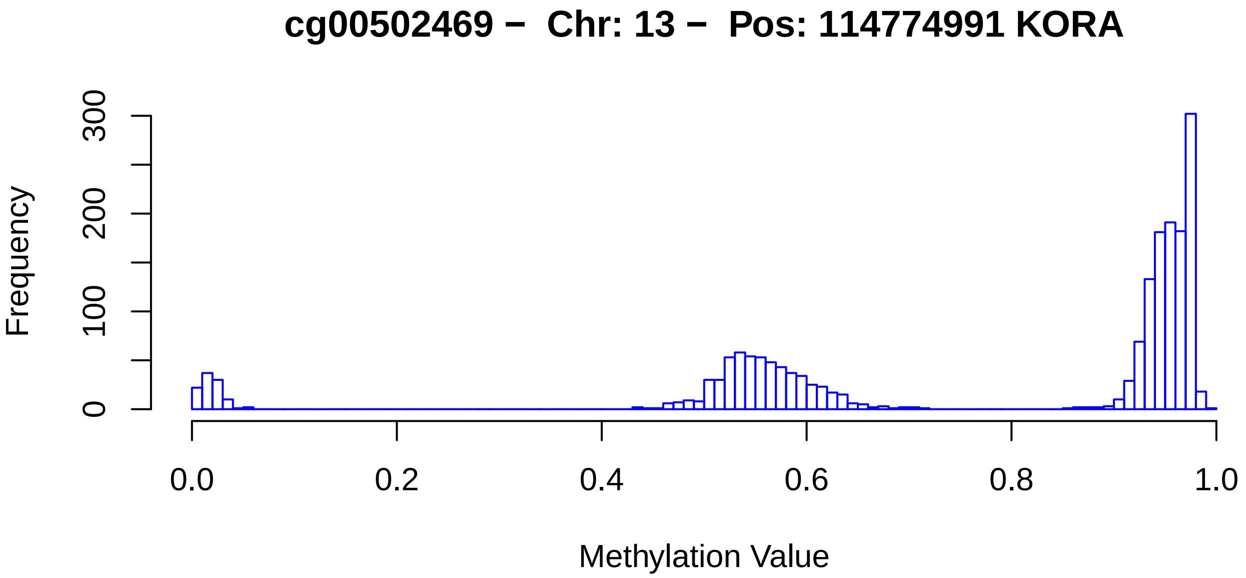
cg00502469 − Chr: 13 − Pos: 114774991 KORA
Frequency
0
100
200
300
0.0
0.2
0.4
0.6
0.8
1.0
Methylation Value

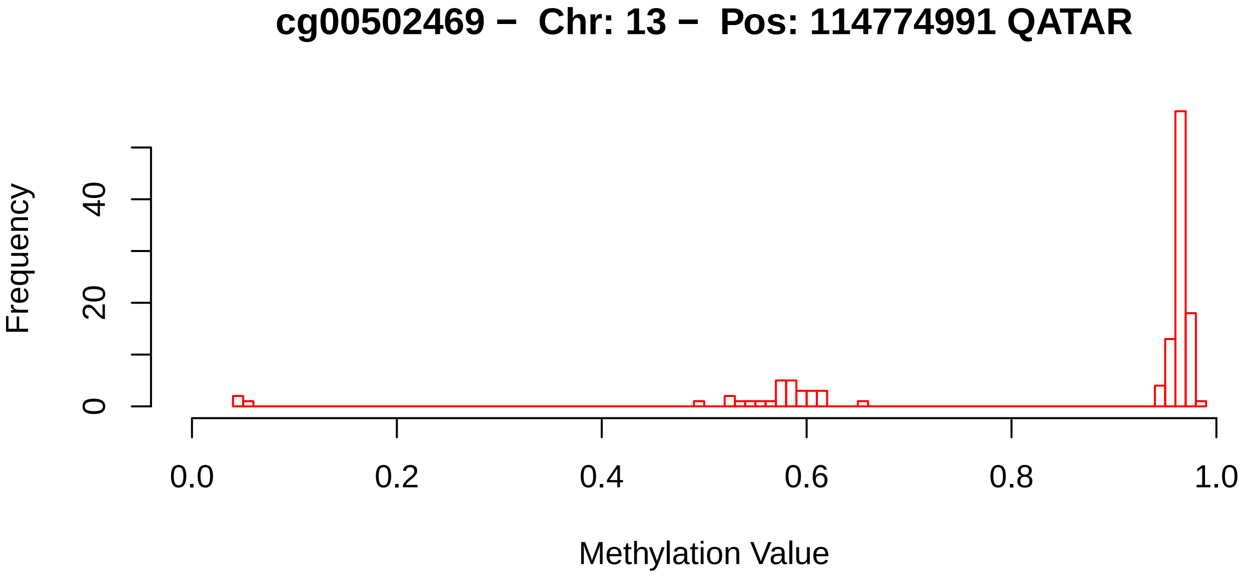
cg00502469 − Chr: 13 − Pos: 114774991 QATAR
Frequency
0
20
40
0.0
0.2
0.4
0.6
0.8
1.0
Methylation Value

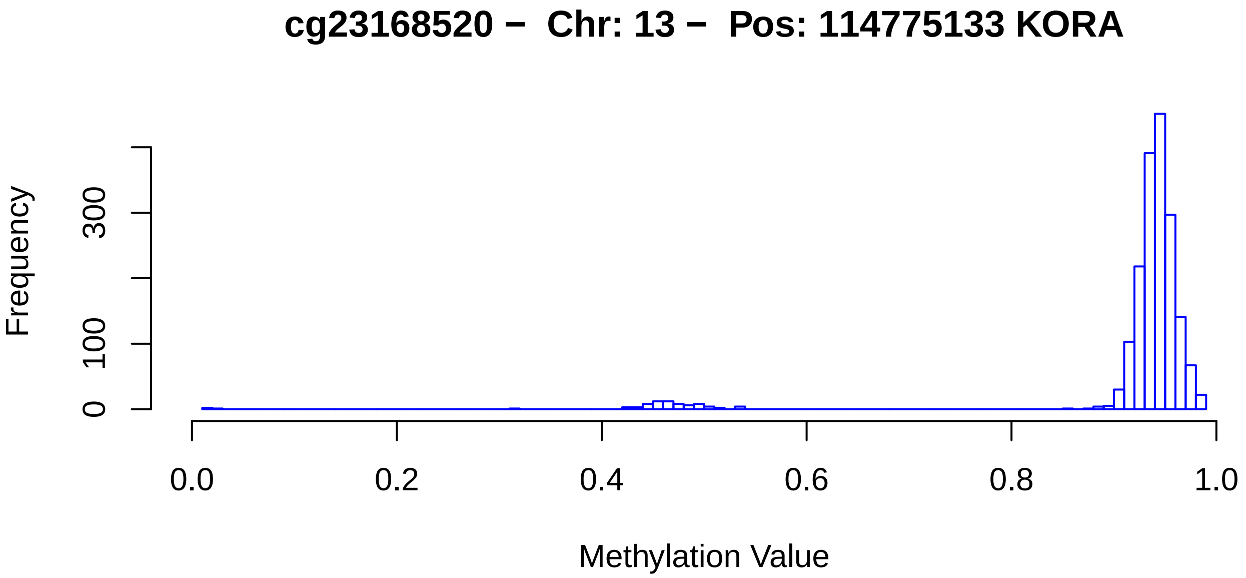
cg23168520 − Chr: 13 − Pos: 114775133 KORA
Frequency
0
100
300
0.0
0.2
0.4
0.6
0.8
1.0
Methylation Value

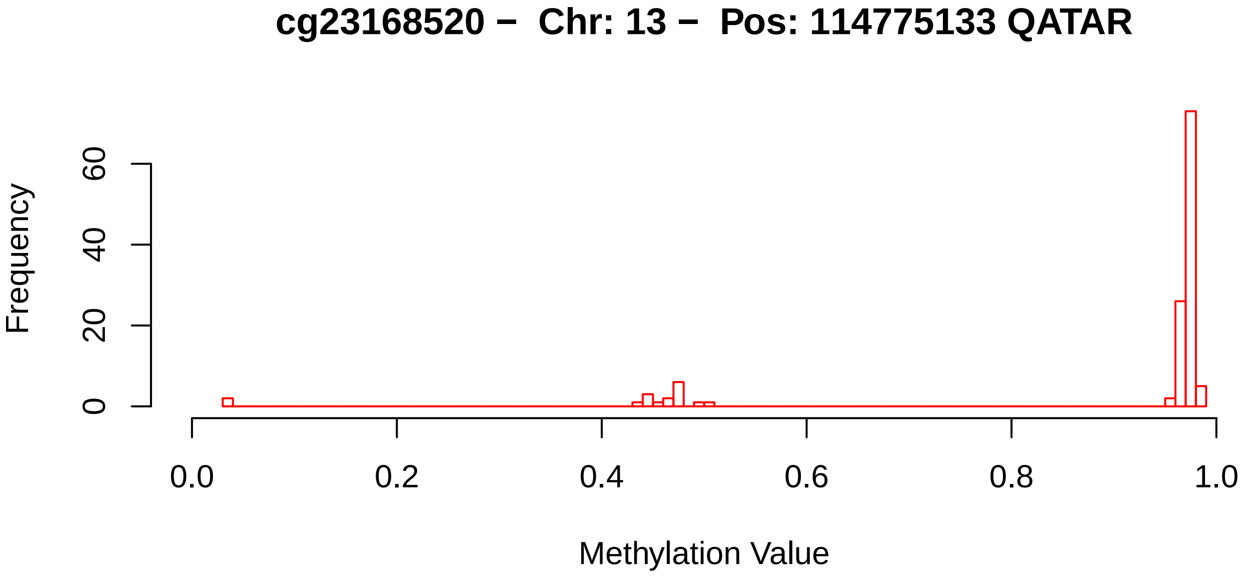
cg23168520 − Chr: 13 − Pos: 114775133 QATAR
Frequency
0
20
40
60
0.0
0.2
0.4
0.6
0.8
1.0
Methylation Value

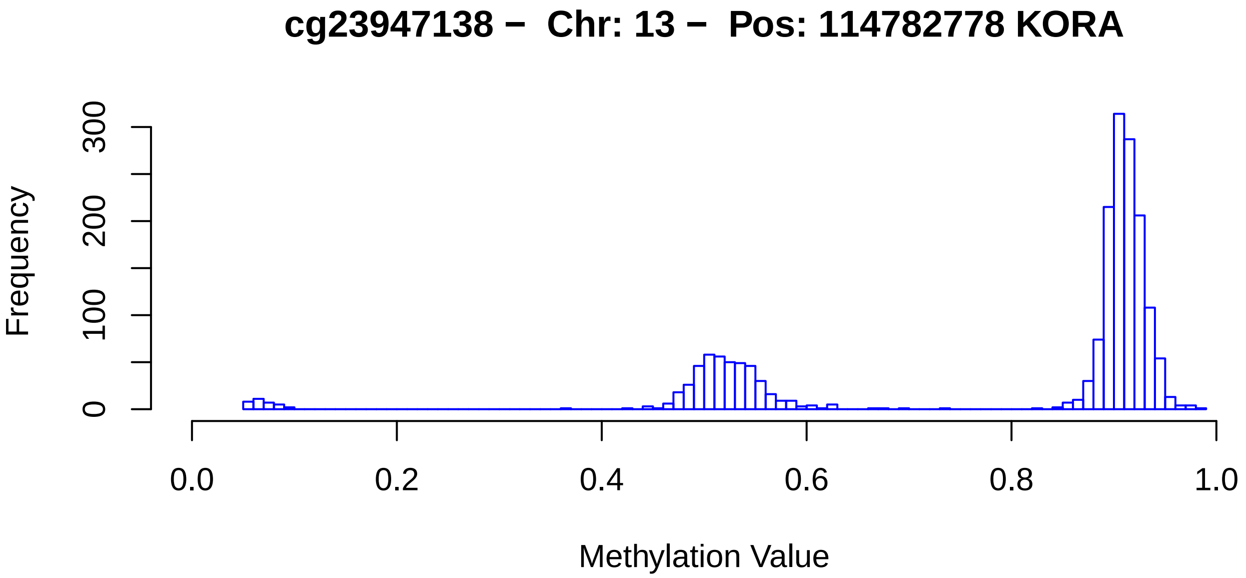
cg23947138 − Chr: 13 − Pos: 114782778 KORA
Frequency
0
100
200
300
0.0
0.2
0.4
0.6
0.8
1.0
Methylation Value

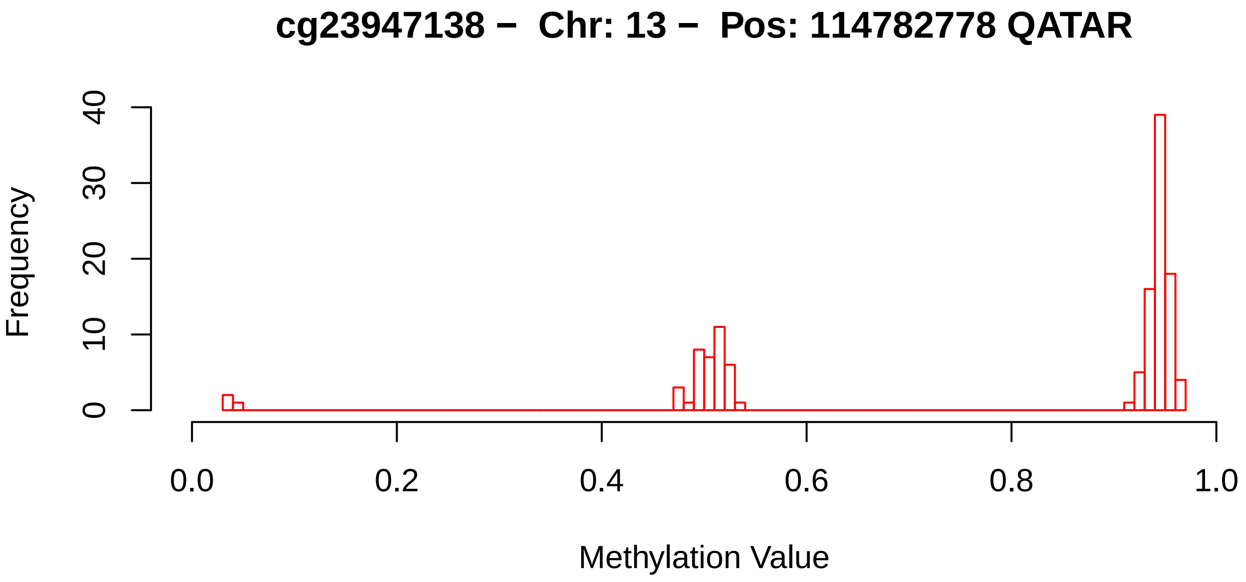
cg23947138 − Chr: 13 − Pos: 114782778 QATAR
Frequency
0
10
20
30
40
0.0
0.2
0.4
0.6
0.8
1.0
Methylation Value

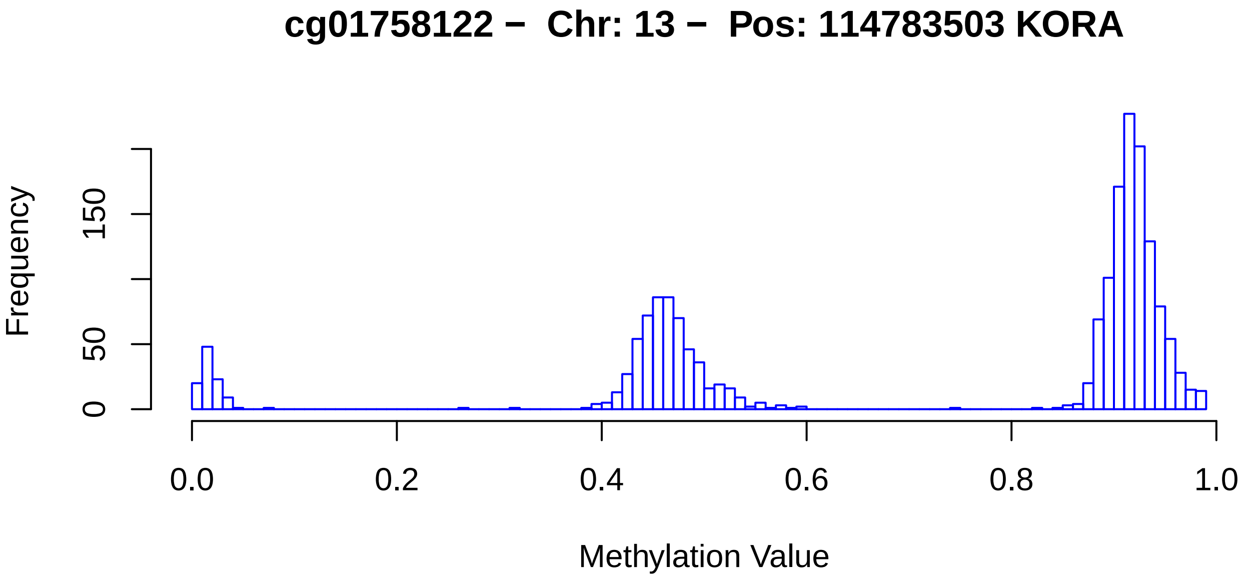
cg01758122 − Chr: 13 − Pos: 114783503 KORA
Frequency
0
50
150
0.0
0.2
0.4
0.6
0.8
1.0
Methylation Value

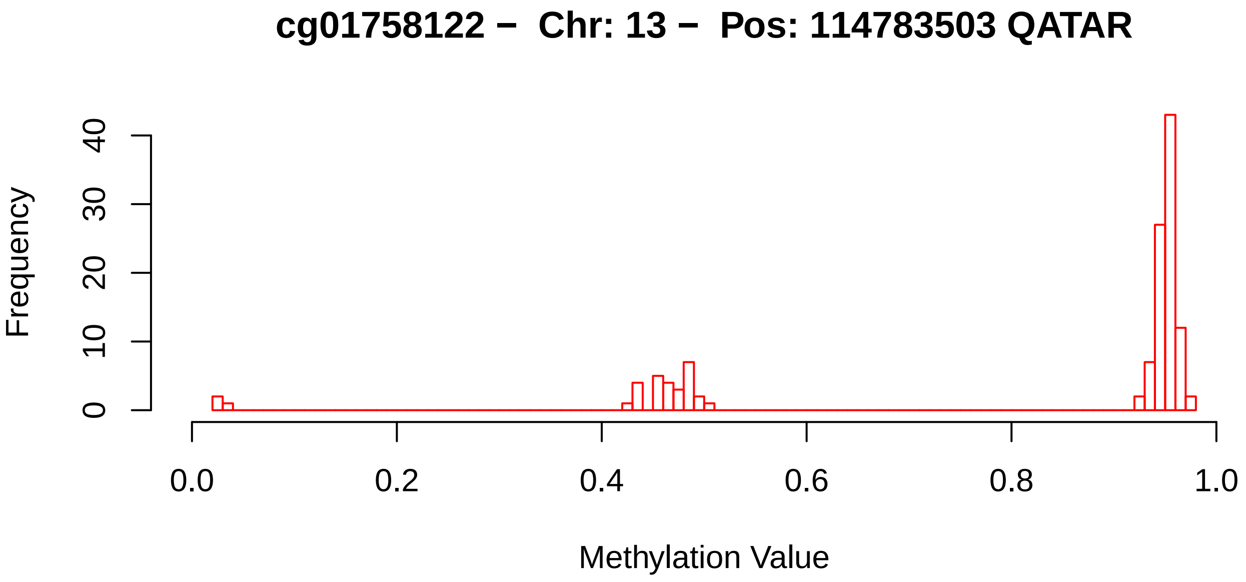
cg01758122 − Chr: 13 − Pos: 114783503 QATAR
Frequency
0
10
20
30
40
0.0
0.2
0.4
0.6
0.8
1.0
Methylation Value

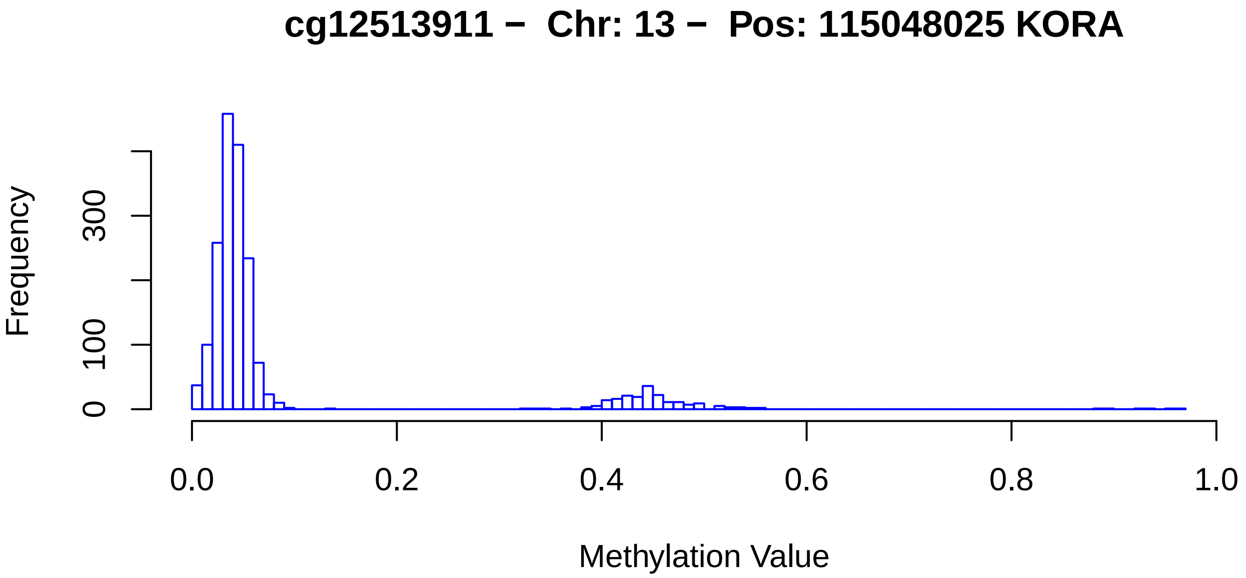
cg12513911 − Chr: 13 − Pos: 115048025 KORA
Frequency
0
100
300
0.0
0.2
0.4
0.6
0.8
1.0
Methylation Value

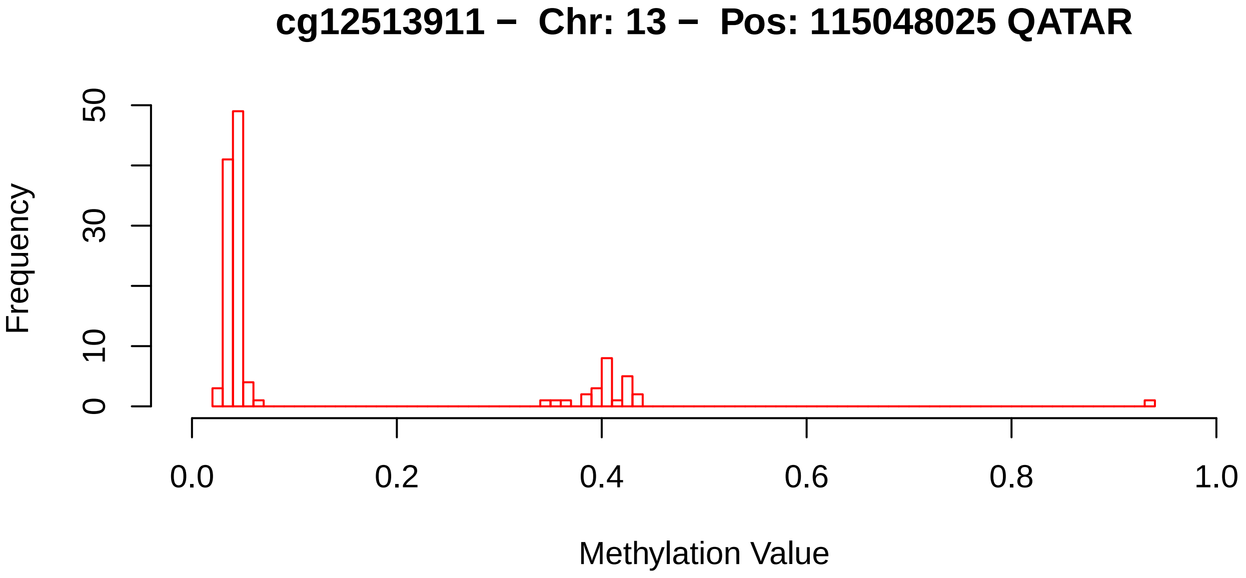
cg12513911 − Chr: 13 − Pos: 115048025 QATAR
Frequency
0
10
30
50
0.0
0.2
0.4
0.6
0.8
1.0
Methylation Value

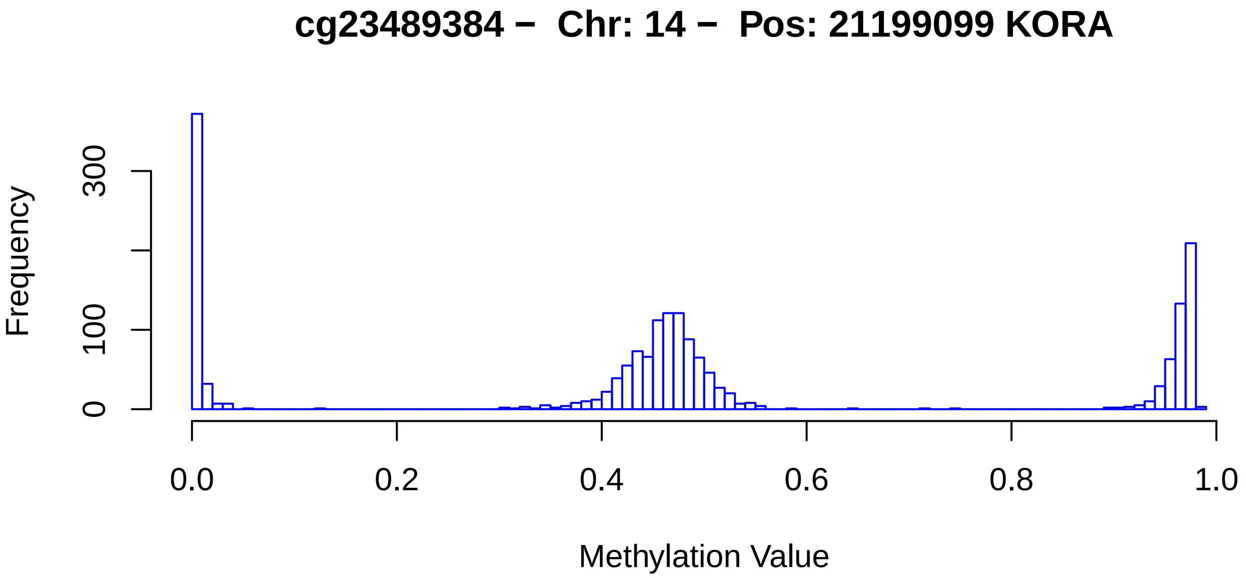
cg23489384 − Chr: 14 − Pos: 21199099 KORA
Frequency
0
100
300
0.0
0.2
0.4
0.6
0.8
1.0
Methylation Value

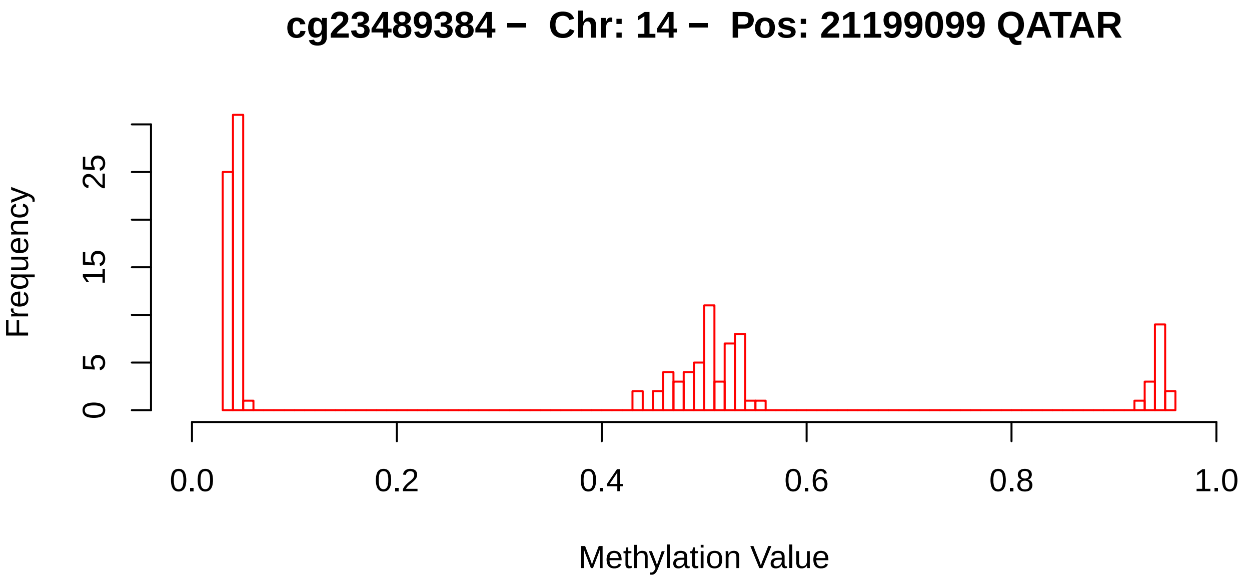
cg23489384 − Chr: 14 − Pos: 21199099 QATAR
Frequency
0
5
15
25
0.0
0.2
0.4
0.6
0.8
1.0
Methylation Value

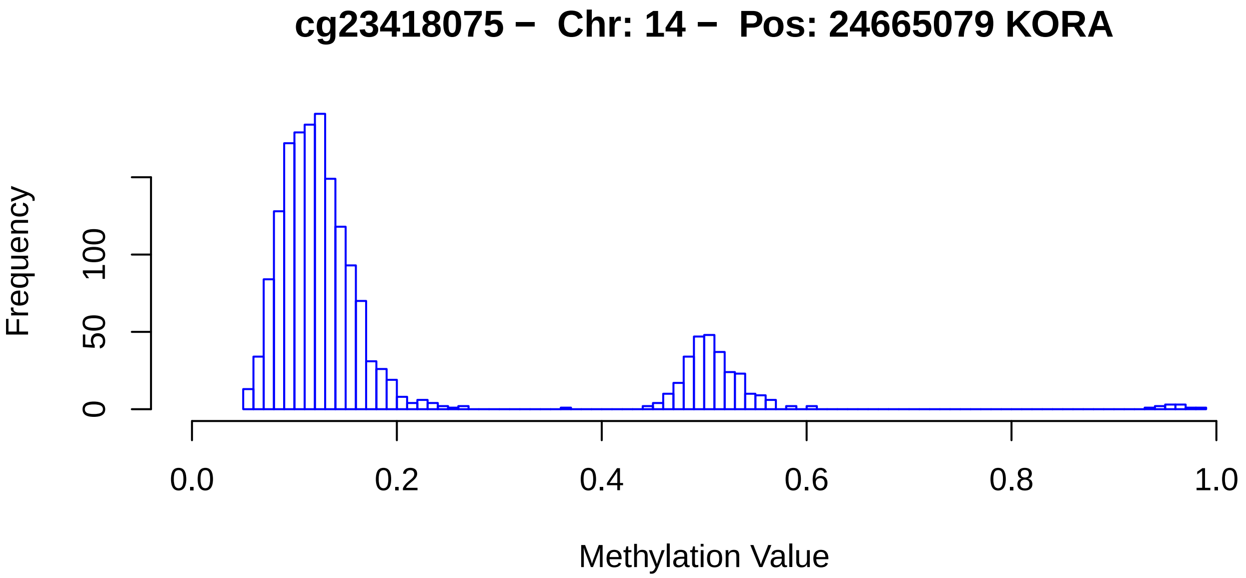
cg23418075 − Chr: 14 − Pos: 24665079 KORA
Frequency
0
50
100
0.0
0.2
0.4
0.6
0.8
1.0
Methylation Value

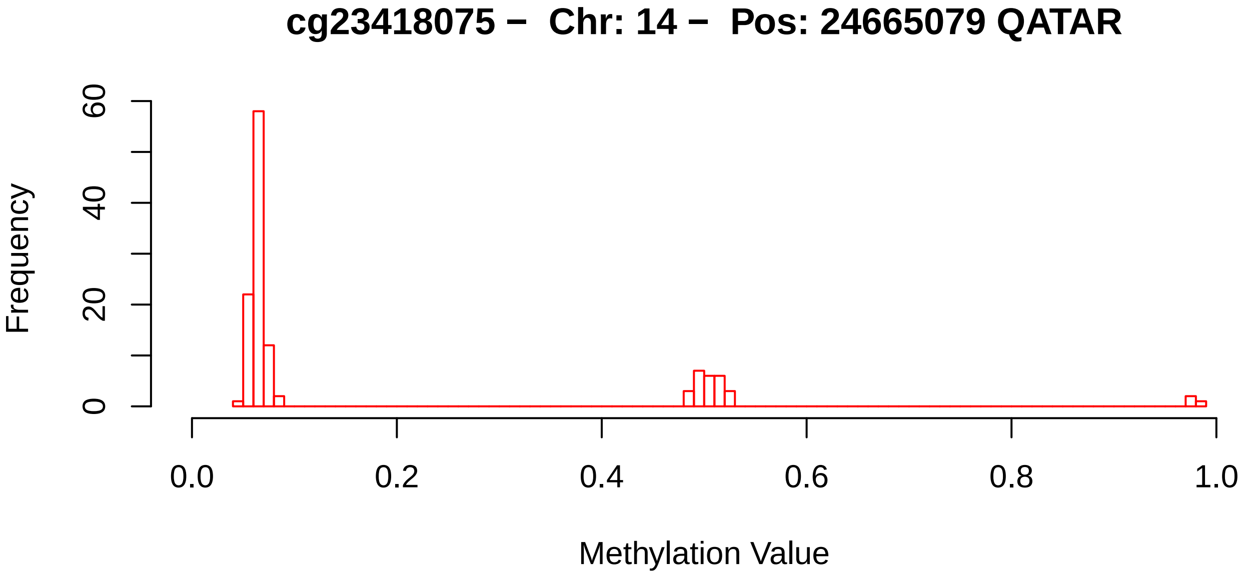
cg23418075 − Chr: 14 − Pos: 24665079 QATAR
Frequency
0
20
40
60
0.0
0.2
0.4
0.6
0.8
1.0
Methylation Value

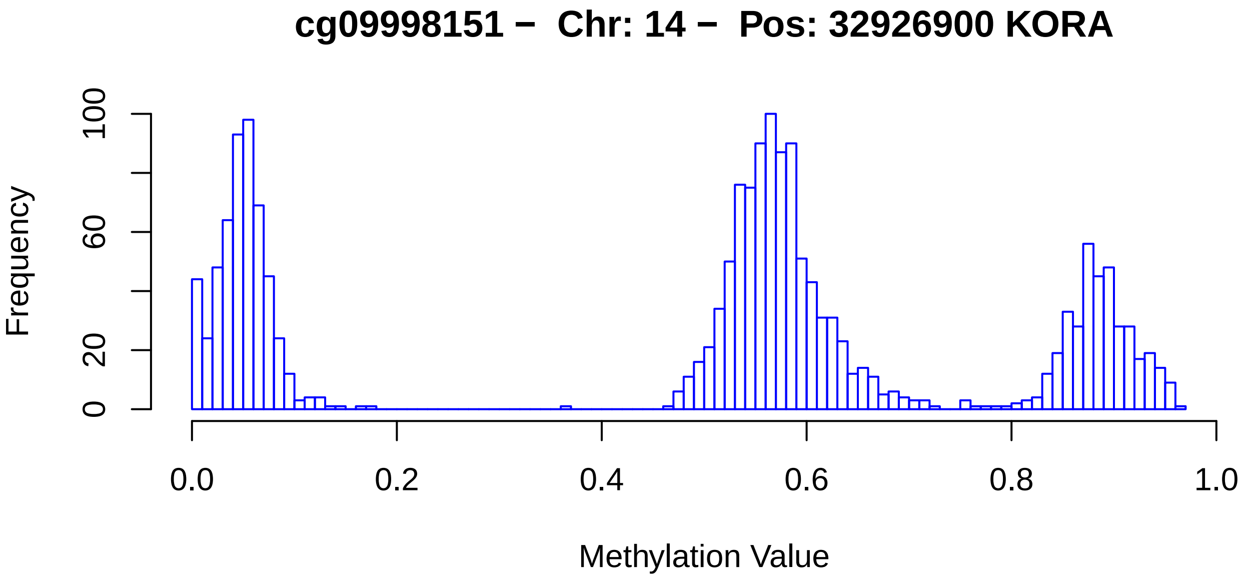
cg09998151 − Chr: 14 − Pos: 32926900 KORA
Frequency
0
20
60
100
0.0
0.2
0.4
0.6
0.8
1.0
Methylation Value

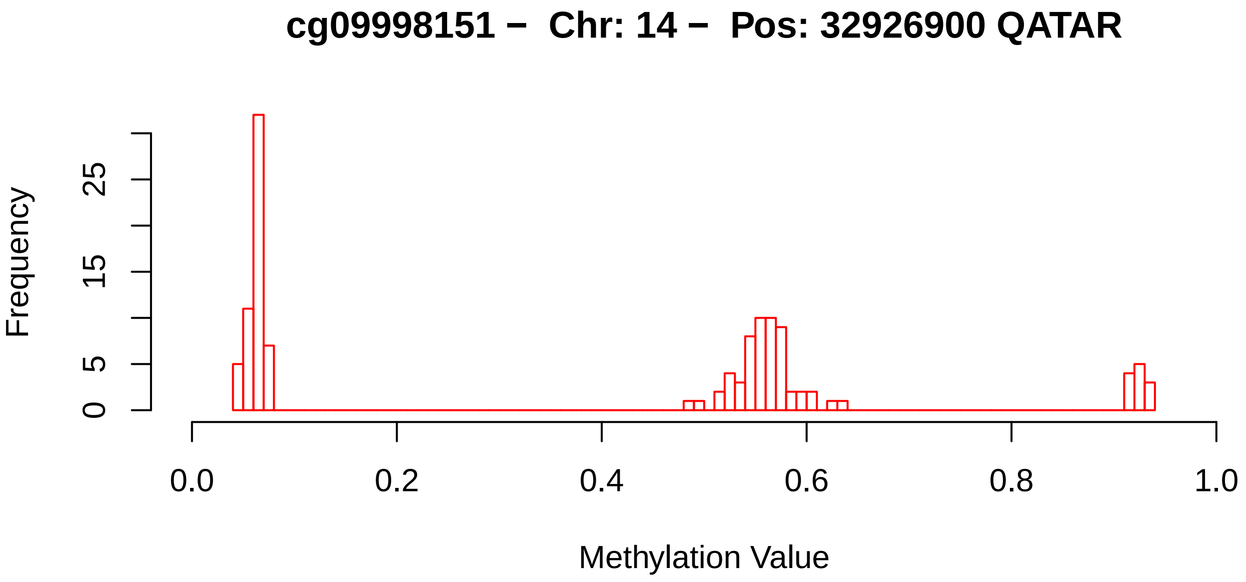
cg09998151 − Chr: 14 − Pos: 32926900 QATAR
Frequency
0
5
15
25
0.0
0.2
0.4
0.6
0.8
1.0
Methylation Value

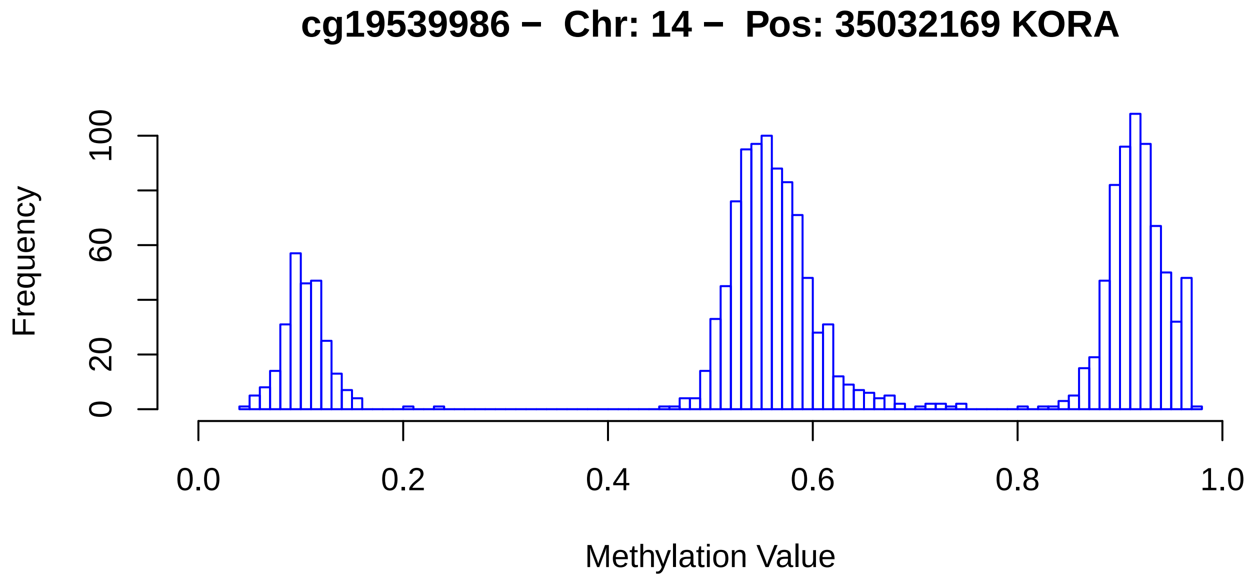

cg19539986 − Chr: 14 − Pos: 35032169 KORA
Frequency
0
20
60
100
0.0
0.2
0.4
0.6
0.8
1.0
Methylation Value

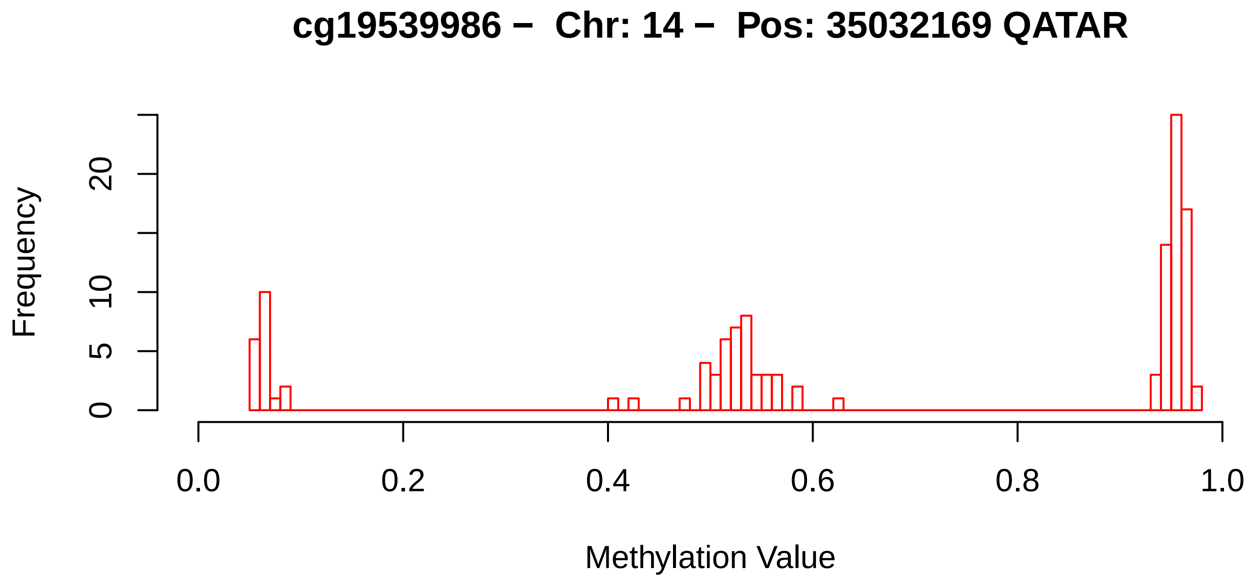

cg19539986 − Chr: 14 − Pos: 35032169 QATAR
Frequency
0
5
10
20
0.0
0.2
0.4
0.6
0.8
1.0
Methylation Value

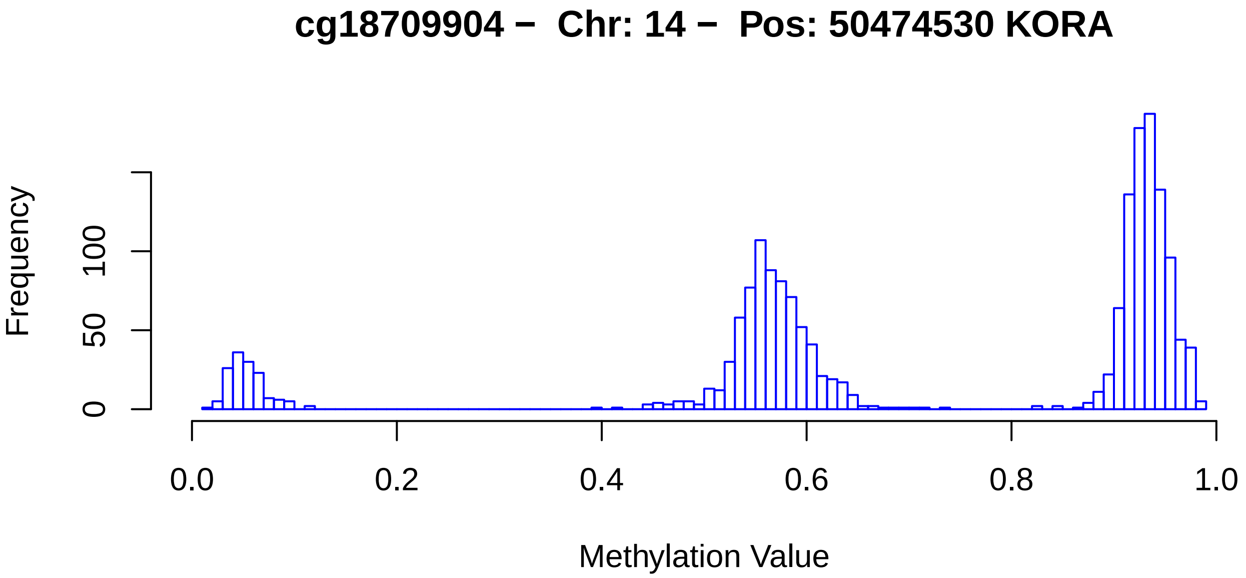
cg18709904 − Chr: 14 − Pos: 50474530 KORA
Frequency
0
50
100
0.0
0.2
0.4
0.6
0.8
1.0
Methylation Value

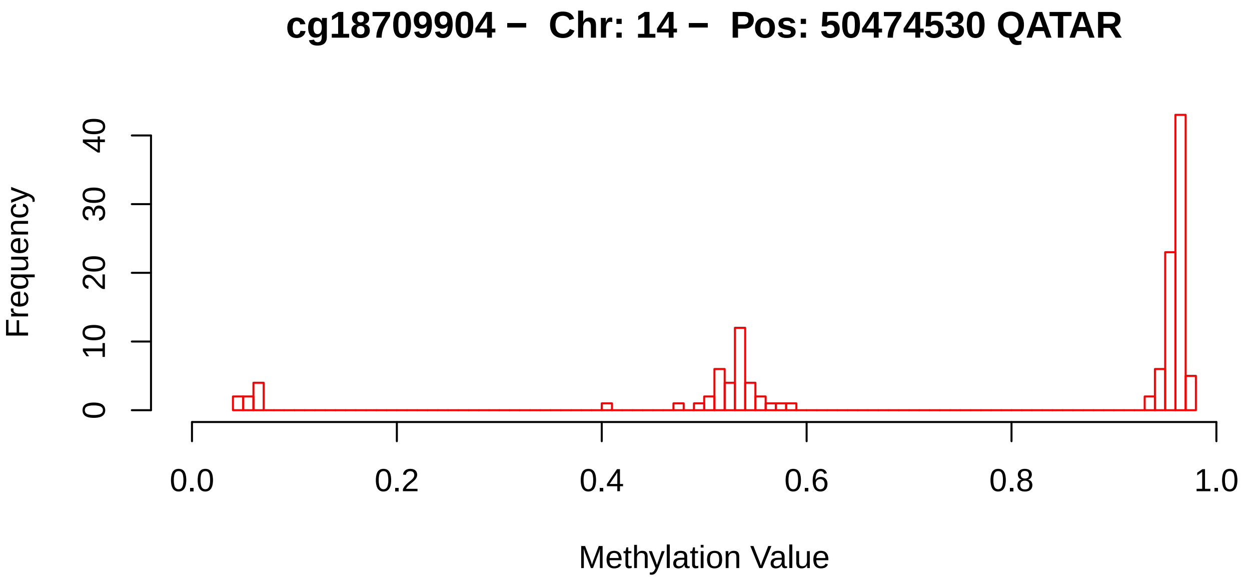
cg18709904 − Chr: 14 − Pos: 50474530 QATAR
Frequency
0
10
20
30
40
0.0
0.2
0.4
0.6
0.8
1.0
Methylation Value

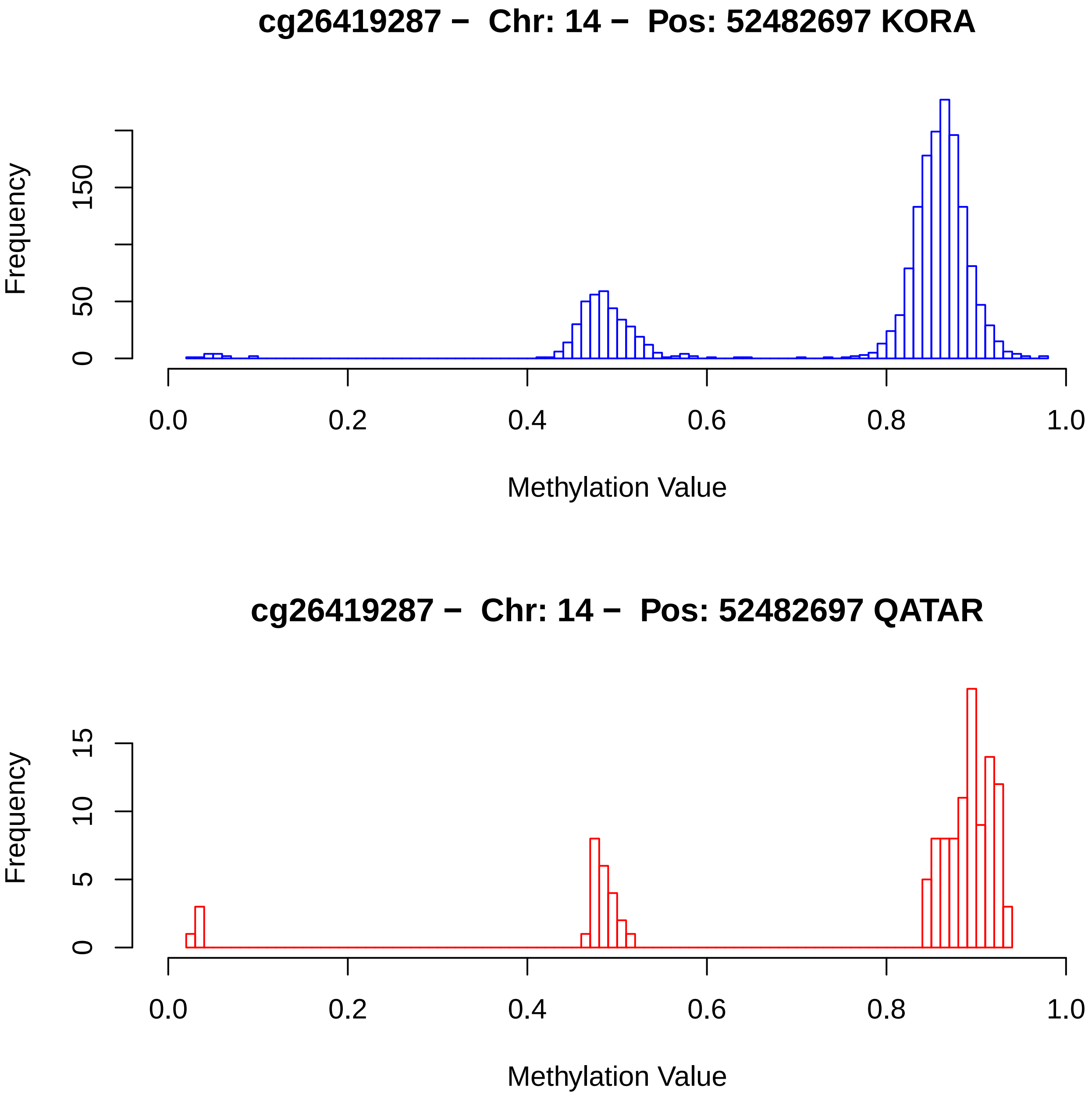
cg26419287 − Chr: 14 − Pos: 52482697 KORA
Frequency
0
50
150
0.0
0.2
0.4
0.6
0.8
1.0
Methylation Value
cg26419287 − Chr: 14 − Pos: 52482697 QATAR
Frequency
0
5
10
15
0.0
0.2
0.4
0.6
0.8
1.0
Methylation Value

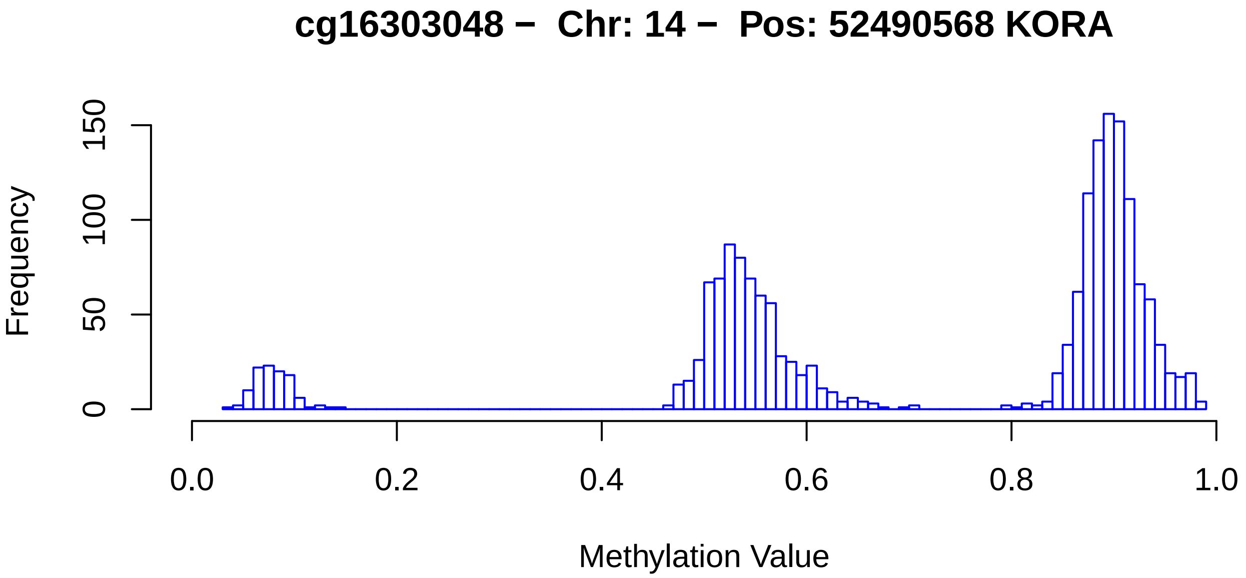
cg16303048 − Chr: 14 − Pos: 52490568 KORA
Frequency
0
50
100
150
0.0
0.2
0.4
0.6
0.8
1.0
Methylation Value

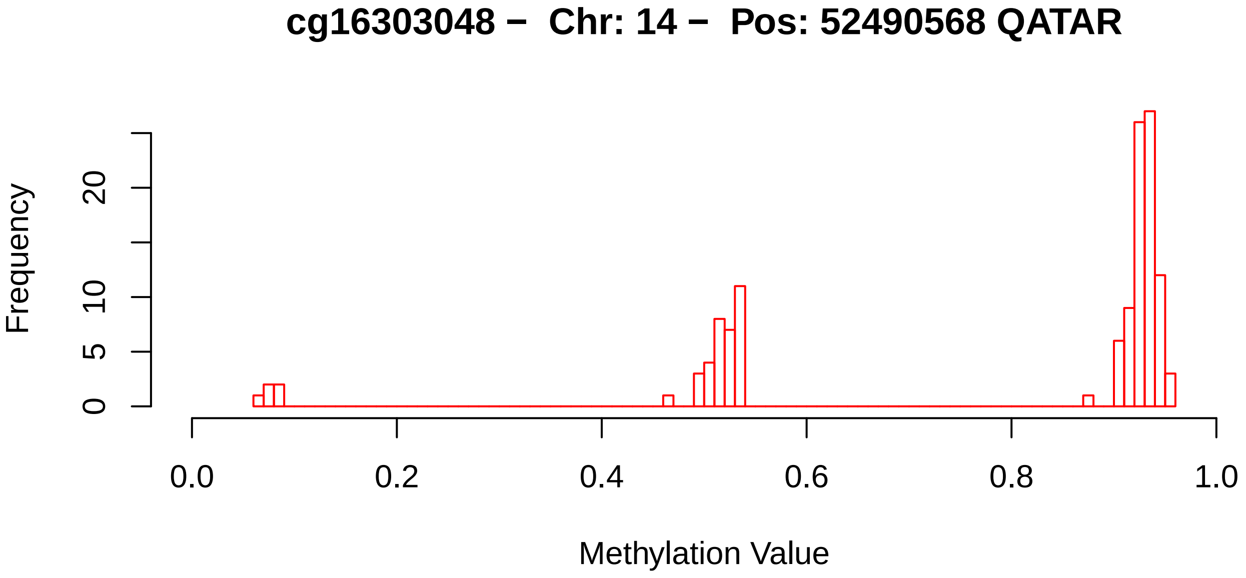
cg16303048 − Chr: 14 − Pos: 52490568 QATAR
Frequency
0
5
10
20
0.0
0.2
0.4
0.6
0.8
1.0
Methylation Value

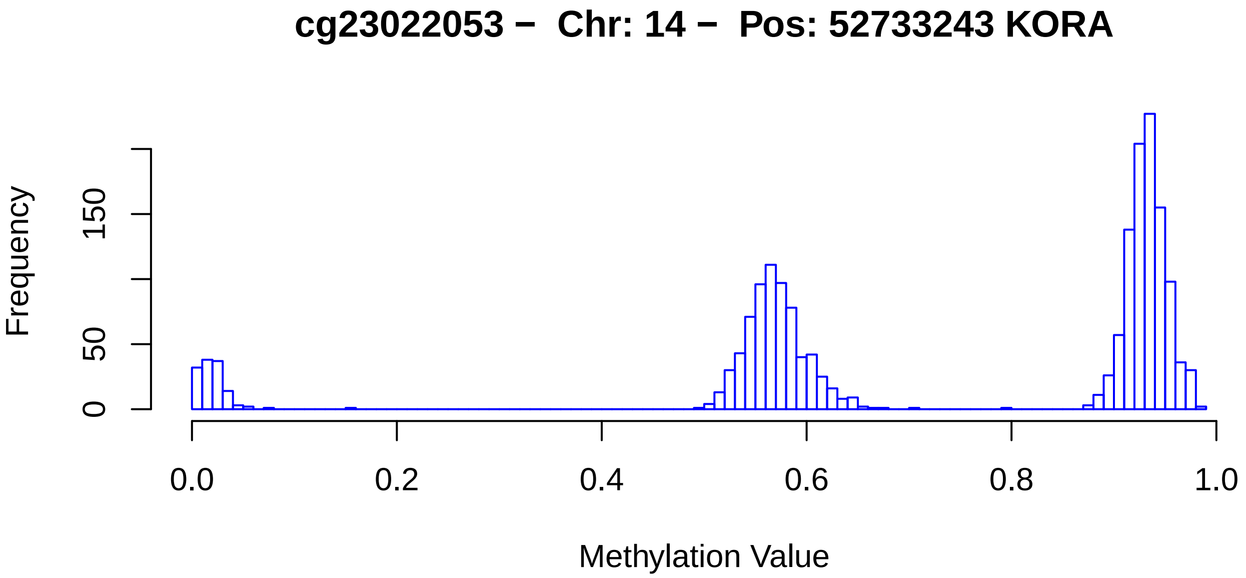
cg23022053 − Chr: 14 − Pos: 52733243 KORA
Frequency
0
50
150
0.0
0.2
0.4
0.6
0.8
1.0
Methylation Value

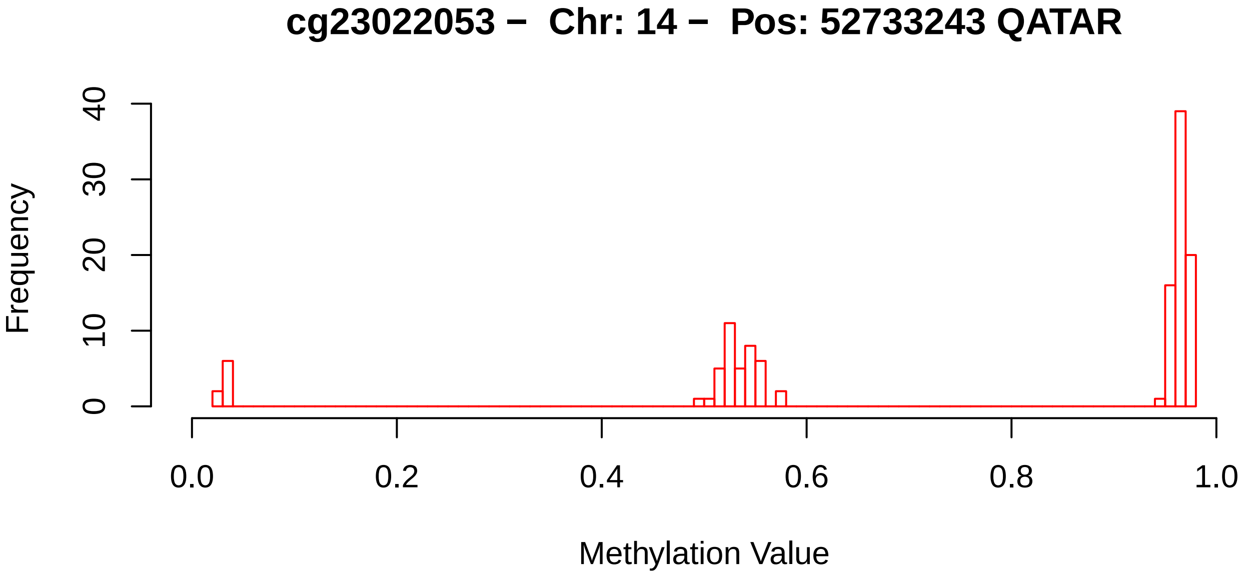
cg23022053 − Chr: 14 − Pos: 52733243 QATAR
Frequency
0
10
20
30
40
0.0
0.2
0.4
0.6
0.8
1.0
Methylation Value

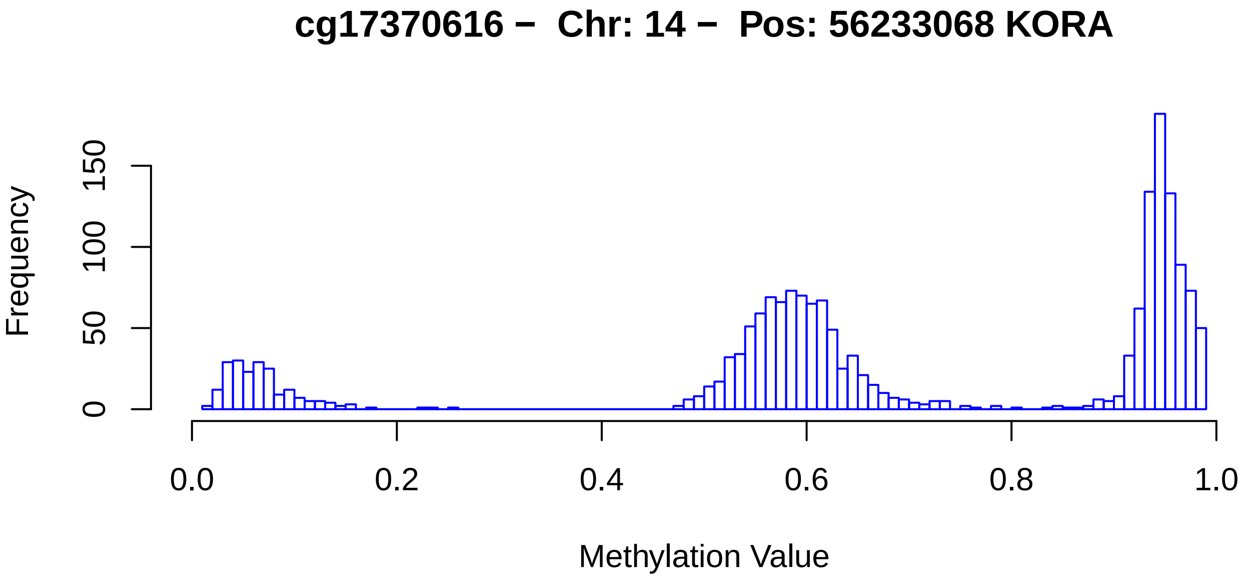
cg17370616 − Chr: 14 − Pos: 56233068 KORA
Frequency
0
50
100
150
0.0
0.2
0.4
0.6
0.8
1.0
Methylation Value

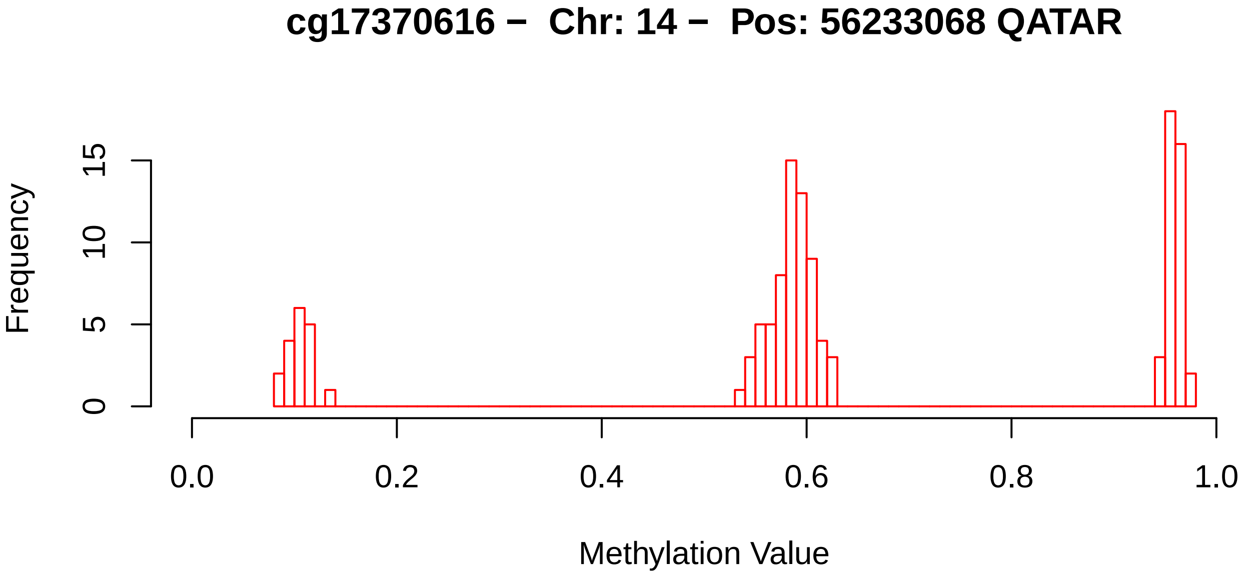
cg17370616 − Chr: 14 − Pos: 56233068 QATAR
Frequency
0
5
10
15
0.0
0.2
0.4
0.6
0.8
1.0
Methylation Value

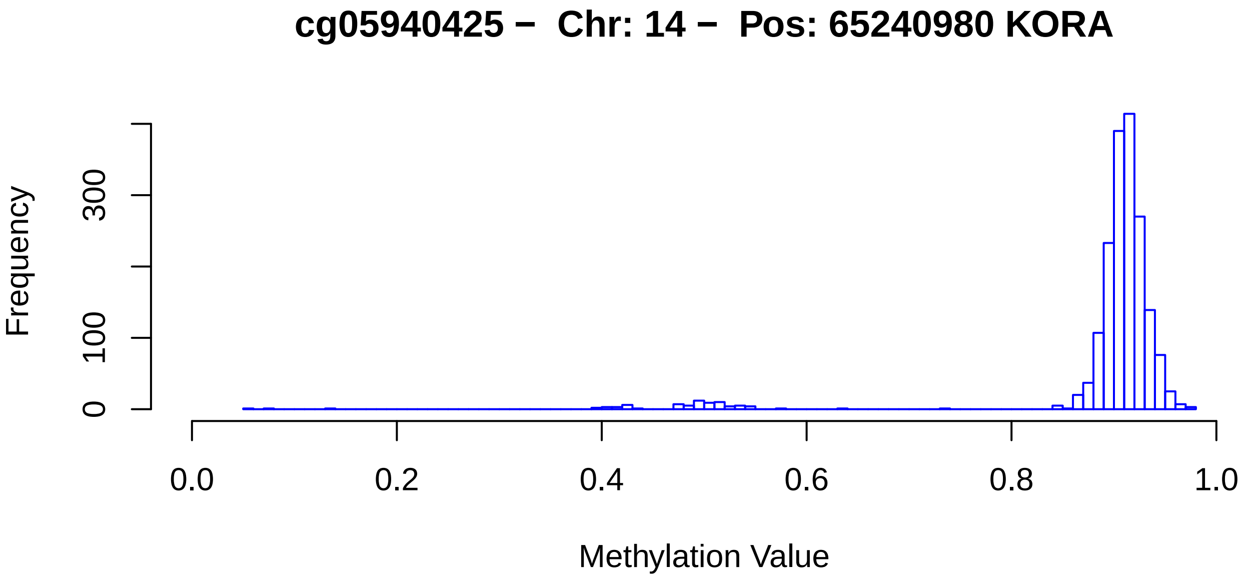
cg05940425 − Chr: 14 − Pos: 65240980 KORA
Frequency
0
100
300
0.0
0.2
0.4
0.6
0.8
1.0
Methylation Value

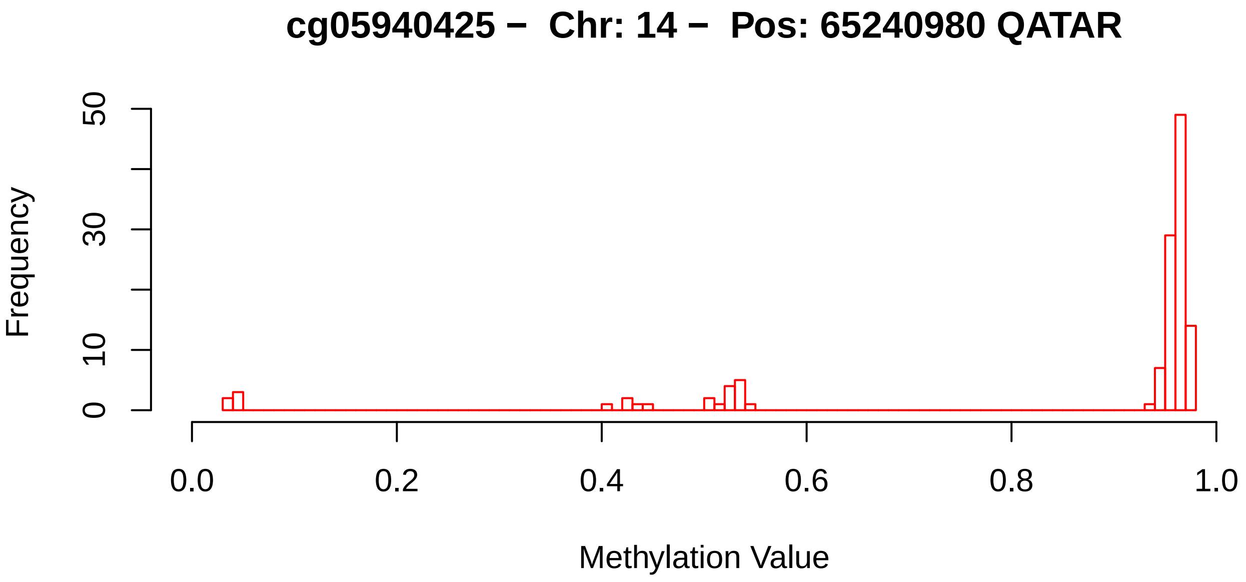
cg05940425 − Chr: 14 − Pos: 65240980 QATAR
Frequency
0
10
30
50
0.0
0.2
0.4
0.6
0.8
1.0
Methylation Value

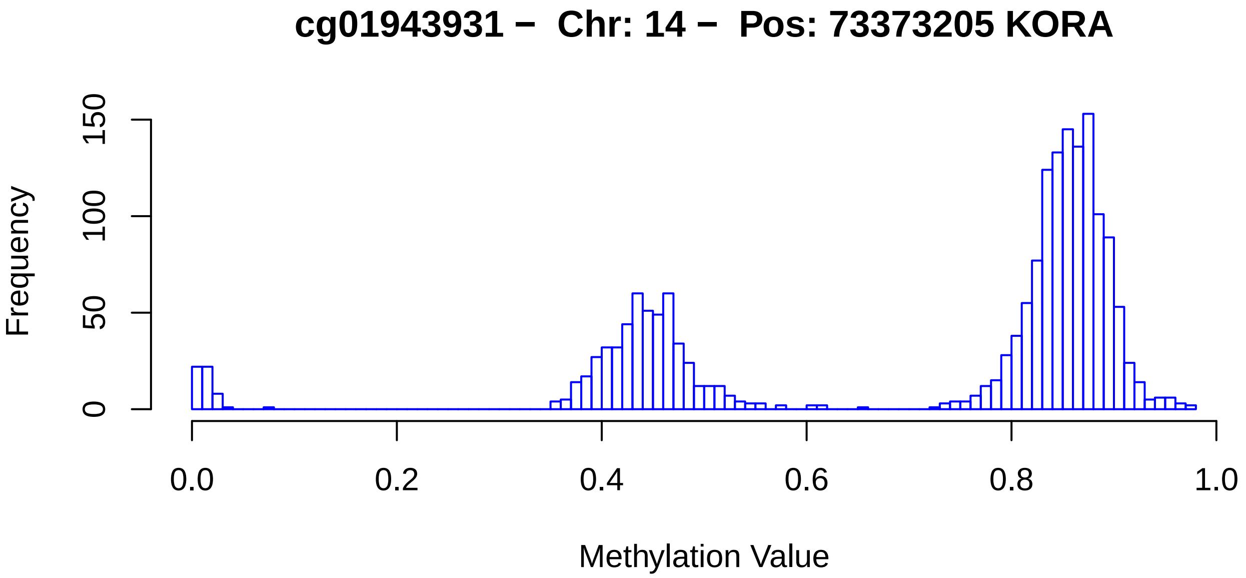

cg01943931 − Chr: 14 − Pos: 73373205 KORA
Frequency
0
50
100
150
0.0
0.2
0.4
0.6
0.8
1.0
Methylation Value

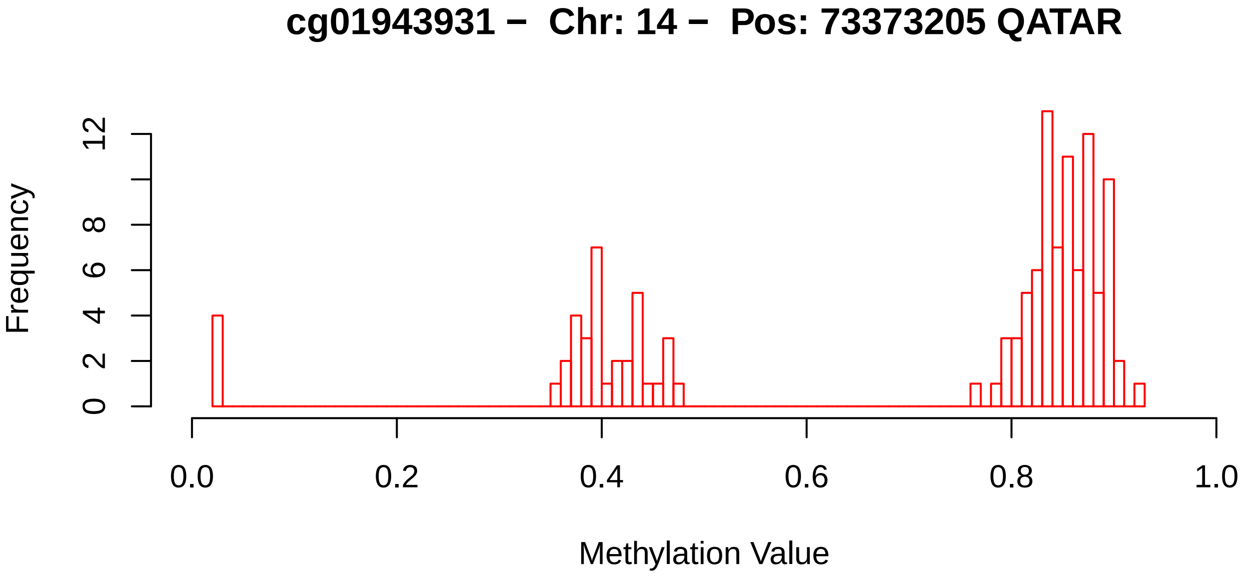

cg01943931 − Chr: 14 − Pos: 73373205 QATAR
Frequency
0
2
4
6
8
12
0.0
0.2
0.4
0.6
0.8
1.0
Methylation Value

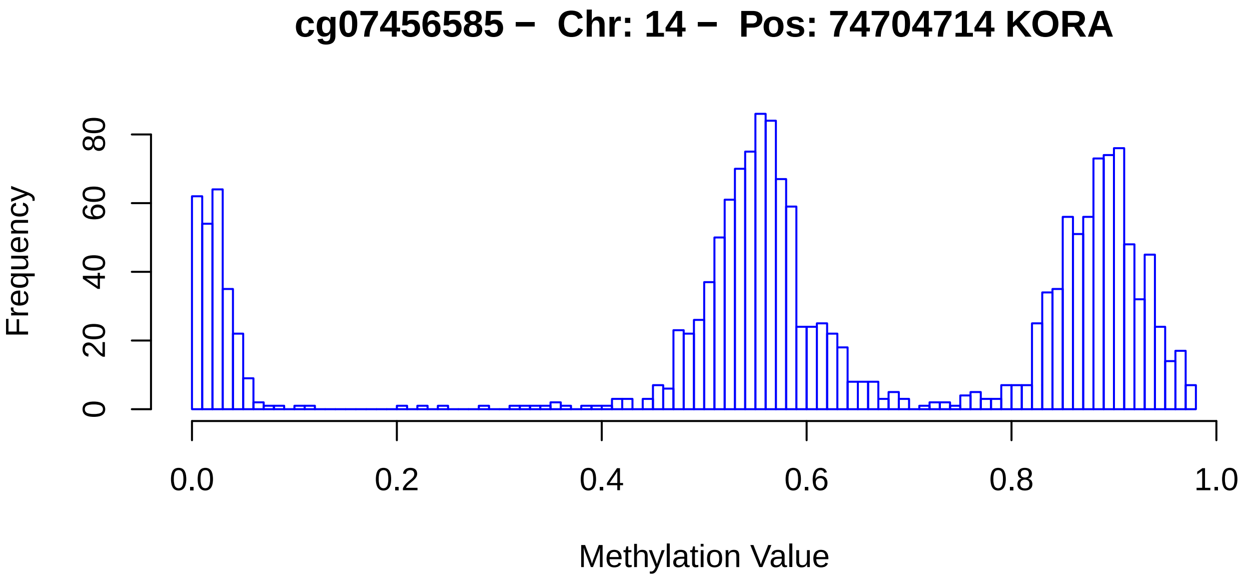
cg07456585 − Chr: 14 − Pos: 74704714 KORA
Frequency
0
20
40
60
80
0.0
0.2
0.4
0.6
0.8
1.0
Methylation Value

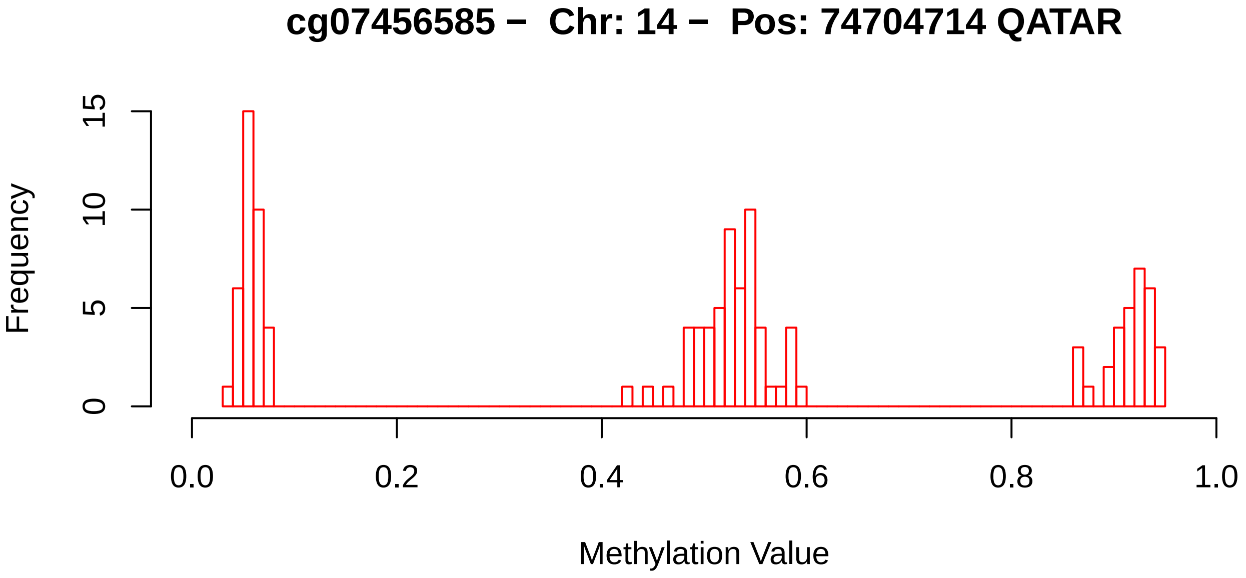
cg07456585 − Chr: 14 − Pos: 74704714 QATAR
Frequency
0
5
10
15
0.0
0.2
0.4
0.6
0.8
1.0
Methylation Value

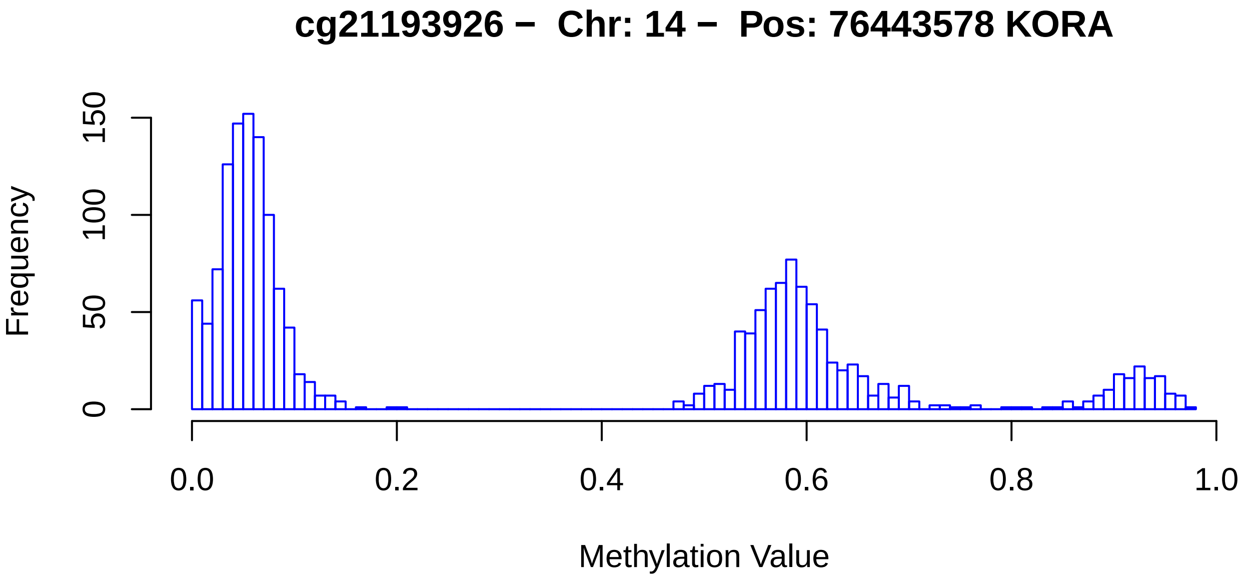
cg21193926 − Chr: 14 − Pos: 76443578 KORA
Frequency
0
50
100
150
0.0
0.2
0.4
0.6
0.8
1.0
Methylation Value

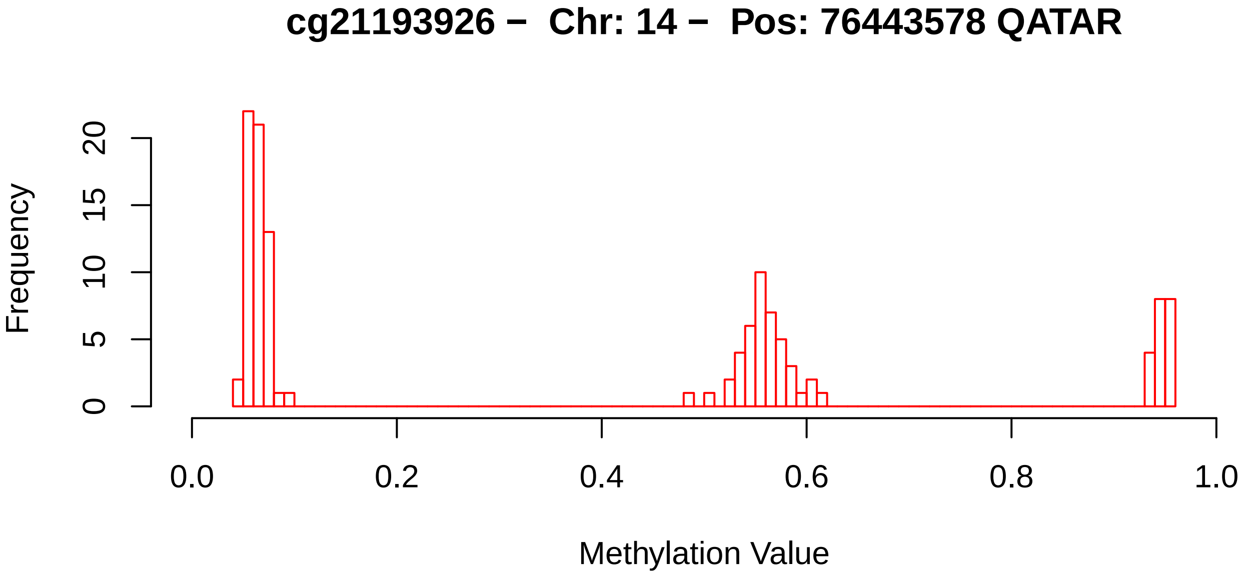
cg21193926 − Chr: 14 − Pos: 76443578 QATAR
Frequency
0
5
10
15
20
0.0
0.2
0.4
0.6
0.8
1.0
Methylation Value

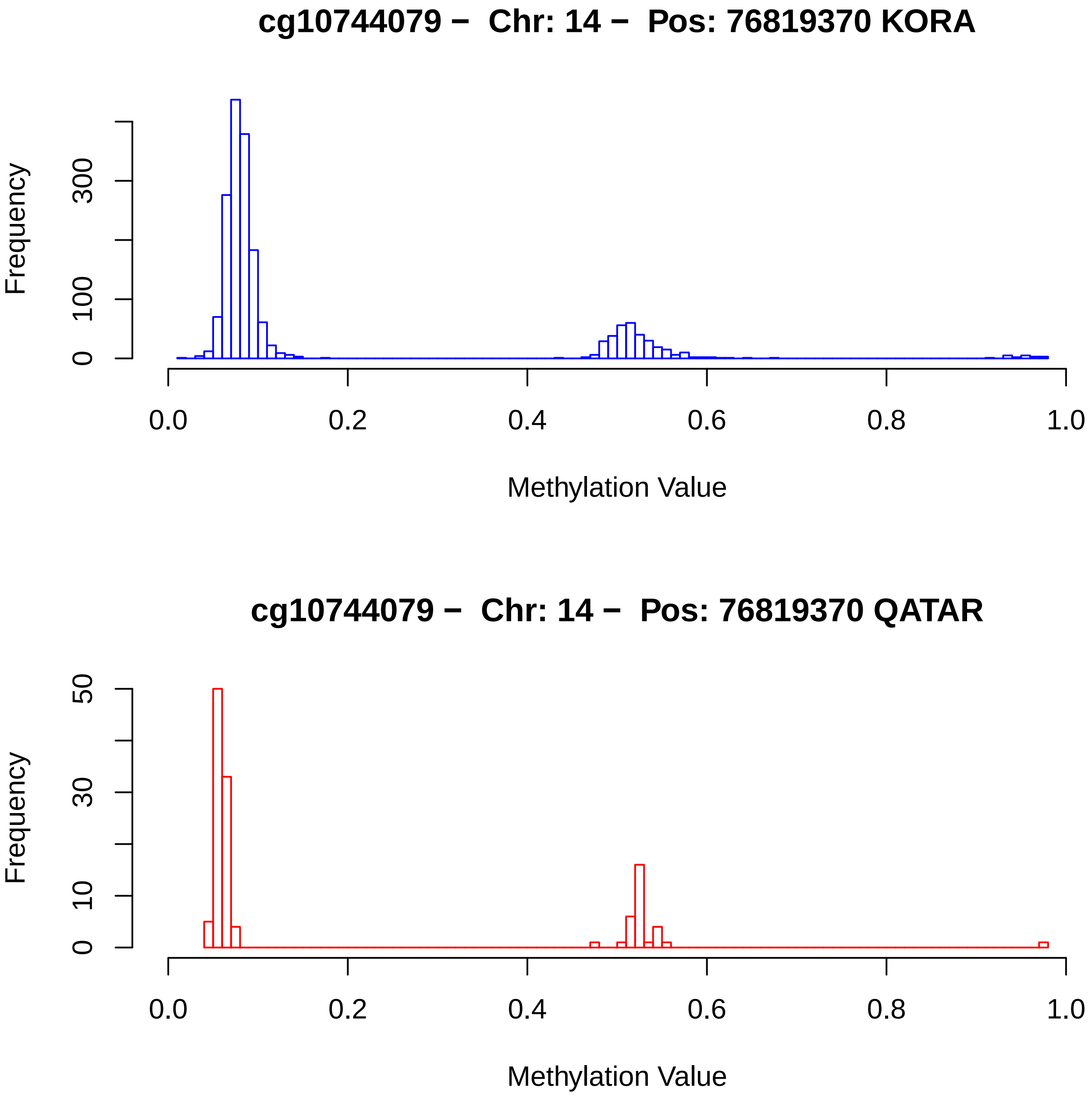
cg10744079 − Chr: 14 − Pos: 76819370 KORA
Frequency
0
100
300
0.0
0.2
0.4
0.6
0.8
1.0
Methylation Value
cg10744079 − Chr: 14 − Pos: 76819370 QATAR
Frequency
0
10
30
50
0.0
0.2
0.4
0.6
0.8
1.0
Methylation Value

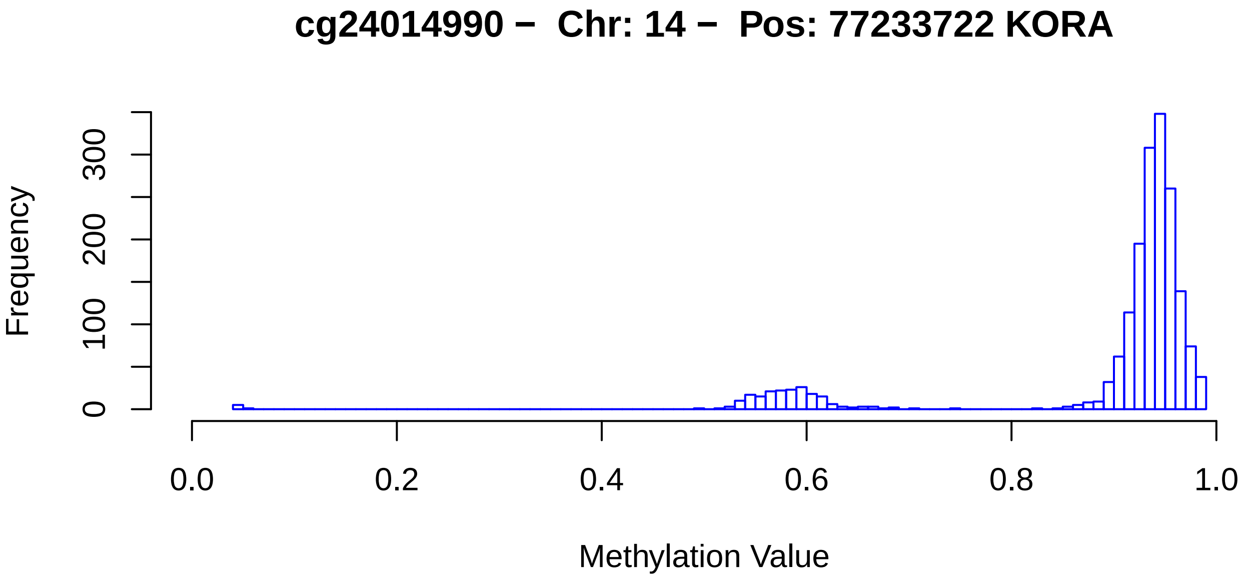
cg24014990 − Chr: 14 − Pos: 77233722 KORA
Frequency
0
100
200
300
0.0
0.2
0.4
0.6
0.8
1.0
Methylation Value

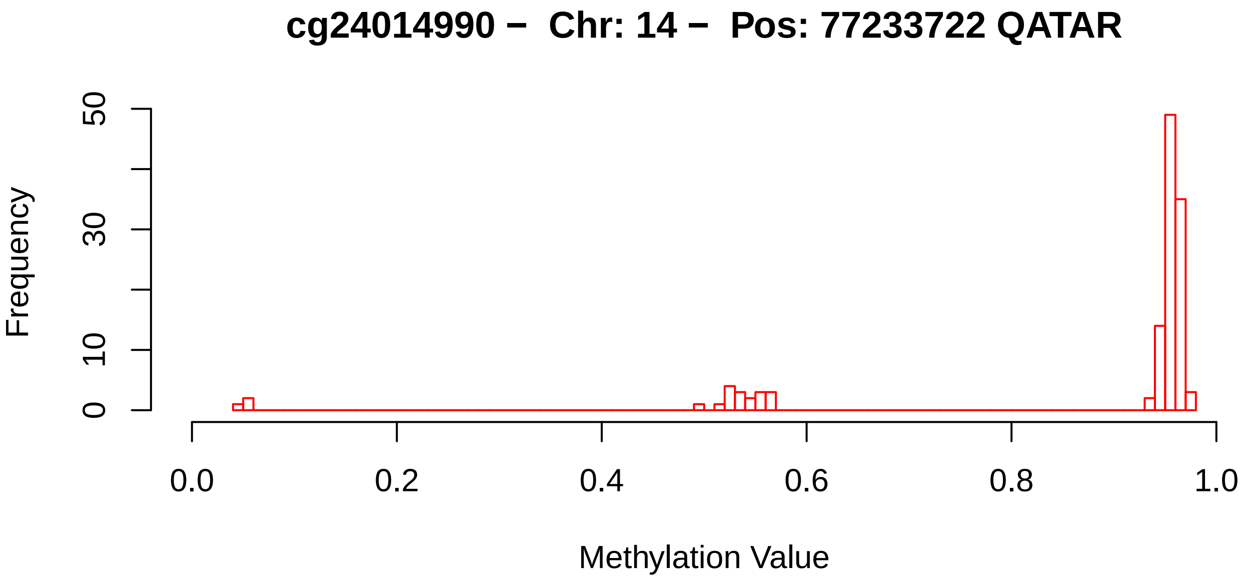
cg24014990 − Chr: 14 − Pos: 77233722 QATAR
Frequency
0
10
30
50
0.0
0.2
0.4
0.6
0.8
1.0
Methylation Value

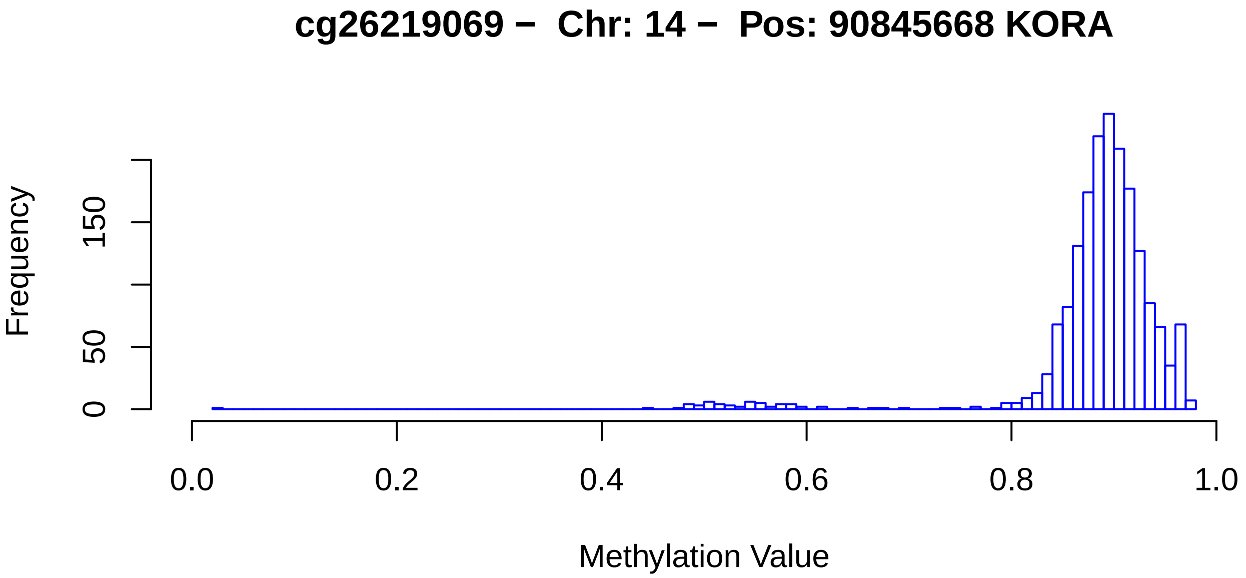
cg26219069 − Chr: 14 − Pos: 90845668 KORA
Frequency
0
50
150
0.0
0.2
0.4
0.6
0.8
1.0
Methylation Value

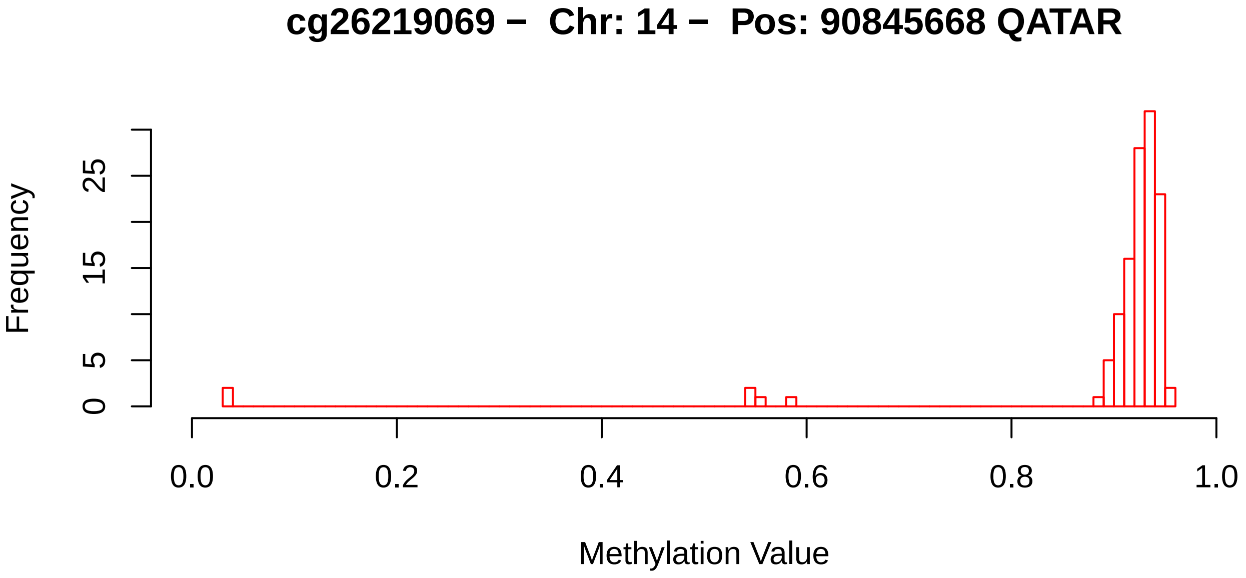
cg26219069 − Chr: 14 − Pos: 90845668 QATAR
Frequency
0
5
15
25
0.0
0.2
0.4
0.6
0.8
1.0
Methylation Value

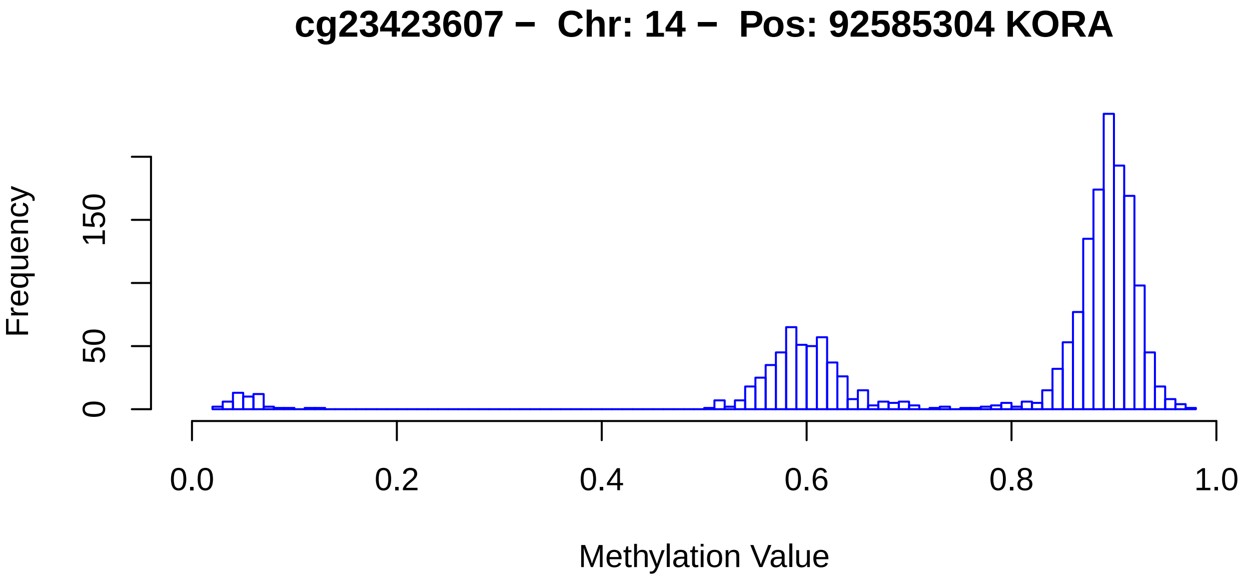
cg23423607 − Chr: 14 − Pos: 92585304 KORA
Frequency
0
50
150
0.0
0.2
0.4
0.6
0.8
1.0
Methylation Value

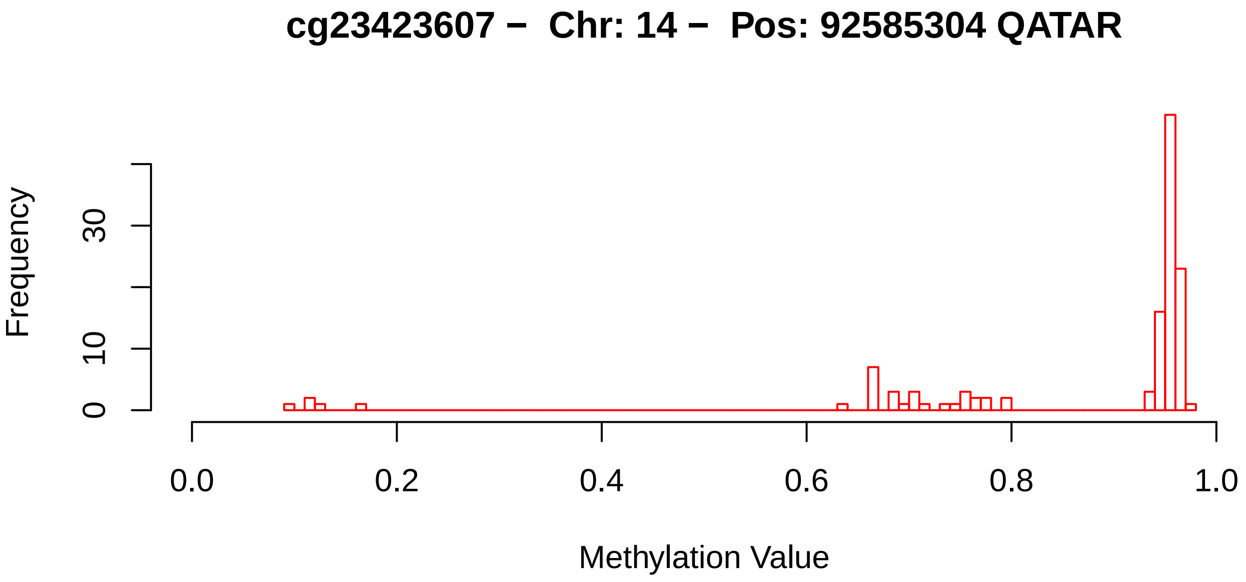
cg23423607 − Chr: 14 − Pos: 92585304 QATAR
Frequency
0
10
30
0.0
0.2
0.4
0.6
0.8
1.0
Methylation Value

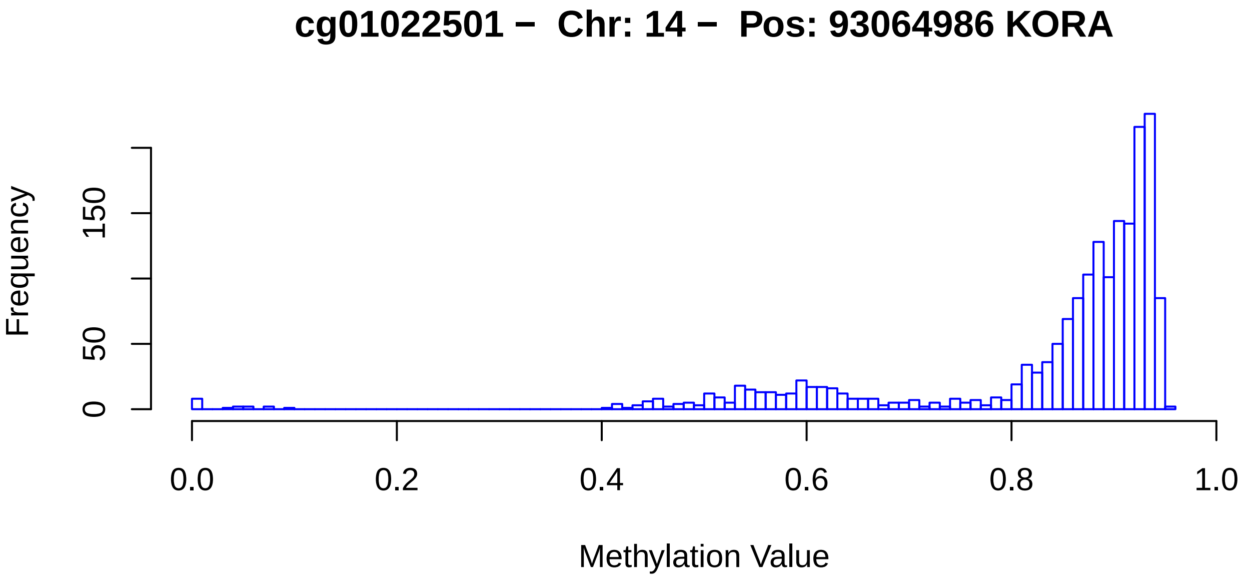
cg01022501 − Chr: 14 − Pos: 93064986 KORA
Frequency
0
50
150
0.0
0.2
0.4
0.6
0.8
1.0
Methylation Value

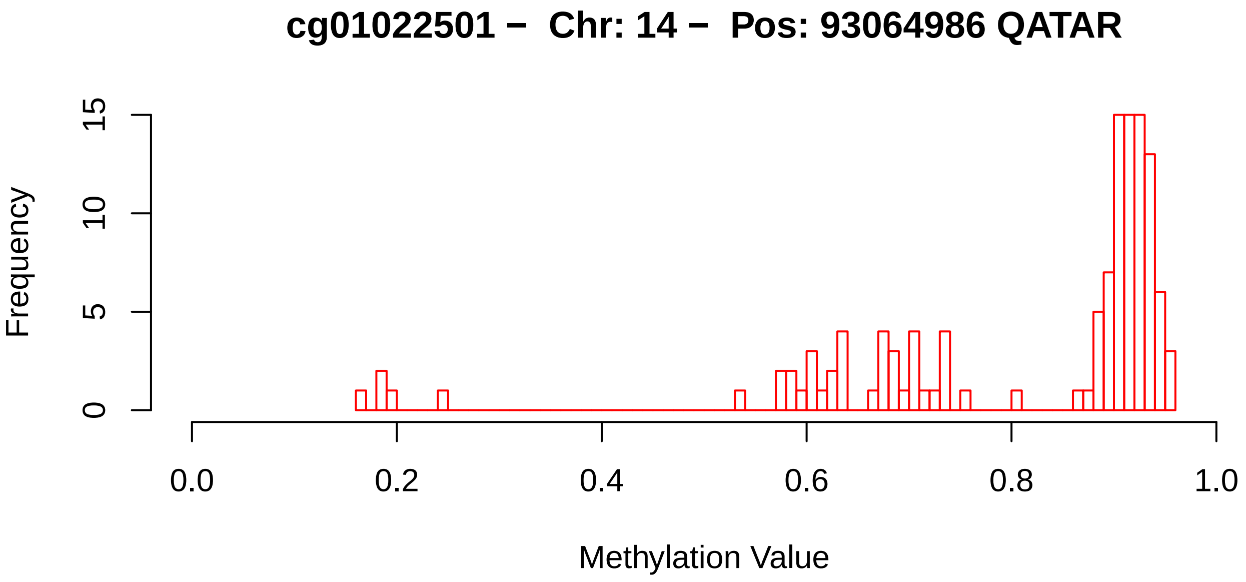
cg01022501 − Chr: 14 − Pos: 93064986 QATAR
Frequency
0
5
10
15
0.0
0.2
0.4
0.6
0.8
1.0
Methylation Value

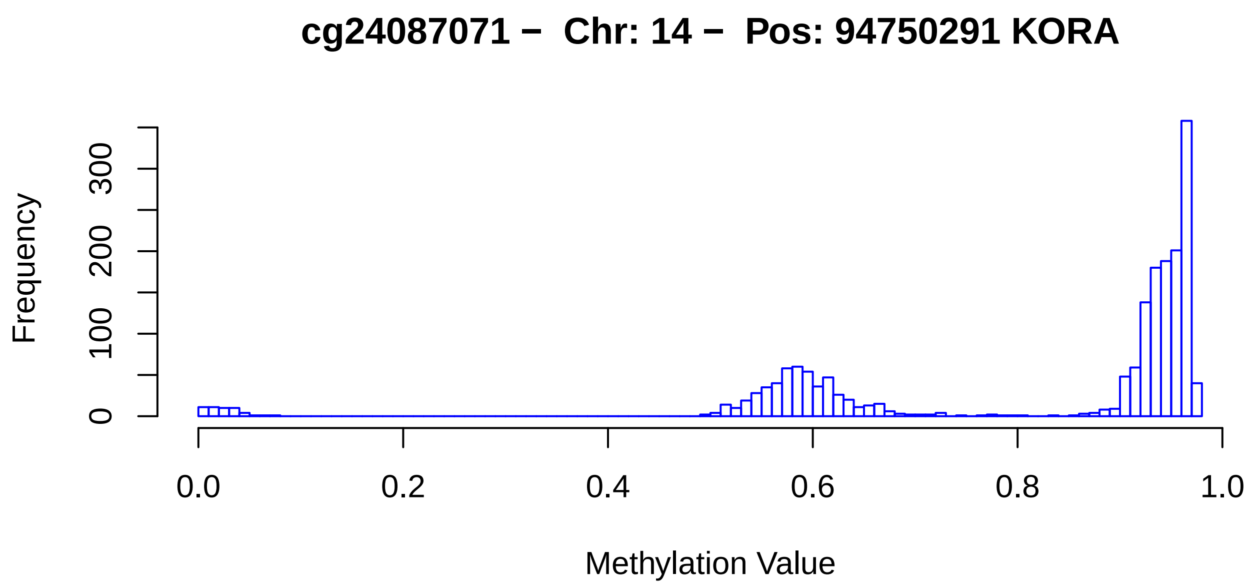
cg24087071 − Chr: 14 − Pos: 94750291 KORA
Frequency
0
100
200
300
0.0
0.2
0.4
0.6
0.8
1.0
Methylation Value

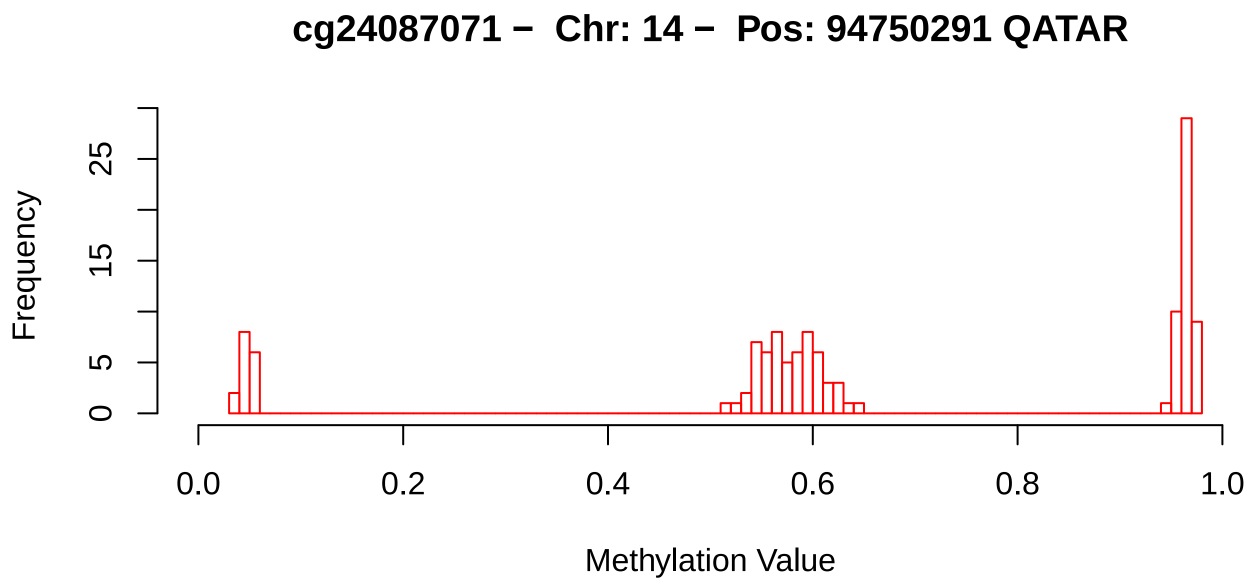
cg24087071 − Chr: 14 − Pos: 94750291 QATAR
Frequency
0
5
15
25
0.0
0.2
0.4
0.6
0.8
1.0
Methylation Value

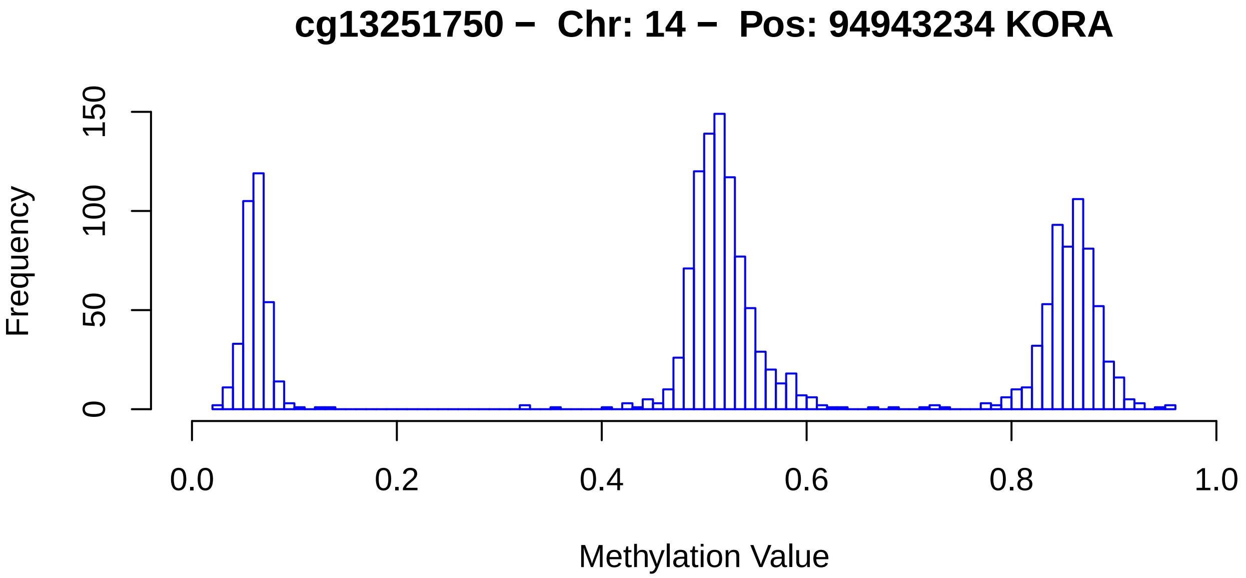
cg13251750 − Chr: 14 − Pos: 94943234 KORA
Frequency
0
50
100
150
0.0
0.2
0.4
0.6
0.8
1.0
Methylation Value

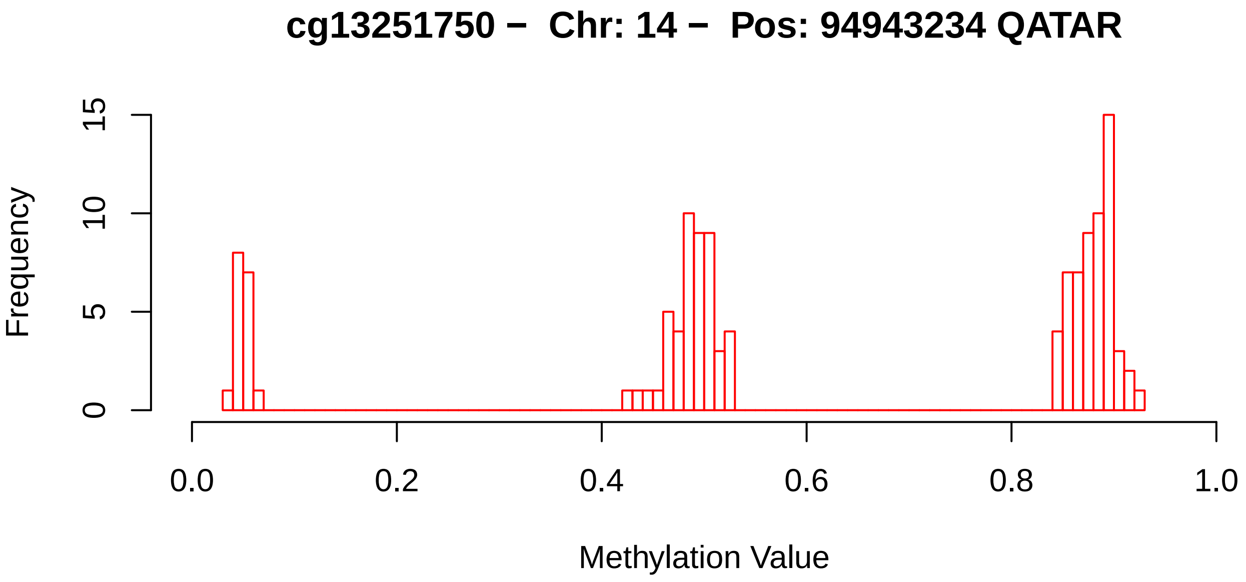
cg13251750 − Chr: 14 − Pos: 94943234 QATAR
Frequency
0
5
10
15
0.0
0.2
0.4
0.6
0.8
1.0
Methylation Value

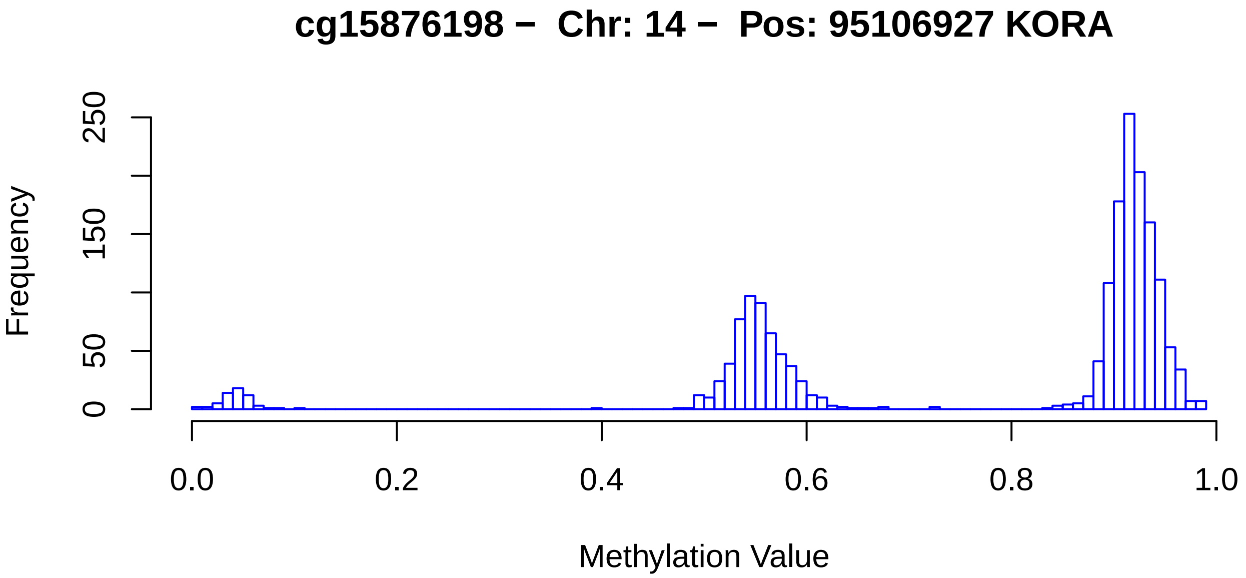

cg15876198 − Chr: 14 − Pos: 95106927 KORA
Frequency
0
50
150
250
0.0
0.2
0.4
0.6
0.8
1.0
Methylation Value

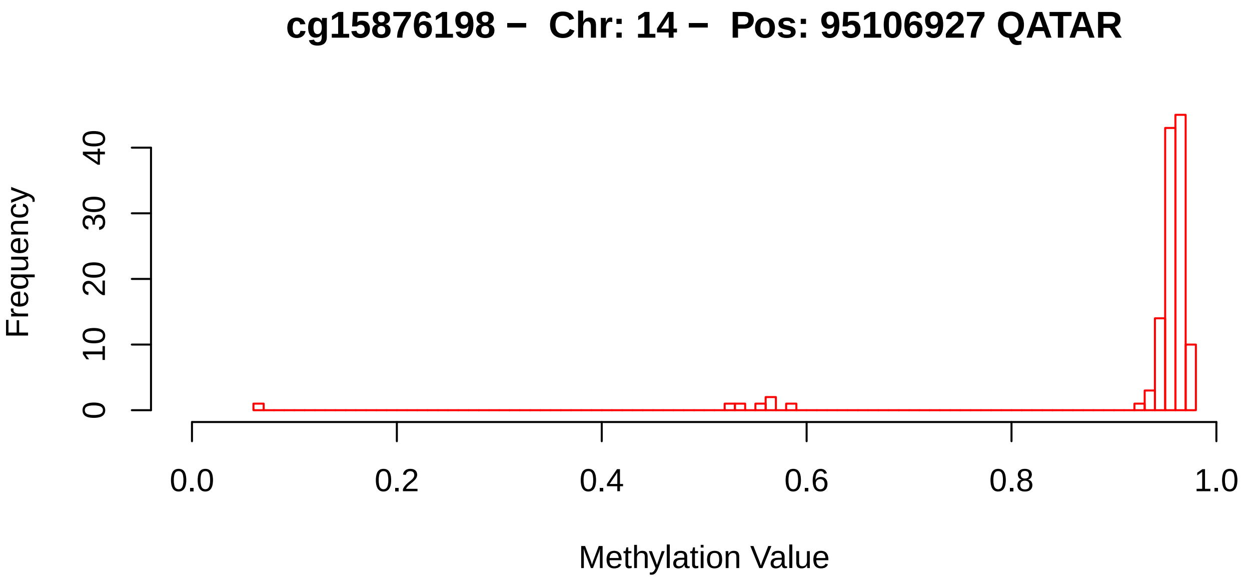

cg15876198 − Chr: 14 − Pos: 95106927 QATAR
Frequency
0
10
20
30
40
0.0
0.2
0.4
0.6
0.8
1.0
Methylation Value

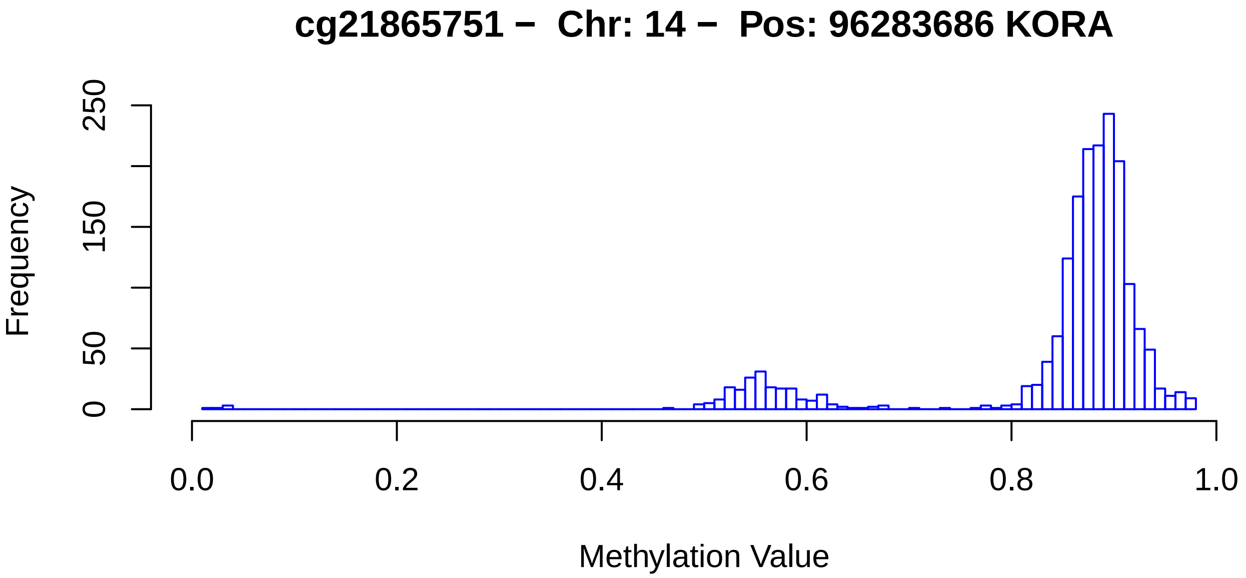
cg21865751 − Chr: 14 − Pos: 96283686 KORA
Frequency
0
50
150
250
0.0
0.2
0.4
0.6
0.8
1.0
Methylation Value

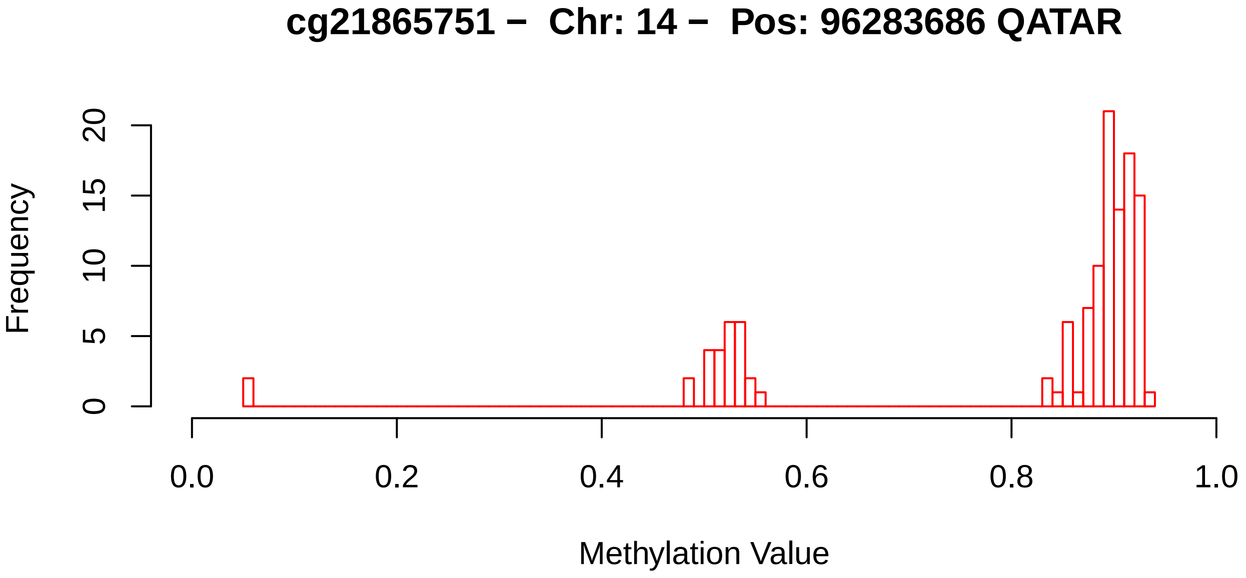
cg21865751 − Chr: 14 − Pos: 96283686 QATAR
Frequency
0
5
10
15
20
0.0
0.2
0.4
0.6
0.8
1.0
Methylation Value

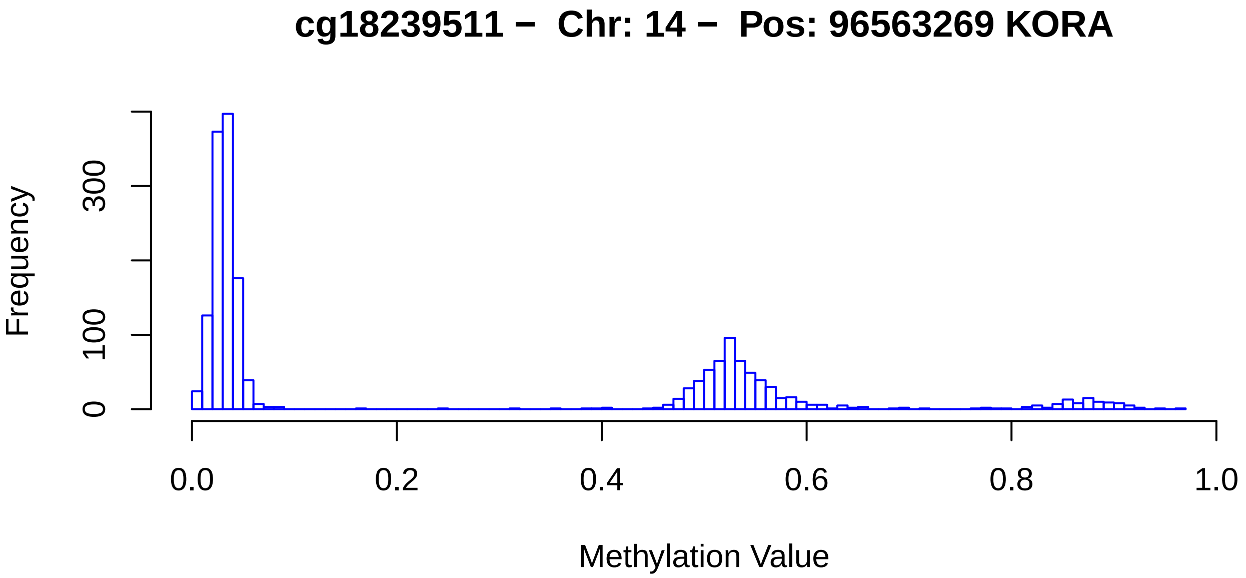
cg18239511 − Chr: 14 − Pos: 96563269 KORA
Frequency
0
100
300
0.0
0.2
0.4
0.6
0.8
1.0
Methylation Value

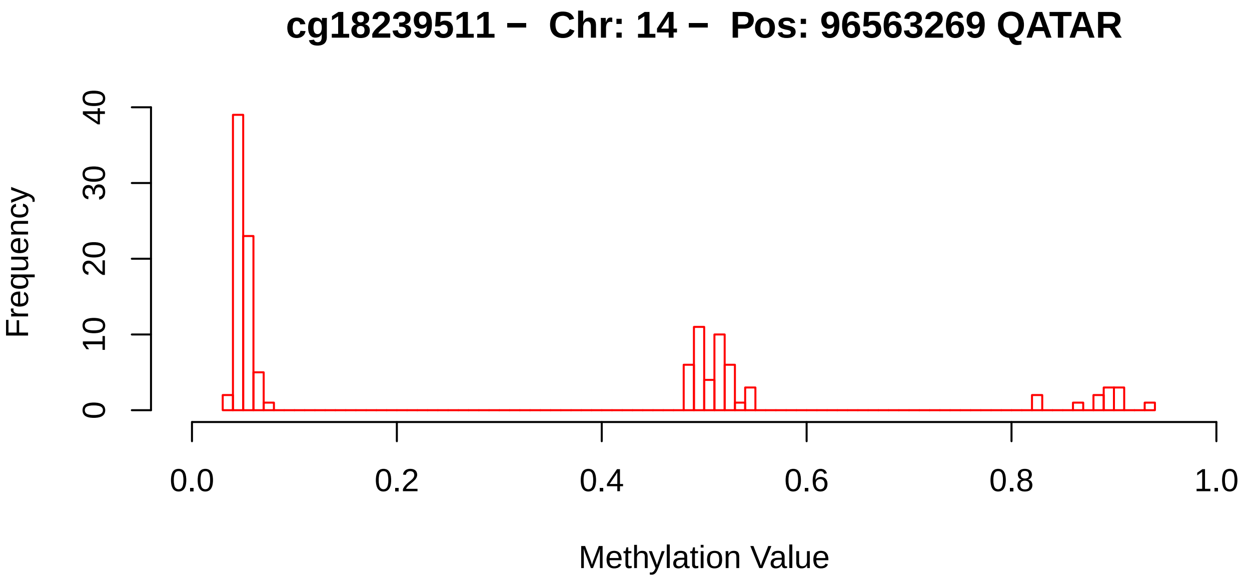
cg18239511 − Chr: 14 − Pos: 96563269 QATAR
Frequency
0
10
20
30
40
0.0
0.2
0.4
0.6
0.8
1.0
Methylation Value

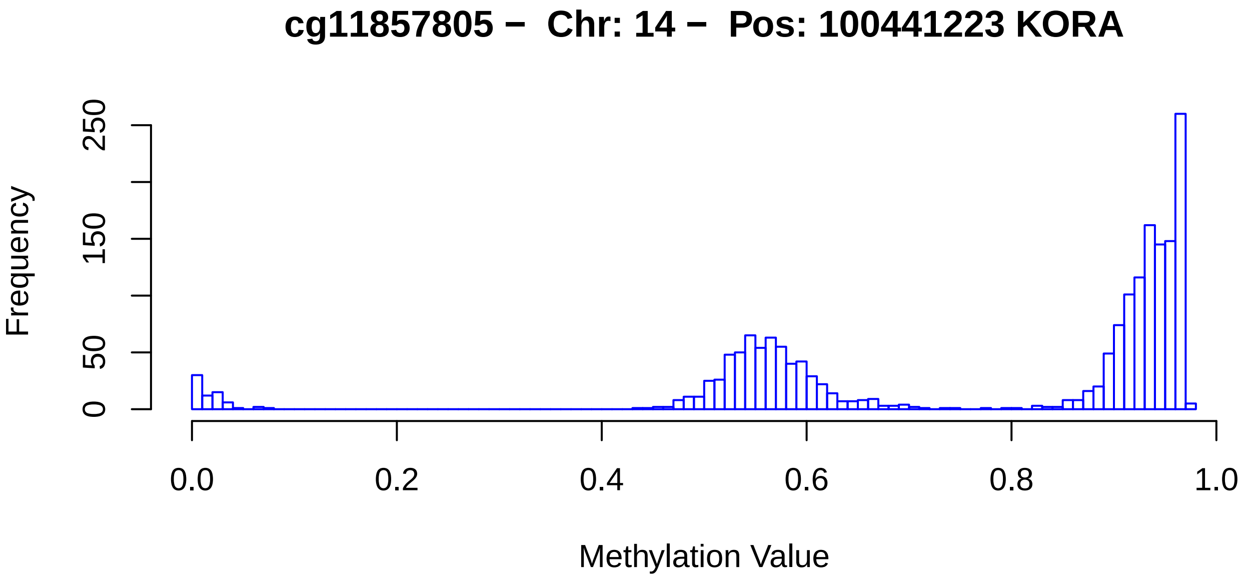
cg11857805 − Chr: 14 − Pos: 100441223 KORA
Frequency
0
50
150
250
0.0
0.2
0.4
0.6
0.8
1.0
Methylation Value

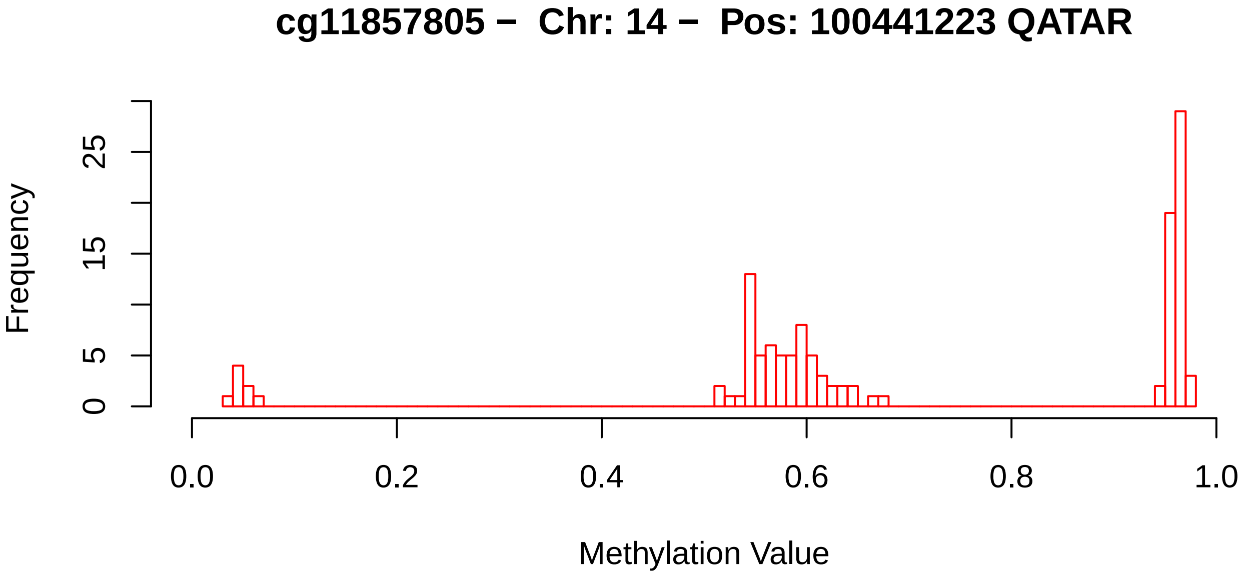
cg11857805 − Chr: 14 − Pos: 100441223 QATAR
Frequency
0
5
15
25
0.0
0.2
0.4
0.6
0.8
1.0
Methylation Value

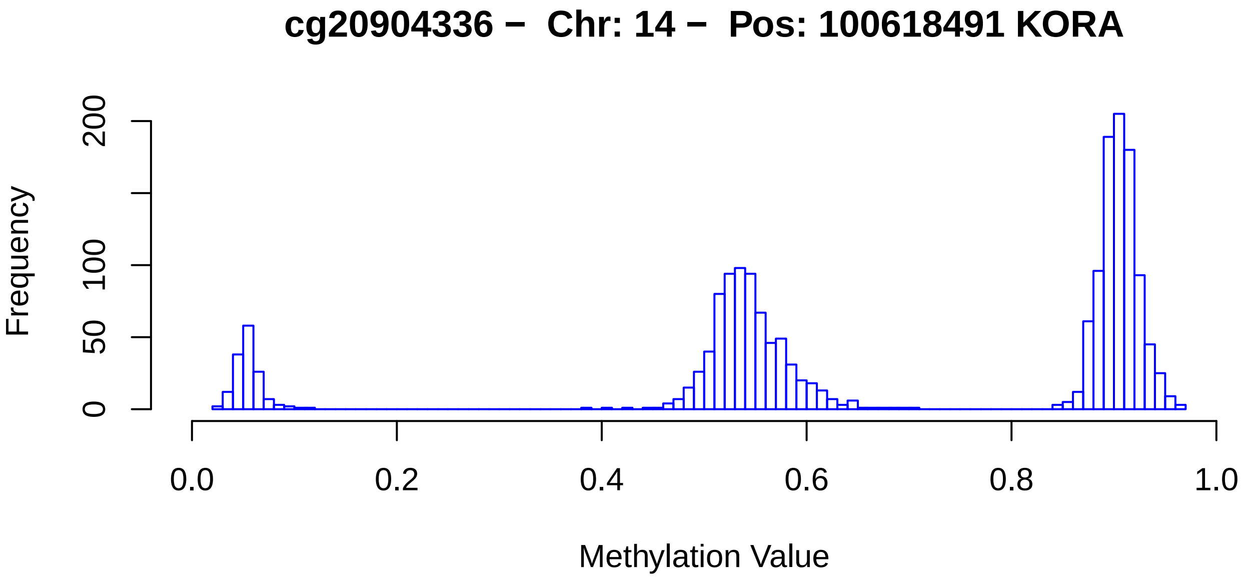
cg20904336 − Chr: 14 − Pos: 100618491 KORA
Frequency
0
50
100
200
0.0
0.2
0.4
0.6
0.8
1.0
Methylation Value

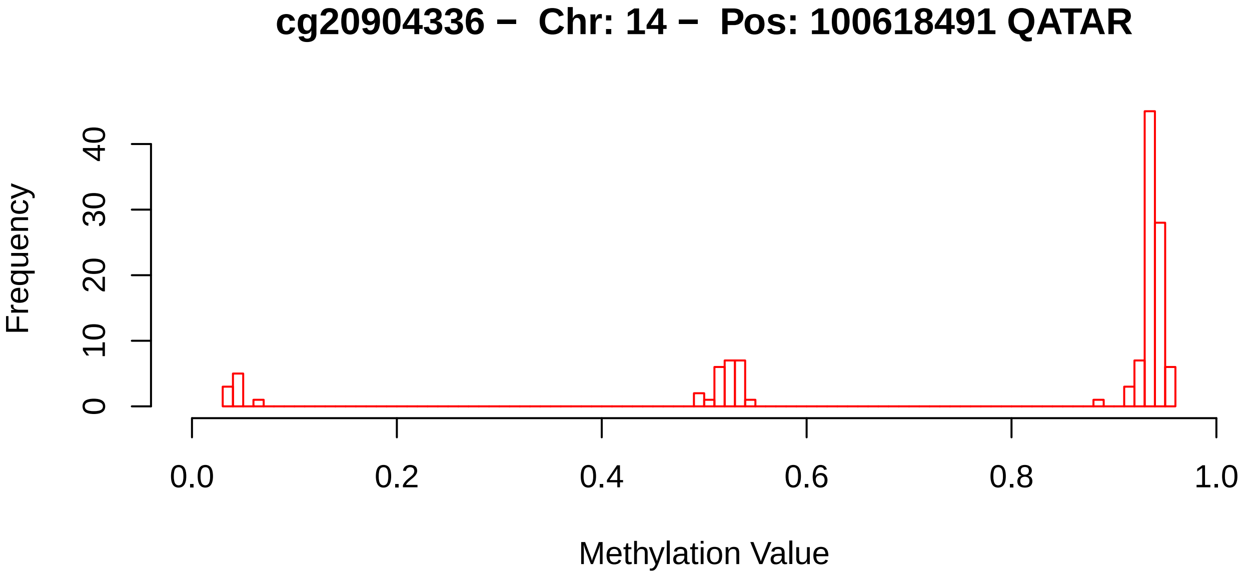
cg20904336 − Chr: 14 − Pos: 100618491 QATAR
Frequency
0
10
20
30
40
0.0
0.2
0.4
0.6
0.8
1.0
Methylation Value

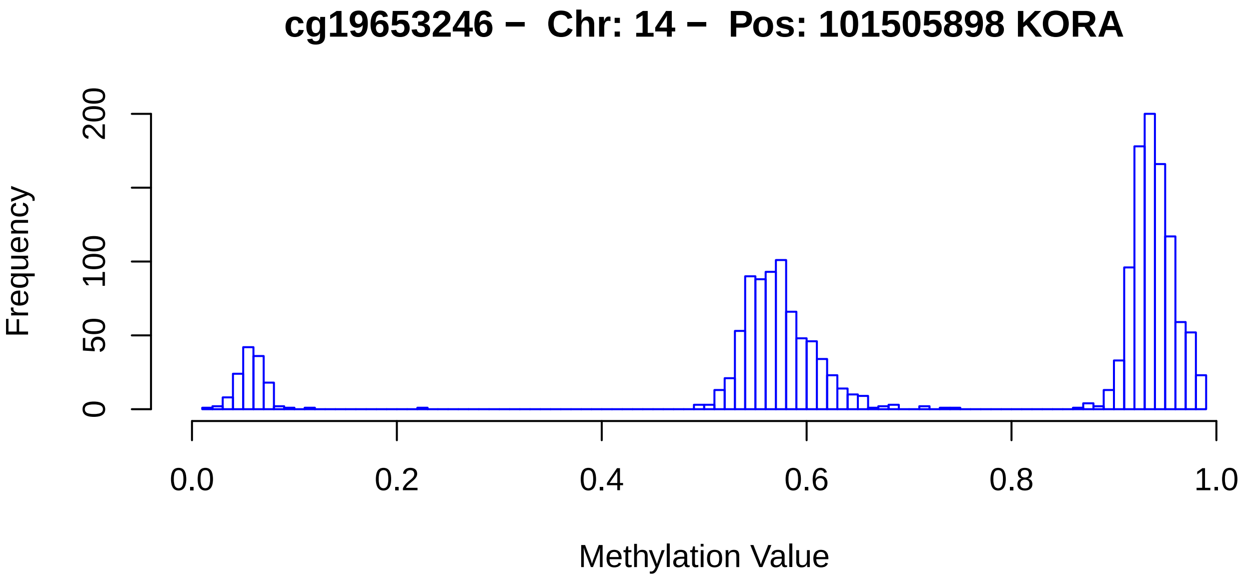
cg19653246 − Chr: 14 − Pos: 101505898 KORA
Frequency
0
50
100
200
0.0
0.2
0.4
0.6
0.8
1.0
Methylation Value

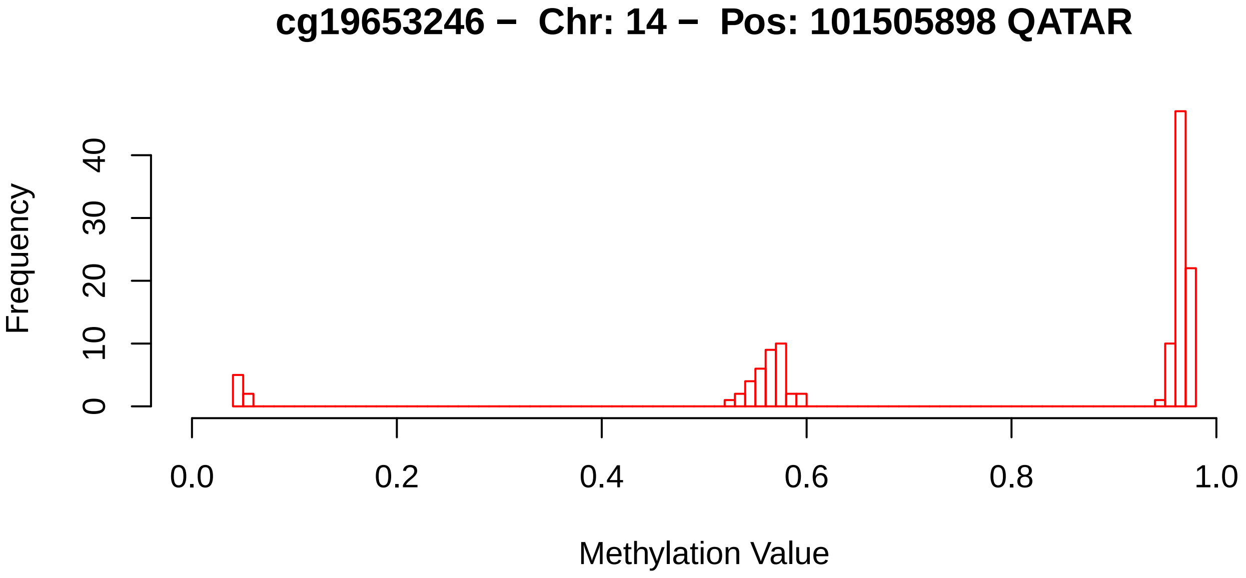
cg19653246 − Chr: 14 − Pos: 101505898 QATAR
Frequency
0
10
20
30
40
0.0
0.2
0.4
0.6
0.8
1.0
Methylation Value

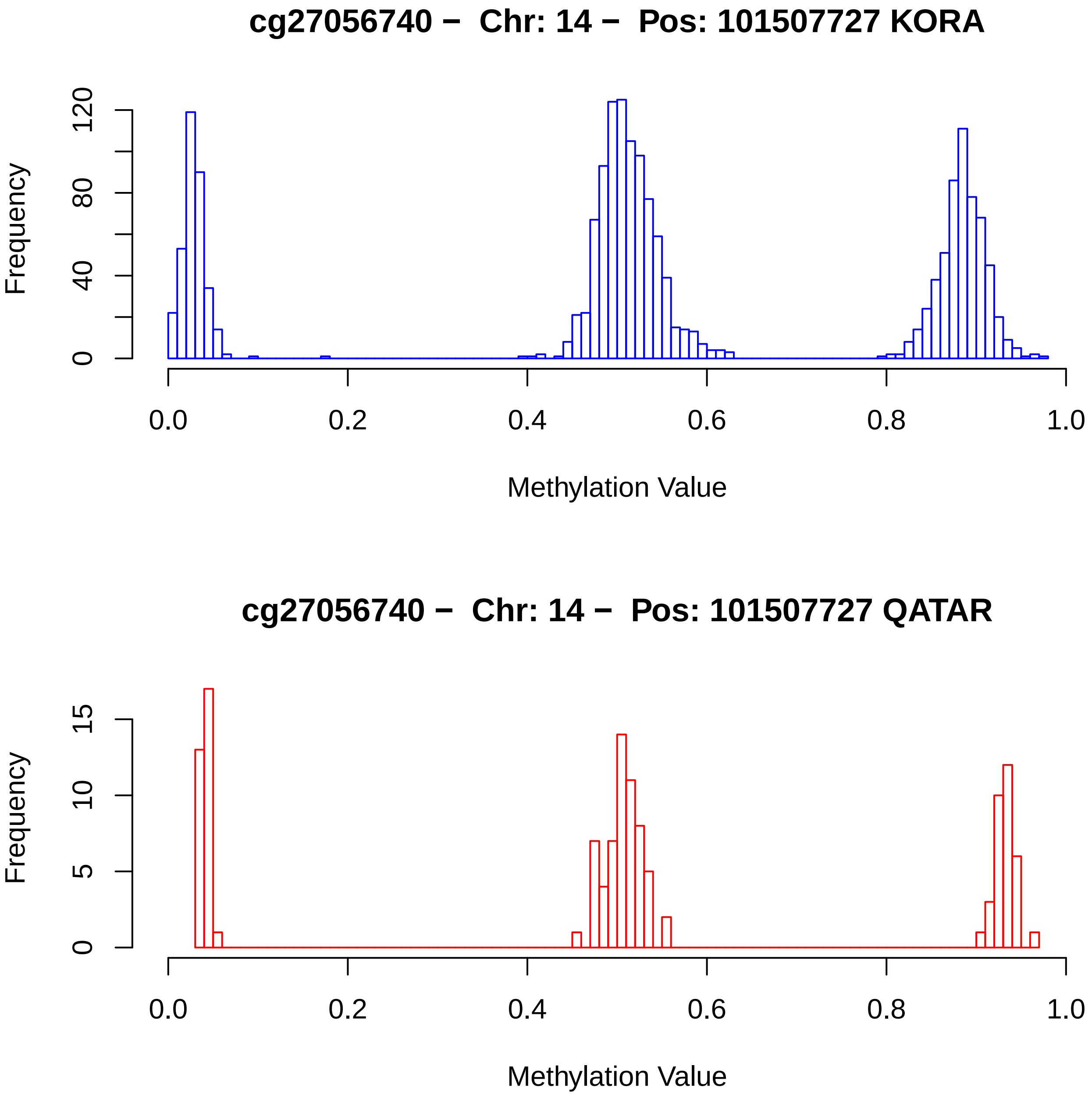
cg27056740 − Chr: 14 − Pos: 101507727 KORA
Frequency
0
40
80
120
0.0
0.2
0.4
0.6
0.8
1.0
Methylation Value
cg27056740 − Chr: 14 − Pos: 101507727 QATAR
Frequency
0
5
10
15
0.0
0.2
0.4
0.6
0.8
1.0
Methylation Value

cg05097165 − Chr: 14 − Pos: 101874838 KORA

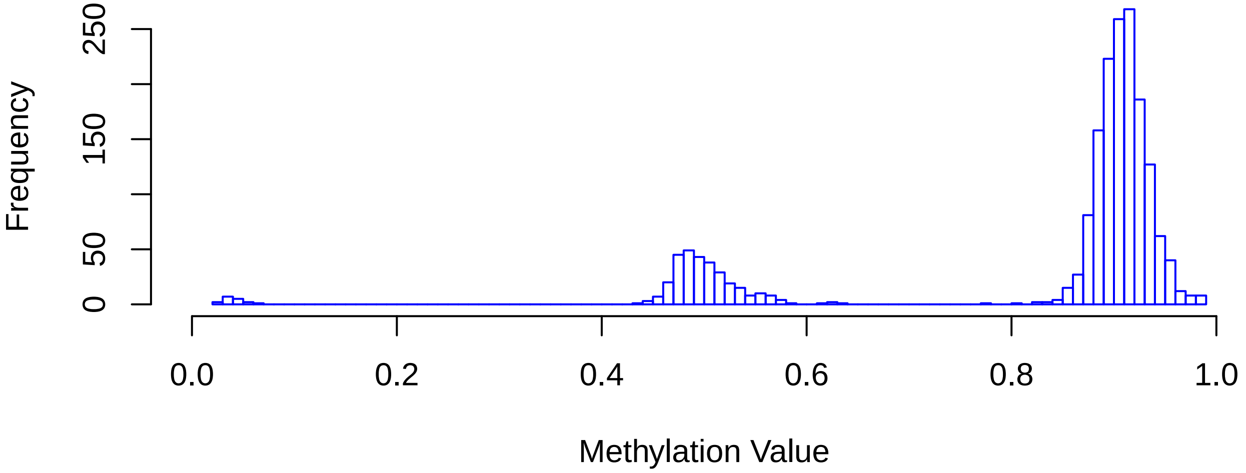

cg05097165 − Chr: 14 − Pos: 101874838 QATAR

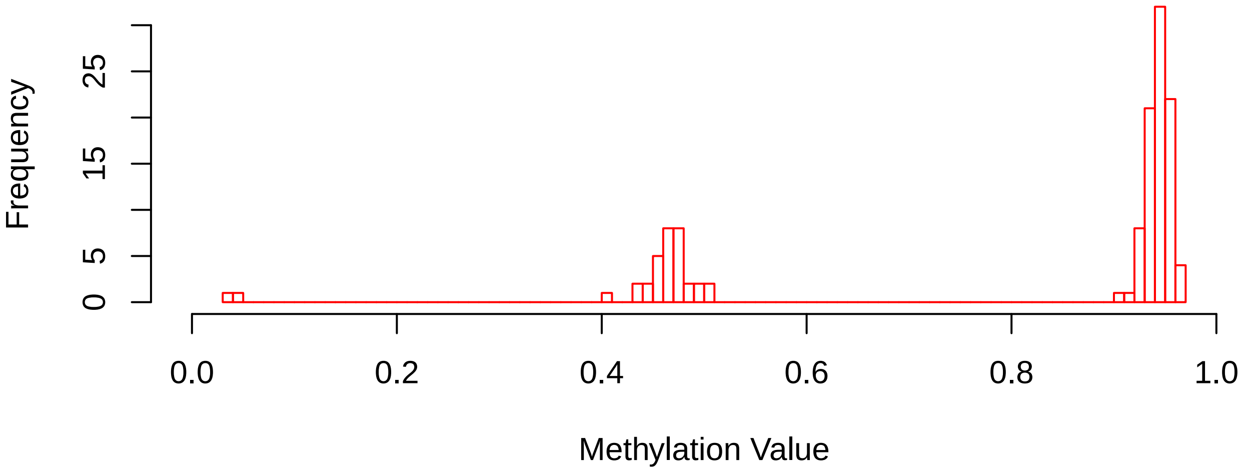

cg01225004 − Chr: 14 − Pos: 101922290 KORA

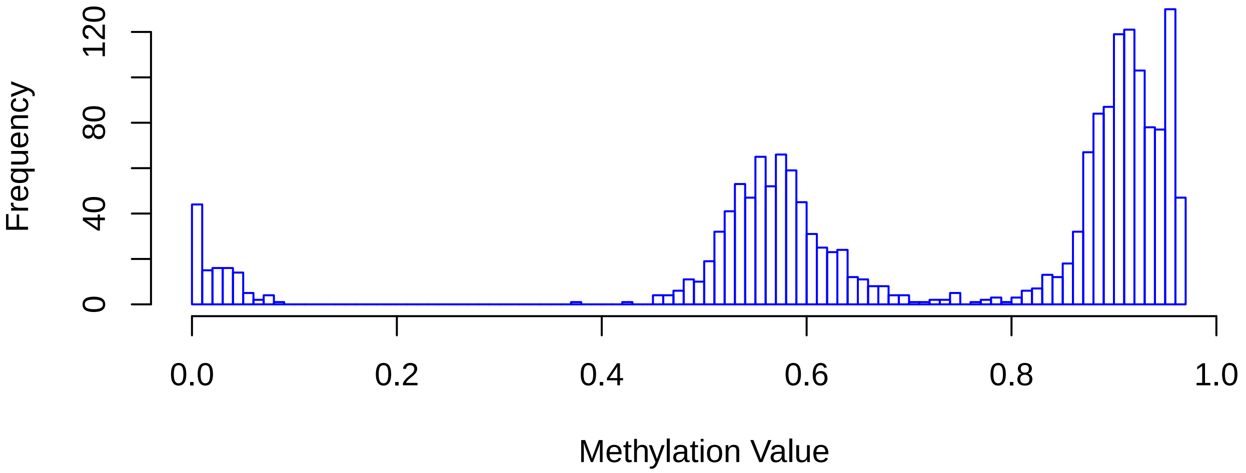

cg01225004 − Chr: 14 − Pos: 101922290 QATAR

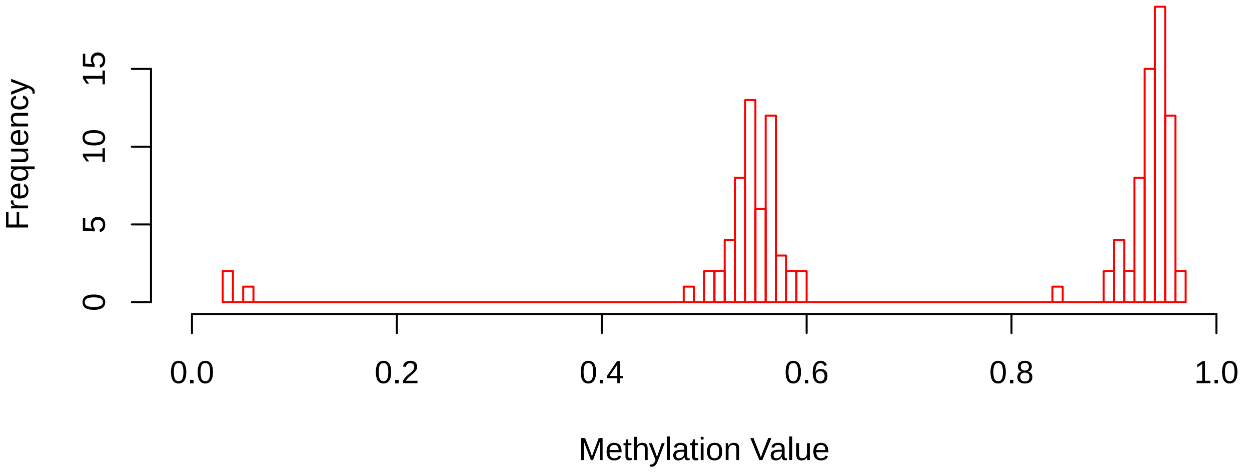

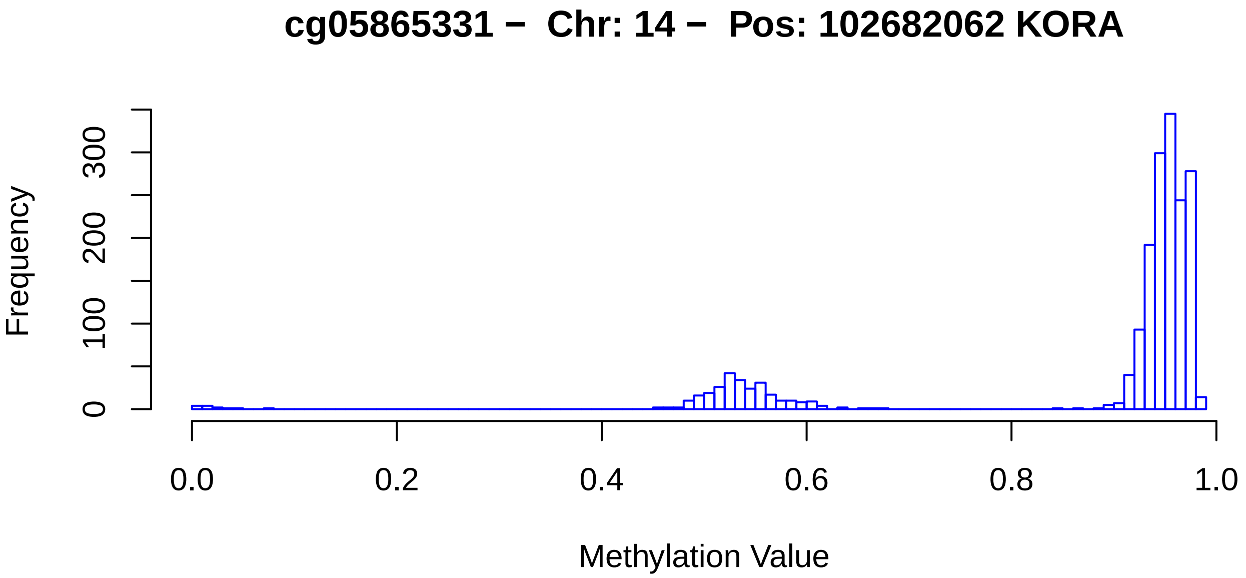

cg05865331 − Chr: 14 − Pos: 102682062 KORA
Frequency
0
100
200
300
0.0
0.2
0.4
0.6
0.8
1.0
Methylation Value

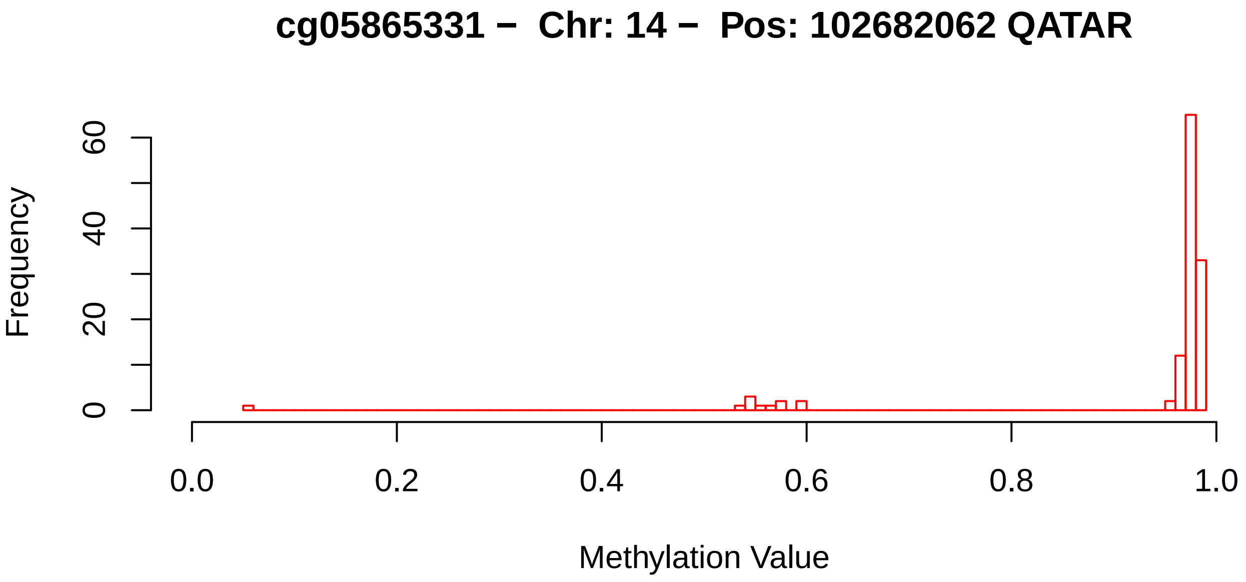

cg05865331 − Chr: 14 − Pos: 102682062 QATAR
Frequency
0
20
40
60
0.0
0.2
0.4
0.6
0.8
1.0
Methylation Value

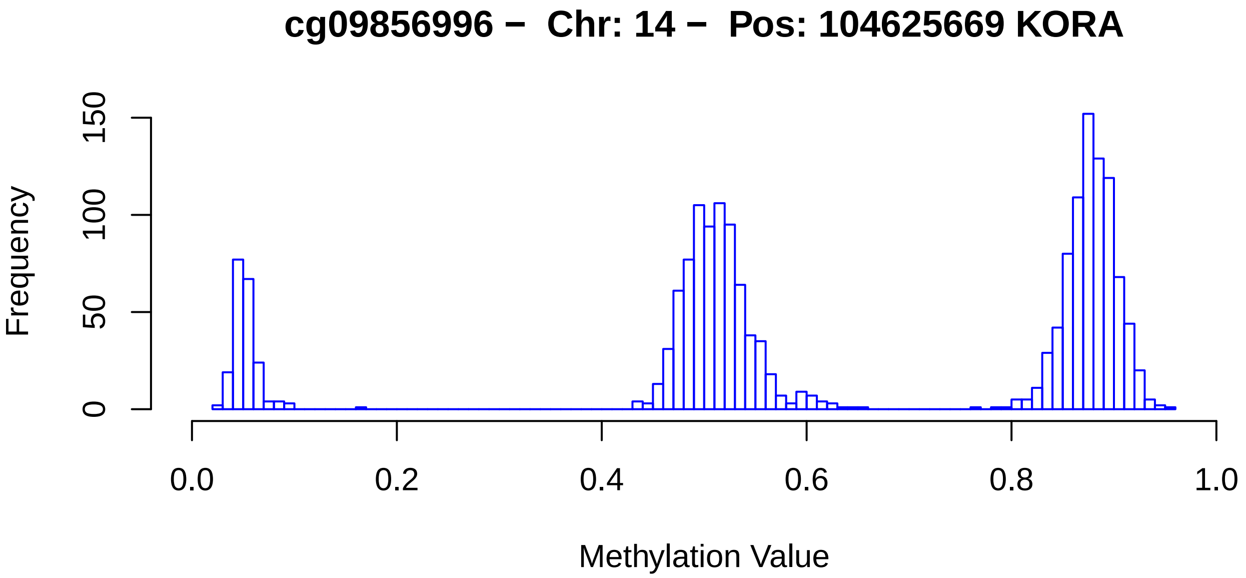
cg09856996 − Chr: 14 − Pos: 104625669 KORA
Frequency
0
50
100
150
0.0
0.2
0.4
0.6
0.8
1.0
Methylation Value

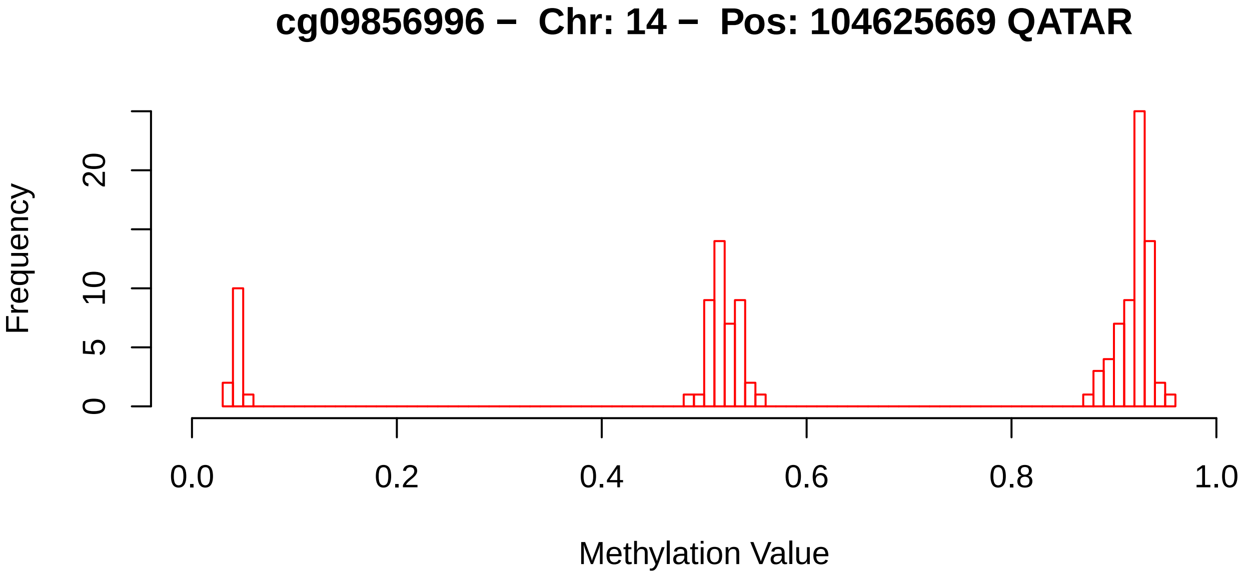
cg09856996 − Chr: 14 − Pos: 104625669 QATAR
Frequency
0
5
10
20
0.0
0.2
0.4
0.6
0.8
1.0
Methylation Value

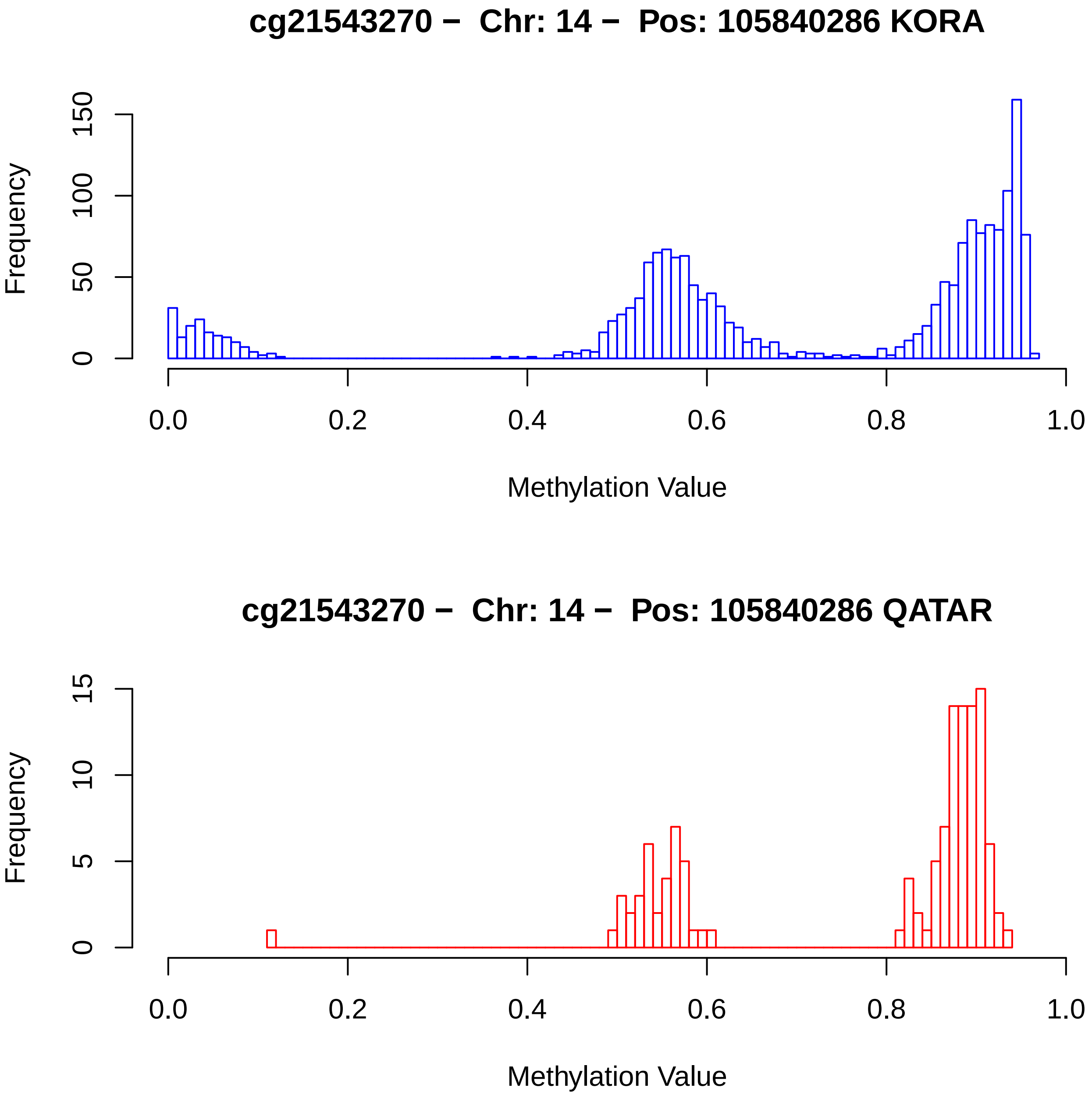

cg21543270 − Chr: 14 − Pos: 105840286 KORA
Frequency
0
50
100
150
0.0
0.2
0.4
0.6
0.8
1.0
Methylation Value
cg21543270 − Chr: 14 − Pos: 105840286 QATAR
Frequency
0
5
10
15
0.0
0.2
0.4
0.6
0.8
1.0
Methylation Value

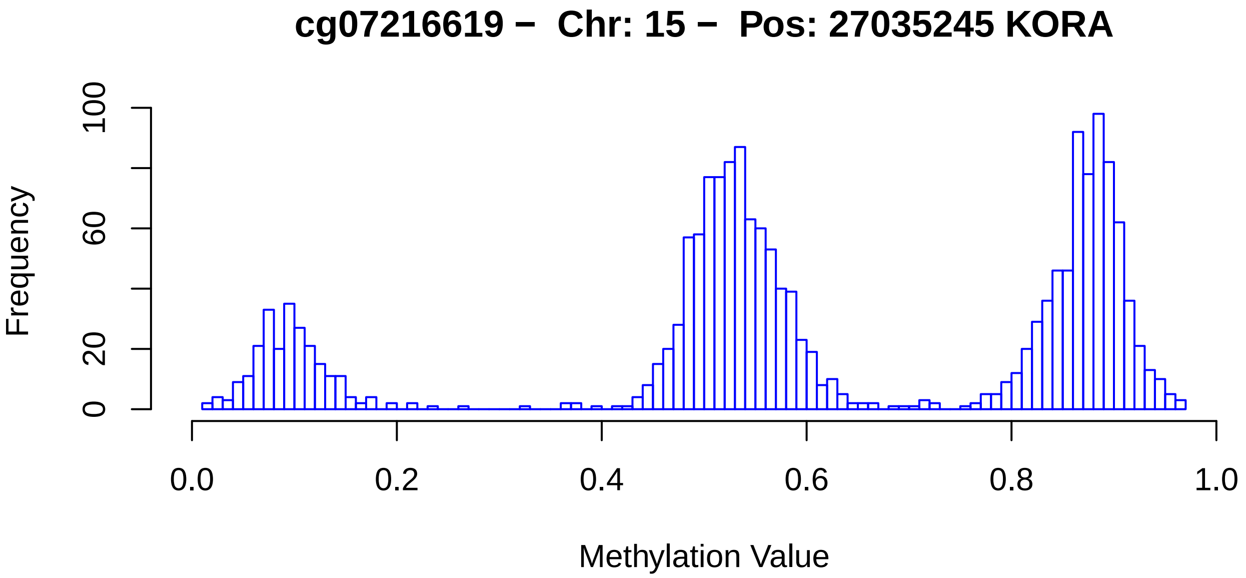
cg07216619 − Chr: 15 − Pos: 27035245 KORA
Frequency
0
20
60
100
0.0
0.2
0.4
0.6
0.8
1.0
Methylation Value

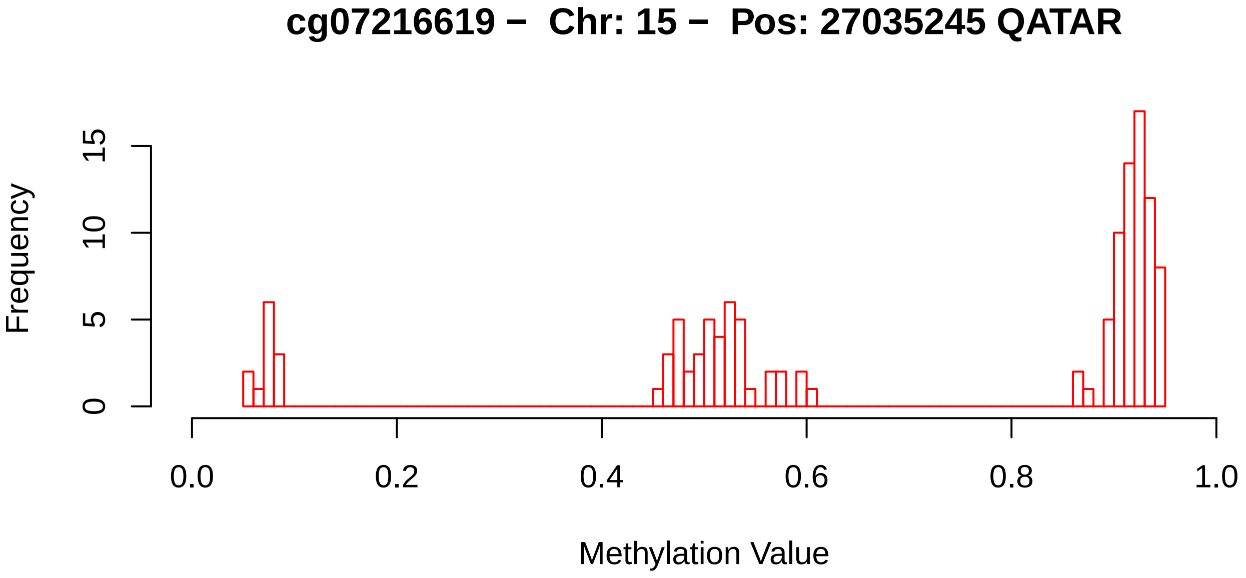
cg07216619 − Chr: 15 − Pos: 27035245 QATAR
Frequency
0
5
10
15
0.0
0.2
0.4
0.6
0.8
1.0
Methylation Value

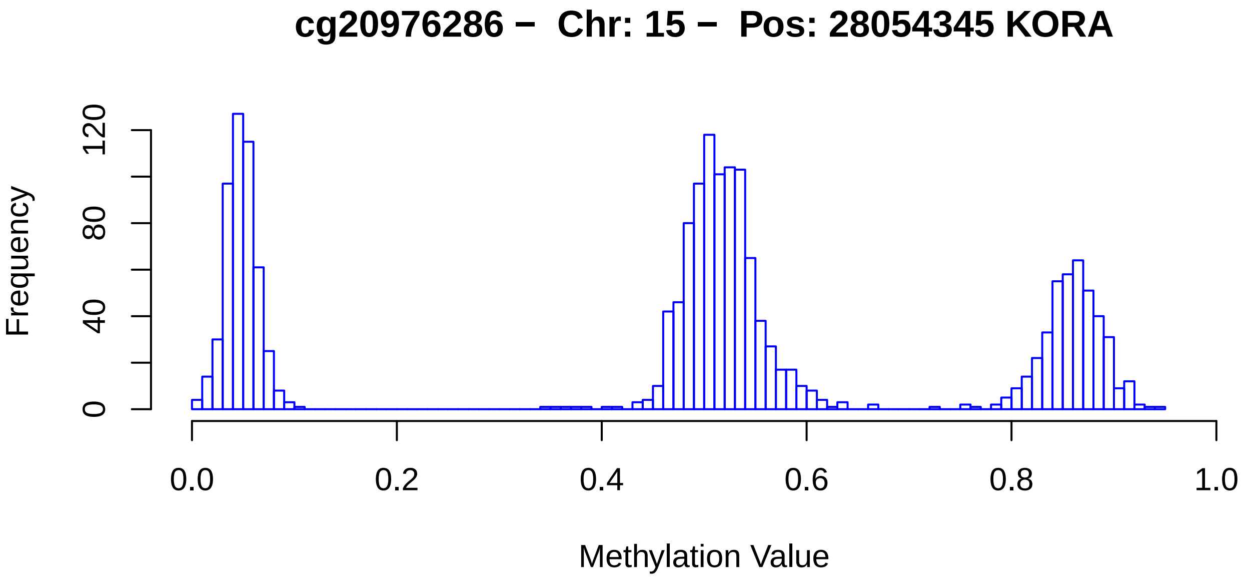
cg20976286 − Chr: 15 − Pos: 28054345 KORA
Frequency
0
40
80
120
0.0
0.2
0.4
0.6
0.8
1.0
Methylation Value

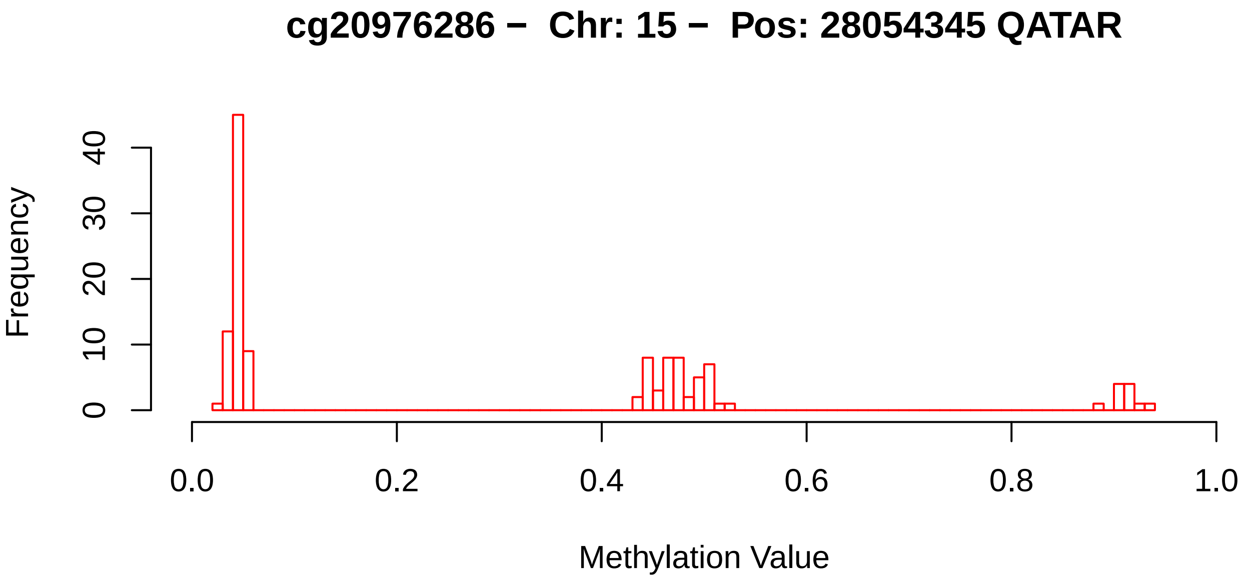
cg20976286 − Chr: 15 − Pos: 28054345 QATAR
Frequency
0
10
20
30
40
0.0
0.2
0.4
0.6
0.8
1.0
Methylation Value

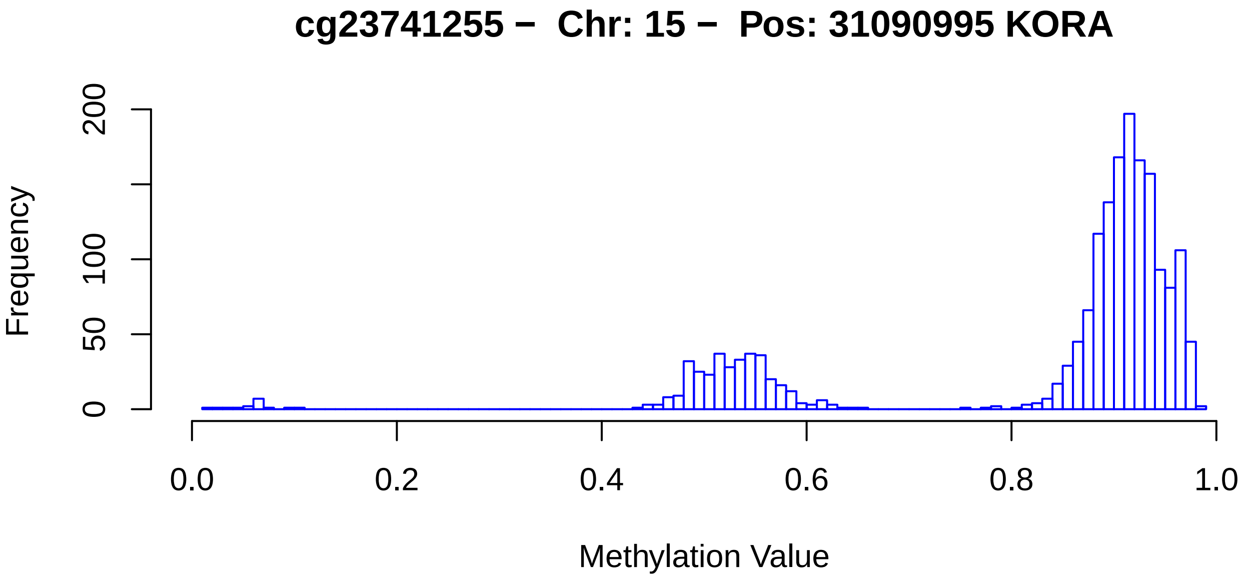
cg23741255 − Chr: 15 − Pos: 31090995 KORA
Frequency
0
50
100
200
0.0
0.2
0.4
0.6
0.8
1.0
Methylation Value

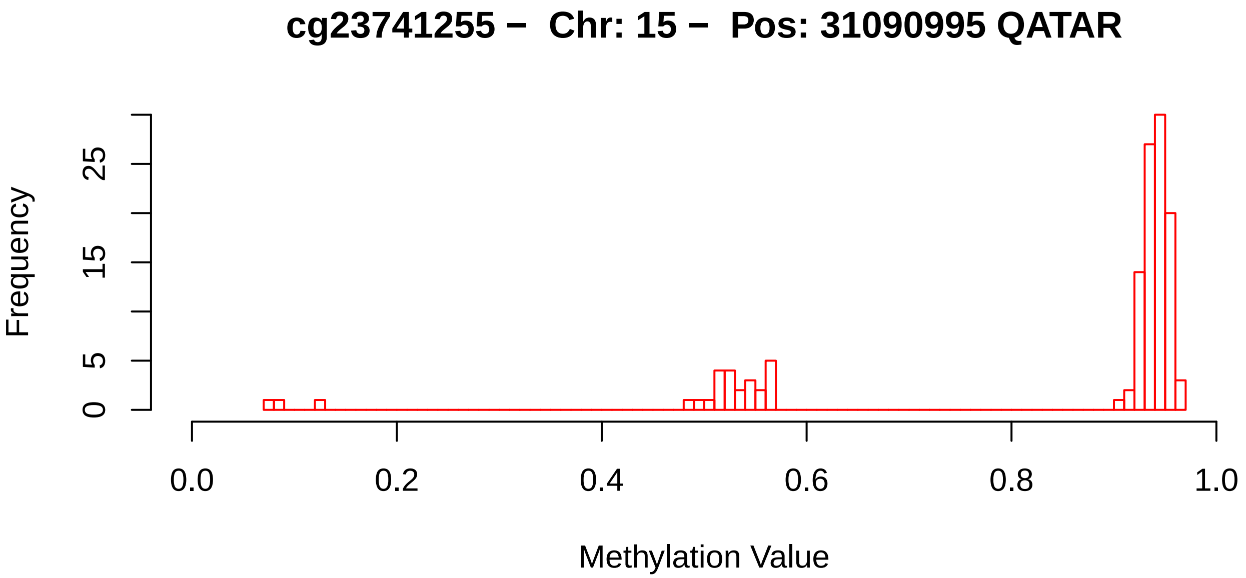
cg23741255 − Chr: 15 − Pos: 31090995 QATAR
Frequency
0
5
15
25
0.0
0.2
0.4
0.6
0.8
1.0
Methylation Value

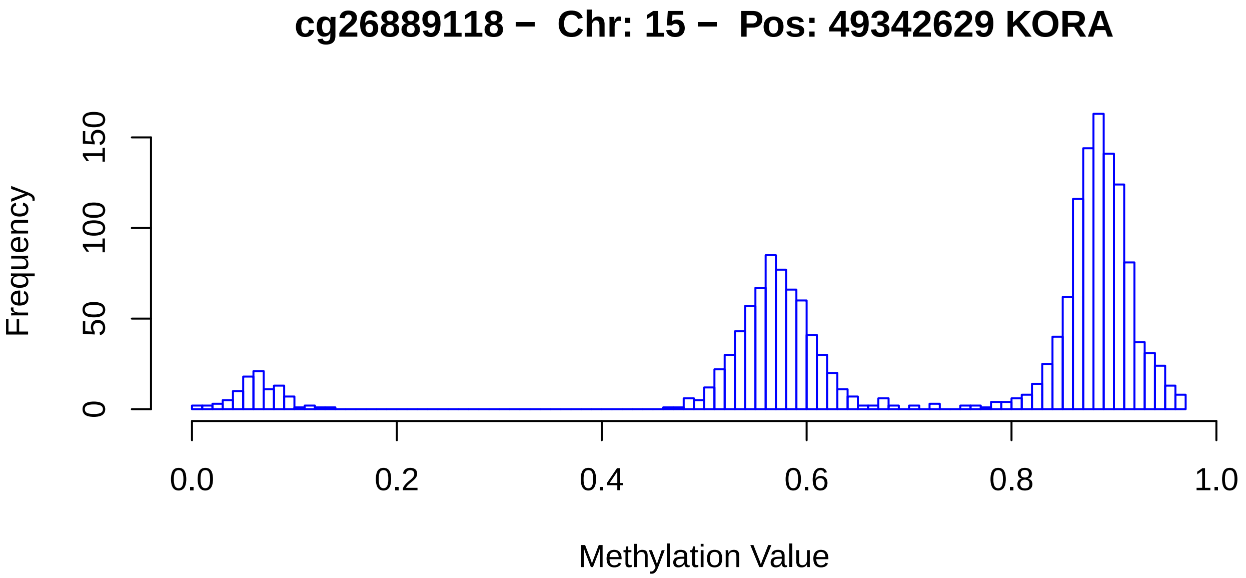
cg26889118 − Chr: 15 − Pos: 49342629 KORA
Frequency
0
50
100
150
0.0
0.2
0.4
0.6
0.8
1.0
Methylation Value

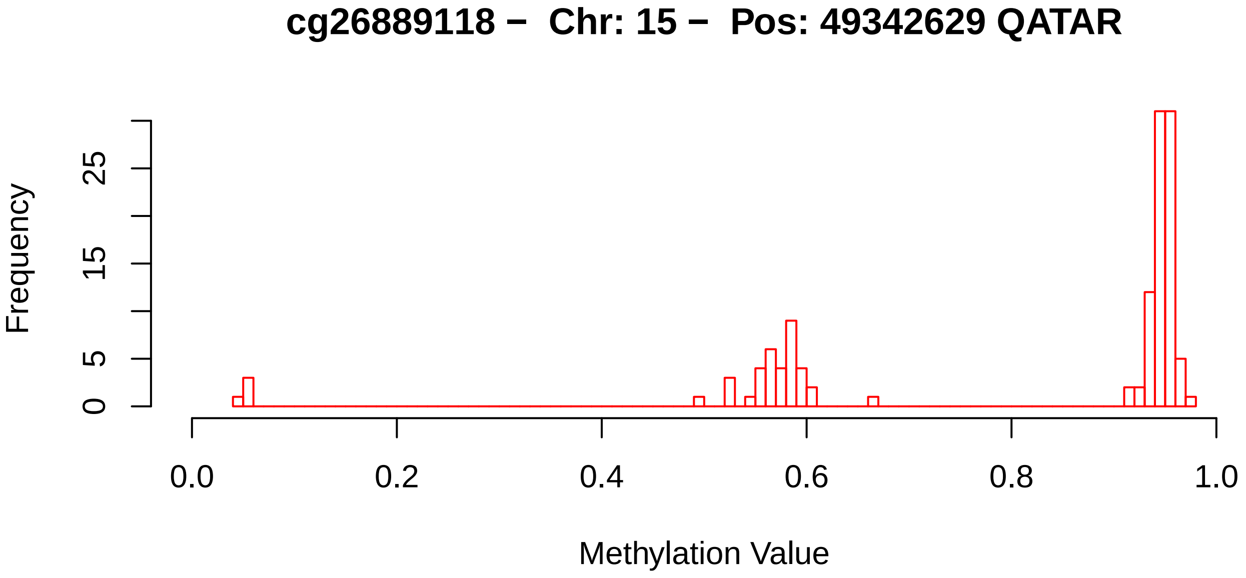
cg26889118 − Chr: 15 − Pos: 49342629 QATAR
Frequency
0
5
15
25
0.0
0.2
0.4
0.6
0.8
1.0
Methylation Value

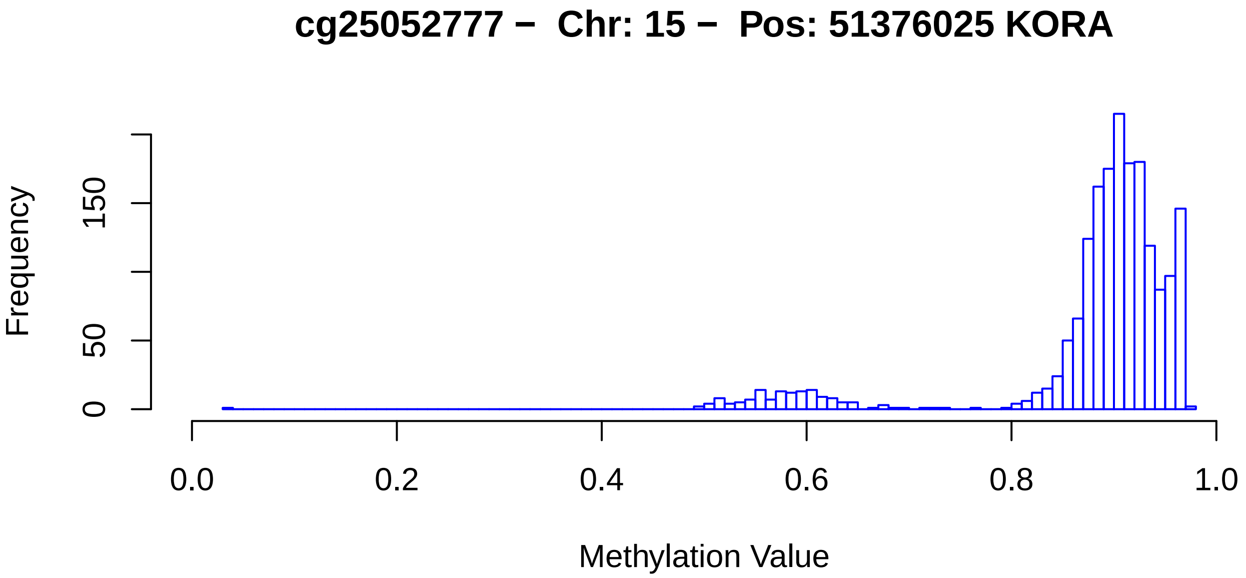
cg25052777 − Chr: 15 − Pos: 51376025 KORA
Frequency
0
50
150
0.0
0.2
0.4
0.6
0.8
1.0
Methylation Value

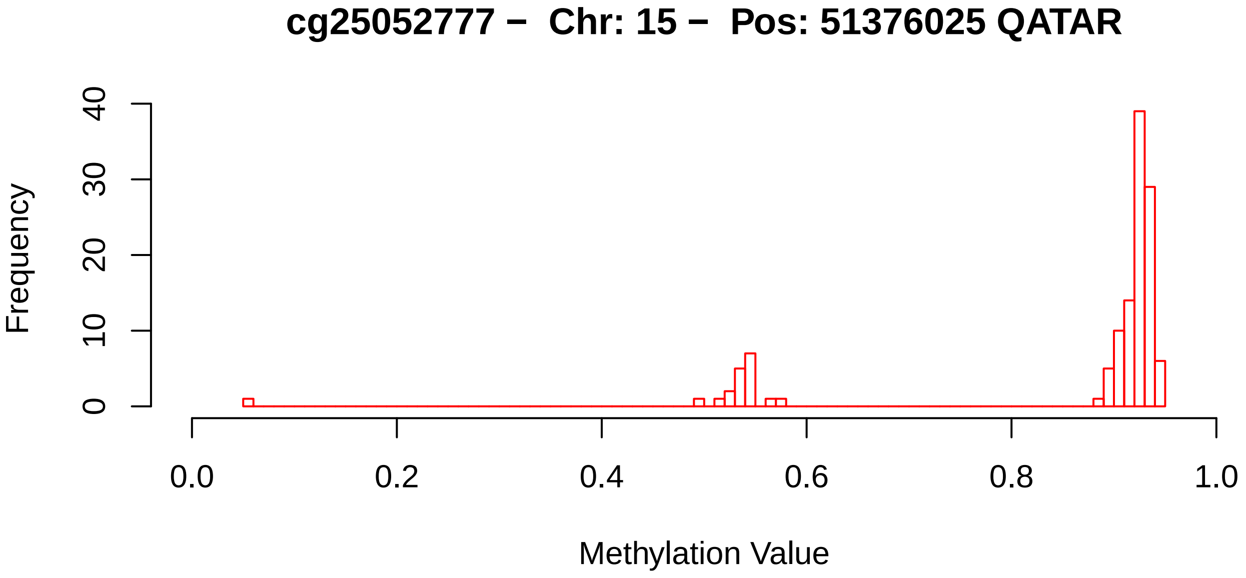
cg25052777 − Chr: 15 − Pos: 51376025 QATAR
Frequency
0
10
20
30
40
0.0
0.2
0.4
0.6
0.8
1.0
Methylation Value

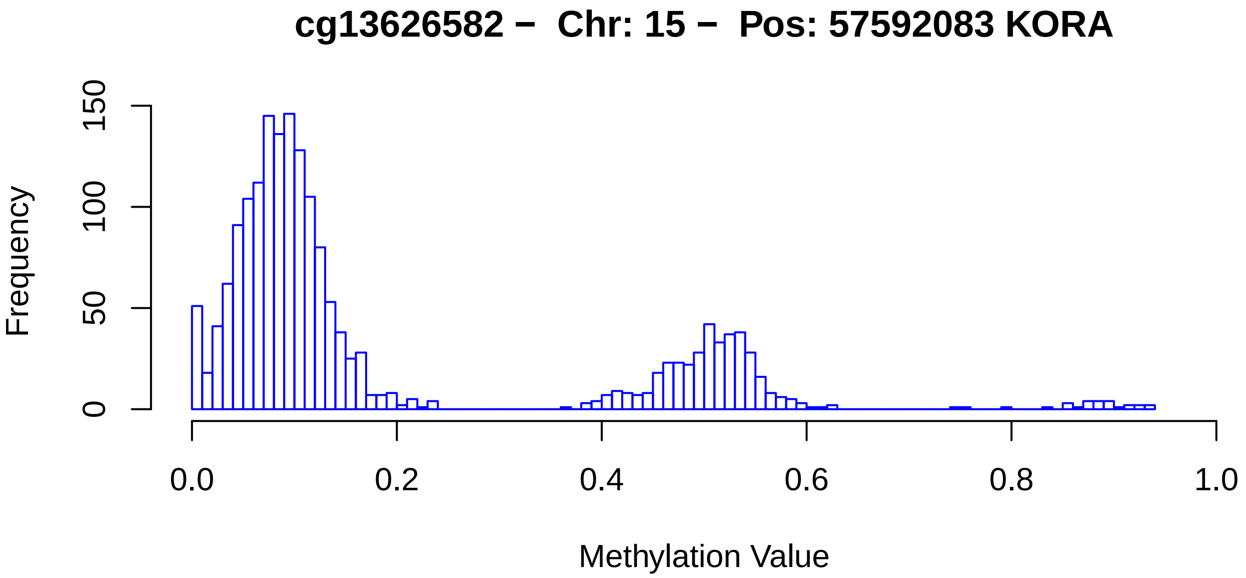
cg13626582 − Chr: 15 − Pos: 57592083 KORA
Frequency
0
50
100
150
0.0
0.2
0.4
0.6
0.8
1.0
Methylation Value

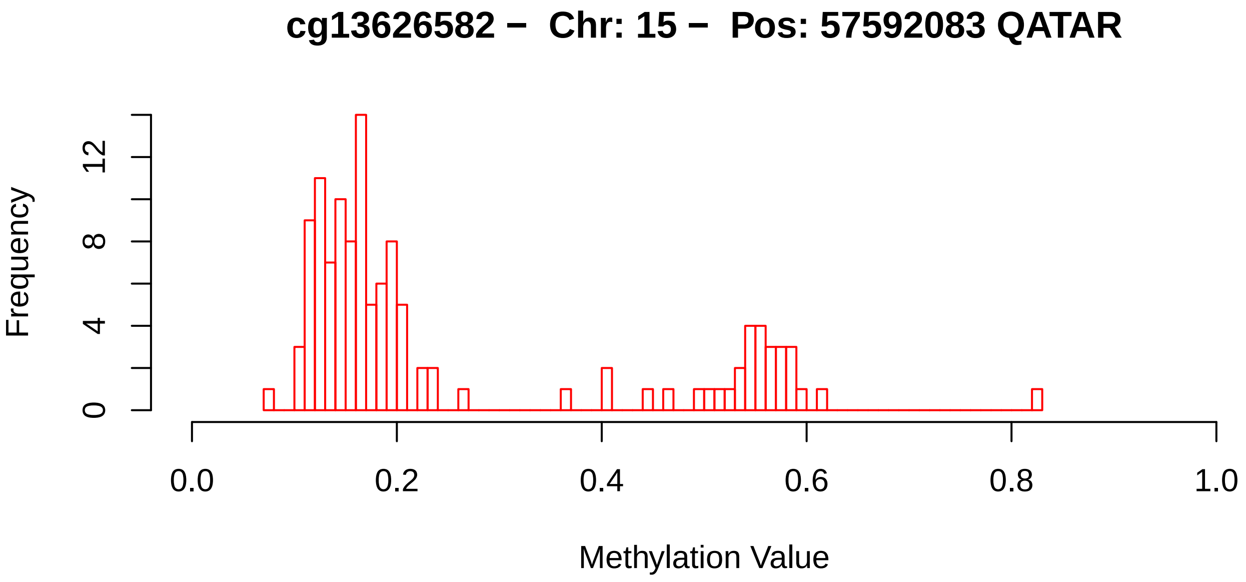
cg13626582 − Chr: 15 − Pos: 57592083 QATAR
Frequency
0
4
8
12
0.0
0.2
0.4
0.6
0.8
1.0
Methylation Value

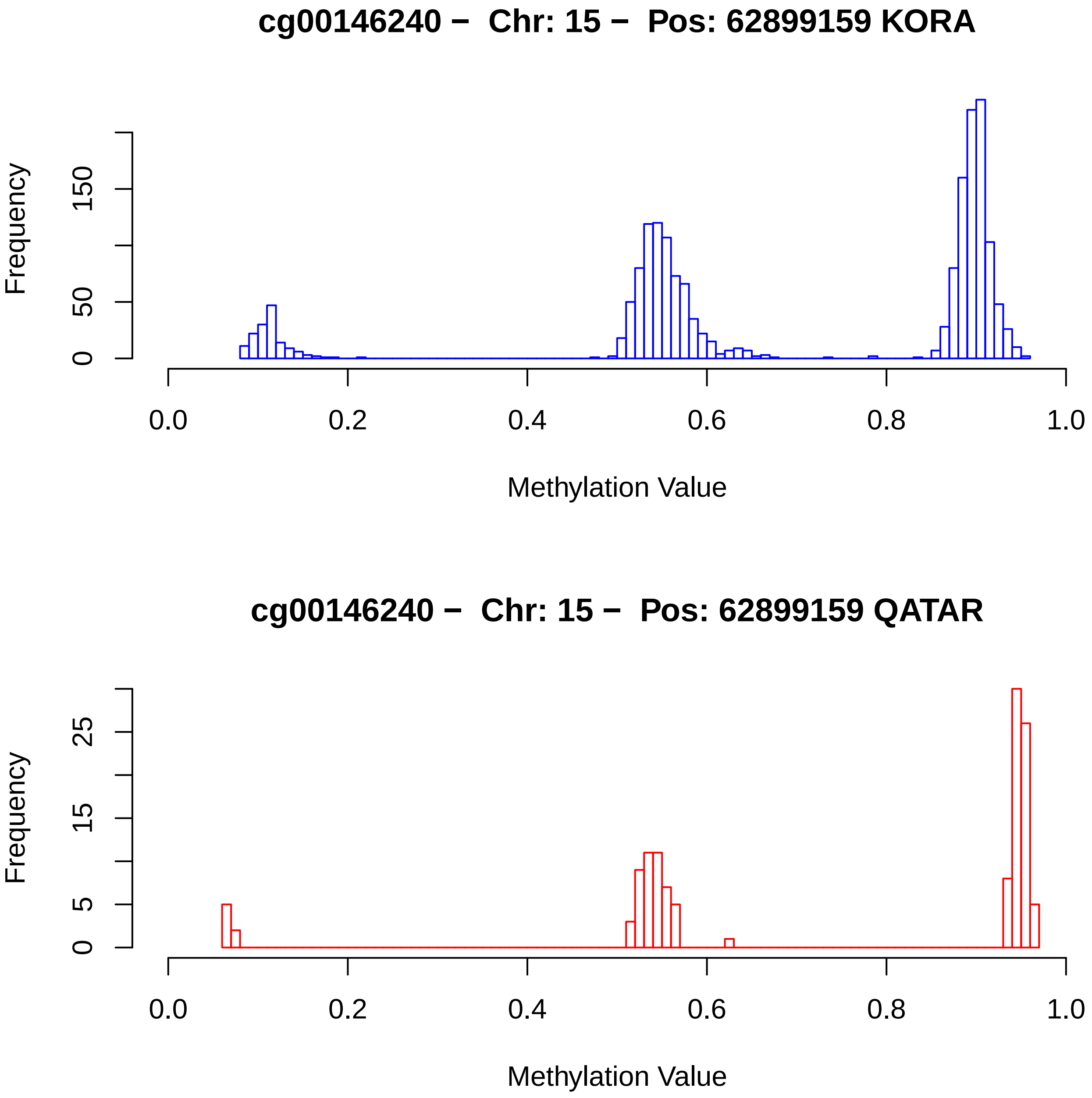
cg00146240 − Chr: 15 − Pos: 62899159 KORA
Frequency
0
50
150
0.0
0.2
0.4
0.6
0.8
1.0
Methylation Value
cg00146240 − Chr: 15 − Pos: 62899159 QATAR
Frequency
0
5
15
25
0.0
0.2
0.4
0.6
0.8
1.0
Methylation Value

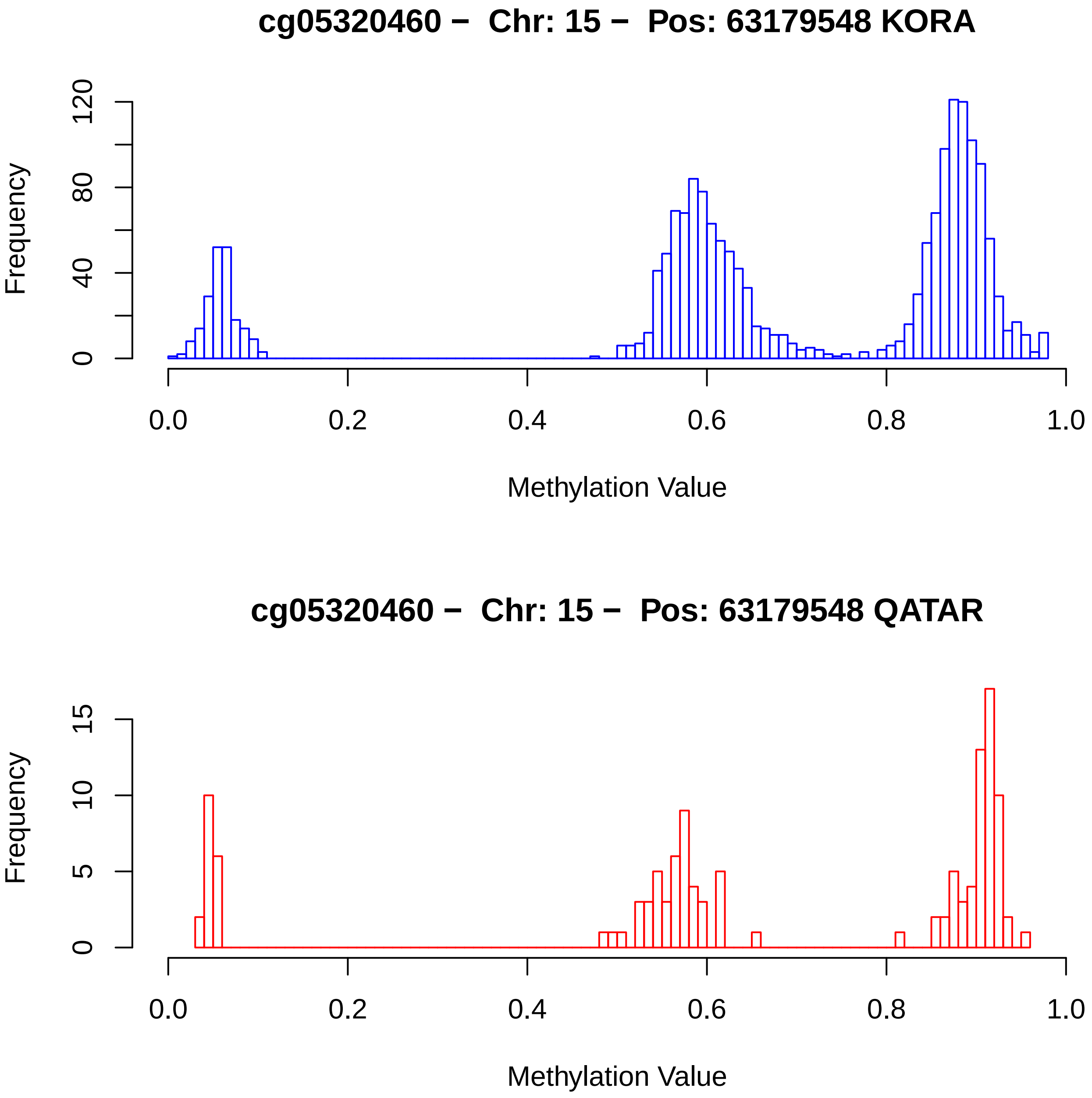
cg05320460 − Chr: 15 − Pos: 63179548 KORA
Frequency
0
40
80
120
0.0
0.2
0.4
0.6
0.8
1.0
Methylation Value
cg05320460 − Chr: 15 − Pos: 63179548 QATAR
Frequency
0
5
10
15
0.0
0.2
0.4
0.6
0.8
1.0
Methylation Value

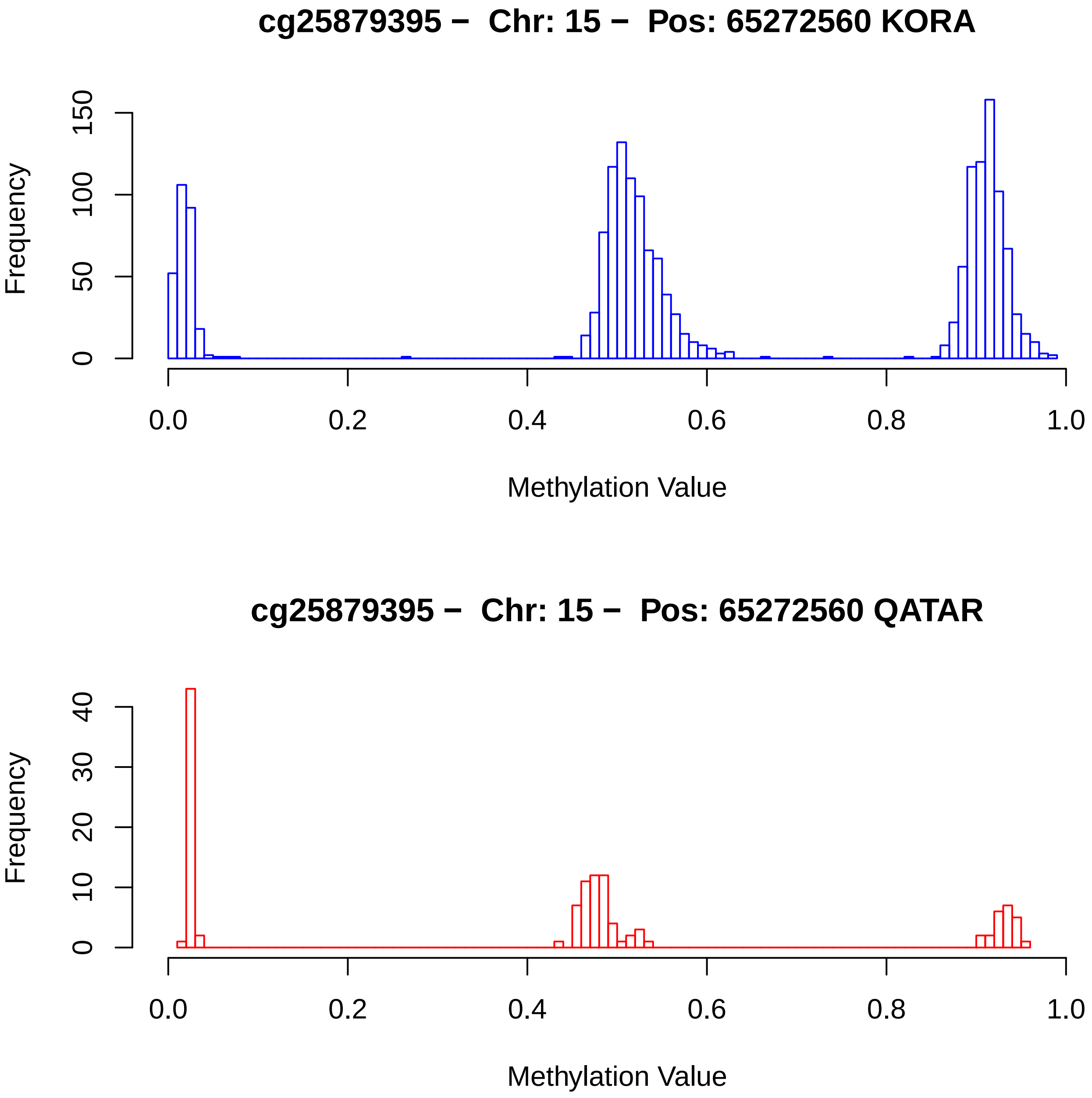
cg25879395 − Chr: 15 − Pos: 65272560 KORA
Frequency
0
50
100
150
0.0
0.2
0.4
0.6
0.8
1.0
Methylation Value
cg25879395 − Chr: 15 − Pos: 65272560 QATAR
Frequency
0
10
20
30
40
0.0
0.2
0.4
0.6
0.8
1.0
Methylation Value

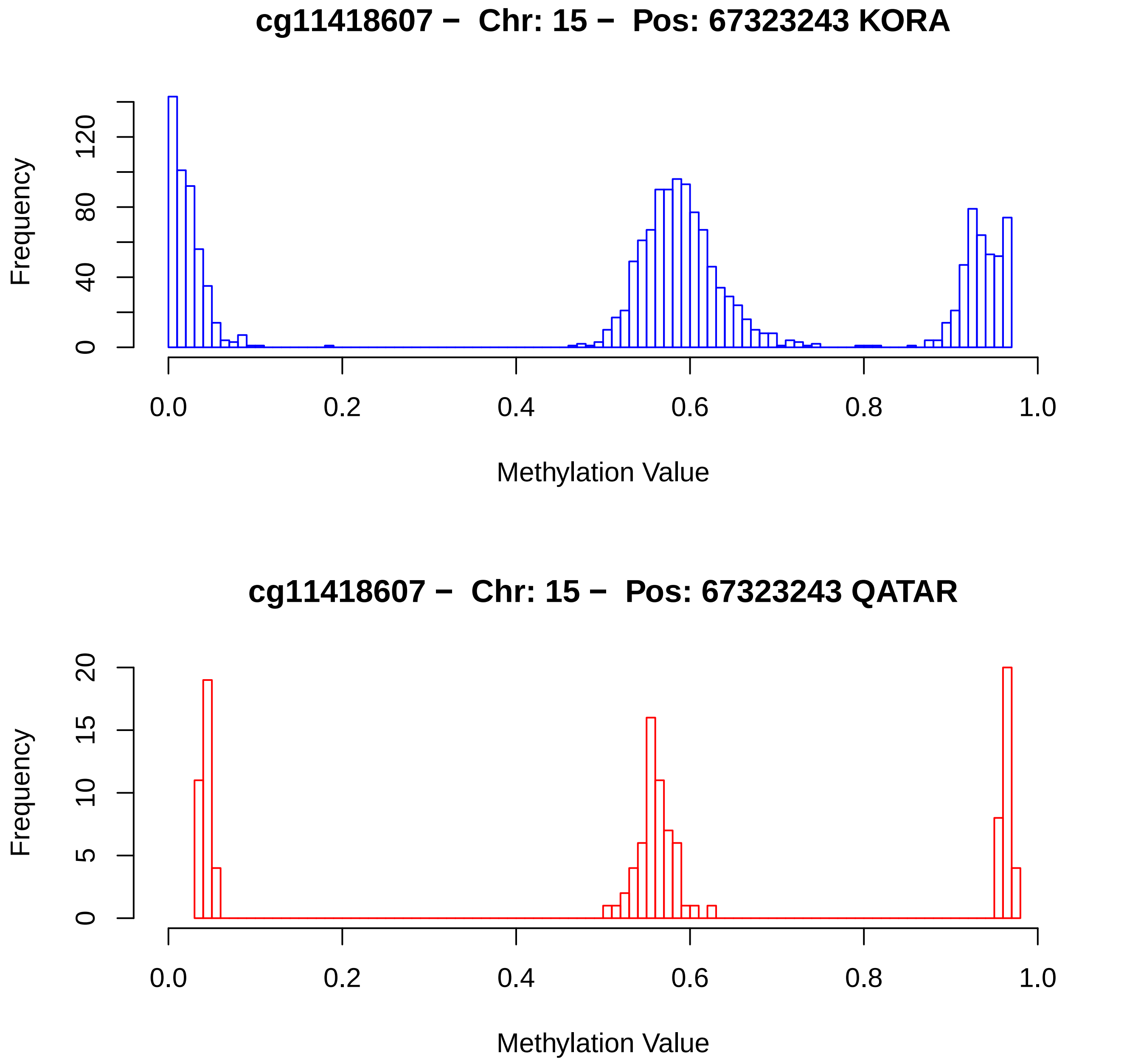
cg11418607 − Chr: 15 − Pos: 67323243 KORA
Frequency
0
40
80
120
0.0
0.2
0.4
0.6
0.8
1.0
Methylation Value
cg11418607 − Chr: 15 − Pos: 67323243 QATAR
Frequency
0
5
10
15
20
0.0
0.2
0.4
0.6
0.8
1.0
Methylation Value

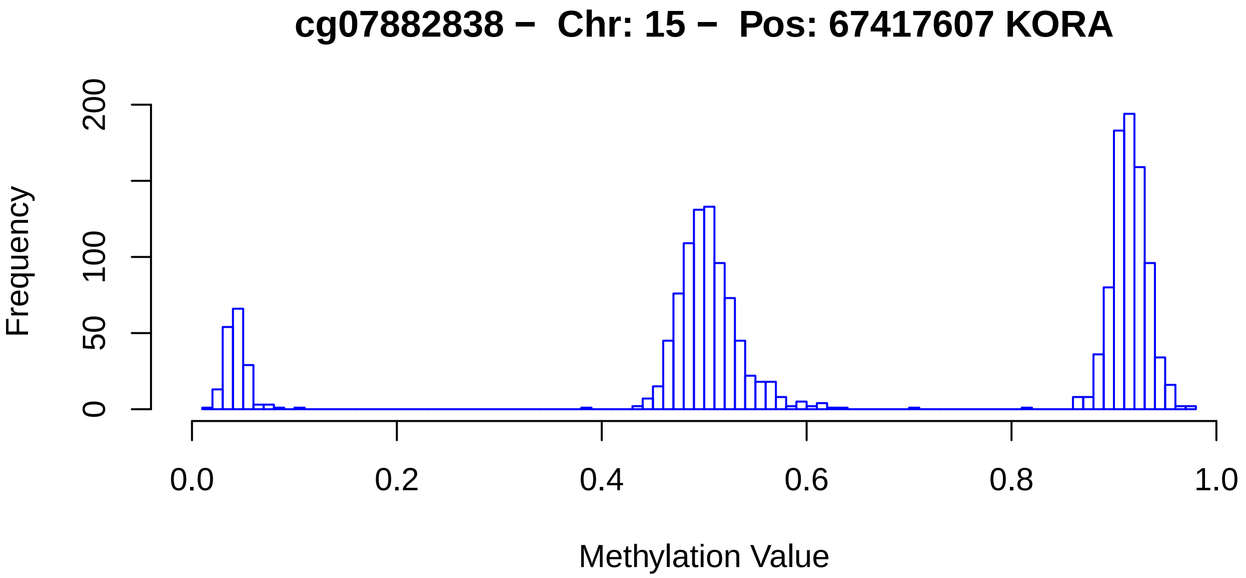
cg07882838 − Chr: 15 − Pos: 67417607 KORA
Frequency
0
50
100
200
0.0
0.2
0.4
0.6
0.8
1.0
Methylation Value

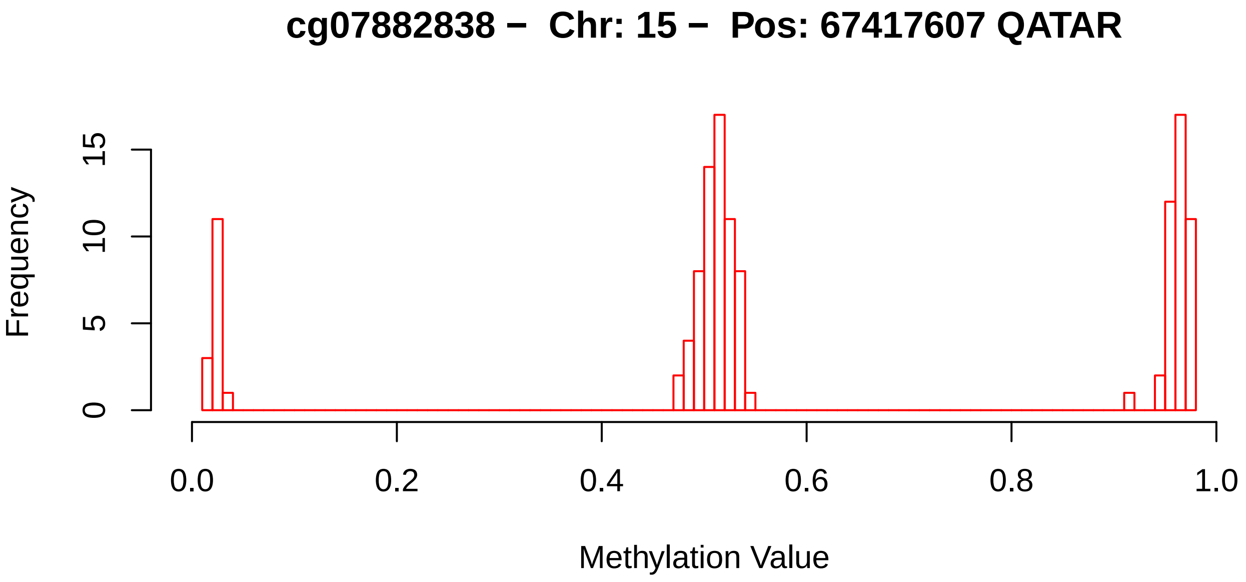
cg07882838 − Chr: 15 − Pos: 67417607 QATAR
Frequency
0
5
10
15
0.0
0.2
0.4
0.6
0.8
1.0
Methylation Value

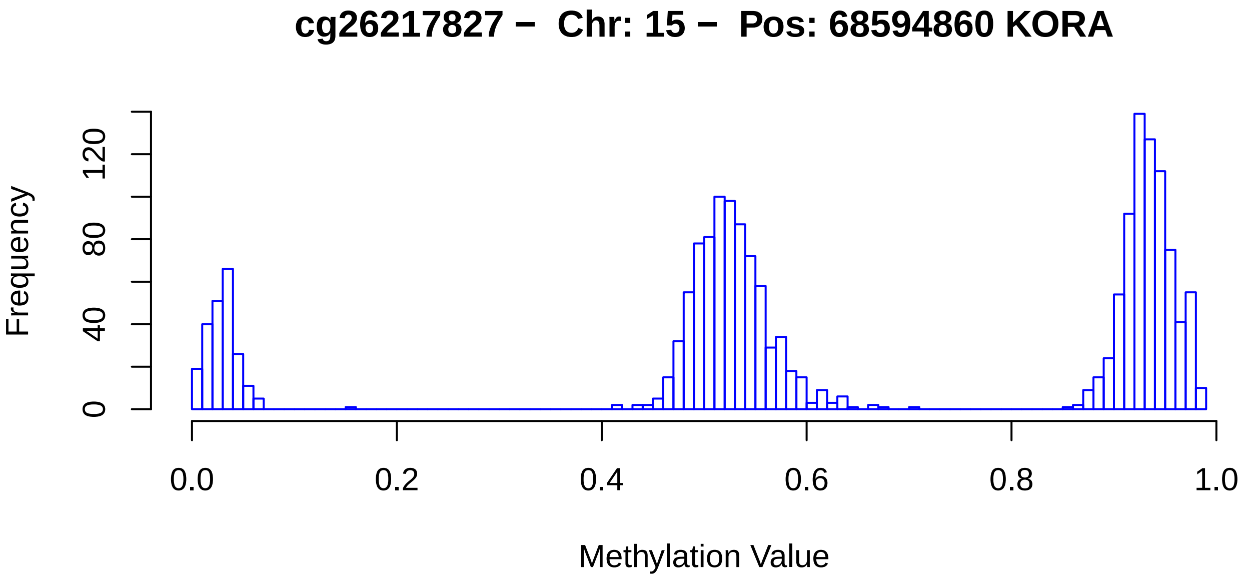
cg26217827 − Chr: 15 − Pos: 68594860 KORA
Frequency
0
40
80
120
0.0
0.2
0.4
0.6
0.8
1.0
Methylation Value

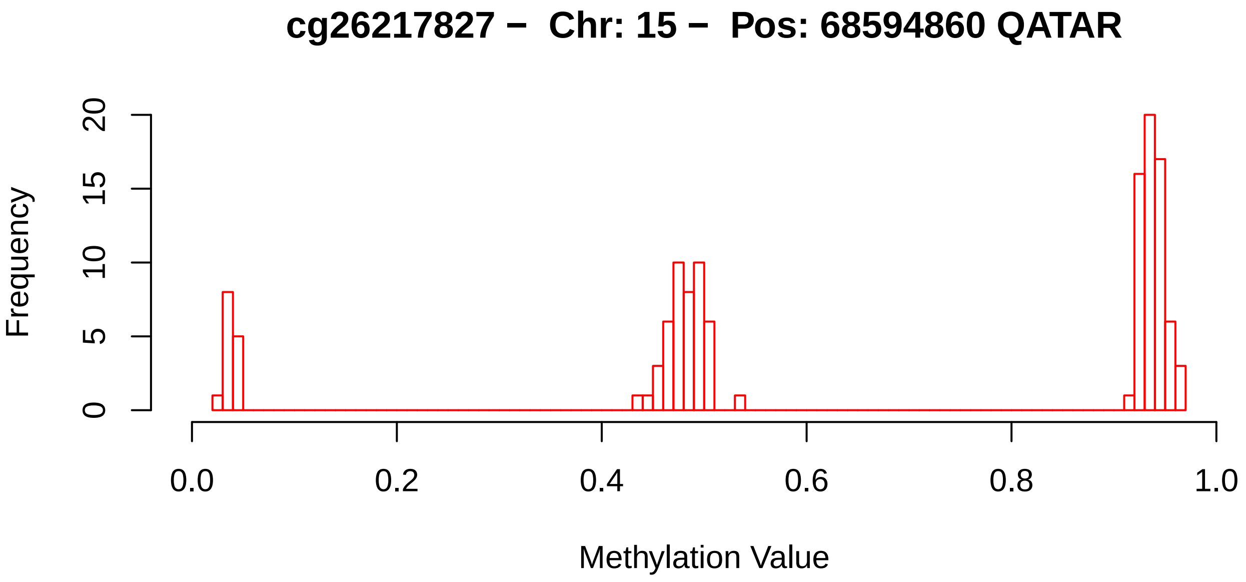
cg26217827 − Chr: 15 − Pos: 68594860 QATAR
Frequency
0
5
10
15
20
0.0
0.2
0.4
0.6
0.8
1.0
Methylation Value

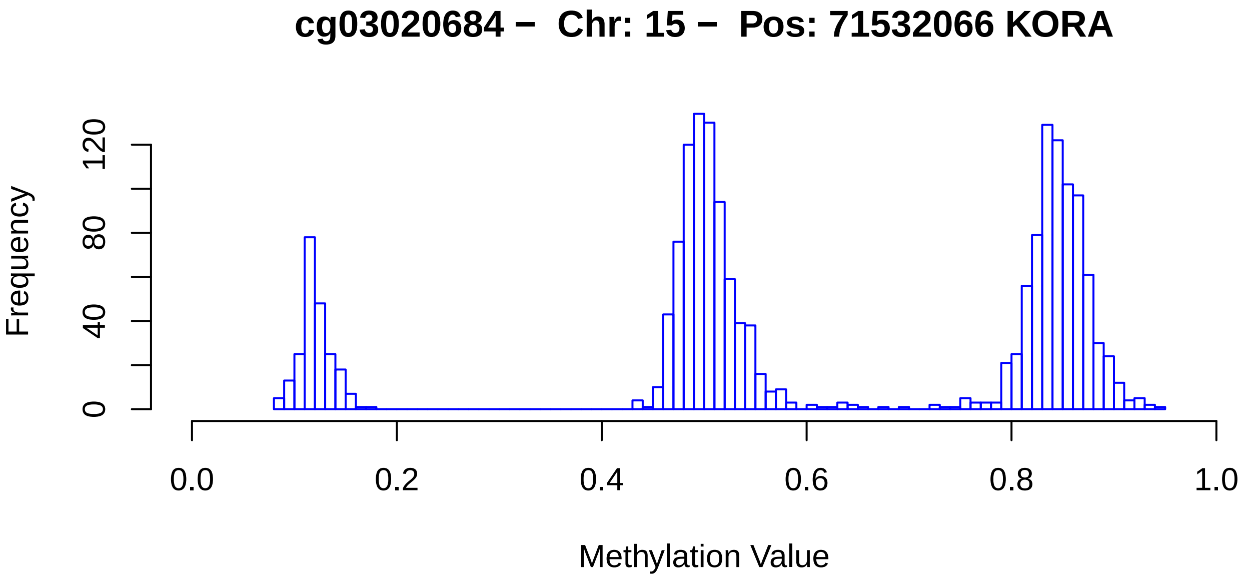
cg03020684 − Chr: 15 − Pos: 71532066 KORA
Frequency
0
40
80
120
0.0
0.2
0.4
0.6
0.8
1.0
Methylation Value

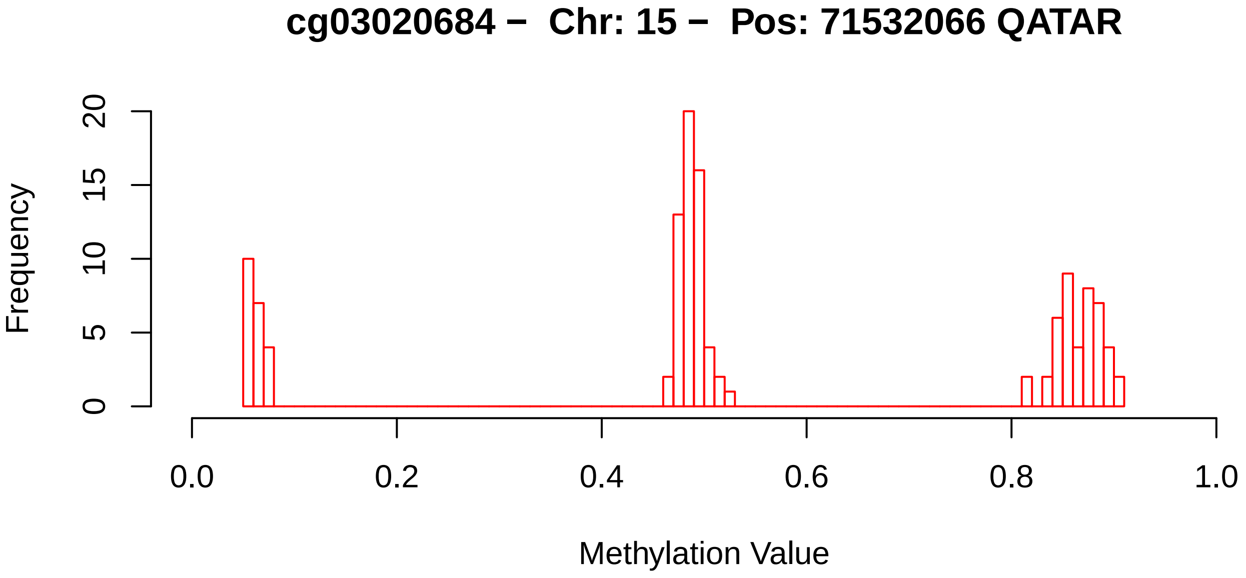
cg03020684 − Chr: 15 − Pos: 71532066 QATAR
Frequency
0
5
10
15
20
0.0
0.2
0.4
0.6
0.8
1.0
Methylation Value

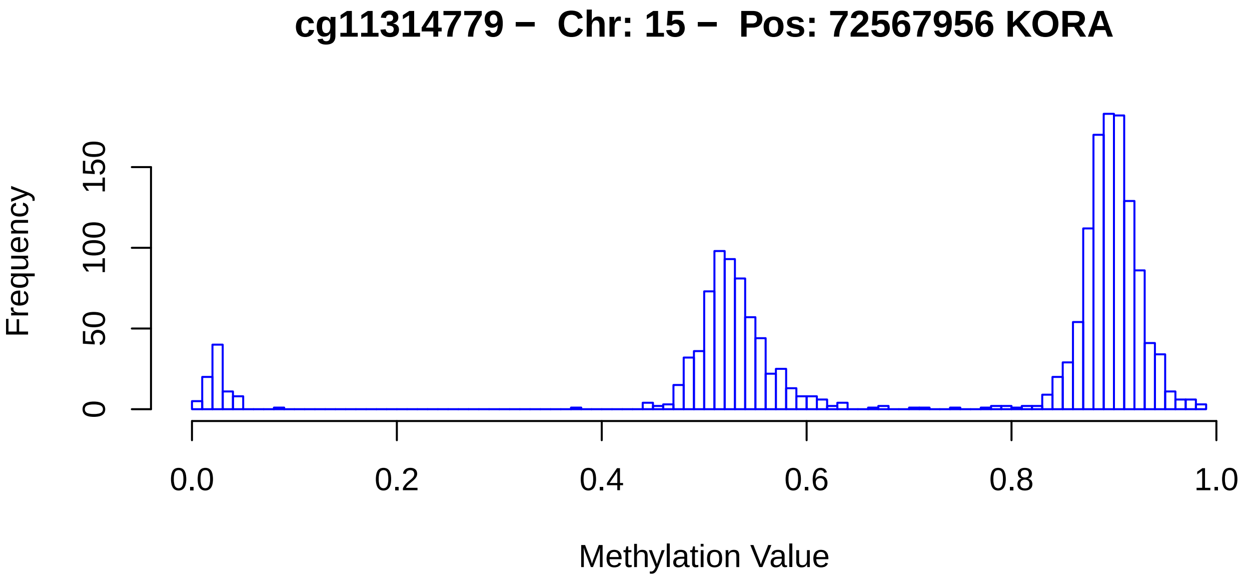
cg11314779 − Chr: 15 − Pos: 72567956 KORA
Frequency
0
50
100
150
0.0
0.2
0.4
0.6
0.8
1.0
Methylation Value

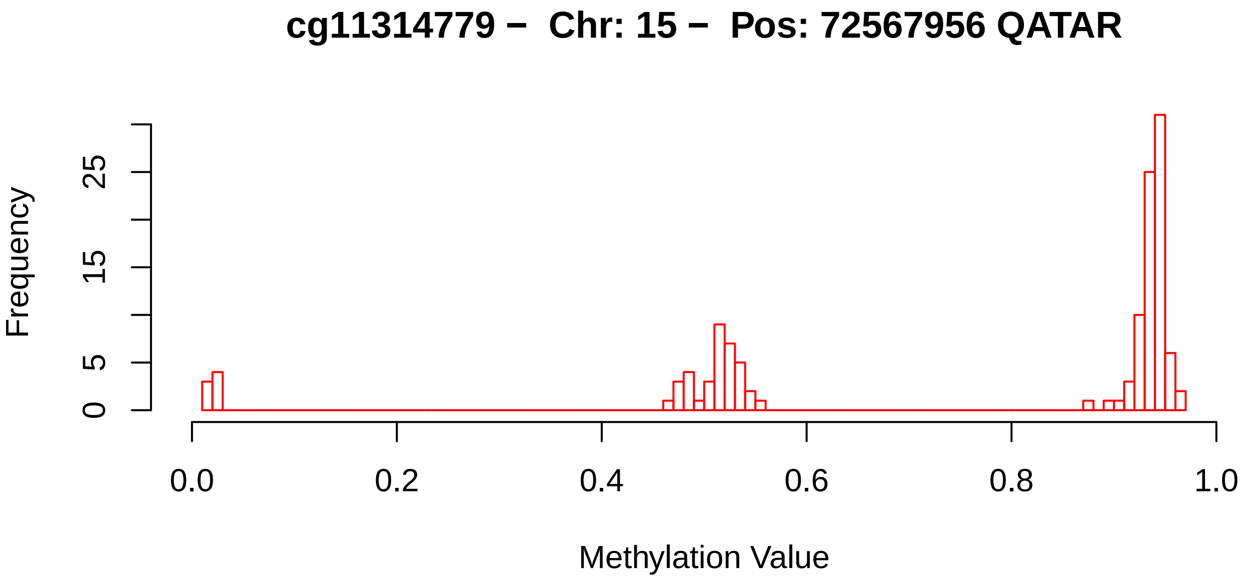
cg11314779 − Chr: 15 − Pos: 72567956 QATAR
Frequency
0
5
15
25
0.0
0.2
0.4
0.6
0.8
1.0
Methylation Value

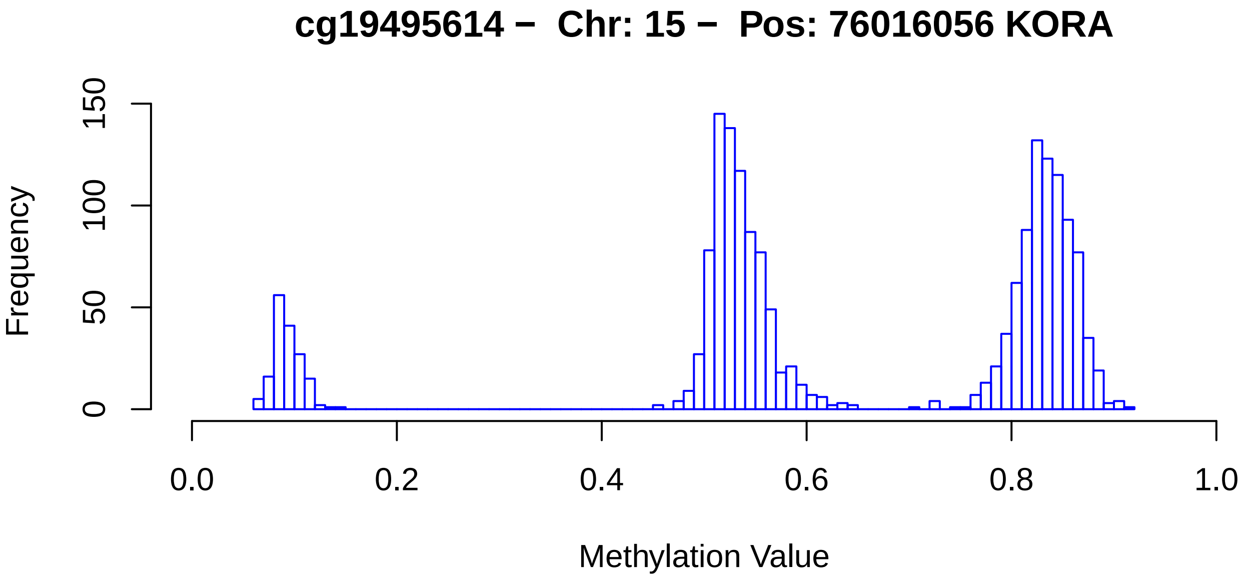
cg19495614 − Chr: 15 − Pos: 76016056 KORA
Frequency
0
50
100
150
0.0
0.2
0.4
0.6
0.8
1.0
Methylation Value

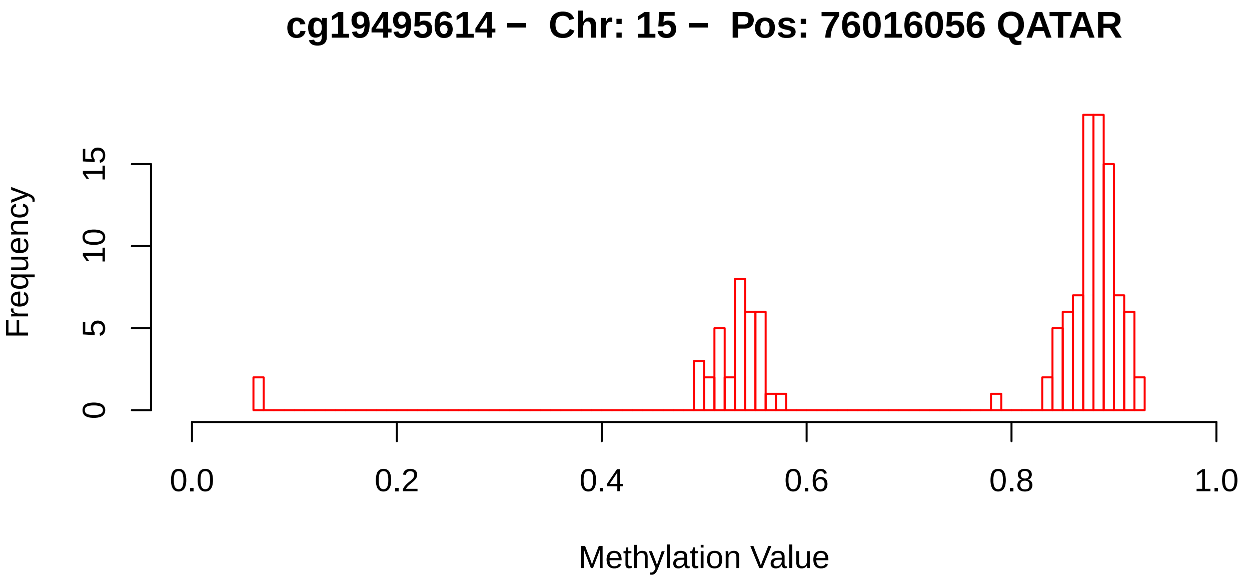
cg19495614 − Chr: 15 − Pos: 76016056 QATAR
Frequency
0
5
10
15
0.0
0.2
0.4
0.6
0.8
1.0
Methylation Value

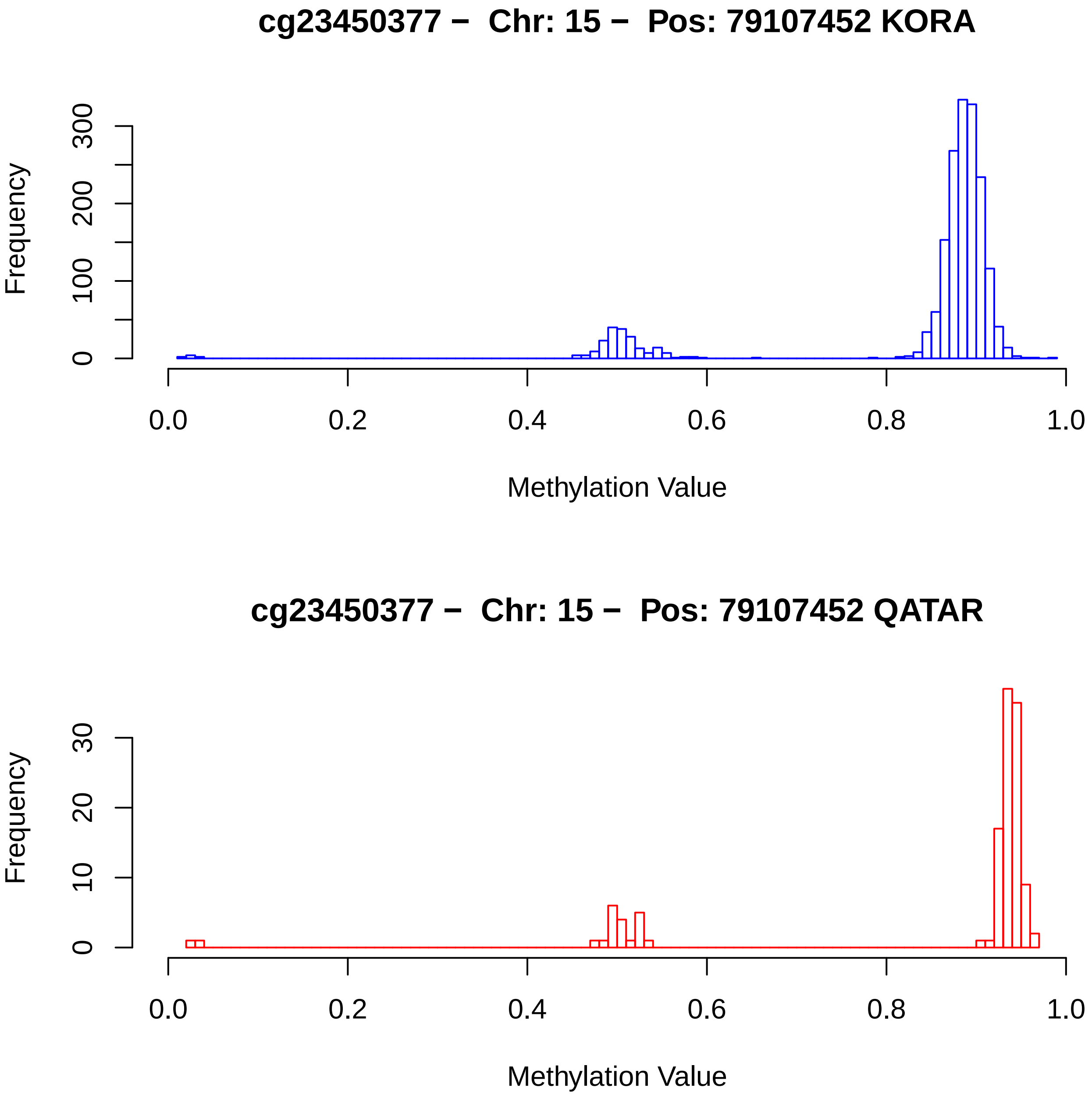
cg23450377 − Chr: 15 − Pos: 79107452 KORA
Frequency
0
100
200
300
0.0
0.2
0.4
0.6
0.8
1.0
Methylation Value
cg23450377 − Chr: 15 − Pos: 79107452 QATAR
Frequency
0
10
20
30
0.0
0.2
0.4
0.6
0.8
1.0
Methylation Value

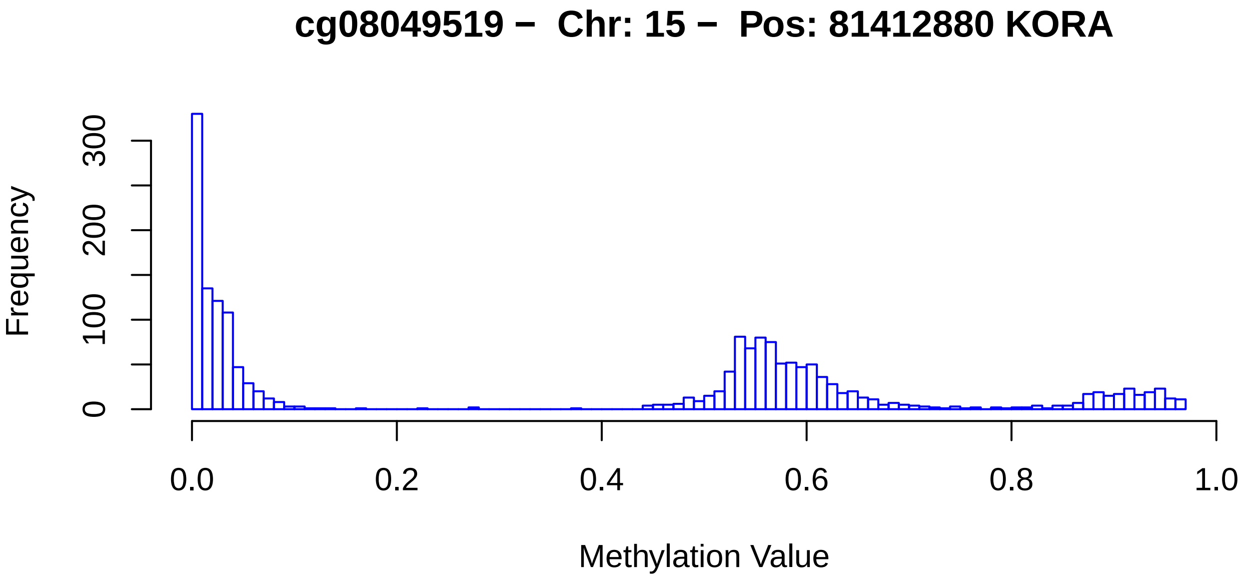
cg08049519 − Chr: 15 − Pos: 81412880 KORA
Frequency
0
100
200
300
0.0
0.2
0.4
0.6
0.8
1.0
Methylation Value

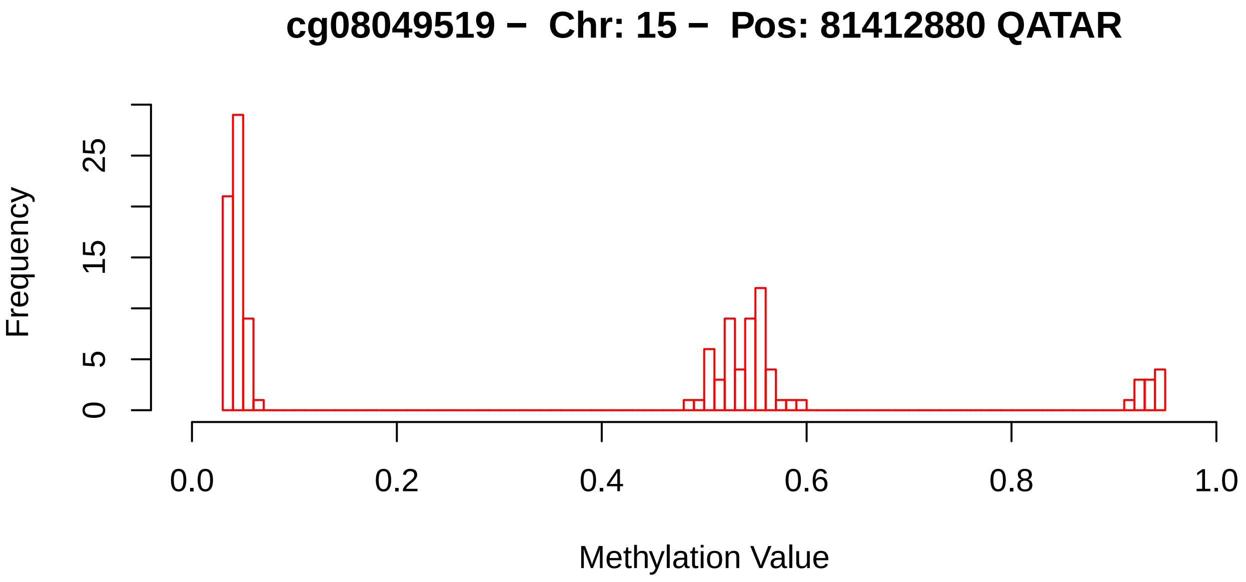
cg08049519 − Chr: 15 − Pos: 81412880 QATAR
Frequency
0
5
15
25
0.0
0.2
0.4
0.6
0.8
1.0
Methylation Value

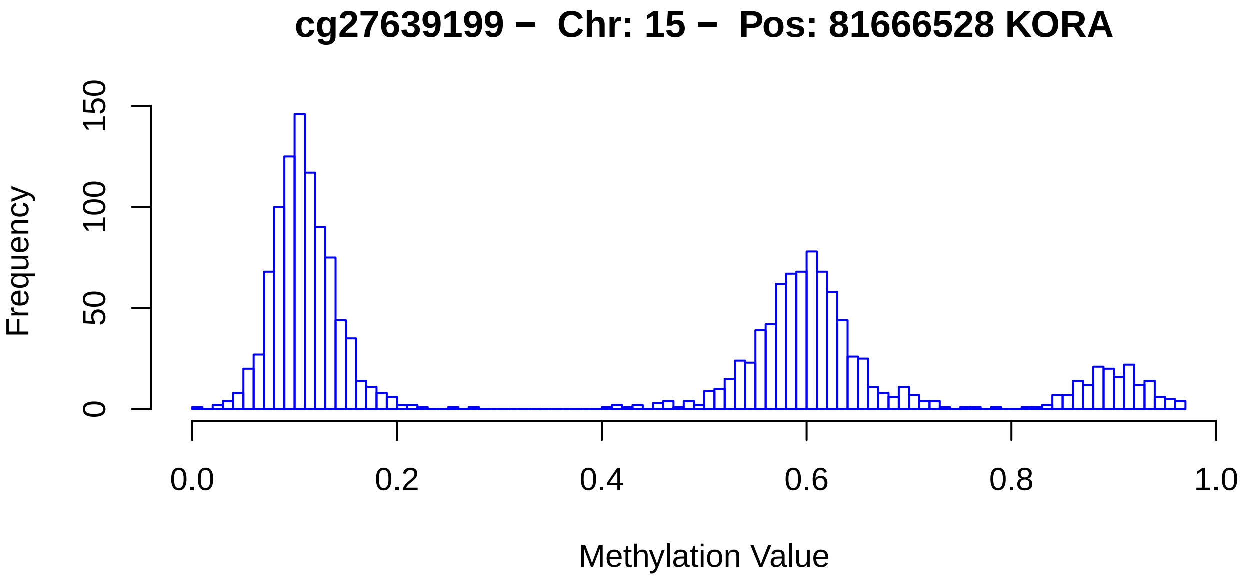
cg27639199 − Chr: 15 − Pos: 81666528 KORA
Frequency
0
50
100
150
0.0
0.2
0.4
0.6
0.8
1.0
Methylation Value

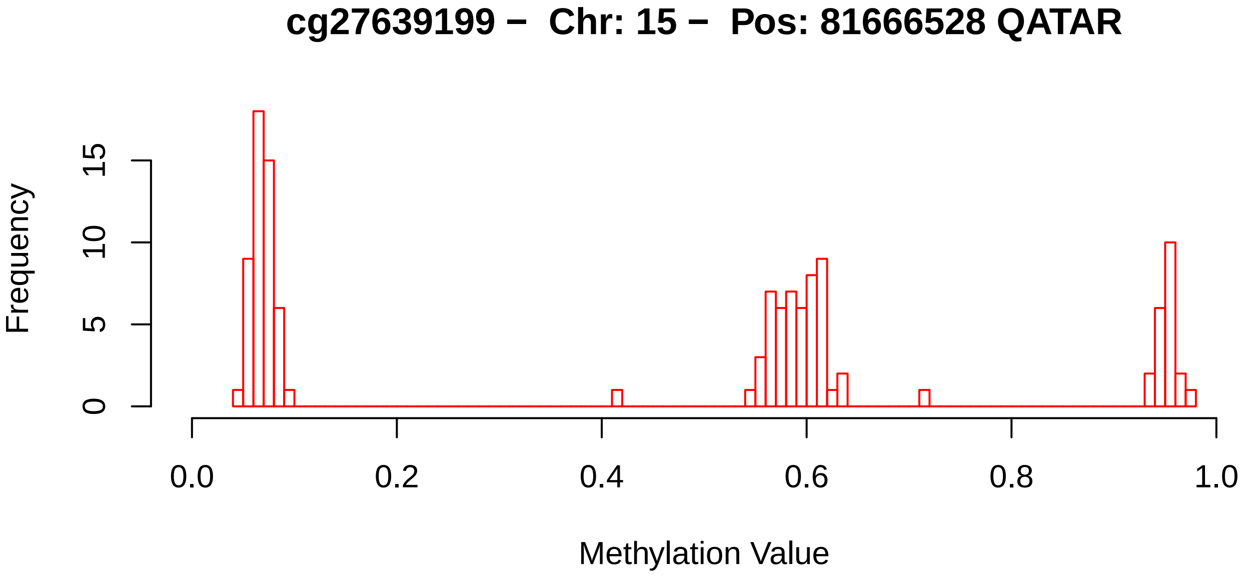
cg27639199 − Chr: 15 − Pos: 81666528 QATAR
Frequency
0
5
10
15
0.0
0.2
0.4
0.6
0.8
1.0
Methylation Value

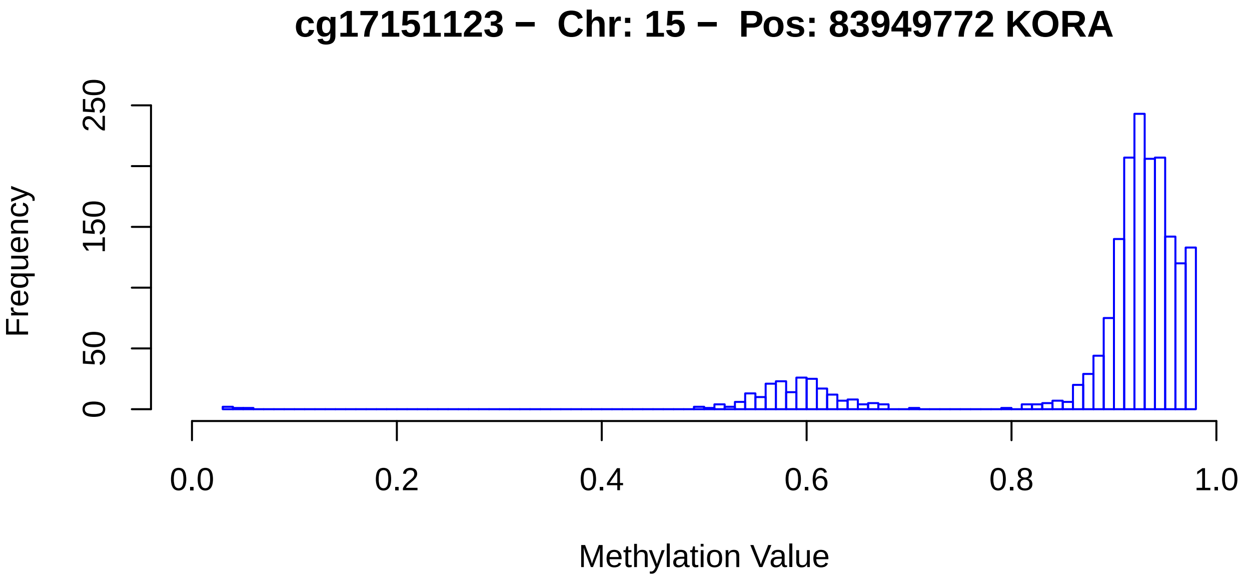
cg17151123 − Chr: 15 − Pos: 83949772 KORA
Frequency
0
50
150
250
0.0
0.2
0.4
0.6
0.8
1.0
Methylation Value

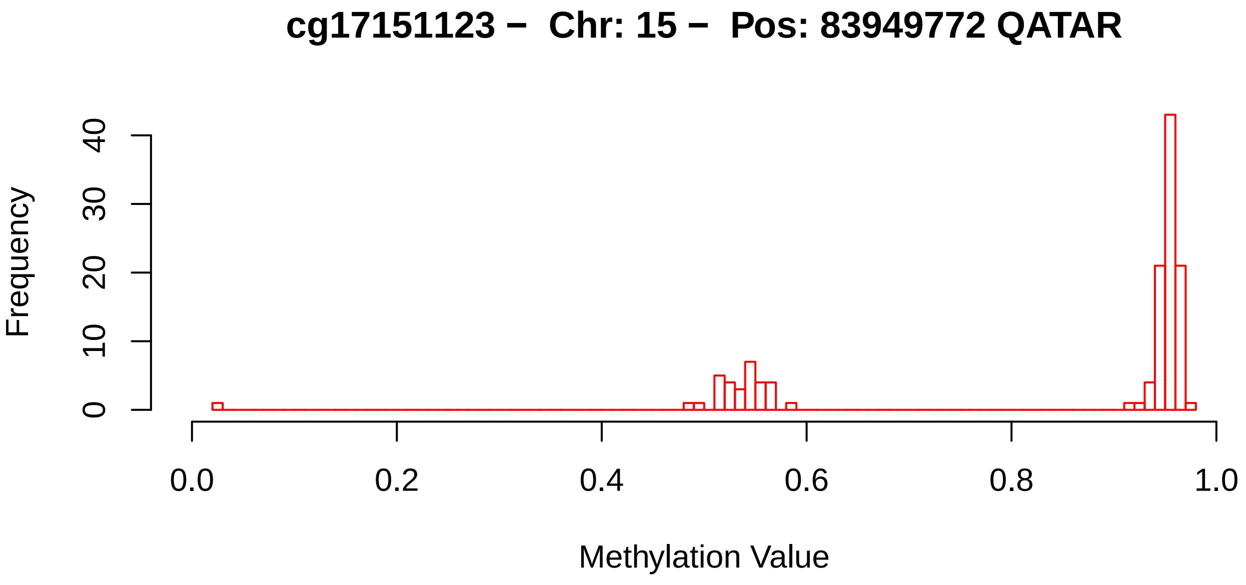
cg17151123 − Chr: 15 − Pos: 83949772 QATAR
Frequency
0
10
20
30
40
0.0
0.2
0.4
0.6
0.8
1.0
Methylation Value

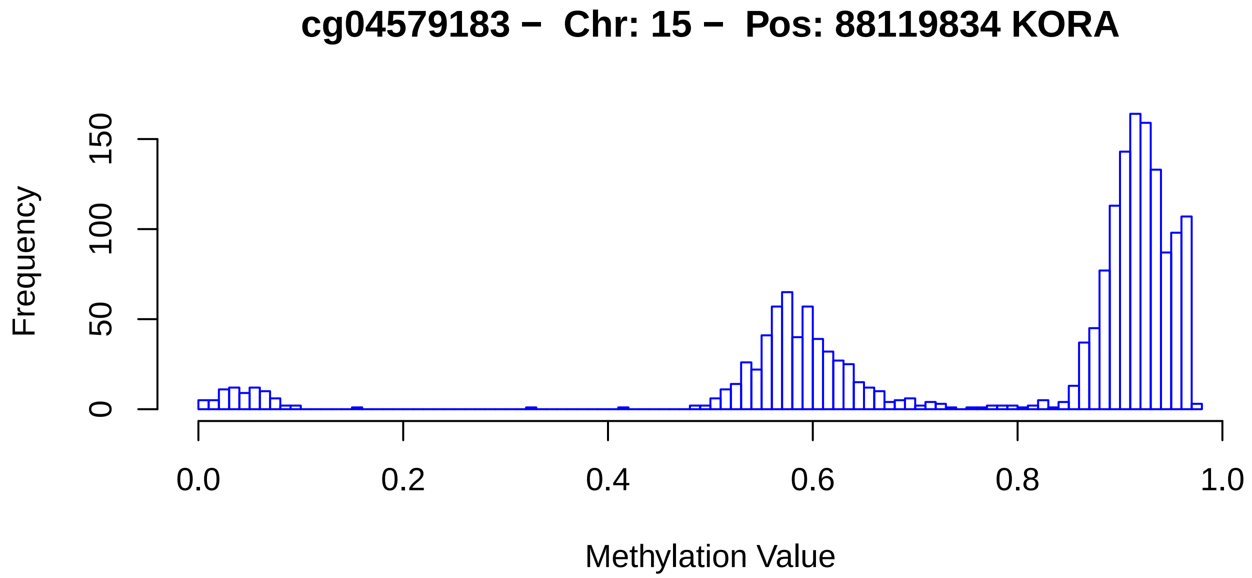
cg04579183 − Chr: 15 − Pos: 88119834 KORA
Frequency
0
50
100
150
0.0
0.2
0.4
0.6
0.8
1.0
Methylation Value

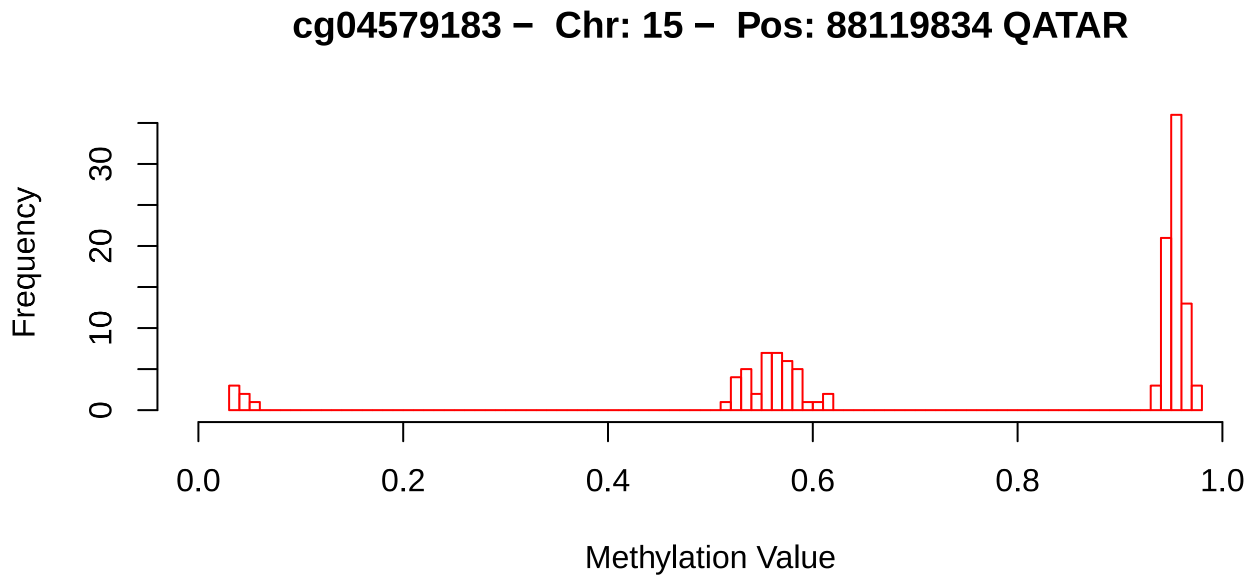
cg04579183 − Chr: 15 − Pos: 88119834 QATAR
Frequency
0
10
20
30
0.0
0.2
0.4
0.6
0.8
1.0
Methylation Value

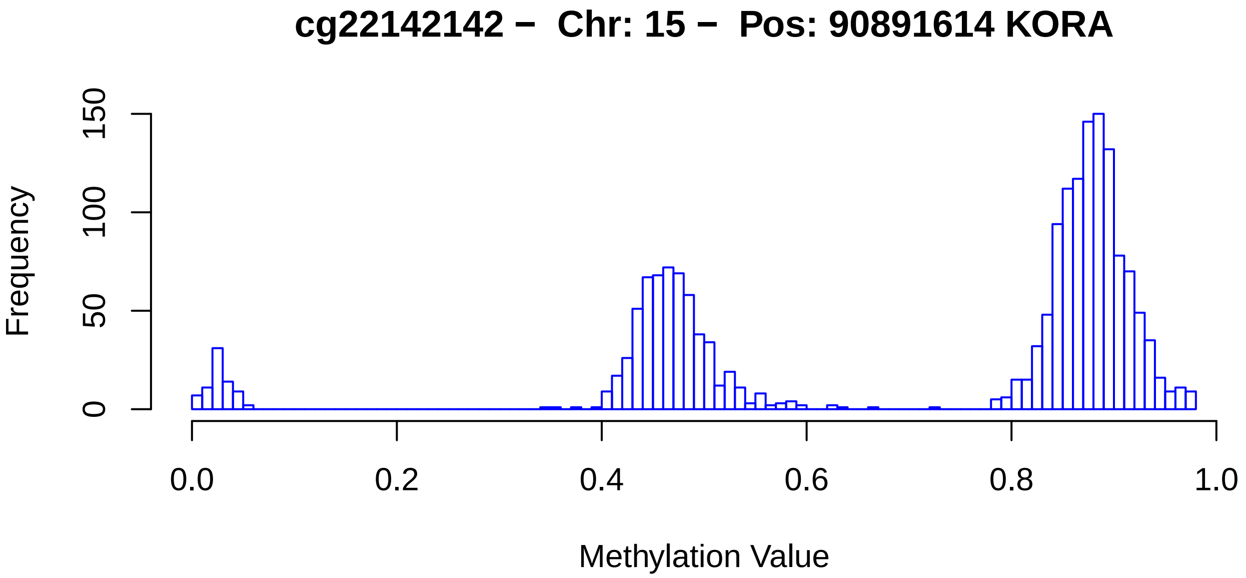
cg22142142 − Chr: 15 − Pos: 90891614 KORA
Frequency
0
50
100
150
0.0
0.2
0.4
0.6
0.8
1.0
Methylation Value

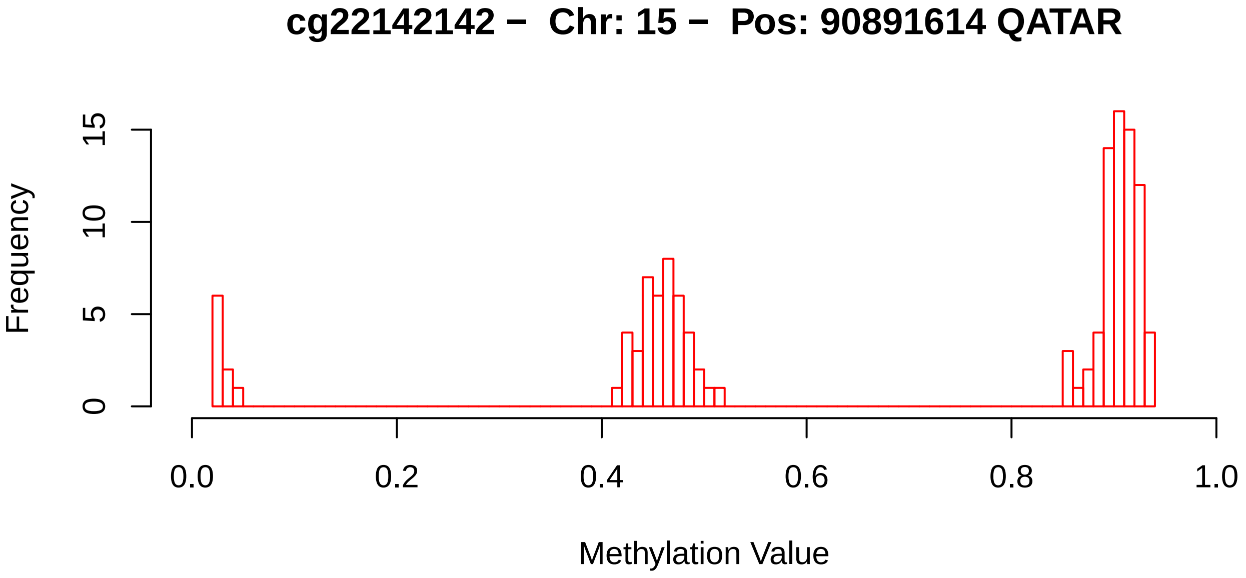
cg22142142 − Chr: 15 − Pos: 90891614 QATAR
Frequency
0
5
10
15
0.0
0.2
0.4
0.6
0.8
1.0
Methylation Value

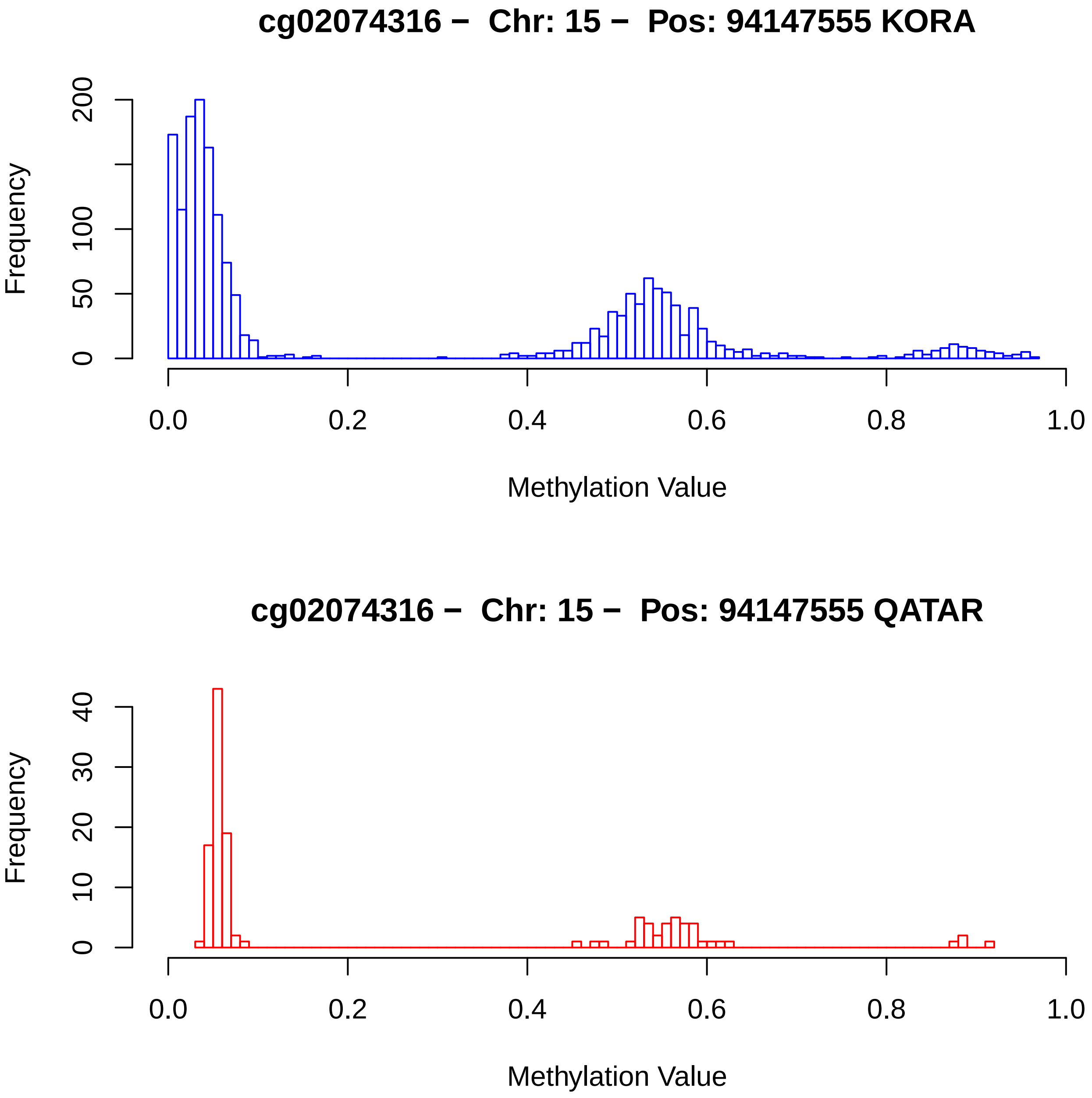
cg02074316 − Chr: 15 − Pos: 94147555 KORA
Frequency
0
50
100
200
0.0
0.2
0.4
0.6
0.8
1.0
Methylation Value
cg02074316 − Chr: 15 − Pos: 94147555 QATAR
Frequency
0
10
20
30
40
0.0
0.2
0.4
0.6
0.8
1.0
Methylation Value

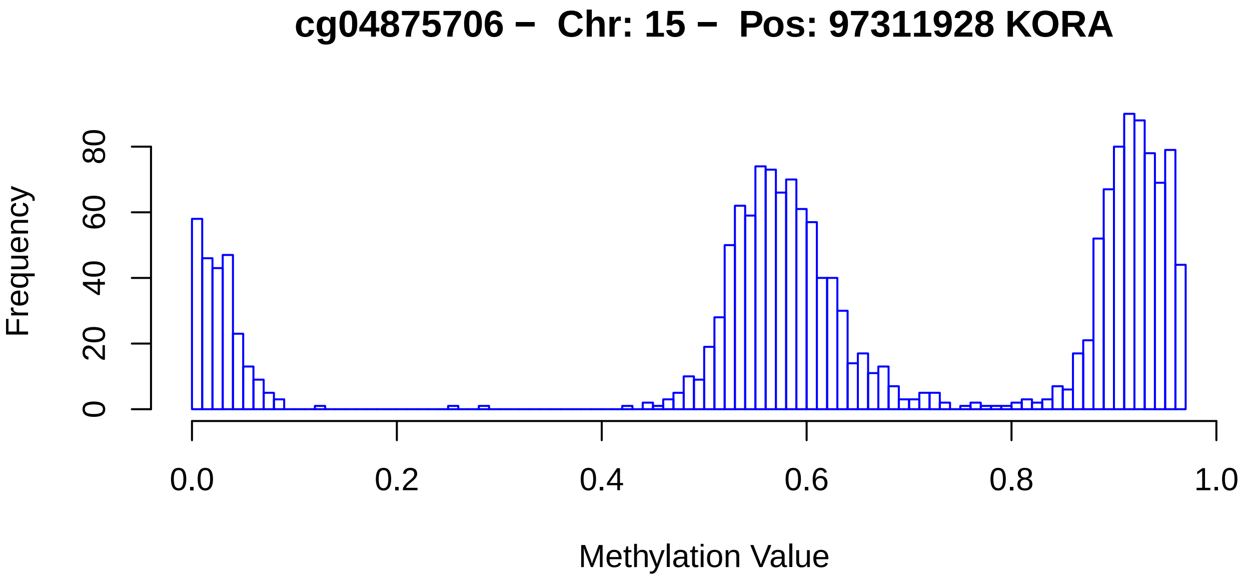
cg04875706 − Chr: 15 − Pos: 97311928 KORA
Frequency
0
20
40
60
80
0.0
0.2
0.4
0.6
0.8
1.0
Methylation Value

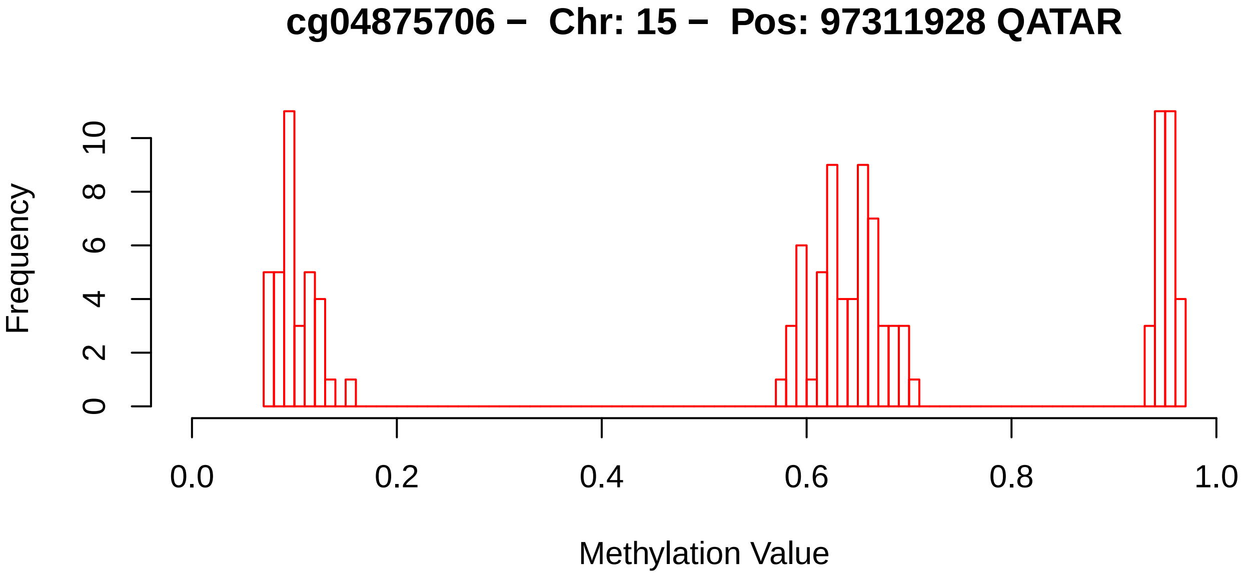
cg04875706 − Chr: 15 − Pos: 97311928 QATAR
Frequency
0
2
4
6
8
10
0.0
0.2
0.4
0.6
0.8
1.0
Methylation Value

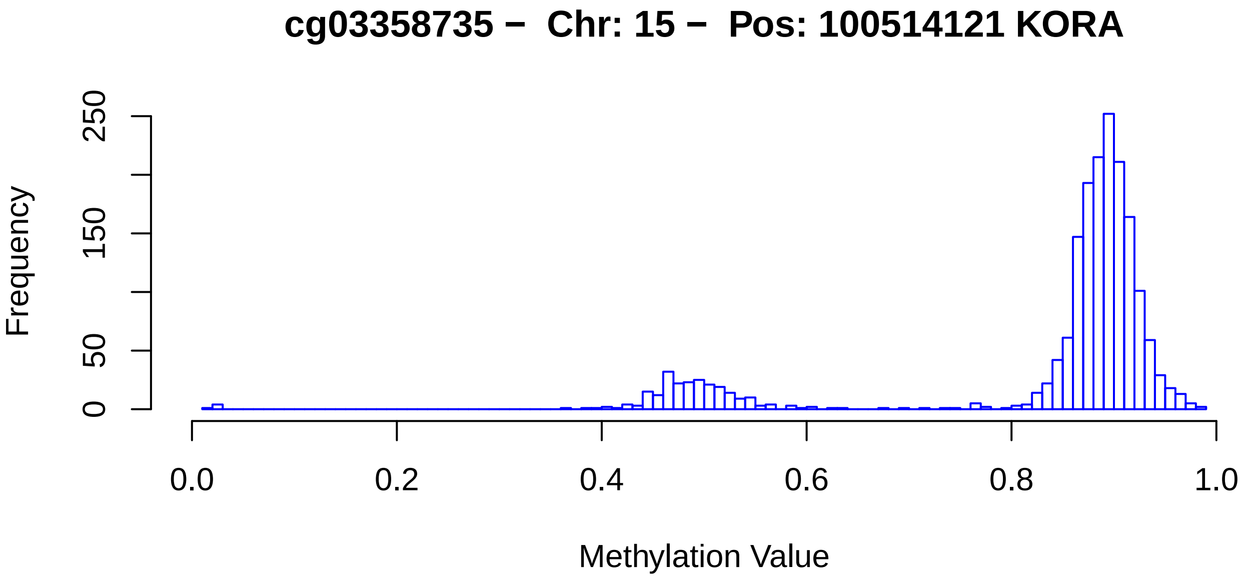
cg03358735 − Chr: 15 − Pos: 100514121 KORA
Frequency
0
50
150
250
0.0
0.2
0.4
0.6
0.8
1.0
Methylation Value

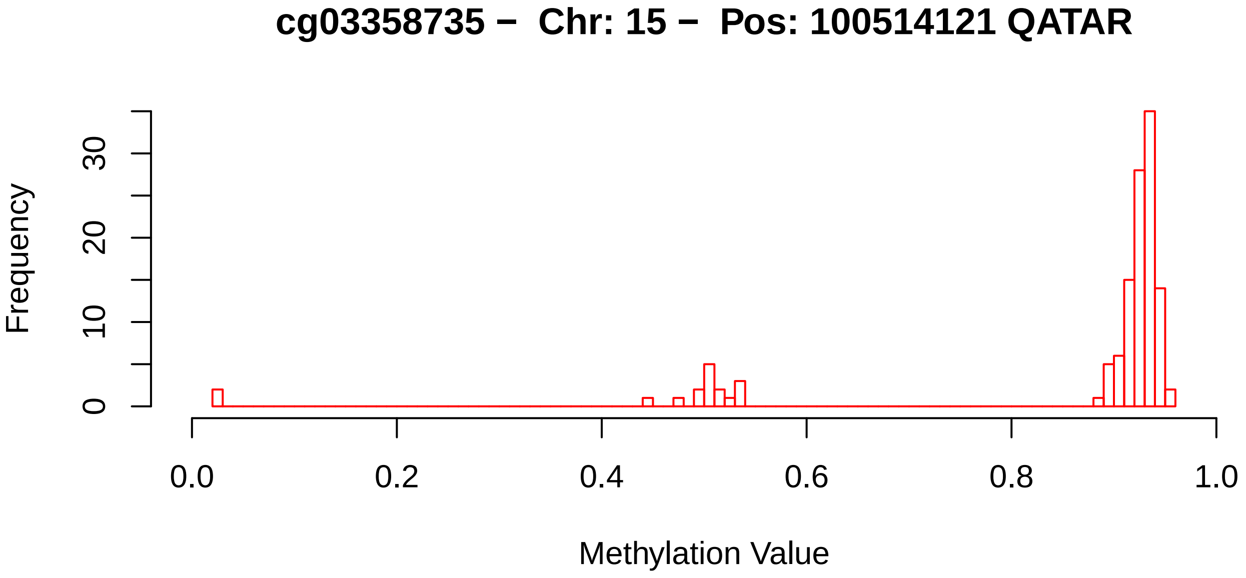
cg03358735 − Chr: 15 − Pos: 100514121 QATAR
Frequency
0
10
20
30
0.0
0.2
0.4
0.6
0.8
1.0
Methylation Value

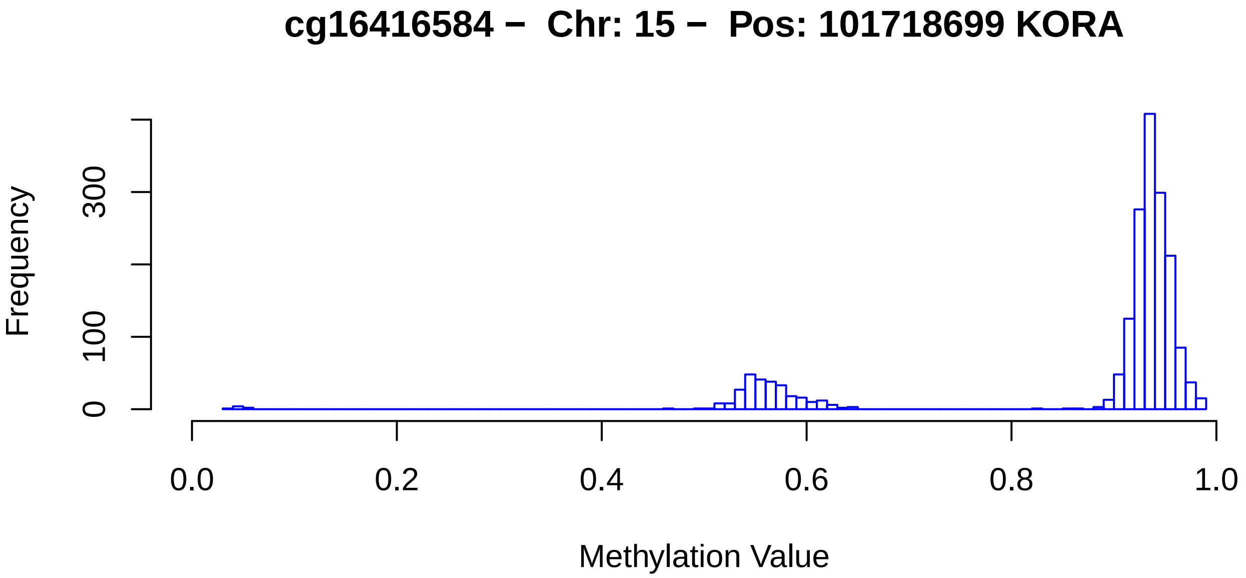
cg16416584 − Chr: 15 − Pos: 101718699 KORA
Frequency
0
100
300
0.0
0.2
0.4
0.6
0.8
1.0
Methylation Value

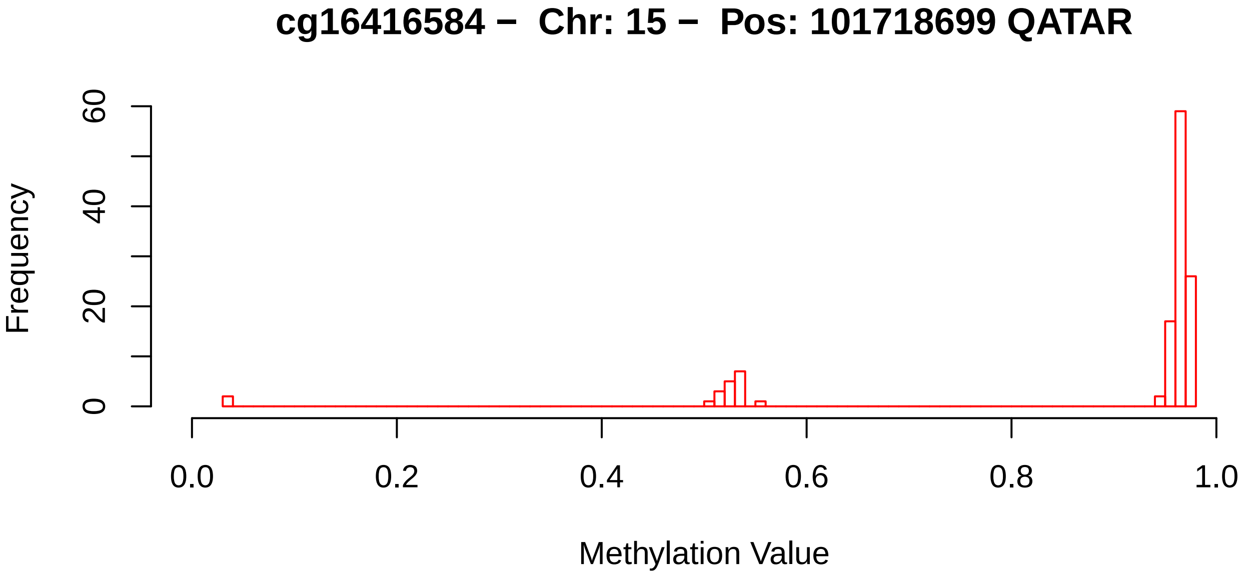
cg16416584 − Chr: 15 − Pos: 101718699 QATAR
Frequency
0
20
40
60
0.0
0.2
0.4
0.6
0.8
1.0
Methylation Value

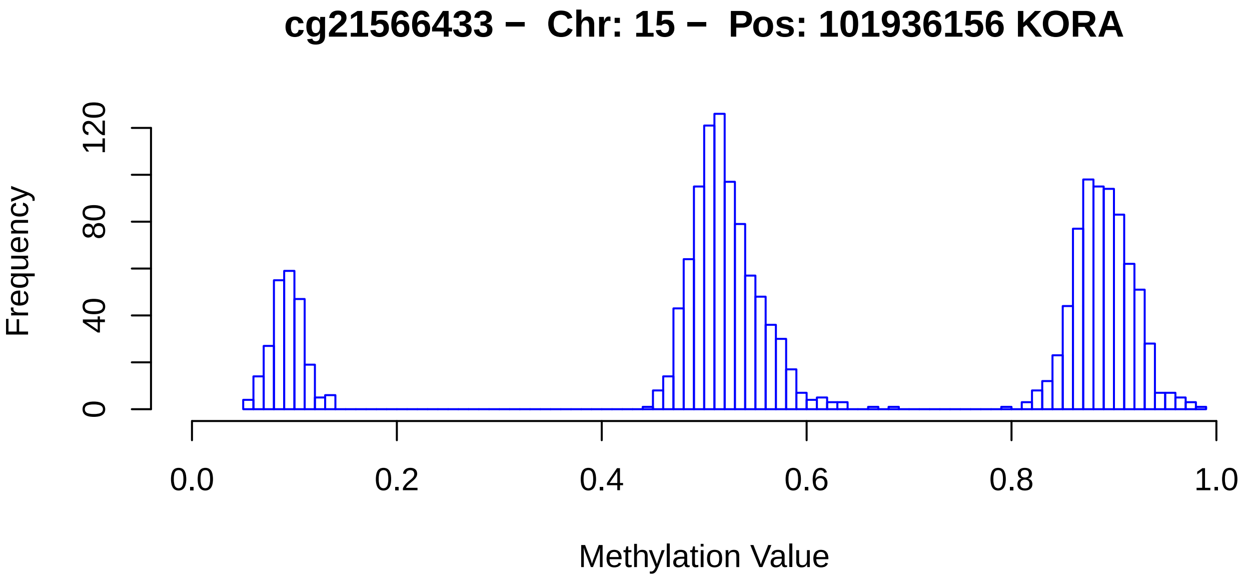
cg21566433 − Chr: 15 − Pos: 101936156 KORA
Frequency
0
40
80
120
0.0
0.2
0.4
0.6
0.8
1.0
Methylation Value

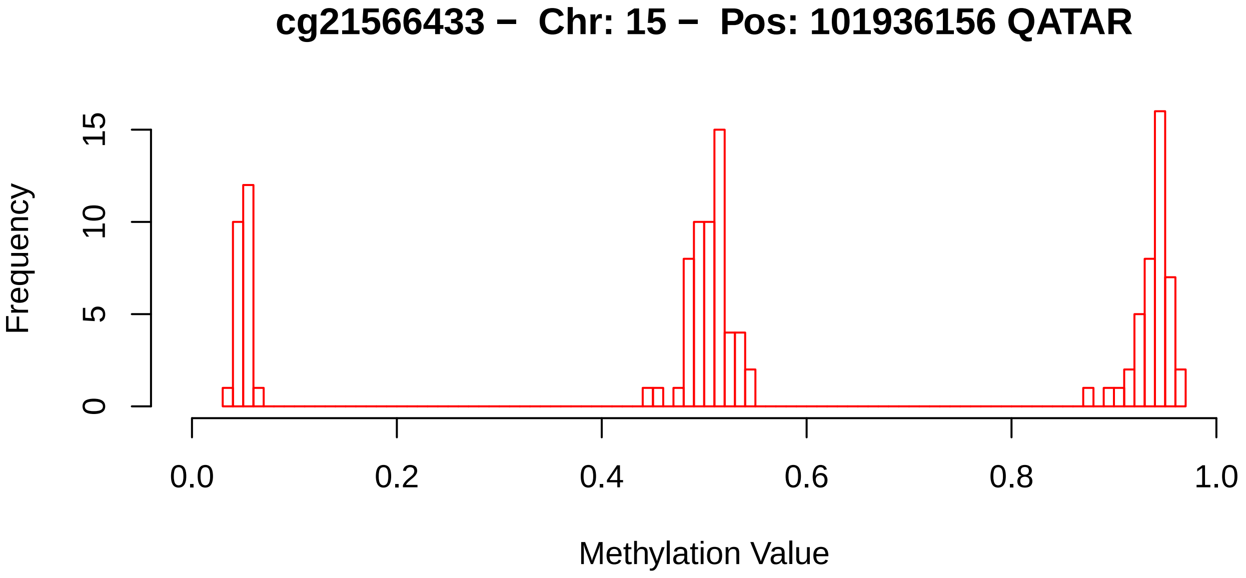
cg21566433 − Chr: 15 − Pos: 101936156 QATAR
Frequency
0
5
10
15
0.0
0.2
0.4
0.6
0.8
1.0
Methylation Value

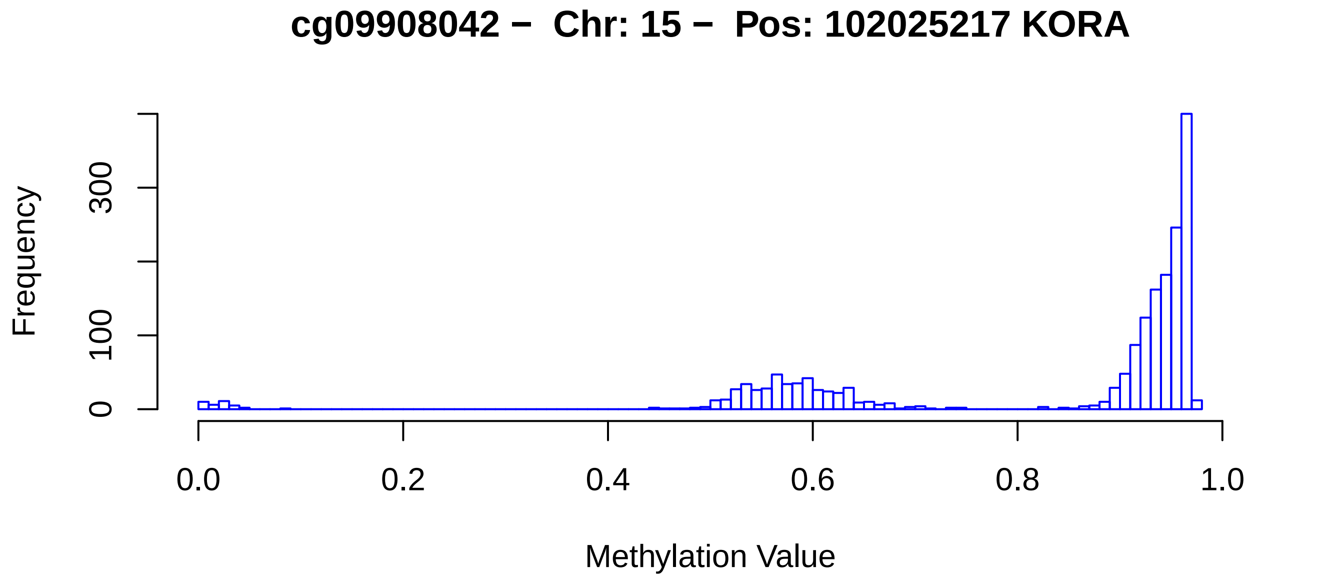
cg09908042 − Chr: 15 − Pos: 102025217 KORA
Frequency
0
100
300
0.0
0.2
0.4
0.6
0.8
1.0
Methylation Value

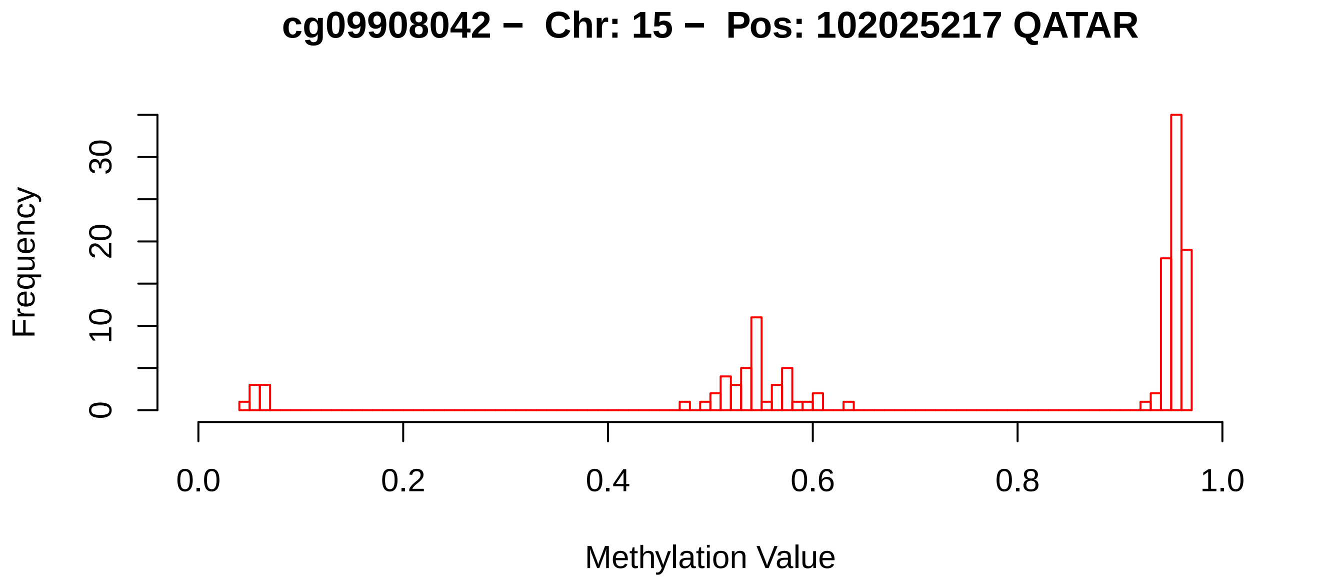
cg09908042 − Chr: 15 − Pos: 102025217 QATAR
Frequency
0
10
20
30
0.0
0.2
0.4
0.6
0.8
1.0
Methylation Value

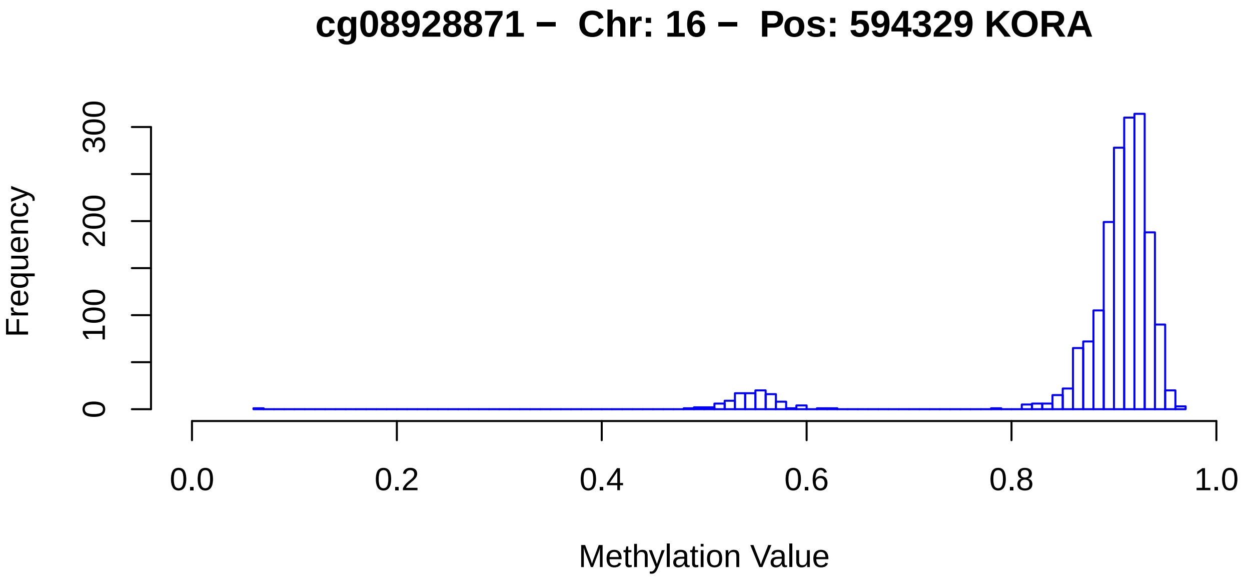
cg08928871 − Chr: 16 − Pos: 594329 KORA
Frequency
0
100
200
300
0.0
0.2
0.4
0.6
0.8
1.0
Methylation Value

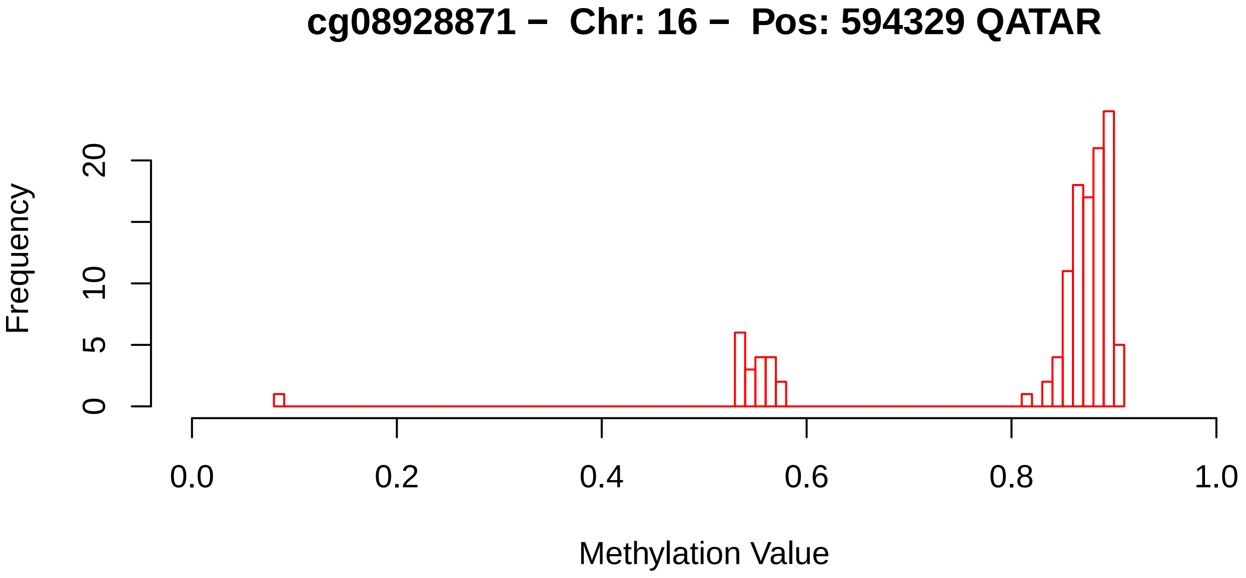
cg08928871 − Chr: 16 − Pos: 594329 QATAR
Frequency
0
5
10
20
0.0
0.2
0.4
0.6
0.8
1.0
Methylation Value

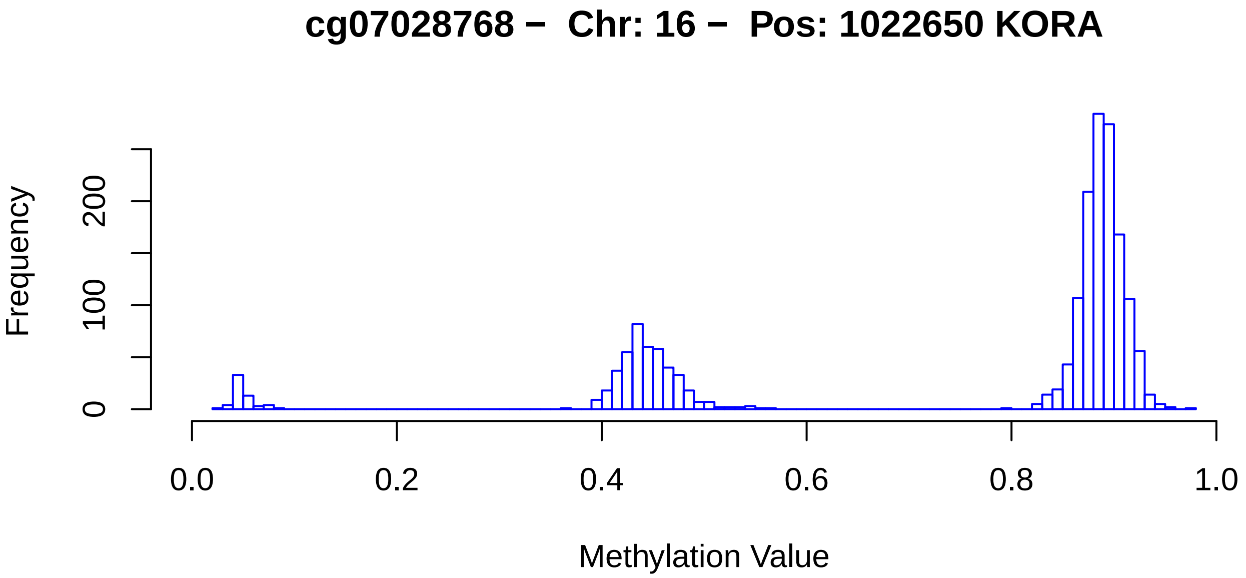

cg07028768 − Chr: 16 − Pos: 1022650 KORA
Frequency
0
100
200
0.0
0.2
0.4
0.6
0.8
1.0
Methylation Value

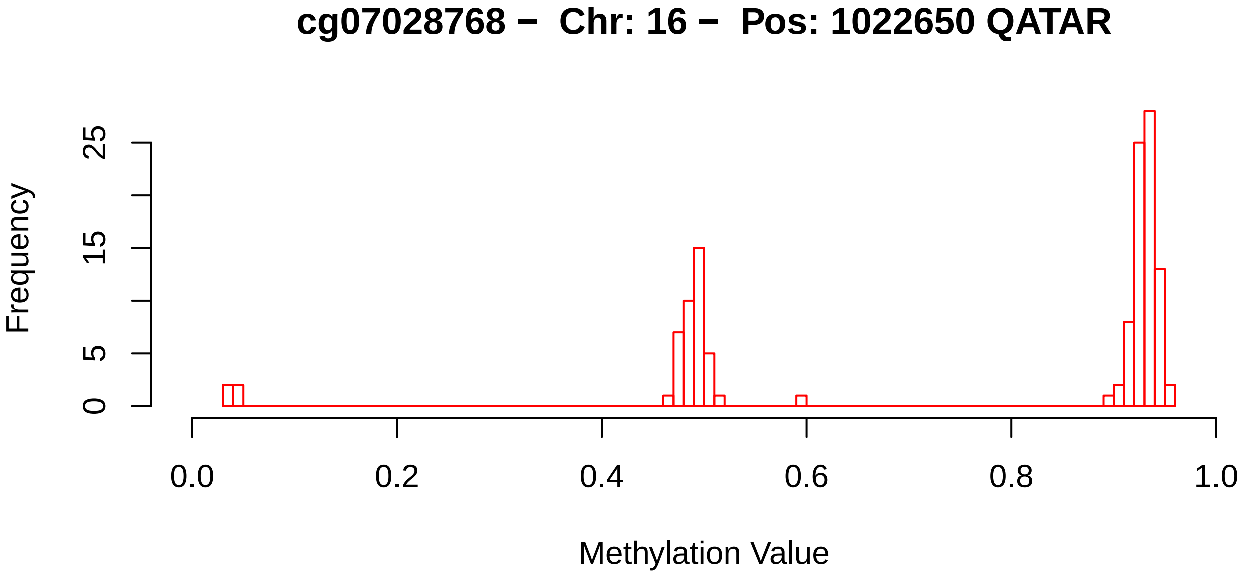

cg07028768 − Chr: 16 − Pos: 1022650 QATAR
Frequency
0
5
15
25
0.0
0.2
0.4
0.6
0.8
1.0
Methylation Value

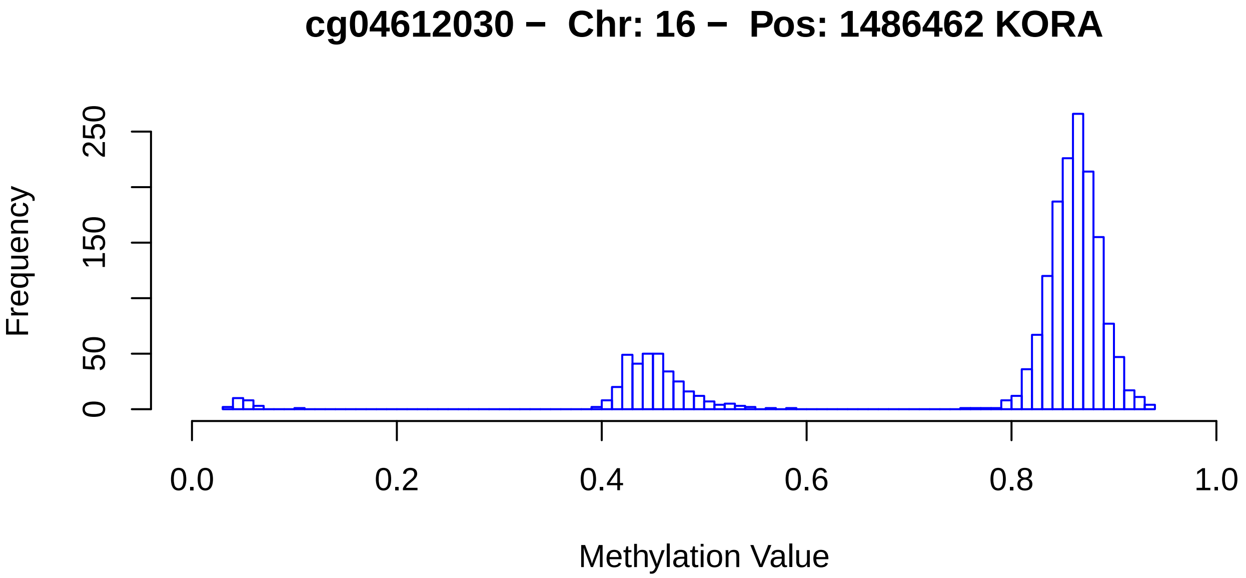
cg04612030 − Chr: 16 − Pos: 1486462 KORA
Frequency
0
50
150
250
0.0
0.2
0.4
0.6
0.8
1.0
Methylation Value

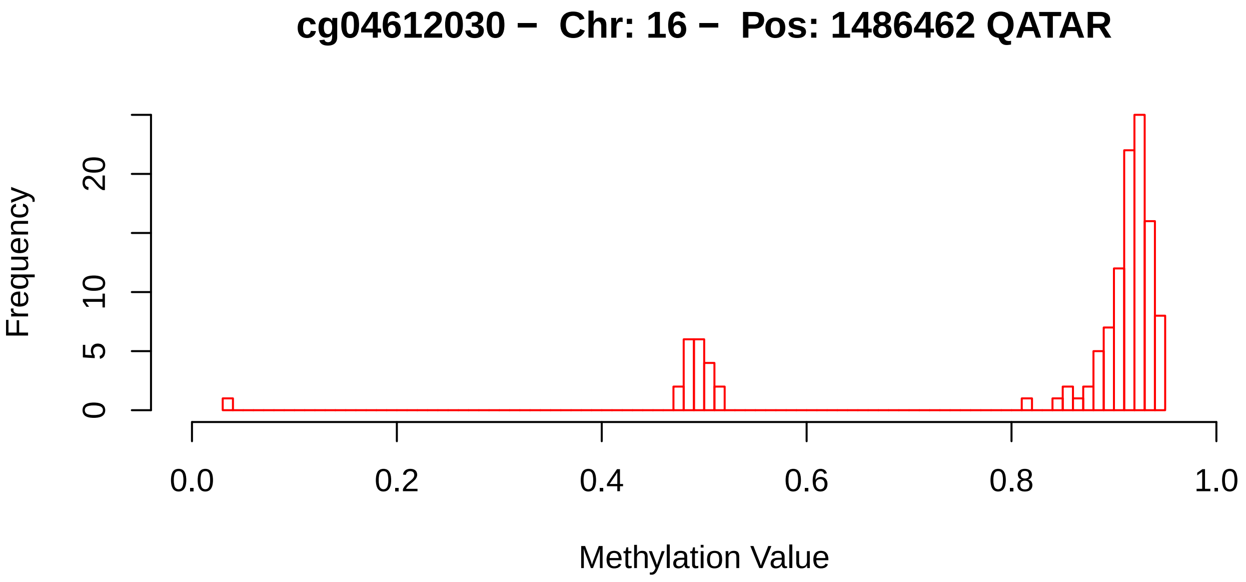
cg04612030 − Chr: 16 − Pos: 1486462 QATAR
Frequency
0
5
10
20
0.0
0.2
0.4
0.6
0.8
1.0
Methylation Value

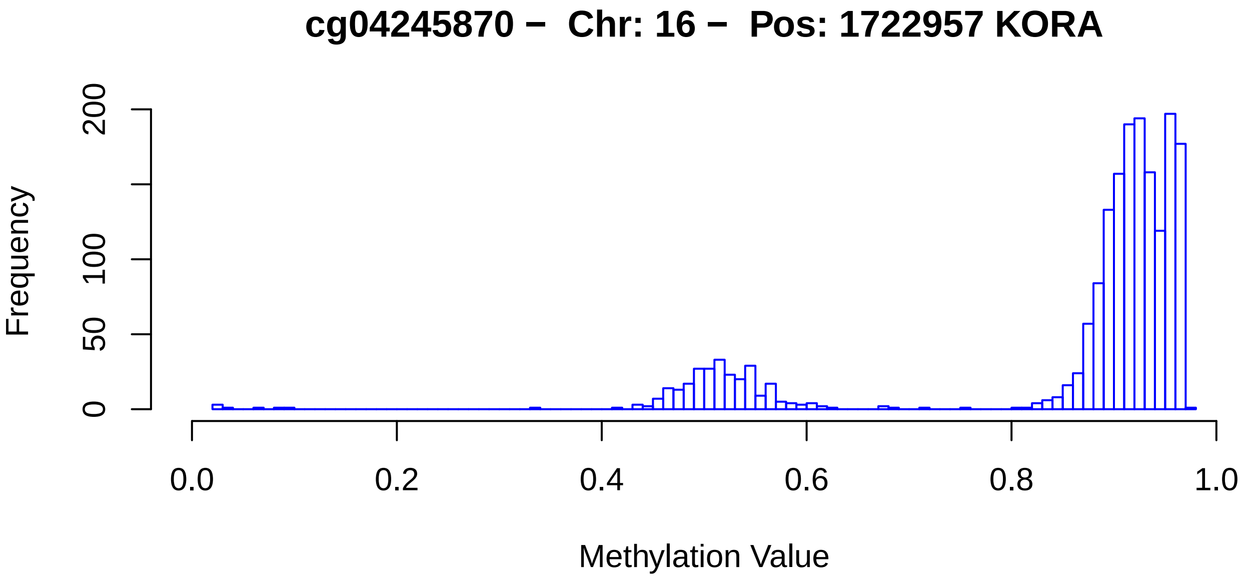

cg04245870 − Chr: 16 − Pos: 1722957 KORA
Frequency
0
50
100
200
0.0
0.2
0.4
0.6
0.8
1.0
Methylation Value

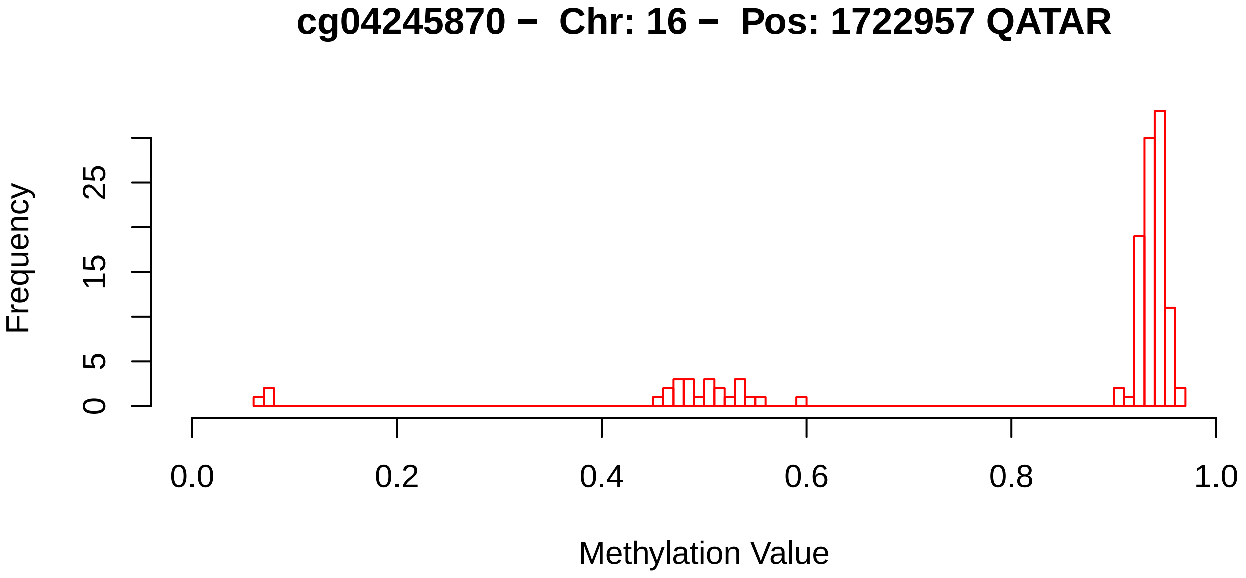

cg04245870 − Chr: 16 − Pos: 1722957 QATAR
Frequency
0
5
15
25
0.0
0.2
0.4
0.6
0.8
1.0
Methylation Value

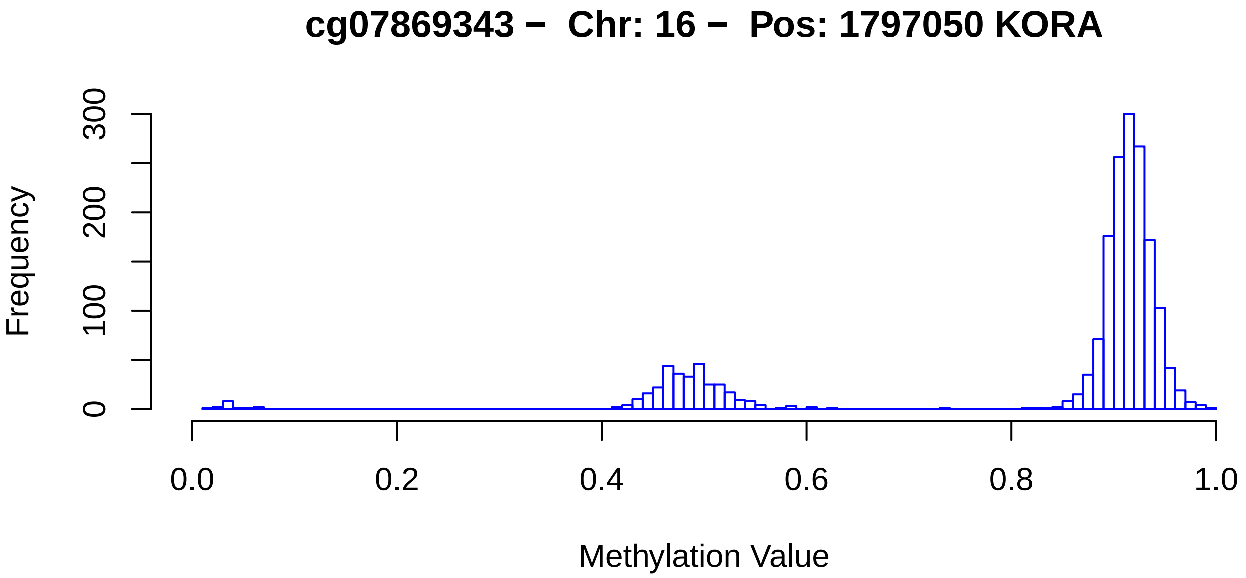
cg07869343 − Chr: 16 − Pos: 1797050 KORA
Frequency
0
100
200
300
0.0
0.2
0.4
0.6
0.8
1.0
Methylation Value

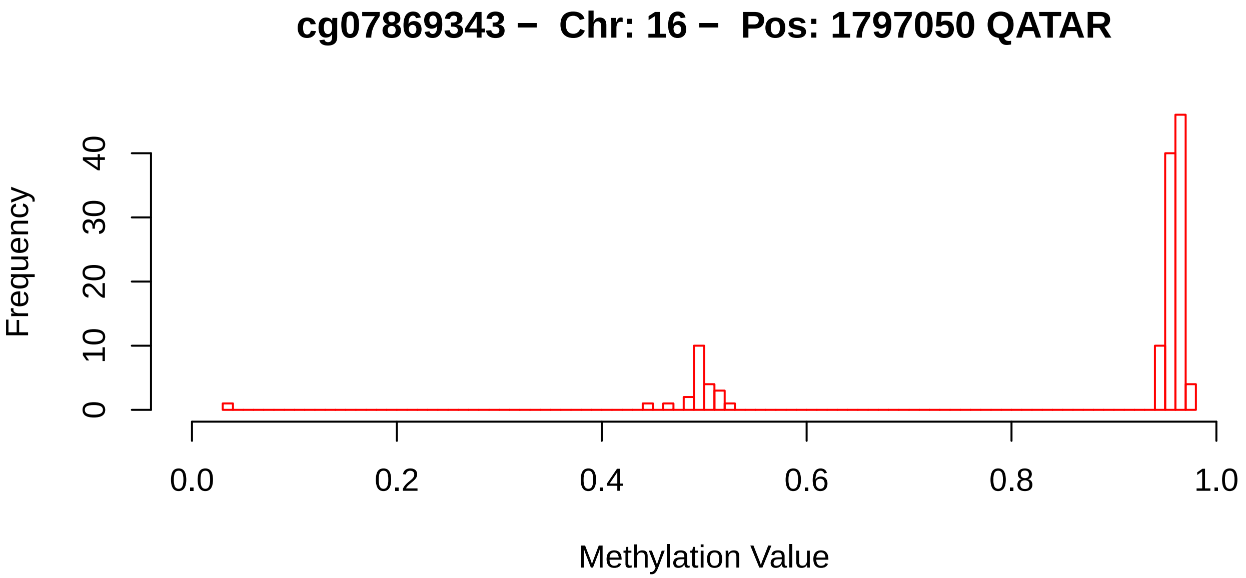
cg07869343 − Chr: 16 − Pos: 1797050 QATAR
Frequency
0
10
20
30
40
0.0
0.2
0.4
0.6
0.8
1.0
Methylation Value

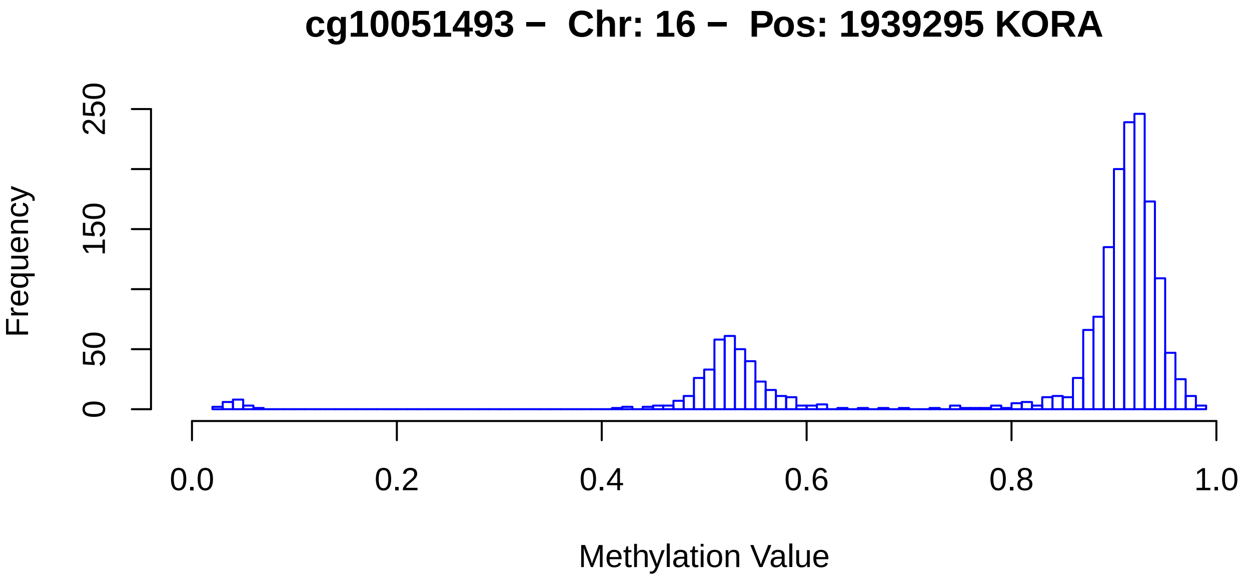
cg10051493 − Chr: 16 − Pos: 1939295 KORA
Frequency
0
50
150
250
0.0
0.2
0.4
0.6
0.8
1.0
Methylation Value

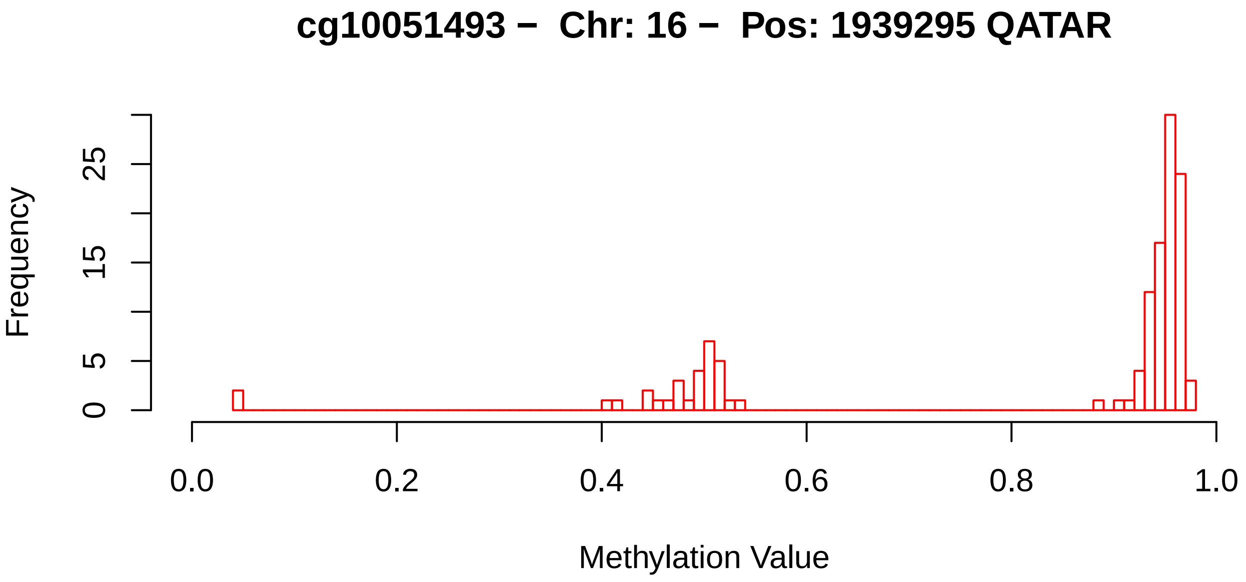
cg10051493 − Chr: 16 − Pos: 1939295 QATAR
Frequency
0
5
15
25
0.0
0.2
0.4
0.6
0.8
1.0
Methylation Value

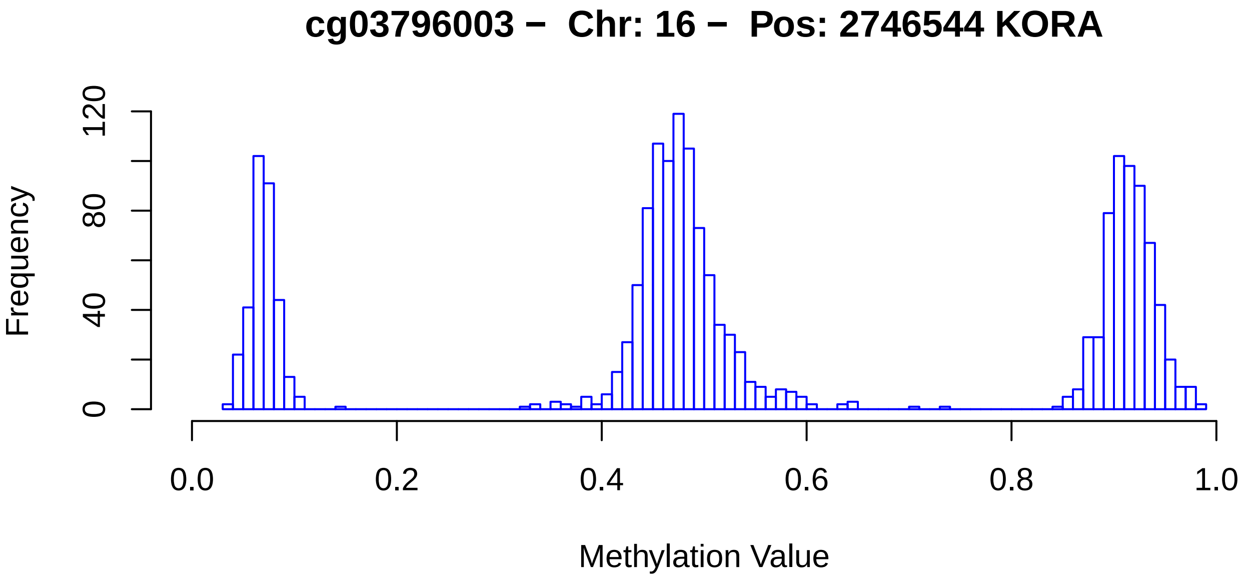
cg03796003 − Chr: 16 − Pos: 2746544 KORA
Frequency
0
40
80
120
0.0
0.2
0.4
0.6
0.8
1.0
Methylation Value

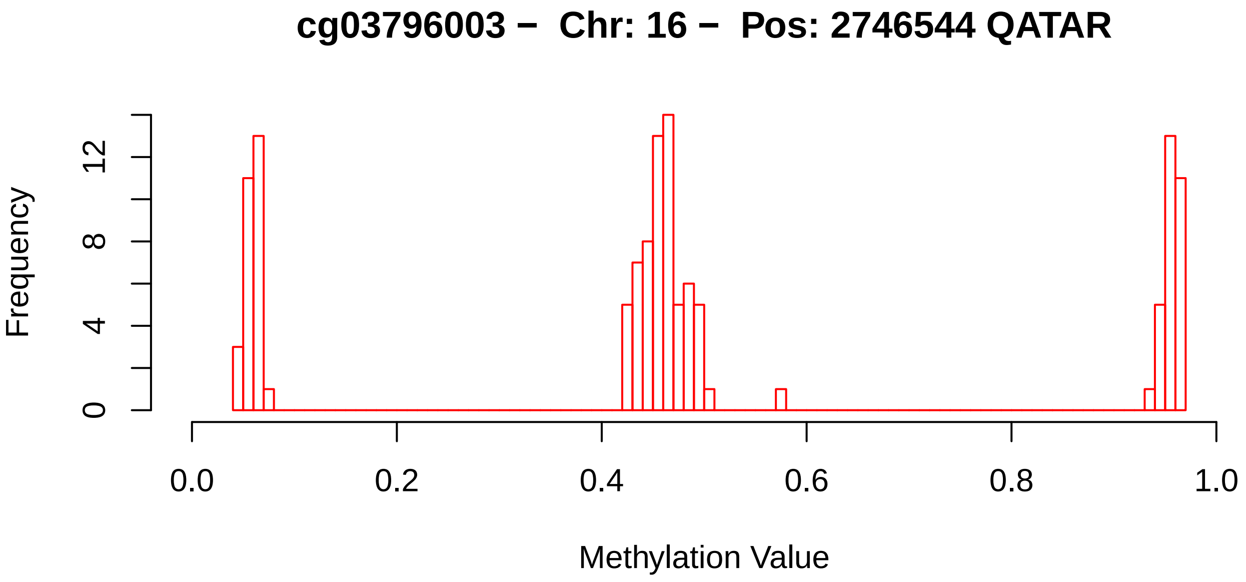
cg03796003 − Chr: 16 − Pos: 2746544 QATAR
Frequency
0
4
8
12
0.0
0.2
0.4
0.6
0.8
1.0
Methylation Value

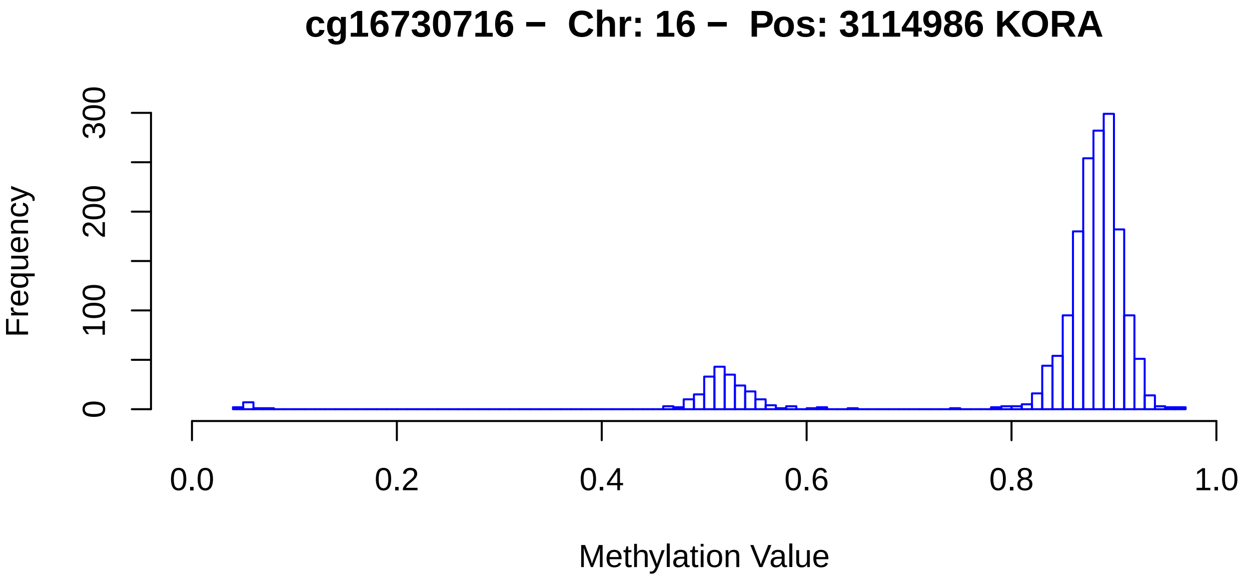

cg16730716 − Chr: 16 − Pos: 3114986 KORA
Frequency
0
100
200
300
0.0
0.2
0.4
0.6
0.8
1.0
Methylation Value

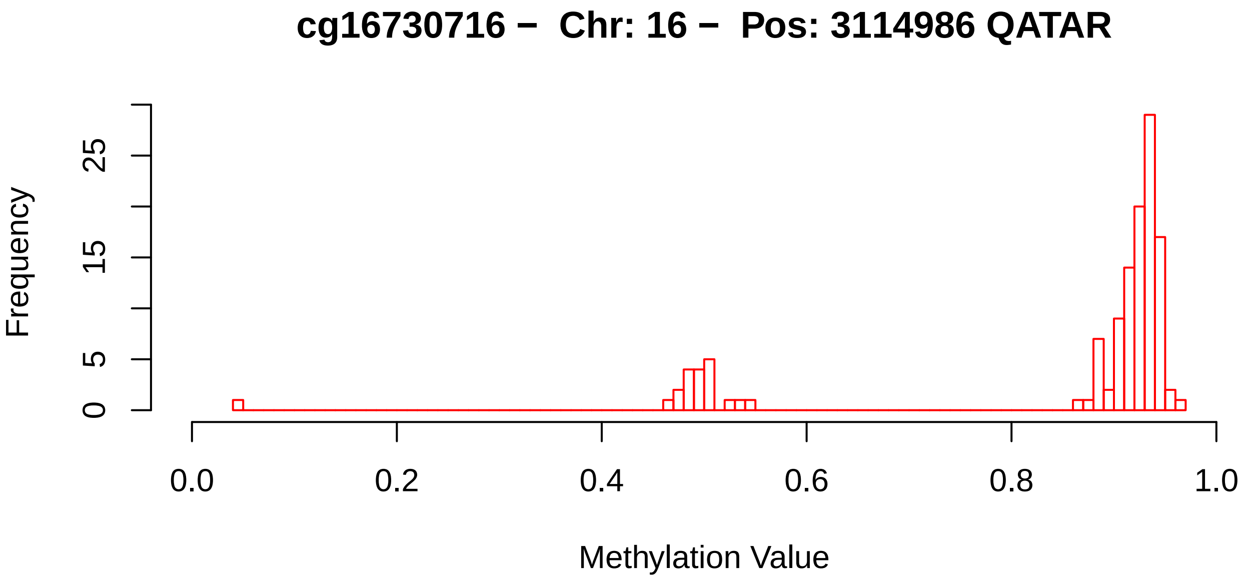

cg16730716 − Chr: 16 − Pos: 3114986 QATAR
Frequency
0
5
15
25
0.0
0.2
0.4
0.6
0.8
1.0
Methylation Value

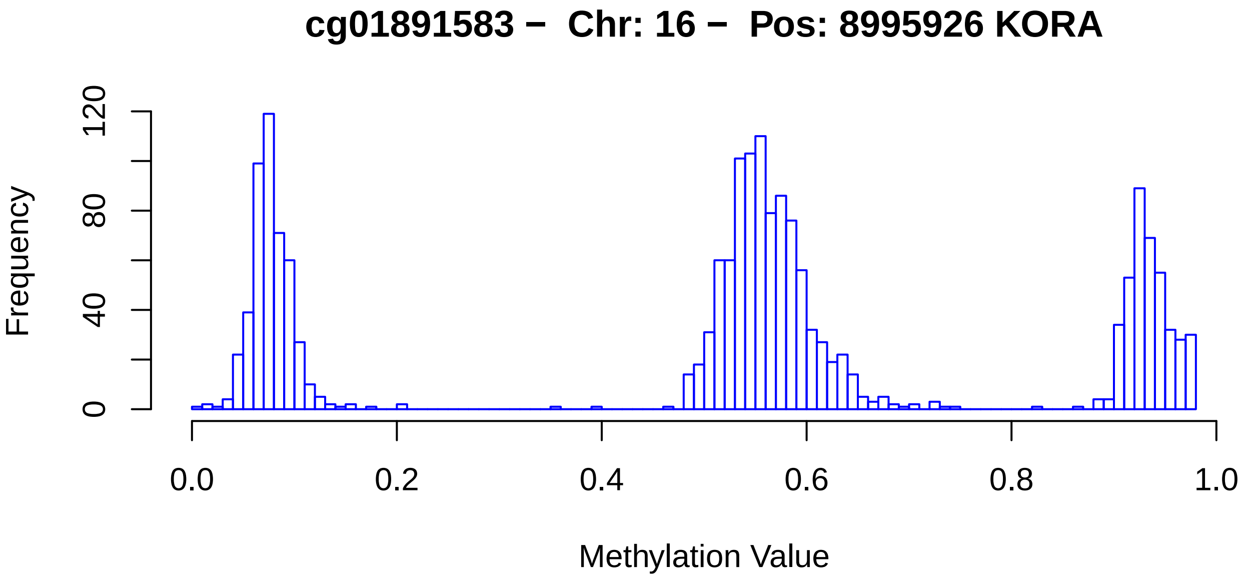
cg01891583 − Chr: 16 − Pos: 8995926 KORA
Frequency
0
40
80
120
0.0
0.2
0.4
0.6
0.8
1.0
Methylation Value

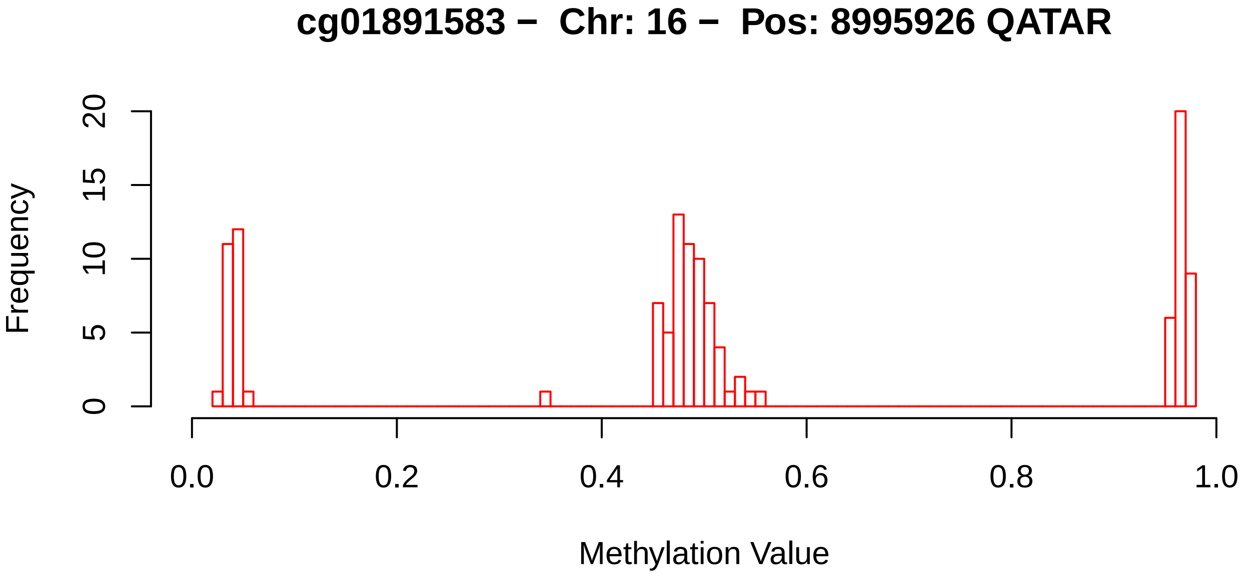
cg01891583 − Chr: 16 − Pos: 8995926 QATAR
Frequency
0
5
10
15
20
0.0
0.2
0.4
0.6
0.8
1.0
Methylation Value

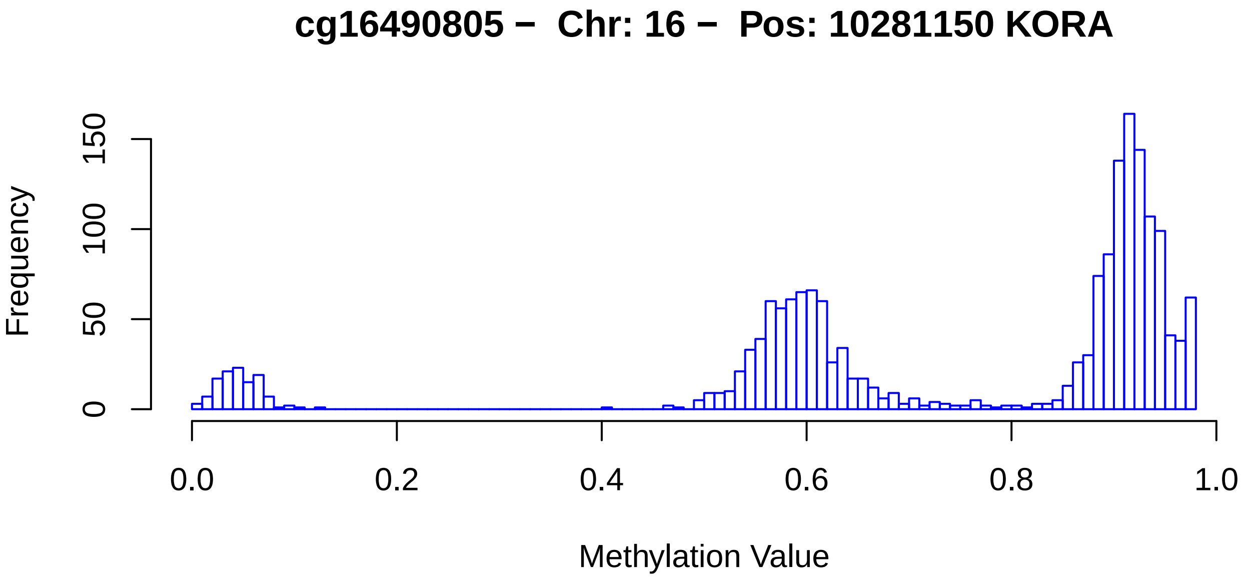
cg16490805 − Chr: 16 − Pos: 10281150 KORA
Frequency
0
50
100
150
0.0
0.2
0.4
0.6
0.8
1.0
Methylation Value

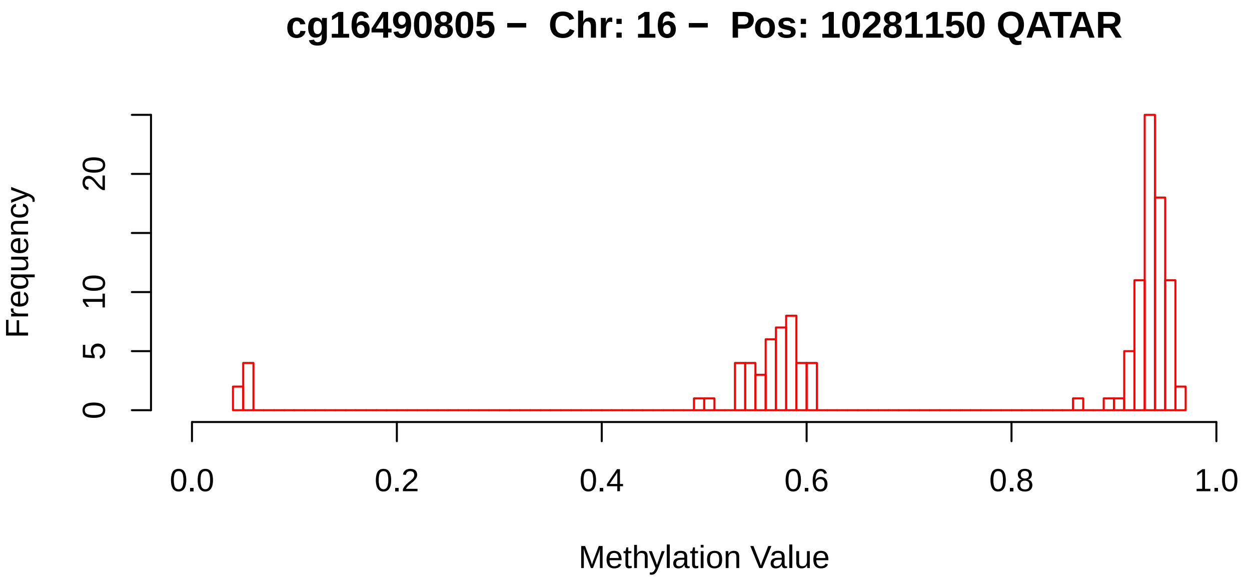
cg16490805 − Chr: 16 − Pos: 10281150 QATAR
Frequency
0
5
10
20
0.0
0.2
0.4
0.6
0.8
1.0
Methylation Value

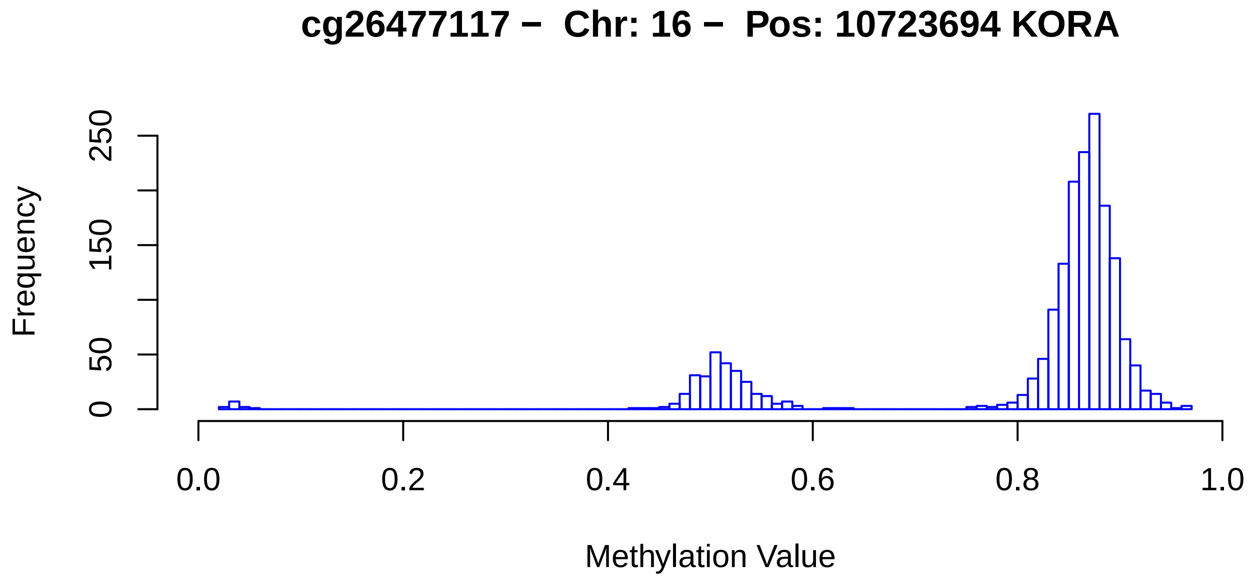
cg26477117 − Chr: 16 − Pos: 10723694 KORA
Frequency
0
50
150
250
0.0
0.2
0.4
0.6
0.8
1.0
Methylation Value

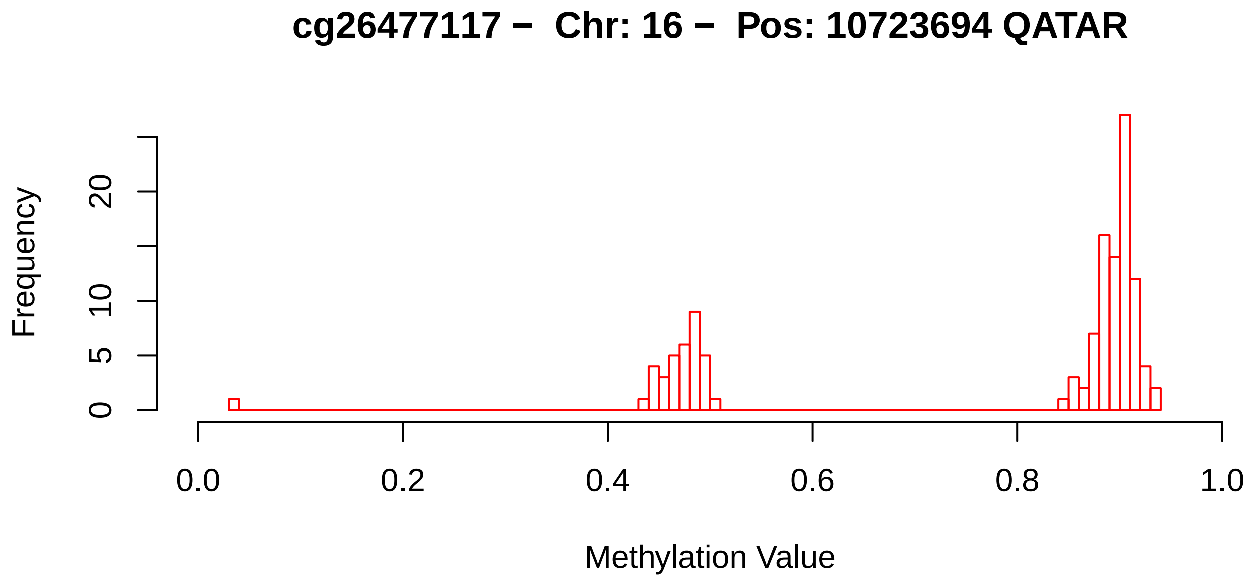
cg26477117 − Chr: 16 − Pos: 10723694 QATAR
Frequency
0
5
10
20
0.0
0.2
0.4
0.6
0.8
1.0
Methylation Value

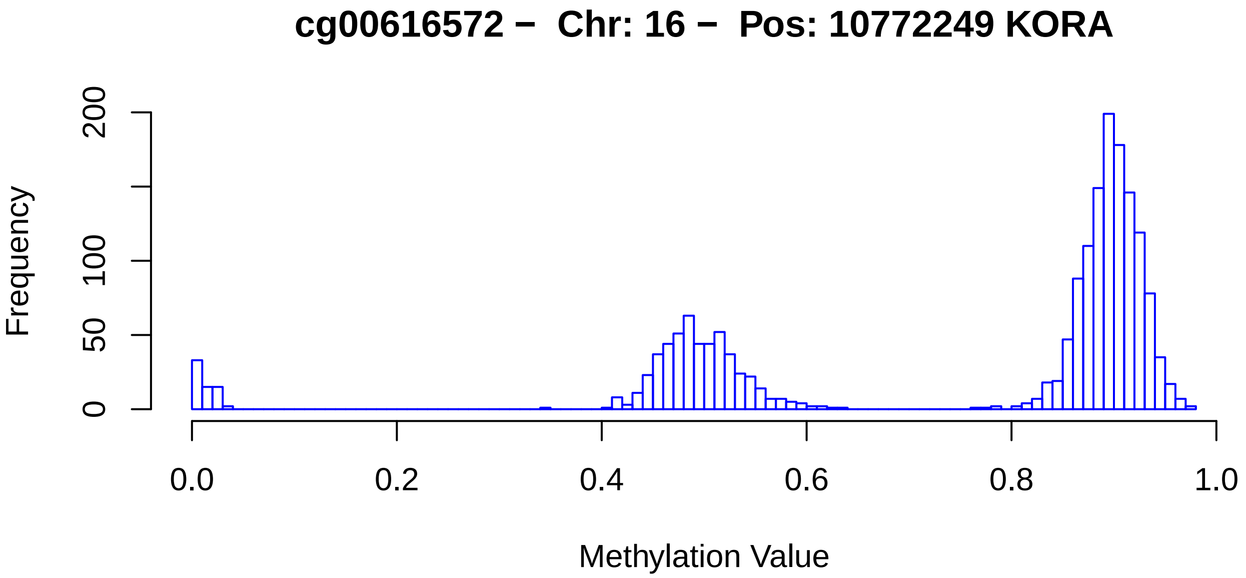
cg00616572 – Chr: 16 – Pos: 10772249 KORA
Frequency
0
50
100
200
0.0
0.2
0.4
0.6
0.8
1.0
Methylation Value

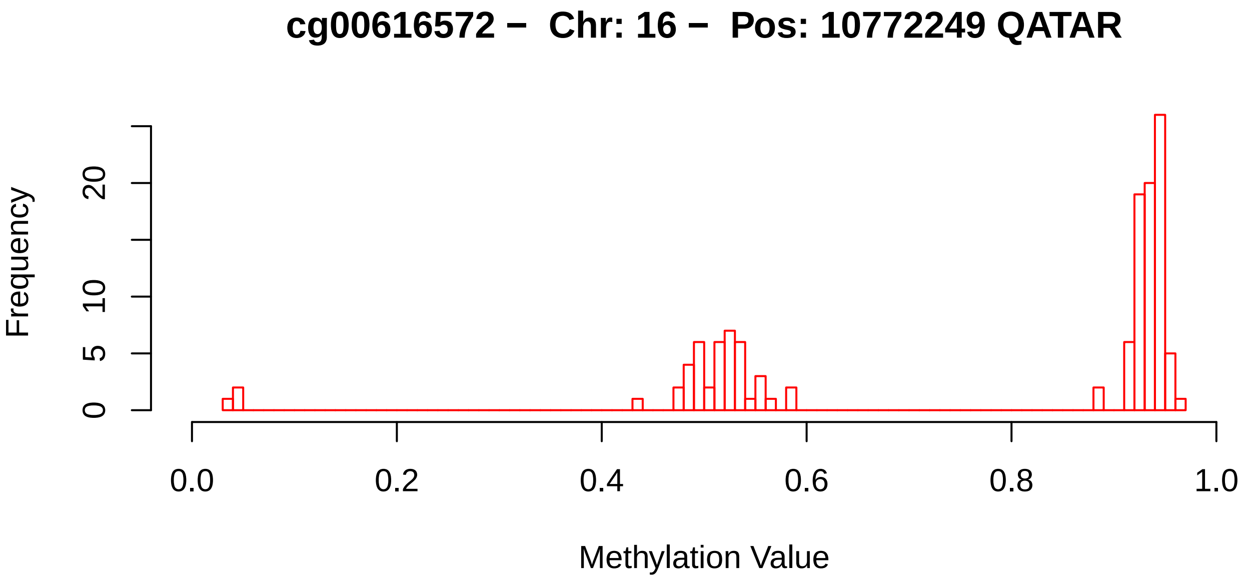
cg00616572 – Chr: 16 – Pos: 10772249 QATAR
Frequency
0
5
10
20
0.0
0.2
0.4
0.6
0.8
1.0
Methylation Value

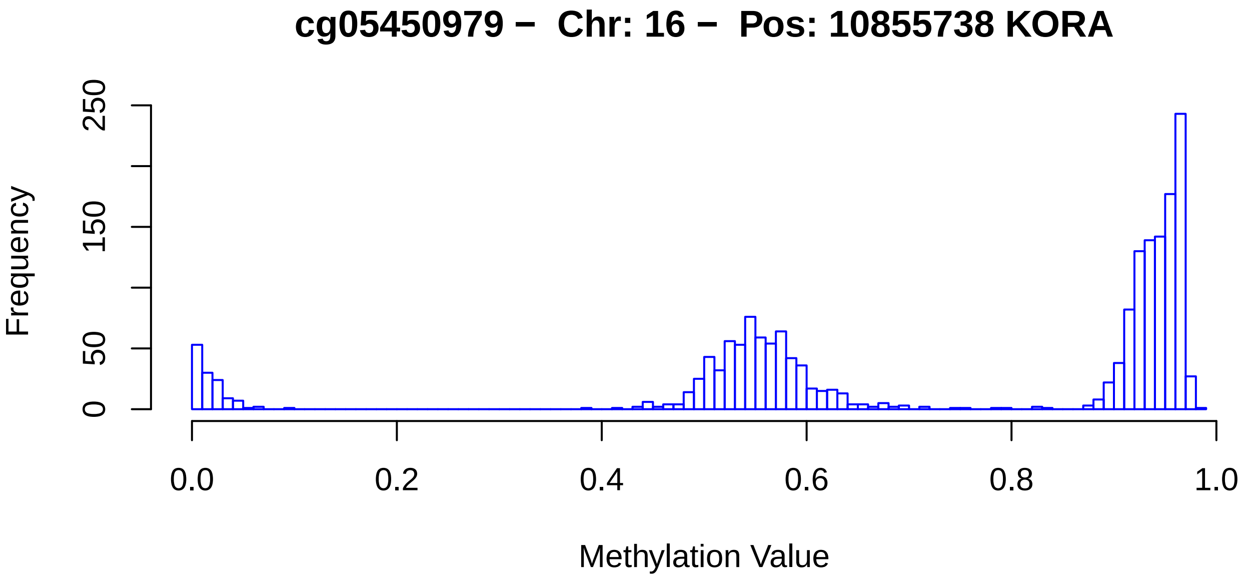

cg05450979 − Chr: 16 − Pos: 10855738 KORA
Frequency
0
50
150
250
0.0
0.2
0.4
0.6
0.8
1.0
Methylation Value

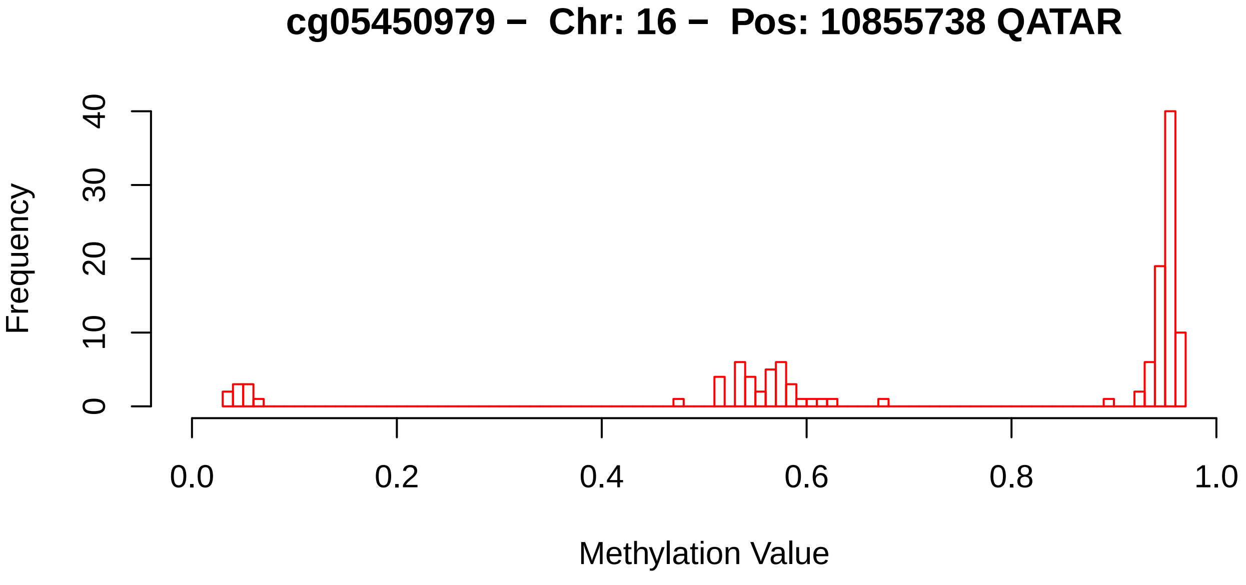

cg05450979 − Chr: 16 − Pos: 10855738 QATAR
Frequency
0
10
20
30
40
0.0
0.2
0.4
0.6
0.8
1.0
Methylation Value

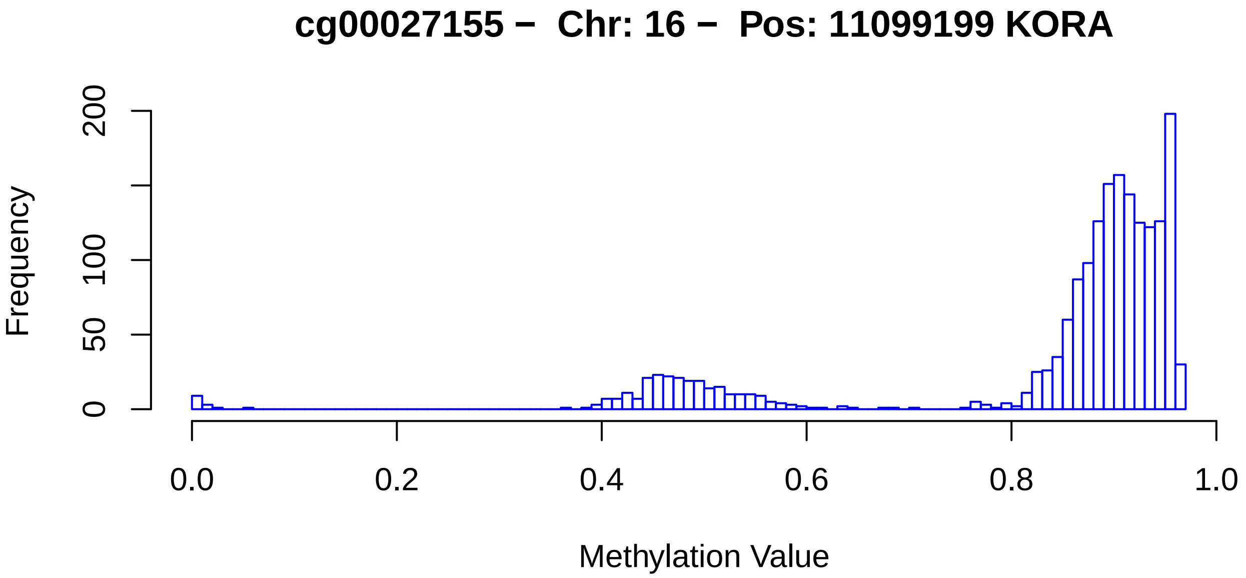
cg00027155 − Chr: 16 − Pos: 11099199 KORA
Frequency
0
50
100
200
0.0
0.2
0.4
0.6
0.8
1.0
Methylation Value

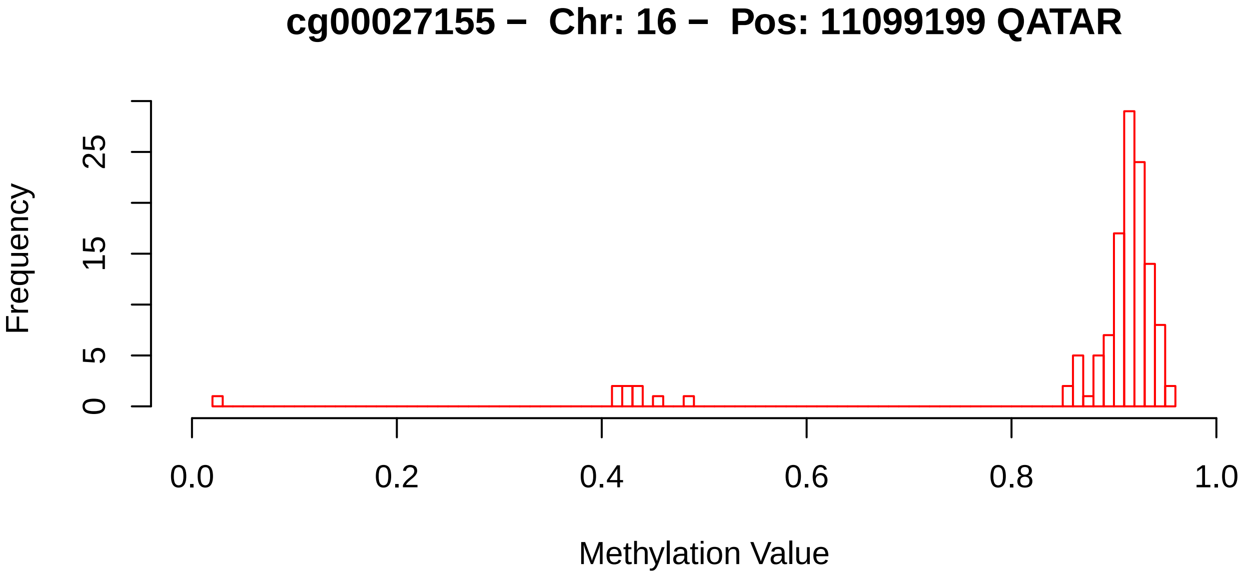
cg00027155 − Chr: 16 − Pos: 11099199 QATAR
Frequency
0
5
15
25
0.0
0.2
0.4
0.6
0.8
1.0
Methylation Value

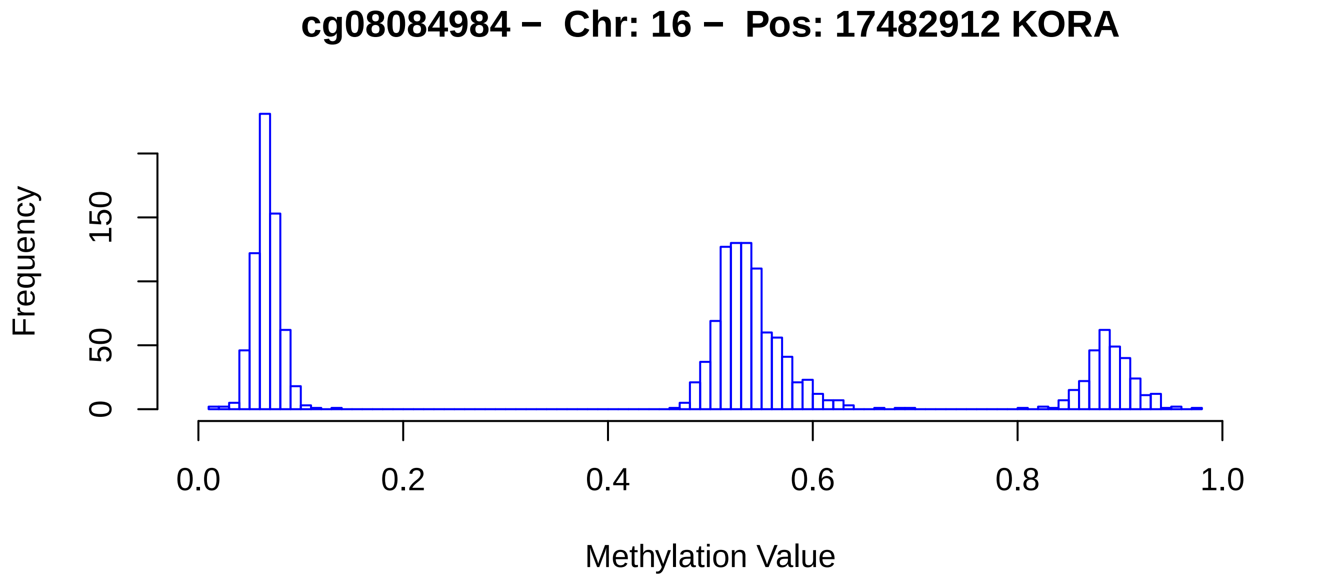
cg08084984 − Chr: 16 − Pos: 17482912 KORA
Frequency
0
50
150
0.0
0.2
0.4
0.6
0.8
1.0
Methylation Value

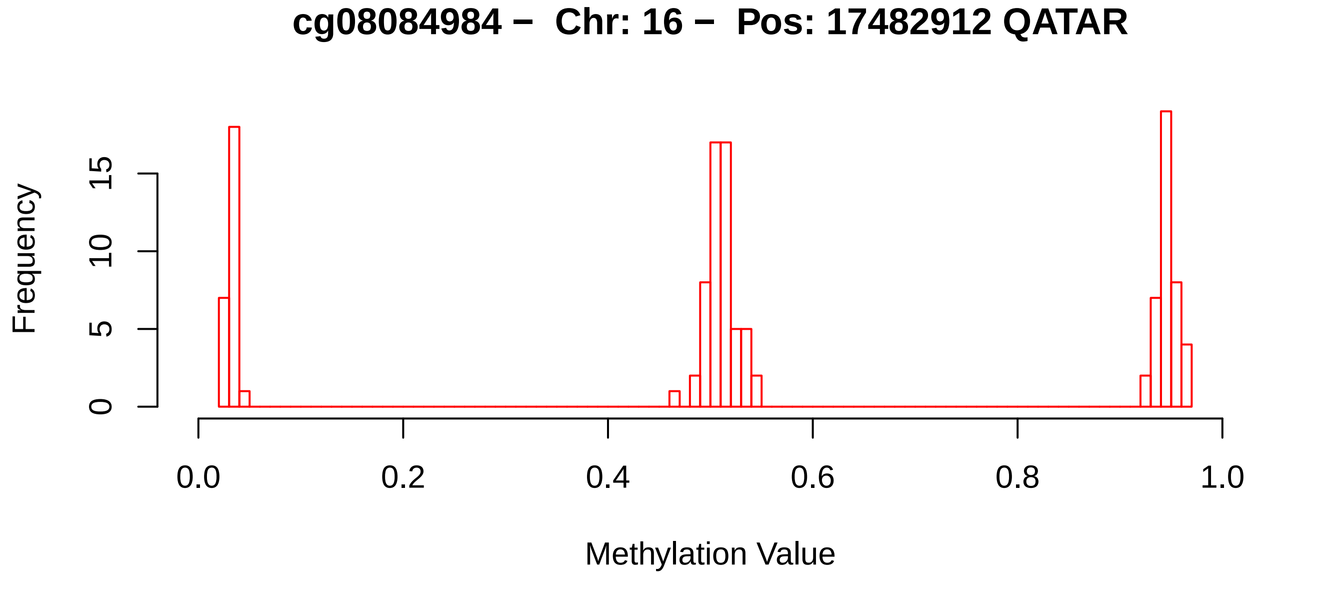
cg08084984 − Chr: 16 − Pos: 17482912 QATAR
Frequency
0
5
10
15
0.0
0.2
0.4
0.6
0.8
1.0
Methylation Value

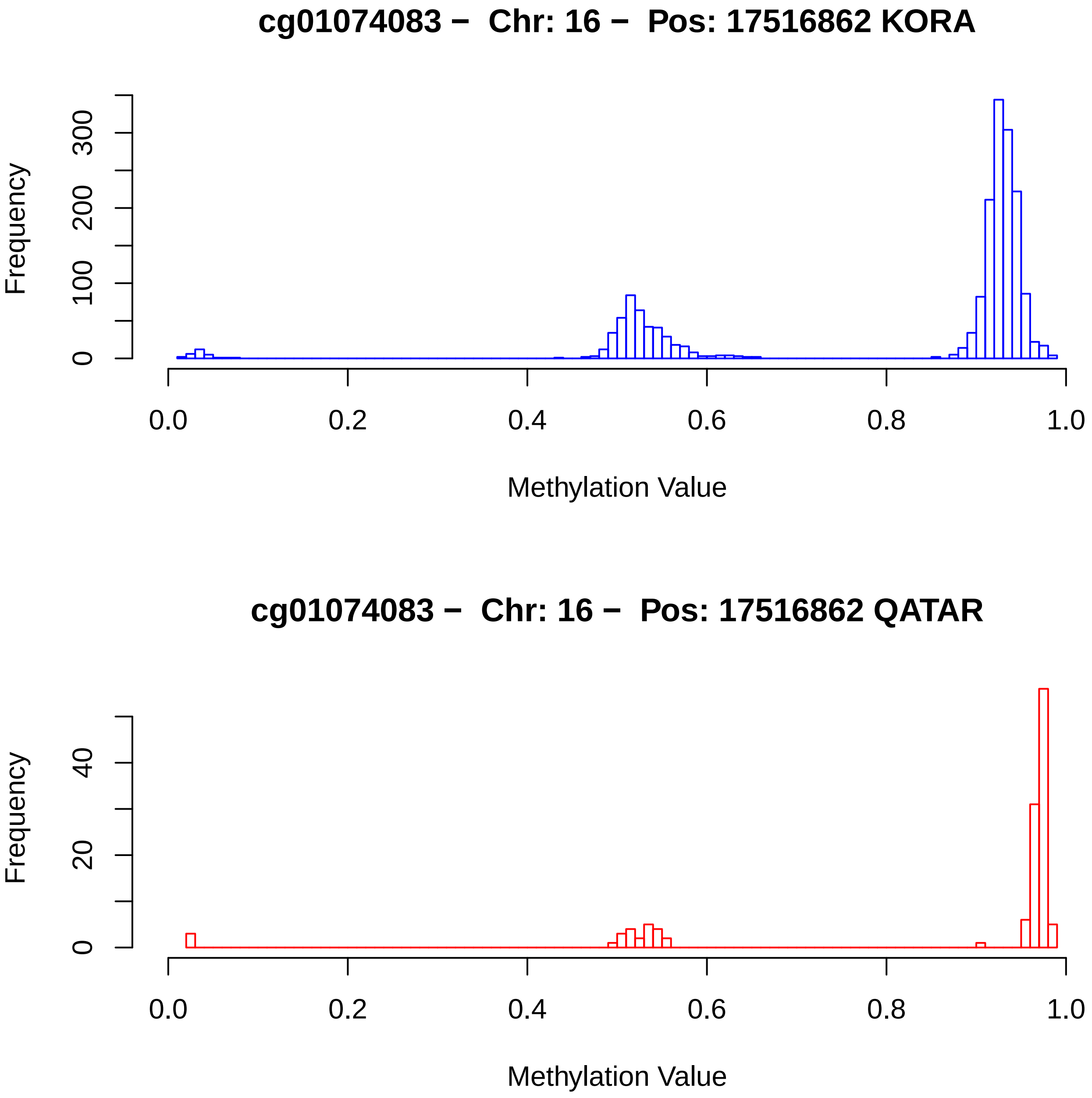
cg01074083 − Chr: 16 − Pos: 17516862 KORA
Frequency
0
100
200
300
0.0
0.2
0.4
0.6
0.8
1.0
Methylation Value
cg01074083 − Chr: 16 − Pos: 17516862 QATAR
Frequency
0
20
40
0.0
0.2
0.4
0.6
0.8
1.0
Methylation Value

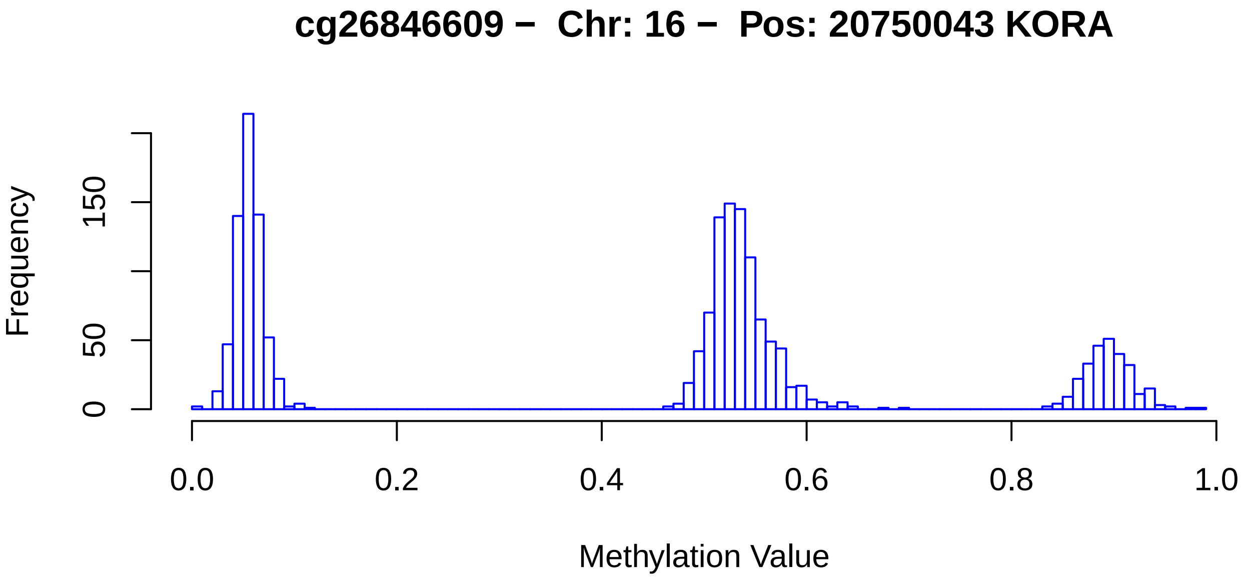
cg26846609 − Chr: 16 − Pos: 20750043 KORA
Frequency
0
50
150
0.0
0.2
0.4
0.6
0.8
1.0
Methylation Value

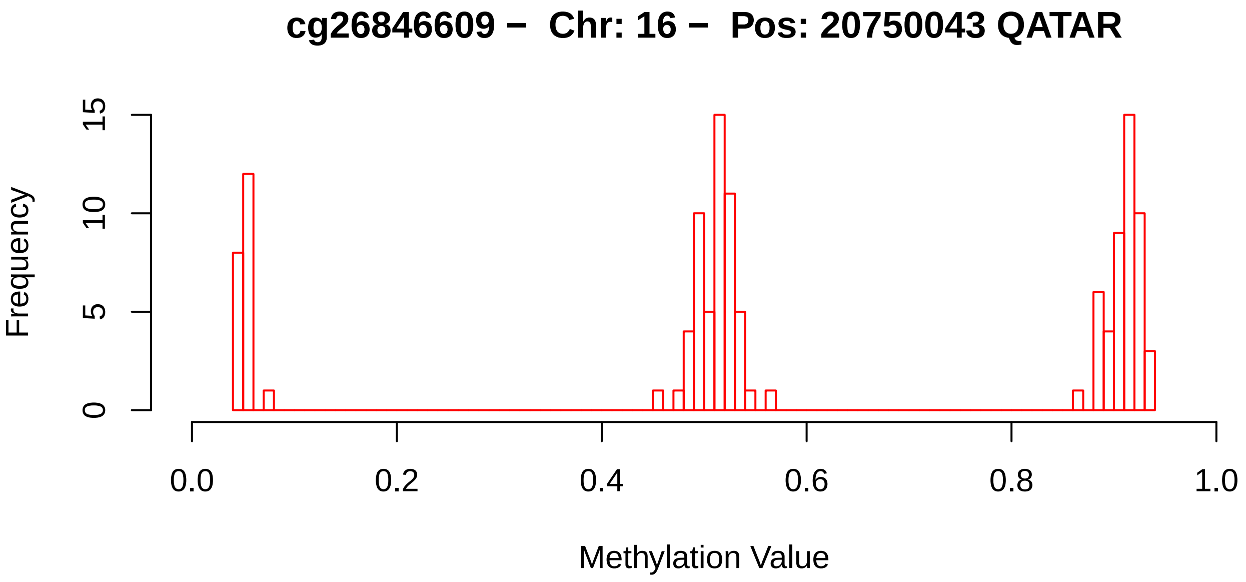
cg26846609 − Chr: 16 − Pos: 20750043 QATAR
Frequency
0
5
10
15
0.0
0.2
0.4
0.6
0.8
1.0
Methylation Value

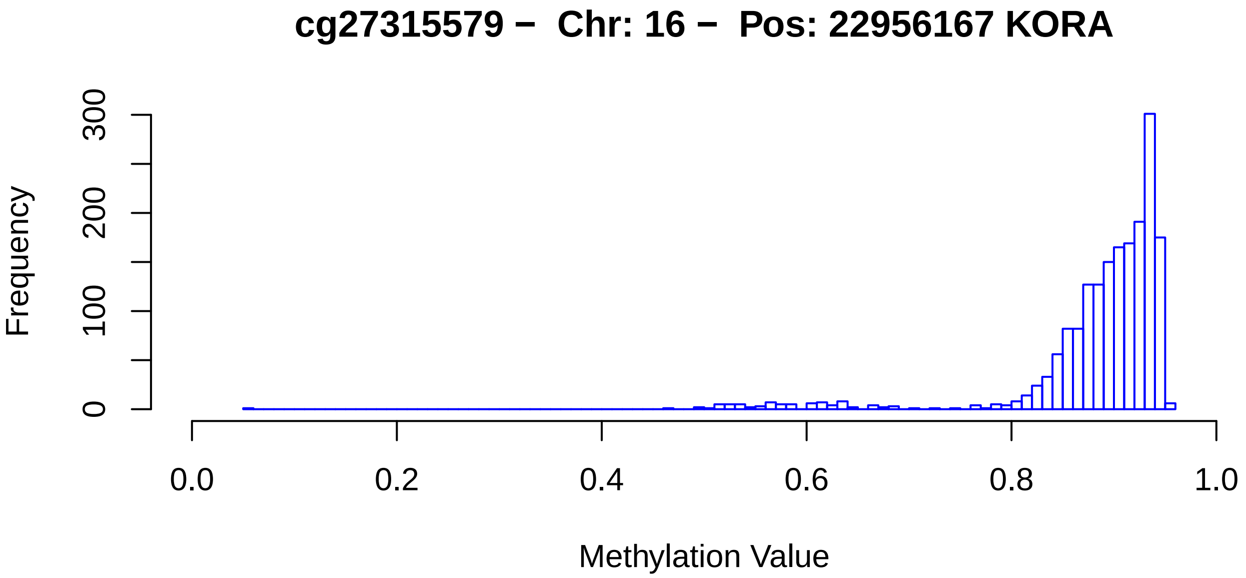
cg27315579 − Chr: 16 − Pos: 22956167 KORA
Frequency
0
100
200
300
0.0
0.2
0.4
0.6
0.8
1.0
Methylation Value

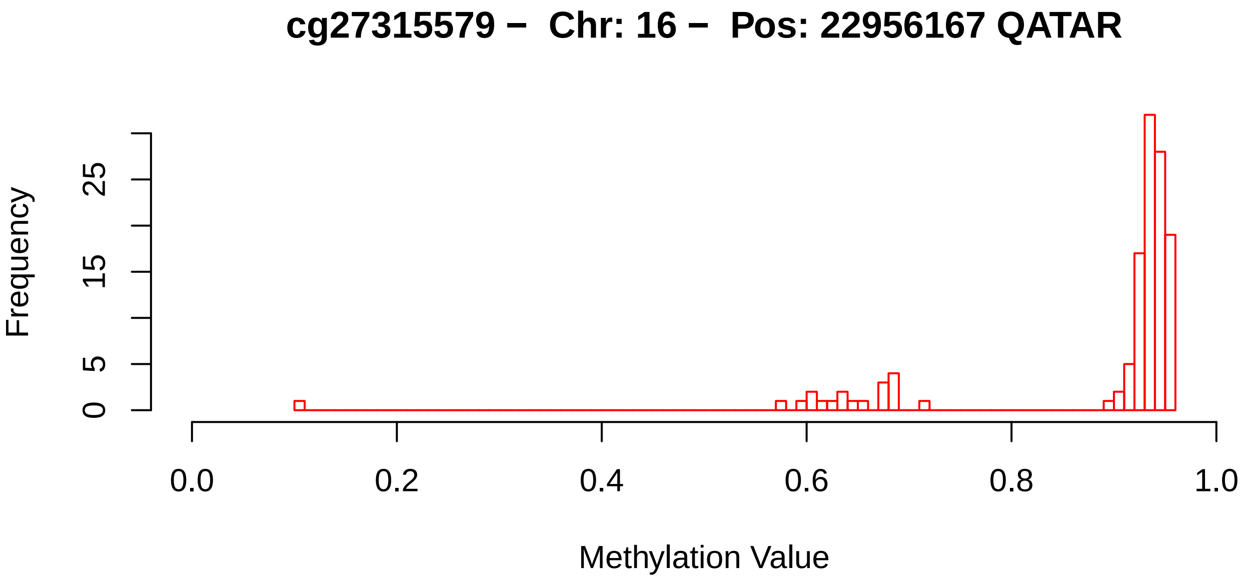
cg27315579 − Chr: 16 − Pos: 22956167 QATAR
Frequency
0
5
15
25
0.0
0.2
0.4
0.6
0.8
1.0
Methylation Value

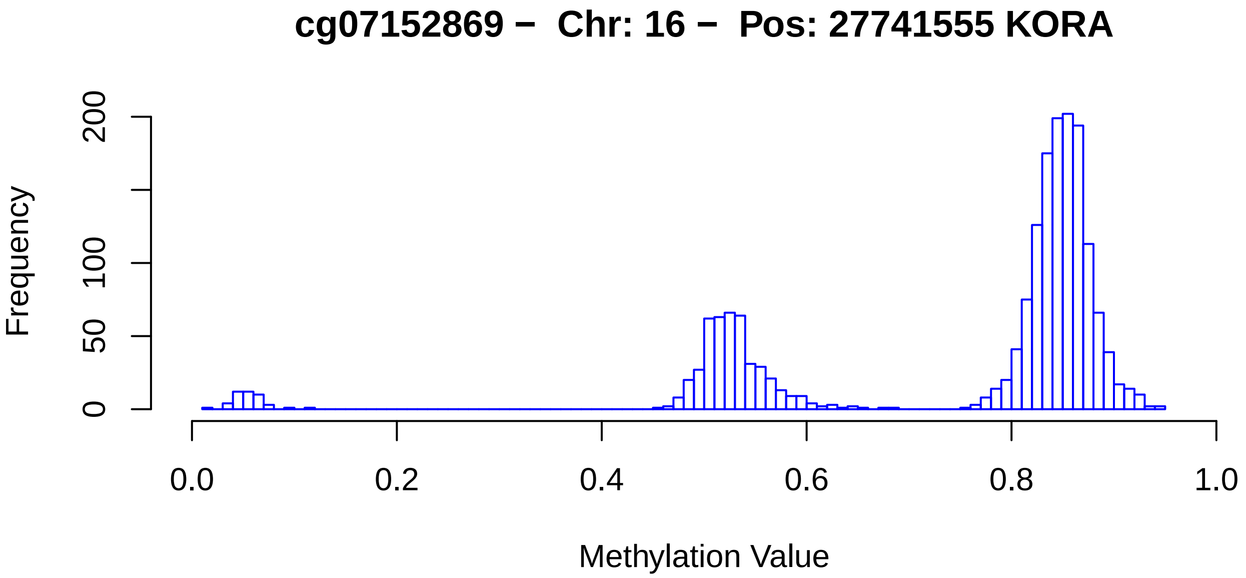
cg07152869 − Chr: 16 − Pos: 27741555 KORA
Frequency
0
50
100
200
0.0
0.2
0.4
0.6
0.8
1.0
Methylation Value

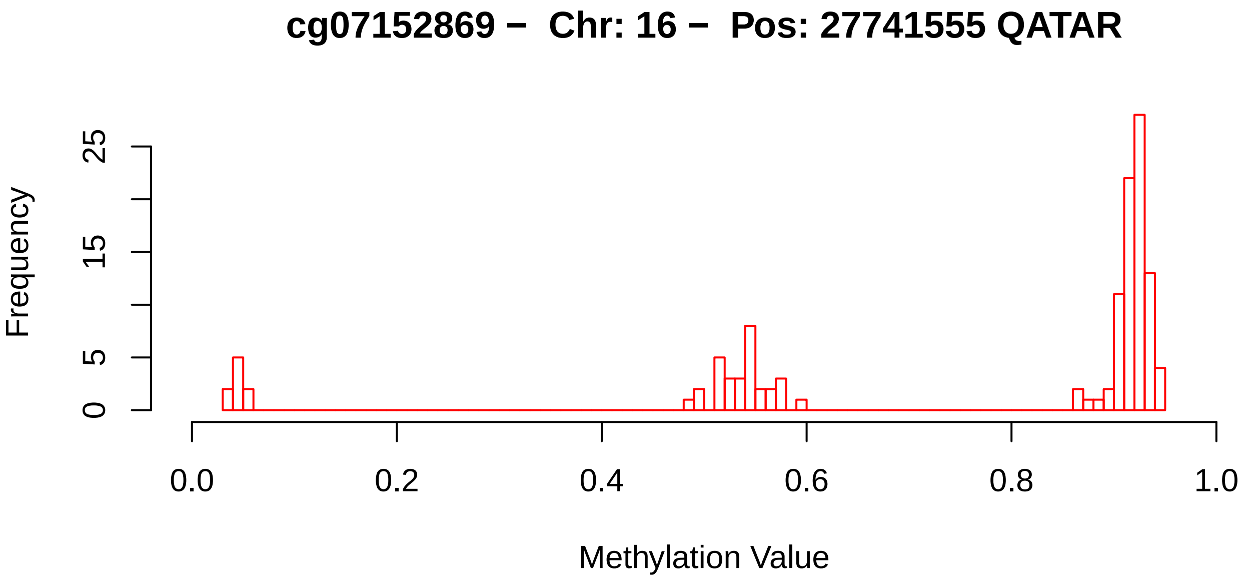
cg07152869 − Chr: 16 − Pos: 27741555 QATAR
Frequency
0
5
15
25
0.0
0.2
0.4
0.6
0.8
1.0
Methylation Value

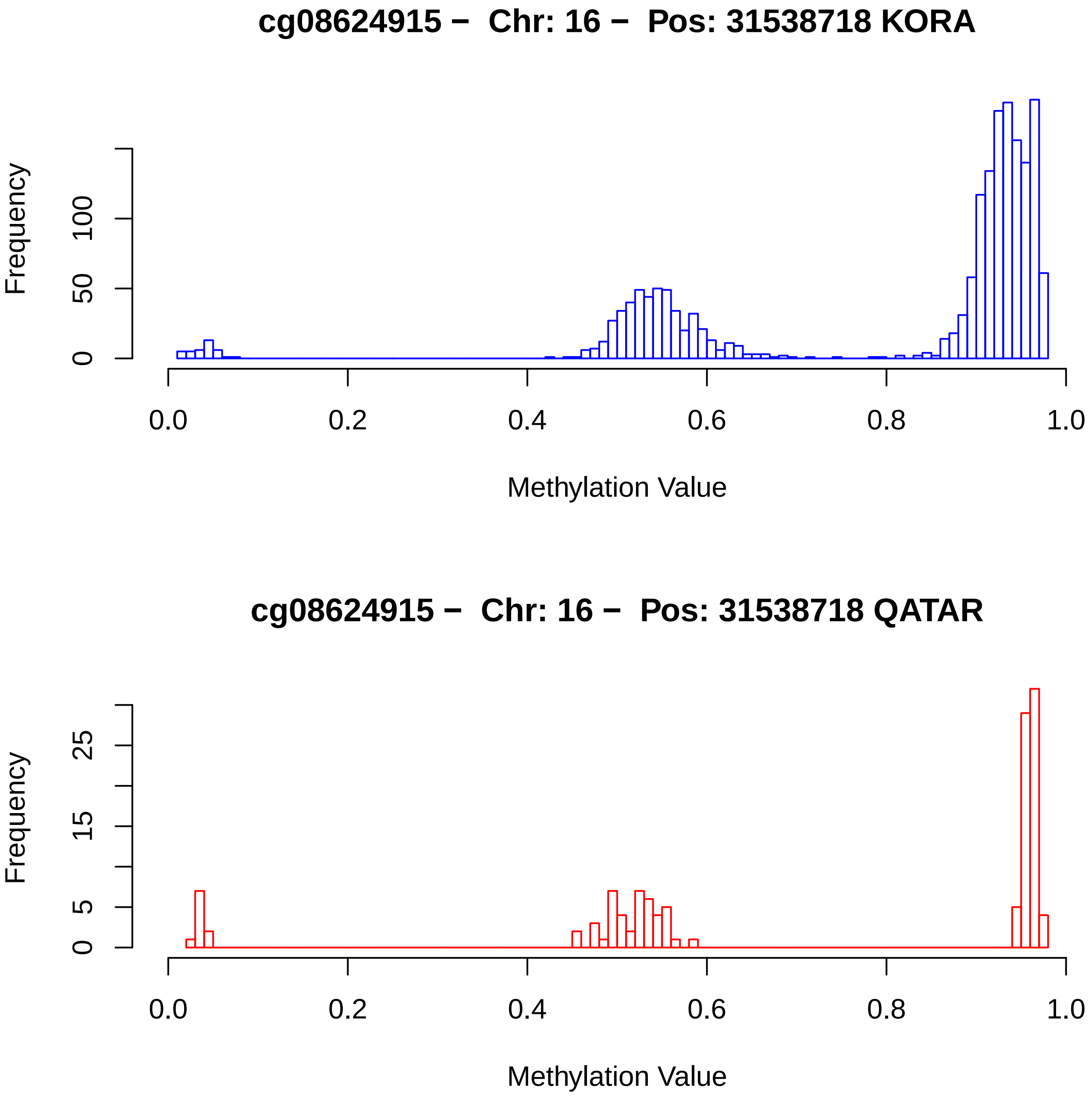
cg08624915 − Chr: 16 − Pos: 31538718 KORA
Frequency
0
50
100
0.0
0.2
0.4
0.6
0.8
1.0
Methylation Value
cg08624915 − Chr: 16 − Pos: 31538718 QATAR
Frequency
0
5
15
25
0.0
0.2
0.4
0.6
0.8
1.0
Methylation Value

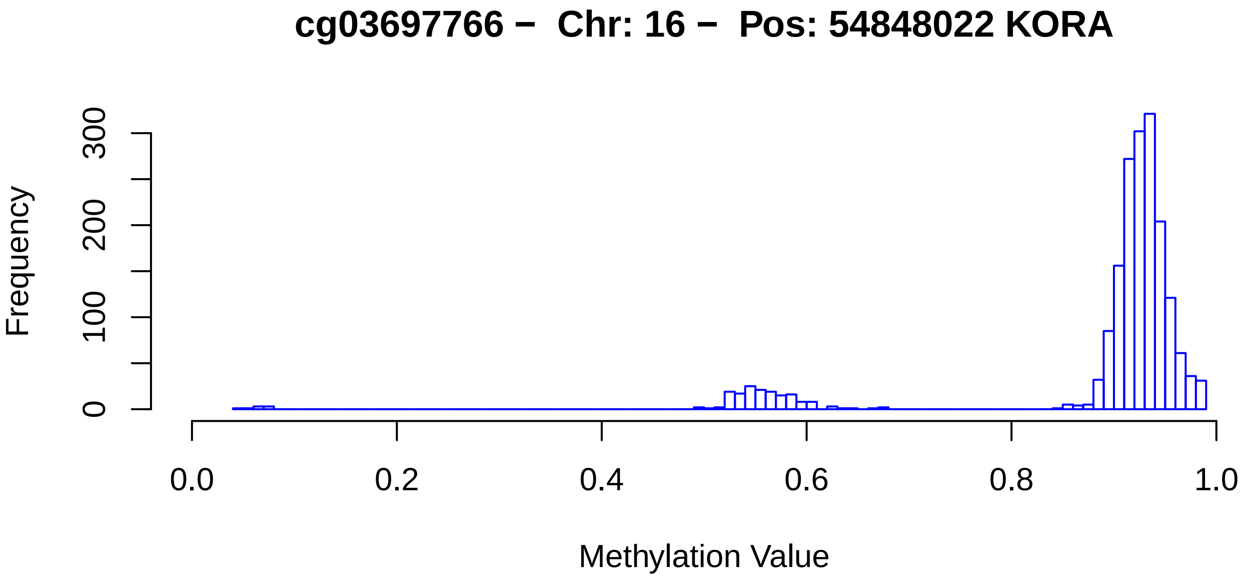

cg03697766 − Chr: 16 − Pos: 54848022 KORA
Frequency
0
100
200
300
0.0
0.2
0.4
0.6
0.8
1.0
Methylation Value

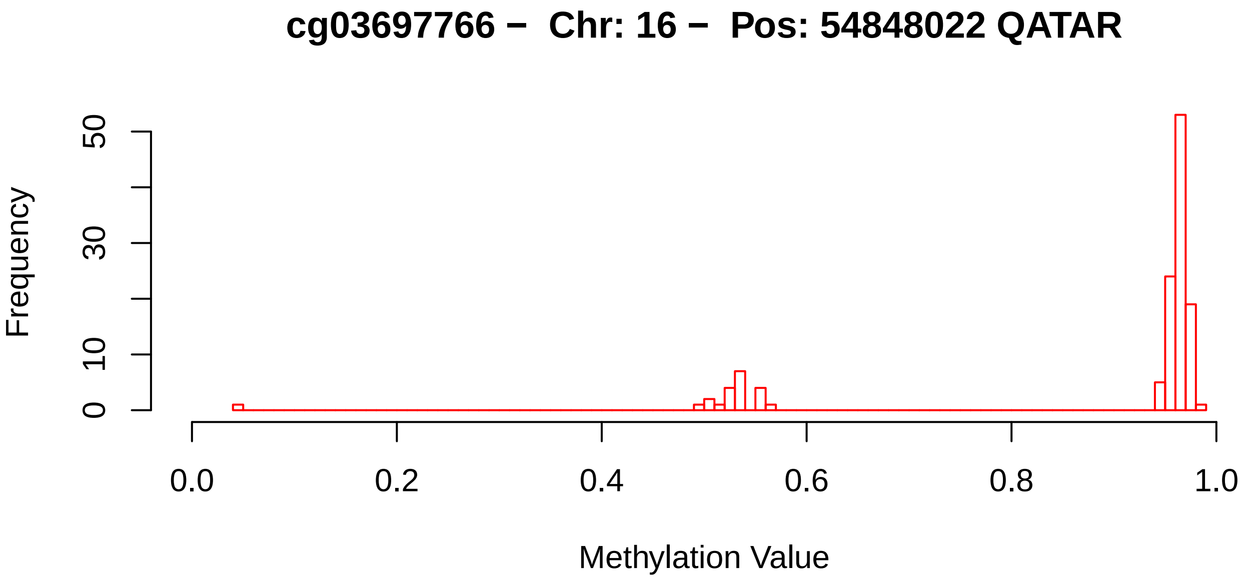

cg03697766 − Chr: 16 − Pos: 54848022 QATAR
Frequency
0
10
30
50
0.0
0.2
0.4
0.6
0.8
1.0
Methylation Value

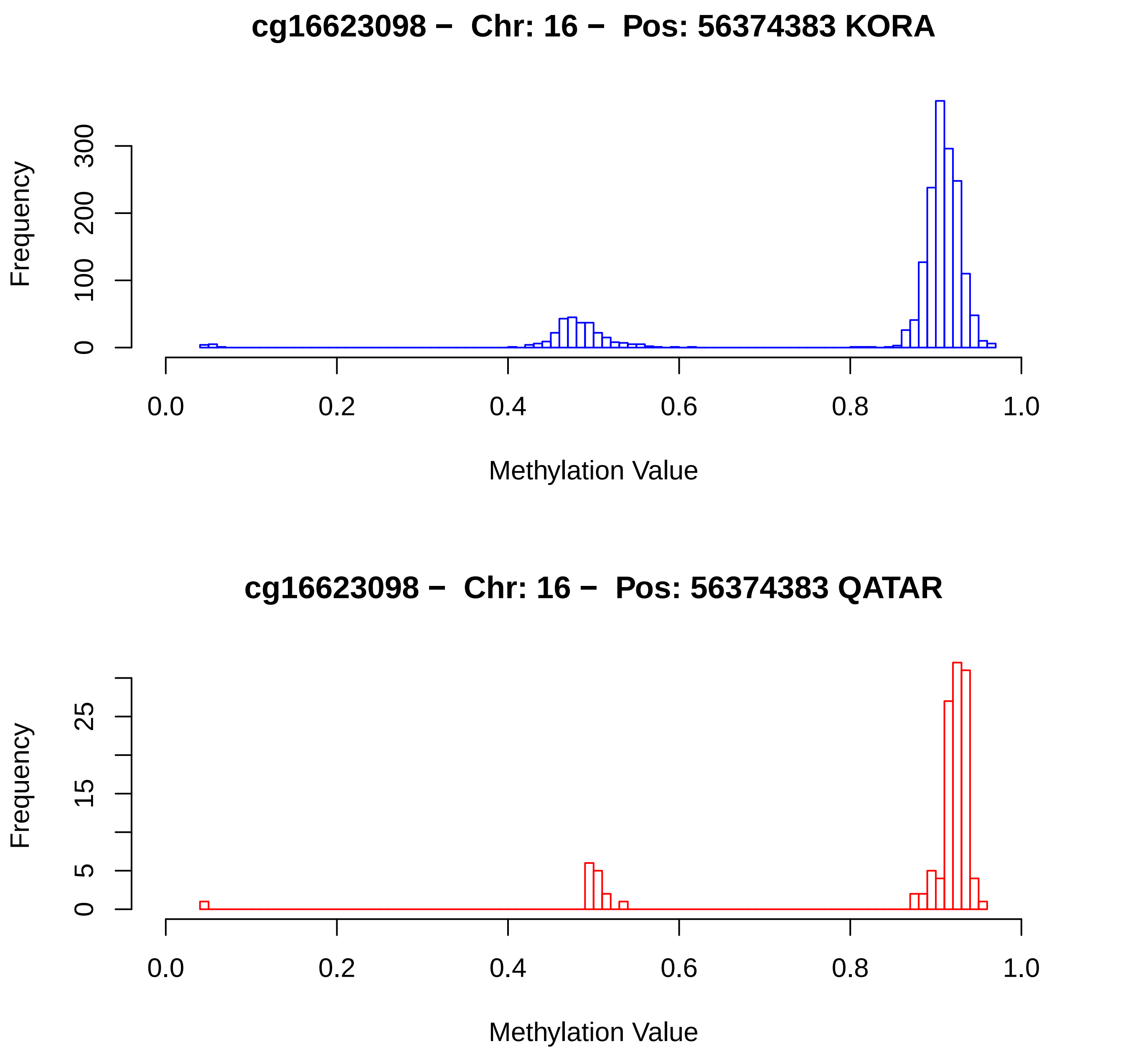
cg16623098 − Chr: 16 − Pos: 56374383 KORA
Frequency
0
100
200
300
0.0
0.2
0.4
0.6
0.8
1.0
Methylation Value
cg16623098 − Chr: 16 − Pos: 56374383 QATAR
Frequency
0
5
15
25
0.0
0.2
0.4
0.6
0.8
1.0
Methylation Value

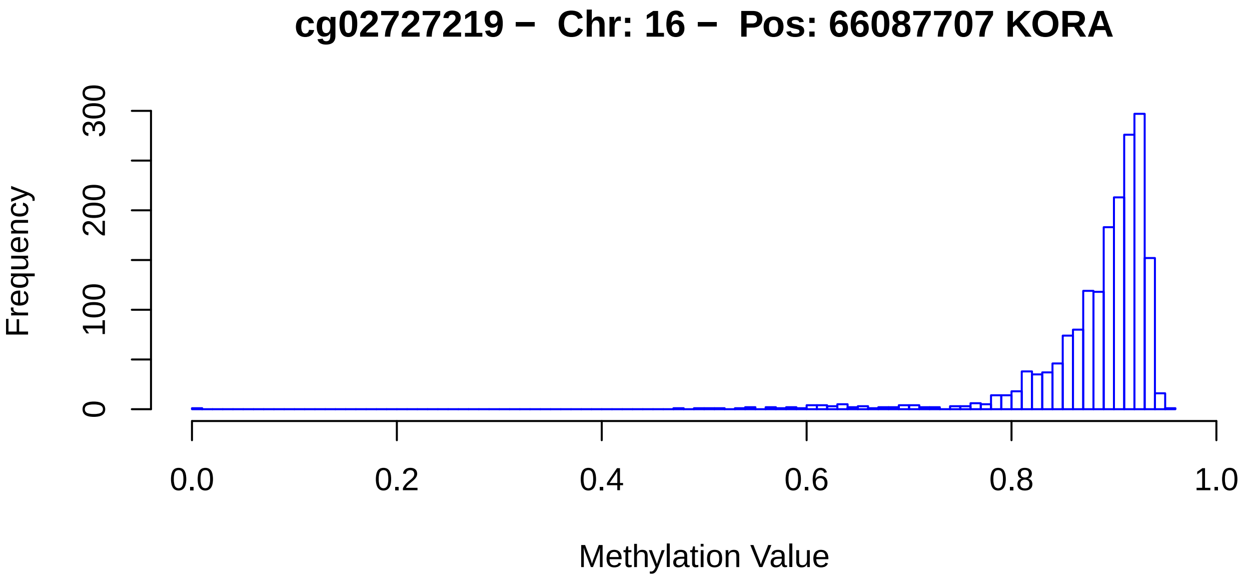
cg02727219 − Chr: 16 − Pos: 66087707 KORA
Frequency
0
100
200
300
0.0
0.2
0.4
0.6
0.8
1.0
Methylation Value

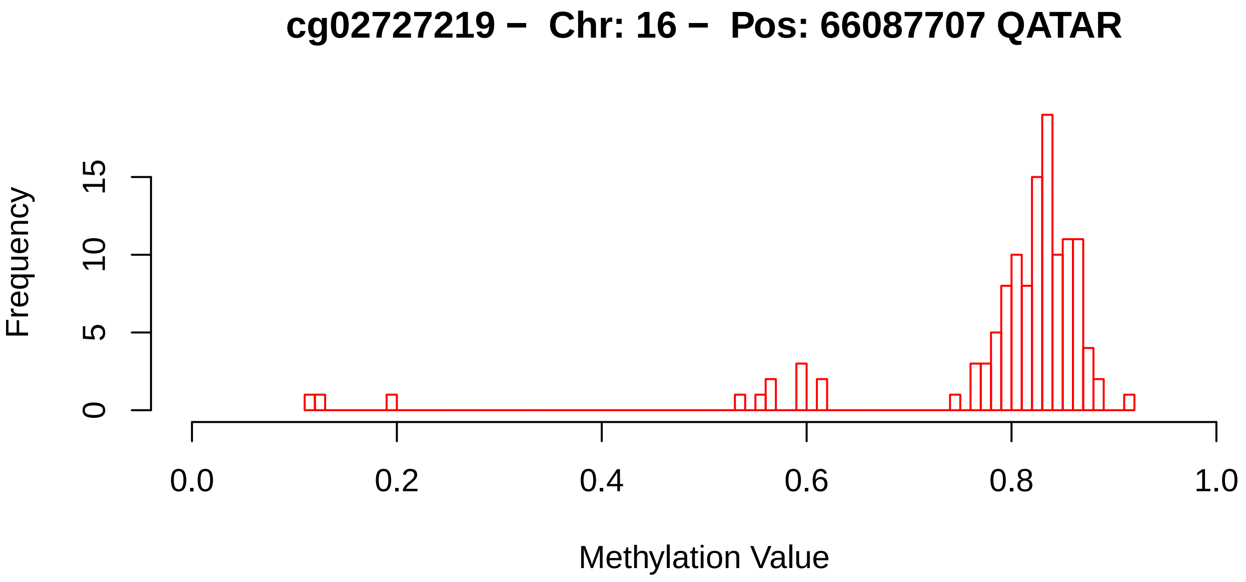
cg02727219 − Chr: 16 − Pos: 66087707 QATAR
Frequency
0
5
10
15
0.0
0.2
0.4
0.6
0.8
1.0
Methylation Value

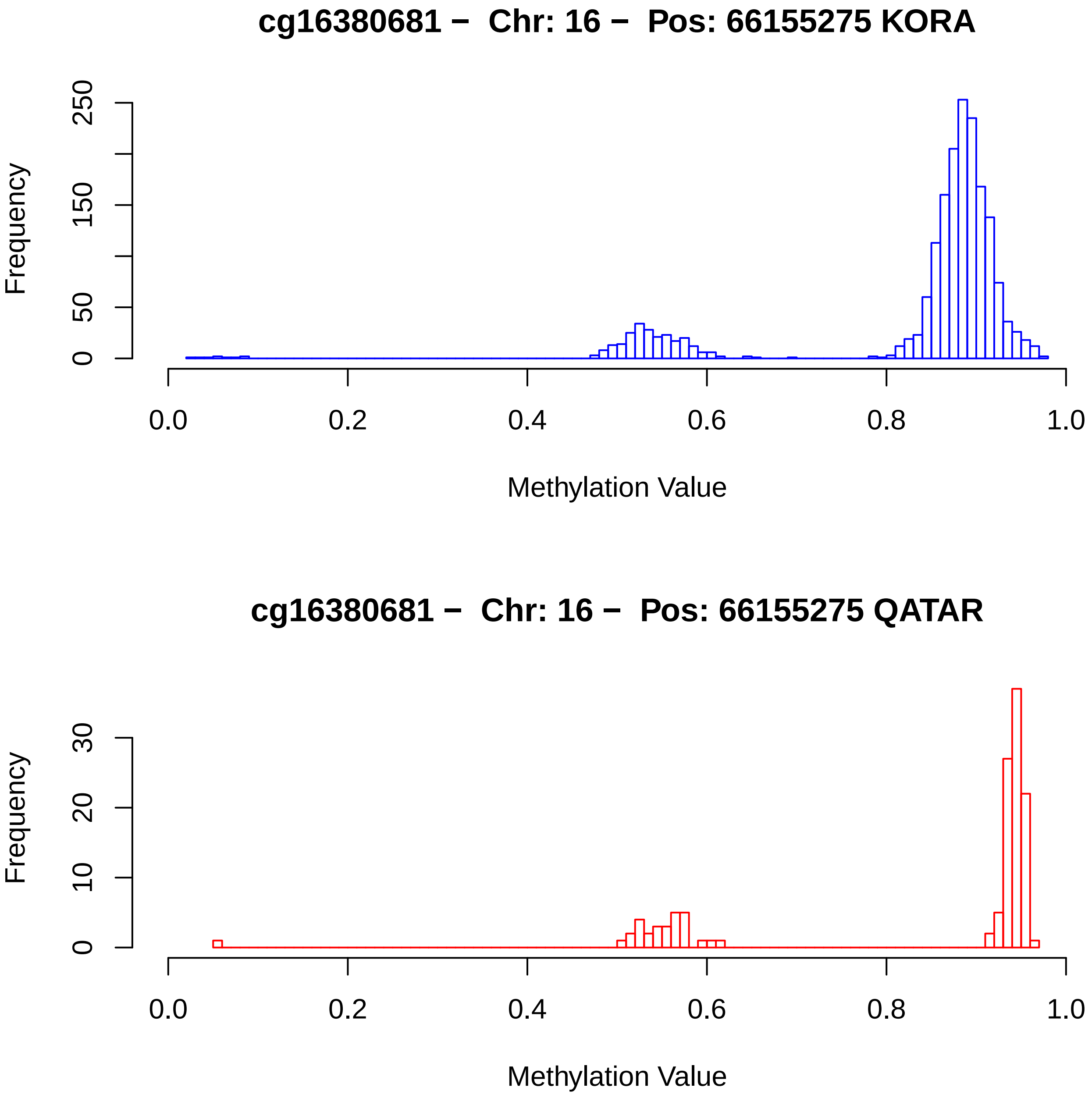

cg16380681 − Chr: 16 − Pos: 66155275 KORA
Frequency
0
50
150
250
0.0
0.2
0.4
0.6
0.8
1.0
Methylation Value
cg16380681 − Chr: 16 − Pos: 66155275 QATAR
Frequency
0
10
20
30
0.0
0.2
0.4
0.6
0.8
1.0
Methylation Value

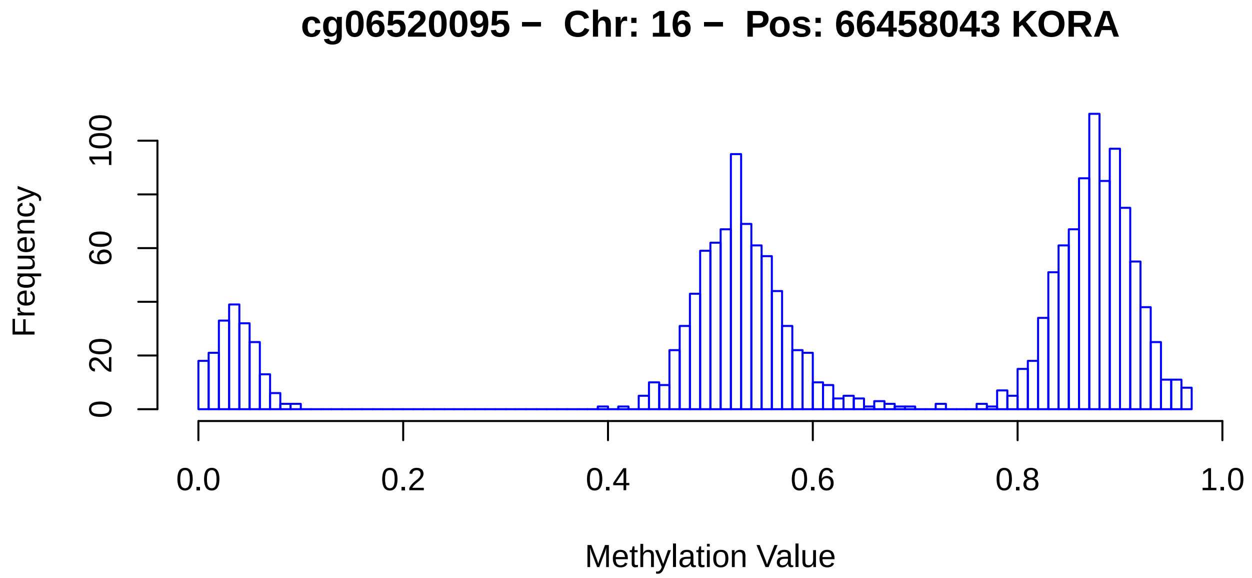
cg06520095 − Chr: 16 − Pos: 66458043 KORA
Frequency
0
20
60
100
0.0
0.2
0.4
0.6
0.8
1.0
Methylation Value

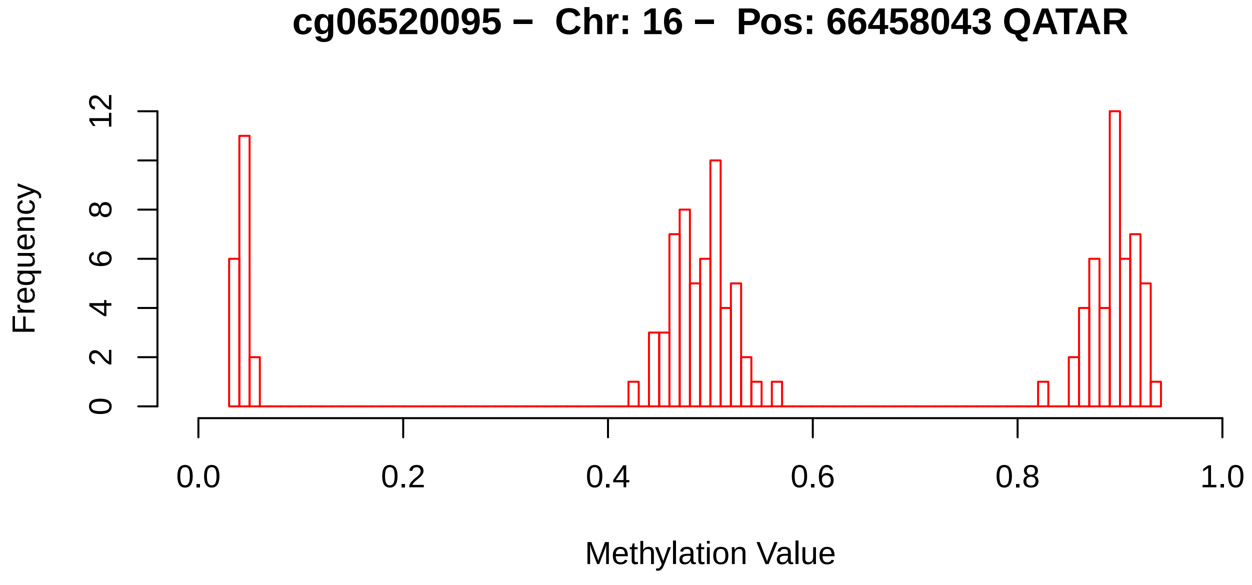
cg06520095 − Chr: 16 − Pos: 66458043 QATAR
Frequency
0
2
4
6
8
12
0.0
0.2
0.4
0.6
0.8
1.0
Methylation Value

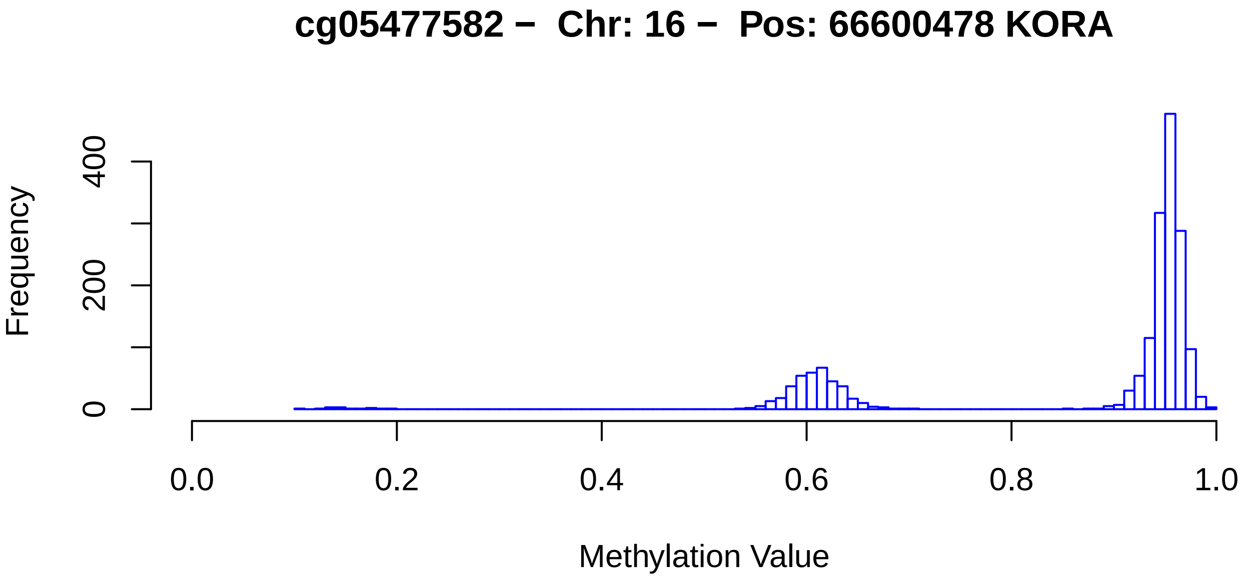
cg05477582 − Chr: 16 − Pos: 66600478 KORA
Frequency
0
200
400
0.0
0.2
0.4
0.6
0.8
1.0
Methylation Value

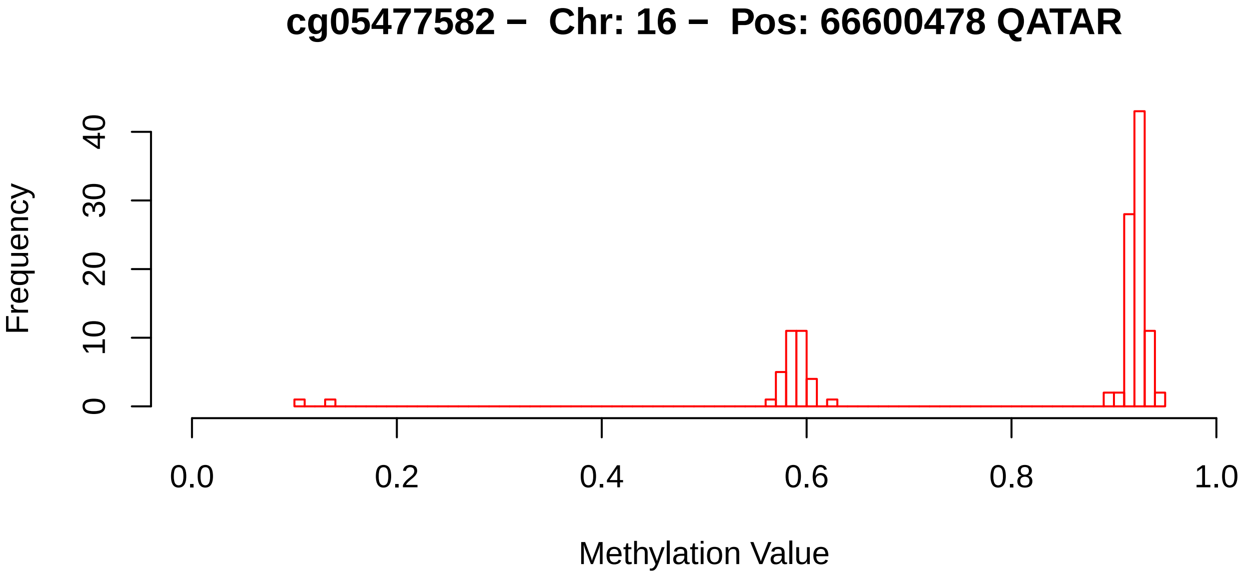
cg05477582 − Chr: 16 − Pos: 66600478 QATAR
Frequency
0
10
20
30
40
0.0
0.2
0.4
0.6
0.8
1.0
Methylation Value

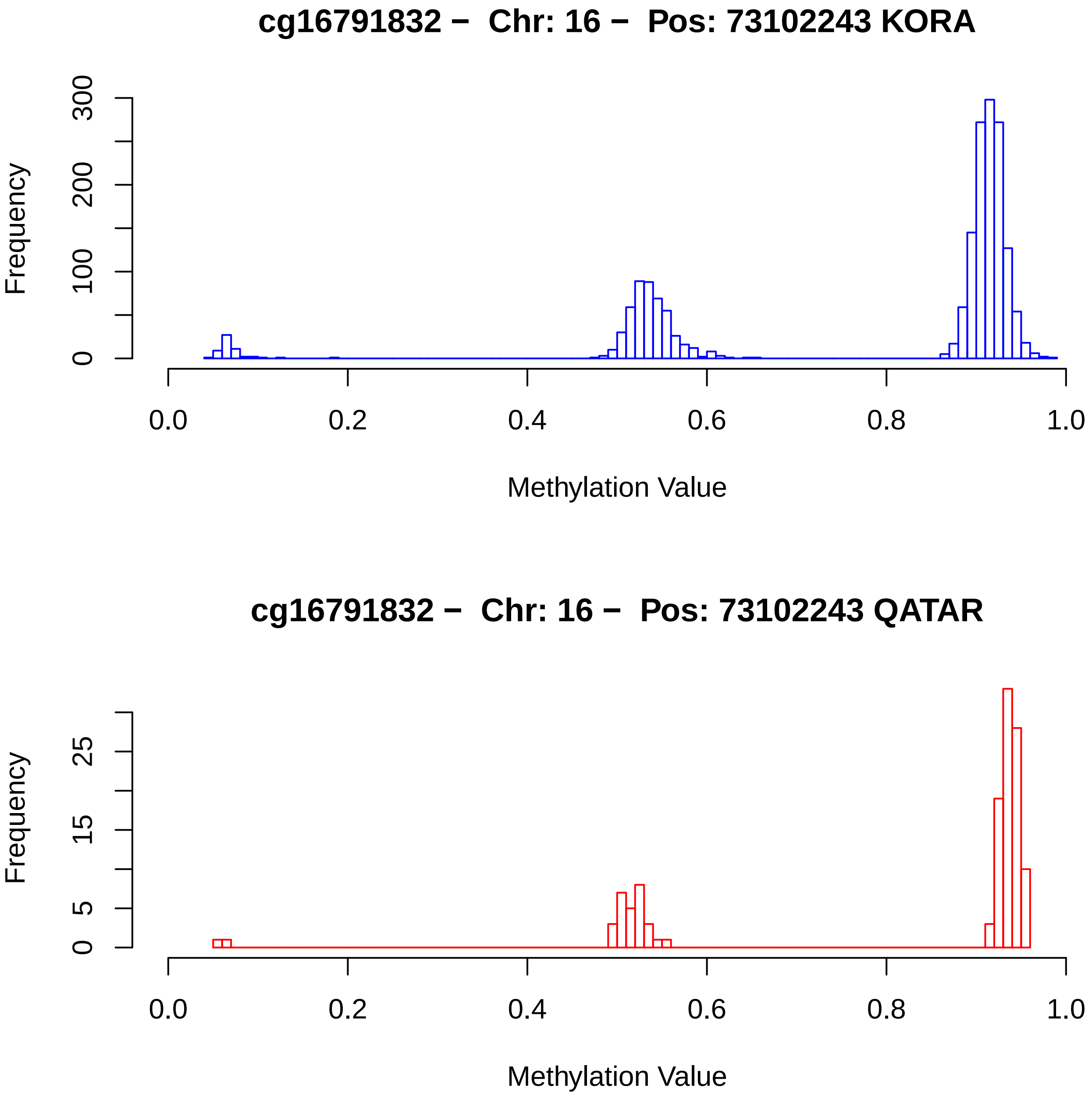

cg16791832 − Chr: 16 − Pos: 73102243 KORA
Frequency
0
100
200
300
0.0
0.2
0.4
0.6
0.8
1.0
Methylation Value
cg16791832 − Chr: 16 − Pos: 73102243 QATAR
Frequency
0
5
15
25
0.0
0.2
0.4
0.6
0.8
1.0
Methylation Value

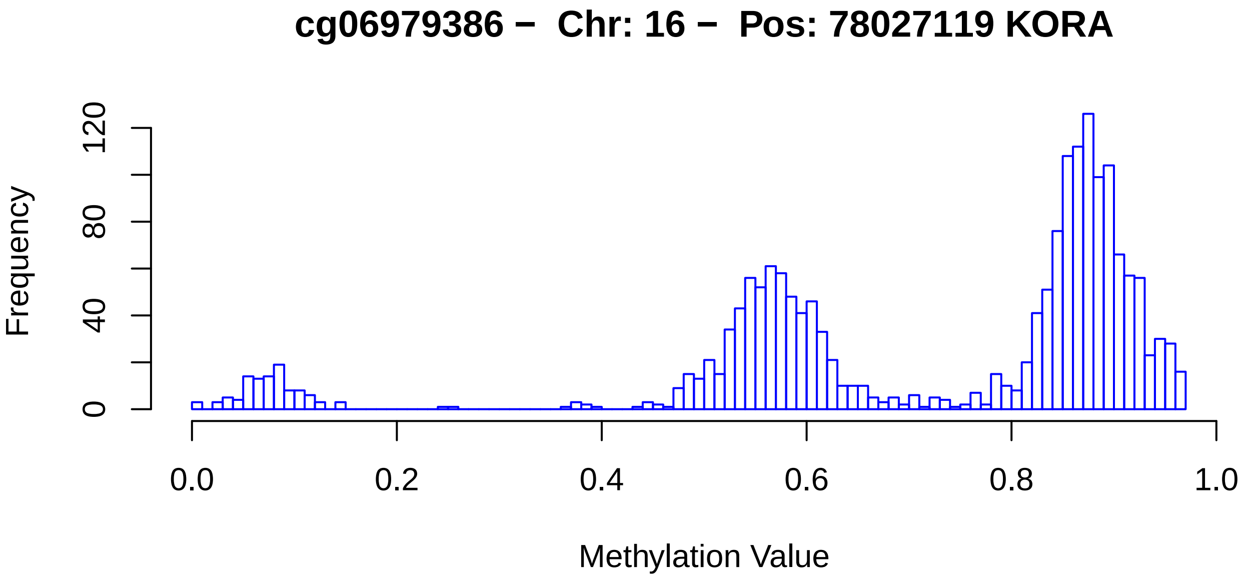
cg06979386 − Chr: 16 − Pos: 78027119 KORA
Frequency
0
40
80
120
0.0
0.2
0.4
0.6
0.8
1.0
Methylation Value

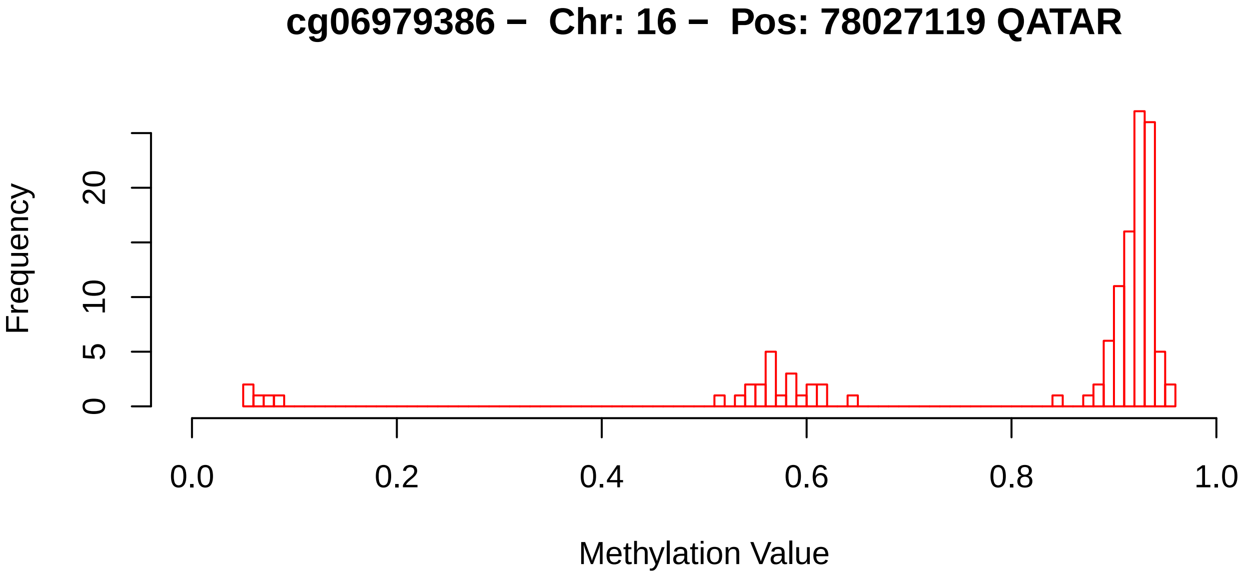
cg06979386 − Chr: 16 − Pos: 78027119 QATAR
Frequency
0
5
10
20
0.0
0.2
0.4
0.6
0.8
1.0
Methylation Value

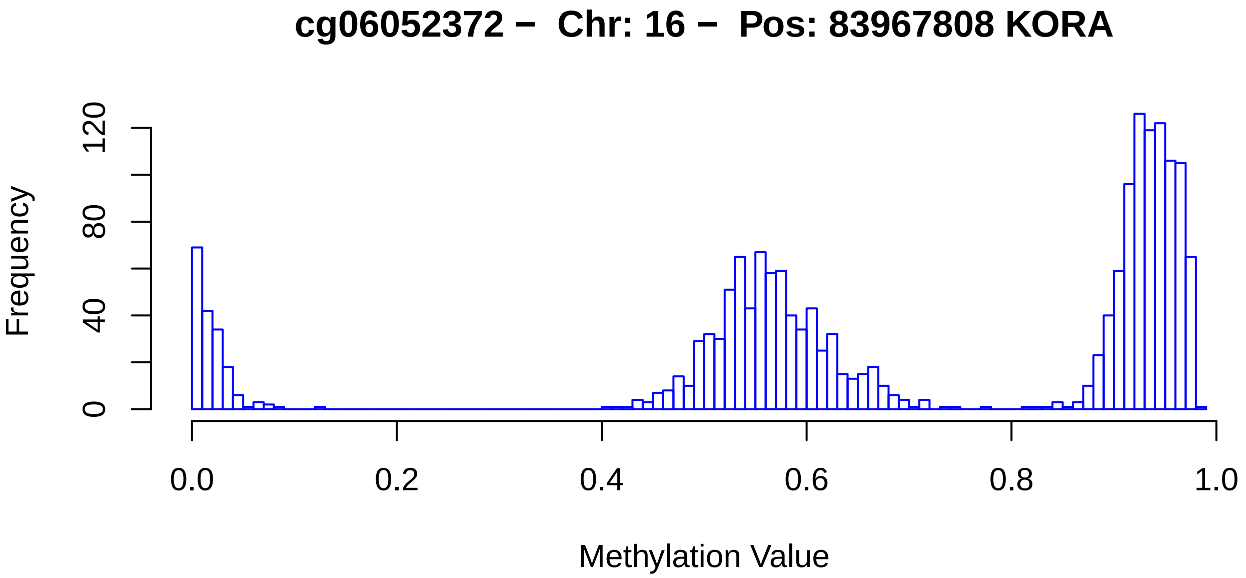

cg06052372 − Chr: 16 − Pos: 83967808 KORA
Frequency
0
40
80
120
0.0
0.2
0.4
0.6
0.8
1.0
Methylation Value

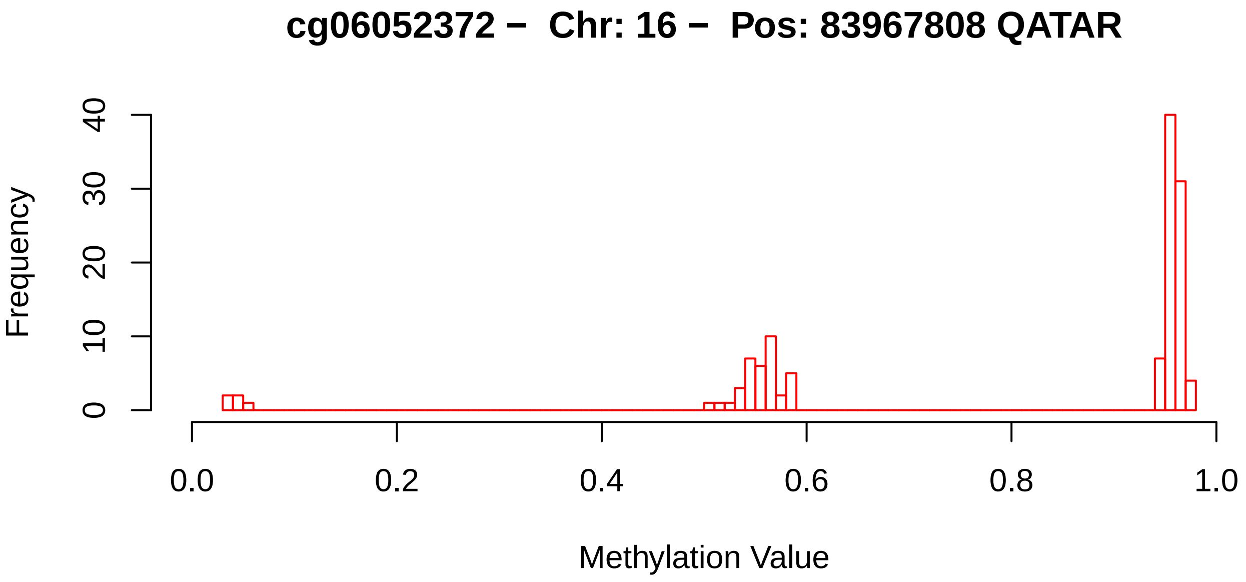

cg06052372 − Chr: 16 − Pos: 83967808 QATAR
Frequency
0
10
20
30
40
0.0
0.2
0.4
0.6
0.8
1.0
Methylation Value

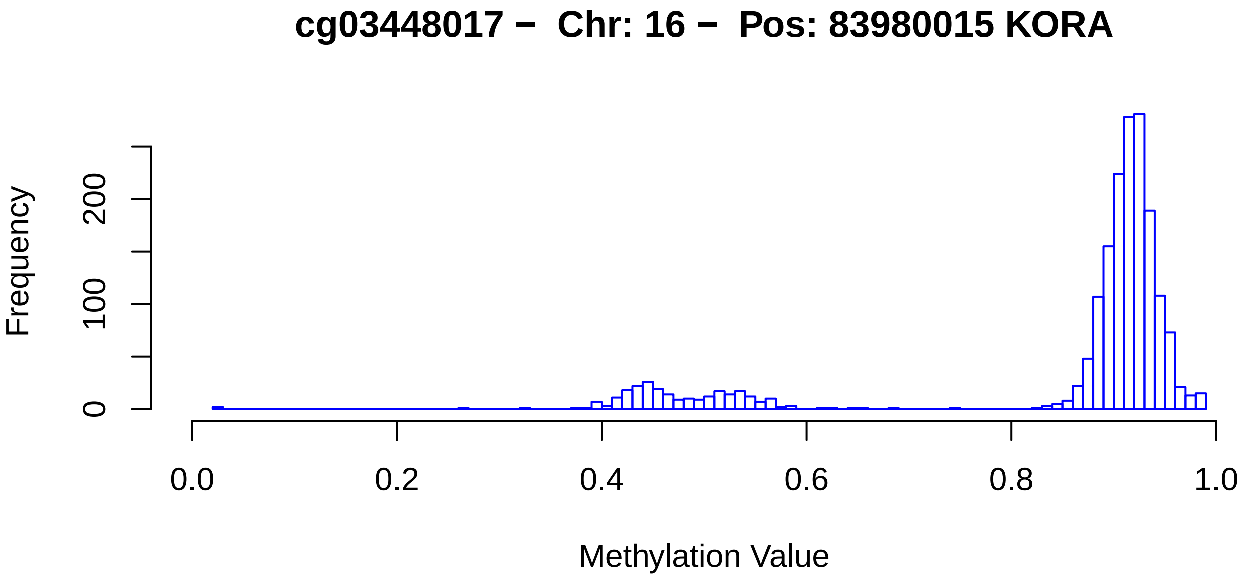
cg03448017 − Chr: 16 − Pos: 83980015 KORA
Frequency
0
100
200
0.0
0.2
0.4
0.6
0.8
1.0
Methylation Value

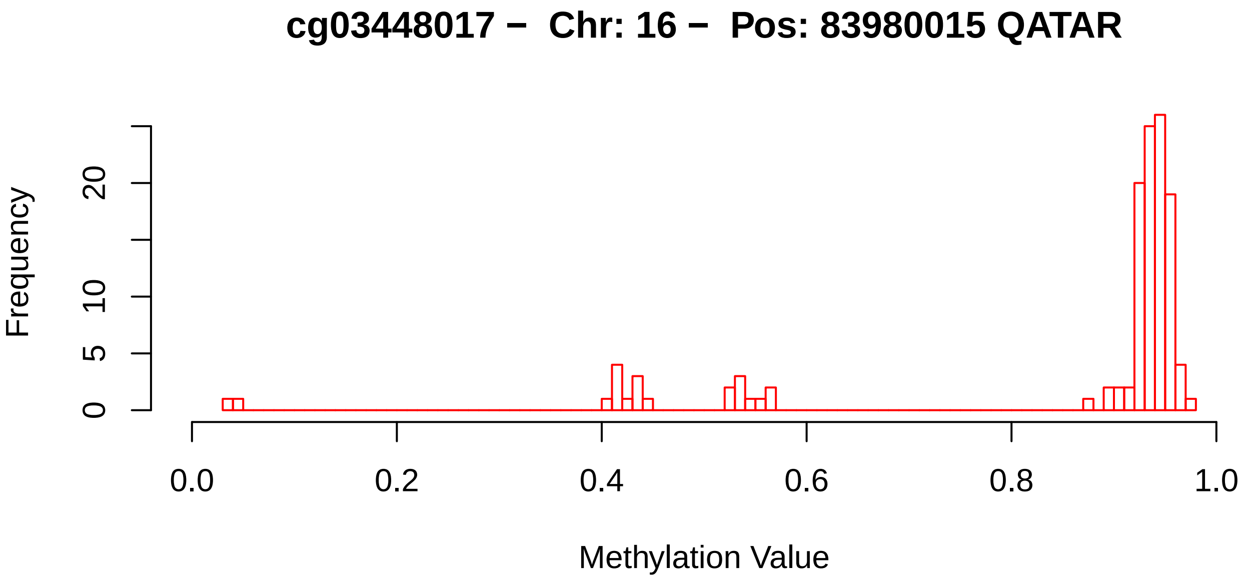
cg03448017 − Chr: 16 − Pos: 83980015 QATAR
Frequency
0
5
10
20
0.0
0.2
0.4
0.6
0.8
1.0
Methylation Value

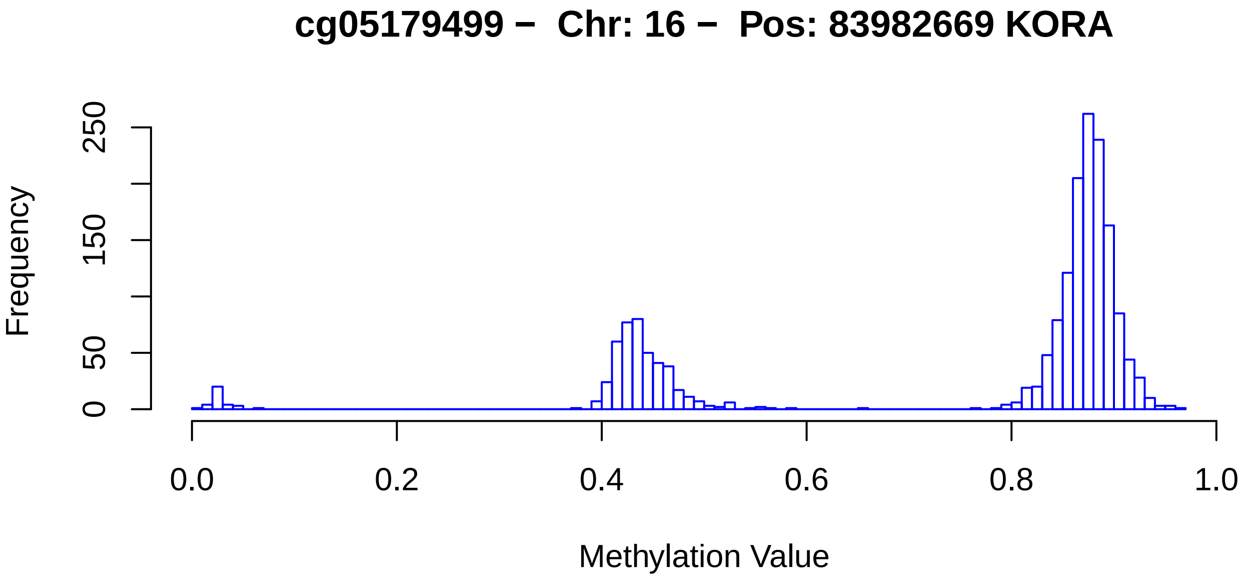
cg05179499 − Chr: 16 − Pos: 83982669 KORA
Frequency
0
50
150
250
0.0
0.2
0.4
0.6
0.8
1.0
Methylation Value

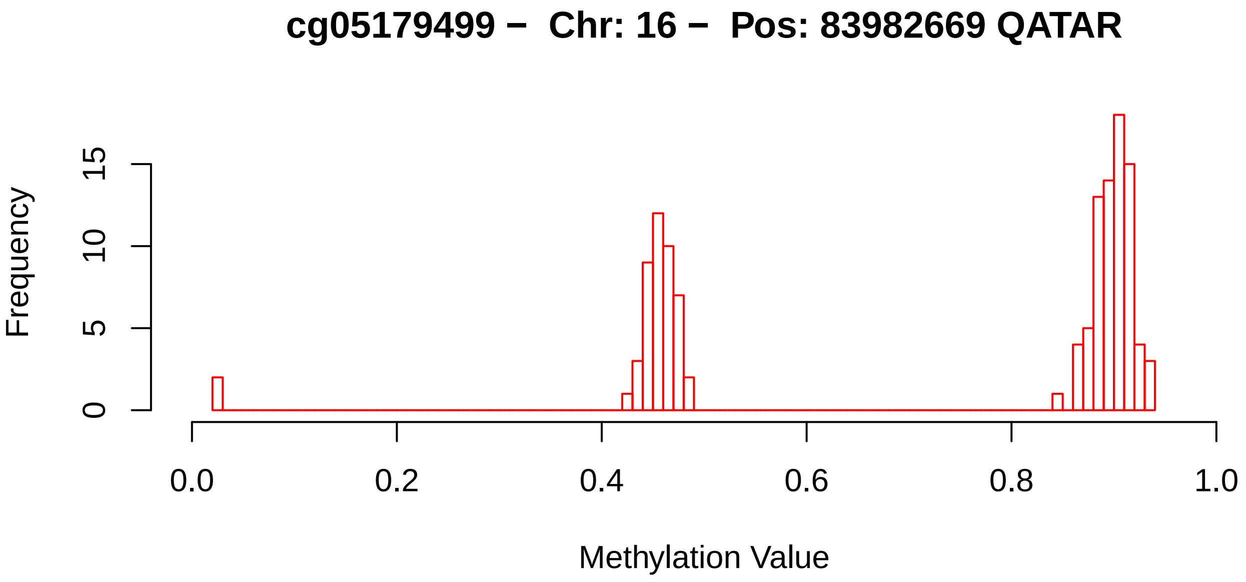
cg05179499 − Chr: 16 − Pos: 83982669 QATAR
Frequency
0
5
10
15
0.0
0.2
0.4
0.6
0.8
1.0
Methylation Value

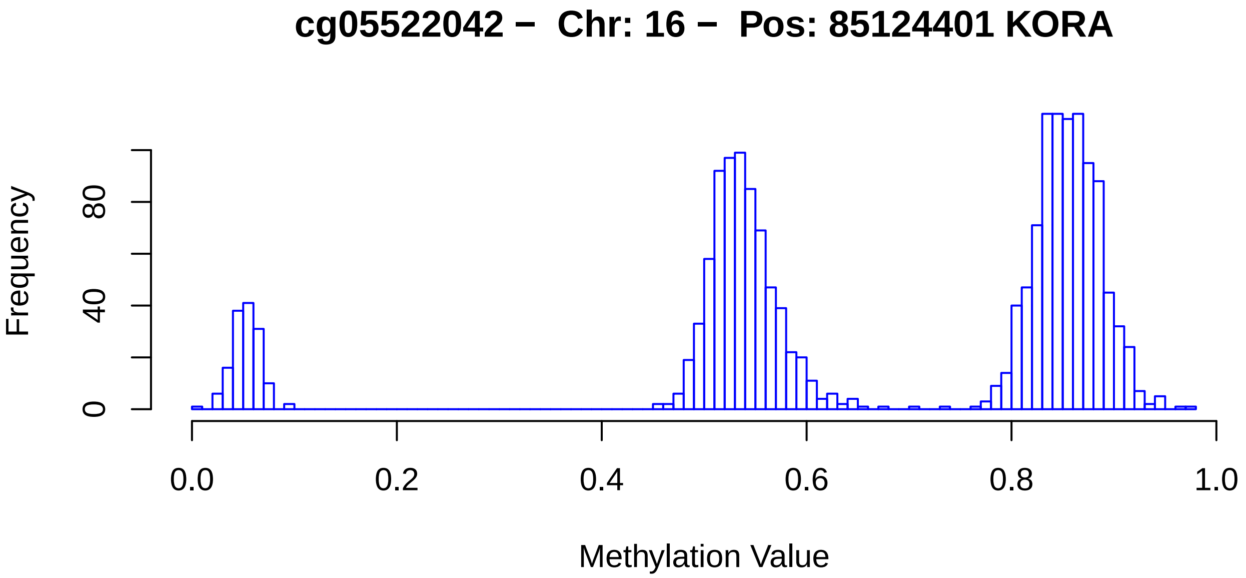
cg05522042 − Chr: 16 − Pos: 85124401 KORA
Frequency
0
40
80
0.0
0.2
0.4
0.6
0.8
1.0
Methylation Value

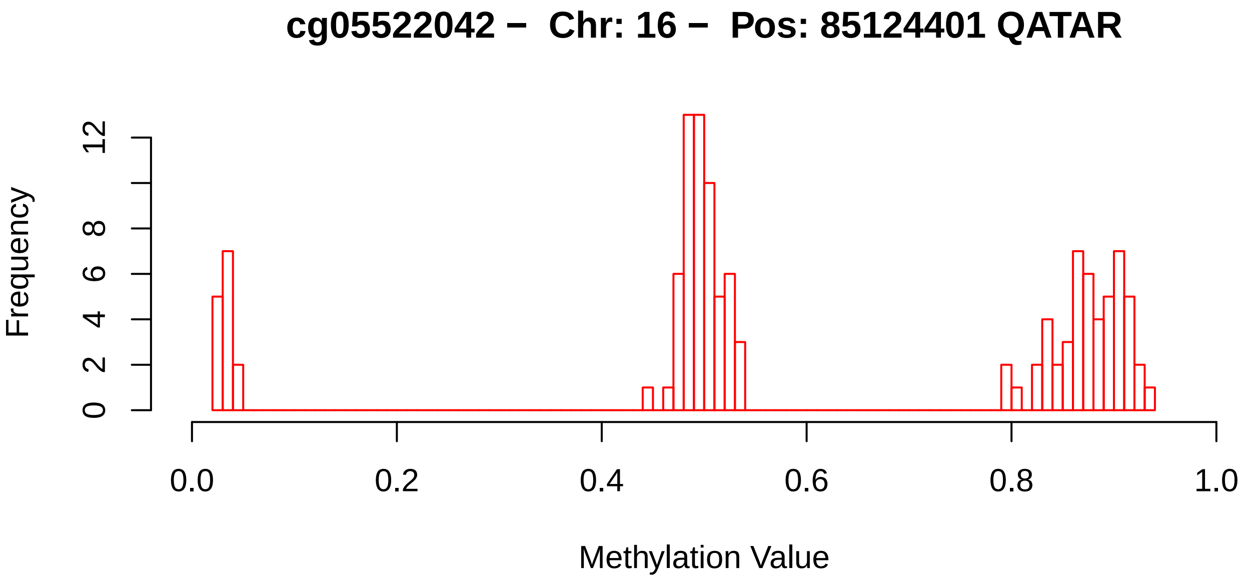
cg05522042 − Chr: 16 − Pos: 85124401 QATAR
Frequency
0
2
4
6
8
12
0.0
0.2
0.4
0.6
0.8
1.0
Methylation Value

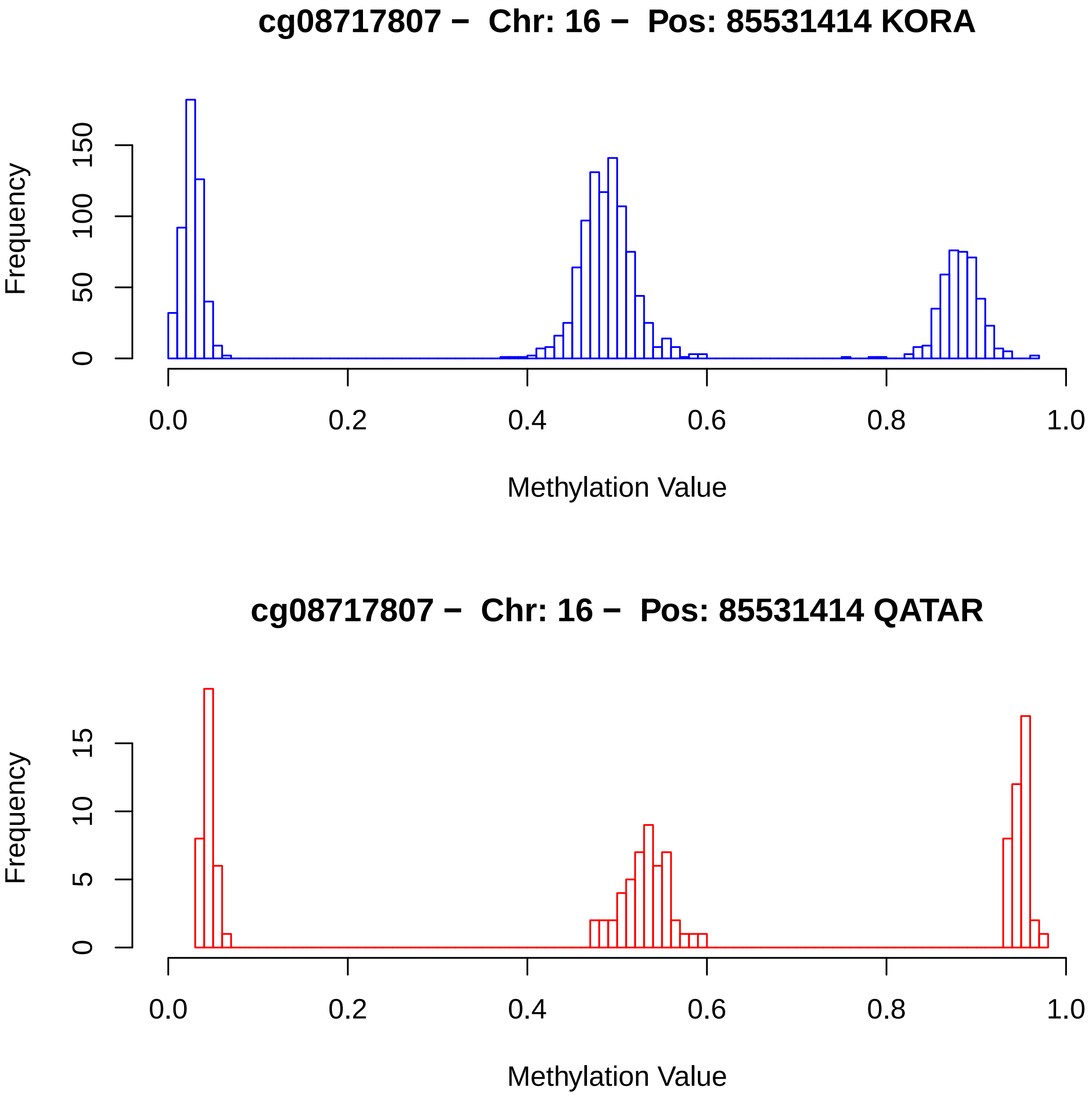
cg08717807 − Chr: 16 − Pos: 85531414 KORA
Frequency
0
50
100
150
0.0
0.2
0.4
0.6
0.8
1.0
Methylation Value
cg08717807 − Chr: 16 − Pos: 85531414 QATAR
Frequency
0
5
10
15
0.0
0.2
0.4
0.6
0.8
1.0
Methylation Value

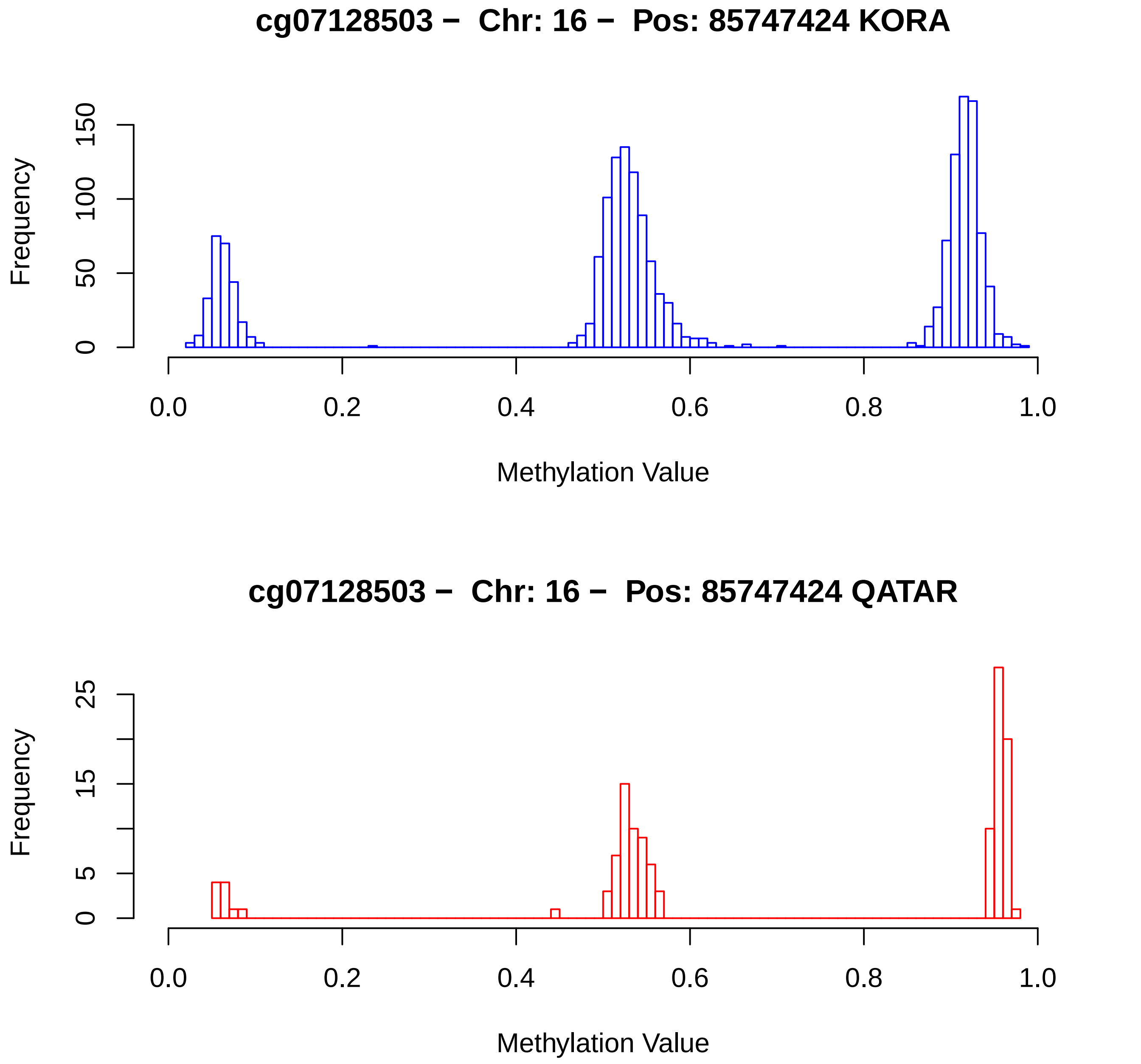
cg07128503 − Chr: 16 − Pos: 85747424 KORA
Frequency
0
50
100
150
0.0
0.2
0.4
0.6
0.8
1.0
Methylation Value
cg07128503 − Chr: 16 − Pos: 85747424 QATAR
Frequency
0
5
15
25
0.0
0.2
0.4
0.6
0.8
1.0
Methylation Value

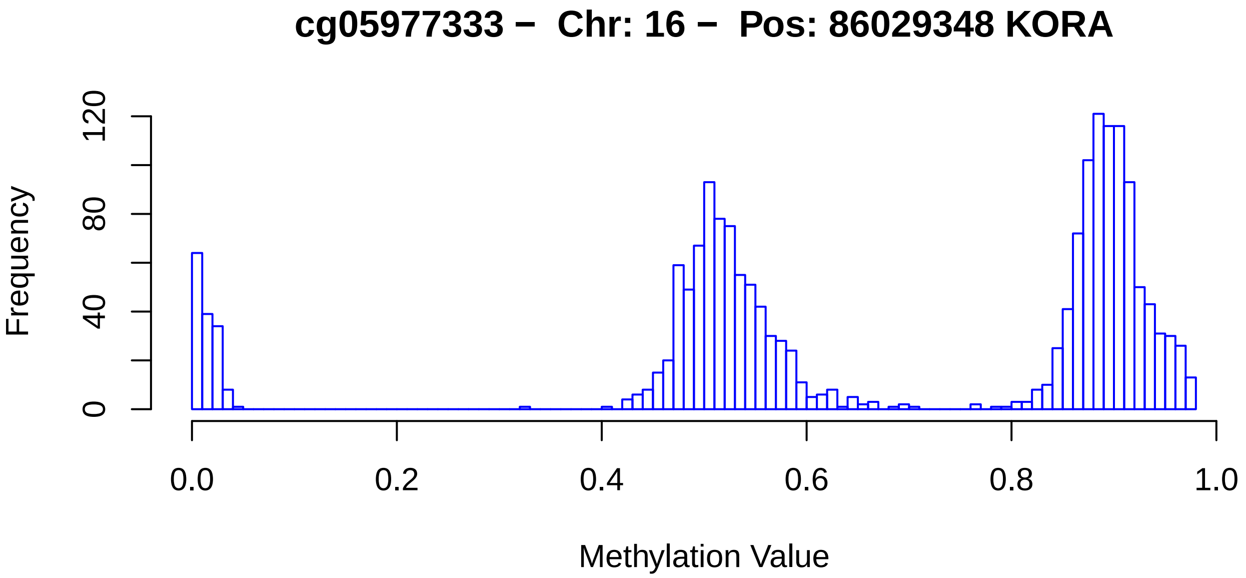
cg05977333 − Chr: 16 − Pos: 86029348 KORA
Frequency
0
40
80
120
0.0
0.2
0.4
0.6
0.8
1.0
Methylation Value

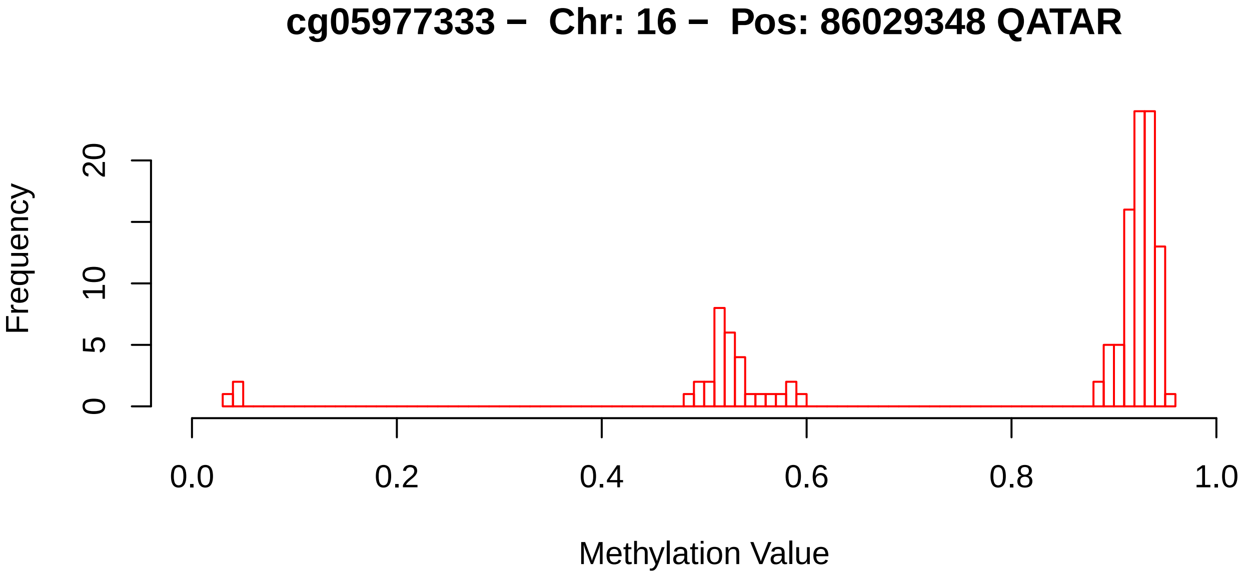
cg05977333 − Chr: 16 − Pos: 86029348 QATAR
Frequency
0
5
10
20
0.0
0.2
0.4
0.6
0.8
1.0
Methylation Value

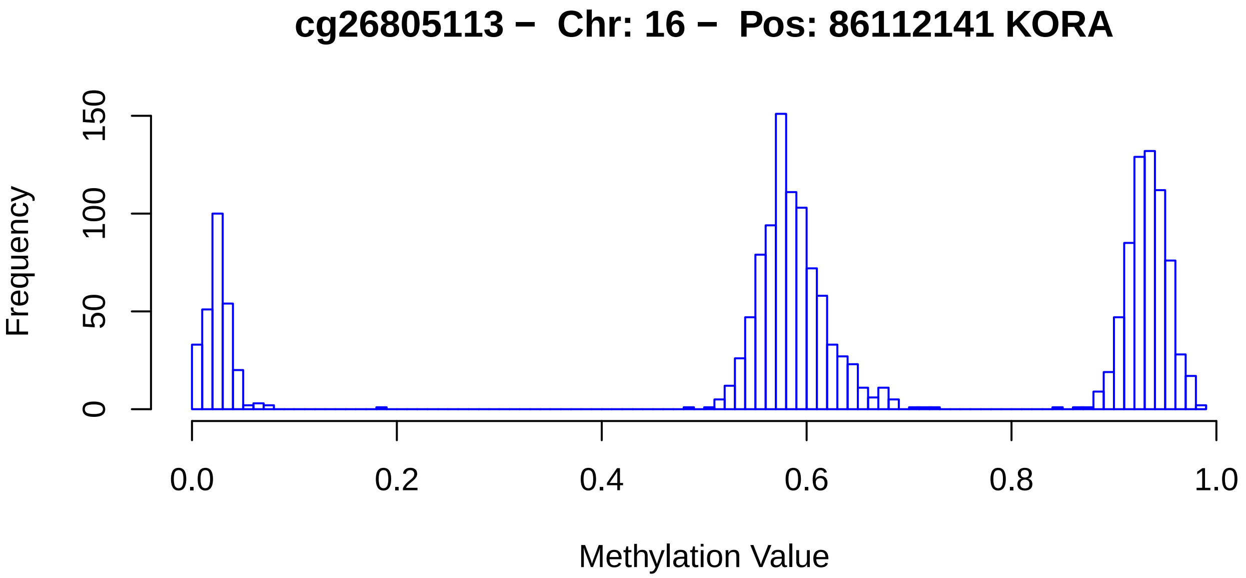
cg26805113 − Chr: 16 − Pos: 86112141 KORA
Frequency
0
50
100
150
0.0
0.2
0.4
0.6
0.8
1.0
Methylation Value

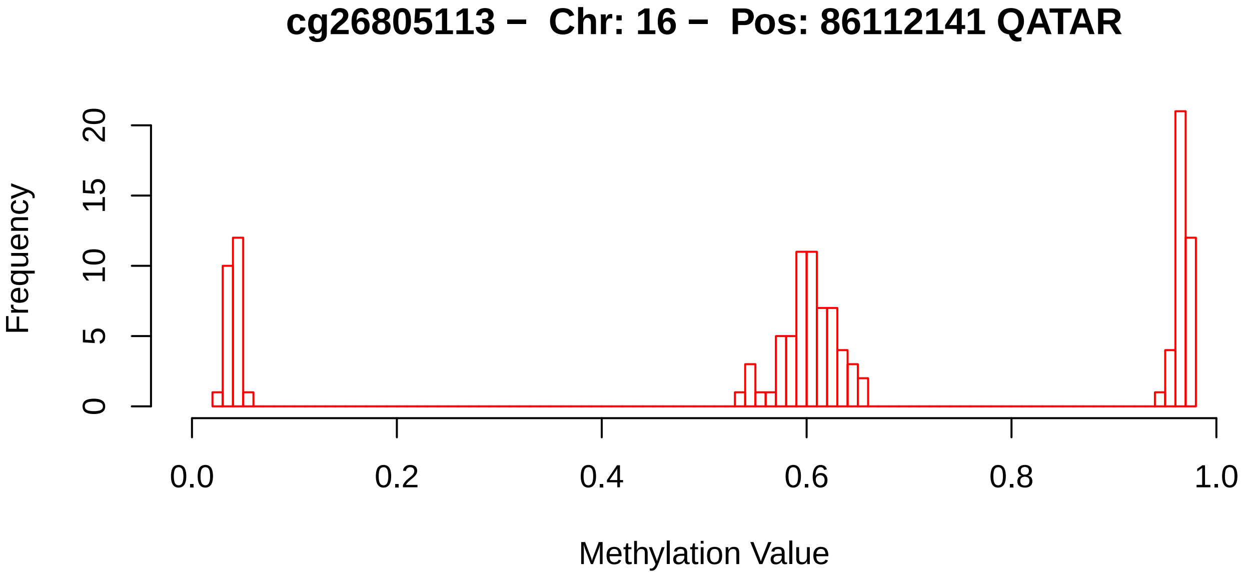
cg26805113 − Chr: 16 − Pos: 86112141 QATAR
Frequency
0
5
10
15
20
0.0
0.2
0.4
0.6
0.8
1.0
Methylation Value

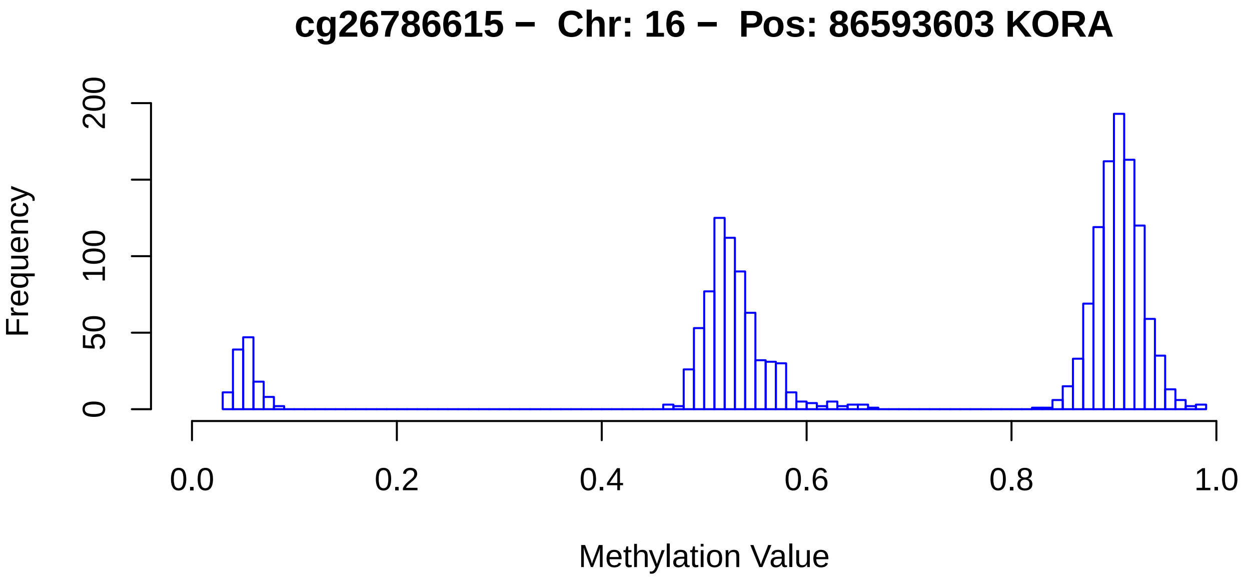
cg26786615 − Chr: 16 − Pos: 86593603 KORA
Frequency
0
50
100
200
0.0
0.2
0.4
0.6
0.8
1.0
Methylation Value

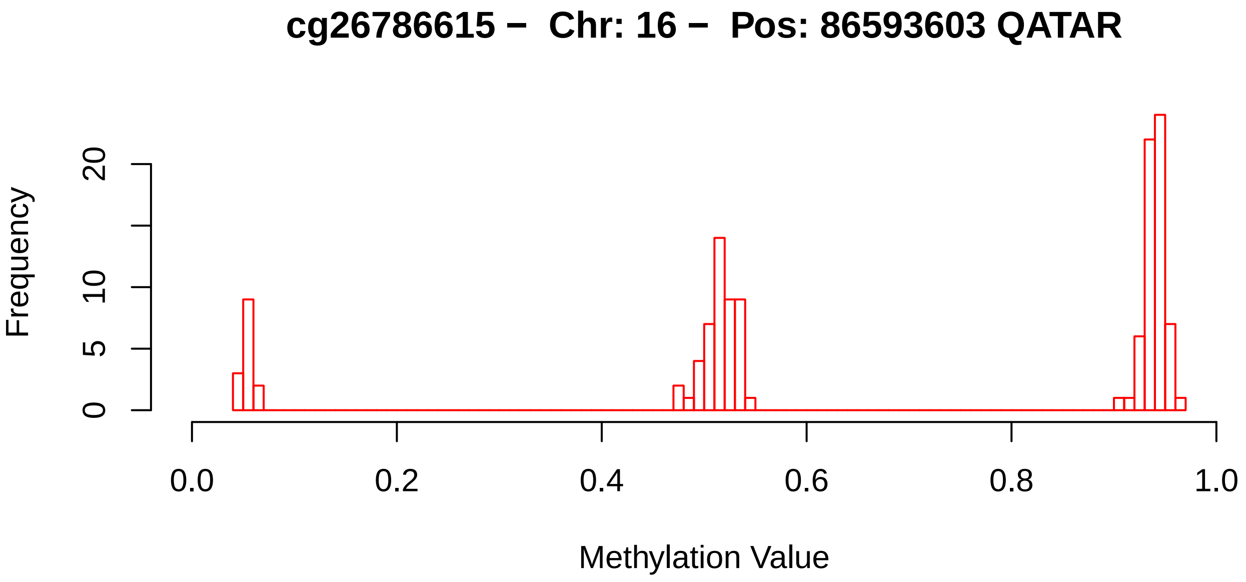
cg26786615 − Chr: 16 − Pos: 86593603 QATAR
Frequency
0
5
10
20
0.0
0.2
0.4
0.6
0.8
1.0
Methylation Value

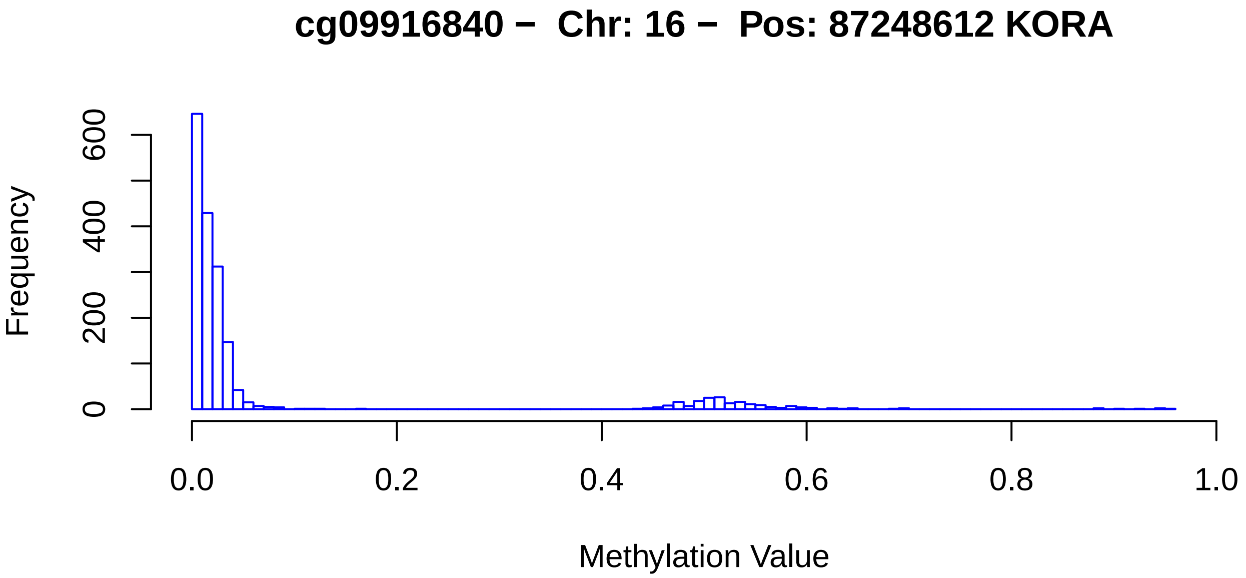
cg09916840 − Chr: 16 − Pos: 87248612 KORA
Frequency
0
200
400
600
0.0
0.2
0.4
0.6
0.8
1.0
Methylation Value

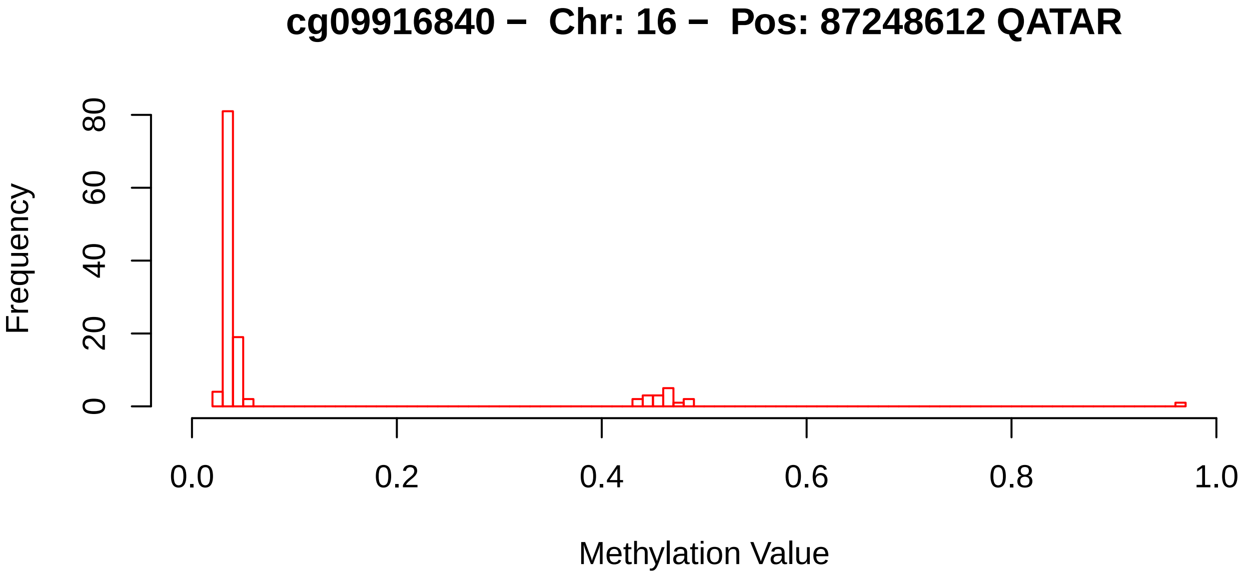
cg09916840 − Chr: 16 − Pos: 87248612 QATAR
Frequency
0
20
40
60
80
0.0
0.2
0.4
0.6
0.8
1.0
Methylation Value

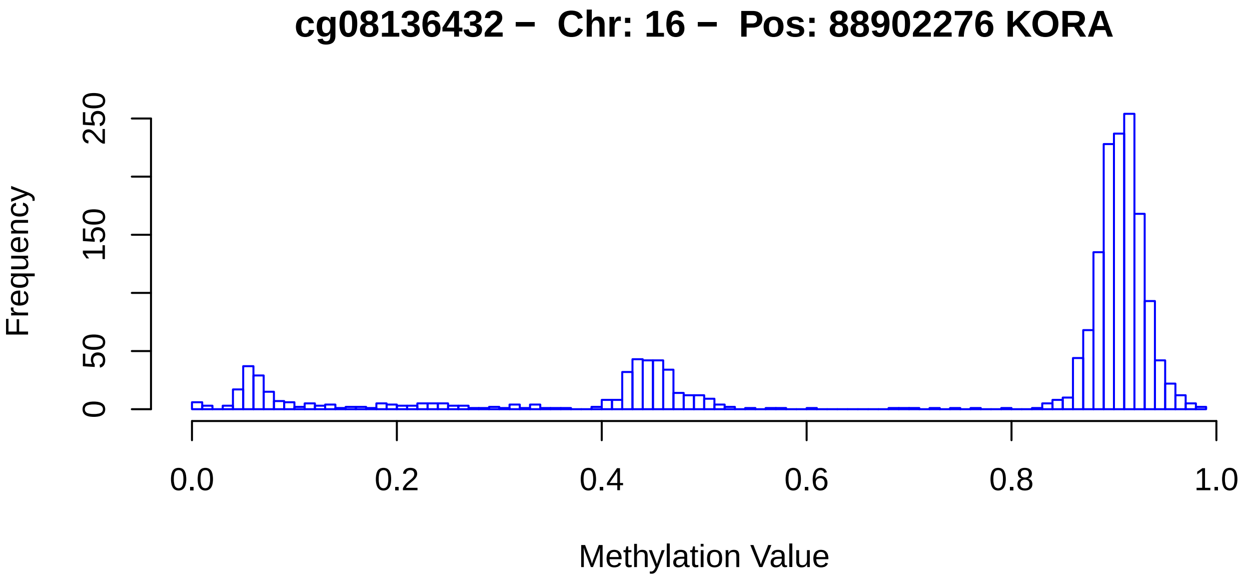
cg08136432 − Chr: 16 − Pos: 88902276 KORA
Frequency
0
50
150
250
0.0
0.2
0.4
0.6
0.8
1.0
Methylation Value

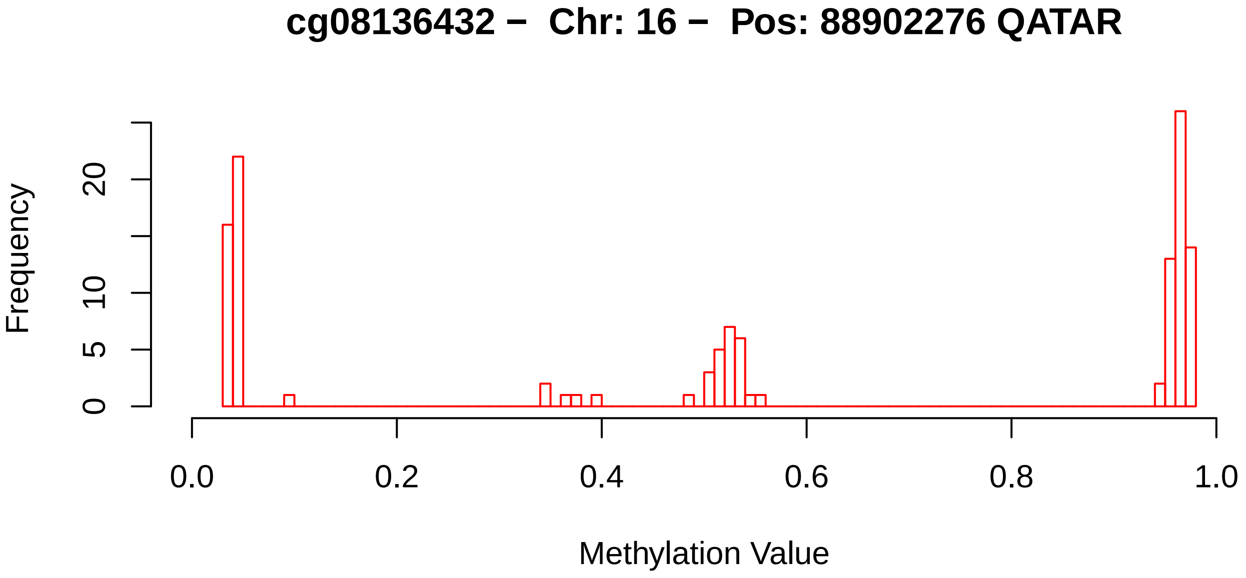
cg08136432 − Chr: 16 − Pos: 88902276 QATAR
Frequency
0
5
10
20
0.0
0.2
0.4
0.6
0.8
1.0
Methylation Value

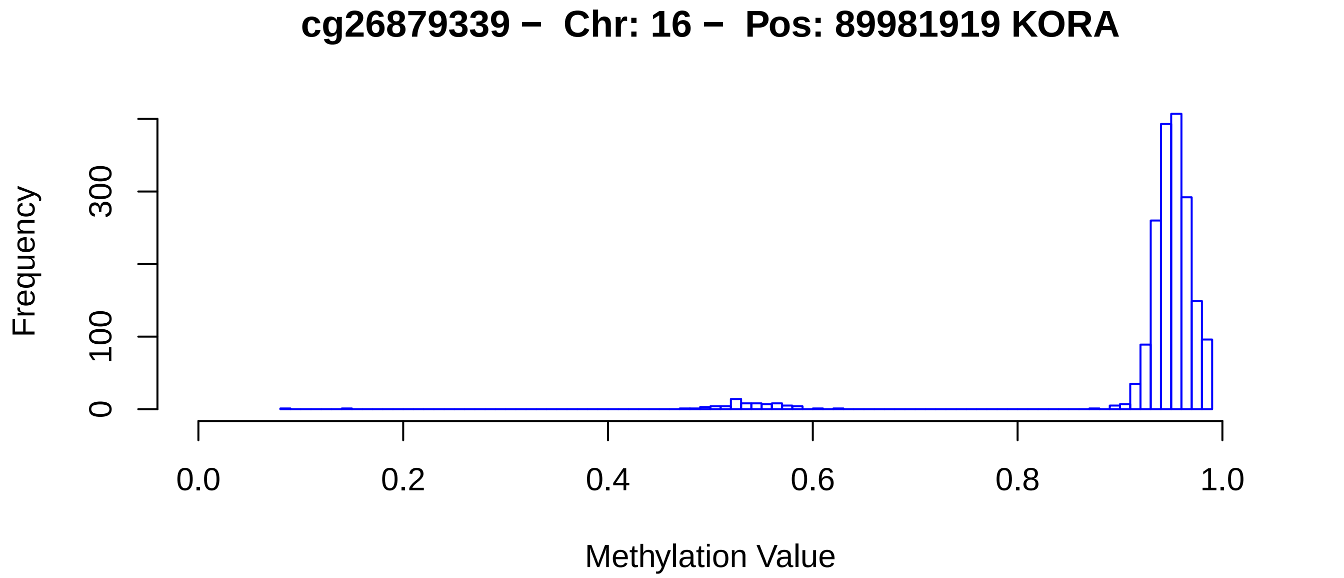
cg26879339 − Chr: 16 − Pos: 89981919 KORA
Frequency
0
100
300
0.0
0.2
0.4
0.6
0.8
1.0
Methylation Value

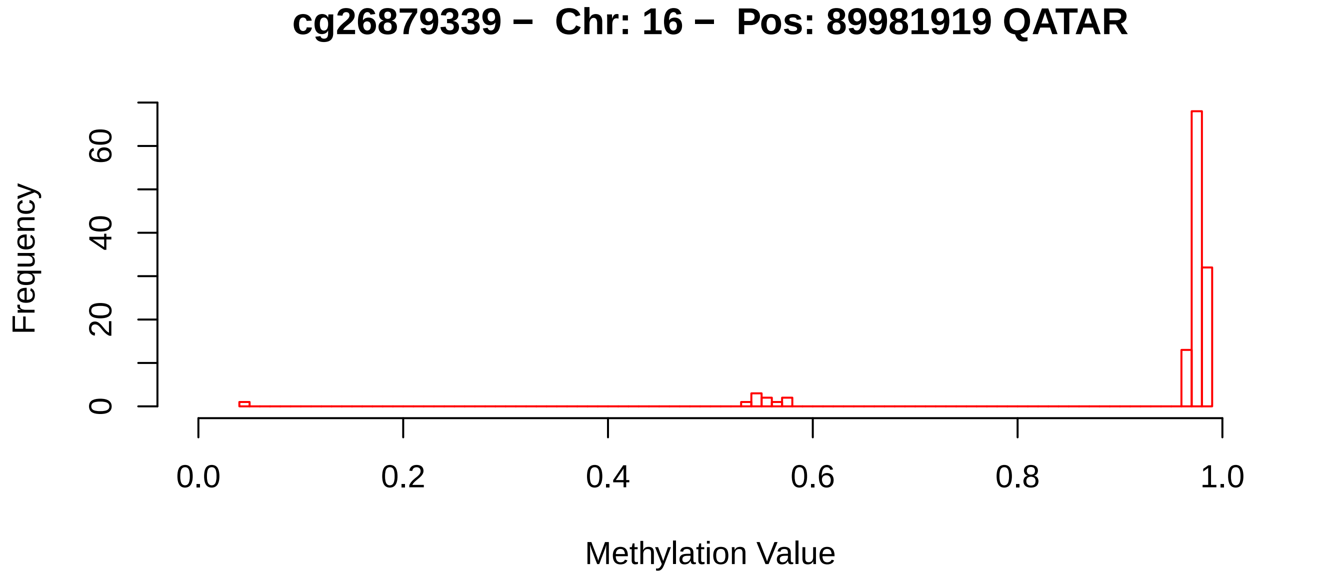
cg26879339 − Chr: 16 − Pos: 89981919 QATAR
Frequency
0
20
40
60
0.0
0.2
0.4
0.6
0.8
1.0
Methylation Value

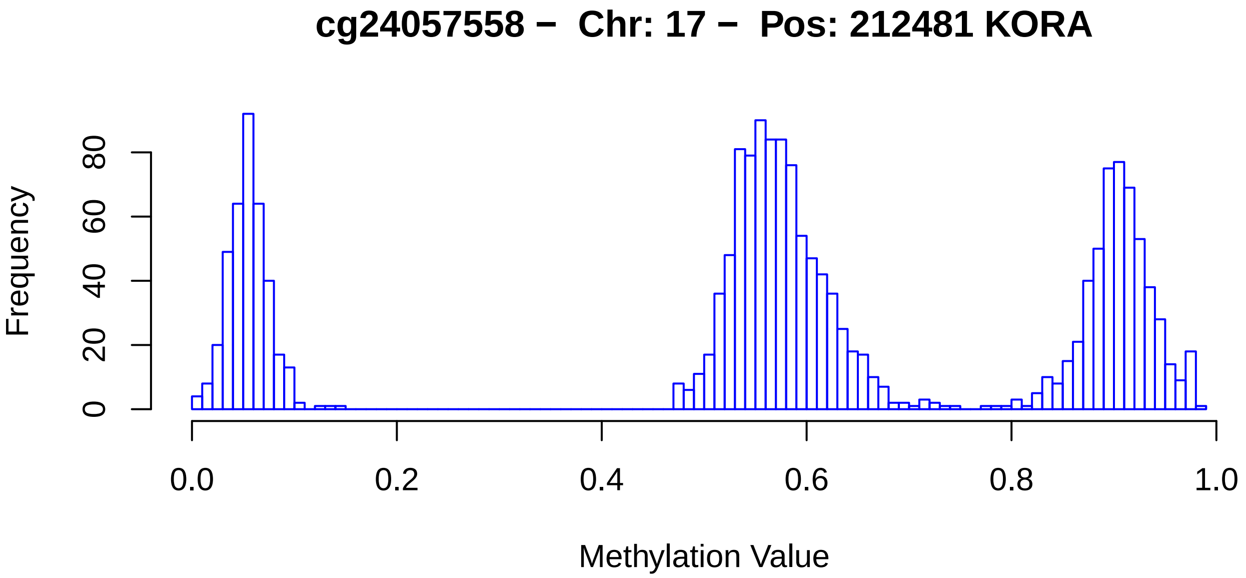
cg24057558 − Chr: 17 − Pos: 212481 KORA
Frequency
0
20
40
60
80
0.0
0.2
0.4
0.6
0.8
1.0
Methylation Value

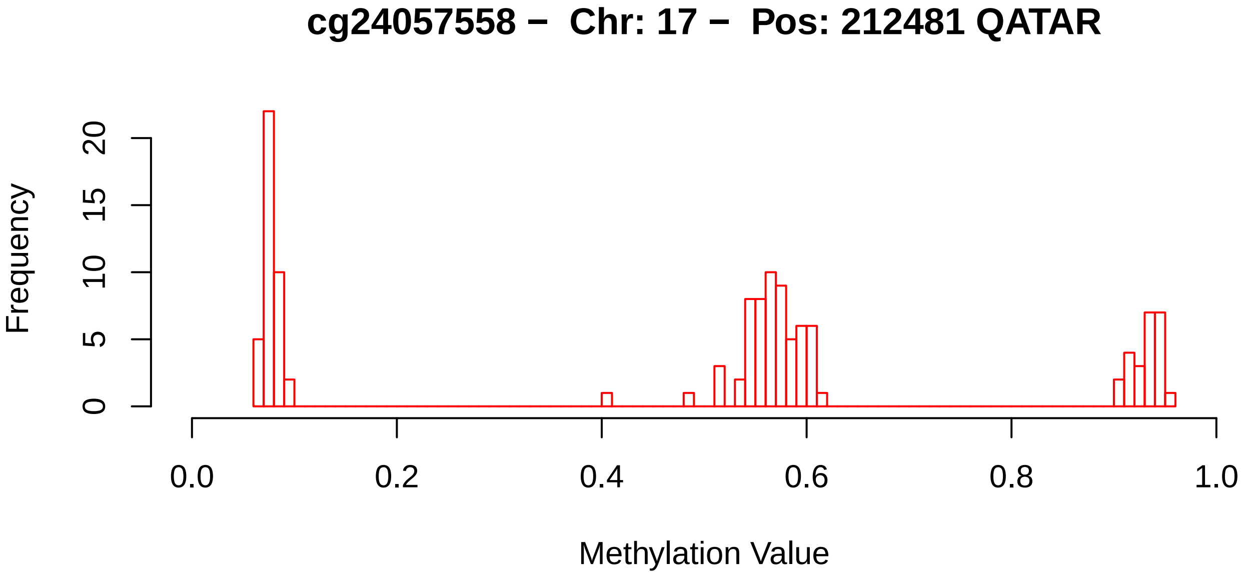
cg24057558 − Chr: 17 − Pos: 212481 QATAR
Frequency
0
5
10
15
20
0.0
0.2
0.4
0.6
0.8
1.0
Methylation Value

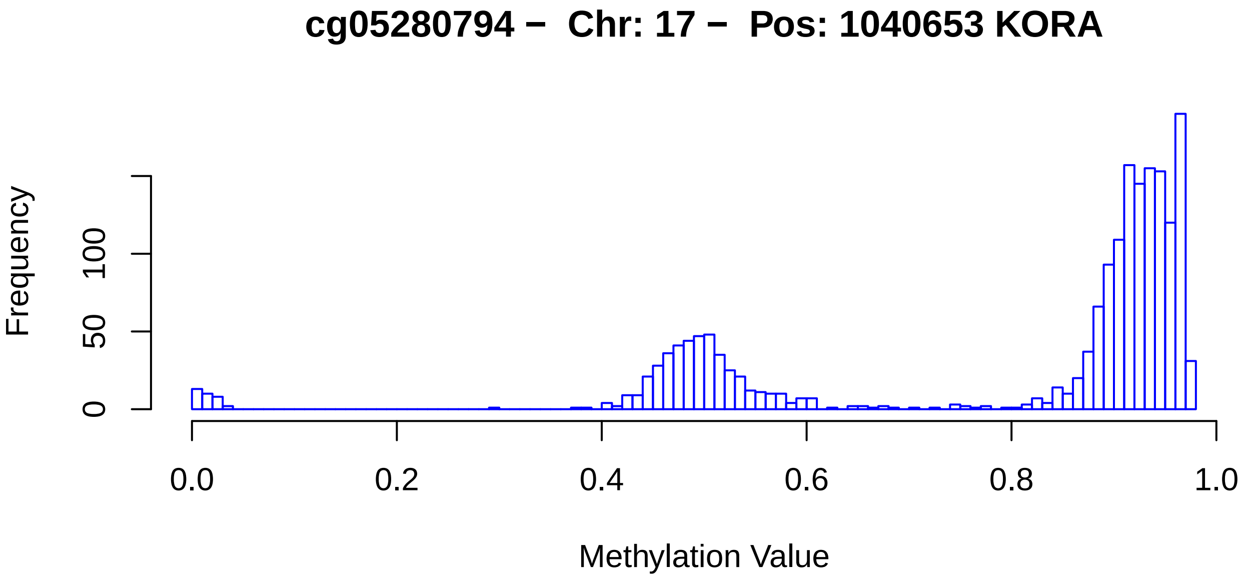
cg05280794 − Chr: 17 − Pos: 1040653 KORA
Frequency
0
50
100
0.0
0.2
0.4
0.6
0.8
1.0
Methylation Value

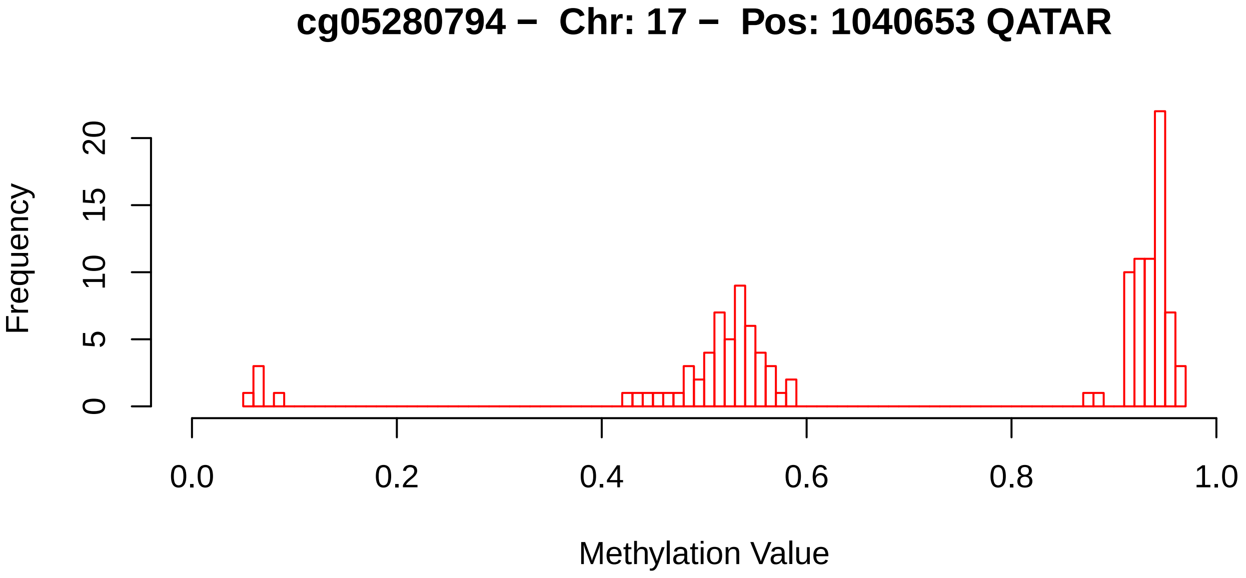
cg05280794 − Chr: 17 − Pos: 1040653 QATAR
Frequency
0
5
10
15
20
0.0
0.2
0.4
0.6
0.8
1.0
Methylation Value

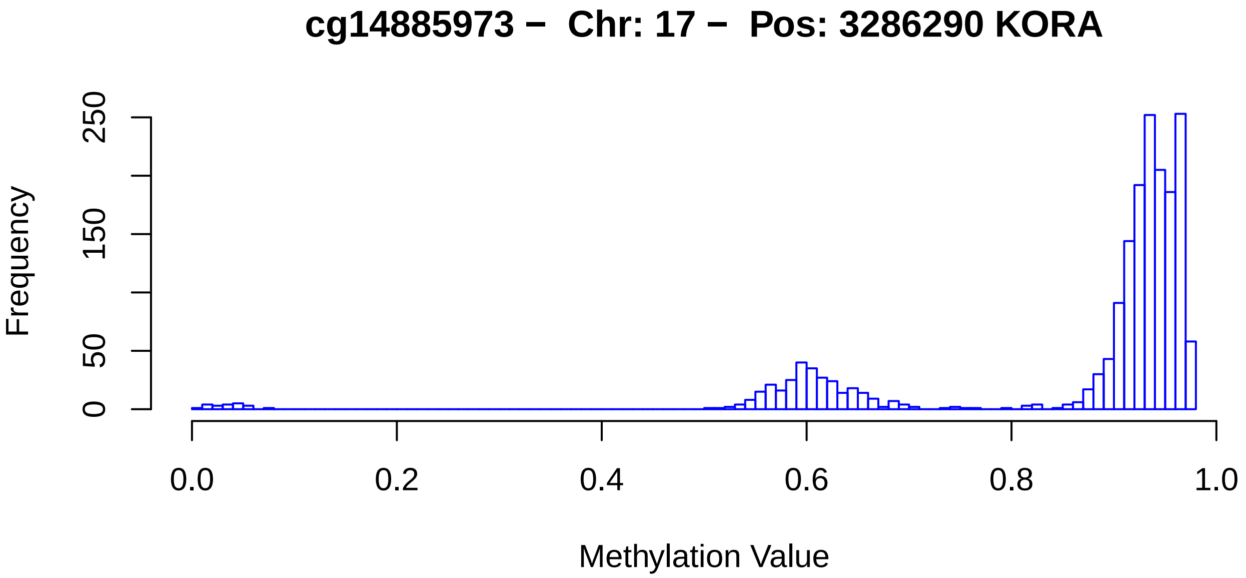
cg14885973 − Chr: 17 − Pos: 3286290 KORA
Frequency
0
50
150
250
0.0
0.2
0.4
0.6
0.8
1.0
Methylation Value

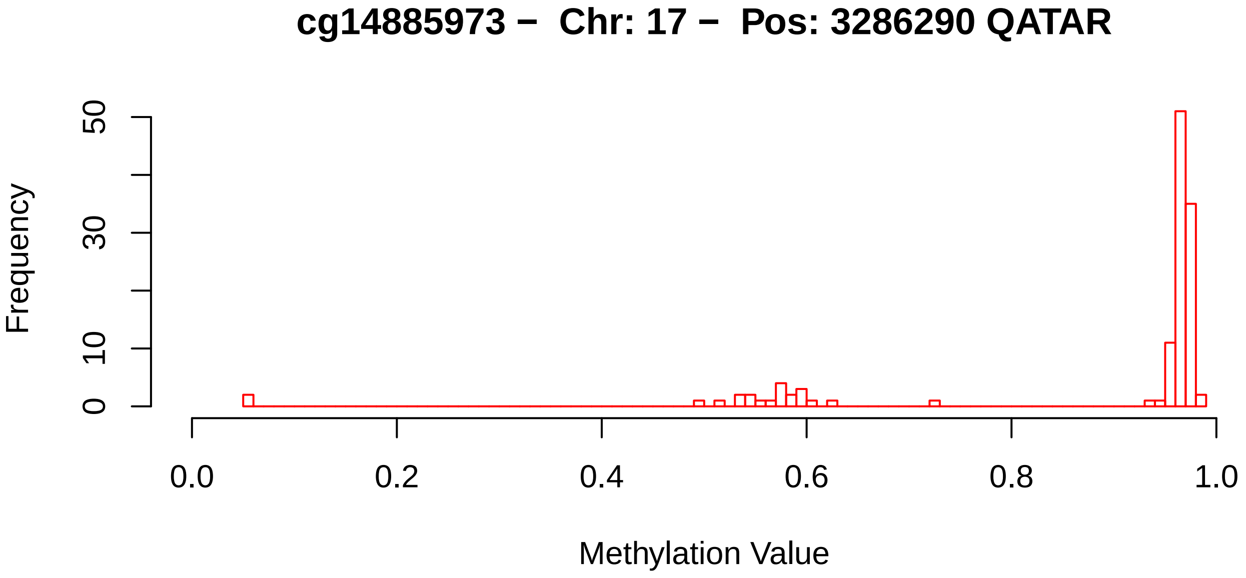
cg14885973 − Chr: 17 − Pos: 3286290 QATAR
Frequency
0
10
30
50
0.0
0.2
0.4
0.6
0.8
1.0
Methylation Value

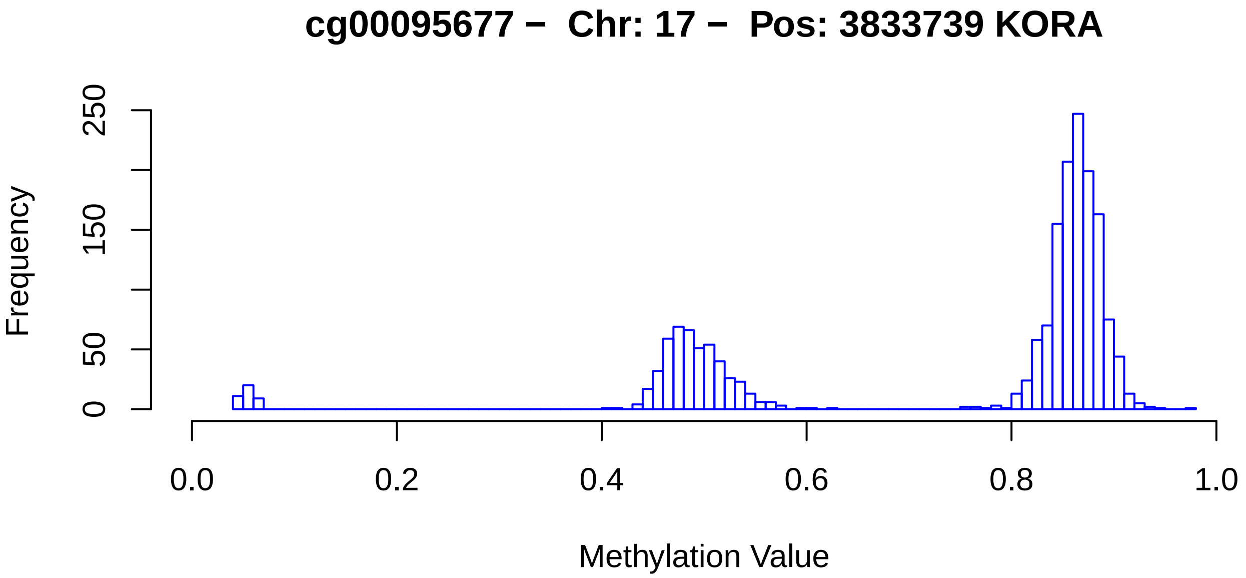
cg00095677 − Chr: 17 − Pos: 3833739 KORA
Frequency
0
50
150
250
0.0
0.2
0.4
0.6
0.8
1.0
Methylation Value

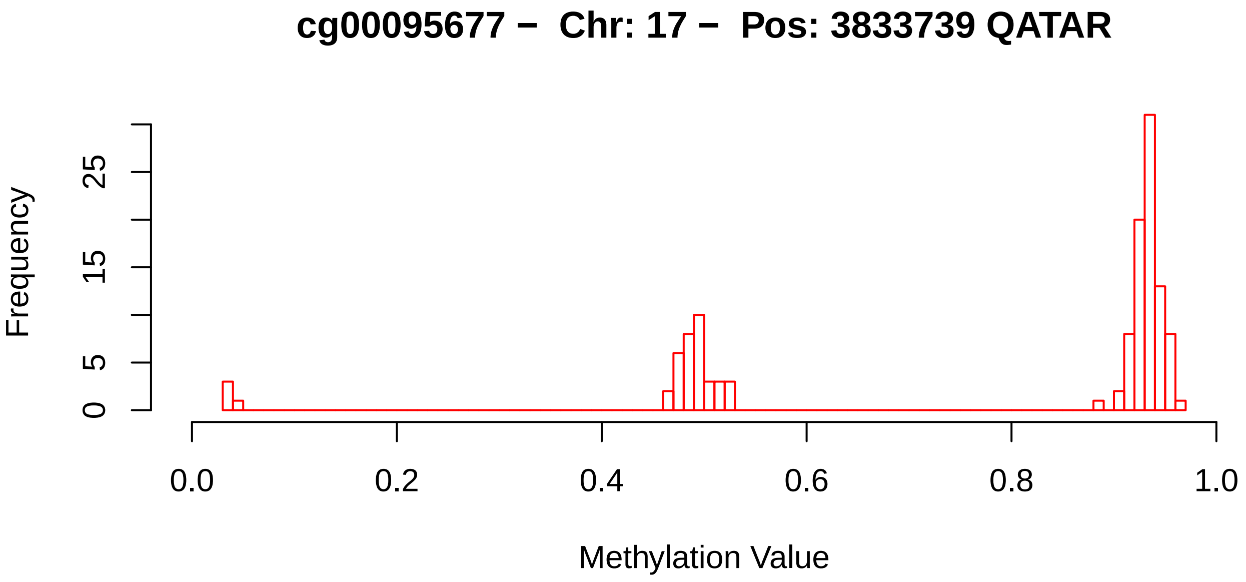
cg00095677 − Chr: 17 − Pos: 3833739 QATAR
Frequency
0
5
15
25
0.0
0.2
0.4
0.6
0.8
1.0
Methylation Value

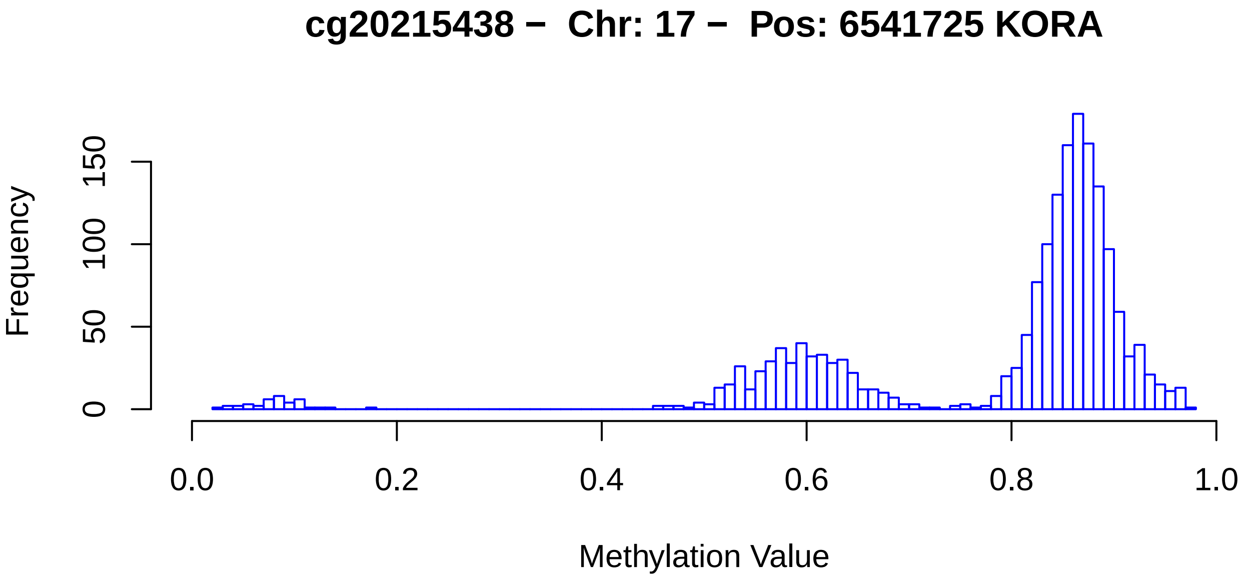

cg20215438 − Chr: 17 − Pos: 6541725 KORA
Frequency
0
50
100
150
0.0
0.2
0.4
0.6
0.8
1.0
Methylation Value

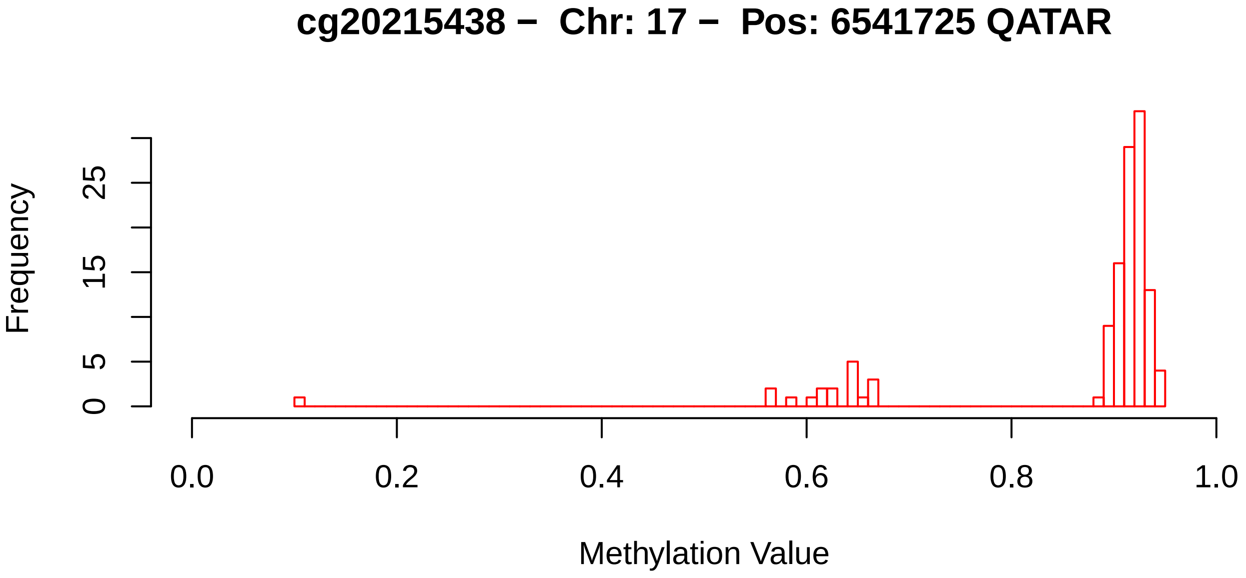

cg20215438 − Chr: 17 − Pos: 6541725 QATAR
Frequency
0
5
15
25
0.0
0.2
0.4
0.6
0.8
1.0
Methylation Value

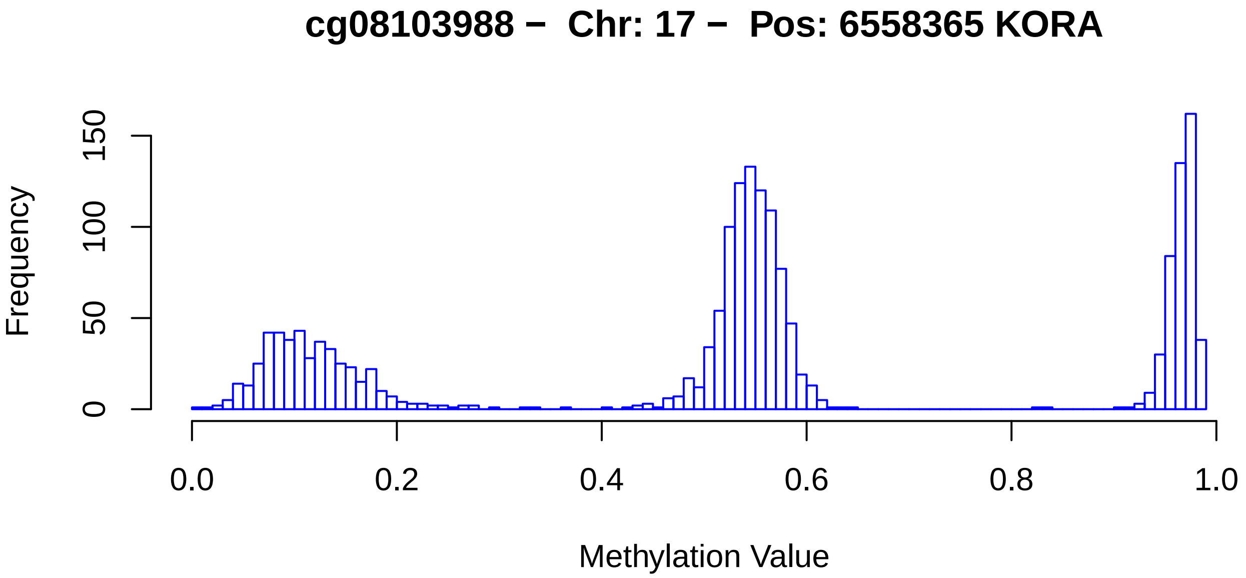
cg08103988 − Chr: 17 − Pos: 6558365 KORA
Frequency
0
50
100
150
0.0
0.2
0.4
0.6
0.8
1.0
Methylation Value

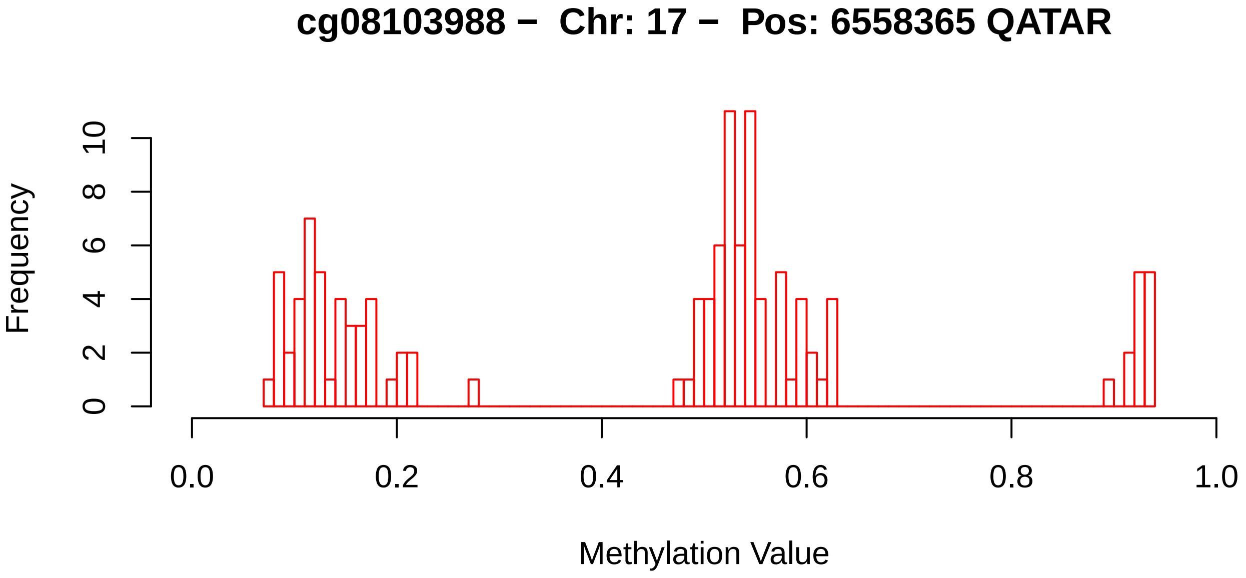
cg08103988 − Chr: 17 − Pos: 6558365 QATAR
Frequency
0
2
4
6
8
10
0.0
0.2
0.4
0.6
0.8
1.0
Methylation Value

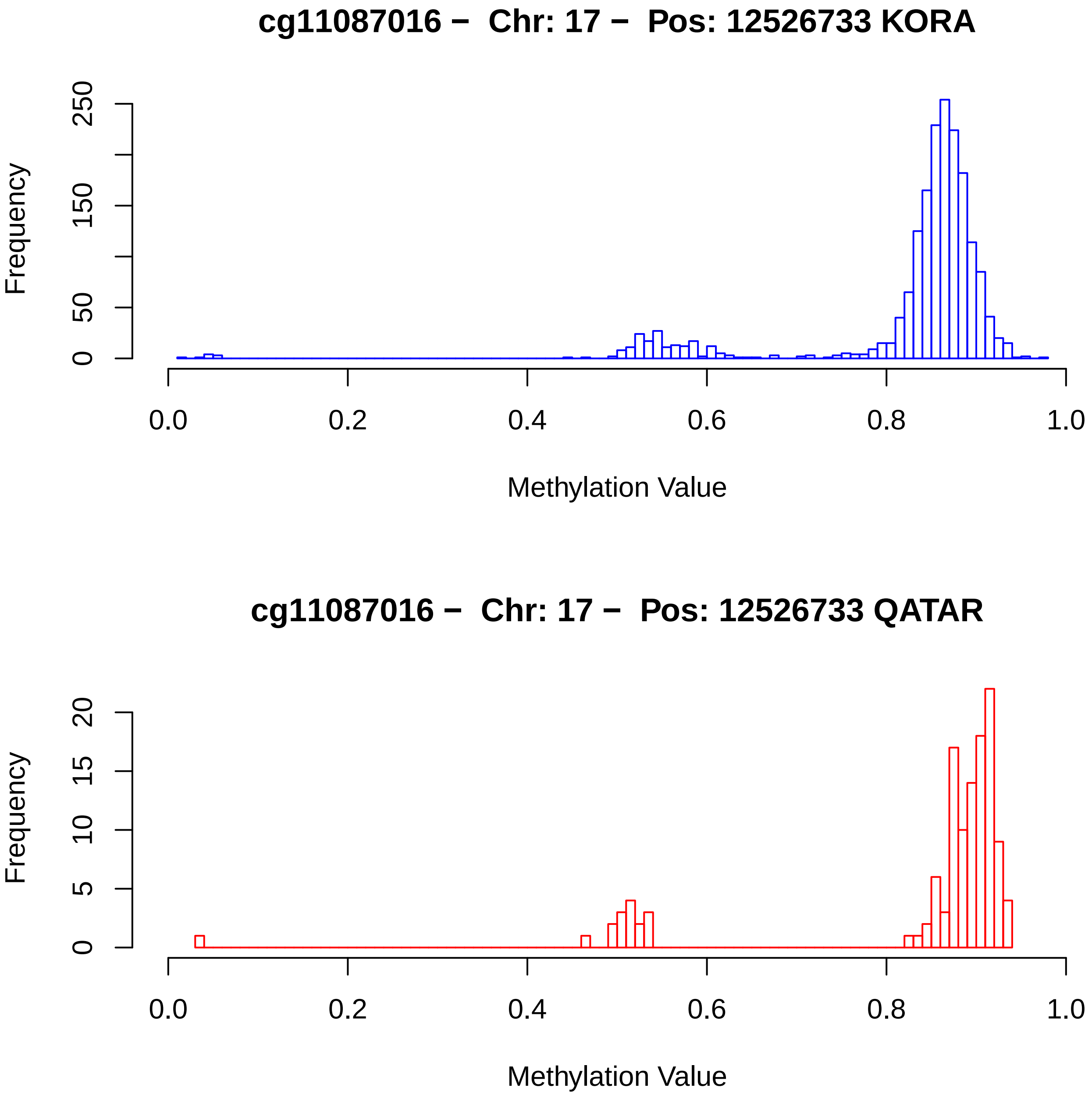
cg11087016 − Chr: 17 − Pos: 12526733 KORA
Frequency
0
50
150
250
0.0
0.2
0.4
0.6
0.8
1.0
Methylation Value
cg11087016 − Chr: 17 − Pos: 12526733 QATAR
Frequency
0
5
10
15
20
0.0
0.2
0.4
0.6
0.8
1.0
Methylation Value

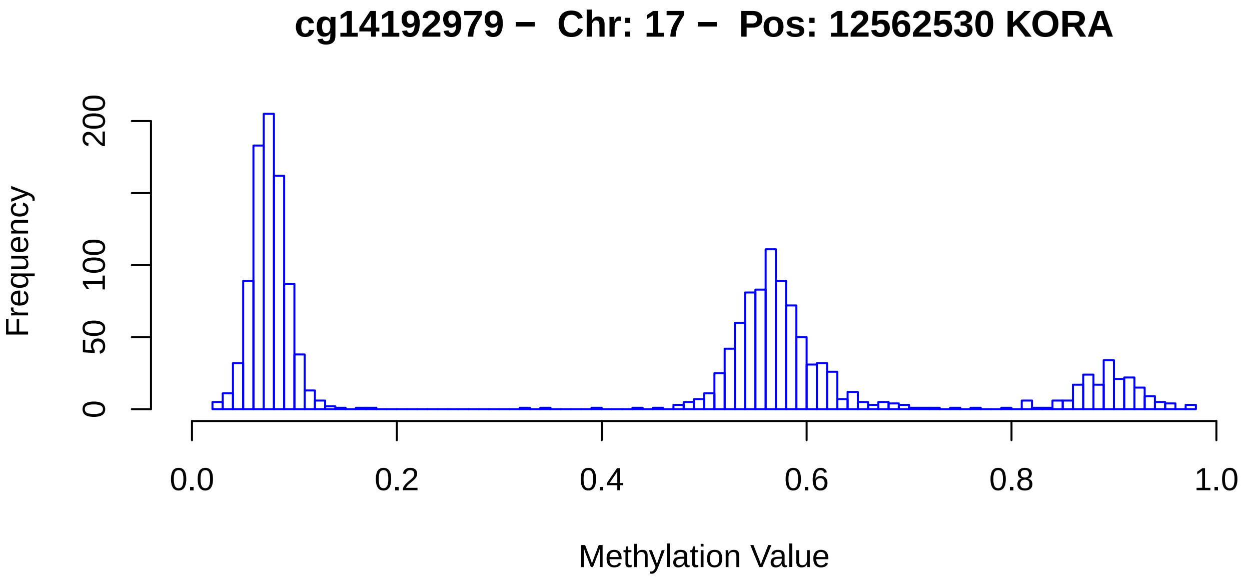
cg14192979 − Chr: 17 − Pos: 12562530 KORA
Frequency
0
50
100
200
0.0
0.2
0.4
0.6
0.8
1.0
Methylation Value

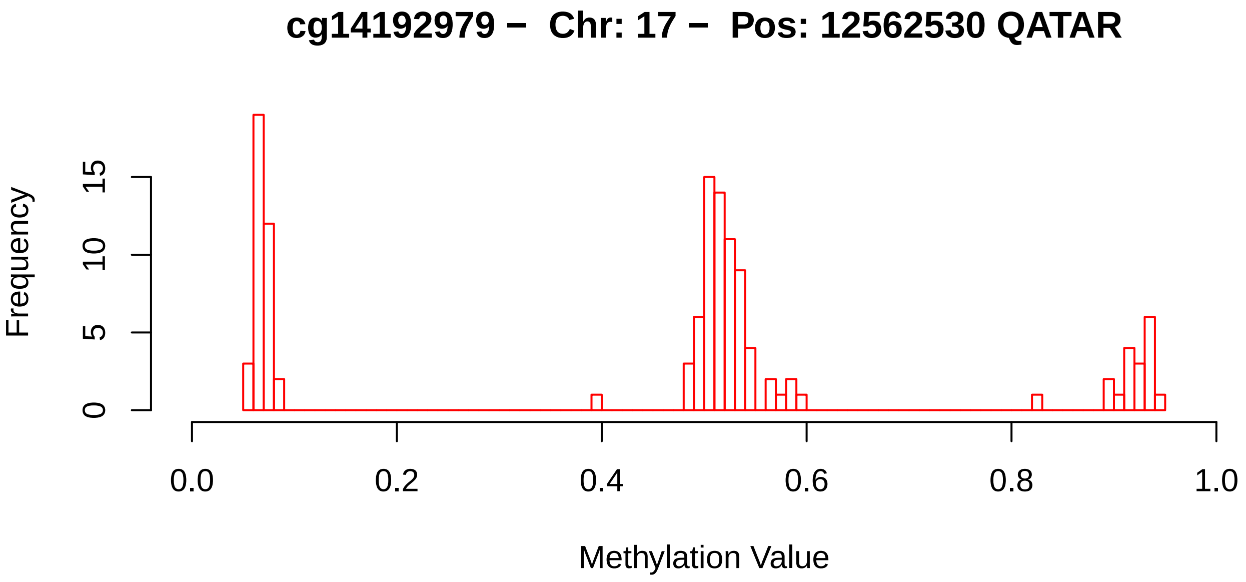
cg14192979 − Chr: 17 − Pos: 12562530 QATAR
Frequency
0
5
10
15
0.0
0.2
0.4
0.6
0.8
1.0
Methylation Value

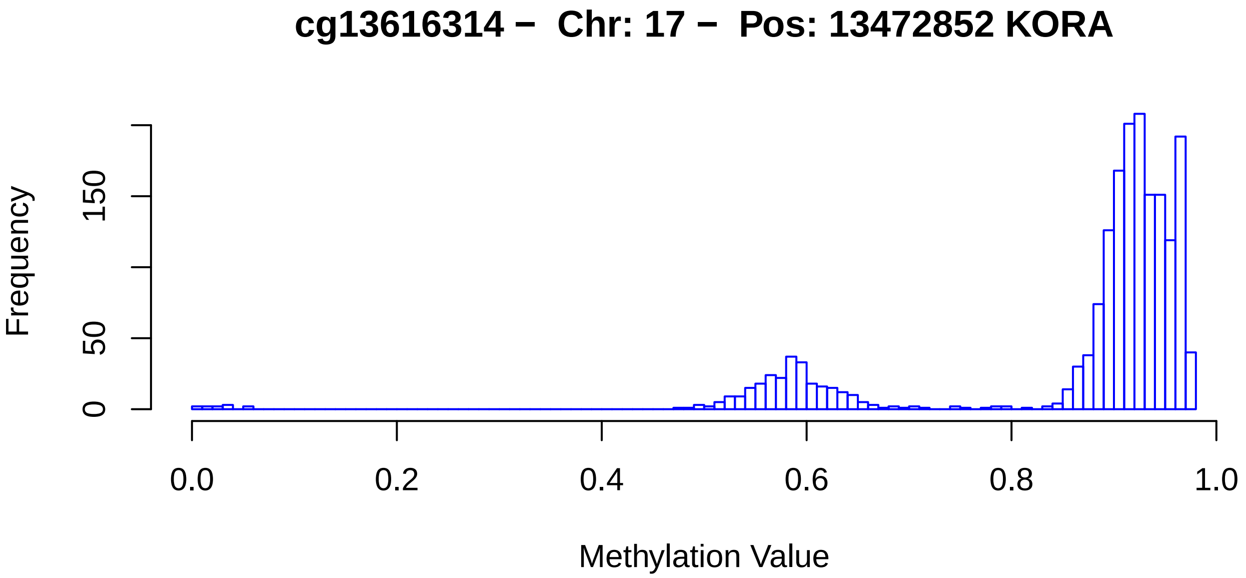
cg13616314 − Chr: 17 − Pos: 13472852 KORA
Frequency
0
50
150
0.0
0.2
0.4
0.6
0.8
1.0
Methylation Value

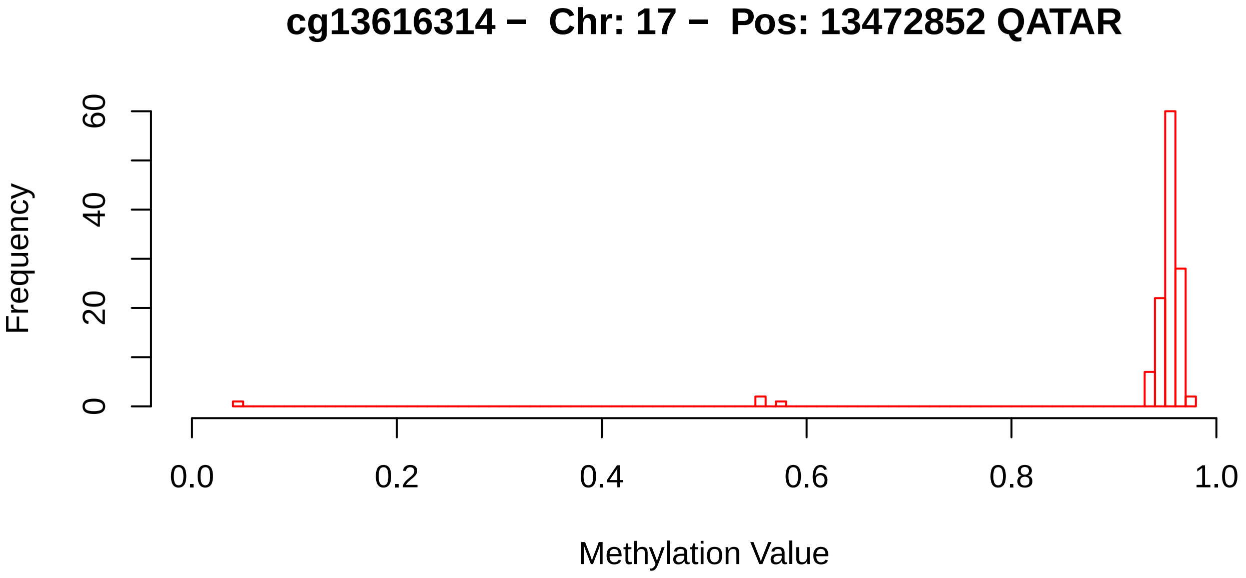
cg13616314 − Chr: 17 − Pos: 13472852 QATAR
Frequency
0
20
40
60
0.0
0.2
0.4
0.6
0.8
1.0
Methylation Value

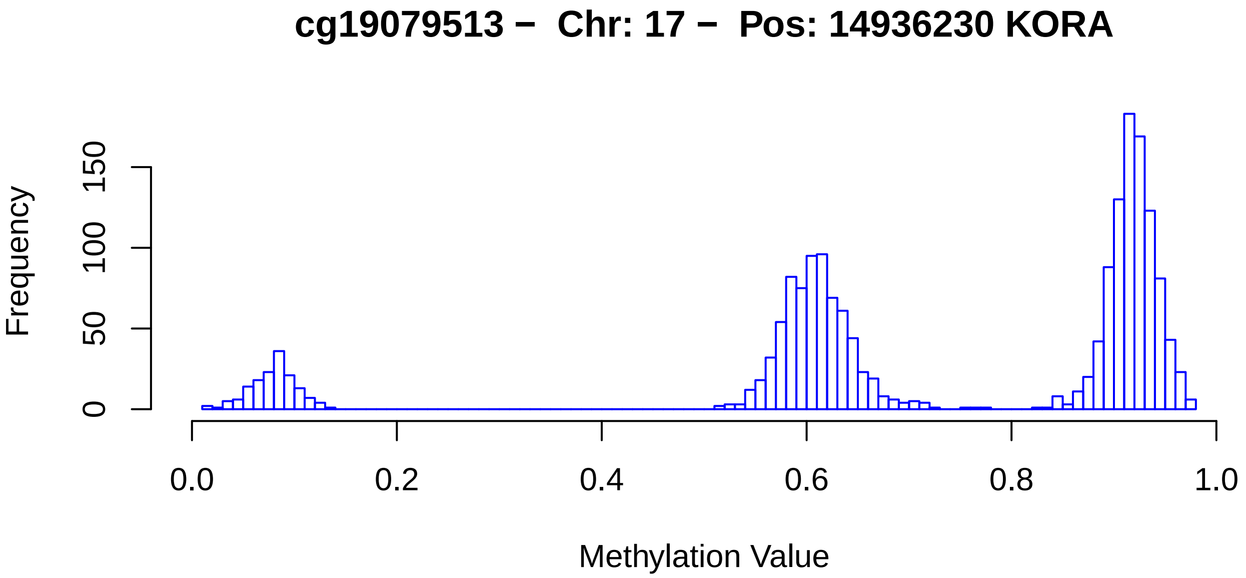
cg19079513 − Chr: 17 − Pos: 14936230 KORA
Frequency
0
50
100
150
0.0
0.2
0.4
0.6
0.8
1.0
Methylation Value

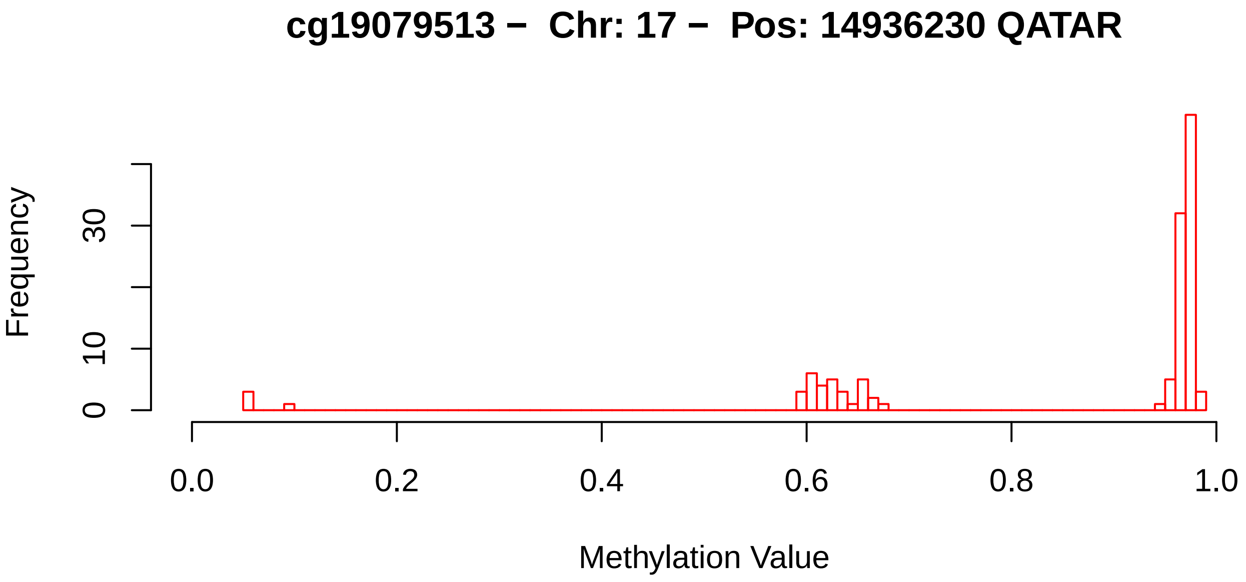
cg19079513 − Chr: 17 − Pos: 14936230 QATAR
Frequency
0
10
30
0.0
0.2
0.4
0.6
0.8
1.0
Methylation Value

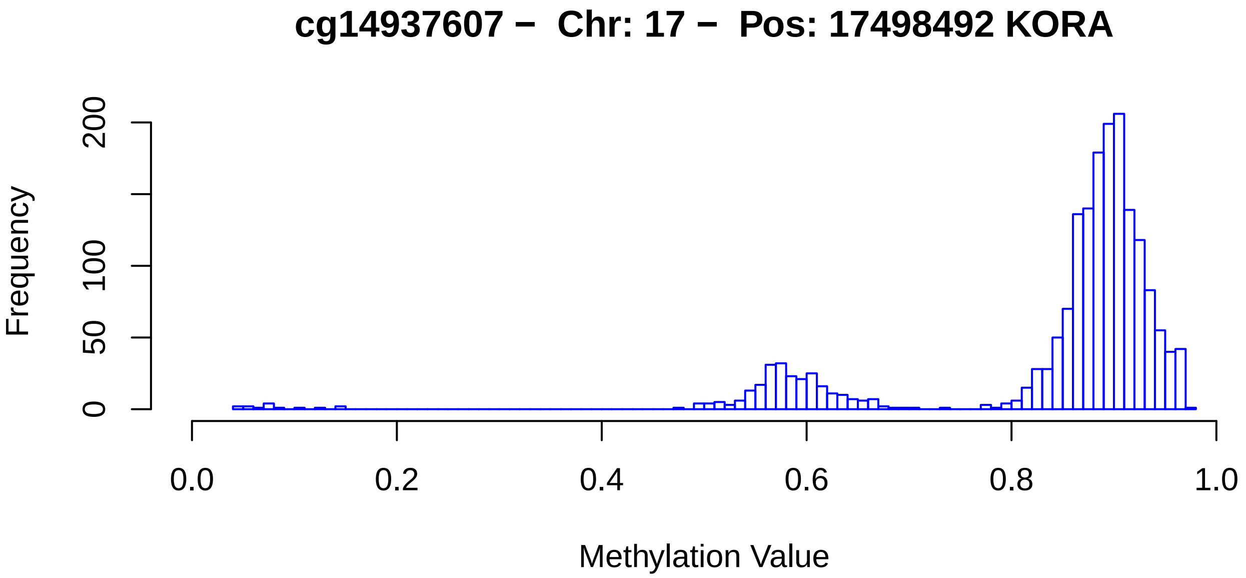
cg14937607 − Chr: 17 − Pos: 17498492 KORA
Frequency
0
50
100
200
0.0
0.2
0.4
0.6
0.8
1.0
Methylation Value

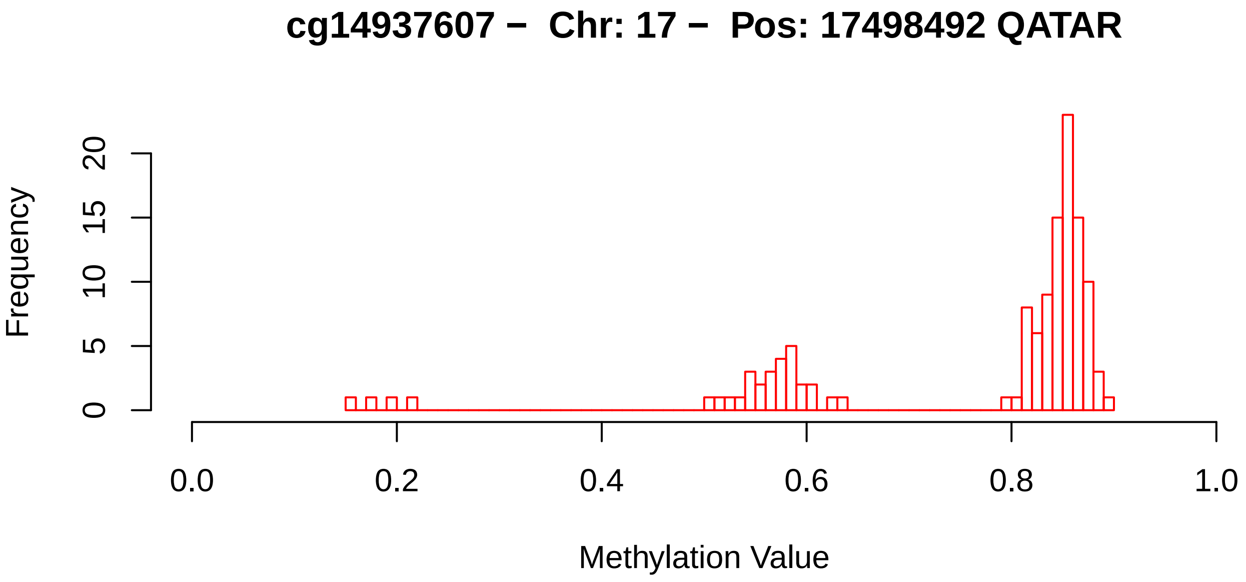
cg14937607 − Chr: 17 − Pos: 17498492 QATAR
Frequency
0
5
10
15
20
0.0
0.2
0.4
0.6
0.8
1.0
Methylation Value

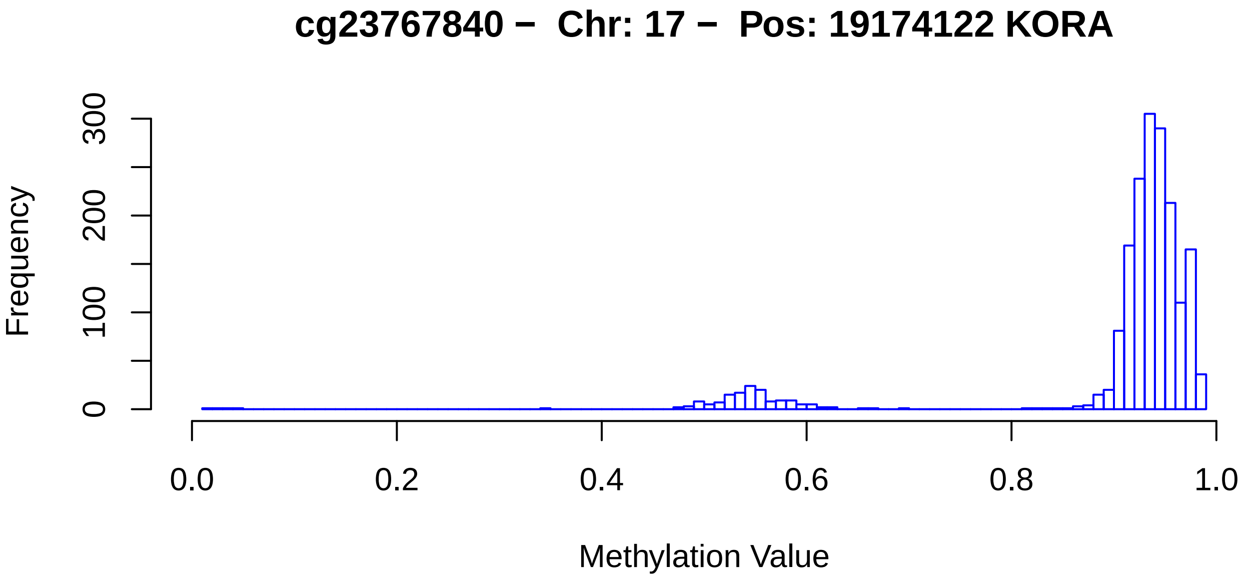
cg23767840 − Chr: 17 − Pos: 19174122 KORA
Frequency
0
100
200
300
0.0
0.2
0.4
0.6
0.8
1.0
Methylation Value

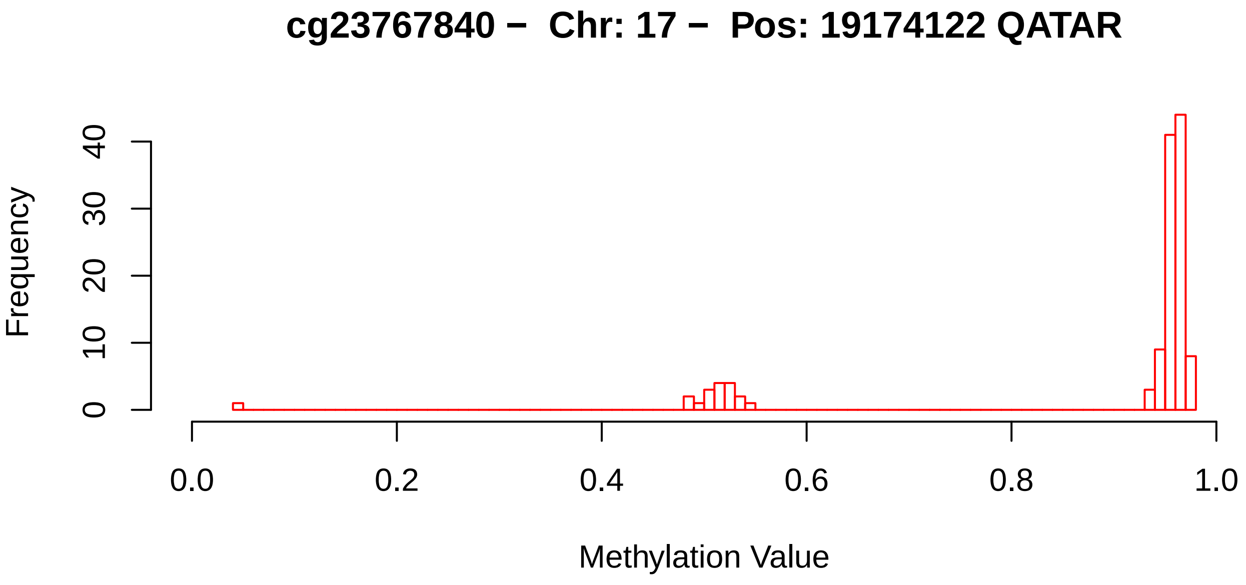
cg23767840 − Chr: 17 − Pos: 19174122 QATAR
Frequency
0
10
20
30
40
0.0
0.2
0.4
0.6
0.8
1.0
Methylation Value

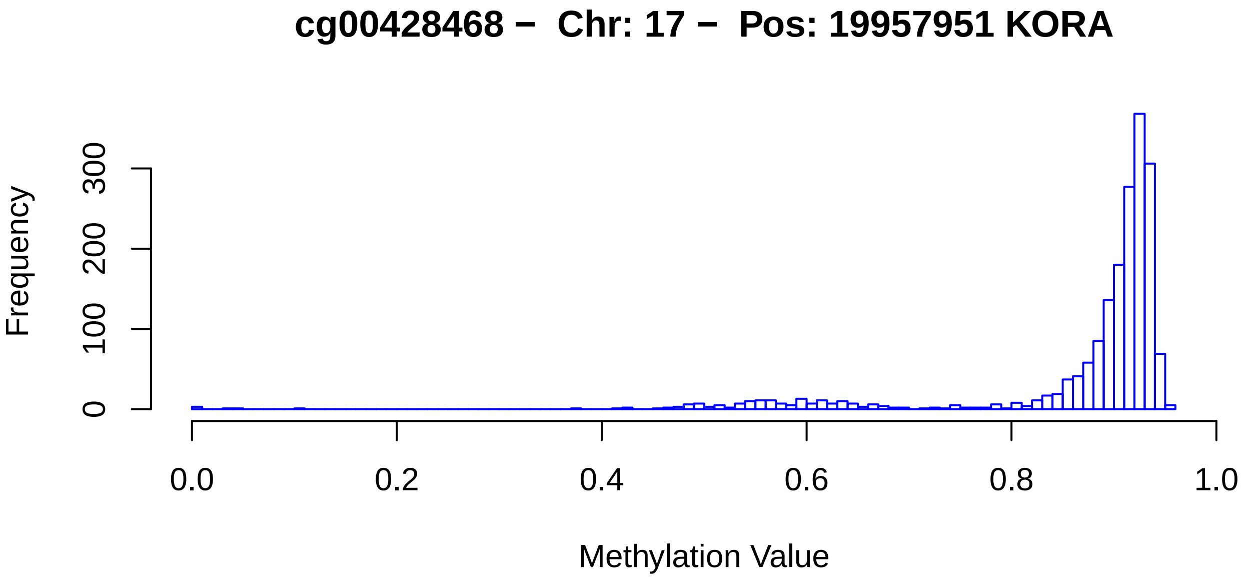
cg00428468 − Chr: 17 − Pos: 19957951 KORA
Frequency
0
100
200
300
0.0
0.2
0.4
0.6
0.8
1.0
Methylation Value

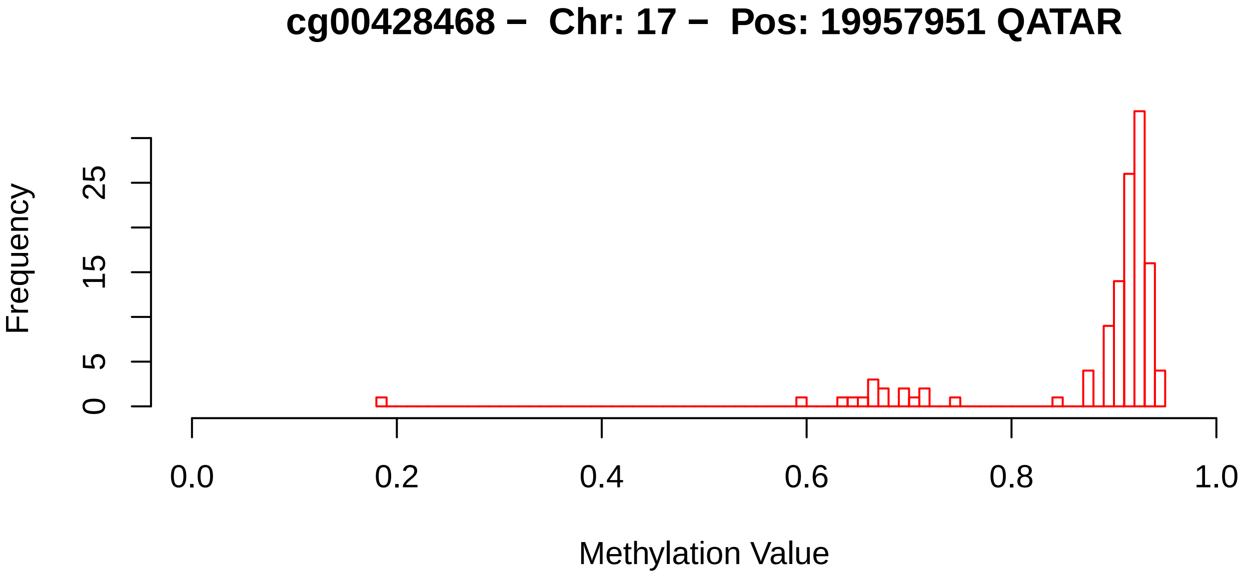
cg00428468 − Chr: 17 − Pos: 19957951 QATAR
Frequency
0
5
15
25
0.0
0.2
0.4
0.6
0.8
1.0
Methylation Value

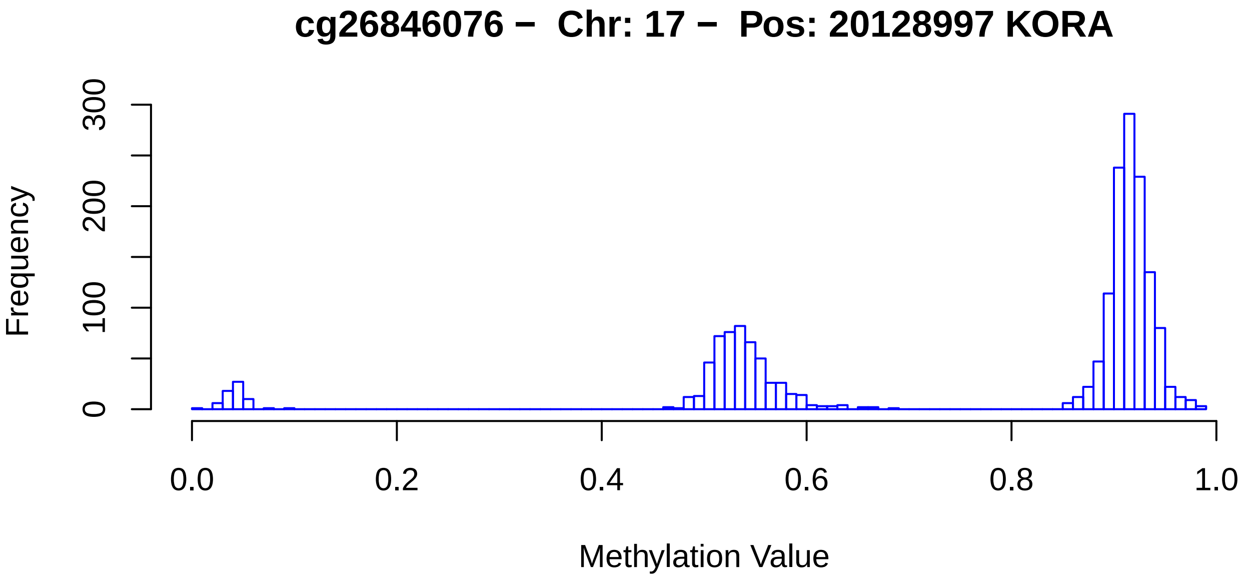
cg26846076 − Chr: 17 − Pos: 20128997 KORA
Frequency
0
100
200
300
0.0
0.2
0.4
0.6
0.8
1.0
Methylation Value

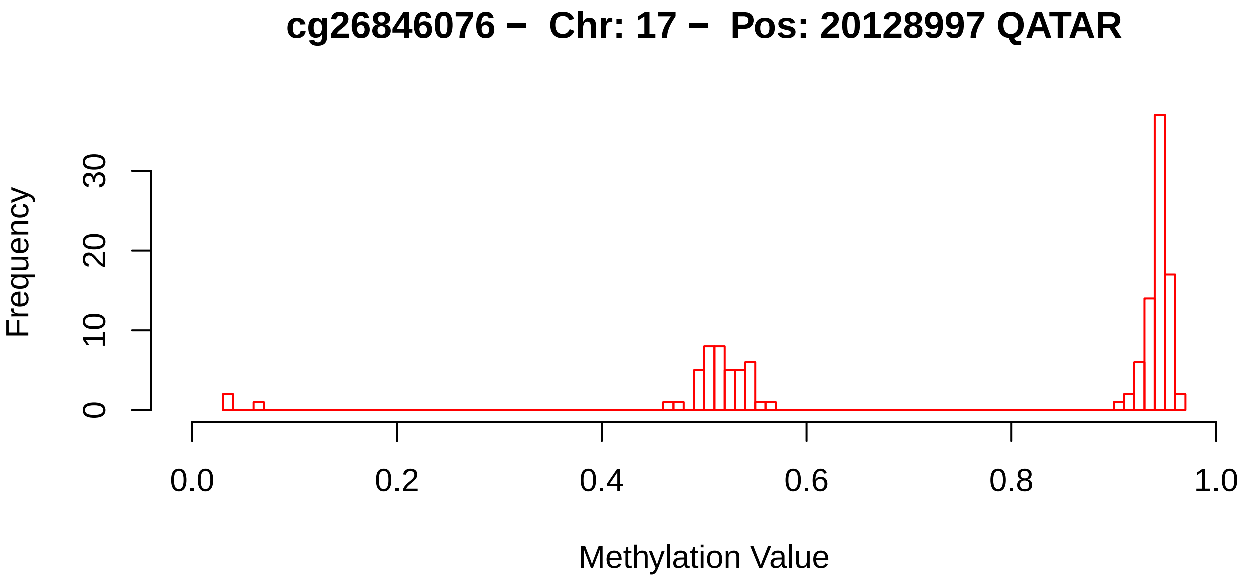
cg26846076 − Chr: 17 − Pos: 20128997 QATAR
Frequency
0
10
20
30
0.0
0.2
0.4
0.6
0.8
1.0
Methylation Value

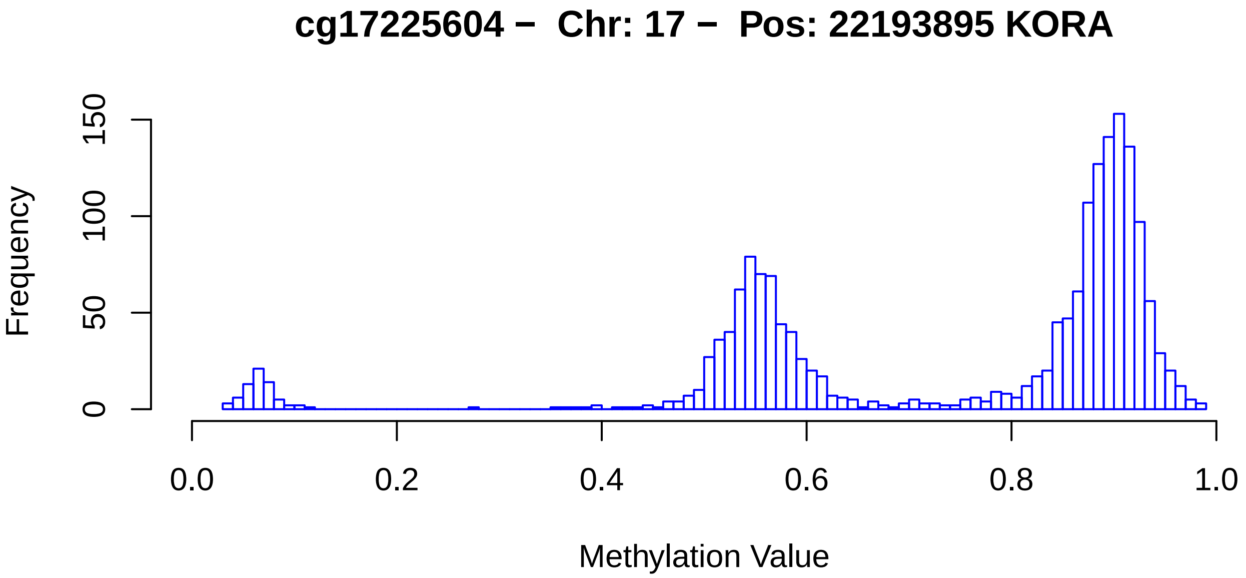
cg17225604 – Chr: 17 – Pos: 22193895 KORA
Frequency
0
50
100
150
0.0
0.2
0.4
0.6
0.8
1.0
Methylation Value

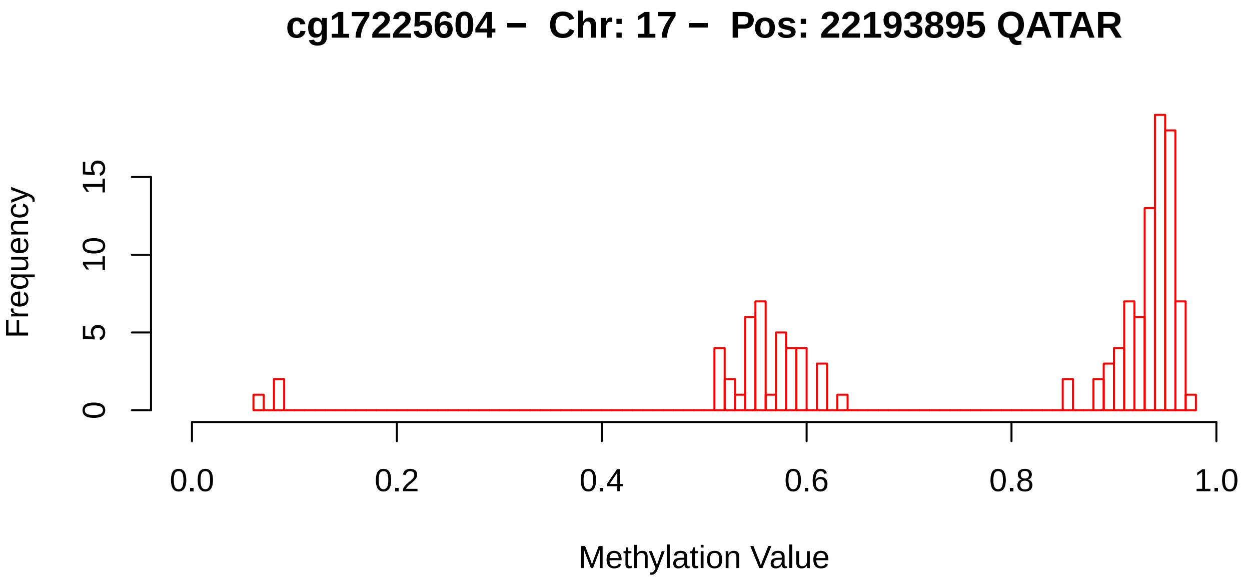
cg17225604 – Chr: 17 – Pos: 22193895 QATAR
Frequency
0
5
10
15
0.0
0.2
0.4
0.6
0.8
1.0
Methylation Value

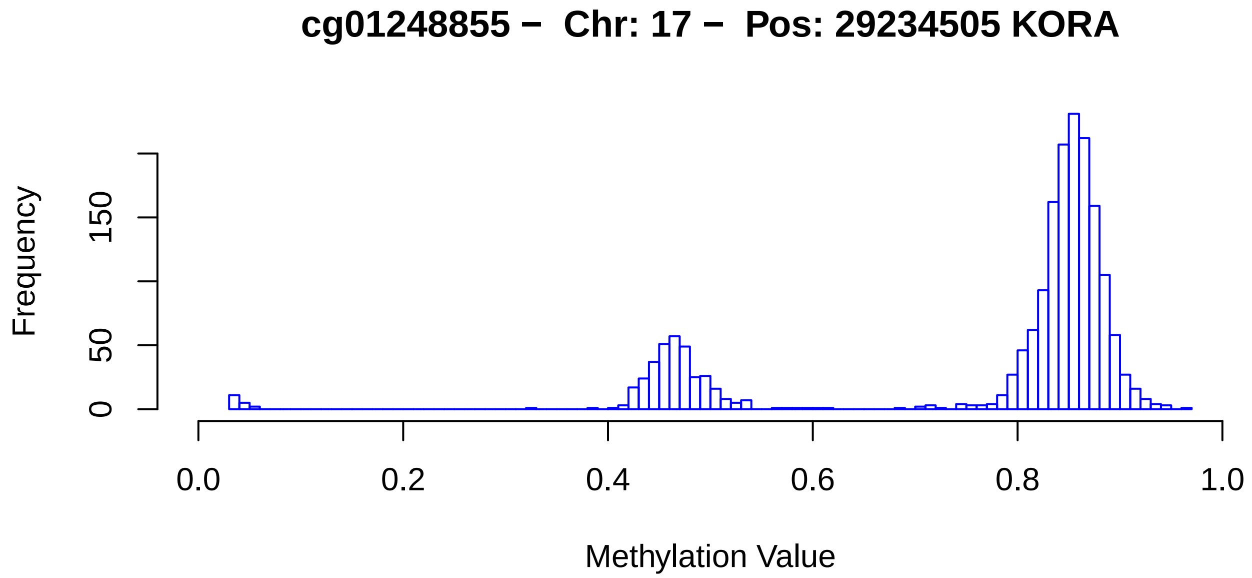
cg01248855 − Chr: 17 − Pos: 29234505 KORA
Frequency
0
50
150
0.0
0.2
0.4
0.6
0.8
1.0
Methylation Value

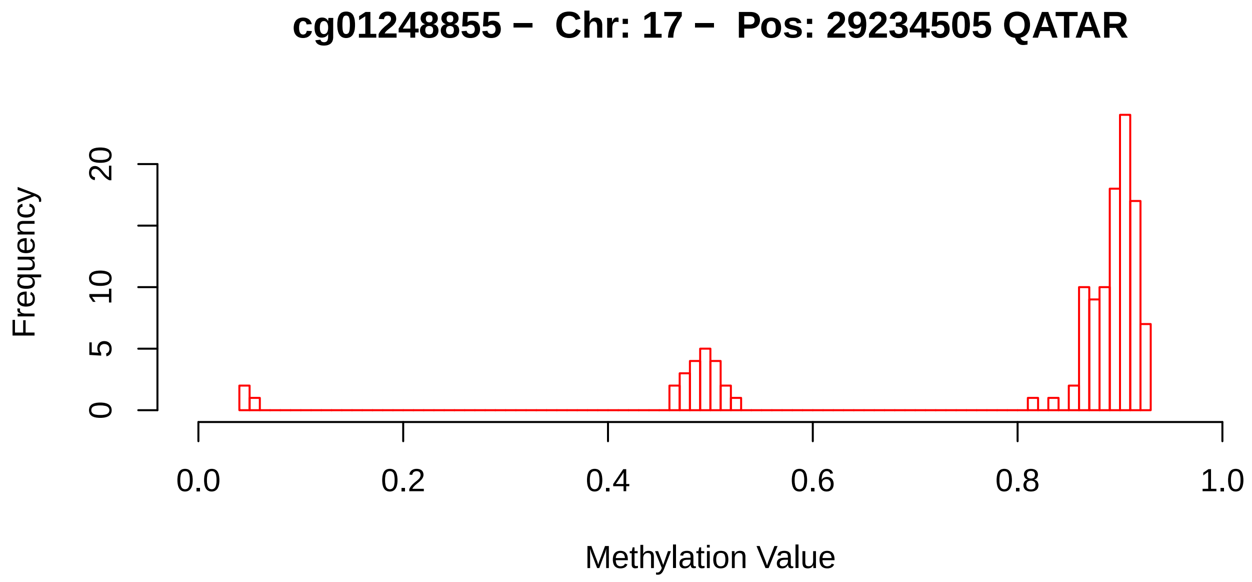
cg01248855 − Chr: 17 − Pos: 29234505 QATAR
Frequency
0
5
10
20
0.0
0.2
0.4
0.6
0.8
1.0
Methylation Value

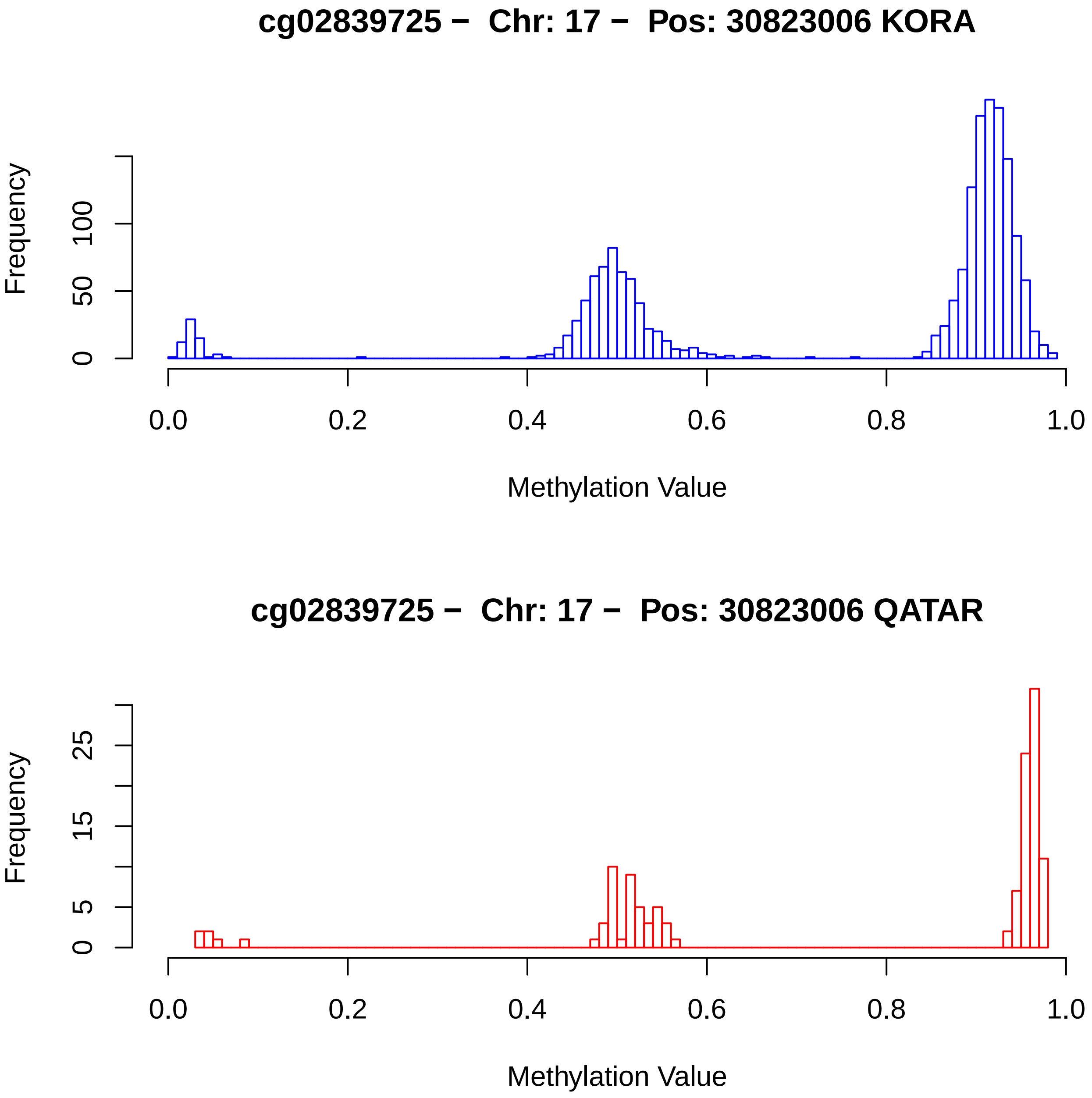
cg02839725 − Chr: 17 − Pos: 30823006 KORA
Frequency
0
50
100
0.0
0.2
0.4
0.6
0.8
1.0
Methylation Value
cg02839725 − Chr: 17 − Pos: 30823006 QATAR
Frequency
0
5
15
25
0.0
0.2
0.4
0.6
0.8
1.0
Methylation Value

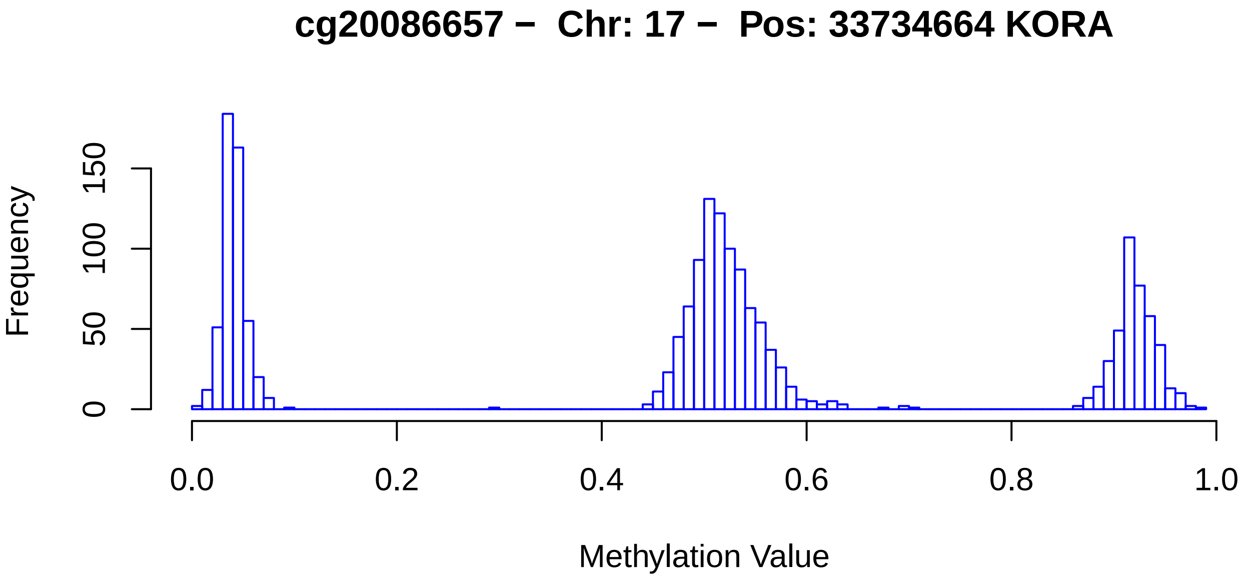
cg20086657 − Chr: 17 − Pos: 33734664 KORA
Frequency
0
50
100
150
0.0
0.2
0.4
0.6
0.8
1.0
Methylation Value

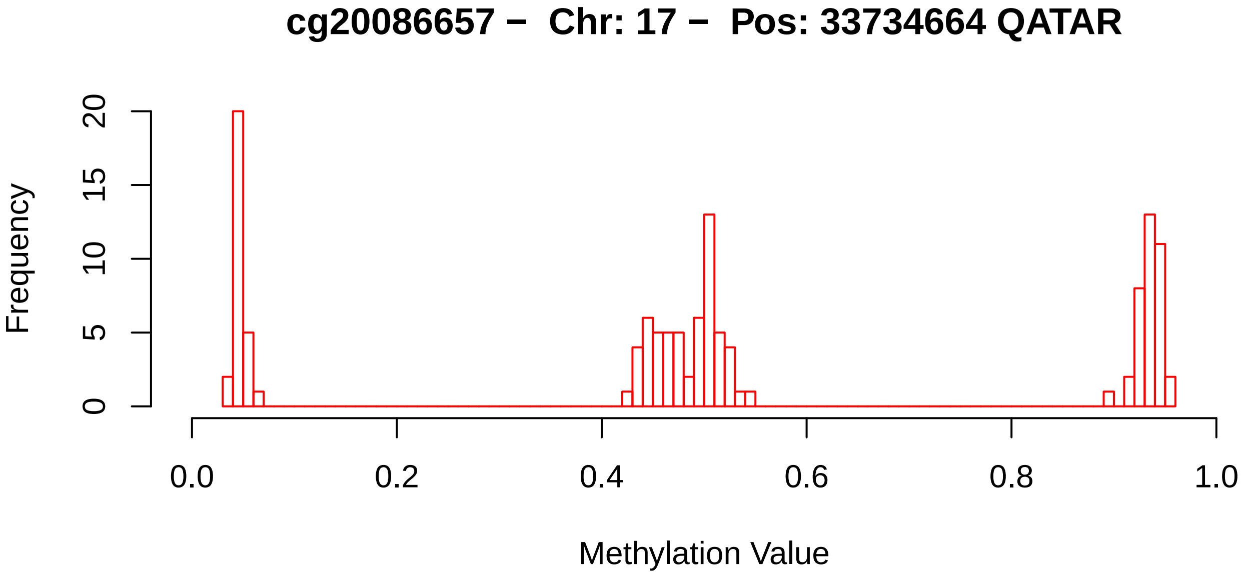
cg20086657 − Chr: 17 − Pos: 33734664 QATAR
Frequency
0
5
10
15
20
0.0
0.2
0.4
0.6
0.8
1.0
Methylation Value

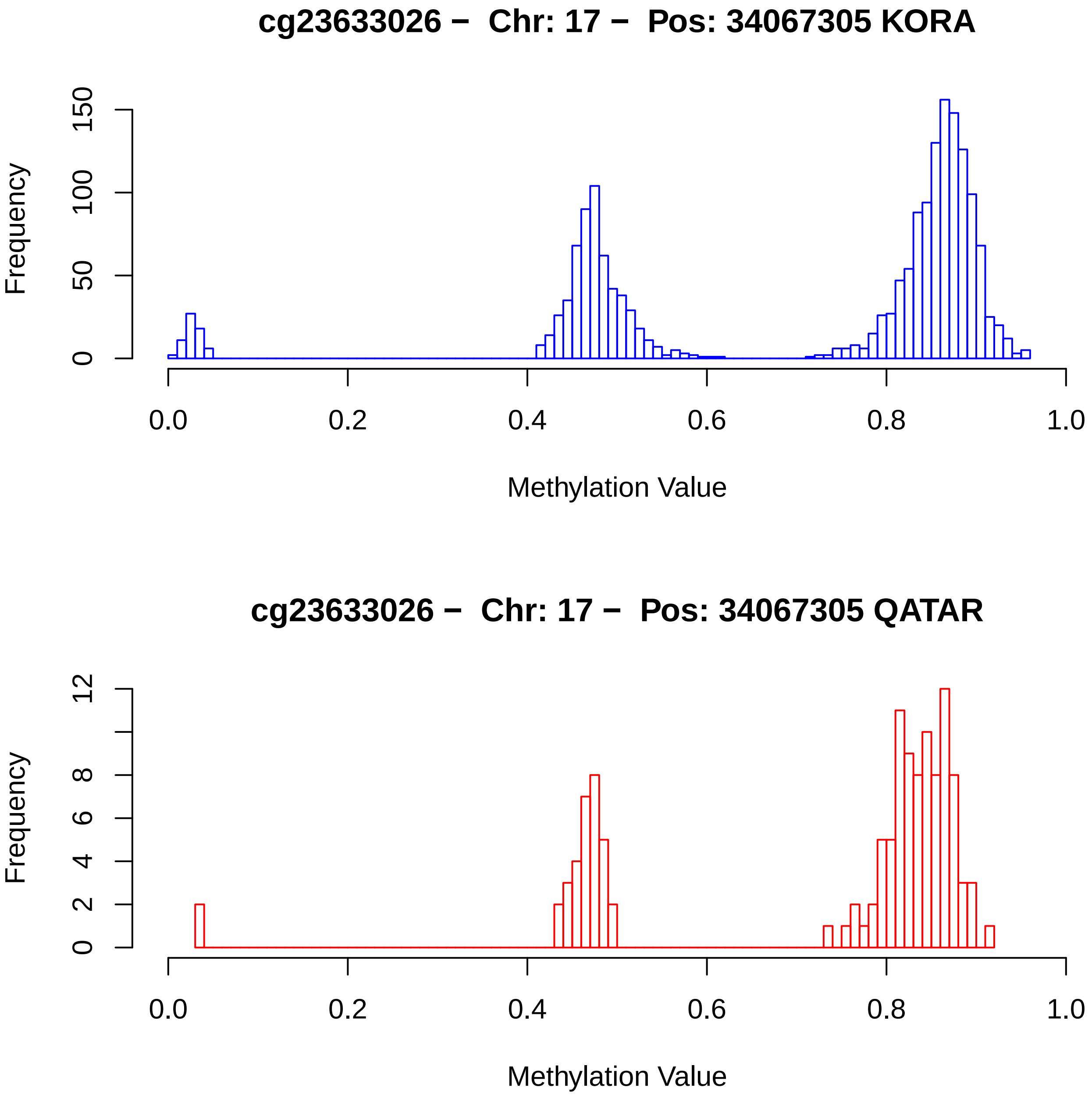
cg23633026 − Chr: 17 − Pos: 34067305 KORA
Frequency
Methylation Value
0
50
100
150
0.0
0.2
0.4
0.6
0.8
1.0
cg23633026 − Chr: 17 − Pos: 34067305 QATAR
Frequency
Methylation Value
0
2
4
6
8
12
0.0
0.2
0.4
0.6
0.8
1.0

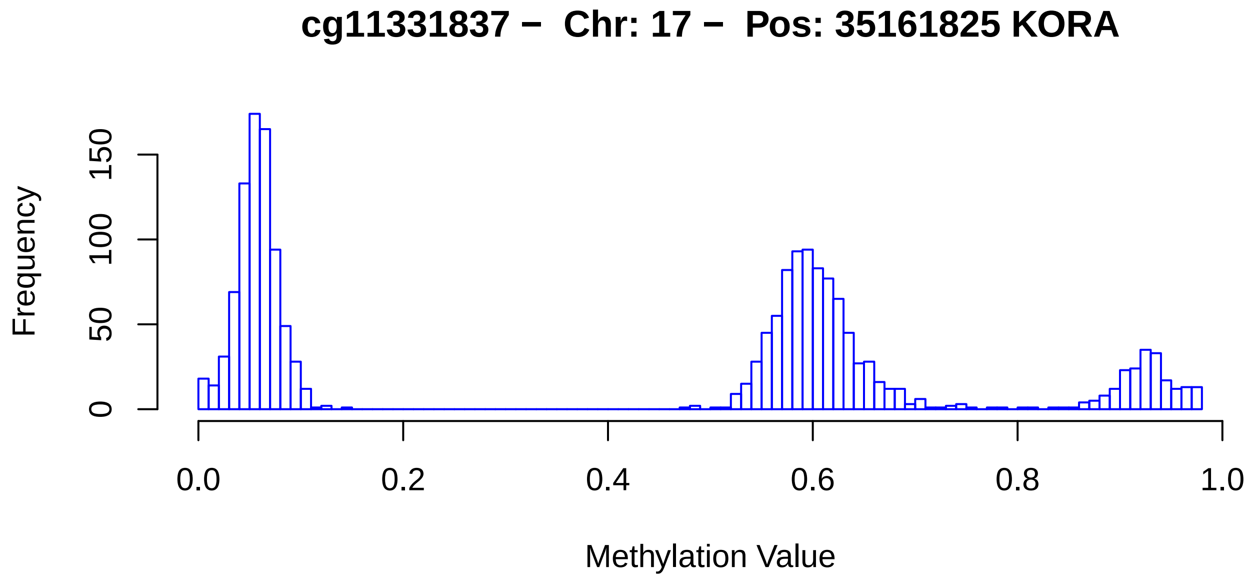
cg11331837 − Chr: 17 − Pos: 35161825 KORA
Frequency
0
50
100
150
0.0
0.2
0.4
0.6
0.8
1.0
Methylation Value

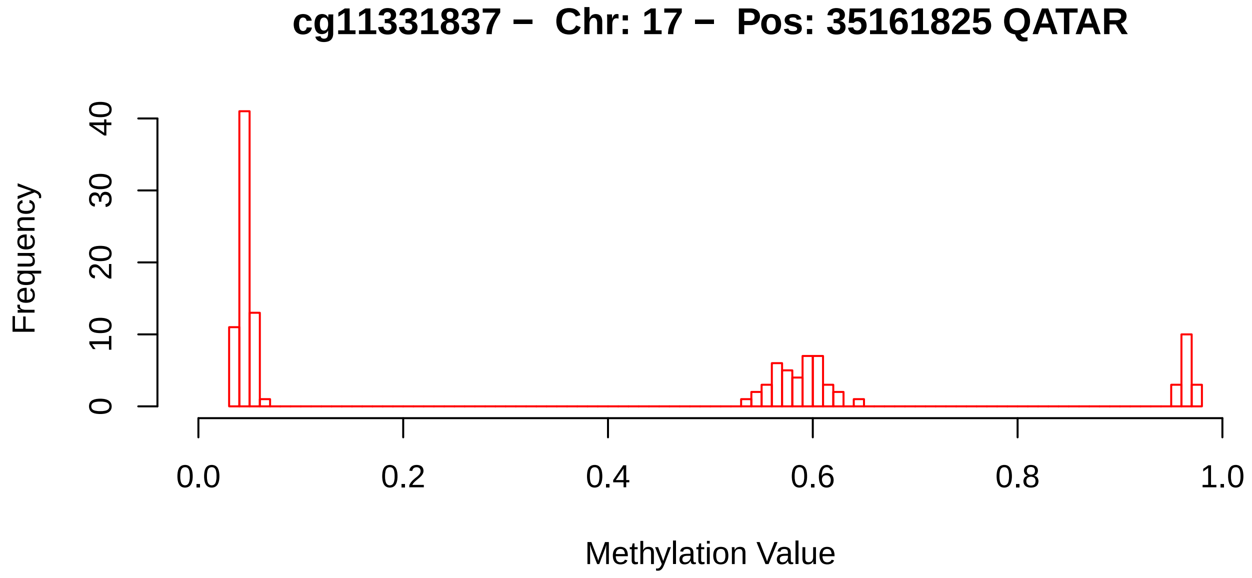
cg11331837 − Chr: 17 − Pos: 35161825 QATAR
Frequency
0
10
20
30
40
0.0
0.2
0.4
0.6
0.8
1.0
Methylation Value

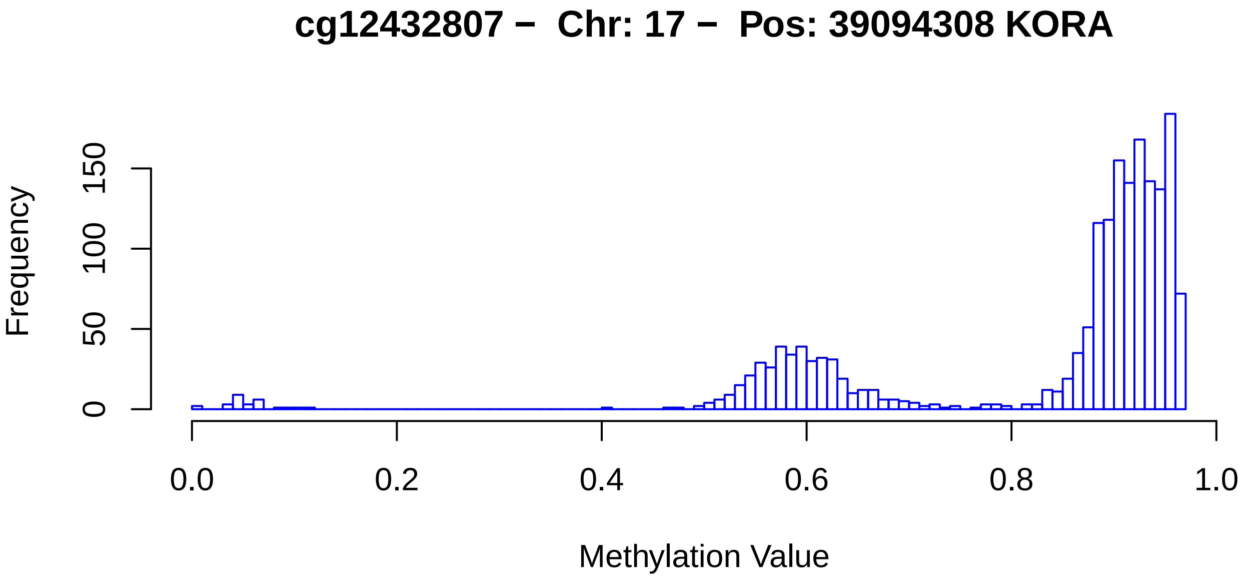
cg12432807 − Chr: 17 − Pos: 39094308 KORA
Frequency
0
50
100
150
0.0
0.2
0.4
0.6
0.8
1.0
Methylation Value

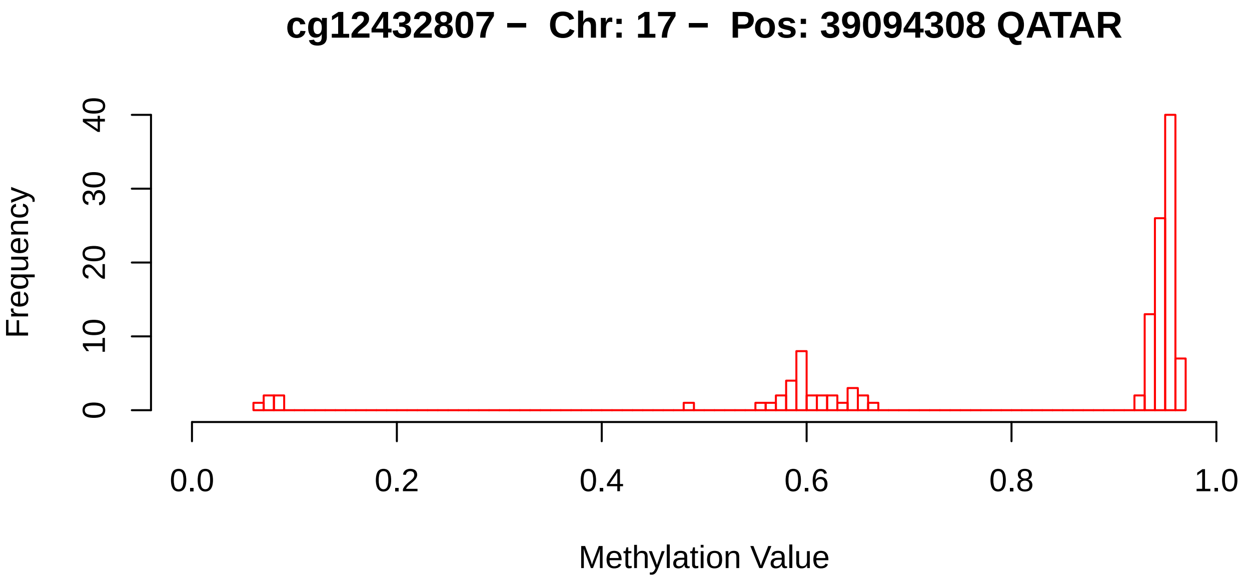
cg12432807 − Chr: 17 − Pos: 39094308 QATAR
Frequency
0
10
20
30
40
0.0
0.2
0.4
0.6
0.8
1.0
Methylation Value

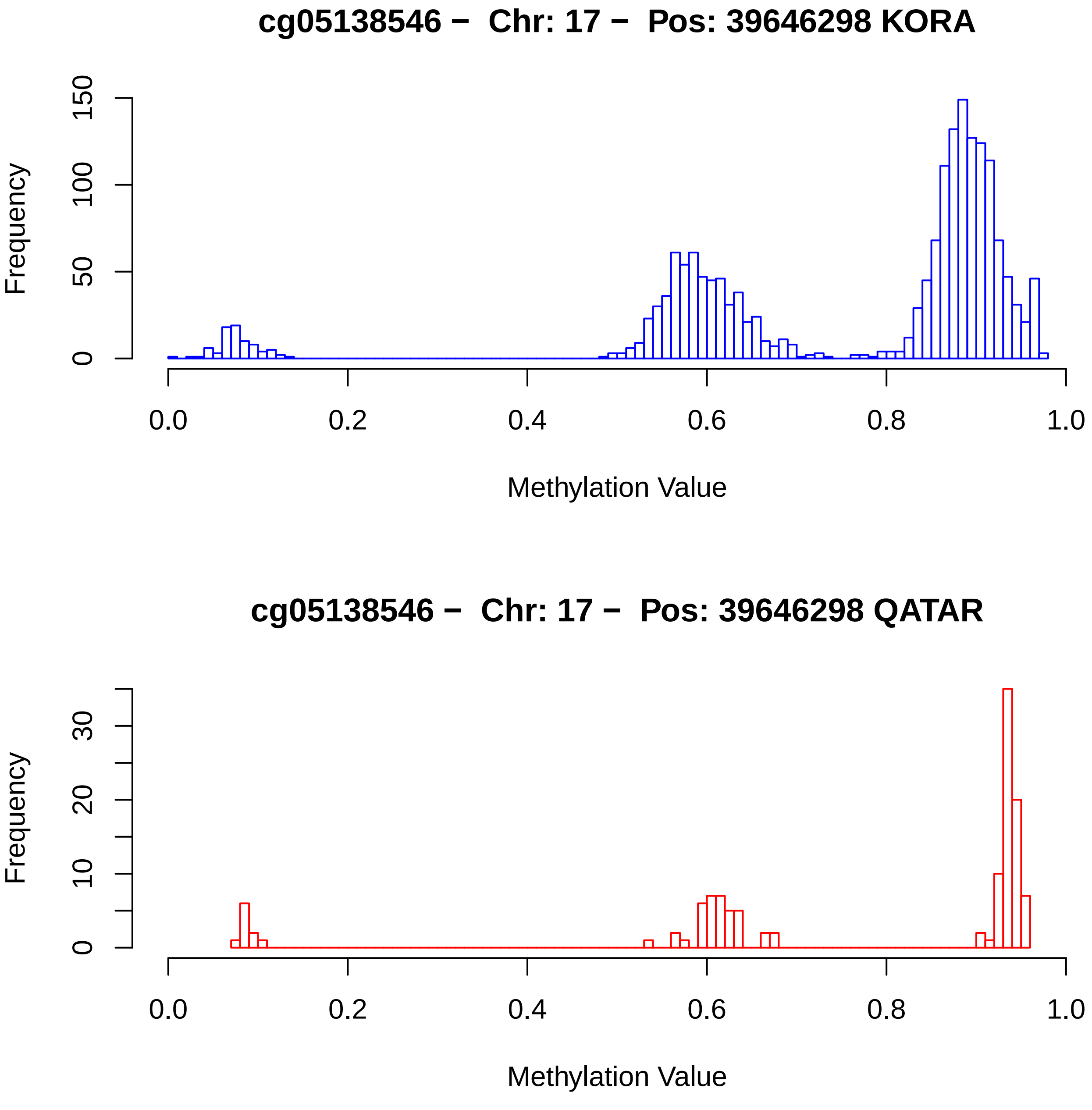
cg05138546 − Chr: 17 − Pos: 39646298 KORA
Frequency
0
50
100
150
0.0
0.2
0.4
0.6
0.8
1.0
Methylation Value
cg05138546 − Chr: 17 − Pos: 39646298 QATAR
Frequency
0
10
20
30
0.0
0.2
0.4
0.6
0.8
1.0
Methylation Value

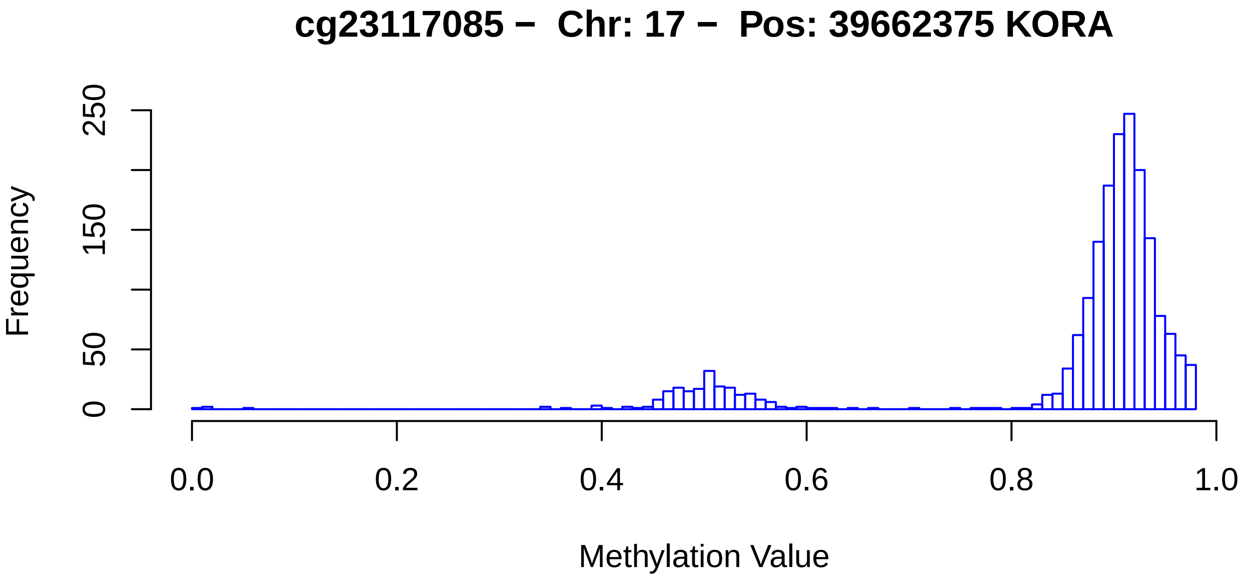
cg23117085 − Chr: 17 − Pos: 39662375 KORA
Frequency
0
50
150
250
0.0
0.2
0.4
0.6
0.8
1.0
Methylation Value

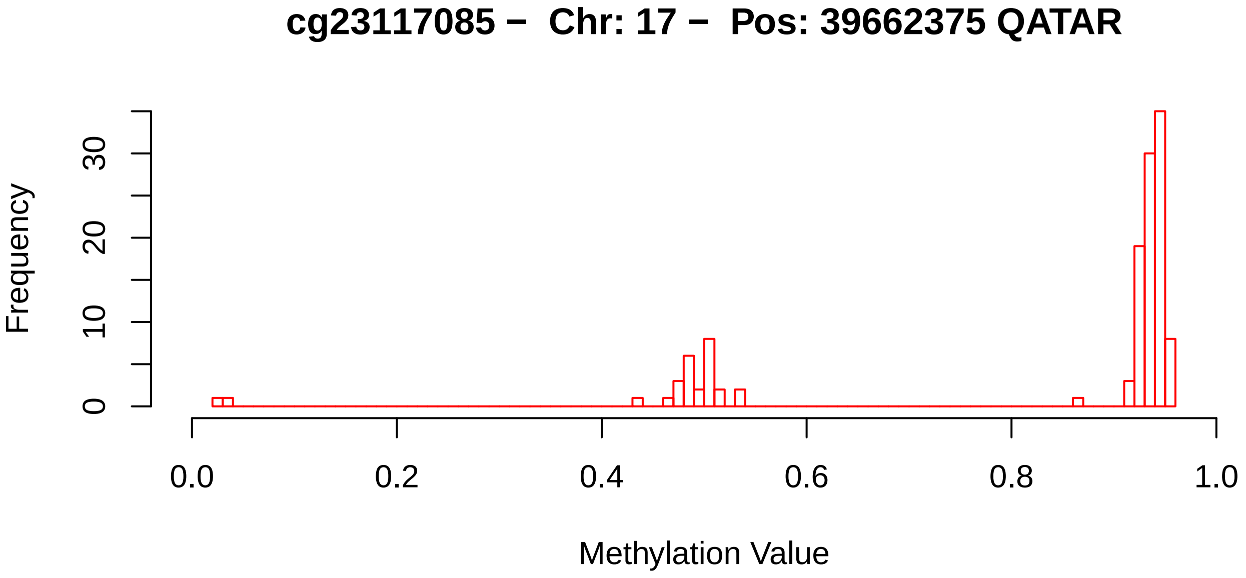
cg23117085 − Chr: 17 − Pos: 39662375 QATAR
Frequency
0
10
20
30
0.0
0.2
0.4
0.6
0.8
1.0
Methylation Value

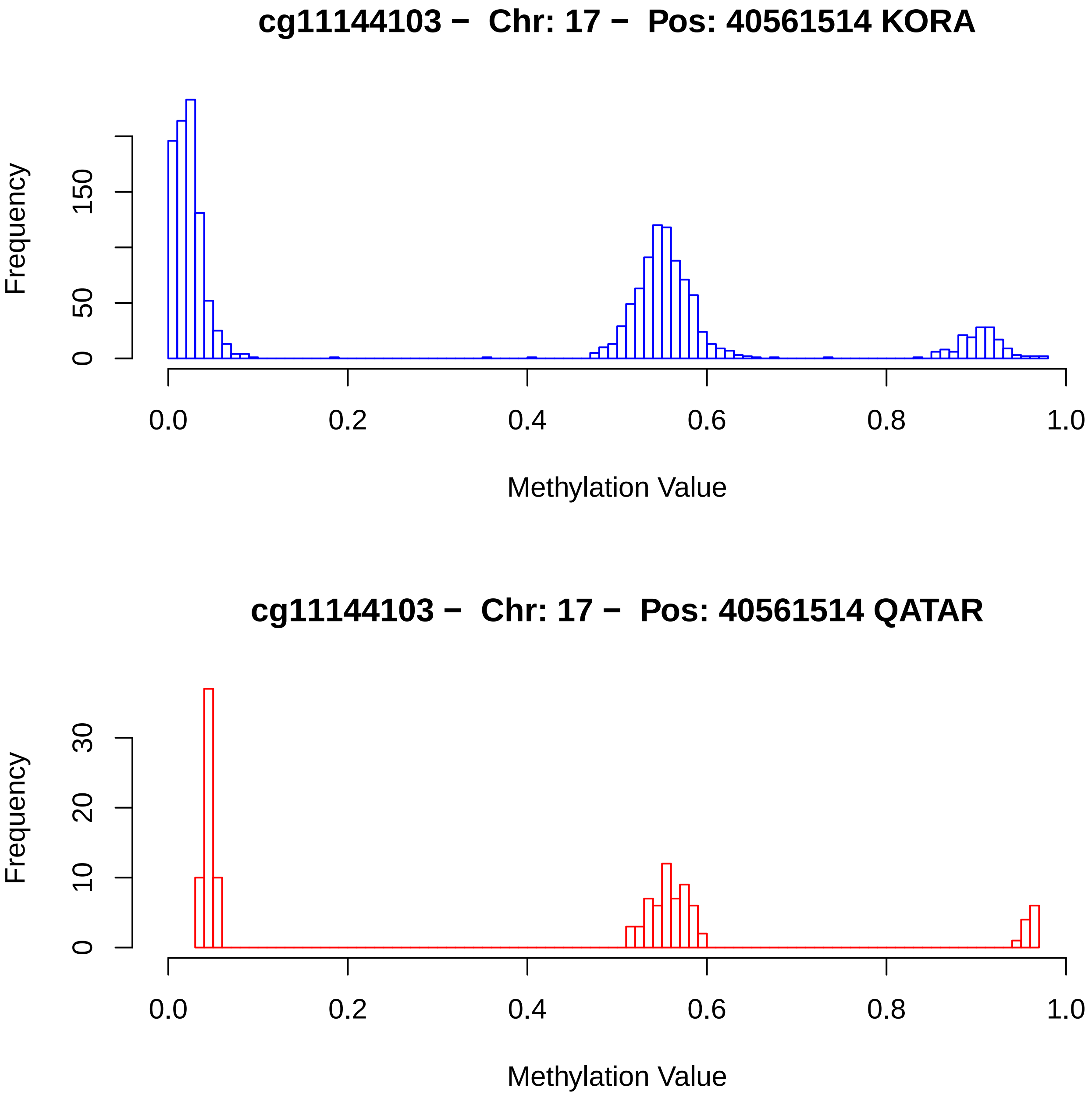
cg11144103 − Chr: 17 − Pos: 40561514 KORA
Frequency
0
50
150
0.0
0.2
0.4
0.6
0.8
1.0
Methylation Value
cg11144103 − Chr: 17 − Pos: 40561514 QATAR
Frequency
0
10
20
30
0.0
0.2
0.4
0.6
0.8
1.0
Methylation Value

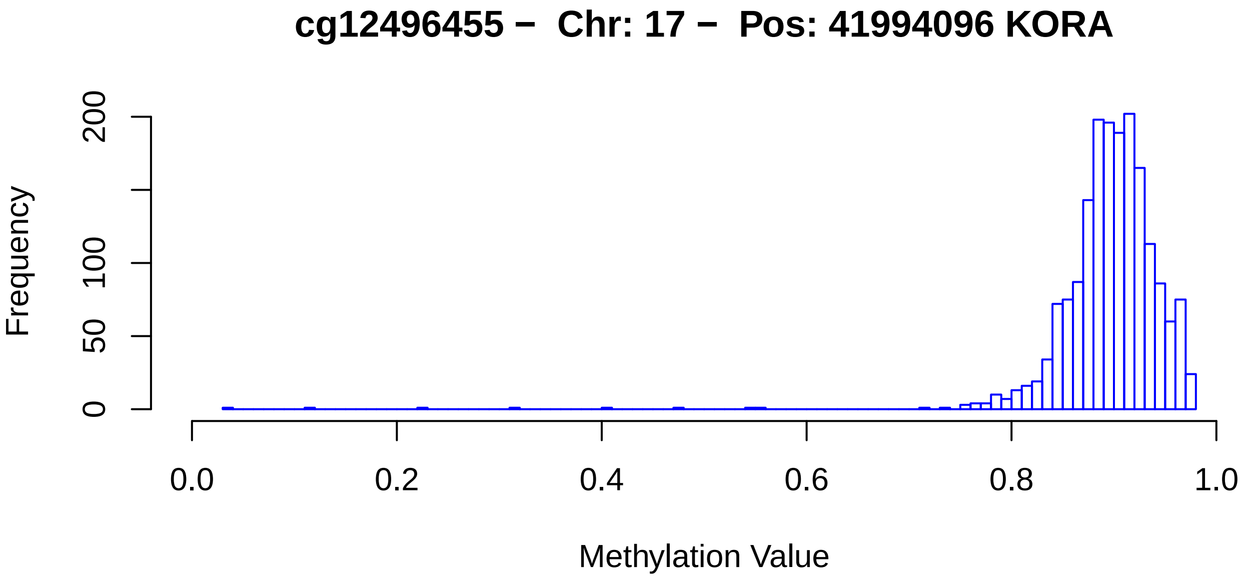
cg12496455 − Chr: 17 − Pos: 41994096 KORA
Frequency
0
50
100
200
0.0
0.2
0.4
0.6
0.8
1.0
Methylation Value

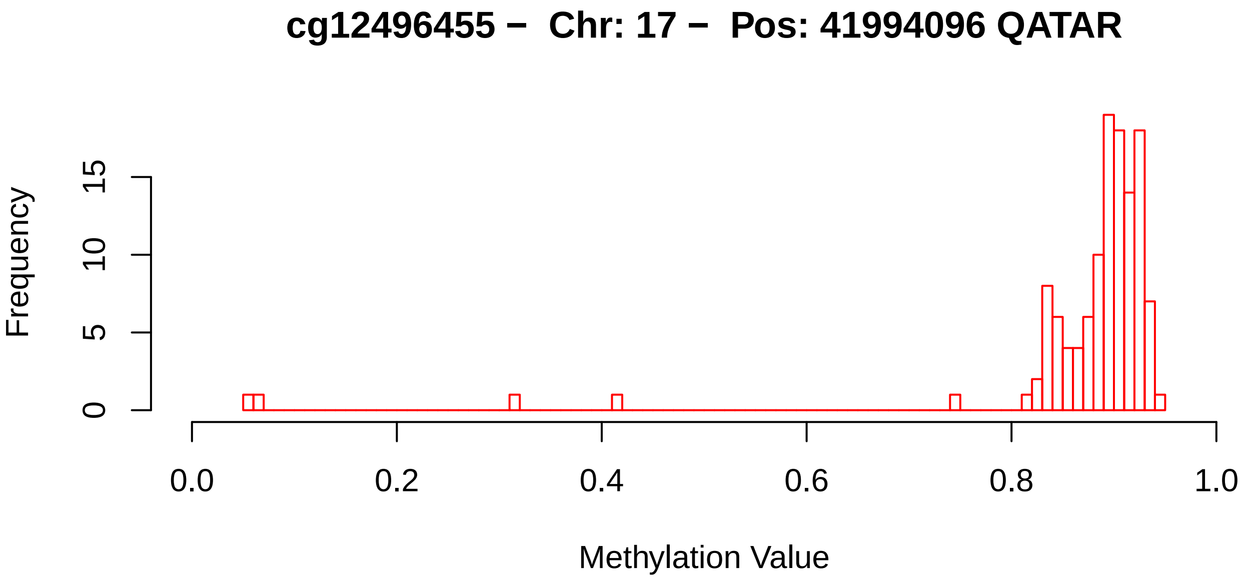
cg12496455 − Chr: 17 − Pos: 41994096 QATAR
Frequency
0
5
10
15
0.0
0.2
0.4
0.6
0.8
1.0
Methylation Value

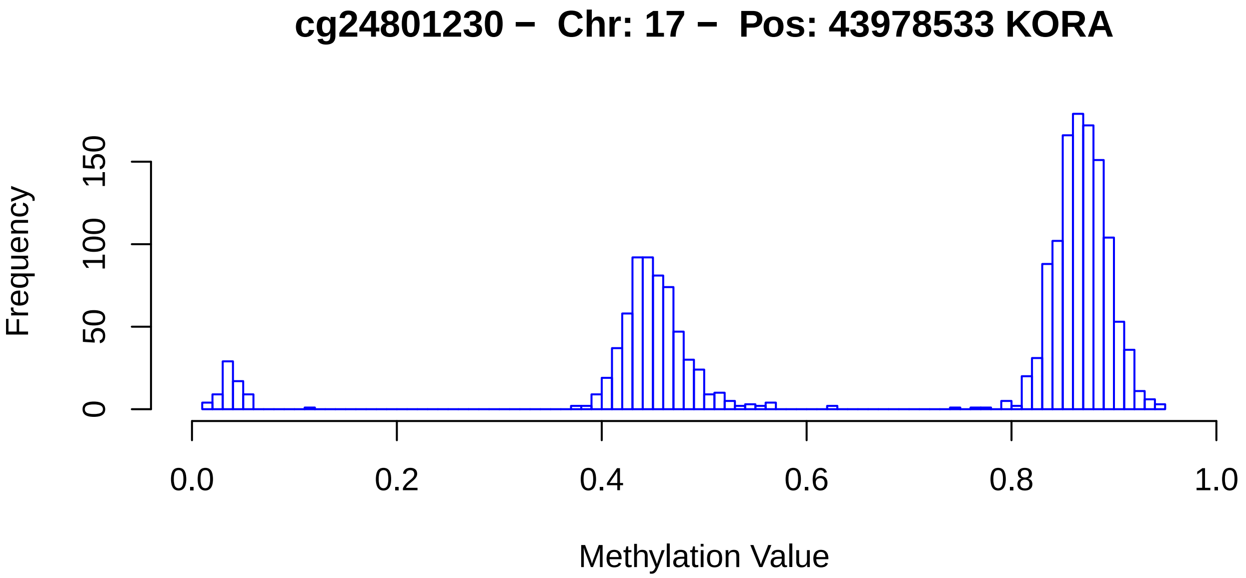
cg24801230 − Chr: 17 − Pos: 43978533 KORA
Frequency
0
50
100
150
0.0
0.2
0.4
0.6
0.8
1.0
Methylation Value

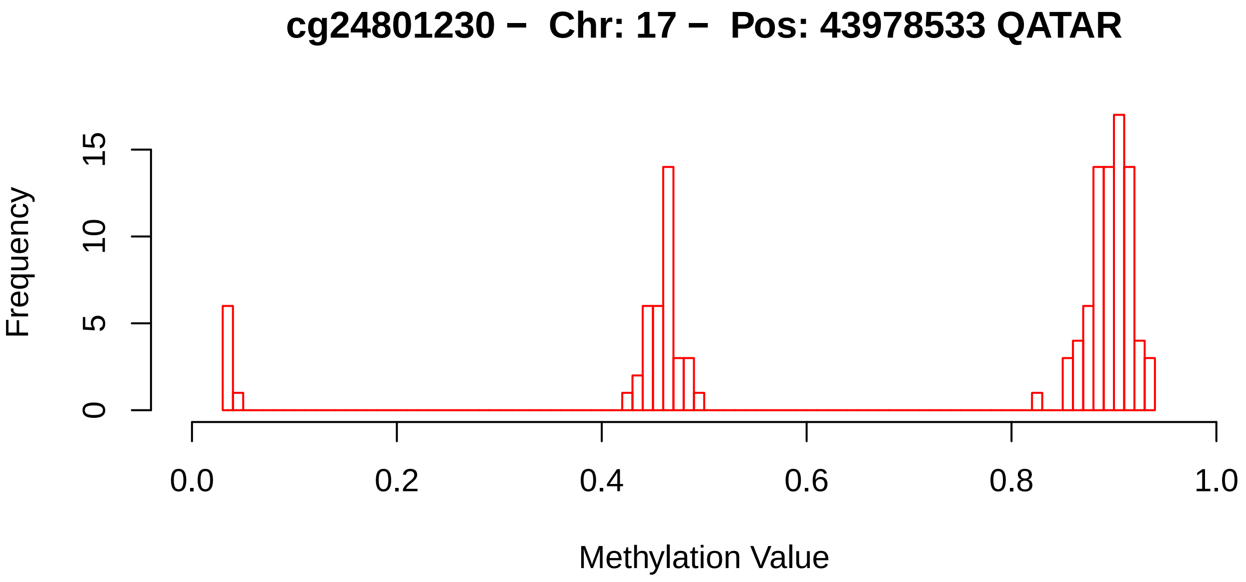
cg24801230 − Chr: 17 − Pos: 43978533 QATAR
Frequency
0
5
10
15
0.0
0.2
0.4
0.6
0.8
1.0
Methylation Value

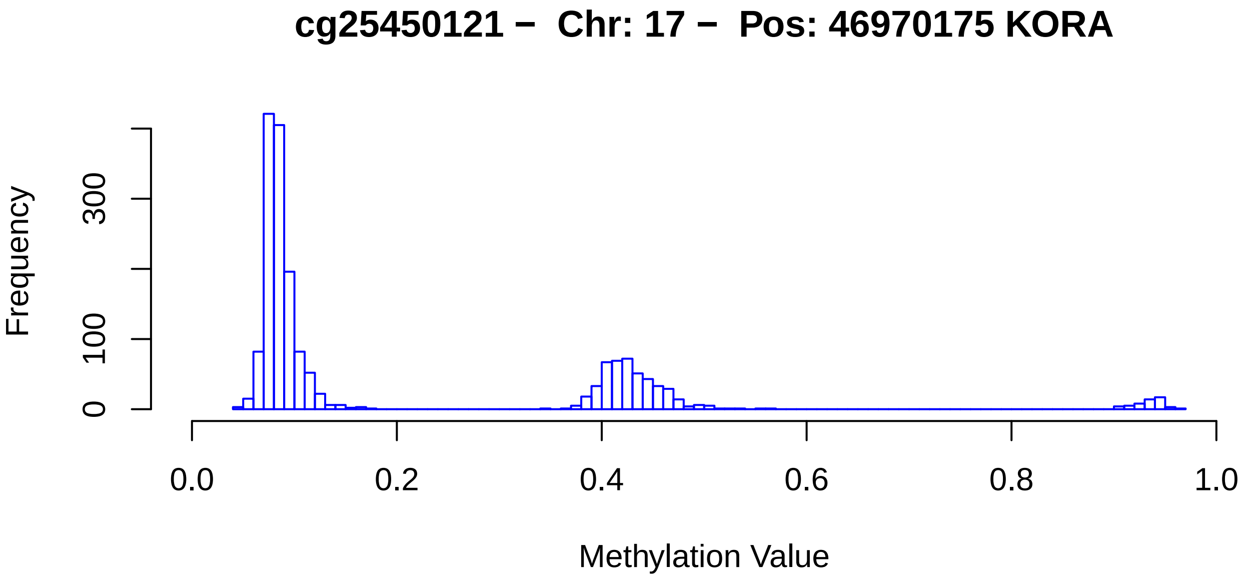
cg25450121 − Chr: 17 − Pos: 46970175 KORA
Frequency
0
100
300
0.0
0.2
0.4
0.6
0.8
1.0
Methylation Value

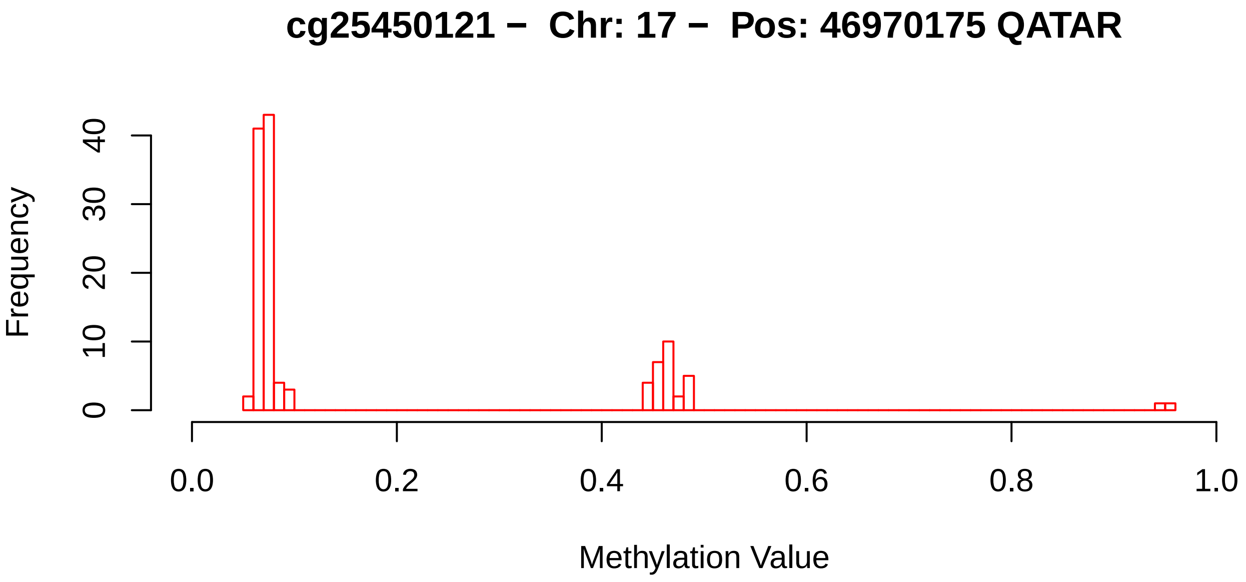
cg25450121 − Chr: 17 − Pos: 46970175 QATAR
Frequency
0
10
20
30
40
0.0
0.2
0.4
0.6
0.8
1.0
Methylation Value

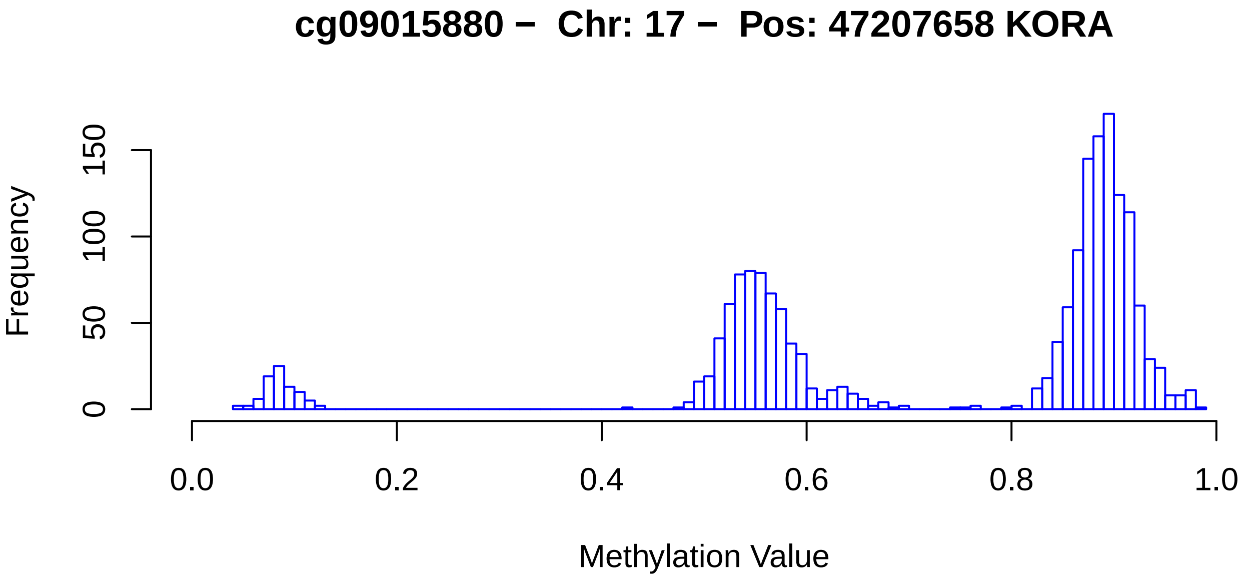
cg09015880 − Chr: 17 − Pos: 47207658 KORA
Frequency
0
50
100
150
0.0
0.2
0.4
0.6
0.8
1.0
Methylation Value

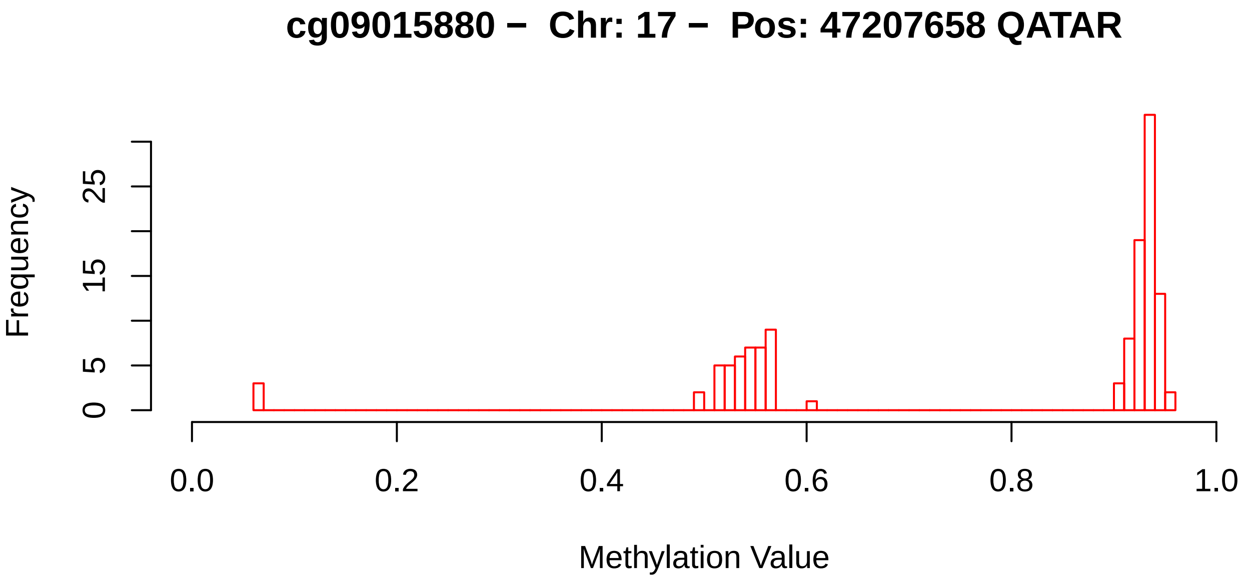
cg09015880 − Chr: 17 − Pos: 47207658 QATAR
Frequency
0
5
15
25
0.0
0.2
0.4
0.6
0.8
1.0
Methylation Value

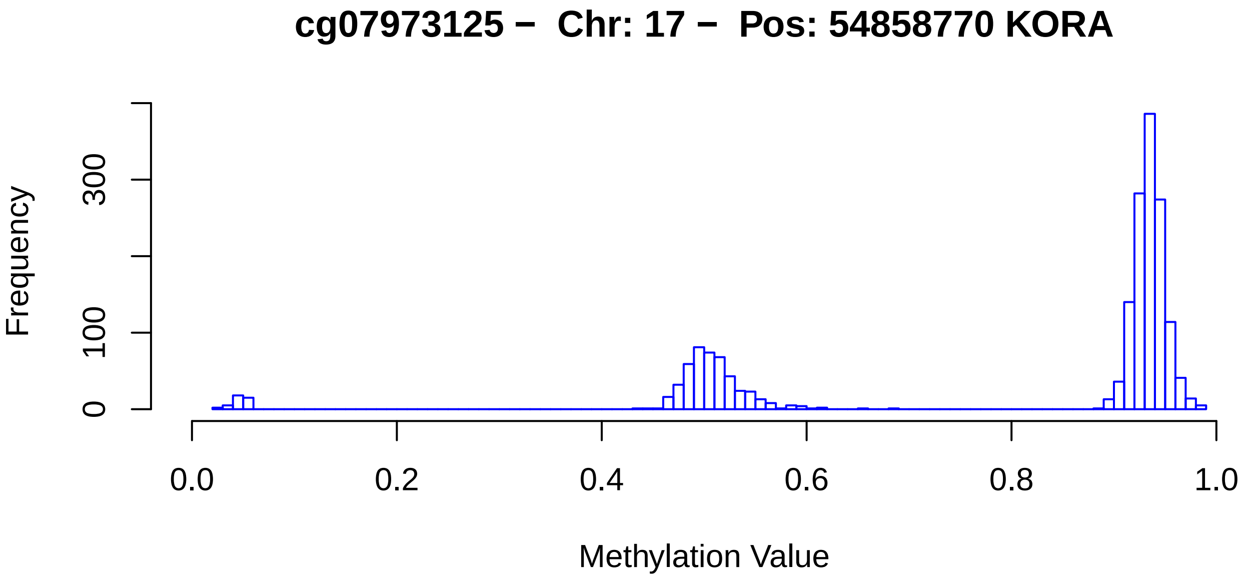
cg07973125 − Chr: 17 − Pos: 54858770 KORA
Frequency
0
100
300
0.0
0.2
0.4
0.6
0.8
1.0
Methylation Value

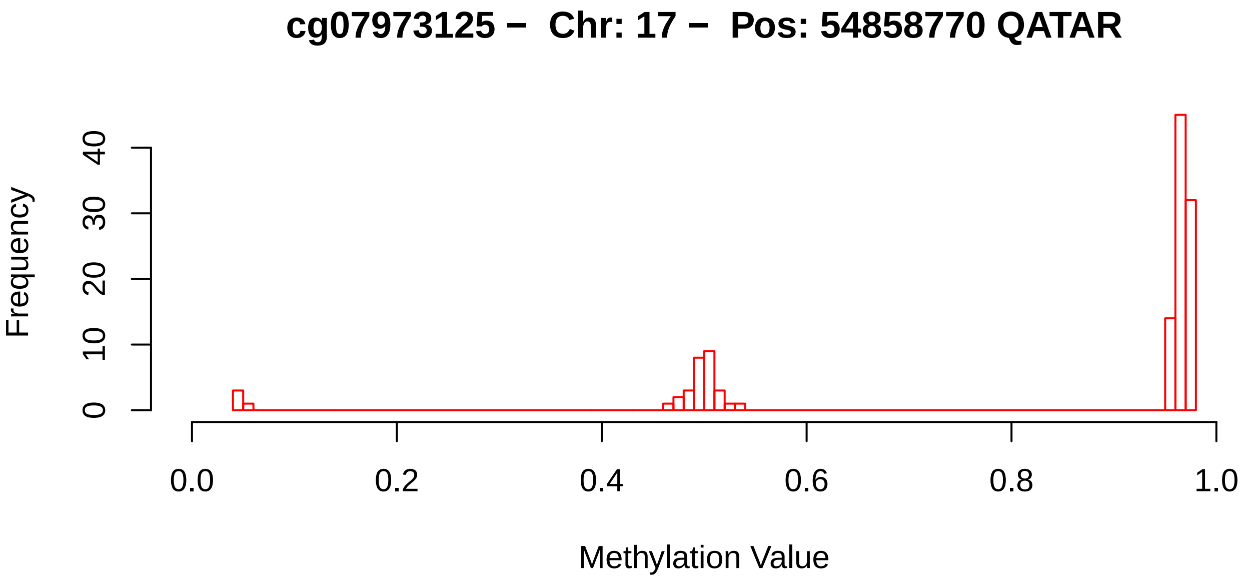
cg07973125 − Chr: 17 − Pos: 54858770 QATAR
Frequency
0
10
20
30
40
0.0
0.2
0.4
0.6
0.8
1.0
Methylation Value

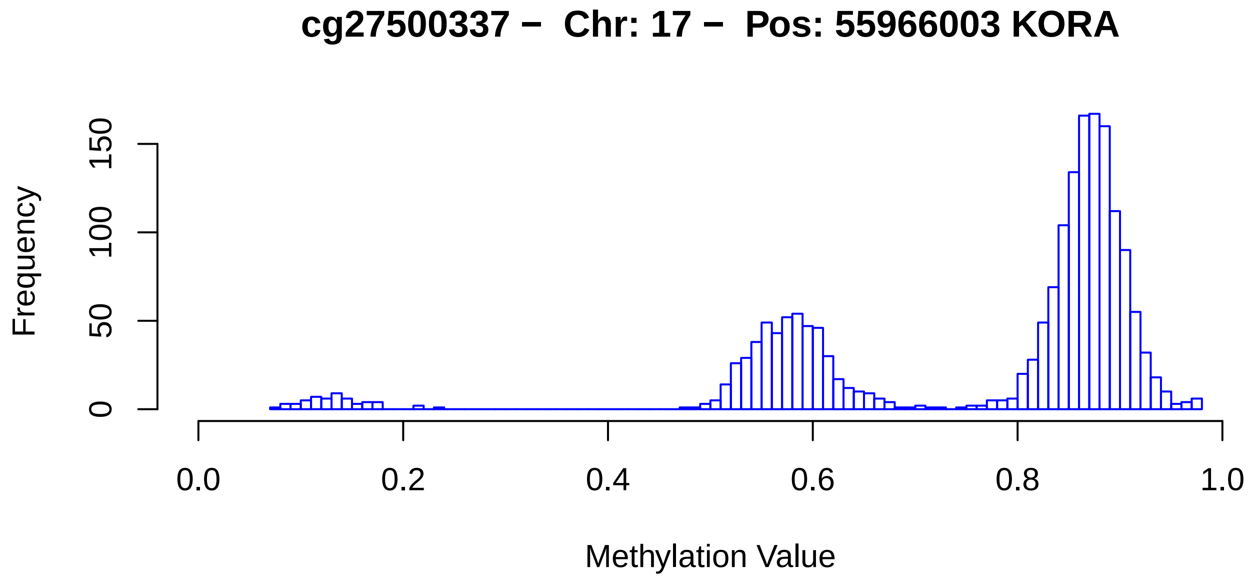
cg27500337 − Chr: 17 − Pos: 55966003 KORA
Frequency
0
50
100
150
0.0
0.2
0.4
0.6
0.8
1.0
Methylation Value

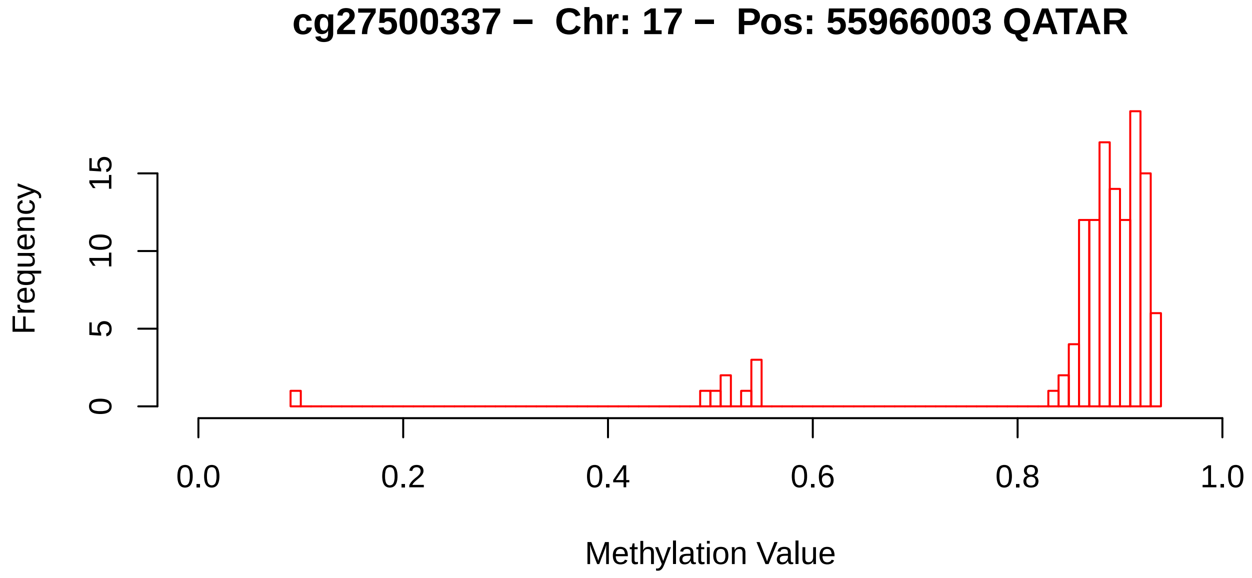
cg27500337 − Chr: 17 − Pos: 55966003 QATAR
Frequency
0
5
10
15
0.0
0.2
0.4
0.6
0.8
1.0
Methylation Value

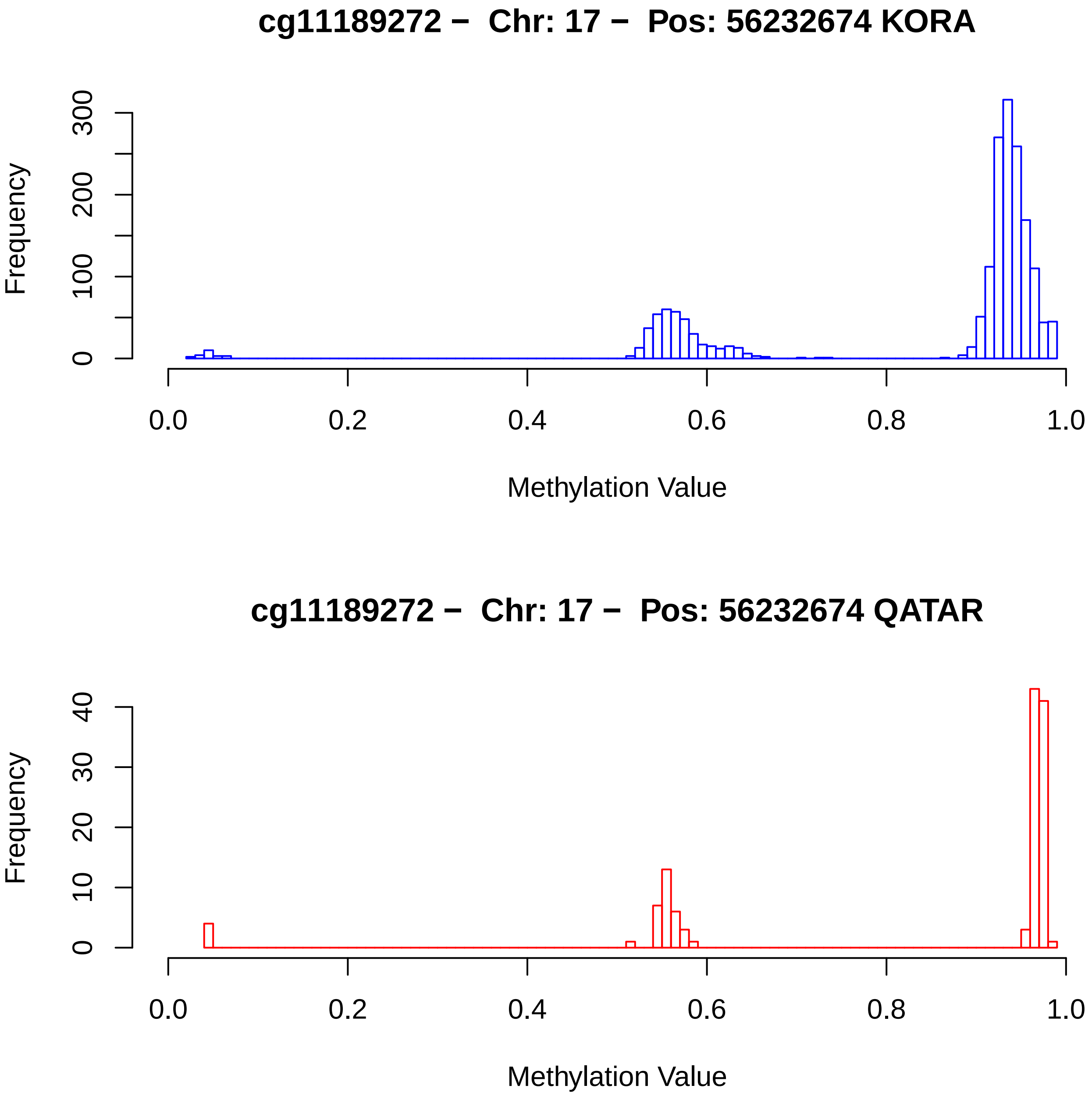
cg11189272 − Chr: 17 − Pos: 56232674 KORA
Frequency
0
100
200
300
0.0
0.2
0.4
0.6
0.8
1.0
Methylation Value
cg11189272 − Chr: 17 − Pos: 56232674 QATAR
Frequency
0
10
20
30
40
0.0
0.2
0.4
0.6
0.8
1.0
Methylation Value

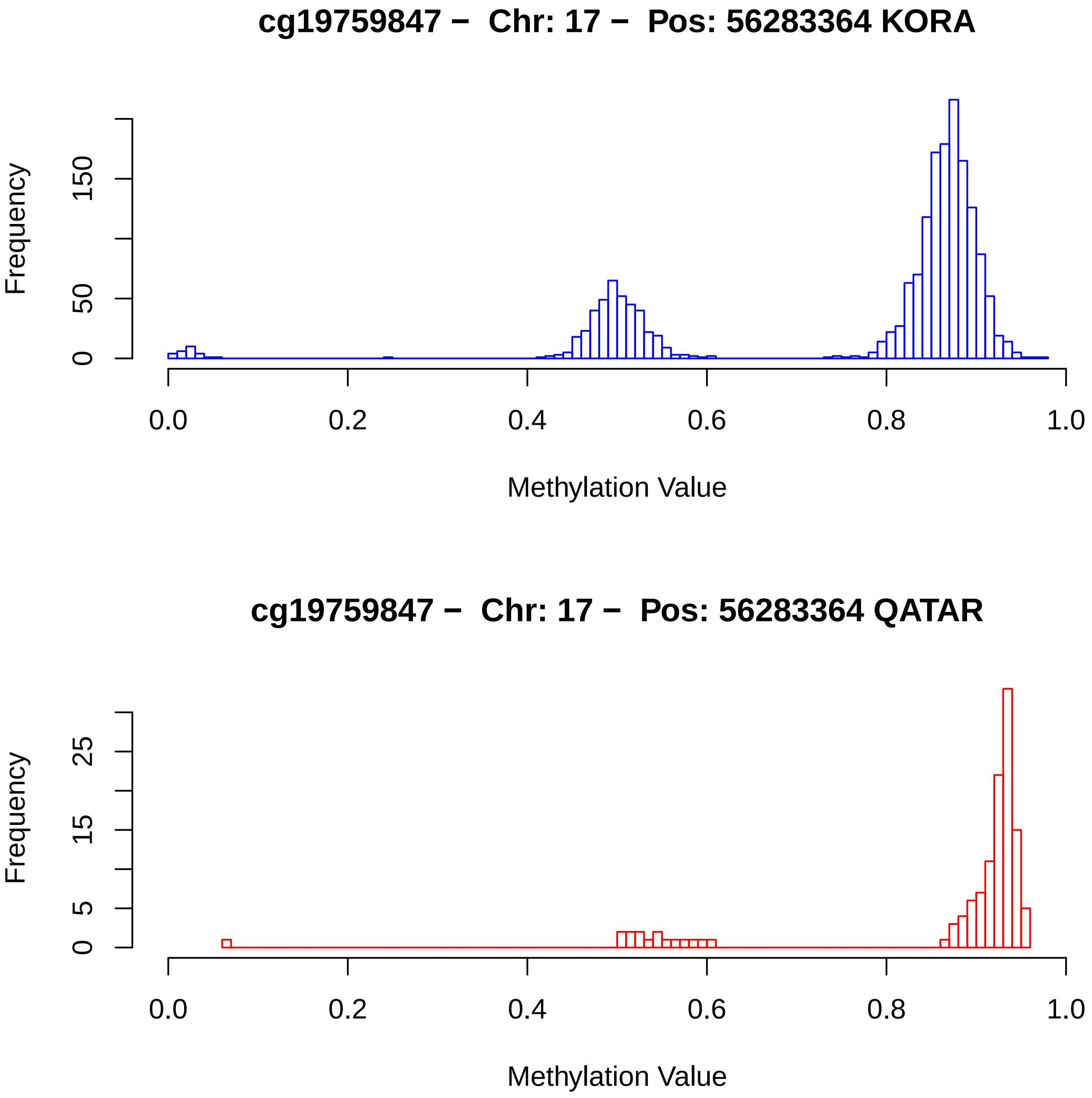
cg19759847 − Chr: 17 − Pos: 56283364 KORA
Frequency
0
50
150
0.0
0.2
0.4
0.6
0.8
1.0
Methylation Value
cg19759847 − Chr: 17 − Pos: 56283364 QATAR
Frequency
0
5
15
25
0.0
0.2
0.4
0.6
0.8
1.0
Methylation Value

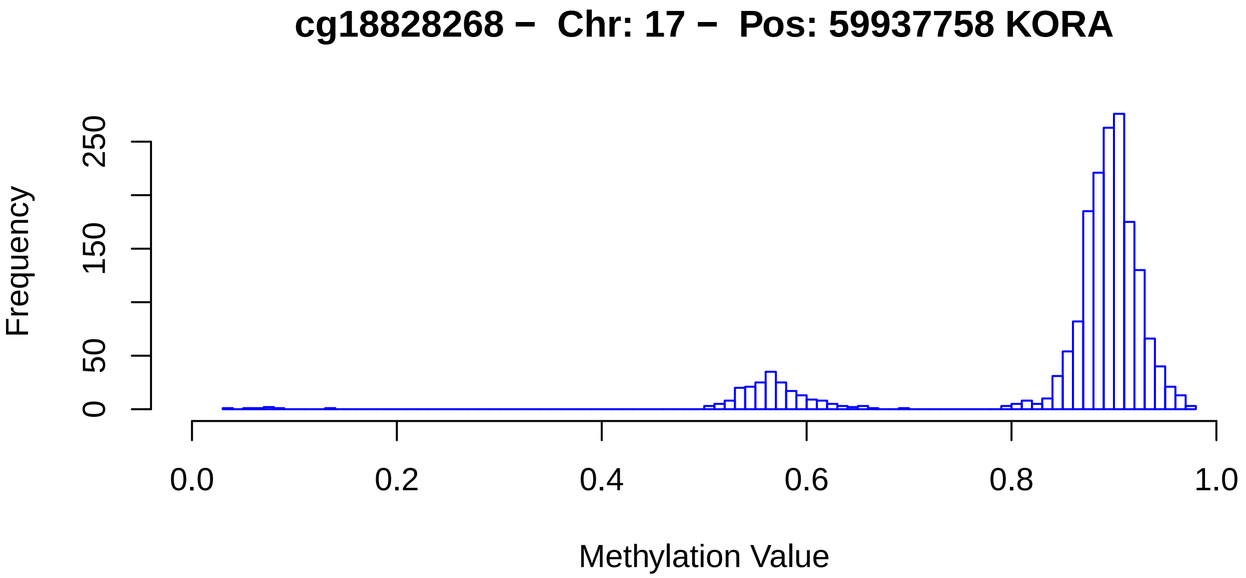
cg18828268 − Chr: 17 − Pos: 59937758 KORA
Frequency
0
50
150
250
0.0
0.2
0.4
0.6
0.8
1.0
Methylation Value

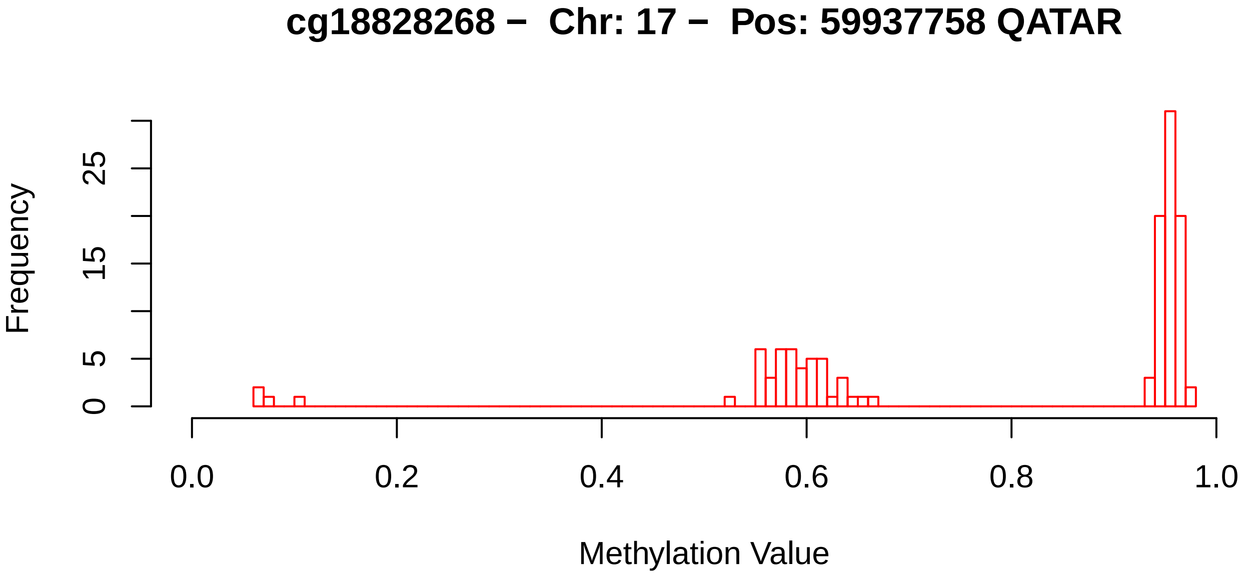
cg18828268 − Chr: 17 − Pos: 59937758 QATAR
Frequency
0
5
15
25
0.0
0.2
0.4
0.6
0.8
1.0
Methylation Value

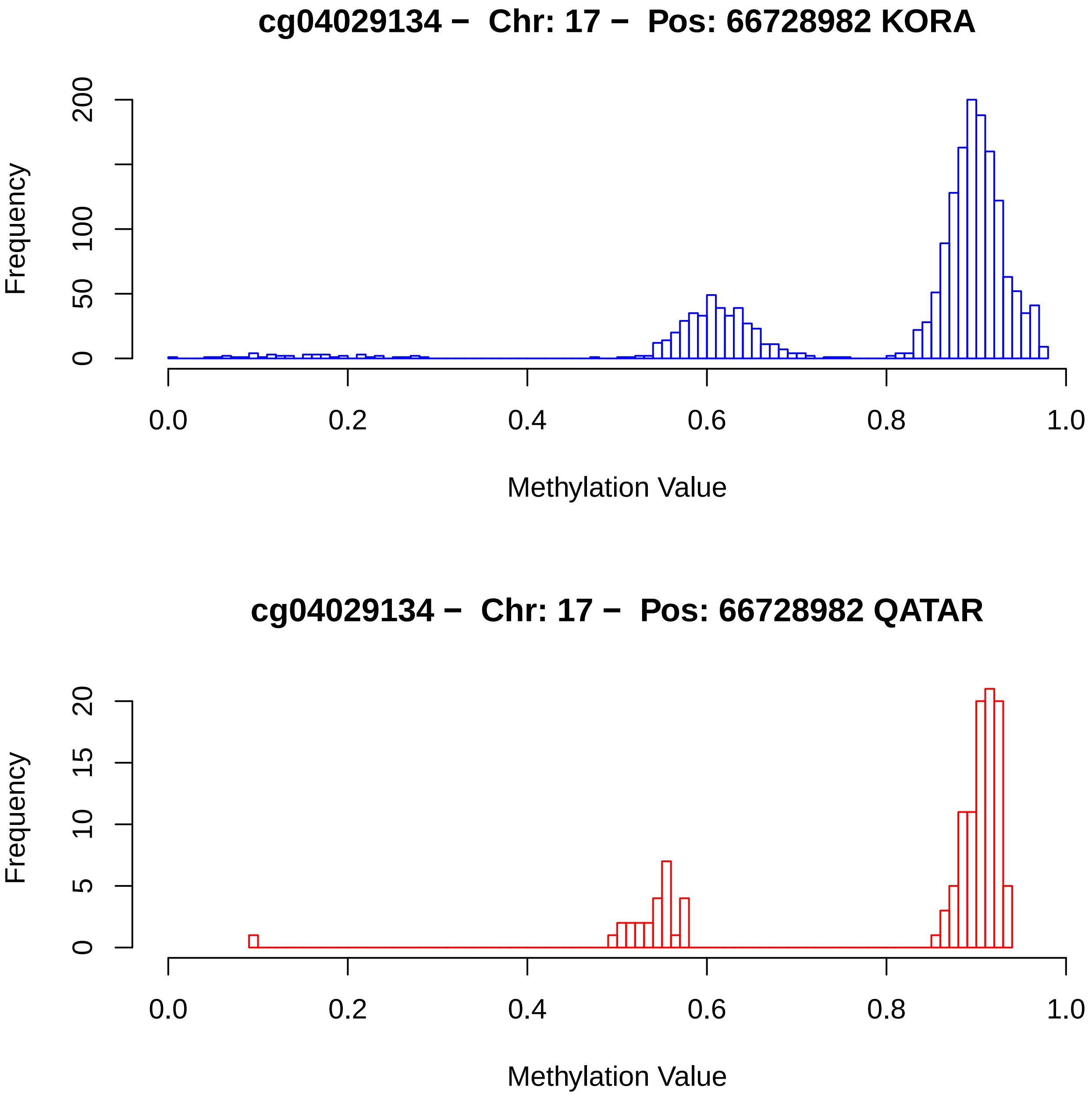

cg04029134 − Chr: 17 − Pos: 66728982 KORA
Frequency
0
50
100
200
0.0
0.2
0.4
0.6
0.8
1.0
Methylation Value
cg04029134 − Chr: 17 − Pos: 66728982 QATAR
Frequency
0
5
10
15
20
0.0
0.2
0.4
0.6
0.8
1.0
Methylation Value

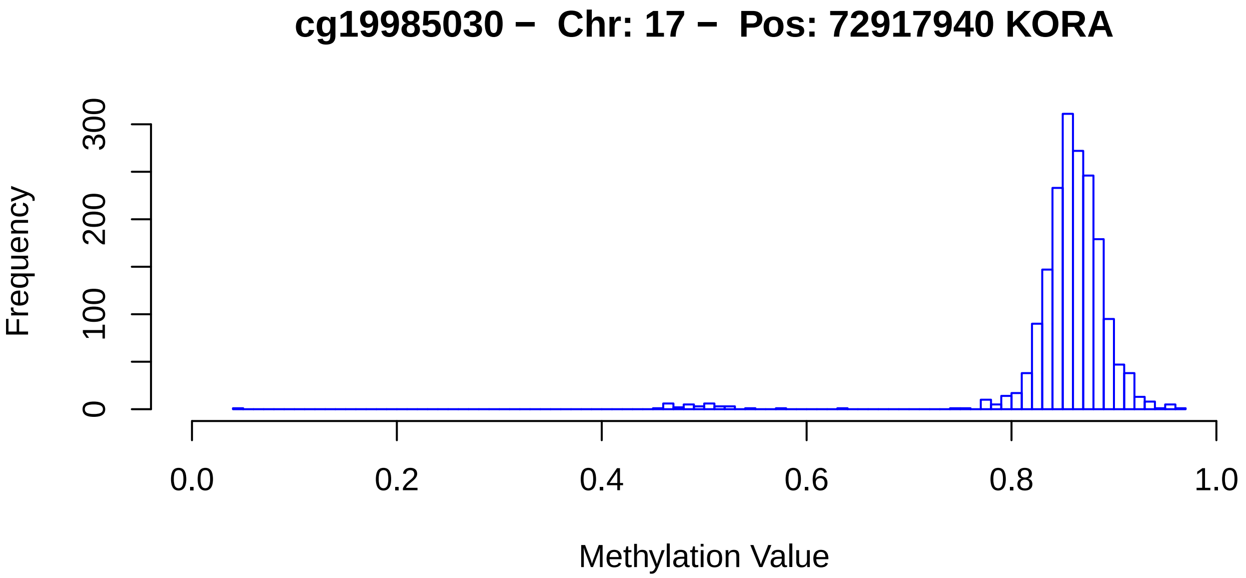

cg19985030 − Chr: 17 − Pos: 72917940 KORA
Frequency
0
100
200
300
0.0
0.2
0.4
0.6
0.8
1.0
Methylation Value

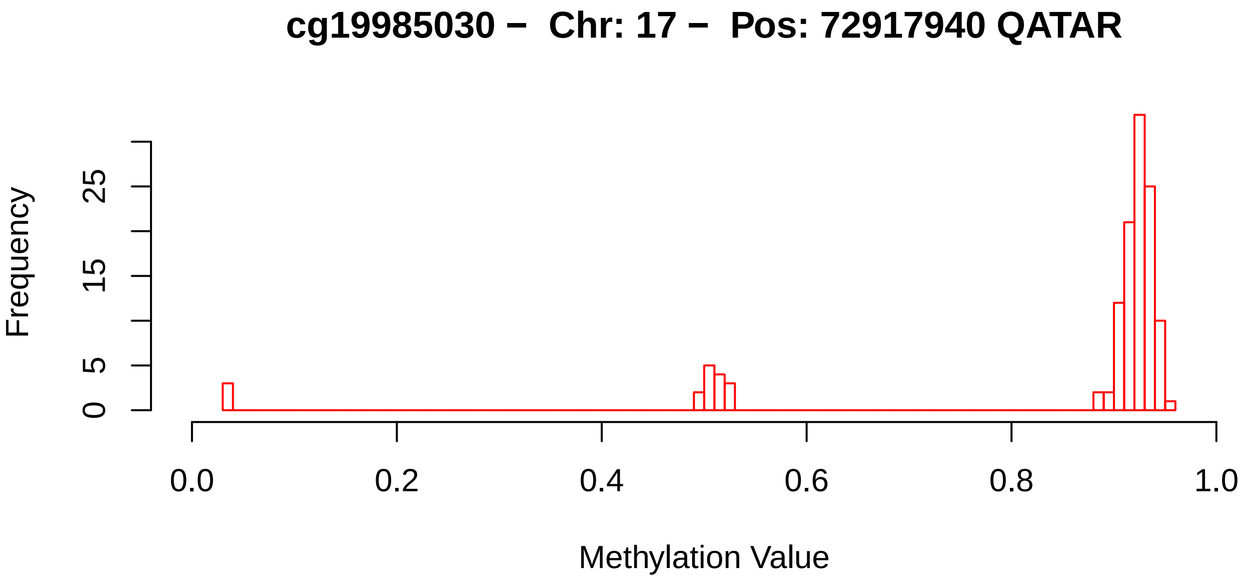

cg19985030 − Chr: 17 − Pos: 72917940 QATAR
Frequency
0
5
15
25
0.0
0.2
0.4
0.6
0.8
1.0
Methylation Value

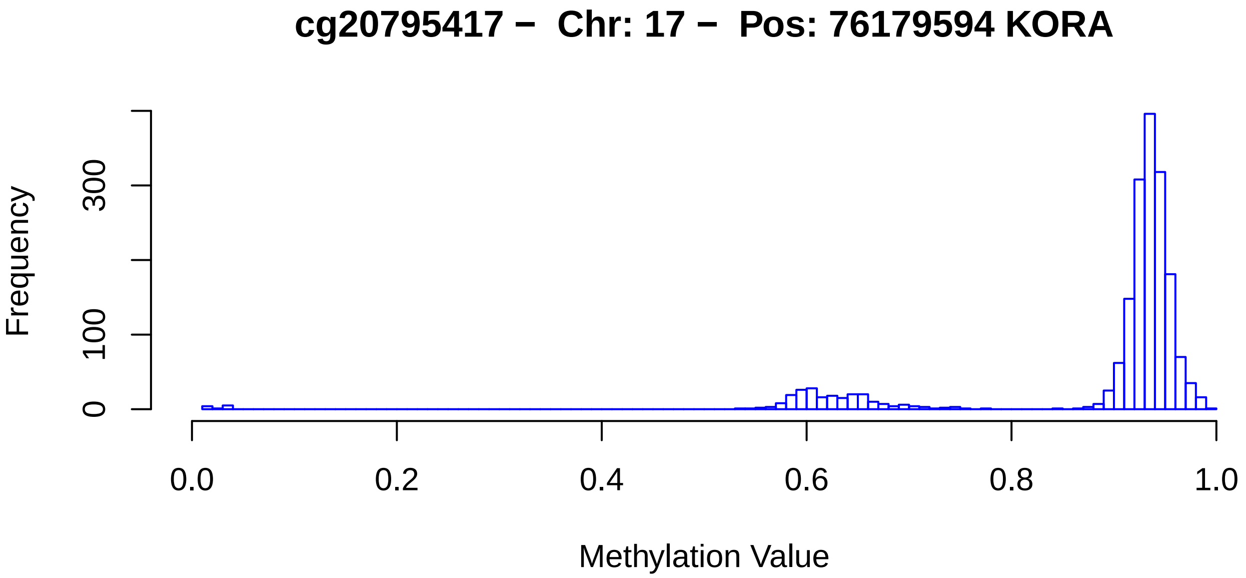
cg20795417 − Chr: 17 − Pos: 76179594 KORA
Frequency
0
100
300
0.0
0.2
0.4
0.6
0.8
1.0
Methylation Value

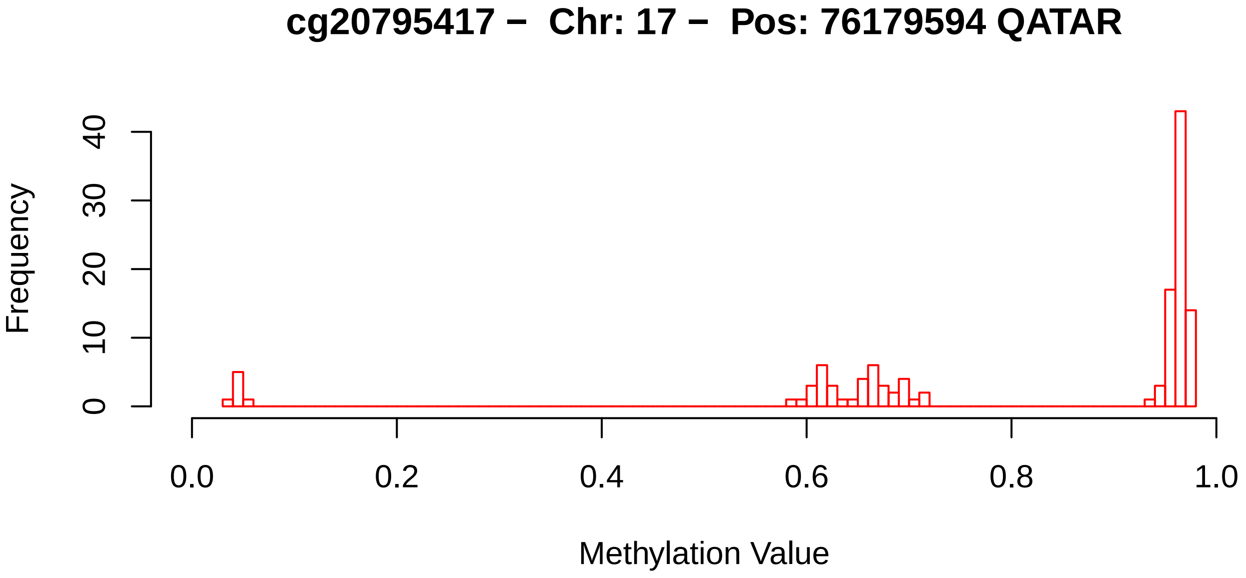
cg20795417 − Chr: 17 − Pos: 76179594 QATAR
Frequency
0
10
20
30
40
0.0
0.2
0.4
0.6
0.8
1.0
Methylation Value

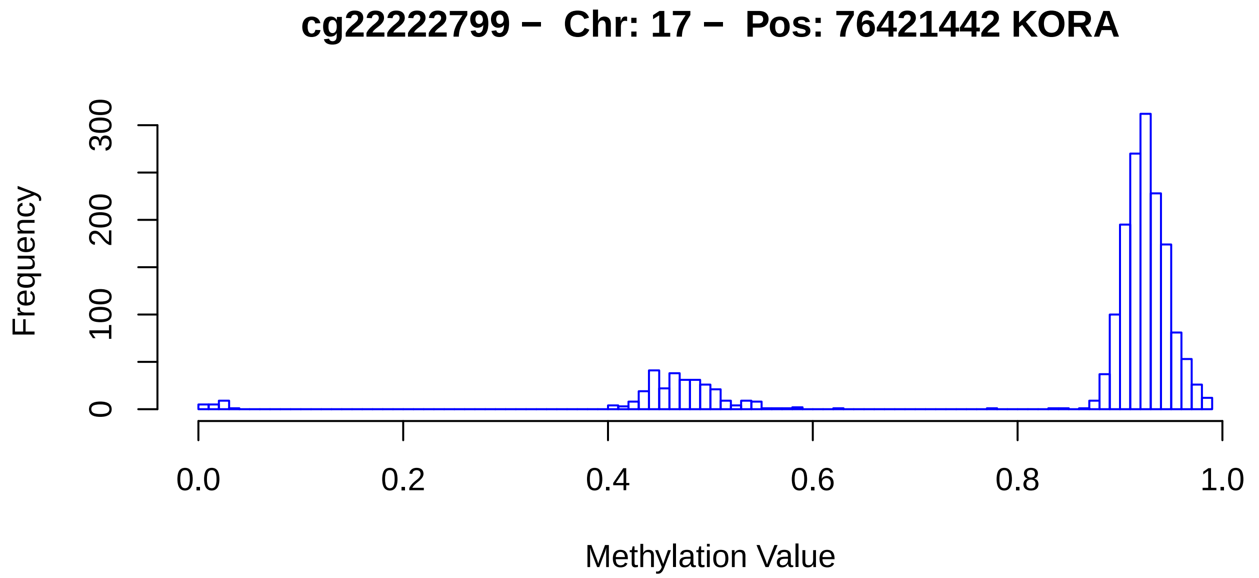
cg22222799 − Chr: 17 − Pos: 76421442 KORA
Frequency
0
100
200
300
0.0
0.2
0.4
0.6
0.8
1.0
Methylation Value

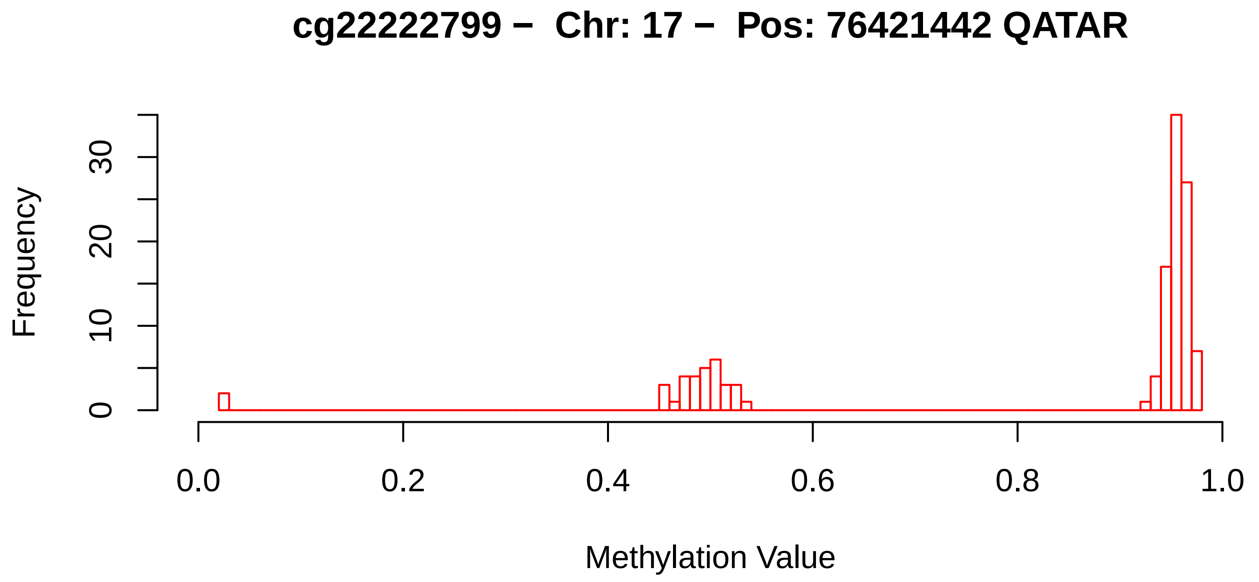
cg22222799 − Chr: 17 − Pos: 76421442 QATAR
Frequency
0
10
20
30
0.0
0.2
0.4
0.6
0.8
1.0
Methylation Value

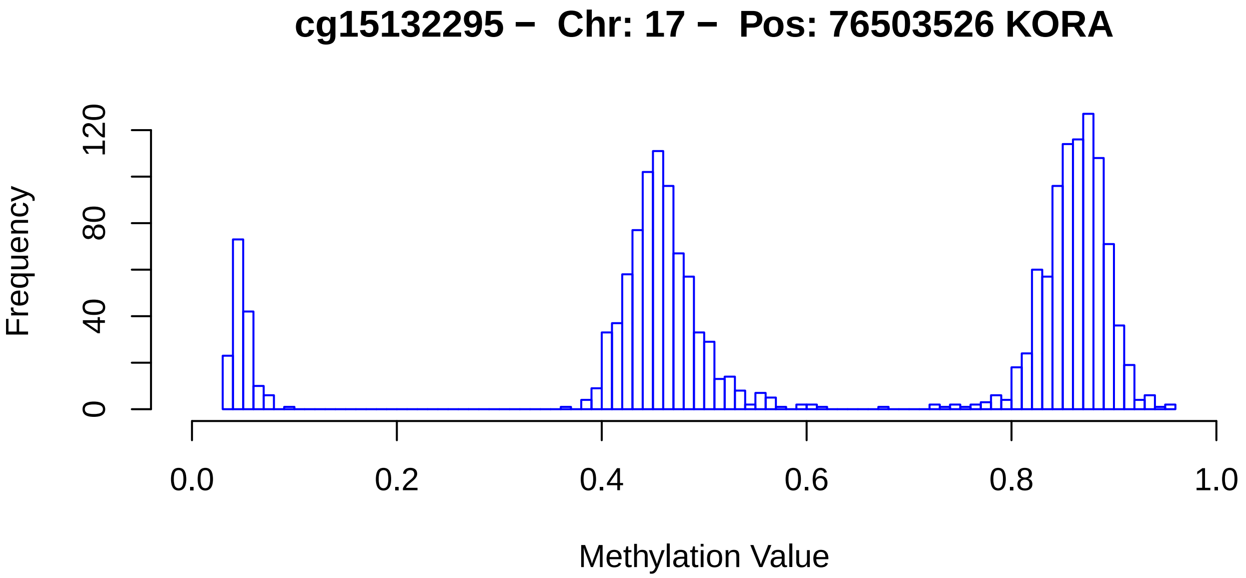
cg15132295 − Chr: 17 − Pos: 76503526 KORA
Frequency
0
40
80
120
0.0
0.2
0.4
0.6
0.8
1.0
Methylation Value

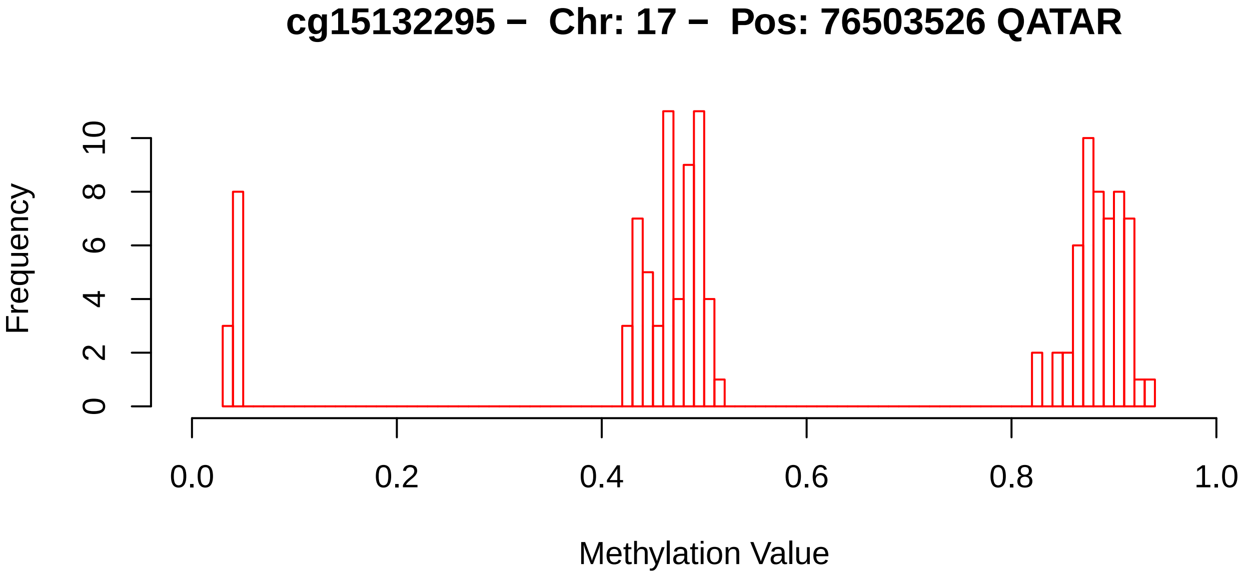
cg15132295 − Chr: 17 − Pos: 76503526 QATAR
Frequency
0
2
4
6
8
10
0.0
0.2
0.4
0.6
0.8
1.0
Methylation Value

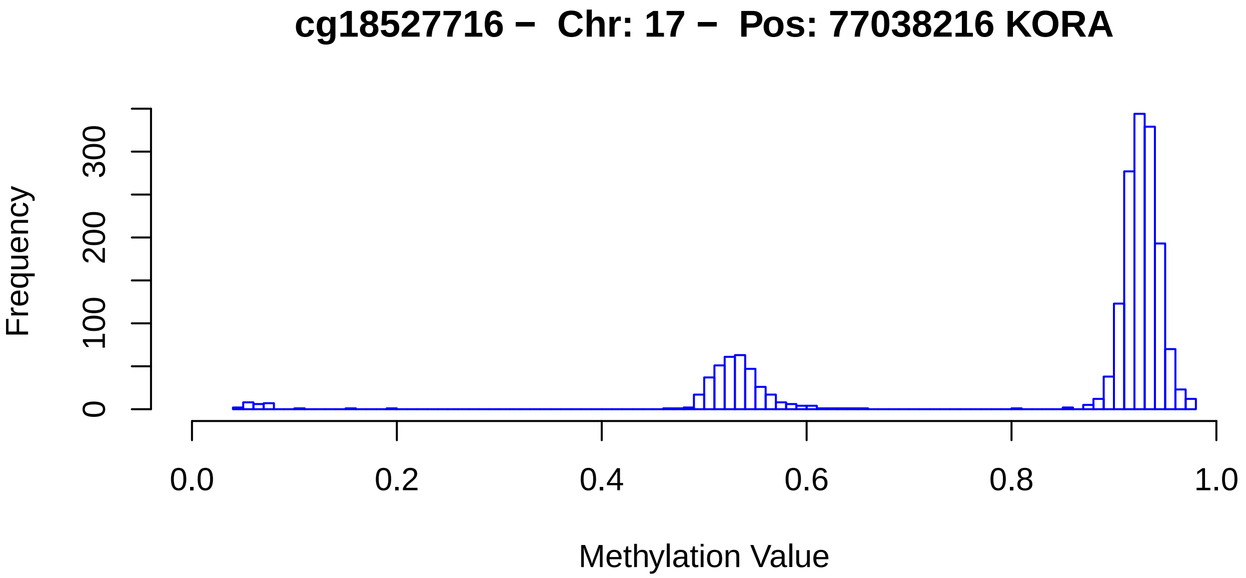
cg18527716 − Chr: 17 − Pos: 77038216 KORA
Frequency
0
100
200
300
0.0
0.2
0.4
0.6
0.8
1.0
Methylation Value

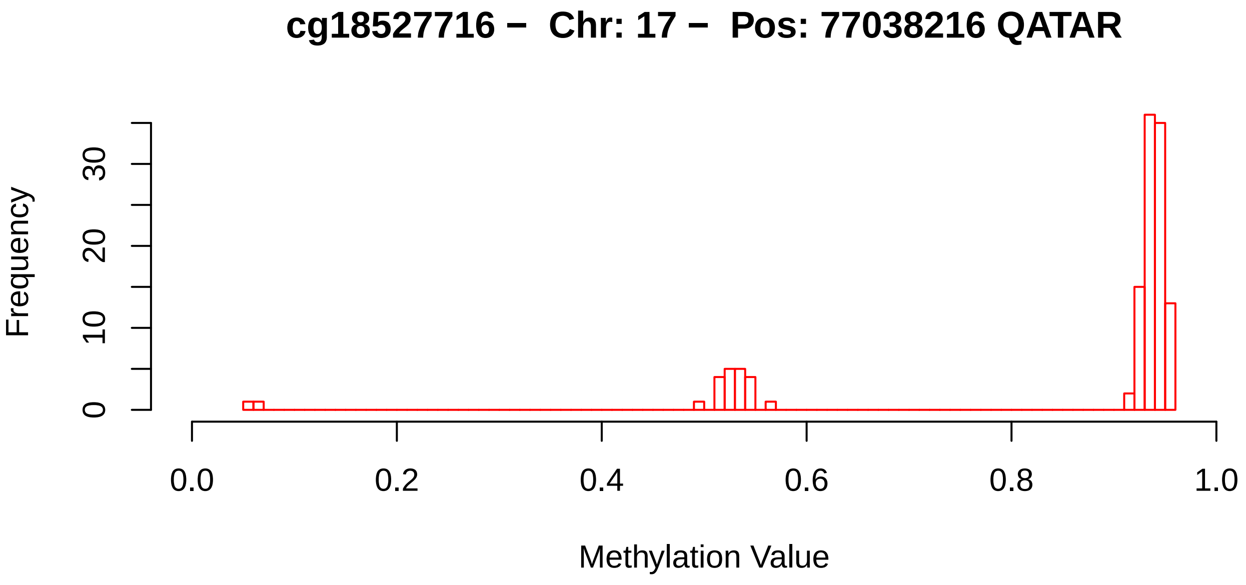
cg18527716 − Chr: 17 − Pos: 77038216 QATAR
Frequency
0
10
20
30
0.0
0.2
0.4
0.6
0.8
1.0
Methylation Value

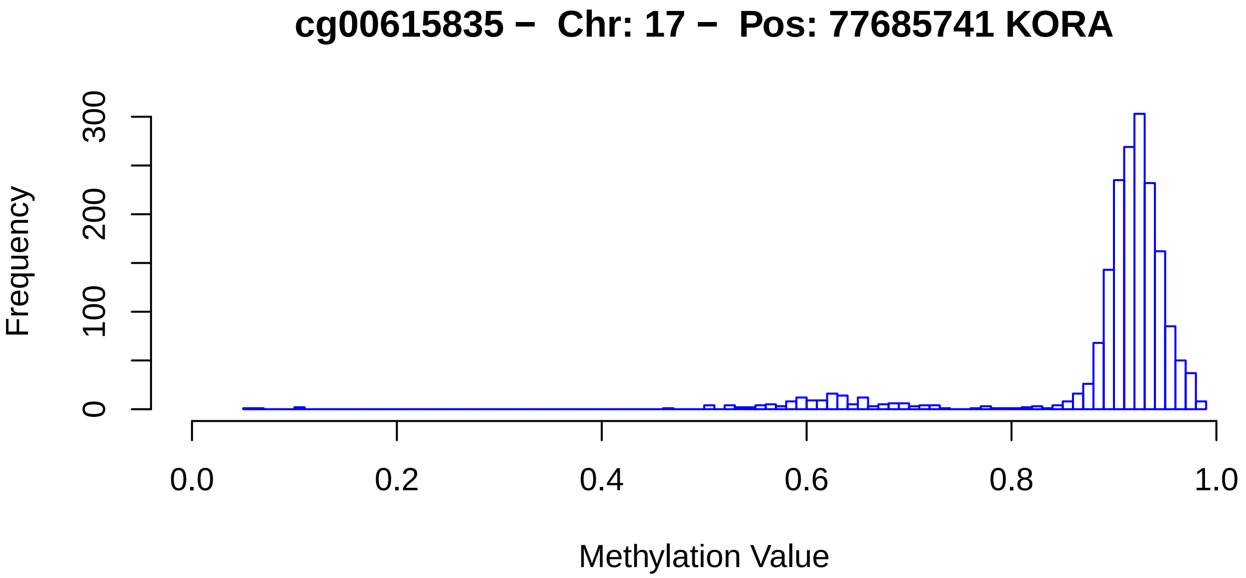
cg00615835 − Chr: 17 − Pos: 77685741 KORA
Frequency
0
100
200
300
0.0
0.2
0.4
0.6
0.8
1.0
Methylation Value

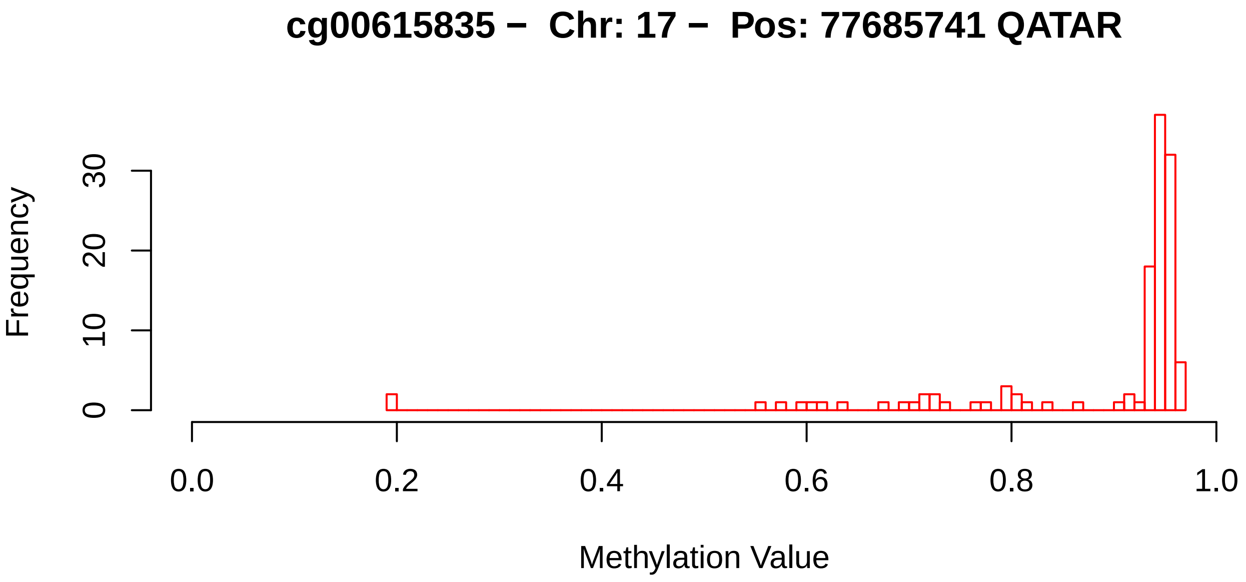
cg00615835 − Chr: 17 − Pos: 77685741 QATAR
Frequency
0
10
20
30
0.0
0.2
0.4
0.6
0.8
1.0
Methylation Value

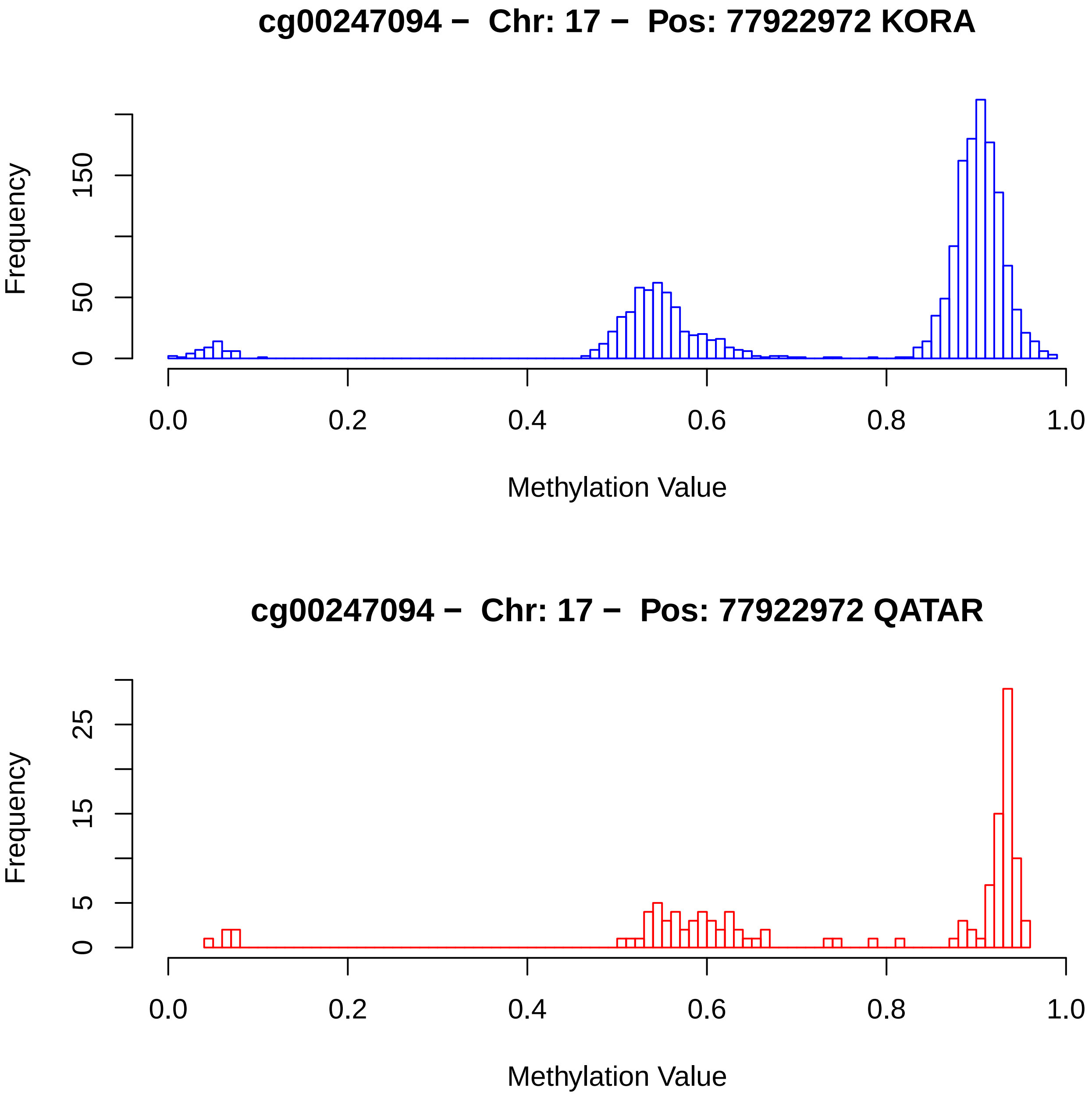
cg00247094 − Chr: 17 − Pos: 77922972 KORA
Frequency
0
50
150
0.0
0.2
0.4
0.6
0.8
1.0
Methylation Value
cg00247094 − Chr: 17 − Pos: 77922972 QATAR
Frequency
0
5
15
25
0.0
0.2
0.4
0.6
0.8
1.0
Methylation Value

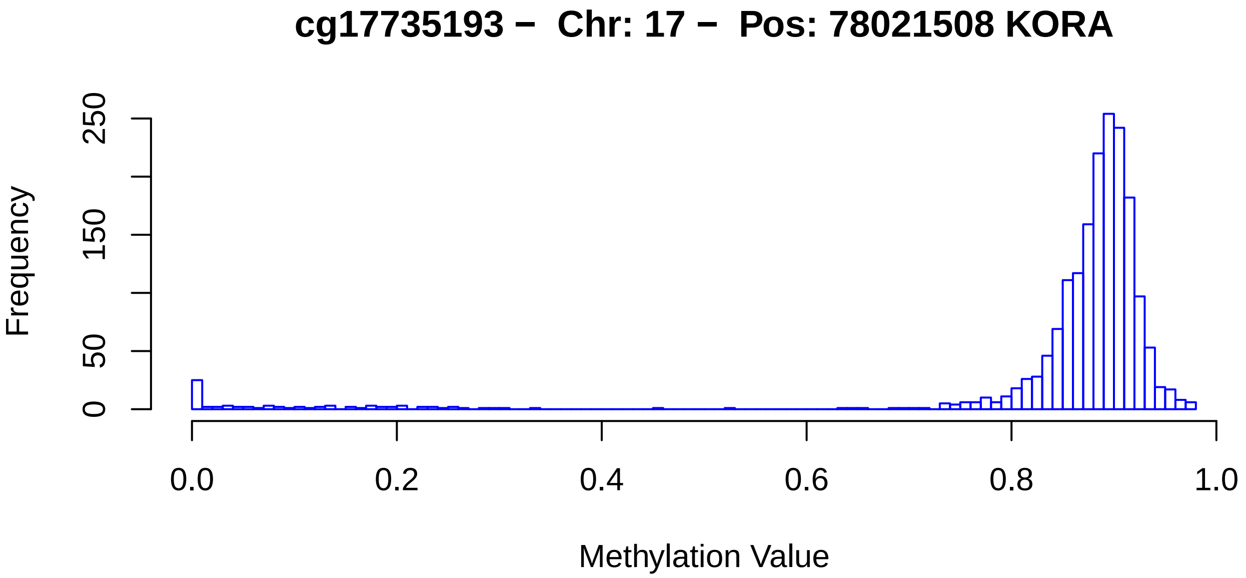
cg17735193 − Chr: 17 − Pos: 78021508 KORA
Frequency
0
50
150
250
0.0
0.2
0.4
0.6
0.8
1.0
Methylation Value

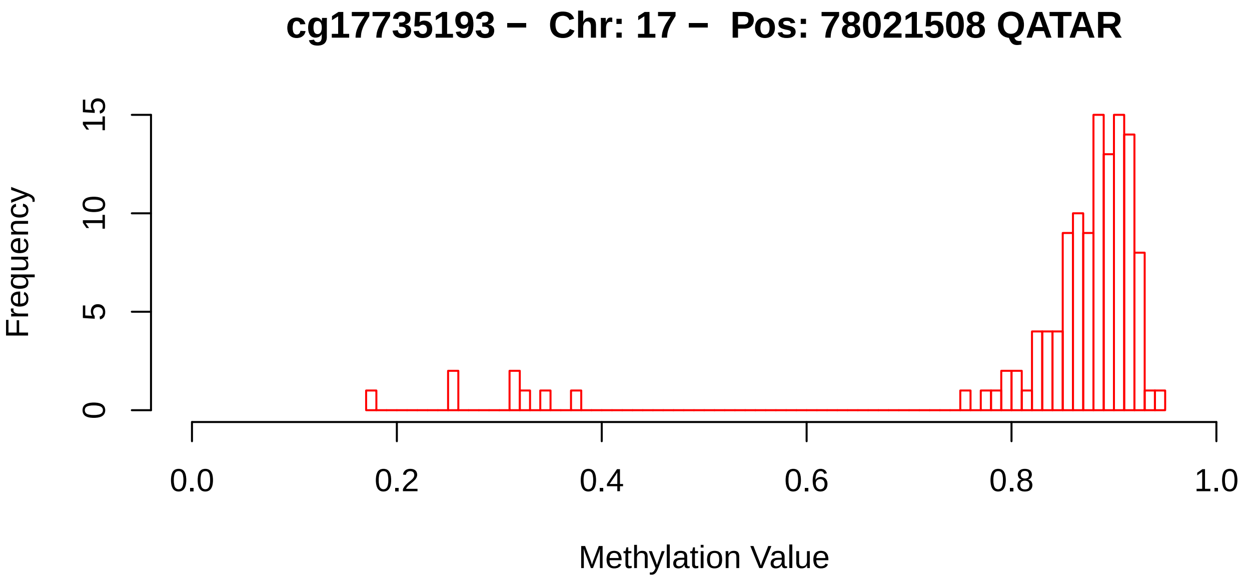
cg17735193 − Chr: 17 − Pos: 78021508 QATAR
Frequency
0
5
10
15
0.0
0.2
0.4
0.6
0.8
1.0
Methylation Value

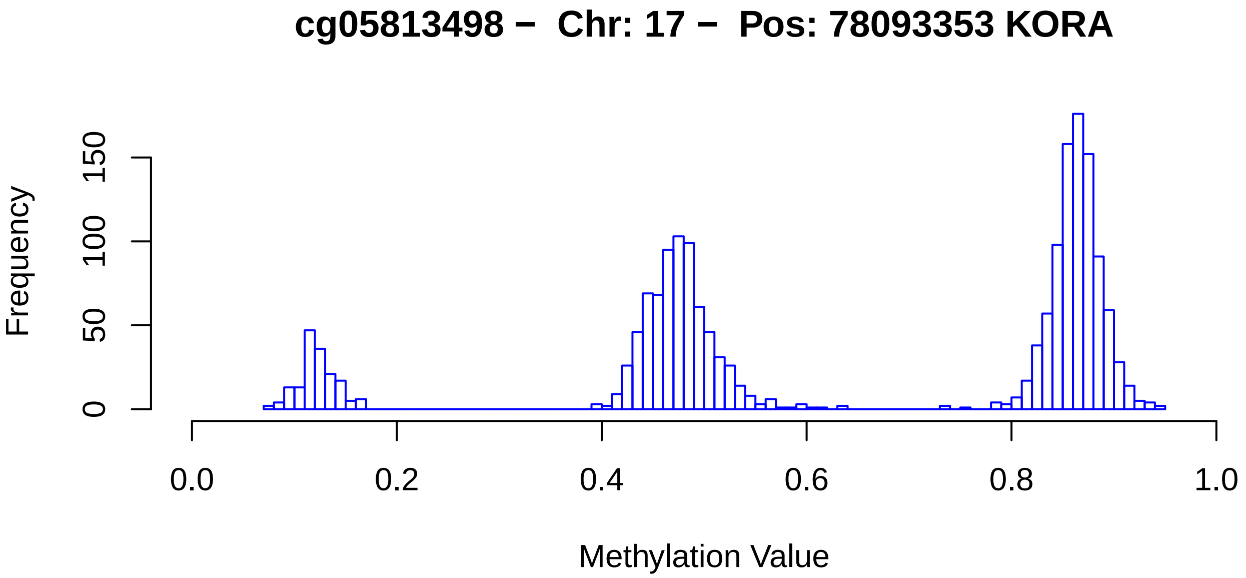
cg05813498 − Chr: 17 − Pos: 78093353 KORA
Frequency
0
50
100
150
0.0
0.2
0.4
0.6
0.8
1.0
Methylation Value

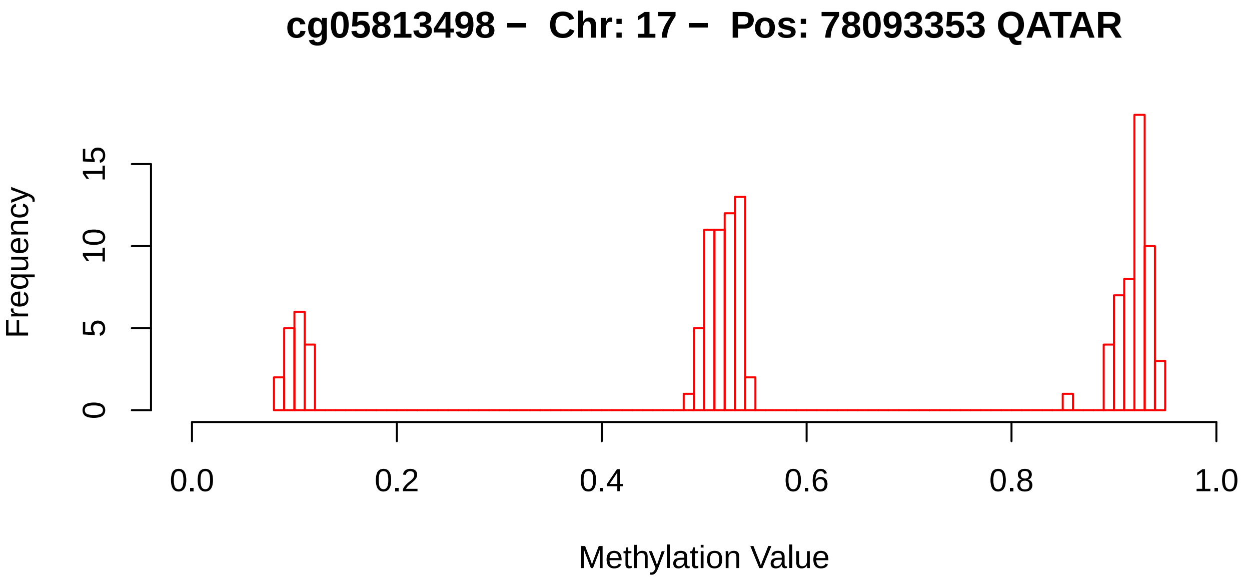
cg05813498 − Chr: 17 − Pos: 78093353 QATAR
Frequency
0
5
10
15
0.0
0.2
0.4
0.6
0.8
1.0
Methylation Value

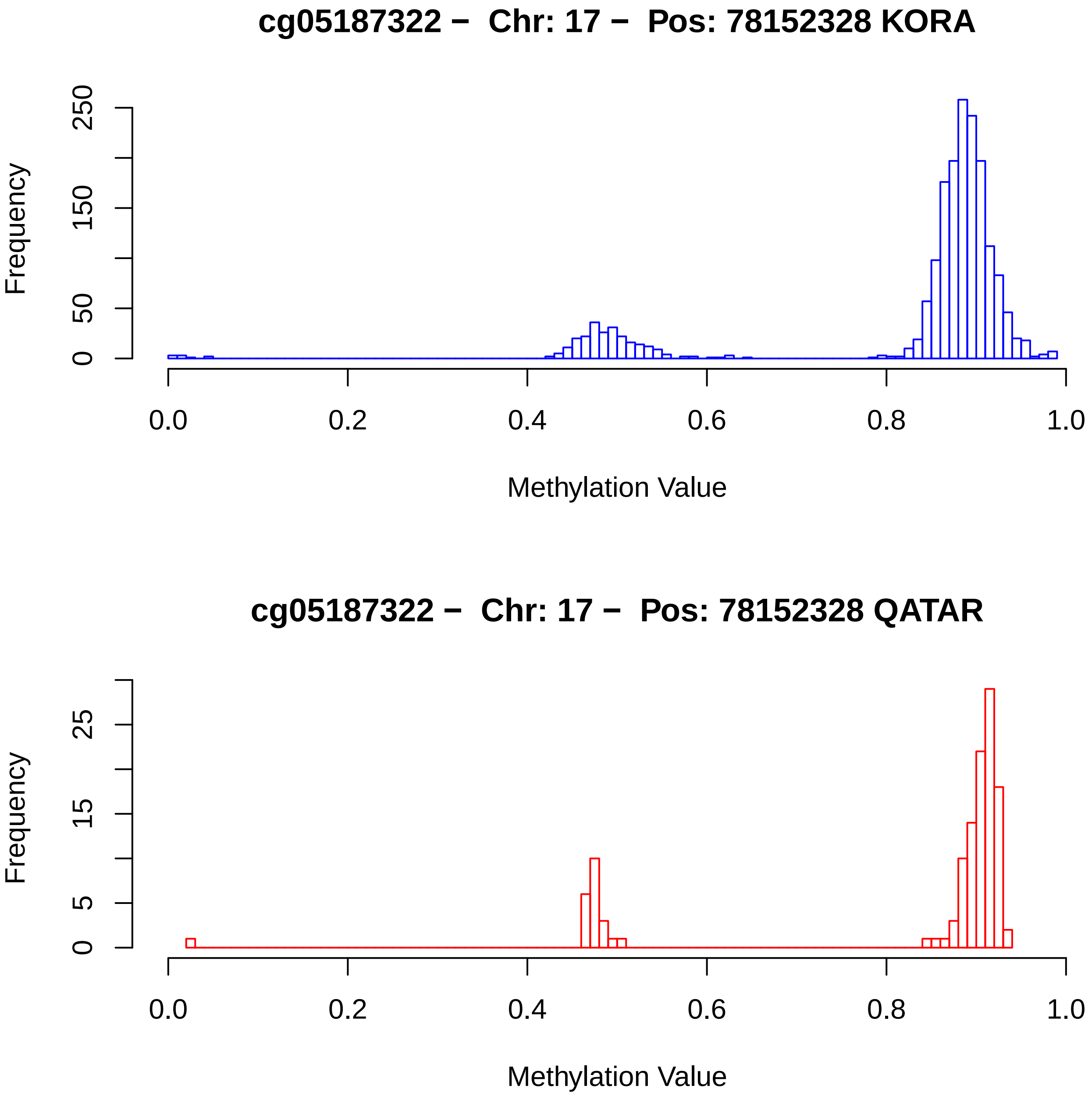
cg05187322 − Chr: 17 − Pos: 78152328 KORA
Frequency
0
50
150
250
0.0
0.2
0.4
0.6
0.8
1.0
Methylation Value
cg05187322 − Chr: 17 − Pos: 78152328 QATAR
Frequency
0
5
15
25
0.0
0.2
0.4
0.6
0.8
1.0
Methylation Value

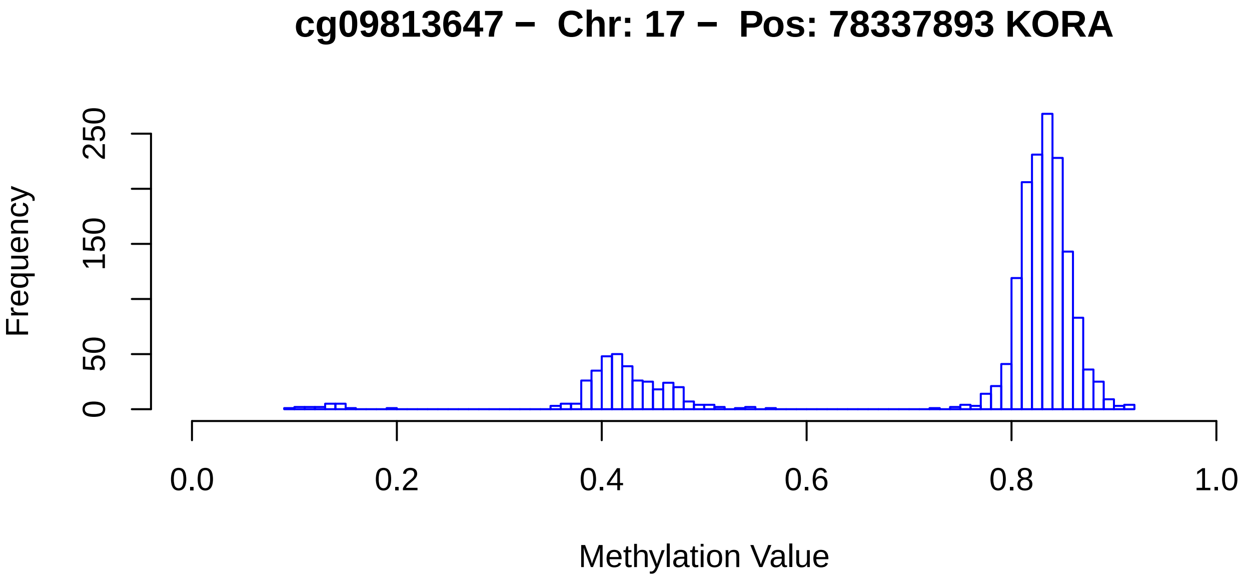
cg09813647 − Chr: 17 − Pos: 78337893 KORA
Frequency
0
50
150
250
0.0
0.2
0.4
0.6
0.8
1.0
Methylation Value

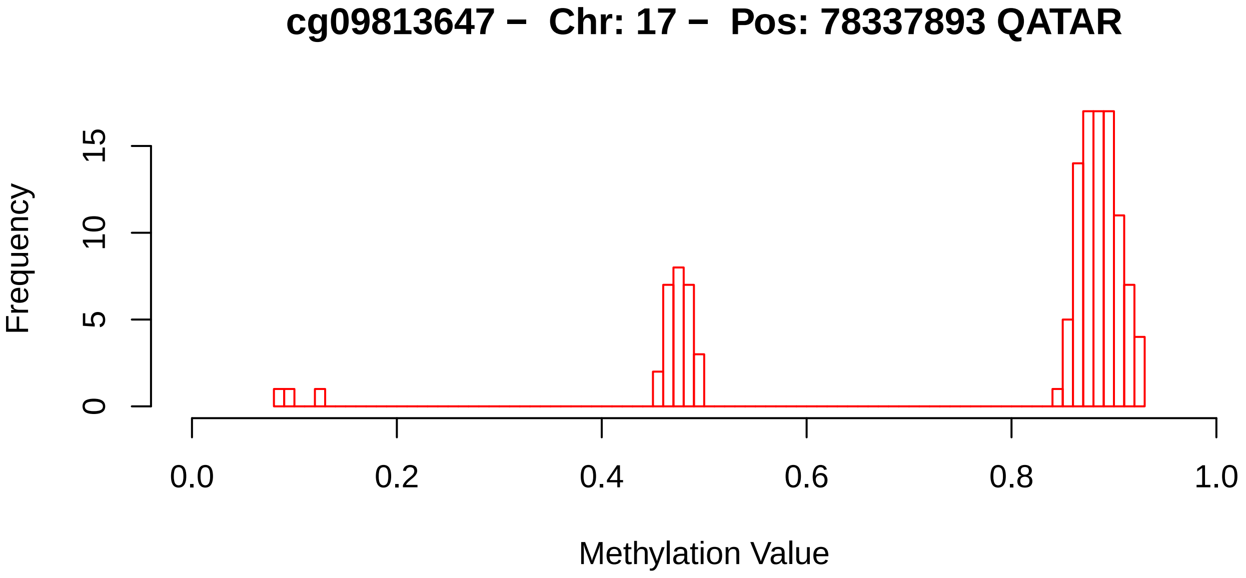
cg09813647 − Chr: 17 − Pos: 78337893 QATAR
Frequency
0
5
10
15
0.0
0.2
0.4
0.6
0.8
1.0
Methylation Value

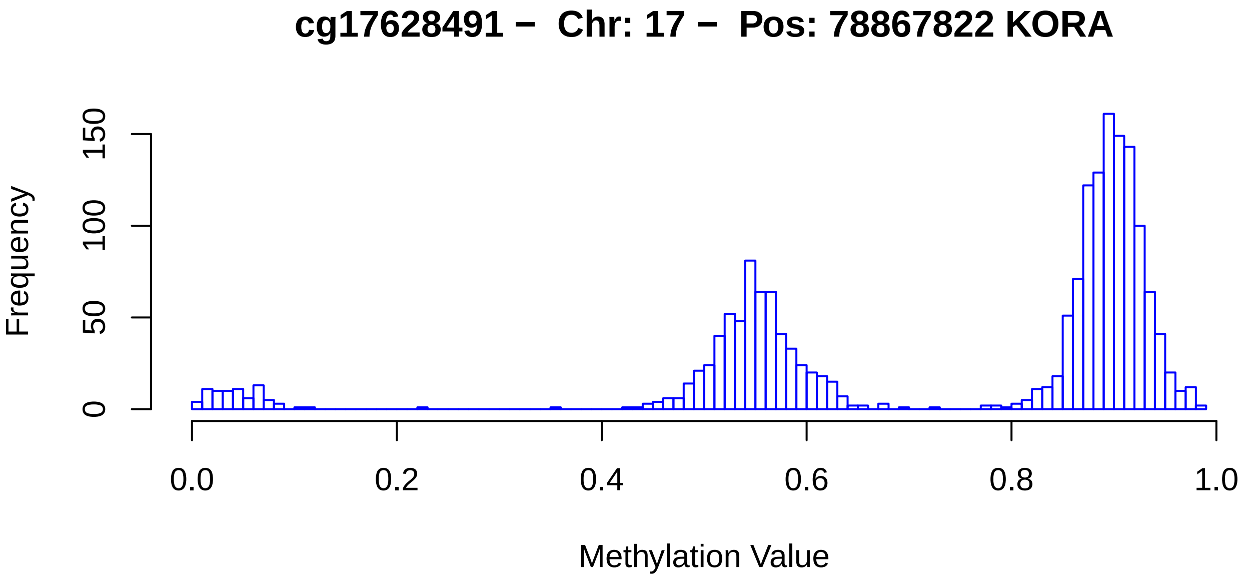
cg17628491 − Chr: 17 − Pos: 78867822 KORA
Frequency
0
50
100
150
0.0
0.2
0.4
0.6
0.8
1.0
Methylation Value

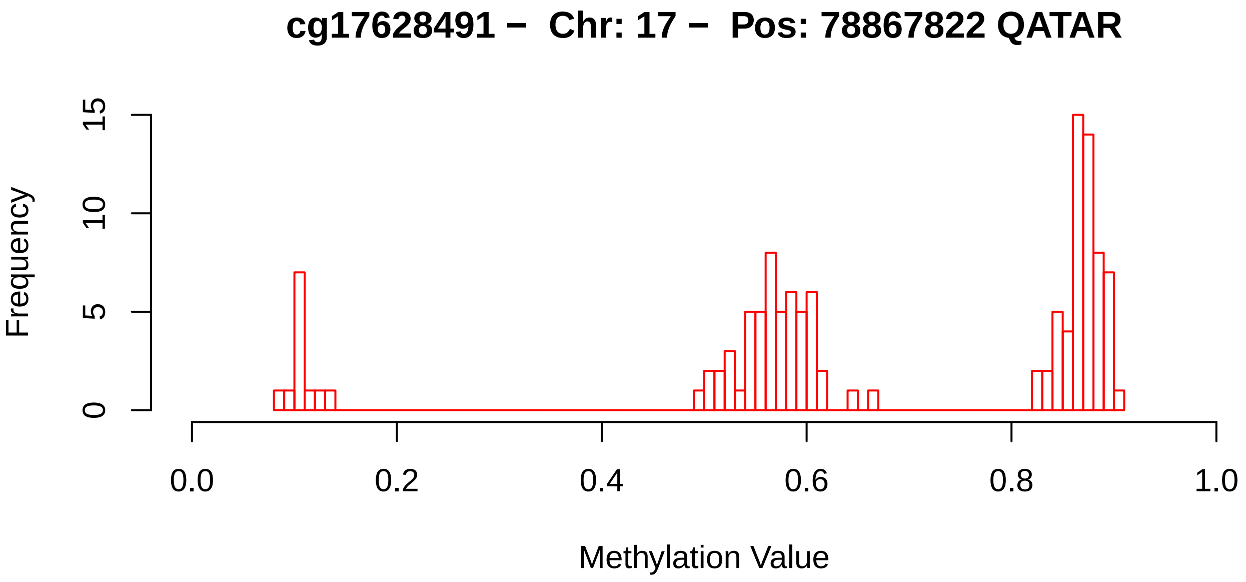
cg17628491 − Chr: 17 − Pos: 78867822 QATAR
Frequency
0
5
10
15
0.0
0.2
0.4
0.6
0.8
1.0
Methylation Value

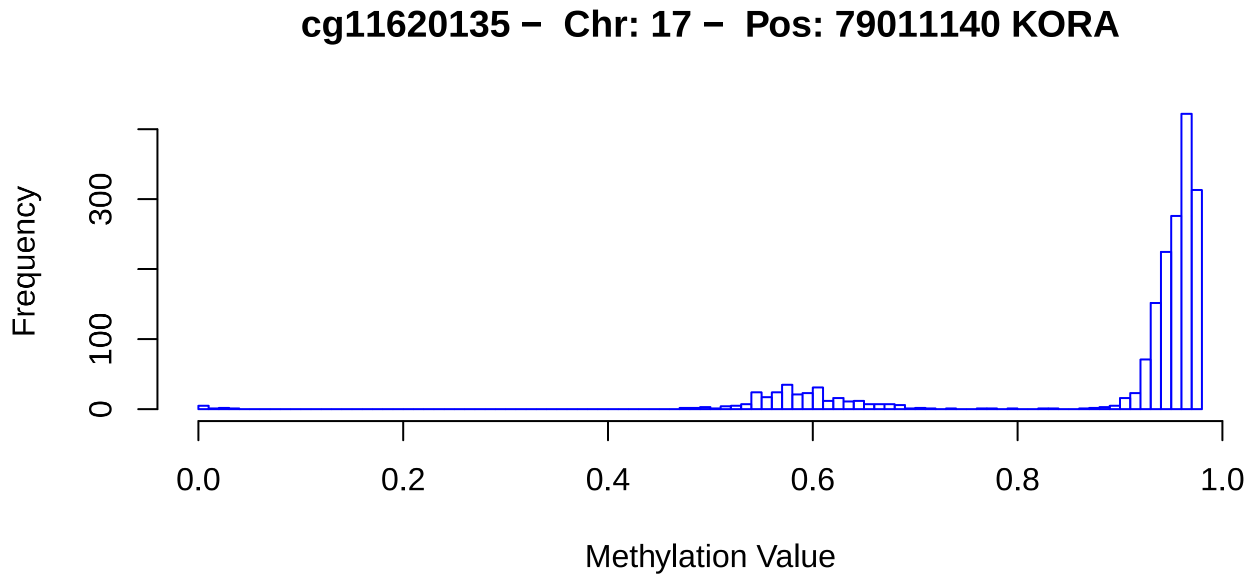
cg11620135 − Chr: 17 − Pos: 79011140 KORA
Frequency
0
100
300
0.0
0.2
0.4
0.6
0.8
1.0
Methylation Value

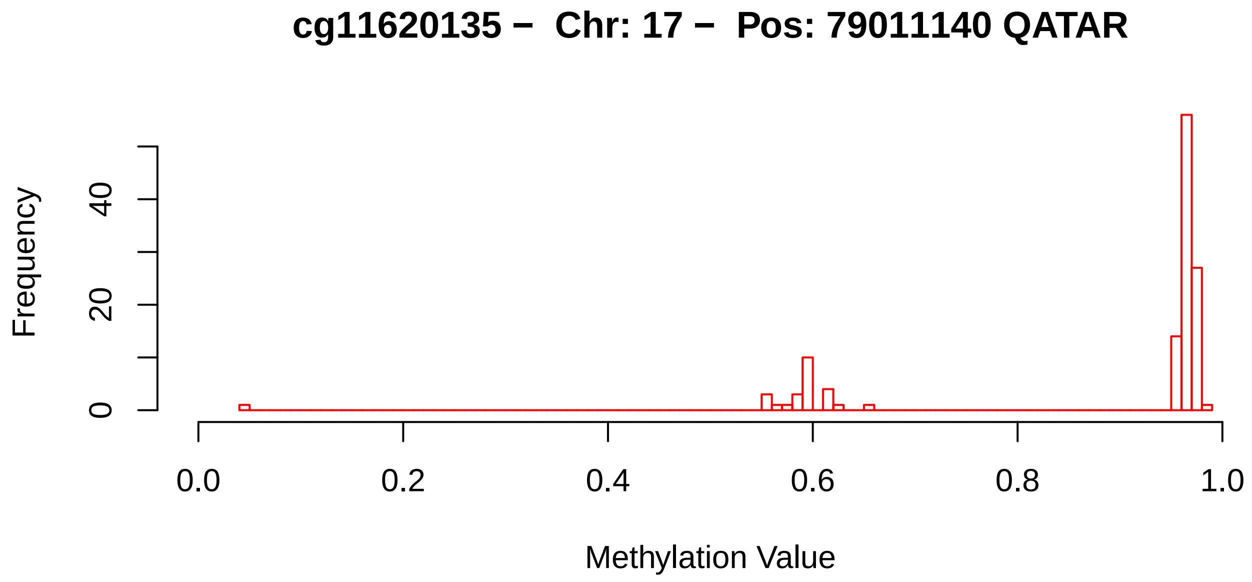
cg11620135 − Chr: 17 − Pos: 79011140 QATAR
Frequency
0
20
40
0.0
0.2
0.4
0.6
0.8
1.0
Methylation Value

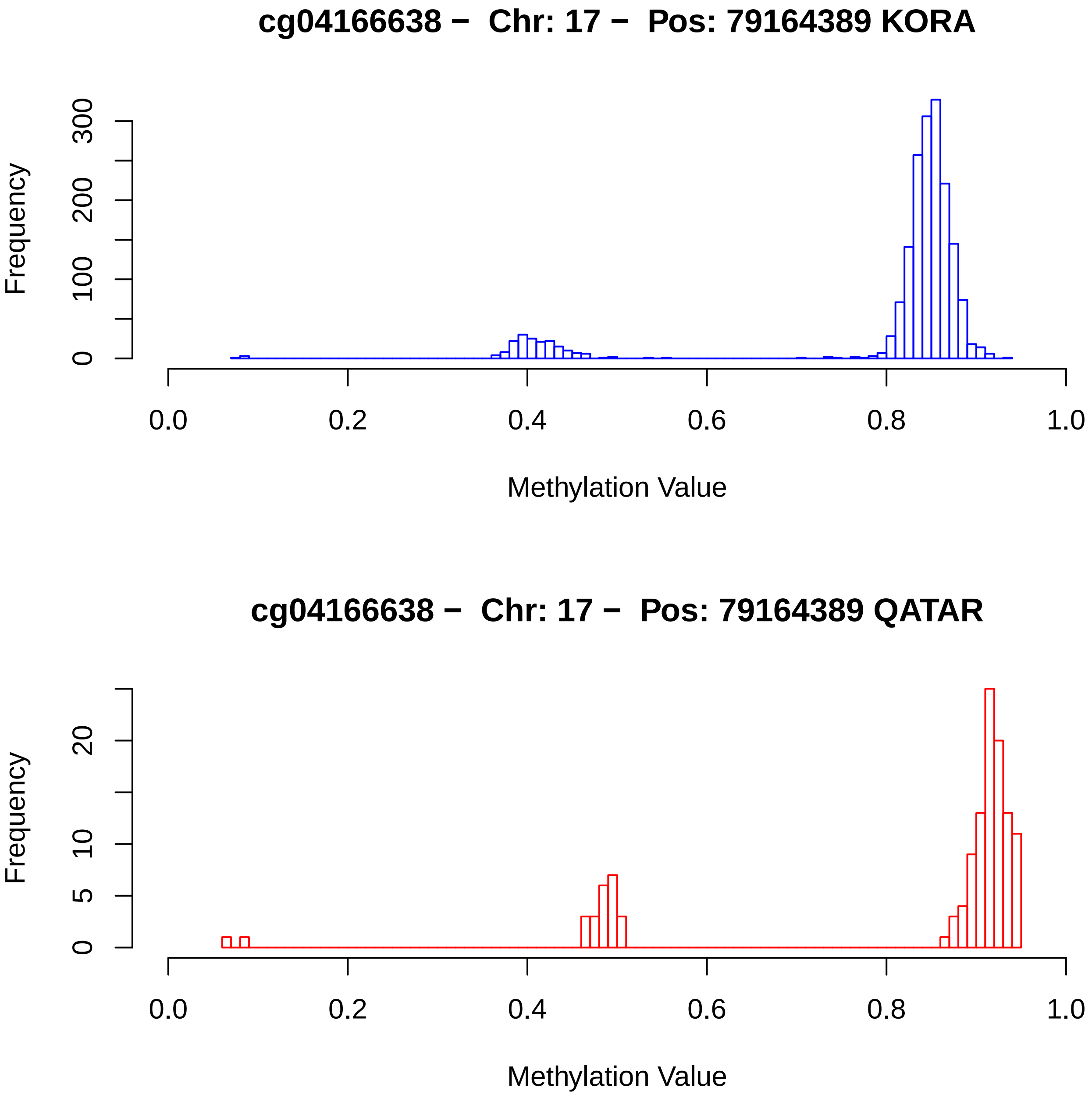
cg04166638 − Chr: 17 − Pos: 79164389 KORA
Frequency
0
100
200
300
0.0
0.2
0.4
0.6
0.8
1.0
Methylation Value
cg04166638 − Chr: 17 − Pos: 79164389 QATAR
Frequency
0
5
10
20
0.0
0.2
0.4
0.6
0.8
1.0
Methylation Value

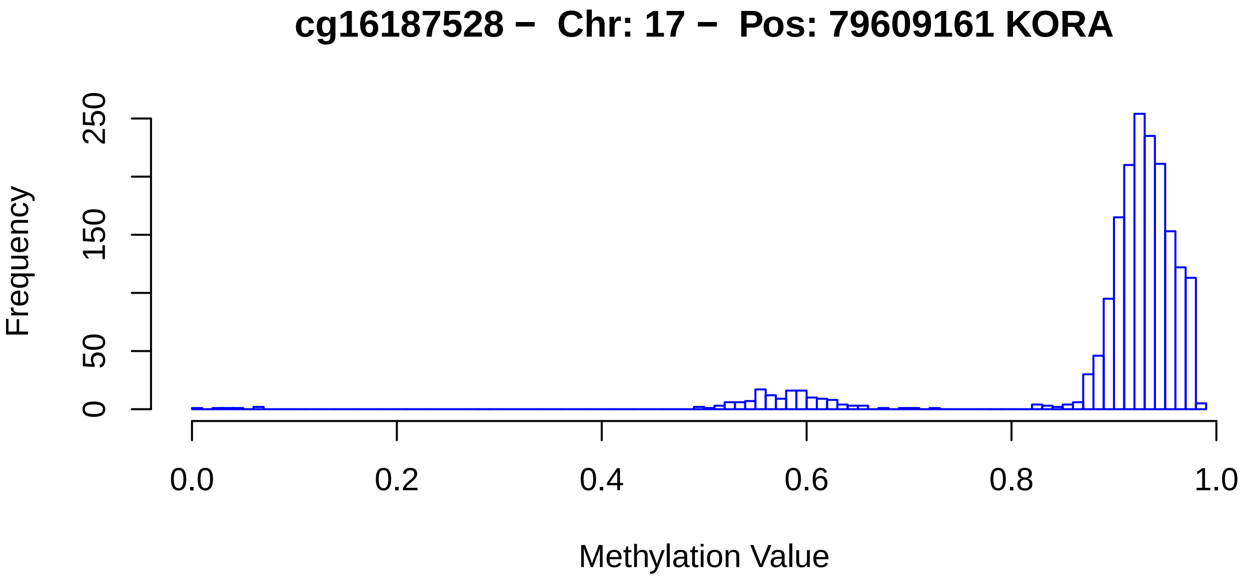
cg16187528 − Chr: 17 − Pos: 79609161 KORA
Frequency
0
50
150
250
0.0
0.2
0.4
0.6
0.8
1.0
Methylation Value

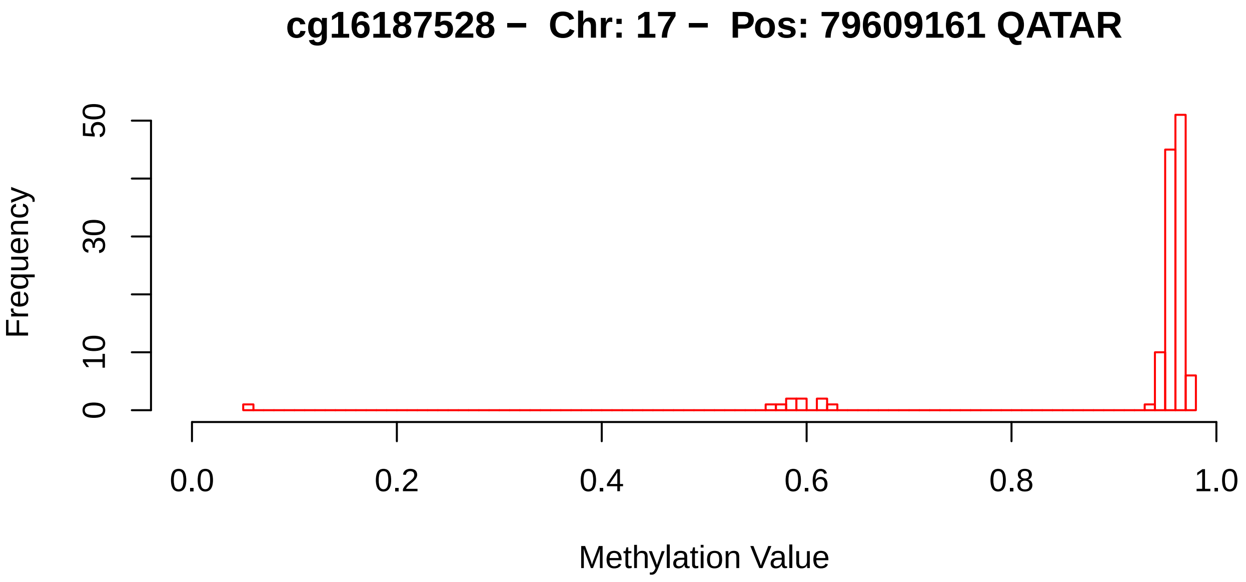
cg16187528 − Chr: 17 − Pos: 79609161 QATAR
Frequency
0
10
30
50
0.0
0.2
0.4
0.6
0.8
1.0
Methylation Value

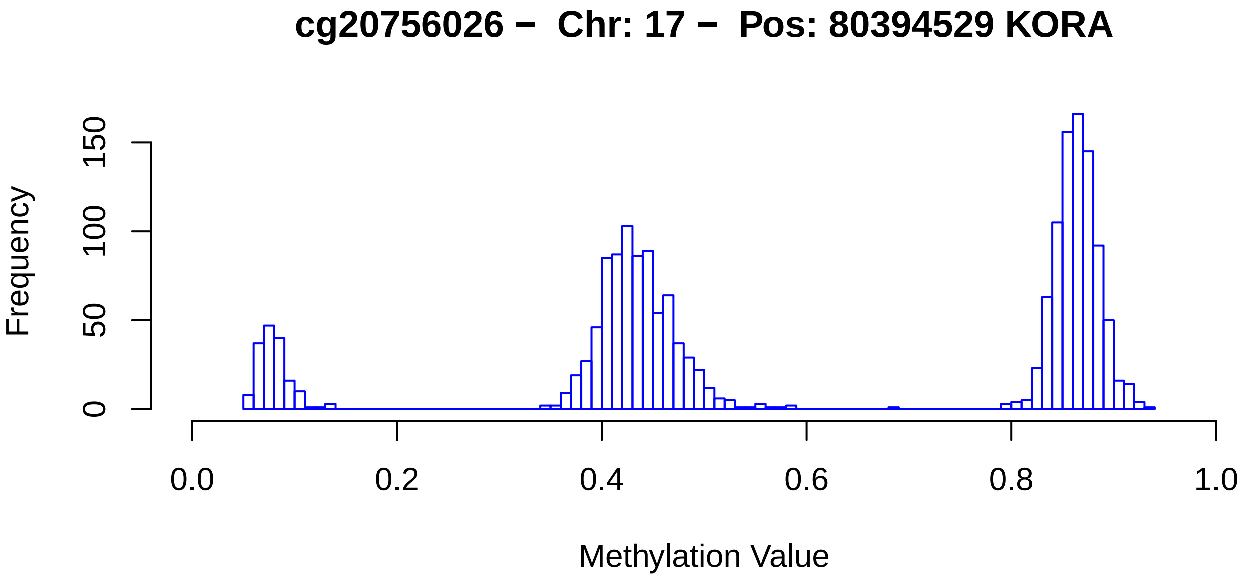
cg20756026 − Chr: 17 − Pos: 80394529 KORA
Frequency
0
50
100
150
0.0
0.2
0.4
0.6
0.8
1.0
Methylation Value

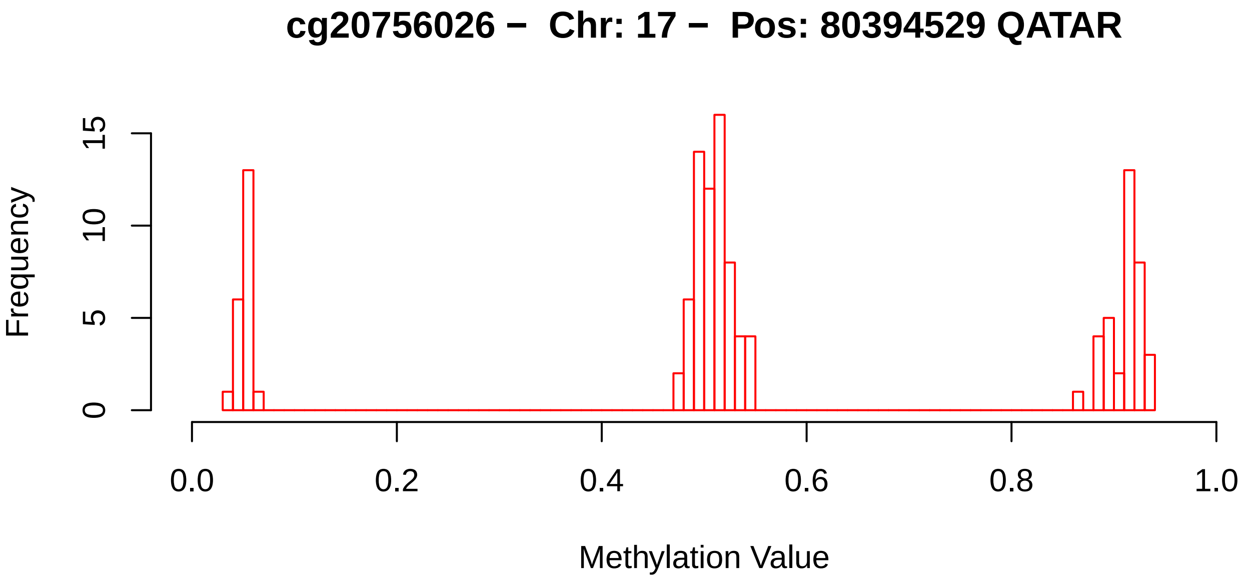
cg20756026 − Chr: 17 − Pos: 80394529 QATAR
Frequency
0
5
10
15
0.0
0.2
0.4
0.6
0.8
1.0
Methylation Value

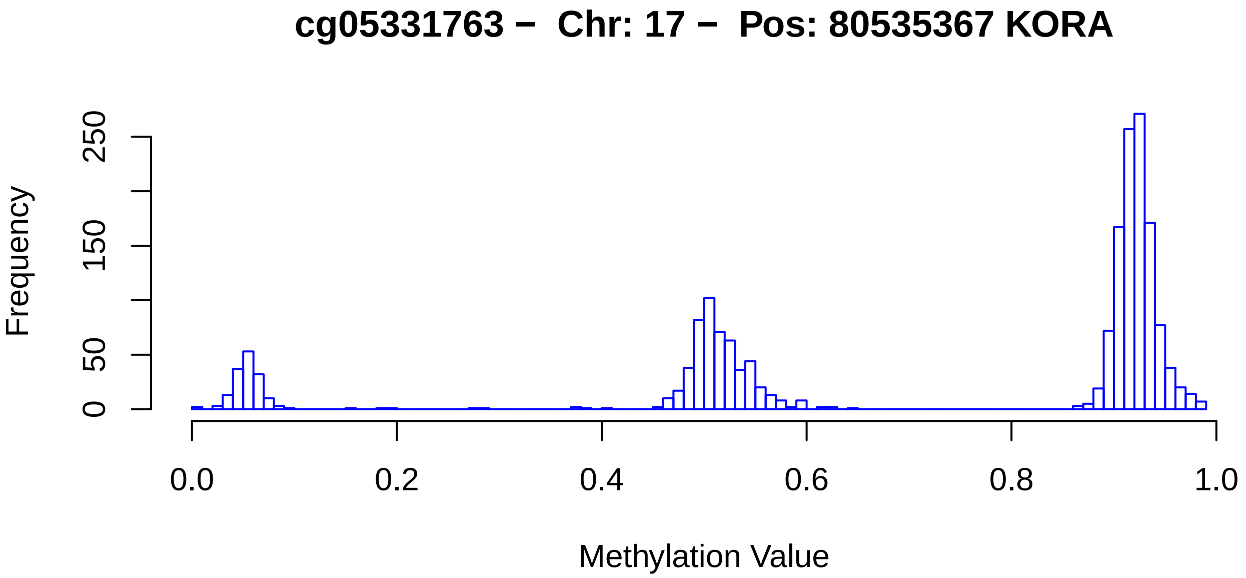
cg05331763 − Chr: 17 − Pos: 80535367 KORA
Frequency
0
50
150
250
0.0
0.2
0.4
0.6
0.8
1.0
Methylation Value

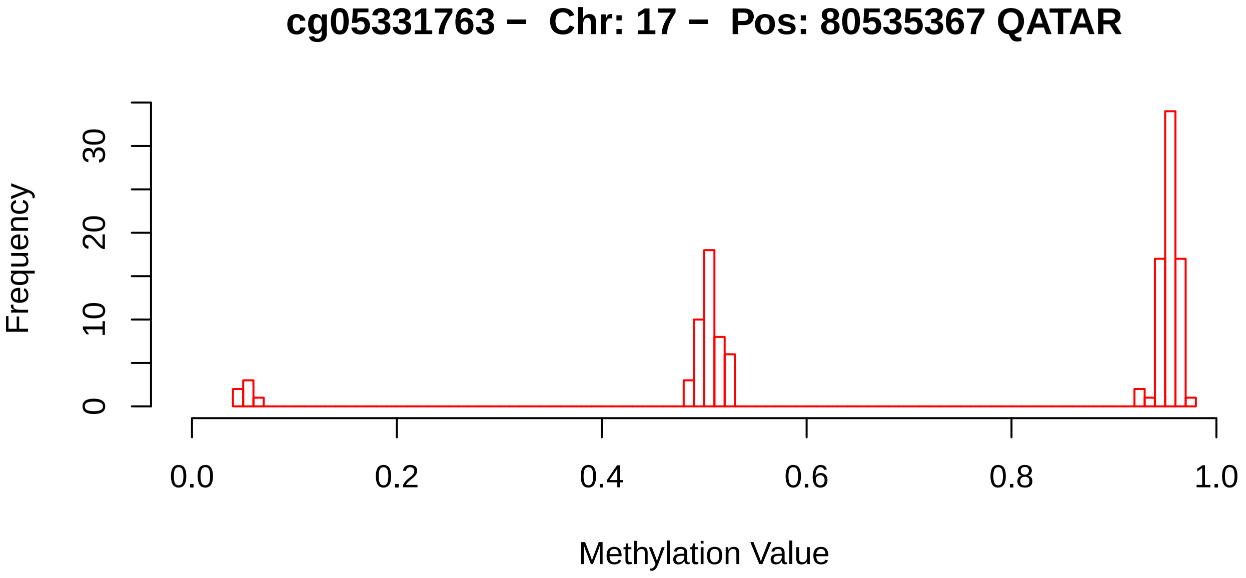
cg05331763 − Chr: 17 − Pos: 80535367 QATAR
Frequency
0
10
20
30
0.0
0.2
0.4
0.6
0.8
1.0
Methylation Value

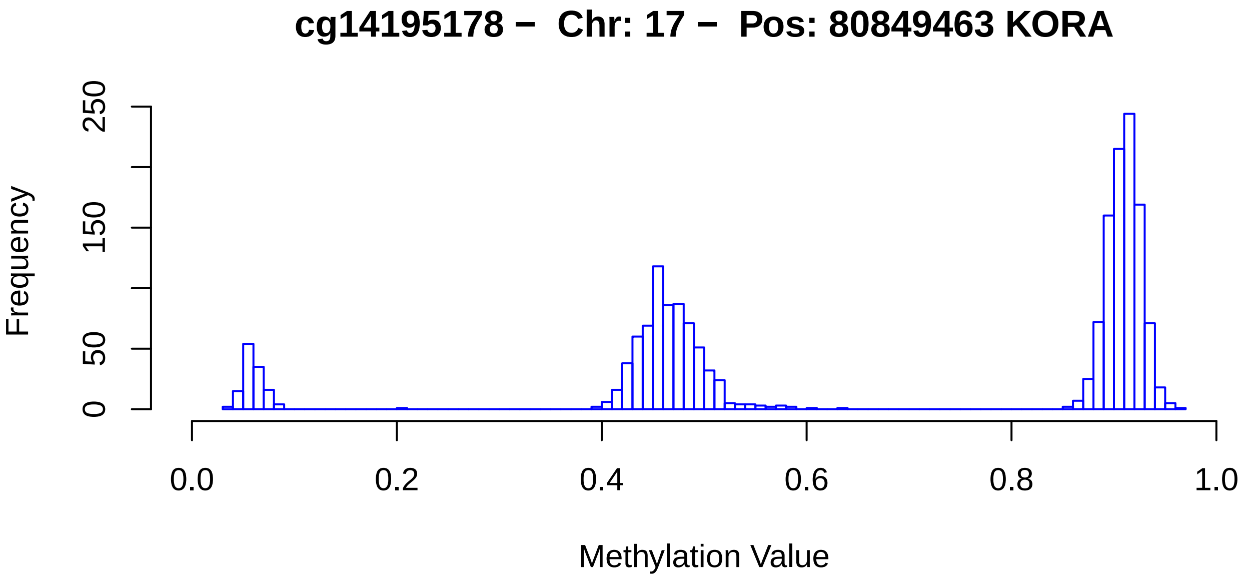
cg14195178 − Chr: 17 − Pos: 80849463 KORA
Frequency
0
50
150
250
0.0
0.2
0.4
0.6
0.8
1.0
Methylation Value

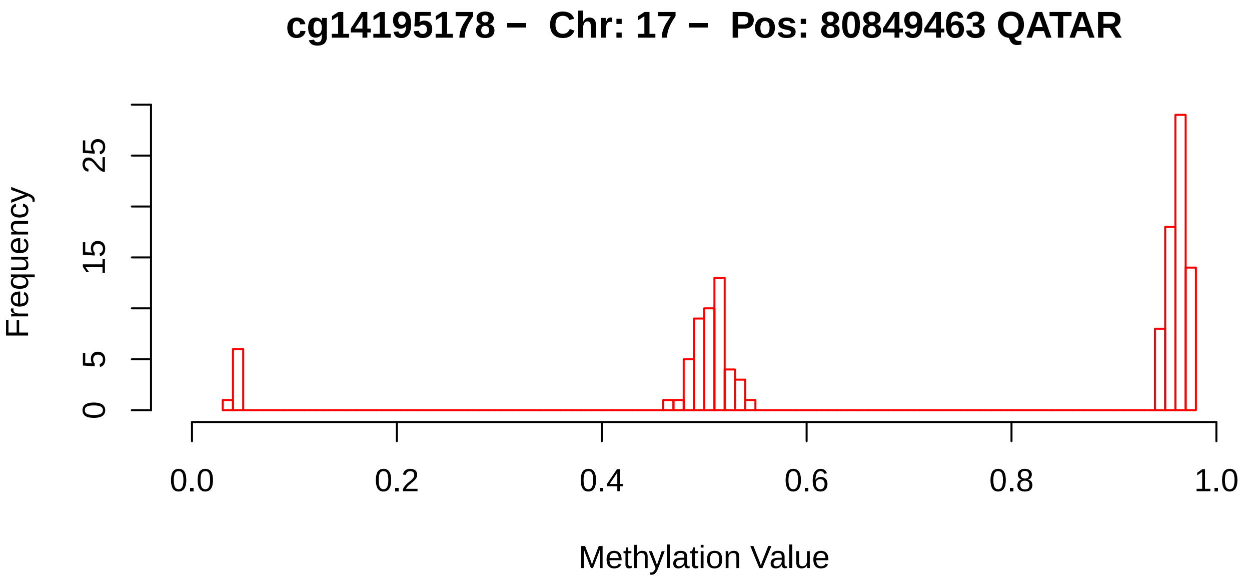
cg14195178 − Chr: 17 − Pos: 80849463 QATAR
Frequency
0
5
15
25
0.0
0.2
0.4
0.6
0.8
1.0
Methylation Value

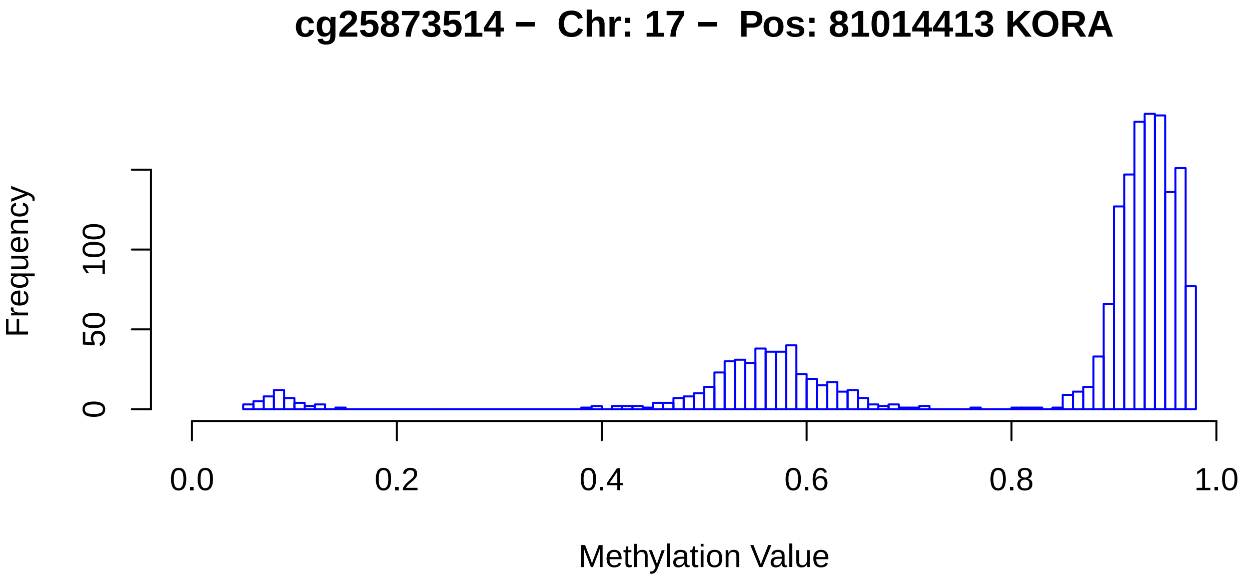
cg25873514 − Chr: 17 − Pos: 81014413 KORA
Frequency
0
50
100
0.0
0.2
0.4
0.6
0.8
1.0
Methylation Value

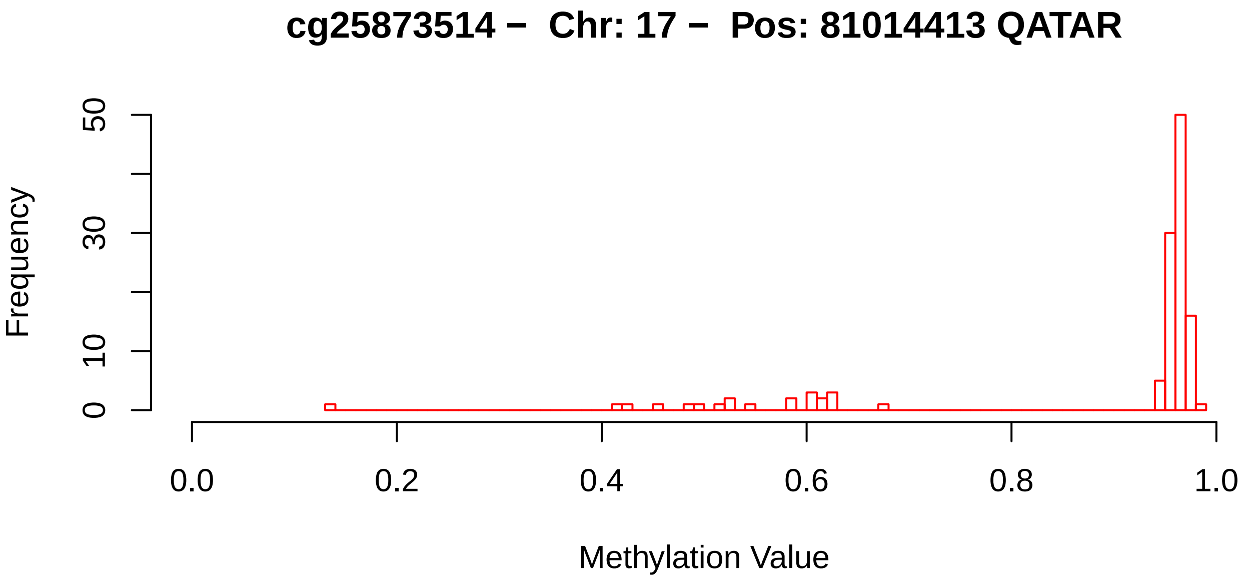
cg25873514 − Chr: 17 − Pos: 81014413 QATAR
Frequency
0
10
30
50
0.0
0.2
0.4
0.6
0.8
1.0
Methylation Value

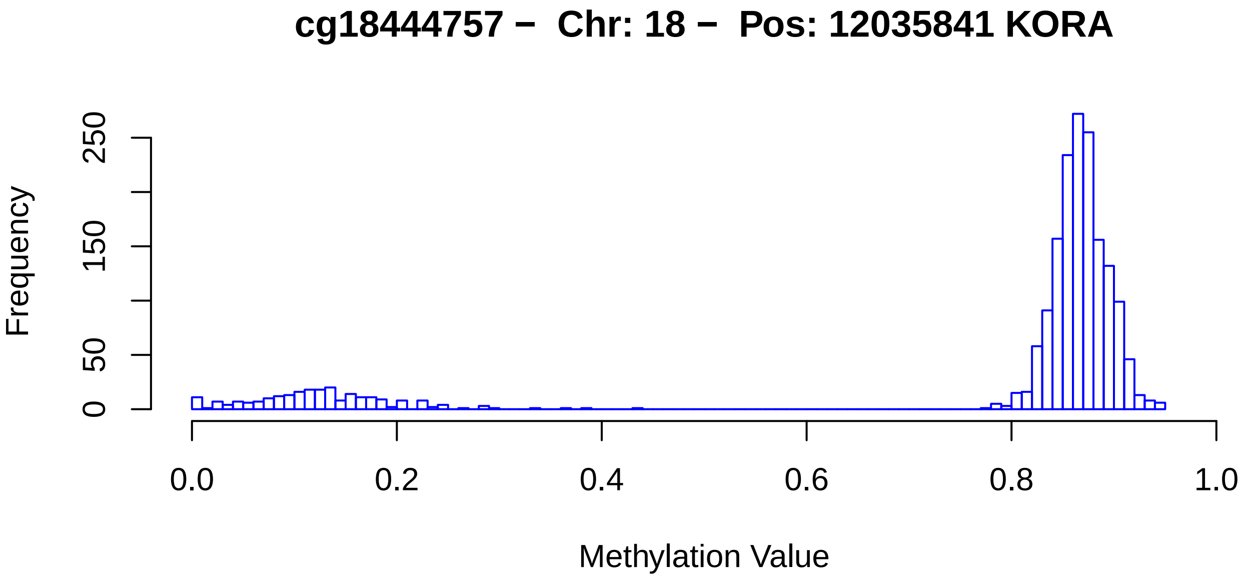
cg18444757 − Chr: 18 − Pos: 12035841 KORA
Frequency
0
50
150
250
0.0
0.2
0.4
0.6
0.8
1.0
Methylation Value

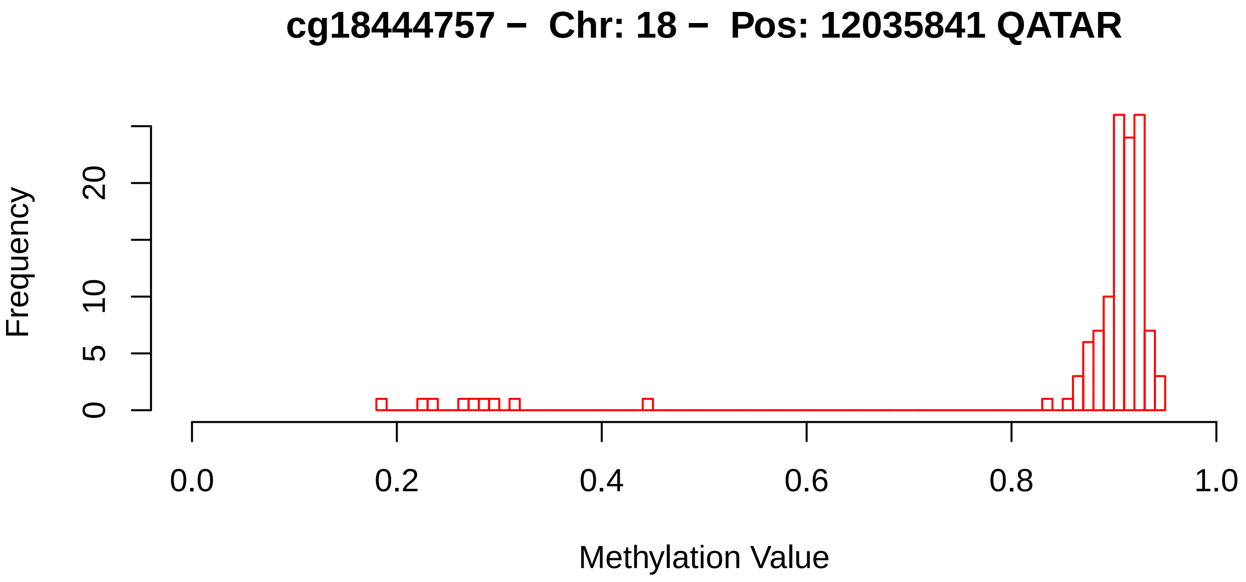
cg18444757 − Chr: 18 − Pos: 12035841 QATAR
Frequency
0
5
10
20
0.0
0.2
0.4
0.6
0.8
1.0
Methylation Value

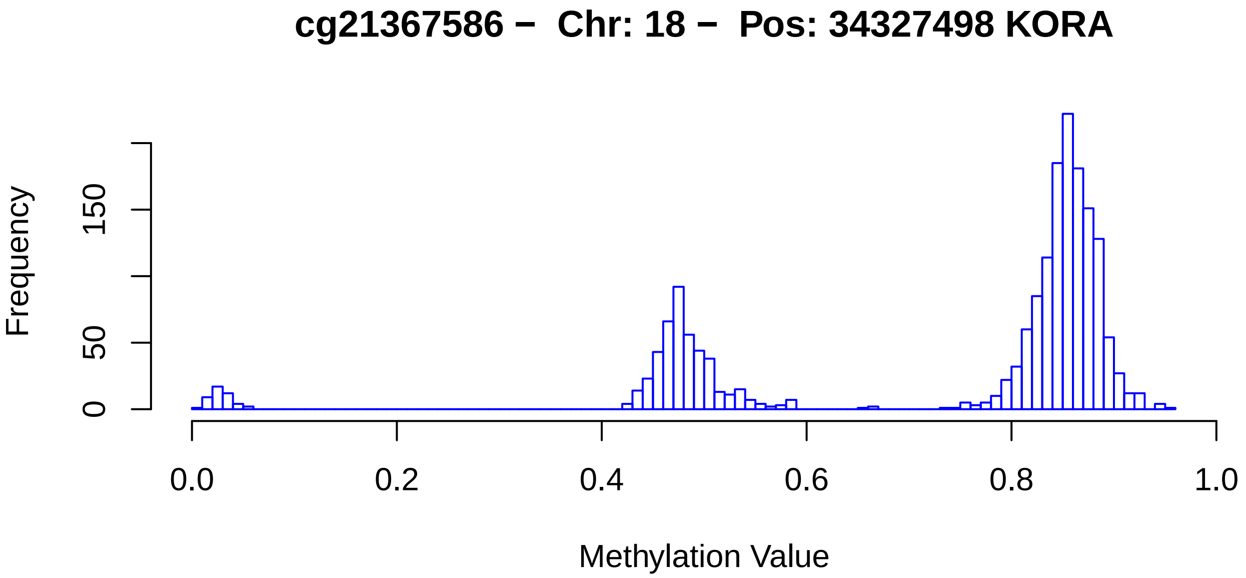
cg21367586 − Chr: 18 − Pos: 34327498 KORA
Frequency
0
50
150
0.0
0.2
0.4
0.6
0.8
1.0
Methylation Value

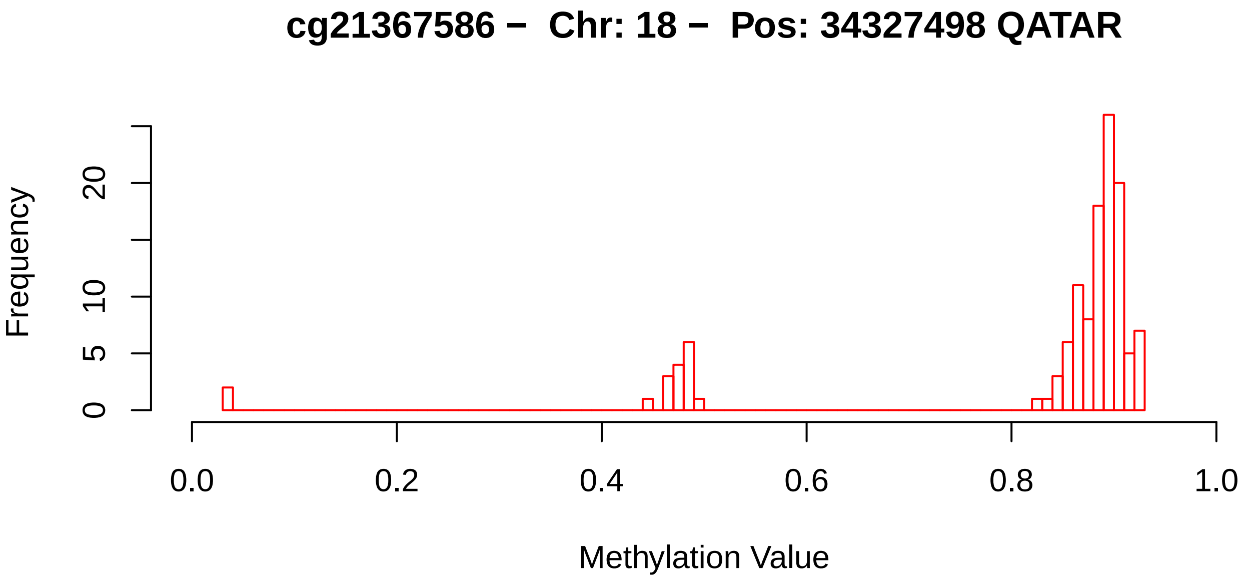
cg21367586 − Chr: 18 − Pos: 34327498 QATAR
Frequency
0
5
10
20
0.0
0.2
0.4
0.6
0.8
1.0
Methylation Value

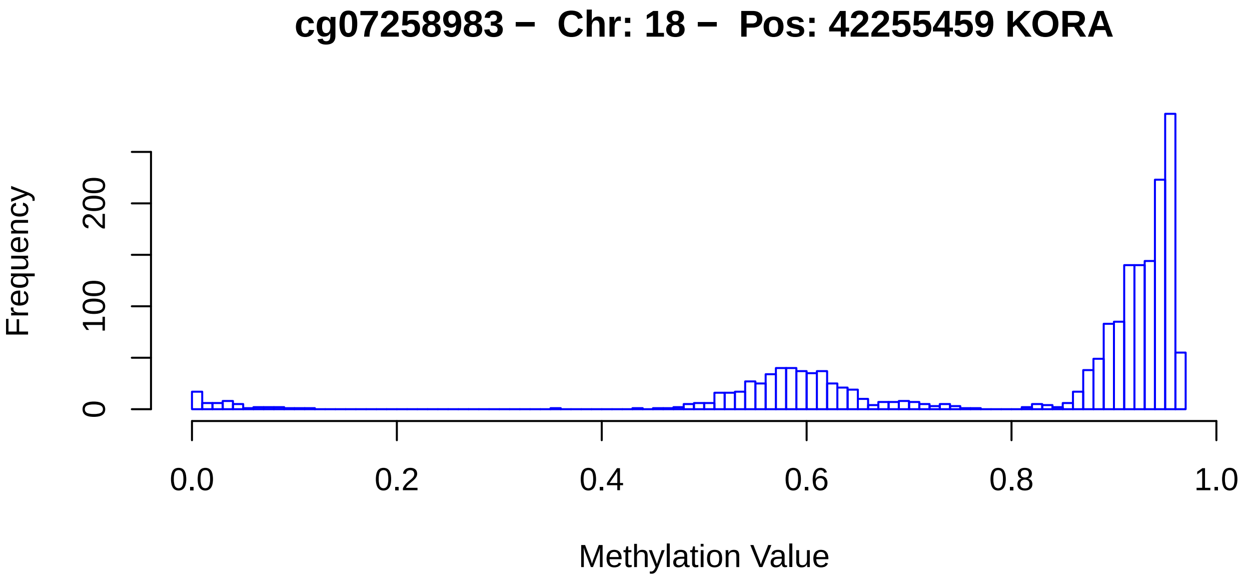
cg07258983 – Chr: 18 – Pos: 42255459 KORA
Frequency
0
100
200
0.0
0.2
0.4
0.6
0.8
1.0
Methylation Value

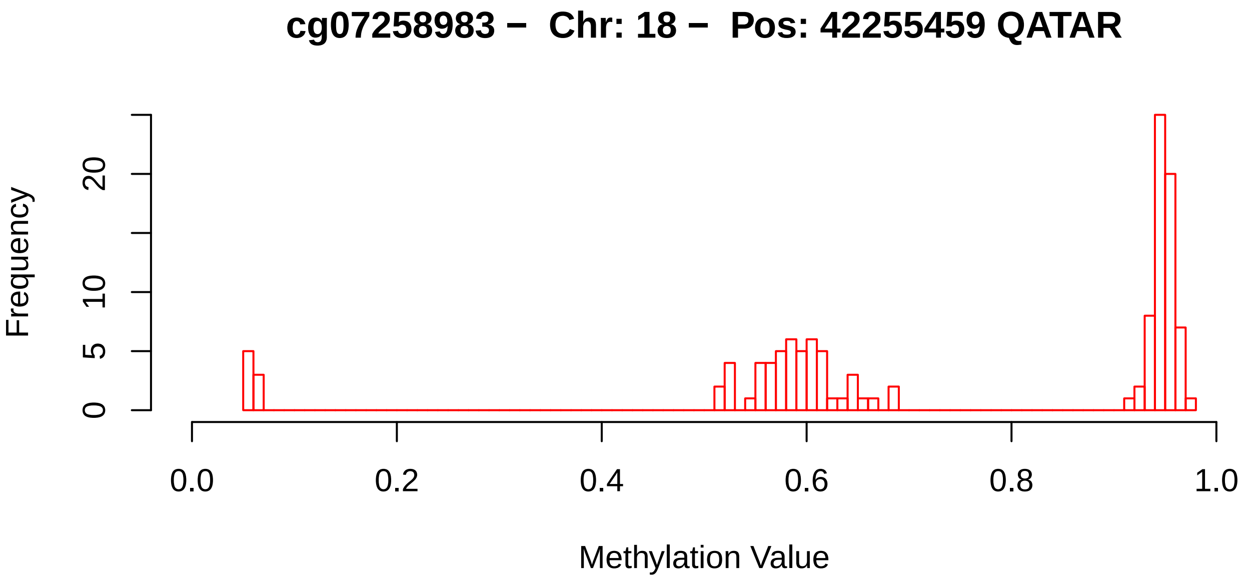
cg07258983 – Chr: 18 – Pos: 42255459 QATAR
Frequency
0
5
10
20
0.0
0.2
0.4
0.6
0.8
1.0
Methylation Value

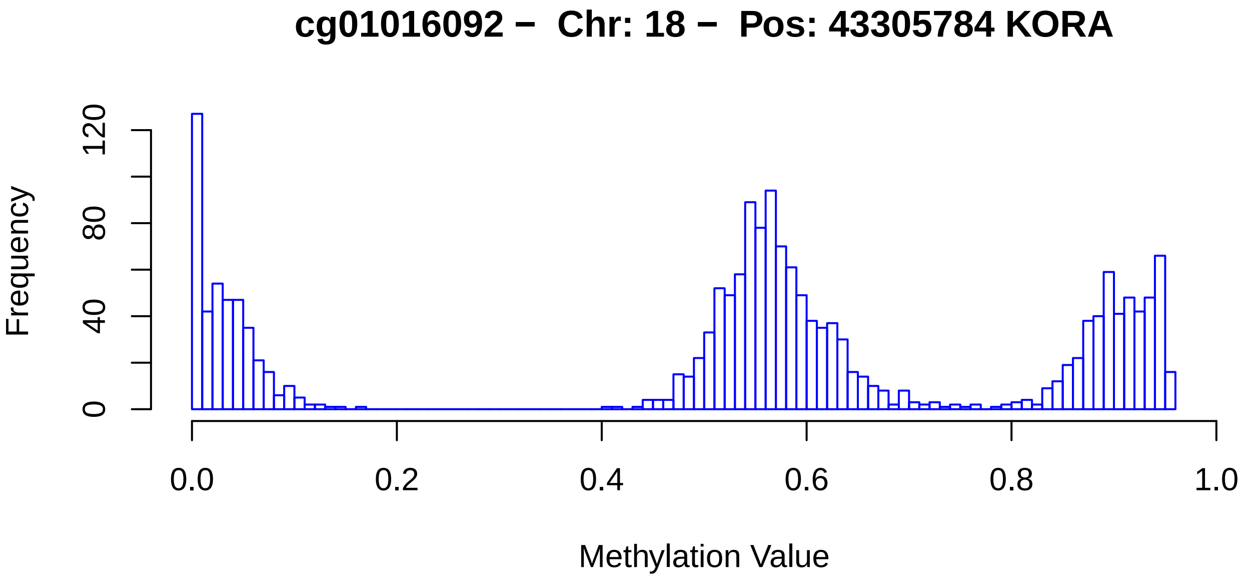
cg01016092 − Chr: 18 − Pos: 43305784 KORA
Frequency
0
40
80
120
0.0
0.2
0.4
0.6
0.8
1.0
Methylation Value

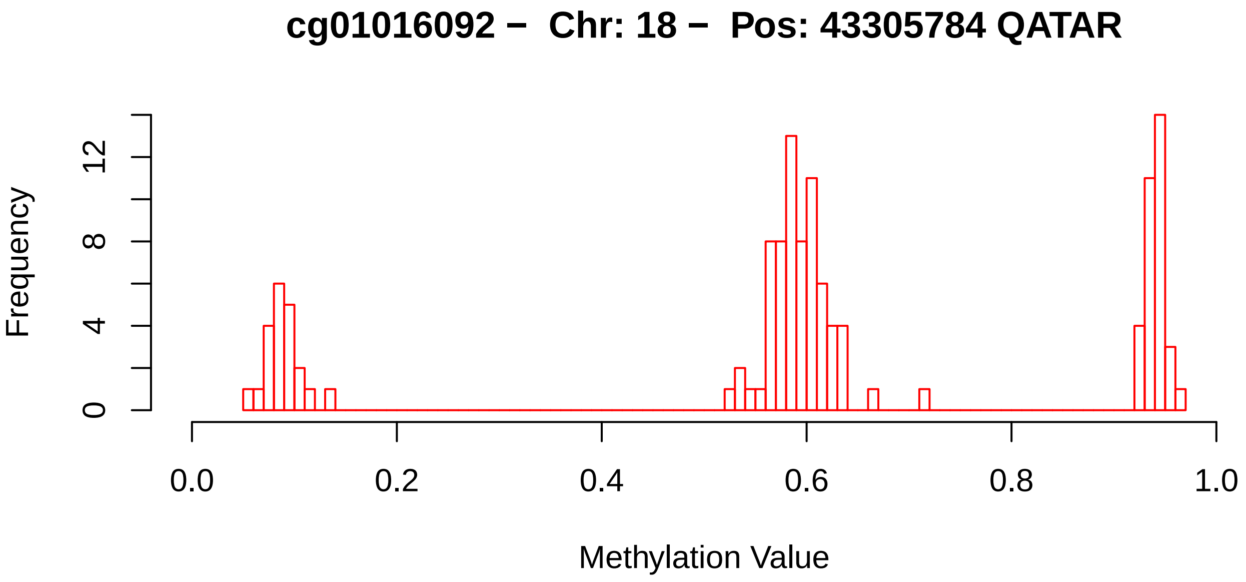
cg01016092 − Chr: 18 − Pos: 43305784 QATAR
Frequency
0
4
8
12
0.0
0.2
0.4
0.6
0.8
1.0
Methylation Value

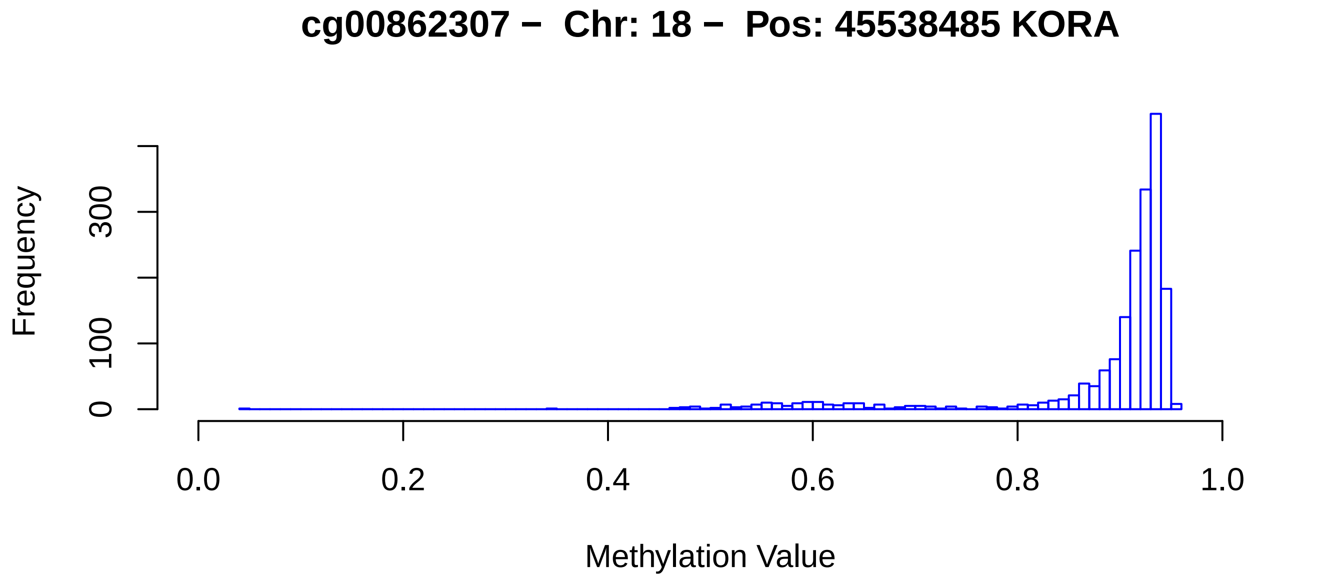
cg00862307 − Chr: 18 − Pos: 45538485 KORA
Frequency
0
100
300
0.0
0.2
0.4
0.6
0.8
1.0
Methylation Value

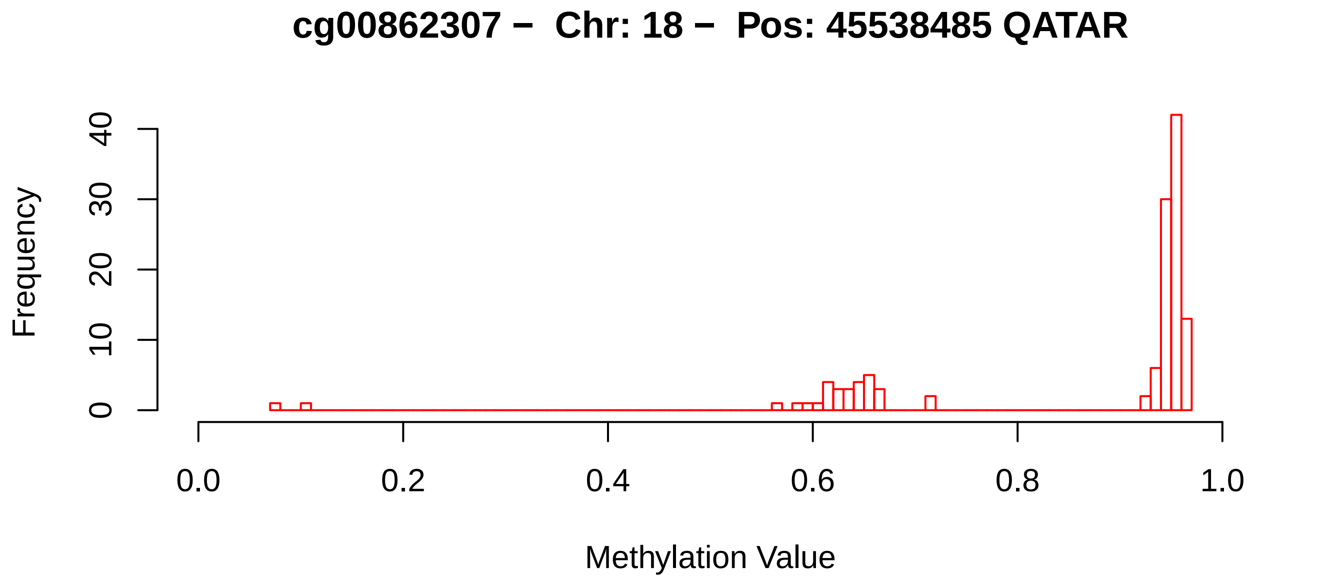
cg00862307 − Chr: 18 − Pos: 45538485 QATAR
Frequency
0
10
20
30
40
0.0
0.2
0.4
0.6
0.8
1.0
Methylation Value

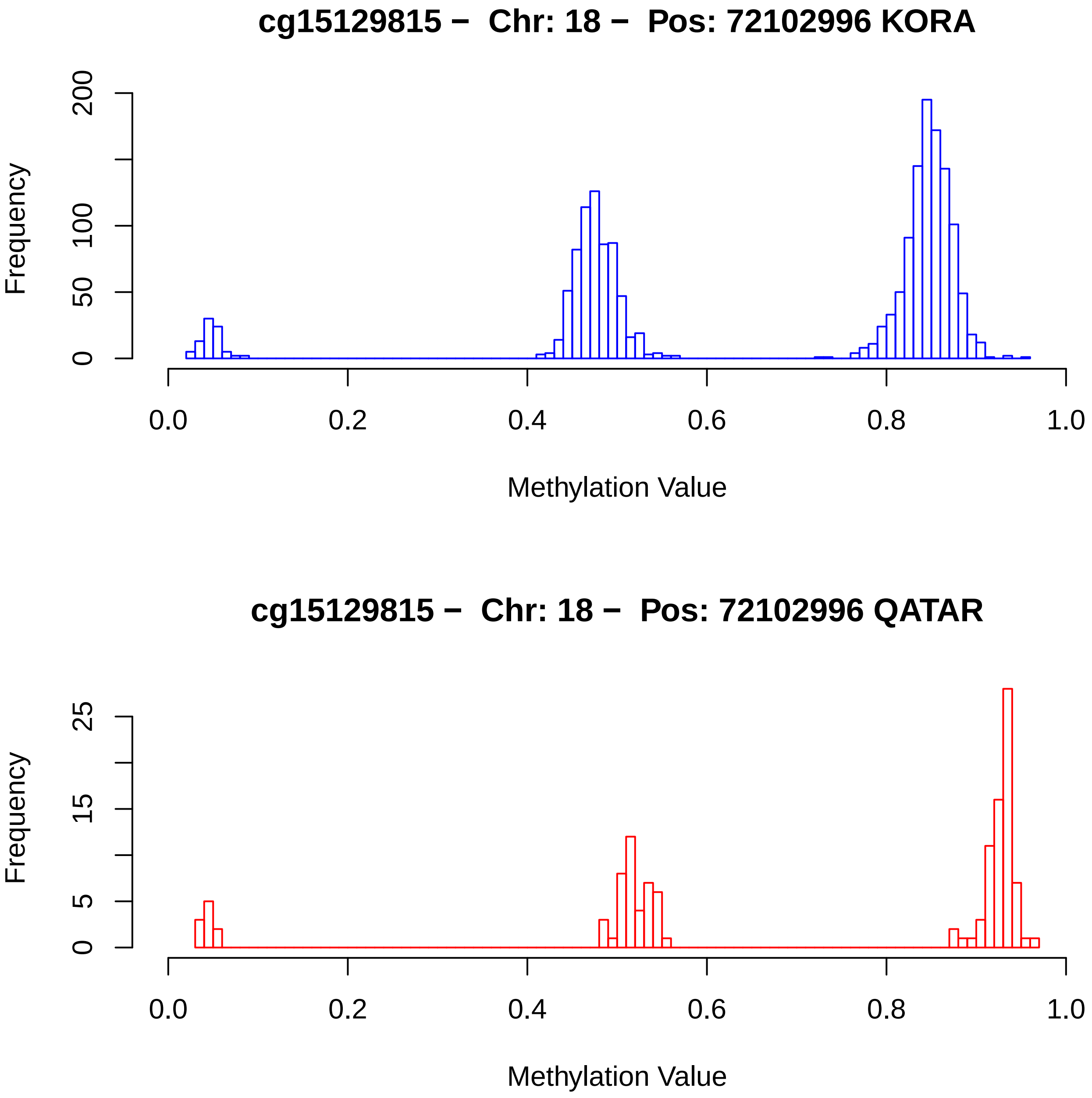
cg15129815 − Chr: 18 − Pos: 72102996 KORA
Frequency
0
50
100
200
0.0
0.2
0.4
0.6
0.8
1.0
Methylation Value
cg15129815 − Chr: 18 − Pos: 72102996 QATAR
Frequency
0
5
15
25
0.0
0.2
0.4
0.6
0.8
1.0
Methylation Value

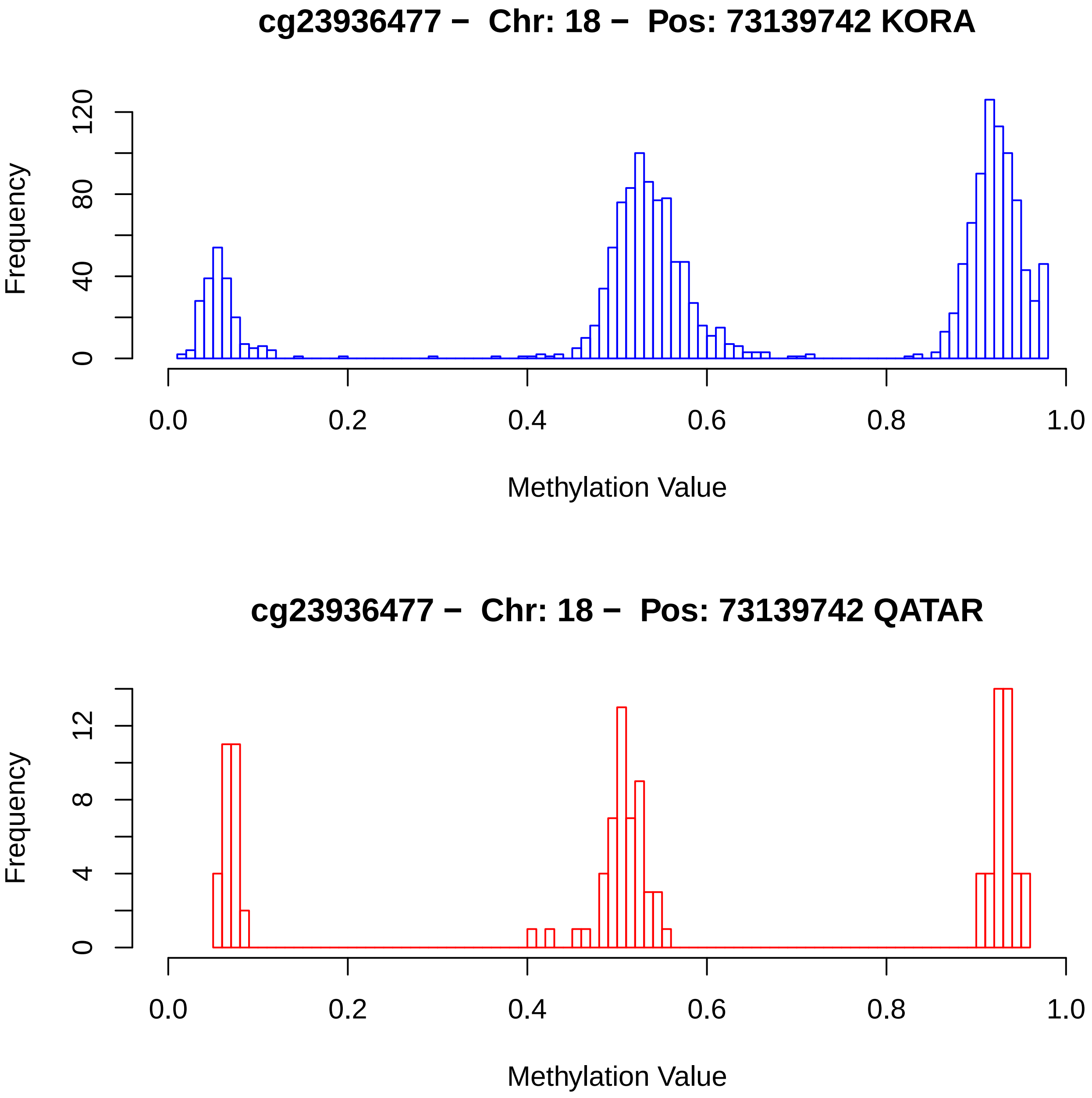
cg23936477 − Chr: 18 − Pos: 73139742 KORA
Frequency
0
40
80
120
0.0
0.2
0.4
0.6
0.8
1.0
Methylation Value
cg23936477 − Chr: 18 − Pos: 73139742 QATAR
Frequency
0
4
8
12
0.0
0.2
0.4
0.6
0.8
1.0
Methylation Value

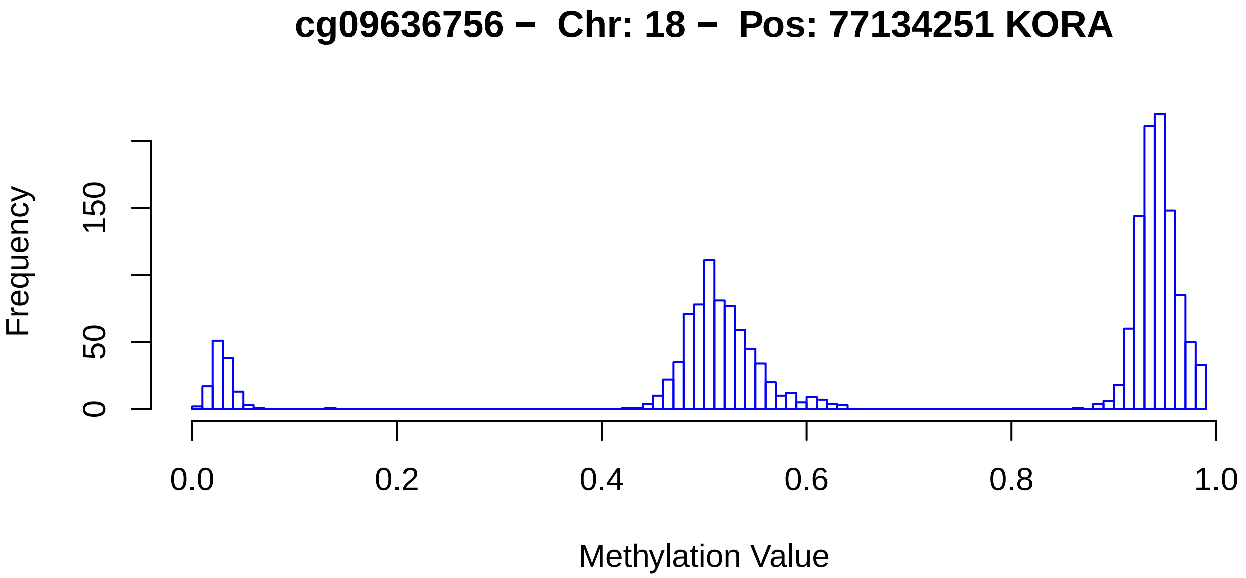
cg09636756 − Chr: 18 − Pos: 77134251 KORA
Frequency
0
50
150
0.0
0.2
0.4
0.6
0.8
1.0
Methylation Value

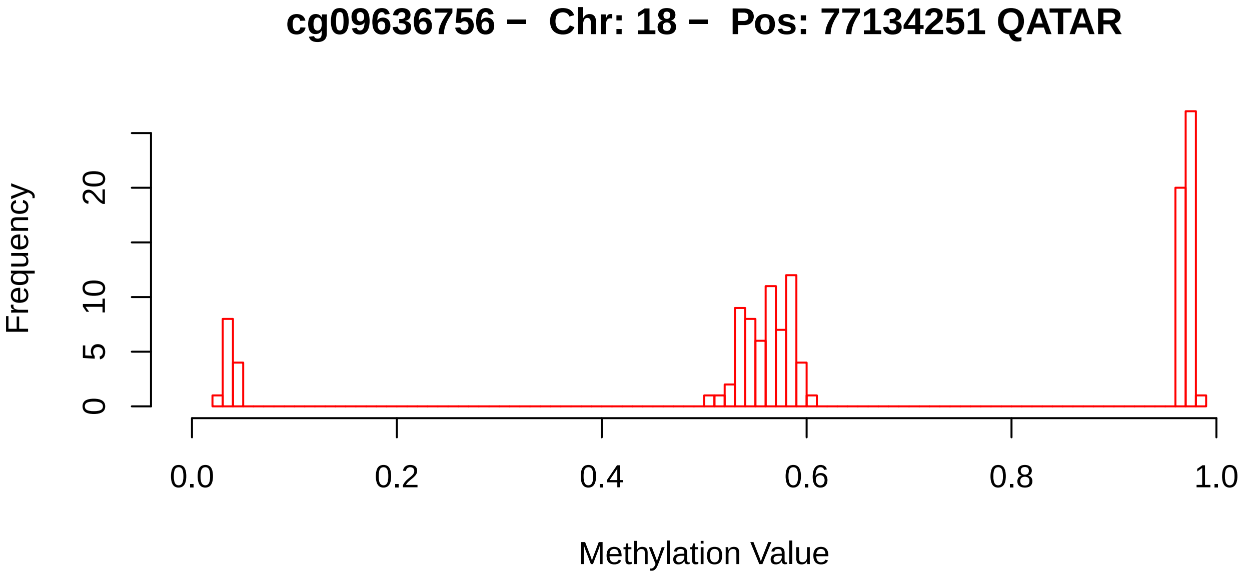
cg09636756 − Chr: 18 − Pos: 77134251 QATAR
Frequency
0
5
10
20
0.0
0.2
0.4
0.6
0.8
1.0
Methylation Value

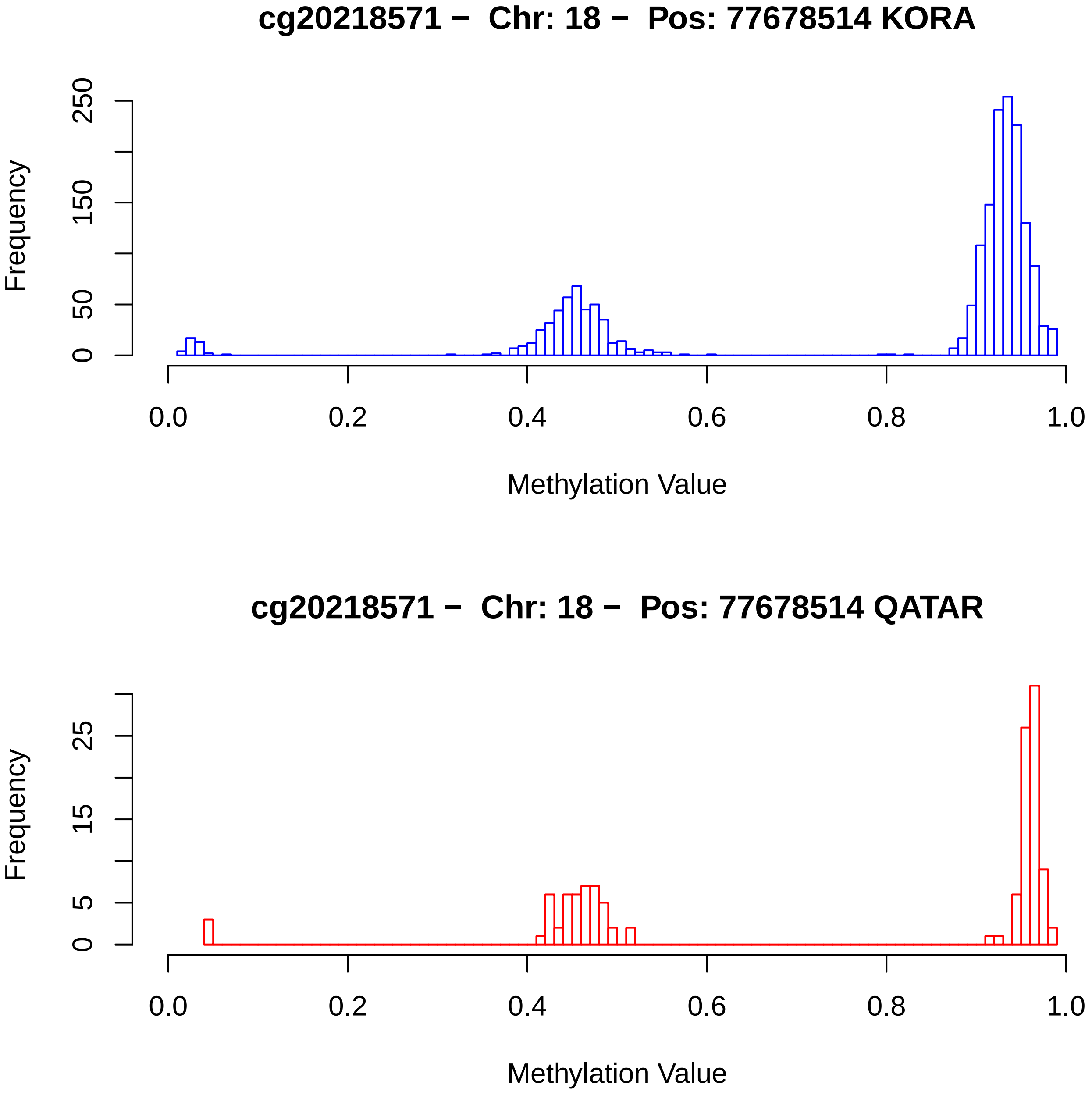
cg20218571 − Chr: 18 − Pos: 77678514 KORA
Frequency
0
50
150
250
0.0
0.2
0.4
0.6
0.8
1.0
Methylation Value
cg20218571 − Chr: 18 − Pos: 77678514 QATAR
Frequency
0
5
15
25
0.0
0.2
0.4
0.6
0.8
1.0
Methylation Value

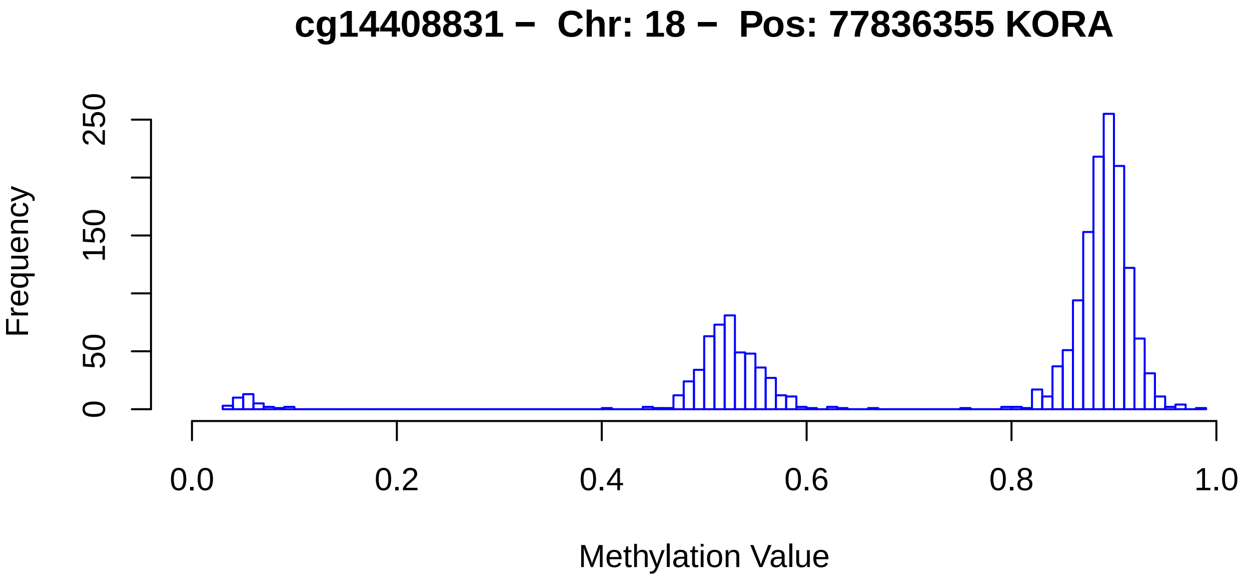
cg14408831 − Chr: 18 − Pos: 77836355 KORA
Frequency
0
50
150
250
0.0
0.2
0.4
0.6
0.8
1.0
Methylation Value

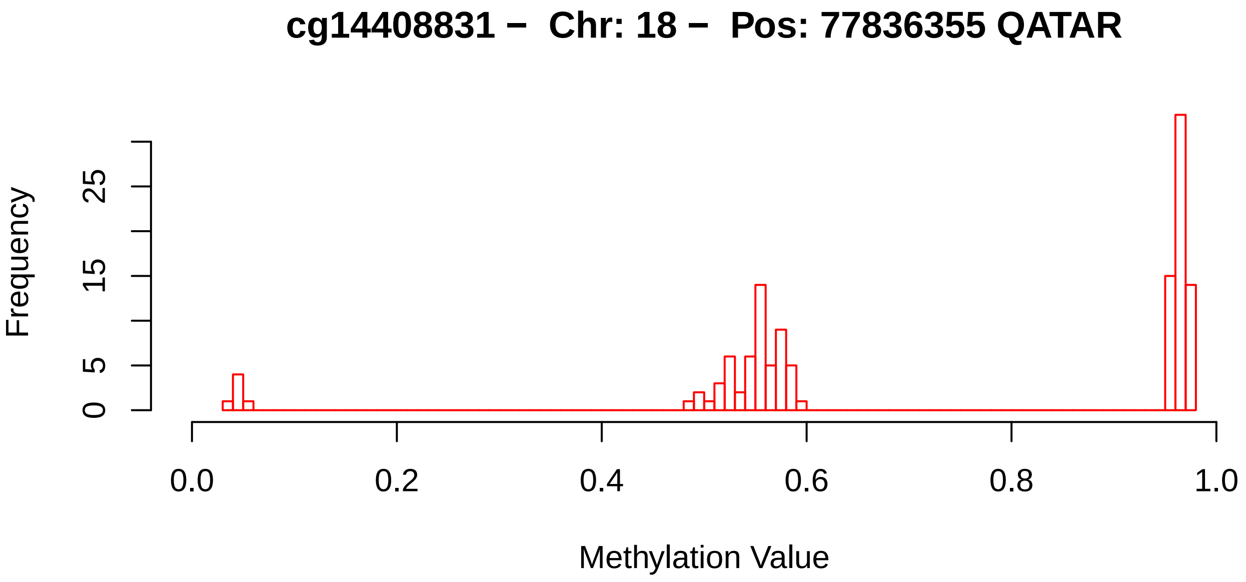
cg14408831 − Chr: 18 − Pos: 77836355 QATAR
Frequency
0
5
15
25
0.0
0.2
0.4
0.6
0.8
1.0
Methylation Value

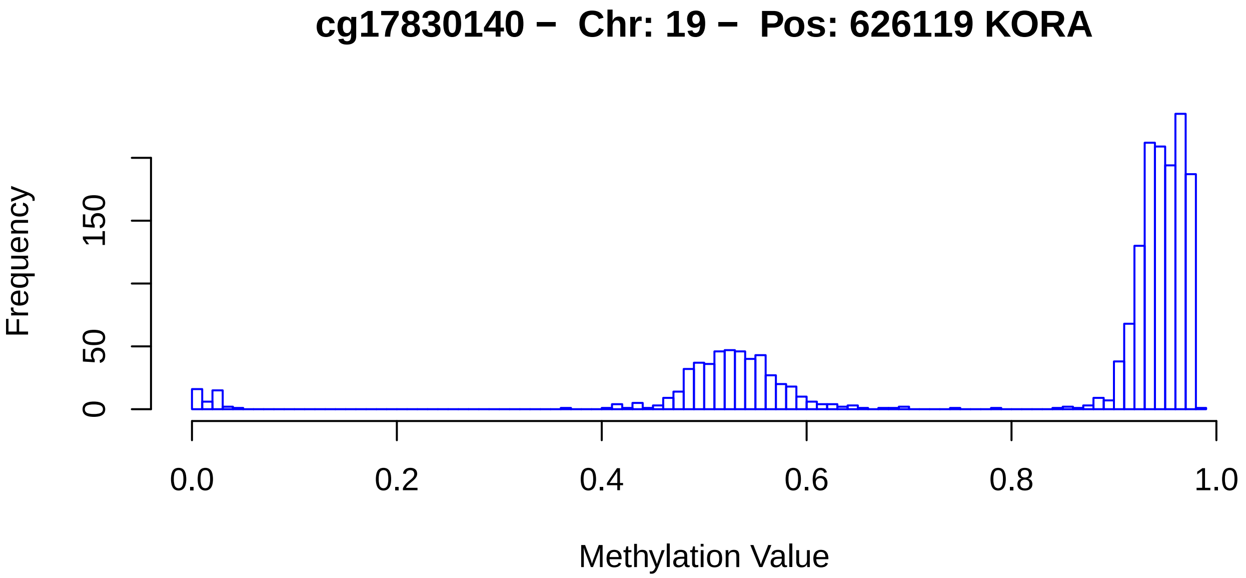
cg17830140 − Chr: 19 − Pos: 626119 KORA
Frequency
0
50
150
0.0
0.2
0.4
0.6
0.8
1.0
Methylation Value

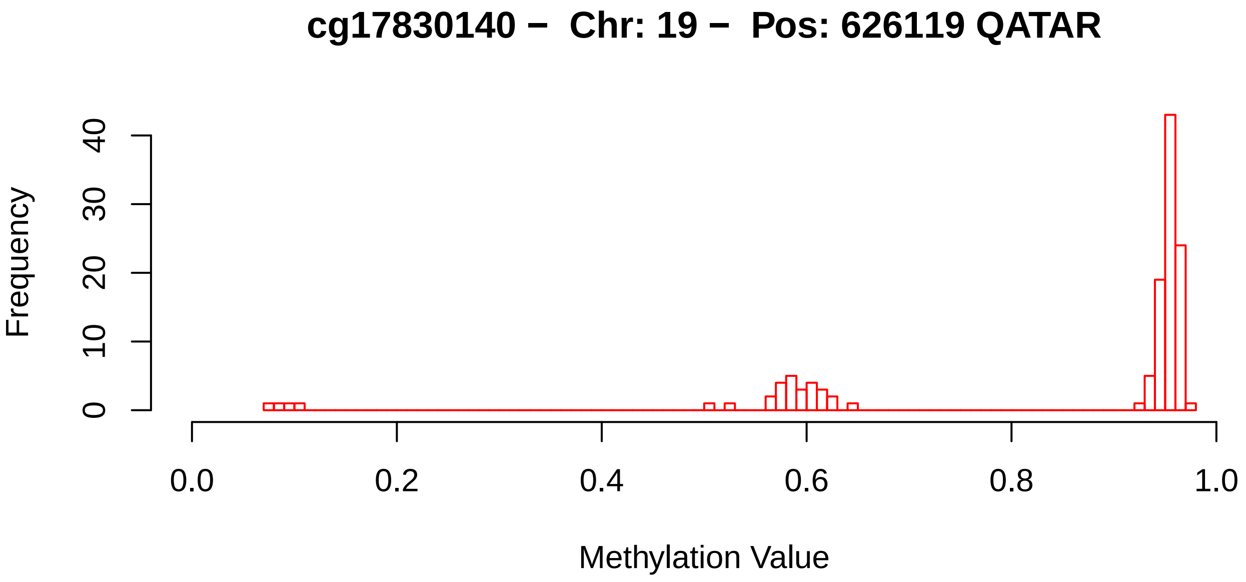
cg17830140 − Chr: 19 − Pos: 626119 QATAR
Frequency
0
10
20
30
40
0.0
0.2
0.4
0.6
0.8
1.0
Methylation Value

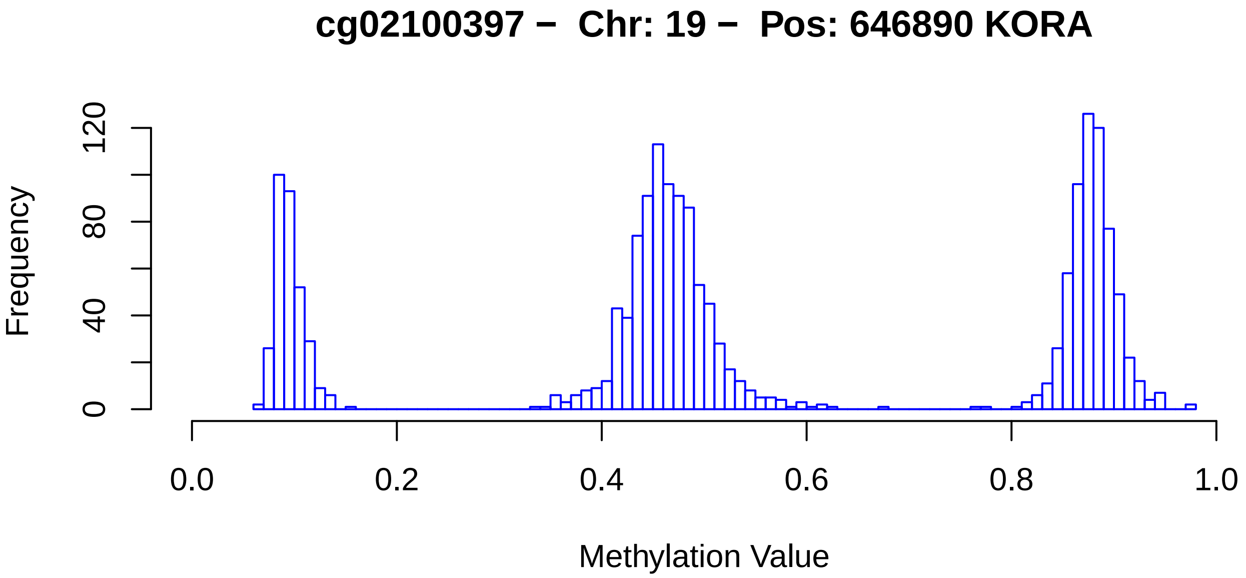
cg02100397 − Chr: 19 − Pos: 646890 KORA
Frequency
0
40
80
120
0.0
0.2
0.4
0.6
0.8
1.0
Methylation Value

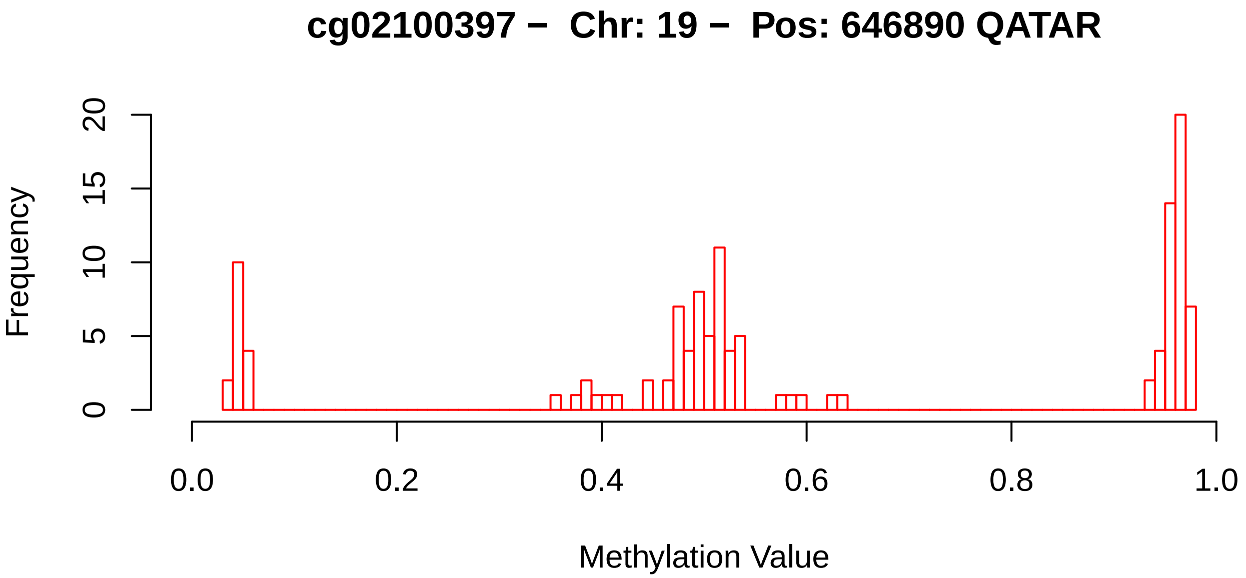
cg02100397 − Chr: 19 − Pos: 646890 QATAR
Frequency
0
5
10
15
20
0.0
0.2
0.4
0.6
0.8
1.0
Methylation Value

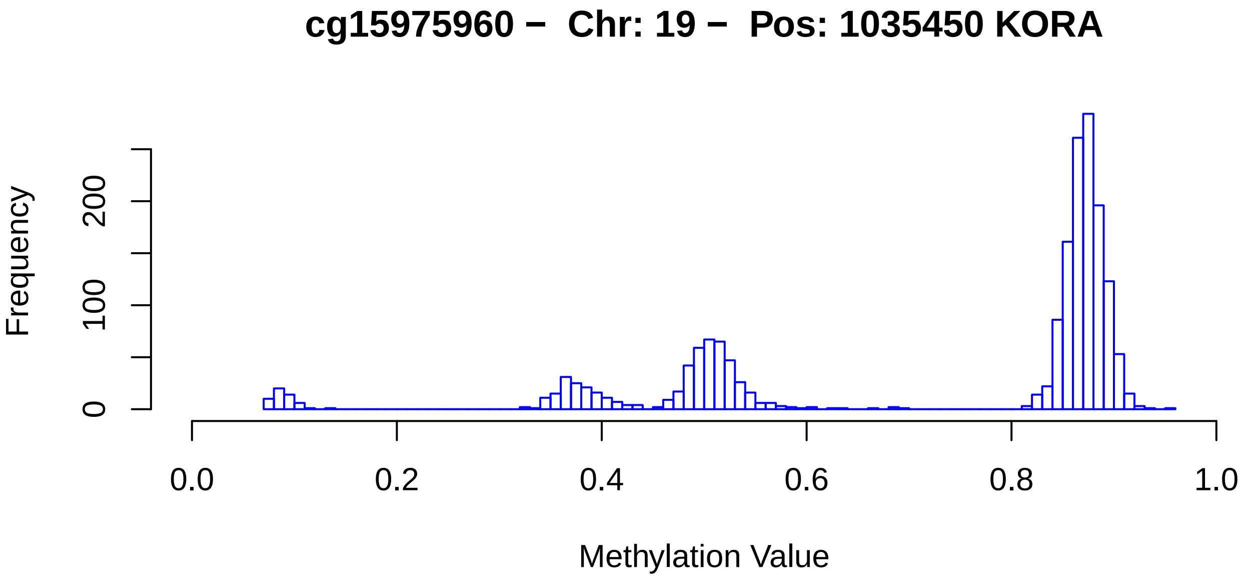
cg15975960 − Chr: 19 − Pos: 1035450 KORA
Frequency
0
100
200
0.0
0.2
0.4
0.6
0.8
1.0
Methylation Value

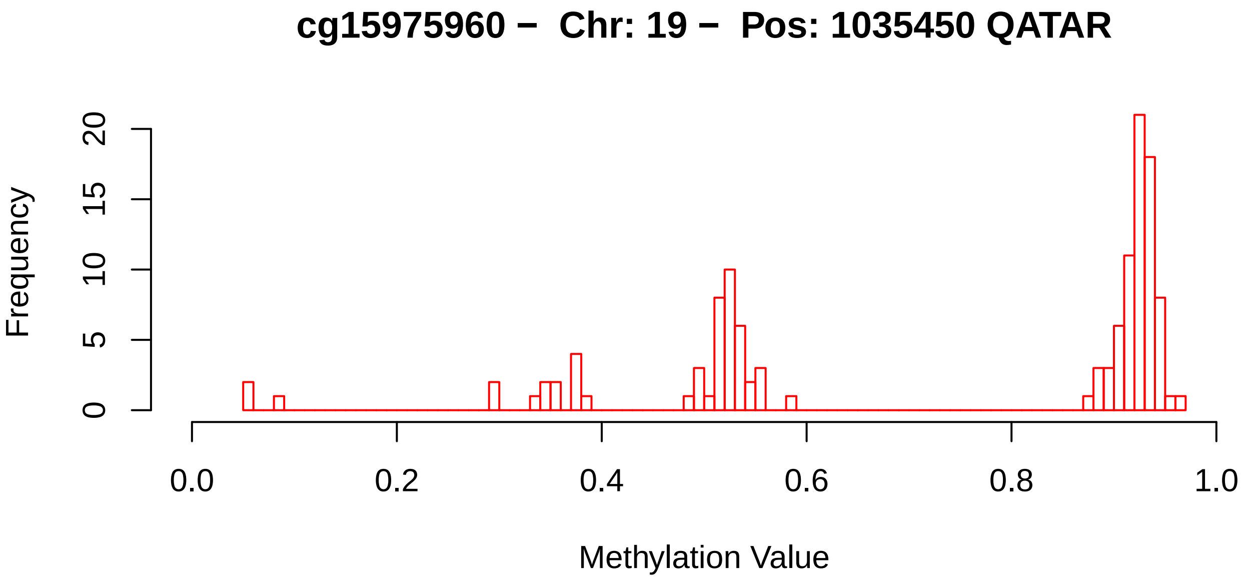
cg15975960 − Chr: 19 − Pos: 1035450 QATAR
Frequency
0
5
10
15
20
0.0
0.2
0.4
0.6
0.8
1.0
Methylation Value

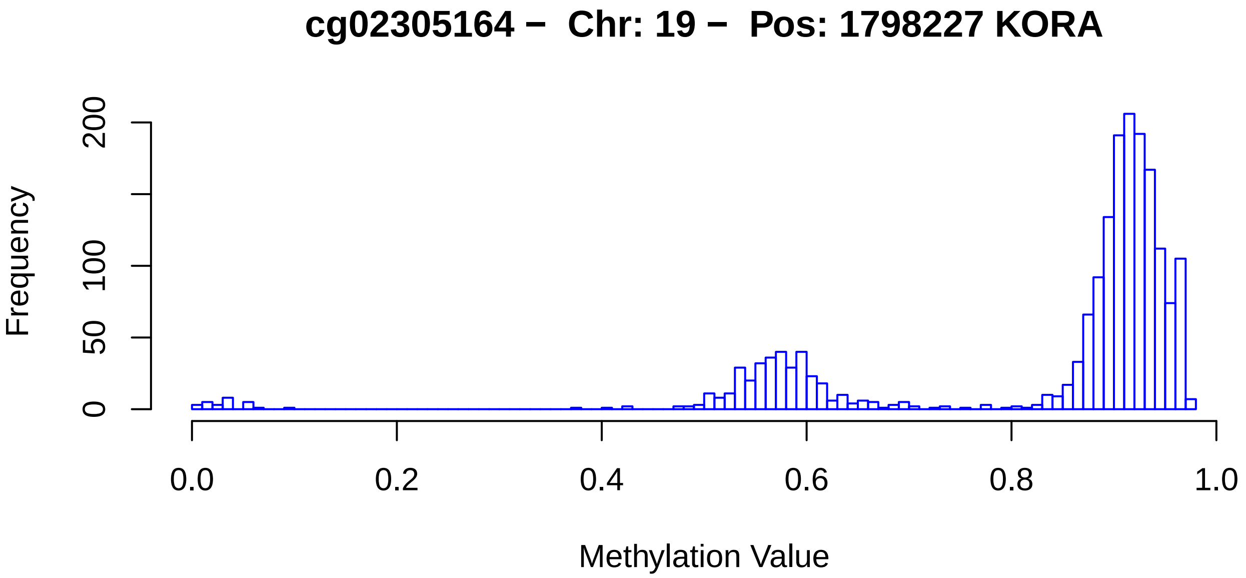
cg02305164 − Chr: 19 − Pos: 1798227 KORA
Frequency
0
50
100
200
0.0
0.2
0.4
0.6
0.8
1.0
Methylation Value

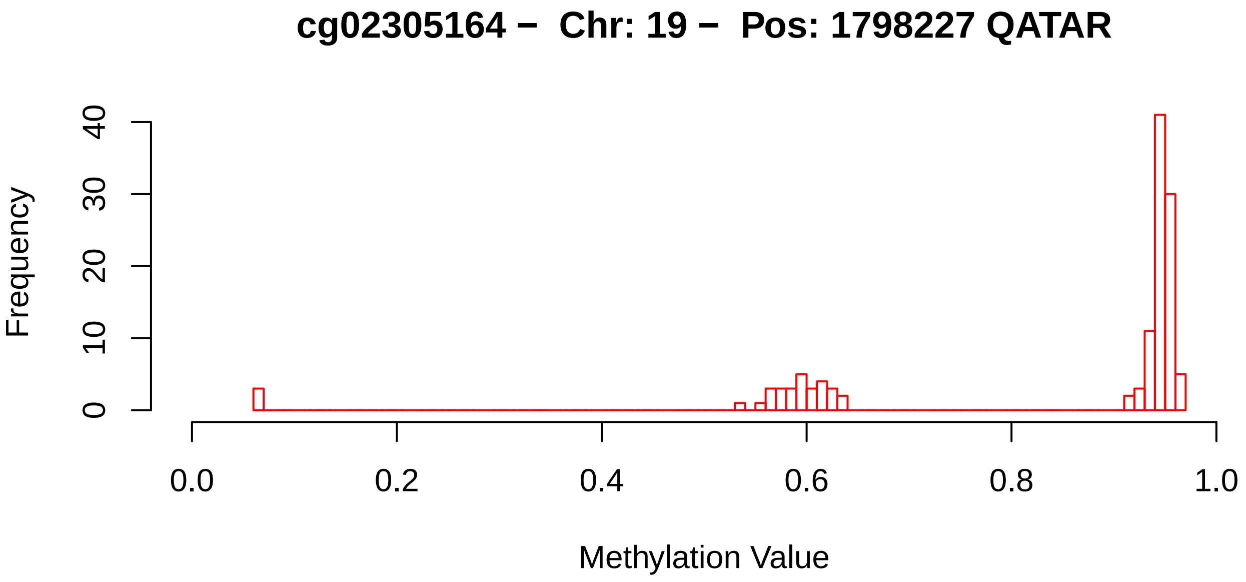
cg02305164 − Chr: 19 − Pos: 1798227 QATAR
Frequency
0
10
20
30
40
0.0
0.2
0.4
0.6
0.8
1.0
Methylation Value

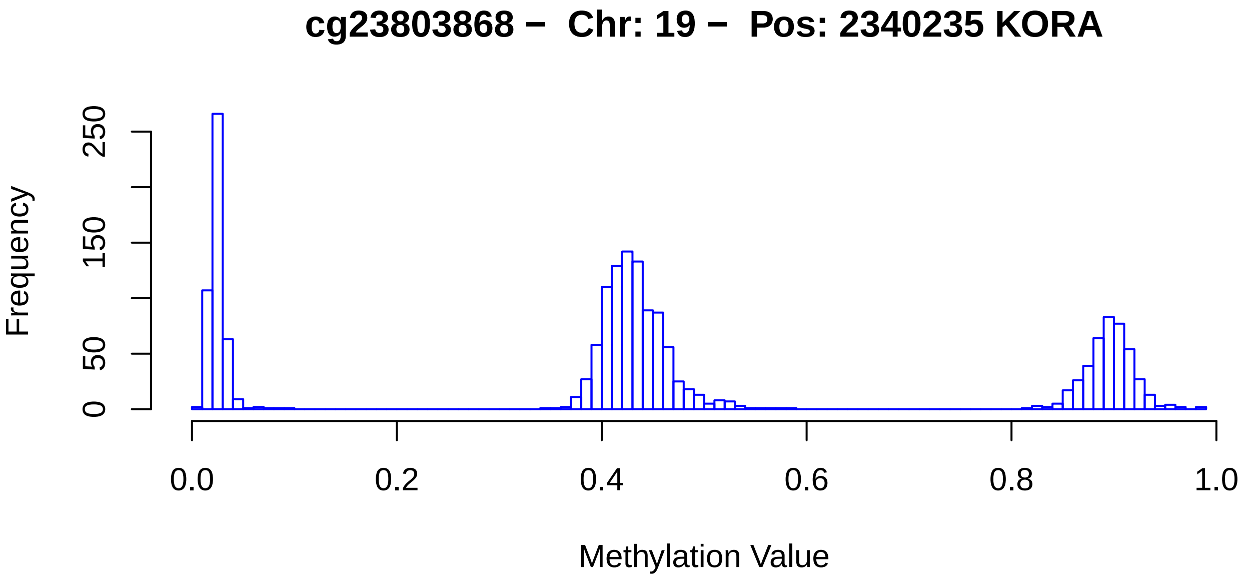
cg23803868 − Chr: 19 − Pos: 2340235 KORA
Frequency
0
50
150
250
0.0
0.2
0.4
0.6
0.8
1.0
Methylation Value

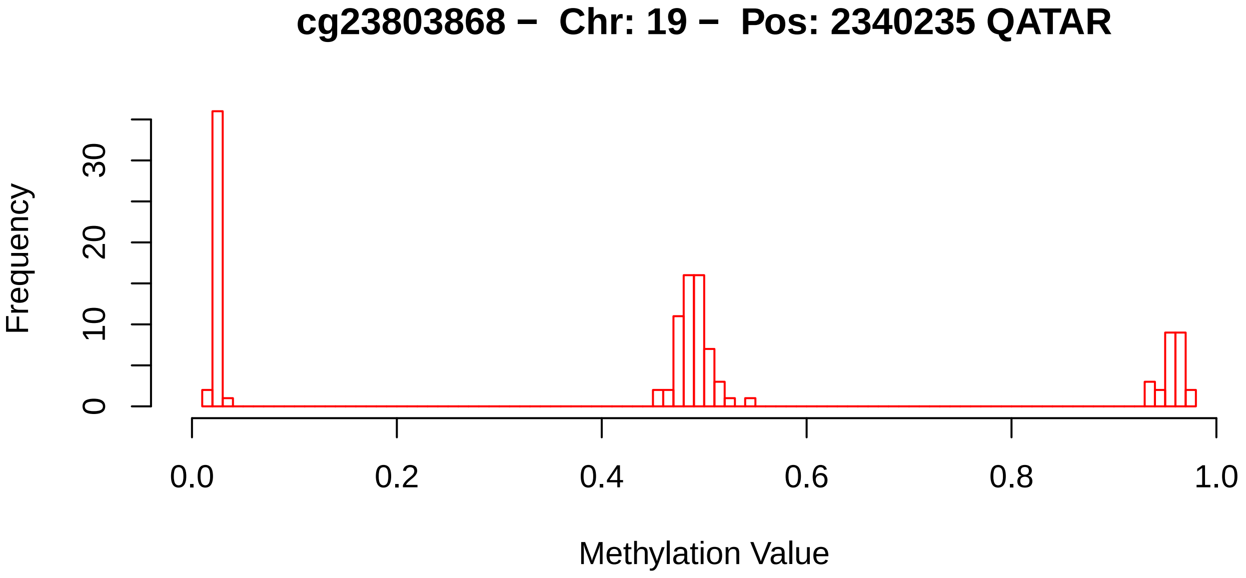
cg23803868 − Chr: 19 − Pos: 2340235 QATAR
Frequency
0
10
20
30
0.0
0.2
0.4
0.6
0.8
1.0
Methylation Value

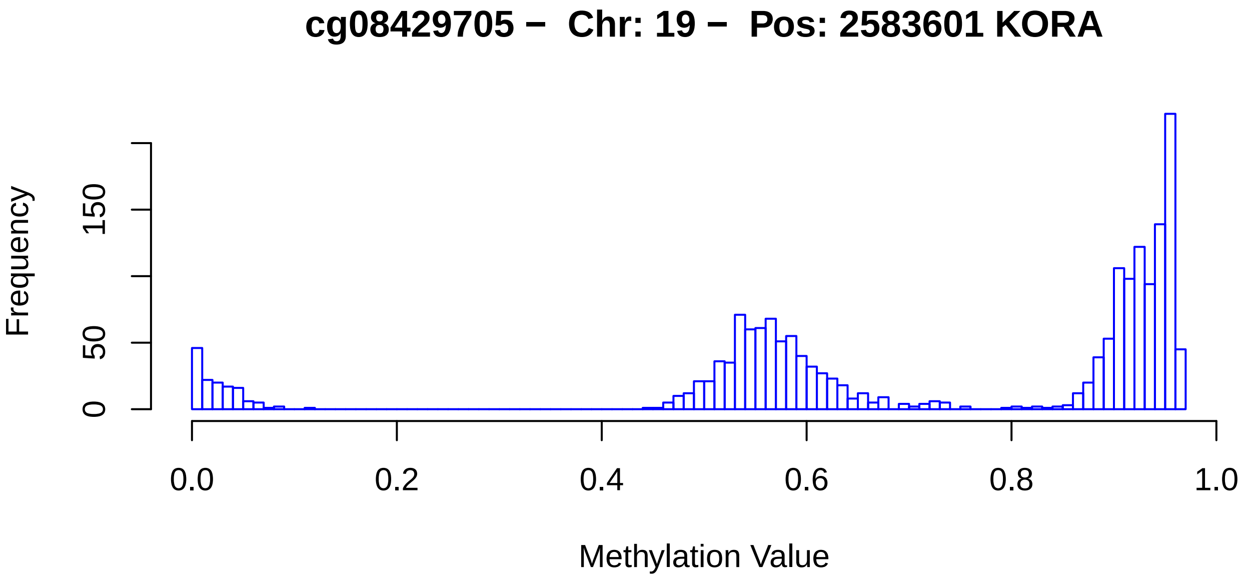
cg08429705 − Chr: 19 − Pos: 2583601 KORA
Frequency
0
50
150
0.0
0.2
0.4
0.6
0.8
1.0
Methylation Value

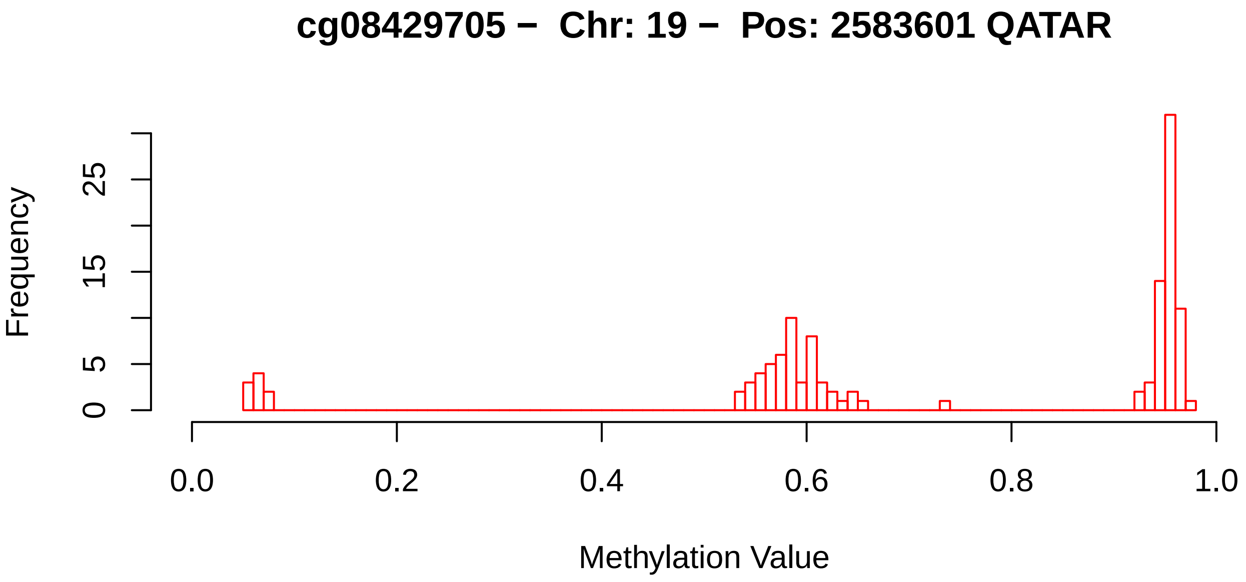
cg08429705 − Chr: 19 − Pos: 2583601 QATAR
Frequency
0
5
15
25
0.0
0.2
0.4
0.6
0.8
1.0
Methylation Value

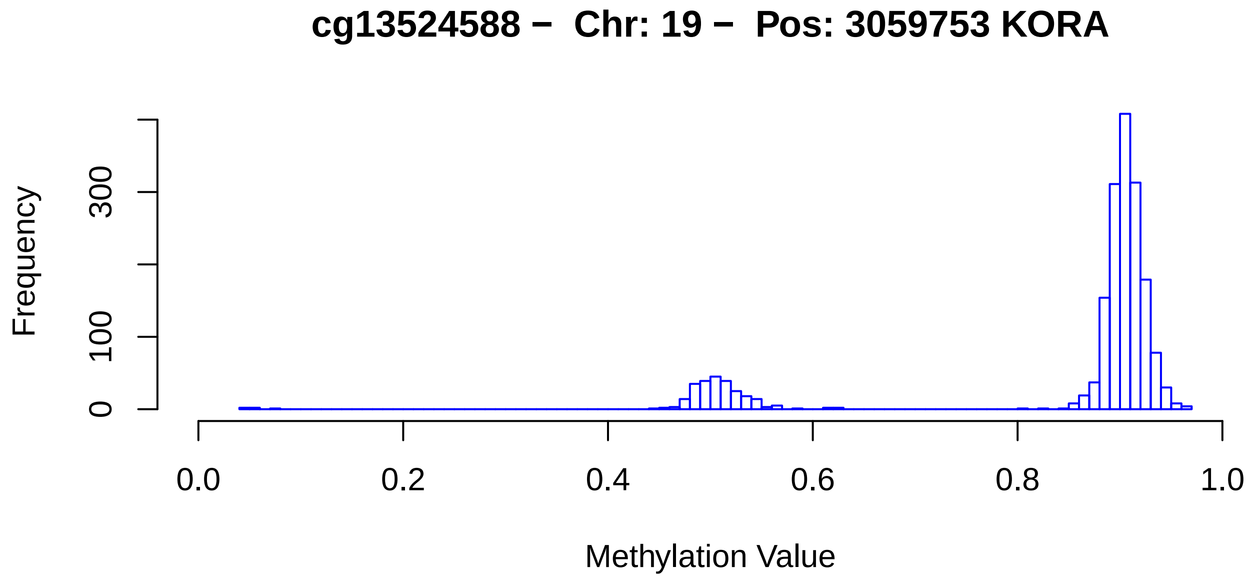
cg13524588 − Chr: 19 − Pos: 3059753 KORA
Frequency
0
100
300
0.0
0.2
0.4
0.6
0.8
1.0
Methylation Value

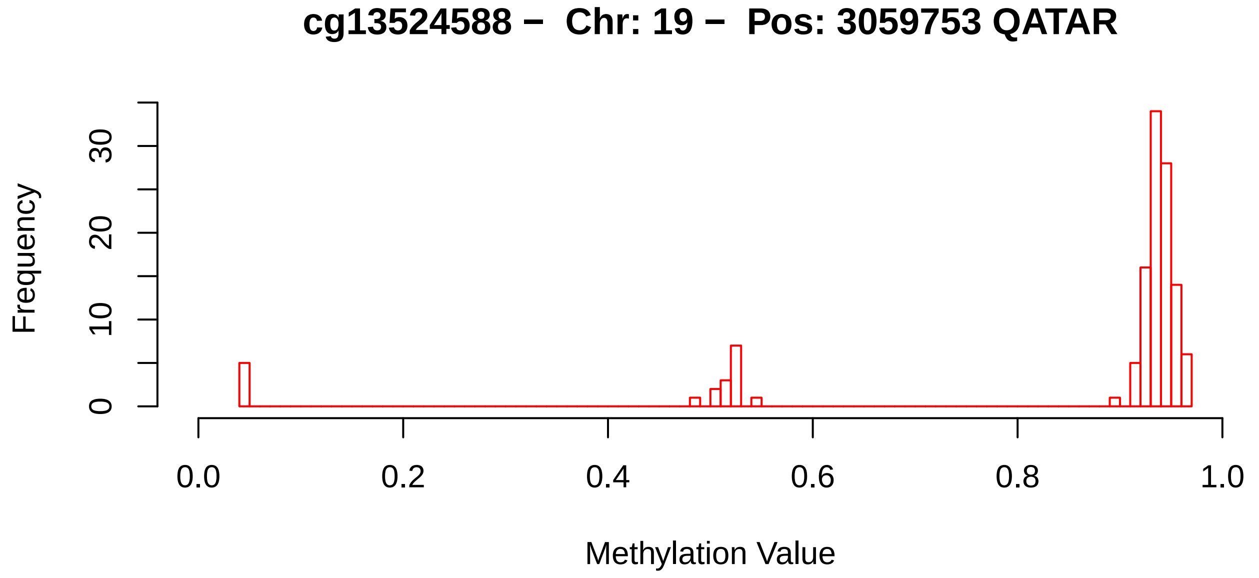
cg13524588 − Chr: 19 − Pos: 3059753 QATAR
Frequency
0
10
20
30
0.0
0.2
0.4
0.6
0.8
1.0
Methylation Value

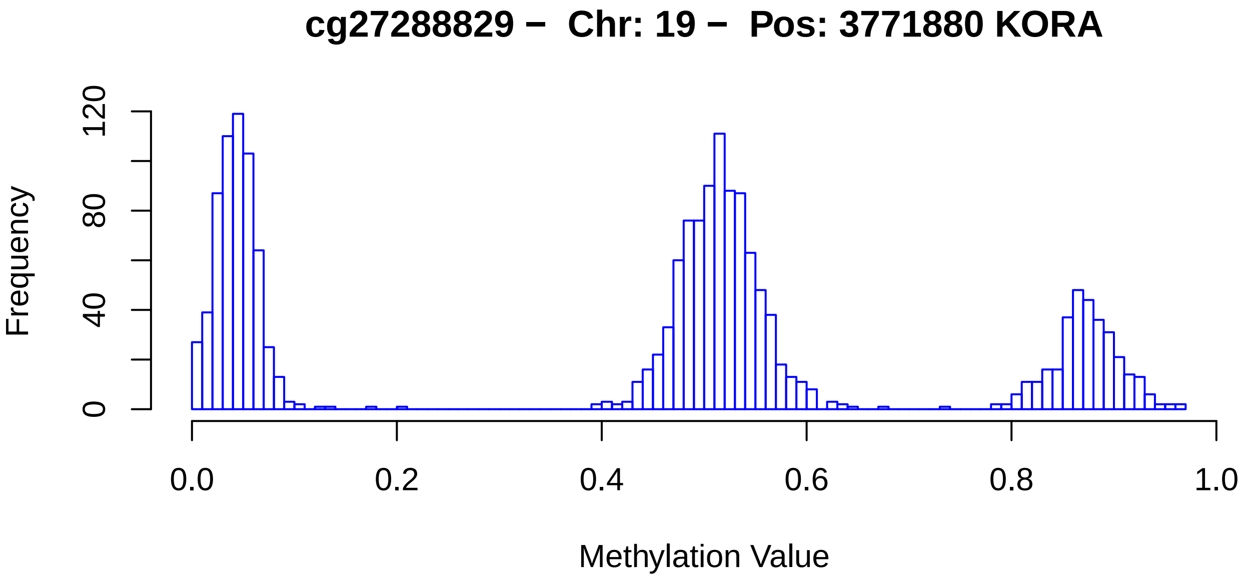
cg27288829 − Chr: 19 − Pos: 3771880 KORA
Frequency
0
40
80
120
0.0
0.2
0.4
0.6
0.8
1.0
Methylation Value

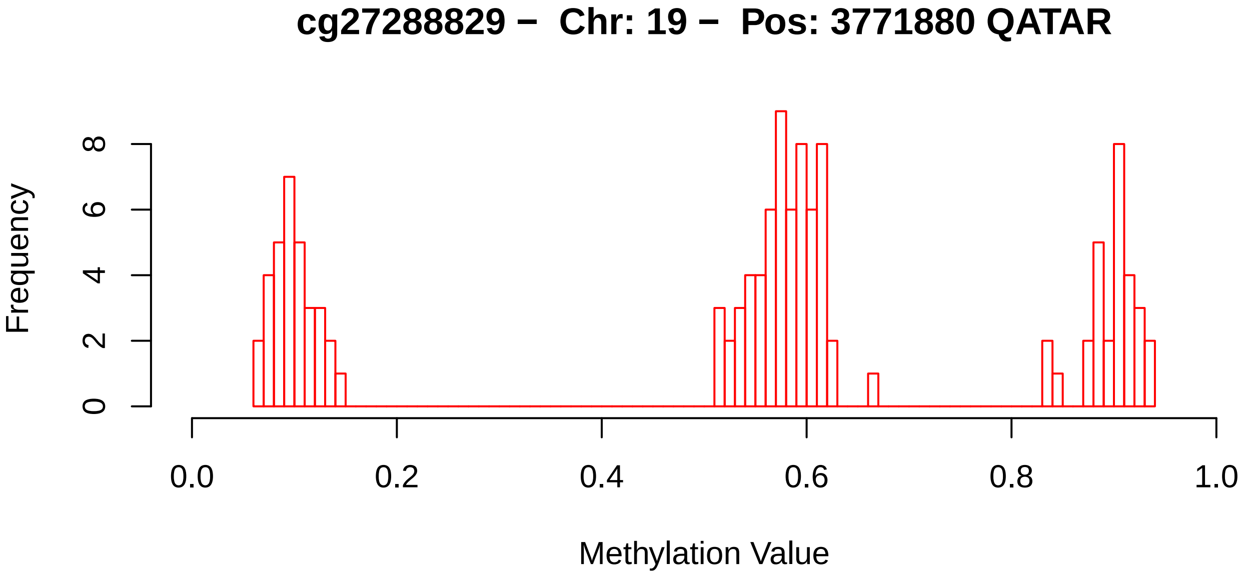
cg27288829 − Chr: 19 − Pos: 3771880 QATAR
Frequency
0
2
4
6
8
0.0
0.2
0.4
0.6
0.8
1.0
Methylation Value

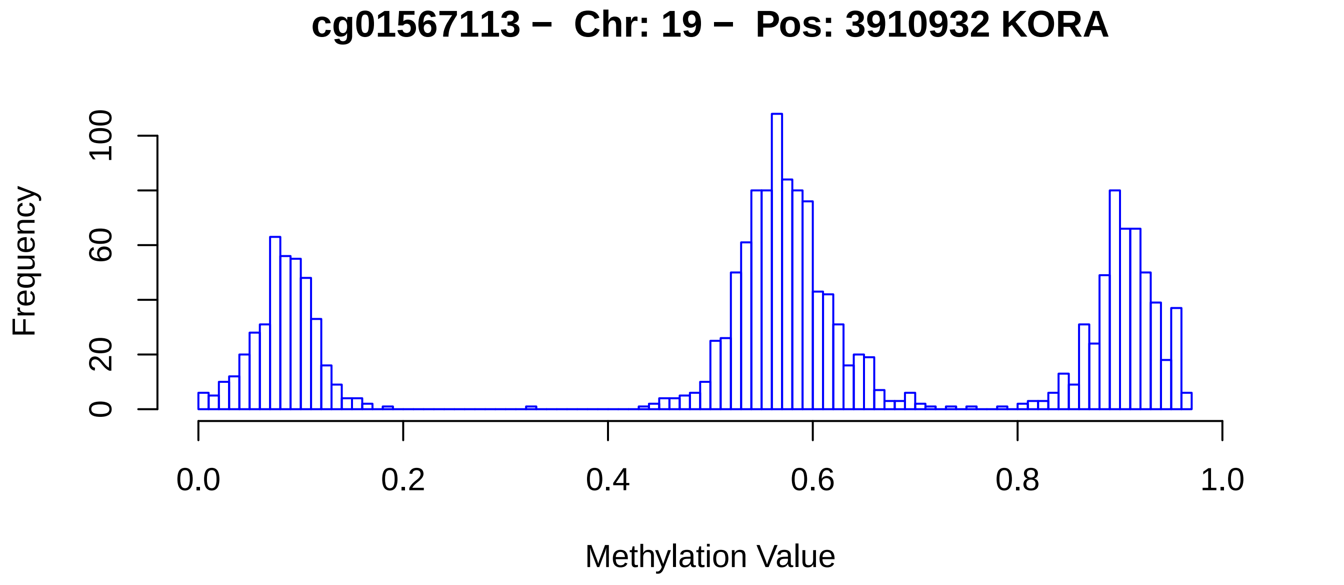
cg01567113 − Chr: 19 − Pos: 3910932 KORA
Frequency
0
20
60
100
0.0
0.2
0.4
0.6
0.8
1.0
Methylation Value

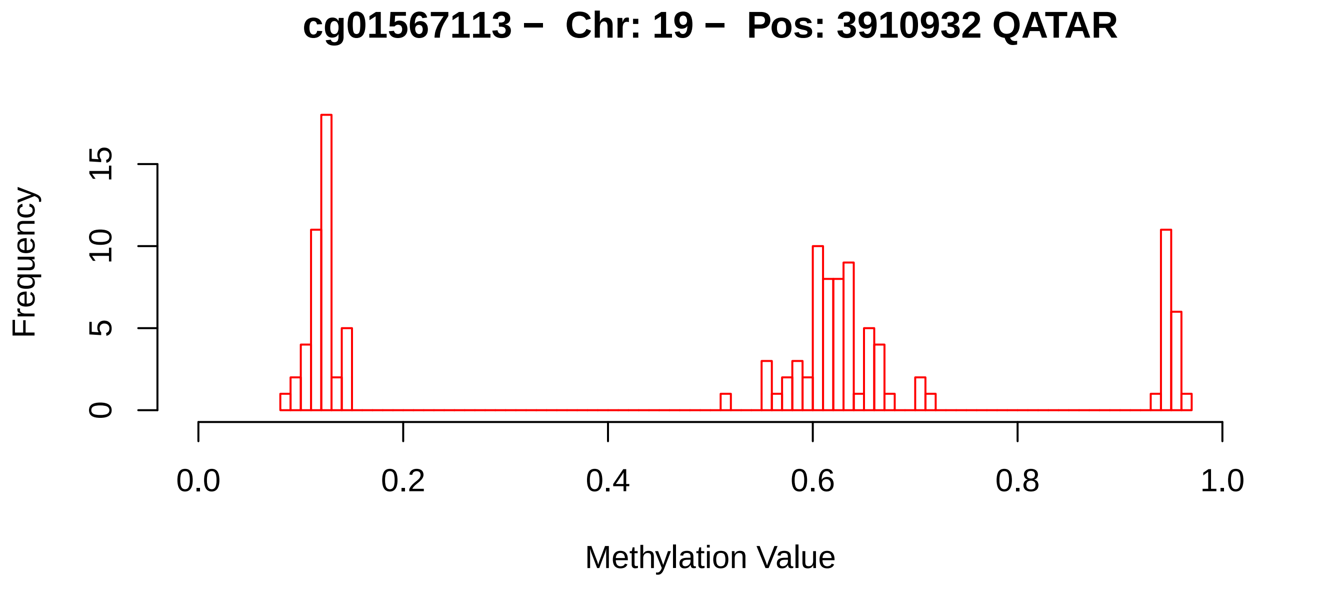
cg01567113 − Chr: 19 − Pos: 3910932 QATAR
Frequency
0
5
10
15
0.0
0.2
0.4
0.6
0.8
1.0
Methylation Value

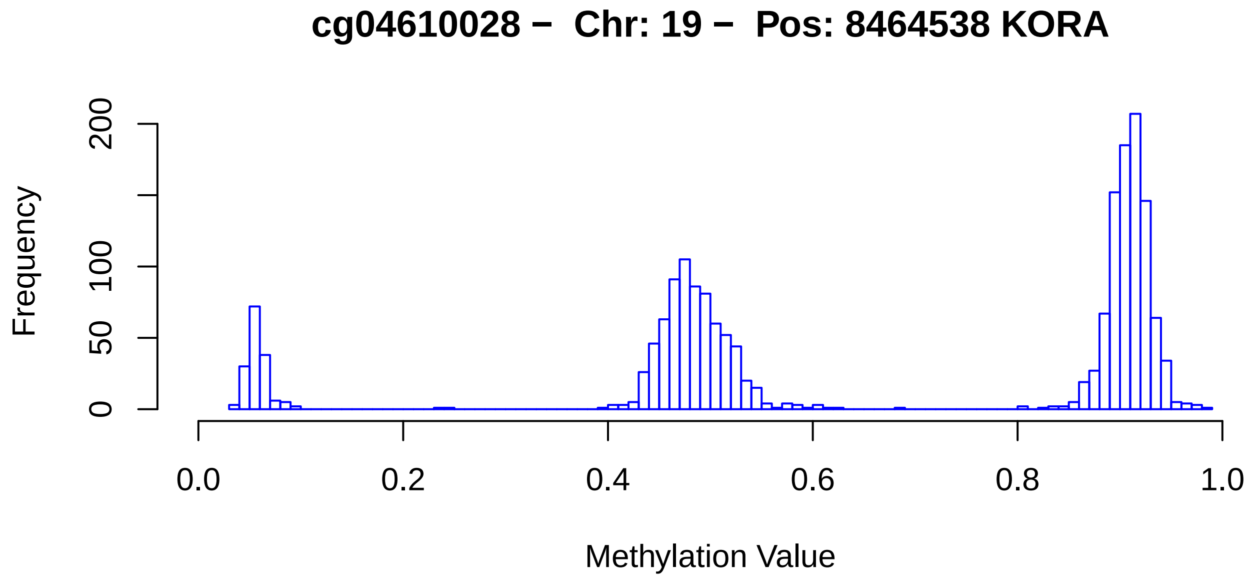
cg04610028 − Chr: 19 − Pos: 8464538 KORA
Frequency
0
50
100
200
0.0
0.2
0.4
0.6
0.8
1.0
Methylation Value

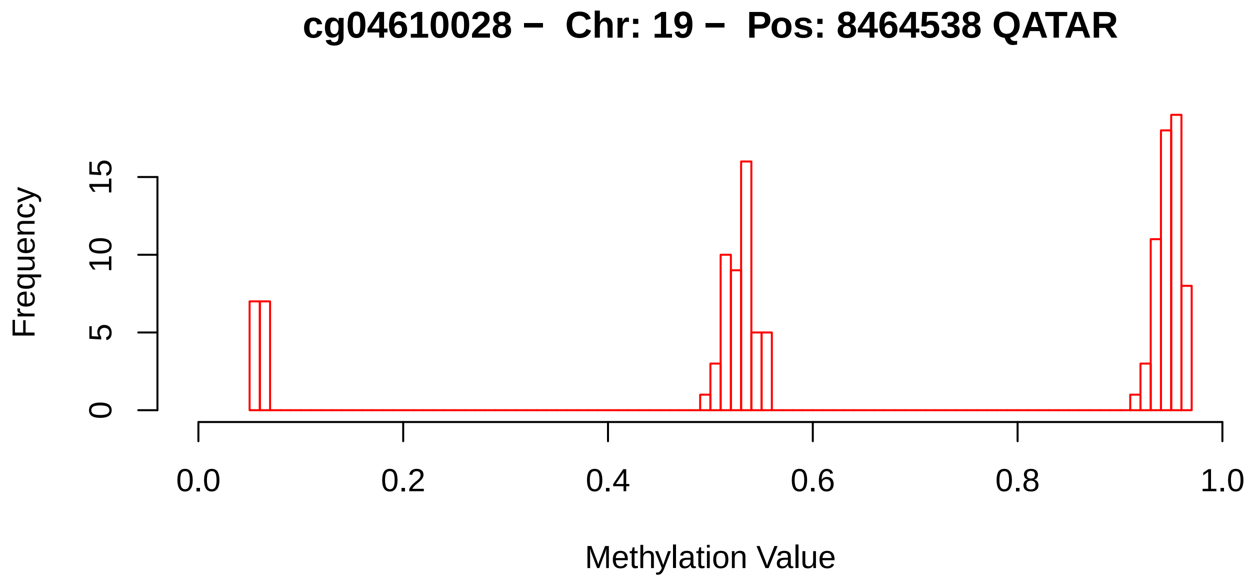
cg04610028 − Chr: 19 − Pos: 8464538 QATAR
Frequency
0
5
10
15
0.0
0.2
0.4
0.6
0.8
1.0
Methylation Value

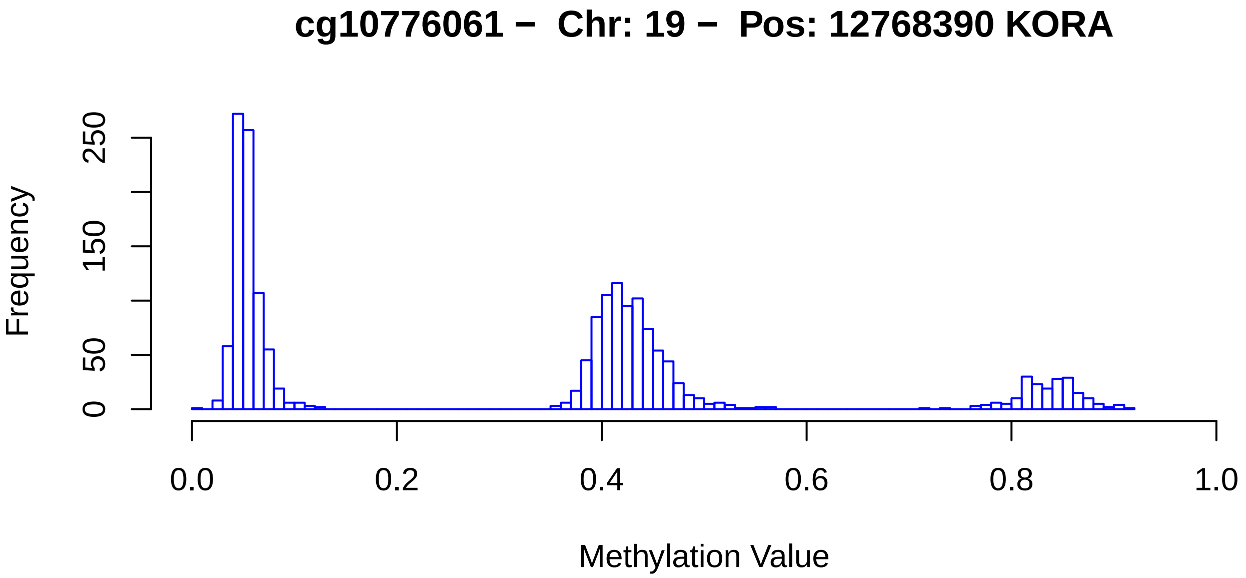
cg10776061 − Chr: 19 − Pos: 12768390 KORA
Frequency
0
50
150
250
0.0
0.2
0.4
0.6
0.8
1.0
Methylation Value

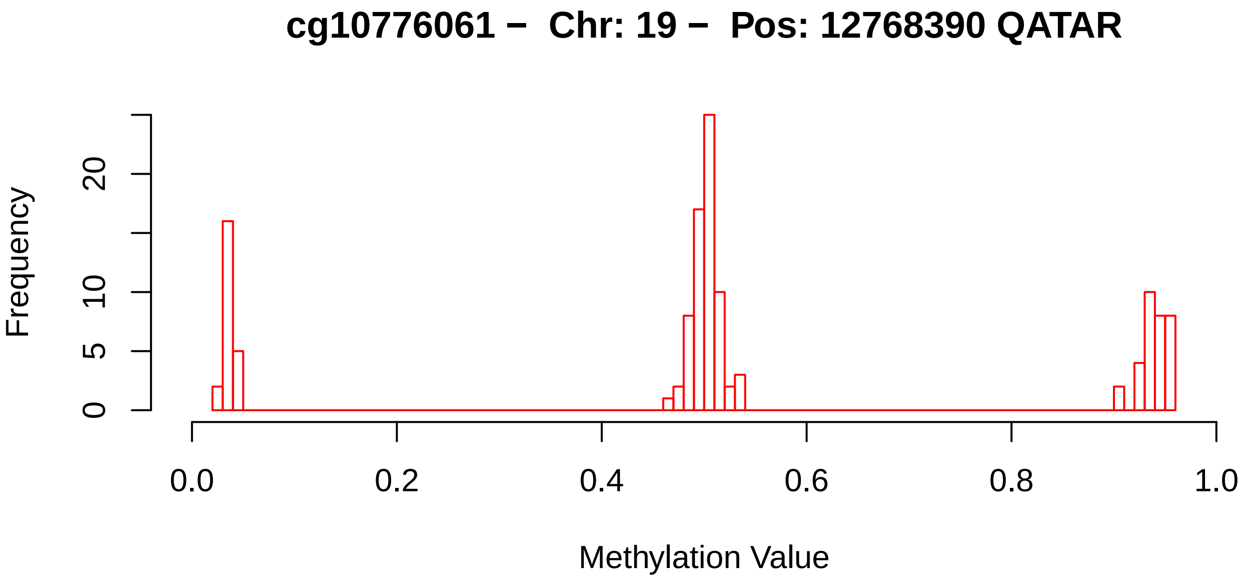
cg10776061 − Chr: 19 − Pos: 12768390 QATAR
Frequency
0
5
10
20
0.0
0.2
0.4
0.6
0.8
1.0
Methylation Value

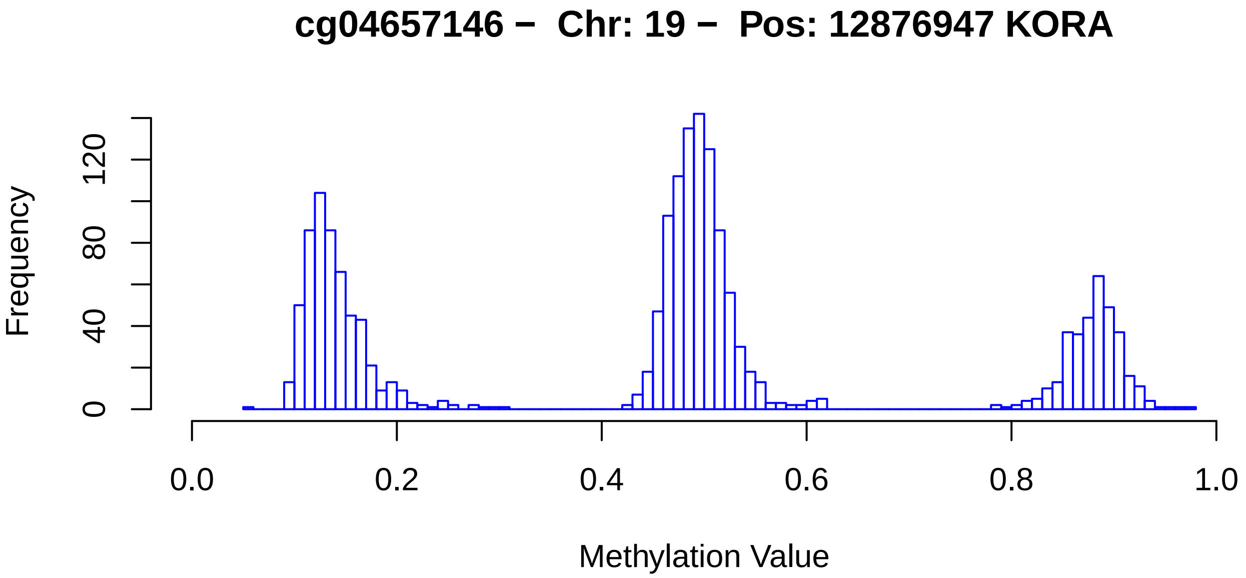
cg04657146 − Chr: 19 − Pos: 12876947 KORA
Frequency
0
40
80
120
0.0
0.2
0.4
0.6
0.8
1.0
Methylation Value

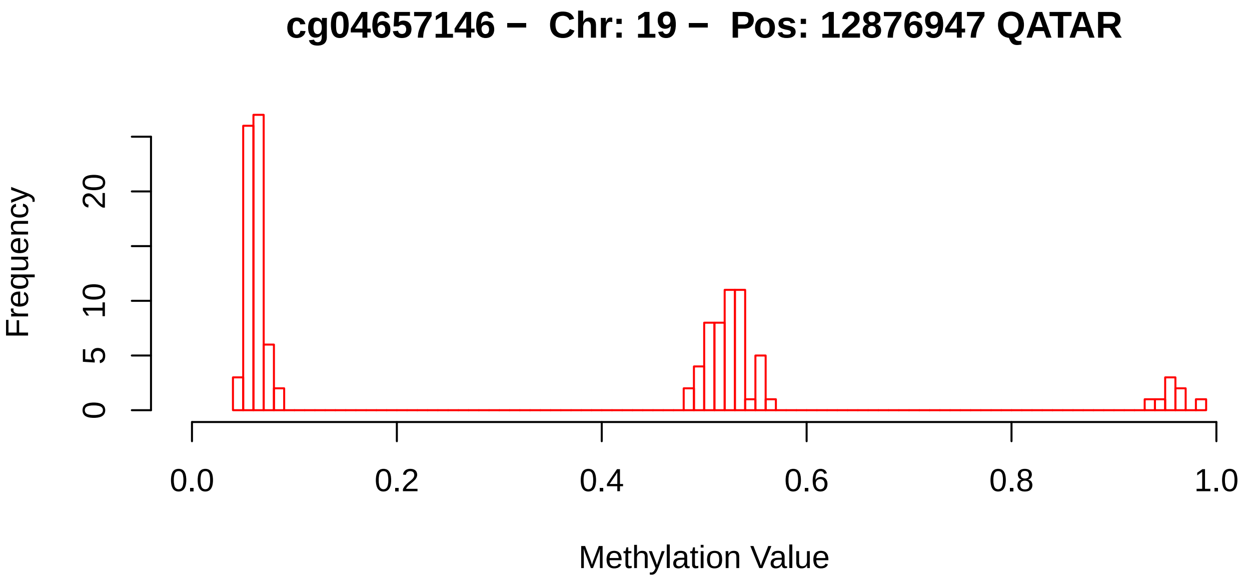
cg04657146 − Chr: 19 − Pos: 12876947 QATAR
Frequency
0
5
10
20
0.0
0.2
0.4
0.6
0.8
1.0
Methylation Value

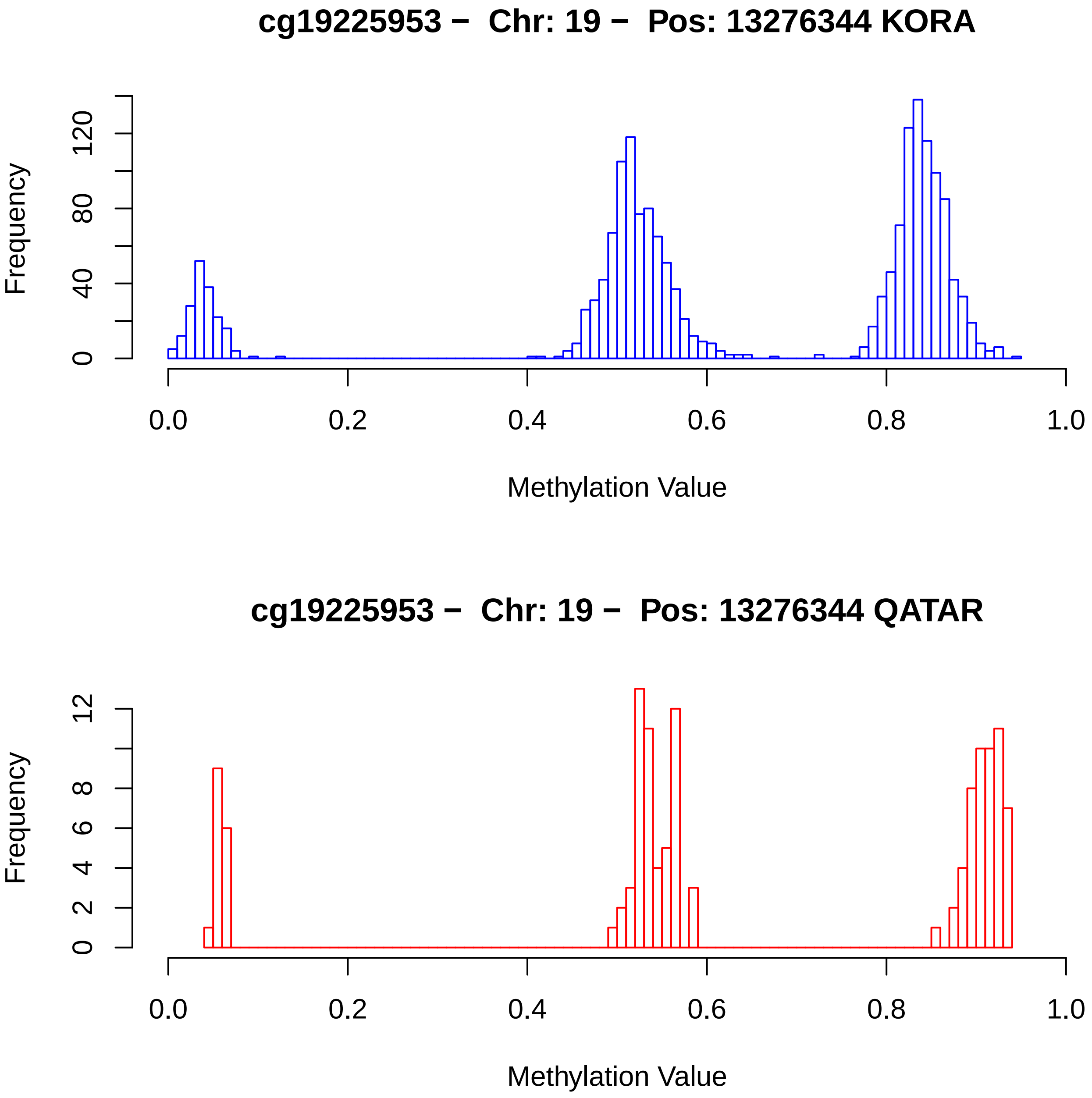
cg19225953 − Chr: 19 − Pos: 13276344 KORA
Frequency
0
40
80
120
0.0
0.2
0.4
0.6
0.8
1.0
Methylation Value
cg19225953 − Chr: 19 − Pos: 13276344 QATAR
Frequency
0
2
4
6
8
12
0.0
0.2
0.4
0.6
0.8
1.0
Methylation Value

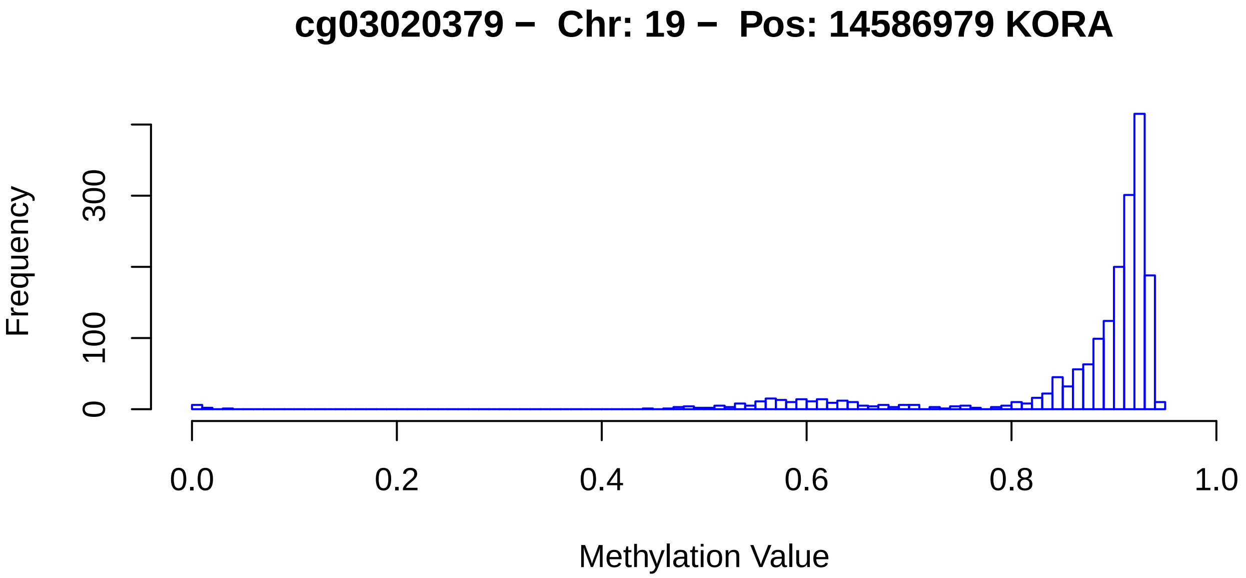
cg03020379 − Chr: 19 − Pos: 14586979 KORA
Frequency
0
100
300
0.0
0.2
0.4
0.6
0.8
1.0
Methylation Value

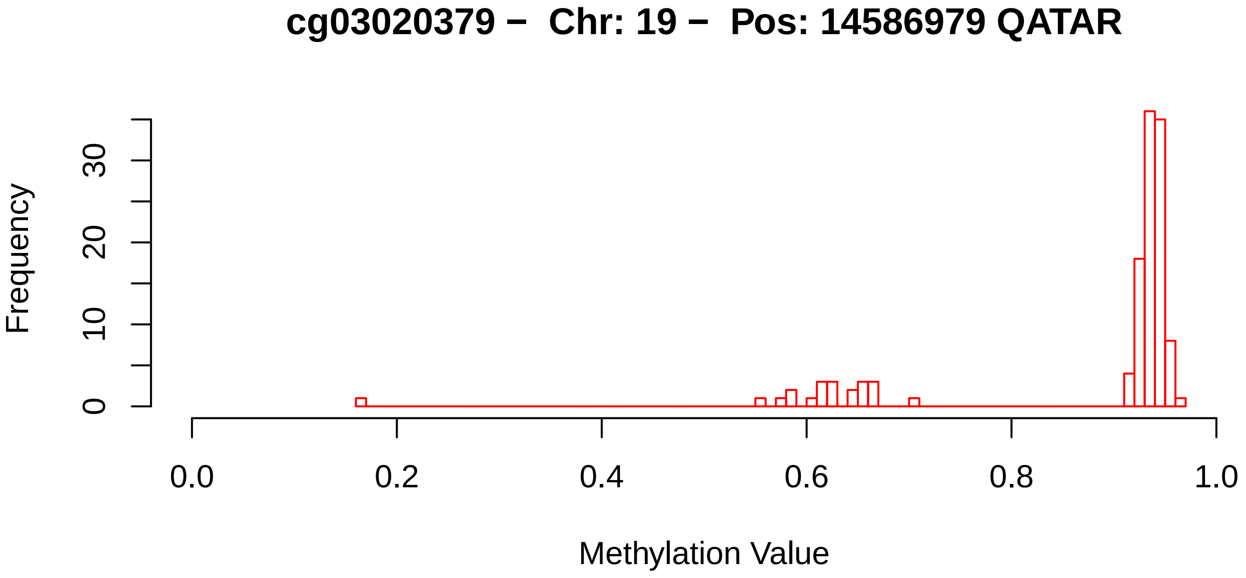
cg03020379 − Chr: 19 − Pos: 14586979 QATAR
Frequency
0
10
20
30
0.0
0.2
0.4
0.6
0.8
1.0
Methylation Value

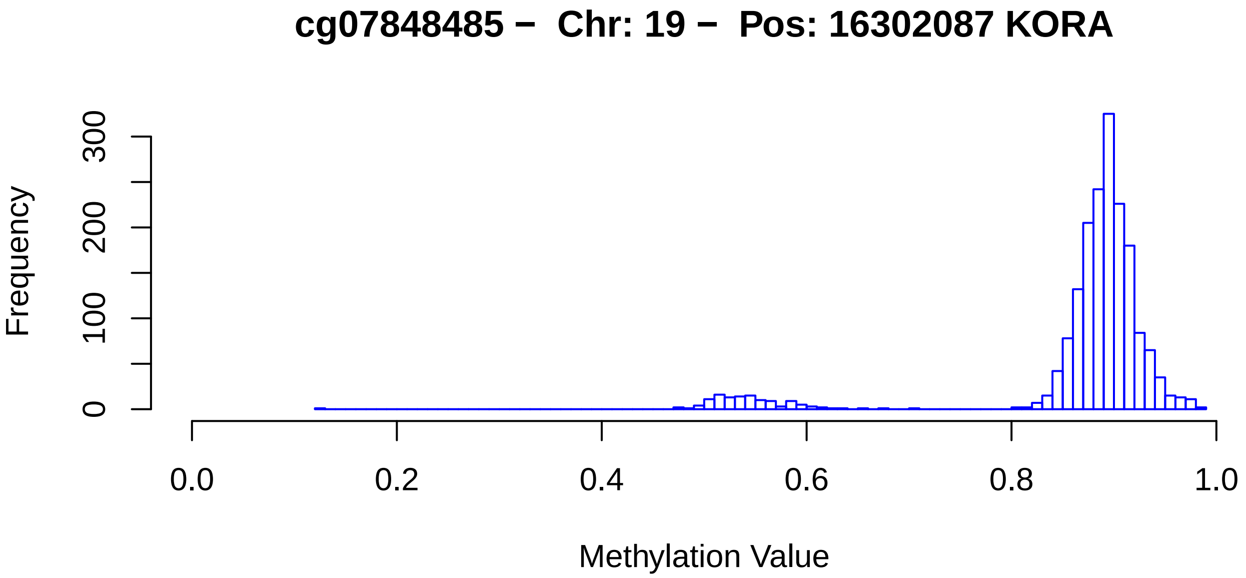
cg07848485 − Chr: 19 − Pos: 16302087 KORA
Frequency
0
100
200
300
0.0
0.2
0.4
0.6
0.8
1.0
Methylation Value

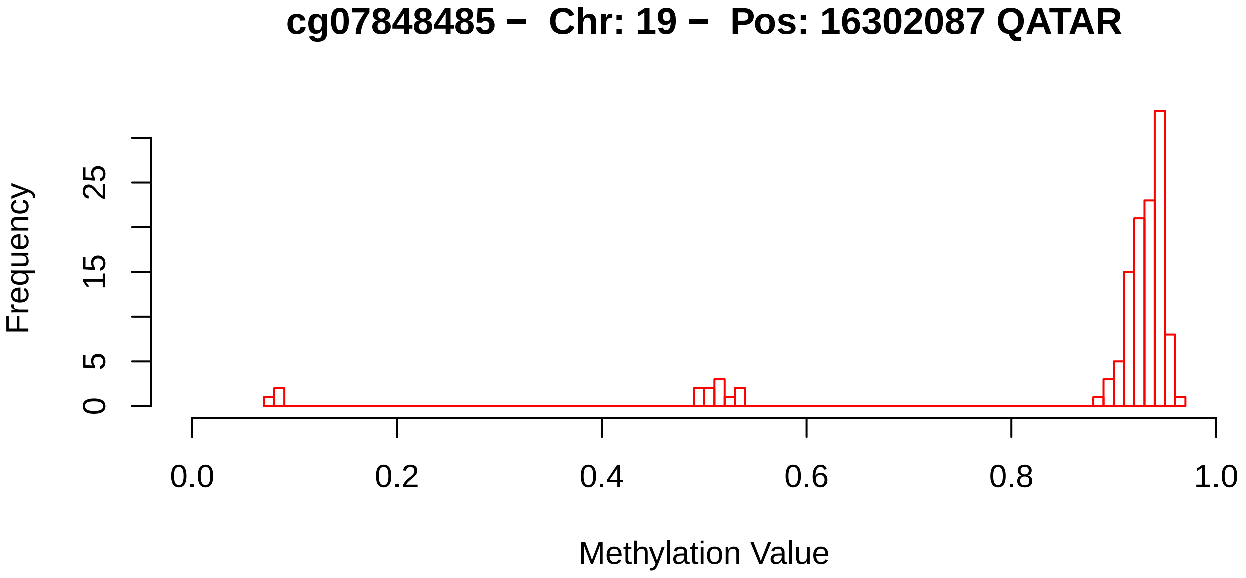
cg07848485 − Chr: 19 − Pos: 16302087 QATAR
Frequency
0
5
15
25
0.0
0.2
0.4
0.6
0.8
1.0
Methylation Value

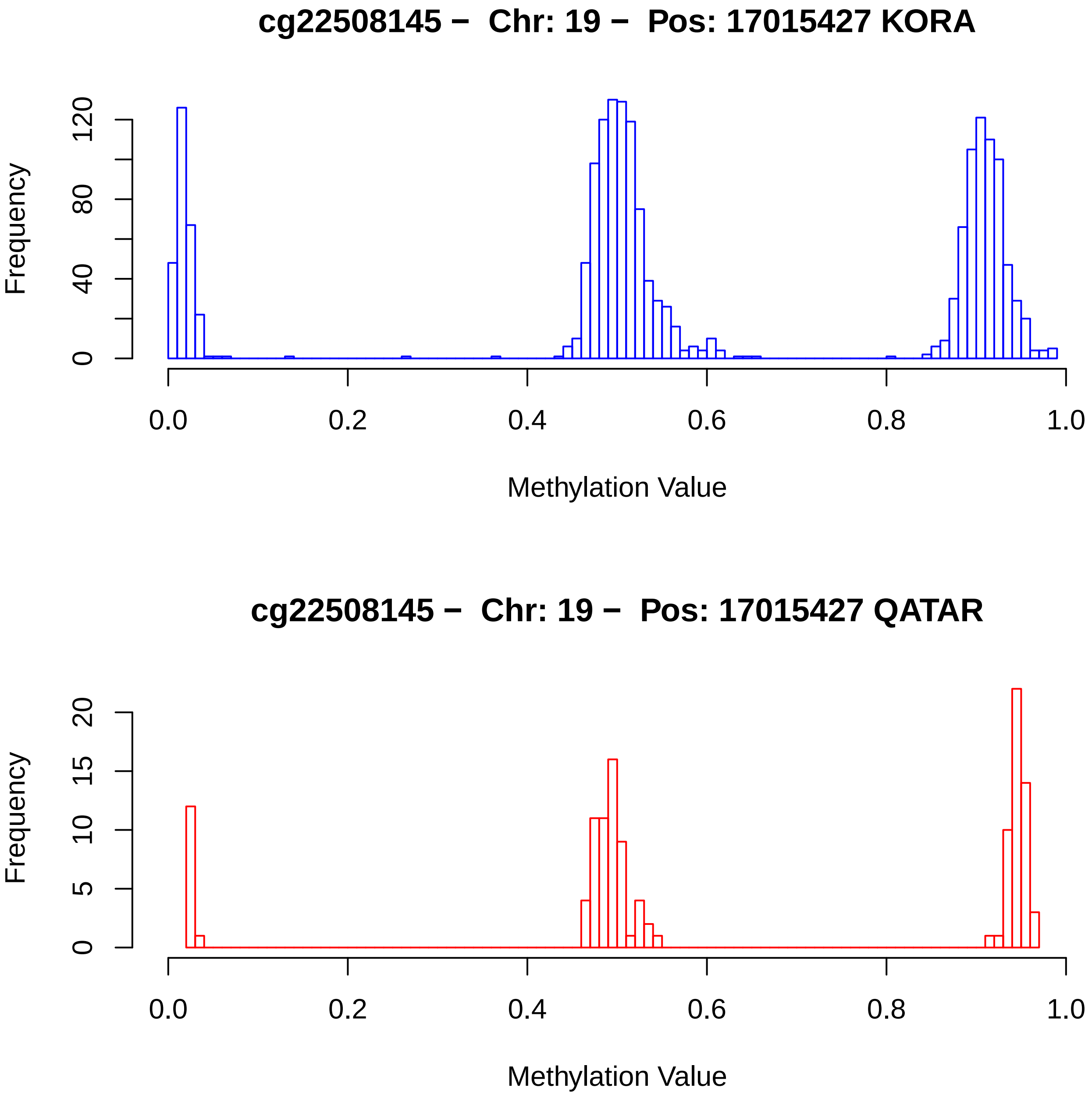
cg22508145 − Chr: 19 − Pos: 17015427 KORA
Frequency
0
40
80
120
0.0
0.2
0.4
0.6
0.8
1.0
Methylation Value
cg22508145 − Chr: 19 − Pos: 17015427 QATAR
Frequency
0
5
10
15
20
0.0
0.2
0.4
0.6
0.8
1.0
Methylation Value

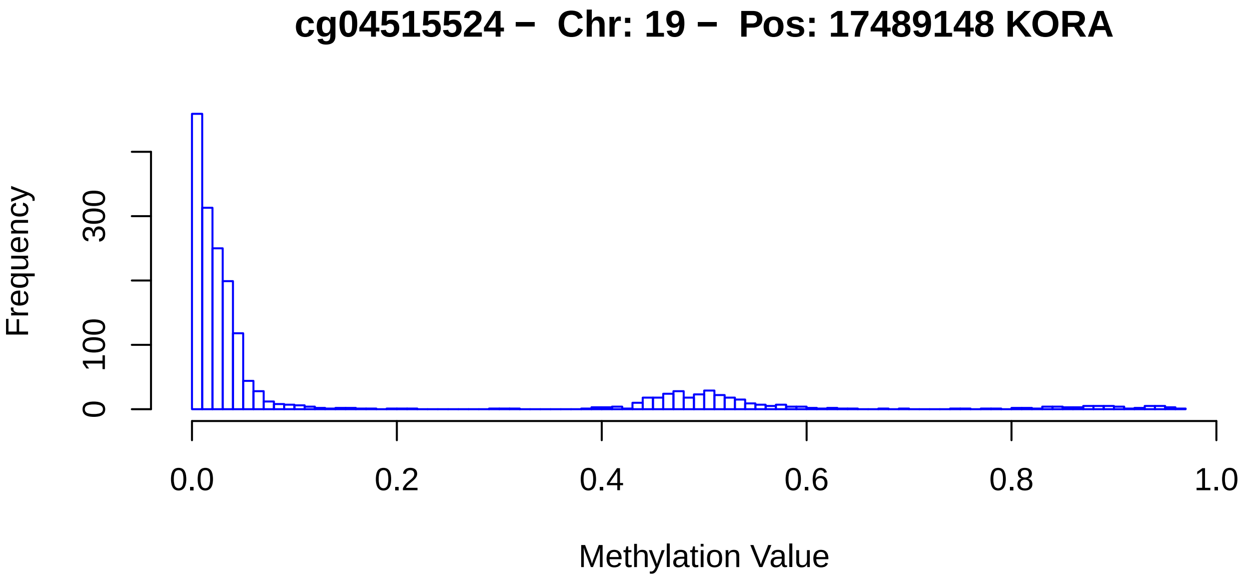
cg04515524 − Chr: 19 − Pos: 17489148 KORA
Frequency
0
100
300
0.0
0.2
0.4
0.6
0.8
1.0
Methylation Value

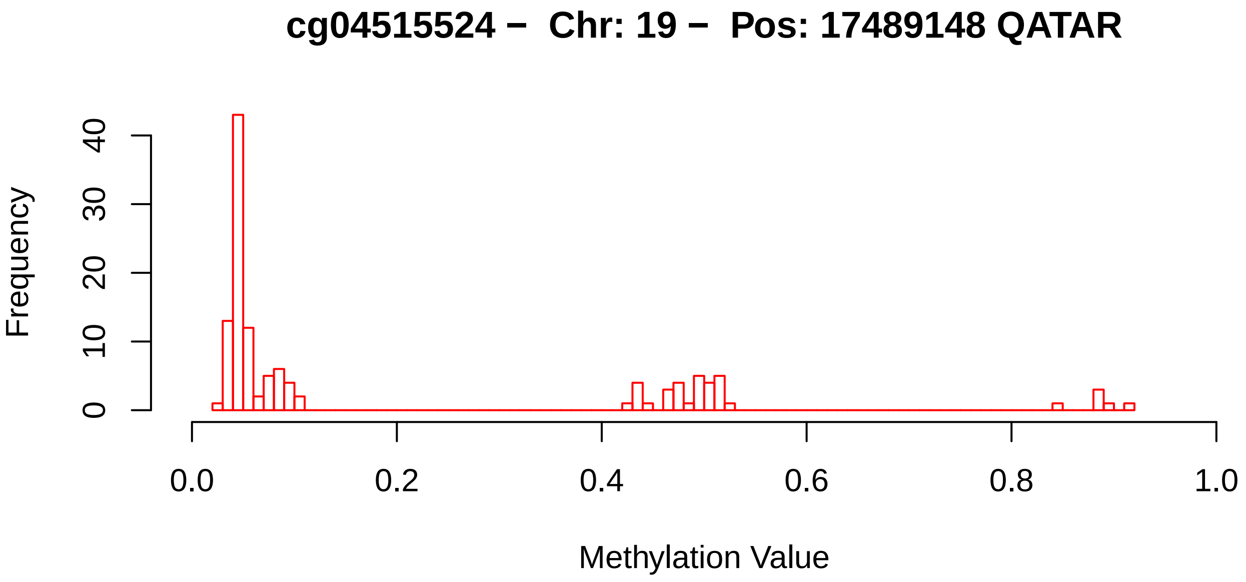
cg04515524 − Chr: 19 − Pos: 17489148 QATAR
Frequency
0
10
20
30
40
0.0
0.2
0.4
0.6
0.8
1.0
Methylation Value

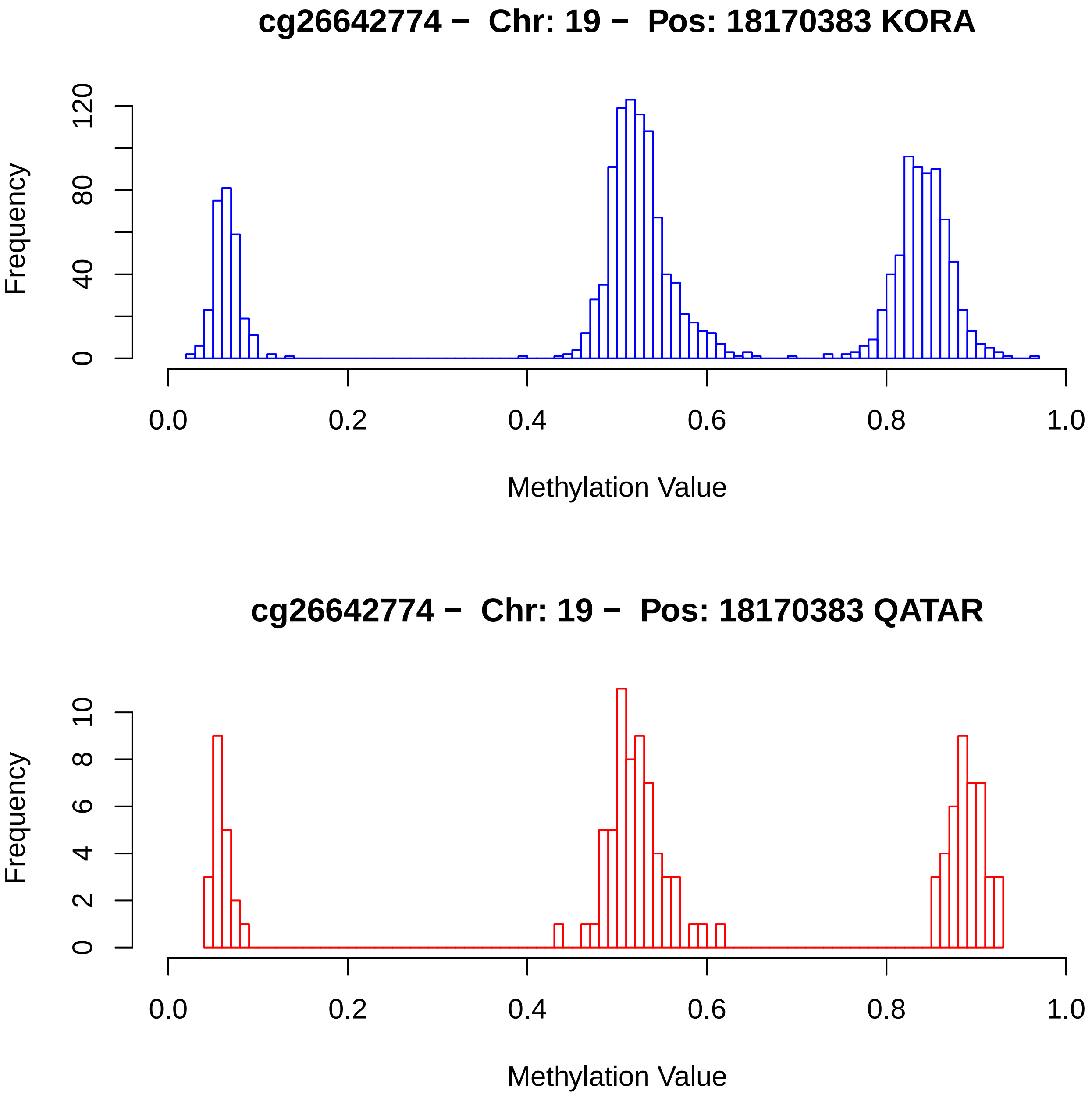

cg26642774 − Chr: 19 − Pos: 18170383 KORA
Frequency
0
40
80
120
0.0
0.2
0.4
0.6
0.8
1.0
Methylation Value
cg26642774 − Chr: 19 − Pos: 18170383 QATAR
Frequency
0
2
4
6
8
10
0.0
0.2
0.4
0.6
0.8
1.0
Methylation Value

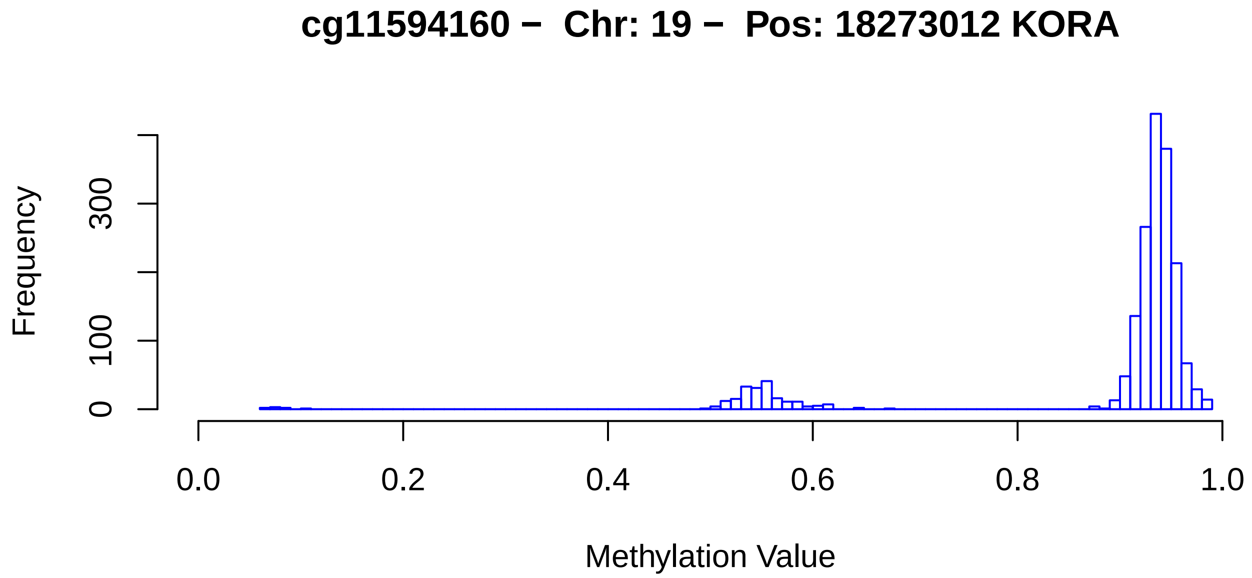
cg11594160 − Chr: 19 − Pos: 18273012 KORA
Frequency
0
100
300
0.0
0.2
0.4
0.6
0.8
1.0
Methylation Value

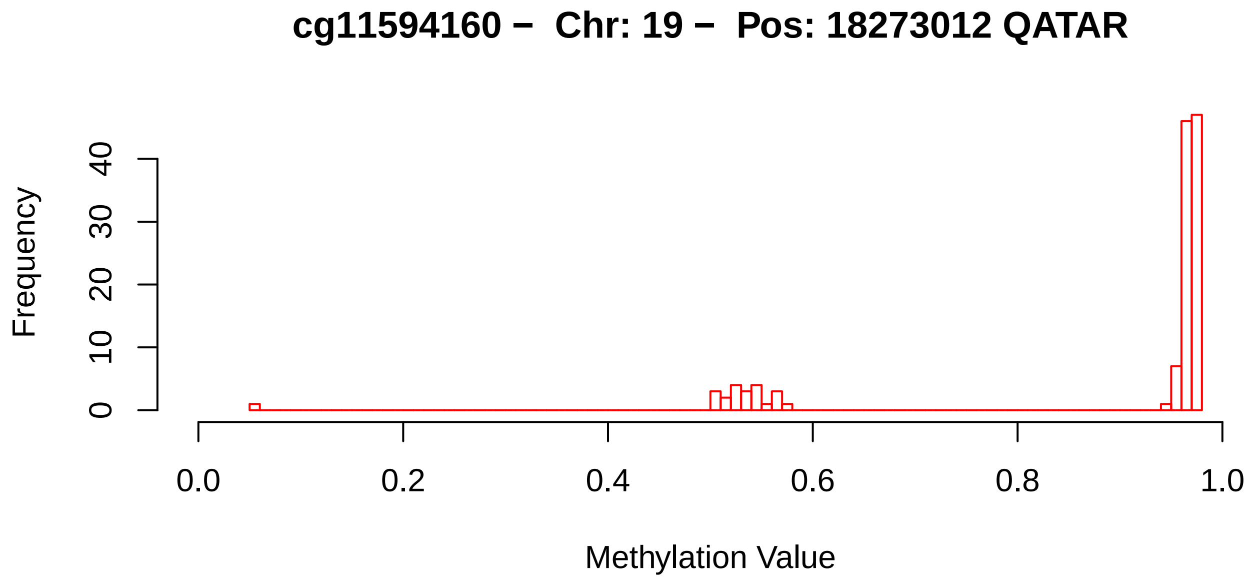
cg11594160 − Chr: 19 − Pos: 18273012 QATAR
Frequency
0
10
20
30
40
0.0
0.2
0.4
0.6
0.8
1.0
Methylation Value

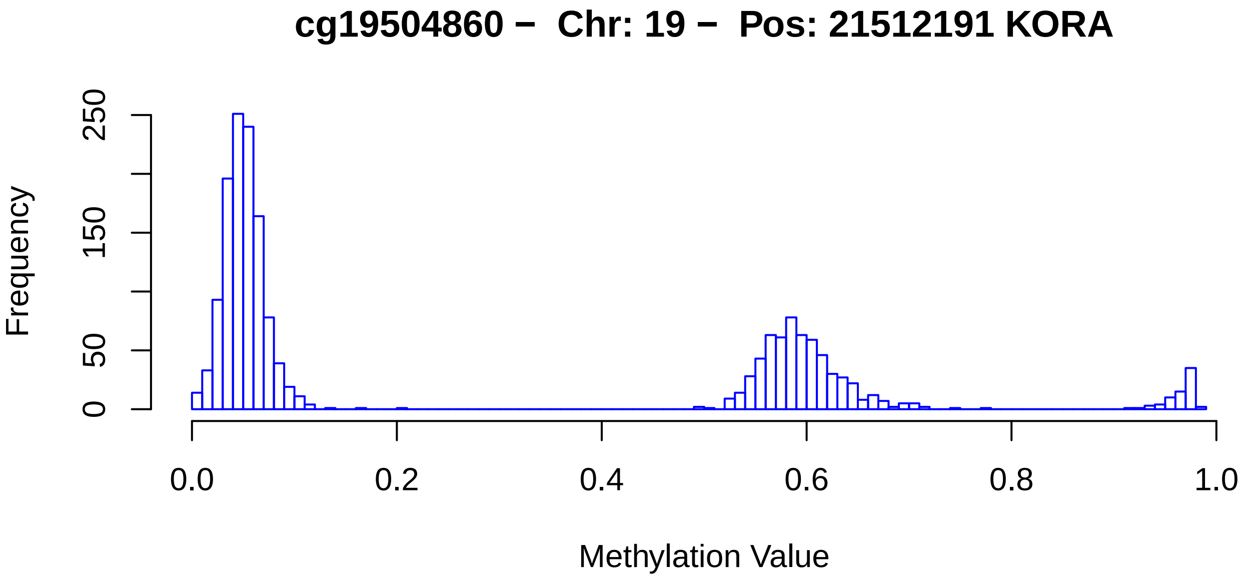
cg19504860 − Chr: 19 − Pos: 21512191 KORA
Frequency
0
50
150
250
0.0
0.2
0.4
0.6
0.8
1.0
Methylation Value

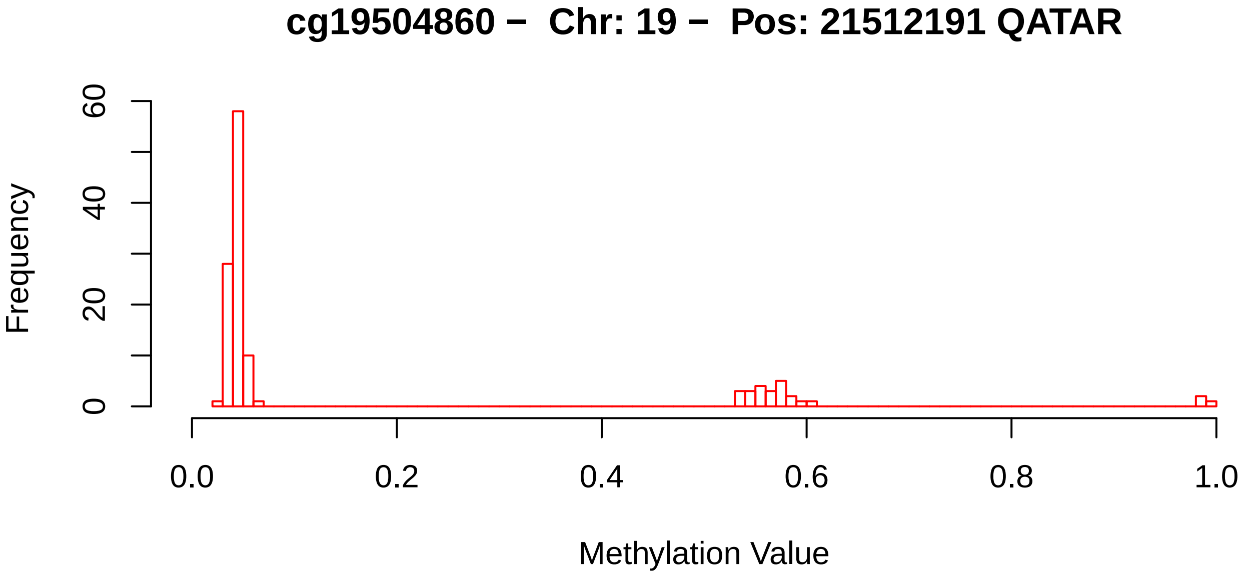
cg19504860 − Chr: 19 − Pos: 21512191 QATAR
Frequency
0
20
40
60
0.0
0.2
0.4
0.6
0.8
1.0
Methylation Value

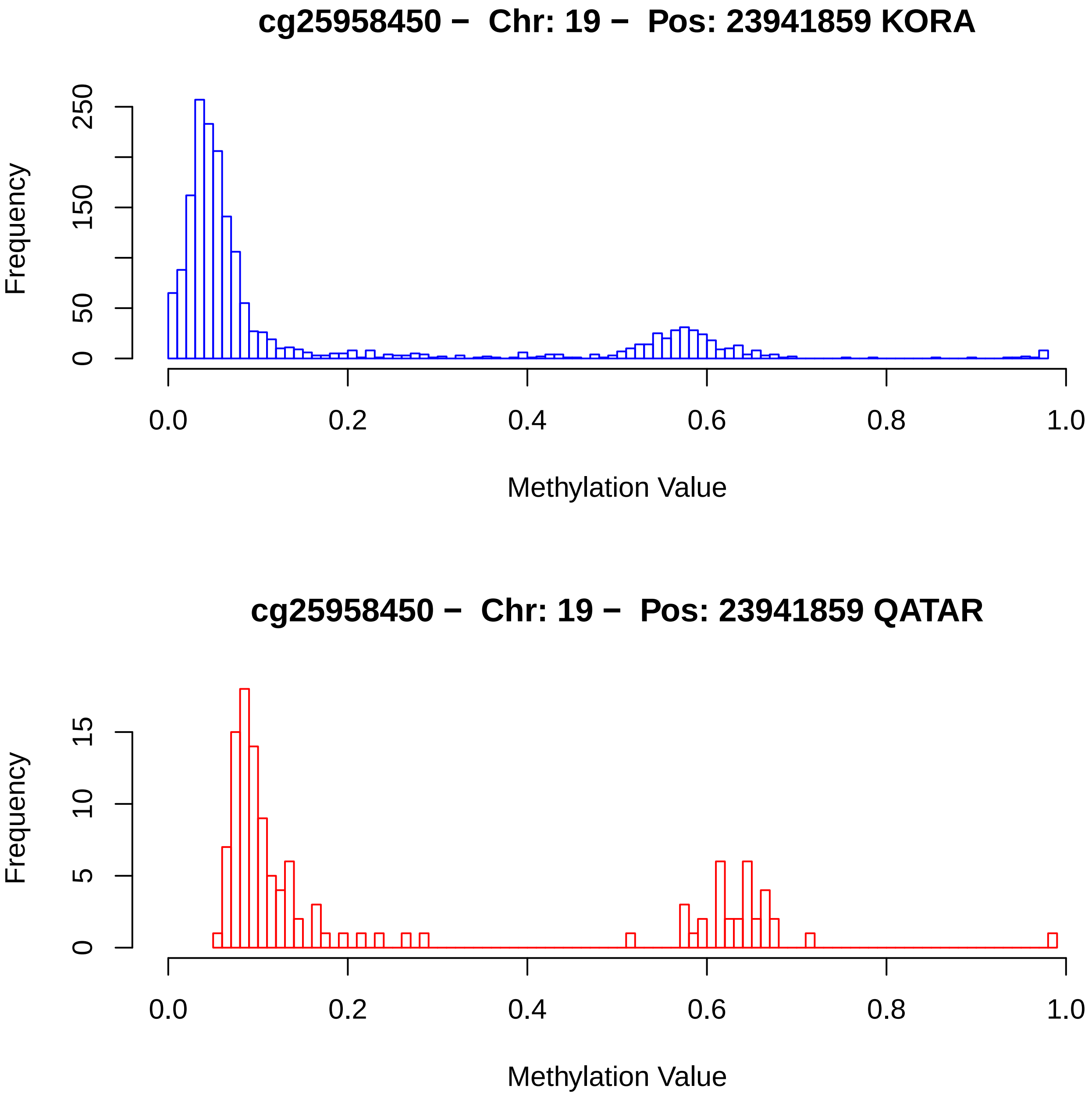
cg25958450 − Chr: 19 − Pos: 23941859 KORA
Frequency
0
50
150
250
0.0
0.2
0.4
0.6
0.8
1.0
Methylation Value
cg25958450 − Chr: 19 − Pos: 23941859 QATAR
Frequency
0
5
10
15
0.0
0.2
0.4
0.6
0.8
1.0
Methylation Value

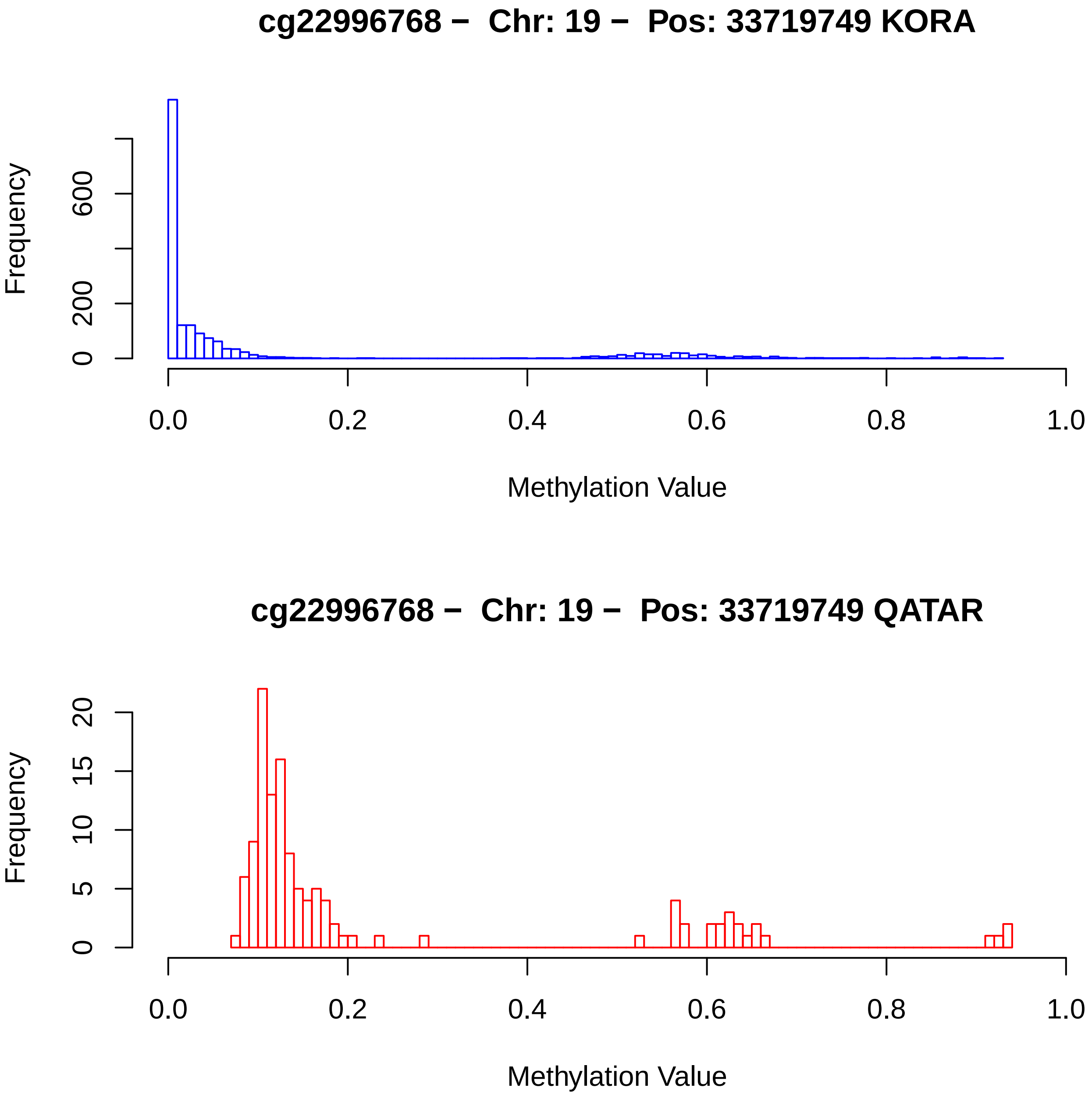
cg22996768 − Chr: 19 − Pos: 33719749 KORA
Frequency
0
200
600
0.0
0.2
0.4
0.6
0.8
1.0
Methylation Value
cg22996768 − Chr: 19 − Pos: 33719749 QATAR
Frequency
0
5
10
15
20
0.0
0.2
0.4
0.6
0.8
1.0
Methylation Value

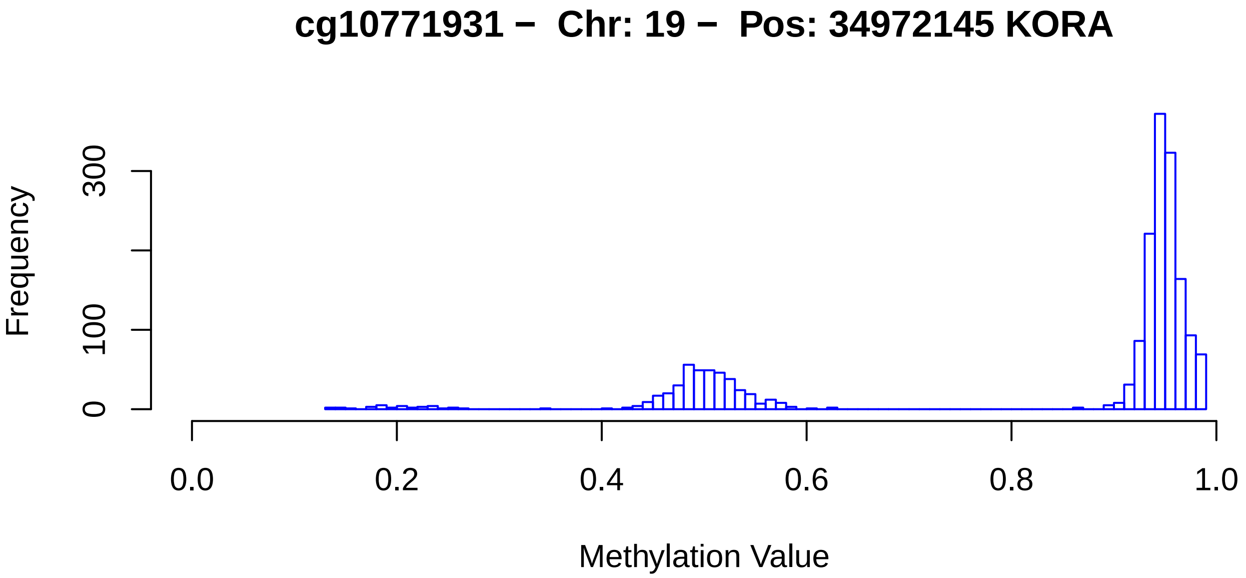
cg10771931 − Chr: 19 − Pos: 34972145 KORA
Frequency
0
100
300
0.0
0.2
0.4
0.6
0.8
1.0
Methylation Value

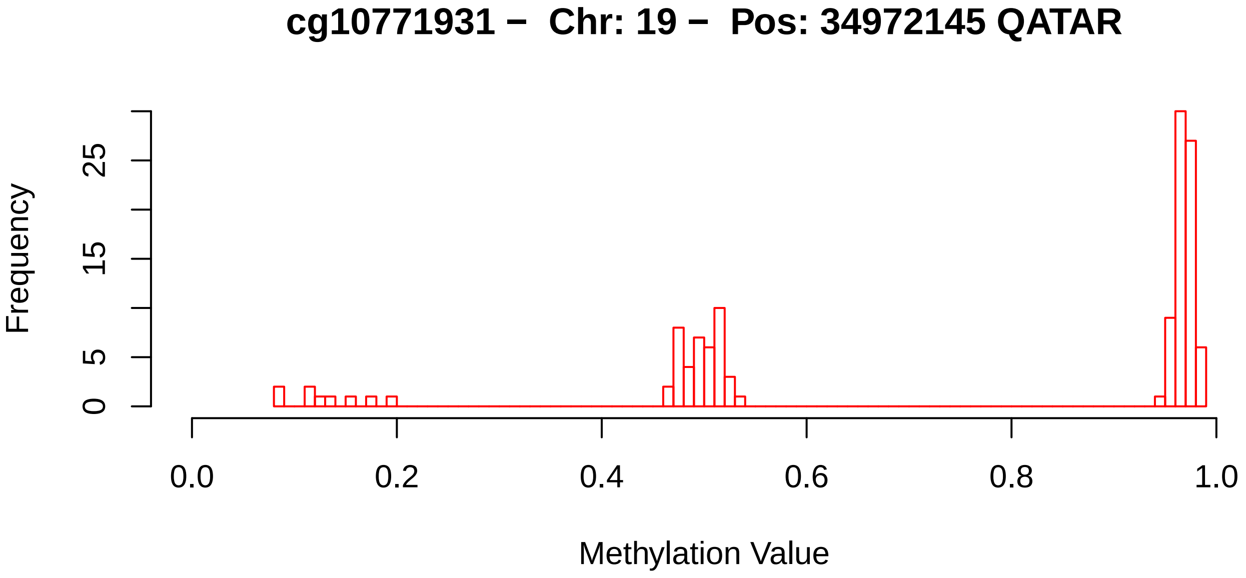
cg10771931 − Chr: 19 − Pos: 34972145 QATAR
Frequency
0
5
15
25
0.0
0.2
0.4
0.6
0.8
1.0
Methylation Value

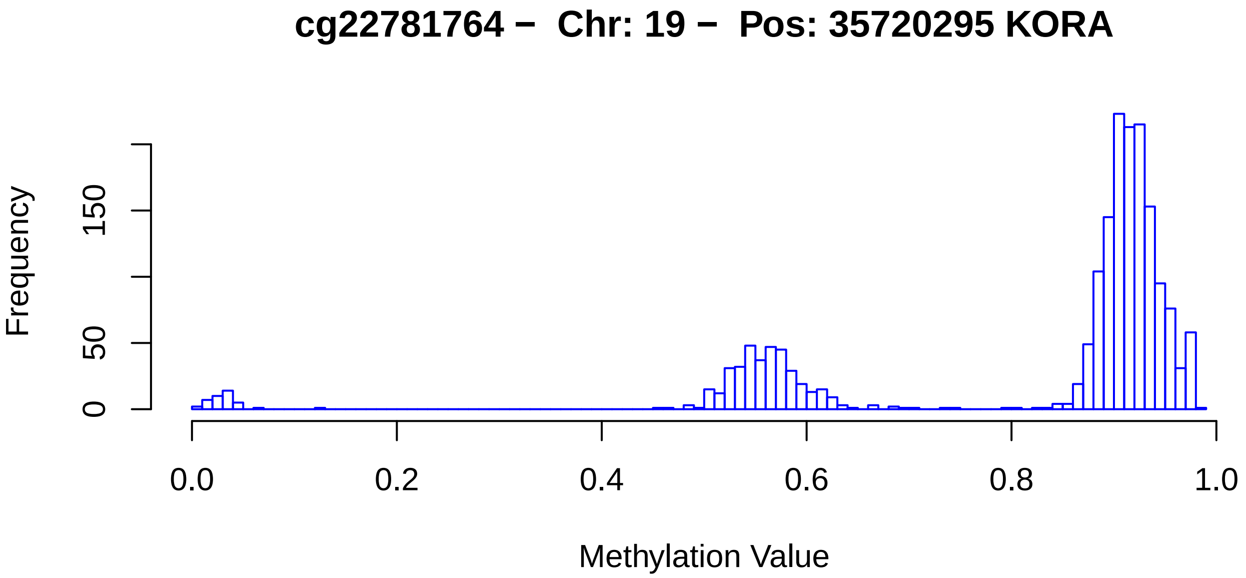
cg22781764 − Chr: 19 − Pos: 35720295 KORA
Frequency
0
50
150
0.0
0.2
0.4
0.6
0.8
1.0
Methylation Value

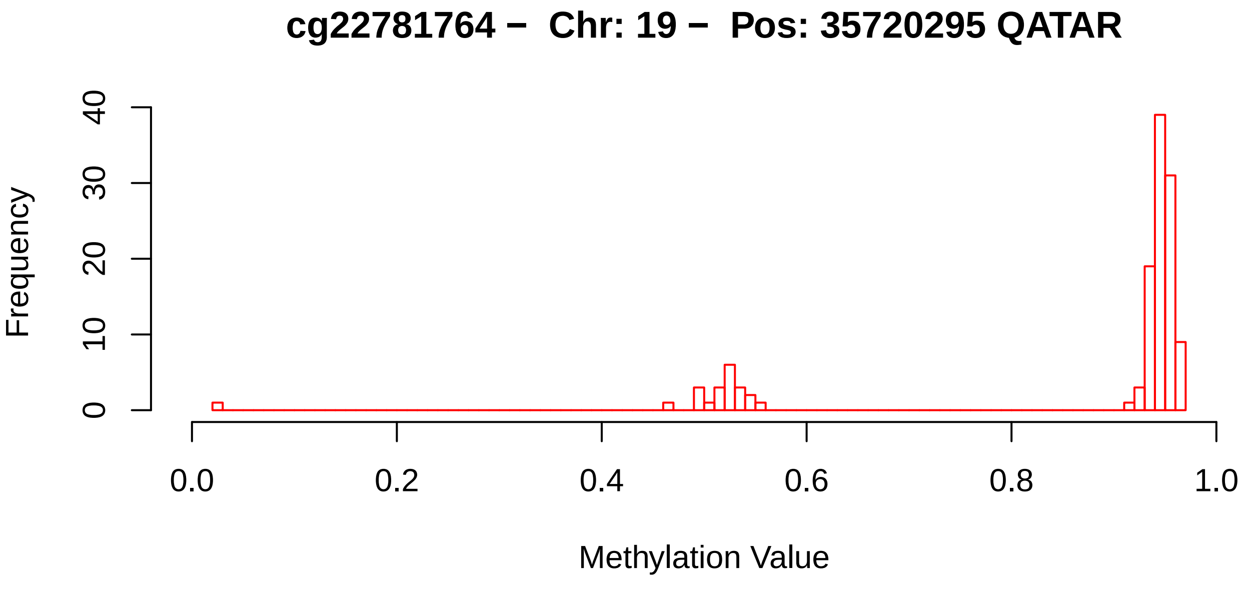
cg22781764 − Chr: 19 − Pos: 35720295 QATAR
Frequency
0
10
20
30
40
0.0
0.2
0.4
0.6
0.8
1.0
Methylation Value

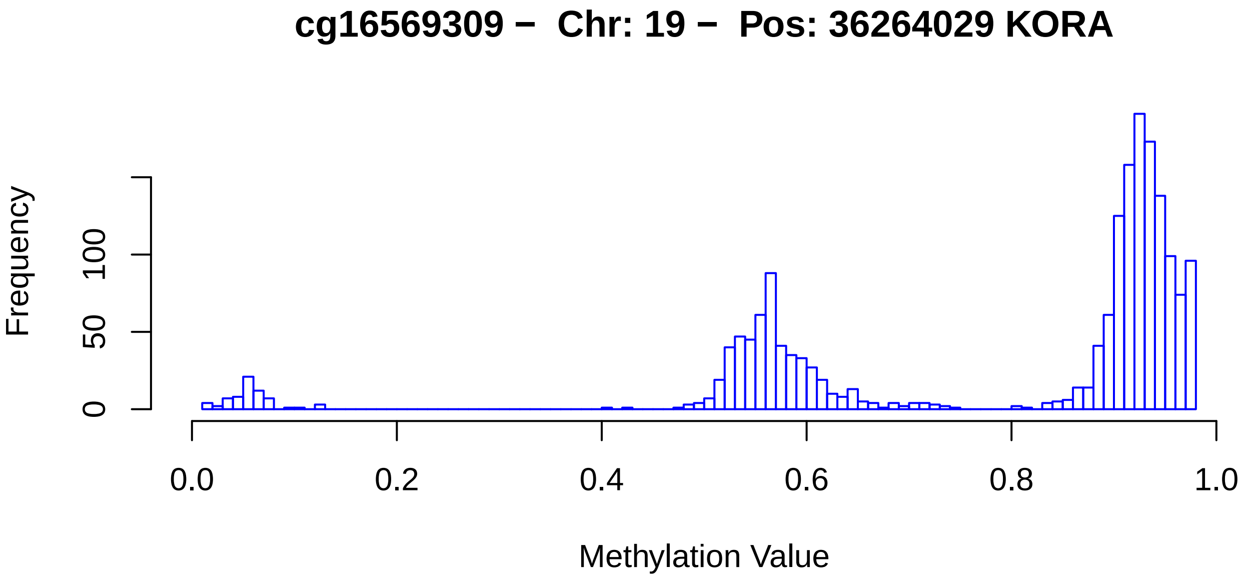
cg16569309 − Chr: 19 − Pos: 36264029 KORA
Frequency
0
50
100
0.0
0.2
0.4
0.6
0.8
1.0
Methylation Value

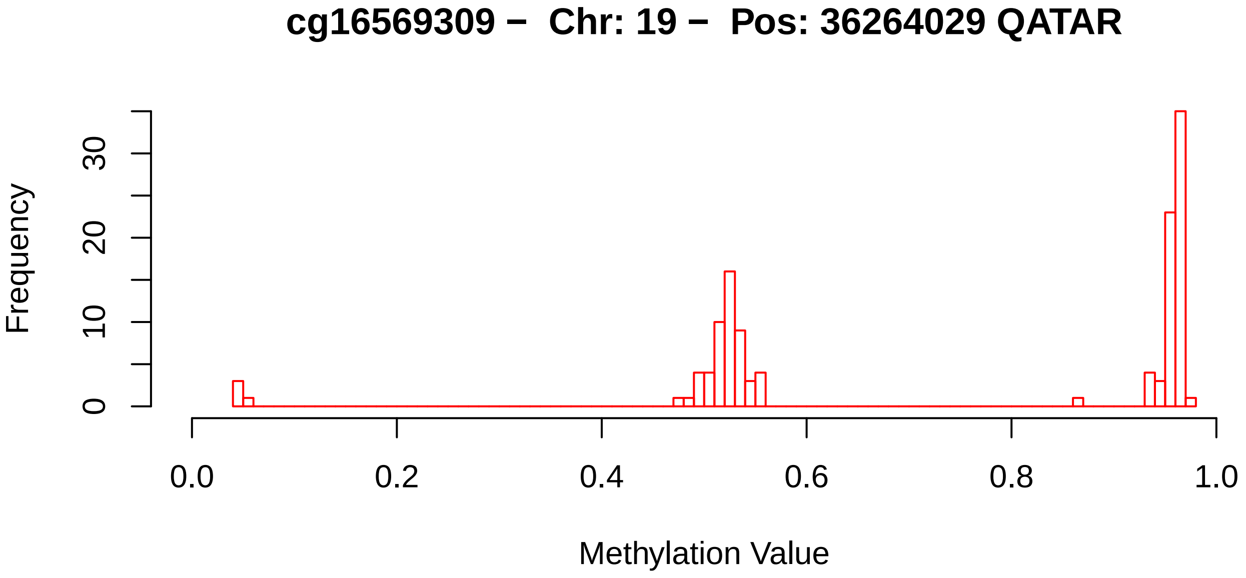
cg16569309 − Chr: 19 − Pos: 36264029 QATAR
Frequency
0
10
20
30
0.0
0.2
0.4
0.6
0.8
1.0
Methylation Value

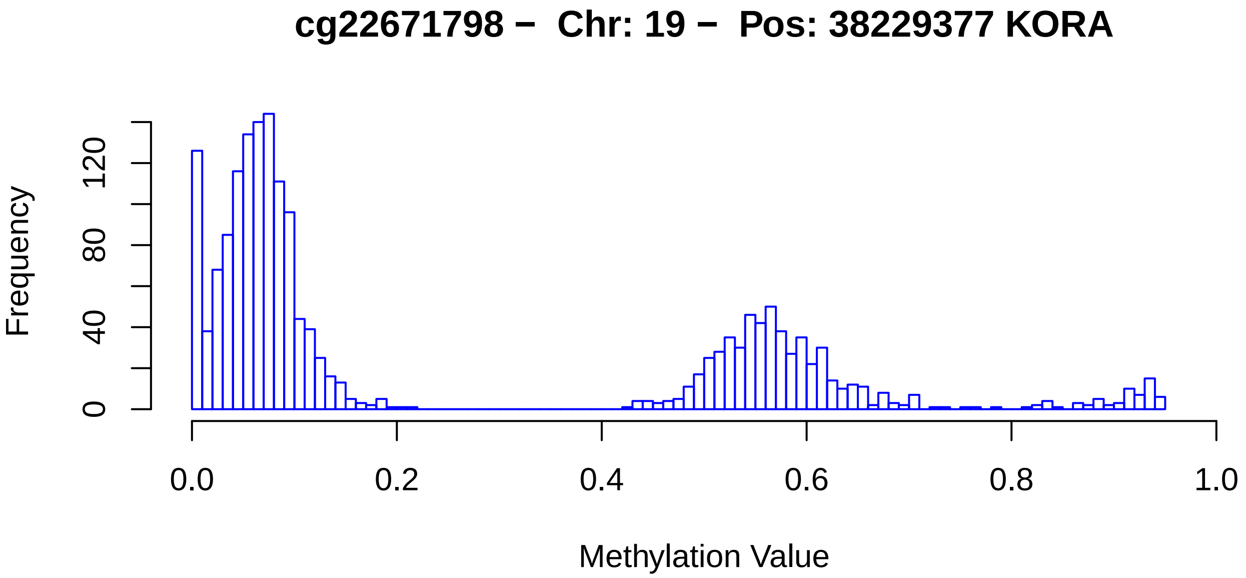
cg22671798 − Chr: 19 − Pos: 38229377 KORA
Frequency
0
40
80
120
0.0
0.2
0.4
0.6
0.8
1.0
Methylation Value

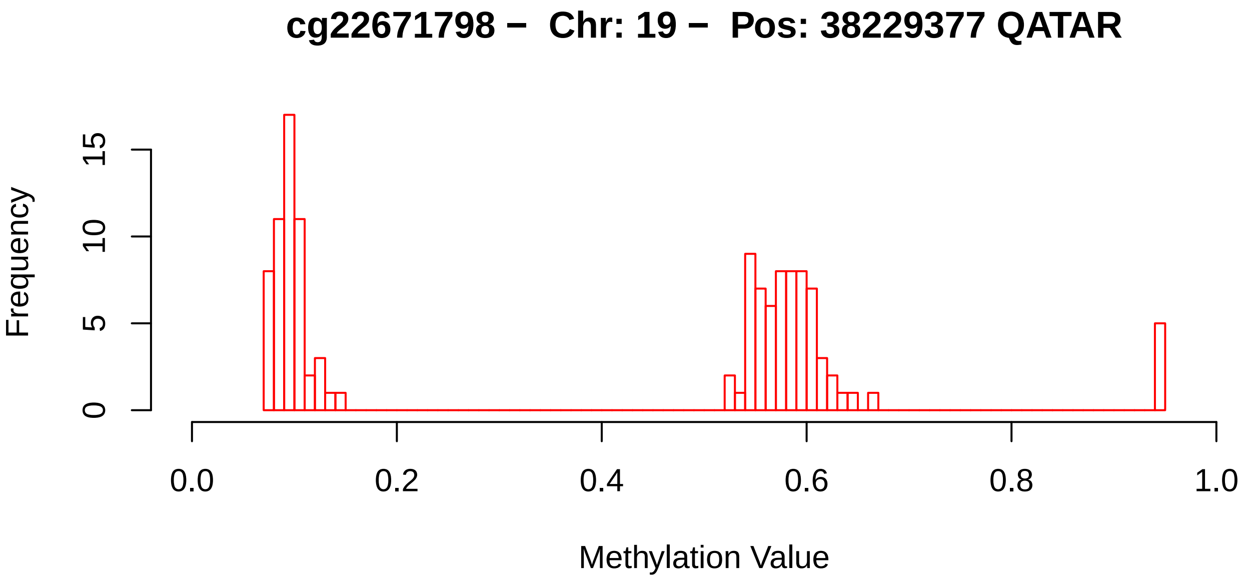
cg22671798 − Chr: 19 − Pos: 38229377 QATAR
Frequency
0
5
10
15
0.0
0.2
0.4
0.6
0.8
1.0
Methylation Value

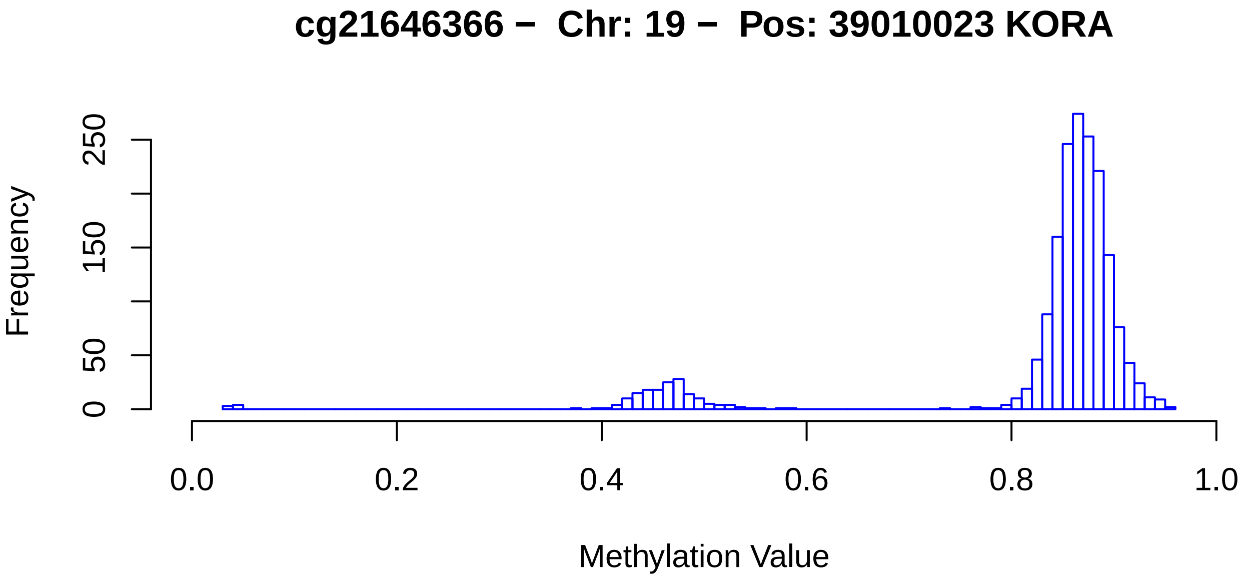
cg21646366 − Chr: 19 − Pos: 39010023 KORA
Frequency
0
50
150
250
0.0
0.2
0.4
0.6
0.8
1.0
Methylation Value

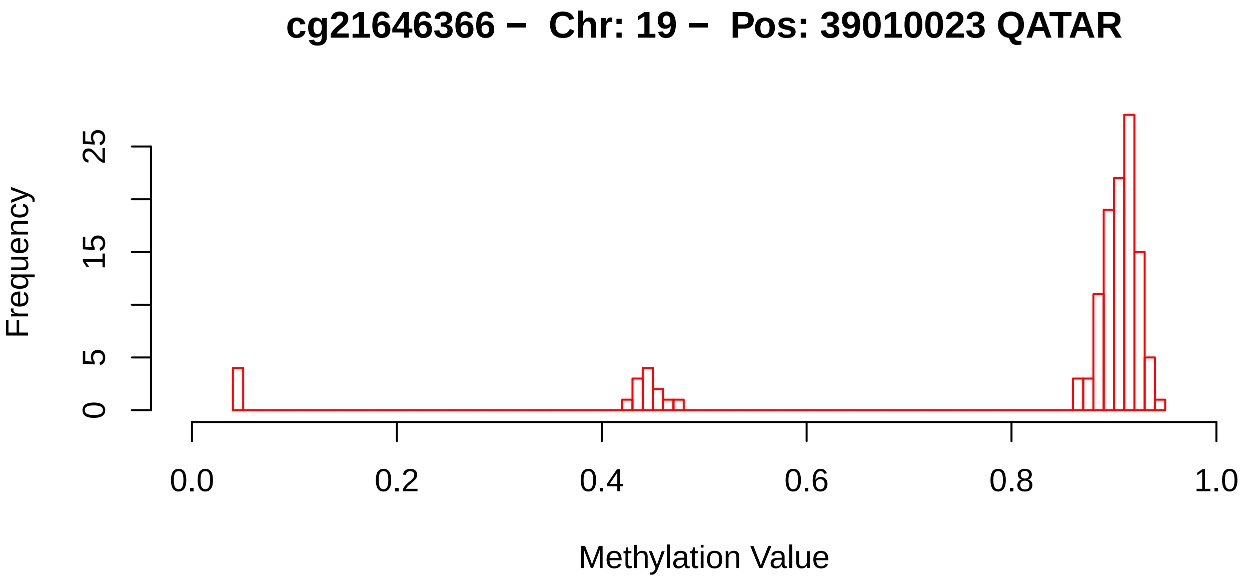
cg21646366 − Chr: 19 − Pos: 39010023 QATAR
Frequency
0
5
15
25
0.0
0.2
0.4
0.6
0.8
1.0
Methylation Value

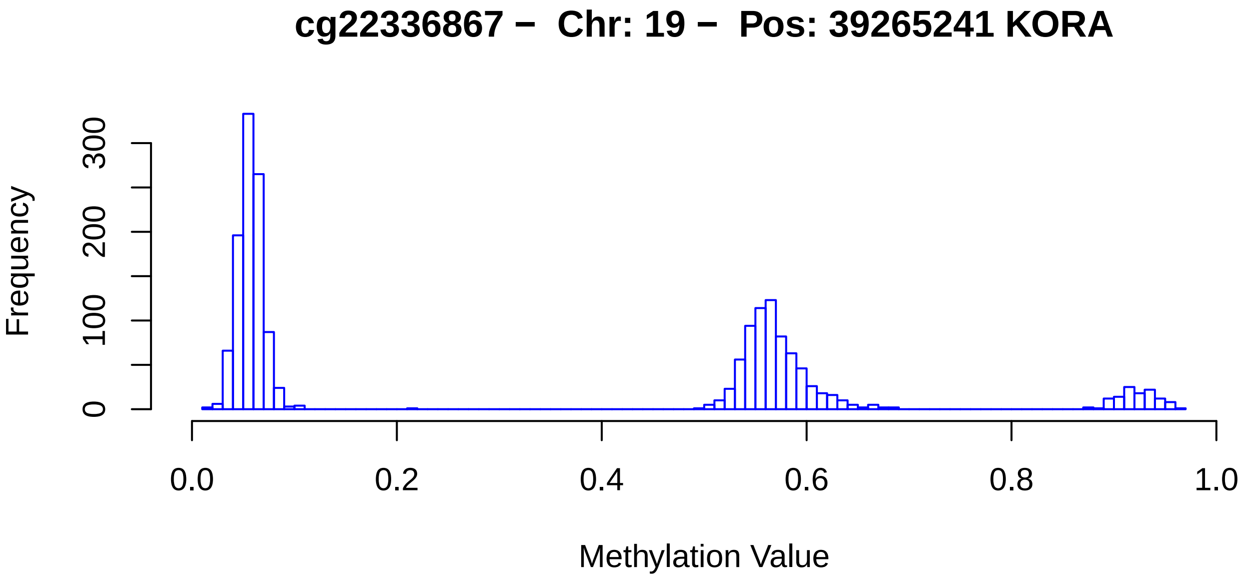
cg22336867 − Chr: 19 − Pos: 39265241 KORA
Frequency
0
100
200
300
0.0
0.2
0.4
0.6
0.8
1.0
Methylation Value

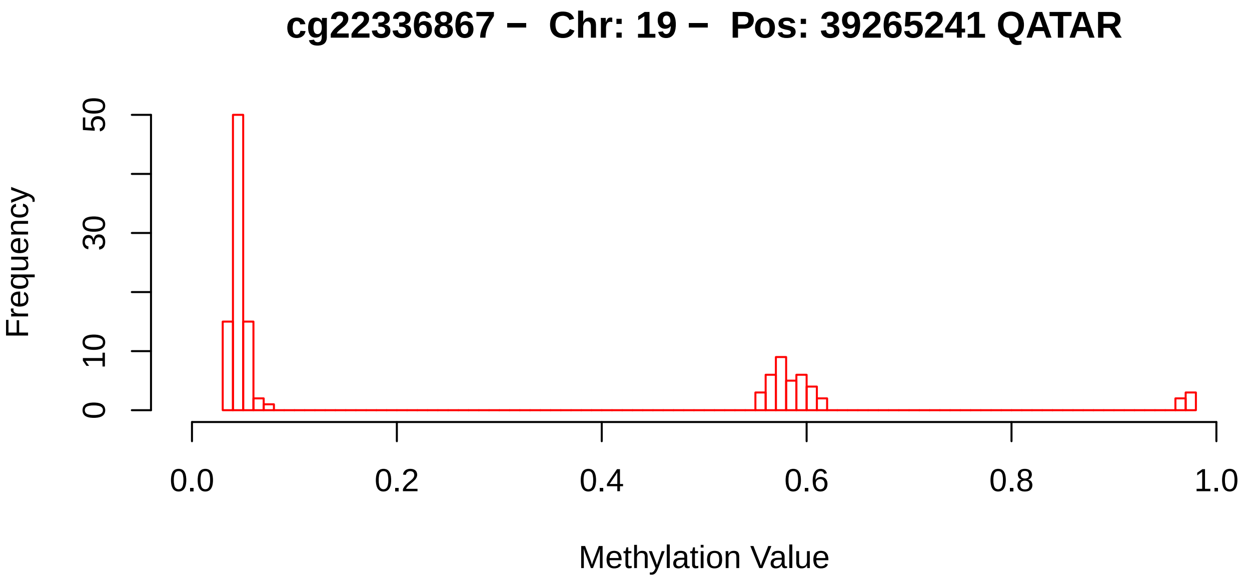
cg22336867 − Chr: 19 − Pos: 39265241 QATAR
Frequency
0
10
30
50
0.0
0.2
0.4
0.6
0.8
1.0
Methylation Value

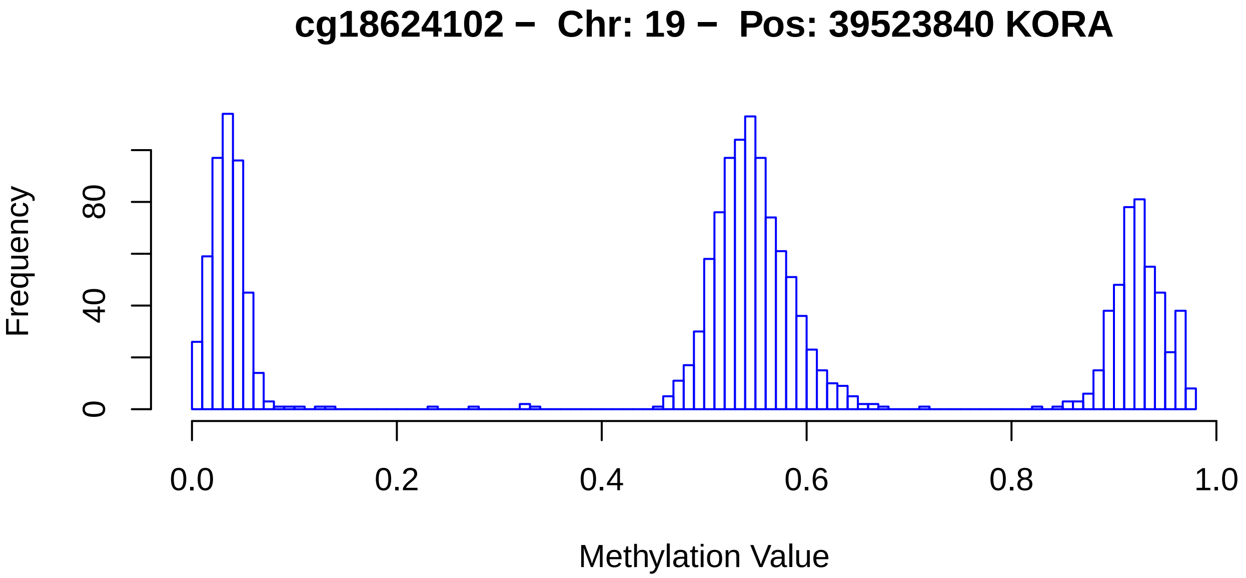
cg18624102 − Chr: 19 − Pos: 39523840 KORA
Frequency
0
40
80
0.0
0.2
0.4
0.6
0.8
1.0
Methylation Value

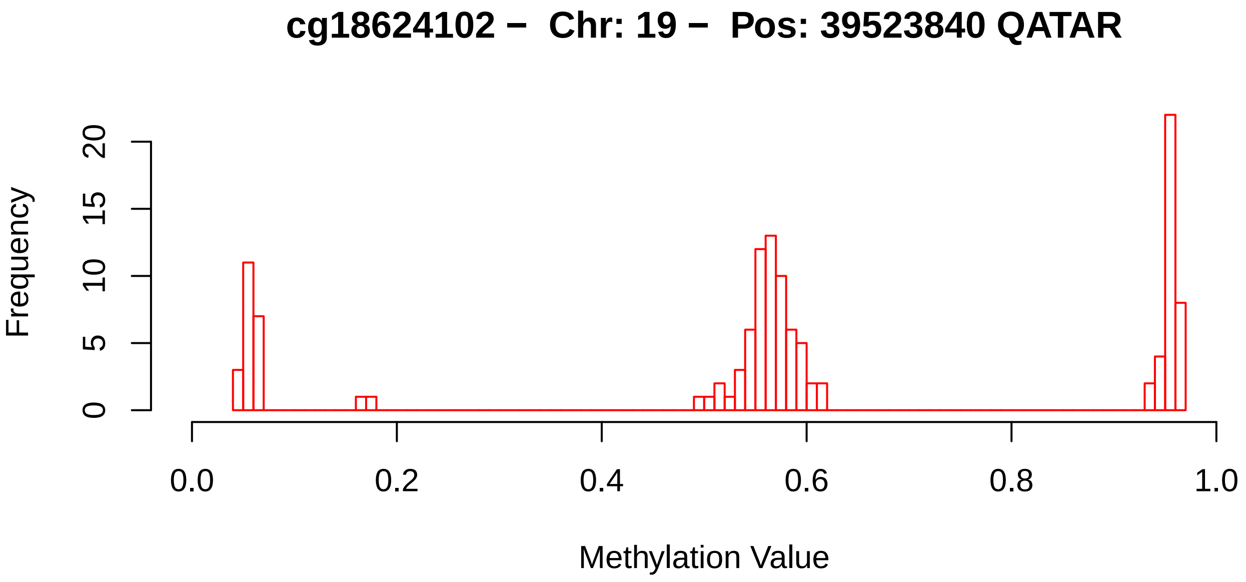
cg18624102 − Chr: 19 − Pos: 39523840 QATAR
Frequency
0
5
10
15
20
0.0
0.2
0.4
0.6
0.8
1.0
Methylation Value

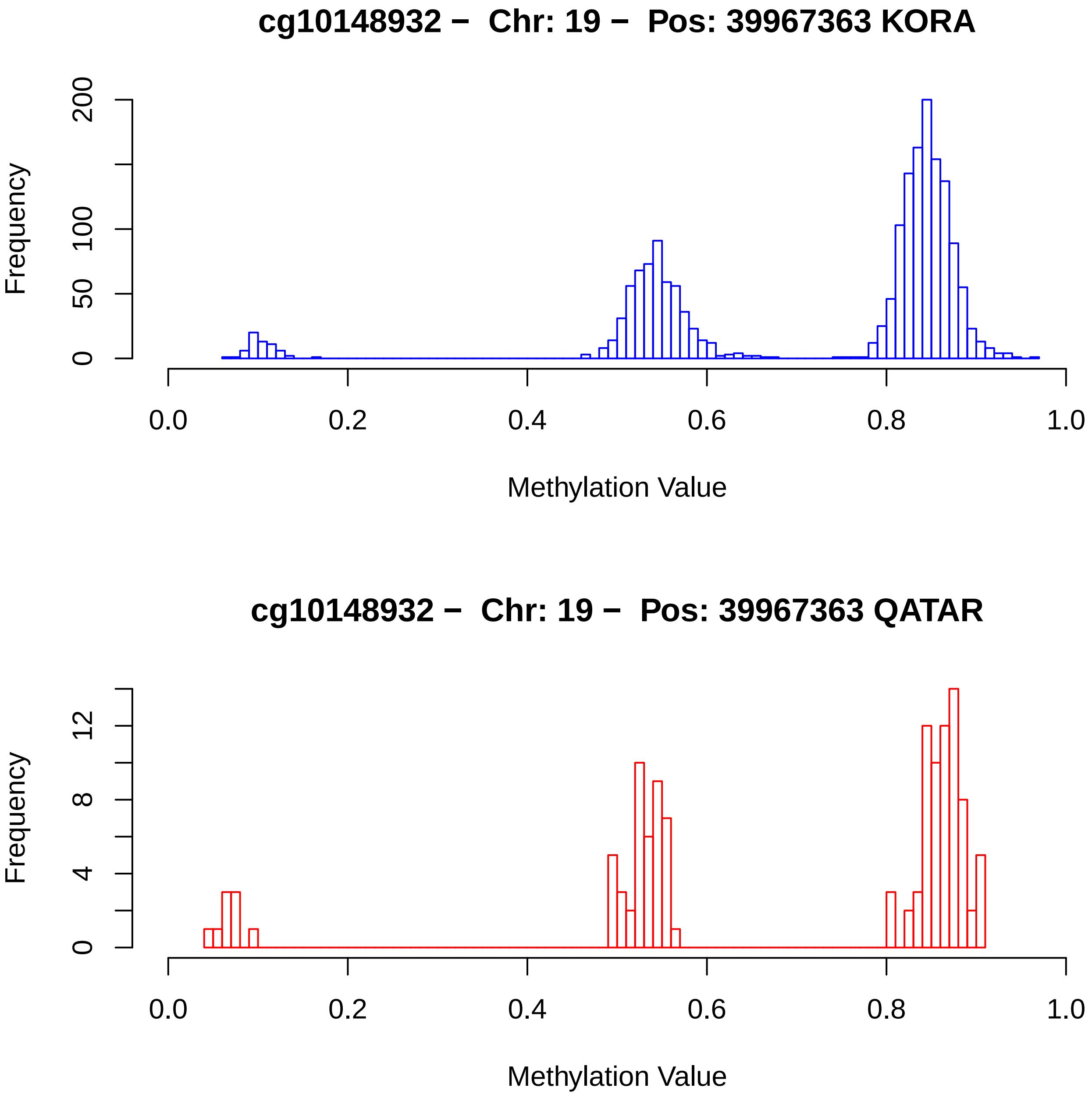
cg10148932 − Chr: 19 − Pos: 39967363 KORA
Frequency
0
50
100
200
0.0
0.2
0.4
0.6
0.8
1.0
Methylation Value
cg10148932 − Chr: 19 − Pos: 39967363 QATAR
Frequency
0
4
8
12
0.0
0.2
0.4
0.6
0.8
1.0
Methylation Value

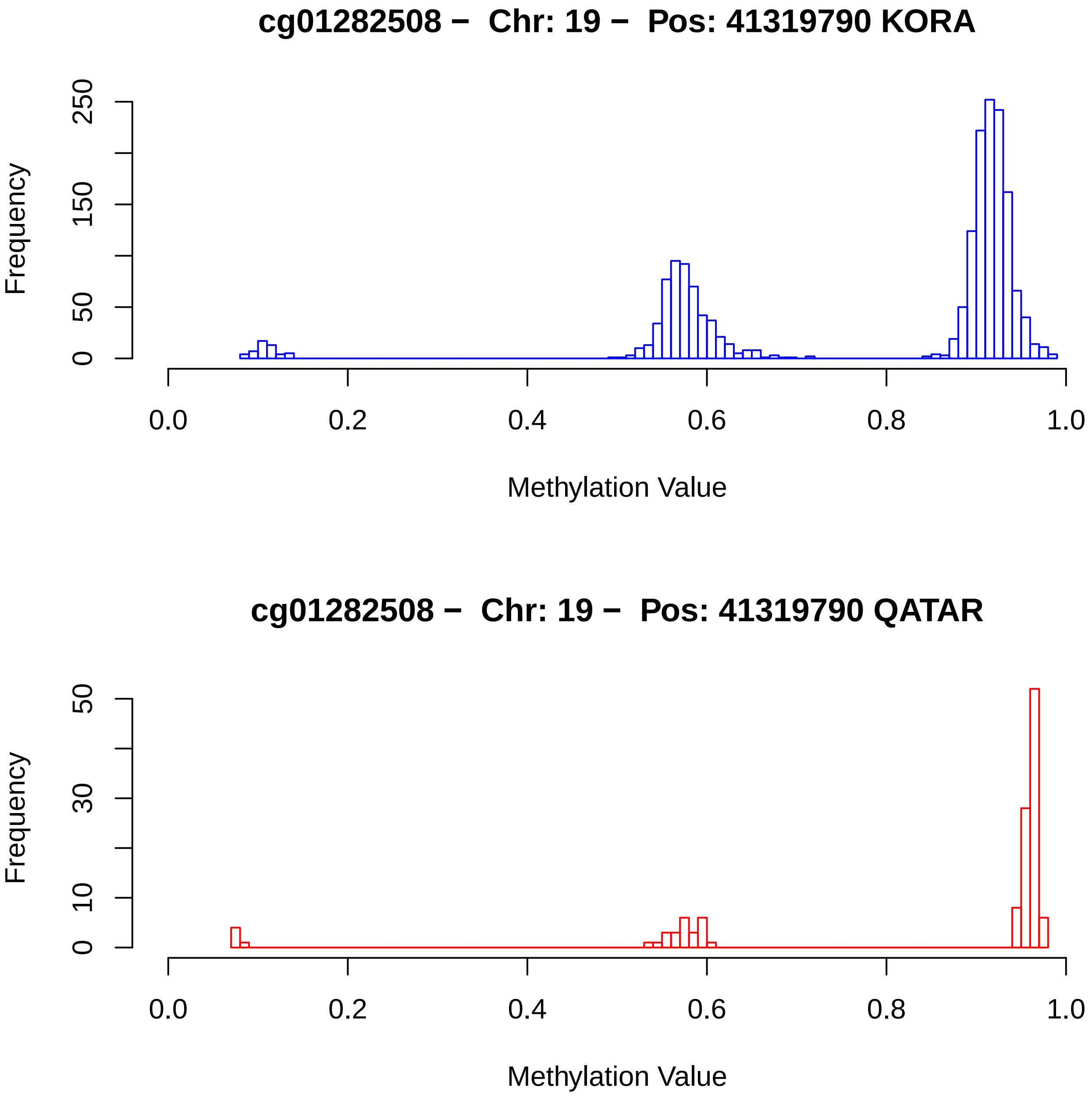
cg01282508 − Chr: 19 − Pos: 41319790 KORA
Frequency
0
50
150
250
0.0
0.2
0.4
0.6
0.8
1.0
Methylation Value
cg01282508 − Chr: 19 − Pos: 41319790 QATAR
Frequency
0
10
30
50
0.0
0.2
0.4
0.6
0.8
1.0
Methylation Value

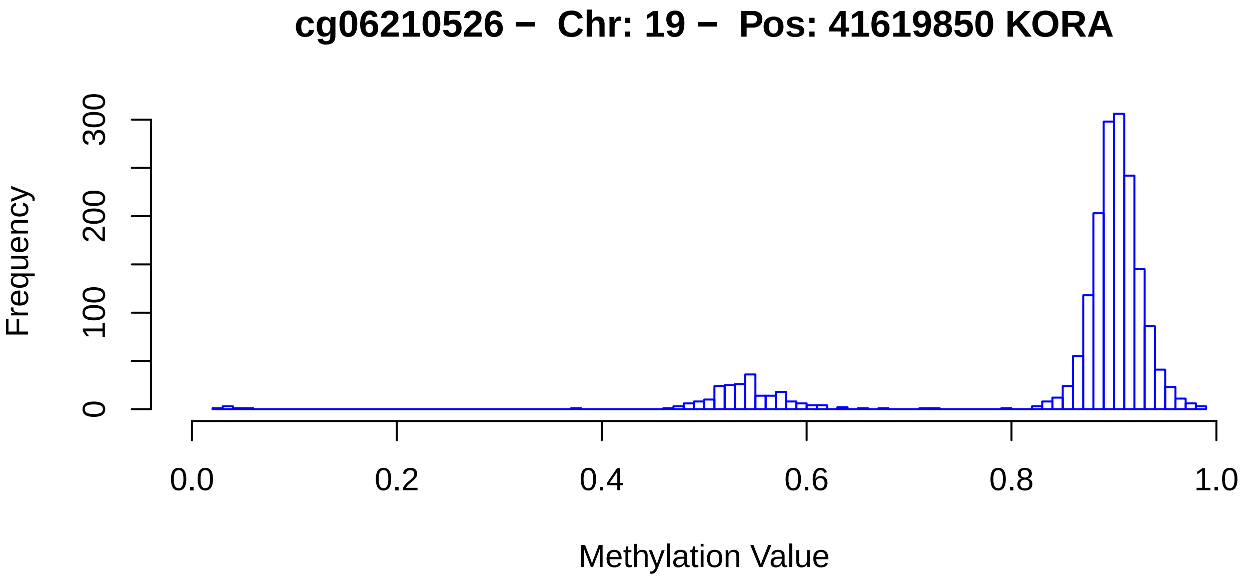
cg06210526 − Chr: 19 − Pos: 41619850 KORA
Frequency
0
100
200
300
0.0
0.2
0.4
0.6
0.8
1.0
Methylation Value

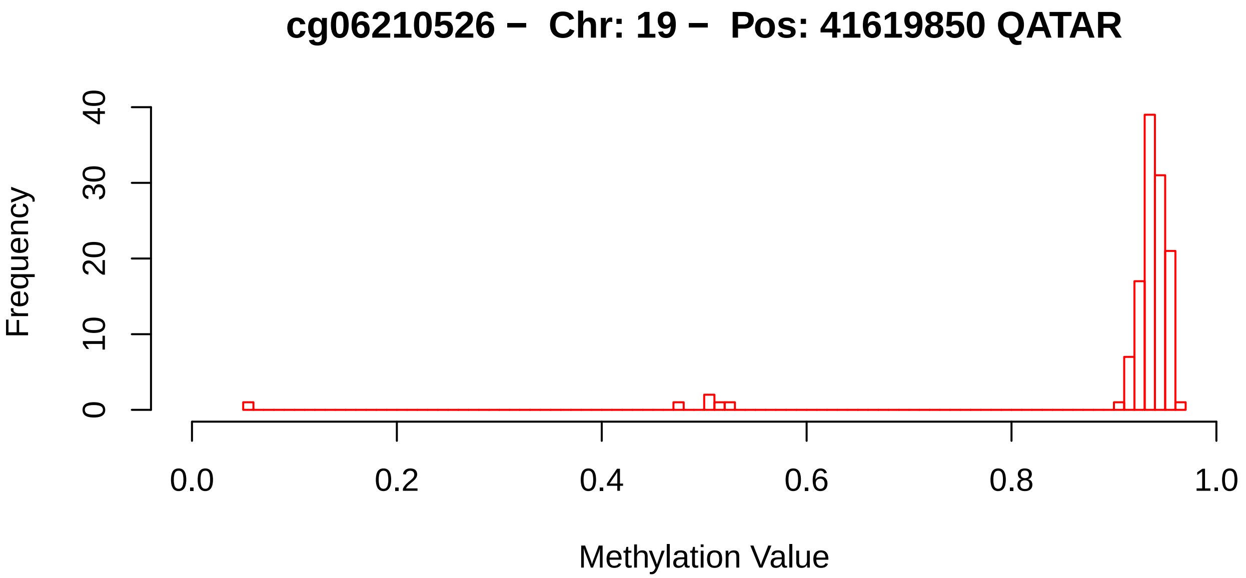
cg06210526 − Chr: 19 − Pos: 41619850 QATAR
Frequency
0
10
20
30
40
0.0
0.2
0.4
0.6
0.8
1.0
Methylation Value

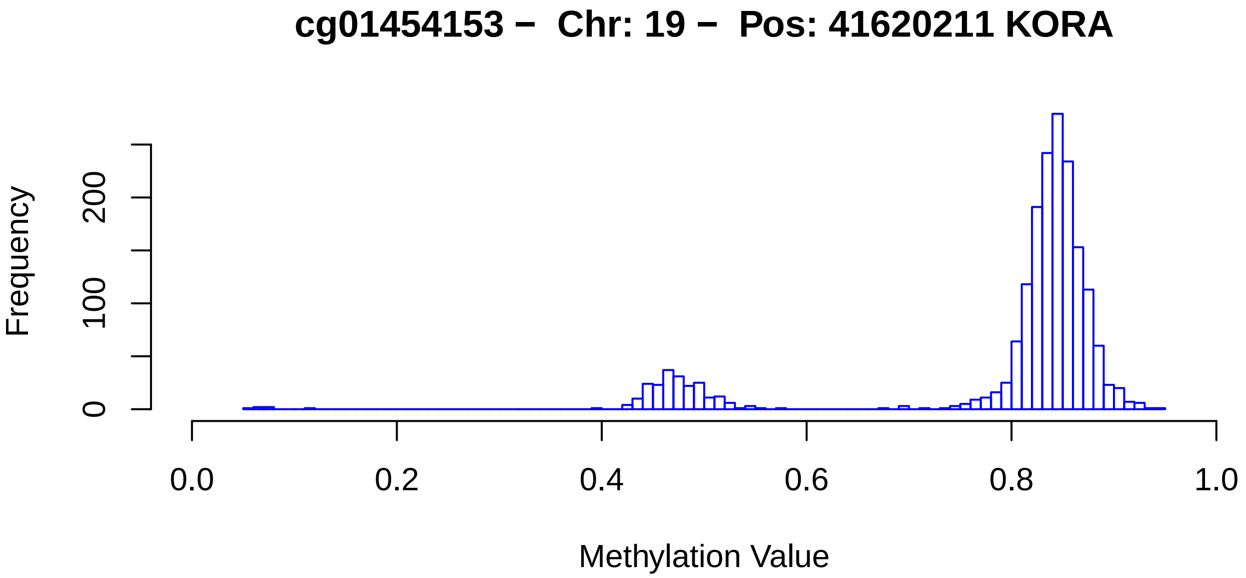

cg01454153 − Chr: 19 − Pos: 41620211 KORA
Frequency
0
100
200
0.0
0.2
0.4
0.6
0.8
1.0
Methylation Value

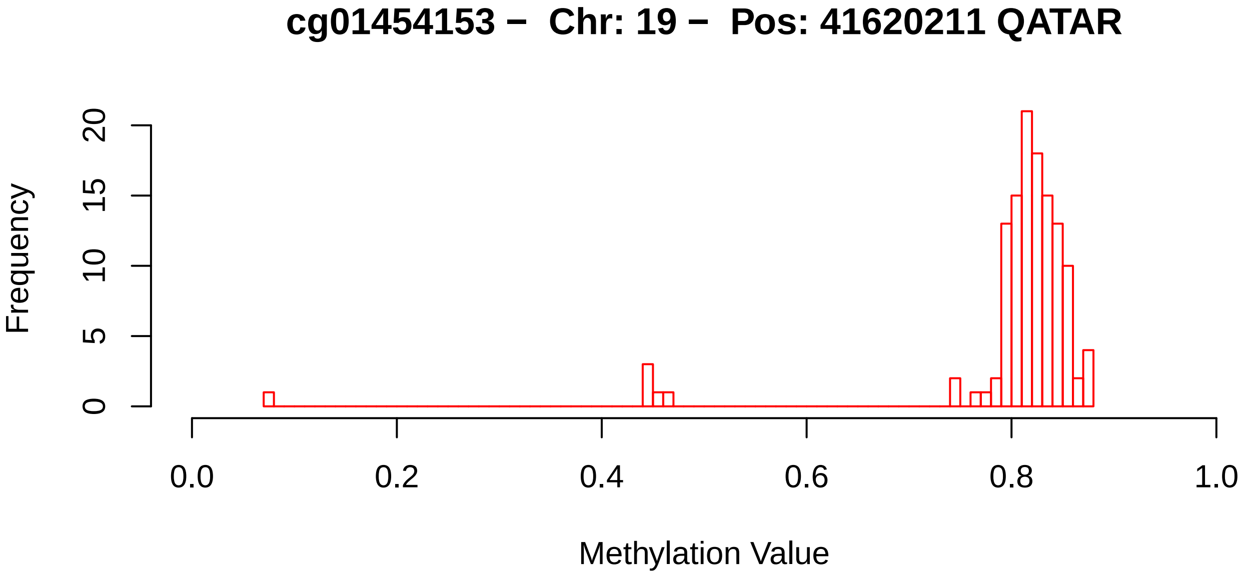

cg01454153 − Chr: 19 − Pos: 41620211 QATAR
Frequency
0
5
10
15
20
0.0
0.2
0.4
0.6
0.8
1.0
Methylation Value

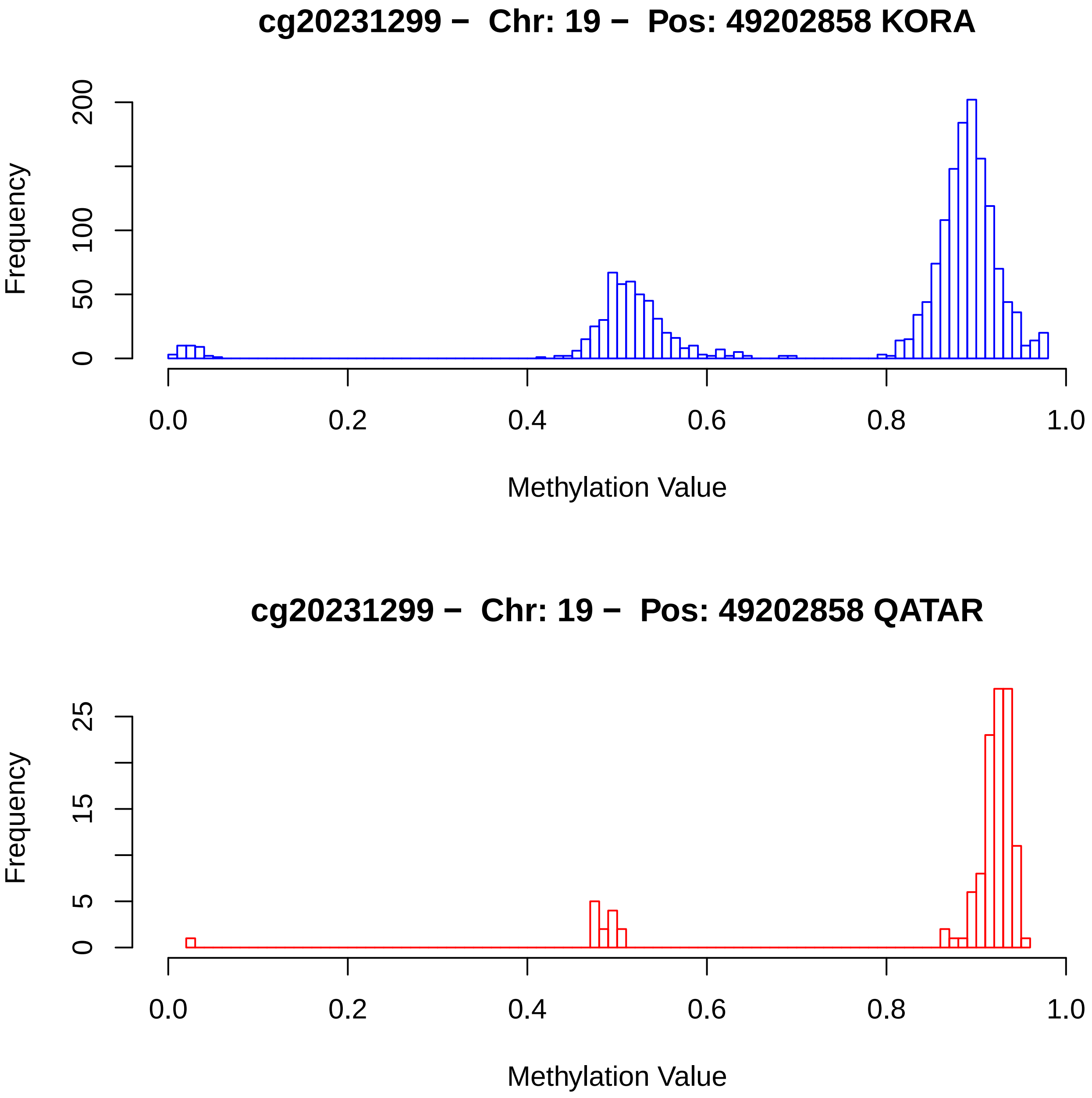
cg20231299 − Chr: 19 − Pos: 49202858 KORA
Frequency
0
50
100
200
0.0
0.2
0.4
0.6
0.8
1.0
Methylation Value
cg20231299 − Chr: 19 − Pos: 49202858 QATAR
Frequency
0
5
15
25
0.0
0.2
0.4
0.6
0.8
1.0
Methylation Value

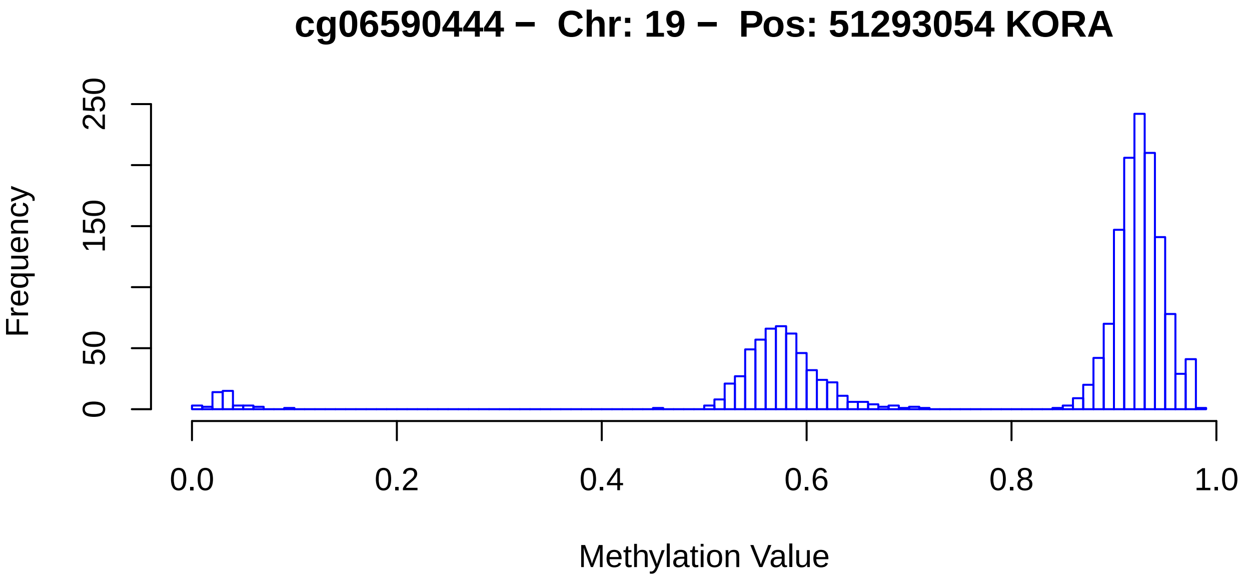
cg06590444 − Chr: 19 − Pos: 51293054 KORA
Frequency
0
50
150
250
0.0
0.2
0.4
0.6
0.8
1.0
Methylation Value

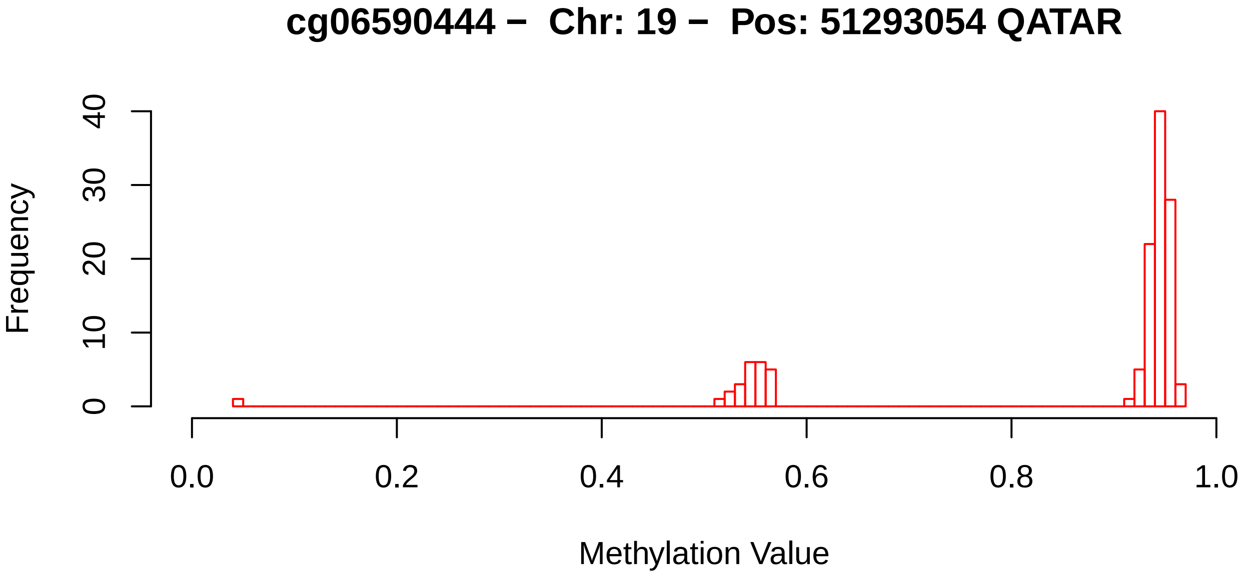
cg06590444 − Chr: 19 − Pos: 51293054 QATAR
Frequency
0
10
20
30
40
0.0
0.2
0.4
0.6
0.8
1.0
Methylation Value

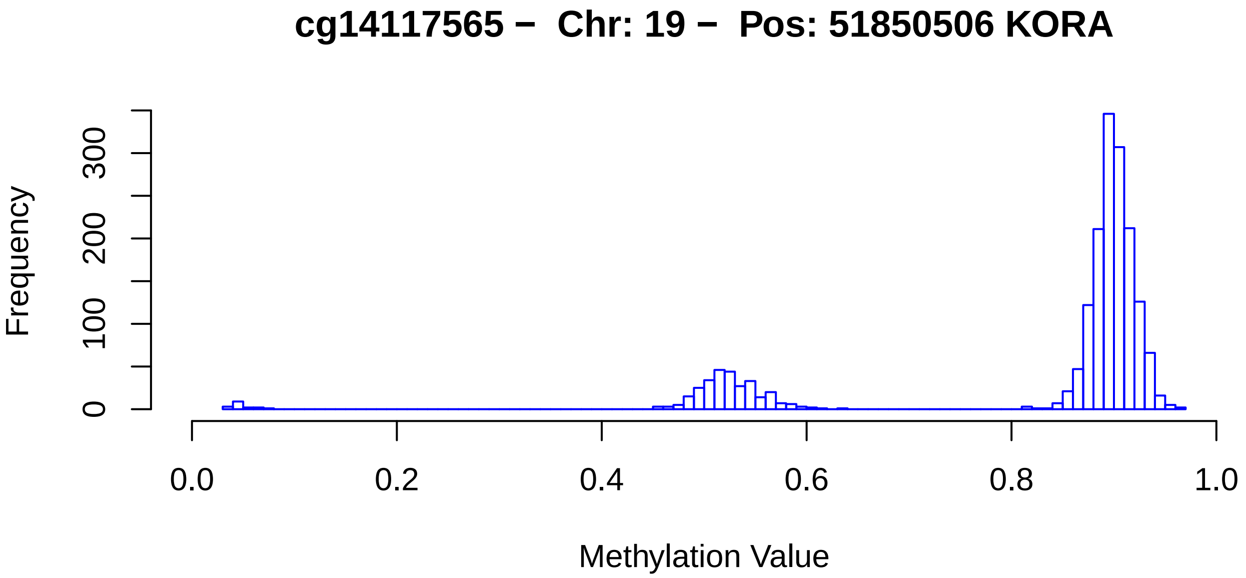

cg14117565 − Chr: 19 − Pos: 51850506 KORA
Frequency
0
100
200
300
0.0
0.2
0.4
0.6
0.8
1.0
Methylation Value

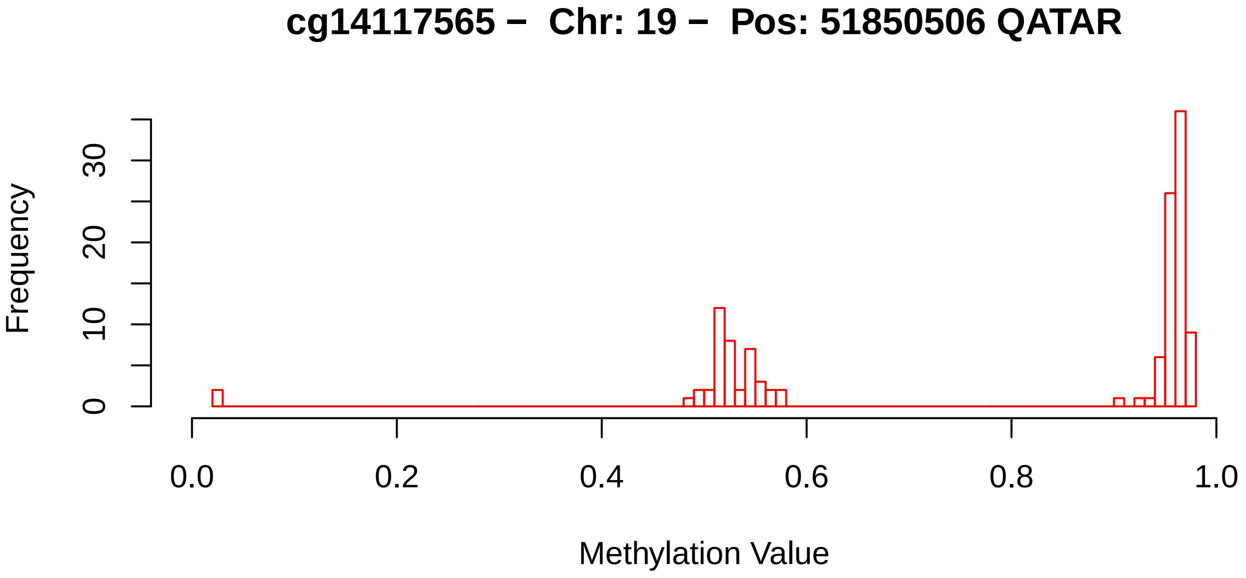

cg14117565 − Chr: 19 − Pos: 51850506 QATAR
Frequency
0
10
20
30
0.0
0.2
0.4
0.6
0.8
1.0
Methylation Value

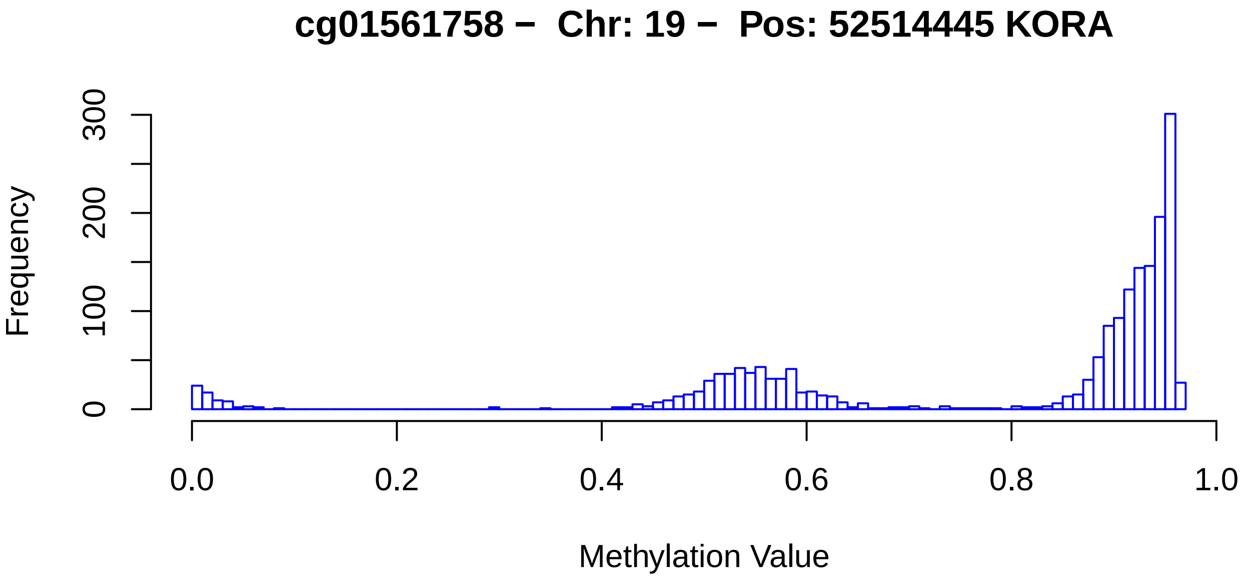
cg01561758 − Chr: 19 − Pos: 52514445 KORA
Frequency
0
100
200
300
0.0
0.2
0.4
0.6
0.8
1.0
Methylation Value

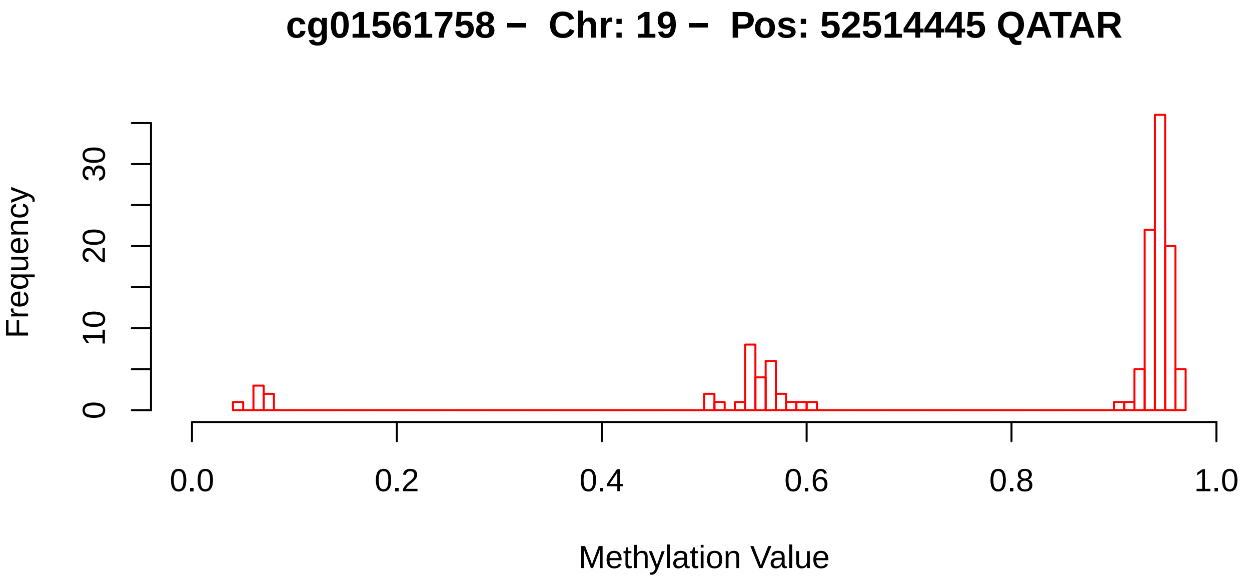
cg01561758 − Chr: 19 − Pos: 52514445 QATAR
Frequency
0
10
20
30
0.0
0.2
0.4
0.6
0.8
1.0
Methylation Value

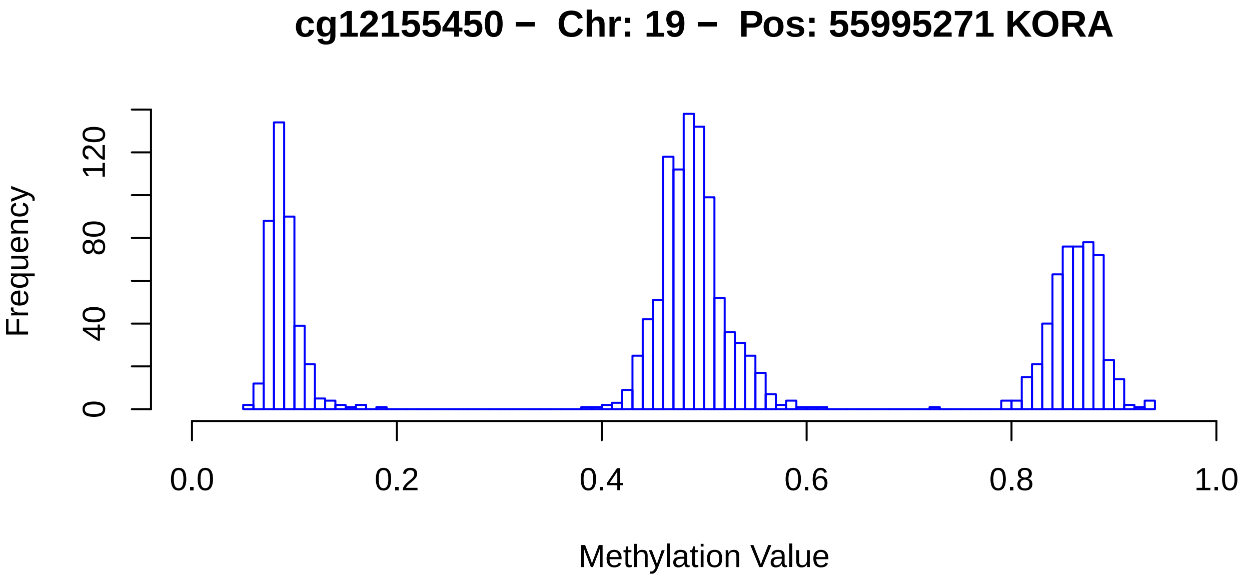
cg12155450 − Chr: 19 − Pos: 55995271 KORA
Frequency
0
40
80
120
0.0
0.2
0.4
0.6
0.8
1.0
Methylation Value

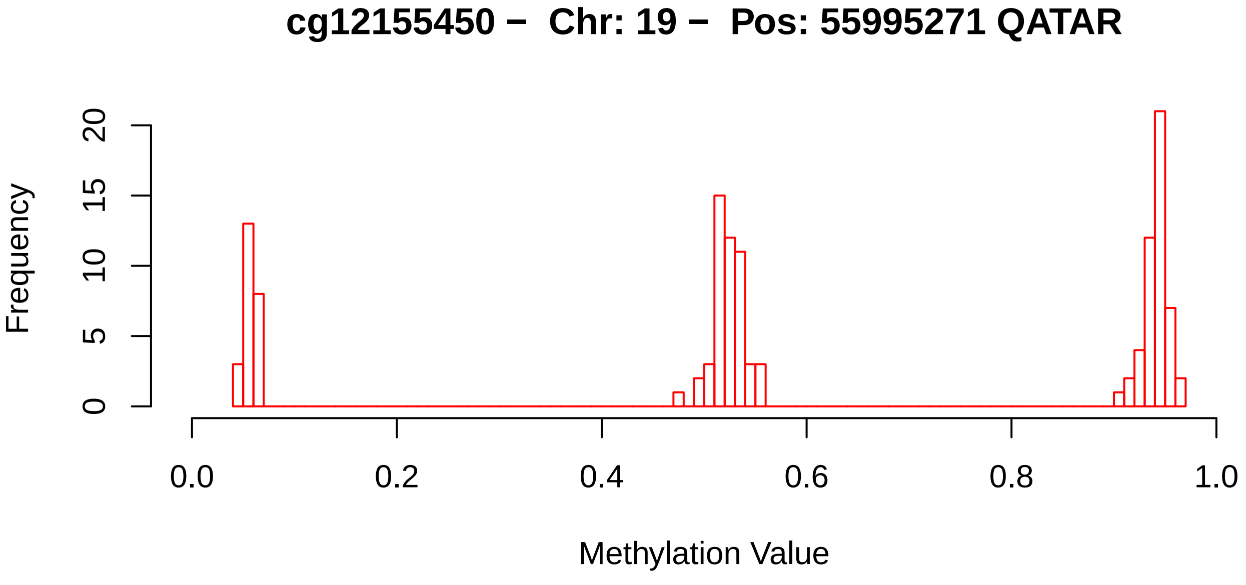
cg12155450 − Chr: 19 − Pos: 55995271 QATAR
Frequency
0
5
10
15
20
0.0
0.2
0.4
0.6
0.8
1.0
Methylation Value

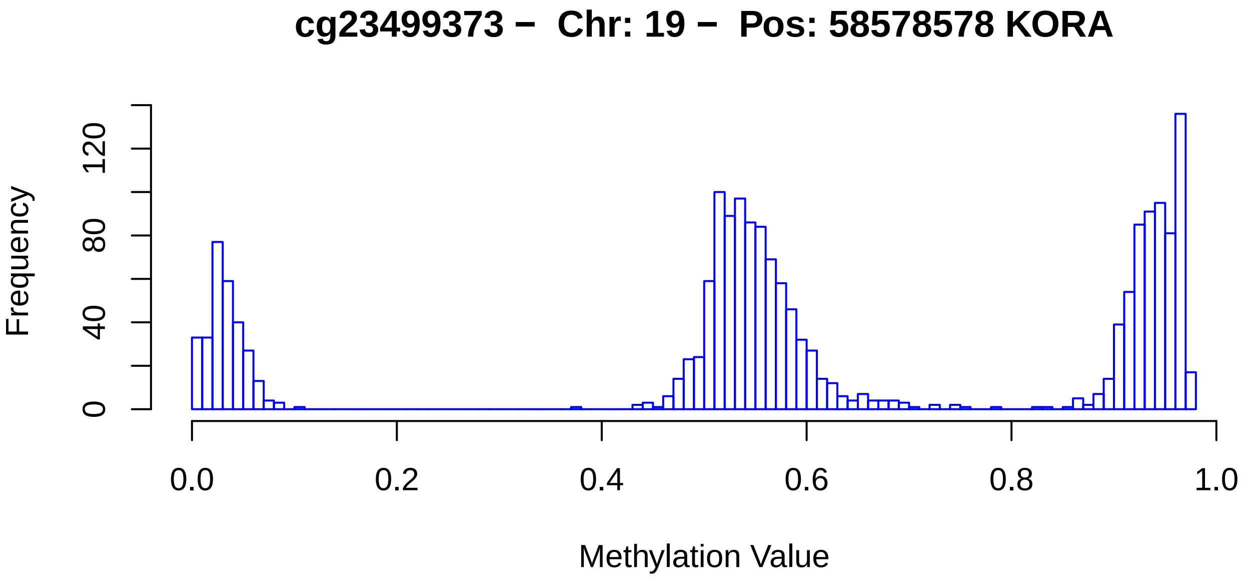
cg23499373 − Chr: 19 − Pos: 58578578 KORA
Frequency
0
40
80
120
0.0
0.2
0.4
0.6
0.8
1.0
Methylation Value

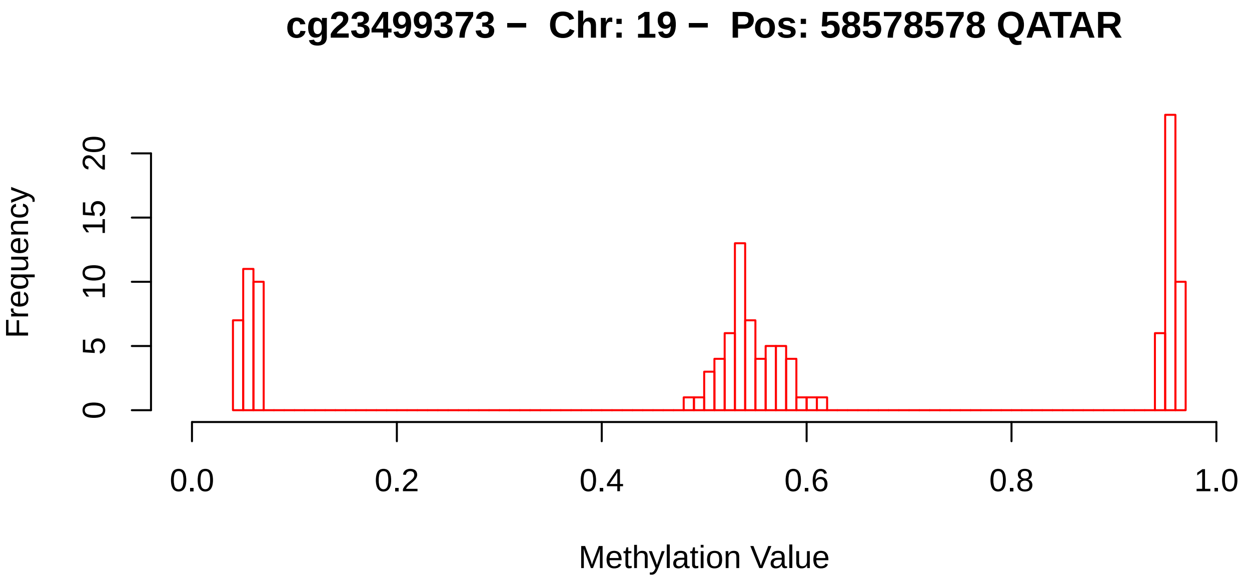
cg23499373 − Chr: 19 − Pos: 58578578 QATAR
Frequency
0
5
10
15
20
0.0
0.2
0.4
0.6
0.8
1.0
Methylation Value

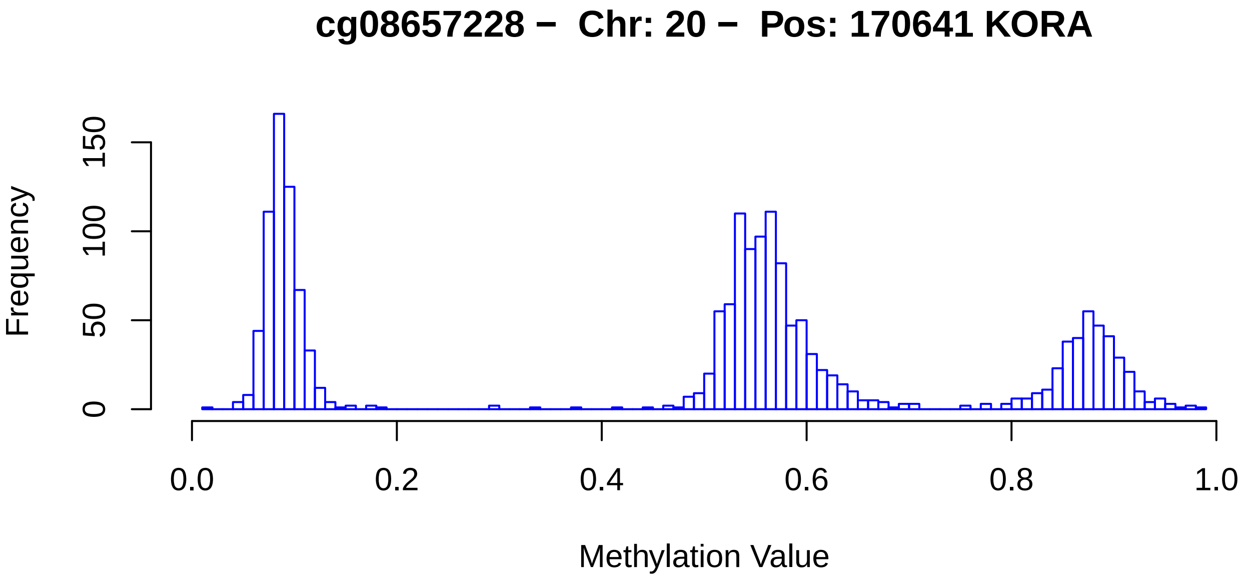
cg08657228 − Chr: 20 − Pos: 170641 KORA
Frequency
0
50
100
150
0.0
0.2
0.4
0.6
0.8
1.0
Methylation Value

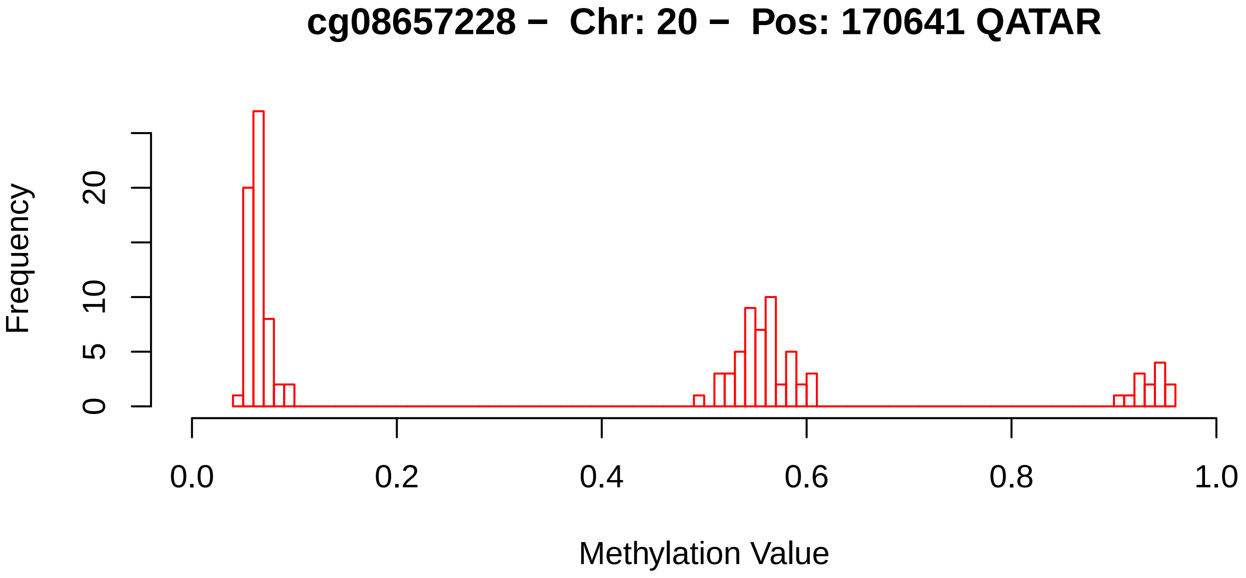
cg08657228 − Chr: 20 − Pos: 170641 QATAR
Frequency
0
5
10
20
0.0
0.2
0.4
0.6
0.8
1.0
Methylation Value

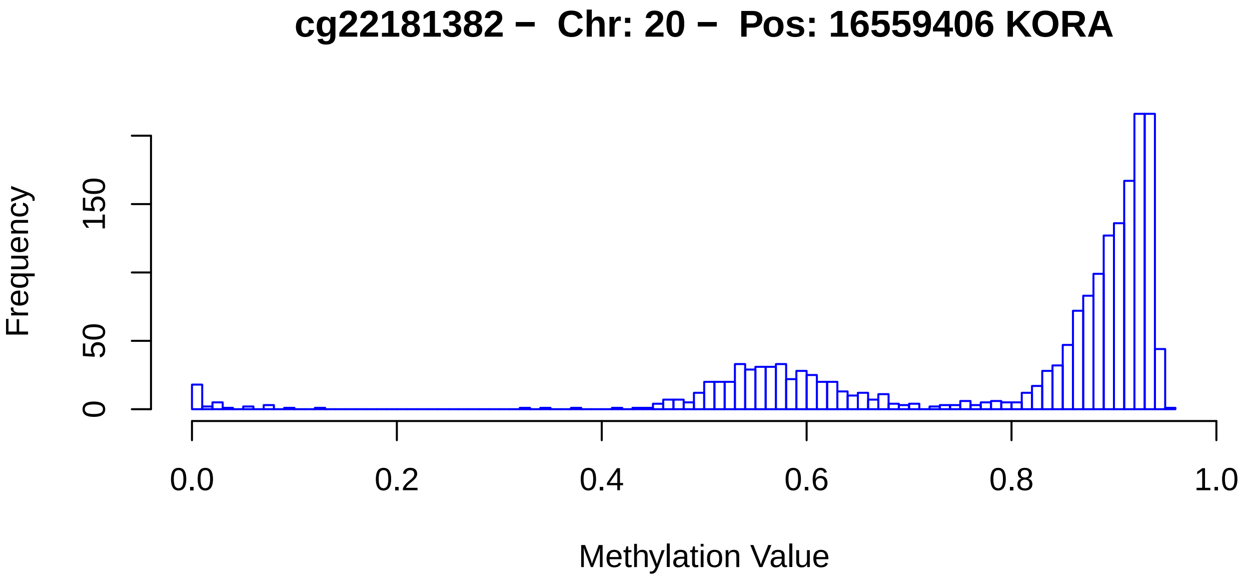
cg22181382 − Chr: 20 − Pos: 16559406 KORA
Frequency
0
50
150
0.0
0.2
0.4
0.6
0.8
1.0
Methylation Value

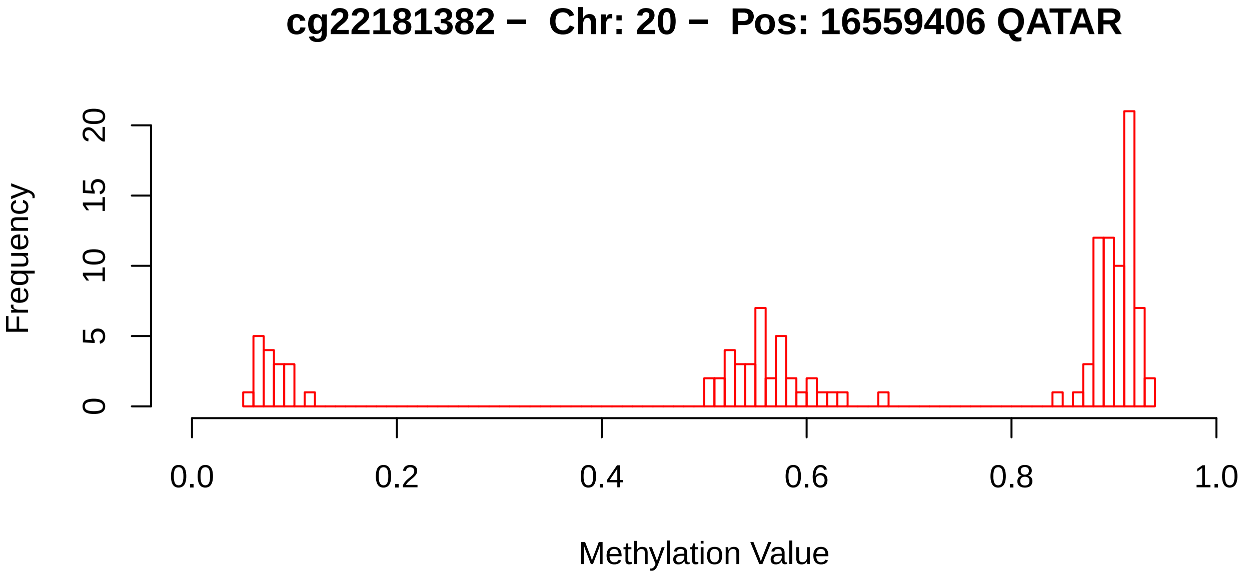
cg22181382 − Chr: 20 − Pos: 16559406 QATAR
Frequency
0
5
10
15
20
0.0
0.2
0.4
0.6
0.8
1.0
Methylation Value

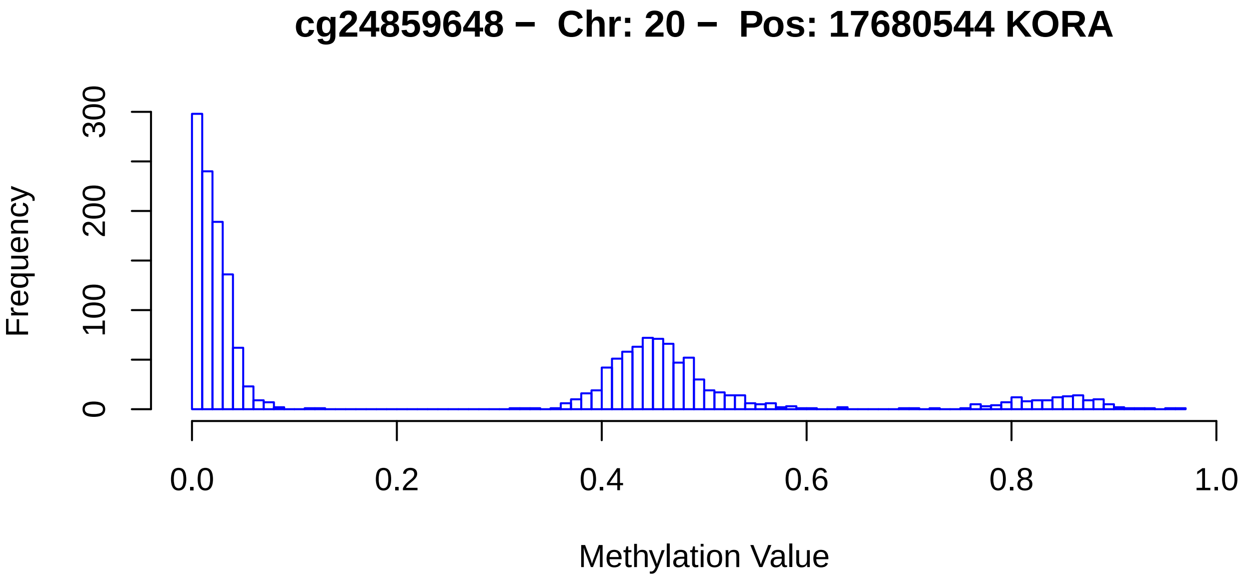
cg24859648 − Chr: 20 − Pos: 17680544 KORA
Frequency
0
100
200
300
0.0
0.2
0.4
0.6
0.8
1.0
Methylation Value

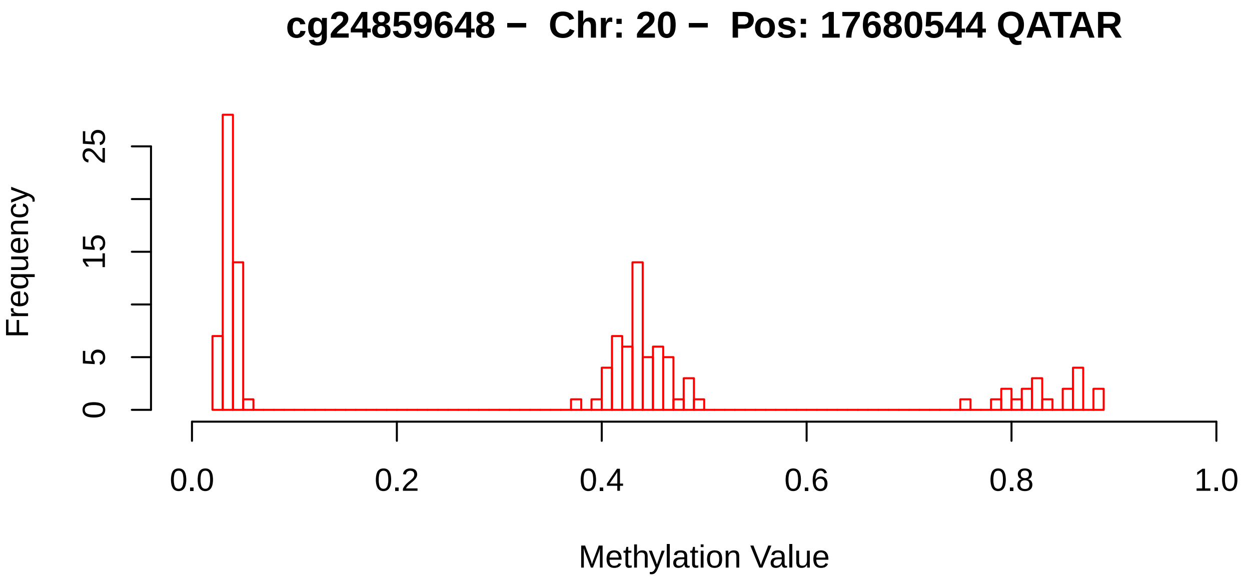
cg24859648 − Chr: 20 − Pos: 17680544 QATAR
Frequency
0
5
15
25
0.0
0.2
0.4
0.6
0.8
1.0
Methylation Value

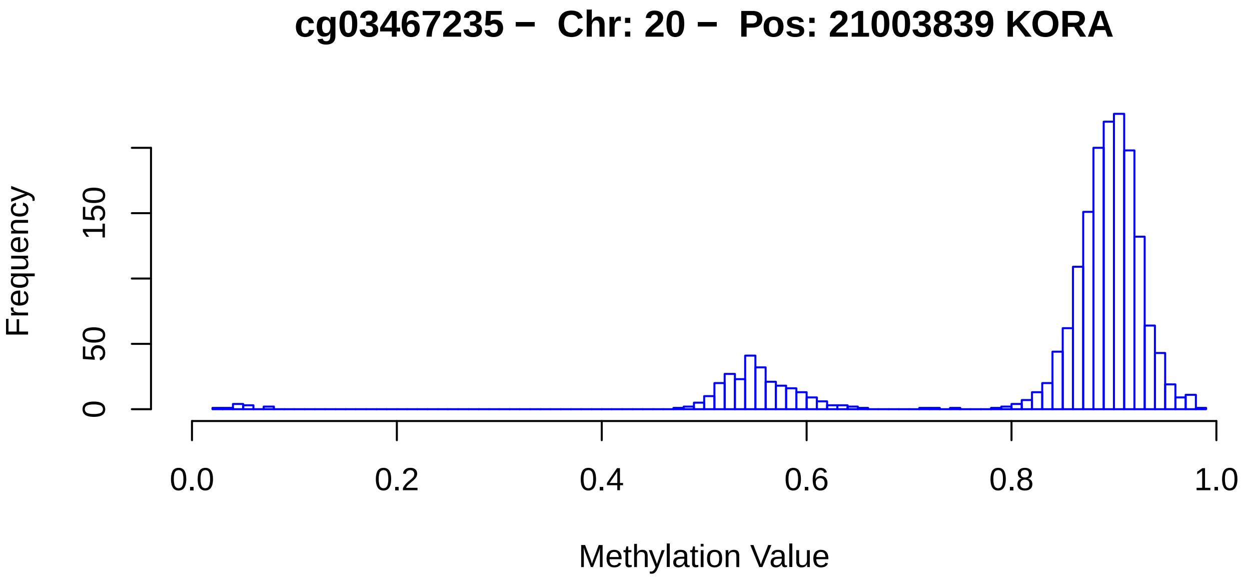
cg03467235 − Chr: 20 − Pos: 21003839 KORA
Frequency
0
50
150
0.0
0.2
0.4
0.6
0.8
1.0
Methylation Value

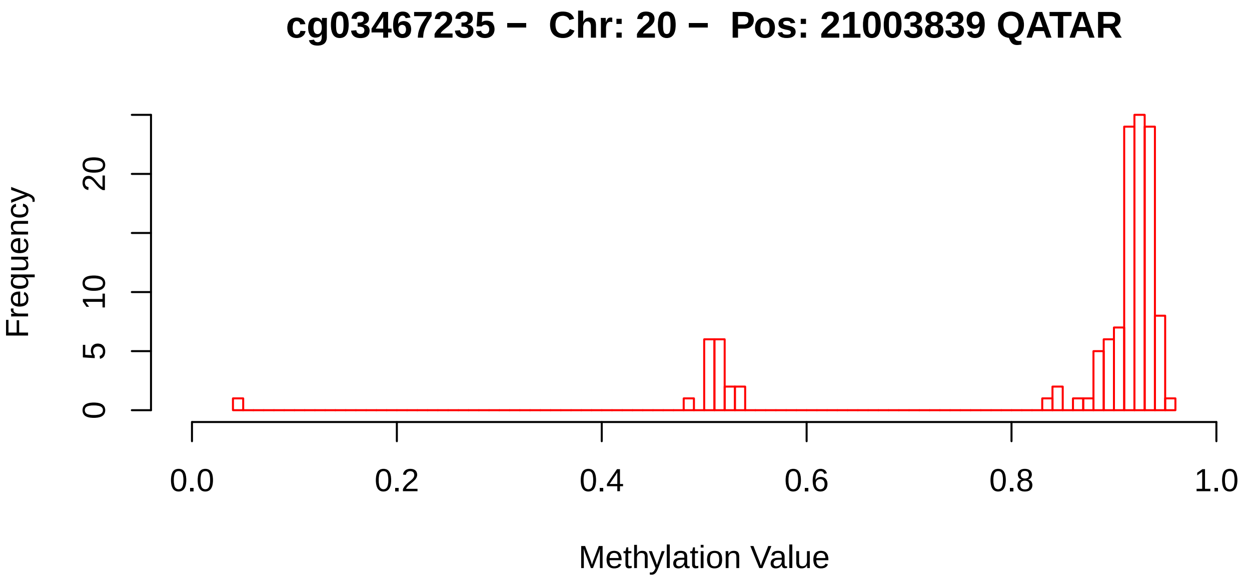
cg03467235 − Chr: 20 − Pos: 21003839 QATAR
Frequency
0
5
10
20
0.0
0.2
0.4
0.6
0.8
1.0
Methylation Value

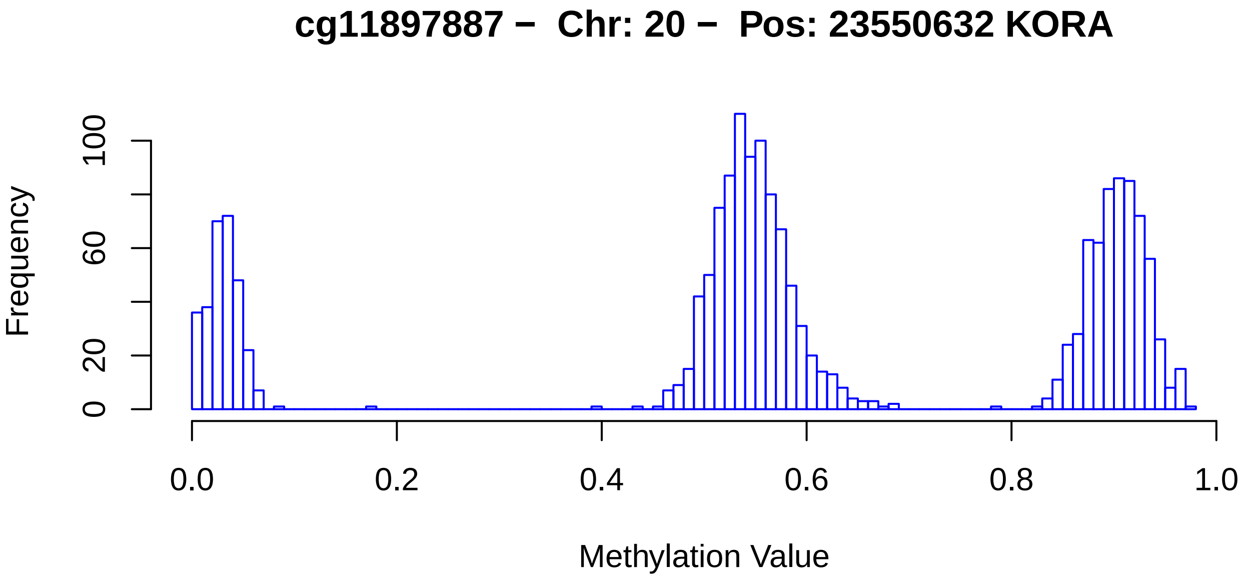
cg11897887 − Chr: 20 − Pos: 23550632 KORA
Frequency
0
20
60
100
0.0
0.2
0.4
0.6
0.8
1.0
Methylation Value

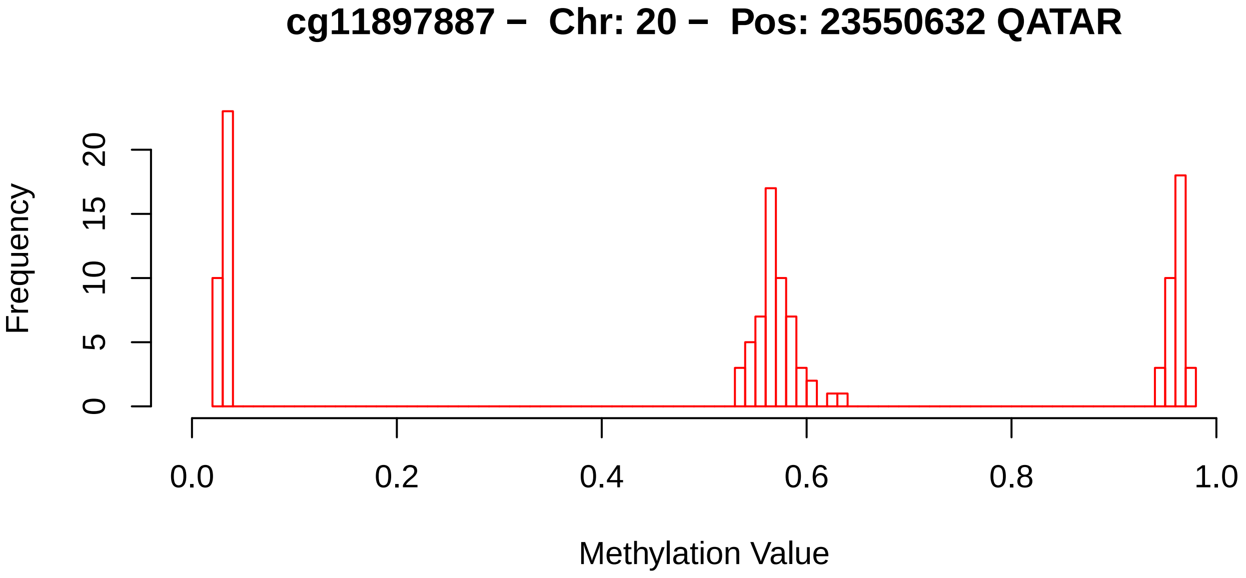
cg11897887 − Chr: 20 − Pos: 23550632 QATAR
Frequency
0
5
10
15
20
0.0
0.2
0.4
0.6
0.8
1.0
Methylation Value

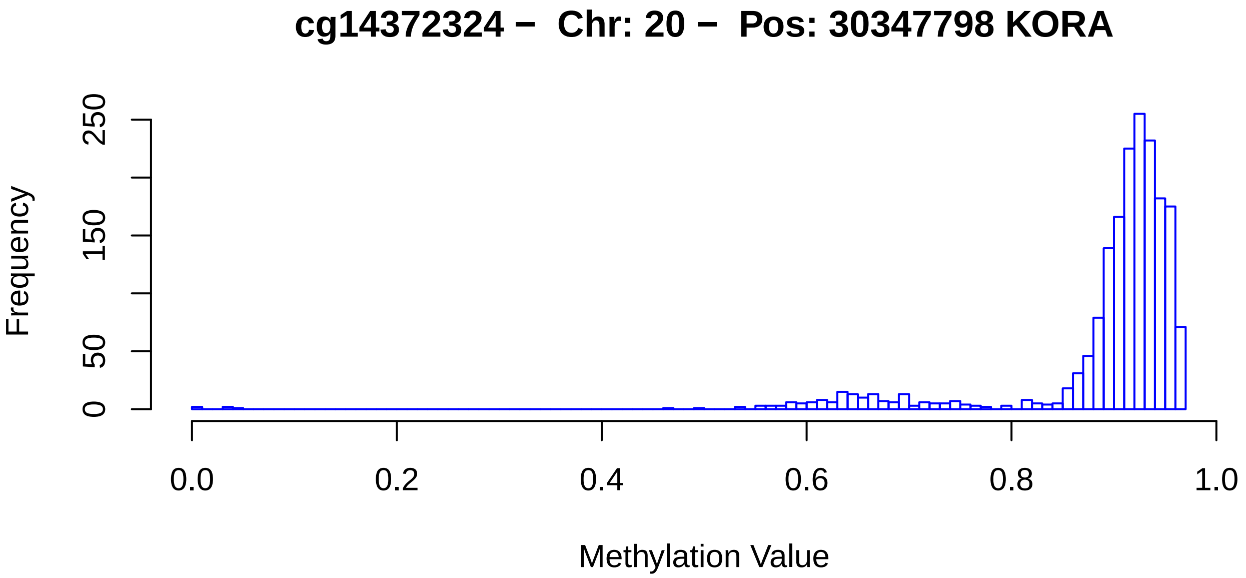
cg14372324 − Chr: 20 − Pos: 30347798 KORA
Frequency
0
50
150
250
0.0
0.2
0.4
0.6
0.8
1.0
Methylation Value

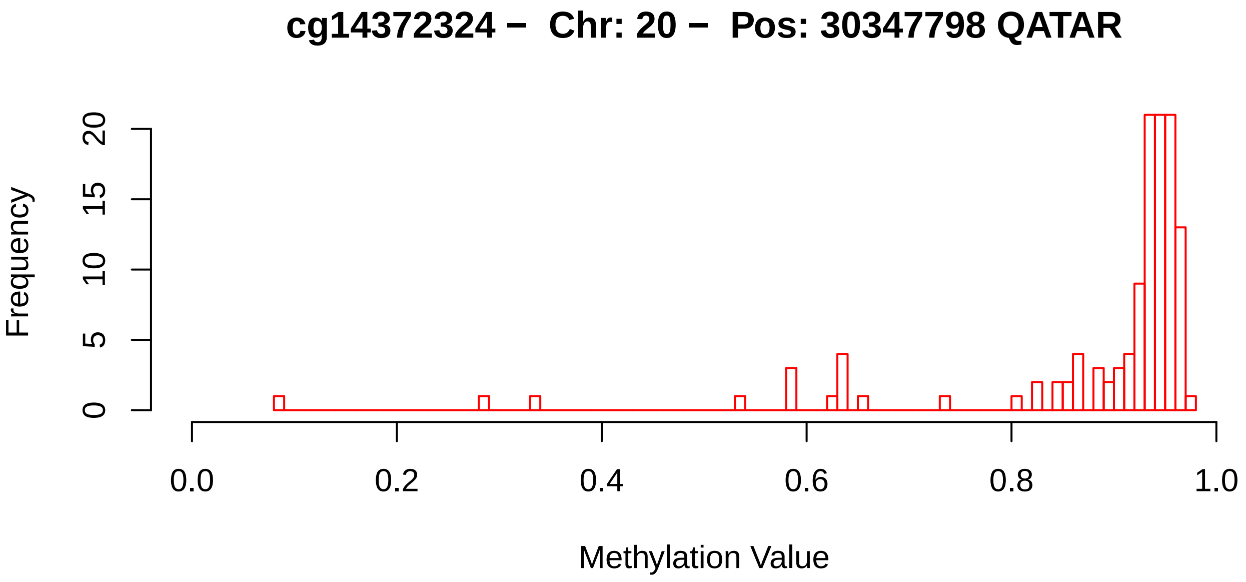
cg14372324 − Chr: 20 − Pos: 30347798 QATAR
Frequency
0
5
10
15
20
0.0
0.2
0.4
0.6
0.8
1.0
Methylation Value

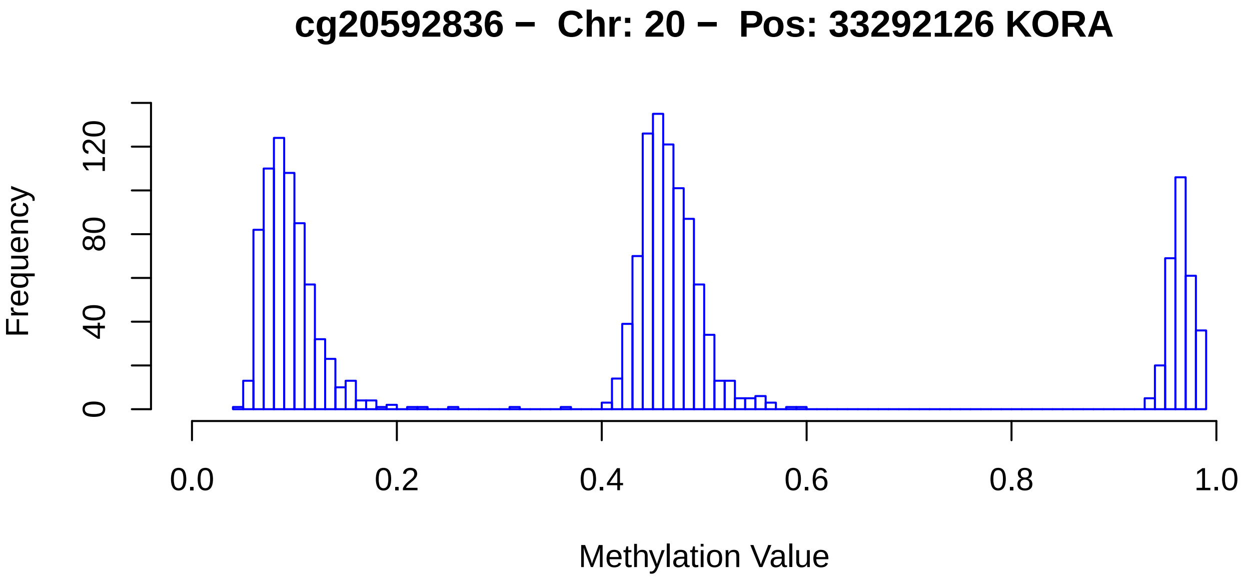
cg20592836 − Chr: 20 − Pos: 33292126 KORA
Frequency
0
40
80
120
0.0
0.2
0.4
0.6
0.8
1.0
Methylation Value

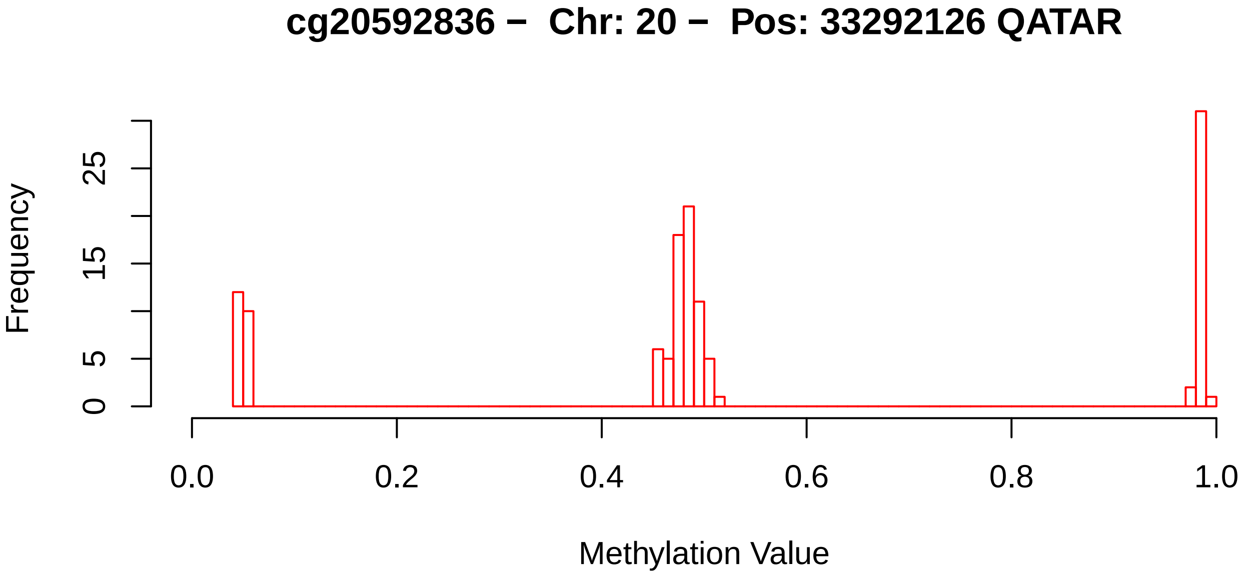
cg20592836 − Chr: 20 − Pos: 33292126 QATAR
Frequency
0
5
15
25
0.0
0.2
0.4
0.6
0.8
1.0
Methylation Value

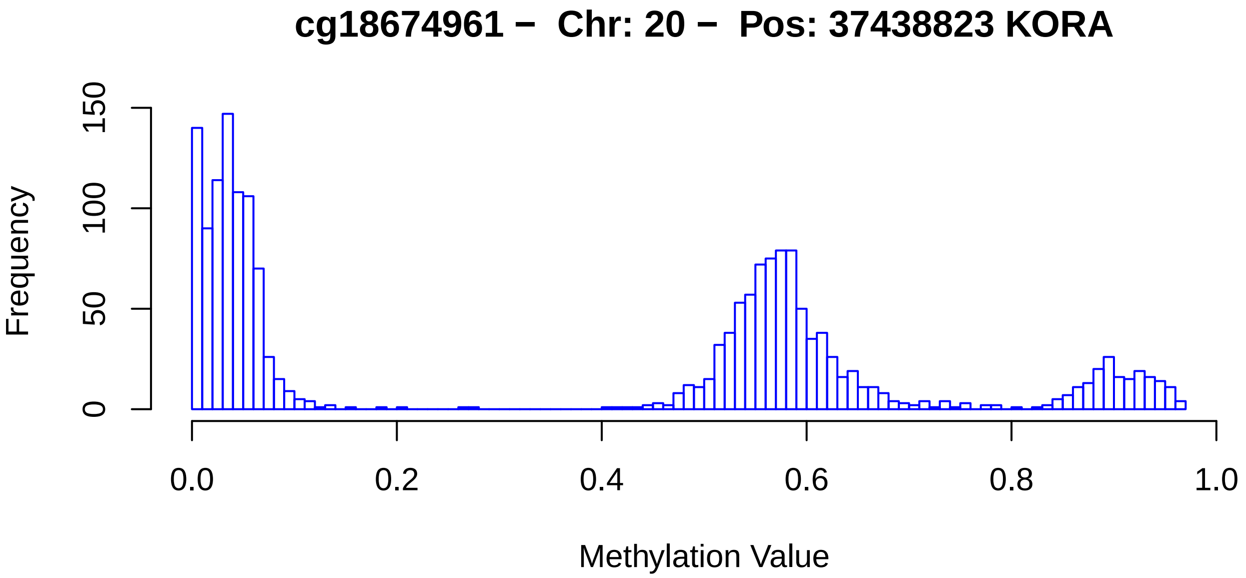
cg18674961 − Chr: 20 − Pos: 37438823 KORA
Frequency
0
50
100
150
0.0
0.2
0.4
0.6
0.8
1.0
Methylation Value

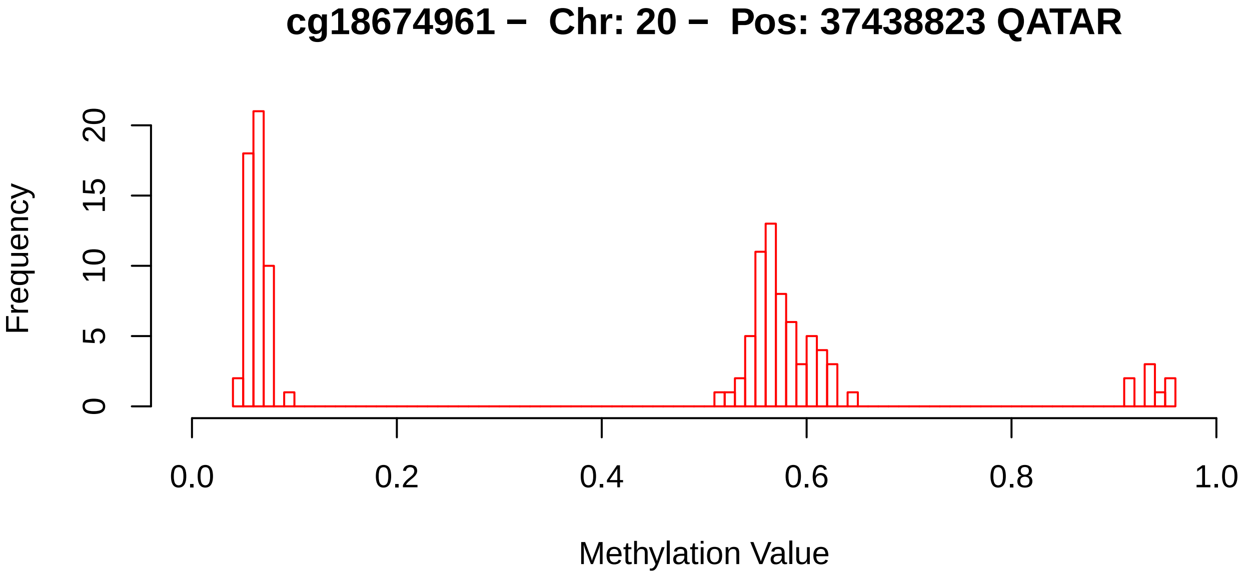
cg18674961 − Chr: 20 − Pos: 37438823 QATAR
Frequency
0
5
10
15
20
0.0
0.2
0.4
0.6
0.8
1.0
Methylation Value

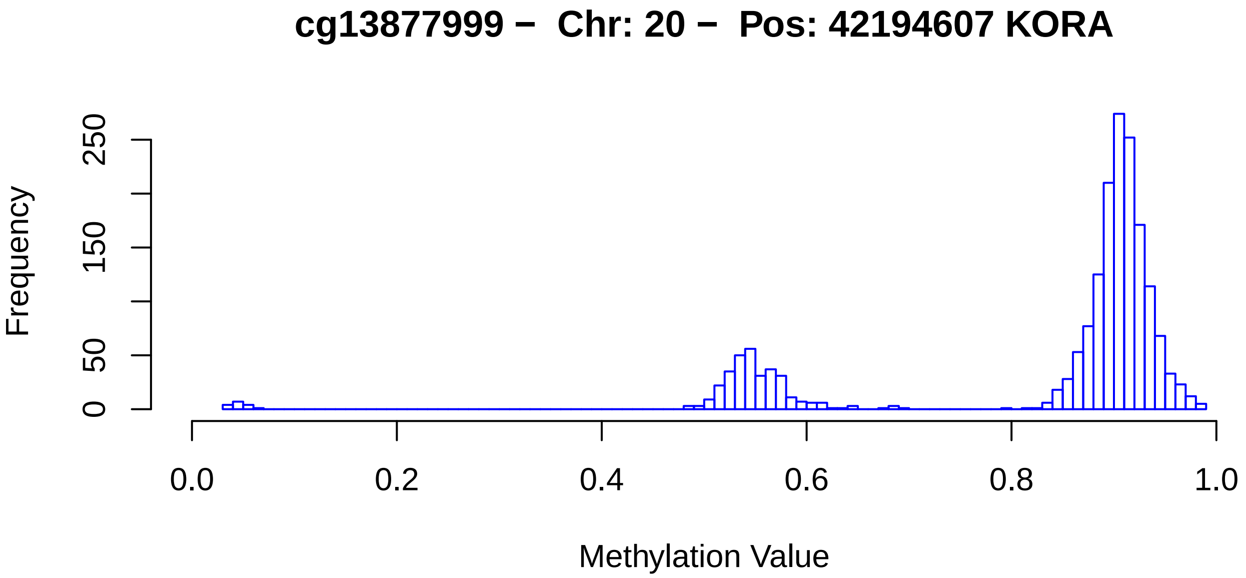
cg13877999 – Chr: 20 – Pos: 42194607 KORA
Frequency
0
50
150
250
0.0
0.2
0.4
0.6
0.8
1.0
Methylation Value

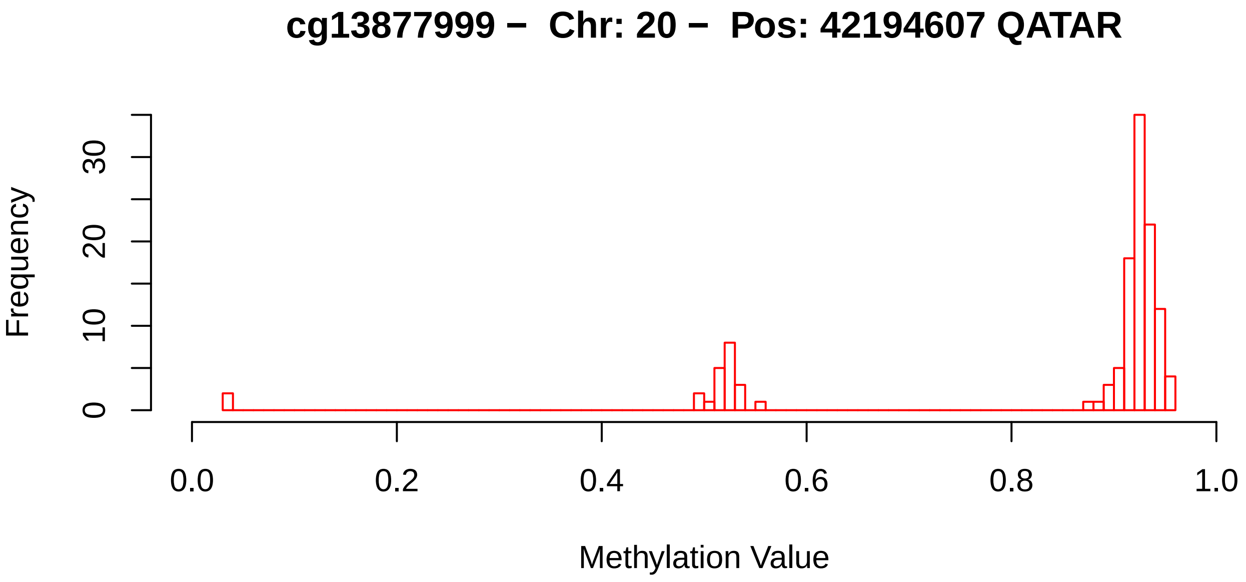
cg13877999 – Chr: 20 – Pos: 42194607 QATAR
Frequency
0
10
20
30
0.0
0.2
0.4
0.6
0.8
1.0
Methylation Value

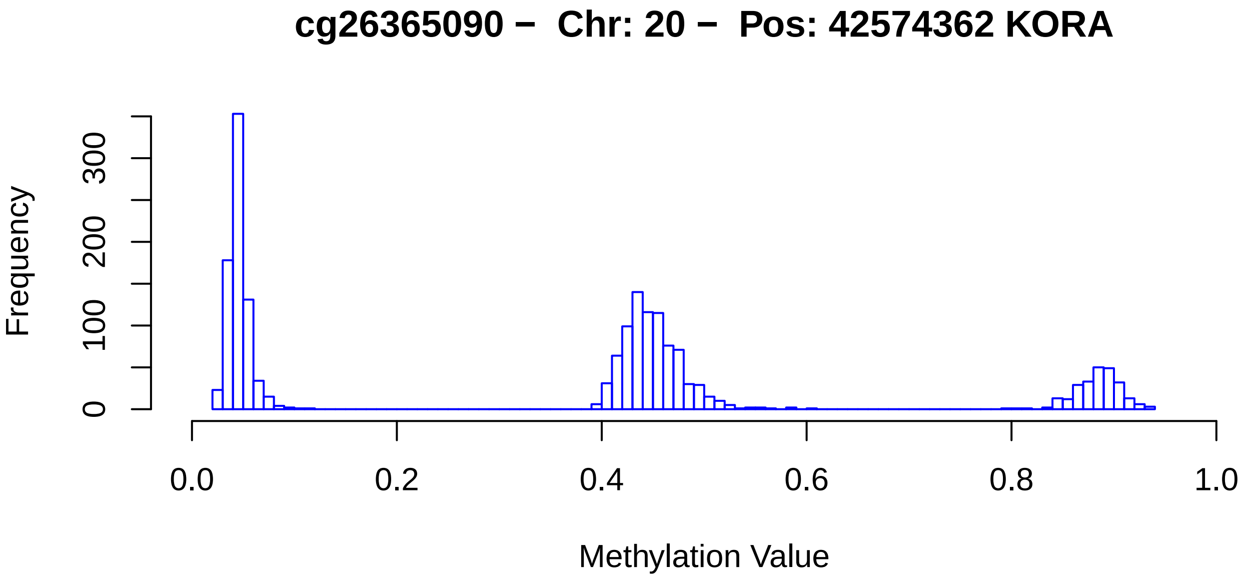
cg26365090 − Chr: 20 − Pos: 42574362 KORA
Frequency
0
100
200
300
0.0
0.2
0.4
0.6
0.8
1.0
Methylation Value

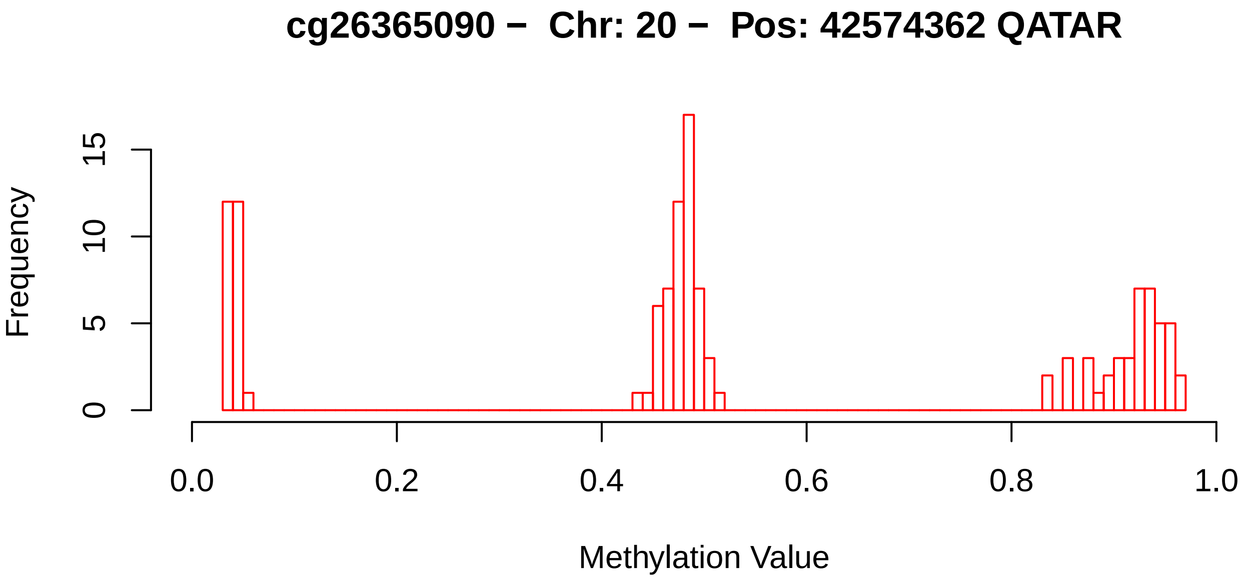
cg26365090 − Chr: 20 − Pos: 42574362 QATAR
Frequency
0
5
10
15
0.0
0.2
0.4
0.6
0.8
1.0
Methylation Value

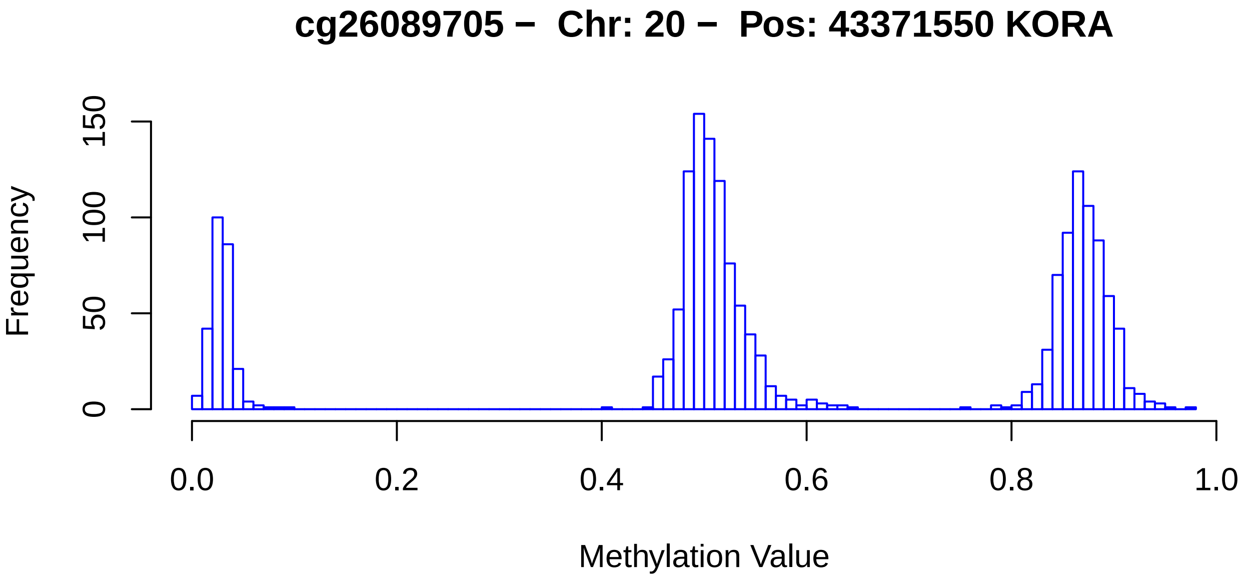
cg26089705 − Chr: 20 − Pos: 43371550 KORA
Frequency
0
50
100
150
0.0
0.2
0.4
0.6
0.8
1.0
Methylation Value

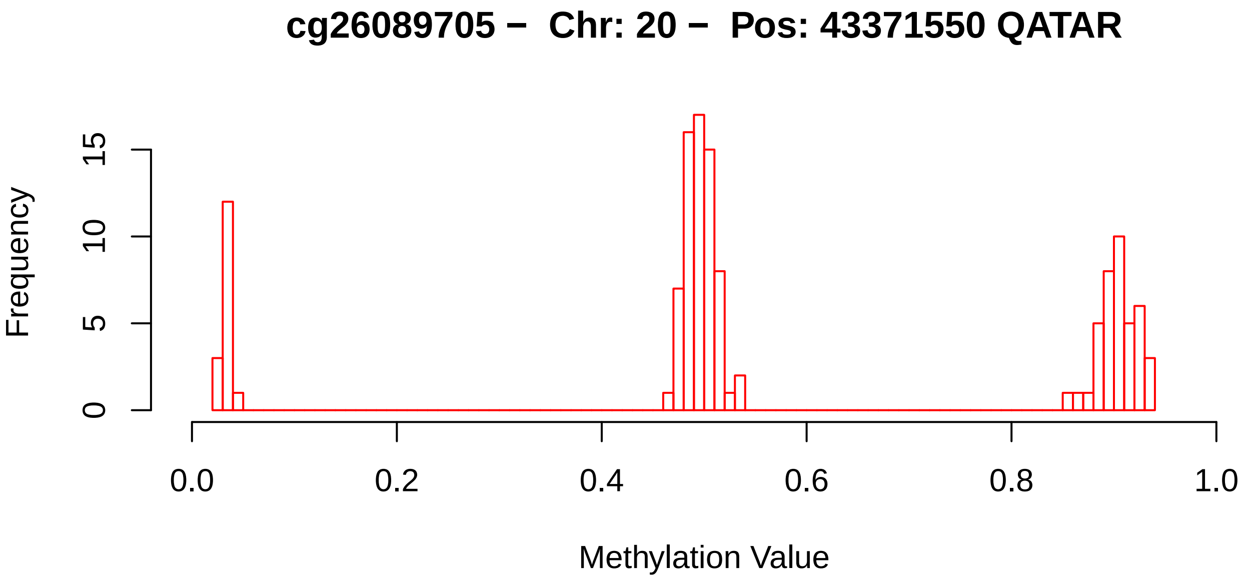
cg26089705 − Chr: 20 − Pos: 43371550 QATAR
Frequency
0
5
10
15
0.0
0.2
0.4
0.6
0.8
1.0
Methylation Value

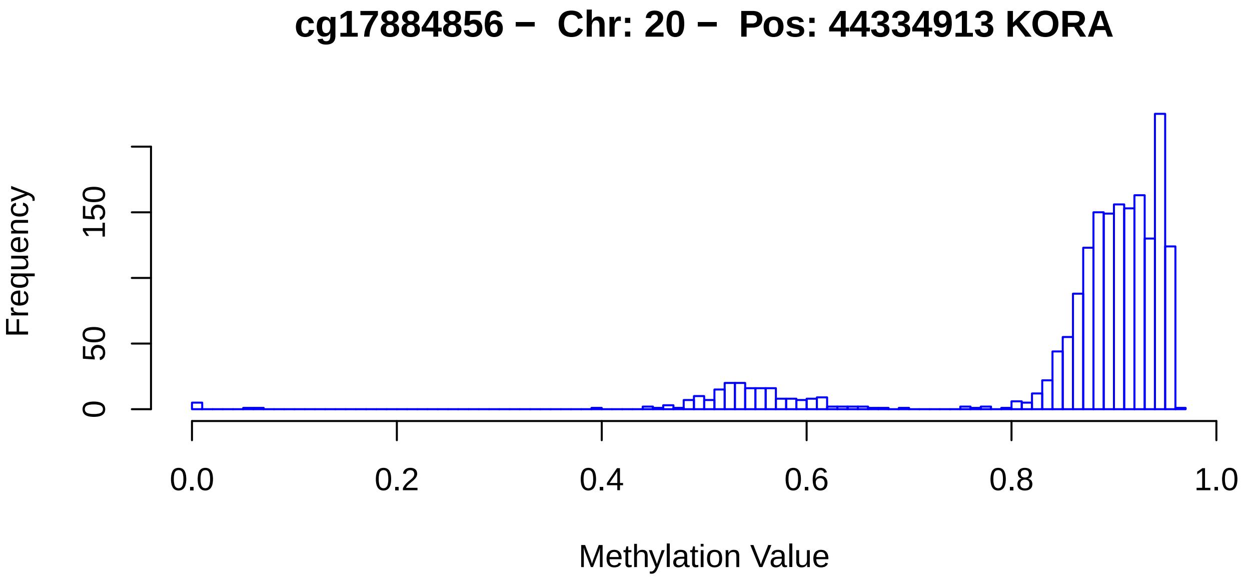
cg17884856 − Chr: 20 − Pos: 44334913 KORA
Frequency
0
50
150
0.0
0.2
0.4
0.6
0.8
1.0
Methylation Value

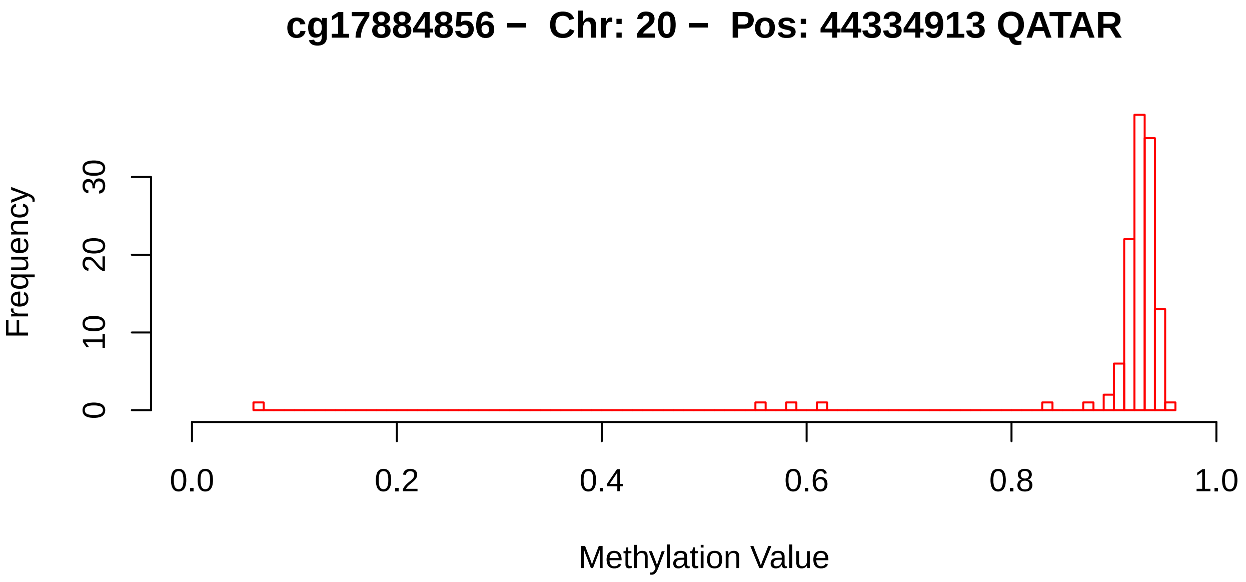
cg17884856 − Chr: 20 − Pos: 44334913 QATAR
Frequency
0
10
20
30
0.0
0.2
0.4
0.6
0.8
1.0
Methylation Value

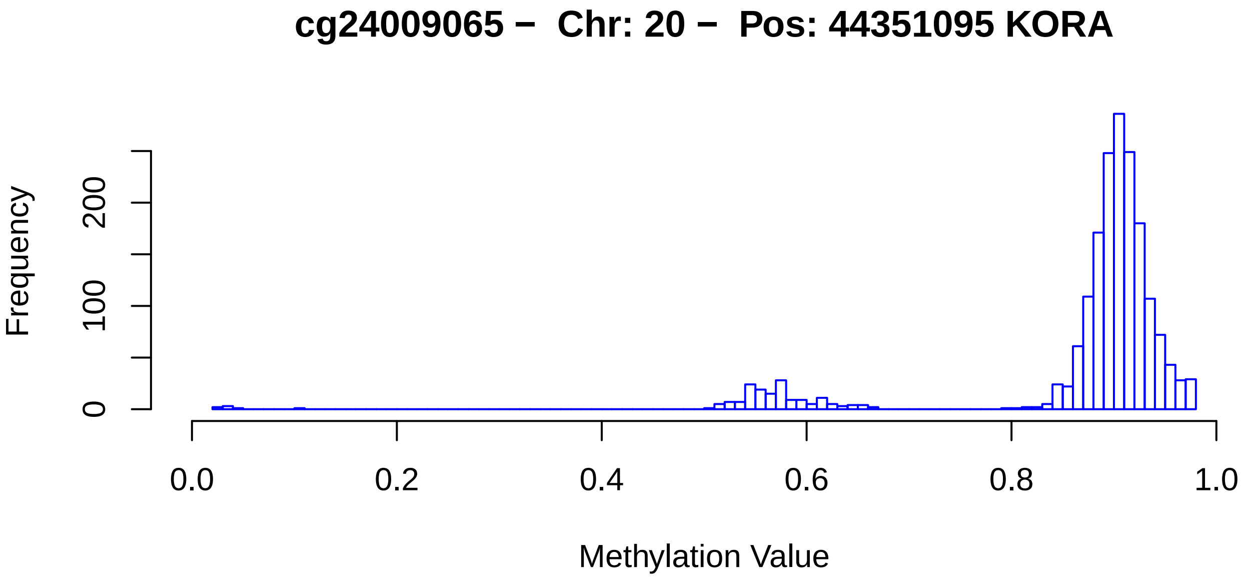
cg24009065 − Chr: 20 − Pos: 44351095 KORA
Frequency
0
100
200
0.0
0.2
0.4
0.6
0.8
1.0
Methylation Value

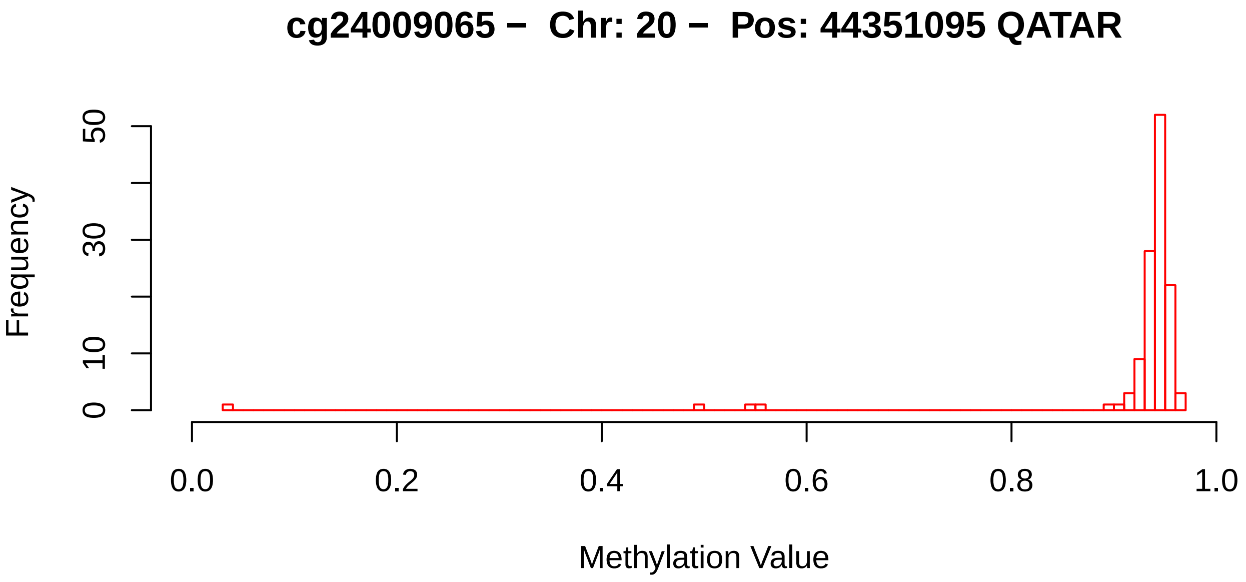
cg24009065 − Chr: 20 − Pos: 44351095 QATAR
Frequency
0
10
30
50
0.0
0.2
0.4
0.6
0.8
1.0
Methylation Value

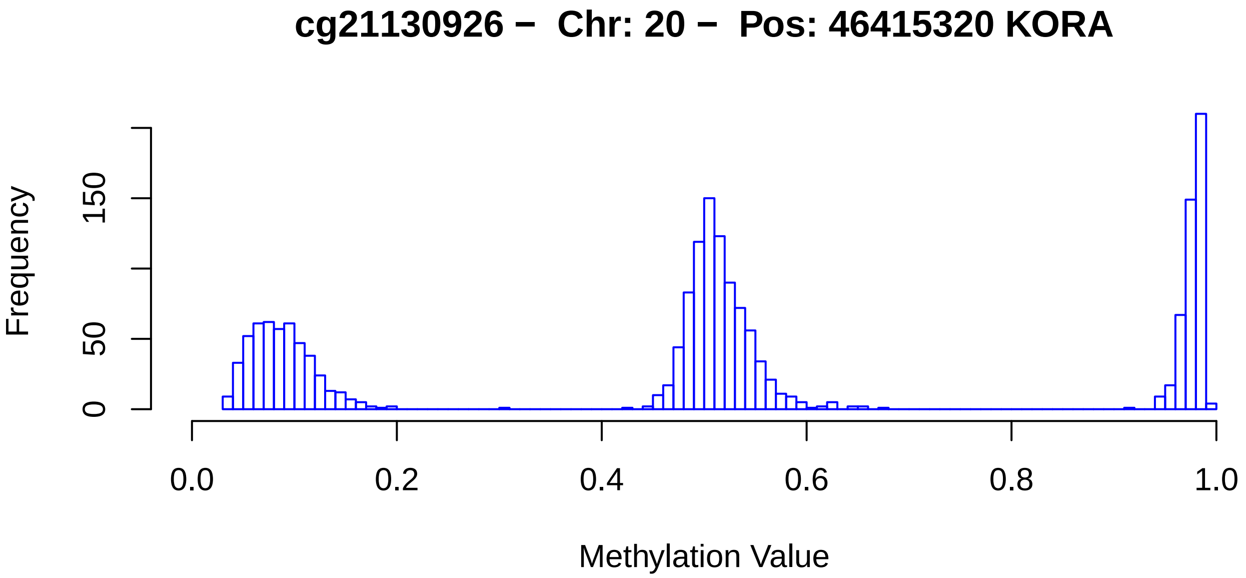
cg21130926 − Chr: 20 − Pos: 46415320 KORA
Frequency
0
50
150
0.0
0.2
0.4
0.6
0.8
1.0
Methylation Value

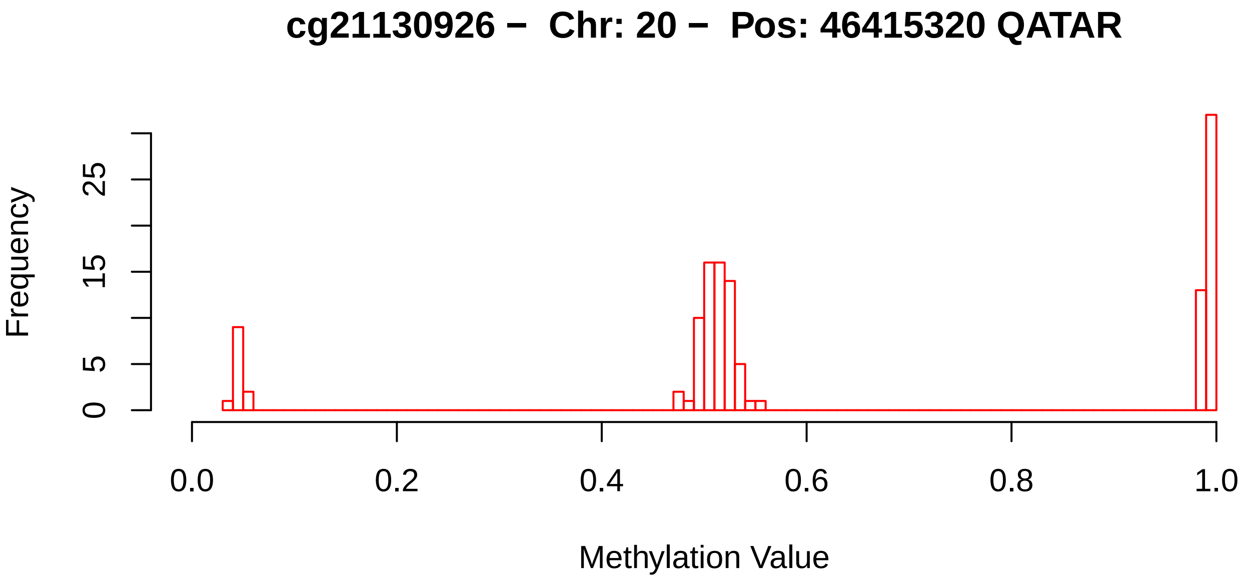
cg21130926 − Chr: 20 − Pos: 46415320 QATAR
Frequency
0
5
15
25
0.0
0.2
0.4
0.6
0.8
1.0
Methylation Value

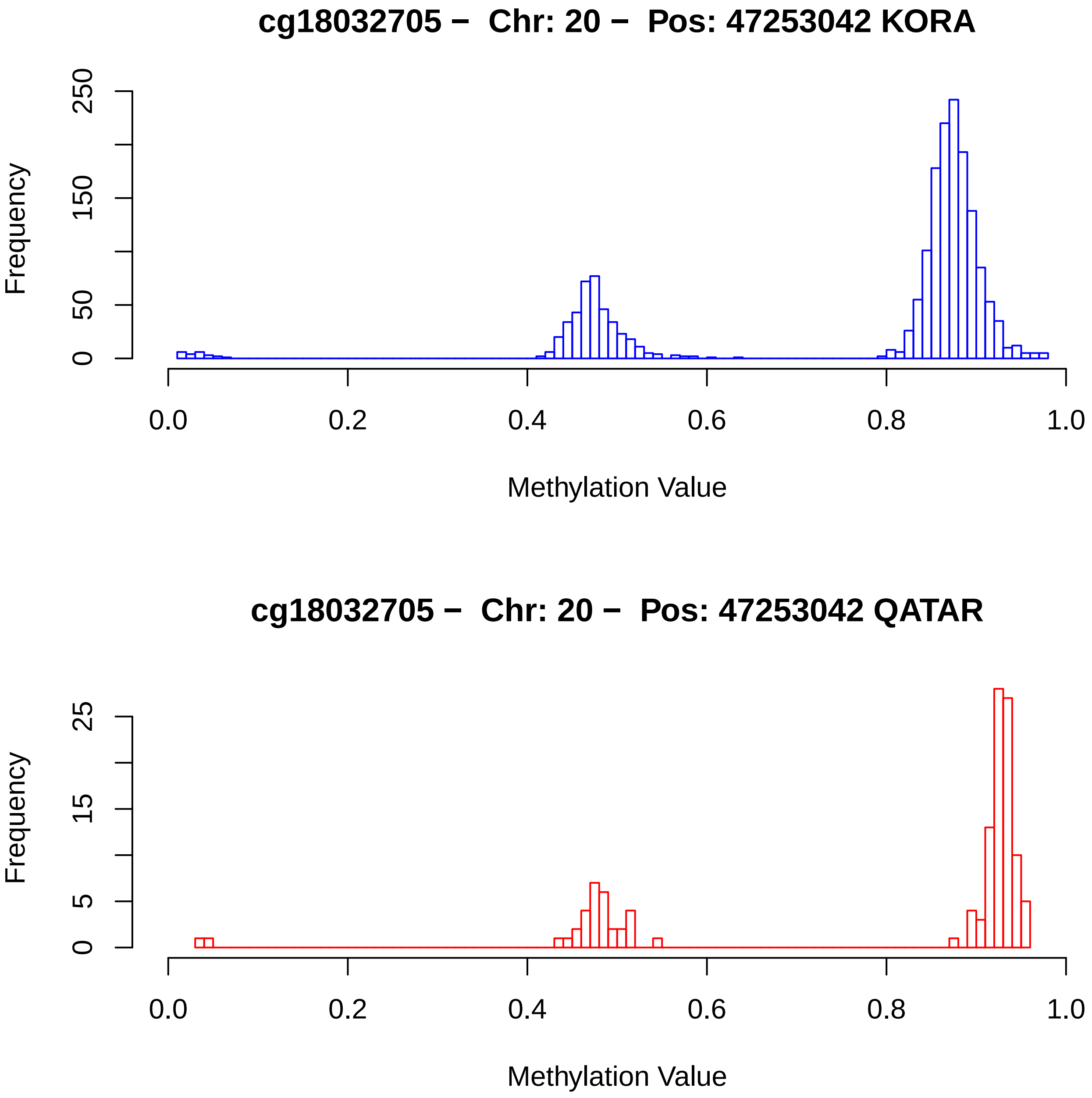
cg18032705 − Chr: 20 − Pos: 47253042 KORA
Frequency
0
50
150
250
0.0
0.2
0.4
0.6
0.8
1.0
Methylation Value
cg18032705 − Chr: 20 − Pos: 47253042 QATAR
Frequency
0
5
15
25
0.0
0.2
0.4
0.6
0.8
1.0
Methylation Value

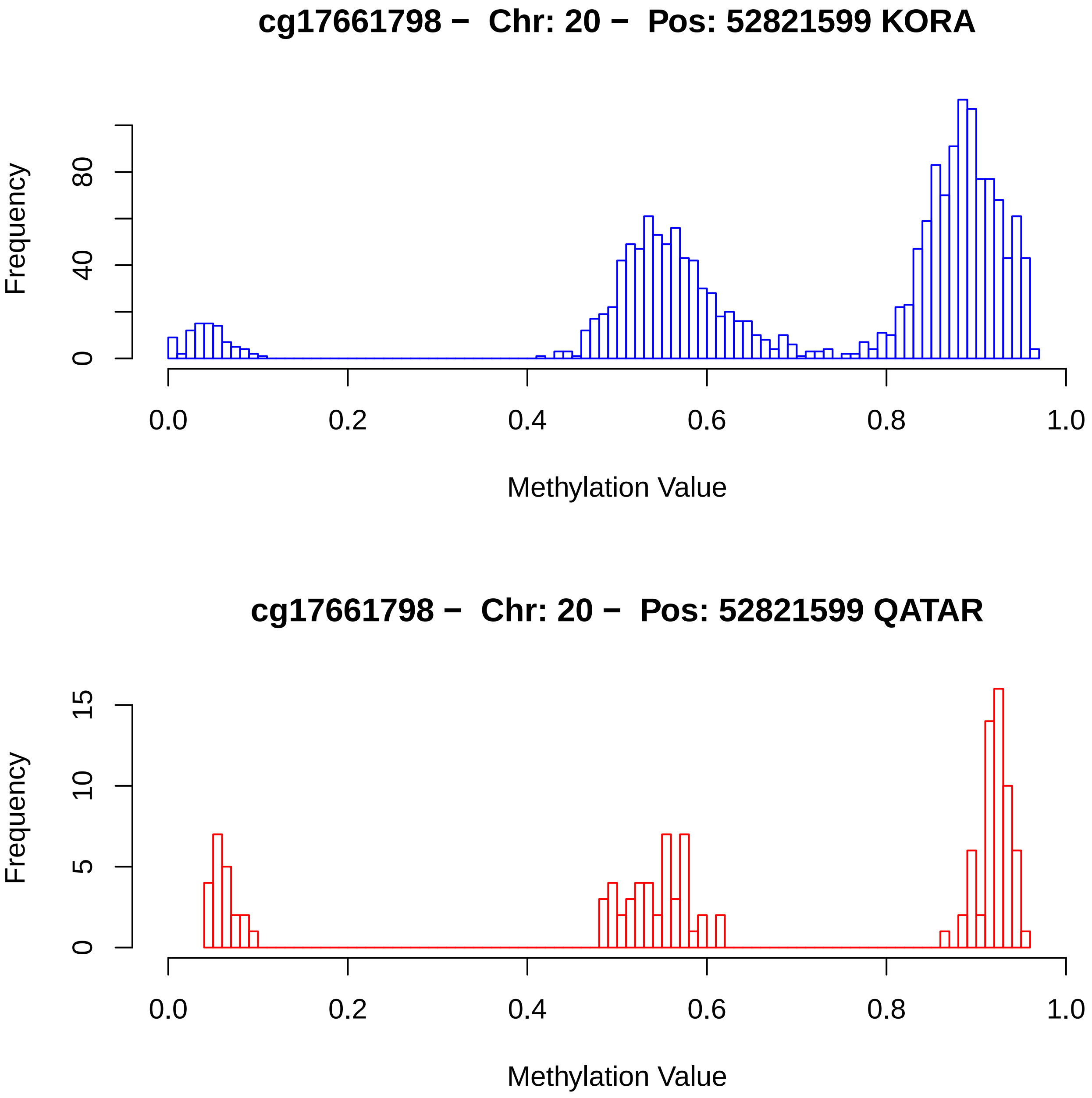
cg17661798 − Chr: 20 − Pos: 52821599 KORA
Frequency
0
40
80
0.0
0.2
0.4
0.6
0.8
1.0
Methylation Value
cg17661798 − Chr: 20 − Pos: 52821599 QATAR
Frequency
0
5
10
15
0.0
0.2
0.4
0.6
0.8
1.0
Methylation Value

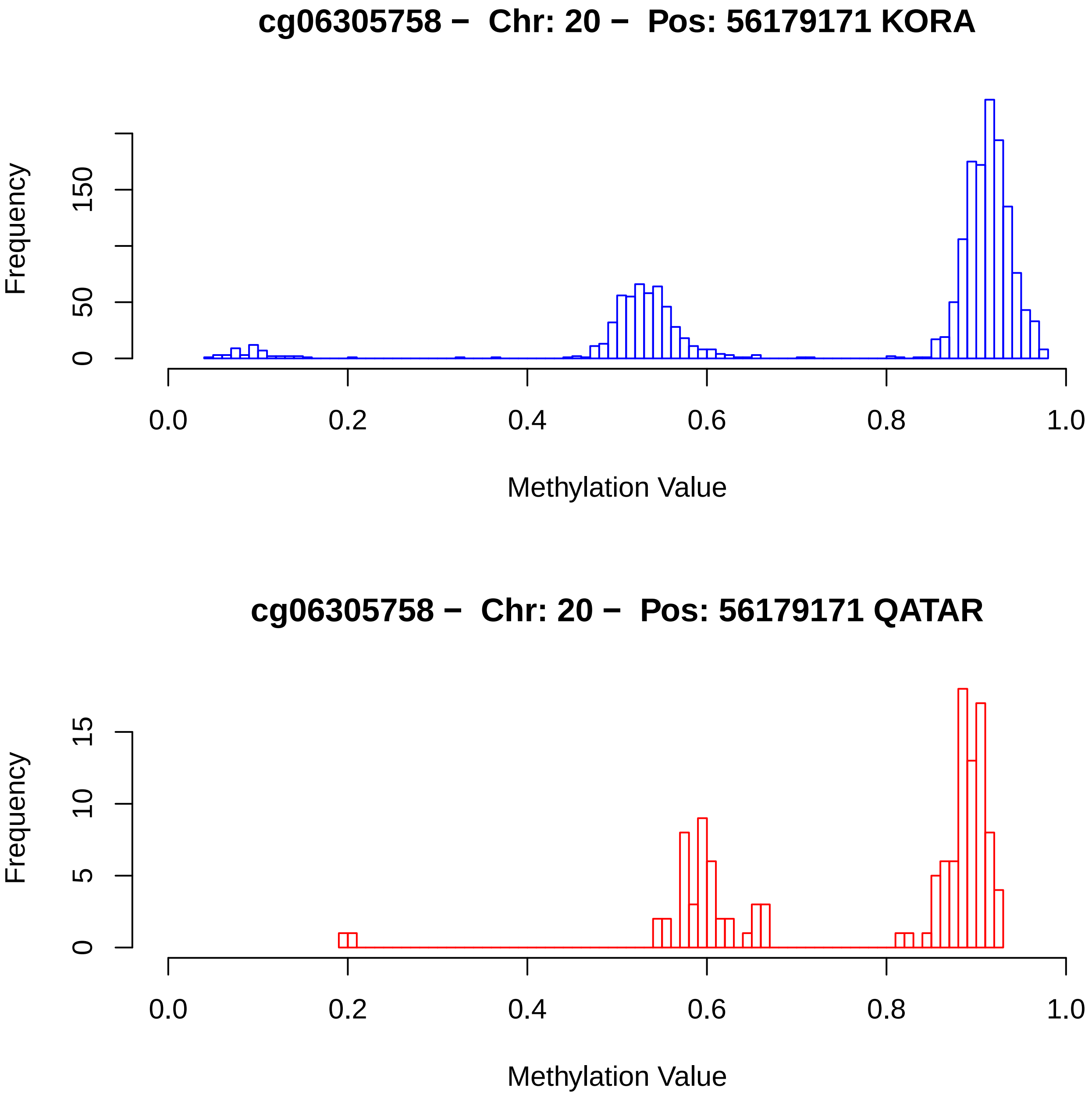
cg06305758 − Chr: 20 − Pos: 56179171 KORA
Frequency
0
50
150
0.0
0.2
0.4
0.6
0.8
1.0
Methylation Value
cg06305758 − Chr: 20 − Pos: 56179171 QATAR
Frequency
0
5
10
15
0.0
0.2
0.4
0.6
0.8
1.0
Methylation Value

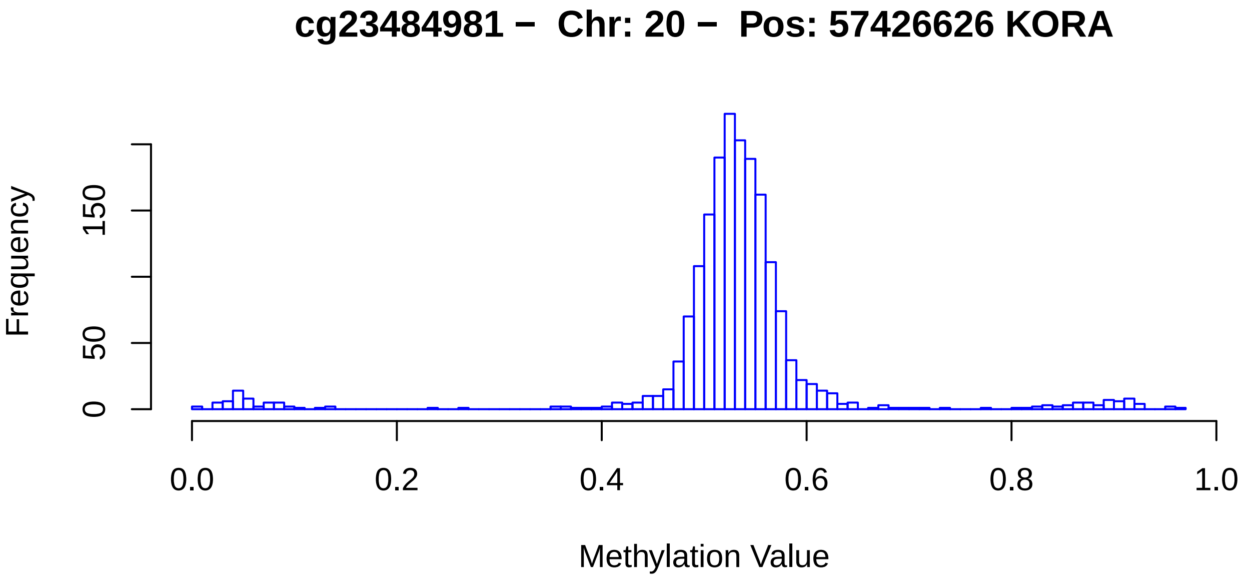
cg23484981 − Chr: 20 − Pos: 57426626 KORA
Frequency
0
50
150
0.0
0.2
0.4
0.6
0.8
1.0
Methylation Value

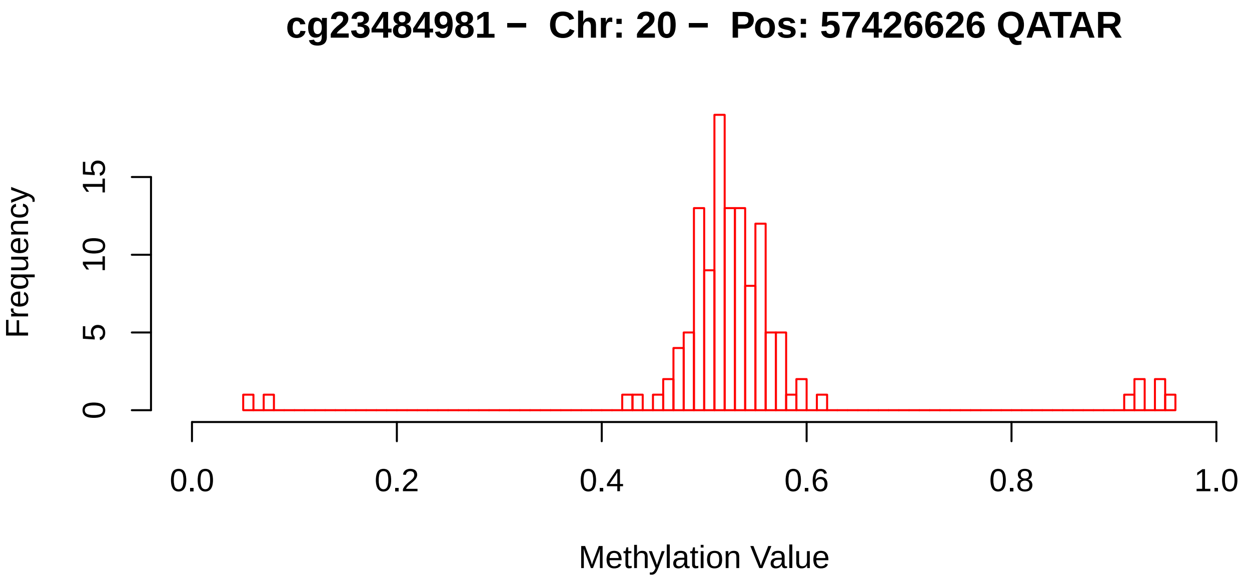
cg23484981 − Chr: 20 − Pos: 57426626 QATAR
Frequency
0
5
10
15
0.0
0.2
0.4
0.6
0.8
1.0
Methylation Value

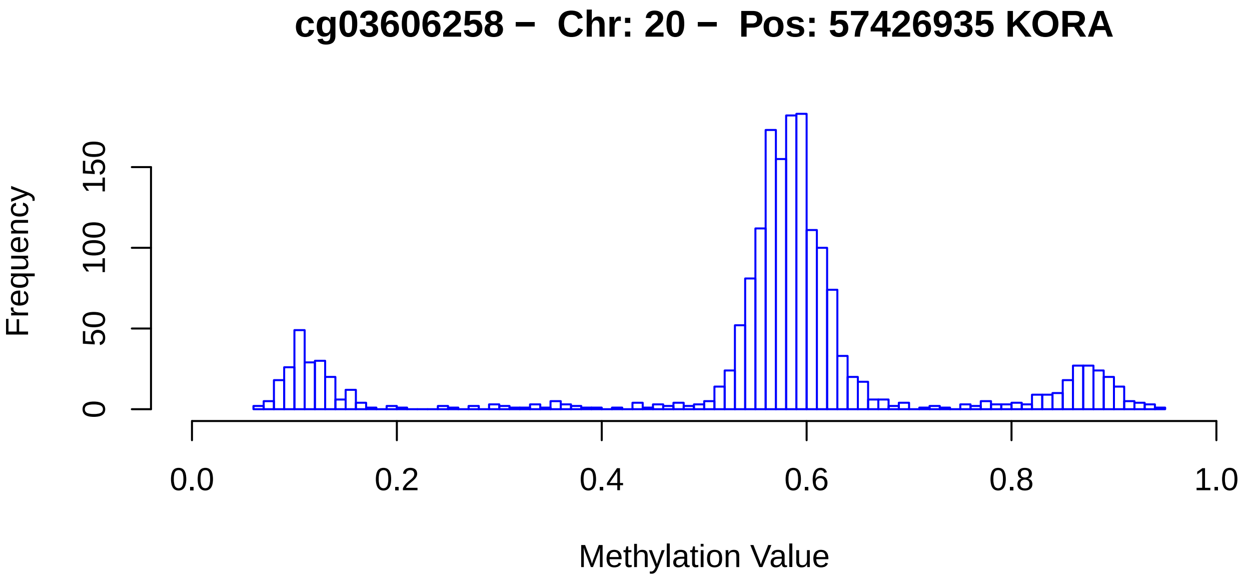
cg03606258 − Chr: 20 − Pos: 57426935 KORA
Frequency
0
50
100
150
0.0
0.2
0.4
0.6
0.8
1.0
Methylation Value

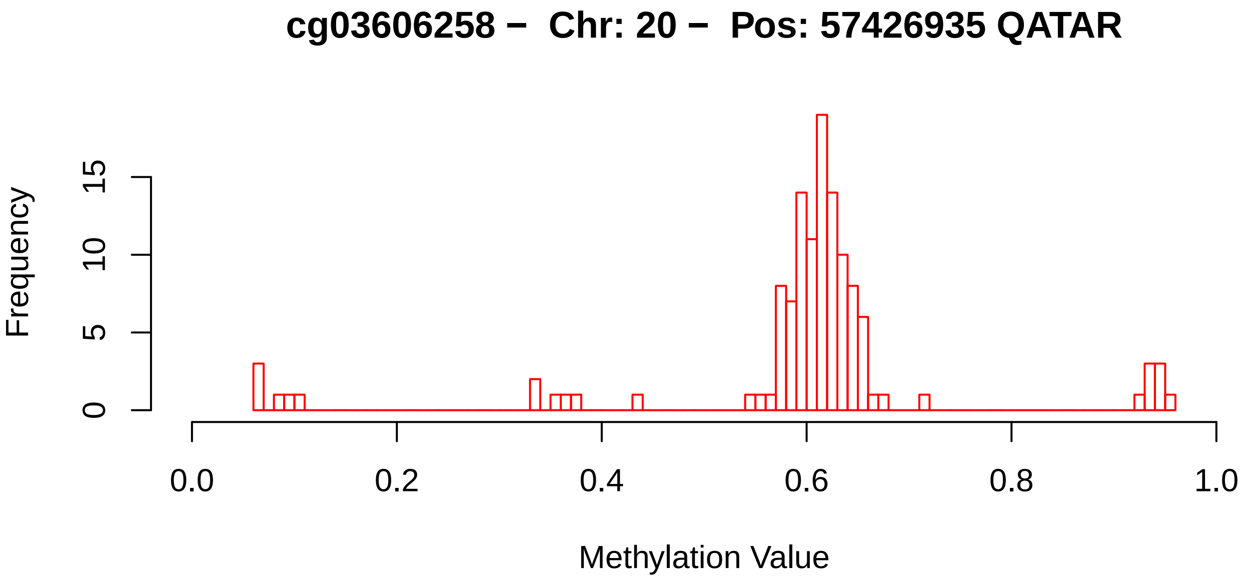
cg03606258 − Chr: 20 − Pos: 57426935 QATAR
Frequency
0
5
10
15
0.0
0.2
0.4
0.6
0.8
1.0
Methylation Value

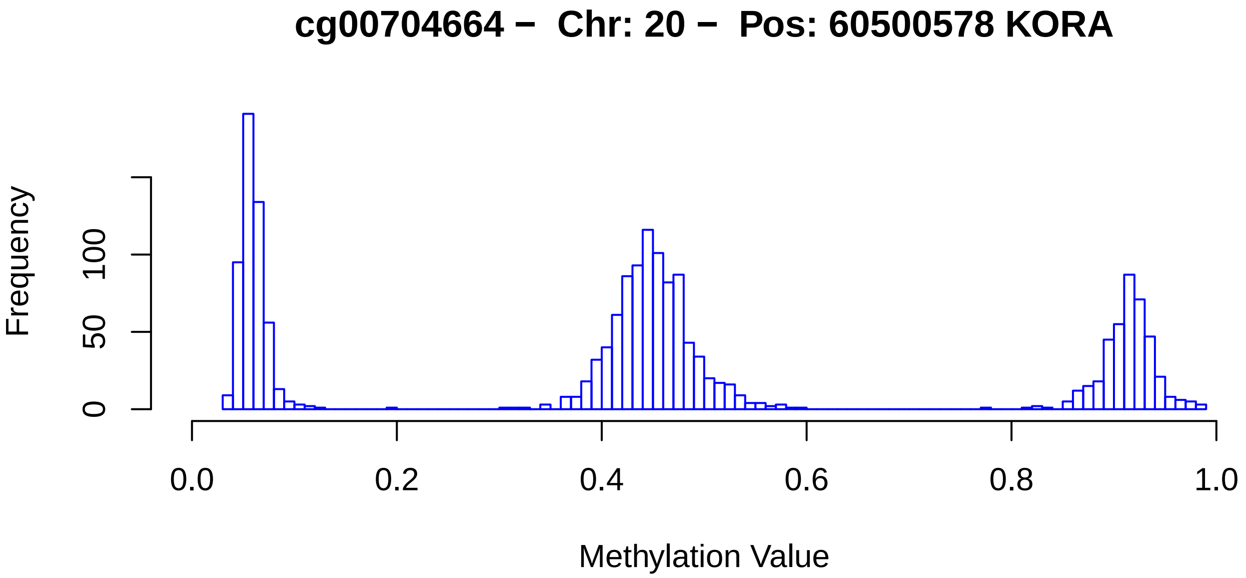
cg00704664 − Chr: 20 − Pos: 60500578 KORA
Frequency
0
50
100
0.0
0.2
0.4
0.6
0.8
1.0
Methylation Value

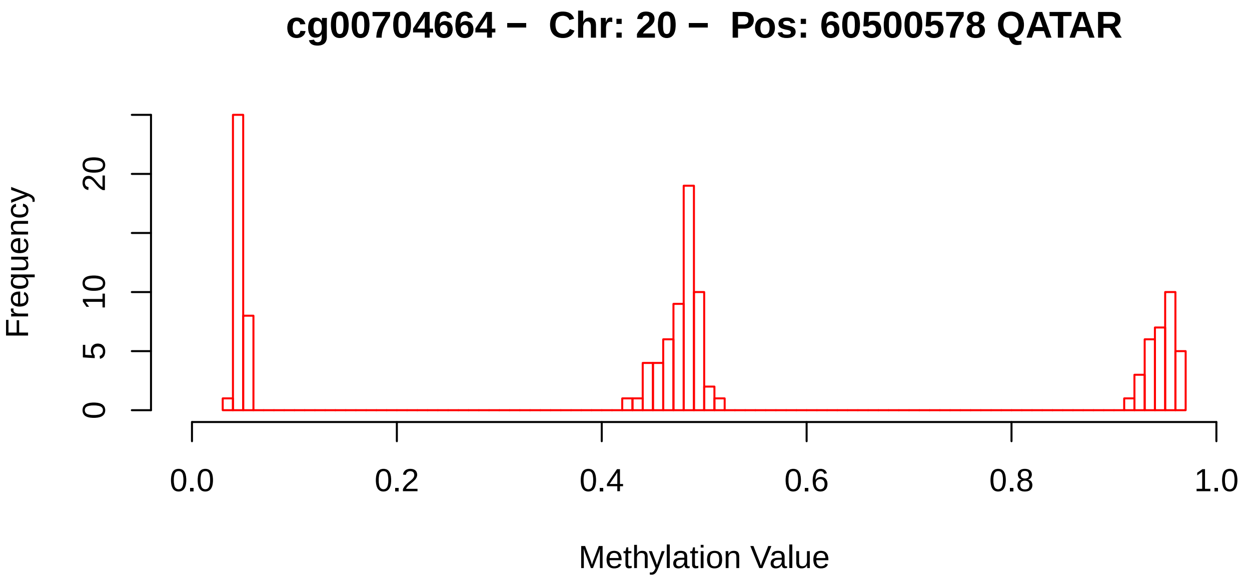
cg00704664 − Chr: 20 − Pos: 60500578 QATAR
Frequency
0
5
10
20
0.0
0.2
0.4
0.6
0.8
1.0
Methylation Value

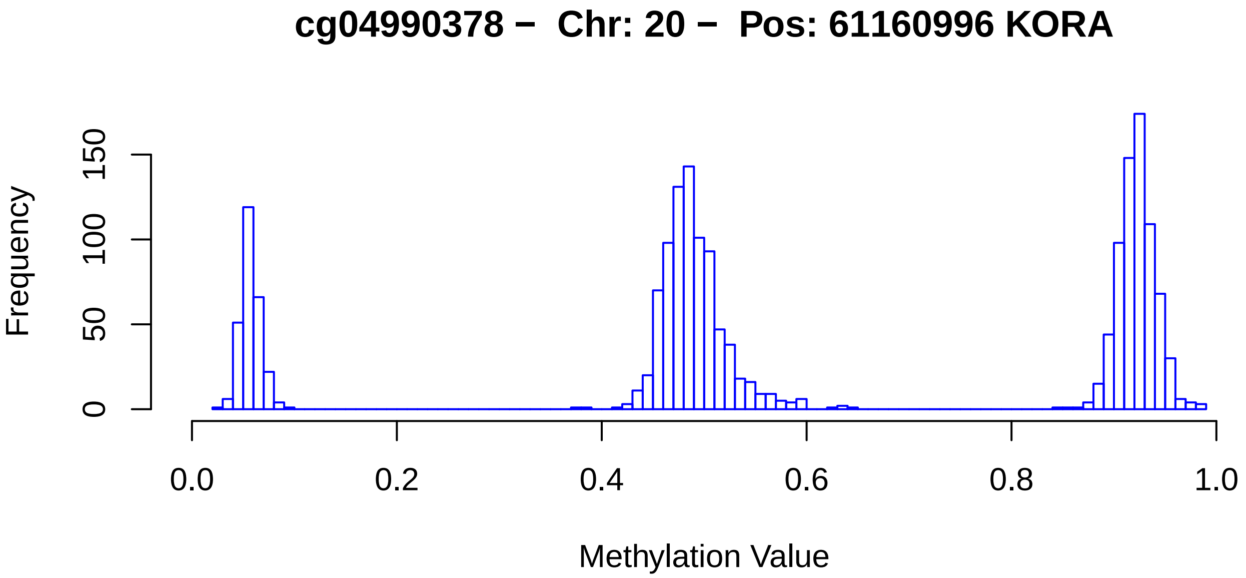

cg04990378 − Chr: 20 − Pos: 61160996 KORA
Frequency
0
50
100
150
0.0
0.2
0.4
0.6
0.8
1.0
Methylation Value

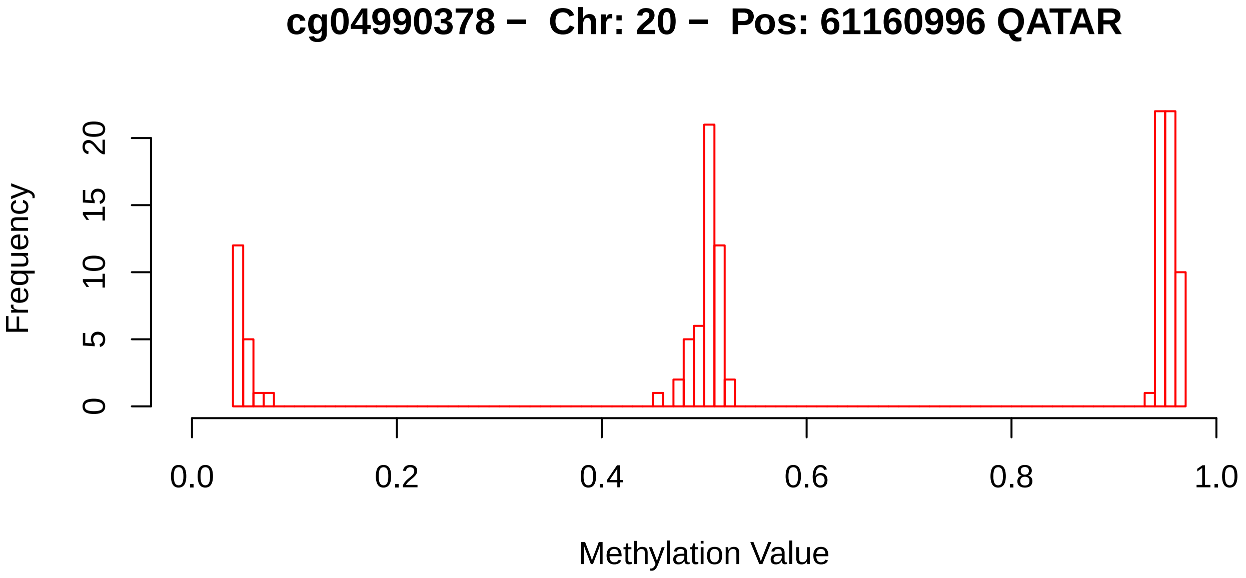

cg04990378 − Chr: 20 − Pos: 61160996 QATAR
Frequency
0
5
10
15
20
0.0
0.2
0.4
0.6
0.8
1.0
Methylation Value

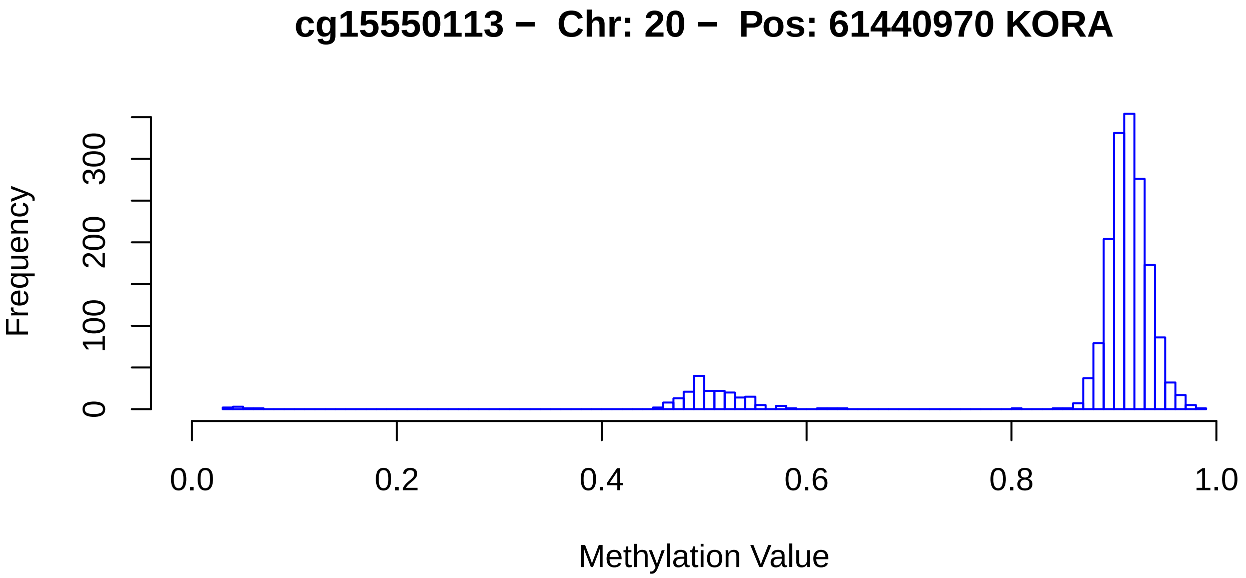
cg15550113 − Chr: 20 − Pos: 61440970 KORA
Frequency
0
100
200
300
0.0
0.2
0.4
0.6
0.8
1.0
Methylation Value

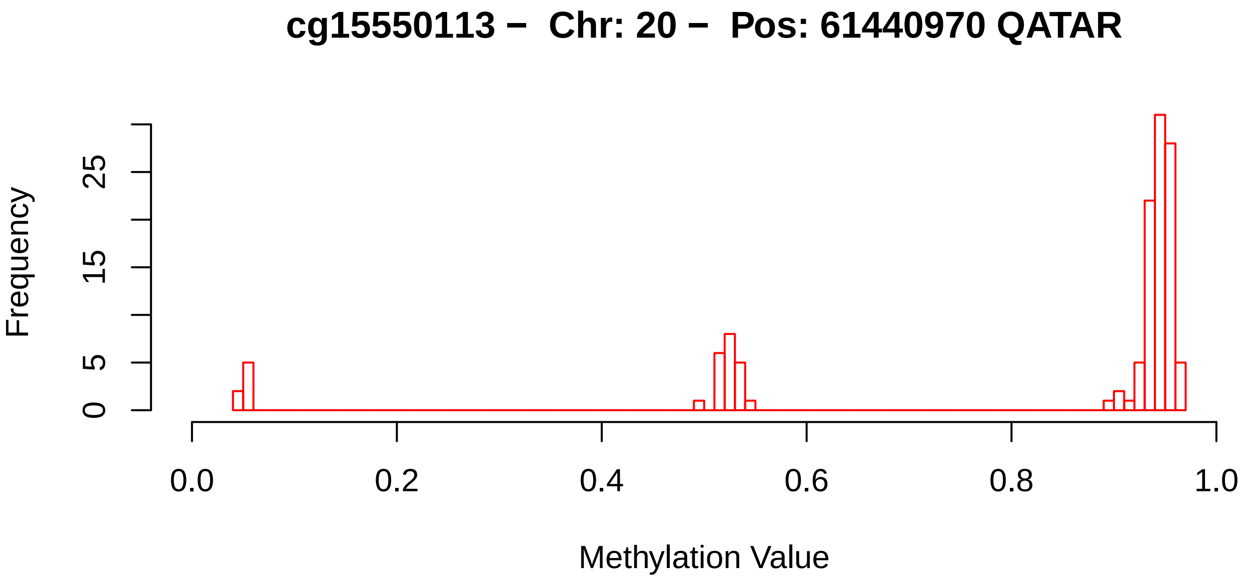
cg15550113 − Chr: 20 − Pos: 61440970 QATAR
Frequency
0
5
15
25
0.0
0.2
0.4
0.6
0.8
1.0
Methylation Value

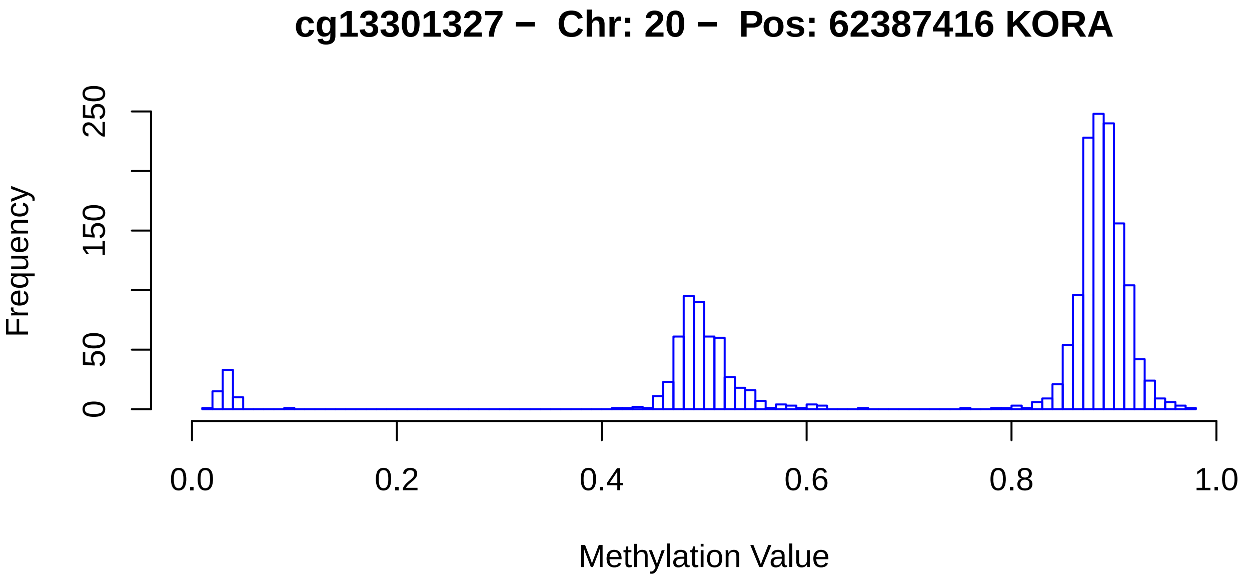
cg13301327 − Chr: 20 − Pos: 62387416 KORA
Frequency
0
50
150
250
0.0
0.2
0.4
0.6
0.8
1.0
Methylation Value

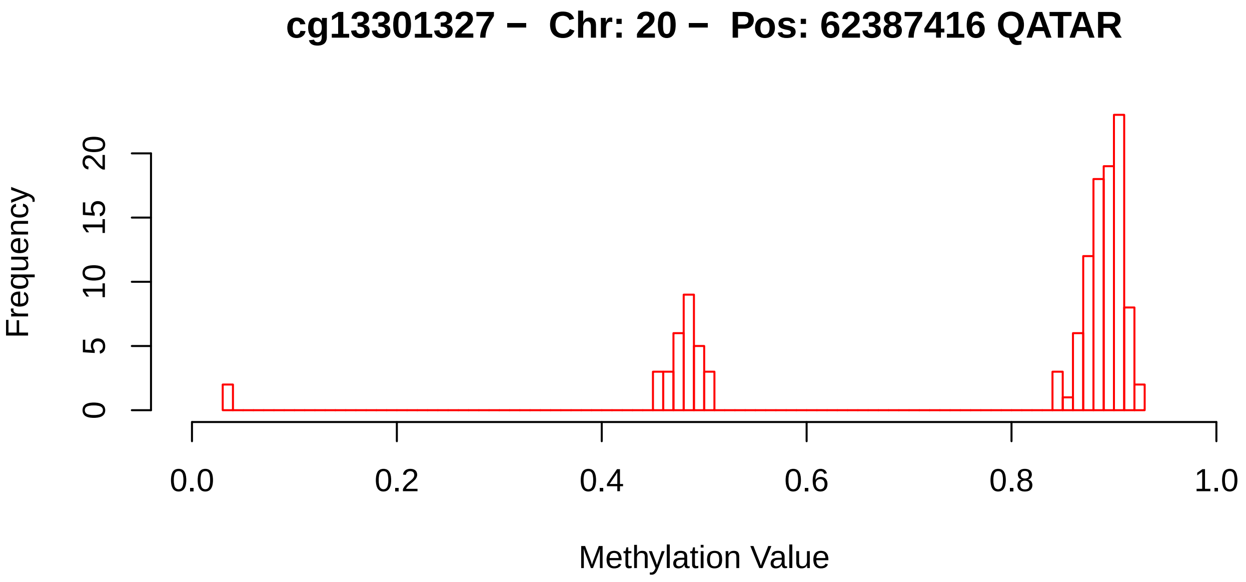
cg13301327 − Chr: 20 − Pos: 62387416 QATAR
Frequency
0
5
10
15
20
0.0
0.2
0.4
0.6
0.8
1.0
Methylation Value

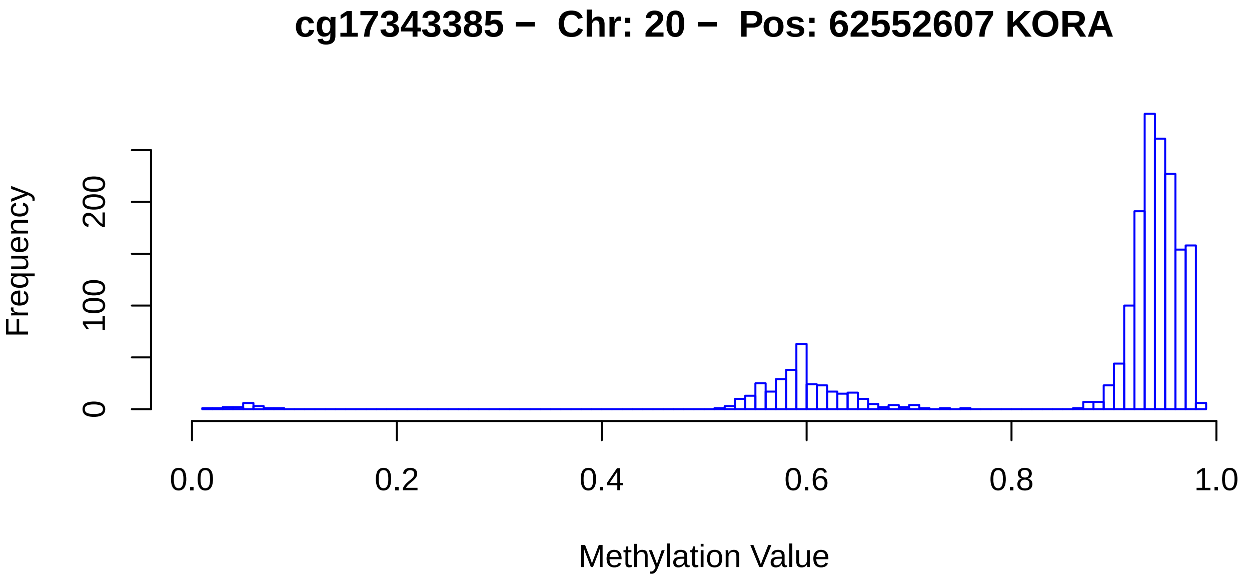
cg17343385 − Chr: 20 − Pos: 62552607 KORA
Frequency
0
100
200
0.0
0.2
0.4
0.6
0.8
1.0
Methylation Value

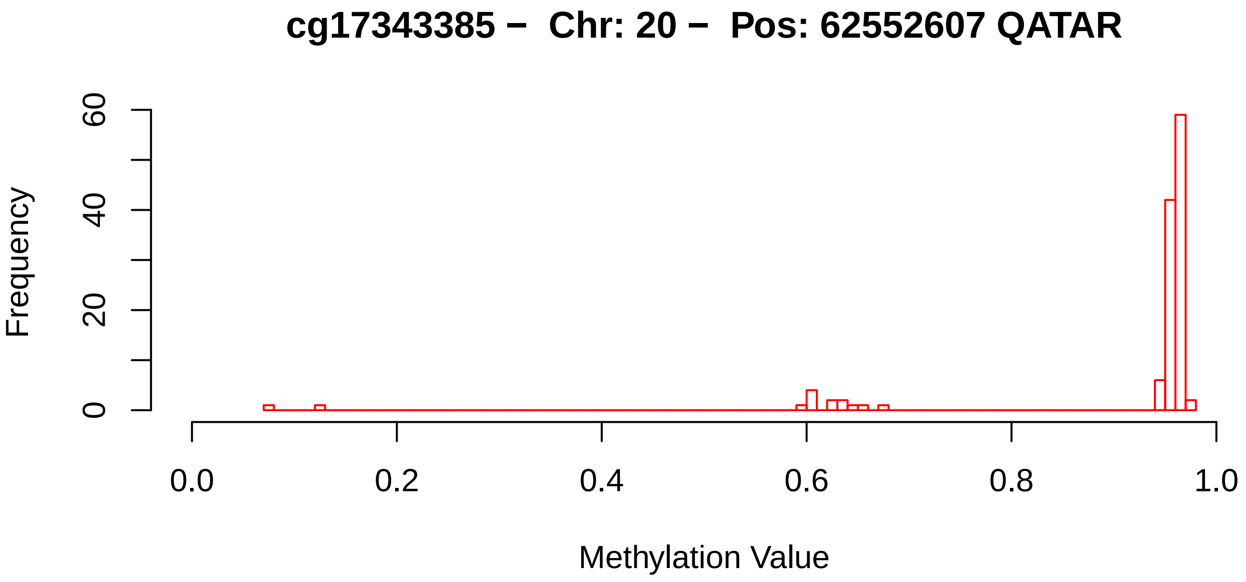
cg17343385 − Chr: 20 − Pos: 62552607 QATAR
Frequency
0
20
40
60
0.0
0.2
0.4
0.6
0.8
1.0
Methylation Value

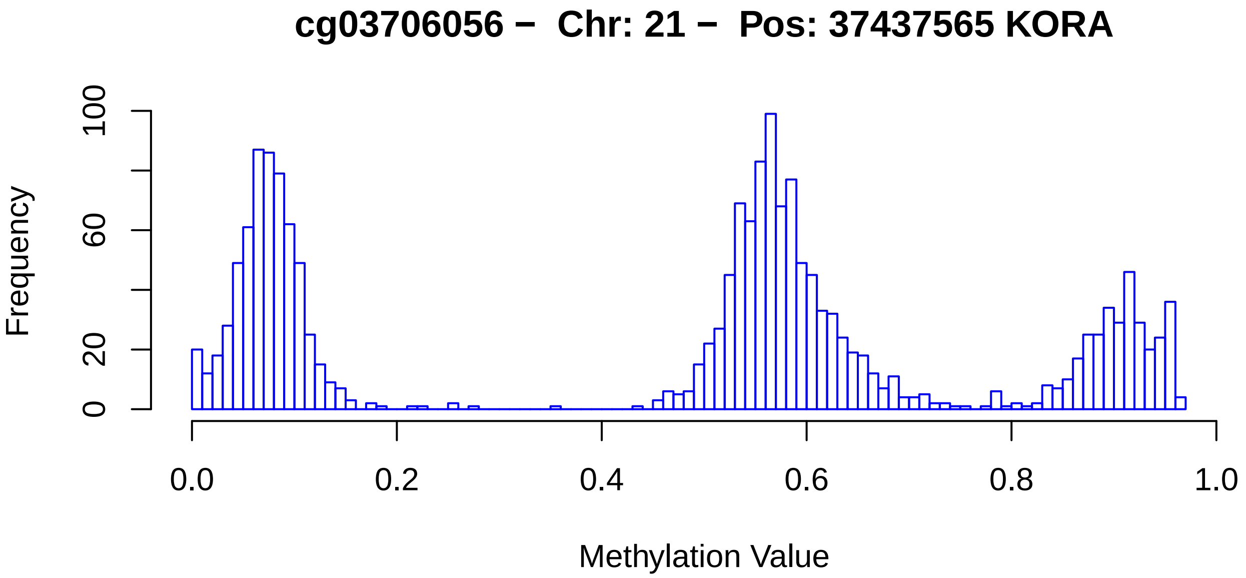
cg03706056 − Chr: 21 − Pos: 37437565 KORA
Frequency
0
20
60
100
0.0
0.2
0.4
0.6
0.8
1.0
Methylation Value

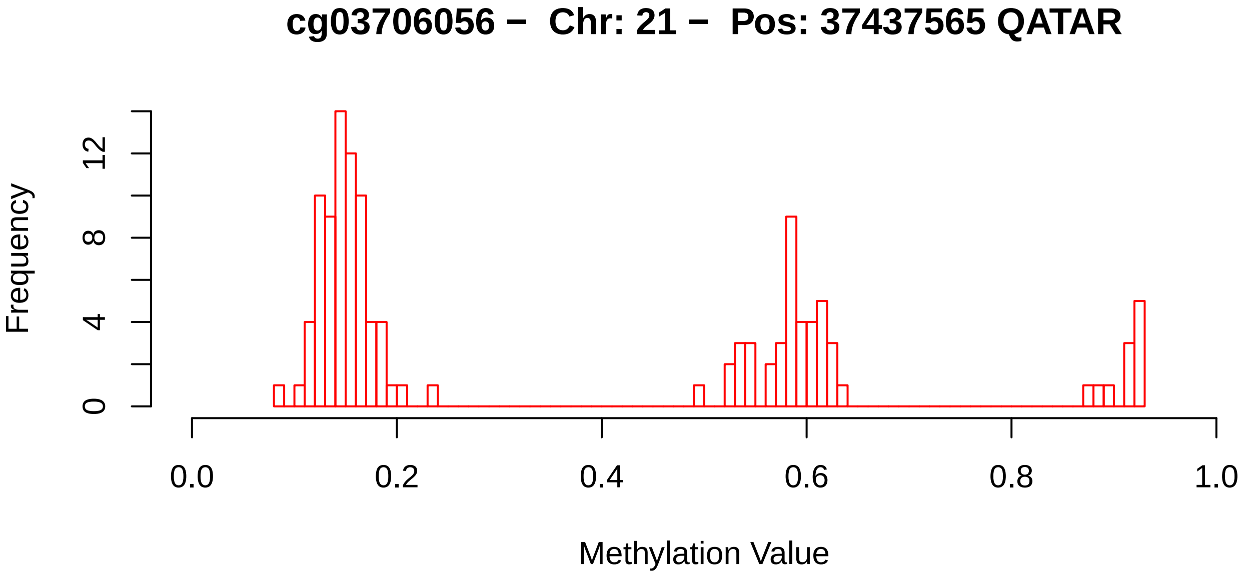
cg03706056 − Chr: 21 − Pos: 37437565 QATAR
Frequency
0
4
8
12
0.0
0.2
0.4
0.6
0.8
1.0
Methylation Value

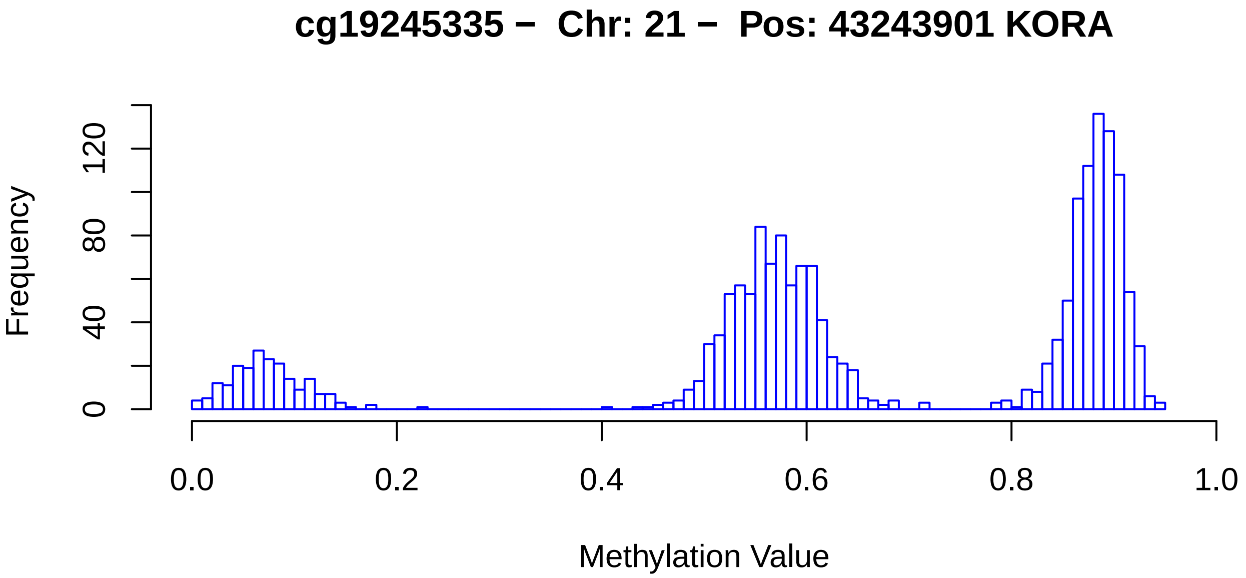
cg19245335 − Chr: 21 − Pos: 43243901 KORA
Frequency
0
40
80
120
0.0
0.2
0.4
0.6
0.8
1.0
Methylation Value

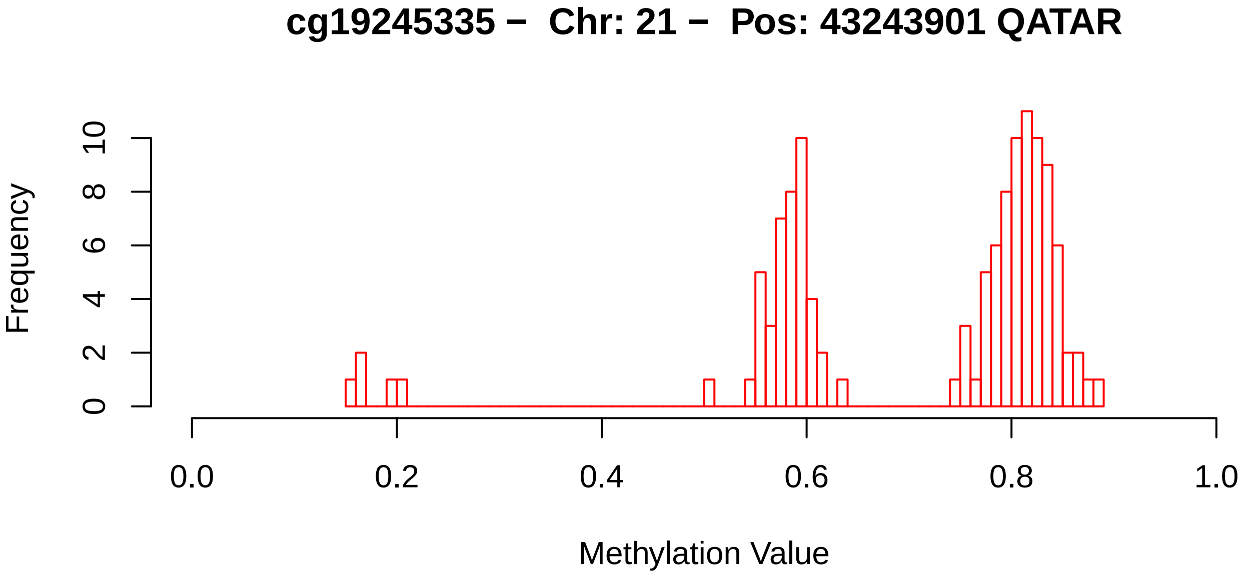
cg19245335 − Chr: 21 − Pos: 43243901 QATAR
Frequency
0
2
4
6
8
10
0.0
0.2
0.4
0.6
0.8
1.0
Methylation Value

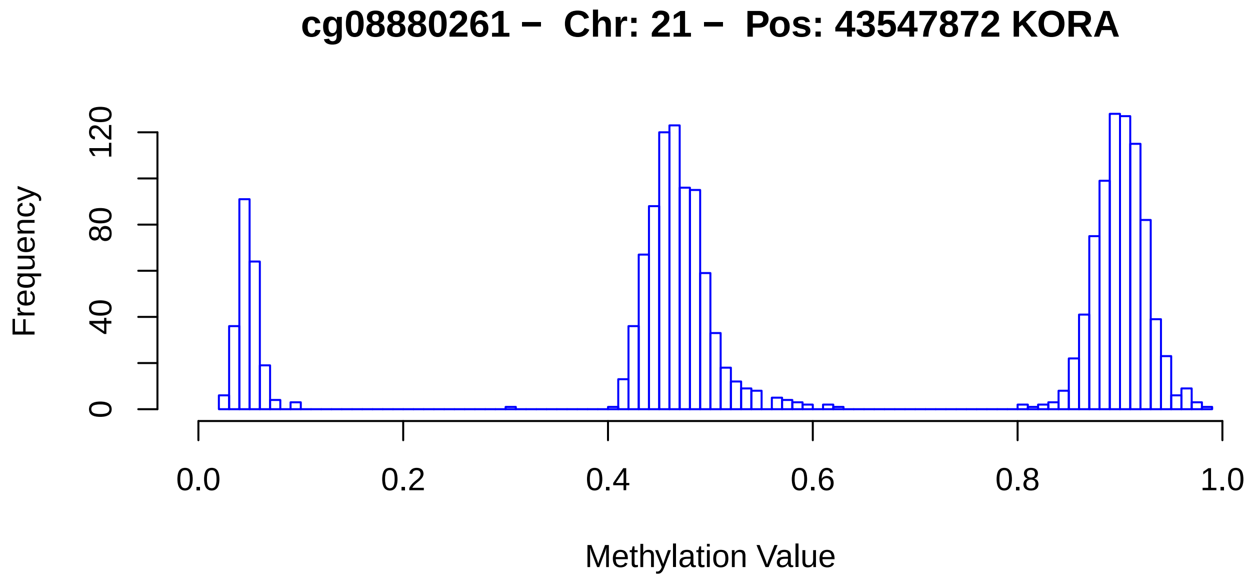
cg08880261 − Chr: 21 − Pos: 43547872 KORA
Frequency
0
40
80
120
0.0
0.2
0.4
0.6
0.8
1.0
Methylation Value

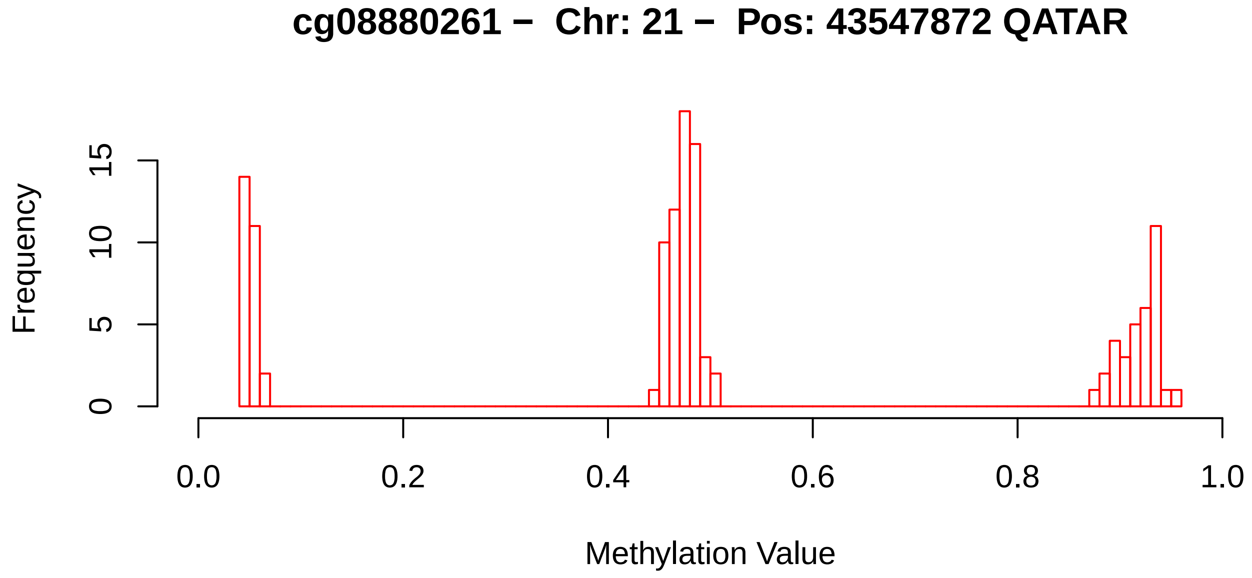
cg08880261 − Chr: 21 − Pos: 43547872 QATAR
Frequency
0
5
10
15
0.0
0.2
0.4
0.6
0.8
1.0
Methylation Value

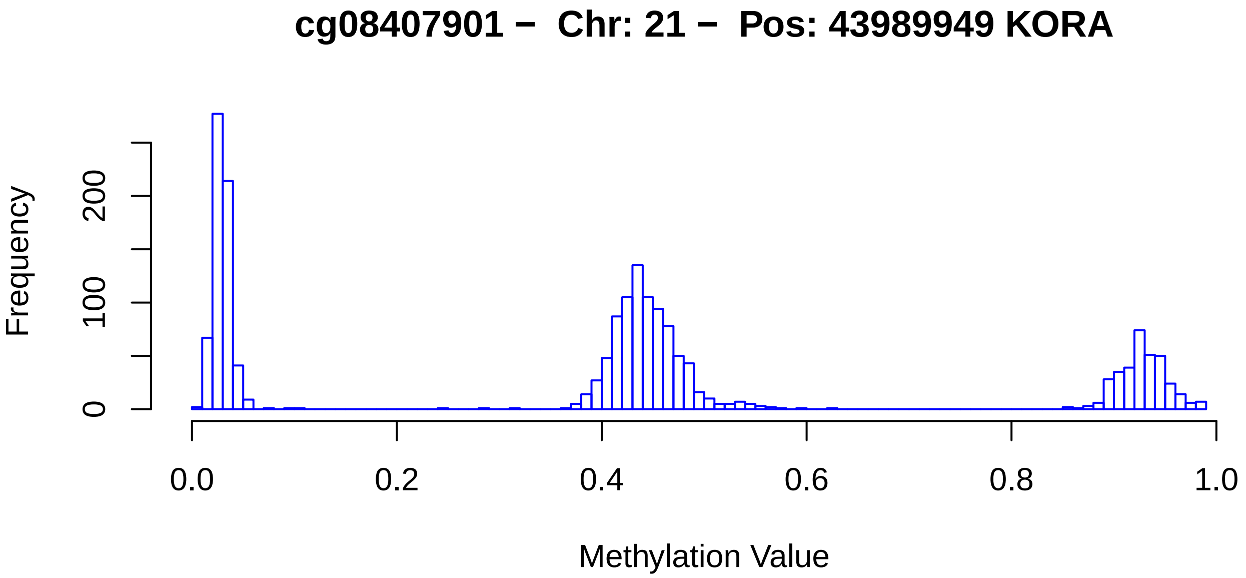
cg08407901 − Chr: 21 − Pos: 43989949 KORA
Frequency
0
100
200
0.0
0.2
0.4
0.6
0.8
1.0
Methylation Value

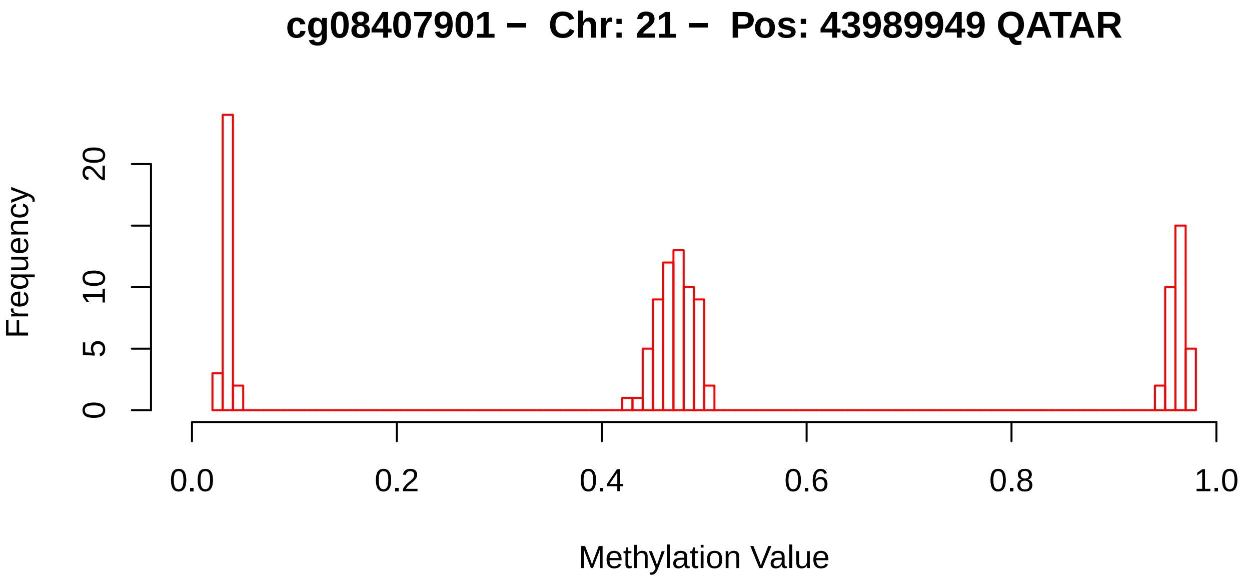
cg08407901 − Chr: 21 − Pos: 43989949 QATAR
Frequency
0
5
10
20
0.0
0.2
0.4
0.6
0.8
1.0
Methylation Value

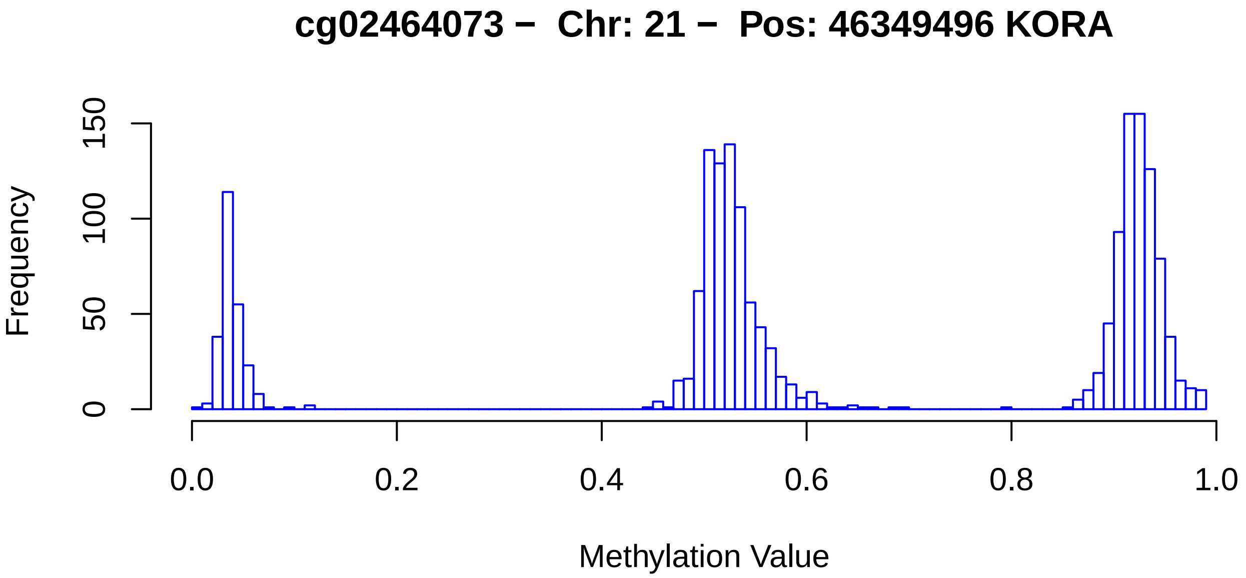
cg02464073 − Chr: 21 − Pos: 46349496 KORA
Frequency
0
50
100
150
0.0
0.2
0.4
0.6
0.8
1.0
Methylation Value

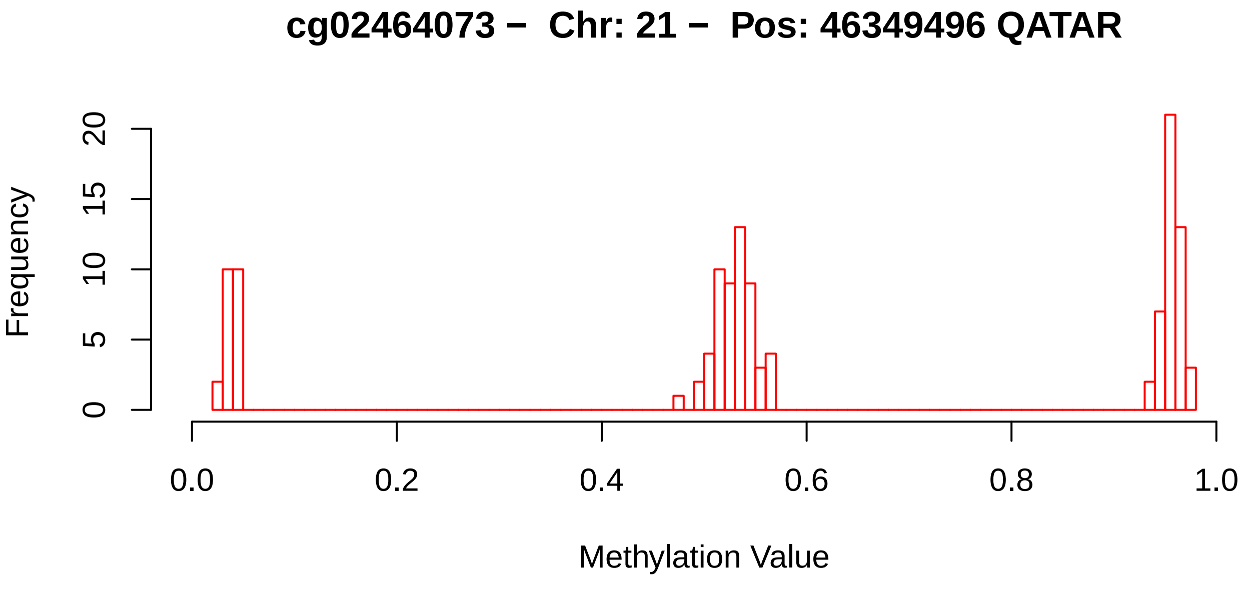
cg02464073 − Chr: 21 − Pos: 46349496 QATAR
Frequency
0
5
10
15
20
0.0
0.2
0.4
0.6
0.8
1.0
Methylation Value

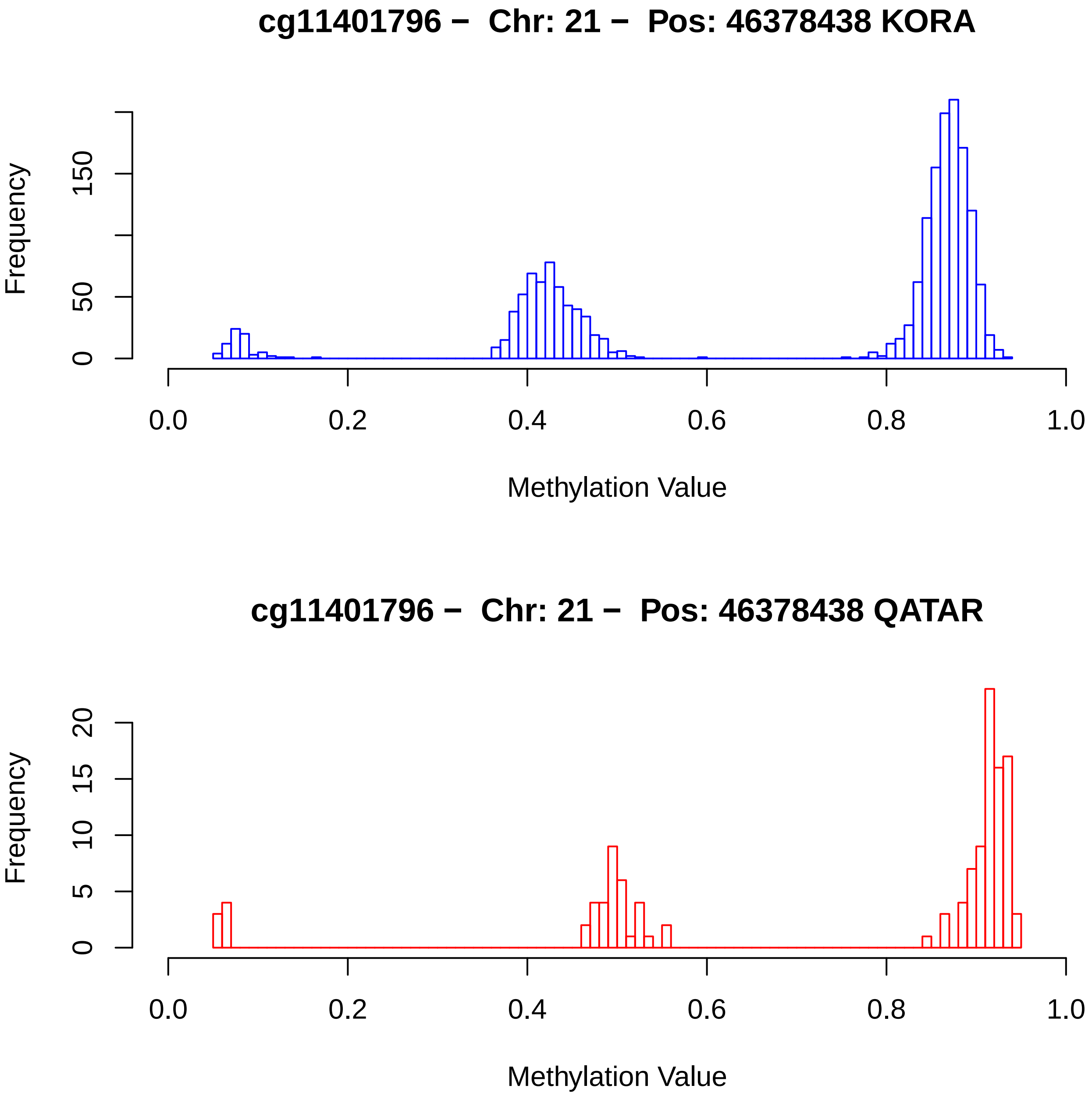
cg11401796 − Chr: 21 − Pos: 46378438 KORA
Frequency
0
50
150
0.0
0.2
0.4
0.6
0.8
1.0
Methylation Value
cg11401796 − Chr: 21 − Pos: 46378438 QATAR
Frequency
0
5
10
15
20
0.0
0.2
0.4
0.6
0.8
1.0
Methylation Value

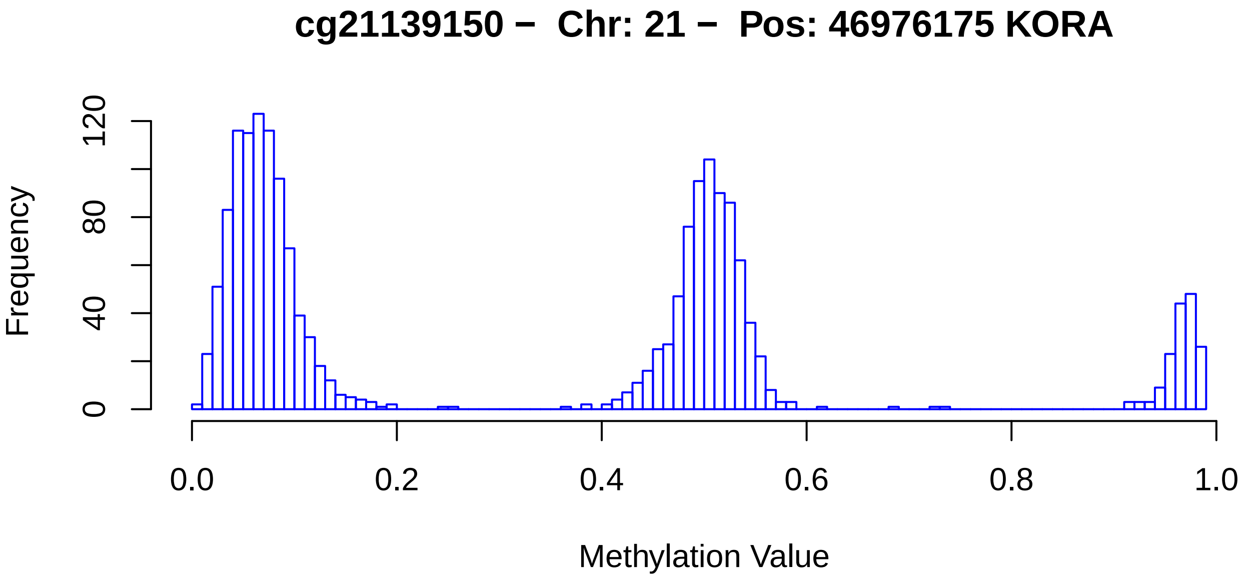

cg21139150 − Chr: 21 − Pos: 46976175 KORA
Frequency
0
40
80
120
0.0
0.2
0.4
0.6
0.8
1.0
Methylation Value

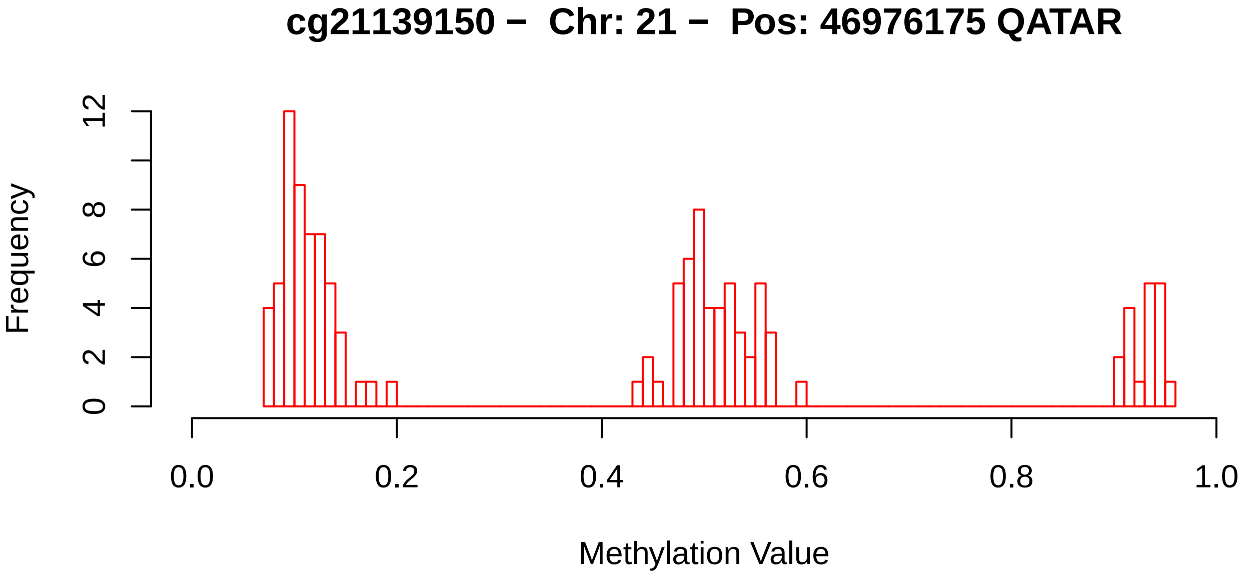

cg21139150 − Chr: 21 − Pos: 46976175 QATAR
Frequency
0
2
4
6
8
12
0.0
0.2
0.4
0.6
0.8
1.0
Methylation Value

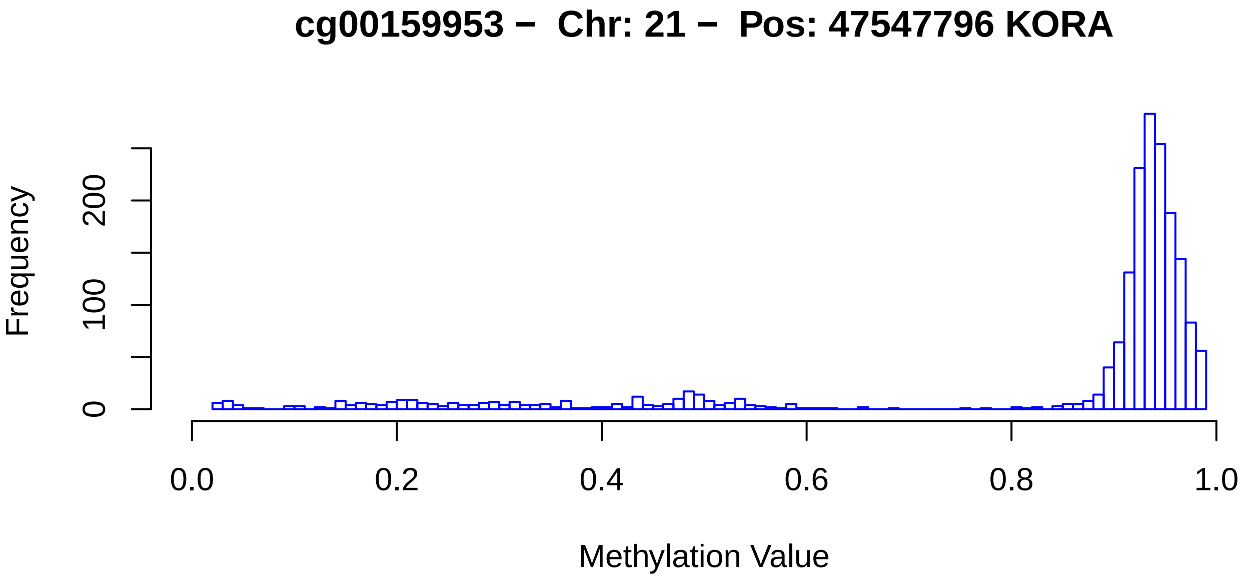
cg00159953 − Chr: 21 − Pos: 47547796 KORA
Frequency
0
100
200
0.0
0.2
0.4
0.6
0.8
1.0
Methylation Value

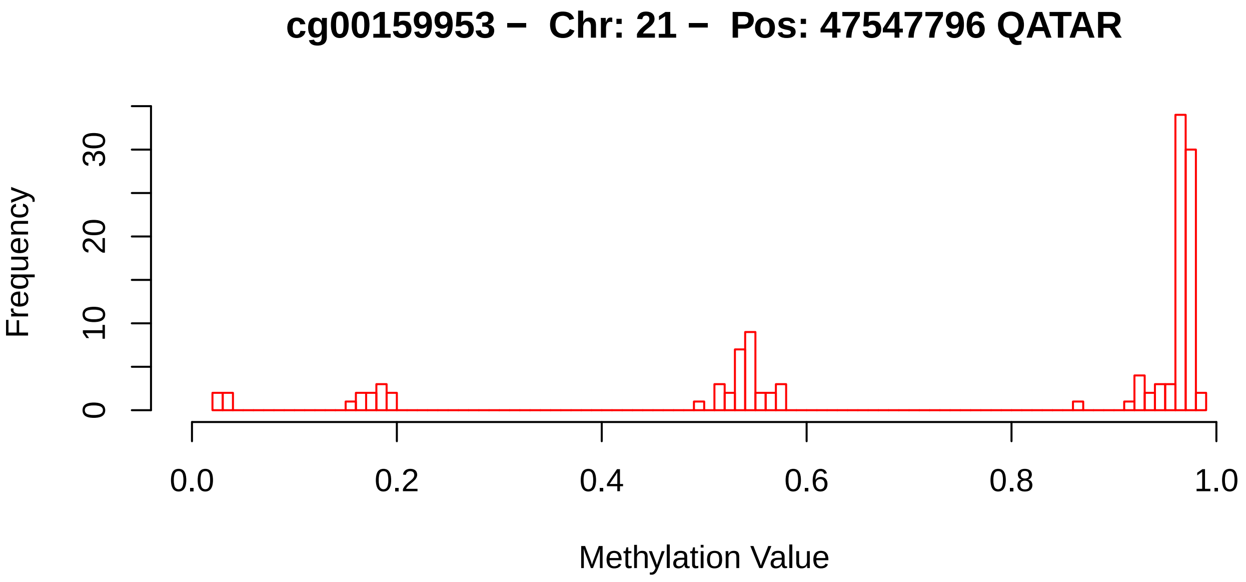
cg00159953 − Chr: 21 − Pos: 47547796 QATAR
Frequency
0
10
20
30
0.0
0.2
0.4
0.6
0.8
1.0
Methylation Value

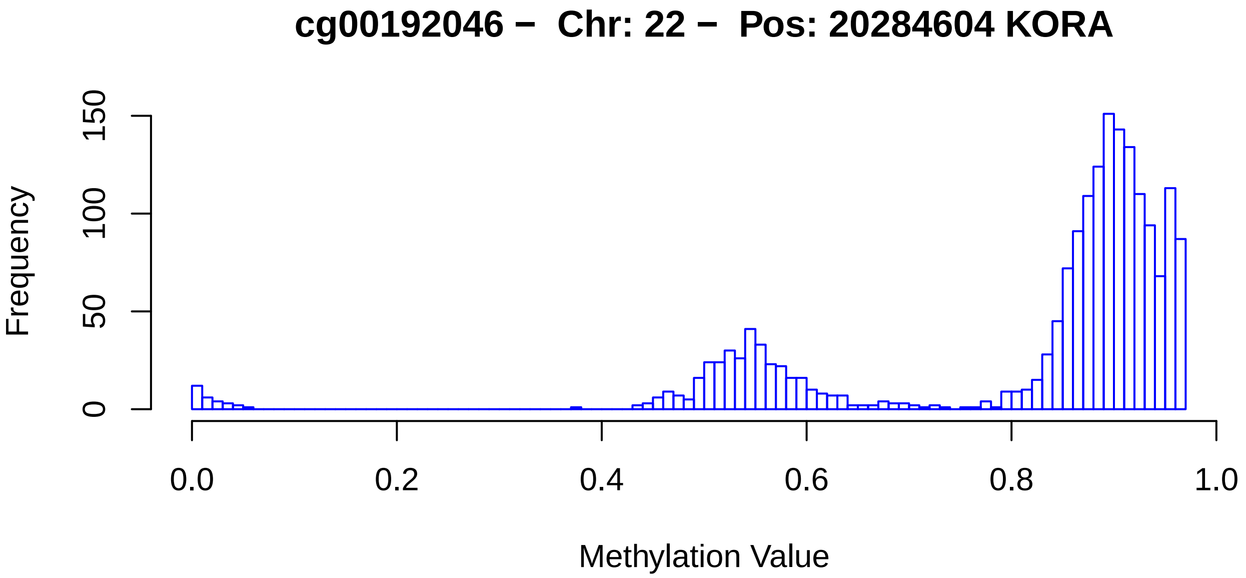
cg00192046 − Chr: 22 − Pos: 20284604 KORA
Frequency
0
50
100
150
0.0
0.2
0.4
0.6
0.8
1.0
Methylation Value

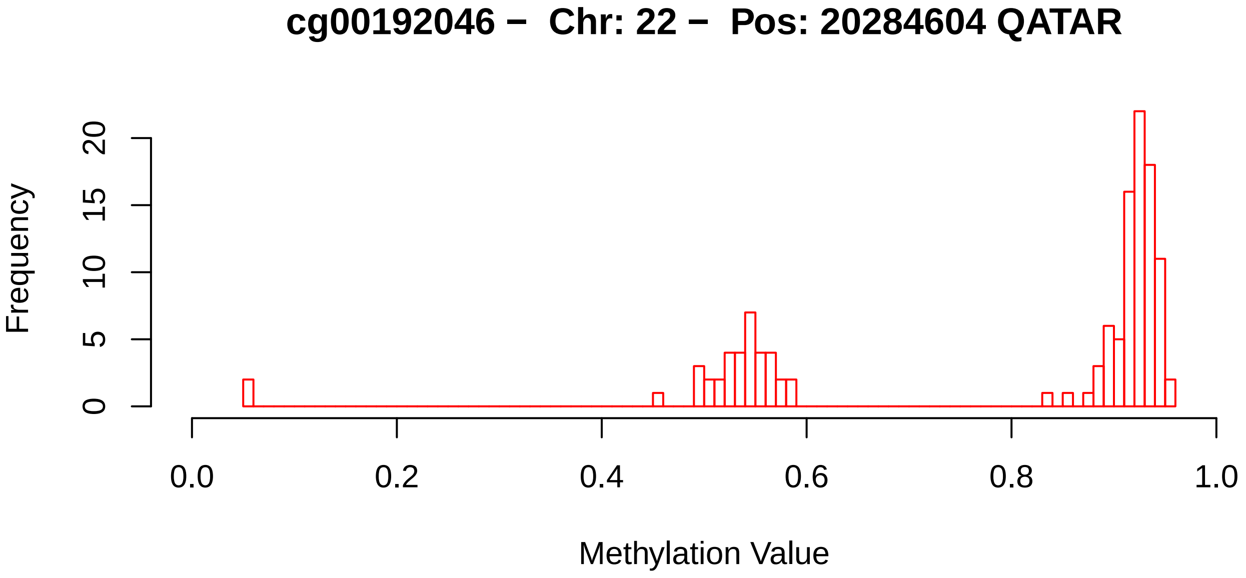
cg00192046 − Chr: 22 − Pos: 20284604 QATAR
Frequency
0
5
10
15
20
0.0
0.2
0.4
0.6
0.8
1.0
Methylation Value

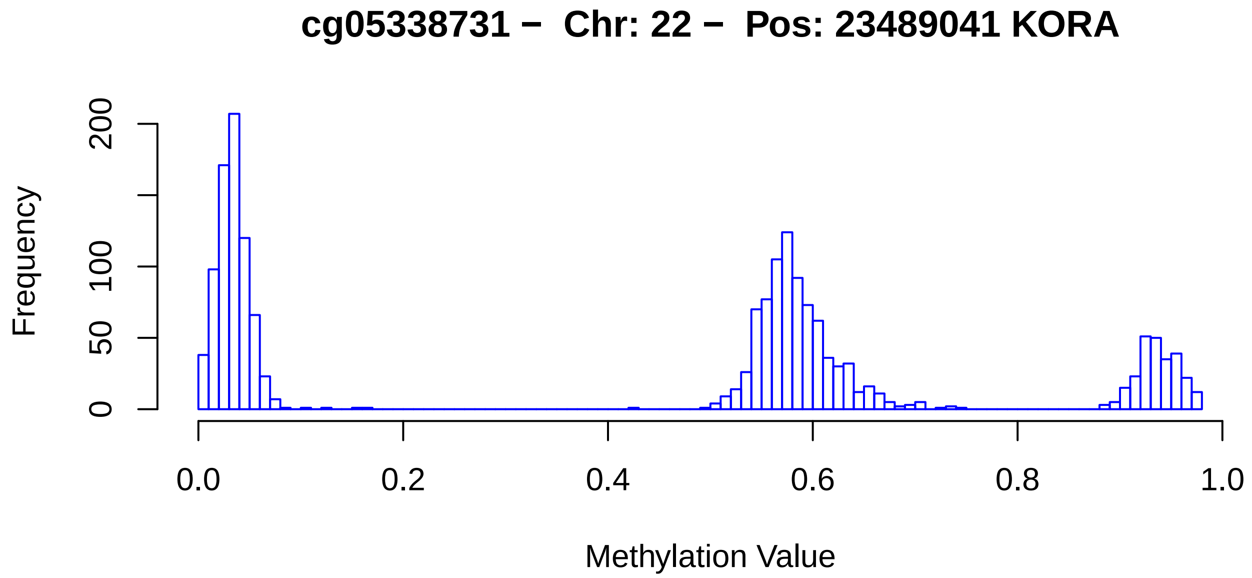
cg05338731 − Chr: 22 − Pos: 23489041 KORA
Frequency
0
50
100
200
0.0
0.2
0.4
0.6
0.8
1.0
Methylation Value

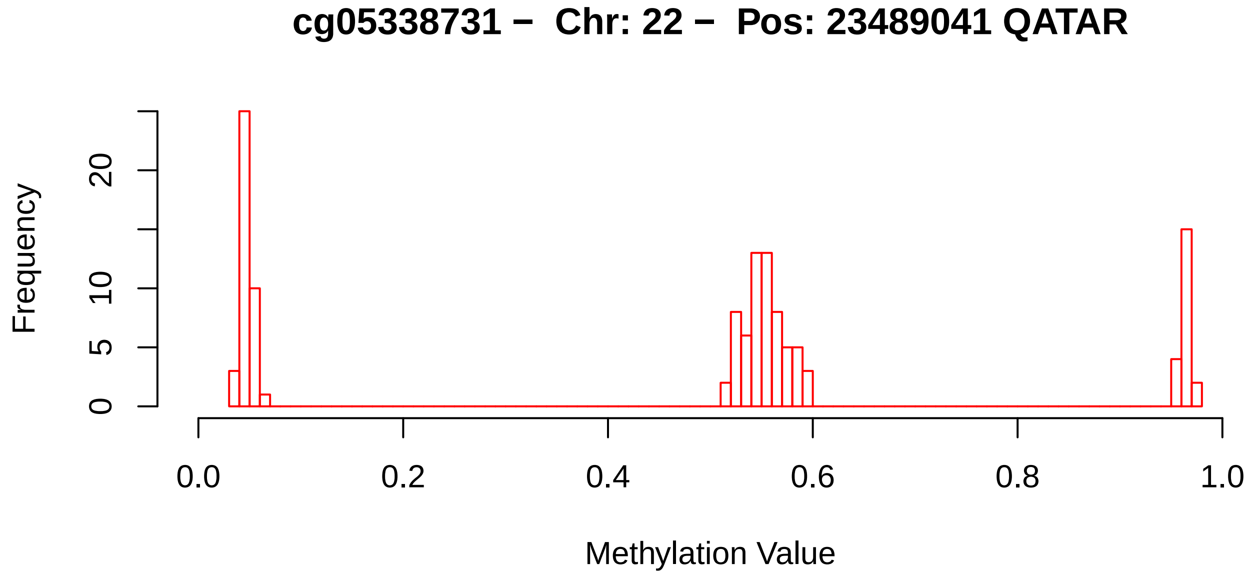
cg05338731 − Chr: 22 − Pos: 23489041 QATAR
Frequency
0
5
10
20
0.0
0.2
0.4
0.6
0.8
1.0
Methylation Value

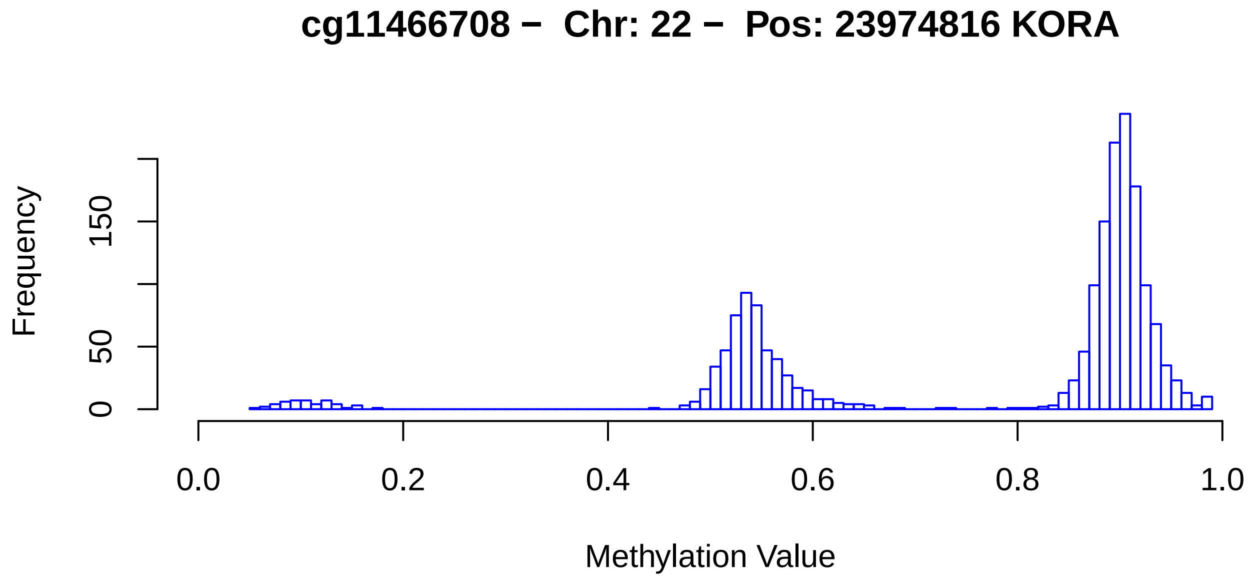
cg11466708 − Chr: 22 − Pos: 23974816 KORA
Frequency
0
50
150
0.0
0.2
0.4
0.6
0.8
1.0
Methylation Value

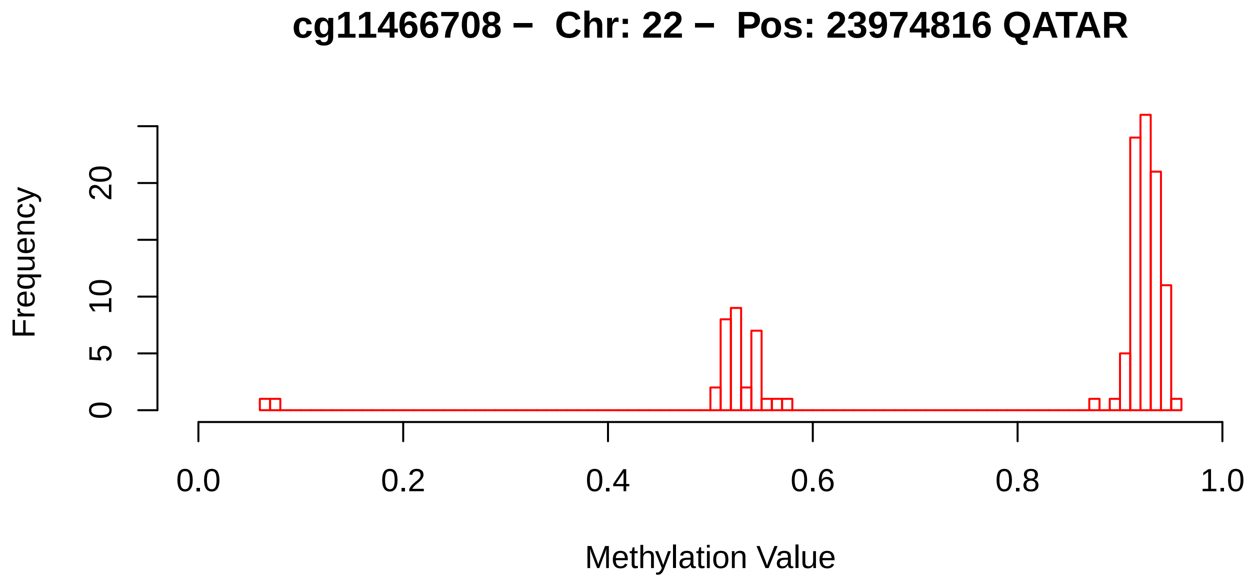
cg11466708 − Chr: 22 − Pos: 23974816 QATAR
Frequency
0
5
10
20
0.0
0.2
0.4
0.6
0.8
1.0
Methylation Value

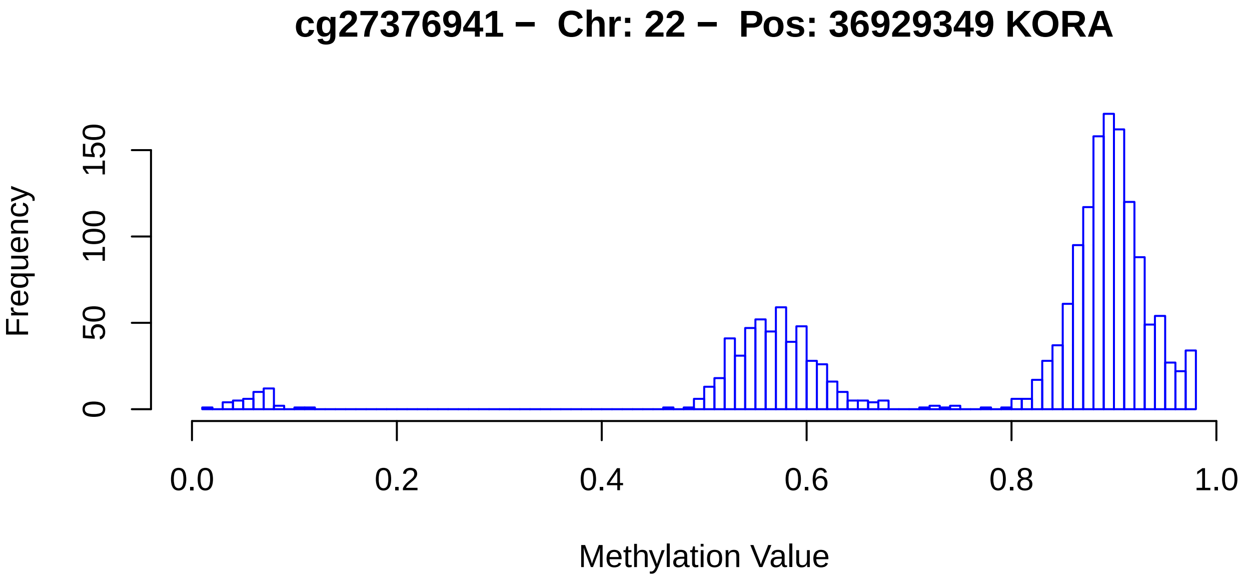
cg27376941 − Chr: 22 − Pos: 36929349 KORA
Frequency
0
50
100
150
0.0
0.2
0.4
0.6
0.8
1.0
Methylation Value

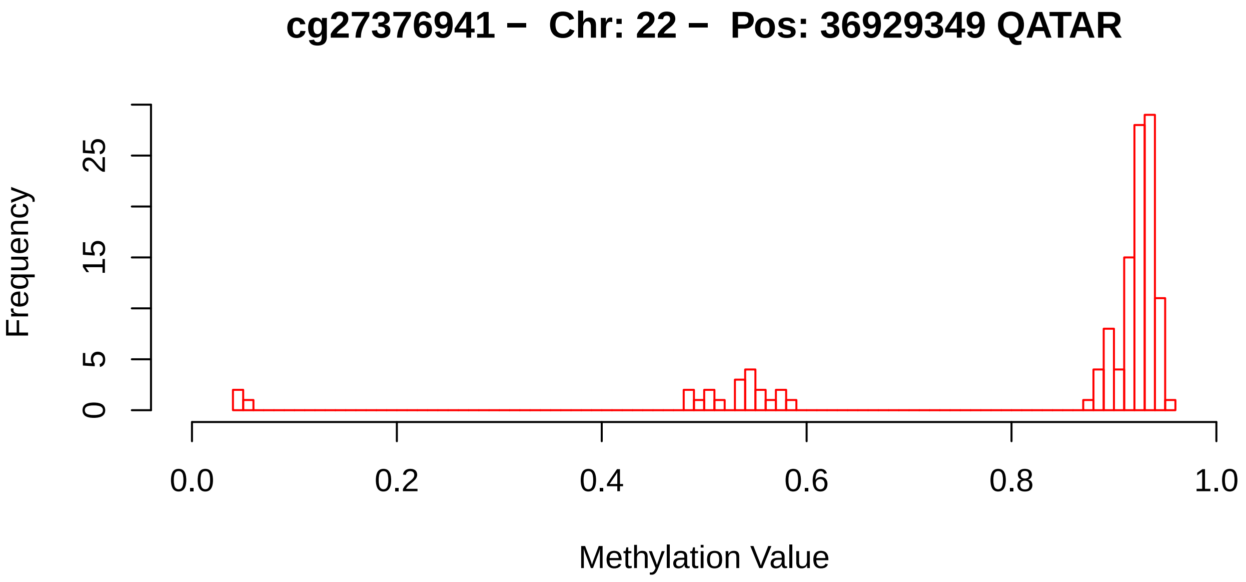
cg27376941 − Chr: 22 − Pos: 36929349 QATAR
Frequency
0
5
15
25
0.0
0.2
0.4
0.6
0.8
1.0
Methylation Value

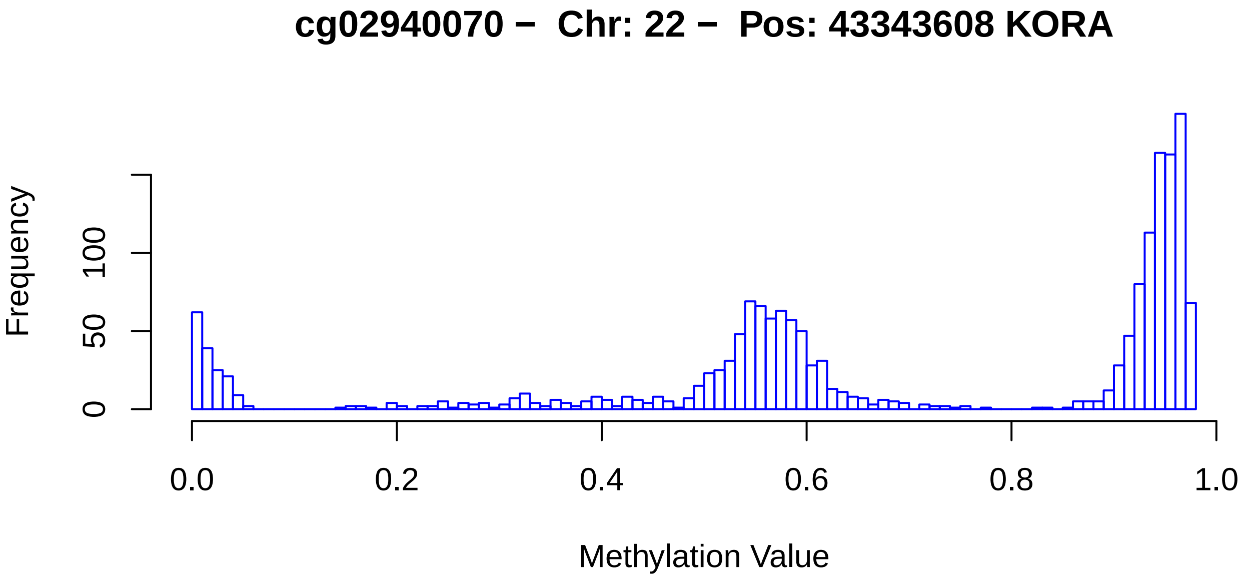
cg02940070 − Chr: 22 − Pos: 43343608 KORA
Frequency
0
50
100
0.0
0.2
0.4
0.6
0.8
1.0
Methylation Value

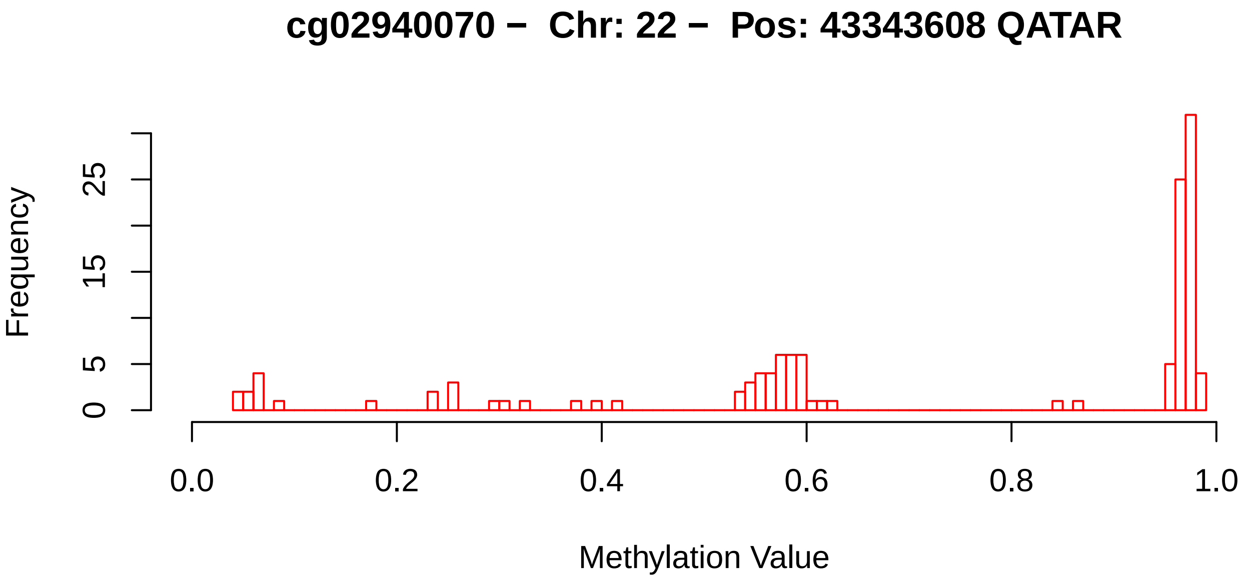
cg02940070 − Chr: 22 − Pos: 43343608 QATAR
Frequency
0
5
15
25
0.0
0.2
0.4
0.6
0.8
1.0
Methylation Value

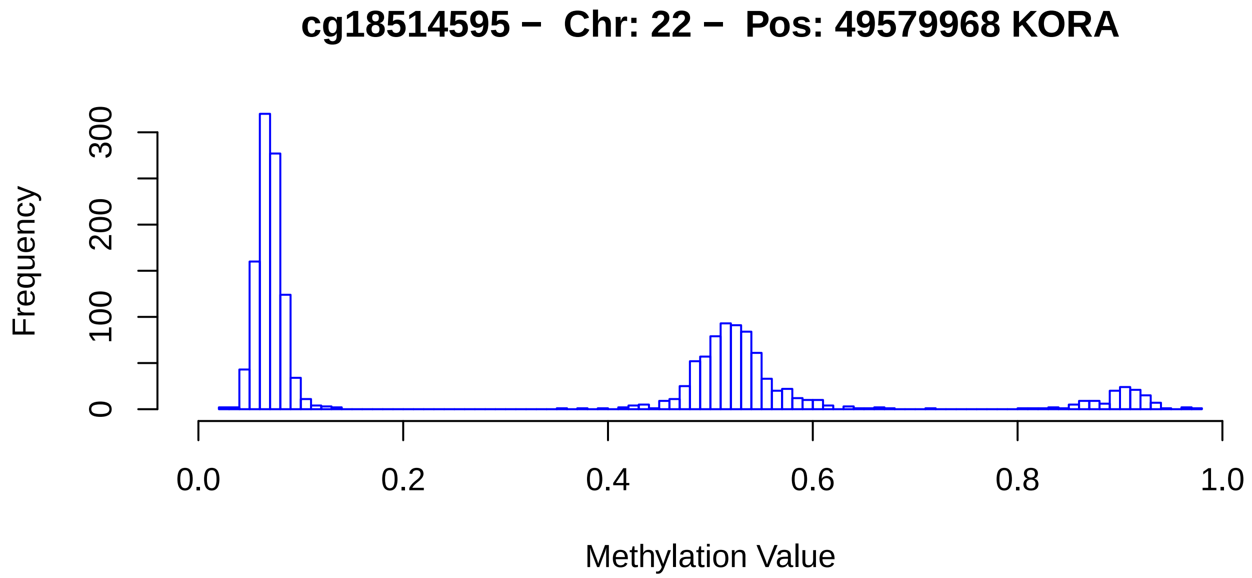
cg18514595 − Chr: 22 − Pos: 49579968 KORA
Frequency
0
100
200
300
0.0
0.2
0.4
0.6
0.8
1.0
Methylation Value

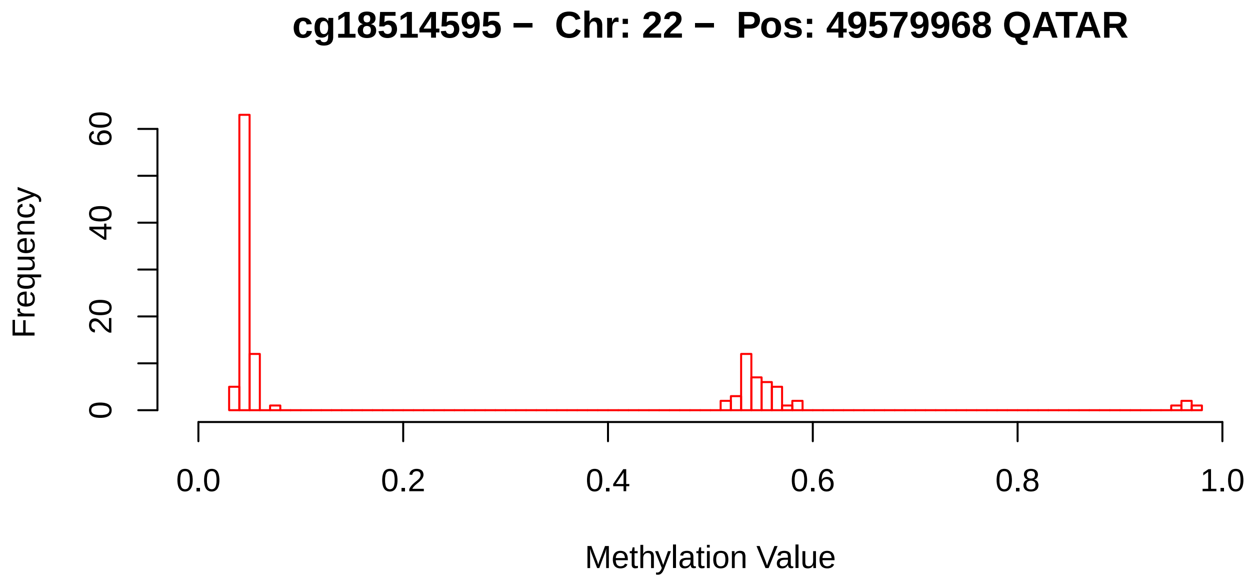
cg18514595 − Chr: 22 − Pos: 49579968 QATAR
Frequency
0
20
40
60
0.0
0.2
0.4
0.6
0.8
1.0
Methylation Value

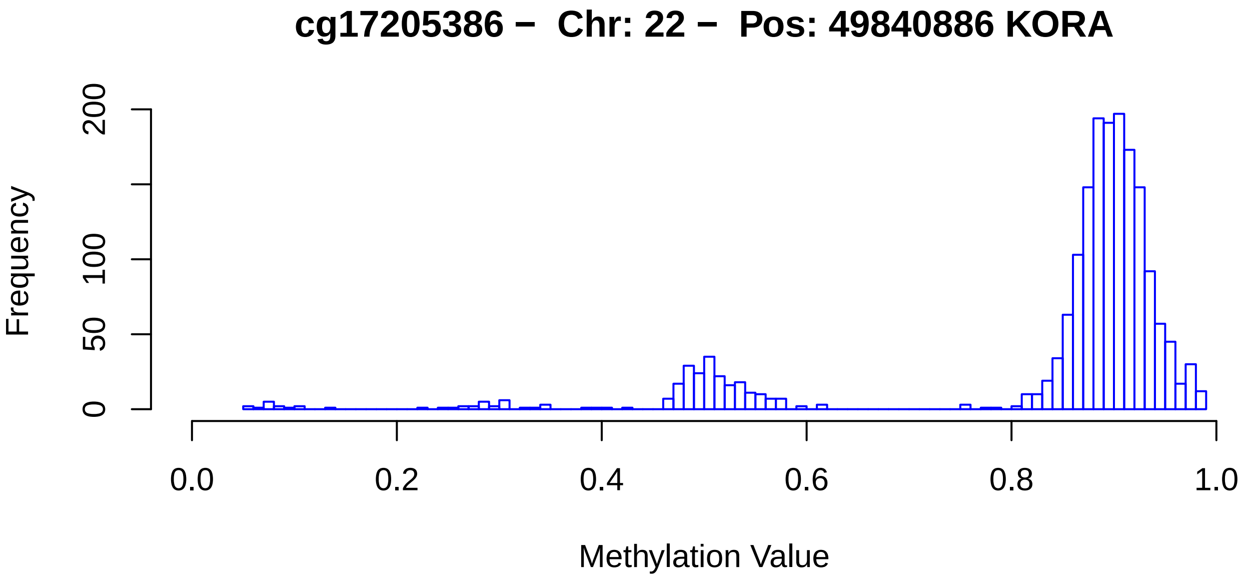
cg17205386 − Chr: 22 − Pos: 49840886 KORA
Frequency
0
50
100
200
0.0
0.2
0.4
0.6
0.8
1.0
Methylation Value

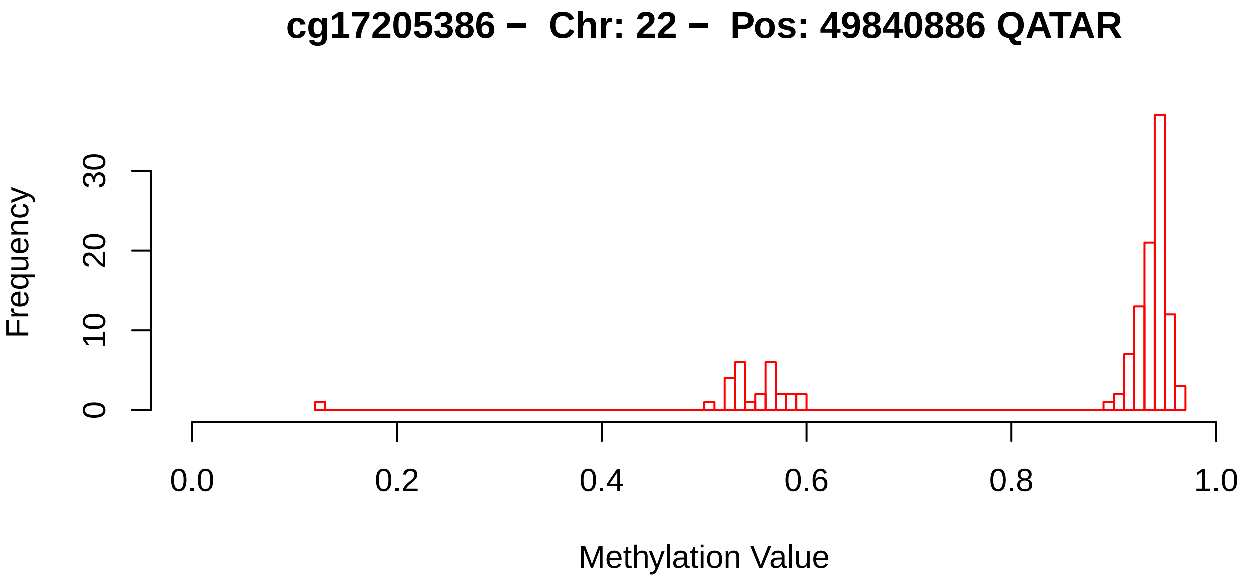
cg17205386 − Chr: 22 − Pos: 49840886 QATAR
Frequency
0
10
20
30
0.0
0.2
0.4
0.6
0.8
1.0
Methylation Value
