# Supplementary material for: Mendelian inheritance of trimodal CpG methylation sites suggests distal cis-acting genetic effects
Source: Clin Epigenetics. 2016 Nov 22;8:124. doi: 10.1186/s13148-016-0295-1 (PMC5120560; doi:10.1186/s13148-016-0295-1)
Supplement: Additional file 3: File S2. — Replication of 28 trimodal CpG sites that follow Mendelian inheritance in both the Qatari trios and the KORA dataset but having no Bonferroni significant underlying SNP association (based on KORA data). (PDF 200 kb) [file 13148_2016_295_MOESM3_ESM.pdf]

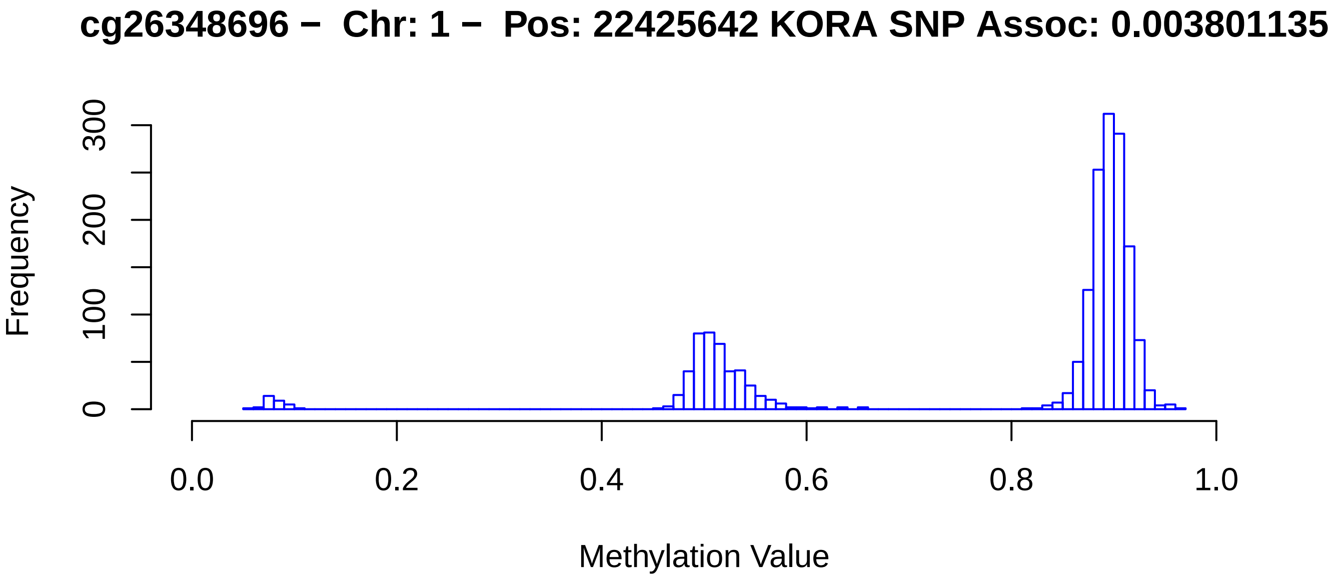
cg26348696 − Chr: 1 − Pos: 22425642 KORA SNP Assoc: 0.003801135
Frequency
0
100
200
300
0.0
0.2
0.4
0.6
0.8
1.0
Methylation Value

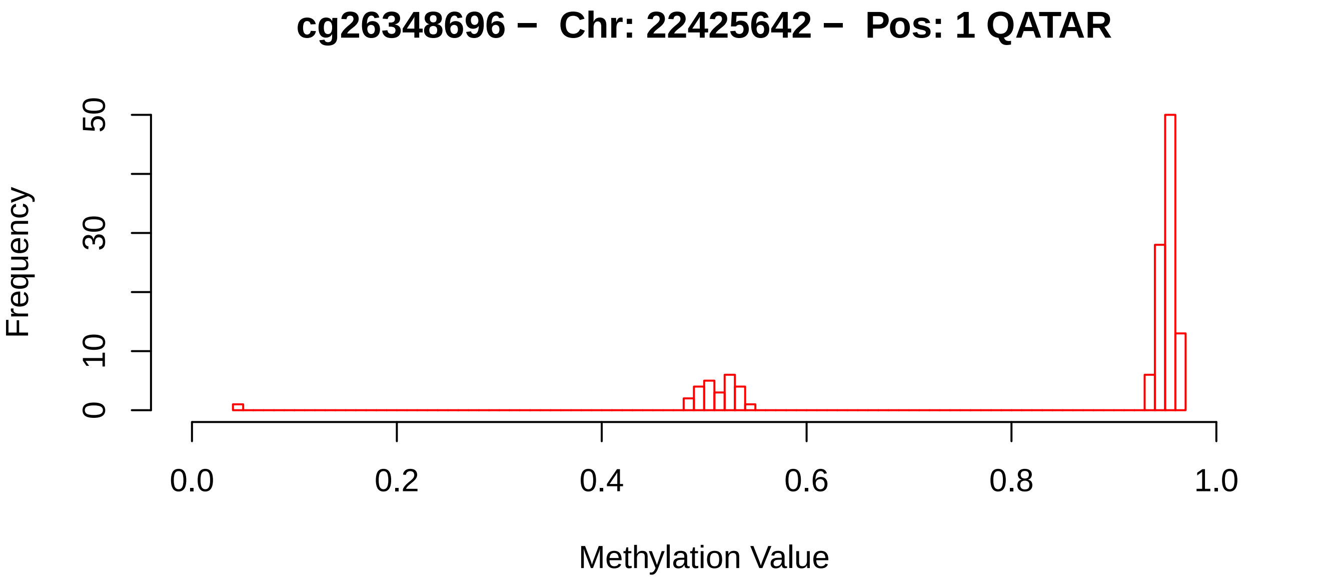
cg26348696 − Chr: 22425642 − Pos: 1 QATAR
Frequency
0
10
30
50
0.0
0.2
0.4
0.6
0.8
1.0
Methylation Value

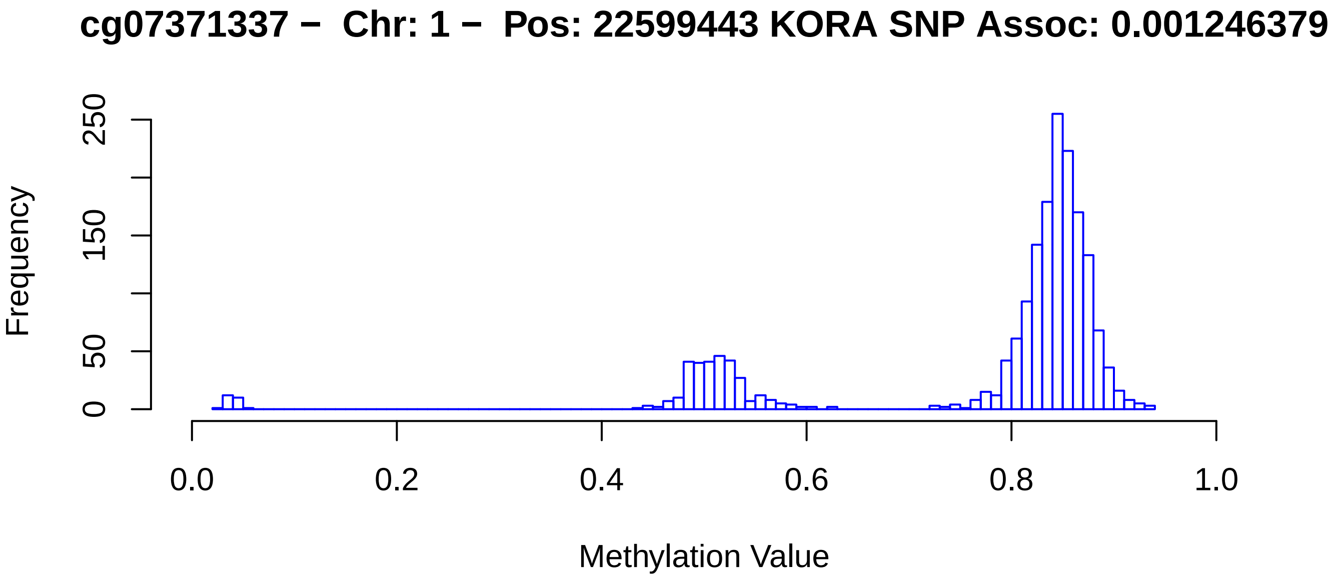
cg07371337 − Chr: 1 − Pos: 22599443 KORA SNP Assoc: 0.001246379
Frequency
0
50
150
250
0.0
0.2
0.4
0.6
0.8
1.0
Methylation Value

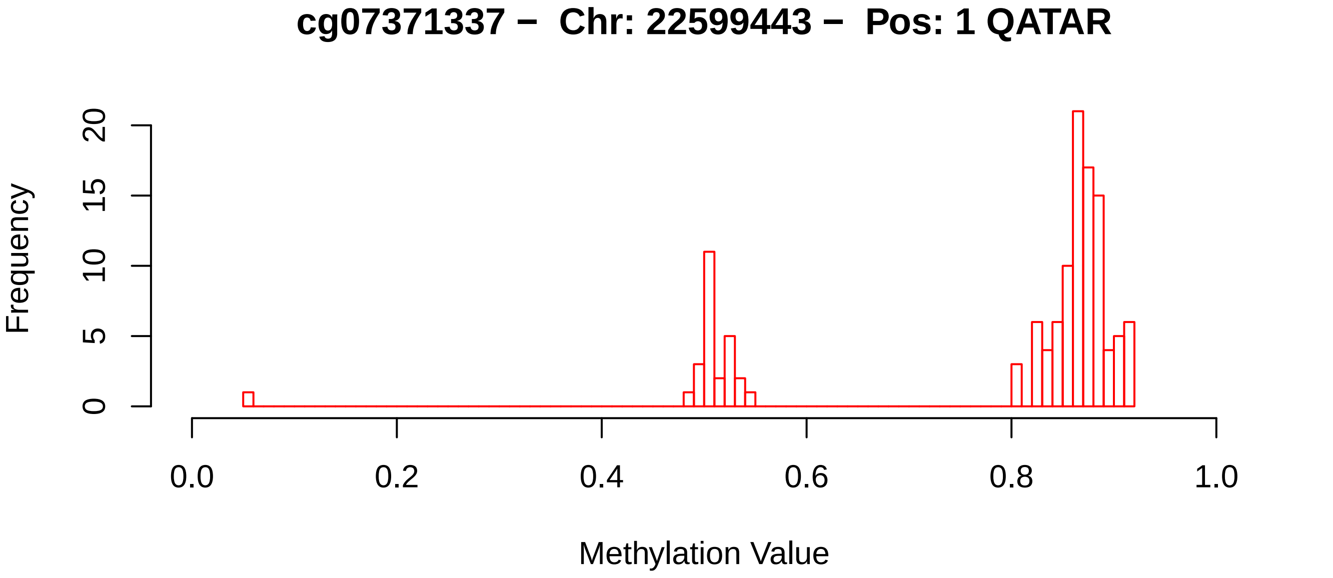
cg07371337 − Chr: 22599443 − Pos: 1 QATAR
Frequency
0
5
10
15
20
0.0
0.2
0.4
0.6
0.8
1.0
Methylation Value

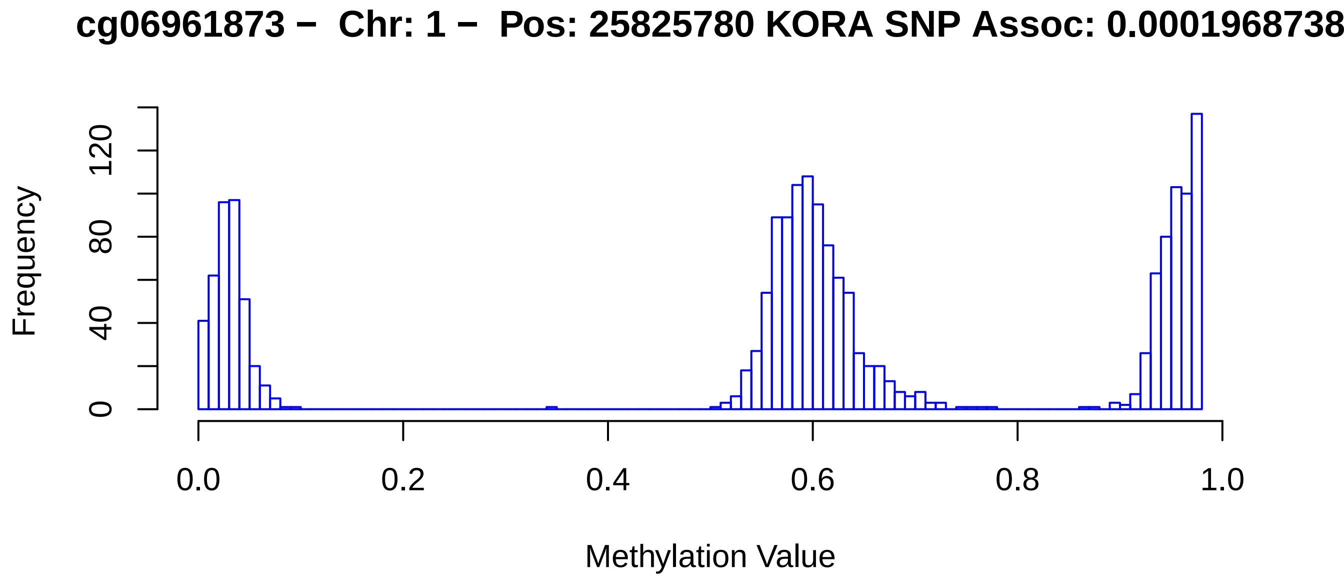
cg06961873 − Chr: 1 − Pos: 25825780 KORA SNP Assoc: 0.0001968738
Frequency
0
40
80
120
0.0
0.2
0.4
0.6
0.8
1.0
Methylation Value

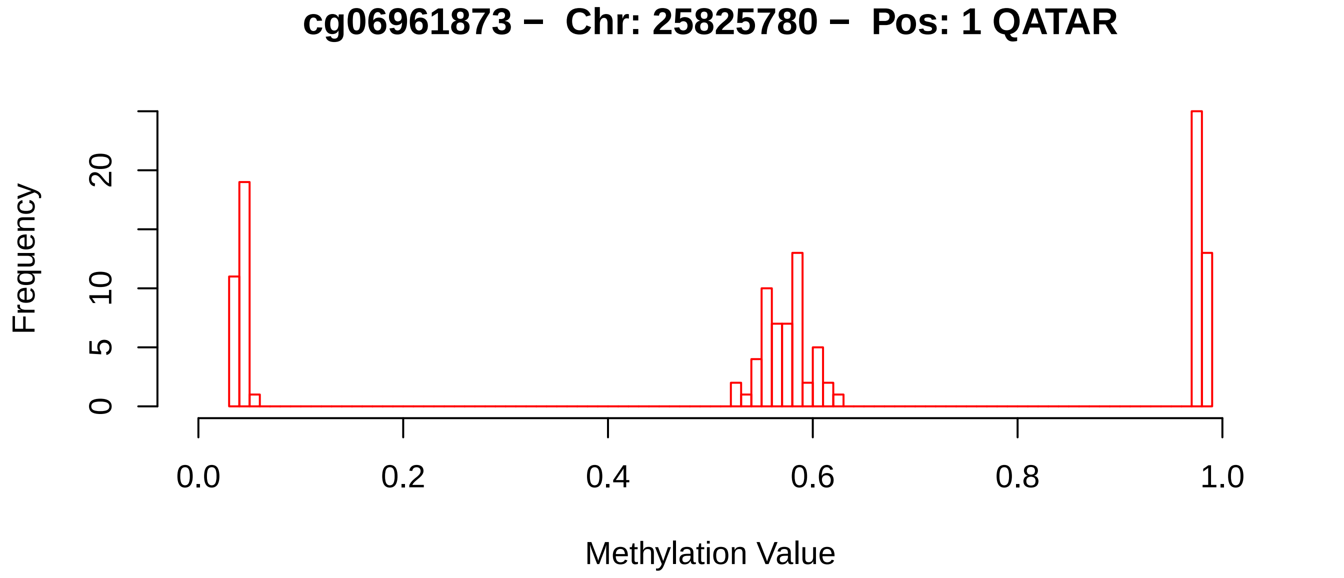
cg06961873 − Chr: 25825780 − Pos: 1 QATAR
Frequency
0
5
10
20
0.0
0.2
0.4
0.6
0.8
1.0
Methylation Value

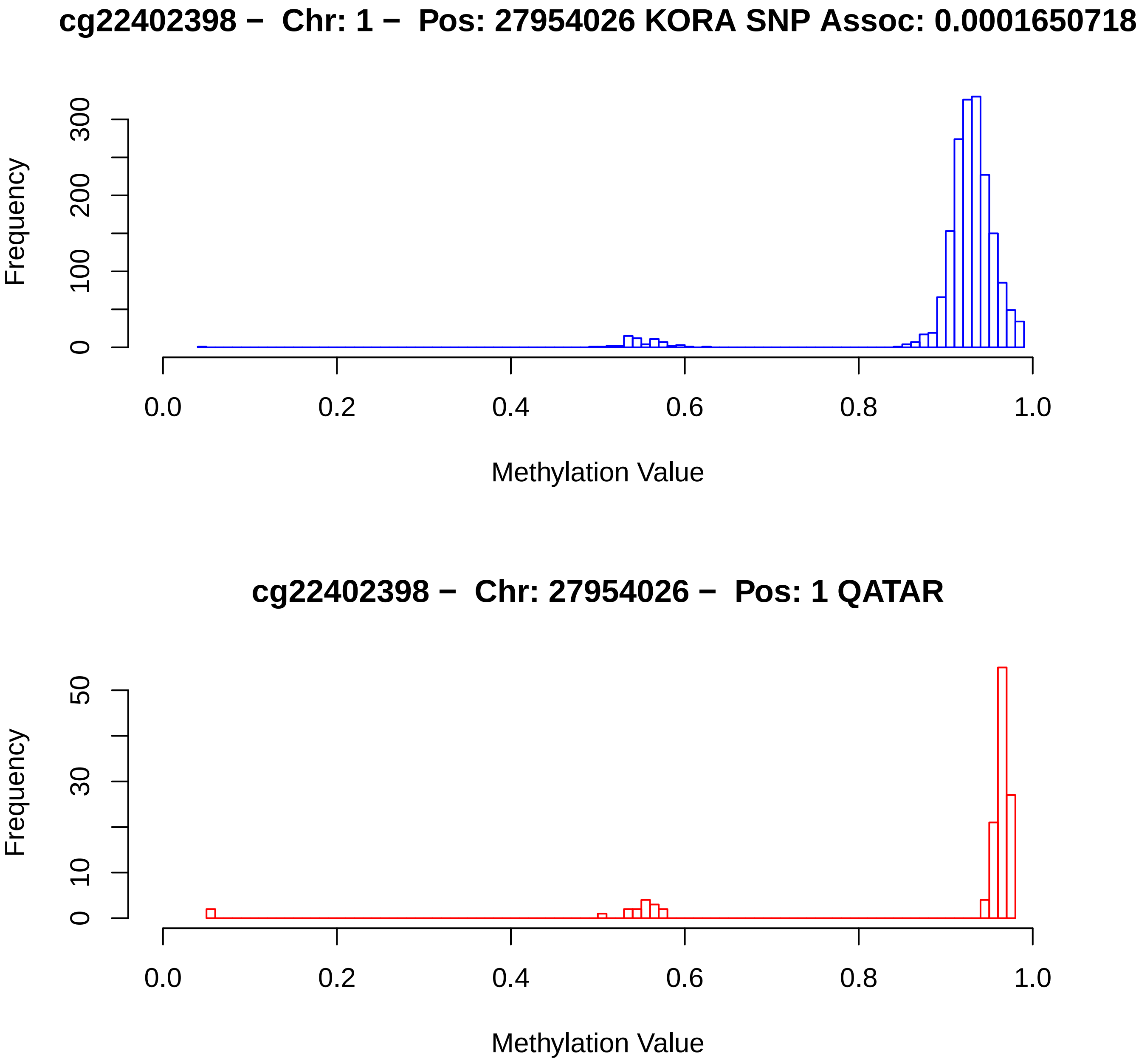

cg22402398 − Chr: 1 − Pos: 27954026 KORA SNP Assoc: 0.0001650718
Frequency
0
100
200
300
0.0
0.2
0.4
0.6
0.8
1.0
Methylation Value
cg22402398 − Chr: 27954026 − Pos: 1 QATAR
Frequency
0
10
30
50
0.0
0.2
0.4
0.6
0.8
1.0
Methylation Value

cg22802014 – Chr: 1 – Pos: 31732891 KORA SNP Assoc: 0.0005013463

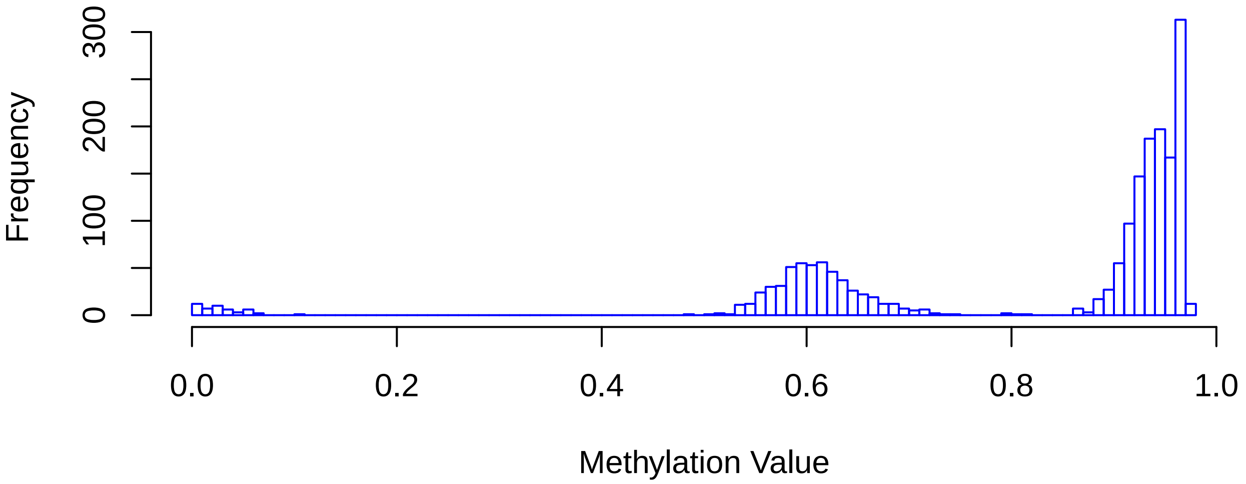

cg22802014 – Chr: 31732891 – Pos: 1 QATAR

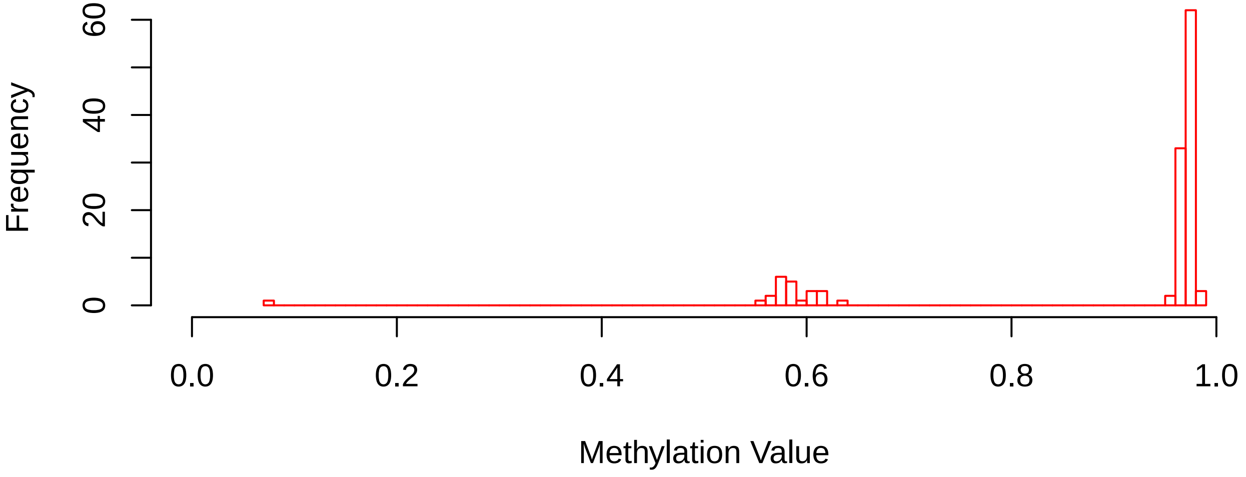

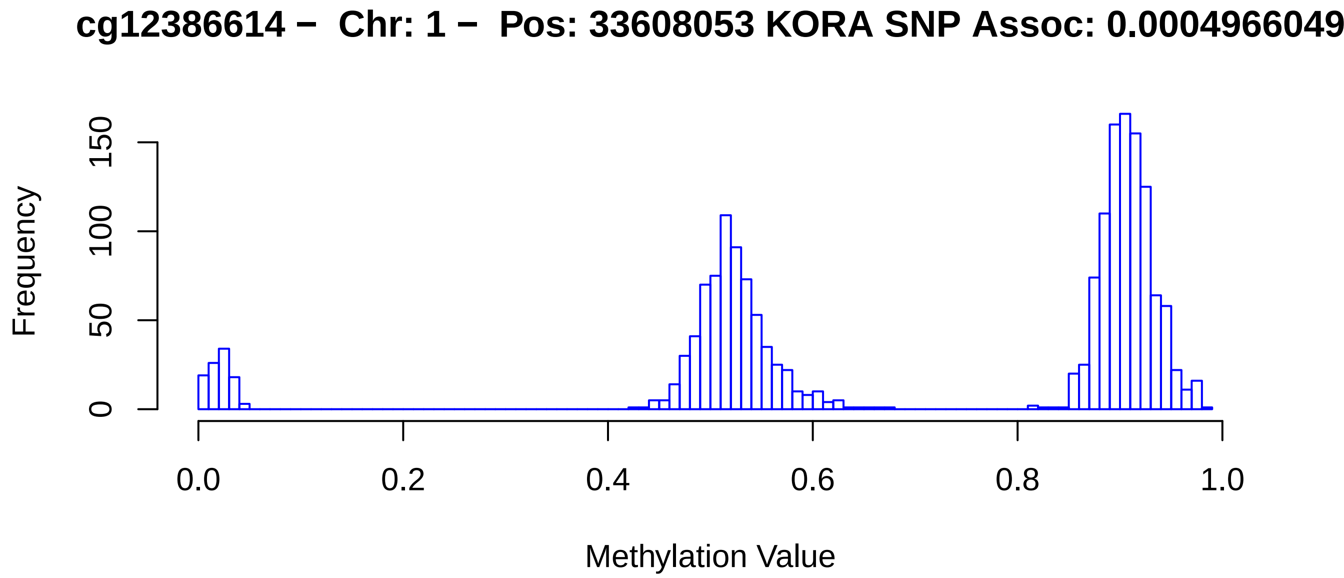
cg12386614 − Chr: 1 − Pos: 33608053 KORA SNP Assoc: 0.0004966049
Frequency
0
50
100
150
0.0
0.2
0.4
0.6
0.8
1.0
Methylation Value

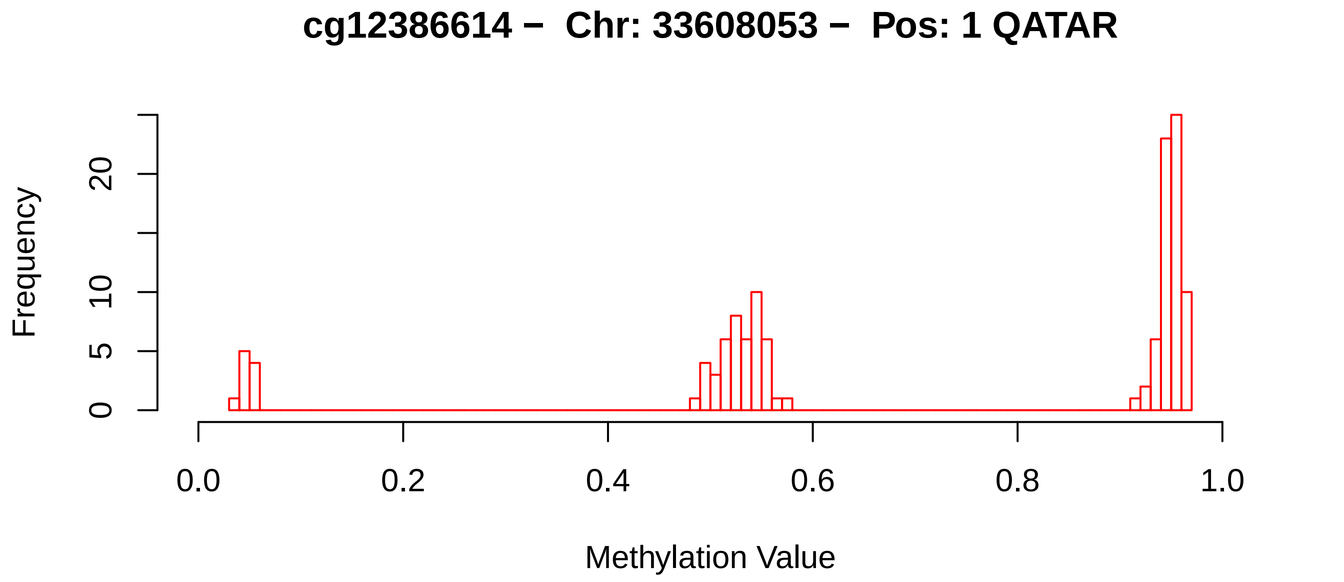
cg12386614 − Chr: 33608053 − Pos: 1 QATAR
Frequency
0
5
10
20
0.0
0.2
0.4
0.6
0.8
1.0
Methylation Value

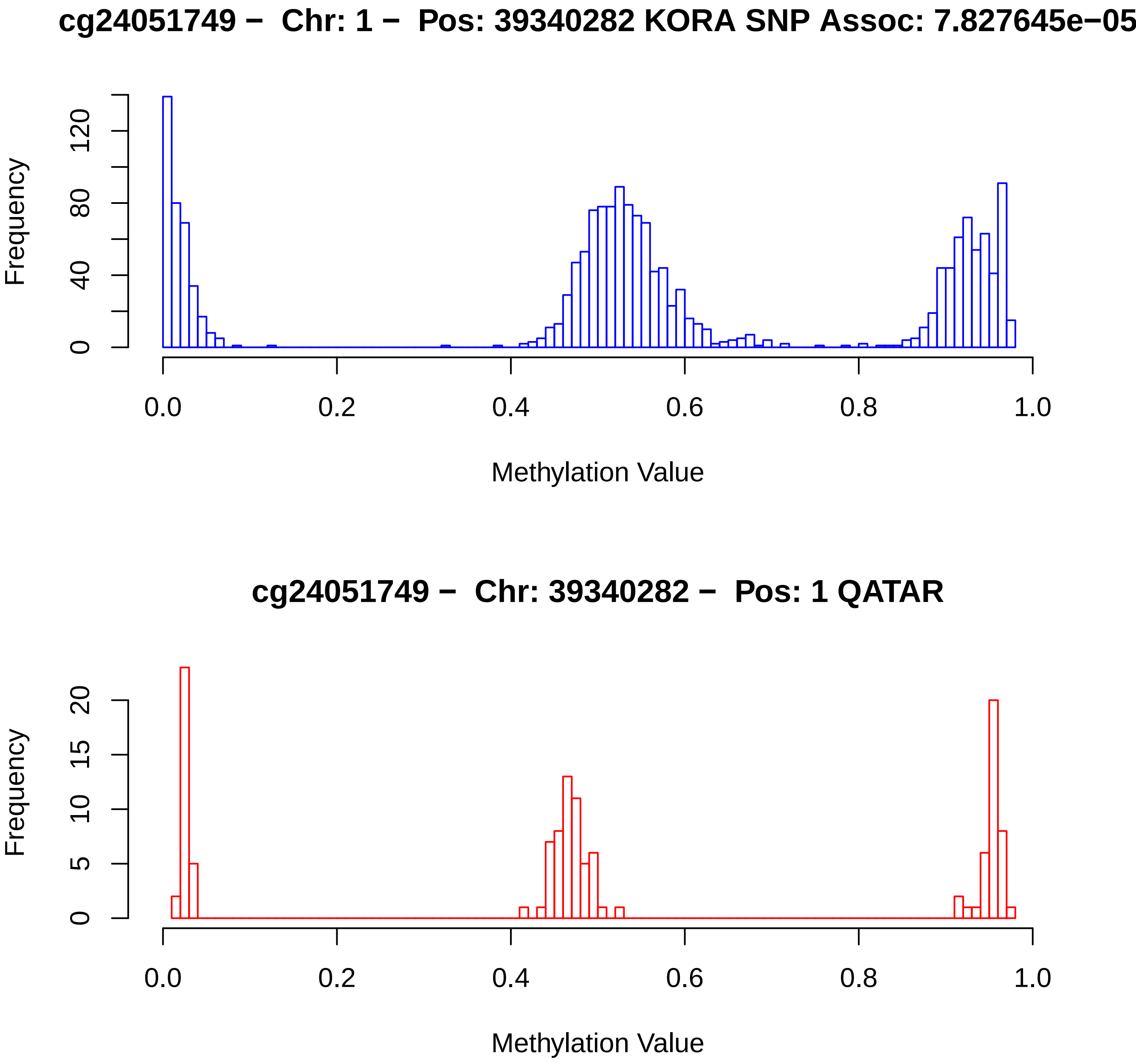
cg24051749 − Chr: 1 − Pos: 39340282 KORA SNP Assoc: 7.827645e−05
Frequency
0
40
80
120
0.0
0.2
0.4
0.6
0.8
1.0
Methylation Value
cg24051749 − Chr: 39340282 − Pos: 1 QATAR
Frequency
0
5
10
15
20
0.0
0.2
0.4
0.6
0.8
1.0
Methylation Value

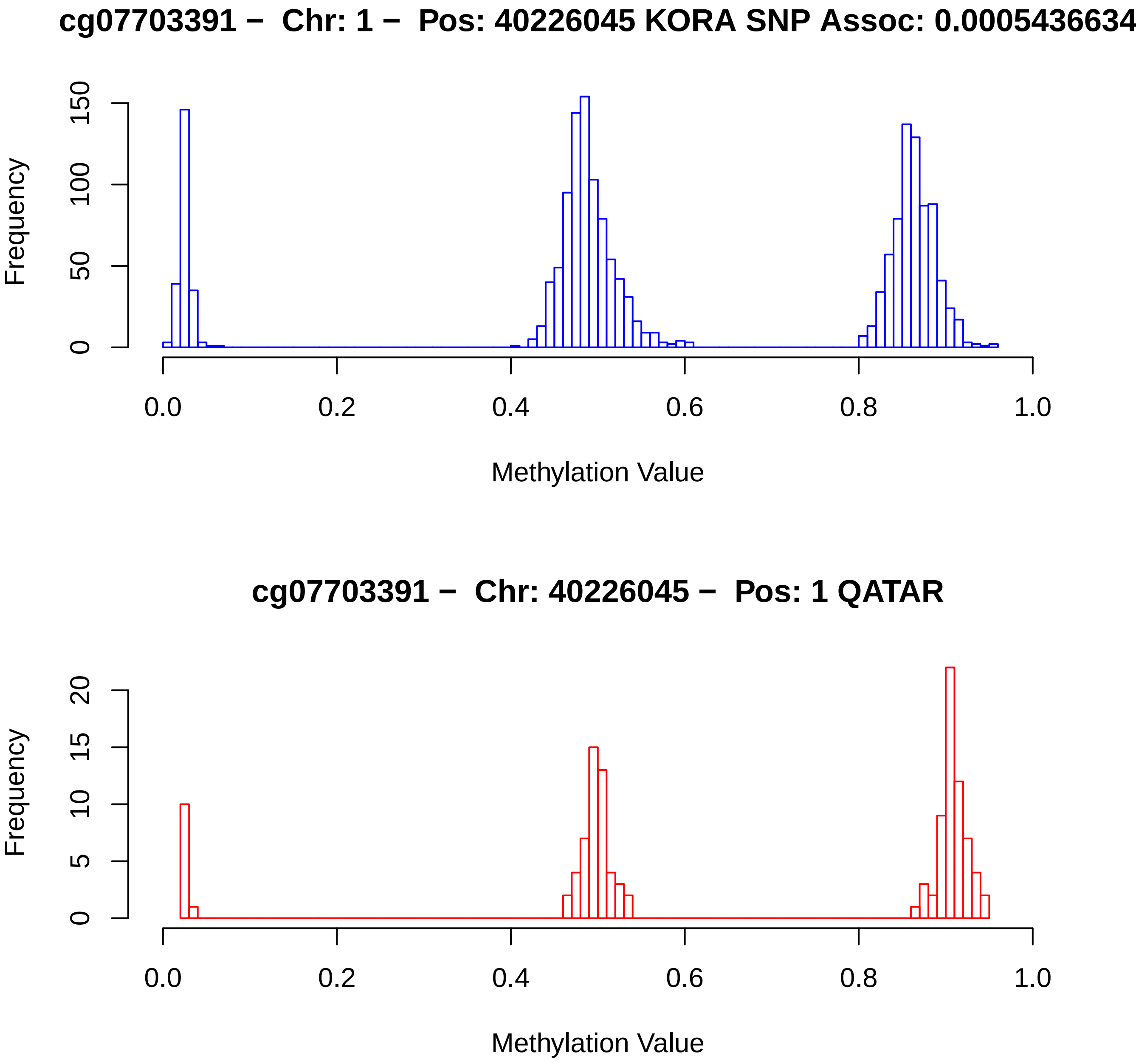
cg07703391 − Chr: 1 − Pos: 40226045 KORA SNP Assoc: 0.0005436634
Frequency
150
100
50
0
0.0
0.2
0.4
0.6
0.8
1.0
Methylation Value
cg07703391 − Chr: 40226045 − Pos: 1 QATAR
Frequency
20
15
10
5
0
0.0
0.2
0.4
0.6
0.8
1.0
Methylation Value

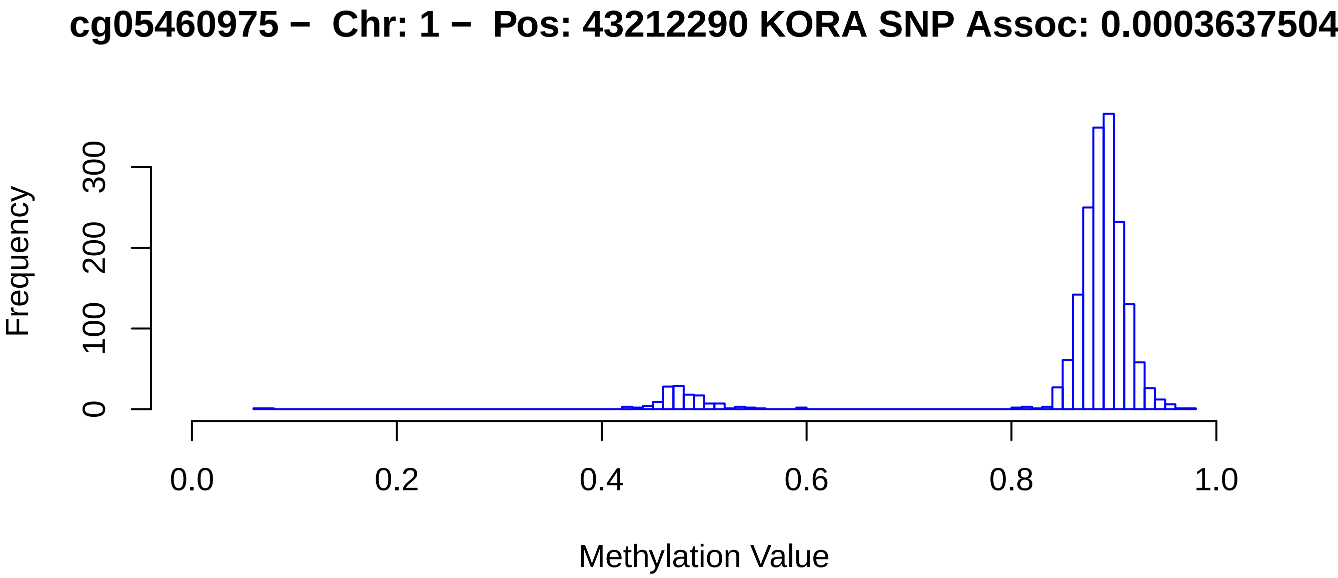
cg05460975 − Chr: 1 − Pos: 43212290 KORA SNP Assoc: 0.0003637504
Frequency
0
100
200
300
0.0
0.2
0.4
0.6
0.8
1.0
Methylation Value

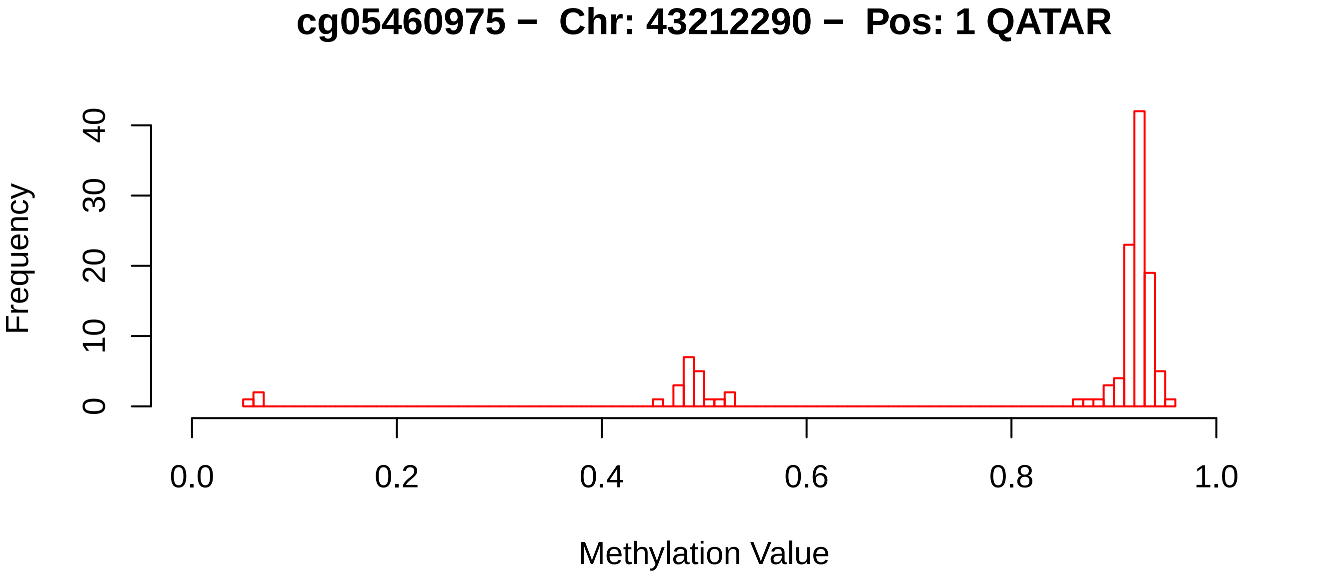
cg05460975 − Chr: 43212290 − Pos: 1 QATAR
Frequency
0
10
20
30
40
0.0
0.2
0.4
0.6
0.8
1.0
Methylation Value

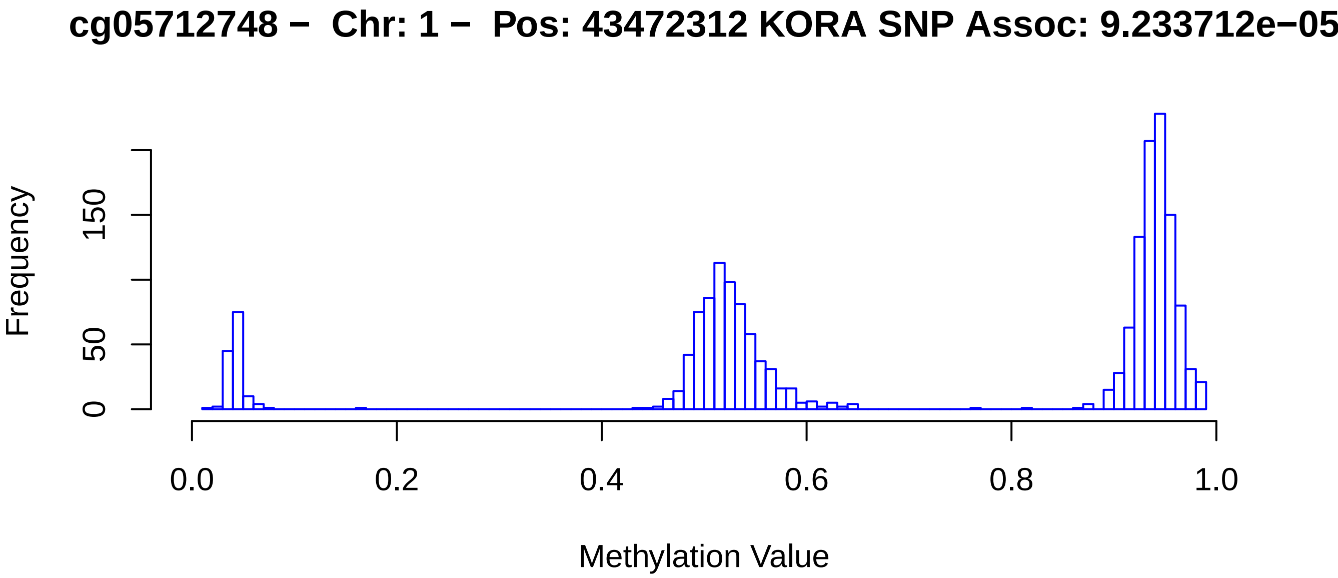
cg05712748 − Chr: 1 − Pos: 43472312 KORA SNP Assoc: 9.233712e−05
Frequency
0
50
150
0.0
0.2
0.4
0.6
0.8
1.0
Methylation Value

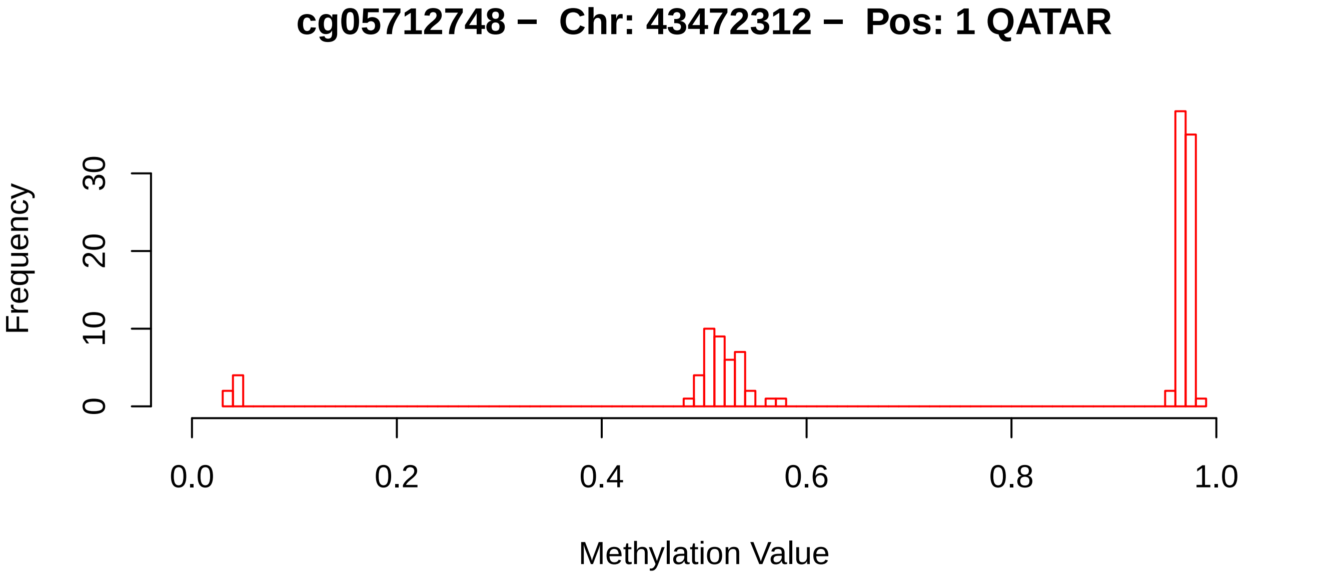
cg05712748 − Chr: 43472312 − Pos: 1 QATAR
Frequency
0
10
20
30
0.0
0.2
0.4
0.6
0.8
1.0
Methylation Value

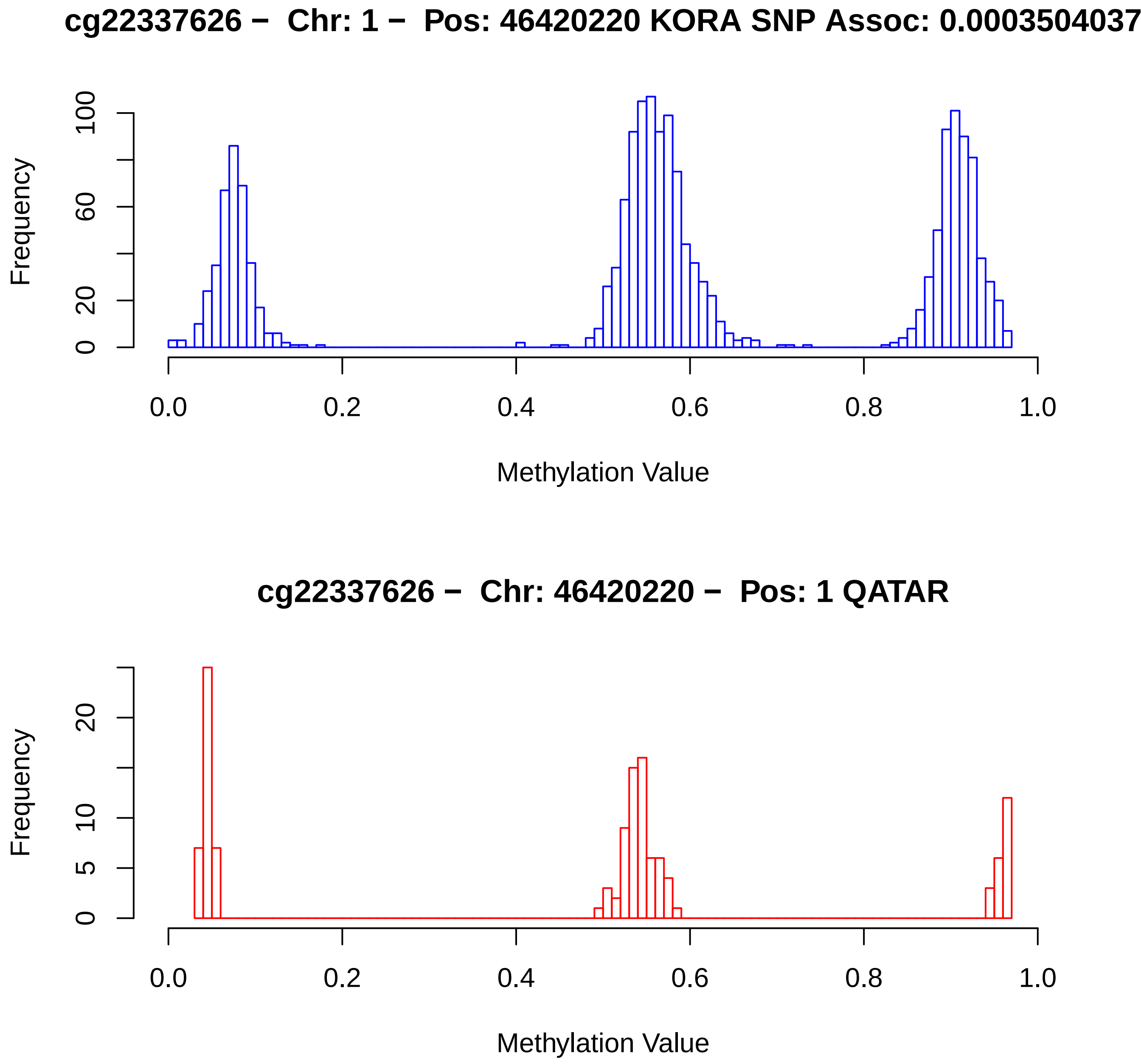
cg22337626 − Chr: 1 − Pos: 46420220 KORA SNP Assoc: 0.0003504037
Frequency
0
20
60
100
0.0
0.2
0.4
0.6
0.8
1.0
Methylation Value
cg22337626 − Chr: 46420220 − Pos: 1 QATAR
Frequency
0
5
10
20
0.0
0.2
0.4
0.6
0.8
1.0
Methylation Value

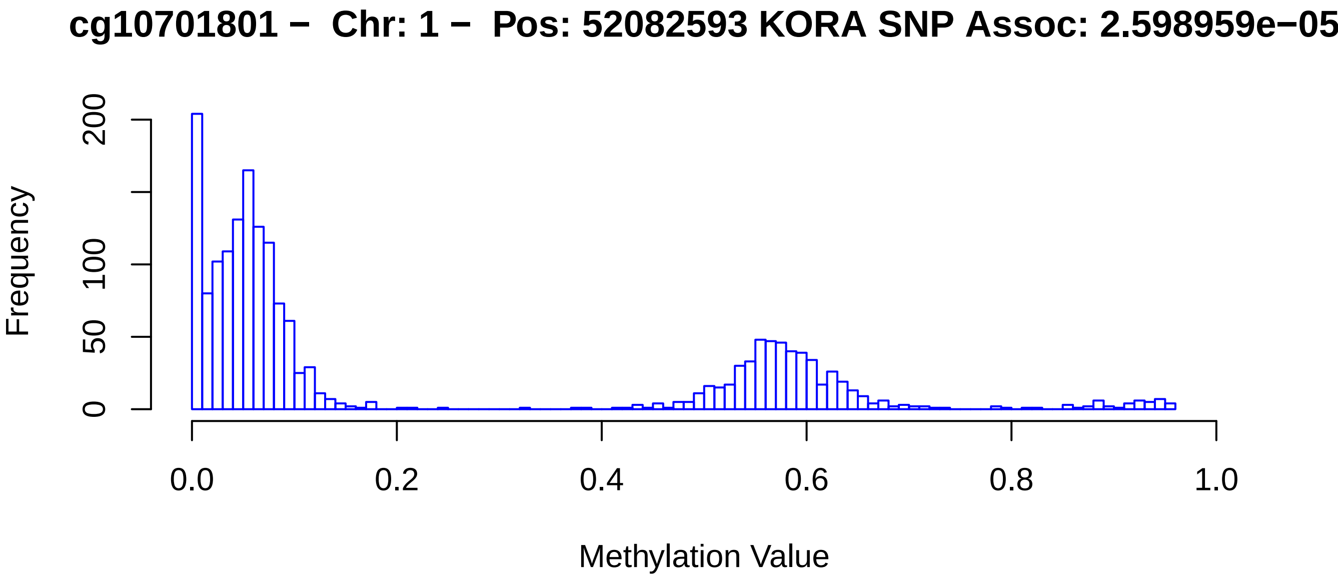
cg10701801 − Chr: 1 − Pos: 52082593 KORA SNP Assoc: 2.598959e−05
Frequency
0
50
100
200
0.0
0.2
0.4
0.6
0.8
1.0
Methylation Value

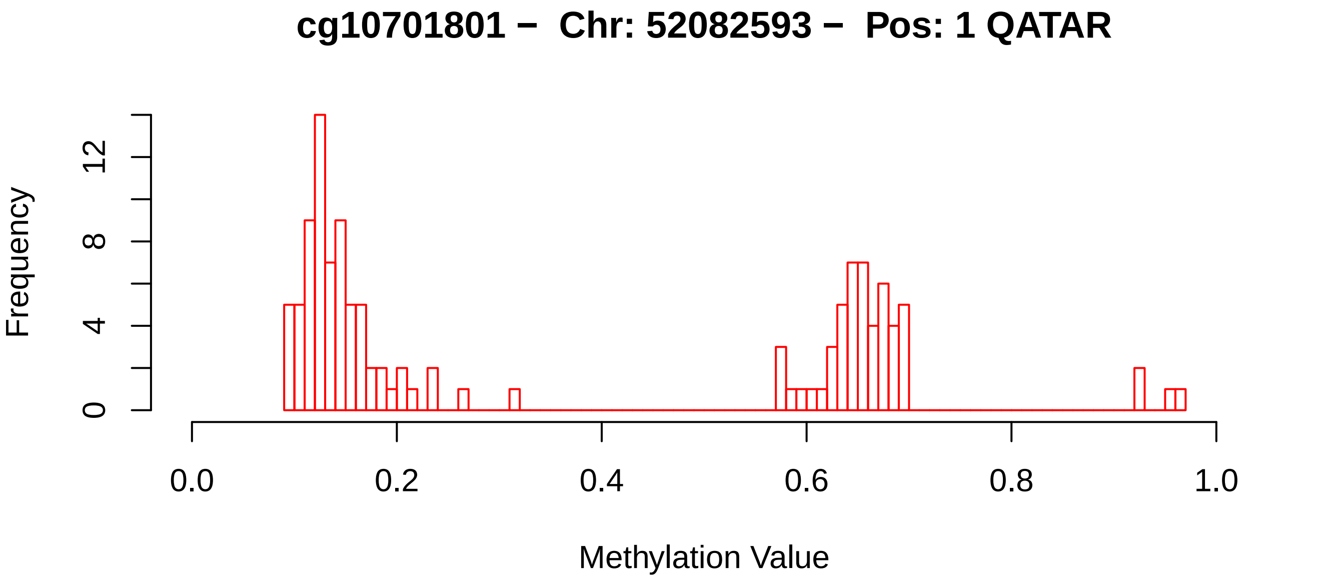
cg10701801 − Chr: 52082593 − Pos: 1 QATAR
Frequency
0
4
8
12
0.0
0.2
0.4
0.6
0.8
1.0
Methylation Value

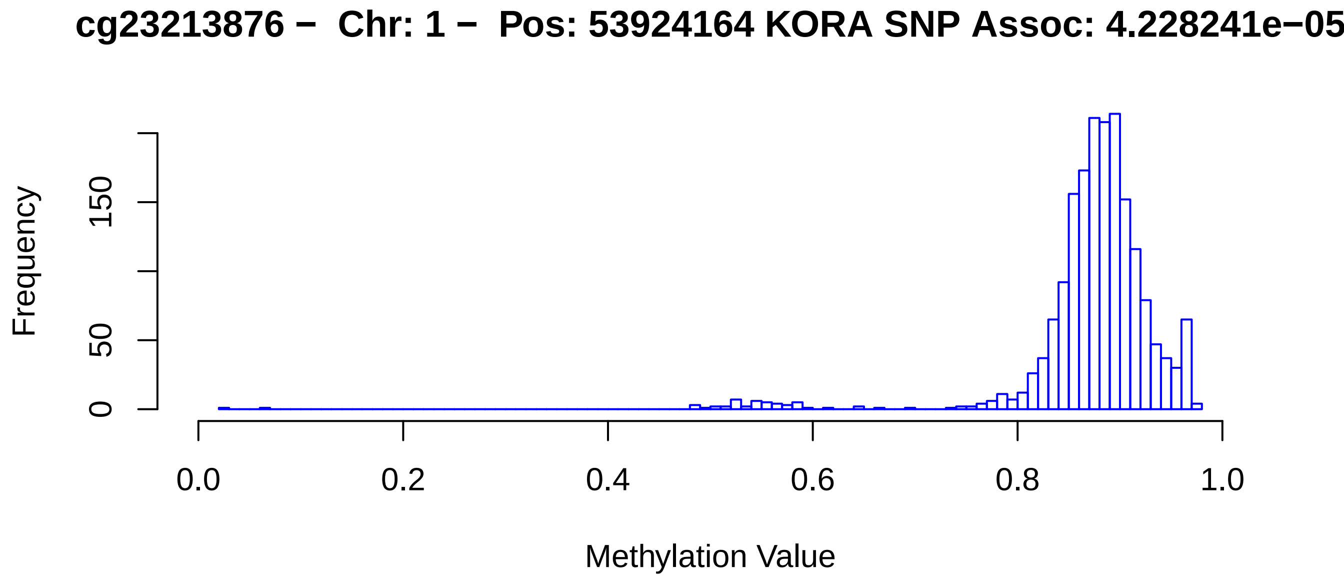
cg23213876 − Chr: 1 − Pos: 53924164 KORA SNP Assoc: 4.228241e−05
Frequency
0
50
150
0.0
0.2
0.4
0.6
0.8
1.0
Methylation Value

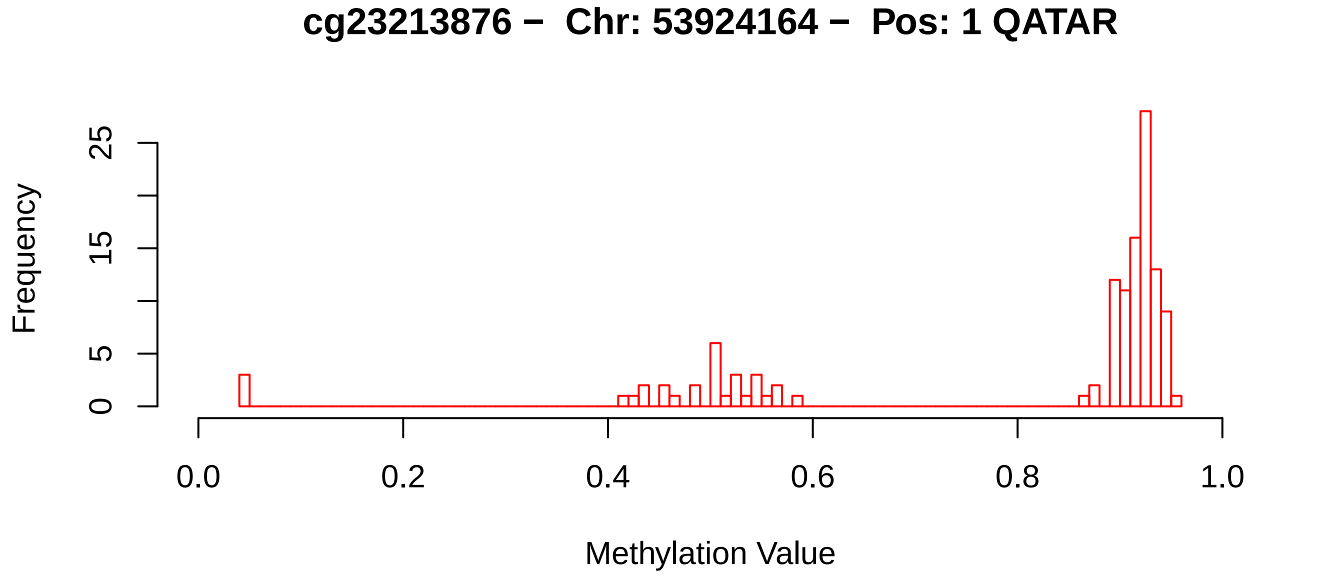
cg23213876 − Chr: 53924164 − Pos: 1 QATAR
Frequency
0
5
15
25
0.0
0.2
0.4
0.6
0.8
1.0
Methylation Value

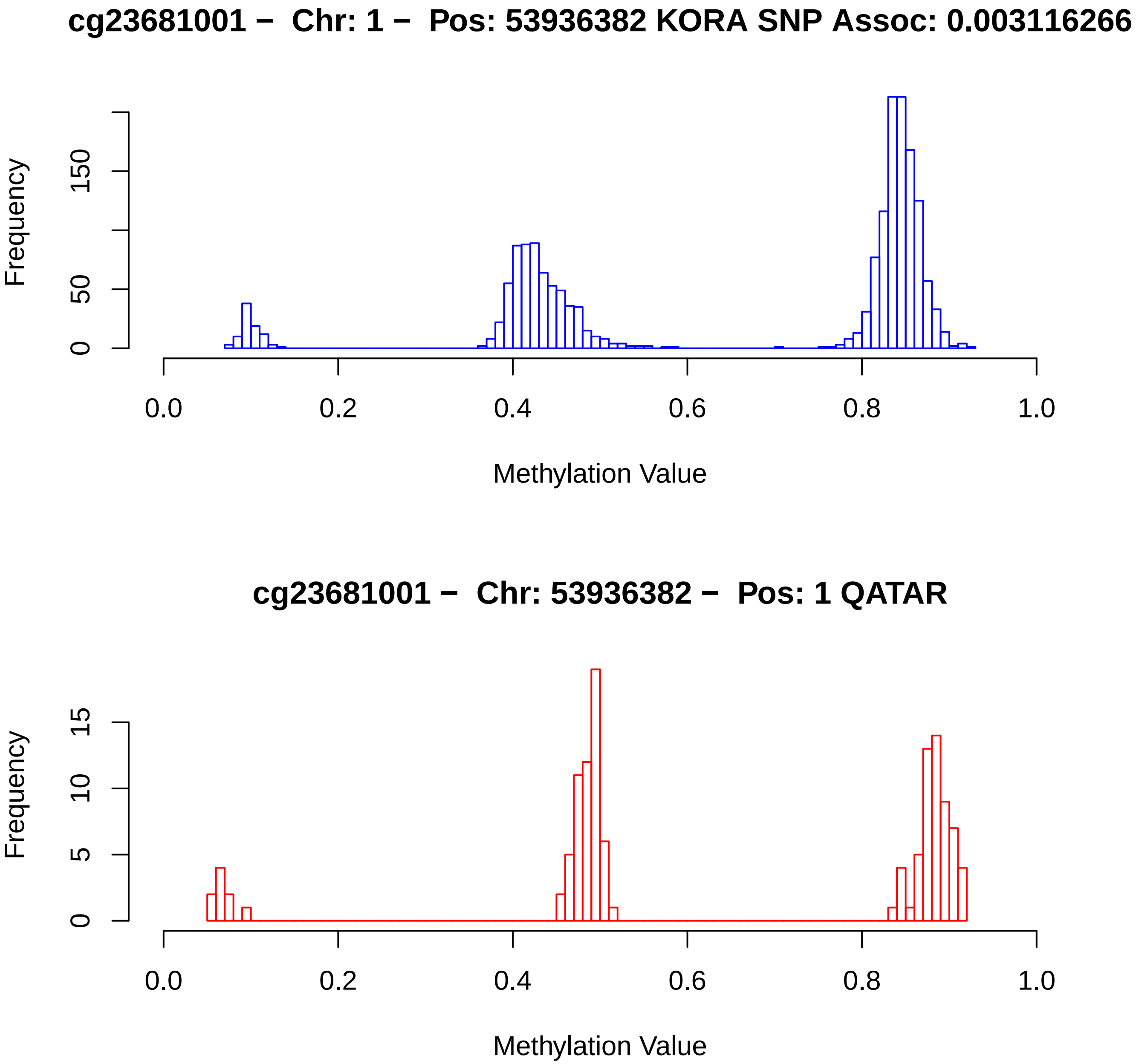
cg23681001 − Chr: 1 − Pos: 53936382 KORA SNP Assoc: 0.003116266
Frequency
0
50
150
0.0
0.2
0.4
0.6
0.8
1.0
Methylation Value
cg23681001 − Chr: 53936382 − Pos: 1 QATAR
Frequency
0
5
10
15
0.0
0.2
0.4
0.6
0.8
1.0
Methylation Value

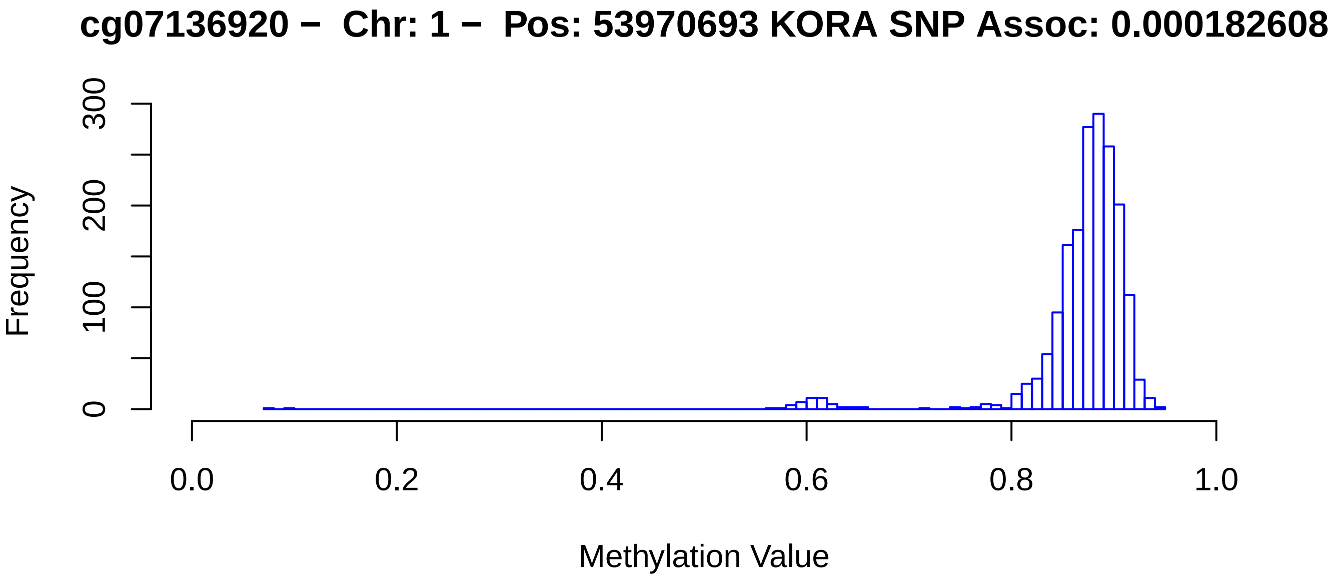
cg07136920 − Chr: 1 − Pos: 53970693 KORA SNP Assoc: 0.000182608
Frequency
0
100
200
300
0.0
0.2
0.4
0.6
0.8
1.0
Methylation Value

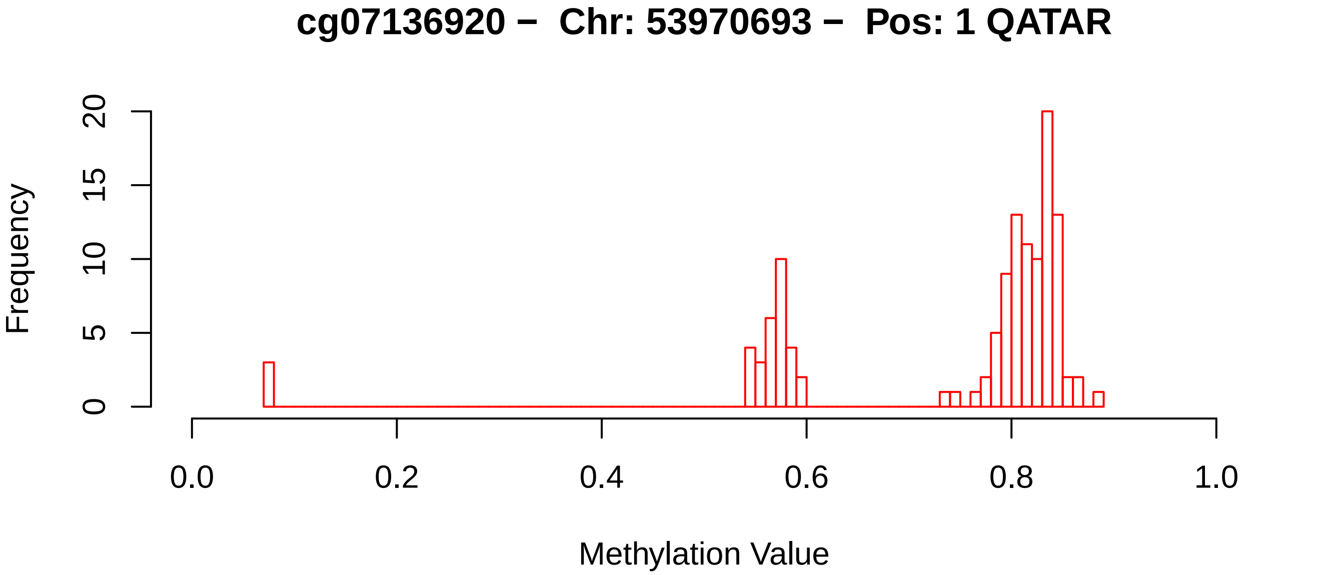
cg07136920 − Chr: 53970693 − Pos: 1 QATAR
Frequency
0
5
10
15
20
0.0
0.2
0.4
0.6
0.8
1.0
Methylation Value

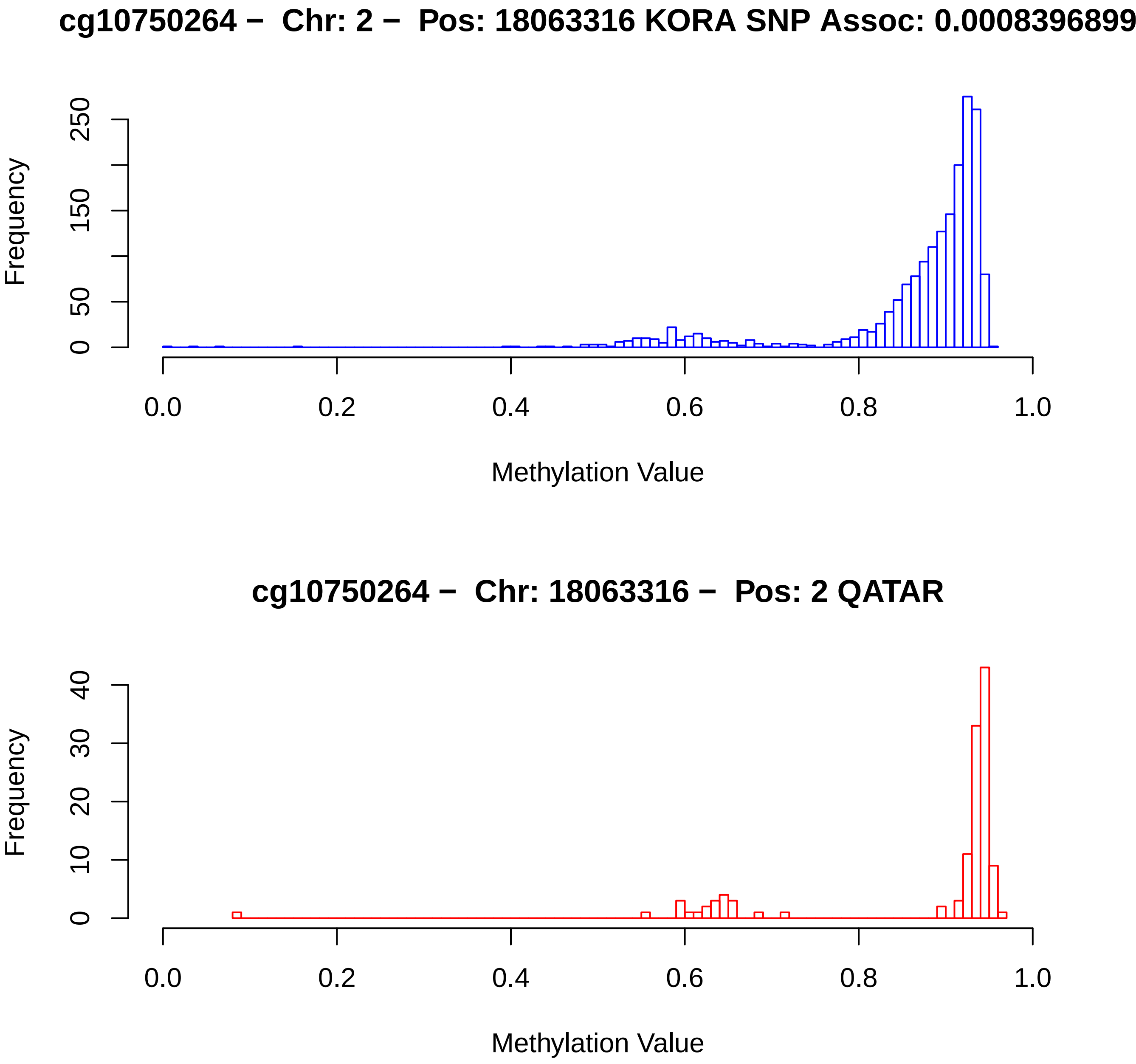
cg10750264 − Chr: 2 − Pos: 18063316 KORA SNP Assoc: 0.0008396899
Frequency
0
50
150
250
0.0
0.2
0.4
0.6
0.8
1.0
Methylation Value
cg10750264 − Chr: 18063316 − Pos: 2 QATAR
Frequency
0
10
20
30
40
0.0
0.2
0.4
0.6
0.8
1.0
Methylation Value

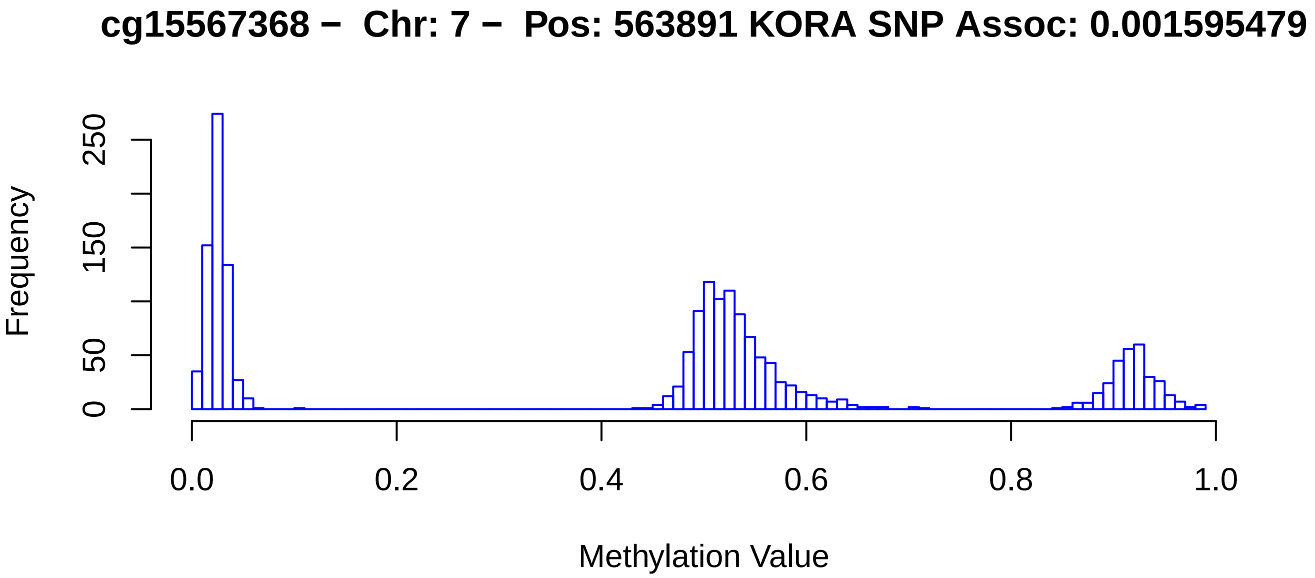

cg15567368 − Chr: 7 − Pos: 563891 KORA SNP Assoc: 0.001595479
Frequency
0
50
150
250
0.0
0.2
0.4
0.6
0.8
1.0
Methylation Value

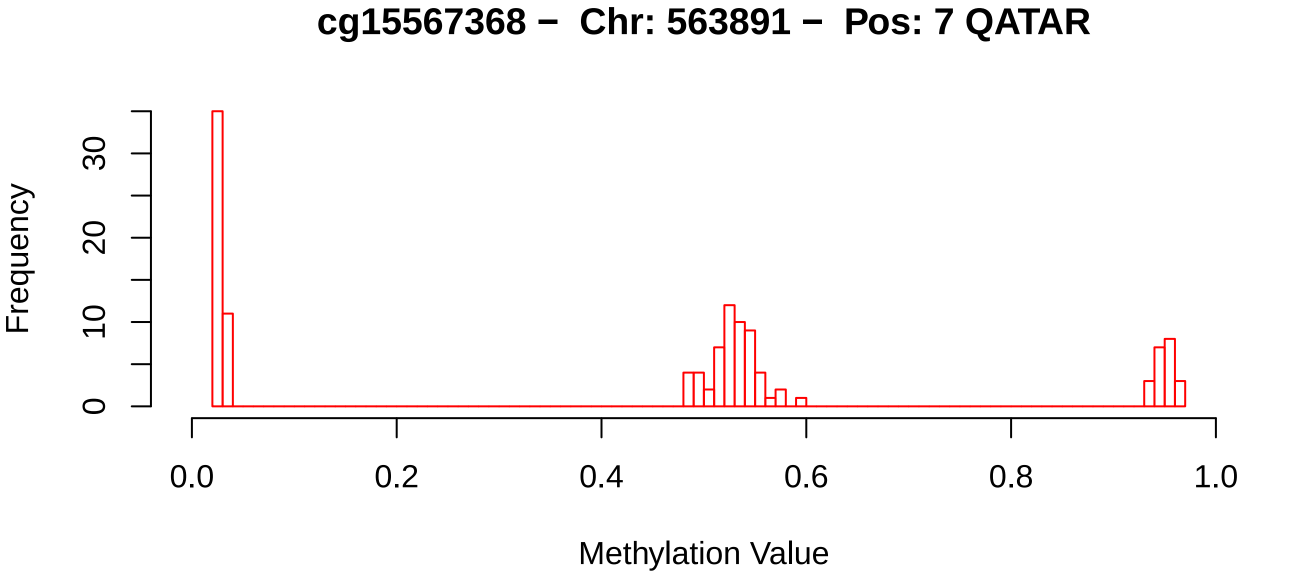

cg15567368 − Chr: 563891 − Pos: 7 QATAR
Frequency
0
10
20
30
0.0
0.2
0.4
0.6
0.8
1.0
Methylation Value

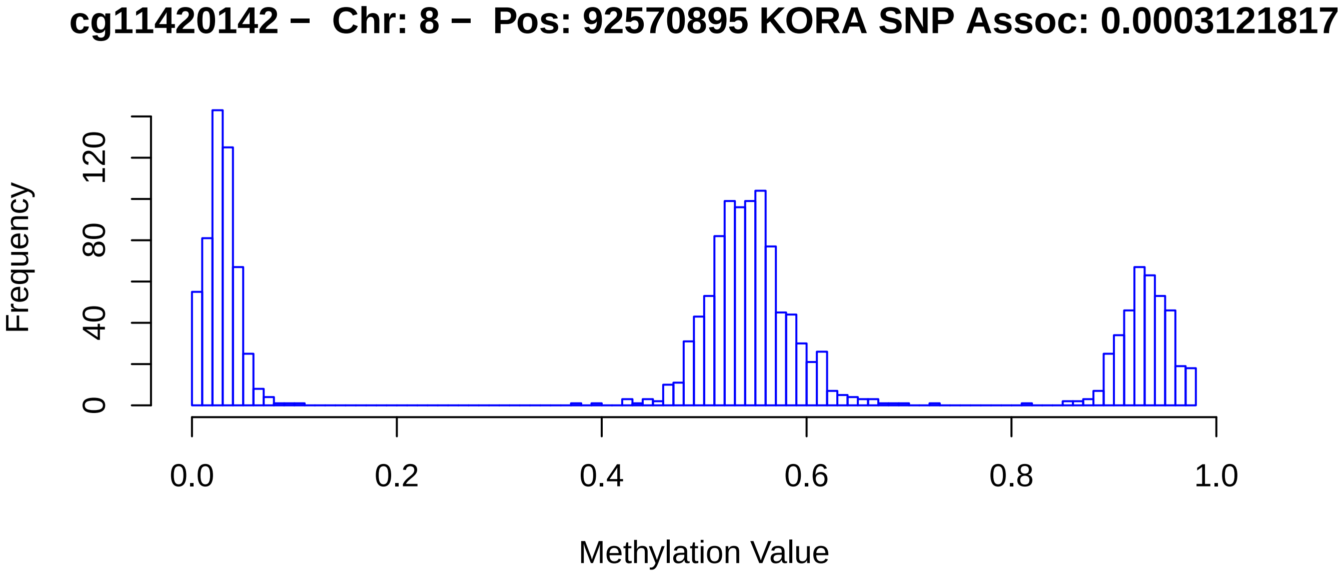
cg11420142 − Chr: 8 − Pos: 92570895 KORA SNP Assoc: 0.0003121817
Frequency
0
40
80
120
0.0
0.2
0.4
0.6
0.8
1.0
Methylation Value

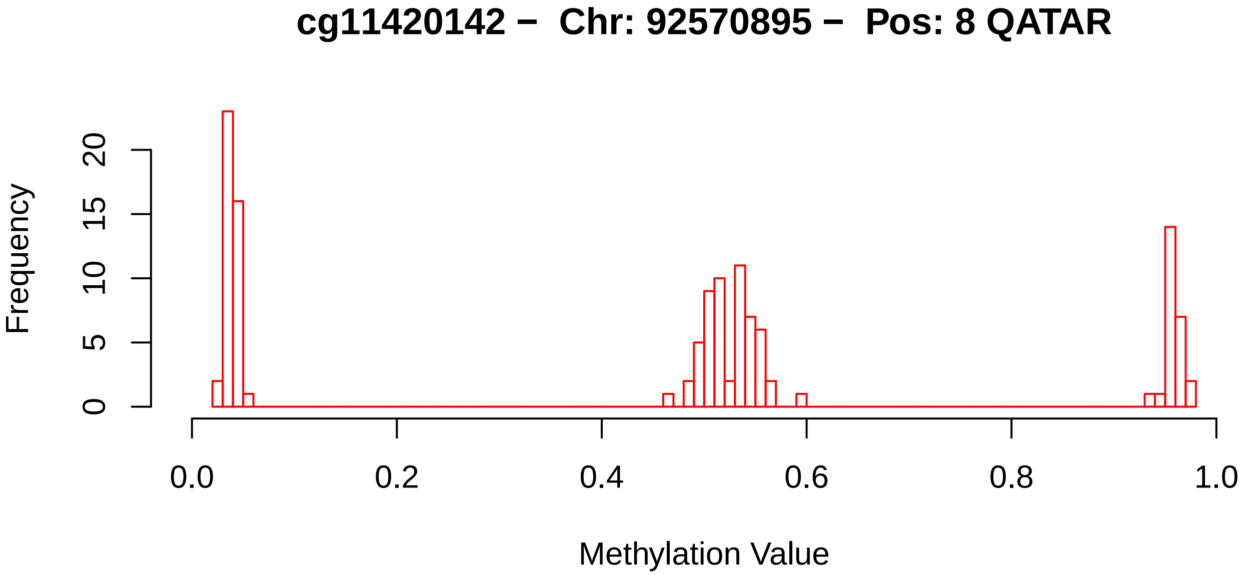
cg11420142 − Chr: 92570895 − Pos: 8 QATAR
Frequency
0
5
10
15
20
0.0
0.2
0.4
0.6
0.8
1.0
Methylation Value

cg10712578 − Chr: 8 − Pos: 96619989 KORA SNP Assoc: 0.0003423382

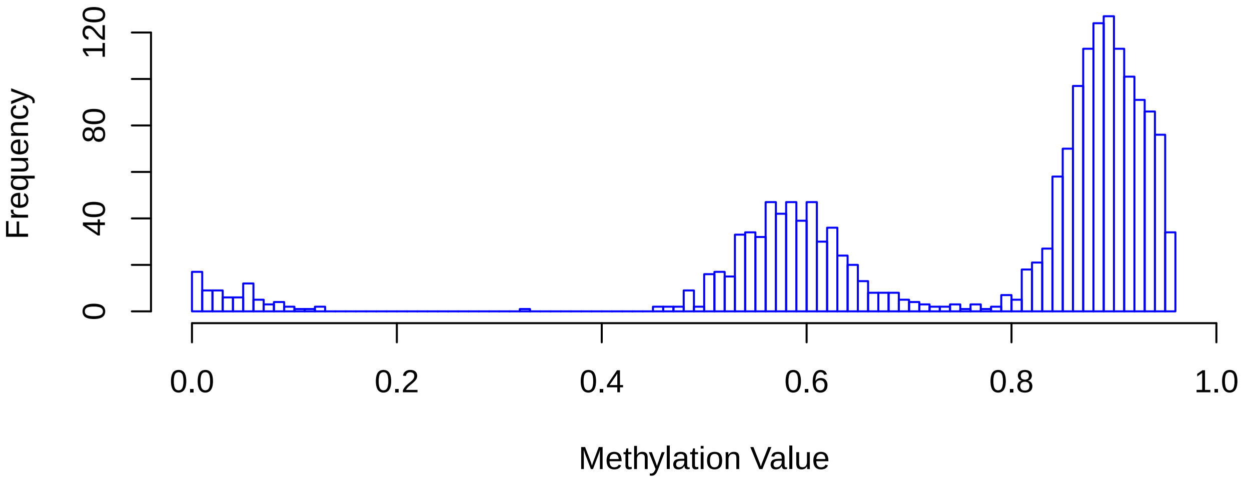

cg10712578 − Chr: 96619989 − Pos: 8 QATAR

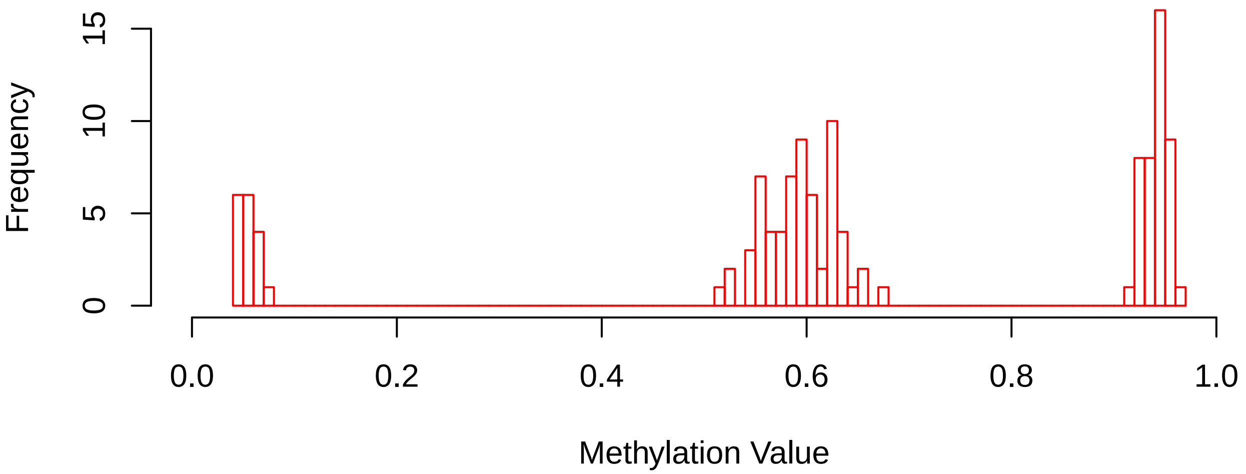

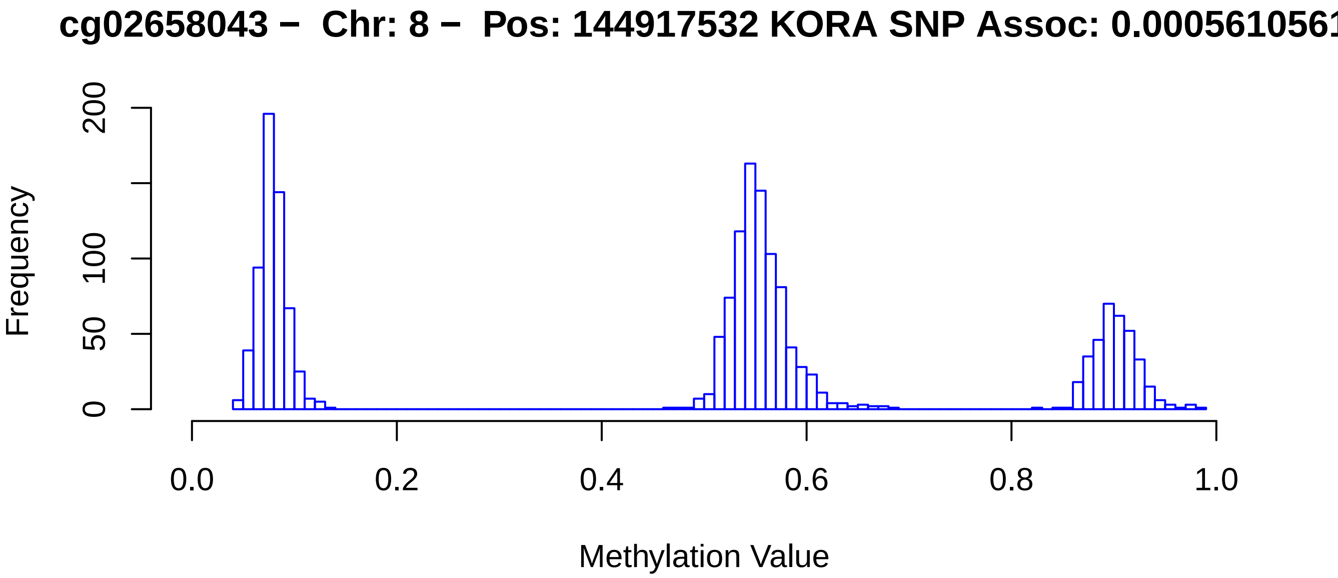
cg02658043 − Chr: 8 − Pos: 144917532 KORA SNP Assoc: 0.0005610561
Frequency
0
50
100
200
0.0
0.2
0.4
0.6
0.8
1.0
Methylation Value

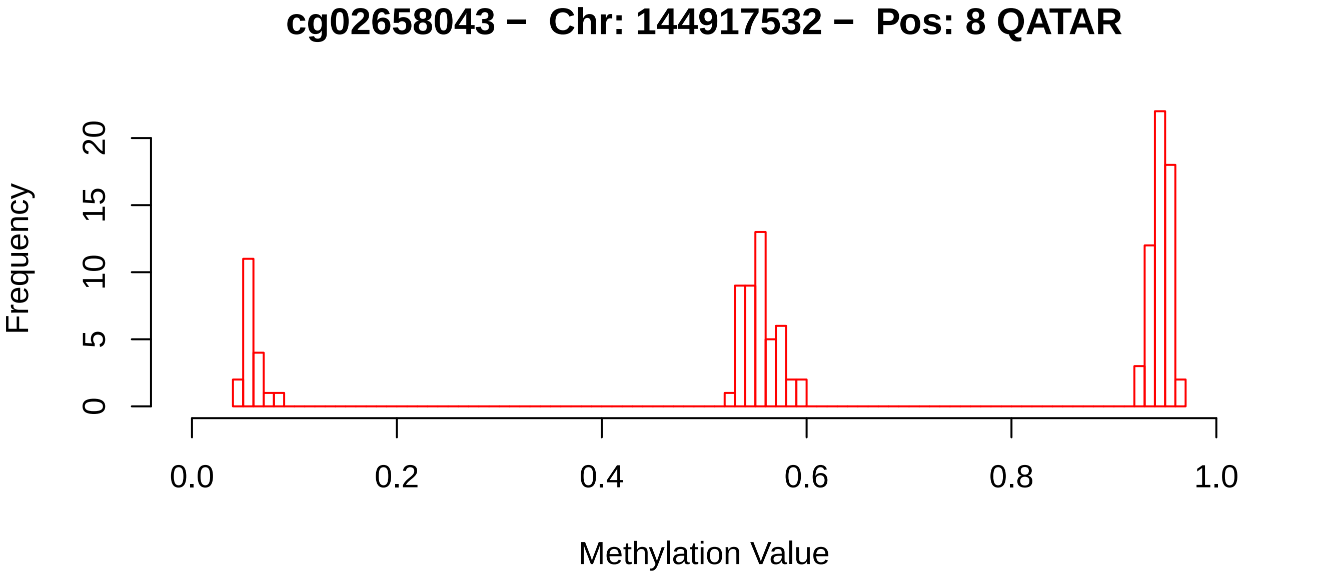
cg02658043 − Chr: 144917532 − Pos: 8 QATAR
Frequency
0
5
10
15
20
0.0
0.2
0.4
0.6
0.8
1.0
Methylation Value

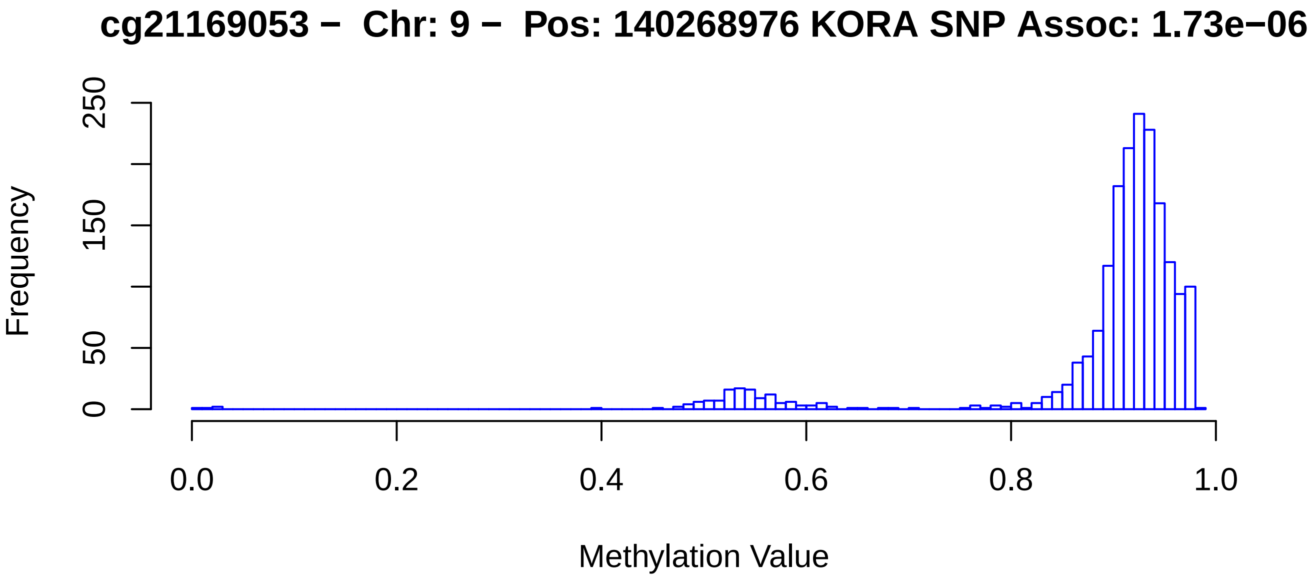
cg21169053 − Chr: 9 − Pos: 140268976 KORA SNP Assoc: 1.73e−06
Frequency
0
50
150
250
0.0
0.2
0.4
0.6
0.8
1.0
Methylation Value

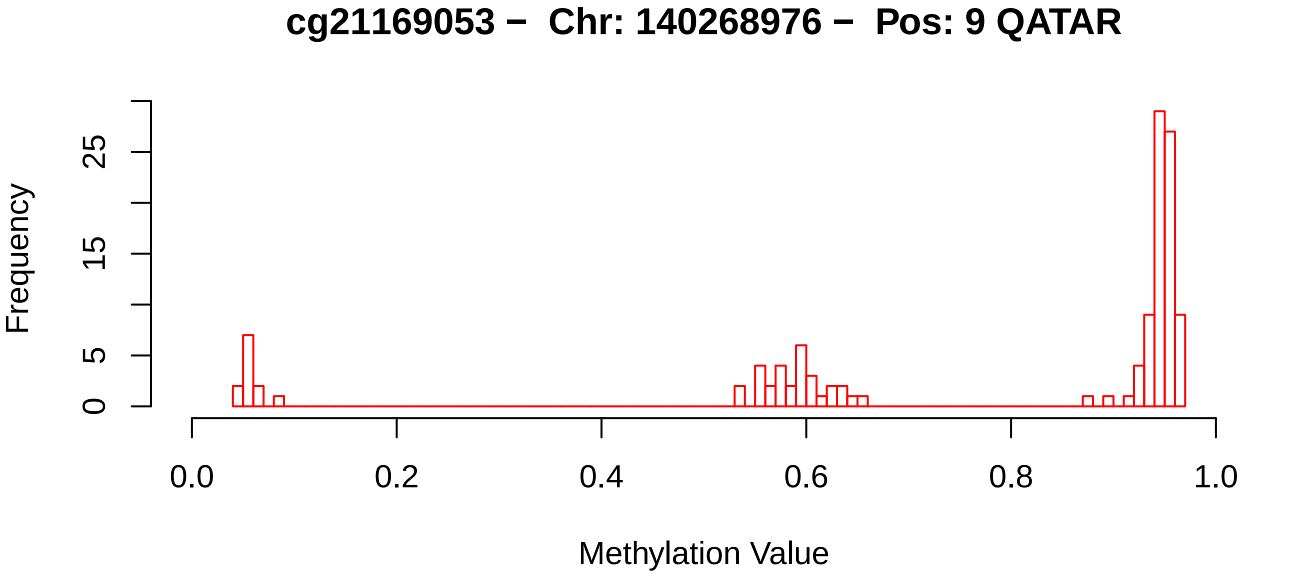
cg21169053 − Chr: 140268976 − Pos: 9 QATAR
Frequency
0
5
15
25
0.0
0.2
0.4
0.6
0.8
1.0
Methylation Value

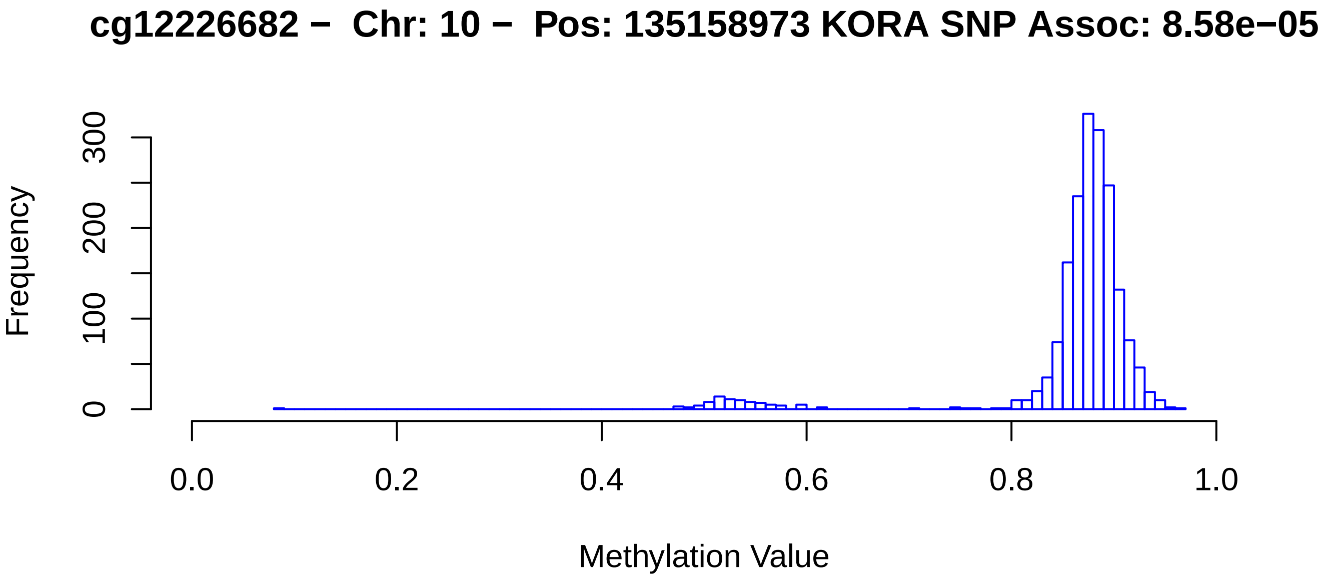
cg12226682 − Chr: 10 − Pos: 135158973 KORA SNP Assoc: 8.58e−05
Frequency
0
100
200
300
0.0
0.2
0.4
0.6
0.8
1.0
Methylation Value

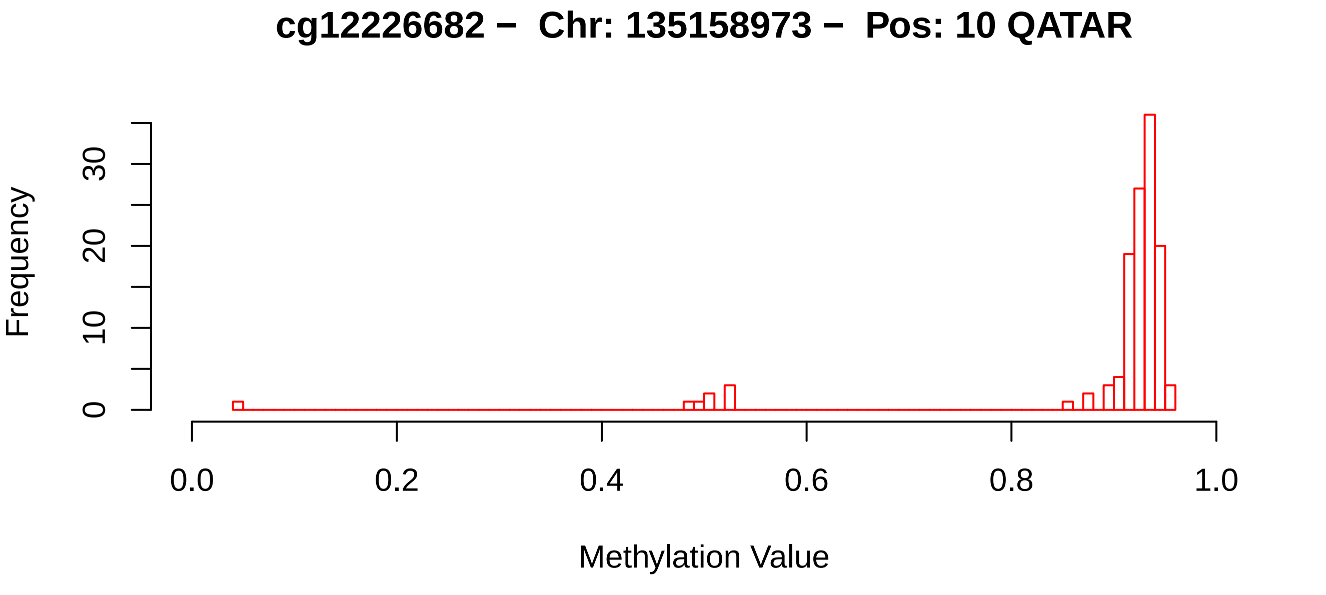
cg12226682 − Chr: 135158973 − Pos: 10 QATAR
Frequency
0
10
20
30
0.0
0.2
0.4
0.6
0.8
1.0
Methylation Value

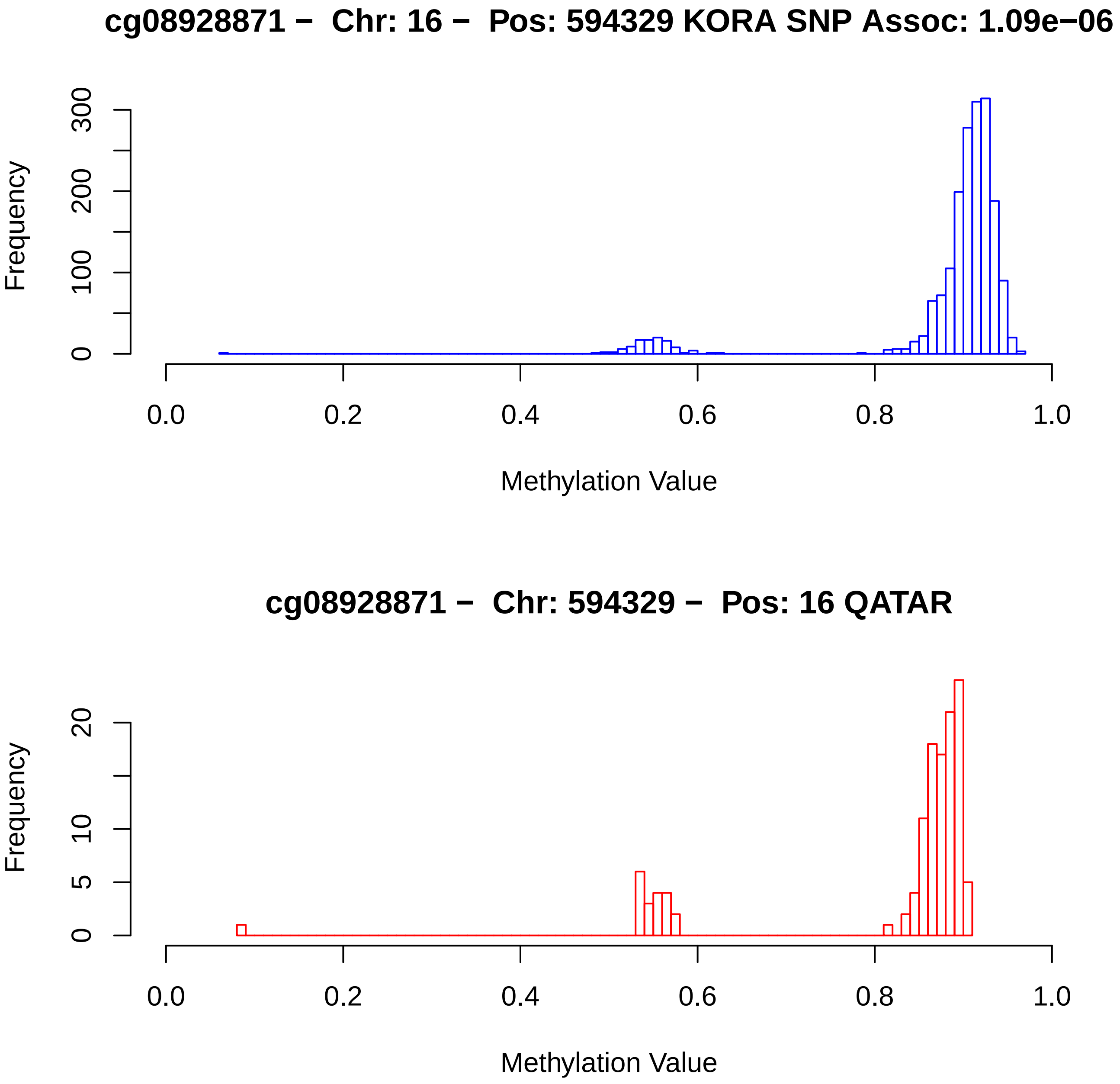
cg08928871 − Chr: 16 − Pos: 594329 KORA SNP Assoc: 1.09e−06
Frequency
0
100
200
300
0.0
0.2
0.4
0.6
0.8
1.0
Methylation Value
cg08928871 − Chr: 594329 − Pos: 16 QATAR
Frequency
0
5
10
20
0.0
0.2
0.4
0.6
0.8
1.0
Methylation Value

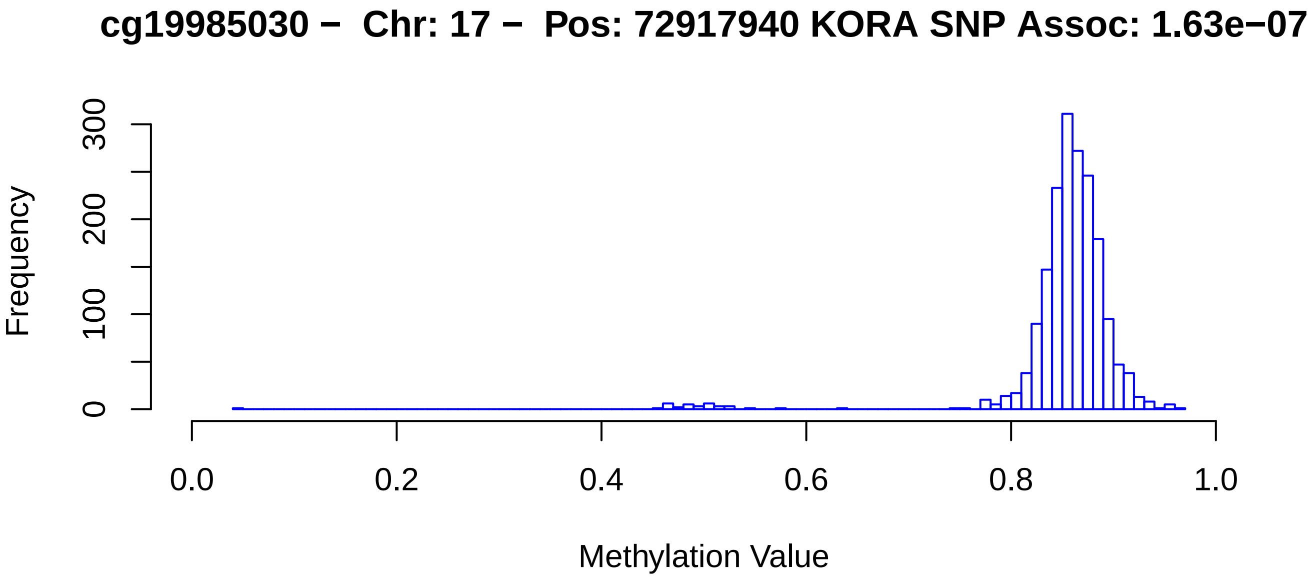
cg19985030 − Chr: 17 − Pos: 72917940 KORA SNP Assoc: 1.63e−07
Frequency
0
100
200
300
0.0
0.2
0.4
0.6
0.8
1.0
Methylation Value

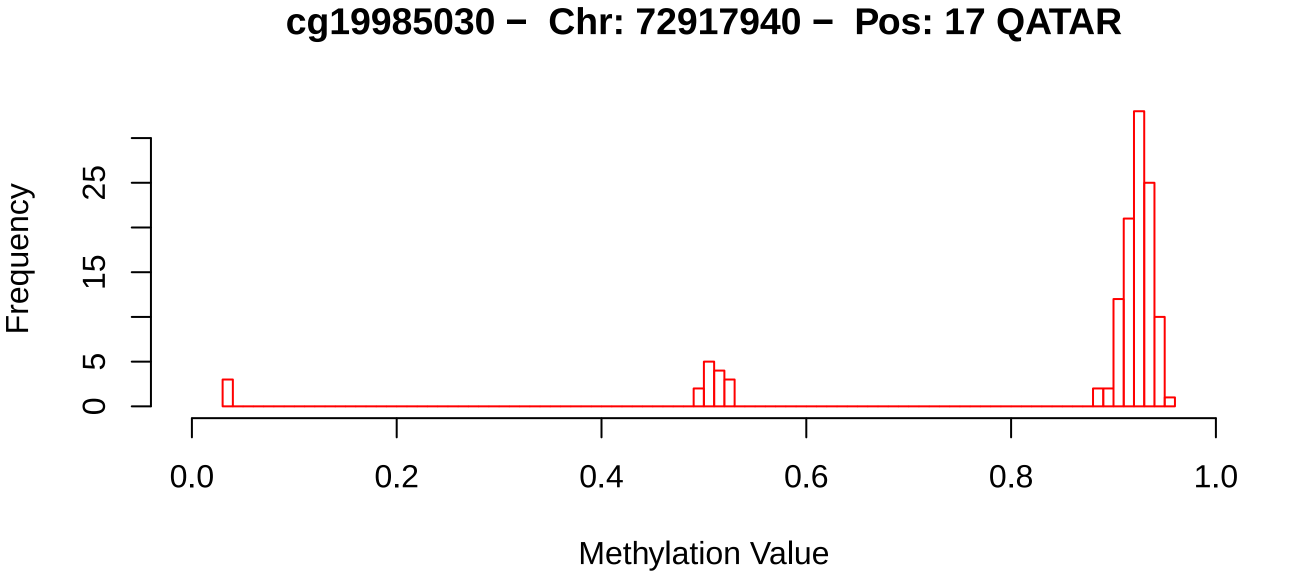
cg19985030 − Chr: 72917940 − Pos: 17 QATAR
Frequency
0
5
15
25
0.0
0.2
0.4
0.6
0.8
1.0
Methylation Value

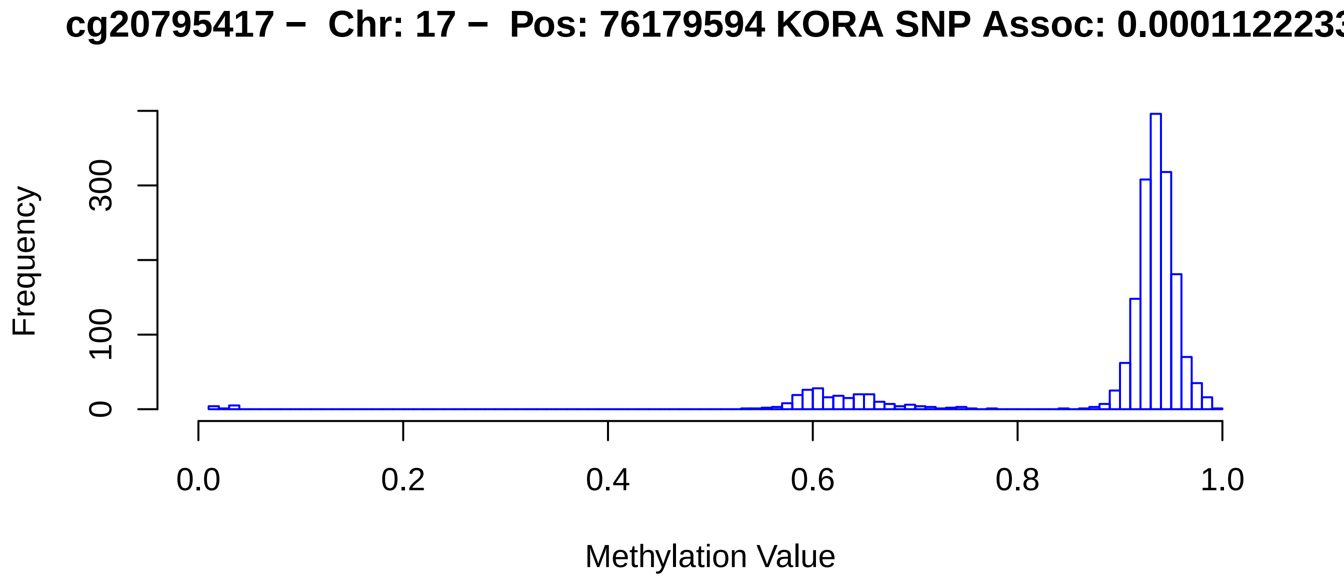
cg20795417 − Chr: 17 − Pos: 76179594 KORA SNP Assoc: 0.000112223
Frequency
0
100
300
0.0
0.2
0.4
0.6
0.8
1.0
Methylation Value

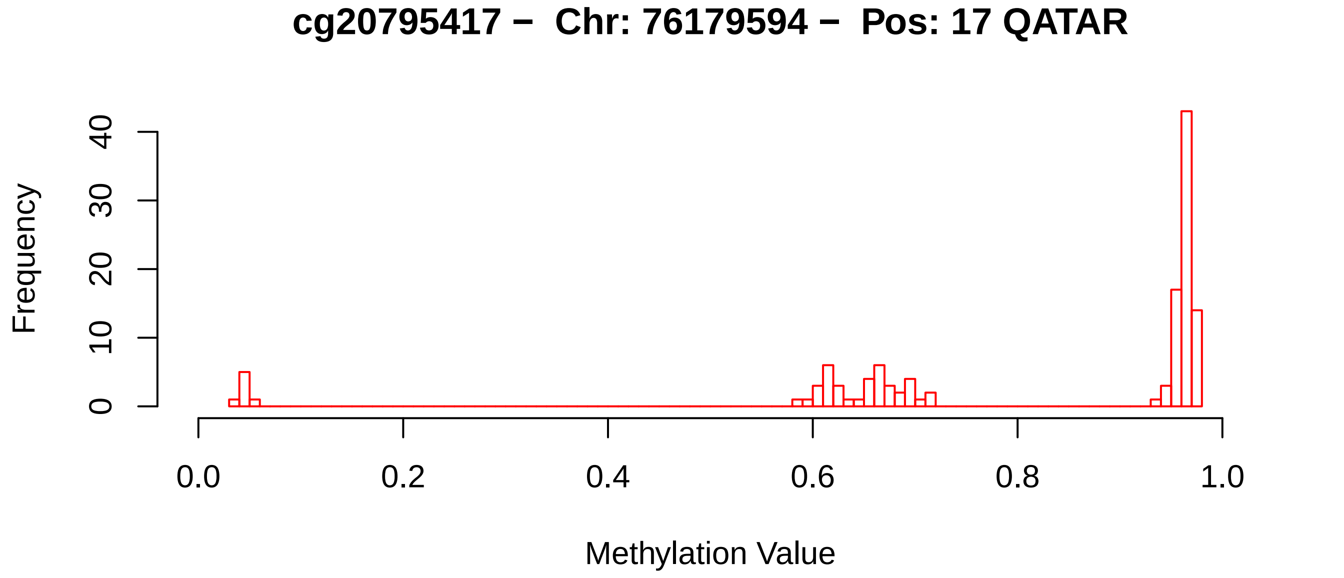
cg20795417 − Chr: 76179594 − Pos: 17 QATAR
Frequency
0
10
20
30
40
0.0
0.2
0.4
0.6
0.8
1.0
Methylation Value

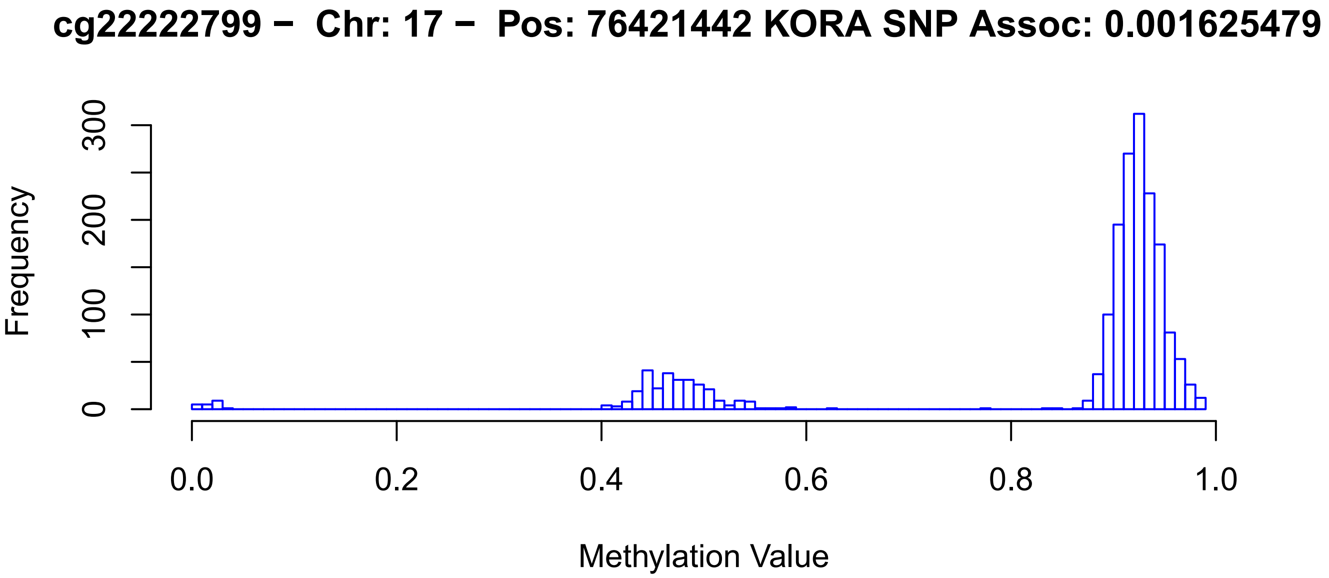
cg22222799 − Chr: 17 − Pos: 76421442 KORA SNP Assoc: 0.001625479
Frequency
0
100
200
300
0.0
0.2
0.4
0.6
0.8
1.0
Methylation Value

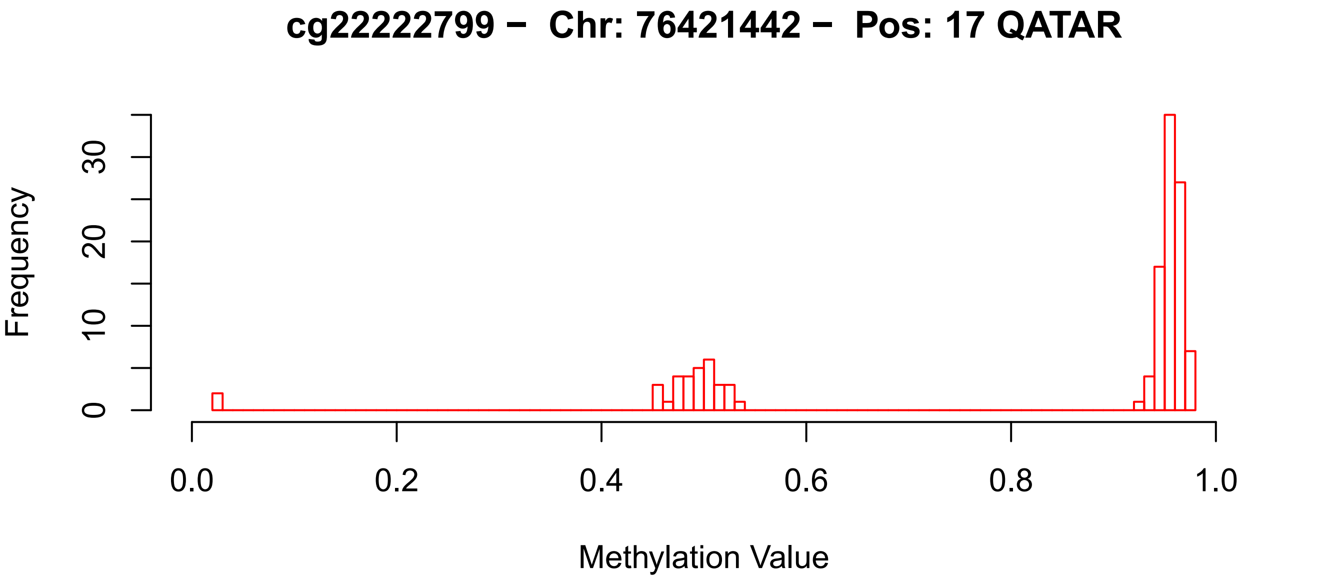
cg22222799 − Chr: 76421442 − Pos: 17 QATAR
Frequency
0
10
20
30
0.0
0.2
0.4
0.6
0.8
1.0
Methylation Value

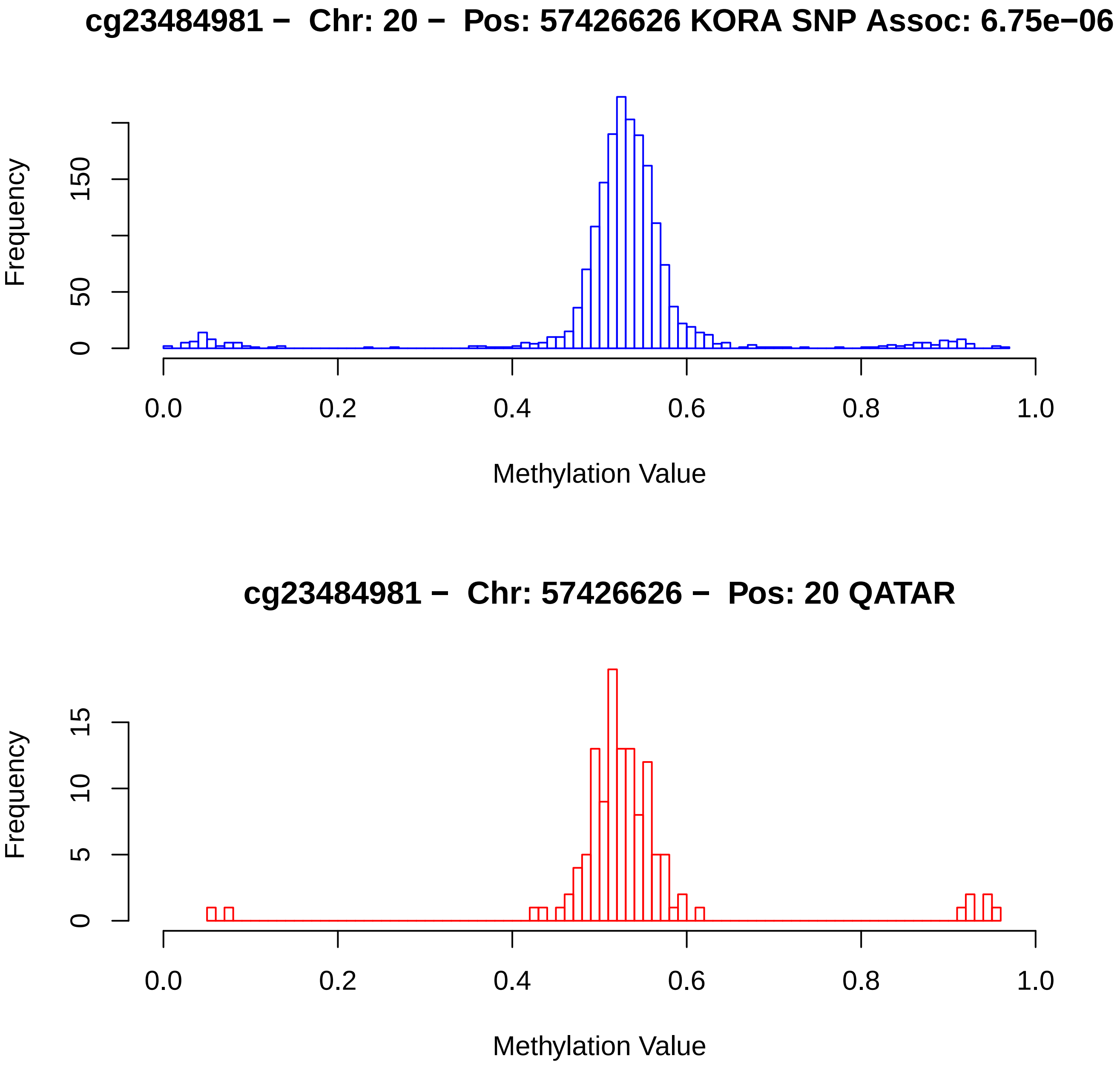
cg23484981 − Chr: 20 − Pos: 57426626 KORA SNP Assoc: 6.75e−06
Frequency
0
50
150
0.0
0.2
0.4
0.6
0.8
1.0
Methylation Value
cg23484981 − Chr: 57426626 − Pos: 20 QATAR
Frequency
0
5
10
15
0.0
0.2
0.4
0.6
0.8
1.0
Methylation Value

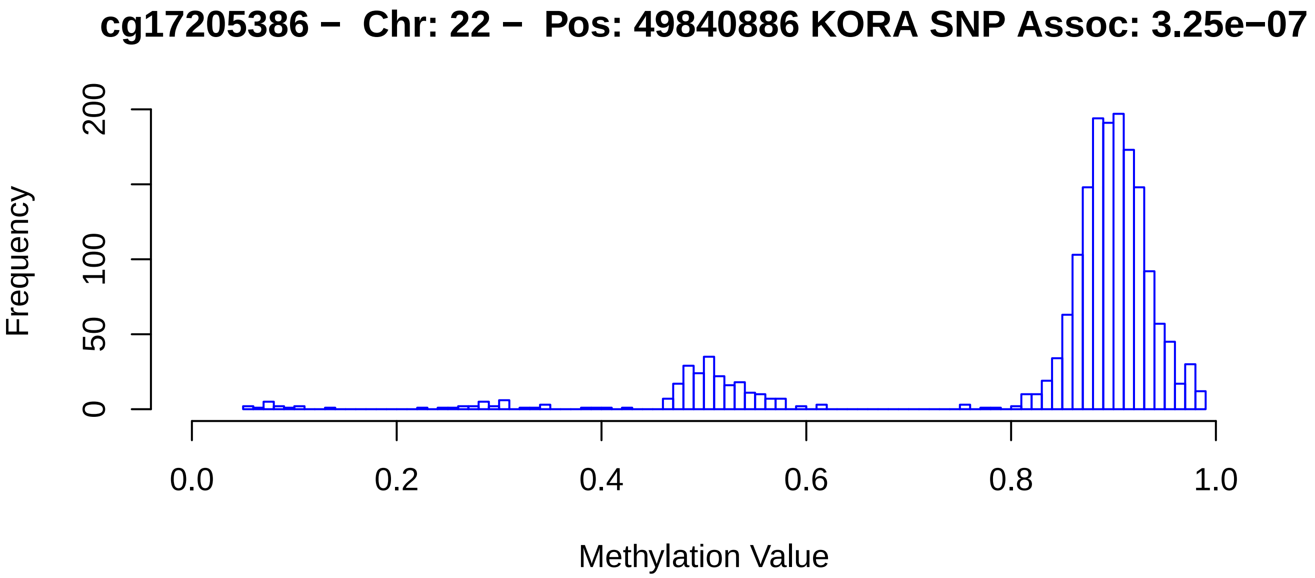
cg17205386 − Chr: 22 − Pos: 49840886 KORA SNP Assoc: 3.25e−07
Frequency
0
50
100
200
0.0
0.2
0.4
0.6
0.8
1.0
Methylation Value

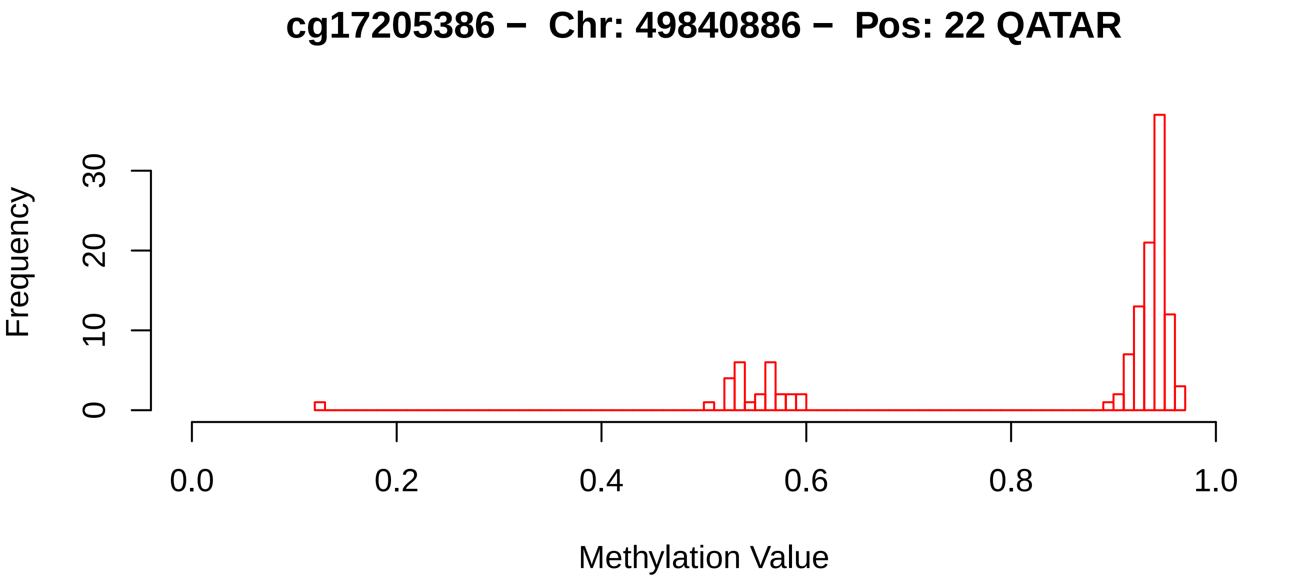
cg17205386 − Chr: 49840886 − Pos: 22 QATAR
Frequency
0
10
20
30
0.0
0.2
0.4
0.6
0.8
1.0
Methylation Value
